# Intron gain and loss in segmentally duplicated genes in rice

**DOI:** 10.1186/gb-2006-7-5-r41

**Published:** 2006-05-23

**Authors:** Haining Lin, Wei Zhu, Joana C Silva, Xun Gu, C Robin Buell

**Affiliations:** 1 The Institute for Genomic Research, Medical Center Drive, Rockville, MD 20850, USA; 2 Department of Genetics, Development, and Cell Biology, Center for Bioinformatics and Biological Statistics, Iowa State University, Ames, IA 50011, USA

## Abstract

**Background:**

Introns are under less selection pressure than exons, and consequently, intronic sequences have a higher rate of gain and loss than exons. In a number of plant species, a large portion of the genome has been segmentally duplicated, giving rise to a large set of duplicated genes. The recent completion of the rice genome in which segmental duplication has been documented has allowed us to investigate intron evolution within rice, a diploid monocotyledonous species.

**Results:**

Analysis of segmental duplication in rice revealed that 159 Mb of the 371 Mb genome and 21,570 of the 43,719 non-transposable element-related genes were contained within a duplicated region. In these duplicated regions, 3,101 collinear paired genes were present. Using this set of segmentally duplicated genes, we investigated intron evolution from full-length cDNA-supported non-transposable element-related gene models of rice. Using gene pairs that have an ortholog in the dicotyledonous model species *Arabidopsis thaliana*, we identified more intron loss (49 introns within 35 gene pairs) than intron gain (5 introns within 5 gene pairs) following segmental duplication. We were unable to demonstrate preferential intron loss at the 3' end of genes as previously reported in mammalian genomes. However, we did find that the four nucleotides of exons that flank lost introns had less frequently used 4-mers.

**Conclusion:**

We observed that intron evolution within rice following segmental duplication is largely dominated by intron loss. In two of the five cases of intron gain within segmentally duplicated genes, the gained sequences were similar to transposable elements.

## Abstract 2

Analysis of over 3,000 co-linear paired genes in rice shows more intron loss than intron gain following segmental duplication.

## Background

Introns are under less selection pressure than exons, and consequently, their sequences diverge faster than exons. However, the position of the intron with respect to the protein sequence is relatively conserved and conservation of intron position has been observed between distinct eukaryotic lineages throughout about 1.5 billion years of evolution such as between animal and fungal genes [1] and between the malaria parasite *Plasmodium falciparum *and other eukaryotes [2]. With respect to intron position within genes, introns within intron-sparse species as well as single intron genes are preferentially located near the 5' end of the gene [3,4], suggesting a biased pattern of intron distribution. Indeed, recent studies on 684 eukaryotic orthologous genes from eight eukaryotic species of animals, plants, fungi, and protists showed preferential intron loss [5,6] and intron gain [6] in the 3' end of genes. This is in contrast to an analysis in fungal species in which no positional bias in intron loss was observed [7].

Introns can be classified into three categories based on location relative to the codon. Introns that do not interrupt the codons are termed phase 0, while phase 1 introns are located between the first and second bases of the codon and phase 2 introns are located between the second and third bases of the codon. It has been reported that eukaryotic genes have more phase 0 introns than phase 1 or phase 2 introns; on average a 5:3:2 ratio of phase 0: phase 1: phase 2 introns is observed, although the specific ratio of intron phase appears to be species specific [8-10]. Several explanations have been proposed for phase bias, including legacy of gene formation in the intron early theory [11,12], phase bias of intron insertion [13], and phase bias of intron loss or selection [5,7].

Discovery of both intron loss and intron gain suggests that these two processes may be ongoing events in evolution. The rates of intron gain and loss seem to differ greatly among species [2,7,14-16] and the underlying mechanism(s) driving intron loss and gain are still unknown. With respect to plants, large-scale computational analyses of intron loss and gain have been focused on *Arabidopsis thaliana*, a model dicotyledonous plant [2,4-6,16-20]. With the availability of the near-complete, high quality rice genome sequence [21] and uniform, high quality gene annotation for the genome [22], we have the ability to examine intron loss and gain within a second plant species that represents the other major clade of angiosperms, monocotyledonous plants. Phylogenetic analysis indicates that date of divergence of *Arabidopsis *and rice is approximately 130 to 200 million years ago (MYA) [23-25]. Interestingly, depending on the completeness and quality of the genome dataset, as well as the methods and parameters employed, the rice genome underwent a segmental duplication that involved 15% to 62% of the genome [25-29] and occurred approximately 70 MYA [25,27], with the exception of the top arms of chromosomes 11 and 12, which underwent a more recent duplication estimated at 5 MYA [27].

Segmental duplication in rice provides the opportunity to study intron gain and loss within a subset of genes that have recently diverged. In this study, we report on the evolution of introns within coding sequences (CDS) after segmental duplication in rice. Through our examination of segmentally duplicated genes, we anticipated that we would identify more intron gain or loss events than for non-duplicated genes due to the accelerated rate of intron loss or intron gain in duplicated versus orthologous genes, as reported previously in two malaria parasites [30]. Other advantages of investigating segmentally duplicated genes are that the age of the duplication is approximately 70 MYA [25,27], which is within the approximately 100 million years divergence limit for investigating recently gained introns [31,32], and that segmentally duplicated blocks are more reliable than individually duplicated genes for this type of analysis. Furthermore, we could exploit the phylogeny of rice with *A. thaliana*, a model dicotyledous plant with a near-complete genome sequence, as the outgroup to readily classify 'intron loss' and 'intron gain' events between the two duplicated rice genes.

## Results

### Rice segmentally duplicated blocks

Previous analyses of segmental duplication in rice used sequence datasets that contained a substantial portion of unfinished genome sequence and lacked refined structural and functional annotation of the genes [25-29]. Thus, we repeated the analysis of segmental duplication using a set of pseudomolecules (about 371 Mb total) that contain 98% finished sequence and had been annotated for genes both at the structural and functional level [22]. Depending on the maximum distance permitted between collinear gene pairs, 25.9% to 53.4% of the rice genome could be identified as segmentally duplicated (Table 1). Using a maximum distance of 200 kb between collinear gene pairs, a total of 149 segmentally duplicated blocks were identified (Additional data file 1). The largest block had 287 pairs of duplicated genes between chromosomes 11 and 12, consistent with the more recent duplicated reported between the top arms of these two chromosomes [27]. These 149 blocks covered 159 Mb (42.8%) of the 371 Mb genome and contained 21,570 of the total 43,719 non-transposable element (TE) related genes (49.3%) in the rice genome. Of these 21,570 genes, 5,567 were retained within the blocks and corresponded to 3,101 pairs of segmentally duplicated genes distributed across all 12 chromosomes of rice (Additional data file 2), with chromosomes 1 and 5 having the largest number of duplicated gene pairs (656 pairs).

An increase in genome coverage within the duplicated regions was observed if the maximum distance permitted between collinear gene pairs was expanded from 200 kb to 500 kb, 1 Mb, or 5 Mb, whereas a much smaller percentage of the genome was covered if the maximum distance was limited to 100 kb (Table 1). Previous studies on segmental duplication in the rice genomes reported that 15% to 62% of the rice genome had undergone segmental duplication [25-29], consistent with our analyses of duplication within the rice genome. As we wished to examine intron evolution within segmentally duplicated genes and there was little difference in percent of the genome identified as duplicated using a maximum distance of 500 kb, 1 Mb, and 5 Mb between collinear gene pairs, we utilized the intermediate estimate of segmental duplication that we obtained using 200 kb as the maximum distance permitted between collinear gene pairs. Thus, our subsequent analyses report on duplicated genes with a maximum distance of 200 kb permitted between collinear gene pairs.

### Conservation of exon-intron structure

**Figure 1:**
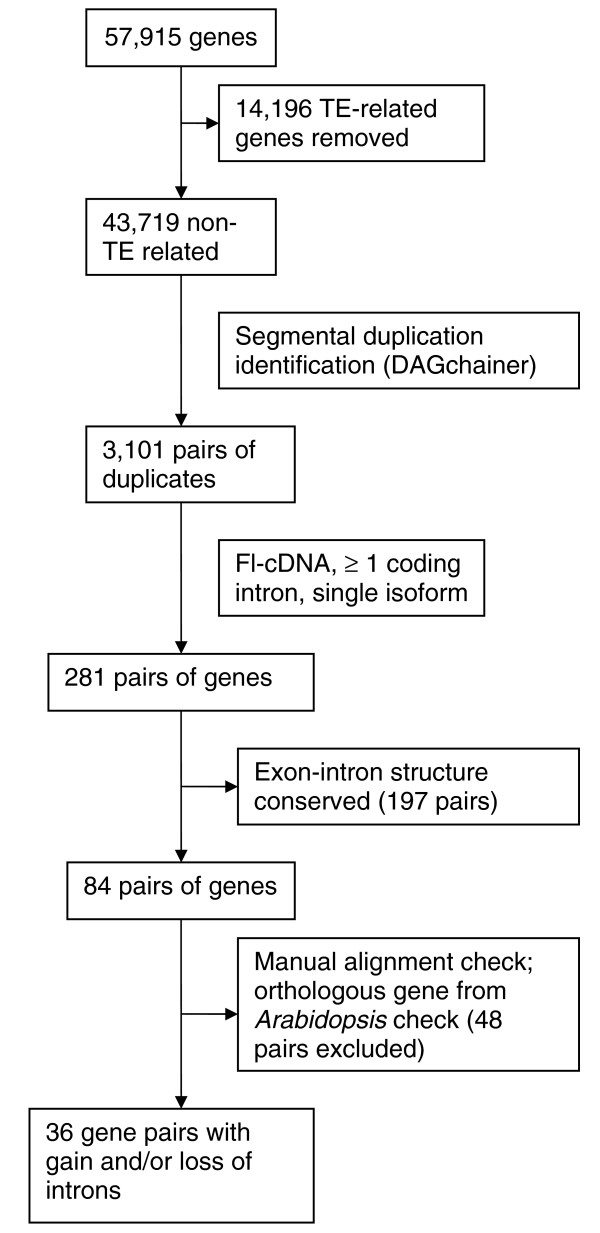
Flow chart for the identification of intron gain and intron loss within segmentally duplicated rice genes. TE, transposable element.

Within the 43,719 non-transposable element-related gene models in rice, 140,827 introns within the CDS are present, with an average length of 385 base pairs (bp; standard deviation (std) 470) and an average GC content of 37.5%. Out of the 3,101 pairs of segmentally duplicated genes, 281 pairs had at least one intron that passed the manual review for full-length (fl)-cDNA support and single isoform. In total, 2,573 introns were present within these 281 gene pairs and had a similar length distribution (average 315 bp) and GC content (36.9% GC) to those found throughout the genome. We found that 197 of the 281 pairs (70%) had completely conserved exon-intron structure in the coding region (958 intron positions in the alignments), that is, the intron number, position, and phase were identical among the duplicated genes (Figure 1). The other 84 pairs (30%) had incongruent exon-intron structure. To eliminate the possibility that the incongruence was due to an aberrant alignment, these alignments were manually checked. Only introns surrounded by reliable alignments and only pairs with a putative orthologous gene from *Arabidopsis *were further investigated. Thus, 48 alignments were excluded and a total of 36 pairs of genes (137 intron positions within the alignments) that showed potential intron loss or intron gain were investigated further.

### Abundance of intron loss after segmental duplication

**Figure 2:**
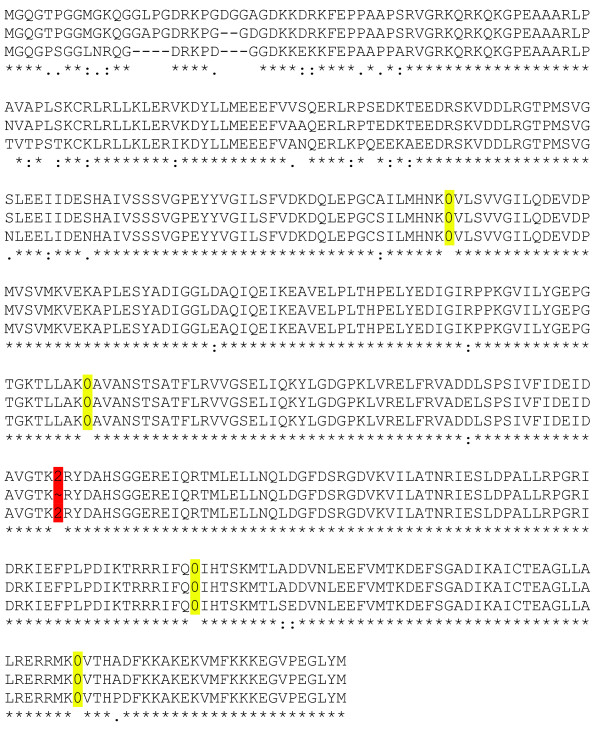
Example of intron loss. Multiple alignment of the two duplicated rice genes (top; LOC__Os03g18690.1, LOC_Os07g49150.1) and their putative orthologous *Arabidopsis *gene (bottom; At4g29040.1) suggests that the third intron of LOC_ Os07g49150.1 was lost. Yellow inserts indicate conserved introns across the three genes while red indicates lost intron. The phase of the intron is inserted into the alignment. All conserved introns are phase 0 whereas the lost intron is phase 2. The two rice genes and putative *Arabidopsis *ortholog encode a 26S proteasome regulatory subunit 4.

**Figure 3:**
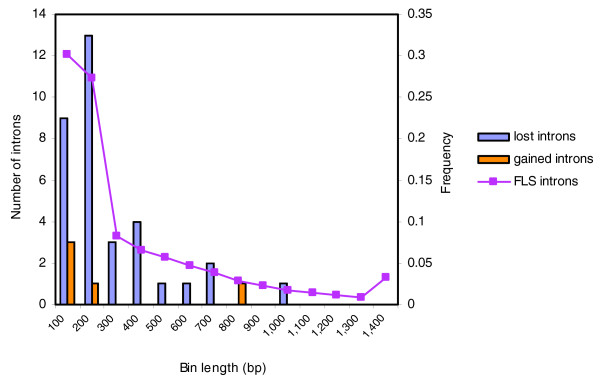
Distribution of the sizes of the lost and gained introns. Intron lengths were binned into 100 bp bins and the number of lost and gained introns in each bin was determined and plotted against the frequency of 33,011 FLS introns within the rice genome.

To determine whether the incongruence was due to intron gain or loss, we used the putative orthologous gene from *Arabidopsis *for the gene pair. From our set of 36 gene pairs with validated alignments, we identified 31 gene pairs with an intron loss(es) (43 intron losses in total), one gene pair with a single gained intron, and four gene pairs in which both intron loss and gain were observed (6 intron losses and 4 intron gains). An example of intron loss is shown in Figure 2. In this example, the third intron of LOC_Os07g49150.1 was lost as shown by the comparison to the duplicated rice gene model LOC_Os03g18690.1 and the putative ortholog from *Arabidopsis *At4g29040.1. Alignments of all of the 36 gene pairs with their orthologs from *Arabidopsis *are displayed in Additional data file 3. The length of the lost introns (226 bp, std 206) was shorter than the average intron length in the rice genome (385 bp, std 470). The distribution of the length of the lost introns and gained introns and the frequency of the length of the 33,011 fl-cDNA supported (FLS) rice introns (see Materials and methods for detail) are shown in Figure 3.

### Intron loss showed no preference at the 3' end of genes

**Figure 4:**
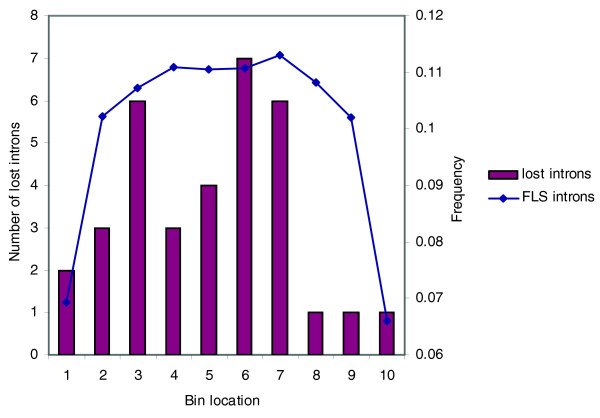
Intron loss along the coding sequence. The positions of the lost introns were inferred from the retained intron of its corresponding duplicated gene. The whole coding sequence was divided into 10 bins. The positions of independently lost introns were placed into the corresponding bin and plotted against the frequency of all 33,011 FLS introns within the rice genome, which had been binned into the same 10 bins.

A single intron loss, termed an independent intron loss, was observed in 31 gene pairs as determined by alignment with the putative *Arabidopsis *ortholog. However, within these 31 gene pairs, 34 introns in total were lost as for 3 gene pairs, both rice genes underwent separate intron loss events. In these 31 gene pairs, we observed no bias in intron loss position at the 3' ends of genes (Figure 4). Neither was there a bias in the position of intron loss in our set of four gene pairs in which multiple intron losses were observed (data not shown). Interestingly, in one gene pair (LOC_Os05g02130.1 and LOC_Os01g74320.1), all seven introns were lost in LOC_Os01g74320.1, and in LOC_Os07g44140.1, multiple consecutive introns at the 3' end of the gene were lost (see Additional data file 3).

### Intron loss rate at phase 0, 1, 2

Previous reports on intron loss suggested a phase bias [5]. To investigate phase bias in intron loss, we first examined intron phase distribution within the rice genome using a set of introns (33,011 total) derived from the coding regions of 6,046 rice gene models that were supported with fl-cDNA evidence, had no alternative splicing isoform, and had at least one intron within the CDS. The phases of the coding introns were distributed as phase 0 (57.3%): phase 1 (21.5%): phase 2 (21.2%), comparable to the distribution reported previously in plants (62: 17: 21) [1].

To examine whether there was a bias in the phase of intron loss in segmentally duplicated genes in rice, we examined the 34 independently lost introns and excluded genes with multiple intron losses. The frequency of intron loss at phase 2 was higher, but not statistically significant, than intron loss at phase 0 and 1 (Table 2; χ^2 ^test *P* value = 0.155). Randomization tests showed that intron loss at phase 2 was unexpectedly high (*P* value = 0.06) and intron loss at phase 0 was unexpectedly low (*P* value = 0.08).

### Rare 4-mers in the exonic sequence at the donor splice site of lost introns

**Figure 5:**
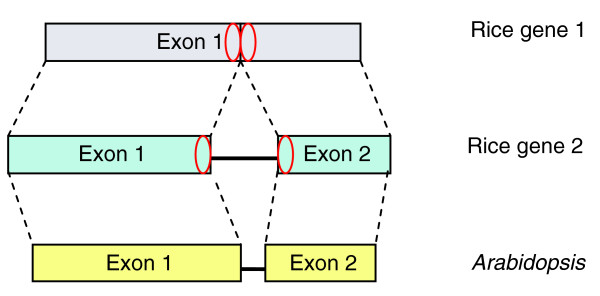
Extraction of the exonic 4-mers at the donor and acceptor splice sites of lost and retained introns. Duplicated rice gene 1 with a single exon and rice gene 2 and *Arabidopsis *orthologous gene with two exons and a single intron are shown in colored rectangles. Dashed lines indicate similar regions. Phylogeny analysis with *Arabidopsis *suggests an intron was lost in rice gene 1. The red ovals show the 4-mers extracted for SoR analysis.

Previous studies indicated sequence composition preferences surrounding splice sites [13,33]. As our sample size was small, we restricted our analysis of nucleotide composition surrounding the splice site to the nearest four nucleotides (4-mers); a total of 31 gene pairs with an independent intron loss (34 total introns) were investigated to determine the exonic nucleotide composition flanking each pair of lost and retained introns (Figure 5). We observed that the 4-mer usage flanking all rice introns was dependent on intron phase (Additional data file 4 and 5). For example, ACAA occurs at the exon donor splice site 70, 17 and 110 times at phase 0, phase 1 and phase 2, respectively. To determine if intron loss is independent of the nucleotide composition of the exon sequence flanking introns, we compared the 4-mers flanking lost introns with those flanking the corresponding retained introns, as well as with the 4-mers flanking all rice introns. To this end, the exonic 4-mers flanking the donor and acceptor splice sites of the lost and retained introns were each attributed a rank, with rank of 1 being the rarest, according to their frequency in the sample of all rice introns (Tables 3 and 4; see Materials and methods).

The sum of the ranks (SoR) of the exonic 4-mers flanking the donor splice site of the lost introns (observed SoR = 6,737) was very significantly lower than expected (expected SoR = 7,647; *P *approximately 0.0007), while that at the acceptor site of the lost introns was within the average range (Table 5). These results reveal a preponderance of rare 4-mers flanking the 5' end of lost introns. This observation is further supported by the fact that the distribution of ranks of 4-mers flanking the donor splice site in lost introns is significantly lower than that in the corresponding retained introns (*P *< 0.013; Wilcoxon's signed rank test). The rank distributions of 4-mers flanking the acceptor splice site did not differ significantly between lost and retained introns (*P* approximately 0.069).

### Source of gained introns

Two out of the five gained introns showed several matches to known rice transposon sequences. The intron of LOC_Os12g02840.1 had a significant hit to a putative Ty1-copia subclass retrotransposon protein (82% identity over the entire intron). A large portion of the other gained intron (LOC_Os12g37660.1; 430 bp out of 741 bp) was highly similar (92% identity) to *Oryza sativa *transposon Rim2-M341 (BK000935) [34]. To ascertain if any of the five gained introns had inserted into other regions of the genome, we searched the five gained introns against our set of 12 pseudomolecules. Three of the gained introns did not match any sequence in the rice genome except itself. For the gained intron in LOC_Os12g02840.1, three high quality matches were detected: to the entire intron of LOC_Os11g03070 (98% identity, putative function of sodium/hydrogen exchanger family protein), which is another segmentally duplicated gene of LOC_Os12g02840.1 from the 5 MYA duplication event; 82% identity to the entire intron of LOC_Os10g05450 (annotated as a hypothetical protein); and 82% identity to the entire intron of LOC_Os06g36500 (annotated as retrotransposon protein, putative, Ty1-copia e subclass). For the second gained intron (LOC_Os12g37660.1), a large portion (approximately 400 bp) matched to numerous regions throughout the pseudomolecules. Of the 64 top alignments to the gained intron within LOC_Os12g37660.1 (approximately 95% identity, approximately 400 bp in length), 54 were in intergenic regions and 10 were within introns of genes, all of which lacked fl-cDNA support (3 hypothetical proteins, 3 expressed proteins, 2 transposable-element related proteins, and 2 known proteins).

We examined these five cases of intron gain further by examining homologous genes from other plant species. With the exception of one case, the gained intron was clearly a straightforward insertion into one of the rice gene pairs (Additional data file 6). For LOC_Os3g16960.1, the gained intron was observed in the maize and sorghum homologs, but absent in the *Arabidopsis *and poplar homologs. Thus, the most parsimonious explanation for the data is a single insertion into one of the rice duplicates prior to the divergence of rice, sorghum, and maize (data not shown).

## Discussion

Intron loss and gain are two important processes in evolution. We observed more genes with intron loss than gain after segmental duplication in rice. We estimated the rates of intron loss and gain after the segmental genome duplication in rice. Allowing *p *to be the proportion of non-conserved introns between duplicated genes, we have *p *= 54/(137 + 958) = 0.0493, where 54 is the number of non-conserved introns, 137 is the total number of the aligned intron positions within the 36 gene pairs that have intron loss and gain, and 958 is the total number of aligned intron positions within the 197 conserved gene pairs. Given that intron loss and acquisition are rare events, the expected rate of intron loss and gain can be estimated under the simple Poisson model and calculated as:

D_int _= -ln (1 - p) = 0.0506

If we estimate t = 70 MYA for the rice genome duplication [25,27], we estimate that the rate of intron gain and loss is:

μ = D_int_/2t = 0.0506/(2 × 70 × 10^6^) = 3.61 × 10^-10 ^per intron per year

As a total of 49 lost introns and 5 gained introns were observed, we estimated the evolutionary rate of intron loss and intron gain after the genome duplication is:

μ_loss _= 3.61 × 10^-10 ^× 49/(49 + 5) = 3.28 × 10^-10 ^per intron per year

μ_gain _= 3.61 × 10^-10 ^× 5(49 + 5) = 3.34 × 10^-11 ^per intron per year

A previous study involving 684 groups of orthologous genes reported an intron loss rate in *Arabidopsis *of 2 to 3 × 10^-10 ^per year and an intron gain rate of 2.2 to 2.9 × 10^-12 ^per year [16]. Our study, which involved segmentally duplicated genes within rice, revealed a similar intron loss rate but a higher intron gain rate, which may be reflective of the reduced evolutionary pressure on duplicated genes. The detection of transposon-related sequences in two of the five gained introns suggests that transposable elements may have a role in intron evolution and is consistent with the increased fraction of transposable elements in the rice genome compared to *Arabidopsis *[21].

It is possible that the rate of intron loss and gain differs within our set of segmentally duplicated genes as it has been previously reported that the segmental duplication between the top arms of chromosomes 11 and 12 is recent (within 5 MYA) in comparison to the bulk of the segmental duplication, estimated at 70 MYA [25,27]. Thus, we determined the *d*_S _for the 233 gene pairs that had a single isoform, were supported by a fl-cDNA, and had been manually validated (197 gene pairs with congruent intron structure and 36 gene pairs with intron loss and/or intron gain). The *d*_S _values ranged from 0.03 to 24.86 with a clear peak between 0.6 to 1.4 (data not shown). Similar rates of intron loss (1.41 × 10^-10 ^per intron per year) and intron gain (0.94 × 10^-11 ^per intron per year) were obtained from the calculations performed using a subset of the 233 gene pairs in which the *d*_S _between duplicates was between 0.6 and 1.4 (117 pairs total with four gene pairs originating from the top arms of chromosomes 11 and 12).

A reverse transcriptase-mediated model in which a segment of the genomic copy of a gene can be replaced by a reverse-transcribed copy via homologous recombination was previously proposed to explain the pattern of intron loss [3,35,36] and has been further supported by recent genomic analysis of several species [5,6,15]. The 3' end bias of intron loss is important evidence for this model as reverse transcriptase is error-prone and, as a consequence, a high frequency of 5'-truncated cDNA fragments are generated. Although we did not observe a 3' end preference of intron loss, we did find examples of multiple consecutive intron loss at the 3' end of genes and even loss of all the introns, which is consistent with the reverse-transcriptase-mediated model. Lack of power due to a small sample size (34 lost introns) might be one explanation for the lack of evidence for a 3' bias of intron loss in rice. Another explanation may be the unusual intron distribution pattern, which is similar to that of *Arabidopsis *(data not shown) in which there is no 5' bias in intron location within single-intron genes [4]. The other explanation is that the reverse-transcriptase-mediated model may not be the only mechanism for intron loss in rice and that intron loss may occur via genomic deletion as proposed by Cho *et al*. [37], who observed no intron loss bias at the 3' end of genes in *Caenorhabditis*. However, according to the genomic deletion model, we would expect some instances of imprecise deletion of introns, which is not the case in our sample. Therefore, an unknown recognition signal may exist that allows the exact deletion of introns in rice.

We did not observe any statistically significant differences in the frequency of intron losses in different phases. Nor did examination of nucleotide compositional patterns in the exons surrounding the splicing site reveal an apparent pattern in the bi-nucleotide sequence of the exon at the boundary other than that shown by canonical splice site consensus sequence (AG|GT) in which '|' represents the intron position (data not shown). Yet conservation of the exon nucleotides adjacent to the exon-intron boundary has been reported to play an important role in correct splicing [38-40]. Within the four nucleotides at the donor splice site, we observed that the exon boundary of lost introns had less frequently used 4-mers than their corresponding retained introns, as well as relative to the sample of all approximately 33,000 introns. Thus, genes with less common exonic sequence at the donor site may experience splicing inaccuracy and inefficiency and, consequently, intron loss at these positions may be strongly favored by selection. Alternatively, it is possible that the less common 4-mers reflect exonic sequences more prone to direct intron loss, in the case of the genomic deletion model. Since we did not have a large sample for each intron phase, our data were insufficient to draw a correlation between intron loss rate at each phase and the nucleotide composition of the flanking exonic sequence.

## Conclusion

We were able to document intron loss and gain in segmentally duplicated rice genes with a rate of loss and gain similar to that observed within orthologous genes across a range of eukaryotes. While we did not observe preferential intron loss at the 3' end of genes, we did observe a nucleotide bias within the exonic sequence flanking the lost introns.

## Materials and methods

### Identification of segmentally duplicated genes

A total of 43,719 non-transposable element-related rice protein sequences from release 3 of the TIGR Rice Genome Annotation [22] were used to identify segmental duplication in rice using an all versus all BLAST search (WU-BLASTP, parameters "V = 5 B = 5 E = 1e-10 - filter seg) [41]. As alternative splicing occurs in rice and some genes have multiple splice forms, the largest peptide sequence was used whenever alternative isoforms existed. Repetitive matches were filtered using perl scripts to remove low scoring matches within multiple alignment regions that were defined by a high-scoring segment pair within 50 kb. Segmentally duplicated blocks were identified using DAGchainer [42] with parameters '-s -I -D 200000'. which primarily includes self comparisons, ignores tandem duplication alignments, and sets the maximum distance allowed between two collinear gene pairs to 200 kb. A minimum of six gene pairs was used to define a block.

### Identification of congruent and incongruent introns

Duplicated genes with at least one intron were checked to ensure that they were supported by a fl-cDNA and that no alternative isoforms existed. Intron positions and phases were retrieved from the TIGR Osa1 genome annotation database [22]. ClustalW [43,44] with default parameter settings was run for each pair to obtain a global alignment. Intron positions and phases were then inserted into the ClustalW alignment using perl scripts. Alignments with incongruent exon-intron structure were manually checked to ensure the introns were supported by reliable alignments. For the ten amino acids flanking the splice site (five amino acids on each side), we required that at a minimum, three amino acids had to be identical and that approximately 60% similarity was observed.

### Identification of intron loss and intron gain

Simple phylogeny analysis was used to determine if the incongruent exon-intron structure was attributable to loss or gain of an intron. We identified putative orthologous genes by searching the predicted *Arabidopsis *proteome (TIGR release 5, [45]) with the predicted rice proteome using blastp (E-value < 1e-10) and selecting the reciprocal best hit. In the event we did not identify an ortholog in *Arabidopsis *via the reciprocal top match method, we used the best *Arabidopsis *match. Using the *Arabidopsis *genes as the outgroup, we aligned the rice duplicated gene models to the orthologous *Arabidopsis *gene model. ClustalW with default parameter settings was run for each triplet (the two rice gene models and their putative *Arabidopsis *ortholog) and intron positions and phases were inserted into the ClustalW alignment (Additional data file 3). Only loss or gain of introns after segmental duplication was examined further. An intron loss was defined if the intron was present at the same position in only a single rice gene and the putative *Arabidopsis *ortholog (referred to as a retained intron). An intron gain was defined if the intron was present in single rice gene but absent in the other rice paralog and the putative *Arabidopsis *ortholog.

### Randomization test for intron loss rate at phase 0, 1, 2

A total of 233 pairs of duplicated genes, among which 197 pairs have completely conserved introns and 36 pairs show putative loss and gain of introns, were used in our randomization test. The total number of conserved intron alignment positions at each phase was counted (P0, 580; P1, 236; P2, 225). The total number of independently lost introns at each phase was counted (P0, 15; P1, 7; P2, 12). A total of 10,000 iterations were simulated. A total of 34 phases were randomly generated in each iteration based on the frequencies of the conserved aligned intron positions at each phase from the 233 gene pairs. The number of lost introns at each phase was then compared with those generated by simulation.

### Nucleotide composition of exonic sequences flanking lost introns, retained introns, and all introns

To determine whether lost introns in duplicated rice genes tend to be flanked by rare nucleotide combinations, we compared the frequency distribution of the four nucleotides (4-mers) in the exonic sequence that flanked lost introns with the exonic 4-mers flanking the corresponding retained introns, as well as with the frequency distribution of the 4-mers flanking all introns in the genome. Comparisons were done independently for 4-mers flanking the donor and the acceptor ends of introns. The small number of lost introns, distributed over three intron phases (34 introns, of which 15, 7 and 12 were from phases 0, 1 and 2, respectively) relative to the total number of 4-mer classes (4^4 ^= 256) precludes effective use of standard tests, such as the chi-square test, to compare the distributions. Instead, tests based on rank distributions were used as described below.

#### Comparison of 4-mers flanking lost introns versus all introns

A total of 33,011 introns within the coding regions from 6,046 rice gene models that were supported with fl-cDNA, had no alternative splicing isoform, and had at least one intron within the CDS were used to determine the 4-mer distribution in exonic sequences that flank the introns. The four nucleotides that flank the donor and acceptor splice sites of each intron were extracted and their frequency calculated. For each intron phase, each 4-mer was given a rank between 1 and 256, to cover all of the 4^4 ^nucleotide combinations, with the lowest frequency having the smallest rank (rank = 1). In this way, three rank distributions, one for each intron phase 0, 1 and 2, and their attached frequency distributions, were generated for each the donor and the acceptor flanking regions.

We devised a statistic that we call 'sum of ranks', SoR, to determine if the 4-mers flanking lost introns are less common than expected by chance. This statistic SoR corresponds to the sum of the ranks of all introns in a sample, as determined by their nucleotide composition and phase. The test was conducted as follows: 10,000 pseudo-replicates were generated by randomly sampling the three rank distribution obtained for all introns, according to their frequency distribution (that is, each rank was selected with probability equal to its frequency). Each pseudo-replicate consisted of 34 sampled introns, 15, 7 and 12 of which were sampled from the rank distribution of phase 0, 1, and 2 introns, respectively, to preserve the characteristics of the observed distribution of lost introns. A SoR value was obtained for each pseudo-replicate to generate the distribution of expected 'sum of ranks'. The SoR for the 34 lost introns was compared against this distribution to determine the probability *P *of obtaining this value by chance. *P *is approximately equal to the fraction of pseudo-replicated with a smaller or equal SoR value.

#### Comparison of 4-mers flanking lost introns versus retained introns, in the corresponding duplicate gene

A rank was attributed to each lost intron, based on the composition of its 4-mer and its intron phase, according to the rank distributions obtained for all 33,011 introns (see above), to obtain a distribution of ranks for the set of lost introns. A distribution of ranks for the set of retained introns was obtained in a similar way. The two distributions were compared using a Wilcoxon's signed rank test. This procedure was done for both donor and acceptor flanking sequences.

### Identification of the source elements of gained introns

Sequences of the five gained introns were searched against the NCBI non-redundant database and were further searched against all the 12 rice pseudomolecules [22]. Significant hits were manually checked. For each case of a gained intron, we examined homologous proteins from three plant species with substantial genome sequence: maize, sorghum, and poplar. Using the protein sequences of the ten rice genes with gained introns, we searched the TIGR Assembled Zea Mays (AZMs) sequences, which are assemblies of gene enrichment sequences [46,47], TIGR Assembled Sorghum Bicolor (ASBs) which are assemblies of gene enrichment reads from sorghum [48], and contigs from the poplar genome project [49]. All of the top hits from maize and sorghum had >70% similarity at the protein level with the rice proteins. Gene models were predicted by running the *ab initio *gene finder FGENESH [50] on the maize, sorghum and poplar genomic sequences. We used ClustalW with default parameter settings to align the six proteins (two rice proteins and the homologous proteins from *Arabidopsis*, maize, sorghum and poplar) and inserted the intron positions/phases into the ClustalW alignment.

### Determination of substitutions per site

The number of synonymous substitutions per synonymous site (*d*_S_) between each of the two rice duplicates was estimated by maximum likelihood, using the codon-based substitution model of Yang *et al*. [51] as implemented in codeml of PAML, version 3.15 [51,52]. Codeml was run using in pairwise mode (runmode = -2), with codon equilibrium frequencies estimated from average nucleotide frequencies at each codon position (codonFreq = 2). Given the estimated age of approximately 70 MYA for the polyploidization event in rice [25], and the estimated substitution rate in synonymous sites of approximately 6.5 × 10^-9^/site/year [53], rice paralogs resulting from this polyploidization event are expected to differ on average by approximately 0.9 synonymous substitution per site.

## Additional data files

The following additional data are available with the online version of this paper. Additional data file 1 lists the segmentally duplicated blocks within the rice genome. Additional data file 2 lists 3,101 pairs of segmentally duplicated genes along with their pairings and their sequence. Additional data file 3 shows the ClustalW alignment of the two rice duplicated genes and their orthologous gene from *Arabidopsis*. Additional data file 4 lists the occurrence of background exonic 4-mers at the donor splice sites of different intron phase. Additional data file 5 lists the occurrence of background exonic 4-mer at the acceptor splice sites of different intron phase. Additional data file 6 shows the ClustalW alignment of the two rice duplicated proteins with putative orthologous proteins from *Arabidopsis*, poplar, maize and sorghum.

## Supplementary Material

Additional File 1 The segmentally duplicated blocks within the rice genome. Click here for file

Additional File 2 The 3,101 pairs of segmentally duplicated genes along with their pairings and their sequence. Click here for file

Additional File 3 The ClustalW alignment of the two rice duplicated genes and their orthologous gene from *Arabidopsis*. Click here for file

Additional File 4 The occurrence of background exonic 4-mers at the donor splice sites of different intron phase. Click here for file

Additional File 5 The occurrence of background exonic 4-mer at the acceptor splice sites of different intron phase. Click here for file

Additional File 6 The ClustalW alignment of the two rice duplicated proteins with putative orthologous proteins from *Arabidopsis*, poplar, maize and sorghum. Click here for file

## Figures and Tables

**Table 1:** Statistics of genome, genes, and regions within segmentally duplicated blocks of the rice genome

	Maximum distance between collinear gene pairs				
Statistics	100 kb	200 Kb	500 Kb	1 Mb	5 Mb

Region covered by duplicated blocks (Mb)	96.04	158.9	193.25	196.35	197.96
Region covered by multiple duplicated blocks (Mb)	7.16	30.6	45.2	45.31	45.74
Number of duplicated blocks	151	149	101	98	96
Genome coverage (%)	25.9	42.8	52.1	52.9	53.4
Non-TE gene coverage (%)	30.3	49.3	59.1	59.7	60
Total number of non-TE genes retained within duplicated blocks	4,377	5,567	5,879	5,894	5,894
Number gene pairs retained within duplicated blocks	2,277	3,101	3,346	3,355	3,355
Total number non-TE genes within duplicated blocks	13,250	21,570	25,819	26,114	26,248

**Table 2:** Distribution of phase of intron loss in segmentally duplicated rice genes

	Phase 0	Phase 1	Phase 2
Intron loss*	15	7	12
Conserved introns^†^	580	236	225
Intron loss rate^‡^	2.5%	2.8%	5.1%

*Multiple consecutively lost introns were excluded from this analysis. ^†^Conserved aligned intron positions within all 235 duplicate gene pairs. ^‡^Intron loss rate was calculated by (intron loss/(intron loss + conserved introns)) × 100.

**Table 3:** 4-mer usage of exonic sequence at donor splice site of lost and corresponding retained introns

Intron lost			Intron retained			
Locus name*	4-mer^†^	Rank^‡^	Locus name^§^	Phase	4-mer	Rank

LOC_Os05g48520.1	CAAG	256	LOC_Os01g48540.1	0	CAAG	256
LOC_Os06g44300.1	CGAG	245	LOC_Os02g08230.1	0	CGAG	245
LOC_Os06g11920.1	CAAG	256	LOC_Os02g51600.1	0	CAAG	256
LOC_Os06g10850.1	GAGG	219	LOC_Os02g52830.1	0	CCAT	211
LOC_Os07g02440.1	CGAC	130	LOC_Os03g55420.1	0	CGAG	245
LOC_Os07g12340.1	CAGG	234	LOC_Os03g60080.1	0	CAGG	234
LOC_Os01g13130.1	CGCC	154	LOC_Os05g14240.1	0	CATG	244
LOC_Os11g01820.1	GCTC	103	LOC_Os05g39600.1	0	CATG	244
LOC_Os12g02840.1	CCTC	172	LOC_Os05g40650.1	0	CCTC	172
LOC_Os02g14430.1	CCAG	251	LOC_Os06g35480.1	0	CAAC	193
LOC_Os09g39720.1	GGAG	246	LOC_Os08g44590.1	0	GGAG	246
LOC_Os02g54640.1	GTTC	28	LOC_Os09g26160.1	0	TTTT	133
LOC_Os08g39370.1	CAAC	193	LOC_Os09g31130.1	0	CAAC	193
LOC_Os08g41880.1	CGAG	245	LOC_Os09g32840.1	0	TGAG	253
LOC_Os03g01820.1	GAGG	219	LOC_Os10g39810.1	0	CAAG	256
LOC_Os05g38420.1	TTCG	225	LOC_Os01g62490.1	1	TTCG	225
LOC_Os06g12960.1	GACG	228	LOC_Os02g50810.1	1	CACG	222
LOC_Os09g26160.1	CATC	54	LOC_Os02g54640.1	1	CACA	171
LOC_Os06g51050.1	ACCG	223	LOC_Os03g04060.1	1	ACAG	250
LOC_Os02g46780.1	GCCG	227	LOC_Os04g50770.1	1	GCAG	251
LOC_Os01g50760.1	GGAG	247	LOC_Os05g46580.1	1	GGAG	247
LOC_Os11g09020.1	GTCG	216	LOC_Os12g08090.1	1	ATCT	194
LOC_Os05g04690.1	CGTG	88	LOC_Os01g18400.1	2	CATG	237
LOC_Os05g48700.1	TGAG	246	LOC_Os01g55240.1	2	TCCG	222
LOC_Os05g39720.1	GGTG	115	LOC_Os01g61080.1	2	GATG	217
LOC_Os07g49280.1	CAAG	254	LOC_Os03g18140.1	2	CCCG	142
LOC_Os07g49150.1	AGAG	251	LOC_Os03g18690.1	2	AGAG	251
LOC_Os07g49000.1	GGAG	245	LOC_Os03g19200.1	2	GGAG	245
LOC_Os09g26360.1	GAAG	249	LOC_Os08g34910.1	2	GAAG	249
LOC_Os08g41730.1	GCGG	208	LOC_Os09g32800.1	2	GCGG	208
LOC_Os12g08090.1	TGCG	115	LOC_Os11g09020.1	2	TGCT	163
LOC_Os01g09540.1	TCGG	225	LOC_Os05g10210.1	2	ATGG	238
LOC_Os05g10210.1	TCCA	175	LOC_Os01g09540.1	2	TAAG	248
LOC_Os03g21820.1	GCCG	195	LOC_Os05g39990.1	2	GCAG	252

*Locus name of the rice gene model with intron loss. ^†^The exonic 4-mer at the donor splice site of the lost intron was inferred from the pair-wise alignment of the coding sequences as illustrated in Figure 5. ^‡^Each 4-mer is associated with an intron phase-dependent rank ranging from 1 to 256 based on the frequency of occurrence calculated from exonic 4-mers at the exon-intron boundary of all 33,011 FLS introns. ^§^The corresponding rice duplicated gene with retained intron.

**Table 4:** 4-mer usage of exonic sequence at acceptor splice site of lost and corresponding retained introns

Intron lost			Intron retained			
Locus name*	4-mer^†^	Rank^‡^	Locus name^§^	Phase	4-mer	Rank

LOC_Os05g48520.1	ACCG	53	LOC_Os01g48540.1	0	ATCG	186
LOC_Os06g44300.1	TACA	136	LOC_Os02g08230.1	0	TACA	136
LOC_Os06g11920.1	GGCT	183	LOC_Os02g51600.1	0	GGTT	222
LOC_Os06g10850.1	GCCA	206	LOC_Os02g52830.1	0	GTGA	255
LOC_Os07g02440.1	GGCT	183	LOC_Os03g55420.1	0	GGAT	201
LOC_Os07g12340.1	CTGG	176	LOC_Os03g60080.1	0	TTGG	169
LOC_Os01g13130.1	GCCA	206	LOC_Os05g14240.1	0	GCGA	178
LOC_Os11g01820.1	GTCG	204	LOC_Os05g39600.1	0	GGCG	152
LOC_Os12g02840.1	GCCG	143	LOC_Os05g40650.1	0	GCTG	251
LOC_Os02g14430.1	GGCT	183	LOC_Os06g35480.1	0	GGGT	178
LOC_Os09g39720.1	ATAC	194	LOC_Os08g44590.1	0	ATAT	215
LOC_Os02g54640.1	GTGT	243	LOC_Os09g26160.1	0	GCAT	223
LOC_Os08g39370.1	GTGC	246	LOC_Os09g31130.1	0	ATCA	230
LOC_Os08g41880.1	ATGA	214	LOC_Os09g32840.1	0	ATGA	214
LOC_Os03g01820.1	GCGG	173	LOC_Os10g39810.1	0	ATGG	232
LOC_Os05g38420.1	GCGA	205	LOC_Os01g62490.1	1	GCGA	205
LOC_Os06g12960.1	AGGT	156	LOC_Os02g50810.1	1	AGGT	156
LOC_Os09g26160.1	GGCA	229	LOC_Os02g54640.1	1	AGGA	226
LOC_Os06g51050.1	GCGG	156	LOC_Os03g04060.1	1	GTGG	255
LOC_Os02g46780.1	GATT	244	LOC_Os04g50770.1	1	GTTT	251
LOC_Os01g50760.1	GAAA	246	LOC_Os05g46580.1	1	GGAA	247
LOC_Os11g09020.1	CCAA	156	LOC_Os12g08090.1	1	CCAA	156
LOC_Os05g04690.1	GAAC	235	LOC_Os01g18400.1	2	GAAC	235
LOC_Os05g48700.1	GGCG	189	LOC_Os01g55240.1	2	GGCC	158
LOC_Os05g39720.1	GAGG	218	LOC_Os01g61080.1	2	GAGG	218
LOC_Os07g49280.1	CTTC	163	LOC_Os03g18140.1	2	GTTC	251
LOC_Os07g49150.1	GTAC	255	LOC_Os03g18690.1	2	GTAT	256
LOC_Os07g49000.1	GTAC	255	LOC_Os03g19200.1	2	GTAC	255
LOC_Os09g26360.1	GTAC	255	LOC_Os08g34910.1	2	GTAC	255
LOC_Os08g41730.1	CACG	97	LOC_Os09g32800.1	2	GACG	158
LOC_Os12g08090.1	CGCC	18	LOC_Os11g09020.1	2	GGCG	189
LOC_Os01g09540.1	GTAC	255	LOC_Os05g10210.1	2	AACT	134
LOC_Os05g10210.1	GCCT	182	LOC_Os01g09540.1	2	GTCG	194
LOC_Os03g21820.1	CGTG	153	LOC_Os05g39990.1	2	GGTG	233

*Locus name of the rice gene model with intron loss. ^†^The exonic 4-mer at the acceptor splice site of the lost intron was inferred from the pair-wise alignment of the coding sequences as illustrated in Figure 5. ^‡^Each 4-mer is associated with an intron phase-dependent rank ranging from 1 to 256 as its based on the frequency of occurrence calculated from exonic 4-mers at the exon-intron boundary of all 33,011 FLS introns. ^§^The corresponding rice duplicated gene with retained intron.

**Table 5:** Sum of the ranks of the exonic 4-mers at the donor and acceptor splice site of lost introns and simulated introns

	Sum of the ranks	
	Donor site	Acceptor site

Lost introns*	6,737	6,410
Simulation average^†^(std)	7,647 (253)	6,679 (337)
*P* value of lost introns^‡^	0.0007	>0.05

*Sum of the ranks of the exonic 4-mers at the donor and acceptor splice site of the 34 lost introns. ^†^A total of 10,000 iterations were generated. In each iteration, a total of 34 ranks were randomly generated according to the frequencies obtained from all the exonic 4-mers at the exon-intron boundaries of 33,011 FLS introns. Standard deviation is listed in the parenthesis. ^‡^The *P* value for the sums of the ranks of the donor and acceptor splice site.
