# Supplementary material for: Intron gain and loss in segmentally duplicated genes in rice
Source: Genome Biol. 2006 May 23;7(5):R41. doi: 10.1186/gb-2006-7-5-r41 (PMC1779517; doi:10.1186/gb-2006-7-5-r41)
Supplement: Additional File 2 — The 3,101 pairs of segmentally duplicated genes along with their pairings and their sequence. [file gb-2006-7-5-r41-S2.pdf]

Additional Data File 2. Peptide sequences of the 3,101 segmentally duplicated gene pairs identified by DAGchainer. In the definition line, the model feat_name, locus identifier, and gene pair information is provided.

```
>11667.m03949|LOC_Os01g41010.1|genepair1-1
MAAESSLVPQTSSESIAQKMGFFRVPDLLVKLSSKCLIELDAVRSPTSPLDLIFFPGLGA
KSPRSSFLGDRVGLGLVDSLTDDSSTPLGSRKVLLGSEMRITDNVTSKNSFTAPVEAGVV
DQKDESMCDDLKGSFMSLDDIVNSEDYTRVVCRGPNPRTTHFFGDHVLEFEGEQLMPDES
KSEESLPPRLEEGMMSFCYFCGEKLEEGKDIYVYQGDKAFCSMECRENFMEDEMEEGEPD
LSAPPSSPVANDGCIFQLIQ*
>11667.m05097|LOC_Os01g52100.1|genepair1-2
MEESNDQGLAQKSTAPTGFFRVPGLFVRLSSKGLNAVDPDSAWSPTSPLDFKNLSSSTGS
TNLKSSGLLGVEADQKFRTNPPRVGLGLVDALTADESSSCLGVTSSFLDSIRPFLELGLP
KAASDAAMQKNGSGSVTLDEIADFALSEEYTCVIEHGPNPRTTHILGDETLEVCKGVPRS
SKKSIFTIEPIGELPSTLACAVSRSCCYCRKRLQQDRDIYMYLGEKAFCSNECRRDYIDE
EIEEVEELMMLDSAGSSALLAW*
>11667.m03953|LOC_Os01g41050.1|genepair2-1
MAAVTPDEHERQQDGHCRARVNLRPAAARPFGAAARAALKDTLFPDDPFRGLGGMPPARR
AWRVARYFVPALDWGAGYSAASFWYDLLAGVTIASLSIPQGISYATLAGIPPVIGLYSCF
VPPLVYAVMGSSRNLGVGPVATSSLLVASIVGGKVRASDDQRLYTQLVFTSAFFTGVLQA
ALGLLRLGILVDFMSRPAITGFMGGTAIVIMLQQLKGFLGMTHFTTKTDIVSVLRYIFHN
THQWQWQSTVLGVCFLIFLVFTEQVRRRRPKLFWVSAMSPLLVVVVGCVFSFLIKGHKHG
IPIVGTLKRGINPSSISQLKFQPEYVGVAMKAGFVSGMLALAEGVAVGRSFAAMKKERID
GNKEMVAFGLMNLIGSFTSCYITTGAFSKTAVNYHAGCRTAMSNAVMSVCMALVLVALAP
LFRHTPLVALAAIITSSMLGLVKHREIRRLYEVDKADFAVCAAALLGVVFSTMITGLGVA
VAISVLRALLHVARPSTSKLGRVSCGSGAGAADDDHAFCDVAQYPGAATAPSILVLQVAG
SPVCFANAEYLRERIARWVEDEEKAVAGEDLLYVVLDIGGVTAIDSPGIEMLREVHGELE
RKGMKMAVTNPRMAVAEKLVLSGLAELVGESWMFLSNGDALAACRYTLQGSKHGGVPPV*
>11667.m05106|LOC_Os01g52130.1|genepair2-2
MGTEAGDVDGRNHRPGRGSSASSRHLHGVGGASAAEVNLSGRRPFAEKLWSDLAETFFPD
DPFRGFGALPPARRAWCAVKYFVPALDWVPRYGLDKFKFDLLAGITIASLAIPQGISYAR
LANLPPIIGLYSSFVPPLMYAVFGSSNNLAVGTVAAASLLLASIIETEVAADENPQLYLQ
LFYTAAFFTGLFQTALGVFRLGLIVDFLSRSTITGFMGGTAMIIILQQFKGLLGMKHFTT
KTDIISVLHSTYHYRHEWKWQSAVLGICFLLFLMSSKHLRKKLPKLFWVSAIAPFMVVVI
GGIFAFLVKGDEHGIPIVGNLNKGINPLSIAQLTFQSRHMKTAVKAGLLSGILALAEGIA
VGRSLAMVKNEQIDGNKEMIAFGIMNIAGSFTSCYLTTGPFSKSAVNFHAGCKTPMSNVV
MSVCIMLVLLFLAPLFKYTPLVALSSIIVVAMIGLVKVKEFCHLYKVDKFDFCICMVAFL
GVVFFTMVAGLSASVGLSVVRALLYVARPATCKLGNIAGSETFRDVKQYPQAKSIPGILV
LQLGSPIYFVNAGYLRERILRWVEDEDNLCKSVGHDLQYLVLDLGGVTSVDNSGVGMLLE
VHKSLERRGITIALTNPRLEVTEKLVLSGYVRDILGDEWVFLTVKDAITACRYALQISRN
KGEDEV*
>11667.m03967|LOC_Os01g41190.1|genepair3-1
MADPTTPADAQPEQAHAAEKPPAAPLTPEEAEAEAETAGSGDDEEYVSDPDDALLPEMRR
REASDDEGSEEGRARIGSDRGDGDDGDGQGAAEVYEDEAYEDDDEEYYDDLAEEEVGEGF
EEEYDGRAEPPKEVAGAQGEDGEKGDVEGEAAVEGDGEEKKEQEPFAVPTSGAFYMHDDR
FQEESRGRRRRMFGGRKLWDAKDDQAWVHDRFEEMNLHEEHYEDKRMSRGRFRGRGGGGR
TRGTGHGFARGGKYRGYNEDINNNHQNRPQKVVRGRGPRRYEAVAKNNRDVVGFQRKQPA
RSRESAASASAVRESGQTLNAQSEMAPPKKNVVNSSLNSASPPFYPSGASNPDFSVPAQR
RDNMQAGGSNKVFPSSMKMDDNAKVQSGPAVRRDYGARDRFQHADGPVRQSPRSGGTSLN
SSGFAASTVNHGQSSVVRTQGGNGIPSNNQSTSSLHQNPRAPTHQQSHTSVVHQKSGQVQ
TQSAMRIPTQQLNHRTGNPSTTQHLPVRSTESVENGLYPSSNKSNASSGAGKTNSQEAGR
GSFMYGGAQVIGAAGAIGLAQGEQNFPGTPALLPVMQFGSQHPGGVGVPTVGMALPGYVA
QQQMGMGNNEMTWLPLLTGAAGAFGGSYPPYIALDPAFYSRSSGQTSSSVPSRESIANKG
ASPPRNDIVNEEVDQRQNKPRRQILRDELQSVKHHAIFLSP*
>11667.m05107|LOC_Os01g52140.1|genepair3-2
MAEKEEAEEEVEDEEEYESDLDDAPLPAVRRRAAASDDEEGGGASGSSAPWSVAGSDLDS
YSDSDGQGAAEMYDDEEEGSEERDELEAGGGGGGGGGGGGVGGGEALEDEGKCADEEALE
DEGRYGDEEADGVVAALGDEGKCDGEEAEVEAAVEGAEVVNKEGEAQAVPTIGAFYMHDD
RFRDPENGRHGSQRKNFGGQKLWYPKDDNVWAHDRFYEMNSHHDRLYETNSHNSPNDSGR
GPRGSFRAWGGDRTHRYDHGYLERTLSQSYYHDDREEYKYVPKEPRTFFATTRDHISFLK
ESNNMYGSANNYKRVPSKFHTYYDHGDTKNFAYVQRESHTYYGNAKDFTSAHDGYRGGVS
NPYVSHWRSDPEICSGQYIRSQNEEASSNAEGGKVSLSNSGFPN*
>11667.m03968|LOC_Os01g41200.1|genepair4-1
MAARGLKKAFRWFPHSNEDHHHLEEDEGSSERRGLLRSHLEQVVPVTDLEDEPNASSSAV
KEPKTVALKVSMHCHCCARKVEKQILKMEGVVSFKVELENKKVTVVGNVSPMEVLESICK
VMKSAQILAAA*
>11667.m05109|LOC_Os01g52160.1|genepair4-2
MGRKLGFEKVLDCFSLALCTNACVCIHSVEDDEEEAIEREALVSSQLEELVKLKDLVGGA
KTLAFHLEPKTVELRVSMHCYGCAKKVQKHISKMDGVTSFEVDLESKKVVVIGDITPYEV
LASVSKVMKFAELWVAPNSQGC*
>11667.m04003|LOC_Os01g41550.1|genepair5-1
MAPPPSLLATALLLLLLAIAGGPNAAAVAEAVRYQTLVATPLSPHPYTATAVEDDGLFQG
SLAADEGGAAASTVGLRVVHRDDFAVNATAAELLAHRLRRDKRRASRISAAAGGAAAANG
TRVGGGGGGSGFVAPVVSGLAQGSGEYFTKIGVGTPVTPALMVLDTGSDVVWLQCAPCRR
CYDQSGQMFDPRASHSYGAVDCAAPLCRRLDSGGCDLRRKACLYQVAYGDGSVTAGDFAT
```

ETLTFASGARVPRVALGCGHDNEGLFVAAAGLLGLGRGSLSFPSQISRRFGRSFSYCLVD
RTSSSASATSRSSTVTFGSGAVGPSAAASFTPMVKNPRMETFYYVQLMGISVGGARVPGV
AVSDLRLDPSTGRGGVIVDSGTSVTRLARPAYAALRDAFRAAAAGLRLSPGGFSLFDTCY
DLSGLKVVKVPTVSMHFAGGAEAALPPENYLIPVDSRGTFCFAFAGTDGGVSIIGNIQQQ
GFRVVFDGDGQRLGFVPKGC*
>11667.m05112|LOC_Os01g52190.1|genepair5-2
MAPLKTLLLFLVVLLPVANACQPPRTLHVPVFHRDALFPPPPGAKRGSLLRQRLAADAAR
YASLVDATGRLHSPVFSGIPFESGEYFALVGVGTPSTKAMLVIDTGSDLVWLQCSPCRRC
YAQRGQVFDPRRSSTYRRVPCSSPQCRALRFPGCDSGGAAGGGCRYMVAYGDGSSSTGDL
ATDKLAFANDTYVNNVTLGCGRDNEGLFDSAAGLLGVGRGKISISTQVAPAYGSVFEYCL
GDRTSRSTRSSYLVFGRTPEPPSTAFTALLSNPRRPSLYYVDMAGFSVGGERVTGFSNAS
LALDTATGRGGVVVDSGTAISRFARDAYAALRDAFDARARAAGMRRLAGEHSVFDACYDL
RGRPAASAPLIVLHFAGGADMALPPENYFLPVDGGRRRAASYRRCLGFEAADDGLSVIGN
VQQQGFRVVFDVEKERIGFAPKGCTS*
>11667.m04009|LOC_Os01g41610.1|genepair6-1
MASRLAQLRSKAAQASELVSKHGCAYYKEVMEKNKQHVVQPPTVEKCQELSKQLFYTRLA
SLPGRYEAFWKEFDGVKQVWKNRKELKVEDLGIVTLFGVELYAWFCVGEIVGRGFTITGY
KV*
>11667.m05113|LOC_Os01g52200.1|genepair6-2
MAALGSKLATLGSAAREAARVAARQGCAYNKAVVEGSRQLQSRACEATRSAAKHGRAFHE
ELMERNKRYVIDPPTIQTCQELSKQLFYTRLASIPGRYESFWKEVDGAKLLWKNRKNLNL
KAEDIGVATLFGIELIAWFAGGEVVGRGFTFTGYHV*
>11667.m04014|LOC_Os01g41660.1|genepair7-1
MAAGVDGDTAAAAAAGGGVVVVFDFDRTIIEWDSDDWVITKLGASDAFRRLRPTMRWNPL
MDRMMVELHAQGRSADDIRDCLKSAPLDAHVLSAITTASALGCDLRVASDANAFFIETVL
EHHGVLGCFSEISTNPARVDGNGRLRISPFHDPDDSSPHGCSLCPENMCKGKIIERIQAT
ANGKRHFIYIGDGRGDYCPSLKLGEGDYVMPKEKYPLWNLISSNKQLLKAEVHPWNNGEE
LEQTLLKLVNKLITPPAQPSQFEYKCDMSNPVSTEVGQALRVPH*
>11667.m05116|LOC_Os01g52230.1|genepair7-2
MAGIVVVFDFDKTIIDVDSDNWVVDGLGATEEFERLLPTMPWNTLMDTMMGELHASGKSL
ADVAGVLRSAPLDPRVVAAIKACYGLGCDLRILSDANRFFIDTILDHHGLTGYFSEINTN
PSAVDAATGRLRIAPYHDFHAGPHGCGLGICPPNMCKGQVLDRIRASAGAAGKRVIYLGD
GRGDYCPSLRLGRDDFMMPRRGFPVWELICEDPSLLHAEVHSWADGAEMEETLLRLVGRV
LLEERNLPPLDCKLESLPAVAVQDGMPMTLRIKN*
>11667.m04019|LOC_Os01g41710.1|genepair8-1
MAAATMALSSPALAGKAAAKVFGEGRITMRKSAAKPKPAASGSPWYGADRVLYLGPLSGE
PPSYLTGEFPGDYGWDTAGLSADPETFAKNRELEVIHSRWAMLGALGCVFPELLARNGVK
FGEAVWFKAGSQIFSEGGLDYLGNPSLIHAQSILAIWAVQVVLMGAVEGYRIAGGPLGEV
VDPLYPGGSFDPLGLADDPEAFAELKVKEIKNGRLAMFSMFGFFVQAIVTGKGPLENLAD
HLADPVNNNAWAYATNFVPGK*
>11667.m05117|LOC_Os01g52240.1|genepair8-2
MAASTMALSSPAALAGKAVANAKVFGEGRVTMRKSAAKPKPAAASGSPWYGADRVLYLGP
LSGEPPSYLTGEFPGDYGWDTAGLSADPETFAKNRELEVIHCRWAMLGALGCVFPELLAR
NGVKFGEAVWFKAGSQIFSEGGLDYLGNPSLIHAQSILAIWGCQVVLMGAVEGYRVAGGP
LGEVVDPLYPGGSFDPLGLADDPEAFAELKVKEIKNGRLAMFSMFGFFVQAIVTGKGPLE
NLADHLADPVNNNAWAYATNFVPGK*
>11667.m04657|LOC_Os01g48060.1|genepair9-1
MVGIDLNTVEEEEDEEEGGATGTVTAPAEARAGGAVCLELWHACAGPVAPLPRKGSAVVY
LPQGHLEHLGAAPGSGPGAAVPPHVFCRVVDVSLHADAATDEVYAQVSLVADNEEVERRM
REGEDGAACDGEGEDAVKRPARIPHMFCKTLTASDTSTHGGFSVPRRAAEDCFPPLDYSL
QRPFQELVAKDLHGTEWRFRHIYRGQPRRHLLTTGWSGFINKKKLVSGDAVLFLRGEDGE
LRLGVRRAAQLKNASPFPALHNQISNTSSLSEVAHAVAVKSIFHIYYNPSCTHRLSQSEF
IIPYWKFMRSFSQPFSVGMRFKLRYESEDASERRRTGIIIGSREADPMWHGSKWKCLVVK
WDDDVECRRPNGVSPWEIELSGSVSGSHLSTPHSKRLKSCFPQVNPDIVLPNGSVSSDFA
ESARFHKVLQGQELLGLKTRDGTVNTASQATEARNFQYTDERSCSINMSNNILGVPRLGV
KTPSGNPGFSYHCSGFGESQRFQEVLQGQEVFRPYRGGTLSDACIRGSGFRQPDGNHAPG
AAFKWLAPQGCDHHGITTSVLPQASSPSSVLMFPQTSSKMPGLEYIYGCLDRNENSRHFK
IGPTQDMTRTDQTLRLWPHLISGKVLDECTRNEKLHSPVSGAEHESNNKCLNTNGCKIFG
ISLTEKAQAGDEVDCGNASYHSRLQSLKPQMPKSLGSSCATVHEQRPVVGRVVDISAVNT
MI*
>11667.m05421|LOC_Os01g54990.1|genepair9-2
MGIDLNNTASGGEEDAPAPGPVCRDLWHACAGPVVSLPRRGSAVVYLPQGHLSAAGAGGG
IRGEVAVALPPHVACRVVDVELCVFERNLHGGGIEREDDMEDGDEERKSRMLHMFCKTLT
ASDTSTHGGFSVPRRAAEDCFPPLDHKQLRPSQELVAKDLHGAKWRFRHIYRGQPRRHLL
TTGWSSFVNKKKLVSGDAVLFLRGDDGELRLGVRRATQLKNEAIFKAFSSESSKMRTLSA
VADSLKHGSVFHICYNPRATASEYVVPYWKFVKSFNHPVCIGMRFKFHFESEDVNERRSG
MIAGVSEVDPIRWPGSKWRSLLVRWEDATDCNSQNRVSPWEIEIVGGSISVAHSLSASSS
KRTKLCPQGNLDVPALYGNGRPDSVETEKFPRVLQGQELMGSRTHRATCSPQSIDITKSK
SFDAWRFLTDTRSCMLGSSTSRLPVQYSGYTHQSVSFGESIGFPEVLQGQEISQTVPPFQ
GMLPDACSAKSRYELKNYVCTPATMNGLSSANEGYCLSLSTVPPSPPSSLMLYQTGVPQL
ELASKNNDKSGNDSQPALRQHKLLSETSWDQFKIGKASTPGNATKPGNGGREVDRTSCRL
FGFSLTEKIIPTDKDGEKEVSYETDCQNPRMLDLFGYNCSTPGALHALCAAPLGI*
>11667.m04673|LOC_Os01g48200.1|genepair10-1
MTTPRASQRRGGAAAGGASPAAEPYNIIPIHDLLAEHPSLRFPEVRAAAAALRAVGGLRP

PPYSAWREGQDLMDWLGAFFGFQRDNVRNQREHLVLLLANAQMRLSSADFSDTLEPRIAR
TLRRKLLRNYTTWCGFLGRRPNVYVPDGDPRADLLFAGLHLLVWGEAANLRFVPECLCYI
YHHMALELHRILEGYIDTSTGRPANPAVHGENAFLTRVVTPIYGVIRAEVESSRNGTAPH
SAWRNYDDINEYFWRRDVFDRLGWPMEQSRQFFRTPPDRSRVRKTGFVEVRSFWNIYRSF
DRLWVMLVLYMQAAIVAWESEGLPWRSLGNRNTQVRVLTIFITWAALRFLQALLDIGTQ
LRRAFRDGRMLAVRMVLKAIVAAGWVVAFAILYKEAWNNRNSNSQIMRFLYAAAVFMIPE
VLAIVLFIVPWVRNALEKTNWKICYALTWWFQSRSFVGRGLREGTFDNVKYSVFWVLLLA
VKFAFSYFLQIRPLVKPTQEIYKLKKIDYAWHEFFGKSNRFAVFVLWLPVVLIYLMDIQI
WYAIFSSLTGAFVGLFAHLGEIRDMKQLRLRFQFFASAMSFNIMPEEQQVNERSFLPNRL
RNFWQRLQLRYGFSRSFRKIESNQVEARRFALVWNEIITKFREEDIVGDREVELLELPPE
LWNVRVIRWPCFLLCNELSLALGQAKEVKGPDRKLWRKICKNDYRRCAVIEVYDSAKYLL
LKIIKDDTEDHGIVTQLFHEFDESMSMEKFTVEYKMSVLPNVHAKLVAILSLLLKPEKDI
TKIVNALQTLYDVLIRDFQAEKRSMEQLRNEGLAQSRPTRLLFVDTIVLPDEEKNPTFYK
QVRRMHTILTSRDSMINVPKNLEARRRIAFFSNSLFMNIPRATQVEKMMAFSVLTPYYNE
EVLYSKDQLYKENEDGISILYYLQQIYPDEWEFFVERMKREGMSNIKELYSEKQRLRDLR
HWVSYRGQTLSRTVRGMMYYYEALKMLTFLDSASEHDLRTGSRELATMGSSRIGSSRREV
GSDGSGYYSRTSSSRALSRASSSVSTLFKGSEYGTVLMKYTYVVACQIYGQQKAKNDPHA
FEILELMKNYEALRVAYVDEKNSNGGETEYFSVLVKYDQQLQREVEIYRVKLPGPLKLGE
GKPENQNHALIFTRGDAVQTIDMNQDNYFEEALKMRNLLEEFNRHYGIRKPKILGVREHV
FTGSVSSLAWFMSAQETSFVTLGQRVLADPLKVRMHYGHPDVFDRLWFLGRGGISKASRV
INISEDIFAGFNCTLRGGNVTHHEYIQVGKGRDVGLNQVSMFEAKVASGNGEQTLSRDVY
RLGHRLDFFRMLSFFYTTIGFYFNTMMVVLTVYAFVWGRFYLALSGLEAFISSNTNSTNN
AALGAVLNQQFVIQLGIFTALPMIIENSLEHGFLTAVWDFIKMQLQFASVFYTFSMGTKT
HYYGRTILHGGAKYRATGRGFVVEHKKFAENYRLYARSHFIKAIELGVILTLYASYGSSS
GNTLVYILLTISSWFLVLSWILAPFIFNPSGLDWLKNFNDFEDFLNWIWFRGGISVKSDQ
SWEKWWEEETDHLRTTGLFGSILEIILDLRFFFFQYAIVYRLHIAGTSKSILVYLLSWAC
VLLAFVALVTVAYFRDKYSAKKHIRYRLVQAIIVGATVAAIVLLLEFTKFQFIDTFTSLL
AFLPTGWGIISIALVFKPYLRSSCVVMAAWTPGDADEDPVQ*
>11667.m05426|LOC_Os01g55040.1|genepair10-2
MSLLRNRRAAAAAGSGEQTVVQAAYNIIPIQDVVMHGDHPSLQVPEVRAAVEALSHASDF
PAPPLARVWDPHRADIFDWLGATFGFQADNVRNQREHLVLLLANAQLRAAPGFPKDHPID
VLHLTVARGIRRKLLKNYTSWCAYLGQKRHFRVPSGGGGGRRTGAATGNDVRMDLLYTAL
YLLIWGEAANLRFMPECLCYIFHYMALDLHHVVEQSIDIETGRPAMPAVCGEDAFLIRVV
TPIYNVLKNEVEASRNGTKPHSAWRNYDDVNEYFWSRRVFKRLRWPLDPSRSFFVEPGKT
GRIGKTGFVEQRSFWNVYRSFDRVWVMHILFFQAAMIVAWDGKTPWVSLRFRDIQVRVLS
VFITWGGLRFVQAMLDAGTQYSLVSRETKTVAVRMVLKVLVAAGWTITFSVLYKRMWDQR
WRDRRWSFAANTRVLNYLEAAAVFVIPQVLAIVLFIIPWIRNFLEKTNWKILYVLTWWFQ
TRTFVGRGLREGLIDNIKYSIFWVCLLVSKFSFSYFLQIKPMVGPTKVIFKLHDIKRNWF
EFMPHTERLAVIILWLPVIIIYLMDIQIWYAVFSSLTGALIGLFSHLGEIRSVEQLRLRF
QFFASAMQFNLMPEEHLDTVHGGIRSKFYDAINRLKLRYGFGRPYRKIEANEVEAKRFAL
VWNEIIQTFREEDIISDKELGLLELPAVVWRIRVVRWPCLLLKNELLLALSQAAELVADD
RTHWNKICNNEYRRCAVIEAYDSIRHLLLEIIKERTNEHIIVNQLFLAFDGAMEYGKFTE
EYRLTLLPQIHKYVISLVEQLLLKDKDQIKIVRTLQDLYDLAVHDFPKIKKDFEQLRREG
LALSRPTESQLLFQDAIKCPDDNDVSFYKQVRRLHTILTSRDSMDDVPKNPEARRRITFF
SNSLFMNMPRAPTVQRMMAFSVLTPYYNEDVLYNKDQLRRENEDGISILFYLQKIYEDDW
KNFLERMQREGMASDDGIWAGKFQDLRLWASYRGQTLARTVRGMMYYYRALKMLAFLDNA
SEVEITEGTKQLASFGSVQYENDVYPMNGGLSQRPRRRLERGTSTVSQLFKGQEDGAAIM
KYTYVVACQIYGNQKKAKDQRAEDILTLMKKNDALRVAYVDEVHPEIGDTQYYSVLVKFD
PVLQREVEIYRIRLPGQLKLGEGKPENQNHAIIFTRGDAVQTIDMNQDNYFEEALKMRNL
LEQYDYYHGSQKPTLLGVREHVFTGSVSSLAWFMSAQETSFVTLGQRVLANPLKVRMHYG
HPDVFDRLWFLTRGGISKASRVINISEDIFAGFNCTLRGGNVSHHEYIQVGKGRDVGLNQ
ISMFEAKVSSGNGEQTLSRDIYRLGHRLDFFRSLSVFYTTVGFYFNTMMVVLTVYTFVWG
RLYLALSGLEAGIQGSANATNNKALGAVLNQQFVIQLGLFTALPMIIENSLEQGFLPAVW
DFFTMQMMFSSVFYTFSMGTKSHYYGRTILHGGAKYRATGRGFVVQHKSFAENYRLYARS
HFIKAIELGIVLTVYAAHSVIARDTLVYIIMMISSWFLVVSWIMAPFAFNPSGFDWLKTV
YDFDDFMNWIWYPGSIFSKAEHSWEVWWFEEQDHLRTTGLWGKILEILLDLRYFFFQYGV
VYQLKIANESRSIAVYLLSWICVAVIFGIFVLMSYARDKYAAKQHLYYRVIQSGVIILAV
LVLIIFLKFTKFQIIDIFTSLLAFIPTGWGLISIAQVIRPFIESTVVWASVVSVARLYEI
LLGVFVMAPVAFFSWLPGFQEMQTRVLFNEAFSRGLQISRILAGKKTIAV*
>11667.m04675|LOC_Os01g48220.1|genepair11-1
MASPARPAAASVSGAFGLPADPAAARCSFDQSRRRPEDLQQEKRMVRTFVNVYGGQENYT
KEAVMAAVEECMKKQAEGLLHSLEGIGGRLSQLELYCYKLERSIGELRSDVMDYHSEGTV
NFRCLEKNLRQVQKSVQILQDKHEISETPNEFSKLQIAHEFPARANEASAFSTFGRENDH
STQVAKHEVAFMPLQQVNAMQSPAVPVQSSNGYILQQLVPVSLSTQPDQQQPSQAAVYYM
QSQNPIKCTESEPSESAVHVIQSQIQNPEARVAVDLSQKSSQVTELYPQPQDQRLHLPAQ
QVESQAWRTQPLVVQPQQYNIQQVPPQLVQQQTSSPQAQSAPQVAVLYPPYSSQKPASAT
TEPLLRNMVVHSPYSSPQQKHHEAMPSFYGQGNTVLLPSTDLNIQHQQPQPLQQHGLSSC
PPQPSKPNHCSVASYAVQGSGQSYSATFKNPSNCAATVVAVLPQHPASGPMAFHHLGPQV
VHNQPFGNMFETASVVGYPRDRVESVALPVVTAAQPADSVAMADKLNAGSNVTSPREWSG
*
>11667.m05428|LOC_Os01g55050.2|genepair11-2
MSGSGVPAGRGSRSFDFGADDVLCSYDDFAAPSEPKRPDPADKQDFHDSRLGRPFGKAYE
QESYGKEDVLFAVEKCMKKYADNLLRSLEGITNRLSQLEIYCYKLERSMGELRSDVLRDE
TDQRLKSLEKHLHEVHRSIQILRDKQELAETQKELAKFQLTHDTSKKKEDMPTPSFPEQK

TLEEKADAPGQQLAIVLPHQVNSSLAPRASQPVQQYKDQTVQQPPSSSVPQQDRYVLSQA
IVYYPQRQAPGIQDTQGQQVQPEVQYLPVRPSATQDVPVHASSQQSQAANQTQPQSFPPY
QQQWPQQSSQPAPAPVAQPQPTFSQPFPPPVQQPQLSNTQQFPPQPMQQPQLSNTQQFAP
QPVQQPNAQQFPPPPVQPQQSNTQLPPQAMQPQQHPPVQNQMRPQTPPNYPHYQPHQSLN
PPPETLPGSMAMQGPYNTVAPAAGSRSEVPYSYGGPGMPPPQHNMQRQQLPPPSQGSFGP
PSKGGYAGPPQYAPQGSSHGYNTAYGYPPSGPSAAQAPQMPPAPGNVGMSHPGSHQMMRG
HPYGEMIEKAITMGYPREQVMNVIQRMTESGQPMDFNTLLDRLNEAGSGAPPRAW*
>11667.m04680|LOC_Os01g48270.1|genepair12-1
MTGADEPSITRWTFEDFEVYYEVRLGIRREPGGDEDGDGDGGGGRGYAPLGSGSAGSTRP
SAAHANGGADLAVFEQFERLERKVELRNGAIEAGPPQKSLLPSFESAEMRNLAETLLRDI
IRGSPDVKWESIKGLENAKRLLKEAVVMPIKYPKYFKGLLSPWKGILLFGPPGTGKTMLA
KAVATECKTTFFNISASSIVSKWRGDSEKLVKVLFELARHHAPSTIFLDEIDAIISQRGE
ARSEHEASRRLKTELLIQMDGLTKTDDLVFVLAATNLPWELDAAMLRRLEKQQEARHAMF
EELLPSVPGTMNIPYDVLVEKTEGYSGSDIRLVCKEAAMQPLRRLMSVLEGRQEEVPEDE
LPEVGPVTTEDIELALRNTRPSAHLHVHRYEKFNQDYGSHVLS*
>11667.m05449|LOC_Os01g55260.1|genepair12-2
MDFKGFWESRFGGKKEQEPEQNGHANGVANGSVRKRTSDLAVYEQFEQQARQTEVRAAAI
RDGNADAIQKPLLPSFESAEMRNLAETLLRDIIRGSPDVKWESIKGLENAKRLLKEAVVM
PIKYPKYFTGLLSPWKGILLFGPPGTGKTMLAKAVATECKTTFFNISASSIVSKWRGDSE
KLVKVLFELARHHAPSTIFLDEIDAIISQRGEARSEHEASRRLKTELLIQMDGLTKTNDL
VFVLAATNLPWELDAAMLRRLEKRILVPLPEAEARHAMFEELLPSTTSKLEVPYDTLVEK
TEGYSGSDIRLVCKEAAMQPLRRLMSVLEARDELVPEEELPEVGPLKPEDIEVALRNTRP
SAHLHAHRYEKFNQDYGSQILSQEQA*
>11667.m04682|LOC_Os01g48290.1|genepair13-1
MANLPSTAAAAAADASGFKLFGKVIQPDGQRGVEESAAAQAPPHPHPPAPPVMEAAAAAA
GTSQTLQAAGGGGGGGGGGGGEPLPCPRCGSRETKFCYFNNYNVRQPRHLCRSCRRYWTA
GGALRRVASASPGRRRPRPSAARSAAAAAASASAASPPAAVPAASEGAESVDSRS*
>11667.m05458|LOC_Os01g55340.1|genepair13-2
MLSHVEMAPAAGGFKLFGKVIMQCGVSEGTQDKAQGFVVAREKVEPEEEEEEEQRVPAAA
TSGQRASIKREAADRDEEQRQGGGDAAGQPTQRRLQDSAEARAAAAAPLPCPRCRSRDTK
FCYFNNYNVNQPRHFCKACHRYWTAGGALRNVPVGAGRRKNRPLGPLAVAHHNHHHRAAA
GFVLGFPNPSSPTSPSPVYTDRWPVTPDRPF*
>11667.m04691|LOC_Os01g48370.1|genepair14-1
MPRETPPPPPPEGGEVHEVVEGEDGQAEDQEERWARLLPELMSEVVRRVEASGGERWPAR
KDVVSCACVCRRWRDAAVAVVRPPAESGKITFPSSLKQPGPREFPMQCFIKRNKKNSTFY
LYLGLTNATVDKGKFLMAARRFRRGPHTEYIVSLDADDLSQGSNAYMGKLRSDFWGTNFK
IYDSKPPYDGAKASSSRSSRRFGSRRISPQVSAGNYEVGQVSYKYNLLKSRGPRRMYCAL
ECPSTQETWENCLKTKFRKPTGNTVLRNKAPRWHEHLQCWCLNFHGRVTVASVKNFQLVA
AADPNDPASSKDEETVLLQFGKVDDNIFTMDYRQPLSAFQAFAISLSSFGTKLACE*
>11667.m05468|LOC_Os01g55430.1|genepair14-2
MVPWRRSSSSSSAPSSRPARRPARTNARVSPDVSSELSPLAGEEGAGEERWSALVPDLLA
DILRCVEAGSERWPPRRDVVACASVCRRWRDVAVAVVQPPLESGKITFPSSLKQPGPRDA
PMQCFIKRNKKNSTFFLYLGLTQELTDDEKFLLAARRCRRGLHKEYAITINSDGLFHGSQ
SCVGNLKSNFTGTKFTIRDWQPPYEGAKAFSSRSGRWFGNKHRCPLVSTGDVEVGEVSYK
YSLLRPRGPRRMSCSVQCPVLKGTAVDPQDGKRLSNSIPSSLVLNSKVPSWHEHLQCWCL
NFHGRVMVASVKNFQLIAPVEPGEPSDKTVVLQFGKIDDDVFTMDYRQPLSAFQAFAICL
SNFGTKLA*
>11667.m04695|LOC_Os01g48410.1|genepair15-1
MVRRHLLRGHSLDRFLPIRSLIMSSSSSFSSSSPSPPPPSSSSSRGSSSGRWCGVSVAEE
DEDDAAVSATTTPPLPPLQKRVLSRSHGSKAKPLGGSVDHLPPVVPPSSKNVRDSGPPSE
IDLVKEKFAKLLLGEDMSGTGKGVSSALALSNAITNLAASVFGEQRRLEPMSADRRARWN
KEIDWLLSVTDHIVEFVPSQQASTDGTSMEVMGTQQRRDLLINIPALRKLDAMLLHYFAV
VLLPVEQEYLDNFKDEQEFWYVKKDADEGEKGDAPRQGDKWWIPTVRVPPEGLPDASKKW
ILHQKDLVGQVLKAAMAINADVLTEMEIPGEYIETLPKNGRSSLGDSIYKIITDDHFDPN
ELLSSVDLSTEHKIVDLKDRIEASVVIWQRKISNKLSWGPGVSLEKREQFEERAQTVLLI
LKHQFPGVPQSSLDISKIQYNKDVGYAILESYSRTLESLAFAVLSRIEDVLHADAIARDP
KRTKSRRRPSLVDIPEIIDNALEEEETVNSIDANSQVTNNSIHWQEQEHEDKGARLRKVH
RMVTKKLLHIEKVDNLGGGLKSFSHR*
>11667.m05479|LOC_Os01g55520.1|genepair15-2
MALWQARHFLIIKHVPLPNYAPPLPRGFFLPRQCGRSIEPPGSWITGTNNNHHHLGINHF
NPPLFVPSRGSLCVGVLQRQSWIIIVCFCTRRSRPQKAASFTEAGHGGVDRLMAASGGGS
LLERRSSVRRSQSMVSEEGRGTPADEDLGGGGTLKIGAVLDKDSAAPKSRLAKDTGEHGG
GGPSEMELMKEKFAKLLLGEDMSGSGKGVPSALAVSNAITNLAASVFGEQRKLEPMAPDR
KGRWKKEVGWLLSVADHIVEFVAKKQVLDNGVEMEVMGTQQRRDLQANIPALRKIDTMLL
DYLDNFKDRNEFWYVKRDSCSDSDEQRSDEKWWIPIVKVPPGGLSPASRGWLQHQKELVN
QVLKAAMAINANCLMEMAIPESYLESLPKNGRASLGDALYRIITDVEFDPDVFLSTVDLT
SEHKILDLKDRIEASVIIWNRKVHNKDGKSAWGSAVSQEKREQFEERAQTLLLIIKHRYP
GIPQSTLDIAKIQENRDVGFAILESYSRVLESLAFNVMSRIEDVLNADDHAREKAKKEAP
PAPAMANDAAEHHHQQAGEVDAPCKMTGSPNGRTLLDFMDDWNGDADRPSPTAPEPAAQE
DGRLMKLPNIMTNLKQTYMDNLFGAHRSPPGRH*
>11667.m01058|LOC_Os01g10890.1|genepair16-1
MEKKASILMNRYELGRMLGQGTFAKVYHARNLASNQSVAIKVIDKEKVLRVGMIDQIKRE
ISIMRLVRHPNIVQLHEVMASKSKIYFAMEYVRGGELFSRVARGRLKEDAARKYFQQLIG
AVDFCHSRGVYHRDLKPENLLVDENGNLKVSDFGLSAFKECQKQDGLLHTTCGTPAYVAP

EIINKRGYDGAKADIWSCGVILFVLLAGYLPFHDSNLMEMYRKISKGDVKFPQWFTTDVR
RLLSRLLDPNPNIRITVEKLVEHPWFKKGYKPAVMLSQPNESNNLKDVHTAFSADHKDNE
GKAKEPASSLKPVSLNAFDIISLSKGFDLSGLFENDKEQKADSRFMTQKPASAIVSKLEQ
IAETESFKVKKQDGLVKLQGSKEGRKGQLAIDAEIFEVTPSFFVVEVKKSAGDTLEYEKF
CNKGLRPSLRDICWDGQSEHPSLAQSSTLTQSSKSISRHAI*
>11667.m05470|LOC_Os01g55450.1|genepair16-2
MLMATVSPARREPTPQAVRASPMPSAAAALVRRGGGGSGGTVLGKYELGRVLGQGSFAKV
YQARHLETDECVAIKVLDKEKAVKGGMVHLVKREINVLRRVRHPNIVQLFEVMASKTKIY
FVMEYVRGGELFSRVSKGRLREDTARRYFQQLVSAVDFCHARGVFHRDLKPENLLVDENG
DLKVSDFGLAAGPDQFDPDGLLHTFCGTPAYVAPEVLRRRGYDGAKADIWSCGVILFALM
AGYLPFHDHNIMVLYRKIYNGEFRCPRWFSKDFTRLITRLLDANPKTRITVPEIIESDWF
KKGYKPVKFYIEDDKLYNLSDDVLNLEPADPVPPPLGLAPPVPPPPQGDDPDGSGSESDS
SVVSCPATLSTGESQRVRGSLPRPASLNAFDIISFSKGFNLSGLFEERGNEIRFVSGEPM
SDIVKKLEEIAKVKSFTVRRKDWRVSIEGTREGVKGPLTIGAEIFELTPSLVVVEVKRKA
GDNEEYEDFCNMELKPGMQHLVHQMLPAPNGTPVSEKVERSSSLQAPLTLKLIGTEGSMS
*
>11667.m01075|LOC_Os01g11060.1|genepair17-1
MRQVSTFGLSLVKLDIRQESERHTDAMDAITTHLGIGSYREWPEERRQEWLVSELRGRRP
LFGPDLPQSEEVADVLGAFRVIAELPADSFGAYIISMATAPSDVLAVELLQRECGVKKPL
RVVPLFEKLADLQQARATMELLFSVDWYKERIDGKQEIMIGYSDSGKDAGRLSAAWQLYK
AQEEIVGVAERHGVKLTIFHGRGGTVGRGGGPSHLAILSQPPNTVNGSLRVTVQGEVIEK
SFGEENLCFRTLQRFTAATLEHGMNPPVSPKPEWRRLLDDMATVATDEYRSIVFQEPRFV
EYFRAATPETEYGRMNIGSRPSKRKPGGGIESLRAIPWIFAWTQTRFHLPVWLGFGAAFR
HAMQQSDAGGGLATLREMYDEWPFFRVTIDLLEMVFAKGDPGIAALYDELLVPDDLRPFG
EQLRANYVETQRLLLQVAGHKDLLESDPYLRQRLMLRDSYITALNVCQAYTLKRIRDGGF
RPETRPPLSKELLGSSAVAEKLVKLNPNSEYDPGLEDTLILTMKGIAAGMQNTG*
>11667.m05459|LOC_Os01g55350.1|genepair17-2
MARNAADKGTSIDAQLRILAPKKLSEDDKLVEYDALLLDRFLDILQDLHGEDIRETVQEC
YELAAEYESKIAYRRRIKLKKGDFADENSATTESNFEETLKRLVGELKKSPHEVFDALKN
QTIDLVLTAHPTQSVRRSLLQKHGRIRSCLTKLYAKDITPDEKQELDEALKREIQAAFRT
DEIRRAPPTPQDEMRAGMSYFHETIWKGVPKFLRRVDTALKNIGINERVPYNAPLIQFSS
WMGGDRDGNPRVTPEVTRDVCLLARMMAANLYYAQIEDLMFELSMWRCSDELRVKADQLH
RCAKKNTTKHYIEFWKQVPPSEPYRVILSNVRDKLYNTRERARHLLASGFSEIPDEATFT
DVEQFLEPLELCYRSLCACGDNSIADGSLLDFLRQVSTFGLSLVRLDIRQESDRHTDVMD
AITQYLGIGSYREWSEEKRQEWLLSELNGKRPLFGPDLPQTDEIADALDTFHVIAELPYD
SFGAYVISMATAPSDVLAVELLQRECHVKKPLRVVPLFEKLADLEAAPAALARLFSVDWY
RNRIDGKQEVMIGYSDSGKDAGRFSAAWELYKAQEELIKVAKQFGVKLTMFHGRGGTVGR
GGGPTHLAILSQPPDTIHGSLRVTVQGEVIEQSFGEEHLCFRTLQRFTSATLEHGMHPPI
SPKPEWRALMDEMAAVATKEYRSIVFQEARFVEYFRLATPELEYGRMNIGSRPSKRKPSG
GIESLRAIPWIFAWTQTRFHLPVWLGFGAAFKHVLQKDIRNLQILQEMYNEWPFFRVTID
LVEMVFAKGDPGIAALYDKLLVSEDLWPFGARLRANYEETKQLLLQVAGHKDLLEGDPYL
RQRLRIRDSYITALNVCQACTLKRIRDPGFHVSPRAHLSKDIMDSGKPAAELVKLNTTSE
YGPGLEDTLILTMKGIAAGMQNTG*
>11667.m01084|LOC_Os01g11150.1|genepair18-1
MVVLAKGELEQIALPAAHPPPADVRAIDLSATGPARAAEARALVAACEEQGFFRVTGHGV
PPGLVRAAEAAAARFFALPQPDKEAAAGAPLGYASKRIGSAGDLGWIEYLLLCLAPAAAA
AALPCAATSPTPPCPLRELLREYSAAVRRVACGVLELMAEGLGVGPADALARLVAREDSD
SILRVNHYPPRPDQLGGGGGPNLTGFGEHTDPQIISVLRSNGAPGLEISLRDGAWASVPH
DGDGDSFFVNVGDTLQVLTNGRFRSVKHRVVVNSEKSRVSMVFFGGPPPGERLAPLPALL
GDGGRSRYREFTWKEYKGSGCKGRLADDRLCRFEN*
>11667.m05447|LOC_Os01g55240.1|genepair18-2
MVVLAGPPAVDHIPLLRSPDPGDVFSGVPVVDLGSPGAARAVVDACERYGFFKVVNHGVA
TDTMDKAESEAVRFFSQTQPDKDRSGPAYPFGYGSKRIGFNGDMGWLEYLLLALDDASLA
DACTVPSCAVFRAALNEYISGVRKVAVRVMEAMSEGLGIAQADALSALVTAEGSDQVFRV
NHYPPCRALQGLGCSVTGFGEHTDPQLVSVLRSNGTSGLQIALRDGQWVSVPSDRDSFFV
NVGDSLQVLTNGRFKSVKHRVVANSLKSRVSFIYFGGPPLAQRIAPLPQLLGEGEQSLYK
EFTWDEYKKAAYKSRLGDNRLAQFEKK*
>11667.m01094|LOC_Os01g11250.1|genepair19-1
METISNIFHNDPLPPLGARANQSIKLRKFIISPYDSRYRTWETFLLVLVVYSAWICPFEL
AYLRNLSWKVSLVDNIIDSFFAIDIILTFFLAYLDQKSYLLVDDPKRIVARLLSMLRLWR
LRRLSELFARLEKDIRLNYYWIRCTKLISVTLFAVHCSGCFNYLIADRYPNPARTWIGAA
IPNYRSQNLWVRYVTAIYWSITTLTTTGYGDLHAENQREMLFSICYMLFNLGLTAYLIGN
MTNLVVQGSCRTRNFRDTIHAASQFAARNQLPGHIKDEMLSHICLRYKTEGLKQKETLDS
LPKGIRSSIACNLFLPVIEKVYLFHGVSFTCMIQLVTEMEAEYYPPREVVILQNEAPRDV
YILVSGAVEERVEIDGTEKVQEVLCNGEIFGEIGVICSIPQPCAFHTIKVSQLLRLNTAV
LKNIIKENSDDRRVILNNLSQKMNQDHRFSTEVMEKSLQMMHQHFGEYNRCSALNQDNEK
NELKANNGHSMALEWKRVTIHMYSQRNKRPEAPLAKVINLPGSLDKLFAIACQKFNNYRL
TKLVNPEFAEIDDITVIRDGLGEFRLVNIYTRKSQQQFILNVSKLYQFDIGTRQGHGAMW
HAAAGGGDDEGDGGARRPMHGVPNKETTASRGSLKVEEKGMDVVVVEGIQEAIVRTRTTP
RPQGQGRLYRSWSQGEDDSTSHRALQSPPPSRIFRDFDRNQINFPIETQFQEGFCGGLSV
TKLYNMLNFQLGLELCRIKRNITDLIP*
>11667.m05443|LOC_Os01g55200.1|genepair19-2
MPRSSRMNLWPHCFPCFDDGDRSGNRFSTVCNFPDDLLPSLGATAHQPPKLRKYLVSPYD
PRYKVWETFLIILVVYSAWICPLEFAFLRYLPSAPFVVDDVVNGFFAVDIMLTFFVPFVD

KKSYLLVNDPKKIAVRYLSSWFVFDVCSTVPFHSISLLFNEHGHDLGFKFLNVLRLWRLR
RVSSMFARLEKDIRFNYAVIRCTKLISVTLFAIHCAGCINYLIADRYPDPRRTWIGAVMP
NFREDGLWIRYVTAMYWSITTLTTTGYGDLHAENAREMLFGICYMLFNLWLTAYLIGNMT
NLVVHSTSRTRDFRDVVQAASEFAARNQLPQQIEEQMLNHICLRYKTDGLKQQETLDVLP
KAMRSSISHYLFFRVVQGAYLFKGVSSRFIQQLVTEMQAEYFAPKEDIILQNDSPSDLYL
LVSGAVDILVFLDGTEQVYRRAAEGELLGEIGVLCNKPQSFTFRTTKLSQILRISRTKLL
GIIQENREDGDIIRSNLQQVNV*
>11667.m01104|LOC_Os01g11350.1|genepair20-1
MAQLPPKIPTMATAWPEFGGGHHHHAAHGHHHQRSPSMGAFLAAPLPPFPLPPPAPANGG
AQQQQQQQQHQPSWVDEFLDFSATKRGAHRRSVSDSVAFLDPVSDDNAGVGAHDFDRLDD
DQLMSMFSDDLQPPPPQQQPAAPAASASSPSDHNSMNDEKQDKGETDEAQSECDGATPGQ
PASPATVDPKRVKRILANRQSAQRSRVRKLQYISELERSVTSLQTEVSALSPRVAFLDHQ
RSLLTLGNSHLKQRIAALAQDKIFKDGGTEEGDREAAANLPPAKPQERGIPTGGRGPGPR
PRQCRPDRQRGGRRGRAMPALVIGRDPDAL*
>11667.m05438|LOC_Os01g55150.1|genepair20-2
MAQLPPKIPVAAPGHHQHWASAGGAGDAAWADEFAEFAASRRGAHRRSLSDSVAFVEVAP
AGCGAGGEFDRLDDDQLMSMFPDEGGSSAPGSDNGGSDSDGGGDKHAAAQSDDGQHAAGE
PTQEQAAATSPTELIRDPKRVKRILANRQSAQRSRVRKLQYISELERSVTTLQNEVSVLS
PRVAFLDQQRTILTVGNSHLKQRIAALAQDKIFKDAHQEALRKEIERLRQVYQQQNTKLS
GGLAADHAHVHGGPPPVRAEKELMS*
>11667.m01124|LOC_Os01g11550.1|genepair21-1
MGDAGGHSHHHQHGFQPQLLSFGGVGHHHHLHQFTAQPQPPAASHTRGRGGGGEIVPATT
TPRSRGGGGGGGGEIVAVQGGHIVRSTGRKDRHSKVCTARGPRDRRVRLSAHTAIQFYDV
QDRLGYDRPSKAVDWLIKNAKDAIDKLDVLPAWQPTAGGAGAGNAAAPPSSSTHPDSAEN
SDDQAQAITVAHTAFDFAGGGSGGTSFLPPSLDSDAIADTIKSFFPMGGTAGGEASSSTT
AAQSSAMGFQSYTPDLLSRTGSQSQELRLSLQSLPDPMFHHQQHRHGGGGGGGNGTTQQA
LFSGAANYSFGGGAMWATEQQAQNQRMLPWNVPDPGGGGGAAYLFNVSQQAAHMQAAAAA
LGGHQSQFFFQRGPLQSSNQPSERGWPETVEADNQMSHHQGGLSPSVSAAIGFAAPGIGF
SGFRLPARIQGDEEHNGGGGGNGDKPPPPSSVSSASHH*
>11667.m05433|LOC_Os01g55100.1|genepair21-2
MSGFTNKDGRQNLASCFNFRSSPFRLTVGERELKLEEDKNQLSKGLDPWTSNPTASASTL
HYLLQEKERAQQAHEQLQIYQQQQGFGSFLQHRIRQPASRGPGGGGGGGDGGGSSGESTP
VEALATAFGAGRIVRSAAGRKDRHSKVCTARGLRDRRVRLAAHTAIRFYDVQDRLGYDRP
SKAVDWLMRNAKAAIDELPDRAEAPPPPAAASTEQPEATEQATSTSYGFGNTTGGTMTSA
ASAAAGSFLPHSLGADRVSDSVKSLFPSSSTASGAASAGHDEYRGSPPDLLSRTTSNQQP
QELCLTLQSNQHQIFSHVSSNHHGMISSAGVPGWPDHSQRMQAWHAPENSTGDGRGGGNG
DGYMFAMPSRQGLDQSQLFSHGEPLQSSGRGWASARAWLDPLAVAAIHHQPSTMAAGQVG
FGHLVGGAGGGGGFMGFLAPAAQRLEGEEEHGSEVIR*
>11667.m06046|LOC_Os01g60730.1|genepair22-1
MGVHTKSMSWYMGTPASPSPGSAGEAQHALSSGGGGGSDASFDTNMVIILAALLFALLFA
LGLNSLARLIIRWARRAATGEGAGGGVEGAGAGGGLKKRALRSIPIEVYGGGGGGSPAAA
AEVCAICLGEFADGEKVRVLPRCGHGFHVRCVDTWLVSHDSCPTCRGSVLHGATTKHKPT
AAAAAAGSRRPGSEVDAPAAAAVTVVIA*
>11687.m00150|LOC_Os11g02430.1|genepair22-2
MRRLGDVVEAPALVLTPASMQQAGGRGSSGALDASMVVILAALLCVVICALGLTSLIRCA
LHCARGLSPTTATPTPSVSTAATAGLKKTELRRIPVEVYGAKQAGVPDGECAICLGDFAD
GDKVRVLPRCHHGFHVRCIDTWLAAHTSCPTCRDSILSVHGVVAGGQT*
>11667.m06048|LOC_Os01g60740.1|genepair23-1
MAPRCATLAVVVVLVAAVVAPPTAVRAAISCSAVYNTLMPCLPYVQAGGTVPRACCGGIQ
SLLAAANNTPDRRTICGCLKNVANGASGGPYITRAAALPSKCNVSLPYKISTSVNCNAYG
GRPDAYVCARASRTRGE*
>11687.m00144|LOC_Os11g02370.1|genepair23-2
MARAQLVLVALVAAALLLAGPHTTMAAISCGQVNSAVSPCLSYARGGSGPSAACCSGVRS
LNSAASTTADRRTACNCLKNVAGSISGLNAGNAASIPSKCGVSIPYTISPSIDCSSVN*
>11667.m06060|LOC_Os01g60850.1|genepair24-1
MESATEKVRRTPSSCLLLRISDICKVRSVGVAPTVREKPKADGSATGESSEDGGAHLKVH
PHHVSDHESVSECSSARCEEAFVERLLDAISGLKLSYVNLQQALVPYDPEEITIADERFT
SELQETAGLKDLYVNMNKWRNPMYQCYVGSRIQEQQKLAVELQAGMCKRDSEIVCLRAEL
DELERKNMELEEKIGQSALQKEGSFAIGMGVSTDMFMELFELSTKSIHDFAKLVVRWMKL
SRWNLGNLTSPIDNSVVYDKRSHKNYAVEAYFACMMLMGHKEEYLSLDVFDYVMSFSDPF
DALMKAPDSCFGRFCREKYLAILPPSMEDSFFGNLDHRSFVENGGHPRTPFYQAFVTMSR
YVWASLTVARSLNPRAEMFYVKGGTEFRSKHMECVPSKITKEGDKVSVGFTVMPGFKIGC
TVIRCRVYLSMVNERNF*
>11687.m00131|LOC_Os11g02270.1|genepair24-2
MESRTPRSSVARSTCNVPGLVLGFSKLCKITKICAAPEFADTKTEFGDYCGGYDQRLIIT
RLFEEIGALKSAYIKLQKAHIPYNPPKIAFADEIITSELDSVTALQSLCSWNGSVGSLIN
DRWSLVQELEAETRKKDSDIMLLRRELDGLKSANSRLNKQISSSKPSVNHHKDYSVVLKK
LTTPSAVLELFKVASTSVHDFAELIFSLISSSDHHCTNNADEHSPYKRYSLEAYLSRTML
AVHDGAEDDDELDLARFDRIMRCCDPLDALMAHPNSSFARFCRTKYLAAVPSEMEAAMFR
NNLDVRAFVSRGGHLRTWFYRAFATMARSAWALQVAVTAHRRCCGRGSVRMLYARRGSRY
AAEYMDSVVAAAAADAGRGGGDGVAFTVTPGMKVGETMVACRVFLCHEQQDTISDETDPK
FR*
>11667.m06067|LOC_Os01g60910.1|genepair25-1
MDGRSILMGRYEVGKQLGQGTFAKVYYARNLTTGQAVAIKMINKDKVMKVGLMEQIKREI

SIMRLVKHPNVLQLFEVMASKSKIYFVLEYAKGGELFNKIAKEGKLSEDSARRYFHQLIN
AVDYCHSRGVYHRDLKPENLLLDENENLKVSDFGLSALAESKRQDGLLHTTCGTPAYVAP
EVLSRKGYDGAKADVWSCGVILFVLVAGYLPFHDPNLIEMYRKICRADFRCPRYFSAELK
DLIHKILDSDPSTRISIPRIKRSTWYRKPVEINAKNSEAATTNSISSGVATTSGSAECST
SEENQGSLSLPNLNAFDIISLSTGFNLSGFFEDTHGHQEERFTTRQPVTTVLGKLKELAK
RLKLKVKKKDNGVLRLAAPKEGKKGFLELDAEIFEVTPSFLLVELKKTNGDTMEYRKLVK
EDIRPALKDIVWVWQGDEHLNSQSILQGEQQQSPLPPELPQDQLQPSLPQQEKQDMPEPP
LLPQVNASTGNHNDHWLVHWLMMKPFRFPPPDSVPSTGRDEEEGSSHHGNGSGAARENPE
NPLPDLALAKSESDRIKAFYQTVLSAIVVFIVAALSGYKDMKPLYSTTNHKKVHLSNLLV
IEGFCMIATFICAAVLMMYEFYTCRDLTVLIAVAGAMLVATDTILVVIANRDNAVFAVLF
VPVLLLVGMAVYTGASMEEQPPNSPHDGGYDEATKSTFDLATVSTMAALGLQGTIVFGYL
KTPEKKSEHDDPPLDLAVCYIASTVCLTVMAFLDGFVVLSVWPEAVALVLYYAAQLCSGR
QPGGGVGSRPWIEFVFRIVATAGFTLMAGLYAAFLGTNHYSVYLKAAMLVLLMAVLSSLS
RLANPVYMPEIDGALLEFCVAGVALAFPAVSLLVACPLVLKVFVDLYIHHSSL*
>11687.m00128|LOC_Os11g02240.1|genepair25-2
MESRGKILMERYELGRLLGKGTFGKVHYARNLESNQSVAIKMMDKQQILKVGLSEQIRRE
ITTMRLVAHKNIVQLHEVMATRNKIYFVMEYVKGGELFEKVAKRGKLTEVVAHKYFQQLI
SAVDYCHSRGVYHRDLKPENLLLDENENLKVSDFGLSALSESKRQDGLLHTTCGTPAYVA
PEVISKIGYDGAKSDIWSCGVILFVLVAGYLPFQGPNLMEMYRKIQHGEFRCPGWFSRKL
QKLLYKIMDPNPSTRISIQKIKESTWFRKGPEENRILKERTLNENTTKNVAPVLGVRRKK
NAHEDVKPMSVTNLNAFEIISFSKGFDLSGMFIVKEWRNEARFTSDKSASTIISKLEDVA
KALNLRVRKKDNGVVKMQGRKEGRNGVLQFDIEIFEVTTSYHIIEMKQTSGDSLEYRQLL
EEGIRPALKDIVLA*
>11667.m06082|LOC_Os01g61060.1|genepair26-1
MVQNFPEDDMAGTPYSDQTSPILTEYHITVPALHDGSMQGSVHHERRLLDCLRATPSVEW
LKNINLCSPLTNFRLPSTGVRRYLHVEVHFVRRINWSSVFSFCKNWLKHPLNIALLAWLL
CVAAAGGMLILLLLGLLNRAFPSKPLRHHWIEIDNQILNALFTLMSIYQHPSLIHHLVLL
CRWRPEDAAELRKVYCKNGDRRPGERAHMSVVVALLHVTCISQYVVCNLYWAYRSRSRSE
FADNFFFVLGVVAPVVAGAYTVYSPLGRDTDDDASGEEAKQQQQHMIEAELPGTRTVVVD
PVWAGGLLDCGEDPAACCLSSLCTFCVFGWNMERLGFGNMYVHTAMFLLLCVAPFWVFNI
TALHIHDYDLSDAVGAAGIALCFLGLLYGGFWRVQMRKRFALPGSRWCCGSASLTDYARW
LFCWPCALAQEVRTGNLYDVEDGGGVFYEKAMDGGDVEGGAASTAATGVVPVSVGGGEGD
GVVGDIKLGMDGEMIPPAQAVMETSGDTQGSGADVAANGDNELSS*
>11687.m00087|LOC_Os11g01850.1|genepair26-2
MLLACDCDDDAQTAENGNAPMLSNYRAQPAPPLAEPLLLDKPQRAHWLRQLKPWRRNAAD
DHLSAGIAINWSSVRSATKDWITNPMNIAMLLWLLCVAVSGAMLVLLLLGLLDGAFPTPA
ARNHWIEINNQVLNALFTLMSLYQHPVLCHHLFLLCRWRPADAADLRAAYFKDGAGPRHG
ERAHMAVVVALLHLTVACQYVLCGLYWGYTKKTRPELVENGFFVLGVVAPVVAVVYTVCS
PLGKDNYGELACPNAFDSVSQHKCTGHAVVEPEWAGGMFDCGGDATAWWLSLSCTFCAFG
WNMERLGFGSMFVHTATFVLLCFAPLWVMGVSALHIHDVVIGDMVGGAGALLCVCGLLYG
GYWRIQMRERFGLPASTACCGSPSVTDYARWLFCWPCALAQEVRTESLYHIDCETFYKKL
PVVDDVEDEKRLPLLASHHVQFHEPPDTMIMAASEGSNDHVVIVHEEMVPPAVQVVVEQV
VVEGDKSEEECSAVHDEKIMGSPLPESVVIVDDDEIPASLSDGSWTVEKVKRLINVVTLV
SLLILLYTRGFIR*
>11667.m06092|LOC_Os01g61160.1|genepair27-1
MASSSSSRLLFLLSCSVLALLAGAEVHHHEFIVQETPVKRLCKTHNVITVNGQLPGPTLE
VREGDTVVINVVNHAQYNVTIHWHGIRQFRTGWADGPEFVTQCPIKPGGSYKYRFTIEGQ
EGTLWWHAHSSWLRATVYGALIIRPRENKTYPFEKPAREVPLILGEWWDADPIQVIREAQ
RTGAAPNISDAYTINGQPGDLYNCSKEETTAVPVKPGETALLRFINAALNQELFVSIAQH
KMTVVGVDASYTKPFTTSVLMIAPGQTTDVLVTMDQAPTRYYLAARAYDSAQGVAFDNTT
TTAVIEYDCGCATDFGPSIPPAFPVLPAFNDTNTATAFAAGIRSPHEVKIPGPVDENLFF
TVGVGLFNCEPGQQCGGPNNTRFTASMNNISFVFPQTTSLLHAHYYGIPGVFTTDFPAYP
PVQFDYTAQNVPRYLWQPVPATKLYKLKFGSVVQIVLQDTSIVSPENHPIHIHGYDFYIL
AEGFGNFDPKKDAKKFNYVDPPQRNTVAVPTNGWAVIRFVADNPGVWLMHCHLDVHITWG
LAMAFLVEDGYGKLETLEAPPVDLPMC*
>11687.m00075|LOC_Os11g01730.1|genepair27-2
MPSRGCSCWLLSLALLCSLAAAKEQYHEFVIRETTVKRLCKSHNIMTVNGQFPGPTLEIN
EGDSLIINLINRGRYNMTLHWHGVRQMRTGWSDGPEYVTQCPVRPGQSYRYRFTVAAQEG
TLWWHAHSSWLRATVYGALLIRPRDGTSYPFDVQPTRELAPILLGEWWDMNPVDVVRAAT
RTGAAPNISDALTVNAQPGDLYSCSSHDTAVFPVTSGETNLLRFINAALNTELFVSLAGH
NMTVVAADASYTKPYTTSLLLLAPGQTTDVLVTFDQPPGRYYLAARAYASAQGVPFDNTT
TTAIFDYGAANNASSAAIAMPTLPAYNDTTAATAFTTNLRGLRKAELPSRVDESLFFTVG
VGLFNCTNATAQQCGGPNGTRFAASINNVSFVLPSSTSILQAHHHGAPGGVFTADFPANP
PVQFDYTAQNVSRALWQPVAGTKVYKLKYGSAVQVVLQGTNIFAGENHPIHLHGYDFYIL
AEGLGNFDAGADTGKFNVEDPPMRNTVGVPVNGWAVIRFVADNPGVWLMHCHLDVHITWG
LAMAFLVDDGVGELQSLEAPPPDLPLC*
>11667.m06093|LOC_Os01g61170.1|genepair28-1
MASAAAPTPQPLLPVTNPAAAGGSAPSSGSALTDAPLATPAFRLFLSEATSRLRRNLAYF
RVNYAAVVAFSLAASLLAHPFSLLVLLAILGGWCFLYVFRAADQPVVLFGRTFTDRETLL
GLVVASVLAFFMTSVASLIISGLLVGGAIVAVHGAFRVPEDLFLDDPSVGSNGNTTSRLL
SFLGAPGSGV*
>11687.m00063|LOC_Os11g01610.1|genepair28-2
MAAASPPLLPTTVLPANATATVSPAPTSVSSADANPAATRAFLARLLDSVKRALSGARPW
PELIDRSALSRPESLSDAGARLRKNLAYFRVNYAAIVALSLAATLLAHPFSLAALLALLA

AWCFLYLLRPSDAPPLAAFGRTFSDRETLGGLIVASAFVVFLTSVGSLIFSALALGAAIV
CAHGAFRIPEDLFLDEPDQANGAASVNLLSFITSATGGRV*
>11667.m06124|LOC_Os01g61460.1|genepair29-1
MANRRKFSQAGGGGGGGVFDPFGTKQAVSSLRKGGRLPVYVAGVFFVIFVIIMYGEDIRS
LTLDPIARAGTTPARIVEPVVTEERHVARVNPPRREVSSAEKAAALPLDVDERPKLATPT
PTEAAKEVPKVEKIRKPKKPKTTKKKPRKPRPAKKTVAAAAGGLLGVPETCDLSKGEWVF
DNTSYPLYREEQCEFLTSQVTCMRNGRRDDTYQKWRWQPKDCSMPRFDAKLFMERLRGKR
FMFVGDSLNRNQWESMVCLVQSAMSPGKKYVTWEDQRVVFHAVEYNATVEFYWAPFLVES
NSDDPKIHSIQHRIIKADAIAAHAQNWRGVDYLVFNTYIWWMNTLNMKIMRPGGQSWEEH
DEVVRIEAYRKVLTTWASWVNDNIDPARTSVFFMSISPLHISPEVWGNPGGIRCAKETMP
LLNWHGPIWLGTDWDMFHAAANVSRTAATRVPITFVDVTTMSERRKDGHTSVHTIRQGKV
LTPEQQADPGTYADCIHWCLPGVPDIWNLILYTRIMSRPQLV*
>11687.m00037|LOC_Os11g01370.1|genepair29-2
MTKPQQQSPPSTTATTTTSPPPPPPSTPPPASSSSSSLAKLPLRLHSLASSSRSLLSALR
RSPVTTLVAAFFLLALFMYGEDVRTLAELSIDDYLYPDADFYNVSALPPLLLPPPTCDLS
RGRWVFDNTSLPAYREKECTFLTKQVSCLANGRPDDLWQYWRWQPNNCSLPTFDARRFME
KMRGKRMMFVGDSLNRNQWESLVCLVQPILSKGRKKIVKRGSFNIFYAKEYRATLEFYWA
PFLVESNSDNPNFHHIDQRIISPERIESHANNWKDVDYLIFNTYIWWMNNEDIKVRRPNS
TSWSDHDEVPRIETYGRVFKTWSTWLEQNVDPARTSVFFMTISPLHNSSKIGIGGRWFNT
DAVITFCQFNIDEPCISATGSLELWNILKYRHRVRWFPAQWGNPNGIKCVKETLPVLNYT
KPLDLNHDMRMYDLVAKVAKNMKNVPVSLIDITRMSDYRKDAHTSLYSIRQGKLLTPEQK
ADPQKYADCIHWCLPGVPDVWNQILYTRILSKSSPPSPHPPLPPQ*
>11667.m05895|LOC_Os01g59360.1|genepair30-1
MGNCCPGSGDAEPASSDASTGNGSSSFKAGASPSSAPAQNKPPAPIGPVLGRPMEDVRSI
YTIGKELGRGQFGVTSLCTHKATGQKFACKTIAKRKLSTKEDVEDVRREVQIMYHLAGQP
NVVELKGAYEDKQSVHLVMELCAGGELFDRIIAKGHYTERAAASLLRTIVEIIHTCHSLG
VIHRDLKPENFLLLSKDEDAPLKATDFGLSVFFKQGEVFKDIVGSAYYIAPEVLKRSYGP
EADIWSVGVILYILLCGVPPFWAESEHGIFNSILRGQVDFTSDPWPRISASAKDLVRKML
NSDPKKRISAYEVLNHPWIKEDGEAPDTPLDNAVMNRLKQFRAMNQFKKAALRVIAGCLS
EEEIRGLKEMFKSMDSDNSGTITVDELRKGLSKQGTKLTEAEVQQLMEAADADGNGTIDY
DEFITATMHMNRMDREEHLYTAFQYFDKDNSGCISKEELEQALREKGLLDGRDIKDIISE
VDADNDGRIDYSEFAAMMRKGNPEANPKKRRDVVI*
>11687.m00331|LOC_Os11g04170.1|genepair30-2
MGQCCTGGGKAVAGDEAEPGTSKAAPPSRGTSSKNGSAKQQPCSPAAKAAATEAAAAASS
SKKPAGPIGEVLERPMEEVRTTYSIGKELGRGQFGVTHLCTHKATGEKLACKTIAKRKLA
NKEDVDDVRREVQIMHHLSGQPNIVDLRGAYEDKHNVHLVMELCAGGELFDRIIARGHYT
ERAAAALLRAIVGIVHTCHSMGVIHRDLKPENFLLLSKGDDAPLKATDFGLSVFFKEGEV
FRDIVGSAYYIAPEVLKRKYGPEADIWSIGVMLYIFLAGVPPFWAESENAIFTAILRGQI
DLASEPWPKISSGAKDLVRKMLNINPKERLTAFQVLNHPWIKEDGDAPDVPLDNVVLNRL
KQFRAMNQFKKAALRIIAGCLSEEEIKGLKEMFKNIDKDNSGTITLEELKNGLAKQGTKF
SDNEIEQLMEAADADGNGIIDYEEFVTATVHMNKMDREEHLYTAFQYFDKDNSGYITKEE
LEQALKEQGLYDANEIKDVITDADSNNDGRIDYSEFVAMMRKGSGCAEATNPKKKRRDLV
L*
>11667.m05914|LOC_Os01g59530.1|genepair31-1
MADQLSEEQIGEFREAFSLFDKDGDGSITTKELGTVMRSLGQNPTEAELQDMISEVDTDS
NGNIEFKEFLGLMARKLRDKDSEEELKEAFRVFDKDQNGFISATELRHVMANIGERLTDE
EVGEMISEADVDGDGQINYEEFVKCMMAKKRRKRIEEKRDHDGGSRTKSAGPSAAPASKR
GQKCVIL*
>11687.m00309|LOC_Os11g03980.1|genepair31-2
METVIQSINQSIACVLGRRSINESRRKCNAGTITSKELGTVMGSLGQSPTEAELKKMVEE
VDADGSGSIEFEEFLGLLARKLRDTGAEDDIREAFRVFDKDQNGFITPDELRHVMANLGD
PLSDDELADMLHEADSDGDGQINYNEFLKAKAEYDGGTWKWRPSVK*
>11667.m05923|LOC_Os01g59620.1|genepair32-1
MALQLMMDNYLLCSAMKGRLSVYSDSQLILVKMNRESNFMPTPDQDVLEVKPLRTLAPMF
PAPLGIDVLNRLTAPPLVFVAPAGQFPGGFGSLNIPAVRSFAAFGGQDASGGKTAGGGDQ
DASGGKTAAGGDQDAGRGETAAFGGQETVRGEFVANGTPNVGASATGPIDATPISACKST
QPSVISLDDDDNDDDEPYGGNQTSASGRKIKRPSHLKGYNVSDGLGTDSSNGTKKRPKTS
NRKAATDNEISLMPPSSDPREVVEVLLMTFEALRRRHLQLDETQETSKRADLKAGAIMLA
SNLRANIGKRIGAVPGVEVGDIFYFRMELCIIGLHAPSMGGIDYMNKFGDEDDSVAICIV
AAGVYENDDDDTDTLVYSGSGGISRSNSEEKQDQKLERGNLALERSLSRKNVIRVVRGYKD
PACLTGKVYIYDGLYKIHESWKERTKTGINCFKYKLQREPGQPDAVAIWKMCQRWVENPA
ARGKVLHPDLSSGAENLPVCLINDVNSEKGPGHFNYITQVKYLKPLRSMKPFQGCRCTSV
CLPGDTSCDCAQHNGGDLPYSSSGLLVCRKLMVYECGESCRCSINCRNRVAQKGVRIHLE
VFRTTNRGWGLRSWDPIRAGSFICEYVGEVVDDTKVNLDGEDDYLFRTVCPGEKTLKWNY
GPELIGEHSINISADTFEPLPIKISAMKMGNVARFMNHSCNPNTFWQPVQFDHGEDGYPH
IMFFALKHIPPMTELTYDYGDIGCESRGVGSRAKNCLCGSSNCRGFFS*
>11687.m00280|LOC_Os11g03700.1|genepair32-2
MENSEDEAESDKLPLDLEPLRSLAPKFPTILGYDVETQSTDPLLVYATPSIPCSSSEQPQ
EAPASFSLPLPKSPVPIKATPISAAFPTPQHEDESSDQDYKPFCKNKKPAMPKRAKRPQQ
AEKSNDANIKRRSIRRNLDNEFNLCSSSSDNPKESVEGILMMFDSLRRRVLQLDEKEDAS
RRADLKAGTLMMQNNLRINNHKMIGHVPGVEVGDIFFFRIEMCIVGLHAPAMGGIDYISS
KNKDETLAVCIISSGGYENDDDDTDILVYTGQGGNSRHKEKHDQKLERGNLALMNSKSKK
NQIRVVRSAQDPFCNSGKIYIYDGLYRIEDTWTDTAKNGFNVFKYKLRRDPGQPDGISLW
KMTEKWKANPATREKAILLDLSSKVEHLPVCLVNDVDDEKGPSHFNYVAGVKYLRPLRKT

KPLQCCKCPSVCLPGDPNCSCAQQNGGDLPYSATGLLAKHTPMVYECSSNCQCSHNCRNR
ITQKGIKLNFEVFWTGDRGWGLRSWDPIRAGTFICEYAGEVIDETKMDIDVEEDKYTFRA
SCPGNKALSWNLGEELLEEKSTAVITKNFKKLPIIIRANNEGNVARFLNHSCSPNLLWQA
VQYDHGDDSYPHIMFFAMEHIPPMTELTYDYGTRGAPPGFEGKPFKACKLKSCLCGSKHC
RGY*
>11667.m05930|LOC_Os01g59670.1|genepair33-1
MTQGSARFVLEAERVHAMALDTKQVARCLKKVLKSSIKDGYRCVSEHPILLTLGVLLYLL
YRSSPGLFAFLLSSSPVIMCTTLLLGILLSYGETNLPEADEDNKITPEISSLKVGNPSSD
FHFEASQRLPVPELRENTTGFKERETKQTVFIRERASEHIELEDNVPLLRRVEHEYDRFD
RHEIPAALTPFPSMVNFHQGSRVGNDLSSNQDINSKGLLSIKDKADGHTSFFEGVRSGLD
EKEAPFGIFSTSKNVNGRGELEENLNQETVFTDSTASRVRDISEEKPTEGEAGTSKSACA
ISTHQSKTLDELRINTSKGFEDNLLDSSLGSPWARVGSEDGVGSDDGSSGFDSDQAESSS
PDASMTDIAPILDEIDPLLGASSTHPDTIPKDDSDTDSHVSSQDHQTDDDSNDETDNNDA
KENGEEKNKEQGKEAAFIWTADDEKNLMDLGYSEMERNRRLELLMARRRSRKNIRFEIDN
DLIGIDNNDGGRGVDDLSRFHVQVPHISVPRRNPFDLPYDSEEAAIPGSAPSVLHTRKNP
FDLPLDQSNDGDVSADNNVNPGELVKASHRDMFFRRHDSFNIGRTDATLERFSRFKPYFV
PETVEGSLSNFQRQFSDKIADQEDHKDLDEKDLPNEHGSPALQRQDSDLADVGSECSDGI
NSVDVELDNSDIDDREIALQHFVFERSQEREAYLSSTKGKGPEDDYLLSSVGNSKTLHPV
ADLFSWEDGNGESSLGVNSSHNMSVEFSDWVSSPKPIAEHDSGPENLQEFLNTEVASSSK
TIVLGARNPAENNGNVDSISYSNNEMPSDNLGHGSMEFPSEFCNESLPVISRDLHPIPEE
RVVENFNVQEKHEAVIFTDSDAALTGFHVIEEHFEVGCDVSPSSEVVPSCLQASDSIQSR
LVENKEVSNPFISIASETNKVDMIDLKEETAAGYPLDSDDDADKIYPEPMEDNVIDESFL
SELDAVGDFRVEATRSDQQMPDVDSHIDNNTSNGVAESSLISPQISSNIFSNMKYASMLE
HEENSPLVDDLNGTGPEFGWSLGASYDDPEQTVYNPRRRILGASRFEETNTEMKPLFDET
EASFVNAPIEANLVVGPSKVDVANESELTKTDTNMIVLDANSLEDIETAFKQASNGVVES
TVDNETPQVSGVDIDPESIESSEQLDVIDAKSVDDIYAALKEHTTAAMNSSFEENEDKHG
CGDTVKFTMHDELPEGTHIEGNTVGDGKEPEPMGITSSMDVIDAKSIDDIYAALKKQSSA
AANSSFEQNEGKNGCGDTVTFTTHDELPEGTHIEDRDNTVEDGKEPEPIGTTSSMDTIEV
KTIDDIDAVFKKLSDGGTKSAAQAVDCENTCEASDESEQH*
>11687.m00274|LOC_Os11g03640.1|genepair33-2
MEARAVRPLLFKPTFYLRVKFKKIYMAFGANDIVLHIKRLMLCPSLFNFLLSSSPVFLLT
ALLLGALLSYGEPAVLVLGENQQTLSFKSKISITDCSIDKVETVAVEEHLDKTTTSNEVY
VRERNFEGNIHDTHWEEKNGTYMTVDTALNDEIHTKDGTSDYDLQDTHREGKGITSVETD
TVPCVAPSSFAYSGVTVETEDVGENSKKNDELQELGSINPESDNNKVQYQYQLGEFMSSC
WEPVMRQEPQDACSDSESDLTESSPDASMTDIIPMLEELHPLIDLQTGHPSLVSRDNLNT
SSDDDEDDLEEEDASTDENQLEGKIDDFANWKDVIDLNYLDMDNNSKLEGMMDLQRAKNI
LKFELDRRLMDLQAADAVQKMEEASRFRVQHRLVSFHGKIYLIFAVNQNMAHDSPLQETW
TPLSYFSARRHRKHGNLYVRHSTSLHHNSFKLEKDEISENDAHNSQSDCDAKQEGNNSKL
FGSLEAHIGEEIKILGAAISDVGVLEVNSGMDSGNQNADFSDDISLSPIQKSRQSTFEAK
EAVHAGIEQLTSCSPYKVNNFEAHIVEADSIDEFNSLFKCRMEEVLVQSISESSISQPLT
VKLEDELSEPLSSDSGTGTHFIDGSSVEDSDPQFAQLKDEALVSATSNATCRNESIEEKS
SEALLAGNEDYSELPNELLKSGDPQFADSSEIQMQVIEATGH*
>11667.m05931|LOC_Os01g59680.1|genepair34-1
MVPHGRVHPAAAASSSDFSGEMNQSAASSDPSSSPPYSFHFEKPLPPTAAAPPPADARRY
QQLLPAPQPGTYVVQMPKDKVFRVPPPENARLFQHYTRRARRRARCSCARVCSWLLLALV
LLAAALAASAAVVYLVFKPRQPDYTLLSLAVSGLGGILGNASSTAAPAPVSFSPEFDATV
RADNPNGKIGVHYEGGGSHVAVSYGGVRLADGAWPAFYQGPRNVTVLVATAKGLGIRFSE
RLLGDIAAAGRLRSVPFDVDVKVPVRLQVGGVRTWAVPVRVRCAVVVDRLAADAKVVSKS
CRVKARFLFWKD*
>11687.m00270|LOC_Os11g03600.1|genepair34-2
MADRVHPMPAPPPPSSSPPLEHAAAATETTPLHPSFRGARPPSPGTYIIQIPKDQVLRVP
PPDRARRYKKLAARPARRRRLRHACCAAFCAALLLLLLAAAFVGAVYLVFRPRAPSFSVA
SLSIRGLDALAVSSLTPQIDAAVRADNGANKKTGIDYRGGGEVTVSYAGERLAAGPWPAF
HQAPRNVTVFSTALAGGGVSFPEEQRKRLAAEQAAGAVPLTVEAIVPVRLRFGKVLRTWT
VDVKTRCEVTVNKLAAAAPPANRGCRVKVRPLWWWW*
>11667.m05941|LOC_Os01g59780.1|genepair35-1
MVSMRKKKKAFAVAAATTLLSPPRSSSSSSSSTASSCIVPPRTESGKKKSKHRKRAKDGTG
GDDDDAAVAAAPRKGSSIYKGVARHRGSGKYEAHLWDKQGWNPNQTRKRGRQGAYDTEEA
AARTYDLAALKIWGSDHVLNFPIDTYRKELERMQRMTREEYLATLRRKSSGFSRGVSKYR
GVAKHHHNGRWEARIGRAVGKKYLYLGTFDTQEEAATAYDLAAIQLRGRSAVTNFDASCY
TYTDHLPPPPPPQPPSVCKTEPELEPPQPAAPPGSESLLRPKMEPCDDWEPPAICPSLRD
ADDADHAIAEILPALCMDRADFEARYPARRARDAAADGWSTSSDDVAAASVDDDVLRSLP
DDVGFVDDVESLFLDAPGPAAAAAAAAMPDDVERAVQRAPSAASRRANAAAVSYAISSLA
SGRWWY*
>11687.m00264|LOC_Os11g03540.1|genepair35-2
MAKRSSPDPASSSPSASSSPSSPSSSSSEDSSSPMSMPCKRRARPRTDKSTGKAKRPKKE
SKEVVDPSSNGGGGGGGGKRSSIYRGVTRHRWTGRFEAHLWDKNCSTSLQNKKKGRQVYL
GAYDSEEAAARAYDLAALKYWGPETVLNFPLEEYEKERSEMEGVSREEYLASLRRRSSGF
SRGVSKYRGVARHHHNGRWEARIGRVLGNKYLYLGTFDTQEEAAKAYDLAAIEYRGANAV
TNFDISCYLDQPQLLAQLQQEPQLLAQLQQELQVVPALHEEPQDDDRSENAVQELSSSEA
NTSSDNNEPLAADDSAECMNEPLPIVDGIEESLWSPCLDYELDTMPGAYFSNSMNFSEWF
NDEAFEGGMEYLFEGCSSITEGGNSMDNSGVTEYNLFEECNMLEKDISDFLDKDISDFLD
KDISISDRERISPQANNISCPQKMISVCN*
>11667.m06401|LOC_Os01g63890.1|genepair36-1

MPKIKTSRVKYPGGWELIEPTIRELDAKMREAENDTHDGKRKCEALWPIFRISHQRSRYI
YDLYYRRKEISKELYEFCLDQGYADRNLIAKWKKPGYERLCCLRCIQTRDHNFATTCVCR
VPKHLREEKVIECVHCGCRGCASGD*
>11686.m00475|LOC_Os12g05410.1|genepair36-2
MGVARGVHSGLPRSPPSATGRHLLYSGPLTSKPYDITSRPYEAQFGPFHVPSRPHRGPDQ
PNPPSGIFFLPPRPPILPPTTASSHLRRRNPKKFLLPLLVRAAEGALRFLIVSPYAARRS
IMPKIKTSGVKYPDGWELIEPTLSELHSKMREAENDPHDGRRKCEALWPIFKINHQRSRY
LYDLYYNRKEISQELYEFCLDQGHADRNLIAKWKKQGYERLCCLRCIQTRDHNFATTCVC
RVPKHLREEQVIECVHCGCKGCASGD*
>11667.m06405|LOC_Os01g63930.1|genepair37-1
MGVVEAEALHGAVEALAGSLQPHVATAFFVFSACTVALAALLAVVRLRPPWWCDCTVCEA
FLTASWAGEFDNLCDWYAHLLRTSPAQTVHVHVLRNVLTANPVTVDHVLRARFDNYPKGA
PFSAILADFLGRGIFNVDGDAWLFQRKLAAAELASPALRAFAARVVASELRCRLIPLLHS
ASREGNGKVLDLQDMFRRFAFDSICKISFGLDPGCLELSMPVSTLVEAFDTASTLSARRA
TVPMQIIWRLKRFLNVGDERKLRDAVRLVDALAAEVIRQRRKLGGAATGSDLLSRFMGSI
DDDKYLRDIVVSFMLAGRDTIASALTAFFLLLSDHPEVATAIRDEVARVTGDGNRTMAAT
FDKLKDMHYVHAAMYESMRLFPPVQFDSKFAAGDDTLPDGTVVAKGTRVTYHAYAMGRME
SVWGPDCAEFRPERWLRDGRFVPESPYRYPVFQAGVRVCIGKELALMEMKAVIVAVVRSF
DIEAIARSSRRPKFAPGLTATFAGGLPVRVRRRRARASGHNPPI*
>11686.m00478|LOC_Os12g05440.1|genepair37-2
MEVELPWGARCAAAAFFVSSLCVAALGVVLLLLRRWPWCGCHVCRAYLAGSWRREFANLG
DWYADLLRRSPTGTVHVHVLGCTVTANPANVEYMLKTRFDNFPKGRPFAALLGDLLGDGI
FNVDGDAWRHQRKMASLELGSVAVRSYAYKIVAQEVEARLMPVLANAADSGAVVDLQDVF
RRFAFDTICKISFGLDPGCLDREMPVSELADAFDAASRLSAMRGAAASPLLWKMKRFLNV
GSERELKKAIKLIDGLAAAMIRERRKLGVANSHDLLSRFMASSGDDARGAADDKFLRDIV
VSFLLAGRDTVSSALTTLFMILSKNPDVAAAMRAEAGAAAGESAAVSYEHLKRLNYTHAV
LYENMRLFPPVQFDSKFCAAADVLPDGTYVDGGARVMYHPYAMGRMPRIWGADCDAFRPE
RWLTGAGGAFVPESLFKYPVFQAGLRVCLGKELAITEMKAVSVAVVRAFDVEVVGENGRC
GGGAAAAPRFVPGLTASISGGLPVKIRRV*
>11667.m06409|LOC_Os01g63970.1|genepair38-1
MKRPLRRPFAVLLFVVLCAAASFPSVLRRSVGPAPVLAPLPPLDPARLNATLLRLAAADP
SEAPLRRDVDDLLEGRLPASSARARAWRLRGDRLHLHLRHHQFPVYRRGHHPDHDHDPLL
HPLPRQELHLDPSLRRALRSWHRLRRHDPGVLRNLPSLLSLPGRIPSCAVVGNSGILLGA
SHGALIDSHAAVFRLNNARISGFAANVGAKTNLSFINSNVLHLCARRPNCFCHPYGDGVP
ILLYICQAAHFLDVASCNASSRSLHAASISVTDPRLDVLCARIVKYYSLRRFVAETGRAA
EEWSSTRDAAMFHYSSGMQAIMVAVGVCDRVSVFGFGKAADAKHHYHSNQKAELDLHDYK
AEYAFYRDLADRPEVVPFLNDAGIAVPPVVFYH*
>11686.m00489|LOC_Os12g05550.1|genepair38-2
MKRRHLPPVLVLLLLSILSLSFRRRLLVLQGPPSSSSSSRHPVGDPLLRRLAADDGAGSS
QILAEAAALFANASISTFPSLGNHHRLLYLRMPYAFSPRAPPRPKTVARLRVPVDALPPD
GKLLASFRASLGSFLAGRRRRGRGGNVAGVMRDLAGVLGRRYRTCAVVGNSGVLLGSGRG
PQIDAHDLVIRLNNARVAGFAADVGVKTSLSFVNSNILHICAARNAITRAACGCHPYGGE
VPMAMYVCQPAHLLDALICNATATPSSPFPLLVTDARLDALCARIAKYYSLRRFVSATGE
PAANWTRRHDERYFHYSSGMQAVVMALGVCDEVSLFGFGKSPGAKHHYHTNQKKELDLHD
YEAEYDFYGDLQARPAAVPFLDDAHGFTVPPVRLHW*
>11667.m06411|LOC_Os01g63990.1|genepair39-1
MVALSKLVLITAVALLGWAYKVARPPPPPILGGPGGPPVSSPRVQLKDGRHLAYREAGVG
REIAKYKIIFSHGFASTKESDFPVSQELAEELGIYLLYFDRAGYGDSDANPKRGLKSDAT
DVEELADKLQLGEKFYVVGTSMGGYVAWSCLNYIPYRLAGVALVVPAVNYWWPMPASVSA
SAYRKLDVGDRRTFWIAHHMPWLFYAWFNQKWFRISPIVEGKPEAFTEKDWEILAEIQRT
GQLDRGRATKQGAYHSLCRDATILFGAWEFDPTAIENPFPNGDGVVSIWQGREDKIVRVE
AQRYVAEKLPWVRYHEHPEGGHLFMCADGLGDKIVRELLLGEEPRDL*
>11686.m00494|LOC_Os12g05600.1|genepair39-2
MVPRILIVLLVVLLGLAFQAILRPPPQKLCGSPGGPPVTSPRIKLRDGRYLAYREDGVQK
DKAKFKIISVHAFDSTKDFPLQVSKELVHELGIYIVGFDRAGYGESDPNPKRDVKSEALD
IEELADQLELGHKFYVLGVSMGGYSIWGCLQYIPNRLAGAAMVVPIINYWWPSFPAELSR
QAFKRLIVPEQRTLWIAHNMPSLLYQWMTQKWLPSSAAAMRHPEIFSKHDLEVLQKMMAM
PLIENKSRQQGIYESTHRDLLVAFGKWEFDPMNITNPFPQNEGSVHIWQGYEDRLVLVEL
QRYIAQRLPWIQYHEFPEGGHMFMLVDGWTDKIIRALLVGEQL*
>11667.m06413|LOC_Os01g64010.1|genepair40-1
MGWGISRLIGLKAAVFLSVAYFFHRLDMTLLSFPLIYASVIAMLVSIASHPSIDLPLLLG
KGSNGSFPVWSWIMFSPFLLFIHLFVLLRRFVKNEPLYTEIADGVYVGGWPSSVERLPPG
EPAVIDCTCELPRSSTISENSYLCVATWDTRAPQPSQIESAVRWALRKRSQNKAVYVHCA
YGHGRSVCVMCALLVALGLAEDWKAAEQMIREKRPSISMNSLHRKSLEEWAKHLLTPSKR
SGESDRRGIVGSLFLKKRGLC*
>11686.m00501|LOC_Os12g05660.1|genepair40-2
MGISKVIGIAGTTLLVTSVGLWKIGLRIVAVPFLATSTIAYIIAVASHNSINIPWILGKN
SKGRFPIWSSVLFGPFLILARVYATVKRHMRKKEAVYNMITEGVYLGGWPFMLKHLPPGD
PSVIDCTCELPRSDFVPTNEYLCVPTWDTRAPTISQIEFAACWACEKRAKGKPVYVHCAF
GHGRSACVVCAVLVALGIAENWKDAENIIREKRKIKMNAVHRKTLDDWSKYRASQKKDK*
>11667.m06414|LOC_Os01g64020.1|genepair41-1
MSSVRYCLGRDFQPAAAAAAYFGELEEALIHGANAGGVVDPGMIRADVHSKSAAAAATAG
YLAARPPTLEIFPSWPMRQQQQLHSGNSQSVGSTTDSSSAQNTMPQMELVSPASIRASSE
HQHQQQQPGQEVMMVTTDDYSYKPGLAAASPSFQQQHQLQHHQQQQLHGGGDHDKRKHGS

TRKDGKSVDAKAYVQNLETSRVRLQQIEQELQRARSQQQLQPASLQLADEGVPRSSHHGA
VKSFAGAAMFDMEYARWLDDDSKRLTDLRGGLQAHLLDTNLGLIVEECMQHYDELFQLKA
ALARSDVFHLLTGTWATPAERCFLWMGGFRPSDLLKILIQQLDPLTEQQMLGIYSLQQSS
EQAEEALAQGLQQLHQSLADTVAAGTLNDGPGVPNYMSLMAIALDKLASLESFYQQVQDC
SDHSAKTSPLRCMVNRSGCVRYTCMRKKADNLRQQTLHQLRRILTTRQAARCFLSIGEYY
RRLRALSNLWSSRPRENFIGTESVSPTGTELQPMHNQPQQNQYSGF*
>11686.m00504|LOC_Os12g05680.1|genepair41-2
MGEARRGQNPHHVLGYGFHGTTLPNSMASANLFEQGGGGGGGAAYFGELEEALVHQVATL
RRRAQQTATTTTSHHGHTTPFSTAAAAATATATARPPATLDIFPSWPMRRSSLPTPKDGC
SNVTADTTDSESSSKNNGDQGAAAADMASQFDQIPQQQQKQHKKMAASSTHSDHRMTKTL
DPKIMRRLAQNREAARKSRLRKKAYIQQLESSKLRLAQMEQDLERARSQGLLLGGSPGGN
TSAGAAMFDAEYGRWLEDGGRRMAELHGGLHAHLPDGDLRAIVDDALAHYDELFRLRAAA
AKADVFHLITGTWATPAERCFLWMGGFQPSDLLKTVAPQLDPLTEQQVVGICSLQQSSQQ
AEEALSQGLEQLHQSLAETVANGGSVVNEASLGSFMGYMALALGKLSNLEGFVIQADNLR
QQTLHQMHRILTIRQAARCFLAIGEYHNRLRALSSLWASRPREILVADEGNCGELSIAAQ
PSESQFSAF*
>11667.m06430|LOC_Os01g64180.1|genepair42-1
MPQVDLESLVCGVAGAGAGDRKVSCETVIAAGESGDASPPRMPPPPPPPDPDFPPESITI
PIGDEVAFSELNPIYDRDDSTKGSTNPKSAAGASSNPIPAKSRSNSTRIAGAPAAATTFF
GLPASIRPAFTRRRPSQGRILPDKRSGSRGGGGGGSSRRGDGEEEPRSPKVSCIGKVLSD
RERYGRSRGRRWWRGLVAVLLCGGGCSCQGGGRRHARKKVALDEDHHDGDDDKQAGIAAM
RRFKSGRRTASWVEEAIAAAEAAGEEEQQQENDAAADDDEKKQEVERYEPTTLNSGPHDR
*
>11686.m00513|LOC_Os12g05770.1|genepair42-2
MAAATAAARDLAGDPPESTLLRVGGEVAWPDVYDRDDSLKENTNPKCILKTYGGGGGGNS
SQRFSGNLKPTAAPIIGLSRKLGHGGGGGGGGGGGFRPPAIFPKKAKTGGGGRTPRAAVP
DHEPGSPKVSCIGKVLSDRERARRHRRWSLETRPRGVGGGCCPGLGSLFVRRSRSRKNVV
ECVDDQSPPPPPPPPPTAAALRRREEKVVLMTEEAAAAPAPALGGMTRFASGRRAADWAA
EMEMDGHVARSGPL*
>11667.m06443|LOC_Os01g64310.1|genepair43-1
MADGGGRRAPGFRFYPTEEELICFYLRNKLDGLRDDIERVIPVFDVYSVDPLQLSEIHHE
MLGGGGEEGEPWFYFCPRQEREARGGRPSRTTPSGYWKAAGTPGVVYSADRRPIGMKKTM
VFYRGRAPSGTKTAWKMNEYRAFHYPDASSASASSAGAAAPPNHLPPQLRSEFSLCRLYT
RSGGIRQFDRRPLAGGGDENPGPSMAAAAASPEENDGSGSSMQQLELMDQGGAVDPDWDQ
WDDLATLTALLYWPRD*
>11686.m00535|LOC_Os12g05990.1|genepair43-2
MVMSGGGGGARIVSDPAATPGFRFYPTEEELIGFYLRHRLAGTRADDVARVIPVVDVYGY
HPSQLAAMAGVATAGDREQWFFFCPRAERELHGGRPARTTPSGYWKATGSPSFVFSSSAA
AAARVIGVKRTMVFYQGRAPSGTKTRWKMNEYKAVAAAAADDDHNAAGVAVQLPPMAPPP
SSSACVRLRNELSVCRVYVSTGTLRSFDRRPLDAPPVISHHQPQLQQQQRQLPSSAAAAA
TNGNLIALAGGYECSHDSSGGSSEDAAIDWSSLITAATDSATAAVDFSFNDDIDFSPAAV
GPWAPQL*
>11667.m06453|LOC_Os01g64410.1|genepair44-1
MVIGRLALSSFFHNKARDTSPSPPPAPATAPPWVWPSCKNPRTQSFRAATAPPPPPGSRT
IASIFLDSAESSFTTSSARHDCSDSLSTASEASAGAEAADTADDAIVRGLRSSDRLLFDP
GASATSSILEEKSSDAAGEASFIGGVAVAFESEDPYVDFRVSMEEMVVAHGVGNWGWLEE
MLGWYLRANGKDTHAAILAAFIDVIVAIADPALASCSSHRRSSTCTITEESSLEVAEKQA
KLAV*
>11686.m00551|LOC_Os12g06150.1|genepair44-2
MGKKGGLTSLFSRLAVAAADSPSCAKNPPHTASFRGFYYVDEPCTTAGGGGGGRSPAAGR
LRKGGDEMYKTVNSVFFDDSADAAHAVADGCAFSGEDDDDDDRFSTTTAADEEWSEAVIR
SLGRRTSTDRFFFDAGPGRPAATNSILATVRPRRPPPPPPPPPPAEEEKEKAAAEAAQLP
GKSSSTSSSQLVEESVAVAVESEDPYGDFRASMEEMVAAHGLRDWDALEELLSWYLRVNG
KHNHPLIVAAFVDLLLALAAVPSSSSSDTTTTTTAAATTTTSDTSCSTASTSTTSNGATS
VTAAATAAEQCGGGGGGGDEEAGCSSSSSCCAASDHDHEEVSAIS*
>11667.m06455|LOC_Os01g64430.1|genepair45-1
MSGRSSRRGSFSLRQPPVVDIGCNCRRPKLFSIFSSSSSSSFRRGGSKPKSPNASSTSTT
TAFTATTGGAGTATSTDSSWGPASFTTNSLFEEPAAAQQEQEQLETRRRRRQQRRRRRRA
GATSFARGGDVGGHDDEQQQLQEQAPYRRVAKESVAVAVESAEPYEDFRESMVQMVVEKE
IYAWDDLNDLLHQFLSLNSPRHHPLILHAFADLWTRNGLFSPPSPCQF*
>11686.m00552|LOC_Os12g06160.1|genepair45-2
MSTAASRARRGGGGGGGRHFPVGGGRWRHVPVVDTGCGCRPRRPRQLLMSLPSFLRPSAL
KPPVPRSTSSHSSSFFPSSASTASFSSSSAATYTTTYSSSSATNQYPYYGKAAAAAAAPV
TVTTNNHGKSSSSSSSSSAAAAARRRPSRKKRYEKMAAAEEEEDVGVAVEKESSDPRADF
RESMVQMVVEMGLCGWDDLRCMLRRLLALNAPRHHAAILTAFAEVCAQLAAPPAPAPPPP
PQPTAAYHYHYYHY*
>11667.m06459|LOC_Os01g64470.1|genepair46-1
MAAYGKPQPPLNDAYYGPPIPPPPAAAYYGAAAPPPAPRRSGAHRLFCCLFRVLAVAVIA
LGTAVLVLWLIYRPSVVKAYADTAALSRFDLTNGGSLLVYNLTVGMRVRNPNRFGINFRS
VDAQASYDGDRFGYAPLQPLYVGRKSDARFDVTLSGSAAIDDRDVERTYRRETAQGSYEV
KVRVYARQGFKVRGFRLNNKSKFTCTLNLPAPNSGNGTASGTPTTVFTRKQPKCDVDY*
>11686.m00559|LOC_Os12g06220.1|genepair46-2
MSSGGNQERTCCGSLFTFIVTGGFVVLIYWAIFQPHHIRATVASADLTNLTVAGAAVSYK
LAVRLNLYNPSLRVNIYYDELDSELRFRGERLGHATGATPAEFYQRRKSSDDVTFEFAGT

GVAVAGDAAGELGKEKGKGSVSLEVAVDGKVRYRFGSIKIRQKPRIWCSLTIPVTADGGG
RLDSGDRCSVKY*
>11667.m06468|LOC_Os01g64560.1|genepair47-1
MAGAAPLRDSLRRLCTDVGWSYAVFWRATRAADSQRLKLVWGDGHYERAAGAPSISGFEA
MDLLLKEKAAALRSGTGRGGGGGEGHAADGAAGHSHDRVDALVHKAMAQQVHVVGEGVIG
QAALTGLHRWIVHDIVDECEEEDEVLLEMKGQFCAGIQTIAVIPVLPRGVIQLGSTKMVM
EEAAFIDHVRSLFQQLGSSTAVVPCGSFVQDSIMRTPFHKSLGVPTSSHSEDLAGGGNTY
NDDMINHQFRHQKSPASTIQSFNPVQQFYAGPTFCRPVTIASRCDLFQPDHGSTFTLNSQ
SEDNRSTALLKNSVSHSKTSNDAFSHAFNPLNEPNVSISGRRECVSIEQHGSCRNGEMEI
TIGRTASSSCTGKTNIINKVDDLLSQDCLVGCQASNATSVNRKFQTMSIVDNTKLQDGSY
AIPHAALVDSTQYSDCFQSLLGTIQGSSSSNSNAIHVDTSHNAVHGKSNFCPLGDRNAAN
SSDLAELLASPIPLELTGGNDLFDVLQLQQKPNGSNNSEVNNRQSMPYGSEQAVKSLIGC
VDDDFTGLITEADPDQLLDAIVSKIITGHKQNVDTSASCSTTVAGFDRPLHSDCHLYTTG
PSSGPIFCNFASVAPVAIKTEGPAAGSRQSSSSIDKSAGCSQTQESYKSQIRLWVENNHS
VGSDSLSTGQASDSLSTGQCKRSDEIGKSNRKRSRPGESARPRPKDRQMIQDRIKELREI
VPNSAKCSIDTLLEKTIKHMLFLQNVAKHADKLKGSGEPKIVSHEEGLLLKDNFEGGATW
AFEVGTRSMTCPIIVEDLNPPRQMLVEMLCKERGIFLEIADQIRGLGLTILKGVMEVRKD
KIWARFAVEANKDVTRMEIFLSLVHLLEPSTGSSILSAGVENTSLPRDSFFPSSIPASGF
SNCL*
>11686.m00571|LOC_Os12g06330.1|genepair47-2
MAVGDALRRLCEEARWSYAVFWKAIGAADPVHLVWEDGFCGHASCSAGSEASEAGCESGG
AVCTLVRKIMASQVHVVGEGTIGRAAFTGNHQWIVHETANDHGLRSEVAAEMNNQFRAGI
KTIAIIPVLPRGVLQLGSTSVILENISSVQQYKKLCCQLNNRSSMVASASAKNDLSQKVQ
SRSLHGLPSIHPYEQCYGHDARALSSSTSANTGRNTSLLKVAQRNDQAIREQVLYAPDMR
FRQQLPYSDRRVDINTHSSAMSSGFISSISASVEKYPLLTNNIGQVEHGNMEESSGPRNV
LLKSLSCRNPVVHENTNTSLFHGGDEVPAFLNSHGSFDFLQAGPRVVEANLYNNGTSSQV
LDQRCSSTSGMAGYKPSVSYKFPHSAQFIVKMENPRRQSFQDPAAPSSGSDVQVSSGLKT
TTRQFNPEHMCQNKKTNEVNDSSAAVSTQDVKNMDRHKILDISNERTSSFVMDPSTENDL
FDIFGTDFHQLHRSLDGDLSWNTAKPQSSDRDAPESSIYLDSSPAFGAQEDEFSYSGIFS
LTDTDQLLDAVISNVNPGGKQISGDSASCKTSLTDIPSTSYCGSKETKQCKSSGAPPLLI
KNELAVSNFVKQPCFLEKAEDGCLSQNNGVQKSQIRLWIESGQNMKCESVSASNSKGLDT
ANKANRKRSRPGESPKPRPKDRQLIQDRIKELRELVPNGAKCSIDALLEKTIKHMVFLQS
VTKHADNLKDSNESKIHGGGENGPLLKDYFEGGATWAFDVGSQSMTCPIIVEDLDRPRQM
LVEMLCEDRGIFLEIADFIKGLGLTILRGVMEARKNKIWARFTVEANRDVTRMEIFLSLM
RLLEPSCDGGGGGVGDNPNNVKIPPGIVQHPVIPATGHLR*
>11667.m06469|LOC_Os01g64570.1|genepair48-1
MASAAAEVPSYFVCPISLQLMRDPVTLPTGISYDRAAIARWLAAPGARRTCPVTRQPLEH
GLELTPNHTLRRLIQSWAASVSPGSAVDEEVAALRPVSSDEVASLLSDAAAAQVGALRRL
RELAAECEDSRAMLESQGGVFDVLSRVVTSGSACSTAREEAVGVLASLRIPEQELIGVST
RHGNLAESLTAVLRSSNLQSRAHAVQLVRTLADAVVPAWVIGLNAELLAEVVGVVRDRVS
ARATKASLHALAALCPYGRHRVKIVGAGAVAALVELLLDEPERRVCELALAVLDRLCTCA
EGRAELVAHAAGVAVVGKKVLRVSEAASERAVRVLRSVARHAATPAVLQEMAQCGVVGKL
CLALRSEQCGVKTKEKAHEVLKLHSRVWRASPCLSPSFLALYPS*
>11686.m00580|LOC_Os12g06410.1|genepair48-2
MEYGTPQHFMCPISLQPMQDPVTSPTGISYDRRAIHRWLAAGHSSCPVTGHPLSLSDLTP
NLTLRRLIHSWHHSTTTPFPVERSTPSPPPLREVDDDDVVERLVMEMEGGGGGSWCPPSC
DLLREAAAVAAGSGVARRRMVGAGVLRRVLRLVVWCGGRGSSSGEAAVMVEMFDACLALF
HALDVSADELRPLVADGHDLVDAVTRVMATLEAGDANATRARESAVRLLEAVTEAADAPV
LERLSPEFFSAATAVVRDRGAVSPGAARAAVRALANACRARASGACRNRALAVDAGAARE
AIELELDAWSSPQAPGGRRATEAVMALLAELCACAEGRAAVASHPAGITVVARRVLRVSA
AADACAVRVLAAVAGRAASPEVLREMARVGAVGKLCCVLQAECDAGVKEAARAVLRMHSG
VWSGSPCVSAYLLSRYL*
>11667.m06487|LOC_Os01g64700.1|genepair49-1
MSFRSIVRDVRDGFGSLSRRSFEVTLASLYGLTGHHKGKTQSSLDELDDSPAIIPESRWA
SLPPELLREVIRRLEADESTWPSRRNVVCFAAVCRTWREMCKETVLSPEFCGKLTFPVSI
KQPGPRDGMIQCYIKRNRSKSTYHLYLCLSNVVTAEGGKFVLAAKRHRKTTCTEYTISMV
SGNISRSSRTNIGKLRSNFLGTKFIIYDTQPPYNGAVVPHVGRTSKRFNSTKVSPKVPSV
TYNIAQVSYELNVLGTRGPRRMRCMMHSIPASSVEPGGIVPGQPEQIVPRALEDSFRSTT
SFSQSFRSTTSFSKSIMDPSMDFSSARFSDISGSIMGGDDNGEIKERPLVLRNKPPRWHE
QLQCWCLNFRGRVTIASVKNFQLVAAPSPPPAGAPTPSQPGPADPEKVILQFGKVARDMF
TMDYRYPLSAFQAFAICLSSFDTKLACE*
>11686.m00602|LOC_Os12g06630.1|genepair49-2
MSFRSIVRDVRDGFGSLSRRGFEVRLVGHRRGRSHSAVHELRDGHAAAAAADVVQSSCWA
NLPPELLRDVIERLEASEAAWPSRKNVVACAAVCRTWRDMCREIVKNPEFCGKITFPVSL
KQPGPRNGAIQCFIKRDKSTQTYNLYLCLSSAVLVESGKFLLSAKRYSRATCTEYTIFMS
ADNTSRSSNMYIGKLRSNLLGTKFVIYDTQPPCNTANVSQSGKTSRRFYSRKVSPKNPSS
TYSIAQVSYELNVLGTRGPRRMNCVMHSIPASSLEAGGTVPCQPDSVLARSLDESFGSIS
FSKSSIMDRSIRFSSSRYSDISVGGPMVGGQALGDSDESKERPLILRNKAPRWHEQLQCW
CLNFKGRVTVASVKNFQLVAATQPAAGAPTPSQPAPPPPPDHDKVILQFGKVAKDMFTMD
YRYPLSAFQAFAICLSSFDTKLACE*
>11667.m05979|LOC_Os01g60110.1|genepair50-1
MSMWRFSRSLLRAAATTTTATAACASASTAGKHAASRRWVTPRELQRCWYASLPAAAAAV
PGKDGEAEVTAEEARRLMRLANVEALKRKLGDGEVIPYAELLRACEEAGAARTRAEAAAL
AGALDEAGVVLLFRDKVYLQPDKIVDLVRKAMPLALTPEDDPRKEELKKLQTQLEDINKL

AHKQVRRILWSGLGFLITQVGLFFRLTFWEFSWDVMEPITFFTTTTGLVVGYAYFLITSR
DPTYRDFMERLFESRQKKLIQRQNFNLDRYLELQRRCKGPLEKMCGTNQTPNPDMAHLHE
LSVNK*
>11686.m00205|LOC_Os12g02880.1|genepair50-2
MWRAAASHLLRRRAAHPPPSPATATGAACALRHVRLFSPPPHPPSSRPAEAEAEVTAAEA
RRLVRLVGVEALKRRLRDGREEVVGYGELLDACVEAGAARTRRDAEALARAMDEAGVVLL
FRDKAYLHPEKVVELVRRAVPLALSPENDSRKEELKKLQEKKEEIDKLAHKQVRRILWSG
LGFFMCQVGLFFRLTFWEFSWDVMEPIAFFTTASGLLVGYAYFLITSRDPTYEDFMERLY
LSRHRKLCAKNSFDMEKYLELQKHCKCPLEGHYPHGPKFHDL*
>11667.m05982|LOC_Os01g60140.1|genepair51-1
MASTVPALIADDLPTNVTSQITDAARPKTTSSVVCYSPMMITTNGIWQGVNPLEFSLPLF
ILQVAVIVVTTRLLVVLLKPFRQPRVIAEILAGVVLGPSVMGQVEVWATMVFPQRSLLTL
ETVAHLGLLYFLFLVGLEMDLDVIRRSGKKALFVAVAGMALPFCIGIATSFIFRHQVSRN
VHQTSFLLFLGVALSVTAFPVLARILAEIKLLNTELGRIAMSAAIVNDMCAWILLALAIA
ISEVNSTALSSLWVLLAGVLFVLFCFYVVRPGMWWLIRRIPEGEVVSDMQVSLILTGVML
AGVCTDAIGIHSVFGAFVYGLVIPGGQLGVALIEKLEDFVTGLLLPLFFAISGLRTNISK
IRDPITVGLLVLVFTMASFAKIMGTIIIAALYTMPFREGIALGFLMNTRGLVEMIVLNIG
RDKEVLDDESFAVMVLVSVAMTTLVTPVVTGVYRPSRRLVGYKRRNLQRIRHDSELRMLI
CVHTTRNVPSVLSLLELSNPTKRSPIFIYALHLVELTGRASNMLAAAAASASKQNRSSSS
STLPPVTEHIFNAFENYERHTGGISIQTLAAVSPYQTMHDDVSVLAEDKHVSLIVVPFHK
QQTVDGAMEPINPSIRGFNESLLSTSPCSVAILVDRGLSAAAARMAALHRVALFFFGGPD
DREALAYAWRMVEHPGVALTVVRFVPPDYRVRSYSNTNYRSVASDADPRSIGIDTEGKTE
LQMDEEYLGDFRTRNIGNDAISYSDKVVANSEETVSAIRNMDDSLHELYIVGRRPGEAGS
PMTASLEDWMECPELGPIGDMLVSSDFSMSVSVLVVQQYVVAAAAPAPATTAPAGNADPV
RQYVSNANQRPSAAYRTSAASTANSRWSGGGTVGF*
>11686.m00201|LOC_Os12g02840.1|genepair51-2
MAAHTVTDPLEELWNHTMSMDKTHLMCFYPSKITMGGVWTGDNPLDFSIPLLLFQILLIT
STTRAATLLLSPLRLPTYISQILAGFLLGPSVLGHLPHFSNLVFPVRSLFVLESMALLGL
VYYTFIVGVEIEVSAITRAGIRSFGFAIGCTLPPFLVGALTGYVALSTDDKHKGDTFLNK
LSFPIFLGSTFSSTAFAVLARNIAELKLAGTDVGQLTLSASLINDTFAWTGLTVATVLGH
SRCTITQTTWTLTSGVVIFGASYLLLRPMLLRLARRAAEGEAVGEDRECWILIGVMVAAL
VADAGGTHAIFGAFVFGLAVPNGPVGVALVEKVEDFVVGALLPLFFALSGLRTDTAKITN
MHSAVLLMVAAMVAAVLKVVAAIGVAGVFGMPLGDGTSIGLLLNTKGIIELVILNIARNK
GIMSDQSFTVLVFVSALITAMVSPFLGMVVKPARRLVFYKRRTVAWAHPESELRVLACVH
VPRDVPALLTLLDVVTPSSRSPVGVLALHLIEFVGRSSALLLINASAPSSSSYDASVHGR
SHTEMQFKHISHAFMAYEEQSVGVSARTMAAVSPYASMHEDITSAAENQHSALILLPFHK
YRSVDGGLEVSHPAIQPLNCSVQSFSPCTVGILVDRGLAAVPGGGYRVVALFFGGSDDRE
VAALATRMVRNPTIDLTLLRFVQKGGSFTASEFDALKERKADEGCLRDFLERANEGGGAT
VEYRERGVFNASEMVGEIQSVEAMGNKDLFVVGKVPGGSGLTAGMAEWSESPELGPIGDL
LASKDFQTTASVLVLQAYGRPAAVVGAGAGAMSVDFGGDSVAMAERTASGRRPWARPGV*
>11667.m05985|LOC_Os01g60170.1|genepair52-1
MAKVHPNVVPVAAAAGPAGGERRGEEEEEAAALTVWRKSLLFNCKGFTVFDAKGNLAYRV
DSYDTESGDEVVLMDAAGAPAFTVRRKRQLSLQGEQWLVFAGEADGRRPPVYAVRRTGRG
GGKSLARVTPCAGAAAAGASAAYEVEGSYARRCCVVYDGERRAVAEVRPKEAVGTDVFRL
VVQPGVGVSLAMAVVVALDQMFGRPSLLRSWSS*
>11686.m00187|LOC_Os12g02720.1|genepair52-2
MAAKVHPNVAAAPQPPCISSIVSQQQEEEEPPVVLTVWRKSLLFNCHGFTVFDAKGNLAF
RLDCYDSTSSRRADLVLMDAAGKPLLTIRRKRMSLSDSWIIYDGDGAATSTATPLLSVRR
RRVGLRASKSKAIAHVTPLSSSLPLPEAYVVEGSYGRRSCAVRDARGDAVAEVRRKESVG
DDVFRLVAQPRLGAPLAMAIVIAIDEMLRGGSSSLLRRTCSA*
>11667.m06025|LOC_Os01g60520.1|genepair53-1
MASPRLKREQSFDFEEASAQEAVGSASASYSPPGGGGVFGISPPESSPRDGRKRRKDRPS
LVKHTFTPHFDGHLWRKYGQKNIKDSAFPRLYYRCSYREDRQCLASKLVQQENDDDPPLY
RVTYTYEHTCNTTPVPTPDVVAEQPPPGAAGDAYLLRFGSSAGGGGGGAHQQQTERERQQ
QNTARRRPFMMLSFDSSSSHQLHEQPHAFPPDGQLPATAAAASPSSFTAAEALAAPPLTT
TMNDGGDLFSTWDALRYGLDYDHGHLGNHVYLPDDCNGGDDNY*
>11686.m00151|LOC_Os12g02400.1|genepair53-2
MAVTESVCLSDEQQAVAVREVAQVYELIKTQQPLLLVHQQPQQLAHGLLNHAMRALNVAL
SVMNQPHASSSSSAAAAAGGHHFPVMTMIKAESTPANSPAADVSDNHVAGKARRSSPAKR
RRINCEDKSSWVYHTVVPHEDGYQWRKYGEKKIQGTHFTRSYFRCTYRDDRGCQATKQIQ
QEDKNDPPMFQVTYSNEHTCTTTRLINNTNNNPAALHSLTANPNGHPDDDSDDTILTKMI
KQEQQAAWLPSPPPDLTTISNNFDETPGLHVSQEVPPCSSNSSAISHYADEFDHHQMGQQ
LETTVMEEALGLGADLDDPYFYDPNLLLIYENLMNCY*
>11667.m06046|LOC_Os01g60730.1|genepair54-1
MGVHTKSMSWYMGTPASPSPGSAGEAQHALSSGGGGGSDASFDTNMVIILAALLFALLFA
LGLNSLARLIIRWARRAATGEGAGGGVEGAGAGGGLKKRALRSIPIEVYGGGGGGSPAAA
AEVCAICLGEFADGEKVRVLPRCGHGFHVRCVDTWLVSHDSCPTCRGSVLHGATTKHKPT
AAAAAAGSRRPGSEVDAPAAAAVTVVIA*
>11686.m00145|LOC_Os12g02350.1|genepair54-2
MRRLVDVVEAPALVLTPAWAATASMQQAGGSSGALDANMVIVLAALLCVVICSLGLSSLI
RCALHCARGLSPSPAMATPAAATTTGGLKKKELRRITVEVYGAKQAGVPDAECAICLGDF
ADGDKVRVLPRCHHGFHVGCIDTWLAAHTSCPTCRDSILSVHAGVTGGQT*
>11667.m06048|LOC_Os01g60740.1|genepair55-1
MAPRCATLAVVVVLVAAVVAPPTAVRAAISCSAVYNTLMPCLPYVQAGGTVPRACCGGIQ

SLLAAANNTPDRRTICGCLKNVANGASGGPYITRAAALPSKCNVSLPYKISTSVNCNAYG
GRPDAYVCARASRTRGE*
>11686.m00140|LOC_Os12g02310.1|genepair55-2
MARAQLVLVALVAALLLAGPHTTMAAISCGQVNSAVSPCLSYARGGSGPSAACCSGVRSL
NSAATTTADRRTACNCLKNVAGSISGLNAGNAASIPSKCGVSIPYTISPSIDCSSVN*
>11667.m06060|LOC_Os01g60850.1|genepair56-1
MESATEKVRRTPSSCLLLRISDICKVRSVGVAPTVREKPKADGSATGESSEDGGAHLKVH
PHHVSDHESVSECSSARCEEAFVERLLDAISGLKLSYVNLQQALVPYDPEEITIADERFT
SELQETAGLKDLYVNMNKWRNPMYQCYVGSRIQEQQKLAVELQAGMCKRDSEIVCLRAEL
DELERKNMELEEKIGQSALQKEGSFAIGMGVSTDMFMELFELSTKSIHDFAKLVVRWMKL
SRWNLGNLTSPIDNSVVYDKRSHKNYAVEAYFACMMLMGHKEEYLSLDVFDYVMSFSDPF
DALMKAPDSCFGRFCREKYLAILPPSMEDSFFGNLDHRSFVENGGHPRTPFYQAFVTMSR
YVWASLTVARSLNPRAEMFYVKGGTEFRSKHMECVPSKITKEGDKVSVGFTVMPGFKIGC
TVIRCRVYLSMVNERNF*
>11686.m00132|LOC_Os12g02230.1|genepair56-2
MESRTPRSSVARSTCNVPGLVLGFSKLCKITKICAAPEFADTKTEFGDYCGGYDQRLVIT
RLFEEIGALKSTYIKLQKAHIPYNRPKIAFADEIITYELDSVTALQSLCSWNGSVGSLIN
DRWSLVQELEAETRKKDSDIMLLRRELDGLKSANSRLNKQISSSKPSVNHHKDYSIVLKK
LTTPSAVLELFKVASTSVHDFAELIFSLISSPDHRCPNNADEHSPYKRYSLEAYLSRTML
AVHDGAEDDDELDLARFDRIMRCCDPLDALMEHPNSSFARFCRTKYLAAVSSEMEAAMFR
NNLDVRAFVSRGGHPRTWFYRAFATMARSAWALRVAVTARRRCCGRGSVRMLYARRGSRY
AAEYMDSVVAAAAAADAGRGEGDGVAFTVTPGMKVGETMVACRVLLCHDQHDTISDETDP
KFR*
>11667.m06061|LOC_Os01g60840.1|genepair57-1
MVDLSGLEIFLAVVALIAAICAISWTVRCLIGRRFEVATGDPNGERQGLLNKEEVVIIDV
AAATEARGDDVALCAICKGRLAVAVADGGGQPCRRLRPCGHVYHAECIGLWLQRGTTCPV
CRADVVASRNEIVGAMA*
>11686.m00131|LOC_Os12g02220.1|genepair57-2
MDAEDIVDCLMWGIIFFFLLACIGVALCFLALTIATVVGLIRRRNDDANNKYDMLIERLL
LRPKDDQDNEQCVICLSESEDDVDDGGGERGRWRMLPGCAHAFHKDCVVKWLRNRTTCPL
CRSDVAVAAADDIISTADNMV*
>11667.m06067|LOC_Os01g60910.1|genepair58-1
MDGRSILMGRYEVGKQLGQGTFAKVYYARNLTTGQAVAIKMINKDKVMKVGLMEQIKREI
SIMRLVKHPNVLQLFEVMASKSKIYFVLEYAKGGELFNKIAKEGKLSEDSARRYFHQLIN
AVDYCHSRGVYHRDLKPENLLLDENENLKVSDFGLSALAESKRQDGLLHTTCGTPAYVAP
EVLSRKGYDGAKADVWSCGVILFVLVAGYLPFHDPNLIEMYRKICRADFRCPRYFSAELK
DLIHKILDSDPSTRISIPRIKRSTWYRKPVEINAKNSEAATTNSISSGVATTSGSAECST
SEENQGSLSLPNLNAFDIISLSTGFNLSGFFEDTHGHQEERFTTRQPVTTVLGKLKELAK
RLKLKVKKKDNGVLRLAAPKEGKKGFLELDAEIFEVTPSFLLVELKKTNGDTMEYRKLVK
EDIRPALKDIVWVWQGDEHLNSQSILQGEQQQSPLPPELPQDQLQPSLPQQEKQDMPEPP
LLPQVNASTGNHNDHWLVHWLMMKPFRFPPPDSVPSTGRDEEEGSSHHGNGSGAARENPE
NPLPDLALAKSESDRIKAFYQTVLSAIVVFIVAALSGYKDMKPLYSTTNHKKVHLSNLLV
IEGFCMIATFICAAVLMMYEFYTCRDLTVLIAVAGAMLVATDTILVVIANRDNAVFAVLF
VPVLLLVGMAVYTGASMEEQPPNSPHDGGYDEATKSTFDLATVSTMAALGLQGTIVFGYL
KTPEKKSEHDDPPLDLAVCYIASTVCLTVMAFLDGFVVLSVWPEAVALVLYYAAQLCSGR
QPGGGVGSRPWIEFVFRIVATAGFTLMAGLYAAFLGTNHYSVYLKAAMLVLLMAVLSSLS
RLANPVYMPEIDGALLEFCVAGVALAFPAVSLLVACPLVLKVFVDLYIHHSSL*
>11686.m00129|LOC_Os12g02200.1|genepair58-2
MESRGKILMERYELGRLLGKGTFGKVHYARNLESNQSVAIKMMDKQQVLKVGLSEQIRRE
ITTMRLVAHKNIVQLHEVMATRNKIYFVMEYVKGGELFEKVAKRGKLTEVVAHKYFQQLI
SAVDYCHSRGVYHRDLKPENLLLDENENLKVSDFGLSALSESKRQDGLLHTTCGTPAYVA
PEVISKIGYDGAKSDIWSCGVILFVLVAGYLPFQGPNLMEMYRKIQHGEFRCPGWFSRKL
QKLLYKIMDPNPSTRISIQKIKESTWFRKGPEENRILKERTLNENTTKNVALVLGVRRKK
NAHEDVKPMSVTNLNAFEIISFSKGFDLSGMFIVKEWRNEARFTSDKSASTIISKLEDVA
KALNLRVRKKDNGVVKMQGRKEGRNGVLQFDIEIFEVTTSYHIIEMKQTSGDSLEYRQLL
EEGIRPALKDIVLAWHGDE*
>11667.m05825|LOC_Os01g58700.1|genepair59-1
MGERRSGYRDRGGGEQGRYWRPPRSHVPRWGNGNFSVPLWEKKFCTDACAIPWGKLCETK
KLMSLYKNVVDWDDSAALETFNDAKARFCAVYHGQPCDIPLPDPNMYIDMVNQDEHVDPE
LVADLEKSRRSYPKRDNTAPDGWDSFIFTNKPVPATGWGDGETSNTPGQQYPTNWNNHVK
QPTEANCMQSSVNWDNYVSQPPQATVQQSSGNWDMYVKQQDQANNWEAPTMPGTWDMKGD
SLDAWKRDSGWGSAAIDSWDNHRENCYVPDSQGWSYGHWKRRNNESSRRNSRGRDRVGPI
SSKAMKPKYHSEERNGANNGWRHCRVRNNMQYSYENPGCNQSLAM*
>11686.m00354|LOC_Os12g04280.1|genepair59-2
MKGHNCHPVPLWEREFCIYVGGISWHRFCDNKRYVCMYKNIEQWDDSEAFDNFKNAKARF
WANYHGQPSDISLPDPDMYIDKIDHNSKIDPELIADLKMVRLPFERDDELLPADGLGSTD
TDNKCQQKQNQSGNWDIYVEKPTEVNKWEQDSRSNMDWGTKHESWNEWSKNCSGWGSALA
DSSALADSSWGNWNNSNNHHSSNNRDSFNGVNRNRYQDPNSISGRKRNSGGHIQQRNSRQ
RNQTEGYQGSTPRW*
>11667.m05831|LOC_Os01g58760.1|genepair60-1
MASGENNVEEELRKTRKPLGNREAVRKYREKKKAHAAFLEEEVKKLRTTNQQLLRRLQGH
ISLEAEVVRLRALLFDIRGKIDAEIGTFPFQKQCSFGSVTCTDHSPCFNTSTEVAVREES
SRPTIVDCGIDGTGIISHELDIPKMVNSVDVIPSFVNSASLTE*
>11686.m00344|LOC_Os12g04190.1|genepair60-2

MDDGEIELSSQMMFPNPEIPTSLDDFLPSIRTTCTHTHTCNPPGPSATEHTHTCYHTHTR
VFSSDDDSCGGDKAKPKKSRKPLGNREAVRKYRQKKKAHTAHLEEEVKRLRVINQQLVKR
LQGQAALEVEVVRLRSLLVDVRSRINGALGSCPIQAQCGVDNVLGCDGMAQCFAGKPELG
VRQSCAPSTVNCHISSDSGQNLVVPHALSPSDVIGSFMVSSTSKDE*
>11667.m05842|LOC_Os01g58870.1|genepair61-1
MAVGKEGGGGAGGEGGMSDSVIRKVLVSYMYVAVWIFLSFTVIVYNKYILDPKMYNWPFP
ISLTMVHMAFCSSLAIALVRLLRVVELPSSPAMTPQLYTSSVLPIGALYSLSLWFSNSAY
IYLSVSFIQMLKALMPVAVYSIGVLFKKENFKSSAMLNMLSISFGVAIAAYGEARFDARG
VALQLAAVAFEATRLVLIQILLTSKGISLNPITSLYYVAPCCLAFLVIPWAFVELPRLRA
VGTFQPDFFIFGTNSLCAFALNLAVFLLVGKTSALTMNVAGVVKDWLLIAFSWSVIRDTV
TPINLFGYGIAFLGVGYYNHVKLQALKAKEAQKKAAQADEEAGSLLQERDSHGERKSDNQ
A*
>11686.m00342|LOC_Os12g04170.1|genepair61-2
MTMEKRTSPQQPPTLLPTNCSTHFLYARAPTTAGVTPRQPHQSFRSYLAQMGGTGDDGAK
APAAAAAMDVVSSSSSSPAPAPSVLKSVLLSYAYVSVWITLSFSVIVYNKYILDPKMYNW
PFPISLTMIHMAFCASLAVVLVRVLRVVAVPASPPMTPSLYAASVVPIGALYALSLWFSN
SAYIYLSVSFIQMLKALMPVAVYSLAVAFRTDSFRRASMLNMLGISAGVAVAAYGEARFD
AFGVMLQLAAVAAEATRLVLIQILLTSKGMSLNPITSLYYIAPCCLVFLTLPWYFVELPR
LRAAAGAAVRPDVFVFGTNSLCAFALNLAVFLLVGKTSALTMNVAGVVKDWLLIAFSWTV
IKDTVTPVNLVGYGIAFLGVAYYNHAKLQGLKAREAERRAASMATAKDGDAEAGARLLPE
KDAGEQKN*
>11667.m05857|LOC_Os01g59020.1|genepair62-1
MELSSTSASLLLILLLTLVYFLYLHQDPKKKPRTHGLKSYPVVGTLPHFINNKDRFLEWS
TGVMKRSPTHTMSFKELGLTGGVITANPANVEHILKANFGNYPKGELAVSLLEDFLGHGI
FNSDGEQWLWQRKAASYEFNKRSLRNFVVDTVRFEVVERLLPLLEYAGRHGRTLDVQDVL
ERFAFDNICRVAFDEDPACLTEESMAAPQSAEFMRAFNDAQNAILDRFNSPAKSLWRIKK
LFNMEPERRMRDSLATIHGYAERIVRERRERREARLERRDDFLSRFAASGEHSDESLRDV
VTNFILAGRDTTSSALTWFFWLLSGRPDVEDKIVREIRAVRQSSAGSEGTRGATFSLDEL
RDMQYLHAAITESMRLYPPVPFDTHSCKEEEFLPDGTFAGKGWLVTYCAYAMGRVEDIWG
ADCEEFRPERWLDEAGAFRPESTFKYPVFHAGPRMCLGKEMAYIQMKSIVACVLEQFSLR
YAGDAKGHPGLVVALTLRMEGGLPMKVTIRE*
>11686.m00335|LOC_Os12g04100.1|genepair62-2
MAMDSYYCSMLFFLPPILYVSYHLTRILADKKKPTTHGLKAHPLLGHLPAFVNNSHRFLD
WTTELIVGSPEMRMGFWIPGMRTGIITGNPADVEHILRTNFANYPKGEHAIGMLEDFLGH
GLFNSDGEQWLWQRKNASYEFSKRSLRRFVVDVVQAEVADRFLPLLRRAAGDGRGGDIVV
LDLQEVLQRFGFDTICMVAFGHDPRCLADGSVMEDARSEYMHTFGEAQDLVVGRFFDPIE
VSWKIKKWLNVGTEHRLRKAIADVHAFAMDIVRTRRQSASVQDRDDVLSRFVASDEHSDE
VLRDIVLSFLIAGRETTASGLSWFFWFLSSRPDVVARIADEVRAVREATGTRPGEPFGFD
ALREMHYLHAALTESMRLYPPAPIDSQSCAADDTLPDGTLLRAGWSVTYSAYAMGRLAAI
WGEDCLEYRPERWLGDDGAFQPASPFRFTVFHAGPRMCLGKEMAYVQMKSIVANVLEEFE
VDVVKEIAGGGVPEHVLSVTLRMKGGLPVKIRRKTEAY*
>11667.m05890|LOC_Os01g59330.1|genepair63-1
MAAEAILGAFMQTLFQKLSEATLDHFISWRGIHGKLESLSSTLSQLQAFLDDAEEKQLTD
ASVRGWLAKLKDIAYDLDDLLDSYSAKSMRMKQRQVIFPTKASFLSSSFLSRNLYQHRIK
HKINIILERLDKIAQERDTIGLQMICEMRRYDTSERPQSSSLVDSSAVFGRERDREEMVR
LVLSDNGHNSCNLCVIPVVGMGGLGKTTLMQMVYHDDRVREHFDLRIWIYVSESFDERKL
TQETLEASDYDQSVASTNMNMLQETLSRVLRGKRYLLVLDDVWNEDLDKWHSYRAALISG
GFGSKIVVTSRNENVGRIMGGIEPYKLQKLSDDDSWSVFKSHAFRDGDCSAHPELEAIGM
EIVKKLKGLPLASKALGSLLFCKTDEEEWKDILQNDIWELPADKNNILPALRLSYNHLPP
HLKQCFAFCSVYPKDYMFRREKLVKIWLALGFIRQSRKKRMEDTGNAYFNELLSRSFFQP
YENNYVMHDAMHDLAKSISMEDCDHLDYGRRHDNAIKTRHLSFPCKDAKCMHFNPLYGFR
KLRTLTIIHGYKSRMSQLPHGLFMKLEYLRVLDMHGQGLKELPESIGNLKQLRFLDLSST
EIETLPASLVKLYNLQILKLSDCNFLREVPQGITRLINLRHLEASTRLLSRIHGIGSLVC
LQELEEFVVQKRSGHNVTELNNMDELQGQLSIRGLNNVPNGQDAVCAKLRNKEHLRTLHL
IWDEDCESNPSEQQEVLEGLQPHLDLKELVIKGFPGVRFPSWLASSFLPKLQTIHICNCR
STRLPALGQLPFLKYLVIAGVTEVTQLSSEFTGFGQPKGFPALEDLLLEDMPNLSEWIFD
VADQLFPQLTELGLIKCPQLKKLPPIPSTLRTLWISESGLESLPELQNNSCPSSPTSLYI
NDCPNLTSLRVGLLAYRPTALKSLTIAHCEGLVSLPEECFRPLISLRSLHIYECPCLVPW
TALEGGLLPTSIEDIRLNSCTPLASVLLNGLSYLPHLRHFEIADCPDINNFPAEGLPHTL
QFLEISCCDDLQCLPPGLHNISSLETLRISNCPGVESLPKEGLPMGLNELYIKGCPQIKQ
QCQEGGEYHAKIAHIRDIEIDGDVIVPEQI*
>11686.m00299|LOC_Os12g03750.1|genepair63-2
MVKAISEAADSRLITNTNLLHWLMRLHAAAQEAKDVLDEFEVDGSNIARKRKASDLILSS
RSLKNLVIADESLKRIKIPSIIFKISRNGKFFSKLSLKNLVIPDESLTRLEHVVKTLTQL
CATSATFIELIKMDDSKTNQLHKAAEASSHLPVDVPVFGRDEVKEFILNHDHPESSIGTG
KVRAARHNILVLPIVGMSGVGKTTLAQVIYNHARVKQHFEHRAWVYVSEDFTIKRTLQEI
LHSFQGHGGAIFNGDESMEATITKLRIKISGGCKFFLVLDNMWEEMCQEWSVLLTALSDE
APLPWESFWPLFQYHTFGGVEVPQQDDNRNMLLIAQGIAKKLDGLPLAAKVIGNLLRCRF
SWDNWRRVAESDWWNLEEALQGILPYLRDRVVQMWLAHDFIQWNEIADVMRPDDVGRQLF
DELVERSFFQPTFVSNNNSVSLHQCFFHGERSTGVSSLAPGNIRHLALQVNSLEQCQELH
KYRNLRTLLIFGRCESDAFFNLLDGMLEKSPSIRVLDLLIRGKPDLLDVDLHHLSYVKAP
GKVWPKDARPLRKLRFLDLSFTKITKLKDLPTNLQVLHLRGYDADRVPQSITKLTNLRHL
YVDGSALSKIQSIGQQTELQELESFIARKGQGFMIRELKNMREFTGRLCIRGIENIRSKD
EAMEARLMDKKHVGALVIEGKRVPKFALEGLQPHTNIQELTIKFFQEQDFPDWVCPDNLV

NLLQVNLESYHFLSTIPPLGHLPLLKLLTLRKLPSVKHANGTSFGGFPSLEEFELHSMEK
WEEWTEPDAAAHAYGLSLFLGHLRKLHLAYCPSLRRFPHLPCFICIERAEDLQAWKLDPG
ITSMFSSLVIPNNLGSRVLQPQRCNVRATVQVPRKY*
>11667.m05923|LOC_Os01g59620.1|genepair64-1
MALQLMMDNYLLCSAMKGRLSVYSDSQLILVKMNRESNFMPTPDQDVLEVKPLRTLAPMF
PAPLGIDVLNRLTAPPLVFVAPAGQFPGGFGSLNIPAVRSFAAFGGQDASGGKTAGGGDQ
DASGGKTAAGGDQDAGRGETAAFGGQETVRGEFVANGTPNVGASATGPIDATPISACKST
QPSVISLDDDDNDDDEPYGGNQTSASGRKIKRPSHLKGYNVSDGLGTDSSNGTKKRPKTS
NRKAATDNEISLMPPSSDPREVVEVLLMTFEALRRRHLQLDETQETSKRADLKAGAIMLA
SNLRANIGKRIGAVPGVEVGDIFYFRMELCIIGLHAPSMGGIDYMNKFGDEDDSVAICIV
AAGVYENDDDDTDTLVYSGSGGISRNSEEKQDQKLERGNLALERSLSRKNVIRVVRGYKD
PACLTGKVYIYDGLYKIHESWKERTKTGINCFKYKLQREPGQPDAVAIWKMCQRWVENPA
ARGKVLHPDLSSGAENLPVCLINDVNSEKGPGHFNYITQVKYLKPLRSMKPFQGCRCTSV
CLPGDTSCDCAQHNGGDLPYSSSGLLVCRKLMVYECGESCRCSINCRNRVAQKGVRIHLE
VFRTTNRGWGLRSWDPIRAGSFICEYVGEVVDDTKVNLDGEDDYLFRTVCPGEKTLKWNY
GPELIGEHSINISADTFEPLPIKISAMKMGNVARFMNHSCNPNTFWQPVQFDHGEDGYPH
IMFFALKHIPPMTELTYDYGDIGCESRGVGSRAKNCLCGSSNCRGFFS*
>11686.m00268|LOC_Os12g03460.1|genepair64-2
MDASALSNPRLQAMLEEEKRKAMANEFVAKLTDVCWDKCITGSIGSSFSNSEASCLSNCA
KRFLELKMLTMQRPQEAPASFSLPLPKSPVPIKATPISAAFPTPQHEDESSDQDYKPFCR
QKKPTLPKRAKRPQQAEKSNDANIKRRSIRRSLDNEFNLCSSSSDNPKESVEGILMMFDS
LRRRVLQLDEKEDASRRADLKAGTLMMQNNLRINNHKMIGHVPGVEVGDIFFFRIEMCII
GLHAPAMGGIDYISSKNKDETLAVCIISSGGYENDDDDTDILVYTGQGGNSRHKEKHDQK
LERGNLALMNSKIKKNQIRVVRSAQDPFCNSGKIYIYDGLYRIEDTWTDTAKNGFNVFKY
KLRRDPGQPDGISLWKMTEKWKANPATREKAILLDLSSKVEHLPVCLVNDVDDEKGPSHF
NYVAGVKYLRPLRKTKPLQCCKCPSVCLPGDPNCSCAQQNGGDLPYSATGLLAKHTPMVY
ECSSNCQCSHNCRNRITQKGIKLNFEVFWTGDRGWGLRSWDPIRAGTFICEYAGEVIDET
KMDIDVEEDKYTFCASCPGDKALSWNLGAELLEEKSTAVTTKNFKKLPIIIRANNEGNVA
RFLNHSCSPNLLWQAVQYDHGDDSYPHIMFFAMEHIPPMTELTYDYGTRGAPPGFEGKPF
KACKLKSCLCGSKHCRGY*
>11667.m05930|LOC_Os01g59670.1|genepair65-1
MTQGSARFVLEAERVHAMALDTKQVARCLKKVLKSSIKDGYRCVSEHPILLTLGVLLYLL
YRSSPGLFAFLLSSSPVIMCTTLLLGILLSYGETNLPEADEDNKITPEISSLKVGNPSSD
FHFEASQRLPVPELRENTTGFKERETKQTVFIRERASEHIELEDNVPLLRRVEHEYDRFD
RHEIPAALTPFPSMVNFHQGSRVGNDLSSNQDINSKGLLSIKDKADGHTSFFEGVRSGLD
EKEAPFGIFSTSKNVNGRGELEENLNQETVFTDSTASRVRDISEEKPTEGEAGTSKSACA
ISTHQSKTLDELRINTSKGFEDNLLDSSLGSPWARVGSEDGVGSDDGSSGFDSDQAESSS
PDASMTDIAPILDEIDPLLGASSTHPDTIPKDDSDTDSHVSSQDHQTDDDSNDETDNNDA
KENGEEKNKEQGKEAAFIWTADDEKNLMDLGYSEMERNRRLELLMARRRSRKNIRFEIDN
DLIGIDNNDGGRGVDDLSRFHVQVPHISVPRRNPFDLPYDSEEAAIPGSAPSVLHTRKNP
FDLPLDQSNDGDVSADNNVNPGELVKASHRDMFFRRHDSFNIGRTDATLERFSRFKPYFV
PETVEGSLSNFQRQFSDKIADQEDHKDLDEKDLPNEHGSPALQRQDSDLADVGSECSDGI
NSVDVELDNSDIDDREIALQHFVFERSQEREAYLSSTKGKGPEDDYLLSSVGNSKTLHPV
ADLFSWEDGNGESSLGVNSSHNMSVEFSDWVSSPKPIAEHDSGPENLQEFLNTEVASSSK
TIVLGARNPAENNGNVDSISYSNNEMPSDNLGHGSMEFPSEFCNESLPVISRDLHPIPEE
RVVENFNVQEKHEAVIFTDSDAALTGFHVIEEHFEVGCDVSPSSEVVPSCLQASDSIQSR
LVENKEVSNPFISIASETNKVDMIDLKEETAAGYPLDSDDDADKIYPEPMEDNVIDESFL
SELDAVGDFRVEATRSDQQMPDVDSHIDNNTSNGVAESSLISPQISSNIFSNMKYASMLE
HEENSPLVDDLNGTGPEFGWSLGASYDDPEQTVYNPRRRILGASRFEETNTEMKPLFDET
EASFVNAPIEANLVVGPSKVDVANESELTKTDTNMIVLDANSLEDIETAFKQASNGVVES
TVDNETPQVSGVDIDPESIESSEQLDVIDAKSVDDIYAALKEHTTAAMNSSFEENEDKHG
CGDTVKFTMHDELPEGTHIEGNTVGDGKEPEPMGITSSMDVIDAKSIDDIYAALKKQSSA
AANSSFEQNEGKNGCGDTVTFTTHDELPEGTHIEDRDNTVEDGKEPEPIGTTSSMDTIEV
KTIDDIDAVFKKLSDGGTKSAAQAVDCENTCEASDESEQH*
>11686.m00263|LOC_Os12g03410.1|genepair65-2
MLWARPKLATQQASGGAAAFALNSGGRRNSGGEDGAGGATEVDAARGWIQRKRNARRSRR
SFISILPSPRPEISAPLLVASIGVPLPRIRPTFYLRVKFKKIYMAFGANDIVLHIKRLML
CPSLFNFLLSSSPVFLLTALLLGALLSYGEPAVLVLGENQQTLSFKSKISITDCSIDKVE
TVAVEEHLDKTTTSNEVYVRERNFEGNIHDTHWEEKNGTYMTVDTALNEEIHTKDGTSDY
DLQDTHREGKGITSVETDTVPCVAPSSFAYSGVTVETEDVGENSKKNDELQELGSINPES
DNNKVQYQYQLGEFMSSCWEPVMRQEPQDACSDSESDLTESSPDASMTDIIPMLEELHPL
IDLQTGHPSLVSRDNLNTSSDDDEDDLEEDASTDENQLEGKIDDFANWKDVIDLNYLDM
DNNSKLEGMMDLQRAKNILKFELDKRLMDLQAADAVQKMEEASRFRVQVPSISTGRQNPF
DSSNGSDEIIELPHVPDSAPSCLLPRKNLFDLAVNQNMAHDSPLQETWTPLSYFSARRHR
KHGNLYVRHSTSLHHNSFKLEKDEISENDAHKSHNSQSDGDAKQERNNSTLFGSLEAHIG
EEIKILGMAISEVGVLEVNSGMVSSNQNADFSDDISSSPFQKPRQSTFEAKDTVHAGIEQ
LTSCSPYKVNNFEAHIVEADSIDEFNSLFKCRMEEVLVQSISKSSISQPLTVKLEDELSE
PLSPDSGTGTHFIDGSSVEDSDPRFAQLNDEALVSSTSNATCRNESIEEKSSEALLAGNE
DYSELPNELLKSGDPQTADSSEIQVQEQQCDTVEHQLQFLGLTVKKQSLHKSPTTTTTRT
RGKSVMSHRDATLFSSDVASLAAPNFLLDGVINFVMAHMTTELGDESLLLVSPSVASLLA
NLQDYEPETVADTAQALLLASRRMVLFPVNNSERLDKADDGSHWSLLVLDNITGRFVHHD
SMDGANLPAATRLADALRPLLPAPPQGPPISGPTPQQSNGYDCGVYLLAVALAICRWWKK
HPRTEEAAPCWFESVMDQVTCERFTRLCIKE*
>11667.m05931|LOC_Os01g59680.1|genepair66-1

MVPHGRVHPAAAASSSDFSGEMNQSAASSDPSSSPPYSFHFEKPLPPTAAAPPPADARRY
QQLLPAPQPGTYVVQMPKDKVFRVPPPENARLFQHYTRRARRRARCSCARVCSWLLLALV
LLAAALAASAAVVYLVFKPRQPDYTLLSLAVSGLGGILGNASSTAAPAPVSFSPEFDATV
RADNPNGKIGVHYEGGGSHVAVSYGGVRLADGAWPAFYQGPRNVTVLVATAKGLGIRFSE
RLLGDIAAAGRLRSVPFDVDVKVPVRLQVGGVRTWAVPVRVRCAVVVDRLAADAKVVSKS
CRVKARFLFWKD*
>11686.m00259|LOC_Os12g03370.1|genepair66-2
MADRVHPMPAPPPPSSSPPPGHDAAAATETTPLHPSFRGAPPPSPGTYIIQIPKDQVLRV
PPPDRARRYKKLAARPARRRRLRHACCGAFCGVLLLLLLAAAFVGVVYLVFRPLAPSFSV
ASLSIRGLDALAVSSLTPQIDAAVRADNGANKKTGIDYRGGGEVTVSYSGERLAAGPWPA
FHQAPRNVTVFSTALAGGGVSLTEEQRKQLAADQAAGAVPLAVDAIVPVRLRFGKVLRTW
TVDVKARCEVTVNKLAAAPANRGCMVKVRPLWWWW*
>11667.m05941|LOC_Os01g59780.1|genepair67-1
MVSMRKKKKAFAVAAATTLLSPPRSSSSSSSTASSCIVPPRTESGKKKSKHRKRAKDGTG
GDDDDAAVAAAPRKGSSIYKGVARHRGSGKYEAHLWDKQGWNPNQTRKRGRQGAYDTEEA
AARTYDLAALKIWGSDHVLNFPIDTYRKELERMQRMTREEYLATLRRKSSGFSRGVSKYR
GVAKHHHNGRWEARIGRAVGKKYLYLGTFDTQEEAATAYDLAAIQLRGRSAVTNFDASCY
TYTDHLPPPPPPQPPSVCKTEPELEPPQPAAPPGSESLLRPKMEPCDDWEPPAICPSLRD
ADDADHAIAEILPALCMDRADFEARYPARRARDAAADGWSTSSDDVAAASVDDDVLRSLP
DDVGFVDDVESLFLDAPGPAAAAAAAAMPDDVERAVQRAPSAASRRANAAAVSYAISSLA
SGRWWY*
>11686.m00251|LOC_Os12g03290.1|genepair67-2
MAKRSSPDPASSSPSASSSPSSPSSSSSEDSSSPMSMPCKRRARPRTEKSTGKAKRPKKE
SKEVADPSSNGGGGGKRSSIYRGVTRHRWTGRFEAHLWDKNCSTSLQNKKKGRQGAYDSE
EAAARAYDLAALKYWGPETVLNFPLEEYEKERSEMEGVSREEYLASLRRRSSGFSRGVSK
YRGVARHHHNGRWEARIGRVLGNKYLYLGTFDTQEEAAKAYDLAAIEYRGANAVTNFDIS
CYLDQPQLLAQLQQEPQLLAQLQQEPQVVPALHEEPQDDDRSENAVQELSSSEANTSSDN
NEPLAADDSAECMNEPLPIVDGIEESLWSPCLDYELDTMPGAYFSNSMNFSEWFNDEAFE
GGMEYLFEGCSSITEGGNSMDNSGMAEYNLFEECNMLEKDISDFLDKDISDFLDKDISIS
DRERISPQANNISCPQKMISVCN*
>11667.m00584|LOC_Os01g06520.1|genepair68-1
MLLLVLLQIQLVAALSLLPPASAPPPAALPCRPDQESPLLRLKSSFSATDMSTAAFRSWR
PGTDCCRWDGVRCGHGDGRVTSLDLGGRQLESRGGLDPAIFHLTSLEYLSLADNDFNGSP
LPSSGFERLTELTHLSLRSTNITGVVPAGIGRLVNLVSLDLSTDFEIIDTFDDVYYVFKMN
SSLDAQQLAVPNLESLVANLSNLRELNLGLVNLSENGARWCNALVDSCPKLQVLRLSCCA
LSGPICATLPRLHSLSVIDLSFNSLPGLIPDFSNFPNLTALQLRRNDLEGFVSPLIFKHK
KLVTIDLYHNPGIYGTLPNFSSDSHLENIYVGGTEFNGIIPSSIAELKSLKNLGLGATGF
SGELPSSIGNLRSLKSLEISGFGLVGSIPSWVANLSSLTVLQFTNCGLSGSIPSSVGNLR
NLGKLLLYNCSFSGKIPSQILNLTQLEILSLHSNNFIGTVELTSMWKLLDLFVLDLSDNN
LVVVDGKGNSSTASIPKLGALRLSGCNVSKFPNFLRFQDEIEYLDLSYNHIDGAIPQWAW
ENWVKMDILSLKNNKFTSVGHDPFLPLSDMKALDLSENMFEGPIPIPRGYATVLDYSGNR
FSSIPFKFTNYLSDVSFFKAGRNNFSGRIPPSFCSAMSLQLLDLSYNSFDGSIPSCLIED
VDKLEVLNLKENKLRGEFPDNIKESCSFEALDFSGNLIEGKLPRSLAVCKNLEVLNIGSN
QINDSFPCWMGTLRKLQVLVLKSNKFFGHVAQSLGEEKGTCEFQSARIVDLASNKFSGIL
PQEWFNKLKSMMIKDSNLTLVMDHDLPRMEKYDFTVALTYKGMDITFTKILRTLVFIDLS
DNAFHGSLPEAIGELVLLNVLNISHNSLTGPIPPQLGRLTQLESLDISSNELSGEIPQQL
ASLDFLTVLNLSYNKLEGEIPESPHFLTFSNSSFLGNDGLCGRPLSKGCINITSLNVIPS
KKNSLDVLLFLFAGLGFGFGFALSIVVIWGIPIRKRSRVRQRAL*
>11686.m01179|LOC_Os12g12130.1|genepair68-2
MRSAYHLMPLLAMLLILVLADHTSSTEAVAPAACLPDQAAALLQLKRSFNATIGDYSAAF
RSWVAVAGADCCSWDGVRCGGAGGRVTSLDLSHRDLQAASGLDDALFSLTSLEYLDLSSN
DFGKSQMPATGFEKLTGLTHLDLSNTNFAGLVPAGIGRLTRLSYLDLSTTFFVEELDDEY
SITYYYSDTMAQLSESSLETLLANLTNLEELRLGMVVVKNMSSNGTARWCDAMARSSPKL
RVISMPYCSLSGPICHSLSALRSLAVIELHYNHLSGPVPGFLATLSNLSVLQLSNNKFEG
WFPPIIFQHEKLTTINLTKNLGISGNLPNFSGESVLQSISVSNTNFSGTIPSSISNLKSL
KKLALGASGFSGVLPSSIGKMKSLSLLEVSGLDLVGSIPSWISNLTSLNVLKFFTCGLSG
PIPSSIGYLTKLTKLALYNCQFSGEIPSLILNLTKLETLLLHSNSFVGIVELTSYSKLQN
LYVLNLSNNKLIVIDGENNSSLVSYPSISFLRLASCSISSFPNILRHLPEITSLDLSYNQ
LQGAIPQWTWETWTMDFSLLNLSHNNLRSIGPDPLLNLYIEFLDLSFNNFEGTIPIPEQG
SVTLDYSNNRFSSMPMPLNFSTYLMNTVIFKVSRNSLSGYIPPTICDAIKSLQIIDLSYN
NLTGSIPSCLMEDVGALQVLNLKGNKLDGELPDNIKEGCALSALDFSDNLIQGQLPRSLV
ACRNLEILDIGNNQISDSFPCWMSKLPVLRVLVLQSNKFIGQVLDPSYTRYGNNCQFTSL
RIADIASNNFSGTLPEEWFKMLRSMMSSSDNGTSVMEHLYPRERYKFTVAVTYKGSHMTF
SKILTSLVLIDVSNNKFHGNIPAGIEELVLLHGLNMSHNVLTGPIPTQFGKLDNLETLDL
SSNKLSGEIPQELASLNFLSILNLSYNMLDGKIPQSLHFSTFSNDSFVGNIGLCGPPLSK
QCGYPTEPNMMSHTAEKNSIDVLLFLFTALGFGICFGITILVIWGGHNRKQPCERAILYI
GRIVW*
>11667.m00599|LOC_Os01g06670.1|genepair69-1
MPAPCLPDQAAALLRLKHSFNMTNKSECTLASWRAGTDCCRWEGVRCGVGIGVGHVTSLD
LGECGLESAALDPALFELTSLRHLNLAWNNFSGSHIPTIGFERLTELTYLNLSNSKFAGQ
IPNTIGRLTNLISLDLSTDFFLIDLDDEFLSVATYSPAWLLVAPNIVSIVANLHNLKELY
MGTIDLSSNSMVQWCSAFSNSTTPQLQVLSLPYCYLEVPICESLSGIRSLSEINLQYNFI
HGPIPESFGDLPSLSVLSLTHNSLEGSFPSRIFQNKNLTSVDVRYNFELSGSLPKNISSN
DILVDLLVSSTNFSGPIPNSVGNIKSLENLGVASSDFSQELPSSIGQLRSLNSLEITGAG

VVGAVPSWIANLTSLTLLDFSNCGLSGKIPSAIGAIKNLKRLALYKCNFSGQIPQDLFNL
TQLRVIYLQYNNFIGTLELSSFWKLPDLFSLNLSNNKLSVVDGEKNNSSWVSINYFYTLR
LAYCNISNFPSALSLMPWVGNLDLSGNQIHGTIPQWAWETSSELFILNLLHNKFDNIGYN
YLPFYLEIVDLSYNLFQGPIPITGPDTWLLDCSNNRFSSMPFNFSSQLSGMSYLMASRNN
LSGEIPLSICDARDILLLDLSYNNLSGLIPLCLLEDINSLSVFNLKANQLHGELPRNIKK
GCALEALDFSENMFEGQLPTSLVACRDLEVLDIGNNQISGGFPCWASMLPKLQVLVLKSN
KFTGEVGSSAIEKDNTCEFANLRILDLASNNFSGTLHHKWLKRLKSMMETSSSATLLMQY
QHNVHSTTYQFSTSIAYKGYEVTFTKILRTLVVIDVSDNALHGSIPKSIGELVLLRGLNM
SHNALTGPIPSQLGALHELESLDLSSNDLSGEIPQELAQLHFLSVLNLSYNGLVGRIPDS
PQFSNNLSYLGNIGLCGFPLSKECSNMTTPPSSHPSEEKHVDVILFLFVGLGVGIGFAVI
IVVTWGIRIKKRSQDSRFPFWKKVLCM*
>11686.m01178|LOC_Os12g12120.1|genepair69-2
MAAPVPCLPDQASALLQLKRSFNTTVGDYSAAFRSWVAGTDCCHWNGVRCGGSDGHITSL
DLSHRDLQASGLDDALFSLTSLEYLDISWNDFSASKLPAIGFEKLAELTHLDLCTTNFAG
RVPVGIGRLKSLAYLDLSTTFFLYEQDEENSITYYYSETMSQLSEPSLETLLANLTNLEE
LRLGMVNMSSNGARWCDAIARSSPKLRVISMPYCSLSGPICHSLSALRSLSVIELHYNHL
SGPVPELLATLSNLTVLQLSNNMLEGVFPPIIFQLQKLTSISLTNNLGISGKLPNFSAHS
YLQSISVSNTNFSGTIPASISNLKYLKELALGASGFFGMLPSSIGKLKSLHILEVSGLEL
QGSMPSWISNLTFLNVLKFFHCGLSGPIPASVGSLTKLRELALYNCHFSGEVAALISNLT
RLQTLLLHSNNFIGTVELASYSKLQNLSVLNLSNNKLVVVDGENSSSVVSYPSISFLRLA
SCSISSFPNILRHLPYITSLDLSYNQIQGAIPQWTWETWTMNFFLLNLSHNNFTSIGSNP
LLPLYIEYFDLSFNNFDGAIPVPQKGSITLDYSTNRFSSMPLNFSSYLKNTVVLKASDNS
LSGNIPSSICDAIKSLQLLDLSNNNLTGSMPSCLTQDASALQVLSLKQNHLTGELPDNIK
EGCALSALDFSGNMIQGQLPRSLVACRNLEILDIGNNQISDHFPCWMSKLPELQVLVLKS
NKFHGKIMDPLYTRDGNNCQFSMLRIADIASNNFSGTLPEELFKMLKSMMTRSDNETLVM
EHQYSHGQTYQFTAALTYKGNDITISKILRSLVLIDVSNNEFDGSIPSSIGELALLHGLN
MSHNMLTGPIPTQFDNLNNLESLDLSSNKLSGEIPQELASLNFLATLNLSYNMLAGRIPQ
SSHFSTFSNASFEGNIGLCGPPLSKQCSYRSEPNIMPHASKKDPIDVLLFLFTGLGFGVC
FGITILVIWGSNNRKQQA*
>11667.m00605|LOC_Os01g06730.1|genepair70-1
MTRYKLLFMTLLLLLLHTQLVVPSSSATSATYTNHTGVPPSAVPCMPDQASALLRLKRSF
SITNKSVIALRSWNAGEDCCRWEGVRCGGGGTAAGGRVTWLDLGDRGLKSGHLDQVIFK
LNSLEYLNLAGNDFNLSEIPFTGFERLSMLTHLNLSSSNFAGQVPVHSIGQLTNLISLDL
SFRFKVTELFDMGYLYTGAYSHEWQLVLPNLTALVANLSNLEELRLGFLDLSHQEADWCN
ALGMYTQNLRVLSLPFCWLSSPICGSLSNLRSLSVIDMQFSGLTGRFPDFFANLSSLSVL
QLSFNHLEGWVPPLIFQKKKLVAIDLHRNVGLSGTLPDFPVDSSLEILLVGHTNFSGTIP
SFISNLKSLKKLGLDASGFSGELPSIIGTLRHLNSLQISGLEVVESFPKWITNLTSLEVL
EFSNCGLHGTIPSSIADLTKLTKLALYACNLFGEIPRHIFNLTQLDTIFLHSNSFTGTVE
LASFLTLPNLFDLNLSHNKLTVINGESNSSLTSFPNIGYLGLSSCNMTRFPNILKHLNKN
EVNGIDLSHNHIQGAIPHWAWENWKDAQFFFLNLSHNEFTRVGHTIFPFGVEMLDLSFNK
FEGPIPLPQNSGTVLDYSNNRFSSIPPNISTQLRDTAYFKASRNNISGDIPTSFCSNKLQ
FLDLSFNFFSGSIPPCLIEVAGALQVLNLKQNQLHGELPHYFNESCTLEALDFSDNRIEG
NLPRSIASCRKLEVLDIQNNHIADYFPCWMSAFPRLQVLVLKSNKFFGQVAPSVGEDSSC
EFPSLCILDLASNKFSGTLSEEWFTRLKSMMIDSVNGTSVMEYKGDKKRVYQVTTVLTYK
GSTMRIDKILRTFVFIDVSNNAFHGSVPKAIGELVLLNTLNMSHNSLTGPVPTQLSHLNQ
MEALDLSSNELSGVILQELASLHFLTTLNLSYNRLVGRIPESTQFSTFLNNSFLGNDGLC
GPPLSKGCDNMTLNVTLSDRKSIDIVLFLFSGLGFGLGFAIAIVIAWGVPIRKWSLLGQR
VP*
>11686.m01159|LOC_Os12g11930.1|genepair70-2
MSSSTKRVTHHLPSLLLTVMHILLPVQATTNLTARTSSSIPPVPCHPDQASALLRLKHSF
DATVGDYSTAFRSWVAGTDCCRWDGVGCGSADGRVTSLDLGGQNLQAGSVDPALFRLTSL
KHLNLSSNNFSMSQLPVITGFERLTELVYLDLSDTNIAGELPASIGRLTNLVYLDLSTSF
YIVEYNDDEQVTFNSDSVWQLSAPNMETLLENLSNLEELHMGMVDLSGNGERWCYNIAKY
TPKLQVLSLPYCSLSGPICASFSSLQALTMIELHYNRLSGSVPEFLAGFSNLTVLQLSRN
KFQGSFPPIIFQHKKLRTINLSKNPGISGNLPNFSQDTSLENLFLNNTNFTGTIPGSIIN
LISVKKLDLGASGFSGSLPSSLGSLKYLDMLQLSGLELVGTIPSWISNLTSLTVLRISNC
GLSGPVPSSIGNLRELTTLALYNCNFSGTVPPQILNLTRLQTLLLHSNNFAGTVDLTSFS
KLKNLTFLNLSNNKLLVVEGKNSSSLVSFPKLQLLSLASCSMTTFPNILRDLPDITSLDL
SNNQIQGAIPQWAWKTWKGLQFIVLNISHNNFTSLGSDPFLPLYVEYFDLSFNSIEGPIP
IPQEGSSTLDYSSNQFSYMPLRYSTYLGETVTFKASKNKLSGNVPPLICTTARKLQLIDL
SYNNLSGSIPSCLLESFSELQVLSLKANKFVGKLPDIIKEGCALEALDLSDNSIEGKIPR
SLVSCRNLEILDIGSNQISDSFPCWLSQLPKLQVLVLKSNKLTGQVMDPSYTGRQISCEF
PALRIADMASNNLNGMLMEGWFKMLKSMMARSDNDTLVMENQYYHGQTYQFTATVTYKGN
DRTISKILRSLVLIDVSSNAFHGAIPDTIGELVLLRGLNLSHNALTGPIPSQFGRLDQLE
SLDLSFNELSGEIPKELASLNFLSTLNLANNTLVGRIPDSYQFSTFSNSSFLGNTGLCGP
PLSRQCDNPEEPIAIPYTSEKSIDAVLLLFTALGFGISFAMTILIVWGSHMRKRH*
>11667.m00621|LOC_Os01g06890.1|genepair71-1
MCTRQARRRYYETSLLVLMLLLLLQARLAQSLPPCSPDQATALLQLKRSFTVNTASATAF
RSWRAGTDCCHWAGVRCDDDDNDAAASGSTGRRATSLDLGGRGLQSGGLDAAVFSLTSLG
YLNLGGNDFNASRLPAVGFERLTELTHLNISPPSFTGQIPAGIGRLTNLVSLDLSTLFYV
INQEDDRADIMAPSFPNWGFWKVDFLRLVANLDNLRELYLGFVYMSNGGEGWCNALVNST
PKDQVLSLPFCKISVNDSVVRSPKVAELSLASCNISKFPNAVKHQDELHVIDLSNNQMHG
PIPRWAWETWKELFFLDLSNNKFTSIGHDSLLPCLYTRYINLSYNMFEGPIPIPKENSDL
ELDYSNNRFSYMPFDLIPYLAGILSLKASRNNISGEIPSTFCTVKSLQILDLSYNILNGS

IPSCLMENSSTIKVLNLKANQLNGELPHNIKEDCAFEALDFSYNRFEGQLPTSLVACKNL
VVLDVGNNQIGGSFPCWMHLLPKLQVLVLKSNKFYGQLGPTLTKDDDCELQHLRILDLAS
NNFSGILPDEWFRKLKAMMSVSSNEILVMKDGDMYGTYNHITYLFTTTVTYKGLDLTFTK
ILKTFVLIDVSNNRFHGSIPETIATLSVLSGLNMSHNALTGPIPNQLASLHQLESLDLSS
NKLSGEIPQKLASLDFLSTLNLSNNMLEGRIPESPHFLTLHNSSFIRNAGLCGPPLSNEC
SNKSTSSEEKSVDVMLFLFVGLGFGVGFAIAVVVSWKPCIGK*
>11686.m01137|LOC_Os12g11720.1|genepair71-2
MLASMTFAYTSSMPLLAMLLILLIQVQSRSTLADGTSSSASAQPAPCLPGQAWALLRLKN
SFDATAGDYSAAFRSWIAGTDCCRWEGIRCGGAQGRAVTSLDLGYRWLRSPGLDDALFSL
TSLEYLDISWNDFSASKLPATGFEKLAELTHLDLCSTNFAGRVPVGIGRLKSLAYLDLST
TFFEDELDDENNVIYYYSDTISQLSEPSLETLLANLTNLEELRLGMVNMSRNGARWCDAM
ARSSPKLRVISMPYCSLSGPICHSLSALRSLSVIELHYNHLSGPVPELLATLSNLTVLQL
SNNMLEGVFPPIIFQLQKLTSISLTNNLGISGKLPNFSAHSYLQSISVSNTNFSGTIPAS
ISNLKYLKELALGASGFSGMLPSSIGKLKSLRILEVSGLELQGSMPSWISNLTFLNVLKF
FHCGLSGPIPASVGSLTKLRELALYNCHFSGEVSALISNLTRLQTLLLHSNNFIGTVELA
SYSKLQNLSVLNLSNNKLVVVDGENSSSVVSYPSISFLRLASCSISSFPNILRHLPNITS
LDLSYNQIQGAIPQWTWETWTMNFFLLNLSHNNFTSIGSNPLLPLYIEYFDLSFNNFDGA
IPVPQKGSITLDYSTNRFSSMPLNFSSYLKSTVVLKASDNSLSGNIPSSICDAIKSLQLL
DLSNNNLTGSMPSCLTQNASALQVLSLKQNHLTGELPDNIKEGCALSALDFSGNMIQGQL
PRSLVACRNLEILDIGNNQISDHFPCWMSKLPELQVLVLKSNKFHGKIMDPLYTRDGNNC
QFSMLRIADIASNNFSGTLPEELFKMLKSMMTRSDNETLVMEHQYSHGQTYQFTAALTYK
GNDITISKILRSLVLIDVSNNEFDGSIPSSIGELALLHGLNMSHNMLTGPIPTQFDNLNN
LESLDLSSNKLSGEIPQELASLNFLATLNLSYNMLAGRIPQSSHFSTFSNASFEGNIGLC
GPPLSKQCSDRSEPNIMPHASKKDPIDVLLFLFTGLGFGVCFGITILVIWGSNNRKQQA*
>11667.m00622|LOC_Os01g06900.1|genepair72-1
MNYTHRCLHSHKLPLLILLIAIVVILIQPYSIHAAANSSNTTIRCLTSQSSALLQLKSSF
HDASRLSSWQPDTDCCRWEGVTCRMASGHVVVLDLSDGYLQSNGLHPALFNLTLLTNLAL
SGNDFMGAQLPDSGFERLSKLVSLDLSATNFAGQIPIGIGNLSNMLALDLSHNPNLYLTE
PSFQTFIANLSNLRELYLDEMDLSSSGATWSSDVAASAPQIQILSFMSCGLSGFIDPSFS
RLRSLTMINVRLNVISGMVPEFFANFSFLTILELSGNAFEGQFPTKIFQLKRLQFIDLYW
NNKLCVQLPEFLPGSRLEVLDLILTNRSNAIPASVVNLKYLKHLGLTTVEASMNSDILLI
RELHWLEVLRLYGGSGQGKLVSFSWIGSLKHLTYLELGNYNFSGLMPSSIINLTNLTSLT
LYNCSMSGPIPSWIGNLIQLNNLNFRNNNLNGTIPKSIFALPALQSLYLDSNQLSGHLED
IPVPLSSSVYDIDLSNNWLHGPIPKSFFCLPNLEYLNLESNHLTGIVELRPFWRLRSLYF
LGFSNNKLSVIDGEDSPSQYLPKIQHLGLACCNLTKLPRILRHLYDILELDLSSNKIGGV
IPGWIWEIWKDTLGSLDLSNNAFTSLENSPSLVTFTHLSHLNLSFNRLQGEIPIPAISLP
YGVVVLDYSNNGFSSILRTFGRYLNKVAYINLSKNKLKGYVPISICSMKKLQFLYLSDNN
FSGFVPSCLVEGRSLRVLNLRGNKFNGMLPKGIKEGCKLETIDLNSNQIEGRLPRTLSNC
KSLELLDVSNNHILDLFPLWLGNLPKLRVLVLRSNQLYGTIKGLHNSDLTRDHFSSLQIL
DLANNTLSGQLPPKWFEKLKSMMANVDDGQVLEHQTNFSQGFIYRDIITITYKGFDMTFN
RMLTTFKAIDFSNNSFVGVIPGTIGSLVSLHGLNMSHNNFTGAIPQQLGNLAQLESLDLS
WNQLSGVIPHELTFLTSLSWLNLSNNNLTGRIPQSNQFLSFSNSSFEGQRGNLLYLLEIS
LNLLCFGFGK*
>11686.m01115|LOC_Os12g11500.1|genepair72-2
MYILLQVQATTNTARTVVPPVRCHPDQASALLRLKHSFNATAGDYSTAFQSWVAGTDCCR
WDGVGCGGADGRVTSLDLGGHQLQAGSVDPALFRLTSLKHLNLSGNDFSMSQLPVITGFE
QLTELVYLDLSDTNIAGEVPGSIGRLTNLVYLDLSTSFYIVEYNDDEQVTFDSDSVWQLS
APNMETLIENHSNLEELHMGMVDLSGNGERWCDNIAKYTPKLQVLSLPYCSLSGPICASF
SALQALTMIELHYNHLSGSVPEFLAGFSNLTVLQLSKNKFQGSFPPIIFQHKKLRTINLS
KNPGISGNLPNFSQDTSLENLFLNNTNFTGTIPGSIINLISVKKLDLGASGFSGSLPSSL
GSLKYLDMLQLSGLQLVGTIPSWISNLTSLTVLRISNCGLSGPVPSSIGNLRELTTLALY
NCNFSGTVHPQILNLTRLQTLLLHSNNFAGTVDLTSFSKLKNLTFLNLSNNKLLVVEGKN
SSSLVLFPKLQLLSLASCSMTTFPNILRDLPDITSLDLSNNQIQGAIPQWAWKTWKGLQF
IVLNISHNNFTSLGSDPFLPLYVEYFDLSFNSIEGPIPIPQEGSSTLDYSSNQFSSMPLR
YSTYLGETVTFKASKNKLSGNVPPLICTTARKLQLIDLSYNNLSGSIPSCLLESFSELQV
LSLKANKFVGKLPDIIKEGCALEALDLSDNSIEGKIPRSLVSCRNLEILDIGSNQISDSF
PCWLSQLPKLQVLVLKSNKLTGQVMDPSYTGRQISCEFPALRIADMASNNLNGMLMEGWF
KMLKSMMARSDNDTLVMENQYYHGQTYQFTATVTYKGNDRTISKILRSLVLIDVSGNAFH
GAIPDTIGELVLLRGLNLSHNALTGPIPSQFCRLDQLESLDLSFNELSGEIPKELASLNF
LSTLNLSNNTLVGRIPDSYQFSTFSNSSFLGNTGLCGLPLSRQCDNPEEPSAIPYTSEKS
IDAVLLLFTALGFGISFAMTILIVWGSHMRKRH*
>11667.m00624|LOC_Os01g06920.1|genepair73-1
MSPRVAQREYEPLLLVLLLLLQTLIASSLPCLPDQAAALLQLKRSFSATTASATAFRSWR
AGTDCCRWAGVRCDGGRVTFLDLGGRRLQSGGLDAAVFSLTSLRYLNLGGNDFNASQLPA
TGFERLTELTHLNISPPSFAGQIPAGIGSLTNLVSLDLSSSIYIVNQGDDDVSIMSNLLP
PWGFSRVNFEKLIANLGNLRELYLGLVYMSNGGEGWCNALANSTPKIQVLSLPLCQISGP
ICQSLFSLRSLSVVDLQGNDLSGAIPEFFADLSSLSVLQLSRNKFEGLFPQRIFQNRKLT
AIDISYNYEVYGDLPNFPPNSSLIKLHVSGTKFSGYIPSSISNLTDLKELSLSANNFPTE
LPSSLGMLKSLNLFEVSGLGLVGSMPAWITNLTSLTDLQISHCGLSGSLPSSIGNLKNLR
RMSLFKSNFTGNIPLQIFNLTQLHSLHLPLNNFVGTVELTSFWRLPYLSHLDLSNNKLSV
VDGLVNDSAVSSPKVKFLSLASCNISKFPNALRHQDKIIFLDLSNNQMNGAIPPWAWETW
KESFFLDLSNNKFTSLGHDTLLPLYTRYINLSYNMFEGPIPIPKESTDSQLDYSNNRFSS
MPFDLIPYLAGTLSLKVSMNNVSGEVPSTFCTVKSLQILDLSYNILNGSIPSCLMENSST
LKILNLRGNELRGELPHNMKEDCAFEALDVSYNWIEGTLPKSLVTCKNLVVLNVANNQIG

GSFPCWMHLLPKLQVLVLKSNKFYGPLGPTLAKDDECELQYLRILDLASNNFSGVLPYEW
FRKLKSMMSVSINETLVMKDGDMYSTFNHITYLFTARFTYKGLDMMFPKILKTFVLIDVS
NNRFHGSIPETIATLSMLNGLNMSHNALTGPIPNQLASLHQLESLDLSSNKLSGEIPQKL
ASLDFLSTLNLSDNMLEGRIPESPHFLTLPNSSFIRNAGLCGPPLSKECSNKSTSNVMPH
LSEEKSADIILFLFVGLGFGVGFAIAIVVRKPCIGK*
>11686.m01102|LOC_Os12g11370.1|genepair73-2
MSSSTKRLVRPHHLAKPLLTMLHILLQVQAIAALTDDATAPVIQCLPDQASALLRLKNSF
NKTAGGYSTAFRSWITGTDCCHWDGVDCGGGEDGRVTSLVLGGHNLQAGSISPALFRLTS
LRYLDISGNNFSMSQLPVTGFENLTELTHLDLSDTNIAGEVPAGIGSLVNLVYLDLSTSF
YIIYYDDENKMMPFASDNFWQLSVPNMETLLANLTNLEELHMGMVDMSGNGERWCDDIAK
FTPKLQVLSLPYCSLSGPICTSLSSMNSLTRIELHYNHLSGSVPEFLAGFSNLTVLQLSK
NKFEGLFPPIIFQHKKLVTINITNNPGLSGSLPNFSQDSKLENLLISSTNFTGIIPSSIS
NLKSLTKLDLGASGFSGMLPSSLGSLKYLDLLEVSGIQLTGSMAPWISNLTSLTVLKFSD
CGLSGEIPSSIGNLKKLSMLALYNCKFSGKVPPQIFNLTQLQSLQLHSNNLAGTVELTSF
TKLKNLSVLNLSNNKLLVLHGENSSSLVPFPKIKLLRLASCSISTFPNILKHLHEITTLD
LSHNKIQGAIPQWAWETWRGMYFLLLNISHNNITSLGSDPLLPLEIDFFDLSFNSIEGPI
PVPQEGSTMLDYSSNQFSSMPLHYSTYLGETFTFKASKNKLSGNIPSICSAPRLQLIDLS
YNNLSGSIPSCLMEDVTALQILNLKENKLVGTIPDNIKEGCALEAIDLSGNLFEGRIPRS
LVACRNLEILDIGNNEISDSFPCWMSKLPKLQVLALKSNKFTGQIMDPSYTVDGNSCEFT
ELRIADMASNNFNGTLPEAWFTMLKSMNAISDNDTLVMENQYYHGQTYQFTAAVTYKGNY
ITISKILRTLVLIDFSNNAFHGTIPETIGELVLLHGLNMSHNSLTGPIPTQFGRLNQLES
LDLSSNELFGEIPKELASLNFLSILNLSYNTLVGRIPNSYQFSTFSNNSFLGNTGLCGPP
LSKQCDNPQESTVMPYVSEKSIDVLLVLFTALGFGVSFAITILIVWGRHMKKQR*
>11667.m01026|LOC_Os01g10590.1|genepair74-1
MSSANSLVLGRVIGDVVDLFSPEVTLRVMYNGVRVVNGEDLRPSAVSARPSVEVMVDPDA
PNPSNPTLREYLHWLVTDIPGTTDANYGREVVCYESPRPAAGIHRVAVVLFRQMARGGVD
QPPLLRHNFSTRGFADDHALGAPVAAAFFTCKPEGGTGGRRFRPPSRHS*
>11668.m01279|LOC_Os02g13830.1|genepair74-2
MANDSLATGRVIGDVLDPFISTVDLTVMYGDDGMPVISGVELRAPAVAEKPVVEVGGDDL
RVAYTLVMVDPDAPNPSNPTLREYLHWMVTDIPASTDATYGREVVCYESPNPTTGIHRMV
LVLFRQLGRETVYAPAVRHNFTTRAFARRYNLGAPVAAVYFNCQRQAGSGGRRFTGPYTS
RRRQA*
>11667.m01027|LOC_Os01g10600.1|genepair75-1
MAGREDGAAAGAMEEGQDSKEVKCESSEDGSSSSSSSRCHGNDVISVQFMQKVHPWCMCM
NKNLLILAEILGTYFMIFAGCGAVVVNQSTGGAVTFPGICAVWGLVVMVLVYTVSHISGA
HFNPAVTVAFATCGRFRWKQVPSYVVAQVLGSTMASLTLRVVFGGGGGGARGEHLFFGTT
PAGSMAQAAALEFVISFFLMFVVSGVATDNRAIGELAGLAVGATVAVNVLFAGPVTGASM
NPARSLGPAMVAGRYGGVWVYVAAPVSGTVCGAWAYNLLRFTDKPLRDIANTASFLRRSS
RRS*
>11668.m01284|LOC_Os02g13870.1|genepair75-2
MAGGDNNSQTTNGGSGHEQRAMEEGRKQEEFAADGQGCGLAFSVPFIQKIIAEIFGTYFL
IFAGCGAVTINQSKNGQITFPGVAIVWGLAVMVMVYAVGHISGAHFNPAVTLAFATCRRF
PWRQVPAYAAAQMLGATLAAGTLRLMFGGRHEHFPGTLPAGSDVQSLVLEFIITFYLMFV
ISGVATDNRAIGELAGLAVGATILLNVLIAGPISGASMNPARSLGPAMIGGEYRSIWVYI
VGPVAGAVAGAWAYNIIRFTNKPLREITKSGSFLKSMNRMNSST*
>11667.m01028|LOC_Os01g10610.1|genepair76-1
MATGGGGGGGGMGGGGVGGGAGAAGVGVGGRMPTWRERENNKRRERRRRAIAAKIFAGLR
AHGGYKLPKHCDNNEVLKALCNEAGWVVEPDGTTYRKGYKPPERMEVIGCSVSPSPCSSY
QPSPRASYNASPTSSSFPSGASSPFLPHPNNMANGVDGNPILPWLKTLSNSPSSKKHPQL
PPLLIHGGSISAPVTPPLSSPTARTPRMKTDWDESNVQPTWTGSNSPCVVNSTPPSPGRT
MLPDPAWLAGIQISSTSPSSPTFSLVSSNPFSVFKDAILVGNNSSRMCTPGQSGTCSPAI
PGMAPHPDIHMMDAVSDEFAFGSSTNGGHQAAGLVRAWEGERIHEDSGSDDLELTLGSSR
TRAAA*
>11668.m01287|LOC_Os02g13900.1|genepair76-2
MMNGGGGGRVPTWRERENNRRRERRRRAIAAKIYAGLRAYGNYTLPKHCDNNEVLKALCN
EAGWTVEPDGTTYRKYWSLDRIEAAHVKKGLVKEAIMETKGCKPPASELADQLGRSPSAS
PCSSYQPSPRGTSSFPSSGSSSQITLGGGGGGEGSSLIPWLKTLSSAGVGIGGGSSSKFP
AHYSYFGGGSISAPVTPPSGSPPRTPRLKTAAWEEYHHHHAGSVLPPWATVGASYAYAAS
SSLPNSTPPSPRRKVAAAAAAAGGGNDAAAWLAGFQISSAGPSSPTYSLVAPPPNPFGAA
AAAAGSSSRVMSGACSPVAGGDVQMADAARREFAFGGEGGKMTGLVKAWEGERIHEECGS
DDLELTLGSSMTRGDR*
>11667.m01040|LOC_Os01g10710.1|genepair77-1
MALPGRLLLLFLTAAHAALQTCGGDQTPGPVANVSRVWSSNTSRDVLLCSVPGNGNEKLS
FAAGFYCSSPCDAILFAVYITSGSGDIPVVVWSANRDLAAHQNATLSFTASGDLVLANAD
GSVVWSTGTSGQFVIGMTITNSGNLVLFNDAYMPVWQSFENPTDSLLPGQMLAEGMMLRP
NSSATNWTTSRQLYFTVRSDGLYAFAGSDQPQPYYRFEFYSSYLVKNESITQYQYKPTFV
TLVNGSLSIPGSDPLETKLPPAHSLQYLRFESDGHLRLYEWEEFKQRWVIAKDIFELNYC
QYPTVCGEYGICLSEGCSTEGMDCSTTECSCPNTTYFKPIDNMRPTLGCAVETEISCQAM
QDHQLVAIPNVTYFHLWGDSRGAPMTDEESCKKDCLSNCSCKAALFSLYLNQTQALLYPD
LSLSMSYLNTCYLLPEVLSLQAYLDPGYYSKDPVNARSTLYVKVQSTHLLPPSKKKNTFG
YAIGATAAALVTLTIISMVIRKRCNRQRADESDFADLPGTITRFTFKMLKAATNDFSSKL
GEGGFGSVFLGKLGNEMVAVKLLDRAGQGKKDFLAEVQTIGNIHHINLVKLIGFCVERSH
RLLVYEYMPRGSLDKWIYYLHSNAPLDWGTRKRIITNVARGLSYLHDECRQRIVHLDIKP
HNILLDDSFNAKVADFGLSKLIEREISKVVTRMKGTPGYMAPEWLTSQITEKVDVYSFGV

VVMEIISGRKNIDYSQSEENVQLITLLQEKAKKGQLEDLVDKNSDEMHLHKEEVIEVMKL
AMWCLQSDSSRRPSMSVVVKTMEGERAVDDNLGYNFFDLSPAISVPVEQLNSSLHPEASI
LSAPR*
>11668.m01294|LOC_Os02g13970.1|genepair77-2
MPWAESLLFLLLLFSLPIPFSHSWSFDYPSPIANINSLWTNNNATIPYSATYPDGSTIRA
ILVRQNPTWYSPFFACGFICTAPCNDFLFAIFSVSVGDPSNPAFNTSSMPRIMWTANRSR
PVKDNASLQFKDGNLILRDFDGSLVWSTNTSDSRVVGLNLAETGNMVLFDAMGKTVWESF
EHPTDTLLLGQSLRQGKRLTSDSLATNWTQGQFYLTVLDNGLYAFIEADPPQLYYQRRFN
ITDAIVQPNMNISSDGAKNYTTYISFLKGSLSAFVSFNNTDINLFDISLPSPSSAQFMSL
ENDGHLRVYRWDGTSWKPQADVLHVDLDDCAYPTVCGDYGICSEGQCSCPSRNSGDEDQF
FRQLDNRQPNMGCSLAIPLSCDLTQYQQLLPLPNVMYFNLGQNWTTDEYSCKEACLKACS
CKAAFFKYNNVSNGSCYLMPKLFSLMNYQPEVVGYNLSAYIKVQMLPPPPRSKQLNPLVY
HVGAPIIVAVICIIILIIRRIMKRKMDDDDPFKGLAGMPTRFSYKQLREATNNFSKKLGQ
GGFGPVYEGKLGNVKIAVKCLRDIGHGKEEFMAEVITIGSIHHINLVRLIGYCSDKFHRL
LVYEHMTNGSLDKWIFRKNPRGTLSWATRYKIILDIAKGLAYLHEECRQKIAHLDIKPGN
ILLDDKFNAKISDFGLAKLIDRDESHVMTKIRGTRGYLAPEWLSSTITEKADIYSFGVVV
LEIVSGRKNLDNNQPEASNNLINLLQEKIKVGQVLDILDNQNEEIQLHGEEMIEVIKLAV
WCLQRDCSKRPAMSQVVKGGFAASCSTRRNHRVIFLGSRQFSPIIYSPARRASSRLSRRE
VIAFAGQQSWDIGRFVRTLYFFNGPPNPLKIVESILSSFTGSAPGEVPKKMESSDVVLVT
GATGGVGRRVVDILRNKGIPVRVLARNEEKARSMLGPDVDLIIGDVTKENSLDPKYFKGI
KKVVNAVSVIVGPKEGDTPDRQKYKQGIKFFEPEIKGPSPEMVEYLGMQNLINAVKNSVG
LTEGKLLFGGTGNLSGKIVWGALDDVVMGGVSESTFQIQPTGSETDGPTGLFKGTVSTSN
NGGFTSIRTKNFTVPEDLSAYDGIELRVKGDGRRYKLIVRTSFEWDTVGYIASFDTTKGE
WQSVKLPFSSLNPVFRARTMPDAAPFDASNVTSLQLMFSKFEYDGKLNPTFTEGSFELPF
SSIRAYINEPITPRFVHVSSAGVTRPERPGLDLSKQPPAVRMNKELGSILTYKLKGEDLI
RESGIPYTIVRPCALTEEPAGADLIFDQGDNITGKISREEIAFICVAALASPNAVEKTFE
VKSTVPFSEPFVVDPSNPPPEKDYDVYFKELKAGITGKEALEGTPAQVLTT*
>11667.m01051|LOC_Os01g10820.1|genepair78-1
MASEKKQANPMREIKVQKLVLNISVGESGDRLTRASKVLEQLSGQSPVFSKARYTVRSFG
IRRNEKIACYVTVRGEKAMQLLESGLKVKEYELLRRNFSETGCFGFGIQEHIDLGIKYDP
STGIYGMDFYVVLERAGYRVARRRRCKSRVGIQHRVTKEDAMKWFQVKYEGVILNKAQAN
TS*
>11668.m01306|LOC_Os02g14050.1|genepair78-2
MASEKKQSNPMREIKVQKLVLNISVGESGDRLTRASKVLEQLSGQTPVFSKARYTVRSFG
IRRNEKIACYVTVRGEKAMQLLESGLKVKEYELLRRNFSDTGCFGFGIQEHIDLGIK*
>11667.m01053|LOC_Os01g10840.1|genepair79-1
MAERVVGTGSFGIVFQAKCLETGETVAIKKVLQDRRYKNRELQLMRAMEHPNVICLKHCF
FSTTSRDELFLNLVMEYVPETLYRVLKHYSNANQRMPLIYVKLYIYQLFRGLAYIHTVPG
VCHRDVKPQNVLVDPLTHQVKLCDFGSAKVLVPGEPNISYICSRYYRAPELIFGATEYTT
SIDIWSAGCVLAELLLGQPLFPGESAVDQLVEIIKVLGTPTREEIRCMNPNYTEFKFPQI
KAHPWHKIFHKRMPPEAIDLASRLLQYSPSLRCTALDACAHSFFDELREPNARLPNGRPF
PPLFNFKHELASASPELIHRLIPDHIRRQHGLNFAHAGS*
>11668.m01314|LOC_Os02g14130.1|genepair79-2
MATLPGGPHPTGGGAAADPMQVDQPRSAAAAAAVAPAGEKHGASLMEGSDPVTGHIISTT
IGGKNGEPKRTISYMAERVVGTGSFGIVFQAKCLETGETVAIKKVLQDKRYKNRELQIMR
SMDHCNVVSLKHCFFSTTSRDELFLNLVMEFVPESLYRVLKHYSNMNQRMPLIYVKLYVY
QIFRGLAYIHTVPGVCHRDVKPQNLLVDPLTHQVKICDFGSAKMLVKGEANISYICSRYY
RAPELIFGATEYTTSIDIWSAGCVLAELLLGQPLFPGESAVDQLVEIIKVLGTPTREEIR
CMNPNYTEFRFPQIKAHPWHKIFHKRMPPEAIDLASRLLQYAPNLRCTALEACAHSFFDE
LREPHARLPNGRPFPPLFNFKQELANLSPELINRLIPEHAR*
>11667.m01054|LOC_Os01g10850.1|genepair80-1
MGWSSSRAMLVARAVALAVVFLAAEAQLSPGYYNATCPGVVSIVRRGMAQAVQKESRMGA
SILRLFFHDCFVNGCDASILLDDTANFTGEKNAGPNANSVRGYEVIDAIKAQLEASCKAT
VSCADIITLAARDAVNLLGGPNWTVPLGRRDARTTSQSAANTNLPPPGASLASLLSMFSA
KGLDARDLTALSGAHTVGWARCSTFRTHIYNDTGVNATFASQLRTKSCPTTGGDGNLAPL
ELQAPNTFDNAYFTDLLSRRVLLRSDQELFGSGAGNGTTDAFVRAYAANATTFAADFAAA
MVRLGNLSPLTGKNGEVRINCRRVNSS*
>11668.m01318|LOC_Os02g14160.1|genepair80-2
MAGAVLVRRMVVVVAIAALVAPGEVAAQLTPTYYDGSCPSLQSIVRSAMAAAVQQEPRMG
ASILRLFFHDCFVNGCDASVLLDDSSTITGEKNAGPNANSLRGFEVIDSIKSQVEAACPG
TVSCADILAVAARDGVNLLGGPTWAVQLGRRDTRTASQSAANSNLPSPSSSAAALVSAFA
SKGLDSRDMVALSGAHTIGAARCATFRARVYNDTNISPGFAVRRRQVCPASGGDGNLAPL
DALSSVRFDNGYFRNLMGRFGLLHSDQELFNGGPVDSIAQQYAANGAAFSRDFVTAVVKM
GNISPLTGSSGEVRSNCRKPN*
>11667.m01081|LOC_Os01g11120.1|genepair81-1
MAAVAGGSRAAPPADSAAVAAVAKEAEYQKGVQKLVDLLSKLNPAAKEFVPSSAAVSSPS
RKALSADAPVFDYNSIGGWNGGGKESGADAYQQRRRRNGYLSQGRRRMNERARHADREDS
IRRTVYVSDIDHTVTEERLADIFANCGQVVDCRICGDPHSVLRFAFIEFADEEGARTALN
LGGTMLGFYPVRVLPSKTAILPVNPKFLPRTEDEKEMVIRTVYCTNIDKKVTQLDVKNFF
EELCGEVSRLRLLGDNVHSTRIAFVEFVHAECAIMALNCSGMILGTLPVRVSPSKTPVKP
RLNRVASN*
>11668.m01379|LOC_Os02g14760.1|genepair81-2
MVAIEAAAAAAAAERGPIRAVAEAEAEAEAKVVDVEGKEEEEEAAKVEEEEEEEREYKSD
MRKLEELMSKLNPRAQEFVPSSRRAPPAAAAQAAGGLSADAPVFVSAAEYFGGAGAGAGQ

LQVGGGGGRDSSSDGSSNGGGQPQNRRRRSGFNQGRRRTGGRTRRADREDSVRRTVYVSD
IDQQVTEQKLAEVFSNCGQVVDCRICGDPHSVLRFAFIEFADDAGARAALTLGGTMLGYY
PVRVLPSKTAILPVNPKFLPRTEDEKEMVSRTVYCTNIDKKVTEEDVKIFFQQLCGKVSR
LRLLGDYVHSTCIAFVEFAQAESAILALNYSGMVLGTLPIRVSPSKTPVRPRSPRVMSN*
>11667.m01094|LOC_Os01g11250.1|genepair82-1
METISNIFHNDPLPPLGARANQSIKLRKFIISPYDSRYRTWETFLLVLVVYSAWICPFEL
AYLRNLSWKVSLVDNIIDSFFAIDIILTFFLAYLDQKSYLLVDDPKRIVARLLSMLRLWR
LRRLSELFARLEKDIRLNYYWIRCTKLISVTLFAVHCSGCFNYLIADRYPNPARTWIGAA
IPNYRSQNLWVRYVTAIYWSITTLTTTGYGDLHAENQREMLFSICYMLFNLGLTAYLIGN
MTNLVVQGSCRTRNFRDTIHAASQFAARNQLPGHIKDEMLSHICLRYKTEGLKQKETLDS
LPKGIRSSIACNLFLPVIEKVYLFHGVSFTCMIQLVTEMEAEYYPPREVVILQNEAPRDV
YILVSGAVEERVEIDGTEKVQEVLCNGEIFGEIGVICSIPQPCAFHTIKVSQLLRLNTAV
LKNIIKENSDDRRVILNNLSQKMNQDHRFSTEVMEKSLQMMHQHFGEYNRCSALNQDNEK
NELKANNGHSMALEWKRVTIHMYSQRNKRPEAPLAKVINLPGSLDKLFAIACQKFNNYRL
TKLVNPEFAEIDDITVIRDGLGEFRLVNIYTRKSQQQFILNVSKLYQFDIGTRQGHGAMW
HAAAGGGDDEGDGGARRPMHGVPNKETTASRGSLKVEEKGMDVVVVEGIQEAIVRTRTTP
RPQGQGRLYRSWSQGEDDSTSHRALQSPPPSRIFRDFDRNQINFPIETQFQEGFCGGLSV
TKLYNMLNFQLGLELCRIKRNITDLIP*
>11668.m01387|LOC_Os02g14840.1|genepair82-2
MTQAHSKSCFHQFWDGLQIKRSSDSFTVELLPSLGATINHSNKLQKFIISPYDPRYRSWE
LFLIVLVVYSAWICPFELAFLRDLPSKLLLVENIVDIFFAIDIVLTFFVAYVDSKTHLLV
DDRKRIAMRNPTFGIEKGPNDSALTLANYDHLQHHFNQSSFYLHTRGMTLLSRLEKDIRF
NYFWTRCSKLISVTLFAVHCAGCFNYMIADRYPNPEKTWIGAVMSTFRSESLWTRYITAL
YWSITTLTTTGYGDLHAENPTEMLFDIVYMMFNLGLTAYLIGNMTNLVVHGTSRTRKFRD
SIQAASEFAARNQLPENIKQQVLSHFCLQFKTEGLNQQVMLDCLPKGIRSSIAYSLFFPI
IRQAYLFNGVSGNFIAELVMEVQAEYFPPKEDIILQNEGEADVYIVVSGAVNIITTIHGN
EQVYEKIAEGEMFGEVGSLCNIPQPFTCRTAELSQLLRISKTRLREIIEENREDSNILMN
NLVQKLKLRESLPDMNQPDRRFLSKYELFHVPREAWLLKKSQLHYTEHTSRDSSNNTPVF
GGDRYSRQLLGEATRSSASENENSSMTDKEENHDEVHTNCEIKKRTEEHCIQINSEDSSS
TYSQRTMNATVQTGSPHKTEENITRRIPDEYYIKEANKRVTIHKYRHNSTVSAAQNGKLI
KLPTSLEELFKIGSQKFQGFHPRKVVSRDYAEIDDVSVIRDGDHLFLLEM*
>11667.m01104|LOC_Os01g11350.1|genepair83-1
MAQLPPKIPTMATAWPEFGGGHHHHAAHGHHHQRSPSMGAFLAAPLPPFPLPPPAPANGG
AQQQQQQQQHQPSWVDEFLDFSATKRGAHRRSVSDSVAFLDPVSDDNAGVGAHDFDRLDD
DQLMSMFSDDLQPPPPQQQPAAPAASASSPSDHNSMNDEKQDKGETDEAQSECDGATPGQ
PASPATVDPKRVKRILANRQSAQRSRVRKLQYISELERSVTSLQTEVSALSPRVAFLDHQ
RSLLTLGNSHLKQRIAALAQDKIFKDGGTEEGDREAAANLPPAKPQERGIPTGGRGPGPR
PRQCRPDRQRGGRRGRAMPALVIGRDPDAL*
>11668.m01394|LOC_Os02g14910.1|genepair83-2
MAQLPPRAPSAAAAAGQEWSAMAAAGEFLGFAAARRGAHRRSASDSAAFLMEAAVPMDDV
IVGVGGGGEFDRLDDEQLMSMFSDVEAPAVSDGGGERGPAGEAHLMDMGDGDDGMGATSP
AGAGAMAAAAAAAAADGIADPKRVKRILANRQSAQRSRVRKLQYISELERSVTTLQMEVS
ALSPRVAFLDHQRSLLTVGNSHLKQRIAALAQDKIFKDAHQEALKKEIERLRQVYHQQQI
KATGGADIATAASMQAKHELLACEGAAMR*
>11667.m01111|LOC_Os01g11420.1|genepair84-1
MGLLAGSTVFLLTLLWGTCVVVGKCDIGPNGVAVDLQNNKGFSLTGTGISTDVQTSYAAR
IMGISVIPFIIAQFPKMLKTHHGQRLAVLLALIVSFSLVLAYCLYQVFQPWIQKRKLAYA
KHKHVISGILRHAQMEALGRLLNEDGTPNEDVIKKLFHKIDMDESQTLSRAELHALIIGI
NFEEVDFDKNDAVDKIMDDFDTSGNDIVEEAEFVSGMKRWLNEAKRSVPTSGAYSNKFIT
DYHARTRQEHDLLVDRSDETVESVENPGWCITKAVGLLLLGSAIAAAFADPLVDAVHNFS
NASHIPSFFISFIALPLATNSSEAVSAIIFASRKKLRTSSLTFSEVYGGVTMNNTLCLGV
FLALIYIRNLTWDFSSEVLIILLVCVIMGLFTSFRTTFPLWTCLVAYMLYPLSLVVVYIL
DFVFGWS*
>11668.m01401|LOC_Os02g14980.1|genepair84-2
MAPPPPPTRCLPVLLLLLLVVAPLLAHGRRPFISDGGNANANANASVLRLPSAAAAAGED
MGCEMSYGFLPCTTTAWGNLFLVLAYGFLMFKSATYLSSGSEMLLQILGPGIVGGLFLPI
LGALPDALLILVSGLSGTKEVAQSQVLIGMGLLAGSTICRTSFYLNKKSVFNLQQGHAGL
MAGSGVSTDKQTSYAARIMAISILPFIIVQIPKIFKLHSGHQITVLIGLIVAALLLLSYC
LYQVFQPWIQRRRLEYTRLKHVMSGLLRHAQKHSIGRLLDDEGRPNVSVIEKLFHRIDQD
NDGKLERGELQAFIVGINFEDIDWNSNLAADQLFCCRVDLSWKDKFQHLTMFQRTRDEQT
GLLDKDEEEGEADGNPTWTCIKAILLLLLGTAMAAASADPLVDAVHNFSNATHIPSFFIS
FIVMPLATNSSEAVSAIIFASRKKKRTLSLTFSEVYGGVTMNNTLCLAVFLALVYVRGLT
WDFSSEVLIILLVCIIMGLFTSFRTDFPLWTCFVAFLLYPLSLIMVYILDYKFGWS*
>11667.m01115|LOC_Os01g11460.1|genepair85-1
MNTPNSWLFADNSKYSTRARLLFMGLSFSIGILSFLVYLAIWYTCTRRRRSRQLRGGGSA
SADQEAPEANSHGMSAAAIAALPTFGYEASAAAAAALDCAVCLGQVDAGEKVRQLPKCGH
LFHAECVDAWLRAHSTCPMCRAAVEGPATAAIAKKASSGGGATDTPPVVAPAPPAAAEAL
PLPPV*
>11668.m01413|LOC_Os02g15100.1|genepair85-2
MSLSSSSNSLPYSTDQGGYSTHDTLVLLGIGFFATAVSVLMIVLCECLCCRRRRRGGGTV
VYVAARPFFLGGGGLSASAVATLPSFVYRREEWAEAAPRGDGSGSGRGGGGGWAQCAVCL
SIVQEGETVRQLPACKHLFHVGCIDMWLHSHSTCPLCRASVEPLGKETPLKDQAPPV*
>11667.m01118|LOC_Os01g11490.1|genepair86-1
MGANDGHLDATSNNRGGLHEQTTTGCLPVDQSCFALSRLSYRRSSSSNPRACCSTTSYLE

VLAISLASLLVIVLVLCAIRCYRMRRAVNRVTVAAASAAAAAAAAGNVTNKKRPAPGLGE
DAIAALPKFEYRGTGDECDRWECSICLCAVADGEVARQLPRCMHLFHRGCVDMWLVAHTT
CPVCRAEVVVNKPPDEDDGRCAETPEDEAAPPASALEPARLENGERDLEAQ*
>11668.m01414|LOC_Os02g15110.1|genepair86-2
MSSHDPSSATAADAVAGGSYRVCDTVVLVCLAFASSIIVFTVAVCFRRAVTLQGYAASAS
ASPSGRGGGAAAAPAAVGGLRGLAPSALAAIPKFAYRRGAAGGGGGWAQCAICLGVVRDG
EAVRRLPECKHLFHVECVDMWLYSHATCPLCRRDVGAAAAAAGDKV*
>11667.m01121|LOC_Os01g11520.1|genepair87-1
MSDTALSGAPPAARSEDSWSQQQSYNFSGRVLLTAVVILFVIAVVFAVTRVLLYYLVVRP
GGGGGGRRRGGLAGGILRSLNSLGVSGRRGLDASALAALPVTAYRKNGGGGGGGEGSNRG
GPGATAADCAVCLSELADGEKVRELPNCRHVFHVECVDAWLRSRTTCPLCRAEAEVPKAR
ASAAATATAQSSSSLGDGGITVVVTIHGGSDEAGGRSTALTGQPGSSNSPSCEAARN*
>11668.m01415|LOC_Os02g15120.1|genepair87-2
MSSTAAGGAPGPAAQRHGGGGGGGGCCSSGVTLELVGAFTAVCLVLYGVILYFNYLYVRW
SGRDGVHRTSGGGGGGGRGAAARKRGGGGGLDKAALAAIPVFRFKASASAAALGGGEAEC
AVCLSGMQDGDAVRALPGCGHAFHAGCVDAWLRAHGTCPVCRARPAVPPPPPAKPPCLKA
PEPAAAAAGRQPVDLESHV*
>11667.m01127|LOC_Os01g11580.1|genepair88-1
MASGPPSSPSSKAFSRKSHAHASGPNSSKAAAAGGGGGGVAAAFDAHNGTHVRTVTFSLS
SSPAARRELRRRLTAELAQVRATCKRLSSLPAPAPSSALSATDPSTPLPPHPPVSKHKSK
KGNPSSNPGLSAEARRKLYAPVFKSCGALLARLMKHKHSWVFNTPVDASALGLHDYHTII
TKPMDLGTVKSRLAAGHYKSPREFAGDVRLTFQNAMRYNPKGQDVHFMAEQLLNMFEEKW
PEIEAEVAQLSPQPPTPSSAAPRKPKEIDNSKVLERSDSTVHAAGMEATPKQNTGRPPVL
KKPKAREPNKREMTFWEKQRLSNNLQELPPEKLDNVVQIIKKRNLSLSQHDDEIEVDIDS
FDVETLWELDRFVTNYKKSISKNKRKAENPVAGQDEMNHDIELEKTEHARLDEVEQDQMP
PVQETLHNPEPESIDIEPPKENTADDNERYVGSSSPVHLEDQKGENAGRSSSSGSSSSDS
GSSSSGKCSHPLQKFILAAFLFAINSIKDIFIYICITYEIYREKHMQIQTQIVHQQMALM
LHSHPERNIYRPGDGFGTVYLVF*
>11668.m01425|LOC_Os02g15220.1|genepair88-2
MASSPPGAGAGGEERPAASPAAPAVAEAVEEGPVTSRWAPEIRVYRRKYPRKNPKPPPNP
SPSSSPLAQTLASIRRSIRRPEDGPAAPRPDPPAAPASSPHPPPPSAPVAPAQQGEPAAP
ASDDVSAGPNRDGGAVPNGHGDVRAAAEEKARKRRARSELRRQLASELDQVRGLSKRLKA
AAEAIAAESAAALALPVVVPPPQLPVGYAHSQFALADPVTPIPGQVAGAIVPVRSVMQRG
PLTVSVTHTESFEKEKRTPKANQLYQNSEFLLAKDKFPPSDSHGRKKPKHHKKKHRSLAS
HGAGYDAEQRLYSHAFKKSMSLLSRLMKHKFGWVFNKPVDAVALGLHDYFAIIKHPMDLG
TIKTRLTHGQYRNPREFADDVRLTFHNAMTYNPKGQDVHFMAEQLLGIFEAQWPEIEAEV
QYLASCPPLPNKFPPPPIDVRFLDRSDSVKHHMVLDSKSRPLSHTPTYSARTPSMKKPKA
KDPDKRDMTIDEKRKLSNNLQNLPPEKLDVVVQIIKNKNLSVRQHDDEIEVEIDSMDTET
LWELDRFVANYKKNLSKQKRKAERAMLARQDAELHAQHVAPQQPSQEPNIGVKSPKQNLI
VDEKLATSVPEQADNNGQNASRSSSSSSSSSDTGSSSSDSDSDSSSSDGSDAANSS*
>11667.m01134|LOC_Os01g11650.1|genepair89-1
MRLSVSVAAVLVVLAALRPPRVAVAQKYAAIFNFGDSLVDAGNLVVDGIPDYLATARLPY
GMTYFGYPTGRCSDGRLVVDFIAQEVGLPLLPPSKAKNATFHRGANFAITGATSLDTPYF
QGRGLGHTVWNSGSLHTQIKWFQDMKASICKSPQECRDLFRRSLFIVGEFGGNDYNSPLF
AFRPLEEKLIEEGAVELVVPGVLPIGCFPVYLSIFRKQPEMYGRRSGCIRDLNTLSWVHN
AALQRKIAELRLKHPGVRIMYADYYTPAIQFVLHAEKYGFLRQTPRACCGAPGVGEYNFN
LTSKCGDPGSYACDDPSNHWSWDGIHLTEASYGHIAKGWLYGPFADPPILETRHH*
>11668.m01426|LOC_Os02g15230.1|genepair89-2
MGAVRGILVVAVVLAVAAILAGAAEGKVNGKAKGKYRALFNFGDSLADAGNLLANGVDFR
LATAQLPYGQTFPGHPTGRCSDGRLVVDHLADEFGLPLLPPSKLKNSSFAHGANFAITGA
TALDTPYFEAKGLGAVVWNSGALLTQIQWFRDLKPFFCNSTKVECDEFYANSLFVVGEFG
GNDYNAPLFAGKGLEEAYKFMPDVIQAISDGIEQLIAEGARELIVPGVMPTGCFPVYLNM
LDEPADGYGPQSGCVRRYNTFSWVHNAHLKRMLEKLRPKHPNVRIIYGDYYTPVIQFMLQ
PEKFGFYKQLPRACCGAPGSVAKAAYNFNVTAKCGEAGATACDDPSTHWSWDGIHLTEAA
YGHIARVRYKGGIGLSKA*
>11667.m05893|LOC_Os01g59350.1|genepair90-1
MEGGRLGGAAASASAAAAADARGGMSGFAAPQHAIHTNLNNVQPTQVTDFGALAQSAGFR
IEDLANLSTNGLFNLKSNAHTIINDPLQFENYVKSISPSNITTTATVTVVDPQTLVPQKG
AQLNLVTIRTGNVENWGESTIADTSPRTDTSTDPDTDERNQMFEQGQLAAPTASDSSDRS
KDKLDHKTLRRLAQNREAARKSRLRKKAYIQNLESSRLKLTQIEQELQRARQQGIFISTS
SDQSHSASGNGALAFDMEYARWLEEHNKHINELRAAVNAHAGDNDLKSTVDSIMAHYNEI
FKLKGVAAKADVFHVLSGMWKTPAERCFMWLGGFRSSELLKLLAGQLEPLTEQQLAGIAN
LQQSSQQAEDALSQGMEALQQSLAETLASGSLGPAGSSGNVANYMGQMAMAMGKLGTLEN
FLRQADNLRLQTLQQMQRILTTRQSARALLAISDYFSRLRALSSLWLARPRE*
>11669.m02070|LOC_Os03g20310.1|genepair90-2
MADASSRTDTSTVLDTDDKNQMVDGQSGAIVPSNSSDRSDRSDKPMDQKVLRRLAQNREA
ARKSRLRKKAYVQQLESSKLKLASLEQEINKARQQGIYISSSGDQTHAMSGNGAMTFDLE
YARWLEEQNKQINELRTAVNAHASDSDLRLIVDGIMAHYDEIFRLKGVAAKADVFHILSG
MWKTPAERCFLWLGGFRSSELLKLLVNQLEPLTEQQLLGLSNLQQSSQQAEDALSQGMEA
LQQSLADTLAGSLGPSGSSGNVANYMGQMAMAMGKLGTLENFLRQADNLRQQTLHQMQRI
LTIRQAARALLAIHDYFSRLRALSSLWLARPRE*
>11667.m05914|LOC_Os01g59530.1|genepair91-1
MADQLSEEQIGEFREAFSLFDKDGDGSITTKELGTVMRSLGQNPTEAELQDMISEVDTDS
NGNIEFKEFLGLMARKLRDKDSEEELKEAFRVFDKDQNGFISATELRHVMANIGERLTDE

EVGEMISEADVDGDGQINYEEFVKCMMAKKRRKRIEEKRDHDGGSRTKSAGPSAAPASKR
GQKCVIL*
>11669.m02076|LOC_Os03g20370.1|genepair91-2
MADQLTDDQIAEFKEAFSLFDKDGDGCITTKELGTVMRSLGQNPTEAELQDMINEVDADG
NGTIDFPEFLNLMARKMKDTDSEEELKEAFRVFDKDQNGFISAAELRHVMTNLGEKLTDE
EVDEMIREADVDGDGQINYEEFVKVMMAK*
>11667.m05923|LOC_Os01g59620.1|genepair92-1
MALQLMMDNYLLCSAMKGRLSVYSDSQLILVKMNRESNFMPTPDQDVLEVKPLRTLAPMF
PAPLGIDVLNRLTAPPLVFVAPAGQFPGGFGSLNIPAVRSFAAFGGQDASGGKTAGGGDQ
DASGGKTAAGGDQDAGRGETAAFGGQETVRGEFVANGTPNVGASATGPIDATPISACKST
QPSVISLDDDDNDDDEPYGGNQTSASGRKIKRPSHLKGYNVSDGLGTDSSNGTKKRPKTS
NRKAATDNEISLMPPSSDPREVVEVLLMTFEALRRRHLQLDETQETSKRADLKAGAIMLA
SNLRANIGKRIGAVPGVEVGDIFYFRMELCIIGLHAPSMGGIDYMNKFGDEDDSVAICIV
AAGVYENDDDDTDTLVYSGSGGISRNSEEKQDQKLERGNLALERSLSRKNVIRVVRGYKD
PACLTGKVYIYDGLYKIHESWKERTKTGINCFKYKLQREPGQPDAVAIWKMCQRWVENPA
ARGKVLHPDLSSGAENLPVCLINDVNSEKGPGHFNYITQVKYLKPLRSMKPFQGCRCTSV
CLPGDTSCDCAQHNGGDLPYSSSGLLVCRKLMVYECGESCRCSINCRNRVAQKGVRIHLE
VFRTTNRGWGLRSWDPIRAGSFICEYVGEVVDDTKVNLDGEDDYLFRTVCPGEKTLKWNY
GPELIGEHSINISADTFEPLPIKISAMKMGNVARFMNHSCNPNTFWQPVQFDHGEDGYPH
IMFFALKHIPPMTELTYDYGDIGCESRGVGSRAKNCLCGSSNCRGFFS*
>11669.m02085|LOC_Os03g20430.1|genepair92-2
MESNQHKASDPQDSMVHLDLDEDKIMVTSALPCPSMSVGKSVMRKRGRPSRHARGTSLSS
VTPEGCKKMEGRSYNLRSDSTILLRNSCLLIADGSTKQKRSWGLDKDDLHIPFFQISDNP
REAVDDILMTFGGLHRRIMQLIDVKMASKQLVFQALNLMRKVGYHVNKDKRVGEVPGVKI
GDIFYSRIEMLLVGLHSNINRGIEFMSGAFINKEDKIATCIVSSGMYENGDDDPYTLVYN
GQGKVHHKLERGNYSLNQSFIRRNHIRLIRSEPNPLVRLGSKEKIYIYDGLYKIEEKYRQ
TTKSRSNLKFNKLVRELGQPNGIVVWKNTQKWRENPSCRDHVIMPDMSNGAEIARVCVVN
NIDSEDAPNNFTYSTKLDNGNHMVSANKMCVCKCTSSCLGEDNCSCLKTNGSYLPYNSSG
ILVCRKTMIYECNDSCACTINCSNRVVQRGSYLHFEVFKTMDRGWGLRSWDPIPAGAFVC
EYVGVVIDKDSLVEEDEYIFEVTRPEHNLKWNYLPELIGEPSFYDMNDTFKKLC*
>11667.m05932|LOC_Os01g59690.1|genepair93-1
MHPRARIHADPAPEFDQFDCLPDPVVLLILNKLEDVRSLGRCAAVSKRFSGLVPLVSDVY
VKIDRVVATDGDADDALNLSSAKPKNIFSHFFKLMLFTIVKPFHSMRNLNGTGRPLFPLA
QHSPVHVLRNFSDVWNLRVELPSGDVGTEEGVLLKWRAEYGSTLRNCVILGGTLVDRKPI
GAEHESSVEDNGSMPESFYTNGGLKLRVVWTISSLIAASTRHYLLRSIIKDHPTLRSLVL
ADADGQGTLYMGMEQLREFRENKLSASACSNRTQVPACNMKLKYAPYLELPGGMTLQGAT
LVVIKPSNDGGSGGHSSRKETEAFVSSAFDEPFRFAVKALMKRRTYLLEMNGF*
>11669.m02092|LOC_Os03g20500.1|genepair93-2
MQSRHRVFAEDLLLPREAEGEDHFDRVPDSLVLLIFNRLADARSLGRCSAVSKRFNSLVP
LVDDACLRIDRVITDAADADDALGLAGPRPAGRGVLSHLLKAMLLAVLKPFGHCDAGVRG
GGGGERAGKHGGGGGGCGAQQHHSPAQVLKNFSSIRNLRMELPVSDVGTDDGVLLRWKAV
FGSTLQSCVILGGTRVDRAAAPAAAAATATAAGDSEASQGDDTGSIPESFYTNGGLKLRV
VWTISSLIAAATRHYLLREIVKEHPTLERVALTDAHGQGTLSMGRDQIREFRDKPLAAAA
AANRTQVPACNMKLRYAPMLELSDGTRIQGATLVVIKPVGEAGGIGGGRKELDEFVADAF
DGPYREAVSALSKRRTYLLEMNGF*
>11667.m05939|LOC_Os01g59760.1|genepair94-1
MASKAGGGGVARRGGGRMRSLGRQGSMYSLTLDEVQSQLGEPLHSMNLDELLRSVFPDGL
AIADGAGATTSSQQHQPGSGLLRQGSITMPPELSKKTVDEVWKGIQAAPKRNAETGGGGG
GGRRRRERQPTLGEVTLEDFLVKAGVVTQGSLKELSDVGNVDPVGRGVTATGTVDLAPGS
HWIEQYKQQIASTDAHHHGQQGVQGAYFPNRLVPQPLNVGPGAILEPSYSDGQTSSGMIG
GMSDSQTPGRKRGMSGDVADKLMERRQKRMIKNRESAARSRARKQAYTNELENKVSRLEE
ENVRLKRQKELDELICAVPVPEPKYQLRRTSSADF*
>11669.m02107|LOC_Os03g20650.1|genepair94-2
MSSHQGSGGVSRQGSLCGLALSEVEGQLHGVNLDDLLRTGGGGAGAGAAAAGRKTVDEVW
RDIQGATGNGFLRPAGAAAGQMTLEDFLSRAGADSGSGGGGGADGARWARAHHHHVGRPV
PRPLGLGAGPVLDALYHDGPVSGSKRAPAAGEGAAAEKTVERRKKRMIKNRESAARSRAR
KQAYTNELENKISRLEEENKRLRMHKVSKPVFVHHFQEP*
>11667.m05950|LOC_Os01g59850.1|genepair95-1
MGSRREEERNEKIIRGLMKLPPNRKCINCNSVGPQYVCTNFWTFICLSCSGIHREFTHRV
KSVSMAKFTTQEVRSLEQGGNQRARDIYLKDWDWQRMRLPDNSNPDRIREFIRAVYVDKK
YAGGKSTDKPVNDSESVKSSENLTRRPSSYHSYSQSPPYDFQYEDRRYGKQVDTLARRPS
DRAFFDGKLGSFLYSPGRLRDQMHEDRFANESTGSRFSDFSASSTGDIRNDVLSPSSQDT
GYSSPSIHHSRNVSSENPPSHRHPNATSQIDFNGVRRSQRTGSSGSFGSFDGSSASNKSV
DSGALPDAPTEKPVNSAVNRQSVAPPMAHSAQLYASQSNINSSVSQTAPTRESVQHGRVH
MVSVAKPPVSTQPTTSTNQDFFDQSMQHPVNSAAPIDLFAGFNQQAPSVPHRAVDVGSHS
VPKETLHDVVVQKAVASSPPVQAEAPSTSHPVHHDLLSLSPLQEPSISSTPPSIDLFAGF
DQQLPPTTSVQQSQPAEPSISSTPQSIDLFAGFDQQLPPTTSVQQSQQAAPLVADEGWAF
FLDTPQHVSPTSISNVQAQVATAIAAFPPSESLAKGTNQSMLPTSPPNALIPQSYPLMMD
QWSLNAEEVKTPVSKETSQPWNAFGASTGNTPNDSFTFNTISQVAPNHFNVPSVPHAEAR
GPQDLPSSEPERLTPGDITPGFNVSPGDMAGPSFRGPLQQQLDIVPSQPAKSTNPFDMAF
ESDVEASNMFMDLTSLQETLPNPHVNTDYSNLTESWIPHNSNMPYISSGPQGGLSYMATQ
VQDSLMLNSAQGSFPPRNPFE*
>11669.m02115|LOC_Os03g20720.1|genepair95-2
MAAAASRKEEERNERVVRGLLKLPPNRRCINCNGLGPQYVCTSFWTFVCISCSGIHREFT

HRVKSVSMSTFTTQEVEALQNGGNQRARESFLKEFDAQKMRLPDSSNVDSLREFIKAVYV
ERRYAGGRFSERPPRDKQNQKNNEQEHRRASSYHSFSQSPPYDYQYEERRNGKQSVMLTR
KPGSDRGHDGKMSGFAYSPQSLHERMSEDRFANENSGPRISDCSGSSISNTFRTTPHSPN
FLDKGCSSPSMQQNQSNIQASSGITQSEVRTISTGNIDSSSTKSSKSSLADMFFESDIAH
RTQQTKDCITPSFTAFSDVANIAQKDLLNEPVAQQQPVTGLDQPVDFFASMPPATPSTDR
MLTAAPSMDNAGWATFDTPPEEKQPGVIGLSGISVMDKHALSGDLFSFEPNNDQPTWLQS
SKTSKNNASVTDQSDVPCKYTSSDASNSQAWSAFEAKSVSTQQASPDLSLMSSIEPKEPI
DENKLQLWHSFDDASETMTLNLSNAQLQTNEHKNVDNNSLTTSNPFTCSITSKESRGNDS
QEIFMGGLAPSEPFAPFPEPSLFATTSVGEASVQQMPLNPFDLPFDADSDSPDMFMDVTA
LQAVLPNADLSTSFVDGLPETWFSNNASAYVPPGSHGGPPCLVEQIPNSALRNITLSAVS
TGNPFA*
>11667.m05952|LOC_Os01g59870.1|genepair96-1
MALTVRLHTAVAAVAVAVVVAMGMAAAQMSPAGAPAPAGGISPACMDAVLNMSDCLTYVM
NGSTARKPDEPCCPELAGLLESKPVCLCQLLAGGASSYDISVDYKRAMALPGICGLAAPP
VTACALLGVPVPMAPSASPMAGLGPSTEPQMPEKSPSASPSESSNHAPGRFTALAAVVLA
VAAAGMGRRTARGLDGSRDQADEALVISQQRALPSPHAPARSRLGFVRLFVGTRSFDADA
EAAASSALDSAGAPKRRPRLHHPVADAGRLTADGGNSTCSHAAEAAHRQDTHRTNYSLNL
NIQ*
>11669.m02119|LOC_Os03g20760.1|genepair96-2
MAMIMLPAASAAAAASVLALLVLTSLQPRAARAQVASSPWAAPAPWPGELDCTGALLNLS
SCLTYVEYRSTLTRPDKGCCGALAGVVDGEAACLCGLVGGYGAYGVRVDAVRALALPTIC
RVDAPPPRLCAALGVPVAEPPGGAVPEESGLSGGMPANAPSTAATGSSGGGGPATHRPTR
RHLILLLLLLVFPASLLLL*
>11667.m06170|LOC_Os01g61860.1|genepair97-1
MKAKSLPFIASEHKRDAYGFAVRPQHLQRYREYANIYKEEEEERSERWKNFLDSQAEYDE
SSGEDQDAKVSPSAEDEEAGKKAEDGRSKLSDEQKVKQQRPHKIQIWSEIRPSLGHIGEM
MSLRVKKKQSSADKENAANELQSANNEEIKPSEDSDDEFYDVEKVDPNQEGPVADSADAD
SGMNVDANQEGHYPWKEELECLVRDGLPMALRGELWQAFVGIGARRVKGYYESLLAADDE
RENSKGSDSPTMEGKPKGSPFSSEKWKGQIEKDLPRTFPGHPALDEDGRNALRRLLTAYA
RHNPSVGYCQRKYSSLYMILLNVFFKAMNFFAGLLLLLMPEENAFWALTGIMDDYFDGYF
SEEMIECQVDQLVLEELVREKFPKLVNHLDYLGVQVAWVTGPWFLSIFMNMLPWESVLRV
WDVLLFDGNRVMLFRTALALMELYGPALVTTKDAGDAVTLLQSLAGSTFDSSQLVLTACM
GYQAVDEARLQELRNKHRPSVISSMEQRAKGLRVWRDTNGLASKLYNFKRDPEPLVSLST
EQLSDLTETSSGSTDDMYSGLTVNTEIDSLPDPKDQVVWLKVELCQLLEERRSAVLRADE
LETALMEMVKQDNRRELSAKVEQLEQELSDLRQALLDKQEQEQAMLQVLMRVEQEQKVTE
DARIFAEQDAAAQKYAAHVLQEKYEEAMASLAQMENRAVMAETMLEATLQYQSSQQKAQL
PSPSPSPRTPTRDASPGQVNQDSSQEFQPRRISLLAPFSLGWRDKNKGKQNISDESTNGN
LNSNTEQMVDTPKKDDEKQGDSPQEGEQRVDTPRRDSEHRLDTPETTIKLEEQLEEIKLD
*
>11669.m01772|LOC_Os03g17540.1|genepair97-2
MMSSRVGKIRNTANDMCGNGEAQLASIEEEERAADKSCRGDPAEESSNPDKVEQAQDREQ
GDSASAALEGGNGESYFPWREELESLVRGGVPMALRGEMWQAFVGVGARKITGYYNKLLD
EGTEELDEKNPEDQELKDQTNAQKKPPEKWKGQIEKDLPRTFPGHPALDEDGRNALRRLL
TAYARHNPSVGYCQAMNFFAGLFLLFMPEEHAFWALVGVIDEYFDGYYTEEMIESQVDQL
VLEEVVRERFPKLAKHMDFLGVQVAWVTGPWFLSIFINMLPWESVLRVWDVILFEGNRTM
LFRTTLALLDLYGPALVTTKDAGDAITLLQSLAGSTFDSSQLVLTACMGFQAVREIGLQE
LRKKHRPDIISAMEERSKDRHSWKDKKGLATKLYSFKHDPLCPQVNSKEGEDDLQVNGEM
QFLDSGSANLETYLTSSALDNELEEGIDLQDQVTWLKVELCKLLEEKRSAELRSEELETA
LMEMVKQDNRHMLSAKVEKLEAEVSELRKSFADKQEQEQAMLQVLIRMEQEQKVAEDARI
AAERDAADKKYAAQLLQEKYDAAMAALRQMEKRAVMAETMLEATKQYQAGQFKANQSFNP
SSPRAAPQSGKPNQDPNQDAPNRRLGLLSRGLGWLEKSKGKSSSTETPEG*
>11667.m06183|LOC_Os01g61940.1|genepair98-1
MARAVHDRLPFLATPPPAPARGRNPPLAEMLRLVGAATVDSDAAAAAADEEANALSLPLP
RGGVTPPPPGGRTIQFRLAFTSLTYSVRAARRARPGGGDGGGGFRLPLQNRCDRVTAAAP
DAHSSRARVLLDGITGEAREGEILAVMGASGSGKSTLIDALANRISRDALKGSVTLNGEP
LTGNVIKSISAYVMQDDLLFPMLTVAETLSFAAEFRLPRALPAAKKRTRVLELIEQLGLR
AAADTIIGDEGHRGVSGGERRRVSIGTDIIHDPILLFLDEPTSGLDSTSAFMVVQVLRNI
AESGSIVITSIHQPSQRILGLLDRLILLSGGRTVFSGPPSAIPAYFAEFGYPVPDDENRA
EFALDLIREFESLPAGTGQLVSFNKTWQVMHAARHNPNDDPWAPTMSLKEAISASISRGK
LVSGSDVAGEAASMHTYANPFWVEMKVLTKRSAINTRRMPELFLIRLGAVVITGAILATV
FYKLDQSPKGAQERLGFFAFAMSTMFYTCADALPVFLHERYIFLRETAYGAYRRTSYVLS
NAIVSFPPLVVLSLAFAFTTFFAVGLAGGVSGFAFYTLAILASFWAGSGFVTFLSGVIPH
VMIGYTVVVAILAYFLLFSGFFINRDRIPDYWIWFHYLSLVKYPFEGVLQNEFGRGGECY
VRGTQMFDNSPLAVLPDTVKTRVLASIGTALGVKIGPNTCVMTGHNVLREAAVTQLGKWE
CLLVTAAWGFFFRLLFYFSLVLGSKNKRR*
>11669.m01752|LOC_Os03g17350.1|genepair98-2
MSRFVDKLPLFDRRPSPMEEAEGLPRSGYLGQLHHHQYYQPHSNMLPLEQSPPTSTKHTS
VTLAQLLKRVNDARSGSSTPISSPRYTIELGGSKPESVSSESDDHHSDDGGSEGQPRALV
LKFTDLTYSVKQRRKGSCLPFRRAAADEPELPAMRTLLDGISGEARDGEIMAVLGASGSG
KSTLIDALANRIAKESLHGSVTINGESIDSNLLKVISAYVRQEDLLYPMLTVEETLMFAA
EFRLPRSLPTREKKKRVKELIDQLGLKRAANTIIGDEGHRGVSGGERRRVSIGVDIIHNP
IMLFLDEPTSGLDSTSAFMVVTVLKAIAQSGSVVVMSIHQPSYRILGLLDRLLFLSRGKT
VYYGPPSELPPFFLDFGKPIPDNENPTEFALDLIKEMETETEGTKRLAEHNAAWQLKHHG
EGRGYGGKPGMSLKEAISASISRGKLVSGATDGTVSVAASDHSAPPPSSSSVSKFVNPFW

```
IEMGVLTRRAFINTKRTPEVFIIRLAAVLVTGFILATIFWRLDESPKGVQERLGFFAIAM
STMYYTCSDALPVFLSERYIFLRETAYNAYRRSSYVLSHTIVGFPSLVVLSFAFALTTFF
SVGLAGGVNGFFYFVAIVLASFWAGSGFATFLSGVVTHVMLGFPVVLSTLAYFLLFSGFF
INRDRIPRYWLWFHYISLVKYPYEAVMQNEFGDPTRCFVRGVQMFDNTPLAALPAAVKVR
VLQSMSASLGVNIGTGTCITTGPDFLKQQAITDFGKWECLWITVAWGFLFRILFYISLLL
GSRNKRR*
>11667.m06187|LOC_Os01g61980.1|genepair99-1
MARHGPQSHDQPLQEEDYIDMDLSSPAAAEAVTASARASLLCYSTAMAASPQNSREFEFH
MSAPVDKWEPAASPADELFYKGKLLPLHLPPRIQMVEKLLESAAEKGLLSASTAPATPYQ
SCNASPANSCYVSGELNAEHYFHECTSAGIGAAEEAAACEKKPWSKKLKFIRHLNLGLKL
KASKAYIKTIFATKAGNPDGKTGTPSANELSNAQFKSWRKNPFGQIRSNRYIASPISNSV
TLGGRLKEDECGHRRSFSSVIIRYSSSNKTSSASSSSCSSSNSSSSILSSTDSGVGPVLR
RSSSASSEMDNPIQGAIAYCKRSQQLASVRKSASDAGFRFMSSSASRIAAESEDPDDIIE
ICGR*
>11669.m01743|LOC_Os03g17260.1|genepair99-2
MARASAQCFGEEEYIDLDLSSCRGFEFRVCRSAAAPPPCGDEMLFRGSRLHKAKHQQEAD
AGGGGGGGGGCGGGRRSTATVAPWHAAAAVAGIRNAQPAARMQRQPDGGATGRRKKGAAG
SVHAKLQASRAFFRSLFARTSCSDEQCHGVGVRATTRSSRAATAASGAAGSVKPAPFGQI
RNSYGSCSGRGAAAAPTTLRSSIEQEKLMDEEEHAASVRQRKSFSGVIKWRPPPAPAAAA
ARPPPPPAFPTRRSSSAASDPPLKRCSSARSESEGLIQGAIAYCKRSQQQLVLARKSVSD
AALCSLQFQIQL*
>11667.m06188|LOC_Os01g61990.1|genepair100-1
MSLEIHEEEGGGTAAAAAVKVMTVSGSGKRGRYVRQVTGRHNDTDLHVAARGGDAGALRR
ALDEAAAAVATGEGREALEEVRRAVAAEPNEAGETPLVAAAERGHLEVVRELLRHLDAEG
VAAKNRSGYDALHVAAREGRHAVVQEMLLHNRLLAKTFGPANTSPLISAATRGHTEVVKL
LLELDDFGLVEMAKDNGKNSLHFAARQGHVEIVKALLEKDPQLARRNDKKGQTALHMAVK
GTNCDVLRALVDADPAIVMLPDKNGNTALHVATRKKRAEIVAVLLRLPDTHVNALTRDHK
TAYDIAEALPLCEESSEIKDILSQHGALRSRELNQPRDELRKTVTEIKKDVHTQLEQTRK
TNKNVHGIAKELRKLHREGINNATNSVTVVAVLFATVAFAAIFTVPGGNANNGVAVVVQA
ASFRIFFIFNAIALFTSLAVVVVQITVVRGETKSERKVVEVINKLMWLASVCTTISFIAS
CYIVLGRHFQWAALLVSLIGGITMAGVLGTMTYYVVKSKRMRKIRKKEKMSRRSGSSSWY
DNTELSETELNQVYAL*
>11669.m01741|LOC_Os03g17240.1|genepair100-2
MSAYSDKGEGDLEIGLASPEAEGGGPGGPALDLSPPRAVRRPGLVMSHSGKRLDQSPAAS
PSPSRPVLVMSHSSNRLDQSPARPVLVMSRSSNRLDQSPASSPASSRGPVLVMSGSGNRL
DSSGPSPSPSPTAAAAAAPVLVLSNSGKRMDQAGRKKYVKQVTGRHNDTELHLAAQRGDL
EAVRQIIAEINAQMTGTGEEFDSEVAEIRAAVVNEPNEVEETALLIAAEKGFLDIVVELL
KHSDKESLTRKNKSGFDVLHVAAKEGHRDIVKVLLDHDPSLGKTFGQSNVTPLITAAIRG
HIEVVNLLLERVSGLVELSKGNGKNALHFAGRQGHVEIVKALLDADPQLARRTDKKGQTA
LHMAVKGTSAAVVRALVNADPAIVMLPDRNGNLALHVATRKKRSEIVNELLLLPDMNVNA
LTRDRKTAFDIAEGLPLSEESAEIKDCLSRAGAVRANDLNQPRDELRKTVTEIKKDVHTQ
LEQARKTNKNVSGIAKELRKLHREGINNATNSVTVVAVLFATVAFAAIFTVPGGNDNNGV
AIAVHAVSFKIFFIFNAIALFTSLAVVVVQITLVRGETKAERRVVEIINKLMWLASVCTT
VAFISSAYIVVGKHFQWAALLVTLIGGVIMAGVLGTMTYYVVRSKRTRSIRKKVKSTRRS
GSNSWQQNSEFSDSEIDRIYAI*
>11667.m06191|LOC_Os01g62020.1|genepair101-1
MKQLHKSSPTHAPAAAHAPASKASKASRPGPRSWVGYLLREQRLLFVLLGALIASSFFLL
RPYLFSLSPSSHVPDRRPLFSFASHTSSASGVPPGFRPPPRRVVVTGGAGFVGSHLVDRL
LEQGDSVIVVDNFFTGRKDNVAHHLRNPSGCSVEILGRRDRFVSCDAQVTNVMGTLNMLG
LAKRIGARFLLTSTSEVYGDPLEHPQKETYWGHVNPIGVRSCYDEGKRTAETLTMDYHRG
GGVEVRIARIFNTYGPRMCLDDGRVVSNFVAQALRRQPMTVYGDGKQTRSFQYVSDLVSN
KQYSYCAFCVCVYSLVTDVVHSFQVCSPGSILLQITCIL*
>11669.m01740|LOC_Os03g17230.1|genepair101-2
MKQLNRQPTLSKQHRPHHHRLPLPRSLASYLLREHRLLFVLLGFLLASSCFLIYPSFTPL
SSSSSPRDTVAARIRRGGGGGGGASSVVVSAAAAASRRLPVGVRKPPLRVVVTGGAGFVG
SHLVDELLARGDSVIVVDNFFTGRKENVARHLADPRFELIRHDVVEPILLEYKTNVMGTL
NMLGLAKRVGARFLLTSTSEVYGDPLEHPQKESYWGHVNPIGVRSCYDEGKRTAETLTMD
YHRGAGVEVRIARIFNTYGPRMCLDDGRVVSNFVAQTLRKQPMTVYGDGKQTRSFQYVSD
LVDGLITLMESEHIGPFNLGNPGEFTMLELAQVVKETIDPSARVEFKPNTADDPHMRKPD
ISKAKSLLHWEPKISLKQGLPRMVSDFQKRIMDEKR*
>11667.m06192|LOC_Os01g62030.1|genepair102-1
MSPLDYLNLNFDSLNKHTSQKNHTSKLPSLVYESKLRAEMQAQTRRRILAQSCVALALLL
TTVHTAAGRRPASNKPTLPSHGSDQTMTLYTTVATPAEAAGVPSSQHPVFAGHGPIGHHS
GGWLRVLTRPGALQPGAAAVVDERFHGKKEFGMPLAGKLQGVLVTGLEDDDDSRIVAVTA
LFSGDGEEDSIRFFGVHRDDQEESHIAVVGGTGRYDGATGFAVVRAADAHKAGRNVSSNS
VLSFRVHLK*
>11669.m01739|LOC_Os03g17220.1|genepair102-2
MVDKAAVWLVVIALAAAANGAFAGRVLEEQPAAAPAPAEAPVAPVDPLPAPTDPPADTVV
APAAVPAGGAAATGNAGVGAAGGGAGAGDHHQLTFFMHDILGGSQPSARIVTGVVASAAA
NGQLPFARPNDNIFPIQGAMPLPQGASNLVNGNNVPYVAGLGGTSSAAIVQGNGNGNGGN
KNIPFVNAGDLPSGATLQNLLFGTTTVIDDELTEGHELGAAVVGRAQGFYVASSQDGTSK
TLVLTAMFDGGGVEAHGDTLSFFGVHRMAAPESHVAVIGGTGKYENAKGFAVIQTLHPGD
EHTTDGVETLLQFSIHLI*
>11667.m06197|LOC_Os01g62060.1|genepair103-1
```

MDDGGGGGGGGDSSPASYIRLVQHLIEKCICYNMNKEECMETLEKHANIKPVITSTVWKEL
EKENSEFFATYKKGQGEEPAESKSSSSSQEAAGSKRSGGDDD*
>11669.m01737|LOC_Os03g17200.1|genepair103-2
MALGDDSPASYIHMVQHLIEKCMTFGMSMEECMETLSKRADVQPVVTSTVWKELEKENKE
FFDKYKQLRSEKGGVSSS*
>11667.m06206|LOC_Os01g62130.1|genepair104-1
MSKRSRSMWDMQEFVGSVDTARVLMLLAQQSQHGLLGGGGFAAGAQPVVVRGGAHDRVFE
CKTCNRQFPTFQALGGHRASHKRPRQQQQHALGGGAGADDAGLCLGRQPTPPRPQPAKPR
VHECPVCGLEFPIGQALGGHMRRHRAEAEAAATTTTTTTKNGDVGKAAAVKACDGGGVCL
DLNLTPSENRAKCRNVVGLGAGGQGVHKALAMLDCFL*
>11669.m01732|LOC_Os03g17150.1|genepair104-2
MAYGKRPRQQAEEAAFSLFDSSDMARIMLLFSGAHGGGGGAAAASPPERMFECKTCNRQF
PSFQALGGHRASHKKPRLADGDPAAEAPAKPKVHGCSICGLEFAVGQALGGHMRRHRAVM
ADGLGLGLSLGLGIGVVGQSDDDGGKKKAAAAAAAAELVFDLNAPAIEEEPDRARPAGLA
VEFPVVVDFPC*
>11667.m06216|LOC_Os01g62230.1|genepair105-1
MAPKAEKKPAEKKPVEEKAEKKPKAEKRVPGAKEGGGEKKGKKKAKKSVETYKIYIFKVL
KQVHPDIGISSKAMSIMNSFINDIFEKLAQEAARLARYNKKPTITSREIQTSVRLVLPGE
LAKHAVSEGTKAVTKFTSS*
>11669.m01722|LOC_Os03g17080.1|genepair105-2
MAPKAEKKPAEKKPAAGEEKSAEKAPAGKKPKAEKRLPASKASSKEGGAGDKKGRKKAKK
SVETYKIYIFKVLKQVHPDIGISSKAMSIMNSFINDIFEKLAQEAARLARYNKKPTITSR
EIQTSVRLVLPGELAKHAVSEGTKAVTKFTSN*
>11667.m06222|LOC_Os01g62290.1|genepair106-1
MAKGEGPAIGIDLGTTYSCVGVWQHDRVEIIANDQGNRTTPSYVAFTDSERLIGDAAKNQ
VAMNPINTVFDAKRLIGRRFSDPSVQSDMKLWPFKVIPGPGDKPMIVVQYKGEEKQFSAE
EISSMVLIKMREIAEAYLGSNIKNAVVTVPAYFNDSQRQATKDAGVIAGLNVMRIINEPT
AAAIAYGLDKKATSSGEKNVLIFDLGGGTFDVSLLTIEEGIFEVKATAGDTHLGGEDFDN
RMVNHFVLEFKRKNKKDISGNPRALRRLRTACERAKRTLSSTAQTTIEIDSLYEGIDFYS
TITRARFEELNMDLFRKCMEPVEKCLRDAKMDKSSVHDVVLVGGSTRIPKVQQLLQDFFN
GKELCKSINPDEAVAYGAAVQAAILSGEGNEKVQDLLLLDVTPLSLGLETAGGVMTVLIP
RNTTIPTKKEQVFSTYSDNQPGVLIQVYEGERARTKDNNLLGKFELSGIPPAPRGVPQIT
VCFDIDANGILNVSAEDKTTGQKNKITITNDKGRLSKEEIEKMVQEAEKYKAEDEEHKKK
VDAKNALENYAYNMRNTIKDDKIASKLSADDKKKIEDAIDGAINWLDSNQLAEADEFEDK
MKELESICNPIIAKMYQGAGADMGGAAGMDEDAPAGGSGAGPKIEEVD*
>11669.m01695|LOC_Os03g16860.1|genepair106-2
MAGKGDGPAIGIDLGTTYSCVGVWQHDRVEIIANDQGNRTTPSYVAFTDSERLIGDAAKN
QVAMNPINTVFDAKRLIGRRFSDTSVQSDAKLWPFKVLPGPGDKPMIGVQYKGEEKQFSA
EEISSMVLNKMKETAEAYLGSTVKNAVVTVPAYFNDSQRQATKDAGVISGLNVMRIINEP
TAAAIAYGLDKKSSSVGEKNVLIFDLGGGTFDVSLLTIEEGIFEVKATAGDTHLGGEDFD
NRMVNHFVQEFKRKNKKDITGNPRALRRLRTACERAKRTLSSTAQTTIEIDSLYEGIDFY
TTITRARFEELNMDLFRKCMEPVEKCLRDAKMDKSTVHDVVLVGGSTRIPRVQQLLQDFF
NGKELCKSINPDEAVAYGAAVQAAILTGEGNEKVQDLLLLDVTPLSQGLETAGGVMTVLI
PRNTTIPTKKEQVFSTYSDNQPGVLIQVYEGERTRTKDNNLLGKFELSGIPPAPRGVPQI
TVCFDIDANGILNVSAEDKTTGQKNKITITNDKGRLSKEDIEKMVQEAEKYKAEDEEHKK
KVDAKNSLENYAYNMRNTIKDDKIASKLPEADKKKIEDAIDGAISWLDSNQLAEAEEFED
KMKELEGVCNPIIAKMYQGAGADMAGGMDEDAPAAAGGSSGPGPKIEEVD*
>11667.m06248|LOC_Os01g62480.1|genepair107-1
MTMAISSALPSPLLLAASLLLLIVQAQGITRHYEFNVQMANATRLCNTKSMVTVNGQCPG
PELVAREGDRVVIRVTNNVAHNISLHWHGVRQVRTGWADGPAYITQCPIQTGQSYVYNFT
VAGQRGTLWWHAHISWLRATVYGALVILPKLGVPYPFPAPHKEVPVIFGEWWNADTEEVV
NQAVQTGGGPNVSDAFTINGLPGPLYNCSAQDTFKLKVKPGKTYMLRLINAALNEELFFA
VANHTLTVVEVDAVYVKPFTVDTLVISPGQTTNVLLTAKPYYPGANFYMSAAPYSTARPG
TFGNTTVAGILEYENPAMSPSAASFVKGLPLFKPTLPQLNDTDFVTNFTDKLRSLATPEY
PAAVPQSVDKRFFFTVGLGTLPCPANMTCQGPNNTQMAASMNNVSFVLPARALLQSHFTG
LSSGVYAPDFPVAPLSPFNYTGTPPNNTNVKTGTKLLVLRYNTSVELVMQDTSILGIESH
PLHLHGFNFFVIGQGFGNYDAVNDPAKFNLVDPVERNTVGVPAGGWVAIRFLADNPGVWF
MHCHLEAHTTWGLRMAWLVLDGSHPNQKLLPPPSDLPKC*
>11669.m01668|LOC_Os03g16610.1|genepair107-2
MGARCLALLLLYGTLLLLLLLPQLPLAGAATRYYTFNVKLQNVTRLCNTRAIPTVNGKFP
GPKIVTREGDRVVVKVVNNIKDNITIHWHGVRQMRTGWSDGPAYVTQCPIQTGQSYVYNF
TINGQRGTLFWHAHVSWLRSTLYGPIIILPKAGLPLPFTEPHKDVPIIFGEWFNADPEAI
VAQALQTGGGPNVSDAYTINGLPGPLYNCSSKDTFRLKVQPGKMYLLRLINAALNDELFF
SVANHTLTVVDVDASYVKPFDTDVVLITPGQTTNVLLRAKPTAEAAGATHLMMARPYATG
RPGTYDNTTVAAVLEYAPPGHIKSLPLLRPSLPALNDTAFAAGFAAKLRSLACPDYPSNV
PRRVDKPFFFAVGLGTTPCPGSNNQTCQGPTNTTKFTASINNVSFDMPTTALLQAHYTGQ
SAGVYTADFPASPLEPFNYTGTPPNNTNVSNGTRVVVLPYNASVEVVLQDTSILGAESHP
LHLHGFDFFVVGQGTGNYDPSKHPAEFNLVDPVQRNTVGVPAGGWVAIRFFADNPGVWFM
HCHLEVHTTWGLKMAWVVNDGPLPEQKLMPPPSDLPMC*
>11667.m06264|LOC_Os01g62630.1|genepair108-1
MPPPPALPVCLLLLLLLLLAVPRPAAAAAAAAATRPLLFELRARQVPAGALPRPASKLRF
HHNVSLTVSLAVGTPPQNVTMVLDTGSELSWLLCAPGGGGGGGGRSALSFRPRASLTFAS
VPCDSAQCRSRDLPSPPACDGASKQCRVSLSYADGSSSDGALATEVFTVGQGPPLRAAFG
CMATAFDTSPDGVATAGLLGMNRGALSFVSQASTRRFSYCISDRDDAGVLLLGHSDLPFL

PLNYTPLYQPAMPLPYFDRVAYSVQLLGIRVGGKPLPIPASVLAPDHTGAGQTMVDSGTQ
FTFLLGDAYSALKAEFSRQTKPWLPALNDPNFAFQEAFDTCFRVPQGRAPPARLPAVTLL
FNGAQMTVAGDRLLYKVPGERRGGDGVWCLTFGNADMVPITAYVIGHHHQMNVWVEYDLE
RGRVGLAPIRCDVASERLGLML*
>11669.m01655|LOC_Os03g16500.1|genepair108-2
MEASLVLLCLCVFLASGGEGRSPAGTVLPLQVRVQEVELEAPAANRLRFRHNVSLTVPVA
VGTPPQNVTMVLDTGSELSWLLCNGSYAPPLTPAFNASGSSSYGAVPCPSTACEWRGRDL
PVPPFCDTPPSNACRVSLSYADASSADGVLATDTFLLTGGAPPVAVGAYFGCITSYSSTT
ATNSNGTGTDVSEAATGLLGMNRGTLSFVTQTGTRRFAYCIAPGEGPGVLLLGDDGGVAP
PLNYTPLIEISQPLPYFDRVAYSVQLEGIRVGCALLPIPKSVLTPDHTGAGQTMVDSGTQ
FTFLLADAYAALKAEFTSQARLLLAPLGEPGFVFQGAFDACFRGPEARVAAASGLLPEVG
LVLRGAEVAVSGEKLLYMVPGERRGEGGAEAVWCLTFGNSDMAGMSAYVIGHHHQQNVWV
EYDLQNGRVGFAPARCDLATQRLGAGA*
>11667.m06265|LOC_Os01g62640.1|genepair109-1
MATFSTAGGIGMDASPIWCFMCSRLHRPDGLSTCPTRAPRAALEEIVEVMDAGEFLQACA
LRRAPVAAAVSSTRQQLPTVTVRDAGRTCAVCLDDLEPGGSAVVTPCDHAYHPQCIAPWL
EAHDTCPLCRRESGLQVVEVEVQVDGMVLSSPDGLVLCELMMPGGRSEYRLGRRVAGRIF
AVRVVDGTGKLVRGGVLRRLGSACHRFAAAAGNLLSLRYRDCVIPNNDLLLGVQC*
>11669.m01653|LOC_Os03g16480.1|genepair109-2
MASSPVSYWCYHCSRFVRVSPSTVVCPECDGGFLEQFPQPPPRGGGGSGRRGAMNPVIVL
RGGSLSGFELYYDDGSGDGLRPLPGDVSHLLMGSGFHRLLDQFSRLEAAAPRPPASKAAV
ESMPSVTVAGSGAHCAVCQEAFEPGASAREMPCKHVYHQDCILPWLSLRNSCPVCRRELP
AAAAPESEADAGLTIWRLPRGGFAVGRFAGGPREQLPVVYTELDGGFSNGVGPRRVTWPE
GDGHVDGGEGRIRRVFRNLFGCFGRSSRPESSSSQSRSG*
>11667.m06269|LOC_Os01g62670.1|genepair110-1
MVVQKMWMPVDLRLPSGPQAALGILAFEAAAAMSKLLSLHRSLSEQEVSRLRSDTMRSPG
VAYLNSTDQAFLLRLACAELVVSLDAAAAAVARLGLRCGIDFGGVYASLKAGAPDARLDP
LVAKGLRVKAKKMERLVASTARLCSEMEALDELEASERKLAFRGWNRLSGPIPMQPVAPS
AAGDSPGADSLQQDLKAQRIKVRRLKEESLWNQSYEKAVGLMARAACAVFSRICTIFGAF
VPGLPPPLPSAATDSVQTRLSKLLNPRAVRAKASSGPITRRDGPSRVHPPVISSSCPIIG
LRPSGQKAAIDWRKLLDAPASTVGGAGLDQQYANVIVSAEQLLQMEAEGRQEEANAERAE
MYEMLPAKLRAAVRSKLRDWWRDPGPLDAGLAQGWKDAVDRIMAWLGPMARDTVQWQAER
NMDRTRRFDGGARVYALQTLRWADKEKAEAALVEVLVALSCVCCQWSEADMPGPRVGLTL
VTESESESEIPDTKTESNIIETENGAKTNRREYGNENLSE*
>11669.m01642|LOC_Os03g16390.1|genepair110-2
MPAGARSWLADLRARFGGGAREEAGLGILAFEAAAAMSRLVSLHRSLSDVEVRRLRADAL
RAEGVARVTSTDQSLLLRLACGEFVADLDHAAGTAARLGARCCAGAPFLHDFDRVYAEAK
RGNGLARLDATVGFYRGAAKRFRKMERHVAATAKLYAEMDALSELEASERRMEQWMRHSG
PIPAQPGPSAKRQVPEPGEKLIRELNSQRQKVRRLMESSLWSVAAHKVSKLMAKSVLAVL
ARISITFGAYVPGLPLLTVGRAWALRRTSGPLQQAASPAAAIRHSAPIFRQKDTAFSASE
SIKPPASTVGGSGMELRYANMIVCAEMLLRQLWPTIHSNEVDAGMDLSKRDELYKMLPVT
IRTAVKAKLRESWRGQPVDEAAAAASMDAVDRMLRWLGPMAHDTVRWHDEHSMERAQRFS
MRPRVLMVQTLHFADRHKAENVIVEVLIGLSCVCWYDDERRRPADWDDDD*
>11667.m06279|LOC_Os01g62760.2|genepair111-1
MAEICCEEAMSPPATATAAVAAAVSASAAAAVSSAIDRRRRRMEMRRIRIASDLELQAGE
DGRPGKRQRLARTASGAPRPDEDSASERPSCGRTEEFPRYGVTAVCGRRREMEDAVSIRP
DFLPASGKFHFYGVFDGHGCSHVATTCQDRMHEIVAEEHNKGASGEVAPWRDVMEKSFAR
MDGEVGNRASTRSDDEPACPCEQQTPSRRDHAGSTAVVAVVSPTQVVVANAGDSRAVISR
AGVPVALSVDHKPDRPDELERIEAAGGRVIYWDGARVLGVLAMSRAIGDGYLKPYVTSEP
EVTVTERTDDDECLILASDGLWDVVTNEMACEVVRACFHNNGPPAPAARPSGVPSSAEAA
ETENGGAASVKGISKAESSDKACSDAAMLLTKLALARRSADNVSVVVVDLRRGL*
>11669.m01619|LOC_Os03g16170.1|genepair111-2
MAEICCEVVAGSSSEGKGPECDTGSRAARRRRMEIRRLRVVAERGAEEETSGKRRRLDGG
GGEASTDEEDREVERARYGFTSVCGRRRDMEDSVSACPGFLPGHHFFGVFDGHGCSHVAT
SCGQRMHEIVVDEAGAAAGSAGLDEEARWRGVMERSFARMDAEAVASSRGSVAPAPTCRC
EMQLPKCDHVGSTAVVAVLGPRHVVVANCGDSRAVLCRGGAAIPLSCDHKPDRPDELERI
HAAGGRVIFWDGARVFGMLAMSRAIGDSYLKPYVICDPEVRVMERKDGEDEFLILASDGL
WDVVSNEVACNVVRACLRSSGRRERNRSSPTSNLSPRQSSSSGDEAPNDGAPSAAAGSES
DEESAAEEDKACAEAAVLLTKLALARQTSDNVSVVVVNLRRRKL*
>11667.m06287|LOC_Os01g62840.1|genepair112-1
MKALILVGGFGTRLRPLTLSFPKPLVDFANKPMILHQIEALKEVGVTEVVLAINYRPEVM
LNFLKDFEDKLGITITCSQETEPLGTAGPLALARDKLVDGSGEPFFVLNSDVISEYPFAE
LIKFHKSHGGEATIMVTKVDEPSKYGVVVMEEVTGMVEKFVEKPKIFVGNKINAGIYLLN
PSVLDRIELKPTSIEKEVFPRIASDAKLFALVLPGFWMDVGQPRDYITGLRLYLDSLRKR
STNRLATGAHIVGNVLVHESAKIGEGCLIGPDVAIGPGCVVEDGVRLSRCTVMRGVHIKK
HACISNSIIGWHSTVGQWARIENMTILGEDVHVGDEVYTNGGVVLPHKEIKSSILKPEIV
M*
>11669.m01617|LOC_Os03g16150.1|genepair112-2
MKALILVGGFGTRLRPLTLSVPKPLVDFGNKPMILHQIEALKEVGVTEVVLAINYQPEVM
LNFLKDFESKLGIKITCSQETEPLGTAGPLALARDKLADGSGDPFFVLNSDVISEYPFAE
LIQFHKSHGGEATIMVTKVDEPSKYGVVVMEDETDKVERFVEKPKVFVGNKINAGIYLLN
PSVLDRIELKPTSIEKEVFPRIAADNGLFAMVLPGFWMDIGQPRDYITGLRLYLDSLRKK
APAKLASGAHVLGNVLVHETAVIGEGCLIGPDVAVGPGCVVEAGVRLSRCTVMRGARVKK
HACISSSIIGWHSTVGMWARVENMTILGEDVHVCDEVYSNGGVVLPHKEIKSSILKPEIV

M*
>11667.m06453|LOC_Os01g64410.1|genepair113-1
MVIGRLALSSFFHNKARDTSPSPPPAPATAPPWVWPSCKNPRTQSFRAATAPPPPPGSRT
IASIFLDSAESSFTTSSARHDCSDSLSTASEASAGAEAADTADDAIVRGLRSSDRLLFDP
GASATSSILEEKSSDAAGEASFIGGVAVAFESEDPYVDFRVSMEEMVVAHGVGNWGWLEE
MLGWYLRANGKDTHAAILAAFIDVIVAIADPALASCSSHRRSSTCTITEESSLEVAEKQA
KLAV*
>11670.m05827|LOC_Os04g58820.1|genepair113-2
MVRKLVASLFLGGGGGGGGGNDACSLSSSSSTASSWQWPSCTQARTLSFARHDAPASADD
DDSKRRRQQEEEEDDYCVYKTSVMNPAFFLDDHSTCRSYSSAASAVNDVVDDDDEVIIRG
LRSSNRRLFFEPESTSSIVVKGRAVDADAAAFDGATAMSIDSADPYGDFRRSMEEMVMSH
MSGGGHDWGWLEEMLGWYLRANGKKTHGFIVGAFVDLVVALASSPSSATASSSAFQLPLQ
KGSQIN*
>11667.m06459|LOC_Os01g64470.1|genepair114-1
MAAYGKPQPPLNDAYYGPPIPPPPAAAYYGAAAPPPAPRRSGAHRLFCCLFRVLAVAVIA
LGTAVLVLWLIYRPSVVKAYADTAALSRFDLTNGGSLLVYNLTVGMRVRNPNRFGINFRS
VDAQASYDGDRFGYAPLQPLYVGRKSDARFDVTLSGSAAIDDRDVERTYRRETAQGSYEV
KVRVYARQGFKVRGFRLNNKSKFTCTLNLPAPNSGNGTASGTPTTVFTRKQPKCDVDY*
>11670.m05830|LOC_Os04g58850.1|genepair114-2
MGSASRAVSCLCCPCKCLACGLFSCLCSILISLLVTLGVLALIFYLIFRPHMIAATVDSA
ALTQFTLSTNSALAYSLTVDLTVRNPNKRVGLYYDNVESLALFDGQRFGYAPLDSFYQST
EASTKLSPAFKGQQPLQGDITAANFRSQQTAGKFDIEVKLNAKLRVKVWAFKVPGPKAKI
SCPITVPASAPNAPAFQRTDCKVWF*
>11667.m06469|LOC_Os01g64570.1|genepair115-1
MASAAAEVPSYFVCPISLQLMRDPVTLPTGISYDRAAIARWLAAPGARRTCPVTRQPLEH
GLELTPNHTLRRLIQSWAASVSPGSAVDEEVAALRPVSSDEVASLLSDAAAAQVGALRRL
RELAAECEDSRAMLESQGGVFDVLSRVVTSGSACSTAREEAVGVLASLRIPEQELIGVST
RHGNLAESLTAVLRSSNLQSRAHAVQLVRTLADAVVPAWVIGLNAELLAEVVGVVRDRVS
ARATKASLHALAALCPYGRHRVKIVGAGAVAALVELLLDEPERRVCELALAVLDRLCTCA
EGRAELVAHAAGVAVVGKKVLRVSEAASERAVRVLRSVARHAATPAVLQEMAQCGVVGKL
CLALRSEQCGVKTKEKAHEVLKLHSRVWRASPCLSPSFLALYPS*
>11670.m05838|LOC_Os04g58920.1|genepair115-2
MEEQQQVEVEVEVPSYFVCPISLQIMRDPVTLPTGITYDRDGIERWLLTAGTCPLTKQPV
PPDCDPTPNHTLRRLIQSWCALHADHGVDLVPTPKPPADRARVADLVSRLRAATSSAALL
DALRELRDVAAESERNRKLLAAVPGAVDVLAAVVVASCRDAKAACDEALEIVCSLELSER
CLARLVERNEELVDALVATLQRTNTTSRAHAALLLEAVTAVMPSNRLVSLPEEVFGEAVQ
LLRDRVSSPATRAALHVLVGTTSWGRNRVKAVDAGAVAVLVDMLLDGPVERRGCELALAA
LDRMCGCAEGRAALVSHGAGVAVVGRKVLRVSEVASEKAVRVLRSVARHAATAAVVQEMG
QTGAVEKLCVVAQSEQCGERTRERARETLRLHARAWRNSPCLQPHLQALYPSC*
>11667.m06483|LOC_Os01g64670.1|genepair116-1
MSEADGGEGAKPKRPAPRLNERILSSLSRRSVAAHPWHDLDTGADAPAVFNVVVEISKGS
KVKYELDKKTGFIMVDRVLYSSVVYPHNYGFIPRTLCEDNDPMDVLVLMQEPVIPGCFLR
ARAIGLMPMIDQGEKDDKIIAVCVDDPEYRHYNDLSELSPHRVQEIRRFFEDYKKNENKE
VAVNEVLPVTAARDAIQYSMDLYAQYIEHLGQ*
>11670.m05850|LOC_Os04g59040.1|genepair116-2
MSEEDTNAAAGQPRRAPKLNERILSSLSRRSVAAHPWHDLEIGPGAPAVFNVVVEITKGS
KVKYELDKKTGLIKVDRVLYSSVVYPHNYGFIPRTLCEDNDPMDVLVLMQEPVIPGSFLR
ARAIGLMPMIDQGEKDDKIIAVCADDPEYRHYNDISELSPHRLQEIKRFFEDYKKNENKE
VAVDAFLPANTARDAIQYSMDLYAQYILQSLRQ*
>11667.m06487|LOC_Os01g64700.1|genepair117-1
MSFRSIVRDVRDGFGSLSRRSFEVTLASLYGLTGHHKGKTQSSLDELDDSPAIIPESRWA
SLPPELLREVIRRLEADESTWPSRRNVVCFAAVCRTWREMCKETVLSPEFCGKLTFPVSI
KQPGPRDGMIQCYIKRNRSKSTYHLYLCLSNVVTAEGGKFVLAAKRHRKTTCTEYTISMV
SGNISRSSRTNIGKLRSNFLGTKFIIYDTQPPYNGAVVPHVGRTSKRFNSTKVSPKVPSV
TYNIAQVSYELNVLGTRGPRRMRCMMHSIPASSVEPGGIVPGQPEQIVPRALEDSFRSTT
SFSQSFRSTTSFSKSIMDPSMDFSSARFSDISGSIMGGDDNGEIKERPLVLRNKPPRWHE
QLQCWCLNFRGRVTIASVKNFQLVAAPSPPPAGAPTPSQPGPADPEKVILQFGKVARDMF
TMDYRYPLSAFQAFAICLSSFDTKLACE*
>11670.m05861|LOC_Os04g59130.1|genepair117-2
MSFRSIVRDFRDSFGTLSKRSFEVKISGFSGRHRGKSIGPSSELDDTPVVAQQSKWAGLP
PELLRDVMKRLEEDDSNWPSRKDVVACASVCTTWRDMCKDIVRNPEFCGKLTFPVSLKQP
GPRDGVIQCFIKRDKSKLTYHLYLCLSSAVLDETGKFLLSAKRSRRTTHTDYIISMDSKN
ISRSSSGYIGKLRSNFLGTKFIIYDTQPPYNARTLCSQERTSRRFSSRKVSPKVPTGCYP
IVQVNYELNVLGTRGPRRMQCAMHSIPASAVEPGGIVPGQPKELLPRLFEESFRSMATSF
SKYSITDHSTDFSSSRFSEFGGGALQGQEQEQDGDDVNKERPLVLRNKAPRWHEQLQCWC
LNFRGRVTVASVKNFQLIAAAPQPSSGAASEPSQAGQAAQQQTQPSQPSSSSSSSSSNHD
TVILQFGKVAKDMFTMDYRYPLSAFQAFAICLTSFDTKLACE*
>11667.m06522|LOC_Os01g64960.1|genepair118-1
MAQSMLVSGANGTVAAASTSRLQPVRPTPFSRLVLSQPSSSLGRAVSVKTVALFGRSKTK
AAPARKAEPKPKFKTEDGIFGTSGGIGFTKENELFVGRVAMLGFAASILGEAITGKGILA
QLNLETGIPIYEAEPLLLFFILFTLLGAIGALGDRGSFVDDQPVTGLDKAVIAPGKGFRS
ALGLSEGGPLFGFTKANELFVGRLAQLGIAFSIIGEIITGKGALAQLNIETGVPINEIEP
LVLFNVVFFFIAAINPGTGKFVSDDDEE*
>11670.m05893|LOC_Os04g59440.1|genepair118-2

MALQQSMAMPMMVVSDLGTAPRSSPMVQLQRMKKHLVVVAAFKSRTKASPKVDKSNKNKS
IVEDGIFGTSGGIGFTKENELFVGRVAMLGFAASLLGEAVTGKGILAQLNLETGIPIYEA
EPLLLFFILFTLLGAIGALGDRGRFVDDATGLERAVIPPGKGFRAALGLSEGGPLFGFTK
ANELFVGRLAQLGIAFSLIGEIITGKGALAQLNIETGVPINEIEPLLLFNILFFFFAAIN
PGTGKFVTDDNDDQ*
>11667.m06523|LOC_Os01g64970.1|genepair119-1
MEKYEAVRDIGSGNFGVARLMRNRETRELVAVKCIERGHRIDENVYREIINHRSLRHPNI
IRFKEVILTPTHLMIVMEFAAGGELFDRICDRGRFSEDEARYFFQQLICGVSYCHHMQIC
HRDLKLENVLLDGSPAPRLKICDFGYSKSSVLHSRPKSAVGTPAYIAPEVLSRREYDGKL
ADVWSCGVTLYVMLVGAYPFEDQDDPKNIRKTIQRIMSVQYKIPDYVHISAECKQLIARI
FVNNPLRRITMKEIKSHPWFLKNLPRELTETAQAMYYRRDNSVPSFSDQTSEEIMKIVQE
ARTMPKSSRTGYWSDAGSDEEEKEEEERPEENEEEEEDEYDKRVKEVHASGELRMSSLRI
*
>11670.m05894|LOC_Os04g59450.1|genepair119-2
MEKYEPVREIGAGNFGVAKLMRNKETRELVAMKFIERGNRIDENVFREIVNHRSLRHPNI
IRFKEVVVTGRHLAIVMEYAAGGELFERICEAGRFHEDEARYFFQQLVCGVSYCHAMQIC
HRDLKLENTLLDGSPAPRLKICDFGYSKSSLLHSRPKSTVGTPAYIAPEVLSRREYDGKL
ADVWSCGVTLYVMLVGAYPFEDPKDPKNFRKTISRIMSVQYKIPEYVHVSQPCRHLLSRI
FVANPYKRISMGEIKSHPWFLKNLPRELKEEAQAVYYNRRGADHAASSASSAAAAAAFSP
QSVEDIMRIVQEAQTVPKPDKPVSGYGWGTDDDDDDQQPAEEEDEEDDYDRTVREVHASV
DLDMSNLQIS*
>11667.m06540|LOC_Os01g65100.1|genepair120-1
MASHAVTDALLLPRSEGAVAGAVDFRGRPASRASTGRWSAAMFVLGVEIAERFAYHGVSA
NLISYLTGPLGESTAGAAAAINLWSGVATMLPLLVACVADAWLGRYRTIVLASLLFVVSM
GMLTLSSALPAFHGDGGGCSYTSKSLSCAPSTAQVAIFYVSLYLVALAEAGHKPCAQAFG
ADQFDQNDAKESVSRSSFFNWWYFGMCSGTAMTTMVSSYIQDNIGWGLGFGIPCLVMAFA
LAMFLLGTRNYRYYVSTQSSPFARLARAFVALIRGSKDDALAVVDDDDGGDHREELRGVL
RLFPIWATCIIYAVIFSQSSTFFTKQAATLDRRIGESFRVPPAALQTFISVTIIAFIPVY
DRAFVPVARRFTRASSGITMLQRIGTGLVLALAAMVVAALVEARRLGVARDAGMVDDPKA
ALPMSLWWMVPQYVLFGLSDVFAMIGLQEFFYDQVPDALRSLGLAFFLSIFGVGHFFSSF
IISAIDGATKKSGASWFANNLNRAHLDYFYWLLAGLCAVELVAFVFVSRVYVYKKRVPHN
GCDVM*
>11670.m05897|LOC_Os04g59480.1|genepair120-2
MDGRPLLPNEEEEEALPGVSDFRGRPVYRATSGGWRSALFVAVLELAGSFAYFGVSANLI
TYLTGPMGQSNASAAAAVNAWSGAACMLPLLGAFLADSFLGRYPSILLACTLYLLGYGML
TVASSVVASKSQVGILYVSLYLVALAQGFDKPCGLALGAEQFDPEHPRESASRSSLFNWW
YFSMATGITVSIATVSYIQENVSWGVGFAIPFAVVSCAFLLFLLATPTYRLSSAAASPLL
SLRHYKQSSEEARRMLRLLPIWATCLAYGVAYAQIMTLFNKQGRTLDRHIGHAGLELPPA
ALQTLGPVTIMVSVPIYDRAVVPMLRRMTGNPRGLTTLQRTGTGMALSLAAVAVAAAVEG
RRLETVREQRPAMSWAWLVPQYVAMGVADVLAVVGMQEFFHGEMPEGMRSLGLALYYSVM
GIGGFISSALISALDGITRRDGGDGWFADDLNRGHLDYFYWLLAGVSAAELAMFLCFARS
YAYRNANKGPLLLVVPSNTPVSPCTNHHHA*
>11667.m06965|LOC_Os01g69080.1|genepair121-1
MASRMALRPNDVTLRLTPPLAAAARRNRRAAAGGVRVYAVASGAVSTKVENKKPFAPPRE
VHVQVTHSMPPQKIEIFKSLDDWARDNILSHLKPVEKCWQPQDFLPDPASDGFHDEVKEL
RERAKEIPDDYFVCLVGDMITEEALPTYQTMLNTLDGVRDETGASPTAWAVWTRAWTAEE
NRHGDLLNKYLYLTGRVDMRQIEKTIQYLIGSGMDPRTENNPYLGFIYTSFQERATFISH
GNTARHAKDFGDLKLAQICGIIASDEKRHETAYTKIVEKLFEIDPDGTVLAFADMMKKKI
SMPAHLMFDGEDDKLFEHFSMVAQRLGVYTAKDYADILEFLVSRWKISDLTGLSSEGNKA
QDYLCTLAARIRRLDERAQSRAKKAGTLPFSWVYGREVQL*
>11670.m02952|LOC_Os04g31070.1|genepair121-2
MAFAASHTASPSSCGGVAQRRSNGMSPVVAMASTINRVKTAKKPYTPPREVHLQVKHSLP
PQKREIFDSLQPWAKENLLNLLKPVEKSWQPQDFLPDPSSDGFYDEVKELRERAKEIPDD
YFVCLVGDMVTEEALPTYQTMLNTLDGVRDETGASPTTWAVWTRAWTAEENRHGDLLNKY
MYLTGRVDMKQIEKTIQYLIGSGMDPGTENNPYLGFLYTSFQERATFISHGNTARHAKEY
GDLKLAQICGTIAADEKRHETAYTKIVEKLFEIDPDYTVLAFADMMRKKISMPAHLMYDG
KDDNLFEHFSAVAQRLGVYTARDYADILEFLVQRWKVADLTGLSGEGRRAQDFVCTLAPR
IRRLDERAQARAKQAPVIPFSWVYDRKVQL*
>11667.m06967|LOC_Os01g69100.1|genepair122-1
MGSCASVHKDLGFPKKLFLASSPTKEKKAANGKGGGGGVSVDLKRKEQQQAAAAGVGVRS
PGSGSKDEMFFDSRAWLDSDCEDDFYSVNGDFTPSRGSTPNYQPRTQTVMSNVFVPDNVQ
NSKSPEPSPTGRRKLAELLQEAMQNGSEDSTDASVPDISKNEKQQFQPVSAAVKPVSESS
STCSTEPTPIREAKNRKEKAWYTGRCCLPAFVHTLDLDERRQKMTPGPCAV*
>11670.m02957|LOC_Os04g31110.1|genepair122-2
MGTWGARRPASTVVRTAPSTVAACTRTHRIRIPPSSSSPQPNNGFGVSLRLPLPPLRVRL
PCSSSPLLSTPPPPPPPPPPPQRVLAFRSRGAGAHMRFRFRNKAAAAGDGSRPASSPPNA
GKFAAPVAGGGGAADVAGESPDQDSTRNGSKDESFFEARPWLDSDSEDDFHSVRGDFTPS
RGTTPDHQRQSPFAGRISVDRSEPSLIEKKQRLLELLQEKQQYDDDSVADVGSEIENGAV
HAEEYLKSSRKGAKANRASKSRGGCFPSSFWKIKFRSCRKKRKEQND*
>11667.m06970|LOC_Os01g69130.1|genepair123-1
MEDGIYSSSAPPSTATAAAAAAAVGSSVIPIVNKLQDIFSQLGSSSTIDLPQVAVVGSQS
SGKSSVLEALVGRDFLPRGSDICTRRPLVLQLVHQPRRPADAEADEWGEFLHLPGRRFYD
FREIRREIQAETDREAGGNKGVSDKQIRLKIYSPNVLNITLVDLPGITKVPVGDQPTDIE
ARIRTMILSYIKHKTCIILAVSPANADLSNSDALQIARNADPDGSRTIGVITKLDIMDRG

TDARNFLLGNVIPLRLGYVGVVNRSQQDIKSDLSIKEALAREESFFRNHPAYNGLAQYCG
IPQLAKKLNQILVQHIKTVLPGLKSRISSQLTTTAKELSFYGDPVESKAGQGAKLLNILA
KYCEAFSSMVEGKNEDISTIELCGGARIHYIFQSIYVKSLEDVDPCEDVTDEDIRMAIQN
ATGPRSALFVPEVPFEVLVRRQISRLLDPSLQCAGFIYDELVKMSHRCLAVELQQFPLLR
RSMDEVIGRFLRDGLKPAQDMIAHIIEMEADYINTSHPNFIGGSKAVEQAQQQVRSSRLA
AVARRALTPGISSFDFVIHYQGLRPAAEAERPGSSGSGSTSFWGSISIFSSTSDDRTHSS
AKDNSSNKSYTASTSHLEHSLSTIQLREPPVVLKPSESQSEQEALEIAITKLLLKSYYNI
VRKNVEDFVPKAIMHFLVNHTKRELHNYLITKLYRDDLFADMLREPDEITIKRRQIRDTL
KVLQQAYKTLDEIPLEADTVERGYSLDADATGLPRAHGLSSSFQDGSSPYSTPKQPRSRK
SSHSGEQLPFNPDASGRREYEADIRDAFFKRKMMTGVLDRCVTPMSDKTAFKQGQGCTVV
LEDCQHFDAASVIPFNQCDQGHIKQITQITHLDK*
>11670.m02968|LOC_Os04g31190.1|genepair123-2
MAEPAAAAAAAEAPATVGQAVIPLVNRLQDIVARLDGGGGGGLELPQVAAIGGQSSGKSS
VLEALVGRDFLPRGPDICTRRPLVLQLVRHSAPEEWGEFLHAPARRFHDFDQIKREIQLE
TDKEAGGNKGVSEKQIRLKIFSPNVLDITLVDLPGITRVPVGDQPSDIESRIRSMIMQYI
KHPSCIILAVTPANADLANSDALQLAKLADPDGSRTIGVITKLDIMDRGTDARNFLLGNV
IPLKLGYVGVVNRSQEDINFKRSVKDALAFEEKFFSTLPAYHGLTHCCGVPQLAKKLNTI
LLKHITYMLPGLKSRINSQLVAVAKEHAAYGDTAESTAGQGVKLLNILRKYCEAFSSMVE
GKNKVSTDELSGGARIHYIFQSIFVKSLEEVDPCKSITDEDIRTAIQNSDGPKGPMFLPE
LPFEILVRRQISRLLDPSLQCANFIYDELVKISRGCLTSELQKYPILKKRMGEAVSNFLR
DGLRPAETMITHIIEMEMDYINTSHPNFVGGNKVVELARQEILPPKAPTSVTIPKDGTAI
SPEIQLTSDRSQKSRAIFARDATRGATSDQGVQPDADTGTSVAGRNQRGHSLVAGSSSSK
SVARVHSLDNLISIIQLREPPITLKPSENQPAQDATEVAIVKLLIKSYYDIVRKSIEDAV
PKAIMHFLVNHTKRELHNVLIRKLYRENLLDEMLRETDEVIIRRQRIQETLQVLEQAHRT
LEEFSLEAEKVEKGYSPAEYATGLPKIHGLSNGDPSIIYASSPNHNRKKASHEDQHGSVA
SYSSTSYPDANGGLLST*
>11667.m06980|LOC_Os01g69230.1|genepair124-1
MAAAPPGGQEKVIAAAQHIVKSLANSKNAADDMIRILSGFDDRLTFMSDLFPPPPPAAAA
VREPSVVEEEEAGPDDAREDEVEEAAELVERWDSPEEGDRLVFDSAKDAGDYLGAAAVLV
GARGARAEAALQAAMARLEDEFRHLLARGMSPLAGEDLHASLLRRLSLTVPSFASSASDL
DCPSFASHTGDGDESGGAGGRASVSDEEISPYLISPDTVGALRGIADVMLRAGYGPELCQ
VYGEMRRDTLMECLAVLGVDKMSLEEVQRVEWGVLDGKMKKWIQALKVVVRGLVAEERRI
CNQIFAADAEAEEDCFTEAAKGCVLQLLNFGDAIAIGKRSSEKLFRILGMYEALDEVLPE
LEGLFSGDARDFIKEEAVGILMRLGDAVRGTVAEFANAIQGETSRRALPGGEIHPLTRYV
MNYVRLLADYSRSLNQLLKDWDTELENGGDNVNMTPLGQCVLILITHLQAKIEEKSKLYE
DEALQNIFLMNNLLYIVQKVKDSELKTLLGDNWIRQRRGQIRRYSTGYLRSSWTRVLACL
RDDGLPQTMGSSSALKASLKERFKNFNLAFEELYKTQTTWKVVDPQLREELKISISEKVL
PAYRSFVGRFRGQLEGGRNSARYIKYNPEDLENQVSDFFEGRRPNA*
>11670.m02984|LOC_Os04g31330.1|genepair124-2
MSAPPAPPHPQPEELEVAEEPPAAAGGVGNDKVLAAAQHIVKSLATSKNAADDMIRILSG
FDHRFSSITADLFPSPLPSSGAGPTPPPPPPPRGAFEAAERLIRQWDATSELLVFEGPEG
DVADYLEAVDVAVDQLLSGVGAAAADAEAEAAGVVVQLAMARMEEELRHLMVRHAVPLDA
SGLFCSLRRLSLESMDDLDTSSEFDPITPHSLEGGPDTARSASLVGNPFDDQVFDLVRPE
AIDDLRSIAQRMDRAGYASELEQVYCGVRRDLLDECLAVLGVERLSIDEVQRMEWKLLND
KMKKWVHGVKTVVRSLLTGERRICDQVLAVSDELRDECFVESTKGCIMQILNFGDAVAVC
SRSPEKLSRILDMYEALAEVIPELKELFFGNSGNDVICDLEGVLERLGDAVKGTLLEFGK
VLQQESSRRPMMAGEIHPMTRYVMNYLRLLVVYSDTLDKLLGDDSAGDVDHSDTHRGGDD
EEEYLESLSPLGRHLVKLISYLEANLEEKSKLYEDGALQCIFSMNNILYIVQKVKDSELG
RILGDHWIRRRRGKIRQNSKNYLRISWTKVLSFLKDDAHGGRSGSGSGSGNSSRIKEKFK
NFNLAFDEIYRSQTLWKVPDPQLREELKISISENVIPAYRAFLGRYGSLVDSGRNSGRYI
KYTPEDLENQLSDLFEGSLGPANHSRRR*
>11667.m06981|LOC_Os01g69240.1|genepair125-1
MQARREVRTKEEQWRGEERVVESPLGSARWSPEAEIGMRVEDIWDSLDQPQLSDRDRLNS
CFDAIPVASFPHTFDGAQVVEIPSDATLAEAVDILSRHRIITAPVRNVDAPDDASWIDRY
IGVVEFAGIAVWLLHQSEAAAARADDLGADELAAKLGTVALEGAAAARAPDQQQSAEGAV
AEAFGALPSSDLFNKTKVKDISGSFRWAPFLALQSSDTFLTMLLLLSKYRMKSLPVVDIG
EGTISNVITQAAVVHMLAECAGLHWFEDWGAKSLTELGLPMIRPSRLVKVRHDEPALKAF
RLMRKRGVGGIPVVDHAGKPTGSIMIKDVKHLLASSDANRDYRTLTAQEFIANARQSSGE
KQMNIVTCKKEESIKEIIFKLDAEKRQRIYVVDEQGNLDGLITLRDIIAKLVYEPPGYFG
DFFNGVFPLPQNSRV*
>11670.m02985|LOC_Os04g31340.1|genepair125-2
MEMESPRSPEAEIGHRVEDLWEVAEPQLSPSEKLNSCFEDIAVASFPRPLGSQVIEIPSN
ASLADTVEILSKNKILSAPIRNVDAPEDASWIDKYIGIVEFAGIAMWLLYQSEAAANGTA
GSAVGSPVANLVSRLGSFTFRRTSSGRVETTTDPESDETASVGGSFFETLTSSEFYKNTK
VGDISGSFRWAPFLALQTSDTFLTMLLLLSKYRMKSLPVVDIGGDKIENIITQSSVVHML
AECVGLPWFESWGTKKLSELGLPLMKPCKLVKVNEDQPVLKAFQLMREKGVGGLPVMDTS
GTKAIGNISIRDVQYLLTAPNIYKDYRTITAKDFLTAVRQHLQEQHEASPLLGSVITCRR
DDEVKDIILKLDSEKIHRIYVIDDKGNTEGVITLRDIISKLVHEPRHYFGDFFDGVVPLP
PNSTV*
>11667.m06984|LOC_Os01g69270.1|genepair126-1
MVGQKRKRSSLPPQYATAGDCCGGGGRRKRLAGGGPDYLDELPDDLVLAVLSKLAASASS
PSDLLSVHLTCKRLNGLGRHDMVFAKASPASLAVKAASWSEPVQRFLKLCADAGNLEACY
ILGMIRFYCLGNRSGGAALLARAAVGGHAAALYSLAVIQFNGSGGAKSDRDLRAGAALCA
RAAALGHVDALRELGHCLQDGYGVRRDPAEGRRFLVAANARELTLALAAAASHRPFAALP

LAGGAAAGAIGCPLLSDFGWSLPEAEPHPANLFMADWWASRGVQATAKKPGLEAPAAATG
DSDGGGELRLCSHVRCGRRETRRHEFRRCSVCGAANYCSRACQALDWKRAHKAQCVPMDR
WLLAAGEAQ*
>11670.m03012|LOC_Os04g31610.1|genepair126-2
MRTRRGACYSPASCQDGRRKRRRIAGGGGEGSAAAAAAVAGGAEGPANDMFEELPDDLVV
SILADVAASARSPGDLAGAMLTCKRFRELGQSKVVLARASPRCLAVRAKAWSDAAHRFLQ
RCADAGNLDACYLLGMIRFYCLGSRGSGAALMAAAAVGGHREALYSLAVIQFNGSGGSKD
DRDLRAGAALCARAASLGHVDALRELGHCLQDGYGVRRSVLDGRRLLIQANARELAAAVA
ASASLLRAATGKPAAAASRRHSCLLSDFGCHAAAPKAGGEAHAANRFLVDWFASRPLAGS
TAAAAAAPTPGSAAEDEAAGLRLCSHALCGRPETRRHEFRRCSVCGVVNYCSRACQALHW
KTAHKAECTPMDRWLDNAAAGAANPNAAAMAAPAP*
>11667.m06997|LOC_Os01g69890.1|genepair127-1
MAPPAASTDWGPIIVAVILFVVLSPGLLFQLPARARVVEFGNMATSAIAILVHAVIFFCL
LTIFVVAIGVHVYAA*
>11670.m03034|LOC_Os04g31820.1|genepair127-2
MADWGPVVVATVLFVLLTPGLLCTVPGRGRVAEFGSFHTSGLAIIVHAVLYFALLTIFLI
AIGVHIYAG*
>11667.m06999|LOC_Os01g69910.1|genepair128-1
MSLPRRFGSIVSDSPFGDSAIDSDSRTGSCLFGSVLPGSELTGRARCSTSKLSLVLSNRA
GGLGFGGNQEGASGPYFLGGAWFLFNRRVLRYFRNDGYEWRKKKNGKTIAEAHERLKVDN
VDALNCYYAHADKNSTFQRRIYWMLDPAYDHIVFVHYRDVQEGSISVSALNDSSTSNQNG
SGSRAEAQSSPGLTSELFAPCLNSCSPGSAEEVSSQIMAINNETNSVSQPDWVQHCNQAA
LRKLKVQLSLEDREDHDVDAKDIPSNSEPITVYGIQNEEPGTCRNLADVFSGLEFSKENH
PEETGLPFSSTIDVLKNSDTWLEEDQIEAILHSASMIVTENQWFNIREVSPEWSYCSEST
KVIIAGDFLRDPSHGSWAIVFGDVKLLFDGHVSEQFLKFGLPFPNLECGLQVSPSEIMKG
ASERLNRDTAVNCVMEVLLNNKFEEWLFSKYEQNSEGNHFLPRQYHGVIHTIAALGYNWA
LKLLLNSGVLVNYRDANGWTALHWAARFGREETVVLLLDAGAAAGALSDPTAQDPAAKTP
ASVASAYGFKGLSAYLSEAELIAHLHSLESKENGSSGDQISRVVGRISDTSAHAQSGSDD
QLALKESLGAMRYAVQAAGRIQTAFRIFSFRKKQQAGLQNRGNHIISIREVGAASHGMLE
KAALSIQKNFRCWKKRKEFLKIRKNVIKIQARVRAHQQHNKYKELLRSVGILEKVMLRWY
RKGVGLRGFHPGAIAMPIDEEDEDDVAKVFRKQRVETALNKAVSRVSSIIDSPVARQQYR
RMLKMHKQNKDDDEKVEVSPASHVYGSGSHHMCWLSHNNKAMH*
>11670.m03042|LOC_Os04g31900.1|genepair128-2
MGSLCLERRKQPDDPLSISQQLHLCLLPLLFCAASKFSAFEGVLAQYPDFSFFFFSASAR
NRGFDINVLHKEARSRWLKPSEVYYILQNHERFPITPEPPKKPPSGSLFLYNRRVNRYFR
RDGHAWRRKKDGRTVGEAHERLKVGNVDALSCYYAHGEQNPCFQRRCFWMLEPAYEHIVL
VQYREVGAAEGRYNSASLLNGPTDSLSVLSYPNATYGNQYLGSTSGVSDGSESLHSNLSS
VTEVSSYSANKDNGILQSIQELSQSTIMGAPALGQSSLEQSIEVRWVDNSNSTNKSGLNR
ALKQIVEQLSLGDDEDDDYIHQAQPFDFITNIEAPDRQRDASRNVSEYQPPGSLYNSDMQ
QISAAKRFLLETEDSIDSPSYNYVPREEGNNGTNTLSVHDYSLQSSLNPDWKKTAPLTLQ
SNLYGSEIPSLLLDHGQFESLSSGENTRLILGQNPRFSIREVSPEWTYCYEITKVIITGD
FLCDPSSSCWAVMFGDSEVPAEIVQAGVLRCHTPLHSSGKLTICVTSGNREICSEVKDFE
FRAKSTASSFLDISPSSRSLKSSEELLLLAKFVRMLLCENGSHANSNGDPQSVQCPKLKM
NDEHWQRLIDELKGGCENPLNVSDWIMEELLKSKLQQWLSVKLQGYDGIACSLSKHEQGI
IHLISALGYEWALSSILSADVGINFRDTNGWTALHWAAYFGREKMVAALLAAGASAPAVT
DPTAQDPVGKTAAFLASERGHLGLAAYLSEVSLTSYLASLTIQESDTSKGSAAAEAERAV
ESISQRNAQLHGGTEDELSLKDSLAAVRNAAQAAARIQNAFRAFSFRKRQQKTARLKDEY
GMTQEDIDELAAASRSYYQSLLPNGQFYDKAAVSIQKKFKGWKGRRHFLNMRRNAVKIQA
HVRGHQVRKKYKTFVSTVSVLEKVILRWRRKGHGLRGFRAEQTAMAEAEEDDEDDDDDDF
NDDEAVKVFRRQKVDESVKEAMSRVLSMVDSPEARMQYRRMLEEFRQATAE*
>11667.m07001|LOC_Os01g69930.1|genepair129-1
MECNRDDAIRSKEIAERKFNENDIAGAKRFALKAKTLFDSLEGIDNMISALDIHIRAQTK
IEGENDLYGILDISASDDDEKIKKQYRKLALQTHPDKNKFSGAESAFKLIQDAWDVLSDK
DKKRSYDQKRFGGSSRVYQNGFAENANATPGSTMSSMNGFFWQNSGRHPSYATDTFWTYC
DSCQMSFQYSREYVNRNLACSFCQTEFVAVETPPPTAPVYYNVTNLMDTSSNMDDPQGTG
VPYSSNKIFDPVLQPVFGSVGGAHASRYPVQQTCKPARKEEVAEVNVARREEATKRKHEQ
ASSSLGSSSSAAKVIHRRKAVTKEMEAEKRRCINNKSKVSGQKNNTNKVVGKSTSSAADG
DSGPQMHPAKRKSASSIGTSGTKRRKMPSDHNSGNARTSFGKVFLQLETEIPGLKMEKMK
LQIRDKLEEFKSRRANVENKGNVHVSLEKKKTWKWKKPATLFVYTRRNRKEHRKEPGVDA
IGAGSSHKHLDGKYSCLDQVPSSDEGSCVMPVPEADFYTFGDHPETSFQNGQIWAAYDEE
DGMPRYYALIQKVLSRHPFKVRLAFLKAKDCSEFVTSNWISYGYSKTCGDFIVGTPKNTD
QLNTFSHVVTWEKGPGGIIRIFPRKGDIWALYQNWSPEWNTCTPDDTIYKYDLVQVLDSY
NPSAGISVMPIVKVPGFVSVFTPLLDPTKSRTIPKEEMLRFSHQVPFHVLTGEEAKNSPK
GCYELDPGSTPKELLQVVPQSDGV*
>11670.m03046|LOC_Os04g31940.1|genepair129-2
MYTYDKKANKRARKNAAAAAAAAAAAAAAAAEATTRPAGVDTFWTSCNRCRMQYEYLRIYL
NHNLLCPNCHHAFLAVETGFPCNGSSSSFSWSTKQQPQNNNSTKHSYGSTSRTSSIPGTG
HGGYQQDGTYDSYNNQSFQWNQYSKTTPAAGTNAYGTQALEKPKRKHEESYSYNYSATGN
SYGHERTNSRRGRFSKRRRHSNDGYTTMDFGGDNRETVAASTETTAFTDVAVAQVNGTSG
EKLRSAVSGRRANVLREISQIDTRALLIEKAKAAIQEKLQEWNITSSSRLAERGKSQGKV
YPSDNNIKQNGGLSDKHVKGLKQCSSRSVDTQAPTVDEKNPEQRRVPVSIDVPDPDFHDF
DKDRTERAFDSDQVWATYDSEDGMPRLYAMVQKVLSMRPFRIRMSFLNSKSNSELAPISW
VASGFQKTCGDFRVGRYQISETVNIFSHKVSWTKGPRGIIRIVPQKGDTWALYRNWSPDW
NELTPDDVIYKYEIVEIIDDFTDEQGLTVIPLLKVAGFKAVFHRHMDPKEARRIPKEELF

RFSHRVPSRLLTGEEGNNAPKGCHELDPAATPVDLLKVITEVTEDTATQPAK*
>11667.m06411|LOC_Os01g63990.1|genepair130-1
MVALSKLVLITAVALLGWAYKVARPPPPPILGGPGGPPVSSPRVQLKDGRHLAYREAGVG
REIAKYKIIFSHGFASTKESDFPVSQELAEELGIYLLYFDRAGYGDSDANPKRGLKSDAT
DVEELADKLQLGEKFYVVGTSMGGYVAWSCLNYIPYRLAGVALVVPAVNYWWPMPASVSA
SAYRKLDVGDRRTFWIAHHMPWLFYAWFNQKWFRISPIVEGKPEAFTEKDWEILAEIQRT
GQLDRGRATKQGAYHSLCRDATILFGAWEFDPTAIENPFPNGDGVVSIWQGREDKIVRVE
AQRYVAEKLPWVRYHEHPEGGHLFMCADGLGDKIVRELLLGEEPRDL*
>11670.m03229|LOC_Os04g33590.1|genepair130-2
MVRKLILALAVFLPALVYQQLQPPPPKICGSPGGPPVTGTRTQLKDGRHLAYLESGVPKD
QAKYKIIFVHGFDSCRYDALPISPELAQELGIYQLSFDRPGYAESDPNPASTEKSIALDV
EELADNLQLGPKFYLMGFSMGGEIMWSCLKHISHRLAGVAILGPVGNYWWSGLPSNVSWH
AWNQQLPQDKWAVWVSHHLPWLTYWWNSQKLFPASSVIAYNPALLSEEDKLIMPKFAFRT
YMPQIRQQGEYSCLHRDMTVGFGKWSWSPLELEDPFAGGKGKVHLWHGAEDLIVPVSLSR
YLSEKLPWVVYHELPKSGHMFPLADGMADTIVKSLLLGDQPPQA*
>11667.m06424|LOC_Os01g64120.1|genepair131-1
MPAPVATCFVPATSGVRCRAFSTPITNYSARGVVADPPKLLSRPGNLQLTSGGARFSGRF
RASAAAVHKVKLIGPDGAESELEVPEDTYVLDAAEEAGLELPYSCRAGSCSTCAGKLASG
EVDQSDGSFLADEQIEQGYVLTCISYPKSDCVIYTHKEEEVH*
>11670.m03233|LOC_Os04g33630.1|genepair131-2
MATTTVTTPILCNLSAKPRDTLRLPTTRSPNDGTRRTTLHLSSSRARGDLIRAAAAVYTV
KLIGPEGQESVIKVPEDTYILDAAEEAGVDLPYSCRAGACSTCAGKVVEGGVDQSDQSFL
DDAQPYVMEINKYYRIGLVLHVLQQQVRPSNAPAELDILGSASHMSEVYVLCARCSRLPQ
ESQSGIETRCASRTMHGSKEAGSKTHAHSLCNLLEKEKVAAPRKGKRCLSRRFGLGDKPA
NHSDPY*
>11667.m06429|LOC_Os01g64170.1|genepair132-1
MELCVRASSLLLGLLLQLLSSVDVVSAAQKFGINYGQIANNLPDPTQVAGLLQSLNVNKV
KLYDADPKVLMAFANTGVEFIIAIGNENLQSMAGNPGAARQWVTQHVQPFLPATRITCIT
VGNEVFSGNDTGMMASLLPAMKAIYAAVGELGLGGQVTVSSAHSVNVLATSFPPSSGAFR
EDLAQYIQPLLDFHGQTNSPFLINAYPFFAYKASPGSVSLPYVLFEPNPGVRDPNTNLSY
DNMLYAQIDAVYAAMKAMGHTDIGVRISETGWPSKGDEDEAGATVENAAAYNGNLMQRIA
MNQGTPLKPNVPIDVFVFALFNEDMKPGPTSERNYGLFYPNGSPVYAINTGAGGVSGRTG
PFDPYSAQMFSSASRLAQSLSCHFIQSRPIACVRPDSKVPSGFESSFQTNGNQPLSRTIE
SFPRVTTIAATIQIE*
>11670.m03234|LOC_Os04g33640.1|genepair132-2
MAAGEQPGTGSPPQPSTRLFLLVLAVILTDQVLAASAQGMSIGINYGQIADNLPSPTRVS
GLLRSMQISKVKLYDADQNVLSAFLDTGVEFVVGIGNENVSAMVDPAAAQAWVQQHVRPY
LPSARITCITVGNEVFKGNDTALKANLLPAMQSVYNAVVALGLQGQVNVTTAHSLDIMGS
SYPPSAGAFRPDAVPYIQPLLNFLSMAGSPFLINCYPYFAYKADPGSVPLEYVLFQPNAG
VTDPNTKLNYDNMLYAQIDSVYAAMQALGHTDVDVKISETGWPSRGDPDEAGATPEYAGI
YIGNLLRRIEMKQGTPLRPSSPIDVYVFALFNENLKPGPASERNYGLFYPDGTPVYDVGL
RGYLPPMDESKSARKAVSVLALIAIASITLILS*
>11667.m06442|LOC_Os01g64300.1|genepair133-1
MDRSRHKNSPSSERFLGSFLPSAAAGDQPGSAAFELDEDDLFASGAGSPERPQPSRRPLI
LSAVRAANPSPLPRLRRPPEGILDALPERRSPFSPPPSSSSNSSTTASPAAAAAAPPRLI
PTIPRPAAALAPHIPQSAPVNVPVAQFRRLSVEALMDKAEDDDDDDEEMLPPHEMVARAR
ARDSPMTTFSVLEGAGRTLKGRDLRQVRNAVWRKTGFLD*
>11670.m03247|LOC_Os04g33760.1|genepair133-2
MDEYRPRRSPASERFIGMFASPSSSPTEPSFVAGDELHEDDFLFSSSPAAPPSSARPGEG
PGSPSRVPQGQVGLLAALHEGDKRLLLLRRGGGGGGGAAAAAAASAGTLLRRKATIAAAAA
SASGGGGSLSPTQSPTSAARAIPMTPRPKSAGPAAPYHQSAPVKVPVRPPRRQEMFKWDE
LDDDDFLRNGDAAMLPPHEMVARASAGGAGPAAPFSMLEGAGRTLKGRDLRRVRDAVLRQ
TGFLD*
>11667.m06453|LOC_Os01g64410.1|genepair134-1
MVIGRLALSSFFHNKARDTSPSPPPAPATAPPWVWPSCKNPRTQSFRAATAPPPPPGSRT
IASIFLDSAESSFTTSSARHDCSDSLSTASEASAGAEAADTADDAIVRGLRSSDRLLFDP
GASATSSILEEKSSDAAGEASFIGGVAVAFESEDPYVDFRVSMEEMVVAHGVGNWGWLEE
MLGWYLRANGKDTHAAILAAFIDVIVAIADPALASCSSHRRSSTCTITEESSLEVAEKQA
KLAV*
>11670.m03258|LOC_Os04g33870.1|genepair134-2
MAKKGLVGILYKLRDVHHRAPPTPTSPSSSSSPHCHGRHQLCYPPAPSSWPWPSCRHPRT
SSFRWPTAPQQGQADDDAAAAAGSVYRTVNTVYDTSSLEHFNPRRSSLDEASSCIADRSF
FAVESEVEVEEEKEKEKELQLRETAVVRGVRSERLFFEPAGAEFLPKQRGFQEMARGKND
DEATAMDVVARKNDDVDEATPMTTPQTGKNEAEAAEAAALKGGAVVLTVESEDPYGDFRS
SMADMVAAHGLRDWEGLEELLAWYLKLNAKGVHGVIVGAFIDMLVSLASSPIPSQSPSSS
CITFEDYSSATMEEES*
>11667.m06469|LOC_Os01g64570.1|genepair135-1
MASAAAEVPSYFVCPISLQLMRDPVTLPTGISYDRAAIARWLAAPGARRTCPVTRQPLEH
GLELTPNHTLRRLIQSWAASVSPGSAVDEEVAALRPVSSDEVASLLSDAAAAQVGALRRL
RELAAECEDSRAMLESQGGVFDVLSRVVTSGSACSTAREEAVGVLASLRIPEQELIGVST
RHGNLAESLTAVLRSSNLQSRAHAVQLVRTLADAVVPAWVIGLNAELLAEVVGVVRDRVS
ARATKASLHALAALCPYGRHRVKIVGAGAVAALVELLLDEPERRVCELALAVLDRLCTCA
EGRAELVAHAAGVAVVGKKVLRVSEAASERAVRVLRSVARHAATPAVLQEMAQCGVVGKL
CLALRSEQCGVKTKEKAHEVLKLHSRVWRASPCLSPSFLALYPS*

>11670.m03274|LOC_Os04g34030.1|genepair135-2
MGEESSPAAAPAVEVLSYFVCPISLEIMRDPVTLSTGITYDRESIERWVFTDGHGECPVT
KQRLAPADREPTPNHTLRRLIQGWCAVHAVERFPTPRPPVDAARVAAIVDAARPLLRRRR
QREELMASLRELADIVAESDRNRRCVQGASGAVEFLLSVVKERASVAGVDDATSAKPEET
TCGGVHDPAKASSPEEAALSILHSLKLSEESFKRVLEGSGGEDFLETMACVLRRPSYLSR
MQGIHLLKSALPAMAPARLTSARAALVDGVVGVVADRPSAKAVKVALHVLCRLCPWARNR
VKAVDAGAVSALVRLLLDEGCGGGGGDRRACELAAVAIDHICRLRGGAPGSGGAPGGAQR
QVACSADRCPHTAGAESAVRALHAVARHSATSAVLQEMLPVGVVARLLFLVQVGASGERT
RARAREMLKMHARVWRDSPCLASHLNASYPR*
>11667.m06474|LOC_Os01g64620.1|genepair136-1
MDVEQLQQHARRLLSNGRVVSAAAAAGASPGPAGRVLEGGAAAAARRPAPFSSLDATVIT
VLSLLLCVLVVGLVLHAIARCAFRVTRRMCYGQEPPGDHGDEAAAERCARVARKKPGRAI
AEKIPAIVCPAGGLDRLAGCGSTECAICLSEFAQGHRVRVLPRCGHGFHARCIDRWLAAR
QTCPTCRREPFAAAAAVQLQVYPDAAGGQHETP*
>11670.m03296|LOC_Os04g34230.1|genepair136-2
MARDAPRPTARCRRAMAVAPPDPDVGRPNPAKEVPDPPPPQAAPPSSPLVVVVDVVSPTP
SSSHQGKGCCSGRPRRRLPGSPLTSSGGGEEEQGRPTQLGEPKIFSCRIPKKAFVHTPRV
EMYSRRILLHTPFSGQPSGPSQPVSGATIVEGGSPGSNFDANIVMILAVLLCALICALGL
NSIVRCALRCSSRMVVDPEPSRVTRLAQSGLRRKALRSMPILLYSTGLKLNTVSPMCAIC
LSDFEDGEHVRVLPKCNHGFHVRCIDRWLLARSTCPTCRQSLFGAPQKASGCSESEGSQA
EPAPARPVLAPLRPEGLVTPYDF*
>11667.m06477|LOC_Os01g64640.1|genepair137-1
MARTKQTARKSTGGKAPRKQLATKAARKSAPATGGVKKPHRFRPGTVALREIRKYQKSTE
LLIRKLPFQRLVREIAQDFKTDLRFQSSAVAALQEAAEAYLVGLFEDTNLCAIHAKRVTI
MPKDIQLARRIRGERA*
>11670.m03297|LOC_Os04g34240.1|genepair137-2
MARTKQTARKSTGGKAPRKQLATKAARKSAPATGGVKKPHRFRPGTVALREIRKYQKSTE
LLIRKLPFQRLVREIAQDFKTDLRFQSSAVAALQEAAEAYLVGLFEDTNLCAIHAKRVTI
MPKDIQLARRIRGERA*
>11667.m00731|LOC_Os01g07890.1|genepair138-1
MAALQRSSQTFRRSGSSGLVWDERLMLDGHSERDQEDGALELRHSRSVGLIGLQRRHGDG
AGHTRCNNSQAFHTRRVPPAQDPPSPKVPGCIFCGIFRKPVLSEPSKPRRF*
>11682.m00804|LOC_Os05g08390.1|genepair138-2
MEIGGLQRSSQTFRRSGSSGLVWDGRLMSEDQNQSDQRATGDAEAGSLESKELRHSRSVG
SSIKVQRRCSDSVERSRSGNQAFRTRHVPPAMDPPSPKVSRCLFCGIFSKEEPSQPPKPR
SLYWQP*
>11667.m00742|LOC_Os01g07970.1|genepair139-1
MAIASGAAFSVRPSPAAARPCVAASASAAGAARFRGDGSGGGGGGGGGGKWWAPLLGWSG
QPDYIDAQPAAREEARPNPMAAAEQRGGGGKRFGVLTEEKARQLRARMMETESFHDCMYH
SAIASRLASAAPADDGKH*
>11682.m00809|LOC_Os05g08420.2|genepair139-2
MAMATSSASTVSFSARPSAASAVRPCAAAGRARARAAAGESGKWWAPLLGWSGKADYIEA
PAPAVVAAAEESEARRRPFVGGLTEEKARELRARMVETESFHDAMYHSAIASRLARSA*
>11667.m00750|LOC_Os01g08040.1|genepair140-1
MDLLRTPFKGVVADIEGRVAWYKHDWVAGFRSGFRILAPTMYIFFASALPVIAFGAQLSR
ETNGILTTVETLASTALCGIIHSILGGQPLLIVGVAEPTIIMYTYLYNFAKNQQALGERL
YLAWAGWVCIWTALMLFLLAMFNASNVISRFTRVAGELFGMLITVLFLQQAIKGIIEEFK
VPRDADHSSPIYQFQWLYVNGLLGVIFSIGLLYTALRSRRARSWVYGQGWLRGFIADYGV
PLMVIVWTAFSYTLPKDVPSGVPRRLFSPLPWESSSLQHWTVAKDLFSVPPAYIFAAILP
ALMVAGLYFFDHSVASQLAQQKEFNLKKPSAYHYDILVLGFMVLLCGLIGIPPSNGVLPQ
SPMHTRSLAVLKGQLLRKKMVQTANEGLMNRASSLEIYGKIQGVFIEMDCEKNVVLYGKC
SSKSTYRSKIVLQTDSVDKELKSLKDAILQEVDKEGTLAEEFDPIKHIEAHLPVRVNEQR
LSNLLQSLLVGACVGAMPVIKMIPTSVLWGYFAYMAIDSLPGNQFWERLRLIFIPSSRRY
KVLEGPHASFMESVPSKTITVFTIFQLVYLLICFGITWIPIAGILFPLPFFLMILIRQHV
LPKFFEPNDLRELDAAEYEELEGVHHDHTLEDGESDSGSCGSRDDAEIFDELTTNRGELK
HRTSSHREERHLQVHSNAIQPRCGDTENLSEC*
>11682.m00810|LOC_Os05g08430.1|genepair140-2
MSSVGETQESPRRLLILVQTALVYSQAWKIWAHDSASFPQRLPRKQIRLSRVQGNGGNKV
PDLELFQMTGTVKAPFEGVVNDFKGRLSCYKQDWIDGFRTGFRILAPTLYIFFASALPVV
AFGEQLSNDTDGALTTVETLASTAICGIIHSILGGQPLLIVGVAEPTIIMYTYIYNFAKN
HPNLGERLFLPWAGWVCIWTAFMLFLMAMFNAAVVINRFTRFAGELFGMLITILFMQEAV
KGMLGEFSVPEGKDHSLPIYQFQWAYVNGLLGIIFSMGLLYTAIRSRSARSSLYGTVWTA
LSYSLPSKIPSGVPRRLFTPLPWEPKSLQHWTVAKVVMQFILIPGSNDLFSVPPPYIFLA
IVPAVMVAGLYFFDHSVASQLAQQKEFNLKNPSAYHYDILVLSFMDTNAMQVLICGLIGI
PPSNGVLPQSPMHTRSLAVLKGQLLRKKMVQTAKEGMMNNASSSEVYGKMQEVFIKMDDK
SNAKSVRKELKELKDAVIPEGNGAGRVSEVFDPEKHIEAYLPVRVNEQRVSNLLQSLLIA
GCVGVMPIIQKIPTSVLWGYFAYMSIDSVPGNQFWERTQLLFISPQRRYKLLEGAHASFM
ESVPIKKISAFTIFQLVYLLIVWGMTWIPVAGILFPLLFFFLIVIRQYILPKFFDPRHLW
ELDAAEYEELEGVRRDPSTDEDASVSRCSDASPEYASEILDEFTTNRGELKHRTKSFRDE
RLIQLNSVKMTRELSRIPTFTPPRS*
>11667.m00754|LOC_Os01g08080.1|genepair141-1
MAAELHFLVVPLIAQGHIIPMVEVARLLAARGARATVVTTPVNAARNGAAVEAARRDGLA
VDLAEVAFPGPEFGVPEGLENMDQLADADPGMYLSLQRAIWAMAARLERLVRALPRRPDC
LVADYCNPWTAPVCDRLGIARVVMHCPSAYFLLATHNLSKHGVYGRLALAAGDGELEPFE

VPDFPVRAVVYTATFRRFFQWPGLEEEERDAVEAERTADGFVINTFRDIEGAFVDGYAAA
LGRRAWAIGPTFGSISHLAAKQVIELARGVEASGRPFVWTIKEAKAAAAAVREWLDGEGY
EERVKDRGVLVRGWAPQVSILSHPATGGFLTHCGWNAALEAIARGVPALTWPTILDQFSS
ERLLVDVLGVGVRSGVTAPPMYLPAEAEGVQVTGAGVEKAVAELMDGGADGVARRARARE
LAATARAAVEEGGSSHADLTDMIRHVGAQ*
>11682.m00815|LOC_Os05g08480.1|genepair141-2
MALSSPSSPPIKSRKPASSEDSVAMAAAPLHFVLVPLPAQGHVIPMMDMARLIAGHGGGG
ARVTVVLTPVMAARHRAAVAHAARSGLAVDVSVLEFPGPALGLAAGCESYDMVADMSLFK
TFTDAVWRLAAPLEAFLRALPRRPDCVVADSCSPWTAGVARRLGVPRLVFHGPSALYILA
VHNLARHGVYDRVAGDLEPFDVPDLPAPRAVTTNRASSLGLFHWPGLESHRQDTLDAEAT
ADGLVFNTCAAFEEAFVRRYAEVLGGGARNVWAVGPLCLLDADAEATAARGNRAAVDAAR
VVSWLDARPPASVLYVSFGSIARLNPPQAAELAAGLEASHRPFIWVTKDTDADAAAAAGL
DARVVADRGLVIRGWAPQVTILSHPAVGGFLTHCGWNSTVESLSHGVPLLTWPHFGDQFL
NECLAVDVLGAGVRAGVKVPVTHVDAVNSPVQVRSGEVASAVEELMGDGAAAAARRARAR
ELAAEARAAMADGGSSARDLADMVWHVARRRDMVVVDPPPPPSPGGIAGGHGKMVSPSVA
SEVA*
>11667.m00757|LOC_Os01g08110.1|genepair142-1
MSSAGHAVDQQRKSTTMKAHFVLVPMMAQGHMIPMTGMARLLAEHGAQVSFVTTPVNAAR
MAGFVTAVEAAGLAVQLVKLPFPATEFGLPDGCENLDMIQSRDLSRNFMEACGALREPLT
ARLRQLCPPPSCIISDMVQWWTGEIARELGIPRLTFDGFCTFASLARYIIFRDKLLDNVA
DEEIVTFSGFPMLLELPKARCPGSLCVPGMEQIRDKMYEEELQSDGNVMNSFQELETLYI
ESFEQITGKKVWTIGPMCLCDRDSNMMAARGNKASVDEAKCLQWLDSKKPGSVIFVSFGS
LASTAPQQLVELGLGLEASKEPFIWVIKAGNKFPEVEEWLADGFEERVKDRGMIIRGWAP
QVMILWHQAIGGFMTHCGWNSTIEGICAGVPMITWPHFAEQFLNEKFVVNLLKIGLEIGV
KGVAQWGSEHKEVRVTRNAVETAVSTLMNDGEAAQEMRMRAKDLGVKARRALEEGGSSYD
NISLLIQEMGNKQNASG*
>11682.m00816|LOC_Os05g08490.1|genepair142-2
MNSLDDVPKPHFVLIPFMAQGHTIPMIDMAHLLAKHGAMVSFITTPVNAARIQSTIDRAR
ELNIPIRFVPLRLPCAEVGLLDGCENVDEILEKDQVMKMTDAYGMLHKPLVLYLQEQSVP
PSCIVSDLCQPWTGDVARELGIPRLMFNGFCAFASLCRYLIHQDKVFENVPDGDELVILP
GFPHHLEVSKARSPGNFNSPGFEKFRTKILDEERRADSVVTNSFYELEPSYVDSYQKMIG
KRGIFFIYNFFY*
>11667.m00759|LOC_Os01g08130.1|genepair143-1
MKASSSSQGQSPRTPSPRATSATAEHTRSSSEPWLVAAAVASTCDDSCVNDVENFARTVA
AAKSRQLVGSSAASRPDMLASVLSHYAAKWLPDVVAASSSSSSPATSASGRFLPPESPTA
TWLKKRLLLESLVAALPPDPPAPAGGGGAADDGITCDFLLKLLRAGSMVGADAALLQELE
SRAARRLDQATLGAVMIPAFGHGYACGTLLDVPLVLRLVRGFLKDAGAGGGGAAKAGGGG
GAAAARVARLVDAYLAEAALEAGLRPAEFEELARAVPAHARPADDALYRAVDTYLKVTHT
YSSFHLH*
>11682.m00820|LOC_Os05g08530.1|genepair143-2
MKPSSPSPSPPHGAAAVKQPASSPDARLTPSSVVAAAAVGIGVTPPRRSCDDAASCVVND
VDAFARTIASIRSKPASAAAAAASSSSDGGGDHLATVLAHYAARWLPDVASSPSGRFLLP
PQSPTATWIRKRLLLESLVAALPPDGGDGDDGGGGVTCDFLLRLLRAGSMAGADAALLAD
LEARAARRLDQASLGAVMIPAFRAAAGDAPGAGATLLDVPLVLRLVRGFLREGGKAGAGG
GGGAAAACRVARLVDAYLAEAALEAGLRPAEFEELARAVPAHARAADDGLYRAVDTYLKA
HPHAGKEERRSLCRLIDARKLTAEAAAHAVQNERLPVRCVVQVLFSEHGSKLTRLAEWTT
GSFRSLQSRSPADLIVTGGGANGGARCPSKREVAAQHHELRRLREDVSRLQVQCHALQAQ
VDRLSSERRRRPGGLFKLLFGGGGGAGATGAVVVDDSDSGLDRTPLSGKKGVVVRATAAA
AAASTPASGTPAVARWRRSHS*
>11667.m00769|LOC_Os01g08220.1|genepair144-1
MPTPSHLKNPLCFDFRAARRVPETHAWPGLDDHPVVDGGGGGGEDAVPVVDVGAGDAAAR
VARAAEQWGAFLLVGHGVPAALLSRVEERVARVFSLPASEKMRAVRGPGEPCGYGSPPIS
SFFSKLMWSEGYTFSPSSLRSELRRLWPKSGDDYLLFCDVMEEFHKEMRRLADELLRLFL
RALGLTGEEVAGVEAERRIGERMTATVHLNWYPRCPEPRRALGLIAHTDSGFFTFVLQSL
VPGLQLFRRGPDRWVAVPAVAGAFVVNVGDLFHILTNGRFHSVYHRAVVNRDRDRVSLGY
FLGPPPDAEVAPLPEAVPAGRSPAYRAVTWPEYMAVRKKAFATGGSALKMVSTDAAAAAD
EHDDVAAAADVHA*
>11682.m00821|LOC_Os05g08540.1|genepair144-2
MQIMTSSSTSPTSPTSPLAAAADNGVAAAYFNFRGAERVPESHVWKGMHEKDTAPVAAAD
ADGGDAVPVVDMSGGDDAAVAAVARAAEEWGGFLLVGHGVTAEALARVEAQAARLFALPA
DDKARGARRPGGGNTGYGVPPYLLRYPKQMWAEGYTFPPPAIRDEFRRVWPDAGDDYHRF
CSAMEEYDSSMRALGERLLAMFFKALGLAGNDAPGGETERKIRETLTSTIHLNMFPRCPD
PDRVVGLAAHTDSGFFTFILQSPVPGLQLLRHRPDRWVTVPGTPGALIVVVGDLFHVLTN
GRFHSVFHRAVVNRERDRISMPYFLGPPADMKVTPLVAAGSPESKAVYQAVTWPEYMAVR
DKLFGTNISALSMIRVAKEEDKES*
>11667.m00781|LOC_Os01g08320.1|genepair145-1
MSVETERSSTESSAASGLDFEDTALTLRLPGSLAAAAAPDPDRKRSSPSSSDAADAADNS
SPLAAAADAPPAPKARVVGWPPVRSFRKNALAAKFVKVAVDGAPYLRKVDLEAYSGYDQL
LRALQDKFFSHFTIPIDGRKVAGKFADDERKLVDAVNGTEYVPTYEDKDGDWMLVGDVPW
KMFVETCQRLRLMKSSEAVNLAPRAAQ*
>11682.m00824|LOC_Os05g08570.1|genepair145-2
MSVETERSSTESSAASGLDFEDTALTLRLPGSSSSSSSSSSSSSSSSSPSEPDRKRASATD
DDPDNRLGSTATESPPSPKARVVGWPPVRAFRKNALAALAAASSSKAKFVKVAVDGAPYL
RKVDLEAYRGYDQLLAALQDKFFSHFTIRKLGNEEMKLVDAVSGNEYVPTYEDKDGDWML

VGDVPWKMFVETCQRLRLMKSSEAVNLAPRSA*
>11667.m00783|LOC_Os01g08340.1|genepair146-1
MGFCLIFFLSPMLSVHIVFLFVQCEGQADSHPLLMGDSGNASHRDHTIDILRNDATFPST
SHQDNHNNLDELHQTRGPLNDVPHVPESSASATPASISRNASFARRDQGHRQPNPLNSGF
WISIELIVSLSQIIAAITVLSVSRNEHPHAPLAQWLIGYTIGCVATLPHLYWRFLHRNRQ
NTEQESTNQVSSERDVYEPNSYVVVSSAHGSEVVDSGNNGGVARIASPRVYALVACFKLA
LDCFFAVWFVVGNVWIFGGRTSLHDAPNLYRLCIVFLAFGFIGYALPFILCTMICCCLPC
IISMMGIHEDLDFNRGATAEAIDALVAYKFQSKKFQDGEAGEDNGGVLAAGTDKERTISA
EDAVCCICLSKFSNNEDLRELPCNHVFHLECVDKWLKINALCPLCKADLGGSTNAPDSSS
RSSHDSNNSRVRNDVESQQ*
>11682.m00831|LOC_Os05g08610.1|genepair146-2
MAVPPVESETRSEETDNFPLLADHMENTGHHAHAVDIPWDSSPSTSRRDNHNGFDQLPRI
LEGSPGTSTPSNSQNGPLARRDDNRGRRQPSPLNSGCWISVELVVNVSQIIAAICVLSVS
RNEHPHSPLFEWVIGYTVGCTATLPHLYWRYLHRNLPTTGQEPTVQNIPPNNTPEANSYG
VTGTNGVSRNNEATVNPRFQAFADHFKMALDCFFAVWFVVGNVWVFGGHSSAHDAPNLYR
LCIAFLTFSCIGYAMPFILCALICCCLPCIISILGFREDLNQNRGASAETINALGTCKFK
SKKTRDGDGNEVGVGVVAAGTNKERVISAEDAVCCICLARYVDNDDLRELPCAHFFHKDC
VDKWLKINALCPLCKAEIDGVSTSAPAIGFGRRHSDNRVGNDIESQL*
>11667.m00787|LOC_Os01g08380.1|genepair147-1
MAAAKSVERLGQRRVVPAEPTPAGPLRLSWLDRYPTQMALIESLHVFKPALDRAIGGDDV
AVGPARTIERALARALVHYYPLAGRLAFSDSGEVCVDCGDAGVWFTEAEASCSLEDVDYL
EYPMMVPKDELLPPTPAGEEERELVLLVQVTAFACGGFVVGFRFSHAVADGPGAAQFMAA
VGELARGAGGVSVDPVWGRDAIPDPAAAVIGSLPDPAGAKRLEYLAVDISADYINHFKNQ
YNAEAHAAAAGVARCSAFEVLIAKAWRSRTRAAGFEPDTTVNLCFAMNARPLLHASLPRG
GAGFYGNCYYIMRVSAPAGKVAGSSVTEVVKIIKDGKRRMPSEFSRWAAGDMAGGDPYQI
TSDYRTLLVSDWTRLGFAEVDYGWGPPAHVVPLTNLDYIATCILVRPWAHKPGARLITQC
VTPDRVAAFHEGLLDLN*
>11682.m00836|LOC_Os05g08640.1|genepair147-2
MAAAAPDKAVERLSQKLVHPSSPTPSAPLRLSWLDRYPTQMALIESLHVFKPDPARDAAG
QGLAPARAIETALARALVEYYPLAGRLAVSRDSGELQVDCCGGAGGHGGVWFIEAAVPCR
LEDVDYLEYPLAISKDELLPHPRPRPTRDEEDKLILLVQVTTFACGGFVVGFRFSHAVAD
GPGAAQFMGAVGELARGGERITVAPSWGRDAVPDPAGAMVGALPEPAGASRLEYLAIDIS
ADYINHFKSQFAAATGGARCSAFEVLIAKAWQSRTRAAAFDPSTPINLSFAMNARPLLLP
RGGAGFYGNCYYIMRVASTAGRVATASVTDVVRMIREGKKRLPSEFARWAAGEMAGVDPY
QITSDYRTLLVSDWTRLGFAEVDYGWGPPGHVVPLTNLDYIATCILVKPWAHKPGARLIT
QCVTPDRVTAFHDAMVDIN*
>11667.m00790|LOC_Os01g08410.1|genepair148-1
MVGGGRRGAAAEEVKLSTGNVFAALETLKKKKKGGDKAKGGSSSSSGRKREQQGAQPSSQ
QQQQQQKEVFWAPAPLTTKSWADVEDDDDDDYFATTAPPRPVWGTGHGDDGAKEEDEEDD
DDAVHAALQEEVESEDEDLDDEVDDGAVDEPEHEVDDTSAEPAGKKAAVPVAPPKDTERQ
LSKKELKKKELAELDAVLAELGISGGSSNAAQDENNAEKKGSNQTGDGDAPAPSESKSSK
KKKNKKAKEAKESQEPADGTEETASAEPDEDTTSVDVKERLKKMASMKKKKSGKESDTAA
KIAAAEAAARSARLAAAKKKEKNHYNQQPVR*
>11682.m00845|LOC_Os05g08730.1|genepair148-2
MAGGGSRRGAAAAAEEARIGTSNVFAALDTLKRRKKPSSSSKKHAEKEKEVLWAPAPLTT
RSWADVEDDDDDYFATTAPPRPVWGTHHHAADAHDDDHDEQAALEQELESEDEEVDDDA
EDEHEHETEDATPAEPAMNKAAAPPAPPKDTERQLSKKELKKKELEELDAILAELELSSK
SNNDAQNETNGKKGAEQAADGENKEGAPAPAESKSSKKKKAKKDKSAKEAKETQELNGGV
EEAAGAEPDEEVASMDVKDRIKKVASMKKKKSSKEMDTAAKIAASEAAARSAKLAAAKKK
EKSHYNQQPVR*
>11667.m00793|LOC_Os01g08440.1|genepair149-1
MPAMEEEAVANEAHHFLVVTYPAQGHINPARHLARRLARAAPGARVTISTAVSACRKMFG
DAAAAGAGGELVDEGGVRYAPYSDGYDDGFDRAVHDSASYMTQVRVVGARTLAAVIEGFR
AAGRPVTRVVYTLLLTWVADVARDHGVPVALYWIQPAAVLAAYFHYFRGTGGVDRDIAAA
AAARDRMAPVRVPGLPPLRLRDLPSFLAIADDDDPYAFVLDAFRDIVAVLSRGDRPTVLA
NTFDAMEPEAVASLRQHGVDVVPVGPVLSFLDAAKSGGGGGAITTTTSNDLFKQNDTGYL
EWLDARPAGSVVYISFGSLSTMSRRQIAEVSRGMAASGRPFLWVLRKDNRGEADDVAIDG
GVVVEWCDQVRVLGHPAVGCFVTHCGWNSTLEAVASGVPAVCVPQWTDQGTNAWLVAERL
GAGVRAAVSEVDGVLEAGELRRCIDAATSEAVRASAAAWREKARAAVADGGSSEKNLQAY
VGKIRAN*
>11682.m00847|LOC_Os05g08750.1|genepair149-2
MAAAMARRQQAHFLIVTYPSQGHVTPARHLARRLVHGAGVRATVCVPVSAFRKMFPADDG
EVVVEEEGGAVAYAAYSDGYDGGFDRAVDDHTRYMAQLSTVGARTVAGVLRRLRGEGRPV
TCAVYTLLLPWVAGVARDHGVGAVAVFWIQPTTALAAYYHYFRGGRDAVVAAAASGDASA
EVNLLPGLPPLRVRDIPSFLAITSDDDPFAFVLSEFAELIDTLERGGGGGGELPTYVLAN
TFDAMERDALASLRPHIDVVAVGPVLSFLHDADETKTASSPNDLFDHDGGGYLDWLGTKP
ARSVVYISFGSSSVMSKNQVAEIAAAMAESKKPFLWVIRKDNCKDDDDDNEAIKKLVAAA
AAADTGGGGMAVEWCDQARVLSHASVGCFVTHCGWNSTVEAVACGVPVVAAPQYSDQGTS
AWVVERIGVGVRAAARAGDGVVEAAELGRCVGAAMSEAVAGRAAAWREEARAAVARGGAS
ERNLSEFVRRFVPK*
>11667.m00796|LOC_Os01g08470.1|genepair150-1
MRGAGFLLLVVFAVAVAGGAVAAAADLDGGDGGGGVAAEWRFPSSRMRDAYVALQTWRRE
AIFSDPGNLTADWVGPDVCNYTGVFCAPLPWDRREVAVAGVDLNHGDIAGYLPPELGLLA
DLALLHLNSNRFCGVLPATLRRLRLLHELDLSNNRFVGRFPEVVLDMPALRFLDLRFNDF

EGGVPRQLFDRPLDAIFLNHNRFRFDLPDNFGNSPVSVIVLAHNSFGGCLPASLGNMSGT
LNEILLINTGLSSCLPPEVGMLREVTVFDVSFNRLAGPLPSAVAGMRKVEQLDVAHNLLT
GAIPQAVCELPRLKNFTFAYNFFTGEPPSCAHAVPRYGDRRNCLPNRPAQRTLRHIETVC
VAVVLKGIGKWWGCGDLEDLGTYD*
>11682.m00849|LOC_Os05g08770.1|genepair150-2
MRKEVVLVAMVVVVVVSWVVAAGGEAAAAAAAVVVDPAWRFPSARLRDAYVALQTWKQTA
IFSDPKNLTADWVGPAVCAYTGVFCAPLPGAGGAPGEVAVAGVDLNHGDIAGYLPAELGL
LTDLALLHLNSNRFCGLVPDALRRLRRLHELDLSNNRLVGAFPSAVLDLPALRFLDLRYN
DFEGAVPRQLFDLPLDAIFLNHNRLRFALPDNFGNSPASVIVLAGNHFGGCLPASLGNMS
ATLNEILLINNGLDSCVPPEVGLLREVTVFDVSFNSLAGPLPPEVTGMRKVEQLDVAHNR
LAGAVPEAVCDLPRLKNFTFSYNYFTGEPPSCARVVPAADGDRRNCLPNRPYQRTPRQCA
AFYAAPPVDCAAFQCKPFVPSPLLPPPPPPAYPGPLPPVYPVPYASPPPPPLYR*
>11667.m00800|LOC_Os01g08510.1|genepair151-1
MGYLLLAPSPAPRPLAFRCRRGGRARRGAAIVASSSASSSSSSGDAGPSHSAAAAGAYVL
ARRGVLLGVSALPLLRAREAAAAAAVATPNSGDLATVYGMSFPQGFIRRKRVDFPRIDHQ
AQTCYKTFKIGRNTVICRPNEAIVDETKDIQKPDEPQPGETQAESPLPEALQPESSLPVT
QEQTPGNPLSGLLNAIAVAASGVLAGLYGTSQQEKKALESVVSSMESKLAENEAAISLMR
ENYEKRLLDQQTAQKKQAMKFQEQEASLLDQLSSTKKTVTSLSEEFRREKTLAEELREEI
RRLESSLAQAGDDKDVLEAKLKEKLGDVNILQEKVSLLSQEIDNKGIRIRELSSLLSSKE
ADYRNLCSFSDQTKESLELAEAKIQQLEEEVHRTRNDLSSKISSIDLLNEELQALNSAKN
EAEEKLSELTKDYTDLKASSEARESRNSELLLEKDNMIKQLDGKLSDALSDSSKDRENIA
ALNKELDATKAMLENEVAAVKSLRESLQSTEEALTDSRSEVSKLSVELDEANRMNQDLVL
QISKLQDEFNEMQEGLTNKLGEVESVSKALSDELVSVKEMVHKGQEELEATSNELASIVE
ARDNLKKELLDVFKKLESTSQELVDERKTVTTLNRELEALVKQLQMDSEARKALEADLDE
ATKSLDEMNRSALSLSKELEETNSRKDTLEAEKKMLSKALAEQQKITTEAHENTEDAQNL
ISRLQTEKESFEMRARHLEEELALAKGEILRLRRQISTSRSQKAKTLPNTNASPEVSQAP
DEQPVNDNQNTSKVAAGSQYTAKRTTRRRKGGAST*
>11682.m00851|LOC_Os05g08790.1|genepair151-2
MAYHHLLLSPPHPHPARLSLVTSRRRPRAGRVAAACSPSPSALAAGRRAVLLVGVSVLPL
LRLRDAAFAAAAARPPSTTTVDLVTDRMDTVKTEETQPEEPSAEESLAEVKVPPAVNPLA
GLLNAIAVIASGVFAGLFGASQREKEALQSTVSTMEIKLAENEAAMSMLRENYEKQIWNE
HAEQKKQARMFQEKEASLLDQLTLTKRTVTSLNEEVRREKELVEQLKQEIHRLKSSIAQA
EDDKHVFEGKLREKLEALDSLQDKVNLLSQEVNAKEEAIRELSSSLSSKEEDYQKLQLIY
NETEASLEYADSKIEQLEEGYSATKDDLNSKMCSIDSLNKEVQTLYTAQTGAEEKISELK
KQYADLAAASELRASCDSELLIEKDNLLNQLEEKLSAALSDTSKNKIIIAELNNELDTNR
TMLDNEAEAHKKLSEILQSTEGALTDYRDKVFNLSEELNRVKISNQQLITQITKLTDESN
IAKQVLTNKIAEAEAVSKVLSDELASVRDVLQKTQEKLDVTSNQLVSTMEAREDLNKELL
DAYKKLESATDELVRERKINATLNRELEALVEQSIVESEARQALQADLDEVTNSQKEVDE
STQFLSERLDSANSRISSIEQEKEMLSEALEQQKRSTMEAQKDMEDAQNLMRMIGTEREN
FETMSKKLEEELATAKGEILRLRRQISASGYLRTELAETSVTSNTSQPEQDVNDPDQSSN
NTGAGDTRSPTRIYRRRKTKRAT*
>11667.m00801|LOC_Os01g08520.1|genepair152-1
MGSSRAEAEAEGEERGGGVLVGFGFGFGLAAMLRRMVVSDTDAGGGGGEAGRVRGGGGGG
AALFAVPRLFVGLAAKRGAGDGGEPASRSPTSPLDPKALLLRSPRSPRTWWDAEPVGLAL
AAAADDDAAKNCLLLSPRVGPLKSFASLPKDCGGGHSPRPGELAKAMSCAAAATATASAA
GGMSVPCGVFFYGDLKSGPEATRSGGAHPNAKRRSFDLGGGKIPGPGSLPASIGGVRRFI
GSVSASEIEQSEDYTCIIARGPNPKTTHIFGDCILEPQTMDASASAAAMDVTEAPTESYW
VVKCDAGAAPASGGGDFLSSCFTCKKKLEGNDIYIYRFKTFFCGANSDLELLKSSNKLLH
CSS*
>11682.m00852|LOC_Os05g08800.1|genepair152-2
MLRRVAVAPPDAGDAADGKPRGGAAALFKVPRLLVGMAAAAAAARPCECDSPVRSPTSPL
DLRAFAAPPLLRSPRSWDARRAGLGGLIDDGLAEPPGAAAMSRLLMPQMRPTKPRPCGPA
QPELGNAVGAAAGMSVPCSSRFYGDVKSGPEVTVAGAAQLRVNGGVHAAAADLGKFPATG
SLPASIGRPLPQYIGSVSATEVEQSEDYTRIIARGPNPKTTHIFGDCILEPCTESYWLVK
LSGSGDELRRLCSSCKKLDGSDLCFYRGEKAFCSGDCREQEILIEDEEESNTAVSSPISI
GSSSSFHDDLFMAGMAVLDMSTSSPHA*
>11667.m00805|LOC_Os01g08560.1|genepair153-1
MSVVGFDLGNESCIVAVARQRGIDVVLNEESKRETPAIVCFGDKQRFIGTAGAASSTMNP
KNSVSQIKRLLGRKYSDPELQRDIAAFPFRVSEGPDGFPLVHARYLGEERVFTPTQLMAM
VLSNLKGIAESNLNTAVVDCCIGIPVYFTDLQRRAVLDAATIAGLCPLRLFHETTATALA
YGIYKTDLPENDQLNVAFVDVGHASMQVCIAGYKKGQLKILSHAYDRSLGGRDFDEVLFK
HFAAKFKDEYKIDVYQNARACIRLRVACEKLKKVLSANPESPMHIECLMDEKDVRGFIKR
EEFEKISASILERVKGPLEKALAEAGLTTENVHFVEVVGSGSRVPAIIKILTDFFGKEPR
RTMNASECVARGCALECAILSPTFKVREFQVNDGFPFSIAMSWKPDSQNGDNQQTVVFPK
GNPLPSVKALTFYRSNTFQVDVTYVDTGDLQISPKISTYTVGPFNPGKGDKAKLKVKVRL
NIHGVVTVESATMLEEEEVEVPVAATTEPPKDSAKMETDDAPNEAASGTDVNMQEAKAPA
DAAADGAENGAPNSEEKSVPMETDAKVEPSKKKVKKTNVPVAELVYGALGTTELQKAVEK
EYEMALQDRVMEETKDKKNAVESYVYDMRNKLYDKYNDFVTAEDKEAFIAKLQEVEDWLY
EDGEDETKGVYVAKLEELKKVGGPIEARYKEWMDRGPSIDQLAYCINSFRDAALSKDPKF
DHIEMEEKQKVINQCSEAEVWLREKIQQQDALPKHANPVLLSSDLKKKAETVDRFCKPIM
MKPKPAPKPQTPPQTPPTETPAGGAQTPEQQPQGAEAAGEASEGGASESTGEQMETDKPE
GTEAA*
>11682.m00856|LOC_Os05g08840.1|genepair153-2
MSVVGFDVGNESGIVAVARQRGIDVVLNEESKRETPAVVCFGDKQRFIGTAGAASSTMNP

RNSVSQIKRLLGRAFADPELQRDLASFPFRVSEGPDGFPLVHARYLGEDRAFTPTQLLAM
VLSNLKGIAEGNLNAAVFDCCIGIPAYFTDLQRRAVADAAAIAGLRPLRLFHETTATALA
YGIYKTDLPEKEWLNVAFIDVGHASMQVSIVGYKKGQLNMLSHAYDRSLGGRDFDEVLFK
HFAEKFKDEYKIDVYQNARACVRLRVACEKLKKMLSANPEAPLNIECLMDEKDVRGFIKR
EEFEQISSPVLQRVKAPLEKALAEAGLTTENVHFVEVVGSGSRVPAIIRIITEFFGKEPR
RTMNASECVARGCALQCAVLSPTFKVREFEVNDGFPFSIALSCKPDSENTESEQTIVFPK
GSPVPSAKTVTFYRSNTFAVDVVSVDADDLQMAKKISSYTIGPFQSSKPEKAKVNVKACL
NIHGIVSIESAMMLEEVDVPVATTNETLKDDTKMDTDDALGDPASGTDENMQESKCSAD
ATHGAAENGKPDSEEISAPMDTDAKVEPLIKNVKKIDVPVSGLVYGALGSEELVKASENE
YEMALQDRVMEETKEKKNAVEAYVYDMRNKLYDKYNDFVMSEYKEGFIAKLQEVEDWLYE
DGEDETKGVYIAKLEELKKVGDPIEIRYKEWAERSSSINQLVHCINGFKEVALSNSQAFD
HIDMSEKQKVLDECSEAEIWLIEKQQQQDALPKHADPVLLISDMKKKAEALDRSCRPIMS
KPKPAPKPQTPPPPTPPTESPTTPEPQTPEQQQQSNGAGEAEEPTSEGGAQDQEPTAEQM
DTDKPDGWAEPSA*
>11667.m00833|LOC_Os01g08800.1|genepair154-1
MGPLWTFILLYPEIFLAIICFFWFSLFRPIRQRQKSNLPVNWPVFGMLPFLVQNLHYIHD
KVADVLREAGCTFMVSGPWFLNMNFLITCDPATVNHCFNANFKNYPKGSEFAEMFDILGD
GLLVADSESWEYQRRMAMYIFAARTFRSFAMSTITRKTGSVLLPYLDHMAKFGSEVELEG
VFMRFSLDVTYSTVFAADLDCLSVSSPIPVFGQATKEAEEAVLFRHVIPPSVWKLLRLLN
VGTEKKLTNAKVVIDQFIYEEIAKRKAQASDGLQGDILSMYMKWSIHESAHKQKDERFLR
DTAVGFIFAGKDLIAVTLTWFFYMMCKHPHVEARILQELKGLQSSTWPGDLHVFEWDTLR
SAIYLQAALLETLRLFPATPFEEKEALVDDVLPNGTKVSRNTRIIFSLYAMGRIEGIWGK
DCMEFKPERWVSKSGRLRHEPSYKFLSFNTGPRSCLGKELSLSNMKIIVASIIHNFKVEL
VEGHEVMPQSSVILHTQNGMMVRLKRRDAA*
>11682.m00857|LOC_Os05g08850.1|genepair154-2
MGGALLSFLLSYPEFILAAACFLAFAAIRRARDARRRAAPVPVSWPVVGMLPFVVAHLGR
LLDAAAAALPELGCTFMFRGPWLVGADFLVTCDPAVFRHCLVANFAGYDKGRDFAEMFDV
VGDGLLVADAASWAAKRHLAASVFTSAAFRGFVLSTVERQTRRLLVPFLDHAGGVVELED
VFMRYSLDVSYTVAFAADLDSLSVASAAEPFPPFGEATRVTGEAVLLRHIAPAGWWKLMR
WLNVGVERRLADAKAVLDEFVYREIANRRSRPAPAVAGGDDLLCMYMASPIDPAMSDQTL
RDAAVGFMFAAKDLIAAALTWLFYMICTHPHVEAKILDELRSLHTTTTAGAVVFDADELR
AATYLHAAVLETLRLYPSAPFEEKEAVGDDVLPGGTAVRKGTRVVFCLYAMGRVEGIWGS
DCREFRPERWLSTGDGDGGAGKVRQEPSYKFAAFNAGPRSCLGKDLGLSNIKIAAAAIVY
NFTVELVAGHVVEPKDSVVLHTKNGLMVRVKRRETA*
>11667.m00851|LOC_Os01g08970.1|genepair155-1
MTDGHLFNNILLGGRAGSNPGQFKVYSGGLAWKRQGGGKTIEIEKSDLTSVTWMKVPRAY
QLGVRTKDGLFYKFIGFREQDVSSLTNFMQKNMGLSPDEKQLSVSGQNWGGIDINGNMLT
FMVGSKQAFEVSLADVSQTQMQGKTDVLLEFHVDDTTGGNEKDSLMDLSFHVPTSNTQFL
GDENRTAAQVLWETIMGVADVDSSEEAVVTFEGIAILTPRGRYSVELHLSFLRLQGQAND
FKIQYSSIVRLFLLPKSNNPHTFVVVTLDPPIRKGQTLYPHIVIQFETEAVVERNLALTK
EVLAEKYKDRLEESYKGLIHEVFTKVLRGLSGAKVTRPGSFRSCQDGYAVKSSLKAEDGL
LYPLEKGFFFLPKPPTLILHEEIEFVEFERHGAGGASISSHYFDLLVKLKNDQEHLFRNI
QRSEYHNLFNFINGKHLKIMNLGDGQGATGGVTAVLRDTDDDAVDPHLERIKNQAGDEES
DEEDEDFVADKDDSGSPTDDSGGEDSDASESGGEKEKLSKKEASSSKPPVKRKPKGRDEE
GSDKRKPKKKKDPNAPKRAMTPFMYFSMAERGNMKNNNPDLPTTEIAKKLGEMWQKMTGE
EKQPYIQQSQVDKKRYEKESAVYRGAAAMDVDSGSGGNESD*
>11682.m00869|LOC_Os05g08970.1|genepair155-2
MTDGHHFNNISLGGRGGNNPGQFKLYSGGLAWKRQGGGKTIEVEKSDITSVTWMAIPRSY
QLGVSTKEGLFYRFFGFREQDISSLTNFMEKNMRITPEEKQLSVGGHNWGGIEINGNMLS
FNVGSKEAFEVSLADVAQTQMQGKTDVVLEFHVDDTTGGNEKDSLMDLSFHVPTSNTQFP
GDENRPSAQVLWQAILNKADVGSSEEAVVTFDGIAILTPRGRYSVELHLSFLRLQGQAND
FKIQYSSILRLFVLPKSNNPHTFVVITLDPPIRKGQTLYPHIVIQFETEAVVQRDLTLSD
EVLAEKYKDRLENSYQGLIHEVFSKVLRGLSGAKVTRPSTFRSCQDGYAVKSSLKAEDGL
LYPLEKGFFFLPKPPTLILHEEIEYVEFERHGAGGASISSHYFDLLVKLKNDQEHLFRNI
QRNEYHNLFNFISGKHLKILNLGEAQGRAGGVTAVLQSTDDDAVDPHLERIRNQTGDDES
DEEDEDFVADKDDSGSPTDDSGEEGSDASLSGGEKEKSSKKEASSSKAPLKKRKPKGGDA
AEGSEKRKPKKKKDPNAPKRAIAPFMYFSKAERANLKNSNPELATTEIAKKLGERWQKMT
AEEKQPYVEQSQVDKKRYAEESAAYRGAAAMDVDSGPASD*
>11667.m00854|LOC_Os01g08990.1|genepair156-1
MALVQVGMGLGRVVLLVGAGMAGSVVIRDGRFADFVAGLQEALRDNDGGGSGSGGGVIDQ
IEEAVKKATMEVNQMISQPVTVITVDPAVVTTLIAPAAAAGALTYGYMRWKGISIASLMY
VTKQNMANAVASMTKHLEQVQSSLAAAKRHLTQRIQHLDDKLDQQKQISGQIKEEVTGAR
LKLQDIGSEMQKIKQVAHGLGGKLDSIEAKQNYSLAGVMYLVEFIEQNGGRLPRSVEHLQ
RTARLSGITGDQKQLQGLGQLLAIESATPVGSGLHCTSARLFKAVA*
>11682.m00870|LOC_Os05g08980.1|genepair156-2
MAMVQAGMGLTRVVVLIGAGMAGSVVLRNGRLSEILGELQEILDKGEKGKDGEGGGGADM
TDALTRQVRNLAMEVKQLASSRGSITVLNGGSGQTGVSGLIVPAATVGALGYGYMWWKGI
SFADLMYVTKRNMANAVSSMTKHLEQVQTSLAAAKRHLTQRIERLDDKLDQQKALSGQIR
DDVTDARLKLENIGSEIKNIKQLVWGLDEKMDSMEAKQNFSCAGVMYLCQFIEQNGGKLP
ERLEGSKMAGKRFGSQNLIQGLQLAIETGNFDKETFNALKNNSDSR*
>11667.m00855|LOC_Os01g09000.1|genepair157-1
MGTGGGEGDKSAVLPLEALLALGLDQRTAENALVNSKVTANLAAVIAEAGISGCDKTVGN
LLYTVATKYPTNALVHRPVLIDYIVSTKIKNPAQLDAALSFLTNTGPDSLDTGKFEEACG
VGVVVSIEEIKSTVNEVLKHNMEAILEQRYHINVGNLCGQVRKRHPWGDAKATKDEIDKK

LAEILGPKTDADNVKPVKKKKEKAAKVEEKKAAVVTAAPPSEEELNPYSIFPQPEENFKV
HTEIFYSDGNIWRAHNSKEILEKHLKATGGKVMTRFPPEPNGYLHIGHAKAMFIDFGLAK
ERNGHCYLRFDDTNPEAEKKEYIDHIQEIVHWMGWEPYKVTYTSDYFQALYEHAVELIRK
GLAYVDHQTAEEIKEYREKKMNSPWRDRPIEESLKLFEDMRRGLIAEGAATLRMKQDMQN
DNKNMSDLIAYRIKFTPHPHAGDKWCIYPSYDYAHCMVDSLENITHSLCTLEFDIRRPSY
YWLLVALGLYQPYVWEYSRLNISNTVMSKRKLNRLVTEKWVDGWDDPRLLTLAGLRRRGV
SSTAINSFICGIGITRSDNSLIRVDRLEYHIREELNKTASRAMVVLNPLKVVITNLEDEK
VIDLDGKMWPDAPADDASSYYKVPFSRIVYIEKTDFRLKDSKDYYGLAPGKSALLRYAFP
IKCTEVVYGDNPDDIIEIRAEYDPSKTTKPKGVLHWVAQPAPGVEPLKVEVRLFDKLFLS
ENPAELEDWLGDLNPNSKEVIKGAYAVPSLATAVLGDKFQFERLGYFAVDTDSTPENIVF
NRTVTLRDSYGKAGPK*
>11682.m00871|LOC_Os05g08990.1|genepair157-2
MATGEAAAAAASPPELASFLAIGLDQRTAENALANRKVTANLTAVIAEAGVSGCDKSVGN
LLYTVATKYPANALVHRPVVIQYIVSSKIKTPAQLDAALSFLSTLGPDPLDTAKFEETCG
VGVVVSTEEIQSMVTDILKENMEAIVEQRYHINVGSLCGQVRKWHPWGDAKFIKEEIDKR
LTEILGPKTEADNVKPVKKKKEKPAKVEEKKTAVAAPAPPSEEELNPYSIFPQPEENLKV
HTEIFFSDGNIWRAHNRKDILEKHLKATGGKVMTRFPPEPNGYLHIGHAKAMFIDFGLAK
ERNGHCYLRFDDTNPEAEKKEYIDHIQEIVRWMGWEPYKVTYTSDYFQELYELAVCLIKK
GLAYVDHQTPEEIKEYREKQMNSPWRDRPIEESLKLFEDMRHGLVAEGKATLRMKQDMQN
DNKNMADLIAYRIKFTPHPHAGDKWFIYPSYDYAHCLVDSLENITHSLCTLEFDIRRPSY
YWLLVSLDQYQPYVWEYSRLNISNNVMSKRKLNKLVTEKWVDGWDDPRLLTLAGLRRRGV
SSTAINSFIRGMGITRSDNSLIRLERLEYHIREELNKVAPRAMVVLHPLKVVINNLDYGT
IIDLDAKKWPDAPGDDASAYYKVPFSRTVYIEQSDFRVKDSKDYYGLAPGKTVLLRYAFP
IKCTEVIYGDNSDNIIEIRAEYDPSKATKPKGVLHWVAEPSPGVNPLKVEIRLFEKLFLS
ENPVELEDWLGDLNPRSKEVIKGAYAVPSLATAALGDKFQFERLGYFAVDSDSTAEELVF
NRTVTLRDSYGKAGPK*
>11667.m00865|LOC_Os01g09100.1|genepair158-1
MAASLGLCHETSYAYSYPASNTSSSLCFPPLMADHIVDGGGGGGCSFGEFLELGHSVYSL
PLPPPPSQPVVVAGGNNDQYGVSSSSSAAATTSRIGFRTRSEVEVLDDGFKWRKYGKKAV
KSSPNPRNYYRCSAAGCGVKKRVERDGDDPRYVVTTYDGVHNHATPGCVGGGGHLPYPTS
AAPPWSVPAAAASPPPAHAQAWGAPLHAAAAAHSSESSF*
>11682.m00875|LOC_Os05g09020.1|genepair158-2
MAASVGLNPEAFFFSNSYSYSSSPFMASYTPEFSAAAIDANLFSGELDFDCSLPAPAQEY
PENENTMMRYESEEKMRARVNGRIGFRTRSEVEILDDGFKWRKYGKKAVKNSPNPRNYYR
CSTEGCNVKKRVERDREDHRYVITTYDGVHNHASPAAAAAALQYAAAAGDYYSPPLSSAG
SPPAAYSAGGSLLF*
>11667.m00881|LOC_Os01g09250.1|genepair159-1
MAPHLHLRLLLVVGVALGSAGLGWGGGGGEGEATDREAPDPYSILTWHDYSPPSPPPPPP
PPVAPAATCEGDLHGKGNFSTRCEVSEEVELGGDVYITGEGSLVLLAGAALTCQRPGCVI
SANLSGEVRLGRGVRVIAGRVSLAAANVTIADTVVVNTTALAGDPPERTSGVPTGTHGDG
GGHGGRGASCYVKDGQTQEDSWGGDAYAWSDLEHPFSYGSKGGSTSVEKDYGGSGGIVW
LYADDLIMNGTVLADGGDSSEKGGGGSGGSIYIKSKTMHGAGKISASGGNGLAGGGGGRV
SINVFSRHDDTQVFAHGGKSSGCPDNAGAAGTLYEAVPKSLVVSNNNLSTQTDTLLLEFP
NQPLWTNVFVKNHAKVAVPLLWSRVQVQGQLSLLSGAILTFGLTRYPYSEFELMAEELLM
SDSTIKVFGALRMSVKMLLMWNSKMLIDGGGDSIVATSLLDASNLIVLKESSVIHSNANL
GVRGQVKSGSGGVHLRIFRAALAGAGVVKYSSHLFLCASIRAPKLNCDDDICPVEIIHPP
EDCNLNSSLSFTLQVCRVEDIDIWGLVQGTVIHFNRARSVSVHTSGTISATGLGCRSGVG
QGKILNSGVSGGGGHGGRGGDGFYNESHAEGGSMYGSADLPCELGSGSGNDTTKLSTAGG
GIIVMGSWEYSLPSLSLYGSVESNGQSSTDVVTNASIGGPGGGSGGTILLFVRALSLAES
SILSSVGGLGNFGSGGGGGGRIHFHWSNIPTGDEYVPVAAVKGSIRTSGGISKGKGFPGE
NGTVTGKACPKGLYGTFCKECPLGTYKNVTGSSKSLCVQCPPDELPHRAIYTSVRGGAYE
TPCPYKCVSDRYRMPHCYTALEELIYTFGGPWLFGLLLSGLLVLLALVLSVARMKFVGTD
ELPGPAPTQQGSQIDHSFPFLESLNEVLETNRAEESHGHVHRMYFMGPNTFSEPWHLPHT
PPEQISEIVYEDAFNRFVDEINTLAAYQWWEGSIHSILCVLAYPLAWSWQQFRRRKKLQR
LREFVRSEYDHSCLRSCRSRALYEGLKVTATPDLMLGYLDFFLGGDEKRPDLPPRLRQRF
PMCLIFGGDGSYMAPFSLHSDSVLTSLMSQAVPSSIWHRLVAGLNAQLRLVRRGSLRGTF
LPVLDWLETHANPSLGVNGVRVDLAWFQATALGYCQLGLVVYAVEEPMSAELDGSPRIKI
EQHSLTQNMHADTQLGHSRIKEALMRKRITGGILDSNSLRTLKDRRDLFYPFSLILHNTK
PVGHQDLVGLVISILLLADFSLVLLTFLQLYSYSMADVLLVLFVLPLGILSPFPAGINAL
FSHGPRRSAGLARVLWLLLAALYTTSHQPKDILVRSHGTLEQFEKVLYQLVLSFPFTSLS
PVFSVDMLVKAQMNRSSRMLVKAISGISLPVGSDKLYSGSADGSVRVWDCNSGKCVDAIK
MGGKIGCMITHGPWIFVGITKSVEAWNTQTGMKSSLREPSGLVCSMTIKDEMLFAGTGDG
RIMAWKIPDKKGDSGPVAILSGHERQVISLGVSVTRLYSGSLDKTIKVWDLKTLQCVQTL
SEHKAAVTSVLCWDEKLLSCSLDKTVKIWAASKSGDLQVIYTHSEEHGWKRAEPARLAVA
RKKLGLAWLGLNLLIALGVFVVVLVVEKHSVFIVVHGYGNHGVRTLFGMHRVGKTPVLFC
SLHNSNCIRLFDLPSFDEMGKLFSKKEVRTIELAAGGLLFTGDGAGELKVWRWAPEEEPA
TPALVKSSM*
>11682.m00909|LOC_Os05g09360.1|genepair159-2
MATHIPLARLRLRLRLPHLALALLAVASGNPSLGGFDRGDAEAEAYSILTFHDYTPPPPP
SLPPPPPAPSATCAGDLRGVGDLDTRCVVPASVRLRGPGVYISGNGTLVLVDGVALTCER
PGCVVSANLSGGIFFGREARVVAGWVSLSATNITLSSDAVIDTTALAGDPPDKTSGVPTG
SYGDGGGHGGRGASCYVKEGQAQEDSWGGDMYAWAELKTPNSYGSKGGSTSVEKDYGGGG
GGVVWLFAKDIMMNGTILANGGDGGTKGGGGSGGSIYLKAKTMQGGGTISACGGDGLAGG
GGGRVSVDVFSRHDDSQFFVHGGRSSGCLDNAGAAGTLYEEVPKSITVSNNNLSTQTDTV

FLEPPYDPLWTNVFIKNHAKVSLPLRWSRIQAQGQISLLSRATLTFGLTHYPYSEFELLA
EELLMSDSTIKVFGALRMSVKMLLMWNSRMLIDGGRESGVATSLLEGSNLIVLKESSVIH
SIGNLGIHGQGILNLSGDGDTIQAQRLILSLFYNIVICRVEDIVVSGLVQGTVINFNRAR
NVTVRSSGTISATGLGCRGGIGRGRMLSSGLSGGGGHGGKGGDAFYSGSHAGGGTAYGSA
DLPCELGSGSGNVSTSSSTAGGGIIVMGSLEQSLPLLSLAGSIEANGGSFAGAVTHAANE
GPGGGSGGTILLFVRALSLEEGSVLSSAGGVGSNGSGGGGGGRIHFHWSDIPTGDDYIPF
ATVNGSILARGGTVDGQGFPGENGTVTGKDCPKGLYGTFCKACPLGTYKNITGSLKSLCS
PCPTNELPHRAVYISIRGGVTETPCPYKCVSDRYRMPHCFTALEELIYTFGGPWLFGLFL
SGLLFLLALVLSIARMKFVGTDELPGPAPTQHSSQIDHSFPFLESLNEVLETNRAEESHC
HVHRMYFMGPNTFSEPWHLPHTPPEQISEIVYEDAFNKFVDEINALAAYQWWEGSIYSIL
CILSYPLAWSWQQWRRRRKLQRLREFVRSEYDHSCLRSCRSRALYEGLKVAATPDLMLGY
LDFFLGGDEKRPDLPPRLHQRLPMSLIFGGDGSYMAPFSLHSDSVVTSLISQGVPSSIWH
RLVAGLNAQLRLARRGNLKATFLPVLKWLETHANPALNTYRVRVDLAWFQATALGYYQFG
LVIHSVGPFSSGLQGGSRMKFDYHAQFQNTDVDSQLDHSRNNDAVMLKRITGRVLDIDNL
RTLKDKRDLFYPLSLILHNTKPVGHQDLVGLVISILLLADFSLVLLTFLQLYSYSMIDVL
LVLFVLPLGILAPFPAGINALFSHGPRRSAGLARVYALWNITSLVNVIVAFACGLVHYKS
SAKRHPSMQPWNLGGDDTSWWLFPTGLVLCKCIQARLVDWHVSILEIQDRAVYSNDPTIF
WQ*
>11667.m00888|LOC_Os01g09320.1|genepair160-1
MLSARAAATAAAAAASPLWKRGEGGSSGSGSGCTSCREVRRRAAAVRVRAAAPRRVEAVA
MESAAETEKKEEVAAAGGGVEDMATEEVPVTPWAFSVASGYTLLRDPHHNKGLAFSEKER
DAHYLRGLLPPAVVSQDLQVKKIMHNLRQYSVPLQRYMAMMDLQERNERLFYKLLIDNVE
ELLPVVYTPTVGEACQKYGSIFRQPQGLYVSLKDKGKVLDVLRNWPERNIQVIVVTDGER
ILGLGDLGCQGMGIPVGKLSLYTALGGVRPSACLPITIDVGTNNEQLLNDEFYIGLRQRR
ATGKEYHELMEEFMSAVKQIYGEKVLIQFEDFANHNAFDLLAKYSKSHLVFNDDIQGTAS
VVLAGLLSSLKVVGGTLAEHTYLFLGAGEAGTGIAELIALEISKQTKAPIEECRKKVWLL
DSKGLIVNSRKESLQAFKKPWAHEHEPVTTLLDAVQSIKPTVLIGTSGVGKTFTKEVIEA
MASFNEGRAVFASGSPFDPVEYNGKIHVPGQSNNAYIFPGFGLGVVISGAVRVHEDMLLA
ASETLADQATQENFEKGSIFPPFTNIRKISARIAASVAAKAYELGLATRLPQPRDLEKYA
ESCMYTPVYRSYR*
>11682.m00917|LOC_Os05g09440.1|genepair160-2
MAGGGVEDTYGEDRATEDQLITPWSFSVASGYTLLRDPRHNKGLAFSEAERDAHYLRGLL
PPSIVSQELQEKKLMHNLRNYTVPLQRYIAMMDLQERNERLFYKLLIDNVEELLPVVYTP
TVGEACQKYGSIYRRPQGLYISLKDKGKILEVLKNWPERSIQVIVVTDGERILGLGDLGC
QGMGIPVGKLSLYTALGGVRPSACLPITIDVGTNNESLLNDEFYIGLKQRRATGEEYHEL
LEEFMTAVKQNYGEKVLVQFEDFANHNAFDLLAKYSKSHLVFNDDIQGTASVVLAGLIAA
LKVVGGTLADHTYLFLGAGEAGTGIAELIALEMSKQTEIPINDCRKKVWLVDSRGLIVES
RKESLQHFKQPFAHEHEPVKTLLEAVQSIKPTVLIGTSGVGKTFTQEVVEAMAAFNEKPV
IFALSNPTSHSECTAEEAYTWTKGSAVFASGSPFDAVEYEGKTYVPGQSNNAYIFPGFGL
GVVISGAIRVHDDMLLAASEALAEQVSEDNFARGLIFPPFTNIRKISAHIAAKVAAKAYE
LGLASRLPRPDDLVKYAESCMYTPAYRCYR*
>11667.m00902|LOC_Os01g09450.2|genepair161-1
MAWRRGFGREEEDAAAAGESGLELCLGLPAYFSSSSSSKPSEGSTAAPAFALRSNGTNAS
KPSGAAAAAPVVGWPPVRSFRRNLASSSSSSSKQAPPPPSSSPQNGDKASKDGGAEKGMF
VKINMDGVPIGRKVDLAAYGGYAQLSAAVDKLFRGLLAAQSAAADGEADAAAAGEMVGGG
EYTLVYEDDEGDRMLVGDVPWQMFIATAKRLRVLKSSDLPPPSLMRAAGSRKRAAADS*
>11682.m00921|LOC_Os05g09480.1|genepair161-2
MAWNGRFGEDGEEERSLELSLALPGYFSSSGLQGNTSTAADGAKGNDGFKASRPAAPVVG
WPPVRSFRRNLASSSSSSKPPRGGRDAAAAATGGKVARFVKVNMDGVPIGRKVDLAAHGG
YGELSAAVDRLFRGLLAAQRDPTMATAAAAAAAGESCTGEEEAIAGLLDGGSGEYTLVYE
DDEGDQMLVGDVPWNMFIAAARRLRVLRSSDLNASTIRAGSRKRAAAE*
>11667.m00903|LOC_Os01g09460.1|genepair162-1
MAAVEAEKVVAELRERCATPASLLRDVAAAMAGEMGAGLEKEGGSRVKMLLSYVDKLPTG
REDGLFYGLDLGGTNFRVLKVHLGGSKKHVVNSESREVSIPPHLMSGTSSELFGFIAGEL
GKFVAEEEEGTDMPNGKKKELGFTFSFPVRQRSVASGTLVKWTKAFSIDDAVGEDVVAEL
QTAMVKQGLDMHVAALINDAVGTLAGARYYDEDVVAGVIFGTGTNAAYVEKANAIPKWEG
ELPNSGDMVINMEWGNFYSSHLPVTEYDEALDKESLNPGEQIYEKLTSGMYLGEIVRRVL
LKLSLQSGIFGSIDNSKLKTCFHLRTPHISAMHHDETPDLKIVAEKLHQILEITHTSLEI
RKMVVEICDIVARRAARLAAAGVAGILMKLGRNGGINNQRSVIAIDGGLFEHYTKFRECL
ESTLGELLGEEASKSVAVKHANDGSGIGAALIAASQSR*
>11682.m00923|LOC_Os05g09500.1|genepair162-2
MVAAAVAAAEQVVAALREECATPAARLLDGVAAAMAGEMAAGLAEEGGSKIKMIVSYVDNL
PNGTEEGLFYALDLGGTNFRVLRVQLAGKEKRVVKRESREVSIPPHLMSGNSSELFGFIA
SALAKFVADEGHNAVFNDRQRELGFTFSFPVRQTSIASGTLIKWTKAFSIDDAVGEDVVA
ELQMAMEKQGLDMRVSALINDTVGTLAAGSYYDEDIVVGVILGTGSNAAYLEKANAIPKL
EGELPKSGNMVINTEWGNFSSSCLPITEYDEALDKESLNPGEQIFEKLISGMYLGEIVRR
VLLKISLQSSIFGNLDQTKLKTRFILRTPDISVMHHDGTPDLRIVAEKLADNLKITDTSL
ETRKMVVEICDIVTRRSARLAAAGIVGILRKIGRGVPGDKRKSVIAIDGGLYEHYTEFRQ
CLETTLTELLGEEASKSVAVKLANDGSGLGAALIAAAHSQYLN*
>11667.m00904|LOC_Os01g09470.1|genepair163-1
MGKAARWFRSLWGGGGGKKEQGREHGRTAAAPPPPDRKRWSFAKSSRDSTEGEAAAAVGG
NAAIAKAAEAAWLKSMYSDTEREQSKHAIAVAAATAAAADAAVAAAQAAVEVVRLTSQGP
PTSSVFVCGGVLDPRGRAAAVKIQTAFRGFLAKKALRALKALVKLQALVRGYLVRRQAAA
TLQSMQALVRAQAAVRAARSSRGAALPPLHLHHHPPVRPRYSLQERYMDDTRSEHGVAAY

SRRLSASIESSSYGYDRSPKIVEMDTGRPKSRSSSVRTSPPVVDAGAAEEWYANSVSSPL
LPFHQLPGAPPRISAPSARHFPEYDWCPLEKPRPATAQSTPRLAHMPVTPTKSVCGGGGY
GASPNCRGYMSSTQSSEAKVRSQSAPKQRPEPGVAGGTGGGARKRVPLSEVTLEARASLS
GVGMQRSCNRVQEAFNFKTAVLSRFDRSSEPAAERDRDLFLQRRW*
>11682.m00927|LOC_Os05g09520.1|genepair163-2
MGKAARWFRNMWGGGRKEQKGEAPASGGKRWSFGKSSRDSAEAAAAAAAAAAEASGGNAA
IARAAEAAWLRSVYADTEREQSKHAIAVAAATAAAADAAVAAAQAAVAVVRLTSKGRSAP
VLAATVAGDTRSLAAAAVRIQTAFRGFLAKKALRALKALVKLQALVRGYLVRRQAAATLQ
SMQALVRAQATVRAHRSGAGAAANLPHLHHAPFWPRRSLVRRWLNLADDIAMYMFDVDVV
CWRWMQQERCAGDDTRSEHGVAAYSRRLSASIESSSYGYDRSPKIVEVDTGRPKSRSSSS
RRASSPLLLDAAGCASGGEDWCANSMSSPLPCYLPGGAPPPRIAVPTSRHFPDYDWCALE
KARPATAQSTPRYAHAPPTPTKSVCGGGGGGGIHSSPLNCPNYMSNTQSFEAKVRSQSAP
KQRPETGGAGAGGGRKRVPLSEVVVVESRASLSGVGMQRSCNRVQEAFNFKTAVVGRLDR
SSESGENDRHAFLQRRW*
>11667.m00911|LOC_Os01g09540.1|genepair164-1
MAPKRLVCFLAVAAALATTCHGWGAGAGDVVSSSAAALSFVDRLRQMMIPAAVGDGDYCD
SWRVGVEANNVRGWTAAPRKCDNYVENYMRGHHYRRDSKVVVDEAAAYAEAAVLSGDPAA
DANATWVFDVDETALSHVKFYKKHGFGYHRTDEPAFMEWLIAGRASALPNTVTLYKKLLL
LGVKIVFLSDRPDTPELRNATATNLIKEGFDCWDELILRSENSTATGSVVEYKSGERKKL
EEEKGMVIIGNIGDQWSDLLGSPEGRRTFKLPNPAYYIDNYKRAGAAVRAAVAITASSSS
SSS*
>11682.m00948|LOC_Os05g10210.1|genepair164-2
MATARLILLLTVAAAAAGSCCFCSAQEVIVGGVGEQLATAPPAAPAPSPPPPYCGSVRTA
VEAHNIIGWKTVPADCAEYVSDYLTGERYGRDSDVVINEAIAYAESLKLSGHGKEIWVFD
VDETALSTLPYQAKHGYGTKPYDHASFVQYVAGGSAPALQGTLRLYRRLLQLGIKPVFLT
DRTEDQRAVTTHNLLSQGYYSWEKLLLQPVGLQTTTQAFKTGERQKLVSAGYVIVGNIGD
QWSDILGSPEGYRTFKYPNPIYYVA*
>11667.m07068|LOC_Os01g70520.1|genepair165-1
MAAAIAVVYLSLLLLLLHGAAPAVLGYTRGDFPEDFVFGSATSSYQYEGGFDEDGRSPSN
WDIFTHQGKMPGRSTADVAADGYHKYKDDLKLMVDTNLEAYRLSISWSRIIPNGRGDVNP
KGLQYYNDIIDGLVKNGIQVHIMLYQLDLPQVLEDEYDGWLSPRILEDFKAYADVCFKEF
GDRVAHWITIDEPNVASIGSYDSGQLAPGRCSDPFGIRKCTVGNSSVEPYIAVHNMLLAH
ASVTKLYREKYQSCGALSRVLRPLVFGDYPQVMKNIVGSRLPSFTKAQSEDVKGSLDFIG
MNHYYSLYVNDRPLGKGTRDFVADISIYYRGSKTDPPPGYGSSNDTVHDNDRVDYLKSYI
GSILTALRNGANVKGYFVWSFVDVFEYLTGYGQSYGLYRVDFADESRPRQARLSARWYSG
FLKNREMDVDQSELAMAAAESRAQQ*
>11682.m02810|LOC_Os05g30350.2|genepair165-2
MAVSSSTSTCSSFSLLLLLLLLLAAAPWRSGEAAAAAARALNFTRQDFPGEFVFGAGTSAY
QYEGATDEDGRSPSIWDTFTHAGKMPDKSTGDMGAGGYHKYKEDVKLMSDTSLEAYRFSI
SWSRLIPRGRGPVNPKGLEYYNSLIDELVERGIEIHVTLYHLDFPQILEDEYHGWLSPRV
IDDFTAYADVCFREFGDRVRHWTTMDEPNVLSIAAYDSGAFPPCRCSPPFGANCTAGNST
VEPYVVAHNSILAHASVTRLYRDKYQATQEGFVGMNIYSFWNYPFSSSSADIAATQRALD
FMVGWILDPLVYGDYPEIMKKKAGSRIPSFTEEQSELIRGSADFIGINHYTSVYISDASN
GETVGPRDYSADMAATFRISRNDTPSGQFVPTRLPRDPKGLQCMLEYLRDTYQGIPVYIQ
ENGFGHFGKDDDSLNDTDRVDYLSSYMGSTLAALRNGANVKGYFVWSFLDVFELLAGYHS
PFGLHYVDFEDPNLPRQPKLSAHWYSKFLRGEIGINIESTISPDEHEHEHADQ*
>11667.m07074|LOC_Os01g70580.1|genepair166-1
MPLPTMTHSSSFLRLPATSSPHPPPADDASAAYAVVVLNQRLPRFAPLLWDRAARLRVCA
DGGANRVFDGMPELLPAEDPDQVRMRYKPDVIKGDMDSIRPEVKEYYSNLGAEIVDESHD
QDTTDLHKCVSFITRNPPGSEESNLYILVLGALGGRFDHEMGNINVLYRFSNIRIVLLSD
DCSIFLLPKTHSHEIHIERSIEGPHCGLIPMGSPSASTTTTGLRWNLDNTSMSYGGLIST
SNIVEEETVRITSDSDLIWTISLRN*
>11682.m02821|LOC_Os05g30460.1|genepair166-2
MAPRPAMSHSSAFLLPSPSAAAAGADADGAAYALLVLNQRLPRFAPRLWDRAQVRVCADG
GANRVFDGMPELFPGQDPDEPLHVIEAPVFVVKSSGVGFLEMGDAQGVTMHMA*
>11667.m07087|LOC_Os01g70710.1|genepair167-1
MASGSEPIECQVLVLRVSIHCEGCKKKVKKVLQHVPGVFRCDVDARSNKVIVTASRNMDA
NILVAKLRKSGKQAEPWPEEPKQQQPPPPPAESQSQETKNQSDESSKPSDQPAEKPGPDK
AEGSAAEPNNPQPSPEPTKSTDETPKPNQEIQEPSNAKANTDANASGNASDETKEAAATG
EQSSEPKGKVKQHRERPIDARVTMEYGGGSHVNYMPQPQPVPVMSYNVARPTASAAYYAA
PPAPAPMSMPMPMARPGPSSQGYIDEEYSPSYYNRSSPYEPYYYPQPSPYRYQHYQQSSA
DDYYYGAPQQRSAFSPPRDAYGEMFNDENSNSCSVM*
>11682.m02832|LOC_Os05g30570.1|genepair167-2
MASESLQCKVLALRVSIHCEGCKKKVKKVLQRVEGVYRCDVDGRSNKATVTVTGKVSADT
LVRKLRRAGKHAEQWPEEQQQQQPNGGQCQEETKNQAAEPGMSGEPAEPEKPASGDAAEP
SDPKVTHEEPKKVAGEGAAAVAPAEDGGTEITDANVSESAGDGGGGGGGVETVKAQQPSE
PKRRRKQQPPQQQKEEKAGEATMATAAAAAASTQGNHTSHHFPAAPLQQQPVHVMSYNVA
RPSSSAAYYAAARPASAARPPPPLPPAPPQEHSYAYSPYYSQSQPSPYRYGGYYSYYYYG
GGGGGGGGGQRTPQRSAASPARNSYGDLFSDDNANSCSVM*
>11667.m07095|LOC_Os01g70790.1|genepair168-1
MAQRTLELTLISAKDLKDVNLLSKMEVYAVVSLSGDRRSRQRIATDRAGGRNPAWNAAPL
RFTVPASGAGSLHVLLRAERALGDRDVGEVHIPLSELLSGAPDGAVPAKFVSYQVRKISS
GKPQGVLNFSYKIGEVTQSGSYPGASPPVAYGQAPPAPAYPPSAAAAAAYPPQSTYPPPT
AYPTAAKADGSAAAAYPPQSAYPPPGKGNEPSTAYPPPAGYPPATGSSKPAKAGEPVTAY

PAAAGPSTAAPYGTAPPPQYGYGYPAQPPPPQAGYGYPPPPPQAGYGGGYGYPPQAGYGG
YQQQAVKPAKKNNFGMGLGAGLLGGALGGLLIGDAISDASAYDAGYDAGFDDGGGFDF*
>11682.m02849|LOC_Os05g30740.1|genepair168-2
MAMACRTLELTLLSASDLRGVNLVSKMEVYAVVYLAGDPRARQRVATDRAGGRNPSWKGK
DATVRLAVPASGAGSGAVRVLLRAERAGLGGDRDVGEVFVPLPDVLAGSGDGPTAAAVAS
YPVRKVGSSRTTHGVLNLSYKLGGVVHPDPAAAAAAACSCKPAAPAAGGSTMAYLAAAAA
AYRAAPPPQPPLYGYRQLPPIPQPVPPMGAVEGGGVRAGR*
>11667.m07101|LOC_Os01g70850.1|genepair169-1
MEISSSSKKHFILVHGLCHGAWCWYRVVAALRAAGHRATALDMAASGAHPARVDEVGTFE
EYSRPLLDAVAAAAPGERLVLVGHSHGGLSVALAMERFPDKVAAAVFVAAAMPCVGKHM
GVPTEEFMRRTAPEGLLMDCEMVAINNSQGSGVAINLGPTFLAQKYYQQSPAEDLALAKM
LVRPGNQFMDDPVMKDESLLTNGNYGSVKKVYVIAKADSSSTEEMQRWMVAMSPGTDVEE
IAGADHAVMNSKPRELCDILIKIANKYE*
>11682.m02851|LOC_Os05g30760.1|genepair169-2
MELGGGGHESVERRRRHQHHFVLVHGLCHGAWCWYKAAAALRRAGHRATALDMAASGAHP
ARVDEVRTFEDYSRPLLDALAALPPAGGDGDDEERVVLVGHSQGGFSVALAAERFPERVA
AVVFLTAAMPPVGRPMSATTVEHVNYVGVEFFLDSMELEQQNADIPGNPVIFGPNFMAQI
LYHLSPQEDLTLGLSLIRPTNKFTGDALMRDPGLLTKERYGSTRRVFVVVEDDRGIPVEF
QRRMIAENPGVEVVDFAGADHMAMISSPAKLAELLVRIADKAHEP*
>11667.m07115|LOC_Os01g70970.1|genepair170-1
MALPTAVVAGIAAAVATLLVAAAVAAAWWWRVARRSRRNSDTGSSETPPTLVEWGRCGRT
LSAPEYQGARQFSLEELAHATKNFSDANLVGAGSFGPVYKGLLLDGTVVAVKRRVASPRQ
DFVDEVKRLSEIWHRNVVTLIGYCQEGGLQMLVFEYLPNGSVCGHLYANTGKESMTRLEF
KQRLSIAIGAAKGLNHLHSLVPPLIHKGFKTSNVLVDENFIAKVADAGIDRLLRGFDGAA
PSHHPSSSCSSIYQDPEVHSLAQLSESSDVYSFGVFLLELITGKEAASLISSEPREPLVH
WMESHFSSNNEVTDPRLGGSFTSEGMKELVGLTLQCVSTSARRRPKMRLIAAELDRILEK
EMSLTTVMGDGTAIVTLGSQLFTS*
>11682.m02857|LOC_Os05g30820.1|genepair170-2
MSLPVAEVAGIAAACVALLAAVAALWCAARRMARRRGRNSDETGSSDPSTLVEWGKGGRS
SSAPEHQGARQFSLDELAQATKSFSEANLVGLGSFGLVYKGLLLDGSVVAIKKRIGAPRQ
EFAEEVRKLSEINHRNIVTLIGYCQEGGLQMLVYEYLPNGSVSRHLYDTGKSSMTRLEFK
QRLSIAIGAAKGLNHLHTLVPPLIHKDFKTSNVLVDENFIAKVADAGLVRLLRGYEDVSP
SHGFSSSVYQDPEVQSVLQFSESSDVYSFGVFLLELITGREAACLISPDSRESLAQWVIS
IDI*
>11667.m07118|LOC_Os01g71000.1|genepair171-1
MSCFLCFGSAQEGEAKKPGADSKDARKDGSADRGVSRVGSDKSRSHGGLDSKKDVVIQRD
GNNQNIAAQTFTFRELAAATKNFRQDCLLGEGGFGRVYKGRLETGQAVAVKQLDRNGLQG
NREFLVEVLMLSLLHHTNLVNLIGYCADGDQRLLVYEFMPLGSLEDHLHDLPPDKEPLDW
NTRMKIAAGAAKGLEYLHDKASPPVIYRDFKSSNILLGEGFHPKLSDFGLAKLGPVGDKT
HVSTRVMGTYGYCAPEYAMTGQLTVKSDVYSFGVVFLELITGRKAIDNTKPQGEQNLVAW
ARPLFKDRRKFPKMADPMLQGRFPMRGLYQALAVAAMCLQEQATTRPHIGDVVTALSYLA
SQTYDPNAPVQHSRSNSSTPRARNLAGWNEDRRSVRSPNHHSPDLRREAARSSRAEVSRT
SSTGDSGRRSGLDDLDMTGSQMGSPAQTGRKRETPRTADRQRAIAEAKTWGENSRERKHP
NGHGSFDSTNE*
>11682.m02862|LOC_Os05g30870.1|genepair171-2
MIQVAPNTRSFSLDKSKSQGGLDSRKDAFIPRDANGQPIAAHTFTFRELAAATKNFRQDC
LLGEGGFGRVYKGHLENGQAVAVKQLDRNGLQGNREFLVEVLMLSLLHHDNLVNLIGYCA
DGDQRLLVYEFMPLGSLEDHLHDIPPDKEPLDWNTRMKIAAGAAKGLEFLHDKANPPVIY
RDFKSSNILLGEGYHPKLSDFGLAKLGPVGDKTHVSTRVMGTYGYCAPEYAMTGQLTVKS
DVYSFGVVFLELITGRKAIDNTKPLGEQNLVAWARPLFKDRRKFPKMADPLLAGRFPMRG
LYQALAVAAMCLQEQAATRPFIGDVVTALSYLASQTYDPNTPVQHSRSNASTPRARNRVG
ANFDQRRLHSPNHQQSPDLRKEGTTTSKYEAEVSRTNSGSGSGRRAGLDSMDVTGSQMGS
PAHAGRKRESSRSTDRQRAVAEAKTWGENSRERKWPNARGSFDSTNE*
>11667.m07119|LOC_Os01g71010.1|genepair172-1
MERRGLLKAALLVCLIVLCSGREIQVIQRHPSTTIYNPKLAKTLVEYASAIYTADLTQLF
TWTCARCGDLIEGFEMMDIIVDVENCLQAYVGFASDINAIVVVFRGTQENSIQNWIEDLL
WKQLDLDYPGMPEAMVHRGFYSAYHNTTMRDGVVSGIQKTRKLFGDVPIMVTGHSMGGAM
ASFCALDLVVNYGLDGVKLMTFGQPRIGNAAFASFFKKYLPHAIRVTHGHDIVPHLPPYF
SFFPQKTYHHFPREVWVHNVGLGSLVYSVEQICDDSGEDPSCCRSVSGSSVQDHIYYLGV
SMHAEAWSSCRIVMDYSKLRYRMDINGNIVLSKHLGLSGDLEHSDQ*
>11682.m02865|LOC_Os05g30900.1|genepair172-2
MERRRWLQAAVLCCLLVLCSGRELKTKHTPIYNSTLARTLAEYTSAVYTADLTQLFSWTC
ERCCDLTEGFEVIELIVDVKNCLQAYVGYASDMNAVVVVFRGTQETSIQNWIEDLFWKQL
DLDYPGMPQAKVHSGFYSAYHNTTLRDGVVNGIKKTREAYGNIPIMVTGHSMGGAMASFC
ALDLVVNYRLKDVTLITFGQPRIGNAVFASHFKCHLPNAIRVTNAHDIVPHLPPYYHYFP
QKTYHHFPREVWVHNVGLGSLVYSIEQICDDSGEDPTCSRSVSGNSVQDHINYLGISMHA
EASGSCRIVTGDNKLQYKMDSDGNIVFSKQPGLSVDQLHSSQ*
>11667.m07123|LOC_Os01g71050.1|genepair173-1
MGQDLAEIHPRELQFTFEVKKQSSCTVHLVNKSNEYVAFKNYIVMLDTYLYGNLTRRLEN
CIFNARLKQHHLKGIVFDQTLESSFQGQHVISQLNCSFLLLILFFTRLNEVTMQAQRTAP
PDMQLKDKFLVQTTVVPYGTSDEDLVPSYFSKESGRYIEESKLRVVLVSASHPFGEQPIN
GIPNTEAAVEVPSLKDTLNIKNEIPVAEKEVHSPLEEDPVVIPAPPYPVKEAPILREVPV
HISPVRETSFLREVPAPVSPVKETPILREVPTPLKETPVILTESPPPPTDTSSITVESLH
PFEQNLASLKESPLEETLPKAAVVLSEQGAVNVQSRQLCHVTEDVQNLKSKLNGLEVKLE

EIILSEVKHKLSDLQAEKMIIKLREESRTTTQERDKLQQEMVVFDLTFVRHQRGTKAEPS
LYRRVANKCKQVGLCITPLEISDKVKSIYKTLQAAIRYLFFIRGCVTILVPCVMAPSNQG
QGLGHPESQVQQEVVGPILQGHLQEGPIPQGHLQEGPNRQGLLPVLQAQIFRGKIQTAPD
LLQLGEYTISFPTASSHGMINQDVNRRWTQNELLGIKLNWWTTTQGIGYNSIQACQKKRK
IWERVLSRLNCNEIDMTHDENEPLLDWWIKRTSQGGKDKAKENKSVHMRVAWEIRCEWNR
RVFRNQEKTLMQLLDKIRCWTRYKIKSSY*
>11682.m02870|LOC_Os05g30950.1|genepair173-2
MGSVDFGLVEIHPGEIRFEFEVKKKSSCYVCLVNKSEEYVAFKVVIMQAQMIAPPDLQIR
DKFLVQTTVVPFGTADEDIAPAFFSKEVGRYIEENKLRVVLVSATQLEEQQLIAGVPSAK
TGVEVRVAKETLNIESEASNVMNEVHHSLKTNFPPLRENPATLNEMPFPVKQTTILAPSK
EVPAISAESAHHWKETPAESLFSSNAVHHSLKTSFPPLRENPATLNEMPFPVKQTTILPP
SEEVPAISAESGHHWKETPAESLFATNALPHSLKTSCLLRENPAILNEIPFPVRQTTILP
PLKEVPVISAESAHHWKETLNVSLESHFSSTETNVVSSECPETLENTSPSKEFAILRDTL
VNAENLHYVTDDVQNLMTKLSNLEAKLEEAESVIVKLREDTRTTIRERDKLKHEMVVLTR
KGASRSQAGFPLLFVVYMAILGASLGYLLHL*
>11667.m07139|LOC_Os01g71200.1|genepair174-1
MAEEGDKATAAAEAGGGAAATAAAGGGGGGGGGGAEEESVKLFVGQVPKHMTEAELLAMF
QEVAIVDEVTVIKDKATKASRGCCFLICPSREEADKAVNAYHNKHTLPGASSPLQVKYAD
GELERLEHKLFIGMLPKNVTDTELTDLFSKYGNIKDLQILRGSQQTSKAGCAFLKYETKE
QAVAAIEALNGKHKIEGSSVPLVVKWADTEKERQARKAQKAQLQSSNMPSASPMQQSSLF
GALQMGYMPQYNGFGYQPPGTYGLMQYPLSPMQNQATFPNMVQPVNQGNSIRGVNPELSP
NSVPRSFNAMQLSSPYPPVPGVQYAGSYPGGLMNNRPFGNSFSSIKVPIVNANSPASSSP
SSNPGGQIEGPPGANLFIYHIPQDYGDQDLSNAFQRFGRVLSAKVFVDKATGSSKCFGFV
SYDSPASAQAAIGVMNGFQLGSKKLKVQLKRDNSKHSKPF*
>11682.m02873|LOC_Os05g30980.1|genepair174-2
MAEDGGGDRDQQNQQQQQEEAAAPAAAAAGVGGGEEQGNGRGEESVKLFVGQVPKQMTED
ELAAMFAAVAVVDEVTLIRDKATKASRGCCFLICPSREEADKAVNAYHNKRTLPGASSPL
QVKYADGELERLEHKLFIGMLPKNVTDAEMTDLFSQYGNIKDLQILRGSQQTSKAGCAFL
KYETKEQALAAIEALNGKHKIEGSSVPLVVKWADTEKERQARKAQKAQFHPSNMSNPNAM
QQSSLFGAMQMGYVPQYNGYGYQPQGTYGLMQYPLSPMQNQAAFPNMVQSVNQGSSIRGV
NSELSPNSAPRSFNSMQLGSPYSPVPSMQYPGSYPGNAINSRPFVNSHNSMKVPNANASS
PTSSSTSSNPGPQIEGPPGANLFIYHIPQEFGDQDLAGAFQGFGRVLSAKVFVDKATGLS
KCFGFISYDSPASAQTAISMMNGYQLGGKKLKVQLKRDNSKHSKTY*
>11667.m07141|LOC_Os01g71220.1|genepair175-1
MGKEAVGEGQHQQQRPPDGAGGGGGGRGGRGGGGGRGGSGRLGWWWCCCCGVGGVVRLKC
VAALVLGVAVLLSAVFWLPPFARRGRGSEGPDPGAGFDADIVASFRLHKMVPELNGNASK
LELDIYEEIGIPNSTVVVNSLQLVGSNWTNVIFSIVPYPKNLTLSSTGLSILRSYFMSFV
VRQSTLQLTESLFGNSSSFEVLKFPGGITIIPPQTAFLPQKPHATFNFTLNFPIYKVQDR
IDELKDQMKTGLLLNSYENLYIKLANLNGSTVDPPTIVETSIFLEVGNHQPSVPRMKQLA
QTITNSSSGNLGLNHTVFGRVKQISLSSYLRHSLHSGGGSEAPSPAPMHHHGHHHHHHHH
HGHEDSRHSAPAQAPVHYPVHEPRYGAPPPSRCPYGTDKPKNKAHVMPAPEPTANGHHFA
SPVALPPHSLSPRNPNVHSRSPIPSPPVLPEPPLPTVSFAHAHPPSEHTSRRDPAGLSAL
APAPHSFWWIVSDQFASEPYKVTGRAFAISFWVETQSSAMTNFMEFC*
>11682.m02875|LOC_Os05g30990.1|genepair175-2
MALSALFLLKPFAAHRARAGAPPPPGPPGDAFAADIVASFMLQKTVSELSGSTSKLEFDI
YGEIGIPNSTVAVNFLQPIGAPNWTNVIFSIVPYPKYSSISSMYLSILRASFMSLVVEQS
TLHLTESLFGDTSLFEVLKFPGGITIIPPQAAFLLQKPYASFNFTLNFPIYKVQGRMNEL
KDQMKAGLQLDPYELSKKSIFLFHGDEELKD*
>11667.m07142|LOC_Os01g71230.1|genepair176-1
MTAAETQEELLRKHLEEQKIEGDEPILEDDDDDEDDDDDEDDKDDDVEGAGGDASGRSKQ
SRSEKKSRKAMQKLGMKTITGVSRVTIKKSKNAHRIVIYHCILLNFSLHYQILFVISKPD
VFKSPNSDTYVIFGEAKIEDLSSQLQTQAAEQFKAPDLSNVISKAEPSAAAQDDEEVDES
GVEPKDIELVMTQATVSRSRAVKALKAANGDIVTAIMELTN*
>11682.m02876|LOC_Os05g31000.1|genepair176-2
MTAAEIGKQEEELRAQIEEQKLNEGDEPVVVEDDDDDEDDDDEDDKDDDDAEGGDASGRS
KQSRSEKKSRKAMVKLGMKSITGVSRVTVKKSKNILFVISKPDVFKSPTSDTYVIFGEAK
IEDLSSQLQTQAAEQFKAPDLSSMLSKPEASTAAQEDDEAVDETGVEPKDIELVMTQATV
SRSKAVKALKAANGDIVTAIMELTT*
>11667.m07146|LOC_Os01g71270.1|genepair177-1
MADSHETDKNIEIWKVKKLIKGLDAARGNGTSMISLIMPPRDQVSRVTKMLGDEYGTASN
IKSRVNRQSVLAAITSAQQRLKLYNRVPPNGLVLYTGTIVTDEGKEKKVTVDFEPFRPIN
ASLYLCDNKFHTEALNELLESDDKFGFIIMDGNGTLFGTLSGNSREVLHKFSVDLPKKHG
RGGQSALRFARLRMEKRHNYVRKTAELATQFFINPATSQPNVSGLIIAGSADFKTELSQS
DMFDQRLQAKILKVVDVSYGGENGFNQAIEISAEELSNVKFIQEKKLIGKYFEEISQDTG
KYVFSVDDTMSALEMGAVETLIVWENLDINRYVLKNSVTGETTVKHLNKAQETDQSNFRD
KATSAELEVIEKTLLLEWFAENYRQFGCSLEFVTNKSQEGSQFVRGFGGIGGILRYQVEI
NAYEDLSDEEYDEDYE*
>11682.m02878|LOC_Os05g31020.1|genepair177-2
MGEGHETDKNIEVWKVKKLIKALDAARGNGTSMISLIMPPRDQVSRVTKMLGDEYGTASN
IKSRVNRQSVLAAITSAQQRLKLYSRVPVNGLVLYTGTIVTDDGKEKKVTFDFEPFRPIN
ASLYLCDNKFHTEALNELLESDDKFGFIIMDGNGTLYGTLSGNSREVLYKFSVDLPKKHG
RGGQSAVRFARLRMERRHNYLRKAAELATQFFINSATNQPNIAGLILAGSADFKTELGKS
EMFDPRLQAKVIKTLDVSYGGESGFNQAIEMSAEVLSDVKFVQEKKLIGKYFEEISQDTG
KYVLGVQDTITALEIGAVDTLIVWENLDVRRYELKNTATGETVIKYLNSDQEADQSNFVD

EATSGEFDVIDKPLLLEWFAENYQQYGCTLEFVTNKSQEGSQFCRGFGGIGGILRYPADV
AAFNDDDDDMLDEADYEDFE*
>11667.m07150|LOC_Os01g71310.1|genepair178-1
MRGAMKPSIVHCLKLLMLLALGGVTMHVPDEDDVVASLGALRLDGHFSFDDAHAAARDFG
NRCSLLPAAVLHPGSVSDVAATVRRVFQLGRSSPLTVAARGHGHSLLGQSQAAGGIVVKM
ESLAAAAARAVRVHGGASPHVDAPGGELWINVLHETLKHGLAPRSWTDYLHLTVGGTLSN
AGVSGQAFRHGPQVSNVNQLEIVTGRGEVVTCSHEVNSDLFYAALGGLGQFGIITRARIA
LEPAPKMVRWIRVLYSDFETFTEDQEKLIASEKTFDYIEGFVIINRTGILNNWRTSFKPQ
DPVQASQFQSDGRVLYCLELTMNFNHDEADIMEQEVGALLSRLRYISSTLFYTDVTYLEF
LDRVHTSELKLRAQGLWEVPHPWLNLLIPRSTVHKFAKEVFGKILKDSNNGPILLYPVNR
TKWDNRTSVVIPDEEIFYLVGFLSSAPSSSGHGSVEHAMNLNNKIVDFCEKNGVGMKQYL
APYTTQKQWKAHFGARWETFERRKHTYDPLAILAPGQRIFPKASLPMSL*
>11682.m02880|LOC_Os05g31040.1|genepair178-2
MRPSLLQYLKLLLLLALGGVTTMHVPKQDVPSSLEELTLDGHFSFHDVSAAAQDFGNLSS
FPPVAVLHPGSVADIATTIRHVFLMGEHSTLTVAARGHGHSLYGQSQAAEGIIISMESLQ
SNTMRVNPGVSPYVDASGGELWINVLHETLKYGLAPKSWTDYLHLTVGGTLSNAGVSGQT
FRHGPQISNVNELEIVTGRGDVITCSPEQNSDLFHAALGGLGQFGVITRARIPLEPAPKM
VRWLRVLYLDFTSFTEDQEMLISAEKTFDYIEGFVIINRTGILNNWRSSFNPQDPVRSSQ
FESDGKVLFCLEMTKNFNPDEADVMEQEVNTLLSQLRYMPSSLFHTDVTYIEFLDRVHSS
EMKLRAKGMWEVPHPWLNIIIPRSMIHKFAKEVFGKILKDSNNGPILLYPVNKSRWDNRT
SVVIPDEEVFYLVAFLSSALGPHNIKHTLDLNYRIIEFSDKAGIGVKQYLPNYTTEQEWQ
SHFGARWDTFQQRKKAYDPLAILAPGQRIFQKASASLPLPS*
>11667.m07151|LOC_Os01g71320.1|genepair179-1
MGRVGLGVAVGCAAVTCAIAAALVARRASARARWRRAVALLREFEEGCATPPARLRQVVD
AMVVEMHAGLASDGGSKLKMLLTFVDALPSGSEEGVYYSIDLGGTNFRVLRVQVGAGSVI
VNQKVEQQPIPEELTKGTTEGLFNFVALALKNFLEGEDDQDGKMALGFTFSFPVRQISVS
SGSLIRWTKGFSIRDTLVVLVNIVNFSMVQARVFVAAGIAVFASDDPSSGGASAIANCMG
EGYPKMAPAPANKDPPAALLQGRLSQRKQVGRDVAQCLNEALANCGLNVRVTALVNDTVG
TLALGHYYDEDTVAAVIIGSGTNACYIERTDAIIKCQGLLTNSGGMVVNMEWGNFWSSHL
PRTPYDILLDDETHNRNDQGFEKMISGMYLGEIARLVFHRMAQESDVFGDAADSLSNPFI
LSTPFLAAIREDDSPDLSEVRRILREHLKIPDAPLKTRRLVVKVCDIVTRRAARLAAAGI
VGILKKLGRDGSGAASSGRGRGQPRRTVVAIEGGLYQGYPVFREYLDEALVEILGEEVAR
NVTLRVTEDGSGVGAALLAAVHSSNRQQQGGPI*
>11682.m02887|LOC_Os05g31110.1|genepair179-2
MEGRAAGWVRVAAVGWAVAACAVAAGMVARRGAARVRWNRAVAVVRDLEERCATPAELLQ
RVVNSLAIEMFAGLASDGGSKVRMLLTCVDALPDGSSHDMTRSEEGISYAIDLGGTSFRV
LKVELGAGSTIINRKVEHQPIPENLTKGTSDDLFNFIASALKNFIEREGGEVEGRALGFT
FSFPVRQTSISSGTLIRWTKEFSIEEAVGKDVAQCLNEALARNGLNMKVNVLVNNTVGTL
ALGHYYDDDTVAAVIIGAGTNACYIERNDAIIKSLGRVTNSERTVVNVEWGSFRPPQIEL
TPYDICFNNETWNYYDQGFEKMISGVYLGEIARLVFQKMAEESDIFGTAVDGLSTPFVLS
TPNLAAIREDDSPDLREVGKILEEHLKLPDVPLKTRKLVARVSDIITRRAARLAAAAIVA
ILQKIGCDGTLCGSTQVRTMRGVRRRTVVAIEGGLFEGYSVFREYLNEALVEILGEEIAA
TVSLRVMEEGSGTGAALLAAAYSSARQKNSE*
>11667.m07168|LOC_Os01g71470.1|genepair180-1
MESETGTAAVGVCWGMSGDNLPPASKVTEMLRENGFTVVRLYAPDSAALAALGGTGIRVV
VGAPNYDLPALAHGGTAAAAAWIRENIQAYPTVLFRFVVVGNEVAGADTQLLVPAMENVH
AALAAAGLGHIKVTTSISQATIGVHIPPSAGEFTDEAKPFMSYVIPFLERTHAPLLANLY
PYFIYSYNPGGMDISFALFTASGAVVQDGEYGYQNQFDATVDALYTAVAKLGGENVRVVV
SETGWPTAGGVGASVENAMTFNQNLVRHVRNGTPRHPGKKTETYVFAMFNENLKEAGVEQ
NWGLFYPSTDRVYPISFHARI*
>11682.m02891|LOC_Os05g31140.2|genepair180-2
MASQGVASMFALALLLGAFASIPQKAEAIGVCYGMSANNLPPASSVVGMYRSNGITSMRL
YAPDQAALQSVGGTGISVVVGAPNDVLSNLAASPAAAASWVRNNIQAYPSVSFRYVAVGN
EVAGGATSSLVPAMENVRGALVSAGLGHIKVTTSVSQALLAVYSPPSAAEFTGESQAFMA
PVLSFLARTGAPLLANIYPYFSYTYSQGSVDVSYALFTAAGTVVQDGAYGYQNLFDTTVD
AFYAAMAKHGGSGVSLVVSETGWPSAGGMSASPANARIYNQNLINHVGRGTPRHPGAIET
YVFSMFNENQKDAGVEQNWGLFYPNMQHVYPISF*
>11667.m07198|LOC_Os01g71770.1|genepair181-1
MATKLVVLGIPWDVDTEGLREYMGKFGPLDDCVVMKERSSGRSRGFGYVTFSSADDAKNV
LECEHVLGNRTLEVKIATPKEEMKSQGSKKATRIFVARIPQSVDESMFRRHFEAYGEITD
LYMPKEHGSKGHRGIGFITFQSAESVDSIMQDSHELDGTTVVVDRATPKDEEVRYPPSRG
ASQGGYGAYNAYISAATRYAALGAPTLYDHPGSAYGSKSSATKITGGGYYGSSQGMGNKK
IFVGRLPQEANTEDLRHYFGKFGRIVDAYIPKDPKRSGHRGFGFVTFADEGVADRVARRS
HEILGHEVAIDTAAPLEGDSGGGYMEPMDLYGAYGSMRTYGRFCSGIDYDFTSKGTVETE
KRTTP*
>11682.m02898|LOC_Os05g31200.1|genepair181-2
MAPVRGERAALPARAACGLRAAAGTPAPIWVPVGGRAVPWAAAAAVEVGCCPPLCPLPCP
SSCQLLKLPVGDDVAGDGAVDAAAQGLNLYLRRTCRIFRRLRLRLRPGCCQLRCRQSGCP
CCCCNGLSCCSPALFCCGELGVLLTPSARGDRTPRPSVGHGAPAGGCCVERGKGKGKEEQ
PVLLLLLAESSTELGKGWNKRCLAYRKDEDVRHPPRRVVQGGYGAYNAYITAATRYAALG
TPTSYDHPGPAYGNRVAQRSHEILGQEVAVDTAEPLEGGSGGGYLEPAEAYGPYGAYGSL
LPYGRFSGSLGYDYGYGPSGNSSRHGNTVYLENNIRLKPGIEHPEVSKPMQIEFHCIFVI
RSAHMAGFSFGDIAMASYGVT*
>11667.m07218|LOC_Os01g71970.1|genepair182-1

MVQDEGSSSSVTSSPLHNFSNMPLHPAAAASPTPPWMVRELRSDERGLCLIHLLLNCAAA
AAAGRLDAANAALEHIASLAAPDGDAMQRVAAAFAEALARRALRAWPGLCRALLLPRASP
TPAEVAAARRHFLDLCPFLRLAGAAANQSILEAMESEKIVHVIDLGGADATQWLELLHLL
AARPEGPPHLRLTSVHEHKELLTQTAMALTKEAERLDVPFQFNPVVSRLDALDVESLRVK
TGEALAICSSLQLHCLLASDDDAAAVAGGDKERRSPESGLSPSTSRADAFLGALWGLSPK
VMVVAEQEASHNAAGLTERFVEALNYYAALFDCLEVGAARGSVERARVERWLLGEEIKNI
VACDGGERRERHERLERWARRLEGAGFGRVPLSYYALLQARRVAQGLGCDGFKVREEKGN
FFLCWQDRALFSVSAWRGRRFD*
>11682.m02916|LOC_Os05g31380.1|genepair182-2
MAVEIPRLALGGGGGGAGGERLPAAGEDSAPAATNAGKRPVVGLGFGSSLAAMAAAAAAG
IQPDAFALGGPAEYPAGDGERDVLMVSFLRSIAAFLADGTCQMQVNDGLSCVVDLAGGDA
DGGGVGEGRSAQRLASAFAEALALRFILPCDGVCRSLHLTRAPPPPAVSAARQGFRAMCP
FVRLAAAAANLSIAEVMEAERAVVHVVDLGGVDANQWVELVRLVAARPGGPPGLLRLTV
VNESEDFLSAVAAYVAAEAQRLDLSLQFHPVLSSIEELSATATGSIGSRLVVIPGQPLAV
VANLQIHRLLAFPDYVDGVASRRPAAEQSGSSQHTMTTATKTKADALLRAIRDLNPKLVV
LTENEADHNVAELGARVWNALNYYAALFDALEASSTPPAAVPPHERACVERWVLGEEIKD
IVVREGTGRRERHETLGRWAERMVAAGFSPVTAARALASTETLAQQMVAAGGGGAGAGVL
RAAHGGGCFPVICWCDVPVFSVSTWTARRVLVPAPPLWPPAAAGGAGPSGSGYGGDGPST
ASSSAAMWWVG*
>11667.m07234|LOC_Os01g72100.1|genepair183-1
MVKIKMPALFRRRSGSKSPPLPQADPASGGGSPAPTPEEEMERVFRKFDANGDGRISRSE
LGALFESLGHAATDDELARMMAEADADGDGFISLDEFAALNATASGDAAAVEEDLRHAFR
VFDADGNGTISAAELARVLHGLGEKATVQQCRRMIEGVDQNGDGLISFEEFKVMMAGGGS
FAKIA*
>11682.m02940|LOC_Os05g31620.1|genepair183-2
MGKVRAFFSRKGRGNSSGRSRSMREAAMNVDWSPRPSDLAAAAAAKPRPPAAEDETERVF
RKFDANGDGRISRAELAALFRSVGHAVTDDEVARMMQEADSDGDGYISLGEFAAISAPPP
GDAAAAEEDLRHAFGVFDADGNGVITPAELARVLRGIGEAATVAQCRRMIDGVDRNGDGL
INFEEFKLMMAAGAGFGRIAS*
>11667.m07243|LOC_Os01g72190.1|genepair184-1
MVQRKQARKKPKDSQLGSNDGLPADHARGGAIVRTALPNYMRATSSSDARGGGREAAAAT
GAPPRRERGAARGKAMVLADGGGPFVSRATCSSAMRGLGRGLGLRGGAAHACPYSYCSFK
CHAADADVVPLRTLVASRRRLIKTQQSMKLKGASPFRKPSNGGGAADGFFVEIYSGAAAA
AAPTVSSGASCSDLSTEDNDAAVREGEYAVFDHRSRGDDEDKARDSDGSVDGSCGSSDVI
SGGSVDLFVTKSRGGKQEIDGGKGIYLDQEAEDFDACKSDISEELDAKYEDGGVDASNGS
SIDDISSAFGGMNFKDACPDPTGAASSQRKRWNIAKRTTSEQGEKMRLFNPRAPNFLPVE
PDPEAEKVDLRHQMMGDRKNAEEWMVDYALRRAVNKLARAQKRKVEMLVQAFETVQQDDD
KKSITLTKSSQPCR*
>11682.m02947|LOC_Os05g31690.1|genepair184-2
MVRSKEVHKKPKDPLMTPPPSSAKKRGFRDDGGGGGGGSRPRNREGAATSLTPASVPNYM
RGTSSSDAKVGRGAASSASPRRRPVRVVARGKVLFPKVSAAAAAAASAGLGRATCSSTMK
EAKFPDALDLAPGATDAEGPAATRVCPYTYCSLNGHAHSPAVPLRSFLASRRRLIKTQQS
MKLRGVSAFRKGAAHQRPEDTNGAGGGARVAPPPPLIDEEALGDFFVEVYAGPRVSTDMS
CSDMSLDEMDATVRKMEFVVFDRCGADESDEKGKNDLDVCGDGGDDDGEARPEERFGAFR
DSTSECSDASTSGEFVEELPWIRYQGYEDDSLDGEFSDEHGIRDEEITRAVVSEFQEDQE
EEGTSGRLGDGCEDEAAQQQEENDEQNISDFACESEIASEHEGVDFRVEACEEQERVSED
NILDAAHQTEVCREQEMQEEKNFAAVCKLGIPEQELAETADNIPDECCKEETSMEQDEGG
DGTNMESESISEVAEHPNVEDEENTQDDGGSEMEISEEIISGFGCEEDFSEEVTSKYVSE
GEISDSGAIVSLHVEMQKQPVENHAFEQDDSSTADNAFHQDDSTADRAFDQDDIRADGYD
DSQKELDIGMREFRVASEEVGIQEANSDDPVDCTEDANMELGVFLCDLQDASEGSGIAQE
SSQEGNSACFNDGAQMVPDITTQTTEDASEESDTAQETTLDDNSTPLAAGAQMELGIGTS
ELIEGSSDVTEQSGIAEETCQDDNAGYFSDDDSQKATVITTCQLQVDYEENVIAQEADDN
STGVRDDAQNEPEQTCELATSEECHFTQKTIQNHGALEESVVIASASEDAHEESDPTQDG
HEEDYSVGINSGAQKEGELDTSESGGASEGTTVHQEDDGHVNTTDLNGSAQKEITVSILD
DSEELCSSEENNQSSNMLIPEFSDNFSAEEPQNQDSVAKESSLDDICNAFSGMHLKGDAY
LDPTESMTCPGNRLIIARRRRTPEEEEYLRGFNPRAPNFLPLELDPESEKVDLKHQMMDE
RKNAEEWMIDYALRRAVNNLGPARKKKVELLVQAFETVLPHDEEEKKGITPTRPVQACN*
>11667.m07247|LOC_Os01g72230.1|genepair185-1
MEGHKPVHQFVDSPKPQRSARRICGMASRIDANDHGNQQGGNPQGPVGTAERLEHLLNQP
ANKICADCGAPDPKWVSLTFGVFICIKCSGAHRSLGVHISKVVSVKLDEWTDEQVDILAD
SGGNAAVNMIYEAFIPENYMKPRQDCSSEGRNDFVRRKYELQQFLSNTQLTCYSQKSGKN
HNRVQHSSSNRHGLGHAFRNSWRRKEHESKSVKKTVEIGMVEFVGLIKVNVIRGTNLAVR
DMMSSDPYVILNLGHQSMKTKVIKSSLNPVWNERILLSIPDPIPMLKLQVYDKDTFTTDD
RMGEAEINIQPLVAAAKAYETSVVADTAQLNRWLAKEGIWIQKDSAISIIDGKVKQEVTV
RLQNVERGHLEMELECVPLTQ*
>11682.m02950|LOC_Os05g31720.1|genepair185-2
MHRQDVRTKEAEEERMEENNLLHFLDSPNAHYRRKCEEYVSAHDDEAHCDASDVDLANAR
ERLEHLLKQPANKFCADCGTPDPKWAALPFGALICIKCSGTHRSLGVHISKVISVNLDEW
TDEEVNCLAGSGGNATVNTRYEAFLPENFKKPRHDCTTEERCNFIRKKYEFQQFVTDPQF
SCPLRLNTKHAPDKNQQQQNCSARHGFGHAFRNSWKRKDTDNKGLKKMTDVGMVEFVGLI
KVDIRRGTNLAVRDVMSSDPYVMLNLGHQTMKTKVIKNTLNPVWNERLMLSIPHPVPPLK
LQVFDKDTFSSDDRMGDVEVDIQPLIAAAREHESSAAIAGSVEVTKLLASDDGTLARDSV
ISVVDGKVKQDIALRLQNVEHGELEIELECVPLSQ*
>11667.m07250|LOC_Os01g72260.1|genepair186-1

MDGTLAPLLLLLLLFLPALLLYLRRRPAAASRINNNHCPHPNPVLGNALPFLRNRHRFLD
WATDLLAAAPTSTIEVRGALGLGSGVATANPAVVDHFLRASFPNYVKGARFAVPFEDLLG
RGLFAADGRLWALQRKLASYSFSSRSLRRFSARVLRAHLHRRLVPLLDAAAGSGEAVDLQ
DVLGRFGFDNICNVAFGVESSTLLEGGDRRHEAFFAAFDAAVEISVARVFHPTTLVWRAM
RLANVGSERRMRDAIRVIDEYVMAIVASEERLRLRRGEDEREHEQHLLSRFAASMEEEGG
ELAAMFGSPGAKRRFLRDVVVSFVMAGKDSTSSALTWLFWLLAANPRCERRVHEEVSSSR
HADPRRADAGEDGHGDGYDELRRMHYLHAAISEAMRLYPPVPIDSRVAVAADALPDGTAV
RAGWFADYSAYAMGRMPQLWGEDCREFRPERWLSDGGEFVAVDAARYPVFHAGPRACLGR
EMAYVQMKAVAAAVIRRFAVEPVQAPASMETPPACEVTTTLKMKGGLLVRIRKREDDAAQ
QKLT*
>11682.m02952|LOC_Os05g31740.1|genepair186-2
METAHLAYVLLFLVTAVLLFHLRRGGRSAPAKLTTAHRPHPNPVLGNTVEFIRNRRRFFD
WYTDLLRASPSGAIEAWGPFGAGHAVTTACPAAVEHLLRGNFGNYVRGASFRAAMSELIG
DGLFAADGRLWSVQRKVASYAFSSRALRRFSDDVLAVHLRGGLLPFLDAAAASGEAVDLQ
DALRRFAFDSICHVAFGVESSTLLETAREDSGRHEALFAAFDAAVEISFRRALAPFTLVR
KLTGLVNVGSSRRLREAIGVIDDYAMSVVESKEAACRDRDSEDVDPDLLSRFMAAMDEED
GGELGAMFPTPEGKRRLLRDVVVSFVLAGKDTTSSALTWLFWLLAANPRCERRVRDEVSR
SPDGDGGGDAKGMHTHYLHAAITEAMRLYPPVPFNGRVAVGDDVLPGGAAVRAGWFANYS
AYAMGRMERLWGEGCLEFSPERWLRDGGEFVAVDAARYPVFHAGPRACLGKEMAYVQMKT
VAAAVLRRFSVEVAAPAPAMESPPAYEMTATMKMKGGLWVRLRKRE*
>11667.m07252|LOC_Os01g72280.1|genepair187-1
MEAKIGRLVGAIGAFFSGGDNVPWCGRDIIAGVEREVAEAATEEHKNVSIMRLSWALVHS
RNTDDVNRGIAMLQASLGGSKSPLEAREKLYLLAVGHYRNGDYPRSRQLLEHCLEIQPGW
GQALSLKKTVEDKIAKDGVIGIGIATTAVGLLVGIAAAVARKN*
>11682.m02958|LOC_Os05g31770.1|genepair187-2
MEAKIGKLVESVGSFFSGGDTIPWCTRDIIAGCEREVAEAANEEQKSDSIMRLSWALVHS
KNQEDVNRGIGMLEASLGQSNSPLQTREKLYLLAVGHYRNGDYPRSRQLVDRCLEVCVQI
FSFHGNVYLVFLVLVLFSCLTYVHNANGIESFNLSRQEHFHIQPDWRQALSLRKAIEDKI
AKDGLIGIGIATTAVGLLVGGIAAIAARKK*
>11667.m07256|LOC_Os01g72320.1|genepair188-1
MPFILCSFLLLCILVEAITALKKGAYLLKYGRRGKPKFCPFRLSNDESVLIWFSGKEEKH
LRLSQVSRIVPGQRTAIFQRYPRPEKECQSFSLISHDRSLDIICKDKDEAEVWFAGLKTL
ISHSHQRKWRTESRSDIISSGATSPRTYTRRSSPLSSPFSSNDSVHKDGSDHYRLRSPFG
SPPKNALDKAFSDVVLYAVPPKGFFPSDSNAGSVHSMSSGHSDNTNGHPRGIPMDAFRVS
YSSAISSSSHGSGHDDGDALGDVFIWGEGTGEGILGGGSSRVGSSSSAKMDYLVPKPLEF
AVRLDVQNISCGGRHAALVTKQGEIYSWGEESGGRLGHGVDCDVAQPKLIDSLAHMNIEL
VACGEYHTCAVTLSGDLYTWGDGTFKFGLLGHGNDVSHWVPKRVDGPLEGIHVSSISCGP
WHTALVTSAGQLFTFGDGSFGVLGHGDRASLSVPREVESLKGLRTVRAACGVWHTAAVVE
VMVGNSSSSNCSSGKIFTWGDGDKGRLGHGDKDSRFVPTCVAALVEPNFCQVACGHCLTV
ALTTSGHVYTMGSAVYGQLGNPQADGLLPVRVEGKLHKNFVEEISCGAYHVAVLTSRTEV
YTWGKGANGRLGHGGTDDKNTPTLVEALKDKQVRSVVCGINFTAAICIHKWVSGSDQSMC
SGCRQPFNLRRKRHNCYNCALVFCHSCSSKKSLKASLAPNPNKPYRVCDSCYSKLTKGLE
TDTNSSTKRGTVVQGFSETNEEELETRSNTQLSRLSSMESFKNMDSRYSKKNKKFEFNST
RVSPVPNGSSHWSGLNISRSFNPVFGSSKKFFSASVPGSRIVSRATSPVSRRTSPPRSTT
PTPTLGGLTSPRVIANDAKPTNDSLSHEVLNLRSQVENLTRKSHLLEVELERTTKQLKEA
IVIAGEETAKCKAAKEVIKSLTAQLKGMAERLPGGVTKNSKLPPLSGFPMPSELSSMATE
SLGSPSSVGEQISNGPNGLLASNGPSSVRIKAGHPEVGKNGSRLPEAESCHEAEWVEQDE
PGVYITLTALPGGARDLKRVRFSRKRFSETQAEQWWQENRTRVYQHYNVRMVEKSASSID
NEIASR*
>11682.m02974|LOC_Os05g31920.1|genepair188-2
MSDASSDLGGARPGAVERDIEQAITALKKGAYLLKYGRRGKPKFCPFRLSNDESILIWFS
GKEEKQLRLSHVSRIIPGQRTAIFQRYPRPEKECQSFSLISHDRSLDIICKDKDEAEVWF
AGLKTLITRSHQRKWRTESRSDMLSSGTTSPRTYTRRSSPLSSPFSSNDSVHKDGVENYR
LRSPFGSPPKVGLEKAFSDIVSYAAPPKPFFPSDSNAGSVHSVSSGQSDNTNLHSRGIPM
DAFRVSLSSAVSSSSHGSGHDDGDALGDVFIWGEGTGEGILGGGNSRVGNSSGVNMDCLI
PKPLEFAVKLDVQNISCGGRHATLVTKQGEIYSWGEESGGRLGHGVDCDVPQPKLIDALA
NMNIELVACGEYHTCAVTLSGDLYTWGNGTSNSGLLGHGNEVSHWVPKRVNGPLEGIHVS
AISCGPWHTAIVTSAGQLFTFGDGSFGVLGHGDRQSLSVPREVESLKGLRTVRAACGVWH
TAAVVEVMVGNSSSSNCSSGKIFTWGDGDKGRLGHGDKDTRLVPTCVAALVEPNFCQIAC
GHCMTVALTTSGHVYTMGSPVYGQLGNPQADGMLPVRVEGKLHKNFVEEISCGAYHVAVL
TSRTEVYTWGKGANGRLGHGDTDDRSSPTLVEALKDKQVRTVVCGINFTAAICIHKWVSG
VDQSMCSGCRLPFNLRRKRHNCYNCALVFCHACSSKKSLKASLAPNPNKPYRVCDSCYSK
LNKGPETDRYSSAKRGSVIQGFNDSVDEELETKSNAQLSRLSSLESFKHMDSRTSKKNKK
FEFNSSRVSPIPNGSSHWSGLNISKSFGSSKKFFSASVPGSRIVSRATSPVSRRASPPRS
TTPTPTLGGLTSPRVVDGVKPNDSISQEVLSLRSQVENLTRKSQLLEADLERTTKQLKEA
ISIAGEETAKCKAAKEVIKSLTAQLKGMAEKLPEGAGLVKNSRLPPLPGISIPTDISVAS
ESLGSPRSSGEPCSNGSNGLLVSNGPTSVRNKLSHPEIPKNGTRLPDSDSKHESEWVEQD
EPGVYITLTALPGGTRDLKRVRFSRKRFSETQAEQWWQENRARVYEQYNVRVVDKSVGSI
DNDVHH*
>11667.m07260|LOC_Os01g72360.1|genepair189-1
MGNGLSPCMHLPATAAAGVKLVYWGGQTRMLDDDGGVCAVAGDVTAELPGDHVVCAADSF
YVGLPIPVVSAGEELMAGRTYFVLPAERFSCFKVLTAAALASLSPAPSKKVSVAGPGQCP
FEYVKGEGGATLIRVLPEFIEKVISCDENGGGARRRKPSTTATATATELCSTPELKRHYA
LLMGRRNQPWSPRLETISECNKTRKLLLRTLARLLSSR*

>11682.m02980|LOC_Os05g31970.1|genepair189-2
MGNGMSPCLCSPPVHGEEAAAEAAAARLVFWGGAASQLVASAATTAGDVMAELPGHLVCA
GDSFFIGLPIPALPAGEELAAGRTYFVLPAARFSCQQALTAASLASLSPSPAAKVSLAGG
ASSPFEYVTGDDGMALIRVLPEFIERAITCVARVAGGGKAGGEAAADDQLCSTPELRKHY
MQLVGARQQRPWSPGLETISEAEKRRRRRSPVRLVALAKAASR*
>11667.m07273|LOC_Os01g72490.1|genepair190-1
MCSSSSSQVTKRKGKRRRVPHLHLIAGAAKSADISVASGRRFPSDTGAFADWVASSAAAA
AAGRGDDLSLGFNATAAAAAPSGASLAAGLWGPASSSRQAALNYGMADVGMVVVTPAASF
HHTHHHHHHHHEAAAAAAAAAAAAAADPIFPLLSAGPCVLDPDKSAASGSAIQFWQPPPQLP
SSAAGGNPNPSSSAFPYLKKPLPMLDTGGGSSGSGGAATCQDCGNQAKKDCGHQRCRTCC
KSRGFDCSTHVKSTWVPAARRRERQQLTGSASSSPATASAAAASKKPRLLTSQTTTSHTS
TSNATTPRSFDTTSSHQDASFRESLPRQVRAPAVFRCVRVTSIDDGEDEYAYQATVTING
HVFKGFLYDQGVDDGRGLAATSNDDSTAGGVPNISELHLGGASISGNAMREGGSSMVHSD
LYGGGGGSGGGPHILGGSSYGVQAIILIYWSSRREGEAGSWWFPAPFTSFFLGEFPYLFL
VPDPIEDDKM*
>11682.m02990|LOC_Os05g32070.1|genepair190-2
MVLVAPAASYHHHRAAAAAAAAAAAAAAAEPVFPLLGTGQCALDADTAKSSGAAAAAGVP
PGSASAIHFWQSQPTTAAGAGGGSADKKPLPMLDYGGIGGPGGSGAATCHDCGNQAKKDC
VHHRCRTCCKSRGFDCPTHVRSTWVPAARRRERQQLAGAASSPPTSSAFPAATTASAKKP
RLLGSQTTTTTSRTSTSNATTPRSFDTSSSHQVASFRDALPRHVRAPAVFRCVRVTSVDD
GDDEFAYQAAVTINGHMFRGFLYDQGADDGRGGMASTSNDESSHGAGAAVPSISDLHLGS
ASAAVPPHLYSGGSGGPLILGGLGYGNTMN*
>11667.m07280|LOC_Os01g72560.1|genepair191-1
MPLSLSSNTRNNGTWLSATYISQTGQKIKEIMAGPKHGCDAKKLNEMLREERGQLRERHQ
TRVVPRRRREGPASVQGQVKVKGWEIVIRGEITSDDMAAAMGLVVGSPFCALAAKREKLL
QQDVHEDESRLNYENSVRRGNAIVIVLHKEHGYAPHINVQSLQLSPSVRTEGPITICAFC
PQPREWKEIILRRGKGTGDIAEQVGGSKRVIYEDRIQTALESEKDQSPHQAVVKSEKDQS
QAAMKSEKADTEKLIQFMEANYEKYVANVDSFEDFYHAIVELIEKFCEERGQVQYKIPSK
KALKEAYEKHHTEQGQLKREEFIKIGKEVIRRDSFTLGKATMDFIMYLFGAPLCALAAKR
ILPGLRWISDDVAIPLATSASVAYLIRTKQL*
>11682.m02997|LOC_Os05g32130.1|genepair191-2
MGQAAAKAKQGGDQAENKTGKDEKVAKPKDAKDLTDFMEKNYDSIKDVTSFEDFYHAFYE
LIEKFCEERGQLQYRIPEKAELQKQYERVNKSPQKGQNLSRKQFMELAGQVIKVNSFTFG
KATMDVLVVLFGAPVCALLAKRVVPGLKSFSDDVVIPVATSGAVVYLAKTNKL*
>11667.m07294|LOC_Os01g72690.2|genepair192-1
MSLDELPHKKLASSEGIRFARSDSRFRSLSPAPIPIPTAPSFRSLVDPVSDERVNHDTVT
SHESEIGSGSISTVSSTVSSVESEKAAYEFLAQTPIKSTDAHLVEFSEAMRTVAKALRRV
AEGKAAAQAEAEEWRRKYELEMAHKQQRKIKGYGSCANNELEKLASQLTLETPASDQAGC
CGNHGICSHEVLQDESPGPNPRSSHKLVSRKASFRLSWGCNGDKNGQHKHDFVSFEKGDI
TTAERSNKQILLKWESSPQTVLFITKPNSNSVHVLCAEMVRWLKEHKKINVVVEPRVSKE
LLTEDSYYNFIQTWDDDEEKKMLHTKVDLIVTLGGDGTVLWAASLFKGPVPPVVAFSLGS
LGFMTPFPSEQYRDCLDNVLNGPFSITLRNRLQCHVIRDAAKDELETEEPILVLNEVTID
RGISSYLTYLECYCDSSFVTCVQGDGLIISTTSGSTAYSLAAGGSMVHPQVPGILFTPIC
PHSLSFRPLILPEYVTLRVQVPHNSRGQAWASFDGKDRKLLSPGDALICSISPWPVPTAC
LVDSTTDFLRSIHEGLHWNLRKSQSFDGPRD*
>11682.m03005|LOC_Os05g32210.1|genepair192-2
MESERAAYAFLPQTPIKSTDAHLVEFSEAMRAVAKTLRQVAEGKAAAQAEAAEWKRKYEL
EKAVKAHRHNTVTKGCSNCDKEKLEQLASQLTLETTSVDPTSCCGNHEICSRQILQDECP
GTNKISHDKIAARKAPFKLSWGCNGDNNGQHKHDFVSFEKGDITTAERSNKQILLKWESP
PQTVLFVTKPNSNSVHALCAEMVRWLKEHNNINIFVEPRVSKELVTEDSYFNFIQTWDND
EEMKTLHTKVDLIVTLGGDGTVLWAASLFKGPVPPVVAFSLGSLGFMTPFSSELYRECLD
HVLKRPFGITLRSRLQCHVIYDSAKNEVDTEEPILVLNEVTIDRGMSSYLTYLECYCDSS
FVTRVQGDGLIISTTSGSTAYSLAAGGSMVHPQVPGILFTPICPHSLSFRPLILPEYVTL
RVQVPINSRGQAWASFDGKGRKQLGPGDALICSISPWPVPTACLVDSTTDFLRSIHEGLH
WNLRKSQSFDGPVA*
>11667.m07296|LOC_Os01g72710.1|genepair193-1
MAFHYAAMPSSSSSSLSGVSSQPPLHLPRLRSPHQASRRLSALPFSRALPLPLRLRLRIP
RPQLPPLPLAFSHGGGGDNDGDDNNNNGGGDGEGDGGAPDNRREALFVLAQLGRKLESLP
SDLAAAVEGGRVTGEIVRRFAEMEGSALLRWLLQFQGFRERLLADDLFLAKLAMECGVGV
IAKTAAEYEKRRENFVKEIDIVIADVVMAIVADFMLVYLPAPTVSLQPPLATNAGHIANF
FHNCPDNAFQIALAGRSYSILQRLGAILRNGAKLFTVGTSASLIGTGVTNALIKARKAVD
KELDDEVEDIPVLSTSVAYGVYMAVSSNLRYQILAGVIEQRMLEPLLHNHKLLLSALCFA
VRTGNTFLGSLLWVDYASLSISSYFPKPLPPRSRRPSFPPLVQQPIAMSSWPPPPPVQLS
MQFLPPRPEPAAAARTSICCTCGVPMAPNAANTCALCIRSRVDIAAGVPRHADVVHCPSC
SSYLHPPRLWLRAAPESPELMSLLLRRVDRHIARLGVALAAAEFVFTEPHSRRLMLRLRL
RGEVLHGSGGGVTLEQGHVVEFAVHDRLCDACAMARARAAEPPDQCGWSAVVQLCPVCRD
DLVFLPKEASRDLGGLGPIVLCVKVTNALALLDTSTLRVVHLGIKEYDRCRLEPALTSRQ
LVEYVVLDVDHEPEPAAGVAYAQVARASDLGKNDTIFTVRTHLGHVLNAGHRALGDDLYG
ANVNNHDVESHGLPDAVLVKKINEKGSTRRQLQDGDGCRRRKRDGDEMEEIAMGIGCIDL
NPPDEKELDELLRGPHNLRASAVTSPCLCQAHMSPHVSDNGTMKSTTEDLNPI*
>11682.m03007|LOC_Os05g32230.1|genepair193-2
MAFPSPNSLSAASHPTSSSSSFHLHLQLQQPVPHLPFPRSLPLNLPVLRLARPLLPPAPL
ASSGSGGIGIGGGGDDDEGRDNAGGGDGGDDDASVNRREALFVLAQLGRKLESLPADLAA
AIEGGRVPGEIVQRFADLEKSGLFRWLLQFGGFKERLLADDLFLAKVAMECGVGIFTKTA

AEYERRRENFVKELDFVIADVVMAIVADFMLVWLPAPTVSLQPPLAVNAGSIAKFFHNCP
DNAFQVALAGTSYSLLQRVGAIMRNGAKLFAVGTSASLIGTGVTNALIKARKAVSKDFEG
ESEDIPIVSTSVAYGVYMAVSSNLRYQILAGVIEQRMLEPLLHHHKLVLSALCFAVRTGN
TFLGSLLWVDYAKWIGIQ*
>11667.m07304|LOC_Os01g72790.1|genepair194-1
MAVAAPGQLNLDESPSWGSRSVDCFEKLEQIGEGTYGQVYMAKETETNEIVALKKIRMDN
EREGFPITAIREIKILKKLHHQNVIQLKEIVTSPGPERDEQGKPIEGNKYKGSIYMVFEY
MDHDLTGLADRPGMRFTVPQIKCYMRQLLTGLHYCHVNQVLHRDIKGSNLLIDNEGNLKL
ADFGLARSFSSDHNGNLTNRVITLWYRPPELLLGSTRYGPAVDMWSVGCIFAELLNGKPI
LTGKNEPEQLSKIFELCGTPDELIWPGVTKMPWYNNFKPQRPMKRRVKESFKHFDQHALD
LLEKMLTLDPSQRISAKDALDAEYFWTDPLPCDPKSLPKYEASHEFQTKKKRQQQRQAEE
AAKRQKLQHPPPHSRLPPIQNPGQPHQIRPGQPMHNAPPVAAGPSHHYAKPRGPGGPNRY
PQGGNQGGYNPNRGGQGGGYGSGPYPQQGRGPPPYPGGGMGGAGGPRGGGGSGYGVGGPN
YQQGGPYGASGPGRGPNYNQGGSRNQQQYGNWQ*
>11682.m03020|LOC_Os05g32360.1|genepair194-2
MAVAAPGQLNLDESPSWGSRSVDCFEKLEQIGEGTYGQVYMARETETQEIVALKKIRMDN
EREGFPITAIREIKILKKLHHQNVIQLKEIVTSPGPERDEQGKPIHGNKYKGSIYMVFEY
MDHDLTGLADRPGMRFTVPQIKCYMKQLLTGLHYCHINQVLHRDIKGSNLLIDNEGNLKL
ADFGLARSFSNDHNGNLTNRVITLWYRPPELLLGSTKYGPAVDMWSVGCIFAELLNGKPI
LPGKNEPEQLSKIFDVCGTPDESNWPGVTKMPWYNNFKPPRQLKRRVKEYFKHFDRLALD
LLEKMLTLDPAQRISAQDALDAEYFWSDPLPCDPKSLPKYESSHEFQTKKKRQQMRQADE
AAKRQKTQHPQPHGRLPPIQQTGQPHPQIRPGQPMNNPHAPMAAGPGHHYAKPRGPGGSS
RYPQGGNQGGGYPNRGGQGGGGSYGNAPYPQQGRGPPPPYPGSGMAGTGGPRGGVGGGYG
GGSNYPQQGGPYGPSGPGRGSNYPQQGGSRNQQQYGNWQ*
>11667.m00911|LOC_Os01g09540.1|genepair195-1
MAPKRLVCFLAVAAALATTCHGWGAGAGDVVSSSAAALSFVDRLRQMMIPAAVGDGDYCD
SWRVGVEANNVRGWTAAPRKCDNYVENYMRGHHYRRDSKVVVDEAAAYAEAAVLSGDPAA
DANATWVFDVDETALSHVKFYKKHGFGYHRTDEPAFMEWLIAGRASALPNTVTLYKKLLL
LGVKIVFLSDRPDTPELRNATATNLIKEGFDCWDELILRSENSTATGSVVEYKSGERKKL
EEEKGMVIIGNIGDQWSDLLGSPEGRRTFKLPNPAYYIDNYKRAGAAVRAAVAITASSSS
SSS*
>11682.m00964|LOC_Os05g10370.1|genepair195-2
MATAARLLLLLFLTAAASAWEMNIRLPTEMLNGGEAVVAPIIHALRPLLGSGGQLAARAG
VACDSWRLGVEAHNVIGWKTVPASLQLSGNGKEIWVFDIDETSLSNLPYYAKHGFGATLY
NDTSFREYVAEGSAPALPETRRLYRRLLQLGVKPVFLTGRTEDQRNITVTNLRRQGYSGW
MELLLKPAVHAAGELQGSAVAYKSGERQKLEDAGFTILGNIGDQWSDILGTPEGARTFKL
PDPMYYIG*
>11667.m00914|LOC_Os01g09550.3|genepair196-1
MNRGHISSSELIDAKLEERRISTAKHCPSCGNKLDCKPDWVGLPAGVKFDPTDQELIEHL
EAKVREEGSRSHPLIDEFIPTIEGEDGICYTHPEKLPGVTRDGLSKHFFHRPSKAYTTGT
RKRRKIQTECDVQKGETRWHKTGKTRPVMVSGRQKGCKKILVLYTNFGKHRKPEKTNWVM
HQYHLGDLEEEKEGELVVCKIFYQTQPRQCSWSSDRGGGAAATASAVTTAAVQQDQQRRR
DSGSGSCSSTRDHEVSATSYSTAGYAVAAAVEMQHLKHAADHFSFAPFRKSFEEVGISGD
QVHSNQLGRSEQQHAGQEQQPHRPLLATTTAVPATAFLISRPTNPVSNIVPPAMQHASVV
LDHDQFHVPAILLHHDKFQQQQQQKLDRRSAGLEELIMGCTSSSTKGEASIPHSQETEWP
YQPYWTPDNQDHHG*
>11682.m00990|LOC_Os05g10620.1|genepair196-2
MSSDGASGSIGMKHDDGDHRPSTGASSRRCPSCGHDPDCNKPFDMVGMPAGVRFDPTDQE
LIEHLEAKVKDGGSTSHPLIDEFIHTIQGEDGICYTHPENLPGVTRDGLSKHFFHRSAKA
YPTGTRKRRKVLADQQPDDHPQASKGRNVAAAETRWHKTGKTREITVRGQPKGCKKILVL
YTSFGKKRKAEKTSWVMHQYHLGELDDEKEGELILSKVFYQTQTRSAAAAEAPVSSGAAM
EVQGQQQQVLKLQADDGHFSSAPTKKRLHQDVVAQVKVDRGHHCMPAQRQVNFNLKVTPV
PTTSSFPVVVDKQLYSPVALFRSEHLHGRTSIHQPQKADWPHQRWPPDNQDQHG*
>11667.m00917|LOC_Os01g09570.1|genepair197-1
MEAVGVAPAPAGVPEKKLLEVKESRKAAPAAPSTSMAAKWAMKKKLVGGDAGYVLEDVPH
LTDYLPELPTYPNPLQDNPAYSVVKQYFVNTDDTVTQKIVVHKTSARGTHFRRAGPRQRV
YFQSDEVNAAIVTCGGLCPGLNTVIRELVCGLYDMYGVTSVVGIEGGYKGFYSRNTVALT
PKSVNDIHKRGGTVLGTSRGGHDTGKIVDSIKDRGINQVYIIGGDGTQKGASVIYEEVRR
RGLKCSVVGVPKTIDNDIAVIDKSFGFDTAVEEAQRAINAAHVEAESAENGIGVVKLMGR
NSGFIAMYATLASRDVDCCLIPESPFYLEGKGGLLEFIEKRLKDNGHMVIVVAEGAGQDL
IAKSMNFVDTQDASGNKLLLDVGLWLSQKIKDHFKKKRNFPITLKYIDPTYMIRAVRSNA
SDNVYCTLLAHSALHGAMAGYTGFTVAPVNGRHAYIPFYRITEKQNKVVITDRMWARVLC
STNQPCFLSHEDVEHLKHDDDEHHLHNTQLLEGESSPVKDSSKCNGTAAPV*
>11682.m00994|LOC_Os05g10650.1|genepair197-2
MEAATVVAAPIPAADAAAKALEKKLLDLELPPFPAPAKKAAAKVVAAAPKKKLAGGAGGY
VLEDVPHLTDYLPNLPSFPNPLQNHPAYSVVKQYFVNADDTVAKKIVVHKGSARGTHFRR
AGPRQRVFFQPDEVSAAIVTCGGLCPGLNTVIRELVCGLHDMYGVTSVVGIEGGYRGFYA
RNTVELTPRSVNGIHKRGGTVLGTSRGGQDTGKIVDSIQDRGINQVYIIGGDGTQKGAAT
IHAEVQRRGLKCAVVGVPKTIDNDIAVIDRSFGFDTAVEEAQRAINAAHVEAESAENGVG
VVKLMGRNSGFIAMYATLASRDVDLCLIPESPFYLEGKGGLLEFAEKRLRENGHMVIVVA
EGAGQDVIARSMRLADAHDASGNKVLLDVGLWLCAKIKDHFKKKANFPITLKYIDPTYMI
RAVPSNASDNVYCSLLAHSAIHGAMAGYTGFTVAPVNGRHAYIPFYRITEKQNKVVITDR
MWARVLCSTNQPCFLSTEDVEKAGQDDEEPIVPLVEGENSLVKAPPLLANAGDRAALCNG
AA*

>11667.m00922|LOC_Os01g09620.1|genepair198-1
MMMMGEGVSSVPPWSHLPVSGVDVLGGGGGGGDEMTPYVIAALRDYLPANDVGVGADEEE
EAAAMAAAVDAYACDEFRMYEFKVRRCARGRSHDWTECPFAHPGEKARRRDPRKYHYSGT
ACPDFRKGGCKRGDACEYAHGVFECWLHPARYRTQPCKDGTACRRRVCFFAHTPDQLRVL
PAQQSSPRSVASSPLAESYDGSPLRRQAFESYLTKTIMSSSPTSTLMSPPKSPPSESPPL
SPDGAAAIRRGSWPGVGSPVNDVLASFRQLRLNKVKSSPSGGWSYPSSSAVYGSPKAATG
LYSLPTTPLASTATVTTASSFMPNLEPLDLGLIGDEEPVQRVESGRALREKVFERLSRDG
AISGDATAFATAGVGLDVDWVSDLIN*
>11682.m00996|LOC_Os05g10670.1|genepair198-2
MVRKRRDTARVNPTAVSGGGLSGLYSRASSSPPLHHSGSRRRLRTNTLPRRSWRRGEELE
SKMMMMGEGAHAPPWQQHVASPVSGVEGGGGRESEVVAAPYHLLDTLRHYLPSNEAAAAE
DEEEAAAVAAAVDAYACDEFRMYEFKVRRCARGRSHDWTECPFAHPGEKARRRDPRRYCY
SGTACPDFRKGGCKRGDACEFAHGVFECWLHPARYRTQPCKDGTACRRRVCFFAHTPDQL
RVLPPSQQQGSNSPRGCGGGGAGAAASPLAESYDGSPLRRQAFESYLTKSIMSSSPTSTL
VSPPRSPPSESPPLSPDAAGALRRGAWAGVGSPVNDVHVSLRQLRLGSPRSAPSCASFLP
AGYQYGSPKSPAAAAAAALYSLPSTPTRLSPVTVTTASGATVTVEPLDLGLIEEEQPMER
VESGRALREKVFERLSKEATVSTDAAAAAAGVAPDVGWVSDLIN*
>11667.m00927|LOC_Os01g09670.1|genepair199-1
MGDSSRSVSIDVERISFGGKEHRVRTRYGSVSVSVFGDEDKPALITYPDVALNYMSCFQG
LFFCPEAASLLLHNFCIYHITPQGHELGAAPISSDVPVPSVDELVDQVADVLDFFGLGSV
MCLGVTAGAYILTLFATKYRDRVIGLMLVSPLCKAPSWSEWLYNKVLLNLLYYYGSRGLV
KECLLQRYFSTEVRGNGQDPESEIVQACRSLLHERQGSNVWRFLQAINERHDLTEALKKL
QCRTLIFVGENSQFHDDAVHMTTKLDRRYCALVEVQACGSLVTEEQPHAMLIPMEYFLMG
YGLYRPSQLDSSPRSTLNPFCISPELLSPESMGVKLKPIKTRISLKV*
>11682.m01004|LOC_Os05g10740.2|genepair199-2
MGDSSGSGSGSGSVSVDVERISFGGKEHQVRTRCGSLSVAIYGDEDKPALITYPDIALNH
MSCFQGLLFCPEVASLLLHNFCIYHINPQGHELGAAPIPSDVPVPSVEDLADQVADVLDF
FGLGSVMCLGVTAGAYVLTLFATKYRERVIGLMLVSPLCRAPSWSEWLYNKVLLNLIYYY
GTRGLVKECLLQRYFSKKVCGSGHYLESDIVQACRNLLDERQGENIWRFLHSINERHDLT
DALRKLQCRTLIFVGENSQFHEDAIHMTTKLDKRYCALVEVQGCGSLVTEEQPHAMLMPM
EYFLMGYGLYRPYQMNSSPRSPLSPCCISPELLSPESMGVKLKPIKTRIAVDY*
>11667.m00930|LOC_Os01g09700.1|genepair200-1
MGRGQLLYYATLSVLLTSSFTESVASYTAIAYKFCTARLIQSERTRENIMGGKLLPAAAF
AGSAPPLSQVATSAAHGEDSPYFAGWKAYDEDPYHAVDNPDGVIQMGLAENQVSFDLLEA
YLRDHPEAAGWSTGGAGAGSFRDNALFQDYHGLKSFRKAMASFMGKIRGGKARFDPDHIV
LTAGATAANELLTFILANPGDALLIPTPYYPGFDRDLRWRTGVNIVPVRCDSANGFQVTV
AALQAAYDEAAAVGMRARAVLITNPSNPLGTTVRRKMLDDILDFVSRNDIHLISDEIYSG
SVFAAPDLVSVAELVEARGGDGIAGRVHIVYSLSKDLGLPGFRVGVVYSYNDAVVTAARR
MSSFTLVSSQTQKTLAAMLSDEAFAGEYIRTNRRRLRERHEHVVAGLARAGVPCLRGNAG
LFVWMDMRRLLLGGGGVGGELRLWEKLLRQAKLNISPGSSCHCSEAGWFRVCFANMSLDT
LDLALHRISRFMDTWNGTKQQASCQQQEQQ*
>11682.m01009|LOC_Os05g10780.1|genepair200-2
MAMSAAHGEDSPYFAGWRAYDEDPYDPITNPQGVIQMGLAENQVSFDLLEEYMREHPEAS
DCGAGVRENALFQDYHGLKSFRKAMASFMETIRGGKARFDPDRVVLTAGATAANELLTFI
LADPGDALLVPTPYYPGFDRDLRWRTGVNIVPVSCDSAAGFQVTAGALRAAYDEAVAAGT
RVRGVLITNPSNPLGTTAARGVLEGILDFVARHDMHLISDEIYSGSVFAAPDLVSVAELV
DERRRARGGAADAEDIARRVHVVYSLSKDLGLPGFRVGVVYSYNDAVVAAARRMSSFTLV
SSQTQRTLAAMLSDAAFAAAYVRSNRDRLRERHARAVAGLRRAGVACLRGANAGLFVWVD
MRRLLGDGEATVAGELRLWRRVVAEAKLNISPGSSCHCREPGWFRVCFANMSLETLDVAL
HRLGCFIKKWEQEQHEN*
>11667.m00943|LOC_Os01g09830.1|genepair201-1
MQAVAAAAGMMRRGSLTIDPAGEEEAPAERVGRLVRESPVVVFARRGCYMAHVMRRLLAA
VGAHATVIELEGGAAEEEEAALGGGAALPALFVGGDPVGGLEGLMGLHLSGRLVPRLREV
GALCT*
>11682.m01024|LOC_Os05g10930.1|genepair201-2
MQGGGGVSCAVAGDAPSSTRGGGGGGMLGLTLFDPPGGEQPAERIGRLVRESPVVIFARR
GCCMCHVMRRLLAAVGAHATVIELDEAAEEAAASAAAAAAVPALFVGGAPVGGLDGLMGL
HLSGRLVPRLREVGALCG*
>11667.m00947|LOC_Os01g09870.1|genepair202-1
MEAEEAAGGGERKRKRDGDAVRDDEEEEEEGVYEGIAEESVAELMRWLEMEISDAAPETE
TKTETESGDDPAGAAAPGFVTINGNEESCGPSFSAAASTVMASVDTRAGAPPAPPVPWPL
PPAADVVPAEVVVDGVGEEWLVELLTSGPAVA*
>11682.m01030|LOC_Os05g10990.1|genepair202-2
MEDEAGAARKRKRRGEGKPRPKATKGGSKAKPKGKAAAAAAAAAAAEEAAAAVEKVAAEP
GVVVEEEEEEDYAEGITEESIAEVMSWLELEIKLASSAAAAGAAATPAPFAPPPPPPPAA
GGGGYMPAAKGVNTSNMEGSCGASFSVSASTVMASVDLRAGAPPPPPLPWPLPGHGGGAT
AAAAAEEAVDDDEWVDQLLTDGPAME*
>11667.m00948|LOC_Os01g09880.1|genepair203-1
MSSNGKPNPPPAASAAAAAGNGAGGPPKMYQRPIYRPQAPAAKRRRGGRSSCRFSCCCCF
FYAVLVVLLLAFVAAVAGGAFYLLYRPHRPAFTLSVARVDKLSLSSSATAPTLTDSIDVT
LTAKNPNKKLVYLYDDFAVTAATAANAVPLGEGSVPGFVHDAGNITVIKATVSASALGVD
PTTAATDIKKSGEFTITLDLETKAGVKVGGLKTKKIGVLVHCEGIKVAAPAPPPPPAKKK
KGGVKLSVSDAPSPAASVDDTTPSPPPATTVARVCQVRIRVKIWKWTF*
>11682.m01032|LOC_Os05g11010.1|genepair203-2

MADRVYPAAKPNPPPAMANAGGGGATASFPAPKSQIGGAGVPADAASAAASAGRCWSSSS
WRSSPPSPAARSTCSTARTAPASPSRPSKLTALNLSSSPTSPSLTDSIQLTVTAKNPNKK
VVYLYDDFSFSASTAANAVPLGAATSPGFTHDAGNTTVFTATIAANAVAVDPAAAASDIK
KSGAFSVAVDAETRAGVRVGSLKTKKIGIQVHCEGIKVTPPPPAALPRPKAVKGKNGTVL
APAPAPADSDTAATTAATVSTAAHSCKVRVRVKIWKWTF*
>11667.m00959|LOC_Os01g09990.1|genepair204-1
MEDCSSWIHGYANANATAGNNGFMCGYAASCSPVEFQQQQQLVGSQIEHHLNQISMQMGM
DDESAVYDGASMVDVLLMASSSPHHHAGAGSFQYSSPTSSSASFRSASVSCSPESSAAAT
THFLGPPAPSAAAAGFHYPEVSSQAPLPLPLPPYEPQHGQYTTVLSPPPPAPELPATTTP
ATGGAFRRYARHLRPRRLPKPGGCGQRMFKTAMSVLTKMHVAATYNRQYYYQQAAAAAAS
ASAAEAPPSGNQLQHMISERKRREKLNDSFLALKAVLPPGSKKDKTSILIRAREYVKSLE
SKLSELEEKNRELEARLASRPAAAAKNDKGETAAAPAPEAGDETKRKDLVEIEVTTSGGG
AGAADAAAAAGGDQETCTLNVDLRGGGGGGGMSTTDVVLRTLQCLREQIGDGASLVAMST
SAGSGGRPPRANLTLQLKV*
>11682.m01039|LOC_Os05g11070.1|genepair204-2
MDGGSWMLKAAAAGDMHGGAGDTMIRCSWSDMATTDQLLRHHEQEPAMTMMMNSQSQAMQ
QQLSQIYMLMDMEEHDHQYATPPSPSSSSFRSFSAGTTTTTTSRDDNSSLMLAAAAASCH
HQTTEVSSQILLPRPGQAARRSSGGHGAAAAATAFRPYSRYLGPKKHLLRRPGAATTATG
GGGGQRAFKKAISVLSKIHAARLAQYYQIMEMAARASPAATAGGGGGENQQLQLQHVLSE
RKRREKLNDSFKALRDVLPPATKKDKASVLMRAKDYVNVLKARIAELEEKNRKLSESQQL
HAGDGDGERDDGPDDDKIEVNTSRSAADQGSSPNKCQELHLKIVLGSSSGCSAMDAVAGI
LQGLNEKRDVSLLATGHNSSSSSSSGRRRLLPRAKSSQQPAVRLQTLCKLSR*
>11667.m00962|LOC_Os01g10010.1|genepair205-1
MSKYGTIPTSSSSSSSGAAPPPLPLGGGGGASPLDFFSRAKARGATALATRRPWRELADP
HALGLPPSLADAYLRVRANLAHYAMNYAIVVLAVVFLSLLWHPASLIVFLVCMVAWLVLY
FLRDEPIVLFGRVVGDGAVLAALAAVTLVLLLLTGATANIVSSLLIGVLLVVLHAALHKA
EENVDDEFNVDTISFTLLC*
>11682.m01045|LOC_Os05g11120.1|genepair205-2
MSKYGTIPTSSSSDGPPPGSSSSSPLDFISRAKARGATALAERRPWRELADPRAASVPRG
LGGAYRRARANLGHFSMNYAIVVLAVVFLSLLWHPVSLIVFLACMVAWLFLYFLRDEPLA
LCGRAVGEGAVLAVLSVLTLVLLLLTGATVNILTSLLVGVVIVLLHAVFHRPADSIDEEA
GRYYTPVPPQPSY*
>11667.m00965|LOC_Os01g10040.1|genepair206-1
MVSAAAGWAAPAFAVAAVVIWVVLCSELLRRRRRGAGSGKGDAAAAARLPPGSFGWPVVG
ETLEFVSCAYSPRPEAFVDKRRKLHGSAVFRSHLFGSATVVTADAEVSRFVLQSDARAFV
PWYPRSLTELMGKSSILLINGALQRRVHGLVGAFFKSSHLKSQLTADMRRRLSPALSSFP
DSSLLHVQHLAKSVVFEILVRGLIGLEAGEEMQQLKQQFQEFIVGLMSLPIKLPGTRLYR
SLQAKKKMARLIQRIIREKRARRAAASPPRDAIDVLIGDGSDELTDELISDNMIDLMIPA
EDSVPVLITLAVKFLSECPLALHQLEEENIQLKRRKTDMGETLQWTDYMSLSFTQHVITE
TLRLGNIIGGIMRKAVRDVEVKGHLIPKGWCVFVYFRSVHLDDTLYDEPYKFNPWRWKEK
DMSNGSFTPFGGGQRLCPGLDLARLEASIFLHHLVTSFRWVAEEDHIVNFPTVRLKRGMP
IRVTAKEDDD*
>11682.m01046|LOC_Os05g11130.1|genepair206-2
MQPLAAGGGVSWPLYAATVAAALLVTAIVLRLAARSTAAACKARPPAGSLGWPLVGETLQ
FISAAYSSRPESFVEKRCRRYGKVFRSHLWGSPAVVSADAEASRAVLQSDASAFVPWYPR
SLMELMGESSILVLGGALQRRVHGLAGAFFKSPELKARVTADMRSRLAAAMDAWRATAAT
GAGAAVRVQDEAKLIVFEILVRALIGLEQGQEMNYLRQQFHIFIAGLISLPIKLPGTQLY
RSLKAKKRMTSLIQNIIQEKRRRIFEGKDLCAVSRDLIDVLMSNGSDELSLTDELISDNM
IDFMIPAEDSVPVLITLAIKYLSECPLALQQLEEENMELKRQKSDVGETLEWTDYMSLTF
TQHVITETLRIGNIISGIMRKAVRDVEVKGQGDVVIPKGWCVLVYFRSVHLDANIYDDPY
AFNPWRWKERDMAAATANSGSGFTPFGGGQRLCPGLDLARLQTSIFLHHLVTNFTWVAQG
DVVVNFPTVRLKRGMPIKVTPKT*
>11667.m00976|LOC_Os01g10150.1|genepair207-1
MPARRGRGKRKQQQPPPPPTGPADARQPEGKQREEERKGMEGSDKGGAGDDGSPAPLPET
VQIGNSPTYKLERKLGKGGFGQVYVGRRISSPTHGNRNSGANALEVALKFEHRTSKGCSY
GAPYEWQVYNTLSGNHGVPRVHYKGKQGGFYIMVMDMLGPSLWDVWNNNSHSMSVEMVAC
IGIEAISILEKMHAKGYVHGDVKPENFLLGPPDTPEGKKLFLVDLGLATKWKDAGTGKHV
EYDQRPDIFRGTVRYASVHAHLGRTGCRRDDLESLAYTLIFLLRGRLPWQGFQGENKGFL
VCKKKMATSPESLCGIGPPPFRQFVEYVVNLKFDEEPNYAKCIALFDGIVGPNPDGRPLN
TDGAQKVGQKRGRLTAAEDEEQPKKKIRMGMPATQWISVYNARRPMKQRYHYNVADDRLA
PHIQKGNEDDLWALIMDAGTGFTAQVHELSHYFLHKEWIMEQWERNYYITSLAGSNNGSS
VVIMSTGTPYAQQSYKVSDSFPFKWINKKWKEGFYVTALATAGSRWAVVMSRNAGFTHQV
VELDFLYPSEGIHQRWDSGYRITATAATCDQVALILSIPRRKPNDETQETLRTSAFPGQH
VKEKWAKNLYLGSICYGRSVS*
>11682.m01047|LOC_Os05g11140.1|genepair207-2
MDDVDSVVGSGDKAGEAPGDDGSSTPLPETVQVANSPTYKLDRKLGKGGFGQVYVGRRIS
SPGVTDRTPGANALEVAIKFEHRTSKGCNYGAPYEWQVYNTLSGIHGVPRVHYKGRQGDY
YIMIMDMLGPSLWDVWNNNSHSMSVEMVACIAIEAISILEKMHSKGYVHGDVKPENFLLG
TPGTLEEKKLFLVDLGLATRWKDTGSGEHVEYDQRPDIFRGTVRYASVHAHLGRTGSRRD
DLESLAYTLVFLLRGRLPWQGYQGENKGFLVCKKKMATSPESLCCFCPQPFRQFIEYVVN
LKFDEEPNYAKCISLFDGIVGPNPDIRPINTDGAQKLIYQVGQKRGRLTMDEDDDEQPKK
KIRMGMPATQWISVYNARRPMKQRYHYNVADGRLAQHITKGNEDGLFISSVASCSNLWAL
IMDAGTGFTSQVYELSQHFLHKEWIMDQWERNFYITSLAGANNGSSLVVMSRGTQYAQQS
YKVSDSFPFKWINKKWKEGFYVTAMATAGSRWAVVMSRNAGFTDQVVELDFLYPSEGIHR

RWDNGFRITATAATWDQAAFILSIPRRKPADETQETLRTSAFPSQHVKEKWSKNLYLASI
CYGRTVS*
>11667.m00979|LOC_Os01g10180.1|genepair208-1
MEPGFGKRLMHVLRAVYHMLRRGLCRKRLMMDLHLLLGRGKLAGRALRDVLLAHQPHGGA
AAVAVMGGGAGVARGGDSSSSPLSASFFHHNPRDVEFSCTTTPSYAPGVFPFRFRGRGGS
RHAGGGASNYGGLDASAVARVFEMLNADAAAAAGAGGETPLSSMPGATPSPLLALSLGRS
PAGTRQLRVTDSPFPVEPPEGAVDGRVDDKATDFIEWFRRQLLQQQASAAPTPDYRG*
>11682.m01049|LOC_Os05g11160.1|genepair208-2
MDLATTPARRPMEAGMARRLWHVVLAVCHMLRRGLCRKRLMVDLHVLLGRGKLAGKALRG
LLAHHAAAGHGHHLAASSSSSAALASFYGRRPREVEFSCTTTPSSYPHYGLFPFKSRGGG
GGRRGGGGGGGEYGGLDAAAVARAFEMMSAEVEGTPSSSAAAVQGGGGGGWATATPSPMVA
WILGRSPAGVRPLRVTDSPFPAVPENGGGGGGEQRVDDAEFEDFINKFYEQLRMQPSAAT
PDCQLRRRGR*
>11667.m00982|LOC_Os01g10210.1|genepair209-1
MAATATATAAPEEEEGKRVMTRALEWEGCVVSPVPTATADEAWALLSDFLAFHRWHPRVA
KCRPASPSAAASTAAAPPGSVVRYCEGTPRGDGAPPDWAHETLLEHDAARRFFRYEMNDN
NMGFGVFVATFRVVPDAGGGDADAPGCELRWEFEGDPVRGTPKEALVARLQAGLDGMAAR
VQEHLMSARAADAAVIAAGGVEAADELNRDKYSIAV*
>11682.m01054|LOC_Os05g11210.1|genepair209-2
MAAAAAAAAARRKQELEWTGRVTAAAPAATADEAWALLSDFLAFHRWHPGVAKCRRVSGS
PRSPGCVRYCEGVPGRAGGVAGAADWAHETLLEHDAAGRALRYEMNDNNMGFGTFFATLS
VAAAGAAAAAGGCELRWEFECEPVAGTAKEALAARLQDGIDGMARRVQEALAGRRGDAAA
AAAVAMEAANSGDVNKLGTSIAV*
>11667.m00984|LOC_Os01g10230.1|genepair210-1
MEEQQRPVQPAGGGGGGGAPPEEQWRGAVEAALPGTPASAAWPHVASFYAAHRYLPGIDV
CERVGDGGEDGGLLLVPGCVRHVASSAAGLWAREELLEAPDHAARRLRYAVVDSNMGFGR
YVATLRVLDGGSGCRIAWAFECDAVCGEGWSEAALVARLAASVDGMAERVQQAVAAEAEA
RAGEEEEDGVAG*
>11682.m01058|LOC_Os05g11250.1|genepair210-2
MADQPQQQQPEPEPEMGAQPQQQWEGAVEARLPSTPAAAAWPHLAAFCSLHRYNPGIDVC
ERVAGDDGVPGCVRYVASRPPPPPPRAKDGGDDDDDDQQPAAAAAGVETWAREELLERDD
ARRRLVYAVVGSNLGFGRYVATMTLVDDDGEDVDVNAPAPAAAAAAGCKLVWAFECEPVK
GWTRDGLLAYLDAAGKGMAERIEAAAAAAVTDIAVEDDAAAARS*
>11667.m00994|LOC_Os01g10310.1|genepair211-1
MEKLIIIKSSGSGSGDARHDDGGGEVETVRCACCGVAEECTAAYIGGVRAAFCGDWLCGL
CSEAVKETARRDPAPGGGVAAALASHAAECRDFNATTRLNPTLSLAGSMRRIARRSFDKR
TSASCQERRLGAAASKAVALARSASCDPRFCSLLAADVINGGAPPGDRCR*
>11682.m01063|LOC_Os05g11300.1|genepair211-2
MQIMAVNTSLEPTNCFLLLSKNKDQTELRRSYSEYSNTTRAADHLVAGAGVVAVASSGGA
SGGGGGGSGSDVETTVRCACCSVTEECTAAYIRHIRAAHYGDWVCGLCAEAVRERMRGGG
GGGGVEAALRWHMEVCRDFNSTTRLNPKLSLAGSMRDIARRSFNRRTTASTSAAATCHDQ
LRAAKTMARTLSCQQRFLA*
>11667.m01004|LOC_Os01g10400.1|genepair212-1
MSDKCGNCDCADKSQKKGTSYGVVIVEAEKSHFEEVAAGEENGGCKCGTSCSCTDCKCGK
*
>11682.m01065|LOC_Os05g11320.1|genepair212-2
MSDKCGNCDCADKSQCVKKGTSYGVVIVDAEKSHFEMAEEVGYEENDGKCKCTTGCSCAG
CNCGK*
>11667.m01014|LOC_Os01g10470.1|genepair213-1
MAPPLLRGVPVLIALVVMAAVALLPAAAADDMGGVVASASGRRKMAAAAATCDGAVGECD
VDDEEEVEEMALMGAAGAASGETLMRRSLAARRPTNRYVSYAALDANKVPCNKRGQSYYQ
NCASQKAANPYRRGCSAITRCARNTN*
>11682.m01066|LOC_Os05g11330.1|genepair213-2
MARLGIGVAAAAAAVVAATLVVSCLRASPAEAYSGGGLGYSQLLTTAHLGAISSSSCGGR
LGRQCSAAVGADGGLLRRALAARKPTNRYVSYSALDANKVPCNKRGQTYYQNCASQQAAN
PYRRGCSAITRCSRNMN*
>11667.m01017|LOC_Os01g10500.1|genepair214-1
MASNGVAAMVQLLGLHLYWETTRGVKKSWWLCVKSTVERYKKANSDTSNSGTVAEVNAQH
YQQESSKLRQQISSLQNANSRTIVGDSINTMSLRDLKQVENRLEKGIAKIRARKNELLYA
EVEYMQKREVELQNDNMYLRSKVVENERGQQPLNMMGAASTSEYDHMVNNPYDSRNFLQV
NIMQQPQHYAHQLQPTTLQLGQQPAFN*
>11682.m01074|LOC_Os05g11410.1|genepair214-2
MSSVKRNNCSSVKETIERYKKANSDTSNASTVAEINAQHYQQEAAKLKQQITNLQNSNRT
LVGDNITTMNHRELKQLEGRLDKGLGKIRARKNELLCAEIEYMQRRETELQNDNMYLKSK
VAESERGLQTVNMMGSASTSEYVQNMIHYDPRNFLQFNIMHQPQYYPEQEDRKAFMSDER
*
>11667.m01018|LOC_Os01g10510.1|genepair215-1
MRESPPQFRQEAAAGEYLAIISASYVTNRNWTVLPVQTKQVSDLQQQEAKLCKQSYIRVT
CITYSETDLSCGPSSMTELTAAAAPAGSGSSAAVAAGSSEKMGRGKIEIKRIENTTNRQV
TFCKRRNGLLKKAYELSVLCDAEVALIVFSSRGRLYEYANNR*
>11682.m01076|LOC_Os05g11420.1|genepair215-2
MVQCHEVGQPRGERRKVQEEEKHQGQYIDGLSPQLPPLHLISSFPNHSLLLEHTPLFLII
ISSLIREELANMHIYKEQEAEPSTGLMMPEPAPVASPGSGGSGGSGSVGAEKIGSRGKIE
IKRIENTTNRQVTFCKRRSGLLKKAYELSVLCDAEVALVVFSSRGRLYEYSNNRGKKWCT

RVMHTLIIHDSFASSSSIGGCPITSALGP*
>11667.m01021|LOC_Os01g10540.1|genepair216-1
MAVVAGFSIREYTASMRGAAGAEGRRLYGLGAGDLPPMEARRFRWWADELAAAPPLPPRS
PSPSPSPPPPPPTKPSRRTLGKARAPKKRSISDLFAAAPPLALPSSDSGGGGNDDDDDDE
ALCAIMRRAREKKRKRRLQEEAAGAASAPVAAAAAAAETRDSEGNFTRKEAHDKTNLPGG
LDTPQASRRPDGVHHARTDEERSPDSKRRKKVKITNLDKNNNKKIDKKRYSESKRATNKV
GKQHDLKKMLPLHSILKKYTKHTSVKMVKEKHGDPKGTEVIEVCRKSVKRVKFSEVNDVL
GINKQNICKLFSDALASSSSSSTDMSSEGDKHIAAESCSSHMPETATKEASKSTDHEDSL
ELTSTQLSSNLFDLNEALPESTDLNYPYVSNPEEPNHEPRQHEPLDSDVQVIDEGGQNQQ
DLSLDSHGLQCQSVPESGLERARSSISPGTFLHGEFMEVSDTFFVGSSRKLTELAESHGD
CSSGSVKDAMTKGKSPCALPNHTVQDSFQQHQRYYAFNLNLGGSQPSNEGEVPPQDCNAS
AGAASSSHAEMGVQQGCRPAPGQTVRLMGKDLAVSTTRGEYVSGTHSYTEDHPTKLFLEL
PRQGRPYLSLQAQSVPNVSANSASPSQSHIRYTAPQNLSHSFPMANALSGDRLQYDDRFS
YLSGSQHHGNVLLGSPSLTSHGSAALRQNLPYVWSRYSDPSSSSTASPSAPILPTTAQHV
TPSSVYHANLPRSYGVVSAGSSVHPHNSPSFTFTRPRRIVEEASGSRRDAACPSRNAENV
AARAAIPEMPSSSSGGRHARRTGPMKLTPGAKHILMPSDTTGDGTSMPVYSCVSFGSKSG
NASATRNMGAGLYKL*
>11682.m01077|LOC_Os05g11430.1|genepair216-2
MPLENADGGMASGAPPTLMPRIGVNEKPAAVLLPLGAPRARNPVPPLVGERQYEKGPPNW
EDVSCLRHEQLRRATETEEAVEGEHRRRVLEPGHTQHVAHAVAVQAGVGGDDELVLVSEL
DVDEVTVVGLRGGDVVAGVDEEELEVEHCWDEGVGDLAGLAQGVEVENVLVSVNKLHPPS
PQTFQVHERQKHINIENMKMYDFEIWKPENSAKVPKNSILRKHTKRSSFTVSINKEKCSN
LKGSEAIELSHKLGKHVTFSGVDDIHIRNKLSSTLPQLQNHCNVYSDKSNEADRLVSAKI
SSHENKEASGRDIYDRWTSESSGAKDPINLIDLNRTLPCIPDFNGAFISGSEVPDLEHTE
NATSDLQIPGDVREEAVLKHNQDLHSKSPRSQCELNSCDLGRIINLRSIASLLPDEAINI
SDRGMIGHPLNSTEVNKFYADYERSSVRDDTMEGKAPYILPQHTVQYTSQFTENWYTNMN
LGNFHHAGREFSSCPCENQLNSEKPMLHSEINVQHEHAVMSQRTMRLMGKDLTVSTTGGK
CIGETAKVHVNSSVSCHHTTNIFLELPRQGHPFLSLQSRSFSNIQVDAPSTSHDYVGYKM
HNLKRRFPEADVFSGNGIECEDRLRDFSYLHCGQNALAGFSPQGGKYNTRSDQNSLSATT
FLPTFIPHAKQSAVYRANSTWKHNPYPANLLVHPPDGTNFRKDQNQIIRGVAEIPSSVNT
MSRDTVWKTRKIDVDNSNISSGVRYISRSGPVKLRPGAKHVLEPRQDTDDGNYPPMYSCV
PFFVIRRGGNILSGQTKSHRKFAVLEPCEKRTQLIWEVLTFASTHQALHPSSLLQQNPGG
KLPFPFFLKENGICRFALKSTPSVLQWTSRSTAAIDVYVLHQWHISPDFIKNSTDQIHVL
SPSTRLSSTSVPPPLIGGGRPGRRAGWPWQNRSLNGDFELKSRISDFRLCAVDPYQEYNR
VEAGTEMLR*
>11667.m01025|LOC_Os01g10580.1|genepair217-1
MKVLCSACEAAEARVLCCADDAALCARCDLHVHAANRLAGKHHRLPLLSSSSSSSSPSPP
TCDICQDAHAYFFCVEDRALLCRACDVAVHTANALVSAHRRFLLTGVHVGLDAAADDDDK
HPPHPLSSSLPRNTAPPPQPPPKRSPSPIYSDDDVIDWATGGHDIGITGNLPDWSLVDEQ
FNTPALPPVVTKTPPKRASRGPVTAGTAAAVFGNLAGGSPDWPLNEFFGFADFSSGFGFA
ENGTSKADSGKIGSMDGSPNGGRSSSSSSSSSAAAAGGGGGGQDFFGQVPEVHWAVPELP
SPPTASGLHWQRDPRYGGGATDASAVFVPDISSPENPFRCFAAAAAGDHTMKRRRRC*
>11682.m01085|LOC_Os05g11510.1|genepair217-2
MSPPPPPYYHHLLLLRSSPTTTGGGARVLAAAELARMKLLCSACEAAEASVLCCADEAAL
CARCDRDIHAANRLAGKHLRLPLLSPASSSSSSAAALAPPPPSPPKCDICQESHAYFFCL
EDRALLCRSCDVAVHTANAFVSAHRRFLLTGVQVGQEQDEHSPDPPEPSPPPPPPPPASK
SDHPAPLYGEGGGGFSWDAADSPAAGGLPDWSAVVDQFGSPPPPRHTDTATVTTPPPTKR
SPRAPAFGGQGGMMDWPLGEFFGGFTDFTGGFGFGFGDSGTSKADSGKLGGSTDGSPYYR
SSSEDDRNADELFGQVPEIQWSVPELPSPPTASGLHWQRHPAATHGGGGGGPDTTAFVPD
ICSPDSCFPATTSKRRRQ*
>11667.m01027|LOC_Os01g10600.1|genepair218-1
MAGREDGAAAGAMEEGQDSKEVKCESSEDGSSSSSSSRCHGNDVISVQFMQKVHPWCMCM
NKNLLILAEILGTYFMIFAGCGAVVVNQSTGGAVTFPGICAVWGLVVMVLVYTVSHISGA
HFNPAVTVAFATCGRFRWKQVPSYVVAQVLGSTMASLTLRVVFGGGGGGARGEHLFFGTT
PAGSMAQAAALEFVISFFLMFVVSGVATDNRAIGELAGLAVGATVAVNVLFAGPVTGASM
NPARSLGPAMVAGRYGGVWVYVAAPVSGTVCGAWAYNLLRFTDKPLRDIANTASFLRRSS
RRS*
>11682.m01090|LOC_Os05g11560.1|genepair218-2
MAGGEHGVNGQHEETRAMEEGSRDHQARCENSEQDGGSKSSSNNHPMFSVQFAQKVIAEI
LGTFFLIFAGCAAVAVNKRTGGTVTFPGICITWGLAVMVMVYSVGHISGAHLNPAVTLAF
ATCGRFPWRRVPAYAAAQVAGSAAASAALRALFGGAPEHFFGTAPAGSDVQSLAMEFIIT
FYLMFVVSGVATDNRAIGELAGLAVGATVLVNVLFAGPISGASMNPARTIGPAIILGRYT
GIWVYIAGPVFGAVAGAWAYNLIRFTDKPLREITMTASFIRSTRRN*
>11667.m01036|LOC_Os01g10680.1|genepair219-1
MDAQMAAAAAHHPLPHQQKAANLARTFTKLLRRKRADAVAAATAVGEPGVPDAAAASVVG
DEYECSVEAAAAGVPSLSKLKLSGNLGAAYSLDAFFRNAAEKKAAGVAGVAVAQTSPQVA
PDVAKDSLLANLFAGVSAVKAAYAQLQLAQFPYDAEAIQAADAALVAELTRLSDTKRRYL
RDPAAAAKNAAAAGHTALYAHAEEQRHLLKTYQITARKLEGELRAKEAEADRARSSLTAE
LRAERAMEARLHPGRTLASLDELHLSGLNPTHFLTALRHTVKSIRSFSKSMLNSMQSAGW
DLAAAAAAVHPGVQLRRAGDTKFVFESYVAMKMFANFHRRDFNLSFLDEREFYDRRRFFE
EFTELKAAPASAFLDARNARWGGFGKFLRAKYLSLVHARMETAFFGRLEQRGIVSAGPGF
PESSWFADFAEMARRVWLLHCLFYAFDGGAEEDGASIFQVRTGARFSEVYMESVSDGRSD
EAAAAAAEERVVGFTVVPGFRVGRTMIQCRVYLSRPGRRP*
>11682.m01100|LOC_Os05g11650.1|genepair219-2

MAAELPQQKAGGGLARRLVRLLRRKRSTSGSVAGGGEYDESSMDSSINSLSKLKLSAAKL
DVLFRSAAQPAASPAVDAAAAHALVASLFAGVSAVKAAYAQLQQAQHPYDSEAIQSADAA
MVAELTKLSDHKRRFARDPAAAAKSAAAGPAALAAHADEQRHLLRTYEITAGKLGRELRA
RDAEAERARAALADDLRAARALEERAHPGRTLAALDGLHLSGLNATHFLTALRHAARSVR
SFAKSMLGEMRRAGWDPVAAAAAAHPGVPLRHPGDAKFALESFVALKMFDGFHRRDFGLS
ALHDRSSYDRRRLFDEFAELKAAPAAEFLDARSSRWGALGEFLRDRYLSVVHERMEAAFF
GSTAQRGAAASAGAALPGTPWFAEFAEMARRVWLLHCLFLAFDDGGASTIFQVAAGARFS
EVYMESVGDGDGDGDDGGAGTAVAAAAAGDRVVGFTVVPGFKVGRTVMQCRVYLSRPARQ
P*
>11667.m01051|LOC_Os01g10820.1|genepair220-1
MASEKKQANPMREIKVQKLVLNISVGESGDRLTRASKVLEQLSGQSPVFSKARYTVRSFG
IRRNEKIACYVTVRGEKAMQLLESGLKVKEYELLRRNFSETGCFGFGIQEHIDLGIKYDP
STGIYGMDFYVVLERAGYRVARRRRCKSRVGIQHRVTKEDAMKWFQVKYEGVILNKAQAN
TS*
>11682.m01106|LOC_Os05g11710.1|genepair220-2
MASEKKQSNPMREIKVQKLVLNISVGESGDRLTRASKVLEQLSGQSPVFSKARYTVRSFG
IRRNEKIACYVTVRGEKAMQLLESGLKVKEYELLRRNFSETGCFGFGIQEHIDLGIKYDP
STGIYGMDFYVVLERAGYRVARRRRCKSRVGIQHRVTKEDAMKWFQVKYEGVILNKAQAN
TS*
>11667.m01053|LOC_Os01g10840.1|genepair221-1
MAERVVGTGSFGIVFQAKCLETGETVAIKKVLQDRRYKNRELQLMRAMEHPNVICLKHCF
FSTTSRDELFLNLVMEYVPETLYRVLKHYSNANQRMPLIYVKLYIYQLFRGLAYIHTVPG
VCHRDVKPQNVLVDPLTHQVKLCDFGSAKVLVPGEPNISYICSRYYRAPELIFGATEYTT
SIDIWSAGCVLAELLLGQPLFPGESAVDQLVEIIKVLGTPTREEIRCMNPNYTEFKFPQI
KAHPWHKIFHKRMPPEAIDLASRLLQYSPSLRCTALDACAHSFFDELREPNARLPNGRPF
PPLFNFKHELASASPELIHRLIPDHIRRQHGLNFAHAGS*
>11682.m01108|LOC_Os05g11730.1|genepair221-2
MDQPAPAPEPMLLDAQPPAAVACDKKQQEGEAPYAEGNDAVTGHIISTTIGGKNGEPKRT
ISYMAERVVGTGSFGIVFQAKCLETGETVAIKKVLQDRRYKNRELQLMRAMDHPNVISLK
HCFFSTTSRDELFLNLVMEYVPETLYRVLKHYSNANHRMPLIYVKLYMYQLFRGLAYIHT
VPGVCHRDVKPQNVLVDPLTHQVKLCDFGSAKTLVPGEPNISYICSRYYRAPELIFGATE
YTTSIDIWSAGCVLAELLLGQPLFPGESAVDQLVEIIKVLGTPTREEIRCMNPNYTEFRF
PQIKAHPWHKVFHKRMPPEAIDLASRLLQYSPSLRCTALDACAHPFFDELREPNARLPNG
RPFPPLFNFKHELANSSQELISRLIPEHVRRQATHNFFNTGS*
>11667.m01058|LOC_Os01g10890.1|genepair222-1
MEKKASILMNRYELGRMLGQGTFAKVYHARNLASNQSVAIKVIDKEKVLRVGMIDQIKRE
ISIMRLVRHPNIVQLHEVMASKSKIYFAMEYVRGGELFSRVARGRLKEDAARKYFQQLIG
AVDFCHSRGVYHRDLKPENLLVDENGNLKVSDFGLSAFKECQKQDGLLHTTCGTPAYVAP
EIINKRGYDGAKADIWSCGVILFVLLAGYLPFHDSNLMEMYRKISKGDVKFPQWFTTDVR
RLLSRLLDPNPNIRITVEKLVEHPWFKKGYKPAVMLSQPNESNNLKDVHTAFSADHKDNE
GKAKEPASSLKPVSLNAFDIISLSKGFDLSGLFENDKEQKADSRFMTQKPASAIVSKLEQ
IAETESFKVKKQDGLVKLQGSKEGRKGQLAIDAEIFEVTPSFFVVEVKKSAGDTLEYEKF
CNKGLRPSLRDICWDGQSEHPSLAQSSTLTQSSKSISRHAI*
>11682.m01114|LOC_Os05g11790.1|genepair222-2
MPEKGTVVMSRYELGRSLGHGTFSKVYQARSLVSGETVAVKVIDKEKALRAGAGMVDQIE
REVAVMRLVGRHPNVVRLHEVMASRSKIYFVMELVRGGELLARLVAGGGRLGEDAARRYF
HQLVAAVDFCHSRGVYHRDLKPENLLVDDDGSGGGGNLKVTDFGLSALSASRRHDGLLHT
TCGTPSYVAPEIIGDKGYDGATADVWSCGVILFLLLAGYLPFFDSNLMEMYKKITNGEFK
VPDWFTPDARSLISRLLDPNPTTRITIDELVKHPWFKKGHTKRPASSNTMKLNEEEKPAN
AAMNMKPASLNAFDIISLSQGFDLSGMFCCHGHSSRTQDQLFVTGKPATAIVSRLEEIAE
TEHFTVKKKQKKRQEEDGMAVKLQGWKEGRKGQLAIDAEIFEVSPSCYVVEVKKTAGDTL
EYQAFCNRDLRPSLNDICWTSPATAASEKNQLPAVSEVSPLSSPRN*
>11667.m00673|LOC_Os01g07370.1|genepair223-1
MKRMQRMPTRKSHSWWWDSHISPKNSKWLAENLEEMDKQVKDMLKLIEDEGDSFAKKAEM
YFERRPLLVTHVENFYRMYRALAERYDNVTGELRKNIPSSLQSQGSLSISESDSETQSAP
PTPKPDSEETTPKQKRKPRAAGFDVFLGSGGSSDISKKGSDGSSSSSSESDSEVDELRED
NGDGSPFALNERIAELEDELQEAREKLEALEEKNTRCQCEKLEEKLKDSHSEISSLQKEL
EGQLAHHDHEIEKCKKELEHVHEKYSHDKSTLETEIIKLQDIVKNFEGDLAKMSQEKLQL
KAQVKELEQASRSLDDSSAQIMKLQEIIKDLQRRLDNDSNEKKMLEERAIEFEQVRKELE
GSRTEVAELQATINNLKADLGRALEEKSQLESRINDLEHTIACNLEEFSQEKSSLGAEIQ
KLKEANASLEGKLTSTESQLQQLHAEKSEASISSEKQISDLNQAIADLETKLELLSSEKT
TVDNKVASLLTDVTARDEKIREMDSHLHQLHLEHVKLIAEADAVTKAVSELRARVSELEE
EVEEQKLMVSDGAEGKREAIRQLCFSLEHYRHGYQQLRQLLQGHHKRPLVMAN*
>11682.m00730|LOC_Os05g07680.1|genepair223-2
MEKMQKTQSRKSSSWWWDSHISPKSSKWLSDNLEVMETQIKETLELIEEGEPSAEKAGVL
ITHVQNFQQMYRVLAKRYGNVTGELRKNIPSSLQSSVSFGISESDSEAQSPSSPERDLQE
KMSQKQKPRSDCFDVSIGSGMSSDVSKKGSDGSSSSSESDLELDEAKEENGNSIFYALSQ
KIIELEDELHEVRGKLDASEEKNMRCHCNFGANSELSEHEEKQQVSDVETSSLQKDLDEV
KSEKEALEAVVLVNKDEIDRLKESMVSAAKQFEVELAHRDTEIDKCKQELEVLSEKYLHD
ISALEAEIGKLQGVIKTFEDDFAKISQEKLMLESRVEELEQSVNSSNYSVSEMVNLQELM
KDTQAELEKVSQEKEVLRERVLEFEQLLGDFENSGMEVAKLPETIKNLGAQIEGTLQEKS
VLQDRIKELEQAVHDSLQNHSLEKSSLSAELSKLSEANASLEAKLASVEAELKQVYDEKA
NESLNSEKEISRLNQELANVKTDLELLLSEKSLVDNKVTTLLTDITTRDEKMKQMDDQLN
QLQLEHSKLMAQADLARKSLSELHARVCELEKEVEMQKLVISESAEGKREAIRQLCFSLE

HYRSGYQELRQLLHDQKRPLVMAT*
>11667.m00674|LOC_Os01g07380.1|genepair224-1
MAAALSSSRRALHALHRRLLLHPLPSRHAPPIPRHHFPSPPSSSSSSSRFFTTTRPDARL
LQLHARRLVVGGARSFAAGVGGGGSSKLAPLGQGVKGLGLGRPLDAAKNAAARYREAVGL
QVEAFWRRNYMLLVGAGAVVVCIALWRVMFGIASTFVGLSEGMAKYGFLALATAMVAFAG
MYTRSRFTINPDKVYRIAMTKLNTSAAILEVMGAPLAGTDVRAYVMSGGGPKLKDFKFRL
GGKRCFLIFPIKGSERKGLVSVEVKKKKGQYDMKLLAVDIPMASGPDQRLFLVGDEQEYK
VGGGLISELRDPIVKAMAAEKEFDYLDEREDAEDEAREREEAERRQQEEEAEALRREEER
LPRQLYTAYISNQIYTAIEIGNLAPQTTQFEIRSIGDLNSGPLGTQVNPMKHELIHGTLL
EPFEEMEVYNSTPKVEFWSNFPQNSCQFARLQAFFGAKSAAQSQFGRSRCIEDGCGYERK
EQVKWGDDYDSVPCRCLEVVSWVDDKMERSDEEDKIDFKGGNVHVISNKENWDHKIAEAN
KDGKIVIANFSAAWCGPCRVIAPVYAEMSQTYPQFMFLTIDVDELMDFSSSWDIRATPTF
FFLKNGEQVDKLVGANKPELEKKVAALADSA*
>11682.m00731|LOC_Os05g07690.1|genepair224-2
MGGCVGKGRRHIEEDKLDFKGGNVHVITSKEDWDRKIEEANKDGKIVVANFSASWCGPCR
VIAPIYAEMSKTYPQLMFLTIDVDDLMDFSSSWDIRATPTFFFIKNEKQVDKLVGANKPE
LEKKVQALADGS*
>11667.m00676|LOC_Os01g07400.1|genepair225-1
MSSQVSTSRGLPSPSKLGTFESPHTWQMSGPTGQAEAGSSDDQDIRLLSVSWNQDFGCFA
AGTSNGFRIFNCDPFKETFRRDLKSGGFGIVEMLFRCNILALVGGGSNAHYPPNKVMIWD
DHRSHCIGEFAFRSDVRAVKLGKDYIVIVLERKIYVYNFTDLKLLHQIETQSNPKGLCCL
SHHSNTSVLACPGVHQGHVRVEHFGLKVTRMISAHDSHISCMALTMDGLLLATASMKGTL
IRIFNTMDGTRLQEVRRGLDKADIYSIALSPNVQWLAVSSDKGTVHIFSLRVRVAGEDAS
NEQRSLEGPRMDHQNSSSSIDPLIQTNTGSNASSSLSFMKGILPKYFSSEWSFAQFHLPE
VTRYIVAFGAQNTVMMVGLDGSFYRCSFDQVNGGQMLQKEYFRFIKADLTPLRTSAP*
>11682.m00736|LOC_Os05g07710.1|genepair225-2
MGHVEEASGSDDDGVELLSVSWNQDNSCFIAATTNGFRVFSCKPFHETMRRMFGPNGGIG
IAEMLFRTSIFGLAGAESNTEFPPTMLQLWDDYNERRIHKYNFTSEIRAVRLSKDYFVVV
LEKTINVYRFKDLRLFYQARTVSNPNGLCCLSHHANASVFACPGTSKGQVLIEHFGLKET
RFIAAHDSPLSCMTMALDGTLLATASVRGTLIRIFNTRDGTCVQEVRRGLDRAEIYSIAL
SPNVQWLAVSSDKGTVHVFSLRVKDAEEDAKKGESATAGAQVNDNCNYGSTVPVTQTKIG
SNTSSSLSFMKGILPKYFSSEWSFAQFRLPEITRYIMAFGDQDTVMMIGLDGSFYRYSFD
PVNGGEMMLKEYHLFLKASKSL*
>11667.m00686|LOC_Os01g07500.1|genepair226-1
MAALRVGTRAVEGRFQASNGGGGGGGGMAPSSRLVAAHREAKPRSSHSAAPWKLPRRRAG
AMPLWRVAVFASVALNVATLALLLHHYATSPPPHHHHHDAGLATRSSDAAVHRRARTASS
MAPSTGKPAVTTDSVINLDHGDPTMFEEFWRETGDAAEVVIPGWQTMSYFSDVTNVCWFL
EPELDRQVRRLHRVVGNAAVDGYHVLVGTGSTQLFMAALYALAPDAAAAAAGEPISVVST
APYYSSYPAVTDFLRSGLFRWAGDADAFKGDSYIELVCSPNNPDGAIREAVLDPKTGNGR
TVHDLAYYWPQYTPITKRASHDIMLFTVSKSTGHAGTRIGWALVKDRAIARKMTKFVELN
TIGVSKDSQMRAAKVLAAVSDGYERRPEQTKETMTTPLRLFDFGRRKMVERWSMLRAAAA
ASGIFSLPEETSGFCNFTKETAATNPAFAWLRCDREDVEDCAGFLRGHKILTRSGAQFGA
DARYVRVSMLDRDDAFDIFINRLSSLK*
>11682.m00737|LOC_Os05g07720.1|genepair226-2
MAAMGSKDGGGGGGGMAAQAGRLGVVASVAFNLAALAFYLRRRYFGGDDAAAVRKKAEAE
VAPSSGKPPVTKDSIINLDHGDPTMYEAFWRGGAGERATIVIPGWQTMSYFSDVGSLCWF
LEPGLEREVRRLHRLVGNAVADGYHVLVGTGSTQLFQAALYALSPPGPSAPMNVVSPAPY
YSSYPAVTDFLKSGLYRWAGDAKMFDGDTYVELVCSPSNPDGGIREAVLKSGDGVAVHDL
AYYWPQYTPITSAAAHDIMLFTVSKCTGHAGTRLGWALVKDRAVAQKMSKFIELNTIGVS
KDSQLRAAKILKAITDGYDRAPAAGDDDDDSSRLFHFARRKMVSRWAKLRAAVAASGIFT
LPDELPGHCTFANETVSAYPPFAWLRCGKEGVDDLEGYLRERKIISRGGGKFGADGRVVR
ISMLDTDEAFAIFVDRLAAMN*
>11667.m00688|LOC_Os01g07520.1|genepair227-1
MNTSQFMDKQILGLAASASTSSGVGVGGGGPELLDLMSPNPQEEGEDRLRRRHSSSNGSA
DDVLPSYDFQPIRTTAPSAAASASAAPASWGSLDSGSKAASASYNLKSAGVLEPHVLKKV
SHEEDRSNFGTVTMADIDRTMKKYSDNLLHALEGVSSRLSQLEGRTHHLENSVDDLKITI
GNYNGSTDGKLRQLENMLREVQAGVQILRDKQEIVETQLHLAKLQPPKSDALASDNVGSS
QTDSHQHAVAPQQAAIQPQHQALTSSQPLALPALPAPNAPPPPPTLQSQPPSQYPGHLPH
SQVPPVPPSAPVPSVPALPRDPYYAPPAQPTETMHQQYQAPPVPQPQAPPAPPQQYQTPP
QFPQYSQPPQSGMVNPSTPLPPAAPQQPEEAMSYAPPQSYPPNVRPPSPYMPPPSGPAPP
FYGQNQSMYEPPVGRPNSGPPPSYGAGGYGPQGGSGFSESYGYSGSPSHRGNAGMKSSPS
PFAPSGPSSGGSGNYGRLPTAQILPQAVPINSSPSGSSGNRVPVDDVVEKVATMGFSREQ
VRATVRRLTENGQNVDLNVVLDKLMNDSDVQQPQKGWFGR*
>11682.m00738|LOC_Os05g07730.1|genepair227-2
MNASQFMDKQILGLAASGAGAAAAAAAASSSPPAAGGGGGGGLFDLMSPDPQEDGGGHAR
RGQQQHGGADEVVPSYDFQPIRAAPAAAAPASAASPWGSLDSKAASSNLKSAGMLESHVL
KKVSHEEERGNFSAVSIADIDRTMKKYADNLLHALESVSSRLSQLEGRTHHLENSVGELK
LIIGNYNGSTDGKLRQFENTLREVQAGVQILRDKQEIVETQVQLSKLQLSKAEDAQSEKA
GVGQADSRQQPTLPQPQHQAPPPSHPPALPALPAPNAPPPPAPQSQPPSQFPGHLPHSQV
QSVPPAPPTPLAPTIPQESYYPPSAVQPTDTTHQQYQAPPAPQSQAPPAPPQHYQTPPQY
AQYSQPPPASANPSTAVPPSVHQQPEEVAAPYGPPPQSYPPNVRLPSPYVPPPSGPAPPF
YGPNPGMYEPPAVRPNSGPPPSYNTGYKPQGGGGFPEPYGYSGSPSHRGNAGMKSPSPFH
PTGSAGSGNYSRLPTAQMLPQAASASSTPSASSGNRVPIDDVVDKVATMGFSREQVRAAV
RQLTESGQNVDLNMVLDKVMNGADAQPQRGWHGR*

>11667.m00692|LOC_Os01g07560.1|genepair228-1
MPPGLSRRRRPDAAALLCCVVAVVAACMVGGALAADAQGAALLAWKRTLRGGDTALPDWN
PADASPCRWTGVRCNANGRVTELSLQQVDLLGGVPDNLSAAMGTTLERLVLAGANLSGPI
PAQLGDLPALTHLDLSNNALTGSIPASLCRPGSKLESLYVNSNHLEGAIPDAIGNLTALR
ELIIFDNQLDGAIPASIGQMASLEVLRGGGNKNLQGALPPEIGNCSKLTMLGLAETSISG
PLPATLGQLKNLNTLAIYTALLSGPIPPELGRCTSLENIYLYENALSGSIPAQLGGLANL
KNLLLWQNNLVGVIPPELGACTGLAVVDLSMNGLTGHIPASLGNLSSLQELQLSVNKVSG
PIPAELSRCTNLTDLELDNNQISGAIPAELGKLTALRMLYLWANQLTGTIPPEIGGCAGL
ESLDLSQNALTGPIPRSLFRLPRLSKLLLIDNTLSGEIPPEIGNCTSLVRFRASGNHLAG
DIPPEVGKLGSLSFLDLSTNRLSGAIPPEIAGCRNLTFVDLHGNAIAGVLPPGLFQGTPS
LQYLDLSYNAIGGAIPANIGMLGSLTKLVLGGNRLSGQIPPEIGSCSRLQLLDLSGNSLT
GAIPASIGKIPGLEIALNLSCNGLSGAIPKGFAGLARLGVLDVSHNQLTGDLQPLSALQN
LVALNISYNNFTGRAPETAFFARLPASDVEGNPGLCLSRCPGDASDRERAARRAARVATA
VLLSALVALLAAAAFVLFGRRRQPLFGRGSTSPADGDGKDADMLPPWDVTLYQKLEISVG
DVARSLTPANVIGQGWSGAVYRASIPSTGVAIAVKKFRSSDEASVDAFACEVGVLPRVRH
RNIVRLLGWAANRRTRLLFYDYLPNGTLGGLLHGGGAAIGAAVVEWEVRLSIAVGVAEGL
AYLHHDSVPAILHRDVKSDNILLGERYEACLADFGLARVADDGANSSPPPFAGSYGYIAP
EYGCMTKITTKSDVYSFGVVLLEIITGRRPIEAAFGEGQTVVQWVREHLHRKRDPAEVID
SRLQGRSDTQVQEMLQALGIALLCASTRPEDRPTMKDVAALLRGLRHDDSAEAQKAGSGS
AIKWADPRQPGSPTKPMAQAQAHSHTSSLAYSTTGSV*
>11682.m00739|LOC_Os05g07740.1|genepair228-2
MASRSPAAAAAAMVMACVVVVLRVSCVLAVDEQGAALLAWKATLRGDGGALADWKAGDAS
PCRWTGVTCNADGGVTELSLEFVDLFGGVPGNLAAAVGRTLTRLVLTGANLTGPIPPELG
ELPALAHLDLSNNALTGTIPAALCRPGSKLETLYLNSNRLEGAIPDTIGNLTSLRELIVY
DNQLAGKIPASIGKMSSLEVLRGGGNKNLQGALPAEIGDCSSLTMIGLAETSITGPLPAS
LGRLKNLTTLAIYTALLSGPIPPELGRCGCLENIYLYENALSGSIPAQLGGLGKLRNLLL
WQNQLVGVIPPELGSCAALAVVDLSLNGLTGHIPPSFGNLSSLQELQLSVNKLSGAVPPE
LARCSNLTDLELDNNQLTGGIPAELGRLPALRMLYLWANQLTGSIPPELGRCGSLEALDL
SSNALTGAIPRSLFRLPRLSKLLLINNNLSGELPPEIGSCAALVRFRASGNHIAGAIPPE
IGMLGNLSFLDLASNRLAGALPPEMSGCRNLTFVDLHDNAISGELPPRLFRDWLSLQYLD
LSDNVIAGGIPPEIGMLTSLTKLVLGGNRLSGPMPPEIGSCTRLQLLDVGGNSLSGHVPG
SIGKIPGLEIALNLSCNGFSGAIPAEFAGLVRLGVLDVSRNQLSGDLQPLSALQNLVALN
VSFNGFTGRLPETAFFARLPTSDVEGNPALCLSRCSGDASEREVEARRAARVAMAVLLSA
LVVLLAAAALVLFGWHRRGGGARGGEDKDGEMSPPWDVTLYQKLEIGVSDVARSLTPANV
IGHGWSGEVYRASMPSSGVTIAVKKFRSCDEASIEAFAGEVSVLPRVRHRNIVRLLGWAA
NRRTRLLFYDYLPNGTLGGLLHGGAMGGGATTTAAVVEWEVRLAIAVGVAEGLTYLHHDC
VPGIIHRDVKADNILLADRYEACLADFGLARVADDGASSSPPPFAGSYGYIAPEYGCMTK
ITTKSDVYSFGVVLLEMITGRRPLDPAFGEGQSVVQWVRDHLCRKRDPAEIIDVRLQGRP
DTQVQEMLQALGMALLCASPRPEDRPTMKDVAALLRGIRHDDGVEARKAGNGVGTDAETR
KRADPRQPISPTKLMALARPAQAQAQLQARANSGSLGLLNDQE*
>11667.m00693|LOC_Os01g07570.1|genepair229-1
MASSGCSSNRLRQTIVVALLLLASSSCLASAKHRGNGTTTTVPFHGKDELRRYRKIMAQV
ARLKKASVKTIQSPDGDVIDCVPAHLQPAFEHPKLRGQKPEAEPEERPKVGGAAAAEAEE
EAVFPQAWTDGGESCPEKTVPVRRTRRRDVLRSSSAVRFGMKQPRAAGVVRRDSTSDGHE
HAVGYVTGDQFYGAKASLNVWSARVATAAEFSLSQIWVISGSFGNDLNTIEAGWQVSPEL
YGDNNPRFFTYWTTDAYQATGCYNLHCSGFVQTNNRIAIGAAISPTSVYNGRQFDISLLI
WKDPRRGHWWLQLGSGPLVGYWPSSLFTHLGGHANMVQFGGEVVNTRPSGSHTPTQMGSG
HFPREGFNRAAYFRNLQVVDWDNNLLPAAALRLVADHPSCYDIQGGYNRAWGNYFYYGGP
GRNVRCP*
>11682.m00742|LOC_Os05g07770.1|genepair229-2
MSSTLMASCCFIISYKRPRPIIATFVPFLLLLFFFAVVVAASSSSNGTAAALHPGEELLR
LERVRAQLARSPDGDVIDCVPSHLQPAFEHPRLRGQKPEEPPSARPTETTRRRRRRRSH
AHGGGGGEHREEEDDGEHGLRQAWWAAGEACPEGTIPVRRTTEADLLRASSAAAAGGRFG
MKPRGVGVVGGAARRDSTSSGHEHAVGYMSGGQFYGAKASLNVWPAKVASPAEFSLSQIW
LISGSFGNDLNTIEAGWQVSPQLYGDNNPRFFTYWTNDAYQETGCYNLHCSGFVQTNSRI
AMGAAISPISSFAGRQFDITLLIWKI*
>11667.m00695|LOC_Os01g07590.1|genepair230-1
MAWRGLARDAAAAYLRRGGAPAHVFSAASRAPGPVVANPGGELRAFGLFRSPMARRADAF
EVPSAAGRHGAQGVWSRSSVPALVRAGAPNSRALPFLVGRVVRGFYPQLSGHKLVKGLGM
GSTLAATFCSQKVAYAEEVAEQPSEGLIGPSTKHQISKLWTIIRKYQLPVGLIALIALGW
QNPLGLFINVLLILYSSRPSPYSIYLFLQEVRHGEMHQNRAFWKEEAVLTRKVDTKDYKL
FSIGTVESADREVLHVIGILGNWWIYRASYGKVENRSFSRGLYWKEFPHLTLSKNLFVMG
GRKIGVAVDFSSCSKAALRWASTNLTRSGDQLVLIHVNSSYHNEQGAVQLWEQSGSPLIP
LAEFSDPHVAKTYAVSPDKETLEILNQMSNQRGVEVLAKILYGDPAKKLYEAVDLVPLNC
LVVGNRGLSTLKRALMGSVSSYIVNNATCPVTVVKENI*
>11682.m00746|LOC_Os05g07810.1|genepair230-2
MICMRDRSRRGVARERMAAVSVGGGGRNIGVAMDFSACSKAALRWAAASLARPGDRLVLV
HVKPSFQYEQGVAHLWEQQGSPMIPLVELADPRVSRIYGVAPDAETIGILTSAANQKGVE
VVAKVYWGEPAKKLTEAAQGIPLHWLVVGNRGLGAVKRVLMGSVSTYVVANHATCPVTVVR
ENLPPPPPPPQPPQPVATAASYY*
>11667.m00698|LOC_Os01g07620.1|genepair231-1
MIARRLKMLVLVSLVPLALRATSLLAGHVAPPCSPESLRPEHQPAGAGDGVTMGGGGASA
SSYRRNRRRMEGGLAAAAVAFHARRFRPHGGGGFEADKRLAPTGSNPLHNLR*
>11682.m00749|LOC_Os05g07840.1|genepair231-2

MVVTLIIVPLALRGASLLGNAVAAAVVPSSSPEQQQQQQRRPRPPPGSKNGASPSSSAHG
QHWKDQSRHAAFTRRRFGTGTGGGGDDGFFSDDKRFSPTGSNPLHNLSSLWLLGYVLVAS
DAT*
>11667.m00699|LOC_Os01g07630.1|genepair232-1
MRMRWWAAPLAAVLAVILLPSSTATLSPAGINYEVVALMAIKTELQDPYNVLDNWDINSV
DPCSWRMVTCSADGYVSALGLPSQSLSGKLSPGIGNLTRLQSVLLQNNAISGTIPASIGR
LGMLQTLDMSDNQITGSIPSSIGDLKNLNYLKLNNNSLSGVLPDSLAAINGLALVDLSFN
NLSGPLPKISSRTFNIVGNPMICGVKSGDNCSSVSMDPLSYPPDDLKTQPQQGIARSHRI
AIICGVTVGSVAFATIIVSMLLWWRHRRNQQIFFDVNDQYDPEVCLGHLKRYAFKELRAA
TNNFNSKNILGEGGYGIVYKGFLRDGAIVAVKRLKDYNAVGGEVQFQTEVEVISLAVHRN
LLRLIGFCTTENERLLVYPYMPNGSVASQLRELVNGKPALDWSRRKRIALGTARGLLYLH
EQCDPKIIHRDVKASNVLLDEYFEAIVGDFGLAKLLDHRESHVTTAVRGTVGHIAPEYLS
TGQSSEKTDVFGFGVLLVELITGQKALDFGRLANQKGGVLDWVKKLHQEKQLSMMVDKDL
GSNYDRVELEEMVQVALLCTQYYPSHRPRMSEVIRMLEGDGLAEKWEASQNVDTPKSVSS
ELLPPKFMDFAADESSLGLEAMELSGPR*
>11682.m00750|LOC_Os05g07850.1|genepair232-2
MDERRAAASLLAVLVLSLVVSASSAAAGVAPAAPPDVLSSPAAAAGEGEAEALLAVKAAL
HDTANVLADWNAGSGGVVVAGGGGGGGPCNWSMVTCSKTGHVSVLDLAHRNLSGTLSPAI
GKLRRLRLLFLQHNAISGPIPDTIGRLKVLQTLDLAYNHFTGTIPSILGHSKGIFLMDLS
FNNLSGPAPVFSANSVLFSALTSVQKVILRGSETFVSRYSGHIFPYQRPEIYLGHLKQFM
IKEIKEATNNFDRRNILGQGGFGIVYKGRLRDGTIVAVKRMKDCFSVCGDDQFHTEVEVI
SLIVHRNLLRLTGFCITDTERLLVYPFMPNGTVSSKLQEYVGGKPTLDWTRRRKIALGAA
RGLVYLHEQCDPKIIHRDIKASNVLLDEYFEAVVADFGLVKLLDHGESHAVTAVRGTMGR
IPPEYLMTGQTSEKTDVYGFGFLLIELITGRKTMELHEDEYQEGGILDWAKELLEGNKLR
SFVDSRLRDNYVIAELEEMVKIALLCTMYNPDQRPSMAEIAGMLQESDGSVVEKWETLKD
AERSKPSTPEFMLSSPVNFASDECNSIQLEAVELSGPREFFFSPEFLVDMFGLLLSEVKS
GLLASSAAVAKCELSRESSGFGRHTLSCSNALVTC*
>11667.m00712|LOC_Os01g07730.1|genepair233-1
MQRAAAASRATAWSTARHGAARVTASASFSGGGGIVAGAALPLRVRGGQLMSLPLLSGGR
AVTARVAAAEAPLPADDADAAAGRERGALAETAQLGAMIVAWYLLNIYFNIYNKQVLQPL
PFPYTITAFQLAFGSFVIFLMWALKLHPAPRISISQLAKIAPLAAGHMLGTVFTNMSLSK
VAVSFTHTIKASEPFFTVLLSAFFLGETPSLLVLGSLVPIVGGVALASLTELSFNWIGFW
SAMASNLLYQSRNVLSKKLLGGEEEALDDINLFSILTILSFLLSLPLMLFSEGVKFSPGY
LRSTGLNLQELCVRAALAGFCFHGYQKLSYLILARVSPVTHSVANCVKRVVVIVASVLFF
RTPISPVNALGTGVALGGVFLYSRLKRTKPKNA*
>11682.m00752|LOC_Os05g07870.1|genepair233-2
MQAVAAATSRAWAASPRRRRHVASCSSPPPPTTTATTTTSSLNRCPVAGAGAPVLPLGIR
GGRMLLAPPLLWNSGAAARKAAVATAAAASPPAEGGGKANGGAVAGGISRTVQLGAMILV
WYLLNIYFNIFNKLVLKSVPFPYTITTFQFASGSFFITLMWLLNLHPKPRLSLGQYAKIL
PLALVHTMGNVFTNMSLGKVAVSFTHTIKAMEPFFSVLLSVLFLGETPSFLVLGSLVPIV
GGVVLASMTEVSFNWIGFWSAMASNLTNQSRNVFSKKLLADKEETLDDINLFSIMTVMSF
LLSAPLMLSVEGIKFSPSYLQSNGVNLQELCMKAALAGTCFHFYQQVSYSLLARVSPVTH
SVANCVKRVVVIVSSVLFFRTPISPINALGTGVALAGVFLYSRFKKAKPKAKTA*
>11667.m00718|LOC_Os01g07760.1|genepair234-1
MAQMLLHGTLHATIFEAASLSNPHRASGSAPKFIRKFVEGIEDTVGVGKGATKVYSTIDL
EKARVGRTRMITNEPINPRWYESFHIYCAHMASNVIFTVKIDNPIGATNIGRAYLPVQEL
LNGEEIDRWLDICDNNREPVGESKIHVKLQYFDVSKDRNWARGVRSTKYPGVPYTFFSQR
QGCKVTLYQDAHVPDNFIPKIPLADGKNYEPHRCWEDIFDAISNAQHLIYITGWSVYTEI
TLVRDSNRPKPGGDVTLGELLKKKASEGVRVLMLVWDDRTSVGLLKRDGLMATHDEETEN
YFHGSDVNCVLCPRNPDDSGSIVQDLSISTMFTHHQKIVVVDHELPNQGSQQRRIVSFVG
GLDLCDGRYDTQYHSLFRTLDSTHHDDFHQPNFATASIKKGGPREPWHDIHSRLEGPIAW
DVLYNFEQRWRKQGGKDLLLQLRDLSDTIIPPSPVMFPEDRETWNVQLFRSIDGGAAFGF
PDTPEEAAKAGLVSGKDQIIDRSIQDAYIHAIRRAKNFIYIENQYFLGSSYAWKPEGIKP
EDIGALHLIPKELALKVVSKIEAGERFTVYVVVPMWPEGVPESGSVQAILDWQRRTMEMM
YTDITEALQAKGIEANPKDYLTFFCLGNREVKQAGEYQPEEQPEADTDYSRAQEARRFMI
YVHTKMMIVDDEYIIIGSANINQRSMDGARDSEIAMGGYQPYHLATRQPARGQIHGFRMA
LWYEHLGMLDDVFQRPESLECVQKVNRIAEKYWDMYSSDDLQQDLPGHLLSYPIGVASDG
VVTELPGMEYFPDTRARVLGAKSDYMPPILTS*
>11682.m00753|LOC_Os05g07880.1|genepair234-2
MAKILLHGTMHVTIFEAESLSNPSRPSSQAPQFLRKLVEGIEDTVGVGKGTSKVYATIGL
DKARVGRTRTLADDTAAPRWYESFHVYCAHLATHVAFTLKAKNPIGASLLGVGYLPVRDV
LAGDEVDRWLPLCDDTDARTPIGDGGGKVHVKLQYFDISKDRSWGRGVRSGKYPGVPYTF
FSQRQGCKVTLYQDAHVPDGFIPRIPLDGGRSYEPHRCWEDIFDAINGARHFIYITGWSV
YTEIALIRDADRPKPGGGVTLGELLKKKAGEGVRVLMLVWDDRTSVGMLKKDGLMATHDE
ETMNYFQGTEVNCVLCPRNPDDSGSIVQDLQISTMFTHHQKIVVVDHDMPSSRHGGGNGG
GRRRVVSFVGGLDLCDGRYDTPFHSLFRTLGTAHHDDFHQPNFATATVAKGGPREPWHDI
HCRLEGPVAWDVLYNFEQRWRKQGGKDLLVQLRDLAETVIPPSPAMFPEDAESWNVQLFR
SIDGGAAFGFPDTPEDAARAGLVSGKDQIIDRSIQDAYIAAIRRARSFIYIENQYFLGSS
YCWKPNDGVKPEDVGALHLIPKELSMKVVSKIEAGERFTVYVVVPMWPEGIPESGSVQAI
LDWQRRTMEMMYTDIAHAIQAKGIDADPKDYLTFFCLGNREAKSAGEYEPPEQAEPDTGY
FHAQQNRRFMIYVHTKMMIVDDEYIIVGSANINQRSMDGARDSEIAMGAYQPHHLAAAGR
PARGQVHGFRMALWYEHLGTVDEAFQRPESLDCVRKVNAMADRCWDLYAGDGPERDLPGH
LLTYPVGVAGDGTITQLPGVEFFPDTQARILGAKSDYLPPILTT*
>11667.m00720|LOC_Os01g07780.1|genepair235-1

MARRRPPPQRHHHLVLLALAAFSCLASGTATPTPTTSSQSQQPQPAGTLLQPALAADLAA
RTCWYTVQIKTSCASPWRTSDAVSLAFGDAYRNEVYAARLAGSSSSPQPSSSAAFERCAT
DTFRVGGPCGYGVCYLYLRRSGRDGWTPQWVRVYEPTSDTPSTFYYGDPLPNAVWYGFNR
CPRLAASAAAQ*
>11682.m00754|LOC_Os05g07890.1|genepair235-2
MVSMAPSSSAAATRRLPLLLLLAAVLLLHPCHAATEASAAARRECTYTVRVKTSCASPAR
TADIVSVAFGDAYRNEAYGARLPAGGASGALDRCAVDAFRVGGQCGYGVCYLYLRRAGRD
GWAPEWVQVFEPGAAAGEKPSTFYFGSPLPDGVWYGHNRCPKASPAMAARRTNTSASPLG
*
>11667.m00727|LOC_Os01g07850.1|genepair236-1
MVNTTAGVKCGGGGAALPLSTLNHVSLVCRSLSTSLTFYRDFLGFVSVRRPGSFDFDGAW
LFNYGIGIHLLQAEDPESMPPNKEINPKDNHISFTCESMEAVQRRLKEMGVRYVQRRVEE
GGVYVDQIFFHDPDGFMIEICTCDKLPVVPLDAAAAHSIFAGRSPPPPVACKIRPVKQPS
ATKLGSVAAGGCVGEVIVVDAINGAAAAGGGGAMS*
>11682.m00759|LOC_Os05g07940.1|genepair236-2
MVNTAAVAAAKGSRGSGLPLASLNHISIVCRSLQESLTFYTDVLGFFPVRRPGSFDFDGA
WLFNYGIGIHLLQAEDPDSLPGKTEINPKDNHISFQCESMVAVERRLKELGIPYIQRCVE
EGGIYVDQIFFHDPDGFMIEICNCDNLPVVPLGADQPLVMAACKRAAVIKQQQQASSSPA
TAAAAAQCAVPSSTKAIHVGEEAHISCA*
>11667.m07344|LOC_Os01g73130.1|genepair237-1
MSSTFNGDEFAPFLGFIGAALALVFSCMGAAYGTARSGVGVAHMGVMRPELVMKSIVPVV
MAGVLGIYGLIIAVIITTGINPTAMPYYHFDGSVHLAAGLATGLCALAAGLAIGVVGDAG
VRANAQQPKLFVGMILILIFAEALGLYGLIVGIILSSRAGQSRAH*
>11682.m00068|LOC_Os05g01560.1|genepair237-2
MSSSTFSGDETAPFFGFLGAASALVFSCMGAAYGTAKSGVGVASMGVMRPELVMKSIVPV
VMAGVLGIYGLIIAVIISTGINPKAKPYFLFDGYAHLSSGLACGLAGLAAGMAIGIVGDA
GVRANAQQPKLFVGMILILIFAEALALYGLIVGIILSSRAGQSRAD*
>11667.m07362|LOC_Os01g73310.1|genepair238-1
MADGEDIQPLVCDNGTGMVKAGFAGDDAPRAVFPSIVGRPRHTGVMVGMGQKDAYVGDEA
QSKRGILTLKYPIEHGIVSNWDDMEKIWHHTFYNELRVAPEEHPVLLTEAPLNPKANREK
MTQIMFETFNVPAMYVAIQAVLSLYASGRTTGIVLDSGDGVSHTVPIYEGYALPHAILRL
DLAGRDLTDSLMKILTERGYSFTTSAEREIVRDIKEKLAYVALDYEQELETAKNSSSIEK
SYELPDGQVITIGSERFRCPEVLFQPSMIGMESAGIHETTYNSIMKCDVDIRKDLYGNVV
LSGGTTMFPGIADRMSKEITALAPSSMKIKVVAPPERKYSVWIGGSILASLSTFQQMWIS
KDEYDESGPAIVHRKCF*
>11682.m00072|LOC_Os05g01600.1|genepair238-2
MADGEDIQPLVCDNGTGMVKAGFAGDDAPRAVFPSIVGRPRHTGVMVGMGQKDAYVGDEA
QSKRGILTLKYPIEHGIVSNWDDMEKIWHHTFYNELRVAPEEHPVLLTEAPLNPKANREK
MTQIMFETFNVPAMYVAIQAVLSLYASGRTTGIVLDSGDGVSHTVPIYEGYALPHAILRL
DLAGRDLTDSLMKILTERGYSFTTSAEREIVRDIKEKLAYVALDYEQELETAKNSSSVEK
SYELPDGQVITIGAERFRCPEVLFQPSMIGMEAAGIHETTYNSIMKCDVDIRKDLYGNIV
LSGGTTMFPGIADRMSKEITALAPSSMKIKVVAPPERKYSVWIGGSILASLSTFQQMWIS
KDEYDESGPAIVHRKCF*
>11667.m07372|LOC_Os01g73410.1|genepair239-1
MHTKGASSDVIRVSTSSAPSTSSHGSAQDDCDSSGDVYVWGEVICDNSVRTGSDTVVRST
VRTDVLRPKPLESNLVLDAYHVDCGVKHSALVTKNGEVFTWGEESGGRLGHGSREDSIHP
RLIESLAVCNVDIVACGEFHTCAVTTAGELYTWGDGTHNVGLLGHGKDVSHWIPKRIAGA
LEGLAVAYVSCGTWHTALITTMGQLFTFGDGTFGVLGHGNRESISCPKEVESLSGLKTIS
VACGVWHTAAIVEVIVTQSSSSISSGKLFTWGDGDKHRLGHGDKEPRLKPTCVASLIDYD
FHRIACGHSLTVGLTTSGKVLSMGNTVYGQLGNPRSDGKIPCLVEEIVGENVVQVACGSY
HVAVLTIKSEVFTWGKGANGRLGHGDIEDRKIPTLVEALRDRSVRHIACGANFTAAICQH
KWVSGAEQSQCASCRQPFGFTRKRHNCHNCGLVHCNACTSRKAVRAALAPNPAKPYRVCD
SCFLKLNNAVDSSAISKKKENVLRESNSDGRLTKAIIPSNLDMIRSLDSKAAKQGKKTDA
LSFLRTPQMNSLLQLRDIALSGGLDLNRPVPRAVRTTAVRSVNTSRAVSPFSRKPSPPRS
TTPVPTTHGLSIGKGAADNLAKTNEMLNQEVERLRAQVDNLRHRCEVQELELQKSAKKVQ
EAMTLVAEESSKSKAAKEVIKSLTAQLKDMAERLPPDQGAYDGNESKQMHFPNGTELHAA
IYSSTNGIHQLQNESISALNTPSLNTGRSLHANGISSQHKSLGSISEHSEVSTHSHRVSS
PHDTELSNRRARISSDELFSASGKSDDSNNRDARSLQNGEDGYKPRGTVSLSSNQVQAEW
IEQYEPGVYITLTTLRDGTRDLKRVRFSRRRFGEHQAENWWNENREKVYERYNCRCKDSE
KFSATIFHPFYVHDADDTK*
>11682.m00074|LOC_Os05g01610.1|genepair239-2
MHTRGASSDVLRASISSAPSTSSHGSAQDDCDSLGDVYVWGEVFCENSVRVGSDTIIRST
EKTDFLLPKPLESRLVLDVYHVDCGVRHAALVTRNGDVFTWGEDSGGRLGHGTREDSVHP
RLVESLAACNVDFVACGEFHTCAVTTTGELYTWGDGTHNVGLLGHGTDAGHWIPKRISGA
LDGLPVAYVSCGTWHTALITSMGQLFTFGDGSFGVLGHGNLTSISCPKEVESLSGLKTIA
VACGVWHTAAIVEVIVTHSSSSVSAGKLFTWGDGDKHRLGHGDKESRLKPTCVASLIDYD
FYRVACGHSLTVCLTTSGKVLSMGNSVYGQLGNPNSDGRLPCLVEDRIAGEHVLQVACGS
YHVAVLTGRSEVFTWGKGANGRLGHGDIEDRKVPTQVEALKDRAVRHIACGANFTAAICL
HKWVSGADQSQCSSCQQPFGFTRKRHNCYNCGLVHCNACTSRKALRAALAPNPGKPYRVC
DSCFLKLKNALDSDSFNKRKDIVSHLAGESNGDTKASKTILSSNMDIIRSLDSKAARQGK
KTDALSFLRTPQVSSLLQLRDIALSGSADMNRSVPRAVRTSAVRSVTTSRAVSPFSRKSS
PPRSTTPVPTTHGLSFSKSATDNLAKTNELLNQEIDRLHAQVDNLRHRCEHQEVELHKSA
KKVQEAMTLVAEESAKSKAAKEVIKSLTAQLKDMAERIPPEQGTYDVSEAKPVHVPNGID
SHIAIYSSINVAHQPQNELLNASNAQSLNSGRSLHPNGISSQHRLLGNATEASEGSAQSH

RITSPCKLDVPHRRAHSNSDDMLTASHRGDDNVSIDAMSLQNGEDGYKPRGTVSSISSSQ
VQAEWIEQYEPGVYITLTTLLDGTRDLKRVRFSRRRFGEHQAEKWWNENREKVYERYNVR
SSERVSSSSAASTRSAY*
>11667.m07423|LOC_Os01g73890.1|genepair240-1
MATFELYRRSTIGMCLTDTLDDMVSSGALSPELAIQVLVQFDKSMTSALEHQVKSKVTVK
GHLHTYRFCDNVWTFILTDAIFKNEEITETINKVKIVACDSKLLETKEE*
>11682.m00084|LOC_Os05g01710.1|genepair240-2
MATFELYRRSTIGMCLTETLDEMVSSGTLSPELAIQVLVQFDKSMTEALENQVKSKVSIK
C*
>11667.m07434|LOC_Os01g73960.1|genepair241-1
MEVEASYSYGFLPSGRHQPYAPPPPHPAEEGELWEYFPCPFCYIEVEVPFICNHLQEEHC
FDTRNAVCPLCADNIGRDMGAHFRVQHSHLLKRRKPSRPSSSWPTPSNNSDPYFEGPPQY
MMNNRTYQDPAPDPLLSQFICSMAQTDTNSDNTNTEIAVSAVSHDQRLSQRVTLTDDASK
LELKERLQRIEFVKEIIMSTIL*
>11682.m00086|LOC_Os05g01730.1|genepair241-2
MEVEAHHGFLPPAAGRQHLYGHPHYQLSGDDEWWEYIPCPFCYIEVEVHFLCDHLQEEHC
FDMKNAVCPICADNLDKDTDEHFRVQHSHLLKRRKSSSFSCKPSSAAADKGSYEEDSYFE
APSHCMGRPAPDSSPDPLLSQFICCSLAPPVDSPRRSEADAEGHGSSSSDDQKRREQGVM
DDASKEELEERLQRIEFVKQMLMTTIAY*
>11667.m07435|LOC_Os01g73970.1|genepair242-1
MAKLPIVGRSAPRKPNESMRLLVVTVIGVMLGFFIGISFPAVSITKLHFPSSIVSYIEDK
NSALTAQAILNHAWTAARNAKGNDTESSSDTAMKIYVPTNPRGAESLAPGIVVPESDFHP
RRLWGNPDEDLPFKPKYLVTFTVGISQKENINRAVKKFSDNFAILLFHYDGRVSEWDEFE
WSKRAIHVSVRRQAKWWYAKRFLHPDIVASYEYIFIWDEDLGVEHFNAEEYIKLVKKYQL
EISQPGLEPDRGLTWQMTKRRGDHQVHKETEERPGWCTDPHLPPCAAFVEIMAPVFSRDA
WRCVWHMIQNDLVHGWGLDFALRRCVDPAHEKIGVVDSQWIVHQVVPSLGNQGQSEHGRA
PWEGVRERCRKEWGIFQTRIAEAEKSYYEMMGVPPPNVTFVH*
>11682.m00091|LOC_Os05g01760.1|genepair242-2
MGKLAAVVRSVLRLTNESMRIVMVTIIGVLLGFFIGISFPSVSITKLHFPSSFVSYIEDR
NSGLTTQALLNHAWTSARNARENSSEPSSNTTLKIYVPTNPKGAERLAPAIVVPETDFHL
RRLWGEPSEDLPFKPKYLVTFTVGYAQKENINRAVKKFSDNFAILLFHYDGRVSEWDEFE
WSKRAIHISARKQTKWWYAKRFLHPDIVAAYEYIFIWDEDLGVEHFNAEEYIKLVKKYHL
EISQPGLEPDRGLTWQMTKRRGDREVHKVTEERPGWCSDPHLPPCAAFVEIMAPVFSRDA
WRCVWHMIQNDLVHGWGLDFALRKCVEPAHEKIGVVDSQWIVHQVVPSLGNQGKSENGRP
AWEGVRARCRKEWGMFQTRMAEAEKAYYKMMGITPPNSTLV*
>11667.m07436|LOC_Os01g73980.1|genepair243-1
MAASMNSKLPLALVLLLLCGGACVAVAMPSELSIVGYSEEDLASHERLMELFEKFMAKYR
KAYSSLEEKLRRFEVFKDNLNHIDEENKKITGYWLGLNEFADLTHDEFKAAYLGLTLTPA
RRNSNDQLFRYEEVEAASLPKEVDWRKKGAVTEVKNQGQCGSCWAFSTVAAVEGINAIVT
GNLTRLSEQELIDCDTDGNNGCSGGLMDYAFSYIAANGGLHTEESYPYLMEEGTCRRGST
EGDDDGEAAAAVTISGYEDVPRNNEQALLKALAHQPVSVAIEASGRNFQFYSGGVFDGPC
GTRLDHGVTAVGYGTASKGHDYIIVKNSWGSHWGEKGYIRMRRGTGKHDGLCGINKMASY
PTKNA*
>11682.m00097|LOC_Os05g01810.1|genepair243-2
METKVTVGVVLLLLCLCGGAACAAGRSGGEFSIVGYSEEDLASHDRLIELFEKWVAKYRK
AYASFEEKVRRFEVFKDNLNHIDDINKKVTSYWLGLNEFADLTHDEFKATYLGLTPPPTR
SNSKHYSSEEFRYGKMSNGEVPKEMDWRKKNAVTEVKNQGQCGSCWAFSTVAAVEGINAI
VTGNLTSLSEQELIDCSTDGNNGCNGGLMDYAFSYIASTGGLRTEEAYPYAMEEGDCDEG
KGAAVVTISGYEDVPANDEQALVKALAHQPVSVAIEASGRHFQFYSGGVFDGPCGEQLDH
GVTAVGYGTSKGQDYIIVKNSWGPHWGEKGYIRMKRGTGKGEGLCGINKMASYPTKDN*
>11667.m07437|LOC_Os01g73990.1|genepair244-1
MSNNKVLTLEEVSKHNTKDDCWLIIGGKVYNVTKFLEDHPGGDDVLLSSTAKDATDDFED
VGHSTTARAMMDEYYVGDIDATTIPTKVKYTPPKQPHYNQDKTPEFIIKILQFLVPLAIL
GLAVAVRIYTKSESA*
>11682.m00098|LOC_Os05g01820.1|genepair244-2
MSNDNKKVYTLEEVAKHNSKDDCWLIIGGKVYNVSKFLEDHPGGDDVLLSSTGKDATDDF
EDVGHSTTARAMMDEYYVGDIDTSTIPARTKYVPPKQPHYNQDKTPEFIIKILQFLVPLA
ILGLAVAIRIYTKSESA*
>11667.m07441|LOC_Os01g74030.1|genepair245-1
MVTEMAMRGEPWRRQEGEQAQAEADRERELDLFRSGSAPPTVEGSMGALHAAAAADVFLE
DELRADPAYHSYYYSNGNINPRLPPPLLSKEDWRSAQRLRPGLGGIGDGRKPRGGGGGRG
GGGAGGMGPGDGLIGMPGLEIGRQNSFSGIFQDDSYQHDTDRQGANCNGTDLLSSSKVQY
GLHRETGAISGLHSDSKAPCLPENQNESSHSYASIIGSSLSRSASPDPELVRRVPSPCLP
PIGVKLGATDKKNNVGSSSFNCSSPNIIESDDLVSALSGMNLSSSRAMNGNTMDQSKLHQ
DVDDVRKFLFDQYMDQTNGNQRHSYMKRSEQGHVKVPQEYSGASMNPSVMRSQINAGGFT
SFNNSSVGSGFASPRLGSRSPGGSLSSRQNLTGASNLPNYVGIGSPTAASAHQMPVDPLY
VQFLRAAEIAALAANCEDPLMDRANLGGSYMDLFGPQKAYLSPLLQSQKQYSYYGNLGVG
LGYAGNSLTSPILPSSPGGPGSPLRHGDRSMRYPSGMRNFGGSFGSWNSDLGGKMEANLV
PSLLEEFKSNKSKSYELSEIAGHVVEFSADQYGSRFIQQKLETASTEEKDMVFAEIMPQA
LTLMTDVFGNYVVQKFFEHGSSAQIKELADQLIGRVLALSLQMYGCRVIQKAIEVVDLDQ
QTKMVAELDGQVMRCVRDQNGNHVIQKCIECIPQHAIQFIVSTFYGQVVMLSTHPYGCRV
IQRVLEHCDDPKTQQIMMDEILQSVCLLATDQYGNYVVQHVLEHGKPHERSAIIEKLIGQ
IVQMSQQKFASNVIEKCLAFGNPVERQVLIGEMLGSSNESEHLEVMMKDQFANYVVQKVL
ETCDDQQREMILTRIKAHLNTLKKYTYGKHIVARVEKLVAAGEKRLGLQPSTAA*

>11682.m00107|LOC_Os05g01910.1|genepair245-2
MATRGEAAAAAAADRDMELFRSGSAPPTVEGAMASAAAAAGDVFLDDELRADPVYQSYYY
SNAHLNPRLPPPLLSKEDWRSAQHRLRSSALGGIGDGRRQPAAAAQGDGLVGLPGIDLDR
QGSFSSIFQFHLIQSKESSYQLDMGKQGADRNISDFLDSSRPQYALHRETSRAMGGLQSD
SNIQSLAEVQNNDSSAHTYASLLGSSLSRSASPDPELVRRVPSPCLPPIGVKVSADDKKN
NGGSSSFRRSSSAIGESDNLIAALSGMNLSSSGAASGQQTVTQSELYQDVDNVRKFLFDR
QGDQSNGNQQHSYMKHPEQGHFKAPDGYSANSPNSSMIRNQINAASFTSFDNLSAGSGFA
SPRIGSRSPGGTLSSRQNLVGGSNFLNYNGIGSPNAATSLQTAIDPSYIQYLQAAEIAAQ
LAASCDDPLMASGHLGSSYMDLLGPQKAYASPLLQSQKNCGYYGNLGFGLGYSGSPLMSP
VLPSSPAAPGSPLRHGERSMRMQSGIRNFGGSFGSWNPDLGGKMNINMMPSLLEEFKSNK
SKSYELSEIAGHVVEFSADQYGSRFIQQKLETASTEEKDMVFSEIMPQALTLMTDVFGNY
VVQKFFEHGSPTQIKELADQLIGRVLALSLQMYGCRVIQKAIEVVGLDQQTKMVAELDGH
VMRCVRDQNGNHVIQKCIECIPQHAIQFIVSTFYGQVVMLSTHPYGCRVIQRVLEHCDDP
TTQQIMMDEILQSVCLLAQDQYGNYVVQHVLEHGKPHERSAIIEKLIGQIVQMSQQKFAS
NVIEKCLAFGNPVERQILISEMLGSTNESEHLEVMMKDQFANYVVQKVLETCDDQQREAI
LTRIKAHLNTLKKYTYGKHIVARVEKLVAAGEKRLGLQPACTSAA*
>11667.m07455|LOC_Os01g74170.1|genepair246-1
MEGGGGGGGGAIELRRRMAAQCLAFERQIADGRERTKAAASAFSAALLSARSLSNHTISQ
REKSNQLKDQLRKLEADFAQALSSHGRSETLQTSSAVQGSNKTKYDLTGQSITNAIATND
QLSCLVTDKRASRDEYANVISSQLEAIEALEAKTDAAGKKNLDEAFMWYKKFLGFQVVGG
EGVKFVFSKIDIQNPDNEYSFCIKLNKDRYNLLQCTPFLKDSEELVKDLNCSNDLFKFVR
IMRERFQAAAINGFLPASSLCPDMSSSITDSSPPALSIDTGRESTTTTSQSHSRSRAKNQ
DNPTKRGARPSNLLSSTRRSPRVADQMLDALPTHDMTGSGRSWGSSDLMPLCGLLWGLK*
>11682.m00117|LOC_Os05g02000.1|genepair246-2
MEAGGTAAGTRGGGGGAELCRQMAAQLAAYQRGMAVERERQASTSANYRAALLSSRSIAR
QTISRQEELSGLNLQLRNLEDGLAEALSVKAGKESKYQLTKETISSTAAINEKLEGMVTD
QRNKRDHHAAVISNHLEAVEALEAKFIEDETRMKKIEEAVIWYSKFLGFQVVGGEGVKFI
FNKIDLQSPDKEYSVTLKLAKDRYNLLQCDPSIKDSEELMKDLNLTNDLFKFVRIVRERF
QAEAATVNGGLLMSSVVCPDASSIPVSPPMLMPLDSRTENVLDKSLSQSKNKGRNLPSKR
GAAALSAASPGSAVSIVRRSPHFVGIR*
>11667.m07458|LOC_Os01g74200.1|genepair247-1
MGNCCGTKISSDAISRNASGSRRRRSGSRRKGTGLSSRVSSSVAAVTPRSEGEILRCANV
RSFAFNELKTATRNFRPDSVLGEGGFGSVFKGWVDENTFLPSRPGTGMVIAVKKLNQDGF
QGHREWLAEVNYLGQLSHPNLVKLVGYCLQDEQRLLVYEFMPRGSLENHLFRRGSHFQPL
SWNLRMKVALGAAKGLAFLHSDKAKVIYRDFKTSNVLLDSNYNAKLSDFGLAKDGPTGDK
SHVSTRVMGTYGYAAPEYLATGTLLEAKIVLSKTHFGFRCSYGSLVGDSLMYADAVGHLS
AKSDVYSFGVVMVEMLSGRRALDKNRPAGEHNLVEWARPYLSSRRRIFRILDARLAGQYS
LAGAHKAAALALQCLSADAKNRPTMHQVVAALEQLQETTTTSHHHRSPQSRMLLGGRGFN
ASGRSSAGAGARPRRLSASPLPA*
>11682.m00120|LOC_Os05g02020.1|genepair247-2
MGNCWGAKISSESPCRSASSPSGGTSKYASNSSVSAASVPPTPRSEDEILEAANVKAFAF
NELRTATRNFRPDSVLGEGGFGSVFKGWIDEKTLAPTKPGTGMVIAVKKLNQEGHQGHRE
WLAEVNYLGQLSHPYLVRLVGYCVEDEQRLLVYEFMPRGSLENHLFRRSTHFQPLSWNLR
MKIALGAAKGLAFLHSDKVKVIYRDFKTSNVLLDANYDAKLSDFGLAKDGPTGDKSHVST
RVMGTYGYAAPEYLATGHLTTKSDVYSFGVVLLEMLSGRRALDKNRPTGEHNLVEWARPY
LMSKRRIFRILDARLGGQYSLAKAQKAATLALQCISVEAKNRPNMEQVVAVLEQLQDSKE
TGANPQLQKKSSSKNAGSNGSKPSSKGKPANARLV*
>11667.m07467|LOC_Os01g74290.1|genepair248-1
MAHSGFSGSLTSPRFDLAVDMGHPFLNRTVDGFLKIGAGTSQSTKLRVQYYCGCICGHGV
WNRKNPWSQGLVCTIDLDVNLHFLGMCVYAIEQKNAMVGGAVTGALVSAASNSHRQNVVK
NAITGGAIATAAEFLNYLT*
>11682.m00124|LOC_Os05g02060.1|genepair248-2
MPRGGFSGSISSPRIDVAIDMGNPFLNRTVDGFLKIGAVGACKVAAEDTFDCLHRGDVSK
HKLEHMLKKMCKEGAYWGTVAGVYVGMEYGVERIRGRHDWKNAMIGGALSGALISAASNN
HKDKIIKDAITGGAVATAVEFINYLT*
>11667.m07470|LOC_Os01g74320.1|genepair249-1
MGHGLSCSRDTDEYDLFRAAQLGDIHALSALLAADPALARRATVYDRFTALHIAAANGRL
QVLSMLLDRDGDVDVLSRKKQTPLMVAAMRGNTECVVRLLRGGANVLTFDSPRARTCLHH
AAYYGHAECLQAILGAAAQAQGPVAASWGFARFVNVRDERGATPLHLAARHARASCVRLL
LDKGAIVSAPTAVYGFPGSTALHLAARAGSMECIRELLAWGADRLQRDSAGRIAYAVAMR
RGHRACAALLNPAAAEPIVWPSPLKFIGELEADAKALLEAALMEANREREKRILHGSDIN
IKGGDEEEESEDEEEACNICFEQACSMEVKECGHQMCAACTLAICCHSKPNPKTLLLHPP
ACPFCRTTISRLVVATTNSNKTNSRRRSRSRSSSFKGGLSSAMGSFSRIGRGSGRLVVDG
SSVGELADKPDHDFSSVAAAAAICDT*
>11682.m00132|LOC_Os05g02130.1|genepair249-2
MGHGVSCARTGDEHDFFRAAQLGDLDALAALLAADPSLARRATLYDRLSVLHIAAANGRI
EVLSMFLDRGAPPDAVNRHKQTPLMLAAMHGKIDCVLKLLQADANILMFDSVHARTCLHH
AAYYGHVDCLQAILAAAQTTPVADSWGFARFVNVRDDHGATPLHLAARQGRPGCVQVLLE
NGAIVSALTGSYGFPGSTSLHLAARSGNLDCIRKLLAWGADRLQRDSAGRIPYSVALKRN
HGACAALLNPTSAEPMVWPSPLKFISELEPEAKALLEAALMEANREREKKILNGTKYSLP
SPSPGDDSADDDACSEVSDTELCCICFDQACTIEVQDCGHQMCAPCTLALCCHNKPNPTT
LTPPSPACPFCRGSISRLVVAQTRSACDPDKPSSLQLTRKRSRRSHNLSEGSSSFKGLPS
AMGSFSKLGRGSSRMADSDSSNLDKPEHDL*
>11667.m07473|LOC_Os01g74350.1|genepair250-1

MAPPLQVATTASSSTREGKAPALNERILSSMSKRSVAAHPWHDLEIGPEAPTIFNCVIEI
PRGSKVKYELDKKTGLVKVDRVLYSSVVYPHNYGFIPRTLCDDSDPLDVLVIMQEPVIPG
CFLRAKAIGVMPMIDQGEADDKIIAVCADDPEYKHYNDIKDLPPHRLAEIRRFFEDYKKN
ENKEVAVNDFMPATSAYETIRHSMDLYATYILEGLRR*
>11682.m00153|LOC_Os05g02310.1|genepair250-2
MAPAVEAVEKKTGSAPVKAPALNERILSSMSRRSIAAHPWHDLEIGPGAPTIFNCVIEIP
RGSKVKYELDKKTGLIVVDRVLYSSVVYPHNYGFIPRTLCEDSDPLDVLVIMQEPVIPGC
FLRAKAIGLMPMIDQGEADDKIIAVCADDPEYKHYNDIKELPPHRLAEIRRFFEDYKKNE
NKEVAVNDFLPASAAYEAIKHSMDLYATYIVEGLRR*
>11667.m07479|LOC_Os01g74410.1|genepair251-1
MRKGPWTEQEDVQLVWFVRLLGERRWDFLAKVSGLQRSGKSCRLRWVNYLHPGLKRGRMS
PEEERMVVQLHAKLGNRWSRIAKSIPGRTDNEIKNYWRTHLRKLKLKQQKQQQSDDHHND
NDDDDDRNSSSSSSSSNSNSNLQQQPQPEDESSASGSLQAQHHEDQHQLFLHPLWNDDII
VDVDCWSSSTNVVAPPPMPASPLWDIDDAFFCSDYSLPLWG*
>11682.m00164|LOC_Os05g02420.1|genepair251-2
MAEAEAEEEMMMRMMCGSGGNEMMKTKMKKKNREGEEEEEVSGGGRMRKGPWTEQEDVQL
VWFVRLFGERRWDFLAKVSGLKRTGKSCRLRWVNYLHPGLKRGRITADEERLILHLHSQW
GSRWSRIARSLPGRTDNEIKNFWRTHMRKIAHHAKKKTNSPSPAPTTSSGSLSSSLTTAT
TTMATAAALQESSSCGGDDEAVDQLVAAATTPASQLLTMDYTMDQLWNDIAAAEADTSCY
DAAAMASPPSPVWEFCTDYSLWRIDDEEYYKKMLDASQ*
>11667.m07487|LOC_Os01g74480.1|genepair252-1
MVQRTSSDSAASAEAMVMDLSPKRPAKSYGGEGGSYFDWSPSELPMLRAASIGAAKLSLA
AGGLALPFYSDSAKVAYVLQGKGTCAVLLPETPSEKILPIKEGDALALPFGVVTWWHNLH
AATTELVVLFLGDTSKGHTAGRFTNMQLTGSTGIFTGFSTEFVARAWDLPQDAAASLVST
QPGAGIVKLKDGFRMPEGCDKDREGMVLNCLEAPLDVDIKNGGRVVVLNTQNLPLVKEVG
LGADLVRIDGHSMCSPGFSCDSAYQVTYIVRGSGRVQVVGIDGTRVLETRAEGGCLFIVP
RFFVVSKIADDTGMEWFSIITTPNPIFSHLAGRTSVWKAISPAVLQASFNTTPEMENLFR
SKRLDSEIFFAPNSNSI*
>11682.m00174|LOC_Os05g02520.1|genepair252-2
MASVDLTPRQARKAYGGDGGTYYEWSPADLPMLELANIGGAKLSLNAGGLALPSFSDSGK
VAYVLQGKGTCGIVLPEASKEKVIAVKEGDSLALPFGVVTWWHNLPESPIELVILFLGDT
SKAHKAGQFTNMQLTGATGIFTGFSTEFVGRAWDLAESDAVKLVSSQPASGIVKIKSGQK
LPEPSAADREGMALNCLEAPLDVDIKNGGRVVVLNTANLPMVKEVGLGADLVRIDGHSMC
SPGFSCDSAYQVTYFIRGSGRVQVVGADGKRVLDTHVEGGNLFIVPRFCVVSKIADASGL
QWFSIITTPNPIFSHLAGKTSVWKAISPEVLEASFNATPEMEKLFRSKRIDSEIFFAPN*
>11667.m07490|LOC_Os01g74510.1|genepair253-1
MLQRAASNAYSWWWASHIRTKQSKWLDSHLQDMEHRVKCMLLLLGEEADSFSKRAEMYYK
RRPEVITQVEEVYRAYRGLADRYDIISGELHKANHTIATAFPDQVQYAMLEEEDDNIPKA
FTPVDPRKIHKSTVDGLMKKKKGGEQPAGSMNKNTTSAPIDKDNAREEISRLQKEILVMQ
TEKEFIKSSYESGIAKYWDLEKQINDMQEQVCHFQDKFDESAVIEDDEARALMTATALKS
CEDTIVKLQEQRKTSASQAMGESERVKVLREKLKAVMEGHGKSLPDSPDPCDKNVRKNHG
FEMEEVQHIKLGEFETQTVLEKIKEHFERDGSISVAEITEHIDELVNKVVDLELMVSSQS
SQIDRLCRENSELESCLQSLEEENVSDPDKVNEKLKKLEEELVRVQALESCFHKDESTIR
SNFSEAISRLSGISEMLQSSEHGGVGGTLAVADGKEEEEDNDAGGIDDVAEPQVQTEAAS
DDVDPAGKSTADVDPAGKSTATQEEAQAVDVGQEKAGGCSRERGSLVRLRHISSDDLGGC
DDEAPAAVDDPDGMRKQKKGQEGEGVEEEKKVILVAEYRALLEENKDAKRRLAEVEKTNQ
ECMHEIRSLRELLSSGSSEAGAAAAGGGGGGGDSSGGRRGHRRTPSYSLGHHRKQSLSSI
SRMIRMGSTIHEGDESEKVKAEELRLPAVATSSSPLENKLRKDIDTLLEENLEFWMKFSS
SLQRVQEFQRKHDELMQQLQPAATDGNSDTKQKQKQEQQLRALKTELQVWSEQNAMLRGE
LQCRFAALCDVQEEITAALEQGGGGGEFTSYQAAKFQGEVLNMQQENNRVSDELQAGQDH
VKGLQAQIEKKLQHGGVTLPDADGPAAGAGAGATTPPPLPLTRVASKSKVPLQSFLFPAK
AKKPSLLARVTPVLQKQQPDLRFLAKLQPR*
>11682.m00202|LOC_Os05g02790.1|genepair253-2
MEDRVKFILFLLGEEADSFAKRAEMYYKRRPEVISSVEEAYRAYRALAERYDHISGELHK
ANHTIATAFPDQVQYSMLEEDDDNLPKAFTAVDPRKIHKSTVEGLMKKKKGEKSGLKDGG
KNSGDKINKENAQEEISRLQKEILVLQTQKEFLKSSYESGMAKYWDLEKQINDMQEEVCY
FQEEFNESAVLEDNEARALMTATALKSCQETIIKLQEQQKLSFSEAMVESERVRSSRDKL
KNIMKVHGKSLPDLGKFFEKTDSVKFANENVTNDGSNTVDVMYSINQEKIELQATVDKIK
EYFQKDSEVSVVEMADKIDALVNKVVDLELMVSSQTAQLNRLCLENTELEKSLHELEEEK
PALNSGPGESYSKFKQAEEDLIRVQNLVSSFHAEGTIVHSNFTETITRFRDVSDMLLSPL
LEHHQDGSAPMPSDETTPSIDMETSSEHDKTNSENEVDELPEHSKELEPADLCDDNHSSS
GYPETKAENCYRGDGSEDLWYSALEDKSSFAAASVNEEESGNADNDSSRDHNNRGEDHAP
EIASDDGSSKQQYTVQSHEKPILERLHHISSNDPGDHNAKKEENEQDLSISDESISEGNS
EQKINKAGNSCITADTAPISRKVDEVGDQEENMIKLQQLLMNGLQDKEKVLLTEYTSILR
NYKNEKRKLTEVETKNQERLNEMSAMISELRSANSMKDEKIRSLLELLNAVLDKDVSGNG
HQMNPTTSFSSISRTFRGHRRTPSFSPGHQRKQSVSSISRIILESPKEDDALYDTVTDQE
SLILEDIKLIDVVKMENASPLEEKFRQDIDALLEENLEFWMKFSTSFQQIQGFQTKYEQL
QPEIGKLTNKDKLKTNNGRADDPSAKGDSNAIEKRLRELKIELQVWLEQNAMLKGELQYR
FASLCSIQEEIEATMEMGADPEEGAHFTSYQAAKFQGEVMNMKQENNKVADELQSGLDHI
KGLQAEIEKVIEKIVERTSLSEAKGSSTWKNAPSRTRVPLRLFLFPAKKKKPSLLACVNP
ALQKQHSDMVFFTK*
>11667.m00557|LOC_Os01g06270.1|genepair254-1
MSSWLRSAVSKAVEAGGRSGVARAVLGYADAVAHHAGQAVAEGAKILNDRMSTQNYKSVK
KMVKRLEEAAVSSRGEDRLQVLRHWLRALQEVEAQLGGLDGAAEQNAHSSEPNTSKPPFA

RVLFYDADIGGAPMNFRDVFLYSQALEGITLSMAMLFFAKALIEVKHSNPYHSQILQAPN
EEEVPLLLEIFGLCLTGGKEINNAIMSSIQDLAKSFSNYHDEVLVKREELLQFTQSAISG
LKRNSDIMRIDAEAVELWKKLNEKETSRVQQTQEDHAKTTERTSATTVEDTTLIPLALQV
DKLKVLATSLSNSSSKAEKRILDHRDLHLAGCRYSGIYQHLLVSELYSLLATTQKHIMLV
ASVVKLCGELVVTFQVVLCHTDNRRQKEEALNFRAKKENEVSAVEKNISACTCNTYIAYV
YIVKGMHMRELTAEISELEKQRDELEARLKKVNISLNAAIGRLKQTREERDQFDEANNQM
IFSLKAKDNELSKSITSCNVEAGVVKTWINFLEDTWQLQSSYNEQKEKRTNDELERCTDN
FLKLTKYHLSTFKEILSPSIERICTYVDNLAVLQSRDVSTEHDNEELSEKTSPQKSLEEE
YLETEKKGCAEHIVVHSRLRRLGRHGTRYQGLIPRYQLDTQGTKYQSIPRYQGYQIVIAF
SITDHMKKLFYSEQGVNSRRDDEDVRNLFSEIEKLRERFESVERPTLDIEVRRAKVPTKE
RAESSPSPVQVPSTPKAETVVSPKSPAKPDQPLDLDSELAKLELEFGQVNKYSPEEISGW
EFDELEEELRADISKSQ*
>11682.m00651|LOC_Os05g06980.1|genepair254-2
MSSWLRSAVSRAGRSGVARAVRGYADAVAHHAGQAVADILQDRTDYKSFKKTVARLEEAA
VSCRGGERVELLRRWLGALQDIEAELSGSDLKDPEDRDPSSETDISKAPLALFYDADIEG
GPMNFRDVFLYSQALEGITLSMVLEAPSEEEVSLLLEIFGLCLTGGKEVNKKIMDTVQDL
AKALSNYKDEVLVKREELLEYTQSVISGLKRNADIMRIDAETLELWKKLDEKEKSRAQIT
EDQDKSSGNISVENIEGLKEALIEVRLCSRVEELVLKKKSISPGDSLEIHSQKIDKLKIL
ADSLANSSSKAEQRILENRRQKEDALNFRVKKENEVSTVEKEVLDEIAELEKQRDELEAQ
LKKVNISLNAAAGRLKKTREERDQFDEANNQIIFKLKTKEDDLSKSIASCNVEANVVKTW
INFLEDTWQLQSTYNEQKEKKTCDELERCVSSFLKLTKHHLSVFKEVLSPSIESIRTYVD
NLVVLNSREETKQDEDDEASEKTNPRISLEEEYLETEKKIIIALSIADHIKKLFYSEQWA
NSRRDDPEVKNLIAEIEKLRGEFESIERPMLSIEANKSKPLPEERSELSPSPIQAPATPK
AAHVDSPKSPMKPEQHLNPDNELANLGAELGSEDRDFSGEEINGWEFDELEEDLKN*
>11667.m00558|LOC_Os01g06280.1|genepair255-1
MIKLRSALGVLEILSVLCISLVAAYTPVDNYLISCGSSVDTPVGQRLFVADDSGTVVLTS
PASDAVKASPSAVSGLRDDAAMYQSARVFKAPSSYSFRIRDPGRHFVRLHFFPFVYLGYD
LATASFKVSTQDAVLLDGFAPAAAARGNASTTTTTATAAAVCEEFLLDVARDTLVVTFVP
LAGRLAFVNAIEVVSVPDDLIGAADSSLSTSDSTGQQLNPAVMPLQTVYRVNVGGQAVAP
DSDTLWREWTSDQQLLVGPAMTKGVSYNRTPNYLPGQATANDAPAIVYATGRELIIMTNS
TDDGMKQMAWQFDVGRSASYLIRFHFCDIVSSVPGRLHMNAYVDSSNAIQDLDLSAIGNG
TLAFPYYRDFVLAASTPSGKLAVYVGSTSQKITTPAAILNGLEIMRILTTAGNVAVVEPT
MPPGTKKKNNLAVVLGSVCGAFGFVSVAAALVIVLRRKEEKEELRTPTTSQPSTAWMPLL
GRISFRSAPPSAVGSRSPSFTIDTNANTPGGGATPGMAAAASSSPSYRFPFAALQDATGN
FDEGLVIGEGGFGKVYAAVLQDGTKVAVKRANPESRQGAREFRTEIEMLSGLRHRHLVSL
IGYCDEQDEMILLYEYMEHGSLRSRLYGGGAATATATALSWAQRLEACAGAARGLLYLHT
ATAKPVIHRDVKSSNILLDDGLTAKVADFGLSKAGPDMDETHVSTAVKGSFGYVDPEYVR
TRKLTAKSDVYSFGVVLLEALCARPVVDPRLPKPMVNLVEWGLHWQRRDELEKIVDRRIA
GTVRPAALRKYGETVARCLADRGADRPAMEDVVWSLQFVARLQEVDGLDASDVSSLNMVH
QLMPPTSLHARQRSAGESETGRTDADEDSSVVDDDYTDASMRGIFWQMVNVRGR*
>11682.m00652|LOC_Os05g06990.1|genepair255-2
MDSTFRKLKLVLALVGIITWIIGTCNAKFTPADNYLVNCGSTVDATVGQRVFVADNSQSI
VLTTPQSQSIAARTTLNSVSGFDNAELFQTARIFTAPSSYSFKMRSSGRHFVRLYFFPFL
YQSYDLASSKFKVSTEDVVLIDNFPQPSNSISVVMEYSLNITRDRLILTFVPEGNSTSFV
NAIEVVSVPDDLITDSAQLLGVGQYLGLAAQPLQTFHRINVGGPKVTAENDTLARTWFAD
QSFFRNPTVAQAVTYQERLNYKDGSATQDDAPDSVYNTARRLVGQRNASSTPNMTWEFNV
DGRSSYLIRFHFCDIVSKAAFQLYFDVYVYNFSAAKDLDLSAREFGTLAAPFYMDIVLPS
SDPSGNLTVSIGPSSLPNATPDGILNGLEIMKMNFSSGSVYVVKPPSAAKQQLPIILGSV
LGGIGAAIIVVVLCVVFRRKKKMKKPQTPLTSRPSSSWTPLSLNALSFLSTGTRTTSRTT
YTSGTNSDTSYRIPFVVLQEATNHFDEQMVIGVGGFGKVYKAVLQDSTKVAVKRGNQKSH
QGIREFRTEIELLSGLRHRHLVSLIGYCDERNEMILVYEYMEKGTLKGHLYGGDQPPLSW
KKRLEICIGAARGLHYLHTGFAKSIIHRDVKSANILLDENLMAKVSDFGLSKTGPEFDQT
HVSTAVKGSFGYLDPEYYRRQKLTDKSDVYSFGVVLLEVICARPVIDPTLPRDMINLAEW
AIKWQKRGELDQIIDKRIAGTIRPESLRKYGETVEKCLAEYGVERPTMGDVLWNLEFVLQ
LQEAGPDMSNIDSMNQISELPSNAQRISSLEISTADESRTAMDYSQMSTSNAFSQLINTE
GR*
>11667.m00564|LOC_Os01g06320.1|genepair256-1
MDRNTNNNSNSSSSSEMPGKKARKPYTITKPRERWSEEEHERFLDALIMYGRDWKKIEEH
VGTKTTIQIRSHAQKYFLKVQKMGLAAGLPPQYPRRRLVMQQQQQQSSPAVSSSVAATAI
LHGQPQCLPPHHNVAVQSSIGWECPGVLPPATNDMQNLEWASTSGTAAWGNHHGLIEPPA
AFVSFPGESSFMGAASFSNTSMDWTGTTSEMATASIVQDETIELPLSPDDLQFAQVYRFI
GDIFDPDSPCPVETHLQKLKSMDDIIVKTILLVLRNLEDNLLSPQFEPIRRLLSTYDPNR
GLSGHL*
>11682.m00654|LOC_Os05g07010.1|genepair256-2
MAAMAAAAAGTKKKARKPYTITRPRERWSAEEHERFLDALILFGRDWKRIEAFVATKTAI
QIRSHAQKHFLKARKFGLAGGLPPPLHPRRATLLRANAAAADMMPPPWLPSAGGGSIGCS
APPSGVQQSMAGRSPACYSTDEASFRPLIHSNDNDCSFIETPSCIGSGGESWIGDDAFFM
QDETIRLPISPDDLGFAQVYKFVGDMFGSGERRPVEAHLRRLQGMDPAISETILLVLKNL
EANLSA*
>11667.m00583|LOC_Os01g06510.1|genepair257-1
MPCRAAARRAAVSALRLVPDLDVEPMLEVSKPGFGDYQCNNAMSVFSRIRGSATNFRNPM
AVGQAIANNLPQSNIIESISVAGPGYINITLSSNWIAQRIQDMLVCGIKTWAPILPVKRA
VLDFSSPNIAKEMHVGHIRSTIIGDTLAHMFEFTNVEVLRRNHVGDWGTQFGMLIEFLFE
QFPDWEDVGNQAVGDLQSFYKASKKRFDDDPDFKERARQAGESFYNPYIPPVLEELTNKG

LIVESKGARVIFVEDHPLIVIKQDGGFNYASTDLAALWYRLNVEKAEWIIYVTDVGQQRH
FHMLFTAAKMAGWLPEQNGKKYPKASHVGFGLVLGSDGKRFRTRCSEVVRLVDLLDEAKA
RSKAQLIKRFTGNGQIADWTDDELDRTSEAIGYGAVKYSDLKNNRLTDYTFSFDQMLSDK
GNTAVYLQYAHARICSIIRKASKDVEKLKMTGAITLGHPYERFLGLHLIQFTEVVEQACA
DLQPHRLCDYLYSLSLTFSKFYTNCQKYYTGENIYRNRPCLPRAPSLRAAGGREGQPR*
>11682.m00656|LOC_Os05g07030.1|genepair257-2
MRALAAAAATATATAAAAAAPSPARFPLRLVVTPRASLGHCRASSSARSPRRACYATTMG
DETSTSVATQSQEPAAVGAGSVKQQLSKLVIASLRTTVPEVEVDPMVEVCTAKFGDYQCN
NAMGLWSRIKGSGTSFKNPNAIGQAIAKNLPPSDIIESTSVAGPGFVNIVLSNSWVAKRI
QDMLVNGIKTWAPILPVKRAVLDFSSPNIAKEMHVGHLRSTIIGDTLARMFEFSNVEVLR
RNHVGDWGTQFGMLIQYLFEKFPNWEEIGSQAIGDLQTFYKASKNRFDGDAEFKDRAQQA
VVRLQGGEERYRAAWNKICEISRNEFDMVYKLLNVKLEEKGESFYNPFIPQVLEELNNKG
LIKESEGAKVIFIEGHQIPLIVVKRDGGFNYASTDLAALWYRLNVEKAEWIIYVTDVGQQ
QHFDMFFNAARMAGWLPDPKEKKYPKTNHVGFGLVLGSDGKRFRTRSTEVVRLIELLDEA
KSRSKSELLQRLTENGKIVDWTEEELEKTSEAVGYGAVKYADLKNNRLTNYTFSFEQMLS
DKGNTAVYLQYAHARICSIIRKSNKDVEELKMSGAISLDHPDERVLGLYLIRFAEVVEEA
CTNLLPNVLCEYLYNLSEMFTRFYTNCQVVGSPEETSRLLLCQATAVVMRQCFELLGITP
VYKL*
>11667.m00586|LOC_Os01g06540.1|genepair258-1
MAGEEFGLAAAAAAAASATVDAASGRSSRVTALRSVEDIFSDFRARRSAIVRALTEDLEK
FAALCNPDLDCLCLYGNSDGTWEVAPPPEMVPPELPEPALGINFSRDTMYRSDWVALLSV
FSDSWLLAVAFFHGARLDRDDRQRRILNLY*
>11682.m00658|LOC_Os05g07040.1|genepair258-2
MDASYRRDGRGGGGGGGGGGSAPRSVEDIFKDFRARRTAILRALTHDVEDFYAQCDPEKE
NLCLYGYANEAWQVALPAEEVPTELPEPALGINFARDGMNRRDWLALVAVHSDSWLVSVA
FYYAARLNRNDRKRLFGMMNDLPTVYEVVSGSRQSKERDRSGMDNSSRNKISSKHTSDVA
RVENNIKEEDEGYDEDDGDHSETLCGTCGGIYSADEFWIGCDVCERWYHGKCVKITPAKA
ESIKQYKCPSCSSKRPRQ*
>11667.m00590|LOC_Os01g06580.1|genepair259-1
MQAMQRLVLCLVVSMAIAASAQGPTAAPAAPAPATPAAPATPAAPAPASTKTTNITGVLA
KAGQFNTLIRLMRSTGAAEQIDNQLNSSRNGLTVFAPTDNAFTSLPSGTLNSLSDQQKNS
LVQYHVLSTLIPMSQFDTVSNPLRTQAGSNSPGQYPLNVTAEGQQVNISTGVVNATVGNA
LYTGDNLVVYQVDKVLLPMAIYSTPAPAPAPLSPATKKKGKTPATSVADAPEAADATPDA
TTPSLAAARVTAGAGVGVVLALASVWLGL*
>11682.m00660|LOC_Os05g07060.1|genepair259-2
MHVAKKSLASMAASATTTILVAAMLVVMAVESPVANGQAAAAAPAPAPAAPKTITAILTK
AGQFTKFLQLLQSTQAGEQINNQIKGKASSSGGLTVFAPPDNAFAALPTGTLNKLSDQQK
TSLVQFHVVSALLPMAQFDTVSNPLRTQAGETAAGKYPLNVTAEGSRVNISTGVVNATVD
NTLYSGDRLVVYQVDKVLLPWALYGPPVPAPAPSPADKAKKKAGPVGVADAPAADTAAGT
TTTTVATASEAAARGTVRRGLVGVAVAVAVAWCGM*
>11667.m00591|LOC_Os01g06590.1|genepair260-1
MVQPNNEQLNQPLPLGQKLFVHAGNDAALKIGPSYHGNVAIRSNDLPSSSRVAQYSGHRV
KNTGTLHNSYVHYPAGSSGGHVSYNPQTEPVITYPHRSEGEFARGSSQIDNRTAAVKRKN
PVIYPEYSINGDGYCAGSSSSTQFSNYPQPAPFSESLHRQMPPSVGPINWNDQSLHMYLN
DYVGASGMHSGEMPYTMGSSNSSVPVPTLQGSSSSAIFASGVFAPRHVHGDTVPSYIHLP
SVASSSSTAIPHEVIIPSYQPATSATTSTPMRASQPLPVRAVASSRHARNVLIGHANSGR
NRRARSSYYGIQPLMIDAQQLIMMQQFALRESREAQDPHRAMRLDIDNMSYEDLLALGES
IGNVCTGLVDEKISGCVREVIYCSSDEQQNDQDDGKCAICLEEYKDNSLLGILKCNHDFH
TDCVKKWLKEKNSCPICKSAAA*
>11682.m00662|LOC_Os05g07070.2|genepair260-2
MAGHQYNNSQMSRMDHMDRLNNEPPPFGQKLFMHPRSDPANGAGSSGYVGNTMRSNDLPS
SSYAGQAYGQQNRAPIHASYSGHAPAGSSSGSYAPYNTQHMPASSYPHGSEDNFIPSSHV
DGRRVALKRRNPIIHPTDGFGVGNYYAGSSSNTQFSRPMPPNPIPPPESCVRMPSHLGSN
HWNDHRYVNHEGSQRNVRGRHDHSTIHLEQSPAAACPSSSINVPPYHPNANGPFGSAPVQ
RDRAPLSVHPRILPPGPDGSSIAFRERPYYPAPQSTNISAPVPTLPISCDSAPFAHGGYA
PRSAHRNNLRTYPPPAFASSSNPGAVSHEPAIPSYPPAAPSYPPATSAASSSVQPFHAEA
AAHLRHPRHVSVGGSGSARSRRMRDSYHGFHHLMIEDNNLGRSAAERFMMLDQLVIHESR
EAFDPHWDMRLDIDDMSYEVERINFLFNTTAALECYLNKCNFFYTQELLALEERIGHVNT
GLADEKISGCVMEVACCSSSHLQDDQDNERCVICLEEYKHEDTLGRLKCGHGFHCNCIKK
WLQVKNTCPVCKAAAADEGS*
>11667.m00592|LOC_Os01g06600.1|genepair261-1
MAGKQGDGREEDGAKVGLPALDISLAFPQATPASIFPPSASDYYQIDDLLTTEEQSIRKN
VRAIMEKEIAPIMATYWEKAEFPFHAIPKLSSLGVAGGTIKGYGCPGLSITASAITMAEI
ARVDASCSTFILVHSSLAMVTIALCGSEVQKQKYLPSLAQLTAVGCWALTEPNHGSDASS
LITTATKVPGGWHIDGQKRWIGNSTFADVLVVLARNANTKQLNGFIVRKGAPGLKATKIE
NKIGLRMVQNGDIVFNKVFVPEEDRLPGVNSFQDISKVLAISRVMVAWQPIGISMGVFDV
CHRYLKERKQFGVPLVAFQLNQEKLVRMLGNIQAMLLVGWRLCKLYESGKMTPGHASLGK
GWTSRMAREVVSLGRELLGGNGILADFLVAKAFCDLEPIYSYEGTYDINSLVTGREITGI
ASFKPAALAKARL*
>11682.m00668|LOC_Os05g07090.3|genepair261-2
MGSLAGGGGGGKVGLPALDVALAFPQATTASQFPPAVSDYYQFDDLLTDEEKTLRKKVRG
IMEREIAPIMTEYWEKAEFPFHAIPKLATLGLAGGTTKGYGCPGLSLTASAISVAEVARV
DASCSTFILVHSSLAMSTIALCGSEAQKQKYLPSLTQFRTIGCWALTEPDYGSDASSLRT
AATKVPGGWHLDGQKRWIGNGTFADVLIILARNSDTNQLNGFIVKKGAPGLKCTKIENKI

GLRMVQNADIVLNKVFVPDEDRLTGINSFQDINKVLAMSRIMVAWQPIGISMGVFDMCHR
YLKERKQFGVPLAAFQLNQEKLVRMLGNIQAMLLVGWRLCKLYESGKMTPGHASLGKAWT
SKKAREVVSLGRELLGGNGILADFLVAKAFCDLEPIFSYEGTYDINSLVTGREITGIASF
KPAALTKSRL*
>11667.m00596|LOC_Os01g06640.1|genepair262-1
MSSSSGSGRSATEARALKIHSEAERRRRERINAHLTTLRRMIPDTKQMDKATLLARVVDQ
VKDLKRKASEITQRTPLPPETNEVSIECFTGDAATAATTVAGNHKTLYIKASISCDDRPD
LIAGITHAFHGLRLRTVRAEMTSLGGRVQHVFILCREEGIAGGVSLKSLKEAVRQALAKV
ASPELVYGSSHFQSKRQRILESHCSIMSI*
>11682.m00671|LOC_Os05g07120.1|genepair262-2
MAACQQQIWQEGKQQQHLHHGGYDDLSSVYRGTVVLPRRQGGLAPEPPPPRPSSSSGRSA
AAQATAMTIHSEAERRRRERINAHLATLRRILPDAKQDSDRNTIVKQRITKHRNCRLIGP
QEKRRNQMREIDSEEMFQEMDKATLLASVVNQVKHLKTRATEATTPSTAATIPPEANEVT
VQCYAGGEHTAAARTYVRATVSCDDRPGLLADIAATFRRLRLRPLSADMSCLGGRTRHAF
VLCREEEEEEDAAAEARPLKEAVRQALAKVALPETVYGGGGRSKRQRLMMESRYSTAVVH
THVDPQYCWYNSR*
>11667.m01949|LOC_Os01g19760.1|genepair263-1
MAATVEEQMVVKAIREECPWESLPKRLQSTLHTKEEWHRRIVDYCIRKRLQWNTCFARRV
CREGEYYEEMMKYLRRNLALYPYHLADYICRVSRISPFRYYCDILFEAMKNEQPYDSIPN
FTAADALRLTGVGRNEFIDIMNKCRSKKLMWKLNKSIAKELLPTQPVDFPIDPWWGVCLV
NFTIEEFKKLSEEETATIDKICKEEANSYVLFDPKVIDGLYKRGLVYFDVPVYPDDRFKV
SRLEGFVSNKDQSYEDPIEELLYAVFVVSSANATVAELAATLLADLYQLQAAASFACRLG
WAVKLMDADSVLEDAPTFPSNILSDDEEGSNASINSEKSGQQLLSVDAGPRKISGTAHVA
LVVDANVTSYLMMGSLSPGLKSHAVTLYEAGKLGDSCIAELCSDLASLEGKKFEGVLEEF
ANHAFSLRCFLECLQSGGVSTNVNIDKAGEAKLTTSSLQDNVTAHLTKINIEDTDEMPQQ
KHSGLNSSDGKMLTSSATLLESGEGMEGNDVEGSGTTELDGSTDINVVKTKRKYRVDILR
CESLASLPPATLERLFLRDYDIIVSMVPLPSSSVLPGSSGPIHFGPPSYSSMTPWMKLVL
YTAGDCGPLSAVFMKGQRIRLLPKPLAGCEKALIWSWDSSVVGGLGGKFEGNLVKGNLLL
HCLNSMLKQSAVMVQPLSVNDLNASGNLVTVDIPLPLKSDDQSIASVIAQTNLPQEQVLN
LASVLKDLSSKFELSTLGYLRLLRLHRLTESDESHLENGSYQWVPLSLEFGIPLFSPKLC
ERICERVVESCILQKEDLAEHYDVMQTVRRRLRELCSEYQATGPVAKLFNKRGSSKDLPR
VLINTISGRWNPVNDPSAPSEHERLKLAGRQRCQTEVVGFDGTFIRSYALPPEHDEAGTK
SLSEEQSSAHDGKPDTEDADSKDVLLPGVNLIFDGAELHPFDISACLQARQPLWLIAEAS
AASSTMI*
>11682.m02691|LOC_Os05g28710.1|genepair263-2
METYVLCAPLYICILLIILAEQPYDSIPNFSAADSLRITGVGRNEFIDIMNKCRSKKIMW
KLSKSIAKELLPALPADLAIEPWWGVRFVNFTLEEFKRLSEAEASAIDKISKDEDNSYVL
FDPKVINGLYKRGMVYFDVAVYPEDRFRVSRLEGFVSNKDQSYEDPIEELLYAVFVVSSE
NATVAELAKTLQADLYQLQAAASFACRLGWAVKLMDADSILRDSNGSTIPSNILSDDDEG
SLTSINSERSGHALLTSDSDGPRRISGASYVGFIVDANVTSYLMMGSLSPGLKSHAVTLY
EAGKLGDSCIADLCKDLASLEGKRFEGVLQEFANHAFSLRCFLECLLSGGTSPDEAIEPN
SQEFCLQENFSKTLSKESIDEGISNVVKSNGGSLETVDTADTDHHNELSQADHPMVDSDV
ADASTSSPSSIVSESKESTDKNDSDTSKTPLPDGSTDSSSLSKTKRSYRINILRCESLAS
LSATTLERLLVRDYDIMVSMIPLPYSSVLLPSTAGLVHFGPPSYSSMTPWMKLALYTSGS
CGPVSAVFMKGHRLRMLPEPLASCEKALIWSWDQSVVGGLGGKFEGNLVKGSLLLHYLNS
MTKHSAVIVQPLSLNDLDETGNLVTVDVPLPLKNADGSIASTVASMDLPEEKILNLSSLL
YNLSSRVELGTVGYLSLVRLHRISKSNEIFSKDENYEWIPLSLEFGIPLFNPKLCEKICE
RVIESHMLQKDDLTEHYEAMQNVRKRLRELCAEYQATGPTARLFNQRGSSKNSPRQLINI
VSGRWSPFHDTVAPTTGGSPRENDRLKVARRQKCFTEVLSFDGSILRSYALAPVYEAATR
SVTEDQPTTPVAKPEPDDADTKDVVLPGVNLIFDGAEIHPFDIGACLQARQPLSLIAEAS
AASLAMK*
>11667.m01953|LOC_Os01g19800.1|genepair264-1
MESPCGCSGSLKYAHRGCVQRWCDEKGSTLCEICLQNFEPGYTAPPKKAQPAHVAVTIRE
SLEVPRPSYEPEDTPLIGEQDYAECAGAAGRSATWCRSVAVTFTAVLLLRHLVAVVTVGA
AHQYAFSLLTIYLLRASGILLPFYVVMRLISTVQKGQRQYQLQLLQEQRRNAARMHRLHG
QEEQNQHAILVR*
>11682.m02693|LOC_Os05g28730.1|genepair264-2
MEKRGRGICHEEEDEWCAAIESPCGCSGSLKYAHRGCVQRWCDEKGSTLCEICLQNFESD
YTIPPKKVQVVETAVTVRAFFCRDEEMLPEELSQEDQEQYAGSEAQTGNGDCSSWCRSLT
ITFTIMLLVWHLIAVVTIEAADHCAFSLVTIFLLRAAGILLPFYAIMRMVRMIQQGQRQF
RLQLLQDQRRRNASNLHSMSGQEQHQQLVINVH*
>11667.m01955|LOC_Os01g19820.1|genepair265-1
MASSVPEDGASAAAAAATADPAAAAAEAPGRRIVVAVDESEESTHALTWCLANVVSSSGG
DTLVLLHARRPRPVYAAMDSSGYMMTSDVMASMDKYAAAVSAAAVGKAKHICAAFPHVTV
ETMVESGDPRDVICDATEKMAADLLVMGTHGYGLIQRYYCKLID*
>11682.m02694|LOC_Os05g28740.1|genepair265-2
MENVTGGGGGGSPRRVVVAVDESEESMHALSWCLSNVVSAAAKSPAAAPPPAVVLVHARP
ARPLYYPVIDGGGYVLTQEVMDSMDRYMATAADSVVAKARDICTAFPNVKVETRVEKGDP
RDVICGAVEKAGADMVVMGSHGYGFLQRTLLGSVSNHCVQHCKCPVVVVKRPGTNAKAS*
>11667.m01957|LOC_Os01g19840.1|genepair266-1
MAKSKNHTAHNQSYKAHKNGIKKPKRHRQTSTKGMDPKFLRNQRYSRKHNKKSGEAESEE
*
>11682.m02695|LOC_Os05g28750.1|genepair266-2
MAKSKNHTAHNQSYKAHKNGIKKPKRHRQTSTKGMDPKFLRNQRYSRKHNKKSGEAESEE

*
>11667.m01973|LOC_Os01g20000.1|genepair267-1
MAPKTTTDPSTSTPPPPGAFRRCSGRVRGAGISCAGCFGDAESKRRRRVAVYKAYAVEGK
VKASLRRGIRWFKRKCSAIFRA*
>11682.m02704|LOC_Os05g28840.1|genepair267-2
MADYHRPYRSDLDLRPPPPPPSSASFPHSNGYYLPSSSSSSPTANGYFSSKGTGGFAANG
DRRIEIYTTAPPPPLPPPPRLALPPPPGRRDGYLGGGGAGGGGGGGGSGGMWCFSDPEMK
RRRRVASYKAYSVEGKMKASLRRGLRWFKGKCSEIFHGW*
>11667.m01997|LOC_Os01g20730.1|genepair268-1
MDHRTYKGGVKAYWKHRGYYRLDAAAAQRRAPLPTAELGGGARRGGAPAQEPRRARRHRG
WRVRHGLGRRVLRALSPRRWLVRLRDAYVSAMLRLASSPAVGFGAGAPYCTAGQESFARP
RQLKEYDEKVLVEIYRSILARGGVPVAVPAGGPAATATAAATTIRLSTAA*
>11682.m02723|LOC_Os05g29000.1|genepair268-2
MAGGDGATYKGGIKAYWKRRGYGRLDPASSAHRRPRLPTAELGDGRGAAGGAGRWRRGWR
VRRRGLGRRILRALSPRRLLARLRDAYVRGMLRLASSAAVAGGGSALYGGPAGGADPFGR
PRPLREYDEKALVEIYRSILARGGGGGGGVVPVAGDAAAVVAVARLPTVAGA*
>11667.m02011|LOC_Os01g20860.1|genepair269-1
MPQEPEGEAAAAGAGSFLRLPESAGAFDELPRARIVGVSRPDAGDITPMLLSYTVEVQYK
QVKEWLQNLGIGEHIPVVHDDDEADDVHVPSQHDEHSVKNRNVPSSAVLPVIRPALGRQQ
SVSDRAKVAMQEYLNHFLGNMEIVNSREVWAVLKPGFLALLQDPFDPKLLDIVIFDVSPH
MDRNGEGQSTLAREIKEHNPLHFAFEIFITDWWLCPELYLRRPFHHHESSRLDILLESRA
KQGVQYLFVCNRFGSIKCLTGSCLHEMIIDKCMKFNFADIGCMHFKIYILLYKEVSLALK
INSMYSKQRLLNIHENVKVLRYPDHFSTGIYLWSHHEKIVIVDNQVCYIGGLDLCFGRYD
TPEHKVVDVPPSIWPGKDYYNPRESEPNSWEDTMKDELDRTKYPRMPWHDVQCALYGPAC
RDIARHFVQRWNYAKRNKAPNEQAIPLLMPQHHMVIPHYMGKIKESNEEVSKQTHVEDIK
GQKLSSLKAPASCQDIPLLLPHEPDHQASNNGELGLNGLDNNHGHSDHPNKTHWKQPIPN
RKAKQDTSLQDLQMKGFVDNLGTPDVSSVIGHYDTSKQNVHHMDNEWWETQERGDQVDYV
LDIGEVGPRATCCCQVVRSVGPWSAGTTQIEGSIHNAYFSLIEKAEHFVYIENQFFISGL
SGDDTIKNRVLEALYRRILRAEKEKRCFRVIIVIPLLPGFQGGIDDGGAASVRAIMHWQY
RTICRGPNSILKNLYDVVGSKAHDYISFYGLRAHGRLGDGGPLVTNQIYVHSKLMIIDDR
MTLIGSANINDRSLLGSRDSEIGMIIEDKEVVSSIMDGRHWEAGKFSLSLRLSLWAEHLG
LHPGEVSQIMDPVDDLTYNNIWMGTAKANTKIYQNVFSCVPNDHIHSRYGTNNLFSRLLA
FQLQYHVLWSQFRQGFAHRKEKIGHTTIDLGVAVEITETHKDGDLAGTDPMEKLQAVRGH
LVSFPLEFMCQEDLRPFFGESEYYTSPQVFH*
>11682.m02728|LOC_Os05g29050.1|genepair269-2
MNVERLVPGGAGGGGGRFRYERMPARGPADDGEGEEEEEEAAVPERRPEVLAASASFRLS
EAARVFEELPRASIVAVSRPDAGDITPMLLSYTIEVHYKQRYLIRHNQFKIKKLTNLEDI
SNIDWSAMFRWRLYKKASQVLYLHFALKRREFLEEFHEKQEQVKEWLQNLGIGEHMPVGH
DEDEADDVNVPAQAEENSIRHRNVPSSAVLPVIRPALGRQHSVSDRAKVAMQEYLNHFLG
NLDIVNSPEVCKFLEVSCLSFLPEYGPKLKEDYVSVGHLPKIQKDHKENCCSCGLFSCCK
SSWQKVWVVLKPGFLALLKDPFDPKLLDVLIFDALPHMDISGEGQISLAKEIKERNPLHF
GLQIFITGWWLCPELFLRRPFQHHGSSRLDALLEARAKQGVQIYILLYKEVALALKINSL
YSKQKLLNIHENVKVLRYPDHFSSGVYLWSHHEKIVIVDNQVCYLGGLDLCFGRYDNSAH
KLSDVPPVIWPGKDYYNPRESEPNSWEDTMKDELDRTKYPRMPWHDVQCALYGPPCRDVA
RHFVQRWNYAKRNKAPNEQGIPLLMPHHHMVIPHYKGIGQEINSEADGKQNHDKDCDVKK
PVSVDSRESCQDIPLLLPQELEPPALPNGDLRVNDLDANHSDHLHKTSFNQPLLNRKAKL
DSSRQDLPMRGFVDNISSLESSSIRHFDSSKEEKYHMDKNWWEMQERGDQVASVLDIGQV
GPRATCHCQVIRSVGQWSAGTTQIEGSIHNAYFSLIEKAEHFVYIENQFFISGLSGDETI
KNRVLEALYRRILRAEREKKRFKAIIIIPLLPGFQGGIDDGGAASVRAIMHWQYRTICRG
PNSILQNLYDVIGPKAHDYISFYGLRAHGRLCEGGPLVTNQIYVHSKLMIIDDRITLIGS
ANINDRSLLGSRDSEIAVVIEDKEVVSSKMNGKPWEAGKFSLSLRLSLWAEHLGLHRGEV
SHIMDPIDDSTFKNIWMATAKTNTMIYQDVFSCVPNDLIHSRAQFRQSFAHCRDKIGHNT
IDLGVAQEKLETYQDGDLKGTDPIERLQMIKGHLVSFPLDFMSQEDLRPYFSESEYYTSP
QVFH*
>11667.m02018|LOC_Os01g20930.1|genepair270-1
MTMRRTGGVGCRNAKGADADATAAGEECGEVGGEAHEDGAREEDAAVSFGIGSLGPVAMS
DRGTSSSVSGDTDADSVPTADAAAPLWAPHGRALTGCLVVVNVALVLLVYLYFWRVFSRK
RAAAAASARSDDDDDDDEASSSASAPPPAAAAVSVRTRDDVLASLPVFVVRSSGGEKAEA
EAECAVCIAELRDGDECRALPRCGHRFHASCVDGWLRLHTTCPLCRASVVALAAAPERKG
GVADTTAAAEDVDARV*
>11682.m02745|LOC_Os05g29710.1|genepair270-2
MSTPTVQMVAPAAAVVEDPSKHWTRHGPVLTACLVGINLLMVLLVFFYFWRFFSGKRGPP
TSSTSTMASGGDDDEEEGASSSSSSADTSPGRHHQDREDIASSLPVFVYSSSAAAPDVGD
AGGNGKAAAAAECAVCIVEFRDGDRARLLPRCGHRFHADCIGAWLQLHSTCPLCRAAVLL
HPAAAEPAKNDQPKDDDCPV*
>11667.m02022|LOC_Os01g20970.1|genepair271-1
MASSPYHAAVGVAVLAILLAAPAAEAGAPTAAPLANYSLEDACKKTGPHYGLCIVTLSAD
RSAKSSDTVGLARVAVLAAQKNASETATYLSSIYDDDSIEKKTVQLQQCLEDCSERYEAA
VEQLTDATVALDTGGYEEAMALVAAGQAEVKMCQRGFKAVPQHRNILTLRNREVDQLCSI
AFTITKLIRVSPSAEE*
>11682.m02748|LOC_Os05g29740.1|genepair271-2
MAASSSVLVVVAACLAAALVCLAANVAPASCARATAALPHASIAETCSFVDDHKLCEESL
SSLPLTARAAADARVLARAAVLLARQNATATAAYLSHLHAAAAAAADDGDDADHRCVGDC
TVRYDRAVAYLGDAAAALDAGEFDEAELLVGAGRTEAELCQKGCEHARLPALLAARNGAV

ERLCNVAMDITRLLHQQH*
>11667.m02023|LOC_Os01g20980.1|genepair272-1
MASFHIATALSATLLLSLLVLGRSDATLPPPATPVPPSTACNGTTDPTFCRSVLPSNGTS
NLYTYGRFSVAKSLANANKFLSLVNRYLSGGRLAAGAVAALQDCQLLSGLNIDFLSAAGA
TLNRTSSTLLDPQAEDVQTLLSAILTNQQTCADGLQAAASAWSVRNGLAVPMSNSTKLYS
VSLSLFTRAWVRPSTKKPRTATPKPPRHGGRGRGLFDATDDEMVRRMALDGAAAAVSTFG
AVTVDQSGAGNFTTVSDAVAAAPTNLDGTKGYFVIHVTAGVYAENVVVPKNKKYVMMVGD
GIGQTVITGNRSVVDGWTTFNSATFAVLGQGFVAVNMTFRNTAGPAKHQAVALRCGADLS
TFYQCSFEAYQDTLYTHSLRQFYRACDVYGTVDYVFGNAAVVFQDCTLYNRLPMQGQSNT
VTAQGRTDPNQNTGTTIQGCAIVAAPDLAANTAFATTNYLGRPWKLYSRTVIMQSVVGGL
IDPAGWMPWDGDYALSTLYYAEYNNSGAGADTSRRVTWPGYHVLNSTADAGNFTVGNMVL
GDFWLPQTGVPFTSGLN*
>11682.m02753|LOC_Os05g29790.1|genepair272-2
MAVRYEKKAMCALLLSLIMVALSVAAAGDGDAPPSTPVSPTTACNDTTDPSFCRTVLPPR
GSSDLYTYGRFSVARSLDSARRFAGLVGRYLARHRGLSPAAVGALRDCQLMSELNVDFLS
AAGATLRSAADALPDPQADDVHTLLSAILTNQQTCLDGLQAASSSWSERGGGGLAAPIAN
GTKLYSLSLSLFTRAWVPTAKGSKHHGGGKKPHQGHGKKQPPAAAASMRRGLFDAADGEM
ARRVAMEGPEATVAVNGVVTVDQGGGGNYTTVGDAVAAAPSNLDGSTGHYVIYVAGGVYE
ENVVVPKHKRYIMMVGDGVGQTVITGNRSVVDGWTTFNSATFAVVGQGFVAMNMTFRNTA
GPSKHQAVALRSGADLSAFYGCSFEAYQDTLYAHSLRQFYRRCDVYGTVDYVFGNAAVCN
TVTAQGRSDPNQNTGTSIQGCSLLAAPDLAAAGDGGRTLTYLGRPWKNFSRTVVMESYVG
GLVDPAGWMPWSGDFALDTLFYAEYNNSGPGADTSRRVAWPGYHVLGAGADAGNFTVTSM
VLGDNWLPQTGVPFTSGFLTSDPPIS*
>11667.m02053|LOC_Os01g21250.1|genepair273-1
MERVASSCLSLLAQRRGYSVAAAVAKGAGRRADEKKVAAAVAKRTMAKAAEEKTAWVPDP
VTGYYRPAGGAKEVDAAELRAKLLSNSKRMAAN*
>11682.m02767|LOC_Os05g29930.1|genepair273-2
MARVAASCVALLAQRRGLSAAITAAEGSAKTIDDKAVKLGTAAKDVATATATTTEEKTAF
WEPDPDTGYYRPVTGTKEVDAADLRAEMLKQRMLHD*
>11667.m02058|LOC_Os01g21300.1|genepair274-1
MEVEYHEEYVRNSRGVQLFTCGWLPAKTSPKALVFLCHGYAMECSGYMRDHELREKGGLY
SQ*
>11682.m02771|LOC_Os05g29970.1|genepair274-2
MTKEMADDVEYHEEFVTNPRGLRLFTCGWLPASSSPKALIFLCHGYGMEVSGFMKGILRP
LAV*
>11667.m02059|LOC_Os01g21310.1|genepair275-1
MEEYRSKSRFLYGESMGGAVALLLHMKDPTFWDGAILVAPMCKISEKVKPHPVVISLLTQ
VEDVIPRWKIVPTKDVIDAAFKDPAKREKIRKNKLIYQDKPRLKTALEMLRTSMYVEDSL
SKVKLPFFVLHGDADTVTDPEVSRALYERAASADKAIKLYAGMWHGLTAGEPDHNVDAIF
SDIVAWLNGRSRTWTVEDRLMKMMASPDRFIRGERGGAADVDGDAKRGPPRRRGGCFSGL
AGRTHHHSEM*
>11682.m02772|LOC_Os05g29980.1|genepair275-2
MVGDIVILAACGVELATAGYGVFGIDYEGHGKSMGARCYIQKFEHLVDDCDRFFKSICEL
EEYRDKSRFLYGESMGGAVALLLHRKDPTFWDGAVLVAPMCKGLALGSNLMAYMISEKVK
PHPLVVTLLTQVEEIIPKWKIVPTKDVIDSAFKDPIKREKIRKNKLIYQDKPRLKTALEL
LRTSISVEQSLSQVSIPFFILHGEADKVTDPEVSRALYERAASADKTIKLYPGMWHGLTA
GEPDHNVHLVFSDIVAWLDRRSHRQDRASITPPAACTDSAAAAAADSPVSPEPPRQGAAG
GFLCGLTGRANPQQCRM*
>11667.m02061|LOC_Os01g21320.2|genepair276-1
MLGLAKRIGAKFLLTSTSEVYGDPLQHPQVETYWGNVNPIGVRSCYDEGKRTAETLTMDY
HRGANLEVRIARIFNTYGPRMCIDDGRVVSNFVAQALRKEPLTVYGDGKQTRSFQYVSDL
VEGLMSLMEGEHIGPFNLGNPGEFTMLELAKVVQDTIDPNARIEFRPNTADDPHKRKPDI
TRAKELLGWEPKVPLREGLPLMVTDFRKRIFGDQEA*
>11682.m02773|LOC_Os05g29990.1|genepair276-2
MASELTYRGGGGATLAGEAEAAVAAGGYSPKPSKPLAWLPRAARYAAGEHRPLFALAGML
VAAAIFSLATPYSSSTPAAAAAGSTAANPLARFSVEPAVSRRQQQLPARQFVGGKVPLGL
KRKGLRVLVTGGAGFVGSHLVDRLVERGDSVIVVDNLFTGRKENVVHHFGNPNFEMIRHD
VVEPILLEVDQIYHLACPASPVHYKWHKTNVVGTLNMLGLAKRINARFLLTSTSEVYGDP
LQHPQVETYWGNVNPIGVRSCYDEGKRTAETLTMDYHRGANLEVRIARIFNTYGPRMCID
DGRVVSNFVAQALRKEPLTVYGDGKQTRSFQYVSDLVEGLMRLMEGEHVGPFNLGNPGEF
TMLELAKVVQDTIDPNAKIEFRPNTQDDPHKRKPDIGRAKELLGWEPKIPLHKGLPLMVT
DFRKRIFGDQDSTATTTGGQQG*
>11667.m02072|LOC_Os01g21420.1|genepair277-1
MAAEASNVEQVEDTDNENDVVIVGSRQRERWDMKESKTATGFGISPMFHKDIDLAGSVGS
WSKIHYEVAKCKSARTQNACTAGIYTYLAETMSRRNSRTIYVGNLPGDIREREVEDLFYK
FEDPRDADDAICGRDGYNFDGYRLRVELAHGGRGQSYSYDRPRSYSSGRRGGVSRRSEYR
VMVTGLPSSASWQDLKDHMRRAGDVCFSDVYREAGATVGIVDYTTYEDMKYAIRKLDDSE
FRNAFSRAYIRVRESVSRSPSPVDERSISRSRTPVSSPSRGRSVSKSPSRSLSRSPSPVK
SD*
>11682.m02788|LOC_Os05g30140.1|genepair277-2
MSRRNSRTIYVGNLPGDIREREVEDLFYKYGRIVDIDLKIPPRPPGYAFVEFEDPRDAQD
AIYGRHGYDFDGHRLRVELAHGGRGPSFDRSSSYSSAGRRGAAKRTDYRVMVTGLPSSAS
WQDLKDHMRRAGDVCFSDVYREGGATIGIVDYTNYEDMKQAIRKLDDSEFRNAFSRAYIR
VREYDSAKRSRSRSRGRSHSRSISRSRSRSRSISRSRSRSYSRSRSPRSRSASRSHSPVK

ERSRSASQSRSPVKERSPSRSPPPATSPPREKSASKSPVKSRSLSRSPSPVRDINQKLCI
FRLQQQRHVWDHDHPPYRCRHEDNIRAAPVLNPECGAPADWKGRYLGIIDYSAILLWVLE
NAEITAVALSAGSATVAGVGMGAVGAVGVAALGAIGAAVAGGLTAEKGVAKDGLTAADHL
GEDFYKVLLQQEHFKSTTVRSIVESYRWSPSVSITLDTSMLTVLPLLPKYRQLTVMEFMK
TIGSTVLDSGNGLVKPPLTCSPDASLGSVIDSIASRITHRIYTSWTVTSRLLVS*
>11667.m02076|LOC_Os01g21460.1|genepair278-1
MAYPSSSMNTLRECRTCQLTSLVQLPDLFGDADEVIPDEEQQEGEDTAPCGEQRLVLLPE
RSVHALVHGLAEPVQHGARRRVMSCTFPFRSSAAASCVAPPGSSSDRSTQLPKYPLYMGN
LINGEIGEVFNMYKLSFRKKKVTAEWRSAVRPMMWRCQWLELRMKDLLSQVSKYDRELAL
INKGKELQQAVNMTNGSRSESAQSSKSRENSCMERRKRRRLEETVNTSLYIKKHEILSYF
FDKQNKGAETDGILIDDDSSGPVGNDVKGGIHTVGLLEPKEYDMVAEQLTLQKFLLTIDG
IRSQVLRLQDRLSKVRSKQENMVSLVDHAHIKVSEKRLRTQKRSFSYKKDRYSKSKKKKN
LNILSKEEDKPAHAVISTLSKRAPDCQTEVTMYSSEEKSGERCQSHKKAITVDLLLPNGH
MGDLCKDNDDVLIDNQAANEGYQPFENAKQPMDKSLELTEKVCETANLRVGSNSSPVEVT
STSAPFRVENASVSLEARSTPGQVVKQEPVFEKPPALKHVYSGKRRRKLKMKEGSGPVSG
LTQSKEASKTPATKKKTESTSPAAKKLKIEETTAPDEGKKAVKTHSTGKKRKAGKSCSST
KNQEAENSSCAARKDISESTPSKPRIEKAVLVAVNSRRSQRVRKPKIY*
>11682.m02795|LOC_Os05g30210.1|genepair278-2
MAAAAGMGGNGNGADDKARDVTDQSKALGGNSCEDRALPSAVRVTVSGDPVGTFGSFGNM
ADYNVHLHQPDEGDDHGDSTECSSSFGPSCSASSDDDDDDTKSEMDGMEVDSPFLGPTRT
GADRASSAPRMVRRRQVTAEWRKIVGPIMWRCQWLELHMKNLLSQVAKYDRELAIINHEK
DLQLEMVKADGPKSEPGKLYPQSHERIIMKRRKRKRDEDTVDTSLYLKRHPALSYYENKN
SGVQTDGPLVNGGFDSSVVEDIESTDDALVENDRVFEQYSLREILLTVDDVQSRILSLQG
RLSNARSKYKKLSQCLDRKQVKVPQKIQNQMTCCKKDGRRSHQKTKCMHTLLQKDDLDRS
LAVVPPVFGRSTDCVLECMKKNDAQEDAVQSDLNGITIEMFCGKDNFLTNAHVGELYKES
ADDVLIDNQAAKEGYQLFEKVKPEEHSELVMPPSKVQKASADIVDYEQVQETAPVAKQII
SGDKRGQKPNKKHGLPVLAKKIKTEKDPGNMKNEKTVLVAVDPRRSTRVRKPKTY*
>11667.m06155|LOC_Os01g61720.1|genepair279-1
MGKKGNWFSAVKKVFSSSDPDGREAKIEKADKSRSRRKWPFGKSKKSDPWTSTVAVPTST
APPPQPPPPPPTHPIQPQPEEIKDVKAVETDSEQNKHAYSVALASAVAAEAAAVAAQAAA
EVVRLTTATTAVPKSPVSSKDELAAIKIQTAFRGYLARRALRALRGLVRLKSLVDGNAVK
RQTAHTLHCTQTMTRVQTQIYSRRVKMEEEKQALQRQLQLKHQRELEKMKIDEDWDHSHQ
SKEQVETSLMMKQEAALRRERALAYAFSHQWKNSGRTITPTFTDQGNPNWGWSWMERWMT
SRPWESRVISDKDPKDHYSTKNPSTSASRTYVPRAISIQRPATPNKSSRPPSRQSPSTPP
SRVPSVTGKIRPASPRDSWLYKEDDLRSITSIRSERPRRQSTGGASVRDDASLTSTPALP
SYMQSTESARAKSRYRSLLTDRFEVPERVPLVHSSIKKRLSFPVADKPNGEHADKLMERG
RRHSDPPKVDPASLKDVPVS*
>11682.m03673|LOC_Os05g38790.1|genepair279-2
MGKKGKWFDAVQRALSTSENDSHENEKKGKRSTLKKILQFSKSSASTSSPPVTSPSARQQ
PHHHHPPPPQAAPPDRQRDDGIKEAKSSDAAAAAAQKTATATAVTRPTTTAPRAPARSAE
ELAAVKIQKACRVYLGRRSQRARGLDRLMLLLEGLAVKRQTYEALYCMQTMTRVQTQIHS
RRVKTEEDKKALKSQVHVKQSLDRIKIGESWDHGHQSKEQIETVLTMKQEAALRRQRALA
YAFSHQETFICASLAASNVHGHRQPQLGMELGGAMDGGGEAVGEPDHAGEQRPCSRGGER
RRQREAGAHVGADTHVDAGVGQVHPPAELPVAVDTHAAVAVEDVGGAAVEPGRQPVPEVG
DGDDRPAPHDEPAAGAAAELRAARGGERQPGPRREGRRRPPLAAAHDEPAVRGAAEEEAEP
RRRRRRPGERRRGAADAELHADDQVREGQGGSAGGDRGPRHRREDGARSPSGDLTVGDQQ
APFPGLRGQAEQRPVAEQGEGREVDAATLAASEPKVLISQQPNVNPVAAMLRSGWLVA*
>11667.m06165|LOC_Os01g61810.1|genepair280-1
MADAGHDESGSPPRSGGVREQDRFLPIANISRIMKKAVPANGKIAKDAKETLQECVSEFI
SFVTSEASDKCQKEKRKTINGEDLLFAMGTLGFEEYVDPLKIYLHKYREMEGDSKLSSKA
GDGSVKKDTIGPHSGASSSSAQGMVGAYTQGMGYMQPQSNFHILVVLQSFAFPYMYQVAQ
IYCNKYEVSREQIWDTPQIMELSPWIPYTINRIWKETHGSQDIRIQGRPREAANSALDWQ
WPSKHSSLASNFYGTRVVGGHHEYQRSTKKDTTHVNFASGLGDLG*
>11682.m03678|LOC_Os05g38820.3|genepair280-2
MADGPGSPGGGGGSHESGSPRGGGGGGGGGGGGGGVREQDRFLPIANISRIMKKAIPANG
KIAKDAKETVQECVSEFISFITSEASDKCQREKRKTINGDDLLWAMATLGFEDYIEPLKV
YLQKYREVRTDVDVWNWGDSLLI*
>11667.m06170|LOC_Os01g61860.1|genepair281-1
MKAKSLPFIASEHKRDAYGFAVRPQHLQRYREYANIYKEEEEERSERWKNFLDSQAEYDE
SSGEDQDAKVSPSAEDEEAGKKAEDGRSKLSDEQKVKQQRPHKIQIWSEIRPSLGHIGEM
MSLRVKKKQSSADKENAANELQSANNEEIKPSEDSDDEFYDVEKVDPNQEGPVADSADAD
SGMNVDANQEGHYPWKEELECLVRDGLPMALRGELWQAFVGIGARRVKGYYESLLAADDE
RENSKGSDSPTMEGKPKGSPFSSEKWKGQIEKDLPRTFPGHPALDEDGRNALRRLLTAYA
RHNPSVGYCQRKYSSLYMILLNVFFKAMNFFAGLLLLLMPEENAFWALTGIMDDYFDGYF
SEEMIECQVDQLVLEELVREKFPKLVNHLDYLGVQVAWVTGPWFLSIFMNMLPWESVLRV
WDVLLFDGNRVMLFRTALALMELYGPALVTTKDAGDAVTLLQSLAGSTFDSSQLVLTACM
GYQAVDEARLQELRNKHRPSVISSMEQRAKGLRVWRDTNGLASKLYNFKRDPEPLVSLST
EQLSDLTETSSGSTDDMYSGLTVNTEIDSLPDPKDQVVWLKVELCQLLEERRSAVLRADE
LETALMEMVKQDNRRELSAKVEQLEQELSDLRQALLDKQEQEQAMLQVLMRVEQEQKVTE
DARIFAEQDAAAQKYAAHVLQEKYEEAMASLAQMENRAVMAETMLEATLQYQSSQQKAQL
PSPSPSPRTPTRDASPGQVNQDSSQEFQPRRISLLAPFSLGWRDKNKGKQNISDESTNGN
LNSNTEQMVDTPKKDDEKQGDSPQEGEQRVDTPRRDSEHRLDTPETTIKLEEQLEEIKLD
*
>11682.m03691|LOC_Os05g38950.1|genepair281-2

MKPKSLPFIAFEHKRDAYGFAVRPQHLQRYKEYAGIYKEEEEERSDRWKNFLERQSEPSG
QEEKGEAAGRVVDSSGIGGSSLLQEKIVQGPHKIEIWKPIRPSLGNIEQIMSVRVEEKQS
PASGNQDTKDVIHPVKVQEGKLSEDSDDEFYDVDKVETSQEMHSSDSANTGIDNRGQEEN
FPSMEELECLVHGGLPMALRGELWQAFVGIGVRRVKGYYDSLLVAEGELEDTSGSRSSTS
DVAGENTEVSSEKWKGQIEKDLPRTFPGHPALDEDGRNALRRLLLAYARHNPAVGYCQAM
NFFAGLLLLLMPEENAFWTLVGIIDDYFDGYFSEEMIESQVDQLVLEELVREKFPKLANH
LDYLGLQVAWVTGPWFLSIFTNVLPWESVLRVWDVLLFDGNRVMLFRTALALLEFYGPAL
VTTKDAGDAVTLLQSLAGSTFDSSQLVLTARMGYQSVDETGLQELRNKHRPSVLSSMEER
AKGLGALDTNGLASKLYNFKHDPEPLVSISDSQDQMSDVGDGNANQSDSGNMDDMYGGLT
VSSEIEALPDPKDQISWLKFELCRLLEERRSAVLRADELETALMEMVKQDNRRQLSAKVE
QSEQELSELRQTLLDKQEQEQAMCQVLLRVEQELKIAEEARISAEQDAAAQRYAVNVLQE
KYEEAMASLAKMENRAVMAETMLEATLQYQTSQQKALLSPLPSPRTSMIDASPGRANHDS
SQEFQPKKINLLSPFSLSWRDKNKGKQNNVDDSAKLTDAHDQREEITNNNDEKQVETPKL
DVLEESMGSPKEDNKSRVEVPKEDSDLASVQVVANDMNGQHEQLQEIKLD*
>11667.m06172|LOC_Os01g61880.1|genepair282-1
MASPYDHQSPHAQHPSGLPRPPGAGAGAAAGGFARGLMKQPSRLASGVRQFASRVSMKVP
EGVGGMRPGGGRMTRMQSSAQVGLRGLRFLDKTSGGKEGWKSVERRFDEMNRNGRLPKES
FGKCIGMGDSKEFAGELFVALARRRNLEPEDGITKEQLKEFWEEMTDQNFDSRLRIFFDM
CDKNGDGMLTEDEVKEVIILSASANKLAKLKGHAATYASLIMEELDPDDRGYIEIWQLET
LLRGMVSAQAAPEKMKRTTSSLARTMIPSRYRSPLKRHVSRTVDFVHENWKRIWLVALWL
AVNVGLFAYKFEQYERRAAFQVMGHCVCVAKGAAEVLKLNMALILLPVCRNTLTTLRSTA
LSHVIPFDDNINFHKVIAATIAAATAVHTLAHVTCDFPRLINCPSDKFMATLGPNFGYRQ
PTYADLLESAPGVTGILMIIMSFSFTLATHSFRRSVVKLPSPLHHLAGFNAFWYAHHLL
VLAYVLLVVHSYFIFLTREWYKKTTWMYLIVPVLFYACERTIRKVRENNYRVSIVKAAIY
PGNVLSLHMKKPPGFKYKSGMYLFVKCPDVSPFEWHPFSITSAPGDDYLSVHIRTLGDWT
TELRNLFGKACEAQVTSKKATLSRLETTVVADAQTEDTRFPKVLIDGPYGAPAQNYKKYD
ILLLIGLGIGATPFISILKDLLNNIKSNEEVESIHGSEIGSFKNNGPGRAYFYWVTREQG
SFEWFKGVMNDVAESDHNNIIEMHNYLTSVYEEGDARSALIAMVQSLQHAKNGVDIVSGS
RIRTHFARPNWRKVFSDLANAHKNSRIGVFYCGSPTLTKQLKDLSKEFSQTTTTRFHFHK
ENF*
>11682.m03694|LOC_Os05g38980.1|genepair282-2
MAGDYVDVPLGGGGQSTLPPVAPLKKQPSRLASGMKRLASMVPDTMKLKRTHSSAQPALR
GLRFLDKTSAGKDGWKNVEKRFDEMSADGRLPQESFAKCIGMADSKEFASEVFVALARRR
SIKPEDGITKEQLKEFWEELTDQNFDSRLRIFFDMCDKNGDGQLTEDEVKEVIVLSAAAN
KLAKLKSHAATYASLIMEELDPDHRGYIEIWQLETLLRGMVTAQGPPEKVKLASASLART
MVPSSHRSPMQRRFNKTVDFIHENWKRIWVLSLWAILNIALFMYKFVQYSRRDAFQVMGY
CVCIAKGAAETLKLNMAVILLPVCRNTLTRLRSTALSKVVPFDDNINFHKVIALTIAIGA
ATHTLAHVTCDFPRLVSCPRDKFEATLGPYFNYVQPTYSSLVASTPGWTGILMILIMSFS
FTLATHSFRRSVVKLPSPLHHLAGFNAFWYAHHLLVIAYILLVLHSYFIFLTKQWYNRTT
WMFLAVPVLFYSCERTIRRVRESSYGVTVIKAAIYPGNVLSIHMNKPSSFKYKSGMYMFV
KCPDVSPFEWHPFSITSAPGDDYLSVHIRTLGDWTTELRNLFGKACEAQVSSKKATLARL
ETTIIADGLKEETCFPKVFIDGPFGAPAQNYKKYDILLLIGLGIGATPFISILKDLLNNI
KSNGDVQSTHDAELGCTFKSNGPGRAYFYWVTREQVPLNGLKA*
>11667.m06178|LOC_Os01g61900.1|genepair283-1
MFRHSSSASSAASYGSDVSFMNHSPAAALPAVPTQIPRVAGGYLDGNVSGGLPHFGGAVS
SSSSSPPSYSSSLPSSYYNNIQRSISSHSLPHHLQLTDHFGGAFFSSSSSSSHQLPLPPP
LSSSPSSSSGDLFEFTSPCPVRRVFSTGDLQGMNGSSPPRPLPSGESCGQDGSGPFSQKV
GRYSAEERKERIERYRVKRHQRNFNKKITYACRKTLADSRPRVKGRFARNGEADAEGDER
EAFDASYSYDYSAAGYSDYRSGSNSSSVNSCYNYNRKDGAATEASVVGGSDNGEWWWRAP
GATTAEAERQRQAGFDVDEEIWATLGDMLSVNLAS*
>11682.m03695|LOC_Os05g38990.1|genepair283-2
MHHALSFSSALPTAPTEIPGSGGGFVDDKGSMFSLPNVAGSAPPPSYYSSLPSFYIHRST
SSHSLLHHRLSDLLNSNAAFSYSSAPACQLQPLPPVSSSTSSSSGDLLEFSSGTLRRVFS
TGDLQAMNVPPSPPPPPPFSGDICIQEVGGPFSQKVGRYSAEERKERIERYRVKRQQRNF
HKKITVLAYKTAFPFPLQAIDHGSYCSSSSSSSPGRFQYACRKTLADSRPRVQGRFARNA
ETEADAEADAVAGLDTEVYGNGYGYCAYSGLTNSISSNCYDNQSQSQWWGTPAGAANWQH
QQQKQQLGFDVAVDGDDEDYELCCGPASLTCAQGPDPELI*
>11667.m06179|LOC_Os01g61910.1|genepair284-1
MLRRAASNAYSWWWASHIRTTQSKWLDNNVQEMEIRVKAMIKLIDIEADTFARKADLYFK
SRPDLINHVEETYRSYQALADRYDRVSGELHKSNHTIATAFPEQVQLSLQDDNGDGFPKG
ITGININRGTSAAPKRTQTHKKISSKMSKDKAQEEIERLQKKILVLQTEKEFFKSSYESS
LNKYLSIERQAAEMQEEVWSLQETFSTSAVIEDNEARALMAAQALISCEDKLASLHCEQK
RSYEETTMEIQRVIDAKKKITIFKSECGYPDDQKDLPNHQDIEFSSIPSSIEDSDLIMKD
CKLELQELSQKVKQKFESSSEASAVHLAGQVDEIVDKVISLEIAASSQNAQINRMKNEAD
ELQKRLDSLEDEKAALIEDSSKLSERLKQVEEVLQTIQRIGKSVHSENGNIHQQLTEVDD
SLNDFVKKLDAHSTDEIVNSSQDDSGIACKSKDEGLLDALDDSSKAHKEEPDETLGWQQL
DLNGLEDKDKILLKDYASILRNYKDTQKQLLEIEKRNREYHLEAMSEMNELKSSSATKDD
EIRSLRRMLSSLQTKLNNSLPQRFVESEESSEANASPSLENKNIAETEEYMKIREHEEPH
DPSLEDKFRAEISRILEENLDFWLRFSTSYHYMQKFQKSFDKAKAEMDKLTDAKAQEGSD
AVPSCQSARKQESAVLEKKLRGLSTDLQVWLEKNVLLQGELESRFSLLCSIEEEISKITA
LGQTDEAHFTPFQAAKFQGEVSSMKQENSKVTKELQTGMDHVRSLQVEVGRALLKLRENI
ELSIGRRNRTQHSFRSLSMKAGVPLRTFLFGSKPKKASLFSCMGPVMPKPVADMRAGPFG
*
>11682.m03696|LOC_Os05g39000.1|genepair284-2

MEDRVKAMLNLIGADGDSFGKKAELYFKSRPELINHVEEMFRSYQALADRYDRISSELHK
ANHTIATAFPDQIQFSMQDADGEGFQKAISGIDLSNFKFPALEGLPMGSRGASRGTSPVP
KRTQMHRRITSHMNKENAQEEIDKLQKQILVLQTEKEFLKTSYDSALGRYLDIEKQVVEL
QDEVCSLQDAFSTGAAIEDNEARALMAARAIVSCEDTLVNLQDQQRRSSEEARTEFKRFI
EAKKKLDTFKAECGQPHTQNDEPDNSDKEYIHAMPSGDVDDSVQNEIRFDLQEVCQKVKE
LIELHPGVSVTDLADKVDRLVEKVIDLELATTSQNAQINRMKTEIDDLHKCLQALEEEKS
ALVADSSKLVDRLKQVEEVLQAVQHLGNSIQNGTQNIHKEMNAACSELAEFVEKLHEPEP
QNSGFMNSSQESSCQEEDSEVTSQYAKKQTSDSIDGSKNEVEKQDKGSEGPLVQQHPDTN
GSDGEDKILLEGYASVLQSYKGTEQKLSEIEKTNQEYHSRSMSELKDLKSANAMKDEEIH
SLRRMLSSLQRKMNAPAPENVDKSEETSKISTTPVTEDKEIAEIEEYMKQCQVEEQLASS
ISEEKFRAEIDRVLENNLGFWLRFSTSYHQIRNFQTSFDKLKTEMDKLIDAQAQCGADGV
PISYQVAKLESAVLEKKFRDLNTDLQVWIEKNVLLKGELENRFSSLCGIQEEISKIATLD
KSDEVHFTPFQAAKLQGEVLNMKQENNKVAKELEAGLDHVRGLQVEVGRVLLKLRENLEL
SIARSNRAQQNFRNLSTKAGVPLRTFLFGTKPKKPSLFSCMGPGVHKHHSGSRAGRR*
>11667.m01118|LOC_Os01g11490.1|genepair285-1
MGANDGHLDATSNNRGGLHEQTTTGCLPVDQSCFALSRLSYRRSSSSNPRACCSTTSYLE
VLAISLASLLVIVLVLCAIRCYRMRRAVNRVTVAAASAAAAAAAAGNVTNKKRPAPGLGE
DAIAALPKFEYRGTGDECDRWECSICLCAVADGEVARQLPRCMHLFHRGCVDMWLVAHTT
CPVCRAEVVVNKPPDEDDGRCAETPEDEAAPPASALEPARLENGERDLEAQ*
>11682.m01121|LOC_Os05g11860.1|genepair285-2
MPSSSSSSVAAAPSGSNGTRRDGGSGSVTGCLPADQACFALSSSASSPGYLHASATTTRR
DASATVARACCTTASYVVVLGISFGSLLAILLILCIIRWYLVWRSARPRRDDGAADEAVG
SAKKRSAGLDDDAIAALPVFAYKQREEGGGGAVGAAEEEEEERECAVCLAVMADGEAARR
LPRCMHVFHRGCVDVWLREHSTCPVCRAEVVVRPAGAARVEKLPESSASRALTSPVPAPA
PRPTGTVVDDGRERDLEAQQ*
>11667.m01129|LOC_Os01g11600.1|genepair286-1
MCCCGGDCRPIGWLLGLPFALLAVVVSFIGAIIWIVGLPISCICPCCLCVTVLLEVAVEL
VKAPLHVMTWFTSKIPC*
>11682.m01125|LOC_Os05g11900.1|genepair286-2
MCCCCGGGGDGDGDGCKCRPLGWLLGLPFALLAVLVSIVGAIIWIIGLPISCICPCCLCV
TLVLEAAVELIKAPLHVMTWFTSKIPC*
>11667.m01131|LOC_Os01g11620.1|genepair287-1
MAANKAVVVVAAVLLLLAVVARRASSQSYNAIYNFGDSITDTGNLCTGGCPSWLTTGQPP
YGNTFFGRPTGRCTNGRVIIDFLGTHDAFSPRIDSAQNFHCKLKDDELNLFARAADRFGL
PLLPPSKASGGDFKKGANMAIIGATTMNFDFFQSLGLGNSIWNNGPLDTQIQWFQQLLPS
ICGNDCKSYLSKSLFIVGEFGGNDYNAPLFGGKSMDEVKGYVPQIIAKITSGVDTLIGLG
AVDIVVPGVMPIGCFPLYLTLYQSSNSDDYDGNGCLKSYNSLSVYHNGLLKQGLAGVQAK
YPAVRLMYGNFYDQVTQMVQSPGSFAKGILVLKSLKVAKYFEGLQYGLKVCCGAGGQGSY
NYNNKARCGMSGASACGDPENYLVWDGIHLTEAAYRSIADGWLSGPYCSPAILH*
>11682.m01126|LOC_Os05g11910.1|genepair287-2
MAALLLLAAAMVVVVFAHAAAAQRYNAIYSFGDSISDTGNLCVGGCPSWLTTGQPPYGKT
FFGRPTGRCSDGRVVVDFLAEHFGLPLPPASKGGGDFKKGANMAIIGATSMDAAFFKSIG
LSDKIWNNGPLDTQIQWFRQLLPSVCGNDCRSYLSKSLFVVGEFGGNDYNAPLFAGRAMT
EVRDYVPQVVSKIIRGLETLIRMGAVDVVVPGVLPIGCFPIYLTLYGTSNGADYDRNGCL
KSYNSLSSYHNTLLKRSLSNLQRTYPHARVMYADFYSQVTAMVRSPQNFGLKYGLKVCCG
AGGQGTYNYNNKARCGMSGSSACADPANYLIWDGIHLTEAAYRSIADGWLKGPYCNPPIL
H*
>11667.m01144|LOC_Os01g11750.1|genepair288-1
MEHSPSNKMTLLLLLLLLLGCTHHGQANMYSGHPKIDSIFSFGNSYSDTGNFVKLAAPVI
PVIAFNNLPYGETFFGHPTGRASDGRLNVDFIAEDFGVPLLPPYLGESKNFSHGANFAVV
GATALDLAFFQKNNITSVPPFNTSLSVQVEWFHKLKPTLCSTTQGCRDYFERSLFFMGEF
GGNDYVFLLAAGKTVDEAMSYVPKVVGVISAGVEAVIEEGARYVVVPGQLPTGCLPIILT
LYASANATDYESGAGCLRRFNELARYHNAALFAAVSLLRGKHPSAAIVFADYYQPVIEFV
RMPENFGFSRSSRLRACCGGGGRYNYNATAACGLAGATACPDPAASINWDGVHLTEAAYG
RIAAGWLRGPYAQPPILAAVRP*
>11682.m01130|LOC_Os05g11950.1|genepair288-2
MEMRRVAVLGLVWLAAAATVAMADPLPSYYNAIFSFGDSFSDTGNFVIINSGKLPNMPKF
PPPYARCSNGRLVIDFLAEAFGLPLLPPSANKGTNFSQGANFAVMGATALDLKYFKDNNV
WSIPPFNTSMNVQLQWFDEVKQTICSSPQECREFFSKALFVFGEFGGNDYSFAWKAEWSL
EKVKTMVPSVVASMAGGIERLLDEGARHVVVPGNLPAGCIPITLTMYATEDRSEYDPRTG
CLKKYNSVALYHNAMLRIALDQLQRRHPDSRIVYADYYTPYIQFARTPHLYGRPATDTNL
TVTSHKTVPIISPTLPHRPCRVSTLGRGDGPELIIVVGELEAEQSRYAGVSGQSNGVCEF
TYQHRGALRACCGGGGPYNYNMSASCGLPGATTCEDPDAHVSWDGIHLTEAPYRFIANTW
IRGPYAHPPLASVVRDDMVY*
>11667.m01170|LOC_Os01g12000.1|genepair289-1
MNPKQRLYSLKLLVKALHKLKKKMMMKPNKGKKIGSSKPSSPAAPAEAASATAAAVAGGG
VEAAIGSSKPKVSPRRAAQGGQRKGVVRVKVVLTKEEAARLLSLTVVSAGAGAGGGRRKT
TAQIIAEIKRMEIRRAMATSSAAVAWRPALASIPEEQHHHSPRRSLGVQQVA*
>11682.m01148|LOC_Os05g12120.1|genepair289-2
MATPRALFLQRKIRDGSEKKQLPAAAGVVRVKVVLTRKQAARLVSLAGEGRRRRTAAQLV
RELRRMEDGAGRVDGSPATATATAWRPVLETISEEWLGSEVR*
>11667.m01177|LOC_Os01g12070.1|genepair290-1
MARGGGAAGVSMAHHLGIALVVLVFAAMAQVARGGGGGHDYGMALSKSILYFEAQRSGVL
PGSQRIAWRANSGLADGKANGVDLVGGYYDAGDNVKFGLPMAFTVTMMAWSVIEYGEEMA

AAGELGHAVEAIKWGTDYFAKAHPEPNVLYAEVGDGDSDHNCWQRPEDMTTSRQAYRLDP
QNPGSDLAGETAAAMAAASLVFRSSNPGYADQLLQHSKQLFDFADKYRGRYDNSITVARN
YYGSFSGYGDELLWASAWLYQASDDRRYLDYLANNADALGGTGWSINQFGWDVKYPGVQI
LAAKFLLQGKAGEHAGVLQGYRRKADFFACSCLGKDAADNVGRTPGGMLYHQRWNNIQFV
TSASFLLAVYSDHLAGGAVRCSGGGGAVAGAAELLAFAKSQVDYILGSNPRGTSYMVGYG
AVYPRQAHHRGSSIASIRASPSFVSCREGYASWYGRRGGNPNLLDGAVVGGPDEHDDFAD
ERNNYEQTEAATYNNAPLMGILARLAAGHGARARGRLGQSLQHGIAANHTSLPHGANHQH
ASPVEIEQKATASWEKDGRTYHRYAVTVSNRSPAGGKTVEELHIGIGKLYGPVWGLEKAA
RYGYVLPSWTPSLPAGESAAFVYVHAAPPADVWVTGYKLV*
>11682.m01151|LOC_Os05g12150.1|genepair290-2
MAKNGGAHGAATLFGLLALASMVKLGFVAGGGHDYAMALRKSILYFQAQRSGVLPPNQRV
SWRASSGLFDGKANGVDLVGGYYDAGDNVKFGLPMAFTVTMMSWSILEYGKQMAAAGELR
NAMDAVKWGTDYFIKAHPEPDVLYGEVGDGDTDHSCWQRPEDMTTSRQAFRVDPQHPGSD
LAAETAAAMAAASIVFRGTYPGYANLLLVHSKQLFEFADKYRGKYDASITVARNYYGSFS
GYGDELLWAAAWLFEATEDRSYLEYLAGNGEALGGTGWSINQFGWDVKYPGVQVLAAKFL
LQGRAGDHAAALQRYRQNAEFFVCSCVGKGAVNVARTPGGMMYHQRWNNLQFVTSASFLL
TVYADFAAISGRGAVHCPAGAAQPFDILKFVKSQVNYILGDNPRGTSYMVGYGASYPRQV
HHRGASIVSIKRDPSFVSCQEGYSSWYGREAGNPNLLDGAVVGGPDEYDDFADERDNYEQ
TEAATYNNAPLLGVLARLAASCGGLKEEEYEQETATPVVNRTSSSSSLPATATAIGIEQN
VTGTWARRRRTYYRYAVTVTNRSRGKTVRELHLGVSGLRGRLWGLEEARYGYVPPRWLPA
LRPGRSLRFVYVQPAPAPANIWVTGYKLV*
>11667.m01178|LOC_Os01g12080.1|genepair291-1
MSRGQENQSYEILCVMPDCLQINIDLGIAVFMSYVAMKDSQDIQSPTELQSSAQGTNEVQ
SNQPNPMATDGPGGDSGSLSIASNDNRKVSREDIELVQNLIERCLQLYMNKGEVVRTLST
RARIEPGFTTLVWQKLEEENSEFFRAYYIRLKLKRQIILFNHLLQHQYNLMKYPAPPNVP
LAPMQNGMHPMPVNNLPMGYPVLQQPMMPAPGQPHIDSMACGLSSGHVVNGIPAAGGYHP
IRMNSGNDMVVDNGAPETAHVGATCSAMSSEMAVSPSSAASSNHAPFTPSEIPGITMDTS
ALDSTFVSDVENTGQLQLGPDGSSRDSIRSLGQLWNFSLSDLTADLTSLGDLEALENYAG
TPFLASDSDILLDSPDQNDIVEYFADAINGPSQSDEEK*
>11682.m01153|LOC_Os05g12170.1|genepair291-2
MAQATLPLAALRNGRAPARPRRHARRHPRLRCRAPRTATATGCPPAADVRVEGKGKPGRG
GGEEKIEDEYLNTSNQLGFAARNGFCPGVALAGCRHVIRHWILAGFRVMWLLGSYAVRSS
SCGSGNHRYGQCNGPMADILNKAHHGKRMVDERARKQLSRHSCLADWWAVSSVSSHFLFF
VYFVPKLYPFCLYSIVHPSWQDKQRKKRSYSIGTNEAGRQETCSLEVSGLKCLPLGVRQR
RGHGCWRRSRFDGDAYMRWVSTSSDEKKARSPADGEEAPSPRRRGEGLQTKSDVQELLEI
LGRFHMAHVLISQLSFGDSNKRILARVSRLWNFADLNDDTKIFHTDLVLLDEMGTSIHAQ
IYPPITKNMKPLLKGRKDIMGVITEVGAVTTIRPKSRNAESLKRTLQIRDASNSTLPVTL
WGERATSFDVDEIYNASQTQAQVVVFVGTLMKDYRGLGLTLTGSSPCKWYINLNIPKVAE
LNERYKVVLIAGDDDANATFILFGRIAQRLLRRPIESLIEENPPNSEYIPSEITSLIGSN
FAWNVSFTRDTVMMSQECLQVNSIISSGASNQPLLLMSPDASQVTSATVSVSSSSSVQIA
PTPSETSNESQQPIQPRQTISTPTKFTITQGTQGTPTSKSNSSTPTKKNIVVSVSRMQSS
RTKPKTDDKPKTDTKAEENTSHDKSSIVVLPDTTENKGKGQTAEEAKQATPSFPTDTQAK
KGALITPRGMPTPTTTPPVAKKLFKDGAQQKGNDSADVVVPRTDVESKAKVHPLVQGDDE
IKGEQPNTDAPLGDSDSLSAASNDNKRVSREDIELVQNLIERCLQLYMNRGEVVRTLSTR
ARIEPGFTTLVWQKLEEENSEFFRAYYIRLKLKKQINLFNHLLEHQYHLMKYPVPQQVPL
TPTQNGIRPMPVNNLPMGYPVLQQPGIPAPVQPHVNSISCGPPGCHVVNGIPAPGGYNPI
RMSSGNGMTENEVPGTAHAGAMSSEMAVSPSSAMSSNHVSFTPDISGMDVDASTVNATFG
DDLGNGGPLQIGPNGGDSSSLGQQIWDFSLSDLSADLTNLGDLAALENYSGNPFLPSDSD
IFESPDDDIVEYFADAINGPSQSDEEK*
>11667.m01184|LOC_Os01g12130.1|genepair292-1
MVSNTIRVAVGILGNAASMLLYAAPILTFRRVIKKGSVEEFSCVPYILALFNCLLYTWYG
LPVVSSGWENSTVSSINGLGILLEIAFISIYTWFAPRERKKFVLRMVLPVLAFFALTAIF
SSFLFHTHGLRKVFVGSIGLVASISMYSSPMVAAKQVITTKSVEFMPFYLSLFSFLSSAL
WMIYGLLGKDLFIASPNFIGCPMGILQLVLYCIYRKSHKEAEKLHDIDQENGLKVVTTHE
KITGREPEAQRD*
>11682.m01169|LOC_Os05g12320.1|genepair292-2
MATLTYSSTQYWSKRGHVPSQAKVLDRLTFKRVIKKASVEEFSCIPYILALFSCLTYSWY
GFPVKQVMLMASLILAVFCMTVFFSSFSIHNHHIRKVFVGSVGLVSSISMYGSPLVAMKQ
VIRTKSVEFMPFYLSLFTLFTSLTWMAYGVIGRDPFIATPNCIGSIMGILQLVVYCIYSK
CKEAPKVLHDIEQANVVKIPTSHVDTKGHNP*
>11667.m01192|LOC_Os01g12200.1|genepair293-1
MAHLLQLPDLAAARPPAARRRIAAAAVVVAEARGGVKQQQQQVAVGRVIRVADPVREGRL
LLLPPPLFSVPVTPSESPAAARRREEDEEERRRYYLNMGYAIRTLREELPDVFSKEPSFD
IYRDDIVFKDPLNKFEGIDNYKRIFWALRFTGRIFFKALWVDIVSIWQPAENLIMIRWIA
HGIPRVPWEAHGRFDGASEYKLDKNGKIYEHKVHNVAMNPPTKFKVLPVHELIRSLGCPS
TPKPTYFETSSQSLSVEPALYIYEDEVVDSISFPQCKPATPMQTVQRKTQNKIRKGTFLH
SDDMKELYTLRKAPSLVKFLILLY*
>11682.m01185|LOC_Os05g12480.1|genepair293-2
MYEEIKEWPLMHASGDDIVFKDPINNFTGIDNYKRIFWALRFTGQIFFKALWIDIISIWQ
PVEDVIMIRWIVHGIPRVLSDGPGRFEGTSEYKFDKNGKIYEHKVDNVAKNTPTKFKVLP
VVELIRSLGCPSTPKPTYFETSSLQLISLLPFCSKFLSRNVGIAEVIPRSWLSFVSCILP
PTWEPSLVVLDQKQGNPPLMTAADMPDSLQRQSSFHPGSSPPPPEPGLFSLDAAVDNGKP
PDDGVLPASQEAAAPPLADAGTTASWVG*
>11667.m01262|LOC_Os01g12870.1|genepair294-1

MAAAAAAEGGGGGGGAVYDPSYVPDSVKTFVAHMYRHVRDKNVYEIHQMYEGGFQRLSDRL
FRDAPWPAAEAVSPYCDGDHVFLLLYRELWYRHAYARASSSSSSSAPLTAGQRAESWANY
CDLFSVVLHGVVNMQLPNQWLWDMVDEFVYQFQSFCQYRAKLKNKSDDELHQLKQFDKAW
NVYGVLNYLQALVEKSMIAQILEREKEGLEQFTATDGYDYQGGSNVLKMLGYYSMIGLLR
IHCLLGDYRTGLKCLAPIDLNQQGVYTIVIGSHISAIYHYGFANLMMRRYAEAIREFNKI
LLYILKYKQYHQKSPQYDQILKKNEQMYAFLAVCLSLCPQHNLIDENVSTQLKEKYNDKM
TKMQRFDEETYAAYDELFSYACPKFITPSPPALDQPLTNYNQDAYRLQLKLFLYEVKQQQ
LLSGIRSYLKLYSTITIAKLAQYMEVDEATLRAMKNLSQYLYRSILMTYKHKMHAVDNNG
KIVSSADFDFYIKEDVIHVMESKPIKRHGDYFLRQILKFEEMIGELEKVQFD*
>11682.m01285|LOC_Os05g13950.1|genepair294-2
MASSAAAAFYRDREDAAPPSSGPGGGGAMAAYDPSYVPDSVKTFVSHLYRHIRDRNVYET
HQMYEGGFTRLSDRHFRDTPWPPAEAVAAHCDGDHVFLLLYRELWFRHAHARVQGLTPAQ
RAESWDNYCSLFSVVLQGVVNMQLPNQWLWDMVDEFVYQFQSFCQYRAKLKNKTHEEIAL
LKQYDQAWSVYGVLNYLKALVEKSMIGEILEREKVGLEQFTATDGYDYEGGSNVLKMLGY
YSMIGLLRVHCLLGDYHTGLKCLAPIDISQQGVYTTVIGSHISTIYHYGFASLMMRRYID
GIREFNKILLYILKCKQYHQNSPQYDQLLKKNEQMYALLAICLSLCPQDKLIDENVGTQL
KEKYGDKMTKMHRYDDEAYAIYDELFSYACPKFITASPPVLREPYTNYNQDAYRLQLKLF
LYEVKQQQLLSGIRSYLKLYSTITIGKLAKYMDVDEVTLRTILMTYKHKMHSIDSDGKVI
SSADFDFYIDEDIIHVVESKLTKNHGDYFLRQILKFEEMITQLDKVQFD*
>11667.m01270|LOC_Os01g12940.1|genepair295-1
MDPVLNSPSFTPTPTLPSYLDIQGRRFRFGAIGGQNVVMVMTGLSMLNAGLTTQLLLSLF
RVKGIVHWGIAGNADEGLQIGDVTIPEHWAHLSLWNWQRYGDGPENELPLEAAGDYTRDL
GFLNFSDYTAAGPSPNELNSIWFQPEEIFPVSGKPEQRQHAFWVPVSSRYFSLAEKLEGM
ELPACVNATTCLPRAPRVTRVRRGCSANVFLDNAAYRQFLRAKFGCTPVEMESAAVALVA
HQHAVPFLTIRSLSDLAGGGSSLGNEAGEFLAIAAQNAVDVMLNFVPLLADGGAAHDAVA
ADM*
>11682.m01287|LOC_Os05g13970.1|genepair295-2
MAARSSSSPAKTTTTLALLLVVAAAAAAVAAEAAAVAADAARGVRQVNRRGPYLGVVVPN
GFEMEPLLRSPAFSPAKKLPYLDVAGRRFRFGSIGEKKVIIVMTGLGMLNSGVTTQLLLT
LFDVEGIVHFGIAGNADPDLHIGDVTVPRYWAHTGLWNWQRYGDGPEKELALESNGDYTR
KYGALNFSDYGVAGGGGGNLLNGVWYQPEEVFPADGTPESRRHEFWVPVDGRYYQLAQKL
EAMPLERCVNRTGTATSPATCLARAPAVARVERGCSASVFVDNAAYRQFLRSRLGVTPID
MESAAVALVAAQQGAPFIAIRSLSDLAGGGSAESNEAGVFAALAAQNAVAVAVKFISLLS
*
>11667.m01276|LOC_Os01g13000.1|genepair296-1
MLISTGDLSKSKSKNLDRISGGLVGDHEKYSVATALLSKGLDDEASMPHEASAAAYDFYG
HGGGAGDGILQASPEASSCKSQLSQMLLQAAASSPRSCVTTSGLGSSMMDFSNTAAVAPA
AEPELTRKHHAGQSDNSSECNSTETGSALKKARVQASSSAQSTLKVRKERLGDRITALHQ
IVSPFGKTDTASVLQETIGYIRFLLGQIEALSYPYLGQCCSANPMQQQTGIMAGERSTDG
LFPEFPAGQDAEKDGKKQQAKKDDDLRSRGLCLVPVSCMPHLAADNDVVVGSDFWAAAGG
GGGGGAPPLAGMNLR*
>11682.m01291|LOC_Os05g14010.1|genepair296-2
MNRGEFQSSLVQQMIWSGSSTGTATGVGSGGGAGSLMGSLKPCHEDQEASPNMPSLSSPS
LIFSQQFQHSSPGLVPMNGTAGAAASLPSLHDGGGGGHESSMPESWSQLLLGGLAGDQER
YSATAALLSKGLENWGDHAAAAAASACMVGGMKEEGSMAQAAATAAAAAYSFYGSHLAGD
HQHEIQAAAAGGGASNKSQLSQMLMASSPRSCITTSLGSNMLDFSNTAAPPELRSHHHNS
DNSSECNSTATGSALKKARVQASSSAQSTLKVRKERLGDRITALHQIVSPFGKTDTASVL
QETIGYIRFLLSQIEALSYPYMGDANGTGPMQNGPVGERNPGLFPEYPGQLLNHNGNTGA
QQPAAQPEQQGANDDGKKDLRSRGLCLVPVSCTSHFGGDNAADYWAPAPLGGILR*
>11667.m01279|LOC_Os01g13030.1|genepair297-1
MSPPLELDYIGLSPPPPPPSSSSAAAARADDVDLKGTELRLGLPGSESPDRRPAAIAAAA
ATATTLELLPAKGAKRVFPDEAALTPPTAAAGKGKAAREGEEVGAEEEDKKVAAPPQPAA
KAQVVGWPPIRSYRKNTMATNQIKSNKEDVDAKQGQGFLYVKVSMDGAPYLRKVDLKTYK
NYKDMSLGLEKMFIGFSTGKEGAENQKDGEYVLTYEDKDGDWMLVGDVPWEMFTDSCRRL
RIMKGSDAIGLGCSQLRLVPLFVPKL*
>11682.m01308|LOC_Os05g14180.1|genepair297-2
MSPPLELDYIGLSPPVPAAADAAADNDLKGTELRLGLPGSHSPDRSPPAATLDLLPAAKG
AKRGFSDEARPLPASAAAAAAAGKGKKAAAGEEDEDAEEEDKKVAAAPQAPAAKAQVVGW
PPIRSYRKNTMATNQLKSSKEDAEAKQGQGFLYVKVSMDGAPYLRKVDLKTYKNYKDLST
ALEKMFIGFTTGKDGLSESRKDGEYVLTYEDKDGDWMLVGDVPWEMFANSCRRLRIMKGS
DAIGLAPRAVDKSKNRN*
>11667.m01291|LOC_Os01g13130.1|genepair298-1
MPLLPMTKLELGHRGEAWEPGCLRAVAGELLFTFLFVFIGVASTITAGKAAGGAGEAAAV
TAAAMAQALVVAVLATAGFHVSGGHLNPAVTLSLAVGGHITLFRSALYVAAQLAGSSLAC
LLLRCLTGGAATPVHALADGVGPVQGVAAEAVFTFTLLLVICATILDPRRAAPPGTGPLL
TGLLVGANTVAGGALTGASMNPARSFGPALATGEWAHHWVYWVGPLAGGPLAVVAYELLF
MDVEDAGGAHQPLPQE*
>11682.m01314|LOC_Os05g14240.1|genepair298-2
MAKEVDPCDHGEVVDAGCVRAVLAELVLTFVFVFTGVAATMAAGVPEVAGAAMPMAALAG
VAIATALAAGVLVTAGFHVSGGHLNPAVTVALLARGHITAFRSALYVAAQLLASSLACIL
LRYLTGGMATPVHTLGSGIGPMQGLVMEIILTFSLLFVVYATILDPRSSVPGFGPLLTGL
IVGANTIAGGNFSGASMNPARSFGPALATGVWTHHWIYWLGPLIGGPLAGLVYESLFLVK
RTHEPLLDNSF*
>11667.m01295|LOC_Os01g13170.1|genepair299-1

MDFGYVGVGQGSSSSSSSASCRAADVAAWGSSTQQQQKRQRCQGSSDDQVGLSTSNSLQM
SEPEPRDFDNGENEEEDYYLDEDDCIYDDGDGYDYEFDGGDYFNQRLADKFDDLDLPPGV
EATVPWLQKIITNEEQSSSKLTVEDESANKSANKSQLFKQFDTVKNFSDHHYAATSGDVT
KRDWVKRIQHDWKLLEKDLPASIYVRVAEDRMDLLRAAIIGPKGTPYHDGLFFFDIQFSN
SYPANPPSVYYHSGGLRINPNLYNNGKVCLSLLGTWAGSGCETWNPSQSTMLQVLVSIQA
LILNEKPYFNEPGYASYANSVSGERIAMEYNDNTFLHSCRTMLYSLRRPPEHFADLVTSH
FRERGHTILAACRYYMEGHKVGSVVPEEKEPEYGDAGASTSSASASAAAAALKPRPDKVD
SVSRRPTFNDNLKTLFEELLMEFNVKGADTAKFLAEKVKKSSGATTTAPVGGARYAAEVV
DEWMD*
>11682.m01320|LOC_Os05g14300.1|genepair299-2
MAAAARLPAVGLGRLPEDARRRGVLLVVVLGFLPRRRRSRVGRGAAAEAAALPGSIDLKL
KLHRLFASLLLLLTKDQFYIDPNQYGSSSNDQVGLSTENNSFQAPEPELQNSDCVEDEEE
DYYLYDEDDVCYDDDGDYEFDETDFNQQLADKFDGLDLPPGVEATVPWLQKKDIADGPST
FKSMAELDADITKKYEFFKQFDVVENFSDHHYADKPVGKTGKDWTKRIQYDWKLLEKDLP
ASIYVRVSENRMDLLRAVIIGPQGTPYHDGLFFFDAQFTSTYPSTPPVVYYHSGGLRLNP
NLYACGKVCLSLLGTWSGSGCEKWNSAHSTMLQVLVSIQALVLNENPYFNEPGYETYANS
ATGQKSAMDYNDTTFQYSCRTMLYSLRRSPQHFDALVAGHFRHRGHAILAACKYYMEGHK
VGSVVPNEDEEDAKQQDDTDAGGSGSSSGAKPQPEKPDLCKGRAASFKTNMAVLFEELLM
EFNVKGADTKKFCDEKLKKNQQAAAAAR*
>11667.m01452|LOC_Os01g14650.1|genepair300-1
MAAARMLVLLASLCALLLTASAAKWTPAFATFYGGSDASGTMGGACGYGDLYGAGYGTRT
AALSTALFNGGASCGACFTIACDTRKTQWCKPGTSITVTATNFCPPNYALSGDAGGWCNP
PRRHFDMSQPAWETIAVYRAGIVPVNYRRVPCQRSGGIRFAVNGHSYFELVLVTNVGGSG
AVAQMWIKGSGTGWMAMSRNWGANWQSNARLDGQALSFRVQADDGRVVTAADVAPAGWSF
GATYTSSAQFY*
>11682.m01854|LOC_Os05g19570.1|genepair300-2
MEKQPAMLLVLVTLCAFACKRSVAQSAFATFYGGKDGSGTMGGACGYGNLYNAGYGLYNA
ALSSALFNDGAMCGACYTITCDTSQTKWCKPGGNSITITATNLCPPNWALPSNSGGWCNP
PRQHFDMSQPAWENIAVYQAGIVPVNYKRVPCQRSGGIRFAISGHDYFELVTVTNVGGSG
VVAQMSIKGSNTGWMAMSRNWGANWQSNAYLAGQSLSFIVQLDDGRKVTAWNVAPSNWFF
GATYSTSWVQF*
>11667.m01453|LOC_Os01g14660.1|genepair301-1
MEKKLLVVLFLSLCCASRLRGEAAQQWTSATATFYGGSDASGTMGGSCGYGNMYSAGYGT
NTTALSSALYGDGASCGACYLVTCDASATRWCKNGTSVTVTATNYCPPNYSESGDAGGWC
NPPRRHFDMSQPAWEAIAVYSSGIVPVRYARTPCRRVGGIRFGIAGHDYYELVLVTNVAG
SGAVAAAWVKGSGTEWLSMSRNWGENWQSNAYLTGQALSFRVQADDGGVVTAYDVAPANW
QFGSTYQSDVNFSY*
>11682.m01857|LOC_Os05g19600.1|genepair301-2
MAMPVVQVLLLCALAYQAVDAQWTPATATFYGGSDGAGTMGGACGYGNLYNAGYGLNNAA
LSSALFNDGAMCGACYTIACDTSQSTWCKPGTSITITATNLCPPNYAKKSDAGGWCNPPR
KHFDMSQPAWTSIAIYQAGIVPVNFKRVPCQKSGGIRFTISGRDYFELVTVFNVGGSGVV
AQVSIKGSKTDWMAMSRNWGQNWQSNAYLNTQSLSFKVKLDDAREVTVWNIAPSNWNFGT
TYTSNINF*
>11667.m01454|LOC_Os01g14670.1|genepair302-1
MAGAAGSSWSSLLLAAVAVALAVAAAPSLAGDPDYLQDICVADLNSEVKVNGFPCKANAT
ADDFFSGVLASPGAAANTTTGAVVTGANVEKVPGLNTLGVSLARIDYAPGGLNPPHTHPR
ATEVVFVLYGELDVGFVTTANKLLSRTISQGDVFVFPRGLVHFQRNTGDKPAAVVSAFNS
QLPGTQSIAATLFAASPAVPDAVLAKAFQIDDEEVDKIKAKFAPKKT*
>11682.m01866|LOC_Os05g19670.1|genepair302-2
MARPSLPCAVVAVLLLALLPTPSTAGDPDLLQDICVADLTSAVKVNGFACKAAVTEDDFY
FKGLAAAGNTNNTYGSVVTGANVEKLPGLNTLGVSMSRIDYAPGGLNPPHTHPRATEMVF
VLQGTLDVGFITTANKLYTKTISAGDVFVFPRGLLHFQKNNGDTPAAVISAFNSQLPGTQ
SLAMTLFAASPEVPDGVLTKAFQVGTKEVEKIKSRLAPKKR*
>11667.m01475|LOC_Os01g14840.1|genepair303-1
MRRALDWTPPLPASLLTTTPRRLLEPSSTFPLPLLRPSPPPPPPISPPPPPPPAAAELPA
ADMDDACAVCAEPLEWVAYGACAHREVCSTCVARLRFVLRDLRCCLCITPCPAVFVTKAM
GDRTKVIPDFSALRGAGGEGKAGEYWHHEATQTWFDDADQYRMISAMCRLSCSVCDSNKE
EEEERTGKAAKAKRKSKIRSVDQLKGHLLDRHGLYMCDLCLEGRKVFICEQKLYTMSQLN
QHIKSGDSEVDGSEVERRGFGGHPMCEFCKSPFYGDNELYTHMTREHFSCHICQRQHSGQ
YDYFRNYDDLEMHFQRDHFLCEDKGCLEKKFVVFESEAELKRHNGVEHGKHMPGAVDSSS
SSMQNGIAAVGHGLGGQSDSSRVPLQSLSISSSSGQSSETRQSFARNRVLQQACVPPLSR
QEVHDARVGSVLQEASFPSLPAQSRKAPAHSQSSRTAARIGDQQFRPLSVTSNRNVALAQ
QGTRTLPENTHVSGLAQYSKRTENMHQAVQPQFLKNNSLIPSGSTSRPVHVPSSAGNERQ
DTFSNSQVLSSVEDILAANKALVEKMRAALGMDQDMFNAFKEIAGEYRQGVINSSEYLSY
VKQFGLLHLVPEMARLLPDAQKQKELADAYYANLRLTSLQENGGGGTDNSKQGNQNKKGK
GGVPDAIGTSNAATDPLKDKLLNTAIKFQSNYMPQEGCCGVQRKEGRTTDGSSQGLPLKG
AWQSRGGQRLFMSKAKK*
>11682.m01897|LOC_Os05g19970.1|genepair303-2
MDDSCAVCADALEWVAYGACGHREVCSTCVVRLRFVMGDKHCCICKTECSSVFVTKAMGD
YTKVINDFNIFPPVATEGKVGEYWYHEDSQAFFDDAEHYKMIRAMCRLSCSVCDKAEDQA
GQAAQVRRKSKFRSIEQLKGHLFHQHRLYMCSLCLEGRKVFICEQKLYTRTQLNQHVKTG
DSEVDGSEVERSGFAGHPMCEFCRSSFYGDNELYTHMSREHYSCHICQRQHPGQYDYFRN
YDDLELHFRKDHFLCEDEACLAKKFVVFQSEAELKRHNAMEHGGRMSRAQRNAALQIPTS
FIYRRNEQDERRGRGRGRNSHRDGSDRDTPSMQNGSAIIGNGFPSRVDNVTGSVSVSSSS

GRGESGQSSGNGRVFEHLSFPPLQDQDIPDARMDSAPDETSFPSLSEQQTRYAHALSQSS
LAAKLGDELLFPPLPGSSSNRGSTSTQQGLQSLAKNTLASRLQQRSKGSVKVLHSARPRP
SENPESVPPVSSSPQMWPTPDQGLLHSSSSQLRIVRENGIMSSADSAWNPGGGASNRMKH
SVSTPNLMSGGSSVQALSTSNGGNKKQQPPQSSQTLPAADDVRAANKTLVERMRSALGMD
EDRYSAFKEIAGEYRQGIIGTSEYLSYVEQFGLSHLVPEMARLLPDPQKQKELADAYHTN
IRFRSLQENSDGLTITSKEGGRKKKGKGKSHDVTETSAAPAKDMKDSLADSFLDTVRKLQ
LNKTQEGEAEVLSRDGYRSSKGKAQLITGGSSSSTPCLDGDHGAISMASCAKDDVGKGGG
SSNSNINKQSKKTSKFLRARLGDNSLATLDLSRPTMSPERPERESQGPQVGLPMRGVWKN
GGGQKLFTGNGRK*
>11667.m01483|LOC_Os01g14900.1|genepair304-1
MPTNLSSSYSSLFEPLLALSSLLLRRLSAAVRHHRGRNTPPASSDVPPPPPPSSSPLELD
RLYGRTVVVDVDAWLLLPAVDAFTFFMVVAVEAGGFLRGLLLLLVYPLLCLLLGDGDGGG
GARARAMATVALVGLEEREVARVGRAVMPRFLMAAAAAEGAEAVRAARRSVAVSATLPRV
MVEAFLREHVGVDAVVGPELRSVAGVVAGIMDDADAARVAARRLRALLGDEMDQGEADGA
GAAVGLVGEGRSGGTVHYLFSRYYCKETFTATEADKRRWRPLPPGGECGGVKPLVFHDGR
LAFPPTPSAALAMYAYLPFGVALAVSRIIALSLLPYGRATFLVGALTGVHYRLVGAGHDA
AAGGGGGRLYVCNHRTLLDPIVVAAALGKPVTAVTYSLSRVSEMIAPIRTARLTRDREED
RRSMAALLARGDLVVCPEGTTCREGYLLRFSPLFAELGADVNPVALDARVDMFHGTSTTP
AAKWMDPFYFMMNPKPSYRVEFLPRAAPAPAAEDGGDSIRVANRVQRQIGEALGFELTGM
TRKDKYMMLAGNEGVVAAAAAAAATIKASR*
>11682.m01912|LOC_Os05g20100.1|genepair304-2
MAKKPCEFPTAVLSFRRFLQRRFLGGRHHHRPTSTTATGAAIPPADKLHNQTVMIDLESW
LLRSPMSTFPYFMIVAIEAGSFLRGLILLLIYPLLWLLLSHDMLLKAMVMVSFFGLPEKE
VVRIGKAVLPKFFLEGMAMEGLEVVRNAKKVVVFSPLFPRVMVEGFLKEYIGVNAVIGRE
VIAVAGRYVGLLVDHIDMDDGGFVDEVMEETKRGKGDGAVGLAGVGSKMHHLFSRYCKET
YVVCDADKKEWQPVPREKYPKPLIFHDGRLAFKLTPCAAVAMYTYLPWGIFLAVFRSLAF
GLLPYRVSVPLAAFTGMRSRLIAGPSPDATRRNSGTAGGRLYVCNHRTLLDPITVAAVLN
KPITAVTYSVSPVSELIAPIRTARLTRDRDEDRRRMEALLARGDLVVCPEGTTCREPYLL
RFSPLFAELTGEVTPVALETRIDMFYGTSTKPAAKWLDPFYFMLNSRPEYHVEFLQPVST
APVDGEAGGHGHSINAANRVQRVLGEALAFELTEQTRKDKYEMLAGNKGNVKGEAKM*
>11667.m01486|LOC_Os01g14930.1|genepair305-1
MARIRRASSGRGLLEDARRKTFLNFLAVVFTLELITRINLIAQTSALNVVAPAISPSQSW
RPVRSMLSKAKVDISISVSEQRRKKLYSSPATLSVHPPMSAPSYSSISGDSDLSFYSSDM
SDNLVQHNRRSEAEISTHVDAAPPDAASNTSAAPSGLVQPPVSPHNACCSHNMVQKRGSQ
DCHCVYPVRVELFLRNVSLTSNWSDEFLGELASQLSLRVTQFEIVNFYVVGASGLNITMY
IAPHTGISFSADQVTAMNYSLSQHTVQINPVLVGDYNLLNLTWFRPLVLAPAPTFTISPK
PSPSQASTVPRHSADTSNEKHMSLITIICIFIGALIAVLVIAMFICFCKLRKGKRKVPPV
ETPKQRTPDAVSAVDSLPRPTSTRFLAYDELKEATNNFDPSSMLGEGGFGRVFKGVLTDG
TAVAIKKLTSGGHQGDKEFLVEVEMLSRLHHRNLVKLIGYYSNRESSQNLLCYELVPNGS
LEAWLHGTLGASRPLDWDTRMRIALDAARGLAYLHEDSQPCVIHRDFKASNILLEDDFHA
KVSDFGLAKQAPEGCTNYLSTRVMGTFGYVAPEYAMTGHLLVKSDVYSYGVVLLELLTGR
RPVDMSQPSGQENLVTWARPILRDKDTLEELADPKLGGQYPKDDFVRVCTIAAACVSPEA
SQRPTMGEVVQSLKMVQRSEFQESIPTPPARPNVRQSSTTYESDGTSSMFSSGPFSGLSP
FETENISRTAFSEDLHEGRASESLTTHLKAGGASDLTSFIGRVRCSPAVALALVVAAAAA
AAATAKLYSPADRILVNCGSTTDGLDAEGRRWVADATNDTWLTDSGKSSIMAAADELETM
LPSSIPYMTARVFTMDTVYNFTVNPRDRHWIRLHFYPSSYNGLEPQDFRFSVFTTTGYTL
LHNFSVYFTTKALTQAYLIREYSLPRVPEGHFGVTFSPSPMMNVTYAFVNGIEVISMPDM
FNNPATMVGFADQTADVSAAAFQTMYRLNVGGAYIPPSNDSGLTRPWYDDTPFVQGPLRG
LVYNAGPHFHIKYPSDAAEYAAPPEVYLGGRSMGRDQRLNQNSNLTWSLHVECNFTYVVR
LHFCELQLIHGNQRVFDIYINNRTAQTDVDVLEMATERGVPVYKDYAVRLSNDTADEHLW
VAVHPSVMLRPQFYDAILNGLEVFKVNNTGGSLASPDPVPYKLLAEKELGWGGPPEFSTD
NPANMASVMGGTAGGAAAAGIVAAICVVVYSNKRSKKLGGGGADSHTSAWLPLYHSHTSG
KSSGHITANIAGMCRHFSFAEIKAATKNFSNDLAIGVGGFGVVYRGVVDGDVKVAVKRSN
PSSEQGITEFQTEVEMLSKLRHRHLVSLIGFCEEDGEMVLVYDYMEHGTLREHLYHNGGK
PTLSWRHRLDICIGAARGLHYLHTGAKYTIIHRDVKTTNILVDDNWVAKVSDFGLSKSGP
TTLNQSHVSTVVKGSFGYLDPEYYRRQQLTDKSDVYSFGVVLFEVLMARPALDPALPRDQ
VSLADYALACKRGGALPDVVDPAIRDQIAPECLAKFADTAEKCLSENGTERPTMGDVLWN
LESAMHFQDAFDAAAGRPVPALDAAAGSSSHLDDGSTASINTLATSSTSHPHEPCVDVVL
EPDDVVAERATFSQLVQPTGR*
>11682.m01917|LOC_Os05g20150.1|genepair305-2
MASRRHVLIAALIMVGVLEFANADKYKPTESILVNCGSDKEGQDIDGRKWLSDKDSKWLI
DGEKSSIMANADFQDPSLPSPVPYMTARVFTKETMYNFSVGEERHWVRLHFYPASYHDLP
AENFFFSVSTSTGITLLKNFSVYITAKALSQAYIIREFTLPPSTTGSLSLIFTPTAMNNA
SYAFVNGIEIISMPNIFSQAAASVDIAGNEVSTTDSSLQTIYRLNVGGSYVAPTNDSGLS
RDWYDDTPYIYGAAVGVTYQANDTVQIKYPKNDPDAEYAAPASVYLTSRSMGPDPKVNKN
YKLTWVFEVDGNFTYIVRLHFCELLLSKPNQRVFDILINNKTAQSGADVIGWGGQFVPVY
KDYATIMPGGAGDKVLWVQLMPNVGSGSEFFDSLLNGLEIFKMSDSSGNLAGPNPDPSKL
LEEAESSAQGKFKSKPSNLKATVIGGAAGGAAAFGIVAAICIVVYQSKKRKVLNNSASHS
SGWLPVYGGNSHTSTSKSSGGRSAALINPNITAMCRHFSFGEIKSATKNFDESLVIGVGG
FGKVYRGVVDGDTKVAIKRSNPSSEQGVLEFQTEIEMLSKLRHKHLVSLIGCCEDEGEMI
LVYDYMAHGTLREHLYKGGKPALSWKQRLEITIGAARGLHYLHTGAKYTIIHRDVKTTNI
LVDEKWVAKVSDFGLSKTGPTAMNQTHVSTMVKGSFGYLDPEYFRRQQLTEKSDVYSFGV
VLFEVLCARPALNPSLPREQVSLADHAMSCQRKGTLHDIIDPLLNGKIAPDCLKKFAETA
EKCLADHGVDRPSMGDVLWNLEFALQMQETFENGGKTEGADSTSDSTTTSVADSMAANAA

ALSLISEDMDEEDIANSVVFSQLVRPTGR*
>11667.m05661|LOC_Os01g57150.1|genepair306-1
MGCCFSKKRRKQLASAAVFSRRCKPPAEARDPPPPPPLPEEEKVKEVLSETPSAKVRAEA
KPVANVAVLEEPEAEKQAPKPSADADVTVSDLGSCMSLATDDRSEAASESSVATSSVTGP
ERSPGKPARRKRPVSVSGELAHAISRRDRAAAAAYGVRSRSARASASPPPRREQRDRSVR
RSPSPAAKRTPEQRRAASPAPSLQRKPPVPVRPSPRRVQEAPPSPLEPPPPPPPQPEEDA
MTADGEPSIADAASAGGDGEGKESLDNPLVSLECFIFL*
>11682.m04069|LOC_Os05g42340.1|genepair306-2
MGCCVSKKSDELLPGSAAAAAGEEVEAPRRRVDQARDPPPTEEEKVKEVLSETPAPSARA
RPRQRRVAGAVAPSVERGGGGGEKVARAKEGGGGGGRVRARRAVGAERATSEKSEAASES
SVATTATGPERSPGKPARKRAVVSGELGRARRDRGPAAAVHGAGRPGGGRASPSPPPPPR
REPGERPTRRSPSPATKRPPDQRRTAASAGAAAGAASGPQRKPPVPPRPCGRASPRRAQE
TNSPASSTTTTQSRGPPPHCSSPPPPQDAGASAAGAGEEVAGGRGEGKESLDNPSVAMEC
FIFL*
>11667.m05669|LOC_Os01g57220.1|genepair307-1
MAGRYDRNPFDEDDVNPFAGGSVPPASNSRMPPLPHEPGFYNDRGATVDIPLDSTKDMKK
KEKELQAKEAELNKRESELRRREEAASRAGIVIEEKNWPPFFPIIHHDISNEIPIHLQRM
QYLAFSSLLGLAACLFWNIIATTAAWIKGAGVMIWLLAIIYFISGVPGAYVLWYRPLYNA
MRTESALKFGWFFLFYLIHILFCIWSAVAPPFPFKGKSLAGILPAIDVIGNNAIVGIFYF
IGFGLFCLESLLSVVVIQQVYMYFRGSGKAAEMKREAARGAMRSAF*
>11682.m04068|LOC_Os05g42330.1|genepair307-2
MAGKHGRNGFEDDDVNPFAGGSVPPANNSRLPPLSHEPADFYNVDIPLDSSKDLKKKEKE
LQAMEAELNKRERELKRKEEAAAQAGIVIEDKNWPPFFPLIHHNISNEIPIHLQRMQYLA
FSSFLGLAACLFWNIIATTTAWVKGEGVIIWLLAIIYFISGVPGAYVLWYRPLYNAMRTE
SALKFGWFFLFYLIHIIFCVWAAVAPPFPFKGKSLAGILPAIDVIGRSAIVGIFYFVGFG
LFCLESLLSIGVIQIMTIDCSLNMQQVYMYFRGSGKAAEMKREAARGALSSAF*
>11667.m05672|LOC_Os01g57240.1|genepair308-1
MAAAANGGGAALFSEEELRDVSGVRRCEDFVEVTCGCTSHRYGDAVGRLRVYASGDLEVS
CECTPGCREDKLTPSAFEKHSGRETAGKWRNTVWVMVQGEKVPLSKTALLKYYSLSHKSA
NGSNKGRNGRLSHRDEFIHCTECGKERRFRLRSKEECRIYHDALAKPNWTCADLTTDRVT
CGDEEERASRKVLRGCSRSTSCTGCVKCVCFGCEICRFTDCGCQTCVDFYHNSKE*
>11682.m04064|LOC_Os05g42290.1|genepair308-2
MAVENGAALFGEEELRDVSGVRVGDRFVEVTCGCTSARYGDAVGRLRLFASGELQVSCDC
TPGCDQDKLSPAAFEKHSGRETAGRWQNTVWVMVKGDKVALSKTCLLRYYHKKLKSSSAN
GGGRRPPCHRDEFVRCAGCGKERRFRLRTKEECRLYHDAMARHDWTCKDMPPAGRCRVRV
SCEEEEERASRKASRGCTRAATCKGCVRTWVLGRQWHSMNVEAVASALCTFTCAADRDGF
DVRTVVCSVQTVQTYRGSLQDIHVSIFSYMEEIK*
>11667.m05684|LOC_Os01g57360.1|genepair309-1
MNGSSGSQGHNVNGGQKKVQHASPLTLNNGSKHRPLTPMRRCRGVACVVIILSTAFTLIV
FIAPITTFLVRLVSVHYSRKATSVLFGMWLSLWPFLFEKINKTNVVFSGESVLPKKRVLL
FANHRTEVDWMYLWDLALRKGYLGYIKYILKSSLMKLPVFSWAFHIFEFIPVERKWEIDE
AIIQNKLSAFKDPRDPLWLAVFPEGTDYTEKKCIKSQEYASEHGLPILKNVLLPKTKGFL
CCLQELKSSLDAVYDVTIAYKHRLPDFLDIIYGTDPSEVHIHIRTVKLCDIPTSEDEVTD
WMIERFRQKDQLLSDFFMQGHFPDEGTEGDVSTPECLANFIAIVSSTGFFLYLSLFSSVW
FKVYVLLSCAYLTFVTYFSIQPPQLICSSEGGTHAKKVL*
>11682.m04062|LOC_Os05g42270.1|genepair309-2
MMNGSNGSQGYHVNGKQVQSVVPSIQNNGPRHRPLTLMRRCRGMLCLVIMPLTAFMMMVY
LSPVTTFLIRLFSVHYSRKSTCFLFGMWLAMWPFLFEKINKTKFVFSGETVPPKERVLLF
ANHRTEVDWMYLWDLALRKGRLQCIKYILKKSLMKLPIFNWAFHIIEFIPVERKWEVDEP
LIRSRLSELKNPKDPLWLAVFPEGTDYTEKKCIKSQEYAKEHGLPILNNVLLPKTKGFHC
CLQELRDSMDSVCDITIAYKHRPPTFMDNVYGIDPSEVHIHVKIIQVSDIPTSEDEVADW
LIERFKLKNKLLSDFSALGHFPNEGTEDDLSTLKCIANFVAVISTTTVLTYLTLFSSVWF
KIFVAFSSAFLTFATLYSIHLPQLICSPEAGTHAKKS*
>11667.m05685|LOC_Os01g57370.1|genepair310-1
MASSSAAAASSAHGVGVVQRLWLEEQERKPPPKRGGGKRRWAWAPLEPRRAGWWAREWDR
AYLLACAAGLMVDPLFLYAVSVSGPLMCVFLDGWFAAAVTVLRCTVDAMHAWNLLMRLRA
AVRPPEEDDGADEEVAAERGAGGNGGGPAPAQVARPVSRKGLMLDMFVILPVMQVIVWVA
APAMIRAGSTTAVMTVLLVSFLFEYLPKIYHAVRLLRRMQNTYVFGTIWWGIALNLMAYF
VAAHAVGACWYLLGAQRATKCLKEQCAQGGSGCAPGALACAAPLYYGGAVGGVGADRLAW
ALDASARGTCLDSGDNYQYGAYKWTVMLVANPSRLEKILLPIFWGLMTLSTFGNLASTTE
WLEIVFNIITITGGLILVTMLIGNIKVFLNAATSKKQAMQTRLRGVEWWMKRKKLPQSFR
HRVRQHERQRWAATRGVDECRIVRDLPEGLRRDIKYHLCLDLVRQVPLFQHMDDLVLENI
CDRVKSLVFPKGEIIVREGDPVQRMLFIVRGHLQSSQVLRTGATSCCTLGPGNFSGDELL
SWCMRRPFLERLPASSSTLVTMESTEAFGLEAADVKYVTQHFRYTFTNDRVRRSARYYSH
GWRTWAAVAVQLAWRRYKHRKTLASLSFIRPRRPLSRCSSLGEEKLRLYTAILTSPKPNP
NQDDLV*
>11682.m04060|LOC_Os05g42250.1|genepair310-2
MSGELSTRASTSSSSSSPPGDARGPEHGGTPRGEVSSKRRLVLRRRQRWRRLGGGAAASW
AAADPRARWVREWNRAYLLACAAGLMVDPLFLYAVSLSGPLMCVFLDGWLAAAVTALRCM
VDAMHAWNIVTQLRVSRAGRERACAAGPDEEQPEAEAAAPAPAADADAAASNKLRDHGRY
RKWLVLDFFVILPVMQVVVWVAAPAMIRAGSTTAVMTVMLVAFMLEYLPKIYHSVVFLRR
MQNQSGHIFGTIWWGIALNLIAYFVAAHAVGACWYLLGVQRATKCLKEQCLLAGLPACAS
STAAVACVDPLYYGAAVASVGGDRLAWGGNATARNVCLSSGDNYQYGAYKWTVMLVSNPS
RLEKMLLPIFWGLMTLSTFGNLESTTEWVEIVFNIMTITGGLILVTMLIGNIKVFLNATT

SKKQAMQTRLRGLEWWMEHKGVPHGFRQRVRQFERQRWAATRGVDECQIVRDLPEGLRRD
IKYHLCLDLVRQVPLFHHMDDLVLENICDRVKSLIFPKGEIIVREGDPVQRMLFIVRGHL
QCSQVMRNGATSWCTLGPGNFSGDELLSWCMRRPFMERLPASSSTLVTAESTEAFGLEAG
DVKYVTQHFRYTFTSDKVRRSARYYSHGWRTWAAVAVQLAWRRYKHRKTLASLSFIRPRR
PLSRCSSLGEEKLRLYTAILTSPKPNQDDDF*
>11667.m05694|LOC_Os01g57450.1|genepair311-1
MAANPSSPSAGGGGDVSASSPTLPPVRLAAAQAASAAAIHPTSPRYFFSSLAGTNASPHR
RIAIAVDLSDESAYAVRWAVQNYLRPGDAVVLLHVRPTSVLYGADWGSIPVSVSDDADGE
VAPAASAEELQKKREEDFDAFTSTKAEDLAQPLVDAQIPFKIHVVKDHDMKERLCLEAER
LGLSAMIMGSRGFGASRKGGKGRLGSVSDYCVHHCVCPVVVVRYPDDAAGADGEAAGPTD
ELHTVPEDEPVYHDAPDVQKEN*
>11682.m04058|LOC_Os05g42230.1|genepair311-2
MATHPASPVAGGEKATPPSTPPPVRLAGGAAAAIQPNSPRFFFSSLAAASASASSPHRRI
AIAVDLSDESAFAVKWSVQNYLRPGDAVVLLHVRPTSVLYGADWGSIPVSVDDDDSAPDA
AQHANAHAATRDEPEEAKKKREEDFDAFTSTKAQDLAQPLVAAQIPFKIHIVKDHDMKER
LCLEAERLGLSAMIMGSRGFGASRRAGKGRLGSVSDYCVHHCVCPVVVVRYPDDGAAAGG
GEAVGDELRTVPEDEPVYHEAPEGQKEN*
>11667.m05706|LOC_Os01g57560.1|genepair312-1
MASSSPLCCYLLLFVVVVVLTGSCRARDTVVPGRPLAANETLVSGGDANFVLGFFTPPGA
NSTYVGVWYNKVSVRTVVWVANREDPLPGDVADNPDATLSVSPTGTLAIVAGNSTVVWSV
TPAAKLASPTARIMDSGNLVIADGAGGGVAWQGFDYPTDTLLPEMRLGVDYVKGRNRTLT
AWKSPSDPSPGPVVMAMDTSGDPQVFIWNGAEKVWRSGPWDGVQFTGVPDTVTYSGFTFS
FINNAKEVTYSFQVHNVSIISRLGLNSTGSYGLLQRSTWVEAAGTWNLYWYAPKDQCDEV
SPCGANGVCDTNNLPVCSCLRGFTPKSPEAWALRDGRAGCVRSTPLDCQNGTDGFVAVEH
AKVPDTERSVVDLGLSLEQCRKACLMNCSCTAYASANVSGGGRGHGAGTGCVMWTTGLTD
LRVYPEFGQDLFVRLAAADLGLTSKSNKARVIIAIVVSISSVTFLSVLAGFLVWTRKKKR
ARKTGSSKWSGGSRSTGRRYEGSSHHDDDLELPIFDLGTIAAATDGFSINNKLGEGGFGP
VYKGKLEDGQEIAVKTLSKTSVQGLDEFKNEVMLIAKLQHRNLVRLLGFSISGQERILVY
EYMANKSLDYFLFARYRIIEGITRGLLYLHQDSRYRIIHRDLKASNVLLDKEMTPKISDF
GMARMFGSEETEINTRKVVGTYGYMSPEYAMDGVFSVKSDVFSFGVLLLEIISGRRNRGV
YSYSNHLNLLGHAWSLWNEGKSLELADETMNGSFDSDEVLKCIRVGLLCVQENPDDRPLM
SQVLLMLATTDATTLPTPKQPGFAARRILMETDTSSSKPDCSIFDSATVTILEGR*
>11682.m04056|LOC_Os05g42210.1|genepair312-2
MAIHQLSFVLLLMLLAPATSRARDSIAPGEPLAGHDTLVSAGAGDGGGFALGFFTPPGSN
DTYVGVWYARVSPRTVVWVANRADPVPGPVDGNAGATLSVSRACELAVADANSTVVWSVT
PATTGPCTARIRDDGNLVVTDERGRVAWQGFDHPTDTLLPGMRIGVDFAAGNNMTLTAWK
SPSDPSPSSVVVAMDTSGDPEVFLWNGPNKVWRSGPWDGMQFTGVPDTITYKNFSFSFVN
SAREVTYSFQVPDASIMSRLVLNSSGGGLVQRWTWVEAAGAWNLYWYAPKDQCDAVSPCG
ANGVCDTNSLPVCSCLRGFAPRSPAAWALRDGRDGCARETPLGCANGTDGFAVVRHAKAP
DTTAATVDYDAGLQLCRRRCLGNCSCTAYANANLSAPPGRRGCVMWTGELEDLRVYPAFG
QDLYVRLAAADLDSTSKSKKKTHIIIAVVVSICALAIILALTGMYIWRTKKTKARRQGPS
NWSGGLHSRELHSEGNSHGDDLDLPLFDLETIASATNGFSADNKLGEGGFGPVYKGTLED
GQEIAVKTLSKTSVQGLDEFRNEVMLIAKLQHRNLVQLIGYSVCGQEKMLLYEFMENKSL
DCFLFGTNRNLPMFSDKSKSKLLDWQTRYHIIEGIARGLLYLHQDSRYRIIHRDLKTSNI
LLDKEMTPKISDFGMARMFGSDDTEINTVRVVGTYGYMAPEYAMDGVFSVKSDVFSFGVI
VLEIISGKRNRGVYSYSSHLNLLARAWSSWSEGNSLDLVDKTLNGSFNQEEVLKCLKVGL
LCVQENPDDRPLMSQVLLMLASADATSLPDPRKPGFVARRAATEDTSSSRPDCSFVDSMT
ITMIEGR*
>11667.m05707|LOC_Os01g57570.1|genepair313-1
MAVKVYVVYYSMYGHVAKLAEEIKKGASSIEGVEAKIWQVPETLHEEVLGKMGAPPKPDV
PTITPQELTEADGILFGFPTRFGMMAAQMKAFFDATGGLWSEQSLAGKPAGIFFSTGTQG
GGQETTPLTAITQLTHHGMVFVPVGYTFGAKMFNMGEVQGGSPYGAGTFAADGSRWPTEM
ELEHAFHQGKYFAGIAKKLKGSA*
>11682.m04054|LOC_Os05g42190.1|genepair313-2
MAVKVYVVYYSMYGHVAKLAEEIKKGASSVEGVEVKIWQVPETLSEEVLGKMGAPPRSDA
PVIAPQELAEADGVLFGFPTRFGMMAAQMKAFFDATGGLWREQSLAGKPAGVFFSTGTQG
GGQETTPLTAVTQLAHHGMVFVPVGYTFGAKMFDMAAVHGGSPYGAGTFAGDGSRWPSEA
ELEHAFHQGKYFAGIAKKLKGASSA*
>11667.m05709|LOC_Os01g57580.1|genepair314-1
MADEWWSASQRSHGTSACSAAPSPLTADRVSCGWTSPAAAAAAESTSSITFQDPSRSSAA
HHQPLSDAASSLGDPHMVDWTQAFLSGRSDASFQAVLQDDMAASTRPFRAQPTAADEAVM
TNPFRDMGVGQGLLLDQASAPLHGLSFDAGEPAVAPATHSITTSFGDYQHSASYDAAAAV
MQFSQTPRAPSLPAAAQMQFLSGSYQLPFGGAPPLPSQLLLQAMQPKPSCSSNANTLLAK
VRKEKLGDRITALQQLVSPFGKTDTASVLHEAIEYIKFLHEQVASLSSPYLKNGNPLQHF
QQKGSESTKDAEQPKPDLRSRGLCLVPVASTYTVASETVPEFWHPTFGGTFR*
>11682.m04053|LOC_Os05g42180.1|genepair314-2
MADEHEWWNSPCSARTGDDEAACSTADADESAVGSTPMSFGHGGQPASLSDAAASSSSSS
FLLAGQHMDYWTQDFMGGRAAAAATASFDTLLQLQLQGGDAASRRLLLGDHAAAPPRHLV
VPGAPYGGGGGDDTAAPPRGLSPTPYEAADNLQQQQSFPGGHHVVSNTDRLHDHHQDAGS
PSPATRSSPGSPAAAKKPRIEAPSPMPTFKVRKEKLGDRITALQQLVSPFGKTDTASVLH
EAIEYIKFLHDQVASLSSPYLRCGRPVQLQHQQGSHKVNGNCEGKQLDLRSRGLCLVPVA
STYTVASETATEFWHPTFGGTFR*
>11667.m05712|LOC_Os01g57610.1|genepair315-1
MPEAPTAKTAPAYGYAPGAHAEALEFIEHVTANAGQVQRRVLGEILAQNAPAEYLRRYGI

PGSPDVVDAFRRLVPLVTYEGLQPDILRIANGDTSPIFSGKPISEFLTSSGTSGGERKLM
PTIADEMNRRSLLYSLLMPVMSQSVSGLDKGKAMYLLFVKAESRTPGGLAARPVLTSYYR
SRQFLDRPRDPYTSYTSPDEAILCVDSYQSMYAQLLCGLVHRADVLRVGAVFASGFLRAI
HFLEKHWARLCHDIRTGELDPEITDRVVRDAVGRVLRADPALADAIEDECARASWEGIIR
RLWPRTKYIDVIVTGTMSQYIPTLEFYGGGLPLTCTMYASSECYFGLNLNPMCKPSDVAY
TLIPTMCYYEFLPVNCNNATAEASHRDLVDLVDVKLGHEYELVVTTYSGLYRYRVGDVLR
VAGFKNKAPMFSFVRRQNVALSVDSDKTDETELHAAVSGAVQHLAPFGASLVEYTSYADA
ATIPGHYVLFWELRAGSTAVPASVFEECCLSVEEALNSVYRQGRACDRSIGPLEIRVVAE
GTFDKLMDYAISRGASINQYKAPRCVRPGPVVELLDARVQGKYFSPKCPKWSPGNKQWNK
SKDLVGKGDA*
>11682.m04050|LOC_Os05g42150.1|genepair315-2
MPEAVAAAVSPAAAAATAMCAEHREKLEHIERVTRNAGQEQRRVLEEILAQNAQAEYLRR
LGVPGDAPGADEAFRRLAPLVTYEDILPDVLRIANGDTSPILSGKPVSEFLTSSGTSGGE
RKLMPTIEEEMERRSGLYSLLMPVMSRQVPGLDKGKAMYLYFVKSEWRTPGGLPARPVLT
SFYRSRYFLERPHDPYTVYTSPDEAVLCEDAYQSMYAQLICGLVHRADVLRVGAVFASGF
LRAIRFLEKHWPSLCRDIRAGELDGGVTDPAVRGAVGRVLRGADPALADAIEAECARPSW
QGIIRRVWPSTKYIDVIVTGAMAQYIPTLEFYGGGLPLACTMYASSECYFGLNLNPMCKP
SEVAYTLIPTMCYFEFLPVNSGANDVAAPEPDHRGLVDLVDVKLGHEYELVVTTYSGLYR
YRVGDVLRVAGFKNAAPMFAFVRRKNVALSIDSDKTDEAELHAAVTEAVQHLAPFGASLV
EYTSYADTATTIPGHYVLFWELRSPAGGTPVPASVFEDCCLAVEEGLNSVYRQCRAADRS
IGPLEIRVVADGTFDKLMDYALSRGASINQYKAPRCVRPGPVVELLDGRVQATYFSPKCP
KWCAGGNKQWISSGAAAKKTTTTCDSLAV*
>11667.m05713|LOC_Os01g57620.1|genepair316-1
MDGHVRRLLNRVSIALAAVATAALLQLFRHSSSSCFVGSPAYSSLSLAPFPRTSCDAASR
RVVDPNLRLAKLRSSPRWRRRSAALSTSVFPRLRRLRLLRRSSRVLCVAAGAGQAVDALH
VAGVGDATGVDLVDFPPLVRRADPHNLPFFDGVFDVVLSDEPMALTGALFPSRFVAEAER
TVRWGGAIALAIERHIDLSTVASLFKKSRVAAAWNATLDGSAATMSFE*
>11682.m04049|LOC_Os05g42140.1|genepair316-2
MDRRVQRLVSGVAAAAATVSLLYLISHASTSCFPGATTLPLARFPRTSCDAASRRVVPPG
RRLAKLRASARWRRRSVALASSSAFASLRGLRLLAGSSRALCLAAGAGHAVDALRAEGVG
DVTGIDFVDFPPLVRRADPHHLPFSDGAFDLIFSDDPAGFSGALFPSRFAAEAERAVRSG
GTIALAVDRHLDPSAVAVLFKRSRIVDQRDLTMDGSQVRMLIFQSNGTTLNSH*
>11667.m05724|LOC_Os01g57730.1|genepair317-1
MALLGVLVGAVIIVVATAAAVSGSGLPVPGYDGLAIGFYHETCPQAEDLVLAEMREIVQE
DRTLAPALLRFMLHDCFVRGCDASIMLKSREKIGERDANSSYSLRGYEQIERIKAKLEDE
CPMTVSCADIIVMAARDAVFLSNGPRYQVETGRRDGKVSCTIDADNDLPPPGSNIVDLKI
YFSVKNLGWKDLVVLSGSHTIGRAQCGSFARDRLYNYSGEGRQDPSLNTAYAPELRKACV
AGDPFDKTYVDMDPGSPYTFDLSYYRDVYRNRGLFVSDQALLNDKWTKQYVERMASADST
DEYFRDYAEAMTNMGRIEVLTGDNGEIRKVCGAYVD*
>11682.m04034|LOC_Os05g42000.1|genepair317-2
MSRELELLLFLLALRASGEVVAGSAEAAAAAAAWPALQVGFYHAKCPVAEDVVLGEMRMI
LEEDPTLAPSLLRMHYHDCFVQGCDGSIMLRSRSGKGERDATPNRSMRGYDAINRIKARL
ETVCPLTVSCADIIAMAARDAVYLSKGPWYDVETGRRDGDVSVAEYAENDLAPPDSNIVD
VKTFFSVKSLNAKDIAVLFGCHSIGTSHCGAFQKRLYNFTGRMDQDPSLDAGYAAKLKKL
CPPGHGHDHDHDGHGGAGGAAKVPMDPGSGFTFDLSYYRHVLATGGLFQSDGSLRDDPVT
RGYVEKLANASSSEEYFADFAAAMVKMGRTDVLTGDLGAVRPTCDSLVD*
>11667.m05745|LOC_Os01g57940.1|genepair318-1
MQTHRTDRTANNGFAKFGSWPPKSQLPPGKKNGSRPQVVARVAVEVSQQIRPAFTRGSAR
GCFPTLASIRAMDLLTSAMKRCCNGKTLLRGGRTCRCSSASAIGSSGVRGKEEASTSAAD
SEPDKKRWRKKRFWRKKKKKAKKDHYGDAAATEHGSERASCRRYENDAVADLVNDISSKS
DVCNVYAAEGILRITHQNIPSMVLTYRQLCNATDSFSPNNLLGEGGFGRVYRGHLEEINE
IVAVKQLDKDGFQGNREFLVEVLMLSLLHHPNLVKLLGYCTDMDQRILVYECMRNGSLED
HLLDLPPKAKPLPWQTRMKIAVGAAKGIEYLHEVANPPVIYRDLKTSNILLDEDFNSKLS
DFGLAKLGPVGDKSHVSTRVMGTYGYCAPEYAMTGKLTKTSDIYSFGVVLLEIITGRRAI
DTSRPTHEQVLVQWAAPLVKDKKRFVRLADPLLEEKFPLKGLYQALAIASMCLQEDASNR
PMISDVVAALSFLAEQKYHPQDGPDQAARKSRDRDCSNPPRKTDMVSEIKADDEIKHRLL
ELAGLSALLQSQVIGANYRYDENDKNDDITISQLYRKCSLPGVRQGEPDRMLPPRVPPPR
GFKKPRELTWVTGVVLAVLTASFGVTGYSLPWDQIGYWAVKIVTGVPDAIPIESWICHIR
IHDQRCLHIQSTRLAVLEPSMIGEPADPFATPLEILPEWKFKIEFGYYNYRWGDYRYRRG
DYHI*
>11682.m04029|LOC_Os05g41950.1|genepair318-2
MGLLSSASRSCCCCSWVRGGCASCCSWIRGFCGGGGGATTSAQDTAASDAKKRKKRKWVV
RGVFGKAAREAEEPLTLETMKKRKSAATSPELEKNKWGTKKNWKKKKGKTQPTGLASLVK
EISLENSTRNRAAAGEILRIGNHNIPSRVFTFRQLADATGSFSPENLLGEGGFGRVYKGF
IPDTKEVIAVKQLDKDGLQGNREFLVEVLMLSLLHHPNLVTLLGYSTECDQRILVYEYMP
LGSLQDHLLDLTPNSSPLSWHTRMKIAVGAARGMEYLHEIANPPVIYRDLKASNILLDGG
FNAKLSDFGLAKLGPVGDKSHVTTRVMGTYGYCAPEYAMTGKLTKMSDIYSFGVVLLEII
TGRRAIDTTKPTREQILVHWAAPLFRDKKKFVKMADPLLDMKFPLKGLYQALAISSMCLQ
EEASSRPLISDVVTALTFLADPNYDPPDDVEPLPIKAPNLDRESSQKEAEGGDNDSDEGG
EEQV*
>11667.m05766|LOC_Os01g58130.1|genepair319-1
MGNCGASQRAVESWADGDEWEDEAAASSSSEDDGHRERMEHVAEVTIRITKRQLHELMER
KGAGNGHGKISRRSTQQLLADIMNSGEVHHHDQHREAHWKPALQSIPEAVES*
>11682.m04025|LOC_Os05g41920.1|genepair319-2

MAAVCNSQFLLSTPCKNCNLLFRLKQKLQIRRLSHERPLRYSEDCKKKMGNCAAAPSDDG
ELGSPEREEMTGVEVRIRISKRQLQELLEMASMTAAGDEKVIAGIINAGEVVDHHQQRHW
QPTLQSIPEAGEP*
>11667.m05775|LOC_Os01g58220.2|genepair320-1
MSDLDVQLPSAFDPFAEANAEDSSVGAGSKDYVHVRIQQRNGRKSLTTVQGLKKEYSYNK
ILKDLKKEFCCNGTVVQDPELGQVIQLQGDQRKNVATFLVQVIYMIPTLIFIFVQIAFI*
>11682.m04022|LOC_Os05g41900.2|genepair320-2
MSDLDVQLPSAFDPFAEANAEDSGAGPGAKDYVHVRIQQRNGRKSLTTVQGLKKEYSYNK
ILKDLKKEFCCNGTVVQDPELGQVIQLQGDQRKNVATFLVQAGIAKKDNIKIHGF*
>11667.m05795|LOC_Os01g58400.1|genepair321-1
MPAQKRRLSSSPSSRPRDHVETNGMTGASKAAAGSGGGGGGGSGGGVPLPPRGGSAAAAA
AKRAADPQPQREGDSDAEFGGGVDGDSESSQSDGDMDEFIIVKLAEIRKEVQCPICLGII
RKTRTVMECLHRFCRDCIDKSMRLGNNECPACRTHCASRRSLRDDPNYDALIAALYPDID
KYEEEELAFSEEERSRNKKIQATIEETIRRQSEAVGKKRSTAKATATVFARKYRRNMRTR
GRGKTIAPDIAPTGSDNEDREEGNAIDTTKESSSADDRSSDLMPKRGRKRPASRASPART
IGSSDHVFEENDELIGGKESFTTSPLRGEMLAWGKNGTRSQTRHGSVGGSNGRMAKGGRV
AKLVDHLRTTDDMDKEFNLYLVLLPLDEQSMPNLDKPYISCRPTLSIRHLVQFIALQLSR
QVEELDIFMRIDHCNGSVTTQDCTTGVAKMRLSDGLERIREDKLLSELHPSFTSHHGDLE
LLYALKTQG*
>11682.m04010|LOC_Os05g41800.1|genepair321-2
MPAQKRPAPEAASAPAGGDGHVEGGGAGGGGGADEDAHRGGERSPKVMNGGGPEKEKERR
DADSDAEEEEEEAGGGGGGGGADEDRDSPSSESDGDMDEFILVKLMDIRKEVQCPICLGI
IRKTRTVMECLHRFCRDCIDKSMRLGNNECPACRTHCASRRSLRDDPNYDALILALYPDI
DKYEEEELAFSEEERTRNKKIQASIAETFRRQSEALVKKRSVAKATGSTITRRTRGNMRA
KRRGRTSSPDIVATDNEDEDRDENGNEGSKESSSVDDRSPDVRQKRVRRWPVPRSSPAKS
IGGIDSSFEDIDDLGSGRDIMSTSPLRGEMLAWGKNGTRSQTRHGNSGGSSGRMAKGGRV
TKLVEYLRNTDEFDNKFNLYLVLLPLNGQSMPKLEKPYLSCQPTFSVRHLCQFVALQLSR
HAKEVEIFIRKNPNNGCFASIDTSADEIKLNHDALERLEEEKSLSELYPSLASGHGDLAP
WTRYGALNNEQMVGCRKNREVIKLLLTGAGLTVCHAMLPLKLADWPSTLTQSRCQSNWPM
SLEVLSESLLCFGATSVTVDDIAAAGNLDEITITSIFAHGEDVGSCVSSAASSAGLEYNP
VYESSVGKQCDWVTVVQETYESTKVIDGLWVIPKWRTPPDPQAINIIINPGLAFGTGEHP
TTKLCLLLLRETVKGGERFLDYGTGTGVLGIAALKMGAALSNGIDIDPQAVTSACENMML
NGIDSNKMLVYLVPTNAQSACFPSNIDKSEENRPTGNLELKSSKGSYDIVAANILLNPLL
ELVEDIVGYAKSGGIVAVSGILSEQVPKVEEAYSRYLENISVSEIDGWACLRGNRRA*
>11667.m05797|LOC_Os01g58420.1|genepair322-1
MAPRAATVEKVAVAPPTGLGLGVGGGVGAGGPHYRGVRKRPWGRYAAEIRDPAKKSRVWL
GTYDTAEEAARAYDAAAREFRGAKAKTNFPFASQSMVGCGGSPSSNSTVDTGGGGVQTPM
RAMPLPPTLDLDLFHRAAAVTAVAGTGVRFPFRGYPVARPATHPYFFYEQAAAAAAAEAG
YRMMKLAPPVTVAAVAQSDSDSSSVVDLAPSPPAVTANKAAAFDLDLNRPPPVEN*
>11682.m04008|LOC_Os05g41780.1|genepair322-2
MAPRTSDKTMSPAAAATGLALGVGGVAGAAAVGTGQHFRGVRKRPWGRYAAEIRDPAKKS
RVWLGTFDTAEEAARAYDAAAREYRGAKAKTNFPYPNGAPAAGVNSGSSNSSTVESFGSD
VQAPMKAMPIPPSLELDLFHRAAAAAAAGAGGMRFPFEGYPVSHPYYFFGQAAAAAAASG
CRMLKIAPAPVTVAALAQSDSDSSSIVDLAPSPPAALAKKAIAFDLDLNCPPPMEV*
>11667.m05813|LOC_Os01g58580.1|genepair323-1
MGRKRALLVGINYPGTKAELKGCHNDVDRMHRCLVDRFGFDEDDIRVLLDRDSSGTQPTG
ANIRRALAQLVGDARPGDFLFFHYSGHGTRLPAETGQNDDTGYDECIVPSDMNLITDQDF
RELVQKVPNGCLFTIVSDSCHSGGLLDSAKEQIGNSTRQNQTQSREREEPSDSGSGFRSF
LKETVRDVFESEGIHLPRSRHSQSHYGGEDQYETYAQPTDGHTKNRSLPLSTLIEMLKEK
TGKDDIDVGSIRMTLFNIFGDDASPKVKKFMKVMLGKFQQGQSGEQGGLMGMVGSLAQEF
LKVKLEGNEEEAFKPAIEQEVHSVDEVYAGTKTWAPNNGILISGCQSNQTSADATTPQGS
SYGALSNAIQTILADKRGNVSNKDLVMKARSLLAKQGYTQQPGLYCSDDHVHVSFIC*
>11682.m03997|LOC_Os05g41670.1|genepair323-2
MGGRKRALLVGINYPGTKAELKGCHNDVARMRRALVDRFGFDEADIRVLADADRSAPQPT
GANIRRELARLVGDARPGDFLFFHYSGHGTRLPAETGQDDDTGYDECIVPSDMNLITDQD
FTELVQKVPDDCLFTIVSDSCHSGGLLDKTKEQIGHSTKQNQAQQIKREERSDSGTGGFR
SFLKETLKETVRDAFESRGVHIPHQSSRRNDDEDEEPHMGSSSHGGDRIKNRSLPLSTLI
EMLKEKTGKDDIDVGSIRMTLFSLFGDDASPKIKKFMKVMLTKLQEGQHGGVMGLVGALA
QEFMKAKLEGNQEADALEPAMKQEVHSVHEAYAGTTARVSNGVLISGCQTDQTSADATTP
KGVSYGALSNAIQTILSEKSGRVTNKELVLRARELLSKQGYTQQPGLYCSDKHTSVAFIC
*
>11667.m05816|LOC_Os01g58610.1|genepair324-1
MARPAARGIVAGAAASTVPLPRAGVASPCPTARSLGFAARGTDPRLAIHVSSRRRAASAS
AGSRLARAVATMAKKSVGDLAAADLEGKRVLLRADLNVPLDASQNITDDTRVIAAIPTIK
HLIGNGAKVILCSHLGRPKGITPKFSLAPLVPRLSELLGIQVQKADDVIGPEVEKSVSVL
PNGSVLLLENVRFYKEEEKNDPEFAKKLASLADLYELDYLVGAVSNPKRPFAAIVGGSKV
SSKIGVIESLLEKCDILLLGGGMIFTFYKAQGFPVGASLVEDDKLELATSLLAKAKEKGV
SLMLPTDVIVADKFAPEANCQAIAKKLAELSGKGVTTIIGGKDSVAAVEKVGVANVMSHI
STGGGASLELLEGKELPGVVALDEA*
>11682.m03991|LOC_Os05g41640.1|genepair324-2
MASAAAPTTSSLSLAARRAAAVGPAAASPLRRGGLAAGCQPARSLAFAAAADPRLATHVA
SRCRQAASSSSRGTRAVATMAKKSVGDLTAADLEGKRVFVRADLNVPLDDNQNITDDTRV
RAAIPTIQYLIKNGAKVILSSHLGRPKGVTPKFSLAPLVPRLSELLGIQVTKAEDVIGPD
VEKLVSELPNGSVLLLENVRFYKEEEKNDPEFAKKLASLADLYVNDAFGTAHRAHASTEG

VTKFLKPSVAGFLLQKELDYLVGAVSSPKRPFAAIVGGSKVSSKIGVIESLLEKCDILLL
GGGMIFTFYKAQGLSVGSSLVEEDKLELATSLLAKAKEKGVSLLLPSDVIIADKFAPDAN
SQVVPASAIPDGWMGLDIGPDSVASFSSTLETTQTVIWNGPMGVFEFDKFAVGTEAIAKK
LADLSGKGVTTIIGGGDSVAAVEKVGVADVMSHISTGGGASLELLEGKELPGVIALDEAV
TVARSKL*
>11667.m05828|LOC_Os01g58730.1|genepair325-1
MDAVLVTAAIFGLLLCGCSVSGVEGIGVNYGMIGNNLPSPDKVIALYRASNITDIRLFHP
DTTVLAALRGSGLGVVLGTLNEDLARLATDASFAASWVQSYVQPFAGAVRFRYINAGNEV
IPGDEAASVLPAMRNLQSALRAAGLGVPVTTVVATSVLGSSYPPSQGAFSEAALPTVAPI
VSFLASSGTPLLVNVYPYFAYSADPSSVRLDYALLSPSTSAAVTDGGVTYTNMFDAILDA
VYAALEKAGGQGLEVVVSETGWPSGGGGAGASVENAAAYSNNLVRHVGRGTPRRPGKAVE
TYIFAMFNENQKPEGVEQNFGLFHPDMSAVYHVDFSA*
>11682.m03988|LOC_Os05g41610.1|genepair325-2
MDSRLRVLALLILLPRFFTLLAISSVFFTSRVIGAEGAIGVNYGMLGNNLPSPAQVISMY
KAKNINYVRLFHPDTAVLAALRNSGIGVVLGTYNEDLARLASDPSFAASWVSSYVQPFAG
AVSFRYINAGNEVIPGDPAANVLPAMRNLDAALKAAGISGIPVTTAVATSVLGVSYPPSQ
GAFSEAASPYMAPIVAYLASRGAPLLVNVYPYFAYAADAERVQLGYALLSASQSASVTDG
GVTYTNMFDAIVDAAHAAVEKATGGQAVELVVSETGWPSGGGGVGATVENAAAYNNNLIR
HVSGGAGTPRRPGKPVETYLFAMFNENQKPEGVEQHFGLFQPDMTEVYHVDFAASS*
>11667.m05829|LOC_Os01g58740.1|genepair326-1
MGGAEDAPRAAAANGHGNGATVEEKLDELRRLLGKADGDPLRIVGVGAGAWGSVFCALMQ
DAYGHLRDKVQVRIWRRPGRAVDRATAEHLFEVINAREDVLRRLIRRCAYLKYVEGRLGD
RTLYADEILRDGFCLNMIDTPLCPLKVVTNLQEAVWDADIVINGLPSTDTREVFGEIGRY
WKERITAPIILSLAKGIEASLDPLPRIITPTQMISNATGVPLENILYLGGPNIASEIYNK
EYANARICGADKWRKPLAKFLRQPHFIVWDNSDLITHEVMGGLKNVYAIGAGMVAALTNE
SATSKSVYFALCTSEMIYITHLLEEEPEKLAGPLLADTYVTLLKGRNAWYGQKLAKGELT
LEMGDSIKGKGTIQGVSAVNAFYELLSQDSLSVMHPEANRSVAPVEMCPILKALYKILIK
RELPPDSILQAIRDETMYDPRERIEMAQGHSLYRPSLLGQPKGDAKA*
>11682.m03986|LOC_Os05g41590.2|genepair326-2
MVGSYAAGGGRGAAVAAVAEGKLDELRRRMGKADGDLLRIVGVGGGAWGSAFCALLQDAY
GRHRDKAQVRVWRRPGRAVDRATAEHLFEVINSREDVLRRLIRRCAYLKYVEARLGDRTL
YADEILRDGFCLNMVDTPLCPLKVVTNLQEAVWDADIVINGLPSTETREVFGEIGRYWKE
RIRPPVIISLAKGIEASIDPVPRIITPTQMISNATGVPLENILYLGGPNIASEIYNKEYA
NARICGADKWRKPLAKFLRQPHFIVWDNSDLITHEVMGGLKNIYAIGAGMVAALTNESAT
SKSVYFSLCTSEMIYITHLLAEDPEKLAGPLLADTYVTLLKGRNAWYGQKLAKGELTLEM
GDSIKGKGTIQGVSAVHAFYELLSQSSLSVTHPEVKKLVAPVELCPILKTLYKILIKREL
ATDSILQAIRDESMYDPRERIEMSQRQCLYRPSLLGLPKVDITQA*
>11667.m05831|LOC_Os01g58760.1|genepair327-1
MASGENNVEEELRKTRKPLGNREAVRKYREKKKAHAAFLEEEVKKLRTTNQQLLRRLQGH
ISLEAEVVRLRALLFDIRGKIDAEIGTFPFQKQCSFGSVTCTDHSPCFNTSTEVAVREES
SRPTIVDCGIDGTGIISHELDIPKMVNSVDVIPSFVNSASLTE*
>11682.m03977|LOC_Os05g41540.1|genepair327-2
MDDVPSQLLFSHPEVPDSFDDFLNNITTCTHTHTCNPPGPSATMHTHTCLHTHTQVFASG
SGEDDIKEDLTKTRRPLGNREAVRKYREKKKAHAAFLEEEVKKLRAANQQLLKRLQGHAA
LEAEVIRLRSILLDVRGKIDMEIGTFPYQKPCSVGSVACTDPGMCFNGNSEIGGVWEECS
RPVGADRMIDKDGSMSQEIDIPGPVHSISMDVVGSLVTSASLSE*
>11667.m05833|LOC_Os01g58780.1|genepair328-1
MPNRATHWCYACRRPIRVSGQDITCPNCNDGFIQEISEIGGSLNTYGIFDPSFDERRDRS
FGMVEAMSDLMRQRMAEMGRNRVLDFHGTRGASSHQGRQPTVRPMLIFGSNAPDRVSSSS
EEADILLRQGRRIGADRPNFSRFLVGPSLEALFEQLLLHNNRQGPPPAPQSAIDSMPVVK
INLRHLRDDPHCPVCTDKFEVGTEAREMPCKHLYHAECIIPWLVQHNSCPVCRHPLPSSS
HRSGSTRSSSTHSNEAISHGVARSDADPVPVARSDDSRNHEMHGSFSFLWPFDSPTPDSS
SYTHEGGVGEPTVHDDAGQMTYSEWHYDY*
>11682.m03975|LOC_Os05g41520.1|genepair328-2
MSNRATHWCYVCRRPVRIRGGSQDVTCPSCDDGFVQEMSEMGRRTASSTLGFVGPDAGDE
FLLRRSPVMEAMSTLMRHAATVGGDEREVDVHDEHGGGDGVPAHARLGVLFRGGPRVGVE
RRGGYYRAGLEALFEQLQNQLGSSRQGPPPAPPSAIDAMPVVTISRRHLRAEPRCPVCQD
EFQLGAEAREMPCAHLYHADCIVPWLVHHNSCPVCRHSLPPPATTASGGGASGGERQVRR
GSRSFLWPFGPTSSTSHSHECEDGSSDTTVYEDPGKVRYIRWHYNH*
>11667.m05840|LOC_Os01g58850.1|genepair329-1
MSGGTRNTRQFFESSSSGGGGRTSIDEGRGVRDGGGGRVAAARGSGVNTGILDEHVLSLV
FRSINWDPQAVCTAACVSRRMRAVAERVLWRELCISRAPRMVASLAGAGAGGAAPPPGRI
VGGWPALAKMLFFCCGAAGPGVPGHFTRMSRFSKTSGRSFLSRRCRSDLLYVSDPCEHAV
AGAGDDLGAYRGVFRGFMRSRTRACLVGRQAALDPRVRCPYCGARVWSMVAAGMVPRTAW
RRLGCLEGRLEYYVCVSGHLHGNCWLARLTSSEGEHDAGSGSDSDASTQGGGSDDDGHVA
L*
>11682.m03972|LOC_Os05g41490.1|genepair329-2
MSMRRLGSGNAGGGAAVAGEWDGGGIAGRMRGVNAGIMDEKVLELVFRALNWDPRELCVV
ARVSRRLRAVAERVLWRELCVSRAPRMVSALSGPTAAVAAAAGRIGGGWPAMAKLLFFCC
GAAGAAVPGHFAPVSRFSKTSGRSFLSRRCAGDLLFVSDPCEHAAGAASDDDVVGAYRGV
YRGFMRSRTRAFLVGHRAPLEPRVRCPYCGARVWSMTAAGLAPRSACRWLGANEGRLEYF
VCVSGHLHGSCWLARLSSSSSSSDGERSADSDSNHSDDETFAAADVSLPLPPAGRVPARR
LRGRPAM*
>11667.m05842|LOC_Os01g58870.1|genepair330-1

MAVGKEGGGGAGGEGGMSDSVIRKVLVSYMYVAVWIFLSFTVIVYNKYILDPKMYNWPFP
ISLTMVHMAFCSSLAIALVRLLRVVELPSSPAMTPQLYTSSVLPIGALYSLSLWFSNSAY
IYLSVSFIQMLKALMPVAVYSIGVLFKKENFKSSAMLNMLSISFGVAIAAYGEARFDARG
VALQLAAVAFEATRLVLIQILLTSKGISLNPITSLYYVAPCCLAFLVIPWAFVELPRLRA
VGTFQPDFFIFGTNSLCAFALNLAVFLLVGKTSALTMNVAGVVKDWLLIAFSWSVIRDTV
TPINLFGYGIAFLGVGYYNHVKLQALKAKEAQKKAAQADEEAGSLLQERDSHGERKSDNQ
A*
>11682.m03970|LOC_Os05g41480.1|genepair330-2
MAGGGGAGGMSESVLRKVLLSYCYVGVWIFLSFAVIVYNKYILDPKMYNWPFPISLTMVH
MAFCSSLAVALVRLLRVVEPPSSPAMTPQLYTSSVVPIGALYAMSLWFSNSAYIYLSVSF
IQMLKALMPVAVYSIGVLFKKETFRSSSMLNMLSISFGVAIAAYGEARFDVRGVALQLAA
VAFEATRLVLIQILLTSKGISLNPITSLYYVAPCCLGFLLVPWVFVELPRLRAVGTFRPD
FFVFGTNSLCAFALNLAVFLLVGKTSALTMNVAGVVKDWLLIAFSWSVIRDTVTPINLFG
YGIAFLGVAYYNHVKLQALKAKEAQKKISQADEEAGSLLQERDGHSDRKSDNQA*
>11667.m05844|LOC_Os01g58890.1|genepair331-1
MRKYRVAGLVAALLVLHSLATPSAQAEAHRAGGEGEEKMSSDGGPVLGGVEPVGNENDLH
LVDLARFAVTEHNKKANSLLEFEKLVSVKQQVVAGTLYYFTIEVKEGDAKKLYEAKVWEK
PWMDFKELQEFKPVDASANA*
>11682.m03968|LOC_Os05g41460.1|genepair331-2
MRASSLFAESVFTTSAAAGRRRCPRLAAVPVTLFFSTGRGSPAMAEEAQQPRGVKVGGIH
DAPAGRENDLTTVELARFAVAEHNSKANAMLELERVVKVRQQVVGGFMHYLTVEVKEPGG
ANKLYEAKVWERAWENFKQLQDFKPLDDATA*
>11667.m05846|LOC_Os01g58910.1|genepair332-1
MASSPCGGFLEKAKPYFAMICLQFGYAGMNVITKVSLNHGMSHYVLVVYRHAFATISIAP
FALLLERKVRPKMTWSVFLQIFVLALLGPVIDQNFYYAGLKFTGPTFACAMSNILPAMTF
VMAVIFRMEKVDLKKVRCQAKVAGTLVTVAGAMMMTLYKGPLMQMAWTSHVQAPHGHGAE
APAAAAAVDPSGREWFLGSLFVIIATLAWASLFILQAHTLKKYSAPLSLTTLICFVGTLQ
AIVVTFAMEHRPSVWAIGFDMNLLAAAYAGIVTSSIAYYVQGLVIQKTGPVFASAFSPLM
MIIVAGMGSFILAEKIYLGGVLGAVLIVVGLYSVLWGKHKETQEQDAAMMELPVASKGND
AEFTAATVVGDDDDAADCKKANGVKKSSSSNEQGASAV*
>11682.m03963|LOC_Os05g41420.1|genepair332-2
MASCCGGFMEKAKPYFAMICLQFGYAGMNVITKVSLNHGMSHYVLVVYRHAFATISIAPF
ALILERKVRPKMTWSIFFQIFILALLGPVIDQNFYYAGLKFTGPTFACAMSNILPAMTFV
MAVIFRMEKLELKKVRCQAKIAGTLVTVAGAMLMTLYKGPLMEMAWSRHAGAGVAEAPAA
AAAAISGRDWFLGSMFVIVATLAWASLFILQTHTIKQYSAQLSLTTLICLVGTLQAVVVT
FAMERRRPSVWAIGFDMNLLAAAYAGIVTSSIAYYVQGLVIQRTGPVFASAFSPLMMIIV
AVMGSFILSEQIYLGGVVGAALIVVGLYSVLWGKHKETQEKQADTKLSLPTSKGAAAAAE
AEEITGAGEDDGDGDDDAERSKNHRSSGGVRSSSDSNGHGASAV*
>11667.m05865|LOC_Os01g59100.1|genepair333-1
MASTAVSRHVVAVPYPGRGHINPMLAACRLLAAADGELTVTVVVTEEWHGLLASAGVPAT
LPPAGRVRLATIPNVIPSEHGRGADPAGFFEAVDAKMGVAVEQLLDRLERRPDAIVADTY
LAWGVPAGAARGIPVCSLWTMAATFFWALYNIHLWPPVDDREGEQDLSRKSLEQYVPGCS
SVRLSDVKIFRSWERSMKLTTEAFVNVRKAQCVLFTSFYELEPCAMDRITQAVPFPVYPV
GPSISDMPLDGGAGKIDDEEHRAWLDAQPERSVLYVSFGSVVSMWPSQLEEVAVALRDSA
VRFFWVARDSASAGDLRRIAGGNGLVVPWCDQLGVLCHRSVGGFLSHCGWNSLLEAVFAG
VPLLALPVVWDQVVDARVVADEWRIGVNLSEQRREEDDGGGVVVGRDAIRAAAARLMDPD
DGESREMRRRAALLREACRGAVQDGPDGGSSRRSLNGFVKDLADGRLNFQ*
>11682.m03961|LOC_Os05g41400.1|genepair333-2
MGSSAEPPPPCHVVAVPYPGRGHVNAMLNLCRILAARDGVTATVVVTEEWLGLLGGAAAA
AAEGGVRLEAIPNVVPSEHGRAGDMLGFVRAVYTRMEAPFERLLDRLALGAAPPPPAAIV
ADTFVLPWAVGVGNRRGLPVCVLSPLSATMFSVHYHFDRLPTATDIADGDEVGNYIPGLK
SIRFSDLEPTHTNKNMVDLILEAYSHARKAQCVIFTSFYELESNAMDALRRDLPYPAFSA
GPCIPYMALQADEHHAGDEEEEPYMAWLDAQPVGSVLYVSLGSFLSVSRPQLDEIAAGLA
DSKVTFLWVLRGDSGARDILRGGGGMVVPWTDQLKVLCHPSVGGFFTHSGMNSTLEAVHA
GVPMLTLPIAFDQPIVARLVADEWRIGYGLRENGDGGGCSGVVGREEIAAAVRRLMVMDS
DAAAAEEAKEMRRRASLMREASRAAVQEGGSSYRDVTSLINYISEFKN*
>11667.m05867|LOC_Os01g59120.1|genepair334-1
MRQQSEGSASSVRKCSRKKVINTLTSVLTARSKVACGITDKPREVIEDIDKLDGDNELAV
VDYIEDIYKFYKVAENECRPCDYIDTQVEINSKMRAILADWIIEVHHKFELMPETLYLSM
YVIDRYLSMQQVQRRELQLVGVSAMLIACKYEEIWAPEVNDFILISDSAYTREQILAMEK
GILNKLQWNLTVPTAYVFIMRYLKAGASADNKSDKEMEHMAFFFAELALMQYGLVASLPS
KVAASAVYAARLTLKKSPLWTDTLKHHTGFTESQLLDSAKLLVTSHSTAPESKLRVVYKK
YSSEQLGGVALRSPAVELCK*
>11682.m03960|LOC_Os05g41390.1|genepair334-2
MALPFPVPRGAHWAVTSTAHVLVGFTGEMARVEQPRAPAPYNAAFLRKKLTLATKMHTTK
SSDSHLDIAGTVVTHSHGAAVAAGKQKAAATAAAGRPGARNRQALGDIGNVLNAHVVDGK
IQLPEGINRPITRSFGAQLLKKAQENAVAANKIVVQNPARKEPAPKPAKKVVPRPENAAK
ASTGAGVNENKKPSESEGAGSSSGGSALKYSRKKVVNTLTSVLTARSKHACGITEKPKEV
VEDIDKLDGDNQLAVVEYIEDIYNFYRTAQICSETDSVVLAQLERRPTDYMSSQVEVNPK
MRAILADWIIDVHYKFELMPETLYLTMYVIDRYLSLQPVLRRELQLVGVAAMLIASKYEE
MWAPEVQDLIHVCDNAYSRQHILAMEKNILNRLQWNITVPTPYVFLLRFIKAAGGDKELE
NMVFFFSEMALKEYGMASLCPSLVAASAVYAAQCTLKRSPLWTSTLKHHTGFTESQLREC
AKVLVNAHAAAPESKLKTAYRKYASEQLGRVSLRPPAVCLA*
>11667.m05890|LOC_Os01g59330.1|genepair335-1

MAAEAILGAFMQTLFQKLSEATLDHFISWRGIHGKLESLSSTLSQLQAFLDDAEEKQLTD
ASVRGWLAKLKDIAYDLDDLLDSYSAKSMRMKQRQVIFPTKASFLSSSFLSRNLYQHRIK
HKINIILERLDKIAQERDTIGLQMICEMRRYDTSERPQSSSLVDSSAVFGRERDREEMVR
LVLSDNGHNSCNLCVIPVVGMGGLGKTTLMQMVYHDDRVREHFDLRIWIYVSESFDERKL
TQETLEASDYDQSVASTNMNMLQETLSRVLRGKRYLLVLDDVWNEDLDKWHSYRAALISG
GFGSKIVVTSRNENVGRIMGGIEPYKLQKLSDDDSWSVFKSHAFRDGDCSAHPELEAIGM
EIVKKLKGLPLASKALGSLLFCKTDEEEWKDILQNDIWELPADKNNILPALRLSYNHLPP
HLKQCFAFCSVYPKDYMFRREKLVKIWLALGFIRQSRKKRMEDTGNAYFNELLSRSFFQP
YENNYVMHDAMHDLAKSISMEDCDHLDYGRRHDNAIKTRHLSFPCKDAKCMHFNPLYGFR
KLRTLTIIHGYKSRMSQLPHGLFMKLEYLRVLDMHGQGLKELPESIGNLKQLRFLDLSST
EIETLPASLVKLYNLQILKLSDCNFLREVPQGITRLINLRHLEASTRLLSRIHGIGSLVC
LQELEEFVVQKRSGHNVTELNNMDELQGQLSIRGLNNVPNGQDAVCAKLRNKEHLRTLHL
IWDEDCESNPSEQQEVLEGLQPHLDLKELVIKGFPGVRFPSWLASSFLPKLQTIHICNCR
STRLPALGQLPFLKYLVIAGVTEVTQLSSEFTGFGQPKGFPALEDLLLEDMPNLSEWIFD
VADQLFPQLTELGLIKCPQLKKLPPIPSTLRTLWISESGLESLPELQNNSCPSSPTSLYI
NDCPNLTSLRVGLLAYRPTALKSLTIAHCEGLVSLPEECFRPLISLRSLHIYECPCLVPW
TALEGGLLPTSIEDIRLNSCTPLASVLLNGLSYLPHLRHFEIADCPDINNFPAEGLPHTL
QFLEISCCDDLQCLPPGLHNISSLETLRISNCPGVESLPKEGLPMGLNELYIKGCPQIKQ
QCQEGGEYHAKIAHIRDIEIDGDVIVPEQI*
>11682.m03950|LOC_Os05g41290.1|genepair335-2
MVIGEAVLSAFMQALFDKVIAAAIGELKFPQDIAEELQKLSSSLSTIQAHVEDAEARQLK
DRAARSWLAKLKDVAYEMDDLLDEYAAETLQSELEGSSRSRHLSKVRSSFCCLWLNNCFS
NHKIVQQIRKIEEKIDRLVKERQLIGPDMSSTMDREEIKERPKTSSLIDGSSVFGREEDK
ENIVKMLLTPNNSNHANVSVLPIVGMGGLGKTTLTQLVYNDPRVKEYFQLRVWLCVSENF
DEMKLTKETIESVASGFSSVTTNMNLLQEDLSKKLEGKRFLLVLDDVWNEDPEKWDRYRC
ALVSGSNGSRIVVTTRNKNVGKLMGGMTPYFLKQLSENDCWNLFRSYAFADGDSSLHPHL
EIIGKEIVKKLKGLPLAAKAIGSLLCTKDTEDDWKNVLRSEIWELPSDKNNILPALRLSY
NHLPAILKRCFAFCSVFHKDYVFEKETLVQIWMALGFIQSPGRRTIEELGSSYFDELLSR
SFFQHHKGGYVMHDAMHDLAQSVSMDECLRLDDPPNSSSTSRSSRHLSFSCHNRSRTSFE
DFLGFKRARTLLLLNGYKSRTSPIPSDLFLMLRYLHVLELNRRDITELPDSIGNLKMLRY
LNLSGTGITVLPSSIGRLFNLQTLKLKNCHVLECIPESITNLVNLRWLEARIDLITGIAR
IGNLTCLQQLEEFVVHNDKGYKISELKTMMSIGGRICIKNLEAVDSAEEAGEALLSKKTR
IRILDLVWSDRRHLTSEEANQEKEILEQLQPHCELRELTVKGFVGFYFPKWLSRLCHLQT
IHLSDCTNCSILPALGELPLLKFLDIGGFPAIIQINQEFSGSDEVKGFPSLKELVIEDMV
NLQRWVSFQDGELLPSLTELEVIDCPQVTEFPPLPPTLVKLIISETGFTILPEVHVPNCQ
FSSSLACLQIHQCPNLISLQNGLLSQKLFSLQQLTITKCAELTHLPAEGFRSLTALKSLH
IYDCEMLAPSEQHSLLPPMLEDLRITSCSNLINPLLQELNELSSLIHLTITNCANFYSFP
VKLPVTLQTLEIFQCSDMSYLPADLNEVSCLTVMTILKCPLITCLSEHGLPESLKELYIK
ECPLITERCQEIGGEDWPKIAHVPVIEIDDDYFIPNRSIRRRLS*
>11667.m05893|LOC_Os01g59350.1|genepair336-1
MEGGRLGGAAASASAAAAADARGGMSGFAAPQHAIHTNLNNVQPTQVTDFGALAQSAGFR
IEDLANLSTNGLFNLKSNAHTIINDPLQFENYVKSISPSNITTTATVTVVDPQTLVPQKG
AQLNLVTIRTGNVENWGESTIADTSPRTDTSTDPDTDERNQMFEQGQLAAPTASDSSDRS
KDKLDHKTLRRLAQNREAARKSRLRKKAYIQNLESSRLKLTQIEQELQRARQQGIFISTS
SDQSHSASGNGALAFDMEYARWLEEHNKHINELRAAVNAHAGDNDLKSTVDSIMAHYNEI
FKLKGVAAKADVFHVLSGMWKTPAERCFMWLGGFRSSELLKLLAGQLEPLTEQQLAGIAN
LQQSSQQAEDALSQGMEALQQSLAETLASGSLGPAGSSGNVANYMGQMAMAMGKLGTLEN
FLRQADNLRLQTLQQMQRILTTRQSARALLAISDYFSRLRALSSLWLARPRE*
>11682.m03949|LOC_Os05g41280.1|genepair336-2
MVYASPGTDASTDPDIDKNIRMTLRRLAQNREAARKSRLRKKAYVQQLEDSRMKLTQLEQ
ELQRARQQGIIISTSGDQQRSTSENEALAFNMEYMRWLEEHNKQINELRSAVHTHAGDDD
LQNIVSSVMAHHEEIFRIKGLAAKADALHVLSATWRTPLERCFLWLGGFRPSDLLKLLAD
QLEPLTEQQLASIYNLQQQSSQQAEETLSQGMEIIQDSLAKTVASQLGRAGSSSPSNAADH
TAAALGKIGDMESLLQQADDLRMQSLQKMQRVLTTRQSARALLLVSDYFSRLRALNSLWI
ARPQQ*
>11667.m05900|LOC_Os01g59410.1|genepair337-1
MDRQRQQSSRGNATATRGGGSSGKGGGGGVGKAAGKKPIKVVYISNPMRVKTSAAGFRAL
VQELTGRNADPSKYSPRASADDDDGGGGGGGGELAAASDGAGEPGPGAAAASPDTGAAAA
SDAADALVAAGHPAAATFDDEGGGGGGGYYDDDDDDIFRSQLLDTSYSVFSPPTLLYDHP
HSKV*
>11682.m03946|LOC_Os05g41250.1|genepair337-2
MDKKQCGAKGGGGGNARRSATGGGAGRMHRKGKHQGDGGGGGGKRREIKVVYIANPMRVT
TSEEGFRALVQELTGRHADPSKYRGGGGGAPVDETSGGGGGGGGGEMMQGAAAMMQPSPGS
TVDSSSTDHGGAGAGGGQGAGLQAAALDDDENSFIAPELIDNRYSVCFSPPTFLYGGGSHT
YDGDDYGL*
>11667.m05903|LOC_Os01g59440.1|genepair338-1
MGAGALGVVAMVAAAVVVAMAGANSEGDALSALRRSLRDPGGVLQSWDPTLVNPCTWFHV
TCDRDNRVTRLDLGNLNLSGHLVPELGKLDHLQYLELYKNNIQGTIPSELGNLKNLISLD
LYKNNISGTIPPTLGKLTSLVFLRLNGNRLTGPIPRELAGISSLKVVDVSSNDLCGTIPT
SGPFEHIPLSNFEKNPRLEGPELQGLAVYDTNC*
>11682.m03943|LOC_Os05g41230.1|genepair338-2
MAPTSAGNLAPATAILVVVVAVVLAAAAASQDGDALTEFRKGMSDPDGALASWDPDLVNP
CTWFRVTCNADNRVIRLDLEEMNLSGHLSADLARLDQLQFMEIASNNIEGPIPPEFGNLE
NLISLDLCNNTISGPIPPSLGKLKSLKFMRIDHNLLTGPIPNELAGLSNLMILNVSNNDL

CGTIPTSGPFDHFPPSSFASNPRLRYPGMDDDDTGR*
>11667.m05914|LOC_Os01g59530.1|genepair339-1
MADQLSEEQIGEFREAFSLFDKDGDGSITTKELGTVMRSLGQNPTEAELQDMISEVDTDS
NGNIEFKEFLGLMARKLRDKDSEEELKEAFRVFDKDQNGFISATELRHVMANIGERLTDE
EVGEMISEADVDGDGQINYEEFVKCMMAKKRRKRIEEKRDHDGGSRTKSAGPSAAPASKR
GQKCVIL*
>11682.m03941|LOC_Os05g41210.1|genepair339-2
MADQLTDEQIAEFKEAFSLFDKDGDGCITTKELGTVMRSLGQNPTEAELQDMINEVDADG
NGTIDFPEFLNLMAKKMKDTDSEEELKEAFRVFDKDQNGFISAAELRHVMTNLGEKLTDE
EVDEMIREADVDGDGQINYEEFVKVMMAK*
>11667.m05921|LOC_Os01g59600.1|genepair340-1
MSSIGTGYDLSVTTFSPDGRVFQVEYATKAVDNSGTVVGIKCKDGIVLGVEKLVTSKMML
EGSNRRIHSVHWHSGLDIILINVMAVAGLAADGRQIVSRAKSEAASYEKVYGEPISVKEL
ADRVASYVHLCTLYWWLRPFGCGVILGGYDRDGPQLYMIEPSGVSYKYFGAALGKGRQAA
KTEIEKLKLSELTCREGIVEVAKIIYGVHDEAKDKAFELELSWICDESNRQHQKVPADLL
EQAKVAAQAALEEMDAD*
>11682.m03938|LOC_Os05g41180.1|genepair340-2
MSSIGTGYDLSVTTFSPDGRVFQVEYAGKAVDNSGTVVGIKCKDGIVLGVEKLVTSKMIL
KGSNRRLHSVHRHSGLAVAGLAADGRQIVSRAKSEAASYEKVYGEPMPVKELADRVASYV
HLCTLYWWLRPFGCGVILGGYDRDGPQLYMIEPSGLSYKYFGAALGKGRQAAKTEIEKLN
LSELTCREGIVEVAKIIYGVHDEAKDKDFELELSWVCDESKRQHEKVPDDLVEQAKAAAQ
AALEEMDAD*
>11667.m05923|LOC_Os01g59620.1|genepair341-1
MALQLMMDNYLLCSAMKGRLSVYSDSQLILVKMNRESNFMPTPDQDVLEVKPLRTLAPMF
PAPLGIDVLNRLTAPPLVFVAPAGQFPGGFGSLNIPAVRSFAAFGGQDASGGKTAGGGDQ
DASGGKTAAGGDQDAGRGETAAFGGQETVRGEFVANGTPNVGASATGPIDATPISACKST
QPSVISLDDDDNDDDEPYGGNQTSASGRKIKRPSHLKGYNVSDGLGTDSSNGTKKRPKTS
NRKAATDNEISLMPPSSDPREVVEVLLMTFEALRRRHLQLDETQETSKRADLKAGAIMLA
SNLRANIGKRIGAVPGVEVGDIFYFRMELCIIGLHAPSMGGIDYMNKFGDEDDSVAICIV
AAGVYENDDDDTDTLVYSGSGGISRNSEEKQDQKLERGNLALERSLSRKNVIRVVRGYKD
PACLTGKVYIYDGLYKIHESWKERTKTGINCFKYKLQREPGQPDAVAIWKMCQRWVENPA
ARGKVLHPDLSSGAENLPVCLINDVNSEKGPGHFNYITQVKYLKPLRSMKPFQGCRCTSV
CLPGDTSCDCAQHNGGDLPYSSSGLLVCRKLMVYECGESCRCSINCRNRVAQKGVRIHLE
VFRTTNRGWGLRSWDPIRAGSFICEYVGEVVDDTKVNLDGEDDYLFRTVCPGEKTLKWNY
GPELIGEHSINISADTFEPLPIKISAMKMGNVARFMNHSCNPNTFWQPVQFDHGEDGYPH
IMFFALKHIPPMTELTYDYGDIGCESRGVGSRAKNCLCGSSNCRGFFS*
>11682.m03937|LOC_Os05g41170.1|genepair341-2
MDRASNFIPGPYQELVDAKPIRSLAPMFPAPLGINVNQSSTPPLVCVTPVGQFPVGFGSG
ILPTFGSTTAFTTTANGVSYTSYTNNGAIDATPISAYKTRPGIVSLDGDEPYSGSASGRK
SKRSSGSAADGSNGVKFKRPKPVYKNFVAGKELAFLPPSSSDPREVVEAVHMTFEALRRR
HLQLDEIQETSKRADLKAGAIMMASNIRANVGKRVGLVPGVEIGDIFYFRMELCIIGLHA
PSMGGIDYMSAKFGSDEDSVAICIVAAGGYENVDDDTDTLVYSGSGGNSRNSEERHDQKL
ERGNLALERSLHRKNEIRVVRGFRDPFCLTGKIYIYDGLYKIQESWKERTKSGINCFKYK
LLREPGQPDGAALWKMTQGWIDNPASRGRVILPDLSSAAEALPVCLVNEVDHEKGPGHFT
YASQVKYLRPLSSMKPLQGCGCQSVCLPGDPNCACGQHNGGDLPYSSSGLLACRKPIIYE
CGDACHCTTNCRNRVTQKGVRFHFEVFRTANRGWGLRCWDPIRAGAFICEYTGEVIDELK
VNLDDSEDDYIFQTVCPGEKTLKFNFGPELIGEESTYVSADEFEPLPIKISAKKMGNVSR
FMNHSCSPNVFWQPVQHDHGDDSHPHIMFFALKHIPPMTELTFDYGVAGSESSELPACTP
RFELWYYSLRMTMGFNMNCEETVTFLSKCTCRSLMMHESDSDPDSDRTHWYMSNSDDESF
VDKDKDCRRSTVALLLKKGPWTSWEDSILEKYIKKHGERNWKLVQKNTGLLRCGKSCRLR
WMNHLRPNLKKGAFSKEEENKIINLHRKMGNKWSRMAADLPGRTDNEIKNYWNTRIKKCK
NNRSPLYPANVCNDALNEDQHESADPNVREKLTNNHLEDTTSMYSAPQFSDASISNILDR
RLASKDYDSIEDQRNQIEVVAKYEIPLPVLKTTNNDIFPSASIFANHGISNGNLSALSTT
DALQMELPLIQFDPNNQFVYSRAYAMHLTNFALLNDQSEELLNDTDVLNYVVMKEELSGG
SLSPTINMPCEAHNSMAASNELVVPQYEGDAPPLQDDFTSYFYLNDTNLSIFEDTNELFL
ENKLDTKGN*
>11667.m05930|LOC_Os01g59670.1|genepair342-1
MTQGSARFVLEAERVHAMALDTKQVARCLKKVLKSSIKDGYRCVSEHPILLTLGVLLYLL
YRSSPGLFAFLLSSSPVIMCTTLLLGILLSYGETNLPEADEDNKITPEISSLKVGNPSSD
FHFEASQRLPVPELRENTTGFKERETKQTVFIERASEHIELEDNVPLLRRVEHEYDRFD
RHEIPAALTPFFPSMVNFHQGSRVGNDLSSNQDINSKGLLSIKDKADGHTSFFEGVRSGLD
EKEAPFGIFSTSKNVNGRGELEENLNQETVFTDSTASRVRDISEEKPTEGEAGTSKSACA
ISTHQSKTLDELRINTSKGFEDNLLDSSLGSPWARVGSEDGVGSDDGSSGFDSDQAESSS
PDASMTDIAPILDEIDPLLGASSTHPDTIPKDDSDTDSHVSSQDHQTDDDSNDETDNNDA
KENGEEKNKEQGKEAAFIWTADDEKNLMDLGYSEMERNRRLELLMARRRSRKNIRFEIDN
DLIGIDNNDGGRGVDDLSRFHVQVPHISVPRRNPFDLPYDSEEAAIPGSAPSVLHTRKNP
FDLPLDQSNDGDVSADNNVNPGELVKASHRDMFFRRHDSFNIGRTDATLERFSRFKPYFV
PETVEGSLSNFQRQFSDKIADQEDHKDLDEKDLPNEHGSPALQRQDSDLADVGSECSDGI
NSVDVELDNSDIDDREIALQHFVFERSQEREAYLSSTKGKGPEDDYLLSSVGNSKTLHPV
ADLFSWEDGNGESSLGVNSSHNMSVEFSDWVSSPKPIAEHDSGPENLQEFLNTEVASSSK
TIVLGARNPAENNGNVDSISYSNNEMPSDNLGHGSMEFPSEFCNESLPVISRDLHPIPEE
RVVENFNVQEKHEAVIFTDSDAALTGFHVIEEHFEVGCDVSPSSEVVPSCLQASDSIQSR
LVENKEVSNPFISIASETNKVDMIDLKEETAAGYPLDSDDDADKIYPEPMEDNVIDESFL
SELDAVGDFRVEATRSDQQMPDVDSHIDNNTSNGVAESSLISPQISSNIFSNMKYASMLE

HEENSPLVDDLNGTGPEFGWSLGASYDDPEQTVYNPRRRILGASRFEETNTEMKPLFDET
EASFVNAPIEANLVVGPSKVDVANESELTKTDTNMIVLDANSLEDIETAFKQASNGVVES
TVDNETPQVSGVDIDPESIESSEQLDVIDAKSVDDIYAALKEHTTAAMNSSFEENEDKHG
CGDTVKFTMHDELPEGTHIEGNTVGDGKEPEPMGITSSMDVIDAKSIDDIYAALKKQSSA
AANSSFEQNEGKNGCGDTVTFTTHDELPEGTHIEDRDNTVEDGKEPEPIGTTSSMDTIEV
KTIDDIDAVFKKLSDGGTKSAAQAVDCENTCEASDESEQH*
>11682.m03935|LOC_Os05g41150.1|genepair342-2
MGVEADHGTPCIRTILRCSIRMSYRYASENWVLLFPVLLLYLLFRSSPGFFAFLLSHSPV
IICAALILGVLISHGSTNVAEIKEERKSVAEVSDPKYADLSRNIHLEANKGFSAKENTAS
LNDGEIKDGLNSSREDAIEVVEMVGKISHDRGSTDSQSDEMKVDSEDKPAGTCKWGRAFS
VRRRKKLSDIKVEPINAAVDSPLDSSLDSPFGRVGCHDGSPGFDHDQTEGTTPGTPRTRI
ASVLDEIDPLSSADSPHPDPIQNDDSDNHMSLQDSRTVRDNNYESDKSKANKNDDKNVST
DPAFLGTVDDDKNVMDLGYSEVERHRRLEILMVKRRSRKNIVFDPDSNLDIDNDKVCKRN
PSDILSCSDETEFPGSAPSVLHTWRNPFDHPFEQSDESDLHEHVAIPHQDMFFTRHESFS
IGSQGRRPSRFKPPFIIEAMDIDEPSASDFQRQFSDKSASTLSTVTESDIISSVADQEDI
SNNIKNDSSREYESPELPTIPTMGSDIICVGGT*
>11667.m05932|LOC_Os01g59690.1|genepair343-1
MHPRARIHADPAPEFDQFDCLPDPVVLLILNKLEDVRSLGRCAAVSKRFSGLVPLVSDVY
VKIDRVVATDGDADDALNLSSAKPKNIFSHFFKLMLFTIVKPFHSMRNLNGTGRPLFPLA
QHSPVHVLRNFSDVWNLRVELPSGDVGTEEGVLLKWRAEYGSTLRNCVILGGTLVDRKPI
GAEHESSVEDNGSMPESFYTNGGLKLRVVWTISSLIAASTRHYLLRSIIKDHPTLRSLVL
ADADGQGTLYMGMEQLREFRENKLSASACSNRTQVPACNMKLKYAPYLELPGGMTLQGAT
LVVIKPSNDGGSGGHSSRKETEAFVSSAFDEPFRFAVKALMKRRTYLLEMNGF*
>11682.m03933|LOC_Os05g41130.1|genepair343-2
MARIHADPVLEADQFDRLPDSLVLVILNNVEDVRSLGRCSAVSKRFYGLVPLVHDVYVKI
DRVVTVDGEAEDALNLSSPKPRNILSHFLKMMLFTIIKPFHSMRGPNGAGRPLFPQLAQH
SPAQVLRNFTHIRNLRVELPSGDVGTEEGVLLKWRAEYGSTLQNCVILGGTQVDRKPVGA
EHELYSEDNGSMPESFYTNGGLKLRVVWTISSLIAASTRHYLLRSIIKDHPTLTSLVLTD
ADGQGTLSMGAEQLKEFRENQLSASACSNRTQVPACNMKLKYAPYLELPGGIALQGATLV
AIKPSPEGSNGGHTSRKETDAFVSGAFDGPFKFAVKALMKRRTYLLEMNGF*
>11667.m05936|LOC_Os01g59730.1|genepair344-1
MRSVSISVYFLIISALLLLHCFLSLQTARLPRDDWASEERFQSRKLLQNVLITSSENPQV
FLEAALLVDNKSKKQPDVEATESLKKETPSKANPIQNYPKIREVWTAGFAEEITNWAGLI
RKVGPKKNAEGINNKLQLVMKSGKYTLGYKTVLKTLRNSKGKLVIVANNCPPLRKSEIEY
YAMLGKVSVYHFNGNNVDLGTACGKYYRVCCLSVVDPGDSDITKQLPESH*
>11682.m03930|LOC_Os05g41110.1|genepair344-2
MAPTKKAKKSTDNINNKLQLVMKSGKYTLGYKTVLKTLRNSKGKLIILANNCPPLRKSEI
EYYAMLGKVSVHHFHGNNVDLGTACGKYYRVCCLSIIDPGDSDIINTTPASQ*
>11667.m05939|LOC_Os01g59760.1|genepair345-1
MASKAGGGGVARRGGGRMRSLGRQGSMYSLTLDEVQSQLGEPLHSMNLDELLRSVFPDGL
AIADGAGATTSSQQHQPGSGLLRQGSITMPPELSKKTVDEVWKGIQAAPKRNAETGGGGG
GGRRRRERQPTLGEVTLEDFLVKAGVVTQGSLKELSDVGNVDPVGRGVTATGTVDLAPGS
HWIEQYKQQIASTDAHHHGQQGVQGAYFPNRLVPQPLNVGPGAILEPSYSDGQTSSGMIG
GMSDSQTPGRKRGMSGDVADKLMERRQKRMIKNRESAARSRARKQAYTNELENKVSRLEE
ENVRLKRQKELDELICAVPVPEPKYQLRRTSSADF*
>11682.m03925|LOC_Os05g41070.1|genepair345-2
MIQAMASHAGGSGGGGGGSGRDAGSAQRGPMQGLARQGSLYGLTLNEVQSQLGEPLLSMN
LDELLKSVFPDGADLDGGGGGGGIAGQSQPALGLQRQGSITMPPELSKKTVDEVWKGIQD
VPKRGAEEGGRWRRERQPTLGEMTLEDFLVKAGVVTDPNDLPGNMDVVGGAAAAAAGTSD
LNAGAQWLQQYHQQALEPQHPSIGAPYMATHLAPQPLAVATGAVLDPIYSDGQITSPMLG
ALSDPQTPGRKRCATGEIADKLVERRQKRMIKNRESAARSRARKQAYTNELENKVLRLEE
ENERLKKQKELDEILNSAPPPEPKYQLRRTSSAAF*
>11667.m05942|LOC_Os01g59790.1|genepair346-1
MGLTFTKLFSRLFAKKEMRILMVGLDAAGKTTILYKLKLGEIVTTIPTIGFNVETVEYKN
ISFTVWDVGGQDKIRPLWRHYFQNTQGLIFVVDSNDRERVVEARDELHRMLNEDELRDAV
LLVFANKQDLPNAMNAAEITDKLGLHSLRQRHWYIQSTCATSGEGLYEGLDWLSNNIASK
A*
>11682.m03924|LOC_Os05g41060.1|genepair346-2
MGLTFTKLFSRLFAKKEMRILMVGLDAAGKTTILYKLKLGEIVTTIPTIGFNVETVEYKN
ISFTVWDVGGQDKIRPLWRHYFQNTQGLIFVVDSNDRERVVEARDELHRMLNEDELRDAV
LLVFANKQDLPNAMNAAEITDKLGLHSLRQRHWYIQSTCATSGEGLYEGLDWLSNNIANK
A*
>11667.m05952|LOC_Os01g59870.1|genepair347-1
MALTVRLHTAVAAVAVAVVVAMGMAAAQMSPAGAPAPAGGISPACMDAVLNMSDCLTYVM
NGSTARKPDEPCCPELAGLLESKPVCLCQLLAGGASSYDISVDYKRAMALPGICGLAAPP
VTACALLGVPVPMAPSASPMAGLGPSTEPQMPEKSPSASPSESSNHAPGRFTALAAVVLA
VAAAGMGRRTARGLDGSRDQADEALVISQQRALPSPHAPARSRLGFVRLFVGTRSFDADA
EAAASSALDSAGAPKRRPRLHHPVADAGRLTADGGNSTCSHAAEAAHRQDTHRTNYSLNL
NIQ*
>11682.m03921|LOC_Os05g41030.1|genepair347-2
MAAQRRWPTSTLAAVAVAVVVLLAASAATTAEAQSAPAAAPGPAGPVLDQACLTALLNMS
DCLTYVQNGSRARRPDKPCCPELAGLVESNPVCLCELLSGAGDSYGIAVDYSRALALPAI
CRVSTPPVSTCAAFGFNVPMGPTPSPSPAAVSPSGEGPQFPGTSPFASPPSTATPSTNAA
AAGRSGDHLVAVGVAIAAAAVVVAGMFRIV*

>11667.m05953|LOC_Os01g59880.1|genepair348-1
MEQEVKGMRLSHFLAQTPNNTTTEPVRIFVATWNVGGKAPTAELNLDDFLPPDDHSDIYV
LGFQEIVPLNAGNVLVIEDNEPAARWLVLINQALNRPAETNANVFQNEPSPSVDSSVSRA
SSSLDTSFSDLAKTSSSSTIFQKSNLKSIRKSFMPVHRKRLKACNCPVEMAKSSYRDACF
GCPKAYAYEIDSSEEDEREEKKGQSRDSNGSVRSEVISPPTARDELKYNLIAWMHISEHD
TAPDKLLFHLQPFGFSKVIWFGDLNYRIALSYADTKNFLMENNWDVLFERDQLKIERDAG
RVFKGWNEGKIFFAPTYKYSYNSDAYASETATSNKKRRTPAWCDRILWRGDGILQLSYYR
GESRFSDHRPVCGTFIVEVEVLNRKAKMRPSNANMKIGAEELLPQGNNKGKGTTLLAHMP
*
>11682.m03918|LOC_Os05g41000.1|genepair348-2
MAFPDDDEKMKGCRPKLFGTKDKKVVKRADYQSCSAVKSGPSSSKSQSSSPFRTLTEVRS
IRLSHLLGHSSSTTKTEPFRIFVSTWNVGGNTPTAELNLDDFLPADDNSDIYVLGFQEIV
PLNAGNVLVVEDNEPAARWLALINRTLNKPVDSNADIFQHKPSSSLDSTSSLSSSNLDAS
FSSRTRTASGSSAIFQKSSLKSIRKPYMPTQRKLLKLCNCSVEMTRKSYKDACFGCPQAY
ANETDSSEDDTDDRSNDPCGYIVDGMNSAASASRDQLKYNLVSCKRMVGIFITVWAKKEL
VHHIGHVRTSCIGRGIMGYLGNKGCISVSMTVHQTSFCFICSHLASGEKEGDELRRNLDV
LEILRLTQFQRICRAGRRIPEKILDHDRVIWLGDLNYRISLSYEDTKKLLTENNWDALFE
KDQLNIERKSGRVFKGWSEEKIYFAPTYKYSSNSDSYAGETATSKKKRRTPAWCDRILWH
GDGIVQLSYFRGESKFSDHRPVCGTFIVDVEIQESRSKRRSSNTNIRIGAEELLPTSKSK
ANKNKGNKGSGT*
>11667.m05961|LOC_Os01g59930.1|genepair349-1
MDLLHGESVQTTVAIAVAVVAVAAGGAFLLLRSRKPKGCLDPENFKKFKLVEKKQISHNV
ARFKFALPTPTSVLGLPIGQHISCRGQDATGEEVIKPYTPTTLDSDLGHFELVIKMYPQG
RMSHHFREMKVGDYMSVKGPKGRFRYQVGQVRAFGMLAGGSGITPMFQVARAILENPNDI
TKVHLVYANVTHDDILLKEELDNMAKTYPDRFKIYYVLNQPPEVWNGGVGFVSQDMIKAH
LPAPAEDIQILRCGPPPMNKAMAAHLDELGYTKEMQFQF*
>11682.m03917|LOC_Os05g40990.1|genepair349-2
MEFLQGQSTETAVAVAVAVVAVAAGAAFLLLLLRSSKKPKGCLDPENFKEFKLVEKRQIS
HNVAKFRFALPTPASVLGLPIGQHISCRGQDATGEEVIKPYTPTTLDSDLGRFELVIKMY
PQGRMSHHFHEMKVGDYLSVRGPKGRFKYQPGQVRAFGMLAGGSGITPMFQVTRAILENP
SDNTKVHLIYANVTYDDILLKEELDSMVETYPDRFKIYYVLNQPPEIWNGGVGFVSMEMI
QTHCPAPAADIQILRCGPPPMNKAMAEHLENLGYTKEMQFQF*
>11667.m05963|LOC_Os01g59950.1|genepair350-1
MADCRSLIEFLRAFEHHRRAADSAAAAGCSSSSSRSRRGGSSLTALCDHSPMAAVDAVVL
LAVVAALGFLVVPYAKMALLEMGALLHPAASCLSAAAFAGAAVAVAAAVLAWELVGHHAR
KCGKPRCRGLKKAVEFDIQLETEECVRGHPAPAARSALLAAAGAHPVELGDAHRELEAEL
RKMAPPNGRTVLIFRSPCGCPKGRMEVWGAKKVRRIKK*
>11682.m03913|LOC_Os05g40950.1|genepair350-2
MADCRSLIEFLRAFEHRHRHHAAAASTDSAACGTAPARPRRGSSPRRGRRRLFTSLCDHS
AMAAVDAVALLAVVSALAFLVTPYVRMVAAEVGGLVSDLDAAGVASSYYAPFAAAGAGAA
IAAVAGVVAWDAVGHRARRCGKPRCRGLRKAVEFDIQLETEECVRGQQQRLLPLPGGRAA
LLAAAGARPVQLGDAHRELEAELRKMAPPNGRTVLIFRSPCGCPKGRMEVWGAKKVRRIK
K*
>11667.m05967|LOC_Os01g59990.1|genepair351-1
MVLKTELCRFSGQKIYPGKGIRFIRADSQVFLFANSKCKRYFHNRLKPAKLTWTAMYRKQ
HKKDIHAEAVKKRRRTTKKPYSRSIVGATLEVIQKKRSEKPEVRDAAREAALREIKERIK
KTKDEKKAKKAEVAKSQKTQSKGGATQRGAKGPKIGGGGGKR*
>11682.m03899|LOC_Os05g40820.2|genepair351-2
MVLKTELCRFSGQKIYPGKGIRFIRADSQVFLFANSKCKRYFHNRLKPAKLTWTAMYRKQ
HKKDIHAEAVKKRRRTTKKPYSRSIVGASLEVIQKKRAEKPEVRDAAREAALREIKERIK
KTKDEKKAKKAEVTKSQKSQSKGAAPRGSKGPKIGGGGGKR*
>11667.m05974|LOC_Os01g60060.1|genepair352-1
MVEMQWLLLLFMLLVSLRLSFSQTNPQDVSALQALMKNWQNEPQSWMGSTDPCTSWDGIS
CSNGRVTEMRLSGINLQGTLSNAIDQLSSLTYLDLSNNLNLGGPLPPSIVNLKQLTTLIL
LGCSFTGDIPEQIGALRQLTFLALNSNKFTGGIPPTLGLLSKLFWLDLSDNQLSGKIPVS
SGSNPGLDQLVNAEHFHFSENQLTGPIDEKLFSEKMNLIHVIFDNNNFTGPIPGSLGRVS
SIQIIRLDHNQFSGPVPGSIANLSRLMELSLASNQLNGTVPDLTSANALTYVDLSNNNFM
SSPAPRWFSTLTSLTTLFMDSDHLTGTIPSALFSFPQLQQISLAKNSFSGELNMSSNISS
LLRVVNLTNNQIFNAEVDPSYTGSLILSGNLICFNNISFCTLKQKQQVPYSTNLGPCGAI
SCPTDQSANPVASQNCACASPFQGLMIFRAPAFSDVTNPKSFQPLEFTLVQNLSLAPGSV
AISNVEFSPGEPLTFTVKVFPESGTSFNHSEVIRISSSLVNQTYKAPAYFGPYSFIASTY
FASPSGKRSSMGKGAIIGIAVAGFLLLVGLILVAMYALRQKKIAKEAVERTTNPFASWGQ
GGKDNGDVPQLKGARYFAFEELKRCTNNFSETQEIGSGGYGKVYKGMLANGQMAAIKRAQ
QGSMQGAAEFKNEIELLSRVHHKNLVSLVGFCYEQGEQMLVYEYIPNGTLRENLKGKGGM
HLDWKKRLQIAVGSAKGLAYLHELADPPIIHRDIKSTNILLDESLNAKVADFGLSKLVSD
TKKGHVSTQVKGTLGYLDPEYYMTQQLSEKSDVYSFGVVMLELITSRQPIEKGTYIVREI
RTAIDQYDQEYYGLKSLIDPTIRDSAKMVGFRRFVQLAMECVEESAADRPTMNDVVKELE
IIIQNEGAQLLNSASLSAQQFGYAKGRDPDPYGDHVPINDDSSSSAFDYNSVYSYSVVEP
K*
>11682.m03893|LOC_Os05g40770.2|genepair352-2
MEPRVLMALALVVVAAGVPAVLCQTNAQDAAALEGLKSQWTNYPLSWNSGDPCGGGWDGI
MCTNGRVTTLRLSSVSLQGTLSSSIGQLGQLTYLDLSFNINLGGPLPAEIGNLGELTTLI
LAGCSFTGNIPIAIGNLRKLGFLALNSNKFSGGIPSSIGVLTNLLWLDLADNQLTGSVPI
STSTSPGLDQLVKTQHFHFNKNQLTGTLTGLFNSNMTLIHILFDSNKFSGSIPAEVGTVS

TLEVLRLDRNGFTGAIPATIGSLVKLNELNLANNKLTGSVPDLSNMTNLNVVDLSNNTFD
PSVAPSWFTSLTSLASVSIVSGSLSGQVPKGLFTLPTLQQVVLSNNQFNGTLEITGNISS
SLQTVNLMDNRIVSTDTASYKKTLLLAGNPFCAEQDPNNRAFCSRQLQNASPYSTSMEKC
GSAQCSDGQNVNPASCGCAFSYNGKMVFRAPFFVDLVSSTPFQLLESTMAAKLNLLPGSV
ALSDIHFNSDNYLQVQVKLFPTSGVTFNLSELTRIGSSLSNQIYKPPANFGPYFFIADPY
APLAVALGGKKSKMSTGAIAGIAVAGGVLVIALIFMSLFALRQKRRAKELKERADPFASW
AAGQKDSGGAPQLKGARFFSFDELKICTNNFSDNHEIGSGGYGKVYRGILGDGTRVAIKR
ADRNSMQGAVEFKNEIELLSRVHHRNLVSLIGFCYEQGEQMLVYEYISNGTLRENLTGSG
MYLDWKKRLRIALGSARGLAYLHELADPPIIHRDIKSTNILLDNNLKAKVADFGLSKLVA
DTEKGHVSTQVKGTLGYLDPEYYMTQQLSEKSDVYSFGVVMLELVSGRQPIEKGRYVVRE
VRLAIDPADHDHHYGLRGIVDPAIRDAARTPVFRRFVQLAMRCVDESAAARPAMGAVVKE
IEAMLQNEPDDAGAGEGDSSADPSANEFDRHRGGGGPPAHPYSDVEISRGSYAGDGASDY
MPYFEVKPK*
>11667.m05976|LOC_Os01g60080.1|genepair353-1
MTTMRASAALLLVAAAVLAAVRAEDPYHFFDWKVTYGTRTIMDVAQKVMLINDMFPGPTI
NCSSNNNIVVNVFNQLDHPLLFNWHGIQQRKNSWMDGMPGTNCPIQPGTNWTYKWQPKDQ
IGTFFYFPSMGMQRAAGGYGIITVHSRLLIPVPFDEPAGDYPVLVGDWYTKDHTVLAKNL
DAGKSIGRPAGLVINGKNEKDASNPPMYTMEAGKVYRFRVCNVGIKTSLNVRIQGHSLKL
VEMEGSHTVQNSYDSLDVHVAQCVSFLVTADQKPGDYLLVASTRFLKEYSAITAIVRYNG
SNTPASPKLPEGPSGWAWSINQWRSFRWNLTASAARPNPQGSYHYGQINITRTIKLCTSK
GKVDGKERFALNGVSHVDDAQTPLKLAEYFNASSGVFEYNLIGDVPPATTVPQKLAPNVI
SAEFRTFIEVVFENPEKSIDSFHINGYAFFAAGMGPGIWTPECRKTYNLLDTVSRHTIQV
YPRSWTAVMLTFDNAGMWNIRSNMWERYYLGAQLYVSVVSPARSLRDEYNMPEIALRCGK
VVGLPMPPSYLPA*
>11682.m03888|LOC_Os05g40740.1|genepair353-2
MTTTTRVAAAAAGVLLVAAALAGVARGEDPYVFFEWKVTYGTKTLLDAPQKVILINGEFP
GPRINCSSNNNIVVNVFNQLDEPLLFTWNGMQHRKNSWQDGLAGTQCPIAPGTNYTYKWQ
PKDQIGSFFYFPSLGMHRAAGGYGGISVVSRLLIPVPFDPPADDHMVLIGDWYTKDHAAM
AKMLDAGKSFGRPHGVVINGKSGKAAADPPMFTVEAGKTYRLRVCNVGIKASLNFRIQGH
DMKLVEMEGSHTVQDMYDSLDVHVGHCLSVLVDADQKPGDYYAVASTRFIHEAKSVSAVI
RYAGSSTPPSPAVPEPPAGWAWSINQWRSFRWNLTASAARPNPQGSYHYGQINITRTIRL
MVSRGHIDGKLKYGFNGVSHVDAETPLKLAEYFNVTDGVFRYNQMTDVPPAVNGPLHVVP
NVITAEFRTFIEIIFENPEKSMDSVHLDGYAFFAVGMGPGKWSAEERKTYNLLDGVSRHS
VQVYPRSWTAIMLTFDNAGMWNVRSNIWERHYLGEQLYISVVSPARSLRDEYNMPENALR
CGKVVGLPLPPSYLPA*
>11667.m05980|LOC_Os01g60120.1|genepair354-1
MDGNFGSEERILWPASVLAGIAMCAAFSALQSLSFSLLVGVKTVTVYDITQKVSSHCFKG
YDNISPMKKVEWNNRGFSTFHALVAAVVSFYLVVISDLFHSNIIIDRNSWLSDAMFGVSI
GYFLTDLVMILWYFPSLGGKEYLLHHGLSMYAICLALLSGKAHMYILMVLFTEATTPFVN
LRWYLEVAGKKTHNLYLYNGLALFVGWLVARVILFIYFFTHMYFHFDQVKSIFPLGFYSI
LTVPPALAVMNLFWFWKIFKGMLKTLSKRRQQSENGKAE*
>11682.m03884|LOC_Os05g40700.1|genepair354-2
MDVRFGPEEQIVWPASVLAGILMCAAVYDITREVSSRCYKGYNGLNELHKLEWNNRGFST
FHALVAAVVSFYLLVISDLFSKDVHGAIIIDRKSWMSDAMFGVSLGYFLTDLLMILWHFP
SLGGKEYLLHHGLSMYAISLALLSGKGHVYILMVLITEATTPFVNLRWYLDLAGRKDSKL
YLYNGVALFAGWLVARVILFVYFFAHVYLHFDQVRTVFPLGFYSMMAVPPAMSAMNLLWF
RKICKGMVKAMSSANRSQCVKTD*
>11667.m05982|LOC_Os01g60140.1|genepair355-1
MASTVPALIADDLPTNVTSQITDAARPKTTSSVVCYSPMMITTNGIWQGVNPLEFSLPLF
ILQVAVIVVTTRLLVVLLKPFRQPRVIAEILAGVVLGPSVMGQVEVWATMVFPQRSLLTL
ETVAHLGLLYFLFLVGLEMDLDVIRRSGKKALFVAVAGMALPFCIGIATSFIFRHQVSRN
VHQTSFLLFLGVALSVTAFPVLARILAEIKLLNTELGRIAMSAAIVNDMCAWILLALAIA
ISEVNSTALSSLWVLLAGVLFVLFCFYVVRPGMWWLIRRIPEGEVVSDMQVSLILTGVML
AGVCTDAIGIHSVFGAFVYGLVIPGGQLGVALIEKLEDFVTGLLLPLFFAISGLRTNISK
IRDPITVGLLVLVFTMASFAKIMGTIIIAALYTMPFREGIALGFLMNTRGLVEMIVLNIG
RDKEVLDDESFAVMVLVSVAMTTLVTPVVTGVYRPSRRLVGYKRRNLQRIRHDSELRMLI
CVHTTRNVPSVLSLLELSNPTKRSPIFIYALHLVELTGRASNMLAAAAASASKQNRSSSS
STLPPVTEHIFNAFENYERHTGGISIQTLAAVSPYQTMHDDVSVLAEDKHVSLIVVPFHK
QQTVDGAMEPINPSIRGFNESLLSTSPCSVAILVDRGLSAAAARMAALHRVALFFFGGPD
DREALAYAWRMVEHPGVALTVVRFVPPDYRVRSYSNTNYRSVASDADPRSIGIDTEGKTE
LQMDEEYLGDFRTRNIGNDAISYSDKVVANSEETVSAIRNMDDSLHELYIVGRRPGEAGS
PMTASLEDWMECPELGPIGDMLVSSDFSMSVSVLVVQQYVVAAAAPAPATTAPAGNADPV
RQYVSNANQRPSAAYRTSAASTANSRWSGGGTVGF*
>11682.m03879|LOC_Os05g40650.1|genepair355-2
MAPIMSGAAAAAGGTGGAVPLIKNATSASQMSRGKAGTGAGAVVCYSPMMVTAYGIWQGA
SPLDFSLPLFLLQVAIIVATTRLLVILLKPFRQPRVIAEILAGVILGPSVMGQVSTWAVK
VFPERSLLTLETVAHLGLLYFLFLVGLEMDVNTIRRSGKKALIIAVAGMALPFCIGTATS
FIFRHQVSKNVHQASFLLFLGVALSVTAFPVLARILAEVKLLNSDLGRIAMSAAIVNDMC
AWILLALAIAISEVNSSAFSSLWVLIAGVAFVLACFYVVRPLMWWIVRRVPEGEAIGDVH
ITLILTGVMVAGVCTDAIGIHSVFGAFVYGLVMPSGPLGVVLIEKLEDFVTGLLLPLFFA
ISGLRTNVTKVRDPITVGLLVLVFVMASFAKIMGTILIAVSYTMTFRDGVALGFLMNTRG
LVEMIVLNIGRDKEVLDDESFAVMVLVSVAMTALVTPVVTTVYRPARRLVGYKRRNLQRS
KHDAELRMLACVHTTRNVPSIISLLELSNPTKRSPIFIYALHLVELTGRASNMLAAHHSA
SNPGGASDHIFNAFESYEEMVGGVSVQALTAVSPYQTMHEDVCVLAEDKHVSLIVLPFHK

QQTVDGGMEPINASLRGFNESILASAPCSVGILVDRGLSAAAARMAAVHHVALLFFGGPD
DREGLAYAWRMVENPGVCLTIVRLIPPGYTAPAISPPQPPMPAAHSRAINVVPEVAKSER
QMDEEYLNEFRSRNLGNDAILYVEQVVANSEETVAAIRSQLDNAHELYIVGRHPGEASSP
LTSALAEWMESPELGPIGDLLVSSEFSKMASVLVMQQYVITAPLPPPVALAGPPTDDPVR
QYLTNANQRPSVAIGGNQMGAAGRGGWSGGAGGY*
>11667.m05985|LOC_Os01g60170.1|genepair356-1
MAKVHPNVVPVAAAAGPAGGERRGEEEEEAAALTVWRKSLLFNCKGFTVFDAKGNLAYRV
DSYDTESGDEVVLMDAAGAPAFTVRRKRQLSLQGEQWLVFAGEADGRRPPVYAVRRTGRG
GGKSLARVTPCAGAAAAGASAAYEVEGSYARRCCVVYDGERRAVAEVRPKEAVGTDVFRL
VVQPGVGVSLAMAVVVALDQMFGRPSLLRSWSS*
>11682.m03877|LOC_Os05g40630.1|genepair356-2
MAAKVHPNLAVPSLIQPPMAPPAAMAAGDSVMKTKAAAAGGDVVLTVWRKSLLFNCRGFT
VFDASGDLVYRVDSYAADSRAEVVLMDAAGVPVLTVRRKKAIGSQLGLGGDQWLVHPGEE
TRLPPLYAVKRTPQYVRGGGSVKTMAHVAPCGVALGAGGGGGYEIEGSYLRRSCAVYDAR
RRAVVAEVQAKEAVGTDVFRLVVRPGMEVSVAMAVVLALEQMFGKPSLLRSWSS*
>11667.m05987|LOC_Os01g60190.1|genepair357-1
MASSGFSWTLPDHPKLPKGKTVALVVLDGWGEANADKYNCIHVAQTPVMDSLKNGAPERW
RLVKAHGTAVGLPSEDDMGNSEVGHNALGAGRIFAQGAKLVDLALASGKIYDGEGFNYIK
ECFDKGTLHLIGLLSDGGVHSRLDQVQLLLKGASERGAKRIRVHILTDGRDVLDGSSVGF
VETLESDLSQLRDKGIDARIASGGGRILSLKNAQNDWDVVKRGWDAQVLGEAPYKFQNAV
EAVKTLRAETKASDQYLPPFVIVDESGKSVGPVVDGDAVVTFNFRADRMVMLAKALEYAD
FDKFDRVRVPKIRETVKFGHVTFFWNGNRSGYFDETKEEYVEIPSDIGITFNVKPNMKAL
EIAEKARDAILSGKFDQVRVNLPNGDMVGHTGDIEATVVACKAADEAVKIILDAIEQVGG
IYLVTADHGNAEDMVKRNKSGQPLLDKNGGIQILTSHTLQPVPVAIGGPGLHPGVKFRSD
IQTPGLANVAATVMNFHGFEAPADYEPTLIEVVDN*
>11682.m03855|LOC_Os05g40420.2|genepair357-2
MGSSDFSWTLPDHPKLPKGKPVAVVVLDGWGEADADQYNCIHVAETPTMDSLKKGAPEKW
KLVKAHGTAVGLPSDDDMGNSEVGHNALGAGRIFAQGAKLVDTALASGKIFEGEGFKYIK
ESFDQGTLHLIGLLSDGGVHSRLDQLQLLLNGASANGAKKIRVHILTDGRDVLDGTSVGF
VETLENDLSQLRAKGIDACIASGGGRMYVTMDRYENDWDVVKRGWDAQVLGEAPHKFQNA
VEAVKTLRSETKANDQYLPPFVIVDDSDKAVGPIVDGDAVVTFNFRADRMVMIAKALEYE
DFDKFDRVRVPKIRYAGMLQYDGELKLPSHYLVSPPEIERTSGEYLVKNGIRTFACSETV
KFGHVTFFWNGNRSGYFDETKEEYVEIPSDSGITFNVKPKMKALEIAEKARDAILSGKFD
QVRVNLPNGDMVGHTGDIEATVVACKAADEAVKMILDAIEQVGGIYLVTADHGNAEDMVK
RNKSGQPQLDKKGEIQILTSHTLQPVPVAIGGPGLHSGVRFRNDVQTPGLANVAATVMNL
HGFEAPADYEPTLIEVVDK*
>11667.m05988|LOC_Os01g60200.1|genepair358-1
MEARRVSANPRPSCVRRVLARKRRRPEATANSARKLQRREISALPCRAFSASTTRERFRN
IQLQEEFDTHDPKEMGSLLPYLMKRSEIIEIVGASDIIFALSQSGVCAAFSRVSNQRICF
LNGRPDEVIRSLFYNKNNDSLITVSVYGSENFSALRCRTTRIEYIRRGKPDAGFPLFETE
SLKWPGFVEFDDVNGKVLTYSAQDSTYKVFDLKNYTLLYTISDKNVQEIKISPGIMLLIY
SRKKGCIPLDILSIEDGKRLKSFKHLLHRNKKVDFIEQFNEKLLIKQEGENLQILDVRNF
QSIEVSRSEFVTPSAFIFLYEMQLFLTFRSRSVSVWNFRGELVTSFEDHMLWHPDCNTNS
IYITSNQDLIISYCKADPNDPSSEENACSINISEILTGKCLAKIKAGNLNKQRVSKFQST
PSEALGDITALYYDEEREEIYTGNRLGLVHVWSN*
>11682.m03853|LOC_Os05g40410.1|genepair358-2
MEARRISASPRPCSGRRVVARKRPRHEAAVNSVRKLQRREISSCRDRAFSMSAAQERFRN
IQLQEEFDTHDPKENSLLLPYLRKRSKIIEIVAARDIVFALSQSGVCAAFSRETNRRICF
LNGSPDEVIRSLFYNKNNESLITVSVYGSENFSALRCRTTRIEYIRRAKPDAGFPLFESE
SLKWPGFVEFDDVNGKVLTYSAQDSTYKVFDLKNYTLLYSISDKNVQEIKISPGIMLLIY
TRTSSSVPLKILSIEDGTVLKSFNHLLHRNKKVDFIEQFNEKLLVKQEGENLQILDVRNF
QLTEVSRTEFMTPSAFIFLYELQLFLTFRNRSVAVWNFRGELVTSFEDHLLWHPDCNTNN
IYITSDQDLIISYCKADSNDSSSEENAGSINISNILTGKCLAKIKASDLCKQKKAWKFQS
TALEALEDITALYYDEERDEIYTGNRQGLVHVWSN*
>11667.m05991|LOC_Os01g60230.1|genepair359-1
MMERSLMEALATAAQGGTVGTSVFDMLKYAVLPIAKVFTVCFMGFLMASKYVNILQPNGR
KLLNGLVFSLLLPCLIFSQLGRAITIEKMLQWWYIPVNIVVGAVSGSLIGFVVASIIRPP
YPYFKFTVIHIGIGNIGNIPLVLIAALCRDPTNPFGDSDKCNEDGNAYISFGQWVGAIIV
YTYVFKMLAPPPGESFDSAEEDILPIKASGDNVVPEKGKYPTSTRTSTVPENEPLLSSEG
DKNVSTSLGSKIMGIVRSMVKFLKDKQLLQPPIIASVFAIAIGVVPVLKNFVLTDDAPLF
FFTDSCLILGEAMIPCILLAVGGNLVDGPGEGSNRLGVRTTVAIIFARLILVPLAGVGII
VLVDKLGFIPKDDKMFKFVLLLQHSMPTSVLSGAVANLRGCGKESAAILFWVHIFAVFSM
AGWIILYLSLLF*
>11682.m03845|LOC_Os05g40330.1|genepair359-2
MMGRSVLEMVVAAAQGGGGAAGESVLGMFRYAVLPIAKVFVVCFMGFLMASKRVGVLKPS
GRKLLNALVFSLLLPCLIFAQLGRSITIDKIMEWWFIPANIALGAVSASLVGLIVALIVR
PPYPYFKFTITHIGIGNIGNIPLVLISALCRDQLNPFGDSNKCTQDGNAYLSFGQWVGAI
IVYTYVFKMLAPPPGQTFDSCDEERDKLPIKAPNTMSSVAKYPSSAHGNTHEEEPLLSIE
EEEEEEGQDVHSLGSKIMIPIKGMVRFLQKKQLLQPPIIASVLAITLGVVPFLKNLILTD
DAPLFFLTDSCLILGEAMIPCILLAVGGNLVDGPGEGSRRLGVRTTVAIIFARLILVPIA
GIGIVSFADKLGFIPKGDKMFKFVLLLQHSMPTSVLSGAVANLRGCGKESAAILFWVHIF
AVFSMAGWIILYLTMLF*
>11667.m05996|LOC_Os01g60260.1|genepair360-1
MEALTNAEKCFSPARAMSPLPLVRPPPSPGAAGQYLAELLQEQQKIGPFVQVLPICGRLL

NQEIMRMSAIVSHLGVREHDRLPIASPNQMHPLPQVPNFCGNGFNPWTGTLPEKNGFPRG
TMGWEGAAHDPSYIVKKIVRLEVPTDAYPHFNFIGRLLGPRGNSLKRVEASTGCRVFIRG
KGSIKDPIKEEQLKGRPGYEHLSDPTHILIEAELPADVIDTRLAQAQEILEDLLKPVEES
QDFLKRQQLRELAVLNSTYREDSPHQNGSASPFSNGSTKLGKQ*
>11682.m03841|LOC_Os05g40290.1|genepair360-2
MDGLHGTDGCFSPGRAMSPQVRPPVPPDAASGGQYLAELLQEHQKLGPFMQVLPICSRLL
NQEIMRVSGMFRQPGVGDFERSQPASPNQMHPSHIVPNFCGNAFGPWNGMRPERVSFSQG
PGWQGAPQSPSSYIVKKILRLEIPTDAYPNFNFIGRLLGPRGNSLKRIEASTGCRVFIRG
KGSIKDPNKEEQLKGRAGYEHLDDPLHILIEAELPANVIDARLAKAQEILEELLKPVDES
QDYYKRQQLRELALLNSPLREESPHPGSASPFSNGGMKRMKQ*
>11667.m05999|LOC_Os01g60280.1|genepair361-1
MANFRGFRRAVIVVVLCICHVNVVRGQSTDPAEVNALRAIKGRLIDPMNNLKNWNSGDPC
TSSWKGVFCDNIPINNYLHVTELQLFKMNLSGTLAPEIGLLSQLKTLDFMWNNLTGNIPK
EIGNIHTLRLIGTFSIKYPNQLLIDSTIREMCRQLIVVAYFHGTLNGNQLSGSLPDEIGY
LQNLNRLQIDQNEISGPIPKSFANLTSMRHLHMNNNSLSGQIPSELSRLPELLHLLVDSN
NLSGPLPPELAETRSLQILQADNNNFSGSSIPAAYENIPTLLKLSLRNCNLQGGIPDISG
IPQFGYLDFQNNSLGNIPAAFEPPEEVTILLYGNPVCTNSTPARAARLCQPTSVTEAPSG
QGSQVSINCSPCPTDKNYEYNPSSPLPCFCAVPLGVGFRLKSPGISDFRPYKEDFQKNLA
HLLVLADYQIYMERYIWEVGPRLNMHLKLFPNNTNLFNTSEVVRLRHLLAGWEITLSNVF
GPYELLNFTLGSYEDEFPTVASSGLKRGALAGILAGTITASIAASVFTTIFIMRRRSKRR
TTSRRSLLSRYSVKVDGVRCFTFDEMAAATNDFTDSAQVGQGGYGKVYKGNLTDGTAVAI
KRAHEGSLQGSKEFCTEIELLSRLHHRNLVSLVGYCDEEDEQMLVYEFMPNGTLRDHLSA
KSRRPLNFSQRIHIALGAAKGILYLHTEADPPIFHRDVKASNILLDSKFVAKVADFGLSR
LAPVPDVDGTMPAHISTVVKGTPGYLDPEYFLTHKLTDKSDVYSLGVVLLELLTGMKPIQ
HGKNIVREVNTAYQSGEIAGVIDERISSSSSPECVARLASLAVKCCKDETDARPSMADVV
RELDAIRSALPEGEELLPEYGDQSATSTSLTATGPLSSSSTTGALFISSGSGGHANSKSG
IPSGTVAPR*
>11682.m03834|LOC_Os05g40270.2|genepair361-2
MINFGGVLCAVTLVVLLPFLEAADGKSTDPSEVSALMAIKGSLVDPMNNLKNWNRGDPCT
KNWTGVFCHDLGDTYLHVTELQLFRRNLSGNLVPEVSLLSQLKILDFMWNNLTGNIPKEI
GNITTLKLILLNGNQLSGLLPDEIGNLQSLTRLQVDQNHLSGAIPKSFANLRSVKHLHMN
NNSLSGQIPSELSRLNTLLHLLVDNNNLSGPLPPELAAAKSLKILQADNNNFSGSSIPTL
YYNMSGLFKLSLRNCSLQGAIPDLSAIPQLDYLDLSWNQLTGSIPTNKLASNITTIDLSH
NMLNGTIPSNFSGLPYLQLLSLKNNLLDGSVPSEIWAGVNPNRNGSLVLDFQNNSLNMLP
AEISPPPPNVTVVLYGNPICENSSETLIINLCRLQSINLEKSKQETSTAMVCGACPTEKN
YEYNPSFSDQCFCAVPLGVGLRLKSPGVTDFHPYENAFKIDLTSLLQLFPYQLYIENYIW
EVGPRLNMHLKLFPSNTSLFNMSEVVRLRHVLAGWEITLLDVFGPYELLNFTLGSYEDEY
PNLASSGLSKAALGGILASTIASAIALSAVVTALIMRRNSRTNRISRRSLSRFSVKIDGV
RCFTYEEMTSATNNFDMSAQVGQGGYGIVYKGILADGTIVAIKRAHEDSLQGSTEFCTEI
ELLSRLHHRNLVALVGYCDEENEQMLVYEFMPNGTLRDHLSGKSKPPLGFGLRLHIALGA
SKGILYLHTDADPPIFHRDVKASNILLDSKYVAKVADFGLSRLAPVPDVEGALPAHVSTV
VKGTPGYLDPEYFLTHKLTDKSDVYSLGVVFLELLTGMKPIEHGKNIVREVKKAYRSGNI
SEIMDTRMGLCSPECVDSFLQLAMKCSRDETDARPSMTEIVRELELILKIMPEGDLIQLE
TPQTYSGRAMSKDPMSKSTSNSTNGNYLASSQTFTSVDASSSGVLSGMVSPR*
>11667.m06004|LOC_Os01g60330.1|genepair362-1
MQDHILTAFLVVSLLFACIPPAKSADLNSDKQALLAFAASLPHGRKLNWSSAAPVCTSWV
GVTCTPDNSRVQTLRLPAVGLFGPLPSDTLGKLDALEVLSLRSNRITVDLPPEVGSIPSL
HSLYLQHNNLSGIIPTSLTSTLTFLDLSYNTFDGEIPLRVQNLTQLTALLLQNNSLSGPI
PDLQLPKLRHLNLSNNNLSGPIPPSLQRFPANSFLGNAFLCGFPLQPCPGTAPSPSPSPT
SPSPGKAKKGFWKRIRTGVIIALAAAGGVLLLILIVLLLICIFKRKKSTEPTTASSSKGK
TVAGGRGENPKEEYSSGVQEAERNKLVFFEGCSYNFDLEDLLRASAEVLGKGSYGTTYKA
VLEDGTTVVVKRLKEVVVGKKDFEQQMEIVGRVGQHQNVVPLRAYYYSKDEKLLVYDYIP
SGSLAVVLHGNKATGKAPLDWETRVKISLGVARGIAHLHAEGGGKFIHGNLKSSNILLSQ
NLDGCVSEFGLAQLMTIPPAPARLVGYRAPEVLETKKPTQKSDVYSFGVLVLEMLTGKAP
LRSPGREDSIEHLPRWVQSVVREEWTAEVFDVDLLRHPNIEDEMVQMLQVAMACVAAPPD
QRPKMDEVIRRIVEIRNSYSGSRTPPEEKQKDESAAP*
>11682.m03824|LOC_Os05g40200.1|genepair362-2
MQHLVLIAFLSASFLFLHIPCARCADLNSDRQALLAFAASVPHGRKLNWTLTTQVCTSWV
GITCTPDGRRVRELRLPAVGLFGPIPSDTLGKLDALQVLSLRSNRLTISLPPDVASIPSL
HSLYLQHNNLSGIIPTSLSSNLTFLDLSYNSFDGEIPLKVQNITQLTALLLQNNSLSGPI
PDLHLPNLRHLNLSNNNLSGPIPPSLQKFPASSFFGNAFLCGLPLEPCPGTAPSPSPMSP
LPPNTKKSFWKRLSLGVIIAIAAGGGLLLLILIVVLLICIFKRKKDGEPGIASFSSKGKA
AAGGRAEKSKQEYSSSGIQEAERNKLIFFNGCSYNFDLEDLLRASAEVLGKGSYGTTYKA
VLEDGTTVVVKRLKEVVAGKREFEQQMEIIGRVGQHQNAVQLRAYYYSKDEKLLVYDYMT
PGSLCAALHGNRTAGRTTLDWATRVKISLEAARGIAHLHAEGGGKFIHGNIKSSNILLSQ
GLSACISEFGLAQLMAIPHIPARLIGYRAPEVLETKRQTQKSDVYSYGVLLLEMLTGKAP
LRSPGREDSIEHLPRWVQSVVREEWTSEVFDADLLRHPNSEDEMVQMLQLAMACVAIVPD
QRPRMEEVVRRIEEIRNSSSGTRLSPEDKLKEEAIQIT*
>11667.m06015|LOC_Os01g60420.1|genepair363-1
MESEQVKRRFGRCPYCRAMIYQDPNAIIYYCSKCRTPIRGKNPEPTDDAEYALSQLEILS
ADTASVFSDDPDTLSRTSSVAYGGGEQPPVRTSSAPYAAFDRGSVRAGSRSGEQSGEERG
GSPMHSRVSELRPTSRRTRRPMSGDMGAFRDDGSSYGSDNDVPTSAAASYRRRASPLTSQ
ELEASSSSMGSSGYQPSGVSSSSMGSSSVYEPSGAARSPLTDPAFQRDLLQALDNLRRVI
AAVEQPYGVDAHLQQAGMPPKSASCNDAATGGSGGGGGAYAAAVTRRNSRLMRRLESQLV

QALPRDGLRRDRSTSSSSSASSSRPGGDRARAAGRKHHCRAVLGGTPFVVCDKCSEILQL
PAAVSANRAARLECGGCGETLSIKLPAAAAAAASGSTDRPKKIFSAPQPAVRRLDDDDAG
EEHASARSNLSGDQRWPASPAEGPLHRMLGYSTVSSVFRSRRYGEQH*
>11682.m03821|LOC_Os05g40170.1|genepair363-2
MQSEEVKMRFGRCPYCRAMIYQNPKAVIFYCSRCRTPIRGKNPEPTDEAEYALSRLEILS
ADTASVFSDEPEDAGSDRRADEVRPLSRRTRRPSSSSDWTTTTDSERSEEAFYTPRNAQE
GRPWQSPSPVSSQELGASGGGGGLPRPPDEPGAVAAARLMDPAFHKELLHALDNLRSLIV
TIEQPRPASGGGGRALTRRDSRLFRRLESQLERALPPQDTASTSASSSSCRGDGGGGRPS
APARREGTDPCRPVLGGAPFVICGKCSELLRTPPPPRPRRRRWTTRIRCGECNEVLELSL
PAGGVPAQHRPIRTCSAPLVSDHHRPLPRRLE*
>11667.m06025|LOC_Os01g60520.1|genepair364-1
MASPRLKREQSFDFEEASAQEAVGSASASYSPPGGGGVFGISPPESSPRDGRKRRKDRPS
LVKHTFTPHFDGHLWRKYGQKNIKDSAFPRLYYRCSYREDRQCLASKLVQQENDDDPPLY
RVTYTYEHTCNTTPVPTPDVVAEQPPPGAAGDAYLLRFGSSAGGGGGGAHQQQTERERQQ
QNTARRRPFMMLSFDSSSSHQLHEQPHAFPPDGQLPATAAAASPSSFTAAEALAAPPLTT
TMNDGGDLFSTWDALRYGLDYDHGHLGNHVYLPDDCNGGDDNY*
>11682.m03811|LOC_Os05g40070.1|genepair364-2
MARRLPKSERSPSPPPPPPGDQRDAAIQELSKGSELATQLMAQLELIPERELDGRRDDAL
ANVRSLSMSLSSSLYALRSERREHYYCGSSSSSGGAGPAAVTSVSGAGGERKTKRRRGKH
GEELIETVFITTTPENDGFHWRKYGEKNILNSEFRKLYYRCGYSDERKCQAKKYVQQENN
KHPPEFRVTLTNEHTCNTVFQDQPSSSSTNSQVLDFTKASISSSLMDSHVGAPILKEEEE
EEVPSIDESTRIMSTIMRNYGSYGDYDESSPQPWNGAGWK*
>11667.m06037|LOC_Os01g60640.1|genepair365-1
MAMLGSSSAVVLELMTMGYQSAAYLGELLRAASPAQAGDEQQELAAEILRCCDRVIAKLN
RGGATGATTGKKRKAAESAAAAAVTSPSLPVTPTKRRARGAEAVREVRSGTTTDGFIWRK
YGQKEINGCKHPRLYYRCAFRGQGCLATRRVQQSQSQDDPAAAFVIAYYGEHTCGGDAAA
AAACRDGELMPPAVINSGASSFAAAWNMASREPASSLAVERRSCDGDAPSETSQGWSPSF
SSEVELDVVGFDLAGADSSASPVWEFLNGSFDWEFVINSL*
>11682.m03810|LOC_Os05g40060.1|genepair365-2
MALIATGATATATAAPVASPAASSMASELMAQGRESAAVLEALLHGASLPPAHGGAHALA
AEILRCCDRALAALRAGGDAESSSADTKRKPATAQPSTRRRRRYIYMYVSSRSCDGARDR
AATQQEIFCRATASGGGAAAAAEPARVEKARTSEDGFLWRKYGQKEIKNSKHPRLYYRCS
YKDDHGCTATKQVQQSEEDPSLYVITYFGDHTCSCQTAAAAAMDDDDDDENSQHFVINFG
PATASRSGSPPLLYDDGDDGDVWRETAATPPSSRQSRCSPEGDGEESGVKMSKEEPVDSC
PGPSAVSSPADVVSCSSPAMEPDLLGCLNWDDDFGDSSFVDADEFMNFDEIDLFQIYS*
>11667.m06040|LOC_Os01g60670.1|genepair366-1
MAAASPLARLLAALLAVAAAATAATALTDDVLALVVFKTGVADPMGRLAAWTEDDDRPCS
WPGVGCDARAGRVTSLSLPGASLSGRLPRALLRLDALASLSLPRNNLSGPVLPGLLAALP
RLRSLDLSSNRLAAPVPAELFAQCRSIRALSLARNELSGYIPPAVTSCASLVSLNLSSNR
LAGPIPDGLWSLPSLRSLDLSGNELSGSVPGGFPGSSSLRAVDLSRNLLAGEIPADVGEA
ALLKSLDVGHNLFTGGLPESLRRLSALRFLGVGGNALAGEVPSWIGEMWALERLDLSGNR
FSGAIPDAIAKCKKMVEADLSRNALAGELPWWVFGLPLQRVSVAGNKLYGWVKVPADAAL
ALRALDLSSNGFSGGIPPQITAFAGLQYLNMSSNSFARQLPAGIGGMRLLEVLDVSANRL
DGGVPPEIGGAVALRELRLGRNSFTGHIPSQIGNCSSLVALDLSHNNLTGSIPSTVGNLT
SLEVVDLSKNKLNGTLPVELSNLPSLRIFDVSHNLLSGDLPNSRFFDNIPETFLSDNQGL
CSSRKNNSCIAIMPKPIVLNPNSSTNPLSQATPTAPSSMHHKKIILSVSTLIAIAGGGTI
IIGVIIISVLNRRARATTSRSAPATALSDDYLSQSPENDASSGKLVMFGKGSPEFSAGGH
ALLNKDCELGRGGFGAVYKTVLRDGQPVAIKKLTVSSLVKSKDDFERQVKLLSKVRHHNV
VALRGFYWTSSLQLLIYDYLPGGNLHKHLHECTEDNSLSWMERFDIILGVARGLTHLHQR
GIIHYNLKSSNVLLDSNGEPRVGDYGLAKLLPMLDRYVLSSKIQSALGYMAPEFACKTVK
ITEKCDVYGFGVLVLEVLTGRRPVEYLEDDVVVLCDLVRSALEEGRLEDCMDPRLCGEFP
MEEALPIIKLGLVCTSRVPSNRPDMGEVVNILELVRSPQDSLEDELV*
>11682.m03809|LOC_Os05g40050.1|genepair366-2
MATATAALLLLTLAAILAAAGAVNDDVLALVVFKSGVSDPGGVLAAWSEDADRACAWPGV
SCDARAGRVDAVALPSAGLSGRLPRSALLRLDALLSLALPGNNLSGPLPDALPPRARALD
LSANSLSGYLPAALASCGSLVSLNLSGNLLSGPVPDGIWSLPSLRSLDLSGNQLAGSVPG
GFPRSSSLRVLDLSRNLLEGEIPADVGEAGLLKSLDVGHNLFTGELPESLRGLTGLSSLG
AGGNALAGELPGWIGEMAALETLDLSGNRFVGAIPDGISGCKNLVEVDLSGNALTGELPW
WVFGLAALQRVSLAGNALSGWIKAPGDNASALQELDLSGNAFSGVIPREIASLSRLQHLN
LSSNTMSGKLPVSIGRMALLEVMDVSRNQLSGGVPPEIGGAAALRKLLMGSNSLTGIIPP
QIGNCRNLIALDLSHNKLTGPIPATIGNLTGLQMVDFSENKLNGTLPVELSKLANLRVFN
VSHNLLSGNLPISHFFDTIPDSFILDNAGLCSSQRDNSCSGVMPKPIVFNPNASSDPLSE
ASPGAPSSQHHKKIILSISTLIAIVGGALIIVGVVTITVLNRRVRSAASHSAVPTALSDD
YDSQSPENEANPGKLVMFGRGSPDFSAGGHALLNKDCELGRGGFGTVYKAVLRDGQPVAI
KKLTVSSLVKSEDEFKRQVKLLGKVRHHNVVTLRGFYWTSSLQLLIYDFVPGGNLYQHLH
ESSAERSVSWMERFDIIIGVARALAHLHRHGIIHYNLKSSNVLLDSNGEPRVGDYGLVKL
LPMLDRYVLSSKIQSALGYMAPEFTCRTVNVTEKCDVYGFGVIVLEILTGRRPVEYLEDD
VVVLCDVVRAALDDGRVEDCMDPRLSGEFSMEEAMLIIKLGLVCTSQVPSHRPDMGEVVS
MLEMVRSSQGTPEDDLV*
>11667.m06042|LOC_Os01g60690.1|genepair367-1
MEPARQLDGGGIAMEIVEEEDAGATRLGRLSLSLDLNGGGGGAFRPSTLLDEYERLAIEA
QLDRAVLRRSYSEPSPSRLAVVAPQDKQEAPPGAAGRRAKEEEKPGAGRAPARRSWLLEA
LKRLLCWLGIGGAWGGGRRRGEEPAAPCPPAPPPRMQLLDYLTTTSTT*
>11682.m03807|LOC_Os05g40030.1|genepair367-2

MERAREEDEVSGGGGGVVARYSLDVSDGCGGRHSALLDEYERMAFEAQLNRAIVLRRCYS
EPSPARFPVAPPQRGGAEGDGCTAPWRSCRLHVVEAVFLRWLEAVKPVLCWLRSAWEQRR
RRMERAAAAPRGPPATVPRVQLMDYFC*
>11667.m06046|LOC_Os01g60730.1|genepair368-1
MGVHTKSMSWYMGTPASPSPGSAGEAQHALSSGGGGGSDASFDTNMVIILAALLFALLFA
LGLNSLARLIIRWARRAATGEGAGGGVEGAGAGGGLKKRALRSIPIEVYGGGGGGSPAAA
AEVCAICLGEFADGEKVRVLPRCGHGFHVRCVDTWLVSHDSCPTCRGSVLHGATTKHKPT
AAAAAAGSRRPGSEVDAPAAAAVTVVIA*
>11682.m03806|LOC_Os05g40020.1|genepair368-2
MASGVAAPAPSVFEAARPALESGGGGGGAPPPGRADASFDTNMVIILAALFFALLFAIGL
NSLARCALRCGGRGAAAAGGGGGGGGGGAAAAGVGCGGIKKRALRSIPVEVYCGGEETAE
TDVCAICLGEFADGEKVRVLPRCRHGFHVRCVDAWLVSHGSCPTCRRQVIGGGGSTPPPD
SDTIAVVVA*
>11667.m06048|LOC_Os01g60740.1|genepair369-1
MAPRCATLAVVVVLVAAVVAPPTAVRAAISCSAVYNTLMPCLPYVQAGGTVPRACCGGIQ
SLLAAANNTPDRRTICGCLKNVANGASGGPYITRAAALPSKCNVSLPYKISTSVNCNAYG
GRPDAYVCARASRTRGE*
>11682.m03805|LOC_Os05g40010.1|genepair369-2
MVPAARSGWPAAAAVLVVVLVLSSPPGTSTVVVARAALSCSTVYNTLLPCLPYVQSGGAV
PAACCGGIRSVVAAARTTADRRAACTCLKNVAAGAAGGPYISRAAGLPGRCGVSVPFKIS
PNVNCNAVN*
>11667.m06053|LOC_Os01g60770.1|genepair370-1
MASRSSALLLLFSAFCFLARRAAADYGSWQSAHATFYGGGDASGTMGGACGYGNLYSTGY
GTNTAALSTVLFNDGAACGSCYELRCDNDGQWCLPGSVTVTATNLCPPNYALPNDDGGWC
NPPRPHFDMAEPAFLQIGVYRAGIVPVSYRRVPCVKKGGIRFTINGHSYFNLVLVTNVAG
PGDVQSVSIKGSSTGWQPMSRNWGQNWQSNSYLDGQSLSFQVAVSDGRTVTSNNVVPAGW
QFGQTFEGGQF*
>11682.m03803|LOC_Os05g39990.1|genepair370-2
MAIAGVLFLLFLARQASAAGYGGWQSAHATFYGGGDASGTMGGACGYGNLYSQGYGTNTA
ALSTALFNDGAACGSCYELRCDNAGSSCLPGSITVTATNFCPPNYGLPSDDGGWCNPPRP
HFDMAEPAFLHIAQYRAGIVPVSFRRVPCVKKGGVRFTVNGHSYFNLVLVTNVAGAGDVR
SVSIKGSRTGWQPMSRNWGQNWQSNAFLDGQSLSFQVTASDGRTVTSNNVAHPGWQFGQT
FEGGQF*
>11667.m06055|LOC_Os01g60790.1|genepair371-1
MTFKRRNGGRNKHGRGHVKYIRCSNCAKCCPKDKAIKRFQVRNIVEQAAIRDVQEACVHD
GYVLPKLYAKVHHCVSCAIHAHIVRVRSRENRRDRRPPERFRRREDRPQGPRPGGGAPAP
GGAAAPAPNVART*
>11682.m03797|LOC_Os05g39960.1|genepair371-2
MTFKRRNGGRNKHGRGHVKYIRCSNCAKCCPKDKAIKRFQVRNIVEQAAIRDVQEACVHD
GYVLPKLYAKVHHCVSCAIHAHIVRVRSRENRRDRRPPERFRRREDRPTGPRPGGAGGPG
APAAAGPGGPPNVART*
>11667.m06057|LOC_Os01g60810.1|genepair372-1
MGRHKFRLSDMIPNAWFFKLRDMRAARGGAGAGGGGASHGGVVTQSSVAVSRAGRACRPL
PNTPRHGALSLPHRASYYYTPRAGDLLVGSPLHPKCSDTQFPPLQLSPPRKSRRRHRRRS
VKLAPSVSGSSVLSSPVSTGCRCGRKPELVVVEAPDTPPCRRDKFVGYNDDDDDEEEEEV
EFKKPTVAVAACDELDGKVITSATDIIIDLRTEKRPDKVLPPIVTKPARRELDGCDLEEK
HIDVVRRASAKKPTTLLEQSKPRRSVSSARRLKTRANTPRIVAKKSKPPPPPPPAAARSP
APTTKPPLAESFAVVKSSRDPRRDFRESMEEMIAENGIRTAADLEDLLACYLSLNAAEYH
DLIVDVFEHIWANLADIKM*
>11682.m03796|LOC_Os05g39950.1|genepair372-2
MGRRKFRLSDMMPNAWFYKLRDMRARGGRGATAMQPPSSSSLMRGSRAAQQQAGTWRLGT
SSSSSSLLPHRASYYYTTRDREVPPLPPPPPPRGVDDQFPSLTLSPPLPTRNSRRRHRVG
RFGSTEMDGGELVLAPSDDHDGCSHQEPPVADASGSSRCRRDMFIGRDGGRGVEFRRRAT
TVDGPEEDAAVDVKVITSDADIIIDLGADDDDDTPERVLRPVVTRPARRELDWCEPAEVK
HVDLAELMTPRASSASASSEKSISTGKPRRSSVSSRRRLKTRTNSPRLAACRKGKPTARA
TTTTPTQPPLAHSFAVVKTSSDPRRDFLESMEEMIAENGIRDAGDLEDLLACYLSLNSGE
YHDLIVEVFEQVWTGLAAACGVMP*
>11667.m06062|LOC_Os01g60860.1|genepair373-1
MAGNPAAAAPSSSGSSSVFLPPPSPSDGELLRPLHRLARDLSAVDTPAPFLRAAFASISR
RSKLLAAAFDDLLLCGAAGELPRSASLCLREVLLVLQRFKAIVADCSARSRMRLLLESDE
MEAELRELNHDLATLLDLLPVVELGLADDVLDVLALASRQCRRCSPAPESEEALKASVLS
LIQEIEREIVPERERLEEILVEVGINDPASCSEEIESLEQEIGDRASEKWTASMIALVGL
LRYAKCVLFSATPRPSDSNSKADVEAEDGEPPVPPSDFRCPISLDLMRDPVVVASGQTYD
RESIDRWFSSGKSTCPKTGQVLANLELVSNKALKNLISKWCRENGVAMEACEASKSEQAQ
AVAANKAALEAARMTASFLVKKLSVSFSPDAANRVVHEIRLLSKSGSENRAFVGEAGAVP
LLVPLLYSEDAGLQLNAVTALLNLSILEANKKRIMHADGAVEAVAHIMSSGATWRAKENA
AAAVLSLASVHSYRRRLGRNQSVVEKLVHLVRTGPTSTKKDALAALLTLAGERENVGKLV
DAGVAEVALSAISKEETAAAVLAALAKRGGAEAIVNIDGAVARLVAEMRRGTDWARENAT
AALVLLCRRLGAPAVTQVMAVPGVEWAIWELMSIGTERARRKAASLGRICRRWAAASAAD
GERGGGCPVATVVPPAMMAS*
>11682.m03794|LOC_Os05g39930.1|genepair373-2
MANARNAAAASPPPPSSSSSYSSSASDGEILRSLHRLARDLAAAEAPAPFLETVFAAVSR
RAKLLAAVFDDLLRCGRLPRSASLCLREVLLVLQRFKAVVADCSARSRMRLLLQADEVAA
RVRELQHDLATLLDLLPVPELGLADDVVDLLALASRQCRRSSPSADAAEHELKTGVLALI

QEVEREIVPERERLEGILEEVGINDPACCSDEIETLEREIGDRVAERWTSAMIALVGLLR
YAKCVLFTAATPRPMDTKVDVDDDDDDDDAEPPSPPPDFRCPISLDLMRDPVVSASGQTY
DRESITRWFGSGKSTCPKTGQVLANLELVPNKALKNLISRWCRENGVAMESSEPSKPEPA
PVVTANKAALEAARMTASFLVKKLSVSFSPAAANRVVHEIRQLARSGNDTRAFIGEAGAV
PLLVPLLHSDDTATQLNAVTALLNLSILDANKKRIMHAEGAVEAICHAMGSGATWRAKEN
AAATVLSLASVHSYRRRLGRNPRVVERVVHLVRTGPSSTKKDAIAALLCLSGERENVGKL
VEAGAAEAALSAISEEETAVAVLASLAKRGGAEAIVNIDGAVVRLVAELRRGTEWSRECA
AAALVLLCRRVGAAVVAQVMSVSGVEWAIWELMATGTERARRKAASLGRACRRWAAAEQT
AEYPATSDVTTTAITAS*
>11667.m06066|LOC_Os01g60900.1|genepair374-1
MASSGGRWRRSGSSMLKLVVALCFFAVAISLCCVCLSSGCGGSGCGGFRRRAVLLRLDFR
TRATAYFGANQDHSSSGRRWRRLLAEGPGSYPPRCTAKCGACVPCYPVHVAVPPGVPVTT
EYYPEAWRCKCGNRLYMP*
>11682.m03789|LOC_Os05g39880.1|genepair374-2
MGSSRRPRRWSGGSKLAVACLAAVAVTSLQLCCLSGCFIAACGGAGRDDDDVRRYSDHFG
RLEGAGAHSGDLLEGGGHYHVGLGRRLLSGGPGSHPPRCTSKCGSCSPCSPVHVSVPPGV
LVTTEYYPEAWRCKCRNRLYMP*
>11667.m06067|LOC_Os01g60910.1|genepair375-1
MDGRSILMGRYEVGKQLGQGTFAKVYYARNLTTGQAVAIKMINKDKVMKVGLMEQIKREI
SIMRLVKHPNVLQLFEVMASKSKIYFVLEYAKGGELFNKIAKEGKLSEDSARRYFHQLIN
AVDYCHSRGVYHRDLKPENLLLDENENLKVSDFGLSALAESKRQDGLLHTTCGTPAYVAP
EVLSRKGYDGAKADVWSCGVILFVLVAGYLPFHDPNLIEMYRKICRADFRCPRYFSAELK
DLIHKILDSDPSTRISIPRIKRSTWYRKPVEINAKNSEAATTNSISSGVATTSGSAECST
SEENQGSLSLPNLNAFDIISLSTGFNLSGFFEDTHGHQEERFTTRQPVTTVLGKLKELAK
RLKLKVKKKDNGVLRLAAPKEGKKGFLELDAEIFEVTPSFLLVELKKTNGDTMEYRKLVK
EDIRPALKDIVWVWQGDEHLNSQSILQGEQQQSPLPPELPQDQLQPSLPQQEKQDMPEPP
LLPQVNASTGNHNDHWLVHWLMMKPFRFPPPDSVPSTGRDEEEGSSHHGNGSGAARENPE
NPLPDLALAKSESDRIKAFYQTVLSAIVVFIVAALSGYKDMKPLYSTTNHKKVHLSNLLV
IEGFCMIATFICAAVLMMYEFYTCRDLTVLIAVAGAMLVATDTILVVIANRDNAVFAVLF
VPVLLLVGMAVYTGASMEEQPPNSPHDGGYDEATKSTFDLATVSTMAALGLQGTIVFGYL
KTPEKKSEHDDPPLDLAVCYIASTVCLTVMAFLDGFVVLSVWPEAVALVLYYAAQLCSGR
QPGGGVGSRPWIEFVFRIVATAGFTLMAGLYAAFLGTNHYSVYLKAAMLVLLMAVLSSLS
RLANPVYMPEIDGALLEFCVAGVALAFPAVSLLVACPLVLKVFVDLYIHHSSL*
>11682.m03788|LOC_Os05g39870.1|genepair375-2
MAADLQTFGALLKYPKVEGPIRKNASAPASACSSPHRRGDPLDADERRLARGGSRSAEFG
AEALILAEGEQASRRVMQGSILVKTESDSPGNAEICAKRSVLMERYVIGRQLGQGTFGKV
YYARNLSSGQSVAIKMIDKEKILKVGLMEQIKREISIMRLVRHPNVLQLFEVMATKSNIY
FALEYAKGGELFHKMARAKLNEESARNYFQQLISAMDYCHSRGVYHRDLKPENLLLDENE
TLKVSDFGLSALAESRRQDGLLHTACGTPAYVAPEVLSRKGYSGSKADVWSCGVILFVLV
ANYLPFHDRNIIQMYRKIAKAEYRCPRHFSAELKELLYGILDPDPSTRMSISRIKRSAWY
RKPIAISALNNETGKKSCTSEAPFSGPTICISSERNQEPPNLHNLNAFDIISLSTGFDLS
GLFGERYGRRESLFTSRKPAAAVLVKLKELAKALNLKVTKTDNGVLKLATTKEGRKGRLE
LDAEVSEVAPFLLVELKKTNGDTLEYQRMMKEDIRPSLKDIIWTWQGNSIELPNDELTVQ
CSEGEVQKQSRLPSIKNFLDRVTVTYSTSRTSWSWTHGHGNCSKNASKNGIKGEAYNADC
VSRRSIEVCAKLLEPDDQMTKLIDRLDRRRCKLIDRFRPKERLEMTD*
>11667.m06069|LOC_Os01g60930.1|genepair376-1
MISTEEDSDSFQDDSEELQMQVTKKTLKRVISLAETFIAIATGILAAAFSAGKDVHLHRH
VLAAGGCFLVVTYLSALLLIYMKLFLSDHRRLRRWHVRSLQLLCVTSGASLVATNSLLLV
LIGEGNGLLSLNLLPVQGIVGVLAYHATPTEGSARDEAFEAQVKSARKVALFAAATAFAV
QTTLVFGAFSNAALQAMGGRRLDLSVSFLASALSVFLVVATCMPLGFRNQGARDKVLSIV
RYLKKGVMAVLAVTAVTLGQEFLGGAAALALFPEITVAAMYYAVSMPADEAAAAADRKME
VLPTVVVATFGFGMLGAAYAALFGTPEYDLYTKALAFTLLTAVVSSLGRVAGPLCNAQRD
KSSAAWVTFLSSILPIVEMLVAVPLAAKVMVDFLAVPGNG*
>11682.m03787|LOC_Os05g39860.1|genepair376-2
MENQPHAHLLPQRNDTQVERATAFGKEAIGITGSAIVAGVSGYKDIGKGAATTLLFKAGG
FSLLVTFVSAVVLMHFQMHQPPAAPRSRCADLSSAVLVSLTGVLLVATNGMFVALMDRDN
DTMLVILVLPVVLVLGMLAGADLPPTEGAVTAAAVAQDEAYEEAMKSSAELATFGATAAF
AIEGALILGYLKYPSSLDGCGRSPPAQVDLAVASFASTVSVLAMAATALPVRTLFPSARA
RAVAVAGHLNRAMLAALVSMATILAVEFLQWWFMLSLLPEAIAVALNVAIMAWTTEGGAN
VVDGSVEAAAAAASAGARERRAKGFRAVATTSFTLMAGTYAVYLGQKKYDVYLRAAMLVM
LTAVVSSLRQMLRPFGRSRARGWWAVAAGAVSLVFPGLALVIAIPLFVKIFVHFYFGHVN
*
>11667.m06070|LOC_Os01g60940.1|genepair377-1
MPPNAAEVARPKSPLRGILPQSPLRIKQDGKFYERLLAKERSAASRSFRHYWAAEPGSVP
FVWESQPGTPKVDVSRMVAGAVPAITPPPSYLLRHGGKAGAAAPAAPRRRPQGKGKAKTG
GKTRYRFKRIKIGFLAGIFRRIALGHVWRRSAASVQVSSLSSRWLFSSAATAPEKAEHLD
HDTPAPPPPPRPNTELSTRARARPSLWMLRFRGFRSWSRDDGWA*
>11682.m03784|LOC_Os05g39830.1|genepair377-2
MASKPQSPLRITHDGEFYARLLTKESSQGNPSFRYYGAGTGAVPFVWESHPGTPKVDASS
PFAEAGGVPAITPPPSYHLRAAAAMSSSPQPHGRSGRRNVVNGRGKYCGYCRLKWIKIGF
IAAVFRRLALGKPSRASSSSSAVQPSPSTRWLFSGSGSVETSDEPQPPAISPASTKQGGL
LCLGVRPSPWMVQLCGVQSIRRVDTGSSWATHGWA*
>11667.m06077|LOC_Os01g61010.1|genepair378-1
MSSPSSAHWLSLVGSIWLQTINGPNSDFPVYSSQLKELKSISQVQLNFLAFASDAGKLFG

WFSGVAALYLPLWVVAFVGAAFGLVGYGIQYMFLDSSGLRYWHLFLLTALAGNGICWINT
VSYLLCINNFASNSRVAVSLATSYLGLSAKVYTSLAETFPGLANSKTKTYLLLNAVVPLF
VTVMVAPSLRVFDLKSAAASSDAAFLVMFAITLATGACAVVGSIGSTANGLSSKEHMISL
GVLLATPILIPVGLKIRETLTKIRETQRENRIHDLGTDESESVESVVVIDVAADANAEVA
KEEDAVVKKPQEEVGGLRLLKSPDFWLYFFSYMFSGTLGLVFLNNLGQIAESRGIGQTST
LVSLSSSFGFFGRLLPAFMDYYSAKSGYSISRTGSMASLMAPMAGAFFLLLNQRDFFLYL
ATAVIGTCTGAITSVAVSATRELFGTKNFGVNHNVVVANIPVGSLCFGYFAAFLYQREAG
ARGTLTCSGAGCYRETFAIWGTTCAVGTLLCAALYARSRNFAGRLPVRIPSCLARLANLV
*
>11682.m03781|LOC_Os05g39800.2|genepair378-2
MPSPSSAHWLSLVGSVWLQTINGPNADFPVYSSQLKEVKGISQVQLNFLAFASDAGKLFG
WFAGVAALYLPLWLVAVVGASFGLVGYGVQFLFLERPGLAYWHLFLLTSLAGNGICWINT
VCYLLCIKNFPSDSRVAVSLATSYLGLSAKLYTTMAEKMPRGATARYSKEKVYLLLNAVV
PMLVTLVAAPSLRVVELTSHRRTDPAFLAMFAITLATGACAVVGSIGSKSIGLSTSEHMI
SLYILLALPVLIPAALKVRESMDKLREAKRENRVHDVAAATDVPETAVSVLEVAEAAENK
EEDDAAAGESGGQDEVGGIRLLRRLDFWLYFLSYMFSGTLGLVFLNNLGQIAESRGLSDP
STLVSLSSSFGFFGRLLPAFLDYYTAKSGYSLSRTASMAALMAPMAGAFFLLLDPRDMFL
YTSTAVVGTCTGAITSVAVSATGELFGRKNFGVNHNVLVANIPVGSLCFGYLAAFLYQRE
ARGASRCAGAACYRGTFLVWGATCAVGTALCTVLYARSRGFAGRLPPPARSTTMPCAGQR
PATNLGDDNKGPEPEVSSTAV*
>11667.m06079|LOC_Os01g61030.1|genepair379-1
MHPAGAVVAPAAGAPARAAPRVEKATSHLLMGPDWAVNLEICDIINADVWQTKDVVKAVK
KRLQNKDPKVQFYALTLLETMMKNCGEYVQLEVAEQHVLQEMVKIIQKKNDMLVRDKILL
LLDSWQEAFGGPGSKYPQYHFAYLEVKRIGAVFPRRPIDAPPIFTPPATHTSQSYGSPRY
EAGSLNEIMSSDVETLSLGDLNNIRNVTELLCDMVHALNPSDHMAVKDEIITDLVSQCRS
NQQKLMQFVSSTGNEQLLKQGLEINDRLQNIISKYDIMASSTHLAVEAPPADNVEAPKED
PAEKPSAPPISTLEEEEEEEDEFTRLAQRKNKSVMTSDDSLSSTGDLALVPIDMESSESS
SSVASNALVPVDPALVSSSPQTKEQDMIDLLSLTLCSPTHEASTDSSTQGPNGPQQPAVT
DGQHNPSGVPQYPSNHQSHPINQEYIRQNRNYVAPWAQSGQYYPAPPWAAPPSVNSNPFQ
SATYQEQPPPVGSVSSTYSAPSASYTSPSMAYVPPSASLPIWNGSTTSNGLSATQAQMNG
NQQPPGSSAAASKPYYIPDNLFSDLIDLKGLSGGNKMGVPTSMGSANGGQPTIGGKK*
>11682.m03776|LOC_Os05g39760.1|genepair379-2
MYPVVTGPPPARLPASTRVDKATSHLLQGPDWAINLEICDTLNADRWQTKDVVKAVKKRL
QHKDPRVQFFTLTLLETMMKNCGEYVHFEVVEQHILQEMVRIVQKKHDTQVRDKVLILLD
SWQEAFGGPGGKYPQYYWSYIELKRSGIMFPRRPVDAPPIFTPPATHHTQSYGSPTYPAG
SLNERMTSDVETLSLGDLNNIRDTTELLCDMVNALNPSDRMAVKDEIISELVTQCRSNQQ
KLMRFVSSTGNEELLKQGLEINDHLQSVLAKHDAIASGAPLPVETPRKDEIPREDPKIKP
SAPPIAPPSAPPVEEDEDDEFAQIARRKNKSVISSDEASSSAGDQALVPVDPVTSEASSS
VASNAVVPVDSSPASGTRTREQDMIDLLSLTLYSPPEASTDSSSQTQNGTQQSVTSNGPE
LPPNYQPAASNGSHYSSNQQAYPTNQAYTPYNNYVVPWAQAGQGTQAGGAYQTQPMQQYG
SSYPAPPWAMPASVNSTNPFQPATYQMQNPPVASVAPTNTYPAPSSPYAAPPRQQVPSPA
TKPMQQYNSFVSQTRSGPAMAQDVRMNGNPRPSETTAAAAKPYYMPDNLFGDLIDVKSYG
GGKMSRTASMPSPNGSGQPLLGGKK*
>11667.m06082|LOC_Os01g61060.1|genepair380-1
MVQNFPEDDMAGTPYSDQTSPILTEYHITVPALHDGSMQGSVHHERRLLDCLRATPSVEW
LKNINLCSPLTNFRLPSTGVRRYLHVEVHFVRRINWSSVFSFCKNWLKHPLNIALLAWLL
CVAAAGGMLILLLLGLLNRAFPSKPLRHHWIEIDNQILNALFTLMSIYQHPSLIHHLVLL
CRWRPEDAAELRKVYCKNGDRRPGERAHMSVVVALLHVTCISQYVVCNLYWAYRSRSRSE
FADNFFFVLGVVAPVVAGAYTVYSPLGRDTDDDASGEEAKQQQQHMIEAELPGTRTVVVD
PVWAGGLLDCGEDPAACCLSSLCTFCVFGWNMERLGFGNMYVHTAMFLLLCVAPFWVFNI
TALHIHDYDLSDAVGAAGIALCFLGLLYGGFWRVQMRKRFALPGSRWCCGSASLTDYARW
LFCWPCALAQEVRTGNLYDVEDGGGVFYEKAMDGGDVEGGAASTAATGVVPVSVGGGEGD
GVVGDIKLGMDGEMIPPAQAVMETSGDTQGSGADVAANGDNELSS*
>11682.m03773|LOC_Os05g39730.1|genepair380-2
MVSNGNEDLKADVELVESTTVDNDTGAPGASTLPTQGVPRQGKQRNGFLNFCNRFSSGDR
FKKLGPSPSFKFRQLALERDEFSRSIHSDSHDNHEHFQFIRKINWGHLWVMCKDWIKEPL
NMALFAWIACVTVSGAILFLVMTGMLNRALPSKSQRDAWFEVNNQILNALFTLMCLYQHP
KRIYYFVLLCRWEQKDVLVLRKTYCKNGTYKPNEWMHMMVVVVLLNLNCFAQYALCGLNL
GYRRSERPPIGVGLTISVAIGAAAFAGLYNIISPLGKDYDTELTEVDQEAQTELTRPATS
RTSLEKRYSFIQSEERRFVESRPEWVGGLMDFWDNISLAYLSIFCSCCVFGWNMQRLGFG
NMYVHIATFMLFCLAPFFIFNLAAVNINNENLREALGLTGLALCFFGLLYGGFWRIQMRK
RFNLPANNFCCRSAEATDCFQWLCCSSCSLAQEVRTADYYDIAEDRSYTEQITARSQHVM
TPLSREDGLPLFRSNPGSPYRSSTASPSIFIMESPSAPRRSPGPSPLGGSPTMGDRTMKA
PTPSVLHRDGEPEL*
>11667.m06084|LOC_Os01g61080.1|genepair381-1
MTTSSSGSVETSANSRLGTFSFASASFTDLLGGNAGAGGGGVSRYKAMTPPSLPLSPPPV
SPSSFFNSPIGMNQADFLGSPVLLTSSIFPSPTTGAFASQHFDWRPEVAAAQSADQGGKD
EQRNSYSDFSFQTAPASEEAVRTTTFQPPVPPAPLGDEAYRSQQQQQPWGYQQQPAGMDA
GANAASFGAAPFQATSSEMAPQVQGGGGYSQPQSQRRSSDDGYNWRKYGQKQVKGSENPR
SYYKCTFPNCPTKKKVERSLDGQITEIVYKGTHNHAKPQNTRRNSGSSAAQVLQSGGDMS
EHSFGGMSGTAATPENSSASFGDDEIRVGSPRAGNGGGDEFDDDEPDSKRWRKDGDGEGI
SMAGNRTVREPRVVVQTMSDIDILDDGYRWRKYGQKVVKGNPNPRSYYKCTTAGCPVRKH
VERASHDLRAVITTYEGKHNHDVPAARGSAALYRPAPPAAAATSSHPYLPNQPPPMSYQP
TGPQPYALRPDGFGGQGPFGGVVGGSSFGGFSGFDDARGSYMSQHQQQQRQNDAMHASRA

KEEPGDDMFFQNSLY*
>11682.m03772|LOC_Os05g39720.1|genepair381-2
MTAAPGSLPLVNSRPVSLSLAASRSSFSSLLSGGAGSSLNLMTPPSSLPPSSPSSYFGGV
SSSGFLDSPILLTPSLFPSPTTTGALFSWITTATATAAIAPESQVQGGVKDEQQQYSDFT
FLPTASTAPATTMAGATATTSNSFMQDSMLMAPLGGDPYNGEQQQPWSYQEPTMDADTRP
AEFTSSAAAGDVAGNGSYSQVAAPAAAGGFRQQSRRSSDDGYNWRKYGQKQMKGSENPRS
YYKCTFPGCPTKKKVEQSPDGQVTEIVYKGAHSHPKPPQNGRGRGGSGYALHGGAASDAY
SSADALSGTPVATPENSSASFGDDEAVNGVSSSLRVASSVGGGEDLDDDEPDSKRWRRDG
GDGEGVSLVAGNRTVREPRVVVQTMSDIDILDDGYRWRKYGQKVVKGNPNPRSYYKCTTA
GCPVRKHVERASNDLRAVITTYEGKHNHDVPAARGSAAAALYRATPPPQASNAGMMPTTA
QPSSYLQGGGGVLPAGGYGASYGGAPTTTQPANGGGFAALSGRFDDDATGASYSYTSQQQ
QQPNDAVYYASRAKDEPRDDGIMSFFEQPLLF*
>11667.m06087|LOC_Os01g61110.1|genepair382-1
MGNSLRCCLACVLPCGALDVVRIVHLSGHVDEFSCPVTAGAVLAAHPNHTLTTTWSSAGV
GCPTKKLVIVSPDSELKRGRIYFLIPSATLPADRRKKSRQSSNKKSKRPSHHHHKSNGAA
TAASTAEQDNYLRELLSEKTAASGGQRRRRSGSRILESHIMQICERWIVSRFVPLSDVGF
LFGLDPVWVLVSLAKWWGIPAVDGISAVKCRSAVSDWICVFDSVFVVEICFWLISLMPRV
DKRKKAAKAVVISSDESHYDDSESSENFFDGVSKYTDSSSDDNMEKLLLKIFKKQIAKKM
KKKFISSSSDVFRKHKAKKAKGVVKSDGSFSRFSAKYFRRVVSSLSPHQMFVIEKYGFKN
LLLFDSGGVPKKFAAWISSKVDLKTSEIILKDRVIPITVESFRDILGLPFGGLSFGKDLN
LLSSYDWCKFVYDWCMNRIKKFQKSKNLGGCLYYWAVSYLDTVDFGERNVPIGFPRMSVW
KEGMIKRYSDFDKMDDDTFGLRPPRVICSTSNSQLHLEKHYLIMQLLNYVIGEALPNYLK
DMISAMESVHAEVHNSTDHIDVGDVHVLDANAHNQFASGAVQNEQQTSPCCNHDVDSYVP
LENDLPDNVPLAGNFDNAGDFNCASGYLPSMNTGNASTSVVNDGIAESFGDGQALVTPDV
GCAKNFKNSSDERFSASAIATAAAAVKHVAIKFKSRLPQFNGSENVDRAVDMFKPSYKNL
FPQDNAGISFHSVEDTPEELIQIKHNHEGTARTPNSGIIKKRVFEDLVNSPDLIIVGESK
FHDRCNNMTAQSDLIYNASILPTSTAHHASSLGGKIPPHGPRRVLAPAKYTSDPFVQLHR
HFPISDVENRYYIAVCRLADSSKWHSYDAVNIDNVKANFYTFGHSLKKSGHEQLVVDPSF
ADAEKVQKSFEGAAKARRLDLCDMLFFPIHYQQHWFLFIVDVKDRMFVFVDSKHEEHSEF
YENLKTFVVDNFQNLWNKFVGSSLDFSVFKTVFPPVPRQDYESDSGVFVMKFMEIWSPRI
LLPNEFSKQNINNIRVKYVNQIFFHAKNKMLHTEIEDAVLNWFNPEKFARQ*
>11682.m03771|LOC_Os05g39710.1|genepair382-2
MGNSLRCCLACMLPCGALDVVRVVHLSGRVDEFTSCPLTAADVLAAHPNHALTAAWSPGG
AGAGGAPCRKIVIVSPDSELKRGRIYFLIPSACSAPAADKMMTRRKKKKRCHGNGNGKGG
SAAAASTAEQDNYLMELLSEKRGTSHRRRRSGARAGVWRPQLESIAEEPSD*
>11667.m06088|LOC_Os01g61120.1|genepair383-1
MLPPAPTRNPGACRFIPLLPPKPLLSPAAAAASSRGGLCVAAASRRDFLLLVPSIAAAST
VLQSLPLSASAADDEKQAASPAPGPAAAPAPTSAGEPEAEALSRVYDATVIGEPQAVGKD
ARRRVWEKLMAARVVYLGEAELVPDRDDRVLELEVVRKLAARCAEAGRSISLALEAFPCN
LQEQLNQFMDRRIDGNNLRLYTSHWAPERWQEYEPLLNYCRDNGVKLVACGTPLEVSRTV
QAEGIRGLSKAQRKLYAPPAGSGFISGFTSISGRSLIDKISAIHGSPFGPSSYLSAQARV
VDDYTMSQKIMKEITNGYPSGMLVVVTGSSHVIYGSRGIGVPARISKKMQKKKQVVVLLN
PERQGIRREGEIPVADFLWYSAAKPCSRNCFDRAEIARVMNAAGRRREALPQDLQKGIDL
GVVSPEILQNFFDLEKYPVMAELIHRFQGFRERLLADPKFLHRLAIEEGISITTTLIAQY
EKRKGRFLEEIDYVLTDTIRGSVVDFFTVWLPAPTISLLSLGDNGSGESLELLKGLLGSL
PDNAFQKGIMGQSWNTNQRFASVLMGGIKLAGVGFISSIGAGVASDVLYAARRVLRPSTS
VETARRRTPIWKSATVYSCFLGTSANLRYQVIAGLVEHRLGEYLMAYYNQPLLANLLSFV
SRTINSYWGTQQWIDLARATGLQTSKKELPSPEISNLPDMPLLECGTTEVQNMDDSNKQQ
PMK*
>11682.m03768|LOC_Os05g39680.1|genepair383-2
MAAEPSPAAASAVEDLETLPLDSSYSSAPATDPLLRPPASPSAASAAAGGDHDPFVIDDD
FLVDEEDDDDVGPPAPTAIPLVAAAARAPEFARIAVSDPKKHAEPTSGAAGVIPGSGSYF
SYLITTRVGEAEVRVRRRFRDVVALADRLAAAHRGLFIPARPDKSVVEGQVMQRHDFVNQ
RCSALQRYLRRLAAHPVVGHSPDLRTFLTEPGAIPAFEGEQPRYWTTTLSSAAPSTPAKA
GRDLFGMFKGLKQTVVNGMVATKPPPVEEETDTEFLAHKARFQDLQQQLTTTSQQPVIYL
IDLKAEALVKARDDLRTTTAHLGMTLIKLAKFEREQATCSPQRRRAADINNFGSSVVKFS
RSQAKLNSEIVKHLGSIHEYLEMMISVHHAFTDRSNALHHVQSLSADLFFLHTRAEKLES
VSSRSRSIDQEWTRHQKLGGLKETISATEAAKSHALKEYENIKENNTIEIKRFDKDRRRD
FVQMLKGFIVNQVSYSDHYANMWAKVADETKVKLELSAAKMSHAPCRNAASFKPLLPSRL
RAKPLAGISSSPRRNVSASAASSQSRRDFLLLIPSLAAASAILRPLPSSAADGEAPPTDS
SSPSPPSAEEAGAVVEEVDESALSRVYDATVIGEPEAVGKDARGRVWEKLTAARVVYLGE
AELVPDPDDRVLELEIMKGLATRCAEAERGVAVALEAFPCDLQQQLDQFMDGRIDGSILK
LYTLHWPQDRWEEYEPLLNYCRDNGIKLIACGTPLEVKRTVQADGIRGLSKAERQEYAPP
AGSGFISGFSSISGRSLIDKISSARDSPFGPTSYLSAQARVVDDYTMSQIILKELNGGDT
SRMLIVITGASHVIYGSRGIGVPARVSKKMPKKDQVVVLLDPERQSIRREGEIPVADFLW
YSAAKPCSRNCFDRAEIARVMNAAGRRPEALPQDLQKGIDLGVVSPEILQNFFDLEKYPV
MSELIHRFQGFRERLLADPKFLQRLAIEEAISITTTLLAQYERRKGRFFEEIDYVLTDTI
RGSVVDFFTVWLPAPTISVLSYADDGSGESLEFVKGLLGSLPDNAFQKNVLGQNWNMKQR
VAAVLVGGLKLASVGFISSVGAGASSDLLYAARGVLKSSVNAEAGRKRSPIWKSAAVYSC
FLGTSANLRYQIIAGLVEHRLGESLATSYNQPLLASLLSFVARTVNSYWGTQQWVDLARY
TGLQKTKEVPPVEATAPPAETSQLATPPAETSQLATPPAETSQLEDGRTEVQNLDDNSNQ
SSG*
>11667.m06093|LOC_Os01g61170.1|genepair384-1
MASAAAPTPQPLLPVTNPAAAGGSAPSSGSALTDAPLATPAFRLFLSEATSRLRRNLAYF

RVNYAAVVAFSLAASLLAHPFSLLVLLAILGGWCFLYVFRAADQPVVLFGRTFTDRETLL
GLVVASVLAFFMTSVASLIISGLLVGGAIVAVHGAFRVPEDLFLDDPSVGSNGNTTSRLL
SFLGAPGSGV*
>11682.m03767|LOC_Os05g39670.1|genepair384-2
MASSAPTPPPLLPVTNPAAAGSSPAATAVGSDAPIATPAFRLFLSKLSDSARRSLSDRRP
WTELVDRSAFSRPDSLSDATSRLRRNLAYFRVNYAAVVAFALGASLLAHPFSLLVLLGLL
AAWCFLYLFRGSDQPVVLFGRTFSDRETLLGLVVASFVAFFFTSVASLIISGLLVGGAIV
AVHGACRMPEDLFLDDADAASGNSAAQGLLSFLGAPGSRV*
>11667.m06094|LOC_Os01g61180.1|genepair385-1
MAEDGEEKLLATVQHIVKTLGRTDTMTEDILKVFSNYDGRLSLDKLYATRAAAAAAAVAA
AGGAGAGEHSVPASPPMPPPPAVPPAVAAMPAVTSLERTVRTLDRQISQFVTMDRLIWAD
SADADAFLEAVDDLIGTVQELDAAGTNRGLLDRADELLSRCMARLEDEFRALIERPDDVA
PPAPGGFASDESEEEDYDADDGYGDEPIPIAKPVSDFDVVIDALPPGSVSDVHQIARRMV
DAGFGRECAEAYAAARRGFIDESVARLGIRARTIDEVHSLPWEELEFDIARWIPAFKMVF
RILIPSERRLCDRVFDGLAPYGDLAFVAAVRTQVLQLISFGDAVSAASRAPERLFRVIDM
YEAVRDLLPDLDPVFADPYSAALRAEVSAVCNTLGSSIKGIFMELENLIRRDPARVSVPG
GGIHPITRYVMNYLRAACGSRQTLEEVMEGDLGAVGGAAIAVDPDRPTSSLAVHIAWIMD
VLHKNLETKSKIYRDPPLASIFLMNNGKYIIHKVNDSELGVLLGDEWMKQMMSRVRRWSL
EYQRGAWAKVMSVLQTGGPGIGSLPAKALLQKLRMFNGYLEEICAIQSEWVIADEQLRED
VRAAITDSVKSAYMGLISRLKSSPEAAQDLFIKHSPEDVEARIQHLFEGVSK*
>11682.m03761|LOC_Os05g39610.1|genepair385-2
MAEDGEEKLLATVQHIVQTLGSSDTMTEDILRVFSNYDGRLSLDKLYAARAAAASAAGGG
GGGGLGGGGEHSMPESPTLPLPPPPAAAVSAAAAAGRPPPVTSMERTVRTLDRQISQFVA
MDRLIWADSADADAFLEAVDDLIGTVQELEAAGTNRGLFDRAEELLSRCMARLEEEFRAL
IERPDDAVPAAPGGFRSDGSDDEEDFGGGDGYGDEPIPIAKPVTDYDVVIDALSPGSVAN
VHQIARRMVDAGFGRECAEVYAAARRGFVDESVARLGVRPRTAEEVHASSWEELEFDIAR
WIPAFNMVFRILIPSERRLCDRVFDGLAPFGDLAFVAAVRTQALQLISFGDAISSSSRAP
ERLFRVVDMYEAVRDLLPDLDPVFADPYSAALRAEVTAVCNTLGSSIKGIFMELENLIRR
DPARVAAQGGGIHPITRYVMNYLRAACGSRQTLEEVMEGDFGAVGGAAAAVDPDRPTSSL
AVHIAWIMDVLHKNLDIKSKIYRDPSLACVFLMNNGKYIIQKVNDSELGVLLGDEWIKQM
TNRVRRWSMDYQRVTWGKVTTVLQTGGPGVGGLPATAMKQKLRMFNTYFQEIYEVQSEWV
IADEQLRVDVRAAVAEAVMPVYTALISRLKSSPEARHDLYIKYTPEDVEACIQHLFEGAA
K*
>11667.m06099|LOC_Os01g61210.1|genepair386-1
MPYAAVRPSPPPQLSRPIGSGAGGGKACPAVPCEVARYHEHAVGAGQCFSTVVQAIAAPA
DAVWSVVRRFDRPQAYKKFIKSCRLVDGDGGEVGSVREVRVVSGLPATSSRERLEVLDDD
RRVLSFRIVGGEHRLANYRSVTTVHEAAAPAMAVVVESYVVDVPPGNTWEETRVFVDTIV
RCNLQSLARTVERLAPEAPRANGSIDHA*
>11682.m03758|LOC_Os05g39580.1|genepair386-2
MPYTAPRPSPPQHSRIGGCGGGGVLKAAGAAGHAASCVAVPAEVARHHEHAAGVGQCCSA
VVQAIAAPVDAVWSVVRRFDRPQAYKHFIRSCRLLDGDGDGGAVAVGSVREVRVVSGLPA
TSSRERLEILDDERRVLSFRVVGGEHRLSNYRSVTTVHETAAGAAAAVVVESYVVDVPHG
NTADETRMFVDTIVRCNLQSLARTAEQLALAAPRAA*
>11667.m06103|LOC_Os01g61250.1|genepair387-1
MAISSALSFLSDRKRPIVVSVFLFLLLSSLFLLFSPAPAALPFFSFPSSHLSSSSSSPIA
ATPPPLTPVSVPANASSPETPVDASGGSNAGATAPTRDAPQPDRSRSTPPAAVGVRGSAG
TNGTSRGVSAGGDGAGAAAAAAAGVAVPSWEVCEVGKGVVAADYIPCLDNVKAVKALKSL
RHMEHRERHCPTAPRPRCLVPLPTGYRSPLPWPRSRDMIWYNNVPHPKLVEYKKDQNWVR
KSGNYFVFPGGGTQFKAGVTKYIRFIQQIMPNIEWGTHTRTVLDVGCGVASFGGYLLDRN
VITMSVAPKDEHEAQIQFALERGIPALLAVIGTQKLPFPDNSFDVIHCARCRVHWYADGG
KPLLELNRVLRPGGYYIWSATPVYRRGKRDEDDWNAMVTLTKSICWRTVVKSKDVNRIGV
VIYQKPTSNSCYFERKQNEPPLCPSREGSHSPWYAPLDSCLLLPAVSSSGEGNSWPISWP
ERLNIKYSTISDNASTQFSQEKFDSDTKHWKDLVSEVYFNEFAVNWSTVRNVMDMNAGFG
GFAASLIHKPLWVMNVVPFDHPEALPIIFNRGLIGVYHDWCESFNTYPRTYDLVHMSYLL
QGLTNRCDIIEVAAEIDRILRPGKWFVLQDTEQVIRKMDPVLRSLHYRTAIVKQQFLVAT
KGFWRPYSAGSESR*
>11682.m03752|LOC_Os05g39520.2|genepair387-2
MAVPTSDQRKRRPFLLSLSLFLLVSALLALAFLFLDPSAQSLSVLSSRLTAPTTTLAPPA
VVAGGAEAGESADATEKAEETASRPDDTAAAVNADAAAGEGGGSSESPRLDADKGAAATE
GVADDDGGGGGDEPAAKVRWETCRPGRGVSSADYIPCLDNMRAIKALRSRRHMEHRERHC
PVAPRPRCLVRVPSGYRSPVPWPRSRDMIWYNNVPHPKLVEYKKDQNWVTKSGDYLVFPG
GGTQFKTGVTRYIQFIEQIMPTIQWGTHTKTVLDVGCGVASFGGYLLDRNVITMSFAPKD
EHEAQIQFALERGIPAFLAVIGTQKLPFPDEAFDVVHCARCRVHWYANGGKPLLELNRVL
RPGGYYIWSATPVYRQEKRDQDDWNAMVKLTKSICWRTVVKSEDSNGIGVVVYQKPASNS
CYLERRTNEPPMCSKKDGPRFPWYAPLDTCISSSIEKSSWPLPWPERLNARYLNVPDDSS
STDEKFDVDTKYWKHAISEIYYNDFPVNWSSTRNVMDMNAGYGGFAAALVDKPLWVMNVV
PVGQPDTLPVIFNRGLIGVYHDWCESFNTYPRTYDLLHMSYLLGSLTNRCDIMEVAAEID
RILRPDRWFVLRDTTEMIKKMRPVLKSLHYETVVVKQQFLVAKKGFWRSGK*
>11667.m06109|LOC_Os01g61310.1|genepair388-1
MDPSGPGPSSAAAGGAPAVAAAPQPPAQLSRYESQKRRDWNTFLQYLRNHRPPLTLARCS
GAHVIEFLRYLDQFGKTKVHASGCAFYGQPSPPGPCPCPLRQAWGSLDALIGRLRAAYEE
SGGTPESNPFAARAVRIYLREVRDSQAKARGIPYEKKKRKRSQAAQPAGVEPSGSSSAAA
AAAGGGDAGSGGGAAATTTAQPGGSGTAPSAS*
>11682.m03749|LOC_Os05g39500.1|genepair388-2

MEGGGGGADGQAQPVAQAPPAMQPMQQLSRYESQKRRDWNTFLQYLKNHRPPLTLARCSG
AHVIEFLKYLDQFGKTKVHASGCAYYGQPSPPAPCPCPLRQAWGSLDALIGRLRAAYEES
GHAPESNPFAARAVRIYLREVRDAQAKARGIPYEKKKRKRTQQQPPPPPPPPPQHQPGA
AAGEASSSSSAAAAAVAAEGSGSSAAAAAATSQTGGGGGGSTTTTTASAAAPTTATRV*
>11667.m06113|LOC_Os01g61350.1|genepair389-1
MWLPWVKTRPSSPSSAAASPSPSTALAAAAASPRLSFSSPSLKDLQALLLSDHSPSPTPP
QLPPNTAPCSPSVRVFHRVRVAASALRALRTLQAPHAAVAEADRRVVLYFTSLHVVRSTY
EDCRAVRAILRGLRASVDERDLAMDPRYLQELGALLPRARGVTLPQVFVGGRHLGGAEEV
RRLHESGELRRVVAGAGATAFAACSRCGGERYVLCGSCNGSHKRYSLKGGGGFRTCAGCN
ENGLVRCPDCSPPAV*
>11682.m03744|LOC_Os05g39450.1|genepair389-2
MWPSWVRTRSSKSKPAAAAADTTSTALVAASKRLTFSSPSLKDLQSLLVPPHAALSSSSP
SPPRVFHRIRVAACALRVLRNLQSAGQQQQPHAAAAIWSEPGGGEGARVVLYYTSLRVVR
GTYEDCRAVRAILRGLRAAVDERDLSMDPAFLPELAALLPHRRHVALPQVFVNGRHLGGA
EEVRRLHESGELRRIVAAANPTPASCGRCAGERYVLCGSCDGSHKRYSHKVGGGFRACAM
CNENGLVRCPDCCLPPA*
>11667.m06115|LOC_Os01g61370.1|genepair390-1
MPMEIVTRRDKEGLRCNGADGEKLPQLLDSPLPTPRRSCASADAASVRCRREASPLRTQV
PFSWESSPGVPKRSSACMHMAQEIMPPPKPPPGRWKQCPGSNWCGYGNSTAASSDDDDAS
FSDALDRVSTPDQRVGSFDRITSKRFEDIFLGRATSFVNDRSSRRAPAEASLATPSSSSG
RGPKHWRRRTTRRDHDGQQPTARQSNDHPVQVQLLPRININGRDEQMSPRACGLMVFFPW
SAKPAVCGFRSPPAQYAPSPLAGASNPSSSQSRRFVTLRDAMQEENKTGSGGRDLPRPRG
EKRSREEWQAASRGWGVSSLLDASKKYCTDARKALSKLSIGLGTDSGSGSGSPRVGSRER
KCGKQDPSSTMPAMATKLTQLRTSRN*
>11682.m03737|LOC_Os05g39400.1|genepair390-2
MQLVHERVAPPPAAELAGRRGGGGGEGMEIVTARVGGCGYGYEEEGGTRRQQRRRRKVSD
GHVVAQLLDSPLPTPRRSCCGSSSAAGTPRSAARCGGGGSPGAPPSSPPLSPQRTHVPFS
WESSPGVPKDAACGRKVVREVLPPRPPPGRGGGGGGSPAHAHARAYFGNATETTSSDDDD
SDDTFSDALDRISASDRFAAFSSRLSSIDGAGSLRLPSFIMDRFLPAANAIATTSADKRP
KKTPRRGARSSKQDEEATASARRRAQSLRRASGREQPKQPPPRHHVSTLQRKESEPPPPP
RQSRDIDEETQSDEMSPRSCGFMLFLPWSVKPVLCGFARSRTSRAADASTTASSPPRRSV
TLGNALEKEKEKEKDKSKLRGGGGDPSRWSDEKSGSGREWSSPGWGTAILGTSKRYCADA
RKALSRLARSATDGRGSPRVTGERRAGKPAAAASPRRSTSGEIPPLSPPSESWLSHARGS
STLSNKR*
>11667.m06120|LOC_Os01g61420.1|genepair391-1
MDADEAAGSSRRMDLNLYLGLPRAPRPRRSDLGSDLALSTPMPSSPSSSAASVDAPPPPP
ELSHPPYSPSHADLSPPLQEVYSLYNPDDPPASETHLPPYAPPPAPVVSELPDDLEFGLH
PPPPLVRASELLGWEDRPSSSTASSSFLPDTAARYWRLLEQTGSRWLRARRFRSDLPPLS
SEAYPAGRDAAAVPVLQHEPMNDTVEHNKVAADGAEVGASEESEEQGRSAATFECNICFD
MASEPVVTSCGHLFCWPCLYQWLNVYSNHKECPVCKGEVTEANITPIYGRGNSCLDAEKA
VEGGKQTGPTIPPRPHGNRLESFRQQFHHLRPISRRLGEAHGLLSSWRRLLDQQIMNTAS
RFEGPPESAVQEMVDTAHAQHTSRLSRLASRMRARRLLREADNPNPPDGGSTSPDSGLIR
NNASDPSRNGPSSLLPDGIDWLRGLTLLGYEDTERFASAMSDFRRITGPSQYGASASSSN
PPNLESTFDRTHVVAAPSADQASNSSTAAVIQGDAGISESAGEPSNAGSSRSLRRRGRSS
ALGSLDADGGGLQRNKRRRIN*
>11682.m03735|LOC_Os05g39380.1|genepair391-2
MAADDAARSSRRMDLNLYLGLPRAPRLRRPDLGSDLALGTPMLSSSPSSSAASADAPPLE
TEPLHPPYSPPPAELVRPPTPLPEPYDPSAPEAHPPYVPPPVPPPEAIPELADDLEFGFS
HPPLLLRPSELLGWVDRPSSSTASSSFRPERVDRYRPPVICLNSRQSRCLRPRRFRSDLP
PLGSEAPALENDAAAQPPPQEPMQDTVEENKVVADGAIVGASEEEPAERGKSVAMFECNI
CFEMASEPVVTSCGHLFCWPCLYQWLHVHSTHKECPVCKGEVTEGNITPIYGRGNSTSDA
EKKVAEEGNVSGPTIPPRPHGNRLESFRQKFHHLRPISRRLGEAHGILSSWRRILDQQIM
NSVSRFEGPPESTVQEMIDHAHHASRLGRITTRMRARRLQREAENSTFVASSAAESGLPA
NSTSDLPRRSSSPFSSERIDLLQHFVDLASTERLASAVSDLRRMVRPSPYGASTSSNPPN
PPNTELLPVDGNHVAVALAADQASNSSTMAVIQEDAAFTESTGEPSNAGSSRSLRRRGRN
DALGSLDVDGVGLHRNKRRRLN*
>11667.m06124|LOC_Os01g61460.1|genepair392-1
MANRRKFSQAGGGGGGGVFDPFGTKQAVSSLRKGGRLPVYVAGVFFVIFVIIMYGEDIRS
LTLDPIARAGTTPARIVEPVVTEERHVARVNPPRREVSSAEKAAALPLDVDERPKLATPT
PTEAAKEVPKVEKIRKPKKPKTTKKKPRKPRPAKKTVAAAAGGLLGVPETCDLSKGEWVF
DNTSYPLYREEQCEFLTSQVTCMRNGRRDDTYQKWRWQPKDCSMPRFDAKLFMERLRGKR
FMFVGDSLNRNQWESMVCLVQSAMSPGKKYVTWEDQRVVFHAVEYNATVEFYWAPFLVES
NSDDPKIHSIQHRIIKADAIAAHAQNWRGVDYLVFNTYIWWMNTLNMKIMRPGGQSWEEH
DEVVRIEAYRKVLTTWASWVNDNIDPARTSVFFMSISPLHISPEVWGNPGGIRCAKETMP
LLNWHGPIWLGTDWDMFHAAANVSRTAATRVPITFVDVTTMSERRKDGHTSVHTIRQGKV
LTPEQQADPGTYADCIHWCLPGVPDIWNLILYTRIMSRPQLV*
>11682.m03732|LOC_Os05g39350.1|genepair392-2
MGLPGRRNPLLSARRAAASLRRSRRLPVYVAAVFFVASVLLMFRDEILYLTTARSPSSSL
PTTGGSAGGAGLARKEELVSVNKPVLLGHGGKPEKHHSVTERHRPKVSAKRRPNKKAAKA
ARKKFMASPSVAAGAEVNVPETCNLSKGKWVFDNATYPLYREQECEYLTAQVTCTRNGRR
DDGYQKWRWQPRDCDLPLAFDARLFMERLRGKRLMFVGDSLNRNQWESMVCLVRPALSPG
KSYVTWWDGQRVVLHAWEYNATVEFYWAPFLVESNSDDPKAHSIRDRVIKPEAIAAHAGD
WVGVDYLVFNTYIWWMNTVNMKVVRPTGKTWEEYDEVGRIEAYRRVLDTWATWVNDNVDP
ARTSVFFMSVSPLHISPEAWGNPGGVRCAKEDAPVQNWHGPLWLGTDWDMFRAARNASRA

AGRVPVTFVDVTAMSELRKDGHTSVHTIRQGRVLTPEQQADPATYADCIHWCLPGVPDVW
NLMLYARILSRPPAAAGHVA*
>11667.m06125|LOC_Os01g61470.1|genepair393-1
MAAAQDASSQALGLRWRYGDVDDGNFAVRGRAVPLLVALLFVLVCFVAVSLYLRWACHCH
RYGRDTTPMPATTSSGFSSSHAAATAPGSASSVTGLDDATIASMPVALYRAVASAAGDGD
DGGAAQCSICLGEFEEGEKVKALPLCGHGFHPECVDAWLRSRPSCPLCRSSLLPAAATTK
PDVAGSDAV*
>11682.m03722|LOC_Os05g39260.1|genepair393-2
MAAQESAAGTTNQVMRWRYGDVDDSNFAVHGRAVYLLVGLLVAVVVFVALCLYLRWACHR
YTPDPEASSSSSAAGAAGAAAAASMHGLDAEAIGGLPVTLYRPRDSSSPPAGKGGGGGVD
DDQAAQCSICISALVAGEKVKALPPCGHCFHPDCVDAWLRSQPSCPLCRSLLLAAAATAA
KPDVNGGDDDDSAV*
>11667.m06133|LOC_Os01g61550.1|genepair394-1
MHLRMASPPQPGPYMPDLPAVPAWLNKGDTAWQLVAATFVGIQSMPGLVVIYGSIVKKKW
AVNSAFMALYAYASTLIVWVLVGFRMAFGDRLLPFWAKAGPALTQDFLVQRAVFPATAHY
GSDGTLETPRTEPFYAEAALVLFEFEFAAITLVLLAGSLLGRMNIKAWMAFTPLWLLFSY
TVGAFSLWGGGFLYQWGVIDYSGGYVIHLSSGVAGFTAAYWVGPRLKSDRERFSPNNILL
MIAGGGLLWLGWAGFNGGAPYAPNVTATVAVLNTNVSAATSLLTWTCLDVIFFGKPSVIG
AVQGMMTGLVCITPGAGLVHTWSAMLMGMFAGSVPWFTMMILHKKSTFLMKVDDTLAVFH
THAVAGILGGVLTGLLATPELCALDCPIPNMRGVFYGSGIGQLGKQLGGALFVTVWNLIV
TSAILLCIGLFIPLRMSDDQLMIGDDAAHGEEAYALWGDGEKFDVTRPETTRTGGAGGAG
REDTMEQRLTNMGARGVTIQL*
>11682.m03720|LOC_Os05g39240.1|genepair394-2
MAAAGAYSASLPAVPDWLNKGDNAWQLTASTLVGIQSMPGLVVLYGSIVKKKWAVNSAFM
ALYAYASSLLVWVLVGFRMAFGDQLLPFWGKAGVALTQSYLVGRATLPATAHGAIPRTEP
FYPEATLVLFQFEFAAITLVLLAGSVLGRMNIKAWMAFTPLWLLLSYTVGAFSLWGGGFL
YRWGVIDYSGGYVIHLSSGIAGFTAAYWVGPRLKSDRERFSPNNILLMIAGGGLLWMGWA
GFNGGAPYAANIAASVAVLNTNVCAATSLLMWTCLDVIFFRKPSVIGAVQGMMTGLVCIT
PGAGLVQTWAAVVMGIFAGSVPWFTMMILHKKSALLMKVDDTLAVFHTHAVAGLLGGILT
GLLATPELFSLESTVPGLRGAFYGGGIKQIGKQLGGAAFVIAWNLVVTTAILLGIGLFIP
LRMPDEQLMIGDDAAHGEEAYALWGDGEKFDATRHDLSRGGGGGDRDGPAGERLSALGAR
GVTIQL*
>11667.m06135|LOC_Os01g61570.1|genepair395-1
MALKPTLAALLFSLAAIHGIVVVAVDAAAAAVSRGGSARRIPAVFAFGDSTLDAGNNNRL
VTAVRADHPPYGQDFPGGAPTGRFCDGKIMSDFLVEALGVKGLLPAYHSGSEVLSDADAA
TGVSFASGGSGLDDRTATNAGVATMASQIADFSELVGRMGAGKAGEVVNKSLFLVSAGTN
DMIMNYYLLPSKYTLDQYHALLIGKLRSYIQSLYNLGARRLLVAGLPPVGCLPVQMTLAA
LRQPPRPQGCIAEQNAEAEKYNAKLRKMLTKFQSTSPGAKAVYADIYTPLTDMVDHPQKY
GFAETGKGCCGTGLLEMGPLCTDLMPTCTTPAQFMFWDSVHPTQATYKAVADHFLRTNML
QFDD*
>11682.m03718|LOC_Os05g39220.1|genepair395-2
MAPSLATVVAIFLAAILLSSPPPCAAASSAPAPAAAGHGVPAVFAFGDSTLDPGNNNRLA
TLVRADHAPYGRDFPGGAATGRFTDGKLITDYIVSSLGIKDLLPAYHSSGLAVADASTGV
SFASGGSGLDDLTANNALVSTFGSQLNDFQELLGHIGSPKSDEIAGKSLYVISAGTNDVT
MYYLLPFRATNFPTVDQYGDYLIGLLQSNLNSLYKMGARKMMVAGLPPLGCLPVQKSLRG
AGSGGCVTEQNEAAERYNAALQKALSKLEADSPGAKIAYVDIYTPLKDMAENPKKYGFTQ
ASLGCCGTGMMEMGALCTSALPQCQSPSQYMFFDSVHPTQATYKALADEIVKSHVPQLMQ
*
>11667.m06136|LOC_Os01g61580.1|genepair396-1
MDRVQLVLLGLPILLFCSDLVTLFGPEQLPTPQPDLPPHPSPDAASDAVQPDDIAADAAA
SAQIAEPQVDGPASGTTVELKFCASCSYRGNAVTVKKMLETSFPGIHVVLENYPPPFPKR
ALSKAVPFLQVGAMATLMAGDQIFPRFGMVPPPWYYSLRANRFGTMATIWLFGNFAQSFL
QSSGAFEVYCNGQLVFSKLSEQRFPSEFELRELIGNRLPDSQFGKNLEKAGFRSANKYYA
CMLEF*
>11682.m03707|LOC_Os05g39110.1|genepair396-2
MDRVQLLLVGLPALLFLSDLSHIFAPPPPHLRHPHHHPPHHHPHPPHHHPPHHPHPPHHP
HPPHHPHPPHHPHPPHHHPHPHPDPAAEAIQANVDGAGYGTTVELQFCASCSYKGTAMTM
KRMLETSFPGIHVILHNYPPPFPKRVLGKLVPILQVGAIATIMAGDHIFPRLGMVPPPWY
YSLRANRFGTMATIWLFGNFAQSFLQSSGAFEVYCNGDLVFSKLAEQRFPSEFELRDLIN
SRLPDSLVGKNVGKSLS*
>11667.m06137|LOC_Os01g61590.1|genepair397-1
MDRDTKELLACKSISKRKLRTAVDVEDVRREVAIMRHLPKSASIVSLREACEDEGAVHLV
MELCEGGELFDRIVARGHYTERAAANVTRTIVEVVQLCHRHGVIHRDLKPENFLFANKKE
NSPLKAIDFGLSIFFKPGEKFSEIVGSPYYMAPEVLKRNYGPEIDIWSAGVILYILLCGV
PPFWAETEQGVAQAILRGNIDFKREPWPNVSENAKDLVRRMLEPDPKLRLTAKQVLEHPW
LQNAKKAPNVPLGDIVKSRLKQFSRMNRFKRRALRVIADHLSAEEVEDIKEMFKAMDTDN
DGIVSYEELKSGIAKFGSHLAESEVQMLIEAVDTNGKDALDYGEFLAVSLHLQRMANDEH
LRRAFLFFDKDGNGYIEPEELREALVDDGAGDSMEVVNDILQEVDTDKDGKISYDEFVAM
MKTGTDWRKASRHYSRGRFNSLSMKLIKDGSVKLVNE*
>11682.m03705|LOC_Os05g39090.1|genepair397-2
MGNCCRSPAAAAREDVKTSHFPASTGGGKKKPHQARNGGGGGGGGGGGGWEKKRLSVLGE
EGSEVNGGIEEKYALDRELGRGEFGVTYLCMDRCSRELLACKSISKRKLRTPVDVEDVRR
EVAIMRHLPRSASIVSLREACEDDGAVHLVMELCEGGELFDRIVARGHYTERAAAAVTRT
IVEVVQLCHRHGVIHRDLKPENFLFANKKENSPLKAIDFGLSIFFKPGEKFSEIVGSPYY

MAPEVLKRNYGPEIDIWSAGVILYILLCGVPPFWAALLFCKFLPSKIAETEQGVAQAILR
GNIDFKREPWPNVSDNAKDLVRQMLQPDPKLRLTAKQVLVRNWLLMNVENSMRCLIVAIN
SRWKQLRRYTQTVIYRLGTQDSCFTSVVAEHTWLQNAKKAPNVPLGDIVKSRLKQFSRMN
RFKKRALRVIADHLSAEEVEDIKDMFKVMDTDNDGIVSYEELKSGIAKFGSHLAESEVQM
LIEAVDTNGRGALDYGEFLAVSLHLQRMANGEHLRRAFLFFDKDGNGYIEPEELQEALVE
DGATDIMEVVKDILQEVDTDKDGKISYEEFVAMMKTGTDWRKASRHYSRGRFNSLSIRLI
KDGSVKLGNE*
>11667.m06141|LOC_Os01g61620.1|genepair398-1
MAAPGLDEVMAFLTDHGFAGAASALRDDVLARAASAAGDAGSDSDAALDPQLPPLRLPAS
TSGGGGAPAAPPASPGSSSDSASSSAFVSMRSSPSGMLNPYGVWSPRHSDTSSSEMEFGT
ARQYDATDFFFQEGWLYDDHLFHSKSELDDDNGEDKEEDKFVLGVHDGSGRIEMGVLSAG
DDHRHEHVGNDGCEGCAEVYTCSSPLCGCCGEGLKNGGLEVVKDSSSTVYGRYQIIDDQT
EILDECGMDGFQLKHPADVVLECHLPRDSGEGDERSELSVVEKELQMLSSFGTRVDADAF
TSPGLVHDITDNAKLDDSIEKNMKNSSDKYLKEGYSIEPFPESSVDDTFEFGDIGPLNTD
AQNSTAAKAEEENPETNVDLALANFHREYEVFELRIVHRKNRTGFEVSKDFPIVINSVIA
GRYYVTEYLGSAAFSKVVQAHDLQTGTDVCLKIIKNDKDFFDQSLDEIKLLKFVNKYDPL
DEYHVLRLYDYFYHQEHLFIVTELLRANLYEFQKYNQESGGEAYFTLPRIQAIARQCLEA
LVYLHHLRIIHCDLKPENILIKSYSRCEIKVIDLGSSCFLTDNLCLYVQSRSYRAPEVIL
GLPYDQRIDIWSLGCILAELYTGEVLFPNEPVPIMLAQMIGIIGPIDMEMLALGEETQKY
FTDDYDLFTKNEETDQFEYLIPEKSSLQHHLQCPDSEFVDFLSYLLQINPRRRPTASEAL
QHQWLSFAY*
>11682.m03704|LOC_Os05g39080.1|genepair398-2
MSVLGAEGAAMGFLPAAEHGFARSAAPSPLREEEEVLDDDPKLPPLRMPTSFAAFPGSSS
GSDSDSFLSMSSTPSGLMNPYGVWSPRAPPSEASSSEMEFGTAREYDTTDLFFGENWLYD
DHLFHTNSDGDEGNGEDKFIVGADSTAQWSETRELDDCGGRHQVHTKSKADAEACAEVYT
CSSAPCSCCYGGRKNDDGLTRDSCSAVYGRYLIMDDQTEVLDECGADAFLFTRDGDAMLK
SEQPIDSKGGDIELLDMSTVEKELQMLSPYLAEADALEKAELEHDFSGNGELDINVVTNE
KTADDKELLKNSYSIHSLPEIGDPLDVYEMEDFGRTDTSVQNSTANKIAEDARTDIDLAL
SRFHEEYEVFELRIFHRKNRTGFEENKEFPIVMNSVVGGRYRITEYLGSAAFSKVVRAQD
LWTGMDVCLKIIKNDKDFFDQSLDEIKLLKFVNKYDPDDEHHILRLYDFFYYQEHLFIVT
ELLRANLYEFQKYNQESGDEVYFSLRRIQAIARQCLEALVYLHHLNIVHCDLKPENILMK
SYSRCEIKVIDLGSSCFLTDNLSLYVQSRSYRAPEVILGLPYDQKIDIWSLGCILAELYT
GEVLFPNESVQIILARMIGTIGPIDMEMLALGQDTQKYFTEDYDLFHKNENLISSEALL*
>11667.m06143|LOC_Os01g61640.1|genepair399-1
MAAAEVYSPTAAAAAQQQQRGKVAAQAWQAVVGWIGFLLQVLLQILRGTPSCAQLLSFVG
FRYPLLSGPADSEQSPSVAFMPLRSEIPADTAPAPTPPPESLQRLTVVLDLDETLVSAYE
SSSLPAIVRTQAVEAGLHCFDMECISTEKDVEGRNKVNHVTVFERPGLHEFLQKTSEFAD
LILFTAGLEGYARPLVDRIDVHNRFKLRLYRPSTVTTEYREHVKDLSCVSKDFCRVVIVD
NNPFSFILQPLNGIPCVPFSAGQHNDNQLMEVIFPLLKHLSIQRDVRPALYERFHMPEWF
QKHGIPQTDQAV*
>11682.m03703|LOC_Os05g39070.1|genepair399-2
MAAAEFLSPEAAAAAGVGPVRQQQHPGEAAWRAVVGWLGFLLQILLQIVRGTPSSWAHLL
SFLGLRHPLLSAAPPQPSPSPSPSPSVAFVRLPSEAPADASSTGPPPPLRRLTVVLDLDETL
VCAYESSSLPAALRAEAVEAGLHCFDMECISAEKARYAKPLVDRIDAHNRFCHRLYRPST
VTTEYREHVKDLSCLSKDFHRIVLVDNNPYSFLLQPLNGIPCLTFSAGQPVDDQLMGVIF
PLLKHLSLQNDVRPALYETFHMPEWFQRHGIPQIDQAA*
>11667.m06148|LOC_Os01g61680.1|genepair400-1
MVRSLDSYPSIKDVTYSCGYCGYALNLSSSTRNTANIGSKYGKQIKKGVVSFFAVDESRF
TQADEVTCVPYFHSRRSWGLFRRRSRLLCRKCGGRIGSAYEEDEPAAAALPACDGPDDLR
TTSSGSSGSASSQKNYVIKINALQPSSDDSDAVAFTL*
>11682.m03702|LOC_Os05g39060.1|genepair400-2
MVRTPERSYSCSSAKEVAYSCGYCGYALNLSSSTRNTANIGSKYGKQIRKGVISFFAIDE
SRFTQTDEVSCMPYFHSRRSWGLFRKRTRLICRKCGGRIGNAYEDEDSTLYDGSDDLHMS
SEGYSMSSGKKYVIKINALQPSTDDSGVPFTL*
>11667.m06170|LOC_Os01g61860.1|genepair401-1
MKAKSLPFIASEHKRDAYGFAVRPQHLQRYREYANIYKEEEEERSERWKNFLDSQAEYDE
SSGEDQDAKVSPSAEDEEAGKKAEDGRSKLSDEQKVKQQRPHKIQIWSEIRPSLGHIGEM
MSLRVKKKQSSADKENAANELQSANNEEIKPSEDSDDEFYDVEKVDPNQEGPVADSADAD
SGMNVDANQEGHYPWKEELECLVRDGLPMALRGELWQAFVGIGARRVKGYYESLLAADDE
RENSKGSDSPTMEGKPKGSPFSSEKWKGQIEKDLPRTFPGHPALDEDGRNALRRLLTAYA
RHNPSVGYCQRKYSSLYMILLNVFFKAMNFFAGLLLLLMPEENAFWALTGIMDDYFDGYF
SEEMIECQVDQLVLEELVREKFPKLVNHLDYLGVQVAWVTGPWFLSIFMNMLPWESVLRV
WDVLLFDGNRVMLFRTALALMELYGPALVTTKDAGDAVTLLQSLAGSTFDSSQLVLTACM
GYQAVDEARLQELRNKHRPSVISSMEQRAKGLRVWRDTNGLASKLYNFKRDPEPLVSLST
EQLSDLTETSSGSTDDMYSGLTVNTEIDSLPDPKDQVVWLKVELCQLLEERRSAVLRADE
LETALMEMVKQDNRRELSAKVEQLEQELSDLRQALLDKQEQEQAMLQVLMRVEQEQKVTE
DARIFAEQDAAAQKYAAHVLQEKYEEAMASLAQMENRAVMAETMLEATLQYQSSQQKAQL
PSPSPSPRTPTRDASPGQVNQDSSQEFQPRRISLLAPFSLGWRDKNKGKQNISDESTNGN
LNSNTEQMVDTPKKDDEKQGDSPQEGEQRVDTPRRDSEHRLDTPETTIKLEEQLEEIKLD
*
>11682.m03691|LOC_Os05g38950.1|genepair401-2
MKPKSLPFIAFEHKRDAYGFAVRPQHLQRYKEYAGIYKEEEEERSDRWKNFLERQSEPSG
QEEKGEAAGRVVDSSGIGGSSLLQEKIVQGPHKIEIWKPIRPSLGNIEQIMSVRVEEKQS
PASGNQDTKDVIHPVKVQEGKLSEDSDDEFYDVDKVETSQEMHSSDSANTGIDNRGQEEN

FPSMEELECLVHGGLPMALRGELWQAFVGIGVRRVKGYYDSLLVAEGELEDTSGSRSSTS
DVAGENTEVSSEKWKGQIEKDLPRTFPGHPALDEDGRNALRRLLLAYARHNPAVGYCQAM
NFFAGLLLLLMPEENAFWTLVGIIDDYFDGYFSEEMIESQVDQLVLEELVREKFPKLANH
LDYLGLQVAWVTGPWFLSIFTNVLPWESVLRVWDVLLFDGNRVMLFRTALALLEFYGPAL
VTTKDAGDAVTLLQSLAGSTFDSSQLVLTARMGYQSVDETGLQELRNKHRPSVLSSMEER
AKGLGALDTNGLASKLYNFKHDPEPLVSISDSQDQMSDVGDGNANQSDSGNMDDMYGGLT
VSSEIEALPDPKDQISWLKFELCRLLEERRSAVLRADELETALMEMVKQDNRRQLSAKVE
QSEQELSELRQTLLDKQEQEQAMCQVLLRVEQELKIAEEARISAEQDAAAQRYAVNVLQE
KYEEAMASLAKMENRAVMAETMLEATLQYQTSQQKALLSPLPSPRTSMIDASPGRANHDS
SQEFQPKKINLLSPFSLSWRDKNKGKQNNVDDSAKLTDAHDQREEITNNNDEKQVETPKL
DVLEESMGSPKEDNKSRVEVPKEDSDLASVQVVANDMNGQHEQLQEIKLD*
>11667.m06182|LOC_Os01g61930.2|genepair402-1
MAAQGGEAAAGSDAKPTAGTGMEQPSPSPYQPSASDHHLWSSSTGAPWNYSMNNSNQNTV
YYDPQRDVSVPGSTENVTSGATHVVQSAMGITGATDSYAPYSNSVQPGYNAPQYPNYYYN
CPQSTNESSVQQGVDQSSGAAYQPLTSFQNSGSYVGPTSNTYYNAGAHQTAPGYATSNNY
YQSNSWTGGSSGDNHVQSYQSYTPSDTNAAQSSSSLPNNSYHYHQQYNQWPYYYDQSAPS
SGGPAVAVSSVSDANTASVSSGYVYPSTQPPPPGTTWRSDAGATAVPPPQAPGTPVFQNQ
HVNQAAGPPGYQNQYVNQAPGTPGFQNQYVNQAPAVPGFQNQYANLAPTYQPGTTYYSQL
PLSNQADQQKASRWQGPISNVSSVNHVSESSQPTFQGSATSDALRVNKIQIPTNPRIAPT
LPMAMPKVEKRNLEADSSKKPAYVSVAVQKNDVKAAQDGHEAVTQGSIPVSLRTYVGRNV
SRCKDDAQRSAVQNILKEIITKATADGILHTKNWDIEPLVPLPENITSTNLTSSAKDLSP
FSFSTSRRSPSRRAKSRWEPVVEEKVANKVELISKESAKTNTYNSSETTKRAGRSWDIGK
FLQSRQAPLSQYNQRPSKKKRIGGNSSLTENGNVSSDSDKEQDLTKYYANAITIANSPEE
KKRREHRSKRFERSQGAASSKSRSSVPDKDGTSNTYARRSMPMLLSRSNGDDVSFAVEDL
DWDALTVKGTCQEIEKRYLRLTSAPDPATVRPEDVLEKALHMVETSQKNYLYKCDQLKSI
RQDLTVQRIQNELTVMVYETHARLALQSGDLPEFNQCQSQLKRLYAEGIKGCHFEFSAYN
LLCVMLHSNNKRDLLSSMASLPKEAKQDRTVKHALAVHSAVSSGNYVLFFKLYKTAPDLN
SCLMGKYDLPLSVI*
>11682.m03667|LOC_Os05g38730.2|genepair402-2
MIHKGMFRFRETIRVWQAVHLIMLSQLWAQKMRLILTCLTQVHFNMGTLLQNMQTIIIVT
HKLQMVLLSSKEEQINIQTAPGYGTSNSYYQNSTWNGGSFENNYAQSYQNYPSSNTNTVQ
HSISVPTNSFSYQQQYNQWPYYYNHTVPNPAGDPVGNSNSIVNTTSSYSYPSIQPPPPGT
TSWKSNSSSSIAPPIQASGGPGPQDQYINQAHAPVLENQYAGQVAGNPRSQNHYASQTPA
CPQSTVNLNPVQQSNHGDQQNTVPRIAPGFSMVIPKSEKKILGADLSKKPAYVSVSMVKN
DARSLPFSLHNYATRNLNCCKDEAQKAACQSMIEEIKNSAIADGTLLTKNWDTEPLLPLV
QNVATIPETSANNSSPSLSTSTNRRRQKSRWEPVVEEKVTDKVEPVKGLVNGTTHNNLEA
KNRMSNNWDSRKFFQSHHATANKVSQRPAKKQKISSYSDQMQNGNASSDSDKEQDLTKYY
ASATALANSPEEKKRREHRSKRFEKNQNSSSKSRNSAASKDVMANIHARRAVSALLARSC
EDGTTLAVEDMDWDALTVKGTCQEIEKRYLRLTSAPDPATVRPEHVLEKALSMVETSQKN
YLYKCDQLKSIRQDLTVQRIQNELTVKVYETHARLAMQAGDLPEYNQCQSQLKRLYAEGI
KGCYFEFSAYNLLCVMLHSNNKRDLLSSLARLSKQAKQDEAVKHALAVHSAVSSGNYVLF
FKLYKQAPNLNSCLMDLYVERMRFEAVKCMSKSYRPTIPVGYVAQILGFSRIDSEASEEC
EMWLKAHGAILSIDNSRDLQLDTKASTTTLYMPEPENAVAHGDASLAVNDFLART*
>11667.m06186|LOC_Os01g61970.1|genepair403-1
MATAAAAARALLLLIAVAGALLRPAAAEIKQESFKDDSRASILFEKFGFSRRGFVSIAIT
GARTSSKLAKAEPDQFGFFLLSDEALFEAIYEQPPPTDLNPNPEPNPGCVLSSPYVKPLF
SFADLDGNGNYKKTFPVTQPDEYSLFFANCAPETAVTMEVRTDMYNTNLDGSKDYLSVGQ
APVPAIYAFFTVCYLVFLAVWLYVTLYRNRLSAHRIHHLMSGLLAARMLYCISAAEDQHY
IRIAGTPHGWDVMFYLFQLVKGVILFAVIALIGTGWSFLKPFLQDKEKKVLMVVIPLQVA
ANIAAAVVGETGPFLQGWVTWNQIFLFVDVACCCAVLFPVVWSMRSLRESSKTDGKAART
LAKLTLFRQFYVVVIGYLYFTRIIVYALKTITNYKYRWVSVAAEEVATVAFYLFMFYMFR
PAERNQYFALDEDEEEAAELALREEEFEL*
>11682.m03665|LOC_Os05g38720.1|genepair403-2
MAAAARRLLLLAIAVAAVSLAPRAAAEIRTEAFREDPRPTILFEKFGFSKTGAVRIVITG
AGISSTFARPDPKQLGFFLLSDESMFQAIYEAQARRPPERREEVAGGGADEPDVSRCILT
SPYVKTLFTFHDLKRGHYNKTFPVTHPDEYSLYFANCAPESLVTMRVRTEMYNGNADGSV
DYLPVGQAPVPAIYGFFAACYAAFLAAWGYLTLSSRDHRAAAHQIHHLMSGLLAARLLYC
LSAAEDQHYIRVTGTPHGWDVAFYLFQLVKGVVLFAVIVLVGTGWSFLRPVLQDREKKVL
MVVIPLQVMANIASAVIGETGPFLQGWVTWNQILLFVDVACCCAVLFPVVWSMRSLRETS
KTDGKAARTLSKLTLFRQFYIVVIGYLYFTRIVVYALKTIASYQFRWVSVLAEEVATLAF
YLFMFYTFRPAERSRYFSFDEDEEEAAEMVLREEEFEL*
>11667.m06187|LOC_Os01g61980.1|genepair404-1
MARHGPQSHDQPLQEEDYIDMDLSSPAAAEAVTASARASLLCYSTAMAASPQNSREFEFH
MSAPVDKWEPAASPADELFYKGKLLPLHLPPRIQMVEKLLESAAEKGLLSASTAPATPYQ
SCNASPANSCYVSGELNAEHYFHECTSAGIGAAEEAAACEKKPWSKKLKFIRHLNLGLKL
KASKAYIKTIFATKAGNPDGKTGTPSANELSNAQFKSWRKNPFGQIRSNRYIASPISNSV
TLGGRLKEDECGHRRSFSSVIIRYSSSNKTSSASSSSCSSSNSSSSILSSTDSGVGPVLR
RSSSASSEMDNPIQGAIAYCKRSQQLASVRKSASDAGFRFMSSSASRIAAESEDPDDIIE
ICGR*
>11682.m03662|LOC_Os05g38690.1|genepair404-2
MASKHQLGQQEAMQEEDYIDMDLTSAAAATAPGEFEFDFHMSGPLGGGGARWEQEPLASP
ADELFYKGKLLPLHLPPRIQMVEELLDGRVVVGGAGRRQLAISTAPATPYDSCTASPANS
CYASGELNVEEYFQEYAARLADAAAAACEKKPWSRKLRFMRQLNLGLKLKASKAYIKTIF
AAKPASSGDDDDKDAILGATRETKELSHGGHHHHRAWRRNPFGQMRSNRCIASSQSGGGR

GSVGGGKHKERDHGGHRRSFSSVIVRYSTSNKTSPAPQSSSCSSSSSVRTSSESDGGAAA
PALRRSSSASSEVENPIQGLIAYCKRSQQLASVRKSASDAGFRFLSSAASKIAAAESDGP
EELVEICRG*
>11667.m06197|LOC_Os01g62060.1|genepair405-1
MDDGGGGGGGDSSPASYIRLVQHLIEKCICYNMNKEECMETLEKHANIKPVITSTVWKEL
EKENSEFFATYKKGQGEEPAESKSSSSSQEAAGSKRSGGDDD*
>11682.m03661|LOC_Os05g38680.1|genepair405-2
MGDSSSSASYIRMVHHLIEKCICFNLNKEECMEALEKHANINPVVTSTVWKELEKENKEF
FETYNKDRAERNIEAETMQRIQKMLSDAAASKGSDDDDDDES*
>11667.m06199|LOC_Os01g62070.2|genepair406-1
MAAAVAGGGEEGEELLLLSAVEAGSFGGGGDGGGAGAAAEKSWRLNFDGFRPPEVQQERR
PPRGLHHHCLGVLSQGPEDVVAEYYQQQVEMLEGFNEMDTLTDRGFLPGMSKEEREKVAR
SETLAIRLSNIANMVLFAAKVYASVRSGSLAIIASTLDSLLDLLSGFILWFTAFSMQTPN
PYRYPIGKKRMQPLGILVFASVMATLGLQIILESVRSLLSDGDEFSLTKEQEKWVVDIML
AVTLVKLALVLYCRTFTNEIVKAYAQDHFFDVITNMIGLVAALLATYIEGWIDPVGAIIL
AIYTIRTWSMTVLENVHSLVGQSASPEYLQKLTYLCWNHHKAVRHIDTVRAYTFGSHYFV
EVDIVLPSSMPLQEAHDIGEALQEKLERLPEIERAFVHLDYEFTHRPEHALSHEK*
>11682.m03660|LOC_Os05g38670.1|genepair406-2
MAAAAGVAAGTGRGSGEGEELLPNAVEGDGGCGGGGTCAGDRPWRLNFDGLRRPEAHQEK
PPRRFHDRLGGLAEDIILKGGKRTHPETCFLKKLLYPFDAVLRNLDISVQSPGDDVAEYY
QQQSELLEGFNEMDTLTDRGFLPGMSKVYILALEECEKVARSEALAIRLSNIANMVLFAA
KVYASIRSGSLAIIASTLDSLLDLLSGFILWFTAFSKKTSNPYRYPIGKRRMQPLGILVF
ASVMATLGLQIILESTRSLFYDGDTFRLTKEQEKWVVDIMLSVTSVKLLLVVYCRSFTNE
ILAIYTIRTWSMTVLENVHSLVGQSASPEYLQKLTYLCWNHHKAVRHIDTVRAYTFGSHY
FVEVDIVLPCDMPLQEAHDIGEAPQEKLESLPEIERAFVHLDYEFTHQPEHARSHDTL*
>11667.m06204|LOC_Os01g62110.1|genepair407-1
MSQRAGRHQRRASQSVFVLPENFASLEDVAADVIGGGGAEQRKPAAAADASSEQQPARMQ
AGRHRRAMSMAVAARDLEMITEDIASYKYGA*
>11682.m03659|LOC_Os05g38660.1|genepair407-2
MSSQRPGRHQRRASQSVFVLPDNFADLDDVPAGAGGAGEDGRKGAAATADAAGGQQQARP
SPAARHRRAMSVAVASSRELEMIKEDMGSYKIGA*
>11667.m06206|LOC_Os01g62130.1|genepair408-1
MSKRSRSMWDMQEFVGSVDTARVLMLLAQQSQHGLLGGGGFAAGAQPVVVRGGAHDRVFE
CKTCNRQFPTFQALGGHRASHKRPRQQQQHALGGGAGADDAGLCLGRQPTPPRPQPAKPR
VHECPVCGLEFPIGQALGGHMRRHRAEAEAAATTTTTTTKNGDVGKAAAVKACDGGGVCL
DLNLTPSENRAKCRNVVGLGAGGQGVHKALAMLDCFL*
>11682.m03655|LOC_Os05g38620.1|genepair408-2
MSKRQFGEMDGGIDTARVLMLLSRRRRQHGDVGHARAARVFECRTCGRRFPTFQALGGHR
ASHKRPRHGAERAPRPAGDDDVGAGAGAALRLVGAASSLSTDEARAGGGGRRTRGAGAAH
GCPVCGLEFAVGQALGGHMRRHRAAAGDVAAPRVKTDDVVVGDECTGGICLDLNLTPSEN
CDKCRHAQLGVAVNSVQRTILLDRPL*
>11667.m06208|LOC_Os01g62150.1|genepair409-1
MDAWFIQKKSNEDLCECDCIIDDIAVPRKPNLQKLKVHQTRMCPGQEKKLNYPAVAVSAT
LNAIKTSRSEDTVLLLKLSDLYPKRKNPLHSPPFSSSVLVLVSRTRSLLPSAIASLAQQS
ATNPPANRGIAPIPSPILQIRFPEMESCPSVKNILVLDSEGKRVAVKYYSDDWPSLSSKQ
AFEKSVFAKTQKTSARTEAEIVMFDSYFVVYKFIQDLHFFVTGGDEENELILASVLQGFS
EAIDYLLRNKVHRRAALENLDLIFLCLDEVVDGGIVLETDAKAILEKVSGHGLEGSGSLT
EQVWQAVASP*
>11682.m03654|LOC_Os05g38610.1|genepair409-2
MESCPSVKNILLLDSEGKRVAVKYYTDDWPTLSAKLAFEKSVFVKTQKATAGAEAEIVMF
DGHIVVYKFIQDLHFFVTGGEEENELILASVLQGFTDAVDIILRNNVDKRTALENLDLIL
LCLDEIVDGGIVLETEGSVIAEKVSAHGIEGATSLAEQTIVQALTTAREHLTKSLLM*
>11667.m06212|LOC_Os01g62190.1|genepair410-1
MESLRVHASALLSLSSPAASASQPTSSSSTTEGVFECKTCSKRFPSFQALGGHRTSHTRL
QAKLLSDPAAAAAAAAERDRARVHECAVCGVEFSMGQALGGHMRRHRGETGTTTVVLADA
DDSGGATVPQPPEPMPDLNYPPLEDAGDGSEPELLNLLV*
>11682.m03653|LOC_Os05g38600.1|genepair410-2
MASGQRAVGEGSGGLRPTTHVAGGGGSARGEYFRCKTCSKTFTSFQALGGHRTGHTRMAA
RQRQEHGAAGAAVVGATNNQRRVVSAHQWHLCAVCGVEFRMGQALGGHMRRHRGEAAAAT
PPPAAAAASAGAVSSMEPPEMIDLNSPPAVEEAGEGDQEVERAEQEPHLLNLLV*
>11667.m06214|LOC_Os01g62210.1|genepair411-1
MGKFRKLGRHAAHRVSMLRTMVSQLVKHERIETTVAKAKEVRRKADQMVQLGKEGTLDAA
RRASAFVRGDDVVHKLFTELAYRYKNRAGGYTRLLRTRIRVGDAAPMAYIEFVDRENELR
EAKPATPQPPQRSPLDPWAKSRASQQWAGPKISEGSRKEGL*
>11682.m03649|LOC_Os05g38580.1|genepair411-2
MGKFRKLGRHAAHRVSMLRTMVSQLVKHERIETTVAKAKEVRTKADQMVQLGKEGTLDAA
RRASAFVRGDNVVHKLFTELAYRYKDRAGGYTRLLRTRIRVGDAAPMAYIEHSKKKSSVS
FTKSRIAEGLSTGRMNFERQNLQHHSHLSGSLLIHGPSHSPANNGQVLKSARTPEQKAYD
KPERSLALFYVVERFF*
>11667.m06216|LOC_Os01g62230.1|genepair412-1
MAPKAEKKPAEKKPVEEKAEKKPKAEKRVPGAKEGGGEKKGKKKAKKSVETYKIYIFKVL
KQVHPDIGISSKAMSIMNSFINDIFEKLAQEAARLARYNKKPTITSREIQTSVRLVLPGE
LAKHAVSEGTKAVTKFTSS*
>11682.m03647|LOC_Os05g38560.1|genepair412-2

MSQRIHFLSRCQFESSQPLDPGVMVPKAEKKPAEKKPAEEKVHPDIGISSKAMSIMNSFV
NDIFEKLEQEAARLARYNKKPTITSREIQTSVRLVLPGELAKHAVSEGTKAVTKFTSS*
>11667.m06217|LOC_Os01g62240.1|genepair413-1
MTFIFSDGGYFNAIMSFPQNYPNSPPTVRFTSEMWHPNVYPDGRVCISILHPPGDDPNGY
ELASERWTPVHTVESIVLSIISMLSGPNDESPANIEAAKEWREKRDDFKKKVRRLVRKSQ
EML*
>11682.m03646|LOC_Os05g38550.1|genepair413-2
MATAASQASLLLQKQLKDLAKNPVDGFSAGLVDDSNVFEWQVTIIGPPDTLYDGGYFNAI
MTFPQNYPNSPPSVRFTSEMWHPNVYPDGRVCISILHPPGEDPNGYELASERWTPVHTVE
SIVLSIISMLSSPNDESPANIEAAKDWREKRDDFKKKVRRIVRKSQEML*
>11667.m06222|LOC_Os01g62290.1|genepair414-1
MAKGEGPAIGIDLGTTYSCVGVWQHDRVEIIANDQGNRTTPSYVAFTDSERLIGDAAKNQ
VAMNPINTVFDAKRLIGRRFSDPSVQSDMKLWPFKVIPGPGDKPMIVVQYKGEEKQFSAE
EISSMVLIKMREIAEAYLGSNIKNAVVTVPAYFNDSQRQATKDAGVIAGLNVMRIINEPT
AAAIAYGLDKKATSSGEKNVLIFDLGGGTFDVSLLTIEEGIFEVKATAGDTHLGGEDFDN
RMVNHFVLEFKRKNKKDISGNPRALRRLRTACERAKRTLSSTAQTTIEIDSLYEGIDFYS
TITRARFEELNMDLFRKCMEPVEKCLRDAKMDKSSVHDVVLVGGSTRIPKVQQLLQDFFN
GKELCKSINPDEAVAYGAAVQAAILSGEGNEKVQDLLLLDVTPLSLGLETAGGVMTVLIP
RNTTIPTKKEQVFSTYSDNQPGVLIQVYEGERARTKDNNLLGKFELSGIPPAPRGVPQIT
VCFDIDANGILNVSAEDKTTGQKNKITITNDKGRLSKEEIEKMVQEAEKYKAEDEEHKKK
VDAKNALENYAYNMRNTIKDDKIASKLSADDKKKIEDAIDGAINWLDSNQLAEADEFEDK
MKELESICNPIIAKMYQGAGADMGGAAGMDEDAPAGGSGAGPKIEEVD*
>11682.m03644|LOC_Os05g38530.1|genepair414-2
MSKGEGPAIGIDLGTTYSCVGVWQHDRVEIIANDQGNRTTPSYVAFTDTERLIGDAAKNQ
VAMNPTNTVFDAKRLIGRRFSDPSVQSDMKLWPFKVVPGPGDKPMIVVQYKGEEKQFAAE
EISSMVLIKMREIAEAYLGSSIKNAVVTVPAYFNDSQRQATKDAGVIAGLNVMRIINEPT
AAAIAYGLDKKATSSGEKNVLIFDLGGGTFDVSLLTIEEGIFEVKATAGDTHLGGEDFDN
RMVNHFVQEFKRKNKKDISGNPRALRRLRTACERAKRTLSSTAQTTIEIDSLYEGIDFYT
TITRARFEELNMDLFRKCMEPVEKCLRDAKMDKSSVHDVVLVGGSTRIPKVQQLLQDFFN
GKELCKSINPDEAVAYGAAVQAAILSGEGNEKVQDLLLLDVTPLSLGLETAGGVMTVLIP
RNTTIPTKKEQVFSTYSDNQPGVLIQVYEGERARTKDNNLLGKFELSGIPPAPRGVPQIN
VCFDIDANGILNVSAEDKTTGQKNKITITNDKGRLSKEEIEKMVQEAEKYKAEDEEHKKK
VDAKNALENYAYNMRNTIKDEKIASKLAADDKKRIEDAIDGAISWLDTNQLAEADEFEDK
MKELEGICNPIIAKMYQGPGADMAGGMDEDAPAGGSGAGPKIEEVD*
>11667.m06228|LOC_Os01g62350.1|genepair415-1
MAPPQPKSGLFVGINKGHVVTKRELPPRPSDRKGKSTKRVNFVRGLIREVVGFAPYEKRI
TELLKVGKDKRALKVAKRKLGTHKRAKKKREEMAGVIRKMRSAGTTDKKK*
>11682.m03643|LOC_Os05g38520.3|genepair415-2
MAPSQPKSGLFVGINKGHVVTKRELPPRPSDRKGKSTKRVTFVRNLIREVAGFAPYEKRI
TELLKVGKDKRALKVAKRKLGTHKRAKKKREEMAGVLRKMRCLVAVTLTPRRRNRVSPSS
*
>11667.m06231|LOC_Os01g62380.1|genepair416-1
MRGGAGALPVPGGLALGVVLLLVLAVLPSRAAGVNVTAALAAFPSFADFARLLESSPVAG
ELAARSSLTLLAVPNNNLPRSPSAFAAASGADIADVLRYHVLLEYLSPSDLARLPASGKL
VTTLFQTTGRAPSDLGAVNLTVGGNSTVVVRSPAPFPGSSATVLGAVTAVPYNLSVLAVG
GLIVPSGLDVAASDSRPAGGVNITHVLADARGFNVAASMLEASGVADEFTADERGAGITV
FVPTDDAFADLPATDRLQSLPADRKATVLRFHVLHSYYPLGSLESIVNPVQPTLATERFE
AGQFTLNITRVNGSVAIDTGIVQASITRTVFDQNPVAVFAVSKVLLPKEMFGRGGADSDV
MAPPPDAMAPDAAENVRTPPTRLSSPPALRGGADSESSSALSTARAVNWWCIRLVLLNLH
LLLLPLV*
>11682.m03639|LOC_Os05g38500.1|genepair416-2
MRGRQIPPPTAAASSRVVVAAVAALLLAAALQPLPVASGVDVAAVLAAFPDLAGFARLLA
SSPVARELAGRSSLTLLAVPNGNLPQSPSAYAAASGADLADVLRYHVLLEYLAPADLRRL
PASGKLVTTLFQTTGRAPADLGAVNVTAAGPSLAVVRSPAPFPGSNATVLGAITAVPYNL
SVLAVDGLIVPSGFDLAASESRPPAAVNITKVLADARAFNVAASMLEASGVADEFEADER
GAGITVFAPTDDAFAGLPAGDRLQSLPAERKAVVLRFHVLHSYYPLGSLESIVNPVQPTL
ATEFTQAGRFTLNITRANGSVAIDTGVVQATITRTVFDQNPVAVFAVSKVLLPKEMFSRS
DSAIVAVASAPPPAALPAESPESAPTKLSSPPALRDTAGNANHTAAAAAVAAAAATTKPT
IGRWCIALLYLLLLPLLLPLICMGLGEEGGEVTGEVRGEERRRVGGGSELAG*
>11667.m06232|LOC_Os01g62390.1|genepair417-1
MQNHAYNRLGSLGGGGGGGSGAVPSPPSSPRRGAGRRSSGKGGSARAGAGAGAAGAVRGG
GAVRRAARVVLAALLRRQAVFLFAPLLYVAAMLLYMGSISLDSVPRIISRPAPGSLYRSP
QLYARLRADMDADNATDAFPNMTSLMVNCVSLNKQTFNSFHWLAASCKFSDIYDEEHFVQ
RLKNDVRVVDKVPEFIMERFGHNLSNVFNFKIKAWSSIRYYKEAVLPKLIEERLIRISPF
ANRLSFDAPSAVQRLRCLANFEALKFSKPITTLSDILVSRMREKSAENNGKYVAVHLRFE
EDMVAFSCCVFDGGEKEKKELDAARERGWRGKFTRPGRVIRPGAIRMNGKCPLTPLEVGL
MLRGMGFSNNTAIYLASGRIYKSEKNMAPLLEMFPLLQTKETLASDEELAPFKNFSSRMA
AIDYTVCVHSEVFVTTQGGNFPHFLLGHRRYIYGGHSKTIKPDKRRLAILFDSPRIGYAI
TVAHELSIKLPSGSGNLGRSCIIRC*
>11682.m03638|LOC_Os05g38490.1|genepair417-2
MPGQTYSRLGRLGGAPSPPPVPPAVAAVASLQRGGGGSGRRTPGKGGSASASAAAGWTGV
GCGCAARRAARAVLAALHRRQAVFLFAPLLYVAAMLLYMGSIPLDVVPRIVARPAPGSVY
RSPQLYARLRADMDADNSTDAICNAVAIAGFLNATLVIPNFHFHSIWRDPSTFSDIYDEA
HFVKRLQNDVRVVEKVPDFIMERFGHNLSNVFNFKIKAWSPIQYYKDAVLPKLIEERLIR

ISPFANRLSFDAPPVVQRLRCLANFEALKFSNPIATLSETLVSRMKEKSTASNGKYIAVH
LRFEEDMVAFSCCVYDGGDEEKKEMNAAREIGWRGKFTKRGRVIRPGVIRMNGKCPLTPL
EVGLMLRGMGFSNNTAIFLASGKIYRAEKNMVPLLEMFPLLQTKETLASAEELAPFKDFS
SRMAAIDYSVCVHSDAFVTTQGGNFPHFLMGHRRYLYAGHSRTIKPDKRKLAILFDNPRI
GILFLGRRIVHGGTPNKARCAQAYPNDAFGYARLHECAA*
>11667.m06234|LOC_Os01g62410.1|genepair418-1
MGAMAMVEQEGCVENRQPLAASSSSVSDGSSYGGGGGGLAQMSPPVSSSANSISGLRRTS
GPIRRAKGGWTPEEDETLRKAVEAYKGRNWKKIAECFPYRTEVQCLHRWQKVLNPELIKG
PWTQEEDDQIIDLVKKYGPTKWSVIAKALPGRIGKQCRERWHNHLNPEIRKDAWTTEEEQ
ALINAHRIYGNKWAEIAKVLPGRTDNSIKNHWNSSLRKKQDMYNTSNNMVVPKLLVHDKF
KDKPKLMAMEGHLDLNKAPIINSKDQPGTAHRSNCSGFLSRSSLPTAQPLTSREASVVDG
SAVTLVAQALESDSVRGKGLEIDSVHEKGLEVNSAPDHTGNSWTIQLEAAPSKGEAELSL
KNEARSLGPLCYQIPNMEDVVPVSSSLFSDHLTGNHTSEHCGDDILSPAGCTTPPPTKGK
LTSQLSVDSILKSAANSFPGTPSILKRRKRDKSTPVSASEMKISGSNTDRFYTPMGMEPA
TATPESFKTTSFLSLGSLDGSVKSFDVSPQYRARSKRMALTKTVEKQLDFSSDGLDTCGS
EILNSSCNNSQSTLSITEAPKLKEKEHAVQLENLTKNFAHTTNLDVT*
>11682.m03635|LOC_Os05g38460.1|genepair418-2
MPAVKVEEEEEERNPVASSPSVSEGSAHAAALASPTAADSIFGRRRKSGPVRRAKGGWTP
EEDEKLRKAVDIYNGKNWKKIAESFSDRTEVQCLHRWQKVLDPELIKGPWTQEEDDVIIN
MVKKHGPKKWSVIARSLNGRIGKQCRERWHNHLDPQIRKEAWTVEEERVLARAHCMYGNK
WAEIAKLLPGRTDNSIKNHWNSSLRKKIDDYNTRDILPVHPPVVGDGLKQLPKRPPADNH
FDLNKEPIICSRDRLGVVHSDPTSHQRASNLKDFKGCADYLSLGQPVTSCEASAADDSAF
DLATQGMRMDSVHDKGTGNNFVCGKVQGINFLGDKGLKINQISDKMGCSRQAKREGEAAI
NGGGSSLQSEAHSVGSLCYQIPKMEDIAPAQSPVFTANYVPEHSRNVMHSPNGYTTPPTH
GKGSDQLSVESILRSAAEKFHGTPSILRRRKRDKPTPAEDNDLKIGRLSSDDFHTPIGKC
TTDSPQSFKTAALLSLGPMDEQGSLDVSPPYRLSSFELPRYRRVTIYQPYHLSLVGCTKP
SPFSTENMLLLFRGEVAEGRSHGRQMAAATAVPGGAATATPGGTATTAPVEDGYRLVAHR
RSLPAAPGEDSFRRHVARRRIDEGGRRRQLQSPGEKAVSGGARGSWQTAPEVAGERRRAR
LQVDWRPAARVTRSGGWCGSGSGWVENLEFDLLT*
>11667.m06249|LOC_Os01g62490.1|genepair419-1
MAISYLLRSSILAVAALLLFSVNLAKGDIREYQFDVKTTNVTRLCSSKSIVTVNGQFPGP
TVFAREGDLVVIRVINHSPYNMSIHWHGIRQLRSGWADGPAYITQCPIQPGGSYVYKYTI
TGQRGTLWWHAHISWLRATVYGPIIILPKAGVPYPFPAPDKEVPVVFGEWWKADTEAVIS
QATQTGGGPNVSDAFTINGLPGPLYNCSAKDTFKLKVEAGKTYMLRLINAALNDELFFSI
AGHTLTVVDVDAVYVKPFTVDTLLITPGQTTNVLLTTKPSYPGATFYMLAAPYSTAMSGT
FDNTTVAGILEYEDPSSHSTAAFNKNLPVLRPTLPQINDTSFVSNYTAKLRSFATAEYPA
NVPQQVDTRFFFTVGLGTHPCAVNGTCQGPNGSRFAAAVNNVSFVLPSTALLQSHYTGRS
NGVYASNFPAMPLSPFNYTGTPPNNTNVSNGTRLVVLPYGASVELVMQGTSVLGAESHPF
HLHGFNFFVVGQGFGNFDPVNDPAKYNLVDPVERNTVGVPAAGWVAIRFLVDNPGVWFMH
CHLEVHVSWGLKMAWVVQDGSLPNQKILPPPSDLPKC*
>11682.m03631|LOC_Os05g38420.1|genepair419-2
MAAASSVLRCCLLVAALMTLSAMGAEAITRQYLFDVQTTSVTRLCSTKSIVTVNGQYPGP
TLFAREGDHVEVTVVNHSPYNMSIHWHGIRQLLSGWADGPSYITQCPIQPGGSYVYRFTI
TGQRGTLWWHAHISWLRATVHGPMVILPPAGVGYPFPAPHEEVPIMFGEWWNNDTEAVIS
QALQTGGGPNISDAYTLNGLPGPLYNCSAQDTFKLKVKPGKTYMLRLINAALNDELFFSI
ANHTLTVVDVDALYVKPFTVDTLIIAPGQTSNVLLTAKPTYPGASYYMLARPYTTTQGTF
DNTTVAGVLEYDDPCPTTAAGKIVPIFSPTLPQINDTNAVSNFTAKLRSLASAGYPAAVP
QQVDHRFFFTVGLGTHPCAVNGTCQGPNGSRFAASINNVSFVLPATALLQSHFAGKSKGV
YASNFPYYPLNPFNYTGTPPNNTNVMNGTKVLVLPYGANVELVMQDTSILGAESHPLHLH
GFNFFVVGQGFGNFDPINDPAKFNLYDPVERNTVGVPAGGWVAIRFHADNPGVWFMHCHL
EVHMSWGLKMAWLVLDGSRPDQKLPPPPLDLPKC*
>11667.m06250|LOC_Os01g62500.1|genepair420-1
MSLSSLSRALARSARSSRQRQGSLLGGHGGLRASSPPLPCGELGFLRSYVTSVIGNRAAV
ASGAGKGGDWRFLLASRQFRRLFSDKSKKNHGKHSEEENKGKGDESDKSDSKKQSSSGDQ
WNFEESIKQFKDMIAPLFLFGLLLLSASASSSEQEISFQEFKNKLLEPGLVDHIVVSNKS
IAKVYVRSSPSIDRIQDSDIHITTSHLPGIESPSSYKYYFNIGSVDSFEEKLQEAQKALE
IDPHYYVPITYTTEAKWFEEVMKYVPTVLIIGLIYLLGKRIQNGFTVGGGPGKGGRSIFS
IGKVQVTKLDKNSKNKVFFKDVAGCDEAKQEIMEFVHFLKNPKKYEELGAKIPKGALLVG
PPGTGKTLLAKATAGESGVPFLSISGSDFMEMFVGVGPSRVRNLFQEARQCSPSIVFIDE
IDAIGRARGRGGFSGGHDERESTLNQLLVEMDGFGTTSGVVVLAGTNRPDILDKALLRPG
RFDRQISIDKPDIKGRDQIFRIYLKKLKLDKEPSFYSQRLAALTPGFAGADIANVCNEAA
LIAARSEGTLITMQHFESAIDRVIGGLEKKNKVISKLERRTVAYHESGHAVAGWFLEHAE
PLLKVTIVPRGTAALGFAQYVPNDNLLMTKEQLFDMTCMTLGGRAAEEVLIGKISTGAQN
DLEKVTKMTYAQVAVYGFSEKVGLLSFPQREDGFEMSKPYSSQTASIIDTEVREWVAKAY
EKTVELIKQHKDQVAQIAELLLEKEVLHQDDLVQVLGERPFKTLEPTNYDRFKQGFQDED
SNRNAELSNADGASSLGEAVAS*
>11682.m03628|LOC_Os05g38400.1|genepair420-2
MSLASLARALSRRSAPSSSRARQGFSLGGLGGTTRSPPPPSSPLPSLHGGEGGGLGLGFV
RGYLTAALGRPAAVKAGTDWRSILANPQFRRLFSDGSKKNYENYYPKGKKEAPKGDGSNK
SDSKQDSSTDDQWNFQETASKQLQNFLAPLLFLGLMLSSLSSSSSDQKEISFQEFKNKLL
EPGLVDRIVVSNKSVAKVYVRSSPQSNSQGQNTDAIITTNDVPSKHTPSRYKYYFNIGSV
DSFEEKLEEAQEALGVDPHDFVPVTYVAEVNWFQEVMRFAPTVFLVGLIYLMSKRMQSGF
NIGGGPGKGGRGIFNIGKAQVTKMDKNSKNKVFFKDVAGCDEAKQEIMEFVHFLKNPKKY
EELGAKIPKGALLVGPPGTGKTLLAKATAGESGVPFLSISGSDFMEMFVGVGPSRVRNLF

QEARQCAPSIIFIDEIDAIGRARGRGGFSGSNDERESTLNQLLVEMDGFGTTSGVVVLAG
TNRPDILDKALLRPGRFDRQITIDKPDIKGRDQIFRIYLKKLKLDNEPSFYSQRLAALTP
GFAGADIANVCNEAALIAARSEETQITMQHFESAIDRIIGGLEKKNKVISKLERRTVAYH
ESGHAVAGWFLEHAEPLLKVTIVPRGTAALGFAQYVPNENLLMTKEQLFDMTCMTLGGRA
AEEVLIGRISTGAQNDLEKVTKMTYAQVAVYGFSEKVGLLSFPQRDDGFEMTKPYSNQTA
SIIDDEVREWVGKAYKKTVELITEHKEQVAKIAEMLLEKEVLHQDDLVRVLGERPFKASE
PTNYDLFKQGFQDEEDSKNQEAAKTPQPDDDGTPSLGEVVPT*
>11667.m06261|LOC_Os01g62610.1|genepair421-1
MELYAVVKPCRLYKKRSSSGGGGKVAMCVRSGGDGGAGKSRPSFTCRVQRAVVKWSYDPT
EMSDEYSLPRSFALCRVRTLQEYTGQLAEEEASAAEEESPIESFPLPRKIRIYKSIKASA
STLSTVHYEGTLAENGEVFDTTHEDNSVFSFEIGEGTVIKAWDIAVKTMKVGEVAKITCK
PEYAYGAAGSPPEIPPEVQSATLTFEVELIACRPRKGSSVESVSEEKARLEVLHFNRELK
KQREIAAAAKEEEKRKREEAKAAAAARVQAKLEAKKGKGKKAK*
>11682.m03625|LOC_Os05g38370.1|genepair421-2
MAEVADLTGDGGVLKTVVRKAKDDAIAPTDSLPLVDVHYEGTLAENGEVFDTTHEDNSIF
SFEIGQGAVIKAWDIALRTMKVGEVAKITCKPEYAYGSAGSPPEIPPNATLIFEVELVAC
RPRKGSSLGSVSDEKARLEELKKQRELAAATKEEEKKKREEAKAAAAARVQAKLDAKKGK
GKGKGK*
>11667.m06262|LOC_Os01g62620.1|genepair422-1
MAGRPGYVTVPILSVLAAIGYVYYTAVFLAIPAWLGLATAAGVANAVAFTALAAACVATY
AVAVSRDPGRVPPAFLPDVEDAESPIHEIKRKGGDLRYCQKCSHYKPPRAHHCRVCKRCV
LRMDHHCIWINNCVGHENYKIFLVFVLYAVVASLYSLVLVIGGAVHSLPKNEQLGSDSSR
TSIIICGVFLCPLALALSILLGWHVYLIFHNKTTIEYHEGVRAMWLAEKAGNLYHHPYDL
GVYENLVSVLGPNALCWLCPISRNTGNGIRFRTSYDIPLSTPPI*
>11682.m03622|LOC_Os05g38360.1|genepair422-2
MGRPGYLTLPILSVLAAIGYVYYTTVFVAVARWLGLATAAGAANAAAITALAAACVATYA
VAVCRDPGRVPPSFAPDVEDAESPLHEIKRKGGDLRYCQKCGHYKPPRAHHCHACKRCVL
KMDHHCIWINNCVGHENYKIFFIFVLYAVTACFYAMILIIGSAMYSVPVDEHSSNDSSRT
SIIICGIILCPLTLALTVLFGWHIYLILQNKTTIEYHEGVRAMWLAEKGGNLYHHPYHLG
VYENLISVLGPNIFCWLCPVSTNTGNGLRFRTSHDIPLSTPSM*
>11667.m06269|LOC_Os01g62670.1|genepair423-1
MVVQKMWMPVDLRLPSGPQAALGILAFEAAAAMSKLLSLHRSLSEQEVSRLRSDTMRSPG
VAYLNSTDQAFLLRLACAELVVSLDAAAAAVARLGLRCGIDFGGVYASLKAGAPDARLDP
LVAKGLRVKAKKMERLVASTARLCSEMEALDELEASERKLAFRGWNRLSGPIPMQPVAPS
AAGDSPGADSLQQDLKAQRIKVRRLKEESLWNQSYEKAVGLMARAACAVFSRICTIFGAF
VPGLPPPLPSAATDSVQTRLSKLLNPRAVRAKASSGPITRRDGPSRVHPPVISSSCPIIG
LRPSGQKAAIDWRKLLDAPASTVGGAGLDQQYANVIVSAEQLLQMEAEGRQEEANAERAE
MYEMLPAKLRAAVRSKLRDWWRDPGPLDAGLAQGWKDAVDRIMAWLGPMARDTVQWQAER
NMDRTRRFDGGARVYALQTLRWADKEKAEAALVEVLVALSCVCCQWSEADMPGPRVGLTL
VTESESESEIPDTKTESNIIETENGAKTNRREYGNENLSE*
>11682.m03617|LOC_Os05g38320.1|genepair423-2
MRWADDGGEGDDDDDLRLPGAGRRSPSLGILAFEAASTMTKLLSLHRSLSEKEVARLRSN
TMRAAGVEYLSSTDQAFLLRLACAEAVAALDAAAAAVARLGARCGLDFAGPYASLKAGAP
DARLDQFVAKGLKVKAKRMERLVAATAKLCAEMEALDKLEAAEQKLARRGWGRLSVPIPS
PAAAAVDAVGSDSLRLGIRAQRARVRRLKEESLWSQSYEKAVILMARAACAVFVRVCVVF
GAHVPGLPPPLPPAEAVHSRLSKLLLHPMSAAAQPRSLSGPIQRRDVPLRIEMSSNSCPI
IRSHCQQPWQTSPPGVDWRKLLEPPPGTVGGAGLDLQYANVITTAERLLLETDHAEGRQR
HEEARAELYAMLPSKLRAAVRAKLRGWWRERGAAAAVAVELDAGLAEGWRSAAGRILAWL
APMARDTARWHAERSLDRQRRFEVGGGGGGSARAWALQTLRWADAEKAEAAVVEVLVALS
CVGWYDERRRVASLRF*
>11667.m06279|LOC_Os01g62760.2|genepair424-1
MAEICCEEAMSPPATATAAVAAAVSASAAAAVSSAIDRRRRRMEMRRIRIASDLELQAGE
DGRPGKRQRLARTASGAPRPDEDSASERPSCGRTEEFPRYGVTAVCGRRREMEDAVSIRP
DFLPASGKFHFYGVFDGHGCSHVATTCQDRMHEIVAEEHNKGASGEVAPWRDVMEKSFAR
MDGEVGNRASTRSDDEPACPCEQQTPSRRDHAGSTAVVAVVSPTQVVVANAGDSRAVISR
AGVPVALSVDHKPDRPDELERIEAAGGRVIYWDGARVLGVLAMSRAIGDGYLKPYVTSEP
EVTVTERTDDDECLILASDGLWDVVTNEMACEVVRACFHNNGPPAPAARPSGVPSSAEAA
ETENGGAASVKGISKAESSDKACSDAAMLLTKLALARRSADNVSVVVVDLRRGL*
>11682.m03614|LOC_Os05g38290.1|genepair424-2
MAAEICREEAAKSMPAAAAGATAIARRRRRVEGFRFAAGSLEPPQEDADAGVARCGKRQR
VAGARAGAGAATAGPCRPSAGAEFGSRWWPRYGVTSVFGRRREMEDAVSIRPDFLRGSTS
SGKHHFFGVFDGHGCSHVARMCQDRMHELVVDAYKKAVSGKEAAAAAPAWKDVMEKGFAR
MDDEATIWAKSRTGGEPACRCELQTPARCDHVGSTAVVAVVGPNRVVVANSGDSRAVLCR
AGVPVPLSVDHKPDRPDELERIKAAGGRVIYWDGARVLGVLAMSRAIGDGYLKPYVTSEP
EVTVTERADDDECLILASDGLWDVVTNEMACEVVRACFRSNGPPSPPGCSRPKAVLPPPA
GASGGGGGDAVVKGVDKAESDKACADAALLLAKLAIARRSADNVSVVVVDLRRPVP*
>11667.m06284|LOC_Os01g62810.1|genepair425-1
MRRRGVKLQPATFSLAASPGSDPHDVASPTGRHVAPPSPGPQTFPEASGRFLARNSPSRS
SGPPSATCLGPSHVGIRRGAPGPGETDTPKSRSSQPHARKASGRRFPTPTKPTPSPPPPP
RGRREARAIETEAATTATEKVAEESAADTGRTGAPEEMNGRGGGGGGGVGVVGAGGEEQM
EMEEDGAGLRARVSAGRKERVVLMWGYLPGVSPQRSPLLGPVPVRLPAAAGGDGWRDVCG
GGCGFAMAISESGKLLTWGSADDMGQSYVTAGKHEMKGMSIHGVGKNVFQQEGSFLTNLL
VELWKKMKDRVRLPLTKVVMITAYLVSFVPKKYNKGNSITVSPRSQVSRTSSGAASGPSE
SRDLGQVWGWGYGGEGQLGLGSRIRTVSSPHPIPCIESALYSKDRPAAMKGNKSAEVQIS

KVMGNCVKAIACGGRHSAVVTASSFADSGALLTFGWGLYGQCGQGNTDDVLSPTCVSSIL
GVKMQDIGAGLWHTVCTSVDGDVYSFGGNQFGQLGTGSDQAETVPKLVDASSLENKNARA
VSCGARHSAIITDEGEVFCWGWNKYGQLGLGDSMDRNVPCSVPVDAYHPLNVSCGWWHTL
VLAESPT*
>11682.m03611|LOC_Os05g38270.1|genepair425-2
MEEDGGGAGRRKERVVLMWGYLPGVSQQRSPLLGPVPVRMPDAAAAAGDAWRDVAGGGCG
FAMAVSEQGKLFTWGSTDDMGQSYVTAGKHEETPEAFPLPSDIAIVRADAGWAHCVAITG
KGDVYTWGWKECVPTGRVIADHSATGAMDCDKRQSGVATDQGNIGNSITVSPRSKVSRTS
TKAASGPAESRSSEDSTKRRRLSSSKHGAESESSSDENLSAPPCVVTFNTGVKIVEVAAG
GRHTLVLSASDFGQVWGWGYGGEGQLGLGSRIRTVSSPHPIPCVQSAFYSKDRTGIVMGS
RTLEGTNDRAATCVKAIACGGRHSAVVTDSGTLLTFGWGLYGQCGQGNTDDILSPTCVSA
IMGVKIQRVAAGLWHTVCTSADGDVYSFGGNQFGQLGTGSDQAEIVPKLVEASKLENNHA
RGVSCGARHSAVITDKGDVFCWGWNKYGQLGLGDSTDRNVPCQVPVEAYNPLNVSCGWWH
TLVLAEYPT*
>11667.m06288|LOC_Os01g62850.1|genepair426-1
MRELAGSPGTWSGLSLRVGQLVFAAASVCATASALGFAAYTAFCYLIASMGLQALWSLGL
ACLDCYALKFKKDLHSAVLLSLFVVGDWASKA*
>11682.m03608|LOC_Os05g38250.1|genepair426-2
MWEVAWWRPGTWGGLAMRVGQVAFAGASIGVMASGAGFANYTAFCYLIASMGLQSLWSLG
LACLDVYALTVKRDLNNALLVSLFVIGDWVTALLSFAASCSAGGVMVLFKRDVLFCRRYP
QLPCGRFELAVALAFLSWALSATSAIIMFCLLAAF*
>11667.m06290|LOC_Os01g62870.1|genepair427-1
MAKHFVLNTGAKIPSVGLGTWQSDPGVVGNAVYAAVKAGYRHIDCASAYNNEKEVGLALK
KLFEEGVVKREDLFITSKLWCDHHAPEDVPEALDATLNDLQLEYLDLYLIHWPFRTKKGS
SIGKPESYLPPDIPSTWAAMEKLYDSGKSRAIGVSNFSSKKLGDLLAAARVPPAVDQVEC
HPGWQQMKLHNFCQSTGIHLSAYSPLGSPGSTFMNGNVLKEPIIISIAEKLGKTPAQVAL
RWNIQMGHSVLPKSVSEERIKQNLDVYDWSIPEDLLAKFSEIKQVRLLRGNFIVNPQSVY
KTHEELWDREI*
>11682.m03605|LOC_Os05g38230.2|genepair427-2
MAGRAGRRRQCRLRRRQGDVALSEFRDKLIFHVSFVLSEFSRRLIFHVLLCCLNCMLLQA
GYRHIDCAQAYFNEKEIGVALKKVFDEGIVKREDIFITSKLWCTNHAPEDVPVALDSTLQ
DLQTDYVDLYLIHWPVRMKKGAGFGGQNVLPTDIPATWAAMEKLHDSGKARAIGVSNFSS
KKLEDLLAVARVPPAVDQVECHPVWQQTKLRKFCTSKGIHLSAYSPLGSPGTASVKAVGN
VLAHPVVVSTAEKLGKTPAQVALRWGIQMGHSVLPKSTHEERIKENIDVYDWSIPEDLFI
KLSEIEQMKLIRGEFWTHPEGVYKSIEELWDGEI*
>11667.m06293|LOC_Os01g62890.1|genepair428-1
MDWSSVTAEDLVDALREVDWSTPPRPVSEFFSRFTAPRSYSKWTSRLKCNLYYYRTNYFI
LIMFILGMGFLRKPVSILAAFSTGLSIAFLNDSFAVTFNEKVTRTVRQFSPHLAAKMRPP
LRPVIRGRPSSKRSIHICGRPRWVFVVLFSVVSCFLWMTSCSLLTVLWALIVGLLATLLH
ASFRTPNLKARLNTFREEFRAVWRNYSEF*
>11682.m03597|LOC_Os05g38160.1|genepair428-2
MDWSAVTAEDLVDALREVDWSTPPRPVPEFFSRFTVPRSYSKWTSRLKCNLYYYRTNYFI
LIMFILGIGFIWKPVAILAAFMTGISIAFLNDSFAVTFNEKVTRTVRQFSPHLAAKMRPP
ITPVLRGRPSSKRSIHICGRPRWLFVLLFSAVSCMLWLTSCSLLTVLWALLIAIFATLLH
ASFRTPNLKARLNTFREEFRAVWRNYSEL*
>11667.m06295|LOC_Os01g62900.1|genepair429-1
MGRGGIGGAGLVAAVAKADVENTDSTRGFVKDVKRIIIKVGTAVVTGPNGRLAMGRLGAL
CEQVKQLNFEGYEVILVTSGAVGVGRQRLKYRKLVNSSFADLQNPQMDMDGKACAAVGQS
VLMAIYDTLFSQLDVTSSQLLVTDRDFMDPSFGNQLRETVNSLLDLKVIPVFNENDAIST
RRQPYEDSSGIFWDNDSLARLLAQELKADLLIMLSDVEGLYSGPPSDPQSKIIHTYVHEQ
HGKLISFGEKSRVGRGGMQAKVAAAFTASSKGIPVVIASGFAIDSIIKVMRGEKIGTLFH
REANQWGCSKEATAREMAVAARDCSRHLQKLSSEERKKILLDIADALEANEDLITSENQA
DLDLAQDIGYDKSLVARMTIKPGKIKSLAGSIREIADMEDPISHTLKRTEVAKDLVFEKT
YCPLGVLLIIFESRPDALVQIASLAIRSGNGLLLKGGKEAMRSNTILHKVITGAIPDVVG
KKLIGLVKNKDEIADLLKLDDVIDLVIPRGSNKLVSQIKAATKIPVLGHADGICHVYIDK
SADMDMAKRIVLDAKVDYPAACNAMETLLVHKDLNRTEGLDDLLVELEKEGVVIYGGPVA
HDTLKLPKVDSFHHEYNSMACTLEFVDDVQSAIDHINRYGSAHTDCIITTDGKAAETFLQ
QVDSAAVFHNASTRFCDGARFGLGAEVGISTGRIHARGPVGVDGLLTTRCILRGSGQVVN
GDKGVVYTHRELPLQ*
>11682.m03595|LOC_Os05g38150.1|genepair429-2
MASVDPSRSFVRDVKRVIIKVGTAVVSRQDGRLALGRVGALCEQVKELNSLGYEVILVTS
GAVGVGRQRLRYRKLVNSSFADLQKPQMELDGKACAAVGQSGLMALYDMLFNQLDVSSSQ
LLVTDSDFENPKFREQLTETVESLLDLKVIPIFNENDAISTRKAPYEDSSGIFWDNDSLA
GLLALELKADLLILLSDVDGLYSGPPSEPSSKIIHTYIKEKHQQEITFGDKSRVGRGGMT
AKVKAAVLASNSGTPVVITSGFENRSILKVLHGEKIGTLFHKNANLWESSKDVSTREMAV
AARDCSRHLQNLSSEERKKILLDVADALEANEDLIRSENEADVAAAQVAGYEKPLVARLT
IKPGKIASLAKSIRTLANMEDPINQILKKTEVADDLVLEKTSCPLGVLLIVFESRPDALV
QIASLAIRSGNGLLLKGGKEAIRSNTILHKVITDAIPRNVGEKLIGLVTTRDEIADLLKL
DDVIDLVIPRGSNKLVSQIKASTKIPVLGHADGICHVYIDKSADMDMAKHIVMDAKIDYP
AACNAMETLLVHKDLMKSPGLDDILVALKTEGVNIYGGPIAHKALGFPKAVSFHHEYSSM
ACTVEFVDDVQSAIDHIHRYGSAHTDCIVTTDDKVAETFLRRVDSAAVFHNASTRFSDGA
RFGLGAEVGISTGRIHARGPVGVEGLLTTRWILRGRGQVVNGDKDVVYTHKSLPLQ*
>11667.m06297|LOC_Os01g62920.1|genepair430-1
MSSAAGGGGYGGGQGGGAEHHHHHHGHAGHLLLHHHPQHVAGAAVAAAAAAAGGQMYHVP

QHSRREKLRFPPDAGDSPPPHGHGHGHAPQQQQQHGSWPPPPAFYSYASSSSSYSPHSPT
LAQAQLVAHGLAPPLPQIPTQNFSLSLSSASSNPPPPQAQPRRQLGGLAQATGPFGPFTG
YAAVLGRSRFLGPAEKLFEEICDVGGAASHVDRTISDEGLLDADPMDGVDHDVVDHDLGG
ADRAAADAGPISGAEQQWKKTKLISMMEEVCKRYRQYYQQVQAVMASFETVAGFSNAAPF
AALALRAMAKHFKCLKSMILNQLRNTSNKVAVKDGLNKEIAVFGLAGGSSGGAGLQRANS
ASAFGQPHNIWRPQRGLPERAVSVLRAWLFEHFLHPYPTDGDKQMLAKQTGLTRNQVSNW
FINARVRLWKPMVEEIHNLEMRQMHKHSVVDKGQHSVHHQAQHSSQCSGNPSVPSDSHPG
QSSSITRNHNTAASQGFPDELSQMSQSIQGQVSFAYNGLTSQHNIASPHHQHQQVGGVGI
GGGNGGVSLTLGLHQNNRVCIAEPLPAALPANLAHRFGLEEVSDAYVMSSFGGQDRHFGK
EIGGHLLHDFVG*
>11682.m03592|LOC_Os05g38120.1|genepair430-2
MSSAAGGGGGYGGGGGEHQHQQQQHHLLLGQAAGQLYHVPQHSRREKLRFPPDHPAESPP
PPPPGSWPLPPAFYSYASSSSSYSPHSPTLAHAQLVAHGMPPGAATSGGAQIPSQNFALS
LSSASSNPPPTPRRQFGGGGGGGGAAGPYGPFTGYAAVLGRSRFLGPAQKLLEEICDVGG
RPAQLDRGSDEGLLDVDAMDAAGSVDHEMDGSDRAVADAVTVSGAEQQWRKTRLISLMED
VCKRYRQYYQQLQAVVSSFETVAGLSNAAPFASMALRTMSKHFKYLKGIILNQLRNTGKG
ATKDGLGKEDTTNFGLMGGGAGLLRGNNVNSFSQPHNIWRPQRGLPERAVSVLRAWLFEH
FLHPYPTDSDKQMLAKQTGLTRNQVSNWFINARVRLWKPMVEEIHNLEMRQLQKNPSLDK
NQLSMQHTQHSSDSSGKPCDPSNSLQGQSSSMTRNHSISASRHIEDGLSQMPHDISGQVS
FAYNGLAAHHSIAMAHHHQPDLIGTGGAANAGGVSLTLGLHQNNNRAYIAEPLPAALPLN
LAHRFGLEDVSDAYVMSSFGGQDRHFTKEIGGHLLHDFVG*
>11667.m06302|LOC_Os01g62970.1|genepair431-1
MEPKRSPALPQPNVETKKSPPRAAAGGGGGGTAAVGGESPLSSLFHQPSHGAKGKEDIYS
IFYKGQNGTAQAGTADGKSQWTPPKSRTVYTKDNKQSNQYDSVDTSCFGSSVNYGGRDYY
GISGHKQSTESNDYKADKKDPSTDSHGDWWQGSFYY*
>11682.m03583|LOC_Os05g38040.1|genepair431-2
MDSRKKSPPSTAAGAAPAAANGYFSTVFSASPTANTKDAKQADLYAMLNKQNSKGQNGGG
FADGKSHSPTKARGAYKDGKQSYPNESSESPYFGSSVHYGAREFYGNTPPKQGDASPGNQ
KEQEQNPDGSLATRGDWWQGSLYY*
>11667.m06304|LOC_Os01g62990.1|genepair432-1
MGSGEEEREARSEAAFTDSADGSSSSSDAASADEWPVTLAAPPRKTAACGRVPGAEVVDS
SKPHAQKRRAPSSEMEMMKERFAKLLLGEDMSGSGKGVCTALAIANAITNLCATIFGQLW
RLEPLPPEKKAMWRREMGWLLCVSDHIVELVPTWQSFPDGTRLEVMTSRPRSDLYINLPA
LRKLDHMLIEILDSFRDPEFWYVEQGICAPDCDGSASFRAAFHRRDEKWWLPVPRVPPGG
LRDKARKQLQHKRDCANQILKAALAINSNALAEMEVPESYLESLPKNGRATLGDIIYRYI
TSDHFSPECLLDCLDLSTEYQALEIANRVEASVYVWRRRIAAKPASVLGRATSGRSSWGM
VKDMIIDTEKRELLAERAEGLLICLKQRFPGLTQTSLDMSKIQYNRDVGKSILESYSRVL
ESLASNIVARIDDLLNIDELNRHAEHFPQGDADCRIACNKAAVPPYQVPASGTPFVTAYA
TPSFSPAQLASPSKKERSPLGAGRRSYSNRGFGAKKALAIDLVNPEVMGVIISGGKMIDV
STTTEL*
>11682.m03579|LOC_Os05g38000.1|genepair432-2
MARGGGGGEEAAEEEREVSEALTADSSADEECRRGSSSSSASSGDASSESYCPPDEWQKV
AIKTCVSSDLVVVSAEPAKEKKPPPPPSSPRVDAAPADKHHRPSEMEMMKERFAKLLLGE
DMSGGGKGVCTALAISNAITNLCATIFGQLWRLEPLLPEKKTMWRREMDWLLCVSDHIVE
LVPTWQTFPDGSKLEIMTSRPRSDLYINLPALRKLDHMLLEILESFRDPEFWYVDQGICP
PDRDGSAPFMLTFHRRDEKWWLPVPRVPPGGVGETTRRQLEHKRDCASQILKAAMAINSN
ALAEMDVPDSYLDSLPKNGRATLGDIIYRYITSDQFSPDCLLDCLDLSSEYQALEIANRV
EASIYVWRRRGTSGAASRAGNKSSWGIVKDMIMDTEKRDDLLADRAEGLLMCLKQRFPGL
TQTSLDTSKIQYNKDVGKSILESYSRVLESLASNIIARIDDLLYVDERSRQAELLPTAGA
GSGKISCMPAMSASSVPAYPVVSTSGTPPPYATAYATPSFSPAQLSSPSNIGRALLVDRR
SHDGRAFDGSMEFMGMAVSNAVFDLPGL*
>11667.m06306|LOC_Os01g63010.1|genepair433-1
MTEAGGERRIGVAMDFSPSSKKALQWAADNLLRKGDTLVLLHIRHHGRDEAKNVLWSHTG
SPLIPLEELMETAVRQRYDIPSDEEVFDMLNAVSREKELSVVLKMYWGEPREKVCEAVGE
LNLESLVMGSRGLGQIQRILLGSVTNYVLSNASCPVTVVKAK*
>11682.m03574|LOC_Os05g37970.1|genepair433-2
MAGGGRADDERRIGVAMDYSASSKRALDWAIANLLRRGDHLVVLHVLHHGGEEAKHALWG
KSGSPLIPLSEFRDPTAMQQYGVHCDAEVLDMLDTAARQLELTVVAKLYWGDAREKLCDA
VEEQKIDTLVMGSRGLGSIQRYNSYFCLQCCCLINQESKLQALTIELAFGPVANIPVQID
KLCRERCTA*
>11667.m06310|LOC_Os01g63050.1|genepair434-1
MAEVCTPGAAEAELWLPDEFLDDDFFTVEEKAAVAAKSESDEEDGLDGLARRMADLLAGE
GGKGTGSKVEVMAGSPQSTLCGLAASGEDSPNGGASQFSSPPSSPLEQPPTDPWDVLSEA
AGQVARLRMNSIPVPQKPHAHAGHGRFVPPARNPSPPVQAQKTAGAFQFAPNNNMLTQRQ
VQVAHFHLLKQRQLLKQQREQQLAAAAAAAWGTHRAGVGVGAPLGLNSSGWPPLQKAHQQ
ASSAAGMRAVFLSPPGGKTERTGTGVFIPRQAGAPAEPKKKPSCSTVLLPARVVQALNLN
VDDLGARPCFPGGFVLDHDALVSRSNAMLTTQKRVQHHLHAATAAPPTLAAAREVNLPQE
WTY*
>11682.m03570|LOC_Os05g37930.3|genepair434-2
MATEWSDGGEEFLLPDEFLDDDFFSEEEKAAVAARSDSSDEEDCLAGLSRRLAGLLGDDG
ERDAPPKAEVTVGSPQSTLCGLPKSGQESPNGGASQVSSPPSSPLEQKPADPWDMLYEAA
GQVARMRVTNSIPVPNNPYGFPAHGGFAAPARKASPPPPVSPPATKVAPAAYYHPLAQLL
TQRQIQAAQFHLLKQQQLLKLQRDRHLAAAAAWGARQTAAAKTAGCGVAASPVDMNPAAW
PPLQKQQHAPAPGVGGGGGGGMRAVFLTPPGAKRERNGTGVFLPRPAGAPAEPKRKTGCS

TVLVPARVVQALNLNLDDLGAQPRYPGGFVLDHDALINRSNAMLASQKRRASPAVPSPAP
APALCHSS*
>11667.m06311|LOC_Os01g63060.1|genepair435-1
MAVYIAREATKLWRKVCAEIAVELQLLFEKWRLLLAGLVFQYIHGLAARGVHYLHRPGPL
LQDLGFMALPELGQDKGYVSESVFTFIFISFLLWSFHPFIYHSKRFYTVLLWRRVLAFLV
ASQFLRIITFYSTQLPGPNYHCREGSKMATLPPPHNVLEVLLINFPRGVLFGCGDLIFSS
HMIFTLVFVRTYHKYGSKRLIKILAWLMAIIQSLLIIASRKHYSVDVVVAWYTVNLVVFF
IDNKLPEMPDRTNGSSLLPVTAKDKDGRTKEELHKLEKDCKMKEEFHKLLNGNTVDSTDR
RQRVQMNGKHGEDINHTLSDATPNGT*
>11682.m03565|LOC_Os05g37910.1|genepair435-2
MTIYIAREASKVWRKVTTETSVELSLLREKWGLLLAGIVFQYIHGLAARGVHYLHRPGPL
LQDLGFMALPEFGQDKGYLSESIFASIFASFVLWTFHPFIYHSKRFYTVLIWRRVLAFLV
ASQVLRIITFYSTQLPGPNYHCREGSKLATLPPPNNVFEVLLINFPRGVLFGCGDLIFSS
HMIFTLVFVRTYHKYGSKRFVKLLAWFMAIVQSLLIIASRKHYSVDVVVAWYTVNLVVFF
VDNKLPEMPDRTNGVPLLPLSTREKDGRLKEEKDSRLKEEFHKLLNGNHGDPTDRRQRAQ
MNGRHDEDINHAHSTLSDAAVNGGT*
>11667.m06329|LOC_Os01g63230.2|genepair436-1
MAAAPPSVASSSSSSSPVLSAAHRRRLNDVERDAFDYGGPCDVDVDHDDDDGGGGVRRGH
GAGVAGVRALFSSARRSKRASVIIDQAWLRNVVACLLGLTVVAGLVLSSHRVSGAGGGRL
VQRMDLGDGEVMGWTEENLTAVARQSPDTPMKIWMTPDSEGYGKCIERPKKHDRMNSATA
GYIIVNANGGLNQMRLGISDMVAVAKLMNATLVIPTLDHKSFWTDPSDFKDIFDVEHFKK
TLEGDISIVDSLPLAYKGLKLYMRAPTSWAKASYYRAFSRTLKKAKVVKFTHTDSRIVNN
GLPPSIQRLRCRANYEALRFHKEIEELSTALVDRLRNGSNHYIALHLRYEKDMLSFTGCS
HNLTHKEADELREMRLNVRHWKEKEINSRERRLQGRCPMTPREVALFLKAMGYPSSTKIY
IVAGEIYGGHSMDSLKAEYPNIYTHYSLATVDELEPFKLYQNRLAALDYNVAVQSDVFVY
TYDGNMAKAVQGHRRFEGFQKTINPDRQKLVGLIDKLDEGTLTWNEFQSEVKIHHENRLG
GPYQRLSGRSPRQEEYFYANPLPGCLCKRMQRIK*
>11682.m03562|LOC_Os05g37880.1|genepair436-2
MATAATTTSSTATTTTTTCSSPSSTTSPIHPAVPHRRRLNDIERVDYAHGAAADDCAACG
GVAPDAALADDDECGHGHPVVGAVTPCGGGGRAALLLARRKRAWVVGAGGQAWMRGVVLC
LLGLVAVVGFLGSHRRGGSGGGGGGAGSGAVGGGGGDDGGRLVKKVEVADADVMGWTEEN
LTALTRRPPDPPISDMVAVAKIMNASLVIPTLDHQSFWTDPSDFKDIFNVEHFKEILKED
IVIVDSLPPTYKRVKPYMRAPTSWSRASYYRDFSRILRKYKVVRFTHTDSRIVNNGLAPS
LQRLRCRANYKALQYRKEIEELGRTLVDRLRNGMDHYIALHLRYEKDMLSFTGCNHNLTV
HEADELTDMRLKVRHWKEKEINSEEKRLQGGCPMTPREAAVFLKAMGYPSTTKIYIVAGE
IYGAHSMDALKLEYPNIYTHYSLATADELEPLELYQNRLAAVDYIVALQSDVFVYTYDGN
MARAVQGHRRFEGFRKTINPDRLKFVELIDKLDEGSMDWNEFQSEVKKHHENRLGGPYDR
LPGESPRQEEYFYSNPIPGCLCRKWHLHLHYPLGGINENRLRKVASPHETFTEAGGSVEA
EVEDGGEEASDAEESGRAEVVGGCECAMTGLSELSEQSESGTDHGHDAAAGFGRGEEYKP
AGIGYPTRPLPGRVAGEPAAIRRRRGALLRHTRRPAAGEALLLPKDTGRASAKVQFQPN*
>11667.m06336|LOC_Os01g63290.1|genepair437-1
MAPPGQLLPLARSLLPLSAPPFVSGRRRRLPTLVLGRALPPPTWLPHGRLSPAHPLPFAP
PRRLSRPPPPATSLPGASPGGGAEAQAVLAEFVTSERVKVAAMLGLALALCNADRVVMSV
AIVPLSQAYGWTPSFAGVVQSSFLWGYLVSPIIGGALVDYYGGKRVMAYGVALWSLATFL
SPWAAARSLWLFLSTRVLLGMAEGVALPSMNNMVLRWFPRTERSSAVGIAMAGFQLGNTI
GLLLSPIIMSRAGIFGPFVIFGLFGFLWVLVWISAISGTPGENAQISAHELDYITRGQKL
VKTQSGGERLRKGYFVILSWMPVYFKTIYHVNLREAAWFSALPWVMMAVLGYVAGVVSDR
LIQNGTSITLTRKIMQTIGFVGPGVALLGLNAAKSPVIASAWLTIAVGLKSFGHSGFLVN
LQEIAPQYAGVLHGMSNTAGTFAAILGTVGAGFFVDRMGSFRGFLILTSLLYFSSTLFWD
IFATGERVDFDGTG*
>11682.m03555|LOC_Os05g37820.1|genepair437-2
MASIRSCVSVNPAAAVTPVKYKSARVGAAGLDPKGLRISCSSSSSSLAAGGGDGCRDAGC
ASSSGRGSGVVGSVGDGWWGRRGGQRERAVAAMCSAGMEGVRHGAAAVASVPAASASALP
ERAKVVALVAAVMLLCNADRVVMSVAVVPFAAQYGWSSSFLGIVQSSFLWGYVFSSMVGG
ALADRYGGKKVMAGAAALWSLATFLTPWAASQSTIMLLAIRALFGLAEGVAFPTMSTFLP
KWFPTHERATAVGISMGGFHLGNVISFLATPIIMSHIGLAGTFAFFASLGYLWLSVWLFN
VESDPLDSRTISKSELQLILAGRSASKIQGSKFPSLREILSKIEMWAIIVANVVNNWGYF
VLLSWMPVYFKTVYNVNLKQAAWFSAIPWAVMALSGYVAGASADFLIKSGFSVALVRKIM
QSIGFIGPGVSLLCLRFAQTPSVAAVLMTIALSLSSFSQAGYFCNVQDIAPKYAGSLHGL
TNGIGTVAAIVSTIGTGYFVQWLGSFQAFLTLTAVLYFSATVFYNTYATGDLIFD*
>11667.m06338|LOC_Os01g63310.1|genepair438-1
MTTLQLDPPVVPARRSAATSCDLHPDETFTGFCTACLRERLAGLEASAAAASAPGRKSTS
AIRSLFARPFGGGAGGSSVAGAGASLPDLRRCKSFSCGRGGDALAAAAAAAAAGTARADE
PQRRSCDVRGHSTLWSLFHQDDRGRVPSSSSAADIAPPHQQPPPPPPRPFIPDDFLDEDI
PVVMEHDEIMPVVEPVIVVDTSGEIETEPNVVAREGKAMKDHMDFESSQPKKPPTKDLKE
SFWVAASVFSKKWQKWRRKQKLKKEAAVSKAAAAAMPPPEKPSKPSFLRRRRLRGEAGSE
LAGGRRSCDTDPRFSLDAGRMSVDDAGFSWDEPRASWDGYLFGAGAGIGLGRAPPPLSRL
PPMMSVLEDTPATIVERSDGQIPVEDDADIDPPGGSLQTRDYYLDSSTRRRRSLERSSSV
RRPSFEVTEPKPAPTTIANGKESPLGGSEFYHFHHAEDLLDRGFSSNSLIEDISASLEAA
LSGPGSAKKPRRWRKAWSLWGFIHRRASGRRSGGPSDIADRSFSEAWPDLRVRGYNPKMQ
RCNSNLSARSSFSSNSGGLGSSRRSHVDVNGSSARRREEHVLERNRSARYSPPGRVDNGM
LRFYLTPMRSGGGGGGARRGGGGGGGGGGLPAKAGRQLTSQSFARSLLRMY*
>11682.m03553|LOC_Os05g37800.1|genepair438-2
MTLQMEPPPPPPPRRSVSTSCDLHPGETFTGFCAACLRERLAGLEASTAAAAAAPGRRSTS

AIRSLFSRPFVAAGGGGGDGGGGGAVPSGSSAAVPDLRRCKSFSCGRGGDVLAGGCGDEP
QRRSCDVRGRSTLWALFHQDDRERVRDGTAFGAFPASSSAAAAALASEVQPQPPPPPPPC
VPEVFLEEEIAMAEESDEITPVVEPILVVDTSGEMETEANGGREAKAMKDHIDLECSQAK
KPQPKDLKEIAGSFWLAASVFSKKWQKWRRKQKLKKQDAAGSKAAAAAMPPPEKPSKPSF
LRRSRLRGEACSEFAGGRRSCDTDPRFSLDAGRMSVDDVGFSWDEPRASWDGYLFGAGTG
IGLGRAPPPLSRLPPILSAMEDSPAGIVERSDGQIPVEDDSQPEPDPDADTPGGSVQTRD
YYDTSSSSRRRRSLERTSSVRRPSFEVTDAKPVLPAAAAITSVKDSPLIGSSEFYHFQHA
EDLLEHHRFSTSSLIEDFPMSLDAAFPGPDKKPRRWRKAWSLWGLIHRRAAGRRGGASDV
ADRAFSEPWPELRVRGCNARMQRCNSNASARSSFSSNSGGLGSSRRSYVDGNGNVVKRRR
EECALERNRSARYSPGHADNGMLRFYLTPMRSASGRRAPGLPAKGGRQLRSQSFARSMLR
LY*
>11667.m06354|LOC_Os01g63460.1|genepair439-1
MMAEALREVLPLPYFPGQPCWYLQERRGAEAWSAEENKVFERALAQVDLDSPNRWEMVAA
MLPRKTVIDVMNHYRDLENDVGSIEAGLVPFPHYSSSLSPASSGFTLQDWDGSDGGFRRG
CYLKRGRAPDQERKKGVPWTEEEHKSFLMGLKKYGRGDWRNISRYFVTSRTPTQVASHAQ
KYFIRLNSGGKDKRRSSIHDITTVNLPEEDTSNPSPSPPSVLTTASDQLGSLVDTKPVPP
PPSLGAQRHFMSPLPGALGVSHHPYGNVKLEPNASFLAGGGTGPGLDDAILLQMQCGHL*
>11682.m03546|LOC_Os05g37730.1|genepair439-2
MMKESYMEVLPPAPAHYFVGQAAAAGGWFLPDRRGGGGAWSQEENKVFEQALAALDRNDP
ERWERVALLLPGKTVADVMTHYDDLENDVCFIEAGLVPFPHYGAAGGGGGSGFTLDWDGG
DDPAGLGFKRSCYMVGGKRARGPDQERKKGVPWTEEEHKLFLMGLKKYGRGDWRNISRNF
VTSRTPTQVASHAQKYFIRLNSGGKDKRRSSIHDITTVNLPDDDHGNPSPSPPPSVLTAH
SSSSAAAVSEQFGVLVDGKPPPPPLGRGAGHHHFMPHPYAQVKIEAGNSHVAGGGRLDDS
VLVQMQCGQLMQPLG*
>11667.m06356|LOC_Os01g63480.1|genepair440-1
MAQMLSAVAMVGAASAASPIVTKSLVGVDAPASGITVVSRQDVRPDGASAVGDLTLSVSD
LPMLSCHYIQKGLFFPAPDVPMASLVSLLMSSLSRALAIFPTLAGRLVTLPDDRVVIRCN
DAGVEFRHAVAPNLSLDDFIVPDADVPTKLTKDLFPMDRTVSYDGHRRPLTSFQVTVLGD
GAVFIGIVANHAVVDGTSFWHFFNTWAALCRGASPKLPDFRRSFFGESTAVLRFPGGVGP
AVTFDADAPLRERVFHFSADAIRELKAITNRRPSGGQDAEVYGKMAHDRKNPEGLSAISS
FQSLCAQIWLSVTRARQRLAPDATSTFRMAVNCRHRLRPAISPVYFGNAIQSAATTATVA
ELASNDLRWAAAKLNASLAAYDDGAIRRAAAAWQGAPRCFPLGNPDGAVITMGSSNRFPM
YEGNDFGWGRPLAVRSGRANKFDGKMSAFPARAGDGSVDIEVCLAPDTMAALLRDSEFMQ
FVS*
>11682.m03539|LOC_Os05g37660.1|genepair440-2
MVEAASAAPATVVVPLTQTLCANAPATSVTVVSKQTVRPDGASAVGDVKLSVSDMPMLSC
HYIQKGLFFPPPGVPIASLVSSLVCALSRALAVFPALAGRLVTLDDGRIVIRCDGVAVEF
YHAVAPALSLGDFLVPNADVPTRLTKDLFPMDRTVSYDGHRRPLTSFQLTVLGDGAVFVG
FVANHAVVDGTSFWHFFNTWAGLCRGTPVQPPDLRRNFFGDSTAVIRFPGGAGPAVTFDA
DAPLRERVLHFSAAAIRELKAKANQWKRSDKFAEANGKHVDETKAHGGYREISSFQSLCA
HIWRAVTRSRRLLAADATTTFRMAVNCRHRLRPAISPLYFGNAIQSVATTATVAELASND
LRWAAARLNATVVAHEDGAIRRAAAEWEAAPRCFPLGNPDGAALTMGSSPRFPMYDGNDF
GWGRAIAVRSGRANKFDGKMSAFPSQAGDGSVDVEFCLAPDTMARLLGDHEFLQYVSRAP
*
>11667.m06366|LOC_Os01g63580.1|genepair441-1
MVSRRFKPVEECSSDGRSEQTVAADFDGTLVRSRSAFPYYLLVALEAGSVLRAVVLLLSV
PFVYVTYIFFSESLAISTLVYISVAGLKVRNIEMVARSVLPKFYAEDVHPESWRVFNSFG
KRYIITASPRIMVEHFAKTFLGADKVVGTELEVGKNGKATGFMVKPGVLVGDHKRQAVVK
ELRDAVPDVGLGDRETDFDFMSICKEIGRLARETSELVLAVPARSTAALAFPFFLPFFQP
EAYLVTSRKYSAVPKNQLLSPLILHDGRLVQRPTPLVALVTFLWMPFGFALALLRVYVNL
PLPERIVFYTYKLMGIRLIVKGNPPPPPKKGHPGVLFVCNHRTVLDPVEVAVALRRKVSC
VTYSISKFSELISPIKAVALSREREKDAENIRRLLEEGDLVICPEGTTCREPFLLRFSAL
FAELTDRIVPVAINTKESMFHGSTVRGFKLMDPYFFFMNPRPTYEITFLNQLPKELTCSG
GKSPIEVANYIQKTLSGQLGFECTAITRKEKYSILAGTDGRVPSKNKEKEKN*
>11682.m03533|LOC_Os05g37600.1|genepair441-2
MSPFKPIEQCSTEGRSQQTVASDLDGTLLLSRSAFPYYLLVALEAGGPLRAVALLMSVPF
VYLTYVTISESLAVRALLYIAVAGLEVRDVESVARSVLPRFYAGDVHPEGWRVFSSFGRR
CVVTASPRVMVEPFARAFLGADRVIGTELEVGEDGRATGFVAKPGVLIREHKRNAVVREF
GDALPDVGMGDRESDFDFMAICKDAYVVTTSRKHRPVPESQLLRTVVLHDGRLAQRPTAI
NTLLVFLWMPVGFALALLRACLSLLLPERVLSYAYKLTGVGLVVRGRPPPPDGSPGVLFV
CNHRTVLDQVAVAAALGRKVICVTYSVPRKTYGMSSRLPEALTASPVKAAVALCRERDRD
ADRVRRLLEEGVDIVAFPEGTTCRGAFLLRFSSLFAELTDRIVPVAIATRETMFHGSTAR
GFKGMDPYFFFMNPRPAYEVTFLSQLPSELTSGGGGKSPVEVANYVQKALAGQLGSEHIG
ITRKEK*
>11667.m06370|LOC_Os01g63620.1|genepair442-1
MGIQVAAVTSSPCAVTTSSSSSLSPSSSAAATTSRRHGVLGGVRLSRGQSSLASWSVGMT
RRRAGGHQMARRALSASIDSIGSDGGDDEEFLRRIQELAVGLHPGAAGCGWPASVERSAS
SVGLPLSLRMLKRKKQQQQQQGRWDERLIDCAGESARGAVGRAFSSMVLIIRELQSFTLQ
MRQALFYEDLQRVLARVHAEMHASFVWLFQHIFSGTPALMVSVMLLLANFTVYSMGDSVA
AAATLPPPQPPAATVAMVDTQHAEQSHSHQRFDHASLKTLSIGRTASVGGNSGGGGKVRP
VAGATGDGRSDEWSNRQSGAVLPQDASQGTPGAGAEEAVPVSEAMAVEETEDELVIWKRI
ADEATRMQASVRAEALMDPDTLGQLVAPVEAKLDTEDTAEFAATEERYERAVSEEPDNSL
LLSNFAQFLYTVQRDHDRAEHYFKRAVRAEPADAEAMGRYATFLWKARNDLAAAEETYQE
AIAAEPSNSHHAAAYAHFLWNTGGDDTCYPLD*

>11682.m03525|LOC_Os05g37520.1|genepair442-2
MGLQVAAVAPSPCARSSASSSPPSTSSSRPALGGAGLARSRAPVNWGAGVMARRRGLRQP
ARCALSASLDGVGGGDAEFLRRIEELAAAVGVQPTGCGWPASVERSASSAGMPLSLRMLK
RKKQQQQLVARQTRWDERLLGSAGDSVGRAFSSMVLIVRELQSFALQQMREAMLGDDLQS
VLARVHGEMHASFVWLFQHIFAGTPALMVSLMLLLANFTVHSMGHSVAAAAAIPPAPPTS
AAVAVVDTQHADPSLPRFDAASVKTFSIGRAASVGGSSGGGGKVRPVAGATGDDRWDESL
ARLSGVAPQQPAPPAGTGAGMAVDEQAIWERMVAEASNMQENARAEELSDPDVLGNLVAP
VEAEIETEGHAEYTRTEQRYELAVSEEPNNPLILANFAQFLYLVQNDHDRAEQYFERAVR
AEPADAEALSRYATFLWKARNDLAAAEDTYQEAIAADPGNAHHAAAYAHFLWNTGGEDTC
FPLD*
>11667.m06388|LOC_Os01g63770.1|genepair443-1
MVPREQAEEAIVADSNGKEEEVGVMGVSAGEHGADDHHGGGGKFSMKNLLWHGGSVWDAW
FSCASNQVAQVLLTLPYSFSQLGMLSGVLLQLFYGFMGSWTAYLISVLYVEYRSRKEKEG
VSFKNHVIQWFEVLDGLLGPYWKAAGLAFNCTFLLFGSVIQLIACASNIYYINDRLDKRT
WTYIFGACCATTVFIPSFHNYRIWSFLGLGMTTYTAWYLAIAALLNGQAEGITHTGPTKL
VLYFTGATNILYTFGGHAVTVEIMHAMWKPAKFKYIYLLATLYVFTLTLPSASAMYWAFG
DELLTHSNAFSLLPKTGWRDAAVILMLIHQFITFGFACTPLYFVWEKVIGMHDTKSICLR
ALARLPIVVPIWFLAIIFPFFGPINSAVGALLVSFTVYIIPALAHILTYRTASARMNAAE
KPPFFLPSWTGMFVLNMFIVVWVLVVGFGLGGWASMVNFIRQIDTFGLFAKCYQCPKPAP
ALAQSPVPLPHH*
>11682.m03520|LOC_Os05g37470.2|genepair443-2
MVPAGDQAEEAIVADAGKEEAEVRAAMGVEQDGKFSMTSLLWHGGSVWDAWFSCASNQVR
PTTNDLVMPLAHISFGILQVAQVLLTLPYSFSQLGMLSGLLLQVFYGLMGSWTAYLISVL
YVEYRARKEKEGVSFKNHVIQWFEVLDGLLGPYWKAAGLAFNCTFLLFGSVIQLIACASN
IYYINDRLDKRTWTYIFGACCSTTVFIPSFHNYRIWSFLGLGMTTYTAWYLAIAAAVHGQ
VDGVTHSGPSKMVLYFTGATNILYTFGGHAVTVEIMHAMWKPQKFKYIYLVATLYVFTLT
LPSASAMYWAFGDALLTHSNAFSLLPRSGWRDAAVILMLIHQFITFGFACTPLYFVWEKA
IGMHGTRSVLTRALARLPIVVPIWFLAIIFPFFGPINSAVGALLVSFTVYIIPSLSHILT
YRSASARLNAAEKPPPFLPSWSGMFVVNVFVVAWVLVVGFGLGGWASVTNFIKQIDTFGL
FAKCYQCPPRAHAGAPLPAPPRH*
>11667.m06393|LOC_Os01g63810.1|genepair444-1
METAAAVVCRGGGLRAPARRGGSDSSTTRAGGVAASPAPATTARRRPLLVASLGEPLITA
QPLSSSLGDGAAVHETLARSDSVIPSLKPSHCVDHSVQVDADEETGSTKTLPPPDDAHDI
FYPPVPTKTVHVKFVLQKRCAFGQRFLVVGDVAALGLWNPAKAAALDWSEDHVWTVKKEL
PAERSIEFKFLLQDRSGHVEWQHGRNRILHVADTSNTLIVCEDWDEAKNQQVSEEIGDAD
GIFSGSDGVFQEDELQLGEEQETNKGVTVGVDDAKSALVTYIYREMMGANDAIQPQLALD
KHHKIPDELSGEANMAAQDGNHTATAAAASGFAGSNGEDAILHKEGDPVENNRLGLASIF
FNDMAWTRKALQQLLRSLGFQIGTRKT*
>11682.m03517|LOC_Os05g37450.1|genepair444-2
MEAAALGVPLVQVRAAVAAYGRGAGKGKGKRRVVGAFHAPPGRRRTALVAALPEPLQPLS
PAQDGAVALASTEADGGEEVHGDVASAEISSPSGVLGKTVRVRFVLKRECTFGQSFHLVG
DDPALGLWDPSKAVPLDWSEGHDWTVEKDLPANKLIEYKFVLQDLSGKLHWQNGRNRSVQ
TGETANILVVYEDWGNANSQTVEEEGKVSIGMEEGKLSVGMEEAVVPDDSESRDDIIVAD
ELQVDDNLAVMQNESSVREDDKKSTVGTVTSVQAELMKLHEANQPELIVDEPQIQEALPE
TADTEPENGGVATCADDRYAESTDDDGVPVGGTDDDGVPVENRWTGAFEHELLWGWKALQ
QLLMSLGFKMDTS*
>11667.m06401|LOC_Os01g63890.1|genepair445-1
MPKIKTSRVKYPGGWELIEPTIRELDAKMREAENDTHDGKRKCEALWPIFRISHQRSRYI
YDLYYRRKEISKELYEFCLDQGYADRNLIAKWKKPGYERLCCLRCIQTRDHNFATTCVCR
VPKHLREEKVIECVHCGCRGCASGD*
>11682.m03510|LOC_Os05g37390.1|genepair445-2
MPKIKTSRVKYPEGWELIEPTLRDLEAKMREAENDPHDGKRKCEALWPIFRISHQKSRYI
YDLYYRRKEISKELYEFCLDQGHADKNLIAKWKKPGYERLCCLRCIQTRDHNFATTCVCR
VPKHLREEKVIECVHCGCRGCASGD*
>11667.m06405|LOC_Os01g63930.1|genepair446-1
MGVVEAEALHGAVEALAGSLQPHVATAFFVFSACTVALAALLAVVRLRPPWWCDCTVCEA
FLTASWAGEFDNLCDWYAHLLRTSPAQTVHVHVLRNVLTANPVTVDHVLRARFDNYPKGA
PFSAILADFLGRGIFNVDGDAWLFQRKLAAAELASPALRAFAARVVASELRCRLIPLLHS
ASREGNGKVLDLQDMFRRFAFDSICKISFGLDPGCLELSMPVSTLVEAFDTASTLSARRA
TVPMQIIWRLKRFLNVGDERKLRDAVRLVDALAAEVIRQRRKLGGAATGSDLLSRFMGSI
DDDKYLRDIVVSFMLAGRDTIASALTAFFLLLSDHPEVATAIRDEVARVTGDGNRTMAAT
FDKLKDMHYVHAAMYESMRLFPPVQFDSKFAAGDDTLPDGTVVAKGTRVTYHAYAMGRME
SVWGPDCAEFRPERWLRDGRFVPESPYRYPVFQAGVRVCIGKELALMEMKAVIVAVVRSF
DIEAIARSSRRPKFAPGLTATFAGGLPVRVRRRRARASGHNPPI*
>11682.m03496|LOC_Os05g37250.1|genepair446-2
MGADATTLHIAVHALASSLQAQVAAVFFVSAACTVALALLLALQRLRPPWWCACPVCEAY
VTASWAREFDNLCDWYAHLLRRAPGRTVHVHVLGNVLTANPATVDHMLRCRFDNYPKGAP
FSAILADFLGRGIFNVDGDGWLFQRKLAAAELASPAIRAFAANVVASELRCRLIPLLRSA
SSYGSERLLDLQDVFRRFAFDCICKISFGLDPGCLELSLPISAFADAFDTASMLSARRAT
VPMHVVWKLKRLRNIGEERELRDAIRLVDALAAEVIRQRRKLGSAASCDDLLSRFMGSIN
DDKYLRDIVVSFMLAGRDTVASGLTAFFLLLSDHPEVAAAIRDEVFRVAGGRDPTAASFD
FDKLKDMHYVHAALYESMRLFPPVQFDSKFAAGDDTLPDGTFVAKGTRVTYHAYAMGRME
SLWGPDCAVFRPERWLSGGRFVPESPYRYPVFQGGVRVCVGKDLAIMEMKAVIVAVVLSF
DVEAVDRSSRRPKFAPGLTATFAGGVPVRVRRRAHCAPS*

>11667.m06410|LOC_Os01g63980.1|genepair447-1
MEGEVVAASPFFEWLKPPPRPASSSSWSSSFSSSSSMASRDQETVVPGEDGGGEIQEDHK
SGMTCLPLLSMLEEGNSKRHEHPVKEEIMSSAHAAGVVEPGVELNIGLPVTGSSAQEVTM
EEDDEEEDDDDVGEEEMDEWKPMHGGCKVEGDEEQYGEAVASVEGSSSITAVGDMFGGVG
AESGVAMSSRYWIPTPAQILVGPVQFICHVCNKTFNRYNNMQMHMWGHGREYRKGPESLK
GTQTLAMLKLPCYCCAAGCKNNVAHPRARPLKDFRTLQTHYKRKHGAKPFRCRRCAKPFA
VKGDWRTHEKNCGKRWFCACGSDFKHKRSLNDHVRSFGAHHLPVAESAAAAATTPADKDR
IISFQR*
>11682.m03490|LOC_Os05g37190.1|genepair447-2
MVLRACTMVEGSSFVVGGGGGVPFFEWLKPRSSPPPSPSSSSTTTTSSSLTAQRQPRGAG
TMLCLPLLGRLGEEPVDADDGGAMNNPPVKEEVSNTTDDYAGVDLNIGLPATTGGGSSED
APMDEDEEDDDDDEEEETEDDEEKAAGLEGCKVEEKEREQVHSEGSKYYVSVGGGEDQSS
NAGDVDAGAACRGRRYWIPTPAQILIGPVQFVCHVCNKAFNRYNNMQMHMWGHGREYRKG
PESLKGTQATATLAMLKLPCYCCAAGCRNNVGHPRARPLKDFRTLQTHYKRKHGAKPFAC
RRCAKPFAVKGDWRTHEKNCGKRWFCACGSDFKHKRSLNDHVRSFGGGHFPVAAAAAAAH
AAAAPPKQQQRIIRFDDAMAQMHGGGLMN*
>11667.m06414|LOC_Os01g64020.1|genepair448-1
MSSVRYCLGRDFQPAAAAAAYFGELEEALIHGANAGGVVDPGMIRADVHSKSAAAAATAG
YLAARPPTLEIFPSWPMRQQQQLHSGNSQSVGSTTDSSSAQNTMPQMELVSPASIRASSE
HQHQQQQPGQEVMMVTTDDYSYKPGLAAASPSFQQQHQLQHHQQQQLHGGGDHDKRKHGS
TRKDGKSVDAKAYVQNLETSRVRLQQIEQELQRARSQQQLQPASLQLADEGVPRSSHHGA
VKSFAGAAMFDMEYARWLDDDSKRLTDLRGGLQAHLLDTNLGLIVEECMQHYDELFQLKA
ALARSDVFHLLTGTWATPAERCFLWMGGFRPSDLLKILIQQLDPLTEQQMLGIYSLQQSS
EQAEEALAQGLQQLHQSLADTVAAGTLNDGPGVPNYMSLMAIALDKLASLESFYQQVQDC
SDHSAKTSPLRCMVNRSGCVRYTCMRKKADNLRQQTLHQLRRILTTRQAARCFLSIGEYY
RRLRALSNLWSSRPRENFIGTESVSPTGTELQPMHNQPQQNQYSGF*
>11682.m03487|LOC_Os05g37170.1|genepair448-2
MIQSDAYTESAGYLAARPPTLEIFPSWPMSHLQEPYSNSQSVGSTTDSSSAQNTMSQAEL
VSPASMRSDSGQEQQQQEVLMVTIDDYNYKQGLGAAIATAPSFQQHAGGLDMRKHGSTRK
DGKLLDAKTERRLAQNREAARKSRLRKKAYVQQLETSRIRLQQIEQELQRARSQGLFPGG
CSAPGDMSSGAVMFDMDYTRWIDDDSKCMAELQGALQAQLPDGNLGAIVEECMRHYDELF
HLRAVLASSDVFHLMTGMWAAPAERCFLWMAGFRPSEILKMLIPQLDPLTEQQLMGMCSL
QQSSEQTEEALAQGLHQLHQSLADAVGGGPLNDGADVANYTGLMALALGRLENLESFYRQ
ADNLRQETLHHMRRILTTRQTARCFLSIGEYNRRLRALSSLWASRPRENFIATENVSPTG
TEFQVIQQSQQNQFSGF*
>11667.m06424|LOC_Os01g64120.1|genepair449-1
MPAPVATCFVPATSGVRCRAFSTPITNYSARGVVADPPKLLSRPGNLQLTSGGARFSGRF
RASAAAVHKVKLIGPDGAESELEVPEDTYVLDAAEEAGLELPYSCRAGSCSTCAGKLASG
EVDQSDGSFLADEQIEQGYVLTCISYPKSDCVIYTHKEEEVH*
>11682.m03483|LOC_Os05g37140.1|genepair449-2
MATATAPRLCFPKPGAAIAPATKSPSFIGYAKQTLNMSGLRISNKFRVSATAVHKVKLIG
PDGVEHEFEAPEDTYILEAAETAGVELPFSCRAGSCSTCAGKMSSGEVDQSEGSFLDENQ
MGEGYVLTCISYPKADCVIHTHKEEELY*
>11667.m06429|LOC_Os01g64170.1|genepair450-1
MELCVRASSLLLGLLLQLLSSVDVVSAAQKFGINYGQIANNLPDPTQVAGLLQSLNVNKV
KLYDADPKVLMAFANTGVEFIIAIGNENLQSMAGNPGAARQWVTQHVQPFLPATRITCIT
VGNEVFSGNDTGMMASLLPAMKAIYAAVGELGLGGQVTVSSAHSVNVLATSFPPSSGAFR
EDLAQYIQPLLDFHGQTNSPFLINAYPFFAYKASPGSVSLPYVLFEPNPGVRDPNTNLSY
DNMLYAQIDAVYAAMKAMGHTDIGVRISETGWPSKGDEDEAGATVENAAAYNGNLMQRIA
MNQGTPLKPNVPIDVFVFALFNEDMKPGPTSERNYGLFYPNGSPVYAINTGAGGVSGRTG
PFDPYSAQMFSSASRLAQSLSCHFIQSRPIACVRPDSKVPSGFESSFQTNGNQPLSRTIE
SFPRVTTIAATIQIE*
>11682.m03481|LOC_Os05g37130.2|genepair450-2
MQSLLPAMQSVHQALLDLGLAGRVNVSTAHSVNILATSYPPSAGAFREDLAQYIQPLLNF
HAEVGSPFLVNAYPFFAYKASPASVSLPYVLFEPNPGVRDPATNLTYDNMLYAQIDAVYA
AMKAMGHADITVRISETGWPSKGDDDEVGATPQNAAAYNGNLMKRIAAGEGTPLKPAVPV
DVFVFALFNEDMKPGPSSERNYGLFYPNGTPVYNIGFDAASFSPSPTTSTFSSSSRPTVT
KKNLNFILLRNFEYANLSFIEQRELLMAMSSLIVLPEKISVRCNG*
>11667.m06443|LOC_Os01g64310.1|genepair451-1
MADGGGRRAPGFRFYPTEEELICFYLRNKLDGLRDDIERVIPVFDVYSVDPLQLSEIHHE
MLGGGGEEGEPWFYFCPRQEREARGGRPSRTTPSGYWKAAGTPGVVYSADRRPIGMKKTM
VFYRGRAPSGTKTAWKMNEYRAFHYPDASSASASSAGAAAPPNHLPPQLRSEFSLCRLYT
RSGGIRQFDRRPLAGGGDENPGPSMAAAAASPEENDGSGSSMQQLELMDQGGAVDPDWDQ
WDDLATLTALLYWPRD*
>11682.m03475|LOC_Os05g37080.1|genepair451-2
MGELPPGYRFYPTEEELVCFYLRHKLDGGRRVPDIERVIPVADVCSLDPWQLPEAHQGAW
TGDGEPWFYFCPRQEREARGGRPSRTTPSGYWKAAGTPGWVYSSDGRPIGTKKTMVFYRG
RAPAGAKTKWKMNEYRAFEEDDDNAAAAAPAQNHYLQTRSDFSLCRLYTRSGCPRQFDRR
PPSSSVAGGGGENRAAPSSTAAAFANEDAAESSGKSQKRKRSAPDDSLDSTSSSDDNGGC
DGSMLQQQQQRQRGTDEELVECSMTDWADLLDWF*
>11667.m06448|LOC_Os01g64360.1|genepair452-1
MDLYGAAAGGGPVARRPWSKVEDKVFESALVLCPEDVPDRWALVAAQLPGRTPQEALEHY
QVLVADIDLIIPAARSGAAAYPGPKTSTGCFSRGWTGTGGETGGTSRGSR*
>11682.m03473|LOC_Os05g37060.1|genepair452-2

MAFYLGSMGGSPSSWGVAEVPVPSSRPWSKAEDKVFESALVAFPEHTHNRWALVASRLPG
RSAHEVWEHYQVLVDDVDLIERGMVASPGCWDDDNNSAGHGRGSGGDERRRGVPWTEEEH
RLFLEGLEKYGRGDWRNISRWSVKTRTPTQVASHAQKFFIRQANASSRGDSKRKSIHDIT
AP*
>11667.m06453|LOC_Os01g64410.1|genepair453-1
MVIGRLALSSFFHNKARDTSPSPPPAPATAPPWVWPSCKNPRTQSFRAATAPPPPPGSRT
IASIFLDSAESSFTTSSARHDCSDSLSTASEASAGAEAADTADDAIVRGLRSSDRLLFDP
GASATSSILEEKSSDAAGEASFIGGVAVAFESEDPYVDFRVSMEEMVVAHGVGNWGWLEE
MLGWYLRANGKDTHAAILAAFIDVIVAIADPALASCSSHRRSSTCTITEESSLEVAEKQA
KLAV*
>11682.m03466|LOC_Os05g36990.1|genepair453-2
MVRKLPLSSVLYTINSARDIPPSSPPPPAATPPAWMWPSCKHPRAHSFRSPSAASAAAAA
KTIASIFLDSGESSFANSSARMHHDCASDSLSTESDVSATAEDMADAIVRGLRSDRLLFE
PRAPSSSILDKKPVRRAAGGGDDDDDGAASFGGGVAVAFDSEDPYEDFRASMAEMLAAHG
VGDWGWLEAMLGWYLRANGKETHAAIVAAFVDLVVSTAARGSSSSRHSSFTLAGTDLESS
SAGGGAAGHISFRLR*
>11667.m06455|LOC_Os01g64430.1|genepair454-1
MSGRSSRRGSFSLRQPPVVDIGCNCRRPKLFSIFSSSSSSSFRRGGSKPKSPNASSTSTT
TAFTATTGGAGTATSTDSSWGPASFTTNSLFEEPAAAQQEQEQLETRRRRRQQRRRRRRA
GATSFARGGDVGGHDDEQQQLQEQAPYRRVAKESVAVAVESAEPYEDFRESMVQMVVEKE
IYAWDDLNDLLHQFLSLNSPRHHPLILHAFADLWTRNGLFSPPSPCQF*
>11682.m03464|LOC_Os05g36970.1|genepair454-2
MAPLMSGSGGGGSARRRPLRQPPVVDDDDVGCSCPKQRLLRSLLSSLVSRARGALGGRAV
SRPKSSAPPSSSASTTTTAAAAFTSTSTTGASATTVDSSKESWGPATYAATNTHTLYEVE
DEVRRQRRKDMRRRRRRRAAAWDEEEEEEGAAAVAVAVEVESAAPYEDFRESMVAMVVEK
EMYAWEELNALLHQFLTLNSPRHHALILHAFADLWAPRSGLFCPPSPCQAL*
>11667.m06461|LOC_Os01g64490.1|genepair455-1
MDSRMDQYEVMEQIGRGAFGAAILVNHKTEKKKYVLKKIRLARQTERCRKSAHQEMALIA
RLQHPYIVEFKEAWVEKGCYVCIVTGYCEGGDMAELMKKANGTYFPEEKLLKWFAQLALA
VDYLHSNFVLHRDLKCSNIFLTKDQDIRLGDFGLAKTLKADDLTSSVVGTPNYMCPELLA
DIPYGFKSDIWSLGCCMYEMAAHRPAFKAFDMAGLISKINRSSIGPLPPCYSPSMKSLIK
SMLRKSPEHRPTASEILKSPYLQPYVNQYRPFADISHPIHSLEKPITSSRSSQKSMSGSQ
CSSISGSDIDSIQSSERNTSGPSTSSNNTIDTEGAEATDHVSVKNCSRSDDVKSNKETVG
PELERQDSSKSIHVDQRPRNEIKQPKIIKKILTTLREESKLRQNNSPIRASRVKLNSPSN
REQLSDDSKHSSDISSSSKSSEVTSRESAKVICEPVKRAQASPPLKHLSPIVEHSPKAKI
KQDEPLQPDPAKQAMEDVDAAVGKVKNRTPPSYSRRLSIPPRRPLGAESPLHADTKRAHN
KVIKERAKSPCRPVHGPDNDIIEPPGFPMAPPSPLGGVQMKVGNARAKSAPPRAVSIKED
SSDCSSSTIAYAENTELSEPSKQDSSAQLVSSCKCSIPDAAIQKHDLTAMPSSELNTTNF
QKSMASNDDVCENLALEPSSDISEQVSIFKDNVPCSKISQSTANAIVQNDEDKFTVQELL
SSVADIAPFVSTKNFALEKGSPPIQSLERTSSPHLNPPIEDVIHVIRHSSFRVCGEQAVA
ENAEMGVQSSDVGKLLNVVREEVDSRSIPSNNLVPHRLPDCAAPKPNISETNTISSKTAC
SDVVKFLTVPEVNSTTTAINNGFKEEASPTKEILDVKSFRQRAEALEGLLELSADLLQHN
RLEELAVVLKPFGKDKVSPRETAIWLAKSFKGMMNDEASRSSM*
>11682.m03463|LOC_Os05g36960.1|genepair455-2
MESRMDQYEIMEQVGRGAFGAAILVNHKIERKKYVLKKIRLARQTERCRKSAHQEMALIA
RLQHPYIVEFKEAWVEKGCYVCIVTGYCEGGDMDELMKKLNGTYFPEEKLLKWFAQLVLA
VDYLHSNYVLHRDLKCSNIFLTKDQDIRLGDFGLAKTLKEDDLTSSVVGTPNYMCPELLT
DIPYGFKSDIWSLGCCMYEMAAHRPAFKAFDMAGLISKINRSSIGPLPACYSSSMKTLIK
SMLRKSPEHRPTASEILKNPYLQPYVNQCRPLSDAPTPIRMPEKPLSTSRSNQRCTSESQ
SSSISCSDIDSTQSSDRSTSGGAPSTDSKLNDIRSIQDADRADSDEKCVTPEDLRGNKNI
SGAELKRQDSSKSVHQHHRGESKQPKIIEKIMTTLREESRLRENNSPVSSSGVKLTSAVS
NKNQAEQSSESSRPHSGVSYSSKFGDISSNGWTNTSDECVDPVQVPLQLKQLSPTVEHCP
KLKNSGSSTPEPAKQIAENGSSASGMSKTKSSPSSSRRPSPQRQTVAGIPIVPFTVSKRA
HIKAESEKTPPRPAHSPNNSLHNLPPLIPISTNLSEENIKLGNSQAMPAPLEFVTAASKE
DISFYSNSVVDCVEKAEPSEVFESNSPAYLTPPWTGPVLDAKGENGLIAIPCSEIHTGTL
QKSMASNDDSSLSSPLDTFYLSFEQEFVCKDDSQSSKHGHSAVTLLSGEDKFTVQELLAS
TPVISPFVSSTSNTLPEDKSSYQSFKKQSDSHSGPPVDVPAQTIRLNSFLVSDEWPTSET
VQGEARDTAASKLLNVVREDFDVRSSSCSTSTQPSGQTPVRSKLNVPETNLASNISIPSI
SEAVRLSTAMDVKPYTSEASNGVKEEASPAKEALDVTSFRQRAEALEGLLELSADLLENN
RLEELAIVLQPFGKNKVSPRETAIWLARSFKGMMNEEGGRLSM*
>11667.m06471|LOC_Os01g64590.1|genepair456-1
MLPYAPRPPSLLVDRRYKQGAEAAPNCPRCDSPNTKFCYYNNYSLSQPRYFCKGCRRYWT
KGGSLRNVPVGGGCRKNRRGKSSSSARSAADAVSSGRDAAFGHRFPGPVRPDMVLEGMVG
NPANPGQAMPDVAAAADGSTIDLAMLYAKFLNHPPTDAGLGAVTPESGGHVDEAFDTFSA
SSDLSPGILAAASAQFDPNQDGFGEWSSPASGNDPTSTATTATTSMLCTDASVQAALGEL
NFAMDQSCFDSLGLPTDVAGAGSLSSWCSIVPSLSTWEEPKYDSLDSFPDDAMSLHECMI
GAPDHDWSVDCQGLEALYMP*
>11682.m03456|LOC_Os05g36900.1|genepair456-2
MLSSHCESMLAYAAAAGRRAVVVDHHQRRYRPNVEVAPNCPRCESPNTKFCYYNNYSLSQ
PRYFCKGCRRYWTKGGSLRNVPVGGGCRKNRRGKAVRAMVGETMTARGGGGGGAAAFSHR
FHGPVRPDMILEGMAGSTAASAGLGEQPGVAAPDEKPAAADGSTIDLALLYAKFLNHHQP
TMAEQGGGAAVPESVDTSSGSSSDRTTSPAAAQPAAAAAYGPGQDGLVGEPISTEEHGAA
AMARCAQALGELNFSVDQISCYTSLGLPTTDGGDLILPSTLDQHAKYEPFDSLPEDALSL
HDIISGDDDVWCNALGCQGLEAALCRP*

>11667.m06474|LOC_Os01g64620.1|genepair457-1
MDVEQLQQHARRLLSNGRVVSAAAAAGASPGPAGRVLEGGAAAAARRPAPFSSLDATVIT
VLSLLLCVLVVGLVLHAIARCAFRVTRRMCYGQEPPGDHGDEAAAERCARVARKKPGRAI
AEKIPAIVCPAGGLDRLAGCGSTECAICLSEFAQGHRVRVLPRCGHGFHARCIDRWLAAR
QTCPTCRREPFAAAAAVQLQVYPDAAGGQHETP*
>11682.m03446|LOC_Os05g36310.1|genepair457-2
MDAPRLHHSARALLLVAPSASPAPAAVPPGVAGALPVAENGRGGPLAVSSLNTNTIVLLA
LLVCGLVAAVALHVVLQCALRVTRRACYGAETAAAAAAGGGGEGGRARRGGGGGGRKRTP
PLSKTIPRVAYTEGLELAGSSRSECVICLAEFARGEHVRVLPGCNHGFHDRCIDRWLAAR
PTCPTCRQAPFAAAAADPVAPPDPAPAAVQVVRVIVLTSQ*
>11667.m06475|LOC_Os01g64630.1|genepair458-1
MAEEDIQPIVCDNGTGMVKAGFAGDDAPRAVFPSIVGRPRHTGVMVGMGQKDAYVGDEAQ
SKRGILTLKYPIEHGIVNNWDDMEKIWHHTFYNELRVAPEEHPVLLTEAPMNPKANREKM
TQIMFETFNCPAMYVAIQAVLSLYASGRTTGIVLDSGDGVSHTVPIYEGFTLPHAILRLD
LAGRDLTDNLMKILTERGYSFTTTAEREIVRDIKEKLAYVALDYEQELDTARSSSSIEKS
YELPDGQVITIGAERFRCPEVLFQPSFIGMEAPGIHEATYNSIMKCDVDIRKDLYGNVVL
SGGSTMFPGIGDRMSKEITALAPGSMKIKVVAPPERKYSVWIGGSILASLSTFQQMWISK
AEYDESGPGIVHMKCF*
>11682.m03443|LOC_Os05g36290.1|genepair458-2
MADEDIQPIVCDNGTGMVKAGFAGDDAPRAVFPSIVGRPRHTGVMVGMGQKDAYVGDEAQ
AKRGILTLKYPIEHGIVNNWDDMEKIWHHTFYNELRVAPEDHPVLLTEAPLNPKANREKM
TQIMFETFNCPAMYVAIQAVLSLYASGRTTGIVLDSGDGVSHTVPIYEGYTLPHAILRLD
LAGRDLTDHLMKILTERGYSLTTSAEREIVRDIKEKLAYVALDYEQELETSRSSSSVEKS
YEMPDGQVITIGSERFRCPEVLFQPSLVGMESPGIHEATYNSIMKCDVDIRKDLYGNVVL
SGGSTMFPGIADRMSKEITSLAPSSMKVKVIAPPERKYSVWIGGSILASLSTFQQMWISK
GEYDESGPGIVHMKCF*
>11667.m06477|LOC_Os01g64640.1|genepair459-1
MARTKQTARKSTGGKAPRKQLATKAARKSAPATGGVKKPHRFRPGTVALREIRKYQKSTE
LLIRKLPFQRLVREIAQDFKTDLRFQSSAVAALQEAAEAYLVGLFEDTNLCAIHAKRVTI
MPKDIQLARRIRGERA*
>11682.m03442|LOC_Os05g36280.1|genepair459-2
MARTKQTARKSTGGKAPRKQLATKAARKSAPATGGVKKPHRFRPGTVALREIRKYQKSTE
LLIRKLPFQRLVREIAQDFKTDLRFQSSAVAALQEAAEAYLVGLFEDTNLCAIHAKRVTI
MPKDIQLARRIRGERA*
>11667.m06480|LOC_Os01g64660.1|genepair460-1
MDHEADAYRTDLMTITRYVLNEQSRNPEARGDLTILLSHIVLGCKFVASAVNKAGLAKLI
GLAGETNVQGEEQKKLDVLSNEVFVKALVSSGRTCVLVSEEDEEATFVDPALRGKYCVCF
DPLDGSSNIDCGVSIGTIFGIYMIKDKENVTLEDVLQPGKNMVAAGYCMYGSSCTLVLST
GNGVNGFTLDPSLGEFILTHPDIKIPKKGKIYSVNEGNAKNWDEPTAKFVEKCKFPKDGS
SPKSLRYIGSMVADVHRTLLYGGVFLYPADKKSPNGKLRYTLSLLSASHFAYGTSNFILI
NNSEFTKSWNYSVLYEVFPMSFLMEQAGGQSFTGKERVLISSFISS*
>11682.m03441|LOC_Os05g36270.1|genepair460-2
MDHAAEAQRTDLMTITRHVLNEQGRHPESRGDFTILLSHIVLGCKFVASAVNKAGLAKLI
GLAGDTNVQGEEQKKLDVLSNEVFVKALVSSGRTCVLVSEENEEAIIVDAPLRGKYCVCF
DPLDGSSNIDCGVSIGTIFGIYMIKDKDNVTLDDVLQPGTDMLAAGYCMYGSSCTLVLST
GNGVNGFTLDPSLGEFILTHPNIKIPNRGKIYSVNEGNAKNWDAPTAKFVEKCKFPQDGS
PSKSLRYIGSMVADVHRTLLYGGIFLYPADQKSPNGKLRVLYEVFPMSFLMEQAGGQAFT
GKQRALELVPRKIHDRSPIFLGSYDDVEDIKALYASESIIG*
>11667.m06483|LOC_Os01g64670.1|genepair461-1
MSEADGGEGAKPKRPAPRLNERILSSLSRRSVAAHPWHDLDTGADAPAVFNVVVEISKGS
KVKYELDKKTGFIMVDRVLYSSVVYPHNYGFIPRTLCEDNDPMDVLVLMQEPVIPGCFLR
ARAIGLMPMIDQGEKDDKIIAVCVDDPEYRHYNDLSELSPHRVQEIRRFFEDYKKNENKE
VAVNEVLPVTAARDAIQYSMDLYAQYIEHLGQ*
>11682.m03440|LOC_Os05g36260.1|genepair461-2
MSSENGENGHGAADEVVEPYQQTPRPGPKLNERILSSLSRRSVAAHPWHDLEIGPDAPAV
FNVVVEITKGSKVKYELDKKTGLIKVDRILYSSVVYPHNYGFIPRTLCEDNDPMDVLVLM
QEPVLPGSFLRARAIGLMPMIDQGEKDDKIIAVCADDPEYRHFNNLSELSPHRLQEIRRF
FEDYKKNENKEVAVNDFLPAPTAREAIQYSMDLYAQYILQSLKR*
>11667.m06487|LOC_Os01g64700.1|genepair462-1
MSFRSIVRDVRDGFGSLSRRSFEVTLASLYGLTGHHKGKTQSSLDELDDSPAIIPESRWA
SLPPELLREVIRRLEADESTWPSRRNVVCFAAVCRTWREMCKETVLSPEFCGKLTFPVSI
KQPGPRDGMIQCYIKRNRSKSTYHLYLCLSNVVTAEGGKFVLAAKRHRKTTCTEYTISMV
SGNISRSSRTNIGKLRSNFLGTKFIIYDTQPPYNGAVVPHVGRTSKRFNSTKVSPKVPSV
TYNIAQVSYELNVLGTRGPRRMRCMMHSIPASSVEPGGIVPGQPEQIVPRALEDSFRSTT
SFSQSFRSTTSFSKSIMDPSMDFSSARFSDISGSIMGGDDNGEIKERPLVLRNKPPRWHE
QLQCWCLNFRGRVTIASVKNFQLVAAPSPPPAGAPTPSQPGPADPEKVILQFGKVARDMF
TMDYRYPLSAFQAFAICLSSFDTKLACE*
>11682.m03433|LOC_Os05g36190.1|genepair462-2
MSFRSIVRDVRDSFGSLSRRSFEVTLAGLSGLTGHHRGKSQSTVHELCDADLIIQESRWA
SLPPELLRDVIRRLEASESTWPSRKDVVSCAAVCKAWREMCKEIVLSPEFCGKLTFPLSL
KQPGPRDGMIQCFIKRDKSKSTYHLYLCLSTAVLADSGKFLLSAKRHRKTTCTEYVISMD
ADNISRSSSTYIGKLRSNFLGTKFIIYDTQPSYNGAVIPPVGRSSRRFNSKKVSPKMPSG
SYNIAQVTYELNVLGTRGPRRMHCVMHSIPASSVEPGGIVPGQPEQIVPRAFEESFRSTT
SFSKSSIMDRSMDFSSSRDFSSARFSDIAGGTINGDEEGQNKERPLVLRNKAPRWHEQLQ

CWCLNFRGRVTIASVKNFQLIAAPAQPPAGAPTPSQPAPPEQDKIILQFGKVAKDMFTMD
YRYPLSAFQAFAICLSSFDTKLACE*
>11667.m06490|LOC_Os01g64730.1|genepair463-1
MMASRVMASSSPSHTASDLARFAAGRGGGGSAGLGSMNVEEILRGIYADMPTPALPLVGG
DRPMSPLPAPDVAAAPRTAEEVWKEITGAGVAAAAGGVVPPAAAAAAAPAVVAGAGAGTG
AEMTLEDFLAREGAVKEDEAVVTDPSAAKGQVVMGFLNGAEVTGGVTGGRSRKRHLMDPM
DRAAMQRQKRMIKNRESAARSRERKQAYIAELESLVTQLEEENAKMFKEQEEQHQKRLKE
LKEMVVPVIIRKTSARDLRRTNSMEW*
>11682.m03430|LOC_Os05g36160.1|genepair463-2
MASSRVMAAAAASSSSSPPPPPPAAAAAGGAADLARFRSTSSGIGSMNMDDILRNIYGEA
APPPGAAGSAPAPPPAGEAAGAPVAEVAARRTAEEVWKEISSSGGLSAPAPAPAAGAAGR
GGGPEMTLEDFLAREDDPRATAVEGNMVVGFPNVTEGVGTAGGGRGGGGGGRGRKRTLMD
PADRAAMQRQKRMIKNRESAARSRERKQAYIAELEAQVAELEEEHAQLLREQEEKNQKRL
KEKWHVFMQIKEQAVAVVIRKKTQDLRRTNSMEW*
>11667.m06493|LOC_Os01g64760.1|genepair464-1
MSHNHGDTIPLHPSSAQSDMDEIESLIYAAPSATVLPARPPSPPRASIPVSTSPAPLPAP
AKPSLPGASVPIIVPQAPPASVSVPIASDGFGPPPNTLTEPVWDTVKRDLARIVSNLKLV
VFPNPYREDPGKALRDWDLWGPFFFIVFLGLTLSWSASVKKSEVFAVAFAVLAAGAIILT
LNVLLLGGHIIFFQSLSLLGYCLFPLDVGALVCMLKDNVILKIIVVTVTLAWSSWAAYPF
MSAAVNPRRKALALYPVFLMYISVGFLIIAID*
>11682.m03429|LOC_Os05g36150.1|genepair464-2
MSHGDTIPLHSSSAQSDMDEIESLIHAAPPSAAAVLPARPPSPPRASIPVSSSPPLLPPP
VAGSKPQLPPFSSSSSVASSSSPPLPSSVSVAIAGDGFGPPPNTLTEPVLDTVKRDLARI
VSNLKLVVFPNPFREDPGKALRDWDLWGPFFFIVFLGLTLSWSASVKKIIMFTRFLCKSE
VFAVAFAVLAAGAIILTLNVLLLGGRINFFQSLSLLGYCLFPLDVGALICMLKDNVLLKI
IAVVVTLAWSSWAAYPFMSAAVNPRRKALALYPVFLMYVSVGFLIIAID*
>11667.m06495|LOC_Os01g64770.2|genepair465-1
MAEPSKVIHIRNVGHEISESELLQVVQPFGTVAKLVMLRAKNQALVQMEDLASAVNVIQY
YNTIQPSVRGRNVYLQYSSHQELTTDQSSHGRNPDQEEPNRILLVTIHHMLYPITIEVLH
QVFSPYGFVEKIVTFQKSAGFQTLIQYQSRQSAIQAYGALHGRNIYDGCCQLDIQYSNLS
ELQVHYNNDRSRDFTNPSLPTEQRSRSSQPSYNDPSSLFGFQQPGDPYAQMSKAAMIAAA
FGGTLPPGVSGINDRCTLLVSNLNTDKIDEDKLFNLFSMYGNIVRIKILKNKPDHALIQM
ADGLQAELAVLYLKGAMLFGKKLEVNYSKYPTVTADPDARDYSTSHLNRFNSNVVKNYRH
CCAPTKMIHISALPQDITEDTIHSLVGEHGTIANSRLFETNGKTQALVLFESVEEATEAL
VEKHASKLDRTNIRISFSQMQNI*
>11682.m03425|LOC_Os05g36120.1|genepair465-2
MAEPSKVIHIRNVGHEIAEADLLQLLQPFGNVSKIVMLRAKNQALLQMQDLHNSVSALQY
YSTVQPSVRGRNVYMQFSSHQELTTDQNSHGRNSDQESEPNRILLVTIHHMMYPITVEVL
HQVFKAYGYVEKIVTFQKSAGFQALIQYQSLQEAMDAFGALHGRNIYDGCCQLDIQYSNL
SELQVHYNNDRSRDFTNPSLPTEQRPRASQQGYPDPGGLYAFQQPGASYAQMGRAAMITA
AFGGTLPPGVTGTNERCTLIVSNLNTDKINEDKLFNLFSLYGNIVRIKILRNKPDHALVE
MADGFQAELAVHYLKGAVLFAKKLEVNYSKYPNITSAPDAHDYTTSSLNRFNSNVIKNYR
HCCAPTKMIHISALPQEITEDAILNHVSEHGSVVNTKLFEVNGKRQALIQFESEEEATEA
LVSKHATSLEGNTIRISFSQMQSI*
>11667.m06500|LOC_Os01g64780.3|genepair466-1
MAEKEESTSIPLSQAAEAVDPEDPAKSPPRPSSPTTSTRKACCAVLQSWVSRKFMTGWYI
FDRNQVKHVTKQKVKNHALFFFPFFFCSVVLFPVAVTFFITWWFIQFVDGFFSPLYAKLG
IDIFGLGFLTSLVFIFLVGIFVSSWVGSTIFWVGEWFIKKMPFVRHIYSASKQVSTAISP
DQNTTAFKEVAIIRHPRIGEYAFGFITSTVVLQTDKGDEELCSVYVPTNHLYIGDIFLVN
SEEIIRPNLSIREGIEIIVSGGMTMPQVIASLEPTPRKSQNIRLNRIMT*
>11682.m03423|LOC_Os05g36110.2|genepair466-2
MPEEKEYAPVPLGQAPEAVDPEDPVKSPPRPTSPANSTRKACFAVLQSWVSRKFMTGCVV
LFPVAVTFFITWWFVKFVDGFFSPLYAKLGFDIFGLGFLTSLLFIFLVGIFVSSWVGSTV
FWIGEWFIKKMPFVRHIYSASKQVSTAVSPDQNTTAFKEVAIISHPRIGEYAFGFITSTM
ILQTDKGDEELCSVYVPTNHLYIGDIFLVRSEEIIRPNLSIREGIEIIVSGGMTMPQVIA
APGQTPHKGQSTRLNRMMSA*
>11667.m06502|LOC_Os01g64790.1|genepair467-1
MTVAGASELMSGYYQAQEMSTMVSALARVVAGGGGGGGDGDGDQWAWSSPSPSSSAAAAA
ARGVQERRREEQAMHELAGYACGGAPSPEFAGSEQSSDTQSASAATMDEHHSPVGGGGNA
EGPDTPRRRYRGVRQRPWGKWAAEIRDPHKAARVWLGTFETAEAAARAYDEAALRFRGSR
AKLNFPEDARLYPTAATTTTAAPPPAPVAAASPSAAIYPGASQSAEYLRYQMLLQGRLTT
ATPNQGTLLPFYGGGGGGGSMTNPYGGGGGGAMSGFLGSYYSFPTPSVSVATVPSSTSSA
PGNYYSSHGGSHQSMSAAEEWNWENALVYPATAASWSESSYHHHPPPPHTQ*
>11682.m03421|LOC_Os05g36100.1|genepair467-2
MADQRRRFRGGGDWQASVDDVVDDGGELEAAAAAAARGSVLSGEYQAQEMSTMVSALTWV
VAAGHDDHGGGQWSGLVDVPATTLAGGGGGDYGHGAQGSYYYYGAAPTSTPEFVAGGQQE
QLSSDVPQGGASLGLAMDEHSPTYTVEASSSADQHGGGGGGRRYRGVRQRPWGKWAAEIR
DPHKAARVWLGTFETAEAAARAYDEAALRFRGSRAKLNFPEDARLSSPPAGAGAGGATAA
AQTVPVAYPASAVSDYLQYQMLLHGGGGGGGGRYPLYYGGGAAAAMSSSLGPYSSIPTSS
VTVASVPSSSSAASSSSGYGAPAEHGEAVQWTSWPDGGGWTYPATTSSWSGSSQYPPPPR
PPQQ*
>11667.m06504|LOC_Os01g64810.1|genepair468-1
MAQPQPQSQRQRVYEAWKGNNRFFFGGRLIFGPDAKSLLFSVALIFVPVAVFCAFVARNL
RHQFPAYNAGYAILAVAIVLAIYVLSLLFITAAQDPGIVPRASHPPEEEFHYDNLSLADT

PGRLVFPRVKDVMVNGVPVKVKYCETCMVFRPPRCSHCSICNNCVERFDHHCPWVGQCIG
KRNYRYFFLFVSSASILCIYVFAMSALYIKILMDGDYPTVWKALKHSPASLALLIYCFIC
LWFVGGLTGFHTYLISTNQTTYENFRYRADGRPNAYDRGCMNNFLEVFYTKVPPSKHKFR
EPIQEEARAPPANRAVEREEEPVGARTKVEDDLDIGGDLLKISQRHNYDGIDIEMGGGDR
NSRNEAVSNSKLISKTDAQAPTVEDEVQHPSRGERSRSSDLASEGITTSAPVSQPAFPFR
REAL*
>11682.m03420|LOC_Os05g36090.1|genepair468-2
MAQPQKRVYEAWKGNNRFLFGGRLIFGPDAKSLLVSVSLIVVPVLVFCVFVARHLRHQFS
TYNAGYAIPAVAVLFMIYVLTLLFITSAQDPGIVPRASHPPEEEFAYGNPLNGGTPGRLQ
FPRVKEIMVNGMLVKVKYCDTCMIYRPPRCSHCSICNNCVERFDHHCPWVGQCIGQRNYR
FFFLFVSSSTLLCIYVFAMSALYIKFLMEEGYPTVWKALKHSPASLVLMIYCFIALWFVG
GLTGFHSYLICTNQTTYENFRYRSDNRPNVYDQGCLNNCLGVFCSKTKPSKHKFRAYVQE
EVRAPVVNFGRQMEEEPAGGPRAKVEDDLEIGSDLLQISQRRNYGDVDLEMGSQDCSEME
GIPNAKLAIGSESQIPAIGSEVRVRHSSWDRRSGNWDMSLDVIGRSASDVIRRSASGHEA
APPFQTETH*
>11667.m06510|LOC_Os01g64860.1|genepair469-1
MAASLAIVAVVVALCVAAAASAETATYIVHMDKSAMPSGGGGGNGSTSLESWYAATLRAA
APGARMIYVYRNAMSGFAARLSAEQHARLSRSPGFLSSYLDAPVTRRDTTHTPEFLGVSG
AGGLWETASYGDGVIVGVVDTGVWPESGSYRDDGLPPVPARWKGYCESGTRFDGAKACNR
KLIGARKFSAGLAAALGRRNITIAVNSPRDTDGHGTHTSSTAAGSPVPGASYFGYAPGVA
RGMAPRARVAVYKVLFDEGGYTTDIVAAIDQAIADGVDVLSISLGLNNRPLHTDPVAIGS
FAAMQHGIFVSTSAGNDGPGLSVLHNGAPWALTVAAGTVDREFSGIVELGDGTTVIGESL
YAGSPPITQSTPLVYLDSCDNFTAIRRNRDKIVLCDAQASSFALQVAVQFVQDANAAGGL
FLTNDPFRLLFEQFTFPGALLSPHDGPAILRYIQRSGAPTAKIAFRATLLNTKPAPEAAA
YSSRGPAVSCPTVLKPDIMAPGSLVLASWAESVAVVGNMTSPFNIISGTSMATPHAAGVA
ALLRAVHPEWSPAAIRSAMMTTAATLDNTGRSINDMARAGHAATPLAMGSGHIDPNRAAD
PGLVYDAVPGDYVELMCAMGYNLSDIRAVTQWSTYAVNCSGASSPDLNYPSFIAYFDRRS
AAAAAAETKTFVRVVTNVGAGAASYRAKVKGNLGGLAVSVTPSRLVFGKKGETQKYTLVL
RGKIKGADKVLHGSLTWVDDAGKYTVRSPIVATTLSSTRL*
>11682.m03412|LOC_Os05g36010.1|genepair469-2
MATAVVPGICHAVALMWLLLVLFCWAPGLTSAADTAAYIVHMDKSAMPRAFASQASWYES
TLAAAAPGADMFYVYDNAMHGFAARVTADELEKLRGSRGFVSCYPDDARAVRRDTTHTPE
FLGVSASSGGLWEASEYGEDVIVGVVDTGVWPESASFRDDGLPPVPARWKGYCESGTAFD
AGKVCNRKLVGARKFNKGLVAATNLTIAVNSPRDTDGHGTHTSSTAAGSPVAGASFFGYA
PGTARGMAPRARVAMYKALWDEGTYPSDILAAIDQAIADGVDVLSLSLGLNDVPFYRDPI
AIGAFAAMQRGVFVSTSAGNDGPDPGFLHNGTPWTLTVASGTGDREFAGIVRLGDGTTVI
GQSMYPGSPSTIASSGFVFLGACDNDTALARNRDKVVLCDATDSLSAAIFAVQVAKARAG
LFLSNDSFRELSEHFTFPGVILSPQDAPALLQYIKRSRAPRASIKFGVTILGTKPAPVVA
TYSSRGPSASCPTVLKPDVLAPGSLILASWPENVSVSTVGSQQLYSRFNVISGTSMSCPH
ASGVAALIKAVHPEWSPAAVRSAMMTTASAVDNTNAPIKDMGRANRGATPLAMGSGHIDP
NRAVDPGLVYDAGADDYVKLMCAMNYTAAQIKTVAQSPSSAVDCAGATLDLNYPSFIAFF
DPGATAPAARTFTRAVTNVGDAPASYSAKVKGLGGLTVSVSPERLVFGRKHETQKYTVVI
RGQMKNKTDEVLHGSLTWVDDAGKYTVRSPIVATTASSAPL*
>11667.m06523|LOC_Os01g64970.1|genepair470-1
MEKYEAVRDIGSGNFGVARLMRNRETRELVAVKCIERGHRIDENVYREIINHRSLRHPNI
IRFKEVILTPTHLMIVMEFAAGGELFDRICDRGRFSEDEARYFFQQLICGVSYCHHMQIC
HRDLKLENVLLDGSPAPRLKICDFGYSKSSVLHSRPKSAVGTPAYIAPEVLSRREYDGKL
ADVWSCGVTLYVMLVGAYPFEDQDDPKNIRKTIQRIMSVQYKIPDYVHISAECKQLIARI
FVNNPLRRITMKEIKSHPWFLKNLPRELTETAQAMYYRRDNSVPSFSDQTSEEIMKIVQE
ARTMPKSSRTGYWSDAGSDEEEKEEEERPEENEEEEEDEYDKRVKEVHASGELRMSSLRI
*
>11682.m03387|LOC_Os05g35770.1|genepair470-2
MDTYEEVRSIGSGNFGVTRLMCNRDTGELVAVKTIPRGNHRINKSAYREIINHRSLRHPN
IIQFIEAILTHTHLAIVMEYASGGELFDRIVDLERFSEDEARYFFQQLIWGVSYCHHMSS
MLHSRPKSAVGTPAYIAPEILNLQEYDGKQKPGTGSTS*
>11667.m06533|LOC_Os01g65070.1|genepair471-1
MEAVAGGGGGGGGESVGELLLRAAAMVPAEHYALAALAVVSVLAYGFLELHFLGDLLRGF
RGGRVELTFHPASEIYHRVASKCRSLHGRYLATPWLASPHLQTLFLGISGRPPSFTYKRQ
LYTVHDGGTIALDWLLATDSKGSDGILSEDASAPLVVIVPGLTSDSAAAYVKHMAYSMAT
KGCNTVVSNHRGLGGVSITSDCLYNAGWTEDLREVINYLHHKYPKAPMLCVGTSIGANIV
VKYLGEEGENTPVAGAASICSPWDLVVGDRFISRKLVQRFYDKALAFGLKGYAKLHEPVL
VRLANWEGIKKSRSIREFDHHATCMVAKYETVDTYYRRCSSASYVGNVSVPLLCVNALDD
PLCTREAIPWDECRANKNIVLATTPNGGHLAFFQGLTAGRLWWVGAVSEFLFALLDSKYM
HQQKAQDHILRSSLESSIDKSPYVNVMEDGMIAPVTDDGPCDDITPSHQVNDIKQDNGDF
TQQNEHTREVDDKNITEVNAMPSQSPEQSAGQQVEEHYVGKFHEAIAPVKRSINQLTRYQ
GKSVWLLAYIAFVTSWPLLGSLAFIAFRKKFRNNLLAKWLRR*
>11682.m03382|LOC_Os05g35730.1|genepair471-2
MGNAAAGDSAAELLLRAAALVPPAHYALAALLLACALLYRFLELHLIGDLLRGLRGGRVA
LTFHPASHVYHRVASKCRSLHGRYLATPWLASPHLQTLFLSISGRPPSFTYRRQLYTVRD
GGTIALDWLLASDCEEEDVGFCDGVISRDDSTPLVVVIPGLTSDSTAAYVKHLVFSMASK
GWNVVVGNHRGLGGISITSDCFYNAGWTEDFREIVNYLHQKYPQAPLFAVGTSIGANILV
KYLGEEGEGTPVAGAVSICSPWDLLVTNRFIQRKLVQRCYDKALAIGLKGYAKLHQPVLA
RLANWEDIRKSRSIREFDRHATCVVAKYETVDTFYRRCSSANYISNVSVPLLCISALDDP
LCTREAIPWDECRANKNIVLATAPNGGHLAFFQGLTAGKLWWVGAVSEFLLALHDSPCMH

RQKAQEHSLHTSLESSIDKSPYVNFMEDGMVTAVTNDDANNSDSDNPISNEMELRNGMVG
VQQDGIATEIQNECDGNRSQENVTPAQGPVGSQEQPKQNIDKIQDAIAPVKISINQLIRS
QGRSVWLLTYIAFVTSWPFLGALGFILFRKKFRNSLPAKRL*
>11667.m06540|LOC_Os01g65100.1|genepair472-1
MASHAVTDALLLPRSEGAVAGAVDFRGRPASRASTGRWSAAMFVLGVEIAERFAYHGVSA
NLISYLTGPLGESTAGAAAAINLWSGVATMLPLLVACVADAWLGRYRTIVLASLLFVVSM
GMLTLSSALPAFHGDGGGCSYTSKSLSCAPSTAQVAIFYVSLYLVALAEAGHKPCAQAFG
ADQFDQNDAKESVSRSSFFNWWYFGMCSGTAMTTMVSSYIQDNIGWGLGFGIPCLVMAFA
LAMFLLGTRNYRYYVSTQSSPFARLARAFVALIRGSKDDALAVVDDDDGGDHREELRGVL
RLFPIWATCIIYAVIFSQSSTFFTKQAATLDRRIGESFRVPPAALQTFISVTIIAFIPVY
DRAFVPVARRFTRASSGITMLQRIGTGLVLALAAMVVAALVEARRLGVARDAGMVDDPKA
ALPMSLWWMVPQYVLFGLSDVFAMIGLQEFFYDQVPDALRSLGLAFFLSIFGVGHFFSSF
IISAIDGATKKSGASWFANNLNRAHLDYFYWLLAGLCAVELVAFVFVSRVYVYKKRVPHN
GCDVM*
>11682.m03372|LOC_Os05g35650.1|genepair472-2
MATYYITDHSARYCVQGLAMLAFSTLISTGGNQCSSAAVAGGKTCPPSTLRVAFFYISLY
MVAVAQGGHKPCVQAFGADQFDPSDPEESVSRSSFFNWWYFGMCGGTAVTLVFLSYVQDN
IGWGLGFGIPCVVMACALAVFLLGTRTYRYYVSGSKKGVVARAGEALAAWRNRAKSIPLL
PPASQECHPTATSAPEFSTGVEEDEQVVGKAGLVEQAKGIVRLFPIWATCLIYAVALAQS
STFFTKQAGTLDRRIGDHIQVPPAALQSFISITIVAIIPVYDRVIVPVARRYTGVPSGIT
MLQRIGAGMVLSLVSMVIAALVETRRLRAARDAGLVDKAGVPVPMSLWWMVPQYVLFGAA
DVFTMVGLQEFFYDQVPDKLRSLGLALYLSIFGVGSFISSALVSGIDRATAARGGSWFSN
NLNRAHLDYFYWLIAALSALELLAYGYFAVTFKYKNKNKGALLATSTSC*
>11667.m06552|LOC_Os01g65210.1|genepair473-1
MAMEAEALLPEPEPESPPLLAVDHLGGPASRGSSGRWPAAFFLIGAEVGERFAYSGIMGN
LVIYLTGPLRQPTAAAAAAVNVWMGTSMLLPLLGSAVADSWLGRYRTIVCASLLYILGLG
MITVSSVLAPEESSESSNLAAHVAFFYFSLYVVAFAQGGHKPCAQALGADQFDENDPGEL
ASRSSFFNWWFFASYGGNTVTVPILNYVQESVSWQLGFAIPCIAMAVSLAIFLIGTRSYR
FYPPKSKGNPFGEVAEWIRRWIASSCSKLPDSSDELLPSSSSEGDVSNSSSEFVPKEAAE
LVKLFPIWASSLIYAAVMAQCITFFTKQASTLDRRVGSLVLPAASNGALFNATIMVFLPI
YDRIFIPVARRYTKNPSGITTLQRIGVGLVLSIITMIVAAMVEMRRLRIARDFGLVDKPE
AVVPMSFLWIVPQNILAAISDMFAVIGLQEFFYGEAPESLRSFSMALFLSIIGVGNFISS
FIVYAIDRVTSSFGDSWFSNNPNRGHVDYFYLLITVLNALSLACFLYFAKMYEHRKKWDS
GCEQHLPMAYKMKGVFKGLKVISQIFVVKEHQMEIGYPTDVKHVTHIGWDSPTGSAASPS
WMNDMKGSPDYSSLNNFGPSTGTSWTSQVCSTDFDHPQDISPFGLYVENAGKEANPPHPD
IPKPPRKSRRKKSKNNSPTASSRSSRSRSKRSFSSTADTVVDNSIQNEVRIV*
>11682.m03367|LOC_Os05g35600.1|genepair473-2
MASGSTDPLLPRAGAARRRPATGGWRSALFIIWVEVAERFAYYGVSSNLISYLTGPFGET
TAAAAAAVNAWSGAASMLPLLGAAVADSWLGRYRTIVASSVLYITVS*
>11667.m06557|LOC_Os01g65260.1|genepair474-1
MAAAAAAATATASSGLIRCATGGAPAHGHHQVFRCSAAKPSPLALRHRAGRPAPLQAFPE
YDRVTPFDYDGEVDGGDGDDHPREECGVFGVVGDPDATSLCYLGLQKLQHRGEEGAGIAA
AGDDGTIKLERGLGLVGDVFGDPARLGKLPGQAAIGHVRYSTAGAAASLRNVQPFLAGYR
FGQLAVAHNGNLVNYQALRNKLEAQGSIFSTSSDTEVILHLIATSLSRPLLARICDACER
LAGAYSLLFLTADKLLAVRDPFGFRPLVMGRRANGAIVFASETCALDLIDATYEREVEPG
EVVVVDRRDMSVSSACLVPHRPRKSCVFEHIYFALPNSVVFGHAVHERRNAYGRALAEES
PAPTADVVIPVPDSGFYAALGFSQTSGLEFQQGLIRWHYSGRSFIQPSQAIRDLAVKLKL
APVHGVIRGKSVVVVDDSLVRGTTSSKIVRLLRDAGAREVHMRIASPPVIGSCLYGIDTP
SEGELISNRMDLEGVRRAIGCDSLAFLSLDKLHTIYGDEAHELCDACFSRNYPVLPTVPE
PVPELVSAFED*
>11682.m03365|LOC_Os05g35580.1|genepair474-2
MAAAAAAASTSRLLLLRHHHHAAADGSKQQQQLRYSSKPSSLALPRLRLLPAAGALLPDR
VTPFSYEEDDESDDHPREECGLVGVVGDPDASSLCYLGLQKLQHRGEEGAGIVAVGGDGK
LKSVTGLGLVADVFGDPARLASLPGPAAIGHVRYSTAGAAASLRNVQPFLAGYRFGQVAV
AHNGNLVNYQALRNKLEARGSIFNTSSDTEVILHLIATSLSRPLLSRICDACERLAGAYS
LLFLTADKMFAVRDPHGFRPLVLGRRRNGTVAFASETCALDLIEATYEREVEPGEVVVVD
RRDMSVSSACLVPHRPRRSCVFEHIYFALPNSVVFSHAVHERRTAFGRALAEESPAAGAD
VVIPVPDSGFYAALGFARASGLEFQQGLIRWHYSGRSFIQPTQAIRDLAVKLKLAPVHGV
IRGKSVVVVDDSLVRGTTSSKIVRLLRDAGAREVHMRIASPPVVGSCLYGIDTPSEGELI
SNRMDLEGVRREIGSDSLAFLSLGKLHSIYGAEAEGYCDACFSRNYPVLPTLPEPVVELE
E*
>11667.m06562|LOC_Os01g65310.1|genepair475-1
MSPDATGEAGGGGGGGGDLFAANLKGSLLAVASSAFIGVSFIVKKKGLLRAGAAGSRAGV
GGYGYLLEPLWWVGMVTMLVGEIANFIAYMFAPAVLVTPLGALSIIVSAVLAHFTLNEKL
QRVGVLGCVLCIVGSTVIILHAPQERTPSSVDEIWHLAIQPDFLCYATAAVAVSLFLMIY
CAPRYGQMNIMVYVGICSVIGSLTVMSIKAVGIAIKLTIEGINQAGYFQTWLFAVISITC
IAVQLVYLNKALDTFNAAVVSPIYYAMFTTLTILASAIMFKDWSGQSASKIASEICGFLT
VLAGTLVLHSTREPDQTLSADLYAPLPPKIYWHIQGNGDIGKQKEDDSLPCDIITVMRQD
YFV*
>11682.m03364|LOC_Os05g35570.1|genepair475-2
MSRAPPDAAGDLFAANLKGSLLAVASSAFIGVSFIVKKKGLRRAGAAGPRAGVGGYGYLL
EPLWWVGMITMLIGEIANFVAYMFAPAVLVTPLGALSIIVSAVLAHFILNEKLQRMGVLG
CVLCIVGSTVIILHAPEEETPSSVEQIWHLATQPAFLCYVAFALVVSLILMAHCAPRYGQ
TNIAVYIGICSVIGSLTVMSIKAVGIAIKLTIEGINQAGYFQTWLFATVSAICIIIQLIY

LNKALDTFNTAVVSPIYYAMFTSLTILASAIMFKDWSGQSISSIASEICGFLTVLSGTVV
LHSTREYDQTISPDLYTPLPPIYWHIQGNGETVKQKEDDSLSADFITVVRQDYFV*
>11667.m06564|LOC_Os01g65330.1|genepair476-1
MGGLCSKGSAVDKSPSDTTLGPDRVVRGHERGGAGVGVGKEERKTVAREAAAKRIQEQQQ
QQRQQPASVQEAPASRAPIDARELPWDGVPNLARLPSQKSGMGVAKASAAKVSEVSSILG
RASTVGLGKAVEVLDTLGSSMANLNINSGFGSGTTTKGNKISILAFEVANTIVKGCNLMR
ALSKESIKHLKEVVLHSEGVQNLISKDMDELLKIYAADKREELKVFSTEVVRFGNRCKDP
QWHNLDRYFDKFASERTPQHHLKEEAESVMQQLVTCVQCTAELYHEMHALDRFEQDYQRK
QQEEDGSSVHQRGENLNILKQEVKSQRKHVKSLQKKSLWSKNLEEVMEKLVDIVHFLHLE
IHNAFGRSDNEESQEPTKRRNRLGPAGLALHYANIISQIDTLVSRSSSIPPNTRDALYQS
LPPTVKSSLRSKVNSFVVNEEVTAAQIKAEMEKTLRWLVPIANNTTKAHHGFGWVGEWAN
TGSEVNCKPTGQMDLTRIETLYHADKEKTETHILELVAWLHHLISRSKSANGERSPIKSP
VRSPTQRGHTITLSPNKASSNSSPLLTQEDQDMLRDVKYRKFIPGISKSQEFETKSRHSK
QSRLSKSNSHSPSSGNMKELLSIRRMLPVIDFEIDRTKAMDLIDRVDNLKSTVRT*
>11682.m03359|LOC_Os05g35530.1|genepair476-2
MGGLCSKVSAVDKSPSDTTLVRDQIVDPEPALTKRAKSPVVEEATAKRVEDQQQSFAFLE
SVVPGLAVYNGADAGQAGSRTPQLARTLSQKAGLGKTKAGAAKVSEVSSLLGRAGTVGLG
KAVEVLDTLGSSMSSLNTSSGFISAAKGDKISILAFEVANTIVKGSNLMRALSKTNIKHL
KEVVLYSEGVQHLISKDMDELHKIAATDKREELEIFSKEVVRFGNRCKNPQWHSLDRYFE
KLASERTPQHRLKEDAESVMQQLIICVQYTAELYHELHTLDRFEQDCRRKQQELDGLGSR
GDSLHMLKQDVKSQTKHVKSLKKRSLWSKNLEEVMEKLVDIVHFLHLEINNAFGLADSEA
PQEPAKHHNRLGPAGLALHYANIINQIDTLVSRSSLIPPTTRDTLYQGLPLTIKSALRSK
LQSFELKEELTASQIKAEMEKTLRWLVPIANNTTKAHHGFGWVGEWANTGSELNCKLSGQ
MDLTRIETLYHAEKEKVDGHILELVVWLHHLISKSKNANGGVRSPIKSPVRSPTQKGITL
MPDKSNSSSPILTQEDKDMLKNVKFRKFVPGISKSQEFDTKSRHSKQIRLIKSNSQSPTS
GSRKDMLSLRRSSMLPVIDFQMDRTKALDLIDRLDGLKKQ*
>11667.m06568|LOC_Os01g65370.1|genepair477-1
MGRSPCCEKAHTNKGAWTKEEDQRLIAYIKAHGEGCWRSLPKAAGLLRCGKSCRLRWMNY
LRPDLKRGNFTDDDDELIIKLHALLGNKWSLIAGQLPGRTDNEIKNYWNTHIKRKLLSRG
IDPQTHRPVSAGSSAAAASGLTTTASTAAFPSLAPAPPPQQHRLHNPVHAAAPSNASFAR
SAASPPSEDGHSSSGGSSDAPRCPDLNLDLDLDLSMSLPSSPPKTPAAASSTTASRHHHH
QQQKTICLCYHLGVRNGDVCSCKAAAPSPAGPRAFRFLRPLEEGQYI*
>11682.m03356|LOC_Os05g35500.1|genepair477-2
MGRSPCCEKAHTNKGAWTKEEDQRLIAYIRAHGEGCWRSLPKAAGLLRCGKSCRLRWMNY
LRPDLKRGNFTDDEDELIIRLHSLLGNKWSLIAGQLPGRTDNEIKNYWNTHIKRKLLARG
IDPQTHRPLLSGGDGIAASNKAAPPPPHPISVPAKAAAAAIFAVAKPPPPPRPVDSSDDG
CRSSSGTTSTGEPRCPDLNLELSVGPTPSSPPAETPTSARPVCLCYHLGFRGGEACSCQA
DSKGPHEFRYFRPLEQGQYI*
>11667.m06569|LOC_Os01g65380.1|genepair478-1
MAEEPQPEAAPAAVAATTEVAVAEKAPVEAEKEKKVEEETPAVEAEAKEEKKDEAAAAAA
AGGDEAGAIEGTGSFKEESNLVADLPDPEKKALDEFKQLIAAALAACEFNLPPPPPPPKA
KVEAAVEETKAEETKAEEEPKAEEPAKEEEPKAEVAAAAAAPPEAGTEEPKAEASSEEAK
TEEPKAEAAADEPAKEESKAEAAPAEEAKPAEPEPEEKTVVVTEEEAATKTVEAIEETVV
PAAAAPAAAATEEAAAPEPEVQAAAAPEPVLIWGVPLVGDDERTDTVLLKFLRAREFKVK
EAMAMLRSAVLWRKRFGIESLLDADLALPELDSVVFYRGADREGHPVCYNVYGEFQDKDL
YEKAFGDEEKRERFLKWRIQLLERGILSQLDFSPSGICSMVQVTDLKNSPPMLGKHRAVT
RQAVALLQDNYPEFIAKKVFINVPWWYLAANKMMSPFLTQRTKSKFIFASPAKSAETLFR
YIAPEQVPVQFGGLFKEDDPEFTTSDAVTELTIKPSSKETVEIPVTENSTIGWELRVLGW
EVSYGAEFTPDAEGGYTVIVQKTRKVPANEEPIMKGSFKVGEPGKIVLTINNPASKKKKL
LYRSKVKSTSESV*
>11682.m03352|LOC_Os05g35460.1|genepair478-2
MAEEAKQETPAAAEVVVVEKADEVVAVEKAVEVEAEEKKLAEQEEEEEKKAEEAEEAAGG
DEAAVIEGTGSFKEESNLVSELPDPERTALAQLKELVAAALAAGEFDLPPPPPPPPAKAE
EPAKEEEPKAAEAPAAEEPKAEAEAEAEAAATEEPKTEEPKTEEPAKEEEPKAAAAAAAE
EPKAEAAAEEAKPAEPETEEKTVVVTEDEGTSKTVEAIEETVVVAAPAAAAEAEAAAPKE
ELIWGVPLTGDDERTDTVLLKFLRAREFKVKEAMAMLKAAVLWRKRFGIDAVLAADLGLP
ELENVVFYRGADREGHPVCYNVYGEFQDKDLYEKAFGDEEKRERFLKWRIQLLERGILDQ
LDFSPSGICSMVQVTDLKNSPPMLGKHRTVTRQALALLQDNYPEFIAKKIFINVPWWYIA
ANKMVSPFLTQRTKSKIIFCTAAKSAETLFRYIAPEQVPVQFGGLYKEDDTEFSTSDAVT
ELPIKPSSKETVEIPATENSTVVWELRVLGWEVSYGAEFTPDAEGGYTVIVQKTRKVPAN
EEPIMKGSFKVGEPGKIVLTVDNAASKKKKQLLYRFKVKSSSESA*
>11667.m06572|LOC_Os01g65410.1|genepair479-1
MDLSRPESSDLSLGFHSLGHARGHAVTGPLRLFDDMEDAKPEKSVGGGGGGGGGGGEEED
GEEGGDQHFSLLGHALCVKRPRRALYGGGGGGGAGGGGGGGGEASSCSSSSSSLHPAKRQ
ATAERGADLEARRGAVRAWGNQALAEADPDVHALMELERDRQVRGIELIASENFVCRAVL
EALGSHLTNKYSEGHPGARYYGGNQHIDGIERLCHERALAAFGLDPACWGVNVQPYSCTS
ANLAVYTGLLLPKDRIMGLEPPSGGHVSHGYYTPSGKKVSGASIFFESLSYKVNPQTGYI
DYDKLEERAMDFHPKILICGGSSYPREWDFARMRLIADKCGAVLMCDMAHISGLVAAKEC
RSPFDYCDVVTSTTHKNLRGPRGGIIFFRRGKNLRRRTGSFSQADENDYDFEDRINFAVF
PSMQGGPHNNHIAALAITLKQVATPEYKAYIIQVKKNAQALASALLRRKCRLVTGGTDNH
LVLWDLRNLGLTGKNFEKVCEACHISINKMPIYGDNGSISPGGVRIGTPAMTTRGCLEDD
FEVIADFLIRATQIASNLMKEHGKMQKEFLRGLQNNKDIIELRNQVENFASQFAMPGFDV
*
>11682.m03349|LOC_Os05g35440.1|genepair479-2

MLDRATGGRRRAAGEGMDHRPDLSLALHPPPPPEGSSAAAAEEVGEEEAEGGEGRFSLMG
QPLFLKRPRPRPPRPPRARGVGDDVDVDVDDDSSPCCSSSCLSPAAKRRAAAGGLEARRA
AVRAWGCQPLTEADPDVHELMERERRRQAGGVELIASENYACRAVLDALGSHLTNKYSEG
LPGARYYCGNQHIDAIERLCCDRALAAFGLDPSRWGVNVQPYSCTSANFAVYTGLLLPND
RIMGLDSPSGGHVSHGYYTPSGKKVSGASIFFENLSYRVNPHTGYIDYDKVEEKAVDFHP
KILICGASSYPRDWDYARMRLVADKCGAVLMCDMAQISGLVAAKECRNPFDYCDIVTSTT
HKSLRGPRGGIIFFRKGKNLRKRVGSLTQVVENDQYDFEDRINFAVFPSMQGGPHNNHIA
ALAIALKQVAMPEFKAYIQQVKKNAQALAMALLRRKCRLVTGGTDNHLMLWDLRTFGLTG
KNFEKVCEACHISLNKTPIYGDNGSISPGGVRIGTPAMTTRGCLESDFEIMAEFLLRAAH
IASIVLKEHGRLQKDFLKGLENNNDIIELRNQVETFALQFAMPGFDV*
>11667.m06575|LOC_Os01g65440.1|genepair480-1
MASGGGEHFLRQLSASNGGYGGAAQYQMGRGVVAEEEVELGGGGRRRGSKRWSKKRAGRG
GYGGGGGGKGDAAAAAAAAAAVAGRKRVMVVVDDTSGAKHAMMWALTHVANKGDFLTLLH
VLPYAGAGRGEETPSLANSLGTLCKACRPEVEVEALVIQGPKLATVLSQVKKLEASVLVL
SQSKPSHFCWLSCILRSSSEEFVEQCINQAECLTLAVRKQSKGVGGYLISTRWQKNFWLL
A*
>11682.m03343|LOC_Os05g35380.1|genepair480-2
MGSSGGSSEHFLRQFSASDGAPLPRELGEEWAAAECGRRGSRRWSRKKARGHHRRGGGGG
GGGGLCRSREEAPAGRKRVMVVVDQSSGAKHAMMWALTHVASKGDFLTLLHVLPHGGGDA
SALANSLGSLCKACKPEVEVEALVIQGPKLGTVLSQVKKLDASVLVLSQCKPSPFCCFMR
SSGEEFVEECINRADCLTLAVRRQSKGVGGYLISTRWQKNFWLLA*
>11667.m06579|LOC_Os01g65480.1|genepair481-1
MGNPPELYHRILNIPRETSPQEIRAAYKSLVKKWHPDKHPPSSKPEAEARFKAITEAYEA
LLDQQENRAVFGVCCNDGRAGEKAMACGVVGGGGAHIARTRSDDFGARMAPGTPAREFKK
VYSSGNSGGRRAFAEFSSSIMRKAPPLERKLDCTLEELCHGCKKEVKFTRDVVTKNGVAG
LITFNGFTILDRSIVKKEVSQMVLVKPGWKKGNKITFEGMGDERPGCLPADAVFVISEKK
HPVFKRVGNDLVLKAEVPLVSALTGWSFSFRLLSGKKVSCSFQDEIICPGYEKIIKGEGM
PIADQKGARGDLRVKFEIAFPKQLTDEQRDGLAQILRGCAWD*
>11682.m03339|LOC_Os05g35340.1|genepair481-2
MGNPPELYYRILNISRDTSPKEIRAAYKTLVRQWHPDKHPPSSKNEAEARFKAITEAYEA
LLDQQENRAAFGARGNVDAVDEKGDRTAAAGGGGGGGATTTGGVGGDGRAPSSAMPRAQG
AEKKKAPSAAAPPPTRTAPCGTPAREFKKPVLYSSTGLGEAAGGGRRRAFAEFSSCVVRK
APPLERRVECTLEELCSGCKKEVKYTRDVVAKNGLVSKKEETKTIRVKPGWKKGMKVTFE
GMGDERPGCLPGDAVFTISERKHKVFKRKGNDLVLKAEVPLVSALTGWSFSFRLIGGEKM
SFTFRDEVISPGYEKVVAGEGMPVVAAGGGGEKAAAARGDLRVKFDVVFPKNLTGEQRAG
LASILRACP*
>11667.m06584|LOC_Os01g65530.1|genepair482-1
MARNPTRTVYIGNLDEKVTERILYEILIQPGRVVDLCIPRDKETSCPKGYAFAEYETEEI
AQYAVQLFSGLVRLYGKTLKFAISGQDKPSSNGNNPVMPKLNPVPLPKQPQFVHHSDMHV
LHTPADPMHYQLDPCIATEGSAMVWRLCFECCWPWSSEATNVVSVLLGTKEGNLPREEAM
KMESPGVQPAAAGEEEGGGGVFFCVAVTSRGRTDRLSYFQAEGDGDDAEEVARATAALCL
DHAPEHHHWHHHTVVGRRTFAFLAGDDGRTYFAVADPTPGSAETVRFLQRVRDAFGSCGG
GGGGGATRRRNQRDDAVDAVVWQFVRALRASAGRGTAALFPGDDSRGGGDASSADGDKDD
EEEEDDRGGEAMAVAADGARRRTRRSWWRYSKVVIGVELVLFLVLFVVWMIVCKGFNCVQ
R*
>11682.m03333|LOC_Os05g35280.1|genepair482-2
MSRNPGCTVFIGNLDEKVPERVLYEILIQVGRVVDLHIPRDKETNRSKGYAFAEYETEEI
AQYAVKLFSGLVRLHNRTLRFAISGQDKQSSNGNIPVTPKVNPIPPPKPAQLMRSSDTPA
SQHTVWDQVCA*
>11667.m06590|LOC_Os01g65590.1|genepair483-1
MMTLSTSERQPQPEKKPPRTRPPLPGKAVAALCVASFVVGLLLSGRVVVPLLPPGSSSPA
SNYKTSFSTGCENKRAKLGESNPTDIMNEVSRTHHAIQSLDKAVSSLEMELAVERARSSA
AVGAGTAVSSLGPQKAFVVIGINTAFSSKKRRDSLRDTWVPRGDKLRRLEKEKGIVIRFV
IGRSGAAAAGDGPLDRAVDAEDAENKDFLRLDHVEGYHELSSKTRVYFTTAVATWDADFY
VKVDDDVHVNLGMLTSRLAKYRTRPRVYVGCMKSGPVLSQKGVKYHEPEYWKFGDEGNKY
FRHATGQIYAVSKDLAAYISINQPILHRFANEDVSLGAWLIGLEVEHVDDRSMCCATPPD
CEWKKRAGNVCVASFDWSCSGVCKSVDRMKHIHRACGEGQGAVWSVAT*
>11682.m03332|LOC_Os05g35270.1|genepair483-2
MSAKAVVVLCATSFFVGLLLSGRMTLLMPPPSGSVGAASSGHGSRLSLFSDDCEHRHKLD
EGNPNDIMNEVSRTHQAIQSLDKSVSSLEMELAVERAKQNGGLGAAVPSKRGRRPPRAFV
VIGINTAFSSKKRRDSLRDTWVPRGERLRRLEEKGVVVRFVIGHSATPGGALDRAIDVED
AETRDFMRLDHVEGYHELSSKTRTYFTAAVATWDADFYVKVDDDVHVNLGMLTSRLARGV
KYHEPEYWKFGDEGNRYFRHATGQIYAISKDLASYISINQPILHRFANEDVSLGAWLIGL
EVEHVDDRSLCCATPPDCEWKKQAGNVCAASFDWSCSGICKSVDRMRAIHSACGEGDGAV
SNNFAAAAAA*
>11667.m06593|LOC_Os01g65620.1|genepair484-1
MVNGYCNLTSSPPVASGAVEAETKWEGVAVGAATLVRNFSSASQRFRPVERSRSTAGGNG
GGLQAVVRRAFSMRRQPSSFADGYWRIHDDMDGDDAAGEHQEQAAFQDGEQKRREEQDHA
TGKKEKITKKKGRIFKACKKLLGF*
>11682.m03329|LOC_Os05g35240.1|genepair484-2
MHGYSNLACSSSSPPPVAAAGNGAGCRARRSLELTNTKETNAWEGLAIGAVTLARTFSTG
SHRISSSSRSGAGERVGRTAGGGGGLPGAVRRAFSMRRHPAGLGKGDGYYWRIHDNMDGD
SDDDGDNPAAAAAEEEERDKKEQLAESADEKKKEQLAEAADEKVSVTATATPKKKKGGRI
MKACKKLLRL*

>11667.m06594|LOC_Os01g65630.1|genepair485-1
MWAAITVEDNLQGAGANLNSEGRRRAGCNLQEAKKRPRQAAMSGRSRPWPGDPSPAPPQP
PVVAAAADAGGEASTSLRDFGTSMDAISFGFAATAILVSMFLLMAIFEHLIKPHVFPPLA
SAALRPARRRHGVSPAGKLRSPPMVETVLQAADLSVLMPGQRYPTYLAQPAPLPPAPCPR
EGVHWPPHDHDVHHSYMPP*
>11682.m03328|LOC_Os05g35230.1|genepair485-2
MSGFAGQRSRPWMGDAAASDQAAAGGVGGGGGDVRDDGDGGAASAAAAKGLGDASTNASA
ISFGFAATAILISMFLLMAIFEHLIKPSLSSSSSSSSSSSSSSRASHGDGDGHGQSSSSH
HAAAAAAGVSPDKLFCTPGKLEVVPAEDLTVLMPGQRYPTFLAQPAPLLPWPREGVRWPP
HGHRHCFVPP*
>11667.m06609|LOC_Os01g65780.1|genepair486-1
MTAPCIAGAIKIATHISSMCSPQWSPVFRHVEADDTAKRRTQKSKSFKEVEKFDVFVLEK
SSGCKFRSLQLLLFAIMSAAFLTLLYTPSVYDHQMQSSSRFVSGWIWDKTIPDPRYVSSL
GVQWEDVYKTVENLNDGERKLKVGLLNFNSTEIGSWTQLLPDSDFSIIRLEHAKESITWQ
TLYPEWIDEEEETEIPSCPSLPDPIFPRGTHFDVVAVKLPCTRAGGWSRDVARLHLQLSA
AKVAVTASRGNRGIHVLFVTDCFPIPNLFSCKNLVKHEGNAWMYKPDLKALREKLRLPVG
SCELAVPLKAKARLYSVDRRREAYATILHSASEYVCGAITAAQSIRQAGSTRDFVILVDE
TISNHHRKGLEAAGWKVRIIQRIRNPKAERDAYNEWNYSKFRLWQLTDYDKIIFIDADLL
ILRNVDFLFAMPEITATGNNATLFNSGVMVIEPSNCTFQLLMDHINEITSYNGGDQGYLN
EIFTWWHRIPKHMNFLKHFWEGDEEEVKVKKTRLFGADPPILYVLHYLGLKPWLCFRDYD
CNWNNPILREFASDVAHARWWKVHDKMPKKLQHYCLLRSRQKAGLEWDRRQAEKANFTDG
HWRRNITDPRLKTCFEKFCFWESMLWHWGESKNSTKENPVPATPTASLTSS*
>11682.m03325|LOC_Os05g35200.1|genepair486-2
MGSLETRYRPAGAPSIWRFDLFVDQPLNLLDDAMVPRGCRLWLMLPSGDLSVLGSSHRLD
DTTKRRTPKSRIYKDVENFGVLVLEKNSGCKFKTLRYLLLAITSATFLTLLTPTFYEHQL
QSSRYVDVGWIWDKPSYDPRYVSSVDVQWEDVYKALENLNDGSQKLKVGLLNFNSTEYGS
WAQLLPGSAVSIVRLEHAKDSITWDTLYPEWIDEEEETDIPACPSLPDPNVRKGSHFDVI
AVKLPCTRVGGWSRDVARLHLQLSAAKLAVASSKGNQKVHVLFVTDCFPIPNLFPCKNLV
KHEGNAWLYSPDLKALREKLRLPVGSCELAVPLKAKARLYSVDRRREAYATILHSASEYV
CGAISAAQSIRQAGSTRDLVILVDDTISDHHRKGLEAAGWKVRVIQRIRNPKAERDAYNE
WNYSKFRLWQLTDYDKIIFIDADLLILRNVDFLFAMPEITATGNNATLFNSGVMVIEPSN
CTFQLLMDHINEITSYNGGDQGYLNEIFTWWHRIPKHMNFLKHFWEGDDDSAKAKKTELF
GADPPILYVLHYLGMKPWLCFRDYDCNWNIPLMREFASDVAHARWWKVHDNMPEKLQSYC
LLRSKLKAGLEWERRQAEKANLEDGHWRRNITDPRLTICYEKFCYWESMLLHWGEKNPTN
NNPVPATISSS*
>11667.m06611|LOC_Os01g65800.1|genepair487-1
MVTGSFSRSTSARLTARGGVGSPRVSAAAAAHRKWWWWAPSGPSFECAALAFFLSSVALV
LACALYLYLFRYQGRGHAVAEFAGDNLESCDVFDGSWVPDRRYPLYNSSDCPFVERGFNC
LANGRKDTGYLKWRWKPRGCDLPRFSARDVLERLRGKRVVFVGDSMSRTQWESFICMLMA
GVENPKTVYEVNGNQISKTIRFLGVRFASFNLNVEFFRSVFLVQQSPAPRSSPKRVRAIL
KLDKMDNISRKWENADVLIFNSGHWWTPSKLFDMGCYFEAGGLLKLGTSINSAFKMALET
WASWVKEKVDLKRTHVFFRTYEPSHWSGSNQKVCEVTEFPTAEAKGDDRSEFGDILAGVV
VNMSVPATILNVTLMGAFRSDAHIVIGAFLESQMLGMN*
>11682.m03324|LOC_Os05g35190.1|genepair487-2
MVSAASSRAGGGPARHAPRGGAGPGSPRVSAQRRRWWWWAAPLPSASGASSLERVALAFF
LASVALVLSCALYLYVFRYLGRGSAVAGFVGRDLEPCDVFDGAWVPDAGYPLYNSSLCPF
AERGFNCLANGRRDTGYLKWRWKPRRCDAPRFTARAALERLRGKRVVFVGDSMSRTQWES
FICMLMAGVDDPKTVYEVNGNEITKTIRSLAVRFASFDLTVEFFRSVFLVQQRPPPRHAP
KRVKSTLRLDRLDNISRKWVNSDVLVFNTGHWWTPTKLFEMGCYFQSGRVLKLGTSVEAA
FRTALETWASWVEKRIDLNRTHVFFRTYEPSHWSDTNQKVCEVTEQPSSEAKGNDKSEFG
TILADVVANMKAPVTLLNVTLMGAFRSDAHVGTWSYPPTVLDCSHWCLPGVPDAWNELVF
SYLLTNGWRKMAR*
>11667.m06617|LOC_Os01g65860.1|genepair488-1
MAPSKLRAALGAVKDRTSVGLARVGGADEVAADLAVAIVKATAHGESVPGDERHVQEILT
LTCYSRARVAACVSAVSRRLGRTRAWAVAVKALALVHRLLADGDPAYEQEVFLATRRGRR
MLDVSHRFPHRSSRSRATWDFHGFVRAYAAYLDDRLKHRMKGRVASQGKWCSDGRRDGFP
DITDGSYEVGEAVAEVWALVPRDTPATGTTTTEELVSKAQHLKHILQRFIGCRPTGKART
NKVVAAALHRLVKESAVMYRELTEVMAMLADRFAELETPCCVRVHSIFTSIAKLFDELDE
FYSWCRSATICRPSEIPEVERVAQKKLDLMDEFIRDRQPASSRWWRCTPPAPSSPLAPIA
SNGDNSSKASPAEPAPAGALVVVDDHMADFLNLGEESTPLSTEEQDRDLTLSLFGDDPAT
PAPKWETFDDDQCDDWETALVQSASKFAATQSATVLALPPPPGATGGEVADPFAASLAVP
PPTYVQMMDMQARQRLLANEQMMWQQFETQQMAAWSYSSLL*
>11682.m03319|LOC_Os05g35160.1|genepair488-2
MPPSKLRKAIGAVKDQTSIGLAKVGSGGAAASELDVAIVKATRHSESFPADERHVREVIA
LTLHSRAYVGACVASLSRRLGRTRSWAVALKTLALVHRLLADGDQAFEQEVFYATRRGTR
MLNMSDFCDHSRTDAWDFSAFVRTYAAYLDDRLEYRMQAKHGGAARPGQPLREQLYASPG
NRFNYDDFIMRDDEADKAVALVARETPTSEMTLEQLLAKAQQLQHLLDRFIACRPVGAAK
TNRVVSVSLYPLVKESVQLYCELTEVMAALVEQFPEMEADDCERVHAVFCGLAKQLDELE
ALYTWCKDAYVCRQSDVPEVELITQKKLELMDEFVRDRRAAAAQSPEPPRREASPVVEDD
VSATKALPAPEEPVAAAQEEQNAGETVPAEPEAPPLIADGDADFLNLKGDAMSGEEHGRQ
LALALFDGNPAGSAPAPDAFDPSSTDWETALVQSASALAHQRAELGGGLSMLVLDGMYSQ
AAAASTAANAQAFSGSASSVAMRPPGAPMLALPAPPGTSGASGDPFAASMAVAPPAYVQM
SDMETKQHQLVEEQMVWQQYGKNGMSGQGALAMLEQQRPPQQQMQMQMLPNGGYNYAGYH
RSS*

>11667.m06619|LOC_Os01g65880.1|genepair489-1
MEHIARFFFGVSGNVIALFLFLSPVVTFWRIIKKRSTEDFSGVPYNMTLLNCLLSACSSY
SPCCRHGQDGCDMWAQEAVVQAHAASRSSVCSSKKWVRNTIRESILRRYGLPFVSPNNIL
VTTINGTGSVIEAIYVVIFLIFAERKARLKMMGLLGLVTSIFTMVVLVSLLALHGQGRKL
FCGLAATIFSICMYASPLSIMRLVIKTKSVEFMPFLLSLSVFLCGTSWFIYGLLGRDPFI
AIPNGCGSFLGLMQLILYAIYRNHKGATPAAAAGKGDAADEVEDAKKAAAAVEMADAKTN
KVVADDADADADGKSADDKVASQV*
>11682.m03317|LOC_Os05g35140.1|genepair489-2
MEDLAKFLFGVSGNVIALFLFLSPVPTFWRIIRRKSTEDFSGVPYNMTLINCLLSAWYGL
PFVSPNNILVSTINGAGAVIETAYVVVFLVFASTHKTRLRTLGLAAAVASVFAAVALVSL
LALHGQHRKLLCGVAATVCSICMYASPLSIMRLVIKTKSVEYMPFLMSLAVFLCGTSWFI
YGLLGRDPFVTIPNGCGSFLGAVQLVLYAIYRNNKGAGGGSGGKQAGDDDVEMAEGRNNK
VADGGAADDDSTAGGKAGTEV*
>11667.m06623|LOC_Os01g65920.1|genepair490-1
MDGEQLMSGELDNSFNALMVSGEGESGQAHHEGTGTTLLGWKDLPMELLLRILSMAGDDR
MVIVGSGVCTGWRDTLEWGVTNLSLSWCQAHMNDLVMSLAQKFTKLQVLSLRQIKPQLED
SAVEAVANNCHDLRELDLSRSFRLSDRSLYALAHGCPHLTRLNISGCSNFSDAALAYLSS
QCKNLKCLNLCGCVRAVSDRALQAIACNCGQLQSLNLGWCDSVTDKGVTSLASGCPELRA
LDLCGCVLITDESVVALANGCPHLRSLGLYYCQNITDRAMYSLAANSRRVRSKGRSWDAA
ARKNAGAGADGLASLNISQCTALTPPAVQAVCDSFPALHTCPERHSLIISGCLSLTSVHC
ACAHHPHRHGRAILSNHAY*
>11682.m03314|LOC_Os05g35110.1|genepair490-2
MVSGRSANGELDACFRSLMLSISSGRGQAEGGGAMPTLSGWKDLPIELLLRIMSIIGDDR
MLVVASGVCTGWRDALGWGLTNLSLSRCQQNMNNLMISLAHKFTKLQVLTLRQNIPQLED
SAVEAVSNYCHDLRELDLSRSFRLSDRSLYALARGCPQLTKLNISGCSNFSDTALTYLTF
HCKNFKCLNLCGCGKAATDRALQAIARNCGQLQSLNLGWCEDVTDKGVTSLASGCPDLRA
LDLCGCVLITDESVIALATGCPHLRSLGLYYCQNITDRAMYSLANSRVKSKRRRWDSVRS
SSSKEEDGLANLNISQCTALTPPAVQAVCDSFPALHTCPGRHSLIISGCLSLTSVHCACA
LHPHRAGRTMVPSHAY*
>11667.m06630|LOC_Os01g65990.1|genepair491-1
MATEASTSAAAGAGGGSWVEGMSADNIKGLVLALSSSFFIGASFIVKKKGLKKAGASGVR
AGVGGYSYLYEPLWWAGMITMIVGEVANFAAYAFAPAILVTPLGALSIIISAVLADIMLK
EKLHIFGILGCVLCVVGSTTIVLHAPQEREIDSVAEVWALATEPAFLFYAVTVLAATFVL
IFRFIPQYGQTHIMVYIGVCSLVGSLSVMSVKALGIALKLTFSGMNQLIYPQTWMFTIVV
VACILTQMNYLNKALDTFNTAVVSPIYYTMFTSLTILASVIMFKDWDRQNPTQIVTEMCG
FVTILSGTFLLHKTKDMVDGLPPTLPIRIPKHDEDGYAAEGIPLSFYVGAKSPNKLVHTL
IQPLPMAEKEECKVLIDQASNEAGDPHQDDDCEDEDDDDSSSLILLTNLILSGTARLNVL
LPTATILAFAIFAPLLTDDGKCTRLNRALTGALMLLCAASCVFFTLTDSFRSPTGRLRYG
IATTSGIRTFCVGGRRRRRGGGKAGPREPERYRLRWSDLFHTALALVAFVTFAASHHDIV
LCYYPGVPRKVVNTVPLVIGFVVSLLFVLFPSKRRGIGYPFLLSTDLVYLRR*
>11682.m03309|LOC_Os05g35060.1|genepair491-2
MGGARLGRWVEGMSADNVKGLLLALSSSLFIGASFIVKKKGLKKAGASGVRAGVGGYSYL
LEPLWWAGMTAMIVGEIANFAAYAFAPAILVTPLGALSIIISAVLAHIILREKLHIFGIL
GCILCVVGSTSIVLHAPPERQIESVAEVWDLATEPAFLLYAAIVLAAAFVLIFHFVPQYG
QTHIMVYIGVCSLVGSLSVMSVKALGIALKLTFSGMNQLVYPQMWVFLLFVVACIVTQMN
YLNKALDTFNTAVVSPIYYTMFTSLTILASVIMFKDWDRQDPTQIVTEMCGFVTILSGTF
LLHKTKDMVDGLPPNLPIRLPKHAEEDGYAAEGIPLRSAADGIPLRSPRATESFRATL*
>11667.m06632|LOC_Os01g66010.1|genepair492-1
MGENVVGTYYYPPSAAAMDGVELGHAAAGSKLFDDDGRPRRNGTMWTASAHIITAVIGSG
VLSLGWAIAQLGWVAGPAVMVLFSLVTYYTSSLLSDCYRSGDPVTGKRNYTYMDAVNANL
SGFKVKICGFLQYANIVGVAIGYTIAASISMLAIGRANCFHRKGHGDPCNVSSVPYMIVF
GVAEVFFSQIPDFDQISWLSMLAAVMSFTYSVIGLSLGIVQVVANGGLKGSLTGISIGVV
TPMDKVWRSLQAFGDIAFAYSYSLILIEIQDTIRAPPPSESAVMKRATVVSVAVTTVFYM
LCGSMGYAAFGDDAPGNLLTGFGFYEPFWLLDIANAAIVVHLVGAYQVFCQPLFAFVEKW
AAQRWPESPYITGEVELRLSPSSRRCRVNLFRSTWRTAFVVATTVVSMLLPFFNDVVGFL
GALGFWPLTVYFPVEMYVVQKKVPRWSTRWVCLQMLSVGCLVISIAAAAGSIAGVMSDLK
VYRPFKGY*
>11682.m03301|LOC_Os05g34980.1|genepair492-2
MGENGVVASKLCYPAAAMEVVAAELGHTAGSKLYDDDGRLKRTGTMWTASAHIITAVIGS
GVLSLGWAIAQLGWVAGPAVMLLFSFVTYYTSALLADCYRSGDESTGKRNYTYMDAVNAN
LSGIKVQVCGFLQYANIVGVAIGYTIAASISMLAIKRANCFHVEGHGDPCNISSTPYMII
FGVAEIFFSQIPDFDQISWLSILAAVMSFTYSTIGLGLGVVQVVANGGVKGSLTGISIGV
VTPMDKVWRSLQAFGDIAFAYSYSLILIEIQDTIRAPPPSESRVMRRATVVSVAVTTLFY
MLCGCTGYAAFGDAAPGNLLTGFGFYEPFWLLDVANAAIVVHLVGAYQVYCQPLFAFVEK
WAQQRWPKSWYITKDIDVPLSLSGGGGGGGRCYKLNLFRLTWRSAFVVATTVVSMLLPFF
NDVVGFLGAVGFWPLTVYFPVEMYIVQKRIPRWSTRWVCLQLLSLACLAITVASAAGSIA
GILSDLKVYKPFATTY*
>11667.m06633|LOC_Os01g66020.1|genepair493-1
MAIAAPLFRLAAAAAVVMGVVSPAVKAAGGGNSSTACPLDLGYVRSFPWDTAPCMPPVAN
QTACCTTLLSVLGVGLAARLRATGHFRLPSANASAACLGAFSDELASPPLSLQDTLVPAC
WPVSSQLAISPSYCAGVTTAKQYVATVGNAAVLGSLNSSCGSDLADLSLCSSCLAAAIDA
SGRLVAAAAKGTNPQNCFYLTVLYAAGVSSSAGPTSPGTANCALGLALSTPSSSSSPASS
SNHTNMAVATAIPVASALLVSVIAALLVWRRRQDSIRSKSRRLSGERRLSRPRPNVGSVL
FSLGELAKATCGFAERNLIGRGGFGVVYRGVLDDGSVVAVKKMLDPDMEGGDEEFTNEVE

IISHLRHRNLVPLRGCCISDDDADEGKQMFLVYDYMPNGSLDHYIFKDGGDGGRRPPPLS
WAQRRGVVLDVARGLEYLHHGVKPGIYHRDIKATNILLGTDMRARVADFGLARRSREGQS
HVTTRVAGTHGYLSPEYALYGQLTEKSDVYSFGVLVLEVMSGRRALDLSDPSGVVLITDW
AWALVRAGRAAEVVAAALREREGPAGVHAMERFVLVGILCAHVTVACRPTMPEALRMLEG
DMDVPDLPERPQPYGQRIAFDEGEANFSASSVLSGPPFMDFGDMLR*
>11682.m03298|LOC_Os05g34950.1|genepair493-2
MPSLALLRAVAMALLLLLARSMEGVDGAGGGGNATCPLDLSYVTTFPWDADLCAGGAGNM
TRCCNTLLSVLAIGLAEQVRATGHFRIPSVGESAACLKDYGAKLSAAPLSLPGASLVQTC
FPSPEQFVSSPSFCAGVTTAAEYRAVVGNDSVAALDSACGDLSSTPHCLRCLDAGIAATS
RLKAAANISANATTDGAATTRNCFYLTVTYAAGISNVAGPTNPPTAACTLGLALSNPPAA
PPKSHDTVIYATAIPVAFLLLASLLAFLVWRRHDKKKKKKKIHEISKEGSAKRRSHPRPN
TGSILYDIAELSKATDAFADRNLVGRGGFGAVYCGVLADGSVVAVKKMLDPDVEGGDEEF
TNEVEIISHLRHRNLVPLRGCCIVDDDAEEGKQKFLVYDFMPNGALEDFIFRDGKRPALT
WAQRRSIIMDVAKGLEYLHYGVKPAIYHRDIKATNILLDGDMRARVADFGLARRSREGQS
HLTTRVAGTHGYLAPEYALYGQLTEKSDVYSFGVLVLEVLSARRVLDMSAPSGPVLITDW
AWAHVKAGQAREVLDGALSTADSPRGGAMERFVLVGILCAHVMVALRPTITEAVKMLEGD
MDIPELPDRPLPYGHSAMFSEAGSNFSASPAFSGPFIDNGDMLR*
>11667.m06634|LOC_Os01g66030.1|genepair494-1
MGRGKIEIKRIENSTNRQVTFSKRRSGILKKAREISVLCDAEVGVVIFSSAGKLYDYCSP
KTSLSRILEKYQTNSGKILWDEKHKSLSAEIDRIKKENDNMQIELRHLKGEDLNSLQPKE
LIMIEEALDNGIVNVNDKLMDHWERHVRTHQQDIALSGSMRDLELGYHPDRDFAAQMPIT
FRVQPSHPNLQENN*
>11682.m03297|LOC_Os05g34940.2|genepair494-2
MGRGKIEIKRIENSTNRQVTFSKRRAGILKKAREIGVLCDAEVGVVIFSSAGKLSDYCTP
KTTSVFPPLSRILEKYQTNSGKILWDEKHKSLSAEIDRVKKENDNMQIELRHMKGEDLNS
LQPKELIAIEEALNNGQANLRDKMMDHWRMHKRNEKMLEDEHKMLAFRVHQQEVELSGGI
RELELGYHHDDRDFAASMPFTFRVQPSHPNLQQEK*
>11667.m06636|LOC_Os01g66050.1|genepair495-1
MEDPVAAPSSSVAAASAAAAPRVAPVAAPLPLAAQQAPAAAAGCRRQVFSVELRPGETTI
VSWKKLLKEAGHAAASPPPAAPAVAVAASDPAFPALPGQPGAVHPPESDPKDPAQPNRFN
AVIEKIERLYMGKHSSDEEDLDDVPDDDQYDTEDSFIDDAELDEYFEVNNLRTKHDGYFV
NKGKLEQIEAGTSANVAPKKRRRKDSSSGYIENNQVAPADYPSIGNMPGKSAARSGAHVG
KKLTSSNIGSYGEYYHDDNRVVKNITGAGVHKRKSMDFSMGSDTAAYTKISSKDMPYASS
ELNKAAGLQPTDYTHRSKTAEAYDYAYSAYRDRDTSMQLDFQQKRAYTGENRDPSNKIHR
KEKHGMGEFSGMATTGALYSGQVMQPITSRDGSGTKPKGTRLERAIRDLQKIAAEYRPPA
IDINEVDPNGQVAVKRRLPPEVKQKLAKVARLSANHGKIQEHELMDRLMGIVGHLVQRRT
LRRNMKEMVESGLSAKQEKADKFQRVKMEINEMIKSRVAAKAKVNEHHSGSADDFQIAND
EKRYLKGKSVMDAALEDRICDLYDLYVEGMDEDKGPQSRKLYVELAELWPEGSMDNVGIK
DAINRSKERRRSLYNQQKVRNEERMKRKRLAAAAKLQDGYPVVMQSALIQQVAQPPITNP
VATYPVTDQGSKSFDRVREISASANPDDINRNTGEMKKKKRKPESDLVDTQANAMKGPSQ
HVEKNKPPKRADEAVETVLCLPFYDQQPS*
>11682.m03292|LOC_Os05g34900.1|genepair495-2
MGEPVVPPRAPAAHAAVAAPKPPGDAVAAEGEARAPPRPPPAVAAGAGAGASGGGRRVFS
VELRPGETTIVSWRKMLKEADLGAALPPPPPAAAAQPAVAPLPGPSGATHPTENDCAQSN
RFNSVIEKIERLYMGKNSSDEEDLDDAPDDDQYDTEDSFIDDDELDEYFEVDNFATKHNG
YFVNKGKLEQIDFDSVQTVEPKKRRRKDSSSSYIENNKEFSPGSSSYMGTPLRDSKRSTL
QTGKSTSNGHKSGANGTFEYPYSAYRDKDAPGHLGLQQKITSNGANQDLSKNMHHKEKYN
AGQFSGLHASSNIYSTETMHLATKIHTEGSGTKTKGTRLERAIRDLQNIVTEYKPQILDV
HEAEANCQVAVKRRLPQEVKQKLAKVARLSANQGKIPEHELINRLMGIVGHLVHRRTLKR
NMKEMVQSGLCAKQEKAGKLQQVKMEIYEMVKARLATKPKGAEHKVESIDGFQDPVTHDD
RMALRGKSVMDAVLEDRICDLYDLYVEGMDEDKGPQSRKLYLELANLWPHGDMDKVGIRD
AISRSKERRNLLYRQRKVRNDQRMKRRRLAAAAKLRDSDPAAPQSAQSLQNMTSTHTMYP
VVNNGNSQSSRSVDKVNEMSVGAGSDGNRSSTSMKKRKIDSEDRQVNPPKATAELHHHGI
EIQKPAKRADEATKVSNLPQTLLAIPSSDSRPSSS*
>11667.m06641|LOC_Os01g66100.1|genepair496-1
MDSTAGSGIAAPAAAAVCDLRMEPKIPEPFVWPNGDARPASAAELDMPVVDVGVLRDGDA
EGLRRAAAQVAAACATHGFFQVSEHGVDAALARAALDGASDFFRLPLAEKRRARRVPGTV
SGYTSAHADRFASKLPWKETLSFGFHDRAAAPVVADYFSSTLGPDFAPMGRVYQKYCEEM
KELSLTIMELLELSLGVERGYYREFFADSSSIMRCNYYPPCPEPERTLGTGPHCDPTALT
ILLQDDVGGLEVLVDGEWRPVSPVPGAMVINIGDTFMALSNGRYKSCLHRAVVNQRRERR
SLAFFLCPREDRVVRPPPSAATPQHYPDFTWADLMRFTQRHYRADTRTLDAFTRWLAPPA
ADAAATAQVEAAS*
>11682.m03288|LOC_Os05g34860.1|genepair496-2
MVYISNAQDASKLIVTAKGGGGEADDAAASSAAVVLDLWRQPAKIPAPFVWPRADVALPP
SSPPTGELDVPVVDLAAALRDAAGMRRAVAQVAAACASHGFFQVSGHGVPPSLARAALDG
AAGFFRLPPAAKQRARRAPGTVTGYTAAHADRFVDNLPWKETLSFGHRHANAAGNNSSTV
ADYFSTLGDDFKHLGEVYQEYCEAMEEVTKAIMAVLGESLGVGGGYYREFFEDSSSIMRC
NYYPPCPEPERTLGTGPHCDPSALTVLLQDGDVDGLQVLVAGAWRPVRPLPGAFVVNIGD
TFMVCTLVLGRDMDLGSIDRRVVSSCPAMMVSHRDLDVRRRLAPLAPPVLLASAVRQRTS
SYSLAS*
>11667.m06643|LOC_Os01g66120.1|genepair497-1
MSGGQDLQLPPGFRFHPTDEELVMHYLCRRCAGLPIAVPIIAEIDLYKFDPWQLPRMALY
GEKEWYFFSPRDRKYPNGSRPNRAAGSGYWKATGADKPVGSPKPVAIKKALVFYAGKAPK
GEKTNWIMHEYRLADVDRSARKKNSLRLDDWVLCRIYNKKGGLEKPPAAAVAAAGMVSSG

GGVQRKPMVGVNAAVSSPPEQKPVVAGPAFPDLAAYYDRPSDSMPRLHADSSCSEQVLSP
EFACEVQSQPKISEWERTFATVGPINPAASILDPAGSGGLGGLGGGGSDPLLQDILMYWG
KPF*
>11682.m03284|LOC_Os05g34830.1|genepair497-2
MSGGGEGAAAAERQELQLPPGFRFHPTDEELVMHYLCRRCAGLPIAVPIIAEVDLYKFDP
WHLPRMALYGEKEWYFFSPRDRKYPNGSRPNRAAGSGYWKATGADKPVGTPRPVAIKKAL
VFYAGKAPKGDKTNWIMHEYRLADVDRSARKKNTLRLDDWVLCRIYNKKGGVEKPSGGGG
GERSNMMSHGETASAGSPPEQKPAVLPPPPPPYAAAAPFSELAAFYDVRPSDSVPRAHGA
DSSCSEHVLTTSASSGGVVERPEVQSQPKIAEWERTFAGAAAPAGAVSTAGPILGQLDPA
AAVAGGGDPLLQDILMYWGKPF*
>11667.m06649|LOC_Os01g66180.1|genepair498-1
MATFSDAPPGDAAAGEKIFRTKCAYCHAVDKAAGHKHGPNLNGLFGRQSGTAPGFSYPSG
DKIVPVIWEENTLYDYLLTPKKHALALPRCGPKLKIVASLELISDTLGSVIPGITHKYTP
AKMGFNGLKQPQDRADLIAYLKNATA*
>11682.m03278|LOC_Os05g34770.1|genepair498-2
MASFSEAPPGNPKAGEKIFKTKCAQCHTVDKGAGHKQGPNLNGLFGRQSGTTPGYSYSTA
NKNMAVIWEENTLYDYLLNPKKYIPGTKMVFPGLKKPQERADLISYLKEATS*
>11667.m06651|LOC_Os01g66200.1|genepair499-1
MAAEVNQTFFAWSQGEPTERDGSQGVSVSQKIDHGSISFGRFELESLSWEKWSVFSDDKR
HEEFGKFNGLVAQKKAYFEEYYRKIRELKAQQQNQQTELILEYSGDGSDSSQTGEYTQGA
ELETPTGSGTIVDDYVEQGAHETTSEQGLTCYDDHENENFNAEFSSSNISSSAVGLQQTG
RDARENVHGDDSAGKMDLEQQNAISGHSLGTAYEVVRAPKRIIEKDSRLRYAPKIVPKSV
KTSSDSPLDRTSVSKRPDSLKLGMSINQKAKTDNDRLLRGPNVAPHKMSGSTERNKLTTK
QTGVRRPSSASSQRPSVGERHRIARESIKKPADVSTPRRPSTAERHPVTTERARKQADVD
TPRRPSTSERRAVNKGSADMTTTHRPSTGERRSVTRESVLKMDVRTPSKTRPTMTQLKGA
TTTVLNLKNMMASDFLEFSSSCRQYQRTGRTFESGQTLCWVKNYGPANCWQTEIKLCPPS
STETIEFQYWRTSTANLYKAKKERCCSAISSIYIEESNDFAHWKRKGEEFKSTPTTTSTA
SAFTDDEQTKWQFFPRGLLYNGIPFLNGMIPVRCQVRYQMIHIRYQTILPRFYDTCEVSD
DTYQVSCDSTTYQVILVRYQVLIPNRYQMILISRRRHPRVVSSSGRGQSISVLTTGDETE
TAAWFSNTLDDALEKDLYTQLWHSVTSDAFPAAAGPSSHHPPTPDLPPSDEATDEERHRV
ELDRQHLFDLLRQ*
>11682.m03277|LOC_Os05g34760.1|genepair499-2
MATEVTQNYFAWSQEESPVQDSSQGTPQVFDHGSISFGRFELESLAWEKWSVFANDRRHE
EFGKFNGLVAKKKAYFEEYFKRIRELKALQQQNQQTELNLDYSGDGSDSSQTGEDVPTAD
QASPSGSGTLLDSMVQTGVQTIFENDLECYDDNDKEMLDKDISPSVGGTCQIEQEFRESA
SGGNHPDRMVDVLQQNTNCGPDDLGRPMESMMTPKRTVKKDSLVGQAAKTMPKTVNMTSS
NIPGHAVVNKGTDSGKSSVVNRRAKPETIQQRLKAVTGNIVDIVGRSKLVVKEVPGIMGV
RRPSSPALQRPSTRERRPVTRDSSRKAPEVATMCRPSTAERRPATRELAPKQANTVVPCR
PSTPNRRPMTRELAPVHSSIATPRRPSTAERRPITRGMAPMHPSIATPVRPSTAERRPTS
KQMAQKHVGMATPSRPSTAERRPITREAARKNADVAILHRPSTAERRPITRETPQKHANV
VALHRPSTAERRPVVREIAPKHADVTLTPARRPSTSERRPVTRETALRHSNFTGSCWPLT
PQRHISRGSAQIHADVSTTPRHPSTGERRPITKESNIKLDEKTPIKLRGMLANPKGAMAT
VVTPQKAITQKLVKSSKPEMKSCAKERTELQAVGKHKASSVNLPPREMFTSNVRANRVPE
SFRKPNKGIQETARSQISSSKSATPAQTRSIKTRAPNPPPPPPPPRRPSQISSKTNTNNL
SVGGRKPKASTPHWH*
>11667.m06658|LOC_Os01g66270.1|genepair500-1
MSRAECGGGEEEERCRYRGVRRRRWGKWVSEIRVPGTRERLWLGSYATPEAAAVAHDTAV
YFLRGGAGDGGGGGATLNFPERAAATYGGGAAVARLSPRSVQRVASDAGMAADAQLVAAR
DAAPAPAPATAYARPDHCAGATTARHDELARRGMYGAHAHAAGANARTSGERQLVCAEEI
SVDDMEILM*
>11682.m03274|LOC_Os05g34730.1|genepair500-2
MGRVAASGGGGGGGEMMRYRGVRRRRWGKWVSEIRVPGTRERLWLGSYATAEAAAVAHDA
AVCLLRLGGGRRAAAGGGGGLNFPARALAAAAAASSYGGAGGLLSPRSVQRVASDAGMAA
DAQLVDLRRDHPPAAAAASSSGSGVAGDGARKQGTRGEVSDTYWCRNGEDGSRSRSSGSE
ELIVYEGLSVDDMEILM*
>11667.m06659|LOC_Os01g66280.1|genepair501-1
MWALTPNSGGSSCLPARRTPPPLAAAGEAGSLAAGPGRWCSWRRRQPAERWPKLAVSASG
RKSKGGRDEGGGDEPKKNKAASSSSSGKGDASAPSGDVSNNELQSNDTMYVPGNLSYWRD
VRASFVVPKVQTVDAHTLPQAATDAPVHCLPRKWAHSIPMPESGCVLVAAEELDGNGTFE
RTVILLLRLGSRDAYDGPFGVILNRPLYTKMKHVNPSFRNQATPFSDCSLFFGGPVDMSI
FLMRTTDDRPIKGFEEVSPGVCFGFRTDLEKASALLKSGAVKPEDLNFYVGYSAWDYDQL
LSEIDQGYWHVTSCSSGLISDSLATDPSCLWTEILKLMGGQYAELSQKPKEDGS*
>11682.m03273|LOC_Os05g34720.1|genepair501-2
MWALNLKAGGGGPCLTPRRRPLAAGEAAPCSVAAAWAGRRVGGRRGMALVAVGASGRRGK
DGPGGGDGGDDEAKSKASSSSGNDDAASTGDSSDGLNQLHNESKSNISNSNYWRDVRANL
VRREQELLVDPSAPAEQKTSSGEPAHQLPQKWAHPITMPEAGCVLVATEVLDDDSIFERT
VILLLRLGSRGTFDSPFGVILNRPLYTKIKNVNPSFQDQATPFGDSPLFFGGPVDMSMFL
VRASDNSRLKGFEEVIPGIRFGFRTDLEKAAVLMKSGAIKSQDLRFFVGHAAWDYEQLLS
EIRAGYWAVASCSTELISDALTGDPSCLWTEILQLMGGHYSELSQKPKEDNQ*
>11667.m06661|LOC_Os01g66300.1|genepair502-1
MDDRIPPPSPLQYSPSPVHSSPHPLSSLRYSSSERERYLAELLAERQKLAPFVQVLPFCT
RLLNQEILRASSLPPNHNFVDPERIEHGSPLRLPGLPVNGQPMDLEGWSGMQTENMRVLQ
ASSMGWNGPPAITGTPVVKKVVRLDVPVDKYPNYNFVGRLLGPRGNSLKRVEASTQCRVY
IRGRGSVKDSVKEDKLRDKPGYEHLNEPLHVLVEAEFPADIIDTRLNQAVTILEDLLKPI

DESMDYYKKQQLRELAILNGTLREESPSPHLSPSVSPFNSTGMKRAKTGR*
>11682.m03267|LOC_Os05g34670.1|genepair502-2
MDERIPPPAFFQFLPSGAHSSPHHQSPLRSPASERERYLAELLAERQKLAPFMQVLPFCN
RLLNQEILRASSLPPNPNFVEPERVNHGSPLRLTGHPMNGQPMDLEGWSGMQTEMGVLQS
PSMGWNVAPGVAGSPVVKKVVRIDVPVDKYPNYNFVGRLLGPRGNSLKRVEATTQCRVYI
RGRGSVKDSVKEDKLRDKPGYEHLNDPLHVLVEAEFPSDIVDVRLNQAVAILEDLLKPVD
ESMDYYKKQQLRELAILNGTLREESPSPHLSPSPSVSPFNSTGMKRAKTGR*
>11667.m06666|LOC_Os01g66350.1|genepair503-1
MAPTVGIKRSAAAVATQTISVPPPDARFAVREAVRATIASPPVEAPPAAAGKAAAPAPAV
EGFLCLEEVDGRRWSYVVDGGQGKGKGRGRGRSGAAVPMGASVRAVPLQSPLPPAEEVMA
FIRSYVVPEGFPDSVTPSYVPYMTWRALKHFFGGAMGVFTTRTLLSSVGVSQSKVTPGAI
AINWILKDGAGRVGKMLFARQGKKFDYDLKQLRFSSDLLLEIGAGIELATAAFPQFFLPM
ACVANVVKNVAAVTSTSTRTPIYKAYARGENIGDVTAKGESVGNIADLVRSVVLNTLNRA
RFTVAVDSFIKTGHIPSLKEGNSQETIFNPPWRHEPVAIGSRFGEAFQEPASFVAIRPLF
EDERYMVTYNPAKDKVYALLKDQAKSDDIIKAAFHAHVLLHFINASHARKQMNSRRSDPY
GNPCNMDFMAHIAESCKIVSSSYGTFKKKAREQGWIMSESLLNPGKARLCPARP*
>11682.m03265|LOC_Os05g34650.1|genepair503-2
MAPAMGMKRPPAAAAATATQTVTLPAPDARAAVRDAVRVAVREAEPPQAPALPPRAPAPA
VAVDGVLCLEEVDGRKWSYVVEGGASPGKAGRVSGSGRGRGRGGAASPMGVTFKAVPLQS
PLPPVEEIMSFIRSYVVPEGFPDSVTPSYVPYMTWRALKHFFGGAMGVFTTRTLLNSVGV
AQSRATSGAVAINWILKDGAGRVGKMLFARQGKKFDYDLKQLRFSGDLLMELGAGIELAT
AAFPQLFLPMACIANVVKNVAAVTSTSTRTPIYKAYAKGENIGDVTAKGESVGNIADLLG
TGLSILISKRNPSLVTSFAFLSCGYLLSSYHEVRSVVLNTLNTARFTVAVDSFIKSGHVP
SLKEGNSQETIFNPPWRHQPVAIGSRFGEAFQEPASFVAIRPLFEDERYIVTYNPTKDKV
YALLKDQAKPDDILKAAFHAHVLLHFINASHANLNARKRMNSNRSYQNANPLNMDFIPHI
AESCKIVTSSYGVFKKKAREQGWIMSESLLNPGRARLCGIVPQ*
>11667.m06673|LOC_Os01g66420.1|genepair504-1
MEGGGGGGGGGGGGGGGGGGGGAPYATRTAEEVFRDLRGRRAGMIKALTTDVEKFYKLCD
PEKENLCLYGYPNETWEVTLPAEEVPPEIPEPALGINFARDGMNEKDWLALVAVHSDSWL
LSVAFYFGARFGFDREARRRLFNMINNLPTIFEVVTGAAKKQAKEKTPNSSSKSNKPSSK
VQSKAESRSKSKLSAPKDEEGSGDDEGEEEEDDHDNTLCGTCGTNDGKDEFWICCDNCEK
WYHGKCVKITPARAEHIKQYKCPDCTNKRARA*
>11682.m03264|LOC_Os05g34640.1|genepair504-2
MDGGSGGPYTSRTAEEVFRDFRGRRAGMIKALTTDVEKFYQLCDPEKENLCLYGYPNETW
EVTLPAEEVPPEIPEPALGINFARDGMNEKDWLALVAVHSDSWLLAVAFYFAARFGFDKE
ARRRLFNMINNLPTIFEVVTGAAKKQTKEKAPNSTNKPNKPSSKMQPRPESHSKAPKPPA
PPKDDDESGDEYADEEEEERDNTLCGSCGTNDGKDEFWICCDSCERWYHGKCVKITPARA
EHIKHYKCPDCGNKRARA*
>11667.m06680|LOC_Os01g66490.1|genepair505-1
MHMRRGRAAGGEGEAAAVVVMNRYDNNGHAAAAAAAAVAGGGGGGGGNKAAGEVDGHEDD
LVMPGFRFHPTEEELIEFYLRRKVEGKRFNVELITFLDLYRYDPWELPGIEHLINKLDGG
AAMAAIGEKEWFFYVPRDRKYRNGDRPNRVTASGYWKATGADRMIRAENNRPIGLKKTLV
FYSGKAPKGVRSSWIMNEYRLPPADTDRYHKTEISLCRVYKRTGIDDGHGQVSTARSSAH
SRGGGAAPVQDNKQGSSSTSTPTPPPTPSKLHLLSSECTSPPAIVTDHAAMVAHKAPSPR
HHQQQQQLHAAKPCGGYLQNSSMASAAGGDQQQQFQQDFAAALYQQYSKNTSGAFASTYS
LLNLVNAASMGSSAAAIDELSSLVGHGTPSYINPAAGSHNYSQFLHLPTTPSSHQPTPAP
LGTTTAAAAATLPMSLAAFSDRIWDWNNPIPEAGGRDYSTSTGFK*
>11682.m03260|LOC_Os05g34600.1|genepair505-2
MSRDDVDDVTAGAAGSGEEAAADQEEAAAAVAGDSHENDLVMPGFRFHPTEEELIEFYLR
RKVEGRRFNVELITFLDLYRFDPWELPAMAVIGEKEWFFYVPRDRKYRNGDRPNRVTASG
YWKATGADRMIRGENSRPIGLKKTLVFYSGKAPKGVRSSWIMNEYRLPPPAADADLFYKS
EISLCRVYKRSGIDDGHGHHQRPAGNVQASSSSAAARPPEQHSGNNTAAGLPACRHRPSP
SSSSTTTAQQHTSFHQLLQGECSAAAAAPPPPPSLPASATTRNSNASQLLMPPPPPRPPC
AAAYTSAAAAPTESAAVLAAASTYSLLAAAGSSSTHIDELSTLLAGHSHGGAYGNNHIVA
GSHHHFPLPPSQLMPQLGTLPISPPLAAVSDKLWDWSSVPDTSTARDYDSSGFSDPK*
>11667.m06682|LOC_Os01g66510.1|genepair506-1
MARAEAAAALEFTPTWIVAAICSLIVLISLAAERCLHYLGKTLKRKNQKALFEALLKVKE
ELMLLGFISLLLTVSQGILQKTCVPPKWTNYLLPCRKMEDQSKQRGPSEAHFVAAGVLGH
LGRRLLADGGTGADHCQNKGKVPLLSLEALHQLHIFIFVLAITHVIFSALTMLLGGAKAY
DSYSSWLYFFQNFIEQIHQWKHWENDIQKDVAQNAPKKVTHVHQFEFIRERFKGIGKDSI
ILSWLHSFVKQFSGSVTKSDYITMRLGFIQTHCRANPKFDFHRYMVRALEADFKKVVGIS
WYLWIFVMIFLLLNVNGWHTYFWISFVPLLLLLAVGTKLEHVITQLAHEVAEKHSAIEGD
LVVNPSDEHFWFGRPKVILYLIHFILFQNAFEIAFFFWILTTYGFNSCIMDHVPFILTRL
IIGAIVQILCSYSTLPIYAIVTQMGSFFKKEIFDEHVQQGLVGWAQKAKKRKGLKESNGA
MAGAGSTNGSSQPSSILQMVRRAAASEEGSSNGGDMRTNQ*
>11682.m03255|LOC_Os05g34550.1|genepair506-2
MAAEAATLEFTPTWIVAAVCSLMVLISLVAERCLHYLGKMLKRKNQKPLYEALLKVKEEL
MLLGFISLLLTVFQGLIQRTCIPPRWTVHMLPCQREAVGPAKEHVAAAQIVGRIGRRLLS
EGGAGAELCQKKGKVPLLSLEAIHQLHIFIFVLAITHVIFSVSTMLLGGAKIHQWKQWEV
EIQKDAVGSGQPGPAKVTHVHQFEFINDHFKGMGKDSKILSWLHSFVKQFYGSVSKSDYT
TMRLGFIMTHCRGNPKFDFHKYMMRVLESDFKKVVGISWYLWVFVVIFLLLNVNGWHTYF
WIAFLPLILLLAVGTKLEHVIAQLAHDVAEKNSAIEGDLVVKPSDDHFWLGRPRIILYLI
HFILFQNAFEIAFFFWILTTYGFSSCIMGQVSFIVPRLVIGVTIQLLCSYSTMPLYAVVT
QMGSFYKKEIFNDHVQQGVLGWAQKVKMRKGLKEGSAGAGVAAEPSNGGGGDTAGPSVKI

EMMRRAAREGNDEAGVSIE*
>11667.m06686|LOC_Os01g66550.1|genepair507-1
MEGLIPFIYKAIKERRSRTYSLCSSDMSAARRFGRVGEEEEDVVAWEEQKQWAVDGGKFA
GGEREMTAHRRHRSLEELAGEVGASPQWRQQGGLARGRSARIFSCISGM*
>11682.m03250|LOC_Os05g34500.1|genepair507-2
MEGLIPLLYKAIKDRRGGRPDGGAAAGVDLYDPEQRRLWLEQEVRSPLHPSAAAAGGQHQ
QQQQPHRRNLSLEELAGEVGLSPGRRLPRVALPKARSVRVFSCIGAGAGVAA*
>11667.m06690|LOC_Os01g66590.1|genepair508-1
MASSSASSVPAPSGSVITIASASASASAAANTAACGTGSPCAACKFLRRKCQPDCVFAPYFP
PDNPQKFVHVHRVFGASNVTKLLNELHPYQREDAVNSLAYEADMRLRDPVYGCVAIISIL
QRNLRQLQQDLARAKFELSKYQQAAAAAAAASASTGTNNGPHSMAEFIGNAVPNGAQSFI
NVGHSAALASVGGAAACFGQEQQFSAVHMLSRSYEGEPIARLGGNGGYEFGYSTSMAGGG
HMSGLGALGGAPFLKSGIAGSDERQGAGQLLTRKLKFPKFWKLKREFACTKGKMAGKRKP
MIRYRKTRCGGLVCLPCFAHDMAHACRECMMHISVAVLASFSLSKALGLLFEEAEGMLQR
VAGPNNTRKRERECVMPSMREGRRAGLPKMSSSMQEIFNAHDPAFMAAYTSALYGHTINS
CNTQADRLVPWLTIAI*
>11682.m03245|LOC_Os05g34450.1|genepair508-2
MAASSSASSLPLSSGSVITVSSSPAVGASSGGGGGTGSPCAACKFLRRKCQPDCVFAPYF
PPDNPQKFVHVHRVFGASNVTKLLNELHPYQREDAVNSLAYEADMRLRDPVYGCVGVISV
LQHQLRQLQQDLSRARFELSKYQAAVAVSSSNGQAAAAAAMVDHFIGSAVQPNCTQNFIS
GGGVAAAAIGIGGGAGGGFVNDHHQFSAVQMLARSYDGESAVARLGGAVNVSGGGGYEFG
YSSAMGGAGGPMSGLGPLGGGTFLKPGTAGGGDRPTAAQ*
>11667.m06696|LOC_Os01g66640.1|genepair509-1
MAPRLSACLVFLLILVLSLQESPLHAADTLTAEQPLSADQKLISQDGKFALGFFQPAAGG
SSSRWYIGIWYNKIPVQTVVWVANRDKPITDPTSSNLTILNDGNIVLLVNHSESPVWSTN
IVNNTIASSPVAVLLDSGNLVVRHESNTSEVLWQSFDDFTDTWLPGNKLSRNKKTGVIKR
MISWKDRADPAPGMFSIQLDPSGATQYILLWNSSSVYWASGNWTGNTYTGVPELSPTNSD
PNSAYTFQFVDNDQETYFNYTVKNDAQLTRGVIDVSGHFQAWVWADAAQAWQLFFAQPKA
KCSVYGMCGTYSKCSENAELSCSCLKGFSESYPNSWRLGDQTAGCRRNLPLQCGNNGSVK
AKQDRFFMISSVKLPDMAHTRDVTNVHNCELTCLKNCSCSAYSYNGTCLVWYNGLINLQD
NMGELSNSIFIRLSASELPQSGKMKWWIVGIIIGGLVLSSGVSILYFLGRRRTIGINRDD
GKLITFKYNELQFLTRNFSERLGVGSFGSVYKGILPDATTLAVKKLEGLRQGEKQFRAEV
STIGNIQHINLIRLLGFCSEGAKRLLVYEYMPNGSLDHHLFQNNSAISSWKRRYQIAIGI
AKGLAYLHDGCRDCIIHCDIKPQNILLDMSFTPKVADFGMAKLLGRDFSRVLTSIRGTIG
YLAPEWISGESITTKADVFSYGMMLFEIISRKRNLTQTETRTEIFFPVLVARKLVQGEVL
TLLDSELVDDVNLEELERACKVACWCIQDDESSRPTMAEVLQMLEGLVDIEVPPAPRYLQ
VLAEGAASKT*
>11682.m03239|LOC_Os05g34390.1|genepair509-2
MAWGGSGAAGSWRGCVGGGRRRHALPREVNDHGEDAAARPSLSTAWVTDAATSSRAWGSS
GVAGSWRRRRRMMEQLRQVGEAIGGVNALLAHAYALAFHAVAAVEVRARRRRRGRRCEVA
ATVGVLEAAVVGAVQGGALGGLMGTLASDSGSQAAARGEANPLAESAAASYALAAASAES
QLPPLPPTPPHRHRSRSAARSALMATAFCERRAGSSSLSPPRSRSRRVEGKTKGGAISSR
PRRSRDGSRTTAAALGGWGGGQGGSRDGMGKGGEREERGGGTHTRWPAVLAGQLWLRTAD
AVEDTITASRPLSGNQKLVSRGEKFALGFFRSAGGHLKKWYIAIWYNKVSIQTAVWIANR
EAPISNLDESQLAISQDGTLVLLNQSRSVVWSSNVPNVTSSNVDSSEAKTVAVLLNTGNL
ALPYLTDGKYDPSTGAFSGIPEMTPIRNSIYAFQYVDNNEEAYFMVTVKNDNILFRLTID
VSSQAKSTVWVADRNQWMLFFLQPKEKLAASELPDSKTKKWRVVSIIIGGFILLVCGVIT
CICFLRKRTMKAIIPIAVDGHLTTLKYSDLQLITKSFSEKLGSGSFGSVFKGALPDKTVV
AVKKLEGFRQGEKQVRAEMSTIRTIHHINLVRLLGFCSHGAQRLLVCEHMQNGSLDRHLF
VNNAGALSWSRRYQIAIGISKGLPYLHERCRDCIIHCDIKPDNILLDASFVPKVADFGLA
KLLGRDFSRVLTSMRGTIGYLAHDVQTLLDPESVDVIDLEELGRACKVACWCVQDEESSR
PSMGEIVQILEGFVDVSIPPVPRYLHVLAERANHVEISINE*
>11667.m06702|LOC_Os01g66700.1|genepair510-1
MAQALSLGLLLAFLAIQSCIAIELTDHIDLWPMPTSVSHGTQRLYVSKDITMSMEGSTYP
DGKGILKDAFQRVVDLMKLNHVVDGANPSSFVLTGVNVVVHSPEDELKFGVDESYNLSVP
TAGYPLRVQIEAQTVFGALHALQTFSQLCYFDFTSKLIELISAPWRISDTPRFPYRGLLI
DTSRHYLPVTVIKKVIDTMAYSKLNVLHWHIVDAQSFPIEIPSYPKLWNGSYSFSERYTT
SDAVDIVRYAENRGVNVMAEIDVPGHALSWGVGYPSLWPSDSCKEPLDVSNNFTFGVIDG
ILSDFSKVFKFKFVHLGGDEVNTSCWTATPHIKKWLDDNQMNVSDAYRYFVLRSQKLAIS
HGYDVINWEETFNNFGDKLDRRTVVHNWLGEDVAPKVVAAGLRCIVSNQDKWYLDHLDAT
WEGFYTNEPLKGIDDPEQQSLVIGGEVCMWGEQIDASDIEQTIWPRAAAAAERLWTPIEK
IAEDPRLVTSRLARFRCLLNQRGVAAAPVAGYGRTAPYEPGPCVRQ*
>11682.m03232|LOC_Os05g34320.1|genepair510-2
MALRLLVVVAVAAVVSCAVAADEGSVVEVWPMPATASKGGQTLHVSRELRMTAEGSKYAD
GEAILKDAFQRMVTLIELDHVINGSSQGLPLLAGVNVVVHLPGDELNFGVDESYNLSVPA
TGSPIYAQIEAQTVFGALHALETFSQLCNFDFTSRLIELQSAPWSITDMPRFPYRGLLID
TSRHYLPVPVIKSVIDSMTYSKLNVLHWHIVDEQSFPIEIPSYPKLWNGAYSYSERYTMD
DAIDIVQYAERRGVNVLAEIDVPGHALSWGVGYPSLWPSATCKEPLDVSSESTFQVINGI
LSDFSKVFKFKFVHLGGDEVNTSCWTSTPRVKAWLAQHGMKESDAYRYFVLRAQKIAKSH
GYEVINWEETFNNFGDKLDRRTVVHNWLGGGVAEKVVAAGLRCIVSNQDKWYLDHLEVTW
DGFYMNEPLRNIKNPAQQKLMRLTFSKPFGHVLQQLQLTPNNLDYNNPKLKETCLTGRPI
DRALAQVASVAGGHAARERSNPRSRHYVNMDNARKFYNLKRRLYSSEEAISASLHEDHSS
ARLKNSFVFSSGYLIVSMFYLQATTNMELTSSSAMWLAMAILAITAALTKIALGGGRRRC
LSESSDLTCKTPPPPPVVNCIALLGLLPALFRGDVPATMQQLYAKFGSVFTVSVAGLLKA

TFLVGPEVSAHFFQGLESEVSHGDLFEFTVPMFGKEVGHGVDNATRIEQGRFFAEALKPV
RLRIHVDPMVQEVEDYFAKWGQHGTVDLKHELEQLLLLISGRCLLGKEVMGTKFDEVCNL
FRDIEGGVNLMSVFFPYTPLIPSNRRRDMARERLHAIFSDIVRSRKQQQGDQEEVNDKDV
LQSFIDSRYKADGRATTEAEVAGLITGVLFAAKHTSTHTSVWTGARLLTHEKFLAAAVDE
QDQIVRKHGIINGRIVTDHYGFLMEMHMLHICIKETLRLHPPAPMIVRTALRQFTVRTRE
GHEYCVPAGHTMASPIVISNRVPYIYKDAHLYDPDRFGPRREEDKVGGKFSYTSFGGGRN
SCVGENYAYMQIKAIWSHLLRNFELKLLSPFPKTDWSKLVPEPQGKVMEKKRKYNSEELA
RYTLLKEGNGGGSESARLPHHQVYVIFSYHLILMYFYITR*
>11667.m06714|LOC_Os01g66820.1|genepair511-1
MADGAKLLLWLLLLSSSPWCFCSELDVQCLETLYRSVIDSNGILQSSWTFVDNGTDGYIC
KFTGVECWHPDENRVLSLRLGNLGLQGPFPQGLQSCTSMIGLDLSSNIFSGPIPADIATQ
VPCLTSLDLSYNSFSGPIPASISNMTYLNTLNLQHNQFSGEIPRQFDSIGRLISFNVADN
LLSGPIPSSLQKFSASNFAENQGLCGAPLDNCPRRRRWRLRLHRINDESSIGAAVGFVVG
FVVAFYFPHWFVFSKRLHPYIFQIWSIPSGNLSANLRRT*
>11682.m03227|LOC_Os05g34270.1|genepair511-2
MTDHYYAPLRLILLLILSATCFGSELDVQCLKTIFQSVTDPNGILKSSWSFVNNGTPGYI
CKFTGVECWHPDENRVLSLRLGNLGLQGPFPAGLQNCTSMTGLDLSSNNFTGLIPQDISQ
QIPYLTSLDLSYNRFSGQIPVNISNMTYLNTLNLQHNQFTGQIPLQFNLLGRLTSFNVAE
NRLSGPIPNNLNKFPSSNFAGNQGLCGLPLDGCQASAKSKNNAAIIGAVVGVVVVIIIGV
IIVFFCLRKLPAKKPKVEEENKWAKSIKGTKTIKVSMFENPVSKMKLSDLMKATNEFCKE
NIIGTGRTGTMYRAVLPDGSFLAVKRLQDSQHSETQFTSEMKTLGQVRHRNLVPLLGFCI
AKRERLLVYKHMPKGSLYDQLNQEEGKDCKMDWTLRLRIGIGAAKGLAYLHHTCNPRVLH
RNISSKCILLDEDYEPKISDFGLARLMNPIDTHLSTFVNGEFGDLGYVAPEYARTLVATP
KGDVYSFGVVLLELITGERPTHVSTAPENFRGSLVEWINYLSNNALLQDAVDKSLIGKGS
DGELMQFLKVACSCTISTPKERPTMFEVYQLLRAIGEKYHFSAEDDLMMLPPLSTDGETL
DELIVAQ*
>11667.m04564|LOC_Os01g47270.1|genepair512-1
MEDRDRDHRGGADEDAPGPAAAAVAAVDGEDEVEDDGGGFSFPVPPFAADAFIVPVYPVF
GRPMSPPPREAVVEEEEEDEPETATLRVPLGRLLLEEREFRARQRESSGTSPVQPQRRRP
DDEGELEGVPPESYCLWAPGGQPSTTPASPRRCRKSGSTGSVLRWRRISERLVRRSHSDG
KEKFVFLNAPGGGAPSPHPPKDNDDANGGGSVGKGDAGRHGWSYYSKGGGGGSGGRRRSY
LPYKQELVGLFANVSGLRRSYHPF*
>11682.m04740|LOC_Os05g49350.1|genepair512-2
MAEADGDRGVAAAAAAAAVVHDDVLESDEEDFTFAAAAAVTCVVGGGRIGAVVYPVFGRP
RSPPPVQEVEEPDTATVRVPLGQLLLEERASAPPSGEQADEDGVLDGVPAETYCLWSPGS
PAPAVSNSPARCQKSGSTGSVLRWRQRLIGRSHSDGKEKFVFLSSGSDVRSKGRTTTTSR
GDAGGRGGGWRYYASGGGNGGGRRPSFLPYKQDLVGLFANAGAFRRSYHPF*
>11667.m04565|LOC_Os01g47280.1|genepair513-1
MDRLQQQRRRHEALTGRRRGGGGGMSRRHGRFRFATGGGDGDGDEECAGVAVVDQADCTA
QSCRSCVAVSLADCIALGCCPCAVVSLLGLAFVKLPLAVARRCARRLRRRQGRLRQKKRV
RDLDAAKNAAGGGHQEPLPGGAAAASKGEDGDVVVVAAAAAASPGSDDAENVWLELYQVG
RWGFGRLSVSAANPPVRPSYVVATARNADCAADGDVS*
>11682.m04739|LOC_Os05g49340.1|genepair513-2
MQLQRPRRWRDGDGDGEEGPTTAGGGGGVGCRCRSCAAVMLADCIALGCCPCALVSMLSL
ALVKAPLAVGRRCVGRLRSRRRTLLHNKRVRDVAATSAAAAGEKKAIAKPEEELEAADIV
ANDDDTASAAAATAGAGAPTDEDLAWLEEMYQMGHWGFGRVSISGKTP*
>11667.m04571|LOC_Os01g47330.1|genepair514-1
MASTALSSAFSLLSLPSSSSPAAAAAAAPRSFAVPSRARPRRAVAVVASTATESPKVLEL
GDAIAGLTLEEARGLVDHLQERLGVSAAAFAPAAVVAAPGAGGAGAAADEAPAEKTEFDV
VIEEVPSSARIASIKVVRALTNLALKEAKDLIEGLPKKVKEGVSKDEAEDAKKQLEEVGA
KVSIA*
>11682.m04737|LOC_Os05g49320.1|genepair514-2
MAMASTAVSSTLPLFHVRTASSAPAALRFTTRGRGGGRHSVACNSTAASSPKVLELGDAI
AGLTLEEARGLVDHLQERLGVSAAAFAPAAVVAAPGAAGGGEDGAPAEKTEFDVVIEEVP
SSARIATIKVVRALTNLALKEAKDLIEGLPKKAKEAVSKEEAEEAKKQLEEVGAKVSIA*
>11667.m04572|LOC_Os01g47340.1|genepair515-1
MLRAGGRRLLAPGLRRVLGGGAAAPVAVGGAKAYHERVVDHYENPRNVGSFENDDPSVGT
GLVGAPACGDVMKLQIRVDESSGKIVDACFKTFGCGSAIASSSVATEWVKGKQMEEVVTI
KNTEIAKHLSLPPVKLHCSMLAEDAIKAAVKDYEAKKAKLAQKGEEKAAEA*
>11682.m04735|LOC_Os05g49300.1|genepair515-2
MLRAAGKRLLGVGLRPAGGGAGEAAAAAAASAVAVVRRRGYHERVVDHYDNPRNVGTFDK
DDPDVGTGLVGAPACGDVMKLQIRVDEESGRIVDACFKTFGCGSAIASSSVASEWVKGKQ
MEDAASIKNSEIAKHLSLPPVKLHCSMLAEDAIKAAVKDYEAKKAKLDKGNE*
>11667.m04575|LOC_Os01g47360.1|genepair516-1
MLHEAAPCTCGLLYGSCGGGCSLLFPAGAPGDHHHHHHYKQYCGAGDGEYPDVPYGGGGS
VDCTLSLGTPSTRRAEAAVAGLPWDQSSLQPSCNGRQEMSGAAAPRTEPSGGAGAAAASA
PRRCANCDTTSTPLWRNGPRGPKSLCNACGIRYKKEERRAAAAAVAPTALASDGGVEYAY
GYPRQQQQWGCYGPAVAKAASFGMFGDAAGEDGPCLPWGLGVMPSSPAFGAVREMPSLFQ
YY*
>11682.m04731|LOC_Os05g49280.1|genepair516-2
MAGGQYYYRQCGGVAEEDSRSPYGGGGAAVDCTLSLGTPSTRRAEAGAYGGGLQPWDVPS
SARPGGGGGGKQDGAGVAPCNKEAPAAGRLPRRCANCDTMSTPLWRNGPRGPKSLCNACG
IRYKKEERRAAAAVAPTPPPSLDTGAGYAYCYSRQPPPPPAPQWGCYGQAAAKSASYAMF
DAADDGPCLSWRLNMMPSSPAFAVGERPGLFQYY*

>11667.m04576|LOC_Os01g47370.1|genepair517-1
MSSRSSSSSRNSNSVNMDSEWSKKENKLFEEALAYYGEGAPDLFHKVSRAMGGTKTADEV
RRHYEILEDDLKLIEARRVPFPKYNTQGAWN*
>11682.m04726|LOC_Os05g49240.1|genepair517-2
MSGSRSSSPNSKSEWSRKENKMFEEALAYYGEDTPNRWDKVASAMGGIKSAEEIRCHYED
LTDDVKTIESGRVQFPKYKTQGYWT*
>11667.m04580|LOC_Os01g47410.1|genepair518-1
MGTRRVGLLLLLLLAAVLLQPLLAAAAAEGVVRIALKKRQVDETGRVGGHLAGEDAQRLL
ARRHGFLTNDAARAASRKARAEAEGDIVALKNYLNAQYYGEIAIGTPPQMFTVIFDTGSS
NLWVPSSKCHLSIACYFHSRYKAGQSSTYKKNGKPASIHYGTGAISGYFSQDSVKVGDVA
VKNQDFIEATREPSITFMVAKFDGILGLGFKEISVGNAVPIWYNMVRQGLVVDPVFSFWF
NRHADEGQGGEIVFGGIDPNHYKGNHTYVPVTRKGYWQFNMGDVLIGGNSTGFCAAGCAA
IADSGTSLLTGPTAIITQINEKIGATGVVSQECKAVVSQYGQQILDQLRAETKPAKVCSS
VGLCTFDGTHGVSAGIRSVVDDEVGKSSGPFSSAMCNACETAVVWMHTQLAQNQTQDLVL
QYIDQLCDRLPSPMGESSVDCSSLASMPDIAFTIGGNKFVLKPEQYILKVGEGTATQCIS
GFTAMDIPPPRGPLWILGDVFMGAYHTVFDYGNLKVGFAEAA*
>11682.m04720|LOC_Os05g49200.1|genepair518-2
MGTRSVALVLLAAVLLQALLPASAAEGLVRIALKKRPIDENSRVAARLSGEEGARRLGLR
GANSLGGGGGEGDIVALKNYMNAQYFGEIGVGTPPQKFTVIFDTGSSNLWVPSAKCYFSI
ACFFHSRYKSGQSSTYQKNGKPAAIQYGTGSIAGFFSEDSVTVGDLVVKDQEFIEATKEP
GLTFMVAKFDGILGLGFQEISVGDAVPVWYKMVEQGLVSEPVFSFWFNRHSDEGEGGEIV
FGGMDPSHYKGNHTYVPVSQKGYWQFEMGDVLIGGKTTGFCASGCSAIADSGTSLLAGPT
AIITEINEKIGATGVVSQECKTVVSQYGQQILDLLLAETQPSKICSQVGLCTFDGKHGVS
AGIKSVVDDEAGESNGLQSGPMCNACEMAVVWMQNQLAQNKTQDLILNYINQLCDKLPSP
MGESSVDCGSLASMPEISFTIGGKKFALKPEEYILKVGEGAAAQCISGFTAMDIPPPRGP
LWILGDVFMGAYHTVFDYGKMRVGFAKSA*
>11667.m04583|LOC_Os01g47430.1|genepair519-1
MSGGGGGGGGGGGGGRGAAAGPVPGSARKLVQGLKEIVNRPDAEIYAALRDCGMDPDEAV
SRLLSQDTFQEVKSKRDKKKEVNKEALEPKSRGAVNSNSRATRAGADRSGRSSSVQSGSS
GADNMSSRSSILGPGVLATNSTQKQTVPSSTVNKDVLHDGSFGAMQPSSGFQNSWCGVPG
QMSMADIVKMGRPQVRSSSKPVASTDNAYSGQTSSFSSVVNQNPNKSASTAPPTTFEQRF
PVLQDPIPQVKNSSHASADNHETQESDWFQQDGTLPGSQLTVPETSRDISLPVASLESST
LVADDANSYNNSHVGESSSAIPSDRHLEIIDGNNHFNNGLLHNSSAYQSRGNHYDDNDDD
DDEAEVSNVDVESASANIQHLSLQTEDLVASKSTEDNPAVIIPDHLQLANADCGHLSFGS
FGSGAFSGLLPSKVHKNGVEEVPVPDESPSVDQEDVRNQDNNVALNSSTNGDVEARIGTS
MENTDEPSVSQPDILTQGAVDVSSLQYNLPSVSDHVYSNTTQPSTMESSQGDIQVQHLPQ
FSSLLQANTLHNNLLGSNLPNLRDFDFSPLLSTQLATKYNPPVPTTSLPAISMQETLKPG
GFSNAQPTQNLPSASIPSGPPLPQQLSVHPYPQPTLPLGPFSNLVGYPYLPQNYYLPSAA
FQQSFSSNGPFHQSAATTGVPGVSMKYSMPQYKGSLPATSPPQPSSVVSGFGGFGSSNNI
PGNFGLNQNVPSAPTTMGFEEALSTQFKDNSQYIALQQNDNSAMWLHGAAGSRAVSAVPP
GNFYGFQGQNQPGGFRQGQQPSQYGGLGYPSFYQSQAGLPQEHPQNLTEGTLNSSQTTPS
QPSHQIWQHIY*
>11682.m04718|LOC_Os05g49180.1|genepair519-2
MTGGGAGGGGGGGGKGGAAAGPVPAASRKLVQSLKEIVNRPEAEIYAALRDCGMDPDEAIL
FKRQCGFKVNLRAWYDINQFHSEANNSKHGWSGTPGQLSMADIVKMGRPQVKQSSSKPAV
TADKGYTGQYPSLPSTVNQNLKQSASTVSPTNPDQGLHSAQDSIHPKDHNHSAAVNKQAY
DNDWLPQDEPPPGNQSALPETSGDQSLYESSLQSSTLVAGVINPHENSHLDENRSAAFSS
ERHLEHHGGDSEYDDGLLQESSTYLPQKNSHAEDEVEGSNSDVALATENFQGLSLHNEEL
VPTKLAEDNPAVIIPDHLQVTGSDCVTLSFGSFESGAFSGLLPVPSRSADDNNVELPVIE
ESVPLDQIDSRDQDYYDSAAVNSSGNENLDTIIGTNMENIDVPSVSQPDVLRQEVLDHSG
LQYNLPSDSSAAYANTTQPSTMESSQGNNQAHTLSHLSNLLQANSLHNSLLGSNIAPLRD
LDFSLSPLLAAQSMTKYNSAAPTTTGPAISMQETLKPGVFSNAQSTQNLPSTSIATGPPL
PQQLVHPYSQPTVPLAPFANMIGYPYLAQNYPAAYLPSAAFQQAYSSNGPFHQSAAAAVP
GAMKYNMNVPQFKNNLSATSLQQQPSSVISGYGGFGSSSNLPGNFTLNQNAASASTNLGF
DEALSTPYKDPSQYMALQQGDNSAMWLHGAGSRATSALPPSHFYGFQGQSQQGGFRQAQQ
PQQHSQFGGHGYPAFYHSQSQEHHQNPAEGGLNGFQNAQSQPSHQGWQQHTGY*
>11667.m04589|LOC_Os01g47490.1|genepair520-1
MSGMPSDEATGQVRLEGDVSDKKEEKTQEQSEASGMPSPQEEEAAIKKKYGGILPKRTPH
ITKDHDRAYFDSADWALGKQGGSHKPKGPLEALRPKLQPTQQHARARRTPYASADNDECM
NLPPEDLIQNGDPIEDKNKEEQ*
>11682.m04716|LOC_Os05g49160.1|genepair520-2
MAICGGWMEAGVRPAARRGGWMEAVLTGEVLGRWGGRDRESRRQRRNGSVVRLAARRGGW
MEAVPAGEERDRRSGDVDPRRRRRGFRRAGVAVGEGVVGARRRMRGAASTVGYMSGKPSD
DTTGQVRPEVDGSDEKVEIANQNEKEVMPSPQEEEAAIKKKYGGIVPRKPALIAKDHERA
YFDSADWALGKPTQQQARSRRFLHASVDNEEGLNSPTEDASQNQESNEVKDDK*
>11667.m04596|LOC_Os01g47530.1|genepair521-1
MAMQTMQTEQQQQQRRKGSPEMDFFSEYGDANRYKIQEVIGKGSYGVVCSAIDQHTGDKV
AIKKIHNIFEHLSDAARILREIKLLRLLRHPDIVEIKHIMLPPSRRDFKDIYVVFELMDT
DLHQVIKANDDLTKEHHQFFLYQMLRALKYIHTANVYHRDLKPKNILANANCKLKICDFG
LARVAFNDTPTTVFWTDYVATRWYRAPELCGSFFTKYSPAIDIWSIGCIFAEILTGKPLF
PGKNVVHQLDLMTDLLGTPSMDTVTRIRNEKARRYLSSMRKKQPVPFSERFPKADPAALK
LLQRLLAFDPKDRPTAEEALADPYFKGLAKAEREPSCQPITKMEFEFERRKVTKEDVKEL
IFREILEYHPQLLKDYMNGTEKTNFLYPSALDNFRRQFANLEENGGKNGDAVPSDRKHVS
LPRTTTVHSAPIPPKDHQNITSQVPQRIPGRTGRGACPVIPFENLSAMGPYNQRRVVRNP

VLPPATTNLSAYAYHRKSDSSERELQQELEKDRMRYQPSEHFMDAKVVSHMSHDLRASSY
YVSKAKSDVADRAALQSNMMQGIGPFNGIAAVGGNYNKVSTVQYGVSRMY*
>11682.m04714|LOC_Os05g49140.1|genepair521-2
MPEANAGGRAAPAAEQRSKNTTEMDFFSEYGDSSRYKIQEIVGKGSYGVVCSAIDQHTGD
KVAIKKIHNIFEHLSDAARILREIKLLRLLRHPDIVEIKHIMLPPSRRDFKDIYVVFELM
DTDLHQVIKANDDLTKEHHQFFLYQMLRALKYIHTANVYHRDLKPKNILANANCKLKICD
FGLARVAFNDTPTTVFWTDYVATRWYRAPELCGSFFSKYSPAIDTWSIGCIFAEILTGKP
LFPGKNVVHQLDLMTDLLGTPSMDAISRALADPYFKGLAKVEREPSCQPISKMEFEFERR
KVTKDDIKELIFREILEYHPQLLKDYMNGSENTSFLYPSAVDNFRRQFAILEENGGKSGA
LDRKHVSLPRATTVHSTSIPPNEGLDATSQVTQRIPTARPGRTVGPVLPFENPGAADPHS
ARRVVRNPMVPPAAANKSGYSYNLKSDYSDRQHQEELEKDRVQYRPAQHLMDAKVAPDTA
PDIRSSQYYFTRSAPRTDLTDRAALQGSMLYGIAPFNGIAAVAGGYSKAKVEERRRETPI
PSQAAIVHLKWQ*
>11667.m04598|LOC_Os01g47540.1|genepair522-1
MPALRMKRNFDDDVFRDEFDSKPTKSMKISHFQVSELEQSAVLNLPYKDPHDELDLTTQL
AGQDIMIMEAASLDDALGGASLLKDLISEVAVSPNMENDMLVNYEDIRSQLNVVNYFDKD
EDANSASYNSCTVNCHEESWGSNEGCSLLDIYNPDDAFSFLLDTPSEFLGSFTALYDEVV
PIDSLVNISGRCGVFPLTESTTEASIGNEPCRSEGDMLFSNSEVLEWLNPHLAEEDLPNL
VDFTELNSHAVSKEQGARKVTLVLDLDETLVHSTTEQCDDYDFTFPVFFDMKEHMVYVRK
RPHLHMFLQKMAEMFEVVIFTASQSVYADQLLDILDPEKKLFSRRYFRESCVFTNTSYTK
DLTVVGVDLAKVVIIDNTPQVFQLQVNNGIPIESWFSDDSDEALPQLIPFLETLASADDV
RPIIAKKFGDKKDIAEIFQ*
>11682.m04712|LOC_Os05g49120.1|genepair522-2
MWDQGAPAAFRHVASRSFFTRRTLVHIRPTAFLPVPPVCHPGVCIIVHIAAAFINMLTLM
MKNNCNDDDIGNVYLRTQIAGQDNKSGEGADFMDDGTFTLLKDLVTESRTSSPYYEKDHQ
LNSLNYVNQDGHGIPTTTNPTWDLTQSCNPLNHSLTEDSVPFNVGTSAGVLTNATSNDFI
PIDELAITSGRYGVLPSIEKTEEGSIAIDEANSFGATATMCYNSVHFSHWIDQNLTGPLP
DLADLPDIYPDSFLPTPRKNITLVLDLDETLIHSSAVDRDGADFSFPMYHGLKEHTVYVK
KRPHVDTFLQKVSEMFKVVIFTASLSSYANRLLDMLDPKNIFFTKRYFRDSCLPVDGSYL
KDLTVIVADLAKVVIIDNSPEVFRLQEENGIPIESWTSDPADKSLVELIPFLEAIAVADD
VRPIIAQMLGRPRSIA*
>11667.m04602|LOC_Os01g47560.1|genepair523-1
MGVAVHWRRAGDSLHMGGEPRARAEAAWAAALPAALVALVRDTATYTRMMHRLRMETPRG
PTCQLDPCCCCCTAHVIPPPPPVSRTHATRRDAEASVPPPPASAAVSSRSDGTGQMAAGV
TLACAAPPPLRAPRASDGGRRRGVVKGGAGTDTCRSPQRLNVRPRERERVRACVRARAKN
HEHGQRRREAAVDPAMSGEYQFQDELAPLFARPGGGAGEMQMLPSSWFADYLQAGTPMQM
DYDLMCRALELPVGEDVKREVGVVDVVAAGGGGAPPLTPNTTSSMSTSSSEGVGGGGGGG
AGAGAGEEESPARCKKEEDENKEEGKGEEDEGHKNKKGSAAKGGKAGKGEKRARQPRFAF
MTKSEVDHLEDGYRWRKYGQKAVKNSPYPRSYYRCTTQKCPVKKRVERSYQDPAVVITTY
EGKHTHPIPATLRGSTHLLAAHAQAAAAAAAAHQLHHHHGHHGHHGMAPPLPLGSGAAAQ
FGRSSGIDVLSSFLPRAAAAHHGMTTMGGAAATTTTSHGLNSAISGGGGVSSETTSAVTV
AASAQPSSPAALQMQHFMAQDLGLLQDMLLPSFIHGTNQP*
>11682.m04710|LOC_Os05g49100.1|genepair523-2
MSGGGGGGGEGFPFHDELASLFAERPPNGAMPGMLQQQQPWSFIDYHHHLMQESAPTTPPL
DYEAFAGEFDDDVAPLEEVKRELVVDGVGLFPGGGASAAAAAAAVAGPMTPNSMSVSSTS
SEACGVGGGAGGDEESAGKCKKEEEGDGGDDDGKEGSSTTKGDGDGEDKNKKGGKGKGKG
EKRPRQPRFAFMTKSEVDHLEDGYRWRKYGQKAVKNSPFPRSYYRCTTQKCPVKKRVERS
YQDAAVVITTYEGKHTHPIPATLRGTAHLLGAAAAAHHHGGLQYHHPGHFAAAVGHRLPP
QPHDALGGGLLAPPHAQHLHAMQHQMQLAAAAAASGGSLHAAAMQQMPQPDHAGLVAIIA
STTGASTTPPPPPATGSAAAATTPLRMQHFMAQDYGLLQDMFIPSPFLHNDDANNNNHR*
>11667.m04603|LOC_Os01g47570.1|genepair524-1
MAAAMGAEAEIAAAVAPAATDECGGKAAAMGGVKQRRGGGGGGGWVKRMMTTTTAVPRRR
GHYTPVGVEVELHGSAAAGADEEKPPRRRGGWLRRMMVPRECVHGRQQRWWKLQAGGGGG
GSSSRLAAGLTRSLSRWKTAGSGGWATAVADAVAFRVMYVVEAVVLGLALSCFFCCCGCQ
I*
>11682.m04708|LOC_Os05g49080.1|genepair524-2
MAVETDTARAAVSSETAPMSPQAEAAPRAATARRGNWLRRLIPRDYLSLSRRWKLGGGAF
AAPGGGAASRRLASLSRSLRWKRRLPGFSLTLRSGSASAVVDAVAFRVMYVVEAVVLGLA
LSCFFLCCGCHL*
>11667.m04610|LOC_Os01g47640.1|genepair525-1
MFVSARKRLFFLFISSLLLSLLAPSDGSSNSTANLNQSHNKTGRTLEMTPKVLFQLKMHA
LFHWSSFGFLMPVGIILARMSSKSKSGRSIRVLFYCHVISQIAAVLLATGGAALSLMNFE
NSFSNSHQRVGLALYGFMWLQPLIGFFRPERGVKVRSLWYFLHWLLGIAICATGITNVYI
GLHTYHERTTKSVKLWTGLLTFELSLLLFFYLLIDRWSYMMKQGNAPIEQLRPTDNRKTY
PTTLRKELGMVQE*
>11682.m04704|LOC_Os05g49040.1|genepair525-2
MLLFGRKRLLAVLGSCCTILSLLTPTQCASSSPDSLNQSYKIVQPLELTPKLSLQLKLHA
FLLWSSVGFLMPIGVLLIRVTSNVKSTRSIRILFYCHVASQIVAVILATAGAVLSISNFE
NAFNNTHQRIGLALYGFIWLQPLIGFLRPDRGVRFRSIWYLTHWLLGIAICVVGVANVYI
GMHTYHERTGRSVRPWTVLLTVEVSAMAFVYLFQDRWNHVVRQQQQQEAAALGDDDDDDD
EQSEEHVYPANDHKEVAVVP*
>11667.m04612|LOC_Os01g47660.1|genepair526-1
MVAFRFHQYQVVGRALPTPGDEHPKIYRMKLWATNEVRAKSKFWYFLRKLKKVKKSNGQM
LAINEIFERNPTTIKNYGIWLRYQSRTGYHNMYKEYRDTTLNGAVEQMYTEMASRHRVRF

PCIQIIKTATVHFKLCKRDNTKQFHNGSIKFPLVYRKVRPPTRKLKTTFKASRPNLFM*
>11682.m04703|LOC_Os05g49030.1|genepair526-2
MAERGCGRQASAAAERWRAVGGADGGRARRRSGGYGRSSRAPWPPSSSPWARRHGAAVQR
HRPGVAVAVHRWLEVPADVAPQSLALLLCRGSGSGGDGSVAGLIPWSSRTPSSRSCSRSV
STLGVPSASWTVVGAGAVTLIVFTDVAVALDVVTHTHIRRFHQYQVVGRALPTPGDEHPK
IYRMKLWATNEVRAKSKFWYFLRKLKKVKKSNGQMLAINEIFERNPTTIKNYGIWLRYQS
RTGYHNMYKEYRDTTLNGAVEQMYTEMASRHRVRFPCIQIIKTATVHFKLCKRDNTKQFH
NSNIKFPLVYRKVRPPTRKLKTTFKASRPNLFM*
>11667.m04614|LOC_Os01g47680.1|genepair527-1
MAGEMPDADGKPRSASSGFQPSAPPQPQAQQYQYGTFGAPSSAPGEVPQPAVGFPQPAPP
PGLRHYPQPPPPSYAVYPPLPPQTYPAAAPYYALGYQAVQGYIPVVEGRPVRMRRLPFCG
LGMGWFLFIIGFFLAAIPWYIGAFVLICVRVHDYREKPGYVACTIAASLAAIAILLGVTR
GEEIW*
>11682.m04702|LOC_Os05g49020.1|genepair527-2
MGGGREEEAAAAASKVGYSSGDLPPSAPPHLQGQDPQQYQYGTFQPPPHHHAASGELARP
PVGFPQPAPPPGFAGASGGGGHYHHHHQQQPYAPAEPYYAQGYQTGPGYGPIAEGRPVRM
RRLPCCGLGLGWLLFIAGFFLAAIPWYVGAFILICVRVHDYREKPGYVACTVAAVIAAIV
IPLGLTKGAHVW*
>11667.m04619|LOC_Os01g47710.1|genepair528-1
MAGSPPSLLSGSSAGSAGGGGYSLKSSPFSSVGEERVPDPKPRWNPRPEQIRILEAIFNS
GMVNPPRDEIPRIRMQLQEYGQVGDANVFYWFQNRKSRSKNKLRSGGTGRAGLGLGGNRA
SAPAAAHREAVAPSFTPPPPILPAPQPVQPQQQLVSPVAAPTSSSSSSSDRSSGSSKPAR
ATSTQAMSVTTAMDLLSPLAAACHQQMLYQGQPLESPPAPAPKVHGIVPHDEPVFLQWPQ
SPCLSAVDLGAAILGGQYMHLPVPAPQPPSSPGAAGMFWGLCNDVQAPNNTGHKSCAWSA
GLGQHWCGSADQLGLGKSSAASIATVSRPEEAHDVDATKHGLLQYGFGITTPQVHVDVTS
SAAGVLPPVPSSPSPPNAAVTVASVAATASLTDFAASAISAGAVANNQFQGLADFGLVAG
ACSGAGAAAAAAAPEAGSSVAAVVCVSVAGAAPPLFYPAAHFNVRHYGDEAELLRYRGGS
RTEPVPVDESGVTVEPLQQGAVYIVVM*
>11682.m04699|LOC_Os05g48990.1|genepair528-2
MASPNRHWPSMFRSNLACNIQQQQPDMNGNGSSSSSFLLSPPTAATTGNGKPSLLSSGC
EEGTRNPEPKPRWNPRPEQIRILEGIFNSGMVNPPRDEIRRIRLQLQEYGQVGDANVFYW
FQNRKSRTKNKLRAAGHHHHHGRAAALPRASAPPSTNIVLPSAAAAAPLTPPRRHLLAAT
SSSSSSSDRSSGSSKSVKPAAAALLTSAAIDLFSPAPAPTTQLPACQLYYHSHPTPLARD
DQLITSPESSSLLLQWPASQYMPATELGGVLGSSSHTQTPAAITTHPSTISPSVLLGLCN
EALGQHQQETMDDMMITCSNPSKVFDHHSMDDMSCTDAVSAVNRDDEKARLGLLHYGIGV
TAAANPAPHHHHHHHHLASPVHDAVSAADASTAAMILPFTTTAAATPSNVVATSSALADQ
LQGRLFRDWTCAWHPSSQKDAFNIASLSGLDFRMHRYASFLAVINLPEGGAAPPPPSATV
VAVSRDDETMCTKTTSYSFPATMHLNVKMFGEAAVLVRYSGEPVLVDDSGVTVEPLQQGA
TYYVLVSEEAVH*
>11667.m04621|LOC_Os01g47730.1|genepair529-1
MAAAAAAAGYRAEEEYDYLFKVVLIGDSGVGKSNLLSRFARDEFSLETRSTIGVEFATKT
VRVDDRLVKAQIWDTAGQERYRAITSAYYRGAVGALVVYDVTRRITFENAERWLKELRDH
TDANIVVMLVGNKADLRHLRAVPAEDARAFAEAHGTFSMETSALEATNVEGAFTEVLAQI
YRVVSRNALDIGDDPAAPPRGRTIDVSAKDDAVTPVNSSGCCSS*
>11682.m04698|LOC_Os05g48980.1|genepair529-2
MAYRADDDYDYLFKVVLIGDSGVGKSNLLSRFTRNEFSLESKSTIGVEFATRSIHVDDKV
VKAQIWDTAGQERYRAITSAYYRGAVGALVVYDVTRHVTFENVERWMRELKDHTDANIVI
MLVGNKADLRHLRAVSTEDAKAFAEKENTFFMETSALESMNVENAFTEVLTQIYHVVSKK
ALDIGDDPAAPPKGQTINVGGKDDVSAVKKSACCSSA*
>11667.m04624|LOC_Os01g47740.3|genepair530-1
MAYRNTVCTPQVIDLETEQGHSHIHSESFNRTGNDSSDQGAQHAVRGVGNATNIGLSDMR
SYYDAGMNHPHQPVHNLPPNLGVDSGFVFPSSMYNPCMSTTSMNQYVSHTQSFGLPSNQV
VLGSMDEGSRNENAGESARGFIKRKNAAVAGSYHCANGFASSSSSHASLNPTHRPWDPSF
ESNVLPNTASYNPSEYHSQTSWPSMEGSSIPSNGFNLMGAHPESAQHGNYAFPTSHISQC
FQPTSNTWISQSANGIADGIPQWEYVNGMNNAPAGRFSRSGMTETVNGSFREYQNGPSTL
CRGPLPYFHQHAGMHAHNLLDHTQVQAPYQQCHNNPVLHGVNHSGNRFHLGPRIPVLFSN
SERTFGPPHHPLLANPVNHRNIRILPPEHATIMDFSRLYEVSNVVDEHRDMRLDIDSMTY
EELLALEEQIGDVNTGLAKSYIVEKLKTSLFVPGSSCMSNKSSESSMENDACIICQEEYQ
VKECIGTLDCGHRYHEDCIKQWLMVKNLCPICKTTALSTGRRSG*
>11682.m04697|LOC_Os05g48970.1|genepair530-2
MVWTHQVASPENQVQPESFYHGGAGSNLSNLSVQVAVGVPGNTDFRSHYESINLQHQHVQ
NPYPHVGVASSSVFPSTMYNPCISTTAVDRYVPPIQSFGLGNPLLLPLYHQLAQGSMDEN
GSSGNFCDSVREFIKRKNALLVGGHHFVNSFASSSSSAYVPPNPLHRSWNASFEANILPS
TGVSNPPEYSSADSLNNSNSMASHPELVHHGNYVFPAGHMSQYNAWIAQASRTGGVPQWE
HGNAAANPPGGFVHSGTIDMPNGGLQGYQAGPFANYYGPLPHFHQNPLNSMQHPALFNHI
QMQVPHQHCLSNNLLHHPSGNGLPLDPRILAISSNSGHTFGPTAQPSLANQVNAGSSRIQ
PYEFLFFKLDNTKFKGYQNLVEKYHLILFLNAPFVDLSRLYEAGVIDEHRDMRLDVDSMT
YEELVALEERIGNVNSGFTESYIEENLKSSSYVPDADCMPDQSSVEKDACIICQEEYEAK
ELVGTLGCGHKYHAMCIKGWLMVKNLCPICKTTALPADRRNG*
>11667.m04626|LOC_Os01g47750.1|genepair531-1
MPTVRFQKLQPAGFTSSPACLRGGKTGSGMLSVRPTISPTVVFANMYQRPDMITPGVDAQ
GQPIDPRQMQEHFEDFYEDIFEELSKFGEIENLNVCDNLADHMIGNVYVQFREEDQAAAA
HTALQGRFYSGRPIIVDFSPVTDFREATCRQLGLGRDLRKKLFGHYRKPQRGRSRSPSPS
PRHRRERHDRDDYRGRDDYSGGGGRRGGSSRHERHDDGGRRRHGGSPPRRARSPVRESSE

ERRAKIEQWNRERDEKQG*
>11682.m04694|LOC_Os05g48960.1|genepair531-2
MAEHLASIFGTEKDRVNCPFYFKIGACRHGDRCSRLHNRPTVSPTIVLANMYQRPDMITP
GVDAQGQPIDPEKMQEHFEDFYEDIYEELSKFGEVETLNVCDNLADHMIGNVYVQFREEE
QAVAAHNALQGRFYSGRPIIVEYSPVTDFREATCRQFEENSCNRGGYCNFMHVKQIGREL
RRKLYGGRSRRSHGRSRSPSPRHRRGNRDRDDFRRERDGYRGGGDGYRGGGGGGGGDGYR
GGDSYRGGGGGGGRRGGGSRYDRYDDGGRRRHGSPPRRARSPVRESSEERRAKIEQWNRER
EEKP*
>11667.m04627|LOC_Os01g47760.1|genepair532-1
MYQAIPYSSTRPWLRPEPAASVVDVVKVETTTAVAGRGGEAEVVGEEEAAEVRRAVAESP
VLVVGRRGCCLIHVVKRLLQGLGVNPAVHEVAGEAALKGVVPAGGEAAALPAVFVGGKLL
GGLDRLMAVHISGELVPILKKAGALWL*
>11682.m04691|LOC_Os05g48930.1|genepair532-2
MYQAIPYNANRAWPAASRPATAAAAPPPPPPRGEEEEVRRAVAECPVVVVGRSGCCLSHV
VKRLLQGLGVNPAVHEVAGEAELAGVVAGGGGVALPAVFVGGRLLGGLDRLMAVHISGEL
VPILKEAGALWL*
>11667.m04629|LOC_Os01g47780.1|genepair533-1
MARSVAVVVLLAMAAVAAAQAPGPAATPAAGATGPPNVTAVLEKGGQYTTFIRLMKETQQ
DTQLNSQLNNSFNGNGYTVFAPTDNAFNNLKPGTLNSLTQQQQVALVQGHVLPQFYSMDS
FQTASNPVRTQASGTDGPYTLNITSTTNNNVNVSTGVVEVTVTNALSAVKPLAVYSVDKV
LLPFELFGVKAPAAAPTASTAKPKKGGSTEAASGPAGAEDAEPTGAASARAVGWGVAGLA
AVVGCLL*
>11682.m04687|LOC_Os05g48890.1|genepair533-2
MAMVARRFLVAAAAVCLALAAVPAAMGQAAAPAPKGAAAAALNVTAILEKGGSYTTFIRL
MKSTQQDTQLNSQLNGTSTGFTVFAPTDGAFSSLKPGTLNSLSAQDQVSLVQAHIVPKFY
SMDAFDTASNPVRTQASGGDGPYTLNITATSTNQVNVSTGVVDTTLGTALRADQPLAVYS
VDKVLLPYALFGPKPPPSPPPAPSKKPAKGDTSASAEAPAGSADHPAGAAPAAARAAGWG
VAALLAAACLL*
>11667.m04657|LOC_Os01g48060.1|genepair534-1
MVGIDLNTVEEEEDEEEGGATGTVTAPAEARAGGAVCLELWHACAGPVAPLPRKGSAVVY
LPQGHLEHLGAAPGSGPGAAVPPHVFCRVVDVSLHADAATDEVYAQVSLVADNEEVERRM
REGEDGAACDGEGEDAVKRPARIPHMFCKTLTASDTSTHGGFSVPRRAAEDCFPPLDYSL
QRPFQELVAKDLHGTEWRFRHIYRGQPRRHLLTTGWSGFINKKKLVSGDAVLFLRGEDGE
LRLGVRRAAQLKNASPFPALHNQISNTSSLSEVAHAVAVKSIFHIYYNPSCTHRLSQSEF
IIPYWKFMRSFSQPFSVGMRFKLRYESEDASERRRTGIIIGSREADPMWHGSKWKCLVVK
WDDDVECRRPNGVSPWEIELSGSVSGSHLSTPHSKRLKSCFPQVNPDIVLPNGSVSSDFA
ESARFHKVLQGQELLGLKTRDGTVNTASQATEARNFQYTDERSCSINMSNNILGVPRLGV
KTPSGNPGFSYHCSGFGESQRFQEVLQGQEVFRPYRGGTLSDACIRGSGFRQPDGNHAPG
AAFKWLAPQGCDHHGITTSVLPQASSPSSVLMFPQTSSKMPGLEYIYGCLDRNENSRHFK
IGPTQDMTRTDQTLRLWPHLISGKVLDECTRNEKLHSPVSGAEHESNNKCLNTNGCKIFG
ISLTEKAQAGDEVDCGNASYHSRLQSLKPQMPKSLGSSCATVHEQRPVVGRVVDISAVNT
MI*
>11682.m04685|LOC_Os05g48870.3|genepair534-2
MTGIDLNTVEEDEEEAAEEVAANGSSPAPARAGAVCLELWHACAGPVAPLPRKGGVVVYL
PQGHLEHLGDAPAAAAAAAAVPPHVFCRVVDVTLLADAATDEVYAQLSLVPEKEEVARRA
DDGEGEDGDGMKQRFARMPHMFCKTLTASDTSTHGGFSVPRRAAEDCFPPLDYSQQRPSQ
ELVAKDLHSTEWRFRHIYRGQPRRHLLTTGWSAFVNKKKLVSGDAVLFLRGDDGELRLGV
RRAAQLKNGSAFPALYNQCSNLGTLANVAHAVATESVFNIYYNPRLSQSEFIVPYWKFMK
SLSQPFSVGLRFKMRYESEDATERRYTGIITGSGDTDPMWHGSKWKCLLVRWDDDAEFRR
PNRVSPWEIELTSSVSGSHLSTPHSKRLKPCLPHVNPEYMVPRGGGCPDFAESAQFHKVL
QGQELLGFKSHGGTAAATSQPCEARHLQYIDERSCSSDASNSILGVPRLGDRAPLGNPGF
SYHCSGFGESHRLQKVLQGQELFRPYRGTLVDASMGSNGFHQQDSPRAPGVVNKWQAQLH
GRAAFHGPPALALPSQSSSPPSVLMFQQANSKMPRLEFGHGQLDKHENDRRVRFGPSEGI
ERREQRIPLQPYPTSGEVIDGQVTVEKSHSPGRHGKDGPDNKAVGTNSCKIFGISLTEKV
PAREELDDGDANYSLQSLKQVPKSLGNSCATVHEQRPVVGRVIDISTMDMMI*
>11667.m04665|LOC_Os01g48130.1|genepair535-1
MTWCNSFNDVRAVENNLATAAAVAAAKKQQQQQQVSQHVNLIKTCPSCGHRAQYEQSQLQ
AAAAATIQDLPGLPAGVKFDPTDQELLEHLEGKARPDARKLHPLIDEFIPTIEGENGICY
THPERLPGTRQPSGHWNWSTGYYDYEATLVVLLTCCCMHARVGKDGLIRHFFHRPSKAYT
TGTRKRRKVHTDEQGGETRWHKTGKTRPVFTGGKLKGYKKILVLYTNYGKQRKPEKTNWV
MHQYHLGSDEEEKDGELVVSKVFYQTQPRQCGGGSAATAKDLSVDLVAGNNIKASNAAAE
HHHNDGVGGGGHGGNNSSMLKEAAGIVDFYNPAAALIGYSQAAPNNRAAASAHLTMPNFE
VHTGGAGFGP*
>11682.m04681|LOC_Os05g48850.1|genepair535-2
MTWCNSFSDVRTAVDSSLSPAAAVAAAAGKKAAASLAVLVKMCPSCGHRARYEQETTTIQ
DLPGLPAGVKFDPTDQELLEHLEGKARPDSRKLHPLVDEFIPTIEGENGICYTHPERLPG
VSKDGLVRHFFHRPSKAYTTGTRKRRKVHSDEVDGGETRWHKTGKTRPVMANGRPRGYKK
ILVLYTNYGKQRKPEKTNWVMHQYHLGSDEEERDGELVVSKVFFQTQPRQCGSTAAAAAA
KEASAAVAAAVVNSNYSIVHGHQGGGGGSFLKEANVVHEFYDPAATMGYRPPAPAAHFAP
NFAVHAARNSFGGP*
>11667.m04670|LOC_Os01g48180.1|genepair536-1
MVGNLPPVHPHAGSRAVHEYQFLPEQPSDRYEGASRSHEGASRSHYYDTPVEASNSRMSS
HTPGSHLLRGSDEAAPGYAFQGQMSGSGHLPQSGRREVLPAVPTDYEMIQSNSDLNSVPV
EGQYGISQVAGIENSLLPSERRAYHDEDGSRVDRKRKHNEEAKIAKEVEAHERRIRKELE

KQDMMKRKREEQMRKEMERHDRERRKEEERLLRERQREQERFLREQRREHERMEKFMQKQ
SRRAEKQRQKEELRKEKEAARQKAANERATARRIAREYMELVEDECLELMELAAQSKGLP
SMLSLDSDTLQQLDSFRGMLTPFPPEPVRLKEPFSIKPWTVSEDNVGNLLMMDKQIDLFD
TLASLHQVWKFSITFADVLGLSSVTFDEFVQSLHDYDSRLLGELHIALLKSIIKDIEDVS
RTPSVALAVNPAGGHPQIVEGAYAWGFNIRSWQRHLNVLTWPEILRQFALSAGFGPQLKK
RNAEDVYYRDDNEGHDGQDVISTLRNGSAAVHAAALMKERGYTHRRRSRHRLTPGTVKFA
AFHVLSLEGSKGLTILEVAERIQKSGLRDLTTSKTPEASIAAALSRDTKLFERTAPSTYC
VKSPYRKDPADSEVVLSSAREKIRAFQNVISDSEAEKEANDAERDEDSECDDADDDPDGD
DVNIDVGDGKDPLIGVKEQDGVPITTIVDSTKREKEKVDALTQSSDLTTSGKEAPKPSLG
KPSSANTSSDSPVRASSEYHEVPPTDAEDKEIDESNQGESWVHGLAEGDYCDLSVEERLN
ALVALVSVANEGNFIRAVLEERLESANALKKQMLAEAQLDKRRSKEEFAGRVQYNSNMNL
KADVNQENATESTPTPFHNVDKHNDGNTGVVDNNNNEIIDHNSNAANASYERNGLGQDIA
ATPDTLSVQQYAYADKTRSQLRAYIGHRAEQLFVYRSLPLGQDRRRNRYWQFSTSASPND
PGSGRIFFECRDGYWRVLDTEEAFDSLVASLDTRGSREAQLHSMLQRIEPTFKEAIKRKK
SAVVEQSAGRYLKNGATEMIRASYRSDFGSPSSNLSGVTSDSAIAYSDSFKIELGRNDVE
KTAISKRADVFIRWMWRECNDCKLTCAMEYGKKRCSELMHSCNYCYQIYLAEERHCSSCH
KNFKSIHNFSDHASQCKDKLRTDHNWKMQTADHSVPIGVRLLKLQLSTIEASIPPEAIQP
FWTDGYRKSWGVKLHSTTSLEEIFQMLTLLEAAIKRDHLSSEFETTSELLNLNTQDNPSQ
NHVGLSGSAAVLPWVPDTTAAIALRMLDLDSAVSYMQNQKMERNGGDFMKPPSRFVAVKN
AQELDPLETTGLDLFDGRWATGSGRRGRGRGSRGGSRGGRGRSRGGRVPRGISISSRIGF
KDENEASRKNTRRGRTRGRGRGRGRRTVRSRQPSEGKGRSIPKENLLGSFSMLSNAKAAT
VEESPRSSGADEWGLENRRPYIDGDENSSGSQLDQSEDNEENGQPMDEEYDEQVPDYSRG
YSGGSRPHGMIDDDVSEEEDEDVEGDDDGEEDDADRAVDDVDAEMDEDDDIGDDGEDGGD
GGDGVEANADEDEGGSSYSSEYSD*
>11682.m04678|LOC_Os05g48820.1|genepair536-2
MDSSGDGGDEGAGAAPAGVEEESPAPASAPAPAPASASAPVPAAVGGGAGASGSSGKVKR
VMKTPYQLEVLERTYTEDPYPNETMRAELSVKLGLTDRQLQMWFCHRRLKDRKPPPKRQQ
LEEEVPVPVMAPPPVLPPPLPHSELTMGTGGMYGEPLLSPSSRRGTGRPSAVPRISASEV
ARRYYEPPQVMLPPLAPMQLTRAEHRVIDSVERLIGEPLREDGPVLGVEFDPLPPGAFGA
PIVPEQQRQPFRSYEAKMFSGHDTKHIKASAFLPSIDPFVPSTVSGKRKSMTGSSSHLGS
RAVREYQFIPEQSSDIYERTTQSRYYDVLTEASDSRMSTGSRFLHGVEKAPGYTFHGQIS
GSSHLSLHGRPPVFSSGSTDYEMDPSNINSSSVPNESQYGIPQVAGFENSVAPSDRMVYH
DDDAFRMERKRKHSEEVKIAKEVEAHEKRIRKELEKQDMLNRKREEQVRREMERNDRERR
KEEERLLREKQKEEERFQREQRREHKRMEKYLLKQSLRAEKIRQKEELRKEKEAARQKAA
NERATARRIAREYMELMEDERLELMELVSRSKGLPSMLSLDSDTLQQLDSFRGMLRQFPS
EIVRLKVPFSIKPWTSSEDNIGNLLMVWKFFITFADVLGIPSFTLDEFVQSLHDYDSRLL
GELHVALLKSIIKDIEDVARTPSVASGMTANPGGGHPQIVEGAYDWGFNILAWQRHLNLL
TWPEILRQFGLSAGLGPQLRKRNAENVNNHDDNEGRNGEDVISILRSGSAAVNAAAKMKE
RGYGNRRRSRHRLTPGTVKFAAFHVLSLEGSQGLTILEVAEKIQKSGLRDLTTSKTPEAS
ISAALSRDSKLFERTAPSTYCVKTPYRKDPADSEAVLAAAREKIRVFQNTISECEEVEKD
VDDAERDEDSECDDADDDPDGDEVNIEEKDVKTSLVKAQDGGMPTAVGDIKKETNSIVNS
LTTPLIHTKSSESSSLRTLDKSVQVRTTSDLPAEISSDNHEGASDSAQDAEIDESNQGES
WVQGLAEGDYCDLSVEERLNALVALIGVATEGNSIRAVLEERLEAASALKKQMWAEAQLD
KRRSREEFSSKMQYDSGMGLKTDVDQQNTLAESNLTPVHNLVKDSNGNGSLVNNELPVDQ
QSQPNACSVVHERNGVRQEFSANPENLSGQQYVTSEKTRSQLKSYIGHKAEQLHVYRSLP
LGQDRRRNRYWQFSTSASPDDPGSGRIFFESRDGYWRLIDSIETFDALVSSLDTRGIRES
HLHSMLQSIEPTFKEAIGRKRCASIEPSAGRVLKNGTSEIISPNHSNEFGSPCSTLSGVA
TDSAMAYSDSFRIELGRNDVEKTAISERADLFIKWMWKECNNHQPTCAMKHGKKRCSELI
QCCDFCYQIYLAEETHCASCHKTFKSIHNISEHSSQCEEKRRTDPNWKMQISDYSVPVGL
RLLKLLLATVEASVPAEALEPFWTDVYRKSWGVKLYSTSSTKEVFEMLTILEGAIRRDFL
SSDFETTTELLNLSTQDSASRNTVPRSGSADVLPWVPDTVAAVVLRLLDLDSAISYTLRQ
KVGSNKERGAGEFMKLPPRYTPAKTKQETEPMGTGFDRQETWLTPSNGRGGRGGRGRGSR
GGSRGGRSRSRGGKVPRGISSSPKIEFRGYSASAVSSEKAPRKYARRGRNRGRGRGLRTV
RPRQPSDIGARSIPKPNLLGSFSMLRNAKHTTAMESPQSSGAEEWGLERRPSYVKDDEDN
SASQSEESSEENSEPMNEEYDELLPDYSRDNSGSSPLQMMDDGSDDNEEDAEGDEDGEED
GEDYDAEQHVDEDNDDAEMGEDDVEDNDDGDGGGGGAENGDEDEDGTSYSSEYSE*
>11667.m04672|LOC_Os01g48190.2|genepair537-1
MDSEHWISSLAAAKRFYAAQLGHVDDMAGIGMEEVEMEMEDDGEGMELELEMQLEEATWP
DVACPYCYEDHDIASLCAHLEEDHPYEPHTSPCPICFEKITRDMLNHITMQHGYLFKSGR
RMRRFDIPESQALSLLSRDLRDAQLQALLGGGHRQRRSNTTATNISADPLLSSFGLGFST
LDSEERSKAPVPIPDDTSIHKDTPAQPWESRIDSSLTSEEREQKRKQATDRATFVQGLVL
STLFED*
>11682.m04674|LOC_Os05g48800.1|genepair537-2
MDSEHWISRLAAAKRFYAAQLGHADRAGMEEVDMDEEVRPEFACPYCYEDHDVVSLCAHL
EEEHPFEPHAAPCPICSDKIAKDMLNHITVQHGYLFKNRRRLRRFVIPGSQALSLLSRDL
REAHLQVLLGGGGHRSNNSSNTTNISADPLLSSFGLSFPTSDTEETSKPPISIPDDASVI
KETPAQPWDSSIDSSLTREEREQKRKQASVRATFVQDLLLTTLFGD*
>11667.m04675|LOC_Os01g48220.1|genepair538-1
MASPARPAAASVSGAFGLPADPAAARCSFDQSRRRPEDLQQEKRMVRTFVNVYGGQENYT
KEAVMAAVEECMKKQAEGLLHSLEGIGGRLSQLELYCYKLERSIGELRSDVMDYHSEGTV
NFRCLEKNLRQVQKSVQILQDKHEISETPNEFSKLQIAHEFPARANEASAFSTFGRENDH
STQVAKHEVAFMPLQQVNAMQSPAVPVQSSNGYILQQLVPVSLSTQPDQQQPSQAAVYYM
QSQNPIKCTESEPSESAVHVIQSQIQNPEARVAVDLSQKSSQVTELYPQPQDQRLHLPAQ
QVESQAWRTQPLVVQPQQYNIQQVPPQLVQQQTSSPQAQSAPQVAVLYPPYSSQKPASAT

TEPLLRNMVVHSPYSSPQQKHHEAMPSFYGQGNTVLLPSTDLNIQHQQPQPLQQHGLSSC
PPQPSKPNHCSVASYAVQGSGQSYSATFKNPSNCAATVVAVLPQHPASGPMAFHHLGPQV
VHNQPFGNMFETASVVGYPRDRVESVALPVVTAAQPADSVAMADKLNAGSNVTSPREWSG
*
>11682.m04670|LOC_Os05g48760.1|genepair538-2
MASPARPAAASVSGAFGLSADPARCSFDQTLRREDFQDNRLLRSLVNIHEQETYSREIIT
EAIESCMKKQADNLVNTLDVISGRLSQLELYCYKLERSIGELRSDVMDYHGEANINFRCL
EKHVKEVQNSVQVLQDKQELAETQKELTKLQILHEESAQKSEGTAPSVLMTKEIDGSMPV
AKHELALVPLHQVNAAQSPAMQFQSCNGLVLQQLVPVSLSTQQDQQHMNQATLYCMQTQA
HVEHRQAQPFQPAPQPVQRHTQNTPQTVVEAQQVTSQAPDFYIQPQQQWAHQTGQQVHQA
RQPQPQVVQQQHYNNIQQVPAQIVQMQTSSPQAQSAPHVTLLYPPYGSQQPACANSEPRS
RSMAMQPSYSTISSSQRNHHEVAPVYVQSNTISVPLAEHSIQSQQPPQLQSFGNGSFKPS
KVSLHGVASYTVQGNAQAYNTAYGNPSNNAATVVAVLPQQAQSSAPMVLHHLGPQSLQNH
PIDMVEKVARMGYFKDQAESMALRMATAGQNVEFKHLA*
>11667.m04685|LOC_Os01g48320.1|genepair539-1
MDGKPPPPNPNLPYREDCWSDGETAALVDAWGRRYVDLNRGSLRQPQWREVAEAVNARPG
ASARRRPPRTDIQCKNRVDTLKKKYKAERARGTPSSWYFYRDLDMLVGPTLSAAAAAGGG
GSAKKPSPPRGLPMMRRRLESPSRSPSPPSPTPAVALPLPNYRQASNLPSAGLLFNKMAA
AAAAESDSEDGYNNPNNNYEDDEDDGSQQSASRSVSSRSGGVAAAGAGGGGVSSSKRKRG
GGGGGGFGELARAVETFAEMYERMEFAKQRHAEEMERQRIKFLKDLELKRMQAFVDVQLQ
LAKAKHGKHPDGATEMLMSLAALPFLSTPAYL*
>11682.m04663|LOC_Os05g48690.1|genepair539-2
MEVREMAVAAAAAAAASSGGGGGGLRMPPPNPNLPYREDCWSEGETEALVRAWGSRYVEL
NRGNLRQKQWQEVADAVNSRRGAAARRRPPRTDVQCKNRVDTLKKKYKAERARVMPSTWS
FFPELDRLVGPTLSASASKRPSPSPSPVPPPPYFAMPIHPSAVRKPPSPSPSPSPPPPMA
LPLPSYRRGSPLPAAALIQQEAAAAAAAAVSDSEDSEGPGDNNNHNAQRSPSQSVSSRSG
NSNKRSRQEVDGGFRELARAIEAFAEMYERVESAKQKQALEIERQRIDFLKQLEVKRMEN
FVDAHVKLARAKRIKKHAGTAPDGIGAAELVSSVAALPFLSTSTYI*
>11667.m04691|LOC_Os01g48370.1|genepair540-1
MPRETPPPPPPEGGEVHEVVEGEDGQAEDQEERWARLLPELMSEVVRRVEASGGERWPAR
KDVVSCACVCRRWRDAAVAVVRPPAESGKITFPSSLKQPGPREFPMQCFIKRNKKNSTFY
LYLGLTNATVDKGKFLMAARRFRRGPHTEYIVSLDADDLSQGSNAYMGKLRSDFWGTNFK
IYDSKPPYDGAKASSSRSSRRFGSRRISPQVSAGNYEVGQVSYKYNLLKSRGPRRMYCAL
ECPSTQETWENCLKTKFRKPTGNTVLRNKAPRWHEHLQCWCLNFHGRVTVASVKNFQLVA
AADPNDPASSKDEETVLLQFGKVDDNIFTMDYRQPLSAFQAFAISLSSFGTKLACE*
>11682.m04661|LOC_Os05g48670.1|genepair540-2
MAAVREPREEAAVGEGEGEEEGRWGGLLPELVEEVVRRVEASGGERWPARKDLVSCACVC
RRWREAAAAVVRPLPESGRITFPSSLKQGSFIAGLQKYQFCEVGFLADQIGIDMVYNLSY
NLKSGFEVSAFALLPGPKDFPIQCFVKRNKKKSMFYLYLGLLNGTMDKGKFLMAARRFRR
GPHTEYVISLDADDLSQGSNAYVGKLRSDFWGTNFKIYDNQPPYDDAKTSSTRSSQRFGS
THRFGSRRICPQISAGNFNVGQISYKYNLLKSRGPRRMFCTMECPSTQETWENSLKTKSL
RCTGTTVLRNKAPRWHEHLQCWCLNFHGRVTVASVKNFQLVATADPSHPDSVGDEETVIL
QFGKVDSNIFTMDYRQPLSAFQAFAICLSSFGTKLACE*
>11667.m04693|LOC_Os01g48390.1|genepair541-1
MWKMVDALLRCSALVALVLHFVVNGCSAVNTEGSALLKFQSRVEEDPHGAMAGWSERDGD
PCSWNGVRCVDGRVVILDSDWKCSLPIRSRIIVAKCSRNLKDLSLRGTLGPELGSLSHLR
ALVLSNNLFDGPIPKEMSDLAMLEILDLSNNNLTGEVPQEIAEMQSIKHLLLSNNNFQWP
LIQNSYRNFDQEIDFDVYDERGDVYQRSENGFESDSSSEENTKDNNNLSAHLSSQFAARN
PTVQLSRRRLLEDTNLAAASANAPVPAVASVPSTGTGSFSAFKEIKVPPPLSPPSSPPMS
SGPPQRSKRWLYAIVISSIALLFIGIACMFLLCRNKSVATIGPWKTGLSGQLQKAFVTGV
PKLQRSELEGACEDFSNIVASYPHYTVYKGTLSSGVEIAVVSTVIATNKDWSKHSEGRFR
KKIDLLSRINHKNFINLLGYCEEENPFMRMMVLEYAPNGTLYEHLHVEGFDHIDWNGRMR
VIMGVAYCIQHMHELNPSITHPDLHSSAILLSEDGAAKVADMSVWQEVISKGKMPKNDDI
VDHHEPVSADPAGNVCSFGLLMLEIISGRPPYSEHKGSLANLAMECIKDDRNISCLLDPT
LKTHKENELEIICELIQECIQSDPKKRPGMREVTTRLREVLAISPEAATPRLSPLWWAEL
EILSVEAT*
>11682.m04659|LOC_Os05g48660.1|genepair541-2
MDSSSSFVLVVLILQAVVIGCSSLDELPVQLSNRRLLHDRNHAALLYKDHFPTVGNMTFP
TSHQLQNDPNYKPLDSSSHPAEASANKGSSKGFKKWLYIVVIPVAGLIMLAGMAWMFLPC
RKKSVATIGPWRTGLSGQLQKAFVSGVPQLQRPELERACEDFSNIVASHPYYTVYKGTLS
SGVEIAVVSTTIKSSKDWSKHCEDCFRKKIESLSRINHKNFINLLGFCEEEEPFTRVMVF
EYAPNGTLYENLHDEAFDHIDWRSRMRIIMGIAYCIQHMHELNPANVHPDLHSSAVFLSE
DCAAKIADLSVWQEVVSDGKKSTANNDHHEPISARLAGNVYSFGILLLEIISGKPPYSEN
EGSLANLALGCIIKGRSIASMLDSVLESHKENELDVICQIIMECIQSDPTKRPGMREITT
RLRETIAISPDAATPRLSPLWWAEVEVLSPVEPR*
>11667.m04695|LOC_Os01g48410.1|genepair542-1
MVRRHLLRGHSLDRFLPIRSLIMSSSSSFSSSSPSPPPPSSSSSRGSSSGRWCGVSVAEE
DEDDAAVSATTTPPLPPLQKRVLSRSHGSKAKPLGGSVDHLPPVVPPSSKNVRDSGPPSE
IDLVKEKFAKLLLGEDMSGTGKGVSSALALSNAITNLAASVFGEQRRLEPMSADRRARWN
KEIDWLLSVTDHIVEFVPSQQASTDGTSMEVMGTQQRRDLLINIPALRKLDAMLLHYFAV
VLLPVEQEYLDNFKDEQEFWYVKKDADEGEKGDAPRQGDKWWIPTVRVPPEGLPDASKKW
ILHQKDLVGQVLKAAMAINADVLTEMEIPGEYIETLPKNGRSSLGDSIYKIITDDHFDPN
ELLSSVDLSTEHKIVDLKDRIEASVVIWQRKISNKLSWGPGVSLEKREQFEERAQTVLLI
LKHQFPGVPQSSLDISKIQYNKDVGYAILESYSRTLESLAFAVLSRIEDVLHADAIARDP

KRTKSRRRPSLVDIPEIIDNALEEEETVNSIDANSQVTNNSIHWQEQEHEDKGARLRKVH
RMVTKKLLHIEKVDNLGGGLKSFSHR*
>11682.m04657|LOC_Os05g48640.1|genepair542-2
MARPLLKIGHGLDRFRWRRSTSSSSSSPLALSLSSSSAAALSDDDPGSPMDPEMPPAARR
ALSRSSGSRGRLSFELPPLAGGPSDKEEAPPRTSSAPAPARPAPAALHEGPPSDAEMVRE
KFSKLLLGEDMSGTGKGVSSALALSNAITNLAASVFGEQRRLQPMAADQKARWRREIDWL
LSVSDHIVEFVPSKQVSEDGSTMEIMITQQRRDLQMNIPALRKLDAMLLEYLDNFKDKQE
FWYVSKDASESEKGNTPRQDDRWWLPTVRVPPGGLSDASRKWVQHQKDLVNQVLKAAMAI
NANVLMEMDVPEAYIESLPKNGKSTLGDSMYKIITEDHFNPEELLGTVDMSAEYNIIDLK
NRIEASVVIWQRKMVHKEGKLSWGHGVKFEKREKFEARAENVLLLIKHRFPGIAQSALDI
SKIQYNRDIGLAILESYSRTLESLAFTVMSRIEDVLHADSLAQASNTRTQESMRMASLSR
YDTDKVVIDAKAEVERLGRMEPVSATLFDFVSPRDQDVVATKMDSKEKGCRGDAHSRKLT
KVSPIATKRYSYLEKLENLSGTRSPISRH*
>11667.m04698|LOC_Os01g48440.1|genepair543-1
MSRRNAGAMQREGSVKDWEEFDPSPSPKLAYSQSYVAMRGLLTSVASLDLVLMSSSLKSA
WAAISSHKHARSLERSRSKGMSLKRAMLQLLVCFMVGIFIGFTPPFSVDLPGKIASENGR
LPFDGDAIDRRQMVERQGTKLEPFVAEAESEASSEPQVEEGPPVPAMLDDEADFVEASPI
VHSVNDSGIVVRKHLIIITTTSVRPHQAYYLNRLAHVLKDVPPPLLWIVAEWPYQSRETA
EILRSSGIMYRHLICNRNTTNIRKIVVCQKNNAIFHIKKHRLDGIVHFADEERAYSADLF
EEMRKIRRFGTWPVAIHVGTKYRVVLEGPVCKGNQVTGWHTNQRRGVSRRFPIGFSGFAF
NSTILWDPQRWNSPTLESIIVHSGGRGGLQESRFIEKLVEDESQMEGLGDNCTRVMVWNF
ELEPPQVNYPIGWLLQRNLDAVVPIT*
>11682.m04653|LOC_Os05g48600.1|genepair543-2
MVSSRRNTGGIQRDGSLRDWSEFVDPSPSPKLLYSQSYVAMRGLLSSLVSMDFALLSSRL
KSAWAAILSQRHTRSPERSKSRGLSCKRLAFHLFVCFMVGIFIGFMPPFFSVDVSQKIVSE
NGRLPFDEGAVDRGMVDGKVKELETIVVEKEVDIIDESEVEESPPVPAMLDDEADFVESA
PAIPDINDLDITVRKLLIIVTITTVRPQQAYYLNRLAHVLKTVQSPLLWLVVEWPDQSFQ
TAEILRSSGVMYRHLICRKNTTSVRKIAVCQRNTAIYHIKKHRLDGIMHFADEERSYMSD
VFEEMRKIRRFGAWPVAIHTGIKYRVVLEGPICKGNRVTGWNTIQNIQKKSAVRRFPVGF
SGFAFNSTMLWDPERWNRPPMDSVIVHSGGRGGLQESRFIEKLVKHERQIEGLPEDCNRV
MVAMRLLSDAFLNKPHTGLHEVAVV*
>11667.m04699|LOC_Os01g48450.1|genepair544-1
MSPPLEPHDYIGLSAAAASPTPSSSSCSSSPNPGGEARGPRLTLRLGLPGSESPEREVVA
AGLTLGPLPPTTTKAASKRAFPDSSPRHGASSGSVAAAAACQDKAAPAAAPPAAKAQVVG
WPPVRNYRKNTLAASASKGKGEDKGTAEGGPLYVKVSMDGAPYLRKVDLKMYSSYEDLSM
ALEKMFSCFITGQSGLRKSSNRDRLTNGSKADALQDQEYVLTYEDKDADWMLVGDLPWDL
FTTICRKLKIMRGSDAAGIAPRSIEQSGQSR*
>11682.m04652|LOC_Os05g48590.1|genepair544-2
MPPPLEARDYIGLAASPASSSSSCCASTPVAEVVGAHLALRLGLPGSESPARAEAEAVVV
DAALTLGPAPPPRGGAKRGFVDSLDRSEGRRAAATAGDDERGVREEEEEEKGLGEAAAGA
PRAAKAQVVGWPPVRSYRKNTLAASATKTKGEDQGKSEVGCCYVKVSMDGAPYLRKVDLK
TYSSYEDLSLALEKMFSCFITGRSSSHKTSKRDRLTDGSRADALKDQEYVLTYEDKDADW
MLVGDLPWDLFTTSCRKLRIMRGSDAAGMAPRSLEQTGQNK*
>11667.m04707|LOC_Os01g48530.1|genepair545-1
MSGSNFGDSMGWGNSGRSSPAGSSRKGKRGGGSGGADKPKQPQRGLGVAQLEKIRLQSEM
AEYFNPLGQPGSLIHRTGSLNLMAYGERGDVRYGEFQTPIMRSPSSSTIYGAPHYTHNPS
ITLPLFEPEESARLRGHHDRSRSADSTSMNSDDPQDVDLELKL*
>11682.m04650|LOC_Os05g48570.1|genepair545-2
MEWPGRGRSFGSSRKGKRSAGNSGSDKPKQPQRGLGVAQLEKIRIQSEMAAGYLQNPPLG
QPPPIHGIDSLNLMAYGGSRSGDIRYGEFQSTSPIIRSPPNHEAIYGAAAHYSHPSSDHT
LPLFEPEESIYLRRHYGLNQPVDHSMNSDDPEEVDLELKL*
>11667.m04708|LOC_Os01g48540.1|genepair546-1
MWPLLLRAAVVYAALLAAGEADGSHDVLDIFGTRSESDYYRNAFQGKQGQAVPLPRGGGL
RREQQELGAAGPGGSGLSKAPPRSAPSKVALDSLKLPVDTSAGFAGGWNLVSENSGVSAM
HLVVMQHGKAIMFDTCTTGRSLMRLPPGRCRPDPRSKQPGAMDCWAHAVEFDYNTGALRS
LKIVTDTWCSSGAFDADGNMVQTGGFFEGDKSVRYLSACGTCDWKEFPKSLADGRWYGTQ
LVLPDGSFIVIGGRRAFSYEFVPAAGRANARATPLRLLRDTTDDVENNLYPFVNLLPDGT
LFIFANDRSIVFNYRTGQVVRELPILPGGSRNYPASAMSTLLPLDLRKGAGLSAEVIICG
GATKNAFKLGETSTFPPALRDCARINPSKPGARWALDQMPSGRVMGDVLILPTGDLLMLN
GAAKGCSGWGFGRQALLSPVLYSPYLRRGKRFRVLNPSNIPRMYHSTSALLPDATVLVAG
SNTNSAYNFSGVDFPTEVRVERFTPPYLSPQLSPNRPAIDAASVPGDGMRYGARFTFRFT
TPAQGVGQGDFKVTMYAPPFTTHGYSMNQRLLILPVTAFAAQGQRHTVTVDAPPKPELAP
PGYYMVYVVAKGVPSKAAWVKMHK*
>11682.m04645|LOC_Os05g48520.1|genepair546-2
MGSSSLPRAAVLAVALLLLLADSGEAFFDLFSIFRPRSDSDYFPFDGSPGQAKRKPKIEQ
EEDGAAPATATGLTKVPPLGAPSKAALDTIVLPVDDSAGHAGSWTIVSENSGVSAMHLAV
MRHGKAIMFDTSTTGRSLMRLPMNNCRADPRAKREGTMDCWAHAVEFDYSTGALRSLKTA
TDTWCSSGAFDADGNLIQTGGYFEGDKAVRRLDACDTCDWREYPNSFAEGRWYATQQVLP
DGRFIVFGGRRAFSYEFVPQPGMTNGQSIKFPLLRETTDDVENNLYPFVNLLPDGNLFVF
ANDRSVVFDHRTGKVVRELPKLAGGGRNHPASAMSAMLPLDLRNLTRGADPEPEVIVCGG
ALKTAFRLGENNTYQPTLRDCARINLGKIDAVWAVEAMPVGRVMGDLLVLPTGDLLMLNG
AAKGSSGWGFARQPILSPILYSPRHPEGSRFRPLAASTVARMYHSTSAVLPDATVLVAGG
NTNAAYNFSGVDFPTEVRVERFAPPYLSRELTGNRAVIDVASVPAGGMRYGTKFTFRFHT
PVAAVEWGDVRVTMYAPPFTTHGYSMNQRLLVLPVAGFSAQGQMYELTVDTPRKPELAPP

GYYLVYVVSKDVPSEAAWVKIQ*
>11667.m04712|LOC_Os01g48580.1|genepair547-1
MPSPSPVPSSTAEGSSKNSPTKGSLSLGDLDAVEVLPASATAGWSSARQKRKWSQVSCDF
YHVSRDTREVSCETYQVPDNFNHVSRDTHEVSCDTCHVLDDFYHVSRDTREVSCDTCQSG
STKDVAGPSSIALKNVDPSDGVGAFPKAMSLADYLKLEGSDINLNINTYYYLVALGEGGI
VVEGQTSAEKPHQHKQMKHVDPKIDEKYVEFKQFDIVGDHSDHFYSNPRERKVQVVNEPG
KDWVKRIQHEWKVLEKDLPDNIFVRVYEDRLELLRAVIIGPSGTPYHDGLFFFDVYFPPQ
YPRNPLLVIYHSGGMRLNPNLYACGKVCLSLLNTWPGDGCEKWNPSNSTLQVLVSIQALV
LNAKPYFNEPGFESYANTPRAEKKSIAYNQETFLLSCKTMLYSLRNPPKHFDDFIIGHFH
KYGHSILIGCNAYMDGAQVGSIIGGVKAIDKGNKGCSTKFKGSLKKLFEELMMEFIGIGV
DCHEFMIDTTLKL*
>11682.m04628|LOC_Os05g48380.1|genepair547-2
MATHAPKGYLCAGSSSFDDPDVVEVTPAAAAAGGWSSGHQKRKRSQVVPHEVIELDADDD
PDGVVIIGEKSSVDKNKQAVGYPIDWLKHAKSSLAGEIAGPSSYPSKNPDILLGGLKIFQ
ENPFYNNIDDYAYEAFEEDYGYDEDEYDDYDYDSTLYESEYNFTLASKFDGLDIPPGVEA
PLPWLQTTAAEMANKTKPVNMMDDKIDEKYSAFKQFDTVDDHSDHYYSKPDLRKVQVVKK
PSKEWAKRIQHEWKVLEKDLPDTIFVRAYEDRMDLLRAVITGPAGTPYHDGLFFFDIYFP
PHYPSVPPLVNYRSGGLRLNPNLYACGKVCLSLLNTWSGSGCERWSPSNSTMLQVLVSVQ
ALVLNAKPYFNEPGYAMHANTPHGEKSSLTYNEDTFLLSCRTMLYSLRNPPKHFEDFIAG
HFRKYGQSILIACRAYLDGAQVGCLVGNGVQDVDEGDKSCSARFKSALKRLFEELLMEFT
VKGAECDKFLAEKAKAAASRAPADTTLRL*
>11667.m04718|LOC_Os01g48640.1|genepair548-1
MAPAKKSPRPCCHVSFLLLQLVLLSVIVFPATAAAASTFRCSNPSPVPNEVPEGNDAREL
LRSFQITTGYFSGGDRLFAPDDDSAYIPRSFALSPYKVAHTTDPAILEVAATLALYGPSS
DHGSGGGARRRRHRYLVSQLVASFVLHGYYSSASGELCVVGGSGSYSVDGGSVEHLRDVN
LHLRVPNAPSLADPFVTGLLDGADFETISLVAYVENDRYVYSEKRPSCPPPMPAHAARGA
LQALEANFSCSHLRELFVSSYRLENTSSDASSPAASTFQFPLSHGGLRMLVNQMHCTANG
SVRAYVVFSNYTDSERRWRRDMVINNRFLVKEEAVVADGYWDSTTSRLCLRACRVAHSSA
AETELKVGEQCGLGMSFWFPAVWTIRDRSIVAGLLWNANQEESGGNKHAGASLSGVMSVS
SIDGDGYNRRRSNLTDVKYNYTMVEKAKKQYLSCKFSKRKTGRFPGNSSMYSYSDFRFDF
IETLGAGGQASPVTIGSVMVDGDQLAAEYMFFRHAMGEMNKSRTTVVRMDHSQLLNVSYD
ISYRVRSANSKARKNSSSLFSHPLSIERREISAEGVYDPKTGILFMVGCQEINGSSTDCQ
ILVTVHFASLDAKGNGHGRGKISSTRDKADRLHFEAMDITLYGMYREQIGESIWRMDLEI
IMAVVSATLSCVFAALQIRHARANPASAPSATSVAMLAVLALGHVTHLALNVDALFVSRR
THYIPISADGWLELNEVMLRVPTLIAFALHLCLLQLVWSSRRSAPRAIAEKWSAAERRSL
WICLPLYLLGGLLAGAVHVINNGRAAAENSLVVRVAGDSGTLWDDLASYAGLVLDGFLLP
QVILNALSRSRARAISAWFYVGVTVLRAAPHVYDALRARGYVPSVRPSSTYVYASPRDDL
FGVAWDVAVPLGAASLALLLFLQQRLGGAFFVRGRRFGEYEMVPTTVSSHQEGDKKMDHE
RGDVIAN*
>11682.m04627|LOC_Os05g48370.1|genepair548-2
MAPPRRDPNPNHLLLVAVLLLLVPATVAAAATYSDHCHGLPSAPDLAGGGGGGEGGADPT
SLRLSLQLNTGYFSGGGARLFGPDLSIPPRSFSFLPSSVVRTTDASLLHVSATLTVSGGR
RRRPPNDGRHLLVEYDGQAHRFRPRLPRFAGRRGSVTFGLEGYYSSASGELCMVGTGSGR
AADGTAVNLLSAVLRVRYPGRANLTRPFVTGSLESTDSPSFFEPVSLVTYAEEGYAYAES
ASCPPPPTGRLDALQVFEGSKFSCAHLSSLFKATFRLDYTNGSSESTASSLGLHQRFMFI
NRMRCADNGAVRAYVVFANQTDVSAYYFMLGEKAMVVEGFWDEKRSRLCLKGCHVVNSGP
SRADLAVGECGIGMSFWSPAVWSLQERSFAAGLVWNTSLKSGEGIAASSNTIAPYFRGSL
SGLKYNYTKVDEAKKYYEKYGLNKKRKGKFPDSNSYRDLTFRFFLQKGGGSGYASPVTIG
SMLYDGNSLVDSDHSYHIMTETNHRLLNVSYDIHYVGNWSLETFRRQHISAEGVYDAKTG
SLCMIACRVVNISLDCEILVTAQFSPLDTKVAQHVKGTIRSLRKKTDPLFFEPLDIASYG
LYIDKVDESMWRMDLESTMALISMTLSCLFIAVQLFHVKKVPEALPAMSITMLVVLSLGY
MIPLVLNFEALFKNSNKQTFPLSGGGWLEVNEVIVRIITMVTFLMQLRLLQLACSARSMD
VSKDQSWAAEKKVLWICLPLYIIGAVAAWVVHMQFNNNRRMLRKVARLPRVNRHAFWEDL
VSYGGLILDGFLLPQVILNACLGSKVKALSPGFYIGSTMIRALPHVYDVFRAKHFVPSLR
PFYRGSRGGFLTHENLHLREVMANAVFG*
>11667.m04731|LOC_Os01g48750.1|genepair549-1
MHVIKPWRILTCSAARRYKDGSAARGTVGVDSATIALSGRAARKAKLRGVVLGCTTSYNG
QSFLASDGVLSLGYSNISFASRAASRFGGRFSYCLVDHLAPRNATSYLTFGPNPAFSSRR
PSEGTASCKPAPAPTPAPAGAPGARQTPLVLDHRTRPFYAVTVKGVSVAGELLKIPRAVW
DVEQGGGAILDSGTSLTMLAKPAYRAVVAALSKRLAGLPRVTMDPFDYCYNWTSPSGSDV
AAPLPMLAVHFAGSARLEPPAKSYVIDAAPGVKCIGLQEGPWPGLSVIGNILQQEHLWEY
DLKNRRLRFKRSRCMH*
>11682.m04621|LOC_Os05g48330.1|genepair549-2
MARGKVVLMVVVCLAVAAAAASAASGGGHSRGRPGKSARLQLVPAAPGASMAERARDDRR
RHEYISARLAASRRRRRAEETSSVSSAGAVAASAFAMPLSSGAYTGTGQYFVRFRVGTPA
QPFVLIADTGSDLTWVKCRGAASPSHATATASPAAAPSPAVAPPRVFRPGDSKTWSPIPC
SSETCKSTIPFSLANCSSSTAACSYDYRYNDNSAARGVVGTDSATVALSGGRGGGGGGDR
KAKLQGVVLGCTTAHAGQGFEASDGVLSLGYSNISFASRAASRFGGRFSYCLVDHLAPRN
ATSYLTFGAGPDAASSSAPAPGSRTPLLLDARVRPFYAVAVDSVSVDGVALDIPAEVWDV
GSNGGTIIDSGTSLTVLATPAYKAVVAALSEQLAGLPRVAMDPFDYCYNWTARGDGGGDL
AVPKLAVQFAGSARLEPPAKSYVIDAAPGVKCIGVQEGAWPGVSVIGNILQQEHLWEFDL
NNRWLRFRQTSCTQ*
>11667.m04734|LOC_Os01g48770.1|genepair550-1
MTKRTKKAGIVGKYGTRYGASLRKQIKKMEVSQHSKYFCEFCGKFAVKRKAVGIWGCKDC

GKVKAGGAYTMNTASAVTVRSTIRRLREQTEA*
>11682.m04620|LOC_Os05g48320.1|genepair550-2
MTKRTKKAGIVGKYGTRYGASLRKQIKKMEVSQHSKYFCEFCGKFAVKRKAVGIWGCKDC
GKVKAGGAYTMNTASAVTVRSTIRRLREQTEA*
>11667.m04740|LOC_Os01g48800.1|genepair551-1
MSITTTSSAPEQLLDMPPSSVAANGARAARRRRRLLLFANYAALLGGSVASSLLSRYYFA
HGGHNRWVATLVQSVGFPFLLVPVYAGRSPSQPRPFAWFTRRLLAACVVIGVLMGVNNLL
FSYSSSYLPVSTSSLLLSTQLAFTLVLAAIIVRHPLNFSNLNAVVLLTLSSVLIALRSSD
SGEQPGGGSRARYFVGFAVTLGAAGLFAAYLPVMELVYRKAVSGGFRMAVEVQVIMQAAA
TALAVAGLAAAGGWKEELARWDLSPAAYWAVLAALVATWQACLMGTAGMVYLTSSLHSGV
CMTAVLTANVIGGVVVFRDPFGADKAVATVLCVWGFSSYLYGEYTTQKKVDGDGKVAAAS
SAAAAASADKIPTGGGAAEGDPVHEAV*
>11682.m04618|LOC_Os05g48300.1|genepair551-2
MAIAAAVAAAMAGPRRRRMLLLMANYAALLVGSVASSLLSRFYFTHGGRNRWVVTLVQSA
GFPLLVAGALAGGRPASAPRPFTWLSRRFLAVCLVIGALMGANNLLFAYSTSLLPVSTSS
LLLSTQLAFTLVLAVVIVRHPVTFVNLNAVVLLTLSSVLLALRSGDSGETAEGGVGGGGR
KSYLVGFVVTLGAAGLFSAYLPVMELVYREAVSGGFVLAVEVQAVMQAMASLIAAVGLAA
SGGVADDVSGWVDGGSSPAVYWAVVATLVLTWQACFMGTAGVIYLTSSLHSGVCMAAVLT
LNVLGGVAVFGDPFGAEKALATALCAWGFSSYLYGEYAKAKKVAAAAAVAAAEEEESASD
GEGSVHKSLTGGGAAGGHVETAV*
>11667.m04744|LOC_Os01g48830.2|genepair552-1
MRGSLEVHAIGRHAASPCALRLKALPALDMMRYQRLSPDCLPLANGGGGGSGSVTRKPAS
RSCKDDDGGMAVAADSSRLSSYLPSSQLDSKPLRARAPQPSSSSAAAWSPARDHAHAHHN
HHHHHHPSDSSDTASPSSNGAGTGGDVLLQWGHNKRSRCRRDASSSANAAPSSSQRRQTA
SAAGKILRRSSAPAEKLMPPPPPSTTTGSYTRGSNLRSASSFPTRSAAAAAVGDAHHHRS
AVEERSGGGYKRSPDKAHKSALDAALHMDSKNNHHHHHHDSSVTANGGAGAGEKIGSERF
ELPRIYISLSRKEKEDDFLIMKGTKLPQRPKKRAKNVDKTLQYVFPGMWLSDLTRGRYEV
REKKCVKKVYSTLHLAFSVHAFCVFLQTRRGKLPRFRASVTQIGWV*
>11682.m04616|LOC_Os05g48280.1|genepair552-2
MNWISKFRIRPENLVLITLPAIGGFYSNTTGSCANHIYTLASRCSRRGGRIENRFQRVSP
DCLPLPNGGGGGGGSRKPAAGPRSSKDDDAPAAAAAADSARTSPYLAAASASLESKPRTR
APPPPPPAPAPAPPSSSSAAAAPGRSPARERERDHGHHPSDISDPTSPSSTGVGGGGAGG
SGSAVPDSAVLLQWGHNKRSRGRRDASASSSSAAAPSPQRRQAAAAAGVKIQRRSSAPAE
KLMPPPSTTPSRSSTLRVTSSLPARGGGGGDAHHHGRSALPHNSSIHRCSGEFAFVAMLE
FDGMMMIRSGEQSAAATKAEKQQQRPAAHRVAEAGGVVMALAKTDSKYQHHTGHGGHGQA
ADHHGPSSSSKAATAAAQKIELPRIYTTLSRKEKEEDFMAMKGTKLPQRPKKRPKLVEKQ
VNFVCPGMWLSDVTRSKYIVREKKSTKKQQKYRGLKGMESMDSDSE*
>11667.m04747|LOC_Os01g48850.1|genepair553-1
MARAGAVLVVVLAAASVLLAPWAATAQTSSCDDALPPALAGNYSGLACRPVWNNFVLRYA
QGKDNVLRVVISSMYSTGWVGMGFSKDGMMVGSSAMVGWVGKTGLSHVKQFSLRGKTPSQ
VVADEGFLQSKDHDHTVVVQQAKIYLAFQLRFQSPLRRQQVLLAFGNAIPVNDRLTEHQG
KTSFTFDFTTGSSSGSSYPDGLKRAHGALNLFAWGVLLPIGAIIARYCRRWDPLWFYLHA
GIQLVGFILGLAGIVAGVSLYNKIQADVPAHRGLGIFVLVLGILQILAFFLRPHKDSKYR
KYWNWYHHWVGRLALFFAAINIVLGIKVGAAGNSWKIGYGFNLAILLITIITLEVLLWTR
WKNNNSSSMPTY*
>11682.m04615|LOC_Os05g48270.1|genepair553-2
MARVSAAAVAVLVVAFAAAAAAQESCNTELPGVLVGNYSGLNCQPVWNNFVLRYHQDKNN
VLRVVLSTMYSTGWVGMGFSRDGLMVGSSAMVGWIGRKGLPHVKQFALRGKTSGKVVVNR
GFLVSNSNNHDHTVVVQQARIYLAFQLQFSYRLTHQHIILAFGSSIPVNNKLTKHQDKTS
FTFDFTTGKAFVDGSFPYGLRRAHGALNLFAWGVLMPIGAILARYFRRMDPLWFYLHVGV
QFVAFIIGLAGVVAGVALYNKIQADIPAHRGLGIFILVLGILQILAFFLRPNTDSKYRKY
WNWYHHWSGRLVLFFAAVNIVLGIHVGGGDNSWKIGYGFNLAIILLAVIGLEFMLRTRWS
KESEPTPTY*
>11667.m04748|LOC_Os01g48860.1|genepair554-1
MWVRTKVNLDDHIIVPDLGPTDTSADPEKAVEDYVSSLSTPSMPMDRSRPLWELHVLGFP
TAEAAATVALRMHHSLGDGVSLLSLLIACTRRADDPDAIPALPSSAAGRRRREGPLHALP
PRPPLAAGALALAAWALSYLVLAWHTVVDVVCFTLTAASLMGDARTVLKGDEGAEFRPRR
FVNRTISLDDVKNIKNAVGCTVNDVLVGLSSAALSRYYFRRTGESEGKKNIKVRTALMVN
LRPTPGLHELAKMMESGKNNGVKWGNRFGYMILPFHLAKHDDPLEYVRKATKVTRRKKSS
MEAIFTYWSADMVVKLFGIKAAASLCYGMFSNTTLSFSNLAGPSEQILFCGNPIVYISPT
SYGHPHALTMHWQSYMNTIKLALAVDETQFPDAHELLDDFTESMRLIREAASRGTDKAQD
GP*
>11682.m04614|LOC_Os05g48260.1|genepair554-2
MDPRSTADPASSSTSTHSTTTTSPTPTPSPSSLRKRVLSIDTSSRDSRGSPGRERESKAR
QEEEEEESVMASAAASEAERERDVEAAVPVPMSPAGRLFRETNFNCYIVAVIGLGARVDV
AAARAGLEATLVRHPRFCSVQVSDEASKRAKPRWVRTTVNLDDHLIFPELDPTATSASPD
QVIEDYMSTLSTQPMDHSRPLWELHVLDFPTSEAAATVAVRMHHSLGDGISLLSLLIACT
RSAADPARLPALPPAPARRDGPVYARRRPPLSAGIVALAAWAWSYLVLALHTLVDVACFV
ATSLFLRDARTPLMGTEGVEFRRKRFVHCTLSLDDVKLVKNAMKCTVNDVLVGVTSAALS
RYYFRKENDTNSEKRTRRKHIRVRSALLVNIRKTPGLHVLAEMMNSNKNNVARWGNLIGY
IVLPFRIAMFHDPLEYIRQGKRTVDRKKSSLEAIFTYWSGNLIVKLFGIKTAAALCHGML
ANTTLSFSSMVGPAEKVEFYGHPIEYIAPSVYGHPHALTVHYQSYMNIIKLVLAVDDAQF
PDAHQLLDDFAESLRLIRQAASTKS*
>11667.m04752|LOC_Os01g48900.1|genepair555-1

MENLISLVNKLQRACTALGDHGEESALPTLWDSLPAIAVVGGQSSGKSSVLESVVGKDFL
PRGSGIVTRRPLVLQLHRINGDREYAEFMHNPTVKYTDFALVRKEIADETERATGHKKQI
SPVPIYLSIYSPNVVDLTLIDLPGLTKVAVEGQSDSIVQDIETMVRSFIEKPNCIILAIS
PANQDLATSDAIKISREVDPKGERTFGVLTKIDLMDKGTNAVDMLEGRSYRLQYPWIGVV
NRSQQDINKSVDMIAARHIEREYFANTTEYKYLAHRMGSEHLAKMLSKHLESVIKSRIPG
IQSLISKAIAELEAELHRLGKPIAADAGGKLYTIMEICRMFDGIYKEHLDGMRPGGEKIY
YVFDNQFPVALKRLQFDKNLSMENVRKLITQADGYQPHLIAPEQGYRHLIESCLVSIRGP
AEAAVDAYGKTIKIKSIESPAYRTLAFVSSFKHELNQFPTLRVEISNAAFESLDRMRNES
KKSTLKLVDMECSYLTVDFFWKLPQDVEKGGNPTNSIFDRYNDSYLRRISTNVLAYVNMV
CSSLRNSIPKSIVYCQVREAKRTLLDRFFTELGAREIKHISKLLDEDPAVMERRANLAKR
LELYRSAQAEIDAVVWAR*
>11682.m04612|LOC_Os05g48240.1|genepair555-2
MENLISLVNKLQRACTALGDHGEESALPTLWDSLPAIAVVGGQSSGKSSVLESVVGKDFL
PRGSGIVTRRPLVLQLHRIDGDREYAEFMHLPRKRFTDFALVRKEIADETDRETGRSKQI
SSVPIHLSIYSPNVVNLTLIDLPGLTKVAVEGQPDSIVQDIENMVRSFIEKPNCIILAVS
PANQDLATSDAIKISREVDPKGERTFGVLTKIDLMDKGTDAVDILEGRSYRLQQQWIGVV
NRSQQDINKNVDMIAARRREREYFSTTPEYKHLAHRMGSEHLAKSLSKHLETVIKSRIPG
LQSLITKTIAELETELNRLGKPIATDAGGKLYTIMEICRMFDGIYKEHLDGVRPGGEKIY
HVFDNQFPVAIKRLQFDKQLAMENVKKLITEADGYQPHLIAPEQGYRRLIESCLVSIRGP
AEAAVDAVHAILKELVHKAINETHELKQFPTLRVEVGNAAFESLDRMRDESKKNTLKLVD
MECSYLTVDFFRKLPQDVEKGGNPSHSIFDRYNDSYLRRIGTTVLAYINMVCSTLRNSIP
KSIVYCQVREAKRSLLDHFFTELGAREIKQLSKLLDEDPAVMERRTNLAKRLELYRSAQA
EIDAVAWSK*
>11667.m04759|LOC_Os01g48950.1|genepair556-1
MGNAAARGMRHDDYATAAAATGGRRRKRCSKNTEAGSVPASVPGRRGAEHSAAAAATGVT
VKVVLRRKDAERLIARLNEQSAMGRKARMAEIKSEIMAGDGGRGAAAASASPGRCRDAWT
HRLAPIQEN*
>11682.m04608|LOC_Os05g48210.1|genepair556-2
MGNSLQLPCPCQEATAVATSGVKKRRKKSPATAAVRSSKAAAAASSRHVVPVIDTPGEEE
EMRMAWPGCHVEPAVDGGDGMRVKVVMKRKDAAELMARLEERCAVERKAKMVELNAGLRG
GHGHGHGGGGARDAWAPRLVAIPEN*
>11667.m04760|LOC_Os01g48960.1|genepair557-1
MSAAQGMAYKLRTDAAPTGAGRRARRSHSSVAAPYRAARLVQGGVSIEGGLVGGCQLTEE
RVAARPPRAAARDAEPVRPLSTLPESSIGLYDPSRERDSCGVGFVAELSGDYKRATVNDA
LEMLERMAHRGACGCEKNTGDGAGILVALPHNFFREVTKDAGFELPQPGEYAVGMVFLPI
DEKRRERSKAEFQKVAESLGHVILGWRRVPTDNSDLGESALQTEPVIEQVFLTKSSSSEA
DFEQQLYILRRLSILSIRAALNLRRGGKRDFYMCSLSSRTIVYKGQLKPCQLKGYYYADL
GHENFTSYMALVHSRFSTNTFPSWDRAQPMRVLGHNGEINTLKGNKNWMKAREGLLECEK
LGLTKDQFSKILPIVDATSSDSGAFDGVLELLIRGGRSLPEAVMMMIPEAWQNDVNMEPE
KKALYEFLSALMEPWDGPALISFTDGRYLGATLDRNGLRPGRFYVTHSGRVVMGSEVGVV
DVPSKDVLRKGRLNPGMMLLVDFENHTVVDDEALKAQYSKAHPYGEWLKRQKIYLKDIVE
SVPETERVAPGISGSLTQKNEKKEHAGVNGIVTPLKAFGYTVEALEMLLLPMAKDGVEAL
GSMGNDTPLAVMSNREKLTFEYFKQMFAQVTNPPIDPIREKIVTSMECMIGPEGDLLETT
EKQCNRLALEGPLVSIDEMEAIKKMNYRGWRSKVLDITYPKKSGRKGLEETLDRICTEAR
GAIKKGYTVLVLSDRGFSSDRVAVSSLLAVGAVHQHLVANLERTRVGLLVESAEPREVHH
FCTLVGFGADAVCPYLAIEAIWCLQNDGKIPPNGDGKPYSKEELVKKYFYASNYGMMKVL
AKMGISTLASYKGAQIFEALGLSSEVIRKCFDGTPSRIEGATFEMLARDALRLHELAFPS
RAPPPGSADAKALPNPGDYHWRKNGEVHLNDPLAMAKLQEAARVNSRAAYKEYSRRIQEL
NKTCNLRGMLKFKDTADMISVDEVEPASEIVKRFVTGAMSYGSISLEAHTALAMAMNKLG
GKSNTGEGGEQPSRMEPLANGSMNPKRSAIKQVASGRFGVSSYYLTNADELQIKMAQGAK
PGEGGELPGHKVIGDIAVTRHSTAGVGLISPPPHHDIYSIEDLAQLIHDLKNSNPRARIS
VKLVSEAGVGVVASGVVKGHADHVLISGHDGGTGASRWTGIKNAGLPWELGLAETHQTLV
ANGLRGRAILQTDGQLKTGKDVAVACLLGAEEFGFSTAPLITLGCIMMRKCHTNTCPVGI
ATQDPVLREKFAGEPEHVINFFFMLAEELREIMSQLGFRTITEMVGRSDMLEVDPEVVKS
NEKLENIDLSLILKPAAEIRPGAAQYCVEKQDHGLDMALDNKLIALSKAALEKEVRVFIE
TPIQNTNRAVGTMLSHEVTKRYHMKGLPAGTIHVKLTGSAGQSLGAFLCPGITLELEGDS
NDYVGKGLSGGKIVVYPPRDSTFIPEDNIVIGNVALYGATIGEAYFNGMAAERFCVRNSG
AQAVVEGIGDHGCEYMTGGTVVILGKTGRNFAAGMSGGIAYVYDIDGKFSVRCNHELVDL
YHVEEEEDITTLKMMIEQHRLNTGSVVARDILSNFDTLLPKFVKVFPRDYKRVLDNMKAE
KAAAKLAKEPKISNGVSVTTKKVQPEQSTNRPTRVSNAKKYRGFISYERESISYRDPNER
VKDWKEVAIESVPGPLLNTQSARCMDCGTPFCHQESSGAGCPLGNKIPEFNELVHQNRWR
EALDRLLETNNFPEFTGRVCPAPCEGSCVLGIIENPVSIKSIECAIIDKGFEEGWMVPRP
PLQRTGKKVAIIGSGPAGLAAADQLNKMGHFVTVFERADRIGGLMMYGVPNMKTDKIEIV
QRRVNLMAEEGITFVVNANVGSDPLYSIERLRSENDAVILACGATKPRDLGIPGRELSGV
HFAMEFLHANTKSLLDSNLEDGRYISAKGKKVVVIGGGDTGTDCIGTSIRHGCTSIVNLE
LLTKPPSKRAADNPWPQWPRIFRVDYGHQEASSKFGNDPRTYEVLTKRFIGDENGNVKAL
EVVRVKWEKVDGRFQFKEIEGSNETIEADLVLLAMGFLGPEATIAEKLGLEKDNRSNFKA
QFGNFATSVDGIFAAGDCRRGQSLVVWAITEGRQAAAAVDKYLSRNEQDAAEDITPSGAG
FVQPVAA*
>11682.m04607|LOC_Os05g48200.1|genepair557-2
MSAAQGLALKLRAAPAAGGVRGEKRRRAASATAAAAARPRHGAMSLEGGFLGGALPAEDR
VAPRASASRQAEAGAGAGAARPPPRSMSKIPESSIGLYDPSMERDSCGVGFIAELSGEYS
RKTVDDAIEMLDRMAHRGACGCEKNTGDGAGILVALPHNFFREANLLEVTKDAGFELPPP
GEYAVGMFFMPTDDKRREKSKLLFREKAELLGHTVLGWRRVPTDNSGLGQSAVDTEPVIE

QVFVTKSASSKADFERQMYVLRRFSVMSIREVLGVKNGGTKDFYMCSLSSRTIVYKGQLK
PSQLKGYFFADLGDESFTSYMALIHSRFSTNTFPSWDRAQPMRVLGHNGEINTLRGNKNW
MKAREGLLKCEGLGLTRDEMLKLLPIVDATSSDSGAIDNVLELLIQSGRSAPEAVMMMIP
EAWQNDVNMDPERKALYEFFSALMEPWDGPALISFTDGRYLGATLDRNGLRPGRFYVTYS
GRVIMASEVGVVDVPPQDVSRKGRLNPGMMLLVDFENHCVVNDDELKKEYSKVRPYGEWL
KRQRIQLTDIIESVNEAERIAPSISGALPITKENKADMGICGILTPLKAFGYTREALEML
MLPMAKDGQEALGSMGNDTPLAVMSNREKLTFEYFKQMFAQVTNPPIDPIREKIVTSMEC
MIGPEGDLSETTERQCHRLTLKSPLLNTNEMEAIKKMNYRGWRSKVLDITYPKKNGRMGL
KQTLDKICAQAREAIHEGYTILVLSDRERVAVSSLLAVGAVHQHLVSHLERTRIGLLVES
AEPREVHHFSTLIGFGADAICPYLAIEAIWRLQIDGRIPPNDGKPYTQEQLIEKYFYASN
YGMMKVLAKMGISTLASYKGAQIFEALGLASEVVSKCFEGTPSRVEGATFEMLAQDALRL
HEIAFPSRTLPPGSADANALPNPGDYHWRKNGEVHLNDPFSIAKLQEAARINSREAYKEY
SRRIYELNKACTLRGMLKFREIPNQISLDEVEPAKEIVKRFCTGAMSYGSISLEAHTSLA
EAMNTLGGKSNTGYITIKNGSKGQLTTQTLTFFRGTVEPFALGRWCCENFVPFNLNTNDA
QSLCAFPKKNNPRISAIKQVASGRFGVSIYYLTNAVEVQIKMAQGAKPGEGGELPGHKVI
GDIAVTRNSTAGVGLISPPPHHDIYSIEDLAQLIHDLKNANPGARISVKLVSEAGVGIVA
SGVVKGHADHVLISGHDGGTGASRWTGIKNAGLPWELGLAETHQTLVANGLRGRAVLQTD
GQMKTGRDVAVACLLGAEEFGFSTAPLITLGCIMMRKCHTNTCPAGIATQDPVLRAKFAG
KPEHVINYFFMLAEEVREIMAQLGFRTVNEMVGRSDMLEIDPKVLEGNEKLENIDLSRLL
KPAAEISPGAVQYCVEKQDHGLDMALDNKLIASSTAALRKGVRVFIETPVRNINRAVGTM
LSHEVTKRYHIHGLPSDTIHIKLNGSAGQSFGAFLCPGITLELEGDSNDYVGKGLSGGKI
VVYPPRNSRFNPQDNIVIGNVALYGATKGEAYFNGMAAERFCVRNSGAQAVVEGIGDHGC
EYMTGGTAVILGKTGRNFAAGMSGGIAYVYDVDGKFSSRCNYELVDLYAVVEEDDITTLR
MMIQQHRLHTQSDLARDILLNFDTLLPKFIKVYPRDYKRVLDKLKEEKAAKEAEQKAREV
VDKKPVEVIQAPNGISVKTEKVMNEEPSSRPSRVSNAVKYRGFIKYEREGTSYRDPNERV
KDWNEVAIELVPGPLLKTQSARCMDCGTPFCHQEGSGAGCPLGNKIPEFNELVHQNRWHE
ALDRLLETNNFPEFTGRVCPAPCEGSCVLGIIDNPVSIKSIECAIIDKGFEEGWMVPRPP
LRRTGKRVAIVGSGPAGLAAADQLNKMGHFVTVFERADRIGGLMMYGVPNMKADKEGIVQ
RRVELMAKEGVQFIVNAHVGSDPLYSVEKLRSENDAIILACGATKPKALSLSRDLPIPGR
ELAGIHFAMEFLHANTKSLLDSNLEDGNYISAQGRKVVVIGGGDTGTDCIGTSIRHGCTN
LVNLELLPEPPRKRAPDNPWPQWPRIFRVDYGHQEATSKFGKDPRSYKVLTKRFIGDENG
NVKALEVIRVEWGKVNGRFQFKEVEGSEEIIEADLVLLAMGFLGPEATVANKLGLEQDMR
SNFKAQFGNFATNVEGVFAAGDCRRGQSLVVWAITEGRQAAAAVDNYLSKDDEGETNGTE
DIAVSSEGLVQPVVA*
>11667.m04767|LOC_Os01g49020.1|genepair558-1
MPVRRRWYPPSSTAAQPSPDGGDVNTDDADACPSSRQQRPPSLPQHSAPIHHRRRVINSI
DASGEVMEYGSSNDQRMQDMEIWPSDGGGVEEYDPWTAWLYKPHTVSVLLAGACLLIWAS
GVLHPEITSSHDKVIPIKRGVWAMIAVFLAYCTLQAPSTILIRPHPAVWRLVHGMAVVYL
VALTFLLFQNYQRDHMVLTAETLFDEFVIAHVLGWWGKAVMIRNQLLLWVLSVGFELMEL
TFRHMLPNFNECWWDSIILDIMICNWFGIWAGMHTVRYFDGKTYEWVGLSRQPSIMGKKS
GCLVCSLVSLIPEFLSNWFILRKLVKFTLSSLVNHFHCGRNPLVVYRLILWWLIAIPAIR
EYNTYLQDRSS*
>11682.m04590|LOC_Os05g48060.1|genepair558-2
MEAKQRTRHRDGEERRLVAAADGGAEEYDPWTAWLYKPHTISVLLVGACLLIWASGALDP
EGASYHSSATSIKRGVWAMIAVFLAYCTLQAPSTILIRPHPAVWRLVHGLAVVYLVALAF
LLFQNRDDARQFMKHLYPDLGVELPERSYGADCRLYVPENPKNKFINIYTLFDEFVVAHI
LGWWGKAVMIRNQLLLWVLSIGFELMELTFRHMLPNFNECWWDSIILDILICNWFGIWAG
MHTVRYFDGKTYEWVGLSRQPSIMGKVKRSLSQFTPAQWDKDQWYPFMGPLRFVQVLFLC
VVFMTVELNTFFLKFCLWIPPRNPLVVYRLILWWLIAIPTIREYNSYLQNSKPVKKVGAF
CWLSLAICIVELLICMKFGHGLFHDPMPTWLIIFWSSVGVALVVFLLAWSWRNHLKYQRK
RL*
>11667.m04777|LOC_Os01g49120.1|genepair559-1
MAAAAREEQPLLLRREEGEEEGEEVGWRRRWGSEAGKLAYLALPMVAIGMASALETLCGQ
AYGAKQYHTLGVHTYRAILTLLVVCIPLSLLWVFMGKILVLIGQDPLISHGAGRYIVWLI
PGLFANALIQPITKFLQSQSLIMPMLVASVATLVFHIPLCWLMVFKTGLGYTGAALSISI
SYWLNVAMLVAYILLSSSCKETRTPPTIEAFKGLDGFLRLALPSALMICLEWWSFELLIL
MSGLLPNPELQTSVLSICLTSITLLFTIPYGLGAGGSTRVANELGAGNPEGARSAVYVVL
SVAVTEALIVCGTLLASRRLLGRAYSSEEEVISFVAMMVPLVCITVVTDGLQGVMSGIAR
GCGWQHLGAYVNLGSFYLLGIPMAILLGFVLHMGAKGLWMGIVCGSISQITLLSAITFFT
NWQKMAENARERVFSEKPTEPSRYHLVE*
>11682.m04588|LOC_Os05g48040.2|genepair559-2
MEERIPLLSKRFPADGTAGVGGGREEEGGDRWWSGLAREAGKVGSMALPMAAMSVAQNAV
QVASNMMVGHLPGVLPLSASAIATSLASVSGFSLLVGMASGLETLCGQAYGAKQYDKLGV
QTYRAIVTLTVVTIPISLLWVFIGKLLTLIGQDPVISHEAGRYIVWLIPGLFAYAVCQPL
TKFLQSQSLIFPMLWSSIATLLLHIPLSWLLVFKTSMGFTGAALAISISYWLNTFMLAAY
IRFSCSCKVTRSPPTIEAFRGVGLFLRIALPSALMLCFEWWSFEILVLLSGLLPNPELES
SVLSICLTTTSLMYTIPYGLGGAASTRVANELGAGNPEGARSAVHLVMSIAGTEAVLVTG
MLFAAQRILGYAYSSDEEVVTYFTSMVPFVCISVAADSLQGVLSGYIS*
>11667.m04780|LOC_Os01g49150.1|genepair560-1
MAVARPPRPLPLCSASPLCSARARSSRRRQGRAAWSRRRPPLPIRSRARLPLPPLSPADA
ASPQPCSPTDAAAPPPRSLPTPPLRLLWPPFWCGGGSFPSGDGGRLNSELGPSRFSVASV
HRVGILDVCLLNIDRHADNILVKKSPESKCASGDSTPTPLDFLYGSGIAKSKYGRYDAEK
LHGSGVAGEDPFARLYASVDADLNECLEKAEAAKQEKNRATVVALNAEIRGTKAKLLEED
LPKLQRLALKKVKGLSKEELAIRGDLVTALPDRIQSIPDGSATSSKKTGLWGSSGSRAGT

GIKFDSTYDLEWKLIYVGSAEDENYDQLLESVLVGPVNVGTYRFVLQADPPDPSKIRKED
IIGVIVLLLTCSYMGQEFIRVGYYVNNDNDDEQLREEPPAKLLIDRVQRNILADKPSVTK
FPINFHPETSAGAGQEQQQQQQSGSPENHPNQGSKPNPDQ*
>11682.m04586|LOC_Os05g48030.1|genepair560-2
MSAVNITNVAVLDNPTAFLNPFQFEISYECLIPLDDDLEWKLIYVGSAEDENYDQQLESV
LVGPVNVGTYRFVLQADPPDPSKIREEDIIGVTVLLLTCSYMGQEFMRVGYYVNNDYDDE
QLREEPPAKLLIDRVQRNILADKPRVTKFPINFHPEPSTSAGQQQQEPQTASPENHTGGE
GSKPAADQ*
>11667.m04781|LOC_Os01g49160.1|genepair561-1
MGRAPCCDKASVKRGPWSPEEDELLRSYVRSHGTGGNWIALPQKAGLNRCGKSCRLRWLN
YLRPDIKHGGYTDQEDRIICSLYNSIGSRWSIIASKLPGRTDNDVKNYWNTKLKKKAMAM
HHHHQPPPPQQQHYHHHHHHRVAGGGARVTLVSPPPAPQSQCASMQPSPASASSSGGDAC
SFGAAAMYSPSPSTQQAPQAATLAVAGYTSVATAAAAAAVAAQRSPLDELICQVPPPPTT
TAADCWASGVTLDDVFLPELVGAGEFPNGDLFGGFGPLLQDRSSMELSACYFPNAAAAEM
WPAATDIVKPAGLCHSLT*
>11682.m04584|LOC_Os05g48010.1|genepair561-2
MGRAPCCDKASVKRGPWSPEEDEQLRSYVQSHGIGGNWIALPQKAGLNRCGKSCRLRWLN
YLRPDIKHGGYTEQEDHIICSLYNSIGSRWSIIASKLPGRTDNDVKNYWNTKLKKKAMGA
VQPRAAASAPSQCTSSAMAPALSPASSSVTSSSGDACFAAAATTTTMYPPPTTPPQQQF
IRFDAPPAAAAAASPTDLAPVPPPATVTADGDGGWASDALSLDDVFLGELTAGEPLFPYA
ELFSGFAGAAPDSKATLELSACYFPNMAEMWAASDHAYAKPQGLCNTLT*
>11667.m04784|LOC_Os01g49190.1|genepair562-1
MATRRALTSVLRSASRLRAASPSPCPRRAPLHPHRRPSPAGFLLNRAAAAYASSAAAQAA
PAPPPASGKATGGGKITDEFTGAGAVGQVCQVIGAVVDVRFDEGLPPILTALEVLDHNIR
LVLEVAQHLGENMVRTIAMDGTEGLVRGQRVLNTGSPITVPVGRATLGRIMNVIGEPIDE
KGDITTNHFLPIHREAPAFVEQATEQQILVTGIKVVDLLAPYQRGGKIGLFGGAGVGKTV
LIMELINNVAKAHGGFSVFAGVGERTREGNDLYREMIESGVIKLGDKQSESKCALVYGQM
NEPPGARARVGLTGLTVAEHFRDAEGQDVLLFIDNIFRFTQANSEVSALLGRIPSAVGYQ
PTLATDLGGLQERITTTKKGSITSVQAIYVPADDLTDPAPATTFAHLDATTVLSRQISEL
GIYPAVDPLDSTSRMLSPHVLGEDHYNTARGVQKVLQNYKNLQDIIAILGMDELSEDDKL
TVARARKIQRFLSQPFHVAEVFTGAPGKYVELKESVQSFQGVLDGKYDDLPEQSFYMVGG
IDEVIAKAEKIAKESAS*
>11682.m04581|LOC_Os05g47980.1|genepair562-2
MATRRALSSLVRAASRLRGASPAPRPRGPLHRPSPSGYLFNRAAAYATAAAAKEAAPPAP
ATGKATGGGKITDEFTGAGAVGQVCQVIGAVVDVRFDEGLPPILTALEVLDHNIRLVLEV
AQHLGENMVRTIAMDGTEGLVRGQRVLNTGSPITVPVGRATLGRIMNVIGEPIDEKGDIT
TNHFLPIHREAPAFVEQATEQQILVTGIKVVDLLAPYQRGGKIGLFGGAGVGKTVLIMEL
INNVAKAHGGFSVFAGVGERTREGNDLYREMIESGVIKLGDKQSESKCALVYGQMNEPPG
ARARVGLTGLTVAEHFRDAEGQDVLLFIDNIFRFTQANSEVSALLGRIPSAVGYQPTLAT
DLGGLQERITTTKKGSITSVQAIYVPADDLTDPAPATTFAHLDATTVLSRQISELGIYPA
VDPLDSTSRMLSPHVLGEDHYNTARGVQKVLQNYKNLQDIIAILGMDELSEDDKLTVARA
RKIQRFLSQPFHVAEVFTGAPGKYVELKESVNSFQGVLDGKYDDLPEQSFYMVGGIEEVI
AKAEKIAKESAS*
>11667.m04785|LOC_Os01g49200.1|genepair563-1
MSSAVKDQLHQMSTTCDSLLLELNVIWDEVGEPDTTRDRMLLELEQECLEVYRRKVDQAN
RSRAQLRKAIAEGEAELAGICSAMGEPPVHVRQSNQKLHGLREELNAIVPYLEEMKKKKV
ERWNQFVHVIEQIKKISSEIRPADFVPFKVPVDQSDLSLRKLDELTKDLESLQKEKSDRL
KQVIEHLNSLHSLCEVLGIDFKQTVYEVHPSLDEAEGSKNLSNTTIERLAAAANRLREMK
IQRMQKLQDFASSMLELWNLMDTPLEEQQMFQNITCNIAASEQEITEPNTLSTDFLNYVE
SEVLRLEQLKASKMKDLVLKKKAELEEHRRRAHLVGEEGYAEEFSIEAIEAGAIDPSLVL
EQIEAHIATVKEEAFSRKDILEKVERWQNACEEEAWLEDYNKDDNRYNAGRGAHLTLKRA
EKARTLVNKIPGMVDVLRTKIAAWKNERGKEDFTYDGVSLSSMLDEYMFVRQEKEQEKKR
QRDQKKLQDQLKAEQEALYGSKPSPSKPLSTKKAPRHSMGGANRRLSLGGATMQPPKTDI
LHSKSVRAAKKTEEIGTLSPSSRGLDIAGLPIKKLSFNASTLRETETPRKPFAQITPGNS
VSSTPVRPITNNTEDDENRTPKTFTALNPKTPMTVTAPMQMAMTPSLANKVSATPVSLVY
DKPEVTLQEDIDYSFEERRLAIYLARQMV*
>11682.m04580|LOC_Os05g47970.1|genepair563-2
MKTICGSLMHELQVIWDEVGEPEAARDRMLLELEQECLEVYRRKVDQANRSRAQLRQAIA
QYEAELAAICSAIGETTVHVRQSNQKACGLRDELGAILPYLEEMKRKKVERWNQFLDVVG
RIKKISSEIRPANFDPFKVSVDQSDLSLRKLEELRVELKSLEKEKGERVKQVMEYLKTLH
SLCVVLGVDFKKTISEIHPSLDEAEGPRNISNTTIEMLAWAIQRLRETKMQRMQKLQDLA
STLLELWNLMDTPFEEQQAYQNITCNIAASEAELTEQNTLSIEFLNYVEAEVLRLEQHKA
SKMKELVLKKKTELEEHRRRAHLVGEEGYATQFTIEAIEAGAIDPSLLLEQIEAYISTVK
EEAFSRKDILERVEKWLNAREEEAWLEDYNKDDNRYNAGRGAHIMLKRAEKARVLVSKIP
GMVDVLETKTRAWETERGNEFTYDGVRLILMLEEYMVVRQEKEQERKRQRDQKKLQDQRK
AEQEALYGSKPSSSKSHSTKKVPRNSTPGVQPPKSEILHSKTIRATKKTEDINTPSPGHK
GLDTVGLPIRKLFPSSNSSTLLEMETPRKPFSQITPGNISSAPVRPISTGGTEENRTPKT
FAPVPTTPMTVSPHMQMAVTPVLTAKAVSVLSYDEPELTSQEDTEYSFEEKRLAVYLAAQ
VA*
>11667.m04786|LOC_Os01g49210.1|genepair564-1
MGNHLASCTMARVPGAARGAKVVLPDGAVRAVKVPAKAAELMLEAPGHFLADARALRAGG
RIAALGADEDLELGGLYAAFPMKRLGAPAAPADMARLAAAVSRDQAAARRSSATAKVAAV
VVAPQHDAAMAEEEEDAARPRLDEMAVGDAAAEAEISELKQRISGGRRSRRPTLETIHEE
SYAPAARC*

>11682.m04579|LOC_Os05g47960.1|genepair564-2
MAKVPGGKGARVILPDDGGLRQVALPATAAELMMDAPGHFLADARAARVGARLAALSADE
ELELGAVYATFPMKRLGTPLAPADMARLAAVATREARRSAKVAAAVVAPPPTPLQAEDAA
PRLRLDEMVDDEAVAADMNVYKHRLSSERSRRPTLETIQEENYMSTN*
>11667.m04789|LOC_Os01g49240.1|genepair565-1
MAPAPAKAQPHVLLVSSPFQSHVNPLLRLGRRLAGKGLSVTFTTALRDGIRVFDDGDGGG
GGVRVERLRGGGMWEPDDPRLRIPGDMARHVEAAGPAALEELIRREAEAGRPVACVVANA
FVSWAVRVAGDVGLPCAILWIQSCAVLSVYYHYVYSLAAFPSGDEADSSGAVTIPGLPEL
DMDELRPLRIYTSDQEMWRQMLVGDLGSMTEKAPWVFVNTFDELEHEAVAGLRKHIPLIP
VGPLVEPDDGGVDDDDVHGCTAWLDAQPRRSVVFVAFGSLVDIGHDEVVEIAEGLASTGR
PFLWVLRDGNRALLPKDALIDACGGDRGKVVPWCEQRRVLAHAAVGCFVTHCGWNSTAEA
LAAGVPMVASPRWSDQRINTRFVVDVYRVGVRAPATPLTREALRLSVEEVTAGPEAEAMA
ARAAILGENARAAVGGGGSSDRGVQAFVDRITSGGAEP*
>11682.m04578|LOC_Os05g47950.1|genepair565-2
MEPHVLLVSFPMQGHVNPLLRLGRRLAATGLLVTFTTVRLAAGGGRLRDVPEDGACADVG
LGRLRFEYLRDDDDDGDERCQQLAPNDVLSHVTAVGPSALAEFIDGQADAGRPVTFVVNN
IFVPWALDVAAGMGIPCAMLWIQPCSVLSIYYHFYESPEAFPTAADPDVPVELPGLPVMA
MVELPFMVRPEYAQCLWGDTLRAQVGAIKRTVSWVLVNSFYELERSAVDALRAHTTVKLA
PIGPLLEHGHDNGGGDDGAPAPALGAEDNDRCVAWLDAQPPRSVVYVAFGSLVNIGRDET
AAVAEGLVATGRPFLWVVRDDSRDLVPEAVLAACRGDKAGKITAWCPQGRVLAHGAVGCF
VTHCGWNSIMEALAAGVPVVGYPWWSDQFANAKFLVEDYKVGVRLPAPVTGGELRACVDR
VMSGPEAAVIRKRAMHWKREAAAAVADGGSSDRSLQDFVDHVRRSKGPEELARLAQDIQI
MNGPVNPVLV*
>11667.m04793|LOC_Os01g49280.1|genepair566-1
MADHFALMAGRLLTESTLQSAVQEALAVASVKIVHDQPDLPVHEDVQDGKAKSGVMVECR
ICQEEGDESYMETPCCCKGSLKKARTFGVSSLMVNADNLSFPIDVLFLPAILTQQISLLSE
YLLELVCHCLQYAHHACIQRWCNEKGDTVCEICLQQFTPNYTAPLKLFRHGRNLISFRRS
GERSDNIDTDRSQEHFAQTSDQAAGTSSFDSQNSSPKGVFYCRVVAISLMALLVLRDAIS
LILGDPEVYSIALFTLLMIRTAGIVIPIYIILVSVTTLLHRYRQHQAVHEATDSEPGGGE
GLRPMPPPQHVISIQ*
>11682.m04572|LOC_Os05g47900.1|genepair566-2
MADHFAVMAGRLLTESTVQSAIDEASAAAMPSSVIASEHNDVQDEREKSGVLVECRICQE
EDDQTYMETPCSCRGSLKYAHRKCIQRWCDEKGDTICEICLQQYTPNYTAPAKLFQHGRN
SIFFRTPGYIQAQVMQNTDRSAAASTSYDPELPNPKGVIYCRIVALTFNAAEQSMQLVSH
CNLTVRKCNCQISDAAAAVNINQQAPQSPRRQHVISIQ*
>11667.m04794|LOC_Os01g49290.1|genepair567-1
MAGAQESLVLAGVMHGHNDVVTAIATPIDNSPFIVSSSRDKSLLVWDLTNPVQNVGEGAG
ASEYGVPFRRLTGHSHFVQDVVLSSDGQFALSGSWDGELRLWDLSTGVTTRRFVGHDKDV
LSVAFSVDNRQIVSASRDRTIKLWNTLGECKYTIGGDLGGGEGHNGWVSCVRFSPNTFQP
TIVSGSWDRTVKVWNLTNCKLRCNLEGHGGYVNAVAVSPDGSLCASGGKDGVTLLWDLAE
GKRLYSLDAGSIIHSLCFSPNRYWLCAATQDSIKIWDLESKHIVQDLKPEIPVSKNQMLY
CTSLNWSADGSTLYAGYTDGTIRIYKISGFSYAG*
>11682.m04571|LOC_Os05g47890.1|genepair567-2
MAGQESLTLAGVLRGHNDMVTAIAAPIDNSPFIVSSSRDKSLLVWDITNPSTAVATDPEA
APPEYGVSYRRLTGHSHFVQDVVLSSDGQFALSGSWDGELRLWDLATGRTTRRFVGHTKD
VLSVAFSVDNRQIVSAARDNTIKLWNTLGECKYTIGGDHGAGEGHTGWVSCVRFSPNPMA
PTIVSGSWDRSVKVWNLTNCKLRTKLEGHNGYVNAVAVSPDGSLCASGGKDGTTLLWDLT
EGKMLYKLDAGAIIHSLCFSPNRYWLCAATEDSVKIWDLESKLVMQDLKPEVQAFKSQML
YCTSLSWSADGSTLFAGYTDGTIRVWKVSGFGGYAI*
>11667.m04796|LOC_Os01g49310.1|genepair568-1
MPESSTNRFCKKVDLEVLPSRSDVGESSYHKHSLWMAHWARSSISPEPQNGQSCSPLKEI
DDVGYSKDCGALPFELMKARVAERLMVGVSHGGVSAGNTRQFSTNMRGVARDVCQEVQCK
NVDQMGSSFESSVMQKNVNLYAAKTVVSERYSVHKISDILVDSRKLCGTENLSSEWNHFP
MFEINRKIDSILNPRRSALVTSSEKIFVPQKSVKINMSTSNVMSFSSKEYQLHTHQVTDE
NRQCKSARGMLSHLDNYTGLNSDHAGKKLKGHLSIEEPCSCSKDDTDSSCSLADEHHARH
YIPNSKKSPHRSCKNSSVYSASKMENQFVEGSLLEHKSEVYGACKKKQHLEGVAFHESAL
HREYQIKSVKTTAITNEGDMDTNGHHVDFGNLLQSDQQYLNKHTEDSAVNLTESCKTPDA
IDSAMILKSKDESLAQEKRTNNKLIDNKRKGPCLFEMFTQPTKSNVKCSIDRTSSGKSCG
NMTSGLLGAQKQFSTKTDTFYSEAHHASKSTAGFASASMQKDLGYPSSAKTEQLVTSSVK
GVSSGSKGNEAVNTSAEHRDFYPKATCANNQEWSMSKTSSMNLDLVLFQISRLKNPIPNA
LNESPACPDPSEKWLKRLQHDTSDSHVPCSKKPKVGDGPLAGGTCTVFGQVFDCDSDSTG
MINHVKNKLICKGLTDQQSQEGSPMSAKSLNRWIGRWCRGGTPVFHGTSNLERQEAKSGM
PSDDLEGQFPSIAAMAMMGRVMNKLRPCELQKRGPSVVWRTEGL*
>11682.m04569|LOC_Os05g47870.1|genepair568-2
MSDPGEPSKHSQWMSHWTKGSSSAEPQVGRSNDSPEDAKYDICEDNSGPSNFEIMKSRLF
ERLMVGISQERASLEHGQKLNSNMKVVVKDARRHAVQNQIDQGDGPIQKSVMQKDVLYAK
AVVSKSLSIQKLSELSVDCQKLAGSDDLSSEWNHFPMLAINRKIDSILNPKRKSAKSTGP
NDVFVPKQTLKLNMTTANLMAFSSQEYELHSHRTTDETMDHCKHAGGIVSRLEDHAGVML
NPAEQKLKGQLLPATSCSCSKDDSNSSDSLLDEQHTSRYIADSDQEPTCRSREKRLKSSE
NNDTNCKIGSCSQNQKSRAPGHHKHKGSAGVMFRTSVPGKEFEAAEINCSDKINQRHLNT
QRIVSAANVTGSCIPDPAADISTVNGRGEAVTQPSSISGDSTKRKAPYLFEMLTIPSKAQ
NMNPEDSLPSGNSTAFGVHMYGTNIGSHLFGANNKSSTETEILSGDSQHVSKSSAGIASL
LAQKAKSEQLATLYMKGASGCNVNEHQGVSSKAIVANKQQCYNPRTARMDLDLMQFQLSR
MRNQESQARTEPGDRWLKRLQIDSKDPHHLPCSKRSKAGDGSGRPVTGGASSMAPRCDGS

NDDDDIVDRDHKEEQGLDEGVEIQGGREASPVPAKSDDRWIGRWCQGGVPVYHEDDHDQR
KEVTKPDLAAGDSGGLEGQFPSIKAMAMMGRAMSKVRPCQEERRGSFMVWKA*
>11667.m04805|LOC_Os01g49390.1|genepair569-1
MAAAAVAGVGRDGSFASQKRPRRVSVRMERSRVGDGCCCSCSGRGGVASTTAVRPSTGMV
VIVGATGTGKTKLSIDAAQELAGEVVNADKIQLYDGLDVTTNKVSLADRRGVPHHLLGAI
RAEAGELPPSSFRSLAAAAAAGIASRGRVPVVAGGSNSLIHALLADPIDAAPRDPFADAD
VGYRPALRFPCCLLWVDVDDDVLDEYLDRRVDDMVGEGMVEELEEYFATTSASERASHAG
LGKAIGVPELGDYFAGRKSLDAAIDEIKANTRVLAARQVGKIRRMADVWGWPIRRLDATA
TIRARLSGAGRAAEAAAWERDVRGPGLAAMRQFVGRADFNAAAVDQLAARSRRQCLRGGM
VAG*
>11682.m04566|LOC_Os05g47840.1|genepair569-2
MTSVATRIATLVRAAAAASRPLRLHRRPGGEDTRMVVIVGATGTGKTKLSIDAAKVIGGE
VVNADKIQLYDGLDVTTNKVSLADRRGVPHHLLGAIRPEAGELPPSSFRSLAAATAASIA
ARRLVPVIAGGSNSLIHALLADHFDASAGDPFSPAAAFRHYRPALRFPCCLLWVHVDEAL
LDEYLDRRVDDMVDAGMVEELREYFATTTAAERAAHSGLGKAIGVPELGDYFAGRKTFSE
AIDDIKANTRVLAAAQVSKIRRMSDAWGWPIHRLDASDTVRARLTRAGSAAESASWERDV
RGPGLATIRSFLADQSPPPRSEGTNDYLYAMETEPEPPPPPTLPPRLLRLPRMQYCDMVG
RICVLFLGEVVIHHIALLLTAALILQSSTFACIIAAVYECQVSWIIASFASGLFCRIAVL
DETVKELDIWKLKIVNGFLKITMCLKDWSGYPLGV*
>11667.m04807|LOC_Os01g49410.1|genepair570-1
MVLMAAVDDHDRHRAGNSSSPSEDSPAPAPAPAPAPPQPSRTRLHSFSFPTLSWGTHR
LLRCSKNPASSPPPAAPDTPSPDKEKAAHRSTDGVGGGCSPQRGPQRPWNLRTRRSATAA
PRPEGSDDAADAAPDRAPSPLAATKKRVFSIVLSKEEIGQDFKAIRGTRPPRRPKKRPRT
VQRQLDLLYPGLCLADLTPETYKIEER*
>11682.m04565|LOC_Os05g47830.1|genepair570-2
MAASASAAASDHHTAQAKSSPSAAPPAGSATRTRLHSFSFPTTFGWGTHRLLRCSKNGDS
APASASPPKQPHTPSPEKGQETSAGGASRPSRPWNLRTRRSATVAPDASRSEAAGKKAAA
AAGGGQALLHPPAPLPVVAKKRGFSVALTREEIVADFIAIRGTAPPRRPKKRPRAVRLEL
DRLYPGLSLADVNLDSYKIVEER*
>11667.m04810|LOC_Os01g49440.1|genepair571-1
MAATADPRAKPPTAHHLKPWVPTPTPRSHRVPSLPAVSGGGGGGARPARDRRRSSSSSSH
RRGGTTTDAGAVDEEEVVEAYDGGLEDLRAKLMGHIKDVADRLHLPQPKPQPRSPEPETP
PAPAPLPPPPPPPPDTSVAAAAAAARPWNLRERKRRPSARGSTAASPTTAWARRADTTRG
GGERPPFAVALAAEEIEEDMYALTGGRPRRRPRKRPRVVQRQLDSLFPGLWLTEITADAY
KVPDD*
>11682.m04563|LOC_Os05g47810.1|genepair571-2
MAATADPRAKPPAATSTHHLEPWGQPPPPPPTPAHRLPPTVLPVVALPAGGDAATRDRRR
SSSHRRGGGGGAAAAAAAVQVVVGEDPFDGGIEELRVKLMGHLRDAADRLRVPQPSPPPP
PPPPPPPPTTTTKPESLPAEADSEPELKAPPPPPPPPPLLPMAQPQADGAARPWNLRQRT
RRRPAASMSWAAAAAVPVPSSSRRRKRAPFSVALTPEEIEEDIYALTGSRPRRRPRKRPR
VVQRQLDSLFPGLWLTEVTADAYRVPDE*
>11667.m04813|LOC_Os01g49470.1|genepair572-1
MATPTPMAGEGTLAAVMPRSPSPTASAAAGSAAEAPMLIFLYFHKAIRAELEGLHAAAVR
LATERAGDVGALAERCRFFVNIYKHHCDAEDAVIFPALDIRVKNVAGTYSLEHKGENDLF
SQLFALLQLDIQNDDSLRRELASCTGAIQTCLSQHMSKEEEQVFPLLTKKFSYEEQADLV
WQFLCNIPVNMMAEFLPWLSSSVSSDEHEDIRSCLCKIVPEEKLLQQVVFAWIEGKTTRK
VTENSTKSNSEATCDCKDASSIDHADNHISSHEDSKAGNKKYAESIDGQVERHPIDEILY
WHNAIRKELIDIAEETRRMQQSGNFSDISSFNARLQFIADVCIFHSIAEDQVVFPAVDSE
LSFVHEHAEEERRFNNFRCLIQQIQIAGAKSTALDFYSELCSHADQIMETIEKHFCDEET
KVLPQARMLFSPEKQRQLLYKSLCVMPLKLLERVLPWLVSKLSDEEASSFLENMRLAAPS
SETALVTLFSGWACKARSEDKSNSGEYLCLTSGEMRCLLDEVDGLEKCRPFCPCASRSNT
DASLHPQTENGSRPGKRGNDAESVPGTNGSDLSQTDDTEARPCSKKPCCIPGLRVETGNL
AISSSLASAKSFRSLSYNSSAPSLYSSLFSWETDASLSCSDGISRPIDTIFKFHKAIRKD
LEYLDVESGKLIDGDESCLRQFIGRFRLLWGLYRAHSNAEDEIVFPALESRETLHNVSHS
YTLDHKQEEQLFGDISDALAELSQLHERLTHPHIEVSEAEKNDFNSSDEIDWTRKYNELA
TKLQGMCKSIRAALTNHVHREELELWPLFDEHFSVEEQDKLVGRIIGSTGAEVLQSMLPW
VTSALTQEEQNMMLDTWKQATKNTMFGEWLNEWWKGAPTSSDSSEEASSAPEDSHLQDKI
DQNDQMFKPGWKDIFRMNQSELEAEVRKVSRDPTLDPRRKAYLIQNLMTSRWIAAQQKLP
EPKSEECSEGAGIPGCAPSYRDQEKQIFGCEHYKRNCKLVAACCNKLFTCRFCHDKISDH
TMERKATQEMMCMVCLKVQPVGPNCQTPSCNGLSMAKYYCNICKFFDDERTVYHCPFCNL
CRLGKGLGVDFFHCMKCNCCLGMKLTEHKCREKGLETNCPICCDFLFTSSAAVRALPCGH
FMHSACFQAYTCSHYTCPICCKSLGDMAVYFGMLDALLAAEELPEEYRDRCQDILCNDCE
RKGRSRFHWLYHKCGSCGSYNTRVIKTDTADCSTPN*
>11682.m04560|LOC_Os05g47780.1|genepair572-2
MATPLADEGSIAAAVMPRSPSPPAAAAGSAAEAPMLIFVYFHKAIRAELERLHAAAVRLA
TERSGDVGELERRCRFLFSVYRHHCDAEDAVIFPALDIRVKNVAGTYSLEHKGENDLFAH
LFSLLKLDVRNDDGLRRELASCTGAIQTFITQHMSKEEEQVFPLLIKKFSHEEQADLVWQ
FLCSIPVNMMAEFLPWLATSVSSDEHQDILNCLHKIVPDEKLLQQVVFAWIGGEAVKTIS
HDFCSPCSKSNVRCKDAIDQTDKYGCSHEHFKTGKRKRAESSYSQLVMHPIDEILCWHNA
IRKELSDIVEETRRIQQSGDFSDISDFNVKLQFIADVCIFHSIAEDQVIFPAVNDQVSFE
QEHAEEERRFNKFRCLIEQIQITGARSTAVDFYSELCSQADQIMEKIERHFKNEETKVLP
QARIHFSSEKQRELLYKSLCVIPLKLLERVLPWFVSKLNDQDAEAFLQNMFLAESFRSLS
LNYSAPSLYSSLFSWETDAAFSGPDNISRPIDTIFKFHKAIRKDLEFLDVESRKLIDGDE
SSLRQFIGRFRLLWGLYRAHSNAEDEIVFPALESKETLHNVSHSYTLDHKQEEELFKDIS

TILFELSQLHADLKHPLGGADAVGANHIHPYNRIDWSKKNNELLTKLQGMCKSIRVTLSN
HVHREELELWPLFDKHFSVEEQDKIVGRIIGSTGAEVLQSMLPWVTSALSLDEQNNMLDT
WRQVTKNTMFDEWLNEWWKRSPTSSGPSSDASHPEEDHFQEKFDQSEQMFKPGWKDIFRM
NQSELEAEIRKVSRDSTLDPRRKAYLIQNLMTSRWIAAQQKSPQPQSEDRNGCTVLPGCC
PSYRDPENQIFGCFVMIKLVTIQWKEHKCREKMLEMNCPICCDFLFTSSAAVKGLPCGHF
MHSACFQAYTCSHYTCPICSKSLGDMTVYFGMLDGLLAAEELPEEYRDRCQSFQLPGPNS
LENQEKPNQMGTKGVSSQGIGGMWGNKIVGTQIWK*
>11667.m04818|LOC_Os01g49520.1|genepair573-1
MSRLCHHGGRVLLPLLLVAAACLGDPTGDTYDTAMCGAQASITCGGVIVRYPFYLSNATR
ALPKYANSSTFCGYPGLEIICDGGGGGKAVMMLGNDSYTVSRIDYASLTVSLADADVANG
TCPVVSHNVTIPPAPSSLHLADTVGMLIFFFRCAFGPAANAPPKPPSIHPLTCGENSEDA
PTQSFLLPASPLPPGDLWHRGCSAVYGVPVLGGSLPSDANDPAWRKDGYIASLRKGFQMS
WDRSDRCSRCELTSGKCGYNQNGKFLGCLCANGLVDSDACSKISDSTLRLAGSNLKTKII
AGVVGGGLSAVFALGLIATVFFVRKRKHKKVNSSSKLLKYSGSGGTPRSMGGDMESGSVK
DLQTHLFSYEELEEATDSFNENRELGDGGFGTVYKGILRDGRVVAVKRLYNNSYRRVEQF
VNEAAILSRLRHPNLVMFYGCTSSQSRELLLVYEFVANGTVADHLHGHRAQERALSWPLR
LNIAVESAAALTYLHAIEPPIVHRDVKTTNILLDADFHVKVADFGLSRLFPLDVTHVSTA
PQGTPGYVDPEYHQCYQLTDKSDVYSFGVVLVELISSKPAVDITRQRNEINLAGMAINRI
QKSQLEELVDLELGYESDPATKKMMTMVAELAFRCLQQNGEMRPPIKEVLEGLKGVQDLC
VMEKDGGKDKKGPDPPLSPDTVHAQWDSRQTTPNTSQ*
>11682.m04559|LOC_Os05g47770.1|genepair573-2
MPPLLFHLLVLSVVVVVVSGGAATAGGGTYDDAICARPIFCGEQVEIKYPFYLSNTTDQV
VVVDGNTRYCGYPWLGIICDHDRAILRLGNYNYTVLEINHGNHTVTVADSDALDGGDCPR
VKHNVTLPEVLTFPSPGNDSITFFFDCNSTANVVLRPPPYIRPINCSTFDFPGRRDTAPS
FVATQPDVAGETEWLGLCKEVVMVPVLKDWLMNEKYYGKLGDDGYGAVLKRGFQLSWDPT
AGMCHECEVSGGRCSYGTKNEFLGCLCSDGHVSKTDCVHHTFEKMHPLCSLPLLIIILLS
SVPPSMQESGAYFRYTNCTPASYQCGSLKFDVDYPFSANGVHRPDYCSYPGYRLICSPDN
KLMIHMNNSTAFQVTDIDYGNKFLAVIDQTQPQEACLDRYHNTTIDESKFMYTDRDQFLTV
YVNCSANFSSLPLIYDLVSCVSGGSSYYRLHKNKDDSLESDILGSCSSTIVVPCNSTMAG
SLAAGNSSLADVIRGGFTARWKVGLGWCSDCKASGGHCGFNGSFPDQYTCYCPYGQAIGS
CSSSGSKKSKKKAIAIATSIASGVLFLLLLVVSFLYIRKRRQYKMTSSSRLLKYTTSGRT
PRSKGSSDKFVESGSFHYLQTHHFAYEELEEATDGFSDARELGDGGFGTVYKGELRDGRV
VAVKRLYNNSCRRVEQFVNEAAILSRLRHPNLVLFYGCTSSRSRELLLVYEFVPNGTVAD
HLHGHRAPERALTWPLRLNVAVEAAAALAYLHAVEPAPIVHRDVKTNNILLDANFHVKVA
DFGLSRLFPRDATHVSTAPQGTPGYVDPEYHQCYQLTDKSDVYSFGVVLVELISSKPAVD
VTRDRDEINLAGMAVNKIQRCQVDQLVDDELGYSSDEATRKTMTMVAELAFRCLQHNGEM
RPPIKEVADVLRGIQDECRAAEKGGKRGSPCSPNTVHAPWDSMSTTPNTSQ*
>11667.m04825|LOC_Os01g49590.1|genepair574-1
MLLSLCLRLLPLLLVLVAASHGDASGDTYDTSMCLQKPTTCGNVSISYPFYFATKTKDIN
GSSNSYCGYPGLAIDCDDGKPILQLNGTEKYKVNYINYGSITNVSLADLEVVDDSSGCPR
VDHNVTIPQISWLFFSGISVDYLVFFLRCSFTTFAPKPANFNPIACGSFINLTRPSFVFP
DELVPPGNWSQLCEETFEVPVLKYQLMEMDSNGNAWNNSGYAQPNLLYPYQYSGYAVMDK
TRYSILMPFSVPNFLHANIIAGTSSVLLLCLLSFACLFGLKKSRYRRISKGTPRIESFLQ
RNGTLHPKRYTYTEVKRMTKSFAEKLGHGGFGAVYRGNLSDGRQVAVKMLKDSKGDGEEF
INEVASISRTSHVNVVTLLGFCLHGSKRVLIYEYMPNGSLERYAFRNNSEGEHSLTWEKL
FDVVVGIARGLEYLHRGCNTRIVHFDIKPHNILLDQEFCPKISDFGMAKLCSNKESIISI
AGARGTIGYIAPEVYSKQFGAISSKSDVYSYGMMILEMVGARERNIDANSESSSHYFPQW
IYEHLDEYCINSSEIDGETTELVDLGLYSSPSYGIIDTFCEDDVVVPYLVDNLKEFLDKL
DCEVVPNICYC*
>11682.m04557|LOC_Os05g47750.1|genepair574-2
MHPLCALPLLITLLLISSVPLSVQESDAFFRYTNCTTASYQCGSLKLDVDYPFSANGVDR
PNYCSYPGYRLICNPDNKLMIHMNSTVFQVTDIDYGNKFLAVIDQTQPQETCPDRYHNTT
IDESRFMYTDLDQFLTVYVNCSAKSSSLPFIYDLLSCVSGGSSYYRLHKNKDDSLESDIL
GSCSSSFVVPFNSTMAGSLAAGNSSLVDVIRGGFTARWKVGVGYRIMFFIRYRLYLLTNL
RASQGSKISKKKAIAIGVSVVSGVILLFLLLMCTLCVKKFWHGLLSSMGKSKEAPNIESF
LQKHEAQHPKRYSYSEVKTMTKSFSHKLGQGGFGTVYMGKMPNGKPIAVKLLKSCKDDGQ
EFMNEVASISRTSHVNVVTLLGYCIVEIIDI*
>11667.m04830|LOC_Os01g49640.1|genepair575-1
MTMKPNEVLVLVLVLAVVTSPGTVCGASRTAPAAATKCDPLALRPCAAAILWGEAPSTAC
CAGLRAQKRCLCRYAKNPDLRKYINSQNSRKVAAACSVPAPRC*
>11682.m04552|LOC_Os05g47700.1|genepair575-2
MAKVALAVAAMAVLLLLAAAAAPGAEAACDALQLSPCASAIIGNASPSASCCSRMKEQQP
CLCQYARDPNLQRYVNSPNGKKVLAACHVPVPSC*
>11667.m04832|LOC_Os01g49660.1|genepair576-1
MPPHFPRDSDSDQNVRMPFHGHKSIHKLLGGGQVEYNIIPLLCQIAILAMLVIFIWSNAA
PLLDRAPPRIPEIIISEHAFREMALTVHYKLTYTVSVLYDIACGKDLKRFLLFWTNNQIV
YDIKVVGSLLVLSAIGSSCSLTSLLYIGFLCAHTLPVLYQRYKTEVDHLVAKGSDDIKKF
YKKVDSNLLNKIPRGPVKTKVK*
>11682.m04551|LOC_Os05g47690.1|genepair576-2
MNVPHVSSDSDDDRPVIRLFHRQKPVHKILGGRKVADIMLWRDRNLSAGILAGATLIWFL
FDVAEYNLVTLLCHIALLGMLVLFIWSNAAPLFDRAPPRIPEVIVSEHAFRELALTLHSK
VAHFSAVLYDISCGKELRKFLAVIGSLWILAVIGETCSFTTLLYVGFLCALTLPALYERY
ETEVDHLVAKGGQDLKKFYKKIDSNVLNKIPRGPVKTKVH*
>11667.m04842|LOC_Os01g49740.1|genepair577-1

MEAAITMDENLGRSGSFRAAMSIFGESINGRKADKNRGTVPAQENLSSEMKQLAQSGLDK
LNERKAYVDKERAGAESELSRARAMAKELERQIEQTTAKATSQRSELQAMWAARTRRKGT
DAPGAERDARYAEVVQELDQAKKELLRLRLEVRLRAIDEMKRRVDEANEEHVLVELARIE
AERERREIEAQRGAEAERFAADIEAARARVRALQKEASRAREMEAKLAVTNSDVEVLQAE
MELVRAMEKSHAKSDEAAEDAARRKKEEAQDKALLKTAEAELDAAKKELETIKAGSFQFM
TSMDCTRTEIMRVAEEIRLLKAKEKKADAQVQQLNTKLLKAKARLEAVTAADERSKAIVS
NLAAAMKQLKAETEAARMEEDLTKLEKRCVIAEAENIEKEIATTEGRIKQSVKELDAAKA
LEAEAMRKLRDTVESTMQARASSAARRQGTMTISRFEYEYLTGRAALVRVVTDKKVAAAQ
AWVQALKASEKEAAARAEAAEREVREMEARAAQVAAEAEKTAAEQKELEQELYDLNAAAE
RDGLQCAYPRRRSSRVSATSRRSKPRRSSVSAGAWNPRSPSFTIKRKRKVMPNLLKLIKN
KRGGKKNTN*
>11682.m04550|LOC_Os05g47680.1|genepair577-2
MEENSVGSGSGGNAMDIFGQSIDVRRPSKSRRRVVSHKNLSPEIEESIGSSRRKLHRRKA
IAEDQEQARVESELSRAMNMAMELERQIEQTNAKARSRRSELQRQRTRASGGGSRRKTAR
GLAAEAAGGAPAHRQEGVGTAYGEVMQELDRVKGELRKLQREVMAAMAAKGTAGRRDAEA
EASTSSAVSSGPRGGGGVERDADGASEEHGVLVELAVGTAATASSDAGSWHSELAVVRAT
DATAMASRGHEVEHEEPSLQAAEAELSSARIELESIKAEGLRFTASIERTRRETARVTDE
IRRLTEQEKAASAHVQQLNAKLLKARSRLEAVTAADERADETISKLAAILRQLEDDAAAA
EKEKTLADTENRRAMSDAENIDAEIAAAEKRIRESVRELGAARASEAAATARLKAIVESA
TLATAAAATPRSSSSGNVTIPRFEYEYLTGRAEVVRAVAEMKAAAAEAWAEARRASEKEI
AMRAEAIERELGEARAADAEATNTTRRMPFSSAATSRMAKSRRMPSSSAAAAARKPRSPS
SSVKRRKRRVLTLNCLKLLAGKCRGQN*
>11667.m04847|LOC_Os01g49770.1|genepair578-1
MAGDRRGGGGVVSADGERRRGIRRLLLPRGEGSSSSSPPQPPPLQAEEGRRKGFASAALR
GLGCTSAAASQAYAPGAGAAAAAAVRSSADWHGRRRRRGKEKRKERGGGGGGGGGGHLVG
GGIGADVWCAPGIPFAAEASSVDCVVARHQMVGRGRGGDAERPHRERPCLSRRVTVQEQI
SSSFMDSPPPPHLDVAPFFGADLLPSGRLRRMRGYRHSPVGLEEEIMMFQTRVLLGGMSM
YDRYQDWRLDVDNMTYEELLELGDKIGYVNTGLREDEIVRNLRKVKHPAFDSSFRYSTEM
EKKCSICQEEFEANEEMGRLDCGHSYHVYCIKQWLSQKNVCPVCKTAVTKT*
>11682.m04549|LOC_Os05g47670.1|genepair578-2
MARRDGVGGDGGASAAEQQRRVALRVLLSRAEASSPPPATVEEEAQRGRSGGGNKGLASA
ALRGLGCTSTAALRAHAPASAVEVASSSERWHGRRRRRKVQERRSARGGGGGGGGGVAPP
GPAPAAAGDVWCTCAPGIPFAAEASSVDCVVVARHHHAHHTAAAMGSGRRGEAERRHRER
PAAPRARRVTMREHISSSLMDSPPFPDMPLLNADLLPPPPSGRHRHGYRHPHVGAAEEEI
MMLRTRLLWGRFGMHDQHQDWRLDVDNMTYEELLDLEDRIGYVSTGLHDDEIARSLRMVK
YSAFNPKHFATEVERNCSICQEEFEANEETGRLICGHSYHVQCIKQWLSRKNTCPVCKTV
VSKT*
>11667.m04852|LOC_Os01g49820.1|genepair579-1
MPAPSASIHLGVPTPYITSHGSKIARLHMYDWIVLILLVVVDGILNIIEPFHRFVGSDMM
TDLRYPMKDNTVPFWAVPIIGIIGPMIIITGIYFKKRNVYDFHHAILGLLFSVLITAVIT
DAIKDGVGRPRPDFFWRCFPDGVPAYDNFTTGVLCHGKASVIKEGHKSFPSGHTSWSFAG
LGFLSWYLAGKIKVFDRRGHVAKLCIIILPLLLAALVAVSRVDDYWHHWQDVFTGGILGL
VVSSFCYLQFFPMPSDENGLWPHAYARHILNPDQLENNAQPTSVDRPNSLPNGSFRSPNG
LEMGNTGQGQALDFMEAGRRYQ*
>11682.m04548|LOC_Os05g47660.1|genepair579-2
MPPPPPATSPAPAAIRLGAPHPYLRTHGTKVARLHLLDWIVLALLVAIDAGLNLIEPFHR
FVGEDMMISLRYPLKRNTVPIWAVPVRLHLPPFLDFRKKKTVPDRLLFSVLITAVLTDAI
KDGVGRPRPNFFWRCFPDGIPKYNNITRGVICHGDKSVIKEGHKSFPSGHTSWSFAGLGF
LSWYLAGKIKAFDRGGHVAKLCIVVLPLLIAAMVGVSRVDDYWHHWQDVFTGGILGLVVA
SFCYLQFFPPPAGEQGGGEHDLHGPRGWASTEAELTDGGALAAEEGVEVRDAIVEPVNGG
WGGGRGKDVAAVVRVCGGGFWPHAYFEHILHPEVENQVQLTATSNHHQGLDMRTNNQSLD
SMEEGRRAR*
>11667.m04854|LOC_Os01g49830.1|genepair580-1
MDSSSCLVDDTNSGGSSTDKLRALAAAAAETAPLERMGSGASAVVDAAEPGAEADSGSGG
RVCGGGGGGAGGAGGKLPSSKFKGVVPQPNGRWGAQIYERHQRVWLGTFAGEDDAARAYD
VAAQRFRGRDAVTNFRPLAEADPDAAAELRFLATRSKAEVVDMLRKHTYFDELAQSKRTF
AASTPSAATTTASLSNGHLSSPRSPFAPAAARDHLFDKTVTPSDVGKLNRLVIPKQHAEK
HFPLQLPSAGGESKGVLLNFEDAAGKVWRFRYSYWNSSQSYVLTKGWSRFVKEKGLHAGD
VVGFYRSAASAGDDGKLFIDCKLVRSTGAALASPADQPAPSPVKAVRLFGVDLLTAPAPV
EQMAGCKRARDLAATTPPQAAAFKKQCIELALV*
>11682.m04547|LOC_Os05g47650.1|genepair580-2
MDSTSCLLDDASSGASTGKKAAAAAASKALQRVGSGASAVMDAAEPGAEADSGGERRGGG
GGKLPSSKYKGVVPQPNGRWGAQIYERHQRVWLGTFTGEAEAARAYDVAAQRFRGRDAVT
NFRPLAESDPEAAVELRFLASRSKAEVVDMLRKHTYLEELTQNKRAFAAISPPPPKHPAS
SPTSSSAAREHLFDKTVTPSDVGKLNRLVIPKQHAEKHFPLQLPPPTTTSSVAAAADAAA
GGGDCKGVLLNFEDAAGKVWKFRYSYWNSSQSYVLTKGWSRFVKEKGLHAGDAVGFYRAA
GKNAQLFIDCKVRAKPTTAAAAAAFLSAVAAAAAPPPAVKAIRLFGVDLLTAAAPELQDA
GGAAMTKSKRAMDAMAESQAHVVFKKQCIELALT*
>11667.m04860|LOC_Os01g49890.1|genepair581-1
MATATASSLSLLFAHPHSSNPRPFAGGPHLRRPLRAAPHRARCASDAATTATRHRRPAEE
NIREEAARLRGPGNDFSAWYVPFPPTPEDDPDERYSLDEVVYRSSSGGLLDVCHDMEALA
RFPGSYWRDLFDSRVGRTAWPYGSGVWSKKEFVLPEIDSDHIVSLFEGNSNLFWAERLGR
EHLGGMTDLWVKHCGISHTGSFKDLGMTVLVSQVNRLRRAPLSRPINGVGCASTGDTSAA
LSAYCAAAGIPAIVFLPADRISLQQLIQPIANGATVLSLDTDFDGCMRLIREVTAELPIY

LANSLNSLRLEGQKTAAIEILQQFDWQVPDWVIVPGGNLGNIYAFYKGFEMCRVLGLVDR
VPRLVCAQAANANPLYRFYKSGWTDFQPRVAETTFASAIQIGDPVSVDRAVVALKATDGI
VEEATEEELMDAMSLADRTGMFACPHTGVALAALFKLRDQRIIGPNDRTVVVSTAHGLKF
TQSKIDYHDRNIKDMLCQYANPPINVKADFASVMDVLQNKLNGKI*
>11682.m04546|LOC_Os05g47640.1|genepair581-2
MAATTHAASLSFLLSHPHPTSPNPNPNPNLPLRRAPHRVRCATDAAATRHRRAADENIRE
EAARHRAPNHNFSAWYAPFPPAPNGDPDERYSLDEIVYRSSSGGLLDVRHDMDALARFPG
SYWRDLFDSRVGRTTWPFGSGVWSKKEFVLPEIDPDHIVSLFEGNSNLFWAERLGRDHLA
GMNDLWVKHCGISHTGSFKDLGMTVLVSQVNRLRRAPLSRPIAGVGCASTGDTSAALSAY
CAAAGIPAIVFLPANRISLEQLIQPIANGATVLSLDTDFDGCMRLIREVTAELPIYLANS
LNSLRLEGQKTAAIEILQQFDWEVPDWVIVPGGNLGNIYAFYKGFEMCRVLGLVDRVPRL
VCAQAANANPLYRYYKSGWTEFTPQVAEPTFASAIQIGDPVSVDRAVVALKATDGIVEEA
TEEELMNAMSLADRTGMFACPHTGVALAALFKLRDQRIIGPNDRTVVVSTAHGLKFSQSK
IDYHDSKIEDMACKYANPPVSVKADFGAVMDVLKKRLKVPCSWHMNTASICSECRPCLSV
VLNFLVTFHPYLVKAGKLIIVECKMHGSWLHLHQCPVAKEIADYTISA*
>11667.m04861|LOC_Os01g49900.1|genepair582-1
MRLLSGASASRIPCPLLSLARARARCLPVPASATACRAASSSAAAAAGDGGALKPWLFVG
LGNPGKVYQGTRHNVGFEMIDVIAEAEGISLSSMQFKAMVGKGRIGDAPIMLAKPQTFMN
ASGESVGQLVSYFKIPLNQVLVMYDDLDLPFAKLRLLPKGGHGGHNGVRSIINHLKQNRD
FPRLRIGIGRPPGKMDPANFVLRPFNRKEQEELDFAFHRGLEAVRIMALEGFNKSATYVN
TAQSSEMLNR*
>11682.m04545|LOC_Os05g47630.1|genepair582-2
MAAAGPACCAPAAAAAAASSSASASAAAAAGDGGGGAQKPWLLIGLGNPGRMYKGTRHNV
GFEMIDAIAEAEGISVSSKQFKSMVGKGLIGDVPVMLAKPQTYMNASGESVAQLVSYFKI
PLSQVLVIYDDLDIPFAKLRLLPKGGHGGHNGMRSIINHLKQSRDFPRLRIGIGRPTGKL
DAIGFVLRSFTKEEQEELNLTINRSLQAVRIMLLEGFNKGATFVNTPQPSEMLNK*
>11667.m04872|LOC_Os01g50010.1|genepair583-1
MSALQSWRKAYGALKDTTTVSLANLNSDFKDLDVAIVKATNHVECPPKERHLRKIAAATS
IGRPRADVAYCIHALARRLAKTRNWIVALKTLVVIHRLLRDGDPTFREEFLTFTQRVRIL
QLSNFKDDSTPVAWDYSSWVRTYGLFLEERLECFRVLKYDIEAERLSKQGQGPEKGHSRT
RELDSPDLLEQLPALQQLLYRLIGCRPEGAANSNYLVQYALALVLKESFKIYCAINDGII
NLVDKFFEMPRHEALKALEIYRRAGQQAGSLSDFYENCRGLELARNFQFPTLREPPQSFL
STMEEYVREAPRMVPIKEPLEFPERLLLTYKPEESEEIPEPVSAEEEKPQIEEPAVAVPS
TEVVPPPPPKPEVVDTGDLLGLSDPTPSVSAIEESNALALAIIPTGGETSTSGTATLQDK
GFDPTGWELALVTTPSTNTNSMAMDSNLGGGFDKLILDSLYDEGTYRQQMQQQQLYGSAA
PNPFMASDPFAMSNQVAPPPSVQMASMTQQPQQMPMMMQPNPFGPPLQPQHAGIAQAPNP
FLDAGFGPFPASNGMHPQANPFGTAQLL*
>11682.m04536|LOC_Os05g47550.1|genepair583-2
MAAMQSWRKAYGALKDSTTVSLANLNSDFKDLDVAIVKATNHVECPPKERHLRKIVAATS
IARPRADVAYCIHALSRRLAKTRNWIVALKTLVVIHRLLREGDPTFREELLNFAQRGRIL
QLSNFKDDSSPIAWDCSAWVRTYGLFLEERLECFRVLKYDVEAERLSKQGQGPEKGHSRT
RELESQDLLEQLPALQQLLYRLVGCRPEGAANNNYLVQYALALVLKESFKIYCAINDGII
NLVDKFFEMPRHEALKALEIYRRAGQQAGSLSDFYENCRGLELARNFQFPTLREPPQTFL
VTMEEYVREAPRMVPVREPLELPERLLLTYKPEEQEEDSVPDPVEEEKPPVEEPVPVPPV
TEAVSPPPPPKTKVADTGDLLGLNDPNPSVSAIEESNALALAIVPADAGASTSSTATWQD
KGFDPTGWELALVTTPSNTNSSAADSQLGGGFDKLILESLYDQGDYRQRQQQQLYGSSAP
NPFMSNDPFVMSNQVAPPPSVQMAAMSQQHQQIPTMMQANPFGPPMQPQHVGMGPATNPF
LDSGFGPFPMANNGHQQANPFGGTQLL*
>11667.m04874|LOC_Os01g50030.1|genepair584-1
MDAAAATAVNGVLEVEERKAQKSYWEEHSKDLTVEAMMLDSRAADLDKEERPEILSLLPP
YEGKSVLELGAGIGRFTGELVKTAGHVLAMDFIESVIKKNESINGHHKNASFMCADVTCP
DLMIEDNSIDLIFSNWLLMYLSDEEVEKLVKRMVRWLKVGGYIFFRESCFHQSGDSKRKV
NPTHYREPRFYTKVFKECQALDQDGNSFELSVLTCKCVGAYVKSKKNQNQICWLWQKVDS
TEDRGFQRFLDNVQYKASGILRYERIFGEGFVSTGGIETTKEFVDRLDLKPGQNVLDVGC
GIGGGDFYMADKYDVHVVGIDLSINMVSFALERAIGRKCSVEFEVADCTKKTYPDNTFDV
IYSRDTILHIQDKPSLFKSFFKWLKPGGKVLISDYCKCPGKPSEEFAAYIKQRGYDLHDV
RAYGQMLENAGFHDVIAEDRTDQFLDVLERELAKVEKNKNEFVSDFSQEDYDAIVNGWKA
KLQRSSAGEQRWGLFIATK*
>11682.m04535|LOC_Os05g47540.1|genepair584-2
MDAVAANGIGEVERKAQRSYWEEHSKDLTVEAMMLDSRAADLDKEERPEVLSVLPSYKGK
SVLELGAGIGRFTGELAKEAGHVLALDFIESVIKKNENINGHHKNITFMCADVTSPDLTI
EDNSIDLIFSNWLLMYLSDEEVEKLVGRMVKWLKVGGHIFFRESCFHQSGDSKRKVNPTH
YREPRFYTKIFKECHSYDKDGGSYELSLETCKCIGAYVKSKKNQNQLCWLWEKVKSTEDR
GFQRFLDNVQYKTTGILRYERVFGEGYVSTGGIETTKEFVDKLDLKPGQKVLDVGCGIGG
GDFYMAENYDAHVLGIDLSINMVSFAIERAIGRKCSVEFEVADCTTKTYAPNTFDVIYSR
DTILHIHDKPALFRSFFKWLKPGGKVLISDYCRNPGKPSEEFAAYIKQRGYDLHDVKTYG
KMLEDAGFHHVIAEDRTDQFLRVLQRELAEVEKNKEAFMADFTQEDYDDIVNGWNAKLKR
SSAGEQRWGLFIATK*
>11667.m04883|LOC_Os01g50100.1|genepair585-1
MDESGRGTGDDHGRETKDAAAAASSSSGKKVPLFSLFRYADRLDVLLMVVGTVGALGNGI
SQPLMTVLFGNVINSFGANTSGSVLRSVTKVVLNFIYLGIGTSVASFLQVSCWTMAGERQ
SARIRSLYLKAVLRQDITFFDTEMTTGEAVSRMSSDTLLIQGALGEKAGGKLVELLSSFI
GGFIIAFTRGWLLTLVMLTSLPLIAIASAVSAQALTRVSSKRQTSYSDAGDTVEQTIGSI
RTVVSFNGEKKAIAMYRNFIKKSYKATIEEGIITGFGMGSVMCVVFGSYGLAFWYGGKLI

IEKGYTGGKIMTILFAVLTGASSLGNATPAVAAVVEGQSAAYNLFKTIERKPEIDSDDNN
GMVLEDMNGDIELKDVYFRYPARPEQLILDGLSLQVASGTTMAIVGESGSGKSTVISLVE
RFYDPQSGEVLIDGISIKKLRLDWIRGKIGLVSQEPLLFMASIKDNIIYGKKDATLEEIK
RAAELANAANFIDKLPNGYDTLVGQRGTQLSGGQKQRIAIARAILKDPKILLLDEATSAL
DVESERIVQEALNRMMVERTTLVVAHRLSTVRNVDCITVVRKGKIVEQGPHDALVKDPDG
AYSQLIRLQETHRDERHKLPDSRSKSTSLSFRRSRTKDFLSKSNRYSFKSPLGLPVDIHE
DGMTSEQQKVDHSDTEYFLFGIAGGKLIQRVRTLSFQRIMHQEVAWFDKPSNSSGALGTR
LSVDALNVRRLVGDNLALIVQAVATLITGFAIAFAADWRLALIITCVIPLVGAQGYAQVK
FLKGFSEESKEMYEDANQVAADAVGSIRTVASFCSEKRVVAIYNKKCEALRKQGIRSGIV
GGIGLSFSNLMLYLTYGLCFYVGAKFVSQGKTTFSDVFKVFFALVLAAVGVSQSSALSTN
ATKARDSAISIFSIIDRKSRIDSSSDEGAIMENVTGSIDFNNVSFKYPSRPDVQIFSDFT
LHIPSQKTIALVGESGSGKSTIIALLERFYDPDSGNISLDGVEIRSLKVSWLRDQMGLVG
QEPVLFNDTIRANITYGKHSEVTEEEITAVAKAANAHEFVSSLPQGYDTVVGEKGVQLSG
GQKQRVAIARAILKDPKILLLDEATSALDAESERVVQDALDRVMVNRTTIVVAHRLSTIK
GADMIAVLKEGKIAEKGKHEALLRIKDGAYASLVQLRSNSE*
>11682.m04531|LOC_Os05g47500.1|genepair585-2
MEEASTARAADGDKRGKEENDRRMAKDGKVAFHHLFKYADSTDVALMLVGTIASLASGMS
QVIMTIIFGQMVDAFGKSSPGNILHQVNKAVLYFVYLGIGSGIVCFLQVSCWSVTGERQA
TRIRSLYLKTILRQDMAFFDKEMTTGQVISSISTDTTLIQGATGEKVGKFLQLVTTFPGG
FVLAFLKGWLLTLVMLSTIPPFIFAAGIVSKMLAKISNEGLASYSKAGDIVEQTVGSIRT
VVSFNGEKKAIGLYNDLIKKAYKGAVKEGFIQGFGMGFLNLIYFSSFGLIVWYGSKLSLS
RGYSGADIMNILFGIMIGARALGDATPCTAAFEEGRIAAYRLFKVIKRKPEIDYDDTSGI
VLEDIKGDIELKDVFFSYPSRSEQLIFDGFSMCVSNGTTMAIVGESGSGKSTVINLVERF
YDPQAGEVLIDGMNIKSLRLEWIRGKIGLVNQEPILFMTSIKDNILYGKENATLEEIKRA
AELANAARFIESMPNGYDTLVGQRGAQLSGGQKQRIAIARAILKNPKILLLDEATSALDL
ESERIVQDALNQIMVGRTTLVVAHRLSTVRNAHCISVVHKGKIAEQGHHDELVKDPNGAY
SQLIRLQEAQQAIDPHLDGPLNKRSQSLKRSLSRNSAGSSSHSLNLPFSLRGATELLEYD
GADGENRNLKNDGKLPKKGSMGRLISLNKPEIAILLFGSLAAAIDGAVFPMIGLVLASAV
KVFYESPDKREKDATFWGLLCVGMGAIAMISKLANILLFAIAGGKLIKRIRALTFRSIVH
QEVSWFDHPANSSGALGGKLCVDALNGYAQVRFLQGFSQDAKIMYEEASQVATDAVGSIR
TVASYCAEKKVMTKYNQKCQASRYQGIRTGIVGGLGFGFSNMMLFMTSALCYYVGAKFVS
QGNSTFGDVFKAFFSLVVAMLGVSSTAAMASDSSKAKDSASSIFAILDRKSQIDSSSNEG
LTLELVKGDIEFTHISFRYPSRPDVQIFSDFTLSIPSGKTVALVGQSGSGKSTAIALLER
FYDPDSGVILLDGVEIKKLEISWLRDQMGLVSQEPVLFNDTIRANIAYGKNEEVTEEEIV
AAAKAANAHEFISSMPEGYSTSVGERGTQLSGGQKQRIAIARAIVKDPRILLLDEATSAL
DAESERIVQDALDHVMVGRTTVVVAHRLSTIQGADIIAVLKDGAIVEKGRHEALMGIASG
AYASLVELRHNVT*
>11667.m04916|LOC_Os01g50400.1|genepair586-1
MEAAVDGRWTRVRTLGRGASGAVVSLAADDRSGALFAVKSAAAAAAAEQLVREGRILSGL
RSPHVLPCLGFRAEAGGECQLFLEFAPGGSLADVVARSGGRLDECAIRAYAADVARGLAY
LHGMSLVHGDVKGRNVVVGADGRAKIADFGCARTVGSDRPIGGTPAFMAPEVARGEEQEP
AADVWALGCTVIEMATGRAPWSDMEDILSAVRRIGYTDAVPEVPEWLSAEAKDFLARCFA
RNPRERWTSSQLLEHPFLASAGCSVKTGEAAPQWVSPKSTLDVAFWESDTDDEEDDMPAS
PAERIKALACPCSALPDWDSDEGWIQVLNESSEACDTAVAKVEAEGKGRVLIEALETINE
SSGGDAECVDPEGTVRLITVASIGQQEVFCLGLINDPLVFSVNKSELTKSLFPQIVSF*
>11682.m04504|LOC_Os05g46760.1|genepair586-2
MAVAVAAAAAVSRQWTRVRTLGRGASGAEVFLAADDASGELFAVKSVGAAGAAALRREQG
VMAGLSSPHVVPCIGGRVGRDGSYQMFLEFAPGGSLADVAARCGGRMEECAVGEYAVDVA
RGLAYLHGMGLVHGDVKARNVVIGGDGRAKLADFGCARWADSGRPIGGTPAFMAPEVARG
EEQSPAADVWALGCTVIEMATGRAPWSDMDDVLAAVHRIGYTEAVPEVPGWLSADAKDFL
ARCLQRRPIDRSTAAQLLEHPFVASAAGDGKPEAAKSKWVSPKSTLDAALWESDTDEEED
DELSQSTAERIGSLACAASSLPDWDSDDGWIDVISTPTEESCETTTSPADEETTTDLNGD
IRTAEFELPHIDVDSGNGNTTHNVGEANAQHIISPSNLVFDQVLCKTPFCNKHIAIEFIP
CFLLTNVFLPLSLLCSYAPHP*
>11667.m04923|LOC_Os01g50470.1|genepair587-1
MFNILKALITLKKGTKLIKYSRRGKPKIRAFRLSSDETSLIWFSHKKEKFLRLSSVTKII
PGQRTAVFGRFLHPEKDYLSFSLIFKNGQRSLDLVCKDQAEVEVWFSALEGLISSFRKKS
LINEHKDRVSFSEFCYTVTILEGKNDSSIVDMHITHKYHNTEVTYYQDRHSYDSTLDIAS
NISRSFNSAGYCGTNSFSFRKSDVGFDRLNMIRTSAADSSRVSISSALSSYSQGSGTDDI
ESLGDVYVWGEVWTDVTPSDGHTSSSCSKVDVLIPKPLESDVVLDVNQIACGTRHVALTT
RQGEVFTWGEEFGGRLGHGTDADISRPKLVESLSLTVVDLISCGEFHTCAVTTSGDLFNW
GDGSYNVGLLGCGTEVSYWLPKKVSGPLEGLQVLSVACGSWHSALTTSSGKLYTFGDGTF
GVLGHGDRETLAYPKEVEALSGFKTIKVACGIWHSAAIVEVTNQTGANVMSKKLYTWGDG
DKNRLGHGDKEPRLVPKCVQALLEYNFHQLACGHNMTVALATSGRVFTMGSSSNGQLGNP
KSDGKQPCLVQDRLASELVEEISCGASHVTVLTSRSEVYTWGMGANGRLGHGDLKDRKKP
CLVEALKDRHVKSISCGSNFTTCICIHKWVSGADQSVCTGCRQAFGFTRKRHDCYNCGLV
HCHACSSRKVLKAALAPTPGKPHRVCDSCFLKLKAAETGSNNSNRRNAVTRRSIDGREKL
ERPEIRPSRTTAPAESVKYTEVDSLKQKCEAQHEQLQISDKKTKTVVSMATEEYTRCSAV
VEFVKFLDNELNGIVHELPSDAAESLKALQNQVQALLREQRSHPSELLNPMDHDGIQLSS
GGNALHDFSNHRSGSTRYLFMSQDASSASGSAISLTSEPPSHRGMEHHAKVPNDFVPKHD
THGEVQLIEQFEPGVYVTLIQLKDGSKVFKRVRFSKKKFAENQAEEWWRENQERVFKKYS
HPTVPQTTSTKTGSSNEEEHHS*
>11682.m04502|LOC_Os05g46740.1|genepair587-2
MASDACSGLHLVRLHAWGMQVVCSTALITLKKGSKLIKYSRKGKPKIREFRLSSDENTLV

WYSHNKEKCLRLSSVSKVIPGQRTAVFRRFLRPEKDYLSFSLIYKNGQRSLDLVCKDQAE
VEVWFSTLESLITSCRLNFLNDGQTDRVSFSEDVTIYQDSTSYDTTLDIASSITRSFNSA
GYSTPNSLNSIRADVGSDRVNMLRASTGDNSRVSISSAPSSSSQSSGLDDIESLGDVYVW
GEVWTEVLPSEGSSNYLCSKTDFLIPKPLESDVVLDVQQIACGSRHIGLTTRQGEVFTWG
EELGGRLGHGTDTDICRPKLVESLAVSNVEYIACGEFHTCVVTASGDLYDWGDGSYNAGL
LGHGTGVSHWLPKRVSGPLEGLQVLSVACGSWHSALTMSSGKLFTFGDGTFGSLGHGDRE
SVAYPKEVEALSGFRAMKVACGVWHSAAIVEISGQASTNAMSRKLFTWGDGDKNRLGHGD
KEAKLVPTCVQALVDHNFHQVACGHSMTVALATSGHVFTMGSSNNGQLGNPKADGKQPCM
VQDKLGNELVEEISCGSNHVAALTSRSEVYTWGMGANGRLGHGSVEDKKKPTLVDALKDR
HVKSISCGSNFTTCICIHKWVSGADQSVCSGCRQPFGFTRKRHNCYNCGLVHCHACSSRK
VLKAALAPTPGKPHRVCDSCFMKLKAADTGVISSYNKRNVITRRSIDIKDKLERPEIRPS
RLATTSPAEPVKYQETKNVRNETKPADPMSMMKASQVPAMLQFKDMAFAGTFGTVPTTVK
SMTMGGQMQMGMPMFSPSPPSKKPSPPPATASPLIGKVDNDGLKKTNELLNQDISKLQSQ
VNKLKQKCETQDEQLQKAERKAKQAASMASEESARRNTVLDFVKHLDSEDILMISQPSIL
QLKVIADRVPGDVADNLKTLQSQSERFLAGQSSNLVEITGLTGHDIGHHRSSSTGSLPVS
QDGSSGNASGSSIAMASDSPCHRIMENNLKAPGDFAPKYGTHGEVQLIEQFEPGVYVTLI
QLRDGTKVFKRVRFSKRRFAEQQAEEWWRENQERVFKKYNHPTN*
>11667.m04938|LOC_Os01g50620.1|genepair588-1
MEIINGPVLPRYAAPATGALTSDAKISGQLLRRVHLRRRACGLQGDHYRAARSYGSSSDG
DGAAAADYDASGEEFVNSSVMEAVELRSVSDGFVIKMRDGKNLRCVQNNPRVLRLRDSAP
HHAIVLKMEDGSDLLLPIIVMETPSIMLLAALRNIRIPRPTIYNVVKEMTERMGYAVRLV
RITEMVHDAYYSRLYLAKIGNEEETISLDLKPSDAINIAFRCKVPIQVNRRIAYNNGLKV
VQPTPSESYVSSDQFQYTRLDRPDDQPCFEAQEFDLVRNMLVAAVEERYKDAGAGFEGIV
HDDDKKEWKSDEDNSEGDKKAKAVSFKNRAISAGNKFRRSLRRKRRRVGDHVASIEDIR
DVKELEAVQRFHQCLHDEGLLPERHDDYHVMLRFLKARKFDIDKAKHMWSEMLRWRKEFG
ADNIEEFDYSELDDVLECYPQFYHGVDKEGRPVYIELIGKVDPNKLVQVTTIDRYVKYHV
KESEKCLQMRFPACSIAAKRHIDSCSTILDVQGVGLKNFSKDARELIMRLQKINNDNYPE
TLHRLYIINAGQGFKMLWGTIKSFLDPQTASKIHVLGSKYQNKLLETIDESELPDFLGGK
CRCEEHGGCIKSDKGPWKDPDIIKRVLNGEANYGRKILAISSVDGKKICYINPRHLTSKL
PGNTSTSGAPPRVEDIPVVDKAVDTCAGPSTSSMAFNSDSFSLRNITMELGGLRNRITAW
LIVLIVSFVAVLRSVPSRVTASLSSQAISRENSTHSSVLRRLGELEEKLQELEAKQSQMP
PDREELLNGAIHRVDALEAELISTKKMLYDALMRLDELLAYADQQKNIQFRKKRFCF*
>11682.m04499|LOC_Os05g46720.1|genepair588-2
MSGPLDRFARPCFEGFTHNDEKKEIRSDADNSEGEKKTKIGSFKKKAINAGNKFRHSLRR
RSKKKNEPRGSIEDIRDVQDLQAVDAFRQCLVDEDLLPQQHDDYHTMLRFLKARKFDVEK
AKSMWSDMLKWRKEFGADNIEEFDYTEADEVMKYYPQFYHGVDKEGRPIYIELIGKVDAN
KLMQVTTIERYVKYHVKEFERCFQMRFPACSIAAKRPIDSSTTILDVQGVGLKNFSKAAR
DLITRLQKIDNDNYPETLRRMYIINAGQGFKMLWSTVKSFLDPKTASKIHVLGSKYQNKL
LEIIDENELPEFFGGKCKCEAFGGCKKSDKGPWKDPNIIKRVLNGEANYGRQIVTISSTD
GKIIRYAGPQYPTRKGSDGSAESGSEVEDGASPMASRNLITNPLLTPVHEESKLAAHGFT
SASPSIIEESIPVVDKVVDDGWGSPRASSSPSRSLPITFDGLWTQVITWLTVLIVSLFAM
VRSVPSRMAKRFSSQSTDHDHSYVEYPQEAEYKEEFRPPSPAPSYTEKDVLSSMVRRLGE
LEEKVQALETKPSEMPFEKEELLNAAVRRVDALEAELISTKKALYEALMRQDELLAYIDK
QDMIKFRKKKFCF*
>11667.m04947|LOC_Os01g50700.1|genepair589-1
MVVDSAYYDVLGVSTDASAAEIKKAYYLKAKLVHPDKNPNNPDAERRFKELGEAYQILSD
PVRKDSYDKHGKEGLPQDNMIDPTAVFGMLFGSDYFEDYVGQFALASVASVEIEEESDNT
EARARIQDKIKELQTEREQKLVQSLKDRLQPYVDGMQDEFGDWAGAEAQRLSQAAFGEAM
LHTIGYIYARQAARELGKSKMYMGVPFIAEWVRDKGHHVKSQVNAAAGAISLIQLQEGIK
KIEGDDKEGQLMKSIEEKKDAMLNSLWKINVVDIESTLSRVCQAVLRENTVSKDVLKVRA
RGLKKLGTIFQPRNILRYFRAFDALECTLLLFTRNLPEIRPQRGAPGAYVPFSGHARRPY
EGTRGLPAGLQIPPVASCFLVQIRVRFRFIIIQFPISSIARITHGSKRVTELETEEEATR
RHQPMAEHATGVYGHPYPRVDQYGNPVPPVDQYGNPVPDEPAPRDTAAGYVAPPDPAVST
GDYGLAGAEAPHPHESAVMSGAAAAAVAPGGEAYTRDGGGVVPPAGEKTFAYEGTVSAAG
VTGASGQLQPTTREEGHTTLGETLRRSGKSSSSSSSSEDDGQGGRRKKKSIKEKIKEKL
PGSHKQEEQKQAGHTAPAAGTGTGTGTHAAGKHEKKGIVEKIKEKLPGHGHH*
>11682.m04489|LOC_Os05g46620.1|genepair589-2
MVKDTAYYDTLGVSVDASPAEIKKAYYLKAKQVHPDKNPGNPDAAQKFQELGEAYQVLSD
PSKREAYDKHGKEGLPQDNMVDPAAVFGMLFGSDYFEDYVGQLALASIASVEVEENLNSQ
EARGKVQEKIKELQKEREQKLIQSLKDRLQPFVDERKDEFVNWANGEAQRLSHAAFGEAM
LTTIGYIYVRQAARELGKSKLYMGVPFIAEWVRDKGHCIKSQVNAASGAIALMQLQEGMK
KMEESDNKEDQIMKSFEEKKDAMLSYLWKINVVDIESTLTHVCQAVLKDASVPKDVLKLR
ARALKKLGTIFQGAKSLYHRENSLQVETSPRQGAATSN*
>11667.m04949|LOC_Os01g50720.1|genepair590-1
MGRHACSAAGVQQKLRKGLWSPEEDEKLYNHIYRYGVGCWSSVPKLAGLQRCGKSCRLRW
INYLRPDLKRGSFSQQEEDAIVGLHEILGNRWSQIASHLPGRTDNEIKNFWNSCLKKKLR
QRGIDPSTHQPISTAAAAAAAALDTSTQDQKPPATADGFALKQQQQVFDPFPVIDSFGSG
FDATGMPLYGHLGGKDAAGFVDYSSVLDVSENLGYGESSSNSSNWNCGVGAPEVNNALES
EPLHWATESKVEPFVGYGEGDAMEHKFGLPCHGQQEQGMTHFDFDVSRSMVVGDFNFEYF
R*
>11682.m04488|LOC_Os05g46610.1|genepair590-2
MMPVSYTPSVSQWLQRCGKSCRLRWINYLRPDLKRGSFSQQEEDLIVALHEILGNRWSQI
ASHLPGRTDNEIKNFWNSCLKKKLRQRGLDPATHKPIAAAAAAATSSESAVTQVDEDHKP
HGAAAAAAAAADGLAANAKQSVFDPFPVTDFGAGFDLGAANMAAALYGSHPDDGAGFVADY

SSVLDVSENLGYGESSSNSSNWTCAEVSNVLDSEVLNWAASAGADAAAKAEPFADMEQQH
SGYGGEYQVEDDATLEHKFSLPCHEQSLAQFDFNLEYF*
>11667.m04954|LOC_Os01g50760.1|genepair591-1
MAAAVVANGASGDSSKAAFAEIYSRLKEEMLEDPAFEFTDESLQWIDRMLDYNVLGGKCN
RGISVIDSFKMLKGTDVLNKEETFLACTLGWCIEWLQAYFLVLDDIMDNSQTRRGQPCWF
RVPQVGLIAVNDGIILRNHISRILQRHFKGKLYYVDLIDLFNEVEFKTASGQLLDLITTH
EGEKDLTKYNLTVHRRIVQYKTAYYSFYLPVACALLLSGENLDNFGDVKNILVEMGTYFQ
VQDDYLDCYGDPEFIGKIGTDIEDYKCSWLVVQALERADENQKHILFENYGKPDPECVAK
VKDLYKELNLEAVFHEYERESYNKLIADIEAHPNKAVQNVLKSFLHKIYKRQK*
>11682.m04485|LOC_Os05g46580.1|genepair591-2
MAAANGSACGGGGVDKEKEEFKQIYGVLKEELLRDPAFEFTDSSRQWIDRMLDYNVPGGK
CNRGLSVVDSYKLLKGTNVLSQEDMFLASTLGWCVEWLQAYFLVLDDIMDDSHTRRGQPC
WFRVPQVGSIAINDGIILRNHITRMLRLHFRGKLYYADLLDLFNEVEFKTASGQLLDLIT
THEGEKDLNKYNIGVHRRIVQYKTSYYSFYLPVACALLLSGEDLTKYGAVEDILVKMGIY
FQVQDDYLDCYGDPKFIGKIGTDIEDYKCSWLVVQALERADESQKSVLFENYGKKDPACV
AKVKSLYRELNLEAVFLDYENESYKKLIADIEAQPSIAVQNVLKSFLHKIYKRQK*
>11667.m04956|LOC_Os01g50770.2|genepair592-1
MAGAVSALFLLDIKGRVLVWRDYRGDVSALQAERFFTKLLDKEGDSEAHSPVVYDDAGVT
YMFIQHNNVFLLTASRQNCNAASILLFLHRVVDVFKHYFEELEEESLRDNFVVVYELLDE
MMDFGYPQYTEAKILSEFIKTDAYRMEVSQRPPMAVTNAVSWRSEGIRYKKNEVFLDVVE
SVNILVNSNGQIVRSDVVGALKMRTYLSGMPECKLGLNDRVLLEAQGRATKGKAIDLDDI
KFHQCVRLARFENDRTISFIPPDGSFDLMTYRLSTQVKPLIWVEAQIEKHSRSRIELMVK
ARSQFKERSTATNVEIEVPVPSDATNPNIRTSMGSAAYAPERDAMVWKVKSFPGGKDYMC
RAEFSLPSITAEEAAPEKKAPIRVKFEIPYFTVSGIQVRYLKIIEKSGYQALPWVRYITM
AGEYELRLI*
>11682.m04482|LOC_Os05g46550.1|genepair592-2
MAAGAVSALFLLDIKGRVLVWRDYRGDVSALQAERFFTKLLDKESDAEVLSPVVHDDAGV
SYMFIQHNNVFLLTASRQNCNAASILLFLHRVVDVFKHYFEELEEESLRDNFVVVYELLD
EMMDFGYPQYTEAMILSEFIKTDAYRMEVTQRPPMAVTNAVSWRSEGIRYKKNEVFLDVV
ESVNILVNSNGQIVRSDVIGELKMRTFLSGMPECKLGLNDRVLLEAQGRTTKGKAIDLDD
IKFHQCVRLARFENDRTISFIPPDGSFDLMTYRLSTQVKPLIWVEAQVEKHSRSRIQITV
KTRSQFKERSTATNVEIEVPVPEDSTNPNIRTSMGSAAYAPERDAMVWKIKSFPGGKEYM
CRAEFSLPSITSEDGMPEKKAPIRVKFEIPYFTVSGIQVRYLKIIEKSGYQALPWVRYIT
MAGEYELRLI*
>11667.m04960|LOC_Os01g50810.1|genepair593-1
MFRHLAALILSAVVLAASTSSGVVDARPVHTGPYHVVIRTAAKPSRRLIGLGGNAAICQE
VHYKALCGTLTTLPGVMTPQQLLDAALRVAESKAMMAEKRLADVMKSRAVKAEGTSMSST
LDTCKGAYSSLADALQKARDTIKSGGSHDDLMTELSSASTFSTDCGEAFDEFPDLTSPIP
GAQRHVNRLVSNCLDLAATIKEN*
>11682.m04480|LOC_Os05g46530.1|genepair593-2
MARFVIISVVVVAAFAAAAVVEARVGPIDVAPTNLITNPLGAIIDNGRKITGAVVDECAW
TCDHVAAGNKKMCNTLRKLPGVSSPKELLTAAVKLSMRKAKAARARFEAAARAAEKGTPM
ESILDTCKEGYDSTVSALQEVQRCIDANDSKASLITKMSAATTFTGDCGNAYEERELEPS
LALKATKNNVNRVVTGALAIAAKLKL*
>11667.m04966|LOC_Os01g50870.1|genepair594-1
MAGLQRSSETFRRSGSSGMVWEDKLQQSGELGGKAEAPAPAARAAAQRSGSSGHGGYKAG
HVQPALDPPSPRVAACGFCNLFGKDKHHQARPRGGASAKSRRR*
>11682.m04477|LOC_Os05g46500.1|genepair594-2
MAGLQRSSETFRRSGSSGLVWDDRHLSGEIKPADGGGGGAAARVERSRSAGHGGYRAAGR
VQPALDPPSPRVAVCGFCRFFGGSGKGGRSGGGAAAAVAGGKAKARRHSSS*
>11667.m04970|LOC_Os01g50910.1|genepair595-1
MASQQERASYHAGETKARAEEKTGRMMGTAQEKAREAKDTASDAAGRAMGRGHGAKEATK
EKAYETKDATKEKAYEAKDAASDATGRAMDKGRGAAGATRDKAYDAKDRAADTAQSAADR
ARDGAGQTGSYIGQTAEAAKQKAAGAAQYAKETAIAGKDKTGAVLQQAGEQVKSVAVGAK
DAVMYTLGMSGDNKNNAAAGKDTSTYKPGTGSDYQ*
>11682.m04475|LOC_Os05g46480.2|genepair595-2
MASHQDQASYRAGETKAHTEEKAGQVMGASKDKASEAKDRASEAAGHAAGKGQDTKEATK
EKAQAAKERASETAQAAKDKTSSTSQAARDKAAESKDQTGGFLGEKTEQAKQKAAETAGA
AKQKTAETAQYTKDSAIAGKDKTGSVLQQASEQVKSTVVGAKDAVMSTLGMTEDEAGTDD
GANKDTSATAAATETTARDH*
>11667.m04981|LOC_Os01g51020.1|genepair596-1
MATIPDSLVWELVKKNNSFLVKQFGNGNAKVQFSKEPNNLYNVHSYKHSGLANKKTVTVQ
PASGKETAVVLSTTKTEKQNKPASLYHKSVMRKEFRKMAKAVKNQVSDNYYRPDLTKPAL
ARLSAVYRSLQVAKSGVKKKNRQAN*
>11682.m04464|LOC_Os05g46430.1|genepair596-2
MATVPEPLIWEIVKKNNCFLVKQFGNSNAKVQFTKEPNNLYNVHSYKHSGLANKKTVTIQ
PSGVKDAAVVLSTTKTKKQNAPAKLYHKSVMRKEFRKMAKAVKNQVSDNYYRPDLTKPAL
ARLSSVYRSLQVSKSGAKKKNRQPTKL*
>11667.m04984|LOC_Os01g51050.1|genepair597-1
MKHRLKIQPLIQVLPVLLRISGDKQVSLMDMQPGHYQQRRMVASSKAIKVGPWGGTAGSP
WDDGAHRGVRSIALTYGRFLESMRVEYDRNGHPVHGEKHGGGGDGRTSRTAEVKLDYPYE
FLTGVGGRCGPVAHGGSTVVRSLTFRTSTGAVHGPFGDASGDGVPFEYPMEGGVVVGFSG
RSGWWHLDAVGLHVAALRPETLCDVVQERGAMAYRSFVYGNGGSSSGAHQLQQKRKPFEW
CYK*

>11682.m04463|LOC_Os05g46420.1|genepair597-2
MEQQPQKKMVVSKKIMKVGPWGGTGGSPWDDGGHTGVRSITLSYDRCIDSIAVEYDRNGV
AVAGERHGGAGGNQTTQIKLGFPEEYLTAVSGHYAAVAQGGAPAVIRWLAFRTNRREYGP
LGGGAAEGTPFAFPVDGGAIVGFWGRSGRQLDAVGLHVAPLRPETMYEKAHKLGLMAYRS
VRQRFGPQQQQQVQVQHHNSFALSQSGRTF*
>11667.m04993|LOC_Os01g51140.1|genepair598-1
METFGWAGGGQLMHHDDIYLPRSVGCGRPFELDDAFLGACFGAQLQCDGGVGGGDGGGCL
QGTSGFGAVAGDPLGLLCSGDVFASVAEGAGGAHDDGLLDAALAFSRNQLGGAACDGSDG
GAVSNGAMLSSYSGTTGGNISSGESNNYSGGGGGYDAEVVSPTSTMSAATQSLHPKRKLY
DDHHHPAGIAAAAAAPPLAPCPRPTTGAVAAKRRASTSATSITFGHQPHHHHAGATTAGY
EPDMEAMAQVKEMIYRAAAMRPVHLGTEAAADKPRRKNVRISSDPQTVAARLRRERVSDR
LRVLQKLVPGGNKMDTASMLDEAASYLKFLKSQVQKLETLGTTTTTSKLPQQYYSGNINS
SNNHHGFLGFAANNNTISAGYANSNAGNATKLL*
>11682.m04458|LOC_Os05g46370.1|genepair598-2
MDAFGWSAPAAPCQPSCGPGGDDDDDVLLAAVLGASFELHSLVDGGGNGAAGAVRSDDAY
GLDVDLPSHQMSLLRCQDGLSALHGDASPTAAAAAFLDSVDVLPVPAIAGATHDDGGLLD
RFAFPNVAETTTVQAAASNTAFSGYSSNTTGGGNISSGESNTYTEVASTPCAVSTTTTTT
ALPPSKRKLPEKYPVVGTSPTTKTTTTSETAAERRSTKRGAGGSSSITFGGGCHGAGAAA
ALLGYGRGYEPDTEAIAQVKEMVYRAAPMRPVTLGGPASASDPSSRPPPPPQRPRRKNVR
ISSDPQTVAARLRRERVSERLRVLQRLVPGGSKMDTATMLDEAASYLKFLKSQLEALETL
GNGNGNGNLLHHGYYTGSRNATATAATGSSNSTVLAFGRDGLAGFVKSNRNLQL*
>11667.m05001|LOC_Os01g51210.2|genepair599-1
MGDNSAAAAAVAAPRGRFGRICVFCGSNAGNRAVFGDAALQLGQELVSRGIELVYGGGSV
GLMGLIAQTVLDGGCGVLGVIPKALMPTEISGASVGEVKIVSDMHERKAEMARQSDAFIA
LPGGYGTMEELLEMITWSQLGIHDKPVGLLNVDGYYDPLLALFDKGAAEGFIKADCRQII
VSAPTAHELLRKMEQYTRSHQEVAPRTSWEMSELGYGKTPEES*
>11682.m04456|LOC_Os05g46360.1|genepair599-2
MGDGAEAAGATAASRFGTICVFCGSNAGRRRVFGDAALDLGHELVRRGVDLVYGGGSIGL
MGLIARTVLDGGRRVVGVIPRALMAVEISGESVGEVIVVQDMHERKAEMARRSKAFIALP
GGYGTMEELLEMITWCQLGIHDKPVGLLNVDGYYDPLLALFDKGEAEGFINSDCRQIFVS
APTASELLTKMEQYTRLHQEVAPATSWEISELGYGRTPGADQS*
>11667.m05003|LOC_Os01g51230.1|genepair600-1
MGASGKWIRTLVGLRPAAEREKERGGGGGKGRKWSRLWRSSSSQRGGGNASASEVYSETS
SSADALSSVVAAVVRAPPRDFRLIRQEWAAVRIQTAFRAFLARRALRALRGIVRLQALVR
GRRVRKQLAVTLKCMQALVRVQARARDRRARISADGLDSQDMLDERGGRVDHVKEAEAGW
CDSQGTADDVRSKIHMRHEGAIKRERARTYAQSHQRCSNHGGRPSSPAVSLKHHGNGATR
SNHSWSYLEGWMATKPWESRLMEQTHTENSTNSRCSESVEEVSVGGPKLSDASSVKIRRN
NVTTRVAAKPPSMISATSSDFVCDESSPSTSSVTPLSANNSLATERRSDCGQVGGPSYMS
LTKSAKARLSGYGSHKPPLQRQRSGDLLHHNNRMAFSSIDVQSTAGSEVSVTSKRLNSLA
LKGRATRSLDKENERRPSSLL*
>11682.m04455|LOC_Os05g46350.1|genepair600-2
MGASGKWIKSLVSLKAAPEGTTKGRRWTRLWRSSSSASASASTAGDASESASSEADAFSS
VVAAVVRAPPRDFRVIRQEWAAVRVQAAFRAFLARRALKALRGIVRLQALVRGRLVRRQL
AVTLKCMNALLRVQERARERRARCSADGRDSQDAVGERDGRADPIKQAEEQWCDSQGSVS
EVRSKIHMRHDAVAKRERAIAYALSHQPRSSKQSARPSSPARSLRNHESNRCNHDWSYIE
GWMATKPWESRLMEQSHAELKCSKNSGELNLAGAQLSNASSVKMRGNRVAAKPPSVLSAS
SSDFPCDVSSASTSSATPARSDGGHGEGPSYMSLTKSAKARQSCNSPFQIQRQRSGGMSS
YKRVALSPLDVQSNACSEFSVTSRKLNSLSLKGRSMTRSLDKENDNLF*
>11667.m05013|LOC_Os01g51310.1|genepair601-1
MDVAADATAWPPGWSLVRGYFSPATLFLLLNVVIGTIALTSRASHRRRQHHHDEHYKAQH
HHDHHDEPQRCHDQYAPPPPAPLERTSSVMERLRSFGLYRFRSGDFPPEYNLSAAGANAI
CDESEKQQAQYTRSRSEPAARPAPPPVEKRGKAENAAAAGAARAKVAVKKSSSSEVRKLE
RAPAQAQRQVLQVQRAQPPPPRAPAPAPARAVKSAAREEVETAQVGLATASSVDARADDF
INKFREQLQLQRLNSLLNYNEMLNRGT*
>11682.m04453|LOC_Os05g46340.1|genepair601-2
MDVAEAASPGVGAWAAIRGYFTPATLFLVVNIVIGTIALTSRTHQRRRRQVYQYHHDGEI
LHHDQLQPPPPLLHQQYYGGGEHQQMMMQETLYAPPPAPAPLARTSSVLDRLRSFGLYRF
RSGDFPPEYATAAAAATSQLHHQRTEEVVSSSPVDEVKLQGHYARSRSEPAAAPAQEERK
AASRLRNSSSEVRRAEVVRAPARVVEAFPEEAAAAVDATAEDDFTPKQQRDLPLQQEYVP
PPAPAPAPLARTSSVLDRLRSFTLYSFRSGDLATDDIPADAAAAATPAQAHYGRTRSEPA
REQDKKAKKQAASEAKTMTKSSSEARKDTAAEDADDGGVDARADDFINKFRQQLQLQRLN
SLLNYKEMLNRGSSKQ*
>11667.m05025|LOC_Os01g51430.1|genepair602-1
MAPNKISSMDAGAAFDDEDASSSNSLQELWPVGEIDPKRARFPCCIVWTPLPIVSWLAPY
IGHAGICREDGTVLDFAGSNLVSMDNFAYGSIARYLQLDRKKCCFPVNLATHVCERSYKH
AEAGTAISWDDALQLGMRSFGHKFYNLFTCNCYSFVANCLNRLAYNGSVKWNVLNVAALV
WLRGQWVDKMSVVRSFFPFLTVTCVGILMAGWPFLIGMAAFSSLLIGWFVFAVYCMKDLV
C*
>11682.m04440|LOC_Os05g46240.1|genepair602-2
MEVEAACGDGVVSSSNEMQELWPLGEVDQKGTRFPCCIVWTPLPVVSWLAPYIGHVGIAR
EDGTVMDFAGSNFVSVDDLAYGSAARYLQLDRRKCCFPANLAAHVCARSYEHSEAGTAIS
WDDALQSGARRFEHKCYNLFTCNSHSFVASCLNRLAYGGSVGWNVLNLAALVWLRGRWLG
KMAVVRSLLPFAAVACVGVLMAGWSFLISMAAFSSLLLGWFVLGVYCFKGLVC*
>11667.m05027|LOC_Os01g51450.1|genepair603-1

MSNPELLSEEKAILVETLKNKLQALAEQHVDVLESLAPVVRKRVDVLIEIQSQHDELEAK
FLEEKAALEANYQKLYGPLYSKRSEIVSGVLEVEGETEEREEKGVPDFWLKAMKNNEILA
EEIHESDEEALKYLKDIKWCRIDDPKGFKFEFFFDTNPFFKNQVLTKTYHMIDEDDEPIL
EKAIGTEIEWHPGNCLTQEVLTKESSESTKPITKTEEYESFFNFFSPPQVPEDDAKIDEN
TAEELQNQMERDYDIASTLRDKIIPHVVSWFTGEAVQDEDYGASWVDDEEDDDDEYSDEE
A*
>11682.m04438|LOC_Os05g46230.1|genepair603-2
MSDGKDSLDLSGLGAAVPNAKELSAEDKANLVESIKNTLQGLAARHTDVLESLEPKVRKR
VEVLREIQSQHDDLEAKFFEERAALEAKYQKMYEPLYSKRYEIVNGVVEVDGVTKEAADE
TPAEQKEEKGVPEFWLNAMKNHEILSEEIQERDEEALKYLKDIKWYRISEPKGFKLEFYF
DTNPFFKNSVLTKTYHMIDEDEPILEKAIGTEIEWFPGKCLTQKVLKKKPKKGSKNTKPI
TKTENCESFFNFFSPPQVPDDDEEIDEDTAEQLQNQMEQDYDIGSTIRDKIIPHAVSWFT
GEAAQDEDFEGIMDDEDDDDEDDDDDEDEDDEGDDEDDEDEKKGGRVPAGEGQQGERPAE
CKQQ*
>11667.m05045|LOC_Os01g51630.1|genepair604-1
MASMLNIVIGSHVWVEDKDSAWVDGEVFRIDGKNAHVRTTKGKTVIANVSDIHPKDTEAP
PDGVDDMTRLSYLHEPGVLDNLAVRYARNLIYTYTGNILIAINPFQRLPNLVDVRTMEKY
KGANLGDLDPHVFAIADVSYRQMMNEGRNNSILVSGESGAGKTETTKLLMRYLAYLGGRS
GTGGRTVEQQVLESNPVLEAFGNAKTVRNNNSSRFGKFVEIQFDKSGKISGAAIRTYLLE
RSRVCQINSPERNYHCFYFLCAAPPEDIKRYKLGDPSSFHYLNQSSCIRVDGINDAEEYL
VTRNAMDTVGIIEQEQEAIFRVVAAVLHLGNINFAKGSEVDSSVIKDDKSRFHLNTAAEL
LMCDCKKLENALIKREINTPEGVITTTVGPSSATVSRDGLAKQIYSRLFDWLVNRINASI
GQDPNSDKLIGVLDIYGFESFKTNSFEQLCINFTNEKLQQHFNQNVFKMEQEEYTREQIN
WSYIEFVDNQDVLDLIEKKPGGIIALLDEACMFPKSTHETFSQKLYEKFKNHKRFTKPKL
SRTAFTIQHYAGDVIYQSDHFLDKNKDYVVAEHQELLNASRCSFVSALFPPASEENTKSS
KSSIATRFKVQLHELMETLSSTEPHYIRCVKPNSVLKPAIFENTNVLQQLRCSGVLEAIR
ISCAGYPTRKLFHDFLHRFRILASEIVKEKNDEKVTCQKVLDKMGLQGYQIGRTKVFLRA
GQMAELDARRTEVRNNAARGVQGQFRTHVAREQFLILRNASVCLQSFVRARLACKLHECL
RREAAAIKIQKNIRCYFAWRTYSQLRLSAITLQTGLRTMAALKEFMFRKQNKATTHIQTQ
WRCHRDNSNYLKLKRAALTYQCAWRRRVARRELRQLRMAARDTQALKVAKEKLEERVEEL
TNRYGATALTYVGTAGCIRIDIETIGHTDLEKSKVAEVSKLQAALNEMEQRMQDVTAMQE
RESAKKAVEEALEQEREKISSLTSEIEGLKALLVAEQEENDLTKKAHANAQERNEELSKE
VEDADGKIKQLSDTVQRLEETIQEREALLLAERQEKEEASAVIAESQARNEAFASKLEDA
EKQIDLLQETVQRFEEAITKLQSSVTIEKQQHEETVVQLAEAQAKIDELLREAGDTDEKS
TQLETTIQRLEESLTEKDALLTTERQETEATKKLLSEAQYKNEELLKKIEDADKSIAHYH
DTTQRLEENVTAVENSLKAERQHNGAIMKQLADAQVEIGELQRNLEDADRRNNQLQDSLQ
RLVEDATTSEALLVAERQENEVTKKTLTEALDQIEELVKEVECAKNSVYQLQDNIQRLEQ
NASAREADLLTERQEKETTSKALAEAQAKIEGLLEEISSANKKTDLLQKTIERLEEGATT
TDALYLTERQEHDQTKKAFSEAQEINQQLYRKIEEAEKNIEQLRENVERLEERIAYIDSL
LAIERRENNETKKELADAQKEIEELLDEMQDNVASIAEHEDTIRRLEENVGAKESLLLTE
REQNASTLKLLAEAHLEIDELIRKLEDSDRKSDSLQSTIKRLEEDGIAKEALLLTEKQAH
EATRMTLTEALEKNEELLKKIHDDDKHILELQFTIQRLEENTAAKENLLLREREQNDATT
KAQIESQERNEQLLKRFVDVDRKIDLLQDTIERIGENSTIKDALLLSERQEKDAIKKELV
EAGERNEELIMKIEDTDKKIEHLQNAIIKLEGDIEAKDISLEAAREENDTIRKSLAEAQE
KNEELLRKISDNEYRIHLLQDTAQKLQVDAISRLSSFVMEKQESDAAKRALTEARERNED
LLKRNEDLLKRNDDLIKKIEESSKTITQLQETLQRLEGKSTNLEAENQVLRQQATATPPS
TAKSSASRSKITRIHRSPENGHILNGDTRQAEIKPSTGTSETIPSIGNPPDLNNEKHVEQ
GEKLQKVLNQKYQSQQPQDDQQWLLTCISQYLGFFGSKPVAALLIYQCLSHWRSFEAMKT
GVFDSILQAINSATEAQNDTRALAYWLSNLSTLTVLLQRSFKTTRTAISTPQRRRFSSER
IFHASQTSNAGLAYLSGQPVVGAAGLPQVEAKYPALLFKQQLVDLIEKVYGMISDSVKKE
LNPLLELCIQDPRTSHSPAKGHANGLGQKNQLGHWLAIVKVLTNYLDVLRANHVPSILVH
KLFTQIFSLIDVQLFNSYRLMRFCLIQFAGSAWDALKHIRQAVDFLVISLKPMRTLKEIR
TDVCPALSIQQLERIVSMYWDDINGSNAISAEFTSSLKSAVREESNTVTTFSILLDDDSC
IPFSLDDIAKTMPIIEVAEDDLLPFVRENPSFAFLLQRGNS*
>11682.m04417|LOC_Os05g46030.1|genepair604-2
MAATLKIVLGSHIWLEDKDLAWIDGEVFRIEGQKAHIRTTNGNMVVASISDIHPKDTEVH
SDGIDDMIRLSYLHEPGVLNNLSVRYAKNIIYTYTGNILIAINPFQRLPHLAEPHTMEKY
KGANFGELDPHVFAIADISYRQMMNERKSNSILVSGESGAGKTETTKMLMRYLAFLGGRS
RTGGRTVEQQVLESNPVLEAFGNAKTVRNNNSSRFGKFVEIQFDKSGKISGAAIRTYLLE
RSRVCQINSPERNYHCFYFLCAAPSEEIRKYNLGDPSSFHYLNQSTCIKVDGISDNEEYL
ATRSAMNTVGITEQEQEAIFRVVAAVLHLGNINFVKGREVDSSVIKDEKARFHLNAAAEL
LMCDHGKLENALIKRKINTPEGVITTTVDPNSATVSRDGLAKQIYSRLFDWLVSRLNASI
GQDENSQYLIGVLDIYGFESFKTNSFEQLCINFTNEKLQQHFNQNVFKMEQEEYNREQID
WSYIEFVDNQDVLDLIEKYSGTGSHIGICSVVCEYNTVWLVTENCKVGSTDCYVLARKPG
GIVALLDEACMFPKCTHESFSQKLYEKFKNHKRFSKPKLSRTAFTIQHYAGEVTYQSDHF
LDKNRDYVVVEHQELLNASTCSFVSGLFPSVQEENTKSSKSSIANRFKGQLHDLMETLSS
TEPHYIRCIKPNNLLKPATFENANVLHQLRCSGVLEAIRISCAGYPTRKLFRDFLQRFRI
IAPDFFKERNDEKVICQKILDKMGLQGYQIGRTKVFLRAGQMAELDARRTEVQNRAARAV
QSRFRTHVAREQFLMLHNTSISFQSFVRAILACKLHLLLRKQAAALKIQKNVRCYFASKS
FSELRSSAITLQTGLRAFGAYNEYIRRKQNKASTDIQTQWRSHRDNSNYLKLKRSVLIYQ
CAWRIQVAKGKLRKLKMAARDTEALKVEKGKLEEHIEELSSRLCLEKKLRSDLENSKATE
ISKLQTTLHEMERRVEEARATQERESAKKVVEEALVLEREKIALLTKEVEELKVLLLKEQ
EEKNATNSAFSIAQERNDDLTKKVEVANENFKQLKDTLKSFEESTKGLETSLMMERQQNE
ANRREVGEAQQRVEELLRLVADANGKSTSLQTTVQRLEQSLIEKEATWLTERQESETTNK

LLIEAHGRNEELLNKIEVAENDISKFRDNIQRFEETATTLETSLLAEKQHSAAIMSQLAE
TKQGNEELQKKLADVNRTNDILQDSLKRFEENVTTRDALYLAERQEHDETKQSLSKSQER
NWELLQKVDEAEKRINKLLENAQRLEKHATARESLLLKTKQSHDSTTKALVEAESRNREL
TKSFEDSDRKINLLEDSVNRLEERIAEKDSLLEIERQENNATKDEVTNAQNKIMELVNES
QQLQDIRKHLEDNIKRLEEDATTREALLISEKQTHEATKRTLTETQLRNEELINKIQDSD
KHALQLELTIERLQENASTMEALLLREREQSNATMKAHSESQERNSQLLKKFEDVDKKIG
LLQGAIQRLGEQTTKDTLLLSERKEKDELKKVLSETEYRNEELVIKIEEENKKVEHLQDT
ITMLKENIAVQAANLEAERQENDRIRKSLVEAQERNDELFKKVSDSEYRAQQLQDTVQKL
RQESDAVRKALAESHGRNEDLIRRNDDLLSRNDDLIKKIEDSGQVVAELQAALERIEGKA
ANLEAENQILRQQAIATPPSTAKSQAAFSKINAFQQRSPENGHILNGNVAYAEKSLTGPA
ETRPSMVVNQGSILNLINQKDYESGDKMQRAHNEVYQHQQPQDDQQLLLQYITQHLGFSG
SKPVAALLLYQCLLHWKSFETAKTSVFDSILQEINSAIEAQHDTRSLAYWLSNLSTLSVL
LQLSFKTTRAAISTPHRRRFSYERIFQASQTSNSGLAYFSAQPVDGPSGLQQIDAKYPAL
LFKQQLVDLIEKVYGMISDKVKKELNPLLELCIQDPRTSHSNQAKASLSSASHLGQQSQL
THWLGIVKILNNCLHLLRANHVPSILIHKLLTQIFSMLKFLVFTQFADSAWEALRHIRQA
VDFLVISLKPIRTWSEICDDVCPALSLQQLERIVGMYWDDMNGTNIISAEFTSSMRTMMK
EESNNATSFSVLLDDDSSIPFSLEDIAKSMPTIEETTENDLLPFVRENQSFAFILHRRD*
>11667.m05051|LOC_Os01g51690.1|genepair605-1
MYMAAAAAGASTPFNFCRHGSHAEYDAVFSGSWMARRPSAAPHGGGASGSGSGSGYGAAS
YVAPTFGAAFRQQHLDLLDYLSDDQGVPAPPPAAVPSASYVTPAPAMAPAEPVVPDAVAA
AGGYPRSVAAAAAAVAGEGRDRTTTDKIAFRTRSDDEILDDGYKWRKYGKKSVKNSPNPR
NYYRCSTEGCNVKKRVERDKNDPRYVVTMYEGIHNHVCPGTVYYAAQDAASGRFFVAGIS
HPDLN*
>11682.m04416|LOC_Os05g46020.1|genepair605-2
MAAVGAHAAVYHHPVSGLSAPAGDAAYSMSSYFSHGGSSTSSSASSFSAALAAATTPPLP
DPSGSQFDISEFFFDDAPPAAVFNGAPTAALPDGAAANATRSAAEAVPAPAPAAVERPRT
ERIAFRTKSEIEILDDGYKWRKYGKKSVKNSPNPRNYYRCSTEGCNVKKRVERDKDDPSY
VVTTYEGTHNHVSPSTVYYASQDAASGRFFVAGTQPPGSLN*
>11667.m05052|LOC_Os01g51700.1|genepair606-1
MSTSRRRTLLKVIVLGDSGFTAVVGTLICLTLVRYVHKKFSQQYKATIGADFVTKEVLIE
DRLVTLQIWDTAGQERFQSLGVAFYRGADCCVLVYDVNSNRSFDTLNTWHDEFLNQEPGG
FGRLISPHLKCFSLSHPLSTAISASDRLTASSELEDQFEIFEHIRVFNVTTVTSTNTKDE
TLLLWSGFLLKDEGEILAEFIENFTASPSDPKTFPFILLGNKIDVDGGKSRVVSEKKAME
WCSSKGNIPYFETSAKEDRNVDSAFLSVAKLALEHERDQDIYFQTVVPDPVPEAEQRSGC
AC*
>11682.m04414|LOC_Os05g46000.1|genepair606-2
MASSRRRTLLKVIVLGDSGVGKTSLMNQYVNKKFSQQYKATIGADFVTKEVLIEDRLVTL
QIWDTAGQERFQSLGVAFYRGADCCMLVYDVNAKRSFNALNTWHDEFLTQASPSDPKHFP
FILLGNKIDIDAGNRRAIPEKKAKEWCVSKGNIPYFETSAKDDYNVDSAFLCIAKLALEH
EHDQDIYFKTVAQPAPDTEHTSGCAC*
>11667.m05059|LOC_Os01g51770.1|genepair607-1
MEAETECKVPGVYSETGIPVEDPAPGLNSDVSKKDAPPAVAAPGPGLYFEIGKKARDLLY
KDFHTDQKFTLTTYTNNGVVSITDFRMLFTGSFLVDILARLLSIVCVWSICQVDVCYDVS
TGFFVRWMVGKSVRFRFGDRRIDWVYLFGPWVVITAASTMKDEAIFSEIQTKLKSNNVML
DVLTTITTEDLGVSGLKQIVSLPFPYQTAGKAELQYLHDYAGISLGVGLTSKPLVNLSGV
FGNKSVAVGADVAVDTSTGDFTKYDAGLTINNSDLAADLTLNNKGDSLTASYYHLVNKES
GTAAGAELTHSFSTKENTLSFGMQHALDPLTTVKARYNNHGMVSALIQHEWRPKSFLTLS
AEVDTKAIDKASKVGLSLVLKP*
>11682.m04409|LOC_Os05g45950.1|genepair607-2
MAAAAPPAGPGLYSDIGKKARDLLYRDYHTDQKFTLTTYAANGAAITVAGTKKNESIFSE
IQSQVKNNNVSVDVKATSDSKLITTFTVHDLGTPGLKGILSIPFPYQKSAKAEVQYLHPH
AGLNAIVGLNANPLVSFSGVFGTGAFAVGTDVAFDTATGDFTKYNAGLSHTTPDLTAALL
LNNKGDSLAASYYHKVSKTSAVGAELAHSFSSNENTLTFGTQHALDELTTVKARFNNFGM
ASALIQHEFRPKSLVTISTEVDTKAIDKSSKVGLSLVLKP*
>11667.m05067|LOC_Os01g51840.2|genepair608-1
MGKSPAKWIKSVLLGKKSAKSNSTKAKDLAKAANNKPVLSEDPPVISEPALVNSHNDGNA
ENCKLPNGVAVEAMGQGVENQNIVGSKAPTSPEKLSEELAAVKAQAAFRGYLARRAFRAL
KGIIRLQALIRGHLVRRQAASTLRVTWLIVKLQALVRGRNVRLSGASIQFVVKSGQHKFL
SDKPSDAWKEKVSSNAYVRKLLSSSIGLEALHLQYDKRDPNSLYNWLERWTISQIWKSSS
QPKKVADGKPQVRKASYAMETESAKLKRNVRKSSAVTVDSFQTNMTVEPEKIKRNSRKFS
SSAADSVPDSQLSELEKVKRNLRKVTNSMAEASKISSSRADASKVSSSMADASKVSSSTA
DASKVSDSVAQIPPSLVNGISDHQDNQCEEAQQNACVSFPPETQELHSGILLEDNSHMNL
LEPDLISNPETPFTSILTWEKFNDSTADAQEVEVLPLQNIDNEDNFPENGVLGKKEKPRS
KEEPLSNGNLKTSKRRSSFSTKSDYPENGAQNTPVPRRKPSYMAATESAKAKLRGQNSPR
LDSDSPADMNGFTRRQSLPSSTNNRAIRAEWRRW*
>11682.m04407|LOC_Os05g45930.1|genepair608-2
MGKSPAKWIKSVLFGKKSSRSGSTKAKDLSKGSNNKGYAAAGKDAGFESSPVISEPVLVT
PHNNEAVQEVGRGENSSLQGEVVVRDVSQDLEKQNTVVSDASNDPERLREEQAAVKAQAA
FRGYLARRAFRALKGIIRLQALIRGHLVRRQAVATLRATWLIVKFQALVRGRNVRLSTNT
IQVNWKLVQQQSGSGKRDAWKEKLSSNAFARKLLASPILVEALHFQYDERDPNSAFNWLE
RWTIGRVWRPISHPKRAAVTDAKPHTRKASYAMETESGKLKRNSRRSSAAPVESSQTNIA
METEKSRRNPRKFTSSTADSVPESQLTELEKVKRNLRKVTNSMAEASKVSTPATEIPERQ
EVQCEKPQRTAEEVPNYPEIQEPQNGNLLENAKTDILVPDLQPEPEVPSYQVETEEKVAE
LTVADPTVETMPLQDIHNEENALVNDMEQRSKEEPLSTESLKSSKRRSSFSTKTEYPENG

SKNSPAVPSYMAATQSAKAKLRGQNSPRLSSDSAEKNGFTRRHSLPSSNGKLNSHSPRTQ
RPTHAGGKEGVKADKSMLSSRDASERPAKAEWKR*
>11667.m05072|LOC_Os01g51890.1|genepair609-1
MAGASSTSASARATPPARSLPPLGASGSQQEPAATASHHAAGAGASSRPMRRKGRKQKQL
WPKTVLRKWLNIRSPESDFSADEGEATGDDDTDSEFEYEEMCHWERQLYDEERRLRGLGA
ETIDSQMEGAPYKLNRRRKSETLRAQYIDIKELRVCVGTWNVAGRLPPDDLDIQDWLDME
EPADIYVLGFQEIVPLNAGNIFGAEDNRPVAMWEHIIRETLNKISPDKPKYKCHSDPPSP
SRFKPSDDVEDELVSESDSESGGEVHPWNEQDFTVDDDSVHSNKYEHSTSGPTETTVNGN
NFSRVPSMKIFDRSHNLSFKDYVSSLEEPIHQKMLTKTLSYSERLGMIWPEQPLDILAQR
LPDSTKPFISEKALRSCLSFKSAHGDSNAFPDDCLVHDFNIKSALVKTKRPYFVRIISKQ
MVGVFISIWVRRSLRKHIQNLKVSTVGVGAMGYIGNKGSIAVSMSIYQTLFCFICCHLTS
GEKDGDELKRNADVQEIHRRTIFNPVSRVSMPKTIYDHERIIWLGDLNYRINLSYEKTHE
FISMKDWNGLFQNDQLKREFKKGHLFDGWTEGVISFPPTYKYKVNSEKYTSDEPKSGRRT
PAWCDRILSFGKGMRLQAYRTVDIRLSDHRPVTAVYTSDVEVFCPKKLQRALTFTDAEVE
DQFSFEEESTSGIFSF*
>11682.m04404|LOC_Os05g45900.1|genepair609-2
MMLCCGVDCWCGALRLWRRVVLRKWLNVGSGSGDSDFSADECDASDGELDGEDRDNESYG
EGTSLDGLGAGTIGRADWLPYVQHKHLFDKTDMAGDEIKSMPYRLRRRKSETLRAQYIDI
RELRICVGTWNLAGKFPPSDLDIQDWLDKEEQADIYVLGFQEIVPLNAGNIFGSEDNSPI
AVWEHIIRETLNKICPDKPQYKCHSDPPSPSRFNPSDYVMVMKDELLSESDSDNYGELHP
LIKQNDDIAIDNDVVHDKTYENFSAASNGRVHKGKDFSRMDSVKTSDQSPNLSYEKDRSK
LEETTKLLYHPERLGMIWPEQPLDMMAQCLRASTSLKALATPASLKSTVNFPNDDLSHQV
NSDNGVIKSKRPCFLRIGSISVSMSIHQTHFCFVCCHLTSGEKDGDELKRNADVEEILRR
TVFNPLPGLSTPKGILGHERIIWFGDLNYRINLSYERAHELISKQDWDGLFENDQLKREL
SKGHTFDGWIEGDISFPPTYKYEFDSEKYVSDEPKSGRRTPAWCDRILSRGKGIRLISYR
RGELKLSDHRPVTAVFMADVEVLCHRKLQKALTFTDAEVEYHLASSSAEGR*
>11667.m05078|LOC_Os01g51920.4|genepair610-1
MVAIENQSQGQRAAEAAAQPRIPREARRLLHEMAASWADVADCRALQVIPLKGAMTNEVY
QVRWLNGAPATADGGEVEAEAAARERREVRKVLVRIYGDGVELFFDREDEVRTFECMSRHG
QGPRLLGRFTNGRVEEFIHARTLSAADLRDPEISALVASKLREFHNLDMPGPKSVLIWDR
LKNWLKTARNLCSSDESKKFRLGSLENEIAALEKEFSGDYHGIGFCHNDLQYGNIMIDED
TNMLTIIDYEYASFNPVAYDIANHFCEMAADYHSEKPHRLDYSKYPDTDEQKRFVKTYLS
NSVSEEPDAEEVENLLQSIEKYTLASHLVWGLWGIISDHVNDIDFDYKEYARQRFEQYWQ
KKQALLTS*
>11682.m04402|LOC_Os05g45880.1|genepair610-2
MVASETNQKAAAAGSSIPEGARQLLHELAAGWGDVADCRALEVVPLRGAMTNEVYQARWP
PAAEAEAAGRRVLVRVYGEGVELFFDREAEVRTFESMSRHGHGPRLLGRFPNGRVEEFIH
ARTLSAVDLRDPEISAIIASKLREFHNLDMPGPKSVLIWDRLRNWLKTAKNLCPSDEAKE
FCLDSMENEITALENELSEDYQCVGFCHNDLQYGNIMIDEETKLLTIIDYEYASFGPVAY
DIANHFCEMAADYHSEKPHILDYTKYPDTDEQKQFVQSYLSSSGEEPDAEKVNNLIKSIE
KYTLASHLIWALWGIISEHVNDIDFDYMGYARQRFEQYWLKKPAILTCQVVE*
>11667.m05107|LOC_Os01g52140.1|genepair611-1
MAEKEEAEEEVEDEEEYESDLDDAPLPAVRRRAAASDDEEGGGASGSSAPWSVAGSDLDS
YSDSDGQGAAEMYDDEEEGSEERDELEAGGGGGGGGGGGGVGGGEALEDEGKCADEEALE
DEGRYGDEEADGVVAALGDEGKCDGEEAEVEAAVEGAEVVNKEGEAQAVPTIGAFYMHDD
RFRDPENGRHGSQRKNFGGQKLWYPKDDNVWAHDRFYEMNSHHDRLYETNSHNSPNDSGR
GPRGSFRAWGGDRTHRYDHGYLERTLSQSYYHDDREEYKYVPKEPRTFFATTRDHISFLK
ESNNMYGSANNYKRVPSKFHTYYDHGDTKNFAYVQRESHTYYGNAKDFTSAHDGYRGGVS
NPYVSHWRSDPEICSGQYIRSQNEEASSNAEGGKVSLSNSGFPN*
>11682.m04395|LOC_Os05g45830.1|genepair611-2
MADREKAEETEEEYESDLDDAPLPAVRRRDAASDNYEEEEEEEEEGARPSPPTKAGSDAE
SDGQGAAEVYDDDDAYEDDEGYEEYGEVYEEFEQGRGVAGGVATGAVAAAGEEAGMGMKG
EAEGEASAAAAEGEEGKKGSEPYAVPTAGAFYMHDDRFQEARGHGRQRRMVGDRRLWNAK
EDQAWVHDRFDEMNLHDFHNDYTRRRPRGRFRGRGGAPVGKVRGGSRDNFRGNRSQTYYR
DGAKNYIYVPKEPHSYHDNTKKVQQVLNDNGKNRTIKPPNPRDGDANNFDFVRKESRPLY
GNAKSNKSAPRVVRGRGSKRYQPHWRSTAEISSEHNNKSQNLENTSSNANLGKHQHQASN
SQPERGFPMKQSFASNLNSASPPFYPSRPSHQELPVSQRGDGQPSTTTRHFSSPIGMEHV
SPTPQYGPLLRGKAFVPSAGHGKLHAEVPIKGMDHPSFHSSTSSSTSQFPIATNQVTGNS
AKSPHPIVQQRLVQSFNQSTPKMPGQMFAAQFASSDKLPSSMQSTSTILTEGTEISSPHG
SNKSNTRLMAKGQHSDQGEEHASFMYGGAQVLGTTGSLGDQNFHGTPALFPVMQFGGQHP
GGTGVPSIGMALPGFVSQQQLGLSNSEMTWLPILAGASGALGATYGSPYITVDGSYYPRT
SEHASSSVSLREPSASSQLKSQEITEALNDELSQRQHKPRRYSEMNFGK*
>11667.m05109|LOC_Os01g52160.1|genepair612-1
MGRKLGFEKVLDCFSLALCTNACVCIHSVEDDEEEAIEREALVSSQLEELVKLKDLVGGA
KTLAFHLEPKTVELRVSMHCYGCAKKVQKHISKMDGVTSFEVDLESKKVVVIGDITPYEV
LASVSKVMKFAELWVAPNSQGC*
>11682.m04394|LOC_Os05g45820.1|genepair612-2
MRRRRLRIGKVLDCFSFSMCCARSSGCLCLRASEEDGDEEAAMERKSLVSSSSQVDQVIR
LRDLVDGTRTLGFHLEPKTVELKVSMHCNGCAKKVQKHISRMEGVTWFEVDLEKKKVVVT
GDVTPLEVLQSISKVKFAQLWPLPPQPAAPATVSSA*
>11667.m05113|LOC_Os01g52200.1|genepair613-1
MAALGSKLATLGSAAREAARVAARQGCAYNKAVVEGSRQLQSRACEATRSAAKHGRAFHE
ELMERNKRYVIDPPTIQTCQELSKQLFYTRLASIPGRYESFWKEVDGAKLLWKNRKNLNL
KAEDIGVATLFGIELIAWFAGGEVVGRGFTFTGYHV*

>11682.m04385|LOC_Os05g45740.1|genepair613-2
MAALASKLAQMQAKACDAARFAAKHGCAYHRALMEKNQKYVADPPTIERCQELSKQLFYT
RLASLPGRYEAFWKEADQVKNLWRNRKDLNVEHAGVAALFGIELYAWFCVGEIVGRGFTF
TGYHV*
>11667.m05115|LOC_Os01g52220.1|genepair614-1
MALLPRTARLALLSAPRAYSAAATGAGAAPARYAGAPPPAPQSKAAEFVISKVDDLMNWA
RRGSIWPMTFGLACCAVEMMHAGASRYDFDRFGVIFRPSPRQSDCMIVAGTLTNKMAPAL
RKSVPPSSPRLLRFRVPVFRSGNLVLPSF*
>11682.m04384|LOC_Os05g45730.1|genepair614-2
MALLPRAARLAALSAPRAYSSATAAAAGAGGSASPAPYGGAPPPAANSKAAEFVISKVDD
LMNWARRGSIWPMTFGLACCAVEMMHTGASRYDFDRFGVIFRPSPRQSDCMIVAGTLTNK
MAPALRKVYDQMPEPRWVISMGSCANGGGYYHYSYSVVRGCDRIVPVDIYVPGCPPTAEA
LLYGVLQLQKKINRRRDFLHWWNK*
>11667.m05118|LOC_Os01g52250.1|genepair615-1
MAACGAAAAEYSALLSLSCGPITRRRFAVSCRARPPGNLSAQQKKKRGKNIAPKQRSSNA
KLLLTTEENGQLPSTSLRTSMERPQKSTSSEDDTNGAISQIDEKIAAIGNEQQERSKDKH
FESDFQLEDFGEMIQNMEKNILLLNQARLQAIEDVDKILTEKEALQKKVDTLEMNLSKAL
ATKGNINTDIPGDHLEKFTKEILIESALSGGNPAHLCESPLFMELTVLKEENMLLKADAQ
FLKAKIVEFAETEEFLFKLEKERSLLDATVRELEARFLVAQTDIWKVVPLQYDVWMEKVE
NLQHMLGCLKNHVEKYAALLDQHDDLHDKIDELEASLKEGKTSEFSPYVVELLQQKLKAA
KSHHQAGHQETNTHIQVYQQLTEEFQDNLGKLIEESGRLEHSANSMPSEFWSHILLMIDG
WFLERKIPNTDARMLREMAWKRDDRICEAYFACKGAKESDVMETFLKLSLSGNSQCSSGL
HIVHIAAEMAPVAKVGGLADVVAGLGKALQTKGHLVEIVLPKYDCMQLDQITNLKVLDVV
IQSYFDGNLFSNNVWTGTVEGLPVYFIEPQHPSKFFWRAQYYGEHDDFKRYSYFSRAALE
LLYQSGKKIDIIHCHDWQTAFVAPLYWDIYATRGFSSARICFTCHNFEYQGTAPAPDLSY
CGLDVEQLDRPDRMQDNAHGRINVAKGGIVYSNIVTTVSPTYALEVRSEGGRGLQDTLKM
HSRKFVGILNGIDTGTWNPSTDRFLAVQYSATDLQGKAANKAFLRKQLGLYSEDASQPLV
ACITRLVPQKGLHLIRHAIYKTAELGGQFVLLGSSPVPHIQREFEGVADQFQKNNNIRLI
LKYDEALSHCIYAASDMFIIPSMFEPCGLTQMIAMRYGSVPIVRQTGGLCDSVFDFDDET
IPVELRNGFTFARTDEQDLSSCLERAFSYYSRKPMVWKQLVQKDMQIDFSWDSPASQYEN
LYQSAVAQARGAAQT*
>11682.m04382|LOC_Os05g45720.1|genepair615-2
MACLAAGAEAAPLLFRRRLAPSPVAARRRLLVSCRARRRGLRTAAELPRKSTSNDKHHNR
VNMQRDEASVSSDKERQEKYGDENGISNLQLEDLIQMIQNTEKNIMLLNQARLQALEHVE
TVLKEKEDLQRKLKILETRLSETDARLKLSAEGQFGTEINDSLPVLELDDIKEENMLLKD
DIQFLKTMLIEVAETENSIFTLEKERALLDASLRELESRFIDAQADMLKSDPRQYDAWWE
KVENLGDLLETATNKVENAAMVLGRNHDLEDKVDKLEASLAEANISKFSCYFVDLLQEKI
KSVEERFQVCNHEMHSQIELYENSIAEFHDILSKLVEETEKRSLEHSASSMPSELWSRIS
LLIDGWLLEKRISYNDANTLREMVRKRDSCLREAYLSCRGMKDREIVDNFLKITLPGTSS
GLHIIHIAAEMAPVAKVGGLADVISGLGKALQKKGHLVEIILPKYDCMQNDQVNNLKVLD
VVVQSYFEGNLFNNKIWTGTVEGLPVYFIEPQHPAKFFWRAQYYGEHDDFKRFAYFSRAA
LELLYQSQKKIDIIHCHDWQTAFVAPLYWEAYANLGFNSARICFTCHNFEYQGAAPAQDL
ACCGLDVQQLDREDRMRDNSHGRINVVKGAIVYSNIVTTVSPTYALEVRSEGGRGLQDSL
KLHSRKFVGILNGIDTDTWNPSTDRHLKVQYNANDLQGKAANKAALRKQLNLSSTNASQP
LVGCITRLVPQKGVHLIRHAIYKTAELGGQFVLLGSSPVPHIQREFEGIADHFQNNNNIR
LLLKYDDSLSHWIYAASDMFIVPSMFEPCGLTQMIAMRYGSVPIVRKTGGLNDSVFDFDD
ETIPKELRNGFTFVHPDEKALSGAMERAFNYYNRKPEVWKQLVQKDMRIDFSWASSASQY
EDIYQRAVARARAAA*
>11667.m05121|LOC_Os01g52260.1|genepair616-1
MTAGQPLRDDPQPRRHSPPALHPAVVPAYPPPESDADESWVWSQIKAEARRDADAEPALA
SFLYATVLSHPSLDRSLAFHLANKLCSSTLLSTLLYDLFVASLAAHPTLRAAVVADLLAA
RSRDPACVGFSHCLLNYKGFLAIQAQRVAHVLWAQDRRALALALQSRVAEVFAVDIHPAA
AIGKGVLLDHATGVVIGETAVIGDNVSILHHVTLGGTGKAVGDRHPKIGDGVLIGAGATI
LGNVRIGAGAKIGAGSLVLIDVPPRTTAVGNPARLLGGKKGDDMPGESMDHTSFIQQWSD
YSI*
>11682.m04381|LOC_Os05g45710.1|genepair616-2
MPAGQPHAHEPDGGGASHRRPQSPPSLPAEVVPAFAPPESEDEESWVWSQIKAEARRDAD
AEPALASFLYATVLSHPSLPRSISFHLANKLCSSTLLSTLLYDLFLASFTAHPSLRAAVV
ADLLAARSRDPACVGFSQCLLNFKGFLAIQAHRVSHVLWAQQRRPLALALQSRVADVFAV
DIHPAAVVGKGILLDHATGVVIGETAVVGDNVSILHHVTLGGTGKAVGDRHPKIGDGVLI
GAGATILGNVKIGAGAKIGAGSVVLIDVPARNTAVGNPARLIGRKNGEVEKDEDMPGESM
DHTSFIRQWSDYTI*
>11667.m05123|LOC_Os01g52280.1|genepair617-1
MEVLLSAVLGDLVSRSISFLVDKYYQQKMGMGVDLQCLRHLLLRIEATVLEVEGRHITNR
AMLQQLQMLREGMYKGYYLVDTIKNGVLQHEMVNDEVGDHSFSFPKLRPTKRLCFSTRTF
TMAFQGEDRKEVGEMLCSLLSITDDMKEFLVFLKGYPHIHRQPYSQHLILEKCMFGRQAE
IERITNFLLRESLGAESLGVLPIIGPARVGKSTLVEHICYDERVRSFFSSIVFCSGSDIG
SKSFADLRDSGIVKHQSCVAHERSLIIIEFIDDGDVDEENWRRLYSSRSCIPHGSKIIIT
SRSERFRNVGTTQPLELSLLPQEAHWYFFKVLAFGSTNPVEHPFLESAAMEMAAEYRCFV
AANFVASLFRANFCTQFWHLFLRCHRNIVEKHVILFGEHPYTLVQKNHGIYLVENFRDPK
FILVNGYKTCLRDDDPKVMLHEVHTGTSKAHGKFEVLVWRSRIPPYHEFVMSCEAQAQQH
IIFKRKRFLA*
>11682.m04380|LOC_Os05g45700.1|genepair617-2
MSNFSGSGYRCAGDWQGNSYWGSYQRSMSFLVGKYCNCNQATAEENLQRLRQLLMRISTI

VEEAEGRHVRNQGMLQQLKILRDEMLKGCYILDNFRYRAIQDKAKDDEVSHSFALSRFNP
AKRLRFPTSKPQQTVFSGGEVEDLQKMVHRLEILIADMKEFIEFLVQYRPMYRQPYSTHL
FLDKCMFNRHMELEHAIEFLLQMEPPGSSNLGVLPIIGPRHIGKSTLVEHVCIDERVRNH
FSLILFYSGNSMKDETPTTLRENFIVKHRGNAFHKRLLLVIELSRVLAWTREHIPNNLNL
YSEYPDDVTMNHPAYIKGIAQPIKHFCMYEPYPKGSLEDDVPDTTVQDMLCGKAKARSNS
EILVWKSQIPPYHNFISRCVPYSDVYLNYKILEAYVGCIMLVHFCASDPGVLQALGPPSI
YATLGGTRHAFMALGDLGTVLLPLGVSCPLLWKNSPPTQANLEVFGTVAPLVVGTSFGWS
PLTAVGQCTVFGVVVYRTRLAVFSVINTTKRLIISWSHARSLASCGGLLLELLGILSVYH
STSNLFILGCITVAKRWPRNIAGGSTPSQHW*
>11667.m05124|LOC_Os01g52290.1|genepair618-1
METFLPAILSDLLGRSISYLVQRYRQQSTVQDDLEKLRLALVRVHVTVEEAEARHITNKA
MLRQLDVLREAMYSGYHMLDALTYRAHADGASFSFAPSRLNAAKRLRLLAADEGAAELRR
TVDSLGRTIADMREFVVFLKGYPRISTQPYSMHLLLDKLMFGRQKEVEQVVGFLLQPDVC
GAGAGAGAGVLHIVGVARVGKSTLVEHVCHDERVRGRFSSIVCLSREDLEDMGDHRALTV
KHGSHASHGSSLVVVDLAEDEEPVGDGAWRRLRSSAMCRARGSRIIVTSRSPETVRGIPA
ARAIELKFLRDDVYWYFFKVLAFGSANPDDHPRLASIAMDISAEQKGGFIGATIASSLMR
ANPDAHYWTLILKNMREYTRKHRAMFGKHPHDLLRNNHPVYLWRLAESSKIFLCHGFYTA
CPAKQEIPRVTFQEVLSGRVTPRGRFEVLAWTSQIPPCRSYLMSCSLDTPPGPHRVLDRK
KRLRQLVT*
>11682.m04377|LOC_Os05g45670.1|genepair618-2
MELFSAILGDLTSRSISYVMDRYCSNQPAAIDDGVRQLRRLLLRTHTIVEEAEGRHITNQ
GMLPQLKAMRDELFRGHYVLDTFRHRADLLQKEEEKEDEQVRSSFAMSRLNPAKRIRFSR
ARTSSFQDLESMIRSLEDAIADTKEFIVFLMSCPPVMYRQPFSTHLYLDKCMFSRQIERE
QVIDFLLRIDPDPHGSCNDIGVLPIIGPALIGKSTLIEHVCRDERVKSHFSLILFYNGDE
LKHETVATFRDRCDVDEDTWKELYYSSENRIPRGSKIIITSRSEVFVFGSADPDEHPKLT
SIAMEIAAELRRSLFCAHVVGALLRVHLDAHFWRRVLEGTREYMQKNLILASEYPHDLKT
DKNHPRYAWIISEPKPIKSLLIYDSYQKGSENAEVPNITIQDLLFGEASGRGKFDILLWK
SQMPPYCSHICSCVV*
>11667.m05140|LOC_Os01g52450.1|genepair619-1
MRKAAALASAAMAAAAVAVVSTVLHQRQRRAAKRSERAEAVLLRDLQERCAAPVELLRQV
ADAMAAEMRAGLAAEGGSDLQMLVTYVDSLPSGGEKGMFYALDLGGTNFRVLRVQLGGKE
RRIIKQDSEGISIPQHLMSSSSHELFDFVAVALAKFVASEGEDCHLPEGTQRELGFTFSF
PVKQKSLASGTLIKWTKSFAIDEMVGKDVVAELNMAIRSQGLDMKVTALVNDTVGTLAAG
RYVNHDTIAAVILGTGSNAAYIDHADAIPKWHGSLPKSGNMVINMEWGNFKSSHLPLTEF
DQELDAESLNPGKQVYEKSISGMYMGELVRRILLKMAQETRIFGDNIPPKLERPYILRTL
DMLIMHHDTSSDLRTVANKLKEVLGIEYTSFTTRKLVLDVCEAIATRGARLAAAGIYGII
QKLGQHSDSPSTRRSVIAVDGGVYKYYTFFSQCMESTLSDMLGQELAPSVMIKHVNDGSG
VGAALLAASYSQYHQAESADSS*
>11682.m04369|LOC_Os05g45590.1|genepair619-2
MRKAAAAAVAAAAAVGVALLVRRQLREAKRWGRADAVLRELEERCAAPPGRLRQVADAMA
VEMHAGLASEGGSKLKMIISYVDALPSGEEKGVFYALDLGGTNFRVLRVQLGGKEGRVIK
QEHDEISIPPHLMTGGSNELFDFIASSLAKFVASEGEDFHLAEGRQRELGFTFSFPVKQT
SIASGTLINWTKGFSIDETVGEDVVTELTKALERQGLDMKVTALINDTIGTLAGGRYDDN
DVIAAVILGTGTNAAYVERANAIPKWHDLLPKSGDMVINMEWGNFRSSHLPLTEFDQALD
AESLNPGEQVYEKLISGMYLGEIVRRVLLKMAEEASLFGDEVPPKLKIPFIIRTPYMSMM
HCDRSPDLRTVGAKLKDILGVQNTSLKTRRLVVDVCDIVAKRAAHLAAAGIHGILKKLGR
DVPNTDKQRTVIAVDGGLYEHYTIFAECVESTLRDMLGEDVSSTIVIKLAKDGSGIGAAL
LAAAHSQYREAEEL*
>11667.m05166|LOC_Os01g52650.1|genepair620-1
MARFAGSLQLYCLSVLLVILTQLGGGSAMGLPRPPPNVNFTIGVEGAVWCKTCRYAGYVK
SKDASPLPNAAALLRCRRGKWALSVWGATDARGYFLIQTGTQVAAFTSKDCRVYVPRSPS
RAACGVALQPGRKTGSPLKFRRFVALPDGLQGRYSAGNFVFGPRDPKKC*
>11682.m04358|LOC_Os05g45480.1|genepair620-2
MALLFVKSFVVLSLAAVLLALDGGGGASAMGLPPPPPTVNFSIGVQGMVWCKTCRYPGYL
AAMDASPLAGAVAYLRCRHGHRRVASIRGVAGSGGYFRIETSQLTSFTSQECRVYVPRSP
SRACAVPGHGRRGLPLKFEEFVKRDNGLQGLYSVGNFVFSPKYPNKCY*
>11667.m05167|LOC_Os01g52660.1|genepair621-1
MRSHTAVVCSALLVLAAALLLPAHHAMAEDVVSSRDNTARAPSSSPSAATATAPAPSPYT
DTAMAPAPSSSNDTAVEPVAPPPLPFIIVEGVVYCKTCKSRGYSSDMDASPLPGATAQLV
CYGKKVVNVTGTVTDANGYFLVMFYDLRNFNPRTCKVFLGSSPTSLCDKPVYPPNKWIGL
SLLKETRTVPPVGLQAIYCPTSVLFYGPANAGQCPSG*
>11682.m04356|LOC_Os05g45460.1|genepair621-2
MVSSKIHCAMAALLLAILLPVSHASGRYVAPAPAPVPVPPPPRTSPPSRIQPVVVVQGTI
YCKSCNLSGYNRYMDASPLPNATAQLVCYGDKVLNMTSTATDKNGYFLVMVYRLDVFRRS
RCRVYLGSSPSPLCAAPFIPSNKWLGLTLERERVASLPKGVRGVYRPKSTLMFGPGTGGK
CPAAAAADAAGVPMM*
>11667.m05174|LOC_Os01g52730.1|genepair622-1
MATMGEELYESDVLWPDHQSPHDVVPPTATATATAPTPARRGQQQITRHCSTASSRPVDI
PRAAQRWSGGDQGDDCGGGTMVPPHVMVSRRRSTEEGDQAFSLRTGTGRARRDLSHLRNS
VLRMTGFIEG*
>11682.m04355|LOC_Os05g45450.1|genepair622-2
MEEFQEADILWPEPAEDNSDDGAVVVTTTPSPVARRPVGSPESSSLSAPVEIAASRRKRR
SRSWASEYNMFDQTNDDDDAVKKKMMNNGVMVAPPHAIVDRRRLRGRTAAYSMCAGKGRT
LKGRDLRNVRNLVLQMTGFIEK*

>11667.m05189|LOC_Os01g52880.1|genepair623-1
MAASVAVFVCLLSVAAAAASMDPAEREALFLVMEAVSSDRDWRSESPDPCGAPWPGLECK
PAAGDAAAALLRVTRLDFGVEPNPSCKDTAAFPPLVFSSLPHLQSLFFVGCFKNPAANTS
LVLPPAANLSTSSLQQLSIRANPSLSGVMPPQLATLRSLQVLTISQNGLIRGEIPQGIGE
LTSLVHLDLSYNSLTGPVPSEISELKSLVGLDLSYNSLSGAIPSRIGELRQLQKLDLSSN
NLTGGIPVSIANLSSLTFLALSSNGLSGHFPPGLSGLRNLQCLIMDNNPMNVPLPSELGG
LPRLQELRLAGSGYSGQIPAAFGQLASLTTLSLEDNNLTGEIPPVLTRLTRMYHLNLSNN
GLGGAVPFDGAFLRRLGQNLDLSGNAGLCLDDRMVVRGVGVGVGACHAGGGGDGPLAPGG
VTGAAATVRGSVDGYPFRLLGHACLVVACLVSLN*
>11682.m04353|LOC_Os05g45430.1|genepair623-2
MPVASVVLLLHLLSMAAAATAALAMDPAERETLLLVMEAVSSDREWRSVGPDPCGSPWPG
LECKPVPAAGNVSSAAARLHVTRLDFGVAPNPTCKDGAAFPHLAFALPHLQSLFLVDCFK
NPAATTAFTLPPSANLTSSRLQQLSVRSNPSLSGTLPPQLSSIRSLQVLTVSQNALIRGE
VPQGIGELKSLVHLDLSYNSLTGTIPSRIGELRSLVGLDLSYNSFSGSIPGQLGDLAMLQ
KLDLSSNNLTGGVPATITGLTSLTFLALSNNGLSGHLPAGLSDLLDLQYLIMENNPMGVP
LPSELGDIARLQELRLANSGYSGSIPETLGRLASLTTLSLENNNLTGRIPAGLSRLKRMY
HLNLSKNGLDGVVPFDGAFLRRLGRNLDLSGNPGLCVDGRAVLQADVGVGVCRRAGDGGD
IASVSAATDVLSVGTLFRRDGQLWLAGGRCLMVTGCDLCVADLAINPAAPAICKQFSGTL
QPELCPAFHCLPLYNPMSVMPLIALSKGTRCSGQVIMYSSVDV*
>11667.m05193|LOC_Os01g52920.1|genepair624-1
MEISFEAWEGVQRHGQDIADRLAQGFTGLLQAQPPQFPWPAASHKRIPFEIDLPVVPFGA
SRGARAGKEFFPAAAVASVIDIGGRLGQAGVEIGASVGGAVQHAVRQLPLPFRNGQIRRR
KLPPQPQAPSPAAAVGEAAVGLSVERAVDRCPLEAAAAAAAAATGSAAASTVSGAVGGDD
VDEEDEGFGCEIGTFGNFKKSKGTVNVSASYNTRNHDIESSVVARGDLWRLEASRSSSTS
GNDSSPLYLVQLGPLLFVRDSTLLLPVHLSKQHLLWYGYDRKSFMDLQFPNGQLTYVAGE
GITASGFLPLFGGLLQAHGKYPGETRVSFSCKNKQGTRFTPMFQWPDKSLSFGVTQALAW
KRSGLMVRPSVQVSLCPTFGGNDPGIGRSKWNGQMGSSGVVVTLETPLNNIGRPSLSVQL
NGGFEI*
>11682.m04347|LOC_Os05g45400.1|genepair624-2
MEISFDAWVGVQRHGQDLADRLAQGFSGLLLHAHPPQLHWSAPALPAKLIPFEIDLPVVP
FVVGGRRAGGGRGAADLPAAAVASFVEIGGRLGQAGSELGACVGGAVQQLARQILVPFLA
ESARGRKWEAIPPQPAAAAAAAATVNEGEVALAVERAEDKGLASERVGDRGPLEVAAAAA
AAATGSATATSVGGAEGGLDEEDDGFGCDIGTIGNFKKAKGTINISATYETRHHDFETSV
VARGDLWRLESSRGGSNSGDENAPLFLVQLGPLLFVRDSTLLLPIHLSKQHLLWYGYDRK
NKMHSLCPAIWSKHRRWMVMSMMCLNPVTCSFMDVQFPNGQLTYVAGEGITASGFFPFFG
GLLQAHGKCPGETRLSFSFKSKQGTRFTPMFQWPDNSLSFGVAQALAWKRSGLMVRPSIQ
VSVCPTFGGSDPGIRAEFVHSLKEELNVMCGFSCARHPSAFTAVSLGRSKWNGQVGSSGL
VITLETPLDNLARPSLSVQLNGGFEL*
>11667.m05203|LOC_Os01g53020.1|genepair625-1
MAPLLSPPLLADSVAKFHCSSTPTPCSGSVRRWAITRFAGAGRRRDWHRRRRTSGRGVLT
VSAVAAESPSSGGGVAEDYYAVLGVMPDATPQQIKKAYYNCMKACHPDLSGNDPDVTNFC
MFINEVYTVLTDPIQRAVYDEIHGYAATATNPFFDDSAPRDHVFVDEFSCIGCKNCANVC
SKVFEIEEDFGRARVYNQSGNAELIQEAIDTCPVDCIHWTSAAQLSLLEDEMRRVERVNV
GLMLAGMGSSVDVFRMASTRWEKRQAKVLEKVRRRVSQEDSGKGSSWSDVWGAPTRYRKN
EDEARERAKRAAAAARRWREYSRKGADKPPTFKLPEAVSNKE*
>11682.m04342|LOC_Os05g45350.1|genepair625-2
MAPLPSPPLLAESLATLRTASPSPPIPCSPRRTRPLVSARFARTAGRRSRSTGGRRDLRS
CAYAAEAEYGSAEDEVADDFYSVLGVMPDATPEEIKKAYYSCMKACHPDLSGDNPEVTNF
CMFINEVYTVLSDPVQRAVYDEIHGYTATATNPFFDDSAPKDQVFVDEFTCIGCKYCANV
CPNVFRIEEDYGSPVNCIHWTSAAQLSLLENEMRRIERVNVGLMSAGMGVSVDVFRLASV
RWEKRQSKVLEKIRTRMTNKKYSDISSSWTDIWGSPTRYQGNDEFRGKFPLAVVW*
>11667.m05208|LOC_Os01g53070.1|genepair626-1
MPTPDRAGAVGATARGGGGGGGHLRGHAHLTNCIHLRHHHAHGGGGGGGGASSSGRRRSP
TSVASAALMRDLLALQRSRSLRDPSTRRSVESSSKVAADPDADTDEDDADLPPKSRRSAS
TTGALKTLLDQLAENPQPKQVRRPPRRFKRGAGRRAGAASKAPDRSAAAAAAAAALSVNS
SSQEAVCGNKYLFRDGEDGLDELQQHVPPQDSRNVCGIPWNWSRLHHRGKSILDMAGRSL
ACGLSDPKSAAGRKSEAGAGASRGHLNGSHSLFPVKSERLASSTSSDSDALPLLVEAAAT
GARNGGIGGSYSGELGIFSNQTSEMESDLLSEGRSGHKSRGSQHGRGRHRSLTQKFAPKT
FKDVVGQSLVVQALSNAVLRRKIGLVYVFYGPHGTGKTSCARVFAKALNCHSPEHPRPCD
SCTSCIAHNLGKSRSVMEIGPVGNIDMDGIVDVLDNVMLSPAPSHYRVFIFDDCDTLPAD
TWSLISKVVDRAPRRVVFILVSPNLDLPHIILSRCQKFFFPKLKECDIVNTLQWISTSES
LDVDRDALKLIASRSDGSLRDAEMTLDQLSLLGQRISLSLVQELVGLVSDDKLVDLLDLA
LSADTVNTVKTLRDITETGVDPLALMSQLATIITDILAGSYTFTRERLRRKFFKRPTLSK
DDMEKLRQALKTLSEAEKQLRVSNDKTTWLTAALLQLAPDKQYVLPSSSSTSLNHGVLAG
SFPDRDMAIHSALEHNGNMASTSYRERRPVEHTSNSHLLSTSAPRANEQSRNSKIENELI
WHAVLESIQSDTLRKIMAKEGRLSSVSLGTAPTVQLIFSSRVNKSKAENYRGQILQAFES
VLRSAIILEIRYESKNDVRASHAPAVFSHGENESSNTTLRRSFTKHSPLSSGGENLRRRL
KKDRASSSKTTWMQSDPHILTEGEIIEVGPSHMHWHDETNNDVHNVNQRRKDNVWEEESL
ASPNSKANQGRNGNKQRRQNSIVKGKVSLAHVIGKAEACSQRGGWSRQKALSIAEKLEQE
NL*
>11682.m04341|LOC_Os05g45340.1|genepair626-2
MPAPAATAAAGMGSGGGGGGSGEHHMRGHAHLTNCIHLRHHHAHGLGAGSASSGRRRSPT
GSSASASAALMRDLLALQRSRSLRDPSTRRSVDSSSRVAAEPQGVGLGLGDDLHDDSSRP
GGALKTLLDQLAENPQPKPSRGPRRRFKGGAGRRAAPASATGGALDRGVDGAAAVSVNSS

SQEAVCGNKYLFGVVGGDDDDDDECGVELQRPQASQDSRNVCGIPWNWSRIHHRGKSFLD
MAGRSFSCGLSDPKSASAARRSIAATSAGSCGHMDGSRSHPHFPVTARLTSTSSDSDSLP
LLVDGVRNGIGGIARSFSGELGIFSNQTSELDSDLASEARSGQRSRGSHRGRHQSLTQKY
SPKTFKDMIGQSLVVQALSNAILKRKIGLVYVFYGPHGTGKTSCARVFAKALNCHSPEHP
RPCDSCASCIAHNLGKSRSLVEIGPVGNIDLDSIVDILDNVMLSPVPTQHRVFIVDDCNT
LPPDTWSVISKVIERAPRRVVFILISPNLDLPHIIVSRCQKFFFPKLKECDIVNTLQWIS
TSEGLDVDRDALKLIASRSDGSLRDAEMTLDQLSLLGQRISMSLVQELVGLVSDDKLVDL
LDLALSADTVNTVKTLRDITETGVEPLALMSQLATIITDILAGTYAFTQERVRRKFFKCP
TLSKDDMEKLRQALKTLSEAEKQLRVSNDKMTWLTAALLQLAPDKQYVLPSSSTSASFNQ
GLLTCPEGDIGRSTAIDHTNIYAGPHGLPRNANLGVDSDNNMAGTRRGKGPAPTVQLMFS
SRVNKSKAERSREQVLHAFESVLASAITLEIRYEPKDDARAGHVPAISPYPEDSTSNVAL
RRSFTRHSSASSRGENLIRRLQKDNVVHGANSNQTRWVQSDPHILTEGEIIEVGPSQVDW
HSEPNNSAGVRKENIVLQGREVVNNEHGRQNSIVRGKVSLAHVINKAETCSQQGGDWSLS
RVYFVGRLQALLDERVHSIGYLDLPLFESMQLLAFSLKETPYIKAAALQPAYALSQVYPR
EKR*
>11667.m05209|LOC_Os01g53080.1|genepair627-1
MGCAGSTPKTDDNSKKLKKPKPWKHNQAITTTQLKQMRDEFWDTAPHYGGQKEIWDALRA
AAEAELSLAQTIVDSAGIIVSNSDMTICYDERGAKYELPKYVLSEPTNLIREG*
>11682.m04339|LOC_Os05g45320.2|genepair627-2
MGCAGSTPKVDENSKKLKKPKPWKHTQAITPTQLSKMREEFWDTAPHYGGQKARCIFTEI
WDALRAAAEAELPLAQTIVNSAGIVVSNSDMTLCYDERGAKYELPKYVLSEPTNLIREP*
>11667.m05221|LOC_Os01g53200.1|genepair628-1
MDPWSEIEGKRAHDPIFQNYFSQNCRQSVDGFCKKRSADAAVARAERCIRVLGPIIVGAG
PSGLAVAACLKEKGVDSLVLERSNCIASLWQLKTYDRLSLHLPRQFCELPLMPFPAYYPI
YPSKQQFVAYLESYAARFGICPTYNRTVVCAEYDEQLQLWRVRTRATGIMGEEVEYVSRW
LVVATGENAEVVLPEIDGLDDFKGTVMHTSSYKSGGAFAGKRVLVVGSGNSGMEVCLDLC
NHNANPHIVVHILPREMLGQSTFGLSMWLLKWLPVHVVDRILLLIAQTMLGDTAQLGLKR
PTIGPLELKSLSGKTPVLDVGTFAKIKSGDIKVRPAIKQISGRQVEFMDTRLEEFDVIVL
ATGYKSNVPFWLKDRELFSEKDGLPRKAFPNGWKGENGLYSVGFTRRGLMGTSVDARRIA
HDIEQQWKARGKHPGVLL*
>11682.m04330|LOC_Os05g45240.1|genepair628-2
MLVWVQGPIVVGAGPSGLAAAACLKEKGIDSLVLERSSCLAPLWQLKMYDRLSLHLPRQF
CELPLFPFPASYPDYPTKQQFVAYLESYAAKFGINPMYNHTVVCAEFDERLMLWRVRTTQ
ATGMMEDDVEYVSQWLVVATGENSEAVLPVIDGLEEFRGSVIHTSAYKSGSKFAGKTVLV
VGCGNSGMEVCLDLCNHNGYPRIVVHILPREMLGQPTFRLAMWLLKWLPIHIVDRILLLV
ARAILGDTSQFGLKRPSLGPLELKSLSGKTPILDIGTLAKIKSGDIKVRPAIRRIAGQQV
KFVDGRSEQFDAIVLATGYKSNVPCWLKVYGNTADVPFAVEEFQYFFS*
>11667.m05227|LOC_Os01g53260.1|genepair629-1
MTSLDWQSLLQTCLQVPPPVLEQQQPAAAAQADQYSGENDHGDLQAAESSGAGNKEKQVM
AKGGAGRPSGTKKKASRPRFAFQTRSDNDILDDGYRWRKYGQKAVKNSKHPRSYYRCTHH
TCNVKKQVQRLAKDTSIVVTTYEGVHNHPCEKLMEALTPILKQLQFLSQF*
>11682.m04329|LOC_Os05g45230.1|genepair629-2
MDGLEAAAGDQQHGRLLIPQLPAAYLASSSMAALSPAGDDWAASLILPDGGSAAAGVGED
DLGGGVMAAAAAESSCGGSSTVTSSGVTEAAAAAATTTRRGRGNGKKAGGGGRTPRFAFH
TRSENDILDDGYRWRKYGQKAVKNSDFPSDDELLLFSDVDNTQTATENLRFIPLGRVYIT
G*
>11667.m05229|LOC_Os01g53280.1|genepair630-1
MNKGKIFKLAKGFRGRAKNCIRIARERVEKALQYSYRDRHNKKRDMRSLWIERINAGTRL
HGVCIHCTQAVKKEISLVVARKDLIIRRSILCLSLSKQTIQDNTTRLTSWSLSSSFLAFD
YQVNYGNFMHGLMKENIQLNRKVLSELSMHEPYSFKALVDVSRSAFPGNRPPVKKEGLAA
IL*
>11682.m04328|LOC_Os05g45220.1|genepair630-2
MPRSHSVSATIVRGSLATVTTLATPMGLSPAATIATASLPFAPMLPRGSRDERTVGGRRA
FPEKKIVQQSRNSKMNKGKIFKLAKGFRGRAKNCIRIARERVEKALQYSYRDRRNKKRDM
RSLWIERINAGTRIHGVNYGNFMHGLMKENIQLNRKVLSELSMHEPYSFKALVDVSRTAF
PGNRPVKKEGLASIL*
>11667.m05231|LOC_Os01g53300.1|genepair631-1
MAVFRLDPRVDQIQEMKSPAISHREQPWLSSVYGSPAAISGSNRFEHKRENVENVLAHPV
LTKQRNGGSEVQGAAETVVDGQRRQNSGVDVAGSTQGANCNFESTGVLEMSHRGCMREES
GGNSPELGVASVLWQLETLLLQKDTYMNYSQALSYTSQALSQNLAGLRKKSSIRKISTSL
SYYFEDNWKRLWVLALWIGIMAGLFTWKFMQYRNRYVFDVMGYCVTTAKGAAETLKLNMA
IILLPVCRNTITWLRSTRAARALPFDDNINFHKTIAAAIVVGIILHAGNHLVCDFPRLIK
SSDEKYAPLGQYFGEIKPTYFTLVKGVEGITGVIMVVCMIIAFTLATRWFRRSLVKLPRP
FDKLTGFNAFWYSHHLFIIVYIALIVHGECLYLIHVWYRRTTWMYLSVPVCLYVGERILR
FFRSGSYSVRLLKVAIYPGNVLTLQMSKPPTFRYKSGQYMFVQCPAVSPFEWHPFSITSA
PGDDYLSIHVRQLGDWTRELKRVFAAACEPPAGGKSGLLRADETTKKILPKLLIDGPYGS
PAQDYSKYDVLLLVGLGIGATPFISILKDLLNNIIKMEEEEDASTDLYPPMGRNKPHVDL
GTLMTITSRPKKILKTTNAYFYWVTREQGSFDWFKGVMNEIADLDQRNIIEMHNYLTSVY
EEGDARSALITMLQALNHAKNGVDIVSGTKVRTHFARPNWRKVLSKISSKHPYAKIGVFY
CGAPVLAQELSKLCHEFNGKCTTKFEFHKEHF*
>11682.m04325|LOC_Os05g45210.1|genepair631-2
MRAGIGSGSGGGTTPVRPRWGSGVTTPRSLSTGSSPRGSDRSSDDGEELVEVTLDLQEDD
TIVLRSVEPAAGGAAVASSSGASPSAVAPPRRAEPPGGVASRSRSPAMRRTSSHRLLQFS
QELKAEAMARARQFSQDLTKRFTRTQSTTTAPPGIESALAARAERRQRAQLDRTKSGAQR

AIRGLRFISGPNKASNAWIEVQANFDRLARDGYLSRDDFPQCIGMTESKEFAMELFDTLS
RRRQMQVDKINKEELREIWQQITDNSFDSRLQIFFDMVDKNADGHITEAEVKEIIMLSAS
ANKLSRLKEQAEEYAALIMEELDPEQLGYIELWQLETLLLQKDTYVNYSQALSYTSQALS
QNLAGLRKRSPIRKISTKLSYYLEDNWKRLWVLALWIGIMAGLFIWKFIQYRHRYVFNVM
GYCVTTAKGAAETLKLNMAIILLPVCRNTITWLRNTRAARALPFDDNINFHKTIAAAIVV
GVILHGGLHLVCDFPRLIGSSEEKYAPLGKYFGETKPTYLTLVKGVEGITGVIMLVCMII
AFTLATRWFRRSLVKLPKPFDKLTGFNAFWYSHHLFIIVYISLVIHGEWLYLIRIWYKRT
TWMYLAVPVCLYVGERTLRFFRSGSYSVRLLKVAIYPGNVLTLQMSKPPTFRYKSGQYMF
VQCPAVSPFEWHPFSITSAPGDDYLSIHVRQLGDWTRELKRVFSAACEPPVGGKSGLLRA
DETTKKALPKLLIDGPYGSPAQDYSKYDVLLLVGLGIGATPFISILKDLINSIIKMEEEE
EASGDLYPPIGRNKAHVDLDTLMRITSKPKRVLKTTNAYFYWVTREQGSFDWFKGVMNEI
AELDQRNIIEMHNYLTSVYEEGDARSALITMLQALNHAKNGVDIVSGTKVRTHFARPNFK
KVLSKIASKHPYAKIGVFYCGAPVLAQELSDLCHDFNGRCTSKFEFHKEHF*
>11667.m05234|LOC_Os01g53330.1|genepair632-1
MERTVVLYPGLGVGHLVPMVELAKVFLRHGLAVTVAAVKPPLDSPDFSAAVARFAECNPA
INFHVLPPPPPPPAPVGSGSDGKSASPIVGMLGFLNAMNAPLRDFLCSLPSVDALIVDMF
CTDALDVASELRLPVYVFFTSAASDLAVFLHLTSMRDSINTSFGELGDSMIHIPGCPPFK
ASELPSDILSDNEASRLILLMFRRHPESRGILVNTFESLETRALRALEDGLCVPGRATPT
VYSIGPIVSGGGGSDKDHDCLRWLDAQPDNSVVFLSFGSLGRFCKKQLEEIAIGLQKSEK
RFLWVVRSPRIDEKNVFEPLAEPDLDALLPAGFMEATRGRGLVVKLWAPQVEVLRHRATG
AFVTHCGWNSTLEGITAGLPLLCWPLYAEQRLNKVFVVEEMKLGVEMRGYDEEVVVAEEV
EAKVRWVMESEGGQALRQRAAAVKDAAAQAIKEGGSSHAAFFKFLEDLPLQVSRGMVSA*
>11682.m04324|LOC_Os05g45200.1|genepair632-2
MMTETVVVNAGLGVGHLAPMVELANLFPRHGLAVTVVLIEPPAKPPSFAAAVSRSMASNP
RITFHVMPSPSCHSNVPELIRAMNAPLREYLRSSVPSARAVVFDMFCACALDVAAELGLP
AYFFQCGGASHLAVGLHLPHVQAEINASFGEIGDEPLLFPSVPPFKPSDLPKAALDRNDE
MYRWILGVFERLPESRGILVNTFQWLETKALRALGDGACVVGRPTPPVCCVGPLVSRSGE
DKKHGCLSWLDAQPEKSVVFLCFGSMGSFPKEQLAEIAIGLERSGQRFLWVVRRPHAGEA
SLSGLLAGCHGTHGELDIDELMPEGFLERTKGRGLAAGSWAPQADVLRHRATGAFVTHCG
WNSVLEGIAAGVPLLCWPLYAEQRLNKVFIMEEVGVGAVMAGYDGEVVRAEEVEAKVRWM
LESNEASPIRERVALAKERAEEATRKSGSSHQSFVKFLIDFGVTK*
>11667.m05244|LOC_Os01g53430.1|genepair633-1
MAMVEKTVLLYPCPAVGHLNPMVQLAEALVRRGVSVTLAVADPPDKGAVLAGAIARIAAV
CPSIGVRLLPIPSCEGKTYSHPVMWIVDALRLANPVLRELLRSFPAAVDALVVDMFCIDA
LDVAAELAVPAYMFYPSAASDLAIYLQVPHVARSAPSSFKDMADTVLSFSGVPTIRALDM
PDTMQDRESDVGTTRIHHCSRMAEARGILVNSFDWLETRALKAIRGGLCLPSGRSVPAIY
CVGPLVDGGKLKENDARHECLEWLDRQPKQSVVFLCFGSRGTFSVSQLSEMARGIENSGH
RFLWAVRSNLGEVDLEALFPEGFLERTQGRGFVVKNWAPQSAVLQHGAVGAFVTHCGWNS
SLEAIMSGVPMICWPLYAEQRLNKAHLVEEMKLGVLVEGYDGELVKADELETKVRLVMES
EEGKRLRERSAMAKEMAADAVKDGGSSDMAFAEFLNNLGTNNVKSGPRDTPVHD*
>11682.m04313|LOC_Os05g45100.1|genepair633-2
MAFFAKKWVTIQRRSHYLACGLRDYIAMTTKTFVMYPSLGVGHLNPMVELAKHLRRRGLG
VVVAVIDPPNNDAVSADAMARLAAANPSITFRLLPAPDSPDVGAHPIKRSHDTLKLANPV
LREFLRSLPAVDALLLDMFCVDALDVAAELAIPAYFFFPSQASALAVFLHLPYYYPNLPT
FMEMGKAALLRFPGMPPIRTVDMPAMLRDKDSEATKVRLYQFKRMTEAKGVLVNSFDWLQ
PKALKALAAGVCVPDKPTPRVYCIGPLVDAGRKSRIGGERHACLAWLDAQPRRSVVFLCF
GSQGAFPEAQLLEIARGLESSGHRFLWTVRSPPEEQSTSPEPDLERLLPAGFLERTKDRG
MVVKNWVPQAEVVQHEAVGAFVTHCGWNSTLEAIMSALPMICWPLYAEQAMNKVIMVEEM
KIAVSLDGYEEGGLVKAEEVEAKVRLVMETEEGRKLREKLVETRDMALDAITEGGSSEMA
FDKFMRDLEESSLENGVRS*
>11667.m05246|LOC_Os01g53460.1|genepair634-1
MESKPSRNVVLYAAMGAGHLLPMVELAKLFLTRGLDVTIAVPATPGSGTTGSPTIAGIAA
SNPSITFHHLPPPPSCADPDPNPLLLMLDVLRRSVPSLASLLRSIPSVAALVLDIFCAEA
VDAAAALHVPAYIYFTSAAGAFAASLGLMHHYSTTTTNLRDMGKALLRFPGVPPIPASDM
PSLVQDREGRFYKARVKLYARAMEASGVLLNTYEWLEARAMGALREGACSPDRPTPPVYC
VGPLVASGEEEGGGVRHACLAWLDAQPARSVVFLCFGSMGSFSAAQLKEIARGLESSGHR
FLWVVRSPRQDPANLLEHLPEPDLAALLPEGFLERTADKGMVVKSWAPQAKVLRHAATAA
FVTHCGWNSTLEGITAGVPLLCWPLYAEQRMNKVFIVEEMKVGVVIDGYDEEMVSAEEVE
AKVRLVMESEEGGKLLERLAVARAKAVEALAEEGPSRVAFDEFIDRLVTSE*
>11682.m04311|LOC_Os05g45080.1|genepair634-2
MEADPNPMVVLHACLGVGHLIPMVELAKLLLRRGLAVVIAVPTPPASTADFFSSSAPAVD
RMAAANPSISFHHLPPPEYPDPDPDAFLQMLDTMRLTVPPLLAFLRSLPSVAALVLDLFC
VDALDAATAAGVPAYFYYTSSAGDLAAFLHLPHHFATTEGSLKDMGKTPLRFPGVPPIPA
SDMPHTVLDRADRTCATRLGHYGRIPEARGILINSYEWLEARSVRALREGACIPDRPTPP
VYCIGPLMAKGEEAANGERHACLSWLDAQPERSVVFLCFGSLGAVSVKQLKEIARGLENS
GHRFLWVVRSPPQDPAKFFLPRPEPDLGMLLPEGFTERTRDRGMVVTSWAPQVEVLRHAA
TAAFVTHCGWNSVLEAASAGVPMLCWPQYAEQRMNKVLLVDGMQLGVVMDGYDEELVKAE
EVEKKVRLVMEFEEGKKLRDRLTMAKEMAAKALADGGSSSLAFTEFLKDLNFGNTTKEKA
*
>11667.m05249|LOC_Os01g53470.1|genepair635-1
MATVGPYGEAEKNEPARPLALPSPSVHPAANDDEEAARAAAAAAGDTAANKRPRSPQYLR
RRRCVIWCGGCCVTSAVVVGIVILVLALTVFKVKDPELTMNRVTLEGLDGDLGTSRHPVS
VNATLNADVSLRNPNVASFRFDRSETDFYYAGETVGVAYAPEGEVGADSTVRMNVTLDVL
ADRISPNVNATDLIFGQGYNLTSYTEISGRVNVLGIYKRNLDIKMNCSITLEVSALSTVQ

SKSTNCVASVS*
>11682.m04310|LOC_Os05g45070.1|genepair635-2
MATAAENKNEPARPFAVPSPSVHPAATGVGEEEEAQAATGWRSMQYLRKRRRALCCCGCC
VTTLVVVGLVILVLALTVFRVKDPRITMNGVWVTAISTGPGTGAGIGSTVATNATLTADV
SVKNPNAASLRFSRSETDVYYKGKTVSVAYVPAGSVGADRTVRMNITLDLLADRLASVLN
GTGLILGQEYDLTTYTAMRARVSVLGIIKKSLDVRMNCSVILDVAGIAGVLLPGDGAKSG
VQTRSVDCVAIVS*
>11667.m05252|LOC_Os01g53500.1|genepair636-1
MSSSSSSLLSSLATLGLGYSIAIALGFLVLLASLLLASYFCFRRGGGGGHFSGVLTPSSS
SSHLSITVPRVLFVAEGSESPDAYSSGVAAASSPVGLDPAAIASYPKVPFYSGAGADADA
MCSICLSEYADGEMLRVMPDCRHRFHVCCLDAWLRRNASCPVCRSSPIPTPVATPLATPL
SELVPLSQYAADRRRSR*
>11682.m04309|LOC_Os05g45060.1|genepair636-2
MASFLDSFSSLGVGYAVAIALGFLVLLASLLLAFYFCSRRGGAGVVRRGGGQGVHSARHA
VSSASSSGHISITVPRVIFVADDSDSPGSSSRGGAGGGAASSPVGLDPAVIASYPKVPFS
RAATGADAEAACSICLCEYKEGEMQRMMPECRHRFHLMCLDAWLRRSASCPVCRSSPIPT
PVSTPLATPLSELVPLSQYAADRRRHR*
>11667.m05255|LOC_Os01g53520.2|genepair637-1
MAEHKEEQSVMEKLSEKLHGDSSSSSSDSDDDKKGSSSSSAAAAMKAKIYRLFGRERPVH
SVLGGGKPADLVLWRNKKISGGVLAGATAIWLLFEIMEYHLLTLVCHCLILSLAVLFLWS
NASTFIHKSPPNIPEVKIPEDLTVNIALSLRYEINRGFATLREIGHGRDLKKFLIVIAGL
WILSVLGSSCNFLTLFYIVFVALYTVPVLYEKYEDKVDAFGEKAEIEFKKYYALFEEKCL
SKIPKGPLKDKKH*
>11682.m04308|LOC_Os05g45050.1|genepair637-2
MAEHKEEESLVESVMDKISDKLHGRGGSSSSSSDSDGERTADLKAKIYRLFGREKPVHSV
LGGGKPADLFLWRNKKISVGVLAGATAIWLLFEVMDYHLLTLLCHCIILTLAMLFLWSNA
STFINKSPPNIPEVKIPEDLAVNVARSLRFEINRGFATLREIGQGHDLKKFLIVVVGLWV
LSVLGSCCNFLTLFYIVFMVLYTVPVLYEKYEDQIDAFGEKAMIELKKYYAIFDEKCLSK
IPKGPLKNKKH*
>11667.m05269|LOC_Os01g53650.1|genepair638-1
MAYETSSDHQLAAAAEFLAALQVHLAGAEASSPTWGGRCAYDEDFMMYEFKVRRCPRSRA
HEWTSCPYAHPGEAARRRDPSHVTYTGEPCPDFRVAARAACPRGSGCPFAHGTFETWLHP
SRYRTRPCRSGMLCARPVCFFAHNDKELRIVGDDAAAATPSPRSPFTTSEDSPPPSPMDM
KQIVLAMQQMDARKATRSVAPKTDMLQQELEEDAPELGWVSDLLM*
>11682.m04304|LOC_Os05g45020.1|genepair638-2
MASREHLLLDPAALAVSWADPAAVEIPPELLAALGEYLSARRSDGEAEADAEAEADDEFM
MYEFKVRRCARARSHDWTACPYAHPGEAARRRDPRRVAYTGEPCPDFRRRPGAACPRGST
CPFAHGTFELWLHPSRYRTRPCRAGVACRRRVCFFAHTAGELRAGSKEDSPLSLSPKSTL
ASLWESPPVSPVEGRRWVDGIDECDADAEMEELMFAMRELGLRKVRPSASSVTPVLPPVT
DEDGPDFGWVSELVM*
>11667.m05274|LOC_Os01g53700.1|genepair639-1
MGFLNLVGNSFGCSASGERLVSAARDGDLQEARALLEYNPRLAWYSTFGGRNSPLHYTAA
QGHHEMGKRTAILHSHFLLLACSNCFSVARIRRQDQPQELQGPGTSDELIPSAYDSMEAR
HDQAHKTRAFPFPQNLRTVRIRPLHVSTRPSSTSAPHRTVAPASPTSPAAAPLISAGRLL
QASATRTAEMYRIAGSHLRSLKRYSYSRFASTSVVKQSSGGLFSWLLGEKSSQLPPLDVP
LPGITLPPPLPDFVEPSKTKVTTLPNGIKIASETSVSPAASVGLYIDCGSIYETPASSGA
SHLLERMAFKSTTNRSHLRLVREVEAIGGNVSASASREQMCYTYDAFKAYVPEMVEVLID
SVRNPAFFNWEIKEQLEKIKAEIAEVSDNPQGLLLEALHSAGYSGALAKPLMAPQSAIHR
LDSSILEEFIAENYTAPRMVLAASGVEHDELVSIAEPLLSDLPSVKRPEEPKSVYVGGDY
RCQADSDKTHIALAFEVPGGWFEEKTAIIVTVLQMLMGGGGSFSAGGPGKGMHSRLYLRI
LNNYHQIESFSAFNSIYNHSGLFGIHATTSPNFASKAVDLAAGELLEVATPGKVTQEQLD
RAKQATKSAVLMNLESRVVASEDIGRQILTYGERKPIEHFLKDLEAITLNDISSTAKKII
SSPLTLASWGDECFLAVIHVPSYESVSQKFFSNTAELLRMCRDFKSMHAEIVLMIFPFMR
RQKATAAVALSRAGGGSGGVQGGDRALQGGWGKRRLPGRAGEAVAVTAPSRSGGGSGGGG
GGALQGGRGKRQRRRSPLGQVGKAAGATAAVADAVAVFFPLVGKLTDARALSAAERHDVP
AFLRLVPSLEAPELPAPLLAVQVTRFVGGGDGGGVAVQHAVPDGQSFWRFMDAWSAPALG
RPSAPAPVFDRSAIVHPMAAAMAQRILRKVTPELPLLLTANLLRRTWQHHDTTTLELERH
RIADIKNLIVELDEATTSPGRVRGAAAAAAATDRRRPPDSPSHPRPPSARATKPCPQLLA
H*
>11682.m04294|LOC_Os05g44920.1|genepair639-2
MWAEPEIAVNYGPRRGGCESSSSRPTLQPAPILRHPFAGGFSPPLPPPLHRRPRNLDTSP
LAGDHHHGASRFASTSVVKQSSGGLLGWFLGGNSSQLPPLDVPLPGVIISPPLPDFVEPS
KTKITTLPNGIKIASETSPIPAVSVGLYIDCGSVYETSSSSGTSHLLERMAFKSTTNRSH
LRLVREVEAIGGNVFASASREQMSYTYDALKCYAPEMVEVLIDSVRNPAFLEWEVKEQLQ
KIKSEISEVSGDPHGLLMEALHSAGYSGALAKPLMASESAVNRLDVATLEEFVSENYTAP
RMVLAASGIEHDELVSVAEPLLSDLPSVKRPEEPKSVYVGGDYHCQADSTSTHIALAFEV
PGGWRQEKTAMIVTVLQVLMGGGGSFSTGGPGKGMHSWLYLRVLNNYGQIESFSAFSSIY
NNSGLFGIHATTNPDFVSSAVDLAARELHEVATPGKVTQEQLDRAKEATKSSVLMDLESR
IVASEDIGRQVLTYGERKPIEYFLKTVEEITLNDISSTAKKIISSPLTLASWGDVACCVF
VQTHANIVLPVLHFNIESDVMASWLSHLPIHRCQLSFPAAQASYATPGIDSALLLDVDDM
AGGDAGLNEGLKKQAKPRLRLPKPAAKRSESEFLRSRPLALLMASSHIILPPDDDDEEQR
RLEEEEDEDPWARSGPPPAAPEHAMKAALPFSATCVRISRDSYPNLRALRNASSVSLADA
AYVKISEGDFGYVLDDVPHLVDHLPDAPTYPNPLQDHPAYSTVKQYFVNEDDTVPQKVVV
QKNSRRGVHFRRAGPRQKVYFESDEVKACIVTCGGLCPGLNTVIRELVCGLAHMYNVSKI

YGIQNGYKGFYSSNYLTLTPKSVDDIHKRGGTVLGTSRGGHDTKKIVDNIQDRGINQVYI
IGGDGTQKGAYEIFKEIRKRGLKVSVAGIPKTIDNDIAIIDKSFGFDTAVEEAQRAIDSA
HVEACSAENGIGLVKLMGRYSERRLKENKHMVIVVAEGAGQDLIAKSLSTSEQQDASGNK
LLLDIGLWLTHKIKDHFKSKKMEMTIKYIDPTYMIRAIPSNASDNVYCTLLAHSAIHGAM
AGYSFTVGNVNGRHAYIPFYRVTSTRNKVKITDRMWARLLSSTNQPSFLSQKDIDEAKEN
DRTANKPPLPTGLSHHVANSFDQSASSSSNSQI*
>11667.m05275|LOC_Os01g53710.1|genepair640-1
MRISELGECDGVRSEGEEEQQVVVEEASVRGGVVVARLSAKRALVGAGARVLFYPTLLYN
VLRNRFEAEFRWWDRVDQYILLGAVPFPSDVPRLKQLGVQGVVTLNEAYETLVPTSLYQA
HGIDHLIIPTRDYLFAPALQDICQAIDFIHRNASEGGITYVHCKAGRGRSTTIVLCYLIK
YRSMSPEAALDHVRSIRPRVLLAPSQWQAVNVFSTLTTGRLPIQSKNLGHFLEAGDDCIT
NSEIDDYYSMELDYEDSGLPLCQVMLPRPTSPTVCTDAVLITEADLEGYDTYADTRKDVV
SLEVIVSRKPIMRRLSCLFGSLKLTNNCEPTPSRFAEGGNVPCYLAHHSLGTCQSLAPVL
PRTTALRGLKYRHLVDNRVVFLLESVPDEIKDSARSLQLASVGFGLGLGRCPGDWVTASF
LPPWRWSLMETFSDVLMESAGFTPAFGKILAGWDYGGSSSPTIGFRCCRVSQMKFPRPEV
VGIRPRAHAPCGKFHSVPDILWMGSHRLPFWLTANIAVAFIPATHPSVNSCI*
>11682.m04293|LOC_Os05g44910.1|genepair640-2
MRIRELRDGAGGSEEAEEDEREEARGGGGGGGGGEVVAVVRLRAKRALVGAGARVLFYPT
LLYNVLRNRFESEFRWWDRIDQYVLLGAVPFSSDVPHLKQLGVRGVVTLNESYETLVPTS
LYQAHGINHLEIPTRDYLFAPSLEDICQAVDFIHRNASQGGSTYVHCKAGRGRSTTIVLC
YLIKYRNMTPEAALDHARSVRPRVLLAPSQWQAVKLFSNLNTRCLSIENSNQTHSAKSCE
ESSEPFSRLASSCHQIQSSNRTHPVRFSEQSSEAIVEAEVDGFTTEFDNEHFVLPLWEGM
LAKPSSPSRSIDAVVVTEEDLEGYETYADASSDTVSVEVVIRQKPMIRRLSCFLGSLKLT
SNCEPSPPRRLAEVRAC*
>11667.m05277|LOC_Os01g53730.1|genepair641-1
MGKFGGAAVLPVYREEEDEDLFETSSSISGDSDDEAQFSDSEEAEAQEDQFAQQPARRMN
SDSLYDLSSMKAQLPVKKGLSKYYDGKSQSFACMSEVRCLEDLRKKENPYKKIKSSKSYV
ALDGNQEACHIPGANSTSIAKKSGSSCANLMARNNTKSMLYRPPPIPVNKSGYHQ*
>11682.m04292|LOC_Os05g44900.1|genepair641-2
MSSSRLGGGDRAGMYEEGEDDELFGESSSVSGGESDDDGEGEDQFSEGGAAAAAALDQME
HRRFAPQPLRRLNSDSIYDMSSMTEQLPAKKGLSRYYEGKSQSFACMSEVRCLEDLRKKE
KPYKSKIKSCNSYAALGGIAKTQSSSSCANLSMMGAGGFRAPPIPVNKNGYHQ*
>11667.m05293|LOC_Os01g53880.2|genepair642-1
MEEGSNKREGLPPQLLDLIPDEKEWKLREALGLGRSRNAGFDGEEDKKLDLKLGLPGFIE
DDEAETLRDYRLQQESPSLSLSFFPKHSKTTSSTTTTTGAKRGFIDTVEDKTEGYNDQKQ
QARAGCGKELAVEEMIAAVSERKKGCCPPPPPPHGAPATPARNRPQTQGRGAAAPVVGWP
PIRSFRRNLASSSSSKHSPEPQNDNANAKVTLTCKKNPLVKINMDGIPIGRKIDLAAYNS
YDGLSSAVKQLFHGFLQAQKDQTNAQIAQQGADDKIFYQLLDGSGEYTLVYEDSEGDRML
VGDVPWKVFVSTAKRLRVLRSSELSHTLVRQARPLKVVSKSLV*
>11682.m04283|LOC_Os05g44810.1|genepair642-2
MEEEFKDKGLPPTLLHLIPDGREWKVKEADGEGSRNTNLDADEDKELELKLGLPGVQQEE
RAADSREKIQQQQRESSSEPSIGCFPTHSKPTTSIGTTGAKRGFFAIVGATLEGYNQSHR
DTEECGKELTLGDENMAGERKKGCCPSPPCSAAAHSSNPQGRGAIPPVVGWPPIRSFRRN
LTNGSSFKQSPERQNDEADDKAKPICKKRPLVKINMDGIPIGRKVDLQIYDSYQKLSSAV
EELFRGFLEAQKDLSCAESGEQGAEDKIFSGLLDGTGVYTLVYEDNDGDRMLAGDIPWKV
FVSTVKRLRVMRRSELPHDMIGADPVK*
>11667.m05301|LOC_Os01g53920.2|genepair643-1
METMAKAPGPVYLLFLLLALPCCLIPHRGAAQPAANEARLLLQIKRAWGDPAVLAGWNDT
AAPAAHCSWPYVTCDTAGRVTNLSLANTNVSGPVSDAVGGLSSLVHLDLYNNNINGTFPT
SVYRCVSLRYLNLSQNYLGGELPADIGVGLGENLTTLVLSGNYFTGTIPKSLSRLQKLEW
LMLDNNNLTGTIPGELGDLTSLTTLTISTNKLGPGQLPESFKNLTKLTTLWARKCQLVGD
MPAYVADMPDLVTLDLAVNNLTGSIPPGIWSLKKLQCLFLFANKLTGDIVVADGAFAAVN
LVFIDLSANPKLGGPIPQDFGLLQKLEVIHLYFNNFSGEIPASIGRLPALKEIHLFNNSL
TGVLPPELGQKSPDLWDLEVDFNKFTGPIPEGLCDGGKLNIFTAANNLLNGSIPERLAGC
TTLQTLFLPNNKLSGDVPEALWTATKLQFVQLQNNGLTGTLPSTMYSNLSSLTVENNQFR
GSIPAAAAALQKFIAGNNNFSGEIPESLGNGMPVLQTLNLSGNQLSGGIPKSVSKLKVLT
QLDLSKNQLSGEIPAELGAMPVLNALDLSSNRLSGGIPSSLASLNLNSLNLSSNQLSGQV
PAKFAIGAYARSFLDNPTLCTSGLGSSYLAGVRSCNAGSPGSASSGGVSPGLRAGLLVAG
AALLLVIVALAFFAVRDIRRRRKRVAQREDWKITPFQTDLGFSEAAILRGLTEENLVGRG
GSGSVYRVAYTNRYTGGDGAVAVKKIRTGAAKVEEKLEREFESEARILGNVRHNNIVRLL
CCVSGDEAKLLVYDYMDNGSLDGWLHGRRAINDGRPVVAAVARARSARGGAPALDWPTRL
RVAVGAAQGLYYMHHECTPPIVHRDVKTSNILLDSEFRAKVADFGLARMLAQAGTPDTVS
AVAGSFGYMAPECGYTRKVDEKVDVYSFGVVLLELTTGKAANDGGEHGSLADWARHHYQS
GESIPDATDQCIRYAGYSDEIEVVFRLGVMCTGATPASRPTMKDVLQILVKCSEQTHQKC
KAESGQEEYEVAPLLLPQRGSRRKQPAKAKGADDDADERSDFDSIV*
>11682.m04278|LOC_Os05g44770.1|genepair643-2
MAFPSGKAYYLHLLLLAILPSLVARRGAAQQGGVDEKQLLLQVKRAWGDPAALASWTDAA
PHCRWVYYVSCDGGGTGRVTSLSLPNVAVAGAVPDAIGGLTALTVLNLQNTSVGGVFPAFL
YNLTAITSIDLSMNSIGGELPADIDRLGKNLTYLALNNNNFTGVIPAAVSKLKNLKVFTL
NCNQLTGTIPAALGELTSLETLKLEVNQFTPGELPGSFKNLTSLKTVWLAQCNLTGDFPS
YVTEMMEMEYLDLSQNSFTGSIPPGIWNIPKLQYLFLYTNQLTGDVVVNGKIGAASLIYL
DISENQLTGTIPESFGSLMNLTNLALMTNNFSGEIPASLAQLPSLVIMKLFENNLTGQIP
AELGKHSPFLRDIEVDNNDLTGPIPEGVCDNRRLWIISAAGNRLNGSIPASLATCPALLS
LQLQDNELSGEVPAALWTETRLITVLLQNNGHLTGSLPEKLYWNLTRLYIHNNRFSGRLP

ATATKLQKFNAENNLFSGEIPDGFAAGMPLLQELDLSRNQLSGAIPVSIASLSGLSQMNF
SRNQFTGDIPAGLGSMPVLTLLDLSSNKLSGGIPTSLGSLKINQLNLSSNQLTGEIPAAL
AISAYDQSFLGNPGLCVSAAPAGNFAGLRSCAAKASDGVSPGLRSGLLAAGAALVVLIGA
LAFFVVRDIKRRKRLARTEPAWKMTPFQPLDFSEASLVRGLADENLIGKGGAGRVYRVAY
ASRSSGGAGGTVAVKRIWTGGKLDKNLEREFDSEVDILGHVRHTNIVKLLCCLSRAETKL
LVYEYMENGSLDKWLHGNKLLAGGATARAPSVRRAPLDWLARVRVAVGAARGLCYMHHEC
SPPIVHRDIKSSNILLDAELMAKVADFGLARMLVQAGTPDTMTAVAGSFGYMAPECAYTR
KVNEKVDVYSFGVVLLELITGREAHDGGEHGSLAEWAWRHLQSGRSIADAVDRCITDSGY
GDDAEVVFKLGIICTGAQPATRPTMRDVLQILVRCEQALQNTVDGKVAEYDGDGAPFLPI
RGGSRRKQLSDTKGIDDGNGSLDSIV*
>11667.m05302|LOC_Os01g53930.1|genepair644-1
MGKGTVVGTAVVVCAAAAAAVGVAVVVSRRRRSKREAEEERRRRAAAVIEEVEQRFSTPT
ALLRGIADAMVEEMERGLRADPHAPLKMLISYVDNLPTGDEHGLFYALDLGGTNFRVIRV
QLGGREKRVVSQQYEEVAIPPHLMVGTSMELFDFIAAELESFVKTEGEDFHLPEGRQREL
GFTFSFPVHQTSISSGTLIKWTKGFSINGTVGEDVVAELSRAMERQGLDMKVTALVNDTV
GTLAGGRYVDNDVAAAVILGTGTNAAYVEHANAIPKWTGLLPRSGNMVINMEWGNFKSER
LPRSDYDNALDFESLNPGEQIYEKMISGMYLGEIVRRILLKLAHDASLFGDVVPTKLEQR
FILRTPDMSAMHHDTSHDLKHLGAKLKDILGVADTSLEARYITLHVCDLVAERGARLAAA
GIYGILKKLGRDRVPSDGSQKQRTVIALDGGLYEHYKKFRTCLEATLADLLGEEAASSVV
VKLANDGSGIGAALLAASHSQYASVE*
>11682.m04277|LOC_Os05g44760.1|genepair644-2
MGKAAAVGTAVVVAAAVGVAVVLARRRRRDLELVEGAAAERKRKVAAVIEDVEHALSTP
TALLRGISDAMVTEMERGLRGDSHAMVKMLITYVDNLPTGNEQGLFYALDLGGTNFRVLR
VQLGGKEKRVVQQQYEEVSIPPHLMVGTSMELFDFIASALSKFVDTEGDDFHLPEGRQRE
LGFTFSFPVSQTSISSGTLIKWTKGFSINDAVGEDVVSELGKAMERQGLDMKIAALVNDT
VGTLAGGRYADNSVVAAIILGTGTNAAYVENANAIPKWTGLLPRSGNMVINTEWGSFKSD
KLPLSEFDKAMDFESLNPGEQIYEKLISGMYLGEIVRRILLKLAHDAALFGDVVPSKLEQ
PFVLRTPDMSAMHHDSSHDLKTVGAKLKDIVGVPDTSLEVRYITSHICDIVAERAARLAA
AGIYGVLKKLGRDKMPKDGSKMPRTVIALDGGLYEHYKKFSSCLESTLTDLLGDDVSSSV
VTKLANDGSGIGAALLAASHSQYAEID*
>11667.m05308|LOC_Os01g53980.1|genepair645-1
MGRIIRWLKKLLTGRKEAHKGLKENHAVSDGAEKEKSRWSFAKHRRSGVDSGRRPSEAAL
AAVAAVAVEPSEVRRPCHCGEVENAIARREKAAMVIQKAYRGYLARKALRALRSLVKLQA
LVRGYLVRKQAATTLHRLQALMRQQASSRAATRASYRKSMEQERISVEETRLKTTTPGHR
RRLSDSADSNYERSPRIVEMDTCHRRSRSSRIAIRHSRDHSSDCLTPAPMPPAPLSCSSP
ISIKQPPRLSIQRSQHHERDTRHAKTAHNTPRLGAPPYGSSPANKSVDGMARARLSHRDA
LGSPRYMAGTASSAARTRCQSAPRQRQAAEAPARSRRSCSRTTQDSGFCFECSEDSRPGY
SEELSDEAARDYYLDRMW*
>11682.m04264|LOC_Os05g44630.1|genepair645-2
MRWFKKVLTGKKEGGDRDRKEHSAAGGANGGVAPPPMERRRWSFAKPRSSFADGSRRPSV
TAVVAGELSQVRPCSCGQQREVEAAVMIQKAFRGYLARRALRALKALVKIQALVRGYLVR
KQAATTLQRLQALMRLQASSRAIKMASSRKSVEQERIVVQMQGGRVKTLTLPVVHRRRVS
DGGDINFDRSPRIVEMDTCQLRCRSSRITSRYAADPPPDGTPGSVPLSSPHLYCYKPPPS
RHLQAEEHEHDARAQPKTTHNTPRLAAALPAGYHGPASPAKGRVVSPRYMADTASSVARA
RCQSAPRQRHGAAGEPRPSLARAGSRKSRPDSAISLKSSEMSRHEDSEFSDDVTRDYYLD
QLW*
>11667.m05309|LOC_Os01g53990.1|genepair646-1
MAVAPVRLVACIVALAAVAPGGVAGHTRGVRPGRAAGKQQPSFPENATRVEAIERQFMEW
VRYMGGLEHSTVHHALARAFPSYSLVVDKNPAFGDFTTIQAAVDSLPIINLVRVVIKVNA
GTYTEKVNISPMRAFITLEGAGADKTIVQWGDTADSPSGRAGRPLGTYSSASFAVNAQYF
LARNITFKNTSPVPKPGASGKQAVALRVSADNAAFVGCRFLGAQDTLYDHSGRHYYKECY
IEGSVDFIFGNALSLFEDCHVHAIARDYGALTAQNRQSMLEDTGFSFVNCRVTGSGALYL
GRAWGTFSRVVFAYTYMDDIIIPRGWYNWGDPNRELTVFYGQYKCTGPGASFSGRVSWSR
ELTDEEAKPFISLTFIDGTEWVRL*
>11682.m04260|LOC_Os05g44600.1|genepair646-2
MAVSVSWSWAPRFLACVAVAVLLVTSGLGGAAAGGGGRRHGHTKGLRPGKAAAKPYYPVN
ATAVEAIERQFTRWVRSMVGRRHSTFQRALYRGLLPTRTLVVDKNPAAGNFTSIQAAVDS
IPLINLARVVIKVNAGTYTEKVTISPLRAFVTIEGAGADKTVVQWGDTADTVGPLGRPFG
TFASATFAVNAQFFVAKNITFKNTAPVPRPGALGKQGVALRISADNAAFLGCNFLGAQDT
LYDHLGRHYYRDCYIEGSVDFIFGNALSLYEGCHVHAIARNYGALTAQNRMSILEDTGFS
FVNCRVTGSGALYLGRAWGTFSRVVFAYTYMDNIIIPRGWYNWGDPTREMTVFYGQYKCT
GPGSNYAGRVAWSRELTDQEAKPFISLSFIDGLEWVKL*
>11667.m05316|LOC_Os01g54040.1|genepair647-1
MAEPDWILERERRQVEQILELDMEELQVEEVDDAGSSSSSEVDTFLRNTHGDGGSRTSEA
LAFNTSVVSLPTCDGEVHDAPGRFAFLDGGVVLCLPMFYLQGVVLFPEAILPIRVVQPRS
LTAVDKAVNHVDAPCMIGVVHVYQHTNDGHHAIASVGTTAEIHHIKQLDDGSSNVVTRGQ
NRFRLRRRWIDADDVQWGEVQIIEEDTPQRTPRDAFGQLATNYIFNQCGTSLLSLGTSCF
RQDDHVNSDQDWDSLSSTSTSSEHSVTDARTYCSSNEDEDLMLEQSWQKYDSVKRNAELE
NPVKHSNTRGKGEPCFQSPKSLPTKNKGAEQRRRFCAAYSSKLALQAPLSFWPRWAYEMY
DSYSLARRVADLWRQIVVNPSMDDYVRKPDILSYHIGSKLPMSCSVRQELLEIDGISYRL
QKEIQLLKAFNIIRCRNCLALISRRSDGPVGAYVKQFSCGQEMMTVYNATGLALRGAPSK
AHSLFPGYTWTIALCAACESNIGWLFRAEKTNLLPKSFWGLRSSQVSDDTQSGHN*
>11682.m04258|LOC_Os05g44590.2|genepair647-2
MADWDGVPARERRQMEEILQLDMEELNVEMVNDDGSSSEEANGGDDGADAFLREGVASTS

GQFTFNTSLASLHTYLGEVDDTRGRVSLLDGGTILSLPMLYLQGVVLFPGATLPLRLIQG
RFVAAVEKALRQVDAPCTIGVVLMYKRHSTRHYANASVGTTAEIRQLGRMDDGSLNVVAR
GQQRFRLKRHWMDVDGVVWGDVQIIEEDTPLRTPRDAFAQLASCNSLRQHTSSPVISLDV
SPIKQRDHVDSELDCGTPSPKSTASNHSAIDSRMCHSDSRSSSSMRSSNEDGIFMHEQFY
SQELHPLKGSAAVQSGENTNMGEEDFCLTSLRSLSSAGTRDTKEQRQYILPKQHFQAPLS
FWPRWAYQMYDSYALARRAADLWRQIITHPSMDDYVRKPDLLSFSIGSKLPVSESVRQKL
LEIDGISYRLRREIQLLKAFNLIKCRCCKSLIAKRSDMVVMSTDGPLGAYVNPHGFVHET
ITVSRATGLALDGNPSTVHSWFPGYSWTIASCTDCESNIGWLFKATKKNLRPRSFWGIRS
SQIADDAQELDQDE*
>11667.m05318|LOC_Os01g54050.1|genepair648-1
MDRYAFLSFYKCTHQVGKFVGSDTWSNVLRESWFFNNWKELLVPVSLSTFLLLGLKGYLD
EQFCQVEDLQDEASPNFVEEVVTLFFKDSGRLMSNIEQALEKYPRDFNRWDTYMQQLKGS
CSSIGASRMKNECMSFRDSCGQGNVEGCMRSFQKVKREHAVLRQKLESYFQLLRQAGPAG
AATRPVM*
>11682.m04255|LOC_Os05g44570.1|genepair648-2
MQSSAAAVEMRRNPEGIIAPLSPIWMPLGKNGLAATTSDKESKHRKGGNTIIALCLCGWS
WEMFYCLLHIEVEMFSFGGWGGKGYLDEQFCQVEDLQDEANPNFAEEVVSLFFKDSTRVM
LNFEQAIEKHPKDFARWDTHMQQLKGSCSRKTLLGEGEPHIASIGASRVKNECTSFRNFC
GEENAEGLAATAAVAVVNGCRLQLVSTAAAAVAVATRASNRPDHYGKTNKLHEILPESEE
GACRPQAEVGVLLPAVATSWSCWDRD*
>11667.m05321|LOC_Os01g54080.1|genepair649-1
MGTVNGEYEDFDAANRRAEVIDWLGGLLPEFDLPLDSSDEELRDYLINGEALCYVADKLM
PGVLEGTWGGYASDQRSNVKKFLSVVAEMGLPGFGVKDLEEGSMSSIVECLLALKDNVAT
QLGGHISNSTAKTPIRRKLELRETDGPVLSVATPGKRYPKSQQRSPLLSGQKINEVVQFK
HGTYTDLPAAKISEMLHSNSLDNAPTQSLLRVVNGILDESIERKRGEIPHRVVHLLRNVI
QEIEHRIGIQADHIRNQNSIIKTREDKYRSKIKALETLVNGTNEENEMAINRLEVVKVEK
SKIDEKRKLGEQDMIRLIREKENAENIIASLHQEMQVMNRMHEQFREQMETKARQMEEHL
TLRAKEAEFCLMQSKKKVEEVEATSQLKSQLWSKKANIFQSFMNNQKLSIKDIKISSQSI
KQEMYALQMTWRDEISNIGHDLKGLVDAAENYHKVLAENQKLFNEVQELKGNIRVYCRVR
PFLPGQDGKLTAIDYIGENGEILIANPSKQGKEGYRMFKFNKVFGTHSSQAEVFSDIQPL
IRSVLDGFNVCIFAYGQTGSGKTYTMSGPGTSREDWGVNYRALNDLFDISLSRKNAFSYE
SNRAVGSTALNERSSRSHSILTVHVRGLDVKNGSTSRGCLHLIDLAGSERVERSEATGDR
LKEAQHINKSLSALGDVIFSLAQKNAHVPYRNSKLTQVLQSSLGGQAKTLMFVQINPDIE
SYSETISTLKFAERVSGVELGAARSNREGKDIKELLEQVASLKDTIARKDMEIEQLQLLK
SKSPNSMTDRNGSNLLRQSTSSTGLSSLPVASQQNQQLSVVTLTPLADTGLAGSVEAEAE
DNASDDGCSVGETEYSPAGASETSAERAYVLILFSFISCAKPSCACDTSGELMIVPVRLR
TGT*
>11682.m04254|LOC_Os05g44560.1|genepair649-2
MGSVDGDFEGLQAADRRAEVIEWLNALLPEYCLPLDSSDDELRELLSDGTVLCHIVNALI
PGVLEESWGAYASSDQHAGHVKKFLAVVADMGLPGFSVKDLEEGSMSGVVDCLLVLRESV
SSGLRDGTSKAPLRKKWRVPETGEPLVPGVAQGKTSPGEDKRNGLPDPKSQQKTPIFNGR
KLREIFQLKRGSYADLPAAKISEMMHSNSLDNAPTQSLLSVVNGILDESIERKKGEIPHR
VVYLLRKVVQEIERRLCIQAEHIRSQNVIIKTREDKYHSKIKALEILVNGTNEENQMAIN
RLQIIKEEKSKIEEKRKLGEQDVARLMKEKEISENTIASLKKEMEVMTSMHEQQLQKIEL
TAKQMEEHLTTKIKEVESLLVQSNKKIEEVEAASLLKSQLWNKKEGIFQKYMNSQQLYVK
GLRISSWSIKNEMHALEMELRDEMSNFGSGLKCLVDAAENYHKVLAENQKLFNEVQELKG
NIRVYCRVRPFLPGQDKKSTTVDYIGENGELLISNPFKQGKDGHRMFKFNKVFSPFSSQA
EVFSDIQPLIRSVLDGFNVCIFAYGQTGSGKTYTMSGPSTSKQDWGVNYRALNDLFDISL
SRRNAFSYEVGVQMVEIYNEQANRAVGSTALNERSSRSHSILTVHVRGLDVKNGSTSRGC
LHLIDLAGSERVERSEATGDRLKEAQHINKSLSALGDVIFALAQKNAHVPYRNSKLTQVL
QSSLGGQAKTLMFVQINPDVESYSETISTLKFAERVSGVELGAARSNKEGKDIKELLEQV
ASLKDTIVRKDTEIEQLQLMKDKVKSPSFAVDINGASMPKNSNSDLRSVLSITTNQQSQL
SDPQSYAEVNRDGGPTSYTDITPTCLDEADFEDNASEDGFSGGTDYSVGCAAGASVFPNS
CSDRTADTSMYSHLPISLTPN*
>11667.m05330|LOC_Os01g54170.1|genepair650-1
MAFMSSASEEESAGLKAPTDARVIRQRYEGDGPILSDDALLPADPGAAAGAAAEQQQQQD
EARSVRCECCGVAEDCTPTYIGRVRERFDGRWVCGICAEAVSELRRRDPALAVREAVASH
AALCAEFNATVRVNPALCLVRGMRDIVRISCRGRSGDSASPSAAPGGGAGARIGRTRSCA
LPYV*
>11682.m04239|LOC_Os05g44410.1|genepair650-2
MPWRLAMARQQAEPPTGAAALQRSNSDGGGGMAAGADQEARSVRCECCGMAEECTPRYIG
RVRERFHGKWVCGLCSEAVKERQKREPALTVDGAVDAHAALCERFNSTVRLNPKPLSLASS
MRDIARKSCQHRATATGADVIPSACSGAGAATMARSTSCALPYV*
>11667.m05335|LOC_Os01g54210.1|genepair651-1
MEVTAEFGGAYYGGAAGREKKALQQGCGDHFAVDDLLVLPYGEEDETTREGEATGGKEEA
AGFGNASADSSTITALDSCSNSFGLADGDFPGELCEPYDQLAELEWLSNYMNEGDDAFAT
EDLQKLQLISGIPSGGFSTASVPSAQAQAASAAASMAVQPGGFLPEAPVPAKARSKRSRA
APGNWSSRLLVLPPPPASPPSPASMAISPAESGVSAHAFPIKKPSKPAKKKDAPAPPAQA
QLSSVPVHSGGSAPAAAAGEGRRCLHCETDKTPQWRTGPMGPKTLCNACGVRYKSGRLVP
EYRPAASPTFMVSKHSNSHRKVLELRRQKEMHQQTPHHHQPQVAAAGGVGSLMHMQSSML
FDGVSPVVSGDDFLIHHHLRTDFRPPI*
>11682.m04238|LOC_Os05g44400.1|genepair651-2
MEVAAADYAGGVRVKKEAGGCGGSGDMFLVDDLLDLPCDEEEEETGLCGAYGGGGAGLGA
GVVGGGGDDRAAGNASADSSTVTAVDSCSNSFSGLADGDFSGGLCEPYEQLAELEWVSTY

MGEETLPTEDLRKLQLISGIPAAPRAPPALAVSAVQLPAGGAGALPTEAPVPGKARSKRS
RVAPCSWSSRLMVLPPPPASPPSPASAVISPSESGTAAPAFPAKKAAKSAKKKDGPSPAP
APNAAAQAAAEGRRCLHCETDKTPQWRTGPMGPKTLCNACGVRYKSGRLVPEYRPAASPT
FVVSKHSNSHRKVVELRRQKEMQLLHHHQQPPPHVGAGGGGAAGGLLHVTSPLLFDGPTS
SAPLFAGADEFLIHNRISPDYRRQAT*
>11667.m05351|LOC_Os01g54340.2|genepair652-1
MVMEDGEMSLSNLVLGFFEEAEREQRWPENGDDDDEGSSGSGAAESKAFWQNQHSQLHEA
LAKTSQVESRIREDTEEAIRKMRAAGAVCSCARRAAAGDCRSCTLRHVDERLRDAGYNSA
ICKSKWTRSPDIPSGEHSYVDVVVQTRSGKAVRVVVELNFRAEFEVARASAEYRALVTAL
PEVFVGRADRLRAVVKAMCAAAKQCMKENNMHMGPWRKHKYMQSKWLGTPERVAAAAAAP
VAVWSPEKQTKFRASMLSFDFARAAVEVA*
>11682.m04225|LOC_Os05g44300.1|genepair652-2
MVVEEAVVAAGNEMSLSNMVLGFYEEAELQSSPPGDCAAAAGDDDDGSDDEGSGGAAKCR
AFWKEQQSQLYEALAKMSSAESRIQADAEEAMRQMRAAAAGACSCASRGAAAAAAGSGGC
RSCTLRFLAERLRDAGYNSAICRSKWPRSPEIPSGEHSYVDVVAPTRSGKAVRVVVEPSF
RGEFEMARGGAGYRALVASLPEAFVGRADRLRGVVRVMCAAAKQCARESGMHMAPWRKQR
YMEAKWLATPERVAPPGNAGGAGDAVAVGSPSSPLSPGMTNRQMQPKFRASMLTLDFGGR
TAVEVV*
>11667.m05353|LOC_Os01g54350.1|genepair653-1
MDEEDYSWVRRTRFSHSVVRSKSGREQFGAFVEQFNRGAARRQRGPGSGFMLHGLNLEPR
ARLPATTSANSSVPRTTGSSNAQPSSETKPASSSSDAKLEQHEKSLNLEPRTRLSATTSA
NSSVPWTTGSSNAQPLSETKTASSSSDAKPEQQEKPRDHQPSQEVSTKQDDKAENDTKAT
ATSGPLEFSFHPDEQTLRLQRACSSPVPFPSNKNMLVADAAARSSSLKVAGEGPKPKPKQ
RARSPPLRRDVPELFKEAKSSSKRFSTPPPRRKPSSPPAPSRSPPHVFATARAHGKPKHK
KDSSANGRAKVAALEVLEKWTVDRSQLLIGHRFASGAHSRLFHGIYKEQPVAVKFIRQPE
DEEDAELAAQLEKQFNTEVTTLSRLNHPNVIKLIGACSSPPVFCVITEFLSGGSLRTFLH
KQEHKSLPLEKIISIGLDIANGIGYIHSQGVVHRDVKPENIIFDSEFCAKIVDFGISCEE
AECDPLANDTGTFRWMAPEMMKHKPYGRKVDVYSFGLILWEMFTGSVPYEDLNPFQAAFA
VFDKNERPVIPSSCPAPLRLLIEQCWASQPDKRPEFWQIVQILDKFKAVLDKDGTLDNMP
CLNLQGTHDHKNWLAHWVQKLKHTHHDLSGPPPPKLM*
>11682.m04223|LOC_Os05g44290.3|genepair653-2
MDDDDYSWVRRTKFSHSIVKSNSGREQFGAFLDPFDSSTAWRQKGFSGELMIHGHNLQPR
AKGAVSKSPRPSIPLAKSPVSQAERKLKAKFSDGELKQRGKISDGSLREASKEKDRKVGH
KGEGLSLDISCPILDRTVQDDSSDTLDFSFHSEEQSLRLLRVCSSPAPFYSKDTTTFGDS
KIHSTSVKIVGEGSKPRRRAKSPMPTRVISEVFKEAKAATKRFSSPQRQRKSSSPHSPRS
PDDSPRFGFPSMRTPGNLKARRASSWPRNYDNGVAKAVALDILERWTIDRSQLLIGQRFA
SGAYSRLFHGIYKEQPVAVKFIRQPDEEEDAELAAKLEKQFTAEVTILARLHHRNVIKLI
GACNAPPVFCVITEFLCGGSLRAFLRKLQRQKLPLEKIICIALDIAHGLEYIHSQRVIHR
DVKPENILFDGECCAKVVDFGVACEEVYCNSLEDDPGTYRWMAPEMYKRKPYGRKVDVYS
FGLVLWELFSGSIPYEEMTPLQAAFAVVNKNLRPVVPSSCPAQLRLLIEQCWSCQPEKRP
EFSQVVQILKNLKEALDRDGTLDKIPSTNCQEPPDQNKKRLSNWIQRLSYSQADFSGPPP
PKLL*
>11667.m05358|LOC_Os01g54400.1|genepair654-1
MEPSTKQLLPMPQQDPNSPSSSTSSSSSSSTSPSHPHHRAPLPPSPRPVPRTIETTPFPT
TFVQADTASFKQVVQMLTGAEQPSKNAATAATAAAGNSSAAGIGGGQGANGPCRPKKPAF
KLYERRSSLKNLKMIAPLAMGALPSPTGRKVGTPEILSPSVLDFPSLKLSPVTPLTGEPF
NRSPASSSEDAERAAISERGFFLHPSPRGAEPPRLLPLFPVTSPRMAAPSE*
>11682.m04219|LOC_Os05g44270.1|genepair654-2
MAHDPNSPSSSTSSSSPSSAAAAASSSPSSHRPPPPPPSSSSQPALPPSPRTVVPRTIDT
TPFPTTFVQADTASFKQVVQMLTGSDTTPPSQRPPAKSNHHQHHHSGAPCRPKKQAFKLY
ERRSGVHKNFKMIAPLAMAAAAAAGASSSPRKAAQHQQQEALSPSVLDFPSLALSPVTPL
VADPFNRSPASASSSASPEEEAAAIAQKGFFLHPSPRSAEPPRLLPLFPVTSPRVASSSS
SSAAAAVAVASPSFE*
>11667.m05363|LOC_Os01g54450.1|genepair655-1
MEELQEADVLWPAEHHRDYERCRHHHHAAQPAVCPVAPLRPSSSSAPVRIPAPTTTTFAG
ARRGYYEDDGTRTDYSAGAGASSRIVPPHVYVAAARRGSEGRTVASSVCVGHGRTLKGRD
LRAVRNAVLHMTGFLGGGPDEY*
>11682.m04218|LOC_Os05g44260.1|genepair655-2
MEEFQEADVLWPDHHQHLRRDDDARRRRHQEQQHGGVDDADDSRGGTGTPSAPVGIPVTR
APTTRRISHGSTAPAAAFVPPHELVAARARRCSEERAAFSVCVGNGRTLKGRDLRDVRTA
VLRMTGFLET*
>11667.m05365|LOC_Os01g54470.1|genepair656-1
MERVSCVPLLRRLLLLAAAGLAGAEPQVPCYFVFGDSLVDNGNNNNIASMARANYPPYGV
DFPGGATGRFSNGLTTADAISRLLGFDDYIPPYAGATSEQLLTGVNFASAAAGIRDDTGQ
QLGERISFSAQLQNYQAAVRQLVSILGGEDAAANRLSQCIFTVGMGSNDYLNNYFMPAFY
PTSRQYTPEQYADVLINQYAQQLRTLYNYGARKVAVFGVGQVGCSPNELAQNSRNGVTCI
ERINSAVRMFNRRVVVLVNQFNRLLPGALFTYINCYGIFESIMRTPVEHGLAVTNRGCCG
VGRNNGQVTCLPYQAPCANRDEYLFWDAFHPTEAANIFVGRRAYSAAMRSDVYPVDLSTL
AQL*
>11682.m04212|LOC_Os05g44200.1|genepair656-2
MAGAWRRWPVVVAAAVVLAVAAPVQAAPQVPCYFVFGDSLVDNGNNNDIVSLARANYPPY
GIDFAGGAATGRFSNGLTTVDVISKLLGFEDFIPPFAGASSDQLLTGVNFASAAAGIREE
TGQQLGARISFSGQVQNYQSAVQQLVSILGDEDTAAAHLSQCIFTVGMGSNDYLNNYFMP
AFYNTGSQYTPEQYADDLAARYAQQLLRAMYSNGARKVALVGVGQVGCSPNELAQQSANGV

TCVERINSAIRIFNQKLVGLVDQFNTLPGAHFTYINIYGIFDDILGAPGSHGLKVTNQGC
CGVGRNNGQVTCLPFQTPCANRHEYAFWDAFHPTEAANVLVGQRTYSARLQSDVHPVDLR
TLASL*
>11667.m05377|LOC_Os01g54560.1|genepair657-1
MMSRSYTNLLDLAEGNFAALGPGGGGGGRRRSGSFGLKRMSRVMTVPGTLSELDGEDDSE
HAATNSVASDVPSSVAGDRVIVVSNQLPVVARRRPDGRGWSFSWDDDSLLLQLRDGIPDE
MEVFFVGSLRAEIPVADQEEVSQALLDRFRCAPVFLPDPLNERFYHRFCKRHLWPLFHYM
LPFSSSASPSPSSSSSSSSSPSSSSGSGHFDRGAWEAYVLANKFFFEKVVEVINPEDDYV
WVHDYHLMALPTFLRRRFNRLRIGFFLHSPFPSSEIYRTLPVREEILKALLNCDLIGFHT
FDYARHFLSCCSRMLGIEYQSKRGYIGLDYFGRTVGIKIMPVGVHMGQLKTVLSLPDREW
RVSELQQQFEGKTVLLGVDDMDIFKGINLKLLAFENMLRTHPKWQGRAVLVQIANPARGK
GKDLEAIQAEIHESCKRINGEFGQSGYSPVVFIDRDVSSVEKIAYYTIAECVVVTAVRDG
MNLTPYEYIVCRQGSDSTSEVNGPKKSMLVVSEFIGCSPSLSGAIRVNPWNIEATAEALN
EAISMSEQEKHLRHEKHYRYVSTHDVAYWSKSFIQDLERACKDHFRRTCWGIGLGFGFRV
VALDPHFTKLNMDSIVMAYERSESRAIFLDYDGTLVPQTSISRTPSAEVLRIINTLCSDR
RNKVFLVSGRRRDKLGEWFSSCPDLGIAAEHGYFLRWTRDEEWQTCTQTSDFGWMEMAKP
VMNLYTEATDGSYIDPKESALVWHHQDADPGFGSSQAKELLDHLESVLANEPVSVKSGQF
IVEVKPQGVSKGVVAEKILVSMKERGKQADFVLCIGDDRSDEDMFENIADTIKKGMVATN
TSLFACTVGQKPSKAKFYLDDTFEVVTMLSALADATEPEPETDLTDEFDELAVSVSSVDI
DDEQTPSDKLIGG*
>11682.m04200|LOC_Os05g44100.1|genepair657-2
MMSRSYTNLLDLAAGNFEALGPAGGGRRRSFGAKRMTRVMTVPGTLSELDDEDDEPAATS
SVASDVPSSAACERLIVVANQLPVVARRRPGAAAGGWAFSWDDDSLLLRLRDGVPDEMEV
LFIGTLRADVPACEQDEVSQSLIDGFGCAPVFLPAGLYDRFYQHFCKGYLWPLFHYMLPF
ASALPAAASGDGRFDRGAWEAYVLANKYFFEKVVEVINPEDDYVWVHDYHLMALPTFLRR
RFNRLRIGFFLHSPFPSSEIYRSLPVREEILRTLLNCDLIGFHTFDYARHFLSCCSRMLG
IEYQSKRGYIGLDYFGRTVGIKIMPVGIHMGQLQSVLRLSEKEKKVAELRQQFEGKSVLL
GVDDMDIFKGINLKLLAFENMLRTHPKWKGRAVLVQIANPARGKGKDLEAVQAEIRESCD
RINKEFGQSGYSPVIFIDQSVPSAVRLAYYTVAECVVVTAVRDGMNLTPYEYIVCREGIP
GSECAPEVSGPKKSMLVVSEFIGCSPSLSGAIRVNPWNIEATAEALNEAISMSEREKQLR
HEKHYRYVSTHDVAYWSKSFVQDLERACKDHFRKPCWGIGLGFGFRVVALDPHFTKLNFD
SIIMSYERSKSRAIFLDYDGTLVPQASLNKNPSEELLRIINTLCADRNNTVFIVSGRSKD
DLSKKLISCPKLGIAAEHGYFLRWTRDEEWQTTAQTSDFGWMQMAKPVMDLYTESTDGST
IETKETALVWHHQDADQGFGSSQAKEMLDHLESVLANEPVSVKSGQFIVEVKPQGVTKGL
IAEKVLTSMKEKGQLADFVLCIGDDRSDEDMFENIADVMKRSIVAPKTPLFACTVGQKPS
KARFYLDDTFEVVTMLSSLADASEPDLMADLEDDLATSVSSIEISDRVVSFSNLRTEGS*
>11667.m05378|LOC_Os01g54570.1|genepair658-1
MKWGLRSSRHNQREKQQRTQEEEHKHEQQEDKGNKSRAAFLSFSPLAWLSKLTAKNNAAA
AKPKPAAPADKSAVAATGGFPSCFFKGASSSTSTSVSLSSSAASQSSLADSSPADHQAPR
RLSVGNDGDTAEAAAARQLYHRRRHYSVGGDRDLQTLRNLIPFSRAASPIPVPAPFVPAL
KTTPPLLPSDTDEEKRPRSRRRRRRSGGGGRRSFSGRTPGARVAATVRVRSPRRVASAAA
AAVSELERFAVVRRTSDPQREFRASMVEMIASKRIGRPEELETLLACYLSLNADEHHDCI
VKVFRQVWFELNPARVAAVAPPRS*
>11682.m04199|LOC_Os05g44090.1|genepair658-2
MGWGIRRQPQQQAAAAAERGGGGKGKAAAFSFSPLSWIAKLTARSSHGKCGGAKHAPAAS
MAGPSCRLPKRAAAAAASSSSVVAAVDDVAAGRSSPPRRSPVDVAPRRLSVGNDSAEAVA
RRLCQQQRRRRRHCSLGGDRDLPPLGHLIPFSLAGSPASQPPENAAAAAAGGATPSDTDA
GAKLRTRRHRRRAHRRRRSSLGGSGRRSFSVSGRMPAVRIRPPRAAASAPELERLAVVRR
TRDPQRAFRESMVEMIASSGGSIAARPEELERLLACYLALNADEHHDCIVKVFRQVWFEY
INLHLHLSRRRRARHC*
>11667.m05380|LOC_Os01g54590.1|genepair659-1
MAAGGGGGYRAEDDYDYLFKTVLIGDSGVGKSNLLSRFTKNEFCLESKSTIGVEFATRSI
QVDGKVVKAQIWDTAGQERYRAITSAYYRGAVGALLVYDVTRRATFDNVARWLKELRDHT
DPSIVVMLVGNKSDLRHLVAVSTEDGKEYAEAESLYFMETSALDATNVENAFAEVLTQIY
QTVSKKTVEASDDGSNAPIKGEKINVKDDVSALKRIGCCSN*
>11682.m04196|LOC_Os05g44070.1|genepair659-2
MSAAAGGYRAEDDYDYLFKVVLIGDSGVGKSNLLSRFTKNEFSLESKSTIGVEFATRSLQ
VDGKVIKAQIWDTAGQERYRAITSAYYRGAVGALLVYDVTRRATFDNVGRWLRELRDHTD
PSIVCMLIGNKSDLRHLVAVSTEDGKEFAEAESMYFMETSALDATNVDNAFAEVLTQIYQ
IVNKKPVEAPEDGSAGPGKGEKINVKDDVSAMKRVGCCSN*
>11667.m05413|LOC_Os01g54920.1|genepair660-1
MGRGKFKGKPTGRRNFSTPEEIAAGTSGRPRTFKKNLAEEEKEEEEDDIEESEEEESEDE
SEGKAKHKGTEGLIQIENPNLVKAKNIKAKEVDLGKTTELSRREREEIEKQKAHERYMKL
QEQGKTEQARKDLERLALIRQQRADAAKKREEEKAAKEQRKAEARK*
>11682.m04186|LOC_Os05g43970.1|genepair660-2
MARGKFKGKPTGERSFSSEEQIAAGTSAGRPKNFKKKQSEKEVYDRRQESDEEYDRSQES
DEEESDNFQKNKHKGTEGLIEIENPNLVKPKNIKAKDIDIGRTSDLSRREREELEKQKSH
ERHMKLQEQGKTEQARKDLERLTLIRQQRAEAAKKREEEKAAKDERKAEARK*
>11667.m05414|LOC_Os01g54930.1|genepair661-1
MGRGPAAGRSSAGAARHQQFRARAKTRVDDLQEMFSGLQSARKEARSTDAAVLEAQLHQM
LREWRAELSGNNRELSDPPSETLRLLQLAAAEEEEDDATSKLVEQQQQHPPSANQAHGHP
QAQGGQDMKPEPPEEAVASPADLTVPQQPQSPGQGVLASGGGMLAPAAAAVFHDQSVKRK
LQSLCELWYNGTLLVGWMWILGNELHGWRELLEVCEVEKEHEHGTCHGCGWDGTKQMYYV
NQELTVEDFLYDDNYKMYLPGYNSDVLNNLESTGQLEYPQFNLPQELPPNAYLDTSNCGQ

NAGDVFLHMSDLLNTMSPVPAAFLRPKCALWDCPRPAQGSERWQDYCSMYHADLAVKEEG
PPGTMPVIRPRGIDLKDGPLFAALSAKIQGKHVGIPVCEGAATAKSPWNAPELFDLYIFE
GESIREWLFFDKPRRAFESGNRKQRSLPDYNGRGWHESRKQVMKDFGGLKRSYYMDPQPS
NSYEWHLYEYEINDCDAFALYRLEFKSSDAKKTAKSKLACNPLNEIQQQMVRLSADSPVE
NKRSARSRTKANPNDINSNIYLVQNTTVQGSIPNAYQAVSQPDQMTYLNGNVVYGPHLPY
GYSTERSDFYWSSNDGA*
>11682.m04184|LOC_Os05g43950.1|genepair661-2
MAGDPAAGGDGGRGSSGGKGSSPSSSRHQQFRNLAKTRVDDLQEMFSGLQSARKESRSAD
AALLEEQNSQSQGNNREASDPPSETLRLLQLAGAEEEDDATSKLVMPRSPMPMQSSHEGH
NLSPVLQGGTMAGGAAELMVPRSPLQQMPSSHQSHGHGQDGGQNLQGEAVMGSTAATAAP
HLGQGMQGDCGGMAGVTNAMFHDQAWAFSQAGHTFLLHTAFFCNCQLFLSIPQKKLIAIL
YVSFLRSTSPQSLTCSMVNLYYIDHELNIDDFLQDDDYKINLPGSNPDGPNTMQGIGQLE
HQQYNLPLDLPPNSFVDANNSAQSSGDVFFHMSDLLTTMCPSPSQYLGPKCALWDCGRPV
RGSDECQHYCNPYHAGLALNDDGLLGTRPVMRPRGIDLKDGPLFAALSAKVQGKNVGIPV
CEGAATTKSPWNAPELFDLSLLEGESLREWLFFDTPRRAFDSGNRKQRSLPDYNGRGWHE
SRKQVMKDFGGLKRSYYMDPQPSSNYEWHLFEYETNDSDALALYRLEYKSSDTKRSVKSK
LASSPLSEIQQQMVRLSADSPVESKRTARSRAKANQKDNNSNAYPALNTPVQVSASNAHQ
TMSVNTPDQVNVSNAYQTMPLNTPNQPGPSNAYHAASQMDQMTFLDGSVVYGPHLPYGYS
TERSDFYWNPSDGT*
>11667.m05419|LOC_Os01g54970.1|genepair662-1
MDASRNSDLAADELLRAQAELWNHIFAYTKSMSLRCAVELGIPDAVHRRGGAVTVPELVA
ELALPRSREPFLRRLMRLLAHGGIFDAAAGAEDAYGLTAVSRLLVSAPGGAGQGLSPFAR
AMLHPIIVSPSISLASWFRAAAADDDDEGADAPRVPFAAVHGGRELWAVAKDDPGFGAAF
NDAMACDGRFVMDVLLHGHHHGGAQLFRGITSLVDVGGGSGGAARASAAAFPHVRCTVLE
LPQVVATVPPGDGGVEFVAGDMFDHVPKADAVLLKWILHGWGDEECVRILRRCREAVPAR
EDGRRVIVMDLVVGSSSSLGDGARDTETQLLWDVMMMGVVGSPERDEREFCKIFHDAGFS
GYKILHVLGIRSVIEVYP*
>11682.m04183|LOC_Os05g43940.1|genepair662-2
MGSLELSKNKCNGGQEVSLLDAQLELYSNTFAVIKSMALKSALDLGIADAVHRHGGAATL
AEIASEVALHPSKIPCLRRLMRALTVSGVFAAAVKPGDGGGGEPVYELTPSSRLLVGSSN
LSGIMSMILHPTLVVPFLGVGEWLRRDREPPEEDPYCIFKQAHGRSLWELAGRDAAFDAL
INDGMVSDSRVIMDYVVREHGEVFRGIASLVDLAGGLGAAAQVISKAFPEVRCSVMDLGH
VVAKAPAGTDVEYIAGDMFESVPPADAVFLKWVLHDWGDNDCIKILKNCKKSITPRDKGG
KVIIMDIVVGAGPSDQKHREVQALFDMYIMLVNGIERDEQEWKKVFVEAGFSGYKIMPIL
GFRSMIEVYP*
>11667.m05421|LOC_Os01g54990.1|genepair663-1
MGIDLNNTASGGEEDAPAPGPVCRDLWHACAGPVVSLPRRGSAVVYLPQGHLSAAGAGGG
IRGEVAVALPPHVACRVVDVELCVFERNLHGGGIEREDDMEDGDEERKSRMLHMFCKTLT
ASDTSTHGGFSVPRRAAEDCFPPLDHKQLRPSQELVAKDLHGAKWRFRHIYRGQPRRHLL
TTGWSSFVNKKKLVSGDAVLFLRGDDGELRLGVRRATQLKNEAIFKAFSSESSKMRTLSA
VADSLKHGSVFHICYNPRATASEYVVPYWKFVKSFNHPVCIGMRFKFHFESEDVNERRSG
MIAGVSEVDPIRWPGSKWRSLLVRWEDATDCNSQNRVSPWEIEIVGGSISVAHSLSASSS
KRTKLCPQGNLDVPALYGNGRPDSVETEKFPRVLQGQELMGSRTHRATCSPQSIDITKSK
SFDAWRFLTDTRSCMLGSSTSRLPVQYSGYTHQSVSFGESIGFPEVLQGQEISQTVPPFQ
GMLPDACSAKSRYELKNYVCTPATMNGLSSANEGYCLSLSTVPPSPPSSLMLYQTGVPQL
ELASKNNDKSGNDSQPALRQHKLLSETSWDQFKIGKASTPGNATKPGNGGREVDRTSCRL
FGFSLTEKIIPTDKDGEKEVSYETDCQNPRMLDLFGYNCSTPGALHALCAAPLGI*
>11682.m04181|LOC_Os05g43920.1|genepair663-2
MGIDLNTVEEEAEEGAAAAVCGELWHACAGPGVALPRRGSALVYLPQAHLAADGGGGEVP
PAGAAAVPPHVACRVVGVELRADAATDEVYARLALVAEGEMLQRNFREGGGEDGAGEMEG
CDAEKKPRMPHMFCKTLTASDTSTHGGFSVPRRAAEDCFPPLVIHLKEKLVTFNDYKTVR
PSQELIAVDLHGTQWKFRHIYRGQPRRHLLTIGWSSFVNRKKLVSGDAVLFLRGDDGQLR
LGVRRAVQLRNEALFEPVNSSDSKLRILSSVASSLENKSVFHICFNPRSGASEFIVPYWR
LLKSLNHPFSIGMRFRVCYESEDANERSAGLISGISEVDPIRWPGSRWKCLLVRWDDSTD
SSHQNRVSPWEIERVGGSVSVTHSLSSGSKRTKLHFPQGSLDTPFLNGNGHPDSMGTENF
HRVLQGQEFRGSRSHGVVCSESPGVPNFQSPDNRRFSADMRGYMMPASGPPQRNTEFTYQ
PIGFSESLGFPEVLQGQEMSQVVPLFRGATFGARTQNDRVVSANSVHRSAAQSGLLASTL
GHPISQFTLSSSKVSSPSSVLMFNQATAPNHETVSGTNNKGMHVSQFASQEMLSETVTWP
GTQRQTPSEITSNQFALARIPAPPSGAESGLPKRDAGRSSCRLFGFSLTGNMLGEDGEGL
DDGAIEAGCENPPVLELFGHSHSTPGALHALCAAAPLGM*
>11667.m05447|LOC_Os01g55240.1|genepair664-1
MVVLAGPPAVDHIPLLRSPDPGDVFSGVPVVDLGSPGAARAVVDACERYGFFKVVNHGVA
TDTMDKAESEAVRFFSQTQPDKDRSGPAYPFGYGSKRIGFNGDMGWLEYLLLALDDASLA
DACTVPSCAVFRAALNEYISGVRKVAVRVMEAMSEGLGIAQADALSALVTAEGSDQVFRV
NHYPPCRALQGLGCSVTGFGEHTDPQLVSVLRSNGTSGLQIALRDGQWVSVPSDRDSFFV
NVGDSLQVLTNGRFKSVKHRVVANSLKSRVSFIYFGGPPLAQRIAPLPQLLGEGEQSLYK
EFTWDEYKKAAYKSRLGDNRLAQFEKK*
>11682.m04177|LOC_Os05g43880.1|genepair664-2
MVVLAKPAALEQISLVRSPSVEDNFGAGLPVVDLAADGAAGEVVRACERFGFFKVVSHGV
GEGVVGRLEAEAVRFFASPQAAKDAHGPASPFGYGSKRIGRNGDMGWLEYLLLAIDGASL
SRSSPAPSSSLRDAANKYVGAMRGMARTVLEMVAEGLGVAPRGALADMVVGDGAASDQIL
RLNHYPPCPPLLQNLMPNCSPTGFGEHTDPQLISILHSNSTSGLQVALHHDADAGDHQWV
TVPPDPASFLVIVGDSLQVMTNGRMRSVRHRVVANKLKSRVSMIYFGGPPLEQRIAPLRQ
LLVAGVGNGEEEEQSRYEEFTWGEYKKAAYLSRLSDNRLAPFHRQPPPVANPLA*

>11667.m05451|LOC_Os01g55280.1|genepair665-1
MPRSRGSELPQRASPRAPLHLKTTACSDANGAHHRTVVDRSSPKLADRHSPRSPLHEKKR
AGTRVAELETKLGKVQDELKKLREQLATAEAAKKDAQVALEEAKKRVGTKGSPASAAAAS
PRSPSPVAVETPKKTEQQLKVTEPPAEEESSINSPATDVFEVVRTESGDKENQSAAGAED
GEEVSCGIKAALAEKELEEEETKKMIEEESIDATAIDSGEKENPEVAELKAKLMAKDMEV
AILTADNAELKKQVGEAAEAAKKAEEEAAAAASPREAQI*
>11682.m04176|LOC_Os05g43870.1|genepair665-2
MLQFQEELALGVKEEQGEEVDSLLVMMLVRTVKKPSKFEDSDTVTAKPARAVFEMPHRTS
PRAPVRSKTTAAAPAAAGSEHHRDRVVVGAGGGAARGTSPRSPQLPEKKPAGGGAAVSRV
AELEAKLGKAEGQLAEMREQLAAAEKARKDARAALVESKKRFSAAKKRVATAGAASSSAA
AEQTPPQAVSDEKCGVISPAGDVPEAAEPGDAQGEETKEMADDDEVNSVTAAIVGDLEGN
KGGQEVEQLRTKLMEKDMEVYELKAKLIAMDAEADDLRASLATKGMEIDELRAKLTSKDA
DIAAVEADNAELMKMAEEASHAVKETATKARDTEHALRESAAREAARVAERLRASERARE
ALEAELQRGRAQSEQWRKAAEEAAAVLAAVEHGAGAPAADVEWRRHSSGAAAGERVAKDT
DEHHVSGGKRNSGGAMRMLSELWKKKAQK*
>11667.m05466|LOC_Os01g55410.1|genepair666-1
MRADRLMAEGGKRIDLGAPLRSVRHADALPYHKADLNSGPVRHPGAVPFVWEQRPGQPKS
VRTRRAPPSPTTASHPQPLEHGVEDEIDGSPYHDALGEHYVGILHGVDASPACSRTGAPA
PAPARDEKRAQVAEAAVLQAKKEVTEKQVVSVAAVLRKGDDDDDDEERFSDALDTLSRTE
SFTVNCSVSGLSGMPEPTSRAAAGAEAGVRGIMMDRFLPAAQAVAIGSPQYTFRKAGAAS
ATSNSGRELARAAGSNASGSSGDDPGRRTPVQLPYQHLPPNYLSCSYPRREEQEDEDDDD
DYDVHSTRGFASKGCGLLPSLCVKGSLLLLNPMPAMKRGKPRGNGRVREFASKGRGRGAP
SPLARSSQNKHLGCASNGSWEDVYKHKLEQKYVRPGEDGRSKLTSESNQLTFWSDSQAGN
GSSPFHHSAGGGMSPYYRDVVLSSSSKADESFGTGVKEDKMSSSNGSSSLGRDHDRGSLL
GSDRSSLKGSSSISSGLDRPVHVESMDHRGDIDSETSHSVLLLDSRTSLDAGGCGSQLGE
QIVGKNPIGKGEDNDPLTERVSEVTECTLLAPSEKLRSVNLDDGKTSGHLEDSSVSKRDM
PLQSLLPLPVPRSPSESWLSRTLPSVTSKPPVPSFLGIQLQSKKQTPWASIQPKENNVKP
PRPRQIRFADVVERPNSLDAEI*
>11682.m04175|LOC_Os05g43860.1|genepair666-2
MVIYLPVVKLSKELDMHRVMLMQLQLKSKENLSERTPEKTLINSLKNLLIEIDISSSKNN
LNHSYESGQTFTQCSGEASDTARVTSGGEARRQLAAPVMADANRRIDLAAPLVSVRRHGG
GAAGEAATRTDGTRPGHPKSVRTRRATMVHATARDEEPARDAMAVVAVAAPVRERDQEAR
FSDALSVADSCLTVNCSSASGLSDAVAQPPRGVGVGGGVMMDRFLPAAHAVAVLSPQCSS
RKASVAAAAARNGHGADALLPPPEPTPTIRTLCIVPREKTDDADAAVDDNGGGGEWDAHS
TRGVSSRRCGLLLPTRCMKSTLLLLNPAPAMRRRGGGRRRDRGAPLLSKIGRSQSLGNPL
VRSAHDTGIMRSWEEVYINSLRRSGRGGRKGIGALLSPELDTTMPSVRELYLEQGDGAVH
PKATHLGFLLVLDRSHDQCHDSHDDPKLLPPPRFPRPAPPKVFDGGKKQRRDAAGAGGGG
GGGGYGWPLLLEDKAAASRDMVPPLPPLPSMKSPSESWLSRALPSVSSNPPATSFLGIHV
QHKKQSPPPRCSSRAPAKLVADGHARPRQMRIHDLQKS*
>11667.m05468|LOC_Os01g55430.1|genepair667-1
MVPWRRSSSSSSAPSSRPARRPARTNARVSPDVSSELSPLAGEEGAGEERWSALVPDLLA
DILRCVEAGSERWPPRRDVVACASVCRRWRDVAVAVVQPPLESGKITFPSSLKQPGPRDA
PMQCFIKRNKKNSTFFLYLGLTQELTDDEKFLLAARRCRRGLHKEYAITINSDGLFHGSQ
SCVGNLKSNFTGTKFTIRDWQPPYEGAKAFSSRSGRWFGNKHRCPLVSTGDVEVGEVSYK
YSLLRPRGPRRMSCSVQCPVLKGTAVDPQDGKRLSNSIPSSLVLNSKVPSWHEHLQCWCL
NFHGRVMVASVKNFQLIAPVEPGEPSDKTVVLQFGKIDDDVFTMDYRQPLSAFQAFAICL
SNFGTKLA*
>11682.m04174|LOC_Os05g43850.1|genepair667-2
MALWRCSSSWLSSVSRSSGGVGGGESKVSPEIAPVSGGEGEGEEEEGEEERWSRLLPELL
TEIMRRVDAGAERWPPRRDVVACACVCRRWRDAAVSVVRPPLECGRITFPSSLKQPGPRD
APMHCFIRRNKKNSTFYLYLSLTQALTDKGKFLLAARRFRNGAHTEYIISYDCDDLFPGS
NSYVGKLRSDFLGTKFIIYDSQPPYDGAKPSRSQSSRRFASKQINPNVSGGNYEVGQVSY
KFNFLKSRGPRRMQCNIQCPVGQSTASDPLKKLISTSSPLALRNKAPRWHEHLQCWCLNF
HGRVTVASVKNFQLVAPAGTSDPWGIADEETVILQFGKIEDDAFTMDYRQPLSAFQAFAI
CLTSFGTKLACE*
>11667.m05470|LOC_Os01g55450.1|genepair668-1
MLMATVSPARREPTPQAVRASPMPSAAAALVRRGGGGSGGTVLGKYELGRVLGQGSFAKV
YQARHLETDECVAIKVLDKEKAVKGGMVHLVKREINVLRRVRHPNIVQLFEVMASKTKIY
FVMEYVRGGELFSRVSKGRLREDTARRYFQQLVSAVDFCHARGVFHRDLKPENLLVDENG
DLKVSDFGLAAGPDQFDPDGLLHTFCGTPAYVAPEVLRRRGYDGAKADIWSCGVILFALM
AGYLPFHDHNIMVLYRKIYNGEFRCPRWFSKDFTRLITRLLDANPKTRITVPEIIESDWF
KKGYKPVKFYIEDDKLYNLSDDVLNLEPADPVPPPLGLAPPVPPPPQGDDPDGSGSESDS
SVVSCPATLSTGESQRVRGSLPRPASLNAFDIISFSKGFNLSGLFEERGNEIRFVSGEPM
SDIVKKLEEIAKVKSFTVRRKDWRVSIEGTREGVKGPLTIGAEIFELTPSLVVVEVKRKA
GDNEEYEDFCNMELKPGMQHLVHQMLPAPNGTPVSEKVERSSSLQAPLTLKLIGTEGSMS
*
>11682.m04173|LOC_Os05g43840.1|genepair668-2
MAATPPSSQHRRPLSSSASAASLAGKPRGGGLLLGRYELGRLLGHGTFAKVYQARSADSG
EPVAIKVLDKEKAMRHGLVPHIKREIAILRRVRHPNIVRLFEVMATKSKIYFVMELVRGG
ELFGRVAKGRLKEDTARRYFQQLVSAVGFCHARGVFHRDLKPENLLVDEHGDLKVSDFGL
SAVADQFHPDGLLHTFCGTPSYVAPEVLARRGYDGAKADIWSCGIILFVLMAGYLPFHDQ
NLMAMYRKIYRGEFRCPRWFSKDLSSLLNRILDTNPETRITVKEVMESRWFQKGFRPVRF
YVEDDQVHSLADGDNDMPELEPSEPPPPPPFPPPPPQQDDDGEESGWESDSSVASCPATL
SSEERRQRPLGSLTRPASLNAFDIISFSKGFDLSGLFEERGSEVRFISAEPMQTIITKLE

EIAKVKSFFVRRKDWRVSIEGTREGLKGPLTIGAEIFELTPSLVVVEVKKKAGDKEEYDD
FCNRELKPGMQHLVHHMGSVPNIPSDTE*
>11667.m05497|LOC_Os01g55700.1|genepair669-1
MDGVARSRLLVPLLPRISARSFSAASPASEPAAAAAAVAASDAAAATDYSSSAGDPSSAP
PPPARKPLGLLKGGVLAVVAAAFGATGYVSYAYSLDEIDQRTREFRKNSKQPIRDDLSGF
EKFQAMAYSAAMKVPVAAIEFYLDTRSQIEDQIRGFSEPSSDKLLPDLLPQEQHVFTLVL
DLNETLVYSDWKRERGWRTFKRPGVDAFLEHLGKFYEIVVYSDQLSMYVDPVIERLDPKG
CVQHRLSRVATKYENGKHYRVW*
>11682.m04166|LOC_Os05g43770.1|genepair669-2
MAAVTRAARSRALLLLPRASAAAPHFSTTASSGAAAAAAAPVEAAAAGASDASAAAAAGA
GEQPAPPPKRWGLLKFGAFAAVCGALGAAGYSSYAYTLEEVDQKTREFRKAMTTPRPVAE
DASEFEKFQAMIYSTAMKAPVAAIEFYMDVRHTIEDHIRGFAEPTSDKLLPDLDPLNQHI
FTLVLDLNETLVYSDWLRERGWRTFKRPGVDAFIEHMGKFYEVVVYSDQMPMYVDPVLER
LDTKGFITGRLSRPATKYQDGKHYRDLSKLNRNPAQVIYISSHALESCLQPENCVQIKPW
KLETDDTQLLDLIPFLEYVAMVRPSDIRTVLASYQGRDVAAEFIERSKEHQRRMQEQKQH
GRIWRQ*
>11667.m05502|LOC_Os01g55750.1|genepair670-1
MILGSNQAAAAAAAAAAAEEEAAELARKHTAAVATSRQWSAQTESRIVRVSRVFGGKDRH
SKVKTVKGLRDRRVRLSVPTAIQLYDLQDRLGLNQPSKVVDWLLNAARHEIDKLPPLQFP
PQDHLCMGHHHHLPSAMPLMHHHGHHHHADDDKYHVAAAAAALAAEKEAAAAGGGGGGGG
DDVDGGGGGGAAHIVGRFPAGGYHRFMGLNNPLGMVNSAAGAAMPFHYAGESWNNGSVQD
SGAGSPQVAAAAAHHTSPFPSLLSLAPGPHHQLVFYSSEAEQFTVDNLGSQGLSLSSARA
FHDQTGS*
>11682.m04165|LOC_Os05g43760.1|genepair670-2
MGAGWQWPAGGGGMEAAGRGLAAARSNHEQLKMTSNNSTNEELGGGGRKAADKPSGGGGA
AAAVASSRHWSASTESRIVRVSRVFGGKDRHSKVRTVKGLRDRRVRLSVPTAIQLYDLQD
RLGLSQPSKVVDWLINAAQAEIDKLPPLQFPPHDHDLVAAAASSMAPPPFANGGDGHHGA
SASSMLEDGDKAAGGGGMKAFMSLSNSLGLLNAATMPATLAAHHHHHHHAAAYYAAAESW
GNGGNGGHHHDVSHGVSPSAHNSPFPSLLSLAPGSHHQFVFYSPEGGGFAVKEAAAEQFP
VDSLDHSQGQLTLSSARSFLHSGSQG*
>11667.m05512|LOC_Os01g55820.1|genepair671-1
MATRLLLLLLLLLGMSLKGSEGAWCVCRPDVAEAALQKALDYACGHGADCAPVTPSGSCY
SPNNVAAHCSYAANSYFQRNSQAKGATCDFGGAATLSSTDPSSGTCKYPATARDKHRNRH
GGCRHRHRYKHEHEHEHFFPGLFNCSHGSGGCARGCSLRGEIGVDARRGVAVVGVRAMRK
LCPNLDRDDSLDTVLEVPIPDEMLINAPGTDKRRGAGGANMRAWLKNQAFDRATVGGPAN
ATAELQLFLNVVGSPLIPCPVPHDRAFSRSIRDSSIQASTAKYIMQQYIAATGGQAALQG
LQSMYAVGKVRMCASEFHLGDQNVTAAQGRAEVGGFVLWQKCPEVWYFELIMAGHKMSAG
SDGKVAWRQSAAENSHASRGPPRPLRRSLQVDRTLLAAAPPLPSLSTSFDITMQGLDPRS
IANLFSDAVCIGEKIITGEECFILKLEASAATLRARSAAAFDIIHHTVWGYFSQRTGLLI
QLEDSHLLRMKSGKGARRSENIFWETTMESTISDYRHIDGINIAHGGHTAVTLFRYGEGS
VNHKRKLEETWTVEEADFNLYGLTTDYFLPPSDLKKDIDEKRAG*
>11682.m04158|LOC_Os05g43690.1|genepair671-2
MAIPLLLLLLLAMSTGSDGAFCVCKPDQSPAAMQKAIDYACWRGADCTQIMQSGACYQPS
TIVAHCSYATNSYFQKNSPIGATCDFGGVATLTNTDPSSGTCKYPATASGVGTGMGTGTS
TGTGTGVGTGGTGTGGAGVGAGTGTGVGTGTGTGAGMGTGAGAGTGITTPGSTTGTQGGA
LSPPFGGAYGPSAGAMNPDYNEAAPARSQLAAATSVLLAAAPFLFHLI*
>11667.m05533|LOC_Os01g56010.1|genepair672-1
MELAEAVRDETAMAMRLLGHLARAPRGGGGDKNLAVSPLSLHAALALLGAGARGETLDQI
IAFLGPAGGPAHAALASHVALCSLADDSGPGDDRGGPKVRFANGVWVDAALRLKAAYARV
VADKYRAEARPVSFRDKLEEARREINEWFESATAGRIKDFLPKDAVDRATPAVLGNALYF
KGDWESKFDARSTSDDVFYLPDGGHVSAPFMSSGKWQYIACRAGYKVLRLPYARGGRGRG
RDTGRLFSMYIYLPDERHGLPDMLRKLCSDPAALIESSAALTEKVPVGAFMVPRFTLSYK
TNAAETLRQLGLRLPFEYPGADLSEMVESSPEAEKIVVSAVYHESFVEVNEEGTEAAAAT
AVVMTLGCAAPSAPVHVVDFVADHPFMFLIKEDLTGVVVFAGQVTNPSSST*
>11682.m04148|LOC_Os05g43590.1|genepair672-2
MAVWVDASLRLNPAFADTAASVFKAAVRSAGNPAAARAEINEWFSSQTGGFVKDILSNSI
DDDDDGSGGGGGAISASVFLANSLYFNAYWDHPFFPHLTEEGDFHVSPDHDVRVPFMAGS
HQHAFMDVGCHPGFNVLRMMYRTGGAAGGDKMFAMYIYLPDDRDGLPELARKLASNPAAF
LRRTIVPAQPVAVGELKIPKFEVSLKVEASRLLREFGLDLPFLPAADNSFSGMLLDPPQG
TAVSSLLHQCFVNVNEEGTVAAAGTVGEIMGFAMPDDQIVDFVADHPFLFFIVEEVSGLV
VFAGQVVNPLLH*
>11667.m05566|LOC_Os01g56310.1|genepair673-1
MMQSSLARPLPRPPIRPACGNPVCRSRPGSVSVARCRAEAAPPAPAPAARRAAGPYTGRD
PEVKKPAWLRQRAAQGEKYARLRESIGELKLNTVCVEAQCPNIGECWNGGGGAGGEGDGI
ATATIMVLGDTCTRGCRFCAVKTSNKPPPPDPLEPLNTALAVASWGVDYVVLTSVDRDDL
PDGGSSHFAQTELKPGILVECLTSDFRGDLEAVSALANSGLDVFAHNIETVRSLQRIVRD
PRAGYDQSLAVLKHAKSCKEGMITKSSIMLGLGETDEEVKQAMIDLRAIGVDILTLGQYL
QPTERHLTVREYVTPEKFQFWKEYGESVGFRYVASGPLVRSSYRAGELFVQNLVRNNKPK
LPASS*
>11682.m04147|LOC_Os05g43580.1|genepair673-2
MAAYCSRVYHHHPVSPSTMQGSLARPSIHAGSASLTFRARPNSVSIVRCDADSPPEGSAV
AGWAPPGPYTGRDPAARKPAWLRQRAAQGEKYARLRESLGELKLNTVCVEAQCPNIGECW
DGGGGAGGDGDGIATATIMLLGDTCTRGCRFCAVKTSNKPPPPDALEPLRTAVAVASWGV
DYVVLTSVDRDDLPDGGSGHFAQTVKALKELKPGILVECLTSDFRGDLEAVSSLASSGLD

VFAHNIETIRSEFSCSETCEKLQRWYGETDEEVKQTMCDLRAIDVDILTLGQYLQPTERH
LRVREYVTPEKFDFWKEYGESLGFLYVASGPLVRSSYRAGELFVQNLFFDESFALKLCAD
DRNLAVTLLKGADWFAILNYTIIVVFPVAGVEDAVVGFVTGKRKATELAHAIWNESSVNG
KCSLGFSGRCVKEYVYTLAIHILSFGFLIRVWRSIVRKGDTVVDATCGNGNDTFAMLKMV
ADERVQGRVYGLDIQESAIASTSSFLKMAVNSHELELVKLFTICHSRMEEVVPKDFPVRL
VAFNLGYLPGGDKTIITVPKTTELALQAASSIVSSGGLISVLVYIGHPGGRDELDVVESF
ASSLPIDTWMSCKFEMLNRPAAPVLILLYKK*
>11667.m05575|LOC_Os01g56380.1|genepair674-1
MAPPSHCHTINGGAPRNGAIPEVETTTSTPAASDTALLLDADEFRRLGHQVVDFIADYYA
GLGDYPVHPSVTPGFLRRQLPADAPSRPEPEAFAAALRDVRDLILPGVTHWQSPRHFAHF
PASSSTVGALGEALAAGINVVPFTWAASPAATELEMVVVDWLGRALHLPESLLFAGGGGG
TILGTSCEAVLCALVAARDRKLAEIGARRIGDLVVYCSDQTHFAFRKAARIAGIPREHCR
EIPTCRDDVFALSPTALHAAMQADVDAGLVPLFLCATVGTTQTTAVDPVRELCAVAARHG
GVWVHVDAAYAGSALVCPEFRDVIAGAEAVDSLSMNAHKWLLANNDCCAVWVAAPSALVA
ALGTEQEYILRDAAAEGHDVVDYKDWGTTLTRRFRALKVWLVLRCYGVEGLRSHVRSHVA
MAAAFEAMVRGDARFEVVAPRRFALVCFRLRSPPERLGVGVGVGGEKAANELNRRLLEEV
NAASSGPYMSSAMVGGVYMLRCAIGSTLTEERHVREAWKVVQERATSILRKRG*
>11682.m04140|LOC_Os05g43510.1|genepair674-2
MAILNHSDAAFPVAATTPLLGRRPLDAGEFRRQGRQVVDFIADYYAGINDYPVRPAVAPG
FLAGKLPATAPSTPEPDALTAGLRDVRELMLPGLTHWQSPRHFAHFSATASNVGALGEAL
AAGLNVNPFTWEASPAATELEVVVTDWLGKALHLPERLLFAGGGGGTLLGTSCEAMLCTI
VAARDEKLAEIGEERIGDLVVYCSDQTHFSFQKAARIAGIRRGNCREIPTCRESGFVLTA
TALQAAVVAADEAAGRVPLFLCATVGTTPTAAVDPLRELCAAVEGRGVWVHVDAAYAGAAC
VCPEFRHAIAGAEAVDSFSTNPHKWLLANMDCCALWVARPAALVAALGTDDDVILKDAAA
AARPARGDHHHHAAVDYKDWQVALSRRFRALKLWLVLRCHGVDGLRAVVRSHVRMAAALK
RMVRADARFEVPVPRQFALVCFRLRGGGAAAQLVGGDELTASNELNRRLLEAVNATGRAY
MSSAVVGGMYVLRCAVGNSLTEEHHVREAWSVVQGQAAAVLATAGAAADTARTKDHAAGD
DHGADQPHAMTTTTTMGCRSGPWEL*
>11667.m05576|LOC_Os01g56390.1|genepair675-1
MEGHEGGEEEGLGDQFERLPDEVLLDVFGRIGDVKALGRCALVSRRFHALVPLVDSVFVR
VDCVISDDPPPSSAGSAQAAAAAEGGPPAARGRGALAHLARLVLGGIVRPIQALGQILSP
AAATVSRRSAAPPAAPAPAAADVSHHSPSEVLRSFKELRRLHIELPTGELGIEDGVLLKW
KADFGSTLGSCVILGASSVTSKPPPPPAAPPPTAADSSAASPDSSREPDELGNIPESLYT
NGGLKLRVVWTISSLIAASARHYLLQPIIADHSTLESLDLTDADGQGVLTMDKRQLQELR
VRPVSASGNSHRTLMPALSMRLWYAPHIELPGGQLLKGATLVAIRPSEDVLREGGGIEVA
GPTGASWILDAFEEPYRTAAQVLLKRRTYSLEMNSF*
>11682.m04137|LOC_Os05g43490.1|genepair675-2
MAEDPAGSRRWRCDAGDEHGCWLSSSAGGGGDDHFDRLPDPLLLVIFNRIGDVKALGRCS
LVSRRFHDLVPLVDSVLVRVDCVIPDDPASSSSSSSSPSAAPSSPTASARARTVFSQIAR
IVLGGIVKPIQALGQILSPANSASVLAASVTSSPSSSSSSSSSPPLPGDVSHHSPSEVL
RSFKELRRLRIELPAGELSMEEGVLLKWKADFGSTLGSCVILGASSAGKDGGAGAAPAVD
CGESDETGSIPESFYTNGGLKLRVVWTISSLIAASARHYLLQPIIADHTTLESLDLTDAD
GQGVLTMDKWQLQELRVKPVSASGGSHRTLMPALSMRLWYAPHIELPGGLVLNGATLVAI
KPTEEATRDTVGSGIAGSAGGCWVSDAFEEPYRTAVGMLLKRRTYSLEMNSF*
>11667.m05582|LOC_Os01g56450.1|genepair676-1
MAIVGEEYCSAEERVLTVRKTSHFSPGDGFAAYDHRTGGLAFRADTYGRGHGGGAASAGE
LALLGPAGEALITVRRRRPSLHQRWEGYLGARADGQKPLFSARRSSILGGAAAGAVVELL
APLPASFSSTHAAAAELLRVDGSFPRRCCRVVAPKAESGGEAAVVAEIRRKVDEGARVVM
GRDVFVLRVGAGFDAAFAMAIVLVLDQIAGDEADGNAGEETNRAMIW*
>11682.m04134|LOC_Os05g43460.1|genepair676-2
MAQQQRMVIVGEEHCGGGEDRELTVRKTTLFCPGDGLEAYDHGTGTLAFRVETYGRGGVC
GGGAAAGDLALLGPEGEPVLTVRRRRPSLHHRWDGFLGDGAASGQKPLFSARRSSILGVG
SGAAAVLVDLLAPGAAGEFRVDGSFPRRCCRVVAVKAAAPAGGGGEEEEEEVVVAEVRRK
VDEDAHVVMGRDVFVLWLRAGFDAAFAMGIVLVLDRITGDELNGDLSEDLAVASSPV*
>11667.m05592|LOC_Os01g56550.1|genepair677-1
MAEEAKNLETARADRSVWLMKCPTVVSRAWQEAATAAASSSSSSDAAAGANSNSNANPNP
VVAKVIVSLDPLRSEDQQLQFKMEMAQTGNGNTPKSYSLNMFKDFVPMCVFSESNQGKLS
CEGKVGHKFDMEPHSDNLVNYGKLCRERTQKSMIKNRKLMVLANDNGMSMRPLPGLVGLM
SSGPKQKEKKPLPVKPSDMKRTRRDRRELENILFKLFERQPNWSLKNLMQETDQPEQFLK
EILNDLCFYNKRGPNQGTHELKPEYKKSTEDADATAT*
>11682.m04133|LOC_Os05g43450.1|genepair677-2
MGEEAKYLETARAERSVWLMKCPPVVSHAWQGAVSSSDAAGSNPNPVVAKVVLSLDLLRS
EEPSLQFKMEMAQTNTGNTPKSYSLNMSKDFVPMCVFSESNQGKLSCEGKVEHKFDMKPH
SDNLVNYGKLCRERTQKSMIKTRKVQVIEDHRMSLIPLPGMVGLIPSGSKEKKKQTPTKP
SDAKRIRRDRRELENIIFKLFERQPNWALKALVQETDQPEQFLKEILNDLCFYNKRGPNQ
GTHELKPEYKKSTGDTDAS*
>11667.m05600|LOC_Os01g56600.1|genepair678-1
MVLAQLGGSISRALAQMSNATVIDEKVLSDCLNEISRALLQSDVQFKMVRDMQSNIKRIV
NLETLAAGTNKRRIIQQAVFTELCNMLDPGKPSFTPKKGKPCVVMFVGLQGSGKTTTCTK
YAYYHQRKGFKPALVCADTFRAGAFDQLKQNATKAKIPFYGSYMESDPVKIAVEGVERFK
KENCDLIIVDTSGRHKQEAALFEEMRQVSEATKPDLVIFVMDSSIGQAAFDQAQAFKQSV
SVGAVIVTKMDGHAKGGGALSAVAATKSPVIFIGTGEHIDEFEVFDVKPFVSRLLGMGDW
SGFMDKIHEVVPTDQQPELLQKLSEGTFTLRLMYEQFQNILKMGPIGQVFSMLPGFSSEL
MPKGHEKESQAKIKRYMTMMDSMTDGELDSTNPKLMTESRILRIARGSGRPVRDVVDMLE

EYKRLAKIWSKMKGLKIPKKGEMSALSRNMNVQHMSKVLPPQMLKQIGGMGGLQSLMKQM
GSKEMGGMFGGMGGDK*
>11682.m04127|LOC_Os05g43390.1|genepair678-2
MVLAQLGGSISRALAQMSNATVIDDKAFADCLHEIARALLQSDVQIRMVSDMRANIRRAV
NLDALPAGTNKRRIIQQAVFAELCNMLDPGKPSFTPTKGKPSVVMFVGLQGSGKTTTCTK
YAHYHQLKGFKPSLVCADTFRAGAFDQLKQNATKAKIPYYGSYMESDPVKIAVEGVERFR
KEKSDLIIVDTSGRHKQEAALFEEMRQVAEATKPDLVIFVMDGSIGQAAFDQAQAFKQSA
SVGAVIVTKLDGHAKGGGALSAVAATKSPVVFIGTGEHMQDFEVFDVKPFVSRLLGMGDL
SGLVNKIKDAMPADQQPELMQRLIEGTFTLRVFYELFQNLLNMGPIGQVLSMIPGFRSEL
MPKGHDKESQAKIKRYMTIMDSMTNAELDSTNPKLMSESRIKRVARGSGRTMKDVTDMLE
EYKRIAKVCSKLKKKLPKNMDRNVMNNKDTLNTINNLIPKQLLNQIGGVNPLQSVMKQMG
LKT*
>11667.m05602|LOC_Os01g56620.1|genepair679-1
MRGRHLLSPLLSPLAYPKPPSSRLAPRRVASHRTVAPPPAARRAPLSEPDVGISLFASAL
PGFRGALKQRYSDFVVHEVALDGSLVRLTSFDLPDGECVDAKEGGMDEAQALESFRLLCG
DADCEALRGFLERVSEGGDSDVSPIILSADADKTHRSEVHDFFKRNFEALLTDTVEHSDG
IQRCIRVRLKPGRRERRDVDGRGRNRKGTGSSGWRDDKPFDSRGSIIWPDHLGKFIRFHL
YKENKDTQEALGKIGKMLGLQPRSFGFAGTKDKRAVTTQQVTVFKVQASRLVALNSKLIG
IKVGDFRSVVTESEDVIKAALDGLITNGFINYYGLQLQRLKKYPGNYLQALMAIPRTLRL
MYVHSYQSYLWNHAASMRVQKYGISRVVEGDLVYKKEAPFEQGALKATSEDDGQTMSSEM
NACCETLPEEMIQSVKIADSEDLSKSLYTFEDVVLPLPGSETLFPGNEVAGIYHEIAMKD
GINLRESVHGVEDFSITRMKGGYRRVIQRPIDFEWDLITYTDEKIPLVETDLDVLSKTKP
LEVNELLSDGISSCTSHDSGLEASLDASESINGASLVVAEAKSIGSSDMLEKLAIKLAFT
LPASSYATMAIRELMKTSTSVAHQKTLNI*
>11682.m04125|LOC_Os05g43370.1|genepair679-2
MARSTSLTEAEAGITCFASSLPGFRGVLKHRYSDFIVHEVARDGSVARLTSFDLPDECVD
VSEEDKAAPSADADHSQALESFRALCGDADCDALKGLLEKASAGAEADVSPVILSPDADK
AHRSEVHNFFKRSFKFLVTDTVEHNDGVQRCIRVRLGSGARGGRGGGGRSGGGRGRKRKN
MGGSDWRDDRPFDSRGSTSWPNHVGKFLRFHLCKENKDTQEALGVIGKMLGLQSRSFGFA
GTKDKRAVTTQQVTVFKISANRLAALNNRLFGIKVGNFSYVKEGLVLGQLMGNRFTITLR
GVVAESEDIIKASVEGLGKNGFINYYGLQRDDIREMREHYKEHGDIDMALRNFPRHLVAE
KAILQCLKKCPGNYLQALKSIPRTLRMMYVHSYQSYLWNHAASARVEKYGISQAVEGDLV
YSKEPPPGEATSVDTSEPCDDQINSSDIDLCSETLPEETIQSVKIVDSEDLLKGVYTFED
VVLPLPGSQALFPGNGIADIYHELAKKDGISLTENAHGVKEFSITSMKGGYRRVFQRPID
FEWELMTYTDDTASLAETDLDVLSRNKPTKAKEVNETISSAISNAQSHDSKVAGPLDSSM
PGHVSENTRLLILLLTSILYGESSKPHLRGVDIVKQHELYCI*
>11667.m05608|LOC_Os01g56680.1|genepair680-1
MATVSAAAATSVVARAVLAGPLGLPQMRARRSERVRCNYSKEAATPAAAVKGAGASLLAM
AATAAPAMALVDERMSTEGTGLSLGLSNNLLGWILLGVFGLIWSLYTIYTSDLEEDEESG
GLSL*
>11682.m04119|LOC_Os05g43310.1|genepair680-2
MATIAAAAAARTAVVGRPSGVAQLRARRGERVRCGYSSRDGKEATPAAAVKGATSMLAAA
VTASSSSAPAAMALVDERMSTEGTGLSLGLSNNLLGWILLGVFGLIWSLYTVYTSTLDDD
DESGGLSL*
>11667.m05616|LOC_Os01g56760.1|genepair681-1
MGLGVEPLGLRAMDRDFAKQIGIEDVSECPHLCALAYGYLKKTEGYEQNLLAFFHNKINS
DALLVLLIEELDKCILGYFSFHWKFATEVITQVLTAEQPRRKLKTMVLEATRKMRFERVS
RKLKVTRLFSTLVEELKVIGVTCNDGQLRDADADVMVPAAHRDRSPVLLLMGGGMGAGKS
TVLKQIMKGLFWSGAAAHAVVVEADAFKESDVIYQAISSRGHHNDMLQTAELVHQSSIDA
AASLLVTALNEGRDVIMDGTLSWEPFVLQTIAMARSVHRQRYRMGVGYKVAADGNATEQY
WEPVEGAPVPSGRRPYRIELVGIICDAYLAVVRGIRRAIISGRAVRVSSQLKSHKRFAGS
FRKYCDLVDSASLYSTNTIAGAKLIGWKDVGSRLLVDVEEIGLLDRVSRINEEANGVHEL
YPDGHPTDGAGSVWEDLVSSPARAAIQRELREAILDSEACFPSP*
>11682.m04117|LOC_Os05g43300.1|genepair681-2
MREYGPAAAPRLLVSDSGRVHEMERFSHYVARQIGFDHVDECPHLCTLAYDYLRKNKGYE
ENIFAFFQNSQDPETLIVKFIEELDKCILGYFSFHWNYATYIISQVLTVEGAPKRKLRNM
VLEATRKQRFERVTRNLKVTRLFSTLVEELKAIGLSSHVEAPRSDVMVPAAHCDRSPVLL
LMGGGMGAGKSTVLKDILKEAFWSGAAANAVVVEADAFKETDVIYRAISSRGHHNDMLQT
AELVHQSSMDAASSLLVTALNEGRDVIMDGTMSWEPFVQQTITMARAVHRQRYRMGVGYK
VTEDGSITEEYWEPVEDSSTDEEGETRNRKPYRIELVGVVCDAYLAVVRGIRRAVITGRA
VRVKSQLKSHKRFATAFHSYCNLVDNARLYCTNSTGAAKLIGWKDGESNLLVDPEEIGCL
ERVSHLNDEADCVHEIYPDGSAAAAWEALVTSPSRAPAQREIMAAVQRSEARFRTTSTPS
*
>11667.m05621|LOC_Os01g56800.1|genepair682-1
MEHDMTVEDFIRTNGLGASGLIETNNQGVSTSSVSDCRSCEHVENGSPSTAPPFWDSDGE
DDDPVTSGPRPSDLFGRYTWRIENFSKEKKREMKSEPFEAGGYKWYILVYPQGCDVSNHL
SLFLCVANHDKLLPGWSHFAQFTIAVGNLDPKKVKYSDTLHKFWKKEHDWGWKKFMELSK
IQDGFLVDDVLEIIAQVQVIREKVDRPFRCLDRPYRRELLRVYMTNIEQIYRRFVEERRS
KLCKLIEDKMRWSSFRAFWLAIDQSTRHRMSTEKSDVILKIIVKHFFVEKEVTSTLVMDS
LYTGLKALECQSKSKSKKGIIDSVDFVELPVPMVHVDVDMFVLAGDVIALLERAALEPLP
CQPVSPKDDKCSQSRTKDGSSGEVNKVSIEREERRLTELGQKIIETFALSHIFSGIEVAY
QEAVALKRQEELIREEEEAWLLENEMKGKRGSTTEKDKRAKKKQAKQKKNNRKVKDKDRE
EKCDSNFPERSQDENTIHDREDSKQAGQISMKVDTSEEGASDVSDNLDGSIEIQKKHSTM
ENKSLSCSSESATMNNAQGKINNLLESKDQISRNRGKARSRSTSNMNITEDVDDLPSSTT

SSDRNTSGCGPAPKLDQETVLLTLKDRLRKLGQRLHEKEIEGRKLLQAHLEKKAAAESAT
GSSSSLSSNSLEETPEVLKSPDQSSVTISDADINASPSKFGASKEVTPVTPTTILSTEPV
PTVASTLSKDEPVLCEDHVSCSTPQIDTPITSNPPQVVKTVTLPSGMLLVGHAIQAPSRS
PAPQVDRVSKAIAAPTKSPAPQVDKVSIAVPTPSKSPATQGEKVAKAILVPPKSLAPQVG
KVAKTIPTPKQPAPLVDKVTSLDPVSKQMSSMSNSEAREAILPKKAAVLSVSQTPAISRP
SSAPLFQVPRSTLPPTPAVQVPPMLSRSMTLAGRSRNEPSPSVPSYTAQTYRNAIIGKSN
LDTASASLDHSTSFGQNVALSQPLSSYASAASAMVPPVGRNGQLPGKQGFMFGQGKSEAI
DNWNPWKGDSNANKYMWKDDSPYHQMTKGDAHTQSWRDNSYQQAGCSGTGEQGEFGGLQY
RQFQREIPTNLVSYQLPGPVGEEFPHLDIINDLLEEEQSSGSMAEPTLHGYHTLGLPYSS
RGNLVDSEVTSISSSGRLNLADHYYDEGYPMAYDRLNALYRLREGQNSTLDAYSNGRMDS
ITSKPWLHNFSNPAVNLGVNPNGFSQQMGNYTNLGSGRVNGEHLYRHANGQW*
>11682.m04115|LOC_Os05g43280.1|genepair682-2
MACAAATDDSAASTAGMRDEDRSLSGESLSEWRSCEQVESESPSTSPPFWDTDGDDDDPG
PKPSDLFGRYTWRIENFSKEKKREMKSEPFEAGGYKWYILVYPQGCDVSNHLSLFLCVAN
HDKLLPGWSHFAQFTIAVGNIDPKKVKYSDTLHKFWKKEHDWGWKKFMELSKIQDGFLVD
DVLEIIAQVQVIREKVDKPFRCLDRPYRRELLRVYTTNIESIYRRFVEERRNKLSKLIED
KMRWSSFCAFWSAIDPSTRHRMSREKTDVILKVLVKHFFVEKEVTSTLVMDSLYTGLKVL
EYQSKGKKGRTIADLDELPAPMIHVDMDMFVLASDVIDLLERAALEPLPCQPVSPKDDKC
SQSRMKDGASGEVNKISMEREERRLTELGRKILETFVLSHIFSGIEVAYQEAVALKRQEE
LIREEEEEAWLLGNEMKGKRGGGANEKDKRAKKKQAKQKKNNRKIKDKERDEKFEAKILE
RLHDETAIDDSDGLSSKQAEEVTTKVENLEEGASDRQGDLDSSEIAHRPDSGDKYPRQMN
GLSDVTGNAQKVKKASSMEANSPVFLADSVAASGTHSRGNNLSDSKNRMTPNRGKNQRNK
GISIISFSEEGEGIPSSSTGGSARCSSSCGTSAKLDQDTVLLTLKDKLRKLGQRLHEKNM
EGRKLLQAHFEAMEAKTSGSSPSSSPLDETPDVVKSPEQSAEGTTDAKANGTPNKDEPVT
NCVAEESVSVMPGTKSTEALSGMALAKTKVEPVSNKDHVPKPTLQANRASANCSKSTPVD
MEKDVPLPSRSPQINKPAPVPPKSPQVGNATPVPPKSPPIEKACPVPPKSPPSAKDTSLP
SVRSLQIDKPVPVPPRLPQVDKAASLSSELPQTSTTSNSEAQEETAAIRVASPSVSEVTV
TASRPSSAPVFPAPRSTVPATQVQVSTLLSRSMSEATRRSGNDPSPSAPAYIPQTYRNAI
IGKHGRGTTSGTTAYQSTSLGQGTALSQPLSTYAPTMSVTMPPAGRNDQFSGRHGLESGL
GKPEARDSWQPWNANRHVDKHLWRDDSTYQQTTNGHAYPQPWKDVNFLQARGTETEIPSR
FGGPQLPRQFQAETHADYLLQQPQGPVAEEFPHLDIINDLLEEEQSNGSMPESIGHDYHT
FGLPLPFLLRGNLADQEMASASSPGRFNLTEPYYDEGYSRAYDMSAFQGTRERQFPSLDA
YSNGLSDMSPSKPWLNGSPNPSMNHAVGTNGYPQQIPDYTNLASELNGASLYHRRYANGR
W*
>11667.m05623|LOC_Os01g56820.1|genepair683-1
MAAVELGACHKDNGRVEGLLVKKDREFVDKVLAVQKNRYFHDESVSDNDVRNIVLSYLMH
NCFKETAETFLSSTGLELPVDYTVDVDKRKAIFSFVLEGNALKAIDLTEELAPNLLENDM
DLHFDLLSLHFIELVRSRKCTEALEFGQKKLTPFGKVPKYVEKLEDFMALLAYEEPEKSP
MFHLLSPEYRQNVADSLNRAVLAHANRPAYSSLERVIQQATVVRQYLQQEVGKDSYPPFS
LKAFLNKDIVSLNQSAVHLLLCLMLGLYKLLSFKGFNGKNPILVGPWGGLGGTLWDDGVH
STVRQVVITHGAAIDSIKIEYDLKGKSVWSEKHGGDGGTKTDQVKLDYPQEILTSVSGYY
GSLGGCIVVRSLTFRSNLSKYGPFGSEEGTPFSLPVAVTGKVIGFHGKSGWFLDSIGCHF
KKEKNATPSSNAPSALRSITRPHDKNGNRYADSNAGYDMVLAVRDRGDSYSVLTSNNPKE
QYPNQSQDATLWNKMVSLPSFYSDNGTMTISTPVRFGPWGGNGGTIFDDGIYTGVRQINL
KRGLGISTMKVLYDRNGQAIWGDKRGSSGAARAEKVVFDFPSEILTHITGYFSSTMIMGS
TVIKSLTFHTTKKSHGPFGDETGTFFSSCLTEGRIVGFHGRDGWYIDSIGVHVLEGKVLS
QRADRALTETSPSRHADMLAVAQREIGDEVTYGVVKEPIPVGPGPWGGEGGKPWDDGVYT
GVKQIYIMRADFIGSVQIEYDRSGQSIWSTRHGNGGQITHRIKLDYPHEVLNCIYGYYNT
CQDEGPRVLRSITLVSNRGKYGPFGEEVGTYFSSATTEGKVVGFHGRSGLYLDAIGVHMQ
HWLGDRNRTAAPSSNKYYISKYLF*
>11682.m04110|LOC_Os05g43240.1|genepair683-2
MRLPACDSGWFPGVVCSGHPINWQSIKSSDGKSPIVVGPWGGTGGYPWDDGVYSTVRQVI
ITHGAAIDSIRIEYDLKGSSVWSETHGSTDGGSETDKVKLDFPDEILVSVSGYYGSVCGT
PVIIRSLTFQSNRSIYGPFGTEDGTPFSLPVSSGKIIGFHGRSGSYLNSIGFYLKQVNVS
DRSNSPVLPQSRSITSAYNKNGYSFPEGASGYDMVLAVRDRGDSYAVYTSNYPNQQYTNP
SPDYNDGIRWNKVPQTSPSLQMVSFPSGYGDRGGAALSSHETYGPWGGSGGTMFDDGMYT
GVWQINLTRAVGITSIKVLYDRNGQAVWGNKHGFSGAVSPDKIVFDFPSEVLTHITGYYG
TTMIMGPTVVRSLTFHTNKRRYGPYGDECGTYFSTSFSDGRIVGFHGREGWYIDGIGVHV
QEGNLAAPRVSSRSTIEMNPSLRYDMLAQSRSETYNEVPYSMVKEPVPMGPGPWGGEGGR
PWDDGVYTGVKQVYVMRGTFIGSIQIEYDRGDQSVWSARHGTSGHITHRIKLDYPHEVLT
CVYGYYNTNREEGPRVLRSITFISNRGKYGPFGEEFGAYFSSAKTEGKVVGFHGRSGQHL
DAIGVHMQHWMGDRRPAPKYVLSKYLF*
>11667.m04123|LOC_Os01g42690.1|genepair684-1
MSYGSSSSGLSLTSSLFSFFASSCLLSPPPACADERSNGMRAGGRGGRRVEYGRTYVVRP
KGRHQATIVWLHGLGDNGASWSQLLDSLPLPNIKWICPTAPTRPVAAFGGFPCTAWFDVE
DTSVDGRDDIEGLDASAAHVANLLSSEPSDVKLGIGGFSMGAAAALHSAACYAHGRFTNG
VAYPVTLSAVIGLSGWLPCSRTLKSKMDSSQTALRRAGALPILLSHGRADEVVTYRNGEK
SADFLRGSGFQYLNFKPYNGLGHYTIPEEMDDVCKWLSSRLGLDRSRG*
>11682.m04921|LOC_Os05g51050.1|genepair684-2
MSYYGSSSSGGRGGQRVEYGRTYVVRPKGRHQATIVWLHGLGDNGASWSQLLDSLSLPNI
KWICPTAATQPVTAFGGFPCTAWFDVEDTSVDGRDDIEGLDASAAHIANLLSSEPPDVKL
GIGGFSMGAAASLYSAACYAHGKFASGIPYPITLSAVISLSGWLPCSRTLRGKMESSHIA
ARRAASLPILLSHGRADEVVSYRNAERLGHYTIPEEMDDVGKWLSSRLGLDRSR*
>11667.m04135|LOC_Os01g42810.1|genepair685-1

MDHSAAAMANKPSLAVAAASRQRWALATSLCALLCLSLVVSAGLLLLGSTRPFRRPLFAA
PQQQREVVGEAPWERYVKLAQAASPGGARDRAPDLGGDEGAEGDDDDAISTAPAPAPSPA
AEEGGDEESCDLFQGRWVRDGAAAGGYPLYEAAECPFLSDQVTCRRNGRPDAEYEQWRWE
PRGCGGGGGGGGGSREAALALALEQCRNRRVVFVGDSLNRNMWESLACLLYTAVPDRSRS
RVLDVASDYRIFRAMDYNCSVEFFWSPFLVTLETKQDRTRALKLDQLPATLEKLRGADVL
VFNTGHWWTHTGNLRAWDHLEADGKPVEMGGEEAFNQALGTWASWVDQNVDSARTRVFFR
SISPEHKSENWCYNQTSPITDETKIVPWFPRGLVSIVERNIRSTRTPVTYLNITRLSELR
VDAHPSVYTITREGKPLSTEQRQQPLVYADCSHWCLPGLPDTWNLLLLASLARSPVNVH*
>11682.m04918|LOC_Os05g51020.1|genepair685-2
MATRKRLAGAALGCLSLFLLSRALLFSQDDPEPVKRPDEASSISLPPDRIAIIAAAPAPS
PATAAASDGSPAPAQDEVRCDLFDGSWVYDPAGYPLYDAGECPFLSDQVTCRRNGRPDSG
YEHWRWQPRRCAAALRLRGGEMLEQCRDKRVVLVGDSLNRNMWESLACILYAAAPDRSRA
TVDDASADHKIFQALDYNCTVEFYWSPFLVDLDDQTRVLKLDRLPATTYRRLAAADVLVF
NTGHWWTHTGKFRAWDHLERNGKKVEMGAEEAFNRALRTWTRWLDRNVDSHKTMVFFRSI
SPEHKNKNWCYNETAPMARAEEYVEAFPRGMVSIVERNVRRARTAVGYLDITRLSELRRD
AHPSVFTPSTRKRDVKKLVQEHSPCQPSTLQADAYTTGLSMKEIERRRKIGAANKGKVPW
TKGRKLSKEHKELIKRRTTEALRDPKVRKKMLGHRQLHRQASKDKIGAALRKIWERRMVA
VKARQEVLRIWSNSIAEAAKYGDYCQDKLDWDSYDRIKSEMISMFLWNKERERIMKKLEK
AEAKIVAKKLQAAERSKLQTRGIKKLQHQKLVLRKSDAQPTRVVVSTRPKLKERLTKWHD
RKKELETMISSRTRKGVGLRRSTPRRKAAERRAEVDLVEELRITCKDRLPREIHHQGETQ
P*
>11667.m04153|LOC_Os01g42960.1|genepair686-1
MSEADRIRVRAAALALDGGGGGAVRDKPDAKADVFADLGSPVSPLRARASVATSSSSSSG
SAKSPAPSNAGALALAGGRSHSGELTAESTPPRLPGHRRCGSGPLIFSGGSSGGSGGGGG
DRGSTASSPMTNALPAGNICPSGRVPVAAAAPPPPRSRPDVLGSGTGNYGHGSIMRGGGG
MAPARSSIDSSSFLGHAPRSPATFPAASSASSGSLQDVTRLGNEWYKKGKHAEALRHYDR
AVALCPESAACRGNRAAALAGLGRLADALRDCEEAVRLDPANGRAHSRLAGLCLRLGMIS
KARRHLTQAGHLHQSDPSEWEKLQEVEMHQGRSIDARKVGDWKSALREADAAIAAGADSS
RLLLAIRSEALLRLHKLEEADSTLASLLKLDSVLLYRMGANPSGMLAESYVSIVRAQVDM
ALGRFDAAVEAADNARFIDPGNAEVGMILNNVKLVAKARAQGNELYKAAKFSDASIAYSE
GLKYEPSNPVLYCNRAACWGKLERWEKAVDDCNEALRIQPNYTKALLRRASSYAKLERWA
DCVRDYEVLHKELPADTEVAEALFHAQVALKTTRGEDVSNMKFGGEVEMVTSVEQLRAAI
GSPGVSVVYFMSIMNQQCTLITPSVNSLCSECPSLNFLKVNVEDSPMVAKAENVRIVPTF
KIYKDGVKVKEMICPSLHVLRYSVRHYAVSSS*
>11682.m04913|LOC_Os05g50990.1|genepair686-2
MSGADRLSIRAAALALDDAAAGRDKPDTKRDVFADLGSPVSPLRLRPGGAAATPSSSSSS
AGSAKSPALCNAGAGVGRGGGGGRGSHSGELVAEGNPPRPPGHRRSGSGPLIFSGGSSSA
GSGGGGGGCGGGSTASSPLTNALPTGNICPSGRVASAAPAPPRRARPDVLGSGTGHYGHG
SIMRGGGGMTPPRSSIDASPYHGSYSRSPAPQGSSGGLQEVTRAGNEWYKKGHYGEALRH
YDQAVALCPDSAACRSNRAAALIGLGRLAEALRECEEAIRRDPASGRAHSRLAALCLRFG
MVERAREHFMLAGQVNQSDPAEFQRLQEVERHLGRCMDARKTGDWKSALREADAAIANGA
DSSQLLLALRSEALLRLNKLEEADSTITSLSKLDIASLSSMSTKLSGMVADSYVHVVEAQ
VNMAFGRFDIAVTMAEKARVIDPGNTEVGRITNNIRLVAQARGQGNELFKAGKFAEASLA
YGEGLKYEPSNPVLYCNRAACWSKLGRWMKAVEDCNEALKVHPGYTKALLRRAASYAKVS
DTCIIVFLIICALWC*
>11667.m04173|LOC_Os01g43100.1|genepair687-1
MAAHGGGGVEEDQAGSSSLCPPAAEAEAAAAAAAIARAARPPRPGRDKRLGVRHPLKHRR
FRAGGKAAVAAGAREVGEATTVAEATATGPPKGSDEDDEARYICGGWTRSSNLQLGLMLF
RLLIGTDLSFARLPSIVDNICSCIQACCDDGRMSCGYSSFRGRRANMEDFYDIKSSKVDD
NQINLFGIFDGHGGSHAAEHLKKHLFENLLKHPSFITDTKSAISETYRKTDSDFLDAETN
INREDGSTASTAIFVGNHIYVANVGDSRTVMSKAGKAIALSSDHKPNRKDERKRIENAGG
VVTWSGTWRVGGVLAMSRAFGNRFLKRFVVAEPEVQEQEIDDDLEFLILASDGLWDVVSN
EHAVAFVKAEEGPEAAARKLAEIAFARGSTDNITCIVVKFLHAKMAVDAASSSERS*
>11682.m04911|LOC_Os05g50970.1|genepair687-2
MVYDGAVKDQESSANPASASAALSEASAAASEVTAAAAAGAGAGAAEEGAAVSGRPPRPP
HDKRLGVRHPLKHRRFRAGGKVMVEPGDPPSAQEVADEEASEVEQEAAPVEREPPQEEGG
DVEVSSAPAEMEVVEGDAMEVSPEPAVAVGESELEGRPGEEEEVSSPVVSQGERKQETAA
AAPVPAVEEKKHKDQENKHKEREREKERERVDEVGYMSGGWKSEDGFLSCGYSSFRGKRA
SMEDFYDIKSSKIDDKQISLFGIFDGHGGSRAAEYLKEHLFENLMKHPEFMTNTKLAISE
TYKKTDSEFLDSESHTHRDDGSTASTAVLVGNHLYVANVGDSRAVISKAGKAIALSEDHK
PNRSDERKRIESAGGVVMWAGTWRVGGVLAMSRAFGNRLLKQFVVADPEIQEQEIDDELE
FLILASDGLWDVVPNEDAVSLVKIEEEPEAAARKLTETAFSRGSGDNITCIVVKFQHDKM
DGDSSPTSDKS*
>11667.m04179|LOC_Os01g43160.1|genepair688-1
MGRQRWWLLLAVAAAAAVVVGAGAQETCSDMVPAAPRRGAWMSLASFGGGGGDGRTLSTA
AFQAAVASIERRRAPGGALLYVPPGVWLTGPFNLTSHMTLFLARGAVIRATQDTSSWPLI
EPLPSYGRGRELPGGRYMSLIHGNGLQDVVITGDNGTIDGQGSAWWDMWKKGTLPFTRPH
LLELMNSSDVVVSNVVFQDSPFWNIHPVYCSNVVIRNVTVLAPHDSPNTDGIDPDSSSNV
CIEDCYISTGDDLIAIKSGWDEYGMAYGRPSSHITIRRITGSSPFAGFAVGSETSGGVEH
VLAEHLNFFSSGFGIHIKTNTGRGGFIRNVTVSDVTLDSVRYGLRIAGDVGGHPDDRYDR
NALPVVDGLTIKNVQGQNIREAGSIKGIATSAFSRICLSNVKLNGGAAVRPWKCEAVSGA
ALDVQPSPCTELTSTSGMSFCTNSL*
>11682.m04910|LOC_Os05g50960.1|genepair688-2
MVASLRLSLAAAAAAALAVAVALLLPPLAAAQGETTCPADVPPRGAWMSVASFGGVGDGR

ALNTAAFARAVARIERRRARGGALLYVPAGVWLTGPFNLTSHMTLFLARGAVIRATQDTS
SWPLIDPLPSYGRGRELPGGRYMSLIHGDGLQDVFITGENGTIDGQGSVWWDMWRKRTLP
FTRPHLLELISSTDVIISNVVFQDSPFWNIHPVYCSNVVITNVTVLAPHDSPNTDGIDPD
SSSNVCIEDSYISTGDDLISIKSGWDEYGIAFGRPSSGITIRRITGSGPFAGFAVGSETS
GGVENVHVEHLNFFGMGVGIHVKTNSGRGGFIRNITVSEVTLNGARYGLRIAGDVGGHPD
ASYDPSKLPVVDGVTIKNVWGQNIRQAGLVRGIRDSVFSRICLSNVKLYGGDSVGPWKCR
AVSGGALDVQPSPCAELTSTSEMSFCTN*
>11667.m04197|LOC_Os01g43320.1|genepair689-1
MAHAAFDAEAGAAAKPPAADAGAAFVLESKGTWWHAGFHLTTAIVGPTVLTLPYALRGMG
WALGLTVLTAVGAVTFYEYSLMSRVLEHCEARGRRHIRFRELAADVLGSGWMFYFVVIVQ
TAINTGVSIGTILLAADCLEIMYTSLSPNGPLKLYHFIIIVAVALAFLSQLPSFHSLRHI
NFASLLLSLGYTILVSAACIGAGLSKDAPGKDYTLSSSKSEQTFNAFLSISILASVYGNG
ILPEIQATLAPPAAGKMMKALVLCYSVIAFAFYIPSITGYWAFGSHVQSNVLKSLMPDTG
PALAPTWLLGLAVLFVLLQLLAIGLVYSQVAYEIMEKSSADATRGKFSRRNVVPRLLLRT
LYLAFCAFMAAMLPFFGDIVGVVGAVGFIPLDFVLPVVMYNIALAPPRRSPMFLANTAIM
VVFSGVGAIGAFASIRKLVLDAGQFKLFSNNVVD*
>11682.m04906|LOC_Os05g50920.1|genepair689-2
MAGTGGSPLLRSPSPVMNTMPTPPSAAVFDVEAASGARRLGIKPAADAGAAFVLESKGKW
WHAGFHLTTAIVGPTVLTLPYALRGMGWALGLVALTAVAAVTFYAYYLMSRVLDHCEAHG
RRHIRFRELAADVLGSGWVFYLVVTVQTAINAGITIGSILLAADCLQIMYSDLAPNGPLK
LYHFIIVVAVVLSLLSQLPSFHSLRYINLGSLLLSFGYTILVSAACIRAGALSDVPEKDY
SLSSSNSEKTFNAFLSISILASVFGNGILPEIQATLAPPAAGKMMKALVLCYTVVLFTFY
LPAITGYWAFGSQVQSNVLQSLMPDKGPSLAPTWLLGLAVVLVLLQLLAIALVYSQVAYE
IMEKSSADAARGRFSRRNVAPRVALRTAYVAACAFVAAMLPFFGDIVGVVGAVGFIPLDF
VLPVVMYNMALAPPRRSPVYLANVAIMVVFTGVGLIGAVASVRKLVLDAGQFKLFSGNVV
D*
>11667.m04198|LOC_Os01g43330.1|genepair690-1
MGSDLKEMKYRRRIGLDERAQCSEQRALDWHALKQDPVELLRKLDELREQITRTCQIVEP
PREHRRAGRRALSLLPENPEPPPMPGYHRSRYGGGRYGHGLPPSPYEPLRPEIGERYSRQ
SSGRYRQYQGRQWDGCGVGHGNYNPSYTCSCPHCLHGQRTAPQEEHIPMARYFAGQHECY
RFERSPSVSSDYDRRSVASSLYSHRSVSKKRAEYFRKKAEHLCRPVYGAAPFVVCSSCYQ
LLQVPMEKCMGRNRLQCGSCSQIVSLKREEKVIPFSPSASFCVPKIEQGSNDQTRRDFEH
QLNEFANSAFYNLNEHSSMQINIDFGDDHSVSSSISHDRTEKGCGSSRSIQLKTDGLLLS
PSRSGDIESPKDILCERDAECQVEPSDARVSPCSPVLEDKLVDPLCSQEKDNNSEDLGMA
NISDVNCKGEHKVNDDDDGSLSMGSEQKRKECDEDSLVDESMCKTHEQKSKDDHSSPEDV
SKTHEFDSTKDNISSAVDGNEKHEFESKKDDTNSLEGESLNKEHEQKSKEDENSGLEGEN
VKKGFDKNNKESENSALEDANAPLEDTRNASDAASLSEISEEKKTEEENGSLDQPFVEDG
NAFAESGGSSFNERTNSGFSRGSSETALEEDQPSTGKSGDSSFFAGFLKKGFKDLSLFNQ
SMDSVKVSINGHPISERALKKAEKKAGPVEPGSYWYDYRAGFWGVMGRECIGIIPPFIRE
FNYPMASNCASGDSGVFVNGRELHQRDLDLLVGRGLPRISGKSYSVEISGNITDEETGKK
LRSLGKLAPTIHLAHDHNHAFSEIKHLIYLKSIMSVNTIDIKDISPF*
>11682.m04905|LOC_Os05g50910.1|genepair690-2
MGSEAKEMKYRRRARVPEPFDYGQCGGDRSGVLDWGALKENPVELLRKLDELRDHITRSC
EITDQPRERHRMSRRTASLRPSHAEPPPLGRGPEHYRSRYTGRYGSGFPHSPNDQLHRSM
HRDRYERQPSGRFRQWPERQWENSGYLGGNHHQSTCQCAQCLHGQRAVMQEEHIPMTRYF
AGQQGSHLFDRSPSVSSELDRRSVASSLYSHFSVSKRRTEFFRKKAESFCRPVRGAAPFV
VCSSCNQLLQLPPGKCTARKQIQVRCGSCSEIVSFKLKEVKIHPLVAPTSFPASKTVGSS
SRQVNKSFGWYQHQDEGNSSFHKLQAQERWQQNKDLADNISVSSTSSYDRIDKECGSNRS
SQLLSVSVRRSRLANIPKDILCQGDAYSQVETSAFNTGNLQAPVIEDKCVDPFSSRLKDC
SGGDRTSKECSLNIMADSVDANVRDERSDVTYEQNSKDHKEGFGEETVSSRHEQKLKEST
SGFCDDGSMGNIDKLTADNDDTSSLEDGDVSKKYEEKIKQDDNNFQSEFITERYSKCSKE
DNNSVIQVETIATICKQDDLDDCYSELLSPNSEHAIMPSKLESSVNERTNSSSRVSSEAE
LDEVQSAATKNGDSKFFAGFLKKGLKDISLFNQSVDSAKVSINGHSISERVLRKAEKKSG
PVGPGSYWYDYRAGFWGVMGHECSGIIPPFIKEFNYPMPKNCAGGNTGVIVNGRELHQKD
FELLAGRGLPRISGKSYSVEINGNVIDETTGKKLRKLGKLAPT*
>11667.m04200|LOC_Os01g43350.1|genepair691-1
MEQLRQLGEAVGSINALMAFEDDLHINPRQCRLLADACARALAAVTGQVRAQLRFDERGA
KWRAIEAPLRELHRAFRDAEAYVRQCLDPRGSWWARAAAMAHGTECVEQHLHNVLWCVAV
ALEAIDAAGEIAGSDPDELARGRLVLARKYDRDMLDPKLFEHAFGKLYLVSQELVARMDM
AWKEDRWVISQMFDEMKGPAASKPLSKNEHRLAELLAAAMGKLHPASVLLGSDYSVRRRL
GGRLKEVHWMGESFAMKHFIGDTDAAGAEVALLCSVAHPNVAHAAYCFHDEEKKEYFVVM
DQLMAKDLGSYVKEVSCPRRRIPFPLVVAVDIMLQIARGMEYLHAKRINHGELNPSNVLV
KPRQPDGGYVHVKVAGYGQPAGITAGGAKASANGNANGNDNSCIWYAPEVLRSDGVADAA
AAGRCTEKADVYSFAMICFELLTGKVPFEDNHLQGDKTSKNICAGERPLFPFQAPKYLTA
LTKRCWHADPAQRLAFASICRVLRYVKRFLILNPEQQQQQQQGQTDDAPKPAVDYLDIEA
QLLKKLPAWQRGGEAPRVADVPFQMFAYRVMEREKAAGAVHVAKDRASDSGSDGNSLYGD
ENGFGAMSPEHTFSAVSNGTLRSRPASSDGRLPTAKKADGKAPRQAGPQPKVKPVNTAAR
TPHSARRALGVKPDDHLQTNGAPTARRRTPEMASE*
>11682.m04897|LOC_Os05g50830.1|genepair691-2
MEQLRQVGEAIGGVNALMAFHDDLRCINPRQCALLAHAYALAFRAVAGELRARLRFHDRL
TKWKPLDDPLRELHRVVRDGEAYIRHCLLLDPAHWWARAAAATHGTECVEHHLHNLLWCV
SVVVEAVENVGEVTGSDPDELARRRLALARDYDKDLLDPKLFRERLGETFLATRELAARM
DMAWKEDRWLLSQLLDERKGPTSSPEPPLTRQEHRLADLLAAPRGKLHPASVLLMSDFHM
RRRLGGNGNLKEVQWLGEAFAVKHVVGVDAEAAAAEVAALASVSPHPNVAHCRYCFHDEE

KRELYMVMDQLMSKDLGSYVKEVNSAKRRAPLPLVVVVDTMLQIACGMAHLHSNKMYHGN
LNPSNVIVKPRHGDAYLHVKVAGFVSGSGTANAANPCIWCAPEVVGNEAAATEKGDVYSF
GMICFELITGKIPFEDNHLQGENMSKNIRAGERPLFPFQSPKYLTSLTRRCWHGEAAQRP
PFHSICRVLRYVKRFLVMNNPEQAAADAAGAGPAVDYLDMEAQLLRRFPEWEGNGVADVP
FEMYAYRVMERDKMSNACRDRSSDSGSDGNSLWGDDSASGGSSTTATDASASSRPLLDRS
GSTRSSPPRKVAIAAAKAGKCRSGIVTRLKPSSKITASSMSVTCAGPPQKSRSMGTVRPP
PVVARRTPRIKSDGHLNRAAIPPTRRRKSGGNASDSELA*
>11667.m04208|LOC_Os01g43410.1|genepair692-1
MGNRTSRHHRAAPEQPPPQPKPKPQPQQQQQWPRPQQPTPPPAAAPDAAMGRVLGRPME
DVRATYTFGRELGRGQFGVTYLVTHKATGKRFACKSIATRKLAHRDDIEDVRREVQIMHH
LTGHRNIVELRGAYEDRHSVNLIMELCEGGELFDRIIARGHYSERAAAALCREIVAVVHS
CHSMGVFHRDLKPENFLFLSKSEDSPLKATDFGLSVFFKPGEHFKDLVGSAYYVAPEVLK
RNYGAEADIWSAGVILYILLSGVPPFWAESEDGIFDAVLRGHIDFSSEPWPSISNGAKDL
VKKMLRQDPKERLTSAEILNHPWIREDGEAPDKPLDITVISRMKQFRAMNKLKKVALKVV
AENLSDEEITGLKEMFRSLDTDNSGTITLEELRSGLPKLGTKISESEIRQLMEAADVDGN
GTIDYAEFISATMHMNRLEKEDHILKAFEYFDKDHSGYITVDELEEALKKYDMGDDKTIK
EIIAEVDTDHDGRINYQEFVAMMRNNNPEIAPNRRRMF*
>11682.m04895|LOC_Os05g50810.1|genepair692-2
MGARASRHRQSPDQSQSQSPSPHHKHHHHHQTTRAPKPKPKPQPPPPQQPRSQPPPPPRH
QPQQAPQQAAAEDGVGRVLGRPMEDVRATYTFGRELGRGQFGVTYLATHKPTGRRYACKS
IAARKLARPDDLDDVRREVHIMHHLTGHRNIVELRGAYEDRHSVNLVMELCEGGELFDRI
IARGHYSERAAAALCREIVSVVHSCHSMGVMHRDLKPENFLFLNKREDSPLKATDFGLSV
FFKPGEQFRDLVGSAYYVAPEVLKRLYGAEADIWSAGVILYILLSGVPPFWAENEDGIFD
AVLQGHIDFSSEPWPSISSGAKDLVKRMLRQDPKERLTAAEILNHPWIREDGEAPDKPLD
ITVISRMKQFRAMNKLKKVALKVVAENLSEEEIVGLKEMFKSLDTDNSGTITLEELRAGL
PKLGTKISESELRQLMEAADVDGNGSIDYVEFISATMHMNRLEKEDHIYKAFEYFDKDHS
GFITVDELEEALTKYDMGDEATIKEIIAEVDTDHDGRINYQEFVAMMKNNSPEIVPNRRR
MF*
>11667.m04209|LOC_Os01g43420.1|genepair693-1
MQQQQAWAAGLGGLAVAGVGEEGGGGGPAPTTVDEASMERSKSFVKALQELKNLRPQLYS
ASEYCEKSYLHSEQKQMVLDNLKDYAVRALVNAVDHLGTVAYKLTDLYEQQASEVSTLEL
KVACLNQQVLTCQTYTDKEGIRQQQMTGTATRHHKHYIVPTLANKRMQAFSEMQTDADID
SRPRPYPSAKTLFWHLASEKNSKTNGARQSEFVLEETKATKPASRGKEPSTSPLPKHLQT
NLASSDFAMHNVGMKDQPGVRHLSSFSSFDNPRGRQIQKAPLRTKSMLAAFFVKHKSGKM
KNVSVR*
>11682.m04894|LOC_Os05g50800.1|genepair693-2
MQPLRPSPAAGGWAGVAGVGPTTVDEASMERSKSFVKALQELKNLRPQLYSASEYCEKSY
LHSEQKQMVLENLKDYAVRAVVNAVDHLGTVAYKLTDLFEQQASEVSTVELKVARLNQQI
LTCQIFTDRAGLRQQKIGGTTFKHHKHYILPSKTLSWHLSSENSISTTGAQKYTFTLGDT
ISSKPASNGSMYLLGKDIPASPMHKPLQPNGNTSFDAKKNVGSKVSISGAIPVDEQNVVI
LHQRPISSMLLLCYSASKKPRLSADNKIHIRNGAAGL*
>11667.m04213|LOC_Os01g43460.1|genepair694-1
MLNACREVERNPGFVNGMEFGNDVRIRMDGGVYGESEQSSGEAALPRLLIEVPSQVIDGF
DCVGGGGDATATATLSEQSKELEMLGEEKDVVISIPAPVYAPRSVSVSAAYEHEGAQIPY
SVSLSMPASPSGFHFSQFGMAAAKAKAVHRDEARVAPAETRFDDAHPPAVGRVQAHSPRL
LLNQTRFHSQPILHLSKNDETTRRCDSTRDKRFDQFKTFSGRLERQLSTLRGRPAQEHMT
NGEGAPEPNIAEEETEQVPGADRYFDALEGPELETLRATETTVLPKDEKWPFLLRFPISA
FGMCLGVSSQAMLWKTLASAPPTSFLHVSPVVNHVLWWISLALMGFVSFIYLLKVVFYFE
AVRREFYHPIRANFFFAPWIACLFLVQGVPRPVTEVHHGVWYALMAPIFCLELKIYGQWM
SGGQRRLSKVANPSNHLSIVGNFVGALLGAKMGLREGPIFYFAVGLAHYMVLFVTLYQRL
PTNVTLPKELHPVFFLFVAAPSVASMAWAKILGEFDYGARIAYFIALFLYMSLAVRINFF
RGFRFSLAWWAYTFPMTGAAIATITYATEVTNVLTRALSIGLSGIATVTVAGLLVTTMFH
AFVLKDLFPNDVSIAITRKKPKFSKILAHFRSSSSDMKEYVLSISKPPSSDSDSSVSSKA
TTVTDPSVTRVKAEP*
>11682.m04891|LOC_Os05g50770.1|genepair694-2
MASTDSAQVAAGWPAAAREHEACPPPLVVGAPSHDADAQPEEEIPYSVSFSVPASPSGMH
LGASVVRVHAAPPSVGEARIDMIHPAEPPPQMLWQQARFHSQPTLTVINGEAAAPVPRSD
STRDRRFDQFKTFSGRLERQFSSLRGMLPQEPAADIETADSKISEEEADGGEVPTADRYF
AALEGPELDTLRTNEDVGVQATEVPVLPEDERWPFLLRFPISAFGMCLGVSSQAMLWKTL
ASEPSTAFLHISLDVNHVLWWVSVALMALVSAIYLLKVVFYFEAVRREFHHPIRVNFFFA
PWIACLFLVKGLPRQVWTIHHVVWFLLMAPILLLDLKIYGQWMSGGERRLSKVANPSNHL
AIVGNFVGALLGARMGLREGPIFFLAVGLVHYIVLFVTLYQRLPTNVQLPKELHPVFFLF
IAAPSVASMAWARLTGEFDFGARIAYFVALFLYMSLAVRVNMFRGFRFSLAWWAYTFPMT
SAAIATVLYASEVTNVATRAMAVGLSGIATVTVTGVLVTTMYHAFVRRDLFPNDVSIAIT
RRKPKFSKILAHLRSSGTDVKELVFSVSSKNGADDSASVSKASNCSSGDQSPVPHAGAGR
GR*
>11667.m04215|LOC_Os01g43480.1|genepair695-1
MEHGSIIASAVGVGVGVGVGIGLVSSRLTGLATGGGATAAEVEAELRCLVVDGRDVGVSF
DDFPYYLSEQSKLALTSTAFVHLSPTILPNHIRVLSASSRTILLCGPSEAYLQSLAKALA
NQFSARLLLLDVIDFACKLHHKYGGPSNTQTRERSMTEAAFDRVSSLVGAFNLFRKKEEP
TGTGPLSRETGILDLRTSTCCPHNTPSVRVQLSLVPPEKDHDPESSKYLASVKPCWSLNE
KVLIQSLYKIIVSASEISPVILYIRDVDDLLGSSEKAYCMFQKMLKKLSGRVIVIGSQFL
DDDEDREDIEESVCALFPCILETKPPKDKVLLEKWKTQMEEDSNNNNNQVVQNYIAEVLA
ENNLECEDLSSINADDDCKIIVAYLEEIITPSVSYHLMNNKNPKYRNGNLVISSESLSHG

LRIFQESNDLGKDTVEAKDETEMVVPDNEYEKKIRPTVIPANEIGVTFDDIGALADIKEC
LHELVMLPLQRPDFFKGGLLKPCKGVLLFGPPGTGKTMLAKALANAAGASFLNISMASMT
SKWYGESEKCIQALFSLAAKLAPAIIFIDEVDSMLGKRDNHSENEASRRVKNEFMAHWDG
LLSKSNERILVLAATNRPFDLDDAVIRRFEHRIMVGLPTLESRELILKTLLSKETVENID
FKELAKMTEGYTSSDLKNICVTAAYHPVRELLQKEKNKVKKETAPETKQEPKEKTKIQEN
GTKSSDSKTEKDKLDNKEGKKDKPADKKDKSDKGDAGETTLRPLNMEDLRKAKDEVAASF
ASEGVVMNQIKEWNELYGKGGSRKREQLTYFL*
>11682.m04888|LOC_Os05g50750.1|genepair695-2
MEQRNLFVSALSVGVGVGLGLASARWAAPGSGEGGGGAGIGVAELEAELRRLVVDGREGD
VTFDEFRYYLSERTKEVLISAAFVHLKQADLSKHIRNLCAASRAILLSGPTEPYLQSLAR
ALSHYYKAQLLILDVTDFSLRIQSKYGSSSKGLAQSQSISETTFGRMSDLIGSFTIFPKS
AEPRESLQRQTSSADVRSRGSEASSNAPPLRKNASMSSDISDVSSQCSAHSVSARRTSSW
CFDEKVLIQSLYKVMVSVAENNPVILYIRDVDQLLHRSQRTYSLFQKMLAKLTGQVLILG
SRLLDSDSDHTDVDERVSSLFPFHVDIKPPEEETHLDSWKTQMEEDTKKIQIQDNRNHII
EVLSANDLDCDDLSSICQADTMVLSNYIEEIIVSAVSYHMIHNKDPEYKNGKLVLSSKSL
SHGLSIFQESGFGGKETLKLEDDLKGATGPKKSETEKSATVPLKDGDGPLPPPKPEIPDN
EFEKRIRPEVIPASEIGVTFDDIGALADIKESLQELVMLPLRRPDLFKGGLLKPCRGILL
FGPPGTGKTMLAKAIANDAGASFINVSMSTITSKWFGEDEKNVRALFSLAAKVAPTIIFV
DEVDSMLGQRARCGEHEAMRKIKNEFMSHWDGLLSKSGERILVLAATNRPFDLDEAIIRR
FERRIMVGLPTLDSRELILRTLLSKEKVAEDIDYKELATMTEGYSGSDLKNLCVTAAYRP
VRELLKREREKEMERRANEAKEKAATAENSESPESKKEKENSENPESKEKEKERKENSEN
KEEKTENKQDNSKAEGGTEGTIDLRPLTMEDLRQAKNQVAASFATEGAVMNELKQWNDLY
GEGGSRKKQQLTYFL*
>11667.m04219|LOC_Os01g43510.1|genepair696-1
MKVLRLLAARRFRRRAVSTITATAPATPCGGGGWYGEDEGPFFDLDLSCCSASAPASSAE
SGSESEDYSSCAGESDFVISLQRSRSASPSYEERVFYVGGGSGWARAAAAAPAHLKFCAS
EPSDAASRYGSGRRGKLRTLSFGSAKAAFYGGRASFSRSSNSARSARLFAAFGYGSPDQG
DEANRRTPSRDVIRRYLSKISRQLRRVAPSAGADLRLRKSRSASAAQTAACQSPPPRRDD
SLLEQQDGIASAIAHCKESLHRASMSEFDSPLLRSRSDPRS*
>11682.m04886|LOC_Os05g50730.1|genepair696-2
MHIFRLLAARRRAVSTITAAAAAAPAASSDAGRDSEDEGPFFDLDFSSVRGASSSGSMSG
SDDSDDDCTDLDFIISLHRSRSASPSYDTLFFAAAASEPSTKASFQPSHHFCAKRRGGGG
LRTLSFGAKKAAFYGGRHSFARSSCSARSLRLFMESPADDDGEEVAEPRRAPPSRDVIRR
YLTKISRRFRRTAPRAAGEARGLRRLRKSRSASAATSLSASSSAAAAPSGRDDSLLEKQD
GIASAIAHCKESLHRASVSECDSSLSRSRSDPGKREADQSCH*
>11667.m04221|LOC_Os01g43530.1|genepair697-1
MSAAEERGDRQERDDGEGGEEERRERGGVGGLVDRAKGFVAEKIAKIPKPDATLDRVSFK
GVSRECITLHSHLDVNNPYSHRIPICELTYTFKSGDKVIASGTMPDPGWIAASGTTNLEL
PVKVPYDFIVSLIKDVSGDWDIDYVLEVGITIDLPVVGSFTIPLATEGEMKLPTFRDLLF
*
>11682.m04884|LOC_Os05g50710.1|genepair697-2
MSSLMDKAKGFVAEKIAHIPKPEASLDSLSFKGMSRECITVHSNVNVSNPYDHRLPICEL
TYTLKCAGNVVASGTMPDPGWIAASDTTKLEIPAKIPYDFLISLVKDVGRDWDIDYQLDV
GLTIDLPIVGNFTIPLSTSGEMKLPTLKDMF*
>11667.m04223|LOC_Os01g43550.1|genepair698-1
MRGPLLLRAVVVVAMEHFNDWDLQAVVRSCSFPQSEPPRVGVGVPAAPGAGGAPVVVAPP
ARAPDGPDQMARASASALYDLEYLDLDHKPFLLPGSSSSSSSSRAVARARGEDDGKGRHE
VMISFPAAAAASTSGAQPRSPSGRKPGIRTPRPKRSKKSQLKKVVYEVPVADGGVSSDLW
AWRKYGQKPIKGSPYPRGYYKCSSMKGCMARKMVERSPAKPGMLVVTYMAEHCHPVPTQL
NALAGTTRHKSAPTGDDDKPTSPGPAAGRAAAGEGVVKCEDVDGNELSAMAADGGAEDTA
AAADDDGELWPEGMGLELDEFLGPMDDDVFEFDHVLEDDGVLGRRLSL*
>11682.m04883|LOC_Os05g50700.1|genepair698-2
MEYSNDWDLQALVRSCGTAVADSEPEPPAAPSTTRRAEAETVFVGRAGGVPEFVGQPVRS
SAASFYDLEYLDLYHERPRAPFLVTAPSTSRERGEGGEHEVLISFPAIASTSGQGRKQPG
RKPGVRTARPKRSKKSQLKKVVCEVPVADGGVSTDLWAWRKYGQKPIKGSPYPRGYYKCS
SLKACMARKMVERSPEKPGVLVITYIAEHCHAVPTQLNSLAGTTRNNKPASPDQQQQQQP
SPGGASTDEAAAAAAKTEDSADTTCSMADDENDLWAPVEMDMNDFFGPFDDDLDHFLDDD
AVLGRRLSL*
>11667.m04230|LOC_Os01g43610.1|genepair699-1
MHTPFVDKSPHRRLDGLGAGGWLKQRLAQILVRSTCTTNTTTTTTTTSSTAFVSLDKTNA
DTHQEPPPPPSPYFCTPCTYQRPKLDAPGRPRRRRRSASLVHISIDCTGGAGATSGRRSV
HSDAPLLPYSSSLPTKDGRKQSRARRKARSSPSTSRRHCPSSSWGRARLPRGAPGQYSSS
SSTVTDDELAPFSTDEEGGEEADTRTLFSSLSFSSDSTSEFYHTNSSSSLARKGHKNAPA
RRPPARRASARITSDPADAFRPVVSVAATKKQHNDMKKKEETAIKRQLGTDDDTAAGAGM
AVVKRSSNPYADFRSSMVEMVVERRICSVPEMEELLGSYLSLNSPQHHPAILAAFEDVWE
AVFGEE*
>11682.m04881|LOC_Os05g50680.1|genepair699-2
MDMSKARRHGVGVGVRLRQRLSQILLHSSCTTTSATAFVTNVAVAAGNAAAARQAPPPAA
ANDAHQPRPKIDGSVRRRRRRSARALVHISIDCSGPTSARSVGAAVMPSPVAPAKDVKAV
IRSKARGGRPRSPSYSCSSSTVTDDELPPFSSSDGEGGEGAETRSSTLFSSLSISSDSTS
DFYNSTGGGSKRHHKNPPRRVPRRAPPRGANAGDAKPHEDNKGGAKKADDKHGGGVVGVA
AAGSMAVVKRSHNPYADFRSSMVEMVVERRICGADAMGDLLMSYLSLNSRRHHPAILAAF
EDVWEAVFATP*
>11667.m04258|LOC_Os01g43890.1|genepair700-1

MASFLSRAAAVSLLAIVLVLTAATSTSPAAAAARFPEEARPTRSGYLNVTSTNSLYFAFY
EATDPVTTQPAAVPLLVWLQGGPGCSSLIGSFAELGPYLLLDSTSALARNDNRWNRRFGV
IFIDNPLGAGFSAPASGDDIPTDERTIAAHLLAALQSFMALDPAFRARPLFLTGESYAGK
YIPAAASHILDANAKLTDDRRVNLQGIAIGNGMTHPVAQVTVHADQAYFAGLINAEQKAK
VEEMQDKTVSLIKSKKWAAARRERNRIIAFLKNATGVATPFNYAREKGYPTRPLRDFLNT
GEAKAALGARSDVEWARCSEAVSAALADDIMRSARGDVEAVFLAPDGVRVLLFQGVFDLH
SGPASVEAWVRELAWPGLGAFLAAERAVWRLGDEQLAGYVQRSGALANAVIVGAGHMAAG
DNRPAAQAMIEGWVLQTGPFDGSGQRVVGSLF*
>11682.m04873|LOC_Os05g50600.1|genepair700-2
MAPPLLLVSLLLIGFVSARAITPSAEAAAVFPKEALPTNSGYLPITTTNASLFFAYYEAT
HPLTPPASTPLLLWLQGGPGCSGLAGNFFELGPYFVNRDALSLSPNPFSWNRRFGLLFID
NPLGTGFSAAPSPAAIPTNQSVVAAHLFAALQSFFALQPGSRSRPFFLTGESYAGKYIPA
AGSYILAVNPTLPTRLRVNLHGVAIGNGLTHPVAQVATHADTAYFMGLINAKQKRELEAL
QARAVELTNAARWSEAADARGLVLSWLENATGLATLFDAAKKRPYETGPVGKFVNRAEVK
AALGARGDVEWEECSDAVGAAMHGDVMKSVKPEVEALLRGTRVLLYQGIRDLRDGVVSTE
AWMRELEWDGLPAFLDADRAVWRIGEELAGYVQRSGPLSHVVVYGAGHLMPADNGRAAQE
MIEDWVLQAGLFGRHGGMKRAA*
>11667.m04260|LOC_Os01g43910.1|genepair701-1
MQQDQRKKSSTEADFFTEYGDASRYKIQEVIGKGSYGVVCSAIDVHTGEKVAIKKIHDIF
EHISDAARILREIKLLRLLRHPDIVEIKHIMLPPSRRDFKDIYVVFELMESDLHQVIKAN
DDLTKEHYQFFLYQLLRALKYIHTANVYHRDLKPKNILANSNCKLKICDFGLARVAFNDT
PTTIFWTDYVATRWYRAPELCGSFFSKYTPAIDIWSIGCIFAEVLTGKPLFPGKNVVHQL
DLMTDLLGTPSMDTISRVRNDKARRYLSSMRKKEPILFSQKFPSADPLALDLLQKLLAFD
PKDRPTAEEALAHPYFKGLAKVEREPSCQPITKMEFEFERRRVTKEDIRELIFREILEYH
PQLLKDYINGTERTTFLYPSAVDQFRKQFAHLEENGGNGPVIPMDRKHTSLPRSTIVHST
PIPAKEQPRIGPSRDKPSDEPYSNPREFDRFSGNAPRTSQAPQRVPTARPGRVVGPVLPY
ENGATKDSYDARRLAMNSGYPPQQQIPQAYGYYQIPGKSACSELSQAERYTLHQQAYTCA
NSATVTDVALDMRAPPFHLSGGPKSDSSERLAAETNLYTRSLNGLAATAAGVAASAHRKV
GVVPYGMSRMY*
>11682.m04869|LOC_Os05g50560.1|genepair701-2
MAVKMRIGRRRAIQQGIAEGGFEWRRVWGCREADSRGALWELVWGERSVRERNAAGAAEE
VIALFIMDEMCDPASNLEYVVEKAKCDVHRTSSAEEFFTEYGDANRYRIQEVIGKGSYGV
VCSAIDLHTRQKVAIKKVHNIFEHVSDAARILREIKLLRLLRHPDIVEIKHIMLPPSRRD
FKDIYVVFELMESDLHQVIKANDDLTKEHYQFFLYQLLRALKYIHTASVYHRDLKPKNIL
ANSNCKLKICDFGLARVAFNDTPTTVFWTDYVATRWYRAPELCGSFFSKYTPAIDIWSIG
CIFAEVLTGKPLFPGKNVVHQLDLMTDLLGTPSMDTISRVRNEKARRYLSSMRKKDPVPF
SQKFPNADPLALKLLQRLLAFDPKDRPTAEEALTDPYFKGLSKIDREPSCQPIRKLEFEF
EQKKLSKEDIRELIFQEILEYHPQLQKNYRNGRERATFLYPSAVDQFKKQFSNLEESNGS
GSAIPMERKHASLPRSTTVHSTPIPPKEQPLAASLKSSRPVSDEPCKNPWVMGGFSGNIP
TSSQVSQVAKPVAPGRPVGSVFPYETGSTNDPYGPRGPVMSSGYPPQQQISQAYGYHQVP
ARMNCVEQSQAMDAYKMHSQSQTQAYAYPNSKVTADVALDMRGSTFHHSAGSKNGSLDRM
VTQTDIYTRSLNGIVAAATSAGVGTNRKVGAVPISTSRMY*
>11667.m04273|LOC_Os01g44040.1|genepair702-1
MEVFGPVTAGQVSFLLGLFPVLIAWIYSEVLEYRKSSSMKVHSDSNLENGTVKEDDKTVL
LEGGLSKSPSTKFRINSTKANLIRFITMDESFLLENRAVLRAMAEFGIVLVYFYICDRTN
IFPESKKSYNRDLFLFLYILLIIASALTSLKKHHDKSAFSGKSILYLNRHQTEEWKGWMQ
VLFLMYHYFAATEIYNAIRVFIAAYVWMTGFGNFSYYYIKKDFSLARFAQMMWRLNFFVA
FCCIVLDNDYMLYYICPMHTLFTLMVYGSLGLFNKYNEIPSVMAMKIVSCFLAVILIWEI
PGVFELLWSPFTFLLGYKDPEPSKANLPLLHEWHFRSGLDRYIWIIGMIYAYFHPNSLCP
SALLHHCLCRMQFIMLWFQVERWMEKLEESETKVRLSIKGTIISISLVAGYLWYEYIYKL
DKITYNKYHPYTSWIPITWLGKITLETYISQIHIWLRSNMPNGQPKWLLSFIPGYPLLNF
MLATAIYLLISYRVFELTGVLKSAFIPSRDNNRLYQNFVAGIAISVCLYFLSIVLLKIPI
V*
>11682.m04864|LOC_Os05g50510.1|genepair702-2
MEVFGPVTPGQVSFLLGLFPVLIGWIYAEILEYRKSLLYGKVHSDANLENETMKEDDKAV
LLEGGQSKSPSTKLRNMSTKANLIRFITMDESFLLENRAVLRAMAEVGIILVYFYICDRT
NIFPETKKSYNRDLFLFLYILLIIASALTSLKKHNEKSAFTGKSILYLNRHQTEEWKGWM
QVLFLMYHYFAATEIYNAIRVFIAAYVWMTGFGNFSYYYIKKDFSIARFAQMMWRLNFFV
AFCCIVLDNDYMLYYICPMHTLFTLMVYGSLGLFNKYNEKPSVMAIKIACCFLTVILIWE
IPGVFEFLWAPFTFLLGYKDPEPSKANLPLLHEWHFRSGLDRYIWIIGMIYAYFHPNVER
WMEKLEESETKVRLFIKGAIVTLSLTAGYLWYEYIYRLDKITYNKYHPYTSWIPITVYIC
LRNCTQQLRSASLALFAWLGKITLETYISQIHIWLRSSTPNGQPKWLLSFVPDYPLLNFM
LTTAIYLLLSYRVFEITGVLKGAFIPSRDNNRLYQNFIAGIAISACLYFCSLILVKITIV
*
>11667.m04279|LOC_Os01g44090.2|genepair703-1
MFCVDFFHMKICGLTCKSIPYIYGFFFQPYQFRLIATGAEHHGAEAAGDASARGASARRG
LATVSVAKPSYPTVTTPMSASTSPSTMPMSSSPSAFPSLATAGGGGGGGGGGSWCVASQS
ASPTALQVALDYACGYGADCSAIQPGGSCFNPDTVHDHASYAFNSYYQKNPVATSCDFGG
TATITNTDPSSGSCQYSASSGGGQNMLPPPSPTTLPPPTPMTPTTPMTPTPTTPDTGTPI
YGGSTTPPDYGSMSPPGGFGSNSPPDYGDVGAAPATMASGRAAVALAGVLIATVSLMSMS
T*
>11682.m04861|LOC_Os05g50490.1|genepair703-2
MDLWRVHCLGWLLVLLLFSHEAAGGGGAEQVATQAHHDASRRLSAQVAHGTPERDVTSPL
ATVPVDNPAANPTVTSTTNPAAMPGTQTTPSLANPVAAGGGGGGGGGSWCVASPSASTAA

LQVALDYACGQGGVDCSAIQSGGGCFNPNTVRDHASFAFNSYYQKNPVQTSCDFAGTAIL
TSTDPNPFTSRCNSAGSSSCKYPSTSTGASVLNTSTPTNPAFGGYDNSPPGFGNNSPPLY
GSMSPPGYNDNIGAAAAMAGSKETLLSLACVVATVSLNLYK*
>11667.m04284|LOC_Os01g44140.1|genepair704-1
MTVAAAAAAAADDAGRPGAAAARLRVMWRVVRAAEALALAVLLSRSLPLLPVAAGAAGAV
LRVGASFLLHPCSVFLLANGIVVLLVALSRRDRPSSSSSSSSSSCSDDHDDVHDQFLSF
AGAHLPPLPAAITGAAAADQEEGAVFEDKQAVHVTPARAAPRRSRSEKVGRGRRPTRAAS
PELRRSESERCRRRRRSLSSSSASLADWGMEEDDGGEKEEEEFRRAVEAFIAKQQTRFHR
EESFVLVAGAGAGDETSAAAAAVEVK*
>11682.m04859|LOC_Os05g50470.1|genepair704-2
MAAAGAALRLRLLYRMLRVGELLALVVFLSWSSSRVPAAAAAVVRLAGSLLLNARFVFVL
GNAIVLLLLALSRHDLSISSNHGTTTTAAAAAVSSDSAGAGSTPASTTAPPAASFPLFIV
PQPSPPPPHATEAPVVAAPPAPVVPCAPSVAPAAPAAAAAFEDKQAVRVNKARAPRRSRS
EKMGSRGAFRRAVSPELRRSESDNGRRRRSSVTARDAEVCWGADDAEEFRRTVEAFIAKQ
TRFHREESMTMTMSIVAGVGHGEVAPAIAGALAVVE*
>11667.m04292|LOC_Os01g44220.1|genepair705-1
MQFMMPLDTNACAQPMRRAGEGAGTERLMERLNIGGMTQEKALRKRCFGDGVTGTARCVF
TSDADRDTPHLRTQSSRKNYADASHVSAVILGGGTGVQLFPLTSTRATPAVPVGGCYRLI
DIPMSNCFNSGINKIFVMTQFNSASLNRHIHHTYLGGGINFTDGSVQVLAATQMPDEPAG
WFQGTADAIRKFMWILEDHYNQNNIEHVVILCGDQLYRMNYMELVQKHVDDNADITISCA
PIDGSRASDYGLVKFDDSGRVIQFLEKPEGADLESMKVDTSFLSYAIDDKQKYPYIASMG
IYVLKKDVLLDILKSKYAHLQDFGSEILPRAVLEHNVKACVFTEYWEDIGTIKSFFDANL
ALTEQPPKFEFYDPKTPFFTSPRYLPPARLEKCKIKDAIISDGCSFSECTIEHSVIGISS
RVSIGCELKDTMMMGADQYETEEETSKLLFEGKVPIGIGENTKIRNCIIDMNARIGRNVI
IANTQGVQESDHPEEGYYIRSGIVVILKNATIKDGTVI*
>11682.m04850|LOC_Os05g50380.1|genepair705-2
MQFSSVFPLEGKACVSPIRRGGEGSASDRLKIGDSSSIKHDRAVRRMCLGYRGTKNGAQC
VLTSDAGPDTLHVRTSFRRNFADPNEVAAVILGGGTGTQLFPLTSTRATPAVPIGGCYRL
IDIPMSNCFNSGINKIFIMTQFNSASLNRHIHRTYLGGGINFTDGSVEVLAATQMPGEAA
GWFQGTADAVRKFIWVLEDYYKHKAIEHILILSGDQLYRMDYMELVQKHVDDNADITLSC
APVGESRASDYGLVKFDSSGRVIQFSEKPKGTDLEAMKVDTSFLNFAIDDPTKFPYIASM
GVYVFKRDVLLNLLKSRYAELHDFGSEILPRALHEHNVQAYVFADYWEDIGTIRSFFDAN
MALCEQPPKFEFYDPKTPFFTSPRYLPPTKSDKCRIKDAIISHGCFLRECTIEHSIVGVR
SRLNSACELKNTMMMGADLYETEDEISRLLSEGKVPIGVGENTKINNCIIDMNARVGRNV
VITNSEGVQESDRPEEGYYIRSGIVVILKNATIKDGKVI*
>11667.m04303|LOC_Os01g44310.1|genepair706-1
MEELAAAAAPPRHRERRRHRRKASSTDAAAAYGDVFGGPPQFAVAFDGVPADYGEVFGGV
AASCSIPYLDLPPAAARDDGAGAGAYGEIFGRFDFGDFAEPYEDLLAEAVALAAEIASSS
ESSRSSVRKESGQLDADPSILHQHYSTVGYDQHFDEDEFSPISSPPDSGKQFSMSYNKAT
RGRPDDIVKMTTCMVEPPISYVVDSRNISNKSAMDQVVVVDCDTFANGEKGSMGLTFPSS
SSLKSASSDSVADQNLHTPICHPISKNDCEDEDYHKRLSTHSASSEEVPSPDYPFLRVSN
NSLHTQPIKVQPPLLAPSKLLNKKESKANGEKGSTGLTFPSSSSVKSASSDPMADQNLHT
PTCHPISKTDCEDEDYHKRLSTHSASSEDVPSPDYPFLRVPNNSLHTQPIKVQPPSKLLN
KKESKANGDSEVSTNSAAAAAAIKEAMEFAEARLKAAKELMERKGDSFKLRKKPGHHRGT
KSTELKESMAPEEVRVYDEKLTMRRIVKEEKTYEETALVNKNGDSSAVNLTHCDHNEKGV
LQPRKPQHTAQSGSKLEQLGKWTSGAEFYVLISPDQKCKTNSVTCEGDNVQTTNPSSKLG
QFEKGKGETTSGDFVGCGKSWDGGDIAELRMEHVNLREYAIGSTEDGCKAPTAPEISFSN
EKPTYQESTETHFKECVGAQNYQERYGDDGAFEISCVDSSKLHAPEIPGASLESCISGGH
CNGNKSPSDASTKETTSLGESNKENNNIEALEVPCADEMQSQILQEYHEFRNENIDEKKA
SQVKVSKLEESVEYYETPNFQKSSSTAHGETETVEKEKMFSFSDELRPQNKNIGITEAPP
ESLIHKEIKKFGTEEKAYITLEGDVVQKSGSLEREANITLESASANENEEAEEANAFVEG
INVMETHVSTYGTSVEDSDQIQDSENRMDGMGDLVSHGNEEAAKDPWLDNSEKSQVEEIF
SHEEGQLSVEGGIDGGPNDAYAGVNAINDGNGNDSETKVIIDDGTDFNTKMSTCSKELSA
SFLESSASMQHLSQIDKSIAAQTSDKSTPLENLGEDCREREFPEENSTALEQGQAIGSKM
EGDDKDKQSKLNVKDQKYFHLDSYIVPKFTENTTLNFVQKLIDETPDGQRIEGRENVKKT
LRETEKEVLHRLDEDKEIYKMEREKEQAKERSRRELEEEKERERERAKDRLAVQRATKEA
HERAFAEARAKAERIALERITLARQRASAEAREKEEKATAEAATEKASREARLKAERAAV
ERATAEARERAIEKAKAAADAKERMERFRSSFKDSFKSTNQDNQLDKQFQKTASNNYERS
TDSSNQVVEFESALRHKARSEREHRTAERAAKALAEKNMRDMLAQREQAERHRLAEYLDP
EVKRWSNGKEGNLRALLSTLQYILGSDNGWQSVPLTDLITATAVKKAYRRATLCVHPDKL
QQRGATIRQKYICEKVFDLLKAVSKQLLTWILKAIIIEIYAAALCLTNLIVTQDFICLLL
FIWSSMVLTSAVCAQLSTCFPVFG*
>11682.m04849|LOC_Os05g50370.1|genepair706-2
MDAPRRERRHQHHHRKAAAQVQAAPAIAAVGVGAGGNGAAARAAYGDVFGGPPRFAAPFG
GAPADYAEVFGGVAATCSIPFLDLPPVPAVGADYGFFGRAGAGDYGEIFGRFDFGDFALP
YEELFAEAEAEAVGEIGAEEIASSTGSSSRSSMIKESSQPDAQSFMLPQHFKEHESSVIS
FPPDNQQFVMSYNKTTQRSDDLVEMTTDPSMDYVVDSCEFPHVPTTNHVATMDSGIEANG
EKRKKSTTTSASVSLRSSESDFTVDQKQHIPAYPPISEKVSANENHKNSNSISTSSNGTP
SPDYAFLRVSDVNVQTQTVKPPPPLKQTSKLLKRREILAKGDVHLENHSCPPASSAHAPS
NTSTSQAERRDDTALFNNEANPSSAAAAMKEAMEYAEARLRAAKELMERKGDSFKLRKKP
SHHRSTRSTEIKVPTESDTFDENLSVKKSTKEEMNSEDSLLDKHQKASAVRTDHCDDSGK
RALSLEKPQHMQSCTAPNQTSSKLGKLGNWTSGDEFYELTGEDQKQKTDAAVGEEDKCEV
TNPVTKLSKEQKCEVTAADSDLERYEKLWEVNDGRDAGVKHVNPREDNTSPMGKDRVSMI
LEASTENIDHEKIYNSHFEGPKVVETSNESHDGEDGAVEIPCKSGITISEPNLMKDMHGS

FMEASSPGEYVADFGKGTTEESPVAGISLEPKTTKEELEAACDAEMQCTTGDSEKLQESS
EVTSIDNSLARQIKSLILEDLEGSSETQAFPGDPGTAGSEAETYGRNLGTTGLETESYGR
EKFSFVEESFMHNANRNVTESPVETPIPEQVENVEIEDRVGSCAHSEESTVDKDSECPEE
GSDITSQNNNLPDHEDSTMLNVFEVASKLIKRDLDQEKQDTLQPGEVETRTVLDSNDKDT
KENPSENSNTIGSEEVLSHGNQEDQKVPEMDKTKGRSDANAQVKLSGVNFYEDGDVTSAA
NNVTTRLTTNSKGQASSSSEMLTGRQHLPQDAGPAISQTSNGTFPSLEKTEEVCKEAGRE
LPTDKSAAFEDENSRACKSKAELKQQQFHSEKSSSLPKSAEGHIPSSADISRKETPGVQR
LKEQGSLRTEREREKDKEASRRLEETKERDKKFEKEREIAEERERKKLEEQERERERERKD
RLAVERATREAHERAFAEARERAEKIALERVTAARQRASAEAREKEERASTEAAAERAAR
IKAERAAVERATAEARERAIEKAKAEKAAAEARERRERYRSSFKESFKSSNLDNRQDTQF
QRAVSSNLMRNPDSYSKGLEVESALRHKARLERHQRTAERVTKALAEKNMRDLLAQREQA
EKHRLSEYLDPEIKRWSNGKEGNLRALLSTLQYILGADSGWQPVPLTELITAAAVKKAYR
KATLCVHPDKLQQRGATIRQKYICEKVFDLLKDAWNKFTSEER*
>11667.m04310|LOC_Os01g44370.1|genepair707-1
MASSSKASSSSSASAASSEWTKQQNKQFERALAVYDTDAPDRWHNVARYMGGAKSAEEVR
RHYERLQADVEQIEAGGVPFSLGYGATPQAGRR*
>11682.m04847|LOC_Os05g50350.1|genepair707-2
MASAAGSKQQQAMMSLPSSRGGGGGGWTQRQNKQFECALAVYDKETPDRWHNIARYMGGA
KSADEVRRHFDHLVEDVSRIESGRVPFPRYSSSSSSRGADDGNRSRYLKYQ*
>11667.m04312|LOC_Os01g44390.1|genepair708-1
MASMSVSSSRAPQWTARQNEQFERALAVYDRDTPERWHNIARAVAGKSADEVKLYYDLLV
EDVKRIETGKVPFPAYRCPQPAIAG*
>11682.m04846|LOC_Os05g50340.1|genepair708-2
MAQQARAQWPQKQNKLFEQALAVYDKETPDRWHNIARAVGGGKSAEDVKRYYEMLEEDIK
HIESGKVPFPAYRCPAAAGYQAERLKHLKI*
>11667.m04316|LOC_Os01g44430.1|genepair709-1
MELSEHEEDAGDVGGGCSSPPTPPHRVLTSAAPETIRCRYHECLRNHAAASGGHVVDGCG
EFMPASTEEPLACAACGCHRSFHRRDPSPGRAGAARLPQLHLPASINSRAPPALLLPPAA
AASKQGLPFPGYGTPSGGTGTTTASSSDERLRPSPVQPRRRSRTTFTREQKEQMLAFAER
VGWRIQRQEEATVEHFCAQVGVRRQALKVWMHNNKHSFKQKQQQENRQEQQQ*
>11682.m04843|LOC_Os05g50310.1|genepair709-2
MEFRGHDEPVDEMGVAYGRTPPSSSSSPAASASAGNGAGAAEVRYHECLRNHAAAMGGHV
VDGCREFMPMPGDAADALKCAACGCHRSFHRKDDGQQQQQLRLLIPSPPTPRVPLLMPPP
QPQPHPHPQHPYLHPPFPYHHTPSGSGGTTTESSSEERGPPSSSAAAAQGRRKRFRTKFT
PEQKEQMLAFAERVGWRMQKQDEALVEQFCAQVGVRRQVFKVWMHNNKSSIGSSSGGGSR
RQPQEQQSQQQQQQQ*
>11667.m04319|LOC_Os01g44950.1|genepair710-1
MCTENYDPYYPDRPVVDQYIPVCAKHPGLGSKPAFIWADDQAGGGSGVSPRETLTYSELD
SAVQRMAAGLLEVLRRGDTVLVLASPGIRLVKLMFACQRAGLVAVPVIPPDPSVIGTPAE
GPAHRHLLRAVSQARPRAAVADAGYVGAVTRSSISALMSLNWVSVDRLERWPAVASTVAA
TGDGVYEGCGPRETYLVQYTSGATGDPKPVVVTAGAAAHNARAARKAYDLRPGSVVVSWL
PQYHDCGLMFLLLTVVSGATCVLTSASAFVRRPRLWLELISEFKATCTPVPSFALPLVLR
RGRSEHGTRPLQLQTLRNLILVNEPIYKSPVDEFLEEFGPAGLRASSISPSYGLAENCTF
VSTAWRGTEVTLPSYKKLLPSARLPQPSLLTEAPDVEIVVVDAETGKLVEDGVEGEIWLS
SPSNGSGYLGDSPASREVFGARLQGRAGPCFVRTSDLGVVHGTERYLYVLGRTTDAIVVA
DSQRRVPAHYIETAAFESSPGRLRGGCIASFTTLPVSPSPVVVVAELQNGGGGGDMAGIC
HGIRQAVWREVGVTVARVVLAESGGVPKTTSGKLQRGAARAKLLAGKLPKVFEARYAGLE
PAAGVLMVKESASRWARLQSPL*
>11682.m04842|LOC_Os05g50300.1|genepair710-2
MSTENYDPCYPDQPVVHRYLPVWAKLPAFAAKPAFVWADDGDTAMSYATLTYSQLDAAVE
RMSSGLLGALRRADTVLVLASPGLRLVKLLFACQRAVLTAVPIIPPDPSRPGAGGAAHSH
LLRAVSQTRPRAAVADARYIDAIKKSNAVVDVAGEPDRLAAMLRSLRWLSVDELEQGGAN
GAAPVTPFVGGEPEDAYLIQYTSGATGAQKPVVITAGAAAHNARAARRAYELHPGSVVVS
WLPQYHDCGLMFLLLTVVAGATCVLASPDAFVRRPRLWLELVTEFRATCTPVPSFALPLV
LRRGRSPDGRRRRPLELGSLENLILINEPIYKSCVDEFVAEFRGDGLLPASISPSYGLAE
NCTFVSTAWRSRRCVDLPSYKKLLPSARLSFHMADEEPEIEIIMVDEETGEPVEDGVEGE
IWVSSPSNASGYLGHPSATREVFCATLPGKGSCYVRTGDRGVVVVRGAERYLYVVGRSAD
VLALDVDGGQRSVCAHYIETAAFGGAPDRLRGGCIAAFATSPVPSTSLVVVVAELLKGSG
GGGGGGDHKDICEGIKRAVWEEEGVRVGWIVLVDSGVVPKTTSGKLRRGAAREKLLAGKL
PILLEARYDGDESSSSVPWDGGEEEMEKCPGMDAAYGSASRRLRLQSFL*
>11667.m04331|LOC_Os01g45060.1|genepair711-1
MASISRMETPQLRVLRLLNRADVATTLVKWLGRIRTLYMAIRPEARRPGPQHSILADPTT
GPCLGLPTGMLGRPGMTDPARHDGSLDGTIVAPTSAKAWDSGLLQWIEFTKLNGVSIQGN
GIINGRGQQWWTYSDTDDDENDDTQYDVEFERMPQVKPTALRFYGSFNVVVAGITIVNSS
QCHLKFDSCQGVMVHDVTISSPENSLNTDGIHLQNSKDVSIHHTNLACGDDCVSIQTGCS
NINIHNVNCGPGHGISIGGLGRDNTKACVSNVTVRDVNMFRTMTGVRIKTWQGGLGLVQD
VRFSNIQVSEVQTPIIIDQFYCDERTCSNQTSAVAVSGVQYENIRGTFTIKPVHFACSDS
SPCSGITLTGVQLRPVQISHYRLNNPFCWQAFGELYTPTVPPIACLHLGKPAGNNLQSYH
DLC*
>11682.m04838|LOC_Os05g50260.1|genepair711-2
MGLGIKGLTFLLLLVLLVLCSNVSLSDARSGKHWRQNRASSSTLLRRKGKGKTNNSHKQY
GKGNQDPYQPSPSTSPNVPVNPSERPVQGKGHPAPTMPPPSSGSGHTLPSPPPPLPPLLP
PPQPPAAQSQNTVFNVVDFGARGDGVTDDTQAFEEAWAAACKVEASTVLVPSELEFVVGP
ISFSGPYCKPNILFQLDGTILAQTSTRVWGSGLLQWLEFTKLSGISIQGSGVINGRGQEW

WTYSDPNDDDNDDVDAYNVELEKMPQIKPTALRFYGSSNVTVTGITIVNSSQCHLKFDSC
QGVMVHDLTISSPENSPNTDGIHLQNSKQVSIHHSNLACGNALINSIKAKPTGFRTKGKL
KTLVQVSEVIFALCDAGDDCVSIQTGCSDINIHNVNCGPGHGISIGGLGRYNTKACVSNV
TVRDVNMFKTMTGVRIKTWQGGSGLVQGIRFSNIQVSEVQTPIIIDQFYCDRTTCRNQTS
AVAVLGVQYENIRGTFTIKPAHFACSDSSPCSEITLTGIQLKPLIVPQYHLYNPFCWQAF
GELSTPTIPPISCLQIGKPSGNNVMSDYDLC*
>11667.m04345|LOC_Os01g45200.1|genepair712-1
MRAALLHGHGGGAAAAAAAGWRPSAGDADVKRTAGGDGGAAGPRTVCVTGGISFVGFAVV
DRLLRHGYTVRLALETQEDLDKLREMEMFGEDGRDGVWTVMANVTDPESLHRAFDGCAGV
FHTSAFVDPGGMSGYTVSAPNSVPNLKILATTTPTYVMCSLMLSQKSLRDRVYYLIRTGN
QKHMASLEAKAAEQVIEACVRTESVRKCVFTSSLLACVWRQNYPHDRRFPTIIDENCWSD
ESFCRDNKLWFALGKTAAEKTAWRAARGRDLKLVTVCPALVTGPGFRRRNSTASIAYLKG
ARAMLADGLLATASVETVAEAHVRVYEAMGDNTAGGRYICYDHVVKRPEEFAELERQLGI
PRRAAAAAAAQDSGDRPARFDLCRQKLARLMSTRRRCTYDDYYSVAFD*
>11682.m04837|LOC_Os05g50250.1|genepair712-2
MGVLRSTQSMEAEVEEMRAALALAPLGRHGAWRSGAAAKREAGAEEGAAPEARTVCVTGG
ISFVGLAVVDRLLRHGYAVRLALETQEDLDKLREMEMFGENGRDGVWTVMANVMDPESLN
QAFNGCVGVFHTSSLIDPGGISGYTKHMAILEARAAEQVVEACVRTESVRKCVFTSSLLA
CVWRQSYPHHRRRFPAIIDESCWSDESFCRDNKLWFALGKTMAEKAAWRAARGRDLKLVT
ICPALVTGPGFRRRNSTPSIAYLKGAHAMLAEGLLATADVERVAEAHVRVYEAMSGGGAA
GGRYICYDHVVRRGEEFAELQRQLGLPITGVAAASRPGYSDDGDVGGDGRFALCNGKLAR
LVSSRRRCTYDVYYPASYD*
>11667.m04350|LOC_Os01g45250.1|genepair713-1
MEERPRDRTVEEAAAFSGDESDGEFEFPFVSRETDAGGVADELFADGRIRAFYPVFGRVL
DDVAVTPAAAAAEERRPPLGRLFLEEGRTSSVGSTSSSSSSTDIAELDGVSPDSYCVWVP
GSSPASSPSRPPRKSGSTGSIARWRRISELVVGRSHSDGKEKFRFLSAPSSPARDHPKPK
PTTKGGAAAATKLHTELDTIAAGHRLSYSPNHKAHGGATRRTFLPYRQDLMGIFANVNGL
SRTHHRPF*
>11682.m04835|LOC_Os05g50230.1|genepair713-2
MEERIPEPQDPTADVAADVLPAEASSSDVTTTGVEEESDSDGEFEFEFPFVSRDSPAGTA
AVADDLFADGRIKPFYPVFGRAGAGGGGDRQQHLAKDDAAATVPPRTRGPLGRLFLEESR
GSFDRWSTSTSSSSSSSAPASDEGGLDGAPPESYCLWTPGAGAGSASASASPRPPRKSGS
TGSMARWRRISELVVGRSHSDGKEKFLFLPIPPPSSKENDVEHFKPKPKPPKPTPASGRK
TAQAAAAEIDTVAAIHRIAYGAKGGGATGTSAGGGTPRRTFLPYREELVGLFANVNGISR
SHPHPF*
>11667.m04364|LOC_Os01g45380.1|genepair714-1
MRHPGAGGGSGDAGFVRADQIDLKSLDEQLERHLGRPAERAASQHGGSGSRRGESARLGL
GEEPPQAPHHQRRREDWEIDPAKLVIRGVIARGTFGTVHRGVYDGQDVAVKMLDWGEDGH
RSEREISSLRAAFAQEVAVWHKLDHPNVTKFIGAIMGARDLNIQTEHGHFGMPSNICCVV
VEYLAGGALKNFLIKNRRRKLAYKVVVQLALDLARGLSYLHSKKIVHRDVKTENMLLDKS
RTVKIADFGVARIEASNPSDMTGETGTLGYMAPEVLNGHPYNRKCDVYSFGICLWEIYCC
DMPYPDLSFSEVTSAVVRQNLRPEIPRCCPSSLANVMKRCWDANPDKRPAMAEVVSMLEA
IDTSKGGGMIPTDQPQGCFSCFGRHRGP*
>11682.m04829|LOC_Os05g50190.1|genepair714-2
MKEGGGDGGFVRADQIDLKSLDEQLERHLSRAWTMEKRKEEAAADQRGSKPPALAAAHYS
QNRRQRREEWEIDPAKLVVRGVIARGTFGTVHRGVYDGHDVAVKLLDWGEDGHRSEQDIA
ALRAAFSQEVSVWHKLDHPNVTKFIGAIMGARDLDIQTESGHLAMPSNICCVVVEYLAGG
SLKGFLIKNRRKKLAFKVVVQIALDLARGLSYLHSKKIVHRDVKTENMLLDKTRTVKIAD
FGVARLEASNPSDMTGETGTLGYMAPEVLNGSPYNRKCDVYSFGICLWEIYCCDMPYPDL
SFSEVTSAVVRQNLRPEMPRCCPSSLANVMKRCWDANPDKRPEMAEVVSMLEAIDTSKGG
GMIPVDQRQGCLSCFRQYRGP*
>11667.m04372|LOC_Os01g45460.1|genepair715-1
MRPVSSAEEVGALLSRSDSSGRRRRSSPVQSASPRPAGCGCGGPRRQSSFRDDVGHAASE
TYLVTRLTFSLLQYLGLGYRWMSQLLALTIYAILLMPGFLQVLSWFTVGYYYFFSSQVRR
SIVYGEQPRNRLDLYIPKDINRPCPVVAFVTGGAWIIGYKAWGSLLGRRLAERGIIVACI
DYRNFPQGTIGDMVSDASQGISYVCNNIASYGGDPNRIYLVGQSAGAHIAACALIEQAVK
ESSGQSISWSVTQIKAYFGLSGGYIGSCCHVLQTFADVLQQAGAQAKLQLYEGKTHTDIF
IQDPLRGGRDPLVEDVLSIIHVDDEITQEKIALAPAPRRLVFEWQLQLARRFSPF*
>11682.m04827|LOC_Os05g50170.1|genepair715-2
MQPASPVSGDAGPVAEAVPPRGAPQVLVRRRSVPFSPDSPLAPGSRGGGERRSTFREDVS
HAAAETYLVTRLAFILLRYLGVGYRWISQLAALIIYAILLMPGFIRVGYYYFFSRQVLRS
VIYGDQPRNRLDLYIPRDPKKPSPVVAFVTGGAWIIGYKAWGALLGRRLAERGIIVACID
YRNFPQGTISDMVSDASDGISFVCETVGAYGGDPNQIYLMGQSAGAHIAACALLEQAAKE
SRGEQISWSVTQIKAYFGLSGGYNIENLVDHFHERGLYRSIFLSIMEGKKSLPHFSPETV
AKKLCPETIALLPQIVLLHGTDDYSIPFSASETFAGVLKQAGAKAKLLLYEGKTHTDVFL
QDPLRGGRDKLVEDVISVIHADDADAREKDALAPIPGRLVSEWQIKLAHRISPF*
>11667.m04377|LOC_Os01g45510.1|genepair716-1
MARSPHESSFSFSRRHFKWPVLGKSSSHGASNAGEDDFIKAEDDEEATMAFSSTCPSFHS
EDFVSPPPCKPLKQQQQQQPQQQRRKGRTAVSRLRTALAAALAGRHRQVGLGARLTGTLY
GHRRGHVHLAFQVDPRACPALLLELAAPTASLVREMASGLVRIALECERAKGGGACAFPT
AAAAPSSSSSSSSSSSAGGRKLVEETVWRAYCNGRSCGYAVRRECGAADWRVLRALEPVS
MGAGVIPAACGGGEGDVMYMRARFERVVGSRDSEAFYMMNPDCGGSGSNNNGGPELSVYL
LRV*
>11682.m04826|LOC_Os05g50160.1|genepair716-2

MRTITARNPHDSLSFSRRHFKWPVLGKSYSHGATRGEEDYMKSSEAEEEEDEATMAFSSV
CPSFHSEDFVSPPKKPPRQQHPQQQHPPQRRKVRTAVSRLRSALAAAVTGRHRQVGLGAR
LTGTLYGHRRGHVHLAFQLDPRACPALLLELAAPTAALVREMASGLVRIALECERAKGGP
APALPTATGGGKRLLEETVWRAYCNGKSCGYAVRRECGAADWRVLRALEPVSMGAGVIPA
ASCGGGEGDVMYMRARFERVVGSRDSEAFYMMNPDSTSTSSNSNSGGPELSVYLLRV*
>11667.m04381|LOC_Os01g45550.1|genepair717-1
MISGHDFYTVMAAVVPLYVAMFLAYGSVRWWGIFTPDQCSGINRFVAIFAVPLLSFHFIS
TNDPYAMNLRFLAADTLQKLLVLAGLAAWSRLPSRTGAPRLDWSITLFSLSTLPNTLVMG
IPLLIAMYGPYSGSLMVQIVVLQCIIWYTLMLFLFEFRAARMLIADQFPDTAASIVSLHV
DPDVVSLEGGHAETEAEVAADGRLHVTVRRSSVSRRSLLVTPRPSNLTGAEIYSLSSSRN
PTPRGSNFNHADFFAMVGGGPPPPTPAAVRGSSFGASELYSLQSSRGPTPRQSNFDEHSA
RPPKPPATTTGALNHDAKELHMFVWSSSASPVSEVSGLPVFSGGGGGGALDVGAKEIHMV
IPADLPQNNGSGKEHEEYGAVALGGGGGGENFSFGGGKTVDGAEAVDEEAALPDGLTKMG
SSSTAELHPKVVDVDGPNAGGGAAGAGQYQMPPASVMTRLILIMVWRKLIRNPNTYSSLL
GLAWSLVAFRWHVSMPAIVEKSISILSDAGLGMAMFSLGLFMALQPSIIACGKSAAVVSM
AVRFLAGPAVMAAASIAIGLRGTLLHVAIVQAALPQGIVPFVFAKEYNVHPAILSTAVIF
GMLIALPITLLYYILLGL*
>11682.m04824|LOC_Os05g50140.1|genepair717-2
MISWHELYMVLSAVVPLYVAMMVAYGSVRWWGVLTPEQCSGINRFVAVIAVPLLSFHFIS
SSDPYAMNLRFVAADTLQKVLVLAALAAWSRFPARFVPPAWPPLDCSITLFSVSTLPNTL
VMGIPLLVSMYGPYSGDLMVQIVVLQSIVWYTLLLFLFEFRAARVLIAAQFPDTAASIAA
VHVDPDVVSLEGSQAEAHAEVAPDGRLRMVVCRSSVSRRSAAAAATPRASNLTGVEIYSI
SSSRNATPRGSTFTLADIPGHQPPNSALRASSFGAADLFSLHSSSRQHTPRPSSFDEHAA
ARARASATVAPTNDLKDTHMIEWSSGASAASEVTGLPVFRSGRETRRLVPSDAPSIASSR
VIRPPPGATGGERAASFNKAVGGQDELAKLEAGAKTEQQTTAVTTTTKGGGAAGAERARG
QQNAPAGVMLRLILTTVWRRLIRNPNTYASLIGLTWSLIAFRFHITMPIIVAKSISILSD
AGLGMAMFSLGLFMATQPKIIACGYSVAAASMGVRFFFGPAIMAAASAAVGIRGTLLRIA
IVQAALPQGIVPFVFAKEYNLHATILCTFCSC*
>11667.m04383|LOC_Os01g45570.1|genepair718-1
MMGATSPSGLELTMAVPGLSSSGSEGAGCNNNNAGGGCNMRDLDINQPASGGEEEEFPMG
SVEEDEEERGVGGPHRPKKLRLSKEQSRLLEESFRLNHTLTPKQKEALAIKLKLRPRQVE
VWFQNRRARTKLKQTEMECEYLKRCFGSLTEENRRLQREVEELRAMRVAPPTVLSPHTRQ
PLPASALTMCPRCERITAATGPPAVRPPPSSAAAAAPSPFHPRRPSAAF*
>11682.m04823|LOC_Os05g50130.1|genepair718-2
MEPMGILKCPSSLKLSVAVPGHALNPFSDGGSGSGSGSGSGCNNNNTRELGIHLDLDRPY
AREELPQQGGSMEVQKGEERGGVRHNSSNHRRLSRVQSKQLDEFYRVNHTVDSTSHLRSR
KLMEHFQKQKKELADRLNLRISQVDAWFRNRRLSHYYVGLTFLLPHMSVSVEDVNPMIPQ
YKSVSSVEQVQSSSSAPVTVMLLLVIPFRLIFTNLQSKQKSTEMECAYLKECFNKLKEEN
HRLQLQVEQLRSTSLQLQLQLQLHSERVATAPTGQQAGTSAAARIFTLPLSGYNPSRGTW
FSPNAH*
>11667.m04388|LOC_Os01g45620.1|genepair719-1
MPPREVPVRPRAGRHRAHGEAPADDRGGPGDEDRGGHRHPERAGEEQERAEDDKRDEGDG
ERDAREDKERREGDSQGEETDEPRGVVRVMLDAPAEREGSGEPEFFSEYGDASRYEVTEV
VGKGSYGVVAAAVDTHTGGRVAIKKINDVFEHISDATRILREIKLLRLLRHPDIVEIKHI
MLPPSRREFRDIYIIFELMESDLHQVIKANDDLTPEHHQFFLYQLLRGMKYIHAASVFHR
DLKPKNILANADCKVKICDFGLARVSFDDTPSAIFWTDYVATRWYRAPELCGSFFSKYTP
AIDIWSVGCIFAEMLMGKPLFPGKNVIRNEKARRYLGNMRKKPRVPFSQKFPGADPMALH
LLERLLAFDPKDRPTAAELCVKALTDPYFTGLANSEREPIAQPISKLEFEFERRKLAKDD
VRELIYREILEYHPQMMQKYLRGGDQSNFLYPRERVVRNGDEPDPTADYCIKLHVGEQPG
HSSVTDGLNKPLLSARNFLKSESIGASQCVVIKEKREKDEESMSEYMNEAADGVPHKIAQ
LKT*
>11682.m04822|LOC_Os05g50120.1|genepair719-2
MGGRARSILRWLRHHRSRRVSSSSFHLTTTGDDTVKDLHDPRREDAEGDGWEEVHEGPES
DPEEYIALVSEDAGTHLPVRTEPRRMDPSKKEPDFFTEYGEANRYKVSEVIGKGSYGVVA
AAVDTQTGERVAIKKINDVFDHVSDATRILREIKLLRLLRHPDIVEIKHIMLPPSRREFR
DIYVIFELMESDLHQVIKANDDLTPEHHQFFLYQLLRGMKYIHAASVFHRDLKPKNILAN
ADCKLKVCDFGLARVSFNDTPSAIFWTDYVATRWYRAPELCGSFFSKYTPAIDIWSVGCI
FAELLTGKPLFPGKNVVHQLDLMTDLLGTPSAESLAKIRNEKARRYLSNMRKKPRVPFTK
KFPGVDPMALHLLERLLAFDPKDRPSAEEALTDPYFNGLANSEREPIAQPISKLEFEFEK
RKLAKDDVRELIYREILEYHPHMLQEYLRGGDQMSFMYPSGVDRFKRQFAHLEEGVSKGE
KSSPQLRQNASLPRERAIGNKHGDDEYHAKLNVGEKPCHASVTDGISKPLMSARSLLKSE
SISASKCIGEKPKQDRDQEDSLTESMDETADEVSEKVAQLKT*
>11667.m04398|LOC_Os01g45720.1|genepair720-1
MAAAVEEAFPIGFTKGIRSYWRSRKYHSVDSSAAGRGTSNLVRLGGGSGSGSDGGAWAVR
LGGMFRTRVKAAAPAATTTAVAKVPARVLGRVRDAYVDAMVGVAKKQAAAALSQPGAGTT
EALWQKRVPVRRSRGQSKKQLRQKADELGQRLVMEMYKSVLASRDLSSMLQASRAQ*
>11682.m04820|LOC_Os05g50100.1|genepair720-2
MEVEAFPIRFTRGIRSHWGRRKYQRLEAANGGGKTRATQQLGGARRGGAGWGLRLRRLLR
VRVRVARAWLSSPARLLARIRDAYVGGMLAVSRKASGMSLPNAPEGLWPRRVPRRKQLPA
ARPGQLTDFEQRLVVEIYKSIVASKELTTMLHHSTAHLPQQHNTAAPASSGQLLVN*
>11667.m04399|LOC_Os01g45730.1|genepair721-1
MQEALVSPVRSALELKPFNFGDQRLASSPRYLPSGDDALYRCSSPFSPSFGFSSPSPLAT
SVSLSPSSSASLVDDGDDGGAAADATGQRLQLARLALQYQEVADRYELCLSHLAEAAEEA
AALRLENAELRVTNSDLALRLALLSGKHTAAVAVADEIRRLRLGEQKVAAATKERTPEKL

AVLPKSISVRSTSYLKLNQQSQAATATSAAPNRKPRTSSNPTNPPNSQRAYDGGKKGDEQ
KAQPADSGAELEVYNQGMFKTELCNKWEETGDCPYGDQCQFAHGVTELRPVIRHPRYKTA
VCRMVLAGDVCPYGHRCHFRHSLTPAERLLLRS*
>11682.m04818|LOC_Os05g50080.1|genepair721-2
MQEALLPPAHPGRFYSDFGPKPFGSGDQRLSSPNLLTNGGDLFYGCYSPFSPTRVLSPPP
PRRAASFSHCSSSSDSVVDDGDGAGAAAATEHRLHLAHLALQYQEMANRFELCLSHLADA
ADEAAALRQENAELRVANNDLACRIAKFGGRQSSAIALAGDLRRLRLPKEQTVPALPPPP
QSPPAALMNPVAVPEKQAVLPKSISIRSTGYQKLNQGGKHRVSKPVNVGSQRVFVGIDGA
EGGEHKVGVKKEEPPMGGLEFEVYNQGMFKTELCNKWEETGACPYGDQCQFAHGVAELRP
VIRHPRYKTQVCRMVLAGGVCPYGHRCHFRHSITPADRFSFGH*
>11667.m04414|LOC_Os01g45860.1|genepair722-1
MDTFPFQWPMDPAASSGLDAGFLPPPAAVAPDDGVGYYDPPAGADVDAAALPEFAAAFPP
CAPDAAAAVLAMRREEEEVAGIRLVHLLMSCAGAIEAGDHALASAQLADSHAALAAVSAA
SGIGRVAVHFTTALSRRLFPSPVAPPTTDAEHAFLYHHFYEACPYLKFAHFTANQAILEA
FHGCDHVHVIDFSLMQGLQWPALIQALALRPGGPPFLRITGIGPPSPTGRDELRDVGLRL
ADLARSVRVRFSFRGVAANSLDEVRPWMLQIAPGEAVAFNSVLQLHRLLGDPADQAPIDA
VLDCVASVRPKIFTVIEQEADHNKTGFLDRFTEALFYYSAVFDSLDAASASGGAGNAMAE
AYLQREICDIVCGEGAARRERHEPLSRWRDRLTRAGLSAVPLGSNALRQARMLVGLFSGE
GHSVEEADGCLTLGWHGRPLFSASAWEAAGDGGGDNNNNSNSNVSGSSGSDSNNSGSSNG
KSSGARDGSSVCL*
>11682.m04803|LOC_Os05g49930.1|genepair722-2
MAQFGGFGGWSAMDVAAAAAAALGNVSGAVYHADPAAAVYASLVPGMAVVPGRAPPSAVQ
IEAARRWKELEKMALRSVNLMVTCAGAIQAGDYAAAGSLSDAREIFAKMPTTRTGIGRV
LTHFADALAERLFPAFPQSAPPPPPPRGEQRELFRGFYEAGPYLKFAHLAANQAILEAFE
GCNSVHVIDFALTDGIQWPSLIQALAVRPGGPPFLRITGIGPHAAGNRDELRDVGLRLAE
FARSCSVPFAFRGIAADQLDGLRPWMFQVAPGEAVAINSVLQLHRLLVDQDAAAAASFPA
PIDGVLDWVASMNPRVFTVVEQEADHNKSSLLERFTNSLFYYASMFDSLEAISRHGGGDG
AGNPLAEAYLQGEIADIVSREGSSRVERHEQMPRWVERLRRGGMTQLPLGATGLWQAAMQ
LREFSGAGFGVQENGGFLTLTWHSQRLYSASAWRATAGKKMTMMASGAADAMEESQNSNT
NGGGGGSSGGGHGALNQIMQ*
>11667.m04435|LOC_Os01g46070.1|genepair723-1
MRPSLMRSASQVLRRRRGYSSASGQPERKVAILGAAGGIGQPLSLLMKLNPLVSSLSLYD
IAGTPGVAADVSHINAPAQVKGFMGDDQLGEALEGSDIVIIPAGVPRKPGMTRDDLFNIN
AGIVKNLCTAIAKYCPNALVNMISNPVNSTVPIAAEVFKKAGTYDEKKLFGVTTLDVVRA
KTFYAGKANVPVTDVNVPVVGGHAGITILPLFSQATPATNALSDEDIKALTKRTQDGGTE
VVEAKAGKGSATLSMAYAGAVFANACLKGLNGVPDVVECSFVQSTVTELPFFASKVKLGK
NGVEEVLGLGQLSDFEKEGLENLKGELKASIEKGIKFANA*
>11682.m04796|LOC_Os05g49880.1|genepair723-2
MRPSLLRYAAQALRRRDYSSAAAAAERKVAILGAAGGIGQPLALLMKLNPLVSSLSLYDI
AGTPGVAADVSHINSPALVKGFVGEEQLGEALEGSDVVIIPAGVPRKPGMTRDDLFNINA
GIVKGLCTAISKYCPNALVNMISNPVNSTVPIAAEVFKKAGTYDEKKLFGVTTLDVVRAK
TFYAGKANVPVTEVNVPVVGGHAGITILPLFSQATPASNALSHEDIKALTKRTQDGGTEV
VEAKAGKGSATLSMAYAGAVFADACLKGLNGVPDVVECSFVQSTVTELPFFASKVRLGKN
GVEEVLGLGQLSEFEKEGLENLKGELKSSIEKGIKFAHAN*
>11667.m04455|LOC_Os01g46250.1|genepair724-1
MASRWRELHGSGHWDGLLDPLDVDLRRCLITYGEMIMATYEAFIGEHRSPNAGMCRYRHA
DLFRRVDVSHPGWYAATRYIYATANADVHGKVLLRPLCREGRATECNWMGYVAVATDEGA
AALGRRDIVVAWRGTQRALEWVADLKLAPASAAGILGPEGADGTDPSVHRGYLSLYTSED
QCSELNKQSARMQNKKLGFDPMRQTLKKVLTEIARLMDKYKDEETSITVIGHSLGATLAT
LNAADIAANSYNTSSLSPSGETRAPVTAVVFGSPRTGDRGFRDAFHRLRDLRMLRVRNRP
DRIPHYPPVGYADVGVELLIDTRLSPFLRRHGSESQSHDLECHLHGVAGWHGDHRGFELV
VDRDVALVNKFDDCLADEYPVPVRWKVHHNKSMVKGPDGRWVLQDHEPDDDDDDDDDD*
>11682.m04792|LOC_Os05g49840.1|genepair724-2
MSSSPMLGGIADRWRELHGQDSWNGLLDPLDLDLRSSILSYGELVQATYDSFNRERRSPH
AGACVYGHGDLLAAAGASAAGSYAVTKFVYATSGLPVPEAFLLLPLPSLLPPAWSRESNW
MGYVAVATDEGVAALGRRDIVVAWRGTVESLEWVNDFDFTPVPAAPVLGAAAAANPRAIV
HRGFLSVYTSSNKDSKYNKASARDQVLEEVRRLMELYKDEVTSITVVGHSLGASLATLNA
VDIVANGANCPPASSSSSQPPCPVTAIVFASPRVGDGFFKAAFASFPDLRALHVKNAGDV
VPMYPPLGYVDVAVKLRISTSRSPYLRSPGTIETLHNLECYLHGVAGEQGSAGGFKLEVD
RDVALANKGVDALKDKYPVPPRWWVSKNRCMVKDADGHWALHDFEQI*
>11667.m04464|LOC_Os01g46340.1|genepair725-1
MVAGRVKAAMGFQRSPKVSKSPAHVGRTPETPGRGSSSGSPAPGGSASKAVSFARSLGVH
FPRSSAQVQPARAPPEVADLLRAIEQLQERESRLRVELLEQKILKETVAIVPFLEAELAA
KSSELEKCKDTAARLESENMRLCAELDAAVLEVTSRKQRIVHMEKEMAELKKQQEAAAAD
ADDCSSTASVSHEQPESASSAANPASLVQRGPPIPPPPPPVPPAAFKSKSYSASSRVSLP
STSAPSPSSSTSTSPTYSCSSSDTVTTPRNRKPELSKLPPIPPPPPMPALSVCGRAAAPP
PPPPPPPARRTSGAASPAASGPRVTRVPEVVEFYHSLMRRDSRSRDGSGGGETANGGGVA
ATRDMIGEIENRSAHLLAIKSDVERQGDFIRFLIKEVEGAAFVDIEDVVTFVKWLDNELS
RLVDERAVLKHFEWPENKEDALREAAFGYCDLKKLEVEASSFRDDARQPCSTALKKMQAL
FEKLEHGVYNLARFRDGATGRYSRFQIPCEWMQPDTGIVSQIKLQSVKLAMKYLKRVSSE
LEAIKGGPDEEELMLQGVRFAFRVHQFAGGFDVDTMRAFQELKEKASMCRIQRQEQNRHL
RRQQKLVARA*
>11682.m04790|LOC_Os05g49820.1|genepair725-2
MVAGRVKAAMGFQRSPATPKTSSSRKAPPPPPPLVQAAAHHAAAGQPETPRRRSSGSPAS

ASASASGSKTGVFARSFGAYFPRSSAQVQPARGAAPEVGELVRLVEELQERESRLRTELL
EHKILKETVAIVPFLENELAAKSSELGRCRDALTRLESENARLRAALDAAAASSRDNEQR
ILEMERQMTELRKRRQRDVATGPDDCSSSASSDNSESSNAATNSAKSAKVAGCSSVRPPP
PPPPPPLPPPMPATFKSKSYFSGSSRASPANSSSSSSSSSAPSTPSCSSDTAASRSRLPE
LSKLPPIPPPPPPPPPPPMPRSRSASPSPSTSSSGSAGPPAPPPPPPPAAKRTSRTSTPA
TTSSSAPASGPCVRRVPEVVEFYHSLMRRDSKRDGGGGGGGAEACPGGGAAAARDMIGEI
ENRSAHLLAIKSDVERQGDFIRFLIKEVEGAAFVDIEDVVTFVKWLDVELSRLVDERAVL
KHFEWPEQKADALREAAFGYRDLKKIEEEASSFCDDPRQPCSSALKKMQALFEKLEHGVY
SLARVRDGAMNRYRGYHIPWEWMQDTGIVSQNTHKDAKNINTYKTYTRDNNKKNLGCNVH
VCQGALIKLQSVKLAMKYLRRVSSELEAIKDGPDEEELMLQGVRFAFRVHQFAGGFDGDT
MRAFQELKEKASTFQSQRECQNQHLQQHKLAGRS*
>11667.m04468|LOC_Os01g46380.1|genepair726-1
MAAATSTSSTALAHPKTLNPASKSAAAGSVSFPAAQPPCPLAASAGGRRRRGAVAAKVSS
PQVIGATMPSLDFETSVFKKEKVSLAGHDEYIVRGGRNLFPLLPEAFKGIKQIGVIGWGS
QGPAQAQNLRDSLAEAKSDIVVKIGLRKGSKSFEEARAAGFTEESGTLGDIWETISSSDL
LLLLISDAAQADNYEEIFSHMKPNSILGLSHGFLLGHLQSVGLDFPKNISVVAVCPKGMG
PSVRRLYVQGKEVNGAGINSSFAVHQDVDGRATDVALGWSVALGSPFTFFATTLEQEYRSD
IFGERGILLGAVHGIVEALFRRYTEHGLDEDSAYKNTVECITGIISKTISKKGMLEVYNS
LTEEGKKQFIEAYSAAYYPCMDILYECYEDVSSGSEIRSVVLAGRRFYEKEGLPAFPMGN
IDQTRMWKVGERVRSTRPAGDLGPLHPFTAGVYVALMMAQIEVLRKKGHSYSEIINESLI
ESVDSLNPFMHARGVAFMVDNCSTTARLGSRKWAPRFDYILTQQAFVTVDKNAPVNQDLI
SNFFSDPVHSAIEVCAQLRPTVDISVPADADFVRPELRQSS*
>11682.m04788|LOC_Os05g49800.1|genepair726-2
MAASTTLALSHPKTLAAAAAAAPKAPTAPAAVSFPVSHAACAPLAARRRAVTAMVAAPPA
VGAAMPSLDFDTSVFNKEKVSLAGHEEYIVRGGRNLFPLLPEAFKGIKQIGVIGWGSQGP
AQAQNLRDSLAEAKSDIVVKIGLRKGSKSFDEARAAGFTEESGTLGDIWETVSGSDLVLL
LISDAAQADNYEKIFSHMKPNSILGLSHGFLLGHLQSAGLDFPKNISVIAVCPKGMGPSV
RRLYVQGKEINGAGINSSFAVHQDVDGRATDVALGWSVALGSPFTFFATTLEQEYKSDIFG
ERGILLGAVHGIVEALFRRYTEQGMDEEMAYKNTVEGITGIISKTISKKGMLEVYNSLTE
EGKKEFNKAYSASFYPCMDILYECYEDVASGSEIRSVVLAGRRFYEKEGLPAFPMGNIDQ
TRMWKVGEKVRSTRPENDLGPLHPFTAGVYVALMMAQIEVLRKKGHSYSEIINESVIESV
DSLNPFMHARGVAFMVDNCSTTARLGSRKWAPRFDYILTQQAFVTVDKDAPINQDLISNF
MSDPVHGAIEVCAELRPTVDISVPANADFVRPELRQSS*
>11667.m04469|LOC_Os01g46390.1|genepair727-1
MAEREGAVVKKGPDEGMKMATALLEEFGLPLGLLPLAEVIEVGFVRATGYMWIAQRKKVE
HQFKMVSKQVSYDVEITGYVKAKCIKKLKGVKAKELMLWPPVNEITVDNPPTGKIHFKSL
AGVTKTFPVEAFAAGQ*
>11682.m04787|LOC_Os05g49790.1|genepair727-2
MAAEREGAVVAKGHEEGMKMAASLLEEFGLPLGLLPLGDVVEVGFARATGYMWIAQRRKV
EHHFRMVGKHVSYDADITGYVKPRCIKKLKGVKAKELMLWPPVNEIAVDEPSTTGKIHFK
SLAGVTKTFPVEAFAAGQ*
>11667.m04487|LOC_Os01g46570.1|genepair728-1
MKYVLVTGGVVSGLGKGVTASSIGVVLKACGLRVTTIKIDPYLNTDAGTMSPFEHGEVFV
LDDGGEVDLDLGNYERFLDIKLTRDNNITTGKIYQSVINKERRGDYLGKTIQVVPHITDE
IQDWIERVAMNPVDGKEGPPDVCVIELGGTIGDIESMPFIEALGQFSYRVGPGNFCLVHV
SLVPVLNVVGEQKTKPTQHSVRGLRGLGLAPDILACRSTEPLEENVKAKLSQFCHVPVSS
IINLHDVTNIWHIPLLLRDQRAHEAILKVLDLQFVGKVPREPKLVEWTERASKFDKLKAT
VKIAMVGKYTGLSDSYLSVLKALLHASVAMGRKLVVEWVPSCDLEDSAAKETPEAHKKAW
KLLKGAEGILVPGGFGDRGVQGKILAAKYARENNVPYLGICLGMQIAVIDFACSIMKLPG
ANSTEFDPDTMSPCVIFMPEGSKTHMGATMRLGSRRTYFHATACKSAKLYGNARFVDERH
RHRYEVNPEMVPEFEKAGLSFVGKDESGRRMEIIELPSHKFFIGVQFHPEFKSRPGKPSP
LFLGLIAAASGQLETLLQPSSNIVNPNPMPRFPIPKKTIYHAKKPLDSLVNGYFANGNVI
HT*
>11682.m04784|LOC_Os05g49770.1|genepair728-2
MKYVLVTGGVVSGLGKGVTASSIGVVLKDCGLRVTSIKIDPYLNTDAGTMSPFEHGEVFV
LDDGGEVDLDLGNYERFLDIKLTRDNNITTGKIYQAVIDKERRGDYLGKTVQVVPHITDE
IQEWIERVAMNPVDGTDEPADVCVIELGGTIGDIESMPFIEALGQFSYRVGAGNFCLVHV
SLVPVLNVVGEQKTKPTQHSVRGLRGLGLIPDILACRSTQPLEENVKVKLAQFCHVPISN
IVNLHDVTNIWHIPLLLRDQKAHESILKVLDLQCVGKVPRAPKLTEWTERASKFDKLKTP
VRIAMVGKYTGLSDSYLSVLKALLHASVALDRKLVVDWVPSCDLEDSAATETPDAYEKAW
DLLKGAHGVLVPGGFGDRGVQGKILAAKYARENNVPYLGICLGMQIAVIEFARSVMKLRG
ANSTEFDPATTTPCVIFMPEGSKTHMGATMRLGSRRTFFQANTCKSAKLYGNASYVDERH
RHRYEVNPEMVPEFEKAGLSFVGRDESGTRMEIIELPTHRFFVGAQFHPEFKSRPGKPSP
LFMGLIAASSGQLDHLLQQSCGVVSSPVRRGNYCNGATKQQKLYQNGHVKNGLVNGCYYA
NGNSILHT*
>11667.m04493|LOC_Os01g46610.1|genepair729-1
MAFEKIKVANPIVEMDGDEMTRIFWQSIKDKLIFPFLDLDIKYYDLGVLHRDATDDKVTV
EAAEATLKYNVAIKCATITPDEARVKEFNLKQMWKSPNGTIRNIINGTVFREPIICKNVP
RLVPGWTKPICIGRHAFGDQYRATDAVLKGPGKLKLVFEGKDEQIDLEVFNFTGAGGVAL
SMYNTDESIRAFAEASMTTAYEKKWPLYLSTKNTILKKYDGRFKDIFQEVYEAGWKSKFE
AAGIWYEHRLIDDMVAYALKSEGGYVWACKNYDGDVQSDFLAQGFGSLGLMTSVLVCPDG
KTIEAEAAHGTVTRHFRVHQKGGETSTNSIASIFAWTRGLAHRAKLDDNARLLDFALKLE
AACVGTVESGKMTKDLALLIHGSSNVTRSHYLNTEEFIDAVAAELRSRLAAN*
>11682.m04783|LOC_Os05g49760.1|genepair729-2

MASTKIKVANPIVEMDGDEMTRVFWKSIKDKLIFPFLELDIKYFDLGLPYRDQTDDKVTV
EAAEATLKYNVAIKCATITPDEARVKEFSLKSMWKSPNGTIRNILNGTVFREPIICKNIP
RLVPGWTKPICIGRHAFGDQYRATDAVIKGPGKLKLVYEGKDEEIELEVFNFTGAGGVAQ
SMYNTDESIRSFAEASMATAYEKKWPLYLSTKNTILKKYDGRFKDIFQEVYEAQWKSKFE
AAGIWYEHRLIDDMVAYALKSEGGYVWACKNYDGDVQSDFLAQGFGSLGLMTSVLVCPDG
KTIEAEAAHGTVTRHYRVHQKGGETSTNSIASIFAWTRGLAHRAKLDDNARLLDFTQKLE
AACIGAVESGKMTKDLALLVHGSSNVTRSHYLNTEEFIDAVADELRSRLAAN*
>11667.m04516|LOC_Os01g46800.1|genepair730-1
MEGSSSDGSRYGGSGRCALVAELVQMQGMVRQLEAEMGTHGGGGGGGGGAAPMAAGADER
CRALVSGLLSSIDRSISIARSCCTEAAAAGRLTQQAGAAPESPPSADGSAGSDLGADSRC
RANAAGPCKKRKTLPKWSKQVKVRSVQDVGPLDDGFSWRKYGQKDILGAKYPRAYFRCTH
RHTQGCHASKQVQRADGDPLLFDVVYHGDHTCAHGVRSAAAAIDGQAAASAEQKHQPTPP
QEQNAVSVAFTSMAVVNASTSSPFVSPAMSDCQISYELGGGSMAGVRNVPDVELASKTNS
SMGDDMEFMFSLDSDFLDTYKYSSYF*
>11682.m04768|LOC_Os05g49620.1|genepair730-2
MVELCGGEGEGQIMLATELAQLRAMARELEAKMDPDRVAARELCRALASSVDRSIRLAAS
CFPPPEHPPPAAGNAGRDAAFKKRKGMAKVRRQVRVTSVQDTASLDDGLSWRKYGQKDIL
GAKYPRAYFRCTHRHTQGCNATKQVQRADGDPLLFDVVYLGDHTCGQAAVAAAAQSAPPE
HAGQEQQRQSSLLAAGTEGIHQQVVAEPMAAPFLFTSTAAGGVDDGYFSFISPANSDCQF
SSDFSAGSVGVDMDHEARFEDLFSSTLEFFQSEIQNL*
>11667.m04532|LOC_Os01g46950.1|genepair731-1
MGHYSEQRPGFEVTKDWNGADQVMIRSPRGASVLVSLHGGQVVSWRNDRGEELLFTSSKA
IFKPPKAMRGGIPICFPQFGNCGTLEQHGFARNRLWAIDDEAPPLNHNDNNGKVSVDLLL
KPSEDDLKCWPHCFEFRLRVSLSTDGDLSLVSRVRNVNGKPFSFSFAYHTYLSVSDISEV
RIEGLETLDYLDNLSQRERFTEQGDAITFESEVDRVYVGSPSVIAVLDHEKKRTFIVRKE
GLPDVVVWNPWDKKSKTMADFGDEEYKQMLCVDAAAVERAITLKPGEEWTGKLELSAVAS
TNCSDHLDYPCS*
>11682.m04748|LOC_Os05g49430.1|genepair731-2
MGHYTNLTDPRTELEVVRDWNGVDQVVLRSPRGAYARVSLHGGQVLSWRNDRGEELLFTS
SKAIFKPPKAMRGGIPICFPQFGNSGTLEQHGFARNRIWALDEEHPPLNQNDNNSKASVD
LILKPSEDDLKCWPHGFEFRLRVSLTKDGNLSLVSRIRNVNGKPFSFSFGYHTYLSVSDI
SEVRIEGLETLDYLDNLSQRERFTEQGDAITFESEVDRVYVSSPNVIAVLDHEKKRTFVI
RKEGLPDVVVWNPWEKKSKNIVDFGDEEYKQMLCVDAAAAERQITLKPGEEWTGKLELSE
VPSTNCSGHLDQPGIII*
>11667.m04534|LOC_Os01g46970.1|genepair732-1
MYQVSFLSLRVLSYFPVPLQPLVPSAFGKPYAAIYPPGGAFSHPFMPLMVSPLSMEPAKS
VNSKDSCSNKKMKEIDGAAVSTGSGNSEKTSGDCSLEGSSDGNNQKASGTPKKRSIDDRP
KSGVETGGALTPNDRPSEQAALPNLCIPVTAIKPDVSTASDFRVIATPVTEVPTKDDKES
KRERRKQSNRESARRSRLRKQAETEELARKVELLTAENTSLRREISRLTESSKKLRLENS
ALMEKLTETGPDEAQEVPPVKTKAQQARGVENFLSMIDKTGTPRSSGHMDHAIATPKLRQ
LLGSGLATDAVAAR*
>11682.m04747|LOC_Os05g49420.1|genepair732-2
MAHDEAVATQKIGKTTSPPKDQPTPCPFPDWSAVQAYYGPGVLPPTYFAPAIASGHAPPP
YMWGPQPIMPPPFGTPYAAMYPHGGAYPHPLMPMMANPLSMEPAKSASSKEKGSNKKLKE
VDGAAVSTGSGDSKKTMTSSGDYSAEGSSDVNDLKVGKTGRKRRLDDGAGAETSAAAKME
NALPPSHILGSTAILPNHSFPAQVIRPSATNVANSRALGTPISPPPGVIVPSHTGVSTEL
LIKDERELKREKRKQSNRESARRSRLRKQAETEDLATQVESLTAENTSLRSEISRLSESS
EKLRLENSALMGKLKDPAASTQAETSLQKTTTASSPRVVENFLSMIDNTNKTSVRHTEHA
EPKLRQLLGSGPATDVVAAS*
>11667.m01785|LOC_Os01g18220.1|genepair733-1
MASPSAAATARKLGSHLPRLRDIIDHDDEDDDFVEEEEEEEEEWEDMSKRMSRLSVEGSD
GGDADDEDDGYLRGEEEEEEEDGDEVRSDVNGEYGDGGGGGGQWHPYGDDDPRNPQAPSS
ASLPGTPDRGAPAPSPWMYSKEYASETEAARWPGGDKRRMRHRRERMMREVWLDRAWQMR
KQRRQMLMQGQGLGADAVTVVVSAAAAGGESPARGVAMDMEEMRACRDLGLDLPCDWTVE
IPCYALSGVDTASSGGNSPASGSWRISSPGDDPKDVKARLKVWAQAVALASASRLGS*
>11682.m00424|LOC_Os05g04830.1|genepair733-2
MATTTTTESLLPIGRVEAAAAEEEVVLGAGDDDSDVESGGESSFSREVRKRVSRLSVEGG
GGGGGGGVRDRRGSSGGGRRVLPPPHAWLAVEETAARKSYGSDPEEQWMRVLQGGAYGGG
GVAAAAAAQRQVQRRSSFSVVRRERAAREAWLDRAWEMKRSWHERNGGAPDADTPVVVVV
GKGPPSSPTSHAAGSVGGGGVAMDMEEVRACRDLGLELPSDCTVEIQCYGLSATSSPTHT
NSGSCSSGAASPSAAAACSLPSPGADDPMDVKARLKVWAQAVALASTTHLGS*
>11667.m01787|LOC_Os01g18240.1|genepair734-1
MGRHSCCYKQKLRKGLWSPEEDEKLMNHITKHGHGCWSTVPKLAGLQRCGKSCRLRWINY
LRPDLKRGAFSQEEEDLIVELHAVLGNRWSQIATRLPGRTDNEIKNLWNSCIKKKLRQKG
IDPNTHKPLAEVDRSKATPTISNDRTSESSDVDPSSGVALHNLSHLLSETAQSSELLPVK
VTKPRTQAPGLARLKVPPKELFLDQLTSGHENLPSCRSSGPIPNFPFQQLLCYNNDFNSM
DVGNRNSLWYNQNESSSSTISTVMPPVSPSTLSTSTGLNPSPDNANSRGTGIHNSQFYWD
TNNPSSSSSTGSSGNNGLGFELQSTSSLLETNIFPWSDLAPEKDSQAQLEEELKWPDLLH
GTFSEMPAPMQNLSQSLYEDVVKAESQFNMEGLCAAWSQNLLPQQHLPVVSDMYDKDLQR
MSLSFENI*
>11682.m00423|LOC_Os05g04820.1|genepair734-2
MGRHSCCYKQKLRKGLWSPEEDEKLMNHITKHGHGCWSSVPKLAGLQRCGKSCRLRWINY
LRPDLKRGAFSQEEEDLIIELHAVLGNRWSQIAAQLPGRTDNEIKNLWNSCIKKKLRQKG
IDPNTHKPLTEADRRGAAPTVSTERTSGSSDVNPSSAGALGNLSHLLSETAQSSMLLPVY

DKNHPETASLPRPKVPPKELFLDQLTAGHESPSSCRSSGPTLYFPFQQPLGYSNECGTGD
GASMNSLWFNQNDFNCSTISTVMPPVSPSALSTSMGLNLPPENPRHGGTGIGNTPFYWDG
SNPSSSGSTGSSGSNSMGFEPQSTTSILENSVFPWTDIGQEKDTRVHLVEELKWPDLLHG
TFAEATTAMQNQSQSLYDDVIKAESQFNMEGICASWFQNQQPQQQLQAASDMYDKDLQRL
PLSFEHI*
>11667.m01792|LOC_Os01g18290.1|genepair735-1
MSDGNDFAELLWENGQAVVHGRKKHPQPAFPPFGFFGGTGGGGGGSSSRAQERQPGGIDA
FAKVGGGFGALGMAPAVHDFASGFGATTQDNGDDDTVPWIHYPIIDDEDAAAPAALAAAD
YGSDFFSELQAAAAAAAAAAPPTDLASLPASNHNGATNNRNAPVATTTTREPSKESHGGL
SVPTTRAEPQPQPQLAAAKLPRSSGSGGGEGVMNFSLFSRPAVLARATLESAQRTQGTDN
KASNVTASNRVESTVVQTASGPRSAPAFADQRAAAWPPQPKEMPFASTAAAPMAPAVNLH
HEMGRDRAGRTMPVHKTEARKAPEATVATSSVCSGNGAGSDELWRQQKRKCQAQAECSAS
QDDDLDDEPGVLRKSGTRSTKRSRTAEVHNLSERRRRDRINEKMRALQELIPNCNKIDKA
SMLDEAIEYLKTLQLQVQMMSMGTGLCIPPMLLPTAMQHLQIPPMAHFPHLGMGLGYGMG
VFDMSNTGALQMPPMPGAHFPCPMIPGASPQGLGIPGTSTMPMFGVPGQTIPSSASSVPP
FASLAGLPVRPSGVPQVSGAMANMVQDQQQGIANQQQQCLNKEAIQGANPGDSQMQIIMQ
GDNENFRIPSSAQTKSSQFSDGTGKGTNARERDGAET*
>11682.m00415|LOC_Os05g04740.1|genepair735-2
MLRGNDTGSDLAELLWDNGAPAPLRPPPPPPFQPFTCSAAATTSPPAHDYLFIKNLMRGG
GAANHHHHDDDDDDDDDVPWLHYHPVVDDDDDADADTAPLPPDYCAALLSGLSDHLPPPA
AAASRVDPDPCSSSHGAVVPSTSAAAAKQARTSGGGGGGVMNFTFFSRPLQQRPSGGETA
SASASAAATSTVPVESTVVQAATNRLRSTPLFSDQRMAWLHPPKPSPRAAAPPPPPPLAP
TTRHRLDTAAATATVAQRLPPSEARAPDAPPPAATATATTSSVCSGNGDRRQLNWRDSHN
NQSAEWSASQDELDLDDELAGVHRRSAARSSKRSRTAEVHNLSERRRRDRINEKMRALQE
LIPNCNKIDKASMLEEAIEYLKTLQLQVQMMSMGTGMFVPPMMLPAAAAAMQHHHMQMQQ
MAGPMAAAAAHFPHLGAAAAMGLAGFGMPAAAQFPCPMFPAAPPMSMFAPPPPPPPFPHAA
ATAVEQTPSPPGAADAGNAPAVKQA*
>11667.m01803|LOC_Os01g18400.1|genepair736-1
MAKPTPAPRATPFLLAAVLSIVVVAASGAEARWYGGGGGGGYSPSPSPVSSIVSEQLYAS
LFLHKDDAACPARGFYTYASFVRAATRFPRFAATGCADARKREVAAFLAQISHETTGGWA
TAPDGPYAWGLCYKEEINPQSSYCDATDKQWPCYPGKSYHGRGPIQISWNFNYGPAGQAL
GFDGLRNPEIVANCSDIAFQTALWFWMTPRDTKPSCHQVMVGEYRPGPADVAANRTAGFG
LVTNIVNGGLECNRAGDARVNNRIGFYRRYCQVLGVDVGPNLDCEHQQPF*
>11682.m00410|LOC_Os05g04690.1|genepair736-2
MAGRRRRPFAAPVINYLLLALWLAASSSFAVAVAVAGHGRGRRSHVSSIVTEEMYNKSLF
IHKDDAACPARNFYTYAAFLRAADQYPSFGGAGGRDTRRREVAAFLAQVSHETTGGWATA
PDGPYTWGLCFKEELKPASNYCDAAVAARWPCFPGKSYHGRGPIQLSWNFNYGPAGEAVG
FDGLREPEVVAGDAVVAFKTALWFWMTPRPPSKPYSCHDVMTGRYRPSRADAAANRTAAG
FGLTTNIINGGLECNNRTGGDPRVEDRIGFFRRYCGALGVDVGDNLDCAHQLPYS*
>11667.m01808|LOC_Os01g18450.1|genepair737-1
MAGSWVRTITSPFRKVFGAQPHKDGGKRPQQPNSGMMVQHVVVDAERSKLHGEVMACAYE
DVQVMWDMLDKARIREFNSS*
>11682.m00409|LOC_Os05g04680.1|genepair737-2
MGSWVRTITSPFRKARTILNQQATNTSGSIDEHHHRHRAKKPRRHSSSSSAAGTPSSSSS
SSTTTATRTGGDVEEEVVMRRSSQLYGDVMACAYEDVQVMWSMLDNKSRLCAAAAAATS*
>11667.m01822|LOC_Os01g18590.1|genepair738-1
MAASDDEAGRGGGDGDASSPSLSNAAGGGGGGNKMRRVGGQDETAAAAPARENGEQQAAA
AAELPCRKPRVSVRARSEAPMISDGCQWRKYGQKMAKGNPCPRAYYRCTMAIGCPVRKQV
TKLIYMHSHNMRFLV*
>11682.m00405|LOC_Os05g04640.1|genepair738-2
MEMMVQKQRHEEGEEERGGLCAREIKELDFFSAAGAGAGRRDDDDVLRADGISSSHAGFM
VSTALDLLTAVNDGDHHEEKKGQSNIHQSKQMDAAATTVEGELRQAGEENRRLRRRLEEL
TSSYGALYHQLVQAQQLHTKHQQQAPIAGVQLLDALAAASPASHRRRAAAAVDGDRTADS
DGGEGDENVSPSLGSKRPAAAATLTRLTPESGSGGENNGGGEQAPAAEMAPCRKARVSVR
ARSEAPMISDGCQWRKYGQKMAKGNPCPRAYYRCTMASQCPVRKQVQRCAEDKSILITTY
EGTHNHPLPPAAAAMAKTTSAAAAMLLSGPAVSRDALFAAHHHVVAPPPFFHHPYAGSTM
ATLSASAPFPTITLDLTQPPPTTTTTAAAAMLQLHRPYAFSSLPFSMYGAGGGSHRPPVV
LPPPSSVVETMTAAITRDPNFTTAVAAALSSIMAGGGAQARTPPRGGSDAAGDINGGGGA
DHATAGARAAAAATQPCGTSPT*
>11667.m01827|LOC_Os01g18630.2|genepair739-1
MGRNHLCLVTCLWILSCAVLLHASPDGLLRISLNKKRLDKKTLDGAKLAREESHRLRADG
LGDDIVPLDNYLDTQYFGEIGIGTPPQNFTVIFDTGSSNLWVPSVKCYFSIACYLHHRYK
SKGSSSYKKNGESCSISYGSGSIAGFFSEDSVLVGDLAVKNQMFIETTREPSLTFIIGKF
DGILGLGFPEISVGGAPPIWQGMKEQQLIEKDVFSFWLNRDPDAPTGGELIFGGVDPNHY
KGSHTYVPVTRKGYWQFEMGDLLIDDYSTGFCSGGCAAIADSGTSLLGGPTTIVAQINHA
IGAEGIVSMECKQVVRDYGDMILEMLIAQASPMKLCSQIGLCAFDGTRSVRNNIESVVDK
EKVGSDLSCTACEMAVVWIQNQLRHNQTRELILQYADQLCERLPSPNGESAVDCDEISNM
PNLSFTIANKTFTLTPEQYVVKLEQQGQTVCISGFMAFDVPPPRGPLWILGDVFMAAYHT
VFDFGKNRIGFAESA*
>11682.m00400|LOC_Os05g04630.1|genepair739-2
MAKRHLLLVTTCLWALSCALLLHASSDGFLRVNLNKKRLDKEDLTAAKLAQQGNRLLKTG
SSDSDPVPLVDYLNTQYYGVIGLGSPPQNFTVIFDTGSSNLWVPSAKCYFSIACYLHSRY
NSKKSSSYKADGETCKITYGSGAISGFFSKDNVLVGDLVVKNQKFIEATRETSVTFIIGK
FDGILGLGYPEISVGKAPPIWQSMQEQELLADDVFSFWLNRDPDASSGGELVFGGMDPKH

YKGDHTYVPVSRKGYWQFNMGDLLIDGHSTGFCAKGCAAIVDSGTSLLAGPTAIVAQVNH
AIGAEGIISTECKEVVSEYGEMILNLLIAQTDPQKVCSQVGLCMFDGKRSVSNGIESVVD
KENLGSDAMCSVCEMAVVWIENQLRENKTKELILNYANQLCERLPSPNGESTVSCHQISK
MPNLAFTIANKTFILTPEQYIVKLEQGGQTVCISGFMAFDIPPPRGPLWILGDVFMGAYH
TVFDFGKDRIGFAKSA*
>11667.m01832|LOC_Os01g18670.1|genepair740-1
MPESWRDAEANASSSASVAAADSSPGNGKGGGGGGGAAVARGERAASASASARVPFHKLF
AFADKTDAALMALGTLGAVANGAALPFMTVLFGNLIDAFGGAMGIHDVVNRVSMVSLEFI
YLAIASAVASFVQVTCWMITGERQAARIRNLYLKTILRQEIAFFDKYTNTGEVVGRMSGD
TVLIQDAMGEKVGKFIQLVVTFLGGFIVAFAQGWLLTLVMMATIPPLVVAGAVMSNVVAK
MASLGQAAYAESSVVVEQTIGSIRTVASFTGEKQAVEKYNKSLKSAYKSGVREGLAAGLG
MGTVMVLLFCGYSLGIWYGAKLILLKGYTGAKVMNVIFAVLTGSLALGQASPSMKAFAGG
QAAAYKMFETINRKPEIDAYSTTGMKPDDIRGDIEFRDVYFSYPTRPDEQIFRGFSLSIP
SGTTVALVGQSGSGKSTVISLIERFYDPQLGDVLIDGVNLKEFQLRWIRSKIGLVSQEPV
LFAASIKENIAYGKDNATDQEIRAAAELANASKFIDKMPQGLDTSVGEHGTQLSGGQKQR
IAIARAILKDPRILLLDEATSALDAESERIVQEALDRVMTNRTTVIVAHRLSTVRNADTI
AVIHQGTLVEKGPHHELLKDPEGAYSQLIKLQEANRQDKSDRKGDSGARSGKQLSINQSA
SRSRRSSRDNSHHSFSVPFGMPLGIDIQDGSSDNLCDGMPQDVPLSRLASLNKPEIPVLI
LGSIASVISGVIFPIFAILLSNVIKAFYEPPHLLRKDSQFWSSMFLVFGAVYFLSLPVSS
YLFSIAGCRLIKRIRLMTFEKVVNMEIEWFDHPENSSGAIGARLSADAAKVRGLVGDALQ
LVVQNTTTLIAGLVIAFVSNWELSLIILALIPLIGLNGWIQMKFIQGFSADAKMMYEEAS
QVANDAVSSIRTVVSFSAEEKVMDLYKKKCEGPLRTGIRTGIISGIGFGVSFFLLFGVYA
ASFYAGARLVEENKTTFPKVFRVFLALAMAAIGVSQSSTLTSDSSKAKSAVSSIFAIVDR
KSRIDPSEDAGVTVETLHGNIEFQHVSFRYPTRPDVEIFRDLCLTIHSGKTVALVGESGS
GKSTAISLLQRFYDPDVGHILLDGVDIQKFQLKWLRQQMGLVSQEPALFNDTVRANIAYG
KEGEATESEIIEAAKLANAHKFISSSHQGYGTTVGERGAQLSGGQKQRIAIARAIVKDPK
ILLLDEATSALDAESERVVQDALDRVMVNRTTVIVAHRLSTIQNADLIAVVKNGVIIEKG
KHDTLMNIKDGAYASLVALHSAASS*
>11682.m00398|LOC_Os05g04610.2|genepair740-2
MFLVFGAVYFLSLPIGSYLFSVAGCRLIRRIRLMTFEKVVNMEIEWFDHPENSSGSIGAR
LSADAAKIRGLVGDALQLVVQNLATLVAGLLIAFISNWELSLIILALIPLIGVNGWIQMK
FIQGFSADAKMMYEEASQVANDAVSSIRTVASFSAEEKVMDLYKMKCEGPLRTGIRTAII
SGIGFGVSIFLLFGVYAASFYAGARLVEDRKTTFPNVFRVFLALTMAAIGVSHTSNLTSD
SSKAKSAVSSIFAIVDRKSRIDPSDDAGVSLEPLRGDIEFQHVSFRYPTRPDVQIFEDLC
LTIQSGKTVALVGESGSGKSTAISLLQRFYDPDAGHILLDGVDIQKFQLRWLRQQMGLVS
QEPALFNDTIRANIAYGKEGDATESDIVSSAQLANAHKFISSLHQGYETMVGERGAQLSG
GQKQRIAIARAIVKDPKILLLDEATSALDAESERVVQDALDRVMMNRTTVIVAHRLSTIQ
GADMIAVVKNGMIIEKGKHDALIGIKDGAYASLVALHVSAAAIS*
>11667.m01839|LOC_Os01g18740.1|genepair741-1
MADGLGAGQFINAVGDYARGLDRPRVSPVWAREAIPSPPKLPPGPPPELKMFQLRHVTAD
LSLDSINKAKSAYFAATGHRCSTFDVAIAKTWQARTRALRLPEPTSRVNLCFFANTRHLM
AGAAAWPAPAAGGNGGNGFYGNCFYPVSVVAESGAVEAADVAGVVGMIREAKARLPADFA
RWAVADFREDPYELSFTYDSLFVSDWTRLGFLEADYGWGPPSHVIPFAYYPFMAVAIIGA
PPVPKTGARIMTQCVEDDHLPAFKEEIKAFDK*
>11682.m00395|LOC_Os05g04590.1|genepair741-2
MKLIMCVLNLQVTEFTCGGFVVGLISVHTIADGLGAGQFINAVADYARGLAKPRVSPVWA
RDAIPDPPRMPAPPPRLELLDLRYFTVDLSPDHIAKVKSAFFESTGHRCSAFDVCVAKTW
QARTRALVAAAAAAGDDDQERRTVRVCFFANTRHLMLKGDGAAAAATGFYGNCFYPVAAV
ASGGEVAGADIVDVVRIVRDAKARLAADVARWAVGGFEEDPYELTFTYDSLFVSDWTRLG
FLDADYGWGTPSHVVPFSYHPFMAVAVIGAPPAPKLGARVMTMCVEEAHLPEFRDQMNAF
AAAN*
>11667.m01840|LOC_Os01g18750.1|genepair742-1
MGFAVVRTNREFVRPSAATPPSSGELLELSIIDRVVGLRHLVRSLHIFSAAAPSGGDAKP
SPARVIKEALGKALVDYYPFAGRFVDGGGGPGSARVECTGEGAWFVEAAAGCSLDDVNGL
DHPLMIPEDDLLPDAAPGVHPLDLPLMMQV*
>11682.m00394|LOC_Os05g04580.1|genepair742-2
MGFTVTRTSRSLVAPSSPTPAETLPLSVIDRVAGLRHLVRSLHVFEAGGRNGGGEPARVV
IREALGKALVEYHPFAGRFVEGDGGGEVAVACTGEGAWFVEATAACSLEEVKLLDHPMVI
PKEELLPEPAPDVQPLDIPLMMQCPTL*
>11667.m01847|LOC_Os01g18800.3|genepair743-1
MVNGEAEAECTRASLLGRYEIGRTLGEGNFGKVKYARHLATGAHFAIKILDRNKILSLRF
DDQIRREIGTLKLLKHPNVVRLHEVAASKTKIYMVLEYVNGGELFDKIAVKGKLSEHEGR
RLFQQLIDAVSYCHDKGVYHRDLKPENVLVDRRGNIKISDFGLSALPQHLGNDGLLHTTC
GSPNYIAPEVLQNRGYDGSLSDIWSCGVILYVMLVGYLPFDDRNLVVLYQKIFKGDTQIP
KWLSPSARDLLRRILEPNPMKRINIAGIKEHEWFQKDYTPVVPYDDDDDNYLDSVLPIKE
QIDEAKQEKPTHINAFQLIGMASALDLSGFFEEEDASQRKIRFTSTHSPKDLFDKIENVV
TEMGFQVQRGNSKLKVMKNGRGSKNLRNPSSFLVCTEVVELGPSLYVVELKKSHGDPILY
RQLCERLSDELGVCKTEQIQRTESLEDDLESFDSGSSLPGF*
>11682.m00391|LOC_Os05g04550.1|genepair743-2
MVKGGREALLGGYEMGRTLGEGNFGKVKYARHLATGGHFAVKILDRGRVVSLRAGDQIRR
EIATLKLLRHPHVVRLHEVAASKTKIYMVLEFVNGGELFERIVSPCFNDLLRYFLAAKIF
MPATMAVKGKLSEKEGRRLFQQLIDGVSYCHDRGVYHRDLKPENVLVDQKGNIKISDFGL
SALPQHLGNDGLLHTTCGSPNYIAPEVLQNKGYDGSLSDIWSCGVILYVMLIGYLPFDDR
NIVVLYQKIFKGDTQIPKWLSHSAQNLLRRILEPNPMKRIDMAGIKSHEWFQKDYIPVLP

YDDDDEDVQFGARLPAKEQINDEPGDKNSHQINAFQLIGMASSLDLSGFFEDEEVSQRRI
RFTSTHPPKDAFDKIESSATELGFQVQRGHSKLKLMRNCKGSKNPESFMVSAEVFELGPS
VNVVELRKSNGDPALYRQLCERISSDMGARNTEQIFATASLEDDLQNSNAGTPLFAL*
>11667.m01849|LOC_Os01g18810.1|genepair744-1
MEIEFTYQVKWEMSEDGSPATYFSFFMVLPFMESSTALQMESEPIVGCDGDGCASGAGTW
PLHHFRRLDGVHCRLCSSCLLLEYRSFYCCCCFLLLGPEPPAHFDDGDPILAPPVPVATC
RLCNEAVAHRYCLQSDDDTFVCAACVAAAHGWRFSYTPTAPPPAALAATTTGGVVSDAPL
DIRATRIMLLASRISLAVLRKAAAAARATAERLFVEAKAEKARAYRALAVALGVDAEVPS
ANHGADEPEPLPMLQAPPPPEDMAPESSSTATNMGALPPSENVAPPESDASSVAMALAMA
PPSENLPSEGNLVAMAMGLDLNAPPPSPAADTIGVGDVAEMTMAAEASSSSPPLPPPPPP
QPRRRPLQLFPDDDM*
>11682.m00390|LOC_Os05g04540.1|genepair744-2
MPPPPPMMPTGGYPGHRGCGGDRCGSGRDAWPLHNVRHQGVFCRLCSSCVLLYHPAAFCS
ACLLLLPPAAAPVAAPPGPVSACSSCGLFVAHHSCVPDPVSFVCPTCADAAVGRVYSYTP
AAAAGRRRTMDERAARVLLVAARLAHESISRAAAAAREEADRAAREAAVARKHARELLDA
ACRAAEPEALEAKKKAEAPVAPAAAPPPPPPQPKKKAPPKSSEASRDRDNKPLKLTATQQ
PALAFAAAAAAAAASSMPLSMPSPMEVDEKPVIEELQGSGDGSLIYDRGSLFGTLQRFASL
SSPCVCDVGIGLGWLDRPTSSEQIF*
>11667.m01852|LOC_Os01g18840.1|genepair745-1
MPISERATTTPSSSYYSLELDPLLSDLAEKKLSLRRSLAWLDAELKDAKIKLASKEQLLA
QESENRKKFAESRARSMEEEVKKLHKCLQDKDEQLRTSICSTEQQYLSSYKLDILRSQIS
VAQATAEASAESAMLARLQCLSLSGGHEKINSLGECELRVKKVEEQLDLVQKFLEAKELS
QLEKNQMTTVHELKKKVLKLECTLKVSRAQLRKLHKMVERRDKPLKKLQSRLPLKQQTAC
DKQKLWESSGFRIIASMSILALAMLSKR*
>11682.m00389|LOC_Os05g04530.1|genepair745-2
MVILTSPPLSLFFIFSLLSLSITGGGAAAAVTAYGGGARRPSAHAPGAFSEKVVPPSSVS
SSDLDPLLKDLTEKKLSFRRNVVSLAAELKDVRNKLASQEQLFVRESQTRKFAETKARSM
EEEISKLQKCLNDKDEQLRSSTGCTEQMHFLNNYSNYVQYFHELDDLRSKLSVTQATAEA
SAASAKSAQSQCLSLLKELNEKDSSLKEHERRVNKLGEQLDHLQKDLESREYSQRQLKDE
VLRIETDIMDAVAKVGSNKDNELLKILSDVSPRNIDNLNKLLNAKDAEIARLRDEIRILS
AHWTNKTKELESQLEKQKRTDQELKKRILKLEFCLQESRSQIRKLQRAGEKRDKQLKELK
DQVGMKQPSVPYRDDKHNFWENQSFKFIASMSMLALVMLTKR*
>11667.m01855|LOC_Os01g18860.1|genepair746-1
MAEVDTFLFTSESVNEGHPDKLCDQISDAVLDACLAEDPESKVACETCTKTNMVMVFGEI
TTKANVDYEKIVRDTCRGIGFVSNDVGLDAEHCKVLVNIEQQSPDIAQGVHGHFTKRPEE
IGAGDQGHMFGYATDETPELMPLSHVLATKLGARLTEVRKNGACAWLRPDGKTQVTVEYQ
NDNGAMVPLRVHTVLISTQHDETVTNDEIAADLKEHVIKPVIPEQYLDEKTIFHLNPSGR
FVIGGPHGDAGLTGRKIIIDTYGGWGAHGGGAFSGKDPTKVDRSGAYIARQAAKSIVANG
LARRCIVQVSYAIGVPEPLSVFVDTYGTGKIPDREILRIVTENFDFRPGMIIINLDLMRG
GNGRYLKTAAYGHFGREDPDFTWEVVKPLKWEEPSA*
>11682.m00386|LOC_Os05g04510.1|genepair746-2
MAALDTFLFTSESVNEGHPDKLCDQVSDAVLDACLAEDPDSKVACETCTKTNMVMVFGEI
TTKANVDYEKIVRETCRNIGFVSADVGLDADHCKVLVNIEQQSPDIAQGVHGHFTKRPEE
IGAGDQGHMFGYATDETPELMPLSHVLATKLGARLTEVRKNGTCAWLRPDGKTQVTVEYR
NESGARVPVRVHTVLISTQHDETVTNDEIAADLKEHVIKPVIPEQYLDEKTIFHLNPSGR
FVIGGPHGDAGLTGRKIIIDTYGGWGAHGGGAFSGKDPTKVDRSGAYVARQAAKSIVASG
LARRCIVQVSYAIGVPEPLSVFVDTYGTGRIPDKEILKIVKENFDFRPGMIIINLDLKKG
GNGRYLKTAAYGHFGRDDPDFTWEVVKPLKWEKPSA*
>11667.m01859|LOC_Os01g18890.1|genepair747-1
MSQANNPSLRGFAVIDRAKRVLERRCRGTVSCADIVAFAARDACGIMGGIDFAVPSGRRD
GAVSAESDVLNNLPPPFFNATQLVAGFAAKNLTADDMVVLSGAHSFGRSHCSAFSFRLYP
QVAPDMDAAYAAQLRARCPPPAAPPATGRRDRVVDLDPVTKLVLDNQYYKNIQRGEVLFT
SDATLVSQSDTAALVDLYARNRKLWASRFAAAMVKMGNLDVLARAVQTEVEKDMVISNWS
QLDSAVSTQMTATGF*
>11682.m00382|LOC_Os05g04470.1|genepair747-2
MAAGSRDGGARRRSCSVLVAAAAIFFGYAATAAGLQVGYYNNSCPGAEDLIQTIVHGAVR
NDAGNGPGLIRLFFHDCFVRGCDASVLLDADPASNGTVEKMAPPNFPSLRGFGVIDRAKR
VVERRCPGVVSCADIVAFAARDASRIMGGIKFAMPAGRLDGRVSSASEALANLPPGSFNL
TQLVARFATKNLTADDMVTLSGAHSIGRSHCSSFSSRLYPQIDPAMNATLGVRSRAKCAA
APGRLDRVVQLDFKTPLQLDNQYYQNVLTHEVVFTSDQSLIDRPDTAALVAQYAGSRKLW
SQKFAAAMVKMGNLDVLTGPPGEIRQYCNKVN*
>11667.m01861|LOC_Os01g18910.1|genepair748-1
MKLILMVAFQAMSLISISTASLQYNFYGSSCPNAEQTISNVVYGLIDADPSMAPALLRLH
FHDCFVMGCDASILLDPTKANGSPEKTAIPLRGYDAVNKIKAAVEAVCPGKVSCADILAF
AARDSVTKSGGFVYPVPSGRRDGDVSSAFSVFSSIPSPFFDADELVQSFAAKGLTVDDLV
ALSEPAVPDGGRLPGRELRGGAAADDGVVNNSPVSPATLGNQYFKNALAGRVLFTSDAAL
LAGRNDTAEKVRENAGDLTAWMARFAASMVKMGGIEVLTGARGEVRGFCNATNS*
>11682.m00380|LOC_Os05g04450.1|genepair748-2
MAMATAACRRPAVMLMAFAMAAAVTMSSVPPASGTTTLQYDFYSSSCPKAEETVRNVVEP
MIFNDPTMGAAFIRLFFHDCFVRGCDASILLDPTSRNTQPEKTAIPLRGYDAVNKIKAAV
EAVCPGKVSCADILAFAARDSAVVNGNFAFAMPSGRRDGTASSASDVARFIPSPAFHLQD
LVDSFAAKGLTADDLVILSGAHSFGLTHCAFVTGRLYPTVDPTMNATFAAALKKLCPPPA
SGGGGRAVSNNQVTDPNVLSNQYFKNVAAGEVMFTSDQTLTSRDDTKAMVDDNAANPVAW
MARFAAAMVKMGGVEVLTGNAGEVRKVCFATNTAS*

>11667.m01867|LOC_Os01g18970.1|genepair749-1
MKVFMAVCMLAVAVRLAAAIVVPSAAPCALTVGFYNGKCGNVSVESVVYDTVKAFLDADK
SKGAALVRLLFHDCFVNGCDGSILLDNSTTNPSPEKFAGANLGIAGLDVIDAVKAKLETA
CPGVVSCADIVVFAGRDASRYMSNGGVNFDVPAGRLDGIVSSSVDAQNTLPDSKADIGKL
IANFAAKGFTPEELVILSGAHSIGKAHCSNFDDRLTAPDSEINADYRDNVLSKTCKSAPN
PTLANNIRDIDAATLGDLASYVVPAVGGDYLDNSYYKNNKNNLVLFNSDWALVGSNATLQ
HVNEYAENGTLWNIDFAQALVKLSKLAMPAGSVRQIRKTCRAINY*
>11682.m00379|LOC_Os05g04440.1|genepair749-2
MVKLVCFVVVVFMAAAAAMAGADRELKVGYYEKTCKDVEKIVNSIVVNSIKDNRGKGAGL
VRLLFHDCFVRGCDASVLLEKSEMNRQPEKESPANIGIRGMDVIDAIKAVLEARCPNTVS
CADIIAYAARDASRYLSHGGVDFPVPAGRLDGVVSRSRDADAFLPDAAANLTDLVRNFRR
KNFTVEELVILSGAHSIGVTHCTSFAGRLTAPDAQINPGYRSLLVSKCGGVSPTPANNHV
VVNNVRDEDGAAVARVMPGFAARVRKARDYLDNSYYHNNLAMAVTFHADWALLTGKEARG
HVVEYAKNATLWNVDFGDALVKLSKLPMPAGSKGEIRAKCSAVNGYHH*
>11667.m01872|LOC_Os01g19020.1|genepair750-1
MRLSVAILCALVAVQAAALLLAGSAAAASELKVGYYHKKCKGVENVIKWHVIKALKQNRR
TGAALVRLLFHDCFVRGCDGSVLLDKSYENPHPEKEAPVNIGLAAFDLLEEIKAAVEKRC
PGVVSCSDILIYAARDAGSILSNGHVHFDVPAGRLDGVVSRADEAQAELPDSTMTVQQLK
DNFAAKGFDTEQLVILSGAHSIGQGHCSSFTGRLSEPPQQITPAYRDLLNYKCSQAANPD
VVNNVRDEDASVVARFMPGFVSRVRKISDFLDNTYYHNNLAKIVTFHSDWQLLTDATSLS
KVHEYADNATLWDSDFSDSLLKLSQLPMPEGSKGEIRKKCSAINHLY*
>11682.m00373|LOC_Os05g04380.1|genepair750-2
MTMRLAAAVVVMVVVAAAAMSVEAELSVDYYKKSCPGVENVVRYHVAKALKANRKEGAAL
VRLIFHDCFVRGCDASVLLDPTAENPHTEKTAPINIGLAAFELIDEIKAAVEERCPGVVS
CADIVIYAARDASILLSNGHVHFDVLAGRLDGRVSRAADAQRDLPDSTFTISELIRNFRR
KNFTIEELVILSGAHAVGVGHCSSLRARLTAPPEQILPGYRSLLAGKCAAGEDPIVPNNV
RDEDPAAVAATIPSFLPKLRKFEFLDNSYYHNNLARIVTFNSDWQLLTEKKARGHVHEYA
DNGTLWDEDFSDALVKLSKLPLPPKAKGEIRRHCRRVNTHHY*
>11667.m01883|LOC_Os01g19130.1|genepair751-1
MVAGAEVMHQVVPLLEASFHRRCSVKGVDEVSPPVEEMSPEAASEAAIEVPELMVKAPVE
SLQFSPNIRSGSFADIGPRRYMEDEHIRIDDLSGHLGSLLMCPAPNAFYGVFDGHGGPDA
AAYMKRHAIRLFFEDSEFPQALEEDESFYESVEKSIHNAFLSADLALADDLAISRSSGTT
ALAALIFGRQLLVANAGDCRAVLCRKGVAVEMSRDHRPTYDAEHERITECGGYIEDGYLN
GVLSVTRALGDWDMKMPQGSRSPLIAEPEFQQTTLTEDDEFLIIGCDGIWDVMSSQHAVT
IVRKGLRRHDDPERCARELAMEAKRLQTFDNLTVIVICFGSELGGGSPSSEQAPIRRVRC
CKSLSSEALCNLKKWLEPNE*
>11682.m00371|LOC_Os05g04360.1|genepair751-2
MVAEAEVMHQPVPVLEVPYHRCVAKGVEEVAAAAAVAPPPVVEVEVAVQVPHMGLESAAG
APSISVDALQFVPSIRSGSFADIGPRRYMEDEHIRIDDLSAHLGSLLVCPLPSAFYGVFD
GHGGLDAAAYMKRHAMRFLFEDSEFPQASQVDETYVQSVENSVRRAFLQADLALADDLDI
SRSSGTTALTALVFGRQLLVANAGDCRAVLCRRGVAMEMSRDHRANYAEECERVAASGGY
IEDGYLNGVLSVTRALGDWDMKMPDGSISPLIAEPEFRQTMLTEDDEFLIMGCDGIWDVM
TSQHAVSIVRRGLRQHDDPERCARELVMEAKRLETADNLTVIVVCFVSELGSPRQEQVGG
QAGVARPRSCKSLSAEALCNLRSWLETDR*
>11667.m01887|LOC_Os01g19150.1|genepair752-1
MTSVGLVDSSSGFPETSTSGATDRLTDDISEMSIRDKEVEAVVVSGNSMDIGHTIVTTVG
GRNGQPKQTISYIAERAVGRGSFGVVFQAKCLETGERVAVKKVLQDARYKNRELQTMQVL
DHPNVACLKHYFCSTTAKEELYLNLVLEYVPETVHRVIRHYNKMSQRMPLIYVKLYMYQI
CRALAYIHNCVGVCHRDIKPQNILVNPHNHQLKLCDFGSAKVLVKGEPNISYICSRYYRA
PELIFGATEYTTAIDVWSAGCVLAELLLGQPVFPGDSGVDQLVEIIKVLGTPTREEIKHM
NPNYTEFKFPQIKAHPWHKIFHKRMPSEAVDLVSRLLQYSPHLRCSALEVLIHPFFDELR
DPNARLPNGRTLPPLFNFKPRELKGASMEFLVKLVPQHAKKQCAFLGL*
>11682.m00369|LOC_Os05g04340.1|genepair752-2
MASVGVVRSSLGFQNETSTSGDADRLPNEMSNMSIRDDNKDIDDIVVNGNGTEPGHVIVT
SIDGRNGQAKQTISYMAERVVGHGSFGTVFQAKCLETGETVAIKKVLQDKRYKNRELQTM
RVLDHPNVVSLKHCFFSKTEKEELYLNLVLEYVPETAHRVIKHYNKMNQRMPLIYAKLYM
YQICRALAYIHNTIGVCHRDIKPQNLLVNPHTHQLKLCDFGSAKVLVKGEPNISYICSRY
YRAPELIFGATEYTTAIDVWSAGCVLAELLLGQPLFPGDSGVDQLVEIIKVLGTPTREEI
KCMNPNYTEFKFPQIKAHPWHKIFHKRMPAEAVDLVSRLLQYSPYLRSTASEALIHPFFD
ELRDPNTRLPNGRFLPPLFNFKPHELKGMPMEFLVKLIPEHARKQCAFVGW*
>11667.m01906|LOC_Os01g19330.1|genepair753-1
MDAPCGWTTSAAAAVAEEEDVRRGPWTVEEDAVLAGYVAANGEGRWNELARAAGLRRTGK
SCRLRWLNYLRPDVRRGDFTPQEQLLILELHFRWGNRWSRIAQHMPGRTDNEIKNYWRTR
VQKHAKQLGCDVNSRQFKDVMRHLWMPRLVERIHAAAASSERAAPPPCAAAPASHSGMCH
SPDPSTTTSSMAGSSVTHGEQFPSSTNHHLMTMASVTTAAADWSSEQCGSGSATSTSVGI
SYDMFEGSWSELLARAYDDDGADSSLLPDFQMADTGDNCWWSNLEDIWSQQPY*
>11682.m00356|LOC_Os05g04210.1|genepair753-2
MEMMRMAMSPAMSSATAAAASEDEGDLRRGPWTVEEDMLLVDYIANHGEGRWNSLARCAG
LRRTGKSCRLRWLNYLRPDVRRGNITADEQLLILDLHSRWGNRWSKIAQYLPGRTDNEIK
NYWRTRVQKHAKQLRCDVNSKEFRDVVRHVWMPRLIERIQADAAAAGEVAAPAPVSAAAT
RSMSSPAGAMYLHHQQIPLAAGAMVVAPAVSSEAYHHHGCGGGGDTSCSEPSQAAVTMSP
DDASSTLRSSSAAAENDTIHGDVLSGSWSELLATTTTTIAATAGLPDFDELGDFEDNLWS
LEDIWLHQQC*
>11667.m01914|LOC_Os01g19410.1|genepair754-1

MESKEVHGRDVLVDLEKGNCLLMREGGDNGTDVNMISSQAKTPWNDLIAMKDDHHIPCCS
SRSQDFAVKSGEDRTSDGEMKVGLLDKSMGEKEKKKRSKKPPRPPRPPSASPLDAADQKL
ISELSELASLKRARIERMKALKKMKNTKPASSIGNLVALIITIIFCLVILWQGVFSKYGA
GIIFHGSPISSGRSHGSLFSIQFYKKNETATSPRSSSSAPKCDSL*
>11682.m00348|LOC_Os05g04150.1|genepair754-2
MDAKGVNDRDALIDLESGNNIVVGEHNNGADANFAVAQERTAPNGSWNGAMGTNGCLKDD
VNHQHMCCCLSSPDAAAKNGDDRKSDGEEKLGLLDTSGGEKTKKKRSKKPPRPPRPPTPT
PLDVSDQKLLNELSELAILKRARIERMKALKKMKNAKHGSSSGNLFPLIITIIFCLVILW
QGFFSRTGSAVSFHGSPESSVRAHSSLISIRFYKNNNSNSRSREAISAAPKNAETPSRLE
IHSKARKITR*
>11667.m01934|LOC_Os01g19610.1|genepair755-1
MEHTNLPSHGAQAAEAEALAAAADLEQGLTRHLMEYHQSEASSDESARQRPRVGRVPPHV
RNLDGGAEAYTPKFVSIGPIHHADATLRRHSHDLKVAYLHALIARRTPDPIDEVAVLAAL
IGYKAGVAAVEDRARRFYKEPVDEHLTAEAFVDLLVLDAAFLLEHMLNLATGYEDPLLHR
THWAPSQLHSDLIRFENQVPFFVVAELLALSPLHRDPELEACRSGRRDFLRSIGVHCLLR
KDDEELKTLPPSDDIHHLLHLYSLSLTEARLRRPHAHAGLGATAWRALWKLPIVTLMPFA
YLLCSGGADDGKGEEEDEAAAVKLPNIPSATDLQRVGIKFKRAPRKPDGGFLDVRLEDGD
TLVIPMVNIEQFTAPQLQNLIALEQATPELPDDCSCYAFFMDNLVANPADVALLESEGIL
KSNLGSHKAVVTYFNKLCKGNKLEVEGNYLRSVFEALMERNRNPMYAWIRTLRKKYFSSP
WGIIAMVVTLFVFASTVLQTYISIVQYYFANNGDY*
>11682.m00339|LOC_Os05g04060.1|genepair755-2
MEEFDGAIARRRAMARRGSGGGNGDGDGITVHVEQMARGLMQRQEAAASDEQHRIMASSH
RVSRVPAHLRDANADAYTPRFVAVGPLHRGDARRLGAGERLKMAYLHSLISRGHSDQARQ
LAVIEEYIRAVAAREREARAFYSEDVDMYAEEFIMMLVLDGCFIIEHLVNVAIGRDEPSL
HATPFAPVQLSVDLILAENQIPFFVLVDLVRITDLPEFASTGHPPPVLIVKLVLYYLAGE
KGRDMVGDALPPAEGVSHILHLLHAMIVAARTKWEPPPRIQDGAVLGTAQDGARLLRRLP
LLLLVPLLYPILPEESKWRASYGREDVPSASDLKRMWVRFKKPRGGGAAAVTGIASVMGP
VPLAVKLAHEDKLRLPQLRVELRTAPLLLNLMAFEQSAAKAEARDVSAYVSFMAKMVQSA
EDAGALAAAEVVAVVHGNGGGEGKEEVVRLFRQVGAASGEVELERSYLGGMVVELRERSR
HPLFMMWADVKRNYFTVPWAVVAEFVAFVTFVSTIVQMYSSFKQKGG*
>11667.m01942|LOC_Os01g19690.1|genepair756-1
MEDLYSIHPGISRVGGAASEASGVGVVVGGGGGSSSSDLTELMKAQIAGHPRYPTLLSAY
IECRKVGAPPEVASLLKEIGRERRAGGGGGGAGQIGVDPELDEFMEAYCRVLVRYKEELS
RPFDEAASFLSSIQTQLSNLCSGATSPPATTATHSGNQHIHYICMHTWQ*
>11682.m00322|LOC_Os05g03890.1|genepair756-2
MEDLYSIHPGISRGGGGGGGGAASEASGVAGGGSSPPHPPPPATTAAAADLTELMKAQIA
GHPSYPSLLSAYIECRKVGAPPEVTTLLEEIGREGRGGGGGATAGGEIGLDPELDEFMET
YCRVLERYKEELTRPFDEAASFLTGIHTQLASLCGGAPPPTDNSALVGQAHFE*
>11667.m01943|LOC_Os01g19700.1|genepair757-1
MVGSSDEDQCSGETDMLDIGQEQSSRLADHELKEMLLKKYSGCLSRLRSEFLKKRKKGKL
PKDARSALLEWWNTHYRWPYPTEEDKLRLAARTGLDPKQINNWFINQRKRHWKPSDGMRF
ALMEGVAGGSSGTTLYFDTGTIGP*
>11682.m00321|LOC_Os05g03880.1|genepair757-2
MVGSSEDEPCSGDADAADFGQEHSSRLADHELKEMLLKKYSGCLSRLRSEFLKKRKKGKL
PKDARSALMDWWNTHYRWPYPTEEDKVRLAAMTGLDPKQINNWFINQRKRHWKPSEDMRF
ALMEGACGPVGGNFKGSYLTMLSSGGLVFGIINIVGNFGTVFVDNGFVYGIIPEKGKPVS
SASDNVRSWWKEKHYDPPQRRFPLEKGVPPPWWPEGTEAWWPESVMPSPSWPPPSFPPSS
TPPSDLDGILSRRRRRTPLPLHRA*
>11667.m03848|LOC_Os01g40070.1|genepair758-1
MRSTSGARPSSGGGGGDDDYAFYYSFFQDAAAAAASPLGLDDAAAMTNGGRKRKRGGGGD
GADGGAPAASRKDGPGGDGEGGNGRKRSIAKILTSLAALEAEEHSDRAGAADASRRELAL
LESNADHKSQAMMDYYAKMEGSFDAAAESDATARSKRSRLAASATTAAVVATEEGAAETA
SASASPSRASGGGGAGHHQRRLWVKDRSRAWWDKCNSPDYPEEEFRRAFRMGRETFDMIC
EALGSAVAKEDTMLRAAIPVRQRVAVCIWRLATGEPLRLVSKRFGLGISTCHKLVLEVCA
AIKSVLMPRFLQWPDEAAAAAFKERFQAAYGVPGVIGAMYTTHIPIIAPKISVAAYFNRR
HTERNQKTSYSITLQGVVGPDGAFTDVCIGWPGSMPDDQVLEKSMLHQRAAAGMMHSACL
VGGASYPLMDWVLVPYTHQNLTWTQHAFNEKVGDLRRVAVDAFARLKARWACLQKRTEVK
LQDLPVVLGACCVLHNICETRGEELEPELRFELVDDETSPETPVRSEAAKRARDNIAHNL
LHRGFAGTTFF*
>11682.m04974|LOC_Os05g51520.1|genepair758-2
MSNRRRKRGESKPQAHDAGDTTPIDNILTSLDDAPPPPPPPRHELPLLQLNDHQEADDAD
ASSSSSPHQQRRLWVKDRSRAWWELCSSADYPEADFRRAFRMSRPTFHFLCDALAAAVAK
EDTALRAAIPVRQRVAVCVWRLATGEPLRVVSKRFGLGISTCHKLILEVCAAIRNLLMPR
FLHWPDHPTSTAYKTRFEATSGVPGVVGAMYTTHIPIIAPKVSVAAYLNRRHTERNHKTS
YSITLQGVVGPDGTFTDVCIGWPGSMSDEQVLRKSALHQRASAAAGSMSWVVGGASYPLT
EWMLVPYAQRNLTWTQHAFNEKVGEVRRVATEAFVRLKGRWACLQKRTEVKLQDLPAVLA
ACCVLHNICETRGEDMDPDLRCDLPPDEEEDDTVLVQSESANKVRDDIAHNLLHRGLAGT
AFF*
>11667.m03850|LOC_Os01g40090.1|genepair759-1
MEDVAVAAALAPAPATAPVFSPAAAGLTLIAAAAADPIAAVVAGAMDGVVTVPPVRTASA
VEDDAVAPGRGEEGGEASAVGSPCSVTSDCSSVASADFEGVGLGFFGAAADGGAAMVFED
SAASAATVEAEARVAAGARSVFAVECVPLWGHKSICGRRPEMEDAVVAVSRFFDIPLWML
TGNSVVDGLDPMSFRLPAHFFGVYDGHGGAQVANYCRERLHAALVEELSRIEGSVSGANL
GSVEFKKKWEQAFVDCFSRVDEEVGGNASRGEAVAPETVGSTAVVAVICSSHIIVANCGD

SRAVLCRGKQPVPLSVDHKPNREDEYARIEAEGGKVIQWNGYRVFGVLAMSRSIEVFWSV
KVKEMKE*
>11682.m04973|LOC_Os05g51510.2|genepair759-2
MEDLALPAAPPAPTLSFTLLAAAAAVAEAMEEALGAALPPLTAPVPAPGDDSACGSPCSV
ASDCSSVASADFEGFAELGTSLLAGPAVLFDDLTAASVAVAEAAEPRAVGATARSVFAMD
CVPLWGLESICGRRPEMEDDYAVVPRFFDLPLWMVAGDAAVDGLDRASFRLPAHFFAVYD
GHGGVQVANYCRKRIHAVLTEELRRAEDDACGSDLSGLESKKLWEKAFVDCFSRVDAEVG
GNAASGAPPVAPDTVGSTAVVAVVCSSHVIVANCGDSRAVLCRGKQPLPLSLDHKPNRED
EYARIEALGGKVIQWNGYRVLGVLAMSRSIGDKYLKPYIIPVPEVTVVARAKDDDCLILA
SDGLWDVMSNEEVCDAARKRILLWHKKNAATASTSSAQISGDSSDPAAQAAADYLSKLAL
QKGSKDNITVVVIDLKAHRKFKSKA*
>11667.m03856|LOC_Os01g40150.1|genepair760-1
MARTASAATGGDEPPPEPHHQAGVQRPPKNDGDGGEEAGGGNVGVPAAAADEDPTAGAVG
AAGRGSRRRRKAGRRAAAPDEEDLDTILAEIDQNMASAALDSMPEMNLAQASASTAMDVA
ATVNDVGAEVKDDEMEAESAAPKRRKRKKEKEKERKTTSKVAEADAKKPPRHVRVMLEVL
AKRKEAEELRKREEELRKRKAEEERLQREEDERMVEEMKMQQKERDKGKTMKKRQDGKTL
TGKQKEEARRLDAMRRQFLGQSRILARSDLGNDGGANERKKRPIYDSKRKKVQSKADEAA
NGDGGHMQELHKVNKEEEECAIMEEQPHYRVEEDGEKIKLEEIKGAESVERINFEERITK
EEKNEAMKSSNEEVVSLVTGWKNRIEEWDVDVDENDKDTRKLTPKRDPAKVDKAEKYTDL
RSPICCILGHVDTGKTKLLDCIRCTNVQGGEAGGITQQIGATFFPIENIRERTKELKAGA
ALHVPGFLVIDTPGHQSFSNLRTRGSSLCDIAILVVDIMRGIQAQTIESLNILKRHKADF
IIVLNKVDRLFGWKRCPNAPIKKALKQQAEGVKMEFDARLTDCTVLEVKVTEGHCTTIDV
VLANGFLREGDQIVTCGMQGPIVTHIRALLTPHPMKELRIKCPYQHHKEIKASQGIKISA
PGLEHSVAGTSLFVVQPGDDQEKSVNKAMAEMVVLMNRIDKNSDALIEFLKSPAVNIPIY
DFNIGPVHRKDVMKASVMLEKKKEYAAIFAFDVKVMPDAREIANESGVKIFVADVIYHLF
DQFTTYIEGLREIEKDEKIVEAVFPCVLKIIPDYVFNLKDPIVLGVDVLEGVAKVGTPLC
LPSNGFARIGNIASIQNSSKQVDVARKGEKVAIKNIVLGVFDRSQEALQMSKRNVSGDLT
LKEWELVRTLKHIFRIP*
>11682.m04971|LOC_Os05g51500.1|genepair760-2
MGRKKNVAIDDDDAFDALAADEDQGEDDDEAPAPAPAPVEPDAAAKSDAEDDDLDFDFSK
AKKKKKKKDKGARPVPLEHDDLDLDKPAPPPPAAAADEADDDEAAAAAASKKPQKKKKKK
GGFTVDNEDIDKLLAEIDDTSPPTEEAEPVEEVPAPDADDALGKKSKKKKKKGGFTVDDE
DVDKILAEFEDQPPPVDDPEPEPEAVKDVGNVAASTSVDDAEGKKSKKKKKKSGRTAQEE
EDLDKLLAELGEGPTPAEKEKEVLPQAPPAAAMVKEDTETAEDGKAGEGEVESAAAKKKK
KKKEKEKEKKAAAKEADAKKEEEKAVEAPKGKVDMKKLPKHVREMQEALARRQEAEERKK
REEEERLRKEEEERLKKEEEERKAEEAKRRKKEREKEKLLKKKQEGKLLTGKQKEEAKRL
EAMRRQFLEQSELQVADGAVPETKKRPIYDSKKKKGQQKTLETAKVVEEQPQEVNETIND
EEEYVLVDQESQLQVEESEKTEPDQDVEELKPEEEEDEDEWDAKSWDDIDVNLPKTSAFE
EEEANPVAKKVAEPVQKQENSKAQSTVATVKKVANSNKGETEDGESSSANARRNRGASKK
GPIKEDETKNGSDLRSPICCILGHVDTGKTKLLDCIRRTNVQEGEAGGITQQIGATYFPT
ENIRERTKELKADATLKVPGLLVIDTPGHESFSNLRSRGSSLCDIAILVVDIMHGLEPQT
IESLNLLKSRDAVFIVALNKVDRLYGWKKCTNAPIGKALRQQNEDVKREFNMRLTDIVTQ
FKMQGVNTALYYKNKEMEDTYNIVPTSAISGEGIPDLLLLLVQWAQKTMEERLTFVDEVQ
CTVLEVKVVEGHGTTVDVVLVNGILHEGDQIVVCGMQGPIVTTVRALLTPHPMRELRVKG
TYQHHKKIRAAQGVKISAQGLEHAIAGTALYVLKPDDDLDRLKDAVMEEMTRVRNRIDKS
GEGVYVVQASTLGSLEALTEFLKSPAVNIPFCDFSIGPVHKKDVMKASVMLERKKEYATIL
AFDVKVMPDARDLAEESGVRIFVADIIYHLFDQFTAYIKNLREEKKKESAEEAVFPCVLK
IMPNCVFNKKDPIVLGVDVLEGIAKVGTPLCIPTKEYIDIGKIASIEINHKQVDMATKGQ
KVAIKIIGSNPDEQQKSFGRHFDMEDELVSRITRRSIDLLKENYRDDLSMDDWKLVVKLK
SILKIP*
>11667.m03878|LOC_Os01g40340.3|genepair761-1
MELLFSFVKSDHPHSTLLSGYFSKVVICLMLRKTAPLMAYVQGHPEIVVQLVDLIGITSI
MEVLIRLIGADETIYSNYGDTLQWLENTDVLEMIADKFSSSDSPEVHANAAEILCAVTRC
APPSLAAKICSPSFVGRLFRHALEESRPKSVLVHSLSVCISLLDPKRLASASYQAFRSNL
SHGALVTASPETVDGMLESLGNLLKLLDTSDAENVLPTTYGCLRPPLGKHRLKIVEFISV
LLTIGSETAEKELIRQSAIKRSVDLFFEYPYNNFLHHHVENIIVSCLEGKRTELVEHVLN
DCDIVGKILAAEKLSSLSTESTGPTIPSDGKSIPKIGNIGHMTRITNKLIQLGSSNGTIQ
IHLQENSEWVDWQTDVLVKRNEVENVYHWACGRPTSLHDRGRDSDDDDFRDRDYDVAALA
NNLSQAFRYGIYSNDDMEENQGTLERDDEDVYFDDESAEVVISSLRLGDDQDGSSLFTNS
NWFTFDGERGITDRLAAVPSSSPNSEEISPDTEETDDGEVVIGTEDHMDTVNLGNGPIEE
AEDAAEFTKHPATSTEDEQLQNAEGIERHLDVSNGDAEASTEAAEAAPVSSAPSSDEVQT
ERTADEPTGSSDSGNSVSEVLPDPEDSSIDPANTAVSSEQTVDNEDVELPTKEVPSVDVE
TKTDEIKANE*
>11682.m04970|LOC_Os05g51490.1|genepair761-2
MFWRMTGLSAASPVDTILDKENFTLEELLDEDEIIQECKALNTRLINFLRDKAQVEQLLR
YIVEEVPEDSEKKRSFKFPFIACEIFTCEIDIILRTLVEDEELMDLLFSFVRPGHPHSTL
LAGYFSKVVICLMLRKTSPLMNYVQEHPDIVVHLVDLIGTTSIMEVLIRLIGADETIYSN
YADTLQWLENTDVLEMIVDKFSSSDSPEVHANAAEILSAVTRCAPPALAAKICSPSFVGR
LFRHALQESRPKSVLVHSLSVCISLLDPKRLASASYQAFRSNLSHGTLVTASPETVDGML
ESLGDLLKLLDISSAENVLPTTYGCLQPPLGKHRLKIVEFISVLLTIGSETAEQELINQS
AVKRSIDLFFQYPYNNFLHHHVESIIISCLEVNRSQLIDHALNECNLVGKILAAERSSSL
STESNTPTLLSEGKVPPKIGNIGHITRIANKLIQLGNSNSIIQSHLQENSEWVEWQTTAL
VKRNEVENVYHWACGRPTSLHDRGRDSDDDDFRDRDYDVAALANNLSQAFRYGMYSNDDI
EEAQVIERDDEDVYFDDESAEVVISSLRLGDDQDSSSLFTNSNWFTFDGDRGINDRLAAS

VPSSSPNSEETSLNTEETDEVQIGEDTSIEPQLESVSLENGPVEEAGDLADVSKQTDSNT
EDEKLLCTEEEDLSKEAEESERHVDVRDGQVDIQAEDAAEGSCGDMGTGIAVDEPVSPSS
EPNNASAGASPDTGDIHTAGSTGSNDSGAELHAKEDSQDGQKTDEPTTTE*
>11667.m03889|LOC_Os01g40450.1|genepair762-1
MESSRGSKAISPLSPIQPAPSFLLPPNPPYATLRSYIPNRPFLTFVPTLIASFPRRLYNL
NHWVPFGVCYAMKRAGSKGVLESSFSSSKKTTRRQKKPPTSSLEELELPNSAMNKIQEVH
DIARDVFFAATPGFVPSSLAEANLTKLLDTLKLEDFGLDASMPYFRADPQGHPKVTYVHF
GDDSLNFSFGVFCLPQSAVIPLHDHLGMTVFSKILHGSMHIKSYDWVKTPNGAHFAKVRT
NTIYDDSSKTTVLYPESGGNLHCFTAETACAVLDVMGPPYSSVEGRDCSYYGVCPSPRGV
SRRITDELSDWLRKERCTFNMNAVLVKPSHSQCVFVAFGLNQKGEYSLSCPLSARPPQEH
VGNLGHGFLVAGGTHARAPHRHVKDGRGRRETSLSIVALHARRARRPGEEGRWMLAGSMG
KVVGLKKLAGFLRPTMPEKGEGRATDGGRGPALSWGRSRPRLLLRQIRTARCPSLPGRFH
AAELALLRSRYQRPSLLTSPSSLPRQVHVADLVGPRRRPRSPSPARAPIPSVAPATPSS*
>11682.m04967|LOC_Os05g51470.1|genepair762-2
MPVQLGGGGKVAEPKDLAATDKDRPSSKKNRRRQKKPATSAAISPPVAAMQTLFDTSREV
FQDSLPGFVPPPQAVARLAALLNDLKPHDVGIEPSMSCFKNADSKGPPRVTYLHFYDCPK
FSFGIFCLPKSAVIPLHNHPGMTVFCKILFGSMHLKSYDWAKSAPDNDNNALETSDGARL
AKVNTDAVFDASSETTVLYPENGGNLHCFTARTACAVLDVMGPPYNRADGRDCSYYDESP
YLSSSGGDARYSWLKENHSTFEMKGVQMPQRFIV*
>11667.m03899|LOC_Os01g40550.1|genepair763-1
MAEQQQQRDGDFEPVYEWLDAGAHYLLRVNVPEFKKEELQVHVDPAGRLTVRGQHGGLR
LNKVFQLPPTCNLDAITGRLEASVLVLTVPKKPATTAAAAALPPKANQEEEKETKKADEH
DVAGKPPPPPPKTDSDQSERRTQLSAREKKEEPPKATAPAAAPPQPAARERHDEEEKARA
EHKARLSREADRRIEAARARLAAQQAASRPAPAPAPEPEKTAAWWKERAAEEGMKLAEAI
GKNKEVVATAVAAFALGVFVSTKLFSRNN*
>11682.m04964|LOC_Os05g51440.1|genepair763-2
MASNKQGGGKQQAAAASPGPDQKDEELDPKFEWLDNANNFLLRLYLTGFKKEDFRVQVDG
TGKLTVRGQRPAAGSKHNTRFHKVFQLPSNANIDDITGRFEASVLTITVPKRPAPTSSAP
APTSVQEIKQKAPTAKQEPQPQFDEAPNKKKQQQEEEEEAAKKKQQQLQEEEEATKKKQQ
EEEEEAAAKKTKKLQEEEAIAKHKPATTTERKQAEPTTAAPLPGHVVDRESLAEKVKRRA
EEECAKAAAAAEEKTATALSRWRERVAGELEHLGDMRWAEGVVETARRNKDVIATAVAAF
SLGFFVSHKFFCRR*
>11667.m03902|LOC_Os01g40580.1|genepair764-1
MGGVTSSTSSSSPATAAGGSSYSAEAAQALCCACVGQSTVAVEEAWGRYDAVLGPGCHFV
PWCVGRRVAGYLSLRVQQLDVRCETKTRDNVFVTVVASVQYRALADRAYDAFYCLTNAHA
QIQSYVFDVIRASVPNMNLDEVFGQKKEVARAVEEELARAMTMYGYEIVQTLIVDIVPDE
VVRRAMNDINAAARLRVAAAERAEADKIQQVKRAEGEAEAKYLAGVGVARQRQAIVEGLK
RFVPNEKDVMDMVLVTQYFDTIRDIGATSRSSTVFIPHGPSAVRDMAAQVRDGLLQATAA
AGGGAATLKAL*
>11682.m04961|LOC_Os05g51420.4|genepair764-2
MMYEHSGTAGSYLRTAAMGNLFCCVQVDQSTVAIREQFGKFDAVLEPGCHCLPWFAGKRI
AGHLTLRLQQLDVRCETKTKDNVFVNVVASIQYRALAGKANDAFYKLSNTRSQIQAYVFD
VIRASVPKLNLDDAFEQKNDIAKAVEDELEKAMSAYGFEIVQTLIVDIEPDEHVKRAMNE
INAAARLRVAANEKAEAEKIVQIKRAEGEAEAKYLSGLGIARQRQAIVDGLRDSVLGFSV
NVPGTTAKDVMDMVLITQYFDTMKEIGASSKASSVFIPHGPGAVRDIATQIRDGLLQGQA
TTTSH*
>11667.m03903|LOC_Os01g40590.1|genepair765-1
MGKEGKRREEEGRRRRKGKGKGAGEMVLQQEEEDAAPAMGCWIRIPRRLGGGCMSSRSKV
DSSTTTSGGGGGGSARVGGESKSANDGCRDHSVQPMASGSTTSSNTGSISPSSIVGEELK
LAFQLRRFTFNELKCATRNFRPESLLGEGGFGCVFKGWIEENGTAPVKPGTGLTVAVKTL
NHDGLQGHKEWVAEVDFLGNLQHPHLVKLVGYCIEDDQRLLVYEFMPRGSLENHLFRRSL
PLPWAIRMRIALGAAKGLAFLHEEAERPVIYRDFKTSNILLDADYNAKLSDFGLAKDGPE
GDKTHVSTRVMGTYGYAAPEYVMTGHLTSKSDVYSFGVVLLEMMSGRRSMDKNRPNGEHN
LVEWARPYLGERRRFYRLVDPRLEGNFSIRGAQKTAQLACACLNRDPKARPLMSQVVEVL
KPLLNLKDMASSSYFFQSMQQERAASLGNPIGSQSMKAQGTFARNGQQPMRSLSYGPHAS
PYRQSPRPNNGKLQ*
>11682.m04957|LOC_Os05g51400.1|genepair765-2
MGAGRSVPARGRDSESSSRKTKAAASSSSTAGCWGRLPLLISSGGIMTSSPPDRSPPYLQ
TTAEPTLYAGTTNNSYKPFLPEEAFSGSISPSLVAADFQLRQFTYADLQRATGYFRPETF
LGVGGFGRVYKGWIQVNETAHGKPRTGIPIAVKTLNYDGLQGHDEWVAEIHYLRNLKHPH
LVKLIGFCMEGDQRQLVYEFMSRGSLENHLFIRSRTPLPWFLRVKIVLGAAKGLAFLHEQ
EMPVIFRDFKTSNILLDEDFNAKLSDFGFARDGPVGDMAHVSTRVLGTYGYAAPEYVLTG
KKAMERNLVEWAHNNANDRSIHRLIDPGLGSNFSMAGAQILARTARSCTRQNPRDRPLMS
EVVHTLETLHTDQRANATTSYSYSQSQPPSPSANPSPSRSPMRSSASSPYGAPYPYGGIG
GHASPLRHGTRRAMA*
>11667.m03909|LOC_Os01g40630.3|genepair766-1
MAMEAAAERSAGAGAAATAAPESGGGGAGERRSRFRRICVYCGSAKGRKASYQDAAVELG
KELVERGIDLVYGGGSIGLMGLVSHAVHDGGRHVIGVIPKSLMPREVTGEPVGEVRAVSG
MHERKAEMARFADAFIALPGGYGTLEELLEVITWAQLGIHKKPVGLLNVDGFYDPFLSFI
DMAVSEGFIAEDARRIIISAPTARELVLKLEEYVPEYEVGLVWDDQMPHSFAPDLETRIT
SS*
>11682.m04956|LOC_Os05g51390.1|genepair766-2
MTLAAAAEAPAPTHAFAIAAEEVAMEPLSTATAPAAMEEESSSSGGGGGVGERRSRFRRI
CVYCGSAKGKKPSYQDAAVDLGKELVERGIDLVYGGGSIGLMGLVSHAVHAGGRHVIGII

PKSLMPREVTGEPVGEVRAVSGMHERKAEMARFADAFIALPGGYGTLEELLEVITWAQLG
IHKKPIWFTLFEYSQTANGMAIGGSYTVGLLNVDGFYNPLLSFIDLAVNEGFITEEARRI
IISAPTAKELVMKLEDYVPEYSIGLVWEDQNQKQNNLVPELDSGITSS*
>11667.m03918|LOC_Os01g40700.1|genepair767-1
MLLAVEGGGFFSSSASGYSHGLALLLLGRKDEEKPVKVSPWNQYRLVDREAEQVYHLASR
KDQAPGKCAPFICFGRAAAGLEGASPPKLSSGNTSGSSSEESSASANEGTNGSLTGNEKK
GCLKSNSRRDSLEHCIVVSEGEEPRESLEEVQTLKSGMERRKVQWTDTCGKELFEIREFE
ASDEGLSDDDTENEGFRKCECVIQ*
>11682.m04954|LOC_Os05g51370.1|genepair767-2
MGVQAISVASILDEFICLQESSCSRRLDEHLRWMLLAVEVGGFFSSSASGYRNGLALLLL
GHKGEEKPVKVTPWNHYRLVGGGEAEPASEENNVPSGKCASFICFGCPPARLKGVSPPKL
ASSDTAGNSSEQSP*
>11667.m04035|LOC_Os01g41870.1|genepair768-1
MAAVANQQARELGPIDQWRGAGGGGGKACFGGVVEGRQGMMVSSTQQQQLQLQRKGKAVA
EKGGAAGAAAAEKVVVAVRAATREISKTALMWALTHVVQPGGSILLLVVVPSHSSGRKFW
GFPLFAGDCASGNKTMLDQKDISELSSQMMDKLKNVYDPNKINVKTKVVSGSPPGVVAAE
SKRAQASWVVIDKELKHEEKHCVEELQCNIVVMKRSQPKVVRLNLVGSPDKDSKVSCSLP
PMLDSSVGKTTTDVKEPRTSIRGPAVTPNSSPDLETTFESTEAGTSSVSSSDPGTSPYSA
SDTNGSMKKEAPATKDRVQHLDINISDSDSETLSPPASFSLQPWMVDILQGSASSRTHGK
GPRKARTATADALLENISKLDLLNEISSMRSRSDLNFRGNVRDAVSLARSAPPGPPPLCS
ICQHKAPVFGKPPRWFTYAELELATGGFSQANFLAEGGFGSVHRGVLHDGQAIAVKQHKL
ASSQGDVEFCSEVEVLSCAQHRNVVMLIGLCVEDRRRLLVYEYICNGSLDSHLYGRNKET
LQWSARQKIAVGAARGLRYLHEECRVGCIIHRDMRPNNILVTHDYEPLVGDFGLARWQPD
GDMGVDTRVIGTFGYLAPEYAQSGQITEKADVYSFGVVLVELVTGRKAVDINRPKGQQFL
TEWARPFLEEYAIDELIDPRLGDRYCENEVYCMLHAAKLCIRRDPHSRPRMSHVLRILEG
DMVVDSGSVSAPSSDSGSRSWRMLNEQQNCRDWSPARQDSHRVVEGKNSYDALRAAWDRN
KQSVSHRY*
>11682.m04936|LOC_Os05g51190.1|genepair768-2
MAVVSGPDKGRGAEAVVVVAVRAAARDISKAALVWALTHVVQHGDTILLLAVMPPPHNSG
KKFWGFPLFAGGCASAHRSVLTQNSDVADLCNQMMLKLRDFYDPNKIITKLKVIPASPGG
VATESKRAQASWVVLDKELKHEEKRCMEELQCNIVAMKRSRPKVLRLNLVRSPPKESKSP
LPPLPELSDSVGETESSINEQRCPIREPAVTPSSSPESETAFGSTDVGTSSVSSSDPGTS
PYSASETNSTFKKEATKDNFQHSDVNVSDSESEASTPPAASSLQPWMADILKGSASSRLA
GNRPRRTRTPTADALLEKISKLDLLAEISAIRSRSDLNFRGNVRDVVSLSRSAPPGPPPL
CSICQHKTPVFGKPPRWFSYAELELATGGFSQANFLAEGGFGSVHRGVLPDGQAIAVKQY
KLASSQGDVEFCSEVEVLSCAQHRNVVMLIGFCVEDKRRLLVYEYICNGSLDSHLYGRNK
ETLEWAARQKIAVGAARGLRYLHEECRVGCIIHRDMRPNNILVTHDFEPLVGDFGLARWQ
PDGDMGVETRVIGTFGYLAPEYAQSGQITEKADVYSFGVVLVELVTGRKAVDINRPKGQQ
FLTEWARPLLEEYAIDDLIDPRLGDRFSENEVLCMLHAANLCIRRDPHSRPRMSHVSHTP
FFSTFLSTYHLSLIYICTNNDVSPRMYEKVLRILEGDMVVESGCVSAPCSEAGSRSRRML
LQQEQQSSPAQQDSQIMVDGKPQSYVARRIAWDRDTQSLSHRF*
>11667.m04036|LOC_Os01g41880.1|genepair769-1
MGTKNQFDLLVDVDNDDPSHLIAAAEKKAAAAAASPKPAAQAKLPTKPPPPAQAVKESRN
YGAPAREGAGRNGPGRGSGGFGGGRIGQRRDFGEGDTNGVEGGYGASGFGDGIVRREEGE
HRPSERGHGPRQPYRGGGRRGGYTGGEAGDESGRAPHRAYERRSGTGRGYGMKREGAGRG
NWGTVTDEALAQESGEAVSIEVSVTVTEENKQEDVPQSDEVEKHKEGESNEEEEKEPEDK
EMTLEEYEKVLEEKRKALLSLKVEERKVVVDKELQSMQQLSVKKDSDEVFIKLGSDKDKK
KENVERDERTRKSLSINEFLKPAEGERYYSPGGRGRGRGRGRGDRGGFRDGYSSRGPVAA
PRIEDQAQFPGLAGRLVH*
>11682.m04935|LOC_Os05g51180.1|genepair769-2
MGSKNQFDLLVDVDNDDPSHLIAAAEKKAAAAAASPKLASSPAPAPAKLPTKPAPPAQAV
REARNYGAPRDGAGRGGPGRGRGGGRGGRGGPRRDFGEGDANGFEGGYGGGGGFGDGGLA
RGEDGEGRQAERGRGPRQPYRGGGRRGGYSDGQSGDDYGRPRRAYERHSGTGRGYELKRE
GSGRGNWGTVTDEGLAQEVAEAVNTEEAPATAEDEKKPEDVPQSEVDKDKESPENEEEEK
EPEDKEMTLEEYEKVLEEKRKALLALKAEERKVEVDKELQAMQQLSVKKANEEVFIKLGS
DKDLKKKEKDEKDERTKKSLSINEFLKPAEGERYYNPGRGRGRGRGRGDRGGFYGGYNGN
GGRRQAAAPVIEDQAQFPSLGGK*
>11667.m04050|LOC_Os01g42010.2|genepair770-1
MGDGEGSTGGGGGGGDPQRLKRIGAAAYDYENDSRWAGYWSNVLVPPHLASRPDVVDHFK
RKFYQRYIDPDLIVEPMSSTSSSQSTKPAARSSATPSNENVRARDSGSSARSTAASQPSQ
TERTANSLWLDGRTIHFSINAWVLVVASLGILPILPKHIASKAYRLSLLGTICSSAYSLY
VTYGKPRAWNMAAIQPWLQSIIVAKDFVHLMFSLMMFTSNVHYKIALLPVLCWALDHVAR
FLRRNFARSSLYRKYLEEPCLWVETNNTTLSLLCSNAEITLGFLMIVSLFSWRRNIIQTF
MYFHLLKLMYHAPVTSGYHQSAWARIGRAVNPYIHRYAPFLNTPISAVQRWWLR*
>11682.m04930|LOC_Os05g51140.1|genepair770-2
MGGGDASGGDGPEQELKRAAAAAYNYEGDARWAEYWSNILVPPHLASRPDVVDHYKRKFY
QRYIDRDLVVEPMSSTGSTQPSGPEVRSSSFSSSENVRARSSGSSSRSAAPPPPPPQTDS
ATNPLRFDARTIHFSINAWVLVVAGLGMLPILPKHLADRACKLSLLGTILSSAYSLYSTY
GKPRAWNMPAVQGWLQAVLGTKDFIHLMFSLMLFTSQLHLKIAALPVFCWALDHVARFLR
RNFARSSFYRSYLEEPCLWVETNNTTLSLLSSNAEIALGFLLIISLFSWQRSIIQTFMYW
QVLKQMYHAPVTASYHQSAWAKIGRIVNPYIHRYAPFLNTPISAAQRWWFR*
>11667.m04052|LOC_Os01g42030.1|genepair771-1
MASYDKAIESYKRAVTTAASLAASAMLVRGVVNELVPYEVRDLLFSGVGYLRSRMSSQHM
VIIEETEGWTNNQLYDAVRTYLATRINTDMQRLRVSRVDETKSMMFSMEEGEEMADVHEG

SEFRWRLVCRDNSSSSNGNGNGRGGNGNYRLEVRSFEMSFHKKHKDKALNSYLPHILATA
KKIKDQDRTLKIYMNEGESWFAIDLHHPSTFTTLAMDHKQKQSVMDDLERFIKRKEYYKK
IGKAWKRGYLLYGPPGTGKSSLIAAMANYLKFDVYDLELTEVNWNSTLRRLLIGMTNRSI
LVIEDIDCTLELQQREEGQESSKSNPSEDKVTLSGLLNFVDGLWSTSGEERIIVFTTNYK
ERLDPALLRPGRMDMHVHMGYCCPESFRILASNYHSIDNHATYPEIEELIKEVMVTPAEV
AEVLMRNDDTDVALEGLIQFLKRKKDVGKEGKAENVEQVVKAEETEKGMMKKNDVPENQD
PQDASK*
>11682.m04929|LOC_Os05g51130.1|genepair771-2
MASYDKAIESYKKAITTAASVAASVMLVRSVVNELVPYEVRDVLFSGLGYLRSQISSQHT
IIIEETEGWSHNHVYNAVRAYLATRINNNMQRLRVSSMDESSEKMVVTMEEGEELVDMHE
GTEFKWCLISRSISADPNNGNGSGQREVRSYELSFHRKHKEKALKSYLPFIIATAKAIKD
QERILQIYMNEYSDSWSPIDLHHPSTFDTLAMDQKLKQSIIDDLDRFIKRKDYYKRIGKA
WKRGYLLYGPPGTGKSSLIAAMANHLKFDIYDLELTGVHSNSELRRLLVGMTSRSILVVE
DIDCSIELKQREAGEERTKSNSTEEDKGEDKVTLSGLLNFVDGLWSTSGEERIIVFTTNY
KERLDQALMRPGRMDMHIHMGYCTPEAFRILASNYHSIDYHVTYPEIEELIKEVMVTPAE
VAEALMRNDDIDVALLGLLELLKSKIKDASETKAESKDANKQTEENKDSKAMENKNDSST
DECT*
>11667.m04060|LOC_Os01g42110.1|genepair772-1
MISPDAARNVVGIIGNVISFGLFLAPVPTFWRICKRKDVEEFKADPYLATLLNCMLWVFY
GIPVVHPNSILVVTINGIGLLVEGTYLLIFFLYSPNKKRLRMCAVLGVELVFMLAVILGV
LLGAHTHEKRSMIVGILCVFFGSIMYFSPLTIMGKVIKTKSVEYMPFFLSLVCFLNGVCW
TAYALIRFDIYVTIPNGLGALFGAIQLILYACYYRTTPKKTKAAKDVEMPSVVVSGTGAA
AAAGGGNTGGGSVSVTVER*
>11682.m04925|LOC_Os05g51090.1|genepair772-2
MVMNPDAVRNVVGIIGNLISFGLFLSPLPTFVTIVKKKDVEEFVPDPYLATFLNCALWVF
YGLPFIHPNSILVVTINGTGLLIEIAYLAIYFAYAPKPKRCRMLGVLTVELVFLAAVAAG
VLLGAHTYDKRSLIVGTLCVFFGTLMYAAPLTIMKQVIATKSVEYMPFTLSLVSFINGIC
WTIYAFIRFDILITIPNGMGTLLGAAQLILYFCYYDGSTAKNKGALELPKDGDSSAV*
>11667.m04079|LOC_Os01g42300.1|genepair773-1
MAGVVTRAVAAAVLVVVVVVAAAELVAAEPPPSERSALLAFLAATPHERRLGWNSSTSAC
GWVGVTCDAGNATVVQVRLPGVGLIGAIPPGTLGRLTNLQVLSLRSNRILGGIPDDVLQL
PQLRLLFLQNNLLSGAIPPAVSKLAALERLVLSSNNLSGPIPFTLNNLTSLRALRLDGNK
LSGNIPSISIQSLVVFNVSDNNLNGSIPASLARFPAEDFAGNLQLCGSPLPPCKSFFPSP
SPSPGVSPADVPGAASSSKKRRLSGAAIAGIVVGAVVLALLLLVAAVLCAVSKRRRGASE
GPKSTTAAAAGAGAAAARGVPPPGSGEGTGMTSSSKEDMGGASGSAAAAVAAVAAEPSRL
VFVGKGAGYSFDLEDLLRASAEVLGKGSVGTSYKAVLEEGTTVVVKRLKDVAVARREFDA
HMDALGKVEHRNVLPVRAYYFSKDEKLLVFDYLPNGSLSAMLHGGGGGACEGVMTARFGL
*
>11682.m04923|LOC_Os05g51070.1|genepair773-2
MPMAALRLLLLALAVACCAVAEPPQQERSALRAFLAGTPHERALAWNASTPACAWVGVTC
DAANATVVALRLPGVGLIGRVPQGTLGALRGLRVLSLRSNRLFGDVPGDLFSLPDLRSLF
LQGNLFSGSVPPDVAKLTALQHLALSHNNLTGAIPFALNGLANLRSLRLDGNRFSGSLPS
LTLPLLEDFNVSYNQLNGSIPASLARFPPESFAGNLQLCGKPLSRPCEPFFPSPAGAPTP
TDGRGSGGGSVPVSEKKKKKLSGAAVAAIAVGGGAAALLALVLLVVCTAASRRRAANGEV
GKTAAARGLTPPSTASGELGEVTSSTSKEIALAAAAATAERSRLVFVGKGAAYSFDLEEL
LRASAEVLGKGSVGTSYKAVLEEGATVVVKRLKEVAASRREFSAHLDSLGKVDHRNLLPV
RGYYFSKDEKLLVCDYLPAGSLSATLHAKAIGAHLHTRHTDYEYDTRNANLIKRVSSVIS
ISIGDAVAISTTNSELTSGSRGTGRRTMDWDARMRAALSAARGVAHLHAAHSLAHGNLKS
SNLLLRPDPDATALSDYCLHQLFAPLSARPNAGGYRAPELVDARRPTFKSDVYSLGVLFL
ELLTGKSPGNASVDGDGAVDLPRWVQSVVREEWTAEVFDVELVRLGGSAEEEMVALLQVA
MACVATAPDARPDTADVVKMIEEIGSGHGRTTTEESEDRSRGTPPAGTTP*
>11667.m02169|LOC_Os01g22370.1|genepair774-1
MAMKCLFLFFAFLVAFFPGAAVGAGLKVGFYNKTCPSAERLVQQAVAAAFKNNSGVAPGL
IRLHFHDCFVRGCDASVLIDGNDTEKTAPPNNPSLRGFEVIDAAKAAVEAACPRVVSCAD
ILAFAARDSVALTGNVTYKVPAGRRDGNVSIAQDALDNLPPPTFNATELVGRFANKSLTA
EDMVVLSGAHTIGVSHCDSFTSRLYNFTGVGDADPAISAAYAFLLRAVCPSNSSQFFPNT
TVDMDVITPAALDNKYYVGVANNLGLFTSDHALLTNATLRASVDEFVKSETRWKSKFVKA
MVKMGGIEVKTGTTQGEVRLNCRVVNKRSANAELELELAAAMDDGDEVAAS*
>11682.m00650|LOC_Os05g06970.1|genepair774-2
MSSAAMKLAVVAALISAAAVGARACLDVGFYDTTCPTAETLIQQVVAAAFRNDSGVAPAM
IRMHFHDCFVRGCDGSVLIDTVPGSTTRAEKDAAPNNPSLRFFDVIDRAKSAVEAACPGV
VSCADVVAFMARDGVVLSGGLGYQVPAGRRDGRTSLEDDALNFLPPPTSTAADLVANFTA
KNLTAEDMVVLSGAHTIGVSHCDSFTNRIYNFPNTTDGIDPSLSKAYAFLLKGICPPNSN
QTFPTTTTFMDILTPTKFDNRYYVGLTNNLGLFQSDAALLTDAALKATVNSFVRSEATFR
LKFARAMIKMGQIGVLSGTQGEIRLNCRVVNPVNVTATAADDHHLTSSSSSSSDEVAAS*
>11667.m02182|LOC_Os01g22490.1|genepair775-1
MQIFVKTLTGKTITLEVESSDTIDNVKAKIQDKEGIPPDQQRLIFAGKQLEDGRTLADYN
IQKESTLHLVLRLRGGAKKRKKKTYTKPKKQKHKHKKVKLAVLQFYKVDDATGKVTRLRK
ECPNAECGAGTFMANHFDRHYCGKCGLTYVYNQQA*
>11682.m00629|LOC_Os05g06770.1|genepair775-2
MQIFVKTLTGKTITLEVESSDTIDNVKAKIQDKEGIPPDQQRLIFAGKQLEDGRTLADYN
IQKESTLHLVLRLRGGAKKRKKKTYTKPKKIKHKHKKVKLAVLQFYKVDDATGKVTRLRK
ECPNNDCGAGTFMANHFDRHYCGKCGLTYVYNQKA*
>11667.m02185|LOC_Os01g22520.1|genepair776-1

MALAILARRRAAEALLRRPLGAAGVSALRASYAAVAGEESDVVVVGGGPGGYVAAIKAAQ
LGLKTTCIEKRGTLGGTCLNVGCIPSKALLHSSHMYHEAKSSFAHHGVKFSNLEVDLPAM
MAQKDKAVAGLTKGIEGLFKKNKVTYVKGFGKLASPSEVSVDLSDGGSTVVKGKNIIIAT
GSDVKSLPGVTIDEKKIVSSTGALCLSEIPKKLVVIGAGYIGLEMGSVWNRLGSEVTVVE
FAPDIVPSMDGEVRKQFQRMLEKQKMKFMLKTKVVGVDTSGDGVKLTLEPAAGGEQSVIE
ADIVLVSAGRVPYTAGIGLESVGVETDKAGRILVDKRFMTNVNGVYAIGDAIPGPMLAHK
AEEDGVACVEFIAGKEGHVDYDTVPGVVYTHPEVASVGKTEEQVKALGIPYRVGKFPLLA
NSRAKAIDDAEGLVKVVAEKETDKILGVHIMAPGAGEIIHEAVLALQYGASSEDIARTCH
AHPTVSEALKEACLQTFTKAIHI*
>11682.m00627|LOC_Os05g06750.1|genepair776-2
MYHEAKASFAHHGIKFSNLEIDLPAMMSQKDKAVAGLTKGIEGLFKKNKVEYVKGFGKFV
SPSEVSVDLLDGGSTTVKGKNIIIATGSDVKSLPGVTIDEKKIVSSTGALALSEIPKKLV
VIGAGYIGLEMGSVWNRLGSEVTVVEFASDIVPSMDGEIRKQFQRMLEKQKMKFMLKTKV
VGVDTSGSGVKLTVEPAAGGEQSVLECDIVLVSAGRVPYTSGLGLNALGVETDKGGRILV
DKRFMTNVKGVYAIGDAIPGPMLAHKAEEDGVACVEFIAGKEGHVDYDTVPGVVYTHPEV
ASVGKTEEQVKASGVAYQVGKFPLLANSRAKAIDDAEGLVKVVAEKETDRILGVHIMAPN
AGEIIHEAVLALQYGASSEDIARTCHAHPTVSEALKEACLQTYLKAIHI*
>11667.m02212|LOC_Os01g22780.1|genepair777-1
MAVAVAAGEISDDGGDQPSPSPSPSASCARRPVVFAFGDSNTDTGGIAAGMGYYFPLPEG
RAFFRRATGRLCDGRLVIDHLCESLNMSYLSPYLEPLGTDFTNGANFAISGAATAPRNAA
FSLHIQVQQFIHFKQRSLELASRGEAVPVDADGFRNALYLIDIGQNDLSAAFSAGGLPYD
DVVRQRFPAILSEIKDAIQSLYYNGAKNLWIHGTGPLGCLPQKLAVPRADDGDLDPSGCL
KTLNAGAYEFNSQLSSICDQLSSQLRGATIVFTDILAIKYDLIANHSSYGFEEPLMACCG
HGGPPYNYDFNVSCLGAGYRVCEDGSKFVSWDGVHYTDAANAVVAGKILSADYSRPKLPF
SYFCSA*
>11682.m00624|LOC_Os05g06720.1|genepair777-2
MASAMNGCGGGGGGGGGGVMKRMGLLRVQYYCVMGFVAAAVVLATLRYMPAPATAPPTVD
GGGATVRSSAATVDSAAAAAAAAPGGGGVERGKGKRKHVWSGEEEEVAAEKKAAAGVVVF
NFGDSNSDTGGVAAVMGIHIAAPEGRAYFHHPTGRLSDGRVILDFICLPFFKKKVFGMDL
EFCLRKGKEERMKEKAGESLNTHHLSPFMRPLGADYNNGVNFAIAGSTATPGETTFSLDV
QLDQFIFFKERCLESIERGEDAPIDSKGFENALYTMDIGHNDLMGVLHLSYDEILRKLPP
IVAEIRKAIETLHKNGAKKFWIHGTGALGCLPQKLATRGEIDRDLDEHGCITRINNVAKR
FNKLLSETCDDLRLQFASSTIVFVDMFAIKYDLVANHTKHGIEKPLMTCCGHGGPPYNYD
PKKSCTANDKDLCKLGEKFISWDGVHFTDAANEIVASKVISGEFSIPRIKLTASVVRPKK
AKNSRL*
>11667.m02221|LOC_Os01g22870.1|genepair778-1
MDQHQVVGENYANPKTCFFHVLFKAGALAFYILSALFVTNFVIIFVITVLLAALDFWVVK
NVSGRILVGLRWWNEIDDEGNSVWKFECLDGESLARMNKKDSWLFWWTLYLTAAAWIVLG
IFSLIRLHADYLLVVGVCLSLSIANIVGFTKCNKDAKKNVADWTRTTLLSSGVRSTIQSA
FGV*
>11682.m00622|LOC_Os05g06700.2|genepair778-2
MGVLSARIQVVTENYANPVTCLFHVLFKAGALVFYILFSLFVKSFVIIFVITVFLAALDF
WVVKNVSGRILVGMRWWNEIDDEGNSVWKFECLDGEALARMNKKDSWLFWWTLYLTAAAW
IILGIFSLIRLEADYLLVVGVCLTLSLANIVGFTRCNKDAKKNIRGYFEGHAQNAITSRI
TSTLQSAFGVI*
>11667.m02225|LOC_Os01g22910.1|genepair779-1
MGWDEPTFRGVWLRGEWDGLVPGEEYSSQIRDQLIGENVADELVPPGTSERRDDAFLPRS
RCSPSGATADGSGDGDSGRLGDGDHAGLGDDDRGGGASAMARRIQWWGGQRRLIRRWGGL
WRWIRRRGGAAAACEEEVEGRRRIRRWGLRWLARRSSRGRSGGGVRGGGRGAAADPAMGG
AATAFPLALAHRRLFLLLLLAAGSSSCSSSGDGEDCGGRSGDRLTESLFVDIGLESDCRQ
VVNDYVEAVRQLACHVLDLLGEGLGLRDPTSLTRLITATDNDSLIRINHYPPSCAAAAGD
HKSGGGPAPTAAIGFGEHTDPQILSVLRANDADGLQLLLPDAAAAGDSVWVPVPPDPSAF
FVNVGDLLQALTNGRLVSIRHRVVVGTGKPRLSTIYFAAPPLHARISALPETVAAGAPRR
YRAFTWAEYKRTMYTLRLSHNRLDLFHAGDGDGDAGVGDDDDHE*
>11682.m00618|LOC_Os05g06670.1|genepair779-2
MVVPSATTPARQETVVAAAPPAAAASGVVGGGGGVTIATVDMSAERGAVARQVATACAAH
GFFRCVGHGVPAAAPVAARLDAATAAFFAMAPAEKQRAGPASPLGYGCRSIGFNGDVGEL
EYLLLHANPAAVAHRARTIDAMDPSRFSEICLHISASEYCSGVMQRSISAIVNEYIEAMK
KLACEILDLLGEGLGLKDPRYFSKLTTNADSDCLLRINHYPPSCNIHKLDHDDQCNIKSL
VSTKASNGGNLMAGGRIGFGEHSDPQILSLLRANDVEGLQVFVPDHEGKEMWVQVPSDPS
AIFVNVGDVLQALTNGRLISIRHRVIATACRPRLSTIYFASPPLHARISALPETITASSP
RRYRSFTWAEYKTTMYSLRLSHSRLELFKIDDDDSDNASEGKA*
>11667.m02232|LOC_Os01g22980.1|genepair780-1
MASCASPRLKSLCHHPLFIILLALSLLQTITAEDEQEADRVAFLPGQPRSPQMSQFSGYI
TVNSQNGRALFYWFFEAQALPSKKPLLLWLNGGPGCSSVGYGAASELGPLMVNGNGTGLE
FNKFAWNNEANLLFLESPVGVGFSYTNTSSDLESIDDRFVAEDTYNFLVNWFKRFPQYKN
HDFYISGESYAGHYVPQLADVVYERNKHVETNQHINLKGFIVGNAETDDYYDYKGLVEFA
WSHSVISDQLYKHVNNVCDFRLSPRSNECNHVMGYIYDQYDMIDIFNVYAPKCNTDDSSL
FSTSYSTADMNAKKRLKGTRMYSGYDPCYSSHIEDYMNKMDVQKSLHANTSGLIKDRKWS
ICSYSIFDNYDITVFSVLPIYSKLIKAGLRIWVYSGDVDGRVPVIGSRYCVEALGLPVKS
QWQPWYLNNQVAGRFVEYQGLTMATVRGAGHAVPQDKPEQALVVINSFLSGRRLPTKNNR
*
>11682.m00617|LOC_Os05g06660.1|genepair780-2
MAVAAAAAARRRDVSCLLLLLCFSSSMAATGGGGGGGEQEADRVARLPGQPASPAVSQFA

GYVGVDERHGRALFYWFFEAQASPAPEKKPLLLWLNGGPGCSSIGYGAASELGPLRVARQ
GAALEFNQYGWNKEANLLFLESPVGVGFSYTNTSSDLSNLNDDFVAEDAYSFLVNWFKRF
PQYKDNEFYISGESYAGHYVPQLADLVYERNKDKRASTYINLKGFIVGNPLTDDYYDSKG
LAEYAWSHAIVSDQVYERIKKTCNFKNSNWTDDCNAAMNIIFSQYNQIDIYNIYAPKCLL
NSTSASSPDRAFFANNQEQFRWRIKMFSGYDPCYSSYAEDYFNKHDVQEAFHANASGLLP
GKWQVCSDQILNSYNFSVLSILPIYSKLIKAGLRVWLYSGDADGRVPVISSRYCVEALGL
PIKTDWQSWYLDKQVAGRFVEYHGMTMVTVRGAGHLVPLNKPAEGLMLINAFLHGEKLPT
SR*
>11667.m02598|LOC_Os01g27040.1|genepair781-1
MAKPVKYDEEEEEVSSSGEEEEEQSDGAGSGSGEEEDEEEEEAPAAAAGEAAGGEEEEVD
EEEIEAVTTGAGADEEEEESGAAAAAPGEGDEESQSTEDDEAVVGEDDDADEAEGGAVVG
KREKARLKEMQKLKKQKIQEILDTQNAAVDADMNNKGKGRLKYLLQQTEIFAHFAKGNQS
KEKKPRGRGRHASKMTEEEEDEEYLKEEEDALAGSGGTRLLSQPSCIKGKMRDYQLAGLN
WLIRLYENGINGILADEMGLGKTLQTISLLGYLHEFRGITGPHMVVAPKSTLGNWIKEIQ
RFCPILRAVKFLGNPEERNHIRENLLQPGKFDVCVTSFEMAIKEKTTLKRFSWRYIIIDE
AHRIKNENSLLSKTMRIYNTNYRLLITGTPLQNNLHELWSLLNFLLPEIFSSAETFDEWF
QISGENDQQEVVQQLHKVLRPFLLRRLKSDVEKGLPPKKETILKVGMSQMQKQYYRALLQ
KDLEVINAGGERKRLLNIAMQLRKCCNHPYLFQGAEPGPPYTTGEHLVENAGKMVLLDKL
LPKLKDRDSRVLIFSQMTRLLDILEDYLMYRGYQYCRIDGNTGGEDRDASIEAFNKPGSE
KFVFLLSTRAGGLGINLATADVVVLYDSDWNPQADLQAQDRAHRIGQKKEVQVFRFCTEY
TIEEKVIERAYKKLALDALVIQQGRLAEQKTVNKDDLLQMVRFGAEMVFSSKDSTITDED
IDRIIAKGEETTAELDAKMKKFTEDAIKFKMDDTAELYDFDDDKFGSLLNSIYILDFLKE
ENKLDFKKLVSDNWIEPPRRERKRNYSESEYFKQALRQGAPAKPREPRIPRMPHLHDFQF
FNNQRLNELYEKEVRYLMQANQKKDTIDGEDEDQLEPLTAEEQEEKEQLLEEGFATWTRR
DFNTFIRACEKYGRNDIRSIAAEMEGKTEEEVQRYAKVFKERYKELSDYDRIIKNIERGE
ARISRKDEIMRAIGKKLDRYKNPWLELKIQYGQNKGKFYNEECDRFMLCMVHKLGYGNWD
ELKAAFRMSPLFRFDWFVKSRTTQELARRCDTLIRLVEKENQEYDEQERQARKDKRMAKN
MTPTKRSALRVSEGETTPSNSFKRRRQSLMDDYVGSGRRKRG*
>11682.m00525|LOC_Os05g05780.1|genepair781-2
MGKPGKYGDGDDDDSEEEQLSPSSSAGEEEEEEVEEEEGEEQQEEQGEEEEGFSGDEEEQ
EVEGEADGEQVEEEEEEEESSVGEEEAEAEGEEEEEEVEEEQGAGEEEEEEVDEEEIEAVT
TGAGGDDDDEEVGDDGGAEEESQSTEDDEVAAGKDGGGEDGDKLEDATGNAEIGKRERAK
LREMQKLKKHKIQEILDAQNKAIDADMNNKGKGRLKYLLQQTEIFAHFAKGNQSTEKKSR
GRGRHASKMTEEEEDEEYLKEEEDALDGAGGTRLVSQPSCIKGKMRDYQLAGLNWLIRLY
ENGINGILADEMGLGKTLQTISLLGYLHEFRGITGPHMVVAPKSTLGNWMKEIQRFCPVL
RAIKFLGNPEERNHIRENLLVPGKFDVCVTSFEMAIKEKTALKRFSWRYIIIDEAHRIKN
ENSLLSKTMRIYNTNYRLLITGTPLQNNLHELWSLLNFLLPEIFSSAETFDDWFQISGEN
DQHEVVQQLHKVLRPFLLRRLKSDVEKGLPPKKETILKVGMSEMQKQYYRALLQKDLEVV
NAGGERKRLLNIAMQLRKCCNHPYLFQGAEPGPPYTTGDHLIENAGKMVLLDKLLPKLKE
RDSRVLIFSQMTRLLDILEDYLMYKGYQYCRIDGNTGGEDRDASIEAFNKPGSEKFVFLL
STRAGGLGINLATADVVILYDSDWNPQVDLQAQDRAHRIGQKKEVQVFRFCTEYTIEEKV
IERAYKKLALDALVIQQGRLAEQKAVNKDELLQMVRFGAEMVFSSKDSTITDEDIDRIIA
KGEEATAQLDAKMKKFTEDAIKFKMDDTAELYDFDDDKDENKLDFKKLVTDNWIEPTSRR
ERKRNYSESDYFKQALRQGAPAKPREPRIPRMPHLHDFQFFNTQRLNELYEKEVKYLVQA
NQKKDTVGEGDDEDQLEPLTVEEQEEKEQLLEEGFSTWTRRDFNTFIRACEKYGRNDIKN
ISSEMEGKTEEEVQRYAKVFQERYKELNDYDRVIKNIEKGEARIYRKDEIMKAIGKKLDR
YKNPWLELKIQYGQNKGKLYNEECDRFMLCMVHKLGYGNWDELKAAFRMSPLFRFDWFVK
SRTTQELARRCETLIRLVEKENQEYDERERLARKDKKNMSPAKRSSSRSLDTPPQSSSKR
RRQSYTEANAGSGRRRRG*
>11667.m02608|LOC_Os01g27140.1|genepair782-1
MDRVNRLAAQRAVVIFSMSSCCMCHTVTRLFCELGVNPTVVELDEDPRGKEMEKALARLL
GRSPAVPAVFIGGRLVGSTDKVMSLHLSGNLVPLLRNAGALWV*
>11682.m00520|LOC_Os05g05730.1|genepair782-2
MDRVTRLASQKAVVVFSKSSCGMSHAVTRLLRELGVDARVVELDEEPAGADMENALAGML
LAGTAANGGGRGRGVVVPTVFIGGRLVGSTDRVMSLHVAGGLVPLLRDAGALWV*
>11667.m02609|LOC_Os01g27150.1|genepair783-1
MATHERKTIDLEQGWEFMQKGITKLKNILEGKPEPQFSSEDYMMLYTTIYNMCTQKPPHD
YSQQLYEKYRESFEEYITSMVLPSLREKHDEFMLRELVKRWSNHKVMVRWLSRFFHYLDR
YFISRRSLPQLSEVGLSCFRDLVYQEIKGKVKSAVISLIDQEREGEQIDRALLKNVLDIF
VEIGLTSMDYYENDFEDFLLKDTADYYSIKAQTWILEDSCPDYMLKAEECLKREKERVAH
YLHSSSEQKLLEKVQHELLTQYASQLLEKEHSGCHALLRDDKVDDLSRMYRLFSRITRGL
EPVSQIFKQHVTNEGTALVKQAEDAASNKKPEKKEIVGLQEQVFVRKIIELHDKYVAYVT
DCFQGHTLFHKALKEAFEVFCNKGVSGSSSAELLATFCDNILKKGGSEKLSDEAIEDTLE
KVVRLLAYISDKDLFAEFYRKKLARRLLFDKSANDEHERSILTKLKQQCGGQFTSKMEGM
VTDLTVARDHQAKFEEFISTHSELNPGIALAVTVLTTGFWPSYKSFDINLPAEMVKCVEV
FKEFYQTRTKHRKLTWIYSLGTCNINAKFEAKTIELIVTTYQAALLLLFNGVDRLSYSEI
VTQLNLSDDDVVRLLHSLSCAKYKILSKEPNNRSISPNDVFEFNSKFTDKLRRLKIPLPP
VDEKKKVVEDVDKDRRYAIDASIVRIMKSRKVLGHQQLVMECVEQLGRMFKPDFKAIKKR
IEDLITRDYLERDKDNPNVYRYLA*
>11682.m00517|LOC_Os05g05700.2|genepair783-2
MAGQERRTIDLEEGWAFMQKGITKLKNILEGKPEPQFSSEDYMMLYTTIYNMCTQKPPHD
YSQQLYDKYRESFEEYITSMVLPSLRDKHDEFMLRELVKRWSNHKIMVRWLSRFFFYLDR
YFISRRSLIPLEQVGLTCFRDLIYQEIKGQVKGAVIALIDKEREGEQIDRALLKNVLGIF
VEIGLGSMECYENDFEDFLLKDTTDYYSLKAQSWILEDSCPDYMIKAEECLKKEKERVGH

YLHISSEQKLLEKVQNELLAQYATPLLEKEHSGCFALLRDDKEEDLSRMYRLFSKINRGL
EPIANMFKTHVTNEGTALVKQAEDSASNKKPEKKDMVGMQEQVFVWKIIELHDKYVAYVT
ECFQGHTLFHKALKEAFEVFCNKGVSGSSSAELLATFCDNILKKGCSEKLSDEAIEDALE
KVVRLLAYISDKDLFAEFYRKKLARRLLFDKSANDEHERSILTKLKQQCGGQFTSKMEGM
VTDLTVARDHQTKFEEFVAAHQELNPGIDLAVTVLTTGFWPSYKTFDINLPAEMVKCVEV
FKEFYQTRTKHRKLTWIYSLGTCNINAKFEAKTIELIVTTYQAALLLLFNGSDRLTYSEI
VTQLNLSDDDVVRLLHSLSCAKYKILNKEPANRSISPNDVFEFNSKFTDRMRRIKIPLPP
VDEKKKVVEDVDKDRRYAIDASIVRIMKSRKVMGHQQLVAECVEQLSRMFKPDFKAIKKR
IEDLITRDYLEREKDNANVYRYLA*
>11667.m02613|LOC_Os01g27190.1|genepair784-1
MAYRLLELTLVSASDLKKVTLFSRMHVYAVASISGSNVPMPMHGTHADRNGGSNPAWNTV
LHFPVPARFDTRGLALHVQLRARRSFGGHRDVGDVFVPLDDLLAGAHDGGEPRPASYQVR
RPMSARAHGTLYFCYRFTDVKHPALEAIEAATATATSSATKQGQYVPMYAQDSDEKATEK
SVSSPVTAYPPPSNAVVAHPPVVPYGAPYGGGYPPHQQQYGYAGQPPYAYNAGPPPPATY
SYAAAQQPAARKGGRMGMGLGLGLLGGAVGGMMLGEMVGDMEADAAYDAGFNDALEF*
>11682.m00509|LOC_Os05g05650.1|genepair784-2
MARRVLEVTLVSAKNLKKVTMFSKMRVYAVASISGGDPRVPTHRTHADREGGRSPMWHAP
LRFPIPDAGADMRAIALHVLLRAERVFGDSDVGEVFVPVKDLVAAAPEGGEHRHLSYHVR
RPVSGRKCGVLHISYQITDEAAEPASAAGARGASSTRHVSGKGLQLSAITAYPSAGRHSG
GRRGAAQSPTLAYHHGSPYGGGGGGAHAQHHHHHHHHYGYGYGPAPYGHGAASHGGGGGM
GMGAGLGMGVVGGAVAGMVLADMLADGEMDTPLDAGMSC*
>11667.m02630|LOC_Os01g27360.1|genepair785-1
MTPVKVFGPAQSTNVARVLLCLEEVGAEYEVVNVDFTVMEHKSPEHLKRNPFGQIPAFQD
GDLYLFESRAIGKYILRKYKTREADLLREGNLREAAMVDVWTEVETHQYNSAISPIVYEC
IINPAMRGIPTNQKVVDESAEKLKKVLEVYEARLSQSTYLAGDFVSFADLNHFPYTFYFM
GTPYASLFDSYPHVKAWWERLMARPSVKKLAAVMAPQGA*
>11682.m00506|LOC_Os05g05620.1|genepair785-2
MVAKVYGVAASPYVATVLVCLEEAGASYELVAVDMAAGENRSRHHLARSPFGKIPAFEDG
EVTLFESRAIQRYVLRNYNKPDLLREGNLEESAMVDMWMEVEAHHYDPAIFHIIRECVIK
PMIGGGARDQAIVDENVEKLRKVLEVYERRLSESEYLAGDFVSVADLNHFPYTYYLMTTE
YATLVESCTNVKAVEIMGI*
>11667.m02659|LOC_Os01g27650.1|genepair786-1
MAAAAPSSARRMSHTQFIELLRRRASCPRHGEALHAWALKSGAASHAPVANSLINFYSSL
PRPLLAAAFAVFDDIPPAARDVTSWNSLLNPLSGHRPLDALSRFRSMLSSSTVLPSPHSF
AAAFTAAARAASAPAGTAAHALACKIPSAVSNVYVCTSLLNMYCKLGIVSDARRVFDGMP
QRNSFSWSTMVAGYAAEKCSEEAFDLFRLMLEECPSEKSEFVATAVLSAVSVPLGLLMGE
QMHGLIVKDGLLDFVSVENSLVTMYAKAGCMGAAFHVFESSRERNSITWSAMITGYAQNG
EADSAVSMFSQMHAAGFTPTEFTFVGVLNASSDLGALAVGKQAHGLMVKLGFEVQIYVKS
ALVDMYAKCGCIADAKEGFDQLYEVDIVLWTAMVSGHVQNGEHEEALTLYARMDKEGIIP
SKSTIASGLRACAGIAALEPGKQLHTQIVKYGLGLGAPVGSALSTMYSKCGNLEDGMSVF
RRIPDRDVIAWNSIISGFSQNGCGNGALDLFEEMKMEGTIPDNITFINILCACSHMGLVD
RGWEYFSLMTKDYGLTPRLDHYACMVDILSRAGMLKEAKDFIESITIDHGTCLWRIVLGA
CRSLRDFDVGAYAGERLMELGTGDSSAYILLSNIYASQRKWNDVERVRHLMRLRGVNKDP
GCSWVELNSRVHVFVVGEQQHPEAENINAQLRRLAKHMKDEGYHSSSKLSFDEELGPLAE
SHEEDQLEWISAAYS*
>11682.m00492|LOC_Os05g05490.1|genepair786-2
MRDASSGLLNYARFSGLLASCGREGDLRLGAALHAAVVKNPAHFRLCASRPWLRHVLVAW
NSLVSMYARCGRREDAARVFDEMRVRDAVSWNSLIAASRGAGDALALFRRMLRSDARACD
RATFTTVLSECARAGAASLPACAMVHGLVVSCGFEAEVPVGNALVTAYFECGSPASAERV
FHGMAEKNVITWTAMISGMARAELYKESFSLFGQMIRTVDANNATYSCALLACARSLAAR
EGQQVHGLVVKAGFEDDLHVESGLMDVYSKCGLMEDALTVFRSCREPDEVFLTVILVGFA
QNGLEEKAFELFAEMVGAGNEIDENTVSAVLGAFGASAPFALGKQIHALVIKRCFGVNTY
VCNGLVNMYSKCGELRESVRVFDEMPSKNSVSWNSIIAAFARHGHGSEVYQLFESMKADG
AKPTDVTFLSLLHACSHVGSAKKGLEILNSMSSQYGVLPRMEHYACVVDMLGRAGLLDDA
KSFIEDGPFTDNALLWQALMGACSFHGNSEVGKYAAEKLLLLDPSCTAAYVLLSNIYSSE
GRWDDRAKVMKRMSEMGLRKDTGKSWIELEKEVHSFVVRSTSHPNSAAAVR*
>11667.m02681|LOC_Os01g27880.1|genepair787-1
MDKLSGSARLIIVSDLDHTMVDHHDEENLSLLRFGALWESVYCQDSLLVFSTGRSPTLYK
ELRKEKPMLTPDITIMSVGTEITYGEAMVPDDGWEEYLNNKWDRNVVVEETAKFSELKLQ
PETEQRPHKVSFFVDKKSAQEVIKSLSGNMEKCGLDVKIIYSGGQDLDILPQGAGKGQAL
AYLLKKLSSCGKPPNNTLVCGDSGNDAELFSIPGVHGVMVSNAQEELLQWYAENAKGNPK
IIHATERCAAGIIEAIGHFKLGPSVSPRDVGFPYVKEDHIKPTDAVVKFYVLYEKWRRAE
VPKSDSVVQYFKNITHANGVIIQPSGLECSLHASVDALSSCYGEKQGKKYRTWVDRLFVS
QSGSDSWLVRFDLWEAEGDARLCCLTSLALNVKPETPAGFLITHVHKTWLKGYSSADEQS
SKL*
>11682.m00470|LOC_Os05g05270.1|genepair787-2
MDKLDGSARLMIVSDLDQTMIDHNDPKNLSLLRFQALWESEFSQDSLLVFSTGRSPISYR
GLRTQKPLITPDITIMSVGTVIAYGEEMIHDVGWAEFLSNKWDRDIVVEETAKFPKLKPQ
PERSQGPHKVSFFVDKEGAREVMDSLPETLNRRGLDVKIIFSSGEALDVLPQGAGKGQAL
LYLLKKFNSDGKPPNSTLVCGDSGNDAELFSVPSVHGVMVSNAQEELLQWYEENARGNPM
MIHATERCAAGIMQAIGHFNLGPNVSPRDLEFPYPKLDAIKPADVVVKFYVLYEKWRQGE
VQKAPFIIQYLKRITVSTFLSYILRITQSSFVHKPLFLKSIIPFVDCYS*
>11667.m05408|LOC_Os01g54870.1|genepair788-1
MGAFRFHQYQVVGRGLPTPTDEHPKIYRMKLWATNEVRAKSKFWYFLRKLKKVKKSNGQI

LAINEIFEKNPTTIKNYGIWLRYQSRTGYHNMYKEYRDTTLNGAVEQMYTEMASRHRVRF
PCIQIIKTATVHFKLCKRDNTKQFHKSDIKFPLVYRKVRPPTRKLKTTFKASRPNLFM*
>11682.m04703|LOC_Os05g49030.1|genepair788-2
MAERGCGRQASAAAERWRAVGGADGGRARRRSGGYGRSSRAPWPPSSSPWARRHGAAVQR
HRPGVAVAVHRWLEVPADVAPQSLALLLCRGSGSGGDGSVAGLIPWSSRTPSSRSCSRSV
STLGVPSASWTVVGAGAVTLIVFTDVAVALDVVTHTHIRRFHQYQVVGRALPTPGDEHPK
IYRMKLWATNEVRAKSKFWYFLRKLKKVKKSNGQMLAINEIFERNPTTIKNYGIWLRYQS
RTGYHNMYKEYRDTTLNGAVEQMYTEMASRHRVRFPCIQIIKTATVHFKLCKRDNTKQFH
NSNIKFPLVYRKVRPPTRKLKTTFKASRPNLFM*
>11667.m05421|LOC_Os01g54990.1|genepair789-1
MGIDLNNTASGGEEDAPAPGPVCRDLWHACAGPVVSLPRRGSAVVYLPQGHLSAAGAGGG
IRGEVAVALPPHVACRVVDVELCVFERNLHGGGIEREDDMEDGDEERKSRMLHMFCKTLT
ASDTSTHGGFSVPRRAAEDCFPPLDHKQLRPSQELVAKDLHGAKWRFRHIYRGQPRRHLL
TTGWSSFVNKKKLVSGDAVLFLRGDDGELRLGVRRATQLKNEAIFKAFSSESSKMRTLSA
VADSLKHGSVFHICYNPRATASEYVVPYWKFVKSFNHPVCIGMRFKFHFESEDVNERRSG
MIAGVSEVDPIRWPGSKWRSLLVRWEDATDCNSQNRVSPWEIEIVGGSISVAHSLSASSS
KRTKLCPQGNLDVPALYGNGRPDSVETEKFPRVLQGQELMGSRTHRATCSPQSIDITKSK
SFDAWRFLTDTRSCMLGSSTSRLPVQYSGYTHQSVSFGESIGFPEVLQGQEISQTVPPFQ
GMLPDACSAKSRYELKNYVCTPATMNGLSSANEGYCLSLSTVPPSPPSSLMLYQTGVPQL
ELASKNNDKSGNDSQPALRQHKLLSETSWDQFKIGKASTPGNATKPGNGGREVDRTSCRL
FGFSLTEKIIPTDKDGEKEVSYETDCQNPRMLDLFGYNCSTPGALHALCAAPLGI*
>11682.m04685|LOC_Os05g48870.3|genepair789-2
MTGIDLNTVEEDEEEAAEEVAANGSSPAPARAGAVCLELWHACAGPVAPLPRKGGVVVYL
PQGHLEHLGDAPAAAAAAAAVPPHVFCRVVDVTLLADAATDEVYAQLSLVPEKEEVARRA
DDGEGEDGDGMKQRFARMPHMFCKTLTASDTSTHGGFSVPRRAAEDCFPPLDYSQQRPSQ
ELVAKDLHSTEWRFRHIYRGQPRRHLLTTGWSAFVNKKKLVSGDAVLFLRGDDGELRLGV
RRAAQLKNGSAFPALYNQCSNLGTLANVAHAVATESVFNIYYNPRLSQSEFIVPYWKFMK
SLSQPFSVGLRFKMRYESEDATERRYTGIITGSGDTDPMWHGSKWKCLLVRWDDDAEFRR
PNRVSPWEIELTSSVSGSHLSTPHSKRLKPCLPHVNPEYMVPRGGGCPDFAESAQFHKVL
QGQELLGFKSHGGTAAATSQPCEARHLQYIDERSCSSDASNSILGVPRLGDRAPLGNPGF
SYHCSGFGESHRLQKVLQGQELFRPYRGTLVDASMGSNGFHQQDSPRAPGVVNKWQAQLH
GRAAFHGPPALALPSQSSSPPSVLMFQQANSKMPRLEFGHGQLDKHENDRRVRFGPSEGI
ERREQRIPLQPYPTSGEVIDGQVTVEKSHSPGRHGKDGPDNKAVGTNSCKIFGISLTEKV
PAREELDDGDANYSLQSLKQVPKSLGNSCATVHEQRPVVGRVIDISTMDMMI*
>11667.m05428|LOC_Os01g55050.2|genepair790-1
MSGSGVPAGRGSRSFDFGADDVLCSYDDFAAPSEPKRPDPADKQDFHDSRLGRPFGKAYE
QESYGKEDVLFAVEKCMKKYADNLLRSLEGITNRLSQLEIYCYKLERSMGELRSDVLRDE
TDQRLKSLEKHLHEVHRSIQILRDKQELAETQKELAKFQLTHDTSKKKEDMPTPSFPEQK
TLEEKADAPGQQLAIVLPHQVNSSLAPRASQPVQQYKDQTVQQPPSSSVPQQDRYVLSQA
IVYYPQRQAPGIQDTQGQQVQPEVQYLPVRPSATQDVPVHASSQQSQAANQTQPQSFPPY
QQQWPQQSSQPAPAPVAQPQPTFSQPFPPPVQQPQLSNTQQFPPQPMQQPQLSNTQQFAP
QPVQQPNAQQFPPPPVQPQQSNTQLPPQAMQPQQHPPVQNQMRPQTPPNYPHYQPHQSLN
PPPETLPGSMAMQGPYNTVAPAAGSRSEVPYSYGGPGMPPPQHNMQRQQLPPPSQGSFGP
PSKGGYAGPPQYAPQGSSHGYNTAYGYPPSGPSAAQAPQMPPAPGNVGMSHPGSHQMMRG
HPYGEMIEKAITMGYPREQVMNVIQRMTESGQPMDFNTLLDRLNEAGSGAPPRAW*
>11682.m04670|LOC_Os05g48760.1|genepair790-2
MASPARPAAASVSGAFGLSADPARCSFDQTLRREDFQDNRLLRSLVNIHEQETYSREIIT
EAIESCMKKQADNLVNTLDVISGRLSQLELYCYKLERSIGELRSDVMDYHGEANINFRCL
EKHVKEVQNSVQVLQDKQELAETQKELTKLQILHEESAQKSEGTAPSVLMTKEIDGSMPV
AKHELALVPLHQVNAAQSPAMQFQSCNGLVLQQLVPVSLSTQQDQQHMNQATLYCMQTQA
HVEHRQAQPFQPAPQPVQRHTQNTPQTVVEAQQVTSQAPDFYIQPQQQWAHQTGQQVHQA
RQPQPQVVQQQHYNNIQQVPAQIVQMQTSSPQAQSAPHVTLLYPPYGSQQPACANSEPRS
RSMAMQPSYSTISSSQRNHHEVAPVYVQSNTISVPLAEHSIQSQQPPQLQSFGNGSFKPS
KVSLHGVASYTVQGNAQAYNTAYGNPSNNAATVVAVLPQQAQSSAPMVLHHLGPQSLQNH
PIDMVEKVARMGYFKDQAESMALRMATAGQNVEFKHLA*
>11667.m05447|LOC_Os01g55240.1|genepair791-1
MVVLAGPPAVDHIPLLRSPDPGDVFSGVPVVDLGSPGAARAVVDACERYGFFKVVNHGVA
TDTMDKAESEAVRFFSQTQPDKDRSGPAYPFGYGSKRIGFNGDMGWLEYLLLALDDASLA
DACTVPSCAVFRAALNEYISGVRKVAVRVMEAMSEGLGIAQADALSALVTAEGSDQVFRV
NHYPPCRALQGLGCSVTGFGEHTDPQLVSVLRSNGTSGLQIALRDGQWVSVPSDRDSFFV
NVGDSLQVLTNGRFKSVKHRVVANSLKSRVSFIYFGGPPLAQRIAPLPQLLGEGEQSLYK
EFTWDEYKKAAYKSRLGDNRLAQFEKK*
>11682.m04664|LOC_Os05g48700.1|genepair791-2
MVAITAPSSIEHIPLVRCPKGANAGPQAVIPCIDLSAPGAAAAVADACRTLGFFKATNHG
VPAGLADALESSAMAFFALPHQEKLDMSGPARPLGYGSKSIGSNGDVGWLEYLLLSAGAA
SSGGAALPAALRAAVEAYTGAVRGVGCRVMELMAEGLGLGASEEGRCVLRRMVVGCEGSD
EMLRVNHYPPCLLPPGRDRDECGVTGFGEHTDPQIISVLRSNCTAGLQILLRGDYSSPAR
WVPVPPDPDSFFVNVGDSLQVLTNGRFRSVKHRVLAPEGEESRLSVIYFGGPAASQRIAP
LEQVMREGEQSLYREFTWGEYKKAAYKTRLGDNRLGPYELQHAAANDEAATKK*
>11667.m05468|LOC_Os01g55430.1|genepair792-1
MVPWRRSSSSSSAPSSRPARRPARTNARVSPDVSSELSPLAGEEGAGEERWSALVPDLLA
DILRCVEAGSERWPPRRDVVACASVCRRWRDVAVAVVQPPLESGKITFPSSLKQPGPRDA
PMQCFIKRNKKNSTFFLYLGLTQELTDDEKFLLAARRCRRGLHKEYAITINSDGLFHGSQ
SCVGNLKSNFTGTKFTIRDWQPPYEGAKAFSSRSGRWFGNKHRCPLVSTGDVEVGEVSYK

YSLLRPRGPRRMSCSVQCPVLKGTAVDPQDGKRLSNSIPSSLVLNSKVPSWHEHLQCWCL
NFHGRVMVASVKNFQLIAPVEPGEPSDKTVVLQFGKIDDDVFTMDYRQPLSAFQAFAICL
SNFGTKLA*
>11682.m04661|LOC_Os05g48670.1|genepair792-2
MAAVREPREEAAVGEGEGEEEGRWGGLLPELVEEVVRRVEASGGERWPARKDLVSCACVC
RRWREAAAAVVRPLPESGRITFPSSLKQGSFIAGLQKYQFCEVGFLADQIGIDMVYNLSY
NLKSGFEVSAFALLPGPKDFPIQCFVKRNKKKSMFYLYLGLLNGTMDKGKFLMAARRFRR
GPHTEYVISLDADDLSQGSNAYVGKLRSDFWGTNFKIYDNQPPYDDAKTSSTRSSQRFGS
THRFGSRRICPQISAGNFNVGQISYKYNLLKSRGPRRMFCTMECPSTQETWENSLKTKSL
RCTGTTVLRNKAPRWHEHLQCWCLNFHGRVTVASVKNFQLVATADPSHPDSVGDEETVIL
QFGKVDSNIFTMDYRQPLSAFQAFAICLSSFGTKLACE*
>11667.m05479|LOC_Os01g55520.1|genepair793-1
MALWQARHFLIIKHVPLPNYAPPLPRGFFLPRQCGRSIEPPGSWITGTNNNHHHLGINHF
NPPLFVPSRGSLCVGVLQRQSWIIIVCFCTRRSRPQKAASFTEAGHGGVDRLMAASGGGS
LLERRSSVRRSQSMVSEEGRGTPADEDLGGGGTLKIGAVLDKDSAAPKSRLAKDTGEHGG
GGPSEMELMKEKFAKLLLGEDMSGSGKGVPSALAVSNAITNLAASVFGEQRKLEPMAPDR
KGRWKKEVGWLLSVADHIVEFVAKKQVLDNGVEMEVMGTQQRRDLQANIPALRKIDTMLL
DYLDNFKDRNEFWYVKRDSCSDSDEQRSDEKWWIPIVKVPPGGLSPASRGWLQHQKELVN
QVLKAAMAINANCLMEMAIPESYLESLPKNGRASLGDALYRIITDVEFDPDVFLSTVDLT
SEHKILDLKDRIEASVIIWNRKVHNKDGKSAWGSAVSQEKREQFEERAQTLLLIIKHRYP
GIPQSTLDIAKIQENRDVGFAILESYSRVLESLAFNVMSRIEDVLNADDHAREKAKKEAP
PAPAMANDAAEHHHQQAGEVDAPCKMTGSPNGRTLLDFMDDWNGDADRPSPTAPEPAAQE
DGRLMKLPNIMTNLKQTYMDNLFGAHRSPPGRH*
>11682.m04657|LOC_Os05g48640.1|genepair793-2
MARPLLKIGHGLDRFRWRRSTSSSSSSPLALSLSSSSAAALSDDDPGSPMDPEMPPAARR
ALSRSSGSRGRLSFELPPLAGGPSDKEEAPPRTSSAPAPARPAPAALHEGPPSDAEMVRE
KFSKLLLGEDMSGTGKGVSSALALSNAITNLAASVFGEQRRLQPMAADQKARWRREIDWL
LSVSDHIVEFVPSKQVSEDGSTMEIMITQQRRDLQMNIPALRKLDAMLLEYLDNFKDKQE
FWYVSKDASESEKGNTPRQDDRWWLPTVRVPPGGLSDASRKWVQHQKDLVNQVLKAAMAI
NANVLMEMDVPEAYIESLPKNGKSTLGDSMYKIITEDHFNPEELLGTVDMSAEYNIIDLK
NRIEASVVIWQRKMVHKEGKLSWGHGVKFEKREKFEARAENVLLLIKHRFPGIAQSALDI
SKIQYNRDIGLAILESYSRTLESLAFTVMSRIEDVLHADSLAQASNTRTQESMRMASLSR
YDTDKVVIDAKAEVERLGRMEPVSATLFDFVSPRDQDVVATKMDSKEKGCRGDAHSRKLT
KVSPIATKRYSYLEKLENLSGTRSPISRH*
>11667.m00979|LOC_Os01g10180.1|genepair794-1
MEPGFGKRLMHVLRAVYHMLRRGLCRKRLMMDLHLLLGRGKLAGRALRDVLLAHQPHGGA
AAVAVMGGGAGVARGGDSSSSPLSASFFHHNPRDVEFSCTTTPSYAPGVFPFRFRGRGGS
RHAGGGASNYGGLDASAVARVFEMLNADAAAAGAGGETPLSSMPGATPSPLLALSLGRS
PAGTRQLRVTDSPFPVEPPEGAVDGRVDDKATDFIEWFRRQLLQQQASAAPTPDYRG*
>11680.m03492|LOC_Os06g36070.1|genepair794-2
MIHQRDGDHLMEESTTMARRLWHVVRAVLFMLRKGMSKRKLAMDLHLLLHRGKIAGNKAL
GKIMNTTTATASHGHGHAADAASTAAGEAAAAAPFSCGRALDPALAVYDPRGAGLEVEFS
CSNTPSYPSSFHLIPTKRRRRNNNGSNGRRRGGGGRGANGGEPGWYNYDAADIARVFEIL
NSSDQLLGDGGAAVAATPSPALWRTSFGGRSPAPVRQLRITDSPFPIRDDGGEDAGAGLV
DLEAEEFINKFYEQLRTQQQSLATATPDYYAGYSRPVTGVAY*
>11667.m00988|LOC_Os01g10260.1|genepair795-1
MAPNGEGAVAAAAAAPAPAPAVARGKRAEKPRRISMEGLQRAMSDLALELTKKQQVADAA
AGGGGGGEKLPEITEQQQVVVEEARCECCGMQEECTPEYARRVRERYCGRWVCGLCAAAA
SAEADRRCRHGGTGWTTATAEEALAAHMAVCGRFNRVGRANPVLMQTEAMREILRKRSRS
NSPRDHGHGGLTRSSSCIPAITKD*
>11680.m03489|LOC_Os06g36040.1|genepair795-2
MAMAAPFIAMASNMQGVVSVDGNAEKRRRTSSDALQRTVSDVSYELHHHVGAKGTTMVDD
AAAAEQKQQLDDIAEVEDARCECCGMSEECTPEYIRGVRARFAGRLVCGLCAEAVAEEAA
RRGGAGGVEAALRAHTAVCKRFNGFGRTHPVLFQAEAMREILRKRSKLGPRSRSSINPRE
ARQAGAAGGGGIARSSSCLPFITDEFSQRVNIN*
>11667.m01001|LOC_Os01g10370.1|genepair796-1
MAASEQSSESSSTASTSSCGKKQQVAGKRKREDVGGGGEQAAAVAYRGVRMRAWGKWVSE
IREPRKKSRIWLGTFPCPEMAARAHDVAALSIKGARAVLNFPDLAPALPRPASLAPRDVQ
AAAALAAVMHHHKHPSSSTSTSSPPAAPPPDEHHPRHEPQQPESSREDDQQQQPAAAAAA
QMAVAELVFDELAPLWVEDVVEFGTSDHCWTAYDALDPIGFQPLLWEY*
>11680.m03485|LOC_Os06g36000.1|genepair796-2
MENLSCLISSPLPMPMPEYELAACSPASPSSSASSTSTPPSPGGEAAARCGEKRGRGGGG
GRHPTYRGVRMRAWGKWVSEIREPRKKSRIWLGTFPTPEMAARAHDAAALVVKGPAAVLN
FPGAAASLPRPASAAPRDVQAAAARAAAMALDAVVPAPPSPPPPLMPPQASPSEAARAHA
LVAQVDQDDDDEELEEIVELPPIDELDAAAELVFASSGATTFHYDPAADQPWYDQPAACL
QDGGGGGIAVHDALGFELDHVWADGVVASGFGALLWNM*
>11667.m01016|LOC_Os01g10490.1|genepair797-1
MAAARMKNTTMGCAFLLAAFAMAAAFVPVAESRTTPVEKTTTTQAEDGVKKPDCVPAFDP
RSFPGHGGTTTPTPIPGHHGGGGSSGTTPSHGGGPSGGALPSPSHGGAAPSHGGGYGASP
PVTPSPGGGYGGGSPAPSHGGGAYGSSPSTPSGGGSSPTPSHGGGAYGGGGAPATPASHD
GHGLIPTTPGTCDYWRSHPMEMWSALGRWPSSVGHFFGSGSGGAGTGMSIQDALANTRGD
GAGELMREGAAALLNSMTRSGFPYTAEQVRDAFAAAAGGGSDGAAAAQAAAFKKANEGGR
A*
>11680.m03482|LOC_Os06g35970.1|genepair797-2

MGSCKILLVTALLVGIASQQSSATRNLHGDHHVAEKKFGGGGGGYGGGGGGGYGGGGGGG
GYSPSPTTGFTGSCDFWKSHPEKIISCIGSLGSIVGSFGDVCSGFFGSKLQTLQDALCST
RKDCYGDLLREGAAAYINSVAAKKQAKFAYTTQQVKSCILLGLTSKAAAIEQAALFKKAN
LACHYT*
>11667.m01026|LOC_Os01g10590.1|genepair798-1
MSSANSLVLGRVIGDVVDLFSPEVTLRVMYNGVRVVNGEDLRPSAVSARPSVEVMVDPDA
PNPSNPTLREYLHWLVTDIPGTTDANYGREVVCYESPRPAAGIHRVAVVLFRQMARGGVD
QPPLLRHNFSTRGFADDHALGAPVAAAFFTCKPEGGTGGRRFRPPSRHS*
>11680.m03479|LOC_Os06g35940.1|genepair798-2
MANDSLTRSHIVGDVLDQFSNSVPLTVMYDGRPVFNGKEFRSSAVSMKPRVEIGGDDFRF
AYTLVMVDPDAPNPSNPTLREYLHWMVTDIPSSTDDSFGREIVTYESPSPTMGIHRIVMV
LYQQLGRGTVFAPQVRQNFNLRSFARRFNLGKPVAAMYFNCQRPTGTGGRRPT*
>11667.m01027|LOC_Os01g10600.1|genepair799-1
MAGREDGAAAGAMEEGQDSKEVKCESSEDGSSSSSSSRCHGNDVISVQFMQKVHPWCMCM
NKNLLILAEILGTYFMIFAGCGAVVVNQSTGGAVTFPGICAVWGLVVMVLVYTVSHISGA
HFNPAVTVAFATCGRFRWKQVPSYVVAQVLGSTMASLTLRVVFGGGGGGARGEHLFFGTT
PAGSMAQAAALEFVISFFLMFVVSGVATDNRAIGELAGLAVGATVAVNVLFAGPVTGASM
NPARSLGPAMVAGRYGGVWVYVAAPVSGTVCGAWAYNLLRFTDKPLRDIANTASFLRRSS
RRS*
>11680.m03478|LOC_Os06g35930.1|genepair799-2
MARREVDDSYTNGSVVEVVSIEEGSKMDKEDDHQNPQAPDGGDVVVCGMPMSFTFLQMLL
AEFLATFFLMFAGLGAITVEEKKGAVTFPGVAVAWGAAVMAMVYAVGHVSGAHLNPAVTL
GFAVAGRFPWRRAPAYALAQTAAATAASVVLRLMFGGRHAPVPATLPGGAHAQSLVIEFV
ITFYLMFVIMAVATDDQAVGHMAGVAVGGTIMLNVLFAGPVSGASMNPARSIGPALVGSK
YTALWVYILGPFAGAAAGAWAYSLIRLTGDRTD*
>11667.m01028|LOC_Os01g10610.1|genepair800-1
MATGGGGGGGGMGGGGVGGGAGAAGVGVGGRMPTWRERENNKRRERRRRAIAAKIFAGLR
AHGGYKLPKHCDNNEVLKALCNEAGWVVEPDGTTYRKGYKPPERMEVIGCSVSPSPCSSY
QPSPRASYNASPTSSSFPSGASSPFLPHPNNMANGVDGNPILPWLKTLSNSPSSKKHPQL
PPLLIHGGSISAPVTPPLSSPTARTPRMKTDWDESNVQPTWTGSNSPCVVNSTPPSPGRT
MLPDPAWLAGIQISSTSPSSPTFSLVSSNPFSVFKDAILVGNNSSRMCTPGQSGTCSPAI
PGMAPHPDIHMMDAVSDEFAFGSSTNGGHQAAGLVRAWEGERIHEDSGSDDLELTLGSSR
TRAAA*
>11680.m03475|LOC_Os06g35900.1|genepair800-2
MTNGAGGGGGGGGLGGTRVPTWRERENNRRRERRRRAIAAKIYAGLRAYGNYNLPKHCDN
NEVLKALCNEAGWTVEPDGTTYRKVIDKYEHNRVPLTCIQGCKPPQAERPDPIGRSASPS
PCSSYQPSPRASYNPSPASSSFPSSGSSSHITIGGNSLIGGVEGSSLIPWLKTLPLSSSY
ASSSKFPQLHHLYFNGGSISAPVTPPSSSPTRTPRLRTDWENASVQPPWASANYTSLPNS
TPPSPGHKIAPDPAWLSGFQISSAGPSSPTYNLVSPNPFGIFKEAIASTSRVCTPGQSGT
CSPVMGGMPAHHDVQMVDGAPDDFAFGSSSNGNNESPGLVKAWEGERIHEECASDELELT
LGSSKTRADPS*
>11667.m01051|LOC_Os01g10820.1|genepair801-1
MASEKKQANPMREIKVQKLVLNISVGESGDRLTRASKVLEQLSGQSPVFSKARYTVRSFG
IRRNEKIACYVTVRGEKAMQLLESGLKVKEYELLRRNFSETGCFGFGIQEHIDLGIKYDP
STGIYGMDFYVVLERAGYRVARRRRCKSRVGIQHRVTKEDAMKWFQVKYEGVILNKAQAN
TS*
>11680.m03458|LOC_Os06g35730.1|genepair801-2
MAAVAWGGALRSLSGVGDAVVREQRDDVNSTRQMTVSLMAPSFLAWRHANSTDSRSSGPP
RKTAVIWVPIFWSSWFLAAVVSSLVDILVGSWKLAPTVHGASSSTLRLSSRQWCSRAPRR
IRRAPASEKKQSNPMREIKVQKLVLNISVGESGDRLTRASKVLEQLSGQSPVFSKARYTV
RSFGIRRNEKIACYVTVRGEKAMQLLESGLKVKEYELLRRNFSETGCFGFGIQEHIDLGI
KYDPSTGIYGMDFYVVLERAGYRVARRRRCKSRVGIQHRVTKEDAMKWFQVKYEGVILNK
AQANTS*
>11667.m01053|LOC_Os01g10840.1|genepair802-1
MAERVVGTGSFGIVFQAKCLETGETVAIKKVLQDRRYKNRELQLMRAMEHPNVICLKHCF
FSTTSRDELFLNLVMEYVPETLYRVLKHYSNANQRMPLIYVKLYIYQLFRGLAYIHTVPG
VCHRDVKPQNVLVDPLTHQVKLCDFGSAKVLVPGEPNISYICSRYYRAPELIFGATEYTT
SIDIWSAGCVLAELLLGQPLFPGESAVDQLVEIIKVLGTPTREEIRCMNPNYTEFKFPQI
KAHPWHKIFHKRMPPEAIDLASRLLQYSPSLRCTALDACAHSFFDELREPNARLPNGRPF
PPLFNFKHELASASPELIHRLIPDHIRRQHGLNFAHAGS*
>11680.m03438|LOC_Os06g35530.1|genepair802-2
MAAMPGGPDLAGAGGAVAVAVDAMQVDDPPRASAEEKHGPTIMGGNDPVTGHIISTTIGG
KNDEPKRTISYMAERVVGTGSFGVVFQAKCLETGETVAIKKVLQDKRYKNRELQIMRSMD
HCNVISLKHCFFSTTSRDELFLNLVMEFVPESLYRVLKHYKDMKQRMPLIYVKLYMYQIF
RGLAYIHTVPGVCHRDIKPQNILVDPLTHQVKVCDFGSAKMLIKGEANISYICSRYYRAP
ELIFGATEYTTSIDIWSAGCVLAELLLGQPLFPGESAVDQLVEIIKVLGTPTREEIRCMN
PNYTEFKFPQIKACPWHKIFHKRMPPEAIDLVSRLLQYSPNLRCTALEACAHSFFDELRE
PHAKLPNGRPFPPLFNFKQELANTHPELVSRLLPEHAQRHSGF*
>11667.m01054|LOC_Os01g10850.1|genepair803-1
MGWSSSRAMLVARAVALAVVFLAAEAQLSPGYYNATCPGVVSIVRRGMAQAVQKESRMGA
SILRLFFHDCFVNGCDASILLDDTANFTGEKNAGPNANSVRGYEVIDAIKAQLEASCKAT
VSCADIITLAARDAVNLLGGPNWTVPLGRRDARTTSQSAANTNLPPPGASLASLLSMFSA
KGLDARDLTALSGAHTVGWARCSTFRTHIYNDTGVNATFASQLRTKSCPTTGGDGNLAPL
ELQAPNTFDNAYFTDLLSRRVLLRSDQELFGSGAGNGTTDAFVRAYAANATTFAADFAAA

MVRLGNLSPLTGKNGEVRINCRRVNSS*
>11680.m03433|LOC_Os06g35480.1|genepair803-2
MATQWVLVVVAVMAVLFAGGAAGGQLSTRYYDGKCPNVQSIVRAGMAQAVAAEPRMGASI
LRMFFHDCFVNGCDASILLDDTANFTGEKNAGPNANSVRGYEVIDAIKTQVEASCNATVS
CADILALAARDAVNLLGGPTWTVQLGRRDALTASQSAANGNLPGPGSDLATLVTMFGNKG
LSPRDMTALSGAHTLGQARCATFRSRIFGDGNVDAAFAALRQQACPQSGGDTTLAPIDVQ
TPDAFDNAYYANLVKKQGLFHSDQELFNGGSQDALVRKYAGNAGMFAADFAKAMVRMGAL
LPAAGTPTEVRLNCRKVN*
>11667.m01058|LOC_Os01g10890.1|genepair804-1
MEKKASILMNRYELGRMLGQGTFAKVYHARNLASNQSVAIKVIDKEKVLRVGMIDQIKRE
ISIMRLVRHPNIVQLHEVMASKSKIYFAMEYVRGGELFSRVARGRLKEDAARKYFQQLIG
AVDFCHSRGVYHRDLKPENLLVDENGNLKVSDFGLSAFKECQKQDGLLHTTCGTPAYVAP
EIINKRGYDGAKADIWSCGVILFVLLAGYLPFHDSNLMEMYRKISKGDVKFPQWFTTDVR
RLLSRLLDPNPNIRITVEKLVEHPWFKKGYKPAVMLSQPNESNNLKDVHTAFSADHKDNE
GKAKEPASSLKPVSLNAFDIISLSKGFDLSGLFENDKEQKADSRFMTQKPASAIVSKLEQ
IAETESFKVKKQDGLVKLQGSKEGRKGQLAIDAEIFEVTPSFFVVEVKKSAGDTLEYEKF
CNKGLRPSLRDICWDGQSEHPSLAQSSTLTQSSKSISRHAI*
>11680.m03399|LOC_Os06g35160.1|genepair804-2
MFRSMGTGTGTKPPAMTTERYEFGPLVGEGNFAKVYLGRHRATGEEVAIKVMDKEKLVRL
GATELIKREIAVMQRLRHPNVVRIHEVMANKRRICVVMEYVRGGALYRYFRRGPSGGAAG
LREHEARRFFQQLVSAVAYCHSRGVFHRDIKLDNLLVDEQGNLKVADFGLSALADMERRE
AHLQTVCGTPLFLAPEVFKRRGYDGAKADVWACGVVLYVLLTGRKPFPDEHVSRLYRLIG
QNQFQCPPSFSPDLARLVRRLLQPDPDRRITIPEIMEMRWFKRGFKEVTYYIDSNDRLRS
LDGLDGEPELYDSDTDTIESSSSSESPTPVAGTPRGMHTSVSAPALSELDRMEDSASLPL
PLPLPPRPRMPRPKSLNAFDIIASSPSFDLSGLFEERGERMRFVSGAPVADIIAKLQEIA
GMVSFTARTKDCQVSIEATRNGQKGALAISAKVFELTRELVMVQVCKKAGDTAEYRRFCD
NELKAGLRGLVVDALPPPVEGGGHGGAAAAAEAE*
>11667.m01081|LOC_Os01g11120.1|genepair805-1
MAAVAGGSRAAPPADSAAVAAVAKEAEYQKGVQKLVDLLSKLNPAAKEFVPSSAAVSSPS
RKALSADAPVFDYNSIGGWNGGGKESGADAYQQRRRRNGYLSQGRRRMNERARHADREDS
IRRTVYYVSDIDHTVTEERLADIFANCGQVVDCRICGDPHSVLRFAFIEFADEEGARTALN
LGGTMLGFYPVRVLPSKTAILPVNPKFLPRTEDEKEMVIRTVYCTNIDKKVTQLDVKNFF
EELCGEVSRLRLLGDNVHSTRIAFVEFVHAECAIMALNCSGMILGTLPVRVSPSKTPVKP
RLNRVASN*
>11680.m03386|LOC_Os06g35030.1|genepair805-2
MVAVAAEAPIRADAVPAAAAAAAAAAPDAAPGEGADAAAAVEAEAAGKDGGEEVREYKSD
ARKLEELFKKLNPSAEEFVPLARRRGGGDGARRLSADAPVFVSPAIDFYSQHPVQQPPPI
QVLPVVVGGGGGAGLDSSSDGSTNGQPNRRVIDRLIALLGSRRSSFNQGRRRMGGRPRRT
DREDSVRRTVYVSDIDQHVTEQKLAEVFSNCGQVVDCRICGDPNSVLRFAFIEFADDVGA
RAALTLGGTVLGYYPVRVLPSKTAILPVNPKFLPRTEDEKEMVSRTVYCTNIDKNVPEDA
VKSFFEGMCGEVARLRLLGDYVHSTCIAFVEFVQADSAILALSCSGMVLGALPVRQANTP
YACSKLVYCNQLRPDCAIDLTLFLALFCRGLGVTRVVARRDSVVVQGENEADSNG*
>11667.m01085|LOC_Os01g11160.1|genepair806-1
MAAVDTPEKPYEGKPAAEARSYWRWQKEDFFPEPSFASWGAYRAALAATPARFRDRFAGR
STDADELGALRRRSENEMRRCLTWWDLTWFGFGSVIGAGIFVLTGQEAHDHAGPAIVLSY
VASGLSAMLSVFCYTEFAVEIPVAGGSFAYLRVELGDVAAFIAAANLILESIIGTAAVAR
SWTSYLASLINKPASALRIQTSLAEGYNELDPIAVVVIAVTATLAILSAKGTSRVNWVAS
AVHVLVIAFVIVAGFLHAKTSNLTPFMPHGVPGVFRAAAIVYFAYGGFDNIATMAEETKN
PSRDIPLGLLGSMSVITAIYCVMALVLSMMQPYTAIDRSAAYSVAFSNVGMRWAQYVVAL
GALKGMTTVLLVGALGQARYTTHIARSHIIPPVFALVHPRTGTPVHATVLIAAAGACIGL
FSSLDVLSSLLSVSTLFIFMMMATALLVRRYYVRGVTSRTHARRLVALLSVVIGSSAGIA
AYWGAAPERWVGYTVLVPAWAAGTLGIQLLVPAARAPKVWGVPLVPWLPSLSIATNLFLM
GSLGAQAFIRFGVCTAIMLLYYVLVGLHATYDVAHGACSGDDVDEEVYADAAHVDDAKAA
AAVAANADVERAGAKI*
>11680.m03366|LOC_Os06g34830.1|genepair806-2
MATAAAAGEGGSGRRYWRWSKADFFPEPSFASWRSYGGALAATVPRLVDRVGSRSSEAAE
AGTLRAVSENPLRRCLSWLDLAFLGFGSVVGSGVFVLTGQEARFDAGPAIPLAYAAAGFS
ALLSSFCYAELATEIPSAGGSFSYLRVELGDIAAFLAAGNILLEAVVGAAGLGRSWTSYL
AALLGRDTDSLRIHVPALAEGFNLLDPIAVVVLVSTSAVAMSGARLTSTLNSLASVVGIA
IIAFVLAAGFSHFDAGNLAPSFFPFGAAGVFRAAGVVYWSYTGFDMVATMAEETKNPGRD
VPLGLISSMSSITVVYCLMSLALVGMQRYTEIDANAAYSVAFAAAGMRWARYVVALGALK
GMTSGLLVGALGQARYTTQIARTHMIPPYFALVHPTTGTPIYATVAVTLGAACVALFSSL
DVLASVSSISTLFIFALVAVALLVRRYHVAGATTPGQLRTFLAFLALVVLSSIGVSAYYN
SRYARRWPGYAAFGCGWAAGAAGLAACAEKQRAPRVYGAPLVPWLPAMSIATNLFLMGSL
GTAAYARFGICTAAMLVYYVLFGVHATYDVAHADDAAADNLEHGKIAAAPAPTTPA*
>11667.m01093|LOC_Os01g11240.1|genepair807-1
MGSQAIEAHREGAEVYHGAALCAEKAVELLAEIHMPLGLLPLAEMEEVGYNRATGFVWLR
QKKAITHTFKQISRQVSFAAEVTAFVEDRRMKRVTGAKTKELLIWVSLSDMFIDKDDASK
ITFKTPTGLGRTYPVDAFAKEDDGKGKVPAAAAAANGKETAVNGKAK*
>11680.m03362|LOC_Os06g34790.1|genepair807-2
MASQLVEEHRSGAEVHTGHELCERKARELLVELGLPDGLLPLPSLEEVGYNRAAGFVWLR
QTQAGGATHTFDTIGKQVWYAGEVTAFVEQGRMHGVAGVKSKELLIWVSISEIVLSPSGT
KLVFRTPAGLGRALPVTAFQLNPAPPEPEKKDAAAADEADAAATN*
>11667.m01115|LOC_Os01g11460.1|genepair808-1

MNTPNSWLFADNSKYSTRARLLFMGLSFSIGILSFLVYLAIWYTCTRRRRSRQLRGGGSA
SADQEAPEANSHGMSAAAIAALPTFGYEASAAAAAALDCAVCLGQVDAGEKVRQLPKCGH
LFHAECVDAWLRAHSTCPMCRAAVEGPATAAIAKKASSGGGATDTPPVVAPAPPAAAEAL
PLPPV*
>11680.m03329|LOC_Os06g34470.1|genepair808-2
MLYLAGARWVLLLLAAVTVLLCLQLSARDNAVDGAAAAAAAAGPAQTADLPLQQPAALPR
EAPAEGDLRGLGASSSPVFVVAVLPAYAWRKKAAVDGDDGDGECAICLGEVRRGQVVKQL
PACTHLFHARCIDKWLITSQGTCPVCRTPVDSAAAALQAVRVADQPP*
>11667.m01119|LOC_Os01g11500.1|genepair809-1
MSSSNGTTTYLPGGEAAGGGGGGNSGCCSTTTLFGALAASFALSFFLITIFICLRALRVA
RRRRDRPLVMEQEQRRPTPPRFGLDAAAIARLPSFPYVRARDDGEVSDSASSSSVECAVC
LSAVDEGETVRQLPACGHVFHRECIDMWLSSRASCPVCRGKAAPADELADAIVARIAVTP
DLVGPRVSMSVVVPVEMLKGEMVGASSTSRAASAPPEQLDARAPGPETDLESK*
>11680.m03327|LOC_Os06g34450.1|genepair809-2
MELPWLDLPFTLLTLLLATRLAYDYYGVVAATFTGSFSLQIFLFYCFARWYRHTIAARAA
ADADGDGGGGAVADEEAAPPVLIPLLEGRGGGGGGAGAASSLANRCFAVVFMVFVPLVIV
VFERSQADVVAYALCLANILVMVVWLSPDAAADPASAAKSFLRLSDDEDEGSCSGSGHGA
AEDKCCVCLAGMREAQALRDLPRCGHRFHAKCIGKWLTAHPTCPVCRTTAVPPPAPLPAS
GDHADDAITPV*
>11667.m01131|LOC_Os01g11620.1|genepair810-1
MAANKAVVVVAAVLLLLAVVARRASSQSYNAIYNFGDSITDTGNLCTGGCPSWLTTGQPP
YGNTFFGRPTGRCTNGRVIIDFLGTHDAFSPRIDSAQNFHCKLKDDELNLFARAADRFGL
PLLPPSKASGGDFKKGANMAIIGATTMNFDFFQSLGLGNSIWNNGPLDTQIQWFQQLLPS
ICGNDCKSYLSKSLFIVGEFGGNDYNAPLFGGKSMDEVKGYVPQIIAKITSGVDTLIGLG
AVDIVVPGVMPIGCFPLYLTLYQSSNSDDYDGNGCLKSYNSLSVYHNGLLKQGLAGVQAK
YPAVRLMYGNFYDQVTQMVQSPGSFAKGILVLKSLKVAKYFEGLQYGLKVCCGAGGQGSY
NYNNKARCGMSGASACGDPENYLVWDGIHLTEAAYRSIADGWLSGPYCSPAILH*
>11680.m03293|LOC_Os06g34120.1|genepair810-2
MRAIASSCRHTQTAAAMAPGSRCSRRQQLLVAVAVVLLLAAAPTGCSAARSKKSYEAIFS
FGDSLSDAGNLIADGIPKSLTTARAPYGMTFFGRPTGRCSNGRLVVDFLAEHFGLPLPPA
SKAHGADFSKGANFAITGATALEYSFFKQHGIDQRIWNTGSINTQIGWLQDMKPSLCKSD
QECKDYFGKSLFVVGEFGGNDYNAPLFSGVAFSEVKTYVPLVAKAIANGVEKLIELGAKD
LLVPGVLPIGCFPLYLTLYNTSSKADYNARTGCLRRYNRLAFHHNRELKQQLDELQKKYP
ETKIMYGDYFKAAMQFVVSPGNFGFSSTMQACCGAGGQGNYNFNLKKKCGEEGASVCSNP
SSYVSWDGIHMTEAAYRYVANGWLNGPYAEPPILK*
>11667.m01134|LOC_Os01g11650.1|genepair811-1
MRLSVSVAAVLVVLAALRPPRVAVAQKYAAIFNFGDSLVDAGNLVVDGIPDYLATARLPY
GMTYFGYPTGRCSDGRLVVDFIAQEVGLPLLPPSKAKNATFHRGANFAITGATSLDTPYF
QGRGLGHTVWNSGSLHTQIKWFQDMKASICKSPQECRDLFRRSLFIVGEFGGNDYNSPLF
AFRPLEEKLIEEGAVELVVPGVLPIGCFPVYLSIFRKQPEMYGRRSGCIRDLNTLSWVHN
AALQRKIAELRLKHPGVRIMYADYYTPAIQFVLHAEKYGFLRQTPRACCGAPGVGEYNFN
LTSKCGDPGSYACDDPSNHWSWDGIHLTEASYGHIAKGWLYGPFADPPILETRHH*
>11680.m03288|LOC_Os06g34070.1|genepair811-2
MARPRRRGGAGAWVAAVLAAALQVLAVSAAGKYRAVFNFGDSLVDAGNLVTDGIPDYLAT
ARPPYGQTYFGYPTGRCSDGRLVVDFIAQEFGLPLLPPSKAKNASFARGANFAITGATAL
DTDFFERRGLGKTVWNSGSLFTQIQWLRDIKPSFCSSTQDCKDFFAKSLFVVGEFGGNDY
NAPLFAGKDLREAYNLMPHVVQGISDGVEQLIAEGARDLIVPGVMPSGCFPVYLTMYKEP
KEGYGSRSSCLKRFNTFSWVHNSMLKRALAKLRAKHPGVRIIYGDYFTPVVQFLLQPEKF
GFYKQLPRACCGAPGTGPYNFNLTAKCGEPGATACADPKTHWSWDGIHLTEAAYGHIARG
WLHGPFGDQPIVQNS*
>11667.m01150|LOC_Os01g11810.1|genepair812-1
MRGRAAVFAAVLVVFLVACTAAAAAAAITISRKHQHRPASGAAAKAACDVFAAGSWVVDE
SYPLYDSATCPFIRAEFDCRRYGRPDKEYLKYRWQPSPPCSTPRFDGVALLRMWSGKKVM
FVGDSLALNQYESLLCMLHAAAPNARTTVTPASGKVDPLTTARFEEFNVTIVYYLTHYLV
DIVNEKAGRVLKLDAIDQARNWLSADVLVFDSWHWWPRSGPTQPWDYIQEGNTVVKDMDR
TEAFSKALNTWARWVDANLLQTNTRVFFQGISPSHYRGQDWGDTASATCMGQTRPVNGTA
YPGGPIPQQAVLRSALAGMAKPVYLLDFTYLSQLRKDAHPTKYNGGIFGDDCTHWCVAGL
PDTWNVLFYAALTGQH*
>11680.m03287|LOC_Os06g34060.1|genepair812-2
MGTALLSRAAGVLVLVVVVAAAAAAPRCDLFQGRWAADESYPLYDASRCPFVPDVFDCRR
NGRPDAAYLNLRWFPSSCRLPRFDGVELLRRWRGKTVLFVGDSLSMNQWASLACMLHAAV
PADGRVSFTSGEPVSSVRFLDYGVSVVLYYSRFLVDVVDDEPGLGRVLKLDSMRDAAAWL
GADVLVFNTWHWWTYRGASQVYVDRRRARTYTTTHDVSFLGTDTVSHVYIYVMCRWDYVQ
EGNKTYRDMDRLTAFSKGLSTWARWVDANIDASRTKVFYQGISPSHYYTSSSSSSNDDGD
GEVAAADGGCYRQTRPLQESTTADGGGGALLPEQVVVRGVVGSMATAVSLLDVTRMSQLR
IDAHPSVYGGPGREGMDCTHWCIAGLPDAWNHIMSSPARREAAASNPVCGRQQQWWEANN
KGSLVVHDGGGEGEGGEGADELELASRPLEGVEEERTWCPQQLCLGFSYGLATLRVGSSP
LLMLILEGHASPPLGIGSPLLPPGHVESGRSCKDGKGAQ*
>11667.m01157|LOC_Os01g11880.1|genepair813-1
MAMAAASASAMARRAASWPRLLLLSRAFAAAAAEPKRVLVPVADGTEPVEAAATADVLNR
AGARVTVATADPAGDDRGLLVEAAFGVKLVADGRVADLEGEAFDLIALPGGMPGSANLRD
CKVLEKMVKKQAEQGGLYAAICATPAVTLAHWGLLKGLKATCYPSFMEKFTAEIIPVNSR
VVVDRNAVTSQGPATAIEYALALVEQLYGKEKSEEVAGPLYVRPQPGVDYVIDEFNSVEW
KCSGTPQVLVPVANGSEEMEALNLIDILRRAGANVTVASVEDKLQVVTRRHKFNLIADIM

VEEAAKREFDLIVMPGGLPGAQKLSSTKVLVDLLKKQAESNKPYGAICASPAYVLEPHGL
LKGKKATSFPPMAHLLTDQSACDSRVVVDGNLITSKAPGSATEFALAIVEKLFGREKAVS
IAKELIFM*
>11680.m03285|LOC_Os06g34040.1|genepair813-2
MATRPLAASTLLPPLRFCSPLKTPPPSPPPPHLRRLQTLTRALASSSSAMASPPAKKVLV
PIASGTEPMEAVITVDVLRRAGADVSVASVDPGSAQVGGAWGVKLAADALLDDLADAEFD
LISLPGGMPGSSNLRDCKLLENMVKKHAGKGKLYAAICAAPAVALGSWGLLNGLKATCYP
SFMDKLPSEVNAVESRVQIDGNCVTSRGPGTAMEYSVVLVEQLYGKEKADEVAGPMVMRP
QHGVEFSLKELNSTSWNVGETPQILVPIANGTEEMEATMIIDILRRAKANVVVASLEETL
EIVASRKVKMVADVLLDDALKQQYDLILLPGGLGGAQAYAKSDKLIGLIKKQAEANKLYG
AICASPAIALEPHGLLKGKKATSFPGMWNKLSDQSECKNRVVVDGNLITSQGPGTSMEFS
LAIVEKLFGRERAVELAKTMVFM*
>11667.m07075|LOC_Os01g70590.1|genepair814-1
MAKIQPLPAASPSAEWHHGDGGGGGVQGKQAVYTVWMKSLVFNGHGCTVYGSDGRVAFRV
DNYGCRGSRDVFFMDTAGTTLIGIQTKSFGMMKRWEASRHHGGEKETTTATVTTPWFRVQ
RGRGPGGAMATVTLHGGVGMAYRIDGCPSKSEYRISRGGGGGGGGDGEVVAEVARKQTAS
GVVLGEDVLTLTVGPGADHLLVLGLVVVCGLISRAM*
>11673.m04704|LOC_Os07g47540.1|genepair814-2
MARVYSSFPPPPPSPPPPSTTSEQRKVFTLWMKSLVLNGRGCTVYDSTGGIVYRVDNYGS
SCSDNVYLMDLRGKIVLNILKKKLAFGKWEGYKWSSGERQRDAAAGAGAGGAWFTVTRPC
SSILFQRRRRPSSTCEFRSGGGGGDDGGRAMRYKMDDCSGGGGGGGKQQAWCRIVDGATG
AVVAEVKGKTTAGGVALGDDVLTLTVEPNVDHALIMGMVLVYGLMNHTM*
>11667.m07087|LOC_Os01g70710.1|genepair815-1
MASGSEPIECQVLVLRVSIHCEGCKKKVKKVLQHVPGVFRCDVDARSNKVIVTASRNMDA
NILVAKLRKSGKQAEPWPEEPKQQQPPPPPAESQSQETKNQSDESSKPSDQPAEKPGPDK
AEGSAAEPNNPQPSPEPTKSTDETPKPNQEIQEPSNAKANTDANASGNASDETKEAAATG
EQSSEPKGKVKQHRERPIDARVTMEYGGGSHVNYMPQPQPVPVMSYNVARPTASAAYYAA
PPAPAPMSMPMPMARPGPSSQGYIDEEYSPSYYNRSSPYEPYYYPQPSPYRYQHYQQSSA
DDYYYGAPQQRSAFSPPRDAYGEMFNDENSNSCSVM*
>11673.m04698|LOC_Os07g47480.1|genepair815-2
MASGEAEPLQYTTTVLRVSIHCEGCKKKVKKVLQNIEGVYKVTIDAAQHKVTVTSSVGAD
VLVRRLHKSGKHATVWPSPPVAAAAKQKPDEVAAAPPPAAAPAPAGERGKGAEKKAKEAA
KEAGTESSEKKPEKEKKAEAKKPAKDDAAKKEGEVPEKEKGSPEPNKESAAAAADEEEGG
EESGGKKGKKKKNKQKDGGGEADAAAPEKPAAKAPPAAPAPSMPAPAPTLSSPERPHGGF
PYYSPQPVMSYNVAHPSASVSSYYAPTPVMSMQPTPMPPPPQMSYGYSPYPPMMMPPPPP
PEYLYGPPGMRSSPPQESYSNMFNEENPSSCSVM*
>11667.m07089|LOC_Os01g70730.1|genepair816-1
MSGVWVFRNGVVKLVENPPASANSGGGGGGGGGGGGGGIRRKALLHMPTGEVVTSYASLE
RKLAALGWERYYSGGGGAAAAAAMMLQFHKRSSVDLISLPKDFSQFGSVHMYDIVVKNRD
AFRVIDV*
>11673.m04695|LOC_Os07g47450.1|genepair816-2
MAGGGVWVFRNNGVMELEEQATSRKALVHVATSEVIRSTEALERRLGALGWERYYEDRAT
LQLHRRDGSADLISIPRDFSRFRSTHMYDVVVKNRDHFKVVDLHT*
>11667.m07094|LOC_Os01g70780.1|genepair817-1
MATPPHDASPAPPNPNPVSDDPPPPPPVTETKPEPEPPLPTTSIDPTPSGDEESGDDSSS
SVSSASSTSPTAAAAAAAAGGGGGGGGERAAPHPAAKDLLHISFNQDYGCFAAGTKSGFR
IYNCDPFREIFRRDLGAAGDNGVGGGGGGIGVVEMLFRCNILALVGGGDAPHYPPNKVMI
WDDHQSRCIGELSFRSPVRGVRLRRDRIIVVLENKIFVYNFADLKLVHQIETAPNPKGLC
AVSQQPGSIVLVCPGAQKGQVRVEHYGARKTKFINAHTSRVACFALSQDGRLIATASTKG
TLVRIYNAAEGNLLQEVRRGADRAEIYSLAFSNNLQYLAVSSDKGTIHVFNLKINVGLTT
NDKPLPAPDPDVPHISPSLSFIKGVLPKYFHSEWSVAQFRLHEGEQYIVAFGHEKNTVAV
VGMDGSFYRCQFDPVNGGEMLQLECYNFLKPSSDQPQ*
>11673.m04691|LOC_Os07g47410.1|genepair817-2
MTATTPPAVAVAGESPPPLVHLAFNLYSTGFIAATATGLRVFSCFSSPLNKVFARDVEVC
PEDDGGCGGGGWKVAIAEMFNEAFAAVVFRREKGGGGGTVDKICFWSIPNGRMYCMHKTL
PFDGAVRGVRLVGEFLLVAGDERAALYELPHASAPPKKVKVVETAANPLGLGAVVQPDGN
ARFVAAAPQRMKGMVQVHRLAEDHVYVRAHYSSLAAIALSADGRLLATAGSKGTLVRIFS
TSDGKLLQATFKLRGLYEESGATAAAGDMRDRNQSTCAVVRRAGAGFEVRRFRPGSLNYS
SRYDSVRIGGGDDDDDVRSVHVHGDRTVVVHAGRVDVFGLDDGRRKAAVLQRRVETGDNR
AGACAVSRGPPGSPFAFACPGVNDGNLRVERWVGGFTPLVIGAHRWRVASVAMSWGAKLV
ATASVKGTIVRVFRVADGELLQEMKRGFDRADIYSIVFSPDSEWLAVSSDKGTVHVFHIN
VCSPSSSKTGCQDTTQSYESYGAKAMKKYVSSIKALLIVKDLLTLGYFDPERSVAQFHLC
DNVKYLVAFGTRPNKNIVLIIGMDGSFYRCQFDPVNGGEMKQLEYTNFLNM*
>11667.m07095|LOC_Os01g70790.1|genepair818-1
MAQRTLELTLISAKDLKDVNLLSKMEVYAVVSLSGDRRSRQRIATDRAGGRNPAWNAAPL
RFTVPASGAGSLHVLLRAERALGDRDVGEVHIPLSELLSGAPDGAVPAKFVSYQVRKISS
GKPQGVLNFSYKIGEVTQSGSYPGASPPVAYGQAPPAPAYPPSAAAAAAYPPQSTYPPPT
AYPTAAKADGSAAAAYPPQSAYPPPGKGNEPSTAYPPPAGYPPATGSSKPAKAGEPVTAY
PAAAGPSTAAPYGTAPPPQYGYGYPAQPPPPQAGYGYPPPPPQAGYGGGYGYPPQAGYGG
YQQQAVKPAKKNNFGMGLGAGLLGGALGGLLIGDAISDASAYDAGYDAGFDDGGGFDF*
>11673.m04690|LOC_Os07g47400.1|genepair818-2
MAYRVLEVTLHSARDLKNVNFISRMEVYAVATISGDPLTRQCTPPDPYGGRHPAWNATLR
FTVPPTAASAAGCLHVLLRAERSLGDRDIGEVIIPLADVLSGPYDLGARPPQFASYQVRK
LHRSETRGVLHLSYRLGPVVAPQSVFAYPAPPPPPPQLFETAPPSPPYVPPPPDAYLRKP

SPPSPPPAKLSPPPPPQTQTQPLAKPPAPATPSRAGGHVAALAPPAVAKADRHVSTPSPA
KADWQMVGTPTATKGASKHGSLEFERGLNAGLVGGAIGGMLVGTEMVSDAAFYHAGYRAG
LADRDGWAVY*
>11667.m07110|LOC_Os01g70940.1|genepair819-1
MDAEAGVGGADQLPWRQHYRNLLLLAYQSFGVVYGDLSTSPLYVYKSTFSGRLRRYQDEQ
TVFGVLSLIFWTFTLIPLLKYVTIVLSADDNGEGGPFALYSLLCRHAKLSFLPNQQSADE
ELSTYYRNGFTSRHGSLPWLRRFMEKHKNARTVLLLIVLCGASMMIGDGILTPAISVLSS
MSGLKVRATGLHDRSVVLLSCIVLVGLFALQHRGTQKVAFMFAPIVVIWLFCIGGIGLYN
IIHWNPRIYQALSPYYIVKFFRTTGKDGWIALGGILLSMTGCEAMFADLGHFTSASVRLA
FITIIYPCLILQYMGQAAFLSKNILDMPTGFYDSIPGPIFWPVFVVATLAAVVGSQAVIS
ATFSIVKQCHSLGCFPRVKVVHTSRWIYGQIYIPEINWILMVLCVAVTVAFRDITLIGNA
YGVACMTVMFVTTFLMALIMIFVWQKNIIFALSFFLLFGSVEVVYLSSSLMKVTQGGWVP
LVLALIFMSVMYIWHYGTRKKYQYDLQNKVSMRYILSLGPSLDVVRVPGIGLIYTELVTG
VPNIFTHFTTNLPAFHEVLVFLCVKSVPVPYVSPDERYLVGRIGPRAYRMYRCIVRYGYK
DVQRDDDNFENMLVMNIGKFIMMEAEDASSSASYDTANEGRMAVITTSDDYDSPLAVRDS
NDLADSMTMRSTKSESLRSLQSSYEQESPNVSRRRRVRFELPEEDDMDQQVKDELLALVE
AKHTGVTYVMGHVYIKARKNSSFFKRFAIDVGYSFLRKNCRGPSVTLHIPHISLIEVGMA
YQV*
>11673.m04684|LOC_Os07g47350.1|genepair819-2
MPSYQYLLSLLFYILDCTDRFSVIVTIHNHRVGVLMIVLLQDQWKSYCRTISLLAFQSFG
VVYGDLSTSPLYVYKSAFSGRLNNYRDETTIFGLFSLIFWTLTLLPLLKYVIIVLNADDN
GEGGTFALYSLLCRHAKFSLLPNQQSADEELSTYYQPGVGGIISSPLKRFLEKHRKLRTC
LLLFVLFGACMVIGDGVFTPAISVLSAISGLKDPGPGGIPDGWVVFIACIVLVGLFALQH
RGTHRVAFMFAPIVVVWLLSIGVIGLYNIIHWNHRIFLALSPHYVIKFFKMTGKDGWLSL
GGVLLAITGTEAMFADLGHFTAASIRLAFVGAIYPCLVLQYMGQAAFLSRNMSAVEDSFY
QSVPRSLFWPVFVIATLAAVVGSQSIISATFSIVKQCLSLGCFPRVKVVHTSRWIHGQIY
IPEINWILMVLCLAVTLGFRDTTVIGNAYGLACIVVMFVTTWLMALVIIFVWQKNILLAL
LFVVAFGSIEVVYLSAAVTKVPQGGWAPIVFAFVFMLVMYVWHYGSRRKYLFDLQNKVSM
KWILTLGPSLGIVRVPGIGLIYTELVTGVPSIFSHFVTNLPAFHQVLVFVCVKSVPVPFV
PEDERYLIGRIGPREYRMYRCIVRYGYKDVQKDDENFENHLVMSIAKFIQMEAEEAASSG
SYESSEGRMAVIHTEDTTGTGLVMRDSNNEASGTSLTRSSRSETLRSLQSIYEQESGSLS
RRRRVRFEIAEEERIDPQVRDELADLLDAKEAGVTYIIGHSYVKARKNSNFLKTFAIDYA
YSFLRKNCRGPAVALHIPHISLVEVGMIYYV*
>11667.m07118|LOC_Os01g71000.1|genepair820-1
MSCFLCFGSAQEGEAKKPGADSKDARKDGSADRGVSRVGSDKSRSHGGLDSKKDVVIQRD
GNNQNIAAQTFTFRELAAATKNFRQDCLLGEGGFGRVYKGRLETGQAVAVKQLDRNGLQG
NREFLVEVLMLSLLHHTNLVNLIGYCADGDQRLLVYEFMPLGSLEDHLHDLPPDKEPLDW
NTRMKIAAGAAKGLEYLHDKASPPVIYRDFKSSNILLGEGFHPKLSDFGLAKLGPVGDKT
HVSTRVMGTYGYCAPEYAMTGQLTVKSDVYSFGVVFLELITGRKAIDNTKPQGEQNLVAW
ARPLFKDRRKFPKMADPMLQGRFPMRGLYQALAVAAMCLQEQATTRPHIGDVVTALSYLA
SQTYDPNAPVQHSRSNSSTPRARNLAGWNEDRRSVRSPNHHSPDLRREAARSSRAEVSRT
SSTGDSGRRSGLDDLDMTGSQMGSPAQTGRKRETPRTADRQRAIAEAKTWGENSRERKHP
NGHGSFDSTNE*
>11673.m04675|LOC_Os07g47270.1|genepair820-2
MSCFPCSGSSGKGGVDAKSVAALSPGPRPAASAAPDRSNSSRGSGIKKDDSVRRGGSSAN
DGPAKIFTFRELAVATKNFRKDCLLGEGGFGRVYKGQMENGQVIAVKQLDRNGLQGNREF
LVEVLMLSLLHHPNLVRLIGYCADGDQRLLVYEYMLLGSLENHLHDRPPGKKPLDWNARM
KIAVGAAKGLEYLHDKANPPVIYRDFKSSNILLGEDYYPKLSDFGLAKLGPVGDKTHVST
RVMGTYGYCAPEYAMTGQLTVKSDVYSFGVVFLELITGRKAIDHTQPAGEQNLVAWARPL
FRDRRKFCQMADPSLQGCYPKRGLYQALAVASMCLQENATSRPLIADIVTALSYLASNHY
DPNAPSAKSSRTCPSTPKAKAHRRTTSVPDAQHAADSLNWNFPDLGRKETTRGEFEQDHS
EGYGSGSSSGRNDGLDVPELLALHNGQNNSEADIYHKSSVKLDAHEKQRSGSGKGSRQF*
>11667.m07119|LOC_Os01g71010.1|genepair821-1
MERRGLLKAALLVCLIVLCSGREIQVIQRHPSTTIYNPKLAKTLVEYASAIYTADLTQLF
TWTCARCGDLIEGFEMMDIIVDVENCLQAYVGFASDINAIVVVFRGTQENSIQNWIEDLL
WKQLDLDYPGMPEAMVHRGFYSAYHNTTMRDGVVSGIQKTRKLFGDVPIMVTGHSMGGAM
ASFCALDLVVNYGLDGVKLMTFGQPRIGNAAFASFFKKYLPHAIRVTHGHDIVPHLPPYF
SFFPQKTYHHFPREVWVHNVGLGSLVYSVEQICDDSGEDPSCCRSVSGSSVQDHIYYLGV
SMHAEAWSSCRIVMDYSKLRYRMDINGNIVLSKHLGLSGDLEHSDQ*
>11673.m04672|LOC_Os07g47250.2|genepair821-2
MMGGWFGCVCRFMERWRCVSVLALVLLLSNASHGRDISVQHSQQTLNYSHTLAMTLVEYA
SAVYMTDLTALYTWTCSRCNDLTQGFEMKSLIVDVENCLQAFVGVDYNLNSIIVAIRGTQ
ENSMQNWIKDLIWKQLDLSYPNMPNAKVHSGFFSSYNNTILRLAITSAVHKARQSYGDIN
VIVTGHSMGGAMASFCALDLAINLGSNSVQLMTFGQPRVGNAAFASYFAKYVPNTIRVTH
GHDIVPHLPPYFSFLPHLTYHHFPREVWVNDSEGDITEQICDDSGEDPNCCRCISTWSLS
VQDHFTYLGVDMEADDWSTCRIITAENVRQLQKDLASNIIVSKHSVDVTIVEPSSQTY*
>11667.m06926|LOC_Os01g68700.1|genepair822-1
MNCGPPDQLPPATAPSCFLNLNWDQSMDAAAGGHLDPALSSMVSSPASNSTGALHGISPQ
PHYGGGTPLSSPPKLNLSMMGQFHHYAAPPQVGGGGGGGGGLPILENLMPMGHLDQFLAD
PGFAERAARLSGFDARGGGGGGGYGGAGPAQFGLPDAGAAGASKEMELGNTRDESSVSDP
APGGAEIPPKGASDGNARKRKASGKGKGKGKDSPMSTSAAKEDSSGKRCKSTEESNAAAEEN
SGKGKAAQSNSENGGGKKQGKDSSSKPPEPPKDYIHVRARRGEATDSHSLAERVRREKIS
QRMKLLQDLVPGCNKVVGKAVMLDEIINYVQSLQRQVEFLSMKLATVNPQLDFNNLPNLL
AKDMHQSCSPLQSSHFPLETSGAPLPYINQPQQGNPLGCGLTNGMDNQGSMHPLDPAFCR

PMGSHHPFLNGVSDAASQVGAFWQDDLQSVVQMDMGQSQEIATSSNSYNGSLQTVHMKME
L*
>11674.m04155|LOC_Os08g41320.1|genepair822-2
MDGGRVGGDYISSLLSSSPRLDFGVPVLDAIVAPGGGGGGGDCGLDKLCGDPGFAERAAR
LSSFNNGGGGVGQRYGGAGAGLFGMPPPAPGDFAGGGSREASSVSDPASSAMKDAAANAK
KRKSTAAAAAAAKGKGKEPPVGEEKESDGKRCKTGNGEKESSVKPKAEQAGSDSSVEDGG
GGGQKQGKGKNAKPVEPPKDYVHVRARRGQATDSHSLAERVRRERISQRMKVLQDLVPGC
NKVIGKALMLDEIINYVQSLQRQVEFLSMKLATVNPLDFSNLPTLLQKDMFQACGPSASS
VFSLESSNSAFRFAEQGDVFQQFAQNSMESQCTLNQLDLALSQATNAAQYAFQDGTAGAN
LQQRNFWEDDLQSVFHIENGQSQENGVSAPNFHGQQQAGHMKMEF*
>11667.m06927|LOC_Os01g68710.1|genepair823-1
MGKRQQQRRLVRFAVAAAVVVAAALILTASAKKSGDVTELQIGVKYKPESCTLQAHKGDK
IKVHYRGSLTDGSVFDSSYDRGDPFEFTLGNGQVIKGWDQGLLGMCVGEKRKLKIPAKMG
YGERGSPPKIPGGATLIFDTELIAVNGKTTGGASNSEL*
>11674.m04163|LOC_Os08g41390.2|genepair823-2
MDDDFEMPPPGADDDLMMGEDGMGDFGGADGPPLKVGEEKEIGKQGLRKKLLKEGEGWET
PEVGDEVEVHYTGTLLDGKKFDSSRDRGTPFKFKLGQGQVIKGWDLGIKTMKKGENAVFT
IPPDLAYGESGSPPTIPASATLQFDVELLSWTSVKDICQDGGIFKKILKEGEKWENPKDL
DEVFVKYEARLEDGTVISKSEGAEFTVKDGFFCPALAKAVKTMKKAEKVLLTVKPQYGFG
ENGRPAAGEEGAVPPNATLLVNLELVSWKTVTEIGDDKKILKKVLTEGTGYERPNEGAVV
KVKITGKLQDGTIFTKKGHDEPEPFEFKTDEEEVIDGIDRAVLNMKNGEVALVTIPPEYA
FGSTESKQDLAVVPPNSTVIYEVELVSFVKDKESWDLNNTEKIETAGAKKEEGNALFKLG
KYVRASKRYEKAAKFIEYDSSFSEDEKKQSKQLKVTCNLNNAACKLKLKDYKQAEKLCTK
VLELDSQNVKALYRRAQAYMQLADLELAEVDIKKALEIDPDNRDVKLTYKNLKEKVKEYN
KKDAKFYSNMFAKMTKQPAEDGKAGSGAESKQGSEPVTAA*
>11667.m06946|LOC_Os01g68900.1|genepair824-1
MERRPELRRTMTLSEQLSAPDQAIRDFLKIPHDGLGGGDGEGGRIPAAAGASGGGGGVGG
MINWKPLRDRLRLRRAASAWMSASPKPAASNSGGGGGGGATLSSSNSGSNHSNKYNYSPG
EAAAAFSRTFSRTRSLRPTPTFSRATSTRLGATAFSSGSRRAAVDGGGHANEDDDEDHTD
DDDDDDEEEQGKDDAPAAQMSLMALLEQSDSQWDSEDEDDQGHGGGGGGGGNGGGGGGGG
ARKNGDGDDDEDDGEGREEEMVHVCCVCMVRHKGAAFIPCGHTFCRLCSRELWVSRGNCP
LCNGFIQEILDIF*
>11674.m04180|LOC_Os08g41520.1|genepair824-2
MEGRVLRRSVTLADQLAAVGPAGTAAATAAAGSCNLRDLLKLRDEDDLAAGRRAAVTLAS
AMAAERLTVAPASSAAAAAAAARTLLDIIRDDQLPSSSGGGGEGGDPLVRRAVSLPAPVT
ATPPPPPPPPETPPRQLPSVSPPTVGEEEEEEQGERVSLMALLEQTERQWSAGSGATVQQ
HLASPSAAASASVSAAAEDEAEAEAGKGAAGGGCCCVCMARAKGAAFIPCGHTFCRTCAR
ELLAGRGRCPLCNAAILDVLDIF*
>11667.m06964|LOC_Os01g69070.1|genepair825-1
MIGWGDVYKVVAATVPLYFALFLGYGSVRWWRIFTREQCDAVNRLVAFFALPFFTFEFTL
HTDPFQVNYRAVAADVISKAVIVAVIGAWARFMSKGGCAVSWSITSFSLSTLTNSLVVGV
PMARAMYGEWAQQLVVQLSVFQAIVWLTLLLFVLEVRKAAIGMYVDGAEAAAAAGKDVEA
AGAAAAAGTVVVAAAAGKPSLWALVKVVAHKLARNPNTYASFVGITWACLANRLHIALPS
AFEGSVLIMSKSGTGMAMFSMGLFMAQQEKIIACGTSFAALGLVLKFALGPAAMAIGSIA
VGLRGDVLRVAIIQAALPQSITSFIFAKEYGLHADVLSTAVIFGMLVSLPLLVGFYIVLE
LIR*
>11674.m04200|LOC_Os08g41720.1|genepair825-2
MAPLYFALGLGYGSVRWWRFFTAEQCAAINTMVVYFSMPFFTFDFVVRTDPFAMNYRVIA
ADAVSKAIAIAAMAAWARTRCGCAAAKAGAQSWSITGFSLAALNNTLVVGVPLLDAMYGR
WAQDLVVQIAVVQSMVWFPLLLMAFELRKAWVVGGGGGVGPAVMSSSSPPEKQSDVEMNG
AVVAAPGGGGGVRLPFWATARTVGLKLARNPNVYASVLGVVWACIAYRWHLSLPGIVTGS
LQVMSRTGTGMSMFSMGLFMGQQERVIACGAGLTALGMALRFVAGPLATLVGAAALGLRG
DVLHLAIIQAALPQSIASFVFAKEYGLHADVLSTAVIFGTLISLPILIAYYAVLGFV*
>11667.m06966|LOC_Os01g69090.1|genepair826-1
MAAVFFHHVVGDLTVGKPEVVELHDTDTLDAAARAIAASPEGAVPVWRPRAAPDEPPSGA
RFLGMISALDIATFVAASGVGDRAMAAVVGEVVQPNPGLLREVDPGTRLIDALDLMKQGV
KRFLVRKNGAWRGISKRFSVLYNGKWLKNMEATSPTSASSSRELSSSTSSTYKFCCLSRE
DILRFLIGCLGALAPIPLSPISSLGAINPHYCHVDASVPAMEAIQKVPPDPSAVAVVETT
PDGTRKILGDISAYKLWKCDYVAAAWALINLSAGQFVIGADDNESTPISAIPVPPISSSL
VEEIGPGRSPRAKKFSSRSIGFLNSQAHQMAFGRMRSMYRGRSAPLMCKSTSSLAAVMAQ
MLSHRATHVWVTDAESEEDGVLVGVVGYTDIFNAVTKSVYPAAS*
>11674.m04202|LOC_Os08g41740.1|genepair826-2
MAHIVFLRASAADLTAGKPPLAGVPASAPLSAAAAAIPASSEAAVAVWRVDGASHHHHHR
APTAAATVVGLLSSIDVVAFLANHPGGAAAAFMTPAGDVVPHEHALVRQVQPDTRLIEIV
ELMKQGARRVLVGKNIKEGCAINKQPFAPFYKAVLKITGTPRRNPSPSPSPATRSPSTTL
GRDRYCCLTREDIVRFLINCLGALAPIPMQSIASLGAISRAYSHVEDSSPAIGAAWELPS
DPRAVAVVRTGHDGSRVILGEISGHKLWKKDYAAAAEAMATMSAMDFATGVDESGPSPTA
TAAAAAGNVGGGGARARMGSVEEEIAPVPRLTRFSSRKIGFSASLANMIMVSHRKNRVLT
CKATSSLAAVMAQMLSHRATHLWVVEDGDADKGAVLVGMIGYMEILRAVTRGVVVPPA*
>11667.m06971|LOC_Os01g69140.1|genepair827-1
MPRGRGETAVISCTNALATGQPMMMGGQQSALNQLVSFLLGVSAAAVLIFFFSSAGGGWS
TTTDLSSWANGTVAATAKETNLTSTAAHVEEKANLTNSQAAAAEAAKEEEEKELEKLLAA
VADEHKNIIMTSVNEAWAAPGSLLDLFLEGFRAGEGIARFVDHLLIVALDDGAFRRCRDV
HPHCYRLAVAGRNFTDEKVFMSEDYLDLVWSKVKLQQRILELGYNFLFTDVDILWFRDPF

EQMSMAAHMVTSSDFFVGGAYNPANFPNTGFLYVRSSRRAVGVMEAWRAARASYPGRHEQ
QVLNEIKRELVERRGVRIQFLDTAHVAGFCSNTRDFATLYTMHANCCVGLGAKLHDLRNL
LEEWRAYRRMPDEQRRQGPVRWKVPGICIH*
>11674.m04208|LOC_Os08g41800.1|genepair827-2
MGKLIPVISFFLGAALTAAFVIATMDINWRLSALASWNNNDSPPAVTDEMKALSELTEVL
RNASMDDRTVIMTSINRAYAAPGSLLDLFLESFRLGEGTEPLLKHVLIVAMDPAALARCR
QVHPHCYLLRRPEGAVDYSDEKRFMSKDYLDMMWGRNLFQQTILQLGFNFLFTDIDIMWF
RNPLRHIAITSDIAVANDYYNGDPESLRNRPNGGFLYVRAARRTVDFYRRWRDARRRFPP
GTNEQHVLERAQAELSRRADVRMQFLDTAHCGGFCQLSRDMARVCTLHANCCTGLANKVH
DLAAVLRDWRNYTAAPPAARRRGGFGWTTPGKCIR*
>11667.m06980|LOC_Os01g69230.1|genepair828-1
MAAAPPGGQEKVIAAAQHIVKSLANSKNAADDMIRILSGFDDRLTFMSDLFPPPPPAAAA
VREPSVVEEEEAGPDDAREDEVEEAAELVERWDSPEEGDRLVFDSAKDAGDYLGAAAVLV
GARGARAEAALQAAMARLEDEFRHLLARGMSPLAGEDLHASLLRRLSLTVPSFASSASDL
DCPSFASHTGDGDESGGAGGRASVSDEEISPYLISPDTVGALRGIADVMLRAGYGPELCQ
VYGEMRRDTLMECLAVLGVDKMSLEEVQRVEWGVLDGKMKKWIQALKVVVRGLVAEERRI
CNQIFAADAEAEEDCFTEAAKGCVLQLLNFGDAIAIGKRSSEKLFRILGMYEALDEVLPE
LEGLFSGDARDFIKEEAVGILMRLGDAVRGTVAEFANAIQGETSRRALPGGEIHPLTRYV
MNYVRLLADYSRSLNQLLKDWDTELENGGDNVNMTPLGQCVLILITHLQAKIEEKSKLYE
DEALQNIFLMNNLLYIVQKVKDSELKTLLGDNWIRQRRGQIRRYSTGYLRSSWTRVLACL
RDDGLPQTMGSSSALKASLKERFKNFNLAFEELYKTQTTWKVVDPQLREELKISISEKVL
PAYRSFVGRFRGQLEGGRNSARYIKYNPEDLENQVSDFFEGRRPNA*
>11674.m04210|LOC_Os08g41820.1|genepair828-2
MADGARDAVVEVAKHMGKSLAVSKNAADDMMRVLSRYEGEAPMFPLSHPEVDQAEEEEVF
AAAEDIIRRCNSVSSPSEMVDYLYAVDDAIAATALQGELASRAAETVQAAMPRLEEEVRA
LLGSSARRLSLDSFEDLDDAGAATTPDGSPPRRDALSPEAAASASGVADRMLRAGYGPEL
AQVYVAVRRDALAESAAHLGVEAVAIEEVLRMEWGVLNQRIRRWSHAVRAVVKTLLAGER
RLCDEVFASDEELGHECFADVARGCLLQLIGFADAVAMSTPATEKLYRMLGMYEALTAVE
PDIESLFTGDARDFFSSEVAGVAAQLGSTIRHTIDQFVNVIHGESSRRPVLGGEIHPMTR
YVLNYCGLLAECRVTLDMVLADNNTSNHDTNDDDHDGGGGGGASSTPSGRCMREILTHLL
RNLDEKSRLYDDAGLKNIFLMNNIYYIVQKMMVEFPALRELLGDDWVRRHRGQIRQYETG
YLRASWMSVLASLRDDASPAAAHGHGGRAALKEKARSFNAAFEELYRSQTAWKVTDPQLR
EELRIAVSERLIPAYRSFVGRSRQLLESGSSSGRHSSSAAKHIKYSLEDLEDYMLDFFEG
VQKFVR*
>11667.m06991|LOC_Os01g69830.1|genepair829-1
MDWDAKMPSWDLGTVVGPSGGGGGGGGGGGALDLKLGAPTSWKTTTTVSAASAAPAAVAP
PPPPPASSSSSAAAAGKRARAGQGQAAVPACSVEGCAADLSKCVRDYHRRHKVCEAHSK
TAVVTVAGQQQRFCQQCSRFHLLGEFDEEKRSCRKRLDGHNKRRRKPQPDPLNPGNLFAN
HHGAARFTSYPQIFSTAASMSPQETKWPANVVKTEAADVFQEPYYHALHLNGAGAAAAAS
IFHHGGNKARKHHFPFLTADHGGGAAAASPLFGCQPFTITPSSESRSSSSSRHSNGKMFA
HDGGLDNCALSLLSDNPTPTAQITIPQPLFAGGGQYGGGGGGDVSLTGLSYVRMAGKDTS
ILAKSATTTATTATTPTTTSAQLQYHGYYHHHVSADQGSSDAAIQALPFSSW*
>11674.m04222|LOC_Os08g41940.1|genepair829-2
MEWDLKMPPAASWELADELENSGGGGVPAAVSSSSAAVGGGVNAGGGGRQECSVDLKLGG
LGEFGGGGAQPRVAVAGEPAKGKGPAAAATGAAAAASSAPAKRPRGAAAAGQQQCPSCAV
DGCKEDLSKCRDYHRRHKVCEAHSKTPLVVVSGREMRFCQQCSRFHLLQEFDEAKRSCRK
RLDGHNRRRRKPQPDPMNSASYLASQQGARFSPFATPRPEASWTGMIKTEESPYYTHHQI
PLGISSRQQHFVGSTSDGGRRFPFLQEGEISFGTGAGAGGVPMDQAAAAAAASVCQPLLK
TVAPPPPPHGGGGSGGGKMFSDGGLTQVLDSDCALSLLSAPANSTAIDVGGGRVVVQPTE
HIPMAQPLISGLQFGGGGGSSAWFAARPHHQAATGAAATAVVVSTAGFSCPVVESEQLNT
VLSSNDNEMNYNGMFHVGGEGSSDGTSSSLPFSWQ*
>11667.m06993|LOC_Os01g69850.1|genepair830-1
MARRGRVQLRRIEDKASRQVRFSKRRAGLFKKAFELALLCDVEVALLVFSPVGKLYEYSS
SSVHIEQDYS*
>11674.m04225|LOC_Os08g41960.1|genepair830-2
MEGGGRRRKRGKVELRRIEDRTSRQVRFSKRRSGLFKKAYELSVLCDAQVALLVFSPAGR
LYEFASSTSSIDTIFGRYWDLLDTTIDLNIEARESRVDCNIQVWIRRQIVHGGLAPLLVG
DAAPPFRGVGVAPRLALCIYDDTPPRIKVAKAGGMEGGMMDVNDGLQEFGLHLMIALVWY
FMRQGSLGNKGSISGTTIIKVSK*
>11667.m07015|LOC_Os01g70050.1|genepair831-1
MAAAARSSTKGVRRTKSLISRTLERCKSGLSSGGGGRSAAAPVAGCFSVYVGPERERFVV
RTEYANHPLFRRLLDDAEREYGYAAQGPLALPCAVDAFLDVLWQMERDGCGGGGDDDDDL
SIGGGGAASPICGLPSCGSKGRAAAAGYRMLSPARMTSILSRSSPMATPSRKR*
>11674.m04250|LOC_Os08g42200.1|genepair831-2
MKERGVRNKQGVIMKALLDRCLSKHRNGGWPEPPEGSFAVYVGGGGGGGGAARERFVVRT
ECVNHPLFRALLEEAEEEYGYVADGPLELPCDAGEFVAVLARIEREMAEERTVGCAGGLV
FRLHPAAHLMLVAPATPPPMIVG*
>11667.m07020|LOC_Os01g70100.1|genepair832-1
MNGASSGGGGGAGAGGDGPLVYQRWKGNNVFVLQGRFIFGPDARSLYVTMFLIIAPASIF
CLFVAKELMNNFSYGLGLPVMISAVVFTAYDLSLLLLTSGRDPGIIPRNAHPPEPEGLDG
NAEVGSNQTPPMRLPRVKDVVVNGITVKTKYCDTCMLYRPPRCSHCSICNNCVERFDHHC
PWVGQCIGLRNYRFFYMFVFSTTLLCLYVFGFCWVYIVKIRNSEQITIWKAMAKTPASIA
LLVYTFIAVWFVGGLSVFHLYLMSTNQTTYENFRYRYDQRANPYNRGVMENIKDIFFSAI
PASKNNFRARVPVPQEQGLRPRPTNGFMSPNMGRAVGDIEMGRKPVAWDEPRMAADIGDL

GAGLGNLLEDKDGRFRSASPDLSRDALAIGGLDDQGSSVMNPGRTSWGVEAGR*
>11674.m04268|LOC_Os08g42370.2|genepair832-2
MYVPQPSRSDAAGRDAGDQPRVYQVWRGSNEFFLRGRFIFGPDVRSIFLTMFLIVAPVVA
FCVFVARHLIDDFPGDWGISVMVVVVVFTVYDLTLLLLTSGRDPGIIPRNTHPPEPEGFD
GSNDSGVQTPQQLRLPRTKDVIVNGISVRIKYCDTCMLYRPPRCSHCSICNNCVERFDHH
CPWVGQCIGLRNYRFFYMFVFSTTLLCLYVFAFCWVYIIKIRNAESLSVWKAMLKTPASI
VLIIYCFLCVWFVGGLSVFHCYLMSTNQTTYENFRYRYDRRANPYNRGVLNNFLEIFCSR
IPPSKNNFRARVTVEQGLQQTRVASRGFMSPNMGKPVGDLEMGRKPVPWDEPRTAADIRD
LEAGLGGFLDEKEGRLTHASPDLSRDDLPADLMKGRAGTHSRRSSWVNRTGTSESE*
>11667.m07021|LOC_Os01g70110.1|genepair833-1
MAAEAASGGGGGYRMLPQAGLPIGFRFRPTDEELLLHYLRRKVMSRPLPADVIPVADLAR
LHPWDLPGEGDGERYFFHLPATSCWRRGGGGSRAGGGGGAWRASGKEKLVVAPRCGKRPV
GAKRTLVFFRRGGARTDWAMHEYRLLPADDHPPEANDVWVVCRVFKKTTTLAHRRSPPSI
RGAPRRRAAAADDDDMPSSPSSCVTDGGDAGEEGEESSSCSVVASNCP*
>11674.m04272|LOC_Os08g42400.2|genepair833-2
MERAAAAAPVVVRHGGVVLPPGFRFHPTDEELVVQYLRRKAFGLPLPAAVIPDLHNLFKL
DPWDIPGASSDGDKYFFAVRPPAARGRRQHVTASGGCWKPAGGRDKPVVVARCGGSHLVG
VKKGMVFVPRQGRKAPAAAAAAGGGCWVMHEYSLALPMHKKGCLAEAEEWVVCRIFQRS
SSGSRSPRRPDNDVRRTMPAVAELGRSPSPSSSSSQSSCVTSSSDQEEVSSG*
>11676.m03951|LOC_Os10g42670.1|genepair834-1
MTTSSWSGLLVISCMLLMSWAAAAVDMSPVRFDAAYMPLFGGDNLVPSPHARTVLLKLDR
FTGSGFVSKSAYHHGFFSASIKLPHDYTAGVVVAFYLSNGDVFPGQHDELDFELLGNRRG
HAWHVQTNMYGNGSTGRGREERYLLPFDPTAAPHSYAIAWTPAAVIFYIDAIPIRELVRC
SSGDYPAKPMSVYATIWDGSAWATDGGRHKVDYAYAPFTAVFSDLVVTGGTDDDHCAAMG
LMTSEVAVMTPAKRGSMRRFRSRHLTYSACYDTVRYNGTGVVFPECDESEQDNFHAWGES
KRVINSRSSSSATYATGSGVRID*
>11668.m04543|LOC_Os02g46910.2|genepair834-2
MALEARFFLAAVFAVAATCLCLSAVASAFAVPSVAFDEGYSPLFGDDNLVRSSDDKSVRL
LLDRRSGSGFISSDYYLHGFFSASIKLPKAYTAGVVVAFYLSNGDVYEKTHDELDFEFLG
SRWGGQWRVQTNAYGNGSTARGREERYLLPFDPTLEAHRYSVLWAPTHIIFYIDDTPIRE
VIRHPGMGGDFPSKPMAVYATIWDGSTWATDGGKYKVNYKYAPFASEFSDLALLGCRADP
VLRAPRDGGGAGCAEPDLLGLLTADYAVMTPRKRAAMRAFRARHMTYTVCYDAVRYAAGP
FPECDVSDVEKESFSAWGESKNVVMKARGRGRRRGRKAGAGAMSRLDVSSS*
>11676.m03953|LOC_Os10g42690.1|genepair835-1
MQQVEGRNCLPAEVRIGLETLKRRRLERMRLTAQNNAGDGPPVPARSGGDALRTPANCGV
RLHANNGTALPSRTTQNKDPFAKRRVDKFDMSSLEWIDKIEECPVYYPTKEEFEDPIGYI
QKIAPVASKYGICKIVSPVSASVPAGVVLMKEQPGFKFMTRVQPLRLAKWAEDDTVTFFM
SERKYTFRDYEKMANKVFAKKYSSASCLPAKYVEEEFWREIAFGKMDFVEYACDVDGSAF
SSSPHDQLGKSNWNLKNFSRLSNSVLRLLQTPIPGVTDPMLYIGMLFSMFAWHVEDHYLY
SINYHHCGAFKTWYGIPGDAAPGFEKVASQFVYNKDILVGEGEDAAFDVLLGKTTMFPPN
VLLDHNVPVYKAVQKPGEFVITFPRSYHAGFSHGFNCGEAVNFAISDWFPLGSVASRRYA
LLNRTPLLAHEELLCRSAVLLSHKLLNSDPKSLNKSEHPHSQRCLKSCFVQLMRFQRNTR
GLLAKMGSQIHYKPKTYPNLSCSMCRRDCYITHVLCGCNFDPVCLHHEQELRSCPCKSNQ
VVYVREDIQELEALSRKFEKDICLDKEISGFDSYKQAEKNEPFFEITRNLRNTEVNLIED
AFSGATAADAAKSSPATSTLTSFAQHDVPVLAEAIVCANQADQLYSTTEQTISSPLVKGT
DAVGANSSSMADANNGTGSCNASAVEYSGNSDSESEIFRVKRRSGVSVKPASDAKTSNLS
DQQVLRRLKKVRPEIQQHNKRPEDYGHCSVPSGRMSMKNLNSSSSCGEEHWRMKRRQLET
QQDESSYSAKQKSYSYPSTSYSFRGEFVEMSRDAAAEVRPKRLKIRLPSSSTNRVVEQGS
SGQRFTRDDKSLGCWPAI*
>11668.m04545|LOC_Os02g46930.1|genepair835-2
MAGNDEVALKPVSCGARLRRSCDASLRFGGSMRDPFLKHKVKKFDLSSLDWIDEIPECPV
FSPSIEEFEDPLVYLNKIAPIAAKYGICKIVSPLCASVPIGPVLMKEQGGLKFTTRVQPL
RLAEWSKDDKFAFFMSGRKYTFRDFEKMANKEFVRRYSSAACLPPRYMEEEFWHEIAFGK
MQSVEYACDIDGSAFSSSPNDQLGTSKWNLKRLSRLPKSTLRLLRAAIPGITDPMLYIGM
LFSMFAWHVEDHYLYSINYHHCGASKTWYGIPGKAAPDFEKVVCEGENAAFDVILGKTTM
FPPNILLRHHVPVYRAIQKPGEFVITFPRAYHSGFSHGFNCGEAVNFAIGEWFPLGALAS
QRYALLKRTPLLPYEELLCKEAALLDHEFSTCDYKDTTTLAGETHSQRCMKVPFVQLMRV
QHRIRWSLMKMGARTHYKADIDATVLCGICRRDCYVAHIMCNCRIDAICLCHEEEIRRCP
CSCDRVVFVRKDIFELETLSKKFEEESGILDAVKKQMARRDGASQHSNFFDCTDHEAEYY
PYCNIHIDPSPEIYSISETNFVGYDLNNPHPAASTVTFSFVPHEYSTQSDECTSSNRRAL
SSSCLENTITPENAISNACQLSTPDQTCLSDKLAAHDTDDSDCEIFRVKRRSGLTPEKRH
MEDGTTNFTGNQVLKRLKKINAHDRQEHKLPELSCGARSEPVHTDDCIHCVDFISENGDD
FIAPTKLKMIHQLDANIVEDEVASSQKYNSCNYQSPSIELGPKRLKIRGPSFPSRISELE
EITSTDCHVAYATK*
>11676.m03963|LOC_Os10g42730.2|genepair836-1
MGDNLMDKVNALGERLKVSGAEVSRKMSAGVSNMSFKMKEFFQGQNMADKIVDEATLETM
DAPDWATNLEICDMVNTGNVNSIELIRAIKRRIMLKNPRVQYLALVLLETVVKNCEKAFS
EIAAERVLDEMVKLIDDPQTVVNNRNKALMLIEAWGESGDELRYLPVYEETYKSLRSRGI
RFPGRDDESLAPIFTPPRSAPSAEPYSAAAQEGYQEIPDESFAPVHVVPAVQVNEAFEVA
RNSVELLSTVLSSSPQKEALKDDLTTTLVQQCQQCQRTIQRIIETAGDNEAQLFEALSVH
DELEKVLSKYKELKEPVVAEPEAEPAMIPVTVEPENSPRTKDGTVGKRAGSGADELLQDL
DDMIFGKKGGTSSQQDRKEQKDDFISF*
>11668.m04548|LOC_Os02g46960.1|genepair836-2
MGKGGELWDDSALVDAFDHAVATFKAMHSKNTQATTSENEEPGDPAVAAPAGEENISAEV

ADELIEKDGSQTEPCEASETPYQTHEERKSTEQAPLQEKDLDKEAHFSEPKIHASDVADA
EQKDTSNQQTWDYNELVKKYYELEEQSRKVLEQLHQTNYWNYQVPGQSSVYQQPQVPAYS
ATAPDPHSSTIQSPCCCANVPLVSVSCCSTGQTSGVSSCMQPSGGCSISLTCDQCPGTSA
TDSTGATCVQQAEKVSTDSDQVAKAAMMTAEGAMNFMRSTISGDLGSFPRTDAASGKESM
PMGMNPNFDTMGADSDLAVVLNAWYAAGFYTGSLPVKMSDNLMDKVSAFGERLKITGSEV
SKKMTAGMSSMSFKMKEIFQGQTPADKIVEEATSENLDGPDWSANLEICDLINTEKVNSV
ELIRGIKKRIMLKDARVQYLSLVLLETIVKNCEKAFSEVAAERVLDEMVRLIDDPQTVVN
NRNKALMLIEAWGESGDELRYLPVYEETYKSLKSRGVRFPGRDNESLAPIFTPARSVAEA
EVDANFSQQTFEDVQVHTYTAEETKEAFDVARNSIELLSTVLSSSPQQDALQDDLTSTLV
QQCYQSQHTIQRMIETAGDNEAMLFEALSVNDEIQKVLSKYEQMKKPAASENAEQRPVVI
PIATEHEDSATVGNEDALVRKPAGSRARSGGDDDILDDLDEMIFGKKGGSSSQEGPKKQD
PKKDDLISF*
>11676.m03980|LOC_Os10g42850.1|genepair837-1
MCDSLFWQSSADQGDLSDVVRASLQLQTAPRHQAASPPYVHLLGGGGGGGEDQLAAVSQH
AEQQQQSMVDASAACDLLHALLPPPPVVQVQQQGASRTRTTIEEDTTGDGEELFAGAHYV
VPPIKRRKSQTKKVVCIPAGASGGGGGEVVPSDLWAWRKYGQKPIKGSPYPRGYYRCSSS
KGCSARKQVERSRADPTMLVVTYTSDHNHPWPTHRNALAGSTRPSSSNSSNIRLQDSTPV
HHQSQTGHDRLTTTHLKQEDVIISPSLLQPDHHQLCTIIDTKHHLLFHQDYPHSFGLFD*
>11668.m04558|LOC_Os02g47060.1|genepair837-2
MCDYFLQRMEGEQAAGDLADIVLRAGGAAAAAVAGGGIPSTEWQLPPAEEEEEEPGLFPL
PPSSSDGSGMSGADAFGDPFAGLPDPFGGDYPSSGGAAAAADFFDAVVAKAGFVDVGVLG
GGGGGGCDGGGVDGGGGGSSLLGMSKPILPRAAMQLPSVSPRAIRPYPVMAGDTVKLGAP
MAGGPCAFDGAAAAGLHMSSSPRGAVGGIKRRKNQARKVVCIPAPAAAGGRTSGEVVPSD
LWAWRKYGQKPIKGSPYPRGYYRCSSSKGCSARKQVERSRTDPNMLVITYTSEHNHPWPT
QRNALAGSTRSHHAKNSSSNSSSSGASSASKNNSSHSGYHHHHHQKPLVKAEPNDQSAAA
TTAATVPVKEEAAMVGTSSEALAKTTQKSMEDAAAAASATAAAVEHSDLMQQMFSQSYRP
MIPEAAAGGHHDDFFADLAELESDPMSLIFSKEYMATNYKPAGDPAGKEMNAVDKGLDPA
YMLDWSSTTVVTRAGGSSFMQGEGGL*
>11676.m03982|LOC_Os10g42870.1|genepair838-1
MGMESGDAQLPLLHHQASASNQHYTKPPFNWKAPALILAFEFLESIAYAGISLNLVVYLG
TVLHGTTASNAANVDTWNGTTFLTPVLGAFLADTYWGKYKTIAISTVFYLIGLLVITASA
VIPSLQPAPCNGSSCPPATGFQYFVFFTALYLISVGTGGVKSALLPFGADQYNDSNLEES
KKKQSFFSLFFIAINLGVFISGTVVVWIQQNVAWSLGFGISSICLVVATVAFLAGTPVYK
VQLPSGSPLKSVVMVFVASFKKRKLEVPADNALLYEGDDADLSNGQSVKLAHTDGFRWLD
KAAVVFEEVEEINKDEGGGGGWLQCSVTQVEEVKILLRMLPIWVTSVLYAASLGQTATTF
VQQGNAMNTKIGSFSVPAASLNSAEVIFMMIWVVFQDTVVVPIARRYTGNPAGLTQLQRM
GVGRLLAVPALAVAAVLETWRLRSVRDGGNLSIAWQLPQFVILACSDVFCGIAQLEFFYS
EAPVSMRSLCSAFSFLALSLGYYVNSLVVSIVAVVTTTSNKGWLPADLNDGHLDYYFWLW
TGISAINFVVYAAFAKNYTVKRLAVPHSH*
>11668.m04561|LOC_Os02g47090.1|genepair838-2
MDAGDAMESGQRAALLPESHGPKTEDDSLQVPLLKDKKRGGSKAPAIVLGFECLESTAFN
GISTNLVVYLETVLHGSNLASASNVTTWYGTSYLTPIFGAIVADTFLGNYNTILISLAVY
LLGMMLVTFSAFLPATAALCAAGATCGTGAAAAQTVAFVGLYLVAVGSGGVRSSLLPFGA
EQFDDDSAADRERKAAFFSWFYLCVDFGLIVSGVLLVWIQQNVSWGLGFGIATACIAVAF
AAFVLATPMYKRRLPTGTPLKSLAQVVVAAFRKVGMKLPADAELLYEVSDKVDSQPKIAH
TSEFTFLDKAAVVSESDLEERPEAASSWKLCTVTQVEELKILLRLLPIWATSIIVSAAYS
QMSTTFIQQGSAMDMHIFSVPVPAASLSSFQVLCVLTWVILYSKVIVPALRGFSSSGAAG
EPSQLQRMGAGRLLMALAMAVAALVETKRLNAAASGEAINIAWQMPQYFFLAGAEVFCYI
AQLEFFFGEAPDTMKSTCTSLALLTIALGSYLSSLIYAVVEAFTATAGGHGWISDDLNQG
HLDYFFWMLAAMCTLNFVVYSGFAKNYKLKTVLS*
>11676.m03992|LOC_Os10g42940.3|genepair839-1
MGQAFRKLFDAFFGNKEMRVVMLGLDAAGKTTILYKLHIGEVLSTVPTIGFNVEKVQYKN
VVFTVWDVGGQEKLRPLWRHYFNNTDALIYVVDSLDRERIGRARAEFQAIINDPFMLNSV
LLVFANKQDMRGAMTPMEVCEGLGLYDLNNRIWHIQGTCALKGDGLYEGLDWLATTLDEM
RASGRITSTSSS*
>11668.m04563|LOC_Os02g47110.1|genepair839-2
MGQAFRKLFDAFFGTSEMRVVMLGLDAAGKTTILYKLHIGEVLSTVPTIGFNVEKVQYKN
VMFTVWDVGGQEKLRPLWRHYFNNTDGLIYVVDSLDRERIGKAKAEFQAIVNDPLMLNSV
ILVFANKQDMKGAMTPLEVCEGLGLYDLKNRIWHIQGSCALKGDGLYEGLDWLASTLKGL
EASGRLPSGGTSLF*
>11676.m03532|LOC_Os10g38880.1|genepair840-1
MKVMTLRRGGAGAGIRIKKKARGFMCGGCGGSKAVSVSDGSDKQSPMATPPPNTSSTTTT
TTTGSAGNKTTAAAGSSSFSPSYDYDYVDTDADTSVGSTPSVAALLRQLGELERSVRSLQ
GAVAEGRGAKNDGRGGGRRHRRTVSDGGGGGSGRVEESVAVVKESADPLFDFRRSMLQMI
VEKEIVGGAELRELLHRFLPLNSPHHHHVILRAFAEIWEEVFAGYERTPDFLVSSRHRRP
TKKKLPASYTAADDDDDDSWNAA*
>11668.m04408|LOC_Os02g45620.1|genepair840-2
MSPGVSAKKRHAGAGFTLGCGCKDAKSVSVSASAAGTPSTTATRRRSAGTNPSGSTTTDT
LTMTSASSSFLWEHSVVEFDHDGGGGCGPESFSGLLRELSELEQSVASWGRKSHHQHHDK
KHSPAPSSPLPPQEDRKEKNGGNGDATDKPGDCRDGGGGGDGVGVALDGSVAVVKQSDDP
LGDFRQSMLQMIVENGIVAGEDLREMLRRFLTLNAPHHHDVILRAFAEIWDGVFAATASL
VHHHHPPPSSRREPVAPAARPPAPRTPPRHRHPSPRAWRV*
>11676.m03549|LOC_Os10g39020.1|genepair841-1
MVGGGGGAGEKPPASNGVHGSGKARFTLLYGLLLYVVMPVLFLYMLVAAATPFYNPRCSP

ESNAAMARFVVAMPNASSVNGSSPSSSSPPPTPVRPMRSADEAPTGLRHIAFGIGASSAL
WKSRKEYIKLWWRPGRMRGFVWMDRPVEEFYSKSSRTGLPPIMVSSDTSKFPYTHGAGSR
SALRISRIVSETFRLGLPGVRWFVMGDDDTVFLPENLVHVLSQYDHRQPYYIGSPSESHI
QNLIFSYGMAFGGGGFAISRALAEELAKMQDGCLHRYPALYGSDDRIHACMSELGVPLTR
HPGFHQCDLWGDVLGLLGAHPVAPLVTLHHLDFLEPVFPTTPSRAGALRKLFDGPVRLDS
AAVAQQSVCYDREHHWTVSVSWGFAVMVVRGVLSPREMETPMRSFLNWYKRADYTAYSFN
TRPVARQPCQKPRVYYMRDSRMDRRRNVTVTEYDRHRGKQPDCRWRIPDPAALVDHIVVL
KKPDPDLWKRSPRRNCCQVVSSPTKAGKNRTMTIEVGVCREGEFAKL*
>11668.m04416|LOC_Os02g45700.1|genepair841-2
MKAAAATGGGGKETLAATLLRYLIILIVPFTVLYILYTLHAILSSTPSCPLDRPIVTSSV
SLSQLSTTRNHTPSSSSLSTPPPAPVSMAATTLQHVVFGIAASARLWEKRKDYIKIWWRP
NAGMRGFVWMDQPVRESGVPDGLPPIKISSNTSGFPYKNRRGHRSAIRISRIVSETFRLG
LSGVRWYVMGDDDTVFLPDNLVAVLQKLDHRQPYYIGYPSESHLQNIFFSYGMAFGGGGF
AISQPLAARLERMQDACIHRYPSLYGSDDRIHACMAELGVPLTRHPGFHQYDVYGDLLGL
LAAHPVAPLVSLHHLDVVRPLFPNARSRPAALRRLFEGPVALDSAGAVQQSICYDARNRW
TVSVSWGFVVMASRGMISAREMELPARTFLNWYKRADYKAHAFNTRPLARRPCEKPSFYY
LSSARRTVARDGETTVTTYQRWRHRNDMRPPCRWKIADPDALLDTVVVLKKPDPGLWDRS
PMRNCCRVLSSPKGQEGNKTMTIDVGVCKDWEFSQV*
>11676.m03568|LOC_Os10g39190.1|genepair842-1
MEFTPISPPTRVAGGEEDSERGAAAWAVVEKEHMFEKVVTPSDVGKLNRLVIPKQHAERY
FPLDAAAGAGGGGGGGGGGGGGKGLVLSFEDRTGKAWRFRYSYWNSSQSYVMTKGWSRFV
KEKRLGAGDTVSFGRGLGDAARGRLFIDFRRRRQDAGSFMFPPTAAPPSHSHHHHQRHHP
PLPSVPLCPWRDYTTAYGGGYGYGYGGGSTPASSRHVLFLRPQVPAAVVLKSVPVHVAAT
SAVQEAATTTRPKRVRLFGVNLDCPAAMDDDDDIAGAASRTAASSLLQLPSPSSSTSSST
AGKKMCSLDLGL*
>11668.m04431|LOC_Os02g45850.1|genepair842-2
MEFTTSSRFSKEEEDEEQDEAGRREIPFMTATAEAAPAPTSSSSSPAHHAASASASASAS
GSSTPFRSDDGAGASGSGGGGGGGGEAEVVEKEHMFDKVVTPSDVGKLNRLVIPKQYAEK
YFPLDAAANEKGLLLNFEDRAGKPWRFRYSYWNSSQSYVMTKGWSRFVKEKRLDAGDTVS
FSRGIGDEAARHRLFIDWKRRADTRDPLRLPRGLPLPMPLTSHYAPWGIGGGGGFFVQPS
PPATLYEHRLRQGLDFRAFNPAAAMGRQVLLFGSARIPPQAPLLARAPSPLHHHYTLQPS
GDGVRAAGSPVVLDSVPVIESPTTAAKRVRLFGVNLDNPHAGGGGGAAAGESSNHGNALS
LQTPAWMRRDPTLRLLELPPHHHHGAESSAASSPSSSSSSKRDAHSALDLDL*
>11676.m03571|LOC_Os10g39210.1|genepair843-1
MADIVSDVFLSFFCCCYYPPGGHRGVGAHNDTALRRRRGGAGRSSSRPPVSLQTVELKVR
MCCEGCERVVRSALANLRGVDSVEVDVAMEKVRVTGYVDRGRVLREVRRSGKKAEFWPSG
GTPRRFTSEKEYFRDGEAYRGSYNYHRRGYGDGDRHGWMREPARGADAVSNMFNDDDVSA
ACAIM*
>11668.m04432|LOC_Os02g45860.1|genepair843-2
MMTRASESYAWMRGDAQRGGGGGGGGARPEAGGVAVSAAGERRLRAQEVQKGLGHGVAGG
RAAAPGSGGASSAVSSGQAAALGAGGAEGRETRRAGGGYGRGRSNGAGDEAGERQGAERS
SGGDKGCVGWFFLASSAQQSTRASAASAVGRHALARLLQPLSAAATPLHSASVAALLRFA
RCVPLCRPRAHRLAASDARLPDAVATRSTPNATSAKPTIYTAMGQTLNGLSPLYVLYRSK
TMIVEMNVHMDCEGCEKRVRKAMSRLEGVSTVEIDMDTQKVTVTGYVDRREVLRAARRTG
RAAEFWPWPYDGEYYPFAIQYLEDDTYMATHKYYVHGYNAPVIGSYPNHAFTHIVDDHAL
AFFHDDNVHACSIM*
>11676.m03572|LOC_Os10g39220.1|genepair844-1
MRRMLEVAADAGSSAAAAAANGAVDWWRDVNESPVWQDRIFHVLAALYGFVSAIALVQLI
RIECRVPEYGWTTQKVFHFLNFLVNGVRSIVFVLRRNVQLIHPEILQHVLLDMPGLAFFT
TYALLVLFWAEIYYQARAMSTDGLRPTFYWINAVIYAIQIILWMVLWWKPVRVMIILSKM
FFAGVSLFAAFGFLLYGGRLFLMLQRFPVESKGRRKKLQEVGYVTTICFTCFLIRCVMMC
LNTFDKAADLDVLNHPILNFFYYLLVEIVPSALVLFILRKLPPKRGITQYHPIH*
>11668.m04433|LOC_Os02g45870.1|genepair844-2
MAAAAPMPVAGAGWWDLVNGSTAWQDGIFLSLAALYGLVAASSFIQVVRIQYRVPEYGWT
TQKVFQLLNFIVNGARCSIFAVRRQVQQVNPEIFQHVILDLPGLAFFTTYAMLALFWAEI
SYQARGLETEGLRSGFYTINGVIYVIQVLLWALLWHNPNPSMIVLSKLFIAGLSFSAALG
FLLYGGRLFFMLKRFPIESKGRQKKLREVGRVATICFLCFLARCIMMCFDAFDKKADLDV
LDHPILNFIYYLIVEILPSSLVLFILRRIPSKLRLAQYHPLNSG*
>11676.m03573|LOC_Os10g39230.1|genepair845-1
MRRRGGGDWGREADPAEPSSSHAFDGGRDGDDDDGGGGWHQAAMKAGAGKGSSSSSSSGD
SLWQWRSQGLSEVVLSWSVDQILNKDLLRDKVAKIPETFSSMEQYMTSFFGPLLEEVRGD
MCSSMEDISKAPYASVLSVNAMRKGKGSYEIKLDKWRGVSHGCAIEGYKPKAADLLLISE
TRPANQSDILKQSKSCVIVWVGKVQGNKMTVKASRRMETGVHGDERQQMGMNRYDKLYAE
GFDKSWEMLDQEAVAPESSNSFMHENGRKEHSKVRKCFEKCSDLQEQNEMGTCGNSSKRW
SFCATYLTNMITYDRVWVVLRRGLTMDSKIVLSMFGKKKKEQRILACAPTNMAVLQVASR
LIELIQDFSSSHCYSFGDIVLFGNKDRLHIGKELSKVYLDDRVHKLLRYFKREDGWKARV
DSVMKFLMNCISRYQMSLDIQQASSDGCNLTFKKYFTSKFSTLVKELARCIDTFFDHLPT
DSLGRNLDRMMFAKSLLDKLQQLLCADDVSDELLFTIFKPSDEPIDSFDSHDQTDDATVD
LHDHDISLDDPLEIKSLCIKTLMDLSKMRLPCEDNESSIRDLCLKRAKLVFCTASSSFEL
FRLQNVMPISILVIDEAAQLKECEALVPLLLPGIEHILLIGDENQLSSLVKSKIAKDADF
GRSLYERLCTMGYRKHLLEVQYRMHPGINKFPNANFYDNRISDGPSVQQEDYMKSYLPGP
IYGAYSFIHIENDMEMLDELGQSSKNMVEVAVATNIVERLAKECSEKRQRTSLGVISPYT
AQVIALQERLGKQFKNHEFLSVTVKSIDGFQGGEEDIILISTVRSNKNGKVGFLSDAGRI
NVALTRAKYCLWILGNGATLLASNSIWAELVHDSKRRGCFFNALDDKNLAEIIMHATKEG

EQRNQREQRSAHNANRWSSGSSRHDIIAAGNSRPMRWSHFPGSGNTRRSNGHDSRLNVCH
TKEDMHRTHFQQRKSYSGDYSQVAPPNQYWHNGNRPSRGSYGYLEVFREHPNHHSGQDSR
TRSYHETMCSTPQTGNGRFPYSGSIQREKSQRQTSILGERQPLGGDGNKGFQDGTSGYPC
RRNSSQIRPNTYEAGAPELQSMNKHREFSSYPQQAPYRTFGGRGRGRPTYHGRGRGGWYE
RTNNHWMEEPHQVQNATCNMPVTMQQGMKRNWCEAEASDSPQQVNAKIRSESADRPHLHD
EHGGYGTASHQLPAIKPEDVSEQQCEMKTDSYKAEASESPNDSTRVRPESVEQPYCQAQG
DSSGAASQEPVIPEQRGMSGDLCEAVCHQSNTSGSPNRGSTEVTLEGAEQPHCQAQPDGS
GVASNEAPVPEQRWTEGDLGEAAEPGQGNAENKAESAEADS*
>11668.m04436|LOC_Os02g45900.1|genepair845-2
MTQALHMLKFFGKLVEPKSEQSLKTLFKLSPDGSISSLFQNFVTYVQDSVSTELKDARAQ
CLQKLKHLSDHFELPNVFDKRSIEDFLVRNAKSILCTASSSSRLHYLPEASPFDLLVVDE
AAQLKECESLIPLQLPGVRHAVLIGYEFQLPALVKSRVCEDAEFGRSLFERLSSLGHPKH
LLDVQYRMHPGISKFPVSSFYENKISDGENVLHRDYERKPLAGPMYGSYSFINVDAGKES
KGKHDKSLMNPIEVAAVTRIVQRLFKGTHCIMPLHSGMASLSSDSLVCAESVDTGRKLCV
GVVSPYKGQVRAIQERLGKAYETHGGFTVKVRSVDGFQGAEEDIIIFSAVRSNTTGSVGF
LSNVNRTNVALTRAKHCLWILGNANTLASSKDHLAAVIELDEVDDLLEMDSLRLSGSRFG
VTCKKRVDVHPRD*
>11676.m03591|LOC_Os10g39410.1|genepair846-1
MSGRGKGGKGLGKGGAKRHRKVLRDNIQGITKPAIRRLARRGGVKRISGLIYEETRGVLK
IFLENVIRDAVTYTEHARRKTVTAMDVVYALKRQGRTLYGFGG*
>11668.m04440|LOC_Os02g45940.1|genepair846-2
MSGRGKGGKGLGKGGAKRHRKVLRDNIQGITKPAIRRLARRGGVKRISGLIYEETRGVLK
IFLENVIRDAVTYTEHARRKTVTAMDVVYALKRQGRTLYGFGG*
>11676.m03592|LOC_Os10g39420.1|genepair847-1
MGNSCQNGTYGNNYQNSNRFQNDRFASRYVDGNDTEDCYSGSSRASLAGALRQGLNLKSP
VLGYKTPNVRELYTLGRELGQGQFGKTYLCTEISTGCQYACKTILKSNLRCVSDIEDVRR
EIQIMHHLSGQKNIVTIKDTYEDEQAVHIVMELCAGGELFSKIQKRGHYSERKAAELIKI
IVGIIETCHSHGVMHRDLKPENFLLLDADDEFSVKAIDFGLSVFFRPGQVFREVVGSPYY
IAPEVLEKRYGPEADIWTAGVILYVLLTGVPPFWADTQSGIYEKVLDGRIDFKSNRWPRI
SDSAKDLIKKMLCPYPSERLKAHEVLKHPWICDNGVATNRALDPSVLPRLKQFSAMNRLK
KLSLQIIAERLSEEEIVGLREMFKAMDTKNRSVVTFGELKGLKRYSSVFKDTEINDLMEA
ADDTTSTINWEEFIAAAVSLNKIEREKHLMAAFTYFDKDGSGFITVDKLQKACMERNMED
TFLEEMILEVDQNNDGQIDYAEFVTMMQSNNFGLGWQTVESSLNVALREAPQVY*
>11668.m04455|LOC_Os02g46090.1|genepair847-2
MGNTCGVTLRSKYFASFRGASQRHDEAGYAPVATSAAAAAAADEPAGKKAPRGSAAAADA
PHAASMKRGAPAPAELTANVLGHPTPSLSEHYALGRKLGQGQFGTTYLCTDLATGVDYAC
KSIAKRKLITKEDVEDVRREIQIMHHLAGHRNVVAIKGAYEDPQYVHIVMELCAGGELFD
RIIERGQFSERKAAELTRIIVGVIEACHSLGVIHRDLKPENFLLANKDDDLSLKAIDFGL
SVFFKPGQVFTDVVGSPYYVAPEVLRKCYGPEADVWTAGVILYILLSGVPPFWAETQQGI
FDAVLKGVIDFDSDPWPVISDSAKDLIRRMLNPRPKERLTAHEVLCHPWICDHGVAPDRP
LDPAVLSRIKQFSAMNKLKKMALRVIAESLSEEEIAGLKEMFKAMDTDNSGAITYDELKE
GMRKYGSTLKDTEIRDLMEAADVDNSGTIDYIEFIAATLHLNKLEREEHLVAAFSYFDKD
GSGYITVDELQQACKEHNMPDAFLDDVIKEADQDNDGRIDYGEFVAMMTKGNMGVGRRTM
RNSLNISMR*
>11676.m03596|LOC_Os10g39450.1|genepair848-1
MLRPPTPTRRLLQSVGGGSNPNRIPGIPPADPPSGGVSSDVVVILAALLCALICVVGLAA
VARCARNRRNSGGAGAGAGSASPSHSPAHAGHFGGGGSGGGGVTTTTAATTAAKGLKKKA
LKALPKLAYADAVAAAAAARGTVVGEEEEGKVEVLAECAICLSEFGEREEVRVMPQCGHG
FHVACVDTWLRSNSSCPSCRRPIVLDDPSPPKRCRKCEAIVLEAVVAASSSSSSSAATAT
GSGGGGGGGGGRFLP*
>11668.m04456|LOC_Os02g46100.1|genepair848-2
MARMLLEAAASGSVEDSLNSDLVVILAGLLCALICVLGLGLVARCACTRRWARAAGGGTA
AGGGGGGAAAANKGVKKEVLRSLPTVTYYVSDGGGGEAEECAICLVEFEDGQAVRVLPQCD
HRFHAACIDTWLRAHSSCPSCRRVLVAAEMPPGERCGRCGARSGGRGIGALLLNYWKAPA
CDAEGPELA*
>11676.m03181|LOC_Os10g35630.1|genepair849-1
MAVRLLANEVSDLCIGKPAVRSLPLSAAAGDLAAALRRGPQQAAGGAAACVAVVGPGRAV
AGRLGLADVLCFLCAAPGALAHPTAALSKPASALLPKDGAGEVRRVDPRASVLEALDAVL
SGAQVLAVPLRSGGRRKQLGGGGGGGGGGDYCWLTQEDLVRYFLNSISLFSHVAGRSVSS
LGLVRADDLLTVRPHEAALSAVPLLRRAIATETAVAVVDDGGHLVGEISPALLASCDETA
AAAIATLSVADLMAYVDYFGAPPEHILRAVKAGLKSKGLDAMLELVENEAVSSFAFSSSS
TSSSSDDEAHGRAARLRRPSSGSYGRRSTEEPVVCSPASSLVAVMMQALAHRASYLWVLD
EDDDCRLAGIVTFADVLTVFREQLQ*
>11668.m04081|LOC_Os02g42640.1|genepair849-2
MAASLLSHVVSDLCIGKPPARVLPPSTPVAAALAALRTGDDPFVFVVDADEALRHSRGKK
IAAGCVVVKVSVADVLCYVCGDADNLSDPAAALGRPVSALAAAVHAGGGDHHGAALRVDS
LTSLLDAIDALLSNDAQTLLVPLHAHAARSRKHHHVHVSGCSPANPAAATDYCVLTREDI
VRHLFSYSISLFAPVAARTVASLGLVRRDVHAVHADDDALDAIPLLRRSIADGTAVAVVA
DDDALVGEICPGVLGSCDIESASAAFAALSAGDVMTYIDCSLSPPEFLLRSIRAQLKGRG
MDAMADLMDAADDAASSLPLSPSSSSSASSDEDSPFGRARRARRSSSGSFRWRSTKDVAA
CHAGSSLVAVMAQALAHRVGYVWVVDEVSGALTGVVSFGDVLAVLREHLRDGDTQMN*
>11676.m03209|LOC_Os10g35870.2|genepair850-1
MAAAVAELWETLKQAIVAYTGLSPAAFFTAVAAAAALYHVVSGIFAGPPPPPPPRPRDEP
EAEPLPPPVQLGEVSEEELRQYDGSDPKKPLLMAIKGQIYDVTQSRMFYGPGGPYALFAG

KDASRALAKMSFEPQDLTGDISGLGPFELDALQDWEYKFMGKYVKVGTVKKTVPVEDGAP
STSPETTETAAAAEPEKAPATEEKPREVSSEEVKEKEDAVAAAAPDEGAKES*
>11668.m04092|LOC_Os02g42740.1|genepair850-2
MGVHSALSVQCISFPTCRLCRRLNHTDQRHIQQAKSVRVGVADTTTQAQAQALHRLVSRG
SPRPAVPCRGEPSISAAPSKHHPKAVQFARPVSHLLCLPLTPYYECPVATTAHKHTRTRR
GCLLSLPAFPLLCSPLPARALGSCIAPPSTEMVAAAEWWEATIAAYTGLSPAAFFTAVAV
AAALYVAVSGLLTRRPPPLPRRQEEARASQPLPPPVQLGEVTEEELRVYDGSDPNKPLLM
AIKGQIYDVTQSRMFYGPGGPYALFAGRDASRALAKMSFELDDLTGDVSGLGPIELEALH
EWEGKFMSKYVKVGTIKKIIPVSEGDAATHGGTSDRGIDVGTIESNRVPEPEENGATSHA
DAVEKSDADVSTHNHEDVVEKSDELLESGVDTRSTHEDAVGKPKEETEDADVQKTISTEV
AGEGKGAPDEDDRNTCSLEDAIEKPKETAYIDVKDTSGHEVAGEPKEAPDVDGNNTSSNQ
DGSG*
>11676.m03215|LOC_Os10g35930.1|genepair851-1
MTFSGTQDKCNACDKTVHFIDLLTADSIPYHKSCFRCSHCKGTLSMCSYSSMDGVLYCKT
HFEQLFKETGTFKKNFPSGTKANSEQAKIPSKLSSVFCGTQDKCTACKKTVYPLEKMTME
GECYHRTCFKCAHGGCLLTNASYASHNGILYCQNHFWQLFKKSGSYDNLLKPASAAAENT
VESEVAVAEPAKEDPETEEAAKEEEEEASPEQVAEAVVEDQEHS*
>11668.m04100|LOC_Os02g42820.1|genepair851-2
MSFTGTQDKCKACDKTVHFIDLLTADGVSYHKTCFKCSHCKGTLSMCNYSSMDGVLYCKT
HFEQLFKETGSFSKKFSQGGKSSEKSDQGRAPSKLSSAFSGTQDKCAACQKTVYPLEKLT
LEGESYHKSCFKCSHGGCILTTSSYAALNGILYCKIHFSQLFKEKGSYNHLIQTAQSKQK
ESEEAAPVAATEASEKEQEVPPQDAT*
>11676.m03223|LOC_Os10g35990.1|genepair852-1
MHHPRARYPPGYTSGGGGGGGGGGGGGRGNGGGGFGGGGGGGGGNHGYYGRGPQPQPQQQ
HYHHQAQQLHQHQQQQQHAQRNSSSQQQQWLRRDQATAAAASGEVAARTAAQLEAVDSSS
EDWKAQLNLPAPDTRYRTEDVTATKGNEFEDYFLKRELLMGIYEKGFERPSPIQEESIPI
ALTGSDILARAKNGTGKTAAFCIPALEKIDPEKNAIQVVILVPTRELALQTSQVCKELGK
YLNIQVMVSTGGTSLKDDIMRLYQPVHLLVGTPGRILDLTRKGICVLKDCSMLVMDEADK
LLAPEFQPSIEQLIHFLPANRQLLMFSATFPVTVKDFKEKYLPRPYVINLMDELTLKGIT
QYYAFVEERQKVHCLNTLFLKLQINQSIIFCNSVNRVELLAKKITELGYSCFYIHAKMLQ
DHRNRVFHDFRNGACRNLVCTDLFTRGIGIQAVNVVINFDFPKTSETYLHRVGRSGRFGH
LGLAVNLITYEDRFNLYRIEQELGTEIKTIPPQIDLAVYCQ*
>11668.m04104|LOC_Os02g42860.1|genepair852-2
MDPRARYPPGIGNGRGGNPNYYNRGPPLQQQHNHHQQQQTSAPHHQQYVQRQPQQHHHHN
HHQQHQQQQQQWLRRNQIAREAAGTDRNSEPKAVAQSPAVDGIDSSSQDWKAQLKLPPQD
TRYRTEDVTATKGNEFEDYFLKRELLMGIYEKGFERPSPIQEESIPIALTGSDILARAKN
GTGKTAAFCIPALEKIDQEKNAIQVVILVPTRELALQTSQVCKELGKHLKIQVMVTTGGT
SLKDDIIRLYQPVHLLVGTPGRILDLTKKGICILKDCSMLIMDEADKLLSPEFQPSVEQL
IRYLPASRQILMFSATFPVTVKEFKDKYLPKPYVINLMDELTLKGITQFYAFVEERQKVH
CLNTLFSKLQINQSIIFCNSVNRVELLAKKITELGYSCFYIHAKMLQDHRNRVFHDFRNG
ACRNLVCTDLFTRGIDIQAVNVVINFDFPKTAETYLHRVGRSGRFGHLGLAVNLITYEDR
FNLLTKLYIANNLTEFQRVFTFSKGNKKTILVNTSSSAHTSEFDLEVEKRGFIMDLLWHM
GTFIFGVLFEMRQTTLPMDTLCLYHFG*
>11676.m03225|LOC_Os10g36000.2|genepair853-1
MDKIHPKYQNCSLFFLPDAEKAAATGGSHERDALLTTVATEKRISLIKAWEENEKAKADN
KAAKKLADIASWENSKVAEIEAEIKKYQEYLERKKAEQVEKLMNGVAKVHRAAEEKRAAT
EARRGEEVVKAEEAAAKYRAKGEPPKKLLFG*
>11668.m04106|LOC_Os02g42880.1|genepair853-2
MAGEALKEAGATPAAANAGEEKAVIPAASTSPVISKTDDDTEPPADDSKALVVFVEKVAD
KPHAEKATATATPTRTSNDRDIALAKVETDKRESLIKAWEENEKAKAENRASKKLLDIIS
WENTKKAVIKTQLKKKEEELERKKAEYAEKAKNKEAIVHKEAEEKRAMVMARRGEEVIKA
EEIAAKYRATGVTPKKHIGCFGA*
>11676.m03228|LOC_Os10g36030.1|genepair854-1
MAVAAPAKAAHALAAAAAGMVLLWCVHFRGGLALSSPTNKGLIFNVHPVLMLIGFIILGS
EAIMGYKIWPWGHDTNKMVHLLLHAIALLLGSVGIYAAFKFHNESGIANLYSLHSWVGLG
TICLYGVQWIFGFVTFFFPGASPSLRRAALPWHVRSGLLVYILALLAAELGFLEKLTFLE
AGGLGRYSSEALLVNFTAVLVILLGSAVVMYVTAPMHNEHSHGYSAVRKP*
>11668.m04107|LOC_Os02g42890.1|genepair854-2
MGLGVRAAPFTYVAHALAVAAATMVLVWCIHFRGGLAFEATNKNLIFNVHPVLMLIGYII
LGSEAIMVYKVLPTWKHDTTKLIHLILHAIALVFGAVGIYCAFKFHNESGIANLYSLHSW
LGIGTICLYGIQWIFGFVAFFFPRASPSVRKGVLPWHILFGLFVYILALATAELGFLEKL
TFLQSSGLDKYGAEAFLVNFTALIVVLFGASVVVAAVSPARVEEPHEYAPIPES*
>11676.m03261|LOC_Os10g36350.1|genepair855-1
MAASCDLVDVDPPELQFPFELDKQISCPLRIANKTERTVAFKVKTTSPKKYCVRPNNGVV
RPRSASVVVVTMQAQIVAPPDLQCKDKFLVQSVVVDDGLSAKDITSQMFLRDENNMVEEV
KLKVSYVMPPEPAMEIAEESDIPKRILVPMQRILDNGRSGSELSSGNVSLRSAEMGTELG
SPLGRFVRNEDMLKTASPVVETRVHAGPDEQYLELSALVAKLTEEKKSALEQNRKLREEL
ELARRQASQHQGGFSLAFVLVIGLLSIILGCLVKK*
>11668.m04112|LOC_Os02g42940.1|genepair855-2
MQEAKKDSIFGGLASGAPVGADRFPFDPREYSPAMSSDSRELLGIDPPELIFPFELKKQI
SCSLHLTNKTDEYVTFKVKTTSPKKYCVRPNNGIVAPQSTSNVLVTMQAQREAPPDMQCK
DKFLVQSAIVTQELTPKDITGDMFTKESGNVVDEVKLKVVYTQPHPTSLNGGSEEGLGSL
SYQEATKGSRESETVTSEPLALISKLKEEKSSAIQQNMKLREELDLLRRQMGSQHGGFSL
VFVLVIAILGILLGFLIKR*

>11676.m03269|LOC_Os10g36420.1|genepair856-1
MSSSSSSSASSAAAAAFRPAVVQREQQVVEEKFPAAAAAMREMVLPPVAAAAADSEQEQL
CYVHCHYCDTVLVVSVPSSSLFETVTVRCGHCSSLLTVNMRGLLLPTTAAAAPPPPPPPP
PPPPPPAAHFPHSLNLAPANPPHHHSLLDEISTANSPTQLLLEQHGLGGLMASAASCRNN
NSPAAAAAPPPPTSQGKAAAKEPSPRTNTAVINRPPEKRQRVPSAYNRFIKDEIQRIKAG
NPDISHREAFSAAAKNWAHFPHIHFGLMPDHQGLKKTSLLPQDHQRKDGLLKEGLYAAAA
AAAAAANMGVAPY*
>11668.m04113|LOC_Os02g42950.1|genepair856-2
MSSSSSSSAVFPLDHLAAPSPTEQLCYVHCNCCDTILAVGVPCSSLFKTVTVRCGHCANL
LSVNLRGLLLPAPAPAPANQLHFGPSLLSPTSPHGLLDEVAFQTPSLLMEQAASASLSSI
TGRSSSSCASNAPAMQMPPAKPVQQEPELPKNAPASANRPPEKRQRVPSAYNRFIKDEIQ
RIKAGNPDISHREAFSAAAKNWAHFPHIHFGLMPDQGFKKTFKPQDGSEDILLKDSLYAA
AAAAAAAAANMGVTPF*
>11676.m03313|LOC_Os10g36810.1|genepair857-1
MDGGTGPGAELLSPGEAEWPPELRLPPPPPPHPPPPPPLEPAPPSTPQLRGEASPPPPPP
PPVGPPGAAVVAAAARKEASASAEGFDDSHFLGSIMGAPAHQHQHQHQQPPAVGPPVVVK
RKRGRPPKNRDGAAPPPPPKPVKKREDDEDVVCFICFDGGNLVVCDKNLLEDLIDDNGTF
SDKITGAFVRIRTPCVGQKQDIYRLVKVLGTHKVAERYSVGKKTTDHALEILNLDKKEVI
TMDTISNQDFTEGDIHEKAKIFQLLRVNDRSCNFWALLKKEHASLMKIQKYMLTHNHRQN
CSATSHHTTTSPPTEGMTHGQGEEGDMEPEKVWHYKDPSGSVQGPFTLLQLSKWAAYFPH
QLVLMMSGPLEATLVNGTIVRIVVEGSHWSSTADSITHDGLQLSLASAKPESCSAVNPIR
DGDSSSASRVPNQSGAHVYSPPHPATTNLSKSEETMNQCKSCEPEASNKSRKPDASHAPV
NQHPKPESDPLLPDTQDFERTHPSPSTEHDTKEPLKDQSRSTSVAPEGSGTMAHGQSSIA
FISEASGPLSSKIVGLQPPKDTSFLVEKDIKDGGSITQTEQQKEESTAFKKENVAVDPIS
DSEAIVSGVLESLTETYNLHEETALENFTPTSAEEEQPQCSTPIALSPWDETSDYQGEAV
DSALWGVQDDQNNEMWSLSSPTPTLQPSGIGADTKGASCAIEEVIVAQGNSGVVEPSPAL
EKKRIEKVPSASIDRGVPEQEC*
>11669.m00138|LOC_Os03g02160.1|genepair857-2
MEGAAAAAGGGEMLSPGEADWPPELRLPPPPPPSAASEGEPPPARAAVGMDDSQFLGSII
GLPAQPPQATAEALAVVGVKRRRGRPPKKRDGAAAATAVVPAARPARRREDEEEVVCFIC
FDGGNLVVCDRRGCPKVYHPACIKRDEAFFQSRSKWNCGWHICSSCEKAVHYMCYTCTYS
LCKVCIKQGKFFSVRGTKGFCDTCYSTILLIESKDEGDTKIVVDFDDQNSWEYLFKLYWV
DLKGKLSLTLEELTSAKARWNAPTTYTRKEKDESSDDLYDANNDDDAGSDCSSGKRKRNS
SRKKGRKRRKPNSDCSIATKKVETVTRDDGTLPNKVPTEEASLPVDTKWASPELLEFVGH
MRDGDQSFISQFDVQALLLDYIKQNNLRDPQRKSQIICDSRLHRLFRKTRVAHFEMLKLL
EMHFIVSEPSAVNDGSQGIINPDSAQIDHASGYNDMAAKFSPDRRRRMHRKMEREPQANP
EDYAAIDMHNINLIYLRRSLMEDLIDDPTLSDKISGAFVRIRISGLGQKQDMYRLVKVVG
THKVSEKYSIGKKMTNFALEIMNLNKKEIITMDTVSNQDFTEEECKRLRQSMKYDLISRL
KVGDIQEKAKIFQFVRVNDLLNTPEERARRINEVLDVHVDSHMDPDYESDDEFGNKKAVE
RSVNWARSDPFVSPVKVKYSNSSQKNGDATRHLKNLSKQNTERKSGAARNFENSHSPVGM
DIPKSGTNVKSTRCETTSPSSHGVVSSDMEPEKVWHYKDPSGNVQGPFTLVQLSKWTSYF
PRDMRVWLTFESEERSLLLTEVLSKQPKDFGQPASVTTSSKSTVADTGQNRNTEIVDLNK
APSPVGYSMLNSFETTVQSTKHSAPERESVNSLDDRLSHSTDSVPPKDANASNSQAMCQI
KHSGSLPSPGSPHQRSDLHHDEVQGGRSGEWNNQHNSELWSPSMPQTSSSAHSNVESHHD
HYPSWSQVQHDPKNSLQAGSGKDLNSRYDIAQKLPSQRITRDVPSPVFAWSPSESRTASS
QHEGSCLSSTTNLCTHDELHSSIASAKAKSFAPATPVEDRGSSSPSGMLSLSERAPICSP
QSAPSASASDTCKMEENMNQQKTLEADISNTSVNQSPQSKILPESSPDNQDAEHEYRSPP
PISESKELSPQSRTTPGSSPDNQDTEREYPSPPPISGSKEISPQSRTILESSPDNQDNGH
EYPSPPPIPESIELSPHSKALPESSPDNQDIEPECPSPPQIPESKELSRQSKILPESSPG
NQDIEPECPSPPQIPESKELSQQSKILPESSPDNHDIKCEYSSPTPIPESKELSLQSKIL
PESSSDNQDIKCEDPSPTPISKSKEVSPQSKILSESYLDNQDVERECPSSILITESKELA
VDLPGSISLAPEKTASTDVGENSSLAFIFPKSTLAGDDALKSVFDMAKAHLECEDSKVKE
ELYVESTVVIRDDMVVNPASGVESIDMSENLLESLMEQSCGTFYMDGTTALEGFLSGSTK
EEPQCSSPIALSTCSSPIALSPWGEHGYYQGDSVGSSLWGVQDDDPIGNIWPLSSQAPAL
QYSSAGSTAHFIDEATVTHGNNGVVLSSTPGEEVNPETNDASVSMIDKNSGLVDSQPSAN
DGSDVGTARNTNHNTNLSLNHETAVPLSRSSGEASRKHGFITDLNVATSEEALGNTKNWN
PYAGNANRGSQRNHHRDRYSQISESWLLSSNYSRSRSDGFGTGGSSRSTPRGQTQRGICK
FHENGYCRKGASCNYLHP*
>11676.m03315|LOC_Os10g36830.1|genepair858-1
MATSSTLSQTQRYAAGALLALALRQAQIHQSVLLGSHGLDDAPAAAAAVPSLADPVDARH
LWTHDSHGLLRPVLRFLEIDHKAWPGVETTAATSEPKHHIGAFLRKVFEDEDDGEAAAAE
RSEQELALAKAVEAMAMGLENDVVAADELIKATGSGGDGDEGWPWLASASSAGGSRTKDY
RKMAVLYMLLSACVADVNMAEEGMGSPRIRRGYDARHRVALRLLATWLDVKWIKMEAIEI
MVACSAMAAAREEEQSEGSESPRSRWENWKRGGIIGAAALTGGTLMAISGGLAAPAIAAG
FTALAL*
>11669.m00139|LOC_Os03g02170.1|genepair858-2
MATTLTPTQRYAAGALLALALRQAQIHQSVLLGAHHHHDDDDEEQGRTSTSSGGGGGSSS
SSSNSGAGADADLWTHDSHGLLRPVFRFLEIDPKAWSGLEETAASSEAKHHIGAFLRIIF
EEDGESSSDRSVQELALAKGVDVMVMSLGNDSEVGNTIKGGDQDALPSSSGTDKSPGESS
HDDQLGINKLTLDDIPANNHRKMALLFALLSACVADKPVSQEEEDRKSTRFRKGYDARHR
VALRLLSTWLDVKWIKMEAIEVMVACSAMAAAKEQEQSQESASPKSKWEKWKRGGIIGAA
ALTGGALLAITGGLAAPAIAAGFGALAPTLGTLVPVIGASGFAAMATAAGSVAGSVAVAA
SFGAAGAGLTGSKMARRIGSVKEFEFKPIGENHNQGRLAVGILISGFAFDEDDFCRPWEG
WQDNLERYILQWESKHIIAVSTAIQDWLTSRLAMELMKQGAMRTVLSGLLAAFAWPATLL

AATDFIDSKWSVAIDRSDKAGKMLAEVLLKGLQGNRPVTLIGFSLGARVIFKCLQELALS
SDNEGLVERVVLLGAPVSVKGERWEAARKMVAGRFVNVYSTDDWILGVTFRASLLTQGLA
GIQAIDVPGVENVDVTELVDGHSSYLSAAQQILEHLELNTYYPVFVPLSAANEETDGTVA
Q*
>11676.m03317|LOC_Os10g36850.1|genepair859-1
MADMVKFTMEWLQDPLSLAIVVTVAVLIMRMQRRRAAPFPPGPKPLPIVGNMAMMDQLTH
RGLAALAKEYGGLMHLRLGRLHAFAVSTPEYAREVLQAQDGAFSNRPATTAIAYLTYDRA
DMAFAHYGPFWRQMRKLCVVKLFSRRRAETWLAVRDESAALVRAVAASRGEAAVNLGELI
FNLTKNVIFRAAFGTRDGEGHDEFIAILQEFSKLFGAFNIGDFIPWLSWADTNGINARLV
AARTALDRFIDKIIDEHMERGKNPDDADADMVDDMLAFLAEAKPHAGKAAAAAAGAGDGA
DDLQNTLRLTRDNIKAIIMFDSIANY*
>11669.m00140|LOC_Os03g02180.1|genepair859-2
MANGVAEYLLMDPWLVLWLVLASMAFALLHLRRRARRGAPPLPPGPRPLPIIGNMLMMDQ
LTHRGLAAMAARYGGLLHLRLGRVHMVVVSSPEHAREVLQVQDGDFSNRPASIAIAYLTY
GRADMAFSHYGHFWRQVRKLSAVRLFSRRRAQSWRAVRDESAKLVGAIARRAGEAVDLGE
LIFGLTKDVIFRAAFGTRDGGGHGELEVLLQEFSKLFGAFNVGDFIPWLAWLDPHGINRR
LRAARAALDSVIDRIIDEHVSNPAGDEDADMVDDMLAFLDEAGRDQTGGGGELQGTLRLT
RDNIKAIIMDFVFGGTETVASAIEWAMAELLHSPGDLRRLQAELADVVGLGRGVEEGDLE
KLPFLRCVAMETLRLHPPIPLLLHEAAADCVVGGYSVPRGARVVVNVWSVGRDAGAWKGD
AGAFRPARFMAGGEAAGMDLRGGCFELLPFGSGRRACPAIVLGMYELELVVARLVHAFGW
APPGGVAPEELDMADGFGLTAPRAARLRAVPTPRLTCPM*
>11676.m03352|LOC_Os10g37190.1|genepair860-1
MWQRYRFLCCGCGGNMAASAAGDRGCDDDADCGGGSGGGGFGEEEEGGKGGVAGAARRLS
WAQVEAMTGGFTSAVVGEGGFSTVYLARVAGALAAVKVHRSSERLHRVFRQELDALLRVR
HPHIVRLLAFCEQQEEGVLVLEFAANGNLHERLHGGGKAAGTMPWARRASVALQVARALE
YLHDRCEPAVVHGDVKASNVLLDEAMSAKLCDFGSARMGFSAAVRPRSSAHTMLGSPGYV
DPHYIRSGMVTKKSDVYSFGVLLLELLTGMEAFCAAEGRLLTAVLAPRLRPASCAACDAR
MLVDERLGSAYDAGEASAVASLAASCVGENPSLRPSMADVVRALEQGAHGSISAVARRSD
GHGKL*
>11669.m00141|LOC_Os03g02190.1|genepair860-2
MLLHRHRHRLLCCGGGVATAPGRFAGDAIADHQQAAAGAKNAAATSSARQLSWAQVEAMT
RGFTSAVVGEGGFSTVYLGRVAGSLAAVKVHRSSERLQRAFRQELDALLRVRHPHIVRLL
AFCDQRDEGVLVLEFAPNGNLHEQLHGGGGGGAMPWARRVAVALQVARALEYLHDRCEPQ
VVHGDVKSSNVLLDAAMGARLCDFGSARAGFSAAVHCPRPRPSALAVLGSPGYVDPHYLR
SGVVTKKSDVYSFGVLLLELLTGTQAFRDGMLLTAAVAPKLKGAAAGDVEKLVDERLGCQ
YNAAEAATVAALAAACIGDNPSLRPSMADVVRTLEKPAQKAGR*
>11676.m03359|LOC_Os10g37240.2|genepair861-1
MAIYYLEREGLTHNMPMHGHFYTPLPLNHWHADTTNAADMTRGPMRMWAPHVSGMNFIFF
ISKEKVLMRVEMVSSPDVLIIPSPPFPSGNTRGGGGGGGGGSSMQQQQPGGGGGVQQFGA
VAPEMSPFSPAGGGGGGRISMAEAASPISSRPPPAQQQFDELGVGGGGGGGGGFDAEALA
AAAVGEEGASGGAGGNRWPRQETLALLKIRSDMDAAFRDATLKGPLWEEVSRKLAEEGYR
RSAKKCKEKFENVHKYYKRTKESRAGRNDGKTYRFFTQLEALHGTAAGVVAAPSPVTSLA
PPPATAVGVSGGVRAPAEPPPAVVMGNVMSFSTSNTEEYSDEEDSDDEGTEDMGGGGGDE
RGKRKRLSEGGAAAGVGGGGGGGGGGSGKMMRFFEGLMKQKITGQTIPMPPIIAAPAITVM
PPPAPSQQPQPPPPPPSHPTPITSVAPAPPPPPPAAAAAASQPSPQATKSPLPATPQTQS
SMDIVMTAAEAHDAGYDGSGGGGGQPSSSRWPKAEVHALIQLRSNLDNRYQEAGPKGPLW
EEISAGMRRLGYSRSSKRCKEKWENINKYFKKVKESNKKRPEDSKTCPYFHQLDALYRNK
AALNSSSSSAAAAAPALPPPEHAEPAVTVAAPISQTPPPPPPQPVTTTKNGNGTSSTNGA
NGEGGGGGSGGMQMQASNGSVVAGNKFFTGAAAKKPEDIMKEMMEQRPQQPAAANNAFNR
TDGGGGGGGVDSDNMDEDDEDDYDDDDDDDDDDVDGNKMQYEIQFQHQHHHQQPPQHRHQ
QSVVRPNAAASAAAGGNPPGTAAPATAAAATTTTGSFLAMVQ*
>11669.m00146|LOC_Os03g02240.1|genepair861-2
MPPFSAAGGEGAPSPISSRPPPPEQAAAAAAEEQLNGSSLEHDGVLGGEEGDRGGSSAGN
RWPRQETLALLKIRSEMDAAFREAALKGPLWEEVSRKLAEMGYKRSAKKCREKFENVDKY
YKRTKDGRAGRGDGKTYRFFTELEALHGAAAATARPPPVSLAPAPVAVAPPATPAGLSAL
RVHASPPPPPVKQHAAPPPPVMDVAACVMTMDDVSFSSGSDTEETAEEGGKRKRRGGGGI
GGGGGGGKAMRMFEGLMRQVMERQEAMQQRLLEAIERRDQERMIREEAWRRQEVARLARE
QDALAQERAIAASRDAAVISFIQRVTGQSIAAVPPPPLQPTPVASAAPPPPPQHHHQQTP
PPIQVQPHHIMPMTPQPQLQPPQPQSKEANTVVRAAPPPQEQHDTAASGGGGGASSSRWP
KAEVHALIQLRTELETRYQDSGPKGPLWEDISAGMRRLGYSRSSKRCKEKWENINKYFKK
VKESNKKRPEDSKTCPYYHQLDALYRTKAANAAAAASASPAPATTTVLAPVPLSQTPPHV
DHGGSNGNGNGWASANNGGGGSSSGGMQTKASNNGTATAGGLPVVSVAGGNGNGNGVAAT
TDNKGSKQVPVAKETAGQRQPQPLAMNHNYGNDRMADDMDSDSMDDDDDDDEFDDDENDD
DIGGGKMQVQYETSSHFQRPQLQNQNVVVGRPNASGGGGGGAPTTPAGPPPPAATSGTSF
LACVQ*
>11676.m03379|LOC_Os10g37430.1|genepair862-1
MKEPAQVRTARGGAADGVEVGVEEEEEPPRSATVKQEEANAVLGAEGSRPFAMRELKEDH
EVAAGSGVKAASGERNGIGSADAQGSSYSQESMQQFSSHHDVAMDLINSVTGVDEEGRSR
QRILSFAAKRYISAIERNHDDPDAYYNWALVLQESADNVDPNSSSSKDALLEEACKKYAE
ATRLCPTLYDAYYNWAIAIADRAKMRGRTKEAEELWKQAILNYEKAVQLNWNSPQALNNW
GLGLQELSAIVPAREKQTIIKTAISKFRAAIQLQFDFHRAIYNLGTVLYGLAEDTMRSGK
PGVSASEFYSQSAIYVAAAHALKPNYSVYRSALRLVRSMLPLPYLKVGYLIAPPENSAIA
PHKEWERSQFVLNHEELQQVNASDQPPSQSPGHVDSGRKLFRIVVADIVSVSACADLTLP
PGAGLRIDTIHGPRFLVADNWETIDSWLDAIRLVYTIFARGRSDVLAGIITG*

>11669.m00149|LOC_Os03g02270.1|genepair862-2
MEDSARPTPADSGELPNGAGMAEDAVVAPDAGEAPEEGEESGIANDSETNAALGAEGEPS
RALTMRELLGELKDSGEPSSGRSTLSEGNGIGSAGAERASQDSLQFSSHHDVAMDLINSV
TGVDEEGRSRQRILSFAAKRYVSAIERNPEDPDAYYNWALVLQESADNVDPDSSSSKDSL
LEEACKKYAEATRLCPTLYDAYYNWAIAIADRAKMRGRTKEAEELWKQAIMNYDKAVQLN
WNSPQALNNWGLGLQELSAIVPARDKQTIIKTAISKFRSAIQLQFDFHRAIYNLGTVLYG
LAEDTMRSAGPDVSPNDLYSQSAIYVAAAHALKPNYSVYRSALRLVRSMLPLPYLKAGYL
TAPPADNTIAPHKHWERSEFVLNHEGLQQVDASESPSSKPLGHMGRSKKYIKVAVEDIVS
VSACSDLTLPPGAGLCIETIHGPLFLVADTWESLDGWLDAIRLVYTIFARGKSDVLAVYR
VNHAGIEVVSPSKTVVPSSINSLFTSFRRTGAE*
>11676.m03384|LOC_Os10g37480.1|genepair863-1
MGRNGSVKRTSSSGAAAAFTANPRDYQLMEEVGYGAHAVVYRALFVPRNDVVAVKCLDLD
QLNNNIDEIQREAQIMSLIEHPNVIRAYCSFVVEHSLWVVMPFMTEGSCLHLMKIAYPDG
FEEPVIGSILKETLKALEYLHRQGQIHRDVKAGNILVDNAGIVKLGDFGVSACMFDRGDR
QRSRNTFVGTPCWMAPEVLQPGTGYNFKADIWSFGITALELAHGHAPFSKYPPMKVLLMT
LQNAPPGLDYDRDRRFSKSFKEMVAMCLVKDQTKRPTAEKLLKHSFFKNAKPPELTMKGI
LTDLPPLWDRVKALQLKDAAQLALKKMPSSEQEALSMSEYQRGVSAWNFDVEDLKAQASL
IRDDEPPEIKEDDDTARTIEVEKDSFSRNHLGKSSSTIENFFSGRTSTTAANSDGKGDFS
FEAFDFGENNVDTKIMPNGYENARSENSSSPSTSKQDPESKYWRSTSGQKQQTSGTPAVH
SGGVNSSTTEKGHGVERDATVQLASDKLRTETRRATNLSGPLSLPTRASANSLSAPIRSS
GGTSRILPLSQFDHPLNKEHQNQGHQNIHHLKFILID*
>11669.m00155|LOC_Os03g02320.2|genepair863-2
MVRSGSVRRTAASSSPAAAAVPTAFTASPGDYRLLEEVGYGANAVVYRAVFLPSNRTVAV
KCLDLDRVNSNLDDIRKEAQTMSLIDHPNVIRAYCSFVVDHNLWVIMPFMSEGSCLHLMK
VAYPDGFEEPVIASILKETLKALEYLHRQGHIHRDVKAGNILMDSPGIVKLGDFGVSACM
FDRGDRQRSRNTFVGTPCWMAPEVLQPGAGYNFKADIWSFGITALELAHGHAPFSKYPPM
KVLLMTLQNAPPGLDYDRDKRFSKSFKEMVAMCLVKDQTKRPTAEKLLKHSFFKNAKPPE
LTVKSILTDLPPLWDRVKALQLKDAAQLALKKMPSSEQEALSMSEYQRGVSAWNFDIEDL
KAQASLIHDDDPPEIKEDVDNDRINEADKEPFSGNHFGQPKILSGKHFSEQTCVTAVSPG
GNMHETSRGLVSEPGDADSERKVDGYRKQGSENESLPSTSKHDSEGQNSSSEVKQKERTC
SGPILCSGVHNKSITESSRIFDREAAVKLASDKQKSCTKRTTNLSGPLALPTRASANSLS
APIRSSGGYVGSLGDKSKRSVVEIKGRFSVTSENVDLAKVQEVPTSGISRKLQEGSSLRK
SASVGHWPVDAKPMSNSHQRKELCNGSVSASVLIPHLRNLVQQTTFQQDLITNLLSSLQQ
NEKADATQYRLGNMDGDTEVETSISEGERSLLVKIFELQSRMISLTDELITTKLQHVQLQ
EELKILYCHEEIIDTREVDNA*
>11676.m03386|LOC_Os10g37500.1|genepair864-1
MTPREGGGGGGGGVVGLVAYAALAVVALRVVLSYKSVAHAVRRMWRWADEWAQAYQYYEV
PRFGGGGGEGVENPLFRKAAAYVAALPSLEDADAACVLSSACKTNDFSLQLGPGHTAHDA
FLGARLAWTNAGPAGDGGGGRERLVLRVRRHDRTRVLRPYLQHVESVADEMELRRRELRL
YANTGGDGAPSPKWTSAPFTHPATLETVAMDPELKARVRADLESFLKGRAYYHRLGRAWR
RSYLLYGPSGTGKSTFAAAMARFLVYDVYDIDMSRGGCDDLRALLLETTPRSLILVEDLD
RYLRGGGDGETSAARTSRMLSFMDGLSSCCGEERVMVFTMSGDKDGVDPAILRPGRLDVH
IHFTMCDFEGFKTLASNYLGLKDHKLYPQVEEGFHAAGARLSPAELGEIMLANRGSPSRA
LRTVINALQHVAPAPAPPQQQPRASSASRPPPRLTARWSGHLDEASAADASAANQSPGGG
GGGFGKDAPMREFKKLYGLIKIRSRKDGGVVPVDDTASANGRGSDVSADKDR*
>11669.m00157|LOC_Os03g02330.1|genepair864-2
MLLGAMSGGGVVVAVAVAYAALAVVALRMALSYKSALYAVRRLWRWADEWAQAYQYHEVP
RFACDGGGAENPLFRKAAQYVAVLPSLEDADAASVLSSASRTNGGFSLQLGPGHTARDAF
LGARLAWTNRGDVLVLRVRRHDRTRVLRPYLQHVESVADEMELRRRELRLFANTGVDGST
GTPRWASAPFTHPATLDTVAMDPDLKARVRADLENFLKGRAYYHRLGRVWRRSYLLYGPL
GTGKSTFAAAMARFLGYDIYDVDLSRAGSDDLRALLLHTTPRSLILVEDLDRFLQGGGAG
DAEARAARVLSFMDGVASCCGEERVMVFTMRGGKEGVDAAVVRPGRLDVHIHFTLCDFEA
FKALASNYLGLKDHKLYPQVEESFHGGARLSPAELGEIMLANRSSPSRALRNVITKLQHV
SGAAAAPRPPHRRNTSWSGAGGPWEEQAARASADAADGGEEAITATAACGVFAKDAPMRE
FKKLYGLIKIRSRKEGSSGFMPLHGGEAPSPANGRGSEHDKER*
>11676.m03388|LOC_Os10g37520.1|genepair865-1
MEVFYYLVFGGLAAVVAGLELGKSGKDRVATTPAFNAFKNNYILVYSLMMSGDWLQGPYV
YYLYSQYGFDKGDIGRLFIAGFGSSMLFGTIVGSLADKQGRKRACITYCISYILSCITKH
SPEYKILMIGRVLGGIATSLLFSAFESWLVAEHNKRGFDPQWLSITFSKAIFLGNGLVAI
IAGLFANLLADNLGFGPVAPFDAAACFLAIGMAIILSSWSENYGDTSDNKDLIAQFKVAA
KAIASDEKIALLGAIQSLFEGSMYTFVFLWTPALSPNDEEIPHGFIFATFMLSSMLGSSI
ASRLLARKLKVEGYMQIVFSISAFTLFLPVVTNFLVPPSSVKGGGISFGGCLQLLGFCIF
ESCVGIFWPSIMKMRSQYIPEEARSTIMNFFRIPLNLFVCVVLYNVNAFPITVMFGMCAI
FLFMAAILQRRLMVVSDLHRSTKAVEMTGEDEPLNP*
>11669.m00162|LOC_Os03g02380.1|genepair865-2
MEVFYYLVFGGLAAVVAGLELGKSAKDRVTTSQAFNSFKNNYVLVYSLMMSGDWLQGPYV
YYLYSQYGFDKGDIGRLFIAGFGSSMLFGTIVGSLADKQGRKRACITYCISYILSCITKH
SPEYKILMIGRVLGGIATSLLFSAFESWLVAEHNKRGFDSQWLSITFSKAIFLGNGLVAI
VSGLFANLLADNLGFGPVAPFDAAACFLAIGMAIIMSSWSENYGDPSESKDLMSQFKVAA
KAIASDEKIALLGAIQSLFEGSMYTFVFLWTPALSPNEEDIPHGFIFATFMLSSMLGSSI
AARLLARKLKVEGYMQIVFTISAFTLFLPVVTNILVPTSSVKGGSISFGGTLQLLGFCTF
EACVGIFWPSIMKMRSQYIPEEARSTIMNFFRIPLNLFVCVVLYNVNAFPITVMFGMCSI
FLFMAAILQRRLMVVSDLHKSSTKAQEMVDEDEPLNP*
>11676.m03390|LOC_Os10g37530.1|genepair866-1

MADPRLFPSGSNSSGDASGPGRRKYNPYHDLSTPYSYQTLYDLPTSPEFLFQEESAAQRR
SWGENLTYYTGVGYLSGAVAGAALGLRDAAAGAEPGDTAKIRANRVLNSCGGGGRRLGNR
LGVIGLMYAGMESAMVAARDRDDWVNSVAAGLGTGALFRAANGPRSAAVAGAIGGVLAGA
AMAGKQLAKRYVPAI*
>11669.m00163|LOC_Os03g02390.1|genepair866-2
MADPRLFSSGSGTRDDRTDASGRRLYNPYQDLNIPYKQLYDLPTSPEFLFQEESLAQRRS
WGENLTYYTGIGYLSGAVAGAAVGLRDAARNAEPGDTAKIRANRVLNSCGSNGRRMGNTL
GVIGLLYAGIESGMVAVRDRDDWINSVTAGLGTGALFRAANGPRSAAVAGAIGGVLAGAA
MAGKQAAKRYVPAL*
>11676.m03407|LOC_Os10g37690.1|genepair867-1
MAFAARRSLASRLSHHLTRRLHPATAPHLLASHSDDDPSPPPELPPFPHSPSPRHPRAGQ
ALDRHLLPFSLHHLAGLRRRGFSSAAGEVDAAAGVLADAAAAAEAVPAPFPGEVAAAAAD
SFFPVAALQHVIDYIHTFTGLNWWACIALATVLIRSATVPLLVNQLKATQKLNAIRPEME
AIKEEMNAMDPKSAKEGKAKMTALFQKHGVSPFTPLKGLLIQGPIFMSFFFAIRNMIDKV
PSMKGGGSLWFTDLTTPDPLYILPVLTALIFLVTVELNLQEGMEGNPMARKMKNFSRGMA
VLTVPFTMSFAKGIFCYWITSNLFTLTYGFVIRRPAVRKFCNLPALEAQSASAKKQMFNL
FGGSKALTTAESPVAITGGPQSSLEQPDAAALGYRVKNPEKKAKSRGKSRRRR*
>11669.m00173|LOC_Os03g02480.1|genepair867-2
MAFSARRSLASGLSRHLSRRLHPSVSHLLPSHHDDHSENPSPPAQPPPLPSALRSPSRSQ
ALGLPLPFGLLHASRRSLSTSPRSNDELDASAEVLSDAASSVSVPADVLADAAASVPVSA
PAPFPGEVAAAAADSFAPVAALQHLIDGVHSLTGLNWWACIALTSLLIRTLTVPLLLNQM
KATAMRPEIEAINLEMRTISSTRIAGNEKSSTRVTDEGSMSTDPQSMLEGKRKLGELFLR
HGVTPLTPLKGLFIQAPIFMSFFFAISNMVEKVPSFKGGGIYWFTDLTTPDELLILPMLT
SLTFLVTVELNMQDGMEGNPMLKTMKNFSRVMAVLTIPFTMSFPKAIFFYWVTSNLFSLG
YGFVLRKPAVRSFLDLPPIETQFAPAQQPTFNLFGASKSVPAAGSSIAESDRSSSVLSQR
FSDLENRAKSRRESQD*
>11676.m03408|LOC_Os10g37700.1|genepair868-1
MPREWGRDEGRNGGGGGAALLGYSSSAASWLYRRAAAPARAYCGAERGPPPVTAARVRLR
DGRHLAYHESGVAREAARVRVVFSHGFTGSRLDGLGASQTFFILVTFL*
>11669.m00176|LOC_Os03g02510.1|genepair868-2
MWVTSMPQVWDEEGVAKGSVVTPAPATALLGSLAGWMSRAVEPPAPRPCGTEGGPPVTAT
RLRLRDGRHLAYCESGVPKEEARFKVVFSHGFTGSREDSVRASQW*
>11676.m03409|LOC_Os10g37710.1|genepair869-1
MGTLVGQLGELLSRAVMPPPPRVCGAPGGPPVTAPRVRLSDGRHLAYEESGVPKEAARYK
IVFSHGFTGSRLDSLRASPEVAEELGVYMVAFDRAGYGESDPNPNRTVKSAALDMAELAD
ALGLGDKFYVVGVSLGSHAVWGALRYIPERIAGAAMMAPVVNYWWPGFPAEDAAAAYGRQ
SYGDQWALRVSHHAPAILHWWMDQSWLPTSTVVDNTTFLPNKRDADIRRTLTADGTLQKK
KEMATQQGINESYYRDMTVMFGKWEFDPMALPEPPCPVHIWQGDEDGLVPVALQRHVAGK
LGWVSYHELPGTGHFLSAVPGLGDTVLRTLFG*
>11669.m00177|LOC_Os03g02520.1|genepair869-2
MELLTTAPMLNCAAKRAGGSALGPLADALGSWIARAVVPPPPPPRICGSPGGPPVAAPRV
RLRDGRHLAYAESGVRKEDARYKVVFSHGFTGSRLDSVRPSPEVAEELGVYMVGFDRAGY
GESDPNPNRSVKSAALDVEELADALGLGPKFYVIGISLGCHAVWGALKYIPERIAGAAMM
APVVNYWWPGFPTDLAAEVYNKQEVGDQWALRVSHHAPSILHWWMEQSWLPTSTVVAGTT
PLPNKRDAEIRKNMKADGSFQKKMDLATQQGIHESYYRDMMVMFGKWEFDPMSLPKPPCP
VHIWQGDEDGLVPVVLQRYLVSRLSWANYHELPGTGHFLSAVPGLGDTVLRTIFG*
>11676.m03414|LOC_Os10g37760.1|genepair870-1
MGALVWDKVVHEHQGWRLVTCIWLHAGVVHLLANMLSLVLIGLRLEQQFGYMRIGIIYLV
SGIGGSVLSSLFIRNSISVGASGALFGLLGAMLSELFTNWTIYTNKAAALVTLLIVIAIN
LAIGILPHVDNFAHIGGFLTGFLLGFIFLMRPHYGWMQRYVLPSSVKYTSKKYLAYQWIL
LAVASVLAVIGFAVGLSMLFRGVNANERCHWCHYLSCIPTSRWTCGN*
>11669.m00178|LOC_Os03g02530.1|genepair870-2
MATSHADVEKGARRKEVGKVPSPLYPQHEGEREWVPWIVPSFLVANIVVFVLTMYANNCP
LHTPPRSGKCIARFLGRFSFQPLHENPLLGPSSATLQKMGALVWDKVVHEHQGYRLITSI
WLHAGVLHLVANMLSLIFIGLRLEQQFGYVRIGAIYLLSGLGGSVLSSLFIRNHISVGAS
GALFGLLGAMLSELLTNWTIYTNKVAAVITLLFVIAVNLALGILPHVNNFAHIGGFLTGF
LLGFVLLMRPHFGWMERYSLPSGSPCSSKKYLVYQWILLAIATALVIVGFAVGMTMLFRG
ENANDSCHWCHYLSCVPTSRWTCSN*
>11676.m03421|LOC_Os10g37830.1|genepair871-1
MGAGVSGLFGLGGDEGETSAAVGGAAAAGLGDLPELCAAEVLLRLDAPEICRLARLNHAF
RGAAGADFVWEAKLPENYRYLMSFVEGGGGGDDGRQLRRRRWRPAGKKEIYARLARPVPF
DGGSKEFWLEKSKGRVCMALSSKSLVITGIDDRRYWQHIPTAESRFYSVAYLQQIWWFEV
VGEIDFSFPVGTYSLYFRIHLGKFYKRFGRRVCSTEHVHGWDKKPVRFQLSTSDGQHSLS
QCSLGEPGSWVLYHAGDFVVSKPDQTIKLKFSMAQIDCTHTKGGLCVDSAFIYPKGFQQE
RMVRSQKCRC*
>11669.m00180|LOC_Os03g02550.1|genepair871-2
MGAGVSDLAAGMEAVGKVAGGAGLGELPELCAAEVLLHLDAPEICRLARLNRAFRGAAAA
DFVWEAKLPENYGYLLDFVDGAMEGGGGGRERSVMGKKEVYARLAKAVPFDGGKKELWLE
KSKGGVCIALSSKSLVITGIDDRRYWSHMPTTESRFHSVAYLQQIWWFEVVGELEFCFPA
GTYSLYFRLHLGKASTRFGRRVCSSEQIHGWDKKPVRFQLSTSDGQHALSQCYLDEPGSW
ILYHVGDFVVASTTEQPIKLKFSLAQIDCTHTKGGLCVDSVLIYPKGFQQEKVISSQK*
>11676.m03426|LOC_Os10g37850.1|genepair872-1
MVSDHEVRCCSFDRGMSLHCCPAAGDPPAPAGTAEELLERARSLVPAALDAARAATGFGG
RWKVIAARLERVPPCLSDLSSHPCFSKNSLCRELLQSVAATLAEAAELGARCREPPRAGK

LQMQSDLDALAGKLDLNLRDCALLIKTGVLSDATVPPVAPAAEAAAQTDVRELLARLQIG
HAEAKHRAVDGLLDALREDEKSVLSALGRGNVAALVQLLTATAPKIREKAATVLCLLAES
GSCECLLVSEGALPPLIRLVESGSLVGREKAVITLQRLSMSPDIARAIVGHSGVRPLIDI
CQTGDSISQSAAAGALKNLSAVPEVRQALAEEGIVRVMVNLLDCGVVLGCKEYAAECLQS
LTSSNDGLRRAVVSEGGLRSLLAYLDGPLPQESAVGALRNLVSSAISPDSLVSLGVLPRL
VHVLREGSVGAQQAAAAAICRVSSSSEMKRLVGEHGCMPLLVRLLEAKSNGAREVAAQAV
ASLMSCLANARDIKKDEKSVPNLVQLLEPSPQNTAKKYAISCLLTLSASKRCKKLMISHG
AIGYLKKLSEMDVAGAKKLLEKLERGKLRNLFSRK*
>11669.m00183|LOC_Os03g02580.1|genepair872-2
MVPPALGAAGAAEGFPGRWKAIAAKLEGLPACLSDLSSHPCFAKNALCRELLQSVAATLA
EAAELAARCREPPAAGKLQMQSAIDALAGKLDLNLRDCALLVKTGVLSDAFTPPPPTDEA
TSTATAAQADVRELLARLQIGHTEAKSLAVDGLLEALNKDEKSVLSVLGRANVAALVQLL
TAPATKVREKAATVICQLAESGGCEGLLVSEGALPPLIRLAESGSLLGREKAVITLQRLS
MSSDTARAIAGHGGARPLIEMCQTGDSISQSAAAGALKNLSAVPEVRQALADEGIVRVMV
GLLDCGTVLGSKEHAADCLQNLTSSSDSFRRAVVSDGGLRSLLVYLDGPLPQESAVSALR
NLVSAVSPDSLVSLGVLPRLAHVLRVGSTGAQQAAAAAICRISTTTDMKRVVGEHGCVPL
LVRMLDAKSNGAREVAAQAMASLVGYPPNAREVRRDGKSVPCLVQLLDPSPANTAKKYAI
ACLLSLAAAKRCKKLMISHGAIGYLKKLSDMDVAGAKKLLERLERGKLRSLFSRD*
>11676.m03442|LOC_Os10g38000.1|genepair873-1
MEQRAAAAAQQRQGRRQSAAACGIRRARAETRHPVYRGVRFRAGKWVSEIRELRKPSRIW
LGTYATPEMAAAAYDAAALALRGRGAALNFPDAARSRPAPASASADDVRAAATAAAAAMA
HQEEDDDSRRQLEDGGGGGGVVDEDDVLEMPRLMVSMAEGLMISPPPVMLGLQADGGGIM
DEGGGVVRLWDHS*
>11669.m00190|LOC_Os03g02650.1|genepair873-2
MDRDESLGTQPLTGRRVRADTRHPVYRGIRLRSGKWVSEIREPGKSSRIWLGTYPTPEMA
AAAYDAAALALRGADAALNFPGTATSRPAPASGSPDDIRAAAAAAAAMIGSGHRGNQRAA
DASTSRAATAAPEAAVAAGAGDQKRVVDEDDVFEMPRLLVSMAEGLMMSPPRLSPSTDGV
GGVSPEDDEDEDGMSLWNHS*
>11676.m03444|LOC_Os10g38020.1|genepair874-1
MAGGSKPLRLKDLLELDCESCSAAGFRCYPRRLCVAGGAAEAAAPPMRHRLVADRSSSAM
RRPKLSSLSKSLSRRLRGGFWRRREEEDEEAAAAAAAPPATASSTAPAVASCCSSSSDSE
TSESSNSTGGRKSRSHSDYSEISSASSDDSLHAAGEPSTTGADHEVMKRGSKEEDEEEEA
DDKEQLSPVGVMDFPFDEDDDDAAAVDEDERVAAGACSFSFSDSLAQLQRRKMQLQPKIR
RLGSMAELSGVDLEARFAASESDRLAGIVPVQHQCITDDVAAAPPRHDDHRNDGVSQKDP
DDDEDSLLDLLADTVSVGVVDDVTERLLLDFFVEAKCSSRNIELHAPTSLLRERRRRENG
ETMRLAKAWLEGTGTPWTLNDVLYHGEDVMAEMERSRRWMHAGEEEREAGVVVAAMAMDE
LLHELVSDLIALPK*
>11669.m00191|LOC_Os03g02660.1|genepair874-2
MAVAAPARRLTPLTLRDFLEQSSSEGFRAYPRFPVADEGVAGGDLAPPVRLLIEAGLRRS
PSRLPSFYNFFHKSPGTLAKISRLSRSLSRRFRDGLWRRRGEDDGEEDDDIAVDERDSLG
LPSPVVSSCSSSECEYMAESEAELATTEEEKCASASSASEYEKTSQSSTGSVAFHGAADA
GGDGHKEDVGDEPVGRKLEMEDKQQLSPVSVLDFPFDDDDGEEGSDAGMCSPSFQQCLAE
LQRSKAELLHKIRRLEGLTQVVVPVDLEAQFTESDSSERTHLNANSTSSSDDTATTAPTT
PRQCTDDQDVVNHGEEEEEEHSLLARLLESVVVTDEVSEWLLLDFFAEGVDRLRSSASSC
PLNDCEEAALLRAAGDWARGAGQRWGVGDVVFSGWAAVADMERSRRWMCVAEEERDVGAE
VDGLVMDALVDELVADLALGGATTVGVEVCTCRR*
>11676.m03447|LOC_Os10g38050.1|genepair875-1
MAALGRASSSAPVLAAAAAVLLSLCLAALSEEQEQLENLRFVRHAQDAPLVSSYNYIVIG
GGTAGCPLAATLSEHSRVLLLERGGLPYANMSSEQHFTDALADTSPASPAQRFISEDGVV
NARARVLGGGSCLNAGFYTRASNEYVRASGWDARLVNSSYRWVERSLVFRPDVPPWQAAL
RDALLEVGVTPDNGFTFDHVTGTKIGGTIFDNSGQRHTAADFLRHARPRGLTVLLYATVS
RILFKSQDGVPYPVAYGVVFSDPLGVQHRVYLRDGDKNEVIVSAGTLGSPQLLMLSGVGP
QAHLEAHGIEVIVDQPMVGQGVADNPMNSVFIPSPVPVELSLVQVVGITRSGSFIEGVSG
SEFGMPVSDGALRWARSFGMLSPQTGQLGTLPPKQRTPEALQRAAEAMMRLDRRAFRGGF
ILEKILGPVSSGHVELRTTDPRANPSVTFNYFREAEDLERCVHGIETIERVIQSRAFSNF
TYANASVESIFTDSANFPVNLLPRHVNDSRSPEQYCMDTVMTIWHYHGGCHVGAVVDDDY
RVFGVQGLRVIDSSTFKYSPGTNPQATVMMLGRYMGVKIQSERWKK*
>11669.m00197|LOC_Os03g02700.1|genepair875-2
MAPGHRHGSVPPVVLLLLVATVLGSLCLSLTASPEAQKGYNFRFARHARDAPLVSYYNYI
VVGGGTAGCPLAATLSERSRVLLLERGGLPYGNRNVSSEYHFADALADTSPRSPAQRFVS
EDGVVNARARVLGGGSCLNAGFYTRASSGYVRAAGWDPRLVNASYRWVERELVFRPDVPR
WQCALREGLLQAGVTPDNGYTLEHVQGTKIGGTIFDRAGRRHTAADFLRRAHPRRLTVFL
RATGTATPVAYGVVFTDPAGVRHHVYLRGGAKSEVIVTAGTLGSPQLLMLSGVGPRGELE
KHGILPVLDQPRVGQGVADNPMNSVFVPSPVPVALSLVQIVGVSRFGTFIEGVSGSQFGI
PLHGRAASRRARSFGMFSPMTGQLGTVPPKERTPEAMRRAAEAMRRLDRRAFRGGFILEK
ILGPMSTGHVALRSADPDANPAVTFNYFRDPRDVERCVRGIETIERVVRSRAFARFTYAN
VTAMEAAVLGRRAGHLPVNLLPRRATDTRPLQQYCRETVMTIWHYHGGCHVGAVVDQDYR
VLGVRGLRVVDSSTFKYSPGTNPQATVMMLGRYMGLKIQKERWTRNDETH*
>11676.m03448|LOC_Os10g38060.1|genepair876-1
MEGGNHGGGGYPYPPPQQYPYPYGQYPYQYPPPQQQPPPPSAYLSPSRSFHGYPSAPPPQ
PQPQPYAHHSAPLQPYPPPPQHHAYPPPQPHPPSPYVVYDPYHAPAAAYPSYPSPNPSPSI
SPSSSFHHHPEPPSPSPSAPSYPSIADGLANMHVSDRHDYPPPPSPAAVPAASSPSVLPP
SASFPGGGSSHGGGGMQMVPYGPPAGGSQHGGMQMVAYGSPAGGSQHGSVRPSLKVVLLH
GTLDVWVYDARNLPNKDLFSKRVGDLLGPRLIGAVGSKMSSANMTSDPYVTIQVSYATVA

RTYVVPNNENPVWTQNFLVPVGHDAAEVEFVVKDNDVFGAQLIGTVSIPAEKLLFGERIN
GIYDVLESNGKPCAQGAVLRLSIQYIPVAQLKMYHHGVIAGPDSLGVPNTYFPMRRGNRV
TLYQDAHVPDGCLPDFCLDHGMRYQHGQCWRDIYDAICQARRLIYIVGWSVFHTIHLIRE
GVEKMPSLGELLKMKSQEGVRVLLLVWDDPTSRSILGIKTDGFMGTRDEETRRFFKHSSV
QVLLCPRSAGKRHSWVKQQETGTIFTHHQKTVILDADAGNHKRKIIAFVGGLDLCGGRYD
TPSHPLFRSLQTVHKEDYYNPNFATVDARGPREPWHDLHSKIDGPAAYDVLQNFQERWLK
ASKRHGIKKLGKSYDDALLSIERIPDFISINDAIYFSDNDPETWHVQVFRSIDSNSAKGF
PKDPREATRKNLVCGKNVLIDMSIHTAYVNAIRGAQHFIYIENQYFIGSSFNWDSNKDIG
ANNLIPIEIALKIANKIKAKERFSAYIVIPMWPEGNPTGAPTQRILYWQHKTMQMMYETI
YRALKEEGLDDLYEPQDYLNFFCLGNREVADSPSTSNSTSTPQEQARKHRRFMVYVHSKG
MIVDDEYVIIGSANINQRSMEGIRDTEIAMGAYQPQYTWASKVSAPRGQIYGYRMSLWAE
HIGVVEEGFNYPETMECMRRVRQIGEQNWERFVDNEVTEMRGHLMKYPVSVDRKGKVKPL
PGCTSFPDMGGNICGSFRAIQENLTI*
>11669.m00201|LOC_Os03g02740.1|genepair876-2
MDVVWPHGHGVWGSSSASRRSAMLHGSLDIWIHEARNLPNMDIVSKTVVDILGTKKKKKA
ANGAMTSDPYVTVQLASATVARTYVVNDDENPVWAQHFLIPVAHEAPAVHFLVKDSDVFG
AELIGEVVVPAEQLEAGEHVEGVYPVLDPAAGKPCAPGAVLRLSVQYIPVARLTMYHHGV
TPGPDFAGVPNTYFPLRRGGRVTLYQDAHVPEGSLPEIRLGNGALYRQGQCWHDVYDAIS
QARRLIYITGWSVFHTIQLVRDGGAGVSLGDLLKRKSQEGVRVLLLVWDDPTSRNVLGIQ
MEGYMGTRDEETRRFFKHSSVQILLCPRSAGKRHSWVKQQETGTIFTHHQKTVILDADAG
NHKRKIVAFVGGLDLCGGRYDTPTHPLFRSLQTLHKDDYYNPNFAVLDAQGPREPWHDLH
SKIDGPAAYDVLTNFEERWLKASKRSGVKKLSKANNDTLLWIGRIPDIASIDDEVYSSDN
DPERWDVQIFRSIDSNSVKGFPKNPREATSKNLVCGKNVLIDMSVQTAYVNAIRGAQHFI
YIENQYFLGSSFNWDSHKDVGANNLIPIEIALKIANKIYANERFSAYIVIPMWPEGNPTG
APTQRILYWQKKTMQMMYEVIHKALKEVGLDNTYEPQDYLNFFCLGNREAGGSPSTCRGS
SSANNPQDQAKKNRRFMVYVHSKGMIVDDEYVIIGSANINQRSMEGTRDTEIAMGAYQPQ
YTWANMLSAPRGQIYGYRMSLWAEHIGAVEESFSCPESLECTRQVRHIGEQNWRQFASSE
VSEMRGHLVKYPVSVARDGKVKPLPGCAAFPDLGGNICGTFLPIQENLTI*
>11676.m03451|LOC_Os10g38080.1|genepair877-1
MEWSSMRWWCLAVVLLLSTPLLAAGYLQERKNYIVHLEPRDEAAAAAGDASVEEWHRSFL
PQVAKLDSDSDGADGGPRIVYSYSDVFTGFAARLTDEEAEAVRATAGCLRLYPEEFLPLA
TTRSPGFLGLHLGNEAFWSHSGFGRGVVIGILDTGILPSHPSFGDDGLQPPPKNWKGTCE
FKAIAGGGCNNKIIGARAFGSAAVNSSAPPVDDAGHGTHTASTAAGNFVENANVRGNADG
TASGMAPHAHLAIYKVCTRSRCSIMDIIAGLDAAVKDGVDVLSFSIGASSGTQFNYDPIA
IAGFKAMERGIVVSCAAGNSGPDPGTVGNGAPWMLTVAAGTMDRAIRTTVRLGNGDEFDG
ESLFQPGNNSAANPLPLVYPGADGSDTSRDCSVLRDAEVTGKVVLCESRGLNGRIEAGQT
VAAYGGAGIIVMNRAAEGYTTFADAHVLPASHVSFDAGTKIAAYVNSTDNPTASIAFKGT
VIGSSPSPAVTFFSSRGPSKASPGILKPDITGPGMNILAAWAPSESHTEFSDGVGLSFFV
ESGTSMSTPHLSGIAALLKSLHPDWSPAAIKSAIMTTSDAVDRTGVPIKDEQYRHATFYA
MGAGYVNPALAFDPGLVYDLHADDYIPYLCGLGIGDDGVKEIAHRPVTCSDVKTITEAEL
NYPSLVVNLLAQPITVNRTVTNVGKPSSVYTAVVDMPKDVSVIVQPPMLRFTELKEMQSF
TVTVRWAGQPNVAGAEGNLKWVSDEHIVRSPIIIPATAA*
>11669.m00202|LOC_Os03g02750.1|genepair877-2
MRTQRILLAVVVSMVVSSAMLAVVSCARERKNYVVHLDPREDGGVADSVELWHRSFLPEA
TPEAAGDDGPRIIYSYSHVLSGFAAQLTDDEAEAMRKKEGCIRLYPEEFLPLATTHSPGF
LGLHLGNDGFWSRSGFGRGVVIGLLDTGILPSHPSFGDAGMPPPPKKWKGTCEFKAISGG
GCNNKIIGARAFGSAAVNATAPPVDDAGHGTHTASTAAGNFVENADVRGNAHGTASGMAP
HAHLAIYKVCTRSRCSIMDIIAGLDAAVKDGVDVLSFSIGASPGAPFNYDLVAIATFKAM
EHGIFVSSAAGNDGPVAATVGNGAPWMLTVAAGTMDRAIRTTVTLGNGQVFDGESLYQPR
NNTAGRQLPLVFPGLNGDSDSRDCSTLVEEEVSGKVVLCESRSIVEHVEQGQTVSAYGGA
GMILMNKPVEGYTTFADAHVLPASHVSYAAGSKILSYIKSTPKPTASVTFKGTVMGSSPA
PSVAFFSSRGPNKASPGVLKPDITGPGMNILAAWAPGEMHTEFADGVSLSFFMESGTSMS
TPHLSGIAAIIKSLHPTWSPAAIKSAIMTSSDVADHDGVPIKDEQYRSASFYTMGAGYVN
PSRAVDPGLVYDLHTNDYIAYLCGLGIGDDGVKEITHRRVSCAKLKAITEAELNYPSLVV
KLLSQPITVHRIVTNVGKANSVYTAVVDMPKNVAVTVHPPLLRFSRAYEKQSFTVTVRWA
GQPAVAGVEGNLKWVSDEHVVRSPIVIPPAKAVV*
>11676.m03592|LOC_Os10g39420.1|genepair878-1
MGNSCQNGTYGNNYQNSNRFQNDRFASRYVDGNDTEDCYSGSSRASLAGALRQGLNLKSP
VLGYKTPNVRELYTLGRELGQGQFGKTYLCTEISTGCQYACKTILKSNLRCVSDIEDVRR
EIQIMHHLSGQKNIVTIKDTYEDEQAVHIVMELCAGGELFSKIQKRGHYSERKAAELIKI
IVGIIETCHSHGVMHRDLKPENFLLLDADDEFSVKAIDFGLSVFFRPGQVFREVVGSPYY
IAPEVLEKRYGPEADIWTAGVILYVLLTGVPPFWADTQSGIYEKVLDGRIDFKSNRWPRI
SDSAKDLIKKMLCPYPSERLKAHEVLKHPWICDNGVATNRALDPSVLPRLKQFSAMNRLK
KLSLQIIAERLSEEEIVGLREMFKAMDTKNRSVVTFGELKGLKRYSSVFKDTEINDLMEA
ADDTTSTINWEEFIAAAVSLNKIEREKHLMAAFTYFDKDGSGFITVDKLQKACMERNMED
TFLEEMILEVDQNNDGQIDYAEFVTMMQSNNFGLGWQTVESSLNVALREAPQVY*
>11669.m00300|LOC_Os03g03660.1|genepair878-2
MGNQCQNGTLGSDYHNRFPREHAVGYVQGDSYLDLKKFDDTWPEVNNFKPTAASILRRGL
DPTSINVLGRKTADLREHYIIGRKLGQGQFGTTYLCTEINTGCEYACKTIPKRKLITKED
VEDVRREIQIMHHLSGHKNVVAIKDVYEDGQAVHIVMELCAGGELFDRIQEKGHYSERKA
AELIRIIVSIVAMCHSLGVMHRDLKPENFLLLDKDDDLSIKAIDFGLSVFFKPGQVFTEL
VGSPYYVAPEVLHKRYGPESDVWSAGVILYVLLSGVPPFWAETQQGIFDAVLKGHIDFQS
DPWPKISDSAKDLIRKMLSHCPSERLKAHEVLRHPWICENGVATDQALDPSVISRLKQFS
AMNKLKKLALRVIAERLSEEEIAGLREMFKAVDTKNRGVITFGELREGLRRFGAEFKDTE

IGDIMEAAHNDNNVTIHYEEFIAATLPLNKIEREEHLLAAFTYFDKDGSGYITVDKLQRA
CGEHNMEDSLLEEIISEVDQNNDGQIDYAEFVAMMQGSNVGLGWQTMESSLNVALRDAPQ
CQAAEATDEPVKVKVKVKCSNQIRRRRRQV*
>11676.m03595|LOC_Os10g39440.2|genepair879-1
MAGAVLVAIAASIGNLLQGWDNATIAGAVLYIKKEFNLQSEPLIEGLIVAMSLIGATIIT
TFSGAVADSFGRRPMLIASAVLYFVSGLVMLWAPNVYVLLLARLIDGFGIGLAVTLVPLY
ISETAPTDIRGLLNTLPQFSGSGGMFLSYCMVFGMSLMPQPDWRIMLGVLSIPSLIYFAL
TIFYLPESPRWLVSKGRMAEAKRVLQGLRGREDVSGEMALLVEGLGVGKDTKIEEYIIGP
DDELADEGLAPDPEKIKLYGPEEGLSWVARPVHGQSALGSALGLISRHGSMVSQGKPLVD
PVVTLFGSVHEKMPEIMGSMRSTLFPNFGSMFSVAEQQQAKGDWDAESQREGEDYGSDHG
GDDIEDSLQSPLISRQATSVEGKEIAAPHGSIMGAVGRSSSLMQGGEAVSSMGIGGGWQL
AWKWTEREGADGEKEGGFQRIYLHEEGVTGDRRGSILSLPGGDVPPGGEFVQAAALVSQP
ALYSKELMEQRLAGPAMVHPSQAVAKGPKWADLFEPGVKHALFVGIGIQILQQFAGINGV
LYYTPQILEQAGVGVLLANIGLSSSSASILISGLTTLLMLPSIGIAMRLMDMSGRRFLLL
ATIPILIVALAILILVNILDVGTMVHASLSTVSVILYFCFFVMGFGPIPNILCAEIFPTT
VRGICIAICALTFWIGDIIVTYTLPVMLNAIGLAGVFGIYAVVCILAFLFVFMKVPETKG
MPLEVITEFFSVGAKQAKED*
>11669.m00302|LOC_Os03g03680.1|genepair879-2
MGYAAADLGAWKNTRQQYSPSSPISLGSSVFSESSLELSTTTADGSTANAVLAAIVASIG
NLLQGWDNAAIAGAIMYIKNEFNLQNDPMMEGLILAMSLIGATIITALSGMITNSIGKRP
LLSVAAILYSISALIMFQASNEYMLLLARLIYGFGSGLVVTYAPLYISETAPTNMRGLLN
TLPQFNGSLGMLLSYIMVFLMSLTLNPNWRIMLGSLSIPSFVFLLLCIFYLPESPVFLVS
KGKIEEAKNVMKRLRGTNEVSSEIAFLIQGLTVDQDNYIEDYMIGHNNDEFDDQSISNTE
TTKLYGHEEGVTWFARPFKGKNVVESDHSPIPNLLDPIVTLFDSIHGNILNTPEFTSSGN
MSNDIEQPKTDLESQEDLDTDYEDDLGHPLLFHQGSYMEGIDDACVNGGWHIAWKFVQRE
NEFGQTQDDFQQIFLQGDILQAGRVSHATALVSTPSFHHSIGPAMVHPSKFNLSTEGQSW
SDLLQPGVKQGLIVGVTIQILQQLAGISGILYYTPQILEQAGAGILLKWFNVSSSSSSIL
TSALTTFTMLPSIGIAMKCMDRYGRRSLLLYTIPMLIVSLIILIVVNVMNLEAIFGAILS
TFGVIIYVCCFVMGFGPIPNVLCSELFPPSCRNRCMSICTLTFWIVSIIVTYAFPVMLSS
IGLIGVCGIYAVVCIVSFIFVLIKVPETKGMPLAVIANSLAVGARLSVKRNENI*
>11676.m03603|LOC_Os10g39520.1|genepair880-1
MTLGFISLLLSVTGRYISRICIPVGAADTMLPCSLRRSSSEQEVPGGGGHGRRHLSGDPT
NFKCAKGMVSLVSADGLHQLHIFVFFLAVFHVAFSAITMSLGRAKTRIWKEWEKETCSLT
YEFSYDPSKFRLTHQTSFVRQHASCWSKSTILLYFVSFFRQFFRSVRRTDYLTLRHGFIA
AHLSLGTRFNFRKYIKRSLEDDFKTVVGISAPLWASALAIMLFNVHGWHNLFWFSTIPLV
VTLAVGTKLQAIIAMMAVEIKERHTVIQGMPVVKLSDEHFWFGKPRLVLHLIHFASFQNA
FEITYFFWIWYEFGLRSCFHDNFELIIARVCLGVVVQFMCSYITLPLYALVSQMGSQMKR
TIFDEQTAKALKKWHKAAVVKKKQQKGSSHEPGSETPGTETTTTTATATEESQRERDAAA
MPVRHLHRYKTIAHVGATGTLSDSDCSDTDTPFASPTRLLIPPTKQRSLDAGRAEVRVDV
DSTPTPTPPERHDSFSFPRLPAHNLQQK*
>11669.m00304|LOC_Os03g03700.1|genepair880-2
MAGGGGGRALPETPTWAVAVVCAVIVLVSVAMEHGLHKLGHWFHKREKKAMGEALEKIKA
ELMLLGFISLLLTVAQTPISKICIPESAANIMLPCKAGQDIVKGLKGKKDHRRRLLWYTG
EEESHRRSLAGAAGEDYCAQSGKVALMSSGGMHQLHIFIFVLAVFHVTYCVITMALGRLK
MKKWKKWELETNSLEYQFANDPSRFRFTHQTSFVKRHLGLSSTPGLRWIVAFFRQFFGSV
TKVDYLTMRQGFINAHLSQNSKFDFHKYIKRSLEDDFKVVVGISLPLWFVAILVLFLDIQ
GFGTLIWISFVPLVILMLVGTKLEMVIMEMAQEIQDRATVIKGAPVVEPSNKYFWFNRPD
WVLFFIHLILFQNAFQMAHFVWTLATPGLKKCFHENMGLSIMKVVVGIFIQFLCSYSTFP
LYALVTQVKPFRQKSLVNALGGLNSNVLNLSVCLQMGSNMKKTIFEEQTMKALMNWRKTA
REKKKLRDADEFLAQMSGDTTPSRGSSPVHLLHKQRVRSEDPPSAPASPGFAGEARDMYP
VPVAPVVRPHGFNRMDPDKRRAASSSAIQVDIADSDFSFSVQR*
>11676.m03613|LOC_Os10g39620.1|genepair881-1
MGGGGGEAGGGDGGESSPAAAAAAAVAGAAALHIRCANGSKFTVRADLDATVGAFKEVVA
GSCDVPAAQQRLIYKGRILKDEQTLESYGVETDHTIHMVRGAGPPAGSAAPAAASPQASA
APSSGPTDGLGSLFPGLGGTGTAGTRPSGLFGSGFPELDQMQQQLSQNPNLMREIMNMPM
MQNLMNNPDLIRNMIMNNPQMRDIIDRNPDLAHVLNDPSVLRQTLEAARNPEIMREMMRN
TDRAMSNIESSPEGFNMLRRMYETVQEPFLNATTMGGEGNTAPNPFSALLGNQGSNQPRD
PATNAPNTGSESTTGTPAPNTNPLPNPWSSNAGGAQGATRAGSTGNARTGATGGLGGLGS
ADLSSLFGGLAGNTGTGATGGLGGLGSADLGSLLGGSPDSSSLSQILQNPVMMQMMQNIM
SDPQSMNQLLNFNPNTRNLMESNTQLREMFQNPEFIRQLTSPETMQQLLSFQQTLLSQLG
QNQPRQDGSQGGNATGMRGNVSLDTLMGMLSGLGAGGGIGVPNTSNVPPEELYATQLTQL
REMGFIDTAENIQALVATAGNVNAAVERLLGNLGQ*
>11669.m00326|LOC_Os03g03920.1|genepair881-2
MSGGDGEEAAAAAAAATLHIRCTNGSKLAVRADLGLSVGAFKAIVAESCDVPAPQQRLIY
KGRILKDEQTLASYGVETDHTIHMVRGAAPPPASTAPPAANNVTPAINATTASNSPAVGF
GGLLHGLGGSGSANSGGLGSFGSGLPELSQMQQQLSENPTLMREIMNMPLMQNILNSPDL
IRNIIMNNPQMREIVDRNPDLAHVLNDPSILRQTVEAARNPELMREMMRNTDRAMSNIES
SPEGFNMLRRMYETVQEPFLNATTMAGEGDRSSNPFSALLGNHGSNQARDPAANSPTTTS
ESTTGSPAPNTNPLPNPWSTTAGAAQGATRPSPVTNARSATAGGLGGLSSTDLGGMLGGG
SDTSFLSQVLQNPTMMQMMQNIMSNPQSMNQLLNINPNVRNMMESNTQLREMFQNPEFVR
QLTSPETLQQLISFQQSLMSQLGQQQAGPERTQSGAGAGNTNLNNLMSMFSGLGAGGGLG
VPSAPNVPPEELYATQLAQLQEMGFFDTQENIRALIATAGNVHAAVERLLGNIG*
>11676.m03619|LOC_Os10g39640.1|genepair882-1
MAVRCCSSMASASVVLFFVVVGMSASMVSGCDRCVRRSKAGFRDSSIALNAGSCGYGSLA

ASFNGGHLAAASPALFRGGVGCGACFQVRCKDGKLCSTAGAKVVVTDEARSTNRTDLVLS
AAAYAAMARPGMAAQLRTRRAVDVEYKRVPCEYAAGRNLSIRVEEKSRPPRELSIRFLYQ
GGQTDIVAVDVATVGSSNWKFMTRDYGPAWSTAQAPAGPLQFRVVVTGGYDGKWVWADGE
VLPRRWTAGRVYDAGVQIADVAQEGCYPCDTQEWK*
>11669.m00339|LOC_Os03g04020.1|genepair882-2
MAVSVRCCFGSSSLSHHARLLLVIVALLAPRLASGCDRCVRRSRAAYYTSSLTLTAGSCG
YGTAAATFNGGGFLAAAGPALYRGGVGCGACYQVRCKDKKLCSNAGARVVVTDRARTNRT
GLVLSSPAFAAMARPGMAASLTELAAVDVEYKRVPCEYRHRSLSVRVDERSRGPNELTIS
FLYQGGQTDIVAVDVAQNLTIVCWELQVGSSSWKFMTREHGPSWSMANAPPGPLQMRLVV
TGGYDGKWVWADREVLPRRWRAGEVYDTGVQITDIAQEGCFPCDTHEWK*
>11676.m03621|LOC_Os10g39660.1|genepair883-1
MAVGFRRTLSTLTSHKAVAPSFLLDCARPKKLSYARVRSTSLPVRLHPLVAGLHDAARAL
LKWADAPAQTGPAWVADGADRAGKVLAGLADLLHHPQAQDALRRPWTEQLLDDLLLLTDL
HGCFRESLVALRQLLAETHAALRRRDGSRLAAALRAQRRSAREVSRLASSARDLSHRAAP
GDDADEATLADAFAAAASSVAAAAAAVFAGVSSASAESAASAAPSPRTPTPYSPARTPAS
PMWLVTDLLRRRRTVSFEDYCNEEEEERKAAMARVRGLEECVAAAESGCEEVYRALVNAR
VSLLNLLTPTF*
>11669.m00341|LOC_Os03g04040.1|genepair883-2
MAAGFRRRLTALTIPKASSYLRRTRHKKLSYSRARSASLPGRFHPVVTGLHESASALLGW
TDEAPAQAGTQWIGEGVGHLARLLAGLTELLHHPQAQDPLRPPRKAAPWTERLLDDLLLL
ADAHGCFREALLALKQLLAEAHAALRRRDATRLAAALRARRRSDRDLSRLASTLRDLSYR
SSSAAATSDSGEAALAEAVGAATCAAAAASASFFAGLASASASSASRSLASPTAASPAKV
AVAPVWWVADLLRWRRRTVSVAACESGAGAKETPLDECIDEEEEERKAAMDRLLRLEECV
VAAEDGCEQVYRALVNARVSLLNVLTPCF*
>11676.m03622|LOC_Os10g39670.1|genepair884-1
MANLIGYCCDGDERLLVAEFMPNDTLAKHLFHWENKAIEWAMRLRVAYNIAEALEYCSNE
ERPLYHDLNAYRVLFDENGDPRLSCFGLMKNSRDGKSYSTNLAYTPPEYLRNGRVTLESV
VFSFGTILIDLLSGKRIPPTLALDMIRSRSIQAIMETNLEGKYSIEEATTLVDLASKCLQ
YEPRDRPDIKKLVSILQPLQTKSEVPSYVMLGVPKPEEVPKAPPAPQHPLSPMGEACSRM
DLTAIHQILVSTHYRDDEGTNELSFQEWTQQMRDMLDARKRGDFAFRDKNFKQAIDCYTQ
FVDVGTMVSPTVYARRSLCHLMCDQPDAALRDAMQAQCVYPDWPTAFYMQAVALSKLNMQ
SDSLDMLNEASQLEEKRQKSIKGP*
>11669.m00342|LOC_Os03g04050.1|genepair884-2
MGCCGSSLQAGTHPEKPPGMAAPPQRPSFSLNQHQAPGSAAAQGVGRGEVPAFAEFSLAE
LRAATGGFAAENIVSESGEKAPNFVYRGRLQRTRRAIAVKKFPKMAWPDPKQFEEEAKGV
GKLRHRRLANLIGYCCDGDERLLVAEFMPNDTLAKHVFHWENQTIEWAMRLRVAHHIAEA
LDYCSSNERPLYHDLNAYRVLFDENGDPRLSCFGLMKNSRDGKSYSTNLAYTPPEYLRNG
RVTPESVIFSFGTVLLDLLSGKRIPPSHALDMIRGKNIQVLLDSHLEGKYSTEEATALVD
LASQCLQYEPRDRPNTGKLVSILDPLQTKLEVPSYEMLGIPKHEEEAPPAPAPAPAPQPQ
HPLSPMGEACSRMDMTAIHQILVATHYRDDEGTNELSFQEWTQQMRDMLDARKRGDFAFR
DKDFKTAIECYTQFVDVGTMVSPTVYARRSLCHLMSDQPDAALRDAMQAQCVYPDWPTAF
YMQAVALSKLNMQSDAMDMLNEASQLEEKRQERLWSKDASAQSPLRLKGLC*
>11676.m03623|LOC_Os10g39680.1|genepair885-1
MTTTTTRFVQLAACAAASLLAVAASGAAAQGVGSVITQAVFNSMLPNRDNSQCPARGFYT
YDAFIAAANSFPAFGTSGGSAELIRRELAAFFGQTSHETTGGTRGSSDQFQWGYCFKEEI
NKATSPPYYGRGPIQLTGQSNYQAAGNALGLDLVGNPDLVSTDAVVSFKTAIWFWMTAQG
NKPSCHDVILGRWTPSAADTAAGRVPGYGVITNIINGGIECGVGQNDANVDRIGYYKRYC
DMLGAGYGSNLDCYNQRNFAS*
>11669.m00343|LOC_Os03g04060.1|genepair885-2
MRRLLPLAGATLLIAAAGGASGQQAGVGSIITRAMFESMLSHRGDQGCQGAFYTYDAFIK
AAGDFPRFGTTGNDETRRRELAAFFGQTSHETTGGWATAPDGPFAWGYCRVNEITPSDPP
YYGRGPIQLTHKYNYQLAGDALGLDLVNNPDLVSSDPVVAFRTAIWFWMTAQSPKPSCHD
VITNQWTPSGDDRSSGRLPGYGMATNIINGGEECGKGYSTDNAKDRVGYYKRYCDMFRVG
YGDNIACRDQKPYGGG*
>11676.m03629|LOC_Os10g39740.1|genepair886-1
MPGAVKVFGSPSSAEVARVLACLFEKDVEFQLIRVDSFRGSKRMPQYLKLQPHGEALTFE
DGNVTLVESRKIIRHIADKYKNQGNPDLIGMGALERSSIEQWLQTEAQSFDVPSADVVYS
LAYLPAATTQPNKGAAAADGGRCEEEKNDDGGRDRQYSSQRQGGAGAGGGRDGQMAAAHR
QKVEEMKQLFEKSSKELSKVLDIYEQRLEEAEYLAGDKFTLADLSHLPNADRLAADPRTL
RMLQSRRNVSRWWADVSGRESWKQVKSLNRPPSAEAPF*
>11669.m00359|LOC_Os03g04220.1|genepair886-2
MAPASVKVFGSPTSAEVARVLMCLFEKDVEFQLVRVDAYRGTQRMPQYLKLQPLGEALTF
EDDNLTLSESRGILRHIAHKYARQGNPDLIGTGALERASIEQWLQTEAQSFDVPSAEMVY
SLAFLPPNMPKQNDNGNGNGNGYGNSNGREVQVANASSKRVVAGATDGKTAASGANGNKQ
QQKEEEMRKVFEKSKKDLEKLLDIYEQRLEEAAYLAGDKFTIADLSHLPNADRLASDPRS
RRMFEARKNVSRWWNNISSRESWEYVKSLQRPPSAAHAGNAQQQQQQQSPSAGNNYQHQQ
GQGQGQQHYRNEQVENYNN*
>11676.m03630|LOC_Os10g39750.1|genepair887-1
MELMDDDGSSSLLEELMAPLRRGTPTTTPEDLWLQAYPMMMSPMCGDGVMLGDLLVGGGN
ARNTLASPPPPSFPLPVPLTTTTPCPPLHEVSFEFDSIDCLGEVCNPYKRSGGAVRATAA
AQVMVAAMDPRREAASSAVAVAAVEEEERCKARRGAGGGGDSGELAPMFVFGGGGGAAAS
VRPRSCRPPQPGAPSKNLMAERRRRKRLNDRLSMLRSVVPRISKMDRTSILGDTIGYVKE
LMDRIKNLQVEAATGDSSSSSTENLSMLKLNTLKPPPSSSSGEETPLIRNSTRFEVERRE
NGSTRIEMACAAIPELLPSTLAALEALGVEIEQCVISCFDDFAMQASCLQDDKKREMTRD

TEEIKQTLFRSAGYGDGCLI*
>11669.m00368|LOC_Os03g04310.1|genepair887-2
MELDEESFLDELMSLRRDGSAPWQAPPYPGGGGGGGGGGMMMSDLLFYGGDGGSAEARGG
MDASPFQELASMAAPPPQHPHEEFNFDCLSEVCNPYRSCGAQLVPSEAASQTQTQLTPLR
DAMVAEEETSGDKALLHGGGGSSSPTFMFGGGAGESSEMMAGIRGVGGGVHPRSKLHGTP
SKNLMAERRRRKRLNDRLSMLRSIVPKISKMDRTSILGDTIDYVKELTERIKTLEEEIGV
TPEELDLLNTMKDSSSGNNNEMLVRNSTKFDVENRGSGNTRIEICCPANPGVLLSTVSAL
EVLGLEIEQCVVSCFSDFGMQASCLQEDGKRQVVSTDEIKQTLFRSAGYGGRCL*
>11676.m03633|LOC_Os10g39780.1|genepair888-1
MLSAAMEYLRSCWGPASSPAGRPRKGSDAAGRQDGLLWYKDAGQLVAGEFSMAVVQANNL
LEDHSQVESGPLSTTDPNLQGTLVGVYDGHGGPETARYINDHLFNHLRGFASEHKCMSAD
VIRKAFRATEEGFFSVVSSQWSMRPQLAAVGSCCLVGVICAGNLYIANLGDSRAVLGRLV
KGTGEVLAMQLSAEHNASFEEVRRELQAAHPDDPHIVVLKHNVWRVKGIIQITRSIGDVY
LKKPEFNREPLHSKFRLQETFRRPLLSSEPAIVVHQLQTTDQFIIFASDGLWEHISNQEA
VDLVQHNPRNGIARRLVKAAMQQAAKKREMRYSDLKKIDRGVRRHFHDDITVVVVFFDSN
AITTANWSRPSVSLRGGGVTLPANSLAPFSVPT*
>11669.m00380|LOC_Os03g04430.1|genepair888-2
MLAAVMDYFRSCWGPRSPAGHRVRGSDVAGRQDGLLWYKDAGQLVTGEFSMAVVQANNLL
EDQSQVESGALSMAEPGPQGTFIGVYDGHGGPETARFINDHMFHHLRRFATEHKCMSTDV
IRKAFQATEEGFLSLVSKQWSLKPQIAAVGSCCLVGVICSGTLYVANLGDSRAVLGRFVK
STGEVVATQLSSEHNACYEEVRQELQASHPDDPQIVVLKHNVWRVKGLIQISRSIGDVYL
KRPEYNREPLHSKFRLRETFKRPILSSEPAIAVHQIQPNDHFVIFASDGLWEHLSNQEAV
DLVQNNPRNGIARRLVKVAMQEAAKKREMRYSDLKKIDRGVRRHFHDDITVIVVFLDSNA
ISKANWSRGPSVSLRGGGVTLPANSLAPFSTPTVLSSTY*
>11676.m03634|LOC_Os10g39790.1|genepair889-1
MFNCAAREKAMVINLEFIRAIVTADEILLLDPLTIDVIPFVEQLTHHLPLKNLVCGNGQP
GGDDHGEKHDDSHGDQVPRLNEATGAEHELPFEFQVLELALETVCSSFDVNVSGLERRAT
PVLEELTKNVSTRNLDRVRTLKSDLTRLLAHVQKVRDEIEHLLDDNEDMAHLYLTRKQLQ
NQQVEALISSAASNSIVPGGTSLSRLNNSFRRSVSIATSMHLDNDVEDLEMLLEAYFMQL
DGIRNRILSVREYIDDTEDYVNIQLDNQRNELIQLQLTLTIASFGIAVNTFIAGAFAMNI
QSKLYSIDDGSFFWPFVGGTSSGCFMICIVLLWYARWKKLLGP*
>11669.m00385|LOC_Os03g04480.1|genepair889-2
MALPCAFLSAAAAANATSFSSSPESRRCRSVHRVPSRPRPPLAPPARVMGKGNSKRKAAN
TRLWMRLDRRGGCEMILCDKSFVARRSGLPARDLRVLSPLLSRSPSILAREKAMVINLEF
VRAIVTADEVLVLEPLAQEVLPFVEKLRKHFPLKSLDVDDVSTHMHTENQDGELAQDVSC
YEVEGANHELPFEFQVLDFALEAVCLSYNSTISDLNRSAIAVLDDLMKSVSTRNLERVWS
LKSSLTRLLASVQKVRDEVEHILDDNEAMAHLCTARKTKGQKDLLNTILFPETRLCRTHS
SIENSTGIRTCVPSDSDAHILDMLLEAYFKQLDGIRNRIFLVRQYIVDTEDYISIQLDNK
RNELLGLQLTLIIASFGIAINTFIAAAFAMNIPHRGYHFVIGVPFGQFVGATSFLCMSIV
ILLFTYAWRNRLLCT*
>11676.m00110|LOC_Os10g02070.1|genepair890-1
MEYSYSYRFMLVCSVLVLCLNTRGARCQLSDDFYDYICPDVYTVVQQHVYAAMRTEMRMG
ASLLRLHFHDCFVNGCDGSILLDGDDGEKFALPNKNSVRGFEVIDAIKEDLENICPEVVS
CADIVALAAGYGVLFSGGPYYDVLLGRRDGLVANQSGADNGLPSPFEPIKSIIQKFNDVG
LDTTDVVVLSGGHTIGRARCTLFSNRLSTTSSSADPTLDATMAANLQSLCAGGDGNETTV
LDITSAYVFDNRYYQNLLNQKGLLSSDQGLFSSDDGIANTKELVETYSADAHKFFWDFGR
SMVKMGNISPLTGDDGQIRKNCRVVN*
>11669.m01288|LOC_Os03g13210.1|genepair890-2
MEYATRGDRTASCLSFLCNIVVLLGLAAAAASGQLTDDYYDYCCPQVYRIVRSRVAAAMK
AEMRMGASLLRLHFHDCFVNGCDASILLDGTNSEKFAAPNNNSVRGYEVIDAIKADLESA
CPGVVSCADIVALAAKYGVLLSGGPDYDVLLGRRDGLVANQTGANSNLPSPFDSISVITA
RFKDVGLNATDVVVLSGAHTIGRSRCLLFSNRLANFSATNSVDPTLDSSLASSLQQVCRG
GADQLAALDVNSADAFDNHYYQNLLANKGLLASDQGLVSSSGDPAVAATKALVQAYSANG
QRFSCDFGNSMVKMGNISPLTGSAGQIRKNCRAVN*
>11676.m00124|LOC_Os10g02210.1|genepair891-1
MRGNLREQKVYHMEGADEERPLIHHLPPQEQCSQYTCDGTVDIDRRPALKHSTGNWRACF
FILGAEFTQCLCFSAVVKNLVRYLTSVLQESNVNAARSVSTWIGTCFFTPLIGAFLADTF
WGRYRTIVICLSVYSIGMLILTTSASLPFLLHDSYNNGDDIRRVVAYLGLYLIALGAGGI
KPCMSALGADQFDGADPVERVTKGSFFNYYYFSNNMGTLLSTTVLVWVQDNIGWGIGFAT
PMLLMGFGLSMFVAGRRVYRYRKLGRSPLTRVSQVVVAAARNHRLKLPDDSSLLHELPSL
TEGGYRIQHTTRFRFLDKAAIPSDSDDNSPVQPDPWRLCTVSQVEELKMLLRVFPVWASL
LVFFVVTAQMSSTLIEQSAAMDGRVGPFTVPPASLATFNVVAVLIWVPVYDAVLVPLARR
ATGNDRGLSHLQRIGVGLALSAVAMAYSAQVERRRRRPAAEEEAMSIMWQAPCYLVLGMA
EVFTSIGMLEFFYERSPGSMKSLGTSLAHLAVATANYLNSGVLGVVVAATTRGGGAGWIP
DNLDEGHLDYFFWMMALVRSDWASAAGQTGGDLMPQESIAGDGSGVNLEIR*
>11669.m01291|LOC_Os03g13240.1|genepair891-2
MEEAAEDRRLQVREEGGDQEPLLLLPQDANLYTGDGSVDIKGRPALKHATGNWRACFFIL
GDECCERLAYYGIAKNLVTYLKTNLHQGNLEAARNVTTWQGTCYLTPLIGALLADSYWGK
YWTIAAFSAIYFIGLVALTLSASVPALQPPKCSGSICPEASLLQYGVFFSGLYMIALGTG
GIKPCVSSFGADQFDDSDPADRVKKGSFFNWFYFCINIGAFVSGTVIVWIQDNSGWGIGF
AIPTIFMALAIASFFVASNMYRFQKPGGSPLTRVCQVVVAAFRKWHTEVPHDTSLLYEVD
GQTSAIEGSRKLEHTSELEFFDKAAIISSDDAKSDSFTNPWRLCTVTQVEELKILIRMFP
IWATTIIFNAVYAHNSSMFIEQGMVLDKRVGSFIVPPASLSTFDVISVIIWIPFYGRVLV
PIARKFTGREKGFSELQRIGIGLALSILAMLSAALVELRRLGIARSEGLIHEDVAVPMSI

LWQIPQYFLVGAAEVFAAIGQVEFFYNEAPDAMRSLCSAFALVTVSLGSYLSSIILTLVS
YFTTQGGDPGWIPDNLNEGHLDRFFSLIAGINFVNLLVFTGCAMRYRYKKA*
>11676.m00140|LOC_Os10g02350.1|genepair892-1
MAAPKLLLGLVMLVGCAAADSHRYRPGDAVPLYANKVGPFHNPSETYRYFDLPFCAPEKV
KDKIEALGEVLNGDRLVDAPYKLDFRVDFDAKSVCSRRLSKDDVVKFRHAVSKDYYFQMY
YDDLPFWGFIGTKPEKADAGDKYYLYRHIIFDILYNKDRVIEINVHTDQNAVVDLTEDKE
LDVEFLYTAKWKETQIPFEKRMEKYSSSSVMPHHLEVHWFSIVNSCVTVLLLTGFLATIL
MRVLKNDFVKYSHDEEEPDDQEETGWKYIHGDVFRFPTNKSLFSAALGTGTQLFALTTFI
FLLALVGVFYPYNRGALFTALVVIYALTSGIAGYSATSFYCQFEGKNWVRNLLLTGCLFC
GPLFLTFCFLNTVAIAYNSTAALPFGTICVIVLIWTLVTFPLLVLGGIAGKNSKNEFQAP
CRTTKFLREVPPLAWYRRTIPQMAMAGFLPFSAIYIELYYIFASIWGHRIYTIYSILFIV
FIILLIVTAFITVALTYFQLTAEDHEWWWRSFLCGGSTGFFVFAYCLYYYRERSDMSGFM
QTSFFFGYMACICYAFFLMLGMVGFRAALLFVRHIYKSIKCQLKQKQQQIEENQEATGFK
AADGSLEEVCRNTDGTIPKLSTEEFSE*
>11669.m01305|LOC_Os03g13380.1|genepair892-2
MYYDDLPLWGFIGKVEKGGKTDPKEWKYYLYRHIIFDILYNNDRVIEINVHTDQSALVDL
TEDKEADVQFLYSVKWKETPTPFEKRMEKYSSSSNLPHHLEVHWFSIINSCVTVLLLTGF
LATILMRVLKNDFVKYAHDEEAADDQEESGWKYIHGDVFRFPKNKSFFSAALGTGTQLFA
LTTFIFLLALVGVFYPYNRGALFTALVVIYALTSGIAGYIATSFYCQLEGTNWVRNLLLT
GCLFCGPLFLTFCFLNTVAIAYSATAALPFGTICVIVLIWTLVTFPLLVLGGIAGKNSKT
EFQAPCRTTKYPREIPPLPWYRQTIPQMAMAGFLPFSAIYIELYYIFASVWGHRIYTIYS
ILFIVFIILLIVTAFITVALTYFQLAAEDHEWWWRSFLCGGSTGFFVYGYCLYYYYARSD
MSGFMQTSFFFGYMACICYAFFLMLGMIGFRAALFFVRHIYKSIKCE*
>11676.m00154|LOC_Os10g02490.1|genepair893-1
MAGGGVGASAAAAATIPEAALRSGKPMPLVGMGTASFPLDAPQLPATVRDAVLRAIDAGY
RHFDTAAAYGTEAPLGEAVLEAVRAGMVASRDDLYITSKLWISDTHPGRVLPALRRTLRN
LQMVYIDLYLIHWPIRLRVEQETPSPVYDNDFVMMDMEGVWKDMEECQRLGLTKAIGVSN
FTCKKLNTLLSFATIPPAANQVEINPYCRQNKLREFCKEKEIQLCAYSPLGASGTIWGSN
AVLDCPVLKHIAVQKGKTVAQVCLRWLHEQGDCIIVKSFNERRMRENLEIFDWELTDADR
QEISALPEFRGNRDFYVHESGPYKTTDEFWDGEITGPQLKTC*
>11669.m01307|LOC_Os03g13390.2|genepair893-2
MSDGGAGAKGAGFGMPRVGMGTAVQGPRPEPIRRAVLKAIEAGYRHFDTAAHYETEAPIG
EAAAEAVRSGAIASRADLFITSKLWCSDAHRDRVLPALRQTLWNLQMEYVDLYLVHWPVS
MKPGRYKAPFTADDFVPFDMRAVWEAMEECHRLGLAKAIGVCNFSCKKLDTLLSFATIPP
AVNQVEVNPVWQQRKLRELCREKGVQICAYSPLGASGTHWGSDSVMASAVLRDIAQSKGK
TVAQARVPEVGVRARGLPDREELRRGADAGEPGHRRMGADGGGEAEDRRHPAAEDQPCPP
LRLRPRALQVARRPLGRRDMSTRRRPLRVSRRRPTCVLL*
>11676.m00182|LOC_Os10g02750.1|genepair894-1
MAVALALLAAMSALSSCTSPATAELTRHEHPVAAGAPLRLLVVGDWGRKGGYNQTRVAEQ
MGKVAEETEIDFVVSTGDNFLENGLAGVDDMAFHDSFMDVYTAQSLHKPWYLVLGNHDYR
GNVLAQIDPALRKIDSRFICMRSFIVSAGIVDFFFVDTTPFQLQYWTDPGEDHYDWRGVA
PRDAYIANLLEDVDAAMKKSTATWKIAVGHHTMRSVSAHGDTQELLELLLPVLKENGVDF
YINGHDHCLEHISSRNSPIQYFTSGGGSKAWRGIFQQNEDKLQFFYDGQGFLSLELSENR
ARFAFYDVFGEALYHWSFSKANLQKVQSSASVTEE*
>11669.m01324|LOC_Os03g13540.1|genepair894-2
MARRSSSRACAAMATAAVFALLAATATASGLVRVEHPAKSDGSLSLLVVGDWGRKGTYNQ
SRVAEQMGKVGEKLNIDFVISTGDNFYEDGLTGVDDQAFEESFTDIYTAKSLQKPWYLVL
GNHDYRGDVLAQLSPVLRKIDQRFICMRSFIVNAEIVDFFFIDTTPFQLKYWTRPKDHHY
DWRGVAPRQKYITNLLKDMDEAMKKSTAKWKIAVGHHTIRSVSDHGDTKELLQLLLPVLK
VNGIDFYINGHDHCLEHISSRDSPIQYFTSGGGSKAWRGVLQPNSDKLQFFYDGQGFMSL
QINQDQADFIFYDVSGNILYKWSKSKANYLQPSTYITEA*
>11676.m00184|LOC_Os10g02760.2|genepair895-1
MAAPGPGWVVPPPPQPQPQQQQQAVVVDGRDGLVAWLRGEFAAANAIIDLLLAHARDAAD
PAGFDAVAAAVQRRRHHWAPVLHLQHYFPVTEVALALHHAAARQGPPPPPPPPRPPSGSA
GAEGDDAAIASGGVKEVETSAEATQNSQLVSHISHATEAQPQKGLHVISNVVPVPTCFVV
NEVIDGRMVNVLEGLKLYKGYVDLTEIGKVLSFVNEAKTMRRKPGLEAGQTVVVAKRPMK
GHGREIIQLGLPITEGPPEDEHLREVKVDPIPGVLQNLFDSLVHQKVVPSSPDYCVIDIF
NEGDYSHPHHHPPWYGRPICTLCLTDCDMVFGHVIAADSRGDHAGPLKLSLSTGSVLVFE
GKSADIAKRALPATSKQRILLSFGKSVSRKHVQSESSLLITPPLTPPPMPWAAPLRPGNI
AIHPSSPKQLVYNPSNRVPAVSTPGLHHIPSNGIQTVFVAPLPITPKAVPFASAVTLPNS
TAAWIAEAAPRPASPRLPLQGTGVFLPPGSGNPPPAQKLGVKHADAKPFFPQESSASSSG
VSARAHKANGSVSSKPTRKDDMAEAKPKCNGSSDGGSSVAHAKATGGLEEQNVVAK*
>11669.m01326|LOC_Os03g13560.1|genepair895-2
MASAAVIPSSAPGAAAAGAAAVESAGWVVDERDGFISWLRGEFAAANAIIDLLLLHLRSV
GEPGEFEHVAAAVQQRRHHWAPVIHMQQFFPVGDVAYALQQAGWRRRAPPHHQQQGPGAS
PSPPPPPPRGRPSFSASHSHHRHGGHHHRSDSVRGGGTGATAGSDKDGREVHNKEEKGMK
EAENVVEAKSSQLESLVSHEGEKTPRPQAVAEGSSKVVPTPVEYTVNDIIDGKTVNAVEG
LKVYEGLVNENEKNKILSLLNETKASFRRGGLEAGQTVIIGKRPMKGHGREIIQLGIPIV
EGPPEDDYPRETKVEAVPGLLHDLFDRLCQKEIIPTKPDYCVIDYYNEGDYSHPHQSPPW
YGRPFCTFCLTDCDMVFGRVISGERGDHRGPLKLSLSTGSLLVLHGKSADVAKRAIPAAC
KQRILLSFGKSLSRKQVPSESVSRFTTPLTPPPMPWGPPRPANMARHSSSPKHFGYAPNS
GVLPAPAIGAHHIPPSDGMQPLFVAPAPVAAAAMPFPSPVPLPNSTTAWMAEAAPRSAPQ
RLPVPGTGVFLPPGSGHALPHQMMTASQSPAEPISSTDSSAYVHNKSTSTMGEMANGDVS
PKSSPAKQSDAVEQKPECNGSSNGGSSLVDKKSAVSKEQQNGGMKKVGSNKVQPNAAK*

>11676.m00185|LOC_Os10g02770.1|genepair896-1
MAASRCFLLLLLLLLSPLLASAGEEEEEAVLAMAARLRRPAAASFREGYTQLFGDSNLAL
HGDGKRVRISLDERTGAGFASQDAYLHGFFSASIKLPPDYAAGVVVAFYMSNGDVYEKTH
DELDFEFLGNIKGREWRVQTNVYGNGSTSVGREERFYIDETPIREVQRTKSMGVQFPSKP
MSLYATIWDGSSWATSGGRYKVNYKYAPFVAEFSELMLHGCAMDTLTRAPMCTPDIANIH
NAVAMSGRQRSAMERFRTKYMTYGYCYDRLRYPTPPSECNVGPEAELFLPTGEARSIDRH
GRARRHRRGPADSAF*
>11669.m01327|LOC_Os03g13570.1|genepair896-2
MAMARCSLLPILAAVLLAASLSLPPRAAAYAAMVDSLLPASATALSFEEGYTQLFGDSNL
MLHGDGKRVHISLDERTGAGFASQGAYHHGFFSASIKLPADHTAGVVVAFYMSNGDVYER
THDELDFEFLGNVRGREWRVQTNVYGNGSTAAGREERYGLWFDPTQDFHRYAIRWSHDTI
IFYVDETPIREVVRTASMGAQFPSKPMSLYATIWDGSSWATSGGRYKVNYKYAPYVAEFT
DLLLHGCPAGSPPPCEGAAASATMPPGQRSAMERFRARHMTYGYCYDRVRYHAPLPECSV
GAEAEAFLPSGEARSTDRRGGRHGKRHRRAGGGVDSAL*
>11676.m00649|LOC_Os10g07290.1|genepair897-1
MPPLILVMAALLLPAAADATSSVLLGINYGRVGNNLPPANAVPPMLSSLGVGRVRLYDAD
PATLRAFANTGVELVVGVPDECLAAVSTPSGAASWVRSVVQPALPATKIAVLTVGNEVLT
GANSSSLSRSLLPAMQCLHDALAQLGLDKQVAVTTAHNLGVLATSYPPSSAYFRKDLLPL
LCPILDFHARTGSPFLVNAYPYFAYAEDPTGVELEYALLEPTYAGVADPSSGLHYPNLLV
AQVDAVYHAIAAANTAAARAVEVRVSETGWPSAGDANETGATPQNAARYNGNVMRLVADG
KGTPLRPSVALRAYMFALFNENMKPGPTSERNYGLFKPDGTPVYELSYRLPKDNTNSGGG
GGGIGGGSGTIGGGGEYNGHGEDGGYYSISASAKPAGWWTWWTQAAVAACVAVLMRMMAL
*
>11669.m01401|LOC_Os03g14210.1|genepair897-2
MAPPSCLLRVGWLPAALCALALALAPASDATSASLVGINYGRVGSNLPPPQAVLPLLEGL
GIGRVRLYDADPAVLHAFAKTGVELFVGVPDQSLAGLADPGGADSWLRSNVMPFLPDTKI
AALTVGNEVLTGNNSAVTRALLPAMQSLHGALAKLGLDKQIAVTTAHNLGVLGTSYPPSS
GAFRRDLLPYICPILDYHARTGSPFLVNAYPYFAYSGDPKGIHLEYALLEAGYAGVPDPN
SGLRYPNLLVAQVDAVYHAIAAANTAAAQVVEVRISETGWPSSGDPGETAATPQNAARYN
SNAMRLVAEGKGTPLKPTVAMRAYVFALFNENLKPGLASERNYGLFKPDGTPVYELSYKL
PRDNSTFGGAGNSGWRFPGGGGGNVSGGYDNNGVNSGYYDISAASPDSAGPCRWSQAAVA
GAMAVLVVAA*
>11676.m00668|LOC_Os10g07480.1|genepair898-1
MKQVRTVKEESEQKLQDVVFAKTKQWEMMKAELEAKLASFEHELIRAGAENDALSRSLEE
REHLMMKVGGEKEQAESQIEVLKGTIQSGEKEISSLKYELHVLSKEFEIRNEEKNMSVRS
ADVATKQHVEDVKKISKLEAECQRLRGLVRKKLPGPAALAQMKLEVDSWGRDHADNRLRR
SPSRSSNFHHPLSPSPDNSLENLQHMQKENEFLTARLLSMEDETKMLKEALSKRNNELQV
SRNTCAKTAGKLRSMDVHMVSARQYKNPTNSNLDVHHDGALSLNGSNPPSLTSMSEDGVD
DATSCAESWANALISELSHIKKDNGGKGSLTENSNQMVLMDDFLEMERLACLSPEGKECG
SFIDKKKATKVHTTLTTITKRESDRDSWPSSQFPDTPSSSEHLPENSPLSKLHSRISSLL
GSHSPQNNVGKVLEGIRNILRDIKEEAESVSAKKNQPDNMNGLADNGSLTKQSKNLRDVD
HGLRHAILEILDFFQPFKRQLSEVQGKSSHQNTILEKIEKFSTIADKVVLNENALAEMVI
ALAEILAGNSAIKLTLPRDSITETESNNLDYVDKVTLLENKVHHEPIKDSLADICSLAPH
SSSDPDFEGSRDAFVVKTTVQMCSMEEYEQLKSEKRKLEMELAKCNETIECRKLEFSLME
KNMEELTSKLSACEKSNSLTETQLKCMAESYKSLELHKLKLESEIEVMHRQINTLRTELA
DERQNHQDDLAKYRDLKEKIERYENEKNTSSVDEDAGVKIKQDKEIAAAAEKLAECQETI
LLLGRQLQTLRPPPAEPLGSVLNQQPVGVFSEDQARTTQGLHFKKLSGQFDTDHTFSSAP
GTGNVSPLNGYRTHKSPSNLDGNTYFASPNNSKRPKHRSRSSSSSSFTNQFTEKQSRGFS
RLFSKSKSEY*
>11669.m01405|LOC_Os03g14250.1|genepair898-2
MDRRSWPWKKKSSDKSSSADASQNSNQAEQDDKAPKYVQISPETYAHLTESEEQVKTLNE
KVKALNEDLSAAQSEITTKDALVKQHAKVAEEAVSGWEKAEAEASALKLQLETVTLAKLA
AEERAAHLDGALKECMKQVRTVKEEGEQKLHDVVFAKTKQWEKIKAEFEAKLLEFEQELI
RAGAENDALSRSLEERGDLLMKIDEEKARAEAEIEVLKNTIQSGEREINSLKYEIHVVSK
ELEIRNEEKNMSVRSADVATKQHMEDVKKISKLEAECQRLRGLVRKKLPGPAALAQMKME
VESLGRDYGESRLRRSPAKNSSFHRPMSPMSPVPDYAFENLQHMQKENEFLTARLLSMED
ETKMLKEALAKRNSELQTSRNMYAKTAGKLRGLEVQMLTGSQRKSTSNPNMDIHFDGALS
QNGSNPPSMTSMSEDGVDDEGSCTESWANALVSELSHIKKEKGAKSSVTEGSNRLELMDD
FLEMEKLACLSSEANGHVSTVEKMKIDDTEASLSGITERDGVKDSQSVLALPGTPSNKLQ
LSDSSPLLKLQSRISSLLDSESPQNNAGNILDSIRNILKDIEDEADSSNDSKTHHGDMVE
VADNGSLMKHSSSGSKHAMDQELVNAILKIQDFVKSLDQEVSKFQGQSSDCDGLCDKIQQ
FSALVEKALSNENVLNDIVMTLSLILSGTSEIKFMMLKENTKEADNNNLDYVDKVTLLEN
KVQLEPLKDSISGPCLPRSSSDPEIEGPTDSGCDVKTAVQICSSEEFEQLKSEKLNLEAE
LSKCNEVIEETKFRFKELEKSLEELTSKLVASEKSNSLAETQLKCMAESYKSLESRKAEL
ENEIKVLQSKIEVLTAELDDERQNHQEDITRYRDLEEKIERYENERNSMCVDEDADTKAK
QEKEIAAAAEKLAECQETILILGRQLQSMRPPAESMGSSPNQRMEDFLQDAAGTTEGVEY
SQKPTGQLDTDQEMHASGNESPVNGYKTHNAPSEADGSPFLSPNGSKRPKHRSRSSSSIS
NQLPEKQNRGFSRFFAKEKI*
>11676.m00671|LOC_Os10g07510.1|genepair899-1
MSSSVVVSASGSGSGGGGGGGGGGAGGGGGGGPCGACKFLRRKCVQGCIFAPYFDSEAGA
AHFAAVHKVFGASNVSKLLQQIPAHRRLDAVVTICYEAQARLRDPVYGCVAHIFHLQHQV
AGLQSELNYLQGHLSTMELPSPPPYVAGPTLAPPQPQPLMPMTAAANFNFSDLPSSSAAN
IPVTADLSTLFDPLPAAQPQWGLYQQQQHHHQQLHHHPYDRMGDGSSSSRGGDDDGSDGG
DLQALARELLDRHGRSSSSSKLEPPPHTQ*

>11669.m01407|LOC_Os03g14270.1|genepair899-2
MSSGGGASSALGGGGGGGGSGGGGGGPSGGGGGGGGPCGACKFLRRKCVSGCIFAPYFDS
EQGAAHFAAVHKVFGASNVSKLLLQIPAHKRPDAVVTICYEAQARLRDPVYGCVAHIFAL
QQQVVNLQAELTYLQAHLATLELPSPPPLMPAPPQMPMPAPFSISDLPSSTSVPTTVDLS
ALFDPPPQPQWASPLQQQQHHHHQHHHHQQQQHQLRQPSYATLARAPSGMTAAAESSGGG
GGGGGGDLQALARELLDRHRSAVKLEQPPPPHSRS*
>11676.m00704|LOC_Os10g08330.1|genepair900-1
MVELLKNTLSMGKACDELAQVVGSRRRIEESEIGQLPYLQAVIKETLRLHPPVPLLPHRA
KMAMQIMGYTIPNGTKILINVWAIGRDKNIWTEPEKFMPERFLDRTIDFRGGDLELIPFG
AGRRICPGMPLAIWMVHVVLASLLIHFKWRLPVEVERNGIDMTEKFGLTLVKAIPLCALA
TPT*
>11669.m01423|LOC_Os03g14400.1|genepair900-2
MTLRLGTVTTVVASSPGAARDILQRHDAAFSARSVPDAARACGHDGFSMGMLPPSSALWR
ALRRVCAAELFAPRSLDAHQRLRRDKVRQLVSHVARLARDGAAVDVGRAAFTASLNLLSS
TIFSADLADFGDARAESSVGDLRDLISEFTIVVGVPNVSDFFPAVAPLDPQRLRRRVARV
FERLQAVFDGHIERRLRDRAAGEPPKNDFLDALLDYRSPEDGRGFDRPTLQFLFTDLFSA
GSDTSAVTVEWAMAQLLQNPPAMAKAREELARVIGSKQEIEESDISQLKYLEAVVKETLR
LHPPAPFLLPHQAETTTQVGGYTVPKGTRVLVNVWAIGRDSKVWSDPDKFMPERFLQSEV
DLRGRDFELIPFGSGRRICPGLPLAVRMVYLMLASLLHRFEWRLLPEVEKNGVDMAEKFG
MILELATPLRAVAIPV*
>11676.m00725|LOC_Os10g08540.1|genepair901-1
MASALFLWLSWLVLSLLSIYLLDLLAHSRRRLPPGPRPLPLIGSLHLLGDQPHRSLAGLA
KTYGPLMSLRLGAVTTVVVSSPDVAREFLQKHDAVFATRSAPDAAGDHTRNSVPWLPPGP
RWRELRKIMATELLATHRLDALHELRQEKVSELVDHVARLARDGAAVDVGRVAFTTSLNL
LSRTIFSRDLTSLDDRGASKEFQQVVTDIMGAAGSPNLSDFFPALAAADLQGWRRRLAGL
FERLHRVFDAEIEHRRRVAGEEHGKVKDDFLRVLLRLAARDDDTAGLDDDTLRSTSVIDN
GIAQLKKPNVTWMAHGWWARLRTLISGIIAQFLPQYCNGGFVSLAFMACALSLLSVYLLD
LLAQSRRRLPPGPHPLPLIGSLHLLGDQPHRSLAGLAKTYGPLMSLRLGAVTTVVVSSPD
VAREFLQKHDAVFATRSAPDASGDHARNSVALLPNSPRWRELRKIMATELFSTSRLDALH
ELRQEKVVELVDHVARLAREGAAVDVGRVAFTTSLNLLSHTIFSRDLTSLDDHGASKEFQ
QVVTDIMGAAGSPNLSDFFPALAAADLQGWRRRLAGLFERLRRVFDAEIEHRRRVVGKEH
GKVKDDFLRVLLRLAARDDDTAGLHDDALQSIFTDLFAAGSDTSSSTVEWAMAELLRNPL
PMAKACDELQRVIGSTRRIEESDIGRLPYLQAVIKETFRLHPPVPFLLPRQATTTIQILG
YTIPKGAKVFINVWAMGRDKDIWPEAEKFMPERFLERATDFKGADFELIPFGAGRRICPG
LPLAVRMVHVVLASLLINFKWRLPVKLLQSKEKDRSDLAYVINAVKDLMGAAAATTATNL
TILPSPRVRKSKCEEAHVMPTTAKLHTPATTKHGLTDYVASCDNEAHIQDNKWPPS*
>11669.m01425|LOC_Os03g14420.1|genepair901-2
MELLLLYAPCVILLVSSLYLLRLFSDARRNLPPGPRPLPLVGNLLELGAKPHRSLARLAE
RHGPLMTLRLGAVTTIVASSPDAARDILQRHDAAFSTRPVPDIVRACGHDRFAMPWLPPS
SPQWRALRKVCSAELFAPRRLDAQQRLRREKARRLVSHVARMAREGAAVDVRRVVFTTLL
NMLSCTLFSADLADLDEGRAGSAGELADTVAEFAGTVGVPNVVDYFPAVAAFDPQRLRRW
LSRVFTRLFAEFDEQIERRMRERDAGEPPKNDFLDVLLDYRTTEDGRQFDRQTLRSRFTD
LFSAGSDTSAVTVEWAMAQLLQSPSSMMKAREELTRVIGSKPEIDESDIDSLEYLQAVVK
ETFRLHPPAPLLLSHRAETDTEIGGYTVPKGATVMVNIWAIGRDSKVWFEPDKFIPERFL
QKEVDFRGRDFELIPFGSGRRICPGLPLAVRMVHLMLASLLHRFEWRLLPEVERNGVNME
EKFGIVMTLATPLQAIATPI*
>11676.m00726|LOC_Os10g08550.1|genepair902-1
MSRIHRNPTLLAPTPSPSSIPPLLEPSRSSPPPPMAATIVSVKARQIFDSRGNPTVEVDV
CCSDGTFARAAVPSGASTGVYEALELRDGGSDYLGKGVSKAVDNVNSVIAPALIGKDPTS
QAELDNFMVQQLDGTKNEWGWCKQKLGANAILAVSLAICKAGAIIKKIPLYQHIANLAGN
KQLVLPVPAFNVINGGSHAGNKLAMQAFMILPTGAASFKEAMKMGVEVYHNLKSVIKKKY
GQDATNVGDEGGFAPNIQENKEGLELLKTAIEKAGYTGKVVIGMDVAASEFYNDKDKTYD
LNFKEENNDGSQKISGDSLKNVYKSFVSEYPIVSIEDPFDQDDWEHYAKMTAEIGEQVQI
VGDDLLVTNPTRVAKAIQEKSCNALLLKVNQIGSVTESIEAVKMSKRAGWGVMTSHRSGE
TEDTFIADLAVGLATLLRIEEELGAAAVYAGAKFRAPVEPY*
>11669.m01428|LOC_Os03g14450.1|genepair902-2
MAATIQSVKARQIFDSRGNPTVEVDICCSDGTFARAAVPSGASTGVYEALELRDGGSDYL
GKGVLKAVDNVNSIIGPALIGKDPTEQTVIDNFMVQQLDGTKNEWGWCKQKLGANAILAV
SLALCKAGAIIKKIPLYQEFMILPTGASSFKEAMKMGVEVYHNLKSVIKKKYGQDATNVG
DEGGFAPNIQENKEGLELLKTAIEKAGYTGKVVIGMDVAASEFYTEDQTYDLNFKEENND
GSQKISGDSLKNVYKSFVSEYPIVSIEDPFDQDDWVHYAKMTEEIGDQVQIVGDDLLVTN
PTFPCWLQYSNQFDLQRVAKAIKDKACNALLLKVNQIGSVTESIEAVKMSKRAGWGVMTS
HRSGETEDTFIADLAVGLSTLLRIEEELGAAAVYAGAKFRAPVEPY*
>11676.m00767|LOC_Os10g08930.1|genepair903-1
MAAAAVYGAGGAMKGGKLGMEEARELQLNRIRITLSSKNVKNLEKVCADLVKGAKDKQLR
VKGPVRIPTKVLHITTRKSPCGEGTNTWDRFEFRIHKRVIDLISSPDVVKQITSITIEPG
VEVEVTIADV*
>11669.m01436|LOC_Os03g14530.1|genepair903-2
MAAAAVYGGMKGGKLGVEEAHELQLNRIRITLSSKNVKNLEKVCADLVKGAKDKQLRVKG
PVRIPTKVLHITTRKSPCGEGTNTWDRFEFRIHKRVIDLISSPDVVKQITSITIEPGVEV
EVTIADV*
>11676.m00784|LOC_Os10g09090.1|genepair904-1
MAATWAATRAATDGPDLPEGRSDGEGGVRPSARAGGLSWPGGPGARRPEEASGGDTARRR
RSRRQRATATQLGGGAEAWQLSSSSARRRGRERGRRGGASPTAIDDDLREELQGVTHAMR

EEVARELAHRVSDGSAGGMPVSVAREAFAAVAGVLWWSMFSEDMDAATTRQLRDVIEEAV
VVAGAPNLSDYFPVIAAADVMGVRRRMDNLVGWVYGIIDVQIDRRRRRRIVCEPRKNDLL
DVAFDMEGEVESEGWVMNQDTMRGMFMDLLVAGSGSTSSTIEWAMAELLQNPKSMIQLPE
ELKGLMGTKTHVAESDISQLPYLQAVIKETLRLHPTVPIAFNKAEATVEIQGYKIPQGTT
VYVNIWAICRRAKIWDDLDKFMPYRFLGRDINFLGTNFEFIPFGAGRRICLGMPLAEGML
HACI*
>11669.m01439|LOC_Os03g14560.1|genepair904-2
MAFFLVACLPWVCFILLSLYVFQLFADARRRLPPGPWPPKPLIGDLLALGKGDQQHRSLA
RLADRYGPVMSLRLGTVLTVVVSTPDAMREIFHKNKDNLAGRPTADAFNAMGHSANSLLG
LEHPGVRWRAIRRFSTAELLAPRRLAALQPLCRDKVRGLVRGVSELAARGEPVHVRRVAL
DMALSLMLSAIYSVDLDPESTAVFRSVVEEAMLLIGTANLSDLFPAIAALDLQGVRRRVA
ELFTITYRQYDEQVARRRPERDAGEAGKNDLLNVVLDMEREWQQKGSVLSHDAMRVLFTD
LYGAGASTTSVLIEWAIADLLQNPESMRKIKEEITNVIGTNAQIQEFDIARLPYLQAVVK
ETLRLRAVAPLVPRRAEATIEVQGFTIPKGTNVILNLWAINRDARAWNDPDKFMPERFIG
NDINYLGQNFQFVPFGVGRRICLGLPLAQKVMYLVLGTLVHQFEWTLPEELKETGIDMTE
KCGMVLCLANPLKVMAKKM*
>11676.m03524|LOC_Os10g38800.1|genepair905-1
MRRSPRAAAAAVLPVLLPLLLLLLNWAAAPVGAATAAETRALLEFKAAVTADPGAVLANW
TLGGDPCRDFGGVSCYPASGAVQRLRLHGEGLEGVLSPSLARLPALESVSLFGNRLSGVI
PASFVGLAATLHKLNLSGNALSGEIPAFLGTFPMLRLLDLSYNAFSGEIPATLFGECPRL
RYVSLAHNALTGRVPPGIGNCVRLAGFDFSYNNLDGELPDKLCAPPEMSYISVRSNSLSG
AIDGKLDGCRSLDLFDVGSNSFSGAAPFGLLALVNITYFNVSSNNFAGEIPSIPTCGDRF
AYLDASRNKLTGSVPETMANCRNLMLLNLGANGQGLTGGIPAALSQLKNLNFLDLSENAL
TGVIPPELGDLSNLAHFNVSFNNLTGSIPSSPLLQQFGPTAFMGNPFLCGPPLDHACPGR
NARRLGVPVIVAIVIAAAILVGICIVSAMNIKAYKNKRRREQQQHDDEEEILVSDSAAIV
SPGSTAITGKLVLFRKNSSASRYEDWEAGTKAVLDRNCLVGVGSVGAVYRASFESGASIA
VKKLETLGRITSQEEFEREMGRLRGLTHPNLVTFHGYYWSPSTQLLLSEFVDNGSTLYDH
LHGSRRRAGPASTGGDGGGLPWERRFRIAVATARALAYLHHDCKPQVLHLNIKSRNILLD
NEHEAKLSDFGLSKLLPEPSNLPGYVAPELASSSMSSRHGGDKCDVFSFGVVLLEMVTGR
KPVSSRHGRQGTVLVVVLRDYVREMVESGTVSGCFDLSMRRFVEAELVQVLKLGLVCTSE
SPSRRPSMAEVVQFLESIRGSS*
>11669.m00291|LOC_Os03g03570.1|genepair905-2
MRQTRSLAAAAAVLVVGVVLHWVGVVDAATAAERGILLEFKAAVTDPNGALASWTAGGDP
CVDFAGVTCDPSSRAVQRLRVHGAGIAGKLTPSLARLASLESVSLFGNGLSGGIPSSFSA
LGPTLHKLNLSRNALSGEIPPFLGAFPWLRLLDLSYNAFSGEIPASLFDPCLRLRYVSLA
HNALTGPVPTAITNCSRLAGFDFSYNRLSGELPDQLCAPPEISYISVRSNSLSGAIAGKL
NACRSIDLLDVGSNHFAGPAPFGLLGLVNITYFNVSSNAFDGEIPNIATCGTKFSYFDAS
GNRLTGPVPESVANCRSLRVLDLGTNALAGDIPPSIGKLRSLSVLRLAGNAGIAGSIPAE
LGGIEMLVTLDLAGLALIGDIPVSLSQCQFLLELNLSGNQLQGVIPDTLNNLTYLKLLDL
HRNHLVGGIPVTLAQLTNLDLLDLSENQLTGPIPSELGNLSNLTHFNVSYNGLSGMIPAL
PVLQSFGSSAFMGNPLLCGPPLNNLCGASRRAKQLAVSVIIVIVAAALILIGVCIVCAMN
IKAYMRRSKEEQEGKEEDEVLESESTPMLASPGRQGSNAIIGKLVLFSKSLPSRYEDWEA
GTKALLDKDCLVGGGSVGTVYKATFENGLSIAVKKLETLGRVRSQDEFEQEMGQLGNLSH
PNLVAFQGYYWSSSTQLILSEFMVNGSLYDHLHGSPHTFSGSSSRVGLSWEQRFKVALGT
ARALAYLHHDCRPQVLHLNIKSSNIMLDKDFEAKLSDYGFGKLLPILGSYELSRLHAAIG
YIAPELASPSLRYSDKSDVFSFGVVLLEIVTGRKPVESPGVATAVVLRDYVRAILEDGTV
SDCFDRSMKGFVEAELVQVLKLGLVCTSNTPSARPNMAEVVQYLESVRTNS*
>11676.m03526|LOC_Os10g38820.1|genepair906-1
MNTTSANELMMHRHVQAAPYAAAPQQQGGKQRAPGLPPTPPPPPAAASSHSSHGDVCMDD
TARGGLLPPRKAHRRSRSDVPFGYFQPLPPPSPKMEAGGGWALPGCGGAGDVDLLNAYMS
LEGMGGADGLNNSDGDSRGSSMRTNGADSSENESEDYVGADSQALLWGGAGGGEAAGKKR
RNNAAGEKAARHARSLSMDSLMGKLSFAANGEPAKFSLEFGSGEFTPAEMKRIMADEKLA
EMALADPKRVKRVLANRQSAARSKERRMRYIAELEQKVQILQTEATTLSAQLTLLQRDSS
GMATQNNELKFRLQSMEQQAQLRDALNEALTAEVQRLKLAANEVCDTSSSSNLAHQIQLR
CQNQMLDLHKQQQQQVEQIPFYQLELPEQQNGTARNHESK*
>11669.m00289|LOC_Os03g03550.1|genepair906-2
MNGGIGDALMQPQHVQVMSSSLPMVASTFVAEPAAAANKPRAAGLPPTPPQVFAAQRAAA
AAGGDVCMEESAQGGGGGLPPRKAHRRSSSDVPFGYLAGQHQLLPPKVEAGWGHLGAGAG
GAAAADDLFNAYLNLEGLDGLNSSDDRHDEGDSRGSSIKTNGADSSENESEECADDTRGG
IRLWSADGGERREGVKRNAAGEPATAPLARHARSLSMDSLIGKFNFTAGTAAAAGNGVAL
GPNRFSLEFGSGEFTPSEMKKIMADEKLAEMALADPKRVKRVLANRQSAARSKERKMRYI
AELEQKVQILQSEATNLSAQLTMMQRDSAGLATQNNELKFRLHAMEQQAQLRDALNEALT
TEVQRLKLATAELGDSCSSSSLAQQIQLNAQNQMFQLQQQQATQIPFYQLQQSQQNGAAK
NNESKE*
>11676.m03527|LOC_Os10g38830.1|genepair907-1
MESCVPPGFRFHPTDEELVGYYLRKKVASQKIDLDVIRDVDLYRIEPWDLQEHCRIGYEE
QSEWYFFSYKDRKYPTGTRTNRATMTGFWKATGRDKAVRERSRLIGMRKTLVFYKGRAPN
GHKTDWIVHEYRLESDENAPPQARSPFISLSINKIDLQFLEKIN*
>11669.m00288|LOC_Os03g03540.1|genepair907-2
MHFAPLHAASAHPCLAGIFSRDVKLRRESSSAPKKLVIPVLSDKESERRIERRLAQVGDH
MDAMESCVPPGFRFHPTDEELVGYYLRKKVASQKIDLDVIRDIDLYRIEPWDLQEHCGIG
YDEQSEWYFFSYKDRKYPTGTRTNRATMAGFWKATGRDKAVHDKSRLIGMRKTLVFYKGR
APNGQKTDWIMHEYRLETDENAPPQEEGWVVCRAFKKRTAYPARSMVETWDYSLHERNIM
SAAAAAAFADPSAAYAQMRRQHRSGRFKQEAELDGAATALLHYSSHLAELPQLESPSAAA

APLQPNPSQLATAGEDDDCKGDNGGRRAKKARAAGDKVATTTDWRALDKFVASQLSPGEC
GSMEATAEAAAAAVAGVSSPLDHGDDDMAALLFLNSDERDEVDRWTGLLGSGAGASGVDG
DLGICVFDK*
>11676.m03531|LOC_Os10g38870.1|genepair908-1
MSKKIVVKLNVHDKAEKQKAMKAVSALIGIDELSMDMASQKMTVIGMVDPVNVVSKLRKS
WAATIESVGPAKEPEKKEEKKDGGGDGKKDGGGDGKKEGEAGDKKDGDAAKKDGDKDGEA
KKEDGDKKPAAPTEQQLFAELMNQYYHRPAAYGYNPYMSVPPHYVVQSMEENPNSCAIC*
>11669.m00284|LOC_Os03g03500.1|genepair908-2
MSKKIVVKLDLHDNKDKQKAMKVVSTLAGIDAISMDMASRKMTVIGTVDPVNVVSKLRKA
SWPAYIESLGPAKEPEKKKEGGGDAKKDGGDGKKEGGGDGKKEGGDGKKEGDGKKGEAAA
AAKKEEGGGEKKVAAVAPMPMPMHQLPPPYMFNAGYMNQYRPPPPPPPAYPYAPPQYYYA
RDMSMEENPNPCAIC*
>11676.m03532|LOC_Os10g38880.1|genepair909-1
MKVMTLRRGGAGAGIRIKKKARGFMCGGCGGSKAVSVSDGSDKQSPMATPPPNTSSTTTT
TTTGSAGNKTTAAAGSSSFSPSYDYDYVDTDADTSVGSTPSVAALLRQLGELERSVRSLQ
GAVAEGRGAKNDGRGGGRRHRRTVSDGGGGGSGRVEESVAVVKESADPLFDFRRSMLQMI
VEKEIVGGAELRELLHRFLPLNSPHHHHVILRAFAEIWEEVFAGYERTPDFLVSSRHRRP
TKKKLPASYTAADDDDDDSWNAA*
>11669.m00282|LOC_Os03g03480.1|genepair909-2
MADQGLPSKKKKKKGSSAAAARGFMCGCGGAKSVSVSVVSRLSAAGNISPATTTPPTMTSSA
TATSAKTTRVTVPTPAPNTVHDADGTPSVGTLLLQLRELERGVRALGVREDRGIRPATPP
PPRHRRSASDASSGGRRRRGRLEEESVPVVTESDDPLGDFRRSMAQMIVENEITATPELR
ELLHRFLSLNSSRHHHLILRAFADVCEELFAGAGEHNHHRPRRRPTKPLPYSATTN*
>11676.m03534|LOC_Os10g38900.1|genepair910-1
MLHGPAHSPPPAAAAVAVAGGGGGGEPLVVTLNCLEDPSMEQEVLAGAAAVEHAPLSALSS
GRVEAAAAVLLTSLAFLPRAAQRRLRPWQLILCLGSPDRAADAAVAAELGLRLVHVDANR
AEEVADTVMALFLGLLRRTHLLSRHASSYSAPPAGWLGSVQPLCRGMRRCRGLVLGIVGV
NAAARCLATRSLAFSMSVLYFDPLHEANGKTKRPSILFPSAARRMDTLNDLLTASDLVSL
HCALTNDTTHILNAERLQHIKPGAFIVNTGSCQLIDDCALKQLLIDGTIAGCALDGAEGP
QWMEAWVREMPNVLILPRSADYSEEVWIEIREKALAILQSFFYDGVVPNNALSDDEEEIT
EAGCEDDQLAKQAKEQVCDGGQQTDESQLTLECDKRRAISHSEEPQASGQSQNRENVVPR
SEGRRSRSGKKGKKRPARRKSQQKRDELLSTLEGGSNYSSRMDDDTVTSGKDQVLSSSSR
FASPEDCKTKLRSSAEFPMEIISENKLTAGLSIKPLERLKDGFVVALRTRDNSGFHVARE
RVAGVGWYLDVVSKATKRDPAAQFLITFRNKDTMGLRSFVAGGKLLQVNKTMELVFASYS
FDVWESWTLEGSLLDCCKLVNRKIPSVVLEVYIEILAAVSEEDGVTRWLD*
>11669.m00280|LOC_Os03g03460.1|genepair910-2
MARSAASGGGGGGQPLVVSLNCLDDPSLEQEGLAGVAGVEHVPLSAVASGRVEAAAAVLL
PSLAFLPRAAQRRLRPWQLLLCLGSPDRAADAALAAELGLRLVHVDANRAEEVADTVMAL
FLGLLRRTHLLSRHASSAPAAVAAGWLGSVQPMCRGMRRCRGLVLGIIGRSAAARCLATR
SLAFRMDVLYFDPRHSANGKAKRPSIVFPSAARRMDTLNDLLAASDLVSLHCTLTNDTMH
ILNADCLQHVKPGAFIVNTGSCQLIDDCALKQLLIDGTIAGCALDGAEGPQWMEAWVREM
PNVLILPRSADYSEEVWMEIREKAITILQSFFFDGAVPSSAISDEDEEISEAGNEDDQLE
EKVSSSQVFYSEQQTDESQLKMEYEKRRAISQHKEPQASARSQHIVPRSEGRRSRSGKKG
KKRPARRRSQQKTDELSAVESGSNYSSRRDDDTAMSGRDQVLSSSSRFASPEDSKYKQKS
PAESPMEITSETKLPTVLRRKYPDTLKDGFVVALRTKDNSGFHVARQRLAGGGGWILDIV
SNATNRDPAAQFLVTFKNKDTMGLRSFVAGGKLLQVLYLYLYLYKLHCTSRYTQPKINRRME
FVFASHTFDVWESWMLEGSLLEGCKLINCRNSSAVLDVCIEILAAASEEDGVTRWLD*
>11676.m03536|LOC_Os10g38920.1|genepair911-1
MATRVKPPPPSRPVVAKSPPRRQPHPPPPPPPPLPRHALHRQHEREGEATMSVWSVGFIN
ARLSQRTPVLGLRLWVLVAAGAAAAVVLALLIVVCLCRRCRRRRCSRLAPAPPHHGRSNR
SLKQQQSMVSDKDIEEAARWPPPPSFQPPIEVIKAEQTAPLIMVEAARTSGETATSSGGS
TRGWSTESGGSDAAEPEASRRGWGRRYTRRELEEATNRFAAENVLGEGGYGVVYKGILRD
NTAVAIKNLHNNRGQAEKDFKVEVATIGRVRHKNLVSLLGYCEGACRLLVYEYMENSNLD
KWLHHGDDEISPLTWDMRMHILLGTARGLAYLHEGLEPKIVHRDVKSSNILLDRHWNARV
SDFGLAKLLCSERSYVTTRVMGTFGYVAPEYARTGMLNERSDVYSFGVLIMEIISGRTPV
DYTRPAPEVNLVEWLKRMVAERRVEEVVDPRLPETPPPKVLKRAVLAALRCVDPDGGQRP
TMGHVVHMLEDDLKFRDELQLARDLSPHASDSYEYEL*
>11669.m00272|LOC_Os03g03410.1|genepair911-2
MAAQRRLLAEAPPHQPHPSRHQQGAPSSVWSAGYLNGWLSQRTAVFGLRLWVLIGIAVGA
AIVLVLVLVFVCLSRRRRRRDDLASNLYPADTKILKQHLQQPTPPKDIQEIVRRQQRQQQ
TPTPTPPQPPPPAAQHGVQLAKAETPPPPQRTQPPVLPAGSTRSTAASGMSATTSGGSER
DGATPRSTASGSAGPEVSHLGWGHWFTLRELEEATDGLAEENVIGEGGYGIVYKGTLQNS
AMVAVKNLLNNRGQAEKEFKVEVEAIGRVRHKNLVRLLGYCVEGAYRMLVYEYVDNGNLD
QWLHGDVGEVSPLTWEVRMNIILGTAKGLAYLHEGLEPKVVHRDIKSSNILLDQQWNAKV
SDFGLAKLLCSERSYVTTRVMGTFGYVAPEYASTGMLNERSDVYSFGVLIMEIITGRSPV
DYTRAPGEVNLVEWLKTMVAERKAEEVVDPKLPEKPSPKALKRALLVALRCVDPDGHKRP
KMGHVIHMLEMDDLLCRDDKKPGRDAPQTSDRHSSRDGGNFSKRENQRYR*
>11676.m03538|LOC_Os10g38940.1|genepair912-1
MAVARLVVITPAVLLGRTARVSPSAVPRLRPIVAGRRAVAAPTRAVLGDGAGVGGEEDAV
VAVVEEDAVARRAARKRSERRTYLVAAVMSSLGFTSMAAAAVYYRFAWQMEAGGGDVPAT
EMVGTFALSVGAAVGMEFWARWAHRALWHASLWHMHESHHRPRDGPFELNDVFAIANAAP
AISLLAYGLLNRGLLPGLCFGAGLGITLFGMAYMFVHDGLVHRRFPVGPIENVPYFRRVA
AAHQIHHTDKFEGVPYGLFLGPKELEEVGGTEELDKEIKKRIKRKEAMDAIR*
>11669.m00268|LOC_Os03g03370.1|genepair912-2

MAVARLVAARAPLLSPAAVAAAHRSPPALLRLAFAPLPARRLAVPLRVAVGEPEPEEDAR
RAVAERAARKQSERRTYLVAAVMSSLGITSMAAAAVYYRFAWQMEVGNTPSPNSRSHLTI
TQFGNSFAAALKFLRVLDQGSGEIPVTEMFGTFALSVGAAVGMEFWARWAHRALWHASLW
HMHESHHRPRDGPFELNDVFAITNAVPAMSLLAYGFFTRGLVPGLCFGAGLGITLFGMAY
MFVHDGLVHRRFPVGPIANVPYFRRVAAAHQIHHMDKFEGVPYGLFLGPKELEEVGGIEE
LEKEIKRRIKRKETLDAIQ*
>11676.m03546|LOC_Os10g39000.1|genepair913-1
MEASPSRSDSFSHGWLRRKARPAAAAASFERLVGGEGDAVDVDGDLGHSFNGSVVSFIDM
DPAELFSMRWTSLTAAAEEEEDHDDDDFDFGMPCVAGAQCSSPLLVGAGRALSDGHHHHL
LLPCEPGVVVARDRTASYADAPSPSPSSPLFHSALSTPASVITTASSRRAGAGSGKARAP
LLATRRILLRYLRFLVPLCRKVRSLPLRVLSPRSAKGSLAAASSSASPARRSTSSSYASA
AEYWCHGNADTAVRDAILYCKKSIGQDM*
>11669.m00263|LOC_Os03g03320.1|genepair913-2
MEVGSLQHLGDSFSYRWLKHAAQAPSFKRLVDDDVGGSSRYFIDMDPADLFSMRWTAPGT
DFDFDFDLPGGDDDAASPIPLLVSASQIFHDGRLLPHELDDDGRFGAQEDGDAARVAHLL
SEPRLSASSPLFHSAQSTPASLSSSSSARSGASKNASAPPLLAAGRRGGGSSPWKILLRY
LRFLMPLYRKVRALPPLRAPRTRVSPASPASARARASTSSIDWCHGIADTAVHDAILYCK
KSSGQNI*
>11676.m03547|LOC_Os10g39010.1|genepair914-1
MAAIVLLLFLVVGLMPVSNGQTTPFSPRFSVYLACGAGGNVVVTSDSPQRTFVPDDGELS
GKSARFSNPDASPPSPLYAAARAGTSGFSYRLSYAADAAPDGNTTLVLRLHFFPFASQSG
DLLSARFSVSAMGRYVLLPPSFSPPRAGVVREFLLPSDGSGEFDVAFTPESGGLAFVNAI
ELFPAPQELLWKFPLTAVNTDVSPSHQALETLYRLNVGGPTVTPTGDTMWRTWLPDDSYL
SPATVSAVASIQGQIIFDRAQGYTQMVAPDAVYKSQRTTNSTTSNVTWTFAVDGNSSYVV
RLHFCAFEELSSVIGEGVDFNVYLMQAMGTRELKAKDYATLSSPTQAFYMDYVAVVPTAG
ENLTVSIGRAASSDSKKAILNGLEIMKLRAVDMTPASSSGKTSKVVVVAVTAAVLGAAVL
AGVALCVLLVRRRQRRATLPVPEEEEKESVGTPWSPFTPDGEGSFGSAVVTPRRMNMKLH
IPLAEIMVATGDFDDANILGVGGFGNVYRGVLRDGTRVAVKRAKRASRQGFPEFQTEILV
LSSIRHRHLVSLIGYCNERSEMILVYELMAHGTLRSHLYGSDAAAATPPPLSWKQRLEIC
IGAAKGLHYLHTGHSDNIIHRDVKSTNILLGDGFVAKVADFGLSRVGPSTGQTHVSTAVK
GSFGYLDPEYFKTRQLTDRSDVYSFGVVLFEVLCARPAIDQSLPPDEINLAEWAMQWSRR
GRFDKIVDPAVAGDASTNSLRKFAETAGRCLADYGEQRPSMGDVVWNLEYCLQLQESQPS
TETALDLDDSGAHLPRDIVVARRVAPLAPDASADAAGDDMSWSETASFTATGNVFSQIMS
RDGR*
>11669.m00259|LOC_Os03g03280.1|genepair914-2
MATVIVILLLLPLLPSTALAAFPYFLACGAASNVSFPGDSPARTFVPDAPFLSSAGRVPA
VTSTGSNTIPPLYAAARAAGSGFSYSFADPDTATVNVSRVLRLHFFPFTSSSSVNLSSAS
FSVSVRDAYTLLSSFSPPRDGVVKEYFVPGDGSGEFRVKFTPDAGSTAFVSAIELFPAPP
ELLWRRPVKPVGALVDSVDVNAWPQQALETVYRLNVGGSKVTAANDTLWRTWLPDDPYFS
SPRGLSQVNSTSTPIIYGTSIGYTREVAPDSVYKTQRAMNMASQQLFLTPGPFNLTWTFA
LPPPAPGSDSDYLVRLHWCDYSLVSSVVATGIVFDVYVAQRLASKDLDRNAADAAEQPNE
AFYLDYAATAPTTGNLTISIGKSDKSDAGGMLNGLEIMKLRRADNLNSAGSHGRRKKILI
GTLSAALGVAVLACALLCLLAVLRRRRQAPTPAPEEKESTQLPWSQHTQDGSSWVDMSNA
SGAGMTGGLHRMSMQLNISLADITAATENFNERNLIGVGGFGNVYSGVLRDGTRVAVKRA
MRASKQGLPEFQTEIEVLSRIRHRHLVSLIGYCNEQSEMILVYEYMEKGTLRSHLYGSEE
PPLSWKQRLEICIGAARGLHYLHTGYSENIIHRDVKSTNILLGDAFIAKVADFGLSRIGP
SFGETHVSTAVKGSFGYLDPEYFKTQQLTDRSDVYSFGVVLFEVLCARTVIDQSLERDEI
NLAEWAVSLQQKGELAKITDPRIAGQVNGNSLRKFAETAEKCLADYGLDRPSMGDVLWNL
EYCLQLQETHVNRDAFEDSGAVATQFPADVVVPRWVPSSTSFLMDDSVTDSGIANSKAFS
QLSSGDGR*
>11676.m03549|LOC_Os10g39020.1|genepair915-1
MVGGGGGAGEKPPASNGVHGSGKARFTLLYGLLLYVVMPVLFLYMLVAAATPFYNPRCSP
ESNAAMARFVVAMPNASSVNGSSPSSSSPPPTPVRPMRSADEAPTGLRHIAFGIGASSAL
WKSRKEYIKLWWRPGRMRGFVWMDRPVEEFYSKSSRTGLPPIMVSSDTSKFPYTHGAGSR
SALRISRIVSETFRLGLPGVRWFVMGDDDTVFLPENLVHVLSQYDHRQPYYIGSPSESHI
QNLIFSYGMAFGGGGFAISRALAEELAKMQDGCLHRYPALYGSDDRIHACMSELGVPLTR
HPGFHQCDLWGDVLGLLGAHPVAPLVTLHHLDFLEPVFPTTPSRAGALRKLFDGPVRLDS
AAVAQQSVCYDREHHWTVSVSWGFAVMVVRGVLSPREMETPMRSFLNWYKRADYTAYSFN
TRPVARQPCQKPRVYYMRDSRMDRRRNVTVTEYDRHRGKQPDCRWRIPDPAALVDHIVVL
KKPDPDLWKRSPRRNCCQVVSSPTKAGKNRTMTIEVGVCREGEFAKL*
>11669.m00258|LOC_Os03g03270.1|genepair915-2
MHAAGKAPKCKASFFLCSLLLYFLLPVLALYVVALAVSPFYSGSSCPEESLASGDVAHLA
AAGDAGNRRNDSSPPSDDAAPTGLGHIVFGIAASSELWKSRREYIRTWWRPEQMSGFVWL
DKPVYEFYSRNASTGLPGIKISGNTTKFPYTHGRGSRSALRITRIVSESFRLGLPGARWF
VMGDDDTVFFPDNLVDVLSRYDHTQPYYIGNPSESHIQNLIFSYGMAFGGGGFAISRALA
AQLAHMQDGCIDRYPALYGSDDRIHACVAELGVPLTRHLGFHQCDLWGDVLGLLGAHPVV
PLVTLHHLDFLQPVFPTTRSRTAALRRLFEGPARLDSAGVAQQSVCYDGDKQWTVSVSWG
FAVVVTRGVLSPREMEMPMRTFLNWYRRADYTAYAFNTRPVARQPCQTPQVYYMRQSRLD
RRRNTTVTEYERRRVAPVKCGWRIPDPAALLDRVIVLKKPDPNLWKRSPRRNCCRVLSSP
RQGKDRKMTIDVGVCRGGEFARIEGS*
>11676.m03550|LOC_Os10g39030.1|genepair916-1
MAHDPSLGYADYFAAEVDGTGATELYGLQQHQQGVGVAEMFGVRGLMPAAHAHEQSKGVG
ALVVGGGGVDDGGATTLPTVHFGGLGELHHHQHRQSQAPLSLSLHRPEAAATSLLMQQQQ
QHLHHQPSPPAGAASTWQLQQGAWHLRGSRFLLPTQQLLQEFCSLPVKSTTSPSSASKAT

KPPQEEAASGGGSSSWTAPTQIQSMDAAELQRLKGKLYTMLEEVDRRYRRYCEQMRALAA
SFEAVAGERAAASYTRLASRTISRHFRSLRDGVVAQLQAVRKQLGEKDTAVPGMTKGETP
RLRVLDQCLRQHKAYQAGMLESHPWRPQRGLPERAVSILRAWLFEHFLHPYPSDVDKHIL
ARQTGLSRSQVANWFINARVRLWKPMVEEMYAEEMKDEEGSGQSTQASNPQNPNPSSYTS
EVRGGGGGGEDRGEQKPSRAQLLHDAGSLASVVSIGHGGAGRTMVDHHHHQSLNFGTMDQ
LDFDAYEAAGGGQGFGAGGGVSLTLGLQQQHADPHDGVNVAFAAAAAPPNSSGVAAEYLF
MGGGEHQQQLPQTAQFGAVMEGDAASHYRGLSATAAGFHLLHDLAG*
>11669.m00257|LOC_Os03g03260.1|genepair916-2
MAHDPNLGFADYFSAADASASSVTTLMPAMDEAAPELFGLQAGMELLGVRGLGMSMMPGA
AGKVAALVADAGDDGGGGSTMRFLSEQHQQPSQAPLSLSLCRPDGVLHLGGAARPQHQLA
PAAPWMTHHDASSSAPQVHGAWHLRSSRFLLPTQQLLQEFCSLPVDSTKRGNGAKAATQQ
EDGRGDGSSSSSASWTPSPQIQAMEALELQRLKDKLYIMLEEVDRRYRRYCEQMRAVAGG
FEAVAGERAAGAYTAVAARTISRHFRSLRDGIVAQLQAARKALGEKDVSAAGTTRGQTPR
LRVIDQCIRHHKSLQGVAAMDSHPWRPQRGLPDRAVTILRAWLFEHFLHPYPSDVDKHIL
ARQTGLSRSQVSNWFINARVRLWKPMVEEMYVEEMKGQDGGDGSGGQGSLNPKPTCSHAS
EARGGQQLVVGDGDGGEQKPTRAQLRHDAGSLASVVNVDVAAGAGGVARLHQAENFGIMD
HLDFDAYDDSHHQQQHGGFGGVSLTLGLQQHGSHGGGGVNIAFGAPGSAHGGAGFLYPGE
QMAPDAMHPGHGHHVVGGQFGVAMDGDAASHAQERYRSLSAGFHLLRDLAG*
>11676.m03556|LOC_Os10g39090.1|genepair917-1
MAEKRLLLLPVALAMAQKHGGGGERVWARPWRWAKTAFFVVAMIASLLLVCAPPVLVVIL
DLALPPALLSARLRGGGGGDDASFVAAVVAQARAFDFRSSLVDLLAVSAARALLILGAYM
ACGGGGAAYLWVVATSAAGSVSYVLAKAAAAVLPRRGVAPAPEGKGPEPMLLLSVALAAA
HLAVAYRTSCRERRRLLVYRIDVEAVRLKGGHQTKQCSV*
>11669.m00251|LOC_Os03g03200.1|genepair917-2
MVEKTKLQLPLVHNERLWARPWRWAKTVFFLVSMLASLLLVCAPPLLIVVLDLLLPPALL
SNFHRAANHPTSLIDQARGFHFRSSLVDLPAVSAARSLLILCAYTACGGGAAYLWVAVAC
SVGSVCYVVAKAAVVFGAAPDRAVLGLQGKGQLVAVEAMFLMSLALAAAHIAMAYRASCR
ERRRLLVYRIDVEAVRLKGGQTPKSLKQQFAV*
>11676.m03558|LOC_Os10g39100.1|genepair918-1
MAASQAYLDKAQLRQSYRNVWHTDLTNAITADFTCCCLSLWCGPCVSYMLRKRALYNDMS
RYVCCAGYMPCSGRCGESNCPEVCLATEVFCCFGNSVASTRFLLQDEFNIQTTQCDNCII
GFMFCLQQFACICSLVACIVGSEELSEASQLISCISNMVYWTVCSCMQTQHKVEMDKRDG
KFGPMTVPPMQQMSRIDQPVPPYVGYAPQAQPAYYR*
>11669.m00249|LOC_Os03g03180.1|genepair918-2
MASSQANLDKMQLRQSYRNLWHSDLTSTIQADFPYCCLALWCGPCVSYMLRKRALYNDMS
RYVCCAGYMPCSGKCGESRCPEFCLATEVFLCFGNSVASTRFLLQDEFNIQTTKCDNCII
GFMFCLQQIACIFSIVAAIVGSEELSEASQILSCLSDMVYCSVCACMQTQHKIEMDKRDG
KFGPQPMAVPPMQQMSRIDQPIPPPVGYTPQQPAYGQPYGGYPPAPPAQGYPPAAYPPAG
YPQGGAYPPPGSYPPPGSYPPQGSYPPPQGYYGK*
>11676.m03560|LOC_Os10g39120.1|genepair919-1
MSGGIARGRLAEERKAWRKNHPHGFVAKPETMADGSANLMIWHCTIPGKQGTDWEGGYYP
LTLHFSEDYPSKPPKCKFPQGFFHPNVYPSGTVCLSILNEDSGWRPAITVKQILVGIQDL
LDQPNPADPAQTDGYHIFIQDKPEYKRRVRVQAKQYPALL*
>11669.m00244|LOC_Os03g03130.1|genepair919-2
MSGGIARGRLAEERKAWRKNHPHGFVAKPETLADGTVNLMIWHCTIPGKQGTDWEGGYFP
LTLHFSEDYPSKPPKCKFPQGFFHPNVYPSGTVCLSILNEDSGWRPAITVKQILVGIQDL
LDQPNPADPAQTDGYHLFIQDPTEYKRRVRLQAKQYPPIV*
>11676.m03561|LOC_Os10g39130.1|genepair920-1
MVRGRTELKRIENPTSRQVTFSKRRNGLLKKAFELSVLCDAEVALIVFSPRGRLYEFASA
PSLQKTIDRYKAYTKDHVNNKTIQQDIQQVKDDTLGLAKKLEALDESRRKILGENLEGFS
IEELRGLEMKLEKSLHKIRLKKTELLEQQIAKLKEKERTLLKDNENLRGKHRNLEAAALV
ANHMTTTTAPAAWPRDVPMTSSTAGAADAMDVETDLYIGLPGTERSSNRSETG*
>11669.m00237|LOC_Os03g03070.1|genepair920-2
MCIVFEIMQVKADADGLAKKLEALETYKRKLLGEKLDECSIEELHSLEVKLERSLISIRG
RKTKLLEEQVAKLREKEMKLRKDNEELREKCKNQPPLSAPLTVRAEDENPDRNINTTNDN
MDVETELFIGLPGRSRSSGGAAEDSQAMPHS*
>11676.m03563|LOC_Os10g39140.2|genepair921-1
MAAEAEQQHQLLSTAVHDTMPGKYVRPESQRPRLDLVVSDARIPVVDLASPDRAAVVSAV
GDACRTHGFFQVVNHGIDAALIASVMEVGREFFRLPAEEKAKLYSDDPAKKIRLSTSFNV
RKETVHNWRDYLRLHCYPLHQFVPDWPSNPPSFKEIIGTYCTEVRELGFRLYEAISESLG
LEGGYMRETLGEQEQHMAVNYYPQCPEPELTYGLPAHTDPNALTILLMDDQVAGLQVLND
GKWIAVNPQPGALVINIGDQLQALSNGKYRSVWHRAVVNSDRERMSVASFLCPCNSVELG
PAKKLITDDSPAVYRNYTYDEYYKKFWSRNLDQEHCLELFRT*
>11669.m00233|LOC_Os03g03030.1|genepair921-2
MVDDFISNPDIHKYGCALVRILRSGRKLSFVYPKTRSNLGPVGQKIKGINHALSPKEIIS
TYCKEVRELGFRLYGAISESLGLEQDYIKKVLGEQEQHMAVNFYPKCPEPELTFGLPAHT
DPNALTILLMDQQVAGLQVLKEGRWIAVNPQPNALVINIGDQLQALSNGRYKSVWHRAVV
NSDKARMSVASFLCPCNDVLIGPAQKLITDGSPAVYRNYTYDEYYKKFWSRNLDQEHCLE
LFRTTPTDTS*
>11676.m03566|LOC_Os10g39170.1|genepair922-1
MAMRGGGATMLSWYLQVAAVSLLAMATGLEAQLRVGFYDNSCPAAEIIVQQEVSKAVSAN
PGLAAGLVRLHFHDCFVRGCDASVLIDSTKGNQAEKDAGPNTSLRGFEVVDRIKARVEQA
CFGVVSCADILAFAARDSVALTGGNAYQVPAGRRDGSVSRSSDTGGNLPPPTASVSQLTQ
MFAAKGLSQREMVALSGAHTIGASHCSSFSSRLYRAGTTAGGAGGGQDPTMDPAYVAQLA

QQCPQSGGAAGGGALVPMDAVTPNAFDEGFFKGVMNNRGLLSSDQALLGDKNTAVQVVAY
ANDASTFQSDFAAAMVKMGAVGVLTGSSGKVRANCRVA*
>11669.m00222|LOC_Os03g02920.1|genepair922-2
MEARGSRGMRLWLLSVAVMAMAMATRSQAQLQVGYYDTLCPAAEIIVQEEVSKAVSGNPG
MAAGLVRLHFHDCFVRGCDASVLLDSTQGNRAEKDAPPNTSLRGFEVIDSAKSRLETACF
GVVSCADVLAFAARDALALVGGNAYQVPGGRRDGNVSVAQETNGNLPPPSANVAQLNQMF
GAKGLTQAEMVALSGAHTIGVSHCSSFSNRLYSSGPNAGQDPSMDPSYVAALTTQCPQQQ
GQPAAGMVPMDAVTPNAFDTNYYAAIVANRGLLSSDQALLADQTTAAQVVGYTNNPDSFQ
TDFAAAMVKMGSIGVLTGNAGTIRTNCRVAS*
>11676.m03568|LOC_Os10g39190.1|genepair923-1
MEFTPISPPTRVAGGEEDSERGAAAWAVVEKEHMFEKVVTPSDVGKLNRLVIPKQHAERY
FPLDAAAGAGGGGGGGGGGGGGKGLVLSFEDRTGKAWRFRYSYWNSSQSYVMTKGWSRFV
KEKRLGAGDTVSFGRGLGDAARGRLFIDFRRRRQDAGSFMFPPTAAPPSHSHHHHQRHHP
PLPSVPLCPWRDYTTAYGGGYGYGYGGGSTPASSRHVLFLRPQVPAAVVLKSVPVHVAAT
SAVQEAATTTRPKRVRLFGVNLDCPAAMDDDDDIAGAASRTAASSLLQLPSPSSSTSSST
AGKKMCSLDLGL*
>11669.m00220|LOC_Os03g02900.1|genepair923-2
MEFITPIVRPASAAAGGGEVQESGGRSLAAVEKEHMFDKVVTPSDVGKLNRLVIPKQHAE
KYFPLDAASNEKGLLLSFEDRTGKPWRFRYSYWNSSQSYVMTKGWSRFVKEKRLDAGDTV
SFGRGVGEAARGRLFIDWRRRPDVVAALQPPTHRFAHHLPSSIPFAPWAHHHGHGAAAAA
AAAAGARFLLPPSSTPIYDHHRRHAHAVGYDAYAAATSRQVLFYRPLPPQQQHHPAVVLE
SVPVRMTAGHAEPPSAPSKRVRLFGVNLDCANSEQDHAGVVGKTAPPPLPSPPSSSSSSS
GKARCSLNLDL*
>11676.m03569|LOC_Os10g39200.1|genepair924-1
MLPYHGDRHRGSPPPYPAAAAHSSLSPSAAPFTVDCPRPAADPRVRNPNPPGLDLPTAPS
LYTTAAAGDWGSSSWMEPPASYMAPSPAAATPPPPAYKGEAPETAPYGIFPGTCQIGNFM
VTRPLRSESSQLTSAKGPGTWLGSSEVLPSGVGPSVFSPQQNTFVHKSEDAEPYPTQRGL
LQYPPQYPAYDKYMTQLSSCSTNVPPPVMWTPPANSSEVVEQMFPVMNKNTGESSSSFSS
YMNPCRINLDYFDCMWNEQKDLGHQTTDKHHGKWSSSASNAGDHLLNSLGADHRAARCFG
NGRPIQESSEMKYDRGSFNSKVSPSEVGYVQSREFSSELPEVNNPTVDSPCWKGAPIAYP
PSFGIMKNTDNSHSVNGVGGYQIEQSPEWSLKYSELFSKHQEVSASESVKSDALKTFKLP
ETRKNTEDNKEVLPVCIGVHNGIGNNASYFPEEQNSRRQKCYDSTGDCKNMIAANQQENL
SVSKAKLLGEDSSNHIGSITEESINKGPSPLGSAPRALVENLSESLHVNVCSQAAGAEEC
TQAQICAKGGQQPRYYSDSGGSMLKTSSESRSKSRAELLKQMHDLSAMLLSTCNSVPLQG
YEEELLQLVIQNLRDASSCISKVQNMSCSRNNLWMAMPEHSLVENDSELKTSISQAVAKL
PEDKTLDDIDVSQLSIYKNLWVEAEASACKLKYELQLTRVKLAAMENHNNTQVPVDLSKG
NKIFISTIPNSKPQNSTAYPANLQCQGADSCDGQLPAVNRSIIDGVDAEVIERLKFLQSN
LKDCRAFCQNNCEEQEEASKKPCAIEDAVMARLRVLNSCPDNIASLKQENNNHHQLDTST
NRADNIDDAVMSRLRILKSRPDNVNPLGQESSKHEPDAATGTNNFIDNAVMSRLRILKSR
PDNANSLGQESSKHEPDASTGTNDLIDNAVMSRLRILKCRDDNINSLDDAIKQHVEACTD
QPNWDEDGVVAKIQAPNGDTVSTADGFQNILHSNNFVRHSEGKDSVSGLDSPGDATCSDE
DNGRKAPSDEVNDKTAVQSEGSFPMNIGWPLSTMDSHICTAGSQETPLISSSVHRYDIFP
PKWEHMLKENFFHPGK*
>11669.m00219|LOC_Os03g02890.1|genepair924-2
MHPVIAETGYAGTANTDGFLFSDSALHNVYPEDQFSSIKQLHPWIPSTTGRSSWLEEKIP
VIHQRTSAAASSEIGSSVLHKPSLFPSIIDCFDKEPVPIHQSDGRYSYDSHLSHLTSCST
SLNYGLSMPSVAASPVVCGMKRIDPSPSDPVLKGRFLQYANPCRFNIGHFDSVQDEQKDH
AGFQTAYRHCSDWNRCTNDTGIVGNYLANSSGETCNVGENSITGRFSQEILCSEVPMSRV
QEPLSHHHSLVQEDLNAFCENITYRCNYHAELIKSMYNLSVALISSCNGDYELDESYQEL
IQSAIQNLSSLSPKRSKNLSIEENKSGNDKDAHVLAYKNLWIEAEASMCKLKYELQLARM
ELALKYHSQQSGAPPTIPLDVQDSSLSKSKSLLCDEVLDDPSKQQNHVKENTICSATLLP
EEGNTGDGQSPKVNRSIANEVEAGVFTQLRVLRSRGDSICSFGEGSDEEQQETSNNKKTN
GFDNTAAVSMDTLKSGDDSMNSVVVEPIKERVESSKTDVDTAAPFYSFVKRLSGSSSSSD
VDFDKFLSSIKKQTDVTVMARHKDFVCDKGNIRSLDDTTNQCQAASNTKQLEDDALRFFQ
SLKIPEGIPEDHSDDGSSDSDYYQTEHYPLRVEPGRLLFIHKVLGSGKEW*
>11676.m03571|LOC_Os10g39210.1|genepair925-1
MADIVSDVFLSFFCCCYYPPGGHRGVGAHNDTALRRRRGGAGRSSSRPPVSLQTVELKVR
MCCEGCERVVRSALANLRGVDSVEVDVAMEKVRVTGYVDRGRVLREVRRSGKKAEFWPSG
GTPRRFTSEKEYFRDGEAYRGSYNYHRRGYGDGDRHGWMREPARGADAVSNMFNDDDVSA
ACAIM*
>11669.m00216|LOC_Os03g02860.1|genepair925-2
MSTVSSALSSFLYCCFSPTGGHRHGHRAGAYYYSSHPTSTNTYYYEGGLAGRRMGRSRPL
SLQTVELKVRMCCSGCERVVKHALMKLRGVDSVEVELEMEKVTVTGYVERQRVLKEVRRA
GKKAEFWPNPDLPLYFTSAKDYFHDEESFRPSYNYYRHGYNGDKHGHLPEPHRGADPVSN
LFNDDDVNACSIM*
>11676.m03572|LOC_Os10g39220.1|genepair926-1
MRRMLEVAADAGSSAAAAAANGAVDWWRDVNESPVWQDRIFHVLAALYGFVSAIALVQLI
RIECRVPEYGWTTQKVFHFLNFLVNGVRSIVFVLRRNVQLIHPEILQHVLLDMPGLAFFT
TYALLVLFWAEIYYQARAMSTDGLRPTFYWINAVIYAIQIILWMVLWWKPVRVMIILSKM
FFAGVSLFAAFGFLLYGGRLFLMLQRFPVESKGRRKKLQEVGYVTTICFTCFLIRCVMMC
LNTFDKAADLDVLNHPILNFFYYLLVEIVPSALVLFILRKLPPKRGITQYHPIH*
>11669.m00215|LOC_Os03g02850.2|genepair926-2
MAASTAAAVAPDWWNDVNNSPMWQDRSFHALATLYGAVSFVALVQLIRIECRVPEYGWTT
QKVFHFMNFIVNGVRSIVFVLRRDVQLVQPEVFQHVLIDFPGLAFFTTYALLVLFWAEIY

YQARAMSTDGLRPAFYTINGVVYAIQIILWMALWWKPVRAMVILSKMFFAATSLFAALGF
LLYGGRLFLMLQRFPVESKGRRKKLNEVGYVTTICFSGFLIRCVMSILQMCLNAFDKEAD
LDVLNHPILNFFYYLLVEIVPSALVLFILRKLPPKRGITQYHPIH*
>11676.m03591|LOC_Os10g39410.1|genepair927-1
MSGRGKGGKGLGKGGAKRHRKVLRDNIQGITKPAIRRLARRGGVKRISGLIYEETRGVLK
IFLENVIRDAVTYTEHARRKTVTAMDVVYALKRQGRTLYGFGG*
>11669.m00205|LOC_Os03g02780.1|genepair927-2
MSGRGKGGKGLGKGGAKRHRKVLRDNIQGITKPAIRRLARRGGVKRISGLIYEETRGVLK
IFLENVIRDAVTYTEHARRKTVTAMDVVYALKRQGRTLYGFGG*
>11676.m02321|LOC_Os10g26990.1|genepair928-1
MDSAARASQSAAPPPPGAGVRVRAPLVESVSCYCRLDTGLKTVVDARKFVPGAKMCMQPD
VKPNKCKSRGSRKERSRTQAPLLPGLPDDLAIACLIRVPRVEHPNLRIVCKRWNRLLSGN
YYYSLRKRNGMAEEWVYVFKRDREGKISWHAFDPLHQLWKSLPPVPAEYSEALGFGCAVL
SGCYLYLFGGKDPLRGSMRRVVFYNARTNKWHRAPDMLRKRHFFGSCVINNCLYVAGGEC
EGIQRTLPSAEVYDPNRNRWACVAEMNNGMVPFIGVVYDGKWFLKGLDSHRQVTSEVYLP
SSNLWSTIDDEMVTGWRNPSITFNGKLYSSDCRDGCKLRVYDPNTGTWAKFMDSKHHLGS
SRAFEAAALVTLNGKLCIVRNNMSITLVDISDPTMSIETDSARMWETVARKGQHRSFVAN
LWSTIAGRNLKSHIIHCQVLQV*
>11669.m00678|LOC_Os03g07160.1|genepair928-2
MDAAAGGAPPRPAAGIRVRVPLVESVSCYCRVDGGLKTVVSARKFVPGAKLCMQPDIKPN
KRKSRSSHKERCRTQAPLLPGLPDDLAITCLMRVPRLEHTNLRLVCKRWNRLLSGNYYYS
LRKKLGMAEEWVFVFKRDRDRKISWHAFDPVHQVWKSLPPVPAEYSEAVGFGCAVLSGCY
LYLFGGKDPVRGSMRRVVFYNARINKWLRAPDMLQKRHCFGSCVINNRLYVAGGECEGIQ
RTLRSAEFYDPNRNRWSYISEMSTGMVPFIGVVYDGKWFLKGLDSHRQVVSEVYMPTSNV
WSVTADEMVTGWRNPSICFNGRLYSAECRDGCKLRVYDRDTRSWTRFMDSRRHLGNSRAF
EAAALVSLNGKICIIRNNMSITLVDVSNTPTVIEINNAHMWDVFARKGQHRSFIANLWFT
IAGRNFKTHIIHCQVLQV*
>11676.m02327|LOC_Os10g27050.1|genepair929-1
MEESVGSRGGGGGGLDAQIEQLMECRPLSEPEVKTLCEKAKEILMEESNVQPVKSPVTIC
GDIHGQFHDLVELFRIGGKCPDTNYLFMGDYVDRGYYSVETVTLLVALKVRYPQRITILR
GNHESRQITQVYGFYDECLRKYGSANVWKIFTDLFDYFPLTALVESEIFCLHGGLSPSID
NLDSVRSLDRVQEVPHEGPMCDLLWSDPDDRCGWGISPRGAGYTFGQDISEQFNHTNNLK
LVARAHQLVMEGYNWAHEQKVVTIFSAPNYCYRCGNMASILEVDDCRNHTFIQFEPAPRR
GEPDVTRRTPDYFL*
>11669.m00677|LOC_Os03g07150.1|genepair929-2
MEPMSVDGGGGCGGLDTQIEQLMQCRPLAEQEVKALCEKAKEILMEESNVQPVKSPVTIC
GDIHGQFHDLVELFRIGGKCPDTNYLFMGDYVDRGYYSVETVTLLVALKVRHPHRITILR
GNHESRQITQVYGFYDECLRKYGNANVWKIFTDLFDYFPLTALVESEIFCLHGGLSPSIE
NLDSVRSLDRVQEVPHEGPMCDLLWSDPDDRCGWGISPRGAGYTFGQDISEQFNHTNNLK
LVARAHQLVMEGYNWAHEQKVVTIFSAPNYCYRCGNMASILEVDDCNSHTFIQFEPAPRR
GEPDVTRRTPDYFL*
>11676.m02333|LOC_Os10g27110.1|genepair930-1
MEMEHIIVPMSEYRQCICTNEKRPRVHADEVSVLTIVHGLDPVTIASVFSAAEHVGEKAV
DGEDCFALRLDVAPSVLSAWGDGAAEVIRHGMTGYFSQRSGLLARLDDSQLTRIQTPGAP
AMYWETTVSSRLGDYRAADGAVVAHAGTSVAHLARFGADVGAARAVTRMEEAWTIDDVAF
NVAGLCPESFIAPEEVRSGGGGGSSSRRYDGGGGAIAKKK*
>11669.m00674|LOC_Os03g07120.1|genepair930-2
MAGACKGCRVAFTPFRHHAFEALKAACRRWLLPNGILSCKQTQHACIKSDYQKFYKNNSR
FPWKFSKISAFPKEYYFGGFTSLGSSLVAPRSYHTNTKQSKAKRDASEDFDFDPPPTRFP
LASLQRCPFPSPALHTRTAAPPSHCRPGPAPSGAHPSLAVASSAQYIIEQFRATTGCGKI
EGAVKSMYAAGRVRLAMLQDPAGGGAGGGGGGGRGHEGSFVMWQLAPSMWIVEMAVAGQH
VAAGSDGRVAWRRTPWVGAHAARGGSRPLRRALQGLDPVTIAAIFSTAEHAGEKLVDGED
CFVLRLDVGPSVLSSWSDGTAEVIRHGLTGFFSQRSGLLVRLEDSQLTRIQSPGAAAMYW
ETTISSSLADYRAVDGGGGVHVAHSGRSTAHLARFGVGVRAARVVTRMEESWTIDDVAFN
VPGLGPDAFIPPEEVRRSRFYDAMAAGK*
>11676.m02339|LOC_Os10g27170.1|genepair931-1
MGLSISYPPDDYLPAMEDNMGRLFIRSLSFDDDMEAAADDSPSTSPMPSPSATLLPAFGS
GGKLIIEGSLSFKRREADPVQMETMISIRSPKSDKESCSSKPNATAGASRFALAGDQTPE
DSPVIAGVASPKHQAAAVRLQKVYKSFRTRRQLADCAVLVEQSWWKLLDFALLKRNSVSF
FDIEKPETAISRWSRARMRAAKVGKGLSKDEKAQKLALQHWLEAIDPRHRYGHNLHYYYH
RWLHCEINQPFFYWLDVGEGKDVNLEEHCPRWKLHKQCIKYLGPKERESYEVIVEDSRLI
YKLSRQIVNTTKSRKGSKWIFVLSTCKTLYIGQKQKGTFQHSSFLAGGATSAAGRLIVED
GILKAVWPHSGHYRPTEQNFQEFMNFLKERNVDLTDVMLNPSEGEDDAEFSLKSSHSRQD
LTELCEPDMQEHEEQVTQHHGADETKTSSDAPTMTSTETMASTPAIRKSTSANKLQGKRP
PRLLISSNNTELPATHCNGRPSPVHKDIDEDSTMFGECLAFCKKNLFAEEGNEEDELVEV
PEEMIMNRINCKKATKSYQLGKQLSFQWSTGAGPRIGCVRDYPSELQFRALEEVSLSPRG
TRSTRFSSPRRKPLTPNSIPVARFGCSPTAQGDNMGLKPRQRCATWTAF*
>11669.m00672|LOC_Os03g07110.1|genepair931-2
MGLSISYPPDDYLPTEEDTDQMFVRSLSSDNLSTIETFESPPALLDSLSSQRPIIKESFN
FKKSEGDPFHVETTVSLMSPKPGKEKSCTHKRTILPRYGSMEYLPPHSPVVGMISPKHQA
AAVRVQKVYKSFRTRRQLADCAVLVEQRWWKLIDFALLKHNSVSFFEEEKPESALSRWSR
ARTKAAKVGKGLSKDEKAQKLALQHWLEAIDPRHRYGHNLHYYYQHWLHCESKQPFFYWL
DVGEGKDVSMEDHCPRWKLLQQCIRYLGPKEREFYEVVIENKKLLYKMSRKIVDTSEGPK
NSKWIFVLSTTRVLYIGTKSKGTFQHSSFLAGGATSAAGRLVVDNGILKAVWPHSGHYRP

TEANFREFMMYLKKRNVDLANIKLSPSEDEEDECLRSRSGRSQLEPTEPGKPEKEEDATA
DDNGTTTVAAQAAPPSTTGGEPATPVMKRSSSGNRLQRKRPPRLTLDKSRLAKGVAEQDA
GSFGDRLDFCKVNLFRGGEEAEEAVVVPQEKILRRLNSRMTMNSYQLGKQLSLRWTTGAG
PRIGCVRDYPPELQFRVMEQISLTPRGGAGPLRLGSTATPRQSPCAPLPSPAPLYAAAGT
PTSLLQHGAA*
>11676.m02345|LOC_Os10g27230.1|genepair932-1
MLSVVRVHLPSEIPIVGCEITPYVLLRLPTGVVSTDDVPEAAPADGYFMRYRWYRIQSDR
KVAICSVHPMEQATIQCLGCVKSKIPVAKSYHCSAKCFSDAWQHHRVLHERASSALNENG
AEEEELFGRFGSTGSGVLSTTGSGSMSNLGQSPGLNNGPVPLYPSGTDKNSGETWYEVGR
TRTYTPTADDIGHVLRFECVSVDAEKKVPVGPPTSIMTSRVIPAPTPTPRRLIQVNGDVL
SHLDLDSQTSFGTFSVLSYNILADAYATSDAYSYCPTWALSWTYRRQNLMREIIGYHADI
ICLQEVQLNHFEDFFSPELDKHGYQALYKKRTTEVYTGAPHAIDGCATFFRRDKFSHVKK
YEVEFNKAAQSLTDAIIPSTQRRVALSRLIKDNVALIAVLEAKFGNHGTDNPGKRQLLCV
ANTHVNVHQDLKDVKLWEVQTLLKGLEKIAVSADIPMLVCGDFNSVPGSSPHGLLAMGKV
DQLHPDLAIDPLGILRPASKLTHQLPLVSAYSSFARMVGVGYDLEHQRRRMDPATNEPLF
TNCTRDFTGTVDYIFYTADSLSVESLLELLDEESLRKDTALPSPEWSSDHIALLAEFRCK
PRIRR*
>11669.m00668|LOC_Os03g07080.1|genepair932-2
MLTILRVHLPSEIPIVGCEITPYVLLRRPDGGISTEDVHEAIPLDGHFMRYKWYRIQSDR
RVAVCNVHPTEQATIQCLGCLKSKIPVAKSYHCSAKCFSDAWQHHRVLHERALSALNENG
NEEEELFGRFGSGNAGIINASLSGSTSNIGQSSSVNNGPTPVYPTGTEKNSGETWFEVGR
SRTYTPTADDIGHALRFECVAVDSETRSSVGAPTSIMTSRVIPAPTPTPRRLIPVNSADV
MGHFDLDSRNSSFGTFTVLSYNILADTYATSDTYSYCPTWALSWPYRRQNLLREIIGYHA
DIICLQEVQSNHFEEFFAPELDKHGYQALFKKRTTEVYTGNLQSIDGCATFFRRDKFSHV
KKYEVEFNKAAQSLTDAIIPAAQRKVALTRLIKDNIALIAVLEAKFGSHGADNPSKRQLL
CVANTHINVHQDLKDVKLWQVNTLLKGLEKIAVSADIPMLVCGDFNATPGSTPHGLLAMG
KVDLMHPDLAIDPLGILRPASKLTHQLPLVSAYSSFARMVGAGYDLEHQRRRMDPATNEP
LFTNCTRDFTGTIDYIFYTADSLSVESLLELLDEDSLRKDTALPSPEWSSDHIALLAEFR
CKPRVRR*
>11676.m02356|LOC_Os10g27330.1|genepair933-1
MEAVAEAVAAAVAAGVEPFPAVDKCDASGREAHAVAADLEGTLLRSRSAFPYYALVAFEC
GGVPRLALLLLLAPLAAALRAAVSEAAAVRVLVFAATAGARVREIESAARAVLPRFYAAD
VHPGAWRVFAACARRRVVLTATPRVMAEPFLVDCLGADAVAGTELATWRGRATGLVDDRR
GGVLVGRRKAQALLDMFAGGDVPDVGLGDRRSDYPFMSLCKEGYIVPRSPAVEAVPMDKL
PRPVIFHDGRLARRPTPLAALLAVLWFPVGFALACVRIAAGALLPMPWVYYAFWALGVRV
VVRGAPPPRAERAAGRRGVLFACSHRTLLDPIFLSAALGRPVAAVTYSLSRLSEFLSPIR
TVRLTRDRAADAAMIGELLDEGDLAICPEGTTCREPFLLRFSALFAELTDEVVPVAMESR
MGMFHGTTARGWKGMDPFYFFMNPSPAYVVTFLGKLPPEHTCGAGGRSSHEVANYIQRLI
AATLSYECTSLTRKDKYRALAGNDGVVDNATGKLPSPATATANNSKDKAC*
>11669.m00666|LOC_Os03g07060.1|genepair933-2
MAVAGGAGGEPFPAVDKCDVDASCRRGGRRAVVVVSDLDGTLLRSRSAFPYYALVAFEAG
GAARLALLLLLAPVLWLLRRAAASESAAVRVLVFAATAGARVSDVESAARAVLPRFYADD
VHPAAWRVFATCGGGRRLVVTATPRVMAEPFLRDYLGADTVAGTELAAWRGRATGMVDAR
RGVLVGERKAEAVREMVGDGEMPDIGLGGRRSDYAFMSLCKVT*
>11676.m02357|LOC_Os10g27340.1|genepair934-1
MAALSSRRRHGSLRLLAVALLVLAGVASSAAAAGSGRGAFDPSRVVQLSWRPRAFLHKGF
LTDAECEHLISLAKDKLEKSMVADNESGKSVMSEVRTSSGMFLEKKQVVFFCSLREMKFD
QLFPIYISIVCKALNHVVIFAIWTSILSNRPVLKLDEVVARIEERIAAWTFLPPDNGESI
QILHYQNGEKYEPHYDYFHDKNNQALGGHRIATVLMYLSDVGKGGETIFPEAEVGKLLQP
KDDTWSDCAKNGYAVKPVKGDALLFFSLHPDATTDSDSLHGSCPVIEGQKWSATKWIHVR
SFDISVKQGASTDGCEDENVLCPQWAAVGECAKNPNYMVGTNEAPGFCRKSCNVCAQ*
>11669.m00660|LOC_Os03g07000.1|genepair934-2
MARLVLLVVLLLLLSVTGETSATGGGGEGGRFDASRTVDVSWSPRVFLYEGFLSDVECEH
LIALAKQGRMERSTVVNGKSGESVMSKTRTSSGMFLIRKQDEVVARIEERIAAWTMFPAE
NGESMQMLRYGQGEKYEPHFDYIRGRQASARGGHRIATVLMYLSNVKMGGETVFPDAEAR
LSQPKDETWSDCAEQGFAVKPTKGSAVLFFSLYPNATFDPGSLHGSCPVIQGEKWSATKW
IHVRSYDENGRRSSDKCEDQHALCSSWAAAGECAKNPGYMVGTSESPGFCRKSCNVCTS*
>11676.m02371|LOC_Os10g27480.1|genepair935-1
MAAKNHARSQDFWRRFWSMLSYACSELCLIILLHVAAGASYLATRLARIHKLKMPCILCT
RMDHALHGKPWFSSDLVCAAHRSEISSLAYCSSHNNLAQCDDLCKRCTVATNDVVDTRRS
KSRQLCSCCSEPFTKARNAHRISETASVSGEINREQIPADHSKDKAFVVGIEEVNESDSS
PRTYEQSTKNNGASGNAGTAKLAPSGSTVPMRVFVDRNSSVKNGFISRANLSSPRPSQII
SAKDSNSTTQQEVKAFLSQMSTVRGIDSSWSDGAPSPGINAQTDESNANGRRPSLERNYS
VIEPSDANLADEVEGESSPENLKRLLELNKKSMSALYKELEEERSASAIAASQAMAMINK
LHEEKAAMQMEALQYLRMMEEQADHDHEAIQNLHDLLTEREKELLDMDAELENFRRLVQN
EQFNGGKHDIAGIMNETDMPFFEVLNDLGYTKNTMSGFEDEMAYILESISRLEDKLCVSTN
RLASDDAKINQEGLIGGADFGSSPTHGESTSDQQDDGNKSVQNHKDNCSCSHPEDGKTSD
ANLKDEVSLLHTRLQALEADQEFLKHVLNSLRCSPDGLQCVQAIASHLLELRRIATQR*
>11669.m00649|LOC_Os03g06890.1|genepair935-2
MAAKARARSPDFARQFWPVLCHAFSECSLIIMLFVTAVVSFTATRFARIWSLRPPCILCS
RLDRLLHGNTWFSEDLICAAHKLEISQLEYCQSHNKLAHSDDLCERCLLSCAGLDETFKK
TRHTHKLPELANGIVPDDVSTVKERSIDMTSVGHSSDEGSEDLSYGGYSKLNVRHDSESE
NRISDDDEDEDGNSMIHKATQRSRDFLFHDSQLQPMISDTNSLSMHPSENVVLAEPMNTA
PVPVSTAAKTDNVATGTNLVSAAKSSEHIAQGSREISLSNVNVSGNNHDVQPKIVPEQVC

AELPKEKTFLVGIEEVDDSAGISGSPDEEVAKGFVASANAGMSSVLDACINRKNSMKSAS
RRRSNLQSPRWSEIISAKDNSSRTNQEVKTFLSQMSSARGFDGPWSEVAASPRITQIDDK
QYDATGSRQFLETNYSNMEPFDVHATSEDEGDTSLEGLKQKVELGKKKMSILYKEFEAER
SASAVAASEAMAMINRLQEEKASMHMEALQYLRMMEEQADHDQEAIERLNDLLTEREKEM
LDLEAELDNYRRLHEPFGCKFDFTDGDMASGVLDSSDFMRDTMFDFEDEKANILKSLSKL
EETLGMSSTDRHNFGGTYDSLQNMSVLHPEHWSGEPVSSQQIDENQSVDSGSCSHLDDGR
ISSMTSVKHEISLLNTRFTALETDQKFLKQILSSLKCSDDGVQCVQEITAHLRELRRIMT
EQRERAVL*
>11676.m02373|LOC_Os10g27990.1|genepair936-1
MTAQTKKRGGGESGNGGAGLGLAAFIANGEDLGPIVRHAFESGKPESLLQGLRSIVKMKE
VEIEELCRVHYEEFILAVDELRGVLVDADELKGMLSGENLRLQEVASSLLLKLDELLELY
SVNKNVEEALGMLKICLQVTSLCQMCNKDIAEARLHSALKSLELIEKDFLQNIPLKLLKK
AVQKQIPIVKLYIEKKVCNEFNEWLVYIRRTAKEIGKVSISQASLARQKNEGIHSQQREA
EDCSRIGFDEHAYALDLDLIGEEEVLEFDLTPVYRANYIHTCLGLGEKFREYYYNNRLMQ
LNLDLQIPTTQPFMESHQHFLAQIAGFFIVEDRVLRTADGLLSDSQVETMWGTAISKVTS
ILEEQFSRMDAANHLLLVKDYVTLLGATMKKYGYQTTSLLEILEKNRDKFYQLLLSDCRK
KIDGIFTKDSYEQMIIKKENEYHMNVSAFQLEPIGVVPDFPYVAPFSSSVPDACLIVRSF
IDDSVNYLSYDPMMDAYDVVKRYLDKLLIEVLNDGLLNLIHGGCLEITQMVQIAGNIAIL
EKSCDMFLFHAAQLCGVPRRLLDKPHSGLTARAVLKASQNAAYNGLIALANSKIDEFMLL
LTSINWTPEETPEHVNDYMNEVVIYLHTLVSTAQNVFPREALYKVVCGAFSHISDSIMTV
FLSDRVKRFNANAAAGIDIDLKKLEEFADDKFHSTGLSELRKETTFRDCLVEIRQLTDLL
LSNQPENFMNPVIREKNFGSLDHKKVSIICDKFRDAPESLFGSLSGRSTVQSARKKSLDV
LKRRLKDFS*
>11669.m00636|LOC_Os03g06760.1|genepair936-2
MTAQPKKRSVIESGDGGLGLGLALFISNGEDLGPIIRHGFDSGKPEALMQNLRSIVKKKE
VEIEELCRLHYEDFILAVDELRGVLVDADELKGMLTGENIHLQKVSSATLLNLDELFELY
SIKKNIGEAITTLKICVKVISLCMACNGYIAEAKFHPALKTLDTIEKGYLKNIPLKLLKK
VVAKHIPLIKMHIEKKVCSEFNDWLVHIRRMAKQIGQVSISQASMARQKDEEMRARKREA
EAHSDARSDEHVYTLDVENTDEESTLNFDLTPVYRAHHMHICLGIGEKFRDYYYKNRLMQ
LNLDMQISTSQPFLESHQPLLAQVAGFFIVEQRVLRSADGLLSESQVESTWETAIAKITS
ILEDQFARMSIASHLLLVKDYVTLLGTTVSKYGYQIAQLIQVIAKYKDKYHQLLLIESRK
QIDDILANDSYEQMIIKKEYEYNMNVTAFHFEPDDVVPEFPYVAPFSSGVPDICRIVRSF
IGDSVSYFSYSARTNIYEIVKGYLDRLLIEVLNDSLLNMVYARSLAMSQMMQLAGNISVL
EQSCDMFLLYSAQLCGIPKRIAQRSHSGLTAKAVLKASQNAAYNSLINLANFKIDEFMVL
LDDVNWIVEEAPDNPNEYMNEVLIYLETLVSTAQEILPLEALYKMVSGAMSHISDSIMTT
LLNEGVKRFTVNAVMGLDIDLKLLEAFAEEKFYRTGLVDLGKETTFADCLVEIRQLVNLL
LSSQPENFMNPVIRGRNYGSLDYKKVAIVCDKYKDFADGLFGSLSNRNKQDARKRSMDVL
KRRLKDFS*
>11676.m02374|LOC_Os10g28000.1|genepair937-1
MATAATLPSSSSSAAAAVQTISRVLSFPRRGGGGFLHLRRHPLAAAAAATRGRGPLLRLG
PRRPFSASASASAGDNGAAAGGDGGGCDYDYDLFTIGAGSGGMRASRVAASLYGARAAVCEM
PFATVASDSLGGVGGTCVLRGCVPKKLLVYASKYSHEFEESHGFGWRYGTEPKHDWSTLM
TNKNLELQRLVGVQTNMLKNSGVTIIEGRGKVVDPHTVSVDGKLYTAKNILIAVGGRPSM
PDIPGIEHVIDSDAALDLPSRPEKIAIVGGGYIALEFAGIFNGLKSGVHVFIRQKKVLRG
FDEEVRDFVADQMSLRGITFHTEETPQAVMKSDDGLLTLTTNKGSINGFSHNLGLEEVGV
KMDKHGAIVVDEFSRTSVDSIWAVGDVTNRVNLTPVALMEGGALARTIFGNEPTKPDYSA
VPSAVFSQPPIGQVGLTEEKAIEKYGDVDVYTSNFRPLRATLSGLPDRVYMKVIVCANTN
KVLGVHVCGEDAPEIIQGIAIAVKAGLMKQNFDATIGVHPTTAEELVTMRSPTRKVRRDA
VDEAKMKDEATSQK*
>11669.m00634|LOC_Os03g06740.1|genepair937-2
MATTATLPFSCSSTLQTLTRTIPLRLRLHRRRFLHHLPSLAALPRLPLPRPPLLPHARRH
VSASAAPNGASSEGEYDYDLFTIGAGSGGVRASRFASTLYGARAAVCEMPFATVASDDLG
GVGGTCVLRGCVPKKLLVYGSKYSHEFEESHGFGWVYETDPKHDWNTLIANKNTELQRLV
GIYKNILNNSGVTLIEGRGKIVDPHTVSVDGKLYTARNILIAVGGRPSMPNIPGIEHVID
SDAALDLPSKPEKIAIVGGGYIALEFAGIFNGLKSEVHVFIRQKKVLRGFDEEVRDFIAE
QMSLRGITFHTEQSPQAITKSNDGLLSLKTNKETIGGFSHVMFATGRKPNTKNLGLEEVG
VKLDKNGAIMVDEYSRTSVDSIWAVGDVTDRVNLTPVALMEGGAFAKTVFGDEPTKPDYR
AVPSAVFSQPPIGQVGLTEEQAIEEYGDVDIYTANFRPLRATLSGLPDRIFMKLIVCATT
NKVVGVHMCGEDAPEIIQGVAIAVKAGLTKQDFDATIGIHPTSAEEFVTMRNATRKVRRS
TTDEVESKDKVVTQN*
>11676.m02380|LOC_Os10g28060.1|genepair938-1
MDRELVRTVKLATKNHAGVLFRRAVRHLPHIVAVTALVAAAPRLSTLLAAAAAGGSTMRW
ARALWSDLAGELGPSAPALAVACWAAALAAYTYAASRPRPVYLIDLAGYKAPREHEASRA
KTIAHFGRCGRFSGESMAFQKRMLERSGLGEATHFPTSLISLPVDMCLRTAREESHAVIF
GVVDEVLRKSGVAAADVGVLIFNSSLLSPTPSFTSLIVNRYGMRPGVVSHNLSGMGCSAG
IIAIDLAKRLLQVHENTYALVVSTENITLNAYMGNNRPMLVTNTLFRVGGAAILLSNRAA
DRRGRAKYQLIHTVRTHRGAHDQSFGCVTQEEDDAGEVGVSLSKELMVVAGEALKTNITT
LGPLVLPISEQLRFLATVVLKRVFRADVKAYLPDFKLALDHFCIHAGGRGVLDELEKSLK
LSPWDMEPSRMTLYRFGNTSSSSLWYELAYCEAKGRIKRGDRVWQIAFGSGFKCNSAVWR
ALRTVDAAGLDAGDNPWMKEVDMLPVDVPKVAPIDETSYQIPN*
>11669.m00629|LOC_Os03g06700.1|genepair938-2
MDSRELLRTVTQAARNHARTLYHRLVGRHLPRILAVTLLLAAARAGGAATTTLDALAREN
ARALVAVAWCCAAAAYAYAMSRPRPVYLVDLAGYKPAASHEATRAESIRRFGLAGDFTGE
SMAFQRRMMERSGLGEATHFPASLFALPVDMCLRTAREESEAVVFGAVDELLAKTGVPPA

DVGVVIVNSSLFSPTPSFTSLVVNRYRLRHDVVTHNLSGMGCSAGIIAIDLAKHLLQVHA
ETYALVVSTENITLNAYMGNYRPMLVTNTLFRMGGAAVLLSNRRAERRRAKYQLMHTVRT
HRGGASDRSYACVTQEEDGAGNVGVSLSKELMSVAGDALRTNITTLGPLVLPLSEQLRFL
ATVVLRRVFGHAAGVKPYLPDFTAALDHFCIHAGGRGVLDELERSLKLSAWHMEPSRMTL
YRFGNTSSSSLWYELSYCEAKGRIRRGDRVWQIAFGSGFKCNSAVWKALRTVDGGAGRDA
GAWAQDIDALPVHVPKVVPIVDDDDGANGGDGDRHDAASHVRPE*
>11676.m02395|LOC_Os10g28210.1|genepair939-1
MRAAAAPDPSPAPARSMLKRLFDRQLLRVSPAERIVAVGGGEKDEVEPSSVCLDGMVRSF
LEDGSGVGAAVERAGGHGARRCNCFHGGGSSDDDDDEDDAAASSDVAETIKGLVHCATLR
ERNLLADVCGHVERHRAGGARRRELLGLVAASLRAAGHDAAVCVSRWDKSPTHPAGEHAY
VDVLLPPASDRGARERVLVDVDFRSAFEVARPTKAYRALLQRLPAVFVGKDDRLRLLVAA
SADAARASLRKRGLHLPPWRKPEYMRAKWLSPYDREPAPPDEASASAAAAEVAGEEAPAA
A*
>11669.m00627|LOC_Os03g06680.1|genepair939-2
MRAAAAAAGQIDPSPGAPAKSRLKRLFERQLLRVSPAERLPSVAGVGEKDESSEPSSVCL
DGMVRSFLEDGVGVERPAGAARCCNCFHGGEASDDDDDGPAAAEAAATSDAAETIKGLVH
CASLRERNLLADVSTLVERHRAAGARKRDLLRLLADSLRAAGHDAAVCISRWDKSSSHPK
GEHAYLDVLLPPASDRAERERILVDVDFRSEFEVARPTKAYRAVLQRLPSVFVGKEDRLR
LLVAAAADAARASLKKRGLHLPPWRKPEYMRAKWLSPYERDVPSPQQPDASAGELAVDGE
GGGAWTQ*
>11676.m02397|LOC_Os10g28230.1|genepair940-1
MAGKGGKGLLAAKTTAAKAAADKDKDRKKAPVSRSSRAGIQFPVGRIHRQLKGRVSANGR
VGATAAVYTAAILEYLTAEVLELAGNASKDLKVKRITPRHLQLAIRGDEELDTLIKGTIA
GGGVIPHIHKSLINKTAKE*
>11669.m00626|LOC_Os03g06670.1|genepair940-2
MAGKGGKGLLAAKTTAAKSADKDKDKKKAPVSRSSRAGLQFPVGRIHRQLKSRASAHGRV
GATAAVYSAAILEYLTAEVLELAGNASKDLKVKRITPRHLQLAIRGDEELDTLIKGTIAG
GGVIPHIHKSLINKTSKE*
>11676.m02409|LOC_Os10g28340.2|genepair941-1
MDPAAAGIVKEEMLESQQQQRQEDGGAAPRPMEGLHEVGPPPFLTKTYDLVEDPATDGVV
SWSRAGNSFVVWDPHVFADLLLPRLFKHNNFSSFVRQLNTYGFRKVDPDRWEFANEGFLR
GQRHLLKTIKRRKPPSNAPPSQQQSLTSCLEVGEFGFEEEIDRLKRDKNILITEVVKLRQ
EQQATKDHVKAMEDRLRAAEQKQVQMMGFLARAMRNPEFFQQLAQQKEKRKELEDAISKK
RRRPIDNVPFYDPGETSQTEQLDSPYLFDSGVLNELSEPGIPELENLAVNIQDLGKGKVD
EERQNQTNGQAELGDDFWAELLVEDFTGKEEQSELDGKIDGIDELAQQLGYLSSTSPK*
>11669.m00618|LOC_Os03g06630.1|genepair941-2
MEKMMPGMVKEEWPPSSPEEGEAPRPMEGLHEVGPPPFLTKTFDLVADPATDGVVSWGRA
GSSFVVWDPHVFAAVFLPRFFKHNNFSSFVRQLNTYFLVRTNYLNKRSHFYSLRFQGFRK
IDPDRWEFANDGFLRGQRHLLKMIKRRRPLSYLPGSQQALGTCLEVGQFGLDEEIDRLKR
DKNILLAEVVKLRHKQQSTKANMRAMEERLQHAEQKQVQMMGFLARAMQNPDFFHQLIHQ
QDKMKGLEDTFSKKRTRSIDIVPFLNPGEVSQGDQLESTLLFDPRPFAELNDEPAKSELE
NLALNIQGLGKGKQDVNRTRNQPRNQASNETELTDDFWEELLNEGARDDAGIPGMERRRP
RYVDALAQKLGYLSNSSQK*
>11676.m02412|LOC_Os10g28350.1|genepair942-1
MENEFQDGKTEVIEAWYMDDSEEDQRLPHHREPKEFIHVDKLTELGVISWRLNPDNWENC
ENLKRIREARGYSYVDICDVCPEKLPNYETKIKSFFEEHLHTDEEIRYCLEGSGYFDVRD
QNDQWIRIALKKGGMIVLPAGMYHRFTLDTDNYIKAMRLFVGDPVWTPYNRPHDHLPARK
EFLAKLLKSEGENQAVEGF*
>11669.m00617|LOC_Os03g06620.1|genepair942-2
MENQFQDGKEEVIEAWYMDDSEEDQRLPHHREPKEFIPLSKLSELGILSWRLNADDWEND
ENLKKIREARGYSYMDICDVCPEKLPNYEAKLKNFFEEHLHTDEEIRYCLEGSGYFDVRD
QNDQWIRVAVKKGGMIVLPAGMYHRFTLDSDNYIKAMRLFVGEPVWTPYNRPHDHLPARK
EYVEKIINRGGTQAVEAR*
>11676.m02419|LOC_Os10g28420.1|genepair943-1
MGKAGRWLRSFLAGKKDGGGRRSGERQQHGGGDATPAVEVAAASTREKKRWSFRRSSASA
SAAAMGKPAAVTAPSTPEPSVSGLASVSERARDVADLEGQSKHAMAVAAVATAAEGDDVS
ASAVEVVAAVMIQATYRGYLARKALCALRGLVKLQALIRGNLVRKQATATLRRMQALLVA
QARLRAQRMRMLEEEEDDDVHGHGHHHHRRSSPHHPRHRRSYVSRARLPSRRRGNFSYVH
ALQEMDRSGEEQAKIVEVDVGEPAPPRRGRSSCSVAASESRERRMAEYGYYAQCSPAPSS
SAFTAAAAASPPRDASYSGHFDDFSPFEPATARSSPYIPPSPGGGGGGGGGEFFPNYMAN
TQSSRAKARSQSAPRQRTEPHLPQPPPPPPHFERQPSRRRGGGVPRSVKMQRSSSHVGVP
AAHGYHHHHLYSYGQYPWSVKQLDRSSASLKDSECGSTTSSVLTAATTVGYCRSLVGLDL
HRGHY*
>11669.m00612|LOC_Os03g06570.1|genepair943-2
MGKAGRWLRSFLAGGKKGGKKGEAMAAALPGEAAKEKRWSFRRPVHGEKAAAEAAAAADG
VVVGEAEAGFDLSASESEFDQKRHAMAVAVATAAAADAAVAAAHAAAAAVRLSSRKAHQL
PASAVEEAAAVRIQATFRGYLARTALCALRGIVKLQALVRGQLVRKQATATLRCMQALLA
AQSQLRAQAQRVRALHEHHRTPPRPRPPSPPQHPRHRRSYEMDRSCEENAKIVEVDSGAG
EPARRGGEYGHHGRWSPAPSAMTEVMSPRAYSGHFEDMAFAATAHSSPHHASASSELLCC
PSYMANTESSRAKARSQSAPRQRTDALERQPSRRKSGGGGGGAKMQRSSSSHAAAAQRGA
QFPWPVIKLDTSSASLKDSECGSTSSVLTAATVYSRTRSLVGFEVSYDFRDIVHVFF*
>11676.m02421|LOC_Os10g28440.1|genepair944-1
MVGQQQVVDAAAAAAAAATTRVPVPPPKPLLRTIGGNLMETFFPDDPFRAVARESGGRRAL
AALRYVFPFLEWLPSYSLAALWSDVVAGVTIASLAVPQGISYAKLGDLPPIMGLYSSFVP

PLVYAVMGSSRELAVGTTAVASLLFAATLGKEAPPGEKPELYAALAFTATFFAGVLQAGL
GVLRLGFLVDLLSHAAIVGFMAGAATIVCLQQLKGMLGLAHFTTSTDVVAVVRSVVTQSH
QWRWQSIVVGCCFLIFLLFARYISKRKPKWFLLSAMAPLASVIAGSVLVYLIHGDRHGIP
VIGYLKKGINPPSARDLLLSSPHTMVALRTGIITGIIGLAEGIAIGRSFAMLKSYNVDGN
KEMIAFGAMNIVGSCTSCYLTAGPFSRAAVNHNAGCKTPMSNAVMAVAVMLTLQFLTPLF
HYTPLVVLSAIIISAMIGIIDYKAAVRLWKVDKIDFCVCVGTYLGVVFGDIQIGLAIAVG
ISILRILLFIARPKTTVLGKMPNSTNFRRMDQYTVAKAVPGLLVLRIDSPIYFANSGYLR
ERIMRWIDHEEDRIKAEGLESLKCVVLDMGAVASIDTSGTKMLEDLKKNLDRSSIQIALA
NPGSEIMRKLDKSNVLGLIGEEWIFLTVSEACYYAQQNCKIGVGMGVVQCVVDPEHMV*
>11669.m00607|LOC_Os03g06520.1|genepair944-2
MGSGSAANGGGGGAGAARVPMPAAKPFLETLGGNMKETFLPDDPFRVVRRERGCGRRAAA
ALRYVFPFMEWAPSYTLGTLKSDLIAGITIASLAIPQGISYAKLANLPPVLGLYSSFVPP
LVYAMMGSSRDLAVGTVAVASLLIGSMLSEEVSAAEDPALYLHVALTATFFAGVFQALLG
VLRLGFIVDFLSHATIVGFMGGAATVVCLQQLKGMFGLDHFTTATDLVSVMSSVFSQTHL
WRWESVVMGCGFLFFLLITRFFSKRRPRFFWVSAAAPLASVIIGSLLVYLTHAENHGIQV
IGYLKKGLNPPSATSLNFSSPYMMLALKTGIITGVIALAEGIAVGRSFAMFKNYHIDGNK
EMIAFGTMNIVGSLTSCYLTTGPFSRSAVNYNAGCKTAMSNVIMSVAVMITLLFLTPLFH
YTPLVVLSAIIMSAMLGLIDYPAAVHLWQVDKVDFCVCLGAYLGVVFGSVEIGLVVAVGI
SILRVLLFVARPRTTVLGNIPNTMIYRRMDQYTAAQRVPGVLVLRVDSPIYFTNASYLRE
RIARWIDDEEDQCKEKGEMGIQYVVLDMGAVGSIDTSGTSMLDELRKTLDRRGLQIVLAN
PGSEIMKKLDSSKVLEAIGHEWIFPTVGEAVAECDFVMHSQKPGMVMGSGAPHENLV*
>11676.m02438|LOC_Os10g28610.1|genepair945-1
MAALVGHDARQYSWWWVSHISPKNSKWLQENLNDMDSKVKAMIKLLNEDADSFARRAEMY
YKKRPELMKLVEEFYRAYRALAERYDQATGALRQAHKSISEAFPNQMPPMSDESPASSGQ
EVEPHTPDLPTFTRLPFDLDDLQKDGVGVSPQQFTSKRNGTHPEEASALPNRKGFDVKVR
KGLSFGSPEVKGSDAISNEMVNLQQEISRLLAESNSMKQQILSESERANKAENEIQVLKD
TILKLNSDKDTSLLQYNQSTERLSTLESELSKAQDDLKKLTDEMATEVQKLSSAEARNSE
IQSELEALDQKVKMQQEELEQKQKELKSFNLTFQEEQDKRMQAESALLSEGKELAQCQEE
VQRLTKEIQMANEKLNELKQTKVNLENAVSELKKEVENLTEQNRSSELLIQELRDEINSL
KDSKNELQNEIQSLRSTISQLNTEKDATLFQHQQSVERVSDLESQLLKLQPELEEIEQKV
QMLMQDLEQKRQEADSAHAQLQDECNRHTQTEADLHRFKNLHSQLEEEVIKLTENLDRST
KELEELENAKLDLENTSRELKSTILDLNSEKDAVLLQQQQSLAKISELELQLSKTQLELK
NSEQKMQLLELEITQKSESMDSLTLSLKDETEKRVQAETSLMSMESMYSQSQEEVNRLHL
EIEKLNFKLNELENLSSELNSTILLLNAEKDATDLKNQQSLVRISDLESELSKLQAQLEK
IEGKVQMLEQELKHKKEEVDSLQISIQDEAHKRSEGEAALLAMTNLNSESQEEVNRLTLE
TKKLKVKLSEVENSNTDLENIVAKHTQDIHVLREKNVSTELMIKELHHELDALKELNVKL
ESEMGLHIGEKEALQRDFACQKEEKQNLEGIHHSLAEEMSTLKSRSAANQKLIEDLQIMN
LKLKEVCAKNEVEKALLSEKVQEVEKLSEEFSLMENSLSDANAEMDSLREKIKVLETSEG
SLKDVISSHVSEKAILTSDLETLGKSYADISEKNSNLDILISDMKAEIENLRTKLTDSEE
TCQAHLANNSALSDEKNNVFSQLESVTVVMKALESKHADLEDKSSSLSREMNLAYDQVRE
LQDQLRVKDEEYEAFVKSHQTQVNDFEEQISSLQKKSYYMNELLEQEQENHMSASINVVI
LENCLADLKDKNVDLFNECQKFAEANHAAEMLISQMKDEARYHQDERKFLLIHTEKLREG
ISQHMKILNICKDLGPANIAEDKIILQTVSDEASNIMKLKEQSEDANRLMYTELTVLATV
MLQVGLELRDLNLQKRALEKELETRAAEFITLQNNNVQMLEWNEQLKQELQQGCEREEVL
KAEILVLQEKLSCSRESYQTSQNEIVSLTEKNETLCKEYQSLIENYNALEDENGTLLSEC
MRLEHLSLFLRGHNNEVATALGSLTDEMALLSVGKDELDCEVQELSRRGMMLESENNNLK
EYFIYLIEILSAQLALSEFDLNINKSICQELASELESCMAQLSQKDDELLEAEDKVHLLQ
GKNRELCGVVGSLQVAIEGAKIVKEELEKKITTLTEEGNTKDGEISLLRQANERLQVEAD
ILKDKEDSLTSSHELLSKEVEQHEGEFVVLMDDAISSSVNAAVYEEKALELMTENTELKA
NLSTHVALIASLSDHVNELEENTLSLSKPYSTESKKEDAEVPFMQERNHGPESHPLPEGT
PELQRLIARMGALQVAIRNAKDLHDQESTKSAATLAAAHRDIQELKARGGSQMEAREIYS
DNEKLNNVEGSKGKQVQMMKDIELDQISTCPPYGTGAALYPLKNGANAGMDDEMLQLWEA
AERSCKNQTSKSSSAEHDIEAVEEVKSEYPSSELARGRDLGINKLEVSTSSVEPHEQWSN
NVLEKLSSDAQRLQSIQVSIKELKRKMGSPSNGKSPMNSEYNTVSTQLLDTEGCVLEQIN
YNNKLTKRVENYPALSDSMNAEQEGYPSRKISGQVQKGSENVGRLELELQKIQYVLLKL
EEEHEYRRLKVSDKRTRVLLRDYLYGRKEKRGGAQKKKKRAPFCGCVQSRTET*
>11669.m00606|LOC_Os03g06510.1|genepair945-2
MEILGPLPMAESSRQAAIDGCTWVNRVATSSAANCSSTTTTTVSTCPCCCNGGGPCEPFS
ARYTLHAADSGDHRERQMGGEMGSCIACCLALPPLATACPPLVTATLRASAMEYHEWEVN
CPQLEGKFGVLFRLLALRFAWKHDIFCCCLNAYFEEVETSFVVTYDLPRCCSILSTKDIE
TDRHIVPLWQLTCSFVPMASLVRHDSNPTQYSWWWVSHISPKNSKWLQENVTDMDVMVKA
MIKLINEDADSFARRAEMYYKKRPELMNLVEEFYRAYRALAERYDQATGALRQAHRTISE
AFPNQMPSMSEDSPSSGQDVEPRTPEVLMPTRAPFDLDDLQDAAGVSPHLLTVKRNGTQP
DDIGFSSSRKGLKQFSDLFAGSDSSQRVNFSDGKVRKGLNFESPDVKGKKDDSNDIMNLQ
HEVSKLLTERQSLKEQISSESQRANKAESEIHSLKDTISCLISEKDTTLLQYNESTRRLS
VLECEISKAHMELKKLSDDMAMEVDKLKCAESQNSAMQSELETLDQKVRVQEQELEQSRK
EIESFHLSLQDEMAKRKQAEDALCSLEKQYAQSQKEINRLTLDMEMANDRLNDFNLVRLN
LENTVCELKKEVTSLEVKIQILVQELEQKREEADVMHAQLQDEHSNHMHKEAALHALENL
HSQSQEDFNLVKLNLENTVGELKKEVTSLELKIQIQAQELEQKREEADTVHAQLQDERSN
HMQKEAALHALENLHSQSQEDFNLVKLNLENTVCELKKEVTSLERKIQIQVQELEQKREE
ADAMHAQLQDERSNHMQKEAALRALENLHSQSQEEVKQMARDVEHSNKKLSDLENNNLKL
HDLSQGLKKTVAELNSMKDSALLQQQKSSEKVSYLEAQVLVVRSEMEKMVQKTQMLDQEL
EHKNKEISELQNSLQEQVQKCILAETSLLRLEDLHTNSQKEAKTLAHDLERLSEQLTEVE
NDRLDLQNISRELKNTISEINSEKDLMLLQQQHSLERQSYLEAQLLDALSEVEENKKEAQ

LLEENLAHKNDELNDLQNNLEEEGHKRMHAEAALSMVENLHSKSQEEVGKLVMDLDKLEN
ELSELQGRNSRMEELSYELQNTISLLNSEKDAALLQQQLSSERACDLMSQLSKIQLELEK
AEEKMQTMEQKLADKNEMVDFLQLSLQDEGKKRVEVETALISSGNLYSQSQEDVNRLTLE
IERLNEMLNDMENKSSEYKSTILLLNSEKDMSVIQYKQSSLRIYELESKLSGVQEELDNA
EQKVQMLDKELKEKREVVETMQASLQDEAQKRMKGEATLLTMTNLHTQSQEEVNRLTPEI
ERLNRKLNEVENVSCELKNTILLLNSERDTTVLQHKQALVRVSDLESELSDVQAELVNAE
KNVQILDKELKQKREEVDSLQASLNEEAQKRIEGEVALLAMENLHSQSQEEVRGLVLKIE
TLHGKLNEMENSNRDLKNMICKHSEEIHVLGEQNLSAELTIKGLHDQLEKFTEMNIGLQN
EVGIHVGEKEVLQQDLARQKEDKDILEKHLCSLEHELKAVNIRVATQQHLIEELQSKNIE
LEEVCNACDVEKTLLLEKLHGMEELSTEYSILKKSFSNAIVEMEDLKEIVKELEASKNSL
KYDVSLHATEKDALALELETLGKRCADVLEEKSILETSFSNVNYELQELRVKYKDSEESS
RSYLADNTALLAEKHKLLSQLESTAVSLKFLEDKHADLRDNHGSLLSEKVLLCNQVKNMQ
DQLGIKNEQHEALLKLHQMQMLEHEQQKCADASISTLILENSLVDARDKNLALFNECQKF
IQATDSAEVLIAQLKEEARKEEEDKKALLNRNEKLRDGISEQIKVLNICKDLGPTDVVHD
EIMLQTMSRETFNHVKHKEETEERNVFMDAELSVLGAILAQTVIGFRALHQQNCELVEEV
ESGAAELLFLQKENHKLIELNEQLEQRLQLGGNREEMLKIEILGLCKELSGLRQSYQTSQ
SEICNLTKKYDSLLQEYKVLVEKYNALDDENAAVIAECIKLDLLSSFFHDLTVESASVLV
SLDNDMAMLSSVRHELDHEVTMLNRRAKILEMDFQHLKCTLENLLEALGSRLVLSEFDSN
TSKIICQELTIECNSSMTQLMQKDDKLRKVDEKVQFLQERNQELCRVLRDLEVAVEDAEG
VKVDLEKKITTLTERGAVQDNETRLLREANNTLQVEVGIHEQKEESLMSTFETMRKEAEQ
HEREITLLVCDTITRSVNTMVLEEQVLEMMMEREVLETRFFTEKDMLMKEISSRDAYVDD
LQKRVASMRGENAGLMAELAAYLPLVASLSDQIRALEELEDGTLLLSELNKEGKLEFVQK
DRHVPESQDDSSGALKLRSLIARVEALHVVILDAKGRRDKEFTESVAQLEAANMEIQELK
ARKGSNAKEECTEDDRQKYDADNSKGKHVQIMKDIELDQVSTCALYGTGATIYPLGGDAN
VELDDEMLQLWETAERDCKNQTAKSSSSENDIQAVEEVKSEYPSFELARGRDPGIDRLEI
SAVSLEPQQLWSKNVLDKLASDAQRLSIVQASIEEIKQKMVGASKGKSTVSSEYSSIRAQ
LQEIDGSVLEQIDFNCNVTKKAENYPAFEVSAELEGYSSRRKISEQVQKGSEKVAKLELE
LQKIQYVLLKLEEEHEYKRVKAPEKRSRVLLRDYMTARKDKNDAGQKTKKKRIPFCGCVR
IKSRTEP*
>11676.m02443|LOC_Os10g28660.1|genepair946-1
MRDGAKKSKLSWSKSLVLKWFNIRGKSYDFHGDDAAAAFGRRGGGGEDEWRSSSFSRRES
CTVKKSRTERASRRSHERSRRSKIDLDAAEATVTLDYRIFVATWNVGGRAPPGSLSLDDW
LRTSPPADIYVLGFQEIVPLNAGNVLGAEDNGPARKWVSLVRRTLNSLAGTGGGGGGGGG
GGMRTPSPAPDPVVEMDDDFEGSSSRQNNPAAFFHRRSFNAGLSRSLRMDGDILGGGGGA
QPRLERRYSVNDRVMYGSRPSDYEANCRWGHPSDDGEIDDGGGESPSAVFSPMSYGYGAP
PYMEESNGGAAHSRYCLVASKQMVGLFLMVWARREIKSDIRNLKVSCVGRGLMGYLGNKG
SISVSMLLHQTSFCFVCSHLTSGQKDGDEHRRNSDVMEILRKTRFPMVYGQYERSPETIL
EHDRIIWLGDLNYRIALSYRSVKALVEMRNWKALLEKDQLRSEQRGGRVFPGWNEGRIYF
PPTYKYSNNSDRYAGDDMNQKEKRRTPAWCDRILWYGRGLSQLSYVRGESRFSDHRPVYS
MFSAEVESINHSRIQKMSCSSSQLDIEELLPYSYGYTDINPYGYTDLNFY*
>11669.m00601|LOC_Os03g06460.1|genepair946-2
MGEIEDGIACWTEGAGCRHPMENLGERCAFQLRLHTFSSPYKSTKSSSSRRGNDQSTTLE
SNTPRATGLLLSDLEYQSLSKERNKSREKKSMKGTKKNKQIRGRSISAPSPKTQEKRLGS
LFGCMSVPLRNNERWQQHYQEEQGGDDEWRGSSFTRREPSTVKKSKTERSSRRSHERSRR
GKIDLDAAEATVTLDYKIFVATWNVGGRSPPNTMSLEDWLHAAPPADIYVLGFQEIVPLN
AGNVLGTEDNGPARRWVSLVRRTLNNLPGTSGNGSFRTPSPAPDPVVEMDDDFEGLSSRQ
NNASFFHRRSFQAGLSRSLRMEGDILAPQPRLERRYSVCDRAIYGRRPSDYEATCRWGGS
SDDENNTGESPSTVYSPMSYGYGNPSSLEESHRPAGHTRYCLVASKQMVGLFLMIWARKD
IRDDIRNLKVSCVGRGLMGYLGNKGSISISMSLHQTSFCFVCSHLTSGQKDGDEMRRNSD
VLEILRKTRFPMVYGQYERSPETILEHERIIWLGDLNYRIALSYRSVKALVEMRNWKALL
EKDQLRIEQRGGRVFVGWNEGKIYFPPTYKYSNNSDKYAGDDMNQKEKKRTPAWCDRILW
YGRGLSQLSYVRGESRFSDHRPVYSVFSAEVESINHSRIQKMSCSSSQLDIEELLPYSYG
YTDINPYGYTDLNFY*
>11676.m02448|LOC_Os10g28700.1|genepair947-1
MEIEAAKCECCGLREDCTVEYIAGVKADFGGRWLCGLCSEAVRDEVAKKGGGGGGGGRQL
EDAVRDHMSFCGKFCRKNPAFRVADGMRQMLRRRSSDISAPSGAS*
>11669.m00598|LOC_Os03g06430.1|genepair947-2
MEVESAECECCELREECTRGYILGVKADFGGRWLCGLCSEAVRDEAAKLGRNRGGGGMEE
AVRDHMSFCGKCRKNPAFRVADGMRQMLLRRRSK*
>11676.m02449|LOC_Os10g28710.1|genepair948-1
MEGLVVISQQRNHHNQHSGGRGKMTGPHFSSPPSSHGFRGMNCRSFHSGVCAGLLPSPPP
PPARTYSSPEPKTPKQQQQLQRRGGKRSRPISISPSTSPPSRPELWAGPAFSNSPPPSSL
PIPKFSLRQNRSISLELPLFERSDEVEVKPHAKSAPSSPVGGSGFDFFNDNETAVATENL
RRILHLDISDH*
>11669.m00596|LOC_Os03g06420.1|genepair948-2
MAALVISKQRSHQQQSSDRRRKPSSHFASPQSMRGFNAVNCRAFHSSVSIGILPSPPPPP
ARTYSSPEPKTPKSQPHHGKKRSRAISISPSTSPPSRPELWAGPAFSNSPPPSSLPIPKF
SLHQKRSVSLELPPAGRSDDVVVPLHAKSAPSSPTAGSGVSFFSDSDTAIATENLRRILH
LKIADH*
>11676.m03636|LOC_Os10g39810.1|genepair949-1
MLPYATAAEAEAALGRAMTAAESLWFRYSAGIPDYVLFWHNILFLFVVFTLAPLPVALLE
LRAPAAVGPFKLQPKVRLSREEFFRCYRDVMRLFFLVIGPLQLVSYPTVKMVGIHTGLPL
PSLGEMAAQLLVYFLVEDYLNYWIHRLLHGEWGYEKIHRVHHEFTAPIGFAAPYAHWAEV
LILGIPSFVGPALAPGHMITFWLWIVLRQMEAIETHSGFDFPFNLTKYIPFYGGAEYHDY

HHYVGRQSQSNFASVFTYCDYLYGTDKGYRYHKAYQAKMKALGQTEGEKADSNGLSYAKL
D*
>11669.m00096|LOC_Os03g01820.1|genepair949-2
MLPYATAAEAEAAVGRGLTWAEAAWFRYSAAIPDYCLYCHNVPILLLVYTLAPLPLALLE
LRRHLPLPHKLQPGVRHPPAAFLRCYAATARVLLLAVGPVQLASFPAVRAVGIRTGLPLP
SAGETAAQVAVYLLVEDYLGYWIHRLLHTPWAYHHIHRVHHEFTAPMGYAAPYAHWAEIL
ILGFPAFAGPAIVPCHMTTFWLWFVLRHLEAIHIHSGFKLPFDPTKYIPLYGGVEYHDYH
HFVGGHSQSNFSSVFTFCDYIYGTDRGYRYHKASLSKLKEMAGNHVEKGDENGFGNGKQD
*
>11676.m03639|LOC_Os10g39840.1|genepair950-1
MARPGSGNIPGSACIPLLILLLLLLLLHPSEAQPSPGYYPSKMFRSMAFYEGYSTLWGPQ
HQTLSQDQKSLTLWMDRSSGSGFKSTRSYRNGYFGASIRVQPGYTAGVNTAFYLSNTEQY
PGHHDEIDMELLGTVPGEPYTLQTNVYVRGSGDGNIVGREMRFHLWFDPTAGFHHYAILW
NPDQILFLVDDVPIRRYEKKVEGTFPEREMWAYGSIWDASDWATDGGRYRADYRYQPFVS
RFADLKVGGCATAAPPACSPVPASSGGGSAALSPQQEAAMAWAQRNAMVYYYCQDYSRDH
TFYPEC*
>11669.m00093|LOC_Os03g01800.1|genepair950-2
MEQKPPAVAANNNQLLLMMIMVVVACSNYMISGAGAQPSPGYYPSKTIRSMAFGEGYDNL
WGGQHQTLSADQTALTVWMDRSSGSGFKSKRSYRNGYFGASIKVPSGYTAGVNTAFYLSN
NELYPGQHDEIDIELLGTVPGEPWTLQTNVYVHGTGDGAIIGREMRFHLWFDPTADFHHY
AILWNPDHIVFLVDDVPVRRYPRAAGNTFPDRQMWAYGSIWDASDWATDGGRYKSDYRYQ
PFVSRYRDLKIAGCEAAAPASCQPVPASPSGATGELSAQQKAAMRWAQQRSMVYYYCQDY
SRNHANYPEC*
>11676.m03640|LOC_Os10g39850.1|genepair951-1
MEEAGDEEGEQGLRLGGVGDGADGEEEVARGVVAPQVLELSSSSSGEEGGSWEEEESQGS
VEVTRGGGGDREARVSESRDSAEVNGGEAESQSLPGCPICMNAWTADGAHRVSCIPCGHV
YGRSCLERWLLQCRKKPATCPQCGRRFKLNNIINLYAPEISVPNNDLEKQVLSLREKNES
LEKQNQELVQEINEHKRQIILQQNFINESSLKRKNEFFLDGARVMGIDASSQIILTSGRA
PGIGAEHVLTKAPGWSCSGDQNSPNHIYAGLQNGMLLTFDIRQTVAPLHSMMGLSTHPVH
TIHSVVDGGGSRKVISASSIGPCIWDVDGSRNRPDLLNGMENHGVCISLACNPPSSDLLV
ASFRPKVELSDDGTSQAGKSQSPTPSASGKLGCHALIRRTSNTSFARDQICRGNVSGLRM
SKSAIIPFTGRSNQQHLFAYGDESLRGVRTWRLPSLQTFADLRPHRQPILDLRFAESSST
GERYLGCLSEDRLQVFRVR*
>11669.m00092|LOC_Os03g01790.1|genepair951-2
MASRPRGRRLPPPHPPLIQEILDDDLFDVEVNYSDEEEEEEEEESDEEESESEDNEPEEV
VPGQESIGEGCGPAIQARVSGAAAEKNACPVCMEAWASQGAHRICCIPCGHIYGRSCLER
WLRHKGNTSATCPQCGKRFRPKDITNLYAPEVAVPNNDLEKEVLYLRGKAESLGETVMKH
EKLIEEMNERLVELTSAQKRQILSEQRLMNVGSSKRQKEFFMDGLRVMAIDAFNQTILAS
GKAPGIGQEHVLYKFSMVSHHEARNIQLPLDTKTVRDICILPSGSAIFTSLGRKLSSFSM
TADRVVLQCDLPCPGWSCSADESSRQICAGLQNGNLIIFDIRQTSRPLHSMVGLSTHPVH
TLHTVIDNNGSRKFLSASSIGPCMWDTDGIQGRPKLLLGTDNQRVCISLACAPSSSDLLV
ASFRPKVETSEDATASQVYLSQTPTPSGGGKLGYHSFIRRAGNSSSFTEDRTCSTLVSEI
RMSKSAIIPYGNNQHLFAYGDESLRGVRTWRLPSFEMHSDLSSLQQPVLDLRYAESSSGG
RYLGYLSTEKLQVFRIR*
>11676.m03641|LOC_Os10g39860.1|genepair952-1
MCRFLISTPFSRRRGERKAEAGRMARSVSYVSAAKLLAMARSNPRVAIIDVRDEERSYQA
HIGGSHHFSSRSFAARLPELARATGDKDTVVFHCALSKVRGPSCAKMFSDYLSETKEESG
TKNIMVLERGFNGWELSGQPVCRCTDAPCKGTCSPEEPEL*
>11669.m00090|LOC_Os03g01770.1|genepair952-2
MARGVSYVSAAQLVPMLRDPRIAVVDVRDEERIYDAHIAGSHHYASDSFGERLPELAQAT
KGKETLVFHCALSKVRGPSCAQMYLDYLSEADEDSDVKNIMVLERGFNGWELSGRPVCRC
KDAPCKGVCS*
>11676.m03647|LOC_Os10g39920.1|genepair953-1
MMSASPEFYKPAPVFSPCSSPLRLLQQQHGEDHQEEYGYGSSGGCCRTPTGGESNLKAPG
TCPPAPRKPRAPAAPCRKRLFEVEVLSLRLEELERLFWRPPPPPPTTQPQPQPQQPPQKR
RRVAKLGS*
>11669.m00083|LOC_Os03g01740.1|genepair953-2
MSASPEFYRPSPPAFSPSCAAGTSTTEVDEYSCCRTPTPGIREPATCPPAPRKPRPVACR
KLLFDPAQQQGKGKAISLRLDELERLFRPITNNANLHLQTNKPTHT*
>11676.m03650|LOC_Os10g39950.1|genepair954-1
MSTELDRRGSSDRLRERVKFRFSGFRAVQVPVVSDRLMLSIVAVDTGKTIAKSTKAAALS
GACQWPDSILESIWFSQDQVSEEFQECQCRFVVSMGSTNSGILGEVFLNLTNYLSSLEST
AISLPLKRCDSGTILQLNIQCLGAKSKTSRTNDDTECTSDGFDSMLNRTTHSLSGNDLGG
SYQDEAGNRDASLSASRSYSGDSTTDRTNMPPSDNLNDELNTQRHNFASPDAIHVSADHV
DEASRSNNSSFSSQTPSRNMLQGNNAQPSASDLSQLSSGVSHASKDVLENAEETIDELRG
EAKMWQRKTRKLKQGLETLKKVSTDKSKQRSEQDLEKMWQRKTRKLKQGLETLKKECADK
SKQQSELELELSISISERDSLRQEIEELKRSLEEVTARQTISRSPRSGDAIELQKEVEDD
VQFLKESNASLATQLNKAQEANIELVSILQELEETIEVQRAEISNLSHTSDLIDHEVSPN
NLLIQEDVEWARKVSLKEDEILMLREKIDRMLHVENPNGEGSGAIYLELEKENDFLKVKI
QELEKDCSELTDENLELIYKLKEVSEVAKGEDPSVPNSEEVSSEGDLSDRLTSKVKYLET
KCADLELKLISFRSESSELEEKLQKSQEELKDRILELSDLRDKLSGFHALEMEEGDTDSA
KSYKLKSEKLDENDNKTELDALRSTVLLKEQEIESLQHSKKEMESFISEIMNEKNKLEEL
LEESLKECSITAACLDEMREELLLLTSSIDSHVSTNNVLETKITELESCKVNLELHISKL
EHENVELSEFISGLESQLTYLANEKELSMLQMDESRSLITNLKDELEQVEAQKVELKLQM

DESRSLITNLKDELEQVEAQKVELKLQMDESRSLITNLKDELEQVEAQKVELKENQLESH
RRLSEVQEDSEALRRSNAKLQATVDHVVEECKSLQTLTADLKKQKLEVHGYASHLEQELE
QSKRKTMDFCKTLESLEAKLSSLQEDISLKEQSLLSELENIFQEHKEHEERIDRVHLLLN
KIEKEKTVELSNLEREVISLTAQLSSTEEERESSTLDTIREVSILRADKAKLEANLEDVN
AQMIHYESQLEDLRESKTKIKDLVDSLNASKQNEEMLTTDVDNMRRSIEAARSNEDNLRK
TLCELELKSKSSDYEKQQIIEEISVLKIQVHKIAGLQDEVLTLQSSLDEAKFEKGKLEGL
IQSLSEECEELKAQKGMLTDKVSCMQDTLNAANEGKQIEISAQTKLVMLGDEPPVKETSD
VLEAELKSELSIIRGANSEYQQKIYSLQEENEDLTRRNQLMEKELDLKTSQNKDENTNKQ
VSLQDEVLMLQSSLDEAKFENGKLEGLLQSLSEECEELKAQKGMLTDKVSCMQDTLNAAN
EGKQIEISAQTKLVMLGDEPPVKETSDVLEAELKSELSIIRGANSEYQQKIHSLQKENED
LTRRNQLMEKELDLKTSQNKDENTNKQGNDANENGDSPVNEVPELQSKIQLLETRLAEAL
EENKLYRGQLKSPMPEGKSASKDGKENDDDKISQLESELKDMQERLLNVSLQYAEVEAQR
EELVMELKTANAKKGRWF*
>11669.m00075|LOC_Os03g01710.1|genepair954-2
MFKLHRHRSSDRVGQRFDFRFSNFRAVQVPAVSDRLFLSIVSVDTGKTVAKSGKAAARSG
ICQWPDSILESIWFSQDEVSKEFDECQYKIVVSVGSIKSGVLGEIFLNLTNFLNLVDPTA
ISLPLKRCNSGTVLQLKVQYLGAKSKSSGVRSWKDLSPRLDDRSPTNDDIDSKSDGSDSV
ANRSVRSSSGNPLGGTTQDELGNREMSFSASGSHRSSNSGDSTADRTNLSPRDSSNGGMH
VGRQDSASSYVSASRGDDGFRSNNSSFSSRASGPNVLQGNTPKSFGNGFGQLSLGTSDSS
KELLEAAEETIEELRDEAKMWERHSRKLKADLEMLKKECSEKSKQQTELEAELSAAHAER
DSYRQEIEELKSSMKEVTTRQKVGGTSKYGDWIDLQKELEDDVKFLKESNANLSIQLKNT
QEANIELVSILQELEETIEEQKAEISKISKVKNVTDADALKKGPLVKQDTEWAKQLSIKE
DEITMLREKLNHVLNIENLGSDAVYLELEKENELLRVKIQELEKDCSELTDENLELIYKL
KEVGGATKGQGPCIPNDSNLQIEELKSQICQLEEELRSKELLHTGSFADASISSSKVLQE
KCADLELKLLNFRSQIYELEEKFQKSQEELEQRNLELSELRQKLDSSHSMAGEGVQTSGA
RGYQFRNGMDSEPETDVLKAKIQLQQQENDDLRCSKVEMESVISKIQAEKSQLEECLEAS
RKESSISSKCLDEVRQDILVLSSSIDSHVSANKVLERKVTELESCKADLELHISDLEQEN
IELSERISGLEAQLTYMTNEKESSELQIHDSKSLIVNLKDKVERQQAEMETQRLEFKQKQ
QEAQRKLSEAQDDSEVLRRSNSKLQSTVESLIEECSSLQNQIAELKRQKLELHGHLTQQE
QELDNSKKRNLDFCKTVEFLEAKLSSLQKDISSKEQSLLSELESIFQEHTEQEEKINRAH
FMLNKIEKEKTLEVENLEREVMSLTAQASSTQEERENATVEAIREVSVLRADKVKLEASL
QDVSAQLRHYESQLEDLRKESKSKIKGLVDSLNASKQSEEMLAADAEHMKKLMEDAKSNE
DKLRKSSGELELKLKANDYEKQQMIEEISGLKLQVQKIMSLQDEVLKLKSSLDEAKFERG
KLEELHRSVTEECEELKAQKAMLTDKMSNMQETLDNGEEEKRSRIAMQAKLVRLESDLSA
VEASHVHEAELKNELNRIKRSNSEYQRKIQSLEQENEDLTSQLEQMAHIKEEDLGKQDIG
GSPVDEESGIHLKIQVLEAKLAEALEENKMYRAQQKSPMPDGQCAAGNGNESSNERVLQL
EGELRDMKERLLNMSLQYAEVEAQRERLVMELKATKKGGGRWF*
>11676.m03653|LOC_Os10g39980.1|genepair955-1
MALASLPKVVMGSVAFGVFWMLAVFPSVPFLPIGRTAGALLGAVLMIVFHVISADDAYAS
IDLPILGLLFATMVVGGYLKNAGMFRHLGRLLAWRSQGGRDLMCRVCVVTALASALFTND
TCCVVLTEFVLELAAERNLPAKPFLLALATSANIGSSATPIGNPQNLVIAFNSKISFISF
LLGILPAMLAGMGINMLMLLCMYWKELDGGACSPDEVAAGKQMEAIEEGRRTALNNNKKD
DGDAATPASPEDDDGGDAESMMSENISTKHRWFMQCSEHRRKLFLKSFAYVVTVGMLVAY
MLGLNMSWTAITTAIALVVVDFRDAEPCLDKVSYSLLVFFSGMFVTVSGFNKTGLPGAIW
NVMAPYSKINHVTGVTVLSVIILLLSNLASNVPTVLLMGDEVAAAAATISPAAVTRSWLL
LAWVSTVAGNLSLLGSAANLIVCEQARRATRNAYDLTFWNHVIFGLPSTLVVTAIGIPLI
GKINI*
>11669.m00074|LOC_Os03g01700.1|genepair955-2
MSELASAPKVALGSIAFAVFWMMAVFPSVPFLPIGRTAGSLLSAVLMVIFHVISPDDAYA
SIDLPILGLLFATMVVGSYLRNAGMFKHLGRLLAWKSQGGRDLMCRVCVVTALASALFTN
DTCCVVLTEFVLELAAERNLPAKPFLLALASSANIGSAATPIGNPQNLVIAFNSKITFPK
FLMGILPAMLVGMAVNMVMLLCMYWRELGGGAELSVDGKQMEAVEEGRSPASAKSTPQLN
GNGNTMMSLEMSENITTKHPWFMQCTEARRKLFLKSFAYVVTVGMVVAYMVGLNMSWTAI
TTALALVVVDFRDAEPCLDTVSYSLLVFFSGMFITVSGFNKTGLPGAIWDFMAPYSKVNS
VGGISVLSVIILLLSNLASNVPTVLLMGDEVAKAAALISPAAVTTSWLLLAWVSTVAGNL
SLLGSAANLIVCEQARRAPRNAYDLTFWQHIVFGVPSTLIVTAVGIPLIGKI*
>11676.m03668|LOC_Os10g40090.1|genepair956-1
MGSLTTNIVLAVAVVAALVGGGSCGPPKVPPGPNITTNYNAPWLPARATWYGQPYGSGST
DNGGACGIKNVNLPPYNGMISCGNVPIFKDGRGCGSCYEVKCEQPAACSKQPVTVFITDM
NYEPISAYHFDFSGKAFGAMACPGKETELRKAGIIDMQFRRVRCKYPGGQKVTFHVEKGS
NPNYLAVLVKFVADDGDVIQMDLQEAGLPAWRPMKLSWGAIWRMDTATPLKAPFSIRVTT
ESGKSLIAKDVIPVNWMPDAIYVSNVQFY*
>11669.m00068|LOC_Os03g01640.1|genepair956-2
MASSCLLLACVVAAAMVSAVSCGPPKVPPGPNITAAYGKQWLEARGTWYGKPKGAGPDDN
GGACGYKDIDKAPFLGMNSCGNDPIFKDGKGCGSCFEVKCSKPEACSDKPVIIHITDMNT
EPIAAYHFDLSGHAFGAMAKEGKDEELRKAGIIDMQFRRVRCKYPGETKVTFHVEKGSNP
NYFAVLVKYVGGDGDVVKVELKEKGSEEWKPLNESWGAIWRIDTPKPLKGPFSLRVTTES
DQKLVANDVIPDNWKANALYKSEIQVD*
>11676.m03680|LOC_Os10g40200.1|genepair957-1
MEYGAATRGALLAATLLAGARRSRLPLSPPPSPPSIQIQNRLYSMSSLPLKARGVRRCEA
SLASDYTKASEVADFDWENLGFGIVQTDYMYITKCGQDGNFSEGEMIPFGPIALNPSSGE
NALRMRTGAERMCMPAPSVEQFVDAVKQTVLANKRWVPPTGKGSLYIRPLLMGSGAVLGL
APAPEYTFIIFVSPVGNYFKEGLAPINLIVEDKFHRATPGGTGSVKTIGNYASVLMAQKI
AKEKGYSDVLYLDAVHKKYLEEVSSCNIFVVKGNVISTPAVKGTILPGITRKSIIDVALS

KGFQVEERLVSVDELLEADEVFCTGTAVVVSPVGSITYQGKRVEYAGNKGVGVVSQQLYT
SLTSLQMGQAEDWLGWTVQLS*
>11669.m00064|LOC_Os03g01600.1|genepair957-2
MELHLTSRGALPLSPPLAGQRRPHLSLSTPSLPIKNHTYSVPPPFSKAHCAIGCQASLAT
NYMETSAVADLDWENLGFGLVQTDFMYIAKCGPDGNFSKGEMVPFGPIELSPSAGVLNYG
QGLFEGLKAYRKTDGYILLFRPEENAIRMRNGAERMCMPAPTLEQFVDAVKQTVLANKRW
VPPTGKGSLYIRPLLMGSGAVLGLAPAPEYTFMIFVSPVGNYFKEGLAPINLIIEENFHR
AAPGGTGGVKTIGNYASVLKAQRIAKQKGYSDVLYLDAVHKKYLEEVSSCNIFIVKGNVI
STPAIKGTILPGITRKSILEVAQRKGFMVEERLVSVDELLEADEVFCTGTAVVVSPVGSI
TYLGQRVEYGNQGVGVVCQQLYTSLTSLQMGHVDDCMGWTVELNQ*
>11676.m03685|LOC_Os10g40250.1|genepair958-1
MGRRSHKRSTEQEENNVGCVWGLMRMLYFRRDAKFLLDTKQVSRRHTFRELADGRHSVKN
SSDFVETDDDDDNKEECASQKRTVKKLMEDELGKVNLLKKIPSNEIQRGLPDLGYDVSLD
GGSEHTNKPVAALNQHTDIFASYLSGSVYSQGSKSLNHSEEYDLESVLANFLGEIYRCHG
ECPHGDCKNKGELCPSLKSLIHNKLNDLNNPHATHGNEQSPESKGEGLLGENSRSNSRAA
QFKEFKDAVEILSSNNELFLKLLQKPNSHILDNIRKYQNSRLTTKLEPDKSLGRSSILEE
KRGSNHELATKAQGKETKHVFFWRKDRSDRKQKPERANRPQPVSKIVILKPNQGRRIDET
ETTSSRYLHQQPCTSQAPEFSGRESSKFSIKEVRRRFKIVTGDSKREKNAIPAENLPGDS
HQLKDSVVEDKDPRHLTEGSLPDKAASNFKNGIKPSASSKQKQQNDSQSEISDHTTGASI
FYEKAKKHLADMLKNTSQSASYPTAQVSKSLEGMLSQPHYNVSPPRSDHRGKCHNAFSPE
EPEVCLVKAVDVEEPAQERSQLHDNSESNAYSTSVAVDDQVAVLEECGIKEDTQEGIIYA
TDEVDTVPVEGVGKLDCSKTICNIQCIPAEQYTDSPLPEILEGTEGKEPVQMFMSSPESM
VENLEQQDPKTPEPKSSPKLPDGCPEQSNEKKEQPSPVSVLDSFDEDDSSPECKTMKKYE
LHEVSCGTLYFPDNESGVKVFWEDKNARLDYIMLVLELSELCAEQNLEVWYLEDELISPC
MFEELQNQGDRIDDMKILFDCICEALTEIQERYFRLSSWLSFVKHDIRTPPVGEKLISEV
DKYVDGYLKCSFPSTLEQIIKRDLEVQAWMDIRSKTEGIVVEIWEFVLDELIDEAVFDLW
I*
>11669.m00062|LOC_Os03g01580.1|genepair958-2
MGKRSQRLARRQENIGCMWGLIGMLYFRRDAKFLLDRKQGSRRHTFGGLSGRRHSRKKSR
DFEETDEYGEDNIEECDTRKQTVKRLMEDELGKVKQVKKIPKEEVQRILADLGHDVCLEK
SSMQSTKQNRAKSHSTSTAMASPSGLLDPSGSKSMKQAEEDDLELSLADFVGELYGYHDD
CKNKSELCPELKSHIHTKLSELKSVPCQRAYEESPDWGQREHFYEKYICNSRSYQSNKLV
DAPDMLSPEKELFLKTLQKPSPHTLEKENTQNNQNRQVVTKLEPRKILEKGENTKNSKQH
EVAIKTHSKEGRNIFFWRKDKSIMKGTSEGTNSSKMVNKIVILKPNPRGIDTTVATASTC
LDQQSCTIQSPKYPATESSKFSIKEVRRRFKIVTGDTRRGRPSVYEDDLQRDSQRINDSV
FKVRKDSKQSDKDNLRPLTSGKQKQRNDGLGEINGDIITSKDTSIFYEEAKKHLTDILEY
NSHTTKHPTVHTSKSLIGMLSLPQRNASSPRSSPRLKGRIDLSPEEINISAIQQDERTEY
AKERDLSDEDSGSVACGNSEVLDGKADQDRHSMKQETAQDGDIMHIEEIDKPACSETICS
EGITLKEQCTCTSSLELIEGAEPGREHAGMLLSYPENVVESLEHQEPKTPRSSASLELIS
QISPEGNHEKQEQPSPVSVLDPFFCEDVDSPDHETMIKCEMHQDMMRPHIPDAISDQWVF
WEDEDARLSYIKAMLELSELCTYQNLEVWYLEDELISPCMIEELHQGNQTDDLKLPFDCI
CEAITIIQETYFRNPPCLSFLMHKIQPPPMGENLIQEINKHIERHLHNQFPRTLNQLVNI
DLEDGTWMNLQLESEEIIVDTWEFILDELLEEVANDLLI*
>11676.m03704|LOC_Os10g40440.1|genepair959-1
MAYSKIAPLLALTILLFAAAAHGCAPYCPGGAPPVIPTPPVVVPTPPAHHHHGGHGHGRC
PIDALKLRVCANVLNGLVGVKIGAGPDDCCPLLSGLADLDAAVCLCTAIKANVLGIINLN
IPVDLSLILNNCGKICPSDFTC*
>11669.m00032|LOC_Os03g01300.1|genepair959-2
MAGKKVQVCALFLALNVLFTMQMGAVVQACEPYCPTPTPPVTPPPSPPSGGGNKCPIDAL
KLSVCANVLNLLKLKIGVPESEQCCPLLGGLVDLDAAVCLCTAIKANILGINLNIPVDLS
LLLNYCHKTCPSDFTCPL*
>11676.m03721|LOC_Os10g40600.1|genepair960-1
MAMVLPETAAEGKALTDAWDYKGRPAGRAATGGWGCAAMILGAELFERMTTLGIAVNLVP
YMTGTMHLGNAAAANTVTNFIGTSFMLCLLGGFVADTYLGRYLTIAIFEAVQATGVMILT
ISTAAPGLRPPACGDPKGASAECVAADGTQLGVLYLGLYLTALGTGGLKSSVSGFGSDQF
DESDVDGERKKMMRFFNWFYFFVSLGALLAVTVLVYVQDNVGRRWGYGICAAGILAGLAV
FLSGTRRYRFKKLVGSPLTQVAAVTAAAWSKRSLPLPSDPDMLYDVDDAAAAGHDVKGKQ
RMPHSKECRFLDHAAIIDRSAAESPATASKWRLCTRTDVEEVKQVVRMLPIWATTIMFWT
IHAQMTTFAVAQAELMDRRLAGGFLIPAGSLTVFLIASILLTVPFYDRLVVPVARRATAN
PHGLTPLQRVFVGLSLSIAGMAVAAAVERHRATASASAAAAAPTVFLLMPQFLLVGAGEA
FTYMGQLDFFLRECPKGMKTMSTGLFLSTCAIGFFFSTLLVTIVHKVTGHGARGGGWLAD
NLDDGRLDYFYWLLAVISAINLVLFTVAARGYVYKEKRLADAGIELADEETIAVGH*
>11669.m00031|LOC_Os03g01290.1|genepair960-2
MAVAARVVEEEDPDVVEMMKRRDDEEEVVGEAWDYKGRGAIRGSTGGWRSAAMILCVELN
ERLTTLGVAVNLVTYLTGTMHLGSAASANAVTNFLGTSFMLCLLGGFLADTYLGRYLTIA
IFTAVQAAGMAVLTISTAAPGLRPPPCSSGGSGCAAANGTQLGVLYLGLYMTALGTGGLK
SSVSGFGSDQFDESDGGERGSMARFFSWFFFFISMGSLLAVTVLVYVQDKVGRRWGYGIC
VVAILTGLLFFLAGTTTYRFKKLLGSPLTQIAAVTAAAWTNRNLPLPSDPSMLNHDDSAH
TTAKPNQKLPHSKQFRFLDHAAIVETTTAEAKKRKRWAACCTVTEVEEVKQLVRMLPTWA
TTILFWTVYAQMTTFSVSQAAVMDRRLSTSFVIPAGSLTVFFVAAILLTVPLYYRLLAPL
VRRLTANPRYDLSPLHRIFVGLLLAALAMVAAALTERARRDAAVTLSVFYLLPQFLLVGA
GEAFTYIGQLDFFLRECPKGMKTMSTGLFLSTLSLGFFFSTALVTVVHKVTAESRRPWLA
DNLDEGRLDNFYWLLGGISVVNLAVFVAAAARGYVYREKRLADAGIIHI*
>11676.m03731|LOC_Os10g40700.1|genepair961-1

MAARMGSKVAAILAILSVLVVHGSCKGHPVNYNVSDASAYGSGWLPARATWYGAPTGAGP
DDNGGACGFKNVNQYPFSSMTSCGNEPIFKDGKGCGSCYQIRCNKDPSCSGNIETVIITD
MNYYPVARYHFDLSGTAFGAMAKPGLNDKLRHSGIIDIQFRRVPCNYPGLKINFHVEEGS
NPVYFAVLVEYEDLDGDVVQVDLMESKSAYGGATGVWTPMRESWGSIWRLDSNHRLQAPF
SLRIRSDSGKTLVANNVIPANWSPNSNYRSIVQFS*
>11669.m00029|LOC_Os03g01270.1|genepair961-2
MAGRSRRRSFWSVGVAAALLCLLAAHGCSAKHHKPKPTPGGISGNASSSSSNSSTPSIPP
PVAPTPTAPTPPIPSPGTGSSNGSSGGGGGGWLNARATWYGAPNGAGPDDNGGACGFKNV
NLPPFSAMTSCGNEPLFKDGKGCGSCYQIRCVGHPACSGLPETVIITDMNYYPVSLYHFD
LSGTAFGAMAKDNRNDELRHAGIIDIQFRRVPCQYPGLTVTFHVEQGSNPVYMAILVEYE
NGDGDVVQVDLMESRYSTGGVDGTPTGVWTPMRESWGSIWRLDTNHPLQGPFSLRITNES
GKTLIADQVIPADWQPNTVYSSIVQFD*
>11676.m03735|LOC_Os10g40730.1|genepair962-1
MGSLSSLAAAAVFLSLLAVGHCAAADFNATDADADFAGNGVDFNSSDAAVYWGPWTKARA
TWYGQPNGAGPDDNGGACGFKHTNQYPFMSMTSCGNQPLFKDGKGCGSCYKIRCTKDQSC
SGRSETVIITDMNYYPVAPFHFDLSGTAFGRLAKPGLNDKLRHSGIIDIEFTRVPCEFPG
LKIGFHVEEYSNPVYFAVLVEYEDGDGDVVQVDLMESKTAHGPPTGRWTPMRESWGSIWR
LDTNHRLQAPFSIRIRNESGKTLVANNVIPANWRPNTFYRSFVQYS*
>11669.m00028|LOC_Os03g01260.1|genepair962-2
MVSGDVGVVVYYLLLVLVVVQGCKGSSAVQGEGRWYNESEAIGGAAAWGNAKATWYGQPN
GAGAADNGGACGFKKVNQYPFMGMTSCGNQPLYKGGKGCGSCYRVRCNRNPACSGNAQTV
AITDMNYFPLSQYHFDLSGIAFGRLAKPGRADDLRRAGIIDVQFARVPCEFPGLKVGFHV
EEGSSPVYLAVLVEYENGDGDVAQVDLKEAGAGGGRWTPMRESWGSVWRLDSNHRLRAPF
SIRIRSDSGKTLVAPDVIPLNWTPNTFYRSFVQYSS*
>11676.m03740|LOC_Os10g40780.1|genepair963-1
MIPFNSAVAEDVRSLVQGVDDSTFDSTHRELCQLADCSPDGCSLLLQVCLDEVLLNADVA
KSSRLKPELLSTVFKYCLDKPYFSTSFCEALKTVHVSDMFLVKLSNELNLSAGERVGVGL
ALSDSGNLGLITKGQKFSIAEIEEICANPAHVLTNDQIHDIVVFLHQTDGLSKHMDSFTN
IISLLNVKEMPFYVPAPIKEGNARPTISSRHMELYTGSLDDDFDSLLSEIGKEISMADII
TELGYGCTSDIAHCKEILSLFEPLDDMGISKLLGAVVCTHVGLGEAHNTYSTFMSAVGNS
QPSDSSQFTAWNIDVLVDSINEIAPRTNWITVVENLDHEGFCIPDEAAFCLLMSIYSRAC
KDPFPLHAVCGSLWKNTEGQLSFLKHAVAAPTDTFTFKHCSRKMVFPEFANRMQGNQAWY
CLDLLEVLCQLAELGYATMVRSMLDYPLIHCPDVLLLGVSHINTPYNLLQYEVLSCVFPM
ILKDTTYSSLMNSLWHVNPYLTLRGFVDSHSDANCLLRTVEICQDLTILSAVLDSTPFAF
SIKLATVAFRQSHSNLEKWLVEKLTAQGETFLEEIMSNTTYEAAEGAVQQPQVMILDICR
ESCPLIIKVLQSHSGQLLSNQLVEELRRVEAVHESRNHGVVGRDAPTSEGGPDDIEAQAN
IYFHQMFSGQISVDAMVQMLARFKESTNKRELSIFNCMVSNLFEEYKFFPKYPDTQLKLA
AVLMGSLIKHQLVAHLGLGIALRSVLDALRKSIDSKMFMFGTTALEQFMDRLIEWPQYCN
HILQISHLRATHAELVAAIERVLAKISSSQNEPNVGSMLSADQHGSSSIGNMEASEASWQ
LINPTPTQLERSHQQRHQGFLGERSKGSTNIIQAKNILSSGQMPLASSPGDLAVNLKAAT
TPSSQASPHHSTTVSAPLQPTGFLRSRSSAPSGIRQPSFTTGFGAALNIETLVAAAERRD
TPIEAPPSEVQDKIFFMINNISTSNMEAKAREFNEVLQEQYYPWFAQYMVMKRASIEPNF
HDLYLKFFDKVNSKSLNKEMVKATYENCKVLLQSDLIKSSSEERSLLKNLGSWLGKFTIG
RNQTLRAKEIDPKILIIEAYERGLMIAIIPFTSKVLFKNLSVDMKDVKPSSLLKDRIRQI
EGNPDFSNKDVSASQAPVVAEVSSGVMPTINHVEPQPEINSTSRATSLPNMLSQYAAPLR
LPTNNMVEDDKSALIMPEQVSSLGLSQVSPSQTPSLSSSSFSLSQLIAAIPRADIYFRIN
EKLSSFGSLQYSKIMDMALDKAIKEIIGPVIQRSVTIASRTTKELILKDYAMEADDSAVS
RSAHLMVGTLAGSLAHVTSKEPLRVALSSHLRSLIQGITNNTESTEQIMLILVNDNLDLG
CALIETVATRKAVEMIDGEIKQPFSQLRRQKELLGSAYYDAFPYTQGLKRVPDALRPKPT
GHLSAAQRRVYEDFITVWHSQSSQNAGGSATATAMAVAPSNSSVPRVYSPNSALTDSSSF
SSHFASASQTTELVHEESDRNAHLSSLSSKIGASDTSTQVIGTTNVASVFPPMVPNDLPV
GEPTTTNKDLVTSAPLSPTTAVDRMGSVFVEPLNTSDALEMYQQVSQKLDTLIAKDGKDA
EIQSVIAEVPDILLRCVSRDEAALAIAQKVFRSLYDNASNSNYVTWLLAALVAIRDVCKL
VVKELTSWVIYSDEDKKFNIDIIIGLIRSELINLGDYNVHLAKIIDGGRNKAATEFAISL
VQTLITQESISIAEVYNVVDALSKLAIRPSSPESLQQLIEIARSFASVKDENIRQSRDKK
VLSGRPLVNKEENNANDVAFTDAVGFQEKVAVSFSEWCNICDHPTMGDSAYTHYIVKLQQ
DGLLKGDDLTDRFYHILTELAVAHSVVSEQVVAPGGISQQPTQQLQISYFSIDSYSKLVA
LVVKYSSVDIGPSKGSLFNKILSVIVRIIQRDAEEKKVSFNPRPYFRLFINLLSELTTSD
LHHDSANFQVLTAFANAFHVLQPLRVPAWSFAWLELVSHRSFMPKLLLCNAQKGWPFFQR
LLVDLFKFMEPYLRNAELGQPIHLLYKGTLRVLLVLLHDFPEFLCDYHFSFCDVIPPSCI
QMRNVILSAFPRNMRLPDPSTPNLKIDLLAEISIPPRIMSDVDGALKSKQMKTQVDEYLK
RPDGSFLTDLKQKLLLPQNEANIAGTRYNVPLVNSLVLYVGMQAVQQLQLNKMNASASAQ
QMNQSQLDVQIETATELFRNLVMNSDTEGRYLLLNAIANQLRYPNNHTHYFSFIILYLFS
EANQEIVQEQITRVLLERLIVNRPHPWGLLITFIELIKNPRYSFWARSFTRCAPEIEKLF
ESVARSCGGKGGDDGVGLPDGGH*
>11669.m00025|LOC_Os03g01240.1|genepair963-2
MVKVGSWALKPTATLIMGRRDYLLSYYNRVQPYNLITTLHHSLLTTLLIIVSNPKTYYYM
DKLVDSGLDGCILLLQVCLDQVLLKPHQLDKSKHDLLSAVFRYCMDKTYFTTCFCEALGP
MPATHGDGFLKTLSNVLELSPAERVGIGLALSDSEDSGLKLKGQQFSIAQIEGLCLNLLQ
SPSNDQIHDIVIFLHQTDGLSKHMDTFNTIISLFKTKETPFFAPTPFNKCDIQSRHLDMC
FGSMDYDSELLLSEIEKEITMADIVTELGYGCTVDTTHCKEILSIFEPLDDVAVSKLVGA
VIGTHNVLVEAHNTYAMFVSAISNMNDSPQLTTWNTDVLVDSINELAPSTNWVHVMENLD
HEGFNVPDEAAFCLLMSIYAHPCKEPFPLHAVCGSLWTNTEGQISFLKHAVSVPTDTFTF
SHCSRKLAFPDLACPIQDNHAWFCLDLMVVLCQLAEVGHTVSVQSMLEYPLQHCPELLIV

GLGHVNTAYNLLQYEVQSRVFPAILKDATKSNVVNYLWQINPSLTLRGFVDAHSDPDCLL
RIVDVCRDLKILSAVLDSTPYAFSIKLAVAASRIDHSHLEKWLIAKLRVCKDDFLQILQY
QSGHLLSNQKLDELRKSYISYELRNHDSVVRGIPTSDNVEIEADAYFHQMFSGQISIAAI
VQMLSRFKNSPEKREQLIFKCMISNMFEEYKFLPKYPDKQLKLSALLFGSLIKHRLVTHL
ELGIALHAVLDALHKSVDSKMFMFGTTALEQFMDRLIEWPDYCNHILQISHLCGAHTEMV
SAIERALARISSSQNELSVNISVSSEQHVTGLAPIEPIERGEWARPRLVVVYGSSFFALA
ADFHDMTSITHPHHHSPSHRLLINHLHYLPLINCPVCHLLCMLLFFHILKVPAQAKAKEC
IEVLPECYYPWFAQSMVMKRASIEPKFHDLYLKFFVKVNSRFLNKEVLKATYENCKILLR
SDLIKSSSEERSLLKNLGSWLGKFTIGRNQALLAKEIDPKVLIVEAYEKGLMIAVIPFTS
KVLFKNLDVDLKDVNPTSLLKDRVCEVEGNPDFSNKDVAASQTQISSGISRSTNHVELQS
VISSTSHAYATPHLPSNSMVEDDNVAFMMPKHVSSHTLTQVSPSETALASQSPFSLTQLV
KLIPHDEIRCKISSKLGSLGRQLQYSKIMDTALDKAIKEILCPVVEKSVGTAIQNTKKLI
LKDYALESDNNTIKSSVHSIARTIAGNLAYANCKEPLCVALTDHLQSQIQTLTSNNKTIK
QLIDVLINDNLDLGCRIIKSVAMCKAIEMIDEEITESFPLQKKQREAAGSAYCDAFTHAQ
GRFAHEPEALHPKHEHLSVAQQVTMFMYGRAIANMLTLHVLVSLELAVKHSLVPNQIVAT
GGVSQKSSQQLKISYFPIDSFSKLVAMVLKYSSAETGPNKCSLLPKILLVAVRIIQRDSE
EKKASFNPRPYFRLFISLLYDLISSDLHSDGANFQVLIAFANAFHALQPLRIPSWSFCDM
IPSSCIQMRNVILDAHPQDMRVVDPASPNLKIDLLPEISMAPQIMSDVEGALKSKLMKTE
VDEYFKKSEGSLFLSDLKQKLLLPQNETSVAGTRYNVPLINSLVLYVGIQGLQQQQTESS
ASGPAIHTAHMDIFRTLMADLDTEGRYLVLNAIANQLRYPNIHTHCFYFIILHLFSEATQ
EIIQDQIMRVILERLVVRRPHPWGLQMTLVELIKNPRYKLWSRPFIRCGPQIDKVLIEFV
DMLDDPRLTSLYSSFRSIN*
>11676.m03775|LOC_Os10g41080.1|genepair964-1
MAAAAEEAGLGHPSRYVQLTRDQDAPADEDIRPGELNLPAHFPQLEQRRCCECGQQLPES
YEAPADEPWTTGICGCAEDTESCWTGSFFPCVLFGHNVEALREDIPWTTPCTCHAVCVEG
GIALAILTVIFPGIDPSTSILIGEGLVFSWWLFATYTGIFRQQLQRKYHLKDSPCDPCLV
HCCLHWCANCQEHRERKGRLADNNANRNTIVNPPPMQEMSVVGNHPSITPENGAA*
>11669.m00022|LOC_Os03g01210.1|genepair964-2
MAEEATLSHPSRYVKLSREHDAPAPAEDIRPGELNQPVHLEGRRCSECGQVLPESYEPPA
DEPWTTGIFACTDDPQTCRTGLFCPCVLFGRNIEALREDIPWTTPCVCHAVFVEGGIALA
ILTAIFHGVDPRTSFLIGEGLVFSWWLCGTYTGIFRQELQRKYHLKNSPCDPCMVHCCLH
WCANCQEHRERTGRLAENSAVPMTVVNPPAVQEMSMAESRGPVSPGMENGAPSNSKGEHE
EPKSDHDDVEVIPL*
>11676.m03784|LOC_Os10g41170.1|genepair965-1
MKVKAIRVHKIGGPEVLTWEEVEIGEPSEGEIRIKNKAIGVNYVDIYYRTGLHQEPLPFV
PGKEAVGVVSAVGPGVTGIEVGDVVGYADTPMGTYTEEQIIPATLAIPIPPSVDHITAAS
VLLKGMTTYVLVKQAFKIQAGHTVLVHAAAGGVGSLLCQWANALGATVIGTVSTQEKAIQ
AAEDGCHHVIIYTEEDFVAQVAEITSRKGVHVVYDAVGKDTFKGSMECLMPRGCMISYGQ
CSGRPDPVPVSDLASKSLILGRPGMRHYTATRDELLHAAGEVFAGVAAGVLRVRVNHVYP
LHEAARAHADLEARRTSGSVVLLPAMPAADS*
>11669.m00020|LOC_Os03g01190.1|genepair965-2
MTYGPVLLCGLCLCLSPLPTRRLLSPSPSASLTTTTVRCSKGEMAVVKCIRVHELGGPEV
LRWEQVEVGEPKEGEIRIKNTAIGVNFIDVYYRQGVYSAPLPFVPGREAVGVVTAVGPGL
TGRKVGDVVAYAGNPMGSYAQEQIIPASVAVPLPPSIDHNTAAAIMLKGMTAHVLLRRVY
KVQSGDCVLVHAAAGGVGSLLCQWANALGATVIGTVSNEEKAAQAAEDGCHHVIIYTKED
VVTRVKEFTAGKGVNVVYDSVGKDTYKGSVECLAWRGMLVSFGQSSGRPDPIPLSDLASK
SLLVTRPSLMHYTATRDELLESAGEVFANVGSGVLRIRVNHTYPLSQAARAHADLQARKT
TGSILLIPDDA*
>11676.m03786|LOC_Os10g41190.1|genepair966-1
MAIGAFVESGGGGGGGGGGGYGGRVTAYVVLTCVVAGSGGILFGYDLGISGGVTSMDSFL
KRFFPDVYQKKQDTRVSHYCAFDSELLTVFTSSLYIAGLVATLFASSVTRRYGRRTSMLI
GGTVFIAGSVFGGAAVNVFMLLINRILLGIGLGFTNQSIPLYLSEMAPPRYRGAINNGFE
LCISLGILFANVLNYCVVKITAGWGWRISLSMAAVPAAFLTIGAVFLPETPSFIIERDGD
TDKARILLQRLRGTTSVQKELDDLVAASNLSRTVQYPFRNIFKRKYRPQLVIALLVPFFN
QLTGINVMNFYAPVMFRTIGLKESASLLSSVVNRLCATFANIMAMIVVDRFGRRKLFLVG
GIQMILSQLAVGAILAAEFKDYGSMDREYAYLVLITMCVFVAGFAWSWGPLTFLVPTEIC
PLEIRSAGQSIVVAVVFLMTFVIGQTFLAVLCRIKSGTFFFFAGWICLMTVFVYFFLPET
KKLPMEQMEQVWRKHWFWKKIVGEEEEKQAEKTALPSM*
>11669.m00018|LOC_Os03g01170.1|genepair966-2
MAIGAFVEGGGSGYSGRVTPFVVLSCIVAGSGGILFGYDLGISGGVTSMEPFLKKFFPDV
YHQMKGDKKKVSNYCRFDSELLTVFTSSLYIAGLVATLVASSVTRRFGRRASILIGGSVF
VAGSVFGGAAVNIYMLILNRVLLGIGLGFTNQSIPLYLSEMAPPQHRGAINNGFELCISI
GILIANLINYGVDKIEGGWGWRISLSMAAVPAAFLTVGALFLPETPSFVIQRSGDVDSAR
ALLQRLRGTAAVHKELEDLVMASEVSKTIRHPLRNMLRRRYRPQLVIAVLVPLFNQVTGI
NVINFYAPVMFRTIGLRESASLMSAVVTRVCATAANVVAMAVVDRLGRRRLLLVGGVQML
VSQVMVGAILAGKFREHGEEMEKEYAYLVLSVMCVFVAGFAWSWGPLTYLVPAEICPLEV
RSAGQSIVIAVIFLLTFLIGQTFLAMLCHLKFATFFLFAACLCVMTLFVFFFLPETKQLP
MEQMDQLWRTHWFWKRIVGDSPQQQVVELHHHHQRSSSE*
>11676.m03790|LOC_Os10g41220.2|genepair967-1
MAIQDKVENNDPTVTVGLAVSSSKSSKYAVQWAVKNFCTNGMVRFVLIHVLQRITTVPTP
MGNYIPIDKVRADIASAYVKEVECKARTMLLFYKNMCDEKAKAEVLVVKGEDVAETISNV
VSMYEIHKLVVGDSSQGNFIRKSKGTRTSSQICRSVPSFCAVYVVSKGGLSAVYSPGFEG
HKSSELFLSSDSSKTEIHSDDKPSLSDATPSRSFRSNLTWENLESLSSADHDRPRSLHEY
LTESTSASVGDNNSNSPCASGQTPRPSNVLISDKAPMTSSPLQELMLSEDMDDVNSELEK

LRLELRHIKGVCKLVQDESINASQHVTDLAAKRAEEEARLSEVYSRINRVNEQAHQEKEQ
LNALEAQCRHVRDLARKEALQKQILQLRTSKEADKMQRLEKLLELDGMSYSTFTWEDIES
ATSSFSEALKIGSGSNGTVYKGNLRQTSVAIKVLTSDDSHRIKHFKQELEVLGKIRHPHL
LLLIGACLDRPCLVYEYMENGSLEDRLQLKGGTAPLPWYQRLRIAWEIALALVYLHSSKP
KPIIHRDLKPANILLDSNFTSKIGDVGLSTLLPLGDALSTTRTIFKDTDLVGTLFYMDPE
YQRTGQVSTKSDTYALGMVLLQLLTGKPPVGLADLVEQAVENGHLVDILDKSAGKWPAQE
AHELAQLGLSCLEMRSKHRPDLKCKVLVELERLKKIASAVSDPVRPVISGPPSHFICPIL
KRIMQDPCIASDGYSYDRVAIEMWLCENDKSPITKSRLPNKDLVPNHALLCAITSWKAEA
RD*
>11669.m00017|LOC_Os03g01160.1|genepair967-2
MDPDPLPLLSLGHVNVSEMMAPPLTVGLALTDSKSSTYALQWALSRFKFTKDDDAPIFLL
IHVLTKLLTVPTPMGNHIPIDKVRTDVADAYFKDVHHQAQQMLLLYKNMCHQNKVKAQVL
LIKGNDVSQTVSTVVSDYQIKILIVGVTARTRKPFGNRTSSKICKNVPSFCTAYLVSKDG
LSSLYSSSLESDLASSSKSDDLSDEMSSSSVTSNNSSDQSPFDSPRLLGSNLPSENPKNS
SPAHRNRSLTLYDYLNGSTSIYPDKDRRVNSSTGTESSKPSELKGSNEVLRQESLLQGML
SDNKDHISTELEKLRLELRLIQGKHKLVQDESDDASRQASKVDLICYQRMAELAAKRMEE
EAQLREIQSRLDKANDNVEKQKAHRYAAEQALNHVQDLVRGEVMQKNMLQVKASRDADKK
LRLEKLFVLQGNSYSTFTWEEIDNATSSFADNLKIGSGANGTVYKGYLNHSAVAIKVLHS
DDNSSNKHFRQELEVLGKIHHPHLVMLLGACVERGCLVYEYMENGSLEERLRCKSGTAAL
PWCDRLRIAWEVASALVFLHSSKPNPIIHRDLKPENILLDGNLVSKVGDVGLSTLVSSGS
GGSSSTMYKKTALAGTLFYIDPEYQRSGQVSVKSDTYALGMVMLQLLTARAPIGLAEVVE
RAVEDGKLRDILDENAGWTWAMEEAQEMADLALRCLEMRGKDRPDLKTRVAVDLDRLKRR
ALPTQPPPEHFICPILKRVMQEPCIASDGYSYERHAIEMWVCDKDVSPVTNVRMPNKTLV
PNRSLLTAITAWKSQGGRKPTNKFVTTSPNHL*
>11676.m03803|LOC_Os10g41340.1|genepair968-1
MMAYALRGAALVGVLLLVVASPALVPVASAVPFIVLHGIGDQCENGGMASFTEMLGEWSG
SKGYCIEIGRGAWDSWLMPLQEQADTVCKKVKKMKELRKGYSIVGLSQGNLIGRAVIEYC
DGGPPVKNFISIGGPHAGTASVPLCGSGIVCVLIDALIKLEIYSNYVQAHLAPSGYLKIP
TDMTDYLKGCKFLPKLNNEIPSERNATYKQRFSSLENLVLIMFEDDAVLIPRETAWFGYY
PDGAFSPVQPPQKTKLYTEDWIGLKALEEAGRVKFVSVPGGHLSISRSDTKKYIVPYLKP
DGSSRFGIRRILSD*
>11669.m00016|LOC_Os03g01150.1|genepair968-2
MAAYSLVFVFVGVAVFVLAPPASAVPFIVLHGIGDQCANHGVAKFTRLLADCEIGSGTWD
SWLMPLQKQADIVCSKVIQLSLLDNTFLRYDDVCIITGLYPFLFQGNLIGRAVVEFCDDG
PPVKNFISLGGPHAGTASVPLCGSGIFCILADSLIKMEIYSDYVQAHLAPSGYMKIPTDI
SDYLKGCRFLPKLNNELPGERNATYKERFSSLENLVLIMFQDDAVLIPRETAWFGYYPDG
GFDPVLPPQKTKLYMEDWIGLKTLDDEGRVKFVSVPGGHLGISKTDMMKYIVPYLKGKDN
GKAWIAATWRAITNTIGGEAFVLN*
>11676.m02594|LOC_Os10g30100.1|genepair969-1
MEEADDAPPPWRPSKPAAAIDGSSGPLAAAAARLSGRSRALPSSRDFHFYNNFPSFRSPV
GAAAARAGASLAALGAAPFYPKRHPPFPGDDLDDAQDWVVGVIDDLAEQFGASMDEFKAA
REEEEATGRRATEEDGFQVVYGKKKKKVMGGGGGEEGVGRGGEAFGGGSGSVKMATRDKS
AAPGAKAKVPFHIPTIPRPQDVHRILVDNSSKPFEHSWLEKSDDGSRVVHPLEKIPMEQL
VDRDFPESEPIKPPALDDTPFTHVEDLKSLEVLATKLKSATEFAVDLEHNHYRSFQGLTC
LMQISTRTEDFIVDTLKLRKYLGDYLREIFKDPTKKKVMHGADRDIIWLQRDFGIYVCNL
FDTGQASRILQMDRNSLEHLLHHFCGVTANKEYAREDTHYLLYIYDLMRLRLVKESSDEN
DLLLEVYKRSKEICLQLYEKELLTHSSYLYIHGLKENEFDARQLSVLANIYKWRDSVARG
EDESTGYILPNKTLLEIAKQMPATTGRLKRIVKSKNSYLERHLGHVISTIRSAIANCDAF
ESIAEQLKKGRLEELAVANMKSNDGDTEMVPADDGNNDDDNVGPSDEHGAVASVENVGAA
SHCTGNVTSGASSVNVQLENPAETKSLGILSGVSGQDMEVLSNGDRKQVAKATVQVSKRP
TAFGALLGKPTSGRRQNLFSGFSSGQNMVDKIKSSVALPFHNFCGGAKSPATSIPLEESV
RPEPESIQYSDPACQTEDVIQLGTETDGPQPPENHNKDGQGHLVPDDMEMSRSPPEHSSA
GAKQRFQSLIESRNQQQNHKPHQEPEFNHQLKPFDYAEARKNITFGERKAERIKDNAVAR
AINKDSGDKGRTSNQFGAGENEGNRQNPRGRQAFPPSGNRSATYH*
>11669.m00568|LOC_Os03g06190.1|genepair969-2
MDEFKGPGEKEKEEEAGDKAAAEEAGDGFQVVRGKKKKRPNGGSASGAGSGTMMAKDKAA
AAAAAEPWTKAKVSFHDPNIPRPQDVYAIRVNNYNVPFDHVWLERTEDGSRPIHPLEKLP
MEQFIDRNVPESEPVKPADLEDTPFTLVEDKNGLADLAKKLKSVNEFAVDLEHNQYRSFQ
GLTCLMQISTRTEDFVVDTLKLRIYIGLYLKEHFKDPTKRKVMHGADRDIMWLQRDFHIY
VCNLFDTGQVQKRSNEICLQLYEKELLTDTSYLHIYGLQEHDLDAKQLAVVYALHQWRDY
IAREVDESTGYVLPNKALIEIAKKMPTDTAELKRMVKSKYPFVDENLDQVVGIIWNATES
SYAFESRAEQLKKERLEQLTDRVQTISSPEMKTSMNLSGQIRSMDKEILSDNIHQQVAQA
TFQELKRPMALGAVGNSTSGGQRDFFGGFSNKSEKMEKAKSYPAFYYPQLPQYSPEVGYG
FQSINRTMAGTAQSPTGNKERDLQNPRRRQSFPPSGNISDTHQ*
>11676.m02603|LOC_Os10g30190.1|genepair970-1
MQPPILLNIGGSRYETTADTLTQRDPGSLLAAALSGAAAHGLPTTEDGAVFVDRDGELFR
HVLNWLRDGAVPALADAEYRQLLREAEYYRLPVYEQCLLPPWLCVWGDFDPNAGLIDCIS
ERIEDWDDKIGRSSEAELTRKDVIKCIQADKVRFRGVNLSGLDLSKLDLSEVDFSCGCIE
ETKFSLANLYKAKFREVQASRSSFNYANLRECEFVGANLQESTLDRANLQSANLQDACLV
KCSFIETDLRSAHLQRADLTGANLTGANLEGANLKGAKLSGSNLQGANLQRAYLREVDLR
ETQLTGAKLGGANLLGAIR*
>11669.m00565|LOC_Os03g06160.1|genepair970-2
MESPPESSSSSPVLLNIGGKRYATTVETLTQREPSSMLAAMFSGRHTLPRHPDTGMVFVD
RDGKHFRHVLNWLRDGAVPDMSESEYQQLLREAEYYQLLGLADCINDRLGWKNDENFSEA

ELTRKDVIKCIQAPRVRFRGVNLSGLDLSKLDLSEVDFSYACIKNANFSSAYLRKAKFRL
TEATCSSFQSANLHECELIGANLEGSVLDKANLQSANLQDACLKQCCFIETDLRSAHLQG
ANLMGANLSGANLEGANLKGAKLAGTNLECANLQRAYLREVDLRETHLTGAKLGGANLLG
AIR*
>11676.m02616|LOC_Os10g30320.1|genepair971-1
MGMTRSLARWFFALRWWRAAACARDRRGGELVGDGDVLRRERRFYQRAFTLRIRLGLDQY
SIGDFDLEYHTDFNFTFAPIYTLILHDLPFHSHNKNIMPLIPFSVPPHMEQPEAPLTAEE
REKKIREQIEYYFSENNLCSDVYLKGWMNQQGWVPLTLVAGFPRVQALTTDYETVQRSVL
SSTEVELQQC*
>11669.m00556|LOC_Os03g06070.1|genepair971-2
MASNADQHAAANGGGGTTPAATGSPPAGKKAAAAAAWKRPGNGAAVPVVVAPGSPIMDAD
SWPALPGLASPPPTTLTPTPMPPKASPKVAPLPPPAEAVIPPISLGNSGAPDANPDHEAP
VRNPPARRALVMPVGDGLDKRAPGSEPSPVYTPNARSNGGGDHHQNGRYGSHPHSRGSGY
GGGGNRRGNGGGGGGGRRGQEHHGGFDGQRRGGGRRDGHGPGHQQRGHHPSYIRAPLAVV
TAAPPPPPPFVNPATPQTPPYGAPIGFPEIAPHVYYFTSPLEGVQALPFVPHPASPPAML
IPQFDPLRAELLKQIEYYFSDDNLCKDIFLRRHMDDQGWVPLPLIAGFNQVKKLTNNVQF
ILETVLQSTVVEVQGDKLRKRERWEIWLLPKQGYSAGNSSGSLSPVTSNIDSLASQFQSV
GLEGAGYHASQGMPGEALLTRSATSVSLGYQAPPLGGLYSNGSGPLFGQKSARSLLRSDT
F*
>11676.m02617|LOC_Os10g30330.1|genepair972-1
MAENLLVLCTILAARMALAAADDWIPATATFYGGNDGSGTMGGACGYGNLYDQGYGLENA
ALSTALFNDGAACGQCYLIVCDTDKAGRWCKPRGAVTVTATNLCPPNWALPSDGGGWCNP
PRRHFDMSQPAWERIGVYRAGIVPVLYRRVRCWRRGGVRFTVGGFDHFELVLVANVAGSG
SVAAVSVRGAGTGWLQMSRNWGANWQSLAGLAGQPLSFGVTTTGGQYILFQDVAPAGWKF
GQTFSTSKQFDY*
>11669.m00555|LOC_Os03g06060.1|genepair972-2
MGNILLQLLAVVALCIAPARSDWLPGTATFYGGADGSGTMVTFVYKIFAKSIPFSSLKNV
RAENERGGACGYGNLYDQRYGINNAALSTPLFNDGASCGQCYLIICDYGKAPDWCKLGKA
ITVTGTNYGGWCNATRPYFDMSQPAWENIGIYSAGIVPILYQQVKCWRYGGVRFIINGFN
YFELVLVTNMAGSGSIVSMSVKGSCTGWIQMTRNWGANWQCLAGLAGQALSFNVTSTGGQ
TIVFDDAVPAGWSFGQTFSTYHQFDY*
>11676.m02618|LOC_Os10g30340.1|genepair973-1
MMVIRFFAVLAAALCITSASAAAAGGWVSGTATFYGGKDASGTMGGACGYGNLYTQGYGV
YNAALSTALFNGGASCGQCYLIMCDASKTPEWCKAGTAVTITATNLCPPNWALANDDGGW
CNPPRPHFDMSQPAWETIGIYRAGIVPVLYQQVKCWRQGGVRFTVSGFNYFELVLITNVA
GSGSVQAMSVKGSKTGWIPLARNWGANWQCNSALVGQALSFRVTSTGGQTLQINSVVPEW
WEFGTTFTSNQQFDY*
>11669.m00550|LOC_Os03g06010.1|genepair973-2
MEYAILFATSLVITVLAASGFAPAHGWNKGTATFYGGADASGTMGGACGYGNLYTAGYGT
NTAALSSVLFNDGWSCGQCYLIMCDAAATPQWCRAGAAVTITATNLCPPNWALPSNSGGW
CNPPRPHFDMAEPAWLQIGIYKAGIIPVLYQQVKCWRQGGIRFTMGGFNFFELVLVSNVA
GSGSVRSVSVKGGSTGWITLNRNWGANWQCNSGLVGQALSFAVTSTGGQTLYIYNVVPSW
WSFGMTFTSNQQFSY*
>11676.m02627|LOC_Os10g30420.1|genepair974-1
MVSGAAHSASTSGGGGGSEGSPTGRAAPGMQGGGSAATPAASASASTPASETTVARRLDG
LDIQGDDAPSSQPATSKKKKRGPGTRATGPDKGGRGLRQFSMKVCEKVESKGRTTYNEVA
DELVAEFADPNNNFASPDPDNPNTPQFDEKNIRRRVYDALNVLMAMDIISKDKKEIQWKG
LPRTSMSDVEELKTEIIGLKGRIDKKNAYLQELEDQFVGLQNLAQRNEQLYGSGNAPSGG
VALPFILVQTRPHATVEVEISEDMQLVHFDFNSTPFELHDDSFVLKALGFSGKEPDDTQA
WVGNGGECSTTPIYHQSPQVARPNGVRLPTSPPIPGILKGRVKHEH*
>11669.m00524|LOC_Os03g05760.1|genepair974-2
MVSGVAHRPDDDGGRAASTFQRPPQPAGARPSLATPPPSGGAQSASTSGGSAGSPSSRSE
QHVPAAAGMAAGAAAASTPISENTFLRLNDLDIHGDDAPSSQAPTSKKKKRGARAVGPDK
GGRGLRQFSMKVCEKVESKGRTTYNEVADELVAEFADPNNSILPPDPDNPNAQQYDEKNI
RRRVYDALNVLMAMEIISKDKKEIQWKGLPRTSINDIEDLQTELVGLKSRIEKKNTYLQE
LQDQFVGMQKLIQRNEQLYGSGNIPSGGVALPFILVQTRPHATVEVEISEDMQLVHFDFN
STPFELHDDSFVLKAMSSCGEEQIDGIHDLISNGGESSSMPNIYRQQVQQPARSTNGTAR
LPSSPPIPGILKGRVKHEH*
>11676.m02630|LOC_Os10g30450.1|genepair975-1
MGEEKKDKASGKDAGEKKDAAGGGEKAAAAAPGPIVLKVELHCAGCASKVKKAIKRAPGV
ETVVTDTAGNKVVVTGAADAAELKERIEARTKKAVQIVSAGAGPPPKKDKEEKKDKDKKG
GGDDKKAEKEKGGGGGDKKAEKEKGGGDKPKEEKKAKEPKEETVTLKIRLHCEGCIDRIK
RRIYKIKGVKDVAVDAAKDLVKVTGTMDAAALPGYLKDKLSRQVEVVAPGKKDGAGGGDK
KDGGGGDKKDKKEGGGGGGDKKDAGGEKTDKDKSAAASASVAPVPLADAGMFQMPPQYGF
NPYHVHPGAAYYGGAPPPNPAAFYHHPNAAAAAAYQPYPYNVHAPQMFSDENPNACSVM*
>11669.m00523|LOC_Os03g05750.1|genepair975-2
MGDEKAAPKAGATADPVVLRMELHCAGCAQKVKKSIKHLAGVESVAADVATNTVVVAGTA
EAAALKARIEAKTKKPVEVVSAGGGGAAAKKPAAEPKAVKDDGGEKKDAQAKEEKGKKQP
PEEKKPKEETVLLRIRLHCDGCADRIRRRIYKIKGVKEVVMDGNAKDEVKVSGTMDVPAM
LTYLTEKLNRAVEAVAPGSKKDEKKKDKGGDADGGEKKKDAAGGDKKDKGKSIEVAGPST
AAAAASMAPAPAEASTYHVSPYGHGYFAYPQQQGPPPSYYQYYGGGNGDGVGYANPNAGG
AGGYYHPHPNDVPTYQPPPSYPPYPYQLDMSPAPQLFSDENPNACSVM*
>11676.m02637|LOC_Os10g30520.1|genepair976-1
MLRVPEQLSHRSSCNLRAETHLHPVAGLPPPPPPLALLRLTRPPAPPDPPAMGCSSSLPA

NNAGGVGTISNENSGTDLKNLRVKLVLLGDSGVGKSCIVLRFVRGQFDPTSKVTVGASFL
SQTLALEDSTTVKFEIWDTAGQERYAALAPLYYRGAGAAIVVYDITSSESFNKAQYWVKE
LQKHGSPDMIMALVGNKADLHDNRSVSSQDAQEYAERNTMFFIETSAKTADNINQLFEEI
AKRLPRPTPS*
>11669.m00522|LOC_Os03g05740.1|genepair976-2
MGCSSSVPARSTGGLNNISNDNSATDSKDLRAKLVLLGDSGVGKSCIVLRFVRGQFDPTS
KVTVGASFLSQTLALEDSTIVKFEIWDTAGQERYAALAPLYYRGAAAAVVVYDITSPESF
SKAQYWVKELQKHGSPDIIMVLVGNKADLHENRHVSSQEAQEYAEKNNMVFIETSAKTAD
NINQVFEEIAKRLPRPTAS*
>11676.m02643|LOC_Os10g30580.1|genepair977-1
MASQGEPSSSADPKGKKDYSTAILERKKSPNRLVVDEATNDDNSVVALHPDTMERLQLFR
GDTVLLKGKKRKDTICIVLADETCEEPKIRMNKVVRKNLRVRLGDVVSVHQCQDVKYGKR
VHILPIDDTVEGITGNLFDAFLKPYFLEAYRPVRKGDLFLVRGGMRSVEFKVIETDPTEY
CIVAPDTEIFCDGEPIKREDEERLDEVGYDDVGGVRKQMAQIRELVELPLRHPQLFKSIG
VKPPKGILLYGPPGSGKTLIARAVANETGAFFFLINGPEIMSKLAGESESNLRKAFEEAE
KNAPSIIFIDEIDSIAPKREKTNGEVERRIVSQLLTLMDGLKARSHVIVMGATNRPNSID
PALRRFGRFDREIDIGVPDEVGRLEVLRIHTKNMKLAEDVDLELIAKDTHGYVGADLAAL
CTEAALQCIREKMDIIDLEDETIDAEILNSMAVTNDHFKTALGTSNPSALRETVVEVPNV
SWEDIGGLENVKRELQEPIYVLEFLQTVQYPVEHPEKFEKFGMSPSKGVLFYGPPGCGKT
LLAKAIANECQANFISVKGPELLTMWFGESEANVREIFDKARQSAPCVLFFDELDSIATQ
RGSSVGDAGGAADRVLNQLLTEMDGMNAKKTVFIIGATNRPDIIDPALLRPGRLDQLIYI
PLPDEQSRLQIFKACLRKSPVAKDVDLNALAKYTQGFSGADITEICQRACKYAIRENIEK
DIERERRSKENPEAMEEDEVDDIAEIKAAHFEESMKYARRSVSDADIRKYQAFAQTLQQS
RGFGSEFRFERTEAGAGAAADPFASAAAVADDDDLYS*
>11669.m00521|LOC_Os03g05730.1|genepair977-2
MASQGEPSSSSDPKGKKDFSTAILERKKSPNRLVVDEATNDDNSVIGMHPDTMEKLQLFR
GDTVLLKGKKRKDTICIVLADDTCEEPKIRMNKVVRKNLRVRLGDVVSVHQCPDVKYGKR
VHILPIDDTVEGITGNLFDAFLKPYFLEAYRPLRKGDLFLVRGGMRSVEFKVIETDPAEY
CIVAPDTEIFCDGEPIKREDEERLDEVGYDDVGGVRKQMAQIRELVELPLRHPQLFKSIG
VKPPKGILLYGPPGSGKTLIARAVANETGAFFFLINGPEIMSKLAGESESNLRKAFEEAE
KNAPSIIFIDEIDSIAPKREKTHGEVERRIVSQLLTLMDGLKARSHVIVMGATNRPNSID
PALRRFGRFDREIDIGVPDEVGRLEVLRIHTKNMKLAEDVDLEHIAKDTHGYVGADLAAL
CTEAALQCIREKMDIIDLEDETIDAEILNSMAVTNDHFKTALGTSNPSALRETVVEVPNV
SWEDIGGLENVKRELQETVQYPVEHPEKFEKFGMSPSKGVLFYGPPGCGKTLLAKAIANE
CQANFISVKGPELLTMWFGESEANVREIFDKARQSAPCVLFFDELDSIATQRGSSVGDAG
GAADRVLNQLLTEMDGMNAKKTVFIIGATNRPDIIDPALLRPGRLDQLIYIPLPDDQSRL
QIFKACLRKSPVAKDVDLNALAKYTQGFSGADITEICQRACKYAIRENIEKDIEMEKRRK
DNPEAMEEDEVDDIAEIKAAHFEESMKYARRSVSDADIRKYQAFAQTLQQSRGFGTEFRF
ADQPASGAGAAADPFASAAAAADDDDLYS*
>11676.m02661|LOC_Os10g30750.1|genepair978-1
MQHRSPAAATASSGPVAAAASAAMAVPGVGGIEPAVTLDQVPRWSDPDQRLYAPSSSSAA
AAGGVEAGEGGGSEPAASAFLSFSDPLTGDDGGGVTGGGRGGASRFPVDHEINSRIYLWR
GHPWNLEVDAVVNSTNESLDESHSSPGLHAAAGSGLAEECSTLGGCRTGMAKMTNAYDLP
ARKVIHTVGPKYAVKYHTAAENALSHCYRSCLELLIENGLESIAMGCIYTEAKNYPREPA
AHVAIRTVRRFLEKQKSKIAGVVFCTVSSSDTEIYKRLLPLYFPRDRQEEEIAVSKLPAD
VGDENGETVIDERKIRIRPLPAGATDRAATTAPIDLPFDSGLASKRSSFKLDSYLDPSFM
SLIKDPDLRRKEQWEKSAQAQKGFNYAKLLGYGDLACPSLSAAEEYSLHSRYLAKANSLN
LSEIAEMKIIYRGGVDSEGRPVMVVVGAHFLLRCLDLERFVLHVVKEFEPLIQKPYSIVY
FHSAASLQPQPDLGFMKRLQQILGRKHQRNLHAIYVLHPTLGLRTAILAMQMFVDGEVWK
KVVYVDRLVHLFRYVPREQLTIPDFVFQHDLEVNGGRGLIVDPRTKHIYQRPSG*
>11669.m00514|LOC_Os03g05660.1|genepair978-2
MQQRSPTAASASASASSVAAAAGVGGVEPAVTLDQVPRWSDADQRLSPSSSPTAAGSDET
PASSFLSFADPLIGDGAAAGAGGRGASRFPVDHEINSKICLWRGHPWNLEVDAVVNSTNE
NLDEAHSSPGLHAAAGPGLAEECTTLGGCRTGMAKMTNAYDLPARKVIHTVGPKYAVKYH
TAAENALSHCYRSCLELLIENGLERLLPLYFPRDKKEEEIASLKLPADVGDENGETIIDE
RKIRIKPLPAGSAINKSAAPAPVDIPLSDSGLTRSSYRGGVDSEGRPVMVVVGAHFLLRC
LDLERFILYVVKLILTGLNLPSLQEFEPLIQKPYSIVYFHSAASLQVRPDLGFMKRLQQI
LGRKHQRNLHVGISYDHTAIYVLHPTLGLRTAILALQLFVDGEVWKKVIYVDRLVQLFRY
VPREQLTIPDFVFQHDLEVNGGKGIIIDPRTKHVYQRPSG*
>11676.m02663|LOC_Os10g30770.1|genepair979-1
MADGQLKVLTTLDHARTQWYHFMAIVIAGMGFFTDAYDLFCISLVSKLLGRIYYTDLAGD
NPGSLPPNVSAAVNGVALCGTLAGQLFFGWLGDKLGRKSVYGFTLVLMVVCSVASGLSFG
RTAKGVVATLCFFRFWLGFGIGGDYPLSATIMSEYANKRTRGAFIAAVFAMQGFGILFGA
IVALVVSAGFRNAYPAPSYADGRAASLVPEADYVWRIILMFGTVPAALTYYWRMKMPETA
RYTALIARNAKQAAADMSKVLDTEIQEDADRAEAVAAGGAGNEWGLFSRQFVRRHGVHLV
ATTSTWFLLDIAFYSQNLFQKDIFSKVGWIPPARTMNAVEEVFRIARAQALIALCGTIPG
YWFTVAFIDVAGRFAIQLMGFAMMTVFMLGLAAPYHHWTTPGNHTGFVVMYGFTFFFANF
GPNATTFIVPAEIYPARLRSTCHGISAAAGKAGAIVGAFGFLYAAQDPHKPEAGYKPGIG
IRNALFVLAGTNFLGMLMTLLVPESKGMSLEEVSKENVADDEEATA*
>11669.m00510|LOC_Os03g05620.1|genepair979-2
MAGGQLNVLSTLDQAKTQWYHFMAIVIAGMGFFTDAYDLFCISLVTKLLGRIYYTDDSKD
TPGALPPNVSAAVTGVALCGTLAGQLFFGWLGDKLGRKSVYGFTLILMVVCSVASGLSFG
SSAKGVVSTLCFFRFWLGFGIGGDYPLSATIMSEYANKRTRGAFIAAVFAMQGFGILFGA
IVALAVSAGFRHAYPAPSYSDNHAASLVPQADYVWRIILMFGTVPAALTYYWRMKMPETA

RYTALIARNAKQAAADMSKVLHTQIEESADRAETVAVGGESWGLFSRQFLRRHGLHLLAT
TSTWFLLDIAFYSQNLFQKDIFSKVGWIPPAKTMNALEELYRIARAQALIALCGTIPGYW
FTVAFIEIMGRFWIQIMGFAMMTAFMLGLAIPYHHWTTPGHHTGFIVMYGFTFFFANFGP
NSTTFIVPAEIYPARLRSTCHGISAAAGKAGAIIGAFGFLYAAQDQHKPEPGYPRGIGIK
NALFVLAGTNFLGTIMTLLVPESKGMSLEVISQEVADGDDEEAAYPK*
>11676.m02665|LOC_Os10g30790.1|genepair980-1
MARQEQQQHLQVLSALDAAKTQWYHFTAIVVAGMGFFTDAYDLFCISLVTKLLGRIYYTD
LAKENPGSLPPNVAAAVNGVAFCGTLAGQLFFGWLGDKLGRKSVYGMTLLMMVICSIASG
LSFSHTPTSVMATLCFFRFWLGFGIGGDYPLSATIMSEYANKKTRGAFIAAVFAMQGFGI
LAGGIVTLIISSAFRAGFPAPAYQDDRAGSTVRQADYVWRIILMLGAMPALLTYYWRMKM
PETARYTALVAKNAKQAAADMSKVLQVEIQEEQDKLEQMVTRNSSSFGLFSRQFARRHGL
HLVGTATTWFLLDIAFYSQNLFQKDIFTSINWIPKAKTMSALEEVFRIARAQTLIALCGT
VPGYWFTVFLIDIVGRFAIQLLGFFMMTVFMLGLAVPYHHWTTKGNHIGFVVMYAFTFFF
ANFGPNSTTFIVPAEIFPARLRSTCHGISAAAGKAGAIIGSFGFLYAAQDPHKPDAGYKP
GIGVRNSLFVLAGCNLLGFICTFLVPESKGKSLEEMSGEAEDDDDEVAAAGGGAAVRPQT
A*
>11669.m00509|LOC_Os03g05610.1|genepair980-2
MGRQDQQLQVLNALDAAKTQWYHFTAIIVAGMGFFTDAYDLFCISLVTKLLGRIYYTDPA
SPTPGSLPPNIAAAVNGVALCGTLSGQLFFGWLGDKLGRKSVYGMTLLLMVICSIASGLS
FSHTPTSVMATLCFFRFWLGFGIGGDYPLSATIMSEYANKKTRGAFIAAVFAMQGFGILA
GGVVTLAMSAGFQAAFPAPAYEVNAAASTVPQADYVWRIILMLGALPAILTYYWRMKMPE
TARYTALVAKDAKQASSDMAKVLQVEIEVEEEKLQDITRGRDYGLFSARFAKRHGAHLLG
TAATWFLVDVAYYSQNLFQKDIFTSIHWIPKARTMSELEEVFRISRAQTLIALCGTVPGY
WFTVFLIDIIGRFKIQLLGFAGMTAFMLGLAIPYHHWTMPGNQVIFVFLYGFTFFFANFG
PNATTFIVPAEIFPARLRSTCHGISAASGKAGAIIGAFGFLYAAQPQDKAHVDAGYKPGI
GVRNALFVLAGCNLVGFLMTWMLVPESKGKSLEEMSGEADDEEASANGGATAVNSSGVEM
V*
>11676.m02671|LOC_Os10g30840.1|genepair981-1
MEDNRSKDTATKYRGVRRRPWGKFAAEIRDPERGGARVWLGTFDTAEEAARAYDRAAYAQ
RGAAAVLNFPAAAAAGRGGGAGGAASGSSSSSSAQRGRGDKIEFEYLDDKVLDDLLDDEK
YRGK*
>11669.m00507|LOC_Os03g05590.1|genepair981-2
MEDDKSKEGKSSSSYRGVRKRPWGKFAAEIRDPERGGARVWLGTFDTAEEAARAYDRAAF
AMKGATAMLNFPGDHHHGAASRMTSTGSSSSSFTTPPPANSSAAAGRGGSDRTTDKVELE
CLDDKVLEDLLAETNYRDKNY*
>11676.m02674|LOC_Os10g30860.1|genepair982-1
MGFLSFAGRVLFAAAFLLSAYQEFSEFGVDGGPAAKALQPKFNTIVANISTRTGLVVPHI
ELKHIVAAMISLKGLGGLLFILSSSLGAYLLLFHLAFITPVVHDFYNYDIESAEFVQLFT
KFAQNCALVGALLFFLAMKNSIPKRQPNRKKAPKPKST*
>11669.m00500|LOC_Os03g05520.1|genepair982-2
MGFVSFAGRVLFASVFLLSAYQEFSEFGADGGPAAKALRPKYNVFTKNISAHLGVAVPHV
ELKHIVAATIGLKGLGGLLFILSSSFGAYLLLIYLAFITPVVYDFYNYDMEKSEFVQLFM
KFTQNLALFGALLFFLGMKNSIPKRQAKKKAPKSKTN*
>11676.m02692|LOC_Os10g31030.1|genepair983-1
MEGSDSSRSRDKRDADDDVDARSSWKEDDEHEDVEDRKNRSGKSTRYIYDDEGDEDDYDV
RRESRVSKVPRRSPEERSERRLSDGYKDRDGDSSRRRREDNNDWDSSRRSGSRTSGHDVS
RSKSRSSDRTSSDRADTRDSRSSADQSKNRSAREAHDYRNELSERWEDTERRKGSARTDK
NDQDRRSIDPRYDSPPARDDRIVDSVDNTRQNTLHYNAKSEESDEKCMDQVEGTNRISDN
VETKDMLPYVDKDGHALSRDGRNKEARHHREKDDGDQGHSDSDNERNISMKEKIRVDAHG
DYKSYRGRDRNRELEGSKEHWGSRQRHDSKEPNDYDVGAEWRHGQERLDGGNFHGRSGYR
KDSRGRYESSKGPSSYGNRYDSSDSIEIRPNRNLDFGRESSVSGRRINMGSLQDLTPGTS
DPSEENKRNYGNGEDAQERYYDDVQNMDGKIPSDSHTGRGGAIASNNSGAGPSGSGSIIS
PTPQQGPKGSRPSRGLRGRPNVRDPQRMGLPVPLMPPPPFGPLGLPPGPMQPIGPNMSHS
PGPLGPGVFIPPFPGHLVWPGARGIDVNMLSVPTNLPIPPVAGPSFTSSVAAGPNHSIHL
NQTGSGLGSPANAPGTGFNPLSTPNHEILQDKPPAGWTPQRGPAGKAPSRGEQNDYSQNF
VDTGMRPQNFIRELELTSVVEDYPKLRELIQRKDDIVSNSASAPMYYKCDLKDHMLSPEF
FGTKFDVILIDPPWEEYVHRAPGITDHIEYWTPDEIMNLKIEAIADTPSFIFLWVGDGVG
LEQGRQCLKKWGFRRCEDICWIKTNKKNATPGLRHDSNTLFQHSKEHCLMGIKGTVRRST
DGHIIHANIDTDIIIAEEPTDGSTKKPEDMYRIIEHFALGRRRLELFGEDHNIRPGWLTL
GKGLSSSNFHKEAYIKNFMDRDGKIWQGGGGRNPPPDAPHLVVTTPEIESLRPKSPPQKS
QQQQSMPPMGSSSSTNRRSVMNSSQIVVTVVGSETMMPSPWSSNPMSGFGMPE*
>11669.m00490|LOC_Os03g05420.2|genepair983-2
MDSRSPKIPRRSPDDSKDKDSDRNRGRDEKNDWDSSRTYGSETDCKEERCDTNKRKGSAM
GEDVGDDSRSVDRSHETEVHVFNDKQDKAVEIKNILHDGVGQSDYGQRQLGLDNERRNGT
VDKSRVDAHIDDKLGSGRDRNWTGKTQEPEGSVDYLRSCKSQDSKEASDSEWKNAQERQD
GGGFHGRVGYRRDFRGRSESTRGSSTYGSRYDTSDSIEIRPNNSLDFGREGSVSGRYDVG
VGAHRDVTYGTNGDKVTNSEPDQSGSASMISQFPQHGHKGDRPSRGGRGRPNGRDSQRVG
VTLPIMPPPFGPLGLPPGPMQHIGPNIPHSPGHPLLPGVFVPPFPGGPLLWPGARGVDVN
MLSVPPNLPIPPPVAGEHSFTPGMGAGPNIHLNQFGSGIGAPTNMSGLSFHQLGTQSREM
VHGKPPVGGGWTPNRNSGPTRKAPSRGEQNDYSQNFVDTGMRPQNFIRELDLTSVAEDYP
KLRELIQRKDEIVAKSASPPMYYKCDLRQHVLSPEFFGTKFDVILVDPPWEEYVHRAPGI
TDHIEYWNGEEIMNLKIEAIADTPSFVFLWVGDGVGLEQGRQCLKKWGFRRCEDVCWVKT
NKKSATPSLRHDSHTILQHSKEHCLMGIKGTVRRSTDGHVIHANIDTDIIIADEPTDGIV
SYVLFKDASCSSYMLVY*

>11676.m02693|LOC_Os10g31040.1|genepair984-1
MPVGRTAGSLLGAMLMVLFRVMTPEEAYAAIDLPILGLLFGTMVVSIFLERADMFKYLGN
MLSWKSRGSKDLLFRVCVVSAVASALFTNDTTCVVLTEFILKVARQNNLPPQPFLLALAS
SSNIGSAATPIGNPQNLVIAVESGITFGQFLLGVFPAMVVGILANTCILLCYFWRLEGGG
GAHGPEVVADEEVTSHRFTPARMSHASSVNDSDCISEPIRRSESMNRADALRSRSYNSEG
DIQVAIRSLRASSLSREMVEVSTVCDRRDVVVDGGGGGGPRKITRSTSHQRSVIIEDAPE
QHQHPAGLFDGEKDKDDEAIGKRRRWKVIVWKYAVYLTTLGMLAALLLGLNMSWTAITAA
LILLALDFTDAQACLEKVSYSLLIFFCGMFITVDGFNKTGIPNTLWELVEPYARIDSPKG
VVLLAIVILVLSNVASNVPTVLLLGTRVAASAAAISPASEKKAWLILAWVSTVAGNLTLL
GSAANLIVCEQARRAQFFGYNLTFWSHLRFGVPSTIIVTAIGLLIVMSY*
>11669.m00485|LOC_Os03g05390.2|genepair984-2
MALAGTSKVVLGCVAFGIFWVLAVFPTVPFMPVGRTAGSLLGAMLMVLFRVISPEDAYAA
IDLPIIGLLFGTMVVSIFLERADMFKYLGNLLSWKSRGSKDLLFRVCIVSAIASALFTND
TCCVVLTEFILKVARQNNLPPQPFLLALATSSNIGSAATPIGNPQNLVIAVESGISFGQF
LLGVFPAMIVGVLTNAAILLCYFWKYLSVEKDQEGGQPAGPEVVADDEVTSHRFTPARMS
HVSSLNPDDMDCISEPIIRSNSVRSTSANENLRSRSVNSEADIQLAIKSLRASSMSHEMV
EVSTVTDRRDEGASSRKFTRTASQQRSVIIEDSPPSPASNGDKEKEDEVAEKRWRVFVWK
TAVYLITLGMLIALLMGLNMSWTAITAALVLLALDFTDAQACLEKVSYSLLIFFCGMFIT
VDGFNKTGIPNTLWELVEPYSRIDSAKGVALLAVVILILSNVASNVPTVLLLGTRVAASA
AAISHDSERKAWLILAWVSTVAGNLTLLGSAANLIVCEQARRAQFFGYNLSFWSHLRFGV
PSTIVVTAIGLLIVTSY*
>11676.m02775|LOC_Os10g31830.1|genepair985-1
MGSPGAGSSGGGGGGHECSFKILLIGDSGVGKSSLLVSFVVAAAAAHLDDDIAPTIGVDF
KIKFLTIGGKKLKLTIWDTAGQERFRTITSSYYRGAQGIILVYDVTKRESFTNLAEVWSK
EIESHSSNKDCIKMLVGNKIDKEDERTVTREEGLAFAEESGCLFLESSAKTRDNVEKCFE
ELALKIMDVPSLLEEGSSSVVKRNILKQKQESQAKHGGGCCQ*
>11669.m00473|LOC_Os03g05280.1|genepair985-2
MASSPASSYDCSFKILLIGDSAVGKSSLLVSFVSASHIDDEIAPTIGVDFKIKFLTVNGK
KLKLTIWDTAGQERFRGITSSYYRGAHGIILVYDVTKRESFTNLADVWAKEIELHSTNKE
CIKMLVGNKVDKNEERMVTREEGLAFAQESGCLFLESSAKTRENVEKCFEELALKILEVP
SLLEEGSSVVKRNSLKQKHENKAKNGGCCQ*
>11676.m02778|LOC_Os10g31850.2|genepair986-1
MELESEQHGCEHYTRGCRIRAPCCGEVFGCRHCHNEAKNSLEIHLNDRHEIPRHEIKKVI
CSLCDKEQDVQQYCSGCGACMGKYFCEKCNFFDDDVSKNQYHCDGCGICRTGGVDKFFHC
DKCGCCYSNVLRDSHHCVEGAMHHNCPVCFEYLFDSTKDISVLHCGHTIHLECLNVMRAH
HHFACPVCSRSACDMSDAWKKLDEEVAATPMPEFYQKKMIWILCNDCGATSNVNFHVLAQ
KCPGCSSYNTRETRGCGRPAAARSTV*
>11669.m00472|LOC_Os03g05270.1|genepair986-2
MASWPTSCTKVYEIIQFSRCKEEASATSSIIDHRTAKVGRRDDGPNMSKVLRATIPENSV
SGSSVRGKLREMEMDLAVEQYGSIGASSILSTLSVPEYSNLAWDETYPKTMLSALSATKN
RMYNRIAPIVGHAWVNISAQNATFMMTMYQRTNFTAMDVAYAGKTGGAENFFHCDKCGCC
YSYVLKDSHHCVERAMHHNCPVCFEYLFDSTKDISALHCGHTIHLECLYEMRSHQQFSCP
VCLRSACDMSHAWQKLDQEVAASPMPVIYQKKMVCKFSYLCSSVQPGNYTLNKSFCGFFG
SVRYGSYATTVGRHRTCNSTYWDTSAPDAALTTPGRQEPPLLPRAPESEF*
>11676.m02802|LOC_Os10g32070.1|genepair987-1
MVVVDGLHDGGGGDLQALLDDAGVDDVAARGGGEVEEEVERPSNEDAFPAVEKMATAAAA
KGLQCRHCGTTETPQWRHGPEGHRTLCNACSMRYRSGKLVPEYRPLRSPTFSPELHSNRH
HRVLQLRRRPGPQSAAPSPAAVARYGGEAKEEEEELAWVSNKDAFATVETTMAPSPRVVE
TPPEHDHRPANTPTTSPEPHSDRPRRVVQLPRRLQEPSASANLAHAVAATARAGRECAHC
GTTKTPAWRLGPDSRRKLCNACGNKYRSGQLNSTTFSQNSQEQKKKSKSSACSRERKRSA
VAATVVVGGGLRDDAAAIADEHLDGGDLQALLDDVALDDVAARGGGDAGEAKEEEEELEW
LSNKDAFPTVETMSPAPPENRTKAPVPPAGWQCRHCGSTETPLWRERDGPAEAEHVRKEE
TPPNITPATKHRRIVDLLRCSTALNTAAKAVERRCTHCGTTKTPAWLSGPDSRGKLCNAC
GKQYRKGRLVPEYRPLNCPTFSPELHSNAHAHRRRRESPVAIAIAGEK*
>11669.m00460|LOC_Os03g05160.1|genepair987-2
MVGDKDAAALAGELTGDAGASLNGFFDHTGLESAVVGEGQGEGEEEEELEWLSNKDAFPS
VDTMAAEVESAAPGAPARAAVGPRTKGLRRRRRVTAPWSLAPLLSRPRQAAAAAADAGAP
RRRCTHCAVDETPQWRLGPDGPRTLCNACGVRFKSGRLFPEYRPANSPTFSPLLHSNSHR
RVMEMRLQSEEDASAASRVNAKARRAERAAARLAGKDKK*
>11676.m02812|LOC_Os10g32170.1|genepair988-1
MKRHNTAEVPVPVSYGGKVEKTMGGAKQGRGGGGGCCSRLWFMVVLSATVTLLVRHCYDS
GVIGHGAAAGGVVRIEPVHRGLYHTRKASPVDRGGGGGGTSFSGHSPSPPDAGGSAKPES
PHDSGVKAPSELTTVEHTKQPSEPASTGTESDDGGKPSSASSSSLPAAAHPFARALAAAG
DKGDRCGGRYVYVQELPPRFNTDMVKNCATLFPWTDMCAFTANGGFGPQMSGGDGGVFQE
TGWYNSDQYTVDIIFHDRIRRYECLTDDPSLAAAVYVPFFAGLEVARHLWGFNVTTRDAM
ALEVVDIITSRSEWRAMGGRDHFFTAGRTTWDFRRLNDGDAGWGSKLFSLPAIKNMTALV
VEASPWHLNDAAIPFPTAFHPASDEAVFVWQDKVRRLERPWLFSFAGAARPGSAKSIRSE
LIAQCRASSVCSLMECADGPSNKCGSPASYMRLFQSSTFCLQPQGDSYTRKSAFDAMLAG
CIPVFFHPGTAYVQYTWHLPRNHADYSVYISEDDVRRNASIEERLRRIAPAAVERMRETV
ISLIPTVVYAQPSSRLDTMKDAFDVAVDAIVDKVTRLRRDIVDGRGEEEKLEMYSWKYPL
LREGQKVEDPHEWDPLFAFG*
>11669.m00455|LOC_Os03g05110.1|genepair988-2
MSAMRRRPVLPTHQDDMEKVGGKPPQSRLCFLATLCAMFWVLIFYFHFFVIANEPGSAGA
DTAAGAAASIARAELPLPEPERVSDPAVPLPPPALVSEPPPTTATVAKVEDEEKPTAVAH

QEAAPRDYAFQRALKTAENKSDPCGGRYIYVHELPPRFNDDMLRECERLSLWTNMCKFMS
NEGLGPPLGNEEGVFSNTGWYATNQFMVDVIFRNRMKQYECLTKDSSIAAAVFVPFYAGF
DVARYLWGHNISTRDAASLDLIDWLRKRPEWNVMGGRDHFLVGGRIAWDFRRLTDEESDW
GNKLLFMPAAKNMSMLVVESSPWNANDFAIPYPTYFHPAKDADVLLWQDRMRSLERPWLF
SFAGAPRPDDPKSIRSQLIDQCRTSSVCKLLECDLGESKCHSPSAIMNMFQNSLFCLQPQ
GDSYTRRSAFDSMLAGCIPVFFHPGSAYVQYTWHLPKNYTRYSVFIPEDGVRKGNVSIED
RLKSIHPDMVKKMREEVISLIPRVIYADPRSKLETLKDAFDVSVEAIINKVTQLRRDIIE
DHEDKDFVEENSWKYDLLEEGQRTIGPHEWDPFFSKPKDKGGDSTNPSTNAAKNSWKNEQ
RGQN*
>11676.m02851|LOC_Os10g32550.1|genepair989-1
MYRAAASLASKARQAGSSARQVGSRLALSRNYAAKDIKFGVEARALMLRGVEELADAVKV
TMGPKGRNVVIEQSYGSPKVTKDGVTVAKSIEFKDRVKNVGASLVKQVANATNDTAGDGT
TCATVLTKAIFTEGCKSVAAGMNAMDLRRGISMAVDAVVTNLKGMARMISTSEEIAQVGT
ISANGEREIGELIAKAMEKVGKEGVITIADGNTLYNELEVVEGMKLDRGYISPYFVTNPK
TQKCELDDPLILIHDKKVSNLHAVVKVLELALKKQRPLLIVAEDVESEALGTLIINKLRA
GIKVCAVKAPGFGENRKANLQDLAILTGGEVITEELGMNLEKFEPQMLGTCKKVTVSKDD
TVILDGAGDKKSIEERAEQIRSAIELSTSDYDKEKLQERLAKLSGGVAVLKIGGASEAEV
GEKKDRVTDALNATKAAVEEGIVPGGGVALLYASKELDKLPTANFDQKIGVQIIQNALKT
PVHTIASNAGVEGAVVVGKLLEQDNTDLGYDAAKGEYVDMVKAGIIDPLKVIRTALVDAA
SVSSLMTTTESIIVEIPKEEKEAPAMGGMGGMDY*
>11669.m00441|LOC_Os03g04970.1|genepair989-2
MYRAAASLASKARQAGSSARQIGSRLALHRNYAAKDIKFGVEARALMLRGVEELADAVKV
TMGPKGRTVVIEQSFGAPKVTKDGVTVAKSIEFSNRVKNVGASLVKQVANATNDTAGDGT
TCATVLTKAIFAEGCKSVAAGMNAMDLRRGISMAVDEVVTNLKGMARMISTSEEIAQVGT
ISANGEREIGELIAKAMEKVGKEGVITITDGNTLYNELEVVEGMKLDRGYISPYFITNQK
NQKCELDDPLILIHDKKVSNLHAVVKVLELALKKQRPLLIVAEDVESEALGTLIINKLRA
GIKVCAVKAPGFGESRKANLQDLAILTGGEVITEELGMNLENFEPQMLGTCKKVTVSKDD
TVILDGAGDKKAIEERAEQLRSAIELSTSDYDKEKLQERLAKLSGGVAVLKIGGASEAEV
GEKKDRVTDALNATKAAVEEGIVPGGGVALLYASKDLDKLQTANFDQKIGVQIIQNALKT
PVHTIASNAGVEGSVIIGKLLEQDNTDLGYDAAKGEYVDMVKSGIIDPLKVIRTALVDAA
SVSSLMTTTESIIVEIPKEEEAAAAAPAMGGMGGMGF*
>11676.m02853|LOC_Os10g32570.1|genepair990-1
MAEEGRSVGEEEPRGKRPAAELRLFDTRTKAAVPFRPRVEGKVAMYVCGVTPYDYSHVGH
ARAYVAFDVLFRYLKYLGYEVNYVRNFTDIDDKIIKRANEAGEDALSLSSRFIDEFHRDM
YELQCLPPTHEPRVTKHIEQIIDLITKIMDNGKAYTIEGDVYFSVDNFPDYLSLSGRKVD
QNRPGTRVAVDARKRNPADFALWKSAKEGEPSWESPWGRGRPGWHIECSAMSAHYLGNIF
DIHGGGKDLIFPHHENELAQSQAAYPESEVKCWMHNGFVNKDGQKMAKADKNFFTIRDII
SLYHPMALRLFLMRTHYRSDVNHSDTALQFASGRLYYIYQTLHDGEETVSLYGEHKLENS
IPADDQKLIEENRSNFLEKMSDDLHTTAALDHLMKLQQKLEHQKKKQSQKQQQQQQKKPE
DYIQALVALQNEVTDKLSILGLMPMSSLAEQLKDKALKRAGMTAEQLQQMIEQRTLARKN
KDFAESDRIRTELSALGIALMGEPTGTLWRPSEPELAEGS*
>11669.m00439|LOC_Os03g04960.3|genepair990-2
MAESAKPTPQLELFNSMTKKKELFEPLVEGKVRMYVCGVTPYDFSHIGHARAYVAFDVLY
RYLKFLGYEVEYVRNFTDIDDKIIKRANEAGETVTSLSSRFINEFLLDMAQLQCLPPTCE
PRVTDHIEHIIELITKIMENGKAYAMEGDVYFSVDTFPEYLSLSGRKLDHNLAGSRVAVD
TRKRNPADFALWKAAKEGEPFWDSPWGRGRPGWHIECSAMSAHYLGHVFDIHGGGKDLIF
PHHENELAQSRAAYPESEVKCWMHNGFVNKDDQKMSKSDKNFFTIRDIIDLYHPMALRFF
LMRTHYRGDVNHSDKALEIASDRVYYIYQTLYDCEEVLSQYRGENISVPVPVEEQDMVNK
HHSEFLESMADDLRTTDVLDGFTDLLKAINSNLNDFKKLQQKLEQQKKKQQQQKQQKQKQ
QQAQKQPEEYIQAMFALETEIKNKISILGLMPPSSLAEALKQLKDKALKRAGLTEELLQE
QIEQRTAARKNKQFDVSDQIRKQLGSKGIALMDEPTGTVWRPCEPESE*
>11676.m02872|LOC_Os10g32740.1|genepair991-1
MAVAKWERAKRALATRLCVRAPRVRAAAAEAEGEGEEGEGRGRSVAASPPAHVASSRRLS
RCGSRSSTKICAICLGGMCSGNGQALFTAECSHKFHFHCISSSVRHGNTVCPICRAVWKE
LPFQGPLPAAAAADASLLGTARVNPHPLDDRHQHQRMAVVRRLSRGDSVTRQWQLPIFRT
LDGGIFDDDEQLDLHPAEDVVGTQDVDSIVADEMAPASVGITTYAAFPAMEESVMVEEFA
VLIHLKAPSSPATVTSRAPIDLVTVLDVSWSMAGTKLALLKRAMSFVIQALGPGDRLSVV
TFSSSARRLFPLRKMTESGRQRALQRVSSLVADGGTNIADALRKAARVMEDRRERNPVCS
IVLLSDGRDTYTVPVPRGGGGGGDQPDYAVLVPSSLLPGGGSARHVQVHAFGFGADHDSP
AMHSIAEMSGGTFSFIDAAGSIQDAFAQCIGGLLSVVAQELRLSVECGDDGVLLTSVRSG
GYASHVDGDGRGGFVDVGDLYADEERDFLVTVRVPAARGVSALITPSCTYRSTATMETVR
VGGDTVTVPRTVDAPVGYDGMSPEVERELHRVQATEDMAAARAAAERGDFELAAAILDER
RGVLESRADDDPQSVALAAELREMQDRVETRQRYEESGRAYMLAGLSSHSWQRATARGDS
TELTSVIHTYQTPFMVDMLQRSQTLQPEVVVAMSRSAPLPAPSLSLSPPPPPSQLRRRSV
RPAMSFPGRRS*
>11669.m00429|LOC_Os03g04890.1|genepair991-2
MAPAWDRAKHALATRLCIRFPARQRAVEDAPAEDEAPPPPAAAAARAVPEEKLKSPSVSV
RRLSSSGSWGKKKVCAICLGGIRTGGQALFTAECSHEFHFHCISSNVNHGNYVCPVCRAE
WKELPFQGTQPGDTAYGRARVSTVNWPQDEGQMSVVRRLSHGYSGNLQQQLAVFRTPEAS
IFNDDENIDPQSETVDDHNAVTNSVEIKTYSEFPAIQKSERRKVFAILIHLKAPKSLDSV
SSRAPLDLVTVLDVSGSMSGIKLSLLKRAMSFVIQTLGPNDRLSVVAFSSTAQRLFPLRR
MTLTGRQQALQAISSLVASGGTNIADALKKGAKVVKDRRRKNPVSSIILLSDGQDTHSFL
SGEADINYSILVPPSILPGTSHHVQIHTFGFGTDHDSAAMHAIAETSNGTFSFIDAEGSI
QDAFAQCMGGLLSVVVKDMRLCIECIDEGVSLTSIKSGSYASQVAGNERSGLVDIGDLYA

DEERGFLVTLHVPAAHGQTVLIKPKCTYLDAITMENVQLDGEEVIIQRPAYCVDCTMSPE
VEREWHRVQATEDMSAARSAAEDGSFSQAVSILESRRRILELHAAHSSDSQFLALIKELR
EMQDRVESRQRYEESGRAYMLSGLSSHSWQRATARGDSTELTTLINTYQTPSMVDMLQRS
QTILPSVVEMLNRSSTVATSKSFSSYLPTSRHIA*
>11676.m02879|LOC_Os10g32810.1|genepair992-1
MALNLAQSAAAAACFATAGDARRAASVVAMPSSSSSATTSLRMKRQAACEPVACRAVARH
VAAAAASSRRNGVPVFVMMPLDTVSKCGSALNRRKAVAASLAALKSAGVEGIMVDVWWGI
VESEGPGRYNFDGYVELMEMARKTGLKVQAVMSFHQCGGNVGDSVNIPLPRWVVEEMEKD
NDLAYTDQWGRRNFEYISLGCDAMPVFKGRTPVECYTDFMRAFRDHFASFLGDTIVEIQV
GMGPAGELRYPSYPESNGTWRFPGIGAFQCNDRYMRSSLKAAAEARGKPEWGHGGPTDAG
GYNNWPEDTVFFRGDCGGWSTEYGEFFLSWYSQMLLEHGERVLSGATSVFGDGAGAKISV
KVAGIHWHYGTRSHAPELTAGYYNTRHRDGYLPIARMLARHGAVLNFTCVEMRDHEQPQE
AQCMPEALVRQVAAAARAAGVGLAGENALPRYDGTAHDQVVAAAADRAAEDRMVAFTYLR
MGPDLFHPDNWRRFVAFVRRMSESGSPREAAESAAHGVAQATGSLVHEAAVALRS*
>11669.m00417|LOC_Os03g04770.1|genepair992-2
MSLNLAHQTGAAAAVAPAAPRTAVVAAAAGTVSAPAVAPAAAPSLQLQTQTVDPAAPAQG
PDLPMAFQALVESLPEEQHPDVGGEERRKVGVPVYVMMPLDTVRKDGNGLNRRKAVEASL
KALKSAGAEGIMVDVWWGIAECEGPGRYNFTGYMELMEMAKKNGLKVQAVMSFHQCGGNV
GDSVTIPLPKWVLEEMDKDQDLAYTDRSGRRNYEYLSLGADAMPVLKGRTPVQCYGDFMR
AFRDHFAAFMGNTIVEIQVGMGPAGELRYPSYPESNGTWRFPGIGEFQCYDRYMLSSLKA
AAEAVGKPEWGNAGPGDSGGYNDWPEDSPFFRREGGWNTPYGEFFMSWYSQMLLEHGERI
LSAASGVYTGTPGVKISVKVAGIHWHYGTRSHAAELTAGYYNTRHHDGYQPIARMLARHG
AVLNFTCVEMRNHEQPQDAQCRPEELVQQVAAAARESGVGLAGENALPRYDETAHDQIVT
TAAEKAEEERMVAFTYLRMGPDLFQPDNWRRFAAFVKRMTESGVRDVCREQVEREAQGVA
HATGSLVHEAAVALSN*
>11676.m02880|LOC_Os10g32820.1|genepair993-1
MPAGHGLRARTRDLFARPFRKKGYIPLTTYLRTYKIGEHVDVKVNGAVHKGMPHKFYHGR
TGRVWNVTKRAIGVEINKQVGNRIIRKRIHVRVEHVQPSRCTEEFRLRKIKNDQLKADAK
ARGEVISTKRQPQGPKPGFMVEGATLETVTPIPYDVVNDLKGGY*
>11669.m00415|LOC_Os03g04750.1|genepair993-2
MPAGHGLRARTRDLFARPFRKKGYIPLTTYLRTYKIGDYVDVKVNGAVHKGMPHKFYHGR
TGRVWNVTKRAIGVEINKQVGNRIIRKRIHVRVEHVQPSRCTEELRLRKIKNDQLKADAK
ARGEVISTKRQPEGPKPGFMVEGATLETVTPIPYDVVNDLKGGY*
>11676.m02883|LOC_Os10g32850.1|genepair994-1
MGSAMDVVDISSDEEGFAAAAVAVATTTKKASVDSLGWIADLLREEDERALSDEFDDLEV
MSELSAPPVMAQQKGGKPDCGGEEDDDDCVVLDGDPDDVVAVAGEKGSEGDGSSDELQIV
AEKGPVACRDFPHSRHLCSNLPFSTTSHVKYCSMCHCFVCDTPAPCNYWGKGTEIYDHCH
ATDKEKKWKAMRHTFKSKGLPTSHPEKRQNVVYPTTTSFVQQDTQCEISLIQSHMTTYFP
NQSHLANVVNQGLTQTRHTSVRVSPSVGRTVSATRTTPATRAGRGMSNAPSIQIPQSRTR
FKRVGATSPGIVTLNDNQFGSAAPNNTQLHQPSSPHASQPAQVAPRTLFGTVQKNPPQRS
LSAPIALQGQQDQSASSYQAASNGTHGTGPQFSRCISLTAQRTQLLPEPALDVYSKSWQD
IIDSLASDLEVPDYNMGAAESQQPDRTISQPLDSITFQGVGLHSEPVVALANLMPCNGQN
VANGMIGSNCLAQTTQILPHLNHQPSLVPNESHLNNSVSSTADGLLMEAAHQRDTQGSDS
LDLLFDFEFEDWDSAEP*
>11669.m00414|LOC_Os03g04740.1|genepair994-2
MGAAGAVVEISSDDEAIPVAAKRPNVPPVSSSHPLPEDCNGVEEGLGDPAALVEFVASML
DDKRSARDVAAADDGDDDDCVMLDGDPDKAVLVVNEQRPGQGGPEEELQIVSEKGELHFR
PYIVFVVIACRDFPHPRHLCVSMPFTSSHADHCAMCHCYVCDSPAPCAFWGKGTEPTDHC
HATDKNAKWTKMRQSLKRKNLPSSNRRGIKNHFQPISATASLQLQQYTGDRFSVPRLSPL
SPVGFHVSRNVSQNQWMMKLIGVPPNVGQPVNLQEATFPRASIPRKRFRSDGSAPPVHLS
TNANHLRHPAPNSVLVQPVSSAAFQTTQSQPASSAVSQNSVSAARPLRVQTTQSQPPSSA
VSQNSVTAARPLRGYSPQNSFSAPVRVQSTSYHQVAPGISQGLQVQSTSYLQVDPGRAVS
AELQLSQCSSLQTQGIQHQHDPSADIYQNIWKEALAKLASELGVSDYNIDPPGRLPSTPQ
PNQLHAQMRPGHQPTQATARQGVQANGGHVAAASQKRTSNGHHLPNHKQFNPGAN*
>11676.m02889|LOC_Os10g32900.1|genepair995-1
MLDDVDDEPFADGISSPIAQHIYDFCDDGNGGDLFAAVNAAAAAGSDMFTASSEDASASS
SSTATAPIAGGGDTLSPPLPSLDSTLSALLEQDEPPGADGELLLPIDDYAFSAVDETQPP
PPPEHQQQQQFTQMPLPVMGGAAAEHPALQAQLSSTATELMQYAGFTDEVFAAALAAGAG
AGAGGYMGLDDPLCPQQQHSGAMLPAAAAAGEAFFSKDAHAAQAAFFAAAGGGGGSMVMS
MMGMDEIGEYQRMMECGGALLGATHAADGADMAFGNAAAAELQMGGGGSSPARLPATGTT
ETTSLEDTSFKTVRLSNEERKEKIHRYIKKRNERNFSKKIKYACRKTLADSRPRVRGRFA
KNDDYCEASRSIGSQNHEEYEQIGGVKGEDMLDSDALAHISGMSSYMYNHTVESWI*
>11669.m00402|LOC_Os03g04620.2|genepair995-2
MLHDDCIGEGLSSPIAAQILDFCDDGLGDDLFAAVATTSEQFAASSEDGSSSSTATPPLC
SNSNDITAVADTAFSPLLSFDSTLSAFLEQEQNPDQDTKLLPSIDETFTAPAYYPAATEA
NIEQFSQIMVPEHTDAPMPPMQTNRTANALLPLASGYDDECFTAALAGGYMGLDGTLYDQ
TGVMIPNCNVETPQVGFFNHNSTSNNGMVMDLNNFGEYQRMMEGEGLTRTYSDTDSMHGA
FNNAAEMQMGENTQHMVTGCNDSPLTLPSTEGSSLEDTPYKGVRLTAEQRKEKISRYIKK
RNERNFSKKIKYACRKTLADSRPRVRGRFAKNDELCEATRSSSQDFEQYEHVVGMKGEDM
LDSSNILAHLSGMNPYGYKYNSTVESWI*
>11676.m02891|LOC_Os10g32920.1|genepair996-1
MSKRGRGGSAGNKFRMSLGLPVAATVNCADNTGAKNLYIISVKGIKGRLNRLPSACVGDM
VMATVKKGKPDLRKKVMPAVIVRQRKPWRRKDGVYMYFEDNAGVIVNPKGEMKGSAITGP
IGKECADLWPRIASAANAIV*

>11669.m00398|LOC_Os03g04590.1|genepair996-2
MSKRGRGGSAGNKFRMSLGLPVAATVNCADNTGAKNLYIISVKGIKGRLNRLPSACVGDM
VMATVKKGKPDLRKKVMPAVIVRQRKPWRRKDGVYMYFEDNAGVIVNPKGEMKGSAITGP
IGKECADLWPRIASAANAIV*
>11676.m02916|LOC_Os10g33170.1|genepair997-1
MENGAGAGDDEYTRDGSVDLRGNPVLRSKRGGWKACSFIVVYELFERMAYYGIASNLVIY
LTEKLHQGTVEAANNVTNWSGTVFITPLIGAVVADAWLGRYWTFVAGSAVYLMGMLLLTL
AVSVPALKPPPCDGGGGAACPRASALQLGVYFGGLYTIALGHGGTKPNISTIGADQFDDF
HPPEKLHKLSFFNWWMFTIFLGILFSTTVLVYLQDNVSWTVGYGIPTLGLMVSVAVFLSG
TPLYRHKVPQGSPLATMGRVVAAAVWKWRVPLPADSKELHELELEHYTTRRGFRMDATVS
MAFLNKAAVKPGEGGGGSVARLPGWTLCTVTQVEETKQIVKLVPLLATMVVPCTLVAQAG
TLFVKQGVTLDRRIGKFHVPPASLGAFVTATMLICIVLYDRFLVPAVRRRTKNPRGITLL
QRISLGMLLQIVTMVVTSVVESQRLGYARRHGLVATGGQLPVTIFILLPQFVLLGVADAF
LVVGQIEFFYDQAPESMKSLGTAMSLTAYGAGNLLSSAILAAVERVTGGGKGRTPWVTNN
LNASRLDYYYAFLATLAAANLLAFVVLSCKYSYRVESTETIDVDVAMDNVAQGGGVVTVK
SEAAPMA*
>11669.m00396|LOC_Os03g04570.1|genepair997-2
MAVTESLPLPAMENGEGKGGGEYTQDGSVDLRGNPVLRSKRGGWTACSFIVVYELFERMA
YYGIASNLVIYLTDKLHQGTVEASNNVTNWSGAVFIMPLLGAYAADAYLGRYWTFVAGSA
IYFLGMCLLTLAVTIPSLKPPPCAGGVCPPASALQLGVYFGGLYIIAFGNGGTKPNISTI
GTDQFDEFDPREKMHKMSFFNWWMFTIFVGILFSSTVLVYLQDNVSWSVGYGIPTLGLLV
SISIFLAGTPLYRHKVPQGSPFTRMGKVVAAAVWKWRVAVPADAKELHELELEEYTRKRK
FRMDSTNAMRFLNKAAVKEDGSPAARWSLCTVTQVEETKQIVKVIPLLATMFVPCTLIAQ
TNTLFVKQGRTMDRHIGRHFQIPPASLGAFVTLSMLVAVVVYDRLFVPAIRKYTKNPRGI
TLLKRMGVGLLLQVVAMATASLMESRRLGYARRHGLDAAAAEVPVPLTIFELLPQYVLMG
VADAFLVVGKIEFFYDQAPESMKSLGTAMSLTAYGVGNVLSSFLLSLVSRVTRERGDAWV
TNNLNASHLDYYYGFLTVLGAINAVVFVALSSRYRYKVESTETIDIAVDVKGDTAKKIQN
*
>11676.m02923|LOC_Os10g33240.1|genepair998-1
MGRHQRSGSLSVTSSATPSSDATELDFAAAADVGCPFGRVDALGPVELRETAYEIFFMSC
RSSSGGNTAGAAEVSSPVAGPRGGGGSRVKKALGLKARRLSSSSAAMVAQPMMVRTLSQT
SGPASPGRGRRPMTSAEIMRQQMRVTEQSDARLRRTLMRAVVGQVGRRPDTIVLPLELLR
QLKPAEFADGEEYHQWQFRQVKLLEAGLILHPSLPLDRLNSAVLRFREVMRATEIRAIDT
AKSSDAMRTLTSAVHALAWRSGVGSGGGDACHWADGYPLNVLLYASLLHAIFDHRDCTVV
LDEVDELLDLIRKTWPTLGVTRPVHNVCLAWAFFQQYVVTGQVEPELAAAALAVLADVAA
DARGTRDAVYGKALLGALGAMQEWSEKRLLDYHDSYEKGIGGAPTEVMEILLSISLAAGK
IIADRDAAADADDAANFAGDRVDYYIRCSMKNAFTKVKFLGSESQFVLHQFMFVLIPDSR
ERNGRRRRRAGGGADAAGEGHGGARRRGAAELQPGAEAVAPGAGGRRGGHPARLLRRRAE
AVPRQGDHPHGGARPRAPVGEQDGEGHGADDGGGRRRLPRRPGQGHRRRHGALRGGFRRY
GLAQGVDGRQVQDHHGLPRQSQRNRAMEMMKLAKYTVEEFSEIPASAKDEVVQDLVDGLE
AIFQEYISFVASCGAKQNYLPPLPPLTRCNQDSGFFKLWRKTVLPSCQAPEGGPRGVGVG
GGSHHVPRPSISRGTQRLYVRLNTLEYVLTHLHAIDKSLVAAPSPRFDGARAAAKSAIAR
VAEVAAFRLVFLDSRHSFYHGLYLRGVADTRIRPALRALKQNLTFLVSVLADRAQPVAVR
EVMRASFEAFLMVLLAGGGDRSFARGDHAMVEEDFRSLRRAFCTCGEGLVPEEVVAREAE
AAERVVELMARPTDALIDAFGVATSESIVAAVGRGGDDGDGGYGGVTPVPPTSRRWDAAD
ANTILRVLCHRDDEAASQFLKRTFQLAKRR*
>11669.m00395|LOC_Os03g04560.1|genepair998-2
MGRLQHHHRSRSASSSSFARSSDTAATDADARSLAADATVDCPFGHVNGLTRSDLREAAY
EVFFMSCRAAGGGRLNYFPAGESGGGDVSPTIGAGPRGGTGMSVVNSRVKRALGLKARRS
SQPTTARVSSMNASSAPGSPGRAMWAMSQPSTPVSPGKGRRPMTSAEIMRQQMRVTEQND
ARLRKTLMRTLIGQVGRKAETIVLPLELLRQVKLTDFADSGEHHQWQRRQLKLLEAGLIA
HPSLPHDRLNAVVLRFREVMQAADTRAIDTGKTSDAMQALCNAVHALAWRSAPGSKAAGG
DACHWADGYPLNVLLYVSLLQAIFDLKEETVVLDEVDELLELMRRTWPTLGITKMLHNVC
FAWVLFQQYVVTGQIEPDLAGAALAMLTEVAADAKQESRDPVYARVLSSILATIHDWSEK
RMLGYHEWFGNGNCGAGGAMAMEGALSLALATTQIISDNAIFTSISTAETEHEDCSVGSF
AGDRVDYYVRCSTRSAFTKVSFLRVWPRHGERLVLICRGNVCQILENGLGQGDSLIIDRH
DDEDPGDILARLAGDTEHIALSERDAFGPVLRRWHPFPGAIAAVTLHGCFGVVLKQYLGK
ATVLSNELVHVLHAAGRLEKALVQMVVEDVADSDDGGKSVVREVVPYDVESIVFGFLRTW
VEERLKICRECMLRAKETESWMPRSKNEPYAQSAVELMKLAKATVDEFFGIPVAVRDDLV
QDLADGMEAIFLEYISFLTSCGSKQSYLPSLPPLTRCNQDSKIIRLWKKAATPCRAPVSS
PRAHGHHQGQGGMASGGQNPRPSTSRGTQRLYVRLNTLHFILSHVHALDKSLSFFSRGRC
SSSPSSAATARLLAPCSHFDRARAAAQSAVGHVAEVAAYRLIFLDSHHSFYDGLYVGGVA
DARIRPALRTLKQNLSLLLSVLVDRAQPVAVREVMKASFQAFLLVLVAGGGDRSFTKEDH
GMVEEDFRSLKRAFCTRGEGVVTEEVVDGEAEAAESVVALMGQTAEQLVEELSIACELNG
TASSAGQRMPLPETTWRWSRTDPDTILRVLCHRDDEVASHYLKRAFQLPKRR*
>11676.m02928|LOC_Os10g33290.1|genepair999-1
MEGRKIERGRLRGFVRRMAMECLCSGEQLRAADEIIRSPESAITKDCSASGYSSQNGEIE
QYLDNGNIEEAELSLREGVCLNYEEARALLGRLEYQRGHVEAALRVFDGIDIPALVPKMK
ISIARKVDRRKTRSQWDSPPMPLHAVSLLMEAIYLKSRALHDLGKFKEAAQECRMILDIV
EAAVPEGLPAGFGKDCKLNEIICKAVELLPELWKLGGFSLEAISSYRRSLLNNWNLDGET
IARIQKEFAIFLLYSGCEARPPNLHSQLDGSFVPRNNMEEAILLLMILLRKFNLKRVERD
PTIMHHLTFALSISGQLKSLAVQFEELLPGMLDKREWSYNVALCYLAEEDDSTALNLLKR
ILKSGDDSDNFKELLLASKACTERSAQTEGASYAQRAIANMQGGCEQMAGVADLLLGVNL
SNQARCATSDTERASWQCEALEVLENAENKMHGKDPRAMYSLSLENADQRKLDAAAFYAK

KLVKLEAGSELRSWLLLARILSAQKQFADAETIIDAALDQTGKWSQGDLLRTKARIQAAQ
GQLRNAVETYTKLLAVIQLRTKSLSAGIFLAKGTKDDISLEIETWYDLALLYLRMSQWRD
AEVCVSKIRTISPYSALAWHVKGKLYEAKGQPKEALGSYFRALDLDRKHVPSLISTASVL
REIGNRPLPSVRCFLTDALQLDRTNHAAWFNLGLLYKEEGGRSAAEAAECFQAAALLEET
APVEPFR*
>11669.m00388|LOC_Os03g04500.1|genepair999-2
MARSSSTSTILILKKLTFPSRREVRLKEARALLGKVENQHGHAEEALRVFSGINMPALIP
KVKMSIIRKVDLQKAQLHSSSPSLPFHAAILLLEIIYFKATALRNLGKIEEATKECSSIL
DVVESALPEGLPDIFGDDCNLKPTLCRAVELLPELYKLGGFHFEAISSYRRALWSNWNLD
EKTIGRIQKEFAVLLLYSGCETCSPNLLSQLDGSFVPRNNLEEAILLLMLLLRKFNLKRL
ERDPTVMHHLTFALSMSGQLKPLAIQFEELLPGVLHNREWSYNVALCYLAEEDDLIALNL
LKRILVSGEDSNNLKELLLVSKICCENSVQGEEGTLYARRALTNLHGGCDQIEVTADLLL
GISLSNQARFATTNTKRASQQREALEVLSISEKKMHGIDFRVLYNLSLENAKQRKLDTAA
RYAKKLLKLEAGSELKTWLLMARIMSAQRRFEDAESIVNAALDQTGKWFQGDLLQIKAKM
QAAQGKFKKAVETYTQLLAVIQLRTKSFNAGISVLKGSKDDRSLEIETWYDLVLLYIRMS
QWRDAELSISKIKAISPYSALAFHATGKLHEAKGFLKEALRAYSTALDLEPRHVPSLIST
AIVLRRLGERPLPAVRCFLTDALQLDRTNHIAWLNLGLLYEDEGGSSALEAAECFQTAAL
LEETNPVEPFR*
>11676.m02931|LOC_Os10g33310.1|genepair1000-1
MGKYMRKAKVVVSGEVVAAAVMELAAAPLGVRTRARSLALQKRQGGEYLELRSRRLEKLP
PPPPPPPRRRATAAAATADATAAESAEAEVSFGGENVLELEAMERIAPFVSLNCVKNTWV
APSPKYPRVGATCLQGTKGVPYERDEQVLRHFWNTRETTPCSLIRDPDTISTPGSTTRRS
HSSSHCKVQTPVRHNIIPASAELEAFFAAEEQRQRQAFIDKYNFDPVNDCPLPGRFEWVK
LD*
>11669.m00386|LOC_Os03g04490.1|genepair1000-2
MGKYMRKGKVSGEVAVMEVGGALLGVRTRSRTLALQRTTSSQKPPEKGEGDPGAGAGAGA
EYLELRSRRLEKPPPHTPPAKEKETARRASAAAAAAVRMPAAPQAAEEFEAEVEVSFGDN
VLDLDGDAMERSTRETTPCSLIRSSEMISTPGSTTKTNTSISSRRRMETSVCRYVPSSLE
MEEFFAAAEQQQHQAFRERYNFCPVNDCPLPGRYEWTRLDC*
>11676.m02262|LOC_Os10g26410.1|genepair1001-1
MSGRRASGRITDDEINELISKLQSLLPESSRRRGATSRSPATKLLKEMCSYIKSLHREVD
DLSERLSELMATMDSNSPQADIIRSLLR*
>11669.m00717|LOC_Os03g07540.1|genepair1001-2
MSSRRGGGGGGGRITDEEINELISKLQALLPESSRSRGASRSSASKLLKETCSYIKSLHR
EVDDLSDRLSELMSTMDNNSPQAEIIRSLLR*
>11676.m02268|LOC_Os10g26470.1|genepair1002-1
MAVDMELDGGGDGKGKAPPQISLSGLFLACMVAGGVQYGWALQLSLLTPYVQTLGIPHAL
TSVMWLCGPIAGLIVQPCVGLYSDKCTSSLGRRRPFILTGCIIICISVIVIGFSSDIGYA
LGDTTEDCKVYRGPRYHAAAAFILGFWLLDFSNNTVQGPARALMADLSGRHGPSAANAIF
CSWMALGNILGYSSGSTNDWHKWFPFLMTRACCEACANLKAAFLVAVVFLGLSTAVTMVF
AREVALDPVAAAKRNEGEASGLLAVFKGMKNLPVGMPSVLIVTGLTWLSWFPFILFDTDW
MGREIYHGRPDGSPAEVTAFQEGVRQGAFGLLLNSIVLGISSFLIEPMCRRLGARAVWVM
SSAVVCVAMAAVSVLSAWSLGDFGGSVQDAARAPAEEGGVRASALALFVFLGLPFAVLCS
VPFAVTAQLAASRGGGQGLCTGVLNISIVVPQMAIALGAGPWDELFGEGNIPAFAMASVF
AAAAAAAGVVLLPKVSVRSVSMAGGH*
>11669.m00711|LOC_Os03g07480.1|genepair1002-2
MARGSGAGGGGGGGGGGLELSVGVGGGGGARGGGGGEAAAAVETAAPISLGRLILSGMVA
GGVQYGWALQLSLLTPYVQTLGLSHALTSFMWLCGPIAGMVVQPCVGLYSDRCTSKWGRR
RPYILTGCVLICLAVVVIGFSADIGYAMGDTKEDCSVYHGSRWHAAIVYVLGFWLLDFSN
NTVQGPARALMADLSGRHGPGTANSIFCSWMAMGNILGYSSGSTNNWHKWFPFLKTRACC
EACANLKGAFLVAVIFLSLCLVITLIFAKEVPFKGNAALPTKSNEPAEPEGTGPLAVLKG
FRNLPTGMPSVLIVTGLTWLSWFPFILYDTDWMGREIYHGDPKGTDPQIEAFNQGVRAGA
FGLLLNSIVLGFSSFLIEPMCRKVGPRVVWVTSNFLVCIAMAATALISFWSLKDFHGTVQ
KAITADKSIKAVCLVLFAFLGVPLAVLYSVPFAVTAQLAATRGGGQGLCTGVLNISIVIP
QVVIALGAGPWDELFGKGNIPAFGLASGFALIGGVAGIFLLPKISKRQFRSCAW*
>11676.m02271|LOC_Os10g26500.1|genepair1003-1
MASNGAAAGAMAPFFPPNFLLQMQQPLPLHHQHLQDHAHGGHGGHHLLPPPPPSLSPFLP
DLAMDAPPPPMYEASGGDGGGGGAASEDEEDGCGGGGGGGGGEKKRRLSVEQVRTLERSF
ESGNKLEPERKAQLARALGLQPRQVAIWFQNRRARWKTKQLEKDFDALRRQLDAARAEND
ALLSLNSKLHAEIVALKGGAAAAGGGGSSCRQEAASELINLNVKETEASCSNRSENSSEI
NLDISRPAPPPPPPPANESPVNRGIPFYASIGRGGAGGVDIDQLLLRGGHSPSPAAVTTP
PPPKMELGITGNGGGADAAAAGAGSFGGLLCGAVDEQPPFWPWADGHHHFH*
>11669.m00708|LOC_Os03g07450.1|genepair1003-2
MASNGMASSPSSFFPPNFLLHMAQQQAAPPHDPQEHHHHHHHGHHGHHHEQQQQQQHHHH
LGPPPPPPPHPHNPFLPSSAQCPSLQEFRGMAPMLGKRPMSYGDGGGGGDEVNGGGEDEL
SDDGSQAGEKKRRLNVEQVRTLEKNFELGNKLEPERKMQLARALGLQPRQVAIWFQNRRA
RWKTKQLEKDYDALKRQLDAVKAENDALLNHNKKLQAEERRSMWVSVLAERLLESVPFSP
PRIVALKGREAASELINLNKETEASCSNRSENSSEINLDISRTPPPDAAALDTAPTAHHH
HHGGGGGGGGGGGMIPFYTSIARPASGGGVDIDQLLHSSSGGAGGPKMEHHGGGGNVQAA
SVDTASFGNLLCGVDEPPPFWPWPDHQHFH*
>11676.m02273|LOC_Os10g26520.1|genepair1004-1
MGNCMDTAAAAAAAPDTNNADPSKAASKTTYSSYPSTTKSGSSWTVPSYKDRSDLPTPRT
EGEILSSSNLKAFTLSELKNATKNFKPDSLLGEGGFGYVYKGWIDEQTLAPARPGSGMVV
AVKKLKPEGFQGHKEWLTEVDYLGQLHHENLVKLIGYCSDGDNRLLVYEYMPKGSLENHL

FRRGADPLSWGIRLKVAIGAARGLSFLHDAENQVIYRDFKASNILLDSEFNAKLSDFGLA
KAGPTGDRTHVSTQVMGTRGYAAPEYVATGRLSVKADVYSFGVVLLELLTGRRALDKSKP
ASEQNLVDWTRPYLGDKRRLYRIMDMKLGGQYPKKGAHAIATIALQCIRSEAKMRPQMSE
VLEKLQQLQDPKYNVTSPQVDTRRRSSSGSVPRSPMRMQPSPRRLSASASPLPAAGSPLP
ACRTAQVH*
>11669.m00706|LOC_Os03g07430.1|genepair1004-2
MGNCMDTTARVDHSMNNGASVKLILVAWDVGLFVLLDRGFIDRSELPTPRTEGEILSSSN
LKAFSFNDLRNATKNFRPDSLLGEGGFGHVYKGWIDEHTLAPSKPGSGMVVAVKKLKPEG
FQGHKEWLTEVNYLGQLHHKNLVKLIGYCSDGDNRLLVYEFMPKGSLENHLFRRGADPLS
WAIRLKVAIGAARGLSFLHDAENQVIYRDFKASNILLDSEFNSKLSDFGLAKAGPTGDKT
HVSTQVMGTHGYAAPEYIATGRLSAKADVYSFGVVLLELLTGRRALDKSKPGIEQNLVDW
AKPHLGDKRRLYRVMDTKLGGQYPKKGAHAIANIALQCICNDAKMRPRMSEVLEELEQLQ
DSKYNMASPQVDIRRTSNAVPKSPMRIQPSPRRSLGAAASPLPGYRTAKVH*
>11676.m02275|LOC_Os10g26540.1|genepair1005-1
MESRKEEEQHGGGGAVGWMTVPAFGEWDMKNGAVPDYSMDFSKIREMRKQNKRELSRASL
GGDDDLLAAQQHKAAAPQPAPNASAAADDHRRPLHAAHDDSPTGRNFALDESNREAAI*
>11669.m00701|LOC_Os03g07380.1|genepair1005-2
MENIKGGHGAAAAGGRNGWMTVPAFGDWDMKNGALPDYSMDFSKIREMRKQNKKELSRTS
LGGDDDLLAQQKQQQPQPQQKPAKANLGRPADDHRHRQLHGRHGSPTYKIGFN*
>11676.m02283|LOC_Os10g26620.1|genepair1006-1
MGECRGGGGGGDGLIKLFGKTIPVQPDAKDVQQHSGSSSSSTESDVQETAAVAVADPSPR
SEVVDGESPPQPGGEAASHQQQQKEMKLKKPDKILPCPRCSSMDTKFCYFNNYNVNQPRH
FCKHCQRYWTAGGAMRNVPVGAGRRKNKNATAAAHFLHRVRACAAAAMPAAPHDATNAT
VLSFGGGGGGHDAPPVTLDLADKMTRLGKEGLVAHARNADAAAACSEVSSNRDDEQIGNT
VAKPANGLQQHPPPPHHHHHSAMNGGGIWPYYTSGIAIPIYPAAPAYWGCMIPPPGAWSL
PWPATVQSQAISSSSPPTSATPSVSSFTLGKHPREGGDHEARDHHGNGKVWVPKTIRIDN
ADEVARSSIRSLFAFRGGDKADDNNDDDGTGVHKLATTVFEPKRDSKTAKHPAITSLPLL
HTNPVALTRSATFQEGS*
>11669.m00699|LOC_Os03g07360.1|genepair1006-2
MGECKVGGGGGGGDCLIKLFGKTIPVPEPGACAAGDVDKDLQHSGSSTTEPKTQENTVQD
STSPPPQPEVVDTEDSSADKNSSENQQQQGDTANQKEKLKKPDKILPCPRCSSMDTKFCY
YNNYNINQPRHFCKNCQRYWTAGGAMRNVPVGAGRRKSKSVSAASHFLQRVRAALPGDPP
LYAPVKTNGTVLSFGSDLSTLDLTEQMKHLKDKFIPTTGIKNTDEMPVGLCAEGLSKTEE
SNQTNLKEKVSADRSPNVAQHPCMNGGAMWPFGVAPPPAYYTSSIAIPFYPAAAAAVAAY
WGCMVPGAWNAPWPPQSQSQSVSSSSAASPVSTMTNCFRLGKHPRDGDEELDSKGNGKVW
VPKTVRIDDVDEVARSSIWSLIGIKGDKVGADHGRGCKLAKVFESKDEAKASTHTAISSL
PFMQGNPAALTRSVTFQEGS*
>11676.m02284|LOC_Os10g26630.1|genepair1007-1
MSAMLVAEAAWMGLASLAAAAARRLRGYGYRWEPMAAPPDVEAPAPAPAEFPMVLVQIPM
YNEKEVYKLSIGAACALTWPPDRIIIQVLDDSTDPFVKFSLVQELVELECKEWASKKINI
KYEVRNNRKGYKAGALRKGMEHTYAQLCDFVAIFDADFEPESDFLLKTMPYLLHNPKIAL
VQTRWEFVNYNVCLMTRIQKMSLDYHFKVEQESGSFMHAFFGFNGTAGVWRVSAINQSGG
WKDRTTVEDMDLAVRASLKGWEFLYVGDIRVKSELPSTFQAYRHQQHRWTCGAANLFRKM
AWEIITNKEVSMWKKYHLLYSFFFVRRAIAPILTFLFYCIVIPLSAMVPEVTIPVWGLVY
IPTAITIMNAIRNPGSVHLMPFWILFENVMAMHRMRAALSGLLETARANDWVVTEKVGDQ
VKDELDVPLLEPLKPTECAERIYIPELLLALYLLICASYDFVLGNHKYYIYIYLQAVAFT
VMGFGFVGTRTPCS*
>11669.m00698|LOC_Os03g07350.1|genepair1007-2
MEGQWGRWRLAAAAAASSSGDQIAAAWAVVRARAVAPVLQFAVWACMAMSVMLVLEVAYM
SLVSLVAVKLLRRVPERRYKWEPITTGSGGVGGGDGEDEEAATGGREAAAFPMVLVQIPM
YNEKEVYKLSIGAACALTWPPDRIIIQVLDDSTDPAIKDLVELECKDWARKEINIKYEIR
DNRKGYKAGALKKGMEHIYTQQCDFVAIFDADFQPESDFLLKTIPFLVHNPKIGLVQTRW
EFVNYDVCLMTRIQKMSLDYHFKVEQESGSSMHSFFGFNGTAGVWRVSAINEAGGWKDRT
TVEDMDLAVRASLKGWQFLYVGDIRVKSELPSTFKAYRHQQHRWTCGAANLFRKMATEIA
KNKGVSVWKKLHLLYSFFFVRRVVAPILTFLFYCVVIPLSVMVPEVSIPVWGMVYIPTAI
TIMNAIRNPGSIHLMPFWILFENVMAMHRMRAALTGLLETMNVNQWVVTEKVGDHVKDKL
EVPLLEPLKPTDCVERIYIPELMVAFYLLVCASYDLVLGAKHYYLYIYLQAFAFIALGFG
FAGTSTPCS*
>11676.m02290|LOC_Os10g26690.1|genepair1008-1
MAAAAAASSGWLRRAAGTVPRIPCGLVTALVPTPPPPAAAVVSEAPALALPSHAAVAMEL
MAVPKKKVSKYKKGLRNGPKALKPVPVIVRCKCCGRVKLPHFYCCSGERGNPGSESS*
>11669.m00692|LOC_Os03g07290.1|genepair1008-2
MAAAAGWLRRAAAAAAAPRLPSGLPILPTPPPAPLTEAQSFVLPGIGTAVAGGMDLMAVP
KKKVSKYKKGLRNGPKALKPVPVIVRCRCCGRVKLPHFYCCSGEKGNPGDSSS*
>11676.m02119|LOC_Os10g25060.1|genepair1009-1
MGDVSLNPPINAEPLTKGDQFLDLMSDGWTNERHSLYISSMEASFMEQLYRHDHHGLDRN
RSHAGGAIGFRVHREGVCDNLRSERNDAHAHDGGMSCFPENPWIRRFRPRDAGVNRKNDA
VGFSVDDDESGTDMVRQRVRVHGREAKSCAGGILADKSTVSDQNFPDEDVEVDSEPCKRR
RRPTNSTATPHDQTT*
>11669.m00834|LOC_Os03g08580.1|genepair1009-2
MGDVSVNRPIKAEPAAGGIAQGNRILDMMSSGWTDERHMHYISSMEASFVEQLYNHEQND
AGNGFKVLRGGVWEKLKFDRTSACSRIGRKYCLPASPWIQHFRPRECSSNARNDAAEALV
GDHESGIQTIQGRTPLSHGREWEACKEEKAVGESTEVSDQNFADDEAEVDAESSKACKRR
KLRSALTRNYQVVPSDKLFASTKADGRKDAPE*

>11676.m02122|LOC_Os10g25090.1|genepair1010-1
MAAWAWPLVFLVSCCCSWTQRRILVAATTDANDVTVLNALFTSLNSPGQLRGWQVNGGDP
CGASWQGITCSGSSVTAIKLPSLGLSGNLAYNMNTMESLVELDMSQNNLGGGQNIQYNLP
NKKLERLNLAGNQFAGNVPYSISTMPKLKYLNLNHNQLQGNMTDVFSNLPSLSTLDLSLN
SLTGDLPQSFTSLSSLKTLYLQNNQFTGSINVLANLPLDNLNVGNNRFTGWIPNELKKIN
SLQTDGNSWSTGPAPPPPPFTAPPPSRNRKKSPGRHSNGSGSSSSSGGNSGLRAGAIAGI
IVALLVIGAVVAFFLIKRKRKGTRQEHVEQRQPFNSYPSNEVKDVKPIPESTKIEVEPLP
SPVAVSLKPPPKIERNQSFDDDDDDFSNKPVAKKSNSASVKATVYSVADLQMATDSFNMD
NLVGEGTFGRVYRAQFSDGKVLAVKKLNSTVLPSQSSDDFFDLVSNISKLHHPNLNELVG
YCMEHGQHLLVYDFHRNGSLHDMLHLPDEYSKPLSWNSRVKIALGSARALEYLHEICSPS
IIHKNFKSSNILLDTEFNPHVSDAGLASSVPDSEFQASDQGSGYSAPEVDMTGQYTLKSD
VYSFGVVMLELLTGRKPFDSARLRTEQSLVRWATPQLHDIDALDRMVDPALKGLYPAKSL
SRFADVIALCVQPEPEFRPPMSEVVQALVRLVQRANMTRRMIDGEEGSRRPDDQDQEFV*
>11669.m00831|LOC_Os03g08550.1|genepair1010-2
MGRADAPWLPLLLLCSSCCFCIWPQKQILVAADTDPNDVTVLNTLFTSLNSPGQLKGWQA
SGGDPCGQSWQGITCSGSSVTAIKLPSLGLSGNLAYNMNTMGSLIEIDMSQNNLGGGQQI
QYNLPTNKLERLNLAGNQFTGNLPYSIFSMSNLKYLNLNHNQLQGNITDVFSSLYSLTTL
DLSFNSLAGDLPQGFTSLSSLKKLYLQNNQFTGYINVLANLPLDDLNVANNHFTGWIPSQ
LKKINNLQTDGNSWSNGPAPPPPPYSAPPPNRPNSPGQNNGGSSSGGSSGIGGGGVAGI
IISLLVVGAVVAFFVIRRRKRRAALEEHFEQHQPFTSFPSNEVKDMKPIEESTTIDVESL
PSPASFSLKPPPKIERHKSFDDDDLSNKPVLKKTNVAPIKATVYSVADLQMATESFSMDN
LVGEGTFGRVYRAQFTGGKVLAVKKLDSTVMPFHSSDDFAELVSDISKLHHPNLNELVGY
CMEHGQHLLVYDFHRNGSLHDLLHLSDEYSKPLSWNSRVKIALGSARALEYLHEICSPSI
IHKNFKSSNLLLDSEFNPHLSDAGLASFISDAEFQAAQQSAGCTAPEVDMTGQYTLKSDV
YSFGVVMLELLTGRRPFDSTRPRSEQSLVRWATPQLHDIDALDRMVDPALKGLYPAKSLS
RFADVLALCVQPEPEFRPPMSEVVQALVRLVQRANMTKRMLDGDTSSRRTDDQEQDFI*
>11676.m02123|LOC_Os10g25100.1|genepair1011-1
MAHTSIHELQSDLWLLKMKKKSCHGRATHDPQHGLSKIAGESTKHQQGNISGSQAAAAVK
SKPWKKRYLTFLSKFQNKMKKHKKPDNIKAHHTRSHKTRSILLSSSQIMEECSNLVQVIR
HTAADCFAAAATAVAAAVDYEEDDHHQPYMQLDQVNYGVMKREAFGPVYLVT*
>11669.m00830|LOC_Os03g08540.1|genepair1011-2
MARVEFSSPKCSSSSSLPSLRLPSISVASPLRPRGPPPSAAGGSFTRRPTAASPPPPAGR
ASRFRQRHGRGRRRRRMMSTTKHFEAQSTLMGLICKPTRELHTCECDIASWTRPDLVEIG
PFATAIANLKCEKDLFSSADTIFIEATITQEEECTQVSNKTTSSSPENMLLSSQGLPDNF
TDEATKDQYLCNSGLQAANRESKKKNSTFLTKFQNRIIASLASESSPCRNAFRRPLLSRE
IVVREYFKLARIIRRTAAACFSPSSDADEDYDYLPHMQLDKVTHAISREAFGPLYLVT*
>11676.m02126|LOC_Os10g25130.1|genepair1012-1
MAAPSVAVDNLNPKVLNCEYAVRGEIVIHAQRLQQQLQTQPGSLPFDEILYCNIGNPQSL
GQKPVTFFREVIALCDHPCLLEKEETKSLFSADAISRATTILASIPGRATGAYSHSQGIK
GLRDAIAAGIASRDGYPANADDIFLTDGASPGVHMMMQLLIRNEKDGILCPIPQYPLYSA
SIALHGGALVPYYLNESTGWGLEISDLKKQLEDSRLKGIDVRALVVINPGNPTGQVLAEE
NQRDIVKFCKNEGLVLLADEVYQENIYVDNKKFNSFKKIARSMGYNEDDLPLVSFQSVSK
GYYGECGKRGGYMEITGFSAPVREQIYKVASVNLCSNITGQILASLVMNPPKAGDASYAS
YKAEKDGILQSLARRAKALENAFNSLEGITCNKTEGAMYLFPQLSLPQKAIDAAKAANKA
PDAFYALRLLEATGIVVVPGSGFGQVPGTWHIRCTILPQEEKIPAIISRFKAFHEGFMAA
YRD*
>11669.m00829|LOC_Os03g08530.1|genepair1012-2
MATGVSVENINPKVLRCEYAVRGEVAIHAQHLQQQLQTQPGSLPFDEIVYCNIGNPQSLG
QKPITFFREVLALCNHPNLLEREEIKSLFSTDAIARAKKILSMIPGRATGAYSHSQGIKG
LRDEIAAGIASRDGFPANADDIFLTNGASPGVHMMMQLLIRNNRDGIMCPIPQYSLYSAS
LALHGGALVPYYLDESSGWGLEVSKLKNQLEDARSKGITVRALVVINPGNPTGQILDEQQ
QYELVKFCKDEELVLLADEVYQENIYVTNKKINSFKKIARSMGYNGDDLQLVSLHSVSKG
YYGECGKRGGYMEVTGFSTPVREQLYKIASVNLCSNITGQILASLIMDPPKAGDASYDLY
EEEKDNILKSLSRRAKAMESAFNSIDGITCNKTEGAMYLFPRIYLPQKAIEAARAVNKAP
DVFYALRLLDTTGIVVTPGSGFGQIIRCYLFTCNSEFLTTQVWSFVKGCRDMAREMHDPA
AGGEDTFDDLPLQGIP*
>11676.m02130|LOC_Os10g25170.1|genepair1013-1
MCGGAILADLIPSPRSGGHTKKNKRRRISDDEDFEAAFEEFDAGDDDSDSDSESEEVDEY
DVVVDDDDSEDGVVVLPPPPPPPPVIPHERHGARRFRGVRKRPWGKWAAEIRDPVRGVRV
WLGTFPTAESAARAYDAAARRLRGAKAKPNFPSAPPPSAAAHRRKKRRAHAATRSPSSPP
ATSEVTAASASASSDVPAPAFASFVGEPGHGGAKSMPTTSHTSQPAPPATVASENVDDPE
VFDPYDVHGGLASYFAGGAYESLESLFAHGGDSAAVDQAASDHWPAALWSFADDGSFCF*
>11669.m00826|LOC_Os03g08500.1|genepair1013-2
MCGGAILAELIPSAPAARRVTAGHVWPGDANKAKKKGARADDFEAAFRDFDNDSDDEEMM
VEEAEEEEATSEHKPFVFRAKKAAAAASSRRRKPAQYRGVRRRPWGKWAAEIRDPVKGIR
VWLGTFTNAEAAALAYDDAARAIRGDRAKLNFPSATTPDTRKRGRATAAAAPAVKATPVI
NLVEEEDEEEVAAAMASIKYEPETSESSESNALPDFSWQGMSASDEFAVAAAALSLDSDD
DLAKKRPRTEPEDTTDSGSGDDTDALFDALLFADQYNHFNGGAYESLDSLFSADAVQTTA
AAAAADQGMGLWSFDDGCCLVDVEASLSF*
>11676.m02131|LOC_Os10g25180.1|genepair1014-1
MELVTDPLSVNTRWLEPPLVETKRDETWWVLKIDRTEPTSLEIEDLVEVIEKKPRESLQR
VQYAKAIDHINNGLSNDKQLMLEQNDVTSASSSTSMNEPKFGIIEILPIVLQKGVLKTNC
IDSRDRTNRAQIVDGLVGLGRQLKALVQTKGLEIHIEEPLSSTLMLFYEEMGDALALQFT
GSAAQNKEFWKQKGQWSAMNKLTRNIQHFVSNAYMDSEKQNSPNIEVVDVCILFQILGTF

PTPTGKASNLEVLAWLAPAKGK*
>11669.m00816|LOC_Os03g08430.1|genepair1014-2
MEAMAGDKFLQKFRLYETRSKFYLIGRDKTRTLWRVLKIDRMEPTELEIEEDHTSYTENE
CQELLWRIHEGNRLTGGLKFVTKCYGIVGFIKFLGPYYMVLITRRRKVGTICSHEIYSVG
KSELIAIPSPIVWPNVAYSRDENRYKRLLCSVDLSKDFFFSYSYNIMRSLQKNITDKNTG
QVVYETMFVWNEFLSRAIRNHLKNTTWTVALIHGFFKQSKLSVAGKEFWLTLIARRSRHF
AGTRFLKRGVNEKGRVANDVETEQIVFEDTPDEIPHQISSVVQHRGSIPLIWFQETSRLN
IRPDIILKPDVDYKTTRLHFENLALRYGNPIIILNLIKTREKKPRESLLRAEFAKAIHYI
NKGLPDDKRLKFLHMDLSKLSRRKGTNVLSLLNKVASDVLDLTDFLHCEITTSKYEDASS
GQGAVANSGDIENIQDQNLCATKLVPLLLQKGVLRTNCIDCLDRTNVAQFAYGLAALGRQ
LHVLQLNETPTIELHAPLADDLMDFYERMGDTLAIQYGGSAAHNKIFCEQRGQWKAATQS
QEFLRTLQRYYSNAYTDPEKQDSINVFLGHFQPQEGKPALWKLDSDQHYNIGRQGTLTEE
TGRSFIKRSLSDGNILCDNTGGPVSDCNVGKNNTSSELLPMQPLEDIREPSDSAPEISIE
PNPCSSTNYSTLSGRHSISEERQNYLRRLGYPELHSSNFLDLDLLSSSGNSCEEEIYERS
SLINSPMDVVSIESTTSYSEQGHNDEQGRDDTDLSRSSSQMSDIRDYSDRFTHWVDGGGM
LCY*
>11676.m02142|LOC_Os10g25290.1|genepair1015-1
MAAAGSSSRFAVTCGLLSQYMRERQQPQPPVTVLEAVAEEEEEEDARTMQLFPPRAAAAD
GVATPSAGTAPLTIFYDGRMVVVDDVPVEKAAELMRLAGSACSPPQPAHAAALPEMPIAR
KASLQRFLQKRKHRITTTSEPYKKAAVASPAPEKSFAVAPVKDEPATWLGL*
>11669.m00803|LOC_Os03g08330.1|genepair1015-2
MEGKSRRFAVACGVLSQYVRAEQKMAAAAGAAPARAVTTLSLMPGAEVVVEEEERREVGE
EEAGPATAPAAPLTIFYGGRMVVFEDFPADKAAEVMRMASSGMAAAPAQREGAALADMPI
MRKASLQRFFAKRKDRLAATTPYARPSPAETKASEPEEKKTPTSWLDLAASASAAARRDS
LTIAL*
>11676.m02171|LOC_Os10g25550.1|genepair1016-1
MGKKTTTSSSPSWSSLFGLGCFTSSHSDGGSAAAKNPGTPLPARPSSCNSNDGVAAAVMP
SPEDLSQSLAGSGVEAFTVEELRRATRDFSVSNFVGEGGFGPVYKGYVDERLKPGVRAQA
VAVKLLDLEGSQGHKEWLAEVIFLGQLRHHHLVKLIGYCYEDEHRLLVYEFMARGSLEKH
LFKKYSASLPWSTRLKIAIGAARGLAFLHEAAKPVIYRDFKTSNILLNSDYEAKLSDFGL
AKDGPQEDETHVSTRVMGTQGYAAPEYIMTGHLTTKSDVYSYGVVLLELLTGRKAVDKKR
PPREQNLVEWARPCLHDSRRLNRVIDKSLNGQYSTRAVQKAAAIAYQCLSVSPKSRPRMS
AVVEALEPLLAMDDGIVEPFVYMAPPESK*
>11669.m00787|LOC_Os03g08170.1|genepair1016-2
MAKQAWSSLFGCFTSHHDGGGKRKKGGGGGKKAKKKVAAAASQKQRKLQSRLSFSDLSFG
GMVSPEDLSLSLAGSNLHVFTIAELRAVTRDFSMTNFIGEGGFGPVYKGYVDDKLKPGLR
AQPVAVKLLDLEGTQGHNEWLTEVIFLGQLRHPHLVKLIGYCYEDEHRLLVYEFMTRGSL
EKHLFKKYAASLPWSTRLKIAIGAAKGLAFLHEAEKPVIYRDFKTSNILLDSDFKAKLSD
FGLAKDGPEDDETHVSTRVMGTQGYAAPEYIMTGHLTAKSDVYGFGVVLLELLSGRKSVD
KSRPAREQNLVEWARPYLTDARRLGRVMDRNLAGQYPAKAAQKAAALAHRCVSLNPKSRP
HMSAVVEALEPLLALDDDCLVGTFVYYVAPPDDVAANGDGSSKRRAGRRRSDGAAAAAAD
GVQRE*
>11676.m02198|LOC_Os10g25810.1|genepair1017-1
MRDMEMRWAAPAPAARGRGRARRRAPDQPSFSSTLLDAICDSMDEGGEDGRTRNAASAAA
KKRQEAANSYHYYYCYKPSLAASYRAAPALGSTADCPGRGYFSSSEVEYSLRRLRPIRTS
AAGGAGDGAAVARKQRHEQPDVEKTAKTKPGSASARACRRPASPGARLASLLNSIFSGKR
PSAQRPACSPDYPEPACSTAPPSSSSSYARRPCHAKTPRTPPTTTTTARARPSRSRTVRF
LDIDGKVAVAAAVAGCRRIPVMEVEADTDDGGEESSDASSDLFELDSLAAIAPAGGRDGS
YGDELPVYGTTGVGIRRDIGRRRPYGHAPCRSWSRAV*
>11669.m00758|LOC_Os03g07920.1|genepair1017-2
MERWAAPKVTAGSARRYVADQPSFSSTLLDAIYKSMDEQPGHGGGATGVEAVAAAAKKQH
EAALHYGNYYKPSLAGSYRARAPGPHATTSSSSECSSYGGFSSSEAESSHHRRLRPIRTT
VPGGAPGPAPEKKAKKPGASIRAKLRDLRKPASPGARLAGFLNSIFAGKRAPATPPSATA
GAESACSTASSYSRSCLSKTPSTRGQAKRTVRFLDSDTESLASSTVVDRRRVPVEAVQQM
LLQRMEMESDEDDDESSDASSDLFELENFAAIAPAGAAYRDELPVYETTRVALNRAIGHG
YGHGRSARVV*
>11676.m02200|LOC_Os10g25830.1|genepair1018-1
MSFGAVDVDDGGAAATAAASGGEEVRAMPAEVSWEMLDKSRFFLLGAALFSGVSAALYPA
VVLKTHLQVSPPPAAAASTTAAAILRRHGPRGFYRGFGASLAGTVPARAVYMAALEATKS
AVGSAAVRLGVAEPAASAAASAAGGVSAAVAAQVVWTPVDVVSQRLMVQTAAAGPPYRGG
ADALRRILRADGVRGLYRGFGVSVLTYAPSSAAWWASYATAQRLIWRALGPAHHDSRASV
VAVQGASAAAAGGAAALVTMPLDTVKTRLQVMDGGGASLASEARALVREGGWGACYRGLG
PRWASMSLSAATMVTAYEFLKRLSTKDTSL*
>11669.m00755|LOC_Os03g07890.1|genepair1018-2
MSFGVVDMDEEGGAAAAADEIRRLPAEVNWEMLDKSRFFVLGAALFSGVSAALYPAVVVK
THLQVAPPPQAATATAAAILRRDGLRGFYRGFGASLAGTVPARALYMAALEATKSSVGSA
AVRLGVSEPAATAAASAAGGVSAAIAAQVVWTPVDVISQRLMVQTSSTCRYRGGVDAFKK
ILLADGVRGLYRGFGLSIVTYAPSNAVWWASYAMAQRFIWRVVGAERSESYPSLMAVQGA
SAALAGGASALVTMPLDTVKTRIQVMETDGAAAARPTLKSTVRGLLKEGGWAACYRGLGP
RWGSMSLSAATMVTTYEFLKRLSAKEGSLD*
>11676.m03532|LOC_Os10g38880.1|genepair1019-1
MKVMTLRRGGAGAGIRIKKKARGFMCGGCGGSKAVSVSDGSDKQSPMATPPPNTSSTTTT
TTTGSAGNKTTAAAGSSSFSPSYDYDYVDTDADTSVGSTPSVAALLRQLGELERSVRSLQ
GAVAEGRGAKNDGRGGGRRHRRTVSDGGGGGSGRVEESVAVVKESADPLFDFRRSMLQMI

VEKEIVGGAELRELLHRFLPLNSPHHHHVILRAFAEIWEEVFAGYERTPDFLVSSRHRRP
TKKKLPASYTAADDDDDDSWNAA*
>11670.m04796|LOC_Os04g48830.1|genepair1019-2
MSPSAAAKMRLGGGGGGGGFMLGCGCRDAKAVAVAVSATSPCSAATETSTATTATWRRAR
THPSASASASTGTLTVPSASSSFLWDDADAEADGEEVDFKRESSATTPSFSGLLRQLNEL
EQSVMTWGWKSPRRGNHFSPPPPPPPPPPLPLRPVVLHRAVDAGGKRSNKEDDAKFSSPP
PSSHCPTTQLHRKVKSVDQRNREDDEAHFAPPAPPPLPLPPQQLRNVKGVDKGGSKEDSK
HCPPPPQAPKHRKTKSCDNNDGFTAGKLDGSLAVVKQSEDPRGDFRRSMLNMIVENRIVT
GDELRELLHRFLELNAPHHHDAILRAFAEIWDEVFAGPDEPRHGPPRPPPRQRTPPRRRH
PLPAWRL*
>11676.m03538|LOC_Os10g38940.1|genepair1020-1
MAVARLVVITPAVLLGRTARVSPSAVPRLRPIVAGRRAVAAPTRAVLGDGAGVGGEEDAV
VAVVEEDAVARRAARKRSERRTYLVAAVMSSLGFTSMAAAAVYYRFAWQMEAGGGDVPAT
EMVGTFALSVGAAVGMEFWARWAHRALWHASLWHMHESHHRPRDGPFELNDVFAIANAAP
AISLLAYGLLNRGLLPGLCFGAGLGITLFGMAYMFVHDGLVHRRFPVGPIENVPYFRRVA
AAHQIHHTDKFEGVPYGLFLGPKELEEVGGTEELDKEIKKRIKRKEAMDAIR*
>11670.m04803|LOC_Os04g48880.1|genepair1020-2
MATGLSGGAMTSFAVKKPLLAAAVRRRSWPPPSGRALPFSPLTRTPRSRGLGTVTCFVPQ
GTESQQAPAPSPPPTVPVPVPSLEEEAAAAAARRIAERKARKLSERRTYLVAAVMSSLGF
TSMAVAAVYYRFHWQLEGGDVPMTEMFGTFALSVGAAVGMEFWAQWAHRSLWHASLWHMH
ESHHRAREGPFELNDVFAITNAVPAISLLAYGFFHRGIVPGLCFGAGLGITLFGMAYMFV
HDGLVHRRFPVGPIANVPYFRRVAAAHKIHHTDKFEGVPYGLFLGPKELEEVGGLEELEK
ELARINRSL*
>11676.m03549|LOC_Os10g39020.1|genepair1021-1
MVGGGGGAGEKPPASNGVHGSGKARFTLLYGLLLYVVMPVLFLYMLVAAATPFYNPRCSP
ESNAAMARFVVAMPNASSVNGSSPSSSSPPPTPVRPMRSADEAPTGLRHIAFGIGASSAL
WKSRKEYIKLWWRPGRMRGFVWMDRPVEEFYSKSSRTGLPPIMVSSDTSKFPYTHGAGSR
SALRISRIVSETFRLGLPGVRWFVMGDDDTVFLPENLVHVLSQYDHRQPYYIGSPSESHI
QNLIFSYGMAFGGGGFAISRALAEELAKMQDGCLHRYPALYGSDDRIHACMSELGVPLTR
HPGFHQCDLWGDVLGLLGAHPVAPLVTLHHLDFLEPVFPTTPSRAGALRKLFDGPVRLDS
AAVAQQSVCYDREHHWTVSVSWGFAVMVVRGVLSPREMETPMRSFLNWYKRADYTAYSFN
TRPVARQPCQKPRVYYMRDSRMDRRRNVTVTEYDRHRGKQPDCRWRIPDPAALVDHIVVL
KKPDPDLWKRSPRRNCCQVVSSPTKAGKNRTMTIEVGVCREGEFAKL*
>11670.m04812|LOC_Os04g48950.1|genepair1021-2
MKGGGGGGKEAVTASILRFLLLLLLPLTALYFFYTLHLLLASAASSSSSSCPPDAASSSS
SVRLSTNGTSAGAAAVTVAAGKKAPAAASTETTLQHVVFGIAASSRFWDKRKEYIKVWWR
PRGAMRGYVWLDREVRESNMSTARTGLPAIRISSDTSGFPYTHRRGHRSAIRISRIVSET
FRLGLPGVRWFVMGDDDTVFFPDNLLTVLNKFDHRQPYYIGSLSESHLQNIYFSYGMAYG
GGGFAISRPLAEALARMQDGCIRRYPALYGSDDRIQACMAELGVPLTKHPGFHQYDVYGD
LLGLLAAHPVAPIVTLHHLDVVQPLFPNAKSRPAAVRRLFDGPIELDPAGIMQQSICYDG
GNRWTVSVAWGFAVLVSRGVMSPREMEMPARTFLNWYRRADYTAYAFNTRPLARSPCQKP
AVYYLSSARRAAALRGGDTTVTRYERWRRANETRPACRWNIADPDAHLDHIVVLKKPDPG
LWDRSPRRNCCRVLSSPKEGKKGGDKTMTIDVGVCRDGEFSQVV*
>11676.m03560|LOC_Os10g39120.1|genepair1022-1
MSGGIARGRLAEERKAWRKNHPHGFVAKPETMADGSANLMIWHCTIPGKQGTDWEGGYYP
LTLHFSEDYPSKPPKCKFPQGFFHPNVYPSGTVCLSILNEDSGWRPAITVKQILVGIQDL
LDQPNPADPAQTDGYHIFIQDKPEYKRRVRVQAKQYPALL*
>11670.m04830|LOC_Os04g49130.1|genepair1022-2
MASGGGIARARLAEERKAWRKNHPHGFVAKPETLPDGSVNLMVWRCIIPGKEGTDWEGGY
FPLTMQFTEDYPTNAPSCKFPSGFFHINVYDSGAVCLSILSTAWKPSITVRQILIGIQEL
FDDPNPNSAAQNISYELYRKDMEEYRKRVRQQAKKYPSAL*
>11676.m03561|LOC_Os10g39130.1|genepair1023-1
MVRGRTELKRIENPTSRQVTFSKRRNGLLKKAFELSVLCDAEVALIVFSPRGRLYEFASA
PSLQKTIDRYKAYTKDHVNNKTIQQDIQQVKDDTLGLAKKLEALDESRRKILGENLEGFS
IEELRGLEMKLEKSLHKIRLKKTELLEQQIAKLKEKERTLLKDNENLRGKHRNLEAAALV
ANHMTTTTAPAAWPRDVPMTSSTAGAADAMDVETDLYIGLPGTERSSNRSETG*
>11670.m04832|LOC_Os04g49150.1|genepair1023-2
MDRSEMGRGRVELKRIENKINRQVTFSKRRNGLLKKAYELSVLCDAEVALIIFSSRGKLY
EFGSAGINKTLEKYNSCCYNAQGSNSALAGGEHQSWYQEMSRLKTKLECLQRSQRHMLGE
DLGPLSIKELQQLEKQLEYSLSQARQRKTQIMMEQVDDLRRKERQLGELNKQLKNKLEAE
ADSSNCRSAIQDSWVHGTVVSGGRVLNAQPPPDIDCEPTLQIGYYQFVRPEAANPRSNGG
GGDQNNNFVMGWPL*
>11676.m03563|LOC_Os10g39140.2|genepair1024-1
MAAEAEQQHQLLSTAVHDTMPGKYVRPESQRPRLDLVVSDARIPVVDLASPDRAAVVSAV
GDACRTHGFFQVVNHGIDAALIASVMEVGREFFRLPAEEKAKLYSDDPAKKIRLSTSFNV
RKETVHNWRDYLRLHCYPLHQFVPDWPSNPPSFKEIIGTYCTEVRELGFRLYEAISESLG
LEGGYMRETLGEQEQHMAVNYYPQCPEPELTYGLPAHTDPNALTILLMDDQVAGLQVLND
GKWIAVNPQPGALVINIGDQLQALSNGKYRSVWHRAVVNSDRERMSVASFLCPCNSVELG
PAKKLITDDSPAVYRNYTYDEYYKKFWSRNLDQEHCLELFRT*
>11670.m04838|LOC_Os04g49210.1|genepair1024-2
MAPAIAKPLLSDLVAQSGQVPSSHIRPVGDRPDLDNVDHESGAGIPVIDLKQLDGPDRRK
VVEAIGSACETDGFFMVKNHGIPEEVVEGMLRVAREFFHMPESERLKCYSDDPKKAIRLS
TSFNVRTEKVSNWRDFLRLHCYPLESFIDQWPSNPPSFRQVVGTYSREARALALRLLEAI
SESLGLERGHMVSAMGRQAQHMAVNYYPPCPQPELTYGLPGHKDPNAITLLLQDGVSGLQ

VQRNGRWVAVNPVPDALVINIGDQIQALSNDRYKSVLHRVIVNSESERISVPTFYCPSPD
AVIAPAGALVDGALHPLAYRPFKYQAYYDEFWNMGLQSASCLDRFRPNDQAV*
>11676.m03568|LOC_Os10g39190.1|genepair1025-1
MEFTPISPPTRVAGGEEDSERGAAAWAVVEKEHMFEKVVTPSDVGKLNRLVIPKQHAERY
FPLDAAAGAGGGGGGGGGGGGGKGLVLSFEDRTGKAWRFRYSYWNSSQSYVMTKGWSRFV
KEKRLGAGDTVSFGRGLGDAARGRLFIDFRRRRQDAGSFMFPPTAAPPSHSHHHHQRHHP
PLPSVPLCPWRDYTTAYGGGYGYGYGGGSTPASSRHVLFLRPQVPAAVVLKSVPVHVAAT
SAVQEAATTTRPKRVRLFGVNLDCPAAMDDDDDIAGAASRTAASSLLQLPSPSSSTSSST
AGKKMCSLDLGL*
>11670.m04840|LOC_Os04g49230.1|genepair1025-2
MEQEQDEEEEEAEASPREIPFMTSAAAAATASSSSPTSVSPSATASAAASTSASGSPFRS
SDGAGASGSGGGGGGEDVEVIEKEHMFDKVVTPSDVGKLNRLVIPKQHAEKYFPLDSAAN
EKGLLLSFEDRTGKLWRFRYSYWNSSQSYVMTKGWSRFVKEKRLDAGDTVSFCRGAAEAT
RDRLFIDWKRRADVRDPHRFQRLPLPMTSPYGPWGGGAGASSCRPRRPPRSTSITAFARA
STSATSTPLCRRGSSSSSAPQGRGFISTRPCHRRRRHLRLLTNSTLRCTTRAP*
>11676.m03573|LOC_Os10g39230.1|genepair1026-1
MRRRGGGDWGREADPAEPSSSHAFDGGRDGDDDDGGGGWHQAAMKAGAGKGSSSSSSSGD
SLWQWRSQGLSEVVLSWSVDQILNKDLLRDKVAKIPETFSSMEQYMTSFFGPLLEEVRGD
MCSSMEDISKAPYASVLSVNAMRKGKGSYEIKLDKWRGVSHGCAIEGYKPKAADLLLISE
TRPANQSDILKQSKSCVIVWVGKVQGNKMTVKASRRMETGVHGDERQQMGMNRYDKLYAE
GFDKSWEMLDQEAVAPESSNSFMHENGRKEHSKVRKCFEKCSDLQEQNEMGTCGNSSKRW
SFCATYLTNMITYDRVWVVLRRGLTMDSKIVLSMFGKKKKEQRILACAPTNMAVLQVASR
LIELIQDFSSSHCYSFGDIVLFGNKDRLHIGKELSKVYLDDRVHKLLRYFKREDGWKARV
DSVMKFLMNCISRYQMSLDIQQASSDGCNLTFKKYFTSKFSTLVKELARCIDTFFDHLPT
DSLGRNLDRMMFAKSLLDKLQQLLCADDVSDELLFTIFKPSDEPIDSFDSHDQTDDATVD
LHDHDISLDDPLEIKSLCIKTLMDLSKMRLPCEDNESSIRDLCLKRAKLVFCTASSSFEL
FRLQNVMPISILVIDEAAQLKECEALVPLLLPGIEHILLIGDENQLSSLVKSKIAKDADF
GRSLYERLCTMGYRKHLLEVQYRMHPGINKFPNANFYDNRISDGPSVQQEDYMKSYLPGP
IYGAYSFIHIENDMEMLDELGQSSKNMVEVAVATNIVERLAKECSEKRQRTSLGVISPYT
AQVIALQERLGKQFKNHEFLSVTVKSIDGFQGGEEDIILISTVRSNKNGKVGFLSDAGRI
NVALTRAKYCLWILGNGATLLASNSIWAELVHDSKRRGCFFNALDDKNLAEIIMHATKEG
EQRNQREQRSAHNANRWSSGSSRHDIIAAGNSRPMRWSHFPGSGNTRRSNGHDSRLNVCH
TKEDMHRTHFQQRKSYSGDYSQVAPPNQYWHNGNRPSRGSYGYLEVFREHPNHHSGQDSR
TRSYHETMCSTPQTGNGRFPYSGSIQREKSQRQTSILGERQPLGGDGNKGFQDGTSGYPC
RRNSSQIRPNTYEAGAPELQSMNKHREFSSYPQQAPYRTFGGRGRGRPTYHGRGRGGWYE
RTNNHWMEEPHQVQNATCNMPVTMQQGMKRNWCEAEASDSPQQVNAKIRSESADRPHLHD
EHGGYGTASHQLPAIKPEDVSEQQCEMKTDSYKAEASESPNDSTRVRPESVEQPYCQAQG
DSSGAASQEPVIPEQRGMSGDLCEAVCHQSNTSGSPNRGSTEVTLEGAEQPHCQAQPDGS
GVASNEAPVPEQRWTEGDLGEAAEPGQGNAENKAESAEADS*
>11670.m04851|LOC_Os04g49340.1|genepair1026-2
MVKKGGRAREGNKKPDDDDLVNTIFSWTLEDVMNQNLFADKVPSISFVFSIRLVNAIPDR
FSGLKSYLDSFRALLLEEIRAEMSSNLETLPNNSSSTKHIQSLVRVPTGLRQCPLYRVTI
SDQRGACAPCIGDIVVLTDTVPRRPSDLASNGRSCCLAHVKDVVNRRTFLIRAAKKIGDA
DSYAFAASLLAFIPYARIWRCLDYDYALKINPPLVMAVAGVALQTTSLAGSSSFHRANGG
TDEITSRLPAFGLNDSQAGAIQSCVSAVQGNGASTTSGRFSLIWGPPGTGKTKTISVLLL
MLMTTATSQSRYRVLTCAPTNTAISQVASRLLALSKQHSAAAAGGLCHGDLLLFGNKDRM
GIDGDLKEVFLDNRVKILQKCFSPESGWRHGLSSLQVFLSFPLALRCQYIQACIALKDGT
ALPESSFVRSRFHDICQKLSRCFQTILSHVPKSVILEKNYNNIILLTTMLENFRKLLSKN
SAAGDEVLVGIFMKEKKPDGSDGGVVHSDLVRNLRQSMTQILGVISTLLRGLQLPATTSP
FKIKKFCLRSASLIFCTVSGSAKLYEQKMDLLLIDEAAQLKECESLIPLQVSGLKHAVLI
GDECQLPATVKSKAADGALLGRSLFERLTLLGHQKHLLNMQYRMHPSISIFPNFSFYDKK
ILDGPNVTHVRHERSFLQGAMFGPYSFINIENGREDPGRNKRNMAEVAAIKKILHNLCKA
CVGTGEGVSVGIICPYAAQVEAIQSGIDANAVRPLDVRVNSVDGFQGSEEDIIILSTVRS
NSTGSIGFLSNRRRANVALTRARHCLWILGDAATLLGSGSVWGELVRDAVDRRCFYDWDD
GGAGLLGVARRGHEDELDDAVEFATAFDTFADEAGCRDDICDALGSLKLA*
>11676.m03591|LOC_Os10g39410.1|genepair1027-1
MSGRGKGGKGLGKGGAKRHRKVLRDNIQGITKPAIRRLARRGGVKRISGLIYEETRGVLK
IFLENVIRDAVTYTEHARRKTVTAMDVVYALKRQGRTLYGFGG*
>11670.m04860|LOC_Os04g49420.1|genepair1027-2
MSGRGKGGKGLGKGGAKRHRKVLRDNIQGITKPAIRRLARRGGVKRISGLIYEETRGVLK
IFLENVIRDAVTYTEHARRKTVTAMDVVYALKRQGRTLYGFGG*
>11676.m03592|LOC_Os10g39420.1|genepair1028-1
MGNSCQNGTYGNNYQNSNRFQNDRFASRYVDGNDTEDCYSGSSRASLAGALRQGLNLKSP
VLGYKTPNVRELYTLGRELGQGQFGKTYLCTEISTGCQYACKTILKSNLRCVSDIEDVRR
EIQIMHHLSGQKNIVTIKDTYEDEQAVHIVMELCAGGELFSKIQKRGHYSERKAAELIKI
IVGIIETCHSHGVMHRDLKPENFLLLDADDEFSVKAIDFGLSVFFRPGQVFREVVGSPYY
IAPEVLEKRYGPEADIWTAGVILYVLLTGVPPFWADTQSGIYEKVLDGRIDFKSNRWPRI
SDSAKDLIKKMLCPYPSERLKAHEVLKHPWICDNGVATNRALDPSVLPRLKQFSAMNRLK
KLSLQIIAERLSEEEIVGLREMFKAMDTKNRSVVTFGELKGLKRYSSVFKDTEINDLMEA
ADDTTSTINWEEFIAAAVSLNKIEREKHLMAAFTYFDKDGSGFITVDKLQKACMERNMED
TFLEEMILEVDQNNDGQIDYAEFVTMMQSNNFGLGWQTVESSLNVALREAPQVY*
>11670.m04870|LOC_Os04g49510.2|genepair1028-2
MGNACGGSLRSKYLSFKQTASQRHDTDDNNNAAAADSPKKPSRPPAAAKTDDHPVSASAP
AAAMRRGQAPADLGSVLGHPTPNLRDLYAMGRKLGQGQFGTTYLCTELSTGVDYACKSIS

KRKLITKEDIEDVRREIQIMHHLSGHKNVVAIKGAYEDQLYVHIVMELCAGGELFDRIIQ
RGHYSERKAAELTRIIVGVVEACHSLGVMHRDLKPENFLLANKDDDLSLKAIDFGLSVFF
KPGQTFTDVVGSPYYVAPEVLLKHYGPEADVWTAGVILYILLSGVPPFWAETQQGIFDAV
LKGFIDFDSDPWPVISESAKDLITKMLNPRPKERLTAHEVLCHPWIRDHGVAPDRPLDPA
VLSRIKQFSAMNKLKKMALRVIAESLSEEEIAGLKEMFQTMDADNSGAITYDELKEGLRK
YGSTLKDTEIRDLMDAADIDNSGTIDYIEFIAATLHLNKLEREEHLVAAFSYFDKDGSGY
ITVDELQQACKEHNMPDAFLDDVINEADQDNDGRIDYGEFVAMMTKGNMGVGRRTMRNSL
NISMRDAPGAL*
>11676.m02965|LOC_Os10g33650.1|genepair1029-1
MEQVVGGKYKLGRKIGSGSFGELYLGVNIHNGEEVGIKLESVRSKHPQLHYESKVYMQMQ
GGNGIPHMKWYGVAGEHNVMVIDLLGPSLEDLFNSCNRKFSLKTVLMLADQIINRVEYMH
SKGFIHRDIKPDNFLIGLGRKANQVYIIDYGLAKKYKDLQTHKHIPYRENKNLTGTARYA
SVNTHLGIEQSRRDDLESVGYLLLYFLRGSLPWQGLKAGTKKQKYDRISEKKMLTPAEVL
CKSYPSEFTSYFHYCRSLRFEDKPDYSYLKKLFRDVFTREGYQLDYIFDWTTSKNPQMGS
TNKLIQVGDI*
>11670.m04206|LOC_Os04g43490.1|genepair1029-2
MERVIGGKFKLGKKIGSGSFGELYLAVNIQNSEEVAVKLESVKSRHPQLHYESKLYMLLQ
GGTGIPHLKWFGVEGEYNVMVIDLLGPSLEDLFNYCNRKLSLKSVLMLADQMIARVEYMH
TRGFLHRDIKPDNFLMGLGRKANQVYVIDYGLAKKYRDLQTHKHIPYRENKNLTGTARYA
SVNTHLGVEQSRRDDLESLGYVLMYFLRGSLPWQGLKAGTKKQKYDRISEKKMLTPVEVL
CKSYPSEFVSYFHYCRSLRFEDKPDYSYLKKLFRDLFIREGYQLDYVFDWTMLKYPQIRD
NKLRPSGKTSGLVGRSAERTERTTGEALARRTGSGSGRNGEPTKHRTLLDSLMSSKATAD
TDKTRPTSLSRNGSTSRRAVVSSSKPNCGDPSDTNRTSRLFSSSSSRPSAAQRALQSAGA
ELRSSSLSKTRKSSRDDPTIRSFEMLSLSADRRK*
>11676.m02978|LOC_Os10g33760.1|genepair1030-1
MAGLREMESTLPPGFRFCPSDEELICFYLRNKVANHRVASGTLVDVDLHAREPWELPEVA
KLTAEEWYFFSFRDRKYATGSRTNRATKTGYWKATGKDRIVHEGTTRAVVGMRKTLVFYL
GRAPNGQKTTWVMHEFRLETPNSQPKEDWVLCRVFDKKKPSTIEAEGGGSSGSDLFIPGA
TDGSTDPSSPTTMAPLLGSSPDPTVVDRFDHRSAAVPPLMVLMQGGGDQMISGSGVHCSN
NDNSGSSSALLNLTMLQYSFLEHRPTGDDMAVGAHFGTCQGGNNDATMALGMGFEEHGMG
EIIEMEPAWRQGGSNCVYRDELYF*
>11670.m04213|LOC_Os04g43560.1|genepair1030-2
MGLREIESTLPPGFRFYPSDEELVCHYLYKKVSNERASQGTLVEVDLHAREPWELPDVAK
LTASEWYFFSFRDRKYATGSRTNRATKTGYWKATGKDREVRSPATRAVVGMRKTLVFYQG
RAPNGVKSGWVMHEFRLDSPHSPPKEDWVLCRVFQKSKGDGEQDNPTSAASPAATFAGSS
QAAVPGQAAYSSDDHTGSSMGFAPRQNEILDSSSHQLLNLAMLQCNSVLDHFPQEVNSSP
MMGLAGSIGIGDEYGFFYDTGFEETASLGGMRFPQGWS*
>11676.m02980|LOC_Os10g33780.1|genepair1031-1
MEFVAHAAAPDSPHSDSGGGGGGMATGATSASAAGASPSRYESQKRRDWNTFGQYLRNHR
PPLSLARCSGAHVLEFLRYLDQFGKTKVHAPACPFFGHPAPPAPCPCPLRQAWGSLDALV
GRLRAAYEENGGRPENNPFGARAVRLYLREVREHQARARGVSYEKKKRKKPPHPSSAAAA
HDDAANGALHHHHHMPPPPPGAAA*
>11670.m04215|LOC_Os04g43580.1|genepair1031-2
MDLSPNPDSPPSGGGNGGGGGSSSSNSSPSMGAGAPQSPSRYEAQKRRDWNTFGQYLRNH
RPPLSLAQCSGAHVLEFLRYLDQFGKTKVHTAACPFFGHPSPPAPCPCPLRQAWGSLDAL
VGRLRAAFEENGGRPESNPFAARAVRLYLREVREHQARARGVSYEKKKRKKPQQQQLQGG
DSSGLHGHQHHPPPPPPAGAAC*
>11676.m02983|LOC_Os10g33810.1|genepair1032-1
MGRAPCCEKEGLRRGAWSPEEDDRLVAYIRRHGHPNWRALPKQAGLLRCGKSCRLRWINY
LRPDIKRGNFTADEEDLIVRLHNSLGNRWSAIAAQMPGRTDNEIKNVWHTHLKKRLDDER
KLAAAGGGGGRRPHTRKQPKAAKSAAAVKREATPSVSVDTSSGVTCSTVTESSPSSADGD
HRRQQQQTQHAAVKEESFSSGGELPAAAAAPTAAADMDESFWSSTEVTGMMAGLGDMDEE
LAIAGTSSAAAARSDDMEFWLKMLLESGDMRDLAVL*
>11670.m04225|LOC_Os04g43680.1|genepair1032-2
MGRAPCCEKMGLKKGPWTPEEDKVLVAHIQRHGHGNWRALPKQAGLLRCGKSCRLRWINY
LRPDIKRGNFSKEEEDTIIHLHELLGNRWSAIAARLPGRTDNEIKNVWHTHLKKRLDAPA
QGGHVAASGGKKHKKPKSAKKPAAAAAAPPASPERSASSSVTESSMASSVAEEHGNAGIS
SASASVCAKEESSFTSASEEFQIDDSFWSETLSMPLDGYDVSMEPGDAFVAPPSADDMDY
WLGVFMESGEAQDLPQI*
>11676.m02992|LOC_Os10g33900.1|genepair1033-1
MRQSRFKRICVFCGSSQGKKRSYHDAAIELGNELVARSIDLVYGGGSIGLMGLVSQAVFD
GGRHVIGVIPKTLMTPEISGETVGEVRPVADMHQRKAEMARQSDAFIALPGGYGTLEELL
EVITWAQLGIHHKPVGLLNVDGYYNSLLTFIDKAVEEGFINTSARRIIVMAPTAEELMDK
LEEYVPYHDRVASKLNWEMGHLGY*
>11670.m04242|LOC_Os04g43840.1|genepair1033-2
MMDTDHTEIIKEGEAVVEAMALLQSRFRRICVFCGSSQGKKKSYQDAAVELGKELVARNI
DLVYGGGSVGLMGLVSQAVYNGGRHVIGVIPKTLMPREITGETVGEVKAVADMHQRKAEM
ARQSDAFIALPGGYGTLEELLEVIAWAQLGIHDKPVGLLNVDGYYNSLLSFIDKAVEEEF
ISPSARHIIVLAPTPKELLEKLEAYSPRHDKVVPKMQWEMEKMSYCKSCEIPGLKEGNKA
TIQAQRGSML*
>11676.m02996|LOC_Os10g33940.1|genepair1034-1
MKEVGEVEEVRCLDPQLWHACAGGMVQMPAPRSRVYYFAQGHAEHADGGGGAAAAAAELG
PRALPPLVLCRVEGVQFLADRDSDEVYAKIRLAPVAPGEAEFREPDELCPLGAAGDAAEP
SPEKPTSFAKTLTQSDANNGGGFSVPRYCAETIFPKLDYRADPPVQTVLAKDVHGVVWKF

RHIYRGTPRRHLLTTGWSTFVNQKKLVAGDSIVFLRTRHGELCVGIRRAKRMACGGMECM
SGWNAPGYGGGGFSAFLKEEESKLMKGHGGGGYMKGKGKVRMADVVEAASLASSGQPFEV
AYYPRASTPDFVVKAASVQAAMRIQWCSGMRFKMAFETEDSSRISWFMGTISSVQVADPN
RWPNSPWRLLQVTWDEPDLLQNVKCVSPWLVELVSSIPPIHLGPFSSPRKKLRVPPHPDF
PFEGHLLNPIFHGNPLGPSNSPLCCYPDTAPAGIQGARHAQFGLPLTDHQLNKLHLGLLH
SGSFNRLDAITPPSRISKGFVVSSAPAHDNISCLLSISTPQVAEKSDDRKTTPHIMLFGK
AIFTEQQITSSGSTETLSPGVTGNSSPNGNAHKTGNASDGSGSSICIGFSSQGHEASDLG
LEAGHCKVFMESEDVGRTIDLSVFGSYEELYGRLADMFGIEKEEIINHLHFRDAAGVVKH
PGEVPFSDFMKAARRLTIIAGDRERIERPLIECLVEQA*
>11670.m04249|LOC_Os04g43910.1|genepair1034-2
MELAGPTEGDGGGSVDSQLWAACAGSMSSVPPVGAAVYYFPQGHAEQASAAVDLSSARVP
PLVPCRVVAVRFMADAESDEVFAKIRLVPLRPGDAVVDVGEAAAAEARREEENSRPRPTS
FAKTLTQSDANNGGGFSVPRFCAETIFPELDYSSEPPVQSVCAKDVHGVEWTFRHIYRGT
PRRHLLTTGWSPFVNKKQLTAGDSIVFMRDEGGNIHVGLRRAKRGFCSIGGDDESLSSIP
GWDQYRGLMRRNATATATGGRTPPKGKVPPENVLTAATRATTGQPFEVLYYPRASTPEFC
VRAAAVRTAMAVQWCPGMRFKMAFETEDSSRISWFMGTVAGVQASDPVRWPQSPWRLLQV
TWDEPELLQNVKRVCPWLVELVSSMPNLHLPSFSPPRKKPRNPPYAELPLEGQIFTGPVF
PPNPMAHDHHHHHGFPFLPFPDSSAQPAGIQGARHAQFASPFPEFHIGNLQPNLMLYAGI
RLPPADRAAPAPRPPRIIISTDLTIGSPGKPDDAACSPSSGGKKIDDTKPRGFLLFGQAI
LTEEQIKNGNSDGRPASPNWDAEKAPNTSEGSDSGVTQGSPTKNTTPSWSLPYFGGNNIS
RASEYELNPGQCKVFVESETVGRSLDLSALSSFEELYACLSDMFSIGSDELRSHLVYRSP
AGEVKHAGDEPFCAFVKSARKLRILTDAGSDNLGD*
>11676.m03003|LOC_Os10g33990.1|genepair1035-1
MAGRSGGGGGMSMVAAHRLFAPPPQQHGAEAVELDEAEVIWGTSSASSSPVEAYLRGGGA
PPTHVAVASSKGGKGKRGGGGGAGAREGGGGGGGGAVAAASLPGERGRSAARWPSDERGG
DEECRGGLGWVPPHELLLCRERAAASFSVREGAGRTLKGRDLRRVRNAIWEKTGFQD*
>11670.m04257|LOC_Os04g43990.1|genepair1035-2
MAGSARSAAAKHAYRMFAPSRGAAARCPGSPGADEFDESDVWGSYGAAGVESSPAELGAR
GRAIPSARAGRKAPLDRAAGSLPVNIPDWQKILGVEYRDHQAAAAEWELQGDGDDDYEYG
KVAGVGGVVIPPHELAWRGRAASLSVHEGIGRTLKGRDLSRVRDAVWKKTGFED*
>11676.m03004|LOC_Os10g34000.1|genepair1036-1
MAISQEEQHAGGGRDYAEPAPQPFLGSSELRRWSLYRAAIAEFVATLLFLYVTVATCTAG
VSGGHVNPAVTLGLLVARKVTLLRAALYVAAQCLGAGLVRALNSAHFARHGGGANVVGDG
YSKGAGLAAEVAGTFVLVYTVFSATDAKRSARDSHIPVLAPLPIGFAVFVVHLATIPITG
TGINPARSFGAAVVYNQPNAWHDQWIFWVGPLVGSAIATLYHEHVLRASTLKALGSFKGA
RQ*
>11670.m04264|LOC_Os04g44060.1|genepair1036-2
MAKDIEAAAAAEGGEYMAKDYSDPPPAPLIDAEELTKWSLYRAVIAEFVATLLFLYITVA
TVIGYKHQSDPGANAADAACSGVGILGIAWAFGGMIFILVYCTAGVSGGHINPAVTFGLF
LARKVSLVRAVLYIVAQSLGAICGVGLVKGFQSAFYVRYGGGANELSDGYSKGTGLAAEI
IGTFVLVYTVFSATDPKRNARDSHVPVLAPLPIGFAVFMVHLATIPITGTGINPARSLGA
AVIYNQHKAWHDHWIFWVGPLIGAAIAAAYHQYVLRASAAKLGSSSSFRG*
>11676.m03028|LOC_Os10g34230.1|genepair1037-1
MEVAMVCTRVNLLILILSLCSPYKFIQSPMDFGPLNLLPTTTTASSDFGRILFHSPSAVL
KPQAPRDISLLLSFLSASPLGKVTVAARGAGHSIHGQAQALDGIVVEMSSLPSEIEFYRR
GEGDVSYADVGGGIMWIELLEQSLKLGLAPRSWTDYLYLTIGGTLSNAGISGQTFKHGPQ
ISNVLQLEVVTGRGEIVTCSPTKDAELFNAVLGGLGQFGIITRARILLQEAPQKVKWVRA
FYDDFATFTKDQELLVSMPVLVDYVEGFIVLNEQSLHSSSIAFPTNVDFNPDFGTKNNPK
IYYCIEFAVHDYQNKNINVEQVVEVISRQMSHIASHLYSVEVSYFDFLNRVRMEEMSLRN
SGLWEVHHPWLNMFVPSAGISDFRDLLMDSISPDNFEGLILIYPLLRHKWDTNTSVVLPD
SGSTDQVMYAVGILRSANPDDGCSHHCLQELLLRHRRLAGAAASGLGAKQYLAHHPTPAG
WRRHFGRRWERFADRKARFDPRCILGPGQGIFPRDSSSSNGAFASYS*
>11670.m04283|LOC_Os04g44230.1|genepair1037-2
MELKAMYLYAAVLAVLLCSSVNFIQSPTDVLGPVALLEPTPSSARDFGAVVSDAPFAVMR
PESPDDIALLLGALSSTAPSPRATVAAVGAGHSLHGQAQARDGIVVETRALPRDVHVVSA
RAHGGDDDATVRAYADVGAGALWVEVLEECLKLGLAPPSWTDYLYLTVGGTLSNGGISGQ
TFKHGPQISNVLQLEVVTGKGEVVTCSPTEIPELFFAVLGGLGQFGIITRARIPLQLAPP
KVRWVRAFYDSFETFTGDQELLVSMPEQVDYVEGFMVLNEQSLHSSSVAFPAQLNFSPDF
GSKGRKKVYYCIEFAVHDFQQDSSRADHVVKLVSAKLSYLRPHVYSVEVSYFDFLNRVRM
EEESLRSRGLWDVPHPWLNVFVPKHGITQFKGLLMDTVSADDFEGPILVYPLLTDKWDGN
TSAVVPAAPDGVMYIFGVLRSTDPARCGRACVDSIMARHRRVADEACRDGGGGGRGIGAK
QYLARQPSPARWRDHFGAGWGRFAARKARFDPLHVLGPGQGIFPRTDSAGSM*
>11676.m03415|LOC_Os10g37770.1|genepair1038-1
MPNAGAARRCGRATHVDLLTVVLAAMLCWASYTLSIWHNSRGAADSSVLGLVVGATVCGD
ADEELDFEARHAADDAGLSVSSGPANSRVRRALSSSGPAPAAAGTTVSRYRAPFPWPASR
GVVWAGNSARGAKAAADAAAAANKWARVDGDMLRFTDAAAVRAYAYVVLRLVAAPVRAAV
DVGAMHGGSWAAELMSRGVVTVSVAAPWGASDGAALVELALERGVPAVLAAAGGAPSRRL
PFPAGAFDMAHCGRCLVPWHLHGKHFPSSRTRRAVLDGDRPRAPARRLLGPLGAPANGTH
ERAAIEAAAASMCWRSVADQNGFTVWQKPVGHVGCDAGENSPRFCAGQNKKFKWDSDVEP
CITPIQEGAAPPREASAAEALRRDSETWTRRVARYKAVATQLGQKGRLRNLLDMNARRGG
FAAALADDPVWVMSVVPATGGGDTDTDTLPAIYDRGLIGAYHDWCEPLPTPALSYDLLHA
DSLFTMYRDRCDMEDILLEMDRILRPGRAVIIRDDIAILARIKNFLTDRMRWDCQIFDGE
DGSDDREKILFAAKTCCNDEDRDQEQ*
>11670.m04727|LOC_Os04g48230.1|genepair1038-2

MAVGATATKLHMPSAGGRRPSLFHLAAVAVLCTVSYLIGIWHHGGFSASPAGGVASSVSI
ATTASVSCVSPTPTLLGGGGGGGDSSSSAPLDFAAHHTAEGMEVASGQVHRTYEACPAKY
SEYTPCEDVERSLRFPRDRLVYRERHCPSEGERLRCLVPAPQGYRNPFPWPTSRDVAWFA
NVPHKELTVEKAVQNWIRVEGEKFRFPGGGTMFPHGAGAYIDDIGKIIPLHDGSIRTALD
TGCGVASWGAYLLSRNILAMSFAPRDSHEAQVQFALERGVPAMIGVLSSNRLTYPARAFD
MAHCSRCLIPWQLYDGLYLAEVDRILRPGGYWILSGPPINWKKHWKGWQRTKEDLNAEQQ
AIEAVAKSLCWKKITLKEVGDIAIWQKPTNHIHCKASRKVVKSPPFCSNKNPDAAWYDKM
EACITPLPEVSDIKEIAGGQLKKWPERLTAVPPRIASGSIEGVTDEMFVEDTKLWQKRVG
HYKSVISQFGQKGRYRNLLDMNARFGGFAAALVDDPVWVMNMVPTVGNSTTLGVIYERGL
IGSYQDWCEGMSTYPRTYDLIHADSVFTLYKDRCQMDNILLEMDRILRPEGTVIIRDDVD
MLVKIKSITDGMRWNSQIVDHEDGPLVREKLLLVVKTYWTLGEEKE*
>11676.m03421|LOC_Os10g37830.1|genepair1039-1
MGAGVSGLFGLGGDEGETSAAVGGAAAAGLGDLPELCAAEVLLRLDAPEICRLARLNHAF
RGAAGADFVWEAKLPENYRYLMSFVEGGGGGDDGRQLRRRRWRPAGKKEIYARLARPVPF
DGGSKEFWLEKSKGRVCMALSSKSLVITGIDDRRYWQHIPTAESRFYSVAYLQQIWWFEV
VGEIDFSFPVGTYSLYFRIHLGKFYKRFGRRVCSTEHVHGWDKKPVRFQLSTSDGQHSLS
QCSLGEPGSWVLYHAGDFVVSKPDQTIKLKFSMAQIDCTHTKGGLCVDSAFIYPKGFQQE
RMVRSQKCRC*
>11670.m04732|LOC_Os04g48270.1|genepair1039-2
MGAGASSMMGPEGYGRGWGQTSLGDMPESCVAAVLLYLDPPEICKVARLNRAFRGAASAD
CVWAAKLPANYRYLAALAAAADDDSGGDGATEGNGSRCSSAAMIKKEIYARLCRPTPFDG
GTKEFWMEKNKGGLCISISSKAMAITGIDDRRYWSHLSTEESRFHHVAYLQQIWWLEVAG
EIDFCFPAGSYSLFFRLQLGRPHKYMGRRVYGYESIHGWNIKPTRFQLSTSDDQQATSQY
YLNEPGNWILYHVGDFVVSSSDQLTNLKFSMMQIDCTHTKGGLCVDSVFIYPKGHRHEDC
TICK*
>11676.m03434|LOC_Os10g37920.1|genepair1040-1
MCEALVDRQLLPPCGCNGGGDVVVVVVPKTSAAPVLEDRPKTSAAAVSKGGEAASILRLS
LPMIMTGLILYIRPMISMLFLGRLGELALAGGSLAIGFANITGYSVLSGLAMGMEPVCGQ
AVGAGNLPLVGATMQRMVLLLLAVSVPVAFLWAWMEPLLLLCGQDAAIAAAAQRYILFCL
PDLLFLSLLHPLRIYLRVQSINLPLTACAALAVAAHLPINHLLVSVLGLGIEGVALASAW
ANLNLVIFLLAFVYVSGVHRDTGGFSLPRKMFKDVDGWVRLVRLAAESCASVCLEWWWYE
IMILLCGLLANPRATVASMGILIQTTSLLYIFPSSLSFGVSTRVSNELGANRPSAARAAA
RAGLALSAVQGLASLAFAVSVRGAWARMFTPDADILALTASVLPILGLCELGNCPQTTGC
GVLRGSARPRDGAHINLGAFYGVGTPVAVGLAFWAGMDFRGLWLGLLAAQAACVAVMLVV
IQRTDWDVQAKLAQVLAGAAASGGDHGVNEAGGNDAVAHVKVAAPHGDEDSSLLITVST*
>11670.m04735|LOC_Os04g48290.1|genepair1040-2
MTTCADDQTGCAFFAPLLSSKGAEVVILVAGDEAEEQQPAPVLTSKPPGRLAKAVNEAWS
VSLGVAFPVTPSMFTCSARGEARSILGLAFPMILTGLLLYLRSMISMLFLGHLGGLALAG
GSLAIGFANITGYSVLSGLAMGMEPICGQAFGAGNYALLGVTMQRTVLLLIAAAIPIGGL
WVQMRPLLLFCGQDAAIAAVAETYIFASLPDLVLQAFLHPVRIYLRTQSINLPLTVCAGL
AIAIHLPINYVLVVVLGLGVKAVALASVLANLNLVLFLLAYIFLKGVHKRTGGFLLSAES
FRGWGELISLALPSCVSVCLEWWWYEIMILLCGLLLNPQATVASMGILIQTTSLIYIFPS
SLSFGVSTRVSNELGAGQPEEASRAATVGLVLGFGFGAFASAFAFLVRNVWASMFTADPA
IVALTASVLPILGLCELGNCPQTTGCGVLRGSARPKDAASINLRSFYLVGTPVALVMAFW
FHLDFRGLWFGLLAAQATCTVRMLLVIGRTDWAAEAKRSKQLTGAGAANMESDDRVAADE
KSRLPVDTDVERSSDHTDRC*
>11676.m03446|LOC_Os10g38040.1|genepair1041-1
MAMAGESRRRRRAAAEAVAWCLALGVVALLLVGSVEKEEEVVVVRGARLAAARPCEEIYV
VEEGETLHSISDRCGDPYILEQNPHVHDPDDVFPGLVIKITPRPGRRN*
>11670.m04744|LOC_Os04g48380.1|genepair1041-2
MASAAATATTRTSLRRAELCAAAASAADAASWCFAVALVALVLLCALRAEAVEVHGPRLG
GAAARPCEEVYVVGEGETLHTISDKCGDPFIVERNPHIHDPDDVFPGLVIALRPTKNSI*
>11676.m03447|LOC_Os10g38050.1|genepair1042-1
MAALGRASSSAPVLAAAAAVLLSLCLAALSEEQEQLENLRFVRHAQDAPLVSSYNYIVIG
GGTAGCPLAATLSEHSRVLLLERGGLPYANMSSEQHFTDALADTSPASPAQRFISEDGVV
NARARVLGGGSCLNAGFYTRASNEYVRASGWDARLVNSSYRWVERSLVFRPDVPPWQAAL
RDALLEVGVTPDNGFTFDHVTGTKIGGTIFDNSGQRHTAADFLRHARPRGLTVLLYATVS
RILFKSQDGVPYPVAYGVVFSDPLGVQHRVYLRDGDKNEVIVSAGTLGSPQLLMLSGVGP
QAHLEAHGIEVIVDQPMVGQGVADNPMNSVFIPSPVPVELSLVQVVGITRSGSFIEGVSG
SEFGMPVSDGALRWARSFGMLSPQTGQLGTLPPKQRTPEALQRAAEAMMRLDRRAFRGGF
ILEKILGPVSSGHVELRTTDPRANPSVTFNYFREAEDLERCVHGIETIERVIQSRAFSNF
TYANASVESIFTDSANFPVNLLPRHVNDSRSPEQYCMDTVMTIWHYHGGCHVGAVVDDDY
RVFGVQGLRVIDSSTFKYSPGTNPQATVMMLGRYMGVKIQSERWKK*
>11670.m04746|LOC_Os04g48400.1|genepair1042-2
MASDMDTTRALAILAATSFVAMLACVQAAGDESYTFMKDAVQSPQVSYYDYIIVGGGTAG
CPLAATLSQRFRVLLLERGGSPYDDERIGNMTRFADTLSDTSPSSPAQRFVSEDGVINSR
PRVLGGGSCINAGFYTRASDEYVRGLGWDLEATTAAYRWVEDVVAFQPELGPWQSALERG
LLEAGIAPQNGFTFDHLGGTKVGGSIFDAEGRRHTAADLLRYARTDGIDVLLRARVAKIL
FNVRAGRRPVAHGVVFHDSEGQMHRAYLSNGRGNEIILSAGAMGSPQLLMLSGVGPADHL
RSFGITLVLNQPAVGQGMSDNPMNAIYVPSPSPVEVSLIQVVGITEVGSYIEGASGANWG
VRRSGSGGDRPHRNFGMFSPQTGQLATVPPKQRTPEAIARAAEAMSQLDDTAFRGGFILE
KILGPLSTGHLELRNRNPDDNPSVTFNYFAHPEDLRRCVAGVSVIERVIRSEAFANFTYP
YFSVETLLNMTAGFPVNLRPRHDNDSTSLEQFCKDTVMTIWHYHGGCQVNRVVDAEYRVI
GVDALRVIDGSTFNASPGTNPQATVMMLGRYMGVKIQNERLGNEGLGRRNL*

>11676.m03452|LOC_Os10g38090.1|genepair1043-1
MATAGALALVAICSYLAVTSNKQKRRRRPPVVGTVFHQLYNVRRIHDYHTALSREHTTFR
MLVPAGGDQIYTCDPAVVEHILKTNFANYGKGPFNHGNAKDLFGDGIFAIDGEKWKQQRK
IASYDFSTRALRDFSCAVFKRNAAKLAGIVSNHAASNQSMDFQGLMLRATMDSIFTIAFG
TDLNTLDGSGEGSRFAAAFDDASEFTMLRYISPLWKLARLLNVGVEAMLKERIKVVDEFV
YRLIRARSDELSNSHDSGSRQDILSRFLQATTSDSVVDYKYLRDIILNIVIAGKDTTAGA
LAWFLYMVCKHPEVQEKICHEAMVATSAGDTASVDEFLQSLTDQALNNMHYLHAALTETL
RLYPSVPMENKQCFSDDVLPNGFNVSKGDIVFFIPYAMGRMESLWGKDAEYFRPERWLDE
NGVFQQESPFKFTAFQAGPRICLGKEFAYRQMKIFAAVLLRFFVLKLRDEKEIVSYRTTL
TLAIDQGLHLTATAR*
>11670.m04753|LOC_Os04g48460.2|genepair1043-2
MESPLSHPAMVALSLLLLVALYLARRAVLGKKRRYPPVAGTMFHQLLNFGRLLEYHTELS
RKYRTFRMLTPTCNYVYTVEPANVEHILKTNFANYGKGPMTHDVLEDLLGDGIFNVDGGM
WRQQRKVASLEFSTRVLRDYSSAVFRDTAAELAGILERGPAAKGRERVDMQDLLMRATLD
SFFRVGFGVNLGVLSGSSKEGLVFARAFDDASEQVLFRFFDLLWKVKRFLNISSEATMKQ
SIRTINDFVYSIIDRKIEQMSREQHEFAKKEDILSRFLLEREKDPGCFDNKYIRDIILNF
VIAGRDTTAGTLSWFLYAVCKNQRVQDKIAREVRDATTGDRDVGVQDFSSFLTEDAINKM
QYLHAALTETLRLYPGVPLDVKYCFSDDTLPDGHAVKKGDMVNYQPYPMGRMKFLWGDNA
EEFKPERWLDDSGMFVAESPFKFTAFQAGPRICLGKEFAYRQMKIVSAVLLYFFRFEMWD
DDATVGYRPMLTLKMDGPFYLRALAR*
>11676.m03215|LOC_Os10g35930.1|genepair1044-1
MTFSGTQDKCNACDKTVHFIDLLTADSIPYHKSCFRCSHCKGTLSMCSYSSMDGVLYCKT
HFEQLFKETGTFKKNFPSGTKANSEQAKIPSKLSSVFCGTQDKCTACKKTVYPLEKMTME
GECYHRTCFKCAHGGCLLTNASYASHNGILYCQNHFWQLFKKSGSYDNLLKPASAAAENT
VESEVAVAEPAKEDPETEEAAKEEEEEASPEQVAEAVVEDQEHS*
>11670.m04367|LOC_Os04g45010.1|genepair1044-2
MSFTGTQDKCTACDKTVHFIDLLTADGVPYHKTCFKCSHCKGILSMCSYSSMDGVLYCKT
HFEQLFKETGSFSKKFAPGCRSTDKELARAPSKICSAFSGTQDKCAACQKTVYPLEKLTL
EGESYHKSCFKCSHGGCILTTSSYAALNGVLYCKIHFGQLFMEKGSYNHMKKKSESQEVL
PEVVPEEQPAPPPPDENREDN*
>11676.m03223|LOC_Os10g35990.1|genepair1045-1
MHHPRARYPPGYTSGGGGGGGGGGGGGRGNGGGGFGGGGGGGGGNHGYYGRGPQPQPQQQ
HYHHQAQQLHQHQQQQHAQRNSSSQQQQWLRRDQATAAAASGEVAARTAAQLEAVDSSS
EDWKAQLNLPAPDTRYRTEDVTATKGNEFEDYFLKRELLMGIYEKGFERPSPIQEESIPI
ALTGSDILARAKNGTGKTAAFCIPALEKIDPEKNAIQVVILVPTRELALQTSQVCKELGK
YLNIQVMVSTGGTSLKDDIMRLYQPVHLLVGTPGRILDLTRKGICVLKDCSMLVMDEADK
LLAPEFQPSIEQLIHFLPANRQLLMFSATFPVTVKDFKEKYLPRPYVINLMDELTLKGIT
QYYAFVEERQKVHCLNTLFLKLQINQSIIFCNSVNRVELLAKKITELGYSCFYIHAKMLQ
DHRNRVFHDFRNGACRNLVCTDLFTRGIGIQAVNVVINFDFPKTSETYLHRVGRSGRFGH
LGLAVNLITYEDRFNLYRIEQELGTEIKTIPPQIDLAVYCQ*
>11670.m04370|LOC_Os04g45040.1|genepair1045-2
MDPRARYPPGIGNGRGGNPNYYGRGPPPSQHQQHQHQHQQPPHPHHHQYVQRQPQPQQTP
HNSQHQQWLRRNQIAAEAAGASEQKAPPVADGIDSSSQDWKAQLKLPPQDTRYRTEDVTA
TKGNEFEDYFLKRELLMGIYEKGFERPSPIQEESIPIALTGSDILARAKNGTGKTAAFCI
PALEKIDQDKNAIQVVILVPTRELALQTSQVCKELGKHLKIQVMVTTGGTSLKDDIVRLY
QPVHLLVGTPGRILDLTKKGVCVLKNCSMLVMDEADKLLSPEFQPSIQELIRYLPSNRQI
LMFSATFPVTVKEFKDKYLPKPYVINLMDELTLKGITQFYAFVEERQKVHCLNTLFSKLQ
INQSIIFCNSVNRVELLAKKITELGYSCFYIHAKMLQDHRNRVFHDFRNGACRNLVCTDL
FTRGIDIQAVNVVINFDFPKSAETYLHRVGRSGRFGHLGLAVNLITYEDRFNLYRIEQEL
GTEIKPIPPQIDRAIYCQ*
>11676.m03225|LOC_Os10g36000.2|genepair1046-1
MDKIHPKYQNCSLFFLPDAEKAAATGGSHERDALLTTVATEKRISLIKAWEENEKAKADN
KAAKKLADIASWENSKVAEIEAEIKKYQEYLERKKAEQVEKLMNGVAKVHRAAEEKRAAT
EARRGEEVVKAEEAAAKYRAKGEPPKKLLFG*
>11670.m04374|LOC_Os04g45070.1|genepair1046-2
MAEEEAKKVEVEVTEAPPAAAAAAETEPAAKDVAEEKAVIPAPAPPAEEEKPPVDDSKAL
AIVEKVADEPPAEKPAQGGSNDRDVALARVETEKRNSLIKAWEENEKTKAENKASKKLSA
ILSWENTKKANIEAQLKKIEEQLEKKKAEYAEKMKNKVAIVHKEAEEKRAMVEAKRGEEV
LKAEEMAAKYRATGHAPKKLIGCFGA*
>11676.m03228|LOC_Os10g36030.1|genepair1047-1
MAVAAPAKAAHALAAAAAGMVLLWCVHFRGGLALSSPTNKGLIFNVHPVLMLIGFIILGS
EAIMGYKIWPWGHDTNKMVHLLLHAIALLLGSVGIYAAFKFHNESGIANLYSLHSWVGLG
TICLYGVQWIFGFVTFFFPGASPSLRRAALPWHVRSGLLVYILALLAAELGFLEKLTFLE
AGGLGRYSSEALLVNFTAVLVILLGSAVVMYVTAPMHNEHSHGYSAVRKP*
>11670.m04377|LOC_Os04g45090.1|genepair1047-2
MAAGLGVKAAPFTYVAHALAVAAAVMVLVWCISFRGGLAFEADNKNLIFNVHPVLMLIGY
IILGSEAIMIYKIFPKLNHDTTKLIHLILHAIAIVLGAVGIYCAFKFHNESGIANLYSLH
SWLGIGTISLYGIQWIFGFVAFFYPGAAPHVRRGALPWHVLFGLFVYVLTLATAELGLLE
KLTFLQSSGLDKYGAEAFLVNFTGLVVALFGAAVVVAAVAPAHVEEPEGYAPIPVN*
>11676.m03245|LOC_Os10g36200.1|genepair1048-1
MAQVSKCNSCLWFNSLIPSAVHAFVSSDDNSQQKVVLRVPTMTDDKIKQKAIEAVADIYG
IDSIAADLKDNKMTIIGEMDTVAIAKKLKKIGKIDIVSVGPAKEEKKEEKKEEKKEEKKE
EKKEEKKEEKKEEKK*
>11670.m04381|LOC_Os04g45130.1|genepair1048-2

MAPQKVILKVSSMSDTKMKQKAMETVADIYGIDSIAADHKDQKMTVIGEVDTVEIAKKLK
KFGKVDIISVGPAKEEKKDDKKGDKK*
>11676.m03261|LOC_Os10g36350.1|genepair1049-1
MAASCDLVDVDPPELQFPFELDKQISCPLRIANKTERTVAFKVKTTSPKKYCVRPNNGVV
RPRSASVVVVTMQAQIVAPPDLQCKDKFLVQSVVVDDGLSAKDITSQMFLRDENNMVEEV
KLKVSYVMPPEPAMEIAEESDIPKRILVPMQRILDNGRSGSELSSGNVSLRSAEMGTELG
SPLGRFVRNEDMLKTASPVVETRVHAGPDEQYLELSALVAKLTEEKKSALEQNRKLREEL
ELARRQASQHQGGFSLAFVLVIGLLSIILGCLVKK*
>11670.m04388|LOC_Os04g45190.2|genepair1049-2
MGSDSKELLGIEPLELRFSFETKKQISCSMQLTNRTDDYIAFKVKTTSPKKYCVRPNSGI
VPPRSTSDVIVTMQAQREAPADMQCKDKFLVQSVVVTEGTTTKDVTGEMFNKGSGNVVDE
RKLKVVYVQPPRPPSPVREGSEEGSSPRASLSEGGNLNYQDTTRESDEPPLLSATRAHKD
AEDVTSDSETSALISRLTEERNSAIQQNNKLLEEMDLVRREISKQNGGFPFVFVVVVALL
GILLGYIMKR*
>11676.m03269|LOC_Os10g36420.1|genepair1050-1
MSSSSSSSASSAAAAAFRPAVVQREQQVVEEKFPAAAAAMREMVLPPVAAAAADSEQEQL
CYVHCHYCDTVLVVSVPSSSLFETVTVRCGHCSSLLTVNMRGLLLPTTAAAAPPPPPPPP
PPPPPPAAHFPHSLNLAPANPPHHHSLLDEISTANSPTQLLLEQHGLGGLMASAASCRNN
NSPAAAAAPPPPTSQGKAAAKEPSPRTNTAVINRPPEKRQRVPSAYNRFIKDEIQRIKAG
NPDISHREAFSAAAKNWAHFPHIHFGLMPDHQGLKKTSLLPQDHQRKDGLLKEGLYAAAA
AAAAAANMGVAPY*
>11670.m04404|LOC_Os04g45330.1|genepair1050-2
MMSSAPETFSLDHLSQHQQQQPPPLAEQEQLCYVHCNFCDTILAVGVPCSSLFKTVTVRC
GHCANLLSVNLRGLLLPAAASTANQLPFGQALLSPTSPHGLLDEVPSFQAPASLMTEQAS
PNVSSITSSNSSCANNAPATSMASAANKATQREPQQPKNAPSANRTSEKRQRVPSAYNRF
IKDEIQRIKASNPDITHREAFSAAAKNWAHFPHIHFGLMPDQGLKKTGIQSQDGAGECML
FKDGLYAAAAAAAAATAASSMGVTPF*
>11676.m03699|LOC_Os10g40390.1|genepair1051-1
MPFGGFWEPRSVLDHRHSPSPSPPTSASTLSSPLADVAALAGANAKNVSVSPPPPGWGTG
GGGGGGEEVVAAKEEWVHQLTPLDMGLGAGEGWDAAGNVLGHGPPVLELDHGGGAARCAR
RVRAVRAAARGKDTKPVVPFAAGHPPPNFLLQHHHHHPQPHAAFFGAHHPSFDAAPPPSK
RHHPMAAAPAPKLPPFPAGGFVPALKPKAEAANDEAAAAVEQLAEAAKLAEAGDAFGARE
ILARLNYRLPAAPTAGTPLLRSAFYFKEALRLTLSPTGDAPAPSASTPYDVVVKLGAYKA
FSEVSPVLQFAHLTCVQAVLDELGGAGCIHVLDFDIGMGEQWASLMQELAQLRPAAALKV
TALVSPASHHPLELQLIHENLSGFAAELGVFFHFTVFNIDTLDPAELLANATAGDAVAVH
LPVGPAHAAATPAVLRLVKRLGAKVVVSVDRGCDRSDLPFAAHLFHSFHSAVYLLESIDA
VGTDPDTASKIERYLIHPAIEQCVVASHRAASAMDKAPPPPWRAAFAAAGFAPVQATTFA
ESQAESLLSKVHVRGFRVEKRAGSLCLYWQRGELVSVSAWRC*
>11670.m04570|LOC_Os04g46860.1|genepair1051-2
MRAAPFSADGNGAAELAGSIAALLWPEDKGGGGGGGGGSLLVEPRSVLDCRGSPSPPNST
STLSSSHGSGAADSISTGVAAVSESSAAAAEATRWAAPGEHGGGGGGELPPIPGALDVGF
VAEESWDAMLGDAAAAAGQEQTFLNWIMAAPGDMEPQAPGLSQQQLLANAAGFGFPLQHH
PGGVSSPAALASDLSSSGGRSLTSSSGSNSKATSAFGLLSPEAALQPPPATTAPFHNGAD
MKPPLLGLPSPTLLLNQHQPTPASTLFMPFPSFSDHQQQPLLQPPPKRHHSVPDNLFLLH
NQPQPPPPAPAQCLPFPTLHSAVPFQLQPSMQHPRNAMKSTAAAAAAQQQHLLDELAAAA
KATEVGNSIGAREILARLNQQLPPIGKPFLRSASYLKDALLLALADGHHAATRLTSPLDV
ALKLTAYKSFSDLSPVLQFANFTVTQALLDEIASTTASCIRVIDFDLGVGGQWASFLQEL
AHRCGSGGVSLPMLKLTAFVSAASHHPLELHLTQDNLSQFAADLGIPFEFNAINLDAFDP
MELIAPTADEVVAVSLPVGCSARTPLPAMLQLVKQLAPKIVVAIDYGSDRSDLPFSQHFL
NCLQSCLCLLESLDAAGTDADAVSKIERFLIQPRVEDAVLGRRRADKAIAWRTVLTSAGF
APQPLSNLAEAQADCLLKRVQVRGFHVEKRGAGLALYWQRGELVSVSAWRC*
>11676.m03711|LOC_Os10g40510.1|genepair1052-1
MAAKAALVLAVSLLVVAVASACTYCPEPPTPKPKPPAPRPPTPGGGAGSCPRDALKLHVC
ANVLGLVKAKIGAVAPYEPCCSLLDGLVDLDAAVCLCTAIKANVLGLNLNIPIDLSLILN
NCGKICPSDYQCA*
>11670.m04565|LOC_Os04g46810.1|genepair1052-2
MAGKASIALFLAVNLVVFSLASACGGRCPTPTPSTPSTPTPTPAAFGKCPRDALKLGVCA
NVLGLIKAKVGVPPAEPCCPLLEGLVDLEAAVCLCTAIRGNILGINLNLPVDLSLILNYC
GKRVPTGFKC*
>11676.m03715|LOC_Os10g40550.1|genepair1053-1
MDLKTSNSPVIADPLPKLALPSAVMTYTTPTSFPSTGLYLNTPKKKPLPGKIEEVRAAGW
LDLMLASSPPRKRQTKDFANDVQADELDLLYRNWVVNHPSALTSFEDIVNLARGKRLALF
LDYDGTLSPIVDNPENAVMSDEMRSAVKHVASLFPTAIISGRSRDKVFDFVKLTELYYAG
SHGMDIMGPVRKSDSSGQHVECIRSTDSEGKEVNLFQPASEFLPMISEVYKKLSESIKDI
DGARMEDNKFCVSVHYRNVAPHDYGEVHQRVTAVLKNYPCLRLTHGRKVLEVRPVIDWNK
GKAVEFLLESLGLCGKEDVLPIYVGDDKTDEDAFKVLKANSIGFGILVSSVPKDTDAFYS
VRDPAEVMEFLKKLASWKEEST*
>11670.m04559|LOC_Os04g46760.1|genepair1053-2
MKISANFLLNNCARTYTKKKTLKKCKRELVEVVDGLVGVMMTSSNREKPDIESGYDGSSD
EDSTENSRAEICPSALCFFDQIVASAQDKKVVLFLDYDGTLSPIVNDPEKAFMSSEMRAT
VKSVAKHFPTAIVSGRSRDKVFDFVKLTEIYYAGSHGMDILASFADSDSTIEKTKETKLF
QPANEFLTMITEVSKSLIEVTKAIKGATVENNKFCVSVHYRNVDKKNWKLVAQVVNNVLK
DFPSLKVSTGRKVLEVRPMINWDKGKAVEFLLRSLELDDSETVLPIYIGDDKTDEDAFKV
LRERKNGCGILVSQVPKKSEAFFMLRGPSEVVILPVMLNFFAALLIMPS*

>11676.m03725|LOC_Os10g40640.1|genepair1054-1
MGWWPPAAWRRWGVVVVVVAALMAVSATAAVEEELGGRESGVVAAAAQGRWPRHRHAYAA
MMYMGTPRDYEFYVAVRVMMRSLARIGADADRVLIASADVPADWVRAMREEDGMRVVLVE
NMKNPYESNLGGINRRFKLTLNKLYAWTLVDYERVVMIDSDNIFLQKTDELFQCGQFCAV
FINPCYFHTGLFVLQPSMDVFKGMLHDLEIGRANSDGADQGFLVGCYPDLLDRPMFHPPE
NGSKLNGTYRLPLGYQMDASYYYLKLHWHVPCGPNSVITFPSAPWFKPWYWWSWPILPLG
LSWHKQRWDDLGYAAEMPVILMEILMYAVIITITRLAKPGMTKLCYNRRPEKQNAMVQGL
IKMSAIVAMLIAYAIPFFIIPRTVHPFMGWSMYLFGALALGVLVSNAFLLPLLAVLTPWL
AIIGMFFVMAFPWYHGGIVRVLAIFGYAFCSAPFLWASLVRVMDSLQTMLEREPFFPRLG
EPAQETEFSKLF*
>11670.m04558|LOC_Os04g46750.3|genepair1054-2
MMYMGTPRDYEFYVATRVMMRSLGRLGSDADRVVIASVDVPPRWVQALKDDGVKVVSVEN
LKNPYEKQGNFNMRFKLTLNKLYAWSLVSYDRVVMLDSDNIFLQNTDELFQCGQFCAVFI
NPCIFHTGLFVLQPSMDVFKNMLHELAVGRDNPDGADQGFLASYFPDLLDRPMFHPPVNG
TKLEGTYRLPLGYQMDASYYYLKLRWSIPCGPNSVITFPSAPWFKPWYWWSWPVLPLGLS
WHEQRRENLGYSSELPVVLIQALFYIGVIAVTRLARPSLSKMCYNRRMEKSTIVLLTTLR
VVAAWSILAAYTIPFFLIPRTVHPLLGWPLYLLGAFSFSSIVINVFLLHPLAVLTTWLGI
IGALFVMAFPWYLNGVVRALAVFAYAFCCAPLIWGSLVKTMSSLQILIERDAFRLGEPNQ
TAEFTKLY*
>11676.m03727|LOC_Os10g40660.1|genepair1055-1
MDKHTCKLCFRRFHNGRALGGHMRSHVMAAAAAAAAYCPSSPAMSLASTSSTEIEMDEKKE
MTKKTEQEKTLTSYVLRENPKRSYKVSAGEFSGGGGGGGGGGGESSVVQDGESDTESSPPR
GGAGSFFAVSRRRSKRARRRRRAPDPEPASSVSDATTEEDVAMSLLMLSRDSWTRSRSEH
ETHHRGASSEAEQNNNNVVNVFDEEDEDARDVAGEDHDEELSYGGGEAAAARHRTSRFQC
GACRKVFRSYQALGGHRASLKRGKGGGCVPPPRPAPASSAAAPAIHECPFCFRVFDSGQA
LGGHKRAHMPSGGARPSPSPSPAKCGESSGSIDLNMPATMEDDFELSAVYDAEFASTRQ*
>11670.m04548|LOC_Os04g46680.1|genepair1055-2
MAKNTCKLCYRRFGNPRALAGHMRSHSVAASRSQISSTSSASTSVAVGDDDGGGDAKRPI
QGYVLREKPKRRVRLAESDFSDRESETEYYSSPPHGKRANTGSGDVEQVSSVSDATSEED
VALSLMMLSRDTWPATPPPPPPYRLRGAGYDDGSDGGDAPPAPAAAAAQKRTRFQCPACK
KVFRSYQALGGHRASHMRGGRGGCCAPPPNPPPSPATPLQPLPECDGGEEEGAKPHPHEC
PYCFRVFASGQALGGHKRSQLCSAAAAAASGDDIPAMIKSNGFIDLNLPAPFDDVELSAV
SDPFLSSKPGS*
>11676.m03734|LOC_Os10g40720.2|genepair1056-1
MAFSISKKAAVAALFSFLVVTCVAGARPGNFSASDFTADPNWEVARATWYGAPTGAGPDD
DGGACGFKNTNQYPFSSMTSCGNEPIFKDGKGCGSCYQIRCVNHPACSGNPETVIITDMN
YYPVSKYHFDLSGTAFGAMAKPGQNDQLRHAGIIDIQFKRVPCNFPGLKVTFHVEEGSNP
VYFAVLVEYEDGDGDVVQVDLMEANSQSWTPMRESWGSIWRLDSNHRLTAPFSLRITNES
GKQLVASQVIPANWAPMAVYRSFVQYSS*
>11670.m04543|LOC_Os04g46630.1|genepair1056-2
MASRFQLILSTFVVIAAVTMLPRPCASIEFHRKLSSWSNGGATWYGAANGAGSDGGACGY
QGAVFQAPFSSMIAAGSPSIYKSGLGCGSCYQVKCTGNSACSGNPVTVVLTDECPGGPCL
SEPVHFDLSGTAFGAMANPGQADQLRAAGVLQIQYNRVPCNWGGVKLTFVVDVGSNPNYF
AVLVKYENGDGDLSGVELMQTGAGAAWTQMQQSWGAVWKLNAGSALQAPFSIRLTSSSGK
TLVASNVIPSGWKPGMSYISTVNF*
>11676.m01769|LOC_Os10g21150.1|genepair1057-1
MVRQVGDLLVLQGFDEMGLRDGFSDNILLDNFGYGGSNLTRVCAPTRSEPSLSTTWSIEI
IQASSSVAYQKMSDHMISNEYAARYIRHISTQEQLSTRVGCGRVHPIIENDLDKCRVVAP
NLPTKTDDLSHSTGHGWTTREKVVNG*
>11674.m01502|LOC_Os08g15390.1|genepair1057-2
MATSNVDSGVPGRGILVLQDFDEMGLRDGLSDNKLLDDFGCGEIQPVQVHLPIRSEPSSS
STTWSIEINQASSFAAYQKMSDRMMSNEYAIRVVAPNLPKKTDDLSHSAGHRWTTREKVV
NG*
>11676.m01783|LOC_Os10g21290.1|genepair1058-1
MENRNTFSWVKEQMTRSISVSIMIYVITRTSISNAYPIFAQQGYENPREATGRIVCANCH
LANKPVDIEVPQAVLPDTVFEAVLRIPYDMQLKQVLANGKKGGLNVGAVLILPEGFELAP
PDRISPELKEKIGNLSFQSYRPNKKNILVIGPVPGKKYSEIVFPILSPDPAMKKDVHFLK
YPIYVGGNRGRGQIYPDGSKSNNTVYNATSTGVVRKILRKEKGGYEISIVDASDGRQVID
LIPPGPELLVSEGESIKLDQPLTSNPNVGGFGQGDAEIVLQDPLRVQGLLFFFASVILAQ
VFLVLKKKQFEKVQLYEMNF*
>11674.m01491|LOC_Os08g15280.1|genepair1058-2
MTRSISVSIMIYVITRTSISNAYPIFAQQGYENPREATGRIVCANCHLANKPVDIEVPQA
VLPDTVFEAVLRIPYDMQLKQVLANGKKGGLNVGVVLILPEGFELAPPDRISPELKEKIG
NLSFQSYRPNKKNILVIGPVPGVVRKILRKEKGGYEISIVDASDGRQVIDLIPPGPELLV
SEGESIKLDQPLTSNPNVGGFGQGDAEIVLQDPLRVQGLLFFFASVILAQVFLVLKKKQF
EKVQLYEMNF*
>11676.m01784|LOC_Os10g21300.1|genepair1059-1
MASFILLGGEPTKRIAFPHARIMLHQPASAYYRARTPEFLLEVEELHKVREMITRVYALR
TGKPFWVVSEDMERDVFMSADEAKAYGLVDIVGDEMLDEHCDTDPVWFPEMFKDW*
>11674.m01490|LOC_Os08g15270.1|genepair1059-2
MASFILLGGEPTKRIAFPHARIMLHQPASAYYRARTPEFLLEVEELHKVREMITRVYALR
TGKPFWVVSEDMERDVFMSADEAKAYGLVDIVGDEMLDEHCDTDPVWFPEMFKDW*
>11676.m01785|LOC_Os10g21310.1|genepair1060-1
MALYELAVFDPSDPVLDPMWRQGVACFGFGAFHVTGLYGPGIWVSDPYGLTGKVQAVNPA

WGAEGFDPFVPGGIASHHIAAAFVVAGTMWYGSATTPIELFGPTRYQWDQGYFQQEIYRR
VSDGLAENLSLSEAWSKIPEKLAFYDYIGNNPAKGGLFRAGSMDNGDGIAVGWLGHPIFR
DKEGRELFVRRMPTFFETFPVVLVDEEGIVRADVPFRRAESKYSVEQVGVTVEFYGGELN
GVSYSDPATVKKYARHSQLGEIFELDRATLKSDGVFRSSPRGWFTFGHATFALLFFFGHI
WHGARTLFRDVFAGIDPDLDAQVEFGTFQKVGDPTTRRQPV*
>11674.m01489|LOC_Os08g15260.1|genepair1060-2
MALYELAVFDPSDPVLDPMWRQGVACFGFGAFHVTGLYGPGIWVSDPYGLTGKVQAVNPA
WGAEGFDPFVPGGIASHHIAAGTLGILAGLFHLSVRPPQRLYKGLRMGNIETVLSSSIAA
VFFAAFVVAGTMWYGSATTPIELFGPTRYQWDQGYFQQEIYRRVSDGLAENLSLSEAWSK
IPEKLAFYDYIGNNPAKGGLFRVGSMDNGDGIAVGWLGHPIFRDKEGRELFVRRMPTFFE
TFPVVLVDEEGIVRVDVPFRRAESKYSVEQVGVTVEFYGGELNGVSYSDPATVKKYARRS
QLGEIFELDRATLKSDGVFRSSPRGWFTFGHATFALLFFFGHIWHGARTLFRDVFAGIDP
DLDAQVEFGTFQKVGDPTTRRQPV*
>11676.m01787|LOC_Os10g21330.1|genepair1061-1
MGIAILSTSRGKIRSSSIRILMGDRVKIEVSRYDSSKGRIIYRLPHKDSKRTEDSKDTED
LKDTKDSKDPQGRVVFWSSAGTCGFKSSRKASPYAGQRTAVDAIRTVGLQRAEVMVKGAG
SGRDAALRAIAKSGEVAGSTQTLQWKCVESRVDSKRLYYGRFILSPLRKGQADTVGIALR
RALLGETEGTCITHAKFGSVPHEYSTIAGIEESVQEILLNLKEIVLRSNLYGVRTASICV
KGPRYITAQDIILPPSVEIVDTAQPIANLTEPTDFRIELRIKRDRGYHTEVRKNTQDGSY
PIDAVSMPVRNVNYSIFACGNGNARYEILFLEIWTNGSLTPKEALYEASRNLIDLFLPFL
HTEEEGTRFQENKNRFTSPLLSFQKRLTNLKKNKKRIPLNCIFIDQLELPSRTYNCLKRA
NIHTLLDLLSKTEEDLMRIDSFRMQDGKQIWDTLEKHLPMDLPKNKF*
>11674.m01488|LOC_Os08g15250.1|genepair1061-2
MGDRVKIEVSRYDSSKGRIIYRLPHKDSKRTEDSKDTEDLKDTKDSKDPQGRVVFWSSAG
TCGFKSSRKASPYAGQRTAVDAIRTVGLQRAEVMVKGAGSGRDAALRAIAKSGEVAGSTQ
TLQWKCVESRVDSKRLYYGRFILSPLRKGQADTVGIALRRALLGETEGTCITHAKFGSVP
HEYSTIAGIEESVQEILLNLKEIVLRSNLYGVRTASICVKGPRYITAQDIILPPSVEIVD
TAQPIANLTEPTDFRIELRIKRDRGYHTEVRKNTQDGSYPIDAVSMPVRNVNYSIFACGN
GNAKYEILFLEIWTNGSLTPKEALYEASRNLIDLFLPFLHTEEEGTRFQENKNRFTSPLL
SFQKRLTNLKKNKKRIPLNCIFIDQLELPSRTYNCLKRANIHTLLDLLSKTEEDLMRIDS
FRMQDGKQIWDTLEKHLPMDLPKNKF*
>11676.m01788|LOC_Os10g21340.1|genepair1062-1
MAEEREVPFGGYPREEIQWRRGGPVAQRIRARGYEPRCRGFESLLAHSLPKGKGLYFPPE
GRKTMIGIADVKLLNLEKYMKKKGGRKIFGFMVKEEKEENWGSVEFQVFSFTNKIRRLAS
HLELHKKDFSSERGLRRLLGKRQRLLAYLAKKNRNPIFLERVEGVGFISGEEAVNWGLSG
PMLRASGIQWDLRKVDLYESYNQFDWKVQWQKEGDSLARYLVRIGEMRESIKIIQQAVEK
IPGGPYENLEVRCFKKAKNSEWNDFEYRFLGKKPSPNFELSKQELYARVEAPKGELGIYL
VGDDSLFPWRWKIRPPGFINLQILPQLVKKMKLADIMTILGSIDIIMGEVDH*
>11674.m01487|LOC_Os08g15240.1|genepair1062-2
MAEEREVPFGGYPREEIQWRRGGPVAQRIRARGYEPRCRGFESLLAHSLPKGKGLYFPPE
GRKTMIGIADVKLLNLEKYMKKKGGRKIFGFMVKEEKEENWGSVEFQVFSFTNKIRRLAS
HLELHKKDFSSERGLRRLLGKRQRLLA*
>11687.m00002|LOC_Os11g01020.1|genepair1063-1
MAADSLDMSLDDLITNKYKRRSRPDPAPSARRFHSRAATRAAAAPYHAITFQAPPTAYTH
PTPVETGTKLHISNLDYAVSNEDIKELFSEVGDVKRYSINYDKSGRSKGTAEVVFSRKSD
ALAAVKRYNNVQLDGKPMKLELIGINIEPPPPAMFGFAAPAGYFDFPPKRGGRGWPRGRG
GFGGRGRGHVGRGRGRGDRGSRKISAEDLDADLDKYHAEGMQMS*
>11686.m00001|LOC_Os12g01010.1|genepair1063-2
MAADSLLDMSLDDLITNKYKRRSRPGPAPSARRSHSRAATRSAAAPYHAITFQAPPTAYV
HPTPAANVETGTKLYISNLDYAVSNEDIKELFSEVGDVKRYSINYDRSGRSKGTAEVVFS
RKSDALAAVKRYNNVQLDGKPMKLELIGINIEPPPPAIFGFAAPAGYFDFPPKRGGRGWP
RGRGGFGGRGRGHVGRGRGRGDRGSRKISAEDLDADLDKYHAEGMQMS*
>11687.m00003|LOC_Os11g01030.1|genepair1064-1
MPPSSAATTGGAVHKPIIMPPESERQINNLPDVLQPRRRWRSSLATGFRSALACTIVGVA
SIYAPLVIRRHLTFPAFSYVVTVIVVTDATLGSSLRGALSAVHATAMGAVPSVLPLWLAH
RTGAGESVLATTAVVALSTFAVAVAGSAGTVAKRIALGQIIIIYVARFREERMRSEAVLL
HPANVVACTALGVVAALLGVLLPCPRLATRDATDKRLAYLEVAAERVRLLADAFQLHFSS
DEAAGDDEERASSCRCRRRRRQCVAACIMSQADRAASAGALLLRRISSAQGDLQWERMPA
LLKRWCSSRWDDDDEQACARLHELIEMPLRGMEMACTHMLQQPCWPNTNTISSICTTPTW
LQHATDHVRLALLTKRIPSCSNTGTGSMEMAKLAPVSVGALEQQQQLAPFLFFLCLDLLL
QGSHPAPQRPPKLLLSVSAHSDAAASQVKVIPAATTKDDDEEQPEQTRKKKHQCPRQTTR
STMRRRLVAAAKCSFSLGLAVLLGLLFSSDHGFWSGLVVATTMATGREWTWALAIARAHG
TALGSVYGALACLVIDRMELRFLALLPWLILTAGFLKRSRAYGPAGAGGVAAAVSGIIIV
GRRYDEPPMAFTVARLVETFIGLACIIVADLVFQPAARPSTKATAQLDRCLAALKGCFSR
GRQTTTKVKVKAVQEQVALLERCVAEAAGEPHFPWSPPFPASCYHKVAGSLGRMAQLLYL
YTQAHPTPIPAADEDATQRFHCLVSASLERSADLLLRLSRISSSSSRDEEDLEAGIRVSS
GSDTCCCDDEDAPEMLVRSFLSQQQQQQDQGVALALASIGFCMGEMAKEALQLEAYMLDL
ILLAH*
>11686.m00002|LOC_Os12g01020.1|genepair1064-2
MPPSSAATTGGAVHKPIIMPPESERQINNLPDVLQPRRRWRSSLATGFRSALACTIVGVA
SIYAPLVIRRHLTFPAFSYVVTVIVVTDATLGSSLRGALSAVHATAMGAVPSVLPLWLAH
RTGAGESVLATTAVVALSTFAVAVAGSAGTVAKRIALGQIIIIYVARFREERMRSEAVLL
HPANVVACTALGVVAALLGVLLPCPRLATRDATDKRLAYLEVAAERVRLLADAFQLHFSS
DEAAGDDEERASSCRCRRRRRQCVAACIMSQADRAASAGALLLRRISSAQGDLQWERMPA

LLKRWCSSRWDDDDEQACARLHELIEMPLRGMEMACTHMLQQPCWPNTNTISSICTTPTW
LQHATDHVRLALLTKRIPSCSNTGTGSMEMAKLAPVSVGALEQQQQLAPFLFFLCLDLLL
QGSHPAPQRPPKLLLSVSAHSDAAASQVKVIPAATTKDDDEEQPEQTRKKKHQCPRQTTR
STMRRRLVAAAKCSFSLGLAVLLGLLFSSDHGFWSGLVVATTMATGREWTWALAIARAHG
TALGSVYGALACLVIDRMELRFLALLPWLILTAGFLKRSRAYGPAGAGGVAAAVSGIIIV
GRRYDEPPMAFTVARLVETFIGLACIIVADLVFQPAARPSTKATAQLDRCLAALKGCFSR
GRQTTTKVKVKAVQEQVALLERCVAEAAGEPHFPWSPPFPASCYHKVAGSLGRMAQLLYL
YTQAHPTPIPAADEDATQRFHCLVSASLERSADLLLRLSRISSSSSRDEEDLEAGIRVSS
GSDTCCCDDEDAPEMLVRSFLSQQQQQQDQGVALALASIGFCMGEMAKEALQLEAYMLDL
ILLAH*
>11687.m00004|LOC_Os11g01040.1|genepair1065-1
MSSSSGGDGGGYNYSEDWVVNSRGMRLFTCAWIPKESSRGVVCLCHGYAVECSVTMRGTA
ERLARAGYAVHGIDYEGHGHSDGLQGYVPDLDALVRDCDSFFSTATASFPRRRFLLGESM
GGAVALLLHRLRPDFWTGAILVAPMCKIAEEMRPHPMVVSVLKVMTSIIPTWRVVPTNDV
IDLAYRMQGKRDEIRGNPLCYKGRPRLKTAYELLRVSILIESTILPHVSLPFLILHGAAD
RVTDPSVSDLLYRSASTTDKTFHLYTGMWHALTSGELPHNIDAVFRDIIDWLHHRTSPTS
ASHVQDHDLSTSFEAERKAKHDDTIHCGKQTS*
>11686.m00003|LOC_Os12g01030.1|genepair1065-2
MSSSSGGDGGGYNYSEDWVVNSRGMRLFTCAWIPKESSRGVVCLCHGYAVECSVTMRGTA
ERLARAGYAVHGIDYEGHGHSDGLQGYVPDLDALVRDCDSFFSTATASFPRRRFLLGESM
GGAVALLLHRLRPDFWTGAILVAPMCKIAEEMRPHPMVVSVLKVMTSIIPTWRVVPTNDV
IDLAYRMQGKRDEIRGNPLCYKGRPRLKTAYELLRVSILIESTILPHVSLPFLILHGAAD
RVTDPSVSDLLYRSASTTDKTFHLYTGMWHALTSGELPHNIDAVFRDIIDWLHHRTSPTS
ASHVQDHDLSTSFEAERKAKHDDTIHCGKQTS*
>11687.m00005|LOC_Os11g01050.1|genepair1066-1
MQRHRRTLSTTVVEETIAAAATLVSKWHPDDHHSSLFLHASSPEADHFLRAAADLHRAML
FFASDPTNAHNGHGLVQAHHLLDTAMRRLQLELPRLLAPPPAGSRDRLRALADTMMSAGY
GKECISTFKEHRRAALAATLRRQHTTVQVQLSKLTWEQVDDNIQSWLAAARIAFSSVFPA
EKELCDTVFAGDASVGDAVFEDVANNQAANLLDVAEAAVARARRAPERLFRVLDVHDALT
EILPEIMSVFGDRSEVAKRGCSALFKAGEAARGALANLEVAIEKEPSKATVAGGGVHPLT
RYVMNYLVFLADYEGALDRINQQQGSPERSWSIGWLVQVLMRKIEAKAGSYREAALRHLF
MANNTHYVARKVAKIPSLGDDDGEAQDAARRHVEAYVRAAWGKVLKAIAAADGVEVEEAV
MQAVAKQEKWVAADEEMGQVLRAAATAAVVPKYRMLYRRHGATLRLTPGDVNAIIAALFG
GIIATPSSC*
>11686.m00004|LOC_Os12g01040.1|genepair1066-2
MMSRLIGDMQRHRRTLSTTVVEETVAAAATLVSKWHPDDHHSSLFLHASSPEADHFLRAA
ADLHRAMLFFASDPTNAHNGHGLVQAHHLLDTAMRRLQLELPRLLAPPPAGSRDRLRALA
DTMMSAGYGKECISTFKEHRRAALAATLRRQHTTVQVQLSKLTWEQVDDNIQSWLAAARI
AFSSVFPAEKELCDTVFAGDASVGDAVFEDVANNQAANLLDVAEAAVARARRAPERLFRV
LDVHDALTEILPEIMSVFGDRSEVAKRGCSALFKAGEAARGALANLEVAIEKEPSKATVA
GGGVHPLTRYVMNYLVFLADYEGALDRINQQQGSPERSWSIGWLVQVLMRKIEAKAGSYR
EAALRHLFMANNTHYVARKVAKIPSLGDDDGEAQDAARRHVEAYVRAAWGKVLKAIAAAD
GVEVEEAVMQAVAKQEKWVAADEEMGQVLRAAATAAVVPKYRMLYRRHGATLRLTPGDVN
AIIAALFGGIIATPSSC*
>11687.m00006|LOC_Os11g01060.1|genepair1067-1
MPREWPKHSPAVGPGRHEHDPRGEMQPNRGGRRWPDLPDVAPHHCRLQFPPPHAPPPAAS
PPAPWLRWSMVRRVPHGSTKKRWYLSSVKGPPCISLPRNLGATNQDKCDFCHRGTDQFSS
SVDAKEAGGGGGEGKGGVKGEGDACVVSARVEGGGGNGYMRIINKEATLRVITSMMPE*
>11686.m00005|LOC_Os12g01050.1|genepair1067-2
MPREWPKHSPAVGPGRHEHDPRGEMQPNRGGRRWPDLPNVAPHHCRLQFPPPHAPPPAAS
PPAPWLRWSMVRRVPHGSTKKRWYLSSVKGPPCISLPRNLGATNQDKCDFCHRGTDQFSS
SVDAKEAGGGGGEGKGGVKGEGDACVVSARVEGGGGNGYMRIINKEATLRVITSMMPE*
>11687.m00007|LOC_Os11g01070.1|genepair1068-1
MPHEVDLLSLDVNPEPSSDKYELLSYKGEPQSLDINPEPSANKDELLYLPINALTEASSH
VPYDGDESLFVEQQEQLSHGNEIIFPVETQEGPVQVSRGESLMTCVLSTLPASCEQHSVA
TNLDNPGNNETVMAHDVQTAAVSSFDLKGIEKHVESLHDAMELDGTESSKMNLIADFTAP
LSSDDSLVCYAIPCSMELSDTSVVNKCPHQSTGFPDYAKACTECDSGLTSCERVDRISQD
HDILSGSSIYSKNKDLYSYSDISVFSETHKDFAEPLELPPCGKDDEAPPDLAAQLHCNSC
KDVMMPPQVISDEVEPVDRGATILVEHTPCGPETALTAFLYDKGSIDTTSKTDELAKQNS
NSLEGDVAKIHEQLLNYSYASGEVEISLTRSEKRTKKLRSQHPIYVPFLGFLKSVSFKKK
ASKVWFFEDTAKHEFLVCYQCDMNNEKQELVLDFFLYCIDRDIVA*
>11686.m00006|LOC_Os12g01060.1|genepair1068-2
MALAGFFDLSILPDDSKSTTTNTSIIARALDLGYSAVALDHPHRGLLADSHAPIASSLLL
PPSASLHHRRHPFLQYTRITLSLDSAAACASALAPSAARLLRTYDIVAARPLTQAAFDHL
CHATFDHLDIVSIDFSHKLPFRLKLPMLKLALQRGLHLEIAYSPLIADAASRRQAVAEAK
LLVEWTKGKNLIISSAAHTASEIRGPYDAINLSSYLLGLSTQRAKAALSVNCRSLISKAL
RKKHFYKKTIRIDRLLPNKQLNSANFKLADWIGWDPMPHEVDLLSLDVNPEPSSDKYELL
SYKGEPQSLDINPEPSANKDELLYLPINALTEASSHVPYDGDESLFVEQQEQLSHGNEII
FPVETQEGPVQVSRGESLMTCVLSTLPASCEQHSVATNLDNPGNNETVMAHDVQTAAVSS
FDLKGIEKHVESLHDAMELDGTESSKMNLIADFTAPLSSDDSLVCYAIPCSMELSDTSVV
NKCPHQSTGFPDYAKACTECDSGLTSCERVDRISQDHDILSGSSIYSKNKDLYSYSDISV
FSETHKDFAEPLELPPCGKDDEAPPDLAAQLHCNSCKDVMMPPQVISDEVEPVDRGATIL
VEHTPCGPETALTAFLYDKGSIDTTSKTDELAKQNSNSLEGDVAKIHEQLLNYSYASGEV
EISLTRSEKRTKKLRSQHPIYVPFLGFLKSVSFKKKASKGLTNHILSRIDYGRGTALLLP

TKLDSIFKTV*
>11687.m00008|LOC_Os11g01080.1|genepair1069-1
MYHHQGWSSSSSSSSSLRRVLSTGGGLVEEERRERIDKYRSKRNQRNFDKKITYACRKTL
ADSRPRVKGRFARNSSDDAAAAAAAQVEVSPATNNNVPEWWPAVQEALARQEQEAAGLHL
CDTADDDLLAAYLGVSSIDLYSPRGH*
>11686.m00008|LOC_Os12g01080.1|genepair1069-2
MYHHQGWSSSSSSSSSLRRVLSTGGGLVEEERRERIDKYRSKRNQRNFDKKITYACRKTL
ADSRPRVKGRFARNSSDDAAAAAAAQVEVSPATNNNVPEWWPAVQEALARQEQEAAGLHL
CDTADDDLLAAYLGVSSIDLYSPRGH*
>11687.m00010|LOC_Os11g01100.1|genepair1070-1
MAPVEPPLLPASSFSQAAGGKAPPMPKRRRPGTKCEAYQKYACRKRLADERARVKGRFVS
SSGGNDNNAPAHELPPSLVNLSDGGAAAAIIPTRSVPEWWPEMQASLARDEMCGGAGMNL
HLCDANEMEQVAAYVGVSSMDLCAYLHCSWPPV*
>11686.m00010|LOC_Os12g01100.1|genepair1070-2
MAPVEPPLLPASSFSQAAGGKAPPMPKRRRPGTKCEAYQKYACRKRLADERARVKGRFVS
SSGGNDNNAPAHELPPSLVNLSDGAAAAAIIPTRSVPEWWPEMQASLARDEMCGGAGMNL
HLCDANEMEQVAAYVGVSSMDLCAYLHCSWPPV*
>11687.m00011|LOC_Os11g01110.1|genepair1071-1
MADRWAEVAPTWRHVGADTGWHRGATWMAQIAPKGDQAATASGRRQMRRRPRVRSGGGDS
VADWGNGAKAGMRRGAAELVVAAARAAVVCGRSSAEGGGDGGVDQWGLEGQPEEQGRGEA
VFREMVVKVSYWELSDGEMAVALLAVVRKRQWAGSTQRETGVNQQWRMQWGTWARCTDTT
SSMPLYALRPSLRLSLLVPFDTSAILKLVDNLARTVLAAPLCASSRVVPRLGKPGVIPCS
PVSPSLSSAHHFTPWSLEPSCARTTTLRCPLCVLWLGYLVISTTDFSYISTAATLCMASS
TMATFYPSL*
>11686.m00011|LOC_Os12g01110.1|genepair1071-2
MASGRRRMRRRPRVRSGGGDSVADWGNGAKAGMRRGAAELVVAAARAAVACGRSSAEGGR
DGGVDQWGLEGQPEEQGRGEAVFREMVVKVSYWELSDGEMAVALLAVVGKRRWAGSTQRE
TGVNQQWRMQWGTWGTSRVEDRRRVLLWKRIRWSVHRCLFFKHVRSTLTTARCTDTTSSM
PLYALRPSLRLSLLVPFDTSAILKLVDNLARTVLAAPLCASSRVVPRLGKPGVIPCSPVS
PSLSSAHHFTPWSLEPSCARTTTLRCPLCVLWLGYLVISTTDFSYIDRSYSMHGFIDHGY
VLPFTLATSITTQKAIICVEHSPRIFL*
>11687.m00013|LOC_Os11g01130.1|genepair1072-1
MPQTPSTRWCPTPEQLMILEEMYRSGVRTPNAAEIQQITAHLAYYGRIEGKNVFYWFQNH
KARERQRLRRRLCARHQQPSPPSSTVPPAPTAAAAGAVVQVHPAVMQLHHHHHHHHPYA
AAAAAQSHHLQQQQQQQAEWPAAVDYCSTASASASATAADMAIPPCCRPLKTLELFPTKS
TSGGLKEDCCSSSKSSSCSTSTN*
>11686.m00012|LOC_Os12g01120.1|genepair1072-2
MPQTPSTRWCPTPEQLMILEEMYRSGVRTPNAAEIQQITAHLAYYGRIEGKNVFYWFQNH
KARERQRLRRRLCARHQQPSPPSSTVPPAPTAAAAGAVVQVHPAVMQLHHHHHHHHPYA
AAAAAQSHHLQQQQQQQAEWPAAVDYCSTASASASATAADMAIPPCCRPLKTLELFPTKS
TSGGLKEDCCSSSKSSSCSTSTN*
>11687.m00014|LOC_Os11g01140.1|genepair1073-1
MERGAPAAAGGRGGMGSTSVSTRGTEGGHGGVERKEQQQQRGYQLPRDSRGSLEVFNPSS
ASSFRTAAAAPKSASPFLAIPDREEDNVVAQQRAAEWGLVLQTDHHTGLPQGVSARPSSG
SARTSSEDNPQQQQSAAAIPRVSEELRAALSVFQQTFVVSDATHPNHPIMYASAGFFNMT
GYTSKEVVGRNCRFLQGSGTDPHEIDKIRQSLANGSNYCGRILNYKKDGTPFWNLLTIAP
IKDEDGRLLKFIGMQVEVSKYTEGKKDTVVRPNGLSESLIKYDARQKDHARSSVSELLLA
LKNPRSLSESSNNTLKRKSQESLSMSMSEVPSKRSSESGSRRNSRSGTRSSLQKINEVPD
QVNRTRKSGLRAFMGFLGMGHGSVEKNMLKPRDEDPLIDSDDERPESFEDEFRRKEMRRG
IDLATTLERIEKNFVITDPRLPDNPIIFASDSFLQLTEYNREEILGRNCRFLQGPETDRA
TVRKIRDAIDNQAEVTVQLINYTKSGKKFWNLFHLQPMRDQKGDVQYFIGVQLDGTEHVQ
DDAAKEGVVLVKKTADNIDEAAKELPDANLRPKDLWANHSKVVLPNPHMKDTASWRAIQK
VLESGESIGLKHFRPVKPLGSGDTGSVHLVELLNTGEYFAMKAMDKSIMLNRNKVHRATA
ERQILDLLDHPFLPTLYASFQTKTHICLITDYCPGGELFVLLDNQPLKVLHEDAVRFYAA
EVVVALEYLHCQGIIYRDLKPENILLHRDGHISLTDFDLSCLTSCRPQVFLPEDADEKKG
RKNGSYPIFFAEPMRASNSFVGTEEYIAPEIITGAGHTSAVDWWALGILLYEMLYGYTPF
RGKTRQRTFANILHKDIRFPASISVSKSSVIFFKLEKLNRFEFSGQPSGEAANV*
>11686.m00014|LOC_Os12g01140.1|genepair1073-2
MERGAPAAAGGRGGMGSTSVSTRGTEGGHGGVERKEQQQQRGYQLPRDSRGSLEVFNPSS
ASSFRTAAAAPKSASPFLAIPDREEDNVVAQQRAAEWGLVLQTDHHTGLPQGVSARPSSG
SARTSSEDNPQQQQSAAAIPRVSEELRAALSAFQQTFVVSDATHPNHPIMYASAGFFNMT
GYTSKEVVGRNCRFLQGSGTDPHEIDKIRQSLANGSNYCGRILNYKKDGTPFWNLLTIAP
IKDEDGRLLKFIGMQVEVSKYTEGKKDTVVRPNGLSESLIKYDARQKDHARSSVSELLLA
LKNPRSLSESSNNTLKRKSQESLSMSMTEVPSKRSSESGSRRNSRSGTRSSLQKINEVPD
QGNRTRKSGLRAFMGFLGMGHGSVEKNMLKPRDEDPLIDSDDERPESFEDEFRRKEMRRG
IDLATTLERIEKNFVITDPRLPDNPIIFASDSFLQLTEYNREEILGRNCRFLQGPETDRA
TVRKIRDAIDNQAEVTVQLINYTKSGKKFWNLFHLQPMRDQKGDVQYFIGVQLDGTEHVQ
DDAAKEGVVLVKKTADNIDEAAKELPDANLRPEDLWANHSKVVLPNPHMKDTASWRAIQK
VLESGESIGLKHFRPVKPLGSGDTGSVHLVELLNTGEYFAMKAMDKSIMLNRNKVHRATA
ERQILDLLDHPFLPTLYASFQTKTHICLITDYCPGGELFVLLDNQPLKVLHEDAVRFYAA
EVVVALEYLHCQGIIYRDLKPENILLHRDGHISLTDFDLSCLTSCRPQVFLPEDADEKKG
RKNGSYPIFFAEPMRASNSFVGTEEYIAPEIITGAGHTSAVDWWALGILLYEMLYGYTPF
RGKTRQRTFANILHKDIRFPASISVSLAARQLMYRLLHRDPANRLGSYEGANEIKGHPFF
RGINWPLIRATAPPKLEIPLFSKDDMEKKGLVTNNRTDMF*

>11687.m00015|LOC_Os11g01150.1|genepair1074-1
MAMAACSCCCHAMVGMPCYLWRTLRSTRRVRRVHCHDDDNVEEGGGGWDSSLSDFAWSSS
KDEEEEGSTSTEGGEYGGHGGGRRERRNQDQMRRSLRLRLMSFRSNYHVSVTSMPRGMKT
*
>11686.m00015|LOC_Os12g01150.1|genepair1074-2
MAMAACSCCCHAMVGMPCYLWRTLRSTRRVRRVHCHDDDNVEEGGGGWDSSLSDFAWSSS
KDEEEEGSTSTEGGEYGGHGGGRRERRNQDQMRRSLRLRLMSFRSNYHVSVTSMPRGMKT
*
>11687.m00016|LOC_Os11g01160.1|genepair1075-1
MAAAARLLPVAAKRLTTTARARRLSTSTSTSPPATAVLYDQHGPPDKVLRVAELPAAEIG
ERDVCVRMLAAPINPSDLNRVEGVYPVRPPLPAAVAGYEGVGQVHALGGAVDSRLLSPGD
WVIPSPPSLGTWQTYIVNPATAWHRVRSDVPPQYVATVTVNPLTALRMLCDFVNLAPGDT
LVQNGATSIVGQCVIQLAKLHGLHTINIIRDRPGSQEAKDKLKQLGADHVFTESQLDIKN
IKSLLGALPEPALGLNCVGGNAASVILKFLRQGGTMVTYGGMSKKPVTVSTSSFIFKDLS
LRGFWLQKWMSSDKAEESRTMIDYLLDLVHEGKLKYEMELTPFSDFHLALDKALGKHGSQ
PKQVLKF*
>11686.m00016|LOC_Os12g01160.1|genepair1075-2
MAAAARLLPVAAKRLTTTARARRLSTSTSTSPPATAVLYDQHGPPDKVLRVAELPAAKIG
ERDVCVRMLAAPINPSDLNRVEGVYPVRPPLPAAVAGYEGVGQVHALGGAVDSRLLSPGD
WVIPSPPSLGTWQTYIVNPATAWHRVRSDVPPQYVATVTVNPLTALRMLCDFVNLAPGDT
LVQNGATSIVGQCVIQLAKLHGLHTINIIRDRPGSQEAKDKLKQLGADHVFTESQLDIKN
IKSLLGALPEPALGLNCVGGNAASVILKFLRQGGTMVTYGGMSKKPVTVSTSSFIFKDLS
LRGFWLQKWMSSDKAEESRTMIDYLLDLVHEGKLKYEMELTPFSDFHLALDKALGKHGSQ
PKQVLKF*
>11687.m00017|LOC_Os11g01170.1|genepair1076-1
MDDYAREMMELKTLVTRTLEKKGVLAKIRAELRASVFEAIEEEDRVVENDDGGNPALLGS
CNDRAKQLHASPSGRLLTALVSEYLEWAQLSHTMKVYLPECNLPKDFWKNELKDFSNKSG
AEGSRSAESGPMLLDVLEGYLKYENLSQTRMAGTGRRIINSESDPALNAEHRNTRRPPSS
SSVTGLPPMGRPMPSSQMSDRRGGSSASNARKDEYNWRYDADDISEEVLRASSALENVQL
DRKARNLTTSWRHPGDGAE*
>11686.m00017|LOC_Os12g01170.1|genepair1076-2
MDDYAREMMELKTLVTRTLEKKGVLAKIRAELRASVFEAIEEEDRVVENDDGGNPALLGS
CNDRAKQLHASPSGRLLTALVSEYLEWAQLSHTMKVYLPECNLPKDFWKNELKDFSNKSG
AEGSRSAESGPMLLDVLEGYLKYENLSQTRMAGTGRRIINSESDPALNAEHRNTRRPPSS
SSVTGLPPMGRPMPSSQMSDRRGGSSASNARKDEYNWRYDADDISEEVLRASSALENVQL
DRKARNLTTSWRLELLPFFFAPSLFAWPFVSDHRISVSRHPGDGAE*
>11687.m00018|LOC_Os11g01180.1|genepair1077-1
MAHGGGEEEEERVLSHGDVVLFRCDLTILRGPHFLNDRIIAFYLAHLAADHDADDDLLLL
PPSIPYLLSNLPDPASVAAVADPLRLASRRLVLLPVNDNPDVSHAEGGSHWTLLVLDNSN
AVSGPRFVHHDSLPPTNLPSARRLAAVLRPLLPASAIPLIEGPTPRQTNGYDCGVFVLAV
ARAICNWWPTRARHSNSDSDWLEAVKREPFNSTSFSPFDCIYQSACHSTRLWDLVRYAKP
QEGCCCEACGFQAFFQIELEDSGTHTAYDQKKADTGKGACWDNLTVSQSVRKPNVSAKNS
LSYDGYSWRKYGQKQVKGSEFPRSYYKCTHPTCPVKRKVEMTPDGRIAEIVYNGEHNHPK
PHPPRKPTLSTSVETLVATNDAGLENKLEGCDQAIGSDAVVEALRGGCHCLDGFRNGNEI
SDCKKRLFRFGVRFHDGSCKRILAQPHNIINLGSHLVAEYVQEKQ*
>11686.m00018|LOC_Os12g01180.1|genepair1077-2
MAHGGGEEEEERVLSHGDVVLLRCDLTILRGPHFLNDRIIAFYLAHLAADHHDDDLLLLP
PSVPYLLSNLPDPASVAAVADPLRLASRRLVLLPVNDNPDVSHAEGGSHWTLLVLDNSNA
VSGPRFVHHDSLPPTNLPSARRLAAVLRPLLPASAIPLIEGPTPRQTNGYDCGVFVLAVA
RAICNWWPTRARHSNSDSDWLEAVKREPFNSTSFSPFDCIYQSACHSTRLWDLVRYAKPQ
EGCCCEACGFQAFFQIELEDSGTHTAYDQKKADTGKGACWDNLTVSQSVRKPNVSAKNSL
SYDGYSWRKYGQKQVKGSEFPRSYYKCTHPTCPVKRKVEMTPDGRIAEIVYNGEHNHPKP
HPPRKPTLSTSVETLVATNDAGLENKLEGCDQAIGSDAVVEALRGGCHCLDGFRNGNEIS
DCKKRLFRFGVRFHDGSCKRILAQPHNIINLGSHLVAEYVQEKQ*
>11687.m00019|LOC_Os11g01190.1|genepair1078-1
MGRTRDMNGRKGLSKQCYVLNLGDSIVDAFSSHRANKQFKPWNKVERGGLDDLEKGKMTM
SDDGDRTCPLCAEDMDITDQQLKPCKCGYEICVWCWHHIIDMAEKEDTEGRCPACRTRYD
KDRIVKMAATCDRTVVEKNVDKKQKTQKVKSKAAVTVEAKKHLASVRVIQRNLVYIIGLP
ANLCNESILERREYFGQYGKVLKVSVSRPTGAPSQQAPTNNSISVLIELRTFAYADVRYI
TYAKEEEAIRCIQAVHNFVLEGKVLRACFGTTKYCHAWLRNMTCGNPDCLYLHDVGSQED
SFTKDEIISAYTRSRVPQMASSVSQRRAGTVLPPPAEDFSYSAVVAAKHPIKNGITNTAN
QSRLSPPNSSSGRSTLPPAASWGHRDLNTRTTATGVASSQSLTKSKTDPQSNSFSSSSTV
SSTKLPSSWNDDTSTVPKMMEGRDSLSKTLKPYKPGIAKETQAVTSLESSLDIDFSTIPS
AWNDDDVTSDGMSKGSDEKQVVNDNGKFECSVSSKPAESGHLTSKSTTSPKKDIAVNSTR
QSPLNCVSSPSISKSEVKDGDGDYQVTNMASKTSTSVIRKDQSNQAAIDTATEDTRSEST
DIDRLSVGVSSVTLSRKDGVQSIAENQQPDAILSTSVVVPFSQNLKLADSNDSTCQPSSD
KHHDWCSDIQSSVSPQLNDIESYAVATDKSHGRVLDAADQASSSPYVHFPNTSPISLWNG
KEINHTSTSDRTSTMMQPGLLSSVDSTSTMLNGHQEGLGTIYAPGKVSEHPRMKNHQPGA
VGAVRIDNIGSFDKAVSVNKDESSIIADILSLEFDPWDESYSTANNFAKMLSASEKNDVL
FDAPSWKTKTSNSESRFSFARQDNQGSFLDSSMRNYKSEQNFSLLSQNSHGNIYQSGIAF
QSPEEGFAKSNSLTMLDMLATGTSKPKVSAPPGFSAPARVPPGFSSGFSSHEGLNPPPGF
SSHNGPNPPPGFSSQGGSNQIYGSAYSETRPFDYLLGINTSHYQPQLARQTSDIEFVDPA
ILAVGKGRMPGISDSGLEMKTSHTFPSQLQTSNDPRFQLLMQQNVPSHQNVGFAEHVQDA
FNPMNDNYLASRLIPQNHGSLSSYTQMSLQQPRSSHLTNGHWDGWGDLRQGNNVSMPDMS

RMLYPTEANNFHMLGSNDLYNRAFGL*
>11686.m00019|LOC_Os12g01190.1|genepair1078-2
MTMSDDGDRTCPLCAEDMDITDQQLKPCKCGYEICVWCWHHIIDMAEKEDTEGRCPACRT
RYDKDRIVKMAATCDRTVVEKNVDKKQKTQKVKSKAAVTVEAKKHLASVRVIQRNLVYII
GLPANLCNESILERREYFGQYGKVLKVSVSRPTGAPSQQAPTNNSISVLIELRTFAYADV
RYITYAKEEEAIRCIQAVHNFVLEGKVLRACFGTTKYCHAWLRNMTCGNPDCLYLHDVGS
QEDSFTKDEIISAYTRSRVPQMASNVSQRRAGTVLPPPAEDFSYSAVVAAKHPIKNGITN
TANQSRLSPPNSSSGRSTLPPAASWGHRDLNTRTTATGVASSQSLTKSKADPQSNSFSSS
STVSSTKLPSSWNDDTSTVPKMTEGRDSLSKTLKPYKPGIAKETQAVTSLESSLDIDFST
IPSAWNDDDVTSDGMSKGSDEKQVVNDNGKFECSVSSKPAESGHLTSKSTTSPKKDIAVN
STRQSPLNCVSSPSVSKSEVKDGDGDYQVTNMASKTSTLVIRKDQSNQAAIDTATEDTRS
ESTDIDRLSVGVSSVTLSRKDGVQSIAENQQPDAILSTSVVVPFSQNLKLAECNDSTCQP
SSDKHRDWCSDIQSSVSPQLNDIESYAVATDKSHGRVLDAADQASSSPYVHFPNTAPISL
WNGKEINHTSTSDRTSTMMQPGLLSSVDSTSTMLNGHQEGLGTIYAPGKVSEHPRMKNHQ
PGAVGAVRIDNIGSFDKAVSVNKDESSIIADILSLEFDPWDESYSTANNFAKMLSASEKN
DVLFDARSWKTKTSNSESRFSFARQDNQGSLLDSSMRNYKSEQNFSLPSQNSHGNIYQSG
IAFQSPEEGFSKSNSLTMLDMLATGTSKPKVSAPPGFSAPARVPTGFSSGFSSHEGLNPP
PGFSSHNGPNPPPGFSSQGGSNQIYGSAYSETRPFDDLLGINTSHYQPQLARQTSDIEFV
DPAILAVGKGRMPGISDSGLEMKTSHTFPSQLQTSNDPRFQLLMQQNMPSHQNVGFAEHV
QDAFNPMNDNYLASRLIPQNHGSLSSYTQMSLQQPRSSHLTNGHWDGWGDLRQGNNVSMP
DMSRMLYPTEANNFHMLGSNDLYNRAFGL*
>11687.m00020|LOC_Os11g01200.1|genepair1079-1
MPSASAAAAALVLLFLAFSLSNAQPGFISLDCGGDDDYTDGIGIQWTSDAKFVSAGQEAN
LLLQNQQLQQYTTVRSFPADNRKYCYTMNVRNRTRYLVRATFLYGNFDNSNVYPKFDLSL
GPTPWTTVVIDDATTPVVQEAIILAAAPTLSVCLSNASTGQPFISTLELRQFNGSLYYTT
DEKQFFLRLSARINFGAESNASVRYPDDPFDRIWESDLVRRANYLVDVAPGTERISTTKP
IFVSTNEEPPQRVMQTAVVGKNGSLTYRIDLEDFPGNAWGVSYFAEIEDLAPNQTRKFKL
VIPGKPEFSKPTVDVEENAQGKYCLYEPGYTNIPLPFVFSFGFKKTNDSSEGPILNAMEI
YKYIEISVGSQDANIMASLVSRYPEAGWAQEGGDPCLPASWSWVQCSSEAAPRIFSISLS
GKNITGSIPVELTKLSGLVELKLDGNSFTGQIPDFTGCHDLQYIHLEDNQLTGALPPSLG
ELPNLKELYIQNNKLSGEVPQALFKKSIIFNFSGNSDLRMGHSNTGRTIVIIVCAVVGAI
LILVAAIVCYLFTCKRKKKSSDETVVIAAPAKKLGSFFSEVATESAHRFALSEIEDATDK
FDRRIGSGGFGIVYYGKLTDGREIAVKLLTNDSYQGIREFLNEVTLLSRIHHRNLVSFLG
YSQQDGKNILVYEFMHNGTLKEHLRGGPDDVKITSWVKRLEIAEDAAKGIEYLHTGCSPT
IIHRDLKSSNILLDKNMRAKVADFGLSKPVVDGSHVSSIVRGTVGYLDPEYYISQQLTEK
SDMYSFGVILLELISGHEPISNDNFGLHCRNIVEWARSHMESGDIHGIIDQSLDAGYDLQ
SVWKIAEVATMCVKPKGVLRPSISEVLKEIQDAIAIELQRELPSSIHHLMSKTSPSEAVN
TTGSVQDLEQNASFDELLMRPGLR*
>11686.m00020|LOC_Os12g01200.1|genepair1079-2
MPSASAALVLLFVAFSLSNAQPGFISLDCGGDDDYTDGIGIQWTSDAKFVSAGQKANLLL
QNQQLQQYTTVRSFPADNRKYCYTMNVRNRTRYLVRATFLYGNFDNSNVYPKFDLSLGPT
PWTTVVIDDATTPVVQEAIILAAAPTLSVCLSNASTGQPFISTLELRQFNGSLYYTTDEK
QFFLRLSARINFGAESNASVRYPDDPFDRIWESDLVRRANYLVDVAPGTERISTTKPIFV
STNEEPPQRVMQTAVVGKNGSLTYRIDLEDFPGNAWGVSYFAEIEDLTPNQTRKFKLVIP
GKPEFSKPTVDVEENAQGKYRLYEPGYTNIPLPFVFSFGFKKTNDSSEGPILNAMEIYKY
IEISVGSQDANIMASLVSRYPEAGWAQEGGDPCLPASWSWVQCSSEAAPRIFSISLSGKN
ITGSIPVELTKLSGLVELKLDGNSFTGQIPDFTGCHDLQYIHLEDNQLTGALPPSLGELP
NLKEFFSGNSDLRMGHSNTGRTIVIIVCAVVGAILILVAAIVCYLFTCKRKKKSSDETVV
IAAPAKKLGSFFSEVATESAHRFALSEIEDATDKFDRRIGSGGFGIVYYGKLTDGREIAV
KLLTNDSYQGIREFLNEVTLLSRIHHRNLVSFLGYSQQDGKNILVYEFMHNGTLKEHLRG
GPDDVKINSWVKRLEIAEDAAKGIEYLHTGCSPTIIHRDLKSSNILLDKNMRAKVADFGL
SKPVVDGSHVSSIVRGTVGYLDPEYYISQQLTEKSDMYSFGVILLELISGHEPISNDNFG
LHCRNIVEWARSHMESGDIHGIIDQSLDAGYDLQSVWKIAEVATMCVKPKGVLRPSISEV
LKEIQDAIAIELQRELPSSIHHLMSKTSPSEAVNTTGSLQDLEQNASFDELLMRPGLRLN
GTGRAAQAASEILNETAILVTSRC*
>11687.m00021|LOC_Os11g01210.1|genepair1080-1
MLRRGLLPDRRAYTAALARLPPSRALRLFDALLHHLRHHHNKTNSLPDTAAFNAALSACA
DAGDCIRFRHLFDQMPAWNAPPDALTYNVLIKMCARAGRKDLVARVLHRILSSGLTPCAT
TFHSLVAAYVGFGDIPTAERIVQAMRERRTDICLLFRAVADDHIISHDQQSCVLEDIVKP
WEQDEVPLLPKAYPPNSRVYTTLMKGYMNAGRVEDVVAMLRAMRREGETSPASRPDHVTY
TTVISTLVAAGDMERARAVLEEMGQAGVAASRVTYNVLIKGYCQQLQAGKAKELLAVDMA
EAGIQPDVVTYNTLIDGCVLTDDSAGAVALFNEMRERGIAPSAVSYTTLMKAFAASGQPK
LAHKVFDEMEKDPRVAVDRAAWNMLVEAYCRLGLLESAKKVVERMKARGVQPDVATYGSL
AKGIAVARRPGEALLLWEEIKEKEVDGEVVEALADVCVRAALFRKALEMVARMEEMGVEP
NKAKYKRMYVDLHSRMFTSKHASQARQDRRRERKRAAEAFKFWLGLPNSYYATDWRLQDD
GLN*
>11686.m00021|LOC_Os12g01210.1|genepair1080-2
MSSSSNALVQHYYSCKPPPPPRLVLVPFFFSGSGSGTNNTASSPAAASSTHTHTHTADAN
AQLDAHLLSLLRDGHTDAAYHLFASNPSLPLSPVSASRLLAQLSYSSFSRASALLHRLRA
RQALHLLDANSLSLASSASARSNNPHLAYSLLLSMLRRGLLPDRRAYTAALARLPPSRAL
RLFDALLHHLRHHHNKTNSLPDTAAFNAALSACANAGDCIRFRHLFDQMPAWNAPPDALT
YNVLIKMCARAGRKDLVARVLHRILSSGLTPCATTFHSLVAAYVGFGDIPTAERIVQAMR
ERRTDICLLFRAVADDHIISHDQQSCVLEDIVKPWEQEEVPLLPKAYPPNSRVYTTLMKG
YMNAGRVEDVVAMLRAMRREGETSPASRPDHVTYTTVISTLVAAGDMERARAVLEEMGQA

GVAASRVTYNVLIKGYCQQLQAGKAKELLAVDMAEAGIQPDVVTYNTLIDGCVLTDDSAG
AVALFNEMRERGIAPSAVSYTTLMKAFAASGQPKLAHKVFDEMEKDPRVAVDRAAWNMLV
EAYCRLGLLESAKKVVERMKARGVQPDVATYGSLAKGIAVARRPGEALLLWEEIKEKEVD
GEVVEALADVCVRAALFRKALEMVARMEEMGVEPNKAKYKRMYVDLHSRMFTSKHASQAR
QDRRRERKRAAEAFKFWLGLPNSYYATDWRLQDDGLN*
>11687.m00022|LOC_Os11g01220.1|genepair1081-1
MASPCRIPAATGRYPISFTSLIISSWTGNKENNISQNRHHVLDCQTPCEGKGAATSKKRK
NKPAGGFNLRKSIAWNPAFFTEQGVLDNTELSMLTGSQVKATRSPASGFSSTFSPLSRFG
KSSNTSVLKEVGENSRGKFPSKCLSAENKGRKLFASSKASEQDERKAPAGTQDKRSAKAV
QKSIPRPPAGYKYPYGDSGIIGYVLFFFLNRIMPLPVPNSSGTAQIQRIPKKSEPSPSVV
SRSRSTSSVTNVPKPTTRPATVTSERTHKVEGLPLESKTERSSVIKSSGPTIGKNMVPTV
TAICQETNGSGKCETFSPYSQDNPSSSVVAPARISAKPSALRMPSPSVGFFTQGKASVSH
SDNAQRNPERCFSGNISSLVKPPSLPVQPATRDPNVLASSLPGVEDTNVRSLKQSLSEST
VPYSEKSGNINYQEMPDDDFSLAGNGATTELSFRDNDGGRNIMPNECSVALSVGQDLNAI
CCSSIEPAEDSCFLKVICSSSEPTVGRNLTTSCISSPGCTPNDLNSQSKSDNGETAVDIE
NSLSGETSGTVCSSEGNNCTSATDSLRKSDSCHQQNMLVQSVHCTDQMPQFDSSTGIKPS
LAYSQLDSNTSLCSEVQLTSSEGPDIDSEMELDTDDAFTVEEPPLLHVGGECDHDYRSAE
CSHMNLAAPSPRVDQKALAGNLTEKVDTADGRTESHHCSTQERRPILSEEQDTEDTIEFD
TKLSSSEGASSIERIKSVGKSRTNTISKDHLKNLVPFTEEWLAVMEAFGEEVLEQKTGAV
QNSPTDKAAPEPSPWSPVKRKAQDVGPFDCTKYSKNVRTSD*
>11686.m00022|LOC_Os12g01220.1|genepair1081-2
MASPCRIPAATGSGNKENNISQNRRHVLDCQTPCEGKGVATSKKRKNKPAGGFNLRKSIA
WNPAFFTEQGVLDNTELSMLTGSQVKATRSPASGFSSTFSPLSRFGKSSNTSVLKEIGEN
SRGKFPSKCLSAENKGRKLFASSKASEQDERKALAVSSSHSILLTYGNAIIKAHAISRFL
LDNCRAHRIKVPNSSGIAQIQRIPKKSEPSPSVVSRSRSTSSVTNVPKPTTRPATARHYF
SVTSECTHKVEGLPLKSKTERSSVTKSSGPTIGKDMVPPTVTTICQETNGSGKCETFSPY
SQDNPSSSVVAPARISAKPSALRMPSPSVGFFTQGKASVSHSGTAQRNPERCFSGNISSV
KPPSLPVQPATSDSTPNVLASSLPGVEDATVCSLKQSLSESTVPYSEKSGNISYQEMPDD
DFSLAGNGATTELSFRDNDDGRNSMPNECSVALSVGQDLNAICCSSTEPAEDSCFLKVIC
SSSEPSVGSNLTTSCISSPGCTPNDLNSQSKSDNGETAVDIENSLSGETSGTVCSSEGNN
CTSATDSLQKSDSCHQQNMLVQSIHCTDQIPQFDSSTGIKPSLAYSQLDSNNSLCSEVQL
TSSEGPDIDSEMELDTDDSFTVEEPPLLHVGDECDHDYRSAECSHMNLAAPSPCVDQEAL
AGNLTEKVDTADGRTESHHCSTQERRPILSEEQDTEDKIGFDTKLSSSEGASSIERIKSV
GKSRTNTISKDHLKNLVPFTEEWLAVMEAFGEEVLEQKTGAVQNSPTDKAAPEPSPWSPD
HFLPLLVTKVKLSRLIDEEPFLVMVLQVKRKAQDVGPFDCTKYSKNVRTSD*
>11687.m00024|LOC_Os11g01240.1|genepair1082-1
MSAADDLAALHHQVSLASSAALSASDHDLAFHLQLSEAIQASLSSNAAAPSHPAPPPPPP
EEPSDASCALAVHAADLARAEQDHRDAQACRAYHARAAASVRVAAHNALFARDLAAIPED
KWAHDGDYFERPLPLEGGGALFRVLFKGMASREVVGPRDRDPGVGVLAVAICGPRGEVVL
RIHKPVQAQAQQGRMMLEAMALVEGLNAALALGIRTLNVLTDNKPLHNHMSGIWRPRQKR
LVDLINEAFSAKQKFEQSEILCVARTQVNYVTKLATDSLHTQIAKAAVVSAGKEKKENCT
ICLEDTDVSKIHAVEGCAHRFCFSCMKEHVKVKLLHGMLPACPQDGCTTKLNCGGFKDVP
ISTVITDHGAAYQGSTNSSNSQDLLPISQVLSLDVHERTDTSNARIILKMTCYDYKRRYR
HARLEDAYLQNLAQQRLWRQCIRCKHMIELAEGCYHMTCVCGYEFCYTCGKEWKEKKATC
SCPLWDERNIIRDDPQGNAAIHDDPEDEYDDYYDEDENNYYVGEGLQYNVDYHRQYDGGD
RHHGHFYQYNR*
>11686.m00023|LOC_Os12g01230.1|genepair1082-2
MSAALHHQLSLASSAALSASDHDLAFHLQLTEAIQASLPSNAAAPAPPEEPSDASCALEI
HAADLARAEHDRRDAQACRAYHARAAASVRVAAHDALFARDLAAIPEDRWAHDGDYFERP
LPLEGGGALFRVLFKGMASREVVGPRDRDPGVGVLAVAICGPRGEVVLRIHKPVQAQAQQ
GRMMLEAMALVEGLNAALALGIRTLNVLTDNKPLHNHMSGIWRPRQKRLVDLINEAFSTK
QKFEQCEILCVARTQVNYVTKLATDSLHTQIAKAAAVSAGKEKKENCTICLEDTDVSKIH
AVEGCAHRFCFSCMKERVKVKLLHGMLPACPQDGCTTKLNCGGFKDVPISTVISDHGAAY
QGSTNSSNSQDLLPISQVFSLDVHERTDTSNASIILKMTCYDYKRRYPHARLEDAYLQNL
ARQRCGYEFCYTCGKEWKEKKATCSCPLWDERNIIRDDPQGNAAIHDDPEDEYDDYYDED
ENNYYVGEGLQYNVDYHRQYDGGDRHHGHFYQYNR*
>11687.m00027|LOC_Os11g01270.1|genepair1083-1
MSGSDHLLDALLAAARAALSHLHLPFPGSNPKPHYSDDLHLLGVGVAHFIHRSPPFTTRA
CFRRTKHPPPPPPGPPPHQLLLCIGIAFHNLLDNHLLHHFHTLLIHANKPHFDAFLSNLP
FAKLKVAPPPQPSASVSPAPPTPAQTGDKEDTDTAANHSPRSTLPVRLLNIPVDRLRSTL
STLSLTDLIDLVARSLPSPDTHPDKKKLFSVHDFFRYAEFEGKRFFEELDRDGDGQVTLE
DLEVAMRKRRLPRRYARDFLRRTRSNLFSKSIGWKQFLSLMEQKEPTILRAYTTLCLSKS
GTLHKNQILTSLKGAGLPANEDNAAAMLRYLNADSEESISYSHFRNFMLLLPSERLEDDP
RNIWFEAATLVAVPPPVEISAGSVLKSALAGGLASALSTSVMHPIDSMKTRVQASSLSFP
DLISTLPQIGLRGLYRGSIPAILGQFSSHGLRTGIFEASKLVLKSVAPTLPDIQVQSLSS
FCSTILGTAVRIPCEVLKQRLQAGIFNNVGEAIVGTMQKDGPKGFFRGTGATLCREVPFY
VAGMCLYAEAKKAAQHVLNRDLEPWETIAVGALSGGLAAVVTTPFDVMKTRMMTAPPGTP
VSMQLIVFSILRNEGPLGLFKGAIPRFFWIAPLGAMNFAGYELAKKAMIQTESDSTDSVH
EKRTTVGSRG*
>11686.m00024|LOC_Os12g01240.1|genepair1083-2
MSGSEHPMVAAARAALAHLQLPLPPAPAGSNMDHHLLPHFLHRSPPSPCFLLLRTQHKPK
PPQPQLHLLLCTAFDSLAHSLQEVLDKHKPKLHLDTLFLHGNLPFAKVGVPPPPPPPAPL
LSPPSVSAHHQHQPATPTETETEDTAANDSPPPRTLPVRLLNIPVDRLRSTLSTLSLTEL
IDLVPHLVARSLPSPDTHPDKKKLFSVHHFFRYAEFEGKRFFEELDRDGDGQVTLEDLEI

AMRKRRLPRRYARDFLRRTRSNLFSKSIGWKQFLSLMEQKEPTILRAYTTLCLSKSGTLH
KNQILTSLKGAGLPANEDNAAAMLRYLNADSEESISYSHFRNFMLLLPSERLEDDPRNIW
FEAATLVAVPPPVEISTGSVLKSALAGGLASALSTSVMHPIDSMKTRVQASSLSFPDLIS
TLPQIGLRGLYRGSIPAILGQFSSHGLRTGIFEASKLVLKSVAPTLSDIQVQSLSSFCST
ILGTAVRIPCEVLKQRLQAGIFNNVGEAIVGTMQKDGPKGFFRGTGATLCREVPFYVAGM
CLYAEAKKAAQHVLNRDLEPWETIAVGALSGGLAAVVTTPFDVMKTRMMTAPPGTPVSMQ
LIVFSILRNEGPLGLFKGAIPRFFWIAPLGAMNFAGYELAKKAMIQTESDSADSVHEKRT
TVGSRG*
>11687.m00028|LOC_Os11g01280.1|genepair1084-1
MASNIKLPLADGEERLSCCGELLAMGKKLEEAVTISGKLPLEEGMGKGKRRPVVAELEDE
EEFKDEELTEQIIARDFRVSWEHRFSPRYSFHDTTTVSPMRYTEGPIPRYACCCDALQIF
SLQVKEAKDGLDWPLHVYGLVATRDSVDQRHNLLFKRTRDNCQILTPQDSHLLLTGPSRA
VVVIDPVTFEVELKHTYMIRRCLSSKHGELELTYAALVGTVEATMVSFQVTEGSWPDHLR
GVVVCKTASVEGGDIVLLDSRDGKMPINCNGAIELSRRVVSAELGGELSVDLVALQANNS
SEIVSRGRVVFTPDEAGRSSGVFDLVFCKVEATVCWSLLATLGQMLSGNPWA*
>11686.m00025|LOC_Os12g01250.1|genepair1084-2
MVSSMKLPLVDGEEQSSYDPMGEKLKMMSGKIISEEQRLVELELELEDEEEVEDEELAEL
ITARDFRVSWEHRFTPRYSFNDTTTVCPMRYTEGPIPRYACCGDTLQIFSLQVKEAKGGL
DWPLHVYGLVATRDSVDQRRNLLFRCTRDNCQILTPQDSHLLLTGPSRAVVVIDPVTFEV
ELKACRLQLIQSRPRSNRFNSLTPINIWMDAISKMQNEQDIFPTSIWDWKGHDMPTLVIV
KYQS*
>11687.m00030|LOC_Os11g01300.1|genepair1085-1
MARSSSQSQSSVGASAGAARPATVGPRGTAAAAAGMRRRRASTAGGSGGFSGGGGSNMLR
FYTDEAPGLRLSPTMVLVMSLCFIGFVTALHVFGKLYRSRTAASASA*
>11686.m00026|LOC_Os12g01260.1|genepair1085-2
MARSSSQSQSSVGAAAGAARPATVGPRGTAAAAAGMRRRRASTAGGSGGFSGGGGSNMLR
FYTDEAPGLRLSPTMVLVMSLCFIGFVTALHVFGKLYRSRTAASASA*
>11687.m00032|LOC_Os11g01320.1|genepair1086-1
MAAEKTKQRRGIEESARMRDKGRLSDETPSEEGHEIIRFRRGWESLYSHPHRSFDATTFA
PMRYTHVPIPKYADCNYGLQIFSVKVNQLLLNEEEEEEEEEGLHWPLHVYGLVATRDSLD
PRRNLLFNRTRDNCQILTQQDPFLLLTGPTRAVVLVIDPVKFEIQLKAKGTNPPFIVRRR
RRCKRSELEFAFTLLVRSVEATISVQVVDGSSWPDDLGVQVAARTASISDEAIKLLDSRS
AHGGRVPICPDDAYPAMRYTFGHIPKSSFVGCDSGLQIFSIKLLLRNTSTTDHQLQWPLH
VYGLVVTRDSLDPRRNLLFNRTRDNCQILTQQDPFLVLTGPSRAIVLIDPVQFEVQLKAK
SNNNTLHDHPDQDQIVNFGVVNSGYLPGPTSHCIGKRNGKMPIDDDGFIQLSRRVVSVEL
AGQLIVQVLAFNSQQQVVDNDNDNKKDEIVAKHEIVFDPKEASLSVETCELQLGGGGGGG
GGPLAVVVRGGREPVPRIDGLGGGGQKLAPRIDGLGSDNSREPMTPIDGLCGEGREPTPL
FDGLLDSNI*
>11686.m00035|LOC_Os12g01350.1|genepair1086-2
MSPSHGGYWVLATWWGEPLLWLWQPFRAGGGAGPLGHLLEISGGLGRIRGWAAMEERGDE
LIRPCVTRLDASRNQVLLAATTDCRSSQSNSSSATDHQLQWPLHVYGLVATRDSLDPRRN
LLFNRTRDNCQILTQQDPFLVLTGPSRAIVLIDPVQFEVQLKAKSNNNTLHDHPDQDQIV
NFGVVDSGYLPGPTRHCIGKRSEVEFTISVLDRSIEATIISVQLVGGSSWPDHLQGKLVS
RTASAIHQEIVLLDSQKQQDGKMPIDDGGFIQLSRRVVSVELAGQLIVQVLAFNSQQQVV
DNDNDNKKDEIVAKHEIVFDPKEASLSVETCELQLGGGGGGPCKLQISVAWSLVDRLPPV
GYF*
>11687.m00033|LOC_Os11g01330.1|genepair1087-1
MPTPVAAARQCLSPAAVPALDAAVASSRRRAHAQTTSLHLISSLLAPPAPPLLRDALARA
RSAAYSPRVQLKALDLCFAVSLDRLPSVSASSSSSGAADEPPVSNSLMAAIKRSQANQRR
NPDTFHFYHQAATAQTPAAVKVELSHLVLAILDDPVVSRVFAEAGFRSGDIKLAILRPAP
PMPLLGRLPTRTRPPPLFLCSFAAADDADVPSPAGNLAGAGEENCRRIAEILSRGRNPML
VGVGAASAADDFAAASPYRIIHVDPNTIDRSDLGVAAAMASATSGLIISIGDLKQLVPDE
DAEAQEKGRRVVAEVTRVLETHSKVGRVWVMGWSATYETYLAFLSKFPLVDKDWDLQLLP
ITAVHAAATAGPAAAAAGLMPPATTVAAFSKPAASLMDSFVPFGGFLCDNYEENSLTANS
CPQALRCQQCNDKYEQEVATIISASGITAEDHHQGGLPSLLQNGSMMGPNNGFDPVKARD
DRMVLNSKILNLRKKWNEYCLRLHQDHQRINRDPYKPFPRYIGVPTDKERSANSSKGSES
VGVQKDVIKPCAVSAVHSSSTARPISSPSVTNKRNEDLVLNLQARHSKSDENLQERGMQS
QHGTLSNVDNPDDHVSPSSAAPVETDLVLGTPRECSSKGSSSTCSKRVEDSERSVHLVPK
KVDDLNLKHPQLSVQPNSCSWSSINVGKTSHSTLHSVASGGFSAFGQWQKRSPLAAQNSD
LSNYKLLVERLFKVVGRQEEALSAICESIVRCRSTESRRGPNRNDIWLCFHGSDSMAKKR
IAVALAELMHGSKDNLIYLDLNLQDWDDSSFRGKTGIDCIVEQLSKKRQSVLFLDNIDRA
DCLVQDSLSDAIKSGRFQDMRGKVVDINDSIVVLSRSMIQGSKNGLEEGLSFSEEKILAT
RGHRLKILVEPGRAITSGCPSGKVVVSPRHFLTKIQASLCSGSISKRKLSISDDQEKLQE
SPSSSKRLHRTSSVPFDLNLPVDEDEPLDADDDSSSHENSYGNTEKSIDALLHSVDGSIN
FKPFDFDKLADDMLQEFSNILRKNLGSECMLEIDVGAMEQILAAAWKSEEDRKPVPTWLE
QVFARSLDELKLKRKHVSSSTLRLVACEDTVPAVKGDGLGVLLPPRIILDC*
>11686.m00037|LOC_Os12g01360.2|genepair1087-2
MPTPVAAARQCLSPAAVPALDAAVASARRRAHAQTTSLHLISSLLAPPAPPLLRDALARA
RSAAYSPRVQLKALDLCFAVSLDRLPSVSASSSSGAADEPPVSNSLMAAIKRSQANQRRN
PDTFHFYHQAATAQTPAAVKVELSHLVLAILDDPVVSRVFAEAGFRSGDIKLAILRPAPP
MPLLGRLPTRTRPPPLFLCSFAAADDADVPSPAGNLAGAGEENCRRIAEILSRGRNPMLV
GVGAASAADDFAAASPYRIIHVDPNTIDRSDLGVAAAMASATSGLIISIGDLKQLVPDED
AEAQENGRRVVAEVTRVLEAHSKVGRVWVMGWSATYETYLAFLSKFPLVDKDWDLQLLPI
TAVHAAPAAAGPAAAGGLMPPATTVAAFSKPAASLMDSFVPFGGFLCDNYEENSLTANSC

PQALRCQQCNDKYEQEVATIISASGITAEDHHQGGLPSLLQNGSMMGPNNGFDPVKVRDD
RMVLNSKILNLQKKWNEYCLRLHQDCQRINRDPYKPFPRYIGVPADKERSANPSKGSESI
GVQKDVIKPCAVSAVHSSSTARPISSPSVTNKRNEDLVLNLQARHSKSDENLQERGMQSQ
HGTLSNADNPDDHASPSSAAPVETDLVLCTPRDCSSKGSSSTCSKRVEDSERSVHLVPKK
VDDLNLKHPQLSVQPNSCSWSSINVGKTSHSTLHSVASGGFSAFGQWQKRSPLAAQNSDL
SNYKLLVERLFKVVGRQEEAVSAICESIVRCRSTESRRGPSRNDIWLCFHGSDSMAKKRI
AVALAELMHGSKENLIYLDLNLQDWDDSSFRGKTGIDCIVEQLSKKRRSVLFLDNIDRAD
CLVQDSLSDAIKSGRFQDMRGKVVDINDSIVVLSRSMIHGSKNGLEEGLSFSEEKILATR
GHRLKILVEPGRAITSGCPSGKVVVSPRHFLTKIQASLCSGSISKRKLSMSDDQEKLQES
PSSLKRLHRTSSIPFDLNLPVDEDEPFDADDDSSSHENSYGNTEKSIDALLHSVDGSINF
KPFDFDKLADDMLQEFSNILRKNLGAECMLEIDVGAMEQILAAAWKSEDKGPVQTWLEQV
FARSLDELKLKYKHVSSSTLRLVPCEDTLPTVKGDGLGVLLPPRIILDC*
>11687.m00034|LOC_Os11g01340.1|genepair1088-1
MAASATQEADCKASEDARLFFDAAKPPPFRIGDVRAAIPAHCWRKTPLRSLSYVARDLLI
VAALFAAAASSIDLAWAWAWPLYWAAQGTMFWALFVLGHDWRISHRTHHQNHGHIERDES
WHPITEKLYWQLETRTKKLRFTLPFTLLAFPVYLWYRSPGKTGSHFLPSSDLFSPKEKSD
VIVSTTCWCIMISLLVALACVFGPVPVLMLYGVPYLVFVMWLDLVTYLHHHGHNDLPWYR
GEEWSYLRGGLTTVDRDYGWINNIHHDIGTHVIHHLFPQIPHYHLVEATKAARPVLGRYY
REPEKSGPLPLHLFGVLLRSLRVDHFVSDVGDVVYYQTDHSLNGTDWAEDAKHK*
>11686.m00038|LOC_Os12g01370.1|genepair1088-2
MAASATQEADCKASEDARLFFDAAKPPPFRIGDVRAAIPAHCWRKTPLRSLSYVARDLLI
VAALFAAAATRIDVSVAWAAWPLYWAAQGTMFWALFVLGHDCGHGSFSDSAMLNNVVGHL
LHSFILVPYHGWRISHRTHHQNHGHIEKDESWHPITEKLYRKLETRTKKLRFTLPFPLLA
FPVYLWYRSPGKTGSHFLPSSDLFSPKEKSDVIVSTTCWCIMISLLVALACVFGSVPVLM
LYGVPYLVFVMWLDLVTYLHHHGHNDLPWYRGEEWSYLRGGLTTVDRDYGWINNIHHDIG
THVIHHLFPQIPHYHLVEATKAARPVLGRYYREPEKSGPLPLHLFGVLLRSLRVDHFVSD
VGDVVYYQTDHSLNGTDWAEDAKHK*
>11687.m00037|LOC_Os11g01370.1|genepair1089-1
MTKPQQQSPPSTTATTTTSPPPPPPPSTPPPASSSSSSLAKLPLRLHSLASSSRSLLSALR
RSPVTTLVAAFFLLALFMYGEDVRTLAELSIDDYLYPDADFYNVSALPPLLLPPPTCDLS
RGRWVFDNTSLPAYREKECTFLTKQVSCLANGRPDDLWQYWRWQPNNCSLPTFDARRFME
KMRGKRMMFVGDSLNRNQWESLVCLVQPILSKGRKKIVKRGSFNIFYAKEYRATLEFYWA
PFLVESNSDNPNFHHIDQRIISPERIESHANNWKDVDYLIFNTYIWWMNNEDIKVRRPNS
TSWSDHDEVPRIETYGRVFKTWSTWLEQNVDPARTSVFFMTISPLHNSSKIGIGGRWFNT
DAVITFCQFNIDEPCISATGSLELWNILKYRHRVRWFPAQWGNPNGIKCVKETLPVLNYT
KPLDLNHDMRMYDLVAKVAKNMKNVPVSLIDITRMSDYRKDAHTSLYSIRQGKLLTPEQK
ADPQKYADCIHWCLPGVPDVWNQILYTRILSKSSPPSPHPPLPPQ*
>11686.m00039|LOC_Os12g01380.1|genepair1089-2
MSKPQQQSPPSTTTTSPPPPPPSTPPPASSSRSLLSALRRSPVTTLVAAFFLLALFMYGE
DVRTLAELSIDDYLYPDADFYNVSALPPLLLPPPTCDLSRGRWVFDNTSLPAYREKECTF
LTKQVSCLANGRPDDLWQYWRWQPNNCSLPTFDARRFMEKMRGKRMMFVGDSLNRNQWES
LVCLVQPILSKGRKKIVKRGSFNIFYAKEYRATLEFYWAPFLVESNSDNPNFHHIDQRII
SPERIESHANNWKDVDYLIFNTYIWWMNNEDIKVRRPNSTSWSDHDEVPRIETYGRVFKT
WSTWLEQNVDPARTSVFFMTISPLHNSPAQWGNPNGIKCVKETLPVLNYTKPLDLNHDMR
MYDLVAKVAKNMKNVPVSLIDITRMSDYRKDAHTSLYSIRQGKLLTPEQKADPQKYADCI
HWCLPGVPDVWNQILYTRILSKSSPPSTHPSLPPQ*
>11687.m00038|LOC_Os11g01380.1|genepair1090-1
MAAANAPIAMREALTLTSLGIAPQFVTFTHVTMESEKYICVRETSPQNSVVIVDMAMPAQ
PLRRPITADSALMNPNTRILALKAQIPGTTQDHLQIFNIEAKTKIKSHQMPEQVVFWKWI
TPKLLGLVTQTSVYHWSIEGDSEPAKMFDRTANLANNQIINYRCDPSEKWLVLIGIAPGA
PERPQLVKGNMQLFSVDQQRSQALEAHAASFASFKVVGNENPSTLICFASKTTNAGQITS
KLHVIELGAQPGKPGFSKKQADLFFPPDFQDDFPVAMQISQKYGLIYVITKLGLLFVYDL
ETAAAVYRNRISPDPIFLTAESSASGGFYAINRRGQVLHATVNDATIVPFVSSQLNNLEL
AVNLAKRANLPGAENLVVQRFQELFAQTKYKEAAELAAESPQGLLRTPETVAKFQSVPVQ
AGQTPPLLQYFGTLLTRGKLNAYESLELSRLVVNQNKKNLLENWLAEDKLECSEELGDLV
KTVDNDLALKIYIKARATPKVVAAFAERREFDKILIYSKQVGYTPDYLFLLQTILRTDPQ
GAVNFALMMSQMEGGCPVDYNTITDLFLQRNMIREATAFLLDVLKPNLPEHAFLQTKVLE
INLVTYPNVADAILANGMFSHYDRPRVAQLCEKAGLYLRALQHYTELPDIKRVMVNTHAI
EPQALVEFFGTLSREWALECMKDLLLVNLRGNLQIVVQAAKEYSEQLGVDACIKLFEQFK
SYEGLYFFLGAYLSSSEDPDIHFKYIEAAARTGQIKEVERVTRESNFYDAEKTKNFLMEA
KLPDARPLINVCDRFGFVPDLTHYLYTNNMLRYIEGYVQKVNPGNAPLVVGQLLDDECPE
DFIKGLILSVRSLLPVEPLVDECEKRNRLRLLTQFLEHLVSEGSQDVHVHNALGKIIIDS
NNNPEHFLTTNPFYDSRVVGKYCEKRDPTLAVVAYRRGQCDDELINVTNKNSLFKLQARY
VVERMDGDLWDKVLQPENEYRRQLIDQVVSTALPESKSPEQVSAAVKAFMTADLPHELIE
LLEKIVLQNSAFSGNFNLQNLLILTAIKADPSRVMDYVNRLDNFDGPAVGEVAVEAQLFE
EAFAIFKKFNLNVQAVNVLLDNIRSIERAEEFAFRVEEDAVWSQVAKAQLREGLVSEAIE
SFIRADDATHFLDVIRAAEEANVYDDLVKYLLMVRQKAREPKVDGELIFAYAKIDRLSDI
EEFILMPNVANLQNVGDRLYDEELYEAAKIIYAFISNWAKLAVTLVKLKQFQGAVDAARK
ANSAKTWKEVCFACVDAEEFRLAQICGLNIIVQVDDLEEVSEYYQNRGCFNELISLMESG
LGLERAHMGIFTELGVLYARYRPEKLMEHIKLFSTRLNIPKLIRACDEQQHWKELTYLYI
QYDEFDNAATTIMNHSPDAWDHMQFKDVAVKVANVELYYKAVHFYLQEHPDLINDLLNVL
ALRLDHTRVVDIMRKAGQLHLVKPYMVAVQSNNVSAVNEALNELYVEEEDYERLRESVDM
HDNFDQIGLAQKLEKHELLEMRRIAAYIYKKAGRWKQSIALSKKDNMYKDCMETCSQSGD
RELSEDLLVYFIEQGKKECFASCLFICYDLIRADVALELAWMNNMVDFAFPYLLQFIREY

TSKVDELVKDRIESQNEVRAKEKEEKDLVAQQNMYAQLLPLALPAPPGMGGPPPPMGMPG
MPPMGGMGMPPMGPGPMPAYGMPPMGSY*
>11686.m00040|LOC_Os12g01390.1|genepair1090-2
MAAANAPIAMREALTLTSLGIAPQFVTFTHVTMESEKYICVRETSPQNSVVIVDMAMPAQ
PLRRPITADSALMNPNTRILALKAQIPGTTQDHLQIFNIEAKTKIKSHQMPEQVVFWKWI
TPKLLGLVTQTSVYHWSIEGDSEPAKMFDRTANLANNQIINYRCDPSEKWLVLIGIAPGA
PERPQLVKGNMQLFSVDQQRSQALEAHAASFASFKVVGNENPSTLICFASKTTNAGQITS
KLHVIELGAQPGKPGFSKKQADLFFPPDFQDDFPVAMQISQKYGLIYVITKLGLLFVYDL
ETAAAVYRNRISPDPIFLTAESSASGGFYAINRRGQVLHATVNDATIVPFVSSQLNNLEL
AVNLAKRANLPGAENLVVQRFQELFAQTKYKEAAELAAESPQGLLRTPDTVAKFQSVPVQ
AGQTPPLLQYFGTLLTRGKLNAYESLELSRLVVNQNKKNLLENWLAEDKLECSEELGDLV
KTVDNDLALKIYIKARATPKVVAAFAERREFDKILIYSKQVGYTPDYLFLLQTILRTDPQ
GAVNFALMMSQMEGGCPVDYNTITDLFLQRNMIREATAFLLDVLKPNLPEHAFLQTKVLE
INLVTYPNVADAILANGMFSHYDRPRVAQLCEKAGLYLRALQHYTELPDIKRVMVNTHAI
EPQALVEFFGTLSREWALECMKDLLLVNLRGNLQIVVQAAKEYSEQLGVDACIKLFEQFK
SYEGLYFFLGAYLSSSEDPDIHFKYIEAAARTGQIKEVERVTRESNFYDAEKTKNFLMEA
KLPDARPLINVCDRFGFVPDLTHYLYTNNMLRYIEGYVQKVNPGNAPLVVGQLLDDECPE
DFIKGLILSVRSLLPVEPLVDECEKRNRLRLLTQFLEHLVSEGSQDVHVHNALGKIIIDS
NNNPEHFLTTNPFYDSRVVGKYCEKRDPTLAVVAYRRGQCDDELINVTNKNSLFKLQARY
VVERMDGDLWDKVLQPENEYRRQLIDQVVSTALPESKSPEQVSAAVKAFMTADLPHELIE
LLEKIVLQNSAFSGNFNLQNLLILTAIKADPSRVMDYVNRLDNFDGPAVGEVAVEAQLFE
EAFAIFKKFNLNVQAVNVLLDNIRSIERAEEFAFRVEEDAVWSQVAKAQLREGLVSEAIE
SFIRADDATHFLDVIRAAEEANVYDDLVKYLLMVRQKAREPKVDGELIFAYAKIDRLSDI
EEFILMPNVANLQNVGDRLYDEELYEAAKIIYAFISNWAKLAVTLVKLKQFQGAVDAARK
ANSAKTWKEVCFACVDAEEFRLAQICGLNIIVQVDDLEEVSEYYQNRGCFNELISLMESG
LGLERAHMGIFTELGVLYARYRPEKLMEHIKLFSTRLNIPKLIRACDEQQHWKELTYLYI
QYDEFDNAATTIMNHSPDAWDHMQFKDVAVKVANVELYYKAVHFYLQEHPDLINDLLNVL
ALRLDHTRVVDIMRKAGQLHLVKPYMVAVQSNNVSAVNESLNELYVEEEDYERLRESVDM
HDNFDQIGLAQKLEKHELLEMRRIAAYIYKKAGRWKQSIALSKKDNMYKDCMETCSQSGD
RELSEDLLVYFIEQGKKECFASCLFICYDLIRADVALELAWMNNMVDFAFPYLLQFIREY
TSKVDELVKDRIESQNEVRAKEKEEKDLVAQQNMYAQLLPLALPAPPGMGGPPPPMGMPG
MPPMGGMGMPPMGPGPMPAYGMPPMGSY*
>11687.m00039|LOC_Os11g01390.1|genepair1091-1
MASSASSVFAAFDKDGDGKVSASELRGCMAAALGEEVSEEEAAAILATADTDGDGLLDHH
EFMRLSAAHQLQEPAEESLRCLREAFDMYAEEEETAVITPASLRRMLRRLGSEHQRLEME
ECRAMICRFDLNGDGVLSFDEFRVMMLMA*
>11686.m00041|LOC_Os12g01400.1|genepair1091-2
MASSASSVFAAFDKDGDGKVSASELRGCMAAALGEEVSEEEAAAILATADTDGDGLLDHH
EFMRLSAAHQLQEPAEESLRCLREAFDMYAEEEETAVITPASLRRMLRRLGSEHQRLEME
ECRAMICRFDLNGDGVLSFDEFRVMMLMA*
>11687.m00040|LOC_Os11g01400.1|genepair1092-1
MKRRKIPNRQRRSSSLGRFTCYHGGGDDDNRCNHTWYDEAARRLPLDDIPELADCLGEAG
CCLGLADPVTNIILTTILITITTCLLAFLKLYFPYVTDHQARRYLHIASYNLLLAIHLLR
HDRRHLPQTQTQTLLLPLPDGGNIKAALRIAAVQADHPAPDDPTTSSNAPTTCSPPMTSG
PSRIYSTPANGRRPTPTSCAANNATCIGGGAFARISIQIMGKYNLSSNSAEASKHPQHTA
LGSFLLSLSGDGEKLDRLRHLLRSITDGSGRVISDADWEQLNAMMIQERLMITKKIGRKR
ALPPPSAAPQDLAELSKRRNQVTNLMSYVEWRNPNPITGEVTMLTSQ*
>11686.m00042|LOC_Os12g01410.1|genepair1092-2
MKRRKIPNPQQQLGQIYVLPRRRQPHRGLHAAPWPPPPRPRRVLGPRLQHHILNALWYHT
AYPLPTHQELPQDISDTRAMSPAPSTASSPSSVPSPPPVTHYPNMRPWSTQCDLTDKLQQ
QHTVTMTKKKNPYAAAAEASKHPQHTALGSFLLSLSGDGEKLDRLRHLLRSITDGSGRVI
SDADWEQLNAMMIQERLMITKKIGRKRALPPPSAAPHDLAELSKRRSAYVKRQRFARSNL
EELLLKYCRQHPWEPRYKLDVICGLEEPKSYHWRSYHANFLASANGTNVLNGGEANPPVR
KLFFAEFWDSQSGRFNKSNSKPICSPVQNYNACFGRLCSFCDEPRTILHPPCATGSHSND
DDDADADVIPDYNVDDAIRMYGSVAPELSEGRDLVESDIIYFDHEKDAANLTQVLNDPSF
KEEDNNLGRRRKQ*
>11687.m00041|LOC_Os11g01410.1|genepair1093-1
MASAAVPEWLNKGDNAWQMLSATLVALQGFPGLALFYVGAVPRKWALTSAFMALYAMAAT
MPCWALWAHNMAFGRRLLPFVGRPAPALAQDYMLSQALLPSTLHLRSNGEVETAAVAPLY
PSASMVFFQWAFAGVTVGLVAGAVLGRMSVKAWMAFVPLWTTLSYTVGAYSIWGGGFLFH
WGVMDYSGGYVVLLAAGVSGYTAAYWVGPRRKEEDEEEMATASGGNLVVMVAGAGILWMG
WTGFNGGDPFSANTDSSVAVLNTHICATTSIVAWVCCDVAVRGRPSVVGAVQGMITGLVC
ITPRSNIKYSFLLVVISDEMPVPDLS*
>11686.m00043|LOC_Os12g01420.1|genepair1093-2
MASVAVPEWLNKGDNAWQMLSATLVALQGFPGLALFYAGAVTRKCALTSAFMALYAMAAT
MPCWALWAHNMAFGHRLLPFVGRPAPALAQHYMLTQALLPFTLHLHSNGEVETAAVAPLY
PSASMVFFQWASAGVTVGLVAGAVLGRMSVKAWMAFVPLWTTLSYTVGAYSIWGGGFLFH
WGVMDYSGGYVVHLAAGVSGYTAAYWVGPRRKEEEEMTMAGGGNLVAMVAGAGILWMGWT
GFNGGDPFSANTDSSVAVLNTHICTTTSILAWVCCDIAVRGRPSVVGAVQGMITGLVCIT
PAAGLVQGWAALLMGVASGTLPCYTMNAAMSFKVDDTLGILHTHAVSGVLGGVLTGVFAH
PTLCDMFLPVTGSRGLVYGVRAGGVQVLKQVAAALFVAAWNVAATSIILVVVRAFVPLRM
TEDELLAGDIAVHGEQAYYFSSGTNCSLSHETIEVGNS*
>11687.m00042|LOC_Os11g01420.1|genepair1094-1
MPKSKRNRPVTLSKTKKKPGLERKGKVVTDIKDAVEHYANAYVFTYDNMRNQKLKDLREQ

LKSSSRIFLAGKKVMQIALGRSSADEAKTGLHKLSKFLQGDTGLFFTNLPRDDVERLFRE
FEEHDFARTGSIVTETVELKEGPLEQFTHEMEPFLRKQGLPVRLNKGAVELVADHIVCEE
GKPISPEAAQTLRLLGMQMATFRLYLVCRWSSDDFEVYKEGLAQLRAEADDSS*
>11686.m00044|LOC_Os12g01430.1|genepair1094-2
MENGEQGSLGLTCGGEEWGCGSTAASTTGGLCRRLAHSQGSLDGGGGIGIGIGGEGSDGS
GIEGGGGGSGERERAGRANIEDDVLLNPRATTPQSNIAAATASRNSTDDEDELRIRGDAE
IQAQPPCSKLVISTKALICAPDSSAVTLSKTKKKPGLERKGKVVTDIKDAVEHYANAYVF
TYDNMRNQKLKDLREQLKSSSRIFLAGKKVMQIALGRSPADEAKTGLHKLSKFLQGDTGL
FFTNLPRDDVERLFREFEEHDFARTGSIATETVELKEGPLEQFTHEMEPFLRKQGMPVRL
NKGAVELVADHIVCEEGKPISPEAAQTLRLLGMQMATFRLYLVCRWSSDDFEVYKEGLAQ
LRAEADDSS*
>11687.m00044|LOC_Os11g01440.1|genepair1095-1
MLVRLGVVVVASVAALTLKRANSGNRDGQARKGKDKTRYSEHGEKEEEKEEVKTISGIIN
SALSDDDDMLSEIESLLSGEIDIPLPSDRFDVKERSWYNSVNSELERLRGLVRELEEREV
KLEGELLEYYGLKEQETDVVELHRQLKIKMVEIDMLKMTINSLQEERKKLQDDVARGTGA
KRELEAARNKIKELQRQIQMEANQTKGQLMLLKNQVIALKSKEEEAAIKDAEVQRKLKKL
KELEVEVVELRRKNKELLYEKRDLIVKLDAAQGKITESDVVSHAREEINKLRHVNEDLTK
QVEGLQMNRFSEVEELVYLRWVNACLRYELRNYQAPSEKISARYLNKTLSPKSRERAKLL
MLEYAGSERGQGDTDLETASSAPSSPRSEDLDNVSVDSSSSRYSFFGKRPNLMQKLKKWG
RGKDDESRLASPTQFFTSDSPKSASQKPKGPLEALMLRNAGDGVGITTFGKREQDPSDIM
DEANVASSFHLMSKTVQGFADDKYPAYKDRHKLATEREKVIKEKAEKARVQRYGGVNSSG
IVPSPRSALPPKLAQIKEKAPTANAESSDQPSDNQNNPLVVTQLKLANIEKRAPRVPRPP
PAPSATANTASALSPPPPRPPGAPPPPPPPGKPGGPPPPPPPPGSLPRNLAGGDKVHRAP
EVVEFYQSLMKREAKKDTTSLGSTTSSAFDVRSNMIGEIENRSTFLLAVKADVETQGDFV
ESLANEVRAASFVNIDDVVAFVNWLDEELSFLVDERAVLKHFDWPESKTDALREAAFEYQ
DLLKLEHKVSSFTDDPKLACEEALKKMYSLLEKVEQSVYALLRTRDMAISRYREYGLPVD
WLSGSGVVGKIKLASVQLAKKYMKRVATELDALQGTEKEPNREFLLLQGVRFAFRVHQFA
GGFDEESMKAFEELRSKMSTQTSAPQISDV*
>11686.m00045|LOC_Os12g01440.1|genepair1095-2
MNRFSEVEELVYLRWVNACLRYELRNYQAPSEKISARDLNKTLSPKSRERAKLLMLEYAG
SERGQGDTDLETASSAPSSPRSEDFDNVSVDSSSSRYSFFGKRPNLMQKLKKWGRGKDDG
SSLASPTQSFTSDSPKSASQKPKGPLEALMLRNAGDGMGITTFGKREQDPSDIMDEANVA
SSFHLMSKTVQGFADDKYPAYKDRHKLATEREKAIKEKAEKARVQRYGGVNSSGIVPSPR
SALPPKLAQIKEKAPTANAESSDQPSDNQNNPLVVTQLKLANIEKRAPRVPRPPPAPSAT
ANTASALPPPPPRPPGAPPPPPPPGKPGGPPPPPPRPGSLPRNLAGGDKVHRAPEVVEFY
QSLMKREAKKDTTSLGSTTSSVSDVRSNMIGEIENRSTFLLAVKVDVETQGDFVESLANE
VRAASFVNIDDVVAFVNWLDEELSFLVDERAVLKHFDWPESKTDALREAAFEYQDLLKLE
HKVSSFTDDPKLACEEALKKMYSLLETVEQSVYALLRTRDMAISRYREYGIPVDWLSDSG
VVGKIKLASVQLAKKYMNRVATELDALQGTEKEPNREFLLLQGVRFAFRVHQFAGGFDEE
SMKAFEELRSKMCTTQTSAPQIS*
>11687.m00046|LOC_Os11g01450.1|genepair1096-1
MWILESPLLRDAGAAVLTGATALAVLRFWEEVGNRALLDQKLCRKLVHITVGLVYFLMWP
LFSADDVYAPFLASLVIAFNIIKVTLIGLGIVKDDGVINSMTRNGDRRELLKGPLYYACA
ITLATVIFWRTSPISIAVICNLCAGDGVADIAGRRFGHVKLPYNPDKSYAGSIAMFLAGF
LASILYMCYFHLFGFVEESWSMVIAFGVTSLAAAIVESLPISTRLDDNLTVPLASVLVGV
LVFYYTGARNLCCMSADSSDISALVQNQMVLDRF*
>11686.m00049|LOC_Os12g01480.1|genepair1096-2
MESQVLRDAGAAVLTGATALAVLRFWEEVGNRALLDQKLCRKLVHITVGLVYFLMWPLFS
ADDVYAPFLASIVIAFNIIKVTLIGLGIVKDDGVINSMTRNGDPRELLKGPLYYACAMTL
ATVIFWRTSPISIAVICNLCAGDGVADIAGRQLGRIKLPYNPDKSYAGSIAMFLAGFLAS
ILYMCYFHLFGFVEESWTMVIAFGVTSLSAAIVESLPISTRLDDNLTVPLASVLIGVLVF
YYIGARNLCCMSADSSDISALVQNQMFLGRF*
>11687.m00049|LOC_Os11g01480.1|genepair1097-1
MGSAGKEEEEEDGGGGAPAAAANNYGSSTTSSTTEEEGSGESRRRTSSSSSVRPYVRSKN
PRLRWTPELHLSFVRAVDRLGGQDRATPKLVLQLMNVRGLSIGHVKSHLQMYRSKKIDES
GQVIGGESWRSDDHLQMQGGGHGGQAYNLGHLSLPALHHRSITAGSGTIFQSRFGNSWSP
WRCHGSYWLPAGHHLLVGSKPYYPPAAEAEAPFRRSSARYVARANTSNHPDFVQGSSSSP
DDNIMNHQRPVLKEMICSEGSNHQEGPLNLDLSLDICPRGEKRKRECSWRKQEEDHDHAT
VAIGADQEAESCATGLSLSLF*
>11686.m00050|LOC_Os12g01490.1|genepair1097-2
MEKTSAGKEEGSESKTAAANNDGSSTSSTTEEEESGESQRRTSSSSSVRPYIRSKNPRLR
WTPELHLSFVRAVDRLGGQDRATPKLVLQLMNVRGLSIGHVKSHLQMYRSKKIDESGQVI
GGGSWRSSDEQQYHHLQMQGGGDGGQAYNLGHLSLPAALHHRHITAGSGTILQSRVANAW
SPWRCHGSYWLRAGHHLLVGSKPYYPPPPAEARANTSSNHPDFVQGSSSSPDDHTMNHQR
PVVLKEMIYNEGSNHQGGPLNLDLSLDICPRGEKRKRELISWRKHEEDHDHTTIAIGGDQ
EAESCATGLSLSLF*
>11687.m00050|LOC_Os11g01490.1|genepair1098-1
MATASSPASVQDYPDLQEDDDDDFQDDDDLDDEDEEDDDDQEPSPSPSDEARLQSVLRRL
TAEEVRIRVHDVEIRGCCRTRRAAVEAAVGSDLPRAATVRDLVRAAAAAADRIRRLGAFD
TVSITLDAAPPGIPGNAAVIVLVDVAEARGRAAGELGIFANKGTRSCSVQGSVKLKNLFG
YCETWDASGDLGLDQTVELSTGVAIPRIGAIPTPLVARISFLSEDWLKSSLREHMMGVSV
GLLSTMNHNLAYNLSWRTITDRALMSSNSIRGQLGHSLLSSIKYAYKVDQRDSRIRPTRG
YAYLFSSQVGGLAPESKDARYIRQELDLRVALPLGVLNGALNAGVAAGIIHPLARGSTGS
ISPLSEQFYLGGNRSLMCRLGGPSSLLGFKKRGLGTDLQSSTPENSENVASTSPELSARG

GDIAVTAFADLSFDIPLKPLRELGIHGHAFVSAGNLAKLTEPDLRKFPLAEFLQTFRSSA
GFGVVVPTRLFRIEVLKKSSNVFLQVQVRQPATMFDKPPNSVIQLFKRKSHQLEELALYQ
IGIIMRKGQIICIAKEIPAEQWGQEERKAKG*
>11686.m00051|LOC_Os12g01500.1|genepair1098-2
MATASSPASVQDYPDLQEDDDDDFQDDDDDDLDDEDEEDDDQEPSPSPSDEARLESVLRR
LTAEEVRIRVHDVEIRGCCRTRRAAVEAAVGSDLPRAATVRDLVRAAAAAADRIRRLGAF
DTVSITLDAAPPGIPGNAAVIVLVDVAEARGRAAGELGIFANKGTRSCSVQGSVKLKNLF
GYCETWDASGDLGLDQTVELSTGVAIPRIGAIPTPLVARISFLSEDWLKSSLKEHMMGVS
VGLLSTMNHNLAYNLSWRTITDRALMSSNSIRGQLGHSLLSSIKYAYKVDQRDSRIRPTR
GYAYLFSSQVGGLAPESKDARYIRQEIDLRVALPLGVLNGAVNAGVAAGIIHPLARGSTG
SISPLSEQFYLGGNRSLMCRLGGPSSLLGFKKRGLGTDLRSSTPENSENVASTSPELSAR
GGDIAVTAFADLSFDIPLKPLRELGIHGHAFVSAGNLAKLTEPDLRKFPLAEFLQTFRSS
AGFGVVVPTRLFRIEVNYCHILKQFDYDLGKAGIQLNFSSP*
>11687.m00051|LOC_Os11g01500.1|genepair1099-1
MEAWTIRVVYFIVFSFFLVSARFGSCAPCSEEGRALLRYSESERDKSTDSLSNRGEGKVI
GRVLNLLLKEKMFSSETPSELKELGTISESVADGTGGFEHCRKCLAK*
>11686.m00052|LOC_Os12g01510.1|genepair1099-2
MEPWAIRVAYFIVFSFLVTARFGSCAPHSEEGRALPGYRESEQDQSIGSLSNWGEGKLIG
RVFNLLLKENMFASETPSESKEHSSISESVPHDSAGFEPCRKCLAKTVHNATPRRLLQAR
ELASNQTQTHPKSQSSPVQSSASHLVPRWAIYALPVAGVLFIAAVATAIYVFFSRRKKDN
TVMPWATGLSGQLKKAFVTGVPSLERTELEAACEGFINVIGTLPECTLYKGTLSSGVEIA
VLSTSVNSSQQWSAQSEEQFRNKISVLSRVNHKNFMNLIGYCACEEPFTRMMVFEYAPCG
SLFEHLHIREAEHLDWKTRLRIIMGVAYCLEHMSQLDPPPLLPTNLSSSSIYLTEDNAAK
IADIEFWKDDINKQDDQESVVYKFGILVLEVISGRRPFSEDDRLLVLWASSYLDGKRPLS
AMADRTLVRSSSAAPEKDVAALCDVVRQCVRRPEAGKRAISMGEVARLVRGIAGLSPEQA
APREKPLWWAELEIASSETA*
>11687.m00052|LOC_Os11g01510.1|genepair1100-1
MLTRKREELAGEVHDLHKKTRADDEPADDNHTMTTGRAPEIDEDLHSRQLAVYGRETMKR
LFASNVLVSGLNGLGAEIAKNLVLAGVKSVNLHDDDNVELWDLSSNFFLTEKDVGQNRAQ
TCVQKLQELNNAVIISTITGDLTKEQLSNFQAVVFTDISLEKAVEFDSYCHNHQPPIAFI
KSEIRGLFGSVFCDFGPEFTVLDVDGEEPHTGIVASISNDNPALVSCVDDERLEFQDGDL
VVFSEVHGMSELNDGKPRKIKNARPYSFTLEEDTTSYGTYVRGGIVTQVKPPKVLKFKTL
KDAIKEPGEFLMSDFSKFDRPPLLHLAFQALDKFRNDLRRFPIAGSSDDVQRLIDFAISI
NESLGDSKLEELDKKLLHHFASGSRAVLNPMAAMFGGIVGQEVVKACSGKFHPLYQFFYF
DSVESLPVEPLEPAELKPENTRYDAQISVFGSNLQKKLEQAKIFMVGSGALGCEFLKNLA
LMGISCNQNGKLIVTDDDVIEKSNLSRQFLFRDWNIGQPKSTVAATAAMAINPKLHVEAL
QNRASPETENVFNDAFWESLDAVVNALDNVTARMYIDSRCVYFQKPLLESGTLGAKCNTQ
MVIPHLTENYGASRDPPEKQAPMCTVHSFPHNIDHCLTWARSEFEGLLEKTPTEVNAFLS
NPGGYATVARTAGDAQARDQLERVIECLEREKCETFQDCITWARLKFEDYFSNRVKQLTY
TFPEDAMTSSGAPFWSAPKRFPRPLEFLTSDPSQLNFILAAAILRAETFGIPIPDWVKNP
AKMAEAVDKVIVPDFQPKQGVKIVTDEKATSLSSASVDDAAVIEELIAKLEAISKTLQPG
FQMKPIQFEKDDDTNYHMDVIAGFANMRARNYSIPEVDKLKAKFIAGRIIPAIATSTAMA
TGLVCLELYKVLGGGHKVEDYRNTFANLAIPLFSMAEPVPPKTIKHQDMAWTVWDRWTIT
GNITLRELLDWLKEKGLNAYSISCGTSLLYNSMFPRHKERLDKKVVDVAREVAKVEVPPY
RRHLDVVVACEDDDDNDVDIPLVSIYFR*
>11686.m00054|LOC_Os12g01520.1|genepair1100-2
MAEVVEARTIGGLSPAAFSLVLAVTVAVGLLGGALHYMLTRKREDLAGEVVHDLHKKTRA
DDEPADDNHTMTTGRAPEIDEDLHSRQLAVYGRETMKRLFASNVLVSGLNGLGAEIAKNL
VLAGVKSVTLHDDDNVELWDLSSNFFLTEKDVGQNRAQTCVQKLQELNNAVIISTITGDL
TKEQLSNFQLPQIPLLLDIWNSIKAVVFTDISLEKAVEFDSYCHNHQPPIAFIKSEIRGL
FGSVFCDFGPEFTVLDVDGEEPHTGIVASISNDNPALVSCVDDERLEFQDGDLVVFSEVH
GMSELNDGKPRKIKNARPYSFTLEEDTTSYGTYVRGGIVTQVKPPKFFYFDSVESLPVEP
LEPAELKPENTRYDAQISVFGSNLQKKLEQAKIFMVGSGALGCEFLKNLALMGISCNQNG
KLTVTDDDVIEKSNLSRQFLFRDWNIGQPKSTVAATAAMAINPKLHVEALQNRASPETEN
VFNDAFWESLDAVVNALDNVTARMYIDSRCVYFQKPLLESGTLGAKCNTQMVIPHLTENY
GASRDPPEKQAPMCTVHSFPHNIDHCLTWARSEFEGLLEKTPTEVNAFLSNPGGYATVAR
TAGDAQARDQLERVIECLEREKCETFQDCITWARLKFEDYFSNRVKQLTYTFPEDAMTSS
GAPFWSAPKRFPRPLEFLTSDPSQLNFILAAAILRAETFGIPIPDWVKNPAKMAEAVDKV
IVPDFQPKQGVKIVTDEKATSLSSASVDDAAVIEELIAKLEAISKTLQPGFQMKPIQFEK
DDDTNYHMDVIAGFANMRARNYSIPEVDKLKAKFIAGRIIPAIATSTAMATGLVCLELYK
VLGGGHKVEDYRNTFANLAIPLFSMAEPVPPKTIKHQDMAWTVWDRWTITGNITLRELLD
WLKEKGLNAYSISCGTSLLYNSMFPRHKERLDKKVVDVAREVAKVEVPPYRRHLDVVVAC
EDDDDNDVDIPLVSIYFR*
>11687.m00055|LOC_Os11g01530.1|genepair1101-1
MLPPRVAPAAAAAAPTYLAAAASTPASVWLPVPRGAGPGAVCRAAGKGKEVLSGVVFQPF
EELKGELSLVPQAKDQSLARQKFVDECEAAINEQINVEYNASYAYHSLFAYFDRDNVALK
GFAKFFKESSDEERDHAEKLIKYQNMRGGRVRLQSIVTPLTEFDHPEKGDALYGELLSAC
PIYVFYSMVASRCNDPQLTDFVESEFLEEQVEAIKKISEYVAQLRRVGKGHGVWHFDQKL
LEEEA*
>11686.m00057|LOC_Os12g01530.3|genepair1101-2
MLPPRVAPSSLAAAAAAAPTYLAAAASTPASVWLPVPRGAGAVAVCRAAGKGKEVLSGVV
FQPFEELKGELSLVPQAKDQSLARQKFVDECEAAISEQINVEFNASYAYHSLFAYFDRDN
VALKGFAKFFKESSDEERDHAEKLMKYQNMRGGRVRLQSIVTPLTEFDHPEKGDALYAME
LALALEKLVNEKLHNLHSVASRCNDPQLTDFVESEFLEEQVKNLLPTWVEAIKKISEYVA

QLRRVGKGHGVWHFDQKLLEEEA*
>11687.m00056|LOC_Os11g01540.1|genepair1102-1
MGTSTSSPSSPPPSPTAGLGTRSSAYSTPRFPPAPTSPRCSTLAHPPAVCCARPPPRHSP
PSPIWSCLAMRYEALLLRQAKYSDDLHLQVSNEEWLTFAKDSLDNGFYTIASKAFANALL
HIDPSHPGYLDSTNSILKKDKINDISGLQNLAKSLSARHSEKGLAYPEYRPVQQCLYFYF
N*
>11686.m00058|LOC_Os12g01540.1|genepair1102-2
MAAAAGDGDEHLLSLFASALSHRRFGDQELRLLDAALSAGADVPSLLHTRSSARCLLRKA
AAQAFSSVPDLGTTLSTADFFARAFALAGDVESCLAMRYEALLLRQAEYSDDLHLQVSNE
EWLTFAKDSLDNGFYTIASKAFANALVRIDPNHPEYLDSTNSILKKDKINDISGLQNLAK
SLSALRSGEHFRAMGILILMSWMGIWDPETMRVSLASSTAVSLGTFIMHGTK*
>11687.m00057|LOC_Os11g01550.1|genepair1103-1
MAGSGSGTPCASCKLLRRRCTSECVFAPYFPAEEAQRFAMVHRVFGASNVSKMLLDVPPP
QRPDAVSSLVYEANARMRDPVYGCVAAISFLQNQVSQLQMQLALAHAETAALQLQLQQQH
QDQDDHHHQQCILENAAAHHQLMLQEAFLKKESMWT*
>11686.m00059|LOC_Os12g01550.1|genepair1103-2
MAGSGSGTPCASCKLLRRRCTSECVFAPYFPAEEAQRFAMVHRVFGASNVSKMLLDVPPP
QRPDAVSSLVYEANARMRDPVYGCVAAISFLQNQVSQLQMQLALAHAETAALQLQLQQQH
QDQDDHHHQQCILENAAAHHQLMLQEAFLKKESMWT*
>11687.m00059|LOC_Os11g01570.1|genepair1104-1
MWSALFSHLREVHKRSGVKEEKLIMKSPAAAGEAAGCHKPQATATNKMTVLQSPLGLRTI
LTSLVAFFIVVSSVSLLFDRSQDAQAQLAVAQHQHQEVQLKQKPASAAVGEQKSVFVDQS
SLRSQEAQVQWTSELQDVATDSGDGGVDGEEECNWSLGRWVYDNSSRPLYSGLKCSFIFD
EVACDKYGRNDTKYQHWRWQPHGCNLPRFNATKFLEKLRNKRLVFVGDSVNRNQWVSMVC
MVEHFIPDGRKMRVYNGSLISFKAFEYNATIDFYWSPLLLESNSDNPIIHRVEYRIIRAD
RIEKHANVWKDADFIVFNSYLWWRKQRDGMTMKVMYGSFEDGDAKLDEVEMVDGYEIALK
KLTEYLGANINKNKTRIFFAGSSPAHSWASNWGGDDNNKCLNETEPIQIEDYRSATTDYG
MMDKAKEIFGTLEPKGIHVQILNITQLSEYRKDAHPTIFRRQYVPLTKEQIANPSIYADC
THWCLPGVPDVWNEFLYAYLMHK*
>11686.m00060|LOC_Os12g01560.1|genepair1104-2
MWSALFSHLREVHKRSGVKEEKLIMKSPPAAGEAGCHKPQATATNKMTVLQSPLGLRTIL
TSLVAFFIVVSSVSLLFDRGQDAQAQLAVEQHQHQEVLLKQKPASAAVGEQKSVVVDQSS
LRSQEAQVQWTSELQDVATDSGDGGFDGEEDCNWSLGRWVYDNASRPLYSGLKCSFIFDE
VACDKYGRNDTKYQHWRWQPHGCNLPRFNATKFLEKLRNKRLVFVGDSVNRNQWVSMVCM
VEHFIPDGRKMRVYNGSLISFKAFEYNATIDFYWSPLLLESNSDNPIIHRVEYRIIRADR
IEKHANVWKDADFIVFNSYLWWRKQRDGMMMKVMYGSFEDGDAKLDEVQMVDGYEIALKK
LTEYLGANINKNKTRIFFAGSSPAHSWASNWGGDDNNKCLNETEPIQIEDYRSATTDYGM
MDKAKEIFGTLEPKGIHVQILNITQLSEYRKDAHPTIFRRQYVPLTKEQIANPSIYADCT
HWCLPGVPDVWNEFLYAYIMHK*
>11687.m00061|LOC_Os11g01590.1|genepair1105-1
MERSTTTTTEQQQQQQQGRRRGAGWRRWAVLVATVWIQAVTGTNFDFSAYSSALKASLGV
SQEALNYLATASDLGKALGWSSGLALIHLPLPAVLLLSAASGLAAYALQYALILDYLHLP
YPLVFLICLVAGCSICWFNTVCFVLCIRSFSSSNRPLALSLSISFNGLSAAFYTLFANAL
SPFSPSVYLLLNAIVPLVVSLVALPAILLCHPHDGHLHVVPKHDKRIFLGLYLLAFITGI
YLVIFGSFNTTNSTAWVVLTGAMVLLALPLIIPASSSCSHVDTHDPEPTVQLNHEDSRKP
LLLNSDHSTESNAMMQKTVEHPMQDCCLGTVLEKGRMLVLCEEHSAKKLIQCVDFWLYYI
AYFCGATVGLVYSNNLGQIAQSFHRESQLTMLLAVYSSCSFFGRLLSALPDFLRRKVSFA
RTGWLAAALVPMPMAFFLMWKLHDVNTLVAGTALIGLSSGFIFAAAVSVTSELFGPNSIG
MNHNILITNIPLGSLLYGQIAALVYDANGLKMSVIDNRNGMVDTMVVCMGPKCYSTTFFV
WGCITFLGLVSSIILFLRTRTAYSAAGGQQVVNTLAKFRLDRTP*
>11686.m00061|LOC_Os12g01570.1|genepair1105-2
MERSTTTTTEQQQQQQQGRRRGAGWRRWAVLVATVWIQAVTGTNFDFSAYSSALKASLGV
SQEALNYLATASDLGKALGWSSGLALIHLPLPAVLLLSAASGLAAYALQYALILDYLHLP
YPLVFLICLVAGCSICWFNTVCFVLCIRSFSSSNRPLALSLSISFNGLSAAFYTLFANAL
SPFSPSVYLLLNAIVPLVVSLVALPAILLCHPHDGHLHVVPKHDKHIFLGLYLLAFITGI
YLVIFGSFNTTNSTAWVVLTGAMVLLALPLIIPASSSCSHVDTHDPEPTAQLNHDDSKKP
LLLNNNHSTESNAMIQKTVEQPMQDCCLGTILEKGHMLVLCEEHSAKKLIQCVDFWLYYI
AYFCGATVGLVYSNNLGQIAQSFHRESQLTMLLAVYSSCSFFGRLLSALPDFLHRKVSFA
RTGWLAAALVPMPMAFFLMWKLHDVNTLVAGTALIGLSSGFIFAAAVSVTSELFGPNSIG
MNHNILITNIPLGSLLYGQIAALVYDANGLKMSVIDNHNGMIDTMVVCMGPKCYSTTFFV
WGCITFLGLVSSIILFLRTRTAYSAANGQQVINTTAKFRVDRTP*
>11687.m00062|LOC_Os11g01600.1|genepair1106-1
MAATSPPMTSVAAAALVLTPSPALNRISFPFSRRHCRSAAPPPRWRPTRCRGKPGVTEVV
AEKETSPDGEEEEVRGRGWFMVDEIGMDILTIALPAVLALAANPITALVDTAFVGHVGST
ELAAVGVSISIFNLVCKLLNVPLLNVTTSFVAEQQAVDAAEIFSPRIGNEISIPQEKASK
QRRFLPAVSTSLALAAGIGLMEMVALILGSGTLMDIVGIPVDSAMRVPAEQFLTLRAYGA
PPVIVALAAQGAFRGFMDTKTPLFAVGVGSLVNALLDAIFIFPLGLGVSGAALATVTSEY
LTAFILLWKLNNKIVLLSWNIIGGDVVRYLKSGALLIARTIAVVLTFTLSTSLAAREGSV
PMAGYEICLQVWLTISLLNDALALAGQALLASEYAKGNYKKARVVLYRVLQIGGITGVAL
ATILFLGFGYLSLLFTDDPAVLDVAQTGVWFVTVSQPINAVAFVADGLYCGVSDFAFAAY
STVQISILVIFHCIVLFAGAVSSAVLLVAAPKFGLGGIWAGLALFMSLRAIADHKDTQHE
QESYGPIVSGGPISSKDRAGVGGRAAEAQPSRKNIRKERLRPEGDDDEGIKGFGIGIGEG
KMPCLNVSTNVNLDGVDTSAVLADASKTVATIIGKPEAYVMVVLKGSVPMAFGGTQEPAA
YGELVSIGGLNPDVNKKLSAGIASILESKLSIPKGRFYLKFYDSKRSDFGWNGTTF*

>11686.m00074|LOC_Os12g01680.1|genepair1106-2
MPCLNVSTNVNLDGVDTSAVLADASKTVATIIGKPEAYVMVVLKGSVPMAFGGTQEPAAY
GELVSIGGLNPDVNKKLSAGIASILESKLSIPKGRFYLKFYDSKVVPIYYVCGGCDDKPK
ISVLADRINQESLAAMRGHCSLDHRRLSVRHLHWRNVVYLSRLLFGASLLG*
>11687.m00063|LOC_Os11g01610.1|genepair1107-1
MAAASPPLLPTTVLPANATATVSPAPTSVSSADANPAATRAFLARLLDSVKRALSGARPW
PELIDRSALSRPESLSDAGARLRKNLAYFRVNYAAIVALSLAATLLAHPFSLAALLALLA
AWCFLYLLRPSDAPPLAAFGRTFSDRETLGGLIVASAFVVFLTSVGSLIFSALALGAAIV
CAHGAFRIPEDLFLDEPDQANGAASVNLLSFITSATGGRV*
>11686.m00075|LOC_Os12g01690.1|genepair1107-2
MAAASPPLLPTTVLPANTTATVSPAPTSVSSADAKPAATRAFLARLLDSVKRALSGARPW
PELIDRSALSHSESLSDSGARLRKNLAYFRVNYAAIVALSLAASLLAHPFSLAALLALLA
AWCFLYLLRPSDAPPLAAFGRTFSDRETLGGLIVASAFVIFLTSVGSLIFSALALGAAIV
CAHGAFRIPEDLFLDEPDQANGAASVNLLSFITSATGGRV*
>11687.m00064|LOC_Os11g01620.1|genepair1108-1
MQGPRRRTPPAPAAKQPAMLMLGPFPAHHRCTLFLLLTVTLLPSLAAAAAAHHHHVHAAG
DGVVISQADYQGLQAIKHDLSDPYAFLRSWNDTGLGACSGAWVGIKCVQGKVVAITLPWR
GLAGTLSERIGQLTQLRRLSLHDNAISGPIPTSLGFLPDLRGVYLFNNRFSGAVPASIGN
CVALQAFDASNNLLTGAIPSSLANSTKLMRLNLSHNTISGDIPPELAASPSLVFLSLSHN
KLSGHIPDTFAGSKAPSSSSLKESITGTYNLAVLELSHNSLDGPIPESLSGLQKLQVVDL
AGNRLNGTIPNKLGSLADLKTLDLSGNALTGEIPASLSNLTTSLQAFNVSNNNLSGAVPA
SLAQKFGPSAFAGNIQLCGYSASVPCPTSPSPSPSAPASPAQSREATGRHRKFTTKELAL
IIAGIVVGILLFLALCCMLLCFLTKKRSGSGGKQTTSSKAAGGGAGAAAGGGRGEKPGSG
AAEVESGGEVGGKLVHFDGPMAFTADDLLCATAEIMGKSTYGTVYKATLEDGSLVAVKRL
REKITKGHKDFESEAAVLGKIRHPNLLPLRAYYLGPKGEKLLVLDFMPNGSLSQFLHGQY
SVNATLLILCI*
>11686.m00076|LOC_Os12g01700.1|genepair1108-2
MLRPFPATAHHRCTLLLLLTVTLLPSLAAAAAAHHHHLHDHAAGDGVVISQADYQGLQAI
KHDLTDPYAFLRSWNDTGLGACSGAWVGIKCVQGKVVAITLPWRGLAGTLSERIGQLTQL
RRLSLHDNAISGPIPTSLGFLPDLRGVYLFNNRFSGAVPASIGNCVALQAFDASNNLLTG
AIPPSLANSTKLMRLNLSHNTISGDIPSELAASPSLVFLSLSHNKLSGHIPDTFAGSRAP
SSSSLKESITGTYNLAVLELSHNSLDGQIPQSLAGLQKLQVMDLSGNRLNGTIPDRLGSL
ADLKTLDLSGNALTGEIPASLSNLTTTLQAFNVSNNNLSGQVPASLAQKFGPSAFAGNIQ
LCGYSVSVPCPASPSPAPSAPASPVQGVETTGRHRKFTTKELALIIAGIVVGILLLLALC
CLLLCFLTKKRSGSGGKQTTSSKAAGGGAGGAAGGGRGEKPGSGAAEVESGGEVGGKLVH
FDGPMAFTADDLLCATAEIMGKSTYGTVYKATLEDGSLVAVKRLREKITKGHKDFESEAA
VLGKIRHPNLLPLRAYYLGPKGEKLLVLDFMPNGSLSQFLHARAPNTPISWETRMTIAKG
TARGLAFLHDDMTIVHGNLTASNVLLDDHSNPKIADFGLSRLMTTAANSNVLAAAGALGY
RAPELSKLKKASAKTDVYSLGVIILELLTGKSPAETTNGMDLPQWVASIVKEEWTSEVFD
LELMRDGDNGPAGDELVDTLKLALHCVDQSPSVRPDAREVLRQLEQIRPGPEGGAGPSDE
GGAGHVAAASAGNE*
>11687.m00065|LOC_Os11g01630.1|genepair1109-1
MMLSDPTTDCRPNWSGCIVHSGSSMLQIFSLKLVSITAPAIGDDPIQVYGFMAVRDHMDC
LRNYVFNRGRDKPFVVSLSDPFILLSGPKRGIGMETPALLEYDIRIKRGDGEDDDLQLID
RAATISETELPLPYAQAYTRWIAGDYGAMNISLALLHNAIEATMHIQITEVRGSGGFDMS
MACRVGQIPNEIKLFESVAIAKLCQLNKRFVLAIVKRGILVLDLKVKRSGALEEEEPVRM
LRGLKGKAHGQVILPMIFDCATILVLQTAVRHRLAKVQGYLKAKERSSRGNLMGKRVDFS
ALPWSIALNLTYPETVTPYNFQGAKYIIWGDAQRLDLCYVKKRTDQYLELGYNVERHLDD
GDFVLFNRQPSLHKMYIMGHRIKIMPYSTFHLNLSATSPYNADFDGDEMNMHVPQSFDGD
EMNMHE*
>11686.m00077|LOC_Os12g01710.1|genepair1109-2
MEGGSGGDSQRFGKMVVESKFRRKRRRGGTTMILAGTTDTEGMMDQEEEEEDDDDQPADV
LEDRKHQPADVLEDRKHRDGSIYRGTDYWSIYYRIADTNETPLKPMMLSDPTTDCRPNWS
GCIVHSGCSMLQIFSLKLVSMAAPAIGDGPIQVYGFMAVRDHMDCLRNYVFNRGRDKPFI
VNLSDPFILLSGPKRGFGMETPALLEYDIRIKRGDGEDDDLQLIDGAATISETELPPPYA
QAYTRRIAGNYGSVNISLALLHNAIEATMHIQITEVRGSGGFNMSMACRVGQIPDEIKLF
ESVAIAKPCQLNKRFVLAIVKRGILVLDLKVKRSGASEEEEPVCMLRGLKAKAHSQVILP
MIFDCATILVLQTAVRHRLAKVQGYLKAKKGSIRGNLMGKRVDFSARTVITPDSNITMNW
*
>11687.m00068|LOC_Os11g01660.1|genepair1110-1
MASRTAEAAAPETSRRGSRTAAAAPEPYRTDGIEDGDGDSSRDLPPQMASRMAVAVAPET
SHHGSRTEAATAALEPSRRRWRRVRRRRLPSPPMWMTPEPSRVDGVEDGGSDSSIDLSLQ
LALRTVAVAPEPSGGSRG*
>11686.m00078|LOC_Os12g01720.1|genepair1110-2
MASRTAVATALETSRRGSRTGAAAPKPSCADGIEDCDGNSSRDLPPQMASRTAVAMAVAL
ETSRHGSRTEAAVAAPEPSRHRWCRGRRRRLLSPPVWMTPEPSRTDGVEDGGSDSSIDLS
LQMASRTAAAAPEPSCGGRGNVRATGGGNPPSLAQERVRGDGAVALGSRGRRRKVC*
>11687.m00075|LOC_Os11g01730.1|genepair1111-1
MPSRGCSCWLLSLALLCSLAAAKEQYHEFVIRETTVKRLCKSHNIMTVNGQFPGPTLEIN
EGDSLIINLINRGRYNMTLHWHGVRQMRTGWSDGPEYVTQCPVRPGQSYRYRFTVAAQEG
TLWWHAHSSWLRATVYGALLIRPRDGTSYPFDVQPTRELAPILLGEWWDMNPVDVVRAAT
RTGAAPNISDALTVNAQPGDLYSCSSHDTAVFPVTSGETNLLRFINAALNTELFVSLAGH
NMTVVAADASYTKPYTTSLLLLAPGQTTDVLVTFDQPPGRYYLAARAYASAQGVPFDNTT
TTAIFDYGAANNASSAAIAMPTLPAYNDTTAATAFTTNLRGLRKAELPSRVDESLFFTVG

VGLFNCTNATAQQCGGPNGTRFAASINNVSFVLPSSTSILQAHHHGAPGGVFTADFPANP
PVQFDYTAQNVSRALWQPVAGTKVYKLKYGSAVQVVLQGTNIFAGENHPIHLHGYDFYIL
AEGLGNFDAGADTGKFNVEDPPMRNTVGVPVNGWAVIRFVADNPGVWLMHCHLDVHITWG
LAMAFLVDDGVGELQSLEAPPPDLPLC*
>11686.m00079|LOC_Os12g01730.1|genepair1111-2
MGSRGCSCWLLSLALLCSLAAAKEQYHEFVIRETTVKRLCKSQSIMTVNGQFPGPTLEIK
EGDSLIINLINRGRYNVTLHWHGVRQMRTGWSDGPEYVTQCPVRPGQSYRYRFTVAAQEG
TLWWHAHSSWLRATVYGALLIRPRDGTSYPFHVQPTRELAPILLGEWWDMNPVDVVRAAT
RTGAAPNISDALTVNAQPGDLYSCSSHDTAFFPVTSGETNLLRFINAALNTELFVSLAGH
NMTVVAADASYTKPYTTSLLLLAPGQTTDVLVTFDQPPGRYYLAARAYASAQGVPFDNTT
TTAIFDYGAANNASSAAIAMPTLPAYNDTTAATAFTTNLRGLRKAELPSRVDESLFFTVG
VGLFNCTNATAQQCGGPNGTRFAASINNVSFVLPSSTSILQAHHHGAPGGVFTADFPASP
PVQFDYTAQNVSRALWQPVPGTKVYKLKYGSAVQVVLQGTNIFAGENHPIHLHGYDFYIL
AEGLGNFDAGADTAKFNMEDPPMRNTVGVPVNGWAVIRFVADNPGVWLMHCHLDVHITWG
LAMAFLVDDGVGELQSLEAPPPDLPLC*
>11687.m00076|LOC_Os11g01740.1|genepair1112-1
MSSNSTLTDSLHERTIVFGLKLWVVIGISVGASLLGVLLILIVCLTLQTWIKRSRRTFKE
IPITQIPSASKDIKEVRAVDEFLPNDFVVHDGLLLAIQNEPVEPVDKDVNQFAQEDKTIQ
GEENSPVPLHYVDNYDVIQSVSTCEQSSSHAPVDSVLLPGLPEFSYLGWGHWFTLRDLEL
ATNCFSKDNVIGEGGYGVVYRGRLSNGTPVAVKKILNNLGQAEREFRVEVEAIGHVRHKN
LVRLLGYCVEGTQSLAYLHEAIEPKVVHRDIKASNILIDDEFNAKISDFGLAKMLGAGKS
HIATRVMGTFGYVAPEYANSGLLNEKSDVYSFGVVLLEAITGRDPIDYDRPTDEVNLVDW
LKMMVAHRRSEEVVDPNLERRPSTKELKRALLTALRCIDLNAEKRPRMDQVVRMLDSNEP
IPQEERRQRQNHISNNSETEPLRGKSSSGKSDAPENEMRPPRYKNRSFPPK*
>11686.m00080|LOC_Os12g01740.1|genepair1112-2
MTQSNKFEDLNGRFASLGTGMTHRSHLWGFTAKKEEAYSGENDERQQILQLIIEEIQPET
SELPDGRIQVHQEIDAAVAELIEQEALDELEQLVGAQLEAPADQQNPVEQHQDEDDKLSL
DNLLEQHRWEGALQRLMRMVRNGEAINGPVPDAPPQDIYVAHPEIVILLMAEEYLRLMRS
GNAAAAMRYYHDKIEIIYSGNTGSAFVNGGVLKEIQDWVNAKRDTPSGKDEATTHMENTC
QAIHDYLKLYFPAYRPQIGGKVRGHQVSKVWEFGERLGKDGGRCLACHKKVKGFNVTQLQ
NHLQGSVKRQGTQCPAINDYILILHQASNLQSASFLFFLRLDLAQVSPLPATPHGSGFRS
LFSIHYRIHFLKANFWFLTIVSNKVLPTQRVVVFLTILLNFHAFVFVAILRTFDRLASLY
VLDDPKMSSNSTLTDSLHERTIVFGLKLWVVIGISVGASLLGVLLILIVCLTIQTWIKRS
RRTFKEIPITQIPSASKDIKEVRAVEEFLPNDFVVHDGLLLAIQNEPVEPVDKDVNQFAQ
EDKTIQGEDNSSSVPLHYVDNYDGIQSVSTCEQSSSHAPADSVPLPGLPEFSYLGWGHWF
TLRDLELATNCFSKDNVIGEGGYGVVYRGRLSNGTPVAVKKILNNLGQAEREFRVEVEAI
GHVRHKNLVRLLGYCVEGTQRMLVYEYVNNGNLESWLHGELSQYSSLTWLARMKILLGTA
KALAYLHEAIEPKVVHRDIKASNILIDDEFNAKISDFGLAKMLGAGKSHIATRVMGTFGY
VAPEYANSGLLNEKSDVYSFGVVLLEAITGRDPIDYDRPPDEVNLVDWLKMMVANRRSEE
VVDPNLERRPSTKELKRALLTALRCIDLNSEKRPRMDQVVRMLDSNEPIPQEERRQRQNH
ISNNSETEPLRGKSSSGKSDAPENEMRPPRYKNRSFPPK*
>11687.m00080|LOC_Os11g01780.1|genepair1113-1
MASPMPTPTHRVKRRRLDLSPPPHLNDLADELLFLILDRAAAHDPRALKSFSLVSRACHA
AESRHRRVLRPFRPDLLPAALARYPALSRLDLSLCPRLPDAALAALPAAPSVSAVDLSRS
RGFGAAGLAALVAACPNLTDLDLSNGLDLGDAAAAEVAKARRLQRLSLSRCKRITDMGLG
CIAVGCPDLRELSLKWCIGVTHLGLDLLALKCNKLNILDLSYTMIVKKCFPAIMKLQSLQ
VLLLVGCNGIDDDALTSLDQECSKSLQVLDMSNYYNVTHVGVLSIVKAMPNLLELNLSYC
SPVTPSMSSSFEMIHKLQTLKLDGCQFMDDGLKSIGKSCVSLRELSLSKCSGVTDTDLSF
VVPRLKNLLKLDVTCCRKITDVSLAAITTSCPSLISLRMESCSLVSSKGLQLIGRRCTHL
EELDLTDTDLDDEGLKALSGCSKLSSLKIGICLRITDEGLRHVSKSCPDLRDIDLYRSGA
ISDEGVTHIAQGCPMLESINLSYCTKLTDCSLRSLSKCIKLNTLEIRGCPMVSSAGLSEI
ATGCRLLSKLDIKKCFEINDMGMIFLSQFSHNLRQINLSYCSVTDIGLISLSSICGLQNM
TIVHLAGVTPNGLIAALMVCGLRKVKLHEAFKSMVPSHMLKVVEARGCLFQWINKPYQVA
VEPCDVWKQQSQDLLVQ*
>11686.m00083|LOC_Os12g01760.2|genepair1113-2
MASPAPTHHAKRRRLALPPPPPPHLNDLADELLFLILDRAAAHDPRALKSFSLVSRACHA
AESRHRRVLRPFRPDLLPAALARYPAISHLDLSLCPRLPDAALAALPAAPFVSAVDLSRS
RGFGAAGLAALVAAFPNLTDLDLSNGLDLGDAAAAEVAKARRLQRLSLSRCKRITDMGLG
CIAVGCPDLRELSLKWCIGVTHLGLDLLALKCNKLNILDLSYTMIVKKCFPAIMKLQNLQ
VLLLVGCNGIDDDALTSLDQECSKSLQVLDMSNSYNVTHVGVLSIVKAMPNLLELNLSYC
SPVTPSMSSSFEMIHKLQKLKLDGCQFMDDGLKSIGKSCVSLRELSLSKCSGVTDTDLSF
VVPRLKNLLKLDVTCCRKITDVSLAAITTSCPSLISLRMESCSLVSSKGLQLIGRRCTHL
EELDLTDTDLDDEGLKALSGCSKLSSLKIGICLRITDEGLRHVSKSCPDLRDIDLYRSGA
ISDEGVTHIAQGCPMLESINMSYCTKLTDCSLRSLSKCIKLNTLEIRGCPMVSSAGLSEI
ATGCRLLSKLDIKKCFEINDMGMIFLSQFSHNLRQINLSYCSVTDIGLISLSSICGLQNM
TIVHLAGVTPNGLIAALMVCGLRKVKLHEAFKSMVPSHMLKVVEARGCLFQWINKPYQVA
VEPCDVWKQQSQDLLVQ*
>11687.m00081|LOC_Os11g01790.1|genepair1114-1
MGICCSKGKEELEEEGFPWKHDAFFHDQLWSAGVSMHTKQGWKGANQDAMTTCQDFAGHK
GQIFCGVFDGHGPLGREVARHVRDVLPVKLSSSLALKTEQDPSSNTDKETLEKSDCTSLS
DTSNEKQLLSTWKNIFVKTFEDVDEDLRQHSGIDCICSGTTAVTVVRQGDHLIIANLGDS
RAVLCTRDSKDRPISVQLTTDLKPNLPSEAERILNSKGRVFAMDDEPDVPRMWLPDQDAP
GLAMARAFGDFCLKSHGLICTPEVYYRKLSAKDDFLVLATDGIWDVLSNKEVIKIVSSAT
DHSKAAKQLVERAVRTWRRKFPTSMVDDCAVVCLFLKPSPSSSESTPGDAKPPQAVSFTG

SFRKVLGGGGGEAEEGTNVWRALEGVARVNSVVRLPRMGAVLSWRRRSTSLEEDDEARID
*
>11686.m00084|LOC_Os12g01770.1|genepair1114-2
MDGVPDAQRTTSPSMIKQQNYFNYPYAFNSILLSTPSFLPSFLPSYLYEVPAAEEAMGIC
CSKGKEELEEGFPWKHDAFFHDQLWSAGVSMHTKQGWKGANQDAMTTCQDFAGHKGQIFC
GVFDGHGPLGREVARHVRDVLPMKLSSSLALKTEQDPSSNTDKEALEKSDCTSLSDTSNE
KQLLSTWKNIFVKTFEDVDDDLRQNSGIDCICSGTTAVTVVRQGDHLIIANLGDSRAVLC
TRDSKDRPIPVQLTTDLKPNLPSEAERILNCKGRVFAMDDEPDVSRMWLPDQDAPGLAMA
RAFGDFCLKSHGLICTPEVYYRKLSEKDEFLVLATDGIWDVLSNKEVIKIVSSATDHSKA
AKQLVERAVRAWRRKFPTSMVDDCAVVCLFLKPSPSSEESTHVDAKAPQVVSFTGSFRKA
LGGGGGGEAEEVEKIYRRSIRTVTRDIWDKVSARLDCDHISTTHNPDETLLDWWERRTEQ
NDKDKTKGTRSIHMLLSWEIWCERNRRVFRNKELAISQLVTKILDEINVWIACGAKNLAR
IVL*
>11687.m00083|LOC_Os11g01810.1|genepair1115-1
MRIASSSGILMDANGKANGSAPSALVAYFLGMGFSREMVFRAIKEIGDTDSEQILELLLT
YQAIGSDPSVGNSSHSACDPQILEEEDEEEDVNWDEDDTVDNFDRATYSDGSGDEDFLQE
MSEKDEKIKSLVSMGFPEDEDTEFSSFGGRKKTKLIDGSKKKRERYRSRPQWNQVPFDGS
HEEPMPLPNSMVGFSLPNDGLRSVHRNLPDQALGPPFFYYENVALAPKGVWTTISRFLYD
IYPEFVYSKYFCAAARKRGYIHNLPIKNRNYTRGVSRTARYRALGNSFQVDTVAYHLSVL
RDIFPNGMNVLSLFSGIGGAEVALHRLGICMKTVVLVEISEVNMTLLRSWWDQTQTGTLI
EIADVQNLTAERIELFIRRFGGFDLVIGGSPCNNLAGSNRYHRDGLEGKHSALFYHYYRI
LDSVKTIMASIFGAKGKLFRHVRKALLLKQSSSLTLKTEQDPSNNSDKDSMDK*
>11686.m00087|LOC_Os12g01800.1|genepair1115-2
MRIASSSGILMDANGKANGSAPSALVAYFLGMGFSREMVFRAIKEIGNDNNNTFPHLLQL
LPFLSGDTDSEQILELLLTYQILEEEDEEEDVNWDEDDTVDNFDRATYSDGSGDEDFLQE
MSEKDEKIKSLVSMGFPEDEAMRAITRCGLDASVDLLVESIYAPASAGNVYFTNLSDYED
TEFSSFGGRKKTKLIDGTKKKRERYRSRPQWNQVPFDGSHEEPMPLPNPMVGFSLPNDGL
RSVHRNLPDHALGPPFFYYENVALAPKGVWTTISRFLYDIYPEFVDSKYFCAAARKRGYI
HNLPIENRSPVLPIPPKTISEAFPSTKMWWPSWDPRRQFNCLQTYVASAKHTERIRCALG
RFGDALPPAVQKSVLEECRKWNLVWVGKNKVATLEPDEMEFLLGYPRNHTRGVSRKRDIE
LLGIHSKLIQLHTTSLC*
>11687.m00084|LOC_Os11g01820.1|genepair1116-1
MAGANASTVKPVVAACYDNNLVNSQGMFLGDEPLRFALPLLLVQVSIILILSAAAHHVLR
RLGQCRFVTHMLVGIFLGPSVLGRNPHLRTALFSERGTYILESVSLVALILFLFSMAVKT
DLTLLRRPTARALAVGLAGSLVPLAVTLPVFHALSPSLPADLRGSSLITELAVRLSLSSF
PVVADALAELDLLNSELGRVALNASLITDVTSWFLRACFAAAFLITQAKSPLFTAKVLAS
FAAFVLFVFFVARPAGRYIARKRTPPGDLLSEGSFVLVVIAALLSALVTDVIGFKFMIGP
MMLGLALPGGMPIGATLTERLDSFFIALFLPVYMALAGYRTDLAELSLIGVSAEHEEKFC
ALELFVALCVAGKMVGCVAAGLFFSMPFREATVLALMLNIRGIVEVAAINNWGDTMKATA
EHYSTLTLSMVVITAVATPLIKLLYDPSGRFARAKRRTMEESRPNAELRVMACLFSEDHA
APLLDLIEASGSSRDAPVSLIVLHLTELVGHAASVLKPHRKSRSSCGNPTPSDRIVNAFR
YFEQQAPLGAVTVSPYVVASPYSSMQHDVCLLAHSRKANLILLPFHKSSDGARSTANNAI
RGINRSVMQYAPCSVGILIDHGVAAGSACATASNSTLQRVALYFLGGADDREALAYVARM
AECGLVAVTVVRLKLRDWVGMGGRDEMRDEEALQEFWQRYSCAGAERVAYVEKTVEDGEG
TASVVRAMSDKFDLLVVGRREGGGDGAEGSSAAALTSGLSEWSEFPELGVLGDMLASADF
AAKVSILVVQQQAATRNDADY*
>11686.m00089|LOC_Os12g01820.1|genepair1116-2
MAGANASTVKPVVAACYDNNLVNSQGMFLGDEPLRFALPLLLVQVSIILTLSAAAHHVLR
RLGQCRFVTHMLVGIFLGPSVLGRNPHLRTALFSERGTYILESVSLVALILFLFSMAVKT
DLTLLRRPTARALAVGLAGSLVPLAVTLPVFHALSPSLPADLRGSSLITELAVRLSLSSF
PVVADALAELDLLNSELGRVALNASLITDVTSWFLRACFAAAFLVTQAKSPLFTAKVLAS
FAAFVLFVFFVARPAGRYIARKRTPPGDLLSEGSFVLVVISALLSALVTDVIGFKFMIGP
MMLGLALPGGMPIGATLTERLDSFFIALFLPRGARGEVLRAGAVRVELFVALCVAGKMVG
CVAAGLFFSMPFREATVLALMLNIRGIVEVAAINNWGDTMKATAEHYSTLTLSMVVITAV
ATPLIKLLYDPSGRFARAKRRTMEGSRPNAELRVMACLFSEDHAAPLLDLIEASGSSRDA
PVSLIVLHLTELVGHAASVLKPHRKSRSSCGNPTPSDRIVNAFRYFEQQAPLGAVTVSPY
VVASPYSSMQHDVCLLAHSRKANLILLPFHKSSDGARSTANNAIRGINRSVMQYAPCSVG
ILIDHGVAAGSACATASNSTLQRVALYFLGGADDREALAYVARMAECGLVAVTVVRLKLR
DWVGMGGRDEMRDEEALQEFWQRYSSAGAERVAYVEKTVEDGEGTASVVRAMSDKFDLLV
VGRREGGGDGAEGSSAAALTSGLSEWSEFPELGVLGDMLASADFAAKVSILVVQQQAATR
NDDDY*
>11687.m00085|LOC_Os11g01830.1|genepair1117-1
MGNILRCFKGDDDGGDHYPYYKPTSRPHYQPPHYHGQPAAPPAPLQQQHLGPHGVTPSTV
GVAALAHDLLNFESTSMVPDGLSQHVVSSRKAQVKWYQKLLEAYKNTTPPPKTPANAAQL
IARALNMIQRADLEGILEFYNLPIPSLPTASSNYQPSLLPEGVQFVLNTLPVYDKCIGDG
DGFTAYVPTTDPRESANVPLEVHELVIARTQARKCRDYQSADALLSSLDEAGYKIISCSD
DEVLARKYRIRMRGIDAPELKMPYGKESRTALVKLIGGKSVKIYVYDLDQFGRYVGDIYC
NNLFIQEQMLKNGHAWHFKTYDKRPEFARWEREARAANRGLWASGNPEKPWDWRRDQRNA
RQDAIQVY*
>11686.m00091|LOC_Os12g01830.1|genepair1117-2
MGILEFYNFPIPSLPSASSNYQPSSLPEGVQFVLNTLPVYDKCIGDGDGFTAYVSTTDPR
ESANVPLEVHELVIARTQARKCRDYQSADALLSSLDEAGYKIISCSDDEVLARKYRIRMR
GIDAPELKMPYGRESRNALVKLIGGKSVKIYVYDLDQFGRYVGDIYCNNLFIQEQMLKNG
HAWHFKTYDKRPEFARWEREAKAANRGLWASGNPEKPWDWRRDQRNARQDAIQVY*

>11687.m00086|LOC_Os11g01840.1|genepair1118-1
MEPQEQHQEPVNDGLQKQEQHKKHKEKKKERLLDFLRAAPSKAPWFSFSGAAFLTRLASL
RTTNNPAASRRLPAFVRSVDWRALRAKCLAWAKHPMNAALLIWLAFVAGGVAFVFLLMTG
ALNSAVPAASRRRRWTEVANQMLNALFTIIDNRPDLAVNLCMALGLGFPIVAALYMVYGP
LGRKIVLIPASTDDEENLNSQVDEANAIAVTAQCDSNRNRAVVAKPEWAGGLFDVGDDPT
VAALSLSCTFCVFGWNMERLGLGNMYVHVFTFALLCAAPVLVFAVAALNVHDDTLRFVVG
AAGALLSVLGLTYGGFWRAQMRRRFGLPAHRWSMCGGRATAADYGKWLCCAPCAWRRRRL
LLAGVAPSNRLPPLTVKLLHARLLRLDLLAGLSSLLLRALTSSALHLHALRVHCLLPNPS
HLTIPIALKSASRLPHPLRAGEQLHARSLKLPSHTNPHVLTSLLSLYAKCGLLHRAQRVF
DEMPHPSTVPWTALITAYMDAGDLREAVHVARNAFANGMRPDSFTAVRVLTACARIADLA
TGETVWRAAEQEGVAQSVFVATAAVDLYVKCGEMAKAREVFDKMRHKDAVAWGAMVGGYA
SNGHPREALDLFLAMQAEGMKPDCYAVAGALSACTRLGALDLGRQAIRMVDWDEFLDNPV
LGTALIDMYAKCGSTVEAWVVFQQMRKKDIIVWNAMILGLGMTGHEKIAFALVGQMEKSG
VKLNDNTFIGLLCSCTHTGLIQDGRRYFHNMTKLYHISPRIEHYGCMVDLLSRAGLLQEA
HQLVDDMPMPANAVILGALLGGCKIHRNTELAEHVLKQLILLEPWNSGNYVMLSNIYSNR
GRWEDAAKLRLDMKAKGVEKVPACSWVEFEGKVHEFRVGDKSHPLSDQIYKKLDELGLEM
KTMGYEPTTEVVMFDVEDEEKEHTLVHHSEKLAIAFNLLITGPGETIRVTKNLRVCSDCH
TAIKLVSRITHREIIVRDNNRFHCFRDGSCSCNDYW*
>11686.m00092|LOC_Os12g01850.1|genepair1118-2
MSGDAIRRLLLAGVAPSNRPPPLTVKLLHARLLRLDLLAALSPLLLRALSSSALHLHALR
LHCLLPNPSHLTFPIALKSASRLPHPLRAGEQLHARSLKLPSHTNPHVLTSLLTLYARCG
LLHRAQRVFDEMPHPSTVSWTALITAYMDAGDLREAVHVARNAFANGMRPDSFTAVRVLT
ACARVADLATGETVWRAAEQEGIAQSVFVATAAVDLYVKCGEMAKAREVFDKMRDKDAVA
WGAMVGGYASNGHPREALDLFLAMQAEGVRPDCYAVAGALSACTRLGALDLGRQAIRMVD
WDEFLDNPVLGTALIDMYAKCGSTAEAWVVFQQMRKKDIIVWNAMILGLGMTGHEKTAFT
LIGQMEKSGVKLNDNTFIGLLCSCTHTGLIQDGRRYFHNMTKLYHISPRIEHYGCIVDLL
SRAGLLQEAHQLIDDMPMPANAVILGALLGGCKIHRNAELAEHVLTQLIRLEPWNSGNYV
MLSNIYSNRGRWEDAAKLRLDMKEKGVEKVPACSWVEFEGKVHEFRVGDKSHPLSDQIYK
KLDELGLEMKTMGYEPTTEVVMFDVEDEEKEHTLVHHSEKLAIAFNLLVTGPGETIRVTK
NLRVCSDCHTAIKLISRITHREIIVRDNNRFHCFRDGSCSCNDYW*
>11687.m00088|LOC_Os11g01860.1|genepair1119-1
MASSSSSSSHLHAHSLKLGTLAHTFNMNHLLIYYARRGLLDSALKVFDEMPQRNLVSWTA
MVSASTGNGAPHLGFRFFVSMIRSGFCPNEFSLATMLTACHSMVAHSSNKLLIALSLHGV
AVRAGLDSNPFVGSSLLLMYAKHGRIAAAQRAFAHIRNKDLTCWNAMLEGYVLNGFGHHA
IRTVLLMHHSGLAPDRYTYISAVKACSISAQWDLGRQLHCLVIHSMLESNTSVMNSLVDM
YFRARQKETAASVFRKIRQKDTVSWNTMISGFAHDEDDKAVFGCLIDMSRIGCKPNEVTF
SVLLRLSGAKENESLGLQIVALAYRHGYTDNVLVANAVINMLSRCGLLNRAYGFFCSLTS
TNIVTWNEMIAGYGLFSHSEETMKLFRSLVCFGERPDEFTYSAVLSAFQEAQGARDHEQI
HATILKQGFASCQFVSTSLIKANVAAFGSVQISLKIIEDAGKMELVSWGVVISAFLKHGL
NDEVIFLFNLFRGDSTNKPDEFILATVLNACANAALIRHCRCIHSLVLKTGHSKHFCVAS
AVVDAYAKCGEITSAESAFTVVSSGTDDAILYNTMLTAYANHGLIHEALNLYEEMTKAKL
SPTPATFVAILSACSHLGLVEQGKLVFSTMLSAYGMHPARANYACLVDLLARKGLLDEAK
GVIDAMPFQPWPAVWRSLVIGCRIHGNKQLGVLAAEQILRMAPSSDGAYISLSNVYADDG
EWQSAEETRRRMVQNHVQKLQGYSRIEM*
>11686.m00098|LOC_Os12g01910.1|genepair1119-2
MASSSSSHLHAHALKLGTLAHTFNMNYLPIYYARRGLLDSALKVFDEMPHRNLVSWTAMV
SASTRNGAPHLGFRFFVSMIRSGFCPNEFSLATMLTACHSMVAHSSNKLLIALSLHGVAV
RAGLDSNPFVGSSLLLMYAKHGRIAAAQRAFAHIRNKDLTCWNAMLEGYVSNGFGHHAIS
TVLVMHHSGLAPDRYTYISAVKACSISAQWGLGRQLHCLVIHSMLESNTSVMNSLVDMYF
RARQKETAASVFRKIRQKDTVSWNTMFSGFAHDEDDKAVFGYLIDMSRTGFKPNEVTFSV
LLRLSGAKENASLGLQIFALAYRHGYTDNVLVANAVINMLFRCGLLDRAYGFFCSLTFRN
IVTWNEIIAGYGLFSHSEDAMRLFRSLVCIGERPDEFTYSAVLSAFQEAHGARDHEQIHA
IILKQGFASCQFVSTSLIKANAAAFGSVQSSLKIIEDSGKMELVSWGAIISAFLKHGLND
EVIFLFNLFRGDSTNKPDEFILATVLNACANAALIRHCRCIHSLVLKTGHSNHFCVASAV
VDAYAKCGEITSAESAFTAVSSATNDAIMYNTMLTAYANHGLIHEALNLYEEMTKAKLNP
TPATFVAILSACSHLGLVEQGKLAFSTMLSAYGMHPARANYACLVDLLARKGLLDEAKGV
IDAMPFQPWPAVWRSLVNGCRIHGNKQLGVLAAEQILRMAPSSDGAYVSLSNVYADDGEW
QSAEETRRRMVQNNLQKVHGYVALEYANSGLLNEKSDVYSFGWFCWKLLQVNLVDWLKMM
VANRRSEEVVDPNLERRPSTKELKRALLTALRCIDLNAEKRPRMDQVVRMLDSNEPIPQE
ERRQRQNHISNNLETEPLRGKSSSGKSDAPENEMRPPRYKNRSFPPK*
>11687.m00089|LOC_Os11g01870.1|genepair1120-1
MASRLVHSSSSSSAAPSAALPNHHTNHLVDDHLPVENGPDPRRDVPDEEPPPPPPPQVAL
LPQVVVLCEQRHEGFDEAAAAAAGPSTSGPVSKWRPKDRMKTGCVALVLCLNISVDPPDV
IKISPCARKECWIDPFSMAPPKALETIGKTLHSQYERWQPKARYKLQLDPTLEEVKKLCN
TCRKFARTERVLFHYNGHGVPKPTANGEIWVFNKSYTQYIPLPITDLDSWLKTPSIYVFD
CSAAGMIVKAFLERLDWSSSSSASSSKDCILLAACEAHQTLPQSAEFPADVFTACLTTPI
KMALHWFCNRSLLRDSMEHNLIDQIPGRQNDRKTLLGELNWIFTAITDTIAWNVLPHDLF
QRLFRQDLLVASLFRNFLLAERIMRSANCSPISYPLLPPTHQHHMWDAWDMAAEICLSKL
PQLIADPNAEFQPSPFFTEQLTAFEVWLDHGSEDKKPPEQLPIVLQVLLSQSHRFRALVL
LGRFLDMGPWAVDLALSVGIFPYVLKLLQTSAMELRQILVFIWTKILSLDKSCQVDLVKD
GGHAYFIRFLDSLDAYPEQRAMAAFVLAVIVDGHRVGQEACANAGLIYVCLRHLQPENPN
DAQTEPLLLQWLCLCLGKLWEDFPEAQLLGLQSNAPEIVICLLSEPQPEVRASAVFALGN
LVDIGSPSLNGADDDSDDDEKVRAEINVVRSLLQISSDGSPLVRSEVAVALTRFAMGHNK
HIKSVAAEYWKPQTNSLLKSLPSLANINSSNVYSPSSLIQGSSGLASHIGPVLRVGSDNS

ATARDGRISTSSPIATNSIMHGSPQSDDSSQHSDSGILLRENASNGGLNYSRSRPIDNGI
YSQFIATMCNVAKDPYPRIASIGKRALSLIGVEQVSMRNSRLSNGGAHPGETSVPPSSNF
GMARSSSWFDMNSGNFSMAFRTPPVSPPQHDYLTGLRRVCSMEFRPHVLNSPDGLADPLL
SSSAAPSNMGLDILPQSLIYRWSCGHFSRPLLTGSDDNEEANARREERERIAMDCIAKCQ
RSSCKMTSQIASWDTRFELGTKASLLLPFSPIVVAADENEQIRVWNYDDALPVNTFENHK
LSDRGLSKLLLINELDDSLLLVGSSDGNVRIWRNYTQKGGQKLVTAFSSVQGYRSAGRSI
VFDWQQQSGYLYASGDMSSILVWDLDKEQLVNTIQSTADSGISALSASQVRCGQFAAGFL
DASVRIFDVRTPDRLVYTARPHAPRSEKVVGIGFQPGFDPYKIVSASQAGDIQFLDVRRA
SEPYLTIEAHRGSLMALAVHRHAPVIASGSAKQMIKVFSLEGEQLTIIRYQPSFMGQRIG
SVNCLSFHRYKSLLAAGAGDNALVSIYAEDNYQVQ*
>11686.m00099|LOC_Os12g01920.1|genepair1120-2
MALGDLMASRLVHSSSSSAAPSAALPNHHTNHLVDDHLPVENGPDPRRDVPDEEPPPPPP
PQVALLPQVVVLCEQRHEGFDEAAAAAAGPSTSGPVSKWRPKDRMKTGCVALVLCLNISV
DPPDVIKISPCARKECWIDPFSMAPPKALETIGKTLHSQYERWQPKARYKLQLDPTLEEV
KKLCNTCRKFARTERVLFHYNGHGVPKPTANGEIWVFNKSYTQYIPLPITDLDSWLKTPS
IYVFDCSAAGMIVKAFLERLDWSSSSSASSSKDCILLAACEAHQTLPQSAEFPADVFTAC
LTTPIKMALHWDAWDMAAEICLSKLPQLIADPNAEFQVLLSQSHRFRALVLLGRFLDMGP
WAVDLALSVGIFPYVLKLLQTSAMELRQILVFIWTKILSLDKFILFLKIQSCQVDLVKDG
GHAYFIRFLDSLDAYPEQRAMAAFVLAVIVDGHRIGQEACANAGLIDVCLRHLQPENPND
AQTEPLLLQWLCLCLGKLWEDFPEAQLLGLQSNAPEIVICLLSEPQPEVRASAVFALGNL
VDIGSPSLNGADDDSDDDEKISSDGSPLVRSEVAVALTRFAMGHNKHIKSVAAEYWKPQT
NSLLKSLPSLANINSSNVYSPSSLIQGSSGLASHIGPVLRVGSDNSATARDGRISTSSPI
ATNSIMHGSPQSDDSSQHSDSGILLRENASNGGLNYSRSRPIDNGIYSQFIATMCNVAKD
PYPRIASIGKRALSLIGVEQVSMRNSRLSNGGAHPGETSVPPSSNFGMARSSSWFDMNSG
NFSVAFRTPPVSPPQHDYLTGLRRVCSMEFRPHVLNSPDGLADPLLSSSAAPSNMGLYIL
PQSLIYRWSCGHFSRPLLTGSDDNEEANARREERERIAMDCIAKCQRSCDGNVRIWRNYT
QKGGQKLVTAFSSVQGYRSAGRSIVFDWQQQSGYLYASGDMSSILVWDLDKEQVNTIQST
ADSGISALSASQVRCGQFAAGFLDASVRIFDVRTPDRLVYTARPHAPRSEKVVGIGFQPG
FDPYKIVSASQAGDIQFLDVRRASEPYLTIEAHRGSLTALAVHRHAPVIASGSAKQMIKV
FSLEGEQLTIIRYQPSFMGQRIGSVNCLSFHRYKSLLAAGAGDNALVSIYAEDNYQTNQW
PLDVLDLLLEDRENAGGSLTFAEIVLGRNSNEDILAVGVENCYVFKSRLQEYAQKTGLQT
PEYHTFKEGPSHEPVFKSTVVINNTSYDSLPGFFNRKAAEQSAAEVALMEIVKSIPANAN
IPAVVHNSTQQETGLCNTGSQQLNLREMHREADHHHHLLATLARHRRLAAAATLFSSTLR
TARALNSLLAAICSSPAFLRFAPKVLLLAAPSVSPDATTFHILTSTLCQAHRPAAAADLL
CCMPSLLLDPDPASCRAVLSSLCQYASAQDAVAFLDKMCHWGISPSRSDYHAVFEALLQE
GKVVEAYEVMKNKMGSNRVAPALAYFKLTMQAFSKCLEFDSVEEVFDEMLLRGLVPDVDV
YNVYIGALCRKGDLARARQMMTCMEHAGCPPDVRTFGVVVAGCMSAGDMGTVRELVQEAT
RRGLQWDPPALSELIGLLQAGGGATQAQELLLEPLFVHDAPQETGLCKNLLQEYAQKMNY
AIPSYICTKSASGLAPFICTVEIGGIQYIGAAARTKKDAEIKAARTALLAIQGQSEGSAN
GATKYIVVPGKRVGKEVEKRPIETPKPLKVKKGGFKKKWNKRKFMKKDGQAVDVEKDEAR
VAGDAHDSDVLMQPTVITQEASCGTLFLQPCEEAKRVEAEPPRDIEMVQPDKENQHSDAA
LVQPDDEARVEQEPSRDISVVQPNEEAISGKQEPSIDAAILQPKEEASSVKQEPFIDTAM
LQACKEAGSVELGPARDTVISQLNEQDRAVKQEPAGDIVVPQPDVHARVVKE*
>11687.m00090|LOC_Os11g01880.1|genepair1121-1
MASATEAEAEERDKPIVVRVKRKPSQTRPDAFWLEINERPVKKAMLDFSSLSVSEPSSAP
NKASEEPRIKKLLVQHIETVHHSEAVQDVLHSLLHSDLDAKEIKSKTKEWNNRTNQDKKQ
DQLRSAARQRHEVCLFSYYSQLHMLKFVVVLTTMNLVDRVYEGNILWIDPKIGETGTLEE
MLALHKFGGVGKETGMKLMNHYEKYAIFMMLSKITSFEEGAILCNFLPLIREHLPSAAEE
IESDIISLAQSEDSDVYDIYTVKEVDDDTTMEGTSSAPYPLLQVDNGDDVCYDDDDPYDT
DDSNAEDNPLYDYPAELSEDEDDDSNSENPFSDLDGSDPEYEKEEDKEPTMEDLPSNLGQ
PSTSASSVDASYSANRIEDSQLFLSVPALNQAASYLAQTASYLTQCLPVSGYTAISDEGQ
ELATLPPASTAGGSSFQASSEQSADSSPGEIDNTGSSSQEITEQMAPLRVFQNGASLFQG
LVERARKTVRGSADDIGWLQQDQSLPPTEDGTARFLEILDAVRQVCCHFGCEFAFWQFSV
LTLAFLLFAGLFSNHGPLYFVKTKSYFSKMGLACHIAKIHSESSVSKNAREIKEYIEEIY
WGSKKRVLLLGHSKGGVDAAAALSLYWPQLKDKVAGLALAQSPYGGSPVASDILREGQLG
DYVRLRKLMEILVSKVLKGDLQALEDLTYERRKEFLRQNPLPPEVPIVSFHTEASITPSV
LTALSHVAHLELPAAADGNPTRIPVVMPLSAAMAACSQLLVARYGEKSDGLVTRKDAEVP
GSVVVRPERKLDHAWMVYSSLKEEPRDQADTSQGMVQQ*
>11686.m00101|LOC_Os12g01930.2|genepair1121-2
MDKEPTMEDLPSTLGQPSTSASSVDARYSADRTEDSQLFLSVPALNQAASYLAQTASYLT
QCLPVSGYTAISEEGQELATLPPASTVGGSSFQASSEQSADSSPGEIDNTGSSSQEITEQ
MAPLRVFQNGASLFQGLVERARKTVRGSANDIGWLQQDQSLPPTEDGTARFLEILDAVRK
NEHKLPDSMVYLLVPGLFSNHGPLYFVKTKSYFSKMGLACHIAKIHSESSVSKNAREIKE
YIEEIYWGSKKRVLLLGHSKGGVDAAAALSLYWPQLKDKVAGLALAQSPYGGSPVASDIL
REGQLGDYVRLRKLMEILVSKVLKGDLQALEDLTYERRKEFLRQNPLPPEVPIVSFHTEA
SITPSVLTALSHVAHLELPAAADGNPTRIPVVMPLSAAMAACSQLLVARYGEKSDGLVTR
KDAEVPGSVVVRPERKLDHAWMVYSSLNEEPRDQADTSQVCEALLTLLVEVAQKRRHEMA
MKDE*
>11687.m00091|LOC_Os11g01890.1|genepair1122-1
MAATVSLSLVVSASGFPYPFPSPLSKTLANPSSSTSLLLAASLSRGATPLPLLRRDVSAA
YGDDDMDDDFGDFDLDDGDGVGDDEDLDNEQDYDVDYDRLLAPVKAPPRPLSGEGDEEEG
DIAMVAAQSFVSTQDSASDTVVDYSVNEDEFHKIRLLHCDFFIRKVPDPDDDVFDFREMY
VTPPDTDIYSIPRVLAPMPQKYVRCMKKNFGRYHVSEPPVEHLRDPLYKTEREIMKVFLT
KHYRNRRCNDPDFFLDFEEIYVIDSKARSITRAKVVVSVPEGKKRDRRNDLLLIRDGGES

FRIIDKTKRDDATTVIQREEWAKSRQDVEKHFRKLRDFDYSNWF*
>11686.m00103|LOC_Os12g01950.1|genepair1122-2
MDDDFGDFDLDDGDGVGDDEDLDNEQDYDVDYDRLLAPVKAPPRPLSGEGDEEEEGDIAM
VAAQSFVSTQDSASDTVVDYSVNEDEFHKIRLLHCDFFIRKVPDPDDDVFDFREMYVTPP
DTDIYSIPRVLAPMPQKYLRCTKKNFGRYHVSEPPVEHLRDPLYKTEREIMKVFLTKHYR
NRRCNDPDFFLDFEEIYVIDSKARSITRAKVVVSVPEGKKRDRRNDLLLIRDGGESFRII
DKTKRDDATTVIQREEWAKSRQDVEKHFRKLRDFDYSNWF*
>11687.m00108|LOC_Os11g02060.1|genepair1123-1
MAERPCRVAAGLGAASVSVSSVELEKKIGFRINVSMEKSQPELLTSVLEAFEELGLDVLD
ADVSCADDTAFRLEALGSSQSEAAETSVDEQMVRHAVLQAIKKCIDGSSI*
>11686.m00110|LOC_Os12g02020.1|genepair1123-2
MSGKDQKKAAALEEKLELLRSVTKSSAANETSILVDASKYIKELKDKVSQEPEQLGSTSS
SMPMPRVSVSSVELEKKRGFRINVSMEKSQPELLTSVLEAFEELGLDVLDADVSCADDTA
FRFEAFGSSQSEAAERSVDEQMVRHAVLQAIKKCMDGSSI*
>11687.m00109|LOC_Os11g02070.1|genepair1124-1
MDCCVCSPMASMYRLPRNAICAACYEGAKAIIAFFNDDDDEHADADQGSVKPSRLTKLNS
TIKGLRDAWEEVKQMRCREEETKQRASFLQEGFAAAWKDGIHTDIAIRPGTGPPIQAHKA
ILATRSEVFRHILAGDDDCKAPAGDSLSLPELTHDELSHLLAFLYTGSLATCAEERHLHA
LLVAGDKYDVPFLRRACEARLAAGVEAGNVLRTLEVAELSSSAALKERAMGTVVEHAEEV
VFSPEYEEFAVRNAALCVQITRTLLANKSLPAKTP*
>11686.m00111|LOC_Os12g02030.1|genepair1124-2
MDCCVCSPMASMYRLPRNAICAACYEGAKAIIAFFNDDDDEHADADQGSVKPSRLTKLNS
TTKGLRDAWEEVKQMRCREEETKQRASFLQQGFAAAWKDGIHTDIAVRPGTGPPIQAHKA
ILATRSEVFRHILAGDDDCKAPAGDSLSLPELTHDELSHLLAFLYTGSLATCTEERHLHA
LLVAGDKYDVPFLRRACEARLAAGVEAGNVLRTLEVAELSSSAALKERAMGAVVEHAEEV
VFSPEYEEFAVRNAALCVQITRALLANKAFPAKTP*
>11687.m00110|LOC_Os11g02080.1|genepair1125-1
MERLSSSVQSWVEEHKLASIGGLWATAVGASVAYGRRKTPQMRLIHARLHAQALTLAVLG
GAALAHHYYNPSSNTNNSSSLDYDFYSQLPPATTDDGQENERWSW*
>11686.m00112|LOC_Os12g02040.1|genepair1125-2
MERLSSSVQSWVEEHKLASIGGLWATAVGASVAYGRRKTPQMRLIHARLHAQALTLAVLG
GAALAHHYYNPSSKTNNSSSLDYDFYSQLPPATTDDGQENERWSW*
>11687.m00111|LOC_Os11g02090.1|genepair1126-1
MEPAAATTTPPPSERHRSPKVKRQRSAAAQPLGDVTNLLLPSTPTNPTTGRPRPLPSDTT
AAASTCSASPSHTPVSKPSSASGETTAPNPISARFVVAIGILLTLAIAAAEERSLVKSAI
STVYTRRNTAQKRRTNDNTPFPAGTSSCPPPATLARKPLWLRVLGLFHPWFLVIDLKRRR
ARGWRIHLQFSSKLQEYINLLQSMTGILLDTLSLIMLEAIDGASCA*
>11686.m00113|LOC_Os12g02050.1|genepair1126-2
MEPAAATTTPPPSERHRSPKVKRQRSAAAQPLGDVTNLLLPSTPTNPITARPRPLPSDTT
AAASTCSASPSHTPVSKPSSATAAEERSLVKSAISTVYTRRNTTQKRRRTNDNTPFPAGT
ASCPPPATLASKNSKTSEAQGTRPISSSAPCHRSKKTKSTRMENTSSGKHMLPEDFVKKQ
RAYFEEVDAFELPEEEASETDLE*
>11687.m00112|LOC_Os11g02100.1|genepair1127-1
MTALSMKGCDGSVLLDDTPTFTGEKTAAPNNNSLRGFDVIDNIKAQVEGICPQVVSCADI
LAVAARDSVFALGGPTWVVQLGRRDSTTASLDTANNDIPAPTLDLGDLTKSFSNKGLSAT
DMIALSGAHTIGQARCVNFRNRIYSETNIDTSLATSLKSNCPNTTGDNNISPLDASTPYT
FDNFYYKNLLNKKGVLHSDQQLFNGGSADSQTTTYSSNMATFFTDFSAAIVKMGNIDPLT
GSSGQIRKNCRKVN*
>11686.m00114|LOC_Os12g02060.1|genepair1127-2
MASPKPFACSAIALLFAANLVSAQLSANFYDKSCPNALPTIRIAGCDGSVLLDDTPTFTG
EKTAAPNNNSLRGFDVIDNIKAHIEGICPQVVSCADILAVAARESVVALGGPTWVVQLGR
RDSTTASLDTANNDIPAPTFDLGDLTKSFSNKGLSATDMIALSGAHTIGQARCVNFRNRI
YSETNIDTSLATSLKSNCPNTTGDNNISPLDASTPYAFDNFYYKNLLNKKGVLHSDQQLF
NGGSADSQTTTYSSNMATFFTDFSAAMVKMGNINPITGSSGQIRKNCRKVN*
>11687.m00113|LOC_Os11g02110.1|genepair1128-1
MEAAGGQLEAALLHIMQRHHHQSLHQRNKTERVKVDAVKSAARVADLLVATVDGGVQELY
INERRIEFEARALLATIARFKKQTDQWLAATNAINSVLKEIGDFENWMKIMDFDCKSINA
AIRNIHQS*
>11686.m00115|LOC_Os12g02070.1|genepair1128-2
MEAAGGQLEAALLLIMQRHHHHSLHQRKITDREKIDAVRSAARVADLLVATVDGGAQELY
INERRIEIEARALLATIARYKKQTDQWLAATNAINSVLKEIGDYENWMKIMDFDCKSINA
AIRNIHHS*
>11687.m00115|LOC_Os11g02130.1|genepair1129-1
MAQPTSSARCSLVVMVVVVLAVAGGSSAQLSPSFYSYSCPGVFDAVKCGMQSAIANEKRI
GASIVRLFFHDCFVQGCDASLLLDDTASFTGEKMANPNNGSVRGFEVIDAIKSAVETICP
GVVSCADILAIAARDSVAILGGPSWDVKVGRRDSRTASLSGANNNIPPPTSGLANLTSLF
AAQGLSQKDMVALSGSHTIGQARCTNFRAHIYNETNIDSGFAMSRQSGCPRSSGSGDNNL
APLDLQTPTVFENNYYKNLVVKKGLLHSDQELFNGGATDALVQSYISSQSTFFADFVTGM
IKMGDITPLTGSNGEIRKNCRRIN*
>11686.m00116|LOC_Os12g02080.1|genepair1129-2
MAQPTWSARRVTAALVVMVVVVLAVAGGSWAQLSPSFYSYSCPGVFNAVKRGMQSAIARE
KRIGASIVRLFFHDCFVQGCDASLLLDDTASFTGEKTANPNNGSVRGFEVIDAIKSAVET
ICPGVVSCADILAIAARDSVAILGGPSWDVKVGRRDSRTASLSGANNNIPPPTSGLANLT
SLFAAQALSQKDMVALSGSHTIGQARCTNFRAHIYNETNIDSGFAMRRQSGCPRNSGSGD

NNLAPLDLQTPTVFENNYYKNLVVKKGLLHSDQELFNGGATDALVQSYISSQSTFFADFV
TGMIKMGDITPLTGSNGEIRKNCRRIN*
>11687.m00120|LOC_Os11g02160.1|genepair1130-1
MTTTTTRSHRLRDAAVASVVAVLPHEISPLISAASTFFFILSAYFVVLPLRDEGAISLGL
STLPGLFAGSLLLTLVAAPVASLAFSLPSIPKPRALVFIHRFFSLSLLVFFVLWFASTPG
HSPSISQSSEDASNKPPGWGNHSWFYILVRISLFLWVSLLNLIAISSTWARVIDVMDNES
GSRLFGFIGAGATLGQLFGSLFAATMAWLGPCFWLIARSSYLMYISLFLWLSAVVSSFFY
FQLHRWKDPPFTIHLHLPYFQKVTIVATTISSPTARRRTFALINSFIAVFILAGQLTLTG
RILTVAGVTVAICASPFIATLNMVALALWPTWVAVAVTETIRKVTTYVLTRPGRELLFTV
VSQDEKYKAKVCIDVVVQRLGDATAAGIYSLLFSSLEKKASMVTLYALPLCFVWLLTAFH
LGRLQTNLVKVQVASFAS*
>11686.m00118|LOC_Os12g02100.1|genepair1130-2
MTTTTTTRHRLRDAAVASVVAVLPHEISPLISAASTFFFILSAYFAVLPLRDEGAISLGL
SSLPGLFAGSLLLTLVAAPVASLAFSLPSIPKPRALVFIHRFFSLSLLVFFVLWFASTPG
HSPSISQSSEDASNKPPGWGNHSWFYILVRISLFLWVSLLNLIAISSTWARVIDIMDSES
GSRLFGFIGAGATLGQLFGSLFAATMALLGPFLLLFSSLLMELAALSSKGICTDDTHGSM
ELSSTIAEQSQNTEADDEMSSLVTSQGLPSQVSESQKTKPEIFVMFEGFWLIARSPYLMY
ISLFLWLSAVVSSFFYFQKVTIVATTISSPTARRRTFALINSFIAVFILAGQLTLTGRIL
TIAGVTVAICASPFIATLNMVALALWPTWVAVAVTETIRKVTTYVLTRPGRELLFTVVSQ
DEKYKAKVCIDVVVQRLGDATAAGIYSLLFSSLEKKASMVTLYALPLCFVWLLTAFHLGR
LQTNLVKVQVASFASSIPR*
>11687.m00121|LOC_Os11g02170.1|genepair1131-1
MGRYIILTGSTGLGSIRLTSPRITGHRHLNVRSPSAAAAVLFRNIPSPPQHSLAGVGVSA
SAPVRNDAHVVSPASDVTQSGADDDEPPLPGMPGIKVPGGVVCDDLPHGGGSAPAGGDSG
RPGTDDDDDDGGDGFGGAARGDGLAPGNPGKRWRLPGKPGKRRTRASDETKAAAFLLGLI
ASDGVAIITEATTTSKTTAARLDAGDAIIFFFA*
>11686.m00120|LOC_Os12g02110.1|genepair1131-2
MGRYIIRTGSTGLGSIRLTSPRITGHRHLNVRSPSAAAAVLFRNIPSPPQHSLAGVGVSA
SAPVRNDAHVVSPASDVTQSVGDEPPPPLPGMPGIKVPGGVVCDDLPHGGGSEPPAGGDS
GRAGTDDDDGGGDGFGGAARGDGLAPGNPGKRWRLPGKPGKRRTRASDETKAAAAFLLGL
IASDGVAIITEATTTSKTTAARLDAGDAIIFFFA*
>11687.m00122|LOC_Os11g02180.1|genepair1132-1
MAAMATAPCFPATPGLPARGAVAARSRMAAGGSRSQRRRSSSGVFLCRSSTTGSSRMEDY
NTAMKRMMRNPYEYHHDLGMNYAIISDSLIVGSQPQKPEDIDHLKDEEKVAFILCLQQDK
DIEYWGIDFQTVVNRCKELGIKHIRRPAVDFDPDSLRTQLPKAVSSLEWAISEGKGRVYV
HCTAGLGRAPAVAIAYMFWFENMDLRTAYEKLTSKRPCGPNKRAIRAATYDLAKNDPHKE
SFDSLPEHAFEGIAGSERSLIQERVRALREA*
>11686.m00121|LOC_Os12g02120.1|genepair1132-2
MAAMATAPCFPATPGLPARGAVAARSRMAAGGSRSQRRRSSSGVFLCRSSTTGSTRMEDY
NTAMKRMMRNPYEYHHDLGMNYAIISDSLIVGSQPQKPEDIDHLKDEEKVAFILCLQQDK
DIEYWGIDFQTVVNRCKELGIKHIRRPAVDFDPDSLRTQLPKAVASLEWAISEGKGRVYV
HCTAGLGRAPAVAIAYMFWFENMNLKTAYEKLTSKRPCGPNKRAIRAATYDLAKNDPHKE
SFDSLPEHAFEGIADSERRLIQERVRALREA*
>11687.m00123|LOC_Os11g02190.1|genepair1133-1
MCILCAVQRWSRRVATMLPWLVLPLILLWALSQLLPAAYRFEVTSPRLACVSVLLLTLFW
YEILLPRLSLWRARRSARLREERRAHALQLHKLRKTATRRCRNCNNPYRDQNPGGGKFMC
SYCGHVSKRPVLDLGPAGTLPTGWPCTQDWPNAAGDPAYWLDLRCSSDNLYSGFSWRLFS
SFCVSMRWFWREVLRFGSSGDGDGLGRDGKRLAKEGENGAKAEESRVEKAKRKAEEKRLA
RLEKEMLEEEERKQREEMAKLVEERRRLRDEKAEAEERSKGATPVGEKDPRKEVERRRQE
RRRKDEKDKGSSKSNSDCEDIERRVTREGERKRDSDRRNEPEKRDATRVGAEGHKPYNFD
ANNQGSKTVQSKAKYFGRMTGGLLSSSRGFGGGSFFGRSAQTSAPQVNKVTKPLVTVTDQ
SNVVKRDTQPPATAKSATAGGTTNSWTNVHRPVSPNVQSQPTGLKKSWHQLFSRSASVSP
CPDVPAAAREMNGQPEPYGAQISNAQIFLSQYPPLDSNPSSSRCMQFPGFPPVNGAPANM
SLSHFPAGHVPYYCEPEPTVFEEPEQFEDPCYDPDAIALLGPVSESLDNFPLDLDSGFIS
SDITKETHTKPSPIESPLSRSRTFEDNPIRHSTGKGPNGSILPEASNEQGGTWQMWGTPL
VQESLGLRGPQTEWLLPNANQFNHGVSHLNGGTRSSVGSGLDDNDLWLQKAPFQQMPLDT
RSLFLSHDVSENAIHNNLDFGSPNKSARLHPIGPPGHSWSKEAVVLNGPQEASKICSSTG
AHVGGGFFSTNPDVQSVWSFNQKETT*
>11686.m00122|LOC_Os12g02130.1|genepair1133-2
MLPWLVLPLILLWALSQLLPAAYRFEVTSPRLACVSVLLLTLFWYEILLPRLSLWRARRS
ARLREERRAHALQLHKLRKTATRRCRNCNNPYRDQNPGGGKFMCSYCGHVSKRPVLDLGP
AGTLPTGWPCTQDWPNAAGDPAYWLDLRCSSDNLYSGFSWRLFSSFCVSMRWFWREVLRF
GSSGDGDGLGRDGKRLAKEGENGAKAEESRVEKAKRKAEEKRLARLEKEMLEEEERKQRE
EMAKLVEERRRLRDEKAEAEERSKGATPVGEKDPRKEVERRRQERRRKDEKDKGSSKSNS
DCEDIERRVTREGERKRDSDRRNEPEKRDATRVGAEGHKPYNFDANNQGSKTVQSKAKYF
GRMTGGLLSSSRGFGGGSFFGRSAQTSAPQVNKVTKPLVTVTDQSNVVKRDAQPPATAKS
ATAGGTTNSWTNVHRSVSPNVQSQPTGLKKSWHQLFSRSASVSPCPDVPATAREMNGQPE
PYGAQISNAQIFLSQYPPLDSNPSSSRCMQFPGFPPVNGAPANMSLSHFPAGHMPYYCEP
EPTVFEEPEQFEDPCYDPDAIALLGPVSESLDNFPLDLDSGFISSDITKETHAKPSPIES
PLSRSRTFEDNPIRHSTGKGPNGSILPEASNEHGGTWQMWGTPLVQESLGLRGPQTEWLL
PNTNQFNHGASHLNGGTRSSVGSGLDDNDLWLQKAPFQQMPLDTRSLFLSHDVSENAIHN
DLDFGSPNKSARLHPIGPPGHSWSK*
>11687.m00124|LOC_Os11g02200.1|genepair1134-1
MCPLRVILIFLSATIAGFFLIRGLNADPDLHDDADASESPRERPPVPLHSKVIKPKKGEK

KPFFLGRSILFLG*
>11686.m00124|LOC_Os12g02150.1|genepair1134-2
MCPLRVILIFLSATIAGFFLIRGLNADPDLLHDDADADASESPRERAPVPLHSKVGSALK
TGFWTMVDMASGKYLWRTLVSPPTKCESEKVQ*
>11687.m00125|LOC_Os11g02210.1|genepair1135-1
MAKSYIDHDGKRPHPGALHFMDRFRNFPCPLLNLQPTPMHHRNERSLDLVTLLGDDETKI
LTADNHGHTVLFDAASYSVVHFPNLNCSKGYDAMAVSINQEPDCLYVLNLRHHPTTSNHC
FEVLSYGVFCERIPIWRSLPPPPFTTTTQTTITSYTTLSTFLPSSVALTPLTRWCLPVVP
KMINKPPGWSSTSPYATHLATIT*
>11686.m00125|LOC_Os12g02160.1|genepair1135-2
MAKTQDATKSYIDHDGKRPHPGALHFMDRFRNFPCPLLNLQPTPMHHRNERSLDCVTLLG
DDETKILTADNYGHTVLFDAASYSVVHFPKLNCSKGYDAMAVSINRAAPQEPDCLYVLNL
RTHPTTSNHCFEVLSYGGFCERIPIWRFLPPPPFTTTTQTTITSYTVVGGDTIYVSSKLC
GTHAFDTLDLISLGSGRFCVAKMFSSMMQDDEIDMEFAVLTGLQMLPPRGTKDDQQVPWM
PPAIPVSYQDEYS*
>11687.m00126|LOC_Os11g02220.1|genepair1136-1
MWDEDSLYVMSQSVDPETKDYCFEVLNYTSSCKDFRGRTPCWSSLQPPPFANYMHADITS
YTVVDSSTIYVSSMEPDATYAFDTVGRQWRRLGCWTMPFDGKAEYVPELKLWFGLSVDHP
YSLCACDLLSDAAKPPTVQQQHTWVDLDIPESWLPYNIDLINLGCGRFCVVKIFRSIAGD
CTLGFSDYDDDDTMDSDPIQGKFAVLTGLQMVGPCGKDGDDQGGVRMIKHKSMYYNFWDY
EIEWVI*
>11686.m00126|LOC_Os12g02170.1|genepair1136-2
MGRRFLNLVVENARSGLYSLRRIPANHLFYPSTRAAEEATAKSQESFNAYVKEHHGRKHP
GLHTLEMLGKLPSPMLNFEPTPWDGQRRYRNLEFASLLGNENRILFADHSGHTIVFDADS
STVFAFPNLISDKGCAAISLSIKNNNTNKNISGGMWDEDSLYVMSQSADPETKDYCFEVL
NYTSSCKDFRGRTPYWSSLQPPPFANYMHADITSYTVVDSSTIYVSSMEPDATYAFDTVG
HQWRRLGCWTMPFDGKAEYVPELKLWFGLSVNHPYSLCACDLLSDAAKPPTVQQQHTWVD
LDIPESWLPYNIDLINLGSGRFCVVKIFRHMADDCTGFSDYDDDDAMDSDLIQGKFAVLT
GLQIVRPCGKDGDDQGGVRMIKHKSMYYNFWDYEIEWVI*
>11687.m00127|LOC_Os11g02230.1|genepair1137-1
MGRRRFVNLVVQGAGGLYSLRRIPANRLFYPSTRAAEEATAKSQESFMEEHGGRKHPGLH
TMEMLEKLPRSTFAFEPAPVDRYHLRSLDFACLLGHESRMLTADNRGNTVVFDADSSSVL
AFPNLISPKRYNAISLSIINNDGSNNNNGLEPPPEDGLYVMTRSPDVHRIKDGCFEVLNY
SSSSADFREMTPHWVSLPPPPFAGCMNAEITSYTVVHGTTIYISCNKPIHSTYAFDTVSR
EWRRLGSWTMPFHGRAEYVPELNLWFGLSADHPYSLCAFDLPSDDSSVAAKPPTVQHTWV
DLVIPQSWLPWNINLINLGCGRFCIAKMFHSISGDGTFCSYSESDDGTIEDSDPIHGSFA
IFTGLHMVRPRGKHDDVQMIKHKFMYYQFFDDYIEWVI*
>11686.m00127|LOC_Os12g02180.1|genepair1137-2
MTRAARMAAVAAAVAAGDGGACGGDGWQAITTEGADASRFRWCIRFEVEAAVSLGGKLGN
DDTVEKSEPPMLFWSTLVTTSYVGLRQWFQLLGAGGLYSLRRIPANRLFYPSTRAAEEAT
AKSQESFMEEHGGRKHPGLHTMEMLEKLPRSTFAFEPAPVDRYHLRSLDFACLLGHESRM
LTADNRGNTVVFDADSSSVLAFPNLISPKRYNAISLSIINNDGSNNNNGLEPPPEDGLYV
MTRSPDVHRIKDGCFEVLNYSSSSADFREMTPHWVSLPPPPFAGCMNAEITSYTVVHGTT
IYISCNKPIHSTYAFDTVSHEWRRLGSWTMPFHGRAEYVPELNLWFGLSARHPYSLSAFD
LLSDDSSVAAKPPTVHHTWVDLDIPQSWLPWNIHLINLGCGRFCIAKCRNMNSAIIKGVA
HET*
>11687.m00128|LOC_Os11g02240.1|genepair1138-1
MESRGKILMERYELGRLLGKGTFGKVHYARNLESNQSVAIKMMDKQQILKVGLSEQIRRE
ITTMRLVAHKNIVQLHEVMATRNKIYFVMEYVKGGELFEKVAKRGKLTEVVAHKYFQQLI
SAVDYCHSRGVYHRDLKPENLLLDENENLKVSDFGLSALSESKRQDGLLHTTCGTPAYVA
PEVISKIGYDGAKSDIWSCGVILFVLVAGYLPFQGPNLMEMYRKIQHGEFRCPGWFSRKL
QKLLYKIMDPNPSTRISIQKIKESTWFRKGPEENRILKERTLNENTTKNVAPVLGVRRKK
NAHEDVKPMSVTNLNAFEIISFSKGFDLSGMFIVKEWRNEARFTSDKSASTIISKLEDVA
KALNLRVRKKDNGVVKMQGRKEGRNGVLQFDIEIFEVTTSYHIIEMKQTSGDSLEYRQLL
EEGIRPALKDIVLA*
>11686.m00129|LOC_Os12g02200.1|genepair1138-2
MESRGKILMERYELGRLLGKGTFGKVHYARNLESNQSVAIKMMDKQQVLKVGLSEQIRRE
ITTMRLVAHKNIVQLHEVMATRNKIYFVMEYVKGGELFEKVAKRGKLTEVVAHKYFQQLI
SAVDYCHSRGVYHRDLKPENLLLDENENLKVSDFGLSALSESKRQDGLLHTTCGTPAYVA
PEVISKIGYDGAKSDIWSCGVILFVLVAGYLPFQGPNLMEMYRKIQHGEFRCPGWFSRKL
QKLLYKIMDPNPSTRISIQKIKESTWFRKGPEENRILKERTLNENTTKNVALVLGVRRKK
NAHEDVKPMSVTNLNAFEIISFSKGFDLSGMFIVKEWRNEARFTSDKSASTIISKLEDVA
KALNLRVRKKDNGVVKMQGRKEGRNGVLQFDIEIFEVTTSYHIIEMKQTSGDSLEYRQLL
EEGIRPALKDIVLAWHGDE*
>11687.m00129|LOC_Os11g02250.1|genepair1139-1
MLFLKELPIWQAFGFSLLTGALAGSTLYAVCVVLLCCVDRQRRHAGAPPPDPKIWLPDHT
HHRRQRDESSECSICLGELEEGERCCTLVACRHEFHKECIYRWLANHNTCPLCRHMPLPP
ASSPPPAPPPHHASPPSNVPPAAHV*
>11686.m00130|LOC_Os12g02210.1|genepair1139-2
MLFLKELPIWQAFGFSLFTGALAGSILYAVCVLLLCCVDRRRRHAGAPPPDPKIWLPDHA
HHRRRRDESSEMECSICLGELEEGERCCTLAACRHEFHKECIYRWLANRNTCPLCRHISL
PPASSPPPAPPPHHASPPPSNVLAAAHV*
>11687.m00130|LOC_Os11g02260.1|genepair1140-1
MDAEDFMDSWLMWGNIFFFFLALAITVEVDLIRLRRNDDGNNKSAHQYDMLIERLLLLRP

KDDQDNEQCVICLSENEDDVDGGGGERGRGRMLPGCAHAFHKDCVVKWLRNRTTCPLCRS
DVAVAAAAAADNMV*
>11686.m00131|LOC_Os12g02220.1|genepair1140-2
MDAEDIVDCLMWGIIFFFLLACIGVALCFLALTIATVVGLIRRRNDDANNKYDMLIERLL
LRPKDDQDNEQCVICLSESEDDVDDGGGERGRWRMLPGCAHAFHKDCVVKWLRNRTTCPL
CRSDVAVAAADDIISTADNMV*
>11687.m00131|LOC_Os11g02270.1|genepair1141-1
MESRTPRSSVARSTCNVPGLVLGFSKLCKITKICAAPEFADTKTEFGDYCGGYDQRLIIT
RLFEEIGALKSAYIKLQKAHIPYNPPKIAFADEIITSELDSVTALQSLCSWNGSVGSLIN
DRWSLVQELEAETRKKDSDIMLLRRELDGLKSANSRLNKQISSSKPSVNHHKDYSVVLKK
LTTPSAVLELFKVASTSVHDFAELIFSLISSSDHHCTNNADEHSPYKRYSLEAYLSRTML
AVHDGAEDDDELDLARFDRIMRCCDPLDALMAHPNSSFARFCRTKYLAAVPSEMEAAMFR
NNLDVRAFVSRGGHLRTWFYRAFATMARSAWALQVAVTAHRRCCGRGSVRMLYARRGSRY
AAEYMDSVVAAAAADAGRGGGDGVAFTVTPGMKVGETMVACRVFLCHEQQDTISDETDPK
FR*
>11686.m00132|LOC_Os12g02230.1|genepair1141-2
MESRTPRSSVARSTCNVPGLVLGFSKLCKITKICAAPEFADTKTEFGDYCGGYDQRLVIT
RLFEEIGALKSTYIKLQKAHIPYNRPKIAFADEIITYELDSVTALQSLCSWNGSVGSLIN
DRWSLVQELEAETRKKDSDIMLLRRELDGLKSANSRLNKQISSSKPSVNHHKDYSIVLKK
LTTPSAVLELFKVASTSVHDFAELIFSLISSPDHRCPNNADEHSPYKRYSLEAYLSRTML
AVHDGAEDDDELDLARFDRIMRCCDPLDALMEHPNSSFARFCRTKYLAAVSSEMEAAMFR
NNLDVRAFVSRGGHPRTWFYRAFATMARSAWALRVAVTARRRCCGRGSVRMLYARRGSRY
AAEYMDSVVAAAAAADAGRGEGDGVAFTVTPGMKVGETMVACRVLLCHDQHDTISDETDP
KFR*
>11687.m00133|LOC_Os11g02290.1|genepair1142-1
MATLLLSSNKKPCSSSSSLLLLVLLLLLVFFAHHGSCSRPLLLPSPTTPMQPQLKHESET
ASADTTTRTEEQVVQQQQLSWLRSMKPRGRPQPSSPSKRTN*
>11686.m00133|LOC_Os12g02240.1|genepair1142-2
MATLLLSSNKKPCSSSSSLLLLVLLLLLVFFAHHGSCSRPLPLPSPTPMQPQLKHESETA
SADTTTTTEEQVMQQQLSWLRSMKPRGRPQPSSPSKRTN*
>11687.m00135|LOC_Os11g02300.2|genepair1143-1
MSGARRCGGRTSERSSVVGDNRNGYVETDPTGRYGRLSEVLGKGAMKTVYRGFDELRGVE
VAWNQATISDVLRTPDALHRMYAEVSLLADLRHDAIIAFHASWVHPSRRTFNFITELFSS
GTLRSYRLRYPRVSRRAVAAWARAILRGLAYLHARGVIHRDLKCDNIFVNGHLGQVKIGD
LGLAAVLRGCASARSVIGTPEFMAPEMYDECYGVGVDVYSFGMCMLEMLTNEYPYSECDN
PAQIYKKVTAGKLPDAFYRLTDADARRFIGRCLVDAAHRPSAEELLLDPFLSPSQNHDDH
NIIAHATAPPPPLPLACSNSSEEQEEAAPAPAAKTTDMAITGKLNKEHDTIFLKVQIGGG
RNVRNIYFPFDVANDTAMEVATEMVKELDIADREPTEIAAMIEQEIVRLVPGYKQHEYSY
ADDNDDDDVSGHPNPFYYLSSSPTSSQGSLCGVGSTSSEGFPGPHGKVDWSRDYCYYPPS
SVSVSDDDDSSTSSLSAAVSASSLHQQQQHCSASSSRLGPASASASEDGGGGHAGRPRQR
EGEEERRRRRMSRNRSMVDMRSQLLHRTLVEELNKRLFFNTVGAVHDIGFRDPTAAASSS
SSSSHHRRRSSNKIDHKHHYMF*
>11686.m00134|LOC_Os12g02250.1|genepair1143-2
MSGARRCGDRRSERSSVVGDNRNGYVETDPTGRYGRLSEVLGKGAMKTVYRGFDELRGVE
VAWNQATISDVLRTPDALHRMYAEVSLLADLRHDAIIAFHASWVHPSRRTFNFITELFSS
GTLRSYRLRYPRVSRRAVAAWARAILRGLAYLHSRGVIHRDLKCDNIFVNGHLGQVKIGD
LGLAAVLRGCTSARSVIGTPEFMAPEMYDECYGVGVDVYSFGMCMLEMLTNEYPYSECDN
PAQIYKKVTAGKLPDAFYLLTDADARRFIGRCLVDAAHRPSAEELLLDPFLSPPQNHDDH
NTIAHATAPPPPLPLACSNSSEEQEEEEAPAAKTTGMAITGKLNKEHDTIFLKVQIGGGG
NVRNIYFPFDVANDTAMEVATEMVKELDIADREPTEIAAMIEQEIVRLVPGYKQHEYSYA
DDDDDDDVNGQPNPFYYLSSSPTSSQGSLCGVGPTSSEGFPGPHGKVDWSRDYCYYPPSS
VSVSDDDDSSTSSLSAAVSAISLQQQHCSASSSRLGPASASASEDGGGHAGRPRQREGEE
ERRRRRMSRNRSMVDMRSQLLHRTLVEELNKRLFFNTVGAVHDIGFRDPTTYGSSSSSSS
SQHRRRSSSKVDHKHHYMF*
>11687.m00136|LOC_Os11g02320.1|genepair1144-1
MVDNQGCSPALEPVPTPPNPDPSSISPEAWDPLEAAAGAVVARIQPNPPSEDRRAAVIAY
VQHLLRCTVGCQVFPFGSVPLKTYLPDGDIDLTAFGHSSDEILAKQVQAVLESEEARKDA
EFEVKDVQYIHAEVKLVKCIVQNIIVDISFNQFGGLCTLCFLEKVDQKFEKYHLFKRSIM
LIKAWCYYESRILGAHHGLISTYALEILVLYIFHLFHGTLDGPLAVLYRFLDYYSKFDWD
NKGISLYGPISLSSLPELVTDSPDTVNDDFTMREDFLKECAQWFTVLPRNSEKNTQVFPR
KFFNIVDPLKQSNNLGRSVSKGNFLRIRSAFDFGARKLGKIIQVPDNFTMDEVNQFFRNT
LKRHCSRVRPDVQEIALDFNGERADNDSSPLYSNNSFGDLSDEFNNISISDSSNHGSLRQ
NGWNYVAENKERKSVSGGLLASKATNPAATNSTGMTNGSDSCEPASPSITGAHSLPSEEG
HDALDLFNESESGTKAGIKYGTNPSHHGMSTVSYAGRSHQSFEEVDNDDRGTIDSNWSDL
TGDYTTNFNNLLYAQGFHQDYPMNQYYPFGPVYYPIPSPPPARYQNRRSSNGHSRNNVYG
YAGTNGIGPAPCPPGYLIMRPYSQIDDSNRARGTGTYFPNPNLSKDRSPSGRGGRGKTHF
LPHNHQRPHHYGRADMSADLTPSEELRHIYDPDANDLGIPSSLRISIPSPSSEAPREIVH
GNGFIQPPAKKLEFGTLGALPLEVTSQELGINRLNTASDSQPSASASPMSLANNPGISSN
QMRNAQPYHLKDNGDFPPLSS*
>11686.m00135|LOC_Os12g02260.1|genepair1144-2
MLIKAWCYYESRILGAHHGLISTYALEILVLYIFHLFHGTLDGPLAVLYRFLDYYSKFDW
DNKGISLYGPISLSSLPELVTDSPDTVNDDFTMREDFLKECAQWFTVLPRNSEKNTQVFP
RKFFNIVDPLKQSNNLGRSVSKGNFLRIRSAFDFGARKLGKILQVPDNFTVDEVNQFFRN
TLKRHCSRVRPDVQEIALDFNGERADNDSSPLYSNNSFGDLSDEFNNISISDSSNHGSLR

QNGWNYVAENKERKSVSGGWLASKATNPAATNSTGMTNGSDSCEPASPSITGAHSLPSEE
GHDALDLFNESESGTKAGIKYDTNPSHHGMSTVSYAGRSHQSFEEVDNDDRGTIDSNWSD
LTGDYTTNFNNLLYAQGFHQDYPMNQYYPFGPVYYQMPSPPPARYQNRRSSNGHSRNNVY
GYAGTNGIGPAPCPPGYLIMRTYSQIDDSHRARGTGTYFPNPNLSKDRSPSGRGGRGKTH
FLSHNHQRPHHYGRADMSADLTPSEELRHIYDPDANDLGIPSSLHISIPSPSSESPREIV
HGNGFIQPPAKKLEFGTLGALPLEVTSQELGINRLNTASDSQPSASASPMSLAHNPGISS
NQMRNAQPYHLKDNGDFPPLSS*
>11687.m00138|LOC_Os11g02310.1|genepair1145-1
MRVLLLVSDDGLLDFARLLGGGRKTKRKRRRRCLCLRLQATVSSFPPATIRWPRGLFLSP
LTKPRPTRRAHQCVPHPRPRLPPTTASCAAGGHQLHALLAKLGLLHHPEFLSALLSRIPP
SPSALSLLLEASPAVLSPSLVCPVIVAFSSSPAPSSALILFNHASSCSLPTPLPTFPALL
KSCARAFNRSSRAGVASVFVSKGMELHCRVLKLGCGKDRYVRNALVSMYGKFGRLGDARK
AFDEMPDKNAVSWNALVGAHRAAADWMGAERVSQAMPERNLSWWNAEIARNVSIGYMDEA
SRLFREMPQRDVVSLNSLISGYTKLGKYTKALEIFQEMKENAIEPTELTLVLILGACAKD
GKLELGTDIHINLQSKGIVSDGLVGNALIDMYAKCGRLDLAKKVFDRMSMRDITCWNAMI
IGFSVHGCSYEALELFDSMKIEPNPVTFLGVLTACSHGGLVNEGRKYFNSMIEDYRIVPD
VKHYGCMIDMLCRYGRIEEAYLMIKENPSTANSVLWKMLLAACRVHGHIDLAYKFFH*
>11686.m00137|LOC_Os12g02280.1|genepair1145-2
MTASYLRLLRRFPRRSAAGGHQLHALLAKLGLLHRSEFLSALLSRLPPSPSALSLLLEAP
PAVLSPSLVCPVIVAFSSSQAPSSALLLFNHASSCSLPTPLPTFPALLKSCARAFNHSSR
ASAASVFVSKGMELHCRVLKLGCGKDRYVRNALVSMYGKFGRLGDARKAFDEMPDKNAVS
WNALVGAHRAAADWMGADRVSQAMPVRNLSWWNAEIARNVRIGYMDEAARIFSEMPERDA
VSWNSLISGYTKLGKYTQALGIFQEMQENGIQPTELTLVLVLGACAKIGKLDLGTNIHRN
LQNKGIVADGLVGNALIDMYAKCGMLDLAKKVFDRMSMRDITCWNAMIVGFSVHGCSREA
LELFDSMKIEPNPVTFLGVLTACSHGGLVNEGRKYFNSMIEDYRIVPDVKHYGCMIDMLC
RYGKIEEAYLMIKENPSTASSVLWKMLLAACRVHGHIDLAYMFFHELRELILTDNGGLVT
ISNVYAEAKRWDDVEHLRMKVRCNSALKHAAHSQMDVM*
>11687.m00142|LOC_Os11g02350.1|genepair1146-1
MAGARRTMALVALVAVVAAAVVAERASAAVSCGDVTSSIAPCLSYVMGRESSPSSSCCSG
VRTLNGKASSSADRRTACSCLKNMASSFRNLNMGNAASIPSKCGVSVAFPISTSVDCSKI
N*
>11686.m00139|LOC_Os12g02300.1|genepair1146-2
MAGARRTMALVALVAVVAAAVVAERASAAVSCGDVTSSIAPCLSYVMGRESSPSSSCCSG
VRTLNGKASSSADRRTACSCLKNMASSFRNLNMGNAASIPSKCGVSVAFPISTSVDCSKI
N*
>11687.m00144|LOC_Os11g02370.1|genepair1147-1
MARAQLVLVALVAAALLLAGPHTTMAAISCGQVNSAVSPCLSYARGGSGPSAACCSGVRS
LNSAASTTADRRTACNCLKNVAGSISGLNAGNAASIPSKCGVSIPYTISPSIDCSSVN*
>11686.m00140|LOC_Os12g02310.1|genepair1147-2
MARAQLVLVALVAALLLAGPHTTMAAISCGQVNSAVSPCLSYARGGSGPSAACCSGVRSL
NSAATTTADRRTACNCLKNVAGSISGLNAGNAASIPSKCGVSIPYTISPSIDCSSVN*
>11687.m00146|LOC_Os11g02390.1|genepair1148-1
MARAQLVLVAVVAALLLAAPHAAVAITCGQVNSAVGPCLTYARGGAGPSAACCSGVRSLK
AAASSTADRRTACNCLKNAARGIKGLNAGNAASIPSKCGVSVPYTISASIDCSRVS*
>11686.m00141|LOC_Os12g02320.1|genepair1148-2
MARAQLVLVALVAALLLAAPHAAVAITCGQVNSAVGPCLTYARGGAGPSAACCSGVRSLK
AAASTTADRRTACNCLKNAARGIKGLNAGNAASIPSKCGVSVPYTISASIDCSRVS*
>11687.m00147|LOC_Os11g02400.1|genepair1149-1
MAALNGKVVVAVMVVAMVVAAPGASAAITCGQVGSAIAPCISYVTGRGGLTQGCCNGVKG
LNNAARTTADRQAACRCLKTLAGTIKSLNLGAAAGIPGKCGVNVGFPISLSTDCSKVS*
>11686.m00142|LOC_Os12g02330.1|genepair1149-2
MAALNFKAVAAVMVMAMVVAAPGASAAITCGQVGSAIAPCISYVTGRGGLTQGCCNGVKG
LNNAARTTADRQAACRCLKTLAGTIKSLNLGAAAGIPGKCGVNVGFPISLSTDCSKVS*
>11687.m00149|LOC_Os11g02420.1|genepair1150-1
MAAVNCKVVVAVIVVAMVVAAPGASAAITCGQVGSAIAPCISYVTGRSGLTQGCCNGVKG
LNNAARTTADRQAACRCLKSLAGSIKSLNLGTVAGVPGKCGVNVGFPISLSTDCNKVS*
>11686.m00143|LOC_Os12g02340.1|genepair1150-2
MAALNGKVVVAVMVVAMVVAAPGASAAITCGQVGSAIAPCISYVTGRSGLTQGCCNGVKG
LNNAARTTADRQAACRCLKSLAGSIKSLNLGTVAGVPGKCGVNVGFPISLSTDCNKVS*
>11687.m00150|LOC_Os11g02430.1|genepair1151-1
MRRLGDVVEAPALVLTPASMQQAGGRGSSGALDASMVVILAALLCVVICALGLTSLIRCA
LHCARGLSPTTATPTPSVSTAATAGLKKTELRRIPVEVYGAKQAGVPDGECAICLGDFAD
GDKVRVLPRCHHGFHVRCIDTWLAAHTSCPTCRDSILSVHGVVAGGQT*
>11686.m00145|LOC_Os12g02350.1|genepair1151-2
MRRLVDVVEAPALVLTPAWAATASMQQAGGSSGALDANMVIVLAALLCVVICSLGLSSLI
RCALHCARGLSPSPAMATPAAATTTGGLKKKELRRITVEVYGAKQAGVPDAECAICLGDF
ADGDKVRVLPRCHHGFHVGCIDTWLAAHTSCPTCRDSILSVHAGVTGGQT*
>11687.m00151|LOC_Os11g02440.1|genepair1152-1
MYCAVGTEIATVEVEGIPFPQEITVSKPLSLLANGITDIEIHFLQIKYNAIGVYLEKDNV
LAHLESWKGKKAEELVQDDGFFQALVSAPVEKLLRIVVIKEIKGSQYGVQLESSVRDRLV
SVDKYEEDEEEALEKVTEFFQSKYFKPNSVITFHFPTTPGIAEISFVTEGKGEAKLTVEN
KNVAEMIQKWYLGGESAVSPTTVKSLADQFAALLSA*
>11686.m00146|LOC_Os12g02370.1|genepair1152-2
MGTEMATVEVEGIPFPQEITVSKPLSLLAHGITDIEIHFLQIKYNAIGVYLEKDNVLGHL

ESWKGKKAEELVQDDGFFQALVSAPVEKLFRIVVIKEIKGSQYGVQLESSVRDRLVSVDK
YEDEEEESLEKVTEFFQSKYFKPNSVLTFHFPNTPGIAEISFVTEGKGEAKLTVENKNVA
EMIQKWYLGGESAVSPTTVKSLADQFAALLSA*
>11687.m00152|LOC_Os11g02450.1|genepair1153-1
MASLRLRHLSPSGATAPPSSFAQLRRPSSCLAGPRPLRSRLTRVYALSSNDIRVGSNLEV
DGAPWKVIEFLHVKPGKGAAFVRTKMRNYITGNTVDKTFRAGSTIPEASISKETKQFTYK
DGAQFVFMDLTTFEESRLNESDVGDRQKWLKEGMDCNLLYWNGRIIDFDLPITVRLTVTD
TDPGQGDSAQGGTKPATVETGAVVTVPSFVNVGDDILIDSRTGQYMNRA*
>11686.m00149|LOC_Os12g02380.1|genepair1153-2
MAFERSWPLVIFVSVHENDRNIAMETEDVEGPSHVGDFVEPSGEDEGNDTGVVQAMGVAD
EGERISTIVDEMKREDSDNEQAEGDASSDEEGDVMPTDWTNEDFSGLVISEEHHADTARA
VNADITSIIVRLDAGLYLSDAEQRITFDRMQEKMHAVMRVFSCCSAMDAVPPPGPVPPCP
RAPSTGVVRPTAGSSHGTAPPRGPRLPSGAFVGGSSSSQRSLIPRPNTQTRGIFASGASS
SHAGGTGPTVRFYDDDLHGAPDQEILGSSQLGGAPKAHTQEQPLTYRTLCNYIEWVDTEN
PQNDRTRPYARSESRSDYLRRKDEHERQIAAEALEWQVNPLGLPTWRERPECRCGDRCQV
IRAIQGGAHSLWIDNVNPTYDGQKITESETQDATSAKLGKKNYRGSSGVYALSSNDIRVG
SNLEVDGAPWKVLEFLHVKPGKGAAFVRTKMRNYITGNTVDKTFRAGSTIPEASISKETK
QFTYKDGAQFVFMDLTTFEESRLNESDVGDRQKWLKEGMDCNLLYWNGRIIDFDLPITVR
LTVTDTDPGQGDSAQGGTKPATVETGAVVTVPSFVNVGDDILIDSRTGQYMNRA*
>11687.m00153|LOC_Os11g02460.1|genepair1154-1
MALRSPNSMLWLALLVWAALLCGSCHGRFVVEKNSLKVTSPSDMKGTYECAIGNFGVPQY
GGTMVGVVAYPKANKKACKSFDDFDISYKAKPGSLPTFLLVDRGDCFFTKKAWNAQNAGA
AAILVADDKTEPLITMDTPEESGNTDYLENITIPSALITKSFGDKLKKAIDNGDMVNVNL
DWRESLPHPDERVEYEFWTNSNDECGPKCDSQIEFVKSFKGAAQVLEKKGYTQFTPHYIT
WYCPDSFILSKQCKSQCINHGRYCAPDPEQDFSKGYDGKDVVVQNLRQVCVYKVAKEHGK
PWLWWDYVTDFAIRCPMKEKKYTKECADGVIKSLGLDHKAIDKCIGDPDADKENPVLKAE
QDAQIGKGSRGDVTILPTLVINNRQYRGKLDKGAVLKAICAGFRETTEPAVCLSEDIQTN
ECLENNGGCWQDKAANISACKDTFRGRVCECPVVKGVKFVGDGYTHCEASGSGRCEINNG
GCWKDSRHGRTYSACTNDGCKCPDGFKGDGVHKCEDIDECKERTACQCKECKCKNTWGSY
ECGCSGGLLYMKEHDTCISKNAATEVGWNFLWVIFFGLVVAGIAGYAVYKYRIRFCLVLA
EKEKSGIPNINWLRDRLFRSIRRRRRRGALAPLAPTLTTAIAMPSCFTLDSASDDGRSTA
QGQGWPAGVGGFLSGFFSAGAARADGGKPSPDWDAHGLAASALPVPLSRLDGKKRYKVSD
LTFLNCRTRAAAAAAAEAPLFDALRPGGVYTRAQLRDELDALATSGMFDHVTLQTKPKPD
GTLGLTVSYAETEWPAVEHFKCINVGGPMARPDGDELELDDDMTARERMEHLRRQEREYQ
QLVRRAKPCVLPEKLQRELQGMVKRQRKVSSGLLKRMAGRIERWYHDEGFHCAQVVSYHG
NLDAGEVVCEVVEGDITKVEYQFLDKLGNVVDGNTSIPLIDRELPQQLRPGHIYNNGAGK
QALKNIDSLGLFSTIEVQPRPDETNQGGVIVAIKLKEHDPKSAQVITDWSIVPGSQGRPT
LASIQPGGTVSFEHRNICGLKRSLIGSVTSSNLLNPEDDLSFKLEYAHPYLDGIDNLSRN
RTFKISCFNSRKLSPIFVAGPNMYEAPPIWVDRIGFKANITESFTKQSKFTYGLIVEEIT
TRDENNNICTHGSRQLPSGALSMIGPPTTLSGTGVDRMAFLQANITRDNTEFVNGATIGD
RCIFQMDQGLGIGSKNPFFNRHQLTVTKFINLNKQEKGSRKPPPAVLALHGRYAGCVGDL
PSYDAFALGGPHSVRGYGMGELGASRNLLEVATELSVPITVKNRHTQVYAFAEHGTDLGS
SKDVEGNPTEFFRRVGHGSSYGVGVKLGAVRAEYAVDHNAGTGAFFLRFGERF*
>11686.m00150|LOC_Os12g02390.1|genepair1154-2
MGLRSPNSMLWLVLLVWAALLCGSCHGRFVVEKNSLKVTSPSDMKGTYECAIGNFGVPQY
GGTMVGVVAYPKANKKACKSFDDFDISYKAKPGSLPTFLLVDRGDCFFTKKAWNAQNAGA
AAILVADDKTEPLITMDTPEESGNTDYLENITIPSALITKSFGDKLKKAIDNGDMVNVNL
DWRESLPHPDERVEYEFWTNSNDECGPKCDSQIDFVKSFKGAAQVLEKKGYTQFTPHYIT
WYCPDSFILSKQCKSQCINHGRYCAPDPEQDFSKGYDGKDVVVQNLRQVCVYKVAKEHGK
PWLWWDYVTDFAIRCPMKEKKYTKECADGVIKSLGLDHKAIDKCIADPDADKENPVLKAE
QDAQIGKGSRGDVTILPTLVINNRQYRGKLDKGAVLKAICAGFRETTEPAVCLSEDIQTN
ECLENNGGCWQDKAANISACKDTFRGRVCECPVVKGVKFVGDGYTHCEASGSGHCEINNG
GCWKDSRHGRTYSACTNDGCKCPDGFKGDGVHKCEDIDECKERTACQCKECKCKNTWGSY
ECGCSGGLLYMKEHDTCISKNAATEVGWNFLWVIFFGLVVAGIAGYAVYKYRIRKNQAFH
TCWWKRMPFTSINWLRDRLFTSIRRRRRRALAPLAPTAIAMPSCFTLDSASDDGRSTAQG
QGWCAGVGGFLSSFFSAGAARADGGKPSPDWDAHGLAASALPVPLSRLDGKKRYKVSELT
FLDRRTRAAAEDPLFDALRPGGVYTRAQLRDELDALAASGMFDHVTFQTKPKPDGTLGPH
RLLRRDPVARGRTPQLEPDDDMTARERMEHLRRQEREYRQLVRRAKPCVLPEKLQRELQG
MVKKQRKVSSGLLKRMAGRIERWYHDEGFQCAQVVGYNGNLDAGEVLRPGHIYNNGAGKQ
ALKNIDSLGLFSTVEVEPRPDETNQGGVIVAIKLKEHDPKSAQVITDWSIVSGSQGTPTL
ASIQPGGTVSFEHHNICGLKRSLIGSVTTSNLLNPEDDLSFKLEYAHPYLDGVDNRSRNR
TFKISCFNSRKLSPIFVAGPNMYEAPPIWVDRIGFKANITESFTKQSKFTYGLVVEEITT
RDENNNICTHGSRQLPSGALSMIGPPTTLSGTGVDRMAFLQANITRDNTEFVNGATIGDR
CIFQYSEQSFLVGYRWIKASVLEARTLFNRHQLTVTKFINLNKQEKGPRKPPPAVLALHG
RYAGCVGDLPSYDAFALGGPHSVRGYGMGELGASRNLLEIATELSVPITVKNRHTQVYAF
AEHGTDLGSSKDVKGNPTEFFRRVGHGSSYGVGVKLGAVRAEYAVDHNAGTGAFFLRFGE
RF*
>11687.m00154|LOC_Os11g02470.1|genepair1155-1
MAVTESACLSYEQEAVAVREVAQVYELIKTQQPLLLVHQQPQQLAHGLLNHALRALNVAL
SVMNQPHASSSAAAAAVPVMSLIKAEAATPANSSSPAADVAADNHVVGKPRRSSSAAKRR
RINGEEYKSSSWSQFTPVPHEDGFQWRKYGEKKIQGTHFTRSYFRCTYRDDRGCQATKQI
QQKDKNDPPMFQVTYSNEHTCTTTRLINNINNPAALHNLTANPNGHHDSDDDDTIFTKMI
KQEEQAAWLPPPPPADLATISNNFDETPGLHVCQEVPPSSSNSSVISHYADEFDHHQMLE
TTVMEEALGLGADLDDPYFYDPNLLLIYESLMNCY*

>11686.m00151|LOC_Os12g02400.1|genepair1155-2
MAVTESVCLSDEQQAVAVREVAQVYELIKTQQPLLLVHQQPQQLAHGLLNHAMRALNVAL
SVMNQPHASSSSSAAAAAGGHHFPVMTMIKAESTPANSPAADVSDNHVAGKARRSSPAKR
RRINCEDKSSWVYHTVVPHEDGYQWRKYGEKKIQGTHFTRSYFRCTYRDDRGCQATKQIQ
QEDKNDPPMFQVTYSNEHTCTTTRLINNTNNNPAALHSLTANPNGHPDDDSDDTILTKMI
KQEQQAAWLPSPPPDLTTISNNFDETPGLHVSQEVPPCSSNSSAISHYADEFDHHQMGQQ
LETTVMEEALGLGADLDDPYFYDPNLLLIYENLMNCY*
>11687.m00155|LOC_Os11g02480.1|genepair1156-1
MALDSVPSYPSDLGSSRARTPQQQRVSPRKEERTWTTDTYAPYDDGHQWRKYGEKKLSNS
NFPSLREDPTYLVCSQELANSCSQFFLQKDSAGFGVTKFYYRCTYKNDMKCPATKQVQQK
DTNDPPLFSVTYFNHHTCNSSSKIVGSTPDSTVQSRKAISICFNSHGQTGEQPTFLSSSA
SLLSPSMQSYSSNQQPDMNTYSRQFQWADTSSSTSNAPVKMEADDYAEASASPSTTGALS
RTLLPIDSSQIFVLE*
>11686.m00153|LOC_Os12g02420.1|genepair1156-2
MALDSVPSYPSDLGSSRARTPQQQRVSPRKEERTWTTDTYAPYDDGHQWRKYGEKKLSNS
NFPRFYYRCTYKNDMKCPATKQVQQKDTNDPPLFSVTYFNHHTCNSSPKIVGSTPDSTVQ
SRKAISICFNSHGKTGELPTFLSPSASLLSPSMQPYSSNQQPDMNTYSRQFHWADTSSST
SYAPVKMEADDYAEASASPSTTGALSRTLLPIGQSRCIEYFHFL*
>11687.m00159|LOC_Os11g02520.1|genepair1157-1
MKILESFGHSDCQVVINMIEHQKALMVELRGMVMPLLPSDNEQAKLALQLLGDILSCSDK
AISMLELGGDTKKLTNLVGGKRKGDKHSMDNHNLEEEAKESVSKRRKNAEHTGSTVAQAP
HNDGHQWRKYGQKWISRAKHSRSYYRCANSKVQGCPATKTVQQMDSSGNGTSKLFNVDYY
GQHTCRGDGIADPYVVDTAHHSMEPINQNECNSPTLEHEAHEVQDERFENLCMVQNMPEY
LIDFELERAFEFIVNSPLGSEHWTFDDSIRCEHSPICIWG*
>11686.m00156|LOC_Os12g02440.1|genepair1157-2
MLELGGDTNKLTNLVGGKRKGDKHIMDNHNLEEEAKESVSKRRKNAEHTGSTVAQAPHND
GHQWRKYGQKWISRAKHSRSYYRCANSKVQGCPATKTVQQMDSSGNGTSKLFNVDYYGQH
TCRGDGIANPYVVDTAHHSMEPINQNECNSPTLEHEAHEVQDERFENLCMVQNMPEYLID
FELERAFEFIVNSPLGSEHWTFDDSIRCEHSPICIWG*
>11687.m00160|LOC_Os11g02530.1|genepair1158-1
MKNSSNKRSLVADQWHPSSVCCDHRAALREIAKGQSLVTQLRAIVLPALHSDERCDLAAQ
MLEGILDCSRKAVSQLQLLLSSPHDDDDHHHVDDKRRVRKIISSSDDDDHCSSKAAEDDN
AKPLRQHKRRRFGDSVSLETPVPHYDGHQWRKYGQKHINNSKHPRSYYRCTYRQEEKCKA
TKTVQQREDLHHANSYNGDHPIMYTVVYYGQHTCCKGPAALADDHVVVEASQISTDSHCQ
SPSSSSDLQAAEVHAGNSSQCSNISVTCSPSVVVEDCNKLLDMMPAADELTADVLLFDMT
AYAPLDLDINWEMDTNALWV*
>11686.m00157|LOC_Os12g02450.1|genepair1158-2
MKNSSNKRPLVADQWHPSSVCCDHRAALREIARGQSLVTQLRAIVLPALHSDERGDLAAQ
MLEGILDCSRKAISELQLQLSSDSPHDDDGHLDDKRRVRKIVSSSSDDDDHSSSKAAEDH
NAKPLRQHKRRRFGDSVSLETPVPHYDGHQWRKYGQKHINNSKHPRSYYRCTYRQEEKCK
ATKTVQQREDLHHANSYNGDHPVMYTVVYYGQHTCCKGPAASADDHVVVEASQISTDSHC
QSPGSSSSELQAAAHAGDSSQCSNISVTCSSSVVVEDCNKLLDMLPAADELTTDVLLFDM
TAYAPLDLDINWEMDTNALWA*
>11687.m00162|LOC_Os11g02550.1|genepair1159-1
MEAPKLRFVRCPGCLQLLVEYPTIAVYQCGGCGTILRAKNQVAPAVNANAESGEHNEFSN
NSTGGSQNNKLICTDGQKIPPSSDAQPGVLQEKITFASEEKTMSSSNSIDSSEHVNIECS
LLDGDASNHDVRTEGINDEDKVTVSNSSLDSVRKVENVETDGNEKGSFTDDGSISNEVAT
TQSMVHMDGAGSDNNFTEVQSAAEGKCALSDANLDSQEIVAICQPDNISVGTKERVQPYE
GFNVESHEDLIEELVRSLSLSDDEEEFVDIAENSELNDALCSQMGSCRFSLGSKMNEGPR
TDPHGRLIEELEMSFSDAEEPLDQNIMVSLNDIEKPTLDEVSKENHILEEDGKESHILDV
DGKESHILDVDGKQNHILDEHGKENHILNEDDKENLILDKGGEDTLDAGGANSYEERVLP
SDDGLLKSGQSFQQCELVAVNMAEKDEGHLEETNMANHAEANSGIAAVLSNLSNDKFCAI
LPPSCDGRKEEKSNIHRGRELCQGLSLDSVDFRSIQNFIESQMDGTSSSLSSGSPSHGDL
EHNRSNRFKKIDRLERLRKMDDLRDQLNRLSSQKGLENRYKNKGPGVLQEQISYRHLEQH
PCGFDGDSILDSDIIDSYYDQGNPPRYPPPDPFSPTHSHYHCGHGQPHIPYNCSAWEFNS
YYQSSYAGSTVLEHVSLRSSYKEQKRAVKKSILRSLSGASPFTICNGCFNLVQVPSDIYV
SKKKIAKFQCGRCSKALMLSFPATNSDDAKLSNKEVNRKPNKPVHNSVVGMEGGYSFSAE
CSRGDPVSISEECGASISRSFSGRTRAAVAASGSGKKVSDSALHRLMGYDSASQLLRRSR
AFEDGYDSFESMVPVSNRVSRRKNL*
>11686.m00160|LOC_Os12g02490.1|genepair1159-2
MEAPKLRFVRCPGCLQLLVEYPTIAVYQCGGCGTVLRAKNRVAPATNTNAESGEHNEFSN
ISTGDSQNNKLICTDGQKISPSSNAQPGVVQEKITFASEEKTVSTSNSIDSNEHVNIECS
LFDGDASNHDVRTEGINDEDKVTVSNSTLDLVRKVENVETDGNEKGSFTDDGSISNEVAT
TQSMVHMDGAGFDNNFTEVQSAAEGKCTLSDANLDSQEIVAICQPDNISVGTKERVQLYE
GFHVESHEDLIEELVRSLSLSDDEEEFVDIAENSELNDALRSQMGSCRFSLGSKMNDGPR
TDPHGRLIEELEMSFSDAEEPLDQNIMVSLNDIEKPILDEVSKENHILEEDGKESHILDV
DGKESHILDVDGKQNHILDEDGKENHILNEDDKENLILDKGGEDTLDAGGANSYEERVLP
SDDGLLKSGQSFQQCELAAVNMAEKDEGHLEETNMANHAEANSGTAAVLSNLSNDKFCAI
LPPSCDGRKEEKSNIHRGRELCQGLSLDSVDFRSIQNFIESQMDGTSSSLSSGSPSHGDL
EHNRSNRFKKIDRLERLRKMDDLRDQLNRLSSQKGLENRYKNKGPGLLQEQISYRHLEQH
PCGFDGDSILDSDIIDSYYDQGNPPRYPPPDPFSPTHSHYHCGHGQPHIPYNCSAWEFNS
YYQSSYAGSTVLEHESLRSSYKEQKRAVRKSILRSLSGASPFTICNGCFNLVQVPSDIYV
SKKKIAKFQCGRCSKALMLSFPATNSEDAKLSNKEVNRKPNKPVHNSVVGMEGGYSFSAE
CSRGDPVSISEECGASISRSFSGRTRAAVAASGSGKKVSDSALHRLMGYDSASQLLRRSR

AFDDGYDSFESMVPVSNRVSRRKNL*
>11687.m00164|LOC_Os11g02570.1|genepair1160-1
MHMDAGDDFSESCRSTEHFVLVHGAGHGAWCWFRLLRLLQDSGHRVSAVDLAGAAGSLVD
PNHVRTFDDYNAPLLDLMASLPAGDKVGDSDYGSLFHQHLLLKVQLQINMFYVSGQPFNL
MHSSLVCQGVPDLSEYGDVYDLTFGLGADRPPTAVALRKEFQRIILYQQSPQEDSALASI
LLRPWPTALSTARFTGDDGGVESFIDRVRRVYIKTANDRMVQPEQQEAMIRRWPPSKVMV
MDTDQSPFFSAPELLFNLILKSL*
>11686.m00162|LOC_Os12g02500.1|genepair1160-2
MHTDAGDDFSESCRSTEHFVLVHGAGHGAWCWFRLLRLLQDSGHRVSAVDLAGAAGSLVD
PNHVRSFDDYNAPLLDLMASLPAGDKVILIGHSAGGLSVVHAMHLFGDRIKQAIFIAATM
LQFGYQTEQDIKDGVPDLSEHGDVYDLTFGLGADHPPTAVALRKEFQRIILYQQSPQEDS
ALASILLRPWPTALSTARFTGDDGGVESFIDRVRRVYIKTENDRMVQPEQQEAMIRRWPP
SKVMVMDTDHSPFFSAPELLFNLILKSL*
>11687.m00167|LOC_Os11g02600.1|genepair1161-1
MEDAHHHHWTMVERRGTQLWASGRPFIIHGFNTYWLMSFAADQATRLRVTAAIAEAGLNV
CCTWAFSDGGYRALQTAPFHYDEDVFRALDFVVSEARRHNMRLILSLCNNWEDYGGKAQY
VRWGKEAGLDLTSEDDFFSDPTIKSYYKAFVEAVVTRINTVTNETYKDDPTILAWELINE
PRCPSDPSGDTLQAWMEEMASYVKSIDPVHLLEIGIEGFYGPSIPELLPVNPDEYSGHAG
IDFIRNHQAPGIDLASIHVYSDIWLPQSIKENHLQFVDKWMQQHIDDAANLLGMPIVVGE
FGVSVKDGKFGNEFREDFMKTIYRIFLSSWKEGVIGGGCLLWQLFPEGAEHMDDGYAVIF
AKSPSTLSLLANHLSVLSFALQGSSTARHGWYDIVCGVAIARRPGLVLVGSHVANVALDQ
SESYPPHQKIGPRERASSVGIRASVAW*
>11686.m00164|LOC_Os12g02520.1|genepair1161-2
MVECSGTQLWASGRPFIIHGFNTYWLMSFAADQATRPRVTAAIAEAAEAGLNVCRTWAFS
DGGYRALQTVPFHYDEDVFQALDFVVSEAKRHNMRLILSLCNNWEDYGGKAQYVRWGKEA
GLDLTSEDDFFSDPTIKSYYKAFVEAVVTRINTVTNETYKDDPTILAWELINEPRCPSDP
SGDTLQAWIEEMASYVKSIDPVHLLEIGIEGFYGLSTPELLPVNPDEYSGHAGTDFIRNH
QAPGIDLASIHVYSDTWLPHSIKENHLQFVDKWMQQHIHDAANLLGMPIVVGEFGVSVKD
GKFGNEFREDFMKTVYRIFLSSWKEGVIGGGCLLWQLFPEGAEHMDDGYAVIFAKSPSTL
SLLANHLRCLEC*
>11687.m00168|LOC_Os11g02610.1|genepair1162-1
MDRTSQWVSSPDIPADLLIRIADDVFPLHKAVMVPKCCYIRKAVAAARGGATATVDLDLS
ALPGAADAFDKVARYCYGANFELSVRNAAALLCAAAFLDMHPTDGGLARRVEEFLAKVGL
RTLPGAVAVLRSCEGLLPAAEEIGVVQRSADAIALRICNEVLFPTRSPPEWWTAELAALS
PASFHKVITALRCRRAEPEVLVAAATAYAELLLAEVLAADGHAADHSGMHRALVESVVAV
LPSTDDAPLPAAFLCHLLHVAITIGASAKTCHDLELRVAAVLDQATAGDLLTVALDGAGE
RVQNVDAVRRIITAFVERDSAASSGGGANGRNRRASLSGAGALQGGGGAMQTVAKTVDEV
AAEIATEESLPISKFVGLAGAVPKEARATHDCLYRAVDIYLKAHPALEEMEREKVCSVMD
PLKLSYQGRLHASQNNRLPLQAVLSALYYDRLKLRSGDEGGGGWDAYGNGVMRSSAAGSA
RKQAKEEASLARENEALRSELARMRAYVSGMQQQSKGSSSSRGKKGSWLRTLSRLNPFKA
GIWGKDTSGIVDGKTDAMNSVKSKRRRFSIS*
>11686.m00165|LOC_Os12g02530.1|genepair1162-2
MDRTSQWVSSPDIPADLLIRIADDVFPLLKGVMVPKCGYIRKAVAAARGGATATVDLDLS
ALPGGADAFDKAARYCYGANFEISVRNAAALLCAAAFLDMHPTDGGLARRVEEFLAKVGL
RTLPGAVAVLRYCEGLLPAAEEIGVVQRSADAIALRICNEVLFPTRSPPEWWTAELAALS
PASFHKVITALRCRRAEPEVLVAAATAYAELLLAEVLAADGHAADHSGMHRALVESVVAV
LPSTDDAPLPAAFLCRLLHVAITICASAKTCRDLELRVAAVLDQATAGDLLTVALDSAGE
RVQNVDTVRRIITAFVERDSAASSGGANGRSRRASLSGAGALQGGGGAMQTVAKTVDEVA
AEIATEESLPISKFVGLAGAVPKEARATHDCLYRAVDIYLKAHPALEEMEREKVCSVMDP
LKLSYQGRLHASQNNRLPLQAVLSALYYDRLKLRSGDEGGGGWDAYGNGVMRSSAAGSAR
KQAKEEASLARENEALRSELARMRAYVSGMQQQSKGSSSSRGKKGSWLRTLSRLNPFKAG
IWGKDTSGIVDGKTDAMNSVKSKRRRFSIS*
>11687.m00170|LOC_Os11g02630.1|genepair1163-1
MDFLACVCLLHLLFLATSRVAAQASSPARALDAMLQDHAYRAFVHPHTGIVYNATVPANL
TGVALSAVRLRSGSLRRKGFSDYFDFTVPTGIVVQPYVERVVLVYHNLGNWSDHYYPLPG
YTYLSPVLGLLLYDAANLSAVGLQELSFVASGSPISINFSDVRSVPAGGPAPRCVWFDLD
GVPQFRDLEASNVCSTYRRGHFSIVVNSSAIPPGPVPSGNITPPIPTPTGRSKGSSKGWK
IAVGVVGGVIALVLLASLVVCLARYKRDKKLELMEQNAETGETLRMAQVGRSQAPVALGT
RTQPVIESEYVA*
>11686.m00167|LOC_Os12g02550.1|genepair1163-2
MDFLACVCLLHLLFLATSRVAAQASSPARALDAMLQDHAYRAFVHPHTGIVYNATVPANL
TGVALSAVRLRSGSLRRKGFSDYFDFTVPTGIVVQPYVERVVLVYHNLGNWSDHYYPLPG
YTYLSPVLGLLLYDAANLSAVGLQELSFVASGSPISINFSDVRSVPAGGPAPRCVWFDLD
GVPQFRDLEASNVCSTYRRGHFSIVVNSSAIPPGPVPSGNITPPIPTPTGRSKGSSKGWK
IAVGVVGGVIALVLLASLVVCLARYKRDKKLELMEQNAETGETLRMAQVGRSQAPVALGT
RTQPVIESEYVA*
>11687.m00171|LOC_Os11g02640.1|genepair1164-1
MAYRRKQGPIAADDRRSSYPQSPQGSSSSYSYTSIKSMNEPKLGLWETLARKAKGILDED
GVAHKSDEYTKEKTPRKFDSSTGAQESQSRWSFENHSKTGDTGSRTRSEALAASVNQLGG
RIRDALEEGLTIVDNKTSNIIEETKKIQIRRKQANSNSYVPNLAFDTLRPPNLSNDQAET
AAQETQLKASRDVANAMAAKAKLVLRELKTVKADLAFAKQRCAQLEEENKFLREAKQKGS
KTEEDDDLIRVQLETLLAEKSRLAQENSMYARENRFLREIVDFHQFTTHDVAPLDDGDME
DSIPGEDSNHTYSEDMFPVVEAYLDREELSPVPSRPESPILSSCESSSPKSSNSKSSAAN
LPSNVSAKCIGT*

>11686.m00169|LOC_Os12g02570.1|genepair1164-2
MAYRRKQGPIAADDRRSSYPQPPQGSSSSYSYTSIKSMNEPKLGLWETLARKAKGILDED
GTAHKSDEYTKQKTPRKFDSSTGAQESRSRWSFENHSRTGDTGSRTRSEALAASVNQLGG
RIRDALEEGLTIVDNKTSNIIEETKKIQIRRKQANSNSYMPNPAFDTLRPPNLSHDQAET
AAQETQLKASRDVANAMAAKAKLVLRELKTVKADLAFAKQRCAQLEEENKFLREAKQKGS
KTEEDDDLVC*
>11687.m00173|LOC_Os11g02660.1|genepair1165-1
MVIATLAFIDQLGAKKAVLVGHSAGCLVAVEAYFEAPERVAALVLVAPAIFVPVFRRKGV
KEYGVGEQEWQNKKDSNGSNLPTNPLNRIWGKFLELCLWIAGFLMNMIRAIGSIVRSLYC
KAVVAVLRSSVGVRLVRLVMDKFGILAVRNAWYDPSKVTDHVIQGYTKPLRSRGWEMALL
EYTISMIMDSISSSKVPVSERLSEISCPVLVVSGDTDRLVPRWNTERVARAIPGAGFEVI
KNSGHLPQEERPEEFVSVVERFLRKAFGRPSEQEKLFQAAA*
>11686.m00172|LOC_Os12g02600.1|genepair1165-2
MPMHLPRSLQYPPSTPHPLHSTAFSHSLRPRRPNGPPPAFASAEFPGSVPDSAQMPPRRR
RRRSVAGIDQDDLLDPDALADPDSSFYEINGVRVHHKVCTHEDSSDQSPDSAITNADQNQ
IGLPIVLLHGFGSSVFSWTHIMRPLARIAGAKVLAFDRPAFGLTSRTIWSGDDTKPINPY
SMAFSVMATLAFIDQLGAKKAVLVGHSAGCLVAVEAYFEAPERVAALVLVAPAIFVPVFR
RKGVKENGVGEQEWQNKKDSNDSNLPTNPLNRIWGKFLELCLWIAGFLMNMIRAIGGVVR
SLYYKSVVAVLRSSVGVMLVRLIMDKFGILAVRNAWYDPSKVTDHVIQGYTKPLRSRGWE
MALLEYTISMIMDSISSSKVPVSERLSEISCPVLVVSGDTDRLVPRWNTERVARAIPGAG
FEVIKNSGHLPQEERPEEFVSVVERFLRRAFGRPNNEQEQVLQAAV*
>11687.m00176|LOC_Os11g02670.1|genepair1166-1
MSDHQFAGQPDVQCLVCTRPFTLDAQVTDTFEALAICRDCKATVLNDVERDEITSTSHHT
RRRRQRSRTASIDSLEDAFSQEFSQLIDLARRQGRETDIDSSSVLPQHASYNATPSHSQR
WHASDDESDGLNYVDSVFGEIESTISFGDYGADSDTSIEEHSVSARRRISIQLDNGSYMN
TDTDIDPMNARLDQWDSDDQEDVEESGFDETINTMTQHQQQSHDIQLSGLSEDESEDGVW
NWSVAVRQRANVTNLLEDMEGPEMRTTFVGNPDDYVDARQFEMLLEQFAEDNSSRRGAPP
AATSFIENLPSVIISTSHQINDDVICPVCKDPIPTRARAKQLPCMHLYHSSCILPWFSSR
NTCPVCRYELPTDDAEYERSKQATTNVRDIQVVEENSDEQEVQVTRQMAVGAIEETNTSE
HNVRVDEQPSSARRRSGWLFIAAAPVVSLIGFALVLCFTNPARSGRRQLYCRSPSATEVH
VGTKKSWWSMF*
>11686.m00174|LOC_Os12g02620.1|genepair1166-2
MSDHRTHASDCHPVASQPDVQCLVCTRPFTLDAQVTDTFEALAICRDCKMTVLNDDERDE
ITSTYRQNTRRRQISRTASIDFLEDAFLQEFSQLIDLARRQGRETDIDSSSVAPQHASFN
STPSQSQRWHASDDESDGLNYVDSVFGEIESAISLGDYGGDSDTSIEEHSVMTRRRISIQ
LDNDSYMNTDTDIDPMNARLDQWDSDDQEDVEESGFDEIVNTMTQHQQQSHDIQLSGLSE
DESEDGVWNWSVSVRQRANVTNLLDDMEEPEMRTTFVGNPDDYVDARQFEMLLEQFAEDN
DSRRGAPPAATSFIENLPSVIISASHQTNGDVICPVCKDPIPTRARAKQLPCMHLYHSSC
ILPWLSSRNTCPVCRYELPTDDAEYERSKQATTNVRDIQVVEEISDEQEVQVTRQMAAGV
IEETNTSEHNVRVDEQPNSARRRSGWLFIAAAPVVSLIGFALVLCFTNPARSGRRQLYCR
SPSATEVHVDTKKSWWSMF*
>11687.m00180|LOC_Os11g02710.1|genepair1167-1
MNLDHNSTDPKITGCLVRFHSDIKNRVGNRTEKPTPDTAISLFPSRIFRASRSSTINMSE
ESGLAAHGSEEGFPSVEIRMLKLEGNRDVCLDIDSGIRVDDDGAGRISSGAGGGAGRRGR
HGRGSSGGSGRHGAEEEAARLGRGWGVARARVRLGRGAKWGRARRGVGQSARRGRRGEQG
AARERQKRVGSIYLYSTGSIQVLNVTDPNMVKELANCKSLDLGKPCYLQKERGALLGMGI
LTSNGDLWVHQRKVIAPELFMERVKGMVNLMMEAAMSMLNSWKNEVEDRGGSAEIVVDEF
LRTFSADKAMAKQSMLIGVPGSRYLPTRSNRGIWNLDSSIRTLILNISKKYEHDSSTSVN
KDLLHSIIQGSKDGPFASCTPEDFIVDNCKNIYFAGHETTSTTAAWCLMLLASHHEWQSR
ARVESLDICQGRPLDFDILRKLKKPPSSPGEALNDMKLAGIDIPKGTNIWIPIAMAHRDP
SVWGPSADKFDPDRFANGIAGTCKPPHMYMPFGVGVRTCAGQNLAMVELKVVLSLLMSKF
EFKLSPNYVHCPAFRLTIEPGKGVPLIFREL*
>11686.m00176|LOC_Os12g02640.1|genepair1167-2
MELFSSQQWLALLPPIILCILLFSYVYIILWLRPERLRQKLRSQGVRGPKPSFLFGNIPE
MRRIQQLAKSAHEQEAGSTDMFSSNYVATLFPYFLHWSRVYGSIYLYSTGSIQVLNVTDP
NMVKELANCKSLDLGKPCYLQKERGALLGMGILTSNGDLWVHQRKVIAPELFMERVKGMV
NLMMEAAMSMLNSWKNEVEDRGGSAEIVVDEFLRTFSADVISRACFGSSFSEGKEIFIKI
RQLQKAMAKQSMLIGVPGSRYLPTRSNRGIWNLDSSIRTLILNISKKYEHDSSTSVNKDL
LHSIIQGSKDGPFASCTPEDFIVDNCKNIYFAGHETTSTTAAWCLMLLASHHEWQSRARV
ESLDICQGRPLDFDILRKLKKLTMVIQETLRLYPPASFVAREALNDMKLGGIDIPKGTNI
WIPIAMAHRDPSVWGPSADKFDPDRFANGIAGACKPPHMYMPFGVGVRTCAGQNLAMVEL
KVVLSLLLSKFEFKLSPNYVHCPAFRLTIEPGKGVPLIFREL*
>11687.m00181|LOC_Os11g02720.1|genepair1168-1
MPSRRCAVVRAAHTNPWRHRVSRCSGRPATRSAPSILWRRRRPRSPCPWSVEKGEEVVLV
HASMGNCFGGRRPHPNRGRCRPNQSRAPIQSCCEVRQQGTHIRFAPLLNLIYSVELEDPS
GLGLDWIDFFFCWFSFCKLQERLSPHYSQIDQGVDKNVTDNSLVSNCDFPVVKKLEKCVD
EEVSVQSPFENKDTRSLGMVYDHENNKSGVAEVIRPDKEAIESSSSMNVADEDPLYGCQT
PRESIFDPFAPGPEELACAPKKNMIKAPELPPRRQLSFDSGDYPIKRLSFEFDDAEEDDQ
FLERICKMFIDLIVSNQALETIGKDLIGSNSPGSCETPSSEPLLTGIADTCPDAPLRRPL
KAVQLSPSICRKLDFDSVSPRCLFVKENK*
>11686.m00177|LOC_Os12g02650.1|genepair1168-2
MAEQGGIVLAAGCLIPIPVGAAQTNLAPQSRAAARSKKLLFFVRQQGTRIRFAPLLIYWV
GLEDPPGLGLDWIDFFFCWFSFCKLQERLSPHYSQMDQGVDKNVTDNSLVSNCDFPVVKK
LEKCVDEEVSVQSSFENKDTRSLGMVCDHENNKSGVAEVITLEKEAIESSSSINVADEDP

LYGCQTPRESIFDPFAPGPKELACAPKKNVIKAPELPPRRQLSFDSGDYPVKRLSFEFDD
AEEDDQFLERICKMFIDLIVSNQALETTGKDLIGSNSLGSCETSSSEPLLTSIAYTCPDA
PLRRPLKAVQLSPSICRKLDFDSVSPRCLFVKENK*
>11687.m00182|LOC_Os11g02730.1|genepair1169-1
MASTTAAAAGNGSGSILPTHTIAATAPPFRTHKDADLESRRRRRRRRCLCCCLLVTLVVL
LVLAITLLVLFLTVLRVRDPTTRLVSTRLIGLSPRLSFPAMSVQLNVTLLITVAVHNPNP
ASFTYATGGHTDLTYRGAHVGDAEIDPGRIPSRGDANVTMALTLQADRFAGDLTQLVTDV
MGGSVALEASTRIPGRVAILGVFKRHAVAYSDCHFVFGVTEMAVRSQQCSDRTKL*
>11686.m00185|LOC_Os12g02700.1|genepair1169-2
MASTTATAAGNGSGSILPTHTTTAPPFRAHKDADLESTTRRRRRRCLCCCLLVTLVVLLV
LAITLLVLFLTVLRVRDPTTHLVSTRLTGLSPRLSFPATSVQLNVTLLITVAVHNPNPAS
FTYATGGHTDLTYRGAHVGDAEIDPGRIPSRGDANVTMALTLQADRFAGDLTQLVSDVMG
GSVALDASTRIPGRVAILGVFKRHAVAYSDCHFVFGVTEMAVRSQQCSDRTKL*
>11687.m00183|LOC_Os11g02740.1|genepair1170-1
MATVTPGVLLKLLQAMHTDDRVAGDHRSPVLQVTAVVPALTASTADSLWPSNGFLLQLSD
GLHSTYVQPSSADADALVSARPQLVGHLVHLDRLRFARPVPRAVGIRPVPSSRSVSFVGN
PEPLVARPAACSRGYVIQPGSHSDSAPPLMPSSSGNAVQSDATDAVKRIVLAPKSVSEAA
PPPAVSAAKRRFSSPAPSKQRDPSPSVKGGASRPSSPSVKGASRASSPAVRGTPRATSPA
PSKCVVPSLVSAKEENRRTAREPAIIVPSRYRQPSPVGGRRGAASPAPGGRRASLSPSSR
RLSGEGSSKKKVGVLVAGISKMTDLTNGSAVKPGRKSWDNTSIAAAAGSVMKSKVKVDKS
TILRTQEAMARRLSDVTTELSSNDDDSSVDEKPKPRKKIESPAVKTKAMAPKIMLHDPKW
TDGSIPLDGVSDVLSKMGKEATERRDAAAIAAADALQEALITESVIRNLSKFSELTSASK
TSNPLPTVDIFLAVYEDTLKWKKIAESISTNRTETASWENSATHWVEAALATDLEVLKLM
NKAPESLSRKRGADKPKAPSVVEAPRTTISKRQSHGTSAKVQSKVLPTSTASCAWNKTQG
VNETAELATTLCREMHTWFLKFVDEAMDLGFHLFEDQNVASRGKQSSHITMVLSQFKKIS
DWLDGVGKIAEEATTKDKVEQLKCKIYGFVINHMGSAFDSSVSISSRN*
>11686.m00186|LOC_Os12g02710.1|genepair1170-2
MATVTPGVLLKLLQAMHTDDRVAGDHRSPVLQVTAVVPALTASTADSLWPSNGFLLQLSD
GLHSTYVQPSSADADALVSARPQLVGHLVHLDRLRFARPVPRAVGIRPVPSSRSVSFVGN
PEPLVARPAACSRGYVIQPGSHSDAAPPLMPSSSGNAAQSDATNAINRTVLAHKNVPEAA
PPPAVSAAKRRFSSPAPSKQRDPSPSVKGGASRPSSPSVKGASRASSPAVRGTPRATSPA
PSKCVVPSLVAAKEENRRTAREPAIIVPSRYRQPSPVGGRRGAGSPAPGGRRASLSPSSR
RLSGEGSSKKKVGVLVAGISKMTDLTNGSAVKPGRKSWDNTSIAAAAGSVMKSKVKVDKS
TILRTQEAMARRLSDVTTELSSNDDDSSVDEKPKPRKKIESPAVKTKAMAPKIMLHDPKW
TDGSIPLDGVSDVLSKMGKEATERRDAAAIAAADALQEALITESVIRNLSKFSELTSASK
TSNPLPTVDIFLAVYEDTLKWKKIAESISTNRTETASWENSVTHWVEAALATDLEVLKLM
NKAPESLSRKRGADKPKAPLVVEAPRTTISKRQSHGTSAKVQSKVLPTSSASCAWNKTQG
VNETAELATTLCCEMHTWFLKFVDEAMDLGFHLFEDQNVASRGKQSSHITMVLSQFKKIS
DWLDGVGKIAEEATTKDKVEQLKCKIYGFVINHMGSAFDSSVSISSRN*
>11687.m00184|LOC_Os11g02750.1|genepair1171-1
MAAKVHPNVAAAPQPPCISSIVSQQQEEEEPPVVLTVWRKSLLFNCHGFTVFDAKGNLAF
RLDCYDSTSSRRADLVLMDAAGKPLLTIRRKRMSLSDSWIIYDGDGAATSTATPLLSVRR
RRVGLRASKSKAIAHVTPLSSSLPLPEAYVVEGSYGRRSCAVRDARGDAVAEVRRKESVG
DDVFRLVAQPRLGAPLAMAIVIAIDEMFRGGSSLLRRTCSA*
>11686.m00187|LOC_Os12g02720.1|genepair1171-2
MAAKVHPNVAAAPQPPCISSIVSQQQEEEEPPVVLTVWRKSLLFNCHGFTVFDAKGNLAF
RLDCYDSTSSRRADLVLMDAAGKPLLTIRRKRMSLSDSWIIYDGDGAATSTATPLLSVRR
RRVGLRASKSKAIAHVTPLSSSLPLPEAYVVEGSYGRRSCAVRDARGDAVAEVRRKESVG
DDVFRLVAQPRLGAPLAMAIVIAIDEMLRGGSSSLLRRTCSA*
>11687.m00185|LOC_Os11g02760.1|genepair1172-1
MEAAYKFGPYKIDAREVFHSTPLSYAMVNLRPLLPVCPKREVKRFADLSSNEISDLWVTA
KEVGIRLEQYHKASSLTFAIQCLQDGPQAGQTVPHVHIHVIPRKKGDFEKNDEIYDALPL
TSGKEQFDWQFGWLCSEVGCDCCTRPEDLVTVVCMLHLHQQEILICKCKSSSFGKKYSAS
GPPADTVHDNDHSHDDRDIDAWPDDEGEHVVAEADRSGHHAGVLPRDPRCLLAVAATDLA
APHGEAAVDSEAGAGRSIRAELFLMEKTCCCDTEGELVLSKPSSSCAL*
>11686.m00188|LOC_Os12g02730.1|genepair1172-2
MEAAYKFGPYKIDAREVFHSTPLSYAMVNLRPLLPVCPKREVKRFADLSSNETSDLWVTA
KEVGVRLEQYHKASSLTFAIQCLQDGPEAGQTVSHVHIHVIPRKKGDFEKNDEIYDAIDV
KERELKEKLDLDIERKDRTMEEMAHEANEYRALFS*
>11687.m00186|LOC_Os11g02770.1|genepair1173-1
MEMEKRKKQLSCVGSNVNYFHQPHQAIASMRRIQIHPSHGGGGGTGGRARRQAADQPVVY
TVWKRSSIGFQGTDGFSVYDSAGKLAFRVDNYSRRRKAFAGDLLLMDGHGTPLLSLRPQV
DHLLIHFITSSLAPLVYCFSSDLFTDRSV*
>11686.m00189|LOC_Os12g02740.1|genepair1173-2
MRRIQIHPSHGGGGGTGGRARRQAADQPVVYTVWKRSSIGFQGTDGFSVYDSAGKLAFRV
DNYSRRRKAFAGDLLLMDGHGTPLLSLRPQILSLHNRWNCYRAQEEEGLDSTSSPSVSQQ
QVFSMRKSSALQSNDEAEVFMSTRTSGDSQPDASPSPSFRIDGCFSMRSCKIRGSNGEEA
ARITRKNAGVMSRPVSLGDDVFTLVVRPGVDVAVVMAMVVVMDRICRRPYTPMACSSSGN
SVVHSGEIIKSKEKYHLNRSSSSLL*
>11687.m00189|LOC_Os11g02800.1|genepair1174-1
MGFQGTDGFSVYDHAGTLAFRVDNYSRRRKLFSGDLLLMDGHGSPLLALTPQIISMHDQW
NCYRASEEGQGKRTRSQQLFSMRKCSVMQSSHEAEVHMSGCTHASSDRTGHVPGFSIEGS
FRRRSCKIRNSVGEEVARITRKKAGAASLSLTLAEDVFSLEVQPNVDCAMIMAFVIALDR
ICWKPYTPMICSS*

>11686.m00191|LOC_Os12g02760.1|genepair1174-2
MSRIHPSYQRQDAAAAAASTAAPRAAVYTVWKRSSMGFQGTDGFSVYDDAGSLAFRVDNY
SRRRKLFSGDLLLMDGHGSPLLALTPQIISMHDQWNCYRASEEGQGKRARSQQLFSMRKC
SVMQSSHEAEVHMSGCTHASSDRTGHVPAFSIEGSFKRRSCKIRNSGGEEVARITRKKAG
AASLSLTLAEDVFSLEVQPNVDCAMIMAFVIVLDRICWKPYTPMICSS*
>11687.m00191|LOC_Os11g02820.1|genepair1175-1
MAKDPHVSISLTGLAMVVFLIFSSSFLQAAQGPDKKMVMKYDVPVKRLMYRPAAIGTEAA
AYEPFELCMGCRCCASSNASSCVDTRCCYAIDCNIPGKPFGVCAFSPHTCDCGATNCTSQ
QP*
>11686.m00196|LOC_Os12g02800.2|genepair1175-2
MAKDPHVSISLTGLAMVVLLIFSSSFLQAAQGSDKKMAMKYDVPVKRLVYRPSAMQAAVI
GTEAAAYEPFELCMGCRCCASSNASSCVDTRCCYAIDCNIPGKPFGVCAFSPHTCDCGAT
NCTSQQQP*
>11687.m00192|LOC_Os11g02830.1|genepair1176-1
MGNCGTREENAVVAAHAQVQQLHLLQHPVKNAVAERKHTRISSDMSDPSTPRKIEDAKNI
SIYNDVIDFTLFELETITRSFRADYVLGEGGFGTVYKGYIDENVRVGLKSLPVAVKVLNK
DGHQGHREWLTEVRFLGQLRHPNLVKLIGYCCEDDHRLLVYEFMFRGSLENHLFRRTATP
LSWATRMSIALGAAKGLACLHNAERPIIYRDFKTSNILLDSDYTAKLSDFGLAKAGPEGD
QTHVSTRVMGTYGYAAPEYVMTGHLTARSDVYSFGVVLLELLTGRKSIDKSRPSREHSLV
DWALPKLNDKRRLLQIIDPKLEGQYSVRAAHKACSLAYYCLSQNPKARPLMSDVVETLEP
LQGSGGSDGAVQSVLGSGLPSYRVNRRLMTNRVHCRALPNPKCSPAVPACRVR*
>11686.m00197|LOC_Os12g02810.1|genepair1176-2
MSDPSTPRKIEDAKNISIYNDVIDFTLFELETITKSFRADYVLGEGGFGTVYKGYIDENV
RVGLKSLPVAVKVLNKDGHQGHREWLTEVRFLGQLRHPNLVKLIGYCCEDDHRLLVYEFM
FRGSLENHLFRRTATPLSWATRMSIALGAAKGLACLHNAERPIIYRDFKTSNILLDSDYT
AKLSDFGLAKAGPEGDQTHVSTRVMGTYGYAAPEYVMTGHLTARSDVYSFGVVLLELLTG
RKSIDKSRPSREHSLVDWALLKLNDKRRLLQIIDPKLEGQYSVRAAHKACSLAYYCLSQN
PKARPLMSDVVETLEPLQGSGGSDGAVQSVLGSGLPSYRVNRRLTTNSVHCRAIPNPKCS
PAVPACRVR*
>11687.m00215|LOC_Os11g03060.1|genepair1177-1
MLKKLLSKPKSKKKEAASSALPTLDRLHETLEMLEKKECFLQKKASAEVEKAKDYTKAKN
KSAAIQCLKKKKLYETQIEQLANFQLRVHDQIIMLESAKATTDTVDALRSGSSAVKAIHQ
SVSIDDIENAIEEANEHTENMRQIQEALATPIGASADFDEDELEAELEDLEEEELDHELP
EPPQSTRMEPSARVTTSSQPANDLAELTKLQAEMAL*
>11686.m00200|LOC_Os12g02830.1|genepair1177-2
MPFSIFLGHLCLFLPTFLKESASNHLSLCFPFPSLNLQSYWRGYIHSSFTSLFPHRLSSP
EIEDQTLEMLEKKECFLQKKASAEVERAKDYTKAKNKSAAIQCLKKKKLYETQIEQLANF
QLRVHDQIIMLESAKATTDTVDALRSGSSAVKAIHQSVSIDDIENAIEEANEHTENMRQI
QEALATPIGASADFDEDELEAELEDLEEEELENDLPEPPQRTSMEPSARVTTTQPANDLA
ELTKLQAEMAL*
>11687.m00217|LOC_Os11g03070.1|genepair1178-1
MSMDKTHLMCFYPSKITMGGVWTGDNPLDFSIPLLLFQILLITSTTRAATLLLSPLRLPT
YISQILASFLLGPSILGHLPHFSNLVFPVRSLFVLESMALLGLVYYTFIVGVEIEVSAIT
RAGIHSFGFAVGCALPPFLVGALTGYVALSTDDKRKGDTFLNKLSFPIFLGSTFSSTAFS
VLARNIAELKLAGTDVGQLTLSASLINDTFAWTGLTVATVLGHSRCTITQTTWTLTSGVV
IFGASYLLLRPMLLRLARRAAEGEAVGEDRECWILIGVMVAALVADAGGTHAIFGAFVFG
LAVPNGPVGVALVEKVEDFVVGALLPLFFALSGLRTDTAKITNMHSAVLLMVAAMVAAVL
KVAAAIGVAGVFGMPLSDGTSIGLLLNTKGIIELVILNIARNKGIMSDQSFTVLVFVSAL
ITAMVSPFLGMVVKPARRLVFYKRRTVAWAHPESELRVLACVHVPRDVPALLTLLDVVTP
SSRSPVGVLALHLIEFVGRSSALLLINASAPSSSSYDASVHGRSHTEMQFKHISHAFMAY
EEQSVGVSARTMAAVSPYESMHEDITSAAENQHSALILLPFHKYRSVDGGLEVSHPAIQP
LNCNVQSFSPCTVGILVDRGLAAVPGGGYRVVALFFGGSDDREVAALATRMVRNPTIDLT
LLRFVQKGGSFTASEFDALKERKADEGCLRDFLERANEGGGATVEYRERGVFNASEMVGE
IQSVEAMGNKDLFVVGKVPGGSGLTAGMAEWSESPELGPIGDLLASKDFQTTASVLVLQA
YGRPVAVVGAGAGAMSVDFGGDSVAMAERTASGRRPWARPGV*
>11686.m00201|LOC_Os12g02840.1|genepair1178-2
MAAHTVTDPLEELWNHTMSMDKTHLMCFYPSKITMGGVWTGDNPLDFSIPLLLFQILLIT
STTRAATLLLSPLRLPTYISQILAGFLLGPSVLGHLPHFSNLVFPVRSLFVLESMALLGL
VYYTFIVGVEIEVSAITRAGIRSFGFAIGCTLPPFLVGALTGYVALSTDDKHKGDTFLNK
LSFPIFLGSTFSSTAFAVLARNIAELKLAGTDVGQLTLSASLINDTFAWTGLTVATVLGH
SRCTITQTTWTLTSGVVIFGASYLLLRPMLLRLARRAAEGEAVGEDRECWILIGVMVAAL
VADAGGTHAIFGAFVFGLAVPNGPVGVALVEKVEDFVVGALLPLFFALSGLRTDTAKITN
MHSAVLLMVAAMVAAVLKVVAAIGVAGVFGMPLGDGTSIGLLLNTKGIIELVILNIARNK
GIMSDQSFTVLVFVSALITAMVSPFLGMVVKPARRLVFYKRRTVAWAHPESELRVLACVH
VPRDVPALLTLLDVVTPSSRSPVGVLALHLIEFVGRSSALLLINASAPSSSSYDASVHGR
SHTEMQFKHISHAFMAYEEQSVGVSARTMAAVSPYASMHEDITSAAENQHSALILLPFHK
YRSVDGGLEVSHPAIQPLNCSVQSFSPCTVGILVDRGLAAVPGGGYRVVALFFGGSDDRE
VAALATRMVRNPTIDLTLLRFVQKGGSFTASEFDALKERKADEGCLRDFLERANEGGGAT
VEYRERGVFNASEMVGEIQSVEAMGNKDLFVVGKVPGGSGLTAGMAEWSESPELGPIGDL
LASKDFQTTASVLVLQAYGRPAAVVGAGAGAMSVDFGGDSVAMAERTASGRRPWARPGV*
>11687.m00221|LOC_Os11g03110.1|genepair1179-1
MGSSSLLLFPSSSSSATHSSYSPSSSSHAITSLLPPLPSDHHLLLYLDHQEQHHLAAAMV
RKRPASDMDLPPPRRHVTGDLSDVTAAAAPSSASAQLPALPTQLPAFHHTDMDLAAPAPP
PPQQQVAAGEGGPPSTAWVDGIIRDIIASSGAAVSVAQLIHNVREIIRPCNPDLASILEL

RLRSLLTSDPAPPPPPPPSHPALLPPDATAPPPPPTSVAALPPPPPPQPDKRRREPQCQE
QEPNQPQSPKPPTAEETAAAAAAAKERKEEQRRKQRDEEGLHLLTLLLQCAESVNADNLD
EAHRALLEIAELATPFGTSTQRVAAYFAEAMSARLVSSCLGLYAPLPNPSPAAARLHGRV
AAAFQVFNGISPFVKFSHFTANQAIQEAFEREERVHIIDLDIMQGLQWPGLFHILASRPG
GPPRVRLTGLGASMEALEATGKRLSDFADTLGLPFEFCPVADKAGNLDPEKLGVTRREAV
AVHWLRHSLYDVTGSDSNTLWLIQRLAPKVVTMVEQDLSHSGSFLARFVEAIHYYSALFD
SLDASYSEDSPERHVVEQQLLSREIRNVLAVGGPARTGDVKFGSWREKLAQSGFRVSSLA
GSAAAQAVLLLGMFPSDGYTLIEENGALKLGWKDLCLLTASAWRPIQASGR*
>11686.m00204|LOC_Os12g02870.1|genepair1179-2
MGSSSLLLFPSSSSSATHSSYSPSSSSHAITSLLPPLPSDHHLLLYLDHQEQHHLAAAMV
RKRPASDMDLPPPRRHVTGDLSDVTAAAAGAPTLSASAQLPALPTQLPAFHHTDMDLAAP
APPAPQQVAAGEGGPPSTAWVDGIIRDIIASSGAAVSVAQLIHNVREIIRPCNPDLASIL
ELRLRSLLNSDPAPPPPPPSHPALLPPDATAPPPPPTSVAALPPPPPAQPDKRRREPQCQ
EQEPNQPQSPKPPTAEETAAAAAAAAAAAAAAKERKEEQRRKQRDEEGLHLLTLLLQCA
ESVNADNLDEAHRALLEIAELATPFGTSTQRVAAYFAEAMSARLVSSCLGLYAPLPSPSP
AGARVHGRVAAAFQVFNGISPFVKFSHFTANQAIQEAFEREERVHIIDLDIMQGLQWPGL
FHILASRPGGPPRVRLTGLGASMEALEATGKRLSDFADTLGLPFEFCPVADKAGNLDPEK
LGVTRREAVAVHWLRHSLYDVTGSDSNTLWLIQRLAPKVVTMVEQDLSHSGSFLARFVEA
IHYYSALFDSLDASYSEDSPERHVVEQQLLSREIRNVLAVGGPARTGDVKFGSWREKLAQ
SGFRVSSLAGSAAAQAALLLGMFPSDGYTLIEENGALKLGWKDLCLLTASAWRPIQASGR
*
>11687.m00223|LOC_Os11g03130.1|genepair1180-1
MWRAAAYHLLLLRRAAHPPPSPATATGAACALRHVRLFSPPPHPPSSRPTEAEAEVTAAE
ARRLVRLVGVEALKRRLRDGREEVVGYGELLDACVEAGAARTRRDAEALARAMDEAGVVL
LFRDKAYLHPEKVVELVRRAVPLALSPENDSRKEELKKLQEKKEEIDKLAHKQVRRILWS
GLGFFMCQVGLFFRLTFWEFSWDVMEPIAFFTTASGLLVGYAYFLITSRDPTYEDFMERL
YLSRHRKLCAKNSFDMAKYLELQKHCKCPLEGHKDAILMPKSKTNIVFLPIKMSTHRKIA
RLAIGKELDE*
>11686.m00205|LOC_Os12g02880.1|genepair1180-2
MWRAAASHLLRRRAAHPPPSPATATGAACALRHVRLFSPPPHPPSSRPAEAEAEVTAAEA
RRLVRLVGVEALKRRLRDGREEVVGYGELLDACVEAGAARTRRDAEALARAMDEAGVVLL
FRDKAYLHPEKVVELVRRAVPLALSPENDSRKEELKKLQEKKEEIDKLAHKQVRRILWSG
LGFFMCQVGLFFRLTFWEFSWDVMEPIAFFTTASGLLVGYAYFLITSRDPTYEDFMERLY
LSRHRKLCAKNSFDMEKYLELQKHCKCPLEGHYPHGPKFHDL*
>11687.m00226|LOC_Os11g03160.1|genepair1181-1
MKGHHSLPPSTPPKRRCTALAAAVPALVVCSILLPLVFLLGLHRPGHGSEERAAVVISTE
LGFSKHKHLDGRMKHKLLKDVSRKKIPGSDGILDEKSGSRSKSKSVSTKSKEKLKGVFSL
VQLKNETRKNKELHTQRRYQLKDLSWRSKDTTIDKKENQDQEVEHENPRSCELEYGSYCL
WSVEYKEVMKDFIVKRLKDQLFMARAHYPSIAKLKNQETFTRELKQNIQEHERMLSDTIA
DADLPPFFAKKLEKMERTIERAKSCEVGCTSVERKLRQLLDITEDEAYFHTRQSAFLYHL
GVQTMPKTHHCLNMRLTVEYFKSTSIHTVQSNKQKLEDPTFHHYVIFSKNVLAVSTTINS
TVMNSKDSGSIVFHLFTDSQNFYAMKHWFDRNMYLEATVHVTDIEDHQKLSKDVDFHDMK
LLRPAEEFRVTFRNHYQSFQKQMKTEYISTFGHSHFLLPDLLPSLNRVVVLDDDLIVQKD
LSSLWNLNMGGKVVGAIQFCEVKLGQLKAYTEERNFGTNSCVWLSGLNVVELKKWRDLHI
TSRYDQLLQKLQKDSVTSFPLKVLPISLLVFQDLIYPLEDSWVQSGLGHDYGVSQTDIKR
SVTLHYNGVMKPWLDLGIHDYKGYWRKYMTNGERFMTECNIH*
>11686.m00208|LOC_Os12g02910.1|genepair1181-2
MGFSKHKHLDGRMKHKLLKGYSLYTVSQGFVAILDVSRKKIPGSDGILGEKSGSRSKSKN
VSTKSKEKLKGVFSLVQLKNETRKNKELQDTTSIHFIVSNVISELHTQRRYQLKDLSWRS
KDTTIDKKENQEVEHEENPKSCELEYGIYCLWSVEYKEVMKDFIVKRLKDQLFMARAHYP
SIAKLKNQETFTRELKQNIQEHERMLSDTIADADLPPFFAKKLEKMEHTIERAKSCEVGC
TSVERKLRQLLDITEDEAYFHTRQSAFLYHLGVQTMPKTHHCLNMRLTVEYFKSTSIHTV
QSNKQKLEDPTFHHYVIFSKNVLAVSTTINSTVMNSKDSGSIVFHLFTDSQNFYAMKHWF
DRNMYLEATVHVTDIEDHQKLSKDVDFHDMKLLRPAEEFRVTFRNHSQSFQKQMKTEYIS
TFGHSHFLLPDLLPSLNRVVVLDDDLIVQKDLSSLWNLNMGGKVVGAIQFCEVKLGQLKA
YTEERNFDNNSCVWLSGLNVVELKKWRDLHITSRYEQLLQKLQKDSVTSFPLKVLPISLL
VFQDLIYPLEDSWVQSGLGHDYGVSQTDIKRSVTLHYNGVMKPWLDLGIHDYKGYWRKYM
TNGERFMTECNIH*
>11687.m00228|LOC_Os11g03180.1|genepair1182-1
MEKELDGESTCAAPCEKKRLIPSCFEWGTVPATKKMKTEEDLEVLLAYPSLGEGKKKKKV
VVKRLGKEEVERLLLNRVVPDWNHPIPDEYHRLDRNMYQNSAVTIRQRQDVIRAQFEAKG
YVHVLAEADDDSDTDQEMHPL*
>11686.m00209|LOC_Os12g02920.1|genepair1182-2
MEMELDGESTCAAPCEKKRLINSKLFRVGYGKKKKKKKKKKKKKVVKRLGKEEVERLLS
YTVVVPTVSNKVLGSETVPARHKEILLQAVDSIKNSAVTILGMDKKLGLVSSARAWLELV
KLGSARRLNEPSPSFLHSSFANRAKPELARKPLGRLVKSVENMST*
>11687.m00229|LOC_Os11g03190.1|genepair1183-1
MAITEEDVPAAKKMKTTTEDDHEEVLLAYPPRKKEDGKKMKKVVKRLGKEEVERLLSVTV
TVPTLSKEAMEEDDEEDVREREMLLRAACARTRRCAANIIRYDFVFTTQEFVYNRIERKT
KNMAMTEEDVPAAKKMKTTTEDDHEEVLLAYRSRKEDGKKRKKVVRRLGKEEVERLLSLK
LAVPTLSEEVVMPMPDDDEDDVWQKEVLLRANRLLRESAIRMRKNQELIRSLFEAKGYVD
VEDEVSDDDDMDMEMQPV*
>11686.m00210|LOC_Os12g02930.1|genepair1183-2
MRGCADGEKRRPHFELVSAAKKKKTTTTEDDHEEVLLAYRPREKEDGKKRKKVVRRLGKE

EVERLLSLKLAVPTLSEEVVMPMPDDDEDDVWQKEVLLRANRLLRESAIRMRKNQELIRS
LFEAKGYVDVEDEVSDNEMDMDMQLV*
>11687.m00230|LOC_Os11g03200.1|genepair1184-1
MLGHLRRSSHRVLIANLAVYSIQSSQIEFIAGWCSNHTTRNFSADTSNRVSNDRGHQSRE
LKPSLVKDDVAIIERIQNSTKELRQGPVGKNLSSAEKRKFLVNTLLDLEDSKEGVYSTLD
AWVAFEQDFPVASIKQALVALEKEEQWHRIVQVIKWMSSKGQGKTIRTYEQLVCALEKDN
RADEACRIWEFKIAHDLQSVPWRFCRLMLGIYYRNNKLDRLVKLFKNLEACGRKPPSKDI
VRKVEDAYEMLGLVEEKKELLEKYKDLFDKPSSNDKKKGRQFKKVEKNIKPA*
>11686.m00212|LOC_Os12g02950.1|genepair1184-2
MPMDILGQSIPTIASLFGLTLHGLCDGPGPQNYPHHYQSLLPLPHTNRIGLGSPSAAAAA
PMLGHLRRSSQRLPVANLAVYSIQSSHIEDDVAIIKHIQNSTKELKQGPVGKNLSSAEKR
KFLVNTLLDLEDSKEGVYSTLDAWVAFEQEFPVASLKQALVALEKEEQWHRIVQVIKWML
SKGQGKTIRTYEQLVCALEKDNRADEACRIWELKIAHDLQSVPWRFCRLMLGIYYRNNRL
DTLVKLFKNLEACGRKPPSKDIVRKVEDTYEMLGLVEEKKELLEKYKELFDKPSSNDRKK
GRQFKKAEKNTKTG*
>11687.m00231|LOC_Os11g03210.1|genepair1185-1
MDHQELEEGAEKMKLLGIWSSPYVVKVIWALRIKHVEYDIEEDLRNKGNLLLECNPVHQK
VPVLIYQGKPSDVIIEFIDDVWKDSGQGRIYSTQLSPPIWKWFTTQGKEQEDA*
>11686.m00213|LOC_Os12g02960.1|genepair1185-2
MWALRIKGVEYDYIEEDLRNKSNLLLECNPVHKKVPVLIYQGKPIAESDVILEFIDDVWK
DLRYRILPEDPYECAMARFWSKFGLDKLSPPIWKWFTTQGKEQEDAYEAAMEQLLVLEKV
LDEKKFFGGERIGFVDLSLGSLSYVIPIYEDITGVRLITSDKFPWLSAWMEGFLGLPLVK
EHLLPLDKLRPSSSTSHC*
>11687.m00232|LOC_Os11g03220.1|genepair1186-1
MGAYEATKVVFARLQALEPNLAPNIIGMLLTKDKNEMDMIHLACGPDNLLQSIIAKVRTD
LTNKPSPPMASWGFPSDIGEEASFSIDKVGCDGGEEFSSKEYDWKLPIGGNHHRSFLSST
VNTPGWKPCLYYQSGMTTHLGSDDMQEYSSRPPQIDQSDLTNNCSARQIYLTFPPDSIFS
KEDVCNYFSMYGMVQDVRIPYQEKRMFGFVTFAYQKTVKLILAKGNPHYICDARVLVKPY
KEKDKVPNKKFQQSDSPSYMNHNRLLYSRVPFDLRRHQIDNKLKNEFAMREIASTAISTE
AKRTVISTEEGKREYGPKAATPNDACGFLESGMEYNLPHSPFSSPTKASNVAATAHTSNI
SSSSSPHKVASSLFLPTCTLELPPTTHASFKRQGKALTDHT*
>11686.m00214|LOC_Os12g02970.1|genepair1186-2
MGAYEATKVVFARLQALEPNLAPNIIGMLLTKDNNEMDMIRLACGPDNLLQSIIAKVRTD
LTNKPSPPMASWGFPSDIGEEASFSVDKVGCDGGEEFSSKEYDWRLPIGGNHHRSFLSST
VDTLGWKPCLYSQSGVTTHLGSDDMQEYSSRPPQIDQSDLTNNCSARQIYLTFPPDSIFS
KEDVCNYFSMYGMVQDVRIPYQEKCMFGFVTFAYQKTVKLILAKGNPHYICDARVLVKPY
KEKDKVPNKFRHVNHCLIPLRIFAHILPFECYIVLGPRILYRDIASHEASFRMKQDEQQH
ATELQRCCLMRLPLLNLQDWGHHLSSPMGSHVLLGQVDNKYNINENDNPTHLEDVTFRDN
KLKNEFAMREIASTAISTAAKRTVISTEEGKREYGPKAATPNDACGFLESGMEYNLPHSP
FSSPTKASNVAATAHTSNISSSSSPHKVASSLFPPTCTLELPPTTHASFKRQGKALTDHT
*
>11687.m00233|LOC_Os11g03230.1|genepair1187-1
MAADHLVVAMLLLLALSPPAVADDTAVLGQKGGVVEGQAAGPGRYAVILDAGSTGTRVHV
FRFDNKLDLLKVGDDIELFAKVDPGLSSYAGRPQDAANSILPLLDKANTVVPTRLMNKTP
LKLGATAGLRLIGDEKANQILEAGSQEGSYMWVALNYLLDKLGGDYFKTVGVVDLGGGSV
QMAYAMLSNTAANAPKVPEGKDPYVVKEYLKGKDYNIYVHSYLHYGGFASRVQILERKDG
PFSNCMLRGFSGNYTYNDKQYDATTAPQGADYHKCREEVVKLLKVNAPCETKNCSFNGVW
NGGGGAGQDDLYVASAFYYIASHVGFIDSDAPSAKSTPATFKAVAEKACKLSVKEAKVEY
PNVRDHAYLCMDLIYEYSLLVDGFGLHPSKEITLVDKVKHGEYYIDAAWPLGTAIEAVSP
KKRLREIYK*
>11686.m00216|LOC_Os12g02980.2|genepair1187-2
MAADHLVVAMLLLLALSPPAVADDTAVLGRKGGVVEGQAAGPGRYAVILDAGSTGTRVHV
FRFDNKLDLLKVGDNIELFAKVDPGLSSYAGRPQDAANSILPLLDKANTVVPARLMNKTP
LKLGATAGLRLIGDEKANQILEAVRDVVHTKSKYQYNPNWINVLEGSQEGSYIWVALNYL
LDKLGGDYSKTVGVVDLGGGSVQMAYAISSNTAATAPKVPEGKDPYVVKEYLKGKDYNIY
VHSYLHYGGFASRAHILERKDGPFSNCMLRGFSGNFTYNGKQYDATAAPQGADYHKCREE
VVKLLKVNAPCETKNCSFNGVWNGGGGAGQDDLYVASAFYYIASHVGFINSDAPSAKSTP
ATFKAVAEKVCKLSVKEAKVEYPNVRDHAYLCMDLIYEYSLLVDGFGLHPSKEITLVDKV
KHGEYYIDAAWPLGTAIEAVSPKKRLREIYK*
>11687.m00240|LOC_Os11g03300.1|genepair1188-1
MPSSGGAMPALPPGFRFHPTDEELIVHYLMNQAASVKCPVPIIAEVNIYKCNPWDLPGKA
LFGENEWYFFSPRDRKYPNGARPNRAAGSGYWKATGTDKSILSTPTSDNIGVKKALVFYK
GKPPKGVKTDWIMHEYRLTGTSANSTTTTKQRRASSMTMRLDDWVLCRIHKKSNDFNSSD
QHDQEPEESTVEQLEDIHDNNSSEQPPAPADMNNQQSDFQPMTAMSMSKSCSLTDLLNTI
DCAALSQFLLDGSSDAIAEPPAPPSPLIYTTPHPNYQTLNYNINSNSSMPHAFESRLDHH
DGYVNNYNVNGLRRKRMMACSATSFDDGSSSNDFVHAVVKKPQLLPSDSRGSGFGGGYCN
QQLSETATGFQFQNGNLLSHPFPLNNHLQMQ*
>11686.m00222|LOC_Os12g03040.1|genepair1188-2
MPSSGGAMPALPPGFRFHPTDEELIVHYLMNQAASIKCPVPIIAEVNIYKCNPWDLPGKA
LFGENEWYFFSPRDRKYPNGARPNRAAGSGYWKATGTDKSILSTPTSDNIGVKKALVFYK
GKPPKGVKTDWIMHEYRLTGTSANNTTTTKQRRASSMTMRLDDWVLCRIHKKSNDFNSSD
QHDQEPEGSTVDEQLEDIHDNNSSSQQPPAPPDMNNQQSDFQPMTAMSMSKSCSLTDLLN
NLDCAALSQFLLDGSSDAIAELPAPPSPLIYPNQTLNYNINNNMPHAFESRLDHHDGYVN
NYNVNGLRRKRMMACSATSFDDGSSSSSSDFLHVAKKPLLLPSDSRGSGFGGGYCNQQLS

ETATGFQFQNGNMLSHPFPLNQQLLLNNHLQMQ*
>11687.m00241|LOC_Os11g03310.1|genepair1189-1
MVEPVKSELGSLFLPPGFRFHPTDAEVILNYLLEKFINPSFTSLPIHEVDLNKCEPWDLP
TARMGNNEWYFSRKDMKYPTGMRTNRATKEGYWKATGKDREIFKPAIYEGSSKNNKQLVG
MKKTLVFYMGRAPKGTRTNWVMHEFRPHANLHNHYPNLRLNPNEWVVCKVFHKKQGDEAI
NNQQQQPAVDQADDDDIFQLDDIFADPSIYDFSNSSANILSAPPNNNAVHSSVSAGTTMT
STTTASSFQHQPNCYSAPLQQHVSSWNNTPGAGGAHGIGSSYYNLQQQQQQAAMVKDLED
IIAVPDYGTLLPSSNKGSSIRSATAGVSQQNPLGVPQYKIENYGDHYISRE*
>11686.m00223|LOC_Os12g03050.1|genepair1189-2
MVESTTSLVKLEQDGGLFLPPGFRFHPTDAEVILSYLLQKLLNPSFTSLPIGEVDLNKCE
PWDLPSKAKMGEKEWYFFSHKDMKYPTGMRTNRATKEGYWKATGKDREIFRQPAAVNTSS
YGGSSNKKKQLVGMKKTLVFYMGRAPKGTKTNWVMHEFRLHANLHNHHPNLRLNPKDEWV
VCKVFHKKQGDEAINNQQQPQYAAVDQYSAETPNSGSSVVQAGDIDGGDDFFQLDDIID
PSIYFVSNSSNILSAPPNNNNAVYSVSASTTTTNTTAVSFQQQPNYYSLINKSSSSSSSN
YSAPLQQHVSSWNITPGAGGAHGIGSSYYNLQQQQAAMVKALENVIAVPNFGTLLPSSNK
LKGLSKSAMAGLTQQNPLGVPQYKIENYGDHYISRQ*
>11687.m00248|LOC_Os11g03380.1|genepair1190-1
MAAAWAAVVAVMLLLAQVSAAAPVMGPAFLWAPKNYGFSSDEAKEIVHYQTVSPKSLVKS
VLEEGGWSNLVCSREDHAKSVDVAVLFLGSKLQSSDISKDKQADSTLVDTLKNSFASSEF
SMAFPYIAMSDDDKLEKSLLSGFAENCNNGFGDNHITYTDTCSVSEDLNKHHNMDSIHGL
VASQTKKNPSGQTDLIVFCDGGFKDNTKSEGELLSELVTLLKKSGAKYTILYASQPFGLL
ENPSNLPLGRYLAEKTNTTKPGRGKCDGECLVKSTLLEGSFVGIVLLIILISGLKCMMGI
DTPSKFDAPPES*
>11686.m00224|LOC_Os12g03060.1|genepair1190-2
MAAAWAAAVAVMLLLAQVSAAAPVMGPAFLWAPNNYGFSSDETKEMVYYQTVSPKSLVKS
VLEEGGWSNLVCSREDHAKSVDVAVLFLGSKLQSSDISKEKQADSTLVDMLKNSFASSEF
SMAFPYVAMSDDDKLEKSLLSGFAENCNNGLGNNHITYTDTCSVSEDLKKHHDMDSIHGL
VASQTKKNPSGQTDLVVFCDGGFKDNTKSEGELLSELVTLLKKSGAKYTILYASQPYGLL
ESPSNLPLGRYLAEKTNTTKPGRGKCDGECLVKSTLLEGSFVGIVLLIILISGLKCMMGI
DTPSKFDAPPES*
>11687.m00249|LOC_Os11g03390.1|genepair1191-1
MEAAVATPSLLFSSPTPRRPSSCLPPPPPCSSSSSSYASHGFKLLQPQLLFINRLTSRNS
NGSSRRSISILSLRCSSSGTDSASSSATSERWVLEPAGDGDWRHIGYRVARPGGFQIASE
AAVTVGRVPEQADIVLSVATVSGTHARLEKKEGSLLVTDLESTNGTYINERRLTPGFPTP
IDPGSLLIFGDIHLAMFRVSKMIVDVSSDTNEAEQEAETAQVSAATQQTN*
>11686.m00227|LOC_Os12g03070.3|genepair1191-2
MSPEIALNTGQTTSILSPLIHKEIKKRTKKARHCISLPLLSSEHKSQTQMEAAVATPSLL
FSSPTPRRPSSCLSLPPPCSSSYASNGAKLQQPRLQFVSQLTSRNSNGSGRRSISILSLR
CSSSGTDSASSSATSERWVLEPAGDGDWRHIGYRVARPGGFQIASEAAVTVGRVPEQADI
VLSVATAVSGTHARLEKKEGSLLVTDLESTNGTYINERRLSPGFPTPIDPGSLLIFGDIH
LAMFRVSKMVVDVPSDASGAEQEAAETAQVSAAAQQTN*
>11687.m00250|LOC_Os11g03400.1|genepair1192-1
MAAALTRPPPGTVQCFGRKKTAVAVSYCKPGRGLIKVNGVPIELIRPEMLRLKAFEPILL
AGRSRFKDIDMRIRVRGGGKTSQIYAIRQAIAKALVAYYQKYVDEASKKEVKDIFARYDR
TLLVADPRRCEPKKFGGRGARARFQKSYR*
>11686.m00230|LOC_Os12g03090.1|genepair1192-2
MAAALTRPPPGTVQCFGRKKTAVAVSYCKPGRGLIKVNGVPIELIRPEMLRLKAFEPILL
AGRSRFKDIDMRIRVRGGGKTSQIYAIRQAIAKALVAYYQKYVDEASKKEVKDIFARYDR
TLLVADPRRCEPKKFGGRGARARFQKSYR*
>11687.m00251|LOC_Os11g03410.1|genepair1193-1
MPPRARTLLMPLAAATLLVASTIFLFAATGARWRPADTGLPVPAADFSAAVIESAVTDAA
AAKEELSFVDENGRPDDPASSSAAAARCDPSHAAVRVFMYDLPPEFHFGLLGWSPPTDGA
ADAAMWPDVGSGAAAPRYPGGLNQQHSVEYWLTLDLLSSSSPPCGAAVRVADSRDADVVF
VPFFASLSYNRHSRVVPPEKVSRDKGLQERLVRYLMAQPEWKRSGGADHVIVAHHPNSLL
HARSVLFPAVFVLSDFGRYHPRVASLEKDVIAPYKHMAKTFVNDSAGFDDRPTLLYFRGA
IFRKEVKIDSWKVSPYSHPNVPIPNYPPNICGYMIHLCSLTEYVYILISKIPKTSKLTDV
PTFELSKGGNIRQELHYMLKDEKDVYFAFGSVQDHGASKASQGMHASKFCLNIAGDTPSS
NRLFDAIVSHCVPVIISDDIELPYEDALDYSKFSIFVRSSDAVKKGYLMRLIRGVSKHQW
TMMWRRLKEVDKHFEYQYPSQKDDAVQMIWQTLARKVPAIRLKSHRSRRFSRYDRGGK*
>11686.m00231|LOC_Os12g03100.1|genepair1193-2
MPPRARTLLMPLAAATLLVASTIFLFAATGARWRPADTGLPVPAADFSAAVLESAVTDTT
AAAKELSFVDENGRPDDPASSSAAAARCDPTHAAVRVFMYDLPPEFHFGILGWSPPTDGA
ADAAMWPDVGSGAAAPRYPGGLNQQHSVEYWLTLDLLSSSSPPCGAAVRVADSRDADVVF
VPFFASLSYNRHSRVVPPEKVSRDKELQEKLVRYLMAQPEWKRSGGADHVIVAHHPNSLL
HARSVLFPVVFVLSDFGRYHPRVASLEKDVIAPYKHMAKTFVNDSAGFDDRPTLLYFRGA
IFRKEGGNIRQELYYMLKDEKDVYFAFGSVQDHGASKASKGMHASKFCLNIAGDTPSSNR
LFDAIVSHCVPVIISDDIELPYEDALDYSKFSIFVRSSDAVKKGYLMRLIRGVSKHQWTR
MWNRLKEVDKHFEYQYPSQKDDAVQMIWQALARKVPAIRLKSHRSRRFSRYDRGK*
>11687.m00252|LOC_Os11g03420.1|genepair1194-1
MGPQQDRSAAKPYANGSTAAAAAAGRKENNKVVRYRECQRNHAASIGGHAVDGCREFMAS
GAEGTAAALLCAACGCHRSFHRREVEAAAAECDCSSDTSSGTGRR*
>11686.m00232|LOC_Os12g03110.1|genepair1194-2
MGPQQDRSAAKPYANGSTAAAAAAGRKENNKVVRYRECQRNHAASIGGHAVDGCREFMAS
GADGTAAALLCAACGCHQSFHRREVEAAAAECDCSSDTSSGTGRRFLQQLVLSWWLSSA*

>11687.m00253|LOC_Os11g03430.1|genepair1195-1
MVRELRLDSFYARLRAAAAASAADASSPLLILPSAADADALCALKVLTHVLSADSIRFSI
YPVASAAAAASLLASFSASHPLCLLLINWGAHRDLRAVLPPAATAFVVDSHRPIHLHNLS
AANDRVVVLFTTDDEHTADLSYDFDVSSLADASDLSAQGEADDHLRVAEEDEDSDASDSD
SDGEGGRRKRRRLSDDAEANGDPERLFGKLRREYYRLGTFHGKPSGCLMYELAHALRKNT
NELLWLACVSLTDQFVHERITNERYQAAVMELEQHINGSGNLDPSGVGAVVTLKDGTKIR
APEASRIAYEDEPRLMLLREWSLFDSMLCSSYVATKLKTWSDNGLKKLKLLLARMGFPLA
DCQKRFQYMSMEVKRKMRDEFDRFLPEYGLTEFYYRSFLRVHGYRSKVSAADVVYGVTAL
LESLNAESKDSKGSSAAEQFWVAYSALSLSNVDQLRKGMQSAIEIQRAILRQGSSAITKT
GFIRSAKKFRWVKLDDPVDTDKLCQPQALTKFCFFLMDALRERGARMKPLICACLAREPE
KVLVVGVCGKPRLGAVKGNAFGNAFRSAAEEIGADYFHDMFESSWIVLDVVAVSSFMIRL
TEKL*
>11686.m00234|LOC_Os12g03130.1|genepair1195-2
MVRELRLDSFYARLHAAAAASAADASSPLLILPSAADADALCALKVLTHVLSADSIRFSI
YPVASAAAAASLLASFSASQPLCLLLINWGAHRDLRAVLPPAATAFVVDSHRPIHLHNLS
AANDRVVVLFTTDDEHTADLSYDFDVSSLADASDLSAQGEADDHLRVAEEDEDSDASDSD
SDGEGGRRKRRRLSDDAEADGDPERLFGKLRREYYRLGTFHGKPSGCLMYELAHALRKNT
NELLWFACVSLTDQFVHERITNERYQAAVMELEQHINGSGNLDPSGVGAVVTLKDGTKIR
APEASRIAYEDEPRLMLLREWSLFDSMLCSSYVATKLKTWSDNGLKKLKLLLARMGFPLA
DCQKRFQYMSMEVKRKMRDEFDRFLPEYGLTEFYYRSFLRVHGYRSKVSAADVVYGVTAL
LESLNAESKDSKGSSAAEQFWVAYSALSLSNVDQLRKGMQSAIEIQRAILRQGSSAITKT
GFIRSAKKFRWVKLDDPVDTDKLCQPQALTKFCFFLMDALRERGARMKPLICACLAREPE
KVLVVGVCGKPRLGAVKGNAFGNAFRSAAEEIGADYFHDMFESSWIVLDVVAVSSFMIRL
TEKL*
>11687.m00254|LOC_Os11g03440.1|genepair1196-1
MGRPPCCDKEGIKKGPWTPEEDIILVSYIQEHGPGNWRSVPINTGLMRCSKSCRLRWTNY
LRPGIKRGNFTAHEEGIIVHLQSLLGNRWAAIASYLPQRTDNDIKNYWNTHLKKKLAAAA
NSTSAASNRHPIFADATFPSAAGSHTVSSNSDVNQMAAIARRSPFADCPSSSYASSMDNI
SKLLDGFMKTNSPSPPPPPLQHYDGGYYDDVKPAVDVVGNPLLSSFDCMSGADLDCCFDV
HQQQPASSFMEYGGYGGGYGDESKQQLMNQAAAPLSSIEKWLFDEAAAEQVADLMDLSDG
CCSVPMMF*
>11686.m00236|LOC_Os12g03150.1|genepair1196-2
MGRPPCCDKEGIKKGPWTPEEDIILVSYIQEHGPGNWRSVPINTGLMRCSKSCRLRWTNY
LRPGIKRGNFTAHEEGIIVHLQSLLGNRWAAIASYLPQRTDNDIKNYWNTHLKKKLAAAA
NSTSSSNRHPIFADATFPSAAGGSYTVSSNSDVTQMAAIARRSPFADCPSSSYASSMDNI
SKLLDGFMKTNSPSPPPPPLQHYDSGYYDDVKPAVDVGGNPLLSSFDCMSGADLDCCFDV
HQQHQQQPASFMEYGGYGGGYGDESKQQLMNQAAPPLSSIEKWLFDEAAAEQVADLMDL
SDGCCSVPMMF*
>11687.m00255|LOC_Os11g03450.1|genepair1197-1
MATDAYPVQLLHRQATAATGGGQWHNLGAAYAAVRFLRPQGRSLVLYAGPDGGAQQRIVF
AYPILPGDAFERMDGETLSWAEPECGDEFALCFLDEAACAAVSGAISPVTESLAALDGLA
ERLAGLRVAREEGGPAGVDIAGRLAAISIGRP*
>11686.m00238|LOC_Os12g03170.1|genepair1197-2
MATDAYPVQLLHRQATAATGGGQWHNLGAAYAAVRFLRPQGRSLVLYSGPDGGAQQRIVF
AYPILPGDAFERMDGETLSWEEPECGDEFALCFLDEAACAAVSGAISPVTESLAALDGLA
ERLAGLRVAREEGAPAGVDIAGRLAAISMGRP*
>11687.m00256|LOC_Os11g03460.1|genepair1198-1
MAAATEQHCGGGGGDKEKDLLSAVVGDIRSYSGSDPLRPWLRGMRKMEAALPPATLRAKL
PRFLQKCAQEFQDDARYRDDPRYLRVWIQLMDYVKDAKPLLKKMEKNRIGLKRSAFYMAY
ALYYEKHKRFEDAENMYRLGTQKYHLPFINVFNSLAEPVGELQKAHEQFIRRMKLYKRRK
SRVQQERMPNKVQSIATSKNEVEGQSRSCTEPKSNPVQRSGSGSNPHLGFPHPLGRPLYR
GTSGDTKSLSRHNSDDTVVVRFVGSALVGKSETEDACHHGLVEPTINTKEAMDAISSMFL
EPLEPETKLKRRSNRDKPSFNQEASAFEIFVDEDEPNKSGPSKLQDKNKLQDKNMKQDNP
KLSQQASAFEIFVDEDDPYCNNQKMVQHRHFNKENTQVNQKASGFEIFVDENEPHGNGRN
AMSHKSTVCPPKPSRDSRQQANFDFQKPFVGGFAILPDDEDEQLEKNDNGVKINSGTMQL
TDDNTSLCSRQTDSKIRCDDLHPAISGLREDTVFHRFVGSAVVGEPKVENACHHGLVEPT
VNLKEAMDDINNMFGIPLNFKGEKPKNKKTTTLSERKAAPLSGFSILADDEPGENPAAQV
KPSNASKFECQSGLFEPTITTRDVMAEINDMFGMPLDF*
>11686.m00239|LOC_Os12g03180.1|genepair1198-2
MAAATEQHCGGGGGDKEKDLLSAVVGDIRSYSGSDPLRPWLRGMRKMEAALPPATLRAKL
PRFLQKCAQEFQDDARYRDDSRYLRVWIQLMDYVKDAKPLLKKMEKNRIGLKRSAFYMAY
AVYYEKHKRFEDAENMYRLGTQNLAEPVGELQKAHEQFIRRVELYKRRKSRVQQERMPNK
VQSIAISKNEVEGQSRSCTKPKSNPVQRSGSGSNPHLGFPHPLGRPLSRGTSGETMSLSR
HNSDDTVVVRFVGSALVGKSETEDACHHGLVEPTINTKEAMDAISSMFLEPLEPETKLKR
RSNRDKPSFNQEASAFEIFVDEDEPNKSGPSKLQDKNMKQDNPKLSQQASAFEIFVDEDD
PYCNNQNMVQHRHFNKENTQVNQNASGFEIFVDENEAHGNGRNAMSHKSSGCPPKPSRDS
KQQANFDFQKPFVGGFAILPDDEDEQLEKNDNGVKINSGTVQLTDDKDTSLCSRQTDSKI
RCDDLRPAISGLREDTVFHRFVGSAVVGEPKVENACHHGLVEPTVNLKEAMDDINNMFGI
PLNFKGDKPKNKKTTALSERKAALLSGFSILADDEPGENPAAQVKPSNASKFECQSGLFE
PTITTRDVMAEINDMFGMPLDF*
>11687.m00257|LOC_Os11g03470.1|genepair1199-1
MAAPVRSVLPVVLLGCGGVGRHLLRHIVSCRPLHANQGVAIRVLGVADSSSLLVADDLHS
NGFDDALLADLCAAKSAGSPLSSLLARGQCQLFNNTEARRKVIDTASVLGKTTGLVLVDC
SATYDTVGMLKDAVDCGCCVVLANKKPLTCAYEDFEKLVSNFRRIRFESTVGASLPVIAS

VTRIIASGDPVSRIVGSLSGTLGYVMSELEDGKKFSEVVKTAKSLGYTEPDPRDDLSGMD
VARKALILARLLGQQISMENINVESLYPSELGPDAMSTKDFLESGLVQLDKSIEERVKAA
SLKGNVLRYVCKIESTGCQVGLEELPKNSALGRLRGSDNVVEIYSRCYESAPLVIQGAGA
GNDTTAAGVLADILDLQDLFHKTA*
>11686.m00240|LOC_Os12g03190.1|genepair1199-2
MAAPVRSVLPVVLLGCGGVGRHLLRHILSCRPLHANQGVAIRVLGVADSSSLLVADDLHS
NGFDDALLADLCAAKSAGSPLSSLLSRGQCQLFNNPEARRKVIDTASVLGKTTGLVLVDC
SATYDTVGMLKDAVDRGCCVVLANKKPLTCAYEDFEKLVSNFRRMRFESTVGAGLPVIAS
VTRIIASGDPVSRIVGSLSGTLGYVMSELEDGKRFSEVVKTAKSLGYTEPDPRDDLSGMD
VARKALILARLLGQRISMENINVESLYPSEFGPDAMSTKDFLESGLVQLDKSIEERVKAA
SLKGNVLRYVCKIESTGCQVGLEELPKNSALGRLRGSDNVVEIYSRCYESAPLVIQGAGA
GNDTTAAGVLADILDLQDLFHKTA*
>11687.m00258|LOC_Os11g03480.1|genepair1200-1
MSASRVASDVIAPLPYARPCFMMVVEIGMASALETLCGQSYGAKQYHMLGIYLQRSWIVL
FCCAVILLPIYLFTTPLLIALGQDPDISVVAGTISLWYIPIMFSYVWGLTIQMYLQSQSK
NMIVTYLSLLNFGLNLFLSWLMVVKFHLGLAGVMGSMVIACWIPIFGQLAYVFFGGCPQT
WTGFSSSAFTDLGAIIKLSISSGVMLCVELWYNTILVLLTGYMKNAEVALDALSICLAYI
FTESQEVVDAVADLAPLLAFSILLNSVQPVLSGVAIGSGWQSVVAYVNVASYYLIGIPIG
AILGYALGFEVKGIWIGMLVGTLVQTLVLLFITLRTNWEKQVEIALERLNRWYTDDNGRS
QNSRGNP*
>11686.m00241|LOC_Os12g03200.1|genepair1200-2
MERTTEDDERLTDPLLEPKPAINGGGGGSNEEEEEVGSLGRRLVEENKKLWVVAGPSICA
RATSFGATVVSQAFIGHIGATELAAYALVSTVLMRLSVGILIGMASALETLCGQSYGAKQ
YHMLGIYLQRSWIVLFCCAVILLPIYLFTTPLLIALGQDPDISVVAGTISLWYIPIMFSY
VWGLTIQMYLQSQSKNMIVTYLSLLNFGLHLFLSWLMVVKFHLGLAGVMGSTVIACWIPI
FGQLAYVFFGGCPQTWTGFSSSAFTDLGAIIKLSISSGVMLCVELWYNTILVLLTGYMKN
AEVALDALSICLAYIFTESQEVVDAVADLAPLLAFSILLNSVQPVLSGVAIGSGWQTIVA
YVNVASYYLIGIPIGAILGYALGFEVKGIWIGMLVGTLVQTLVLLFITLRTDWEKQVEIA
RERLNRWYTDDNGRSQNSRGNP*
>11687.m00260|LOC_Os11g03500.1|genepair1201-1
MERPGDEHDDCRTVPLLEPKHAHGEGSNNKQEEDEEEVGSLGRRVLVESKKLWVVAGPSI
CARFSTFGVTVISQAFIGHVGATELAGYALVSTVLMRFSGGILLGMASALETLCGQSYGA
KQYHMLGIYLQRSWIVLLCCAVLLLPIYLFTTPLLIFLGQDPKIAAMAGTISLWYIPVMI
SNVGNFTLQMYLQAQSKNMIVTYLAMLNLGLHLFLSWLLTVQFYLGLAGVMGSMLAFVFF
GGCPLTWTGFSFAAFTELGAIVKLSLSSGVMLCVELWYNTILVLLTGYMKNAEIALDALS
ICLNINGWEMMISIGFLSAKGVRVANELGAGSARRAKFAIFNVVTTSFSIGFMLFVLFLI
FRGRLVYIFTESTVVADAVAELSPLLAFSNLLNSIQPVLSGVAVGSGWQSVVAYVNVTSY
YLFGIPIGVILGYVLGFQVKGIWIGMLLGTLVQTIVLLFITLRTDWEKQVEIARQRLNRW
SMDENGRQQNPGENP*
>11686.m00247|LOC_Os12g03260.1|genepair1201-2
MERPGDEHDDCRTAPLLEPKHAHGEGSNNDKQEEDEEEVGSLGPRVLVESKKLWVVAGPS
ICARFSTFGVTVISQAFIGHIGATELAGYALVSTVLMRFSGGILLGMASALETLCGQSYG
AKQYHMLGIYLQRSWIVLLCCAVLLLPIYLFTTPLLIFLGQDPKIAAMAGTISLWYIPVM
ISNVGNFTLQMYLQAQSKNMIVTYLAMLNLGLHLFLSWLLTVQFHLGLAGVMGSMVIAYW
IPVFGQLAFVFFGGCPLTWTGFSSAAFTELGAIVKLSLSSGVMLCVELWYNTILVLLTGY
MKNAEIALDALSICLNINGWEMMISIGFLSATGVRVANELGAGSARRAKFAIFNVVTTSF
SIGFMLFVLFLIFRGRLAYIFTESKVVADAVAELSPLLAFSILLNSIQPVLSGVAVGSGW
QSVVAYVNVTSYYLFGIPIGVILGYVLGFQVKGIWIGMLLGTLVQTIVLLFITLRTDWEK
QVEIARQRLNRWSMDENGRQQNPGENP*
>11687.m00262|LOC_Os11g03520.1|genepair1202-1
MELLPLLLLLLLRIVASAPASPLATALFVLGDSTASCAATTLPLNLSLTSSSGNCLFPSA
HRLLPDLLAAKMGLPSPPLITTLNGTATEVARGVNFAGEDGGRGAIFRLGAVGQQLRMAT
ETLQLLRLEAPTPQDADAAAGGAVFVLSFGTDAYARVLSRGAGADASAPKHGRRGLARLL
ADRVARAVEELYEAGARRTAVMGVAPLGCAPRVMWEGLHVVDGRSCVEEANELVQGYNAR
VAARLAALRPRLAGADVVFCDIYKGIMDIITHPARYGN*
>11686.m00248|LOC_Os12g03280.1|genepair1202-2
MALLPLLLLLLLRIVASAPASLPATALFVLGDSTASCAATTLPINLSLTSSSGTCLFPSA
HRLLPDLLAAKMGLPPPPLISTLNGTAAEVARGVNFAGEDGGRGAIFRLGAVGQQLRLAT
ETLQLLRLEAPTPQDADAAAARAVFILSFGTDAYARVLSRGSEADASAPKHGRRGLARLL
ADRVARAVEELYEAGARRTAVMGVAPLGCAPRVMWEGLHVVDGRSCVEEANELVQGYSAR
VAARLAALRPRLPGADIVFCDIYKGIMDIITHPARFGKQDWSKFNNSIANY*
>11687.m00263|LOC_Os11g03530.1|genepair1203-1
MASKAIKRKPYTADIDRSEKQMETIIPDSVREPLLGNRTHESKSERHEPNMQPNLWDGKG
QERLGWMHVISTFIAQSVRKIGNALSQFGPLLAKFFSRSCASHGSHDEQAVLLDLSPLQE
ERLRFLRQRLNVPFDSSSVKHQDALKELWRLAYPSRQLPPLKSDLWKEMGWQNSDPATDF
RIPFTVYYTRRMVRDLNGNIPLQ*
>11686.m00249|LOC_Os12g03270.1|genepair1203-2
MASKAIKRKPYTADIDRSEKQMETIIPDSVREPLLGNRTHESKSERHEPNMQPNLWDGKG
QERLGWMHIISTFIAQSVRKIGNALSQFGPLLAKFFSRSCASHGSHDEQAVLLDLSPLQE
ERLRFLRQRLNVPFDSSSVKHQDALKELWRLAYPSRQLPPLKSDLWKEMGWQNSDPATDF
RAGGFMSLENLIYFARNYPDSFHSLLHKADGKRSEWEYPFAVAGVNISYMLVQMLDLQSG
KMGTKVSSQFVQLLREDEMAFDNLFCMAFQMLDAQWLTRQASYMEFNEVLKSMRIQLEQE
LTIGSISCVQEMPSFRLLKR*
>11687.m00264|LOC_Os11g03540.1|genepair1204-1

MAKRSSPDPASSSPSASSSPSSPSSSSSEDSSSPMSMPCKRRARPRTDKSTGKAKRPKKE
SKEVVDPSSNGGGGGGGGKRSSIYRGVTRHRWTGRFEAHLWDKNCSTSLQNKKKGRQVYL
GAYDSEEAAARAYDLAALKYWGPETVLNFPLEEYEKERSEMEGVSREEYLASLRRRSSGF
SRGVSKYRGVARHHHNGRWEARIGRVLGNKYLYLGTFDTQEEAAKAYDLAAIEYRGANAV
TNFDISCYLDQPQLLAQLQQEPQLLAQLQQELQVVPALHEEPQDDDRSENAVQELSSSEA
NTSSDNNEPLAADDSAECMNEPLPIVDGIEESLWSPCLDYELDTMPGAYFSNSMNFSEWF
NDEAFEGGMEYLFEGCSSITEGGNSMDNSGVTEYNLFEECNMLEKDISDFLDKDISDFLD
KDISISDRERISPQANNISCPQKMISVCN*
>11686.m00251|LOC_Os12g03290.1|genepair1204-2
MAKRSSPDPASSSPSASSSPSSPSSSSSEDSSSPMSMPCKRRARPRTEKSTGKAKRPKKE
SKEVADPSSNGGGGGKRSSIYRGVTRHRWTGRFEAHLWDKNCSTSLQNKKKGRQGAYDSE
EAAARAYDLAALKYWGPETVLNFPLEEYEKERSEMEGVSREEYLASLRRRSSGFSRGVSK
YRGVARHHHNGRWEARIGRVLGNKYLYLGTFDTQEEAAKAYDLAAIEYRGANAVTNFDIS
CYLDQPQLLAQLQQEPQLLAQLQQEPQVVPALHEEPQDDDRSENAVQELSSSEANTSSDN
NEPLAADDSAECMNEPLPIVDGIEESLWSPCLDYELDTMPGAYFSNSMNFSEWFNDEAFE
GGMEYLFEGCSSITEGGNSMDNSGMAEYNLFEECNMLEKDISDFLDKDISDFLDKDISIS
DRERISPQANNISCPQKMISVCN*
>11687.m00265|LOC_Os11g03550.1|genepair1205-1
MASSAFKSTTRRTLHPAADDRPPARPRKAPPPCPRRSRSASVEPRARGIGEYAAGNTRTN
PLFDDSASPPPPQPQVDTEAAGCRGGEARRERGREVARNGSCAGGSGRARSVSLAPRGRG
ADSSPSWGNGDGGGGRRASRAPSVAVDLQPYRGDEKRREIETRSLHSISTNPLLASSVLA
KFEQSDAEIRDMSIEFQFWESMHSVSSSVEVIWQSNHSNVPVQQVIEIPPEFDPDSSEFV
SDISDYTTEFKKEEILHIPFEFDLDRADLAPDIEHHSIELQREQMEIPLDFDPDSAELSP
DITEYTTKLKQSHERARKLRADLAVEEQREQELSRVLKGIVTTPNFTEAHKRRPRRKSSV
ERLKVSKHLAEEAMNYFEECVSISTLDSTDFSSLEDPQINSVVNIPQKSRNTSFNKGGSS
IAEIHYPTDRHWHNEESDNQTQCSVSLTGSDVSGGRTFSHNMMTPVSRTTNNSSDDLDGF
DTPKSRSSCFSFTHEPTKTVEGDDVQQYLRSFGRGISKDLREIRLTYGYCDI*
>11686.m00254|LOC_Os12g03320.1|genepair1205-2
MAIVLHWESRNYFFMGQREYAATSSVERLKVSKHLAEEAMNYFEECVSISTLDSTDFSSL
EDPQINSVVNIPQKNRNTSFDKGGSSIAETHYPTDRHWHNEESDNQTQCSVSLTGSDVSG
GHTYSHTMMTPVSRTTNSSSDDLDGFDTPKGRSSCFSFTHEPTKTVEGDDVQQYLRSFGR
GISKDLREIRSNYCDDDYVFQKMNADLIMDTVTFKNMVNFGGLLICNIRRY*
>11687.m00266|LOC_Os11g03560.1|genepair1206-1
MKKKALHRVFAAIFLLHLLLSVTAAASPTIGTDLLRDGNNNAVAAAAARSSRRLLLQQQQ
PTAAAPAAMATNTFRVNGVHQANGEPKVEFDASMKHNPGTNFNPRHN*
>11686.m00255|LOC_Os12g03330.1|genepair1206-2
MKKKALHRMFAAIFLLHILLAVTAAASPKTSTGLLRDGNNNAVAAVAARSSRRLLLQQQQ
PRAAAPTAAMATNTFRVNGVHQANGEPKVEFDASMKHNPGTNFNPRHN*
>11687.m00267|LOC_Os11g03570.1|genepair1207-1
MADTKADAKAEAKAETIGGGGSGSFSEQAFVEKLNKLNNTATRIQSILSSAALSNWCIFH
RKRARKVVDTWEKQYNSANKDKKVSFLYLSNDILQNSKRKGGEFVNEFWRVLPGLLKDFY
VNGGEDGKKVVGRLIDIWDERKVFGTRIESLKDDILGGSTHTMGNNGNSSNPSSHPSSVS
KAVRKDSGTVTKKLTIGGMPEKIVTAYQSVLDQHFDEDTALNKCNNAVSVLERMDKDVDD
ACTQGIQQGSSLISDLQGQETVLKQCIEQLESVNMARITLINKLREALSEQEAKSELLRN
QLHNHMLDLSISYAMSVTQLQWNVKVARAKAEHAMQLKQRLGSALNNGAGSSSSPLMVTL
PPGQTAAMMQNSAAMPIFPHYQPLHPATSLPATSSAVGDEPKKTAAAMADKLASLSAPEK
VLSSIFSSLAAEQARNSGSTSGDLSAGPPGFESNKKPRLDNPIHVSDMGAPPFFGQVPQV
QPQIGATAALGGTQPPTQANQATGSFPPPPPPLPLMPQFVQNTGGMFGMGPFGMVSGSAP
PPPLLPNIMSAGFPRLSAPPPLPLPTQPQNQSQPQQQQSPQAPQQSPTSTGFFQPPGAGF
FPPVQVQQSPSAQRQ*
>11686.m00256|LOC_Os12g03340.1|genepair1207-2
MADTKAEAKAETIGGGGGGSGSFSEQAFVEKLNKLNNTATRIQSILSSAALSNWCIFHRK
RARKVVDTWEKQYNSANKDKKVSFLYLSNDILQNSKRKGGEFVNEFWRVLPGLLKDFYVN
GGEDGKKVVGRLIDIWDERKVFGTRIESLKNDILGGSTHTMGNNVNSSNPSPNPSSVSKA
ARKDSGTVTRKLTVGGMPEKIVTAYQSVLDQHFDEDTALNKCNNAVSVLDRMDKDVDDAC
TQGQRGGKIVVSPFQPQYHPETPQNPQIPPPSQELNAILPPLRAVDEAGCKIVAYVGRHI
VGIQQGSSLISDLQGQETVLKQCIEQLESVNMARITLINKLRKALGEQEAKSELLRNQLL
VARAKAEHAMQLKQRLGSALNNGAGSSSSPLMVTLPPGQTAAMMQNSAAMPIFPQFQPLH
PATSLPATSSAVGDEPKKTAAAMADKLASLSAPEKVLSSIFSSLAAEQARNSGSTSGDLS
AGPPGFESNKKPRLDNPIHASDMSAPPFFGQLPQVQPQIGATAALGGTQPPTQANQAAGS
FPPPPPPLPLMPQFVQNTGGMFGMGPFGMVSGSAPPPPPLPNIMSAGFPRLSVPPPLPLP
TQSQNQSQPQQQQSPQAPQQSPTSTGFFQPPGAGFFPPVQVQQSPSAQRQ*
>11687.m00268|LOC_Os11g03580.1|genepair1208-1
MAHSHSAASLHAARLLPQQRTPTAPRTLLPAGGGLLLRRPHPPLHQQRRSRSSSRPDLRC
RRRLLTARGDYDFYENYADEEGDEEEESEVIGGSFDAAVALFNGGEFHACHDVVEELWYT
AEEPTRTLLHAILQCAVAFHHLFNQNHRGAMMELGEGLCKLRKLRLDDDTTSPFSRFQEE
VAAALNFIYRTQKELAACTDDLCLTMDGSATSYQLLGNFAAGQKLYRLETTTSADGDGVP
TIIFSASSRLVRVKLPTLSATEHHLAALQCTSEYI*
>11686.m00257|LOC_Os12g03350.1|genepair1208-2
MAPALAPCRSAASLHAARLLPQQRTPTAPRILLPAGGLLLRPQPPLHHPQRRSSSRRDLR
CRRRLLTARGDYDFYENYADEEGDEEEESEVIGGSFDAAVALFNGGEFHACHDVVEELWY
TAEEPTRTLLHAILQCAVAFHHLFNQNHRGAMMELGEGLCKLRKLRLDDDDDTTSPFSRF
EEEVAAALNFIYRTQKELAACTDDLCLTMDGSATSYQLLGNFAAGQKLYRLETATGADGD
GDGVPTIIFSASSRLVRVKLPTLSATEQHLAALQCTSEYI*

>11687.m00269|LOC_Os11g03590.1|genepair1209-1
MAPLSWRHHTLLQALLSRGPLSERDFHALFSAISGGKNPATHRQLFDDTLLKINKELTYL
QFELRAGINQYDGTVYYGVVNNIADEESKLGSKFSVPQIAFYKGLLEAIVHEAGNDGSIT
NIDALNTRIENQVVIADASQGSQSRLPTSITNFSLSQKEKTLNELIQDRWLSYTPTGKIG
LGIRSFLDLRSWLRSNDIPSCEVCNEACIKASCCPNEECNVRIHGYCLKKKFSQRKASRA
CGCGTEWPRLEGEDDGAEDEDVNEPEEDQVPSANQHSRTRRRGVKSELVEENERAGPSAR
MTRRALRSSKAEAVEAAQEVPSAAGPSQSTRASKRRKN*
>11686.m00258|LOC_Os12g03360.1|genepair1209-2
MAPLSWRHHTLLQALLSRGPLSERDFHALFSAISGGKNPATHRHLFNDTLLKINKELAYL
QFELRAGINQYDGTVYYGVVNNIADEESKLGSKFSVPQIAFYKGLLEAIVHEAGNDGSIT
NIDALNTRIENQVVIADASQGSQSRLPTSITNFSLSQKEKTLNELIQDRWLSYTPTGKIG
LGIRSFLDLRSWLRSNDIPSCEASSCPNEGCNVRIHVYCLKKKFSQRKASRACGCGTEWP
RLEGEDDGAEDVNEPEEDQVPSANQHSRTRRRGVKSELVEENERAGPSARMTRRSLRSSK
AEAVEAAQEVPSAAGPSQSTRASKRRKN*
>11687.m00270|LOC_Os11g03600.1|genepair1210-1
MADRVHPMPAPPPPSSSPPLEHAAAATETTPLHPSFRGARPPSPGTYIIQIPKDQVLRVP
PPDRARRYKKLAARPARRRRLRHACCAAFCAALLLLLLAAAFVGAVYLVFRPRAPSFSVA
SLSIRGLDALAVSSLTPQIDAAVRADNGANKKTGIDYRGGGEVTVSYAGERLAAGPWPAF
HQAPRNVTVFSTALAGGGVSFPEEQRKRLAAEQAAGAVPLTVEAIVPVRLRFGKVLRTWT
VDVKTRCEVTVNKLAAAAPPANRGCRVKVRPLWWWW*
>11686.m00259|LOC_Os12g03370.1|genepair1210-2
MADRVHPMPAPPPPSSSPPPGHDAAAATETTPLHPSFRGAPPPSPGTYIIQIPKDQVLRV
PPPDRARRYKKLAARPARRRRLRHACCGAFCGVLLLLLLAAAFVGVVYLVFRPLAPSFSV
ASLSIRGLDALAVSSLTPQIDAAVRADNGANKKTGIDYRGGGEVTVSYSGERLAAGPWPA
FHQAPRNVTVFSTALAGGGVSLTEEQRKQLAADQAAGAVPLAVDAIVPVRLRFGKVLRTW
TVDVKARCEVTVNKLAAAPANRGCMVKVRPLWWWW*
>11687.m00271|LOC_Os11g03610.1|genepair1211-1
MEDNVLVSSKIHGRSKSTTAKRNCIICGQRISKRRRTQHNFQKISRGQLNLQRTRPCLLN
FQSLPKDIVLRVMSKLTLKEVAQLSVVSTNWRQAWTFHPNLYFGIKTALGNNAKRKGTSS
DLNCRISSGNKFIKRVDAILEKHCGTMGCHLQRMELHAPNLTTFEYDGSLALVTLNECSN
IKASTIRLFDEKTLQNILTGIPSVLPHVETLYVEVHVKTQMSGFTQSPLKFTQLKCLTLE
ITFERGSFDRNSVFQLTNLFVAAPFLEDLYLDMYCSLNRCPLDLDDIVDQPHYHLKMVCI
FGFCGNTGQVELAKCILRNALILEQMIIDPKGRYRLDGYFGRQEADEKLVPEDIDGVLTI
L*
>11686.m00261|LOC_Os12g03390.1|genepair1211-2
MEDNVLVSSKIHGRSKSTTAKRNCIICGQRISKRRRTQHNFQKISRGQLNLQRTRPCLLN
FQSLPEDIVLRVISKLTLKEVARLSVVSTNWRQAWTFHPNLYFGIKTVLGNNAKRKGTSS
DLNCRISSANKFIKRVDAILEKHCGTMVNKFAVKFGLSNEHANHVNGWVAFAIASKARVI
ILDFSPDWKSHENNYDFPCHIFDKHNGSYLEALRLDSVTLNPPLDFCGFANLKLLALDNV
RLQHLEQLISKCHVLEWLSIQSCNQLHNLHVSEPLCRLQCLSIQGCHLQRMELHAPNLTT
FEYDGSLALVTLNECSNIKASTIRLFDEKTLQNILTGIPSVLPHVETLYVEVNVKTQV*
>11687.m00272|LOC_Os11g03620.1|genepair1212-1
MDRSTAATIFWHRFYKVADTRETLKEAMMLSNPTNCRPHMWACNIHEEQFMMQIFSLKLS
NITATVDGPVHLYGYFAVRDHLDPLRNYIFNRTRDDPFIMGQDNGVDSDNSLIPMPGPKR
GIGNQARVLIEFDMKIKNGETRDDDFQLIDGAIICSEFVLPNRVFTQRIEGDCGAVDISL
ALLHSTVEATVQVSISQVHGNGFSLSLYSYTSRIPEKIQLFDGFISKPCDLNRFVVAVVV
NTPLILIFKIDKRDGSDHVPGCCAFKARTHGYEYDMQELKLGCTNILVKLSWSTLE*
>11686.m00262|LOC_Os12g03400.1|genepair1212-2
MAKRRAESELELVEAEAEAEAEAEAEKNGRRAAGSQDGVNRAFILECSKHSDGSIYSGDD
FWHRFYKVADTRETRMEAMMLSNPTNCRPHMWACKAHSVQFMMQIFSLKLSNITAAVDGP
VHLYGYFAVRDHLDPLRNYIFNRTRDDPFIMGQDNGVDSDNSLIPMSGPKRGIGNQVRVL
IEFDMKIKNGETQDDDFQLIDGAIICSEFVLPDRVFTQRIEGDCGAVDISRALFHEAVEA
TIQVSISQVHVNGLSLSLYSYTSRIPEKIRLFDGVISKPCDLNRVVAVVENTPLFLIFRA
VHRDGSDYDIPKYCPLVFKVDQGDGSYRVSEYCPFKARRHGYDMKELKLGGARVLLKVSW
STLK*
>11687.m00274|LOC_Os11g03640.1|genepair1213-1
MEARAVRPLLFKPTFYLRVKFKKIYMAFGANDIVLHIKRLMLCPSLFNFLLSSSPVFLLT
ALLLGALLSYGEPAVLVLGENQQTLSFKSKISITDCSIDKVETVAVEEHLDKTTTSNEVY
VRERNFEGNIHDTHWEEKNGTYMTVDTALNDEIHTKDGTSDYDLQDTHREGKGITSVETD
TVPCVAPSSFAYSGVTVETEDVGENSKKNDELQELGSINPESDNNKVQYQYQLGEFMSSC
WEPVMRQEPQDACSDSESDLTESSPDASMTDIIPMLEELHPLIDLQTGHPSLVSRDNLNT
SSDDDEDDLEEEDASTDENQLEGKIDDFANWKDVIDLNYLDMDNNSKLEGMMDLQRAKNI
LKFELDRRLMDLQAADAVQKMEEASRFRVQHRLVSFHGKIYLIFAVNQNMAHDSPLQETW
TPLSYFSARRHRKHGNLYVRHSTSLHHNSFKLEKDEISENDAHNSQSDCDAKQEGNNSKL
FGSLEAHIGEEIKILGAAISDVGVLEVNSGMDSGNQNADFSDDISLSPIQKSRQSTFEAK
EAVHAGIEQLTSCSPYKVNNFEAHIVEADSIDEFNSLFKCRMEEVLVQSISESSISQPLT
VKLEDELSEPLSSDSGTGTHFIDGSSVEDSDPQFAQLKDEALVSATSNATCRNESIEEKS
SEALLAGNEDYSELPNELLKSGDPQFADSSEIQMQVIEATGH*
>11686.m00263|LOC_Os12g03410.1|genepair1213-2
MLWARPKLATQQASGGAAAFALNSGGRRNSGGEDGAGGATEVDAARGWIQRKRNARRSRR
SFISILPSPRPEISAPLLVASIGVPLPRIRPTFYLRVKFKKIYMAFGANDIVLHIKRLML
CPSLFNFLLSSSPVFLLTALLLGALLSYGEPAVLVLGENQQTLSFKSKISITDCSIDKVE
TVAVEEHLDKTTTSNEVYVRERNFEGNIHDTHWEEKNGTYMTVDTALNEEIHTKDGTSDY
DLQDTHREGKGITSVETDTVPCVAPSSFAYSGVTVETEDVGENSKKNDELQELGSINPES

DNNKVQYQYQLGEFMSSCWEPVMRQEPQDACSDSESDLTESSPDASMTDIIPMLEELHPL
IDLQTGHPSLVSRDNLNTSSDDDEDDLEEEDASTDENQLEGKIDDFANWKDVIDLNYLDM
DNNSKLEGMMDLQRAKNILKFELDKRLMDLQAADAVQKMEEASRFRVQVPSISTGRQNPF
DSSNGSDEIIELPHVPDSAPSCLLPRKNLFDLAVNQNMAHDSPLQETWTPLSYFSARRHR
KHGNLYVRHSTSLHHNSFKLEKDEISENDAHKSHNSQSDGDAKQERNNSTLFGSLEAHIG
EEIKILGMAISEVGVLEVNSGMVSSNQNADFSDDISSSPFQKPRQSTFEAKDTVHAGIEQ
LTSCSPYKVNNFEAHIVEADSIDEFNSLFKCRMEEVLVQSISKSSISQPLTVKLEDELSE
PLSPDSGTGTHFIDGSSVEDSDPRFAQLNDEALVSSTSNATCRNESIEEKSSEALLAGNE
DYSELPNELLKSGDPQTADSSEIQVQEQQCDTVEHQLQFLGLTVKKQSLHKSPTTTTTRT
RGKSVMSHRDATLFSSDVASLAAPNFLLDGVINFVMAHMTTELGDESLLLVSPSVASLLA
NLQDYEPETVADTAQALLLASRRMVLFPVNNSERLDKADDGSHWSLLVLDNITGRFVHHD
SMDGANLPAATRLADALRPLLPAPPQGPPISGPTPQQSNGYDCGVYLLAVALAICRWWKK
HPRTEEAAPCWFESVMDQVTCERFTRLCIKE*
>11687.m00276|LOC_Os11g03660.1|genepair1214-1
MLTAAMHMLDSSTSSSPWLPPDLMPPPPSAATLHRHFRGAAAAASTSRRIAKRRPRPSRR
LPTTYISADPANFRRMVHQVTGADDLPPPPPPSLSPTTTELLRHAAPAGSPGPAGALMLP
TLDTSAFLLGRRAEPTAAAAPCDVSVALVGGAVLINTPSNSTTAPAPSH*
>11686.m00264|LOC_Os12g03420.1|genepair1214-2
MHMLDSSTSSSPWLPPDLMPPPPPPPPAAATLHHHFRAAAAAPGRRRIAKRRPRPSRRLP
TTYISADPANFRRMVHQVTGADDLPPPPPSLSLQQTTELLLPAVHAAPAGSPGAAGALML
PTLDTSAFLLGRRAEPAAAAPPCDESVALVRGGAGNYGSNNNSISSSSSSGNCGGGFPTL
DSWDLL*
>11687.m00277|LOC_Os11g03670.1|genepair1215-1
MAMASTLPLLLVHRSTPPTPRPTAPPLLHSRRLTLPSRLASLPATIAVVHPRKGVRLSKL
HAASCCDSASAAGVTTGGGAGGGGAKGAMDWRLLLAWYLLALDKHPITTKAVTSAVLTLT
GDLICQLAIDKVPKLDLKRTFVFTFLGLVLVGPTLHVWYLYLSKLVTINGASGAIARLLL
DQFIFSPIFIGVFMSLLVTLEGKPSLVVPKLKQILCSTADAVAVADMWVREWLSSVIANW
QLWIPFQFLNFYFVPQKFQVLAANFVALAWNVILSFKAHKEVTVK*
>11686.m00265|LOC_Os12g03430.1|genepair1215-2
MAMASTLPLLLVHRSTPPTPRPTAPPLLHSRRLALPPRPASLPATTAVVHPRKGVRLSKL
HAASCCDSASAAGVAAGGGGGGGGAKGEMDWRLLLAWYLLALDKHPITTKAVTSAVLTLT
GDLICQLAIDKVPKLDLKRTFVFTFLGLVLVGPTLHVWYLYLSKLVMINGASGAIARLLL
DQFIFSPIFIGVFMSLLVTLEGKPSLVVPKLKQEWLSSVIANWQLWIPFQFLNFYFVPQK
FQLDFPKATQTTFLYLLQFKMMLIGAKGAVCSFVTHEEQPVVSLEAKEMDKVK*
>11687.m00278|LOC_Os11g03680.1|genepair1216-1
MAEMVRLPEQQRRRPLTIANLPEEILSEILLLLPPKSILQCRAVCKVWRDVTSDRAFLLP
HHCRQPPQRLLTFIRDVGSRHDDLDILDYCVEAVDFRTHQFQSLARFTGQDYDCSLEDSP
FTVHASCDGLLLMSYNNYLHLCNPTTRQWLWVFPPALQHDTVLGLYSHGHSSEYRVLYYR
EIGLGPEFYISIVGSGKERSIWPHSSSASLRKWLAKGKEETQFNEPFLFHGNLHWLPHLG
GQNKIVVFDTLDEVFRWLHVPFKMHNMSSLLEIEGSLAMSNSHIGSSKVDLWLLQDYKHM
VWVHKYRIELPVIEIRRFEEDDGWYLHIVSQEGDVLVDGFDWQFHYDIKGNLLEKFQCSG
RMLNITPHILQESLVPHEVCQILNNESRHEPHFFRGL*
>11686.m00266|LOC_Os12g03440.1|genepair1216-2
MAMAETVRLPEQQRRRPLTIASLPEEIMSEILLLLPPKSILQCRAVCKAWRDVTSDRAFL
LTHHRRQPPQRLLTFIRDVGSHHDDLDILDYCVEAVDFRTHQFQSLARFTGQDYDCSLED
SPFTVHASCDGLLLMSYNNYLHLCNPTTRQWLWVSPPALQHDKVVGLYAHGHPSEYRVLY
YREFGLGRTFYITTVGSRKERRIWPHSSSESLRKWLTKGTEDTEFNKPFLFHGNLHWLPQ
SGRQNKIVVFDTLDEAFRWLHVPFKMHNVSSLLEIEGSLAMSNSHIGSSKVDLWLLQDYK
HMVWVHKYRIELPVIEIRRLVEDDVWFLHIVSQEGDVLVDGSYWQFHYDMKGNLLEKFQC
SGRMLNITPHILQESLVPHEVFQILDNESRHAPHFFRGL*
>11687.m00279|LOC_Os11g03690.1|genepair1217-1
MTNVSLPVAAQVHMGPATAAAAAAEAALARRAADPLPALRRRDALPLPLPARLFAQLHGL
LLTAGLARHSPNFSLLLRLASPLLPVPHRLRLLLSSPLPPTTFLANSFLLASSSPGCLPS
ALSLYALLFLSSSSPPLLRPNAFTYPPLFRAAPPALALALATHSIKFLGAHAASCDRVLG
AALLDVFARCGRIASCRKVFDRIANPDLPAWNALLSAYARLRARDVACASSAADAILEMF
VRMLSLAIKPNEITLVAVIGACGELGAVSHGVWAHTYAVKRRLAVNCIVATALVEMYAGC
GRLDLAEQVFAAASDRDTRCYNAMLHGLAVHGHGRAALSLFDRMHGEGVPVDGVTVLSVM
CACAHAGLVDEGLDYFDRMEIEFGIEPSIEHYGCMVDMLSRAGRLNDAEKLIHGMPIVPN
AAIYRSLIRACGIHGKLELGKKMIAELMRLEPDDSGNHVLISNFYATTNRWDDAKKARKE
MKSMGIDKSPGSSFVDINGVLHEFLVGDKTHPASKEIYAMVEDIETRLSECGHRSSTSSA
LFDVEEEDKADALSYHSERLAIAFALIASNPGAPIRIIKNLRVCADCHESAKLVSRVYGR
EIVMRDRTRFHHFRDGVCSCGDFW*
>11686.m00267|LOC_Os12g03450.1|genepair1217-2
MGPATAAAEAALARRAADPLPALRRRDALPLPARLFAQLHALVLTAGLARHSPNFSLLLR
LASPLLPVSHRLRLLLSSPLPPTTFLANSLLLASSSSRCLPSALSLYALLFLSSSPPLLR
PNAFTYPPLFRAAPPALALALATHSVKFLGAHAASCDRVLGAALLGVFARCGRIASCRRV
FDRIAHPDLPAWNALLSAYARLRARDVACATSAADAILELFVRMLSLAIEPNEITLVAVI
GACGELGAVSHGVWAHTYAVKRRLAVNCIVATALVEMYAGCGRLDLAEQVFAAASDRDTR
CYNAMLHGLAVHGHGRAALSLLDRMHGAGVPVDGVTVLSVMCACAHAGLVDEGLDYFDRM
EIEFGIEPRIEHYGCMIDMLSRAGRLNNAEKLIHEMLIVPNAAIYRSLIRACGIHGKLEL
GEKMIAELMRLEPDDSGNHVLISNFYARTNRWEDAKKARKEMKSMGIDKNPGSSLVDING
VLHEFLVGDKTHPASKEIYTMVEEIETRLIECGRRSSTSSALFDVEEEDKADTLSYHSER
LAIAFALIASNPGAPIRIIKNLRVCADCHESAKLVSRVYGREIVMRDRTRFHHFRDGVCS
CGDFW*

>11687.m00280|LOC_Os11g03700.1|genepair1218-1
MENSEDEAESDKLPLDLEPLRSLAPKFPTILGYDVETQSTDPLLVYATPSIPCSSSEQPQ
EAPASFSLPLPKSPVPIKATPISAAFPTPQHEDESSDQDYKPFCKNKKPAMPKRAKRPQQ
AEKSNDANIKRRSIRRNLDNEFNLCSSSSDNPKESVEGILMMFDSLRRRVLQLDEKEDAS
RRADLKAGTLMMQNNLRINNHKMIGHVPGVEVGDIFFFRIEMCIVGLHAPAMGGIDYISS
KNKDETLAVCIISSGGYENDDDDTDILVYTGQGGNSRHKEKHDQKLERGNLALMNSKSKK
NQIRVVRSAQDPFCNSGKIYIYDGLYRIEDTWTDTAKNGFNVFKYKLRRDPGQPDGISLW
KMTEKWKANPATREKAILLDLSSKVEHLPVCLVNDVDDEKGPSHFNYVAGVKYLRPLRKT
KPLQCCKCPSVCLPGDPNCSCAQQNGGDLPYSATGLLAKHTPMVYECSSNCQCSHNCRNR
ITQKGIKLNFEVFWTGDRGWGLRSWDPIRAGTFICEYAGEVIDETKMDIDVEEDKYTFRA
SCPGNKALSWNLGEELLEEKSTAVITKNFKKLPIIIRANNEGNVARFLNHSCSPNLLWQA
VQYDHGDDSYPHIMFFAMEHIPPMTELTYDYGTRGAPPGFEGKPFKACKLKSCLCGSKHC
RGY*
>11686.m00268|LOC_Os12g03460.1|genepair1218-2
MDASALSNPRLQAMLEEEKRKAMANEFVAKLTDVCWDKCITGSIGSSFSNSEASCLSNCA
KRFLELKMLTMQRPQEAPASFSLPLPKSPVPIKATPISAAFPTPQHEDESSDQDYKPFCR
QKKPTLPKRAKRPQQAEKSNDANIKRRSIRRSLDNEFNLCSSSSDNPKESVEGILMMFDS
LRRRVLQLDEKEDASRRADLKAGTLMMQNNLRINNHKMIGHVPGVEVGDIFFFRIEMCII
GLHAPAMGGIDYISSKNKDETLAVCIISSGGYENDDDDTDILVYTGQGGNSRHKEKHDQK
LERGNLALMNSKIKKNQIRVVRSAQDPFCNSGKIYIYDGLYRIEDTWTDTAKNGFNVFKY
KLRRDPGQPDGISLWKMTEKWKANPATREKAILLDLSSKVEHLPVCLVNDVDDEKGPSHF
NYVAGVKYLRPLRKTKPLQCCKCPSVCLPGDPNCSCAQQNGGDLPYSATGLLAKHTPMVY
ECSSNCQCSHNCRNRITQKGIKLNFEVFWTGDRGWGLRSWDPIRAGTFICEYAGEVIDET
KMDIDVEEDKYTFCASCPGDKALSWNLGAELLEEKSTAVTTKNFKKLPIIIRANNEGNVA
RFLNHSCSPNLLWQAVQYDHGDDSYPHIMFFAMEHIPPMTELTYDYGTRGAPPGFEGKPF
KACKLKSCLCGSKHCRGY*
>11687.m00283|LOC_Os11g03730.1|genepair1219-1
MASRESFMSTIFFFLLLFSLGCKCIASELHLHATQTAVLKVDASPQLARQIPDTLFGIFF
EEINHAGAGGIWAELVSNRGFEAGGPHTPSNIDPWSIIGDDSSIFVATDRTSCFSRNTVA
LRMEVLCDNCPAGGVGIYNPGFWGMNIEDGKIYNLVMYVKSPETVELTVSLTSSDGSQNL
ASTTIPLVPFLSILGQENKDVCPFPLVLKGLYCCRVSGASNWTKLEQKLVAQGTNRTSRL
QITTNKKGVVWFDQVSLMPADTYKGHGFRTELISMMLDLKPRFLRFPGGCFVEGEWLRNA
FRWRESIGPWEERPGHFGDVWHYWTDDGLGYYEFLQLSEDLGAAPIWVFNNGISHNDEVD
TAAIAPFVKDVLDSLEFARGNADSTWGSVRAAMGHPEPFPVKYVAIGNEDCGKKFYRGNY
LKFYNAIREAYPDIQMISNCDASSSPLDHPADLYDFHVYTDSKTLFSMKNTFDRSSRNGP
KAFVSEYAVWRSDAGRGSLLASLAEAAFLTGLEKNSDVVQMASYAPLFVNNNDQTWNPDA
IVFNSWQQYGTPSYWMQTLFGESSGAMFHPVTITSSYSGSLAASAITWQDSENSFLRVKI
INFGSDPVSLTISATGLQARVNALGSTATVLTSSNVMDENSFSNPNKQSRCKSRLLLTLS
PHSILRLPSPNSWQRCEAATQGILTSHLHCGKLSREQIQLVVDKIMMGEKIGEGNFVSFA
LMRTVSVTPDKEINHAGSGGLWAELVSNRGFEAGANTSNIDPWSIIGDESSVHVTTDRSS
CFSQNPVAVRIEVVCDDCPAGGVGIYNPGFWGMNVEERKTYNLVMHIRSLESVELTASLT
CSNGSQNLASNFVRETDLLNWTKIELQLLAHGTCRTSRLELTTRRRGVIWLDQVSLMPSE
TYKGHGFRQELMYMLLDLKPRFLRFPGGCFVEGNWLKNAFRWKETIGPWEERPGHYGDVW
HYWTDDGLGYYDFLQDAIDSLEFARGSKESTWGSVRAAMGHPEPFPLKYVALGNEDCEIF
KPTYQENYPKFYNAIREAYPDIQIISNCDGSSRPLDHPADLYDFHTVKITIDYAIVSLQV
FVSEYAVNNDKGGDAGNGSLLASLAEAAFLTGLEKNSDVIQMASYAPLFVNENDRTWNPD
AIVFNSWQQYGTPSYWMQTYFRESSGSVIHPVTISSSYFDLLAASAITWQDNEDIFLRVK
IVNFGPSAVNLTISSSGLQAGVNAAKSTVTVLTSSNLLDENSFSEPNKVVPVTRELPNAG
QEMQFLLLPFSLTSFDLAMF*
>11686.m00270|LOC_Os12g03470.2|genepair1219-2
MGSREPFLSAIFFFFFLLLFCLGCKCIASELHLHTTQTAVLKVDASLQHARQIPDTLFGI
FFEEINHAGAGGIWAELVSNRGFEAGGPHTPSNIDPWSIIGDDSSIFVATDRTSCFSRNT
VALRMEVLCDNCPAGGVGIYNPGFWGMNIEDGKTYNLVMYVKSPETVELTVSLTSSDGSQ
NLASSTIPVSGASNWTKLEQKLVAKGTNRTSRLQITTTKKGVVWFDQVSLMSADTYKGHG
FRTELISMMLDLKPRFLRFPGGCFVEGEWLRNAFRWRESIGPWEERPGHFGDVWHYWTDD
GLGYYEFLQLSEDLGAAPIWVFNNGISHNDEVDTAAIAPFVKDVLDSLEFARGSADSTWG
SVRAAMGHPEPFPVKYVAIGNEDCGKKFYRGNYLKFYNAIREAYPDIQMISNCDASSRPL
DHPADLYDFHVYTDSKTLFSMKSAFDRSSRNGPKAFVSEYAVWRSDAGRGSLLASLAEAA
FLTGLEKNSDVVQMASYAPLFVNNNDQTWNPDAIVFNSWQQYGTPSYWMQTLFRESSGAM
FHPITITSSYSGSLAASAITWQDSENSFLRVKIINFGSDPVSLTISATGLQARVNALGST
ATVITSSNVMDENSFSNPNKVVPVKSQLSNAAEQMQVTLAPHSFSSFDLALAQSKLVAEM
*
>11687.m00286|LOC_Os11g03760.1|genepair1220-1
MASRRNVGGYAPLPTEDRDDSNLTDDVDLRFTYTPKSLRKIPWKSIALALFLLLLGCSLL
FLSYFIFTGHMEGDNSQAYGLLFLGILAFLPGFYETRVAYYSSRGAPGYTFASIPDY*
>11686.m00274|LOC_Os12g03500.1|genepair1220-2
MASRRNVRGYAPLPTEDRDDSNLTDDVDLRFTYTPKSLRKIPWKSIALALFLLLLGCSLL
FLSYFIFTGHMEGDNSQAYGLLFLGILAFLPDSRQTSLLLYPFSEKDYIVLTLFCRFL*
>11687.m00287|LOC_Os11g03770.1|genepair1221-1
MSLVVFATRAQHNGLPEAFGSMECQRRGKRRDGLVGDRSRFHNGLLPWRHQSLFLFAIVL
VAASQVQLAINTDPFMSGACKTVAGSNGGVISVTFCMDALGSDSRSLNANHYSDLAIVAI
DLLTSNATSANAKIDSILKDDGGGMKPDDATTVSLQMCQAAYAGVCRIKQAD*
>11686.m00275|LOC_Os12g03510.1|genepair1221-2
MVSSHGIVNAFFLFAVALVAASQAQHAANADSFMSGACKIVAGSSSGVISVTFCMDALGS

DSRSLSASYYSDLAIIAIDLLTSNTTSTKAKIDNILKDDGNGLKPGDATTVCFQSCQAAY
ASVLQGQLGIFYNVQAGRFPEAMSALEKAANMVEECEKGFGKSNVKSLLTTENHDSFELA
KLGALLLNEEH*
>11687.m00290|LOC_Os11g03790.1|genepair1222-1
MPSFPPPGAVTICEINRDLGVLSPLPAPPLSAVSKLIPSLCVAVAADAVSDDRAKDAYGD
VLGMVFSPIPFQPDAIVATHEPPAATEAAEPAEIVPRTSLASTIAESFKQMIFPSCDPNL
LQEIDTQKVSWNPHKHCLAFVSGKNQVTVHDFEEPDNKESYILTSDHQKDVKAVEWRPNS
GKMIAVGCKGGICLWSASYPGNVASVKSGVTSSSFGAFPRGLGMPIRRGLSSISLVQWSP
SGDYLLTAKLDGTFHLWETNTWTSEPWSSSNGYVSGANWDPEGRIVLLSFSNSTTLGSIH
FSSKPPSLDAHLLPVEHPEISSLIVSRGIDKLAWDSSGERLALSFKDGNEMYHGLVAVYD
VRRSPLVSVLLVGFIRGPGEGAKPLAFAFHSKFKQGPLLSVCWSSGWCCTYPLILRPH*
>11686.m00278|LOC_Os12g03540.1|genepair1222-2
MPSFPPPGGVTVCEINRDLVVADALSDDRAKDAYGDVLGMVFSPIPFQPDATVATHEPPA
VTEAAEPAEVVPRTSLASTIAESFKQMLFPSCNPNLLQEIDTQKVSWNPHKHCLAFVSGK
NQVTVHDFEEPDNKESYILTSDHQKDVKAVEWRPNSGKVIAVGCKGGICLWSASYPGNVA
SVKSGVTSSSFGAFPRGSGGQWILVDILRGSSAELGANWDPEGRTALLSFSNSTTLGSIH
FSSKPPSLDAHLLPVELPEISSLIVSRGIDKLAWDSSGERLALSFKDGNEMYHGLVAVYD
VRRSPLVSVSLVAGAVAGVVHIPSYFVLIDSLRGVRKARWVKR*
>11687.m00292|LOC_Os11g03810.1|genepair1223-1
MRSLLAFFAASILLASTTTTVHGVHIDPNDIIKTIESECGDIIDCVDIYKQPSLKNPLLK
DHKILFKPSVDRPKIVEKMVVLGGNNSFKFAEQAWHRSEVYPQFFGDDLPRLYIYSTNDG
GVKLKCFNLECSFVQTSKKHAIGAKYDKFSTVGGTTYFTHVVIYRDDGPAVWWVSLMDEP
IGYFHESAFAAPFIESFHNEMGGHVLDRRPGGRHTLTPMGSGMYPSDGLQNAACIHAYLA
IAYTGADQVDDPVNTIVTHPKCYDIKGDGPDLYRPGINVAFGGPGGYDCDHN*
>11686.m00287|LOC_Os12g03630.1|genepair1223-2
MGGHVLDRRPGGRHTLTPMGSGMYPSDGLQNAACIHAYLAIAYTGADQVDDPVNTIVTHP
KCYDVKDDGPDLYRPGINVAFGGPGGYDCDHNYTQRRWKKPIDSARTRLEGRIRDHKLDK
LMIQLKNLRLALDLHEFISQQRNGYASLQLLSRWRHEVGLNIEIGAFLKKYPHIFDIYVH
PIKNNQCCKVTPKMADLIAEEDAAIWENEPAIVKRLKKLLMLSTDGTLNMHALWLIRREL
GDENLAVADVEEWRVKEYTEKWLAESETKYSFPINFPTGFKIEKGFREKLGNWQRLPYTK
AYENNELHPIHNVEQLEKHIVGILHELLSLTVEKMIPLERFSHFRRPFDMEVNLRELILK
HPGIFYISTKGSTQTVLLRESYSKGCLVDPNPVYNITCKGNYNPTPISVATLRWLTSGPA
NGLGVHSPDRETTAPIKGGGSGLGAMSRALEPLSWLMGLWGPRTGAGASGGPWLGSGILR
AFRLASNFPEPTALFGLGRLPRPSEWVLPTETGSAGCWRRASGRRPYLLRSHQGVNGARS
QSFPQQ*
>11687.m00293|LOC_Os11g03820.1|genepair1224-1
MASIAFGQRNLRVFPLRRHIWDAVFQHGPYNCTIQRRWKKPVDSARTRQEGRTRDHKLDK
LMIQLKNLRLALDLHELISQQRNGFASLQLLSRWRHEVGLNIEIGAFLKKYPHIFYIYVH
PVKRNECCKVTPKMAELIAEEDAVIRENEPAIVKRLKKLLMLMKDGTLNMHALWLIRREL
GLPDDYRCSILTNHQSDFSLGSPDTLTLVTRDETGFKIENGFREKLGNWQRLPYTKAYDK
NDLHPIHNVGRLEKRIVGILHELLSLTVEKMIPLERLSHFRRPFGMEVNLRELILKHLGI
FYISTKGSTQHVLLRESYSKGCLVDPNPVYNVFQREGALHHRLRAVLSLNYEQAAVEYSK
EVRKRWDIIWKRQQMPTERELYVNNGILMSNSHDCFRFVEVDYEFGLPKTCITLTKLDCS
FQANIIYSRCILGMFVLAIYSSSDHLSKTATMDAPFLSTSLAVLATLFLLALPLSAATHD
ILPLKSSLFVEEYETNILQSSDGTFSCGFYNITNAYNITSAFTFSIWYSNSADKAIVWSA
NRGRPVHSRRSEITLRKDGNIVLTDYDGTVVWQTDGKFPNVRYVQLLNTGNLVLKNSSGN
IVWQSFDSPTDTLLPTQRILATTKLVSTTGLQVPSHYTFRFSDQSILSLIYDDTNVSGVY
WPDPDYQYYENNRNLYNSTRIGSLDDYGEFFSSDLAKHQARVASDRSLGIKRRLTLDYDG
NLRLYSLNNSDGTWTISWIAQPQTCMTHGLCGPYGICHYSPTPRCSCPPGYKMRNPGNWT
QGCKPIVEIACDGKQNVTFLQLRNTDFWGSDQQRIEKVPWEVCWNTCISDCTCKGFQYQE
GNGTCYPKSFLFNGRTFPTPFVRTMYIKLPSSLDVSKKPIPQSSIHDYTLSGLDCDHLNT
ITTEAVRNMNKIGGEEPKWFYFYGFIGVFFIVEVFFFAFAWFFVLRKEMRSSQVWIAEEG
YRVMTSHFRMYSHRELVKATERFKHELGWGGSGVVYKGILDDDRAVVIKKLENVTQNREE
FQDELHVISRINHMNLVRIYGFCSERFHRLLVLEYVENGSLANVLFNSKILLDWKQRFNI
ALGVAKGLAYLHHECLEWVIHCNLKPENILLDENLEPKITDFGLAKLLSRSGSNQNVSRA
RGTIGYIAPEWISGLPITAKVDVYSYGVVLLELVSGRRVFDLIVGEDKTKVHEMLKKFIK
MICYRLDNEKSLWLAEFVDFRVGDEFNYLQAKTLVKLAVSCLEEDRKKRPTMESIVESLL
SVDLARS*
>11686.m00288|LOC_Os12g03640.1|genepair1224-2
MTHGLCGPYGICHYSPTPRCSCPPGYKMRNPGNWTQGCKPIVEIACDGKQNVTFLQLRNT
DFWGSDQQHIEKVPWEVCWNTCISDCTCKEFQYQEGNGTCYPKSFLFNGRTFPTPFVRTM
YIKLPSSLDVSKKPIPQSSIHDYTPSRLDCDRVNTITTEAVRNMNKIGGEEPKWFYFYGF
IGVFFIVEVFFFAFAWFLVLRKEMWSSEVWAAEEGYRVMTSHFRMYSYRELVKATERFKH
ELGWGGSGVVYKGILDDDRAVVIKKLENVTRNREEFQDELHVISRINHMNLVRIYCFCSE
RFHRLLVLEYVENGSLANVLFNSKILLDWKQRFNIALGVAKGLAYLHHECLEWVIHCNLK
PENILLDENLEPKIADFGLAKLLSRSGSKQNVSRARGTIGYIAPEWISGLPITAKVDVYS
YGVVLLELVSGKRVFDLIIGEDKTKVHEMLKKFIKMICYRLDNEKSLWLAEFVDFRVGDE
FNYLQAKTLVKLAVSCLEEDRKKRPTMESIVESLLSVDLARS*
>11687.m00295|LOC_Os11g03840.1|genepair1225-1
MSKNCSRAAATVPLLAAVVVFLSLSRPFPCEARRDSLPRGASIDVEDHATDLLLSPDGTF
AAGLYGVSPTVFTFSVWFARAADRAVVWSANRGRPVHGARSRLALDGRRGALVLTDYDGE
VVWNSTVANATAARARLHDSGNLAIEDASRNILWQSFDHPTDTLLPTQRIVAAGEVMVSA
GKLLAAGFYSFRFSDYAMLSLVYDNHKMPSSIYWPNPYYSYWQNNRNIYYNFTREAFFDA
SGHFLSSDNATFDAADLGEDAGVRFRRLTLDTDGNLRLYSLDETAGTWSVSWMAFVNPCV

IHGVCGANAVCLYSPAPVCVCVPGYARADPRDWTRGCQPTFNYTNGGGGGGRPPAMKLVA
LPHTDFWGFDINSSAHLSLHECAARCMSEPSCVVFEYKQGTGECYTKGLMFNGRTHPAHL
GTAYLKVPADLDMPELHVHQWQTHGDGHSLAIEEDIAGCSGSSSSEFLLNVSDMSSSSSN
NQGKSIWFYFYGFLSAIFVIEVFLIAMGCWIFSNKGVFRPSQVSVLEEGYRIVTSHFRAY
RYSKLERGTKKFNNKIGHGGSGIVYKGSLDDERVVAVKVLQDVRQSEDVFHVELSVIGRI
YHMNLVRMWGFCSEGTHRILVYEYIENGSLAKVLFDRRDSSKFLGWKQRFNIALGVAKGL
AYLHNECLEWIIHCDMKPENILLDEDMEPKITDFGLSKLLNRDGSSSEMSRIRGTRGYMA
PEWVSSLPITEKVDVYSYGVVLLELVKGRRITEWVVDGKDGVETDVRSVVKMVVDKLDSK
DESWIMDLIDDQFGGEFNHLQAQLVIKLAISCLEEDRNKRPSMKYIVQMLISVEDEAHAF
T*
>11686.m00289|LOC_Os12g03650.1|genepair1225-2
MGKSCSRATATVLLLLVVVVFLSSSRPFPCEARRDSLPRGASIAVEDHATDVLLSPDGTF
AAGLYGVSPTVFTFSVWFARAAGRTVVWSANRGRAPVHGARSRVALDGRRGALVLTDYDG
EVVWNSTVANATAARARLHDSGNLAIEDASGNILWQSFDHPTDTLLPTQRIVAAGEAMVS
AGKLLAAGFYSLRFSDYAMLSLVYDNHKMPSSIYWPNPYYSYWQNNRNIYYNFTREAFFD
ASGHFLSSDNATFDAADLGEGAGVRRRLTLDTDGNLRLYSLDEMAGTWSVSWMAFVNPCV
IHGVCGANAVCLYSPAPVCVCVPGYARADASDWTRGCQPTFNHTDGGGGRPRAMKLVALP
HTDFWGFDINSSAHLSLHECTARCMSEPSCVVFEYKQGTGECYTKGLMFNGRTHPAHLGT
AYLKVPADLDMPELHVHQWQTNGLAIEEDIAGCSGSSSSEFLLDVSDMSSSSSNNQGKSI
WFYFYGFLSAIFVIEVFLIAMGCWIFSNKGVFRPSQVSVLEEGYRIVTSHFRAYRYSELE
RGTKKFNNKIGHGGSGIVYKGSLDDERVVAVKVLQDVSQSEDVFQAELSVIGRIYHMNLV
RMWGFCSEGTHRILVYEYIENGSLAKVLFDRRDSSKFLGWKQRFNIALGVAKGLAYLHNE
CLEWIIHCDMKPENILLDEDMEPKITDFGLSKLLNRDGSGSEMSRIRGTRGYMAPEWVSS
LPITEKVDVYSYGVVLLELVKGRRITEWVVDGKDGVETDVRSVVKMVVDKLDSKNESWIM
DLIDDQFGGEFNHLQAQLVIKLAISCLEEDRNRRPSMKYIVQMLISAEDEAHAFT*
>11687.m00297|LOC_Os11g03860.1|genepair1226-1
MSTSCSGALRAATTKALLLLVVAVFVVSLSRPFPCAAAARDSLLRGASIAVEDHATDVLL
SPDGTFACGFYVVSPTVFTFSVWFARAADRAVVWSANRGRPVHSKRSRLKLNGRRRALVL
TDYDGEVVWNSTVSANATATATAARARLHDSGNLAVEDASGNVLWQSFDHPTDTLLPTQR
IAAGEAMVSADKLLAAGFYSFRFSDYAMLSLVYDNHEMSSIYWPNPYYSYWQNSRKIYNF
TREAFFDASGHFSSSDNATFDASDLAKNVAVRRRLTLDTDGNLRLYSLDEVTGTWSVSWM
AFSNPCIIHGVCGANAVCLYSPAPVCVCAPGYGRAEPSDWSRGCRPTFNSSDDGGQPRAM
KMVPLPHTDFWGFDINSSENLSLDECSTRCMSEPSCVVFQYKQGKGECYPKSLMFNGRTF
PGLPGTAYLKVPADLDMLEIHIHQWQADSDGHGIAAIQEDIVGCGGMSSPEFLLNVSNAS
SSKSNQGKSIWFYFYGFLTAFFVIEVFVIAFGCWLFSNKGVFKPCQVSALDEGYRMVTNH
FRTYSYAELQKGTRKFQSEIGRGGSRCYKM*
>11686.m00291|LOC_Os12g03670.1|genepair1226-2
MSTSCSSSGALRAAATKALLLVVFVSLSRPFPCTARDSLLRGASIAVEDHATDVLLSPDG
TFACGFYGVSPTVFTFSVWFARAADRAVVWSANRARPVHSKRSRLKLSGRRGALVLTDYD
GEVVWNSTVSASATAARARLHDSGNLAIEDGSGNVLWQSFDHPTDTLLPTQRIAAGEAMV
SADKILAAGFYSFRFSDYAMLSLVYDNHEMSSIYWPNPYYSYWQNSRKIYNFTREAFFDA
SGHFSSSDNATFGAADLGKNVAVRRRLTLDTDGNLRLYSLDEVAGTWLVSWMAFSNPCII
HGVCGANAVCLYSPAPVCVCAPGYARADPSDWSRGCRPTFNSGDGGGRPRAMKLVALPHT
DFWGFDINSSENLSLDECSTRCMSEPSCVVFQYKQGKGECYPKSLMFNGRTFPGLPGTAY
LKVPADLDMPEIHIHQWQKDGDRHAIAIQEDIVGCGGMSSPEFLLNVSNASSSKSNQGKS
IWFYFYGFLTAFFVIEVFVIAFGCWLFSNKGVFRRCQVSALDEGYRMVTNHFRAYSYVEL
RNGTRNFQSEIGRGGSGVVYKGILDDERTVAVKVLQDVKQGEDVFQAELSVIGRIYHMNL
VRMWGFCSEGIHRILVYEYIENGSLAKVLFQGRDSGMFLGWKQRFNIALGVAKGLAYLHN
ECLEWIIHCDMKPENILLDEDMEPKITDFGLSKLLNRDGSGSDMSWIRGTRGYMAPEWVS
SLPITEKVDVYSYGVVLLELVKGVRISDWVLDGKEELEAELRSVVKMVVSKLESNIESLV
ADLMDDRLHGEFNHLQARLLMQLAVSCLEEDKNKRPTMKYIVQMLISAEDDAHAFT*
>11687.m00299|LOC_Os11g03880.1|genepair1227-1
MSSRLATFALLVIITLSSSSRPCPRRVDAAREWLARGASIAVEDHATDVLRSPDGTFAAG
FYDASPTVFTFSVWFARAADRAVVWTAARARPVHSKGARVTLDARHGALVLTDYGGEVVW
NSSTPAAGGSGGARVRLHDSGNLVVEDAGGKTLWQSFDFPTDTLLPTQRLTAATRLVSRD
RLLSAGYYSLGFSDYAMLSLFYDNGNFSSIYWPNPYFSYWQNNRKIYNFSREAAMDALGQ
FLSSDGTTFEAADLGAAGVRRRLTLDTDGNLRAYSLDGATGAWSVSWMAFGNPCNIHGVC
GANAVCLYSPAPVCVCAPGHERVDASDWSRGCRPTFRIECGRPAKLVALPHSDFWGYDLN
DGEVMPLGDCANKCLDNCACVVFQYKEHMECYLKSVLFNGKTFPGLPGTVYIKVPADFDV
PEFHVHQWQRGGDGGGGGLAIQEDITGCAAAATGDSNRKVLLNVSSSLSSHDAGKPVWPY
LYGFLSALLVVEAIVIGFGCWLFSSKGLFRHSRVYAIDQEGYKLITTHFQRFTYVDIKKA
TANFTGVIGRGGSGVVYKGVLDDERVVAVKVLKNVSWQSEEEFQAELSVIGRIYHMNLVR
MWGCCSQAKHRILVSEYIENGSLAQRLFDHGFDDDVLDWNQRFKIALGVAKGLAYLHSEC
SEWIVHCDMKPENILLDKDLEPKITDFGLSKLLNRDGSHAILTRIRGTRGYMAPEWVTNL
PVTEKVDVYSYGVILLELVKGIRISEWVIHGIKVCEMNIRMVVRATRQMMGSNEERSIED
LVDYRLNGDFNHVQVKLMLEIAVSCLEEDRSKRPNMNSVVQALISVEG*
>11686.m00293|LOC_Os12g03690.1|genepair1227-2
MMNSRLATFALLVIITLSSSPRPCPRRVDAAREWLARGASIAVEDHATDVLRSPDGTFAA
GFYDASPTVFTFSVWFARAADRAVVWTAARARPVHSKGARVTLDARRGALVLTDYGGEVV
WNSSTPAAGGSGGARVRLHDTGNLVVEDACGKTLWQSFDFPTDTLLPAQRLTAATRLVSR
DRLLSAGYYSLGFSDYAMLSLFYDNGNFSSIYWPNPYFSYWQNNRKIYNFSREAAMDALG
QFLSSDGTTFEAADLGAAGVRRRLTLDTDGNLRAYSLDDATGTWSVSWMAFGNPCNIHGV
CGANAVCLYSPAPVCVCAPGHERVDASDWSRGCRPTFRLECGRPAKLVALPHSDFWGYDL
NDGEVMPLGDCANKCLDNCACVVFQYKEHMECYLKSVLFNGKTFPGLPGTVYIKVPADFD

VPEFHVHQWQRGGDGGGGGLAIQEDIAGCAAAATGDSNRKVLLNVSSSLSSHDAGKTVWP
YLYGFLSALLVVEAIVIGFGCWLFSSKGLFRHSRVYAIDQEGYKLITSHFQRYTYADIKK
ATANFTGVIGRGGSGVVYKGVLDDERVVAVKVLKNLSRQSEEEFQAELSVIGRIYHMNLV
RMWGCCSQAKHRILVSEYIENGSLAQRLFDHGFDDDVLDWNQRFRIALGVAKGLAYLHSE
CSEWIVHCDMKPENILLDKDLEPKITDFGLSKLLNRDGSDAILTRIRGTRGYMAPEWVTN
LPFTEKVDVYSYGVILLELVKGIRISEWVIHGIKVCEMDIRMVVRATCQKMESNEKRSIE
DLVDYRLNGDFNHVQVKLMLEIAISCLEEDRSKRPNMNSVVQSLISVEG*
>11687.m00300|LOC_Os11g03890.1|genepair1228-1
MYGHRGAMMGGGGVSDGYEGSKRPRMIESNPYFAVTAGSPLDVSKRARMMEPAPPYFGAM
GSSAAGGTSAFYQPYGANLPGAGANSAIQNFPGVRLRGLPFDCDDLDIFKFFVGLDIVDC
LLVHKNGRFTGEAFVVFPSAMQAEFALHRNRQNMGRRYVEVFRCKKQEYYSAIAAEVNQG
GFFDSEYRHSPPPRPKKPAEDKSSMEYTEVLKLRGLPYSATTEDIIKFFVEYELTEENVH
IAYRPDGKATGEAFVEFPTAEVAKTAMCKDKMTIGTRYVELFPSTPEEASRAKSRARQ*
>11686.m00295|LOC_Os12g03710.1|genepair1228-2
MGSSAAGGTSAFYQPYGTNLPGAGANSAIQNFPGVRLRGLPFDCDDLDICKFFVGLDIVD
CLLVHKNGRFTGEAFVVFPSAMQAEFALHRNRQNMGRRYVEVFRCKKQEYYSAIAAEVNQ
GGFFDSEYRHSPPPPRPKKPAEDKSSMEYTEVLKLRGLPYSATTEDIIKFFVEYELTDEN
VHIVYRPDGKATGEAYVEFPTAEVVKTAMCKDKMTIGTSMHHVATVPQPVGVVTL*
>11687.m00301|LOC_Os11g03900.1|genepair1229-1
MCGIFAYLNYNVSRERRYILEVLFNGLRRLEYRGYDSSGIAVDADVPSCASTSAVPPYAG
APPLVFRQEGKIENLVRSVYSAILGLVILAREILLFALCIFGIAPKILDLLGKEVDEKDV
NLDAAFNVHAGIAHTRWATHGVPAPRNSHPQSSGAGDEFLVVHNGIITNNEVLKETLIRH
GFTFESDTDTEVIPKLAKFVFDKAHDEEGDVTFSQVVMEVMRQLEGAYALIFKSPHYPNE
LIACKRGSTLILGVNELSGQNSGKPFHDVKALTTNGKPKELFFSSDLFAIVEHTKNYLAI
EDDEIVHIKDGSVSILKFDHEKEKPASVQRALSVLEMEVEQIKKGSYDHFMQKEIHEQPH
SLTTTMRGRVKDSGVLLGGLKEKEYLKTIRRSRRLVFIGCGTSYNAALAARPFVEELTGI
PVTMEVASDLLDRQGPIYREDTAFFVSQSGETADTLLALDYALENGALCVGITNTVGSTL
SRRTHCGVHINAGCEIGVASTKAYTSQIVVMVMVALAVGSDQISTQVRRQAIISGLSNLP
SNVSEVFKLDTEMKELASSLIDSESLLVFGRGYNYATALEGALKVKEVALMHSEGMLAGE
MKHGPLALVDENLPIIVIATRDACFSKQQSVIQQLLSRKGRLIVMCSKGDASAVCPSGSC
RVIEVPEVADCLQPVINIIPLQLLAYHLTVLRGFDVDQPRNLAKSVTTQ*
>11686.m00296|LOC_Os12g03720.1|genepair1229-2
MCGIFAYLNYNVSRERRYILEVLFNGLRRLEYRGYDSSGIAVDADVPSCASSSSSTAVPA
YAGASPLVFRQEGKIENLVRSVYSEVDEKDVNLDAAFNVHAGIAHTRWATHGVPAPRNSH
PQSSGAGDEFLVVHNGIITNYEVLKETLIRHGFTFESDTDTEVIPKLAKFVFDKAHDEEG
DVTFSQVVMEVMRQLEGAYALIFKSPHYPNELIACKRGSTLILGVNELSGQNSGKPFHDV
KALTTNGKPKELFFSSDLFAIVEHTKNYLAIEDDEIVHIKDGSVSILKFDHEKEKPASVQ
RALSVLEMEVEQIKKGSYDHFMQKEIHEQPHSLTTTMRGRVKDSGVLLGGLKEKEYLKTI
RRSRRLVFIGCGTSYNAALAARPFVEELTGIPVTMEVASDLLDRQGPIYREDTAVFVSQS
GETADTLLALDYALENGALCVGITNTVGSTLSRRTHCGVHINAGCEIGVASTKAYTSQIV
VMVMVALAVGSDQISTQVRRQAIISGLSNLPSNVSEVLKLDTEMKELSSSLIDSESLLVF
GRGYNYATALEGALKVKEVALMHSEGMLAGEMKHGPLALVDENLPIIVIATRDACFSKQQ
SVIQQLLSRKGRLIVMCSKGDASAVCPSGSCRVIEVPEVADCLQPVINIIPLQLLAYHLT
VLRGFDVDQPRNLAKSVTTQ*
>11687.m00302|LOC_Os11g03910.1|genepair1230-1
MASSQEFEANDPQQLHAKPKRLYQVWKGNNIFLCGGRLIIGPDAASLLLSMFLILGPAIV
FSYQMESTIHRSQQRMRRAAQLIVIITTAADLFFLFMTSARDPGIVPRNTRAPPEADEFL
GSTTPSMEWSSGRTPRMRFRRAKDVTVNGFTVKVKFCETCLRYRPPRSSHCSICNNCVEK
FDHHCPWVGQCIGLLKKTHQSSKLNYTRLNRNYRYFFLFVATSTFLCIIVFIFSWVNVYY
ERGDDGGSIWKALRKETYSFVLIIYTFIVVWFVGGLTVFHLYLISTNQTTYENFRYHYNK
KDNPYRKSVAANFVEVFFTKIPPPQNNFCSWVGEGALEAGFYTPYIALDLTDPREKIDLE
MGNKDILVGGMQIPTVLQNIDYGSFEDNPDDKNRNEDDRLVPFASTWVQQANEGARTSEI
ATVEYKDEISEDGGKEIISSNTSSEQTSIEANAAASEDESNEGIAGKSNSSDRSSTQNLG
DVN*
>11686.m00297|LOC_Os12g03730.1|genepair1230-2
MASSQEFEANDPQQLHAKPKRLYQVWKGNNIFLCGGRLIIGPDAASLLLSMFLILGPAIV
FSYQMESTIHRSQQRMHRAAQLIVIITTAADLFFLFMTSARDPGIVPRNTRAPPEVDEFL
GSTTPSMEWSSGRTPRMRFRRSKDVTVNGFTVKVKFCETCLRYRPPRSSHCSICNNCVEK
FDHHCPWVGQCIGLRNYRYFFLFVATSTFLCIFVFIFSWVNVYYERGYNGGSIWKALRKE
VYSFVLIIYTFIVVWFVGGLTVFHLYLISTNQTTYENFRYHYNKDNPYRKSIAANFVDVF
FTKIPPPQNNFRSWVGEGALEAGFYTPYIALDLTDPREKIDLEMGNKDILVGGIQIPTVL
QNIDYGSFEDNPDDKNRNEDDRLVPFASTWAQQANEGARTSEIATVEYKDEISEDGGKEI
ISSNTSSEQTSIEANAAASEDESNEDNAGKNCSTAFMVLQRRRLYASRNGSNLVVVDAGE
EVGVIPGEPQPRSGVGVRASGAAELGGDELAEVLLGVPPPTCFCTALSTRRTPDDAFVIS
WASTRCQHSRPEAIDASHIILARNSNSITYNIGPGMVFSPIPFQPDAIVATHEPPAVTEA
AEIVPRTSLASTVAESFKQMLFPSCDGGICLWSASYHDNVAFVKSGTFPRGSSGQGMLVD
ILYCSSAEGSSLVGKDPMAYKILYTRFNKL*
>11687.m00308|LOC_Os11g03970.1|genepair1231-1
MSTTKVKRRVGKYELGRTIGEGTFAKVKFARDTETGDPVAIKILDKEKVLKHKMVEQIKR
EISTMKLIKHPNVVRIYEVMGSKTNIYIVLEYVTGGELFDTIVNHGRMREDEARRYFQQL
INAVDYCHSRGVYHRDLKPENLLLDSYGNLKVSDFGLSALSQQIKDDGLLHTTCGTPNYV
APEVLEDQGYDGAMADLWSCGVILFVLLAGYLPFEDSNLMTLYKKISNAEFTFPPWTSFP
AKRLLTRILDPNPMTRITIPEILEDEWFKKGYKRPEFDEKYDTTLDDVDAFVNDSEEHHV
TEKKEEPEALNAFELISMSAGLNLGNLFDSEQEFKRETRFTSKCPPKEIVRKIEEAAKPL

GFDVQKKNYKICSPCLTTICMNIPFKLRLEKVKAGRKGNLNVATEILQVAPSLHMVEVRK
AKGDTLEFHKFYKNLSRTLKDVVWKSDDLQNQLS*
>11686.m00306|LOC_Os12g03810.2|genepair1231-2
MSTTKVKRRVGKYELGRTIGEGTFAKVKFARDTETGDPVAIKILDKEKVLKHKMVEQIKR
EISTMKLIKHPNVVRIYEVMGSKTKIYIVLEYVTGGELFDTIVNHGRMREDEARRYFQQL
INAVDYCHSRGVYHRDLKPENLLLDSYGNLKVSDFGLSALSQQIKDDGLLHTTCGTPNYV
APEVLEDQGYDGAMADLWSCGVILFVLLAGYLPFEDSNLMTLYKKISNAEFTFPPWTSFP
AKRLLTRILDPNPMTRVTIPEILEDEWFKKGYKRPEFDEKYDTTLDDVYAVFNDSEEHHV
TEKKEEPEALNAFELISMSAGLNLGNLFDSEQEFKRETRFTSKCPPKEIVRKIEEAAKPL
GFDVQKKNYKLRLEKVKAGRKGNLNVATEILQVAPSLHMVEVRKAKGDTLEFHKVDHAN*
>11687.m00310|LOC_Os11g03990.1|genepair1232-1
MGRKIKVKKKKASSKKAEASSSRVPSAPAKVWQPGVDTLEEGEELQFDPQAYNYLRGFNI
GWPCLSFDVVRDQLGLVRSEFPHTLYGVAGTQAERATWNYIGIFKICNINGKKREPIPAS
AIDGDSDMDSESSSDEEDEAVNEDTMPILHLKKVAHAGCVNRIRSMNQEPHICATWGDTG
HVQVWDFSSFLNSLAESGAVAHNEDDRIHNHVPVKIFGSHKDEGYAIDWSPLVTGRLVSG
DCNKCIHLWEPTSNSWNVDTNPFVGHTASVEDLQWSPTEADIFASCSADRTISIWDIRTG
KKPCISVRAHNADVNVISWNRLASCMIASGCDDGSFSIRDLRLIKDDSLVAHFEYHKHPI
TSVEWSPHEPSTLAVSSADHQLTIWDLSLEKDAEEEAEFRARMREQADAPEDLPPQLLFV
HQGQKDLKELHWHPQIPSMIISTAADGFNMLMPSNIDTTIREADA*
>11686.m00307|LOC_Os12g03820.1|genepair1232-2
MGRKIKVKKKKASSKVWQPGVDTLEEGEELQFDPQAYNYLRGFNIGWPCLSFDVVRDQLG
LVRSEFPHTLYGVAGTQAERASWNYIGIFKICNINGKKREPIPASAIDGDSDMDSESSSD
EEDEAANEDTMPILHLKKVAHAGCVNRIRSMNQEPHICATWGDTGHVQVLIFDSRGTESK
LYQRLEWTCEVWDFSSFLNSLAESGAVAHNEDDRIHNHVPVKIFGGHKDEGYAIDWSPLV
TGRLVSGDCNKCIHLWEPTSNSWNVDTNPFWSPTEADIFASCSADRTISIWDIRTGKKPC
ISVRAHNADVNVISWNRLASCMIASGCDDGSFSIRDLRLIKDDSLVAHFEYHKHPITSVE
WSPHEPSTLAVSSADHQLTIWDLSLEKDAEEEAEFRARMREQADAPEDLPPQLLFVHQGQ
KDLKELHWHPQIPKNLLLVLLIIFEGRQLMDHLTKEQIAEFREAFNLFDKDGDGTITSKE
LGTVMGSLGQSPTEAELKKMVEEVDADGSGSIEFEEFLGLLARKLRDTGAEDDIRDAFRV
FDKDQNGFITPDELRHVMANLSDPLSDDELADMLHEADSDGDGQINYNEFLKAKAEYDGG
TWKWRPSVK*
>11687.m00313|LOC_Os11g04020.1|genepair1233-1
MSEEAPPSPVMRPVFYDGCPGCAMERKLESSQGIPYKEFFFVGITTIASSLPISSLFPFL
YFMIEDLHVAKKEQDIGLYAGFLGASYMIGRCFASLFWGVVADRIGRKPIIKFSILSVVI
FNTLFGLSVKYWMAIATRFLLGALNGMLAPIKAYSIEVCRPEHQALGLSIVSTGWGVGLV
VGPAIGGYFAQPAKQYPNVFSEKSIFGRFPYFLPCLCISLIALVVLISCIWLPETLHKHK
DTEGEIEMIDNSRSTLEEDSHKQKSLYKNWPLISSIIAYCVFTLHDTAYSEIFSLWAVSE
KRYGGLSFSSKEVGQVLAVAGAGLLVYQLFIYRSVHKFLGSINSSRIASALSVPILATYP
FMTHLSGFRLGIALYLGTILKGVLSITIITGTSLLQNNAVSQSQRGAANGISTTAMSFFK
AIAPAGAGALFSWAQERQNEAFFPGDQMIFFILNVIELIGLALTFKPFLAIPN*
>11686.m00308|LOC_Os12g03830.1|genepair1233-2
MRAVFYDGCPGCAMERKLESSQGIPYKEFFFVGITTIASSLPISSLFPFLYLMIEDLHVA
KKEQDIGLYAGFLVADRIGRKPIIEFSILSVVIFNTLFGLSVKYWMAIATRFLLGALNGM
LAPIKAYSIEVCRPEHQALGLSIVSTGWGIGLVVGPAIGGYFAQPAKQYPNVFSEKSIFG
RFPYFLPCICISLIALVVLISCIWLPETLHKHKNTEGEIEMIDNSRSTLEEDSHKQKSLY
KNWPLISSIIAYCVFTLHDTAYSEIFSLWAVSDKRYGGLSFSSKEVGQVLAVAGAGLLVY
QLFIYRSVHKFLGSINSSRIASALSIPILAAYPFMTHLSGLRLGIALYLGTILKGVLSSQ
SQRGAANGISMTAMSFFKAIAPAGAGALFSWAQERQNAAFFPGDQMIFFALKWIVPVLLA
VKL*
>11687.m00316|LOC_Os11g04030.3|genepair1234-1
MAEPPATKVYHDGCPGCAMEQRKEEHKGIPYKEFLFVAITTLASSLPISSLFPFLYFMIR
ELHISRTEEDIGFYAGFLGASYMIGRGFASILWGMVADRIGRKPVIIFSIFAVIVLNTLF
GLSVKYWMAVTTRFLLGALNGLLAPIKAYSIEVCRAEHQALGLSIVSTAWGIGLVVGPAI
GGYLAQPVKQYPHLFHEKSIFGRFPYLLPCLCISLFALLVLISCIWLPETLHKHKGLETG
VEAAEASTTQGSAESHKKSLFRNWPLMSSIITYCVFSLHDTAYSEIFSLWTVSDRKYGGL
SFSSKDVGQVLAVAGASLLVYQLFIYRWVDKILGPINSTRIASVLSIPIIAAYPFMTHLS
GIRLGVALYSAAMIKSVLAITIITGTSLLQNKAVPQGQRGAANGIATTAMSLFKAVAPAG
AGVLFSWAQKRQHAAFFPGDQMVFLLLNLTEVIGLMLTFKPFLAVPQQYK*
>11686.m00311|LOC_Os12g03860.1|genepair1234-2
MAEPPATKVYHDGCPGCAMEQRKEEHKGIPYREFLFVAITTLASSLPISSLFPFLYFMIR
DLHVARTEEDIGFYAGFLGASYMIGRGFASILWGMVADRIGRKPVIIFSIFAVIVLNTLF
GLSVKYWMAVTTRFLLGALNGLLAPIKAYSIEVCRAEHQPLGLSIVSTAWGIGLVVGPAT
GGYLAQPVKQYPHIFHEKSIFGRFPYLLPCLCISLFALLVLLSCIWLPETLHKHKGLEVG
VETAEASTTQESAESHQKSLFRNWPLMSSIVTYCVFSLHDTAYSEIFSLWTVSDRKYGGL
SFSSKDVGQVLAVAGASLLVYQLFIYGWVDKILGPIHSTRISAALSVPIIAAYPFMTHLS
GIRLGVALYSAAMIKSVLAITIITGTSLLQNKAVPQGQRGAANGIATTAMSLFKAIAPAG
AGVIFSWAQKRQHVAFFPGDQMVFLLLNLTEVIGLMLTFKPFLAVPQQYK*
>11687.m00320|LOC_Os11g04060.1|genepair1235-1
MDEMKGIDHEHALPITCLFPFLYFMVRDLQVAQTEEDIGNYAGFLGASYMVGRSFAAIFW
GVVADRIGRKPVIMFSILSVVIFNTLFGLSTKYWMALTTRFVLGALNGLLAPIKVNTAWG
LGLVVGPALGGYLAQPVEKYPHIFSKESVFGRFPYLLPCLGVSLFAAIVLISCIWLPETI
HKHKSPEKDIKRIKELPLQQAYWDSPRKKSLLQNWPWMSTMISYCFFGLHDTAYSEILSL
WAVSDRKYGGLSFSSEDIGQVLAAAGASLLAYQLIFYHWVHKFLGPIISLRIASALSILI
LSTYPFMTYLSGTGLSFALYSAAMMKSALAITISTGISLLQNNAVLQEHRGTANGVSTTA

MSFFKAIAPVGGGVLFSWAQKRQDAFFFPGDQVVFLMLNVVELIGLIFTFEPFMVLPAAS
DECS*
>11686.m00312|LOC_Os12g03870.1|genepair1235-2
MGSGSEEAAPLLLPVAAATATAEERCPGCVQERRKASRGGRIPYTELFFVAVTTLASSLP
ITCLFPFLYFMVRDLQVAQTEEDIGYYAGFLGASYMVGRSFAAIFWGVVADRIGRKPVIV
FSILSVVIFNTLFGLSTKYWMALTTRFVLGALNGLLAPIKVNTAWGLGLVVGPALGGYLA
QPVEKYPHVFSKESVFGRFPYLLPCLGVSLFAAIVLISCIWLPETIHKHKSPDKDIKRIK
ELPLQQAYWDSPRKKSLFQNRPWMSTMISYCFFGLHDTAYSEILSLWAVSDRKYGGLSFS
SEDIGQVLAVAGASLLAYQLIIYHWVHKFLGPIISLRIASALSILILSTYPFMTYLSGAE
LSLAFYSAAMMKSALAITISTGICLLQNNAVLQEHRGTANGVSTTAMSFFKAIAPVGAGV
LFSWAQKRQDALFFPASAAWTQEPAAKQLAALASELTFKEEIRRD*
>11687.m00321|LOC_Os11g04070.1|genepair1236-1
MAIKRTKAEKKVAYDKKLCQLLDEYTKVLIAVADNVGSNQLQEIRKGLRGDSIVLMGKNT
LIRRCIKVHADNTGNKDFLELMPLLVGNVGLIFTKGDLKEVREEVAKYKVGAPARVGLVA
PVDVVVPPGNTGLDPSQTSFFQVLNIPTKINKGTVEIITPVELIKKGDKVGSSESALLAK
LGIRPFSYGLVITNVYDSGSVFSPEVLDLTEEDLMEKFASGVSMVASVSLAISYPTIAAA
PHMFLNGYKNVLAVAVETEYSYPHADKIKEYLKDPSKFAVAAAVAAADSGAAAPAASKEE
EKKEEPEEESDGDLGMSLFD*
>11686.m00313|LOC_Os12g03880.1|genepair1236-2
MAIKRTKAEKKVAYDKKLCQLLDEYTKVLIAVADNVGSNQLQEIRKGLRGDSIVLMGKNT
LIRRCIKVHADNTGNKDFLELMPLLVGNVGLIFTKGDLKEVREEVAKYKVGAPARVGLVA
PVDVVVPPGNTGLDPSQTSFFQVLNIPTKINKGTVEIITPVELIKKGDKVGSSESALLAK
LGIRPFSYGLVITNVYDSGSVFSPEVLDLTEEDLMEKFASGVSMVASVSLAISYPTIAAA
PHMFLNGYKNVLAVAVETEYSYPHADKIKEYLKDPSKFAVAAPVAAADSGAAAVASSKEE
EKKEEPEEESDGDLGMSLFD*
>11687.m00325|LOC_Os11g04110.1|genepair1237-1
MGDHGRRRQAEAAAPLLEKKGSTGTGEGDGSCIEGCPGCVVDRRKAASSGIPYGSFLFVW
IVTLCTDLCTSTNR*
>11686.m00315|LOC_Os12g03900.1|genepair1237-2
MGDHGRRRQAAAPLLEKKGSTGTGVDDGYCIEGCPGCVVDRRKAASYGIPYGSFLFVWIV
TLCTDLCTSYNRSILAMAYSRGF*
>11687.m00326|LOC_Os11g04120.1|genepair1238-1
MLTLMFFLSRTFFPAVLLFRNSVCLRTCTSRQAIKFLGSVLAQLLLFCFDFFLCIAMASI
GDGAPGAVGNGSAPGGGGNGSAPGDNTNEGNTNASTSSALFSGGEEADHGRRRQAEAPLL
EKKGSTGIGNGYCIERCPGAASTGVRPRAPASPTGASSSSGSSRSAQIREPQPTIEFGHD
TEGFFGEILNNYSFSHNQKLSK*
>11686.m00316|LOC_Os12g03910.1|genepair1238-2
MAHQELEGTAMLQETTLTEAIQMTQPPCTVFWVEAPLLEKKGSTGIGDGYCTERCPGAAS
IGVRPRAPASPTGASSLSGSSRSAQIRAPQPTIEFGHDTKGFFGEILNSFSHNQKLSKQF
SFSLIF*
>11687.m00328|LOC_Os11g04140.1|genepair1239-1
MTLGVLIKLFFCALIGITLGVNLYHDGVCEAEELFRHTKVTSVAICLAGVFTIAFFTGPS
ISPINHHRAFASDTSSKTVVPRGVWIKWTFLMVVANMCWSLWIIFQAAVQKEYPDKMVVT
VTQCLFSTVQSFVVAVVAERDFSRWKLRFDISLLAILYSGVMVTGVSYYLQTWCLEMRGP
MFFASWTPLCFVFTIFCSSFFLGEIVHLGSILGGILLVGSLYTMLWGKSKEGNETDDVTD
DDIEKSTHIYPGEQQHTTTDQAKESTLTSSTALHVQEL*
>11686.m00317|LOC_Os12g03920.1|genepair1239-2
MVKASMKPYFVAIVVQLIYTGMFVISKAAFNHGMNIYIFVFYRQAVGSLILLPAALLQRR
SARPAMTLGVLIKLFFCALIGITLGVNLYHDRTEYVKLRSSSGIAKVTSVALCLAGVFTI
VFFTGPSISPINHHRAFASDTSSKTVVPRGVWIKWTFLMAAVQKEYPDKMVVTVTRCLFS
TMQSFVVAVVAERDFSRWKLRFDISLLAILYSGVMVTGVSYYLQTWCLEMRGPMFFASWT
PLCFVFTIFCSSFFLGEIVHLGNILGGILLVASLYTMLWGKSKEGNETDDVTDDDIEKST
HIYPREQQHTTTDQAKESMLTSSAALHVQEL*
>11687.m00329|LOC_Os11g04150.1|genepair1240-1
MHVEAYERGNMTEKSESSRSRLSKSILGLPISSLFPFLYFMIRDLHVAKRVEDIGFYAGF
VAYYNRALQAYAVEVCRPEHQSIGLSLVSTSWAIGLIVGPAIGGYLAQPSEKYPILFPAN
SLFGRFPYFLPCLCISIFCFVILISCIWLPETLHKHATERNGDCKIGSLSTHLVDSEEFV
KQHTGPAKDKSLFKNWPLMSSIVLFCIVSFDDMAYTEIFSLWSESDKQFGGLNFSSEDVG
QVLAITGASILIYQTFIYPHIVKVLGIINTSRVAVILSMAILCSYPPMTYLSRPWLSIVV
NIASMLKNNFVVTINTCSFILQNNSVPQHQRATANGLATTLMSFFKAFAPAGAGILFSWA
QKRQHAFFFPGDQMVFFILIIIEFLELIWTFKPFLAVP*
>11686.m00320|LOC_Os12g03950.1|genepair1240-2
MANGLPGSAWPKHGCAWAEKREKKMHVEAYERGNMTEKSESSRSRLSNSFLGLPISSLFP
FLYFMIRDLHVAKRVEDIGFYAGFVSTSWAIGLIVGPAISGYLAQVPIFSTLFMYIDLLL
RYSYKLHLAPEFVKQHTGPAKDKSLFKNWPLMSSIVLFCIVSFDDMAYTEIFSLWSESDK
QFGGLNFSSEDVGQVLAITGASILIYQTFIYPHIVKVLGIINTSRVAVILSMALLCSYPP
MTYLSRPWLSIVVNIASMLKNNFVCKLPVYMVVQVFLGTKTPTCVFLSR*
>11687.m00330|LOC_Os11g04160.1|genepair1241-1
MASQRRLQPSQLLPLLLLAVPELLTPGFVREDDRKRALVSRLLQYSLVHHALGIPFHQIR
INRTLEGKPYLQNKNANFPGFNFNTSHQGEYVGIASEPLCLVGLDIVCISKPQRETALEF
INNFTSYLTDHEWNCIVTAGSHDGMLTEFYRYWCLKEAFVKATGAGVGFGLQRLEFHHMN
WTNISLRIDGEEDRKWRFWLFKIDEKHLVICLRLSTPLQASIAKGHPEDAIDSFRRTLSD
VVIQEGELHTAIEIPEEAFTLLTVEQLIQLHD*
>11686.m00321|LOC_Os12g03960.1|genepair1241-2

MMEEGEKQSSGGRWRCRWLVDVGRWRPSPAEFQAAAAVLPPHDRPAIHRFVREEDRKRAL
VSRLLKYSLVHHALGMPFHQIRINRTLEGKPYLNKNVNLPGFNFNTSHQGEYVGIASEPL
CLVGLDIVCISKPQRETALEFINNFTSYLTDHEWNCIVTADSHDGMLTEFYRYWCLKEAF
VKATGAGVGFGLQRLEFHHMNWTNISLCIDGEEARKWMFWLFKIDEMHLASIAKGHPEDA
IDSFRRTLSDVVIQEGELHTAIEIPEEAFTLLTVEHLIQLHD*
>11687.m00331|LOC_Os11g04170.1|genepair1242-1
MGQCCTGGGKAVAGDEAEPGTSKAAPPSRGTSSKNGSAKQQPCSPAAKAAATEAAAAASS
SKKPAGPIGEVLERPMEEVRTTYSIGKELGRGQFGVTHLCTHKATGEKLACKTIAKRKLA
NKEDVDDVRREVQIMHHLSGQPNIVDLRGAYEDKHNVHLVMELCAGGELFDRIIARGHYT
ERAAAALLRAIVGIVHTCHSMGVIHRDLKPENFLLLSKGDDAPLKATDFGLSVFFKEGEV
FRDIVGSAYYIAPEVLKRKYGPEADIWSIGVMLYIFLAGVPPFWAESENAIFTAILRGQI
DLASEPWPKISSGAKDLVRKMLNINPKERLTAFQVLNHPWIKEDGDAPDVPLDNVVLNRL
KQFRAMNQFKKAALRIIAGCLSEEEIKGLKEMFKNIDKDNSGTITLEELKNGLAKQGTKF
SDNEIEQLMEAADADGNGIIDYEEFVTATVHMNKMDREEHLYTAFQYFDKDNSGYITKEE
LEQALKEQGLYDANEIKDVITDADSNNDGRIDYSEFVAMMRKGSGCAEATNPKKKRRDLV
L*
>11686.m00322|LOC_Os12g03970.1|genepair1242-2
MGQCCTGGGKAVAGDEAEPGTSKAAPPSRGTSSKNGSAKQQPCSPAAKAAATEAAAAASS
SKKPAGPIGEVLERPMEEVRTTYSIGKELGRGQFGVTHLCTHKATGEKLACKTIAKRKLA
NKEDVDDVRREVQIMHHLSGQPNIVDLRGAYEDKHNVHLVMELCAGGELFDRIIARGHYT
ERAAAALLRAIVGIVHTCHSMGVIHRDLKPENFLLLSKGDDAPLKATDFGLSVFFKEGEV
FRDIVGSAYYIAPEVLKRKYGPEADIWSIGVMLYIFLAGVPPFWAESENAIFAAILRGQI
DLASEPWPKISSGAKDLVRKMLNINPKERLTAFQVLNHPWIKEDGDAPDVPLDNVVLNRL
KQFRAMNQFKKAALRIIAGCLSEEEIKGLKEMFKNIDKDNSGTITLEELKNGLAKQGTKF
SDNEIEQLMEAADADGNGIIDYEEFVTATVHMNKMDREEHLYTAFQYFDKDNSGYITKEE
LEQALKEQGLYDANEIKDVITDADSNNDGRIDYSEFVAMMRKGSGCAEATNPKKKRRDLV
L*
>11687.m00332|LOC_Os11g04180.1|genepair1243-1
MPHLVRERLFFGDINDAIAALTTTAADTGGFTHLLSVVSSASISFITDCRPGLSIPTEEV
RRVVAGEEGAPPVSAVAPGRLLRVVERAGVGLRVTRMAVPLRDTEEENLLDHLEPCLDFI
DEGRKEGNVLVHCFAGVSRSATIIVAYLMRTEQKSLEEALESLKEVNESACPNDGFLEQL
KLFEEMGFKVDTSSPLYKRFRLKLLGQSYKIGEKIGSYVFEDDPGLSGQPNSSTQDLPNK
QTQQTAYRCKKCRRIVAVQGNVVSHTPGEGESCFQWQNKRKGERSYSKEQDCSSLFVEPL
KWMTPVEDGALEGKLSCIHCGARLGYFNWSGIQCNCGSWITPAFQISKSKVDISTT*
>11686.m00324|LOC_Os12g03990.1|genepair1243-2
MPHLVRERLFFGDINDAIAALTTTTAEAGGFTHLLSVVSSAFISFITDCRPGLSIPTEEV
RRVVAGEEGAPPVSAVAPGRLMRVVERAGVGLRVTRMAVPLRDTEEENLLDHLEPCLDFI
DEGRKEGNVLVHCFAGVSRSATIIVAYLMRTEQKSLEEALESLKEVNESACPNDGFLEQL
KLFEEMGFKVDTSSPLYKRFRLKLLGQSYKIGEKIGSYVFEDDPGLSGQPNSSTQNLPNK
QTQQPAYRCKKCRRIIAVQGNVVSHTPGEGESCFQWQNKRKGERSYSKEQDCSSLFVEPL
KWMTPVEDGALEGKLSCIHCGARLGYFNWSGIQCNCGSWITPAFQISKSKVDISTT*
>11687.m00334|LOC_Os11g04190.1|genepair1244-1
MITVVDLYHVLTAVVPLYVAMTLAYASVRWWRIFSPDQCSGINRFVALFAVPLLSFHFIS
TNNPFAMNLRFLAADTLQKLIVLALLALWCRLSARGSLDWLITLFSLSTLPNTLVMGIPL
LKGMYAAAADVDSGSLMVQIVVLQCIIWYTLMLFLFEYRGARLLVMEQFPDTAASIVSFR
VDSDVVSLAGGGGGAAELQAEAEVGDDGRMRVTVRKSTSSRSEAACSHGTQSHSQSMQPR
VSNLSGVEIYSLQSSRNPTPRGSSFNHAEFFNIVGNGKQGDEEKGAAGGGGHSPQPVVGK
RKDLHMFVWSSSASPVSERAAAAAAGAVHVFGGGGADHGDAKGAQAYDEYSFGNKNEKDG
PTLSKLGSNSTAQLRPKDDGEGMAAAMPPASVMTRLILIMVWRKLIRNPNTYSSLLGVIW
SLVSYRWGIEMPAIIARSISILSDAGLGMAMFSLGLFMALQPRIIACGNSLASYAMAVRF
LVGPAVMAAASIAVGLRGVLLHIAIVQAALPQGIVPFVFAKEYNVHPNILSTAVIFGMLI
ALPITLVYYILLGL*
>11686.m00325|LOC_Os12g04000.1|genepair1244-2
MITVVDLYHVLTAVVPLYVAMTLAYASVRWWRIFSPDQCSGINRFVALFAVPLLSFHFIS
TNNPFAMNLRFLAADTLQKLIVLALLALWCRLSARGSLDWLITLFSLSTLPNTLVMGIPL
LKGMYAAAGAAAGADSGSLMVQIVVLQCIIWYTLMLFLFEYRGARLLVMEQFPDTAASIV
SFRVDSDVVSLAGGGGGAAELQAEAEVGDDGKMRVTVRKSTSSRSEAACSHGTQSHSQSM
QPRVSNLSGVEIYSLQSSRNPTPRGSSFNHAEFFNIVGNGKHGDEEKGAAGGGGHSPQPV
VGKRKDLHMFVWSSSASPVSERAAAAAAAGAVHVFGGGGADHGDAKGAQAYDEYSFGNKN
EKDGPTLSKLGSNSTAQLRPKDDGEGRAAAMPPASVMTRLILIMVWRKLIRNPNTYSSLL
GVIWSLVSYRWGIEMPAIIARSISILSDAGLGMAMFSLGLFMALQPRIIACGNSLASYAM
AVRFLVGPAVMAAASIAVGLRGVLLHIAIVQRDIRDADSSPHHIGLLHTAGALKSSPSLN
STVQELDVTDSLIRIYGQLSDARWTIIRELLGSSDGQPRFLIED*
>11687.m00335|LOC_Os11g04200.1|genepair1245-1
MAMDLDEPLDAEKGEAALRRLRDADPALYLSPSADLAAAAREASKHLYASLVPFSPAQPP
PLSNLLAGPAFDAEQIWSQIELLSRPLIPHLRRQLRRLEQQPPSQPPPRTESKSADAEEK
SEEEEDGEGDEELEELDDVDDEEESEEEEKEELGLEGEAGNEVEDEFFKIKDLDKFMVKG
EEAEYGGGAKQGKKKKKTENWMEEDSDSEDEEYLDEDEEDDDEHLDLEDFDDEEEEEEGA
VGDIMKDDFSRYKDFFQEADDQKVRKKGGSKKVQFKDEPDKPEVDDKNDDGNVSQDEQGL
STHEKARLKMHAKIEEMEKANLEPSTWTMQGEVNASSRPKNSALEVDLDFEHNVRPAPVI
TEEVTASLEEMIKKRIIEGHFDDVEKPSPLQFKSPKEQKDLDESKSKKGLAELYEDDYAQ
KAGLAPAPLSISDELKNEAKTLFKRICLKLDALSHFHFAPKPVIEDMSIQANVPALAMEE
IAPVAVSDAAMLAPEEIFEGKGDVKEEAELTQAERKRRRANKKRRYAGSHKERPAKMQKD
*

>11686.m00326|LOC_Os12g04010.1|genepair1245-2
MAMDLDEPLDAEKGEAALRRLRDADPALYLSPSADLAAAAREASKHLYASLVPFSPAQPP
PLSNLLAGPAFDAEQIWSQIELLSRPLIPHLRRQLRRLEKQPPSQPPPRTESKSADAEEK
SEEEDEEDGEGDEELEELDDVDDEEESEEEEEEEEEDKEGLEDKAGNQVEDEFFKIKDLD
KFMVKGEEAEYGGGAKQGKKKKKTENWMEEDSDEEEEEDLDEDEDNEDEEDDDEHLDLED
FDDEEEEEEGAVGDIMYKDFFEEADDQKVRKKGGSKKVQFKDEPDEPEVDDKNDDGNDEQ
GLSTHEKARLKMHAKIEEMEKANLEPSMWTMQGEVNASSRPKNSALEVDLDFEHNVRPAP
VITEEVTASLEEMIKKRIIEGHFDDVEKPSPLQFKSPKEQKDLDESKSKKGLAELYEDDY
AQKAGLAPAPLSISDELKNEANTLFKRICLKLDALSHFHFAPKPVIEDMSIQANVPALAM
EEIAPVAVSDAAMLAPEEIFEGKGDVKEEAELTQAERRRRRANKKRRYAGSHKERPAKMQ
KD*
>11687.m00336|LOC_Os11g04210.1|genepair1246-1
MSSLEEPLGLGDLPKLSINRLERFSPNACRASADDRSTSNYKHHNGGNNQTIFHSSSHSW
HMQGQYTDSSCNGVDMEFRALPRKVLWELPRFVKIVEVGPRDGLQNEKSTVPASVKIELI
HKLVASGLSVVEATSFVSPKWVPQLADAKDVLQGIRHVPDVRFPVLTPNLRGFEAALAAG
AKEVAVFASASESFSKSNLNCTIKESLVRYRDVVTSAKKHGMRIRGYVSCVVGCPVEGTI
HPSKVAYVAKELYDMGCSEISLGDTIGVGTPGSILAMLEAVMSFVPVDKLAVHFHDTYGQ
ALANILVSLQLGINIVDSSVSGLGGCPYAKGATGNVATEDVVYMLHGLGIETNVDLNKLM
DAGDYISKHLGRQSGSKTTTALRKLTT*
>11686.m00327|LOC_Os12g04020.1|genepair1246-2
MTATPTITSIAMVATIRRSFTAVLIHGICKANILIHPAMGWIWSSELFHGRFHQTTSNRR
NELTCSIMTVAMQHVLWDLPRFVKIVEVGPRDGLQNEKNTVPTSVKIELIHKLVASGLSV
VEATSFVSPKWVPQLADAKDVVEGIKHVPDVRFPVLTPNLRGFEAAVAAGAKEVAVFASA
SESFSKSNLNCTIKESLVRYHDVVTSAKKHGIRIRGYVSCVVGCPVEGTIHPSKVAYVAK
ELYDMGCSEISLGDTIGVGTPGSVLAMLEAVMSFVPVDKIAVHFHDTYGQALANILVSLQ
LGINIVDSSVSGLGGCPYAKGATGNVATEDVVYMLHGLGIETNVDLNKLMDAGDYISKHL
GRQSGSKTTTALRKLTT*
>11687.m00337|LOC_Os11g04220.1|genepair1247-1
MAETSSAAAAAQTDAEREEALDRMLTRLALAEDARLAPLLARVLPYAITSLASATASVRK
LVMEILSHINKRVKHRPEISLPMLDLWRIYTESTSSTIVRNFCIVYIEMAFERLLSEDKG
SIAPDLLINISNVTEQHQGIILRLVVKAIGECNTHKVGDNVASKYQSISGSNDGLVFADF
CFHTVLYQTPPQGVGCPAGLSVAQSDRVTGKQPLKGDTLTSRKLGILNVIEAMQLAPEIV
YPLYLAAASDSQESVTKRGEELLKRKASAVNLEDSNLMKKLFTLFNGTASPENIAAELKV
APAHSSLRVRLMGVFCRSIAAANAFPYTLQCIFGCIYGNGTTSRLKQLGMEFTVWVFKHA
ANDQLKLIGPVILSGILRSLDGSSTTEADSSSRDIKIFAYQAIGLLATRMPNLFSNKTDM
AIRLFTALRLEEQSLRLTIQEAATALATAYKLINDLMRNKLTSFVDEAHEWIVTPHGPNM
PLENVFGTEGRYKKANVLLSVDIFGFYILGASVVILKDIEALLLENSQMEQSEVRFSAVR
WATTLYDMKHCPSRYICMLGASDVKLDIREMALTGLNLLNDERESFAIATDSNYPDIADM
LVSSRYVNRLLWLRTLLGHVDADAREATSRLLGITSSALSSTAALDLLSELTSTFDQNRP
SRFENYHGLLCAIGYITAGCLKESYITEEIVQKSIDVLVKVVESEGSALASTAMEALGHI
GLHCLLPSINRNSSQAALLTILNEKLAKLLSENDTKAIQKILISLGHLSWNELSFAHLNN
ALDLIFSLSRSKVEEVLFAAGEALSFIWGEVPVTTDVILETNFVSLSQATNYLTGDAPVL
VSSNSNKGSDCEEAHAMAREEIIKRLFDTLIYSSRKEERCAGTVWLVSLTMYCGQHPKIL
ELLPQIQEALTHLLGDQNDLTQDLASQGMSIVYELGDASMKEQLVHALVNTLSGAAKKKR
AIKLMEDSEVFQEGTIGNNPTGGKLSTYKELCSLANEMGQPDLIYKFMDLANYQAAINSK
RGAAFGFSKIAKQAGEALQPHLHTLIPRLVRYQYDPDKNIQDSMAHIWKLIVADPKKTID
EHYDLIVEDLLVQSGSRLWRSREASCLALADIIQGRRYGQVSKHLRKIWITTFRAMDDIK
ETVRNAGDSLCRAVSLLTVRLCDVSLTTSSDANETMNIVLPYLLSEGILSKVSSVQKASI
SLVMKLAKGAGPALKPHLSELVSCMLECLSSLEDQRLNYVEMHAGNAGIQTEKLESLRIA
VAKDSPMWETLDICLKVVDKESLDLLVPRLAQMVKSAVGLNTRVGVASFITLLVQKVMVE
IKPYAAALLRLLYSAVLEEKSSAAKRAFASSCAAVLKYASPSQAQKLIEDTTSLHLGEKN
AQLSAAILIKSYLSNAADILSGYNAVVLPVIFASRFDDDKDIGALYGELWEDIPSSERVT
LQLYLPEIISLLCDSMSSSSWAGKRKSAKAIKKLCDALGESLSVHHNNILESLLKELPGR
FWEGKDAILDALAALCSSCHTAMSAEDSGMPSVILNAVCAACSRKSKLYREAAFSCLQQV
ITAFKDPGFFNIVFPMLYEVSNRSVICKTRNSSSLTASSSAEQDETEGVSVSLDKVLNCV
ASLITVAFLQDIINQRKNILEIILNSLSPEESWQIKLSSFLCIKELCYKFQNPDGNNTWP
EETTYLVEELFHSTAPKVVDVIRLVKIAQITALLLYVTVHMLVFGFTDFRTNSYKVHTAA
SECLLELSKLYRDFPLVDRKGPKFSGELAELCESEKSEQAKAFLKQCMDILKDFEDATGL
AMEMD*
>11686.m00328|LOC_Os12g04030.1|genepair1247-2
MAETSSPAAAAAAAAQTDAEREEALDRMLTRLALAEDARLAPLLARVLPYAITSLASPAA
SVRKLVMEILSHINKRVKHRPEISLPMLDLWRIYTESTSSTIVRNFCIVYIEMAFERLLS
EDKGSIAPDLLINISNVTEQHQGIILRLVVKAIGECNTHKVGDNVASKYQSISGSNDDLV
FADFCFHTVVYQTPPQGVGCPAGLSVAQSDRVTGKQPLKGDTLTSRKLGILNVIEAMQLA
PEIVYPLYLAAASDSQESVTKRGEELLKRKASAVNLEDSNLMKKLFTLFNGTASAENIAA
ELKVAPAHSSLRVRLMGVFCRSIAAANAFPYTLQCIFGCIYGNGTTSRLKQLGMEFTVWV
FKHAANDQLKLIGPVILSGILRSLDGSSTTEADSSSRDIKIFAYQAIGLLATRMPNLFSN
KTDMAIRLFTALRLEEQSLRLTIQEAATALATAYKLTNDLMRNKLISFVDEAHERIVTPH
GPNMPLENVFGTEGRYKKANVLLSVDIFGFYILGASVVILKDLEALLLENSQMEQSEVRF
SAVRWATTLYDMKHCPSRYICMLGASDVKLDIREMALTGLNLLNDERESSAIATDSNYPD
IADMLVSSRYVNRLLWLRTLLGHVDADAREATSRLLGITSSALSSTAALDLLSELTSTFD
QNRPSRFENYHGLLCAIGYITAGCLKESYITEEIVQKSIDVLVKVVESEGSALASTAMEA
LGHIGLRCLLPSINRNSSQAALLTILNEKLAKLLSENDTKAKQKILISLGHLSWNELSFA
HLNNALDLIFSLSRSKVEDVLFAAGEALSFIWGEVPVTTDVILETNFVSLSQATNYLTGD

APLLVSSNSNKGSDCEEAHAMAREEIIKRLFDTLIYSSRKEERCAGTVWLVSLTMYCGQH
PKILELLPQIQEALTHLLGDQNDLTQDLASQGMSIVYELGDASMKEQLVHALVNTLSGAA
KKKRAIKLMEDSEVFQEGTIGNNPTGGKLSTYKELCSLANEMGQPDLIYKFMDLANYQAA
INSKRGAAFGFSKIAKQAGEALQPHLHTLIPRLVRYQYDPDKNIQDSMAHIWKLIVADPK
KTIDEHYDLIVEDLLVQSGSRLWRSREASCLALADIIQGRRYGQVSKHLRKIWITTFRAM
DDIKETVRNAGDSLCRAVSLLTVRLCDVSLTTSSDANETMNIVLPYLLSEGILSKVSSVQ
KASISLVMKLAKGAGPALKPHLSELVSCMLECLSSLEDQRLNYVEMHAGNAGIQTEKLES
LRIAVAKDSPMWETLDICLKVFDKESLDLLVPRLAQMVKSAVGLNTRVGVASFITLLVQK
VMVEIKPYAATLLRLLYSAVLEEKSSAAKRAFASSCAAVLKYASPSQAQKLIEDTTSLHL
GEKNAQLSAAILIKSYLSNAADILSGYNAVVLPVIFASRFDDDKDIGALYGELWEDIPSS
ERVTLQLYLPEIISLLCDSMSSSSWAGKRKSAKAIKKLCDALGESLSVHHNNILESLLKE
LPGRFWEGKDAILDALAALCSSCHTAMSAEDSGMPSVILNAVCAACSRKSKLYREAAFSC
LQQVITAFKDPGFFNIVFPMLYEVSNRSVICKTRNSSSLTASSSAEQDETEGVSVSLDKV
LNCVASSITVAFLQDIINQRKNILEIILNSLSPEESWQIKLSSFLCIKELCYKFQNSDGN
NTWPEETTYLVEELFHSTAPKVVDVIRLVKIAQITALLLYVTVHMLVFGFTDFRTNSYKV
HTAASECLLELSKLYRDFPLVDRKGPKFSGELAELCESEKSEQAKAFLKQCMDILKDFED
PTGLAMEMD*
>11687.m00338|LOC_Os11g04230.1|genepair1248-1
MVYDVVRYRIHRMRKKLKDGLDDRDTVQHYVCPNCKRRYSAFDALQLVSDMDDYFHCEHC
KGELRPESEKLTLDEIVCGGGNAIKHTHDKLKDMQQRMEEQLKPLIAVLDRVKDLPFPSF
MSLQDWERATIGASANGAVGSSQNSEGRYSSKPMPFLGETEVEVNFLGSTGAQEGVESGM
ESIKPQHSWMNRKRTVLAGEHKEENNNTANLDQSSEAKSDKKQLSEEDEMKSIQEAYAKA
YYEAIQKRQEDEGKRAIQEESLACISDQPFASDAQFERRLGAKSKRDDGGESGDDGIELK
VRQSTGNIEEVYKFADLNVETQELVEKNCIPPAE*
>11686.m00331|LOC_Os12g04060.1|genepair1248-2
MDDYFHCEHCKEQLLPESEKLTLDEVVCGGDNAIKHKHDKLKDMQQRMEEQLKPLIAVLD
RVKDLPFPSFMSLQDWERATMEASANGAVGSSQNSEGRYSSKPMPFLGETEVEVNFLGST
GAQEGVESGMESIKPQPSWMNRKSTVLTGEHKGEISNTADLDQSSEAKSDKKQLSEKDEM
KSIQEAYAKAYYEAIQKRQEDEDKRMIQEESLTCISDQPFASDAQFERRLGAKSKRDDGG
DSGDDGIEMKVEQPTGNIGEVYKLADLDVETQESIDDDDDDDLVWVEG*
>11687.m00342|LOC_Os11g04270.1|genepair1249-1
MEVQVKRTLVVPPPPTCETEEVPLTVFDLVAPTYHVTVLFAFSPPNPTTRALLDALSAML
PHFPLLTARLERRGARRRPFFVTGRGGAGALVVEAEVSSELADHLPLAPSPELARLHPPV
NTDAPTPHVLLVQINRLAEEWKVTYQWQEFLPIDAEEILKIRIHKRPVDDVVAWHFEKTV
SSLRRDRPSYKYNWQAEVPPKVKILHGGWLVMNTVATNTFSGSVMEDAMEDNLSCSG*
>11686.m00333|LOC_Os12g04080.1|genepair1249-2
MEVQVKRSSVVPPPPRETEETPLTVFELVAPTYHVTVLFAFSPPNPTTRALLDALSATLP
HFPLLTARLDRRGARRRPFFVTGRGGAGALVVEAEVSSDLADHLPLAPSPELARLHPPVN
TDAPTPHVLLVQINRFACGGLVVVSSAHHQAADGFSMSTFFHAWTDAVRRNGAPLLDRPV
PYGPGALSPRRPPRCEFEHRGKEFLPHDGVTSRQGQGADTGAVRIDPSEVANVLLHYPSE
FVAELKRRAQGKYTTFETVSAHVWKKITAVRGLDAGARTSVNVSVNGRARLGTGTVPNGF
FGNLIINASSGPTARELTTGTLADAAALIRAGIRAVDRRYFQSFIDFGALHVDGGRDEEE
PLQPANVDEPGVLSPDVDSDSWLHLELHRLDMGLGGRLAGILPAKVPEDGVVVVMPSLRK
SGGVEVFVALWEKHANELTSIAYTMD*
>11687.m00343|LOC_Os11g04280.1|genepair1250-1
MDPPARRLMDSRAAQLAAPLPDPYSAETFEDLLRELGVDPSIHTIVRSAGRWMDPAAAAA
RVPVRFHRTLQRLGIDPNSDARSIRDMLQEFYRVVYHGEVYWAGRVIRPRPSSMPTPVIG
RRRRAADGDAPMQPPSKYARVHAVSRDVLLGLALTKACDARQEECAVCLSDFEEKDRLRT
MPCNHSFHENCLFRWLRDSCLCPLCRYALPKQQQVQSC*
>11686.m00334|LOC_Os12g04090.1|genepair1250-2
MDPPARRLMDSRAAAQLAAPLSDPYAAEKFEDLLRELGVDPSIHTVVRSAGRWMDPAAAA
ARVPMRFHRTLQRLGIDPNSDARSIRDMLQEFRRGVYQGEDDVAAPPTGMPRCKMSRDVL
LGLALTKACDARQEECAVCLRDFEEKDMLRTMPCNHSFHEICLFRWLSESCLCPLCRYAL
PKQQQVQSC*
>11687.m00344|LOC_Os11g04290.1|genepair1251-1
MNTPLASIHRSTCRQPSLLPSRSMAMDTYYYSMLFVLPPILYMSYHLTRILADKKKPTTH
GLKAHPLLGHLPAFVNNSHRFLDWTTELIVGSPEMRMGFWIPGMRTGIITGNPADVEHIL
RTNFANYPKGEHAIGMLEDFLGHGLFNSDGEQWLWQRKNASYEFSNRSLRRFVVDVVQAE
IADRFLPLLRRAAGDGGGDGDVVVLDLQEVLQRFGFDTICMVAFGHDPRCLADGGVMEDA
RSEFMHTFGEAQDLVVGRFFDPIEVSWKIKKWLNVDTERRLRKAIADVHAFAMDIVRARR
QSASVNDRDDVLSRFVASDEHSDEVLRDIVLSFLIAGRETTASGLSWFFWFLSSRPDVVA
RIADEVRAVREATGTRPGEPFRFDALREMHYLHAALTESMRLYPPAPIDSQSCAADDTLP
DGTLLRAGWSVTYSAYAMGRLAAIWGEDCLEYRPERWLGDDGAFQPASPFRFTVFHAGPR
MCLGKEMAYVQMKSIVANVLEEFEVDVVKDVAGGGVPEHVLSVTLRMKGGLPVKIRRKTE
AY*
>11686.m00335|LOC_Os12g04100.1|genepair1251-2
MAMDSYYCSMLFFLPPILYVSYHLTRILADKKKPTTHGLKAHPLLGHLPAFVNNSHRFLD
WTTELIVGSPEMRMGFWIPGMRTGIITGNPADVEHILRTNFANYPKGEHAIGMLEDFLGH
GLFNSDGEQWLWQRKNASYEFSKRSLRRFVVDVVQAEVADRFLPLLRRAAGDGRGGDIVV
LDLQEVLQRFGFDTICMVAFGHDPRCLADGSVMEDARSEYMHTFGEAQDLVVGRFFDPIE
VSWKIKKWLNVGTEHRLRKAIADVHAFAMDIVRTRRQSASVQDRDDVLSRFVASDEHSDE
VLRDIVLSFLIAGRETTASGLSWFFWFLSSRPDVVARIADEVRAVREATGTRPGEPFGFD
ALREMHYLHAALTESMRLYPPAPIDSQSCAADDTLPDGTLLRAGWSVTYSAYAMGRLAAI
WGEDCLEYRPERWLGDDGAFQPASPFRFTVFHAGPRMCLGKEMAYVQMKSIVANVLEEFE

VDVVKEIAGGGVPEHVLSVTLRMKGGLPVKIRRKTEAY*
>11687.m00347|LOC_Os11g04320.1|genepair1252-1
MSERTIPPPVSSHGEDFAEVVVVRHGETSANALCIIQGQTDIELNEAGRQQAVMVARRLA
KEAKPVAVYSSDLKRAAETAQTIATACNVSNLVLSPALRERHMGDLHGLKFEDAVRSKPD
AYKAFSSEDRSQEIPGGGESLDQLSERCVSYLNTIAGKHKGERVIVVSHGASIEELCRHA
DPTSSVRRRIPNTSICVFNISGSTGHWILERFGDVAHLNEDDFP*
>11686.m00337|LOC_Os12g04120.1|genepair1252-2
MDEKTFYVVSVAAWQIADASTNHNDSMSERTIPPPVSSHGEDFAEVVVVRHGETSANALC
IIQGQMDIELNEAGRQQAVMVARRLAKEAKPVAVYSSDLKRAAETAQTIATACNVSNLVL
SPALRERHMGDLHGLKFDDAVRSKPDAYKAFSSEDRSQEIPGGGESLDQLSERCVSYLNT
IAGKHKGERVIVVSHGASIEELCRHADPTSSVRRRIPNTSICVFNISGSTGHWILERFGD
VAHLNEDDFP*
>11687.m00348|LOC_Os11g04330.1|genepair1253-1
MPAAESCHSRSLSWLVKSCIPADPARHIAVPVLCPTPQPPPPSSPPAPPISTLPDDLLLE
CLARVPRASLPPLPAVSRRFATLLASDAFLHLRRAHAHLRPSLLALSVSDNGCVPQALLR
FESSVPVLEVAPLPLPPTLLHCGGSVFAHARAVVLGRDVFLIGRGATLRVDALTGAARAC
APTLFPRKKFAAAAVGDRIYVAGGSARTAAVEEYDPEVDAWRVVGEAPRRRYGCAGASAG
GVFYVAGGVAVSGEGARALEAHVCAGSVDALHVASGTWARPRALPGGGCVVGACGVGDHL
YVVASHAVELSFWRWSGATGRGGDGRGWGGWVALEAPPMPRGSVGLGMAVRVAMAGLGTN
RVAAVLRLKFCHAIFNSYAARIAARSCHYPAASSTSLQPQSPSIPFDLPTLLLRMHASIS
FALAPASSPDAASFAPTAADVGGAVCLGYGIAIAVGVLVFISTVMLASYICVRAKAGAAA
VLLADDDGGGAPAASAVVVLGLDGPAIDALYPKLLHVGVGDDDDACAGAQCAICLGEFVA
GDALRRGHGCGHRFHAECAERWLRVSATCPVCRDSPLPSPMATPLAEAVPLAAHAR*
>11686.m00338|LOC_Os12g04130.1|genepair1253-2
MPAAESCHSRSLSWLVKSCIPADPARHIAVPVLCPTPQPPPPSSPPAPPISALPDDLLLE
CLARVPRASIPPLPAVSRRFATLLASDAFLHLRRAHAHLRPSLLGLSVSDNGCIAQALLR
FESSVPVLEVAALPLPPTLLHCGGSVFAHARAVVLGRDVFLIGRGATLRVDALTGAARAC
APTLFPRKKFAAAAVGDRIYVAGGSARTAVVEEYDPEADAWRVVGEAPRRRYGCAGASAG
GVFYVAGGVAVSGEGARALEAHVCAGSVDALHVASGAWARPRALPGGGCVVGACGVGDHL
YMVASHAVELSFWRWCGATGRAGDGRGWGGWVALEAPPMPRGSVGLGMAVRVAMAGLGTN
RVAAVVSAAAVRGHNAGGGALEGMVLVYDIAGGKWSRAPDLPPGFRRAALQFAMWSSSCF
INHDEREPRDCTNRFGSASTNSALNFVTPFRLIRRSHRSSFVPLSSRKPHFPAAAVSISS
ILFAHTEMHARLLLRMHAPISFAPASSPDAASFAPAAADVGGAVCLGYGIAIAVGVLVFI
STVMLASYICVRAKAGAAAVLLADDDDDGGAPAASAVVVLGLDGPAIDALYPKFLHVGVG
DDDNACAGAQCAICLGEFVAGDALRRGPGCGHRFHAECVERWLRVSATCPVCRDSPLPSP
MATPLAEAVPLAAHAR*
>11687.m00350|LOC_Os11g04350.1|genepair1254-1
MPSTTTAPETDPSKTVVEEVTGWLRLYSDGTVERLTPPDAEPFTVIVPPYTEPRNGVTVH
DVTTARGVDVRLYLPAEPPAAAPRPRRRRPLLLHLHGGGFCLSRPSWALYHNFYAPLAAK
LDVAGIVSVFLPLAPEYRLPAAIDAGHAALLWLRDVACGDEGNLDPAVERLRDEADFSRV
FLIGDSSGGNLVHLVAAHAAAKDDGAGADLHAVRLAGGVLLNPGFAREEKSRSELENPPS
LFLTEDMVDKLLALGVPLGMNKDSPYTSPSLVAEAVARLHMPPMLLVVAEKDLLHDPQVE
YGEAMARVGKTVETVVSRGAVAHVFYLNFFAVESDPLTAERTRELIDTIKTFIDRY*
>11686.m00339|LOC_Os12g04140.1|genepair1254-2
MPSTTTAPETDPSKTVVEEITGWLRLYSDGTVERLTPPGAEPFTVIVPPYTEPRNGVTVH
DSIVGSLPQLLRASLTTKLDVAGIVSVFLPLAPEHRLPAAIDAGHAALLWLRDVACGEDE
NNNGAAHHLDPAVERLRDEADFARVFLIGDSSGGNLVHLVAAHAAAKDDGAGADLHPVRL
AGGVLLNPGFAREDKSRSELENPPSLFLTEEMVDKLLALGVPLGMNKDSPYTSPSLAAEA
VARLHMPPMLLMVAEKDLLHDPQVEYGEAMARVGKTVETVVSRGAVAHVFYLNFFAVESD
PLTAERTRELIDTIKTFIDRY*
>11687.m00351|LOC_Os11g04360.1|genepair1255-1
MATLPQMAATKERQEAANPTTTTRTLVESVTNWIRVYSDGSVDRLGPPEAAAFMVLVPPY
DDPRDGVTVHDVATDHGVDVRLYLTTTAPARRRPVLVHFHGGGFCLSHAAWSLCHRFYAR
LTVDLDVAGIVSVVLPLAPEHRLPAAIDAGHAALLWLRDVASGGSDTIAHPAVERLCGAA
DFSRVFLIGDSAGGVLVHNVAARAGEAGAEALDPIRLAGGVQLHPGFILPEKSPSELENP
PTPFMTQETVDKFVVLALPPTKDTEKFICRADVYGSEPSDLAGKFAPVPRCEKGGRLFFT
SCKRHKGSSTRKERTAGDGTWVRQNSKGVKNKAGVKVGETQNFRFKKDGSYTDWLMEEHH
CCRQQAVAGDEEPVICRMYVSPRAPPDSAARQESAAFVQQQPAPQVSEPPCDKKKRDDVA
EEAPAAA*
>11686.m00340|LOC_Os12g04150.1|genepair1255-2
MATLPQMAATKERQEAANPTTTTRTLVESVTNWIRVYSDGSVDRLGPPEAAAFMVLVPPY
DDPRDGVTVHDVATDHGVDVRLYLTTTTPAGRRPVLVHFHGGGFCLSHAAWSLYHRFYAR
LAVELDVAGIVSVVLPLAPEHRLPAAIDAGHAALLWLRDVACGTSDTIAHHAVERLRDAA
DFSRVFLIGDSAGGVLVHNVAARAGEAGAEALDPIRLAGGVLLHPGFILPEKSPSELENP
PTPFMTQETVDKFVMLALPVGTTSRDHPYTSPAAAVTAAEGAQLPPMLVMVAEEDMLRDA
QVEYGEAMARAGKAVETVLNKFPAQCIRVFVSPIRLRNFDLMEKHVEKMLSLGFRFNPSA
EDLITFYLPRLIAGKPMKDTEKFICRADVYGSEPSDLAGKFAPVPRCEKGGRFFFTSCKR
HKGSSTKKERTAGAGTWVRQNSKEVKNKAGVKVGETQNFRFKKDGSYTDWLMEEHHCCRQ
QAVAGDEEPVICRMYVSPRAPPDSAARQESAAFVQQQPAPQVSEPPCDKKKRDDVAEEAP
AAA*
>11687.m00352|LOC_Os11g04370.1|genepair1256-1
MGWKAAEKLIRHWKILRGDNVMIIRGKDKGESGLIKRVIRSQNRVIVEGKNLVKKHIKQG
EGHTGGIFSIEAPLHVSNVQVLDPVTGKPCKIGYKYLEDGTKVRFARGMNASGAVIPRPE
ILKERRKPRPTSPGPKDTPIEHVLEKTYDAKAGIGMPDL*

>11686.m00341|LOC_Os12g04160.1|genepair1256-2
MACRPAMCSPSALAPLRHRLFLLGYVMIIRGKDKGESGLIKRVIRSQNRVIVEGKNLVKK
HIKQGEGHTGGIFSIEAPLHVSNVQVLDPVTGLLVKGKFIHTTLATGNHVRLDTSIWKME
LKSAGPKDTRIEHVLEKTYDAKAGIGMPDL*
>11687.m00353|LOC_Os11g04380.1|genepair1257-1
MGAAGDNAAKAPAAAAGSNGKGTAASMDVVSSSSSSSPAPAPSVLKSVLLSYAYVSVWIT
LSFSVIVYNKYILDPKMYNWPFPISLTMIHMAFCASLAVVLVRVLRVVAVPASPPMTPSL
YAASVVPIGALYALSLWFSNSAYIYLSVSFIQMLKALMPVAVYSLAVAFRTDSFRRASML
NMLGISAGVAVAAYGEARFDAFGVMLQLAAVAAEATRLVLIQILLTSKGMSLNPITSLYY
IAPCCLVFLTLPWYFVELPRLRAAAGAAARPDVFVFGTNSLCAFALNLAVFLLVGKTSAL
TMNVAGVVKDWLLIAFSWTVIKDTVTPVNLVGYGIAFLGVAYYNHAKLQGLKAREAERRA
ASMATAKDGDAEAGARLLPEKDAGEQKN*
>11686.m00342|LOC_Os12g04170.1|genepair1257-2
MTMEKRTSPQQPPTLLPTNCSTHFLYARAPTTAGVTPRQPHQSFRSYLAQMGGTGDDGAK
APAAAAAMDVVSSSSSSPAPAPSVLKSVLLSYAYVSVWITLSFSVIVYNKYILDPKMYNW
PFPISLTMIHMAFCASLAVVLVRVLRVVAVPASPPMTPSLYAASVVPIGALYALSLWFSN
SAYIYLSVSFIQMLKALMPVAVYSLAVAFRTDSFRRASMLNMLGISAGVAVAAYGEARFD
AFGVMLQLAAVAAEATRLVLIQILLTSKGMSLNPITSLYYIAPCCLVFLTLPWYFVELPR
LRAAAGAAVRPDVFVFGTNSLCAFALNLAVFLLVGKTSALTMNVAGVVKDWLLIAFSWTV
IKDTVTPVNLVGYGIAFLGVAYYNHAKLQGLKAREAERRAASMATAKDGDAEAGARLLPE
KDAGEQKN*
>11687.m00354|LOC_Os11g04390.1|genepair1258-1
MPRTDNAASANSVEPDKSEECLEFDDEEEEVEEEEIEYEEIEEEIEEEEEVEEDEDVVEE
VEEVDEEEDEEEDEESDETEGVSKTKGVHQKDVTEKGKHAELLALPPHGSEVYVGGISSD
VSSEDLKRLCEPVGEVVEVRMMRGKDDSRGYAFVNFRTKGLALKVVKELNNAKLKGKRIR
VSSSQAKNKLFIGNVPHSWTDDDFRKAVEEVGPGVLKADLMKVSSANRNRGYGFVEYYNH
ACAEYARQEMSSPTFKLDSNAPTVSWADPKNNDSVSTSQVKSVYVKNLPKNVTQAQLKRL
FEHHGEIEKVVLPPSRGGHDNRYGFVHFKDRSMAMRALQNTERYELDGQVLDCSLAKPPA
ADKKDDRVPLPSSNGAPLLPSYPPLGYGIMSVPGAYGAAPASTAQPMLYAPRAPPGAAMV
PMMLPDGRLVYVVQQPGGQLPLASPPPQQAGHRSGSGGRHGGSGSRYGGGGGSSGSSRPE
EFVSETRLYMRIYQYFPISQIAESEEALYRQFTSVSHMEYEISGQESEVDSEVWLGWVAQ
RRVCSRLETRLPLPKLNTSCCLVTLSRGGRASNLSRGRRLRPVLLLDEPPPPGNLVQGGA
GPPLQIPVDRESTKHRTHGDDTAIRRSWVLAAGESSDDDSCGGDKAKPKKGRKPLGNREA
VRKYRQKKKAHTAHLEEEVKRLRAINQQLVKRLQGQAALEAEVVWLRSLLVDVRSRINGA
LGSYPFQAQCGVNNVLGCDGMAQCFAGKPELDTLSNFMGGWVWGYSLGVVNYALVLLL*
>11686.m00343|LOC_Os12g04180.1|genepair1258-2
MPRRTDNAASANSVEPEKSEECLEFDDDEEEEVEEEEIEYEEIEEEIEEEEVEEDEDVVE
EVEEVDEEEDEEEEEESDETEGVSKTKGVHQKDVTEKGKHAELLALPPHGSEVYVGGISS
DVSSEDLKRLCEPVGEVVEVRMMRGKDDSRGYAFVNFRTKGLALKAVKELNNAKLKGKRI
RVSSSQAKNKLFIGNVPHSWTDDDFRKVVEEVGPGVLKADLMKVSSANRNRGYGFVEYYN
HACAEYARQEMSSPTFKLDSNAPTVSWADPKNNDSASTSQVKSVYVKNLPKNVTQAQLKR
LFEHHGEIEKVVLPPSRGGHDNRYGFVHFKDRSMAMRALQNTERYELDGQVLDCSLAKPP
AADKKDDRVPLPSSNGAPLLPSYPPLGYGIMSVPGAYGAAPASTAQPMLYAPRAPPGAAM
VPMMLPDGRLVYVVQQPGGQLPLASPPPQQAGHRSGSGGRHGGSGGRYGGGGGSSGSSRP
EECVSETRLYMRILPVSQSAESDGALYRQFTPVSHMDYEISGPESEVDSEVWLGWVAHDV
CAQD*
>11687.m00355|LOC_Os11g04400.1|genepair1259-1
MAGIAALANCQSDERSSSCGAALLEFMGPAAPPSEAAAAADDVVLPYISRILMEEDIDDD
MFFCLYPDHPALLEAQQPFAQILSSSSSIAGEVNSAPMEDSAALMMQGSGNGRGRKGSKH
GGDELEAEVGRASKLMATPEEEEDDDDGVGEMLEKMMLNGDEDEAFHGETNAPRVPAEKK
CGKAARRRRRQAKGEVVDLRELLMSCAQAVASGNRRSAGELLEQIKRHSSPTGDATERLA
HYFADGLEARLAGAASLERRLVASAEERASAMELLEAYQVFMAACCFKWVAFTFANMAIL
RAAEGRNRLHIVDYGGQYHGLQWPSLLQRLAEREGGPPEFRAVAAARWETVTAEDVVGVD
PDDEAAVVVNDVLSLGTLMDESGVFDDPSPRDTVLGSIRDMRPAVFVQAVVNGAHGAPFF
PTRFREALFFFSALFDMLGATTPEEGSHLRVVLERDVLRRAAVGVIAGEGAERVERPETY
RRWQARNRRAGLRQAAVEGDVVEAVRRRVRRRHHEEFVIEEDAGWLLQGWKGRILYAHSA
WVVAEDGAH*
>11686.m00345|LOC_Os12g04200.1|genepair1259-2
MGPAAPPSEAAAAADDVVLPYISRILMEEDIDDDMFFCLYPDHPALLEAQQPFAQILSSS
SGIAGEVNSAPMEDSAALMMQGSGNGRGRKGSKHGGDELEAEVGRASKLMATPEEEDDDD
DGVGEMLEKMMLNGDEEMNAPRVPAEKNGGKAARRKRRQGKGEVVDLRELLMSCAQAVAS
GNRRSAGELLEQIKRHSSPTGDATERLAHYFADGLEARLAGAASLEHRLLASAEERASAM
ELLEAYQVFMAACCFKWVAFTFANMAILRAAEGRSKVHIVDYGGQYHGLQWPSLLQRLAE
REGGPPEVRMTLVGHPQPGFRPARRLERTGRRLSNCARAFGLPFKFRAVAAARWETVTAE
DVVGVDHDEAAVVVNDVLSLGTLMDESGVFDDPSPRDTVLGSIRDMRPAVFVQAVVNGAH
GAPFFPTRFREALFFFSALFDMLDATTPEEGSHLRAVLERDVLRRAAVGVIAGEGAERVE
RPETYRRWQARNRRAGLRQVAVEADVVEAVRRRVRRRHHEEFVIEEDAGWLLQGWKGRIL
YAHSAWVVAEDATSVGELTVVTTSLVAIILSAASAQDELPLSTNE*
>11687.m00359|LOC_Os11g04440.1|genepair1260-1
MSLRRLAASALRRGGANDGGVLAAVRAEIAHELSSSPSSSPPSLQSQDIPDFSTVSDAPR
GQEVLLRRRDASEEVLVSAVLAPLRFEGEEPLPRDALMKVFVSKPDVKPVMRFDCRAFAA
EGDGGSADYDVTAVCYHPFAGECDAGEDKYEGPEFRILLHPTLDGGILKGTLRNLLVENA
LCHKGIADLFGAVGKAAIAPPSYPASAFFPEAARVPAAPVPSPPSQVGLVAFKDLFMSIN
NIVVISTIFDHKWTHLLKYLAACKTPLFFAAEIHMKQFSLKPMKALFNASTVSYDVKEIE

RPEEKMVKRMTGCHARRENLDPQLQVALKGYMVARGVNSKLASLLHHHLVEKERWQYMNW
LKTLEEMFSKDH*
>11686.m00346|LOC_Os12g04210.1|genepair1260-2
MSKSYITALSNDDDNNDTPIINENPTVCLNFLPGNRCFFDASWKDNLTGFGLFLHNPINH
QAIFVQGKSSLYKSPMQAELAAFKLALFICDFLKIANPLFLSDNQELTLLLQNTDYINQN
CHWSLLPLLALLKIFAGLANLIYGPSTVFGSRTDRQLANDSGVMLLRRLAASALRRGGAN
GGVLAAVRAEIAHELSSSTPSSPPSLQSQDIPDFATVSDPPRGQEVLLRRRDASEEVLVS
AVLEPLRFEGEEPLPRDALMKVFVSKPDVKPVMRFDCRAFADEGDGGSADYDVTNACYHP
FAGDAGEDKYEGPEFSGLINLEFLQEIWKNKHFHGILVELCNSSDR*
>11687.m00361|LOC_Os11g04460.1|genepair1261-1
MEKLDRYLQEHFDVPAKNPSEEAQRRWRQAVGTIVKNRRRRFRWVPDLDRRSLDKAKVRS
TQEKIRVALYVQQAALIFSDDELALITSKHDSKALKMHGGVDGISKKVRSSFDHGICASD
LDTRQNIYGVNRYAEKPSRSFWMFVWDAFQDMTLIILMVCALLSVAVGLATEGWPKGMYD
GLGIILSIFLVVMVTAVSDYKQSLQFKELDNEKKKIFIHVTRDGRRQKISIYDLVVGDIV
HLSIGDQVPADGLYIHGYSLLIDESSLSGESDPVYVSQDKPFILAGTKVQDGSAKMIVTA
VGMRTEWGKLMSTLSEGGEDETPLQVKLNGVATVIGKIGLVFAILTFLVLLVRFLIDKGM
TVGLLKWYSTDALTIVNYFATAVTIIVVAVPEGLPLAVTLSLAFAMKKLMNDKALVRHLS
ACETMGSAGTICTDKTGTLTTNYMVVDKIWISEVSKSVTSNTISGELNSVVSSRTLSLLL
QGIFENTSAEVVKEKDGKQTVLGTPTERAILEFGLGLEGVHDAEYSACTKVKVEPFNSVK
KKMAVLISLPSGTSRWFCKGASEIILQMCDMMVDGDGNAIPLSEAQRKNILDTINSFASD
ALRTLCLAYKEVDDDIDDNADSPTSGFTLIAIFGIKDPVRPGVKDAVKTCMSAGITVRMV
TGDNINTAKAIAKECGILTEDGVAIEGPEFHSKSPEEMRDLIPNIQVMARSLPLDKHTLV
TNLRGMFDEVVSVTGDGTNDAPALHEADIGLAMGIAGTEVAKESADVIVLDDNFTTIINV
ARWGRAVYINIQKFVQFQLTVNIVALVINFVSACITGSAPLTAVQLLWVNMIMDTLGALA
LATEPPNDEMMKRPPVRKGESFITKVMWRNIMGQSLYQLFVLGALMFGGESLLNIKGADS
KSIINTLIFNSFVFCQVFNEINSREMQKINVFRGIISNWIFIAVIAATVAFQVVIIEFLG
TFASTVPLNWQHWLLSVGLGSISLIVGVILKCIPVGSGETSATPNGYRPLANGPDDI*
>11686.m00347|LOC_Os12g04220.1|genepair1261-2
MEKLDRYLQENFDVPAKNPSEEAQRRWRQAVGTIVKNRRRRFRWVPDLERRSLDKAKVRS
TQEKIRVALYVQQAALIFSDGAKKKEYKLTGDIIKAGYAINPDELALITSKHDSKALKMH
GGVDGISIKVRSSFDHGIYASELDTRQNIYGVNRYAEKPSRSFWMFVWDALQDMTLIILM
VCALLSVAVGLATEGWPKGMYDGLGIILSIFLVVMVTAVSDYKQSLQFKELDNEKKKIFI
HVTRDGRRQKISIYDLVVGDIVHLSIGDQVPADGLYIHGYSLLIDESSLSGESDPVYVSQ
DKPFILAGTKVQDGSAKMIVTAVGMRTEWGKLMSTLSEGGEDETPLQVKLNGVATIIGKI
GLVFAILTFLVLLVRFLIDKGMTVGLLKWYSTDALTIVNYFATAVTIIVVAVPEGLPLAV
TLSLAFAMKKLMNDKALVRHLSACETMGSAGTICTDKTGTLTTNHMVVDKIWISEVSKSV
TSNTISGELNSVVSSSTLSLLLQGIFENTSAEVVKEKDGKQTVLGTPTERAILEFGLGLK
GDHDAEYRACTKVKVEPFNSVKKKMAVLISLPNGTSRWFCKGASEIILQMCDMMVDGDGN
AIPLSEAQRKNILDTINSFASDALRTLCLAYKEVDDDIDDNADSPTSGFTLIAIFGIKDP
VRPGVKDAVKTCMSAGITVRMVTGDNINTAKAIAKECGILTEDGVAIEGPEFHSKSTEEM
RDLILNIQVMARSLPLDKHTLVTNLRGMFDEVVSVTGDGTNDAPALHEADIGLAMGIAGT
EVAKESADVIVLDDNFTTIINVARWGRAVYINIQKFVQFQLTVNIVALVINFVSACIIGS
APLTAVQLLWVNMIMDTLGALALATEPPNDEMMKRPPVRKGESFITKFMWRNIMGQSLYQ
LFVLGALMFGGERLLNIKGADSKSIINTLIFNSFVFCQVFNEINSREMQKINVFRGIISN
WIFIAVIAATVAFQVVIIEFLGTFASTVPLNWQHWLLSVGLGSISLIVGVILKCIPVGSG
ETSATPNGYRPLANGPDDI*
>11687.m00362|LOC_Os11g04470.1|genepair1262-1
MAEEDDKKQKGPDVTVPSGYFFVPKPEQLIRDYLNHWITGRPSEELRDIVREADVYGSDP
ATLTEAHSAYGHDGKSWREKTTGASQQNIFTISRRGGFEGGGTWHNSQRRRVIEGYGDRQ
AFEYRAPGNKKTDWLMEEIASNLPAAITDEGIMVICKVYLSPRAKEATANEEERQETNVV
PGPKRLREAEATGYDAPAPPQPDVGYSYSGGGETSQATASMDYCCSTTTHTADDTANAAY
YHGDADAIKPDAYDGGDYGIGFNADGELVLCGNGHGGIGTQGQTPLAMQNTNGEMTLFSP
MNGYGVGFNEEVRQEPQVEGEVEMDNFFNDLFVDFDGAGDLNPNPNGGGDSHGHILCE*
>11686.m00348|LOC_Os12g04230.1|genepair1262-2
MAEEDDKKQKGPDVTVPSGYFFVPKPEQLIRDYLNHWITGRPIEELRDIVREADVYGSDP
ATLTEAHRAYGHDGKSWYFLTVAKWKGGRGGAGTAGRLNRCVEGGGTWHNSQRRRVIEGY
GDRQAFEYRAPGNKKTNWLMEEIASNLPAAITDEGIMVICKVYLSPRAKEATADEEERQE
TNVVPGPKRLREAEATGYDAPAPETPQPDVGCSYSGGGETSQATASMDYCCSTTTHTADD
TANAAAYYYGDVDAIKPDAYDGGDYGIGINADGELVLCGNGHGGIGTQGQMPLAMQNTNG
EMTLFSPMNGYGVGFNEEVRQEPQVGGEVEMNDFFNDLFVDFDGAGDPNPNPNEGGDSHG
HILCE*
>11687.m00363|LOC_Os11g04480.1|genepair1263-1
MAGYPPNPGSGYPYGGAGGYGAPPPPYGSSPAPSAPPYGEKPPKEGKTSSSSAPYYGGGG
GYGAPPSTQPYGSGGGYGAPPSTQRPQSYGGGYGAPPSSQPYGAPYGAPPPSSAPYGAPG
GYGSPFASLVPSAFPPGTDPNVVACFQAADRDGSGMIDDKELQSALSGYSQSFSLRTVHL
LMYLFTNTNVRKIGPKEFTSVFYSLQNWRSIFERFDRDQSGKIDATELRDALLSLGYSVS
PTVLDLLVSKFDKTGGKNKAIEYDNFIECCLTVKGLTEKFKEKDTAFSGSATFTYEAFML
TVLPFLIA*
>11686.m00349|LOC_Os12g04240.1|genepair1263-2
MAGYPPNPGSGYPYGGAGGYGAPPPPYGSSPAPSAPPYGAKPPKEGKTSSSSAPYYGGGG
GYGAPPSTQPYGSGGGYGAPPSSQPYGAPYGAPPPSSAPYGAPGGYGSPFASLVPSAFPP
GTDPNVVACFQAADRDGSGMIDDKELQSALSGYSQSFSLRTVHLLMYLFTNTNVRKIGPK
EFTSVFYSLQNWRSIFERFDRDRSGKIDATELRDALLSLGYSVSPTVLDLLVSKFDKTGG
KNKAIEYDNFIECCLTVKGLTEKFKEKDTAFSGSATFTYEAFMLTVLPFLIA*

>11687.m00364|LOC_Os11g04490.1|genepair1264-1
MATVVRACMPLPPAAVASSSAAPSTDAQAQRRSSSSSARVLVLGGTGRVGGSTATALSKL
RPDLNILIAGRNLEKGESLASKLGDESEFVQVDIRDRNMLEEVLQDVDLVVHAAGPFQRE
NECTVLQAAIATKTAYIDVCDDTDYSWRAKGFHEQAKDCGIPAITTAGIYPGVSNVMAAE
LVHAARSENAGEPERLRFFYYTAGTGGAGPTILTTSFLLLAEDVIAYNKGEEIKLKPYSG
ALSIDFGKGVRKKDVYLLNLPEVKSAYKVLGVPTVSARFGTAPFFWNWVMQAFANFLPVE
FLRDKNKVLKLVGFVDPFVRAIDGIAGERVSMRQLLYRKFLPVCFRSTSSFQGFKLFPEL
TAYSFKLWPEQVDLDCSNGKNTIGLFSHRKLSVSVGYATAAFVLAVLEGSTQPGVWFPEE
PEGVAIESRKVLLERASQGTTIFVMNK*
>11686.m00351|LOC_Os12g04260.1|genepair1264-2
MATVVRACMPLPPAAVASSSAAPSTDAQAQRSPSSSSARVLVLGGTGRVGGSTATALSKL
RPDLNILIAGRNREKGESLASKLGEESEFVQVDIRDRNMLEEVLQDVDLVVHAAGPFQRE
NECTVLQAAIATKTAYIDVCDDTDYSWRAKGFHEQAKDCGIPAITTAGIYPGVSNVMAAE
LVHAARSENAGEPERLRFFYYTAGTGGAGPTILTTSFLLLAEDVIAYNKGEEIKLKPYSG
ALSIDFGKGVRKKDVYLLNLPEVKSAYKVLGVPTVSARFGTAPFFWNWGMQAFANFLPVE
FLRDRNKVLKLVGFVDPFVRAIDGIAGERVSMRVDLDCSNGKNTIGLFSHRKLSVSVGYA
TAAFVLAVLEGSTQPGVWFPEEPEGVAIESRKVLLERASQGTTIFVMNK*
>11687.m00365|LOC_Os11g04500.1|genepair1265-1
MLCVAMAARPVSSTTSTCRPCLPAQVSASKPSTSSSPGTGVLVGVPRERGSSVSKAAIRG
ARLEAAARCSLVRQRPMLLATVAVGSLVAAGAANATEIGDSLLGSSGLALADLSVGDWFG
NLLYSAGQQANEAVQDQLSALSFTSLAVIFGAGLVTSLSPCTLSVLPLTLGYIGAFGSGK
DRSEVVGNSVAFSLGLATTLAILGVAASFAGKAYGQVGQGLPVAASGLAVIMGLNLLEVI
ELQLPSFFSDYDPRAAAANLPSSVQAYLAGLTFALAASPCSTPVLATLLGYVATSRDPIV
GGSLLLTYTTGYVAPLLIAASFAGALQSLLSFRRYSAWINPISGAFLLGGGVYTLLDRLF
PATSMVM*
>11686.m00352|LOC_Os12g04270.1|genepair1265-2
MAARPVSSTTSTCRPCRPAQAAASKPSTSSSPGTGVLVGVPRERGSSVSKAAIRGARLEA
AARCSLVRQRPMLLATVAVGSLVAAGAANATEIGDSLLGSSGLALADLSIGDWFGNLLYS
AGQQANEAVQDQLSALSFTSLAVIFGAGLVTSLSPCTLSVLPLTLGYIGAFGSGKDRSEV
VGNSVAFSLGLATTLAILGVAASFAGKAYGQVGQGLPVAASGLAVIMGLNLLEVIELQLP
SFFSDYDPRAAAANLPSSVQAYLAGLTFALAASPCSTPVLATLLGYVATSRDPIVGGSLL
LTYTTGYVAPLLIAASFAGALQSLLSFRRYSAWINPISGAFLLGGGVYTLLDRLFPATSM
VM*
>11687.m00366|LOC_Os11g04510.1|genepair1266-1
MLVFWDLTRPKSAGHNCHPIPLWEREFCIYVGGISWHRFCDNKRYVCMYKNIEQWDDSEA
FDNFKNAKARFWANYHGQPSDISLPDPDMYIDKVDHNSKIDPELIADLNMVRLPFEMDDE
LLPADGLGSTDTDNKCQQRQNQSRNWDIYVEKPTEVNKWEQDSRSNMDWGTKHESWNEWS
KNCSGWGSALADSSWGNWNNSNNHHSSNNRASFNGINRNRYQDPSSISGRKRNSGGYIQQ
RNSRQRNQIEGYQGSRW*
>11686.m00354|LOC_Os12g04280.1|genepair1266-2
MKGHNCHPVPLWEREFCIYVGGISWHRFCDNKRYVCMYKNIEQWDDSEAFDNFKNAKARF
WANYHGQPSDISLPDPDMYIDKIDHNSKIDPELIADLKMVRLPFERDDELLPADGLGSTD
TDNKCQQKQNQSGNWDIYVEKPTEVNKWEQDSRSNMDWGTKHESWNEWSKNCSGWGSALA
DSSALADSSWGNWNNSNNHHSSNNRDSFNGVNRNRYQDPNSISGRKRNSGGHIQQRNSRQ
RNQTEGYQGSTPRW*
>11687.m00368|LOC_Os11g04520.2|genepair1267-1
MVEVEEVSNKMQVQMRLHPAAAAEEEDADLPLPALFDKASHLHSLASSSSLDQEGIRKGV
DLLRRCDEMVSKVGLFSSNETKDDISTANLKYLLVPYYLGEMTERVAQEDRIPVLKASQD
HLKEFISICEALELISEDELEISRQKQPDTMANRRAQKVARFKRQKAAETKLLEIKERKE
RRRRSLRAAALSAPIEAGEEDAFEDDGEEEREAWLATISLALCKAFDLLDMLKKEEEMLP
AVKERKAKDGNAFAREMLDERTKRAEAWHHNAANRAPYSKPADPITCATFAQDVIEGRAS
VSQAHEHKHQPLIFGPASLVGGGLTSERERMAAQVFQPSYRLPTMSIEEAGLREMKMMEK
WQERTAKMIQESNSAWHKDGSRSAQEDEDAEEEKARGWDDWKDDNPRGAGNKKLTPCG*
>11686.m00355|LOC_Os12g04290.1|genepair1267-2
MVEVEEVSNKMQAQMRLHPAAAAEEEDADLPLPALFDKASHLHSLASSSSLDQEGIRKGV
DLLRRCDEMVSKLGLFSSNETKDDVSTANLKYLLVPYYLGEMTERVAQEDRIPVLKASQD
HLKEFISICEALELISEDELELSRQKQPDTMANRRAQKVARFKRQKAAETKLLEIKERKE
RRRRSLRAAALSAPIEAGEEDAFEDDGEEEREAWLATISLALCKAFDLLDMLKKEEEMLL
AVKERQAKDGNAFAREMLDERTKKAEAWHHNAANRAPYSKPADPITCATFAQDVIEGRAS
VSQAHEHKHQPLIFGPASLVGGGLTSERERMAAQVFQPSYRLPTMSIEEAGLREMKMMEK
WQERTAKMIQESNSAWHKDGSRSAQEDEDAEEEKARAWDDWKDDNPRGAGNKKLTPCG*
>11687.m00371|LOC_Os11g04550.1|genepair1268-1
MAPSFPLSFAPQSADEATAHKEIYDQLRQAVETFPTAPNSSTSFTYSRHPDGWYTFPEGV
VSAMVIKSHLTARTTDIFMVTFPKSGTTWLKALLHSALHRRADDLAAHSPHQLVPFLETQ
VFIKDRIPDLSSLPAPRLLMTHIPSQSLPDSVADSSCKVVYLCRDPKDCFISLWHFLNRF
RPWDINEAHRNFCDGVSLFGPYWEHVLGYWRWHVKRPSQVLFLTYEELTTDTLGQLRRLA
EFVGRPFMVKEQEIEVDRKIVEACAMESLSRLEVNQSGTTDMVDKTYANNIFFRRGVVGD
WRNHLTPEMARRIDEITEIKFKGSGLLLHPQFLQAKRE*
>11686.m00358|LOC_Os12g04320.1|genepair1268-2
MAPSFRLSSAPESADEATAHKEIYDQLRRVAETFPSAPSLIGLPCSRHPDGWYTFTNGVV
SSMVIKEHLTARATDIFLTTFPKSGTTWLKVLLYSTLHRGTDELVAHSPHQLVPFLESQV
FVNDRIPDLSSLSSPRLFMTHIPSQSLPNSVATSGCKVVYLCRDPKDCFVSLWHFWNRFM
PWDIDEAHRQFCDGVSQFGPFWEHILGYWRWHVEKPNQVLFLTYEELAADTLGQLRRLAE
FVGCPFTTEEQKHGVDRNIVEACALENMSGLEVNRSGTITIVDSTVPNNTFFRRGVVGDW

RNHLTPEMARRIDEITKSKFKGSGLLLHPQFLQVKRE*
>11687.m00372|LOC_Os11g04560.1|genepair1269-1
MMKLAHLFGSSSSSSSSSSKKRRKKRRSGAKSCSFGSTTSSSSSSLAASSSDDSAATTPR
SVLPASAAASSSGTKKPAAVAVTREDLEVALRRIVSSKEELAAMLAEAECAGELVLEEIA
AAAADEGELKETFAVFDADGDGRISAEELRAVLASLGDELCSVDDCRRMIGGVDTDGDGF
VCFDEFARMMMYGCA*
>11686.m00362|LOC_Os12g04360.1|genepair1269-2
MMKLAHLFGSSSSSSSSKKENKVSSKKRRSGAKSCSFGSTTSSSSLAASSSDDSAATTPR
SVLPTSAAASSSGTKKPAAAAVTREDLEVALRRIVSSKEELAAMLAEADYAGELVLEEIA
AAAADEGELKETFAVFDADGDGRISAEELRAVLASLGDELCSVDDCRRMIGGVDTDGDGF
VCFDEFARMMMCGRA*
>11687.m00373|LOC_Os11g04570.1|genepair1270-1
MMHGLWVQDQGVVDHLAQLVPLLHECASHVTEGSFEKADFSFKKIRMLTIADGPLQRLST
IIVDSLAHRLLSSIQGLPGALIDPSDYFEKSTLRAARHNFFKLNPYLSTGFVTINWAIME
AMEDEKVDLQVVHIVDLSCSAAHPWQWPKLLDDFHGRPGGAPELYLTVLHDDNDFLADMQ
SLLSKKAESLGVSFHFISVIGRLETLDFSNLRSTFQIKFGVAVAISCALQMHRLLLVDDN
LSSTSIAQLQKMANFTQPKQMASSVCSPASTLNYLQTPSPRTPKLLARLLSAIRALKPNI
MLIMEQDADHNTLLFRDRFNEVLNYYAALFDCFHAVAAANPGRTDERLRVDRMILREEIK
NILVCEGVHRHERHERLDQWAMHMEESGFHNVQLSFSAIREAYVWQLKVQADNLRLCCTD
RGMFQDDMLSSATSSPASSVYSPSPSPSNGSWVQELSHDQQSVRLIGLLYQCAAEVSAGS
FDRANLCLEHITQLASLDAPHALQRLAAVFADALARKLLNLILGLSRALLSSANSADAHL
VPVARRHMFDVLPFLKLAYLTTNHAILEAMEGERFVHVVDFSGPAANPVQWIALFHAFRG
RREGPPHLRITAVHDSKEFLANMAAVLSKEAEAFDIAFQFNAVEAKLDEMDFDALRHDLG
VRSGEALAVSVVLQLHRLLAVDDGRRHAAAGCLTPVQIIARSSPRSFGELLERELNTRLQ
LSPDASVVSSLSPHSPAAATAAHPTTSTPKLGSFLSAVRSLSPKIMVMTEQEANHNGGAF
QERFDEALNYYASLFDCLQRSAAAAAERARVERVLLGEEIRGVVACEGAERVERHERARQ
WAARMEAAGMERVGLSYSGAMEARKLLQSCGWAGPYEVRHDAGGHGFFFCWHKRPLYAVT
AWRPAASRRGHTRS*
>11686.m00363|LOC_Os12g04370.1|genepair1270-2
MMHGLWVQDQGMVDHLAQLVPLLHECASHVTEGSFEKADFSFKKIRMLTIADGPLQHLSK
IIVDSLDHRLLSSIQGLYGALINPSDYFEKSTLPGCPAHNFFKLNPYLSTGFVTINRAIM
EAMEDEKNFLEIKVKSNLCSILKLSHYNFKTLQGAIYVERSLMFNMVELQVVHIVDLSCS
AAHPWQWLKLLDDFHGRPGGAPELYLTVLHDDNDFLAEMQSLLSKKAESLEVSFRFISVI
GRLETLDFSNLRSTFQIKFGVAVAISCALQMHRLLLVDDNLSSTSIAQLQKMANFTQPKQ
MASSVCSPASTLNYLQTPSPRTPKLLARLLSAIRALKPNIMVIMEQDADHNALLFRDRFN
EVLNYYAALFDCFHAVAAANPGRTDERLRVERMILREEIKNILVCEGVHRHERHERLDQW
AMHMEESGFHNVQLSFSAIREGKENLLSFGLKNCQNKEDRGCLLLSWGSTNLYSISLRLC
CTDRGMFQDDMLSSATSSPASSVYSPSPSPSNGSWVQELSHDQQSVRLIGLLYQCAAEVS
AGSFDRANLCLEHITQLASLDAPHALQRLAAVFADALARKLLNLIPGLSRALLSSANSAD
AHLVPVARRHMFDVLPFLKLAYLTTNHAILEAMEGERFVHVVDFSGPAANPVQWIALFHA
FRGRREGPPHLRITAVHDSKEFLANMAAVLSKEAEAFDIAFQFNAVEAKLDEMDFDALRH
DLGVRSGEALAVSVVLQLHRLLAVDDGRRHAATGCLTPVQIIARSSPRSFGELLERELNT
RLQLSPDASVVSSLSPHSPAAATAAHPTTSTPKLGSFLSAVRSLSPKIMVMTEQEANHNG
GAFQERFDEALNYYASLFDCLQRSAAAAAERARVERVLLGEEIRGVVACEGAERTERHER
ARQWAARMEAAGMESVGLSYSGAMEARKLLQSCGWVGPYEVRHDAGGHGFFFCWHKRPLY
AVTAWRPAASRRGHTRS*
>11687.m00375|LOC_Os11g04590.1|genepair1271-1
MEKATRCLARATGLAAAAAAGDGPRKRLAVAMVDCLARRLLRPVQAITDALIDPSVYLDR
RSVRAARRGFFELSPFPKVAFVVGNRAIVEAVENESLVHVVGMSGPFTQPCQWIQLLHEL
RRRPEGPPRVVRLTVVHDDGELLAKMEELVSDEAEELGMEFQFHGVVGQLEDLDFSNLRN
VLEIKSGEALVVSCTLQLHRLLAADDDAMYSSRSAHLNQMASIAQLQHMAVNSCPSSSGG
GSVQYKDDDPYRSPATPLTFVSPPVSTPHFQTPAALASFLSAVRALSPKILVVAEQDADH
NGVSFRKRFCEALHHYAAVFDSLDAAAATTSAASRLWSPDERAQVERVVVGEEIKGVLLR
DGAHRREWHDRLRQWAARMEMAGFTGVPLSYAAIKKGNDMVRRCGLRRCENKECGGCLLL
CWSSRPLYSISAWRPAASRGSGSGSERSEYVHVGAEPDDR*
>11686.m00364|LOC_Os12g04380.1|genepair1271-2
MEKATRCLARATGLAAAAAAGDGPQKRLAEAMVDCLARRLLRPVQAITDALIDPSVYLDR
RSVRAARRGFFELSPFPKVAFVVGNRAIVEAVENESLVHVVGMSGPFTQPCQWIQLLHEL
RRRPEGPPRVVRLTVVHDDGELLAKMAEVLSDEAEELDMEFQFHGVVGQLEDLDFSNLRD
VLEIKSGEALVVSCTLQLHRLLAADDDAMYSSRSAHLNQMASIAQLQQMAVSSCPPSTGG
GGSVQYKDDDDDPYRSPATPLTFVSPPASTPHLQMPAALANFLSAVRALSPKIVVVAEQD
ADHNGVSFRKRFCEALHHYAAVFDSLDDAAAATTSAASHLWSPDERAQVERVVVGEEIKG
VLLRDGAHRRERHDRLRQWAARMEMAGFTGVPLSYAAIRKGNDMVRRCGLRRCENRECGG
CLLLCWSSRPLYSISAWRPAASGGAGSGSERSEYIHVGAEADDR*
>11687.m00376|LOC_Os11g04600.1|genepair1272-1
MSSEDSLKSLSLDYLSLLINGQAFSDVAFSVEGRLVHAHRCVLAARSLFFRKLFCGLDPN
HQPPPPPPPLNWPMAGGGGGGSGGGGRGGAGGGGGAPATPELVIPVSSIRYEVLVLVLQF
LYSGQASVAAPKSGPLPGCGARGCWHTRCGAAVDLALDTLAAARSFGVEQLALLVQKQLE
SMVKEASVDDVMKVLMASRKFEMQELWATCSHLVARSGLSADLLAKHLPIDVVAKIEEIR
AKSPLAAVAAPRSPFLTHHYLPMNPASSAADRDNKIRRMRRALDAADIELVKLMVMGEGL
DLDDALAVHYAVQHCNRDVVKALLELGAADVNSRAGPTGKTALHLAAEMVSPDMVSVLLD
HHADPNSRTLDGVTPLDVLRSLTSEFLFKGAVPGLTHIEPNKLRLCLELVQSAVMVTTRD
DGAPVTGGAEAGGSDGGNFPRSDADDSLVSLTMNSTLMYQGQEMAAAVAAGEGRKSNNGR
GSPPPAMYFPNGFA*

>11686.m00367|LOC_Os12g04410.1|genepair1272-2
MSSEDSLKSLSLDYLNLLINGQAFSDVAFSVEGRLVHAHRCVLAARSLFFRKLFCGLDPN
HQPPPPPPPPLNWPTAGGGGGGSGGGGRGGAGGGGGAPATPELVIPVSSIRYEVLVLVLQ
FLYSGQASVAAPKSGPLPGCGARGCWHTRCGAAVDLALDTLAAARSFGVEQLALLVQKQL
ESMVKEASVDDVMKVLMASRKFEMQELWATCSHLVARSGLSADLLAKHLPIDVVAKIEEI
RAKSPLAAAAAPRSPFLTHHYLPMNPASSAADRDNKIRRMRRALDAADIELVKLMVMGEG
LDLDDALAVHYAVQHCNRDVVKALLELGAADVNSRAGPTGKTALHLAAEMVSPDMVSVLL
DHHADPNSRTLDGVTPLDVLRSLTSEFLFKGAVPGLTHIEPNKLRLCLELVQSAVMVTTR
DDGAPVTGGEAGGSDGGNFPRSDADDSLVSLTMNSTLMYQGQEMAAAVAAGEGRKSNNGR
GSPPPAMYFPNGFA*
>11687.m00381|LOC_Os11g04650.1|genepair1273-1
MKASLVVLAAAVAAAAALLVSLDPRSDDVPVLEIRERDVELITVDAGGAVGPESVAFDGD
GEGPYTGVSDGRVLKWLPLERRWVEHSSAVIEPQLSTTVVVCTRWDHGMVMGHRT*
>11686.m00368|LOC_Os12g04420.1|genepair1273-2
MARAAAAFVFVSLTLLCNASSQGEGAAAAAACRAADLVVRQRATGRVVEGKPEYAVEVAN
RCRWVTLRLHPVGPPLLVPHHARQDTVVAGYDVPADARVLVHVRAIAPDPASWPDRTDAF
LPERFLPGAGGCDGGVDVHGQHFELLPFGSGRRISPATNLAKKMVALGVASLLQGFAWRL
PDRGREHGGAGQAVHTPEGAARHRRRAQAAATSLRRRRRMNVVIHIGSGLLVPMKARLVV
LAAAVAAAALLVSLDPRSDDVPVLEIWERDVELITVDAGGAVGPESVAFDGDGDGPYTGV
SDGRVLKWLPLERRWVEHSSAVIEPHMSTMDHGMVMGHRT*
>11687.m00382|LOC_Os11g04660.1|genepair1274-1
MNTTAKLLALAVFAAAAILSLDSRSDVRQLEIRDGDVELIPLLDGAAGPESIVFGDAGDG
PYTSVSDGRILKWLPPPERRWVEHSCSVPELLDSCRGSKDTKREQECGRPLGLKFNSKTG
ELYVADAYLGLRVVSPGENVSRPLVPKRTGSPFSFSNGVEIDHETGVIYFTETSTRFQRR
EFLNIVITGDNTGRLLKYDPKENKVEVLVDGLRFPNGLAMSIDGSYLLLAETTTGKILRY
WIKTPKASTIEEVAQLPGFPDNIKMSPRGGFWVGLHAKRGKIAEWSISYPWLRKLIFKLP
AQRIQRITSFLTGFGRQVIALRLSEDGKTIEAMSVHGDVRKLFKSISEVEEKDGNLWIGS
VLSPFLGLYRI*
>11686.m00369|LOC_Os12g04430.1|genepair1274-2
MSTTAKFLALAVFAAAAILSLDSRSDVRQLEIRDGDVELIPLLDGAAGPESIVFGDAGEG
PYTSVSDGRVLKWLPPPERRWVEHSCSVPELLDSCRGSKDTKREQECGRPLGLKFNSKTG
ELYVADAYLGLRVVSPGENVSRPLVPKWTESPFSFSNGVEIDHETGVIYFTETSTRFQRR
EFLNIVITGDNTGRLLKYDPKENKVEVLVDGLCFPNGLAMSNDGSYLLLAETTTGKILRY
WIKTPKASTIEEVVQLHGFPDNIKMSPRGGFWVGLHAKRGKIAEWSISYPWLRKVILKLP
AQRIQRITSFLTGFGRQVIALRLSEDGKTIEAMSVHGDVRKLFKSISEVEEKDGNLWIGS
VLSPFLGLYRI*
>11687.m00383|LOC_Os11g04670.1|genepair1275-1
MASSLLSSPKPSSFSSANPTSTPRPRAQTLSPFRAAAPRFSHGLATAAAAANPSASRRCY
HRAFARPVRASMAQPRRPEYVPNRIDDPNYVRIFDTTLRDGEQSPGATMTSAEKLVVARQ
LARLGVDIIEAGFPASSPDDLDAVRSIAIEVGNTPVGEDGHVPVICGLSRCNKRDIDAAW
EAVRHARRPRIHTFIATSEIHMQHKLRKTPEQVVAIAKEMVAYARSLGCPDVEFSPEDAG
RSNREFLYHILEEVIKAGATTLNIPDTVGYTLPYEFGKLIADIKANTPGIENAIISTHCQ
NDLGLATANTLAGAHAGARQLEVTINGIGERAGNASLEEVVMAIKCRRELLGGLYTGINT
QHITMSSKMVQEHSGLHVQPHKAIVGANAFAHESGIHQDGMLKYKGTYEIISPDDIGLTR
ANEFGIVLGKLSGRHAVRSKLVELGYEITDKEFEDFFKRYKEVAEKKKRVTDEDIEALLS
DEIFQPKVFWSLADVQATCGTLGLSTATVKLIGPDGEEKIACAVGTGPVDAAYKAVDDII
QIPTVLREYSMTSVTEGIDAIATTRVVVTGDVSDSKHALTGHSFSRAFSGSGAALDIVVS
SVRAYLSALNKMSSFVGAIKASSEVSESQRVQTTE*
>11686.m00370|LOC_Os12g04440.1|genepair1275-2
MASSLLSSPKPSSFSSANPTSTPRPRAQTLSPFRAAAPRFSHGLATAAAAANPSASRRCY
HRAFARPVRASMAQPRRPEYVPNRIDDPNYVRIFDTTLRDGEQSPGATMTSAEKLVVARQ
LARLGVDIIEAGFPASSPDDLDAVRSIAIEVGNTPVGEDGHVPVICGLSRCNKRDIDAAW
EAVRHARRPRIHTFIATSEIHMQHKLRKTPEQVVAIAKEMVAYARSLGCPDVEFSPEDAG
RSNREFLYHILEEVIKAGATTLNIPDTVGYTLPYEFGKLIADIKANTPGIENAIISTHCQ
NDLGLATANTLAGAHAGARQLEVTINGIGERAGNASLEEVVMAIKCRRELLGGLYTGINT
QHITMSSKMVQEHSGLHVQPHKAIVGANAFAHESGIHQDGMLKYKGTYEIISPDDIGLTR
ANEFGIVLGKLRLNIAHLTLRKALPGLLGYEITDKEFEDFFKRYKEVAEKKKRVTDEDIE
ALLSDEIFQPKVFWSLADVQATCGTLGLSTATVKLIGPDGDEKIACAVGTGPVDAAYKAV
DDIIQVTVAIFFIVLALVYQFMGWNMDVFIQYIPTVLREYSMTSVTEGIDAIATTRVVVT
GDVSDSKHALTGHSFNRAFSGSGAALDIVVSSVRAYLSALNKMSSFVGAIKASSEVSESQ
RVQTTE*
>11687.m00384|LOC_Os11g04680.1|genepair1276-1
MASDQSIVMSEQQRAVFCLTLMAVTYGHCRQPADAELLHRLQRADQPPPIRGARVVENTE
PNVLFAYTPHNADDDEAYSNGGFGAVPALSEAIVSLPEMAVGCGCGGGGEAREKECGVCL
EGFEEGEKLRKMPCEHYFHESCVFKWLQGPSYVPHGVESAYIHINRDIEEDDDTYSDDGF
CAVPASSDAIAALPVPETTVSETETREEEACAVCLEGFKEGDRVKKMPCSHDFHANCISE
WLRVSRLCPHCRFALPAERDSEQKNPEEA*
>11686.m00371|LOC_Os12g04450.1|genepair1276-2
MAGDQSIVMSEQQRAEFCLTLMAVMYGHTRQPADAELLRRPQRRRAAAAADPPPHIRNAS
VVENTEPDVLFAYTPHDGLSPEFDDDSGGVADYFAADDDEAYSNGGFGAVPALSEAIVSM
PELSVGEAREKQCGVCLEGFEEGDKLRKMPCEHYFHESCVFKWLQGPSYVPHGVESAYIH
INRDIEEDDDAYSEDGFCAVPASSDAIAALPETTEKKGRRKGRKEVRHRSLAKRAIAHQA
PPL*
>11687.m00385|LOC_Os11g04690.1|genepair1277-1

MAGEEPWSSEASDDVPDTSHMSDEQFQQFIDQYWAEQGFNIWSWIRASRTSSSSTPGPTR
RTAASWQAVTFDGDGVARFSGNSDRSGGLDDQATSGFSIVDLLDGILQADDDGNGGGATP
ASSMAIVNLPEITVGDEKGEAKDCPVCLQGFEEGDKLRKMPCADSHCFHEQCIFSWLLIN
RHCPLCRFPLPAETEEDEEVVQAENDDDDDDEETILCLHRLFADAADEYTS*
>11686.m00372|LOC_Os12g04460.1|genepair1277-2
MSDEEPWSRDASDDVPDTSHMSDEQFQQLIDQYWAEQGFNIWSWIRASRTSSSSTPGPTR
RTAASWLAVTFDGDGVARFSGNSDRSGGLDDQATGGFSIVDLLDGILQPDDDGNGGGATP
ASSMAIVSLPEITVGDEKGEAKDCPVCLQGFEEGDKLRRMPCADSHCFHEQCIFSWLVIN
RHCPLCRFPLPAETEEEEEVAQAENDDDDDDGEETILCLHRLFANAAD*
>11687.m00387|LOC_Os11g04710.1|genepair1278-1
MAAAALLLLAAAAAIVVVAMVLRWLLLLGGPAAGRLGKRALMPPGSTGLPLIGETLRLIS
AYKTPNPEPFIDERVARHGGVFTTHVFGERTVFSADPAFNRLLLAAEGRAVHSSYPSSIA
TLLGARSLLLTRGAAHKRLHSLTLTRLGRPASPPLLAHIDRLVLATMRQWEPAATVRLMD
EAKKITFNLTVKQLVSIEPGPWTESLRREYVKLIDGFFSIPFPLANLLPFTTYGQALKAR
KKVAGALREVIKKRMEEKAENGGSIGDDEGKKEKKDMVEELLEAEGGSFSEEEMVDFCLS
LLVAGYETTSMLMTLAVKFLTETPAALAELKEEHANIRDMKGKKQPLEWSDYKSMPFTQC
VINETLRVGNIISGVFRRANTDIHYKDYTIPKGCKIFASFRAVHLNNEHYENARTFNPWR
WQINNKLQNAVGANIFTPFGGGPRLCPGYELARVVVSIFLHHLVTRFSWEETEEDRLVFF
PTTRTLKGYPINLRLLSESIC*
>11686.m00374|LOC_Os12g04480.1|genepair1278-2
MAAAALLLLAAAAAAVVVAMALRWLLLLGGPAAGRLGKRARMPPGSTGLPLIGETLRLIS
AYKTPNPEPFIDERVARHGGVFTTHVFGERTVFSADPAFNRLLLAAEGRAVHSSYPSSIA
TLLGARSLLLTRGAAHKRLHSLTLTRLGRPASPPLLAHIDRLVLATMRQWEPAATVRLMD
EAKKITFNLTVKQLVSIEPGPWTESLRREYVKLIDGFFSIPFPLAYFLPFTTYGQALKAR
KKVAGALREVIKKRMEEKAENGGSIGDDEGKKEKKDMVEELLQAEGGSFSEEEMVDFCLS
LLVAGYETTSVLMTLAVKFLTETPAALAELKEEHANIRDMKGKNQPLEWSDYKSMPFTQC
VINETLRVGNIISGVFRRANTDIHYKDYTIPKGCKIFASFRAVHLNNEHYENARTFNPWR
WQINNKLQNAVGANIFTPFGGGPRLCPGYELARVVVSIFLHHLVTRFSWEETEEDRLVFF
PTTRTLKGYPINLRLLSESIC*
>11687.m00389|LOC_Os11g04720.2|genepair1279-1
MAVAIEAPFHVLAVDDSLPDRKLIERLLKTSSFQGLAFVCVVTTVDSGSKALEFLGLHDH
EDSPISTQSDQQEVAVNLIITDYCMPGMTGYDLLKKIKESSYLRDIPVVIMSSDNIPSRI
NRCLEEGADEFFLKPVRLSDMSKLKPHILKSRCKEHYQQEQNLQSNSESNNSSNPTSENS
SSSTSSNSHKRKAVDEEILPHTIRPRHS*
>11686.m00376|LOC_Os12g04500.1|genepair1279-2
MAVAIEAPFHVLAVDDSLPDRKLIERLLKTSSFQVTTVDSGSKALEFLGLHDHEDSPIST
QSDQQEVGVNLIITDYCMPGMTGYDLLKKIKESSYLRDIPVVIMSSDNIPSRINR*
>11687.m00390|LOC_Os11g04730.1|genepair1280-1
MAAEMEVDDVDVHEVPECIASMIDRGSVESHRLFLARRTAMEMLRDRGYSVPEAEIARTL
PEFRAWWAEKPGIERLAFTTTLVSDPSKKVQLVFCPPEPVKIATIREIYLQTKEENLSRL
VLILQSKILSRAREAIKEIFKFKVDIFQATDLLVNITKHVLKPKHEVLSADQKAKLLKEY
NVEDSQLPRMLETDAVARYYGFDKGTVVKVIYDGELTGKRVAYRCVF*
>11686.m00377|LOC_Os12g04510.1|genepair1280-2
MAAEMEVDDVDVHEVPECIASMIDRGSVESHRLFLARRTAMEMLRDRGYSVPEAEIARTL
PEFRAWWAEKPGIERLAFTTTLVSDPSKKVQLVFCPPEPVKIATIREIYLQTKEENLSRL
VLILQSKILSRAREAIKEIFKFKVDIFQATDLLVNITKHVLKPKHEVLSADQKAKLLKEY
NVEDSQLPRMLETDAVARYYGFDKGTVVKVTYDGELTGKRVAYRCVF*
>11687.m00391|LOC_Os11g04740.1|genepair1281-1
MRRLLLAGILRRASSSPSSHHHLHLVRALSASSPLPASDADLRKYAGYALLLLGCGAATY
YSFPLPPDALHKKAVPFKYAPLPDDLHAVSNWSATHEVHTRVLLQPDSLPALHDALAAAH
GECRKLRPLGSGLSPNGLALSRAGMVNLALMDKVLGVDAKKKTVTVQAGIRVAELVDALR
EHGLTLQNFASIREQQVGGIIQVGAHGTGARLPPIDEQVISMKLVTPAKGTIELSREKDP
DLFYLARCGLGGLGVVAEVTLQCVERHQLIEHTFVSNADEVKKNHKKWLSENKHIKYLWI
PYTDTVVVVQCNPPSRWRTPKFTSKYGKDEAIQHVRDLYHESLKKYRTKAESNDPEVDQL
SFTELRDRLLTLDPLDKDHVIRINKAEAEYWKKSEGYRMGWSDEILGFDCGGQQWVSETC
FPAGTLAKPNMKDLDYIEELLQLIEKEDIPAPAPIEQRWTACSRSPMSPASSSQEDDIFS
WVGIIMYLPTSDARQRKEITEEFFNYRSKTQTNLWDGYSAYEHWAKIEVPKDKDELAELQ
ARLRKRFPVDAYNKARMELDPNKVLSNAKLEKLFPVTEVQHEK*
>11686.m00378|LOC_Os12g04520.1|genepair1281-2
MRRLLLAGILRRASSSPSSHHHLHLVRALSASSPLPASDADLRKYAGYALLLLGCGAATY
YSFPLPPDALHKKAVPFKYAPLPDDLHAVSNWSATHEVHTRVLLQPDSLPVLHDALAAAH
GERRKLRPLGSGLSPNGLALSRAGMVNLALMDKVLDVDAKKKTVTVQAGIRVAELVDTLR
EHGLTLQNFASIREQQVGGIIQVGAHGTGARLPPIDEQVISMKLVTPAKGTIELSREKDP
DLFYLARCGLGGLGVVAEVTLQCVERHQLIEHTFVSSADEVKKNHKKWLSENKHIKYLWI
PYTDTVVVVQCNPPSRWRTPKFTSKYGKDEAIQHVRDLYRESLKKYRTKAESNDPEVDQL
SFTELRDRLLALDPLDKDHVIRINKAEAEYWKKSEGYRMGWSDEILGFDCGGQQWVSETC
FPAGTLAKPNMKDLDYIEELLQLIEKEDIPAPAPIEQRWTACSRSPMSPASSSQEDDIFS
WVGIIMYLPTSDARQRKEITEEFFNYRSKTQTNLWDGYSAYEHWAKIEVPKDKDELTELL
ARLRKRFPVDAYNKARMELDPNKVLSNAKLEKLFPVTEVQHVK*
>11687.m00392|LOC_Os11g04750.1|genepair1282-1
MAPKRKPDDDDDVGESTDEFANPFDDTMQFTKPVYLVAVRDDDQAAAYSVLKIDAAAVAG
NDEPRRVRAVAVLTTGTEPGMSFVTARSRHGSWIVGVGGGLRAGTIIFDPGTIITRLGYP
KHKPVLISHGSEPELVVGTYAFHVVNKIWEKIHEKNLPFVGQAVHLGGSLFAACPISNTA
STSTSASVFHMSIKISSSIPSLSIQKFKVMASVDKITFPLFCPMGMGSFCCIRLGPSRLR

HRRKTNYRRWRSPKTSCLKEVHVISTAFRIENIEAIMTHCQSQESKAKDQLLALQVKEQM
HSCESKEIHGLLGSGIPVVAALSM*
>11686.m00379|LOC_Os12g04530.1|genepair1282-2
MAPKRKPDDDDDVGESTDEFANPSGDTMEFAKPVYLVAVRDDDQAAAYSVLKIDAAAVAG
NDEPRRVRAVAVLTTGTEPGMSFVTARSRHGSWIVGVGGGLRAGTIIFDPGTIITRLGYP
KHKPVLISHGSEPELVVGTYAFHVVNKIWEKIHEKNLPFVGQAVHLGGSLFAACPISNTA
STSTSASVFHMSIKISSSIPSLSIQKFKVMASVDKITFPLFCPMGKGSFCCIRLGPSRLR
HRRKTNYRRWRSPKTSCLKEVHVISTAFRIENIEAIMTHCQSQESKAKDQLLALQVKEQM
HSCESKEIHGLLGSGIPVVAALSM*
>11687.m00393|LOC_Os11g04760.1|genepair1283-1
MGSEKRKTRDRHGARGKASYDKPKPSITDPELEETEDADQFESGHLGIIGGDSDEDQADC
DQPMQEATEDLNQPGIVGDELDEGRGRSVYLVACHWDWSRYSKPYSVYNVGVTATATATS
SPPQAKRKRLRRITRLPTAAGGKSFTSVRSIHRAWIVGVGGDPGDTIIFDTRTEKVIHGP
ALNSAKWCPALMAVGDKVYAMSKSPSWIADPDFPPWFELLDLSQSKVVAATAGRGYHLEG
CSWIKLPHPPCFPWKLRPVDYTLLPVVIVMSYVVVDAYILVSFNQPWGTYAFDTNSIKWH
KVDNKKLPFTGCAAPHGSVFLGLSKDNGPINAYRINVTTSDKNHDPCLSIVVLPVKYMEH
EVDAGSCFFSLEDGLFCSLSFSLDSNSVILSKNLDFFPTKAHVDLRTYQTENTSPLEAPE
ETLLAVKPEVTVCNQ*
>11686.m00380|LOC_Os12g04540.1|genepair1283-2
MQEATEDLNQPGIVGDELDEGRGRSVYLVACHWDWSRYSKPYSVYNVGVTATATATSSPP
QAKRKRLRRITRLPTAAGGKSFTSVRSIHRAWIVGVGGDPGDTVIFDTRTEKVVHGPALN
SAKWCPALMSVGDKVYAMSKSPSWIADPDFPPWFELLDLSQSKVVAATAGRGYHLEGCSW
IKLPHPPCFPWKLRPVDYTLLPVVIVMSYVVVDAYILVSFNQPWGTYAFDTNSIKWHKVD
DKRLPFTGCAAPHGSVFLGLSKDNGPINAYRINVTTSDKEYDPCLSIVVLPVKYMEHEVD
AGSCFFSLEDGLFCSLRFSLDSSSVIRSKNLEVFPTKAHVDLRTYQTENTSPLEAPEETL
LAVKPEVTVCSQWEQAFKISCSSHGFSPFAFALLSI*
>11687.m00394|LOC_Os11g04770.1|genepair1284-1
MTKVFLPLFQPLNFLLCDKHNTHSTCPSEHSETKARTELTHGDVAVVMAALGLSFNAEGN
EVEDEALVLLEEKQASWEELEEAFSVFDGDGDGFISPLELQNVMRRLGLQHDAGHEECER
MLKVFDRDGDGMINFDEFKVMMQGVV*
>11686.m00384|LOC_Os12g04580.1|genepair1284-2
MAALGLRVNGEGDERSLVEDEALVLLEEKQASWEELEEAFSVFDGDGDGFISPLELQNVM
RRLCLQRDAGHEECERMLKVFDRDGDGMINFDEFKVMMQGVV*
>11687.m00397|LOC_Os11g04800.1|genepair1285-1
MEAPGELLLLSMAAFATAHPGERASIQAALGSLPRAPSSTMYYRFGSHGLQVLGENREPS
ASGGGGQAQATQQQQGTSSGVADNRREHAFDFGVGSSSSSSGRRHAMGAAPSVATGISGS
KRVAREEGGHSCGFEFEGSDQGRHAARGQACGLGGLDQGPSGGSAHDEDARGYGFGDARC
VGQRKDAAPTHQLRIGAGRDQGRSVRLRRDAREDGRGRRLEEGNRAGDGYVAASVQAHGD
ACVEDQGAGSSSVQQLPSSVDGSDGMIAFEVGKELTLHGSVVSSPWNELWLGKPLIFHDL
DLVRAMKDQGGMPNERIDLSGKITSILLSHPGPVSYFRIDSSVINNGAQQKIEEWCDVLR
KKNVETVVMANCQWPSHPIEFPLQSLNCSSLRTLHLCFFNIPDMYLDHVSSLAVIDLACC
RISDENLFALVCQCVSLRELDIGMFSEGKERIRSESLKFLQIWRSSVSHITIQWAPKLEK
VIIGAAQGMKSFSSRTSSSTWISILGAPMLREVWFNLSSQTRSIDNVYLDVGHVPITSLR
KLELSIAFKERKGRHALLNFFRSCTELKELVLWREDKVYFEEECDVHSDDWSSALKDIAC
LKSHLQVLKLFDYGGGETEIAIASAVLEHGASIENLTIMSTTSNADDILSQAKQKLEKVE
RDSQITGDKKKYSIRDSQENWRQKIQIIQRVAVKQALIPREFIQSINSPRGLRFLLRGWG
SSRVTDPAGAVAQDAELMESVGAGHRPDVVALLEILEAHRAALLCRLAHSQVVKIAAGEC
MFHGVRGGGGGGGGGILAKVAGLHTMHWRIPYHPTRDLLDEVALGICRGGCGIHACANAS
RSRG*
>11686.m00388|LOC_Os12g04610.1|genepair1285-2
MADDVSKRCSPEEEVIADILFALPRGPRPVLLRRRGEINRTGGDSTRDGDSAEKSRTGRG
HEIRGRAGGVEAEAARGGYSREDLATKGGSVGSRDGGGEAFQSKREGALRYRCTIEDLST
SKGPGGGGGYDYELVGRGGVAAAERDTRCRYSVQDLSTVGKSGGMYDGGNGPGFKEGAQE
TVDCVQGFKTHPELGLPANDSGTDTETVDVSVSEELIRLQTTISKPKTNTATDDSADDSA
AEIEAFDVSEELIRFQTVVSKPWDAIWREAPLVFCDSFLCPTGDRSGLSQSELKDAVASI
LLNHKGVVSYFRIDSSRSLNLQTLETWFNILSEKKVKEMVLFNCSGPQKLIEFPMDILDG
SQVEVLRICFFKIPEVYAFDLSKLHLLDFSYCKFDTEHLLHFVEACPNIRELHLGYYDGN
VRIRSDKLEIFQVWCSTMKSVNIEHAPELRKLTIAAFPGKYSSSLSVRVINSLYLEHITC
NISNQWITINGSNIQTDDKVLLNVRKLYIGLSMSKRRQREQLSNILNCLTHLEDLTIWRM
DTVANNEDYDAALEDWSPKLRVKTCLKSLQICKMEGYQGGKLESDFASAVLVRANRLKRL
IIESDKEDVFKKAVGILQKVKWTSPDVSVERRLNPLVSN*
>11687.m00399|LOC_Os11g04820.1|genepair1286-1
MAIELVGYFAMPCVTSMIGVAKCDDKDARYEDCEKMIHVFDKDGDGRISLDEFRAV*
>11686.m00395|LOC_Os12g04680.1|genepair1286-2
MGWLPLISEPPGPLFQAPEKRENGDRVDWVFCNAVRHVDDWVRVAKCDDKDARYEDCEKM
IHVFDKDGDGRIRLDEFRAV*
>11687.m00400|LOC_Os11g04830.1|genepair1287-1
MAADAYADAAPPRRAPAPATSTVAKEAEFLWELRKYVLLLATLAATVTYTAGLDPPGGFW
TDNVGELLAGDPVLQKTYPRRYKAFFYCNATAFVASLVIVNLLLVRFLCRRRWWLRALQA
AMTLDMFGLMGAYAAGSSREAAMSAYILVLVILVCSYVSAHVLLYGLTAQVSAPDAPERV
ERARKYLLIFATLAATVAYQAGLSTPGGFWLGSLDNQHLAGDPMLRGNHPYRFMVFFYFN
TTAFVASLVTIMLLMSRTVSRHGFRSSALWVCVGAAMVGLMGAFAAGSCRSFKTSIYVIA
LVGAVLLYIAIQAMVFFSEPVKDWLHRAGETLQKCLKLDELEQRNQQQITLSNQGNGDAY
LLLKKSRMYLLLLGILAASVTYQAGLNPPGGFWQSDGTDGYRHYLAGDPVLHITYHRRYM

VFFYSNATAFIASLVILILLLSNMISTQGIKYCALQVAMILNLFGLIGAYAAGSCRQASK
SVYVSVLVLPVFLYVGIHVLVFMLEVSPTWATWRGRVREKLKQRMPEWLKNLLELEKHVE
EEEEEWKLEKRRKLLLLVAILAASLTYEAGMNPPGGFWQEGKSGHVGDPVLNNDNYRHRY
LAFFFCNTTAFVASLAIIMLLVNRKLSARGIRSYALRVCVILVLVGLMGAFAAGSCRKVK
TSVYVFVLVLAVLLCIAFQVALVVSGSLRRLVNSLLSKLGAPLEEDAGERLPHTAADEPR
DLWDEKLPKYLLLLAALAAAVTYQAAMSPPGGLWDDGQTEHIVGDPVLLTNYARRYKAFF
YCNATSFMASLVIMVLLLIKRVSNTQPALLALHAAMILDLFGLMGAYAAESCRRVTTSAY
ILALLVGVSAYIVVLVVVSIGVARWMKKVMDKVGEKLTHCFSFEDL*
>11686.m00396|LOC_Os12g04690.1|genepair1287-2
MRVLMVDGDAWRRAPAPATSPAAEEAEFLWELRKYVLLLATLAATVTYTAGLDPPGGFWT
DNVGELLAGDPVLQKTYPGRYKAFFYCNATAFVASLVIVNLLLVRFLCRRRWWLRALQAA
MILDMFGLMGAYAAGSSREAAMSAYILVLVILVCSYVSAHVLLYGLTTAQVSAPDAPERV
ERARKYLLIFATLAATVAYQAGLSTPGGFWLGSLENQHLAGDPMLRGNHPSSALWVCVGA
AMVGLMGAFAAGSCRSFKTSIYVIALVGAVLLYIAIQFMVFISEPVKDWLHRAGETLQKC
LKLDELEQRNQQQITLSNQGNGDAYLLLKKSRMYLLLLGILAASVTYQAGLNPPGGFWQS
DGTDGYRHYLAGDPVLHITYHRRYMVFFYSNATAFIASLVILILLLSNMISTQGIKYCAL
QVAMILNLFGLIGAYAAGSCRQASKSVYVSVLVLPVFLYVGIHVLVFMLEVSPTWATWRV
EVREKLKQCMPEWLKNLLELEKHVEEEEEEWKLEKRRKLLLLVAILAASLTYEAGMNPPG
GFWQEKKSGHVGDPVLNNDNYRHRYLAFFFCNTTAFVASLAIIMLLVNRKLSARGIRSYA
LRVCVILVLVGLMGAFAAGSCRKVKTSIYVFVLVLAVLLCIAFQVALVVSGSLRRLVNSL
LSKLGAPLEEDAGERLPHTAADGGDGEPRDLWDEKLPKYLLLLAALAAAVTYQAAMSPPG
GLWDDGQTEHIVGDPVLLTNYARRYKVFFYCNATSFMASLVIMVLLLIKRVSNTQPALLA
LHAAMILDLFGLMGVYAAGSCRRVTTSAYILALLVGVSAYIVVLVVVSVGVARWMKKVMD
KVGEKLTHCFSFEDL*
>11687.m00401|LOC_Os11g04840.1|genepair1288-1
MNPLPPPASRLWEASIRKLKYSTILRGSVVPSGGTFDGAAAAAATGGDPVTLTPSLSVIS
STSNTIYQYEDGDDDIDSVLDDVDTDDDDVDEASLGEPSHSDQLLPSGDFYQGDLRGDLP
HGAGKYLWTDGSMYEGSWRGGRAAGRGKFSWSSGAIYEGDLAGGYMHGQGTYIGELGDTF
AGLWANNLRHGRGTQAYVNGDVYDGHWRDGLQDGHGRYIWRGGHEYIGTWKAGEMHGRGT
VIWADGDRYDGAWEDAKPKGQGTFRWSDGGMYIGLWCQESGETQGKGVYYPPSGGPAVPL
PREPKEVITKLLEELEMSEGKTVSLLPSQKVLTWPGVEPVTKKPVWRPPEVAADQGMWRP
PEVGADQGRNSMSSDIDSLVEGEDGGEESRNDRSWVRTPSCMRAPTLPKPGKKQGETISK
GHKNYELMLNLQLGIRHAVGRQSAPASLDLKSSAFDPKEKVWTRFPPEGSKHTPPHQSCD
FRWKDYCPLVFRTLRKLFDVDPGDYMLSICGDDALLELSSPGKSGSFFYFTNDDKYMIKT
MKKAEVKVLLRMLPAYYKHVRSFDNTLVTKFFGLHCVKITGAIQKKVRFVIMGNLFCSNY
SIHRRFDLKGSSHGRTTDKPIDQIDETTTLKDLDLNFIFRLEGSWYEDFCRQLDKDYSSS
PDNETTQTALEDEERRKAPVKLGISMPSRVENVVKNPESESQLIGEPTGEFQDVILFFGI
IDILQDYDISKKLEHAYKSMQYDPNSISAVDPKQYCKRFRDFIYRAFSEDLQ*
>11686.m00397|LOC_Os12g04700.1|genepair1288-2
MNPLPPPASRLWEASIRKLKYSTILRGSVVPSGAAFDGAAAAATGGDPVTLTPSLSVSSS
TSNTIYQYEDGDDDIDSVLDDVDTDDDDVDEASLGEPSHSDQLLPSGDFYQGDLRGDLPH
GAGKYLWTDGSMYEGSWRGGRAAGRGKFSWSSGAIYEGDLAGGYMHGQGTYIGELGDTFA
GLWANNLRHGRGTQAYVNGDVYDGHWRDGLQDGHGRYIWRGGHEYIGTWKAGEMHGRGTV
IWADGDRYDGAWEDAKPKGQGTFRWSDGGMYIGLWCQESGETQGKGVYYPPSGGPAVPLP
REPKEVITKLLEELEMSEGKTVSLLPSQKVLTWPGVEPVTKKPVWRPPEVAADQGMWRPP
EVGADQGRRSSRRNSMSSDIDSLVEGEDGGEETRNDRSWVRTPSCMRAPTLPKPGKKQGE
TISKGHKNYELMLNLQLGIRHAVGRQSAPASLDLKSSAFDPKEKVWTRFPPEGSKHTPPH
QSCDFRWKDYCPLVFRTLRKLFDVDPGDYMLSICGDDALLELSSPGKSGSFFYFTNDDKY
MIKTMKKAEVKVLLRMLPAYYKHVRSFDNTLVTKFFGLHCVKITGAIQKKVRFVIMGNLF
CSNYSIHRRFDLKGSSHGRTTDKPLDQIDETTTLKDLDLNFIFRLEGSWYEDFCRQVDKD
CEFLEQERIMDYSLLVGVHFKDRCKDISSPDNETTQTALEDEEKRKAPVKLGIGMPSRVE
NVVKNPESESQLIGEPTGEFQDVILFFGIIDILQDYDISKKLEHAYKSMQYDPNSISAVD
PKQYCKRFRDFIYRAFSEDVQ*
>11687.m00402|LOC_Os11g04850.1|genepair1289-1
MDFEPLPSAAGDATADETPPGRAVCSSGCGRPSTVCLCPYLPATPLPTSTTVVVLHHPHA
LRRNPLSTLPLLARSLSNLRLIPGRRLLPSSGPLIPNPVLLLFPSPGAADLASWCRSTPP
AARANPTLLLLDGTWKQAKEMHAASLPFLSSFAVPVSLPVDCGVDGDSMFEGKLVVKKEP
HKGCVSTMEAVARALRLLEPEGRGAEIEETMVGVLRAMVAFQAEHLQHRPMKPRVKMRKK
KDIKREEEMKRDARLE*
>11686.m00415|LOC_Os12g04860.1|genepair1289-2
MDFEPLPSAAGDATADETPPGRAVCSSGCGRPTTVCLCPYLPATPLPTSTTVVILHHPHA
LRRNPLSTLPLLARSLSNLRLIPGRRLLPSSAPVISRSPVLLLFPSPDAADLASWCRSTP
PPARANPTLLLLDGTWKQAKEMHAASLPFLSSFAVPVSLPVDCGVDGDSMFEGELVVKKE
PHKGCVSTMEAVARALRLLEPEGRGEEIEETMVGVLRAMVAFQAEHLQHRPMKPRVKMRK
KKDIKREEEMKRNTLDWSD*
>11687.m00405|LOC_Os11g04880.1|genepair1290-1
MIEVVLNDRLGKKVRVKCNEDDTIGDLKKLVAAQTGTRPEKIRIQKWYNIYKDHITLKDY
EIHDGMGLELYYN*
>11686.m00422|LOC_Os12g04920.1|genepair1290-2
MARRRGVEDPNWRSDSCVYLLPHWCQRPLCLCGDRCQLMASRNPDIRGRRFFRCPNYDRE
TRTTACAYIEWVDTENPVFDLTTCLQEGRWYFASESTEQYLQRKAAYERQCREQQSDWRV
LTTALPPWEARPRCRCGDRCQVLRSIKPTTLGRRFFVCPNILDDDFMLELPEQYRVTKAR
FERGEGYSRRGGSRREFEEEEEEEKMIEVVLNDRLGKKVRVKCNEDDTIGDLKKLVAAQT
GTRPEKIRIQKWYNIYKDHITLRDYEIHDGMGLELYYN*

>11687.m00410|LOC_Os11g04930.1|genepair1291-1
MADAVAAYDALPAPPSPPSPALPPPFQPPPPDTPISDATPSAAETPNLPDTPASASPFSD
AALAADASDADASAVAAPPDDDGTNPLGGAMKHMALAPPPPPNKKSKKKNSNSVWTRPNS
RKGKKKAKQPANALAGGSAGANGRLPKPSSGDDELVLTPAPRFAAERNDDAPDLPVLLSR
VFKSDKVEVSDDRLTAGSTKGYRMVRATRGVAAGAWYFEVKVLHLGSTGHTRLGWATNNA
DIHAPVGYDVFGFGYRDMDGTKVHKAWRANYADQGYGEGDVLGFYIHLPDGELYEPKQPF
LVHYKGLPFRAEAPKAAEQKTPDPVPGSEICYFKNGVCQGTAFVDIPGGRYYPAASMYTL
PDQPNCEVRFNFGPNFEFFPEDFGGRSVPQPMNNVPYRPYQLANEVPAENGTAEKTIKLQ
*
>11686.m00423|LOC_Os12g04930.1|genepair1291-2
MADAAADDDAPPSPPPSAFHPASADTPMSDATPSAADTPNLPDTPASASAEPETPFSDAA
LADASDADASGVAAPPDDDGTNPLGGAMKHMALAPPAPPSKKSKKKNSNSVWTRPNSRKG
KKKAKQPANALAGGSGGANGRLPKPSSGEDELVLTPAPRFAAERNDDAPDRPVLLSRVFK
SDKVEVSDDRLTAGSTKGYRMVRATRGVAAGAWYFEVKVLHLGSTGHTRLGWATNNADIH
APVGYDVFGFGYRDMDGTKVHKAWRANYAEQGYGDGDVLGFYIHLPDGELYEPKQPFLVH
YKGLPFRAEAPKAAEQKTPDPVPGSEICYFKNGICQGTAFVDIPGGRYYPAASMYTLPDQ
PNCQVRFNFGPNFEFFPEDFGGRLIPRPMSDVPYRPFELANGGPAENGTAEKNI*
>11687.m00412|LOC_Os11g04950.1|genepair1292-1
MAPSKQYDEGGQLQLMDAERIEEEEECFESIDKLISQGINSGDVKKLQDAGIYTCNGLMM
HTKKSLTGIKGLSEAKVDKICEAAEKLLSQGFMTGSDLLIKRKSVVRITTGSQALDELLG
GGIETLCITEAFGEFRSGKTQLAHTLCVSTQIIYARAYTYEHQYNLLLGLAAKMAEEPFR
LLIVDSVIALFRVDFSGRGELAERQQKLAQMLSRLTKIAEEFNVAVYITNQVIADPGGGM
FITDPKKPAGGHVLAHAATIRLMLRKGKGEQRVCKIFDAPNLPEGEAISFCIVHTCLLKL
LVQLVTLVQHTAHSSSPAPAAVTSRRPCMALREAGSNPGRLSAASLEGLLDKMSSKKNYY
KEKMMRRKEEKKEEPETPRYRDRAKERREDQNPDYEPTELGSFHAVAPPGADLRLADAHK
ISIEKSKYLGGDLEHTHLVKGLDYALLHKVRSEIEKKPEAEDGKDTQSRSTKEDQAVSFR
TAAAKEDGLTNDIPTTLHRSKADCSVPEEMVTVSVDGSVLDRIAKIMSYLRLGSSGKVLK
KKKKERDTKGKNSLASGDYDEVARPGQTNGSALKHQFEKDMPPPPPPRNNNLSKNEKPSA
PVARADEDDIFVGDGVYYSVPNKEMSQSPVSEDMDESPHNHQKQSYFTEEKPIYGPIPPS
DPAQAWPQPNAYDAIQAQMVAAGYQGEWSGYQYGEQQMAYPEQYMQQSAQDCDVLADPNI
TQDPRLMTQADKDRGLGSVFKRDDERLKQLREKDAREKDPNFISDSYSECYPGYQEYNHE
IAGSDEEDDLSKMDMGGRAKGRLHRWDFETEEEWATYNDQKEAMPKAAFQFGVKMQDGRK
TRKQNKDQKLTNDLHKINKILARKKGDKDGGHDGGHYDDDMPSGKKQRA*
>11686.m00424|LOC_Os12g04940.1|genepair1292-2
MSSKKNYYKEKLMRRKEEKKEEPETPRYRDRAKECREDQNLDYEPTELGSFHAVAPPGAD
LRLADVHKISIEKSKYLGGDLEHTHLVKGLDYALLHKVRSEIEKKPDAEDGKDTQTRSTK
EDQAVSFRTATAKEDGLTNDIPTTLHRSKADCAVPEEMVTVSVDGSVLDRIAKIMSYLRL
GSSGKVLKKKKKERDTKGNNSLASGDYDEVARPGQTGSSLKHQFQKDMPPPPPPRNNNLS
KNEEQSIPVARADEDDIFVGDGVDYSVPNKEMSHSPVSEDMDESPHNHQKQSYFTEEKPV
YGPIPPSDPAQAWPQTNGYDAIQAQMVAAGYQGEWSGYQYGEQQMPYPEQYMQQSTQDYD
VLADPNIAQDPRLMTQADKDRGLGSVFKRDDERLKQLREKDSREKDPNFISDSYSECYPG
YQEYNHEIAGSDEEDDLSKMDMGGRAKGRLHRWDFETEEEWAKYNDQKEAMPKAAFQFGV
KMQDGRKTRKQNKDQKLTNDLHKINKILARKKGDKDGGDDGGHYDDDLPSGKKQRA*
>11687.m00415|LOC_Os11g04980.1|genepair1293-1
MRGNGRGREEEGNSPMGRRLRAVSAHLLPPASTTTTTGGVDLAANPTAGEYAHVQGYSAV
LPEKLQTGKWNVYRNAKTPLRLIDRFPDTPDIATLHDNFVYAVETFRDCRYLGTRICADG
TVGDYKWMTYGEASTNRTAIGSGLIYHGIPEGACIGLYFINRPEWIIVDHACAAYSFVSV
PLYDTLGPDAVQFIVNHATVEAIFCVPQTLSTLLSFITQMPCVRLIVVIGGDNANMPSTP
TSTGVEIIIYSRLLNQGKMSSRPFRPPKPEDVATICYTSGTTGTPKGVVLSHRNLIANVA
GSSVVIKFYPSDVYISYLPLAHIYERVNQIALLHYGVAIGFYQGDNLKLMDDLAALRPTV
FSSVPRLYNRIYAAITNAVKESGGLKERFFHAAYNAKRQAIMNGRNLSPMWDKLVFNKIK
ARLGGRVRLMSSGASPLSADVMEFLRVCFGCLVIEGYGMTETSCVIATMDCDDRLIGHVG
PPNPSCEIKLVDVPEMNYTSEDQPYPRGEICVRGTTIFFGYYKDEIQTREVIDEDGWLHT
GDIGLWLPGGRLKIIDRKKNIFKLAQGEYIAPEKIENVYAKCKFIAQCFIYGDSLNSSLV
AVVAVEPEVLKAWAASEGIQYEDLRQLCADTRARAAVLADMDSIGKEAQLRGFEFAKAVT
LVAEPFTLENGLLTPTFKIKRPQAKAYFAKEIADMYAQLREAESTKSKL*
>11686.m00429|LOC_Os12g04990.1|genepair1293-2
MARQQQQREEERAPMGAAQRRLRAVSGHLQPPTESGGVDLAANPTAGEYAHVQGYSAVLP
EKLQTGKWNVYRSAKSPLKLINRFPDNPDIGTLHDNFVYAVETFRDCRYLGTRVCVDGTV
GDYKWMTYGEASTSRTAIGSGLIYHGIPEGARIGLYFINRPEWTIVDHACAAYSYVSVPL
YDTLGPDAVQFIVNHATVQAIFCVPQTLSTLLSFITQMPCVRLIVVIGGDNANMPSTPTA
TGVEIITYSRLLSQGKMSSQSFRPPKPEDIATICYTSGTTGTPKGVVLSHGNLIANVAGS
SLVIKFYPSDVYISYLPLAHIYERVNQVSVVHYGVAIGFYQGDNLKLMDDLAALRPTVFP
SVPRLYNRIYAAITNAVKESGGLKERLFHAAYNAKRQAIINGRNPSPMWDRLVFNKIKAR
LGGRVRLMTSGASPLSADVMEFLRICFGGEVLEGYGMTETSCIISAMNIGDRLIGHVGSP
NPSCEIKLVDVPEMNYTSEDEPYPRGEICVRGPTIFCGYYKDEIQTREAVDEDGWLHTGD
IGLWMPGGRLKIIDRKKNIFKLAQGEYIAPEKIENVYAKCKFIAQSFIYGDSLNSSLVAV
VAVEPDVLKAWAASEGIQHEDLRQLCADPKARSAVLAEMDSIGKEAQLRGFEFAKAVTLV
AEPFTLENGLLTPTFKVKRPQAKAYFAKEIADMYAQLRQAEQIKPKL*
>11687.m00416|LOC_Os11g04990.1|genepair1294-1
MASADLLRREEEFYASLFDSAKGGDAVKSRGQMIERKIEVLEDMAAKVSNRRSRRWLNDR
LLIELVPRLHVEEIKGLFAPPPWGEELPVSAFCRTSVGEWDAFRSIDMDVEARLVQQMKQ
SSTKQKNHLDRDELVALNSWHHIDRQTREAIKRNFLPDLLEIYEERVRTFIEDTSGKDML
VLNVQDPFQRLLLHGVCEFYNVSSTTTTTVRDGKLCKTTAIKKRSGTGAPSRITLVSFLR

MKKKSH*
>11686.m00432|LOC_Os12g05000.1|genepair1294-2
MADLLRREEEFYASLFDSPKGGDAVKSRGQMIERKIEVLEDMATKVSNRRSRRWLNDRLL
IELVPRLHVEEIKGLFAPPPWGEELPVSAFCRTSDGEWDAFRSIDMDVEARLMQQMKQSS
TKQKNHVDSDELIALNAWHRIDRQTREAIKRNFLPDLLDIYEDRIRAFIEDASGKDVLVL
NVQDPFQRLLLHGVCEFYNVSSTTTTTMRDGKLWKTTTIKRRPGTGAPSRLTLVSFLRMK
KNGSQ*
>11687.m00418|LOC_Os11g05010.1|genepair1295-1
MSKEEVLKIQTCVLKVNIHCDGCQKKVKKILHKIEGVYQTSIDAEQGKVTVSGLVDPATI
IKKLNKAGKPAELWGSKVGVAAVNNQFQKLHLDGGGGKGQPKDGGGKGQPKDAGGKGQKG
GGGGGGGGNGGGGSKDVKMMMPPQMPQPTPQQIQQLQQQLQMKGLKLPQFMDAAKMAPFA
AAAPIKDPKSVKFNLPPPEDDFGDDGSEFDDEFDEFDDEDDFDDDGLDDDYFDDPKMMKQ
MAMPPPNAGGGGDKKGGNNGGGAGNGGKKGGGGNEIPVQIKGNANNAAGGGKKDSGAKQN
QGGGGKNGGGQPNNAKGGGGAPNGGGNHPAQGKKGGGGQGGGVGGPMGGMPAQQQAMMMR
PNMMGGSAGFPGMGQMGGGPMTMPMGHHPHMGAGAVQGMPPAAFYQGGGGMPSGPEMLQA
AAAAGNPMAQQQYMAMMQQQQQQQQMMMNGHGHHGHHGHHGHGGGAAPAGYPAMGYGYG
RPPMPYPMHYPMQPHPHADPYNYFSDENPNSCSVM*
>11686.m00437|LOC_Os12g05040.1|genepair1295-2
MSKEEVLKIQTCVLKVNIHCDGCQKKVKKILHKIEGVYQTSIDAEQGKVTVSGLVDPATI
IKKLNKAGKPAELWGSKVGVAAVNNQFQKLHLDGGGGGKGQPKDGGGKGHPKDAGGKGQK
GGGGGGGNGGGGSKDVKMMMPPQMPQPTPQQLQQLQQQLQMKGLKLPQFMDAAKMAPFAA
AAPIKDPKSVKFNLPPEDDFGDDGSEFDDEFDEFDDEDDFDDDGLDEDYFDDPKMMKQMA
MPPPNAGGGGDKKGGGKKGGGGNEIPVQIKGNANNAAGGGGKKDAGGKQNQGGGGGNGKN
GGGGGQPNNAKGGGAPNGGGNHPAQGKKGGGGGGGGQGGGVGGPMGGMPAQQQAMMMRPN
MMGGGAGFPGMGQMGGGPMTMPMAHHPHMGSAQGGGAGAVQGMPTAAFYQGGGGGGGGM
PSGPEMLQAAAAAGNPMAQQQYMAMMQQQQQMMMNGHGHHGHGHHHGHHGHGGGAAPAG
YPAMGYGYGRPPMPYPMHYPMQPHPHADPYNYFSDENPNSCSVM*
>11687.m00423|LOC_Os11g05050.1|genepair1296-1
MLAVFSGAVVEVPAELVAAGSRTPSPKTRASELVGRFLAAAEPAVSLQLGDLGHLAYSHA
NQSLLRPRSFASKDDIFCLFEGVLDNLGRLSQQYGLSKGANEVLLVIEAYKTLRDRAPYP
ASFMLSQLTGSYAFVLFDKSTSSLLVASDPEGKVPLFWGITADGSVAFSNDIDLLKGSCG
KSLAPFPQGCFYSNALGGLKCYENPKNKVTAVPANEEEICGATFKVESATAILTALH*
>11686.m00438|LOC_Os12g05050.1|genepair1296-2
MLGVFSGDVVEVPAELVAAGSRTPSPKTRASELVSRFLGGAEPAVSVQLGDLGHLAYSHA
NQALLRPRSFAAKDDIFCLFEGVLDNLGNLNQQYGLSKGANEVLLVIEAYKTLRDRAPYP
ASFMLSQLAGSYAFVLFDKSTSTLLVASDPEGKVSLYWGITADGSVAFSDNIDLLKGSCG
KSLAPFPQGCFYSNALGVGGLKCYENPKHKVTAVPAKEEEICGATFKVEGSTILTALH*
>11687.m00426|LOC_Os11g05080.1|genepair1297-1
MAGLAVAVAAAAALVLLLCAASLRCSAAVGLALSAAPGKLWSGGVSIAAEAAAVEARAEG
EEEEECDLFDGEWVWNDSYPLYHSTDCPFLDVGFRCSENGRPDASYSKWRWRPSRCDLPR
FDARNMLEKLRNKRVVFVGDSIGRNQWESLLCMLSVAVPDKSSIFEVNGNPITKHMGFLI
FKFRDYNCTVEYYRSPFIVLQGRAPPGAPGVVKYTIRVDAMDWLSDRGQWRDADVLILNT
GHWWNYEKTIRSGTYFQEGDAVKMDMTVGDAYKRSIQTLFGWLHNEVNSSKTHVIFRTYA
PVHFRGGDWKTGGNCHLETHPDVTPVKSLEQWADFLNPVNDVLGNSFRPKLLGLDILNVT
QMTAQRKDGHVSVHLSPSGPVPLYRQDCSHWCLPGVPDTWNELVYNLLLKRQSMIGQNVP
LVGTKTLKAGWRKLNKYNLTI*
>11686.m00441|LOC_Os12g05080.1|genepair1297-2
MRRPLEKAGAAGLVAMLAAAAALLLLVCAASLRCSAAVGSALAAPKKLLSGGVSIEEPRG
GGAGGGGGGEECDLFDGEWVWDDGYPLYASRDCPFLDVGFRCSENGRPDDSYTKWRWRPS
RCDLPRFNARNMLEKLRNRRVVFVGDSIARNQWESLLCMLAAAVPDKSSIFEVNGNPITK
HMGFLIFKFREYNCTVEYYRSPFIVLQGRAPAGAPAVVKYTIRVDAMDWMSGRGKWKDAD
LLIFNTGHWWNHEKTIKGGAYFQEGNEVKMEMTVRDAYQRSMQTLFHWLRNEVNTNKTQI
IFRTYAPVHFRGGDWKTGGTCHLETLPDVTPIKSLEPWADLLEPINHVLGNSITTKLVGL
DILNVTQMTALRKDGHLSVYLSPSGPASHHRQDCSHWCLPGVPDAWNELLYALFLRRKMV
MPHNVSSVGAKRLNTG*
>11687.m00427|LOC_Os11g05090.1|genepair1298-1
MAVVEEEEGSPPAPAAAADPASSGSSDNEITVEEASFVHTEPPQDGSVPPVVSSNMEVLH
DKVKKQVIKEGHGKKPSKFATCFLHYRAWVQGSLHKFEDTWQEQHPIELVIGKEKKQMSG
LGIGVGNMRSGERALLHVGWELGYGKEGSFSFPNVPPMADLLYEVELIGFDDVKEGKARS
DMTVEERIEAADRRKIEGNEYFKEKKFEEAMQQYEMAIAYMGDDFMFQLFGKYRDMALAV
KNPCHLNMAACLIKLKRFDEAIAQCSIVLAEDENNVKALFRRGKARAELGQTESAREDFL
KAKKHSPEDKEIQRELRSLAEQDKALYQKQKELYKGLFGPRPEPKPKASNSIVRFWQWLV
SLIGYLIKLFKPKNE*
>11686.m00442|LOC_Os12g05090.1|genepair1298-2
MAVLEEDPAPVPAADSTSGASDDEITVEETSFVHTEPPQDGTAPPVVTSDMEVLNDKVKK
QVIKEGHGKKPSRFATCFVHYRAWVQGSSHKFEDTWQEQHSIELVLGKEKKEMTGLGIGV
SNMRSGERALLHVNWELGYGKEGSFSFPNVPPMADLVYEVELIGFDDVKEGKARSDMTVE
ERIEAADRRKIEGNEYFKEKKFEEAMQQYEMAIAYMGDDFMFQLFGKYRDMALAVKNPCH
LNMAACLIKLKRFDEAIAQCTIVLSEDENNVKALFRRGKARAELGQTESAREDFLKAKKY
SPEDKEIQRELRSLAEQDKALYQKQKELYKGLFGPRPEPKPKASNFLVLFWRWLVSLIGY
LVKLFKRKDE*
>11687.m00428|LOC_Os11g05100.1|genepair1299-1
MDAVAFPPPPAPFLDDDLDFGDFAFADPQPAAAAAFDAFGAYDDDWGDFVDSRLGSNPDG
GSSAAAPAEKPPSWEKPRGPLPLSLFGADEDEEEEGPAELPPTAADQRGASHASSNGSK

PADLKDLIAGLYGSHPQPSSTDAAEVGTQEGSAVAAAEEEEEDGDGFEDDGWEFKAAAPS
SSSDAVQDGGRQAHGDLTQDVPKSMSSDQEGWSLFTSVSENLNNVQTTDHVGTRESAGQS
VKAFSYFPPNNAAILDLYKESEPIDAVHIMQCSSESVQSSSDMFSNTEMNSSFGTDENHS
IKSASDRILIDFYHKLREESLTVISQYKKDLKESQKNSMLSDEKNEVMTETEREIQEICK
ELQDSSLAKGFCKDEHPSKDVCISELLNSAKEDHLKDFDKEYHLTEIIAMALEDMSSAVK
LYKHSVSILRTLEIASKEEQCDYVSAWYSMLLSCAQELQHGAMIWQESCHANVGETVISQ
GAHYFIALGEIYRVAQILHISMLSFKPWVLADPGMLSKMLVCWNSCVNSWTSGLGMALTM
VVDSKNLHAPVAKVLLESIININDIEVPNLQSFLPSDKMACKLTLLPTSLVPGMEVIIWD
ADHYFVKVANLWANQISSDPPQFSVSRVA*
>11686.m00443|LOC_Os12g05100.1|genepair1299-2
MAGSGLGGAGSAASPHIPAVAAFDHAGGELRHHLVHVAPRRENATKKASPPPSLLAAGFA
AGKLRRRREDWSLFTSVDENLNHVRTTNHIETCKSTGPNDAAILDLYKETELVDAVHMTQ
SSSESVQSPSDMFSNNEMNSSFETDENHSIKSSSDRTLIDFYHKLREETLTVIFRNGKDF
KEICEKLPEGFCIEEHTAKDVFISELLDSAREVHLKDFEEEYHLIEKIPMHGVVLWQESC
QSNVCSVVISQGGQFFIALGEIYRVAQILNLSLQSFKPWVLADPGMEWK*
>11687.m00429|LOC_Os11g05110.1|genepair1300-1
MHSTNLLLEEPIRMASILEPSKPSFFPAMTKIVGTLGPKSRAVDTISSCLKAGMSVARFD
FSWGDAEYHQETLENLKLAIKSTKKLCAVMLDTVGPELQVVNKSEAAISLEANGTVVLTP
DQGQEASSELLPINFSGLAKALKPGATIFVGQYLFTGSETTSVWLEVSEVKGDDVVCVIK
NSATLAGSLFTLHCSQIHIDLPTLSDEDKEVIRRWGAPNKIDFLSLSYTRHAEDVRQARE
FLSKLGDLSQTQIFAKIENVEGLNHFDEILQEADGIILSRGNLGIDLPPEKVFLFQKSAL
HKCNMAGKPAVVTRVVDSMTDNLRPTRAEATDVANAVLDGSDAILLGAETLRGLYPVETI
SIVGKICAEAEKVFNQDLYFKRTVKYVGEPMTHLESIASSAVRAAIKVKASVIICFTSSG
RAARLIAKYRPTMPVLSVVIPRLKTNQLRWSFTGAFEARQSLIVRGLFPMLADPRHPAES
TSATNESVLKVALDHGKASGVIKSHDRVVVCQKVGDSSVVKIIELDD*
>11686.m00444|LOC_Os12g05110.1|genepair1300-2
MHSTNLLLEEPIRMASILEPSKPSFFPAMTKIVGTLGPKSRSVDTISSCLKAGMSVARFD
FSWGDAEYHQETLENLKVAIKSTKKLCAVMLDTVGPELQVVNKSEASISLEENGTVILTP
DQGQEASSQVLPINFAGLAKAVKPGDTIFVGQYLFTGSETTSVWLEVSQIKGDDVVCVIK
NTATLAGSLFTLHCSQIHIDLPTLSDEDKEVIRKWGAPNKIDFLSLSYTRHVEDVRQARE
FLSKLGDLSQTQIFAKIENVEGLNNFDEILQEADGIILSRGNLGIDLPPEKVFLFQKSAL
HKCNMAGKPAVVTRVVDSMTDNLRPTRAEATDVANAVLDGSDAILLGAETLRGLYPVETI
SIVGKICAEAEKVFNQDLYFKRTVKHVGEPMTHLESIASSAVRAAIKVKASVIICFTSSG
RAARLIAKYRPTMPVLSVVIPRLKTNQLRWSFTGAFEARQSLIVRGLFPMLADPRHPAES
TNATNESVLKVALDHGKVSGVIKSHDRVVVCQKVGDSSVVKIIELDD*
>11687.m00430|LOC_Os11g05120.1|genepair1301-1
MALSTSRPHHLLRPLLRGFHATSQAMARPEPHEFSKPSDYLGSWEPAGDPREAWARLERL
RKGYARDVRQLRRQYSYEMQLLEAERQRKAEARAEAARLANEERKATKAAAAQTRAAERR
AFEKDFRQALMKERAEKLESWREKEKLKVQKKADHRELLRKKSSMWVAEDKLETTILDAI
KNTTAL*
>11686.m00451|LOC_Os12g05170.1|genepair1301-2
MALSSSRPRHLLRPLLRGLHATAQALARPEPHEFSNPSEHLGSWGEPAGDPREAWARLER
LRKGYARDVRAAGAMKVRGEKLENWREKEKLKADKRAEDRELLGRKSSVWIADNELENRI
LKAIKFTTPL*
>11687.m00435|LOC_Os11g05160.1|genepair1302-1
MHMEGGEGIAVAGAGGGHEAGFGLFRAADVTMTEAQEAAKEYQSSPSSPSTSPTPSPPPV
AASGHGGEAAATPTMWSLGGEKMPSEAAGDNGMQMSGHSEHASLSSGRRRGRPKGSGRRQ
ILATLGEWYALSAGGSFTPHVIIVGTGEDVAGRIMSFSQKGPRSICILSANGTISNVALS
QPGSSGSTFTYEGRFEILQLTGSFTMAEEGGRRRTGGLSVSLAGPDGRVVGGVVAGMLRA
ASPIQVIVGSFLPNSLKQHQRRMGLQQQPSAAPALPPPMAPPPVLTAAMPISQAAPGTNG
CHAPQVSSMHPQAHTGVMEHSATASGAMNLNSSSSTGFTMVGWPVSSQSMGHRPSPDINV
CLTPQE*
>11686.m00454|LOC_Os12g05200.1|genepair1302-2
MKQQQRQQATACSSEQQIVPWLKQLHNKKPQQQRKGEWYALSAGGSFTPHVIIVATGEDV
AARIMSFSQKGPRSVCILAANGTISNVVLNQPGSSGSTFSYEGCFEILQLTGSFTIAEEG
VRRRTGGLSVSLAGPDGRVVGGVVAGMLRAASPIQVIVGSFLPNNLKQHQRRMGLHPQPS
AAPAFPAPMAPLHPPPVLTAAMPISQAAPGNNGCRSPQVSISSMPPQAHAGVEQSRGAMN
LNSSSSSTGFAMVGWPAAASSQSMVHRPSPDINVCLTPQE*
>11687.m00436|LOC_Os11g05170.1|genepair1303-1
MAYMRVTHRDEEGKKVTEKVPIPETRRPDTARHFERKLEEQGFHRLERHPANGPARAGIG
APPPKSGRGGKFTWEGPDGPVDAQLQPAPPAVDPNDPNYDEGDGAGVDEEVAKEVVIGEV
EVAKVAEARDGVDVVAPAPLLQQEQQ*
>11686.m00455|LOC_Os12g05210.1|genepair1303-2
MMYMRATHRDEEGKKVTEKVAVPETRHPDTARHFERKLDQQGLHRMERHPANGSRGIGAP
PPKSGRGGKFTWEGPDSIVDSQLDPLPAAIDRNDPNYEEEEGDHEREADVVGEVEVAKVA
GDARDGVARVDVVAPPQLHEKLQLQPQ*
>11687.m00438|LOC_Os11g05190.1|genepair1304-1
MSTTRGVSSSSAAAALALLLLFALCFFSFHFAAAARAVPRDEHQENGGVKAVAAVAADQL
VLQLEGDTGNGDEVSELMGAAEEEAAACEEGKNNDECVQRRLLSDAHLDYIYTQHKNKP*
>11686.m00460|LOC_Os12g05260.1|genepair1304-2
MRPTGRRSSPPVAAALALLLLLVLFFFSHCASAARPLPASAAAELVLQDGATGNGDEVSE
LMGAAEEEAAGLCEEGNEECVERRMLRDAHLDYIYTQKRNRP*
>11687.m00442|LOC_Os11g05230.1|genepair1305-1
MATATAFDNLVTLLEAYQRLGDSDDDDGDGGIDRPAAAANGDGGDGEGDGEEEDSDALSQ

YASFLLGNGDNGGGSGQGGAEHGEVRNGDDDDGGGFAMGAVESHSYEDTIIVGSTDDAGS
SLHHGDDELPVPLPPAELPPPGSAGNAPPAPLEAMTMSFLQEAAMRRRQGTTNGDGQTIL
IQRLGELLRAYRANAAGGANGGGAPRSGRQRPASAAAVAALEKRKHGCGGGGGAAAQCVI
CIEDYEVGDDISVMPCSYGHSFHHACLADWLARSRFCPLCRHKLPAADDDDQDDAPDGQA
P*
>11686.m00461|LOC_Os12g05270.1|genepair1305-2
MEADNDVSTAALTDGGGGQRHRFTFLLPSQNDDEDATMPPPPTSTDDEDDDFSVDDVAQI
LSFLLVNGVISGETALLLQILTVALHFDLGGGGGGGHGENDDEDAMMAAPLPSIDDEDDD
DGSPLLDQVLCYLLLNGIISGERALQILQNANMPLDLDLDDGGANMPLDLDDGGGFRGVP
ASAAAVAGLEKQVFHQFDHHGGDDDDDDEAKDSAAGCVICMEEFVAGDEVCAIPCAGNHS
FHHHCITEWLGRSNVCPLCRHALPVEEQDEGVVASST*
>11687.m00448|LOC_Os11g05280.1|genepair1306-1
MVSASSLLLPSSVRDFASCIGDGAVRVADVAACTAPSSTRVSTCSSSSSTTASSSSPTLS
VTVSYRATLLAAPAPPLQLRLTWGHSPLGPTLSFAPSPTARAIQLRRRRGSRSLPSGSSS
GDENGGGGDESGTTTPPPPPPLALFWDLTAARYDPAASSPEPVSGYYVVAVASAEVVLAV
GDLAAEFVKAKFEGQIPRARVAPVSRVERVVVSDPAAMHAARVRFAEGGPEHEVSVSCAP
AAPGSGGGGDELWVCVDGKRAVQACRLRWNFRGNQTVFVDGAPVDVMWDLHGWWFREPPG
CAVVMLRARSALESRLWLEEEAAAPGFSLIVQAFKSPP*
>11686.m00470|LOC_Os12g05360.1|genepair1306-2
MVSASSLLLPSSVRDFASCIGDGAACTAPSSTRTRRGSAAVQAQPSTLSVTASYRVALAS
SSSPPLQLRLTWAHSPLGPTLSFSPSASGRKVLVRRRRGSCSVLSSGEDEAVESESELSA
SSPRLALFWDLTAARFDPGGVAGAGVRVVLALGDLAAEFVKAKFEGTTQIPMAAPFARGE
RVVVAVSSDAAAAVTHTARARFAEGGAEHEVSVGCAPGGGGGGGGDELWVSIDGKRAVQA
RRLRWNFRGNQTVFVDGQPVDVMWDLHGWWFRREPPAPGWAVVMLRARSALESRLWLEEE
AAAPAFSLLVEAFKSPP*
>11687.m00450|LOC_Os11g05300.1|genepair1307-1
MASGVSTSMVLTLLGFCGSVLFIVFVCTRLACSLLRRHRRRRARLPAASSHFLSSVYVV
DHHRHLPPSGLDPATVAAFPTRAFLGAAPRGHASASSSSSSSDAAAQCVVCLADYEEKDV
LRILPYCGHNFHALCIDIWLMQHSTCPVCRISLCDYPDSKQTMSPLPSEVIIPPCSPEPS
RSDQCNCLFVGTGHSPRTSQVLINEPDQSNRTLYSPSVEGDNNLPSSEVNPPGEINNQTM
KKHVENHRIQDRS*
>11686.m00471|LOC_Os12g05370.1|genepair1307-2
MAAAGVSRSMVLTLLGFCVSVLFIVFVCSRLACALLRRRRGRARLRRASPLAVSGVLSIY
VDRHGHHQPSSAAGAASGTGGLDPAAVAAFPTRAFSPAASSSASASTQCVVCLAEYEEKD
VLRVLPYCGHGFHVACIDIWLMHHSTCPVCRISLCDYPDSKHTMSPVPSAVIIPLPPCSP
EASRSDQCNCLFVGTGHSPRTSQVLRNEPDQVKLPVILETSTQGDPWIAVIGSDGDMISL
LKPGASSGRRTAYMFVHAWMSAGPIMAHTIDLNFVCSKHQQQQWFFFPAATGKSRVEPRD
ADAGLHSCLLPDLRELALKI*
>11687.m00452|LOC_Os11g05320.1|genepair1308-1
MPPGGEPDPAADELQSLSFASSDRSRSRSASTVSTATTTSTTTTTTTPPRLGAVALSDIR
FLKRLGAGDIGSVYLAEVRGAATALVAAKVMDRKELEGRNKEGRARTEREILEAVDHPFL
PRLFGVAEGDRWSCLLTEFCPGGDLHVLRQRQPHRRFSESAVRFYAAEVVAALEYVHMVD
IVYRDLKPENVLVRADGHIMLTDFDLSLKCDPTAPTPAHVISDPIALAGGQSSSSSSSSC
IIPSCIVPAVSCFQLFPGRGRHRRRRWRGRKKPSSGGGGNGGSSFPSGGLELEFVAEPVE
LRSMSFVGTHEYLAPEIVSGEGHGSSVDWWTLGVFVFELLYGVTPFKGHDNEMTLANIVA
RALEFPREPPVSAAAKDLVTSLLAKDPARRLGATVGAAVIKRHPFFSGVNWALLRCATPP
YVPPPFSVATATAANAAAANADMSYDDDSCPGTPVEYY*
>11686.m00474|LOC_Os12g05400.1|genepair1308-2
MANEWVDSEEINMGVVGFYAAEVVAALEYIHMMDIVYRDLKPENVLVRADGHIMLTDFDL
SLKCDPTAPTPAHVISDPIALAGSHYSASSSCIIPSCIVPAVSCFQLFPGRGRRRRHRRK
KKTASGGGGGISGSSFPAGGLELEFVAEPVELRSMSFVGTHEYLAPEIVSGEGHGSSVDW
WTLGVFVFELLYGVTPFKGYDNEMTLANIVARALEFPRDPPVSSAAKDLVTSLLAKDPTR
RLGATVGAAAIKRHPFFSGVNWALLRCATPPYVPPPFSVAAATAAAAAADMSDDDSCPGT
PVEYY*
>11687.m00457|LOC_Os11g05370.1|genepair1309-1
MKRNPRVTSSRRKCRKAHFTAPSSVRRVLMSAALSTELRHKYNVRSIPVRKDDEVQVVRG
SYKGREGKVVQVYRRRWVIHVERITREKVNGSTVNVGIHPSKVVVTKLKLDKDRKAILDR
KASGRAADKAKGKFTAEDVAAAGAAGASLQEID*
>11686.m00477|LOC_Os12g05430.1|genepair1309-2
MKRNPRVTSSRRKCRKAHFTAPSSVRRVLMSAALSTELRHKYNVRSIPIRKDDEVQVVRG
SYKGREGKVVQVYRRRWVIHVERITREKVNGSTVNVGIHPSKVVVTKLKLDKDRKAILDR
KASGRAADKAKGKFTAEDVAAAGASLQEID*
>11687.m00458|LOC_Os11g05380.1|genepair1310-1
MDATTTVSMEMELPWGARCAGLAFFAFSVCLAALGVVLLVARRWPWCSCHVCRAYLTGSW
AREFTNLGDWYAHLLRRSPTGTVHVHVLGCTVTANPANVEHMLRTRFDNFPKGRPFAALL
GDLLGDGIFNVDGHAWRHQRKMASLELGSVAVRSYAYKIIAQEVEARLMPVLADAADRGA
VLDLQDVFRRFAFDNICKISFGLDPGCLDREMPVSELADAFDAASRLSAMRGAAASPLLW
RAKRFLNVGSERELRKAIKVVDELAAAMIRERQKLGVGSSHDLLSRFMASTGVDDAAADD
KFLRDIVVSFLLAGRDTVSTALTTLFMLLSKNPEVAAAMRAEAEAGDGGETGAAITYEHL
KGLHYTHAVLHENMRLFPPVQFDSKFCAAADVLPDGTYVGGDARVMYHPYAMGRMPHIWG
ADYAAFRPARWLTGPGASFVPANPYKYPVFQAGQRVCLGKELAVTEMKAASVAVVRAFDV
EVVGENGRSGGAAAAPRFVPGLTASISGGLQVRVRRRVHT*
>11686.m00478|LOC_Os12g05440.1|genepair1310-2
MEVELPWGARCAAAAFFVSSLCVAALGVVLLLLRRWPWCGCHVCRAYLAGSWRREFANLG

DWYADLLRRSPTGTVHVHVLGCTVTANPANVEYMLKTRFDNFPKGRPFAALLGDLLGDGI
FNVDGDAWRHQRKMASLELGSVAVRSYAYKIVAQEVEARLMPVLANAADSGAVVDLQDVF
RRFAFDTICKISFGLDPGCLDREMPVSELADAFDAASRLSAMRGAAASPLLWKMKRFLNV
GSERELKKAIKLIDGLAAAMIRERRKLGVANSHDLLSRFMASSGDDARGAADDKFLRDIV
VSFLLAGRDTVSSALTTLFMILSKNPDVAAAMRAEAGAAAGESAAVSYEHLKRLNYTHAV
LYENMRLFPPVQFDSKFCAAADVLPDGTYVDGGARVMYHPYAMGRMPRIWGADCDAFRPE
RWLTGAGGAFVPESLFKYPVFQAGLRVCLGKELAITEMKAVSVAVVRAFDVEVVGENGRC
GGGAAAAPRFVPGLTASISGGLPVKIRRV*
>11687.m00461|LOC_Os11g05410.1|genepair1311-1
MAMAMTNTALAFFLLVAAASLLSLPPPSLAVTSPYVRPKPRATLSLLKDDDDGRKPEQVH
ISAVGSDKMRVTWITGGDAPATVEYGTTSGQYPFSATGSTNTYSYVLYHSGNIHDVVIGP
LQPSTTYFYRCSNDTSRELSFRTPPASLPFKFVVAGDLGQTGWTESTLRHIGGDDYDMLL
LPGDLSYADLYQPRWDTYGRLVEPLASARPWMVTQGNHEVERIPLVEPHAFKAYNARWRM
PFDAGASPSGSNLYYSFDVAGGAVHVIMLGSYADYAAGSAQHRWLRRDLAAVDRARAAFV
VALVHAPWYNSNEAHRGEGDAMRAAMEELLRGARVDAVFAGHVHAYERFARVYGGKEDPC
GAVHVTIGDGGNREGLAGSYVDPQPAASAFREASFGHGRLEVVNATHALWTWHRNDDDEA
VVADQAWITSLASNPACNK*
>11686.m00488|LOC_Os12g05540.1|genepair1311-2
MASVAALRLVVLLAAAVPLLPPPAASLAVTSTYVRPTARATLSVLHDGDGRTPQQVHISA
VGSDKMRVTWITDDDAPATVEYGTVSGEYPFSAAGNTTTYSYVLYHSGNIHDVVIGPLKP
STTYFYRCSNDTSRELSFRTPPASLPFKFVVVGDLGQTGWTASTLRHVAADVYDMLLLPG
DLSYADFYQPRWDTFGRLVEPLASARPWMVTEGNHEVERIPVIHPRPFTAYDARWRMPHD
AGASPSGSNLYYSFDVAGGAVHVVMLGSYAGYAAGSAQHRWLRRDLAGVDRAKTAFVVAL
VHAPWYNSNRAHRGEGDAMRAAMEELLYGARVDAVFAGHVHAYERFARVYGGGEDACGPV
HVTVGDGGNREGLATRYVDPQPAASAFREASFGHGRLEVVNATHALWTWRRNDDDEAVVA
DEVWITSLASNPACNKKYSISLY*
>11687.m00467|LOC_Os11g05470.1|genepair1312-1
MSRSVEPLVVGRVIGEVLDTFNPCMKMIVTYNSNKLVFNGHELYPSAVVSKPRVEVQGGD
LRSFFTLVMTDPDVPGPSDPYLREHLHWIVTDIPGTTDASFGREVISYESPKPNIGIHRF
IFVLFKQKRRQTVIVPSFRDHFNTRRFAEENDLGLPVAAVYFNAQRETAARRR*
>11686.m00493|LOC_Os12g05590.1|genepair1312-2
MSRSVEPLVVGRVIGEVIDSFNPCTKMIVTYNSNKLVFNGHEFYPSAVVSKPRVEVQGGD
MRSFFTLVMTDPDVPGPSDPYLREHLHWIVTDIPGTTDASFGREIISYESPKPSIGIHRF
VFVLFKQKRRQAVVVPSSRDHFNTRQFAEENELGLPVAAVYFNAQRETAARRR*
>11687.m00468|LOC_Os11g05480.1|genepair1313-1
MGEASSSSGHPRQNPHVLGYGFHGAMPNSLPSANLFEQQGGANYFGELEEALMQQVATLR
RTQQTATTTSTLHHGDTTPFSTTATAAATARPPPTLDIFPSWPMRSLHTPKEGSNVTADT
TDSESSSKNNSNQNASSDQHVLVGDMAGQFDQIPQQEQHKKMATNSPTHSSKTGKALDPK
TMRRLAQNREAARKSRLRKKAYIQQLESSKLKLAQMEQDIHRARSQGLLLGAPGGNTSSG
AAMFDVDYARWLEEDSQRMAELHGGLHAHLPDSDLRAIVDDTLTHYDHLFNLKGMAAKAD
VFHLITGMWATPAERCFLWMGGFRPSELLKTLTPQLDPLTEQQVVGICNLQQSSQQAEEA
LSQGLDQLHQSLAETVAGGSPLDDPNVGSFMGHMAIALGQLSNLEGFVIQADNLRQQTIH
QMHRILTVRQAARCFLAIGEYHNRLRALSSLWASRPREILVADEGNCGELSIAAHPSESQ
YSAF*
>11686.m00504|LOC_Os12g05680.1|genepair1313-2
MGEARRGQNPHHVLGYGFHGTTLPNSMASANLFEQGGGGGGGAAYFGELEEALVHQVATL
RRRAQQTATTTTSHHGHTTPFSTAAAAATATATARPPATLDIFPSWPMRRSSLPTPKDGC
SNVTADTTDSESSSKNNGDQGAAAADMASQFDQIPQQQQKQHKKMAASSTHSDHRMTKTL
DPKIMRRLAQNREAARKSRLRKKAYIQQLESSKLRLAQMEQDLERARSQGLLLGGSPGGN
TSAGAAMFDAEYGRWLEDGGRRMAELHGGLHAHLPDGDLRAIVDDALAHYDELFRLRAAA
AKADVFHLITGTWATPAERCFLWMGGFQPSDLLKTVAPQLDPLTEQQVVGICSLQQSSQQ
AEEALSQGLEQLHQSLAETVANGGSVVNEASLGSFMGYMALALGKLSNLEGFVIQADNLR
QQTLHQMHRILTIRQAARCFLAIGEYHNRLRALSSLWASRPREILVADEGNCGELSIAAQ
PSESQFSAF*
>11687.m00469|LOC_Os11g05490.1|genepair1314-1
MGNCLVIQDDRDKEIKIMGMDGGEILKLHRDAAVSDSDDPAADMAKAAAAAAAAAAADTA
GGVVRVKLVISKQQLKKMLHKDGVSLDDMVSLMQREASEQEMISCRGWRPALKSIPEGSD
C*
>11686.m00505|LOC_Os12g05690.1|genepair1314-2
MGNCMVIQDRNREIKIMSMDVDGEILKLPPPPPPLNGVSSDDDDEALRPATADMADDPPG
GAVVRVKLVVRKQELKKMLLHNDAAAISLNDMVSLMQKQAEADELLHQQESCGSVWQPTL
QSIPEGSVF*
>11687.m00472|LOC_Os11g05520.1|genepair1315-1
MAQMEFEPDKVGTVTVNGKVYSFRRVHWHAPSEHTINGEKHPLELQMVHAAADGSLAVIA
ILYKYGAPDSFYFQLKRKLAELAADGCSFGEENAQVALGLVHLRSLQKRTGSYFRYAGSL
TAPPCTEDVFWSVLGKIRQISQEQVALITALLPAGGARPTQPLNGRTVQFYNPPNSTISF
KV*
>11686.m00509|LOC_Os12g05730.1|genepair1315-2
MVSLRAAIVLVVAASSVAVAFSHAEGNEGPDFTYIEGAMDGPSNWGKLSPEYRMCGEGRS
QSPIDINTKTVVPRSDLDTLDRNYNAVNATIVNNGKDITMKFHGEVGQVIIAGKPYRFQA
IHWHAPSEHTINGRRFPLELHLVHKSDADGGLAVISVLYKLGAPDSFYLQFKDHLAELGA
DECDFSKEEAHVAAGLVQMRSLQKRTGSYFRYGGSLTTPPCGENVVWSVLGKVREISQEQ
LHLLMSPLPTKDARPAQPLNGRAVFYYNPPGSAVSFQEFAK*
>11687.m00473|LOC_Os11g05530.1|genepair1316-1

MASSARDLSGDPPESTRLRIGDDIAWSDVGGVYDRDDSLKENTNPKCILKNHLPGAHNGG
SQRFSGNLKPTAAPIIGISGKLGQGGKNRHHPPAMFPKKVAVTGGGGRNPKAAVPEHEPT
SPKVSCIGKVLSDRERARRGRRPAGRMVPAGGCCPGLGGLFRRSHSRKKNAVECVDQSPP
PLPPWASRRGEPKEVKEATPAAAAAMAPGLGGMMRFASGRRAADWATEMEPDGRVARSGP
L*
>11686.m00513|LOC_Os12g05770.1|genepair1316-2
MAAATAAARDLAGDPPESTLLRVGGEVAWPDVYDRDDSLKENTNPKCILKTYGGGGGGNS
SQRFSGNLKPTAAPIIGLSRKLGHGGGGGGGGGGGFRPPAIFPKKAKTGGGGRTPRAAVP
DHEPGSPKVSCIGKVLSDRERARRHRRWSLETRPRGVGGGCCPGLGSLFVRRSRSRKNVV
ECVDDQSPPPPPPPPPTAAALRRREEKVVLMTEEAAAAPAPALGGMTRFASGRRAADWAA
EMEMDGHVARSGPL*
>11687.m00475|LOC_Os11g05550.1|genepair1317-1
MAADGGALKRLFEKPLPENPTLLEALSAWNHTHRKKLVDPASFTEIFGELHFQEKPPVVD
SLARAAARPSPSPPPPPPPPRRTVSWLDITDAATADNDNDKSKDDSSLDALLKPPRPASGG
ATVKRSASFCLKSSTSLLLCTEGLGSESTVDADDMVKDGDGSGAVVDSGMDVDDDASDVA
AAVAGDDAFGAGGKENRPPPPPPSFPPPIRSIGRGGKPSVCFRSVRAEGRFVLMEVVIPG
KDLLRATREGGRLRLQFSNAAAAAAAAVGVIDDEEMHGQEAAACVGGDTFA*
>11686.m00525|LOC_Os12g05890.1|genepair1317-2
MAADGGALRRLFEKPLPVPENPTLLEALSACNHVHHRKKLVDTASFTEIFGELHFQEKPV
DVAAAARFLPPPPPPVRAASWIDVADDKSKDGSSLDALLRPKSSAVKRSASFCLKSSESS
LLLCTEGLGTESTVDADDMVKDGDGDGEAIRRGEEETDGVEEDDGAGREKRGTPTLAPTP
PTFPPPIRSIGRGGKPCVCFRSFRAEGRFVLMEVVIPGKELLQATREGGRLTLRFANAAA
VGGGGEADDDVNDDDVDGGETKNACAARDDMLANNCTC*
>11687.m00479|LOC_Os11g05590.1|genepair1318-1
MGVAASNCGLQRRRRRCPSSDDRLEGELGRNLVGEVVAVPPAGAGDDDDGRGSPWSDLPP
ELAGLVFCRLLSHGDRRRFRAVCSDWRLAAREQVAVTTGPSSSSLQLPPSLPWLALDRRT
YQSLPDGEVHRFADGPGIMVCRGSFDGWLLYHRNGYRDIRSSFLWNPFSGAVLDLPSRCD
DAAGGEPMCFVNAIKRKIVVCSPDLVAAAVEYTSLIFHLPNKHSSWARTNPNICCHDIAF
HHGKLYSINNNDALFVHEFFTTTAADRGGGSARVTASSDWAAVTDARPPREHLGNHGYHL
RFTSYLVASLAGKLLLVRWSLPDELFSGEGGRLAFSLLSNLITVRVFEADMEARRWTEVT
DIGDDQALFVSATCSRALRLPDNNGGGRHGFLRGNRVFIVGSDLGRRCGGGGGGIGCCCC
SCGVYDMSNGRFSTVSLKRWRAGHEQRSRDTSE*
>11686.m00532|LOC_Os12g05960.1|genepair1318-2
MSNDALWCISSHNDGWLMHMDVFKPCIMACDNRHFLKNSFSNATIDIPCRFDRPINAFVV
DNRYSTWPERFTLHKIIVCSPDLIVAASRLIDNNIVSFRPGIDTSWSVLPYDDDDGDHKR
MYEDIALYRGKLYALTSNGDLLVHEIRDNNTLSRAELVIRGAAAEPPLSHLQWQYSIDDI
VHQYRVISCKYLVISCSGTLLMFRCTISPMLGTSANVDDYEIKFKVFEADLEGGQWLEVK
SLDGQVVFLRSLLQGDPLF*
>11687.m00480|LOC_Os11g05600.1|genepair1319-1
MERLLHFRPPKPTSANAVAAAATADGDLLELDVLWPASRAPGLLAALPEEDGKRRRKRGG
SGSGSVAVRSAARPIPETAALTLVPSSSAAAMAKSAPVRIPSEAAAARRGVMWAAQAGGE
DGEAAMVPPHEIVARRAAAHSSVLEGSGRTLKGRDLHRVRNAVLRRTGFLD*
>11686.m00534|LOC_Os12g05980.1|genepair1319-2
MDRLLLFRPRKPASSSSVAAVADGDLLERDVLWPASSAPGLLAALPDDEGSKKKKRAGPA
AVRSASRPVPETAPLTPTAGAARSAPVRIPSEAAAAAGRRGRWAAAQSSVGGEDGDAAMV
VPPHEVVARRAAAHSSVLEGAGRTLKGRDLRRVRNAVLRRTGFLD*
>11687.m00481|LOC_Os11g05610.1|genepair1320-1
MATTRSGVGGAISDPFATPGFRFYPTEEELLGFYLRHRLAGTRPDVERVIPVVDVYGYHP
SQLAALAGEASARDTEQWFFFCPRAERELHGGRPARTTPSGYWKATGSPSCVISSATNRV
IGVKRTMVFYQGRAPTGTKTRWKMNEYKAVADDADAAAAAMLHPMAPPRLRNELGVCRVY
ISTGTLRSFDRRPLDNQAAAPTQQQVMPSLTAAAAVNTNLCGGGGGVVFAGAQGDSSRDC
SSSSGSRELAGGADGSEDDAIDWNSLISSATADDLGFNTVVGFDPSIVGSWPQV*
>11686.m00535|LOC_Os12g05990.1|genepair1320-2
MVMSGGGGGARIVSDPAATPGFRFYPTEEELIGFYLRHRLAGTRADDVARVIPVVDVYGY
HPSQLAAMAGVATAGDREQWFFFCPRAERELHGGRPARTTPSGYWKATGSPSFVFSSSAA
AAARVIGVKRTMVFYQGRAPSGTKTRWKMNEYKAVAAAAADDDHNAAGVAVQLPPMAPPP
SSSACVRLRNELSVCRVYVSTGTLRSFDRRPLDAPPVISHHQPQLQQQQRQLPSSAAAAA
TNGNLIALAGGYECSHDSSGGSSEDAAIDWSSLITAATDSATAAVDFSFNDDIDFSPAAV
GPWAPQL*
>11687.m00484|LOC_Os11g05640.1|genepair1321-1
MALADMAGAAPGQPEASPPPHATQEAAAAAVAKGEQEDEAEMASTRLSLELGKVGIQSSP
PCSSSSSAGHPAMQPAAAATAAPGYGPRPRHMLTEAEKEAKRLRRVLANRESARQTILRR
QAIRDELARKVADLSSQNETMKKVAIRTIKKAAVAEPMDTAPPAQQQAETAAAVAPPTTA
TPPSTAPQPSFLYTAAPAGVAPVPYVWGSWPPCGPTGYEPPPPLCLPPCAWYYPVVADPR
VSSPPTSTYPQSYQEQPTSSPGGGTAEEDTDDDPCSLTLAIDVDKRSAPGAGGSAAGAGQ
HASISDREKATAAAEARKRRKELTKLKQMHGGGGGSRPGGGGEHW*
>11686.m00537|LOC_Os12g06010.1|genepair1321-2
MAGAAVKPAMQLQLQPTAAQEEDEEMATTRLSLQLGNNVGSIIQSSSCSSSGSSAGLPAP
PPPPPPATAYGTKPLHMLTEEEKEAKRLRRVLANRESARQTILRRQAIRDELARKVADLS
TQNESMKKERETVMQEYLSLKETNKQLKEQAQHHLSLSLF*
>11687.m00485|LOC_Os11g05650.1|genepair1322-1
MPPPPPPQPQPTTTATTSANGGKVTPNLTMDAEGTRLLNLTVLQRLDPAVEDILITAAHV
TLYDFNIDLNQWSRKDVEGSLFVVKRNTQPRFQFIVMNRRNTDNLVEDLLSDFEYELQPP
YLLYRNAAQEVNGIWFYNQHDCEAVASLFGRILNAYAKVPPKPKVPSTKSEFEELEAVPT

SAAIDGPLEPSPATTTIVSDAPDESLVNYFSSAASIGSVSNAPMAGRAHPSSESVATPHV
PLIIPSATPTHQIPPPLVGSSAPPLPLHDTNVHTARSANLVTPAFFTPPSSSSTSMVPPA
SSMMPTAPPLHPTSASAQRATYGTPLLQPFPPPTPPPSLTPSYNEGPIISRDKVKEALLR
LVQNDQFIDLVYRELQNAHM*
>11686.m00538|LOC_Os12g06020.1|genepair1322-2
MRPPAPATTAGAGGKVTPNLAMDAEGTRLLNLTVLQRLDPAVEDILITAAHVTLYDFNIE
LNQWSRKDVEGSLFVVKRNKQPRFQFIVMNRRNTDNLVEDLLSDFEFELQPPYLLYRNAA
QEVNGIWFYNQQDCDAVAGVFGRILNAYAKVPPKPKVPSTKSEFEELEAVPTSAAIDGPL
EPSSSSTVLVSNAPDESLTDYFSGAVNVGNVSITPMVGRTHQPIESVASSHVPLIIPSAA
PTHQMGVPSGAASAPPLPVIDTNVHSSHSTNLVTPAFFAPPSSSSASLVAPASSFMPTAS
PFHPTSADAHRPAYGHGTPLLQPLPPPTPPASLTPVHNDEPVISRDKVKDALLRLVQLWL
LFISDVREEIVQLCLDALLM*
>11687.m00486|LOC_Os11g05660.1|genepair1323-1
MDELRAPACGGGVGTVIEDLPTDVLALVLRRLDGASLAAVGCACSSFRELAVDQETWRGL
CLALWPSVRDVLGCGGGGGGDGYRAFFADAFPFPEAAAAVASAAPAPVPGSLPSRLVSAV
DLHHGGVCIMSRVVETDTSSAWFLGSPFRVDALVQEGFSAPSPITPSSLTLSWILIDPAS
GRAMNASSRRPVAVDRKWLTGETVARFTVMLGGGVALDAAVTCDDRYGHVREVSLCVEDG
EGGGVSGQDGLGAVAAAMAGARRGKGAEAAARQRYEEFVKGKRARKEWKARREGIVDLCC
SGVGAAAFVGFLVMLTFR*
>11686.m00539|LOC_Os12g06030.1|genepair1323-2
MASVCSAAAVSLDGAVAGATTTTAIEDIPGDVLSLVLRRLDDGASLAALGCACSAFRELA
ADAETWRGLCLATWPSLRDVDGLDECGGVTGGGGYRALFADAFPLPAEARGLVPSSASLL
PARLVSAVDLHHGEVCLMSRVVETDASSEWFLGSPFRVDALVQEGFSAPAPITPAELTLS
WILIDPATGRAVNASSRRPVSVDREWLAGETVARFTVVLGGGVVALDAAVTCDDRHGHVR
EVSLRAEDGDGGGVSGRGVLAAVAAAMEGARHGRGAEAAAWRRYEAFARGRAARKVKKAR
RDGAVDFFCSGVAAAAFVGVLSTLTLR*
>11687.m00489|LOC_Os11g05690.1|genepair1324-1
MARRLRWHDLVGLGLGGMVGAGVFVTTGRATRLYAGPGVVVSYAIAGLCALLSAFCYTEF
AVDMPVAGGAFSYLRVTFGELAAFLTGANLIMEYVFSNAAVARSFTAYLGTAVGVDAPSK
WRIAVPGLPKGFNEVDLIAVGVILLISVCICYSTKESSVVNMVLTAVHVAFILFIIVMGF
WRGDTRNLTRPVDLAHNPGGFFPHGAAGVFNGAAMVYLSYIGYDAVSTMAEEVERPSRDI
PVGVSGSVVLVTLLYCLMAASMSMLLPYDAIDTEAPFSGAFKGSSGWGWVSNVIGAGASL
GILTSLMVAMLGQARYLCVIGRSGVMPAWLAKVHPCTATPVNASAFLGVFTAALALFTEL
DVLLNLVSIGTLFVFYMVANAVVYRRYVAADDDDADHRRAWPTLVFLAAFSLVALCFTLL
WQFAPAGRARTGLLAACGAAAVATVGAFRALVAEARRPELWGVPAMPWVPAASVFLNVFL
LGSLDRPSYVRFGFFTAAAVLVYVLYSVHASYDAEEGGGAGAGAAALDGAKVQDEAADFK
V*
>11686.m00542|LOC_Os12g06060.1|genepair1324-2
MARALRWPDLVGLGLGGMVGAGVFVTTGRATRLYAGPAVVVSYAIAGLCALLSAFCYTEF
AVDMPVAGGAFSYLRVTFGELAAFLTGANLIMEYVFSNAAVARSFTAYLGTAVGVDAPSK
WRIAVPGLPKGFNEVDLVAVGVILLITVCICYSTKESSSVNMVLTAVHVLFIMFVIVMGF
WRGDTRNLTRPADPEHNPGGFFPHGAAGVFNGAAMVYLSYIGYDAVSTMAEEVERPSRDI
PIGVSGSVVLVTLLYCLMAASMSMLLPYDAIDTEAPFSGAFKGSSGWGWVSNVIGAGASL
GILTSLMVAMLGQARYLCVIGRSGVMPAWLARVNPRTATPVNASAFLGVFTAALALFTEL
DILLNLVCIGTLFVFYMVANAVVYRRYVAAAEDDEEGRRRGAVPTLAFLLAFSLVALCFT
LVWKLAPRGGARTGLLVACGAAAAAAVAAFRALVPQARRPELWGVPAMPWVPAASVFLNV
FLLGSLDRPSYVRFGFFTAAAALVYVLYSVHASYDADEAAAALDGAKVLDEGCKV*
>11687.m00494|LOC_Os11g05740.1|genepair1325-1
MAMNHPLFSQEQPQSWPWGVAMYANFHYHHHYEKEHMFEKPLTPSDVGKLNRLVIPKQHA
ERYFPLGAGDAADKGLILSFEDEAGAPWRFRYSYWTSSQSYVLTKGWSRYVKEKRLDAGD
VVHFERVRGSFGVGDRLFIGCRRRGDAAAAQTPAPPPAVRVAPAAQNAGEQQPWSPMCYS
TSGGGSYPTSPANSYAYRRAADHDHGDMHHADESPRDTDSPSFSAGSAPSRRLRLFGVNL
DCGPEPEADTTAAATMYGYMHQQSSYAAMSAVPSYCRHGWGGFRKHQVEKERGIEETGRE
LCNYAMLPFVAIQ*
>11686.m00544|LOC_Os12g06080.1|genepair1325-2
MAMHPLAQGHPQAWPWGVAMYTNLHYHHHYEREHLFEKPLTPSDVGKLNRLVIPKQHAER
YFPLGGGDSGEKGLLLSFEDESGKPWRFRYSYWTSSQSYVLTKGWSRYVKEKRLDAGDVV
HFERVRGLGAADRLFIGCRRRGESAPAPPPAVRVTPQPPALNGGEQQPWSPMCYSTSGSS
YDPTSPANSYAYHRSVDQDHSDILHAGESQREADAKSSSAASAPPPSRRLRLFGVNLDCG
PEPEADQATAMYGYMHHQSPYAAVSTVPNYWGS*
>11687.m00497|LOC_Os11g05770.1|genepair1326-1
MAKKKGFTSIFSRLLVVTGGGDDERGGGAAATTSPSPPPWPWPSCGNRPRTVSSRRDGGC
STSAAAAASAAAARRIAGEMYKTVNSVYFDYSAADGYSCFDDDDGRVVDDDDDGDSFSTT
TASEEWSEAVIRSLGRTSTDRFFFDPGPPTNSILAAAAVPETKPVAAAAVFHDEEKEKLP
EPPASLVEESVAVAVESEDPYSDFRSSMEEMVAAHGLRRWDALEELLVWYLRVNGKHNHA
LIVAAFVDLLVGLAAAATTTGTTPTTTTTTSSGRSTASTSTACDITTTTTITTSSATATM
EPCPCGGGSSDDLEEEEEEARVMISLGASSCSLAPSTFANS*
>11686.m00551|LOC_Os12g06150.1|genepair1326-2
MGKKGGLTSLFSRLAVAAADSPSCAKNPPHTASFRGFYYVDEPCTTAGGGGGGRSPAAGR
LRKGGDEMYKTVNSVFFDDSADAAHAVADGCAFSGEDDDDDDRFSTTTAADEEWSEAVIR
SLGRRTSTDRFFFDAGPGRPAATNSILATVRPRRPPPPPPPPPPAEEEKEKAAAEAAQLP
GKSSSTSSSQLVEESVAVAVESEDPYGDFRASMEEMVAAHGLRDWDALEELLSWYLRVNG
KHNHPLIVAAFVDLLLALAAVPSSSSSDTTTTTTAAATTTTSDTSCSTASTSTTSNGATS
VTAAATAAEQCGGGGGGGDEEAGCSSSSSCCAASDHDHEEVSAIS*

>11687.m00498|LOC_Os11g05780.1|genepair1327-1
MARRGGGGGGGGGRHQFPVGRRRRHVPVVDSGCGCRPRRPRLLSLASFIWPSSMSSACKQ
AAAAAVAVPARVGGGGSKSKSSPYYFRSSSSAASFSSSSAATTYSTASYYSSSRGGGGGA
KKKKQEEEPPYLAPPKGKAAAKSPSRRKKKTAEEDDGGGGVGVAVEKESSDPRADFRESM
VQMVVEMGLCHWDDLRSMLRRLLALNAPAHHAAILTAFAEVCAQLAAPSPPPPPYGGHHR
RS*
>11686.m00552|LOC_Os12g06160.1|genepair1327-2
MSTAASRARRGGGGGGGRHFPVGGGRWRHVPVVDTGCGCRPRRPRQLLMSLPSFLRPSAL
KPPVPRSTSSHSSSFFPSSASTASFSSSSAATYTTTYSSSSATNQYPYYGKAAAAAAAPV
TVTTNNHGKSSSSSSSSSAAAAARRRPSRKKRYEKMAAAEEEEDVGVAVEKESSDPRADF
RESMVQMVVEMGLCGWDDLRCMLRRLLALNAPRHHAAILTAFAEVCAQLAAPPAPAPPPP
PQPTAAYHYHYYHY*
>11687.m00500|LOC_Os11g05800.1|genepair1328-1
MLGELISKVLLVLFGYAMPAFECFKTLETRPDDAHMLRFWCQYWIIVSMVIACESFVSWM
PMYGEIKLAFFVYLWYPKTKGSDVVYDSFIRPTVMQYEPNIEQRLEHLRANSGQLIAFYI
KNFADKGTAFFMDFLRYVVSERPEAAANSEPQRSSWSSWNPFASRRQEPSPPPSAPPRER
RFSGADPDDEPPAIADVFRASLGGGAMNRRPHNNNNN*
>11686.m00554|LOC_Os12g06180.1|genepair1328-2
MVIAFESLISWMPMYGEIKLAFFVYLWYPKTKGSDVVYDTFLRPIVMQYEPNIEQRLLHL
RAKSGQLLSFYMKNFADKGTAFFMDVLRYVVSDKPEGSNQEDSDAQPAAAACSVVGKKNH
PAGNVGGQFLPDDPQQRNKKSGGWSPFATKRRPPSPPRPPQESLFESNPEAAAVAEVLKA
TINPRPRRGAQNGKNYY*
>11687.m00501|LOC_Os11g05810.1|genepair1329-1
MITAAVAAVVESASPSPSPARKRCRLGGPVVANGEGGAASNTTTSSEFQLRHWRPAAAGK
RAGLGMRRRWAPPEIEIPGGGSGVGVGARGYTSLRDILSSPEYAASSKSSSPADGGSGGG
GGGGGGGGDVHMIRHPLVKHAAYAYLQLTPSARDAADAAGRRRRRSRGPLCRLLLGCLGF
VGALFGR*
>11686.m00555|LOC_Os12g06190.1|genepair1329-2
MPAAAAAAATGMTESSSPSSSPSPPRKFRRVRSPLANGGAAGDFELRHWRTPPKRARSSA
APPWAPPEIEIPCGGGEAAGRGGGYTSLRDILMSPGYAASCSPAACGGGGGGGSCGDIHM
IRHPLVKHAAYAYLQMTPSARDDPGRRHRRRWRGPLCRLLLGCLSFIGALFRP*
>11687.m00508|LOC_Os11g05860.1|genepair1330-1
MGKDCGNHGDDDIRQACRRLLTILFGLALIVAIIALIVYLVLRPTHPRFFLQDATLRQLD
LSNSSTSGVLSTALQVTVASRNPNDRVGVYYDRLDVYASYKYQQITLAASLPPVYQGHGD
VDVWSPVLSGPDVPFAPYLGDALAKDVAAEYLILQVKIDGRVRWKVGSWISGHYHLFVTC
PAFFIASGGNGYPGANGLKFQTATYCRVEV*
>11686.m00563|LOC_Os12g06260.1|genepair1330-2
MGKDCGKHGDDDFRQGCRRFITVLVVLAILVGIIALIVYLVLRPTHPRFYLQDATLRQLD
LSNSSSTAGGVLSTTIQVTVASRNPNDRVGVYYDRLDVYASYKYQQITVAASLPPVYQGH
GDVDVWSPVLAGPSVPFAPYLADAISQDCQAGYLILQVKIDGRVRWKVGSWISGHYHLFV
TCPAFLVTAGGNGSPGASGFRFQTTTYCHVEV*
>11687.m00524|LOC_Os11g06010.1|genepair1331-1
MAVGDALRRLCEEVGWSYAVFWKAIGAADPVHLVWEDGYCGHASCPAGSDPSEALPTDVG
CAAAADTMTMCSLVNKVMASQVHVVGEGTVGRAAFTGNHQWIIHGTANDHGIPSEVAAEM
SYQFRVGIQTIAIIPVLPRGVLQLGSTGVIISKPIFHAIVNSECAGAKLWQLPVISSSEK
QIKNSSSQQGRSRPLHGASNVQSTENRSKLFSQFPVTCEQYNHPDTMAVSGSTSLNACMN
GSLLKIAQLNGQAVREHIVYSKPDVRFIQQVYRDGQLGSNAQSIAMSSDLISSSLRSVQK
QPLLMNNISQLEYGDGAETSADLRKNVLLKPPVCLDPFIHDRNINISHGITEVSNVINDH
GNFDFLSGGARVVRANLCTSATSQVLDRRSHSVSGMLLHREPIVSCEVPQSSEFSTKMGS
LERGSFQISSAPSSESDVQISNGLNTSISRENQLSVSNHICQDQKINGVNDLSATLSTER
MNNMDGCKPPGLSLERTSPLFMEQSVENDLFDILGPQFHHLCHNAGADLVPWTDAKPESS
DRDVPESSIHADSAPLFSSRDNELYSGIFSLTDTDQLLDAVISNVNPAGKQSSDDSASCK
TSLTDIPATSYLCSKEMKQCGSSGVPSVLIKNESAQFIKQPCLAENAEDGCLSQNNGMHK
SQIRLWIESGQSMKCESASASNSKGLDTPSKANRKRSRPGESPKPRPKDRQLIQDRIKEL
REMVPNGAKCSIDALLEKTVKHMLFLQSVTKHADKLKDSTESKILGNENGPVWKDYFEGG
ATWAFDVGSQSMTCPIIVEDLDRPRQMLVEMICEDRGIFLEIADFIKGLGLTILRGAMEA
RKSKIWARFTVEANRDVTRMEIFLSLVRLLEPNCDSSGAAENANNVNMPLGLVHQPVIPA
TGRIQ*
>11686.m00571|LOC_Os12g06330.1|genepair1331-2
MAVGDALRRLCEEARWSYAVFWKAIGAADPVHLVWEDGFCGHASCSAGSEASEAGCESGG
AVCTLVRKIMASQVHVVGEGTIGRAAFTGNHQWIVHETANDHGLRSEVAAEMNNQFRAGI
KTIAIIPVLPRGVLQLGSTSVILENISSVQQYKKLCCQLNNRSSMVASASAKNDLSQKVQ
SRSLHGLPSIHPYEQCYGHDARALSSSTSANTGRNTSLLKVAQRNDQAIREQVLYAPDMR
FRQQLPYSDRRVDINTHSSAMSSGFISSISASVEKYPLLTNNIGQVEHGNMEESSGPRNV
LLKSLSCRNPVVHENTNTSLFHGGDEVPAFLNSHGSFDFLQAGPRVVEANLYNNGTSSQV
LDQRCSSTSGMAGYKPSVSYKFPHSAQFIVKMENPRRQSFQDPAAPSSGSDVQVSSGLKT
TTRQFNPEHMCQNKKTNEVNDSSAAVSTQDVKNMDRHKILDISNERTSSFVMDPSTENDL
FDIFGTDFHQLHRSLDGDLSWNTAKPQSSDRDAPESSIYLDSSPAFGAQEDEFSYSGIFS
LTDTDQLLDAVISNVNPGGKQISGDSASCKTSLTDIPSTSYCGSKETKQCKSSGAPPLLI
KNELAVSNFVKQPCFLEKAEDGCLSQNNGVQKSQIRLWIESGQNMKCESVSASNSKGLDT
ANKANRKRSRPGESPKPRPKDRQLIQDRIKELRELVPNGAKCSIDALLEKTIKHMVFLQS
VTKHADNLKDSNESKIHGGGENGPLLKDYFEGGATWAFDVGSQSMTCPIIVEDLDRPRQM
LVEMLCEDRGIFLEIADFIKGLGLTILRGVMEARKNKIWARFTVEANRDVTRMEIFLSLM
RLLEPSCDGGGGGVGDNPNNVKIPPGIVQHPVIPATGHLR*

>11687.m00525|LOC_Os11g06020.1|genepair1332-1
MAAYYHGGAGTDIQSGTDGLQTLYLMNPSYAGYGDAAAAAAAPGAAANMMLLNSAVTSMT
PVSFGHQPSPSSSSAAQHFVGIPLQAPPASGYNLWTPAAATGAGDMSPPTPQHQHQQAHG
GGAAGVSAVLSLSSREAAPPVTVAAVVAAGDEGKYLQAVAQGAASHGQMVMSSKYLKAAQ
ELLDEVVSVSKGVDDVKAAAAAKSPASVKKKEDSEGVSGGGTEDGGGAKSGGAPPPPEMS
TAERQELQMKKGKLINMLDEVEQRYRQYHQQMQVVVASFEAVAGGGSARTYTALALRTIS
RQFRCLRDAIAGQVRAASRALGEAVDADGGCGRTVGSRLRYIDHQLRQQRALQQLGMMQS
SAWRPQRGLPERSVSILRAWLFEHFLHPYPKDSDKIMLAKQTGLTRSQVSNWFINARVRL
WKPMVEEMYLEETKDQDGGGGAGAGDEGSKPGGSKGGGAGVNGGVVDSAAKMDSKAAHME
SGGGVHPSLLELAGDHQAQAGFYDDDDEDGGAAAALQQKLKKARTEEQQQAAFHVSDVAT
LHAHAAAAAAAARHDEVSHRELLMKFMESGSAGAGAGAAARDHHHEHHGGVGYSLFAPAPY
GQFATEQFAFAGHGGGGGGGGVSLTLGLPHGAEQTASFLMTSSNGSDGAGHVAGGGGYDM
NMQSTKSFAAQLMRDFVA*
>11686.m00572|LOC_Os12g06340.1|genepair1332-2
MAAYFTGGGAGTDVVQAAGTDGLQTLYLMNPSYVGFTDAAAAPGGGAAAANMVFLNSAVS
TLTPASFSHHHQPTPAAQHFVGIPLQSGYNLWGPDATGGNDVSPPRHGAQQQAPAAAGTS
AAAVSPVLSLSSREAAPPVTVAAAAAAAAVPGGTDQEKVVMRSRYLKAAQELLDEAVSVS
KGAATAVKKKEDSEGGVSGGGGGAEDGGGSKSGAAAEMSTAERQELQMKKSKLLNMLDEV
EQRYRQYHRQMQGVAAAFEAAAGAGSATTYTSLALRTISRQFRCLRDAIAAQVRAASRGL
GEDCGDDEGGGGGGRTTVGSRLRFIDHQLRQQRAMQQLGMVHAAAAGGRRRRRVAAAAWP
PRARRLRPPRLALRAFPPPIPQGFGQGHARQANRPHQEPGTYVYVYVQYYVYVCLKYVYV
FVGVELVHQREGEAVEANGGGDVRRGDQGQGGGGGGARRGGGRRRRPWRRGGAGSE*
>11687.m00537|LOC_Os11g06130.2|genepair1333-1
MNVLCEVCGDVGWEELILHCNKCKNATRHQYCFDPVIYDGSLVEWLCDDCLPNGNEVGNL
LDISNQKKSSQTELGFSITKETNVKKMKLTKGLWSWGHHRNRSFKARCDGSDSKTKHFAS
GNAFSSSEVVTGEISKINDCEMEGRGKNEYSSHSALDHASRVEQHINIQNPMGIIKPSLN
SIKRLNLSNEKDDRFSSSDHVEGSIPQVNHVERAHLMIEDGSNPTFTCVEHMDLVHKKQL
LQPSSLERNSIGTSIPCSENMDVLHKMQLLKPSPLDKKYVDRSIPNSENMDVVLMRRSCT
LNNSMGSCEKQVVTKVDPIEPSRQFDRACLEVSSNAHEIHEADDGSKGAQSIKNGKPKKQ
RRLILPYEEDKDAEPIQVDDMNRQSCGINGQVKKPVEIVASLGDINAGCGQNVCSQLALP
TIAVKGQCGLSSTPFIPKYFCVQPIDEPNWTYVSFPPFHSDDLYNVFLCAANIFVLYVDS
GIMKIGTNYIPVGAHFSNKACKKVCELSMSLPQIMKVTELPKLKAWPKSWEKASVPSAES
IGLFFFSQNTRSNKEFDDLVKHVIDYDIVLETDVSFAKLLVFPSVVLPAEYRVFQGKHYL
WGVFKRSKDMAERDALVEQNCTTCLADEDVPEQNALDIVPCKALDQEMALVVSDIHHDNQ
PSLTTSQVVESEASSDKGPSHPVINSPERPMYLILDTSCKVLKKWSCERMDTKLSML*
>11686.m00587|LOC_Os12g06480.1|genepair1333-2
MALCPDCGAITVCEVCGVLGYKNLLLSCKNCNGAAVHRYCLDKVDFDGTVDWSCDECHPR
HGKGTNRRSLEVTLDDKTVVGKQPENQSSLDTNHDKPGMNGGNDDYVSDDLMLERNKERF
QLHDEANNDIHLKSMSANVPRSSTLHENSVARNVSSSANTGLPMDSNCVPSAHIDIGNPR
DSSVRLILTGENRRESSMLLDGACSGSLSKDSSKEKIDREANSSHMEPSDAVKNFCKDNP
MKRRKLMLLDDDDDDVGVELSDTVQNVVKDNPSKRRKLILPDDDDVEVELCNTVQSIAKD
NPGKTIQLIVLDDDDDKQEDAENLNHLSLEFEGPIEKHKIHIGYATGQRCLEDDEHGLLD
SLDHRSLENTCPTKKRRRYICPSDDEEEEVIKGSTTTGCAPNDVANTASQHVDAKDHHLQ
SRMAFASDFTKHQYYIYSEPMGEPAWSGIFMTDSNVPIMLAAHLSTKACQRVSEFARSLQ
PVVEVIKLPRLKAWPERWDKSGPTDDSIGLFFFPHSMRPNEELDKLVKEVIESDVVLKAV
LGTVELLIFPSILLPEQYHEFQGKYYLWGVCKARKHNPDTAVLVEEQCGLVSASEEGSSD
KESYVMKHVEDRLPADCNPEAREGDIKTALGEGCFSPDSCLSSNKASPVKGGSPCFMQPG
LGDKPHEPGVADQKEDEQDFTSLPRRNDQNATNPPIDSLPSATRLFGFVTARSERCQQLI
QEMVKEGALLFSVPEDMTINRSTISKSNGVGAAQAPDSGCQHVQERCEPIEFVPIDHNDP
DSASEACLDLFPVRHEQIGLTSQVDVKEVELDLSLGAFRRAPSELP*
>11687.m00539|LOC_Os11g06140.1|genepair1334-1
MMPPKPAAEDVADEQPEPPDEDPDVAEADPTGRYLRYREIIGSGSSKTVYKAFDAVDGIE
VAWGKVEINERIMGSSKELQRLRTEIQLLKSLQHKHILKLYASWVDTNRRTVNIVTELFT
SGNLREYRTKHKKVDMKAMRRWAKQILTGLEYLHSQKPPIIHRDLKCDNIFINGNHGKVK
IGDFGLAMVMQQRKTRSIQGTIEFMAPELFGENYNELVDIYSFGMCMLEMVTCECPYSEC
KGFIQIYKKITEGVKPAALSKVKDAEVRGFIESCLASVSDRLPASELLKSPFLQSDDANH
RSSNSVQEPVKFPENNFTKDEPIFVSLAPNNGTVNGKEQSFILVLQKSDFLLEGNMSTTN
PVMLFLRFPGPDGKFKNVQFPFDMEKDTSLSVSTEMVEQLELPEWNNPVLAELIDAFLLH
ILPSWKPCVKVGKMLPSSS*
>11686.m00588|LOC_Os12g06490.1|genepair1334-2
MDLVEAEAEEQPPDEDGDEEGYVEADPAGRFIRYDEIVGSGAVKTVYKAFDKLEGVEVAW
SQSRIDDSVMGSSKKMKQLNTEIQLLKTLKHKNIEKMFASWVDGEKKTVNIITELFTSGS
LTQYRRKHKKVNMKAMKRWAIQILTGLEYLHSQKPAIIHRDLKCDNIFINGNHGKVKIGD
FGLATFMQQQKKSIKGTLEFMAPELLTGHYNELVDIYSFGMCMLEMVTCEYPYSECQGMA
HIFKKIDEGKKPAAFYKIKDAEVRSFIENCLAPVENRMSATELLKSSFLQDDDLISVSLV
KNMSEDGQQPVSCMLRKGEFLLTGNVDVASHVDLWLRFPDPSGCFKSVEFPFNLTEDTSL
SVAVEMVEQFGLTQDSRPIIAQLIDAFLVILIPEWTPCVAIRQVVSEGANGLTIEKR*
>11687.m00542|LOC_Os11g06170.1|genepair1335-1
MDPRFPPPAPSGGAPPRGHHRRAHSETFIRLPDADLLLDPDGEFGFSDLDFPSLSDDSPA
ASDPTPPPPPPALPQAAPRPPGGAHLRSLSLDAAFFDGLAFQGGGGGGGAGSGSSGGGAG
HKRSGSMDGESSLFEGESAPPDYAKKAMPADRLAELALLDPKRAKRILANRQSAARSKER
KIKYTGELERKVQTLQTEATTLSTQLTLLQVCIIAQACGASATTTIKVRLVLYLIS*
>11686.m00591|LOC_Os12g06520.1|genepair1335-2
MDGRFPLPPASGGGRGHHRRAHSETFLRLPDADLLLDPEGDFSFSDLDFPSLSDDSPAIS

DPTPPPPPPMAATPAPAPRPPGGAHMRSLSLDTAFFEGFSLQGGGGGGGGGGGSGGSGGHK
RSGSMDGVNSPFEGESALSGGLPDYAKKAMPAERIAELALIDPKRAKRILANRQSAARSK
ERKIKYTSELERKVQTLQTEATTLSAQLTLLQRDTSGLTAENRELKLRLQSMEEQAKLRD
ALNEALREEVQRLKIAAGQAPNMNGNPFNGGLQQQIPPYYTQQQQQQQMPYLGGHHAQQ
RHPSHQQSSSNGGQSLSGQSLNDSMDFM*
>11687.m00544|LOC_Os11g06180.1|genepair1336-1
MVLDSNELHQHDAPDVDVSINCDDRIFGQESVNLAAIQEELLEEDSLSDLLLAGAEAVEA
GDSILASVAFSRLDDFLSGIPENGAASSFDRLAYHFDQGLRSRMSSASTGCYQPEPLPSG
NMLVHQIIQELSPFVKFAHFTTNQAILDAIIGDMDVHVVDLNIGEGIQWSSLMSDLARCG
GKSFRLTAITTYADCHASTHDTVVRLLSEFADSLELPFQYNSICVHNEDELHAFFEDCKG
SVIVSCDTTSMYYKSLSTLQSLLLVCVKKLQPKLVVTIEEDLVRIGRGVSPSSASFVEFF
FEALHHFTTVFESMASCFIGSSYEPCLRLVEMELLGPRIQDFVVKYGSVRVEANASEVLE
GFMACELSACNIAQARMLVGLFNRVFGVVFKKISLLMVYYISLGKNDLREPNKVIWSSLA
AGCGSHGIVVLAFYAADKLLEFKPKGIETYIHPAVERAHFHKEDRMWQEYEN*
>11686.m00593|LOC_Os12g06540.1|genepair1336-2
METMSYPCSLLIPFSTQFEEISSSSLLLWSPQAEENPHENANMYEFDADHSHDQIHQDHQ
FLDMMVIQESANEFDGNHSHDQIHQDHEFLETMVIQESANEFDGDHSHDQIHQDHEFLEM
MAIQESANDLLQLQDDFSVPNADPLAASFEFDERLAVAGHENGNVVATQEESAGDLLLAG
AMAVDAGDAVHASAIMSRLDDLLADIAGRRSCEATSPVDHLAYYFARGLKLRISGAATPA
SSPPPPAANWSSPAYRMLQELTPFVKFAHFTANQAILEATADDLDVHVVDFNVGEGVQWS
SLMSDLAAAGRHRCSTSRTLKLLLLGTITILQPKLVILIEDELSRISKNPPSPSLAAPPP
FPEFFSDAVAHFTAVMESTASCLVSYDDEAWLSLRRVGEEVVGPRVEDAVGRYGSLAGGA
QMMEGLRAREVSGFSVAQGKMLAGLFGGGFGVVHQEKGRLALCWKSRPLISVSLWCPK*
>11687.m00567|LOC_Os11g06390.1|genepair1337-1
MADGEDIQPLVCDNGTGMVKAGFAGDDAPRAVFPSIVGRPRHTGVMVGMGQKDAYVGDEA
QSKRGILTLKYPIEHGIVSNWDDMEKIWHHTFYNELRVAPEEHPVLLTEAPLNPKANREK
MTQIMFETFNVPAMYVAIQAVLSLYASGRTTGIVLDSGDGVSHTVPIYEGYALPHAILRL
DLAGRDLTDSLMKILTERGYSFTTTAEREIVRDIKEKLAYVALDYEQELEAAKSSSSVEK
SYELPDGQVITIGAERFRCPEVLFQPSFIGMEAPGIHETTYNSIMKCDVDIRKDLYGNIV
LSGGSTMFPGIADRMSKEITALAPSSMKIKVVAPPERKYSVWIGGSILASLSTFQQMWIS
KGEYDESGPAIVHRKCF*
>11686.m00606|LOC_Os12g06660.1|genepair1337-2
MTQIMFESFNVPAMYVAIQAVLSLYASGRTTGIVLDSGDGVSHTVPIYEGYALPHAILRL
DLAGRDLTDALMKILTERGYSFTTTAEREIVRDIKEKLAYVALDYEQELNAAAAAKNSSS
VEKSYELPDGQVITIGAERFRCPEVLFQPSLVGMEAAGIHETTYNSIMKCDVDIRKDLYG
NVVLSGGSTMFPGIADRMSKEITALAPSSMKIKVVAPPERKYSVWIGGSILASLSTFQQM
WISKAEYDESGPAIVHRKCF*
>11687.m00577|LOC_Os11g06440.1|genepair1338-1
MPATDYQGSSSTHSHPSPFSSFGRSLLSLRRDSPAAAAGASPAMASGEEADLEAFQRHVA
AHLADLRGGEDELLSIEWIRRLLEAFLLCQEEFRVVVALARRRGALSAAAEKMVGDFYER
AVKALDVCNAARDGVDQVRRWERLAGIAASVLLAPGEIHEGQLRRARKALSDLSVLLIDD
AAAAAGGGGVASFLSSHRNRSFGRARASPSRSATLASSSSSSSSSSSSHFRSLSWSVSR
NWSAARQLQAIGSGLAAPRAHEGGLVAPVYSMGCLLHLAAWALVAAVPCPDRAAALQAHH
LPAAPPRAAFPWAPPLLALQERLTEEGKRKDRRNSCGLLKEIHALEKCTQRLAEAIDAAP
VPLSGEREAEVREAAAELAAVCAAMRAGLEPLERQVREVFHRIVRSRMEGLDSPMLNAD*
>11686.m00619|LOC_Os12g06780.1|genepair1338-2
MPVTEHQGSSSSPSTFSFGRSLLSLRRDHVAMPSGEEADLEAFQRHFAASLGELLPGEVE
GGGGGGGGGGGGGGEEILSVAWIRRLLEAFILCQEEFRVFVAQARRRGALPAAGEKLVVE
FHERAVKALDVCNAARDGVDQVRRWERLADIAASVLLAPGEIHEGQLRRARKALSDLSVL
LVDDTAASGSGGVASFLASHRNRSFGRARASPSRASFAGATSSSHFRSLSWSVSRAWSAS
RQLQAIGAGLAAPRAHEAGLAAPVYAMGCVLHLVAWALVAAVPCPDRSTALQAHHLPAAP
ARAAFPWAPPLLTLQERLAEEGKRKDRRNSCGLLKEIHVLEKSTQKLTDAIDAAPIPLFG
DREADVREAAAELAAVCAAMRDGLEPLERQVREVFHRIVRSRVEGLDSSMRNAD*
>11687.m00603|LOC_Os11g06690.1|genepair1339-1
MDFFLTVTDSSIGPPGAAQYANGLLGLDARGAAHCWKTRIGRGEGGKWVAARRRGGAAGR
GGRWPISPALVERSGVGEASPAAGRRRGGWEAVMETAHEVAIYIDRFHNLDLFQQGWYRL
KISAAWEEDEYRAPVSPARVVQYEVPDIGSKGAFGLWKIDDVDNSFYTQPFQIKYSRQDI
YLSVMVSFYIPNTEDEGPATSSVILKFELIYVPTLEDRTEIEDPSDIYVPVHEFRIPYKA
LLGLHAYFPVHFDAFHPVLVDLTMHIVYLKAGVTKSSQKASEQGLCSKSCYIIKALLSSR
EILLEEVMKMSAGIGKTLEDLDDADLTLGKHEPIDSSKAGLPKYSKGLYIPTKCIGHLTG
VLHDLIERSDNVVRSTNDILLYTLSKEDLLELFQAPVQNSSSRAELHRRSIAQMKVGSQS
MLKTETICITPKKRILTQIILQINARFIQDMQIYANPSEIPVVHIEQHVMVVPQHGSSKR
LGHHLDLRLIRNQWILCDPGAECLLSQTNEDRTCGDFKEMGRRLSNEVVAFLKRKIDRYS
RNGGCKDLKLSFVGHSIGNIIIRSALADPKMQPFLKNLYTYMSISGPHLGYWYSSNSLFN
SGLWLMKRLKGVQCMHQLTFSDEQDPQNTFFYKLCKLNTLDNFKNIILVSSPQDGYVPYH
SARIDSCPASSSDNSKKGQVFTEMLNNCLDQLRAPSSETRVFMRCDVSFDQSSHGRRRSL
NTMIGRAAHIEFLENDLYARFIMWSFPDFFR*
>11686.m00621|LOC_Os12g06800.1|genepair1339-2
MFGRMRCLVGGGVEDSPRGAVRRVSPALRRVHNANASAAAAAGAEGKSGLPFRSPDVMET
VHEVAIYIHRFHNLDLFQQGWYQMKISATWEEGGSKTPASPARVVQYEASDVGADDALGI
WKIDDADNSFYTQPFRIKYARQDIYLSVMVSFNIFNSEEEGPAASSVILKFELIYAPTLE
NGSDIQASSATSSAAVHEFRVPRRALLGSHSYCPVHFDAFHSVLVDLTLHIVYLKAGATK
SSLKIPDQGLGPTSHHIVKALLTSREMLLEELKKISDAIGKTVEDLDVADLSLGKYEAVQ
PAKSGLPNSNKVFPATTKGVGHLAGILHDFLEKPNSAVDGANDAMLYTLPKEELLELFLT

VSSQLSLLWNAFLKFHRINKTKILDYLRDIWALDRKSEWSIWTVHSKIEIPHRYLRSTDD
ESSHRHSLLRVSGSRKFHDDPVQNSASRAELHRKSIAQMKINTLSVQDMQIYADPSRVPV
VLIEQHVMVVPQHGSSKDLATNSSEQKDTIVLPKLQGDSLALKSSAGKKGRILRAVIFVH
GFQGHHLDLRLVRNQWLLLDPGAECLMSEANEDKTSGDFKEMGGRLAGEVVAFLKKKVDK
LAKYGGCKELKLSFVGHSIGNVIIRTALAEPALQPYLKNLYTYMSISGPHLGYWYSSNSL
FNSGLWLLKKLKGAQCIHQLTFSDDQDPQNTFFYKLCKLKTLENFKNIILLSSPQDGYVP
YHSARIELCPAASSDNSRKGQVFTEMLNNCLDQMRAPTSETRIFMRCDVNFDQSAQGRNL
NTMIGRAAHIEFLETDIYAKFIMWSFPELFR*
>11687.m00604|LOC_Os11g06700.1|genepair1340-1
MDRIPPQKSSSFSPATFREERLGRNLSLGAIKISEHAPAVRVKEEAEEGRGGVDNAGAGE
GEGAAGEEAAPPEEAAEPDLAMLSAEVDAFLAGREGDAPTSISEVTLDKFASAVEQEMAQ
SEGDDDKWAVGENGEAAPLLAAIRRISALAAALTAVPEGSKFTIGVHRVTGVLHRAMAFV
EDEFHTMLEDPRVAKAAQNGDTRSATGKSMRRGPSFNHAGGDPASDGGGGGGGGDTPPP
FPPETVDRLRAMADAMIAVGYMTECTQVFLVARRNALDASLQNLGYEKASIDDVVRMAWE
SLESDVATWIKAFHHTINVGLSAEHDLCARVFAGCDAAVGRAIFVDLARCAMLQMLNFTE
AVAMTKRAAEKLFKVLDMYEAVRDAAPVIDAFIAACSTTDAAADEPDTTTDALTDIKTEL
ASVRSRLGESAAAIFCDLESSIRADAGKQPVPGGAVHPLTRYLMNYLKYACEYKNTLEQV
FHEHHRTDIDADDEGSDPFAAQLMEVMELLHDNLEAKSRLYKDPALCSIFLMNNGRYMLQ
KIRGSPEINAVVGEAWSRKRSTDLRQYHKNYQRETWSRVLTLLRDDGVITVKGSVQKPVL
KERFKQFNAAMDEIQRTQGAWVVSDEQLQSELRVSIAAVVVPAYRSFLGRFSQSFSAGRQ
AEKYIKLSAEDLEAIIDELFDGNAVSMPRRRN*
>11686.m00626|LOC_Os12g06840.1|genepair1340-2
MPLRRRDSDSRSSHHAPHKSSSFSQPSARDDKPRDAIDRNLSLGSAGHHHHHDGRRLDLH
HPPPAGDAIKEEYEEEERGGAGAPCGGGDGGGGDGGGGGDGGGGDGGVPDLAALSVEIDA
FVAAGQDGGEALSDATLERFAAAVEMEIAQSESAVDKWATGANGEPRALLAAISRIAALA
AALAKAPEGKHATAGAHRVTAVLHRAMAFLEDEFLALLDDPRVPKATTFDQVQHEVDRCV
LPASVDVGAGVGESAPPYPPETVDRLRSMADAMVTAGYVTECTQMFLVARRNASDASLRA
LGYEKASIDDVVKMTWEALEAEIATWTKAFRHTINVGLSTEHDLCARVFAGRHAAVGRGM
FADLARCVMLHMLNFTEAVTMTKRAAEKLFKVLDMYEATRDASPVIDAFLTADDGNNSTA
LTDLKHELNSVRSRLGEFAAAIFRELESSIRADAGKQPVPGGAVHPLTRYVMNYLKYACE
YNSTLEQVFREHGAHGGGGGGDGENPFAAQLMEVMELLHGNLEGKSRLYKDPSLSNIFLM
NNGRYMLQKIRGSPETNAMLGEAWARKQSTNLRQYHKNYQRETWSRVLGLLRDDGVLTVK
GSVQKPVLKERFKQFNAAMDEIQRTQGAWVVSDEQLQSELRVSIAAVVVPAYRSFLGRFA
QTFSAGRQSEKYVKLSADDVEAIIDELFDGNATSMTRRRT*
>11687.m00607|LOC_Os11g06730.1|genepair1341-1
MALAVKLAVLLLLAAAAAGGSSTTTVPPLEERLGAAFDGMAAAAEGGGGGGWMMECWSAV
TKLGSCTNEIVLFFVNGESYLGPDCCVAIRTVTRRCWPAMLASIGFTAQEADILRGFCDA
ELAAPPPPSTNASSAAPAPAPASA*
>11686.m00643|LOC_Os12g06970.1|genepair1341-2
MASLLSVAVVLVVVSAQALAAVAVADAARVNAGAAAFSPAVPLGGRLDGGGGGLVECWSA
VAELRSCTDEIVLFFLNGETTQLGAGCCRAVRAATRDCWPAMLAAVGFTAEEADVLRGLC
DAEAAAAAADSTSPAPSAA*
>11687.m00609|LOC_Os11g06750.1|genepair1342-1
MSHRKFEHPRHGSLGFLPRKRSSRHRGKVKSFPKDDVNKPCHLTSFVGYKAGMTHIVREV
EKPGSKLHKKETCEAVTIIETPPIVVVGLVAYVKTPRGLRSLNSVWAQHLSEEVRRRFYK
NWCKSKKKAFTKYALKYDSDAGKKEIQMQLEKMKKYASVVRVIVHTQIRKMKGLKQKKAH
LMEIQINGGTIADKVDYGYKFFEKEIPVDAVFQKDEMIDIIGVTKGKGYEGVVTRWGVTR
LPRKTHRGLRKVACIGAWHPARVSYTVARAGQNGYHHRTEMNKKVYKIGKSGQESHAACT
EFDRTEKDITPMGGFPHYGVVKGDYLMIKGCCVGPKKRVVTLRQSLLKQTSRLALEEIKL
KFIDTSSKFGHGRFQTTDEKQRFFGKLKA*
>11686.m00647|LOC_Os12g07010.1|genepair1342-2
MSHRKFEHPRHGSLGFLPRKRSSRHRGKVKSFPKDDVSKPCHLTSFVGYKAGMTHIVREV
EKPGSKLHKKETCEAVTIIETPPLVIVGLVAYVKTPRGLRSLNSVWAQHLSEEVRRRFYK
NWCKSKKKAFTKYALKYDSDAGKKEIQMQLEKMKKYASIVRVIAHTQIRKMKGLKQKKAH
LMEIQINGGTIADKVDYGYKFFEKEIPVDAVFQKDEMIDIIGVTKGKGYEGVVTRWGVTR
LPRKTHRGLRKVACIGAWHPARVSYTVARAGQNGYHHRTEMNKKVYKIGKSGQESHAACT
EFDRTEKDITPMGGFPHYGVVKGDYLMIKGCCVGPKKRVVTLRQSLLKQTSRLALEEIKL
KFIDTSSKFGHGRFQTTDEKQRFFGKLKA*
>11687.m00622|LOC_Os11g06880.1|genepair1343-1
MNPYFIGFAIPFLASLLFTKRKGEKKRGVPVDVGGEPGYVIRNHKFERPVETHWEGVSTL
AELFEQSCEQYVYMPLLGTRKLISREMEAARDGRSFEKLHLGQYEWKSYADAFKTVCNFS
SGLVRIGHQRDERVAIFADTRAEWQIALQACFRQSITVVTIYSSLGEGALCHSLNETEVT
TVICGRKELKKLLDISGQLDTVKHVIYVNEEGVSSEVSLAQKCTSWRVESFEEVERLGLE
TPVEAKLPLPSDTAVIMYTSGSTGMPKGVMMSHRNVLAVVSAVMTIVPALGKKDVYLAYL
PLAHILELAAETVMSAVGASIGYGSPLTLTDTSNKIKKGTQGDASALKPTLMTAVPAILD
RVRDGVRKNVDAKGGAAKKLFDIAYSRRLAAINGSWFGAWGLEKHLWDMLVFQKVEHLCL
EILRDLSIYALGFGLCCCRAPIGQGYGLTETCAGGTFSEYDDNSVGRVGAPLPCSYIKLI
DWAEGGYLTNDSPMPRGEIVIGGPNVTKGYFKNEAKTNEVYKDDERGMRWFYSGDIGRLH
PDGCLEIIDRKKDIVKLQHGEYVSLGKVEAALSVCPYVDNIMIHADPFHNYCVALVVVAH
SELKSWASQQGITYSDVSDLCEKQETVKEVLQCLAKAAKQARLEKFEIPAKVKLVPEPWT
PESGLVTAALKLKREAIKKAYEDDLAALYS*
>11686.m00657|LOC_Os12g07110.1|genepair1343-2
MNPYFVGLLVPIAVSLLLRKRRMVGKMRALPVDVGGEPGYAIRNYRFKQPVETHWEGVTT
LAELFEQSCKDYVNMPLLGTRKLISREQESSLDGRSFEKLHLGEYDWKCYAEVFKSVCNF

ASGLIRLGHQKTDRVAIFAETRAEWQIALQACFRQNITVVTIYASLGEEALCHSLNETEV
TTVVCGQKELKKMIDISGQLDTVKRVIYINEEGISAEVSLAQKSTSWIIEPFEDVGRLGD
TAPVDANMPLPSDVAVIMYTSGSTGLPKGVMMTHRNVLATLSAVMTIVPEIGKKDVYLAY
LPLAHILELAAEALIAAVGASIGYGSPLTLTDTSNKIKKGTLGDASALKPTLMTAVPAIL
DRVRDGVRKKVDTKGGVAKQLFDVAYNRRLAAVNGSWLGAWGLEKLLWDMLVFKKVRAVL
GGKIRFVLSGGAPLSGDTQRFINICLGVPIGQGYGLTETCAGGTFSEYDDPSVGRVGAPL
PCSYIKLIDWSEGGYLTSDSPMPRGEIVIGGPNVTKGYFKNEAKTNEVYKDDEKGMRWFY
SGDIGRFHPDGCLEIIDRKKDIVKLQHGEYVSLGKVEAALIVSPYVENIMIHADPFHSYC
VALVVAAHNELENWASQQGVTYTDFVDLCQKPEAVKEVLGSLSKAAKQARLEKFEIPAKI
KLISEPWTPESGLVTAALKLKREVLRKTYEDDLAKLYA*
>11687.m00623|LOC_Os11g06890.1|genepair1344-1
MSSVFSGDETAPFFGFLGAASALIFSCMGAAYGTAKSGVGVASMGVMRPELVMKSIVPVV
MAGVLGIYGLIIAVIISTGINPKAKPYYLFDGYAHLSSGLACGLAGLAAGMAIGIVGDAG
VRANAQQPKLFVGMILILIFAEALALYGLIVGIILSSRAGQSRAD*
>11686.m00661|LOC_Os12g07140.1|genepair1344-2
MSSVFSGDETAPFFGFLGAASALVFSCMGAAYGTAKSGVGVASMGVMRPELVMKSIVPVV
MAGVLGIYGLIIAVIISTGINPKAKPYYLFDGYAHLSSGLACGLAGLAAGMAIGIVGDAG
VRANAQQPKLFVGMILILIFAEALALYGLIVGIILSSRAGQSRAD*
>11687.m00624|LOC_Os11g06900.1|genepair1345-1
MEYYISCGMVALYRTLESSPIVEQAKVFVLLELSSAHQLPSMGVFSSAPKVYKPASEVNL
GADSNEFYISPNVKAPRVAGLLVKIFAWVLEAPIIGSIVLYILKRDNLVNKLVSDAEIPE
PPLFTAAHTWQDIPEQNVSLTKPDMSPAERVQEAVVCLPARLESVLADPPSPGFRRWTIR
DFTSAYISGEITPVMVARRFLAAVKECSGPDLNMALFISCNPQDVIRQAEASTLRYQQGA
PLSAMDGVLVAVKDEIDCLPYPTTGGTRWLQRMRPCVQDAAVVAQLRACGAVLAGKTNMH
ELGAGTSGINPHHGSTRNPYNTGKVAGGSSGGSAAVVCAGLCPVALGADGGGSVRMPAAL
CGVVGLKPTAGRFSKDGLLPLNWTVGMPGILAATVEDALIAYAAIADQSQPSHLQANART
LYFLLHVWMFSETEFAEDLIFFVCLQPELNLPLLKAASSMPTIRLARYAKWFNDCSEDIR
SCCYKAVHTLRTRYGWEVTADVTIPEIEEMRLAHYVTMGSECTASFDKYLKKLSKSEIGW
DVRIALSAYGSFSSRAYLNSQRIRNRQMYFHDKIFETFDVIVTPMTGVTAHELQDNAGHT
GELDYINGAALVRYSIAGNFLGLPAITVKVGYDREGLPVGLQFIGRPWSEATLLHLAYAM
QEACGKNYRKPMVYYDLLNKNKY*
>11686.m00663|LOC_Os12g07150.1|genepair1345-2
MAAKVYKPAAEVNLGPDSDEFYISPNVKAPRVAGLLVKIFVWILEMPIIGSMVLYILKKD
NLINKLVQDAEIPEPPLFTSTHSWEDIPEQNVCLTKPDLSPPERVQEAVSCLPASLESTL
AGSPPSSPKRWTIRDFNRAYSSGEVTPVQVAKRFLAAVKECSGPGLNMAFFISYSPEDII
RQAEESTLRYQRGTPLSAMDGILVAVKDEIDCLPYPTTGGTRWLGRARACAADAAVVAQL
RACGAVLAGKTNMHELGAGTSGINPHHGSTRNPHNPGRVSGGSSSGSAAAVCAGLCPVAL
GVDGGGSVRMPAALCGVVGFKPTAGRLSNAGVLPLNWTVGMPGILAGTVEDAAVAYSAIV
DQSQPSYLRPELNLPLLKSSLSIKNIKLAKYAKWFNDSSEDIRSCCDKSLQMLHAHYGWE
TLDVTIPEIEEMRLAHYVTIGSECTASLAKYLDKLKRSEIGWDVRVALGVYGSFSSRAYL
NSQRLRNRQMYFHKEIFKTADVIVSPMTGVTAYKLQDDALKSGELDYINGAALVRYSIAG
NFLGLPAITVMVGYDKAGLPIGLQFIGRPWSEATLLHIAFAMQEACKKHYKKPEVFYDLL
KKD*
>11687.m00625|LOC_Os11g06910.1|genepair1346-1
MREEGGIASPGEKPIPNGASPNHSQSPKICSRITDNETQGTATAKSLNEKLVLETVSDDS
STQHCQSPQPDVFTNVKDEDMQDSVKSLSEKLASALLTINAKDDLVKQHTKVAEEAVAGW
EQAEAEVSTLKRLLEASTQKNASLDDQVNHLDDALKECVRQLRQAREEQEEKIRDAVAKK
TQELDSHKSELQNHIYELKQQLEAAKLEAATVAVQHDLQDKLQAAEKENKGLKIELLTLA
KDLKRLSLERDLSNEAAETASKQHLESVKKIARVEAECRKLRHLTRRTSLANDSRPAPNN
ACMESLTDSQSDSGERMLTVDSEMRNSDSWASALIAELDQFKNSSASSRDVVNNHVEIDL
MDDFLEMEKLAALSEVERVSSSFGTETDSDQAVAIDKASKVETETLKSQVTDLQAKVEKL
ETEKRDLEMALAEARVQLDASCDALMAANNKLAELQMQFNLANESKIAALGQADQLDAER
GSLALQLESKSIEVEKLQAVVASLEESTDKKELESQLESTSVELADLCKTVASLQEQIDA
ERTLSLQHKAYADMADADKKSLEAQLQSAHADIGKLRGSIETLESELQKEKTMYEELVVQ
MESMKIESEKKLGVESAKEALEARLLVVNSEIAKLHGTVNDLECDAAKEKAFSSELKMQL
EAVEGIRKMLESELESSHQETMKLQEKISLLEVRLKDQTALLVEFTAKAEDAAVGRKAME
GQLEGAKLEITKLTNRVSLLQGKIEQEKLLSEEYEAKCRKLEAQLSRDSREARLWRLANT
NGDLKVKQDKELSSAAGKLVECQKTIANLGRQLKSLTDLDSVTAEPEKLESGDALLDFRE
PDVEVPPAGFANGLYDLDLPKSNGSCLSPIPRVQSSSTHSQTSVFSGGLSSLGSYRNKTR
K*
>11686.m00664|LOC_Os12g07160.1|genepair1346-2
MVMDRTSWLWRRKPSDKSPGGAENTVSVSSHSEHYSDDQEVLRPVSNNASPHLGQSPGMP
SRVRDDGTQEIGVTKPSNEKLALGFKLNDSSPRHGQSSEPQSSSNVRDEDVKENLKSLND
KLAAAFLTINAKEELVRQHAKVTEEAVLGWEQAESEVAALKKLLEASAQKNGSLEVQVSH
LAEKNASLEVQVSRLDEALKECVRQLHLAQEDQAEKVHDVVTKSQELESENSKLQNRITE
LKKQLETTKLEASNMSIDHDLQEKFQAIKKENMDLKSKLLVQSKDLKILSLERDLSNQAA
ETASKQHLENVKKIARLEAECRRLHHLTRKATLINDSRPLANNTCVESLTDSQSDSAERM
AAVDNELRNSDSWASALVAEFDQFKNGNADEKNLVNNPVVIDLMDDFLEMERLAALPESD
RTSSTFDMETDSDKAVTRNSSSKLETEELRNQVADLHAQVEKIESEKKELEMALMEARNQ
LDISCNALVAAKNRLVEMQMELDLANDSKHDALRDFEGLNSEKKALEFQLESKSVRVEEL
LQVVASLEENTDKKELESQLELLSAEAKELRLTVTSLLEKIEAERSLSVQHQAEAVAACN
AKESLEEQLYSANTEVERLHVIVKELEDEVEKEKMRQEELVAELEMKMETAVEAIKESLE
AQLCAANTEVERLNSIVQALENDIEKEKALHKELTAQLEVKFEEEKARSVQTVKESMEAQ
LCSSNTEVLKLRDIVKALENEVEKEKALHEDLSAQLEAKIEAERTFSVEAIKESFQSELQ

SVNSEVVELRGMVTALEHEVVKEKTFSAELQMQLEALEAIKRVLESEIESAHQDNRKLND
KVKSFEAKLKKQVSSAVDFTAKEEAMQSERRAMKQQLEAAKMEVGKLTNKVSLLQGEVLQ
ERLLSEEFEQEYHKLEARLSRDSRDAKLWRLANSNGGLKAKQEKELANAAGKLAECQKTI
ASLGRQLKSLTDIDNTIVEPERLEPREIREMPLDFRNSDADFAVFADELYDFDLPKVNSS
CFSPLPSIQPSSPPSEMSVFAGGLSSLSSFRSKRRK*
>11687.m00627|LOC_Os11g06930.1|genepair1347-1
MSAAGVALNRRTRSRPPSVASSQKSDDPAAAVAAISTAEATPSPSHAAGERTVKKLRLTK
AVTIPEGTTVAEACQRMAARRVDAVLLTDANGLLSGIVTDKDIAKRVIAEGLRVEQTITS
KIMTRTPVYVMSDTLAIEALQKMVQGKFRHLPVVENGEVIAMLDIAKCLYDAISRLEKAA
EQGSALAAAVEGVERQVGDNLPDHSSVIETLRERMLKPSLSTIISENTKVAIVSPWDPVC
VAARKMHELRVNSVVITAGNSLQGIFTSKDVLMRVVTQNLSPELTHVEKVMTAHPECATL
DTSILDALHIMRDGKFLHIPVVDGEGRVVACLDVLQITHAAISMVSLHMRIFSLLIPCFF
LMVSVHLTSFCVKVEGGPETTNGVANTIMQKFWDSALALEPPDEEFDSRSEISLLMPSEA
GDGRSSINPPVVGNSFVFKIEDQKGRMHRFACGSESLHELVSSVVQRLGIDGEKGTVQLL
YDDDEGDRVLLTTDTDLTGAVLHAKSSGLKSLRLYTDESNSSSEVTKHSSEVTKHASEVT
KHTSEVTKQPPELTSSHTSQLTPAHYGLMADFSQM*
>11686.m00667|LOC_Os12g07190.1|genepair1347-2
MSTASATAPPSRRTRSRPPSASSRKSDDPSAAAANGNGKASSKPTSPGQLTGERTVRKLR
LSKALTIPEGTTVSEACRRMAARRVDAVLLTDAQGLLSGIVTDKDVATRVVAEGLRVEQT
IMSKIMTRNPTYAMSDTLAIEALQKMVQGKFRHLPVVENGEVIAMLDIAKCLYDAISRLE
KAAEQGSAIAAAVEGVERQLGGNFSAPYAFIETLRERMFKPSLSTIVTEGTKVAIVSPSD
PVYVATQKMREFRVNSVVVATGNTLQGIFTSKDLLMRVVAQNLSPELTLVEKVMTVNPDF
ATLDTTILDALHIMHDGKFLHIPVLDREGQIAACLDVLQLTHAAIQLVEGGNDTVNDVAN
TVMQRFWDSTLALESPDEECDSRSEVSLLLASETGDGKSSIYPPVIGNSFAFKLQDQKGR
VHRFTCGSESLNELASSIKQRLSITDEEGIMQLLYEDDEGDRVLLTTDADLAGAVLHAKS
SGLKVLKLHIDLSESSTEVTKPSQQLAAARRSRLSPVRVGLMAGVVALSGAAVMVYLKRA
KV*
>11687.m00638|LOC_Os11g07020.3|genepair1348-1
MASATLLKSSFLPKKSEWGATRQAAAPKPVTVSMVVRAGAYDDELVKTAKTIASPGRGIL
AMDESNATCGKRLASIGLENTEANRQAYRTLLVTAPGLGQYISGAILFEETLYQSTVDGK
KIVDILTEQKIVPGIKVDKGLVPLAGSNNESWCQGLDGLASREAAYYQQGARFAKWRTVV
SIPNGPSELAVKEAAWGLARYAAISQDNGLVPIVEPEILLDGEHGIDRTFEVAQKVWAET
FFYMAENNVMFEGILLKPSMVTPGAECKDRATPEQVSDYTLKLLHRRIPPAVPGIMFLSG
GQSEVEATQNLNAMNQGPNPWHVSFSYARALQNTCLKTWGGQPENVKAAQDALLLRAKAN
SLAQLGKYTSDGEAAEAKEGMFVKNYVY*
>11686.m00669|LOC_Os12g07210.1|genepair1348-2
MVVRAGAYDDELIKTAGDLGDGRVERDVREEAGVDRNRQAYRTLLVTAPGLGQYISGAIL
FEETLYQSTVDGRRIVDVLAEQGIVPGINVDKGLVPLAGSDPESLEDGLVPIVEPEILLD
GEHRVERTFEVAQKVWADTFFHMSENNVMLEGILLKPSMVTPGAESKDGAATPEQVAAAS
RRRCPASCSSPAASRRWRRRGNLNAMNQAAPSANPWRWRVSFSYARALQNTCLKTWGSRR
ENVAAAQGELAGAARQVHQRRRGRRGQRGHVRQELHLLKIIFD*
>11687.m00641|LOC_Os11g07040.1|genepair1349-1
MQPDPSGSGGDGNANAKAKLAPPPVTAAGGRPVSVLPHKTANVRDHYRIGKKLGQGQFGT
TYLCVDKASGGEFACKSIPKRKLLCREDYEDVWREIQIMHHLSEHPNVVRIRGAYEDALF
VHIVMELCAGGELFDRIVAKGHYTERAAAQLIRTIVAVVEGCHSLGVMHRDLKPENFLFA
SAAEDAPLKATDFGLSMFYKPGDKFSDVVGSPYYVAPEVLQKCYGPESDVWSAGVILYIL
LCGVPPFWAETEAGIFRQILRGKLDFESEPWPSISDSAKDLVRNMLCRDPTKRLTAHEVL
CHPWIVDDAVAPDKPIDSAVLSRLKHFSAMNKLKKMALRVIAESLSEEEIGGLKELFKMI
DTDDSGTITFDELKEGLKRVGSELTEHEIQALMEAADIDNSGTIDYGEFIAATLHMNKLE
REENLVSAFSFFDKDGSGFITIDELSQACREFGLDDLHLEDMIKDVDQNNDGQIDYSEFT
AMMRKGNAGGAGRRTMRNSLQLNLGEILNPSNS*
>11686.m00671|LOC_Os12g07230.1|genepair1349-2
MQPDPQPHGRGREKAAGAGPRLPPPVTAPSVGRPASVLPHKTANVRDHYRIGKKLGQGQF
GTTYLCVGKPDGGEYACKSIPKRKLLCREDYEDVWREIQIMHHLSEHPNVVRIRGAYEDA
LFVHIVMELCAGGELFDRIVAKGHYTERAAALLIRTIVGVVEGCHSLGVMHRDLKPENFL
FASTAEDAPLKATDFGLSVFYKPGDKFSDVVGSPYYVAPEVLQKIYGPEADVWSAGVILY
ILLCGVPPFWAETESGIFRQILRGKLDLESDPWPSISDSAKDLVRNMLIRDPTKRFTAHE
VLCHPWIVDDAVAPDKPIDSAVLSRLKHFSAMNKLKKMALRVIAESLSEEEIGGLKELFK
MIDTDNSGTITYDELKNGLKRVGSDLMEPEIQALMDAADIDNSGTIDYGEFLAATLHMNK
LEREENLVSAFTFFDKDGSGFITIDELSQACEQFGLSDVHLEDMIKDVDQNNDGQIDYSE
FAAMMRKGNAGGANAGGVTSTGGTGRRTMRNSLRVNLGDILKPNEN*
>11687.m00685|LOC_Os11g07460.1|genepair1350-1
MIKDLRTLESWAKEKPEIEQPALQAVVGGGGLRAAAAAAEGGMEQQAAPSSSTSTSTNSS
RSTSDHHAAAAAAAAAAAAQVAHQHHPFYYAAAQGGANTMPAPASFMGSLAIVPAAAAPG
GGGGQVQAAAAPVASSEKKAVVAAGAGAKRPTKDRHTKVEGRGRRIRMPALCAARVFQLT
RELGHKTDGETIEWLLQQAEPAIVAATGTGTIPANFSSLAVSLRSAASHSSSPRAAPFHH
LQQQQQHDVAAMLGFHHHHHHQLLPPPPPHQHPEPTPQDPGAGEFMRKRYREADDLFKDTS
RQDPVDGATGEAEQKARAAAAAAAAPPPTAPSAMWAVGPNTTGATAAFWMQPAWAFPHGAG
AGAAGNTVQAPLQFMSRSSFPTAMNVTMADNNNSSNNNLGMLAALNAGGGGRSGEHQHQH
EGQSPAEMDHQRRANGGGGEAGGAASSQ*
>11686.m00699|LOC_Os12g07480.1|genepair1350-2
MDHGGGGGGGGAAPSSSNIGGGSGGGGGGGGGRENHPHHPFYYSGPAAAAAAAAAAQQQQQ
TFMGALAITPVVAEQPQGSSGGGEKKVVAPTTPAAAGAAATTTLAKRPSKDRHTKVDGRG
RRIRMPALCAARVFQLTRELGHKSDGETIEWLLQQAEPAIIAATGTGTIPANFSSLNISI

RSGAASASSASNPNRASPFPALALHPHHQQHDVSAMLGYHHHLLPPPQQQQESPQDPGGA
GAFMRKRYREDLFKEDDDRQDPSAPKAREQQPTTPQAAAAAAAAAAMWAVAPNTAAPGGA
FWMLPVSASSAAAARPTEQPMWSFAGGGGGAATVQAPLQFMSTRVNYPGSAGAGMSDTNL
GMLAALNAYNRGGAGDQQPQQQPEMDQQGRNDDDDDDGDDSGDEDNGGANNNNNNSSQ*
>11687.m00690|LOC_Os11g07510.1|genepair1351-1
MPVVVVVVVVAVMRGDGGGLKGARGRQGRGAAVAGRWRRVAVILLALAYAASMLVVFLGG
GAGGVAVAGAGAGALRQRGAPAPAGSVYRSHLVLDRLLPELRASSASRPHPLMTPQNKKS
GKRWAPCITKKLRRSELPPSNGFLIIEANGGLNQQRISICDAVAVASLLNATLVTPAFHL
NSVWRDSSKFGDIFDEDHFIGSLRKYIRVVKKLPEDAFVNFDHNISMIPNMRTKAFSSES
YYLQKGCAYCSFLKQIGPFGSTEYPGVEDMLAFSCCIYDGGWRESIEMENARERSWRGKF
HRPGRVINPEANRRNGKCPLTPLEVGMMLQGMGFGNTTSLYVASGKIYNAEKYMTPLRQL
FPLLQTKDTLASPEELAQFKDQTTIGLETIFISMRLPSTQTPTYALSGALI*
>11686.m00705|LOC_Os12g07540.1|genepair1351-2
MTVASLRRRRAAAAAAPQPGSVYRSHLVFERLWPDIRDDASSASAAASSLSSTSWRRSML
MTSHYQNPGELWMPCVNRKLIRPELPPSNGYLMIEANGGLNQQRLSICDAVAVASLLNAT
LVIPAFHFNSVWRDHSKFGDIFDEDHFIETLKEHVRVVKELPVDVLTRFDHNISSIPNMR
TKAYSSPNHYMQKVLPKLLELGAVRIAPFSNRLAQSVPSNLQALRCFVNYQALRFAEPIR
VLAEDMVERMVKRSTLTGGKFVSVHLRFEEDMVAFSCCTYDGGLKEKTEMENARERSWRG
KFHRHGRVINPEANRRDGKCPLTPLEVGMMLRGMGFDNTTSLYVASGKIYNAEKYMAPLR
QMFPLLATKDTLALPEELAEFEGHSSRLAALDYSVCLPSEVFVTTQGGNFPHFLMGHRRY
LFGGNARTIKPDKRKLVLSFDDPNIRWNRFKRHMQDILHHSDMRGTALRKPNDSIYTFPM
PECMCQQDGMM*
>11687.m00708|LOC_Os11g07690.1|genepair1352-1
MAASSSSFVFAAALLVLAAATAAQAQRETKLRVFWHDVVSGGPNSTVAQVAEAPTTNASA
TGFGAVVVIDDPLTDGPNLTASRLVGRAQGMYVAAGKDALSLMMAMNFVFAGDGPYNGSS
LAILGANPAERAVREMPVVGGTGVFRFARGYCQATTWWFNATTGDATVEYNIHLRLD*
>11686.m00709|LOC_Os12g07580.1|genepair1352-2
MAAMLISRRSIQLVLVVAAVVAIAGAVHAAAGETTATTTHIKVYWHDVVSGPSPTAVQVA
RAATTNSSASFFGAVVVIDDPLTSGPDLNASSPVGRAQGTYVSAGKDTVALLMNMNFVFQ
SGRYNGSTVAIMGRNEVFAAVREMAVVGGTGVFRWARGYAQARTHTLDMKTGDATVEYNL
YINH*
>11687.m00724|LOC_Os11g07850.1|genepair1353-1
MPFDETRVRLKPSASDHPSSNEYINASLIETDDQGQSHTKFISTQGPLVKTFGDFWQMVY
ENQCPVIVMVTKFDGAKCDRYLPTNEGEERDYGKFSVKITKFKCDGVLELRGLEVQQNES
LTVRHVLHILYSDWPDHGVPHDSAFVRKILKRLYGIPKEHPIVAHCSAGIGRTGAYITIH
NTIERILLGDMSALDLSKTVKKFRSQRPGMVQTEDQYKFCYMAIVSELNDLLSNSKH*
>11686.m00710|LOC_Os12g07590.1|genepair1353-2
MAAARFAGVPRRAAQSSSSSSQAPLDEPPPPALTPEQVGLCREALEYFEGMCGRPEAMSD
EFRRLQDTRHELMRSSNEARNAANREKNRYIDVVPFDTTRVRLKRSTTSQTSSNDYINAS
FIKVTEDNRVAKFISTQGPLAKTFDDFWEMVYEYQCPVIVMLTQFDSLKCDEYLPLRKQR
EAYGKYNVKITNAKRDSHQLWLRDVMVQCNESSRVHSVRHIEYPDWPDHGVPTNTDAVRQ
IRKWLQNTPMEHPIVVHCSAGIGRTGAYITIHSTIERLLLGDKSSYHLDETVKTLRTQRV
GMVQTEKQYMFCYRAIADELKDLLESNR*
>11687.m00730|LOC_Os11g07910.1|genepair1354-1
MHRRGGHHLHLLLAAAVVLLLTVAGLPLASASESDHKYKAEETVKLWVNKVGPYNNPQET
YNYYSLPFCQPSENPAHKWGGLGEVLGGNELIDSQVDIKFLKNVEKGPICTIELDDNKIQ
QFTDAIERSYWFELFIGFVGETDKNNENKHYLYTHKNIVVKYNGNRIIHVNLTQESPKLL
EAGKKLDMTYSVKWVQTNVAFARRFEVYLDYPFFEHQIHWFSIFNSFMMVIFLTGLVSMI
LMRTLRNDYAKYAREDDDLESLERDVSEESGWKLVHGDVFRPPRSLVFLSAFVGIGTQLA
ALILLVIVLAIVGMLYVGRGAIITTFIVCYALTSFISGYVSGGLYSRNGGKNWIKSMILT
ASLFPFLCFSIGLVLNTIAIFYRSLAAIPFGTMVVIFVLWAFISFPLVLLGTVVGRNWSG
APNNPCRVKTIPRPIPEKKWYLTPSVISLMGGLLPFGSIFIEMYFVFTSFWNYKVYYVYG
FMLLVFVILIIVTICVTIVGTYFLLNAENYHWQWTSFFSAASTALYVYLYSIYYYHVKTK
MSGFFQTSFYFGYTLMFCLGLGILCGTVET*
>11686.m00719|LOC_Os12g07670.1|genepair1354-2
MLLPTSASACRGSGRALLLALAAALLALATPRPASASESDHKYKVEEPVKLWVNKVGPYN
NPQETYNYHSLPFCQPSENPAHKWGGLGEVLGGNELIDSQIDIKFLRNEERGSICTLELD
SKKVQQFSDAIDNSYWFEFFMDDLPLWGFVGETDKNNENKRYLYTHKSILVKYNDNRIIH
VNLTQESPKLLEAGKKLDMTYSVKWLQTDVTFARRFEVYLDYPFFEHQIHWFSIFNSFMM
VIFLTGLVSMILMRTLRNDYAKYAREDDDLESLERDVNEESGWKLVHGDVFRPPRSLAFL
SAVVGIGTQLAALILLVIVLAIVGMLYVGRGSIITTFIVCYALTSFISGYVSGGLYSRNG
GKNWIKAMILTASLFPFLCFAIGFVLNTIAIFYRSLAAIPFGTMVVMFVLWAFISFPLVL
LGTVVGRNWSGAPNNPCRVKTIPRPIPEKKWYLTPSVISLMGGLLPFGSIFIEMYFVFTS
FWNYKVYYVYGFMLLVFVILLIVTICVTIVGTYFLLNAENYHWQWTSFLSAASTALYVYL
YSIYYYHVKTKMSGFFQTSFYFGYTLMFCLGLGILCGAIGYLGSTLFVRRIYRNIKCD*
>11687.m00731|LOC_Os11g07920.1|genepair1355-1
MVEEGRSLAETPTWSVATVTTLMVAACFLVERGISRFAKWLRKTKRKAMLAALEKIREVL
LLARCLIVFLTKGNPCLIAELMLLGVISLLLSQTARWISEICVPSSLFTSRFYICSETDY
EDLVVGGKRSTMEMNQTVVPNGLFGIQSQNVCSEGHEPFVSYEGLEQLHRFLFILGITHV
LYTFVTVVLSMIKIYSWRKFETQACQLPTEQLQARRTKVMQRQSTFVFHHTSHPWSKNKI
LIWMYHKLPHSYNFHKYMVRSMEDDYNGSVGISWPLWAYAIICIFVNIHGLNIYFWISFA
PAILVLLVGTELQHVIAQLALEVVGATAPYVGTQLKLRDDLFWFGKPRVLWWLIQFISFQ
LELSAQSCFMKNHYMIVLRLTSGILVQFWCSYNTLPLNVIITQMGSKFKKSLVSESVRES
LHSWCKRVKDKNRHNLASRSVCSLDTTYEETDHETATVGTLSRTVSATSLDEELTVATVE

DNDDDEEMSRIEQEIDRSLGGTRFPWFCQADEFLIWGLFTGGDRVAVWDGCSRLLRAEVG
CCGHSPGAAAAAARVVKAIANPDPAVELPLTAENVEIVLDEVRPYLMADGGNVALHEIDG
NVVRLKLQGACGSCPASVTTMKMGIERRLMEKIPEIVAVEPIADEETGLELNQENIEKVL
DEIRPYLSGTGGGELEFVAIEEPIVKVRLTGPAAGVMTVRVALTQKLREKIPKIAAVQLL
SRKKRNAGATNGIGRETARVLARRGAEVIIPARTMESGNAVKQSIAEEVPGSRLHVMEMD
LASLDSVRRFATAFDSSHTHLNILINNAGIMGCPFKLSKDGIELQFATNHVGHFLLTNLL
LDKMKSTARKTGVQGRIVNVSSIAHKRSDGSCFDLNKLNDKSRSAMCSFLLLPRTLSQCV
MVSILSVGNLFLKNTQQGAATTCYLALHPELKDVSGKYFADCKEATPRPAARDAELAKRL
WDFSEQLVDTNRRGEFNRQK*
>11686.m00722|LOC_Os12g07700.1|genepair1355-2
MQTTTVPMAAAAAVAPSTTTSSSASFKVAAYAWSSCRSSSSPATRLVAAPNHQRPPLVVG
AIAGLDPVTAVQLPLTAGNVESVLDQVRPYLTADGGDVALHEIAGNVVRLKLQGACGSCP
SSLITIKRGIERRLMEKIPDVAAVEPVTDKETGLELNEENVEKVLNEIRPYLAGTGGGGL
QFLMIKGPIVKVRLTGPAAVVRTVRIAVSKKLREKIPSIQIVQLLS*
>11687.m00747|LOC_Os11g08080.1|genepair1356-1
MPRKASSTSDSRLKWRKWKRNPTASPSPSNRSSAAAAAADHSDDSDSAAVNEDDDSAVPE
DADDETLAGAEDPVLDLREAEVLPSAEPVSAFPVATRRVVNRPHPSVLAVIAAERSACAG
EGSAAVAAAPVLENISYGQQQVLSGVLPDHASLATDTDKPSTYVCTPPNLMEGHGVTKQF
QGRLHVVPKHSDWFSPGIVHRLERQVVPQFFSGKSPGNTPEKYMLLRNKVIAKYLENPSK
RLAFAECQGLVANTAELYDLSRIVRFLDTWGIINYLASGSVHRGLRMATSLLREEPTGEL
QLLTAPLKSIDGLILFDRPKCNLQAEDISSLASNSEVVDFDAGLAELDGKIRERLSESSC
SYCLQPLTSLHYQSLKEADIALCSDCFHDARYITGHSSLDFQRIDGDNDRSENDGDSWTD
QETLLLLEGIEKYNDNWNNIAEHVGTKSKAQCIYHFIRLPVEDGLLENIEVPDASVPFRA
ETNGYPHLDCNGSTSGNLPQKIPPDNQLPFINSSNPVMSLVGFLASAMGPRVAASCASAA
LSVLTVDDDSRVNSEGICSDSRGQGPHPNFRDHNGGVSSSISPEKVKHAAMCGLSAAATK
AKLFADQEEREIQRLTATVINHQLKRLELKLKQFAEVETLLLKECEQVERIRQRIASDRV
RIVSTRLASPGNSLPGGSTSTMSSNPMSMSPRPMGVPGSMPQSSMPAPFANNMQGHGHPQ
MAFLQQQRQQMLSFGPRLPLSAIQTQPSPQTSNIMFNPGMPNSVTPNHHQLLRSSSGNN
SSVG*
>11686.m00726|LOC_Os12g07730.1|genepair1356-2
MPPRKATSSADARAKWRKRKRNANTSAADHSDDSDSAVAAAANDDNDDDDAALHAATAAA
NGGGGTLGGGDDDPVVDLREAEVHPTAIERVSAFPPAFRRVVNRLHPSVLAVMAAERAAA
AAGAGAGGGGAAVPALENISHGQLQVLSSVLPDHPSLSNDPDKPSSYVCTPPLLMECRGV
AKQFDGKLLMVPKHSDWFLPMTVHRLERQVLPQFFSGKSPGHTPEKYIMLRNRVITTYLE
RPARRLAFSECQGLVTSTPELYDLSRIVRFLDAWGIINYLAAGSVQRGLRMAATLIREEP
TGELHLMSAPLKSIDGLILFDRPKCSVRAEDIASGASLSSSPGMENGDAGFDEKTLLERL
SESFCSFCAQPLPSLHYESQKEADIALCSDCFHDARFVTGHSSLDFQRVDGKKDGLDNDG
DSWTDQETFLLLEGIDKYKENWNAVAEHVGTKSKIQCLHHFLRLPVEDGLLENIKVPEAS
FSSKFWLMQNASGSVFKLPQLFALGSLPQSGEAGDLPFINTANPVMSLISIMSLALLFRD
DLQIAFLASSLGPRVAASCASEALIVLTGGDSRISSIGNDVMGHAARPNCGDYLIPKINS
SLAVSSENVRHAARCGLSAAATKCKLFADQEEREIQRLSATIINHQLKRLELKLKQFADI
ETYLLRDSEQSERMRQGLQAQRIRMMSGLRLASPRGNTMASNPLSQANIRPPGIPGSMPQ
AGTPAFYSNNMQVHPQMAFLQQQMQQQQQKQQQQQQMQLQQQQQQMQLQQQQQRQAFLQQ
QQQQMQQQQQQLQQQQQRQLQMLSFGGRLPLSAMNAPSTSAAPNVMFDNPDMPGPSNQG*
>11687.m00750|LOC_Os11g08100.1|genepair1357-1
MAAAAARWAPVVGLLGLLLLPFAPAPAGAATPARSPPSSASSAVFLLSGDVYPTGHYYVT
MNIGDPAKPYFLDVDTGSDLTWLQCDAPCQSCNKVPHPLYRPTKNKLVPCANSICTALHS
GSSPNKKCTTQQQCDYQIKYTDKASSLGVLVMDSFSLPLRNKSNVRPSLSFGCGYDQQVG
KNGAAPATTDGLLGLGRGSVSLLSQLKQQGITKNVLGHCLSTSGGGFLFFGDDMVPTSRV
TWVSMVRSTSGNYYSPGSATLYFDRRSLSTKPMEVVFDSGSTYTYFSAQPYQATISAIKG
SLSKSLKQVSDPSLPLCWKGQKAFKSVSDVKKDFKSLQFIFGKNAVMDIPPENYLIITKN
GNVCLGILDGSAAKLSFSIIGDITMQDQMVIYDNEKAQLGWIRGSCSRSPKSIMSSFP*
>11686.m00732|LOC_Os12g07780.2|genepair1357-2
MAAGWARPAGFALLVVVVLAAAASADRPARGGLSVTAGAEESSAVFPLYGDVYPHGLYYV
AMSIGNPPRPYFLDVDTGSDLTWLQCDAPCVSCSKVPHPLYRPTKNKLVPCVDQMCAALH
GGLTGRHKCDSPKQQCDYEIKYADQGSSLGVLVTDSFALRLANSSIVRPGLAFGCGYDQQ
VGSSTEVSATDGVLGLGSGSVSLLSQLKQHGITKNVVGHCLSTRGGGFLFFGDDIVPYSR
ATWAPMARSTSRNYYSPGSANLYFGGRPLGVRPMEVVFDSGSSFTYFSAQPYQALVDAIK
GDLSKNLKEVPDHSLPLCWKGKKPFKSVLDVKKEFRTVVLSFSNGKKALMEIPPENYLIV
TKYGNACLGILNGSEVGLKDLNIVGDITMQDQMVIYDNERGQIGWIRAPCDRIPNDNTIH
GFEDGYCWPQFPNIIGYQNEQSAVCYSSITK*
>11687.m00771|LOC_Os11g08300.1|genepair1358-1
MAEEEVAAVVGELRGSFRSGRTRAAEWRAAQLRGIVRMVEEREGDISDALHSDLAKPRME
SYLHEISLAKAACTFALKGLKNWMKPEKMLIMLTLINDDQVPAALTTFPSTAQIVSEPLG
VVLVISAWNYPFLLSIDPVIGAIAAGNAVVLKPSEIAPATSALFAKLLPEYVDSSCIKVV
EGGVPETTALLEQKWDKIFYTGSGNVGRIVMAAAAKHLTPVALELGGKCPAIVDSNTDLH
VTMKRLAVGKWGCNNGQACIAPDYVITTKSFAPELVDSLKRVLKRFYGEDPLQSEDLSRI
VNSNHFRRLTNLIEDKKVAQKIVYGGQTDEKQLKIAPTVLLDVPLDTTLMAEEIFGPLLP
IVTVDKIEDSIQFINSRTKPLAAYLFTKDKKLQEEFVSNVPAGGMLVNDVALHLANPHLP
FGGVGDSGIGSYHGKFSFDCFTHKKAVLIRGFGGEATARYPPYTIEKQKILRGLINGSFF
ALILALLGFPKERR*
>11686.m00735|LOC_Os12g07810.1|genepair1358-2
MAAARSVGMEAEVAALRGRFAAGGTRGAEWRAAQLRGILRMAAEAEAEVCRALHADLAKP
YTESYVHEIALVKSSCKFALKNLKKWMKPQKVTAPLMTFPSTARVAAEPLGVVLVISAWN

YPFLLSIDPIIGAIAAGNAVVLKPSEVAPATSSLLAELLPRYVDGSCIKVVEGGVAETTT
LLEQKWDKIFYTGNGKVGRIVMASAAKHLTPVVLELGGKCPVVVDSNVNLHVTAKRIAAG
KWGCNNGQACISPDFIITTKSFAPKLLEALEKVLEKFYGRDPLRSSDLSRIVNSNHFNRL
KKLMDDENVSDKIVFGGQRDEHQLKIAPTIFMDVPLDSGIMKEEIFGPLLPIITVDKIHE
SFALINSMTKALAAYLFTKDSKLQEQYEAAISAGGMLVNDTAVHLTNQYLPFGGVGESGM
GAYHGRFSFEAFSHKKAVLVRRFAGEAAARYPPYSPAKLKILRGVLKGNLGAMIKAILGF
PRGK*
>11687.m00772|LOC_Os11g08310.1|genepair1359-1
MALFDRNQKQRSSLCSTATVVVFVALCLVGLWMISSPETIPAAAANVSKKPDVVAVKEED
SSLDATNNVKQNSANVVAETAAADEAAAADEDDNPAKPAAGEKAAAAAASSKDQTFDDEN
GRTEGGALVKPESGGGDEAASDVKEIGSLEQAAIDMKDTTEHSVGDTTKEPGVVQDKSSE
EITMAASDARESSDGGGGGGAAKNKQTFDDENGKLDGVNLVKDVENKTMSEEGAKPLPEE
TTTVSSKNSIVAAAAMSDEKLTDNNGEQAQPVEALPNGQAELLTERAAQNGSFTTQAAES
IKEKKKRAEKKKKKKKVKAASVAAAAEEEGGGGGAASLGWRLCNTSAGADYIPCLDNEA
AIKKLKTTAHYEHRERHCPASPPTCLVPSPEGYRDPIRWPRSRDKIWYHNVPHSELAAYK
GHQNWVKVSGEYLTFPGGGTQFKHGALHYIELIQSSFPEVAWGRRSRVALDVGCGVASFG
GYLFDHDVLTMSLAPKDEHEAQVQFALERGIPAISAVMGTRRLPFPSNVFDAVHCARCRV
PWHIEGGMLLLELNRLLRPGGFFVWSATPVYQELPEDVEIWGGLRRWRDDGAEMVKLTKA
MCWEMVSKTSDTVDQVGLVTFRKPADNACYMKRRQKEPPLCEPSDDPNAAWYQLAPFHNI
RIL*
>11686.m00738|LOC_Os12g07840.1|genepair1359-2
MPLFDRDRYQRLDGNGGGGGGGRRSSPSSCSTATIVLFVALCLVGAWMMSSTGNVPMAVS
PEDKPPAVVKEDDASSIDVTDGVKGRGGGGDGGDTPRTTDEAADDVGKKVQDAGDTAKTT
DDVGDTGVKGGGVDTQTATDAVAKTTTGGTGAGESGKPAGDKVGDGETTTTSKNQTFSDE
NGKTEGGEVVSPEDPDKQSADDAPTDGKDTGDQASGDADEAPSTDTKGKKNSTAEEPRDT
KDAGENADEASTETKADKSSDDTPTDAKATGDGGTPSKNQTSFDDENGKMDGVETVAEDG
KVTEKSSEQVPTNGDDGGGGGEAQTTDDDTATGASSNNQTISDMNDDSNTTTTTTTTLAA
VDSSNGTVSQTTEDDAPANSAAAAAAATEKINPAAEQELLPSGQAELLNETASAVAQNGS
FPTQASESSAEKKARDRNKNKSNGSDTAAAAAVAVAHGWKLCNVSTGEDYIPCLDNEAAI
KKLKTTKHYEHRERHCPAAAPTCLVPLPGGYRRPIPWPYSRDKIWYHNVPHTKLASYKGH
QNWVKVSGEHLTFPGGGTQFINGAAHYIDLIEEAVPAVAWGRRSRVVLDVGCGVASFGGF
LFDRDALTMSLAPKDEHEAQVQFALERGIPAISAVMGTKRLPFPGGAYDAVHCARCRVPW
HIWGGKLLLEVNRLLRPGGLFVWSATPVYRKTPEDVQIWHDMAALTKSMCWKMVKKTNDT
VDETAMVIFKKPTSNGCYSNREKPEPPLCDADDDPNAAWNITLRACMHRLPTNKSVRGAR
WPELWPERMSAAPYWLSHSQVGVYGKPAPDDFAADEEHWNHVVNSSYLAGVGIDWSNVRN
VMDMRAVYGGFAAALKDMNVWVMNVVPVDSADTLPIIYERGLFGMYHDWCESFSTYPRSY
DLLHADHLFSKLKKRCKLLPVMVEVDRILRPEGKLIVRDGRDTAAEVESILRSLHWEVRM
TVSKQGEVMLCAEKTMWRPKEVEKAATTAS*
>11687.m00781|LOC_Os11g08400.1|genepair1360-1
MTREHDGDGEEVATAVHGDAGAEEDGDRNVVDKSEFSDAVHVVVDRDDEEPEFPSDDDEG
GDDDVRVSFATAVGDSDEHLREEQGELDLDDDDEEDVSRYEYDYGMWMEAEPMSIQERRR
RLLQGMGLASSRDLLRSRSARMRPILPPNIPRCASRRQPPPQCPAAAADDAPSTSTAATV
KRQRNAVLTRCRSDSRLAVRGGGAARKPPTFRRVYSVPHSLHGSPVHKALRAAARSRSPL
PLPAPKDERENTVRKLDDGKEFVVSGGQPAAGGSRGALSDLKTGVQLSLDEFERFIGYTPF
VKQLMRRSQSQPVAAGAANGDAKPGKKKPRWLKNIKLVASAAGLIQEKYKESNCGGGGCG
RSSSSSSSSAEQAHQPGVTMSKSASTNAATMASSSSSLERPKVHSFGKTARELTGMYFRQ
EVRAHEGSIWSIKFSPDGRFLASGGEDRVVHVWHVVDDGAPPSSMSPELLSSSQSLPPLA
PHGDGGLAAQLSRKLRARRWKTCKDVLPEHVVVPETAFALADEPACSLEGHLDDVLDLAW
SMYSQLLLSSSMDKTVRLWDTEAKACLKLFPHNDYVTCVQFNPVDDGYFISGSLDSKVRI
WSVAERQVVDWSDLDDMVTAACYTPDGQAAIVGSHKGSCRFYKTADCKLNQEAQIDMNIS
KKRKSHAKKITGFQFAPGNPSEILVTTADSQIRVFNGITVLQKFKGFKNTSSQISASYSG
DGRYVVCSSEDSNVYVWRRATSPGGAAGGGVAVKAKTWRTSRAYECFFCKDVSAAVPWPL
SPCLPPTRGGGGGGDDDERASSSVRGAVVGGDASASRSPARQLGSLPLRPKSGPMTYSGE
KQLGVPREPSSRWHGGAEGGNAWGMVVVTASLAGEIRVYQNFGMPLSLFRKT*
>11686.m00742|LOC_Os12g07880.1|genepair1360-2
MATPPESLSPAAAAVEDDWEEARGLLYEAYNELQGLAAELGGAAAPAPAVVVVGHQTDGK
SALVEALMGFQFNHVGGGTKTRRPVALHLRFNPRCHAPRCRLLAGSGAGDDEDEEAGVAG
RAMPLADIQAYIEAENMRLENDPSQFSEKEIIIRIEYKHCPNLTIIDTPGLILPAPGRKN
RVLQSQACAVETLVRAKIKHKETIILCLEDCSDWSNATTRRVVMQVDPDLARTVLVSTKL
DTKILQFARASDVEVFLHPPTCALDGSLLGDYPFFTSVPSGRVGSCHEAVFRSNEEFKKA
ISLRELEDVASLEDKLGRGLTKEEKNRIGVSNLRLFLEELLRKRYIESVPLIIPLLEKEH
RGATRKLREVNQEISDLDEAKMKEKARLFHDSFLSKLSLLLKGMVVAPPDRFGETLINER
INGGTFTGSENFLIPNKLMPNAGMRLYGGAQYHRAMAEFRLVVGSIRCPPITREEIVNAC
GVEDIHDGTNYSRTACVLAVAKARDTFEPYLHQVIIHNEETDSDICFSVGEFQQKDGENC
SSHDVLVKRCQSCQFDRVCMNLLSNHVVKEFSVVSKCMEDLVSTTRYVTWSLHNKFNCFL
LMPIVDKLPALLREDLESAFEDDLDSIFDVTQLRHSLGQRKRDLEIELKRIKRLKEKFAE
INKKLNSLQVRQYYPTPPLSLPRKPWWLAPRIGHRQERGVVDDMTGGDKDHRRGGSGGGK
ETFLRSLDRVPSGLHIDADFPSDDDDDDDDEEVRVSFASTMGDHKMYSFRHHQAAVLEEE
EEDDDDDEFSKYDMDEDMSIQERRRRLHQGLGLASSRDLALRRHSTRRRLFRNAMAKRDL
PTAAVPPTTPPPAAAAAAAAPVDKGRTDDGGGKDRDDATKNQESSKEVAVVAAPKDAAPA
SNTQTGVQLGLEEIEKFIGNTPIVKHLMRRGQSQHHSGQLASPSGGAPPKAEKPAGGKKK
GGWLKNIKSVAIGFIDSGGNSKSTTSTTTSSAGANATSSSSSSASSTERLKVHQSGKSCK
ELTGLYMCQEIMAHEGSIWSIKFSTDGRWLASAGEDHVVRIWQVVEANSPACLPNDGHSG
PLPPHPPGAAPADGTSSSSTPALSQLSKKSVKGKSGRDTLPEHLVVPDKVFALADQPACV

LEGHQDDVLDLTWSKTDQLLSSSMDKTVRLWDTTTKACLKVFAHNDYVTCIQFNPADDRF
FISGSLDAKVRLWSIPDRQVVDWTDLNEMVTAASYTPDGQGAIIGSHKGSCRFYKTTDCK
LDQEAQIDIETKKRKSQAKKITGFQLLKFKSLARTWPNCSSWPWPWTPQFAPGNPSEVLV
TSADSQIRVFDGVTMVQKFRGFKNTSSQISAAYTSDGRYVVCPSEDSHVYLWRAARGAPP
AAAAIGSIGGIGMKPKTWCTIRSFENFYCKDVSAAVPWPLAPSGAGGDGSTSGSSPSRRQ
GGVSCTDDVCSMPAKSGELGSAGTPLTHSGQLGSPAPGGGKGGADGNAWGLVVVTASLQG
EIRVYQNFGMPFRIRGQGNLFY*
>11687.m04458|LOC_Os11g47460.3|genepair1361-1
MVTVREEMRKGPWTEQEDLQLVCTVRLFGDRRWDFVAKVSGLNRTGKSCRLRWVNYLHPG
LKHGRMSPKEEHLIIELHARWGNRWSRIARRLPGRTDNEIKNYWRTHMRKKAQERRGDMS
PSSSSSSLVYQSCLLDTVPIISMDGGDIHDDRSCMARVLKSTQSVMDGYTMDQIWKEIEA
PGAPSLLGIDEGKDKACSNLPCPLLTSTMSDYSCPEVFWKIDNEETRMLATQSGYGK*
>11686.m03740|LOC_Os12g37970.1|genepair1361-2
MAKSRRIAAAHLTCMEIFNRALDWALERELHTSRRGADDDRQILKLVHAATARGRRSRGR
GSTSPPRKGGMESSVPMIMADKKASAFHIRRTGKNIWKKPDRDFSKIPFPMAGVHQGSHP
EAGANKHTEVEEEQEDRLRADTRVINMVTVREEIRKGPWTEQEDLQLVCTVRLFGERRWD
FIAKVSGLNRTGKSCRLRWVNYLHPGLKRGRMSPHEERLILELHARWGNRWSRIARRLPG
RTDNEIKNYWRTHMRKKAQERKSNMSPSSSSSSLTYQSCHPETPSMIIGIEEQELHGGSG
CITSIMKSTPVDMDGYPMDQIWMEIEAPNVLPGPCFDEAKDSASNSLSGPLLPYPMWDYY
CPETCLRMDDEIKVAPQFGYGKGVGPCY*
>11687.m04461|LOC_Os11g47490.1|genepair1362-1
MWPADVTRATNPFENRRFKYKKLKFITDGFKTTIGKGGFGPVYIGYLENGTPVAVKMRSQ
TSSQGNTEFLAEARVHHRNRVSLIGYCKDKKHLALVYEYMDGGSLADHLKAISSILLNSE
QLADSIFFFFTGLEYMHRSCSPPLISITIDP*
>11686.m03741|LOC_Os12g37980.1|genepair1362-2
MEEAAAAVHRRWRRRLLPKLMLLLCAVVHGQQPDSLGFISIDCGIPDGGGYSDESTRGLR
YVPDAGFLDAGAGLSAGINPPYTDRDLAARYLTVRYFPGAASAAGERGGCYTLRQLSPGG
RYLVRATFYYGNYDGAIAMLPVVFDLHLGANRWTAVNVTAADAIYIFEAVVSPPADFLQV
CLVNIGKGTPFISGLDLRPLKPELYPEATANQSLLLLNHDRPPARFAFNRYQFWRPASYY
KLFRYPFDPYDRLWQPYGDDPSWTNITVAAAVDVTNISRSDDPSPILRSAATPANATVRR
LDFPWSSDDAATTTYLLLLYFAELQRLPAGAARRFDVLVDGDASAGGGRRGYTPRYLAAE
VVRSTVRAARPGQRHVVSLVAAPDSALPPIVNGLEIYSVQPMPELATNDRDAKAMMEIRD
NYELKKNWMGDPCAPKAFAWVGLNCGYSSSDPALVTALNLSSSVLIGPVNLSFGDLKSLQ
YLDLSNNSLSGPIPDFLVQMPALKFLDLSSNKLSGSIPSDLLQKRENGSLVLRIGNNANL
CYNGANNTCAPESKQSKRILVIAIAVPIVAATLLFVAAKFILHRRRNKQDTWITNNARLI
SPHERSNVFENRQFTYRELKLMTSNFKEEIGKGGFGTVFLGYLEDGTPVAVKMCSKTSSE
GDKKFLAEAQHLTRVHHRNLVSLIGYCKDKKHLALVYEYMQGGNLEDRLRGEASIAAPLT
WHQRLKIALDSAQGLEYLHKSCQPPLIHRDVKTRNILLSGDLDAKIADFGLTKVFAGDVV
THVTTQPAGTLGYLDPEYYHTSRLSEKSDVYSFGVVLLELVTGRPPAVPLGDGDGGGGES
VHLAVWARQRLAEGDIESVADAAMGGCFEVNSAWKVAELALRCKERPSRERPAMADVVAE
LKECLELEASRALGRGYSCYSSGSGGGSSVATTTTTSGAANISAAASAASVSDAQIGELR
QESVLELGPR*
>11687.m04480|LOC_Os11g47680.1|genepair1363-1
MKASMAFTVLALAVVAVSGAAAAWTTTFTMHNLCPYTVWPLVTPNAGQPAIITGGATIRL
DPNGLASLAFPAAAGWSGRVVPRTGCTGAATCATGDAPPATVAQVSVNAAGGLAEYSVSL
VDGFNVPATITPHAFDGSQTCPVLGCAADINAACPADARVGAGCRASPQFFKEMCPEART
TATDVEATPQKCFGPGELKVVFCPTN*
>11686.m03755|LOC_Os12g38120.1|genepair1363-2
MASALAFVAVLLAAAAAATSPAAVAATTLTIQNLCPHPVWPLVTPTSGQPISDNTARLDP
NSLISLAFPPTPWSGRVAARTGCDAAASPPAGCETGASPPSTVAQLSVHGGGDVATYSVS
LVDGFNVPVVVSPQAVGGGQCPALGCVVDLNCDCPLGQRFSDGAACRGPPEYFKGRCPQT
RTTPGDVEPVPQSCRSPGELKVIFCPPTMLTAAAAAAASDMLIRTVVASS*
>11687.m04489|LOC_Os11g47760.1|genepair1364-1
MAGKGEGPAIGIDLGTTYSCVGVWQHDRVEIIANDQGNRTTPSYVGFTDSERLIGDAAKN
QVAMNPINTVFDAKRLIGRRFSDASVQSDIKLWPFKVIAGPGDKPMIVVQYKGEEKQFAA
EEISSMVLIKMREIAEAYLGTTIKNAVVTVPAYFNDSQRQATKDAGVIAGLNVMRIINEP
TAAAIAYGLDKKATSVGEKNVLIFDLGGGTFDVSLLTIEEGIFEVKATAGDTHLGGEDFD
NRMVNHFVQEFKRKNKKDITGNPRALRRLRTACERAKRTLSSTAQTTIEIDSLYEGIDFY
STITRARFEELNMDLFRKCMEPVEKCLRDAKMDKSSVHDVVLVGGSTRIPRVQQLLQDFF
NGKELCKNINPDEAVAYGAAVQAAILSGEGNEKVQDLLLLDVTPLSLGLETAGGVMTVLI
PRNTTIPTKKEQVFSTYSDNQPGVLIQVYEGERTRTRDNNLLGKFELSGIPPAPRGVPQI
TVCFDIDANGILNVSAEDKTTGQKNKITITNDKGRLSKEEIEKMVQEAEKYKSEDEEHKK
KVESKNALENYAYNMRNTIKDEKIASKLPAADKKKIEDAIDQAIQWLDGNQLAEADEFDD
KMKELEGICNPIIAKMYQGAGADMAGGMDEDDAPPAGGSGAGPKIEEVD*
>11686.m03761|LOC_Os12g38180.1|genepair1364-2
MAESSAIWYLTHNHFSDYMSRNMAENAKRLIGRRFTDASVQSDIKLWPFKVIAGPGDKST
IVVQYKGEEKQVAAEQIDDETISPSVCYADPYDGGLCASYADAKRLIGQRFTDASVQSDI
MLWPFKVIAGPGDKSMIVVQYKGEEKQFAAEEISSMVLIKMREIAEAYLGTTIKKAVVTV
PAYFNYSQRQATKDARVIAGLNVMRIINEPTAAAIAYGLDKKASSVGEKNVLIFDLGGAG
DTHLGGEDFDNRMVKHFVQEFKRKSKKDITGNPRPVGRLRTACEWAKRTLSPPLPRPPTI
EIDSLYEGIDFYSNITCARFEELTMDLFRKCMRGYQDGQRAACTMFILVGGSTRIPRVQQ
LLQDFFNGKELCKNINPDEAVAYGAAVQAPILVWRRPEE*
>11687.m04495|LOC_Os11g47810.1|genepair1365-1
MPVANWKLRLASFYVLKELEDDGPVTGHTSGQLKQTSWQRSIKPIVVSVPLKDSKFSHDE

CYKEETVRRKKYPDLEEKSSSTKATVVLGVAPEKKAQQFEAAAESGETAHGCSCGSSCRC
NPCNC*
>11686.m03773|LOC_Os12g38300.1|genepair1365-2
MSCGGSCNCGSCGCGGGCGKMYPDLAEKITTTTTTATTVLGVAPEKGHSEGVGKAAESGE
AAHGCSCGSSCRCNPCNC*
>11687.m04497|LOC_Os11g47830.1|genepair1366-1
MRSRSPSKRRRHGSRGRSPPSSRHGCAKDKEGAAVSLFVSNLPRSCRPEDVQVPFQKFGP
VRDVYLPKDYNTGEPRGFAFVEFAHSSDASKARYHMNRKMLSGREISVAFAVQTRKRPEE
MRRIIGARHNSPQRKEECRTNSPGQPKGHDEKRKRRSYTPKYKDRQYADIGRDETPPAPD
SERPWALCRSPRPSPPGQSHSRSYSRSHSLHLHDHARTRSCSPAPGRQDDQYASPQRKEH
QTKSSGQTKGHDDMRRSYTPEYNECQDADNGFDETPPAPDGERSSVLGRSPRPSPPGRSH
CHSHSRSRSPELRGHARSRSCSPATGRQDNQSTSPQRREKHQTKSSGQAKEHDEKRRSYT
PEYNDRRDADNGYDQTPPAPDGERSWALGRSPQPSPPGRSHFHSHSRSRSPELRGRARSR
SCSPAPGRQGDDQYASPQRKEEQQTKSSRQTKEHDEERRSCTPEYSDRRDAFIGHDETPP
SAEWGSKLGTVQVISNTAASSN*
>11686.m03786|LOC_Os12g38430.1|genepair1366-2
MRRYSPPYRSPPRRGYGGRGRSPPRRGYGGRREQGSGSLLVRNIPLSCRGEDLRVPFERF
GPVRDVYLPKDYYSGEPRGFAFVEFVDPYDASEAQYHMNRQVFFGREITVVLAAESRKRP
EEMRSRARVRGYSGNEGRRSSYYGRSRSRSRSPHYRGRPRSRSYSPAPRRRDDYSASPPR
KDTHPTKSPRRQPKEHDEEKKRRSYSPASRDGDPRDADNGYEKRSPPPDSDGSPPHRRSP
RHSSGSPPGSRSRSADVSPARSD*
>11687.m04501|LOC_Os11g47870.1|genepair1367-1
MAAAAPQLEELVDLEPFSPSLFLDLPPTPHSDDPNDDDLILPFISRMLMEDDIDDKFFYQ
FPDHPALLHAQQPYAQILDAPSDDTTTNSSDDSASATTNNTTNSAAAANASWPYDPIELS
QLLQSPPHPVSDNHDADVGDTRSAPEDDKDLKLLFSAADNMEMLNMAFLKGREEANKLVP
TNNTLFAGFDGASLLKTEPAVDEPTLMFGRSGGSGRGRKNRHGEEDDLEAETGRSSKLMV
PPQEATAAASEMFDEIMFNWYEVIMKGMEELRVAMDSEAEKKARNGGGAGRRAARAKAAV
VDLHTLLIHCAQAVATSDRRSATELLKQIKQNSSARGDATQRLACCFAEGLEARLAGTGS
QVYKSLVAKCTSTVDFLKAYKLFAAACCIKKVSFIFSNKTILDAVAGKRKLHIVDYGLSY
GFQWPGLFKCLSEREGGPPEVRITGIDFPQPGFRPADQIEETGRRLSNCARQFGVPFRFQ
AIAAKWETVRREDLHLDREEEEEEEEEVLVVNCLHFLNALQDESVVVDSPSPRDMVLNNI
RDMRPHVFVQCVVNGAYGAPFFLTRFRETLFFYSSQFDMLDATIPRDNDERLLIERDILG
RWALNVIACEGADRVDRPETYKQWLVRNHRAGLTQLPLQPQVVELVRDKVKKLYHKDFVI
DVDHNWLLQGWKGRILYAMSTWVADRDHKSLF*
>11686.m03793|LOC_Os12g38490.1|genepair1367-2
MGSSAADSFPACGDDAIRDVYGIGGGGEEDDPSLFLYLSDLAPVSPSAYLDLPPSPPPPT
TTATTMVKEGEEAPEDLVLPFISRMLMEEDIDDKFFYDYPDNPALLQAQQPFLEILSDPS
SNSRSSNSDDPRLSPTSSSDTSAAINSYDAAATATAVAAAAVPVPQYESIELDPAAFFAA
ANSDLMSSAFLKGMEEANKFLPTENKLIIDLEASSENNYLRGLEEAKRFLPSDDKLQVGF
AAAAAPVVSVKKEAVDVVVATASGGGGRGRKNPYDDEELELEGGRSSKQTAVQGDDVAAR
AMFDKVMMPSHENCTEMMEKLRIAMKEEAAKNEASAGGKGGNGKVKGGRRGGRDVVDLRT
LLIHCAQAVATDDRRSATELLKQIKQHAKPTGDATQRLAHCFAEGLQARIAGTGSLVHQS
LVAKRTSAVDILQAYQLYMAAICFKKVSFIFSNQTIYNASLGKKKIHIVDYGIQYGFQWP
CFLRRISQREGGPPEVRMTGIDLPQPGFRPTERIEETGHRLSKYAQEFGVPFKYNAIAAV
KMESVRKEDLNIDPDEVLIVNCQYQFKNLMDESVVIDSPRDIVLSNIRKMQPHVFIHAIV
NGSFSAPFFVTRFREALFFYSALFDVLDATTPRESEQRLLIEQNIFGRAALNVIACEGID
RVERPETYKQWQVRNQRAGFKQLPLNPEIVQVVRNKVKDCYHKDFVIDIDHQWLLQGWKG
RILYAISTWTPNDALSYF*
>11687.m04271|LOC_Os11g45220.1|genepair1368-1
MSPRIPTRANLALAALLILLTIAATTLASAKCIAKNKPEGEEGEPSGAGAAAAHASPEKK
PGSNGGLTTMSVGESVPEIKKDSSDDGAAVNESKKPKSSGGLTTLSVDDSQAEPADSIAE
PVEDGTDDGEDESEKKKKKKKKKSKSKSSDDDDDAEKKSKKKSKNSDDDEDDKKKSKKK
PKNPDDDEDDKKKSKKKSSDEDNDGAKKKKKKKSKGKSSDEEDDEKPKKKSKSKSKSSSS
DEEDEKKSKSEGQAAAKPKEEDEEGGSASASASTSAPKNEHHSGTMSLPDPDMIAQPVMQ
ALNPVVKALCGKTDHADLCESSIGQLPQQPPAQLDDIGVLRLSMNALRAKVQEAISVATN
RMGAASGDEVSKDAMGDCLQMYDDMKSNLDSADAALKKGDKDTAHTMLDSARTDVDTCED
GFSEREGLKPIMGDLDKILAELSSNTIAIASAIIE*
>11686.m03684|LOC_Os12g37480.1|genepair1368-2
MASSPPLVACVLLAAVVFTAVAPPPAGAVCVPRNGKAAPGKPGMSPAPPKKLTPAPPTTP
PPKAKPILPGPGGDLVKALCAKTDYPVVCQMTVVPPPAAGAAAKLDATAVLRLAMGAVRA
KAAAAKKAAGALAADARTPALAKPVLRDCMDSYDDIAYSLDEADKAMAAGDKDTTGTMLD
TARTDVDTCDQGFEERDGDIPPLMSKQDAELAKLASNCIAIAVAAGLRSSS*
>11687.m04279|LOC_Os11g45280.3|genepair1369-1
MEAAELVKKIRELEEGQAELKREISRIVPERGGARRPPTLPAQQRRTALPQAAAAAPSSR
LLQRVGRAGLPDRHYVRILHSLGQAVHVISLDGKLMYWNRYAEHLYGYSVPEAVGQDALE
LIVHPSDYGAANDIIQNIFMGKCWRGKFPVKHKSGERFNIVASNTPLYDDDGSLVGLICL
STDTRTLEEILGHSTSGKVYPSSAKPRVQLNGSKSGLLNKVSCDSQQPLQSAITSRITNL
ATRVTNRVRSRVKTGQNCDDQFGGACESHYSEHDAREEQTSSEGSTPSGDVLHGAFVSED
NYSGKSSKTNSDDSGEGKLGLHKILSSTAEALWANRGIPWPWRGHGNDDAGKNRTNLPQF
HEIQENGQSHKEVPEPIILPDCQDTEFVQEVKYEVSGSWWSFNASTSSMSSTGSTNSSAI
ERADREADCLDFEILWEDLAIGEQVGQGSCGTVYHALWYGSDVAVKVFSKYEYSEDMILT
FRQEVALMKKLRHPNVILFMGAVASLQRLCIVTEFLPRGSLFRLLQKNAGKLDPRRRVHM
AIDIARGMNYLHNSSPPIVHRDLKSSNLLVDKNWTVKVADFGLSRLKLETFLTTKTGKGT
PQWMAPEVLRNEPSNENYGVILWEIATQKIPWDNLNTMQVVGAVGFMDHRLDIPSDVDPH

WASMIESCWDSDPQRRPSFQELLDQLRDLQKQYNLQAQLQRTAAAKMSVDDC*
>11686.m03696|LOC_Os12g37570.1|genepair1369-2
MEASGEELLKKIRELEVGQAQLKQEMSKLGGAAAAAERRRSQSVSPRRGAAPPPPHQPPL
PARRLSGGFEGGARAWARGSASFPHSSPLQREGRAAAAAGGGLTEKQYTRVLQSLGQSVH
ILDLEGKIMYWNRSAEKLYGYPASEALGQDGLMLLIDSCDINVVNDIFRRISLGESWTGK
FPVKNRAGDRFSAVATNTPFYDEDGSLVGIVCVSSDLRTMEEIISGPSICARPHPESSRT
YCEASCSNSNRKASLLSRSPFDSQQPLQSTIASKITNLATKVTNKVRSRVRADENGIERE
GGSGESHCSDRDAKEEPTSSGTTTPRGDAPRGAFATEESSPGKTAKMNSDESEGKVGFHR
ILSSKAEALLNKKGISWPWKGRDNDGPDVKNQATWPWLHGEQDGSQNHQKISDSAITQDG
QGAEYNQPNKNEASGSWSSFNNNSTSSASSTGSTNSSALYKVDHEADCLDYEILWEDLVI
GEQIGQGSCGTVYHALWYGSDVAVKVFSKQEYSEEVIQTFRQEVSLMKKLRHPNILLFMG
AVTSPQRLCIVTEFLPRGSLFRLLQRNNTKLDWRRRVHMALDIARGMNYLHHFSPLIIHR
DLKSSNLLVDKNWTVKVADFGLSRLKRETFLTTKTGKGTVSTSMHNVISRRAFPCSLP*
>11687.m04292|LOC_Os11g45400.1|genepair1370-1
MPKKKLSHRLFSALVSLLLHGKPISRSSSNTNTTLPHPSLLHKSSSSFPPMEKLAAKTLV
LDVEGGLLRSSSLFPYFMLVALEAGGFLRGLVLLLLYPLLCVMGSDMALKVMAMVSFCGL
RASRFRAGRAVLPKWFLEDVGEEGFDVMRSAMRRVCVTKMPRIMVEGFLKEYLEVEVVSG
REMKVIWGFFTGIMEEEEGGDQEEVLLEEKKMLVDVVGFSTSLEFLQHHLSHCCKLVQEV
YLVTREEKARWSALPRDKYPKPMVFHDGRLAFRPAAGDTLAMFTWLPFGAALAVARLAVA
LAVPYRYSTPILAATGLSWRLKGEAPTPLAGAGDGARRRGQLFVCNHRTLIDPVYYVSVAL
DRPVRAVSYSLSRLSELISPIGRTVRLTRDRDSDGRAMARLLDGGDLVVVCPEGTTCREP
CLLRFSPLFAELSDDVVPVGIAVDTAMFYATTAGGLKCLDPLYYIANPRTCYAVQFLERV
DTSPARERRAPSTDVANLVQRRMGDALGYRCTMLTRKDKYLMLAGNDGVVNTTQDNHSAP
GKKKMQ*
>11686.m03699|LOC_Os12g37600.1|genepair1370-2
MAKTKLFPALFSLLLHGGAATLPPPRVPAVTVHGCTPPAARLSAGGEKTVTMVVDVEGAL
LRSSSSRSLFPYFMLVAVEAGSFLRGLLLLLYPVISLLAGAGGGDVAVRAMAAVAFCGL
RESRFRAGRTVLPRWLLDDVGKEAVDAIVTLTRRRSSPAATATVVWASSMPRVMVEPFLR
EYMAAAEGGGEVVVAAREMKVVWGFYTGVMEDGGEVAAASPEVRRAMEGVDDVVGFSGGS
MDLLRSPLVSFCKEVYVVSHEEKSKWRPLPRRREYPRPLVFHDGRLAFLPTPLAAAAMLV
WLPFGAALAATRLAVALALPYRHATLLLAATGQSWRLRGSPPPTPTPPPRRATGERRRGQ
LYVCNHRTLIDPVYVSIALDRPVRAVSYSLSRVSDLLSPIGATVRLARDRAHDGAAMARL
LEAGAHVVVCPEGTTCREPYLLRFSPLFAELADGVVPVALAAEAAAFHGTTAGGWKSMDA
LCYLANPRMCYTVEFLPAVDASPVREGKAASTELANAVQRRVAEALGYESTMLTRKDKYL
MLAGNDGVVRRRGDVGAK*
>11687.m04322|LOC_Os11g45700.1|genepair1371-1
MALVIADEIKAKAEVYYDDEICQQCTRLLLKEAGLPNGLLPLKDIMECGYVEETGFVWLK
QKKRIDHVFQSLGRLVSYGTEITAFAEKGRIKKVKGIKTRELMVWVPVEEIALDEQKTGK
LICKSIAGITKTFPASAFQIPEKEEKITCAIPKPVVLMERAHQVIKNN*
>11686.m03706|LOC_Os12g37650.1|genepair1371-2
MTLTIPDDVRAKAEVYVGDEAGQEKTRLLLEETGLPSGLLPLRDIIECGYVEETGFVWLK
QRRKVDHFFAKAGRHVSYAAEVSAVAEKGRLRKITGVKAKELLIWVTLHEIAVDDPPTGK
LTCKAIGGLSRSFPVDAFEAPPPPPKNPSPAAGDTTKVDEEKKKEEEVAGDAAAAAIDEI
EGKMKEMNSKEVQVQAEGVAAKN*
>11687.m04326|LOC_Os11g45720.1|genepair1372-1
MDRPNLAVAVVGLLAVVAATLPAPSWQFFDLFLPAGPSHRSSGGGFGKWVLMNHEEYVEK
KSLYAMKAAGDIGGKTIDASLSAAEEAKVTWVVDPKGTPGDTTFTTIAAALEKVPEGNTK
RVILDLKPGAEFREKLLLNITKPYITFKSDPANPAVIAWNDMAATRGKDGKPVGTVGSTT
VAVESDYFMAYGVVFKNDAPLAKPGAEGGQAVALRLFGTKAAIYNCTIDGGQDTLYDHKG
LHYIKDSLIMGSVDFIFGFGRSLYEGCTIVSVTKEVSVLTAQQRTKTIEGAIESGFSFKN
CSIKGQGQIYLGRAWGDSSRVVYSYTDMSKEVVPIGWDGWNIAKPESSGIYYGEFKCTGP
GSDAKKRVGWALDLTADQAKPFIGTHYIYGDSWILPPPDGKSAASTSTASKSTASAIPRN
STAPATATESNSTAPATPSNSTAPVTASNSTAPATASSSNPPATKSYSGPPATPSASSTP
AKASR*
>11686.m03707|LOC_Os12g37660.1|genepair1372-2
MEIISPSSSSNNNSPVLATFLVVLVVLLASSRPASSQNQQSFTINPGGAAAARPGGGKGG
GGGGGPGSFSDFLTQNVQHYVLSEQKYAGKVKALDAELSAAEAGAARYVVSGDGKGKFRT
ITEAIKAVPEYNKKRVILDIRPGTYKEKLLIPFTKPFITFVGNPRSPPTIMWDDRAATHG
KDGQPMGTMLSATVAVEADYFMASSIIFKNNAPMAAPGAHGGQAVALRVFGSKVAMYNCT
IDGGQDTLYDHKGLHYFKNCLIRGSVDFIFGFGRSLYADCTIESVTKEVAVVTAQQRSKN
IAEAIDTGFSFLRCKISGIGQIYLGRAWGDSSRVVYSYTTMGKEVVPIGWDGWEVQKPEH
SGIYYGEYKCSGPGALPSKRIGWSLVLSDIQAKPFTGSHFVYGDSWILPPPKSM*
>11687.m04329|LOC_Os11g45740.1|genepair1373-1
MEMVLQRTSHHPVPGEQQEAAAELSSAELRRGPWTVDEDLTLINYISDHGEGRWNALARA
AGLKRTGKSCRLRWLNYLRPDVKRGNFTAEEQLLILDLHSRWGNRWSKIAQHLPGRTDNE
IKNYWRTRVQKHAKQLNCDVNSKRFKDAMKYLWMPRLAERIHARAGAVDDSGDYSNNDLS
CVSGVTMATVANCFDGSPSMVTSSSSDSFTSESQDLKKINLHVHGDDEKMNSEDWMQEVD
HEFWSTEIQPNNEQFQDQQLNGWVQGFSEGLSETLWSLEDIWKMQ*
>11686.m03710|LOC_Os12g37690.1|genepair1373-2
MAASQRSRSTAAQLDVDDQAAADQLMTMMRRPAAVLQDEAAAEEEASAAAADLQLELRRG
PWTVDEDLTLVNYIADHGEGRWNSLARAAGLKRTGKSCRLRWLNYLRPDVKRGNFTADEQ
LLILDLHSRWGNRWSKIAQHLPGRTDNEIKNYWRTRVQKHAKQLNCDANSARFKDAMRYL
WMPRLADASQLGDHHGYNSTTAMGDAHGMPVMTSSSSDSFATSESYDGGLYANVQDNEMV
NGGDYWMQGANKGFCSNYESEQLHPHEHSQFQDPDLVGWVQGFSEGISENFWSLEDIWKM

*
>11687.m00836|LOC_Os11g08940.1|genepair1374-1
MIIPVRCFTCGKVIGNKWDLYLDLLQADYTEGDALDALGLVRYCCRRMLMTHVDLIEKLL
NYNTLEKTETTAGN*
>11686.m00753|LOC_Os12g07980.1|genepair1374-2
MAISRSSSRRRRWVGSARGRCAEESASAAGRRRSRSRPVGVIWSGVDGGGVGGGCGWAAA
TSPAGASGRDASAMSGIKTTTTDFLSSRDKTEKVIGNKWDLYLDLLQADYTEGGSGRQRG
EERYWGLMAATEMVCYGGGGRRLADGDADLSVHMWDALDALGLVRYCCRRMLMTHVDLIE
KLLNYNNDGSDITSGQSTSGCV*
>11687.m00837|LOC_Os11g08950.1|genepair1375-1
MRLLVPDGARGGGGGGGGGLLPAAAASPAVGRGRRTVVVGIRRDAASRELLTWALVKVAN
AGDRIVALHVAAAAAAAAGGGGGGGGGAVGLEERSDAAEDSLVSVLAVYDGFCNLKQINL
ELKVCGGSSIRKTLVKEAASYGAAHLILGVAKNSLSFSRSSSISVAKYCAKRVPTGCSVL
AVNNGKILFHKDAVQQEPYHSASTMTETPRRSYRKLLTSVIGEKLRDECEQDNRSIFRAV
TMPPSSPAPTREVSLALVPMKVHRRESPEVATGWSFLRKKFLPDRKPASHDRSKMSVVQW
AMRLPSRYSSASPVCSEYRTTTPDGITSASRILRDRVAVPSRSNSGKSSVVIEELDNSSD
KEIPEELIALREKFPSVYSTFSHSELAKITSDFSPECIVGQGGTSQVYKGCLENGKELAV
KILKYSDEVLKEFVSEIEIVSSLSHKNIISLAGFCFKDTDLLLVYEYLRRGSLEEILHGE
KGCDNLFGWTERFNVALGVAHALDYLHGSGNNRPVIHRDVKSSNILISQDFEPKLSDFGL
ALWDTDATSQITCNDVAGTFGYLAPEYFMHGKVNDKIDVYAFGVVLLELISGKKPLCTGC
PKGQESLVMWANSIIQGGKLTQLVDPNLPTEDHANKVERMTLAASLCIRPTPQRRPHIAV
VLKLLNGDNGILKWARSEVGLSYESDGDEPVVTLPENNRNIQSYINLAFDVDDDSASVSS
NDFIAANTSLEEYLRGRWSRSSSFD*
>11686.m00754|LOC_Os12g07990.1|genepair1375-2
MSRRAPPTMLALPAPPRLGWGGRTVVVGVRDDAEGRELLTWALAMVAFAGDRVVALHVAT
PAAAAGADQEGAMRMAARRIRATESLAALLRAYDGFCDLNQISLELRICHGSSIKKALVN
EASSYGAAHLILGVTNNSRSHLRPSSSPSSSSAAVAKYCAKRVPPSCSVLAVGNGRVVYR
RDAAQQQLNQCISPLVETPRRIYRKIYRKLVRAATTITREKSQDDAAIAGGGRHLRRNIS
TPTSAPVSPVVAFTPRQAEVAAGWPLLSPDLKSALPEWTEMSVARWAMQLPSRCPAPSPL
NPRNNSGDQATSPAITASETPSPATDEAAEQVAQELASLRNKYSSKYTMFSYSELARITS
NFSPDRIIGKGGASEVYKGCCDDGKEVAVKVLKSSDKVMEELVSEMEIVSSVRHGNAMPL
TGFCLDGGGGAAKIMLVYDYMARGSLEEILHGEKEGKDLFGWPERFKVAAGVARALVYLH
GGDGDGRPVIHRDVKSSNILVSEDFQPKLCDFGLALWAAEAASPVTGDDVAGTFGFVAAA
ASSHLLEEFKYLAPEYFMHGKVSDKIDVYAFGVVLLELVSGRKPVSSGGGKGKESLVMWA
NTIIQGGKLTDLVDPSLPTDGSGVAGEVERMTLAAALCIRRSPQRRPSIANVLKLLDGDG
DAVRWVRSQAGLSAGDDTDDGDGASPEKKDIQSYINLALLDVVDDSASVSSGGGDFTAAN
VSLEEYTKGRWSRSSSFD*
>11687.m00844|LOC_Os11g09020.1|genepair1376-1
MGKAAAMEVSASAAAEAGMMVGHGEWRDDDGRARRMGTVWTASAHIITAVIGSGVLSLAW
AIAQLGWVAGPAVMLLFAFVIYYTSTLLAECYRSGDPCTGKRNYTYMDAVRANLGGSKVR
LCGVIQYANLFGVAIGYTIAASISMLAIKRADCFHEKGHKNPCRSSSNPYMILFGVVQIV
FSQIPDFDQIWWLSIVAAIMSFTYSTIGLSLGIAQTVANGGFMGSLTGISVGTGVTSMQK
VWRSLQAFGDIAFAYSYSIILIEIQDTIKAPPPSEAKVMKRATMVSVATTTVFYMLCGCM
GYAAFGDKSPDNLLTGFGFYEPFWLLDVANAAIVVHLVGAYQVFVQPIFAFVERWAAARW
PDGGFISRELRVGPFSLSVFRLTWRTAFVCATTVVSMLLPFFGDVVGLLGAVSFWPLTVY
FPVEMYIAQRGVRRGSARWLCLKVLSAACLVVSVAAAAGSIADVVDALKVYRPFSG*
>11686.m00764|LOC_Os12g08090.1|genepair1376-2
MASGQKVVKPMEVSVEAGNAGEAAWLDDDGRARRTGTFWTASAHIITAVIGSGVLSLAWA
IAQLGWVAGPAVMLLFAFVIYYTSTLLAECYRTGDPATGKRNYTYMDAVRANLGGAKVTF
CGVIQYANLVGVAIGYTIASSISMRAIRRAGCFHHNGHGDPCRSSSNPYMILFGVVQIVF
SQIPDFDQIWWLSIVAAVMSFTYSGIGLSLGIVQTISNGGIQGSLTGISIGVGVSSTQKV
WRSLQAFGDIAFAYSFSNILIEIQDTIKAPPPSEAKVMKSATRLSVATTTVFYMLCGCMG
YAAFGDAAPDNLLTGFGFYEPFWLLDVANVAIVVHLVGAYQVFVQPIFAFVERWASRRWP
DSAFIAKELRVGPFALSLFRLTWRSAFVCLTTVVAMLLPFFGNVVGLLGAVSFWPLTVYF
PVEMYIAQRGVPRGSARWVSLKTLSACCLVVSIAAAAGSIADVIDALKVYRPFSG*
>11687.m00845|LOC_Os11g09030.1|genepair1377-1
MAPPMQEGEERPGGSACVWMVTTLLLLSVLAGGGCLAGYVVLPPHEAPHWLPAVGLALVA
LPWAFWVATCSYRCVRRRAADRQAMGSAAVAPAATGSMRSCADS*
>11686.m00770|LOC_Os12g08150.1|genepair1377-2
MGQQRQAAADQAGRAAGGACVWAVAAALFLAVLAGGGCLVFYLALPPAEVPEWLPVAGLS
LVALPWAFWIATCAYRLCCCCFSSSSAPEKANAAAAAAGHVERQPSSAAVAPLPSSTNLK
SAVRSAMGSYSHSGTRRVHFGDSTVLGEKAAGAGGGEPAVVEEVEEEEEKECSSATSSHE
SEAPIAQSMHSS*
>11687.m00846|LOC_Os11g09040.1|genepair1378-1
MATDRVEVDTARPFRSVREAVAVFGERILVGDGYSRRPSNGNAAAAAVVDIAIAKHEASG
DSDDATVSSPDAMEAEPEVEEDAAPAVVPMMYSAPSSPQSSPPPPNDGGDADDERDGGVV
DEGVTVAMMRSVKKLEAEVAETRQEVAQLKKRGSEMEMAVASLNAQLHRGLSKLAEMEAG
DTAAAARRSVGGETDVSSTVATFRSERWGGVGGGGAAVSRATSCEYLPSFSHALSLGEVD
DGELVGRRRKARKVKPIVPLIGDIIFSKRKSTKDKGGDGFYGNNGDLYSVLG*
>11686.m00771|LOC_Os12g08160.1|genepair1378-2
MAATAASPERARAEVDTSSAFRSVKEAVAVFGERILVGENRNGGGGYGGGDRRAGREGRT
RSNTLAIAASFAKLEGGGGGDGVRVSNHSKPNAIGVNAKLPVASDATPPAMYLVPSSSPP
FFASSPSLANDDDGVSAASASDAMVMGSIRKVEEEAARARQEVVQLKKRLAETELAMATL
SAKLHRALSKLAHMEADRAAAERARIQRRDGRDMALAVWAASGGGDRRRGVATAAAHAAA

TARRQPLGELLRLGEADVVGARRRRRRARW*
>11687.m00855|LOC_Os11g09130.1|genepair1379-1
MEVISRHRAPASSSSLCLLFLTFLLSVSMAAATEKSPMQLNKAQENIMRDILGLVSSAMD
SSLTKSWNTSSNPCEWSGVHCTSAASSSFVTRLSLPGYGLSNATILASICLLDTLHSLNL
SRNSFTDLPSQFSPCPMKAELQVLDLSYNRLSSHLGNFSGFHELEVLDLSFNSLNDNIST
QLNYLPKLRSLNLSSNGFEGPIPTSMVTSLEELVFSGNNFSGRIPMGLFRYGNITLLDLS
QNNLVDDVPDGFLSFPKLRILLLSENNLTGKIPQSLLNVTTLFRFASNENKLSGSIPQGI
TKNIRMLDLSYNMLNGEMPSDLLSPDSLETIDLTANRLEGLIPGNFSRSLYHLRLGCNLL
SGSIPESIGNAIRLAYLELDDNQLSGPIPSQLGKCNNMVLMDLSTNKLQGVVPDELRNLQ
QLEVIKLQTNNFSGYIPRIFSGMTNMEVLNLSANSFSGEIPSTLVLLSKLCYLDLHGNNF
SGVIPPSISSLQFLSTLDLGNNQLTGTIPTMPTKIGALILSHNHLQGSIPSSIGALSNLL
LLDLSDNHLSGQVPSSFANLKGLIYLSLCYNQLSGPMPELPRGVKVDVSGNPGLTICTED
SDSQYNMASTEDDFRSTTWVATVSFVVGFIISFYWAGIRKYCY*
>11686.m00773|LOC_Os12g08180.1|genepair1379-2
MKAGLRSLNLSSSQLSMPLSNFSGFPLLEVLDLSFNSFSGDVRTQLSSLLKLRSLNLSSN
NLAGDVPTSMTPSLEELVLSINNFSGSIPIALFNYQNLTMLDLSQNNLNGDVPDEFLKLP
KLKTLLLSGNQLSGNIPVSVSNVASLARFAANQNNFTGFIPSGITKNVKMLDLSYNELSG
VIPSDILSPVGLWTVDLTHNKLEGPIPSSLSPTLYRLRLGGGNSLNGTIPATIGDASTLA
YLELDSNQLTGSIPLELGRCKSLSLLNLASNKFQGPVPDAISSLDKLVVLKLQMNNLDGP
IPSVFSNLTSLITLNLSGNSFTGGIPREIGKLPKLSILNLQCNKISGTIPDSLHLLTSLI
ELNLGNNILTGTIPTMPTKLSTVLNLSHNNLSGSIPSNIDLLSDLEILDLSYNNLYGEVP
ASLAKLESLTQLVLSYNHLSGSIPIFRQHVDIATNGNPDLTNGTRNYDNAPTSGKRRTHN
TVIIVVAITGALVGLCLLAAIVTISYSKRIYRVEDEGPSTEDVARIINGHLITMNSIHTS
AIDFVKAMEAVSNHSNIFLKTRFCTYYKAVMPNGSTYSLKQINCSDKIFQIGSQGKVAHE
LEVLGKLSNSNVMVPLAYVLTEDNAYIIYEHVHKGTVFDFLHAGRSDVLDWPSRYSIAFG
LAQGLTFLHGCTQPVLLLDLSTRTVHLKSMNEPQIGDVELYKIVDTLKSSGSLSTIAGTV
GYIPPEYAYTMRLTMAGNVYSFGVILLELLTGKPSVSDGIELAKWALSLSGSPDQREQIL
DTRVSRTSAAVHSQMLSVLNIALACVALSPDARPKMRTVLRMLFNAK*
>11687.m00467|LOC_Os11g05470.1|genepair1380-1
MSRSVEPLVVGRVIGEVLDTFNPCMKMIVTYNSNKLVFNGHELYPSAVVSKPRVEVQGGD
LRSFFTLVMTDPDVPGPSDPYLREHLHWIVTDIPGTTDASFGREVISYESPKPNIGIHRF
IFVLFKQKRRQTVIVPSFRDHFNTRRFAEENDLGLPVAAVYFNAQRETAARRR*
>11668.m03104|LOC_Os02g32950.1|genepair1380-2
MSRVLEPLIVGKVIGEVLDNFNPTVKMTATYGANKQVFNGHEFFPSAVAGKPRVEVQGGD
LRSFFTLVMTDPDVPGPSDPYLREHLHWIVTDIPGTTDASFGREVVSYESPRPNIGIHRF
ILVLFRQKRRQAVSPPPSRDRFSTRQFAEDNDLGLPVAAVYFNAQRETAARRR*
>11687.m00472|LOC_Os11g05520.1|genepair1381-1
MAQMEFEPDKVGTVTVNGKVYSFRRVHWHAPSEHTINGEKHPLELQMVHAAADGSLAVIA
ILYKYGAPDSFYFQLKRKLAELAADGCSFGEENAQVALGLVHLRSLQKRTGSYFRYAGSL
TAPPCTEDVFWSVLGKIRQISQEQVALITALLPAGGARPTQPLNGRTVQFYNPPNSTISF
KV*
>11668.m03112|LOC_Os02g33030.1|genepair1381-2
MGTAKKSAIFVALLCTHILVNHACDSDVVFGYSGSTGPEHWGSLSPNFTTCSKGTYQSPI
NILKDDAVYNPKLEPLEMDYTAANTTIVDNVFNIALRYNDTAGTVKVDGKKYKLRQLHWH
SPSEHTINGQRFAVELHMVHHSDDGNITVIAVLYRHGKPDPFLFQIKDKLAALYLEGCKA
EKGEPLPVGLVDMRELKKGADRYFRYVGSLTTPPCTENVIWNIFGEIREMTKEQAAALRA
PLHGSYRHNRRPTQPLNGRTVQLNYM*
>11687.m00473|LOC_Os11g05530.1|genepair1382-1
MASSARDLSGDPPESTRLRIGDDIAWSDVGGVYDRDDSLKENTNPKCILKNHLPGAHNGG
SQRFSGNLKPTAAPIIGISGKLGQGGKNRHHPPAMFPKKVAVTGGGGRNPKAAVPEHEPT
SPKVSCIGKVLSDRERARRGRPAGRMVPAGGCCPGLGGLFRRSHSRKKNAVECVDQSPP
PLPPWASRRGEPKEVKEATPAAAAAMAPGLGGMMRFASGRRAADWATEMEPDGRVARSGP
L*
>11668.m03115|LOC_Os02g33060.1|genepair1382-2
MPPLTLDSLLHGGGGEPEDECEDEFSGSDDDGEDGGGGSEEWGGDVDGEYDPYSPAESLW
LRIGEDIDWSEVGAVLEREDSTKGASNPKSAAACSCAGAPAARMPTCAGGGGTAKAVVIA
GLPAAARKASREHERRRRLGRARARARVFAGDAVEVAEPGSPKVSCLGGVRSRARAQQPC
CPAAAAAGRRRWWCAPWLVSAACRRCWSAPPPRV*
>11687.m00475|LOC_Os11g05550.1|genepair1383-1
MAADGGALKRLFEKPLPENPTLLEALSAWNHTHRKKLVDPASFTEIFGELHFQEKPPVVD
SLARAAARPSPSPPPPPPPRRTVSWLDITDAATADNDNDKSKDDSSLDALLKPPRPASGG
ATVKRSASFCLKSSTSLLLCTEGLGSESTVDADDMVKDGDGSGAVVDSGMDVDDDASDVA
AAVAGDDAFGAGGKENRPPPPPPSFPPPIRSIGRGGKPSVCFRSVRAEGRFVLMEVVIPG
KDLLRATREGGRLRLQFSNAAAAAAAAVGVIDDEEMHGQEAAACVGGDTFA*
>11668.m03120|LOC_Os02g33090.1|genepair1383-2
MPRPPPPLFGQAGQDQRRRYLGLCTEGLGSESSESSGGDVDLGTGGGDDTGNDGVGRALP
CKRQHRPIDDEEEEEEKTVVPAALAPPLPAWTRAAFPPPISVIGAGGKPWLYLRAHRGDG
RLVLREVRIPSRELLHGRREDGRFKLHFAHPDEQLQQQLLLLADDQDPAEKNE*
>11687.m00486|LOC_Os11g05660.1|genepair1384-1
MDELRAPACGGGVGTVIEDLPTDVLALVLRRLDGASLAAVGCACSSFRELAVDQETWRGL
CLALWPSVRDVLGCGGGGGGDGYRAFFADAFPFPEAAAAVASAAPAPVPGSLPSRLVSAV
DLHHGGVCIMSRVVETDTSSAWFLGSPFRVDALVQEGFSAPSPITPSSLTLSWILIDPAS
GRAMNASSRRPVAVDRKWLTGETVARFTVMLGGGVALDAAVTCDDRYGHVREVSLCVEDG
EGGGVSGQDGLGAVAAAMAGARRGKGAEAAARQRYEEFVKGKRARKEWKARREGIVDLCC

SGVGAAAFVGFLVMLTFR*
>11668.m03136|LOC_Os02g33240.1|genepair1384-2
MAATTTTVEDLPGDVLACALRRLDGPSLAAAGCATSGLRALADDPDTWRALCLSRWPSLA
AAEQRCVLSAAGAVSPRRLFADAFPFPCVDDAAAAAPLDGDDQRLPGELVSAVDVYHGGA
AVVSRVVETSTSSSWFLASPFRVDAVEGKSPAPAPASVASSWSPAELELSWILLDPSTGR
AVNVSSRRPVAVERHWYTGDTLVRYAVVLAGCKFEATVSCSEEAGQITEVSLAADDADGA
AISGEGCLRLLAAAMAGPRKGGRGQEGEAKRRYDEFVRRKRGRKESKARREVLVDLCCSA
VSAVAVISFLAAVVLR*
>11687.m00508|LOC_Os11g05860.1|genepair1385-1
MGKDCGNHGDDDIRQACRRLLTILFGLALIVAIIALIVYLVLRPTHPRFFLQDATLRQLD
LSNSSTSGVLSTALQVTVASRNPNDRVGVYYDRLDVYASYKYQQITLAASLPPVYQGHGD
VDVWSPVLSGPDVPFAPYLGDALAKDVAAEYLILQVKIDGRVRWKVGSWISGHYHLFVTC
PAFFIASGGNGYPGANGLKFQTATYCRVEV*
>11668.m03168|LOC_Os02g33550.1|genepair1385-2
MSKEKHHKREHHLRRCCGGMAACILALVLVVGFIALVVYLALRPSKPSFYLQDLQLRSVD
LGDPSLSATAQVTLASRNPNDHVGVHYRRLDVFVTYRDEPVTVPVSLPPTYQGHRDVTIW
SPVLSGESVPVAGFVADALRQDVAAGYVALQVKVDGRVKWKVGSWVSGSYHLFVSCPAML
ASAGPGGVGPMPLGGASAAVVNGTGAGAVASLRFTQPTGCSVEV*
>11687.m00521|LOC_Os11g05990.1|genepair1386-1
MASYGVDTRPAAAAAGGGGAGAGAAGEGALSFLSRGLREDLRLIRARAGELETFLTAPVP
EPELLARLRRAYSSSAGTTRLDLSAIGKAFGTGVVGRGSRGARWGWEEVQEAEEWEPIRM
VKARLREMERRRQWQATDMLHKVKLSLKSMSFVPEASEEVPPLDLGELLAYFLKQSGPLF
DQLGIKRDVCDKLVESLCSKRKDHLAYNSFPASEPSAFSNDNAGDELDLRIASVVQSTGH
NYEGGFWNDGHKYETADKRHVAIVTTASLPWMTGTAVNPLFRAAYLAKSSKQDVTLVVPW
LCKSDQELVYPNSMTFSSPQEQEAYMRSWLEERVGFKTDFKISFYPGKFQKERRSIIPAG
DTSQFIPSKEADIAILEEPEHLNWYHHGKRWTDKFNHVVGVVHTNYLEYIKREKNGVIQA
FFVKHINNLVARAYCHKVLRLSGATQDLPKSMICNVHGVNPKFLEVGERIAAERESGQHS
FSKGAYFLGKMVWAKGYRELIDLYAKHKSDLEGIKLDIYGNGEDSHEVQSAAMKLNLNLN
FHKGRDHADDSLHGYKVFINPSISDVLCTATAEALAMGKFVVCADHPSNDFFRSFPNCLT
YKTSEDFVAKVKEAMARDPQPLTPEQRYNLSWEAATQRFMEHSELDKVLSSSNRDCTTST
SGCGKSGDNKMEKSASLPNMSDMVDGGLAFAHYCFTGNELLRLSTGAIPGTLNYNKQHSL
DLHLLPPQVQNPVYGW*
>11668.m03171|LOC_Os02g33580.1|genepair1386-2
MEARASPSPSPQRPASMGRGGGGGGGGGDASAALSFIYKGWREVRDSASADLRLMRARAD
SLRTLADRELEHLLVSASTTVAAPAPPVAAGAPIAEVEFVRNRIQPKISELRRQYAASGD
WELGLGRRVLEGWVAPPPPRGATTARVDLSGITAIRNALVPEVAGGGGASTAWWSGDEME
EEEKEWEVVRMIRGGLKELERRSQSSGEILGGIPGPSELVEKFKSRLKSFNMEPLGSKE
VPPLDLTEIMANLVRQSGPFLDQLGLRRELRDKLVETLYSRQNHSLSADSSLLGDDNSTD
ELDLRIASVLQSTGYHTDDGLWNEPSKYEVSDNKRHVAIVTTASLPWMTGTAVNPLFRAA
YLARNSKQDVTLVVPWLCKSDQELVYPNSMTFSSPEEQETYIKKWLEERLGFESNFKISF
YPGKFSKERRSIIPAGDTSQFISSREADIAILEEPEHLNWYHHGNRWTDKFNHVVGVVHT
NYLEYIKREKNGALQAFLVKHINNWVTRAYCDKVLRLSAATQDLPKSIICNVHGVNPKFL
KIGDKIMADRENGQQSFSKGAYFLGKMVWAKGYRELLDLLDKRKSDLQGFKLDVYGSGED
SQEVQSTAKKLNLNLNFFKGRDHADDSLHGYKVFINPSISDVLCTATAEALAMGKFVICA
EHPSNEFFMSFPNCLTYRTPEEFVARVNEAMAREPQPLTPEQRYNLSWEAATERFMEYSD
LDKVLSQPVTEGVHRSKTRRTIQSNLSDAMDGGLAFAHHCLTGSEVLRLATGAIPGTRDY
DKQHCVDMGLLPPQVQHPVYGW*
>11687.m00034|LOC_Os11g01340.1|genepair1387-1
MAASATQEADCKASEDARLFFDAAKPPPFRIGDVRAAIPAHCWRKTPLRSLSYVARDLLI
VAALFAAAASSIDLAWAWAWPLYWAAQGTMFWALFVLGHDWRISHRTHHQNHGHIERDES
WHPITEKLYWQLETRTKKLRFTLPFTLLAFPVYLWYRSPGKTGSHFLPSSDLFSPKEKSD
VIVSTTCWCIMISLLVALACVFGPVPVLMLYGVPYLVFVMWLDLVTYLHHHGHNDLPWYR
GEEWSYLRGGLTTVDRDYGWINNIHHDIGTHVIHHLFPQIPHYHLVEATKAARPVLGRYY
REPEKSGPLPLHLFGVLLRSLRVDHFVSDVGDVVYYQTDHSLNGTDWAEDAKHK*
>11669.m01827|LOC_Os03g18070.1|genepair1387-2
MARLVLSECCGLTPLRLRGRGAIALPAPPSLAAGPRRPVSAAAAGGAIHREWALRVSAPT
RLTSVVEEDNRGEEVVEEEARGSLAAAEAAAGEVGGDGDGFDPGAPPPFGLAEIRAAIPK
HCWVKDPWRSMSYVLRDVVVVLGLAAAAARVDSWLVWPLYWAAQGTMFWALFVLGHDCGH
GSFSSNAKLNSVVGHILHSSILVPYHGWRISHRTHHQNHGHVEKDESWQPLSERLYNSLD
YMTKKLRFTMPFPMLAFPLYLFARSPGKKGSHFNPSSDLFQPNEKKDVITSTASWLAMVG
ILAGLTFVMGPLKMLKLYAVPYVIFVMWLDFVTYLHHHGHEDKLPWYRGKEWSYLRGGLT
TLDRDYGWINNVHHDIGTHVIHHLFPQIPHYHLVEATEAAKPVLGKYYKEPEKSAPLPFH
LLVGHWAMGILLSLSVKLPPLSSRFEAYHPHSDEDADMKYDVDRMIKPLMNAFVWCMHMR
DTSIVGIDGADNRTLQHVRSSGDGAESVIPLIHALLAYFIHLALFRMQQPDTLLVIDDAS
LSIID*
>11687.m00037|LOC_Os11g01370.1|genepair1388-1
MTKPQQQSPPSTTATTTTSPPPPPPSTPPPASSSSSSLAKLPLRLHSLASSSRSLLSALR
RSPVTTLVAAFFLLALFMYGEDVRTLAELSIDDYLYPDADFYNVSALPPLLLPPPTCDLS
RGRWVFDNTSLPAYREKECTFLTKQVSCLANGRPDDLWQYWRWQPNNCSLPTFDARRFME
KMRGKRMMFVGDSLNRNQWESLVCLVQPILSKGRKKIVKRGSFNIFYAKEYRATLEFYWA
PFLVESNSDNPNFHHIDQRIISPERIESHANNWKDVDYLIFNTYIWWMNNEDIKVRRPNS
TSWSDHDEVPRIETYGRVFKTWSTWLEQNVDPARTSVFFMTISPLHNSSKIGIGGRWFNT
DAVITFCQFNIDEPCISATGSLELWNILKYRHRVRWFPAQWGNPNGIKCVKETLPVLNYT
KPLDLNHDMRMYDLVAKVAKNMKNVPVSLIDITRMSDYRKDAHTSLYSIRQGKLLTPEQK

ADPQKYADCIHWCLPGVPDVWNQILYTRILSKSSPPSPHPPLPPQ*
>11669.m01835|LOC_Os03g18140.1|genepair1388-2
MQQRRKSVFASAPFAMKQAALGAGVAARRNGAPLSLAAVVFALFVFATFLYNEDIKSIAD
FPFGAGALRAKSPDLHVLQETVGAAHLAAGSIAKRGEEVIVRVLDAPASTAMAAAAGSSS
NNSTIEVAKANANANANAADAGVKVDEGQERERDVTLPSVKEGGADEARRREDEEAAEKE
SSAKAAAATAALRTVVSVPDTCDLYRGNWVYDEVNAPVYKESQCEFLTEQVTCMRNGRRD
DSYQKWRWQPTDCDLPRFDARLLLERLRNKRLMFVGDSLNRNQWESMVCLVQSVIPKGKK
TLTKFVNGGNSNIFYAHEYNATVEFYWAPFLVESNSDNPQVHSVPDRVIQWHSIAKHAHN
WLGVDYLIFNTYIWWLNTLDMKVLKGSFDQGATEYVEVDRPVAYKEVLKTWAKWVDRNID
PNRTTVFFMSMSPNHITPEAWGNYGGIKCAMETLPITNRTTSLDVGTDWRLYAGAQEVLQ
TFRRVPVHLVDITALSELRKDAHTSVHTLRQGKLLTPEQQSDPKTYADCIHWCLPGLPDT
WNQFLYARIASAPWSSDQ*
>11687.m00044|LOC_Os11g01440.1|genepair1389-1
MLVRLGVVVVASVAALTLKRANSGNRDGQARKGKDKTRYSEHGEKEEEKEEVKTISGIIN
SALSDDDDMLSEIESLLSGEIDIPLPSDRFDVKERSWYNSVNSELERLRGLVRELEEREV
KLEGELLEYYGLKEQETDVVELHRQLKIKMVEIDMLKMTINSLQEERKKLQDDVARGTGA
KRELEAARNKIKELQRQIQMEANQTKGQLMLLKNQVIALKSKEEEAAIKDAEVQRKLKKL
KELEVEVVELRRKNKELLYEKRDLIVKLDAAQGKITESDVVSHAREEINKLRHVNEDLTK
QVEGLQMNRFSEVEELVYLRWVNACLRYELRNYQAPSEKISARYLNKTLSPKSRERAKLL
MLEYAGSERGQGDTDLETASSAPSSPRSEDLDNVSVDSSSSRYSFFGKRPNLMQKLKKWG
RGKDDESRLASPTQFFTSDSPKSASQKPKGPLEALMLRNAGDGVGITTFGKREQDPSDIM
DEANVASSFHLMSKTVQGFADDKYPAYKDRHKLATEREKVIKEKAEKARVQRYGGVNSSG
IVPSPRSALPPKLAQIKEKAPTANAESSDQPSDNQNNPLVVTQLKLANIEKRAPRVPRPP
PAPSATANTASALSPPPPRPPGAPPPPPPPGKPGGPPPPPPPPGSLPRNLAGGDKVHRAP
EVVEFYQSLMKREAKKDTTSLGSTTSSAFDVRSNMIGEIENRSTFLLAVKADVETQGDFV
ESLANEVRAASFVNIDDVVAFVNWLDEELSFLVDERAVLKHFDWPESKTDALREAAFEYQ
DLLKLEHKVSSFTDDPKLACEEALKKMYSLLEKVEQSVYALLRTRDMAISRYREYGLPVD
WLSGSGVVGKIKLASVQLAKKYMKRVATELDALQGTEKEPNREFLLLQGVRFAFRVHQFA
GGFDEESMKAFEELRSKMSTQTSAPQISDV*
>11669.m01851|LOC_Os03g18300.1|genepair1389-2
MMREGDACVALLRSKLHGLVERNRSLEEENKQLRHQVSRLKGQVSSLEGQDTDRKMLWKK
LDNSSTGNSYLKEKQFVPNNDAKEAMDLNSTSCYSRQQFSRAPLVRSRAPRVPNPPPSPT
YTQPIVNARKEGGMAPPPPPPPLPSRLLKSTKAVQRVPDVVELYRLLVRREGKNDAKSGS
MGIPAATNSREMIGEIENKSAYVLAIKSDVENQSEFINFLAVEVKNAAYKEIADVEEFVK
WLDGELSYLVDERAVLKHFPNWPEKKADTMREAAFTYRDLKKNLESEASSFHDDRRVATPM
ALKRMQALQDKIEQGIHNTERARDSASGRYKDLKIPWEWMLDSGIISQLKMASLKLAREF
MNRVVNALKSDPFTNDEELLLQGVRFAFRIHQLAGGFDEGCRKAFQELKMYASKSD*
>11687.m00052|LOC_Os11g01510.1|genepair1390-1
MLTRKREELAGEVHDLHKKTRADDEPADDNHTMTTGRAPEIDEDLHSRQLAVYGRETMKR
LFASNVLVSGLNGLGAEIAKNLVLAGVKSVNLHDDDNVELWDLSSNFFLTEKDVGQNRAQ
TCVQKLQELNNAVIISTITGDLTKEQLSNFQAVVFTDISLEKAVEFDSYCHNHQPPIAFI
KSEIRGLFGSVFCDFGPEFTVLDVDGEEPHTGIVASISNDNPALVSCVDDERLEFQDGDL
VVFSEVHGMSELNDGKPRKIKNARPYSFTLEEDTTSYGTYVRGGIVTQVKPPKVLKFKTL
KDAIKEPGEFLMSDFSKFDRPPLLHLAFQALDKFRNDLRRFPIAGSSDDVQRLIDFAISI
NESLGDSKLEELDKKLLHHFASGSRAVLNPMAAMFGGIVGQEVVKACSGKFHPLYQFFYF
DSVESLPVEPLEPAELKPENTRYDAQISVFGSNLQKKLEQAKIFMVGSGALGCEFLKNLA
LMGISCNQNGKLIVTDDDVIEKSNLSRQFLFRDWNIGQPKSTVAATAAMAINPKLHVEAL
QNRASPETENVFNDAFWESLDAVVNALDNVTARMYIDSRCVYFQKPLLESGTLGAKCNTQ
MVIPHLTENYGASRDPPEKQAPMCTVHSFPHNIDHCLTWARSEFEGLLEKTPTEVNAFLS
NPGGYATVARTAGDAQARDQLERVIECLEREKCETFQDCITWARLKFEDYFSNRVKQLTY
TFPEDAMTSSGAPFWSAPKRFPRPLEFLTSDPSQLNFILAAAILRAETFGIPIPDWVKNP
AKMAEAVDKVIVPDFQPKQGVKIVTDEKATSLSSASVDDAAVIEELIAKLEAISKTLQPG
FQMKPIQFEKDDDTNYHMDVIAGFANMRARNYSIPEVDKLKAKFIAGRIIPAIATSTAMA
TGLVCLELYKVLGGGHKVEDYRNTFANLAIPLFSMAEPVPPKTIKHQDMAWTVWDRWTIT
GNITLRELLDWLKEKGLNAYSISCGTSLLYNSMFPRHKERLDKKVVDVAREVAKVEVPPY
RRHLDVVVACEDDDDNDVDIPLVSIYFR*
>11669.m01860|LOC_Os03g18380.1|genepair1390-2
MLPTKRANGAEAESSSDAPAKKARVGASASEAEAMVAGEAGGGGGGVSGNGSEVAEIDED
LHSRQLAVYGRETMRRLFASNVLVSGLNGLGAEIAKNLALAGVKSITLHDMGNVEMWDLS
GNFFLSEDDIGKNRAVACTAKLQELNNAVLISTLTEDLTNEHLSKFQAVVFTDISLDKAF
EFDDYCRNHQPSISFIKAEVCGLFGSVFCDFGPKFTVLDVDGEEPHTGIIASISNDNPAM
ISCVDDERLEFQDGDLVVFSEVQGMTELNDGKPRKIINARPYSFCIQEDTSKFGIYAKGG
IVTQVKEPINLEFKSLRDSIREPGNFLLSDFSKFDRPPLLHFAFLALDKFRKEFGRFPGA
GCDQDAQRFIEFVASVNEATIDYKMDELDGKLLRNFASGSRAVLNPMAAMFGGIVGQEVV
KACSGKFHPQYQFFYFDSAESLPTYPLDSKDLKPLNSRYDAQISVFGSKLQKKMRDANVF
VVGSGALGCEFLKNLALMGVSCGLKGKLTITDDDIIEKSNLSRQFLFRDWNIGQAKSTVA
AAAASAINSSLHINALQNRACPETEHVFHDKFWEGLDVIINALDNVNARMYMDMRCLYFQ
KPLLESGTLGPKCNTQMVIPHLTENYGASRDPPEKQAPMCTVHSFPHNIDHCLTWARSEF
EGLLEKTPNEVNSFISNPAQYAAAMRKAGDAQARELLERVCECLDKERCDGFEDCIAWAR
LKFEDYFANRVKQLTFTFPEDAVTSTGAFFWSAPKRFPRPLQFSTVNSSHIHFILAASIL
RAVSFGISIPDWAKNTSNLVDAVSKVVVPEFEPKSGVKIETDEKASNISSASVDDASVIE
DLLTKLEASAKKLPPGFQMKAIQFEKDDDTNFHMDLIAGLANMRARNYGIQEVDKLKAKF
IAGRIIPAIATSTAMATGLVCLELYKVLAGGHPVEDYRNSFANLAIPMFSMAEPLPPKVI
KHQDMRWTIWDRWSIEGNITVAELLKWLSDKGLSAYSVSCGTSLLYNTMFPRHKDRVNKK

LVDVAKEVAKVDVPAYRRHLDVVVACEDDDGNDVDIPLISIYFR*
>11687.m00064|LOC_Os11g01620.1|genepair1391-1
MQGPRRRTPPAPAAKQPAMLMLGPFPAHHRCTLFLLLTVTLLPSLAAAAAAHHHHVHAAG
DGVVISQADYQGLQAIKHDLSDPYAFLRSWNDTGLGACSGAWVGIKCVQGKVVAITLPWR
GLAGTLSERIGQLTQLRRLSLHDNAISGPIPTSLGFLPDLRGVYLFNNRFSGAVPASIGN
CVALQAFDASNNLLTGAIPSSLANSTKLMRLNLSHNTISGDIPPELAASPSLVFLSLSHN
KLSGHIPDTFAGSKAPSSSSLKESITGTYNLAVLELSHNSLDGPIPESLSGLQKLQVVDL
AGNRLNGTIPNKLGSLADLKTLDLSGNALTGEIPASLSNLTTSLQAFNVSNNNLSGAVPA
SLAQKFGPSAFAGNIQLCGYSASVPCPTSPSPSPSPSAPASPAQSREATGRHRKFTTKELAL
IIAGIVVGILLFLALCCMLLCFLTKKRSGSGGKQTTSSKAAGGGAGAAAGGGRGEKPGSG
AAEVESGGEVGGKLVHFDGPMAFTADDLLCATAEIMGKSTYGTVYKATLEDGSLVAVKRL
REKITKGHKDFESEAAVLGKIRHPNLLPLRAYYLGPKGEKLLVLDFMPNGSLSQFLHGQY
SVNATLLILCI*
>11669.m01889|LOC_Os03g18630.1|genepair1391-2
MAAVPAGGQPSDGVVIAQADLQGLQAIRQALVDPRGFLRGWNGTGLDACSGGWAGIKCAQ
GKVVAIQLPFKGLAGALSDKVGQLTALRKLSLHDNALGGQLPASLGFLPELRGVYLFNNR
FAGAVPPQLGGCALLQTLDLSGNFLSGAVPASLANATRLLRLNLAYNNLTGAVPSSLTSL
PFLVSLQLSSNNLSGEVPPTIGNLRMLHELSLSYNLISGSIPDGIGSLSGLHSLDLSNNL
LSGSLPASLCNLTSLVELKLDGNDIGGHIPDAIDGLKNLTKLSLRRNVLDGEIPATVGNI
SALSLLDVSENNLTGGIPESLSGLNNLTSFNVSYNNLSGPVPVALSSKFNASSFAGNIQL
CGYNGSAICTSISSPATMASPPVPLSQRPTRKLNKRELIFAVGGICLLFLLLFCCVLLFW
RKDKQESESPKKGAKDATAKAAAGKSGGGGGGSGGAGGDGGGKLVHFDGPLSFTADDLLC
ATAEILGKSTYGTVYKATMENGTFVAVKRLREKIAKNQKEFEAEVNALGKLRHPNLLALR
AYYLGPKGEKLLVFDFMTKGNLTSFLHARAPDSPVDWPTRMNIAMGVARGLHHLHAEASI
VHGNLTSNNILLDEGNDARIADCGLSRLMNATANSNVIAAAGALGYRAPELSKLKKANAK
TDIYSLGMIMLELLTGKSPGDTTNGLDLPQWVASVVEEEWTNEVFDLELMKDAAAAGSET
GEELVKTLKLALHCVDPSPAARPEAQQVLRQLEQIKPSVAVSASSSFTGEPSQTTATATT
ITDDTKSTITE*
>11687.m00075|LOC_Os11g01730.1|genepair1392-1
MPSRGCSCWLLSLALLCSLAAAKEQYHEFVIRETTVKRLCKSHNIMTVNGQFPGPTLEIN
EGDSLIINLINRGRYNMTLHWHGVRQMRTGWSDGPEYVTQCPVRPGQSYRYRFTVAAQEG
TLWWHAHSSWLRATVYGALLIRPRDGTSYPFDVQPTRELAPILLGEWWDMNPVDVVRAAT
RTGAAPNISDALTVNAQPGDLYSCSSHDTAVFPVTSGETNLLRFINAALNTELFVSLAGH
NMTVVAADASYTKPYTTSLLLLAPGQTTDVLVTFDQPPGRYYLAARAYASAQGVPFDNTT
TTAIFDYGAANNASSAAIAMPTLPAYNDTTAATAFTTNLRGLRKAELPSRVDESLFFTVG
VGLFNCTNATAQQCGGPNGTRFAASINNVSFVLPSSTSILQAHHHGAPGGVFTADFPANP
PVQFDYTAQNVSRALWQPVAGTKVYKLKYGSAVQVVLQGTNIFAGENHPIHLHGYDFYIL
AEGLGNFDAGADTGKFNVEDPPMRNTVGVPVNGWAVIRFVADNPGVWLMHCHLDVHITWG
LAMAFLVDDGVGELQSLEAPPPDLPLC*
>11669.m01890|LOC_Os03g18640.1|genepair1392-2
MRLCFFSAAALFLLCFLVPAAVAEERFYEFVVQETLVKRLCNTQKIITVNGQFPGPTIEV
YDGDTVAIRAVNMARYNVTLHWHGLRQLRNGWADGPEFVTQCPIRPGGSYTYRFAIQGQE
GTLWWHAHSSWLRATVHGALLIRPRPGVPYPFPKPHSEFPIILAEWWRRDPIAVLRQSMI
TGAPPNVSDAILINGQPGDFLECSAQVPDLVKPTKATFGLEKSIPTVVYSIGRRDERCIV
LFRCLQRDKMKRQRFSRLLTMHAVAVTETSIIPVAAGETTLLRIINAAMNTELFVSLAGH
KMTVVAADAMYTKPFETTVVLLGPGQTTDVLVTAHAAPGRYYLAARAYASAQGVPFDNTT
ATAIFQYKGGAGCPTTAGGAGAAGAVAGAGVGAGAAGGAGAVAGTGAGAGTFNGSLGRSK
YSGGNPGRAGPAPMLPYLPAYNDTNTATAFSNSIRSPAPVKVPGPVTQEVFTTVGFGLFN
CMPGPFCQGPNNTRFGASMNNVSFQLPNTVSLLQAHYHHIPGVFTDDFPPMPPVFFDFTS
QNVPRALWQPVKGTKLYRVRYGAVVQIVFQDTGIFAAEEHPMHIHGYHFYVLATGFGNYD
PVRDAHKFNLVDPPSRNTIGVPVGGWAVVRFVADNPGVWLVHCHIDAHLTGGLGMALLVE
DGEAELEATMAPPLDLPLCAL*
>11687.m00087|LOC_Os11g01850.1|genepair1393-1
MLLACDCDDDAQTAENGNAPMLSNYRAQPAPPLAEPLLLDKPQRAHWLRQLKPWRRNAAD
DHLSAGIAINWSSVRSATKDWITNPMNIAMLLWLLCVAVSGAMLVLLLLGLLDGAFPTPA
ARNHWIEINNQVLNALFTLMSLYQHPVLCHHLFLLCRWRPADAADLRAAYFKDGAGPRHG
ERAHMAVVVALLHLTVACQYVLCGLYWGYTKKTRPELVENGFFVLGVVAPVVAVVYTVCS
PLGKDNYGELACPNAFDSVSQHKCTGHAVVEPEWAGGMFDCGGDATAWWLSLSCTFCAFG
WNMERLGFGSMFVHTATFVLLCFAPLWVMGVSALHIHDVVIGDMVGGAGALLCVCGLLYG
GYWRIQMRERFGLPASTACCGSPSVTDYARWLFCWPCALAQEVRTESLYHIDCETFYKKL
PVVDDVEDEKRLPLLASHHVQFHEPPDTMIMAASEGSNDHVVIVHEEMVPPAVQVVVEQV
VVEGDKSEEECSAVHDEKIMGSPLPESVVIVDDDEIPASLSDGSWTVEKVKRLINVVTLV
SLLILLYTRGFIR*
>11669.m01905|LOC_Os03g18790.1|genepair1393-2
MGRRGAQLQALVLSARKPHAVLKSPSARCCLEQEPGADMVPDGGEGHEIVEVAGEPGAPS
STMRLMDFIPIYIPTVETGALSRSVRKRRFLDFLRAHPSRDWFLRSTFVGRLRHRGQGQA
ASGDDEEVDSGGGRRRPRRRFRVPFVRKIKWGKLWSYAVSWCRKPENFAMIIWLAFVAAG
LLMLFMLMTGMLDSAIPDDEQRKKWTEVINQILNALFTIMCLYQHPKIFHHLVLLLRWRP
GAGADREEIRKVYCKDGAPRPHDRAHMLVVVVLLHATCLAQYFCCALFWSYARKERPDWA
LNIGYGLGTGCPVIAGLYAAYGPLGRKQHEDSDEESAAAQAGGGNRPAENDREVEIKIYN
RRVVVSSPEWSGGLFDCCDDGTVCALSATCTFCVFGWNMERLGFGNMYVHAFTFILLCVA
PFLIFSVTALNVHDDDIRDTVVSVGVLLGLCGFLYGGFWRTQMRKRYKLPASGCGCGCEC
GAGGQGHACRAAVSDCAKWLFCWSCALAQEVRTANFYDVEDDRFVFHGARNEDGRAVLVP
LPREASTATAHSRSMSCPPKIDAVAALSGASPLGVQMAAINMERSATYSGEHHPAAMRPP

VPPLMQMDQE*
>11687.m00226|LOC_Os11g03160.1|genepair1394-1
MKGHHSLPPSTPPKRRCTALAAAVPALVVCSILLPLVFLLGLHRPGHGSEERAAVVISTE
LGFSKHKHLDGRMKHKLLKDVSRKKIPGSDGILDEKSGSRSKSKSVSTKSKEKLKGVFSL
VQLKNETRKNKELHTQRRYQLKDLSWRSKDTTIDKKENQDQEVEHENPRSCELEYGSYCL
WSVEYKEVMKDFIVKRLKDQLFMARAHYPSIAKLKNQETFTRELKQNIQEHERMLSDTIA
DADLPPFFAKKLEKMERTIERAKSCEVGCTSVERKLRQLLDITEDEAYFHTRQSAFLYHL
GVQTMPKTHHCLNMRLTVEYFKSTSIHTVQSNKQKLEDPTFHHYVIFSKNVLAVSTTINS
TVMNSKDSGSIVFHLFTDSQNFYAMKHWFDRNMYLEATVHVTDIEDHQKLSKDVDFHDMK
LLRPAEEFRVTFRNHYQSFQKQMKTEYISTFGHSHFLLPDLLPSLNRVVVLDDDLIVQKD
LSSLWNLNMGGKVVGAIQFCEVKLGQLKAYTEERNFGTNSCVWLSGLNVVELKKWRDLHI
TSRYDQLLQKLQKDSVTSFPLKVLPISLLVFQDLIYPLEDSWVQSGLGHDYGVSQTDIKR
SVTLHYNGVMKPWLDLGIHDYKGYWRKYMTNGERFMTECNIH*
>11669.m02177|LOC_Os03g21250.1|genepair1394-2
MKATPPPAKRRRGPRLAVLALVFCSLLVPIAFLFNRFPAVYVTDERPQQEIDLPSFGRMG
LERSGGVTVVKPEDGSGANAETKDTPEMIHRGGINSHHHSDNVPSKVSANPKVPPPPKIE
PLKPKAKSVPVPVQRTEVISGNNMKPAKVQNADDVEKAKACQLEFGSYCLWSIEHKEVMK
DTIVKRLKDQLFVARSYYPSIAKLKGKEALTRGLKQNIQEHERVLSESIVDADLPSFIKS
KIEKMDQTIGRAKACTVDCSNVDRKLRQILHMTEDEAHFHMKQSAYLYNLGVHTMPKSHH
CLNMRLTVEYFKSAPLDSDDSAVHKFNVPDHRHYVILSKNVLAASVVINSTVSNSEETEN
VVFHILTDAQNFYAMKHWFGRNSYRESAVHVINYEHIILENLPEFSSQQLYLPEEFRVFI
SNLERPSEKTRMEYLSVFSHSHFFIPEILKDLKKVIVLDDDVVVQRDLSFLWNIDMGDKV
NGAVKFCGLRMGQLRNLLGKATYDPQSCAWMSGVNVIDLEKWREHNVTENYLQLLKKFQH
NDDEASVRAAALPISLLAFEHLIYPLDERLTISGLGYDYAIKEELVRNSVSLHYNGNMKP
WLELGIPDYRKYWKRFLTRDERFMDECNVSP*
>11687.m00232|LOC_Os11g03220.1|genepair1395-1
MGAYEATKVVFARLQALEPNLAPNIIGMLLTKDKNEMDMIHLACGPDNLLQSIIAKVRTD
LTNKPSPPMASWGFPSDIGEEASFSIDKVGCDGGEEFSSKEYDWKLPIGGNHHRSFLSST
VNTPGWKPCLYYQSGMTTHLGSDDMQEYSSRPPQIDQSDLTNNCSARQIYLTFPPDSIFS
KEDVCNYFSMYGMVQDVRIPYQEKRMFGFVTFAYQKTVKLILAKGNPHYICDARVLVKPY
KEKDKVPNKKFQQSDSPSYMNHNRLLYSRVPFDLRRHQIDNKLKNEFAMREIASTAISTE
AKRTVISTEEGKREYGPKAATPNDACGFLESGMEYNLPHSPFSSPTKASNVAATAHTSNI
SSSSSPHKVASSLFLPTCTLELPPTTHASFKRQGKALTDHT*
>11669.m02165|LOC_Os03g21140.1|genepair1395-2
MDAYEATKVVFSRIQALDPDHAAKIMGLLLIQDHGDKEMIRLAFGPEALLHSVMAQARKE
LALLPPPPPPSSSSPTVPAAHSPFLLSRQNSGRGPAPSPSPLSASSPSSWAQAQPFSRSN
GSVDEVVGAGEELISPANSGGGAAANAPPFFPRGGDVLLDDFQLQEQLAFLNEGGVNPSH
PLQGFDGAECRSPGPGEGGGMFPYGLGWANGGPGHRRSASVNELCLGGGSSDGFGWKPCL
YYARGFCKNGSSCRFVHGDDAAALTGAAMDAATAEQQQCQDFLLRSKSQRLGPAAFPYSP
TGSLPGSPSAATKCLSLLLQQQHNDNQRAAAAAALMLGGSDEAHKFMGRPRLDRVDFASM
MNPGSRQIYLTFPADSTFREEDVSNYFSIYGPVHDVRIPYQQKRMFGFVTFVYPETVKLI
LAKGNPHFICDARVLVKPYKEKGKVPDKYRKHQGDFSGCTTPTGLDGRDPFDLHQLGARM
LQHSNSTNEMMLRRKLEEQQQAAELQQAIELHSRRLMDLQLLDLKNRAAAAVTTAMAMTI
PTANAFGSSQPLATTMVESPPDSGEQLKGTGYFTEERKMVNGGGDKEESAGEASLNADSD
QSLEHNLPDSPFASPTKSSVSAHQSFTTTDTGVVATSSCSASHVGISAGTNAGGGINHLR
PSTLDIPSPRDFFSVSRLASDHGAIGM*
>11687.m00237|LOC_Os11g03270.1|genepair1396-1
MAAHHVMSIAAAAVAMLLLMASSPAVAGTAVLGRKGGAMTDDDVVGGQAATGPGKYAVIL
DAGSTGTRVHVFRFDRMMDLLKIGDDIEVFAKNKTLINKYRILIGYYFVVKINVVPGLSS
YAGRPREAANSIQPLLDKAIHVVPNWLMKKTPLKLGATAGLILIGDEKANQILEAVRDVV
HTKSKFQYNPNWINVLTGSQEGSYMWVALNYLLDRLGEDYSKTVGVIDLGGGSVQMAYAV
SSSIAANAPEVPNGQDPYITEEYLKGRDYNIYVHSYLHYGAQASRVEILKRKNGPFSNCM
LRGFKGKFTYNGEQYEAMAAPQGADYHKCRQDVVKALNLDSPCETKNCSFNGVWNGGGGV
GQDEIYVTSSFYYIASGIGFIDSEAPSAKSTPAAYKAASEKVCILSIEEAKAAYPIARDH
AYLCMDLIYQYTLLVDGFGLEATKEITLVEKVKHGESYIEAAWPLGTAIEATTGPRLIGD
EKANQILEAILKRKNGPFINCMLRGFNGLDPNKKIMLVNKVKHGEYYIDAAWPLGTAIEA
VSPKKGLQ*
>11669.m02163|LOC_Os03g21120.1|genepair1396-2
MRRFSAAAGARQQQQQGEAVSDRVLRFRGVLVVVLAPVLLISLVLLLMPRAPASATVEGS
AGELVAAAGRRWGPRAVSGLGDGSTRYAVIFDAGSSGSRVHVYCFDGNLDLLPIGKEIEL
FKQKKPGLSAYAMDPQEAAKSLVSLLEEAEKVIPVELREQTPVRVGATAGLRALGTEKSE
EILQAVRDLLQDKSSFRSQPEWVTVLDGSQEGAFQWVTINYLLGNLGKPYSHTVGVVDLG
GGSVQMAYAISEKDAGKAPPVAEGEDSYVKELLLKGTTYYLYVHSYLRYGLLAARAEILK
AGEGNDYRNCMLEGHHGQYRYGDDIFEASGLSSGASYSKCRAVAVRALKVDEPACTHMKC
TFGGVWNGGGGDGQKNLFVASFFFDRAAEAGFVNPKAPFAKVKPSDFEEAARRVCKLNVK
DAQATYPDVSEENVPYLCMDLVYQYTLLVDGFGVDPYQDITLVKKVPYSNSFVEAAWPLG
SAIEVASSS*
>11687.m00240|LOC_Os11g03300.1|genepair1397-1
MPSSGGAMPALPPGFRFHPTDEELIVHYLMNQAASVKCPVPIIAEVNIYKCNPWDLPGKA
LFGENEWYFFSPRDRKYPNGARPNRAAGSGYWKATGTDKSILSTPTSDNIGVKKALVFYK
GKPPKGVKTDWIMHEYRLTGTSANSTTTTKQRRASSMTMRLDDWVLCRIHKKSNDFNSSD
QHDQEPEESTVEQLEDIHDNNSSEQPPAPADMNNQQSDFQPMTAMSMSKSCSLTDLLNTI
DCAALSQFLLDGSSDAIAEPPAPPSPLIYTTPHPNYQTLNYNINSNSSMPHAFESRLDHH
DGYVNNYNVNGLRRKRMMACSATSFDDGSSSNDFVHAVVKKPQLLPSDSRGSGFGGGYCN

QQLSETATGFQFQNGNLLSHPFPLNNHLQMQ*
>11669.m02156|LOC_Os03g21060.1|genepair1397-2
MVLSNPAMLPPGFRFHPTDEELIVHYLRNRAASSPCPVSIIADVDIYKFDPWDLPSKENY
GDREWYFFSPRDRKYPNGIRPNRAAGSGYWKATGTDKPIHSSGGAATNESVGVKKALVFY
KGRPPKGTKTNWIMHEYRLAAADAHAANTYRPMKFRNTSMRLDDWVLCRIYKKSSHASPL
AVPPLSDHEQDEPCALEENAPLYAPSSSSAASMILQGAAAGAFPSLHAAAAATQRTAMQK
IPSISDLLNEYSLSQLFDDGGAAAAAPLQEMARQPDHHHHQQQQHALFGHPVMNHFIANN
SMVQLAHLDPSSSAAASTSAGAVVEPPAVTGKRKRSSDGGEPTIQALPPAAAAAKKPNGS
CVGATFQIGSALQGSSLGLSHQMLLHSNMGMN*
>11687.m00251|LOC_Os11g03410.1|genepair1398-1
MPPRARTLLMPLAAATLLVASTIFLFAATGARWRPADTGLPVPAADFSAAVIESAVTDAA
AAKEELSFVDENGRPDDPASSSAAAARCDPSHAAVRVFMYDLPPEFHFGLLGWSPPTDGA
ADAAMWPDVGSGAAAPRYPGGLNQQHSVEYWLTLDLLSSSSPPCGAAVRVADSRDADVVF
VPFFASLSYNRHSRVVPPEKVSRDKGLQERLVRYLMAQPEWKRSGGADHVIVAHHPNSLL
HARSVLFPAVFVLSDFGRYHPRVASLEKDVIAPYKHMAKTFVNDSAGFDDRPTLLYFRGA
IFRKEVKIDSWKVSPYSHPNVPIPNYPPNICGYMIHLCSLTEYVYILISKIPKTSKLTDV
PTFELSKGGNIRQELHYMLKDEKDVYFAFGSVQDHGASKASQGMHASKFCLNIAGDTPSS
NRLFDAIVSHCVPVIISDDIELPYEDALDYSKFSIFVRSSDAVKKGYLMRLIRGVSKHQW
TMMWRRLKEVDKHFEYQYPSQKDDAVQMIWQTLARKVPAIRLKSHRSRRFSRYDRGGK*
>11669.m02129|LOC_Os03g20850.1|genepair1398-2
MAAAAASASASCRRRPIAWFFAIAALLFFFSWYLLLDSAAVTPEPLLAARGQGLRVGSSG
RKCDPATAALRVFMYDLPAEFHFGLLDWEPQGGGGGGGGGVWPDVRGGGVPEYPGGLNLQ
HSIEYWLTLDLLASEQGAPTPCGAVRVRHAAAADVVFVPFFASLSFNRHSKVVPPARASE
DRALQRRLLDYLAARPEWRRSGGRDHVVLAHHPNGMLDARYKLWPCVFVLCDFGRYPPSV
AGLDKDVIAPYRHVVPNFANDSAGYDDRPTLLYFQGAIYRKDGGFIRQELYYLLKDEKDV
HFSFGSVVGNGIEQATQGMRASKFCLNIAGDTPSSNRLFDSIVSHCVPIIISDEIELPFE
DVLDYSKFCIIVRGADAVKKGFLMNLINGISREDWTRMWNRLKEVERHFEYQYPSQNDDA
VQMIWKAIARKAPSIRLKVNRLRRFSRFETNRTDETPTRSSWLENQPS*
>11687.m00263|LOC_Os11g03530.1|genepair1399-1
MASKAIKRKPYTADIDRSEKQMETIIPDSVREPLLGNRTHESKSERHEPNMQPNLWDGKG
QERLGWMHVISTFIAQSVRKIGNALSQFGPLLAKFFSRSCASHGSHDEQAVLLDLSPLQE
ERLRFLRQRLNVPFDSSSVKHQDALKELWRLAYPSRQLPPLKSDLWKEMGWQNSDPATDF
RIPFTVYYTRRMVRDLNGNIPLQ*
>11669.m02109|LOC_Os03g20670.1|genepair1399-2
MSMTNLRRRLHHGDVDGRKNEHVDISSVDSLNEPLLGKSSSDTGGSEVYDPRRQDLWDDD
RKKEQLHWSFLFSNLIAQWAQWLANIIVGSGSLFGRLFPFSLDNQNSSPVYLSPLQEDRL
NTLRSRLQIPFDGSRVEHQDALRQLWRLAYPNRDIPPLKSELWKEMGWQGTDPSTDFRGG
GFISLENLIFFARNYPGSFQALLNKVQGQRADWEYPFAVAGINISFMLIQMLDLQSSVPS
SKSGVRFVELLGRDENAFDHLYCIAFRLLDAQWLVKRASYMEFNEVLKSTRTQLERELVL
EDVLEVKDLPSYTMLDK*
>11687.m00280|LOC_Os11g03700.1|genepair1400-1
MENSEDEAESDKLPLDLEPLRSLAPKFPTILGYDVETQSTDPLLVYATPSIPCSSSEQPQ
EAPASFSLPLPKSPVPIKATPISAAFPTPQHEDESSDQDYKPFCKNKKPAMPKRAKRPQQ
AEKSNDANIKRRSIRRNLDNEFNLCSSSSDNPKESVEGILMMFDSLRRRVLQLDEKEDAS
RRADLKAGTLMMQNNLRINNHKMIGHVPGVEVGDIFFFRIEMCIVGLHAPAMGGIDYISS
KNKDETLAVCIISSGGYENDDDDTDILVYTGQGGNSRHKEKHDQKLERGNLALMNSKSKK
NQIRVVRSAQDPFCNSGKIYIYDGLYRIEDTWTDTAKNGFNVFKYKLRRDPGQPDGISLW
KMTEKWKANPATREKAILLDLSSKVEHLPVCLVNDVDDEKGPSHFNYVAGVKYLRPLRKT
KPLQCCKCPSVCLPGDPNCSCAQQNGGDLPYSATGLLAKHTPMVYECSSNCQCSHNCRNR
ITQKGIKLNFEVFWTGDRGWGLRSWDPIRAGTFICEYAGEVIDETKMDIDVEEDKYTFRA
SCPGNKALSWNLGEELLEEKSTAVITKNFKKLPIIIRANNEGNVARFLNHSCSPNLLWQA
VQYDHGDDSYPHIMFFAMEHIPPMTELTYDYGTRGAPPGFEGKPFKACKLKSCLCGSKHC
RGY*
>11669.m02085|LOC_Os03g20430.1|genepair1400-2
MESNQHKASDPQDSMVHLDLDEDKIMVTSALPCPSMSVGKSVMRKRGRPSRHARGTSLSS
VTPEGCKKMEGRSYNLRSDSTILLRNSCLLIADGSTKQKRSWGLDKDDLHIPFFQISDNP
REAVDDILMTFGGLHRRIMQLIDVKMASKQLVFQALNLMRKVGYHVNKDKRVGEVPGVKI
GDIFYSRIEMLLVGLHSNINRGIEFMSGAFINKEDKIATCIVSSGMYENGDDDPYTLVYN
GQGKVHHKLERGNYSLNQSFIRRNHIRLIRSEPNPLVRLGSKEKIYIYDGLYKIEEKYRQ
TTKSRSNLKFNKLVRELGQPNGIVVWKNTQKWRENPSCRDHVIMPDMSNGAEIARVCVVN
NIDSEDAPNNFTYSTKLDNGNHMVSANKMCVCKCTSSCLGEDNCSCLKTNGSYLPYNSSG
ILVCRKTMIYECNDSCACTINCSNRVVQRGSYLHFEVFKTMDRGWGLRSWDPIPAGAFVC
EYVGVVIDKDSLVEEDEYIFEVTRPEHNLKWNYLPELIGEPSFYDMNDTFKKLC*
>11687.m00288|LOC_Os11g03780.1|genepair1401-1
MGFEETYFSAFCYLLLFSVSWKCQAAELDMAQTAVLEVDASWNLSRKIPDTLFGLFFEEI
NHAGAGGIWAELVSNRGFEAGGPHTPSNIDPWSIIGDESSIYVTTDCSSCFRQNIVALRM
EILCDNCPAGGVGIYNPGFWGMNIEEGKAYNLVMYIRSLESVELTASLTCSDGMQNLASV
SIQGIDLSNWTKIELQLLAEGTCRTSRLELTSMKKGIIWFDQVSLMPSDTYKGHGFRKEL
LYMLLELKPQFLRFPGGCFVEGDWLRNAFRWRETIGPWEQRPGHFGDVWNYWTDDGLGYY
EFLQLSEDLGAAPIWVFNVGISHHDEVDTTIIEPFVKDVLDSLEFARGRAESTWGSVRAA
MGHPERFPLKYVAIGNEDCDKGFYRGNYLKFYDAIRKAYPDIQMISNCDGSSRPLDHPAD
LYDFHVYTSAANLFIMKNKFDRTSRIGSKVFVSEYAVNEQRDAGKGSLLASLAEAAFLTG
LEKNSDLVQMASYAPLFVNDNDRTWNPDAIVFNSWQQYGTPSYWMQTYFRESSGSVIHPI
TIGSSYSDSLAASAITWKDTHDIFLRIKIVNFGPNAVNLAISSRGLQAGVNTAKSTVTVL

TSGNLLDENSFAEPNKVVPVKSELPDASEEMEAALSPYSFTSFDLALDQYSKLVAEM*
>11669.m02084|LOC_Os03g20420.1|genepair1401-2
MGVRRGRLDSSLFHMLVLVCALSQVLFVGLVTGQTAQLSVDASSQNGRTIPDKMFGIFFE
ELNHAGAGGLWAELVSNRGFEAGGINTPSNIDPWLIIGDESNIIVETDRSSCFASNPIAL
RMEVLCGATGTNACPSGGVGIYNPGYWGMNIEKTKVYKVSMYIRSSDSVDLAVSLTSSDG
LQNLATHTITAEKGDFAGWTKVEFDLQSSERNTSSRLQLTTTKNGIIWFDQVSVMPSDTY
MGHGFRKDLATMLANLKPRFLKFPGGNYVMGNYLLNAFRWSETIGPWEERPGHFNDVWNY
WTDDGLGFFEFLQLAEDLDACPVWVINDGASLNEQIPSATIAAFVKDVVDGIEFARGDPK
TTWGSVRAAMGHPEPFPLYYISVGNQECSKPYYKEKYVKFYSAIKASYPDIKIISSCDIS
SISAVNPADLYDVHVYTSSGDMFSKTRMFDSTPRSGPKAFVSEYAVTGNDAGRGTLVAAL
AEAAFLIGLERNSDVVEMASCAPLFINDNDRGFSPDAIVFNSWQHYGCPNYWMLHFFKDS
SGATLHPLTIQVSNYDQLAASALTWQNSNDGNTYLKIKVVNFGNKAVNLNIAVAGLENGI
QEFGSIKTVLTSGWLRDENSFQQPDKVVPAASPITNAGEKMGVIVDPYSLTSFDLLLDTN
TDKYPLLESSFHSSM*
>11687.m00308|LOC_Os11g03970.1|genepair1402-1
MSTTKVKRRVGKYELGRTIGEGTFAKVKFARDTETGDPVAIKILDKEKVLKHKMVEQIKR
EISTMKLIKHPNVVRIYEVMGSKTNIYIVLEYVTGGELFDTIVNHGRMREDEARRYFQQL
INAVDYCHSRGVYHRDLKPENLLLDSYGNLKVSDFGLSALSQQIKDDGLLHTTCGTPNYV
APEVLEDQGYDGAMADLWSCGVILFVLLAGYLPFEDSNLMTLYKKISNAEFTFPPWTSFP
AKRLLTRILDPNPMTRITIPEILEDEWFKKGYKRPEFDEKYDTTLDDVDAVFNDSEEHHV
TEKKEEPEALNAFELISMSAGLNLGNLFDSEQEFKRETRFTSKCPPKEIVRKIEEAAKPL
GFDVQKKNYKICSPCLTTICMNIPFKLRLEKVKAGRKGNLNVATEILQVAPSLHMVEVRK
AKGDTLEFHKFYKNLSRTLKDVVWKSDDLQNQLS*
>11669.m02079|LOC_Os03g20380.3|genepair1402-2
MYRAKRAALSPKVKRRVGKYELGRTIGEGTFAKVRFAKNTENDEPVAIKILDKEKVQKHR
LVEQIRREICTMKLVKHPNVVRLFEVMGSKARIFIVLEYVTGGELFEIIATNGRLKEEEA
RKYFQQLINAVDYCHSRGVYHRDLKLENLLLDASGNLKVSDFGLSALTEQVKADGLLHTT
CGTPNYVAPEVIEDRGYDGAAADIWSCGVILYVLLAGFLPFEDDNIIALYKKISEAQFTC
PSWFSTGAKKLITRILDPNPTTRITISQILEDPWFKKGYKPPVFDEKYETSFDDVDAAFG
DSEDRHVKEETEDQPTSMNAFELISLNQALNLDNLFEAKKEYKRETRFTSQCPPKEIITK
IEEAAKPLGFDIQKKNYKMRMENLKAGRKGNLNVATEVFQVAPSLHVVELKKAKGDTLEF
QKFYRTLSTQLKDVVWKCDGEVEGNGAAA*
>11687.m00309|LOC_Os11g03980.1|genepair1403-1
METVIQSINQSIACVLGRRSINESRRKCNAGTITSKELGTVMGSLGQSPTEAELKKMVEE
VDADGSGSIEFEEFLGLLARKLRDTGAEDDIREAFRVFDKDQNGFITPDELRHVMANLGD
PLSDDELADMLHEADSDGDGQINYNEFLKAKAEYDGGTWKWRPSVK*
>11669.m02076|LOC_Os03g20370.1|genepair1403-2
MADQLTDDQIAEFKEAFSLFDKDGDGCITTKELGTVMRSLGQNPTEAELQDMINEVDADG
NGTIDFPEFLNLMARKMKDTDSEEELKEAFRVFDKDQNGFISAAELRHVMTNLGEKLTDE
EVDEMIREADVDGDGQINYEEFVKVMMAK*
>11687.m00460|LOC_Os11g05400.1|genepair1404-1
MMMATAAMAASSCDRGDTRKKLQITVVFLVRTLLLACIIARGVLALIRVAFRVAVVAPAR
SLVAVAGAAFSAVNARCAWCLEQAALGRSCTGTVLGDAVVGAMASSWRLLLQGITSLVFL
CARGADEYVRPPPSPLVLTAHGKPASHPQQVHISMVGEKNMRISWVTDDLNAPSVVEYGT
SPGKYTASATGDHTTYRYFLYKSGAIHHATIGPLEASTTYHYRCGKAGDEFTLRTPPARL
PVEFVVVGDLGQTKWTASTLSHIGGGGGDYDVLLLPGDLSYADTQQPLWDTFGRLVQPLA
SARPWMVTEGNHEIEALPVVGIAPFAAYNARWRMPREESGSPSNLYYSFDAAGGAAHVVM
LGSYAEFEEGSPQRAWLERDLAGVDRRRTPWLLALVHAPWYNTNEAHQGEGERMRRAMES
LLYEARVDVVFAGHVHAYERFTRIYDNEADSRGPMYITIGDGGNREGLALKFIKGHKSAH
LSEFREASFGHGRLRVLNETSAVWTWHRNDDQFATVRDEVWLHSLAAGEPAATVASAAGG
GGGHPADEL*
>11670.m03223|LOC_Os04g33530.1|genepair1404-2
MTRRADDLLVAGTLVISIVFFRCAAAVAATEYVRPPPGRVIFTEHTKPASHPQQVHVSLV
GANHMRVSWITEDKHVKSVVEYGKVSGNYTASATGEHTSYRYFLYSSGKIHHVKIGPLDP
GTVYYYRCGMAGDEFGLRTPPAALPVELAVAGDLGQTEWTASTLSHVGRSDYDVLLVPGD
LSYADAQQPLWDSFGRFVQKYASRRPWMVTEGNHEVEAAMALPGWPRPFTAYAARWRMPY
EESGSGTSLYYSFDAAGGAVHVVMLGSYADFNSSSEQYRWLARDLAAVDRGATPWVVVLL
HAPWYNTNAAHEGEGEAMRKAMERLLYEARVDIVFAGHVHAYERFTRVYNNEANPCGPVH
ITIGDGGNREGLAFDFRKNHKLAPLSLMREASFGHGRLSVVNATAARWTWHRNDDADSTV
RDEIWLESLAANGACQQSSSAAAAADSQNDEL*
>11687.m00467|LOC_Os11g05470.1|genepair1405-1
MSRSVEPLVVGRVIGEVLDTFNPCMKMIVTYNSNKLVFNGHELYPSAVVSKPRVEVQGGD
LRSFFTLVMTDPDVPGPSDPYLREHLHWIVTDIPGTTDASFGREVISYESPKPNIGIHRF
IFVLFKQKRRQTVIVPSFRDHFNTRRFAEENDLGLPVAAVYFNAQRETAARRR*
>11670.m03227|LOC_Os04g33570.1|genepair1405-2
MSRVLEPLVVGKVIGEVIDNFNPTVKMTATYSSNKQVFNGHELFPSAVVSKPRVEVQGGD
LRSFFTLVMTDPDVPGPSDPYLREHLHWIVTDIPGTTDASFGREVVSYESPKPNIGIHRF
VLVLFKQKRRQAVTPPSSRDYFSTRRFAADNDLGLPVAAVYFNAQRETAARRR*
>11687.m00472|LOC_Os11g05520.1|genepair1406-1
MAQMEFEPDKVGTVTVNGKVYSFRRVHWHAPSEHTINGEKHPLELQMVHAAADGSLAVIA
ILYKYGAPDSFYFQLKRKLAELAADGCSFGEENAQVALGLVHLRSLQKRTGSYFRYAGSL
TAPPCTEDVFWSVLGKIRQISQEQVALITALLPAGGARPTQPLNGRTVQFYNPPNSTISF
KV*
>11670.m03237|LOC_Os04g33660.1|genepair1406-2

MAASHGNAIFVLLLCTLFLPSLACDSGGVKFGYTGSIGPDFWGNLSADFTRCSNGKQQSP
IDIDTNNLVHELNMEPLHRNYTAANATLVDNIFNVALRYEEAAGVLSINGVKYTLKQMHW
HSPSEHTINGFRFPLELHMVHTNENGNITVLAFLYRFGRPDPFFEQDKLAALNAEGCKAE
KGSPVPAGSVSLLTMRQHVHIYYRYVGSLTTPPCAENVIWNIPAMPREMTPQQAADLMAP
LDEGYRRNSRPTQQMNGRTVQLYHRFWGKKKRRSSP*
>11687.m00475|LOC_Os11g05550.1|genepair1407-1
MAADGGALKRLFEKPLPENPTLLEALSAWNHTHRKKLVDPASFTEIFGELHFQEKPPVVD
SLARAAARPSPSPPPPPPPRRTVSWLDITDAATADNDNDKSKDDSSLDALLKPPRPASGG
ATVKRSASFCLKSSTSLLLCTEGLGSESTVDADDMVKDGDGSGAVVDSGMDVDDDASDVA
AAVAGDDAFGAGGKENRPPPPPPSFPPPIRSIGRGGKPSVCFRSVRAEGRFVLMEVVIPG
KDLLRATREGGRLRLQFSNAAAAAAAAVGVIDDEEMHGQEAAACVGGDTFA*
>11670.m03242|LOC_Os04g33710.1|genepair1407-2
MAPPPPPPPLHCYKAPEEQRLGPRPATQSRPPPHLLPYSGGLDLLTEALGAESFDPDDDD
DATAASPAMEDVGAAVAAVDFLAPPCKRPHHVLLSSSSEGVGHDDDDNQHAVMVLRRTRS
GRAFPPPISVIGKGGRPWLSLRAHREAGRLVLREMRLPSQELLQPCKEDGRFKLLIHPEA
GRRSGGAGAGPRVGSGREGHGALES*
>11687.m00486|LOC_Os11g05660.1|genepair1408-1
MDELRAPACGGGVGTVIEDLPTDVLALVLRRLDGASLAAVGCACSSFRELAVDQETWRGL
CLALWPSVRDVLGCGGGGGGDGYRAFFADAFPFPEAAAAVASAAPAPVPGSLPSRLVSAV
DLHHGGVCIMSRVVETDTSSAWFLGSPFRVDALVQEGFSAPSPITPSSLTLSWILIDPAS
GRAMNASSRRPVAVDRKWLTGETVARFTVMLGGGVALDAAVTCDDRYGHVREVSLCVEDG
EGGGVSGQDGLGAVAAAMAGARRGKGAEAAARQRYEEFVKGKRARKEWKARREGIVDLCC
SGVGAAAFVGFLVMLTFR*
>11670.m03253|LOC_Os04g33820.1|genepair1408-2
MAPGREEEKMVATTTIEDLHADVLARALRRLDGRSLAAASCATAGLRALAADPETWRALC
LAEWPSMAGHPRLLSVVPPRRLFADAFPFPRPDAGELGGGGGGPLPSELVSAVDVYYRGA
PLLSRVVETPASSPWFLGSPFRVEAVECKKPAAEAALSPAELELSWVVVDPARGRAVNVS
SRRAVAVDRHWYTGETLVRFAVVLGGCKFETTVTCSEGAGNISEVSLAVQDADGAAASGE
RSLRLLAAAMEEQRIGGGRERDEAKRRYDEFVKSRKGRKESKARREALIDLCCSAASAMA
VLSFVAAVVLR*
>11687.m00497|LOC_Os11g05770.1|genepair1409-1
MAKKKGFTSIFSRLLVVTGGGDDERGGGAAATTSPSPPPWPWPSCGNRPRTVSSRRDGGC
STSAAAAASAAAARRIAGEMYKTVNSVYFDYSAADGYSCFDDDDGRVVDDDDDGDSFSTT
TASEEWSEAVIRSLGRTSTDRFFFDPGPPTNSILAAAAVPETKPVAAAAVFHDEEKEKLP
EPPASLVEESVAVAVESEDPYSDFRSSMEEMVAAHGLRRWDALEELLVWYLRVNGKHNHA
LIVAAFVDLLVGLAAAATTTGTTPTTTTTTSSGRSTASTSTACDITTTTTITTSSATATM
EPCPCGGGSSDDLEEEEEEARVMISLGASSCSLAPSTFANS*
>11670.m03258|LOC_Os04g33870.1|genepair1409-2
MAKKGLVGILYKLRDVHHRAPPTPTSPSSSSSPHCHGRHQLCYPPAPSSWPWPSCRHPRT
SSFRWPTAPQQGQADDDAAAAAGSVYRTVNTVYDTSSLEHFNPRRSSLDEASSCIADRSF
FAVESEVEVEEEKEKEKELQLRETAVVRGVRSERLFFEPAGAEFLPKQRGFQEMARGKND
DEATAMDVVARKNDDVDEATPMTTPQTGKNEAEAAEAAALKGGAVVLTVESEDPYGDFRS
SMADMVAAHGLRDWEGLEELLAWYLKLNAKGVHGVIVGAFIDMLVSLASSPIPSQSPSSS
CITFEDYSSATMEEES*
>11687.m00500|LOC_Os11g05800.1|genepair1410-1
MLGELISKVLLVLFGYAMPAFECFKTLETRPDDAHMLRFWCQYWIIVSMVIACESFVSWM
PMYGEIKLAFFVYLWYPKTKGSDVVYDSFIRPTVMQYEPNIEQRLEHLRANSGQLIAFYI
KNFADKGTAFFMDFLRYVVSERPEAAANSEPQRSSWSSWNPFASRRQEPSPPPSAPPRER
RFSGADPDDEPPAIADVFRASLGGGAMNRRPHNNNNN*
>11670.m03259|LOC_Os04g33880.1|genepair1410-2
MSVEFLTKALTALFGYAMPALECFKAIEQRPGRTDHLRIILVILVIFDDIAGVLTSKIPM
YSELRLAFLVYLWYPQTRGTDIVYDTFLRPLVMQYQPNIEERLRYLRANAGDILIFYLKN
FTDRGYDLFLRGMEYIRSQTSRGSRTRDSGNVTVQSKNSGYVTVQRWFSFGGDRAERSSY
VDDYVAGGGDRRSTARHRRPRDDY*
>11687.m00508|LOC_Os11g05860.1|genepair1411-1
MGKDCGNHGDDDIRQACRRLLTILFGLALIVAIIALIVYLVLRPTHPRFFLQDATLRQLD
LSNSSTSGVLSTALQVTVASRNPNDRVGVYYDRLDVYASYKYQQITLAASLPPVYQGHGD
VDVWSPVLSGPDVPFAPYLGDALAKDVAAEYLILQVKIDGRVRWKVGSWISGHYHLFVTC
PAFFIASGGNGYPGANGLKFQTATYCRVEV*
>11670.m03270|LOC_Os04g33990.1|genepair1411-2
MGKDKHHRDWILRRCCGSIAACILTLAVLVGFIVLVIYLAIHPSKPSFYLQDVQLRNIDL
SDPAISLNLQVTIASRNPNDRVGVYYKTLHVFTTYREEPITVPVELPAIYQGHKDVSVWS
PVMSGESVPVGQYVADAMRQDIAAGYVLLHVKVDGRVKWKVGSWVSGGYHLFVTCPALLA
ASGGNVGGAFAMSATAGGGAGGNATVSLKFAQAADCTVDV*
>11687.m00521|LOC_Os11g05990.1|genepair1412-1
MASYGVDTRPAAAAAGGGGAGAGAAGEGALSFLSRGLREDLRLIRARAGELETFLTAPVP
EPELLARLRRAYSSSAGTTRLDLSAIGKAFGTGVVGRGSRGARWGWEEVQEAEEWEPIRM
VKARLREMERRRQWQATDMLHKVKLSLKSMSFVPEASEEVPPLDLGELLAYFLKQSGPLF
DQLGIKRDVCDKLVESLCSKRKDHLAYNSFPASEPSAFSNDNAGDELDLRIASVVQSTGH
NYEGGFWNDGHKYETADKRHVAIVTTASLPWMTGTAVNPLFRAAYLAKSSKQDVTLVVPW
LCKSDQELVYPNSMTFSSPQEQEAYMRSWLEERVGFKTDFKISFYPGKFQKERRSIIPAG
DTSQFIPSKEADIAILEEPEHLNWYHHGKRWTDKFNHVVGVVHTNYLEYIKREKNGVIQA
FFVKHINNLVARAYCHKVLRLSGATQDLPKSMICNVHGVNPKFLEVGERIAAERESGQHS
FSKGAYFLGKMVWAKGYRELIDLYAKHKSDLEGIKLDIYGNGEDSHEVQSAAMKLNLNLN

FHKGRDHADDSLHGYKVFINPSISDVLCTATAEALAMGKFVVCADHPSNDFFRSFPNCLT
YKTSEDFVAKVKEAMARDPQPLTPEQRYNLSWEAATQRFMEHSELDKVLSSSNRDCTTST
SGCGKSGDNKMEKSASLPNMSDMVDGGLAFAHYCFTGNELLRLSTGAIPGTLNYNKQHSL
DLHLLPPQVQNPVYGW*
>11670.m03271|LOC_Os04g34000.1|genepair1412-2
MGGERPPPISGGGAFAFISKGWREVRDSASADLRQMRARADRELEHLLASASALAGPPLP
PVAAGAPIAEVEFVRKRIQPKIMELRRQYSSTVRDAGWAPKAAGASLRVDLSGITAIRNA
IVAEGGGGGGGGGRWGLVRWKGHADDEGRKEWEVVRMIRSGLKEFERRSLSSEVFGGFRG
RGEFVEKFKLSLKSLNKESQESKEVLPLDLTEILAYLVRQSGPFLDQLGIRRDLCDKIVE
TLYSKHNGRLIYHSLSADRSLIGNENMTDELDLRIARVLESTGHHTEESFWKDHAKYKLS
DNRRHVAIVTTASLPWMTGTAINPLFRAAYLARSTKQKVTLVVPWLCKSDQELVYPNNIT
FSSPEEQENYIRNWLQERLGFEANFKISFYPGKFSKERRSIIPAGDTSQFISSSEADIAI
LEEPEHLNWYHHGKRWTDKFKHVIGVVHTNYLEYIKREKNGALQAFLVKHINNWVTRAYC
HKVLRLSAATQDLPRSVVCNVHGVNPKFLKVGEKIAADKEHGQQSFTKGAYFLGKMVWAK
GYRELIDLLSKHKSDLEGFNVDVYGNGEDSQAVQMAARKLNLSLNFFKGRDHADSSLHGY
KVFINPSVSDVLCTATAEALAMGKFVICADHPSNEFFKSFPNCLTYKTSEEFVARVKEAM
ASEPSPLTPEQRYSLSWEAATERFMEYSELDKVLNNKIGYSGQDGKRSKVRKIPLLPRLS
EVVDGGLAFAHHCLTGNEILRLATGAIPGTRDYDKQQCMDLNLLPPQVQHPVYGW*
>11687.m00037|LOC_Os11g01370.1|genepair1413-1
MTKPQQQSPPSTTATTTTSPPPPPPSTPPPASSSSSSLAKLPLRLHSLASSSRSLLSALR
RSPVTTLVAAFFLLALFMYGEDVRTLAELSIDDYLYPDADFYNVSALPPLLLPPPTCDLS
RGRWVFDNTSLPAYREKECTFLTKQVSCLANGRPDDLWQYWRWQPNNCSLPTFDARRFME
KMRGKRMMFVGDSLNRNQWESLVCLVQPILSKGRKKIVKRGSFNIFYAKEYRATLEFYWA
PFLVESNSDNPNFHHIDQRIISPERIESHANNWKDVDYLIFNTYIWWMNNEDIKVRRPNS
TSWSDHDEVPRIETYGRVFKTWSTWLEQNVDPARTSVFFMTISPLHNSSKIGIGGRWFNT
DAVITFCQFNIDEPCISATGSLELWNILKYRHRVRWFPAQWGNPNGIKCVKETLPVLNYT
KPLDLNHDMRMYDLVAKVAKNMKNVPVSLIDITRMSDYRKDAHTSLYSIRQGKLLTPEQK
ADPQKYADCIHWCLPGVPDVWNQILYTRILSKSSPPSPHPPLPPQ*
>11682.m03732|LOC_Os05g39350.1|genepair1413-2
MGLPGRRNPLLSARRAAASLRRSRRLPVYVAAVFFVASVLLMFRDEILYLTTARSPSSSL
PTTGGSAGGAGLARKEELVSVNKPVLLGHGGKPEKHHSVTERHRPKVSAKRRPNKKAAKA
ARKKFMASPSVAAGAEVNVPETCNLSKGKWVFDNATYPLYREQECEYLTAQVTCTRNGRR
DDGYQKWRWQPRDCDLPLAFDARLFMERLRGKRLMFVGDSLNRNQWESMVCLVRPALSPG
KSYVTWWDGQRVVLHAWEYNATVEFYWAPFLVESNSDDPKAHSIRDRVIKPEAIAAHAGD
WVGVDYLVFNTYIWWMNTVNMKVVRPTGKTWEEYDEVGRIEAYRRVLDTWATWVNDNVDP
ARTSVFFMSVSPLHISPEAWGNPGGVRCAKEDAPVQNWHGPLWLGTDWDMFRAARNASRA
AGRVPVTFVDVTAMSELRKDGHTSVHTIRQGRVLTPEQQADPATYADCIHWCLPGVPDVW
NLMLYARILSRPPAAAGHVA*
>11687.m00050|LOC_Os11g01490.1|genepair1414-1
MATASSPASVQDYPDLQEDDDDDFQDDDDLDDEDEEDDDDQEPSPSPSDEARLQSVLRRL
TAEEVRIRVHDVEIRGCCRTRRAAVEAAVGSDLPRAATVRDLVRAAAAAADRIRRLGAFD
TVSITLDAAPPGIPGNAAVIVLVDVAEARGRAAGELGIFANKGTRSCSVQGSVKLKNLFG
YCETWDASGDLGLDQTVELSTGVAIPRIGAIPTPLVARISFLSEDWLKSSLREHMMGVSV
GLLSTMNHNLAYNLSWRTITDRALMSSNSIRGQLGHSLLSSIKYAYKVDQRDSRIRPTRG
YAYLFSSQVGGLAPESKDARYIRQELDLRVALPLGVLNGALNAGVAAGIIHPLARGSTGS
ISPLSEQFYLGGNRSLMCRLGGPSSLLGFKKRGLGTDLQSSTPENSENVASTSPELSARG
GDIAVTAFADLSFDIPLKPLRELGIHGHAFVSAGNLAKLTEPDLRKFPLAEFLQTFRSSA
GFGVVVPTRLFRIEVLKKSSNVFLQVQVRQPATMFDKPPNSVIQLFKRKSHQLEELALYQ
IGIIMRKGQIICIAKEIPAEQWGQEERKAKG*
>11682.m03736|LOC_Os05g39390.1|genepair1414-2
MATAADQNPNDAEHREAAAGANAAAEEYEEEDEEEEEVELDGPAAVAAEREKVQAVFKRL
SSDPVGIRVHDVIIKGNAKTKEELIEAEVAELLRAAPTVQDLLRNASIASARLRQLDVFD
SVNITLDAGPPELPGTTNVVVEVVEAANPITGSAGVYSKPEARSWSLEGSVKLKNLFGYG
DIWDASGAYSWDQTSEVGIGVSLPRFKSISTPLMARASLSSQDWLKFSSYKERLLGLSFG
LISTMQHDLSYNLTWRTLTDPSQVSSKSIRRQLGHNLLSALKYTYKIDQRNSHLRPTKGY
AFLSTSQVGGLWDSKGLRFFRQEFDVRGAVPLGFYNSALNVGLGVGAILPLGRGFMNLSS
SVPDRFYLGGHSSPVCSLSGLSSLLGFRTRGIGPTEPRRLVPSESEDGSAASPGRDYLGG
DLAVSAFADLSFDLPLKIFRDAGIHGHAFLTAGNLAKLSEGEYKKFSLSEFGRTFRSSAG
VGIILPTKLFRVEVNYCYILKQAEHDSGRTGIQFSFSSPL*
>11687.m00084|LOC_Os11g01820.1|genepair1415-1
MAGANASTVKPVVAACYDNNLVNSQGMFLGDEPLRFALPLLLVQVSIILILSAAAHHVLR
RLGQCRFVTHMLVGIFLGPSVLGRNPHLRTALFSERGTYILESVSLVALILFLFSMAVKT
DLTLLRRPTARALAVGLAGSLVPLAVTLPVFHALSPSLPADLRGSSLITELAVRLSLSSF
PVVADALAELDLLNSELGRVALNASLITDVTSWFLRACFAAAFLITQAKSPLFTAKVLAS
FAAFVLFVFFVARPAGRYIARKRTPPGDLLSEGSFVLVVIAALLSALVTDVIGFKFMIGP
MMLGLALPGGMPIGATLTERLDSFFIALFLPVYMALAGYRTDLAELSLIGVSAEHEEKFC
ALELFVALCVAGKMVGCVAAGLFFSMPFREATVLALMLNIRGIVEVAAINNWGDTMKATA
EHYSTLTLSMVVITAVATPLIKLLYDPSGRFARAKRRTMEESRPNAELRVMACLFSEDHA
APLLDLIEASGSSRDAPVSLIVLHLTELVGHAASVLKPHRKSRSSCGNPTPSDRIVNAFR
YFEQQAPLGAVTVSPYVVASPYSSMQHDVCLLAHSRKANLILLPFHKSSDGARSTANNAI
RGINRSVMQYAPCSVGILIDHGVAAGSACATASNSTLQRVALYFLGGADDREALAYVARM
AECGLVAVTVVRLKLRDWVGMGGRDEMRDEEALQEFWQRYSCAGAERVAYVEKTVEDGEG
TASVVRAMSDKFDLLVVGRREGGGDGAEGSSAAALTSGLSEWSEFPELGVLGDMLASADF
AAKVSILVVQQQAATRNDADY*

>11682.m03760|LOC_Os05g39600.1|genepair1415-2
MDKIDCYVVPQTTGTGRNIFQGGSPLSASLPLLGVQLVLIVAVTRVLYFLLKPLKQPRVV
SEIMGGIILGPSVLSRHAAFREVVFPARGEPVLNTVATFGLMYVIFLIGVRMDPRLVVRS
GRKGVVIGLSGFLLPLAMTGAGSSGEAMATEPDVSRRSTFLFALATSLSVTSFAVLSPIL
SELSLLNSDLGRIAMSASMTTDGIAWIIMVVYILAEAFLVSPATSIWAFLSLAVLAAFIL
FVVRPVALRVIERTPPGKPVEETYVFVFLLVVLLVGFYSDVIGTNSFHGALMLGLAIPDG
PPLGTALGEKIEAMVSGLILPLYYAMTGLSTDVWRMHWGRLQVVMFLAWVGKLVGVMVSS
LYLEIPLRDAVSLSLFMNSKGIVEVITFNFFLTNKLIGKNTFSILICLSVAITAVSVPVA
GWLYDPARRYAVYKRRTVQHLKADADLRILACVHDQSHVPGTLAVLEASNATPQTPLSLY
LLQLVEIAGRSAPVFIPHKLRHSASRAGAAAASAAPPTDSDRIINAFFQYELKHPEGAVS
VHPFTTISPYSSMHDEVCRLAVEKRTSLILLHYHKRHMLAGGVRAAVGLRVVNRKVLEVA
PCSVAVFVDRNAGSVGLCAFIPGPVQDQSTSSRSRSGGHAASGSGQGFKAAVASLFFGGG
DDREALAYATRMARHEGVAVGVIRFLPARGIKDEPSDRRIDNRAIEEVKALAARSRNIKV
QEELVGDMERIVQVLRGLDEAGYDLVLVGMRHRWYPVMPANGMSDWSECPELGVIGDLLA
SSDFDTPYSVLIMKQQDQGGLNAAVPGAQDVWHVNDGGAEDAPAPPRNMSTAGS*
>11687.m00087|LOC_Os11g01850.1|genepair1416-1
MLLACDCDDDAQTAENGNAPMLSNYRAQPAPPLAEPLLLDKPQRAHWLRQLKPWRRNAAD
DHLSAGIAINWSSVRSATKDWITNPMNIAMLLWLLCVAVSGAMLVLLLLGLLDGAFPTPA
ARNHWIEINNQVLNALFTLMSLYQHPVLCHHLFLLCRWRPADAADLRAAYFKDGAGPRHG
ERAHMAVVVALLHLTVACQYVLCGLYWGYTKKTRPELVENGFFVLGVVAPVVAVVYTVCS
PLGKDNYGELACPNAFDSVSQHKCTGHAVVEPEWAGGMFDCGGDATAWWLSLSCTFCAFG
WNMERLGFGSMFVHTATFVLLCFAPLWVMGVSALHIHDVVIGDMVGGAGALLCVCGLLYG
GYWRIQMRERFGLPASTACCGSPSVTDYARWLFCWPCALAQEVRTESLYHIDCETFYKKL
PVVDDVEDEKRLPLLASHHVQFHEPPDTMIMAASEGSNDHVVIVHEEMVPPAVQVVVEQV
VVEGDKSEEECSAVHDEKIMGSPLPESVVIVDDDEIPASLSDGSWTVEKVKRLINVVTLV
SLLILLYTRGFIR*
>11682.m03773|LOC_Os05g39730.1|genepair1416-2
MVSNGNEDLKADVELVESTTVDNDTGAPGASTLPTQGVPRQGKQRNGFLNFCNRFSSGDR
FKKLGPSPSFKFRQLALERDEFSRSIHSDSHDNHEHFQFIRKINWGHLWVMCKDWIKEPL
NMALFAWIACVTVSGAILFLVMTGMLNRALPSKSQRDAWFEVNNQILNALFTLMCLYQHP
KRIYYFVLLCRWEQKDVLVLRKTYCKNGTYKPNEWMHMMVVVVLLNLNCFAQYALCGLNL
GYRRSERPPIGVGLTISVAIGAAAFAGLYNIISPLGKDYDTELTEVDQEAQTELTRPATS
RTSLEKRYSFIQSEERRFVESRPEWVGGLMDFWDNISLAYLSIFCSCCVFGWNMQRLGFG
NMYVHIATFMLFCLAPFFIFNLAAVNINNENLREALGLTGLALCFFGLLYGGFWRIQMRK
RFNLPANNFCCRSAEATDCFQWLCCSSCSLAQEVRTADYYDIAEDRSYTEQITARSQHVM
TPLSREDGLPLFRSNPGSPYRSSTASPSIFIMESPSAPRRSPGPSPLGGSPTMGDRTMKA
PTPSVLHRDGEPEL*
>11687.m00128|LOC_Os11g02240.1|genepair1417-1
MESRGKILMERYELGRLLGKGTFGKVHYARNLESNQSVAIKMMDKQQILKVGLSEQIRRE
ITTMRLVAHKNIVQLHEVMATRNKIYFVMEYVKGGELFEKVAKRGKLTEVVAHKYFQQLI
SAVDYCHSRGVYHRDLKPENLLLDENENLKVSDFGLSALSESKRQDGLLHTTCGTPAYVA
PEVISKIGYDGAKSDIWSCGVILFVLVAGYLPFQGPNLMEMYRKIQHGEFRCPGWFSRKL
QKLLYKIMDPNPSTRISIQKIKESTWFRKGPEENRILKERTLNENTTKNVAPVLGVRRKK
NAHEDVKPMSVTNLNAFEIISFSKGFDLSGMFIVKEWRNEARFTSDKSASTIISKLEDVA
KALNLRVRKKDNGVVKMQGRKEGRNGVLQFDIEIFEVTTSYHIIEMKQTSGDSLEYRQLL
EEGIRPALKDIVLA*
>11682.m03790|LOC_Os05g39890.1|genepair1417-2
MYRKIAKAEYRCPHHFSAELKELLYGILDTDPSTRMSISRIKRSAWYRKPIAISVLNSEI
GKNSCTSEAPFSGPTTCISSERNQEPPNLHKLNAFDIISLSTRFDLSVLFGERYGQREAR
FKSRKPATIVLVKLKELAKALKLKVTKTDNGVLKLATMKE*
>11687.m00144|LOC_Os11g02370.1|genepair1418-1
MARAQLVLVALVAAALLLAGPHTTMAAISCGQVNSAVSPCLSYARGGSGPSAACCSGVRS
LNSAASTTADRRTACNCLKNVAGSISGLNAGNAASIPSKCGVSIPYTISPSIDCSSVN*
>11682.m03805|LOC_Os05g40010.1|genepair1418-2
MVPAARSGWPAAAAVLVVVLVLSSPPGTSTVVVARAALSCSTVYNTLLPCLPYVQSGGAV
PAACCGGIRSVVAAARTTADRRAACTCLKNVAAGAAGGPYISRAAGLPGRCGVSVPFKIS
PNVNCNAVN*
>11687.m00150|LOC_Os11g02430.1|genepair1419-1
MRRLGDVVEAPALVLTPASMQQAGGRGSSGALDASMVVILAALLCVVICALGLTSLIRCA
LHCARGLSPTTATPTPSVSTAATAGLKKTELRRIPVEVYGAKQAGVPDGECAICLGDFAD
GDKVRVLPRCHHGFHVRCIDTWLAAHTSCPTCRDSILSVHGVVAGGQT*
>11682.m03806|LOC_Os05g40020.1|genepair1419-2
MASGVAAPAPSVFEAARPALESGGGGGGAPPPGRADASFDTNMVIILAALFFALLFAIGL
NSLARCALRCGGRGAAAAGGGGGGGGGGAAAAGVGCGGIKKRALRSIPVEVYCGGEETAE
TDVCAICLGEFADGEKVRVLPRCRHGFHVRCVDAWLVSHGSCPTCRRQVIGGGGSTPPPD
SDTIAVVVA*
>11687.m00154|LOC_Os11g02470.1|genepair1420-1
MAVTESACLSYEQEAVAVREVAQVYELIKTQQPLLLVHQQPQQLAHGLLNHALRALNVAL
SVMNQPHASSSAAAAAVPVMSLIKAEAATPANSSSPAADVAADNHVVGKPRRSSSAAKRR
RINGEEYKSSSWSQFTPVPHEDGFQWRKYGEKKIQGTHFTRSYFRCTYRDDRGCQATKQI
QQKDKNDPPMFQVTYSNEHTCTTTRLINNINNPAALHNLTANPNGHHDSDDDDTIFTKMI
KQEEQAAWLPPPPPADLATISNNFDETPGLHVCQEVPPSSSNSSVISHYADEFDHHQMLE
TTVMEEALGLGADLDDPYFYDPNLLLIYESLMNCY*
>11682.m03811|LOC_Os05g40070.1|genepair1420-2

MARRLPKSERSPSPPPPPPGDQRDAAIQELSKGSELATQLMAQLELIPERELDGRRDDAL
ANVRSLSMSLSSSLYALRSERREHYYCGSSSSSGGAGPAAVTSVSGAGGERKTKRRRGKH
GEELIETVFITTTPENDGFHWRKYGEKNILNSEFRKLYYRCGYSDERKCQAKKYVQQENN
KHPPEFRVTLTNEHTCNTVFQDQPSSSSTNSQVLDFTKASISSSLMDSHVGAPILKEEEE
EEVPSIDESTRIMSTIMRNYGSYGDYDESSPQPWNGAGWK*
>11687.m00171|LOC_Os11g02640.1|genepair1421-1
MAYRRKQGPIAADDRRSSYPQSPQGSSSSYSYTSIKSMNEPKLGLWETLARKAKGILDED
GVAHKSDEYTKEKTPRKFDSSTGAQESQSRWSFENHSKTGDTGSRTRSEALAASVNQLGG
RIRDALEEGLTIVDNKTSNIIEETKKIQIRRKQANSNSYVPNLAFDTLRPPNLSNDQAET
AAQETQLKASRDVANAMAAKAKLVLRELKTVKADLAFAKQRCAQLEEENKFLREAKQKGS
KTEEDDDLIRVQLETLLAEKSRLAQENSMYARENRFLREIVDFHQFTTHDVAPLDDGDME
DSIPGEDSNHTYSEDMFPVVEAYLDREELSPVPSRPESPILSSCESSSPKSSNSKSSAAN
LPSNVSAKCIGT*
>11682.m03843|LOC_Os05g40310.1|genepair1421-2
MAYRRKHGIQRSATFVEDHRQQPPQPGDTSSPAIASPRATRFADDSRRPDRSLAAASSSP
QPDGSTPDPVTQLYTSARGAKGNETKHGFWGVLAQQAIVMLDENGGTDDNHSVTSQSRWS
YDRVRKPENPPLDIGCKIKTALEVAFSSRLAFRDFMNAMINIGLTKVEGSSRTGDGVHGR
KLHIRRKACSMDLRNSSMGLSSPEAMSPTMSDTESPQIKASRDVASAMAAKVKLLQRELK
TVKADMAFSRERCAQLEEENRMLRDGKHDADEDLIRQQLETLLAEKARLANENTVYAREN
RFLREIVEFHQLNMQDVVDLDDEDMAGDGDGEEGDDDHQQYGCHLRAHEAAHGLWAGGGL
GTPPQSPLGHAGRMGMSRSNSRAAESPTMRRSLKEENVDEPETPPTRRSLKEKADVDAPP
ETPPTRRSLKEADVDEPDTPPTRRSIKEDADDAPETPTTKQDIGSPETATTPARRSSNDD
LGAAETTTPTRRSFKDDNGVTEMKNEH*
>11687.m00180|LOC_Os11g02710.1|genepair1422-1
MNLDHNSTDPKITGCLVRFHSDIKNRVGNRTEKPTPDTAISLFPSRIFRASRSSTINMSE
ESGLAAHGSEEGFPSVEIRMLKLEGNRDVCLDIDSGIRVDDDGAGRISSGAGGGAGRRGR
HGRGSSGGSGRHGAEEEAARLGRGWGVARARVRLGRGAKWGRARRGVGQSARRGRRGEQG
AARERQKRVGSIYLYSTGSIQVLNVTDPNMVKELANCKSLDLGKPCYLQKERGALLGMGI
LTSNGDLWVHQRKVIAPELFMERVKGMVNLMMEAAMSMLNSWKNEVEDRGGSAEIVVDEF
LRTFSADKAMAKQSMLIGVPGSRYLPTRSNRGIWNLDSSIRTLILNISKKYEHDSSTSVN
KDLLHSIIQGSKDGPFASCTPEDFIVDNCKNIYFAGHETTSTTAAWCLMLLASHHEWQSR
ARVESLDICQGRPLDFDILRKLKKPPSSPGEALNDMKLAGIDIPKGTNIWIPIAMAHRDP
SVWGPSADKFDPDRFANGIAGTCKPPHMYMPFGVGVRTCAGQNLAMVELKVVLSLLMSKF
EFKLSPNYVHCPAFRLTIEPGKGVPLIFREL*
>11682.m03851|LOC_Os05g40390.1|genepair1422-2
MGKPKYLQKGQEPLFGGGVLKANGACWARQRKVIAPEFYMARVRAMVQLMVDAAQPLIAS
WESRIDAAGGAAAAEVVVDGDLRSFSFDVISRACFGSDYSRGREIFLRLRELSGLMSETS
VIFSIPSLRHLPTGKNRRIWRLTGEIRSLIMELVRERRCAARAAREHGGKAAPPSPPERD
FLGSIIENSGGQPRPDDFVVDNCKNIYFAGHETSAVTATWCLMLLAAHPEWQDRARAEVL
EVCGGDGAAAPAAPDFDMVSRMRTVGMVVQETLRLFPPSSFVVRETFRDMQLGRLLAPKG
TYLFVPVSTMHHDVAAWGPTARLFDPSRFRDGVAAACKHPQASFMPFGLGARTCLGQNLA
LVEVKTLVAVVLARFEFTLSPEYRHSPAFRLIIEPEFGLRLRIRRAGGQDATSQVDTSTA
PVHSSHN*
>11687.m00182|LOC_Os11g02730.1|genepair1423-1
MASTTAAAAGNGSGSILPTHTIAATAPPFRTHKDADLESRRRRRRRRCLCCCLLVTLVVL
LVLAITLLVLFLTVLRVRDPTTRLVSTRLIGLSPRLSFPAMSVQLNVTLLITVAVHNPNP
ASFTYATGGHTDLTYRGAHVGDAEIDPGRIPSRGDANVTMALTLQADRFAGDLTQLVTDV
MGGSVALEASTRIPGRVAILGVFKRHAVAYSDCHFVFGVTEMAVRSQQCSDRTKL*
>11682.m03852|LOC_Os05g40400.1|genepair1423-2
MSGSGDAAPARHNAGHGRRRRRVLVWASFAALVLLLVAAAAAIAALAVLRPRDPTTELLS
VNATGATPRVAALPAVSVQLNVTFLLVVRVRNPNRAEFRHGAATTALLYRGAEVGAAGVP
AGTVPSRGAATLRLNMTVRADRVVAAAGVGGLLADVLAGEMEFEARTEVRGRVKLLGLVR
RSAVARSLCRVVIGVADVKVRRQECHNESKL*
>11687.m00184|LOC_Os11g02750.1|genepair1424-1
MAAKVHPNVAAAPQPPCISSIVSQQQEEEEPPVVLTVWRKSLLFNCHGFTVFDAKGNLAF
RLDCYDSTSSRRADLVLMDAAGKPLLTIRRKRMSLSDSWIIYDGDGAATSTATPLLSVRR
RRVGLRASKSKAIAHVTPLSSSLPLPEAYVVEGSYGRRSCAVRDARGDAVAEVRRKESVG
DDVFRLVAQPRLGAPLAMAIVIAIDEMFRGGSSLLRRTCSA*
>11682.m03877|LOC_Os05g40630.1|genepair1424-2
MAAKVHPNLAVPSLIQPPMAPPAAMAAGDSVMKTKAAAAGGDVVLTVWRKSLLFNCRGFT
VFDASGDLVYRVDSYAADSRAEVVLMDAAGVPVLTVRRKKAIGSQLGLGGDQWLVHPGEE
TRLPPLYAVKRTPQYVRGGGSVKTMAHVAPCGVALGAGGGGGYEIEGSYLRRSCAVYDAR
RRAVVAEVQAKEAVGTDVFRLVVRPGMEVSVAMAVVLALEQMFGKPSLLRSWSS*
>11687.m00217|LOC_Os11g03070.1|genepair1425-1
MSMDKTHLMCFYPSKITMGGVWTGDNPLDFSIPLLLFQILLITSTTRAATLLLSPLRLPT
YISQILASFLLGPSILGHLPHFSNLVFPVRSLFVLESMALLGLVYYTFIVGVEIEVSAIT
RAGIHSFGFAVGCALPPFLVGALTGYVALSTDDKRKGDTFLNKLSFPIFLGSTFSSTAFS
VLARNIAELKLAGTDVGQLTLSASLINDTFAWTGLTVATVLGHSRCTITQTTWTLTSGVV
IFGASYLLLRPMLLRLARRAAEGEAVGEDRECWILIGVMVAALVADAGGTHAIFGAFVFG
LAVPNGPVGVALVEKVEDFVVGALLPLFFALSGLRTDTAKITNMHSAVLLMVAAMVAAVL
KVAAAIGVAGVFGMPLSDGTSIGLLLNTKGIIELVILNIARNKGIMSDQSFTVLVFVSAL
ITAMVSPFLGMVVKPARRLVFYKRRTVAWAHPESELRVLACVHVPRDVPALLTLLDVVTP
SSRSPVGVLALHLIEFVGRSSALLLINASAPSSSSYDASVHGRSHTEMQFKHISHAFMAY
EEQSVGVSARTMAAVSPYESMHEDITSAAENQHSALILLPFHKYRSVDGGLEVSHPAIQP

LNCNVQSFSPCTVGILVDRGLAAVPGGGYRVVALFFGGSDDREVAALATRMVRNPTIDLT
LLRFVQKGGSFTASEFDALKERKADEGCLRDFLERANEGGGATVEYRERGVFNASEMVGE
IQSVEAMGNKDLFVVGKVPGGSGLTAGMAEWSESPELGPIGDLLASKDFQTTASVLVLQA
YGRPVAVVGAGAGAMSVDFGGDSVAMAERTASGRRPWARPGV*
>11682.m03879|LOC_Os05g40650.1|genepair1425-2
MAPIMSGAAAAAGGTGGAVPLIKNATSASQMSRGKAGTGAGAVVCYSPMMVTAYGIWQGA
SPLDFSLPLFLLQVAIIVATTRLLVILLKPFRQPRVIAEILAGVILGPSVMGQVSTWAVK
VFPERSLLTLETVAHLGLLYFLFLVGLEMDVNTIRRSGKKALIIAVAGMALPFCIGTATS
FIFRHQVSKNVHQASFLLFLGVALSVTAFPVLARILAEVKLLNSDLGRIAMSAAIVNDMC
AWILLALAIAISEVNSSAFSSLWVLIAGVAFVLACFYVVRPLMWWIVRRVPEGEAIGDVH
ITLILTGVMVAGVCTDAIGIHSVFGAFVYGLVMPSGPLGVVLIEKLEDFVTGLLLPLFFA
ISGLRTNVTKVRDPITVGLLVLVFVMASFAKIMGTILIAVSYTMTFRDGVALGFLMNTRG
LVEMIVLNIGRDKEVLDDESFAVMVLVSVAMTALVTPVVTTVYRPARRLVGYKRRNLQRS
KHDAELRMLACVHTTRNVPSIISLLELSNPTKRSPIFIYALHLVELTGRASNMLAAHHSA
SNPGGASDHIFNAFESYEEMVGGVSVQALTAVSPYQTMHEDVCVLAEDKHVSLIVLPFHK
QQTVDGGMEPINASLRGFNESILASAPCSVGILVDRGLSAAAARMAAVHHVALLFFGGPD
DREGLAYAWRMVENPGVCLTIVRLIPPGYTAPAISPPQPPMPAAHSRAINVVPEVAKSER
QMDEEYLNEFRSRNLGNDAILYVEQVVANSEETVAAIRSQLDNAHELYIVGRHPGEASSP
LTSALAEWMESPELGPIGDLLVSSEFSKMASVLVMQQYVITAPLPPPVALAGPPTDDPVR
QYLTNANQRPSVAIGGNQMGAAGRGGWSGGAGGY*
>11687.m00221|LOC_Os11g03110.1|genepair1426-1
MGSSSLLLFPSSSSSATHSSYSPSSSSHAITSLLPPLPSDHHLLLYLDHQEQHHLAAAMV
RKRPASDMDLPPPRRHVTGDLSDVTAAAAPSSASAQLPALPTQLPAFHHTDMDLAAPAPP
PPQQQVAAGEGGPPSTAWVDGIIRDIIASSGAAVSVAQLIHNVREIIRPCNPDLASILEL
RLRSLLTSDPAPPPPPPPSHPALLPPDATAPPPPPTSVAALPPPPPPQPDKRRREPQCQE
QEPNQPQSPKPPTAEETAAAAAAAKERKEEQRRKQRDEEGLHLLTLLLQCAESVNADNLD
EAHRALLEIAELATPFGTSTQRVAAYFAEAMSARLVSSCLGLYAPLPNPSPAAARLHGRV
AAAFQVFNGISPFVKFSHFTANQAIQEAFEREERVHIIDLDIMQGLQWPGLFHILASRPG
GPPRVRLTGLGASMEALEATGKRLSDFADTLGLPFEFCPVADKAGNLDPEKLGVTRREAV
AVHWLRHSLYDVTGSDSNTLWLIQRLAPKVVTMVEQDLSHSGSFLARFVEAIHYYSALFD
SLDASYSEDSPERHVVEQQLLSREIRNVLAVGGPARTGDVKFGSWREKLAQSGFRVSSLA
GSAAAQAVLLLGMFPSDGYTLIEENGALKLGWKDLCLLTASAWRPIQASGR*
>11682.m03885|LOC_Os05g40710.1|genepair1426-2
MQDSLGLMQFLDHHQYLYSSSSSNLPLQQPLLSHHHRFLEANEGCAGEDDSPEFVEPPAA
AAAAGTFEQRPELGACKEVYSEEGGAAEERTGVAMAGADVEQVAVEDEEEAHGVRMIALL
MECAAAMSVGNLAGANGALLELSQMASPYAASCGERLVAYFARAMAARLVGSWVGVVAPM
APPPSCGAINAAFRALYNVAPFARLAYLACNQAILEAFHGKRLVHIVDLDVVPGGALQWL
SLLPALAARPGGPPVIRVTGFGMSASVLHDTGNQLAGLARKLCMFFEFYAVAKRPGDADA
VADMPGRRPGEAVAVHWLRHAMYDAAGDDGASMRLVRWLEPAAVTLVEQERAHGGGGGHG
RFLDRFVSALHHYSAVFDAMGASRPDGEDASRHLAEHGVLGREIANVLAVGGPARSSGRE
GPGSWREVLARHGFAHAGGGGGGRAQLVAAACPGGLGYTVAGDHDGTVRLGWKGTPLYAV
SAWTWCSPPHARA*
>11687.m00226|LOC_Os11g03160.1|genepair1427-1
MKGHHSLPPSTPPKRRCTALAAAVPALVVCSILLPLVFLLGLHRPGHGSEERAAVVISTE
LGFSKHKHLDGRMKHKLLKDVSRKKIPGSDGILDEKSGSRSKSKSVSTKSKEKLKGVFSL
VQLKNETRKNKELHTQRRYQLKDLSWRSKDTTIDKKENQDQEVEHENPRSCELEYGSYCL
WSVEYKEVMKDFIVKRLKDQLFMARAHYPSIAKLKNQETFTRELKQNIQEHERMLSDTIA
DADLPPFFAKKLEKMERTIERAKSCEVGCTSVERKLRQLLDITEDEAYFHTRQSAFLYHL
GVQTMPKTHHCLNMRLTVEYFKSTSIHTVQSNKQKLEDPTFHHYVIFSKNVLAVSTTINS
TVMNSKDSGSIVFHLFTDSQNFYAMKHWFDRNMYLEATVHVTDIEDHQKLSKDVDFHDMK
LLRPAEEFRVTFRNHYQSFQKQMKTEYISTFGHSHFLLPDLLPSLNRVVVLDDDLIVQKD
LSSLWNLNMGGKVVGAIQFCEVKLGQLKAYTEERNFGTNSCVWLSGLNVVELKKWRDLHI
TSRYDQLLQKLQKDSVTSFPLKVLPISLLVFQDLIYPLEDSWVQSGLGHDYGVSQTDIKR
SVTLHYNGVMKPWLDLGIHDYKGYWRKYMTNGERFMTECNIH*
>11682.m03886|LOC_Os05g40720.1|genepair1427-2
MKGQGGGAGAPAKRRWRSVAAAAAALALLFLSVGVPLAVLLGLHQRFPSMYLADESAVSV
FGGSEGGGWEPNTSQENDRLPVNDTNKFPPSIEKSAFAISWILFHMALFCISTAIKALFL
SFWHDWSKTNTGNSDAESNGTNNQPSIDKPISNTSIHPGLPIKQIVIFDDISLLSNTDAD
PKDNFEQGLPGDESIKSCQLEFGSYCVWSVEHKEVMKDSVVKRLKDQLFVARAYYPSIAK
LEGMEKLSHEMKQSIQEHEHMLSEAICDADLPAFHGANMAKMEKTIAAAKSCLIECTNFE
KKLRQLLDMTEDEAHFHARQGAYLYRLGVQTLPKSLHCLSMRLTVDYFKSFADMEYSNVQ
KLENPVLRHYVIFSTNLLASSMTVNSTVINSEESANVVFHLVTDAQNFYAFKNWFIRNSY
KEATIGVLNFEDFQATHLDNRRVEHLSPYEEFRIASHSNARIPNTQMRTEYISVFGHSLF
LLPELFSNLKRVIVLEDDTIVQRDLSHIWNLDLKGKVIGAVQSCRVRLRHLRPYLVDFPY
DASSCIWMSGVSVIDLNKWREHDVTAVRNRVLQKLQHGPEASWRAAVLPAGLLAFQNLVH
PIEAQWIQSGLGHDYGVNHGAIKKAGILHYNGNMKPWLELGIRRYRKYWRRYLPRDDPFL
IDCNVNP*
>11687.m00108|LOC_Os11g02060.1|genepair1428-1
MAERPCRVAAGLGAASVSVSSVELEKKIGFRINVSMEKSQPELLTSVLEAFEELGLDVLD
ADVSCADDTAFRLEALGSSQSEAAETSVDEQMVRHAVLQAIKKCIDGSSI*
>11673.m04752|LOC_Os07g47960.1|genepair1428-2
MSRERKKAAALHEKLQILRSITHSHALSNTSIITDASEYIKELKQKVVRLNKEIACAEAA
ALRQNSIPTVTVETLGHGFLINVFSDKSCPGLLVSILEAFDELGLNVLEATASCDDTFRL
EAVGGENQVDEHVIKQTVLQAISNSNCSESSGDQEG*

>11687.m00112|LOC_Os11g02100.1|genepair1429-1
MTALSMKGCDGSVLLDDTPTFTGEKTAAPNNNSLRGFDVIDNIKAQVEGICPQVVSCADI
LAVAARDSVFALGGPTWVVQLGRRDSTTASLDTANNDIPAPTLDLGDLTKSFSNKGLSAT
DMIALSGAHTIGQARCVNFRNRIYSETNIDTSLATSLKSNCPNTTGDNNISPLDASTPYT
FDNFYYKNLLNKKGVLHSDQQLFNGGSADSQTTTYSSNMATFFTDFSAAIVKMGNIDPLT
GSSGQIRKNCRKVN*
>11673.m04758|LOC_Os07g48010.1|genepair1429-2
MASSLSVAVLLCLAAAAAAQLSPTFYDTSCPRALATIKSAVTAAVNNEPRMGASLLRLHF
HDCFVQGCDASVLLADTATFTGEQNALPNKNSLRGFNVVDSIKTQLEGICSQTVSCADIL
AVAARDSVVALGGPSWTVGLGRRDSTTASMDSANNDLPPPFFDLENLIKAFGDKGFSVTD
MVALSGAHTIGQAQCTNFRGRIYNETNIDAGYAASLRANCPPTAGTGDSNLAALDTTTPY
SFDNAYYSNLLSNKGLLHSDQVLFNGNSTDNTVRNFASNRAAFSSAFSSAMVKMANLGPL
TGSQGQIRLSCSKVN*
>11687.m00128|LOC_Os11g02240.1|genepair1430-1
MESRGKILMERYELGRLLGKGTFGKVHYARNLESNQSVAIKMMDKQQILKVGLSEQIRRE
ITTMRLVAHKNIVQLHEVMATRNKIYFVMEYVKGGELFEKVAKRGKLTEVVAHKYFQQLI
SAVDYCHSRGVYHRDLKPENLLLDENENLKVSDFGLSALSESKRQDGLLHTTCGTPAYVA
PEVISKIGYDGAKSDIWSCGVILFVLVAGYLPFQGPNLMEMYRKIQHGEFRCPGWFSRKL
QKLLYKIMDPNPSTRISIQKIKESTWFRKGPEENRILKERTLNENTTKNVAPVLGVRRKK
NAHEDVKPMSVTNLNAFEIISFSKGFDLSGMFIVKEWRNEARFTSDKSASTIISKLEDVA
KALNLRVRKKDNGVVKMQGRKEGRNGVLQFDIEIFEVTTSYHIIEMKQTSGDSLEYRQLL
EEGIRPALKDIVLA*
>11673.m04768|LOC_Os07g48100.1|genepair1430-2
MAEQRGNMLMKKYEMGKLLGQGTFAKVYHARNTETSESVAIKMIDKEKVLKGGLMDQIKR
EISVMKLVRHPNIVQLYEVMATKTKIYFVLEHVKGGELFNKVQRGRLKEDAARKYFQQLI
CAVDFCHSRGVYHRDLKPENLLLDENSNLKVSDFGLSALADCKRQDGLLHTTCGTPAYVA
PEVINRRGYDGAKADIWSCGVILFVLLAGYLPFHDKNLMDMYKKIGKAEFKCPSWFNTDV
RRLLLRILDPNPSTRISMDKIMENPWFRKGLDAKLLRYNLQPKDAIPVDMSTDFDSFNSA
PTLEKKPSNLNAFDIISLSTGLDLSGMFEESDKKESKFTSTSTASTIISKIEDIAKGLRL
KLTKKDGGLLKMEGSKPGRKGVMGIDAEIFEVTPNFHLVELKKTNGDTLEYRKVLNQEMR
PALKDIVWAWQGEQPKQQQQPTC*
>11687.m00136|LOC_Os11g02320.1|genepair1431-1
MVDNQGCSPALEPVPTPPNPDPSSISPEAWDPLEAAAGAVVARIQPNPPSEDRRAAVIAY
VQHLLRCTVGCQVFPFGSVPLKTYLPDGDIDLTAFGHSSDEILAKQVQAVLESEEARKDA
EFEVKDVQYIHAEVKLVKCIVQNIIVDISFNQFGGLCTLCFLEKVDQKFEKYHLFKRSIM
LIKAWCYYESRILGAHHGLISTYALEILVLYIFHLFHGTLDGPLAVLYRFLDYYSKFDWD
NKGISLYGPISLSSLPELVTDSPDTVNDDFTMREDFLKECAQWFTVLPRNSEKNTQVFPR
KFFNIVDPLKQSNNLGRSVSKGNFLRIRSAFDFGARKLGKIIQVPDNFTMDEVNQFFRNT
LKRHCSRVRPDVQEIALDFNGERADNDSSPLYSNNSFGDLSDEFNNISISDSSNHGSLRQ
NGWNYVAENKERKSVSGGLLASKATNPAATNSTGMTNGSDSCEPASPSITGAHSLPSEEG
HDALDLFNESESGTKAGIKYGTNPSHHGMSTVSYAGRSHQSFEEVDNDDRGTIDSNWSDL
TGDYTTNFNNLLYAQGFHQDYPMNQYYPFGPVYYPIPSPPPARYQNRRSSNGHSRNNVYG
YAGTNGIGPAPCPPGYLIMRPYSQIDDSNRARGTGTYFPNPNLSKDRSPSGRGGRGKTHF
LPHNHQRPHHYGRADMSADLTPSEELRHIYDPDANDLGIPSSLRISIPSPSSEAPREIVH
GNGFIQPPAKKLEFGTLGALPLEVTSQELGINRLNTASDSQPSASASPMSLANNPGISSN
QMRNAQPYHLKDNGDFPPLSS*
>11673.m04775|LOC_Os07g48170.1|genepair1431-2
MAAYRGRRDYGGGWWSASAGSGRMTLAAVMATRAPRPAFIRREAIRAAEAAADEVVLRVQ
PTEEAERTRQGIIGYLKLLFGTALGCEVFAFGSVPLKTYLPDGDIDITILGNTAPDSTFI
SEVRGILELEEQEDGADVAITGLQFIDAEVKLIKCVIDNIVVDISFNQIGGVTTLCLLEL
VDHEVGNDHLFKRSIMLIKAWCYHESHILGAHRGLISTYALEVLVLYIFNIFHKSLHSPL
EVLYKFLEYFSKFDWDKYCISLNGPVPLSSLPNLTGESYSVEPSGIHDELLFGPNGSCDR
LIVLKKDSDGSNMNFRPKYLNIIDPIKSSNNLGRSVSKGSFYRIRGAFSFGAQNLSQILM
LPTDLIPTEIFGFFVNTLKSHGRGKRSDVGNNGSFEPSLDPESEYALWEDSSDVKESDMS
EDENRSPDLQRTSDSCFYNKVSGDSFSSHSPFSQEKGNNMKRHYDCAREEYLPLGRSSME
QHIYANNQSQILTPSTRINTLDISNSCPAETNRSDLHEEKLPLSHFSPSNLLDLSGDLDL
HLECLRKVQYHLESMFDWLIQEASFSGAVNNDSFNIPTQSSFSNTDGRALRPLLVSSAYT
QRGNLSRVYCSHSTREISQKSVSRTEVQVNAVCQQNVALPSGTNNRLALPPSPVADSEKS
PVSPLHNTVDIVGTHGAGMHTLNNVSLLSGTDVLSNAFAQLSFPAVNSVDYKYCWSYTTT
NNRATSSQKTNRGKGGTGTYIPRMNYHTYKERIFYYNGRSQREMLPDRPFKIKTNPIGYI
RRRSSPEMGCSSSSNGGITFENTSHTPSKKQDHSSKSTVTAEGSFAQERAPASQEWNICT
NMNMVDSQKPGNDEDLVRPNNESRELRTLHPSEVQNREMTASSSSSVELPHCVGNGLQES
NTSQPSSPATEASSPIKTSLVEGLEFGSFEPILGTSFLCEKFCEEFPPLPARKWPAVAAV
STPVTVSSSPAETGSKPEGLYQLRDEADFPPLKAGARNGFNHRVGR*
>11687.m00153|LOC_Os11g02460.1|genepair1432-1
MALRSPNSMLWLALLVWAALLCGSCHGRFVVEKNSLKVTSPSDMKGTYECAIGNFGVPQY
GGTMVGVVAYPKANKKACKSFDDFDISYKAKPGSLPTFLLVDRGDCFFTKKAWNAQNAGA
AAILVADDKTEPLITMDTPEESGNTDYLENITIPSALITKSFGDKLKKAIDNGDMVNVNL
DWRESLPHPDERVEYEFWTNSNDECGPKCDSQIEFVKSFKGAAQVLEKKGYTQFTPHYIT
WYCPDSFILSKQCKSQCINHGRYCAPDPEQDFSKGYDGKDVVVQNLRQVCVYKVAKEHGK
PWLWWDYVTDFAIRCPMKEKKYTKECADGVIKSLGLDHKAIDKCIGDPDADKENPVLKAE
QDAQIGKGSRGDVTILPTLVINNRQYRGKLDKGAVLKAICAGFRETTEPAVCLSEDIQTN
ECLENNGGCWQDKAANISACKDTFRGRVCECPVVKGVKFVGDGYTHCEASGSGRCEINNG
GCWKDSRHGRTYSACTNDGCKCPDGFKGDGVHKCEDIDECKERTACQCKECKCKNTWGSY

ECGCSGGLLYMKEHDTCISKNAATEVGWNFLWVIFFGLVVAGIAGYAVYKYRIRFCLVLA
EKEKSGIPNINWLRDRLFRSIRRRRRRGALAPLAPTLTTAIAMPSCFTLDSASDDGRSTA
QGQGWPAGVGGFLSGFFSAGAARADGGKPSPDWDAHGLAASALPVPLSRLDGKKRYKVSD
LTFLNCRTRAAAAAAAEAPLFDALRPGGVYTRAQLRDELDALATSGMFDHVTLQTKPKPD
GTLGLTVSYAETEWPAVEHFKCINVGGPMARPDGDELELDDDMTARERMEHLRRQEREYQ
QLVRRAKPCVLPEKLQRELQGMVKRQRKVSSGLLKRMAGRIERWYHDEGFHCAQVVSYHG
NLDAGEVVCEVVEGDITKVEYQFLDKLGNVVDGNTSIPLIDRELPQQLRPGHIYNNGAGK
QALKNIDSLGLFSTIEVQPRPDETNQGGVIVAIKLKEHDPKSAQVITDWSIVPGSQGRPT
LASIQPGGTVSFEHRNICGLKRSLIGSVTSSNLLNPEDDLSFKLEYAHPYLDGIDNLSRN
RTFKISCFNSRKLSPIFVAGPNMYEAPPIWVDRIGFKANITESFTKQSKFTYGLIVEEIT
TRDENNNICTHGSRQLPSGALSMIGPPTTLSGTGVDRMAFLQANITRDNTEFVNGATIGD
RCIFQMDQGLGIGSKNPFFNRHQLTVTKFINLNKQEKGSRKPPPAVLALHGRYAGCVGDL
PSYDAFALGGPHSVRGYGMGELGASRNLLEVATELSVPITVKNRHTQVYAFAEHGTDLGS
SKDVEGNPTEFFRRVGHGSSYGVGVKLGAVRAEYAVDHNAGTGAFFLRFGERF*
>11673.m04783|LOC_Os07g48240.1|genepair1432-2
MTMMGCAVLLVVVASMAGEAAGRFVVEKNSLRVTSPAGLRGVYECAIGNFGMPQYGGTMH
GVVVYPKANKKACRSFDDFDLSFKPKPGGLPIFLLVDRGDCYFTTKGWNAQTAGAAAVLV
ADDRLEPLITMDSPESSGTDYIEKITVPSALVTKKFGDDLKKALENGDMVNVLLDWRESL
PHPDERVEYEFWTNSNDECGAKCDMQMNFVRNFRGTAQVLEKRGYTQFTPHYITWYCPEA
FVLSKQCRSQCINHGRYCAPDPEQDFNIGYDGKDVVLQNLIQICLFKVGNETHKPWVWWD
YVHDFSIRCPMKEKKYTRECANGVIKSLGLDLERINKCVGDPEADEENPVLKAEQDAQIG
QGSRGDVTILPTLVVNNKQYRGKLEKSAVLKAVCSGFEETTEPDVCLSQGYKTF*
>11687.m00162|LOC_Os11g02550.1|genepair1433-1
MEAPKLRFVRCPGCLQLLVEYPTIAVYQCGGCGTILRAKNQVAPAVNANAESGEHNEFSN
NSTGGSQNNKLICTDGQKIPPSSDAQPGVLQEKITFASEEKTMSSSNSIDSSEHVNIECS
LLDGDASNHDVRTEGINDEDKVTVSNSSLDSVRKVENVETDGNEKGSFTDDGSISNEVAT
TQSMVHMDGAGSDNNFTEVQSAAEGKCALSDANLDSQEIVAICQPDNISVGTKERVQPYE
GFNVESHEDLIEELVRSLSLSDDEEEFVDIAENSELNDALCSQMGSCRFSLGSKMNEGPR
TDPHGRLIEELEMSFSDAEEPLDQNIMVSLNDIEKPTLDEVSKENHILEEDGKESHILDV
DGKESHILDVDGKQNHILDEHGKENHILNEDDKENLILDKGGEDTLDAGGANSYEERVLP
SDDGLLKSGQSFQQCELVAVNMAEKDEGHLEETNMANHAEANSGIAAVLSNLSNDKFCAI
LPPSCDGRKEEKSNIHRGRELCQGLSLDSVDFRSIQNFIESQMDGTSSSLSSGSPSHGDL
EHNRSNRFKKIDRLERLRKMDDLRDQLNRLSSQKGLENRYKNKGPGVLQEQISYRHLEQH
PCGFDGDSILDSDIIDSYYDQGNPPRYPPPDPFSPTHSHYHCGHGQPHIPYNCSAWEFNS
YYQSSYAGSTVLEHVSLRSSYKEQKRAVKKSILRSLSGASPFTICNGCFNLVQVPSDIYV
SKKKIAKFQCGRCSKALMLSFPATNSDDAKLSNKEVNRKPNKPVHNSVVGMEGGYSFSAE
CSRGDPVSISEECGASISRSFSGRTRAAVAASGSGKKVSDSALHRLMGYDSASQLLRRSR
AFEDGYDSFESMVPVSNRVSRRKNL*
>11673.m04787|LOC_Os07g48280.1|genepair1433-2
MAISDARVVRHVRCPKCFSVLQEPTGAPVYQCGGCGTTLRAKNRTGNSQEVISAPSSLGS
GLPPHSKHLGSSDVASTSGSTPEAQISSGQQGADMTSRRETDDLVSARNNAPEPERVVPA
EKEEEHVQSTSQQAVGNSEDLTRGDAATAADAQCSDRASEGKVQFSESREDSNTELQDVQ
RSDQTESDAEGKKSSEETSQSPRRDVVELPPSSVQTPDSQPAPAVLKREDDPATSPPHGH
ARRSPESLAPLQKRILKTVDNLKDDLSELFSKSPELNKPRTHARPPRLPRQEGYAPRDAA
MAAAASIQAIRARHAAVHRPGYIARAGKPGQLAAPPPPRGLPSRRYRRCRADHPCCHDAR
HGPSCHHGCCPPHHGKQACTSCRGQHCCRPRTQESPAPRRPAAAAAKEVVKRRAPPRNHC
RPVLKGAPFIICSSCFKLVQVPADFAVSTKTVRKLRCGSCSAVLSYSYRDPDRKKHGDQY
SADGSPAAPRGHGRRGDKFAFLDDFGHVDVSYSTEDEQPLHVSRNSSFNTVDEMAAAATQ
QHGSSLHRLMGYGSASDLLFRQHSPDLYESFSERTTPEAAALYDRKGKGVCVDLDDDGGD
DDSDEDCSGALKRSRLRGSGWPLPGILNSKGTTGMGAIRIKS*
>11687.m00180|LOC_Os11g02710.1|genepair1434-1
MNLDHNSTDPKITGCLVRFHSDIKNRVGNRTEKPTPDTAISLFPSRIFRASRSSTINMSE
ESGLAAHGSEEGFPSVEIRMLKLEGNRDVCLDIDSGIRVDDDGAGRISSGAGGGAGRRGR
HGRGSSGGSGRHGAEEEAARLGRGWGVARARVRLGRGAKWGRARRGVGQSARRGRRGEQG
AARERQKRVGSIYLYSTGSIQVLNVTDPNMVKELANCKSLDLGKPCYLQKERGALLGMGI
LTSNGDLWVHQRKVIAPELFMERVKGMVNLMMEAAMSMLNSWKNEVEDRGGSAEIVVDEF
LRTFSADKAMAKQSMLIGVPGSRYLPTRSNRGIWNLDSSIRTLILNISKKYEHDSSTSVN
KDLLHSIIQGSKDGPFASCTPEDFIVDNCKNIYFAGHETTSTTAAWCLMLLASHHEWQSR
ARVESLDICQGRPLDFDILRKLKKPPSSPGEALNDMKLAGIDIPKGTNIWIPIAMAHRDP
SVWGPSADKFDPDRFANGIAGTCKPPHMYMPFGVGVRTCAGQNLAMVELKVVLSLLMSKF
EFKLSPNYVHCPAFRLTIEPGKGVPLIFREL*
>11673.m04792|LOC_Os07g48330.1|genepair1434-2
MAAASLCCGVAAYLYYVLWLAPERLRAHLRRQGIGGPTPSFPYGNLADMRSHAAAAAGGK
ATGEGRQEGDIVHDYRQAVFPFYENWRKQYGPVFTYSVGNMVFLHVSRPDIVRELSLCVS
LDLGKSSYMKATHQPLFGEGILKSNGNAWAHQRKLIAPEFFPDKVKGMVDLMVDSAQVLV
SSWEDRIDRSGGNALDLMIDDDIRAYSADVISRTCFGSSYVKGKQIFDMIRELQKTVSTK
KQNLLAEMTGLSFLFPKASGRAAWRLNGRVRALILDLVGENGEEDGGNLLSAMLRSARGG
GGGGGEVAAAAEDFVVDNCKNIYFAGYESTAVTAAWCLMLLALHPEWQDRVRDEVQAACC
GGGGRSPDFPALQKMKNLTMVIQETLRLYPAGAVVSRQALRELSLGGVRVPRGVNIYVPV
STLHLDAELWGGGAGAAEFDPARFADARPPLHAYLPFGAGARTCLGQTFAMAELKVLLSL
VLCRFEVALSPEYVHSPAHKLIVEAEHGVRLVLKKVRSKCDWAGFD*
>11686.m00472|LOC_Os12g05380.1|genepair1435-1
MAVTGGGRPAVRQQAARGKQMQRTFNNVKITLICGFITLLVLRGTVGINLLTYGVGGGGG
SDAVAAAEEARVVEDIERILREIRSDTDDDDDDEEEEPLGVDASTTTTTNSTTTTATAAR

RRSSNHTYTLGPKVTRWNAKRRQWLSRNPGFPSRDARGKPRILLVTGSQPAPCDDAAGDH
YLLKATKNKIDYCRIHGIEIVHSMAHLDRELAGYWAKLPLLRRLMLSHPEVEWVWWMDSD
ALFTDMAFELPLARYDTSNLVIHGYPELLFAKRSWIALNTGSFLLRNCQWSLELLDAWAP
MGPKGRVRDEAGKVLTASLTGRPAFEADDQSALIHILLTQKERWMEKVYVEDKYFLHGFW
AGLVDKYEEMMERHHPGLGDERWPFVTHFVGCKPCGGYGDYPRERCLGGMERAFNFADNQ
VLRLYGFRHRSLASARVRRVANRTDNPLVNKEAALKMDAKIES*
>11668.m03084|LOC_Os02g32750.1|genepair1435-2
MGQEGMGYNNGKGGGGGGGGLPMTAPRPRGASPLSSHGHHHRSRKIHRTFNNVKITVLCG
LVTILVLRGTIGLNLSLPNQPTDADALAGAKAVEDIDRILREIRSDGGADDDAAAAGDLA
GSFNATALNATEAAAAYASAVERYALGPKISDWDGQRRRWLRQNPGFPSTVAGGKPRILL
VTGSQPGPCDNPLGDHYLLKTTKNKIDYCRLHGIEIVHNLAHLDTELAGYWAKLPLLRRL
MLSHPEVEWIWWMDSDALFTDMAFELPLSRYQDRNLIIHGYQDLLFEKHSWIALNTGSFL
FRNCQWSLDLLDAWAPMGPKGFIRDEAGKILTANLKGRPAFEADDQSALIYLLLSQKEKW
MNKVFIENSYYLHGFWAGLVDKYEEMMENHHPGLGDERWPFVTHFVGCKPCGSYGDYPVE
RCLRSMERAFNFADNQVLRLYGFAHKGLESPKIKRVRNQTTKPIDDKENLDVKAKISTTS
*
>11686.m00493|LOC_Os12g05590.1|genepair1436-1
MSRSVEPLVVGRVIGEVIDSFNPCTKMIVTYNSNKLVFNGHEFYPSAVVSKPRVEVQGGD
MRSFFTLVMTDPDVPGPSDPYLREHLHWIVTDIPGTTDASFGREIISYESPKPSIGIHRF
VFVLFKQKRRQAVVVPSSRDHFNTRQFAEENELGLPVAAVYFNAQRETAARRR*
>11668.m03104|LOC_Os02g32950.1|genepair1436-2
MSRVLEPLIVGKVIGEVLDNFNPTVKMTATYGANKQVFNGHEFFPSAVAGKPRVEVQGGD
LRSFFTLVMTDPDVPGPSDPYLREHLHWIVTDIPGTTDASFGREVVSYESPRPNIGIHRF
ILVLFRQKRRQAVSPPPSRDRFSTRQFAEDNDLGLPVAAVYFNAQRETAARRR*
>11686.m00494|LOC_Os12g05600.1|genepair1437-1
MVPRILIVLLVVLLGLAFQAILRPPPQKLCGSPGGPPVTSPRIKLRDGRYLAYREDGVQK
DKAKFKIISVHAFDSTKDFPLQVSKELVHELGIYIVGFDRAGYGESDPNPKRDVKSEALD
IEELADQLELGHKFYVLGVSMGGYSIWGCLQYIPNRLAGAAMVVPIINYWWPSFPAELSR
QAFKRLIVPEQRTLWIAHNMPSLLYQWMTQKWLPSSAAAMRHPEIFSKHDLEVLQKMMAM
PLIENKSRQQGIYESTHRDLLVAFGKWEFDPMNITNPFPQNEGSVHIWQGYEDRLVLVEL
QRYIAQRLPWIQYHEFPEGGHMFMLVDGWTDKIIRALLVGEQL*
>11668.m03106|LOC_Os02g32970.1|genepair1437-2
MADSGKKRDGSANGAEKKASPTPKPPSSSTSGFSKNVPLLVFVLLLGLLYRQLQPPVPKI
CGTPGGPPVTGPRLQLKDGRHLAYHEYGVPKDQAKHKIIFVHGFDSCRYDALQVSPELAE
ELGVYMVSFDRPGYGESDPHPGRTEDSIAFDIEGLADGLQLGPKFYLIGYSMGGEIMWSC
LKNIPHRLAGVSILGPVGNYWWSGYPSNVSTEAWYVQLPQDQWAVRVAHHAPWLAYWWNT
QKLFPASSVISFNPAILSREDLTVIPKFAYRTYAGQVRQQGEHESLHRDMLVGFGKWGWS
PLEMENPFPAGEAAVHLWHGAEDLIVPVQLSRHIAQRLPWVRYHELPTAGHLFPITEGMP
DLIVRSMLLTDE*
>11686.m00509|LOC_Os12g05730.1|genepair1438-1
MVSLRAAIVLVVAASSVAVAFSHAEGNEGPDFTYIEGAMDGPSNWGKLSPEYRMCGEGRS
QSPIDINTKTVVPRSDLDTLDRNYNAVNATIVNNGKDITMKFHGEVGQVIIAGKPYRFQA
IHWHAPSEHTINGRRFPLELHLVHKSDADGGLAVISVLYKLGAPDSFYLQFKDHLAELGA
DECDFSKEEAHVAAGLVQMRSLQKRTGSYFRYGGSLTTPPCGENVVWSVLGKVREISQEQ
LHLLMSPLPTKDARPAQPLNGRAVFYYNPPGSAVSFQEFAK*
>11668.m03112|LOC_Os02g33030.1|genepair1438-2
MGTAKKSAIFVALLCTHILVNHACDSDVVFGYSGSTGPEHWGSLSPNFTTCSKGTYQSPI
NILKDDAVYNPKLEPLEMDYTAANTTIVDNVFNIALRYNDTAGTVKVDGKKYKLRQLHWH
SPSEHTINGQRFAVELHMVHHSDDGNITVIAVLYRHGKPDPFLFQIKDKLAALYLEGCKA
EKGEPLPVGLVDMRELKKGADRYFRYVGSLTTPPCTENVIWNIFGEIREMTKEQAAALRA
PLHGSYRHNRRPTQPLNGRTVQLNYM*
>11686.m00525|LOC_Os12g05890.1|genepair1439-1
MAADGGALRRLFEKPLPVPENPTLLEALSACNHVHHRKKLVDTASFTEIFGELHFQEKPV
DVAAAARFLPPPPPPVRAASWIDVADDKSKDGSSLDALLRPKSSAVKRSASFCLKSSESS
LLLCTEGLGTESTVDADDMVKDGDGDGEAIRRGEEETDGVEEDDGAGREKRGTPTLAPTP
PTFPPPIRSIGRGGKPCVCFRSFRAEGRFVLMEVVIPGKELLQATREGGRLTLRFANAAA
VGGGGEADDDVNDDDVDGGETKNACAARDDMLANNCTC*
>11668.m03120|LOC_Os02g33090.1|genepair1439-2
MPRPPPPLFGQAGQDQRRRYLGLCTEGLGSESSESSGGDVDLGTGGGDDTGNDGVGRALP
CKRQHRPIDDEEEEEEKTVVPAALAPPLPAWTRAAFPPPISVIGAGGKPWLYLRAHRGDG
RLVLREVRIPSRELLHGRREDGRFKLHFAHPDEQLQQQLLLLADDQDPAEKNE*
>11686.m00539|LOC_Os12g06030.1|genepair1440-1
MASVCSAAAVSLDGAVAGATTTTAIEDIPGDVLSLVLRRLDDGASLAALGCACSAFRELA
ADAETWRGLCLATWPSLRDVDGLDECGGVTGGGGYRALFADAFPLPAEARGLVPSSASLL
PARLVSAVDLHHGEVCLMSRVVETDASSEWFLGSPFRVDALVQEGFSAPAPITPAELTLS
WILIDPATGRAVNASSRRPVSVDREWLAGETVARFTVVLGGGVVALDAAVTCDDRHGHVR
EVSLRAEDGDGGGVSGRGVLAAVAAAMEGARHGRGAEAAAWRRYEAFARGRAARKVKKAR
RDGAVDFFCSGVAAAAFVGVLSTLTLR*
>11668.m03136|LOC_Os02g33240.1|genepair1440-2
MAATTTTVEDLPGDVLACALRRLDGPSLAAAGCATSGLRALADDPDTWRALCLSRWPSLA
AAEQRCVLSAAGAVSPRRLFADAFPFPCVDDAAAAAPLDGDDQRLPGELVSAVDVYHGGA
AVVSRVVETSTSSSWFLASPFRVDAVEGKSPAPAPASVASSWSPAELELSWILLDPSTGR
AVNVSSRRPVAVERHWYTGDTLVRYAVVLAGCKFEATVSCSEEAGQITEVSLAADDADGA
AISGEGCLRLLAAAMAGPRKGGRGQEGEAKRRYDEFVRRKRGRKESKARREVLVDLCCSA

VSAVAVISFLAAVVLR*
>11686.m00556|LOC_Os12g06200.1|genepair1441-1
MAAAGAGSSEVAARVLLQRYQPFAPPPGEYHQFGSGGAAAAGDMTEAVLIRTPLKRKHDR
EENEAAESNDWMMSPGYTNPAGSPVPTPLSGKGSKAFAKSKAAKGQKSCPQTPLCASSPG
NPVTPVGGCRYDSSLGLLTKKFLNLLKGAPGGIVDLNNAAETLEVQKRRIYDITNVLEGI
GLIEKKLKNNIRWKGIDDSRPGEVSDDMSILQADIEALSLQEHSVDQQISEMRDKLRGLT
EDENNQKWLYVTEDDIKSLPCFQNQTLIAIKAPHGTTLEVPDPDEVNDYPQRRYRIVLRS
TMGPIDVYLVSQFEEMSGMETPPRTVQPVSMDSLENPRTPLAAEPNKAAESQPNIQDGLL
MPSDAPSSSQDIGGMMKIVPSELDTDADYWLLSDAGVSITDMWKTAPEVEWEGIEKFNAE
DFLEVSTPRQQDKPSSDIMDGDSCIS*
>11668.m03156|LOC_Os02g33430.1|genepair1441-2
MAGSGRPPAAQKKILQSLRPPLPFAASSRSPFAAPNDYHRFPAGGAAAAAASGSGGIGAG
GAGGGGDIEEGLVIRTPQKRKAPEESDVAESSDCMITSPGFAVSPMLTPVSGKAVKTSKS
KTKNNKAGPQTPTSNVGSPLNPPTPVGTCRYDSSLGLLTKKFINLLKQAPDGILDLNNAA
ETLEVQKRRIYDITNVLEGIGLIEKTLKNRIRWKGLDDSGVELDNGLSALQAEVENLSLK
EQALDERISDMREKLRGLTEDENNQRWLYVTEDDIKGLPCFQNETLIAIKAPHGTTLEVP
DPDEAGDYLQRRYRIVLRSTMGPIDVYLVSQFDEKFEDLGGGATPSGHANVPKHQPTEVF
NTTNAGVGQCSNSVAVDNNIQHSQTIPQDPSASHDFGGMTRIIPSDIDTDADYWLISEGD
VSITDMWKTAPDVQWDESLDTDVFLSEDVRTPSSHNQQPSAVGGPQMQVSDMHKP*
>11686.m00563|LOC_Os12g06260.1|genepair1442-1
MGKDCGKHGDDDFRQGCRRFITVLVVLAILVGIIALIVYLVLRPTHPRFYLQDATLRQLD
LSNSSSTAGGVLSTTIQVTVASRNPNDRVGVYYDRLDVYASYKYQQITVAASLPPVYQGH
GDVDVWSPVLAGPSVPFAPYLADAISQDCQAGYLILQVKIDGRVRWKVGSWISGHYHLFV
TCPAFLVTAGGNGSPGASGFRFQTTTYCHVEV*
>11668.m03168|LOC_Os02g33550.1|genepair1442-2
MSKEKHHKREHHLRRCCGGMAACILALVLVVGFIALVVYLALRPSKPSFYLQDLQLRSVD
LGDPSLSATAQVTLASRNPNDHVGVHYRRLDVFVTYRDEPVTVPVSLPPTYQGHRDVTIW
SPVLSGESVPVAGFVADALRQDVAAGYVALQVKVDGRVKWKVGSWVSGSYHLFVSCPAML
ASAGPGGVGPMPLGGASAAVVNGTGAGAVASLRFTQPTGCSVEV*
>11686.m00580|LOC_Os12g06410.1|genepair1443-1
MEYGTPQHFMCPISLQPMQDPVTSPTGISYDRRAIHRWLAAGHSSCPVTGHPLSLSDLTP
NLTLRRLIHSWHHSTTTPFPVERSTPSPPPLREVDDDDVVERLVMEMEGGGGGSWCPPSC
DLLREAAAVAAGSGVARRRMVGAGVLRRVLRLVVWCGGRGSSSGEAAVMVEMFDACLALF
HALDVSADELRPLVADGHDLVDAVTRVMATLEAGDANATRARESAVRLLEAVTEAADAPV
LERLSPEFFSAATAVVRDRGAVSPGAARAAVRALANACRARASGACRNRALAVDAGAARE
AIELELDAWSSPQAPGGRRATEAVMALLAELCACAEGRAAVASHPAGITVVARRVLRVSA
AADACAVRVLAAVAGRAASPEVLREMARVGAVGKLCCVLQAECDAGVKEAARAVLRMHSG
VWSGSPCVSAYLLSRYL*
>11668.m03173|LOC_Os02g33590.1|genepair1443-2
MEEAAAEVPSYFLCPISLEIMRDPVTLATGITYDRSSIERWMFGGGGGDGGKGTCPVTRR
QLAPAEREATPNHTLRRLIQAWCAAHAVERFPTPRPPVDSCRVAALVDEGTTTMLGGGGR
QRQLAALREIKAIAAESDRNKRCVEATPGAVEFLVSVVVQSHAAASTSASSDDDDLFDSV
IDSPMSTSSPEEEALGVLYSLKPSEPTLRRVLGKDNGVGFLDTLASVLRRPSYRSRAYAI
LLLKAVTSAMPPERLMAVSPELVEEVVRVVSDGVSSKAVKAALHVLCRLCPWGRNRVKAV
EAGAVAALVELLLDEEGGGGRRRAAELAVVAIDHLCGCAEGRSELVAHPAGLAVVSKRAM
RVSPAATESAVRALHAVARNAATPAVLQEMLAVGVVAKLLLVLQADGGERARARAREMLR
ANARVWKDSPCLQAHLKASYPS*
>11686.m00603|LOC_Os12g06640.1|genepair1444-1
MEGNNLPSGSLMRSNSGQMHAPNPGKQGFDHTQMPGNLSMHVNQSTDSDHLSEFQFGELG
KVDHHHHHHHRQHAKNGMSDDEEHGVNEDATDSQSGKGKKGAAWQRMKWTDSMVKLLITA
VSYTGEDPGADSGAGKRNSAIMQKKGKWKAISKVMGERGCSVSPQQCEDKFNDLNKRYKR
LTDILGRGTACKIVENHALLDCMSNLSDKMKDDARKILSSKHLFYEEMCSYHNNNRVSLP
EDPALQRSLQLALRCKDEHDLRRGTSGDADEDDQSVDSDSEEENDEENYTLQGDKSALPM
HKRLRLMTDQEDVGFGNSSSSHGCSRRSDSHGISLDINKAFPDGTNLALAQKDLATQSAD
LEEQRLQIEVQAVYLAKQRLKWERFSKNKDRELEQMRLENEKMRLENKRLELEVRHKELE
LELKQKGSGNHA*
>11668.m03192|LOC_Os02g33770.1|genepair1444-2
MEGNLPPRGALVHGHGGGVGAFDLEATMQPPPPFHFAQDPHLHHHQGMVPVRGNPMLDLG
NVVKTSPSDEEDVDDGHHHGGGGGSGKEASQWHRVKWISGMVKLLVSAVAYIDEDVDMDY
GTGSAARRKHAMLKRKGKWRLVSAAMTERGFPVSPQQCEDKFNDLNKRYKRMTEILGRGT
ACQVVEHPELLEGMRLSGKLKEEARKHLNSKHLHYEEMCSYHNRNKMCLFDDPALQKSLR
LALRSGEEHAKKNPFGYDDEDFSDDDDEDEEFDDLEVSAEDHHHGIHGAKRLKHDQEETH
FGSNLSEVAVIDMNKMLSEGSGGPTAEKSPSTPGMRDIRLEKRRLKIKAQMLKIEQKHFK
WLRFSKEKDRELEKMRLENEKMKLENERLELELKLKEIEMGIKPKKIFSD*
>11686.m04046|LOC_Os12g40790.1|genepair1445-1
MKYAPIYLLTLSCLHYAVCRKCIYKKINDEELEHCPVCKIDLGCAPLEKLRADHNIQDVR
SKIFPLKRKKVNAEEVESPIAPPAKRKERSISSLVVNTPEITPKSLTGRRTRASTRKSAA
ALRDLGPIIPPVKKDSDNTNKNADNSSLLDSLSKVPQTRRQVLSNAETSSHPSSKDKGGD
DKDLDKSELWRPLNCLVEAASKTKSYRSSSAARGNQPTESPSSANASRTKAREYLLKSKV
QDEKKEVPVATVPFKRKGPGRGRKPAQPPAAAVSSHSASKHEKLLTPVWFSLIASFDQKG
APPLPQIPTHYLRIKDDNMPASSIQKYIMQKLSLPSETEVEISCCGQPVNPIQPLRNLIE
RWLRFGPARTLQTVVGSSGGDYVMVISYGRPKAA*
>11669.m04308|LOC_Os03g43360.1|genepair1445-2
MQPAPASPPKADGGEDEEEECSRAVVKEEPHHQQEEDDDDAAAAADGGEDEKEKVEEEEV

EERGRRRRGRPGRKRGRRSGGGGGSAAAAAAARGGVVMVKRELLARCMTCPLCGRLLRDA
TTVSECLHTFCRKCIYEKLNDEEVESCPVCKIDLGCTPVEKLRADHNLQDVRSKIFPFKR
KKISADEVAAPVLLPSKRKERSISSLVVDTPTVTPTGLTGRRTRAVTRKAAALRGLGPGI
DDPVKKEIDNGEKHAQNSSLPTNLGKVPQTRRQMSSNAEASNHSSNKDTEGDRKDLADKT
DELWRPLNCLVEAANRTKSSRSSSQSPFVKREQLSDSPGSTSVNKTKSREYMQKSKIEDD
KKDVPLLKRKNQRTGRRRELHAQSDSKPEAAATQNEKKFSSIWFSLVASFEQEGDPPLPQ
IPSHYLRIKDGNIPASSIQKYLMQKLGLPNEAEIMETFIELSQLHQGRNKLLRAASEPYT
TPVQFSRGVVEGAINTDNTDHDRLPCQGVCNGANLWAAKGHHAMNGTTNTRLKPFFA*
>11686.m04054|LOC_Os12g40860.1|genepair1446-1
MASSSDASSALAAAAAVGKKRGSYNCGRCGLPKKGHVCAVAGEEQKPPRRALHFDEAAPP
PPPEKKVKVEVVEVDSSSSEEEEREARGWVEVGGGRRVPGEVVVEVMRRLPPRGVAASAA
VCRGWRGCARRVWRAADELRLRAAGVRPVGALLPRCPALSRLVLRMESDIDATMLACVAF
SCPNLQYLEISMVGSAANRMTGDELTRFVSEKRSLSVLKLDGCSNLNFLNISSSSLSTLW
LSDLSSLSKSVINCPNLNELSLGFTQQNNDSTDLISLMDSLGRTCSNLRNLHISSIHLCN
EAVFSLESANLRGLCMLSLILGSKITDAAVASIVRSYASLDLLDLSGSSITDNGLGMICK
AFPHTLTRLLLALCPNITSCGVQVATSQLPLLQLMDCGKSLCANSQPEAERSYFGEIYGG
IKFCSKLPIQRKQQPNYQKLIIKHANLKKLSLWGCSALDALYVNCPELSDLNLNCCTNLH
PGAMSLPENAERLLLQCPSLKDVHASGCRDMLIGAIRNQVLNEFASAEPRVPCKRLADGS
KRVQVPHFMLEQQFEEEKWGSGSKRSQCTVHLS*
>11669.m04311|LOC_Os03g43390.1|genepair1446-2
MASLPKPPPPPRPKTRGSYNCGRCGLPKKGHVCAAGGPAPTPSPSSSSGAATTTTSGGGG
GGGEGTKLRRALSFEDAATASASATPSSPEKKPRVVPDADAVGRGGEGEVEMVEGQGGEE
ELKEEEEAVELGGRAVPRELMAEVLRRLGPRGVMAAAAVSRGWRDCAGRVWRAAEELRLR
AAGVSLVGALLPRCPALSRLSLRMESDVDATILACLAFSCPSLGTLEISMSDNAVNRMTG
EELSRFVSEKHSLSVLKIGGCCNLGFLNLNSSSLSILWLSDLCSLSKSVINCPNMSEISL
CFTQQSNDCTDLVTLMDGLGRTCPNLKNMHISSAQLSNEAVFALEGASLRGLCMLSLILG
SKITDAAVASIVRSCASLELLDLSGSSISDNGVGMICKAFPHTLSRLLLALCPNVTTCGI
QFATAQLPLLQLMDCGMTLCASLQNEKQGPYFGEINGAIRFCPKLPTSKKQSTNQKLIIK
HGNLKKLSLWGCSAIDALYLNCPELNDLNLNSCTNLNPERLLLQCPKLKNVHASGCHDML
IGAIRNQKLINRKTT*
>11686.m04058|LOC_Os12g40890.1|genepair1447-1
MAADLAFEATELRLGLPGGGGDGDAAAAAARSSSGKRGFAETIDLKLKLEPAAAAVDDDD
DKEEAAADDREKKVDIVGADNDDASPPAAAAAGGMKRSPSQSSVVTAAADPEKPRAPKAQ
VVGWPPVRSYRKNILAVQADKGKDAADGGGDKSGAGAAAAAFVKVSMDGAPYLRKVDLKM
YKSYLELSKALEKMFSSFTIGNCGSHGVNGMNESKIADLLNGSEYVPTYEDKDGDWMLVG
DVPWEMFVESCKRLRIMKGSEAIGLAPRAMEKCKNRS*
>11669.m04312|LOC_Os03g43400.1|genepair1447-2
MAGLGFDETELRLGLPGAGELAARSSGKRGFAETIDLKLKLQPAAPAAVSGEEGAQEDKE
DADAAAAAADEKMSMKRSASQSSVVTAEPDPDKPRAPKAQVVGWPPVRSFRKNVLAEKCK
AAALVKVSMDGAPYLRKIDVAMYKSYPELSMAFQNMFTSFTIGKCGSHQQLKESNKLRDD
LEYVPTYEDKDGDWMLVGDVPWEMFVESCKRLRIMKGSEAIGLAPRAVEKCKS*
>11686.m04059|LOC_Os12g40900.1|genepair1448-1
MENLKATELRLGLPGTEEEAAPPPSTPRAGSKRALAGEPDQAKIKPAAAAKAQVVGWPPV
RSYRKSCLQPTTTTTKSKPPPAAAAAETQQKEDVAGAGGLFVKVSMDGAPYLRKIDLKVY
KGYRELREALEAMFLCFSGGAAADAAVNPSDFAVTYEDKDGDLMLVGDVPFEMFISTCKR
LRIMKGSEARGLGATRG*
>11669.m04313|LOC_Os03g43410.1|genepair1448-2
MEAAVGYAADSLIKATELRLGLPGTADDLPSTPRGKKRAAAAEDNNANAAAADDDEHDAV
EAAPPVAKAQVVGWPPVRSYRKSCFQQQSAAASKSKAAVSSCNNKDEPITKNAAPAPAAS
SAAAANGGSLVKVSMDGAPYLRKIDLRMYKGYRELREALEAMFVCFSGAADGANPSEFAI
TYQDKDGDLMLVGDVPFDMFTSTCKKLRIMKRSEATGLGSPRQMKI*
>11686.m04079|LOC_Os12g41090.1|genepair1449-1
MAMAAMDARKKKSGGGGGEPLLGKYELGRMLGRGTFAKVYLARAVAGGEAVAVKVIDKAE
VMGTAGMAPRVLREVAAMRRLRHPHVLRLHEVLATRARIYLVMELATGGDLLSRLAALPR
RRLPESAARRVFVQLVDALSYCHARGVAHRDVKPQNVLLDGDGNLKVSDFGLAALPGHAP
RRRPPPHRVRHAGAHRREYELPRWVSQPARRLVSRLLDPNPDTRVAVESLAAHHPWFKRS
LSVDSQLDGLLNGEPERAVAFQAAPPPPLNAFDIISMSPGLDLSGLFGEHDKSLREKRFT
TTASPEKTLEQLGLAGGKLGYVVVVGKKGVECLPLAGGRLSSGIAAMSVEMSEVAPPLLL
VELRLEVAAGDVDGGDGEVKGFGWEQLRMELGDVVRAWHSCEDLCEI*
>11669.m04316|LOC_Os03g43440.1|genepair1449-2
MAATKSKAAKKGAPLLGKYELGRLLGRGTFAKVYHARSLAPGADPVAVKVLDKPDLAAAG
AGMATRVLREVAAMRRLRHPNVLRLHEVLATRSKVYLVMELAPGGDLLSRLASLPSRRLP
EHAAQRVFLQLVSALIYCHARGVSHRDVKPQNVLLDAHGNLKVSDFGLAALPDSLRDDGR
LHTACGTPAFAAPEVLRRKAYDGAKADAWSCGVILFVLLAGHLPFDDSNIADMCRKAHRR
EYALPRWVSQPARRLVSRLLDPNPATRLAVAELATHPWFKRSLSLDSQLGSLLGGQPERE
LAFQAPPPLNAFDIISMSPGLDLSGLFGESKRRREKRFVTTASPERTVERLGQAGAKLGY
FMVGKKGVERLPLGGLSGLVAMSMEMSEVSPSMMLVELRLEGGDDGDGDGGAEEFGWEEL
RAELGDDVVMAWHGCDGGKKDKEGILL*
>11686.m04084|LOC_Os12g41140.1|genepair1450-1
MSESGRVTAGGGRGELRPPELPRDPALEFLSRSWSASGAADVSRRALAAAAFPAAAASAA
VIAEDVSGELDVDGSASGSSFSFASAATSQLIMDRIMSQSIDTENLLVSCFMEQQEVSPL
TSGRLSHSSGPLNGGGSLSDSPPVSPDVDDSKAHMLQKYYTPKSHCRIISDQQEEVFDLM
VFGGCLFCRAVSTPKPQPYRGVVGGGGKTVGRWLKERKEKKKEEARAHNAQVHAAVSVAA
VAAAVAAVAAATAASGGGRDDRAARTDMAVASAATLVAAQCVEAAESLGAERDHLAAAIA

SAVNVRTPGDIVTITAAAATALRGAATLKARALKDVWNVAAVIPVEKNAIAAATTGGGHH
KHNAQKQQHHHRHHGNGSNTSSSFSDEVAAVDDDDDDDNNFLTICSQELLARGTELLKRT
RKGALHWKVVSVYIHRTGVVMLKMKSRHVAGTLTKKKKNVVVDVCRDVAAWPGRHLLEGG
EHRRYFGLRTAEHRVIEFECGSQREHDMWTKGVARLLATIDGRRKRFA*
>11669.m04323|LOC_Os03g43510.1|genepair1450-2
MGDHHHHHHPRRGSRHAAAGDLRPPEPPLDPLEFLSRSWSASASALDAPRPPPPAPSPSA
VLGIGPIAEDASSAATAACEVVDDGSAFAAAGSSFSFASAATSQLIMERILAQSEVAPLT
SGRLSHSSGPLTGGGSITDSPPVSPEIDDAKYCRAASTPKPQMYRGGNKTVGRWLKDRKE
KKKEETRAHNAQVHAAVSVAAVAAAVAAVAAATAAASGSGKDDRAARTDMAMASAATLVA
AQCVEAAESMGAEREHLEAVIGSAVNVRTPGDIVTVTAAAATALRGAATLKARALKEVWN
IAAVIPVEKGTMGGGHHHKQNMQKQHRKLESNGSSISDDLSLEEENNFLGICSQELLARG
TELLKRTRKGALHWKVVSVYINRMGLVMLKMKSRHVAGTITKKKKSVVIDVCKDVAAWPG
RHLLEDGEHRRYFGLRTAEHRVIEFECTSQREYEMWTKGVARLLIIASERKRAP*
>11686.m04086|LOC_Os12g41160.1|genepair1451-1
MAHCSAACSLLLQHCSGLQEFAVLQIYLTECMCKKKKKKSKMGKAGRWLRSFLTGGKKDR
KGKDGGQPPAPPSPAPPSAKEKRRWSFRRPPAQATTNTSSLCFSDVHAVSPAPEAESSAA
ADVAEENEAAAAAAVRIQAAFRSYLARKALCALRGMVKLQAMVRGQLVRRQASTTLRRMQ
ALVAAQRRARAERLCLLDDDKDKHARSPRPPTTTSRRSSPQHHRSRKPLEVVDRGSEENV
RVVEVDNGGGGGGAARGCGRRSTCGAAAAAAKGELYQKVSPTPSALTEASARTMSGRLDD
YSFSAAASEASGRHHSVPAAAVAGGDHAAALQQLFPKNYMSNTESSRAKARSQSAPRQRH
DQPISAAASPSPSPSCGEWTTTPGDRRRRASLDPRDLAAPASAAGVGVGARMERCASQAR
ASASASAACPWAVRMDRSTASLAGGSDGGSSAATAVTAATAARVTS*
>11669.m04332|LOC_Os03g43580.1|genepair1451-2
MGRAGRWLRSFLPGKKDRARAPAPEKEQAVAVVTTPGAKEKRRWSFRRPAVKDGGGGFLE
PRVDPDQHAVAVAIATAAAAEAAVAAKQAAAAVVRLAGSSRRGVVVVGIEEAAAIKIQCV
FRSYLARKALCALRGLVKLQALVRGHLVRRQASNTLRCMQALVAAQHRARAARLRLLDDD
KEKPLLHTPRMMPTRRSPHHPRFRHQQQQQEAEENVKIVEVDTGFGGGGGSGEAHCTPRT
SSRRSSCYATPLCRTPSKVELYQKVSPTPSALTDASARTYSGRYDDFSFSTARNSPWHHH
HASDAPCKPHHPHHGNGDHPLFFPNYMANTESSRAKARSQSAPRQRASVSSSASEASSVR
WERQASARRRASLEAQAASAPPNKCGAAMMARVQRCPSQASAPASCPWGSRLPDHDSECG
STSTVLTAATTTYCWSLATDNAAAAMA*
>11686.m04088|LOC_Os12g41180.1|genepair1452-1
MDQYEVLEQIGKGAFGSALLVRHKLEKKKYVLKKIRLARQTDRTRRSAHQEMQLIATVRN
PFIVEYKDSWVEKGCYVCIVIGYCEGGDMAEAIKRANGTYFSEEKLCKWLVQLLMALDYL
HANHILHRDVKCSNIFIARDQSIRLGDFGLAKILTSDDLASSVVGTPSYMCPELLADIPY
GTKSDIWSLGCCIYEMTALRPAFKAFDMQALINKITKSIVSPLPTKYSGAFRGLIKSMLR
KSPEHRPSAAQLLKHPQLQPYVLQVQLKSSPTRNILPIHQSLTDKVKKMTFPSDVVDSAR
RRMARRNSLGNERTVTFSKPSPERNSVSSTRSIKEYTTTQSVKGLSVDSSEAGDEVTSKA
IITKTSSILRTPKSLPAKTYTARNQLEPPKTSYNRTYRSELPSKTTPNKIARPARRASLP
LSTYETPTKRSISILEQLDSPDVSVNAPRIDRIAEFPLASSEDPLLPIHNKLSPGHGSCS
TPPFINRSITKDKCTIQVLRTDGDNGSDSSGRNATAASSRGSNDSRQQRFDTSSFQQRAE
ALEGLLEFSAQLLQQERYEELGILLKPFGPEKASPRETAIWLTKSFKETAS*
>11669.m04333|LOC_Os03g43590.1|genepair1452-2
MEQYEVLEQIGKGAFGSALLVRHKVEKKKYVLKKIRLARQTDRTRRSAHQEMQLIATVRN
PFIVEYKDSWVEKGCYVCIIIGYCEGGDMAEAIKRATGDHFSEEKLCKWLVQLLMALDYL
HANHILHRDVKCSNIFLTRDQSIRLGDFGLAKILTSDDLASSVVGTPSYMCPELLADIPY
GTKSDIWSLGCCIYEMTALRPAFKAFDMQALISKITKSIVSPLPTRYSGAFRGLIKSMLR
KSPEHRPSAAELLKHPHLQPYVLQVHLKSSPARNIIPSHQSPIDKVKKMTFPTESMCRSK
GRRNSLGNERIVTFSKPSPERKFTSSIQSIKDYSTTRSVKDLSIDVSLVEEVSSKTTFTT
RTSSIVKTPKRTPSKTITTPQLEPPKVSYNRVNRSELLSRTPVNRSARVIRRASLPLPLP
SSETPKRGVSSISILEQLESPDVSVNSPRIDRIAEFPLASSEDPPFLKLHGRRSPTPTPQ
HCVIDQSITKDKCMVEAFHIIDVDDDDGRSDSSSGRNNAAAAASSRAGSSESTRQRRFDT
SSYQQRAEALEGLLEFSAQLLQQERYDELGVLLKPFGPEKVSPRETAIWLTKSFKETGL*
>11686.m04090|LOC_Os12g41200.1|genepair1453-1
MEMMSPTNPMRKYSWWWDSHISPKNSKWLQENLTDMDSKIKRMIKIIDEDADSFARRAEM
YYRRRPELMSLLEELYRAYRALAERHDHAAGELRSAQRKMAEAFPDEFQLDLDDDLPAET
LSTETEADSRDMTPFFLSFINSGDSKKRAKDDQEHEKLQKEISSLSQENQELKKKISSVL
ENSDRAESEVASLKEALAQQEAEKEAAFSQCQQSSDRLQALKSEILQTQEEFKRLKEEMQ
NGLENLSTAEERCLLLERANQNLYSELDKLKNDSKERHGELNEKHVELEKLSISIQEEQL
KSMQAEMTRLSLEKQLAQAKEKLRILTLEKNGEASKFNDIEASKVRLQNDLDKIREENRK
LEEQNNSSISAIIRLQDEVISLKNAQRLLEEEVSRHVEEKKVLQYELSQLKDDKGDSERK
HFSIKEQIQVVNFNVESLQALAQEVRDGNVELKETIKHHEGVKALYVDNLMQLERTLERN
AHLERSLSAATTEVEELREKKVALEESCKHLNSKINGFQSERSMFIARIEGISHTMEKLS
EKNVFLENLLSENNTELEILRRKLNDSEESTHALLNQNSVLRSEKRTLVREVDSMNGALL
NLEAQFTELEGHHLDLQQEKNKASSEVIMLQEMLRLEREAHKELNYSGKTQFSAVQKQLS
FLLEEGRRRENQLQDEEHKIVEAQMEIFVLQKCLGDMAEANSDVSGQLQKQKELCEIQEE
KLTFLTENNQRLTEGIGSVMEELHLDDKYGSLDLMKLDVIVQLILHEIKCLLNTISDAQD
VKQNQILEKSLVVTLLEHFGREVADLRSERSVLRQEWQAKSEELLQLQNERHDLMKISCE
LRKEMEARNRRVEEMKGEAKFLVRQLSELQESRQSLQAEVIKLIEENSSLSGKLYDSREK
EKTANDDFNTLLGEAISTDILGVVFKSLHDERTSQLQSLHEDFGSLHAAGNELYQEIKLM
NKKLGDLQLENNYLEKELSKTMSICDSSGSEIGAGRRRTMRRDTKLLKSGRKSQQESTVN
IEQRKEIDHAGLEKSNELLREKLHKLQSEVQALRSKEQPVIDVKSCDAEITKLLTNMQMA
TANAALFKEKVLELIASCESFEISEMVQKEVLKEEITRRNSYVNALKDKLNAVEIENSRL
KVDLNGDFTLLGALQTEVSALEKQTMSLAKDCLPSNKLRMEEFSVSPQLSKIAVKPIHGE

PNATKMVKDMELEKLHGTIKALQKVVTDTGVVLEQERLDFNANLLDARRQIDLLRLRDDM
AAAVDDSDAASDPAAAAYDRRLLKDIQLDLVQTTTPTNRSRAATATATAAASSQRHHRRR
NGGSTEAPPLGLWSVVRASRRRQQEEGGDGDDDDLRPPQSEASAERGRRSCSSEVSQLTV
VKDLSVDKQELLLPPRPPPPAMAEAPHREWKKKVIERLTADAQRLVDLQSIVGELRASAE
AAPELDDVTAQMVDAESAVAQLIDTNGKLLRKAEEFTSADAAGGAAGDDLRSRSQRKILE
RVRKMSEKIARLEQETQRFQHALLRHEEERATRRAAAAAATAAASSGKSSAAVQRRSSRV
QLVEYLYGRRRDSRRQRRGPSCCMRAKAIDD*
>11669.m04342|LOC_Os03g43680.1|genepair1453-2
MDSKIKMMIKIIEEDAESFAKRAEMYYRRRPELMALLEELYRAYRALAERYDHAAGELRQ
AHRKIAEVFPEQVLVDLDDDLPAETASIETEMDNPDMAPYFLSFINASDSKKQAKDNQDN
ERLQKELESLSEENKDLKSRISSLLEQTNKAELEVVCLKEALAQQNTEKEAIVLQCQQST
ARLQNLKSEILHTQEKFNRLKEEMQSGFQPFTTADERSVLLEKANQEMNLELNKLKHMLK
QKHEELNEKQAELEKLNISTEEEHLKCMQAEMAQLSLEKQLILAQDKMRLLALEKQIEVS
KAKDTETEKVMLEKELEKIQKESTSLNDQIHSSSSMIIRLQDEIITMKNAQRRLEEDVCR
HVDEKKTLQNELCHLKEDRSDLDKKHSSIKEQIQAVDLNVESLQALVQELKDGNVELKGI
IRNHESTEVLHIENLRRLERMSEKNSYLEKSLSAVTTELEVLREKKAELEESCKHLSSKI
SSHQSERAVLVAQIEAISQTMAELFEKNVFLENSLSDANAELESLRGKLKELEESSEALY
SQNSALQHEKSTLACQVDRISDTLQNLEAHYAELEKRHSDLQEEKGSVLDEVIKLQEQIR
FERKEHNDLEHSRKSQLDALHEKINVLSQEGWNREEQLEEEEQNIVKAQTEIFIWKQCLE
DIADANSDFLAQLKMKQEVCQVLEEKMEYLSENNQKLTKCIGSVLKVLHLEEKYESLDQM
KLDSIVHLILHEINCLLNTISDAQDVKQNELVEKSLVVTLLEHFGQEVADLRSERNTLKQ
EQQAKSEELLQLQREKQELVNITDEFWEEVETRNRKVDELRAEAKFLVGQLSELQGSRRS
LQSEIIKLIQENSMLSDELCDSREKERVFEDDFSILISEVMSKDILSVVFRSLHEERTLQ
LVSLHSDFAQLQAAGSELYQDIKMMNMKLGDLEKESNECNKELSRTISICNSTSTENAIG
SGYPVGRDTDHLNSGRSQLEYHVNMETGEIEVDMAGLEKSNEMLQEEVHKMQSEMEVLTS
KENSAIDIKSCDEDIKRLLANMQMAIVNAALFKEKVLELIITCESFEISSMVQKEVLKEE
ITRRNSYVDELKDKLNAVEIENRRLKVDLNGDFTVLGSLQNEVSALEKQTLSLANDCLQS
NKLRMEENALSTQVLKTNMRSSGDQNTVRTVKDMELQKLHGTIKALQKVVTDTAVLLDQE
RLDFNANLQEARKQIEVLKLKEILDDDLIEMNYEQMLKDIQLDLIQISSGNKTGSLGQAN
KTVAQANEKMLDSHGIVGASSSHVRNDLRPPQSESFERDNYKRPPSELMVVKELSIDKQE
LPRSITTEPHQEWKNKVIERLASDAQRLNALQSSIQELKTNTEASEGLELESVRYQIREA
EGFITQLIDSNGKLSKKAEEFTSEDGLDGDNIDLRSRHQRKIMERARKMAEKIGRLEVEM
QKVQEALLKYEEQTSTRTSKTMHRRSKVQLVDFLYGRRRDSRKQQRCSPCGCMKANAIDD
*
>11686.m04093|LOC_Os12g41230.1|genepair1454-1
MDTPDRPPRAAATAAVEDSPVFNFINSLSPIPPPKPSDSAHNVQLFKSSDLAPVSSIFAS
PHVNPAKESKLPIREDSVQLSRESHSPNSVRTRTGATSSIIRMIRCKNIVSENCSITCYL
NDSTSSKASQPIQLCGGSAESDTNQNTDGKKDPTTEQDRTDIEFVLLDQCGPEKMDSSQS
GNNACENQLSEQHKDELGSFDGGYMFAHQPHSDMLRLAPPFESETQLVNETLQTDNVYCE
TLLTDGPSGSYTQNSAPDPHLYWAGAVEGCATDYTPQMLPGACQSQLVPNDQINNKLNEP
SDYMPMDHNVSSQNLRGMRRRCLFNEKSGAANEGAKNSSARHSTNSTTPRRKISSSDNNL
KTLRTPPCALPGIGLHLNALATVPKDKMVPHNDIQSSLNQASNVPSAVGSSPPTDDPHTI
NDDSSQTAVVAYVGESSQGSPKKKRHKFDNGDGTSCKRCSCKKSKCLKLYCECFHAGVFC
SEPCSCQGCLNMPSNMETVLSTREQIESRNPLAFAPKVIRTEPGQELADDSNKTPASSRH
KRGCNCKKSSCLKKYCECYQGGVGCSVSCRCEGCKNAFGRREGVALLGIEEAKRGCEEKD
GGVKEETTDNDKQLVIYQDSINLTPAESVLATPSVVDYRPLVALPPLSSKKPRSSTKLGG
YSSRLEGHLKSDILLSPFESYAEMMLGDGTSNVLKGESSPQTSVKVVSPNKKRVSPPRIG
TGLSPICKSGRKLILKSIPSFPSLGVDITNEDPNTSSLAP*
>11669.m04351|LOC_Os03g43730.1|genepair1454-2
MGSETNKEGARGGGQFEVVATTCEVTIVVGCMKEMSAGQFYCKGEERVDGGAGIKRRGAP
QRKGKSPKKVEGKTYKYKGFVIEGCELDSTIIMIKSSDLVHIPSIFTPPEVNSQKGSKTS
ICIDRSLSQAYHDSPDNASILPTNLAQRIQLSSDTLGSDKRHGIAGKTDHETAQKHAKLS
CFDQRCLDKMKQLTSGMNVQKRDLAKTHNDEITACDWDYLGTQYDSSVVPESDLRFETAE
LLLETPKNGDAMPGKSFLPIVEANLENSRRKLFQGSADCYSQSAVDNIHAYCTSRGKEVA
TNHVSGILPCPRESQLIPDHHFSDSLEVPSDYMAMNPSAVSQHLRGLHRRSLFNDKVRDP
TMGVQSVSNLGASTCATRHRSIPDDNYSKLVGSPVCALPNVDLHLVRMTEEMVPYNMTCT
VNSTLSISEHNTEISDYSSQATMPTSAGNSGQENPKRKRQKCQNDNVDSCKRCSCKKSKC
LKLYCECFASKVYCSESCSCRGCFNDHSHEETVLSTRNRIESRNPLAFAPKVIRTCGPGL
EFGEDSNATPASSRHKRGCNCKRSYCVKKYCECFQSGVGCSMSCRCENCKNSFGVRKGIE
RFETKEIERADRKMKDHPKEEQSEMDKYHALCEIWGVRSTENLFTTPSMDSRRAFALFPS
ECPKSSLTSSTRTSSHLHSPTRTDVLLSPFGSYTQMLLGNEASDMLLQQGDSSCTASLRI
ASPNKKRVSPLRTGNTLSPTCRKELGLKSIIPPFPSLTGDANSELQ*
>11686.m04099|LOC_Os12g41260.1|genepair1455-1
MASSCTMVPADLVEEKAAAAAVEEDAGSYLRADQVDLMSLDFEIEERMADRFRKLNSGGV
ERGDEGPKAAWEIDLSKLEIGHVVEHGDHGTLFRGKYYSQDVAVKLLDWGAEGDSSEDQI
AHFRTSLKEVVAVWHEFNHPNITKFIGASMGTTNLNIPKDIPDHSSRKGARTDLPDRACC
VVVEYLTGGTLKQHLIKHYRKNKKLLYEEVVRLALDLARGLSFLHSKKIVHRDVKSENML
LDPQLNLKIADFGVARLVEAQDPKDLTRTTGTLGYMAPEVLDGKPYNRKCDVYSFGICLW
ETYCCDMPYGPYSDLSFADFSSFVVHKNLRPEIPDCCPSAMASIMRRCWDANPEVRPEME
EVVRLLESLDTSNGGGMLLEKKKKKHPGGGCFCFFVPRAA*
>11669.m04354|LOC_Os03g43760.1|genepair1455-2
MASSTSDIPEGREKLKRSGSLGSSDTAYVRADKIDLTSLDIQLEKQLTKTWGKANLKAHG
PKEDWEIDLAKLEIRYVIAQGTYGTVYRGTYDGQDVAVKLLDWGEDGFATEAETAALRTS
FKQEVAVWHKLSHPNVTKFVGASMGTTDLKIPTNNSNAGARTNLPARACCVVVEYLAGGT

LKQYLIKNSRRKLAYKVVVQLALDLARGLSYLHSRKIVHRDVKTENMLLDTQRNLKIADF
GVARVEAQNPKDMTGATGTLGYMAPEVLDGKPYNRKCDVYSFGICLWEIYCCDMPYPDLS
FADVSSAVVHQNLRPDVPRCCPSAFANIMRKCWDANPDKRPDMDEVVQLLEALDTSKGGG
MIPDGQSSGCLCFTKARGP*
>11686.m04140|LOC_Os12g41630.1|genepair1456-1
MASQSAGLLFALLSFLIFPVIWAVPESLITAELSTAMPGNGGFVVWADRAFGPFSGSLMG
AAAEIAIGKLARTKGGANGHRRGGGGGGGGAVGRHGRRLPRRDLPAAAARRRGRGGAAGV
PVVARRGARRVAVAGARPPRRRRGGGVSWRGSPLAAAFAARYGVRRFTFAGYLRLCVARA
AGRAAELALPPLLGAPDLDLVSLRCTELRRVALPALSAADDARLPGLVARWRRLEHLELE
HRPASFPATAARVGAGCPGFSSLKMAGAIRDVDAAAMAASLPRLKRLCLDGCYLPRHELL
AVIHGCLELESLSAKHCVGFDEGDEEVAREAAMIGRLEVGGSRLVDKFDQRDVDGLDDDT
SSYVDVM*
>11669.m04355|LOC_Os03g43770.1|genepair1456-2
MADRTTRSTPPPQWAELPTDCLVHVFRRLDLDELASAAPLVCRGWRRAAADPSLWRALDL
RRDHLARFMPWGALAGALARLHGVHRFTLAGFLRLCVARAAGTVADLALPPLLSSSELDH
VAAECPALRRLALPELPPADDARLPSLLPRWRRLTHLELDSKPSSFPAVAAALALHCPDL
AVLRVTSGSVKPEDAAAMAAASPLRGRLRSLCLDRCYLPRQELLAILAGCGGAAPLREFT
ARFCVGFDDKDEEVLARGAAIERFDIGGSRLLDEPDGDATNGDDYCDSSYVDVI*
>11686.m04144|LOC_Os12g41650.3|genepair1457-1
MNQFVPDWNTTSMGDGFAPLGEDDGLVELLWCNGHVVMQSQAPRKPPRPEKTTAAAAAAM
AEDESASWFQYPVDDVLEKDLFTELFGEMTAAGGGGGDVRRAACKEERGAVAAFQSRMMP
PPWPARGKAEFGDVDDVCGVSEVVMAKMDGAAAAETVGESSMLTIGSSICGSNHVQTPPV
GNGKAGAGTAGAARRAHDTATVASSSMRSRSCTAKAEPRDVAAAGVGGKRKQRGGAAMES
GSPSEDVEFESAAATCSPAQKTTTAKRRRAAEVHNLSERRRRDRINEKMKALQELIPHCN
KTDKASMLDEAIEYLKSLQLQLQMMWMGGGMAPPAVMFPAAGVHQYMQRMGAVGMGPPHM
ASLPRMPPFMAPPPAAVQSSPVVSMADPYARCLAVDHLQPPPPMQHYLQGMSFYQLAAAK
NLQQQQNTAEAPPPPPAGGNRAAADS*
>11669.m04359|LOC_Os03g43810.1|genepair1457-2
MNQFVPDWSNMGDASRTLGEDDNLIELLWCNGHVVMQSQNHHRKLPPRPPEKAAAAAVQE
DEAGLWFPFALADSLEKDIFSDLFYEAPVAATAEAAPAGPGAGADGEGKTCKGDAAMAEE
ERGGPGAASEAPRELMPPPKSTNASCSRQQTMSLADGGDNAGDLSELVRARRSSGGAARR
KAEAGGGGGGASSSMLSAIGSSICGSNQVQVQQRTASEPGRRGAPPSAVGSANAIPCGGR
DHGHGHEATTVASSSGRSNCCFGTTTTTEPTSTSNRSSKRKRLDTTEDSESPSEVGVLPS
NTNRDAH*
>11686.m04149|LOC_Os12g41700.1|genepair1458-1
MQSQIVCHGCRNILLYPRGAPSVCCAVCHAVSSTAPSPGMDIAHLICGGCRTLLMYTRNA
TSVRCSCCDTVNLVRPVSSIAHLNCGQCQTVLMYPYGAPSVKCAICNFITNTGMNTMRHL
PPNGTSYTAPSTSAPTTQSQNVTVVVENPMTVDAKGKLVSNVVVGVTTGGKK*
>11669.m04362|LOC_Os03g43840.1|genepair1458-2
MQSQIVCHGCRSVLRYPSGAPSVCCALCQAITTVPPPAPVMEMAHLICGGCRTLLMYTRN
ADTVRCSCCSTVNLVRPVNNIAHVSCGQCRTTLMYPYGAPSVKCAICHYITNTGMNTVAP
TPSPMPTSSGSSYNAPPSGSSYNAPPPTSAPTSRPQNVTVVVENPMTVDEKGKLVSNVVV
GVTTGK*
>11686.m04151|LOC_Os12g41720.1|genepair1459-1
MASPPTPQPPPPVDVDLGKLSYEIFSLLESNFLFGAGAGGGGGVCSLPGTPGRALLGGKV
RVLAIDGCGPGPGDALLAAAALVRLETALREKSGDGDARVADFFDAAAGAGAGGVLAAML
FLKGADGRPRYTAADALAFVAASLGKGGWRGGGGGGGGRRWWGVAALFRRGSSAERSSLR
RVFGDAHAGTTVAPLLVPCYDLATAAPFLFSRADAVESGSFDFRLRDVCAATCAGGAAAT
AVRSVDGRTAIAAASGGVAAMGNPTAAAITHVLHNKQEFPLAAGVDDLLVVSIGSGSSSA
ATPSTAAGWRTPLPSRSPSPAEMVRLTAEGVADMVDQAVAMAFGHTCGRNYVRIQAASPA
CKTKALSSVDAKKAAAIADGMLTQRNVEAELFRGRRLSEKSNREKLDAFAAELVKEHERR
RASPGLPNVVIKQVAAAAAAVTPARLSSATTTSSATATTARTTVSSMPSPAASLDSGRH*
>11669.m04366|LOC_Os03g43880.1|genepair1459-2
MASSPMAMDADKLSYEIFSLLESKFLFGAGGGGCLSSGPCTPARPFLGGGGGMDGRVRVL
AIDGCGSSGAGDALLAAAALARLEAGLRKRTGDSDARVADFFDVAAGAGAGGVLAAMLFL
RGPDGRPRYTAEEALEFVAASVGRDWAGRRGRWARLFRGGARGAERSFRRVFGDATLKDT
VAPLLVPCYDLATAAPFMFSRADAVESDSYDFALRDVCAATCAAGSTAAAVRSVDGRTAI
AAASGGVAAMGNPAAAAITHVLHNKQEFPLATTVDDILVLSIGTGASTSATATPMPTRSP
SPREMARVTAEGVADMVDESVAMAFGHTSGSSSNYVRIQASKAATALHGAAAAGAMLSQR
NVESVLFRGRRMSERTNAEKVDAAAAEVVKEHERRRRSPLPNVVIKQVGTPRVSSATTAS
SGTARTAASTLASPASYGSRQ*
>11686.m04166|LOC_Os12g41860.1|genepair1460-1
MAAAAVGGRGERLSSSSPTAAAPQVDAGKYVRYTPEQVEALERVYTECPKPSSLRRQQLI
RECPILSNIEPKQIKVWFQNRRCREKQRKEASRLQTVNRKLNAMNKLLMEENDRLQKQVS
RLVYENGYMRTQLHNPSAATTDTSCESVVTSGQHHQQQNPAVLHPQRDANNPAGLLAIAE
ETLAEFMSKATGTAVEWVQMVGMKPGPDSIGIIAVSHNCSGVAARACGLVSLEPTKVAEI
LKDRPSWYRDCRCVDIIHVIPTGNGGTIELIYMQTYAPTTLAAPRDFWTLRYTSGLEDGS
LVICERSLTQSTGGPSGPNTPNFIRAEVLPSGYLIRPCEGGGSMIYIVDHVDLDAWSVPE
VLRPLYESPKILAQKMTIAALRHIRQIAHESSGEIPYGAGRQPAVFRTFSQRLSRGFNDA
VSGFPDDGWSLLSSDGSEDITISVNSSPNKLVGSHVSPNPLFSTVGGGILCAKASMLLQN
VPPALLVRFLREHRSEWADPGVDAYSAASLRASPYAVPGLRTSGFMGSQVILPLAHTLEH
EEFLEVIRLEGHGFSHDEVLLSRDMYLLQLCSGVDENATSASAQLVFAPIDESFADDAPL
LPSGFRVIPLDTKMDGPSATRTLDLASALEVGPGGASRASVEASGTCNRSVLTIAFQFSY
ENHLRESVAAMARSYVRAVMASVQRVAVAIAPSRLGPQIGMKHPPASPEALTLASWIGRS

YRAHTGADIRWSDTEDADSPLALLWKHSDAILCCSLKPAPMFTFANNAGLDILETTLVNL
QDISLEMILDDEGRKALCSEFPKIMQQGFTYLPGGVCKSSMGRQASYEQAVAWKVLSDDD
APHCLAFMLVNWTFM*
>11669.m04372|LOC_Os03g43930.1|genepair1460-2
MAAAMVAAVHGVGRQDRSSPGGGGAPQVDTGKYVRYTPEQVEALERVYGECPKPSSLRRQ
QLIRECPILSNIEPKQIKVWFQNRRCREKQRKEASRLQTVNRKLTAMNKLLMEENDRLQK
QVSRLVYENGYMRQQLHNPSVATTDTSCESVVTSGQHHQQQNPAATRPQRDANNPAGLLA
IAEETLAEFLSKATGTAVDWVQMVGMKPGPDSIGIIAVSHNCSGVAARACGLVSLEPTKV
AEILKDRPSWYRDCRCVDVLHVIPTGNGGTIELIYMQTYAPTTLAAPRDFWILRYTSGLE
DGSLVICERSLTQSTGGPSGPNTPNFVRAEVLPSGYLIRPCEGGGSMIHIVDHVDLDAWS
VPEVLRPLYESPKILAQKMTIAALRHIRQIAHESSGEMPYGGGRQPAVLRTFSQRLSRGF
NDAVNGFPDDGWSLMSSDGAEDVTIAFNSSPNKLVGSHVNSSQLFSAIGGGILCAKASML
LQNVPPALLVRFLREHRSEWADPGVDAYSAAALRASPYAVPGLRAGGFMGSQVILPLAHT
LEHEEFLEVIRLEGHSLCHDEVVLSRDMYLLQLCSGVDENAAGACAQLVFAPIDESFADD
APLLPSGFRVIPLDGKTDAPSATRTLDLASTLEVGSGGTTRASSDTSSTCNTRSVLTIAF
QFSYENHLRESVAAMARQYVRTVVASVQRVAMAIAPSRLGGQIETKNPPGSPEAHTLARW
IGRSYRFHTGADLLRTDSQSTDSSLKAMWQHSDSIMCCSLKAAPVFTFANQAGLDMLETT
LIALQDISLEKILDDDGRKALCTEFPKIMQQGFAYLPGGVCVSSMGRPVSYEQAVAWKVL
SDDDTPHCLAFMFVNWSFV*
>11686.m04170|LOC_Os12g41890.1|genepair1461-1
MAEAAAGGEVRRRGGCCGGGGGGGGLFPEESFASWGAYGRALMETGPRLVERPTARSAAA
VEVNEVRGRSGAEMKRNLTWWDLAWFGVGAVIGAGIFVLTGQEARDAAGPAVVLSYAVSG
VSAMLSVLCYTEFAIEIPVAGGSFAYLRVELGDFVAFIAAGNILLEYCIGGAAVARAWTS
YFATLLNHRPNDFRIHAASLAADYSRLDPIAVAVIAVVCALSVLSTKASSRFNYALSIAH
LAVLVFIVTAGLSRARLSNLTADFAPFGARGVFAASAVLFFAYVGFDAVSTMAEETRDPA
RDIPAGLVGAMAVTTAAYCALAATLCLMQPYREIDPDAPFSVAFSAAGMGWARYVVAFGA
LKGMTTVLLVSAVGQARYLTHIARAHMAPPCLARVHPRLGTPVNATVAMLAATAAIALFT
DLGVLANLLSISTLFIFMLVAVALLVRRYYATGETARGDRNRLAGCLAVIVASSVATAAY
WGLGGDGGGWAAYAVAVPAWLAATLFLQLRVPMARTPEKWGVPLVPWLPSASIFINIFLL
GSIDGRSFMRFGVWTAALLAYYFFFGLHASYDTAKALAAEVAAGKVEEGGSKPAVGGAAG
N*
>11669.m04376|LOC_Os03g43970.1|genepair1461-2
MAFVAAGNILLEYCIGGAAVARAWTSYFATLLNHHPNDFRIHAASLAADYSRLDPIAVAV
IAIICLLSVLSTKASSRFNYVLSVLHVAVIAFIIVAGLTKADAANLTRDFMPYGPRGVFA
ASAVLFFAYIGFDAVSTMAEETRDPARDIPVGLVGAMALTTALYCALAVTLCLMVPYGEI
DPDAPFSVAFADRGMGWAKYVVAFGALKGMTTVLLVSAVGQARYLTHIARTHMMPPWLAR
VHPGTGTPVNATVAMLLATAVIAFFTDLNVLSNLLSISTLFIFMLVAVALLVRRYYVSGE
TSRADRNRLAACIAAILASSVATATCWGLDRGGWVPYAVTVPAWLAATASLWALVPQARA
PKLWGVPMVPWLPSASIAINVFLLGSIDSKSFMRFGIWTAALLVYYLFVGLHASYDTAKA
LAADAVAGKVEDGDAKTSAPPM*
>11686.m04181|LOC_Os12g41960.1|genepair1462-1
MKRARSSEVFLGGRGRARRRVAPLLAAVAFVYLLFVSFKLSGLAGIADPAAVTRPASGGA
GEVVMPRRLEDPAPRARGDGDGVAVAGYGRITGEILRRRWEAGGRGRRRWGRGGNFSELE
RMADEAWELGGKAWEEACAFTGDVDSILSRDGGGETKCPASINIGGGDGETVAFLPCGLA
VGSAVTVVGTARAARAEYVEALERRGEGNGTVMVAQFAVELRGLRAVEGEEPPRILHLNP
RLRGDWSHRPVLEMNTCFRMQWGKAHRCDGNPSKDDDQVDGLIKCEKWDRRDSVDSKETK
TGSWLNRFIGRAKKPEMRWPYPFSEGKMFVLTIQAGIEGYHVSVGGRHVASFPHRMGFSL
EDATGLAVTGGVDVHSIYATSLPKVHPSFSLQQVLEMSDRWKARPVPEEPIQVFIGIISA
TNHFAERMAIRKSWMQFPAIQLGNVVARFFVALSHRKEINAALKTEADYFGDVVILPFID
RYELVVLKTVAICEFGVQNVTAEYIMKCDDDTFVRLDVVLKQISVYNRTMPLYMGNLNLL
HRPLRHGKWAVTYEEWPEFVYPPYANGPGYVISIDIARDIVSRHANHSLRVRLILKLHCT
HFSLHIPLMLSDCHLTGTAVVQDGRSDTISQELFYAKISIISVGYLLLHLPELMCMCWFF
ECANSIAVQRLRFNLMDSDDKKFGKGPRELTGAVDLISHYKLLAHHDFFCKKPLPLAISD
THYLDNVVGDTEIRKGEGMELDQLVQNAYLRDKPAYIQPFDMETLGQAFQLRETAPVDLP
SAEKGIPTISGKLKSESKDKEKKHKKHKDKDRDKDKEHKKHKHRHKDRSKDKDKDKDKDK
KKDKSGHHDSGGDHSKKHHEKKRKHEGMEDSADVHKHKKSKVAHTAIMILAGIANFLIE*
>11669.m04380|LOC_Os03g44010.1|genepair1462-2
MSGFNRMGSDGNFGKGPRELTGAVDLISRYKLLNHHSFFCKKPLPLAISDTNYLHNVVGD
TEIRKGEGMELDQLFQDAYLREKTSYIQPFDMETLGQAFQLRETAPIDLPSAEKGTPTIS
GKSKIKSKDKVKKHKRHKEKDKDKYKDQKKHKHRHKDRSKDKEKEKEKEKEKEKKKDKSA
HHDSGADRSKKHHEKKRKQEGLEDLASGHNPKKVQKRKNQ*
>11686.m04187|LOC_Os12g42020.1|genepair1463-1
MVAAVRAPVKPEMVELSPAAMERYSSDADTTAPNSSLSSAASSTGSLARCSSLSRLSFDC
SPSAAVAAAATSCSPPRASVLLRPHRSGDVAWAAIRAASTTSAAPLGPRDFKLVRRIGGG
DIGTVYLCRLRSSPERESPCMYAMKVVDRRAVARKQKLGRAAAEKRILRQLDHPFLPTLF
ADFDATPHFSCAVMEFCPGGDLHSLRHRMPSRRFPLPSARFYAAEVLLAIEYLHMMGIVY
RDLKPENVLIRADGHIMLTDFDLSLQSTTSPSLDGDTDTDDEASGGASCFPDHLLRFKRR
RNAVAAPRPRFVAEPVDARSCSFVGTHEYVAPEVASGGAHGAAVDWWAYGVFLYELIYGR
TPFAGATNEATLRNIVRRPLAFPSGSGSCGPADADARDLIARLLAKDPAARLGSRRGAAD
VKSHPFFKSLNLALLRSSRPPVVPGAGAGAAPLHRSQSCKAAPTTPPPPTTTKPANATAR
FDLF*
>11669.m04381|LOC_Os03g44020.1|genepair1463-2
MAAAAASPTSSSSLPPKPPNSAAMLVEQQPLSYHDVDAASTPSSSVSSSSTASVGGRSST
FSLDSAATATPTSSPPRPHRAADVAWAPIRAAAAPLGPRDFTLVRRVGAGDIGTVYLCRL

DGKRGAGSPSPCEYAMKVVDRRALAKKGKLGRAAAEKRVLRRLDHPFLPTMFADFDAGQD
YSCVVMEFCPGGDLHSLRHRVPGRRFPVASARFYAAEVLLALEYLHMMGIVYRDLKPENV
LIRADGHIMLTDFDLSLESTASPALEDARNGADDDPATPTCLPEVQLFRLRRWRRRAAPR
RRPRFVAEPVDARSSSFVGTHEYVAPEVARGGGHGAGVDWWAYGVFLYELLYGRTPFVGA
TNEATLRNIVRRPLEFPPDAAGGGSPHDAAARDLIARLLDKDPRSRLGSRRGAADVKSHA
FFKGLNFALLRSSAPPVVPPPAVAAAQCSKAADVPQLFDLF*
>11686.m04192|LOC_Os12g42070.1|genepair1464-1
MALPGCPDKCGNISIPYPFGIGPSCAATSISSYFNLTCNNTFNPPRPMVGDSEALVEVTD
ISLEHGEMRVLSPVYYICFTANTTFTRFTEGYELKHTPFLPSPSRNRFTVIGCNTLGLIG
GYKGTVSHYVTGCYSYCESINSTSDGAPCAGMGCCEAAIPTDLTAWGAMFEMNQSKVWSF
NPCFYAMVSEVGWYSFQQKDLVGHLGFIDDRAQRGAPVVADWAIRNGSCPEEGKGIPGDY
ACISANSYCMDANNGPGYLCQCSKGYEGNPYLLNGCQDVDECALRKQDPKYEDIYPCRKG
VCHNTPGGYLCKCKLGKRSDGTNYGCRPLRTTAEQVVIGTSVSAIALMALTCVLAMQIQR
KRHKKDKDEYFKQNGGLKLYDEMRSRKVDTIRILTEKDIKKATDNYSEDRVLGIGGHGMV
YRGTLDDNKEVAIKKSKVINDEWREEFVNEIIILSQINHRNIVRLIGCCLDVHVPMLVYE
FVSNGTLSEFLHGTDHRSPIPLDIRLKIATQSAEALAYLHSSTSRTILHGDFKSANILLD
GQHNAKVADFGASALKSMNESEFIMFVQGTLGYLDPESFISHCLTDKSDVYSFGVVLLEL
MTRKRAIFANSINEKESLSYSFLLMFDQNIHRNMLDREIMDKETMVVLEKLSILAANCLR
PRGDDRPTMKEVAECLQMIRRHPMHAASDHKGDSSAHHNYEGSSSPSMSAHFDETIYKSI
EASRLVQDLVR*
>11669.m04384|LOC_Os03g44050.1|genepair1464-2
MSLPGCPDKCGDVSIPYPFGIGDRCAAAGLSRFFNLTCDGSRSPPVPMLGDPGAQADVID
FSPERGELRLYAGLSYACYASSATSPSTNATFAFSLVGTPFRVSPSRNRLTVVGCSALGL
VVGTASGGGGDDDDLYATGCFTYCAELNAAGADGAPCAGAGCCQVPISPDIPYLGAAFRT
GNWTNTAWRFNPCFYAMVAEDGWYSFRRRDLVGVLAYYNETVDAGRGVPVVIDWAVRDGW
CPATAEERARRKYACVSGNSYCVNSSNGMGYTCNCSRGYEGNPYLAGGCQDINECVLREQ
DPKYEEMYPCRHGICINTPGLSACAILAMALSCLLVIQLQRRKHIQEKQQYFKQNGGLRL
FDEMVSRQVDTVRVLTEDELKKATNNFSDDQVIGCGGHGTVYRGTLDDLREVAIKRSKAA
VDGRGGGGCEDEFVNEIIVLSQINHRHVVRLLGCCLEVHVPMLVYEFVPNGTLFDLLQGG
TAARRRPVSLGLRLKIAAQSAEALAYLHSSASRAILHGDVKSLNILLDGALDAKVADFGA
SVLRSAMGEGESFIEYVQGTLGYLDPESFVSRHLTDKSDVYSFGVVLAELATRRKAVYDD
DDASCSGHGGQKRSLSTAFLAALRHGELWSVLDRELVRRPDDDGDGDDKAAVDVVRELAE
LAARCLGPSGDERPAMKEVAERLQVLRRRAEMRAVAGAGRDSNGGEVDRSWIMCGGGGGA
VGRGHLDTNTTASYQSTETDKMPLTLSVNDLAR*
>11686.m04208|LOC_Os12g42220.1|genepair1465-1
MLPLLNMQAEAAGGDGGDAEKKSGNNKQMMVAAKVSISILVMSLPVLYVSFLRIPPATLF
RDTTFWFLMSNSIIIVIAADSGMLFFAARPASSSGELQAMVVTDVSLSHALVVAPPRGGQ
EDDDGVVVAGEPAEEESTMMLVPYYGGEVVQAAARPTRLTASSEAERTTTMARRRRRSRS
HSSHHALMMTPPVVQEKSIVVVREEKLRRTATERPPEPEEEMTTTSSSEYSRLSDEELNR
RVEEFIARFNMEIRLQLEKEQEQAAAA*
>11669.m04404|LOC_Os03g44250.1|genepair1465-2
MRQQQQKRKVASVSSALVMSLLPILYASLLRLPPAALARDTTFWFLLSNCIIAIIAAADS
AAAAPITTSSSSSHGHDDVDEPALLAAAVVPAAPPPAAGDDQLPAEAPVAVRNDDEINEP
PSAPAVTSSATPSSDNPPAFIASDDVATEGERPDQQPQEAATDGETHGEAVKGDDDEDET
ASDKTTTTNKSLPSSSSEELAIVTSNNDDDYDDGGDSASFGEEDEGKVVPWGMPAPATTT
GGGGGGKQYWQLSDEELNRKVEEFITRFNREMRLQILQEAGV*
>11686.m04210|LOC_Os12g42230.1|genepair1466-1
MATAAAASLQYALHGAASASAKPRSAAPGRSVRVVAARRSVRARGGAVVARAAVTASADA
TAESKSGGHEVLLFEALREALIEEMKEDPTVCVFGEDVGHYGGSYKVTKGLAEMFGDLRV
LDTPIAENSFAGMGVGAAMKGLRPIVEGMNMGFLLLAYNQISNNCGMLHYTSGGQFKIPI
VIRGPGGVGRQLGAEHSQRLESYFQSIPGLQMVACSTPYNAKGLMKAAIRSENPVVLFEH
VLLYNLKEKIPDEEYICCLEEAEMVRPGEHVTILTYSRMRYHVMQAAKTLVNKGYDPEVI
DIRSLKPFDLHTIGNSIKKTHRVLIVEECMRTGGIGASLRSAIIDNFWDYLDAPIMCLSS
QDVPTPYAATLEDATVVQPAQIVAAVEQICQ*
>11669.m04409|LOC_Os03g44300.1|genepair1466-2
MAAASSLHAAPRVGSSSSFSSSSSAGRRSASAARSVRVAAAAGSCAARRAGGRMVARAAV
ASKAESPASAASSKSDGHEVLLFEALREALIEEMKEDPTVCVFGEDVGHYGGSYKVTKGL
AEMFGDLRVLDTPIAENSFTGMGVGAAMKGLRPVVEGMNMGFLLLAYNQISNNCGMLHYT
SGGQFKIPIVIRGPGGVGRQLGAEHSQRLESYFQSIPGLQMVACSTPYNAKGLMKAAIRS
ENPVVLFEHVLLYNLKEKIPDEEYVLCLEEAEMVRPGEHVTILTYSRMRYHVMQAAKTLV
NKGYDPEVIDIRSLKPFDLHTIGNSIKKTHRVLIVEECMRTGGIGASLRSAIIDNFWDYL
DAPIMCLSSQDVPTPYAAPLEDATVVQPAQIVAAVEQICQ*
>11686.m04213|LOC_Os12g42260.1|genepair1467-1
MDLRRPTTRSSSGGVEPRYRQVGFVTTTAEPAAAAASSSSSPRGSDVYPGSLSPVMIPPP
RIPDHLTALSPAPVSLLPSSPPPPSSSRLDAAESDLDDDDDDDVDVSWARPPPPALLESK
EGLPDTQNEGAPAPAPAPVQQKQKLSKAERRAIQEAQRAAKASAKEAGLSRKSVATVSTSNA
AMSKQLKTVKTPPKKDLPQLTPPVASEKKTSEHPPDRDRKKDVPPPRMQFDNVHRVEKTK
KHSLVNQAEAQNRVELFRHLPQYVHGTQLPDLESKFFHLDLMHPSVYKVGLQFLSGVISG
GNACCVAMLLAFREAIKDYSTPSTKTLNRDLTAKISSYVSFLIECKPLSISMGNAIRFLK
NRIAKLLLTLSESEAKASLQSDIDRFINEKIIIADHVIVSHAIAKVRDDDVLLTYASSSV
VEMIFDHANELGKKFRVVVVDSRPNNEGQALLHRLVAKGISCTYTHINAISYIMHEVTRV
FLGASSILSNGAVYSSVGTAAVAMVAHAFGVPVLVCCEAYKLHERVQLDSICSNELGDPD
VISRVPEREDLCHLKNCTYNENLQLLNLKYDTMPSDYVSMIITEYGMLPPTSVPVIVREY
RREHIWI*

>11669.m04411|LOC_Os03g44310.1|genepair1467-2
MDLRRPPRSSSGGVEPKIRQVGFVTPDASSSAPPAEPPAAAAAAVAAAAAAASSAAAAAA
QAQASSEGSPPASELSPGSLSPVMIPPPRHADHLAPGSPSPAASPSELGDDESWSRAPSA
AELESNNNDLAEIRNDNVPASIPQKQKTSKAERRAIQEAQRAAKAAAKEAGLSGKSAGTG
SGANPAMPKQAKSSKVSQKKDVPQAASTAASEKKVTERPSERDRKKDAPHPRMQFDDVHR
VEKAKKRAIVNQSEARNRVELFRHLPQYVHGTQLPGLESKFFQLEPMHPSVYKVGLQYLS
GEVSGGNGRCIAMLLAFQVAIKDYSTPPKKTLSRDLTAKISSYVSFLIECRPLSISMGNA
IRFLKNRIAKLPLTLSESEAKASLQSDIDRFINEKIVVADKVIVSHAITKVRDNDVLLTY
GSSSVVEMILDHAHELGRKFRVIVVDSRPKLEGQGLLRRLVEKGINCTYTHINAISYIMH
EVTRVFLGASSILSNGTVYSRVGTASVAMVAHAFGIPVLVCCEAYKFHERVQLDSICANE
LGDPDVILKVPGKADLGHLKNLADNENLQLLNLTYDATPSDYVSMIITDYGMLPPTSVPV
IVREYRKEQLWI*
>11686.m04216|LOC_Os12g42280.1|genepair1468-1
MPTTFTPNSPASSCSIHHRASPSRGARNSVRFTRPRAAAAATNSVLSAPSSVPPAYVPPP
PPPPTKMFPEAGDAAAAKAAARRCGKKKDGLNFFQRAAAVALDAFEEGFITNVLERPHAL
PRTADPAVQIAGNFAPVGEQPPVRSLPVSGRIPPFINGVYARNGANPHFEPTAGHHLFDG
DGMVHAVRIRNGAAESYACRFTETARLGQERALGRAVFPKAIGELHGHSGIARLALFYAR
GLCGLVDPSHGTGVANAGLVYFNGRLLAMSEDDLPYQVRVTADGDLETVGRYDFDGQLGC
AMIAHPKLDPVSGELFALSYDVIKKPYLKYFYFDADGTKSPDVEIELEQPTMIHDFAITE
NFVVVPDHQVVFKLGEMFRGGSPVVLDREKTSRFGVLPKHATSSLEMVWVDVPDCFCFHL
WNAWEEAESGEVVVVGSCMTPADSIFNESDEHLESVLTEIRLNTRTGESTRRAVLPPAAQ
VNLEVGMVNRAMLGRKTRYAYLAVAEPWPKVSGFAKVDLATGELTKFEYGEGRFGGEPCF
VPMGGAGAAASPARGEDDGYILSFVRDEAAGTSELLVVNAADMRLEATVQLPSRVPYGFH
GTFINAGELATQA*
>11669.m04418|LOC_Os03g44380.1|genepair1468-2
MATITTPGYAHIQRQHGRCSTTAGRRGASNSVRFSARAVSSVPHAAAASSAPAFLPVPFV
PGADAPSPSGKSAIGVPKAPRKGEEGKRLNFFQRAAAMALDAFEEGFVANVLERPHGLPS
TADPAVQIAGNFAPVGETPPARALPVSGRIPPFINGVYARNGANPHFDPVAGHHLFDGDG
MVHAVRIRNGAAESYACRFTETARLRQERAMGRPMFPKAIGELHGHSGIARLALFYARAA
CGLLDPSHGTGVANAGLIYFNGRLLAMSEDDLPYQVRVTADGDLETVGRYDFDGQLGCAM
IAHPKLDPATGELHALSYDVIKKPYLKYFYFAPDGTKSADVEIPLDQPTMIHDFAITENY
VVVPDHQVVFKLQEMLRGGSPVVLDKEKTSRFGVLPKHAADASEMVWVDVPDCFCFHLWN
AWEEADTDEVVVIGSCMTPADSIFNESDDRLESVLTEIRLNTRTGESTRRAILPPSSQVN
LEVGMVNRNLLGRKTRYAYLAVAEPWPKVSGFAKVDLATGELTKFEYGEGRFGGEPCFVP
MDAAAATPRGEDDGYILSFVHDERAGTSELLVVNAADMRLEATVQLPSRVPYGFHGTFIT
GDELTTQA*
>11686.m04219|LOC_Os12g42310.1|genepair1469-1
MDVDSRMTTESDSDSDAAAQGGGGGGFGSETSSASPSAPGTPTAMGAGGGAAPIAAAAIA
AAASAAVVAGPRPRAGVHGGERGDGEEGGRARVPVRPHAHRGAGRRGGGRAGVLLVLLRM
CTATMFHRISGAVNVVRLTPVGEPPSPRAAHVATAVGTMVVIQSSSYVSFIVRPALTCSQ
LQGGIGPAGLSAEDLHVLDLTQQRPRWHRVVVQGPGPGPRYGHVMALVGQRFLLTIGGND
GKRPLADVWALDTAAKPYEWRKLEPEGEGPPPCMYATASARSDGLLLLCGGRDANSVVFV
NARLHVSGGALGGGRMVEDSSSVAVLDTAAGVWCDTKSVVTTPRTGRYSADAAGGDASVE
LTRRCRHAAAAVGDMIYVYGGLRGGVLLDDLLVAEDLAAAETTNAANQAAAIAAASDIQA
GREPGRYAYNDEQTGQPATITSPDGAVVLGTPVAAPVNGDMYTDISPENAVIQGQRRMSK
GVDYLVEASAAEAEAISATLAAVKARQVNGEAEHSPDREQSPDATPSVKQNASLIKPDYA
LSNNSTPPPGVRLHHRAVVVAAETGGALGGMVRQLSIDQFENEGRRVIYGTPESATAARK
LLDRQMSINSVPKKVIASLLKPRGWKPPVRRQFFLDCNEIADLCDSAERIFSSEPSVLQL
KAPIKIFGDLHGQFGDLMRLFDEYGAPSTAGDIAYIDYLFLGDYVDRGQHSLETITLLLA
LKVEYPLNVHLIRGNHEAADINALFGFRIECIERMGERDGIWTWHRMNRLFNWLPLAALI
EKKIICMHGGIGRSINHVEQIENLQRPITMEAGSVVLMDLLWSDPTENDSVEGLRPNARG
PGLVTFGPDRVMEFCNNNDLQLIVRAHECVMDGFERFAQGTANNAGAILVLGRDLVVVPK
LIHPLPPAITSPETSPEHHLEDTWMQELNANRPPTPTRGRPQAANNDRGSLAWI*
>11669.m04431|LOC_Os03g44500.1|genepair1469-2
MDVDSRMTTESDSDSDAAATAAASASVAAQGGLASETSSSSSASAPSTPGTPTVAPAPAA
AGATGPRPAPGYTAVSAVIEKKEDGPGCRCGHTLTAVPAVGEEGTPGYIGPRLILFGGAT
ALEGNSATPPSSAGSAGIRLAGATADVHCYDVLSNKWSRLTPQGEPPSPRAAHVATAVGT
MVVIQGGIGPAGLSAEDLHVLDLTQQRPRWHRVVVQGPGPGPRYGHVMALVGQRFLLTIG
GNDGKRPLADVWALDTAAKPYEWRKLEPEGEGPPPCMYATASARSDGLLLLCGGRDANSV
PLASAYGLAKHRDGRWEWAIAPGVSPSPRYQHAAVFVNARLHVSGGALGGGRMVEDSSSV
AVLDTAAGVWCDTKSVVTTPRIGRYSADAAGGDAAVELTRRCRHAAAAVGDQIFIYGGLR
GGVLLDDLLVAEDLAAAETTTAANHAAASAAATNVQSGRTPGRYAYNDERARQTAPESAQ
DGSVVLGTPVAPPVNGDMYTDISPENAVLQGQRRLSKGVDYLVEASAAEAEAISATLAAV
KARQVNGEMEQLPDKEQSPDSASTSKHSSLIKPDSILSNNMTPPPGVRLHHRAVVVAAET
GGALGGMVRQLSIDQFENEGRRVSYGTPENATAARKLLDRQMSINSVPKKVIASLLKPRG
WKPPVRRQFFLDCNEIADLCDSAERIFSSEPSVLQLKAPVKIFGDLHGQFGDLMRLFDEY
GAPSTAGDIAYIDYLFLGDYVDRGQHSLETMTLLLALKVEYPQNVHLIRGNHEAADINAL
FGFRIECIERMGERDGIWTWHRMNRLFNWLPLAALIEKKIICMHGGIGRSINHVEQIENL
QRPITMEAGSVVLMDLLWSDPTENDSVEGLRPNARGPGLVTFGPDRVMEFCNNNDLQLIV
RAHECVMDGFERFAQGHLITLFSATNYCGTANNAGAILVLGRDLVVVPKLIHPLPPAITS
PETSPEHHIEDTWMQELNANRPPTPTRGRPQVAANDRGSLAWI*
>11686.m04225|LOC_Os12g42370.1|genepair1470-1
MAGGGGKKEKGEGLGRALIRQRNKAAAAVKERGDALAHARRRAQPLESVIEVSDIDAVLE
RAAEADRLHSALADSVSSSDLVIDLDATGETDEERRRMQKEQEALHAGSLRVPRRPPWNS

RMTVEELDENERRAFLVWRRNLARLEENDKLVLTPFEKNIDIWRQLWRVLERSDLLVMVV
DARDPLFYRCPDLEVYAKEIDEHKRTMILVNKADLLPMNIRKKWADYFKAHDILYVFWSA
KAATATLEGKILSGYSEQDSASLDLDTKIYGRDELLKKLQTEAEFIVAQRRAAAIKEDSR
ATSSDSVSSVAKHVVVGFVGYPNVGKSSTINALVGEKKTGVTHTPGKTKHFQTLIISEEL
TLCDCPGLVFPSFSSSRHEMVSCGVLPIDRMTKHREAIQVVADRVPRSVLEQIYKITLPK
PKPYESQSRPPTAAELLRAYCASRGHVSHAGLPDETRAARQILKDYIDGKIPHFELPPGM
VDTENEHEETSGLEGPTTSAYNESGGSDSDERDDTVDPAQPDMRHVLSDLESFDLATEGS
KPAGKKKKEASHKQHKKPQRKKDRSWRVGNDGGDGTAVLRVYQKPAVNLSAVSASDKT*
>11669.m04434|LOC_Os03g44530.1|genepair1470-2
MGGGGGGGGRKDRGEGLGRALTRQRNKAAAAKERGHALALARRARQPLESVIEVSDIDA
VLQRAAEEYLLLGGGGGDGAGDVALSASLGSGLIDLDGTVETEEERRWLREEQEALHAGS
LKVPRRPPWTPQMTVEELDANEKRAFLEWRRNLARLEENEKLVLTPFEKNIDIWRQLWRV
LERSDLLVMVVDARDPLFYRCPDLEVYAQEIDEHKRTLLLVNKADLLPLNVRQRWAEYFK
QHDILYLFWSAKAATADLEGKKLSSYSMENWNTADLDTKIYGRDELLVRLQGEAEYIVNQ
KGALRAEEGHESSRSDSVSTRPKHVVVGFVGYPNVGKSSTINALVGQKRTGVTSTPGKTK
HFQTLVISEELILCDCPGLVFPSFSSSRHEMVACGVLPIDRMTKHRGAIQVVANRVPRNV
LEQIYKITLPKPKAYEQLSRPPTAAELLRAYCTSRGHVSHAGLPDETRAARQILKDYLDG
KIPHFELPPGDTDSETDPEETTDLEGSDTAVGATADHCASDEQDEEISQADPNISHVLSD
LESFDLASEVSKNSTKKKKEASYKHHKKPQRKKDRSWRVGNDGADGSAVVRVFQKPAVNF
ATVTG*
>11686.m04228|LOC_Os12g42400.1|genepair1471-1
MMSFNKSQEGFGQVAAVATLASNGGGSLPWLLYGEPLGQGKPAMSPEGVVPRAQTPLDPP
QVPAMDRGVPEILNFSMVPGKGEKCSEHSTTIALQSPFAEYNGCFELGLGQSVVPSNYPY
ADQHYGLLSPYGVRPTPSGRILIPPNMPADAPIYVNAKQCSAIIRRRHARAKAERENRLV
KARKPYLHESRHLHAMRRARGSGGRFLNTKKETNGKTTGGGRKVMDIIIPPLCPAASPSS
EQCNPSSVSSLSGSEVSSIYEHEDMDHFHSFDHLRTHFFTPLPSLMDVEHGAGNPFKWTA
ASDGCCDLLKA*
>11669.m04435|LOC_Os03g44540.1|genepair1471-2
MLSFKQSHEGFGHVAAAGAGPQQQQQPWWAGSQLLYGEASPEEAALRDGGQFQVVPGGRA
ALDPAAPEPEKTAVPAMPKRGGGGGAPEVLKFSVFSGNLEPGDTGEKNREHSATIAMQSP
LPEYNGHFELGLGQSMVSPNYPCIDQCYGLMTTYAMKSMSGGRMLLPLNAPADAPIYVNA
KQYEGILRRRRARAKAQRENRLVKGRKPYLHESRHRHAMRRARGSGGRFLNTKKEATAAG
CGGSSKTPLASLVSPADVAHRPGSGGRASSLSGSDVSSPGGVMYDHHRHDDADAADHYNS
IDHHLRTPFFTPLPIIMDSGGGGGDHASHSAAAVAAPFRWATAAGDGCCELLKA*
>11686.m04230|LOC_Os12g42420.1|genepair1472-1
MALPHAASPYVLSLLLLLSIPAVFLLAPRLLPPKTLPSIPDADETDDLALFRRAVLLSAA
PDSSSASAGAASLFGRRPQPKVAFLFLTNSDLVFSPLWEKYFAGNHHLLNLYIHADPSAA
VDLPATASFRGHVIRGTKATARASATLISAARRLLATALLDDPSNHFFALLSQSCIPLHP
FPTFYRTLLSDSDNNGGSPRRPRRRRSFIEILDNEPTLHDRYYARGDDVMLPEVPYDSFR
VGSQFFVLVRRHAVMVVRDRRLWNKFKLPCLTKRKDSCYPEEHYFPTLLDMQDPQGCTKF
TLTRVNWTDSVDGHPHTYRPDEVSGELIRELRKSNGTHSYMFARKFAPDCLKPLMEIADS
VILRD*
>11669.m04438|LOC_Os03g44580.1|genepair1472-2
MTSPATTYASPFVLSVLLLVSIPVIFLLAPRLLPPKTLPAIPDADESDDLALFRRAILSS
SSPSSATPTPSSAASYFFRRRPAPKVAFLFLTNSDLVFSPLWEKFFRGHHHLFNLYVHAD
PFSALTMPPTPSFRGRFVPAKATQRASPTLISAARRLIATALLDDPSNQFFALLSQSCIP
LHPFPTLYNTLLSDNAGPHGRHRSFIEIMDDAYMIHDRYYARGDDVMLPEVPYDQFRFGS
QFFVLTRKHAIMVVRDMKLWRKFKLPCLIKRRDSCYPEEHYFPTLLDMQDPEGCTGYTLT
RVNWTDQVEGHPHTYRPGEVSASLIKELRKSNGTYSYMFARKFAPECLEPLMEIADSVIL
RD*
>11686.m04231|LOC_Os12g42430.1|genepair1473-1
MGISAKWIKSLVGIKKHEKAQTSESSGVRSSTAQLLHKRKHSIDTESAAAVEELSVQTEP
LACDTNIQAISNITSSPGKKGKTVVNAVPSIMCDKVLDNLSPVKMARRALRALKGLVRLQ
ALVRGHAVRKQAAETLQCMQALVRAQARVRARRVRISLESQVTQKKASEQNVHEDHVWEI
EERWCDGIGSAEQMQAKVLKRQEAAAKRERAMAYALTHQWQAGSRKQKAATLQGLEVDEN
QWSQNWLERWMAARPWENRLLDTNAKESAPTGDDNHADENEAKAPNKPKGKVSISTTHPN
GSNKKNGANHKKSHSDISGSSSGQSAGVLPTNSLGSSKLKPKPSDEISEEVNSQPSNLAS
RSTSNPKERPTQVNASSKKRLSLPNNGTMGGGVGKGATNGRTNQSMSSKNAAKGSSKLES
KQQQRPNPPNTTVKRVEYFLFALLQFCYISGDNNLVMSKII*
>11669.m04444|LOC_Os03g44610.1|genepair1473-2
MDTCAGHASINDFLPTPPHLLLPMELSFTGGSLNYIKRLFVMGISSKWFKSLVGIRKQEK
ARNAEKQEKAQNAESCETSTPAAQLLHKRKHSLDTERAILVEELAVQSEPLTDDTNTQTV
SDSISSDSTLLGVHISQTEEHKTKEDVAATLIQSAFRAFLARRALRALKGIVILQALVRG
HIIRKQTSETLQCMQALVRAQARVRARQVRVSLENQVARKKVPEQDDHENHVREIEERWC
GSIGSVEELQAKVLKRQEAAAKRERAMAYALTHQRQAGSRQQKPTTPQGLELDDSHWGSN
WLERWMAVRPWENRLLDSNTKETMPLCDDKQDMETKSQITPKGKVQVSSALSNGSNKKKG
INHKKSYSDVTCASFGRSPNIPSTSLGSCKQKSKLSDEALEEVSSQPTDLASLSTCQPKA
KLVQANTPVKKWLSLPTNVGGGAAKGATNSNSICRSTSAKSDPKPRANASNQSRKQVELQ
A*
>11686.m04232|LOC_Os12g42440.1|genepair1474-1
MAAPGCNSDNSRYYDLLGVPRGADGDEIRRAYRRAAVTHHPDKGGDEEAFKEVARAYQVL
GDPALREVYDVYGEDGVNGGVGAAAAGFGRYDDAFDEFVETFRYLVAAGGADRAFGDAVE
MLRHLVAGVAAGGGADDGGKAFDEVIVGMFKNMMSGGDSSVEFVDLSLEEFYNGATKKFT
LSRDVTCIPCKGTGSTLASPATCAACSGAGYKVVSQLMRLRRRGSEPCAACGGRGEVSRG

LKRCSACRGSKVATDTKVLELAVEKGVPDGHRITFPGEADVKENGVAGDLVMGLRQKKHG
KFTRKGDDLVYEHELSLAEALCGFQFVITHLDGRRLLVTSGAGEVIRPGQLKAIDGEGMP
VHGMPFAKGTLYVAFRVAFPGTMTPALRDAVAAAFPAATKAAAVEDGGGCEETTTTTRDV
GGEEEMKLNAKGEQSPTTRMEHGAGGEDEYVHVHGHVDEEEEDNEEM*
>11669.m04445|LOC_Os03g44620.1|genepair1474-2
MFGRAPKKSDNTKYYEILGVPKTASQDDLKKAYRKAAIKNHPDKGGDPEKFKELAQAYEV
LSDPEKREIYDQYGEDALKEGMGGGGSHVDPFDIFSSFFGPSFGGGGSSRGRRQRRGEDV
IHPLKVSLEDLYNGTSKKLSLSRNVLCAKCKGKGSKSGASMRCPGCQGSGMKITIRQLGP
SMIQQMQQPCNECKGTGESINEKDRCPGCKGEKVIQEKKVLEVHVEKGMQHNQKITFPGE
ADEAPDTVTGDIVFVLQQKDHSKFKRKGDDLFYEHTLSLTEALCGFQFVLTHLDNRQLLI
KSNPGEVVKPDQFKAINDEGMPMYQRPFMKGKLYIHFTVEFPDSLAPEQCKALEAVLPPK
PASQLTEMEIDECEETTMHDVNNIEEEMRRKAQAAQEAYDEDDEMPGGAQRVQCAQQ*
>11686.m04245|LOC_Os12g42570.1|genepair1475-1
MVARMWPWPPPARKFRVRLVVRRAEGLTATATATASSSPVAEAKVAVEVRWKGPKASPLG
SLRRVMHSNRTRLESAAEAAVAWEEEFERVETFTATSHRKSGAAFHPWDLAFSVFVNDSN
KGPKGELILGTASLNLAEYTSASEEVEIILPLSVPNGSSESSPSLHLTLSLVELGPPHQS
PDASQRSAVTAPLSPSSGDSVPSSKDEVSSVIKAGLRNLKILTDLVSTRRSKKTNRDDDG
SEDKCYVHSDGAEYPSDTDSLDEDLDDRERDDGLGGSTVRKSFSYGSLQSVNYAGGLLYA
HARIDGEHEDWIYYSHRKSEAGYSVEQEASSTAEEPVVSVSRRSLLPWKKKRKLNLRLLK
VLKNKGEPLLKKGNDEEGGDDIDYDRRLLTTSDGNALEGSDSSINSMVSIFGDDNFVVGN
WESKEVLSRDGHLRLSTQVFFASIDQRSERAAGESACTALVAVIADWFEANQDLMPIRSQ
FDSLIREGSLEWRKLCENETYRERFPDKHFDLETVLHAKIRPLTVSPNRSFIGFFQPEST
EDGSGFDFLDGAMSFDNIWDEISRAAECSTEKPTLYIVSWNDHFFVLKVEAGAYYIIDTL
GERLYEGCSQAYILKFDDNTTIHKVPAEKKEANPDSSGRLKDSSDSSSTDQDSGTDTEEC
ELVSKGKESCKEYIKSFLAAIPIRELQADIKKGIIASTPLHHRLQIEFHYTESCPEEIPL
PAPLPAIEAPFEFSWPEPPPAMEVTLAPAVAAI*
>11669.m04451|LOC_Os03g44670.1|genepair1475-2
MRWPRPPAARKFRVRLVVRRAEGLQQPCAAAAEHAASAEAEAPPTRVAAEVRWKGPKASP
LSSLRRTAVRRNRTREAEAEAAAVVACATAADDAAAAAGAGDAAGVVVAAWEEEFESTVT
LAAASHREHAAFQPWELAFSVFTAANRGPKIKPSILGTASLNLADYASAAEENIEIILPL
SVPNGSAESAPSLHLTLGMVELRAFQETSDASQRSAMAAPLSPSSGDSAPVGKDEVSVIR
AGLRKVKILTDLVSTRRSKKTSQDDESSEEKCYVNSDGAEYPCDIESLDDDLDDRAQQDE
VGDSTVRKSFSYGSLQSVNYVGGLVYAHAKIDGEHEDWIYYSHRKSDAGYHVEGKPSSTV
EETMLPTVKRSILPWRKRKLSLRSLKAKGEPLLKKAYGEEGGDDIDYDRRLLTSSDGSVS
EGSRGEDGSINGMVSEFGDDNFVVGNWELKEIVSRDGHLKLSSHVFFASIDQRSERAAGE
SACTALVAVIADWFQSNQDIMPIQSQFDSLIREGSLEWRNLCENLMYRERFPDKHFDLET
VLQAKIRPLTVSSSKSFIGFFQPEGADDMHRFDFLDGAMSFDSIWAEISKAAEYSSSDNP
NLYIVSWNDHFFLLKVERDAYYIIDTLGERLYEGCNQAYILKFDNDTMIHKLPEKAPSSP
NSSGPLKDSSRSSSVEQDSEDGTEENILVSKGKESCKEYIKSFLAAIPIRELQVDIKKGL
MASTPLHHRLQIEFHYTASSPKEITSAPQILTIEAPFEFSWPEPPPAMEIALAPAVAVV*
>11686.m04249|LOC_Os12g42610.1|genepair1476-1
MSAQIAPAEQVCYVHCNFCNTILAVSVPGNSMLNIVTVRCGHCTNLLSVNLRGLMHSAPA
LQDHHHHHLQESGLSGCFRDQSGYPEFGFSAASSSSKLRLPPAAAAMVSYSQQNQQLEQA
LHARPPEKRQRVPSAYNRFIKEEIRRIKANNPDISHREAFSTAAKNWAHYPNIHFGLSPG
HEGGKKLVDVDPIPTAPSSKKIQGFYS*
>11669.m04455|LOC_Os03g44710.1|genepair1476-2
MSAQIVPAPEHVCYVHCNFCNTIFAVSVPSNSMLNIVTVRCGHCTSLLSVNLRGLVQALP
AEDHLQDNLKMHNMSFRENYSEYGSSSRYGRVPMMFSKNDTEHMLHVRPPEKRQRVPSAY
NRFIKEEIRRIKANNPDISHREAFSTAAKNWAHFPNIHFGLGSHESSKKLDEAIGAPSPQ
KVQRLY*
>11686.m04253|LOC_Os12g42650.1|genepair1477-1
MAAVAPIAMPARVHHHHHHHHRRALAASPAALAAAGNGLSATRRVRRSPAVEMRRERERRR
AREQQPRCGEVAGGTAAECAAVFCCFPFAVVELVVLAAVRAPAALCRRAVRGGRRRRVRS
TKPKETGAMDIASPRSLAAAAAKARKVDADFPATPKAEHLVDMEKEVWASFYGGGFWRSP
SQREDRR*
>11669.m04459|LOC_Os03g44750.1|genepair1477-2
MAGGRSSSSSRHHHGHHHHHHHHHRRHRSETSCPPTSVAVAAARAGDAHAPDHPLRRSQAF
PPRRQPPHQQQQQQQRMQPQRWDSEQVRQPRCGEVAGGTAAGCAAVCCCLPCAVVEVVVL
ATVRAPAALCRRAVRGRRGGRGTRRSASAGQAGEIYELLVDEGGAVDSGEKKAPVVWPVA
AITAAAVPSEEAGELEKEVWARFYGAGFWRSPSQLSDHMR*
>11686.m04273|LOC_Os12g42830.1|genepair1478-1
MTAAAVNASRVMRRRAAGEDLGDGGDGDGDFWAGGAPRLYDFSQQEQKPFLPAPAPAPPS
PAPVPASPPSPAAESVAPCLLTLQCSGVGWGVRKRVRYVGRHHHLARHHAPERAVDAARD
DDEASSAKAKNESPKEEAAAAEEDDDNVEHKVAVPTTSEEKKRRRRRKRGRGRVGGHGVA
KRPKKEEEEEETKLSAPKAEQLEEEEGAAVAAPSGMIDRWKATRYATAEASLLAIMRARG
ARAGKPVPRGALREEARAHIGDTGLLDHLLRHIADKVAPGGAERFRRRHNAGGGLEYWLE
PAELAAVRRNAGVADPYWVPPPGWKPGDPVSPEGYLLEVRKQVEKLAVELAGVRRHMDHL
SSNVSQVGKEIKSEAEKSYNTCQEKYACMEKANGNLEKQLLSLEEKYENATHANGELKEE
LLFLKEKFVSVVENNTRLEHQLTALSTSFLSLKEELLWLEKEEADLYVKEPWEDDDEKQE
HDAGKEAKDDDVAGVSAANDQPDVDGDGTTTTTTTSSNGGSGKRTSRKCSVRISKPQGAF
QWPTPSLPFSPELAAPPSPPLTPTAPVVAGAANFATMDELYEYMMAGGLPTPPSTTSNAG
KLPSLPAATACATTPPVKTADAAGDVGTELALATPAY*
>11669.m04460|LOC_Os03g44760.1|genepair1478-2
MDAEMAAPALAAAHLLDSPMRPQVSRYYSKKRGSSHSRNGKDDANHDESKNQSPGLPLSR

QSLSSSATHTYHTGGFYEIDHEKLPPKSPIHLKSIRVVKVSGYTSLDVTVSFPSLLALRS
FFSSSPRSCTGPELDERFVMSSNHAARILRRRVAEEELAGDVMHQDSFWLVKPCLYDFSA
SSPHDVLTPSPPPATAQAKAPAASSCLLDTLKCDGAGWGVRRRVRYIGRHHDASKEASAA
SLDGYNTEVSVQEEQQQRLRLRLRLRQRREQEDNKSTSNGKRKREEAESSMDKSRAARKK
KAKTYKSPKKVEKRRVVEAKDGDPRRGKDRWSAERYAAAERSLLDIMRSHGACFGAPVMR
QALREEARKHIGDTGLLDHLLKHMAGRVPEGSADRFRRRHNADGAMEYWLEPAELAEVRR
LAGVSDPYWVPPPGWKPGDDVSAVAGDLLVKKKVEELAEEVDGVKRHIEQLSSNLVQLEK
ETKSEAERSYSSRKEKYQKLMKANEKLEKQVLSMKDMYEHLVQKKGKLKKEVLSLKDKYK
LVLEKNDKLEEQMASLSSSFLSLKEQLLLPRNGDNLNMERERVEVTLGKQEGLVPGEPLY
VDGGDRISQQADATVVQVGEKRTARKSSFRICKPQGTFMWPHMASGTSMAISGGGSSSCP
VASGPEQLPRSSSCPSIGPGGLPPSSRAPAEVVVASPLDEHVAFRGGFNTPPSASSTNAA
AAAKLPPLPSPTSPLQTRALFAAGFTVPALHNFSGLTLRHVDSSSPSSAPCGAREKMVTL
FDGDCRGISVVGTELALATPSYC*
>11686.m03927|LOC_Os12g39660.1|genepair1479-1
MESYLEENFGGVKAKNSSEEALRRWRKLCGVVKNPKRRFRFTANLDKRGEAQAIKHANHE
KLRVAVLVSKAALQFIQGLSLRSEYVVPEEVKAAGFQICADELGSIVEGHDSKKLITHGG
VTGIADKLATSPADGLSTAEESIKRRQDVYGLNKFTESEVRSFWVFVWEALQDTTLIILA
VCAFVSLVVGIAMEGWPKGAHDGLGIVASILLVVFVTATSDYRQSLQFKDLDKEKKKIQV
QVTRNGFRQRLSIYDLLPGDVVHLAIGDQVPADGLFISGFSLLINESSLTGESEPVVVNE
DNPFLLSGTKVQDGSCKMLITTVGMRTQWGKLMATLSEGGDDETPLQVKLNGVATIIGKI
GLFFAVITFIVLSQGLISKKYHEGLLLSWSGDDALEMLEHFAIAVTIVVVAVPEGLPLAV
TLSLAFAMKKMMNDKALVRHLAACETMGSATTICSDKTGTLTTNHMTVVKACICGNIKEV
NNPKNASDLCSELPETVVKTLLESIFNNTGGEVVIDQDGKYQILGTPTETALLEFALSLG
GNFKAKRDETKIVKMEPFNSTKKRMCVVLKLPGGGCRAHCKGASEIVLAACDKFMDETGA
VVPLDKTTADKLNGIIESFANEALRTLCLGYREMEEGFSVEEQIPLQGYTCIGIVGIKDP
VRPGVRESVATCRSAGIMVRMVTGDNINTAKAIARECGILTEDGLAIEGPEFREKSLDEL
LKLIPKIQVMARSSPLDKHTLVKHLRTTFNEVVAVTGDGTNDAPALHEADIGLAMGIAGT
EVAKESADVIILDDNFSTIVTVAKWGRSVYVNIQKFVQFQLTVNVVALLVNFSSACFTGN
APLTAVQLLWVNMIMDTLGALALATEPPNDDLMKREPVGRTGKFITNVMWRNILGQSFYQ
FIVMWYLQTQGKSMFGLDGPDAEVVLNTIIFNSFVFCQVFNEISSREMEKINVLRGILKN
YVFLGVLTSTVVFQFIMVQFLGEFANTIPLTRLQWIASVLLGLIGMPISAIIKLLPVGSS
*
>11669.m04168|LOC_Os03g42020.1|genepair1479-2
MHSGVNGCCPLRLPAAAAVHGRRIPPLLPPRGAWPGCIAAPALHRKPGRGGGGALSSCRR
ASHHEKLQVAALPSKATLEFEHGVSLRSAYIVPEDVQAAGFQIDADELASIVESRDTKKL
TVHGQLNGIADKLGTSLTNGIVTDKDLLNQRQDIYGVNKFAETEIRSFWEFVWEALEDTT
LIILSACAIFSLVVGITTEGWPQGAHDGVGIVASILLVVSVTGTSNYQQSLQFRDLDKEK
RKILVQVTRNGLRQRVLIDDLLPGDAVHLAVGDQVPADGLFISGFSVLVDESSLTGESEP
VFVNEDNPYLLSGTKVLDGSCKMLVTAVGMRTQWGKLMAVLTDGGDDETPLQTRLNGVAN
TIGKIGLFFAVLTFIVLSQGIIGQKYLDGLLLSWSGDDVLEILDHFAVAVTIVVVAVPEG
LPLAVTLSLAFAMKKMMNDKALVRQLAACETMGSATVICSDKTGTLTTNRMTVVKACICG
NTIQVNNPQTPNMSSNFPEVAVETLLESIFNNTSGEVVTNQDGKYQILGTPTETALLEFA
LLLDGDCKEKQLGSKIVKVEPFNSTKKRMSTILELPGGGYRAHCKGASEIVLAACDKFID
ERGCIVPLDDKTSSKLNDIIKAFSSEALRTLCLAYREMEEGFSTQEQIPLQGYTCIGIVG
IKDPVRPGVRQSVATCRSAGISVRMITGDNIDTAKAIARECGILTKDGIAIEGAEFREKS
AEELHDLIPKMQVLARSSPLDKHTLVKHLRTAFNEVVAVTGDGTNDAPALREADIGLAMG
IAGTEVAKESADVVILDDNFSTIVTVAKWGRSVYVNIQKFVQFQLTVNVVALLVNFTSAC
FTGDAPLTAVQLLWVNMIMDTLGALALATEPPNNNLMKKAPVGRKGKFITNVMWRNIVGQ
SLYQFAVMWYLQTQGKHLFGLEGYHADIVLNTIIFNTFVFCQVFNEISSREMEDINVLRG
MAGNSIFLGVLTGTIFFQFILVQFLGDFANTTPLTQQQWLISILFGFLGMPIAAAIKLIA
VEPHEKADTRRTP*
>11686.m03945|LOC_Os12g39830.1|genepair1480-1
MSMEEAEECSAACGFSLTCQEDGADLGDGVVDDDDDGDVFLFYNAVAAADDEEEEEEYVE
QMVSKEASFCCSSSSSLFDAAAGDGYGDGDGDGDWFRQARLAAVKWILETRGYFGFGHRT
AYLAIAYFDRFCLRRRVDREAMPWAARLLSIACVSVAAKMEEYQSPALSEFDAGGGRVFC
SDSIRRMELLVLSTLGWRMGAVTPFDFLPCFSSRLHRHHHGGAGAAGHGAAAAARVALNA
VGFIFATAEAGSVLDYRPSTVAAAAILAASYGAPLTKEALESKMSNLSPSCLIDKENVHA
CYSMMVGDMNNNRRSSKRPLQCSDSNEITTTSTYDSVLVDDVTDTAAFAATAMNKRLRPE
PPRIR*
>11669.m04173|LOC_Os03g42070.1|genepair1480-2
MEAEDEYSAGCSFSLMCQEDSTDLDDDGGGGGCFAGDGRADLLLVYNAAAAADDEDEEEV
EEYMDHLVSKESSFCSSSSSTSSSSCCFSDAGGESAAAAAPMDWFALARRATVKWILETR
GCFGFCHRTAYLAIAYFDRFCLRRCIDRSVMPWAARLLAVACVSLAAKMEEYRAPALSEF
RAGVGDDGYEFSCVCIRRMELLVLSTLDWRMAAVTPFDYLPCLSSRLRRHVGGGGGAGAS
AALIFSAAEAASVLDHRPSTVAAAAVLAATHGALTREALESKMSGLSPSFLLDKEDVFAC
YSAMLSQPTSPASKSTTTTTGKRSSSSSCSESTDAASSYDATAASFPAAASCGSKRMRLE
LPGGILR*
>11686.m03947|LOC_Os12g39850.1|genepair1481-1
MEGGGLIADMSWTVFDLPSHSDESEMMAQLFSAFPIHGEEEGHEQLPWFDQSSNPCYYSC
NASSTAYSNSNASSIPAPSEYEGYCFSDSNEALGVSSSIAPHDLSMVQVQGATEFLNVIP
NHSLDSFGNGELGHEDLDSVSGTNKRKQSAEGEFDGQTRGSKCARKAEPKRAKKAKQTVE
KDASVAIPNGSCSISDNDSSSSQEVADAGATSKGKSRAGRGAATDPQSLYARKRRERINE
RLKTLQNLVPNGTKVDISTMLEEAVHYVKFLQLQIKLLSSDEMWMYAPIAYNGMNIGLDL
NIDT*

>11669.m04176|LOC_Os03g42100.1|genepair1481-2
MESGGVIAEAGWSSLDMSSQAEESEMMAQLLGTCFPSNGEDDHHQELPWSVDTPSAYYLH
CNGGSSSAYSSTTSSNSASGSFTLIAPRSEYEGYYVSDSNEAALGISIQEQGAAQFMDAI
LNRNGDPGFDDLADSSVNLLDSIGASNKRKIQEQGRLDDQTKSRKSAKKAGSKRGKKAAQ
CEGEDGSIAVTNRQSLSCCTSENDSIGSQESPVAAKSNGKAQSGHRSATDPQSLYARKRR
ERINERLKILQNLVPNGTKVDISTMLEEAMHYVKFLQLQIKLLSSDEMWMYAPIAYNGMN
IGIDLNLSQH*
>11686.m03961|LOC_Os12g39990.1|genepair1482-1
MEAPLHQSPVPLLPPPPPPPRVVGVQQQQQQEAVVPPPPAMAAAAGGGGREQCPRCASRD
TKFCYYNNYNTAQPRHFCRACRRYWTLGGSLRNVPIGGSTRKRPRPSRPARAAVAAAIAA
AAAASASGSQIAAQQQQAPPVVMSQHEAAAAAAAAASGGGGGDGLLVSLLGAAPVLEGRLG
GGIGVDLLGGEQLGFGAMAMPPAPLLWPARVLEGGDAWKSAAAAAGVSYSPFPALWQELA
AAAPVELAGGGGLLRHGGGGAPQLM*
>11669.m04187|LOC_Os03g42200.1|genepair1482-2
MPGQVMEAALQQLPASMASGSLLLPPACLQHPLPAAAAASGGVGGSSREQCPRCASHDTK
FCYYNNYNTSQPRHFCRACRRYWTLGGSLRNVPIGGSTRKRPRPPVRRPPVHFTAAAAAA
AAAAPPHHHHHHHGGPAAAVATSSSSSAAAPLLWPTGLLDSSSNNAETWRMAAGGMWPEF
TAAAAQVGGLMHGGAQQQPQLL*
>11686.m03969|LOC_Os12g40070.1|genepair1483-1
MGDQKRSFINVMIGDFVAVPTKFANFIRGQISEVVKLEVPNGKTYDVQVAKEHNELVLRS
GWGAFARDYELKQCDILVFAYSGSSRFKVRIFNPSGCEKELSCVMMNNTPCGHEGSMSYH
DNHLQSPSESSSFFNISLPPHSPFQELSGVDSTSLLVSDPPNMQQFCLRCSWTNPKRLAK
PSLAIASLSHQHLAFDKTRCMFILKIENDTLKTILKMFAKNVQGLISGVAKLEVPDGKTY
DVEISKEHNELVFRSGWEVFAIAYELEQGDILAFGYSGNSHFKVQIFNPSNCEKELSCVV
MNRSISDDNHRQSPRRERMNKPSTTCMDCITNHYWLHMDDRERYFFKVMMSVSDIKDELA
IPKKFAANVRGKIPEQVRLEVSDDVPSSEDIKDPMSSGGLQKSKKSCYVLPMLYNMTSAQ
ESEVLALEKKIQPQIPLYITAMDKTSVASGSLVFCKDYAVRYLLDQNRTIKLCQSGGSKT
WDISLDMDTDDLYALSTGWLDFFRCNLLQEGDICVFEASKSKRGVALTFHPFKESHCPKS
SEYTLSTKSPTRRVPKRDYFATNLTNLTDQQERKCFSVKYASKYLPHKDQNMRLRLPETK
YKCKAALHIDTSTNLHKLLKGWGKFVNDNKLEIHDICLFQLMKNKKKLTMTVHIIRKGEC
S*
>11669.m04205|LOC_Os03g42370.1|genepair1483-2
MAPFLASPIRLHPCPLLFSHPHLFGSDYFAFSVKGRGAHLESMRVENKRGNRSWGTDAKR
THPNSIFNQLVRYGYIGTGFPDMNCSLTWSLIGGPSTLSLPSFFPSAHLLLSLALFFFLC
IQSNHRMSKSGCERCRGRGFWDTDDQDTYFFKVMIGGFRRQMTIPYKFAENFRDQIQGTI
KLKARNGNTCSVLVDKCSNKLVLTKGWAEFANSHDIKMGDFLVFRYTGNSQFEVKIFDPS
GCVKAASHNAVNIGQHAQNMQGDPIEILSCSDEHLRAQSLTTERQNQPEKDVIDNCNKKM
KTEHASSSEDDQETPTAEVHRMKVEEMVRAIHSNHPVFVAVMKKSNVTRQPCYVAISRKY
ANEYFPGGDQMLTLQRHGKRWQVKFCISKRKLRMLSKGWRKFTRDNELQHWAPLALFITL
FFSPHFRKMKKCGQKMRKLNTRSTARDDQEKYFFKVMIGDFHKRMTIPDKFARHFKGVIS
KTIKLEPRSGYTFDVQVTKKLNILVLGSGWESFVNAHDLNMGDFLVFKYNGDFLLQVLIF
DPSGCEKSTSCSMENAIDHVGQGWKEHNDISTSYHDQPKGNKHWMQKDSSSKGNKIGNTR
SSNTPSKFSGCILPRGTCLPVVQEKKMKEKIQAIHSKTPMYGNVMTKCNVSGSPCVLEIT
QLYDDAYLPFNNGQELMLRHRDKSWKVRFYRFKNKSRKLTQASSLYKMRRPGARCREGHA
HFNGNHIDGQYKNFFKVMIGRFRERMIIPNEFLQYFRGKIPRTIKLQLRDGCTYDVQVTK
NLGKISLQSGWKAFVTAHDLQMGDFLVFSYDGISKLKVLIFGPSGCEKVHSRSTLKNATH
CGEKWEEPLHISSNSHDLPVKSPQNVSKSEKQWDSSEQENDTANIEEVALQGDDLQGHPV
LNCILPKHTRLTDMQKQQLESKVGAIHSEIPIYGCILRKSRVHGKSQTVDICREYADVYL
PFKELNMTLQRHGKNWEVLCRTKDTRTKRLSTGWSRFAQENNLQVGDICLFELLKKKEYS
MNVHIIPKK*
>11686.m03974|LOC_Os12g40120.1|genepair1484-1
MGEKGCESCREWQEHCYREHMDVSRIRFFRLMTGDFAHGISIPEKVADRFSGQITKGFNL
KAPSGETWRVSVEKVADELILMSGWEDFAKAHELQENDLLFFTCNGRCNGSFSFDVLIFD
ASGCEKVSCFFIGKKNSIGGQTQNAEQYHLSDSEDTSTPSTFLVGSPHKASTSKKLNGKT
KTNPNKDDPFSTGKEPEDPNSSRSHVKHEMIEEEKSDDDDEHADYEHADYYYSRFANYLT
GEEREEIFSLVSLQPGNPVFVTVLQAPQIHRKGLLIVPSGFAADHLDSRSQEILLMRPNK
KEKWYVKYYHASTTRGFNCQRWIKFIRENRLREGYICIFELMKGARRVTMTVHVIGKVDD
RFVLLG*
>11669.m04211|LOC_Os03g42430.1|genepair1484-2
MGMLKIGKNCSVCKEWQEHCYWSHMADHSKHFLKHMVGDFTESMTVPARFANNFNGHISE
EVNLRSPSGETWSIGVANSDAGELVLQPGWKEFVDGNGIEEGDCLLFRYSGVSSSFDVLI
FDPSGCEKASPHFVGSHGFGRAENSAGAEQGGRNGRRTPPIVDGDNGHRHHLEMTLHRNS
CRSIPRACKRSLFSDETEAKENDGEDEDVVAAAEGGRYGEYYFSRHGRVAEYNLREEDRE
EISRVPVPVQPGNPVFVQVIHSSHVRSSKYCIVGVSPEFAGKYLGAVEREVVLERASRGG
EWHVPFVHRQNTRGFYGAGWRQFAGDNRLVAHDVCLFELTMVDAAASGGGNRRRRWSRRP
TMTVHVLRRVRGRFVLLR*
>11686.m03988|LOC_Os12g40260.1|genepair1485-1
MGNHKKLLQFLRPADTAVAAARLSDDDDDGGAPSSVPPSPMSTWSGRTSAAASPSPYVMS
PWVNLPGFGVGGGDEMVAGGGGTGLLGSLVKEDGHVYSLAAAGELLYTGTDSKNVRVWRH
RREFAGFRSGSGLVKAIVVAGDGRIYTGHQDGKVRVWRASADDPAVHRRVGSLPGLGDVL
RSAVRPSRYVETRRRHSALWLRHFDAVSCLSLDAAAGLLYSGSWDKTFKVWRVSDSRCLE
SVRAHDDAVNTVAAAGFDALVFTGSADGAVKVWRREPGKGGATRHAMERVLRKGESAVTA
IAVAAEARVVYYVGSSDGAVTHWQWRRGGAGVAGPPRNGGALRGHRMAVLCLAVAGRVVVS
GSADRTISVWRREEGADHARLAVLAGHTGPVKCVAMDEEDDTAGDKRWVVYSGSLDGSVK

VWRVSSTPDAAAARTPAHGWKATPSPLGAWTPYAATPARKRMAAA*
>11669.m04240|LOC_Os03g42710.1|genepair1485-2
MGSGHRKLIHLLRAEQAAAAASAASASFSPKSFSSSSASDDDGCSSSSWQTNDGAGGYGS
AASSPSRCSASTPPKSPWAAHLPGLGGGGVGTGAGATGLVASLVKEDGHVYSLAAAGDVL
YTGTDSENVRVWRDRRELAGFRTGSGLVKAIVVADDGRIFTGHQDGKVRVWRADAGDPAV
HRRVGSLPRLADYVRSSVNPSSYVETPRRRRGRRREVWLRHSDAVSCLSLDEGAGLLYSA
SWDGSFKVWRVSDSRCLESVCAHDDAINTVAAAGFDGVVFTGSADGTVKVWRREEEPAAS
GGEAKTRHVLETVLREDESAVTAIAVSAEGRVVYVGSSDGDVTYWHWIDGEARYGGALRA
HGTAVMCLAVAGNVVVSGSADRTLCAWRRGGGEHSRLAVLAGHTGPVKCVAVDEEETSSC
SSDGERRFVVYSGSLDGSVKVWRISDIEPTNPPPRLPSPHVWKREDQPAAATAAAARAWS
PYQTSEMNSVAAA*
>11686.m04013|LOC_Os12g40490.1|genepair1486-1
MPLRSGHGPWKCRRCRPNKRKGRLSLAAYLATCHSYHCSANIKHVGNDLLIGVGVHRGVL
GWGEIKNRSCPWPVVRLSRARLVTLCLGSWCVVNRDYRELEFLVGVSGSLRGESGLSGRR
GDRRRTEFLVCCRLKLWFSEVLETFSIGEKFVRFLSSGYRKFDPQITSSHGLGAYDESDN
EDIDRAIALSLSEEQNKGKAVDIDYNLEEDEQLARALQESLNADSPPRQNIPVENVPSEP
PRELPPILFASSGSRTCAGCKNPIGHGRFLSCMDSVWHPQCFRCFACNKPISEYEFAMHE
DQPYHKSCYKDFFHPKCDVCKNFIPTNRNGLIEYRAHPFWMQKYCPSHEDDGTPRCCSCE
RMEPMDIKYITLDDGRKLCLECLNSSIMDTPECQQLYMDIQEFFEGLNMKVEQQVPILLV
ERQALNEALETEKNGHHLPETRGLCLSEEQIVRTILRRPIIGPGNRIIDMITAPYKLERR
CEVTAILILYGLPRLQTGSILAHEMMHAYLRLKGFRSLSPQVEEGICQVLSHMWLESEII
FGSSIDISATSVASSSSSSSTPTTSKKGAKTEFEKKLGAFIKHQIETDSSEAYGDGFRAA
NRAVESYGLRSTLNHMKMTGSFPY*
>11669.m04252|LOC_Os03g42820.1|genepair1486-2
MPKRLDMYKDFRLPILENMGWLSKIFKGSVNRVSRGHYNGNTHEGHSAWHTKAYEHDSDH
EDIDRAIALSLSEEDQRKGKAVDEVDIDHRLHEDEQLARALQESLNDEPPRQNVPVKDVH
SESTPATFMPPYIFPSTGLRVCAGCKTPIGQGRFLSCMDSVWHPQCFRCFACDRPISEYE
FAVHEGNPYHRSCYKELFHPKCDVCKNFIPTNKDGHIEYRAHPFWMQKYCPAHETDRTPR
CCSCERMEPKDSKYITLDDGRKLCLECLNTSIMDTDECQPLYIDIQEFYEGLNMKVEQQI
PLLLVERQALNEAMEAEKTGHHLAETRGLCLSEEQIVRTILRRPVIGPGNKIVDMITGPY
KLVRRCEVTAILILYGLPRLLTGSILAHEMMHAYLRLKGYQTLDPKVEEGICQVLAHMWL
ESEITSGSSSIIASIAASSSSSSSSAPSSKKGVQTDFEKKLGEFFKHQIETDPSDVYGD
GFRDGIKAVERYGLRKTLDHMKLTGVFPC*
>11686.m04015|LOC_Os12g40510.1|genepair1487-1
MATHRSKIETPGTGSGRGMGGRVSVSEVEALYELFKKISSAVIDDGLINKEEFQLALFKT
SKKESLFADRKNSCMIDVSADFAFISVLPHVLNILPALTGFDEFARALSVFHPSAPLDEK
IDFSFQLYDLKQQGYIERQEVKQMVVATLAESGMNLSDEIIESIIDKTFEEADTKHDGRI
DKEEWRNLVLRHPSLLKNMTLQYLKDITTTFPSFVFHSQVDDT*
>11669.m04255|LOC_Os03g42840.1|genepair1487-2
MLQCLEGVKQLCGVLLKCCDLDLKQPKGLEDPEILARETVFSVSEVEALYELFKKISSAV
IDDGLINKEEFQLALFKTNKKESLFADRVFDLFDTKHNGILGFEEFARALSVFHPNAPLD
EKIDFSFQLYDLKQQGFIERQEVKQMVVATLAESGMNLSDEVIESIIDKTFEEADTKHDG
KIDKEEWRNLVLRHPSLLKNMTLQYLKDITTTFPSFVFHSQVDDT*
>11686.m04022|LOC_Os12g40560.1|genepair1488-1
MDGNIENPAEDMSGIASNLDNEEQAIPLSDVPEQYKEDPENTYDEETKDSSYEESGIPYN
EDQVNINDGNVGHQHEEDQAIPSEEGHAYGGEAQGEQQANAVTDEKKWPGWPGESVFRIL
VPAQKVGAVIGRKGEFIKKMCEESRARIKVLDGPPGVPDRAVMISAKDEPDAPLPPAVDG
LLRVHKRITDGLDGESDQPQRAAGTVGPTRLLVPASQAGSLIGKQGATIKSIQDASKCVL
RILESVPPVALSDDRVVEIQGEPLDVHKAVELIASHLRKFLVDRSVLPLFEMQMKVHNAH
REQPMPPPQTWGPPPPWGHPSNVPPGGPGYGGNPQFMPPRPQDHYYPPPDVPPVEKQPHY
GISSYGRDAPPTGAPPASGNQHPPHGSSQITHSMQVPLSYADAVIGAAGASISYIRRHSG
ATISIQEGVPGEMTVEISGSASQVQTAQQLIKQHLLQNFMAEASPQGPPQAPAPPAQPVD
TGYSSYPPYGGTSYGSPPGGAGPHNGGSYGAVTGTPCSMPVHVLQDPGTRDLQRQPRGFG
GA*
>11669.m04262|LOC_Os03g42900.2|genepair1488-2
MDGLVENFDADDLGEMPQNHYNEEQLIPYSDVSHPYNEEPDNMDNVEEGNPYIQQVSLYS
EEPENQYNEEPSNPYQEESDNAYNGEVKQQDSLPVEADKKWPGWPGESVFRILIPAQKVG
AIIGRKGEFIKKMCEESKARIKILDGPPGVPERTVMISAKDEPDAPISPAMDGLFRVYKR
ITDGSDGDSGQPERNISNVGPTRLLVPASQAGSLIGKQGATIKSIQDSSKSIVRIVETLP
LVALNDDRVVEIQGEPVGVQKALESIASHLRKFLVDRSVLPLFEGQMKMHNAQREQAMAA
PQPWGPPQPWGPPPSHLPPGGPGYGGHPQFMPPRPQDNYYPPPDVPSMEKQPHYGISAYG
REAPTGVSASGNQPPSHVASQVTHNMQIPLSYADAVIGAAGASISYIRRHSGATVTIQES
RGAPGEMTVEIIGSASQVQTAQQLVQNFMAEAPQGPPPPASNPPAPAVDLSYGSYPPPYG
ASYGSAASGAGPAPHNGGSYGGTTYPSYGY*
>11686.m03862|LOC_Os12g39100.1|genepair1489-1
MMRMDKLVRQCDMEVMKMAMLKHEETFRQQVHELHRLYRIQRQLMSDLTMAELSSGHRRR
QPRRSSKQPRRALNLQLPADEYIVSADAADDNDDTAELDLTLAVGGGRSSRKCNAAIAAA
AAAAGGSSPFASDCSGSGLSSSPSSAEYSDGAAMFLHAPPPMPPPCQRAMAFDLAMGDAM
KQQQSPWLVQCQYLSLRMT*
>11669.m04037|LOC_Os03g40770.1|genepair1489-2
MEYICTVGDPEDLRKKLISLVNLIRTCANEFVWKSIICTGGILPLAEIPQNFMVEKESRQ
IMQQDDHGTWKSAHVHVHGTVSEYHQLADLSQGQEHNKNKVKLKMMVQMEKLVRQCDMEV
MKMAMLKHEETFKQQVYELHRLYRVQKQLMSDLNRSPPELTCRRRQRRKQHTRRRALNLQ
LPADEYIVVADAGGQATPLPPPPPSSREDELALTLAVGGGGAAGRRNNKRRESSPFTSNC

SGGSLTTATSTSTSSSTDSDGSLRQPPPCPRAMAFDVLHDGSTAAAAAAAPWLQQRLSLR
MA*
>11686.m03869|LOC_Os12g39160.1|genepair1490-1
MDKKQGFFSALREEVARGLSPARARRRSQAAAAAEVAAALRYAGGGGGGEMLAPLMEGPD
PESGDGGRSSSSAAAARREGWGRWVRGQLSRAPSTAGAAVVAAGGGGGAARRNDLRLLLG
VMGAPLAPVHVAAGEPLPHLSIKDTPIETSSAQYILQQYLAASGGQKLLSSVRNSYAMGK
VRMVATEFETGGRVVRNRMAARATESGRFVLWQMAPEMWYIELAVGGSKVHAGCNGKLVW
RHTPWLGAHSAKGPVRPLRRALQGLDPLTAASMFAGARCIGERKVNGEDCFILKLCADPE
TLRARSEGLAEIIRHVLFGYFSQKTGLLVHLEDSHLTRIQSTTGGDAVYWETTINSFIED
YRPVEGIMVAHAGRSAVTLFRFGEVAMSHTKTRMEEAWSIEEVAFNVPGLSIDCFIPPTD
IKSGSISETVELPHGEKSKVGLLQCHSAKVAALEKADDNVAWSGALQRDFK*
>11669.m04053|LOC_Os03g40930.1|genepair1490-2
MRMSGGGGGGGEMLAPLMEGPDPESGDGEGGGGGGGGGGGGGGGGGRGARGRRGEGWGQWV
RGQLSRTPSSVAAAAAGAGAARNDLRLLLGVMGAPLAPVHVSAAEPLPHLSIKDTPIETS
SAQYILQQYLAASGGQKLLASVRNAYTMGKVRMVATEFETAGRLVKNRNAARCAEPGRFV
LWQMAPEMWYIELAVGGSKVHAGCNGKLVWRHTPWLGAHAAKGPVRPLRRALQGLDPLTT
ASMFAGARCIGERKVNGEDCFILKLCTEPETLKARSEGLAEIIRHVMFGYFSQRTGLLVH
IEDSHLTRIQSTTGGDAVYWETTINSFIEDYRPVEGIMIAHSGRSAVTLFRFGEVAMSHT
KTRMEEAWSIEEVAFNVPGLSMDCFIPPTDIKSGSISETVELSHGEKSKVGPPPGHRAKV
AALEKAVDGKVAWSGTILEDHN*
>11686.m03871|LOC_Os12g39180.1|genepair1491-1
MAPLPAAATATASSAATPADDEAHSLLPSTPSNEEDDDDLEERAYEATEKVIVSISDFPD
ADDDEEESGLATSTAASGIPPFSWRKLWLFTGPGFLMSIAFLDPGNLEGDLQAGAVAGDT
LLWLLLWATSMGLLVQLLAARVGVATGRHLAELCRDEYPSWARRALWLMAEVAMVGADIQ
EVIGSAIAIKILSRGFLPLWAGVVITALDCFIFLSLENYGVRKLEAVFAILIATMAVSFA
WMFTDTKPNMKNLFIGILVPKLSSRTIRQAVGVVGCVIMPHNVFLHSALVQSRKIDPNKE
HQVREALRYYSIESTIALAVSFMINLFVTTVFAKGFYGTKEAGNIGLENAGQYLQEKFGG
GFFPILYIWGIGLLAAGQSSTITGTYAGQFIMGGFLNLKLKKWIRSLITRSFAIVPTIIV
ALFFDKSDSLDVLNEWLNVLQSIQIPFALIPLITLVSKEKVMGVFKIGRNTQAVTWTVAT
LLITINGYLLLDFFSSEIRGLLSGSILCVAVLAYASFVLYLILRGTELPNQIITTIRKSF
S*
>11669.m04067|LOC_Os03g41070.1|genepair1491-2
MAAAAAAAADADAAGERAAGAGLGSRSAAEERSLLHPSLFSAYSDEDLVERAFEPAQKIV
VSISSDPDDPDADDEHLYYGGHAPPFSWRKLWLFTGPGFLMSIAFLDPGNLEGDLQAGAT
AGDTLLWLLFWATAMGLLVQLLAARLGVATGKHLAELCRDEYPDWARRALWLMAEVSMVS
ADIQEVIGSAIAIKILSNGFLPIWVGVVITALDWLM*
>11686.m03874|LOC_Os12g39200.2|genepair1492-1
MPISSGILAPTRGASSSSSAVGRKLLLLGARHPPSSVAVAGRGVWRRGLAGVGVAAAAAS
SSSPDELHARGRPLRGGAYEEVSERAQPRGLNSMFLLHVIRNRRIEAKRSALWNLIKDIE
PLDLSIIQKDVPSETVDAMKRTVSGMLGLLPSDQFHVVIESLWNPFFKLLASSIMTGYTL
FNAQYRLSLERTLEFSEEETECKKRDSCEEIHSVGRPSMFLSLPEDVGLTIESEMADEKL
CGNMDGLGSLSIEAKKLILGMQSRLDSMEKELHELKKKNSSQMQQFAGEEKNELLYYLR
SLSPEKVVELSESSCPGVEEAVYSVVHGLLATLSPKMHTNRSPTSENMAGGAVNFGMEED
DEFTELVEDVSLPFQPLISIPRDRLARLLFWCMMLGHYIRGQECRLELMHLLAVSSDAHS
*
>11669.m04068|LOC_Os03g41080.1|genepair1492-2
MPTASCLRPPLPHSSAAASASAARGLRVPLPPPPPPPQQQLFQAALRLPRRRLAGVGVVA
ASASPFDELYARGRPAHGSSKKSILWNLIQDIEPLDLSVIQKDVPPETVDAMKRTISGML
GLLPSDQFRVVVEALWNPFFKLLVSSIMTGYTLRNAEYRLSFERNLELSEEDSEGQNRDI
SEDNHHNINLGSPVTIFRLSEEDMLQDTEKNDEELPCETVGEDLGNLTPQAEDYIIQLQS
RLDAMKKELHDLRRKNSALQMQQFVGEEKNDLLDYLRSLTPEKVAELSESTSPGVQEAIH
SVVHGLLATLSPKIHSKAPPPLGNASGGVLNLGGEDDDCAELVENASLPFQPLISVPRDY
LARLLFWCMLLGHYIRGLEYRLELAQLLRISTDVESFPSGDDLII*
>11686.m03875|LOC_Os12g39210.1|genepair1493-1
MADKENSTPASAARLTRSSAAAGAQAKRSAAAGVADGGAPPAKRKRVALSDLPTLSNAVV
VAPRQPHHPVVIKPSSKQPEPAAEAAAPSGGGGGSPVSSASTSTASPSSGWDPQYASDIY
TYLRSMEVEARRQSAADYIEAVQVDVTANMRAILVDWLVEVADEYKLVADTLYLAVSYLD
RYLSAHPLRRNRLQLLGVGAMLIAAKYEEISPPHVEDFCYITDNTYTRQEVVKMESDILK
LLEFEMGNPTIKTFLRRFTRSCQEDKKRSSLLLEFMGSYLAELSLLDYGCLRFLPSVVAA
SVVFVAKLNIDPYTNPWSKKMQKLTGYKVSELKDCILAIHDLQLRKKCSNLTAIRDKYKQ
HKFKCVSTLLPPVDIPASYLQDLTE*
>11669.m04070|LOC_Os03g41100.1|genepair1493-2
MAGKENAAAAQPRLTRAAAKRAAAVTAVAVAAKRKRVALSELPTLSNNNAVVLKPQPAPR
GGKRAASHAAEPKKPAPPPAPAVVVVVDDDEEGEGDPQLCAPYASDINSYLRSMEVQAKR
RPAADYIETVQVDVTANMRGILVDWLVEVAEEYKLVSDTLYLTVSYIDRFLSAKSINRQK
LQLLGVSAMLIASKYEEISPPNVEDFCYITDNTYMKQEVVKMERDILNVLKFEMGNPTTK
TFLRMFIRSSQEDDKYPSLPLEFMCSYLAELSLLEYGCVRLLPSVVAASVVFVARLTLDS
DTNPWSKKLQEVTGYRASELKDCITCIHDLQLNRKGSSLMAIRDKYKQHRFKGVSTLLPP
VEIPASYFEDLNE*
>11686.m03876|LOC_Os12g39220.1|genepair1494-1
MEQADESSVNKESSEQQQLTSEQDDDGATWLSLTLATQGSPEEATAEAEETEAANCSESE
APKPSSAPHKVFSCNFCMRKFFSSQALGGHQNAHKRERSAAKRSYHAQRMMMGLPLEAHA
AFVHSLRVNQSSVIQKASQQAQIRTAPRFHEGSISWPPIAYEEVPNSTWPGSFRLRSQPS
DQPSEQSKIDLNLRL*

>11669.m04071|LOC_Os03g41110.1|genepair1494-2
MRVDREMERGDEASVNRGWLELRLGVNGGGGVETVPVTAADSSASSEAGEADTVTPSQQQ
QQQQQGSPSSPAASSAPNKVFSCNFCMRKFFSSQALGGHQNAHKRERSAAKRTPSSSPYH
LHHHRMMMAGAGLPLEAHAAFMRAALRVNPAGSAIHKQQQQHQPPPPITQDATAPRFHDG
AAVAAAAAVTPWAPVAPLAYDEVLSSSASSWPGSFRFRTQPEPPPSSEQEPPSEQSKKID
LSLRL*
>11686.m03895|LOC_Os12g39400.1|genepair1495-1
MAVEAVLEASRSSSEEEAEVIVTHGGGGGGGGGGQVEGWGKRKRSRRRRPQLPPSEEEY
LALCLLMLARGRRDGDDVAASASAAAAAVEHRCSVCGKAFASYQALGGHKASHRKPPPPA
MVDDDEVVVETKPAAIATPSSSASGVSGGGGGRAHECNVCGKAFPTGQALGGHKRCHYDG
TIGSAAGAGASKPAAKTTVAVAASRGFDLNLPALPDVAAAADQRCAAEDDEVLSPLAFKK
PRLMIPA*
>11669.m04100|LOC_Os03g41390.1|genepair1495-2
MAVEEVLDGAAPMLSSSPAASGEEVGARKPQQRCGGAEGWSKRKRSRRRHRDRAAAPPPH
GSEEEHLALSLLMLARGHRDPSPAPQEQHGCSVCGRVFSSYQALGGHKTSHRPRTPPTMA
AVVVVDEPAATTASPAASSSNSGSGSGGGGGNKVHECSVCKKTFPTGQALGGHKRCHYEG
PIGSGGGAAVAGRGFDLNLPAVALPDIMTERCLPAAAEEEEVLSPLASFKKPRLMIPA*
>11686.m04358|LOC_Os12g43620.1|genepair1496-1
MDDSSFFMQWAMDTLHQLPSDSTAAAAYATDVAGDSGAFPSLQALRNASAAGGGGGFRDL
TVQVDQVHRANSWSSSDSPGGGAATAAAGWSPHVTGGGGRGHRPMSWNFSAASAQPTTED
SGGGGGGGVVPAPLQAMETTATARAAVKKGGGGGSSSSAAAPGYVQDHIIAERRRREKIN
QRFIELSTVIPGLKKMDKATILGDAVKYVKELQEKVKTLEEEDGGGRPAAMVVRKSSCSG
RQSAAGDGDGEGRVPEIEVRVWERSVLVRVQCGNSRGLLVRLLSEVEELRLGITHTSVMP
FPASTVIITITAKASSLSNHPALLCLYICIKLPCVISS*
>11669.m04689|LOC_Os03g46860.1|genepair1496-2
MEDSSLFMEWAMETLQHLHPLPATPPPAGGGYAGDNATFPSLQALRESSVSQNGMAPPEP
TAHEGHRASNSWSSGDTDSVSGGGGGAVMEHDGWSTSPNSVRCAAGGGGGGGGGGLWPVS
WNFSSAMTQPCNDQATPSNPPTTTRARYGGGGVRYLPAAVSPSPSAQTRRASSKGNGGGG
SGSSSAAPYAQEHIIAERKRREKINQRFIELSTVIPGLKKMDKATILSDAVRYVKEMQEK
LSELEQHQNGGVESAILLKKPCIATSSSDGGCPAASSAVAGSSSSGTARSSLPEIEAKIS
HGNVMVRIHGENNGKGSLVRLLAAVEGLHLGITHTNVMPFSACTAIITIMAKVEDGVSVT
AEDIVGKLNTVLQQNSRNSARETKS*
>11686.m04369|LOC_Os12g43720.1|genepair1497-1
MKISGLLTSAGINIALSVLFISLYSVLRKQPANVRVYFGRRIAEEHNRLREAFILERFVP
STGWIVKALQCTEEEILAAAGLDAVVFNRILVFSLRIFSLAAILCVFGILPLNYFGQDIH
HVRIPSESLDIFTIGNVKVRSRWLWVHCVALYIISGVACILLYLEYKHIARLRLRHLTCA
MPNPSHFTVLVRGIPKETKESCSNAIDDFFTKYHGSSYLFHQVVYKVGKVQKIMTGAKKA
YRKFKHFTDSTIDQRCRAISYRCCLCGASSNSFQLLATGLEQNQGKSDLQDSSLKLDDQE
CAAAFVYFRTRYAALVASEILQTSNPMKWVTDLAPEPDDVYWSNLWLPYKQLWIRRIATL
LGSIVFMLFFLIPVTFIQGLSQLEQLQQRLPFLKGILEKKYMSQLVTGYLPSVILQIFLY
AVAPIMILFSTLEGPISHSERKRSACCKVLYFTVWNIFFGNVLSGTVISQLNVLSSPKDI
PVQLARAIPVQATFFITYVLTSGWASLSSELMQLFGLIWNFVRKYILRMPEDTEFVPSFP
YHTEVPKVLLFGLLGFTCSVLAPLILPFLLVYFFLGYIVYRNQLLNVYRTRYDTGGLYWP
IAHNAVIFSLVLTQIICLGVFGLKESPVAAGFTIPLIILTLLFNQYCRNRLLPLFRTTPA
QDLIDMDREDERSGRMDEIHHRLHSAYCQFHDTEDIPLEKIQTVGSDEEQGCSSDKSNGK
ESFEEPRAELSHPTLNGLPVSRLRHAVKSITFLVRLQKRGLSE*
>11669.m04711|LOC_Os03g47070.2|genepair1497-2
MKVGALLTSAGINISLCILFLSLYSVLRKQPQNVKVYFGRRIAEENSRLREAFILERFVP
SASWILRSLRCTEDELLATAGLDAVVFNRILVFSIRIFSLAAFLCVLGVLPLNYFGQDML
HVRIPSASLETFTIGNMQERSRWLWVHCVALYIISGVACLLLYLEYKHIARLRLLHVSRA
STNPSHFTVLVRGVPKSTKESISCTVESFFTKYHVSSYLSHQIIYKVGKLQKIVTGAKKA
YKKFKHFKGTTVDQRCGPITYRCGLCGASSKSFELLPVEPEQEMKKHDVKDSELSLPDKD
CGAAFVFFKTRYAALVVSEIVQTSNPMEWVTSLAPDRDDVYWSNLWLPYKQLWIRRIVTL
SGSIVFMFLFLIPVTFIQGLTQLEQLQQRLPFLNGILKKKYITQLVTGYLPSVILQIFLY
TVPPTMMFFSTLEGPVSHSERKRSACCKVLYFTIWNVFFVNVLSGSAISQVNALSSPKDI
PMVLARAVPVQATFFTTYVLTSGWASLSSELMQLFGLTWNFIMKYVLRMKEDSYFVPSFP
YHTEVPKVLLFGLLGFTCSVLAPLILPFLLVYFFLGYVVYRNQFLNVYCTKYDTGGLYWP
IAHYTTIFSIVLTQIICLGVFGLKESPVAAGFTVPLIILTLLFNQYCSNRLRPLFKTLPA
QDLIDMDREDEQSGRMDDIHHRLHSAYCQFADTDDIPLKGVHVDRDADASGSSGESSCKE
DTNQPTTSDISHPTLEGLPVNRLRHAVRSLSSIIRLQKRGLSPQPAGPSADVNPQTA*
>11686.m04375|LOC_Os12g43780.1|genepair1498-1
MSVLDLDINSDDESSSMLGVGHREKDDESPSTSGKSSPPPPPEQNPLDAAAAPCFTFYEA
GKNCKAETGDEEEYETTRSKLEEQMIISSWERISGDISRIPALVELDLTGSLQQWRRLRE
EEASQVGASIEAMIFEEMRVEAVRDMMLVA*
>11669.m04723|LOC_Os03g47190.1|genepair1498-2
MAAARRLSELLQEQQEPFLIEAAKIRRLRRGGGRGGGGGGACCPVAACRRLLRLCNHGFK
KRRGCGGVGGGGGVGGGGVRGLRSALSKALCGKAVRRVLRWDSLGCFPGGVDREFRRLRR
STGDSGECDPRAMDFSGHSNDERPPGRWKAPGIGMDMDVDESSRQLSPVSVLDLHSDDGD
SPVHCRWEDAKPSTSGSSPPSEGFIGATSPCFTYNIHGKIIPMEVEEDEEEGDEEEEEMA
RAGKSIEQQISSWERIAEDISNIPRMVEMDFSQSIQQWGELKLEAAMEIGTRIETLIFDE
IRRETVCDMLASHCTLAAATSC*
>11686.m04379|LOC_Os12g43820.1|genepair1499-1
MATMAPRLLIVVVSVLFTAGIIASAAAARDLTADPAGQAPPAYDFGIPAGFFVPGTNNPY
NGDPAAWAAGYGSAAAGDVGGFGNGGAEAPSMVCSDKGPCNGKKLTCPKKCFVSFSRSGN

GYAAGGGGGGGCSFDCSTKCEATC*
>11669.m04731|LOC_Os03g47270.1|genepair1499-2
MASTARVAAALFLLLLGLSATAPLAARDLMSAAPSAKKQPAGRKPSVQPGYPGTNPGGGG
GGGIPTIPGFGSIPGMGGGMGGFNVPGMGGGWGGGYGTPSGGYSRGGVVVPTVVCSDKGP
CYRKKVTCPKKCFSSYSSSGKGYGGGGGGGGCTIDCKTKCTAYC*
>11686.m04390|LOC_Os12g43930.1|genepair1500-1
MAANVGESTSGSSSGGADSGGSFECNICFELPQEPIVTLCGHLFCWPCLYKWLHIHSHSP
ECPVCKAVVEEDKLVPLYGRGKDRVDPRSKNVPGADIPNRPAGQRPATAPQANPNTHFPN
ANPNPWFMGGGIPLANARWGNYTFSAAFGGLFPLLSFQVHGFPDATAYGQPAGFPYGYGH
GHGHGHGHAFHGGHAHAAAAPRHGPPGQQQQADVYLKALLILVGFLVIASLITF*
>11669.m04754|LOC_Os03g47500.1|genepair1500-2
MAANVGESTSSGTNGDAAGGSFECNICFELPQEPIVTLCGHLFCWPCIYRWLHIHAHSPE
CPVCKAVVEEDKLVPLYGRGKDRVDPRSKNIPEADIPNRPTGQRPATAPQADPNNNFAHA
NPNANANPWFMGTGVPLANARWGNYAFSAAFGGLFPMLSFQVHGFPDANPYAQPAGFHYG
YGHGHGFHGGHMGHAAHGVPRQGPLEQPQQADIYLKALLIMVGFLVVASLLAF*
>11686.m04391|LOC_Os12g43940.1|genepair1501-1
MDDLDMALPQAFELLLGRDRDAWPRGAAFLVAAHYGDVREMKRIAKELDEDGKGIEATVA
NTSFLGMNALHALGWLGKVPAYRYLVEEVKMDVNKADTAQGFTPLEHAVYHGRLPATRYL
LDHGADVHQIRSTGNVSLLHSAAVKGFSEVAKFLLSRGVNVDAESEMGTPLALAAFRGYD
STVKVLLEHNADPNKVTNKALGAPLDLALTSSSVSCVKLLVQAGAEVKVEGPNNHLVRAA
EKGLTEAIKCMLEAGANPNVPDRLGRMPIELAAEYGTREDVEILFPFSSAIPTVANWSVD
GIINHVQSEIKQLEDDNFIKKRRSDMKQQGDAAFKKQDYLNASVFYTQALKVDPFDGTLF
SNRSLCWLRMGDGERALDDANACEKLRPKWAKSYYRQGAALMFLKEYERAHRALGRALEL
DPESEEIENLYWESMELCG*
>11669.m04772|LOC_Os03g47650.1|genepair1501-2
MPRGRVPGGSTANFSTHLIAAQLGMQCRMGPAAAEQMRRVIASSDCDTGRVMRMFGAMEG
GDSDSDSDDSAETEKVGPLHEAASAGKMDTCKHLVEQLGFDINAEASDDLGMTPLACAVS
KGKAIAVRYFLDKGADPNKQDNIGFTPLHYATKEGYDGLARLLLSKGASVDVISSKGTAL
HLAASSWKSGIMKILLEHNADPNKVSADSETPLAATLIASDGLNEPAVLKCIKLLVKAGA
NLNRAIPDTPLVIATNKDFVECVEYLLEAGANANIPTNNGGKTPIEIAAKSGRRKLVEIL
FPCTLPIKGVSNWTVEGIITHVKSKKSKKKACAQDKESGTDKKAQLKSLGASAVQGKDYV
GASKFYSEAIQLDPTDATLHSNRSFCYLKSGEAREALVDAKTCIGLKPDWPKGYYRKGAA
LMSLKEYKEACDAFMDGVKLDPASGEMHEAFWEAAAALKKKHLAGKTVSSFD*
>11686.m04392|LOC_Os12g43950.1|genepair1502-1
MATYYSSPGSERDSQTMYSRDPGSASYPMSSALGNLLYLNNPSSGPYTEFSGILQPQQNC
MEMPGPGHASAMSQDPSSRESDMLSSHQGQRSFSHVKDMKNEMLMHMMDGAQGSGSELIH
DDAHTGSQLEFGVLNNHNSSSVPSMQSQGLSLSLNTQIMAPSLPYWSIKPDMLTPQSYHD
NLRGEDMRMKNLQSEASRAIRNSRYLKAAQELLDEVVSVWKSIKQKAQKEKVESGKADGK
ETDGGPKSEGVSSNPQESGANAAPELSTAEKQELQNKMAKLMAMLDEVDRKYKHYYHQMQ
TVVSSFDVVAGPGSAKPYTAVALQTISRHFRCLKDAINDQINVIRKKLGEEENSSGKEGK
LTRLRYIDQQLRQQRAFQQYGMIPQNAWRPQRGLPENSVTILRAWLFEHFLHPYPKDSEK
LMLARQTGLTRSQISNWFINARVRLWKPMIEDMYKEEIGDLEQDSNSSSDNAPRSKDKMA
SSEDKEDLKNSRARICETSQLSESRTSIGAMNVGGAPVGFQNEPNPDDSFMNLMLKDQRS
NEVDGGLLLHNTVAQHSDENARFMAYHLAELGRYGNGNVSLTLGLQHSSSNLVPNAQPGF
PGVNEDDIYNATAPLGVTVASSDYDSMNQMDQRQRFEHSPLLHDFVA*
>11669.m04781|LOC_Os03g47740.1|genepair1502-2
MATYYSSPGNERDSQAMYPADSGNSSYPVPSAIGNMLYPGNGSSGPYTEFSGIIQHQQNF
MELPGHPTAISQDSSSREPNMVASYMDQRSFGPAKDMRNEMLMHLMDGAHNAGADLIHND
THSSAQIEFGLLNNHNSMSVAPAPGQGLSLSLNTHILAPSYPYWSAKTELLTPHSYHGDD
NRMKNMQSEASQAIRNSKYLKAAQELLDEVVSVWKSIKQKAQKDQAEAGKSDNKEAEGGS
KGEGVSSNPQESTANAAPEISAAEKQELQNKMAKLMAMLDEVDRKYKHYYHQMQIVVSSF
DMVAGSGAAKPYTAVALQTISKHFRCLKDAINDQINVIRKKLGEEESSSGKEGKLTRLRY
IDQQLRQQRAFQQYGLLQQNAWRPQRGLPENSVSILRAWLFEHFLHPYPKDSEKLMLARQ
TGLTRSQISNWFINARVRLWKPMIEDMYKEEIGEADLDSNSSSDNVPRSKDKIATSEDKE
DLKSSMSQTYQPSQLGESKANIGMMSLGGAPAGFHNEGNQDDSFMNLMLKDQRPGEAEGS
LLHDAVAHHSDENARFMAYHLSGLGRYGNSNVSLTLGLQHPDNRLSVQNTHQPGFAGAGE
EIYNSTASLGVAAASSSDYESTNQIDQRQRSSCRIEAAVWLPQAQGLGSCIFFVRVNAPC
CCHVGPGTMASLWVR*
>11686.m04398|LOC_Os12g44000.1|genepair1503-1
MTSSSSPSRKALSKIACNRLQKELAEWQVSPPSGFKYRVSDNLQRWVIEVTGAAGTLYAG
ETYQLQVDFPEHYPMEAPQVIFLNPAPMHPHIYSNGHICLDILYDSWSPAMTVSSVCISI
LSMLSSSPAKQRPQDNDRYVRNCRNGRSPKETRWWFHDDKV*
>11669.m04785|LOC_Os03g47770.1|genepair1503-2
MTSSSSPSRKVLSKIACNRLQKELAEWQVNPPSGFKHKVTDNLQRWVIEVAGAAGTLYAG
ETYQLQVDFPEHYPMEAPQVIFLHPAPMHPHIYSNGHICLDILYDSWSPAMTVSSVCISI
LSMLSSSPAKQRPADNDRYVRNCRNGRSPKETRWWFHDDKV*
>11686.m04405|LOC_Os12g44070.1|genepair1504-1
MPMAAAAAFAAHVLRGRWFMAYGSFLIMSAAGATYIFAIYSKDIKSTLGYTQEQLNTVGF
FKDVGANVGIHAGLIAEVTSPWFILAIGAAMNLGGYLMLYLSVTGRVGAKTPLWLVCLYI
AVGANSQAFANTGALVTCVKNFPESRGVILGLLKGFVGLSGAIFTQLYLAFYGGGNTKPL
ILLVGWLPAAVSLAFLGTIRIIRTPRSPAAARREYRAFCGFLYVSLALAAYLMVAIILQK
RLRFTRAEYGVSAAVVFAMLLLPFTIVVREEAALFKNKSPEEEEADDVPRALSVVTAPAK
PAAQPSPESQRPTTATARILQALRPPPRGEDYTILQALVSVDMVLLFTATVFGVGGTLTA
IDNMGQIGESLGYPQRSVATFVSLISIWNYLGRVAAGFASEALLARHRLPRPLILAVVLL

LTAPGHLLIAFGVPGSLYAASVVVGFCFGAAQPLILASVSELFGFKYYSTLYNFCGTASP
VGSYILNVRVAGRMYDREAARQGHGVAAAAGKKALTCIGVRCYRESFLVMTAVTVAAAAV
AAVLAWRTRVFYAGDIYAKFKDGKTELGVDSNGSGTAKE*
>11669.m04789|LOC_Os03g47810.1|genepair1504-2
MAFGVSGPGSGDGVAAAARFGAHVVRGRWFMFFASILIMAAAGGTYIFGIYSKAIKTSLG
YDQQTLNTLSFFKDVGANVGVLPGLINEVTPPSVVLAAGAAMNLAGYLMIYLAVSGRTPR
PPVWLMCLYIAVGANSQSFANTGALVTAVKNFPEDRGVVLGLLKGFVGLSGAIFTQLYRA
IYGADDDGASLVLLMAWLPAAISLLFIPTIRIMPRDAAAAGADARRRRERKAFFYFLYAS
IVLAVYLLVMNVVELEVVGFPKPAYYVTATVLLLLIFFPLVIVVKQELNTYLQPPPPPTT
TSSTVDEKKEHDGGGGEDDKPVACMQDVFRPPARGEDYTILQALFSVDMAVLFVATICGI
GGTLTAIDNMGQIGQSLGYPQRSISTFVSLVSIWNYAGRVAAGFGSEYVLAAYKLPRPLA
LTAVLLLATAGHLLIALGVGNGLYAASVILGFCFGAQWPLLFAIISEVFGLKYYSTLYNF
GAVASPVGSYILNVRVTGHLYDREAERQLAAAGGGAAARRGSRDLTCAGVRCFRVSFLII
AAVTLLGAAVSLLLAWRTRKFYRGDLYGKFREVAMAGGEEGGARQVKVDDEASGSSGGGG
NGTTKV*
>11686.m00038|LOC_Os12g01370.1|genepair1505-1
MAASATQEADCKASEDARLFFDAAKPPPFRIGDVRAAIPAHCWRKTPLRSLSYVARDLLI
VAALFAAAATRIDVSVAWAAWPLYWAAQGTMFWALFVLGHDCGHGSFSDSAMLNNVVGHL
LHSFILVPYHGWRISHRTHHQNHGHIEKDESWHPITEKLYRKLETRTKKLRFTLPFPLLA
FPVYLWYRSPGKTGSHFLPSSDLFSPKEKSDVIVSTTCWCIMISLLVALACVFGSVPVLM
LYGVPYLVFVMWLDLVTYLHHHGHNDLPWYRGEEWSYLRGGLTTVDRDYGWINNIHHDIG
THVIHHLFPQIPHYHLVEATKAARPVLGRYYREPEKSGPLPLHLFGVLLRSLRVDHFVSD
VGDVVYYQTDHSLNGTDWAEDAKHK*
>11669.m01827|LOC_Os03g18070.1|genepair1505-2
MARLVLSECCGLTPLRLRGRGAIALPAPPSLAAGPRRPVSAAAAGGAIHREWALRVSAPT
RLTSVVEEDNRGEEVVEEEARGSLAAAEAAAGEVGGDGDGFDPGAPPPFGLAEIRAAIPK
HCWVKDPWRSMSYVLRDVVVVLGLAAAARVDSWLVWPLYWAAQGTMFWALFVLGHDCGH
GSFSSNAKLNSVVGHILHSSILVPYHGWRISHRTHHQNHGHVEKDESWQPLSERLYNSLD
YMTKKLRFTMPFPMLAFPLYLFARSPGKKGSHFNPSSDLFQPNEKKDVITSTASWLAMVG
ILAGLTFVMGPLKMLKLYAVPYVIFVMWLDFVTYLHHHGHEDKLPWYRGKEWSYLRGGLT
TLDRDYGWINNVHHDIGTHVIHHLFPQIPHYHLVEATEAAKPVLGKYYKEPEKSAPLPFH
LLVGHWAMGILLSLSVKLPPLSSRFEAYHPHSDEDADMKYDVDRMIKPLMNAFVWCMHMR
DTSIVGIDGADNRTLQHVRSSGDGAESVIPLIHALLAYFIHLALFRMQQPDTLLVIDDAS
LSIID*
>11686.m00039|LOC_Os12g01380.1|genepair1506-1
MSKPQQQSPPSTTTTSPPPPPPSTPPPASSSRSLLSALRRSPVTTLVAAFFLLALFMYGE
DVRTLAELSIDDYLYPDADFYNVSALPPLLLPPPTCDLSRGRWVFDNTSLPAYREKECTF
LTKQVSCLANGRPDDLWQYWRWQPNNCSLPTFDARRFMEKMRGKRMMFVGDSLNRNQWES
LVCLVQPILSKGRKKIVKRGSFNIFYAKEYRATLEFYWAPFLVESNSDNPNFHHIDQRII
SPERIESHANNWKDVDYLIFNTYIWWMNNEDIKVRRPNSTSWSDHDEVPRIETYGRVFKT
WSTWLEQNVDPARTSVFFMTISPLHNSPAQWGNPNGIKCVKETLPVLNYTKPLDLNHDMR
MYDLVAKVAKNMKNVPVSLIDITRMSDYRKDAHTSLYSIRQGKLLTPEQKADPQKYADCI
HWCLPGVPDVWNQILYTRILSKSSPPSTHPSLPPQ*
>11669.m01835|LOC_Os03g18140.1|genepair1506-2
MQQRRKSVFASAPFAMKQAALGAGVAARRNGAPLSLAAVVFALFVFATFLYNEDIKSIAD
FPFGAGALRAKSPDLHVLQETVGAAHLAAGSIAKRGEEVIVRVLDAPASTAMAAAAGSSS
NNSTIEVAKANANANANAADAGVKVDEGQERERDVTLPSVKEGGADEARRREDEEAAEKE
SSAKAAAATAALRTVVSVPDTCDLYRGNWVYDEVNAPVYKESQCEFLTEQVTCMRNGRRD
DSYQKWRWQPTDCDLPRFDARLLLERLRNKRLMFVGDSLNRNQWESMVCLVQSVIPKGKK
TLTKFVNGGNSNIFYAHEYNATVEFYWAPFLVESNSDNPQVHSVPDRVIQWHSIAKHAHN
WLGVDYLIFNTYIWWLNTLDMKVLKGSFDQGATEYVEVDRPVAYKEVLKTWAKWVDRNID
PNRTTVFFMSMSPNHITPEAWGNYGGIKCAMETLPITNRTTSLDVGTDWRLYAGAQEVLQ
TFRRVPVHLVDITALSELRKDAHTSVHTLRQGKLLTPEQQSDPKTYADCIHWCLPGLPDT
WNQFLYARIASAPWSSDQ*
>11686.m00052|LOC_Os12g01510.1|genepair1507-1
MEPWAIRVAYFIVFSFLVTARFGSCAPHSEEGRALPGYRESEQDQSIGSLSNWGEGKLIG
RVFNLLLKENMFASETPSESKEHSSISESVPHDSAGFEPCRKCLAKTVHNATPRRLLQAR
ELASNQTQTHPKSQSSPVQSSASHLVPRWAIYALPVAGVLFIAAVATAIYVFFSRRKKDN
TVMPWATGLSGQLKKAFVTGVPSLERTELEAACEGFINVIGTLPECTLYKGTLSSGVEIA
VLSTSVNSSQQWSAQSEEQFRNKISVLSRVNHKNFMNLIGYCACEEPFTRMMVFEYAPCG
SLFEHLHIREAEHLDWKTRLRIIMGVAYCLEHMSQLDPPPLLPTNLSSSSIYLTEDNAAK
IADIEFWKDDINKQDDQESVVYKFGILVLEVISGRRPFSEDDRLLVLWASSYLDGKRPLS
AMADRTLVRSSSAAPEKDVAALCDVVRQCVRRPEAGKRAISMGEVARLVRGIAGLSPEQA
APREKPLWWAELEIASSETA*
>11669.m01859|LOC_Os03g18370.1|genepair1507-2
MELPCPRLLLLLRLLVFLVAVSWPLCGAGTEGLGVGEEGLSSIGASSGSRNVRRLLQTGG
VNQGAPAPLPLEQSPSASGPVSSPSPSPWVSPPKGSPSPSPSSKIIAHRSPHHPFTTPPQ
LVRPKPTTRRAEHDHSVETTGRSWFKRSWTTYGFIAAGIAALLIISAAGAFYCRAKKMGT
VRPWATGLSGQLQKAFVTGVPALKRSELETACEDFSNIIGCTSTCTLYKGTLSSGVEIAV
ASSLVTSADDWSKECESRYRRKITSLSKVSHKNFMNLLGYCEEEQPFTRVMVFEYAPNGT
LFEYLHVREAEKLDWMTRLRISMGIAYCLEHMHQLKPPVVPRNFDSTTIYLTDDFAAKVS
DLEFWSGAKEPNPATSNSSSSSDLENTVRKYGMVLLEMLTGRVPDSEEDGPLERLASRYF
DGETRLAELIDPSIGSFSEEAARSLCEVVRSCIDPDPKRRPTMAEVAARMREITALGPDG
ATPKVSPLWWAELEIMSSES*

>11686.m00054|LOC_Os12g01520.1|genepair1508-1
MAEVVEARTIGGLSPAAFSLVLAVTVAVGLLGGALHYMLTRKREDLAGEVVHDLHKKTRA
DDEPADDNHTMTTGRAPEIDEDLHSRQLAVYGRETMKRLFASNVLVSGLNGLGAEIAKNL
VLAGVKSVTLHDDDNVELWDLSSNFFLTEKDVGQNRAQTCVQKLQELNNAVIISTITGDL
TKEQLSNFQLPQIPLLLDIWNSIKAVVFTDISLEKAVEFDSYCHNHQPPIAFIKSEIRGL
FGSVFCDFGPEFTVLDVDGEEPHTGIVASISNDNPALVSCVDDERLEFQDGDLVVFSEVH
GMSELNDGKPRKIKNARPYSFTLEEDTTSYGTYVRGGIVTQVKPPKFFYFDSVESLPVEP
LEPAELKPENTRYDAQISVFGSNLQKKLEQAKIFMVGSGALGCEFLKNLALMGISCNQNG
KLTVTDDDVIEKSNLSRQFLFRDWNIGQPKSTVAATAAMAINPKLHVEALQNRASPETEN
VFNDAFWESLDAVVNALDNVTARMYIDSRCVYFQKPLLESGTLGAKCNTQMVIPHLTENY
GASRDPPEKQAPMCTVHSFPHNIDHCLTWARSEFEGLLEKTPTEVNAFLSNPGGYATVAR
TAGDAQARDQLERVIECLEREKCETFQDCITWARLKFEDYFSNRVKQLTYTFPEDAMTSS
GAPFWSAPKRFPRPLEFLTSDPSQLNFILAAAILRAETFGIPIPDWVKNPAKMAEAVDKV
IVPDFQPKQGVKIVTDEKATSLSSASVDDAAVIEELIAKLEAISKTLQPGFQMKPIQFEK
DDDTNYHMDVIAGFANMRARNYSIPEVDKLKAKFIAGRIIPAIATSTAMATGLVCLELYK
VLGGGHKVEDYRNTFANLAIPLFSMAEPVPPKTIKHQDMAWTVWDRWTITGNITLRELLD
WLKEKGLNAYSISCGTSLLYNSMFPRHKERLDKKVVDVAREVAKVEVPPYRRHLDVVVAC
EDDDDNDVDIPLVSIYFR*
>11669.m01860|LOC_Os03g18380.1|genepair1508-2
MLPTKRANGAEAESSSDAPAKKARVGASASEAEAMVAGEAGGGGGGVSGNGSEVAEIDED
LHSRQLAVYGRETMRRLFASNVLVSGLNGLGAEIAKNLALAGVKSITLHDMGNVEMWDLS
GNFFLSEDDIGKNRAVACTAKLQELNNAVLISTLTEDLTNEHLSKFQAVVFTDISLDKAF
EFDDYCRNHQPSISFIKAEVCGLFGSVFCDFGPKFTVLDVDGEEPHTGIIASISNDNPAM
ISCVDDERLEFQDGDLVVFSEVQGMTELNDGKPRKIINARPYSFCIQEDTSKFGIYAKGG
IVTQVKEPINLEFKSLRDSIREPGNFLLSDFSKFDRPPLLHFAFLALDKFRKEFGRFPGA
GCDQDAQRFIEFVASVNEATIDYKMDELDGKLLRNFASGSRAVLNPMAAMFGGIVGQEVV
KACSGKFHPQYQFFYFDSAESLPTYPLDSKDLKPLNSRYDAQISVFGSKLQKKMRDANVF
VVGSGALGCEFLKNLALMGVSCGLKGKLTITDDDIIEKSNLSRQFLFRDWNIGQAKSTVA
AAAASAINSSLHINALQNRACPETEHVFHDKFWEGLDVIINALDNVNARMYMDMRCLYFQ
KPLLESGTLGPKCNTQMVIPHLTENYGASRDPPEKQAPMCTVHSFPHNIDHCLTWARSEF
EGLLEKTPNEVNSFISNPAQYAAAMRKAGDAQARELLERVCECLDKERCDGFEDCIAWAR
LKFEDYFANRVKQLTFTFPEDAVTSTGAFFWSAPKRFPRPLQFSTVNSSHIHFILAASIL
RAVSFGISIPDWAKNTSNLVDAVSKVVVPEFEPKSGVKIETDEKASNISSASVDDASVIE
DLLTKLEASAKKLPPGFQMKAIQFEKDDDTNFHMDLIAGLANMRARNYGIQEVDKLKAKF
IAGRIIPAIATSTAMATGLVCLELYKVLAGGHPVEDYRNSFANLAIPMFSMAEPLPPKVI
KHQDMRWTIWDRWSIEGNITVAELLKWLSDKGLSAYSVSCGTSLLYNTMFPRHKDRVNKK
LVDVAKEVAKVDVPAYRRHLDVVVACEDDDGNDVDIPLISIYFR*
>11686.m00076|LOC_Os12g01700.1|genepair1509-1
MLRPFPATAHHRCTLLLLLTVTLLPSLAAAAAAHHHHLHDHAAGDGVVISQADYQGLQAI
KHDLTDPYAFLRSWNDTGLGACSGAWVGIKCVQGKVVAITLPWRGLAGTLSERIGQLTQL
RRLSLHDNAISGPIPTSLGFLPDLRGVYLFNNRFSGAVPASIGNCVALQAFDASNNLLTG
AIPPSLANSTKLMRLNLSHNTISGDIPSELAASPSLVFLSLSHNKLSGHIPDTFAGSRAP
SSSSLKESITGTYNLAVLELSHNSLDGQIPQSLAGLQKLQVMDLSGNRLNGTIPDRLGSL
ADLKTLDLSGNALTGEIPASLSNLTTTLQAFNVSNNNLSGQVPASLAQKFGPSAFAGNIQ
LCGYSVSVPCPASPSPAPSAPASPVQGVETTGRHRKFTTKELALIIAGIVVGILLLLALC
CLLLCFLTKKRSGSGGKQTTSSKAAGGGAGGAAGGGRGEKPGSGAAEVESGGEVGGKLVH
FDGPMAFTADDLLCATAEIMGKSTYGTVYKATLEDGSLVAVKRLREKITKGHKDFESEAA
VLGKIRHPNLLPLRAYYLGPKGEKLLVLDFMPNGSLSQFLHARAPNTPISWETRMTIAKG
TARGLAFLHDDMTIVHGNLTASNVLLDDHSNPKIADFGLSRLMTTAANSNVLAAAGALGY
RAPELSKLKKASAKTDVYSLGVIILELLTGKSPAETTNGMDLPQWVASIVKEEWTSEVFD
LELMRDGDNGPAGDELVDTLKLALHCVDQSPSVRPDAREVLRQLEQIRPGPEGGAGPSDE
GGAGHVAAASAGNE*
>11669.m01889|LOC_Os03g18630.1|genepair1509-2
MAAVPAGGQPSDGVVIAQADLQGLQAIRQALVDPRGFLRGWNGTGLDACSGGWAGIKCAQ
GKVVAIQLPFKGLAGALSDKVGQLTALRKLSLHDNALGGQLPASLGFLPELRGVYLFNNR
FAGAVPPQLGGCALLQTLDLSGNFLSGAVPASLANATRLLRLNLAYNNLTGAVPSSLTSL
PFLVSLQLSSNNLSGEVPPTIGNLRMLHELSLSYNLISGSIPDGIGSLSGLHSLDLSNNL
LSGSLPASLCNLTSLVELKLDGNDIGGHIPDAIDGLKNLTKLSLRRNVLDGEIPATVGNI
SALSLLDVSENNLTGGIPESLSGLNNLTSFNVSYNNLSGPVPVALSSKFNASSFAGNIQL
CGYNGSAICTSISSPATMASPPVPLSQRPTRKLNKRELIFAVGGICLLFLLLFCCVLLFW
RKDKQESESPKKGAKDATAKAAAGKSGGGGGGSGGAGGDGGGKLVHFDGPLSFTADDLLC
ATAEILGKSTYGTVYKATMENGTFVAVKRLREKIAKNQKEFEAEVNALGKLRHPNLLALR
AYYLGPKGEKLLVFDFMTKGNLTSFLHARAPDSPVDWPTRMNIAMGVARGLHHLHAEASI
VHGNLTSNNILLDEGNDARIADCGLSRLMNATANSNVIAAAGALGYRAPELSKLKKANAK
TDIYSLGMIMLELLTGKSPGDTTNGLDLPQWVASVVEEEWTNEVFDLELMKDAAAAGSET
GEELVKTLKLALHCVDPSPAARPEAQQVLRQLEQIKPSVAVSASSSFTGEPSQTTATATT
ITDDTKSTITE*
>11686.m00079|LOC_Os12g01730.1|genepair1510-1
MGSRGCSCWLLSLALLCSLAAAKEQYHEFVIRETTVKRLCKSQSIMTVNGQFPGPTLEIK
EGDSLIINLINRGRYNVTLHWHGVRQMRTGWSDGPEYVTQCPVRPGQSYRYRFTVAAQEG
TLWWHAHSSWLRATVYGALLIRPRDGTSYPFHVQPTRELAPILLGEWWDMNPVDVVRAAT
RTGAAPNISDALTVNAQPGDLYSCSSHDTAFFPVTSGETNLLRFINAALNTELFVSLAGH
NMTVVAADASYTKPYTTSLLLLAPGQTTDVLVTFDQPPGRYYLAARAYASAQGVPFDNTT
TTAIFDYGAANNASSAAIAMPTLPAYNDTTAATAFTTNLRGLRKAELPSRVDESLFFTVG

VGLFNCTNATAQQCGGPNGTRFAASINNVSFVLPSSTSILQAHHHGAPGGVFTADFPASP
PVQFDYTAQNVSRALWQPVPGTKVYKLKYGSAVQVVLQGTNIFAGENHPIHLHGYDFYIL
AEGLGNFDAGADTAKFNMEDPPMRNTVGVPVNGWAVIRFVADNPGVWLMHCHLDVHITWG
LAMAFLVDDGVGELQSLEAPPPDLPLC*
>11669.m01890|LOC_Os03g18640.1|genepair1510-2
MRLCFFSAAALFLLCFLVPAAVAEERFYEFVVQETLVKRLCNTQKIITVNGQFPGPTIEV
YDGDTVAIRAVNMARYNVTLHWHGLRQLRNGWADGPEFVTQCPIRPGGSYTYRFAIQGQE
GTLWWHAHSSWLRATVHGALLIRPRPGVPYPFPKPHSEFPIILAEWWRRDPIAVLRQSMI
TGAPPNVSDAILINGQPGDFLECSAQVPDLVKPTKATFGLEKSIPTVVYSIGRRDERCIV
LFRCLQRDKMKRQRFSRLLTMHAVAVTETSIIPVAAGETTLLRIINAAMNTELFVSLAGH
KMTVVAADAMYTKPFETTVVLLGPGQTTDVLVTAHAAPGRYYLAARAYASAQGVPFDNTT
ATAIFQYKGGAGCPTTAGGAGAAGAVAGAGVGAGAAGGAGAVAGTGAGAGTFNGSLGRSK
YSGGNPGRAGPAPMLPYLPAYNDTNTATAFSNSIRSPAPVKVPGPVTQEVFTTVGFGLFN
CMPGPFCQGPNNTRFGASMNNVSFQLPNTVSLLQAHYHHIPGVFTDDFPPMPPVFFDFTS
QNVPRALWQPVKGTKLYRVRYGAVVQIVFQDTGIFAAEEHPMHIHGYHFYVLATGFGNYD
PVRDAHKFNLVDPPSRNTIGVPVGGWAVVRFVADNPGVWLVHCHIDAHLTGGLGMALLVE
DGEAELEATMAPPLDLPLCAL*
>11686.m00114|LOC_Os12g02060.1|genepair1511-1
MASPKPFACSAIALLFAANLVSAQLSANFYDKSCPNALPTIRIAGCDGSVLLDDTPTFTG
EKTAAPNNNSLRGFDVIDNIKAHIEGICPQVVSCADILAVAARESVVALGGPTWVVQLGR
RDSTTASLDTANNDIPAPTFDLGDLTKSFSNKGLSATDMIALSGAHTIGQARCVNFRNRI
YSETNIDTSLATSLKSNCPNTTGDNNISPLDASTPYAFDNFYYKNLLNKKGVLHSDQQLF
NGGSADSQTTTYSSNMATFFTDFSAAMVKMGNINPITGSSGQIRKNCRKVN*
>11669.m02258|LOC_Os03g22010.1|genepair1511-2
MGYSYSSAAVAVSVLVVALAAAASGQLSTTFYASSCPTALSTIRSAVNAAGCDASILLAD
NATFRGEQGAFPNVNSLRGFEVISSIKMQLEASCRQTVSCADILAVAARDSVVALGGPSY
PVELGRRDGMTTNQTMANTNLHPPTTDLGNFVTSFAGKGLSPTDLVVLTEISNLRKNQSW
KNKSAVVQQQEAATFFLGDGGQDLFTECDQVTNSGAHTVGVAQCTNFRSRLYGESNINAP
FAASLRASCPQAGGDTNLAPLDSTPNAFDNAFFTDLIAGRGLLHSDQELYRGDGSGTDAL
VRVYAANPARFNADFAAAMVRMGAIRPLTGTQGEIRLNCSRVN*
>11686.m00135|LOC_Os12g02260.1|genepair1512-1
MLIKAWCYYESRILGAHHGLISTYALEILVLYIFHLFHGTLDGPLAVLYRFLDYYSKFDW
DNKGISLYGPISLSSLPELVTDSPDTVNDDFTMREDFLKECAQWFTVLPRNSEKNTQVFP
RKFFNIVDPLKQSNNLGRSVSKGNFLRIRSAFDFGARKLGKILQVPDNFTVDEVNQFFRN
TLKRHCSRVRPDVQEIALDFNGERADNDSSPLYSNNSFGDLSDEFNNISISDSSNHGSLR
QNGWNYVAENKERKSVSGGWLASKATNPAATNSTGMTNGSDSCEPASPSITGAHSLPSEE
GHDALDLFNESESGTKAGIKYDTNPSHHGMSTVSYAGRSHQSFEEVDNDDRGTIDSNWSD
LTGDYTTNFNNLLYAQGFHQDYPMNQYYPFGPVYYQMPSPPPARYQNRRSSNGHSRNNVY
GYAGTNGIGPAPCPPGYLIMRTYSQIDDSHRARGTGTYFPNPNLSKDRSPSGRGGRGKTH
FLSHNHQRPHHYGRADMSADLTPSEELRHIYDPDANDLGIPSSLHISIPSPSSESPREIV
HGNGFIQPPAKKLEFGTLGALPLEVTSQELGINRLNTASDSQPSASASPMSLAHNPGISS
NQMRNAQPYHLKDNGDFPPLSS*
>11669.m02242|LOC_Os03g21850.1|genepair1512-2
MARAAPGAAAIPAGAVARAEEAAGEVVRRVRPTEASERRRAAVVGYARRLVGTALGCEVF
AYGSVPLKTYLPDGDVDLTVLGNTSYGSTLIDDIYHILQSEEQNCDAEFEVKDLQLINAE
VRLIKCTIENIVVDISFNQTGGICALCFLELVDRKVGKNHLVKNSIILIKAWCYYESRLL
GAHHGLISTYALETLILYIFNLFHKSLHGPLEVLYRFLEYFSKFDWDNYCISLNGPVALS
SLPNQIVEATNTPGSDLLFDKEFLNNSVQKTDSNACNTEFRSKYLNIIDPLKEHNNLGRS
VNKASFNRIRTAFSYGAQKLGQVLLLQPELIPDEIYGFFKNTLNRIGSGVRPDIGDESYN
DAFRCESFLGPGKALWDEMSSMKISCNNQDENRGPHHLSKCLVNNDSYATLNVPTHFHGD
HMVASSTDLSLKSSCFIQETPNQYPLFYLEDGNGSSEQYLDHEMVEQASCCTAETCHANE
EPSMHPQVYPNNTLHTFYSSLANNLEYSKSGQSDMTNSSINVAHEEKQKFSPSPLSLVDL
SGDLDLQLRCLRQVQYHLEYMFDGFLQSVQEASSDCKVARDSFEIPAVNITSNSDVVLPG
LLSPSSTETDERRLSPVSSSHSTEDSSQQSHDESNWDNSVQLYDSSDDISNMHETDQHIL
QKHMVSLGQNKTLINRQVRVKSNQASVPKGKFSICKEQITQDTATKDIKLSRHLRVKDSE
HEYISTAKKISSYNCDTCLECVKPESEAMIPRHYKHARSSKNSFEHRIYDIDMGFARSGS
SRNQMPKYQSLKNQDMSSLNVQKEHEINWPRKQMPSELLKLQNSLRGRACSNKKLAAKQI
NNNHKEHLSFVRDPEQMPYNQVNSNKEFETVGKSSQLLPRVQLSLHNDRSLTASTCQSSF
PVTKGSTQFNDLEMPSLENIEFGTLGSFSLTLVSPKSNKNPNTHSTSHQDSTKLEMKITS
HLSVLGLAETFGTYTDDTVDHRFRELKDCSLPRSNLEQWPQYHDKMDCVAVQITTGSAPL
CFSSEFWNLNTCTQQSSLLTLHHLTPRTSLSDFSFRQELTHCAFLIKRQFILCSSERLSF
STSFFGPIGSCRKAMPVPFASLFW*
>11686.m00150|LOC_Os12g02390.1|genepair1513-1
MGLRSPNSMLWLVLLVWAALLCGSCHGRFVVEKNSLKVTSPSDMKGTYECAIGNFGVPQY
GGTMVGVVAYPKANKKACKSFDDFDISYKAKPGSLPTFLLVDRGDCFFTKKAWNAQNAGA
AAILVADDKTEPLITMDTPEESGNTDYLENITIPSALITKSFGDKLKKAIDNGDMVNVNL
DWRESLPHPDERVEYEFWTNSNDECGPKCDSQIDFVKSFKGAAQVLEKKGYTQFTPHYIT
WYCPDSFILSKQCKSQCINHGRYCAPDPEQDFSKGYDGKDVVVQNLRQVCVYKVAKEHGK
PWLWWDYVTDFAIRCPMKEKKYTKECADGVIKSLGLDHKAIDKCIADPDADKENPVLKAE
QDAQIGKGSRGDVTILPTLVINNRQYRGKLDKGAVLKAICAGFRETTEPAVCLSEDIQTN
ECLENNGGCWQDKAANISACKDTFRGRVCECPVVKGVKFVGDGYTHCEASGSGHCEINNG
GCWKDSRHGRTYSACTNDGCKCPDGFKGDGVHKCEDIDECKERTACQCKECKCKNTWGSY
ECGCSGGLLYMKEHDTCISKNAATEVGWNFLWVIFFGLVVAGIAGYAVYKYRIRKNQAFH
TCWWKRMPFTSINWLRDRLFTSIRRRRRRALAPLAPTAIAMPSCFTLDSASDDGRSTAQG

QGWCAGVGGFLSSFFSAGAARADGGKPSPDWDAHGLAASALPVPLSRLDGKKRYKVSELT
FLDRRTRAAAEDPLFDALRPGGVYTRAQLRDELDALAASGMFDHVTFQTKPKPDGTLGPH
RLLRRDPVARGRTPQLEPDDDMTARERMEHLRRQEREYRQLVRRAKPCVLPEKLQRELQG
MVKKQRKVSSGLLKRMAGRIERWYHDEGFQCAQVVGYNGNLDAGEVLRPGHIYNNGAGKQ
ALKNIDSLGLFSTVEVEPRPDETNQGGVIVAIKLKEHDPKSAQVITDWSIVSGSQGTPTL
ASIQPGGTVSFEHHNICGLKRSLIGSVTTSNLLNPEDDLSFKLEYAHPYLDGVDNRSRNR
TFKISCFNSRKLSPIFVAGPNMYEAPPIWVDRIGFKANITESFTKQSKFTYGLVVEEITT
RDENNNICTHGSRQLPSGALSMIGPPTTLSGTGVDRMAFLQANITRDNTEFVNGATIGDR
CIFQYSEQSFLVGYRWIKASVLEARTLFNRHQLTVTKFINLNKQEKGPRKPPPAVLALHG
RYAGCVGDLPSYDAFALGGPHSVRGYGMGELGASRNLLEIATELSVPITVKNRHTQVYAF
AEHGTDLGSSKDVKGNPTEFFRRVGHGSSYGVGVKLGAVRAEYAVDHNAGTGAFFLRFGE
RF*
>11669.m02229|LOC_Os03g21720.1|genepair1513-2
MRGPVRWAVVVVVMAMVEAAAGRFVVEKNSLRVTSPEGIKGKYECAIGNFGVPQYGGTLH
GWVEYPKSNQKACKSFEDFDISFKSTRSGGRPKFVLIDRGQCYFTTKAWNAQNAGAAAVL
VVDDKSEPLITMDNPDDAGTEHLENITIPSVLITKKLGDDLKKSAENGDMVSVLLDWRES
LPHPDERVEYEFWTNSNDECGPKCDMQMDFVKSFRGTAQILEKKGYTQFTPHYITWYCPE
AFVVSKQCKSQCINHGRYCAPDPEQDFSQGYDGKDVVVQNLHQICVFKAANESGKPWLWW
DYVHDFSIRCPMKEKKYTPECAVHVIKSLGLDVEKIKKCVGDPEADEENPVLKAEQDAQI
GHDKRGDVTILPTLVINNRQYRGKLDKSAVLKAVCAGFEETTEPAICLSEDVQTNECLEN
NGGCWQDRDNNVTACKDTFRGRVCECPVVKGVKFVGDGYTNCEASGIGRCEIKNGGCWKE
TRNGKTISACSNEVSEGCKCPPGFKGDGIKSCEDIDECKEKLYCQCKGCSCENTWGSYEC
SCGGNNMLYMREHDTCLSKEATSAVGWSFLWVIFFGLVLAGVGAYAVYKYRLRSYMDSEI
RAIMAQYMPLDNQEAANQHHVAHAGDDI*
>11686.m00160|LOC_Os12g02490.1|genepair1514-1
MEAPKLRFVRCPGCLQLLVEYPTIAVYQCGGCGTVLRAKNRVAPATNTNAESGEHNEFSN
ISTGDSQNNKLICTDGQKISPSSNAQPGVVQEKITFASEEKTVSTSNSIDSNEHVNIECS
LFDGDASNHDVRTEGINDEDKVTVSNSTLDLVRKVENVETDGNEKGSFTDDGSISNEVAT
TQSMVHMDGAGFDNNFTEVQSAAEGKCTLSDANLDSQEIVAICQPDNISVGTKERVQLYE
GFHVESHEDLIEELVRSLSLSDDEEEFVDIAENSELNDALRSQMGSCRFSLGSKMNDGPR
TDPHGRLIEELEMSFSDAEEPLDQNIMVSLNDIEKPILDEVSKENHILEEDGKESHILDV
DGKESHILDVDGKQNHILDEDGKENHILNEDDKENLILDKGGEDTLDAGGANSYEERVLP
SDDGLLKSGQSFQQCELAAVNMAEKDEGHLEETNMANHAEANSGTAAVLSNLSNDKFCAI
LPPSCDGRKEEKSNIHRGRELCQGLSLDSVDFRSIQNFIESQMDGTSSSLSSGSPSHGDL
EHNRSNRFKKIDRLERLRKMDDLRDQLNRLSSQKGLENRYKNKGPGLLQEQISYRHLEQH
PCGFDGDSILDSDIIDSYYDQGNPPRYPPPDPFSPTHSHYHCGHGQPHIPYNCSAWEFNS
YYQSSYAGSTVLEHESLRSSYKEQKRAVRKSILRSLSGASPFTICNGCFNLVQVPSDIYV
SKKKIAKFQCGRCSKALMLSFPATNSEDAKLSNKEVNRKPNKPVHNSVVGMEGGYSFSAE
CSRGDPVSISEECGASISRSFSGRTRAAVAASGSGKKVSDSALHRLMGYDSASQLLRRSR
AFDDGYDSFESMVPVSNRVSRRKNL*
>11669.m02220|LOC_Os03g21640.1|genepair1514-2
MASVNTGGLRLVRCPKCYNILPEPPSVEVYKCGGCDTILRVKIRPSNGQNVATKQVRQDS
DDFSVATTASNGVHPQKKDIAFSGATMDRSRTPDAPSTDTEHASNGTSSNDNGHAMSVEN
NASEVADTDNKEDCNLDGQNTSGRIEGPSEEIPPNANGMDIDSDREETYNVEGIAENSED
CRVRGGGDIDTECNLSLPEHELPLHQESKSDSELKEATKTEDEATKKGHLVRVQSRSCDL
RESHRASAGSSMDFHSARTSLQSKSFRASEPLQSKIMKTVDELRGDLSEFFSKPEEEGED
DDDRKPKTAAYPPRPSKQDGYSKPRAPFTSSVPLTAYHPAAKHSGHVSRLSRSGQVPPPP
HHHRELSSLRYRRRRRAYSCCHSDQMETMRRPCSHDCCHYHSCRPPPCHHHDRPWKSQEG
AMQRPPVQETTRRRAPPRHHCRPVLRGAPFVVCSGCNRLVQLPTDFAVPSKGTRRLQCGS
CSEILSYSYRDPAKKKLQSPSGDGEECQYSTDDYEIHQAAGDADPFSYSEEYGVISYSTE
EEQQPLHVSRNSSFDTVDDERSAKLHRLMGYSSASELLRLRRSPDLYESFGERTPAARTS
DTKGKAICVADEEEHPSAKVRRGRGLPLPGILKKGIHGLESLKLR*
>11686.m00176|LOC_Os12g02640.1|genepair1515-1
MELFSSQQWLALLPPIILCILLFSYVYIILWLRPERLRQKLRSQGVRGPKPSFLFGNIPE
MRRIQQLAKSAHEQEAGSTDMFSSNYVATLFPYFLHWSRVYGSIYLYSTGSIQVLNVTDP
NMVKELANCKSLDLGKPCYLQKERGALLGMGILTSNGDLWVHQRKVIAPELFMERVKGMV
NLMMEAAMSMLNSWKNEVEDRGGSAEIVVDEFLRTFSADVISRACFGSSFSEGKEIFIKI
RQLQKAMAKQSMLIGVPGSRYLPTRSNRGIWNLDSSIRTLILNISKKYEHDSSTSVNKDL
LHSIIQGSKDGPFASCTPEDFIVDNCKNIYFAGHETTSTTAAWCLMLLASHHEWQSRARV
ESLDICQGRPLDFDILRKLKKLTMVIQETLRLYPPASFVAREALNDMKLGGIDIPKGTNI
WIPIAMAHRDPSVWGPSADKFDPDRFANGIAGACKPPHMYMPFGVGVRTCAGQNLAMVEL
KVVLSLLLSKFEFKLSPNYVHCPAFRLTIEPGKGVPLIFREL*
>11669.m02194|LOC_Os03g21400.1|genepair1515-2
MGNVVFLHVSRPDVVRDINLCVSLDLGKSSYLKATHEPLFGGGILKSNGEAWAHQRKIIA
REFFLDKVKGMVDLMVDSAQTLLKSWEEGIDKNGGTIDIKIDDDIRAYSADVISRTCFGS
SYIKGKNIFLKIRELQKAVSKPNVLAEMTGLRFFPIKRNKQAWELHKQVHKLILEIVKES
GEERNLLRAILLSASSSKVELAEAENFIVDNCKSIYFAGYESTAVTAAWCLMLLGLHPEW
QDRVREEVQELTMVIQETLRLYPAGAFVSRQALQELKFGGVHIPKGVNIYIPVSTMHLDP
NLWGPDVKEFNPERFSNAQPQLHSYLPFGAGARTCLGQGFAMAELKTLISLIISKFVLKL
SPNYEHSPTLKLIVEPEFGVDLSLTRVQGKMSMDKWFMSTSYGYVVSTKRQHILETLKKI
PFSSSGYHLKVNKSMMD*
>11686.m00208|LOC_Os12g02910.1|genepair1516-1
MGFSKHKHLDGRMKHKLLKGYSLYTVSQGFVAILDVSRKKIPGSDGILGEKSGSRSKSKN
VSTKSKEKLKGVFSLVQLKNETRKNKELQDTTSIHFIVSNVISELHTQRRYQLKDLSWRS

KDTTIDKKENQEVEHEENPKSCELEYGIYCLWSVEYKEVMKDFIVKRLKDQLFMARAHYP
SIAKLKNQETFTRELKQNIQEHERMLSDTIADADLPPFFAKKLEKMEHTIERAKSCEVGC
TSVERKLRQLLDITEDEAYFHTRQSAFLYHLGVQTMPKTHHCLNMRLTVEYFKSTSIHTV
QSNKQKLEDPTFHHYVIFSKNVLAVSTTINSTVMNSKDSGSIVFHLFTDSQNFYAMKHWF
DRNMYLEATVHVTDIEDHQKLSKDVDFHDMKLLRPAEEFRVTFRNHSQSFQKQMKTEYIS
TFGHSHFLLPDLLPSLNRVVVLDDDLIVQKDLSSLWNLNMGGKVVGAIQFCEVKLGQLKA
YTEERNFDNNSCVWLSGLNVVELKKWRDLHITSRYEQLLQKLQKDSVTSFPLKVLPISLL
VFQDLIYPLEDSWVQSGLGHDYGVSQTDIKRSVTLHYNGVMKPWLDLGIHDYKGYWRKYM
TNGERFMTECNIH*
>11669.m02177|LOC_Os03g21250.1|genepair1516-2
MKATPPPAKRRRGPRLAVLALVFCSLLVPIAFLFNRFPAVYVTDERPQQEIDLPSFGRMG
LERSGGVTVVKPEDGSGANAETKDTPEMIHRGGINSHHHSDNVPSKVSANPKVPPPPKIE
PLKPKAKSVPVPVQRTEVISGNNMKPAKVQNADDVEKAKACQLEFGSYCLWSIEHKEVMK
DTIVKRLKDQLFVARSYYPSIAKLKGKEALTRGLKQNIQEHERVLSESIVDADLPSFIKS
KIEKMDQTIGRAKACTVDCSNVDRKLRQILHMTEDEAHFHMKQSAYLYNLGVHTMPKSHH
CLNMRLTVEYFKSAPLDSDDSAVHKFNVPDHRHYVILSKNVLAASVVINSTVSNSEETEN
VVFHILTDAQNFYAMKHWFGRNSYRESAVHVINYEHIILENLPEFSSQQLYLPEEFRVFI
SNLERPSEKTRMEYLSVFSHSHFFIPEILKDLKKVIVLDDDVVVQRDLSFLWNIDMGDKV
NGAVKFCGLRMGQLRNLLGKATYDPQSCAWMSGVNVIDLEKWREHNVTENYLQLLKKFQH
NDDEASVRAAALPISLLAFEHLIYPLDERLTISGLGYDYAIKEELVRNSVSLHYNGNMKP
WLELGIPDYRKYWKRFLTRDERFMDECNVSP*
>11686.m00214|LOC_Os12g02970.1|genepair1517-1
MGAYEATKVVFARLQALEPNLAPNIIGMLLTKDNNEMDMIRLACGPDNLLQSIIAKVRTD
LTNKPSPPMASWGFPSDIGEEASFSVDKVGCDGGEEFSSKEYDWRLPIGGNHHRSFLSST
VDTLGWKPCLYSQSGVTTHLGSDDMQEYSSRPPQIDQSDLTNNCSARQIYLTFPPDSIFS
KEDVCNYFSMYGMVQDVRIPYQEKCMFGFVTFAYQKTVKLILAKGNPHYICDARVLVKPY
KEKDKVPNKFRHVNHCLIPLRIFAHILPFECYIVLGPRILYRDIASHEASFRMKQDEQQH
ATELQRCCLMRLPLLNLQDWGHHLSSPMGSHVLLGQVDNKYNINENDNPTHLEDVTFRDN
KLKNEFAMREIASTAISTAAKRTVISTEEGKREYGPKAATPNDACGFLESGMEYNLPHSP
FSSPTKASNVAATAHTSNISSSSSPHKVASSLFPPTCTLELPPTTHASFKRQGKALTDHT
*
>11669.m02165|LOC_Os03g21140.1|genepair1517-2
MDAYEATKVVFSRIQALDPDHAAKIMGLLLIQDHGDKEMIRLAFGPEALLHSVMAQARKE
LALLPPPPPPSSSSPTVPAAHSPFLLSRQNSGRGPAPSPSPLSASSPSSWAQAQPFSRSN
GSVDEVVGAGEELISPANSGGGAAANAPPFFPRGGDVLLDDFQLQEQLAFLNEGGVNPSH
PLQGFDGAECRSPGPGEGGGMFPYGLGWANGGPGHRRSASVNELCLGGGSSDGFGWKPCL
YYARGFCKNGSSCRFVHGDDAAALTGAAMDAATAEQQQCQDFLLRSKSQRLGPAAFPYSP
TGSLPGSPSAATKCLSLLLQQQHNDNQRAAAAAALMLGGSDEAHKFMGRPRLDRVDFASM
MNPGSRQIYLTFPADSTFREEDVSNYFSIYGPVHDVRIPYQQKRMFGFVTFVYPETVKLI
LAKGNPHFICDARVLVKPYKEKGKVPDKYRKHQGDFSGCTTPTGLDGRDPFDLHQLGARM
LQHSNSTNEMMLRRKLEEQQQAAELQQAIELHSRRLMDLQLLDLKNRAAAAVTTAMAMTI
PTANAFGSSQPLATTMVESPPDSGEQLKGTGYFTEERKMVNGGGDKEESAGEASLNADSD
QSLEHNLPDSPFASPTKSSVSAHQSFTTTDTGVVATSSCSASHVGISAGTNAGGGINHLR
PSTLDIPSPRDFFSVSRLASDHGAIGM*
>11686.m00216|LOC_Os12g02980.2|genepair1518-1
MAADHLVVAMLLLLALSPPAVADDTAVLGRKGGVVEGQAAGPGRYAVILDAGSTGTRVHV
FRFDNKLDLLKVGDNIELFAKVDPGLSSYAGRPQDAANSILPLLDKANTVVPARLMNKTP
LKLGATAGLRLIGDEKANQILEAVRDVVHTKSKYQYNPNWINVLEGSQEGSYIWVALNYL
LDKLGGDYSKTVGVVDLGGGSVQMAYAISSNTAATAPKVPEGKDPYVVKEYLKGKDYNIY
VHSYLHYGGFASRAHILERKDGPFSNCMLRGFSGNFTYNGKQYDATAAPQGADYHKCREE
VVKLLKVNAPCETKNCSFNGVWNGGGGAGQDDLYVASAFYYIASHVGFINSDAPSAKSTP
ATFKAVAEKVCKLSVKEAKVEYPNVRDHAYLCMDLIYEYSLLVDGFGLHPSKEITLVDKV
KHGEYYIDAAWPLGTAIEAVSPKKRLREIYK*
>11669.m02163|LOC_Os03g21120.1|genepair1518-2
MRRFSAAAGARQQQQQGEAVSDRVLRFRGVLVVVLAPVLLISLVLLLMPRAPASATVEGS
AGELVAAAGRRWGPRAVSGLGDGSTRYAVIFDAGSSGSRVHVYCFDGNLDLLPIGKEIEL
FKQKKPGLSAYAMDPQEAAKSLVSLLEEAEKVIPVELREQTPVRVGATAGLRALGTEKSE
EILQAVRDLLQDKSSFRSQPEWVTVLDGSQEGAFQWVTINYLLGNLGKPYSHTVGVVDLG
GGSVQMAYAISEKDAGKAPPVAEGEDSYVKELLLKGTTYYLYVHSYLRYGLLAARAEILK
AGEGNDYRNCMLEGHHGQYRYGDDIFEASGLSSGASYSKCRAVAVRALKVDEPACTHMKC
TFGGVWNGGGGDGQKNLFVASFFFDRAAEAGFVNPKAPFAKVKPSDFEEAARRVCKLNVK
DAQATYPDVSEENVPYLCMDLVYQYTLLVDGFGVDPYQDITLVKKVPYSNSFVEAAWPLG
SAIEVASSS*
>11686.m00222|LOC_Os12g03040.1|genepair1519-1
MPSSGGAMPALPPGFRFHPTDEELIVHYLMNQAASIKCPVPIIAEVNIYKCNPWDLPGKA
LFGENEWYFFSPRDRKYPNGARPNRAAGSGYWKATGTDKSILSTPTSDNIGVKKALVFYK
GKPPKGVKTDWIMHEYRLTGTSANNTTTTKQRRASSMTMRLDDWVLCRIHKKSNDFNSSD
QHDQEPEGSTVDEQLEDIHDNNSSSQQPPAPPDMNNQQSDFQPMTAMSMSKSCSLTDLLN
NLDCAALSQFLLDGSSDAIAELPAPPSPLIYPNQTLNYNINNNMPHAFESRLDHHDGYVN
NYNVNGLRRKRMMACSATSFDDGSSSSSSDFLHVAKKPLLLPSDSRGSGFGGGYCNQQLS
ETATGFQFQNGNMLSHPFPLNQQLLLNNHLQMQ*
>11669.m02156|LOC_Os03g21060.1|genepair1519-2
MVLSNPAMLPPGFRFHPTDEELIVHYLRNRAASSPCPVSIIADVDIYKFDPWDLPSKENY
GDREWYFFSPRDRKYPNGIRPNRAAGSGYWKATGTDKPIHSSGGAATNESVGVKKALVFY

KGRPPKGTKTNWIMHEYRLAAADAHAANTYRPMKFRNTSMRLDDWVLCRIYKKSSHASPL
AVPPLSDHEQDEPCALEENAPLYAPSSSSAASMILQGAAAGAFPSLHAAAAATQRTAMQK
IPSISDLLNEYSLSQLFDDGGAAAAAPLQEMARQPDHHHHQQQQHALFGHPVMNHFIANN
SMVQLAHLDPSSSAAASTSAGAVVEPPAVTGKRKRSSDGGEPTIQALPPAAAAAKKPNGS
CVGATFQIGSALQGSSLGLSHQMLLHSNMGMN*
>11686.m00231|LOC_Os12g03100.1|genepair1520-1
MPPRARTLLMPLAAATLLVASTIFLFAATGARWRPADTGLPVPAADFSAAVLESAVTDTT
AAAKELSFVDENGRPDDPASSSAAAARCDPTHAAVRVFMYDLPPEFHFGILGWSPPTDGA
ADAAMWPDVGSGAAAPRYPGGLNQQHSVEYWLTLDLLSSSSPPCGAAVRVADSRDADVVF
VPFFASLSYNRHSRVVPPEKVSRDKELQEKLVRYLMAQPEWKRSGGADHVIVAHHPNSLL
HARSVLFPVVFVLSDFGRYHPRVASLEKDVIAPYKHMAKTFVNDSAGFDDRPTLLYFRGA
IFRKEGGNIRQELYYMLKDEKDVYFAFGSVQDHGASKASKGMHASKFCLNIAGDTPSSNR
LFDAIVSHCVPVIISDDIELPYEDALDYSKFSIFVRSSDAVKKGYLMRLIRGVSKHQWTR
MWNRLKEVDKHFEYQYPSQKDDAVQMIWQALARKVPAIRLKSHRSRRFSRYDRGK*
>11669.m02129|LOC_Os03g20850.1|genepair1520-2
MAAAAASASASCRRRPIAWFFAIAALLFFFSWYLLLDSAAVTPEPLLAARGQGLRVGSSG
RKCDPATAALRVFMYDLPAEFHFGLLDWEPQGGGGGGGGGVWPDVRGGGVPEYPGGLNLQ
HSIEYWLTLDLLASEQGAPTPCGAVRVRHAAAADVVFVPFFASLSFNRHSKVVPPARASE
DRALQRRLLDYLAARPEWRRSGGRDHVVLAHHPNGMLDARYKLWPCVFVLCDFGRYPPSV
AGLDKDVIAPYRHVVPNFANDSAGYDDRPTLLYFQGAIYRKDGGFIRQELYYLLKDEKDV
HFSFGSVVGNGIEQATQGMRASKFCLNIAGDTPSSNRLFDSIVSHCVPIIISDEIELPFE
DVLDYSKFCIIVRGADAVKKGFLMNLINGISREDWTRMWNRLKEVERHFEYQYPSQNDDA
VQMIWKAIARKAPSIRLKVNRLRRFSRFETNRTDETPTRSSWLENQPS*
>11686.m00249|LOC_Os12g03270.1|genepair1521-1
MASKAIKRKPYTADIDRSEKQMETIIPDSVREPLLGNRTHESKSERHEPNMQPNLWDGKG
QERLGWMHIISTFIAQSVRKIGNALSQFGPLLAKFFSRSCASHGSHDEQAVLLDLSPLQE
ERLRFLRQRLNVPFDSSSVKHQDALKELWRLAYPSRQLPPLKSDLWKEMGWQNSDPATDF
RAGGFMSLENLIYFARNYPDSFHSLLHKADGKRSEWEYPFAVAGVNISYMLVQMLDLQSG
KMGTKVSSQFVQLLREDEMAFDNLFCMAFQMLDAQWLTRQASYMEFNEVLKSMRIQLEQE
LTIGSISCVQEMPSFRLLKR*
>11669.m02109|LOC_Os03g20670.1|genepair1521-2
MSMTNLRRRLHHGDVDGRKNEHVDISSVDSLNEPLLGKSSSDTGGSEVYDPRRQDLWDDD
RKKEQLHWSFLFSNLIAQWAQWLANIIVGSGSLFGRLFPFSLDNQNSSPVYLSPLQEDRL
NTLRSRLQIPFDGSRVEHQDALRQLWRLAYPNRDIPPLKSELWKEMGWQGTDPSTDFRGG
GFISLENLIFFARNYPGSFQALLNKVQGQRADWEYPFAVAGINISFMLIQMLDLQSSVPS
SKSGVRFVELLGRDENAFDHLYCIAFRLLDAQWLVKRASYMEFNEVLKSTRTQLERELVL
EDVLEVKDLPSYTMLDK*
>11686.m00270|LOC_Os12g03470.2|genepair1522-1
MGSREPFLSAIFFFFFLLLFCLGCKCIASELHLHTTQTAVLKVDASLQHARQIPDTLFGI
FFEEINHAGAGGIWAELVSNRGFEAGGPHTPSNIDPWSIIGDDSSIFVATDRTSCFSRNT
VALRMEVLCDNCPAGGVGIYNPGFWGMNIEDGKTYNLVMYVKSPETVELTVSLTSSDGSQ
NLASSTIPVSGASNWTKLEQKLVAKGTNRTSRLQITTTKKGVVWFDQVSLMSADTYKGHG
FRTELISMMLDLKPRFLRFPGGCFVEGEWLRNAFRWRESIGPWEERPGHFGDVWHYWTDD
GLGYYEFLQLSEDLGAAPIWVFNNGISHNDEVDTAAIAPFVKDVLDSLEFARGSADSTWG
SVRAAMGHPEPFPVKYVAIGNEDCGKKFYRGNYLKFYNAIREAYPDIQMISNCDASSRPL
DHPADLYDFHVYTDSKTLFSMKSAFDRSSRNGPKAFVSEYAVWRSDAGRGSLLASLAEAA
FLTGLEKNSDVVQMASYAPLFVNNNDQTWNPDAIVFNSWQQYGTPSYWMQTLFRESSGAM
FHPITITSSYSGSLAASAITWQDSENSFLRVKIINFGSDPVSLTISATGLQARVNALGST
ATVITSSNVMDENSFSNPNKVVPVKSQLSNAAEQMQVTLAPHSFSSFDLALAQSKLVAEM
*
>11669.m02084|LOC_Os03g20420.1|genepair1522-2
MGVRRGRLDSSLFHMLVLVCALSQVLFVGLVTGQTAQLSVDASSQNGRTIPDKMFGIFFE
ELNHAGAGGLWAELVSNRGFEAGGINTPSNIDPWLIIGDESNIIVETDRSSCFASNPIAL
RMEVLCGATGTNACPSGGVGIYNPGYWGMNIEKTKVYKVSMYIRSSDSVDLAVSLTSSDG
LQNLATHTITAEKGDFAGWTKVEFDLQSSERNTSSRLQLTTTKNGIIWFDQVSVMPSDTY
MGHGFRKDLATMLANLKPRFLKFPGGNYVMGNYLLNAFRWSETIGPWEERPGHFNDVWNY
WTDDGLGFFEFLQLAEDLDACPVWVINDGASLNEQIPSATIAAFVKDVVDGIEFARGDPK
TTWGSVRAAMGHPEPFPLYYISVGNQECSKPYYKEKYVKFYSAIKASYPDIKIISSCDIS
SISAVNPADLYDVHVYTSSGDMFSKTRMFDSTPRSGPKAFVSEYAVTGNDAGRGTLVAAL
AEAAFLIGLERNSDVVEMASCAPLFINDNDRGFSPDAIVFNSWQHYGCPNYWMLHFFKDS
SGATLHPLTIQVSNYDQLAASALTWQNSNDGNTYLKIKVVNFGNKAVNLNIAVAGLENGI
QEFGSIKTVLTSGWLRDENSFQQPDKVVPAASPITNAGEKMGVIVDPYSLTSFDLLLDTN
TDKYPLLESSFHSSM*
>11686.m00488|LOC_Os12g05540.1|genepair1523-1
MASVAALRLVVLLAAAVPLLPPPAASLAVTSTYVRPTARATLSVLHDGDGRTPQQVHISA
VGSDKMRVTWITDDDAPATVEYGTVSGEYPFSAAGNTTTYSYVLYHSGNIHDVVIGPLKP
STTYFYRCSNDTSRELSFRTPPASLPFKFVVVGDLGQTGWTASTLRHVAADVYDMLLLPG
DLSYADFYQPRWDTFGRLVEPLASARPWMVTEGNHEVERIPVIHPRPFTAYDARWRMPHD
AGASPSGSNLYYSFDVAGGAVHVVMLGSYAGYAAGSAQHRWLRRDLAGVDRAKTAFVVAL
VHAPWYNSNRAHRGEGDAMRAAMEELLYGARVDAVFAGHVHAYERFARVYGGGEDACGPV
HVTVGDGGNREGLATRYVDPQPAASAFREASFGHGRLEVVNATHALWTWRRNDDDEAVVA
DEVWITSLASNPACNKKYSISLY*
>11670.m03223|LOC_Os04g33530.1|genepair1523-2
MTRRADDLLVAGTLVISIVFFRCAAAVAATEYVRPPPGRVIFTEHTKPASHPQQVHVSLV

GANHMRVSWITEDKHVKSVVEYGKVSGNYTASATGEHTSYRYFLYSSGKIHHVKIGPLDP
GTVYYYRCGMAGDEFGLRTPPAALPVELAVAGDLGQTEWTASTLSHVGRSDYDVLLVPGD
LSYADAQQPLWDSFGRFVQKYASRRPWMVTEGNHEVEAAMALPGWPRPFTAYAARWRMPY
EESGSGTSLYYSFDAAGGAVHVVMLGSYADFNSSSEQYRWLARDLAAVDRGATPWVVVLL
HAPWYNTNAAHEGEGEAMRKAMERLLYEARVDIVFAGHVHAYERFTRVYNNEANPCGPVH
ITIGDGGNREGLAFDFRKNHKLAPLSLMREASFGHGRLSVVNATAARWTWHRNDDADSTV
RDEIWLESLAANGACQQSSSAAAAADSQNDEL*
>11686.m00490|LOC_Os12g05560.1|genepair1524-1
MERVNLKLYLENVYIMEENERLRRKAQALNQENKALLAKLNTNHAAASSTSTTTQHRPPT
AASAAGAGASSTLKPGKQQPK*
>11670.m03226|LOC_Os04g33560.1|genepair1524-2
MDRLNAKLYLQNCYIMKENERLRKKALLLNQENQALLTELKQRLAKTKAAAAAAAATKAN
GNGNMPAGGGRASLPDLNSAPPAHGHDKAVPKSKKTAAK*
>11686.m00493|LOC_Os12g05590.1|genepair1525-1
MSRSVEPLVVGRVIGEVIDSFNPCTKMIVTYNSNKLVFNGHEFYPSAVVSKPRVEVQGGD
MRSFFTLVMTDPDVPGPSDPYLREHLHWIVTDIPGTTDASFGREIISYESPKPSIGIHRF
VFVLFKQKRRQAVVVPSSRDHFNTRQFAEENELGLPVAAVYFNAQRETAARRR*
>11670.m03227|LOC_Os04g33570.1|genepair1525-2
MSRVLEPLVVGKVIGEVIDNFNPTVKMTATYSSNKQVFNGHELFPSAVVSKPRVEVQGGD
LRSFFTLVMTDPDVPGPSDPYLREHLHWIVTDIPGTTDASFGREVVSYESPKPNIGIHRF
VLVLFKQKRRQAVTPPSSRDYFSTRRFAADNDLGLPVAAVYFNAQRETAARRR*
>11686.m00494|LOC_Os12g05600.1|genepair1526-1
MVPRILIVLLVVLLGLAFQAILRPPPQKLCGSPGGPPVTSPRIKLRDGRYLAYREDGVQK
DKAKFKIISVHAFDSTKDFPLQVSKELVHELGIYIVGFDRAGYGESDPNPKRDVKSEALD
IEELADQLELGHKFYVLGVSMGGYSIWGCLQYIPNRLAGAAMVVPIINYWWPSFPAELSR
QAFKRLIVPEQRTLWIAHNMPSLLYQWMTQKWLPSSAAAMRHPEIFSKHDLEVLQKMMAM
PLIENKSRQQGIYESTHRDLLVAFGKWEFDPMNITNPFPQNEGSVHIWQGYEDRLVLVEL
QRYIAQRLPWIQYHEFPEGGHMFMLVDGWTDKIIRALLVGEQL*
>11670.m03229|LOC_Os04g33590.1|genepair1526-2
MVRKLILALAVFLPALVYQQLQPPPPKICGSPGGPPVTGTRTQLKDGRHLAYLESGVPKD
QAKYKIIFVHGFDSCRYDALPISPELAQELGIYQLSFDRPGYAESDPNPASTEKSIALDV
EELADNLQLGPKFYLMGFSMGGEIMWSCLKHISHRLAGVAILGPVGNYWWSGLPSNVSWH
AWNQQLPQDKWAVWVSHHLPWLTYWWNSQKLFPASSVIAYNPALLSEEDKLIMPKFAFRT
YMPQIRQQGEYSCLHRDMTVGFGKWSWSPLELEDPFAGGKGKVHLWHGAEDLIVPVSLSR
YLSEKLPWVVYHELPKSGHMFPLADGMADTIVKSLLLGDQPPQA*
>11686.m00509|LOC_Os12g05730.1|genepair1527-1
MVSLRAAIVLVVAASSVAVAFSHAEGNEGPDFTYIEGAMDGPSNWGKLSPEYRMCGEGRS
QSPIDINTKTVVPRSDLDTLDRNYNAVNATIVNNGKDITMKFHGEVGQVIIAGKPYRFQA
IHWHAPSEHTINGRRFPLELHLVHKSDADGGLAVISVLYKLGAPDSFYLQFKDHLAELGA
DECDFSKEEAHVAAGLVQMRSLQKRTGSYFRYGGSLTTPPCGENVVWSVLGKVREISQEQ
LHLLMSPLPTKDARPAQPLNGRAVFYYNPPGSAVSFQEFAK*
>11670.m03237|LOC_Os04g33660.1|genepair1527-2
MAASHGNAIFVLLLCTLFLPSLACDSGGVKFGYTGSIGPDFWGNLSADFTRCSNGKQQSP
IDIDTNNLVHELNMEPLHRNYTAANATLVDNIFNVALRYEEAAGVLSINGVKYTLKQMHW
HSPSEHTINGFRFPLELHMVHTNENGNITVLAFLYRFGRPDPFFEQDKLAALNAEGCKAE
KGSPVPAGSVSLLTMRQHVHIYYRYVGSLTTPPCAENVIWNIPAMPREMTPQQAADLMAP
LDEGYRRNSRPTQQMNGRTVQLYHRFWGKKKRRSSP*
>11686.m00525|LOC_Os12g05890.1|genepair1528-1
MAADGGALRRLFEKPLPVPENPTLLEALSACNHVHHRKKLVDTASFTEIFGELHFQEKPV
DVAAAARFLPPPPPPVRAASWIDVADDKSKDGSSLDALLRPKSSAVKRSASFCLKSSESS
LLLCTEGLGTESTVDADDMVKDGDGDGEAIRRGEEETDGVEEDDGAGREKRGTPTLAPTP
PTFPPPIRSIGRGGKPCVCFRSFRAEGRFVLMEVVIPGKELLQATREGGRLTLRFANAAA
VGGGGEADDDVNDDDVDGGETKNACAARDDMLANNCTC*
>11670.m03242|LOC_Os04g33710.1|genepair1528-2
MAPPPPPPPLHCYKAPEEQRLGPRPATQSRPPPHLLPYSGGLDLLTEALGAESFDPDDDD
DATAASPAMEDVGAAVAAVDFLAPPCKRPHHVLLSSSSEGVGHDDDDNQHAVMVLRRTRS
GRAFPPPISVIGKGGRPWLSLRAHREAGRLVLREMRLPSQELLQPCKEDGRFKLLIHPEA
GRRSGGAGAGPRVGSGREGHGALES*
>11686.m00539|LOC_Os12g06030.1|genepair1529-1
MASVCSAAAVSLDGAVAGATTTTAIEDIPGDVLSLVLRRLDDGASLAALGCACSAFRELA
ADAETWRGLCLATWPSLRDVDGLDECGGVTGGGGYRALFADAFPLPAEARGLVPSSASLL
PARLVSAVDLHHGEVCLMSRVVETDASSEWFLGSPFRVDALVQEGFSAPAPITPAELTLS
WILIDPATGRAVNASSRRPVSVDREWLAGETVARFTVVLGGGVVALDAAVTCDDRHGHVR
EVSLRAEDGDGGGVSGRGVLAAVAAAMEGARHGRGAEAAAWRRYEAFARGRAARKVKKAR
RDGAVDFFCSGVAAAAFVGVLSTLTLR*
>11670.m03253|LOC_Os04g33820.1|genepair1529-2
MAPGREEEKMVATTTIEDLHADVLARALRRLDGRSLAAASCATAGLRALAADPETWRALC
LAEWPSMAGHPRLLSVVPPRRLFADAFPFPRPDAGELGGGGGGPLPSELVSAVDVYYRGA
PLLSRVVETPASSPWFLGSPFRVEAVECKKPAAEAALSPAELELSWVVVDPARGRAVNVS
SRRAVAVDRHWYTGETLVRFAVVLGGCKFETTVTCSEGAGNISEVSLAVQDADGAAASGE
RSLRLLAAAMEEQRIGGGRERDEAKRRYDEFVKSRKGRKESKARREALIDLCCSAASAMA
VLSFVAAVVLR*
>11686.m00551|LOC_Os12g06150.1|genepair1530-1
MGKKGGLTSLFSRLAVAAADSPSCAKNPPHTASFRGFYYVDEPCTTAGGGGGGRSPAAGR

LRKGGDEMYKTVNSVFFDDSADAAHAVADGCAFSGEDDDDDDRFSTTTAADEEWSEAVIR
SLGRRTSTDRFFFDAGPGRPAATNSILATVRPRRPPPPPPPPPPAEEEKEKAAAEAAQLP
GKSSSTSSSQLVEESVAVAVESEDPYGDFRASMEEMVAAHGLRDWDALEELLSWYLRVNG
KHNHPLIVAAFVDLLLALAAVPSSSSSDTTTTTTAAATTTTSDTSCSTASTSTTSNGATS
VTAAATAAEQCGGGGGGGDEEAGCSSSSSCCAASDHDHEEVSAIS*
>11670.m03258|LOC_Os04g33870.1|genepair1530-2
MAKKGLVGILYKLRDVHHRAPPTPTSPSSSSSPHCHGRHQLCYPPAPSSWPWPSCRHPRT
SSFRWPTAPQQGQADDDAAAAAGSVYRTVNTVYDTSSLEHFNPRRSSLDEASSCIADRSF
FAVESEVEVEEEKEKEKELQLRETAVVRGVRSERLFFEPAGAEFLPKQRGFQEMARGKND
DEATAMDVVARKNDDVDEATPMTTPQTGKNEAEAAEAAALKGGAVVLTVESEDPYGDFRS
SMADMVAAHGLRDWEGLEELLAWYLKLNAKGVHGVIVGAFIDMLVSLASSPIPSQSPSSS
CITFEDYSSATMEEES*
>11686.m00554|LOC_Os12g06180.1|genepair1531-1
MVIAFESLISWMPMYGEIKLAFFVYLWYPKTKGSDVVYDTFLRPIVMQYEPNIEQRLLHL
RAKSGQLLSFYMKNFADKGTAFFMDVLRYVVSDKPEGSNQEDSDAQPAAAACSVVGKKNH
PAGNVGGQFLPDDPQQRNKKSGGWSPFATKRRPPSPPRPPQESLFESNPEAAAVAEVLKA
TINPRPRRGAQNGKNYY*
>11670.m03259|LOC_Os04g33880.1|genepair1531-2
MSVEFLTKALTALFGYAMPALECFKAIEQRPGRTDHLRIILVILVIFDDIAGVLTSKIPM
YSELRLAFLVYLWYPQTRGTDIVYDTFLRPLVMQYQPNIEERLRYLRANAGDILIFYLKN
FTDRGYDLFLRGMEYIRSQTSRGSRTRDSGNVTVQSKNSGYVTVQRWFSFGGDRAERSSY
VDDYVAGGGDRRSTARHRRPRDDY*
>11686.m00556|LOC_Os12g06200.1|genepair1532-1
MAAAGAGSSEVAARVLLQRYQPFAPPPGEYHQFGSGGAAAAGDMTEAVLIRTPLKRKHDR
EENEAAESNDWMMSPGYTNPAGSPVPTPLSGKGSKAFAKSKAAKGQKSCPQTPLCASSPG
NPVTPVGGCRYDSSLGLLTKKFLNLLKGAPGGIVDLNNAAETLEVQKRRIYDITNVLEGI
GLIEKKLKNNIRWKGIDDSRPGEVSDDMSILQADIEALSLQEHSVDQQISEMRDKLRGLT
EDENNQKWLYVTEDDIKSLPCFQNQTLIAIKAPHGTTLEVPDPDEVNDYPQRRYRIVLRS
TMGPIDVYLVSQFEEMSGMETPPRTVQPVSMDSLENPRTPLAAEPNKAAESQPNIQDGLL
MPSDAPSSSQDIGGMMKIVPSELDTDADYWLLSDAGVSITDMWKTAPEVEWEGIEKFNAE
DFLEVSTPRQQDKPSSDIMDGDSCIS*
>11670.m03266|LOC_Os04g33950.1|genepair1532-2
MSSGGGRPPAAQHIVRSVRQRFVPLPPPLARAPFAAAPGDYHRFAAASRGGEIEEGIVIR
RTPLKRKTPCGESEAAESSERMMTSPGFTEGVGSPLMTPVSGKTSRTTKSMAKFNKAGPQ
TPISNAGSPGNPSTPASSRYDNSLGLLTRKFINLLKQTQDGILDLNDAAKILDVRKRRIY
DITNVLEGTGLIEKKLKNRIRWRGSDDSGTNLDSDISCLKTEVENLYIQEQALDRSISEI
REKMEELTEDESNHRWLFVTEDDIKGLPCFQNEALIAIKGPRGTTVEVPDPDEAGDYLQR
RYRILLRSTMGPIDIYLVR*
>11686.m00563|LOC_Os12g06260.1|genepair1533-1
MGKDCGKHGDDDFRQGCRRFITVLVVLAILVGIIALIVYLVLRPTHPRFYLQDATLRQLD
LSNSSSTAGGVLSTTIQVTVASRNPNDRVGVYYDRLDVYASYKYQQITVAASLPPVYQGH
GDVDVWSPVLAGPSVPFAPYLADAISQDCQAGYLILQVKIDGRVRWKVGSWISGHYHLFV
TCPAFLVTAGGNGSPGASGFRFQTTTYCHVEV*
>11670.m03270|LOC_Os04g33990.1|genepair1533-2
MGKDKHHRDWILRRCCGSIAACILTLAVLVGFIVLVIYLAIHPSKPSFYLQDVQLRNIDL
SDPAISLNLQVTIASRNPNDRVGVYYKTLHVFTTYREEPITVPVELPAIYQGHKDVSVWS
PVMSGESVPVGQYVADAMRQDIAAGYVLLHVKVDGRVKWKVGSWVSGGYHLFVTCPALLA
ASGGNVGGAFAMSATAGGGAGGNATVSLKFAQAADCTVDV*
>11686.m00130|LOC_Os12g02210.1|genepair1534-1
MLFLKELPIWQAFGFSLFTGALAGSILYAVCVLLLCCVDRRRRHAGAPPPDPKIWLPDHA
HHRRRRDESSEMECSICLGELEEGERCCTLAACRHEFHKECIYRWLANRNTCPLCRHISL
PPASSPPPAPPPHHASPPPSNVLAAAHV*
>11682.m03795|LOC_Os05g39940.1|genepair1534-2
MPFSLLLFTGAVAGVAMLVLPWWCAVGEDDDTYIDGVVDDSYDSDSWYTDYSDEDDDDVG
GDNKDGLTPDQLRRLPWFAYCGGGGRSCSICLEEMRDGERCRRPGRCRHAFHAACVDEWL
TTRRTCPCCRELVLVPPAARLAAPTYR*
>11686.m00140|LOC_Os12g02310.1|genepair1535-1
MARAQLVLVALVAALLLAGPHTTMAAISCGQVNSAVSPCLSYARGGSGPSAACCSGVRSL
NSAATTTADRRTACNCLKNVAGSISGLNAGNAASIPSKCGVSIPYTISPSIDCSSVN*
>11682.m03805|LOC_Os05g40010.1|genepair1535-2
MVPAARSGWPAAAAVLVVVLVLSSPPGTSTVVVARAALSCSTVYNTLLPCLPYVQSGGAV
PAACCGGIRSVVAAARTTADRRAACTCLKNVAAGAAGGPYISRAAGLPGRCGVSVPFKIS
PNVNCNAVN*
>11686.m00145|LOC_Os12g02350.1|genepair1536-1
MRRLVDVVEAPALVLTPAWAATASMQQAGGSSGALDANMVIVLAALLCVVICSLGLSSLI
RCALHCARGLSPSPAMATPAAATTTGGLKKKELRRITVEVYGAKQAGVPDAECAICLGDF
ADGDKVRVLPRCHHGFHVGCIDTWLAAHTSCPTCRDSILSVHAGVTGGQT*
>11682.m03806|LOC_Os05g40020.1|genepair1536-2
MASGVAAPAPSVFEAARPALESGGGGGGAPPPGRADASFDTNMVIILAALFFALLFAIGL
NSLARCALRCGGRGAAAAGGGGGGGGGGAAAAGVGCGGIKKRALRSIPVEVYCGGEETAE
TDVCAICLGEFADGEKVRVLPRCRHGFHVRCVDAWLVSHGSCPTCRRQVIGGGGSTPPPD
SDTIAVVVA*
>11686.m00151|LOC_Os12g02400.1|genepair1537-1
MAVTESVCLSDEQQAVAVREVAQVYELIKTQQPLLLVHQQPQQLAHGLLNHAMRALNVAL

SVMNQPHASSSSSAAAAAGGHHFPVMTMIKAESTPANSPAADVSDNHVAGKARRSSPAKR
RRINCEDKSSWVYHTVVPHEDGYQWRKYGEKKIQGTHFTRSYFRCTYRDDRGCQATKQIQ
QEDKNDPPMFQVTYSNEHTCTTTRLINNTNNNPAALHSLTANPNGHPDDDSDDTILTKMI
KQEQQAAWLPSPPPDLTTISNNFDETPGLHVSQEVPPCSSNSSAISHYADEFDHHQMGQQ
LETTVMEEALGLGADLDDPYFYDPNLLLIYENLMNCY*
>11682.m03811|LOC_Os05g40070.1|genepair1537-2
MARRLPKSERSPSPPPPPPGDQRDAAIQELSKGSELATQLMAQLELIPERELDGRRDDAL
ANVRSLSMSLSSSLYALRSERREHYYCGSSSSSGGAGPAAVTSVSGAGGERKTKRRRGKH
GEELIETVFITTTPENDGFHWRKYGEKNILNSEFRKLYYRCGYSDERKCQAKKYVQQENN
KHPPEFRVTLTNEHTCNTVFQDQPSSSSTNSQVLDFTKASISSSLMDSHVGAPILKEEEE
EEVPSIDESTRIMSTIMRNYGSYGDYDESSPQPWNGAGWK*
>11686.m00169|LOC_Os12g02570.1|genepair1538-1
MAYRRKQGPIAADDRRSSYPQPPQGSSSSYSYTSIKSMNEPKLGLWETLARKAKGILDED
GTAHKSDEYTKQKTPRKFDSSTGAQESRSRWSFENHSRTGDTGSRTRSEALAASVNQLGG
RIRDALEEGLTIVDNKTSNIIEETKKIQIRRKQANSNSYMPNPAFDTLRPPNLSHDQAET
AAQETQLKASRDVANAMAAKAKLVLRELKTVKADLAFAKQRCAQLEEENKFLREAKQKGS
KTEEDDDLVC*
>11682.m03843|LOC_Os05g40310.1|genepair1538-2
MAYRRKHGIQRSATFVEDHRQQPPQPGDTSSPAIASPRATRFADDSRRPDRSLAAASSSP
QPDGSTPDPVTQLYTSARGAKGNETKHGFWGVLAQQAIVMLDENGGTDDNHSVTSQSRWS
YDRVRKPENPPLDIGCKIKTALEVAFSSRLAFRDFMNAMINIGLTKVEGSSRTGDGVHGR
KLHIRRKACSMDLRNSSMGLSSPEAMSPTMSDTESPQIKASRDVASAMAAKVKLLQRELK
TVKADMAFSRERCAQLEEENRMLRDGKHDADEDLIRQQLETLLAEKARLANENTVYAREN
RFLREIVEFHQLNMQDVVDLDDEDMAGDGDGEEGDDDHQQYGCHLRAHEAAHGLWAGGGL
GTPPQSPLGHAGRMGMSRSNSRAAESPTMRRSLKEENVDEPETPPTRRSLKEKADVDAPP
ETPPTRRSLKEADVDEPDTPPTRRSIKEDADDAPETPTTKQDIGSPETATTPARRSSNDD
LGAAETTTPTRRSFKDDNGVTEMKNEH*
>11686.m00176|LOC_Os12g02640.1|genepair1539-1
MELFSSQQWLALLPPIILCILLFSYVYIILWLRPERLRQKLRSQGVRGPKPSFLFGNIPE
MRRIQQLAKSAHEQEAGSTDMFSSNYVATLFPYFLHWSRVYGSIYLYSTGSIQVLNVTDP
NMVKELANCKSLDLGKPCYLQKERGALLGMGILTSNGDLWVHQRKVIAPELFMERVKGMV
NLMMEAAMSMLNSWKNEVEDRGGSAEIVVDEFLRTFSADVISRACFGSSFSEGKEIFIKI
RQLQKAMAKQSMLIGVPGSRYLPTRSNRGIWNLDSSIRTLILNISKKYEHDSSTSVNKDL
LHSIIQGSKDGPFASCTPEDFIVDNCKNIYFAGHETTSTTAAWCLMLLASHHEWQSRARV
ESLDICQGRPLDFDILRKLKKLTMVIQETLRLYPPASFVAREALNDMKLGGIDIPKGTNI
WIPIAMAHRDPSVWGPSADKFDPDRFANGIAGACKPPHMYMPFGVGVRTCAGQNLAMVEL
KVVLSLLLSKFEFKLSPNYVHCPAFRLTIEPGKGVPLIFREL*
>11682.m03851|LOC_Os05g40390.1|genepair1539-2
MGKPKYLQKGQEPLFGGGVLKANGACWARQRKVIAPEFYMARVRAMVQLMVDAAQPLIAS
WESRIDAAGGAAAAEVVVDGDLRSFSFDVISRACFGSDYSRGREIFLRLRELSGLMSETS
VIFSIPSLRHLPTGKNRRIWRLTGEIRSLIMELVRERRCAARAAREHGGKAAPPSPPERD
FLGSIIENSGGQPRPDDFVVDNCKNIYFAGHETSAVTATWCLMLLAAHPEWQDRARAEVL
EVCGGDGAAAPAAPDFDMVSRMRTVGMVVQETLRLFPPSSFVVRETFRDMQLGRLLAPKG
TYLFVPVSTMHHDVAAWGPTARLFDPSRFRDGVAAACKHPQASFMPFGLGARTCLGQNLA
LVEVKTLVAVVLARFEFTLSPEYRHSPAFRLIIEPEFGLRLRIRRAGGQDATSQVDTSTA
PVHSSHN*
>11686.m00185|LOC_Os12g02700.1|genepair1540-1
MASTTATAAGNGSGSILPTHTTTAPPFRAHKDADLESTTRRRRRRCLCCCLLVTLVVLLV
LAITLLVLFLTVLRVRDPTTHLVSTRLTGLSPRLSFPATSVQLNVTLLITVAVHNPNPAS
FTYATGGHTDLTYRGAHVGDAEIDPGRIPSRGDANVTMALTLQADRFAGDLTQLVSDVMG
GSVALDASTRIPGRVAILGVFKRHAVAYSDCHFVFGVTEMAVRSQQCSDRTKL*
>11682.m03852|LOC_Os05g40400.1|genepair1540-2
MSGSGDAAPARHNAGHGRRRRRVLVWASFAALVLLLVAAAAAIAALAVLRPRDPTTELLS
VNATGATPRVAALPAVSVQLNVTFLLVVRVRNPNRAEFRHGAATTALLYRGAEVGAAGVP
AGTVPSRGAATLRLNMTVRADRVVAAAGVGGLLADVLAGEMEFEARTEVRGRVKLLGLVR
RSAVARSLCRVVIGVADVKVRRQECHNESKL*
>11686.m00187|LOC_Os12g02720.1|genepair1541-1
MAAKVHPNVAAAPQPPCISSIVSQQQEEEEPPVVLTVWRKSLLFNCHGFTVFDAKGNLAF
RLDCYDSTSSRRADLVLMDAAGKPLLTIRRKRMSLSDSWIIYDGDGAATSTATPLLSVRR
RRVGLRASKSKAIAHVTPLSSSLPLPEAYVVEGSYGRRSCAVRDARGDAVAEVRRKESVG
DDVFRLVAQPRLGAPLAMAIVIAIDEMLRGGSSSLLRRTCSA*
>11682.m03877|LOC_Os05g40630.1|genepair1541-2
MAAKVHPNLAVPSLIQPPMAPPAAMAAGDSVMKTKAAAAGGDVVLTVWRKSLLFNCRGFT
VFDASGDLVYRVDSYAADSRAEVVLMDAAGVPVLTVRRKKAIGSQLGLGGDQWLVHPGEE
TRLPPLYAVKRTPQYVRGGGSVKTMAHVAPCGVALGAGGGGGYEIEGSYLRRSCAVYDAR
RRAVVAEVQAKEAVGTDVFRLVVRPGMEVSVAMAVVLALEQMFGKPSLLRSWSS*
>11686.m00201|LOC_Os12g02840.1|genepair1542-1
MAAHTVTDPLEELWNHTMSMDKTHLMCFYPSKITMGGVWTGDNPLDFSIPLLLFQILLIT
STTRAATLLLSPLRLPTYISQILAGFLLGPSVLGHLPHFSNLVFPVRSLFVLESMALLGL
VYYTFIVGVEIEVSAITRAGIRSFGFAIGCTLPPFLVGALTGYVALSTDDKHKGDTFLNK
LSFPIFLGSTFSSTAFAVLARNIAELKLAGTDVGQLTLSASLINDTFAWTGLTVATVLGH
SRCTITQTTWTLTSGVVIFGASYLLLRPMLLRLARRAAEGEAVGEDRECWILIGVMVAAL
VADAGGTHAIFGAFVFGLAVPNGPVGVALVEKVEDFVVGALLPLFFALSGLRTDTAKITN
MHSAVLLMVAAMVAAVLKVVAAIGVAGVFGMPLGDGTSIGLLLNTKGIIELVILNIARNK

GIMSDQSFTVLVFVSALITAMVSPFLGMVVKPARRLVFYKRRTVAWAHPESELRVLACVH
VPRDVPALLTLLDVVTPSSRSPVGVLALHLIEFVGRSSALLLINASAPSSSSYDASVHGR
SHTEMQFKHISHAFMAYEEQSVGVSARTMAAVSPYASMHEDITSAAENQHSALILLPFHK
YRSVDGGLEVSHPAIQPLNCSVQSFSPCTVGILVDRGLAAVPGGGYRVVALFFGGSDDRE
VAALATRMVRNPTIDLTLLRFVQKGGSFTASEFDALKERKADEGCLRDFLERANEGGGAT
VEYRERGVFNASEMVGEIQSVEAMGNKDLFVVGKVPGGSGLTAGMAEWSESPELGPIGDL
LASKDFQTTASVLVLQAYGRPAAVVGAGAGAMSVDFGGDSVAMAERTASGRRPWARPGV*
>11682.m03879|LOC_Os05g40650.1|genepair1542-2
MAPIMSGAAAAAGGTGGAVPLIKNATSASQMSRGKAGTGAGAVVCYSPMMVTAYGIWQGA
SPLDFSLPLFLLQVAIIVATTRLLVILLKPFRQPRVIAEILAGVILGPSVMGQVSTWAVK
VFPERSLLTLETVAHLGLLYFLFLVGLEMDVNTIRRSGKKALIIAVAGMALPFCIGTATS
FIFRHQVSKNVHQASFLLFLGVALSVTAFPVLARILAEVKLLNSDLGRIAMSAAIVNDMC
AWILLALAIAISEVNSSAFSSLWVLIAGVAFVLACFYVVRPLMWWIVRRVPEGEAIGDVH
ITLILTGVMVAGVCTDAIGIHSVFGAFVYGLVMPSGPLGVVLIEKLEDFVTGLLLPLFFA
ISGLRTNVTKVRDPITVGLLVLVFVMASFAKIMGTILIAVSYTMTFRDGVALGFLMNTRG
LVEMIVLNIGRDKEVLDDESFAVMVLVSVAMTALVTPVVTTVYRPARRLVGYKRRNLQRS
KHDAELRMLACVHTTRNVPSIISLLELSNPTKRSPIFIYALHLVELTGRASNMLAAHHSA
SNPGGASDHIFNAFESYEEMVGGVSVQALTAVSPYQTMHEDVCVLAEDKHVSLIVLPFHK
QQTVDGGMEPINASLRGFNESILASAPCSVGILVDRGLSAAAARMAAVHHVALLFFGGPD
DREGLAYAWRMVENPGVCLTIVRLIPPGYTAPAISPPQPPMPAAHSRAINVVPEVAKSER
QMDEEYLNEFRSRNLGNDAILYVEQVVANSEETVAAIRSQLDNAHELYIVGRHPGEASSP
LTSALAEWMESPELGPIGDLLVSSEFSKMASVLVMQQYVITAPLPPPVALAGPPTDDPVR
QYLTNANQRPSVAIGGNQMGAAGRGGWSGGAGGY*
>11686.m00204|LOC_Os12g02870.1|genepair1543-1
MGSSSLLLFPSSSSSATHSSYSPSSSSHAITSLLPPLPSDHHLLLYLDHQEQHHLAAAMV
RKRPASDMDLPPPRRHVTGDLSDVTAAAAGAPTLSASAQLPALPTQLPAFHHTDMDLAAP
APPAPQQVAAGEGGPPSTAWVDGIIRDIIASSGAAVSVAQLIHNVREIIRPCNPDLASIL
ELRLRSLLNSDPAPPPPPPSHPALLPPDATAPPPPPTSVAALPPPPPAQPDKRRREPQCQ
EQEPNQPQSPKPPTAEETAAAAAAAAAAAAAAAKERKEEQRRKQRDEEGLHLLTLLLQCA
ESVNADNLDEAHRALLEIAELATPFGTSTQRVAAYFAEAMSARLVSSCLGLYAPLPSPSP
AGARVHGRVAAAFQVFNGISPFVKFSHFTANQAIQEAFEREERVHIIDLDIMQGLQWPGL
FHILASRPGGPPRVRLTGLGASMEALEATGKRLSDFADTLGLPFEFCPVADKAGNLDPEK
LGVTRREAVAVHWLRHSLYDVTGSDSNTLWLIQRLAPKVVTMVEQDLSHSGSFLARFVEA
IHYYSALFDSLDASYSEDSPERHVVEQQLLSREIRNVLAVGGPARTGDVKFGSWREKLAQ
SGFRVSSLAGSAAAQAALLLGMFPSDGYTLIEENGALKLGWKDLCLLTASAWRPIQASGR
*
>11682.m03885|LOC_Os05g40710.1|genepair1543-2
MQDSLGLMQFLDHHQYLYSSSSSNLPLQQPLLSHHHRFLEANEGCAGEDDSPEFVEPPAA
AAAAGTFEQRPELGACKEVYSEEGGAAEERTGVAMAGADVEQVAVEDEEEAHGVRMIALL
MECAAAMSVGNLAGANGALLELSQMASPYAASCGERLVAYFARAMAARLVGSWVGVVAPM
APPPSCGAINAAFRALYNVAPFARLAYLACNQAILEAFHGKRLVHIVDLDVVPGGALQWL
SLLPALAARPGGPPVIRVTGFGMSASVLHDTGNQLAGLARKLCMFFEFYAVAKRPGDADA
VADMPGRRPGEAVAVHWLRHAMYDAAGDDGASMRLVRWLEPAAVTLVEQERAHGGGGGHG
RFLDRFVSALHHYSAVFDAMGASRPDGEDASRHLAEHGVLGREIANVLAVGGPARSSGRE
GPGSWREVLARHGFAHAGGGGGGRAQLVAAACPGGLGYTVAGDHDGTVRLGWKGTPLYAV
SAWTWCSPPHARA*
>11686.m00208|LOC_Os12g02910.1|genepair1544-1
MGFSKHKHLDGRMKHKLLKGYSLYTVSQGFVAILDVSRKKIPGSDGILGEKSGSRSKSKN
VSTKSKEKLKGVFSLVQLKNETRKNKELQDTTSIHFIVSNVISELHTQRRYQLKDLSWRS
KDTTIDKKENQEVEHEENPKSCELEYGIYCLWSVEYKEVMKDFIVKRLKDQLFMARAHYP
SIAKLKNQETFTRELKQNIQEHERMLSDTIADADLPPFFAKKLEKMEHTIERAKSCEVGC
TSVERKLRQLLDITEDEAYFHTRQSAFLYHLGVQTMPKTHHCLNMRLTVEYFKSTSIHTV
QSNKQKLEDPTFHHYVIFSKNVLAVSTTINSTVMNSKDSGSIVFHLFTDSQNFYAMKHWF
DRNMYLEATVHVTDIEDHQKLSKDVDFHDMKLLRPAEEFRVTFRNHSQSFQKQMKTEYIS
TFGHSHFLLPDLLPSLNRVVVLDDDLIVQKDLSSLWNLNMGGKVVGAIQFCEVKLGQLKA
YTEERNFDNNSCVWLSGLNVVELKKWRDLHITSRYEQLLQKLQKDSVTSFPLKVLPISLL
VFQDLIYPLEDSWVQSGLGHDYGVSQTDIKRSVTLHYNGVMKPWLDLGIHDYKGYWRKYM
TNGERFMTECNIH*
>11682.m03886|LOC_Os05g40720.1|genepair1544-2
MKGQGGGAGAPAKRRWRSVAAAAAALALLFLSVGVPLAVLLGLHQRFPSMYLADESAVSV
FGGSEGGGWEPNTSQENDRLPVNDTNKFPPSIEKSAFAISWILFHMALFCISTAIKALFL
SFWHDWSKTNTGNSDAESNGTNNQPSIDKPISNTSIHPGLPIKQIVIFDDISLLSNTDAD
PKDNFEQGLPGDESIKSCQLEFGSYCVWSVEHKEVMKDSVVKRLKDQLFVARAYYPSIAK
LEGMEKLSHEMKQSIQEHEHMLSEAICDADLPAFHGANMAKMEKTIAAAKSCLIECTNFE
KKLRQLLDMTEDEAHFHARQGAYLYRLGVQTLPKSLHCLSMRLTVDYFKSFADMEYSNVQ
KLENPVLRHYVIFSTNLLASSMTVNSTVINSEESANVVFHLVTDAQNFYAFKNWFIRNSY
KEATIGVLNFEDFQATHLDNRRVEHLSPYEEFRIASHSNARIPNTQMRTEYISVFGHSLF
LLPELFSNLKRVIVLEDDTIVQRDLSHIWNLDLKGKVIGAVQSCRVRLRHLRPYLVDFPY
DASSCIWMSGVSVIDLNKWREHDVTAVRNRVLQKLQHGPEASWRAAVLPAGLLAFQNLVH
PIEAQWIQSGLGHDYGVNHGAIKKAGILHYNGNMKPWLELGIRRYRKYWRRYLPRDDPFL
IDCNVNP*
>11686.m00110|LOC_Os12g02020.1|genepair1545-1
MSGKDQKKAAALEEKLELLRSVTKSSAANETSILVDASKYIKELKDKVSQEPEQLGSTSS
SMPMPRVSVSSVELEKKRGFRINVSMEKSQPELLTSVLEAFEELGLDVLDADVSCADDTA

FRFEAFGSSQSEAAERSVDEQMVRHAVLQAIKKCMDGSSI*
>11673.m04752|LOC_Os07g47960.1|genepair1545-2
MSRERKKAAALHEKLQILRSITHSHALSNTSIITDASEYIKELKQKVVRLNKEIACAEAA
ALRQNSIPTVTVETLGHGFLINVFSDKSCPGLLVSILEAFDELGLNVLEATASCDDTFRL
EAVGGENQVDEHVIKQTVLQAISNSNCSESSGDQEG*
>11686.m00114|LOC_Os12g02060.1|genepair1546-1
MASPKPFACSAIALLFAANLVSAQLSANFYDKSCPNALPTIRIAGCDGSVLLDDTPTFTG
EKTAAPNNNSLRGFDVIDNIKAHIEGICPQVVSCADILAVAARESVVALGGPTWVVQLGR
RDSTTASLDTANNDIPAPTFDLGDLTKSFSNKGLSATDMIALSGAHTIGQARCVNFRNRI
YSETNIDTSLATSLKSNCPNTTGDNNISPLDASTPYAFDNFYYKNLLNKKGVLHSDQQLF
NGGSADSQTTTYSSNMATFFTDFSAAMVKMGNINPITGSSGQIRKNCRKVN*
>11673.m04758|LOC_Os07g48010.1|genepair1546-2
MASSLSVAVLLCLAAAAAAQLSPTFYDTSCPRALATIKSAVTAAVNNEPRMGASLLRLHF
HDCFVQGCDASVLLADTATFTGEQNALPNKNSLRGFNVVDSIKTQLEGICSQTVSCADIL
AVAARDSVVALGGPSWTVGLGRRDSTTASMDSANNDLPPPFFDLENLIKAFGDKGFSVTD
MVALSGAHTIGQAQCTNFRGRIYNETNIDAGYAASLRANCPPTAGTGDSNLAALDTTTPY
SFDNAYYSNLLSNKGLLHSDQVLFNGNSTDNTVRNFASNRAAFSSAFSSAMVKMANLGPL
TGSQGQIRLSCSKVN*
>11686.m00129|LOC_Os12g02200.1|genepair1547-1
MESRGKILMERYELGRLLGKGTFGKVHYARNLESNQSVAIKMMDKQQVLKVGLSEQIRRE
ITTMRLVAHKNIVQLHEVMATRNKIYFVMEYVKGGELFEKVAKRGKLTEVVAHKYFQQLI
SAVDYCHSRGVYHRDLKPENLLLDENENLKVSDFGLSALSESKRQDGLLHTTCGTPAYVA
PEVISKIGYDGAKSDIWSCGVILFVLVAGYLPFQGPNLMEMYRKIQHGEFRCPGWFSRKL
QKLLYKIMDPNPSTRISIQKIKESTWFRKGPEENRILKERTLNENTTKNVALVLGVRRKK
NAHEDVKPMSVTNLNAFEIISFSKGFDLSGMFIVKEWRNEARFTSDKSASTIISKLEDVA
KALNLRVRKKDNGVVKMQGRKEGRNGVLQFDIEIFEVTTSYHIIEMKQTSGDSLEYRQLL
EEGIRPALKDIVLAWHGDE*
>11673.m04768|LOC_Os07g48100.1|genepair1547-2
MAEQRGNMLMKKYEMGKLLGQGTFAKVYHARNTETSESVAIKMIDKEKVLKGGLMDQIKR
EISVMKLVRHPNIVQLYEVMATKTKIYFVLEHVKGGELFNKVQRGRLKEDAARKYFQQLI
CAVDFCHSRGVYHRDLKPENLLLDENSNLKVSDFGLSALADCKRQDGLLHTTCGTPAYVA
PEVINRRGYDGAKADIWSCGVILFVLLAGYLPFHDKNLMDMYKKIGKAEFKCPSWFNTDV
RRLLLRILDPNPSTRISMDKIMENPWFRKGLDAKLLRYNLQPKDAIPVDMSTDFDSFNSA
PTLEKKPSNLNAFDIISLSTGLDLSGMFEESDKKESKFTSTSTASTIISKIEDIAKGLRL
KLTKKDGGLLKMEGSKPGRKGVMGIDAEIFEVTPNFHLVELKKTNGDTLEYRKVLNQEMR
PALKDIVWAWQGEQPKQQQQPTC*
>11686.m00150|LOC_Os12g02390.1|genepair1548-1
MGLRSPNSMLWLVLLVWAALLCGSCHGRFVVEKNSLKVTSPSDMKGTYECAIGNFGVPQY
GGTMVGVVAYPKANKKACKSFDDFDISYKAKPGSLPTFLLVDRGDCFFTKKAWNAQNAGA
AAILVADDKTEPLITMDTPEESGNTDYLENITIPSALITKSFGDKLKKAIDNGDMVNVNL
DWRESLPHPDERVEYEFWTNSNDECGPKCDSQIDFVKSFKGAAQVLEKKGYTQFTPHYIT
WYCPDSFILSKQCKSQCINHGRYCAPDPEQDFSKGYDGKDVVVQNLRQVCVYKVAKEHGK
PWLWWDYVTDFAIRCPMKEKKYTKECADGVIKSLGLDHKAIDKCIADPDADKENPVLKAE
QDAQIGKGSRGDVTILPTLVINNRQYRGKLDKGAVLKAICAGFRETTEPAVCLSEDIQTN
ECLENNGGCWQDKAANISACKDTFRGRVCECPVVKGVKFVGDGYTHCEASGSGHCEINNG
GCWKDSRHGRTYSACTNDGCKCPDGFKGDGVHKCEDIDECKERTACQCKECKCKNTWGSY
ECGCSGGLLYMKEHDTCISKNAATEVGWNFLWVIFFGLVVAGIAGYAVYKYRIRKNQAFH
TCWWKRMPFTSINWLRDRLFTSIRRRRRRALAPLAPTAIAMPSCFTLDSASDDGRSTAQG
QGWCAGVGGFLSSFFSAGAARADGGKPSPDWDAHGLAASALPVPLSRLDGKKRYKVSELT
FLDRRTRAAAEDPLFDALRPGGVYTRAQLRDELDALAASGMFDHVTFQTKPKPDGTLGPH
RLLRRDPVARGRTPQLEPDDDMTARERMEHLRRQEREYRQLVRRAKPCVLPEKLQRELQG
MVKKQRKVSSGLLKRMAGRIERWYHDEGFQCAQVVGYNGNLDAGEVLRPGHIYNNGAGKQ
ALKNIDSLGLFSTVEVEPRPDETNQGGVIVAIKLKEHDPKSAQVITDWSIVSGSQGTPTL
ASIQPGGTVSFEHHNICGLKRSLIGSVTTSNLLNPEDDLSFKLEYAHPYLDGVDNRSRNR
TFKISCFNSRKLSPIFVAGPNMYEAPPIWVDRIGFKANITESFTKQSKFTYGLVVEEITT
RDENNNICTHGSRQLPSGALSMIGPPTTLSGTGVDRMAFLQANITRDNTEFVNGATIGDR
CIFQYSEQSFLVGYRWIKASVLEARTLFNRHQLTVTKFINLNKQEKGPRKPPPAVLALHG
RYAGCVGDLPSYDAFALGGPHSVRGYGMGELGASRNLLEIATELSVPITVKNRHTQVYAF
AEHGTDLGSSKDVKGNPTEFFRRVGHGSSYGVGVKLGAVRAEYAVDHNAGTGAFFLRFGE
RF*
>11673.m04783|LOC_Os07g48240.1|genepair1548-2
MTMMGCAVLLVVVASMAGEAAGRFVVEKNSLRVTSPAGLRGVYECAIGNFGMPQYGGTMH
GVVVYPKANKKACRSFDDFDLSFKPKPGGLPIFLLVDRGDCYFTTKGWNAQTAGAAAVLV
ADDRLEPLITMDSPESSGTDYIEKITVPSALVTKKFGDDLKKALENGDMVNVLLDWRESL
PHPDERVEYEFWTNSNDECGAKCDMQMNFVRNFRGTAQVLEKRGYTQFTPHYITWYCPEA
FVLSKQCRSQCINHGRYCAPDPEQDFNIGYDGKDVVLQNLIQICLFKVGNETHKPWVWWD
YVHDFSIRCPMKEKKYTRECANGVIKSLGLDLERINKCVGDPEADEENPVLKAEQDAQIG
QGSRGDVTILPTLVVNNKQYRGKLEKSAVLKAVCSGFEETTEPDVCLSQGYKTF*
>11686.m00160|LOC_Os12g02490.1|genepair1549-1
MEAPKLRFVRCPGCLQLLVEYPTIAVYQCGGCGTVLRAKNRVAPATNTNAESGEHNEFSN
ISTGDSQNNKLICTDGQKISPSSNAQPGVVQEKITFASEEKTVSTSNSIDSNEHVNIECS
LFDGDASNHDVRTEGINDEDKVTVSNSTLDLVRKVENVETDGNEKGSFTDDGSISNEVAT
TQSMVHMDGAGFDNNFTEVQSAAEGKCTLSDANLDSQEIVAICQPDNISVGTKERVQLYE
GFHVESHEDLIEELVRSLSLSDDEEEFVDIAENSELNDALRSQMGSCRFSLGSKMNDGPR

TDPHGRLIEELEMSFSDAEEPLDQNIMVSLNDIEKPILDEVSKENHILEEDGKESHILDV
DGKESHILDVDGKQNHILDEDGKENHILNEDDKENLILDKGGEDTLDAGGANSYEERVLP
SDDGLLKSGQSFQQCELAAVNMAEKDEGHLEETNMANHAEANSGTAAVLSNLSNDKFCAI
LPPSCDGRKEEKSNIHRGRELCQGLSLDSVDFRSIQNFIESQMDGTSSSLSSGSPSHGDL
EHNRSNRFKKIDRLERLRKMDDLRDQLNRLSSQKGLENRYKNKGPGLLQEQISYRHLEQH
PCGFDGDSILDSDIIDSYYDQGNPPRYPPPDPFSPTHSHYHCGHGQPHIPYNCSAWEFNS
YYQSSYAGSTVLEHESLRSSYKEQKRAVRKSILRSLSGASPFTICNGCFNLVQVPSDIYV
SKKKIAKFQCGRCSKALMLSFPATNSEDAKLSNKEVNRKPNKPVHNSVVGMEGGYSFSAE
CSRGDPVSISEECGASISRSFSGRTRAAVAASGSGKKVSDSALHRLMGYDSASQLLRRSR
AFDDGYDSFESMVPVSNRVSRRKNL*
>11673.m04787|LOC_Os07g48280.1|genepair1549-2
MAISDARVVRHVRCPKCFSVLQEPTGAPVYQCGGCGTTLRAKNRTGNSQEVISAPSSLGS
GLPPHSKHLGSSDVASTSGSTPEAQISSGQQGADMTSRRETDDLVSARNNAPEPERVVPA
EKEEEHVQSTSQQAVGNSEDLTRGDAATAADAQCSDRASEGKVQFSESREDSNTELQDVQ
RSDQTESDAEGKKSSEETSQSPRRDVVELPPSSVQTPDSQPAPAVLKREDDPATSPPHGH
ARRSPESLAPLQKRILKTVDNLKDDLSELFSKSPELNKPRTHARPPRLPRQEGYAPRDAA
MAAAASIQAIRARHAAVHRPGYIARAGKPGQLAAPPPPRGLPSRRYRRCRADHPCCHDAR
HGPSCHHGCCPPHHGKQACTSCRGQHCCRPRTQESPAPRRPAAAAAKEVVKRRAPPRNHC
RPVLKGAPFIICSSCFKLVQVPADFAVSTKTVRKLRCGSCSAVLSYSYRDPDRKKHGDQY
SADGSPAAPRGHGRRGDKFAFLDDFGHVDVSYSTEDEQPLHVSRNSSFNTVDEMAAAATQ
QHGSSLHRLMGYGSASDLLFRQHSPDLYESFSERTTPEAAALYDRKGKGVCVDLDDDGGD
DDSDEDCSGALKRSRLRGSGWPLPGILNSKGTTGMGAIRIKS*
>11686.m00176|LOC_Os12g02640.1|genepair1550-1
MELFSSQQWLALLPPIILCILLFSYVYIILWLRPERLRQKLRSQGVRGPKPSFLFGNIPE
MRRIQQLAKSAHEQEAGSTDMFSSNYVATLFPYFLHWSRVYGSIYLYSTGSIQVLNVTDP
NMVKELANCKSLDLGKPCYLQKERGALLGMGILTSNGDLWVHQRKVIAPELFMERVKGMV
NLMMEAAMSMLNSWKNEVEDRGGSAEIVVDEFLRTFSADVISRACFGSSFSEGKEIFIKI
RQLQKAMAKQSMLIGVPGSRYLPTRSNRGIWNLDSSIRTLILNISKKYEHDSSTSVNKDL
LHSIIQGSKDGPFASCTPEDFIVDNCKNIYFAGHETTSTTAAWCLMLLASHHEWQSRARV
ESLDICQGRPLDFDILRKLKKLTMVIQETLRLYPPASFVAREALNDMKLGGIDIPKGTNI
WIPIAMAHRDPSVWGPSADKFDPDRFANGIAGACKPPHMYMPFGVGVRTCAGQNLAMVEL
KVVLSLLLSKFEFKLSPNYVHCPAFRLTIEPGKGVPLIFREL*
>11673.m04792|LOC_Os07g48330.1|genepair1550-2
MAAASLCCGVAAYLYYVLWLAPERLRAHLRRQGIGGPTPSFPYGNLADMRSHAAAAAGGK
ATGEGRQEGDIVHDYRQAVFPFYENWRKQYGPVFTYSVGNMVFLHVSRPDIVRELSLCVS
LDLGKSSYMKATHQPLFGEGILKSNGNAWAHQRKLIAPEFFPDKVKGMVDLMVDSAQVLV
SSWEDRIDRSGGNALDLMIDDDIRAYSADVISRTCFGSSYVKGKQIFDMIRELQKTVSTK
KQNLLAEMTGLSFLFPKASGRAAWRLNGRVRALILDLVGENGEEDGGNLLSAMLRSARGG
GGGGGEVAAAAEDFVVDNCKNIYFAGYESTAVTAAWCLMLLALHPEWQDRVRDEVQAACC
GGGGRSPDFPALQKMKNLTMVIQETLRLYPAGAVVSRQALRELSLGGVRVPRGVNIYVPV
STLHLDAELWGGGAGAAEFDPARFADARPPLHAYLPFGAGARTCLGQTFAMAELKVLLSL
VLCRFEVALSPEYVHSPAHKLIVEAEHGVRLVLKKVRSKCDWAGFD*
>11686.m00208|LOC_Os12g02910.1|genepair1551-1
MGFSKHKHLDGRMKHKLLKGYSLYTVSQGFVAILDVSRKKIPGSDGILGEKSGSRSKSKN
VSTKSKEKLKGVFSLVQLKNETRKNKELQDTTSIHFIVSNVISELHTQRRYQLKDLSWRS
KDTTIDKKENQEVEHEENPKSCELEYGIYCLWSVEYKEVMKDFIVKRLKDQLFMARAHYP
SIAKLKNQETFTRELKQNIQEHERMLSDTIADADLPPFFAKKLEKMEHTIERAKSCEVGC
TSVERKLRQLLDITEDEAYFHTRQSAFLYHLGVQTMPKTHHCLNMRLTVEYFKSTSIHTV
QSNKQKLEDPTFHHYVIFSKNVLAVSTTINSTVMNSKDSGSIVFHLFTDSQNFYAMKHWF
DRNMYLEATVHVTDIEDHQKLSKDVDFHDMKLLRPAEEFRVTFRNHSQSFQKQMKTEYIS
TFGHSHFLLPDLLPSLNRVVVLDDDLIVQKDLSSLWNLNMGGKVVGAIQFCEVKLGQLKA
YTEERNFDNNSCVWLSGLNVVELKKWRDLHITSRYEQLLQKLQKDSVTSFPLKVLPISLL
VFQDLIYPLEDSWVQSGLGHDYGVSQTDIKRSVTLHYNGVMKPWLDLGIHDYKGYWRKYM
TNGERFMTECNIH*
>11673.m04798|LOC_Os07g48370.1|genepair1551-2
MKATLLPPPPPPAKRRRGPRVAVLALFLCSLLVPLAFLFDRSQSGYVTTDERRRQEVVLP
EFHHVEKADGDGTVNGLNQDAPKKTPKVNSGGLQKHKQTDRHTSRISTKPKVLPSPKVDP
SEAVKESTQGTREVSKVRKRLDKGTNTDEVENEKACQLEFGSYCLWSREHKVVMKDSIVK
RLKDQLFVARSYYPSIAKLEGQEELTVLMKQNIQDHERVLSVSTVDADLPSFINKKMEQM
EQTIARAKSCTVDCRNVDRKLRQILDMTEDEAHFHMKQSAFLYNLGAQTLPKSHHCLSMR
LTLEYFTSSSLGSNDSSARKFSAAHGRHYVILSKNILAASVVINSTVNSSKDPKKIIFHI
LTDAQNFYAMKYWFDKKSYREAAIHVVNYEDIIKEKLTKFNVRHLYLSEEFRVLVRSTEQ
PAGKTRMEYLSLFSHSHFFIPEIFKDLNKVVVLDDDVVVQRDLSFLWSLDMGDKVNGAIE
FCGLRLGQVRNLLGSTTVDTKSCAWMSGINVINLDKWRKHKVTENYLLLLKKFLTKDETS
LRAAAFPLSLLSFQHLIYPLDERLILSGLGYDYAIDEDVARSSAALHYNGNMKPWLELGI
PSYRRYWKRFLTRDDKFMDECNIIP*
>11686.m00214|LOC_Os12g02970.1|genepair1552-1
MGAYEATKVVFARLQALEPNLAPNIIGMLLTKDNNEMDMIRLACGPDNLLQSIIAKVRTD
LTNKPSPPMASWGFPSDIGEEASFSVDKVGCDGGEEFSSKEYDWRLPIGGNHHRSFLSST
VDTLGWKPCLYSQSGVTTHLGSDDMQEYSSRPPQIDQSDLTNNCSARQIYLTFPPDSIFS
KEDVCNYFSMYGMVQDVRIPYQEKCMFGFVTFAYQKTVKLILAKGNPHYICDARVLVKPY
KEKDKVPNKFRHVNHCLIPLRIFAHILPFECYIVLGPRILYRDIASHEASFRMKQDEQQH
ATELQRCCLMRLPLLNLQDWGHHLSSPMGSHVLLGQVDNKYNINENDNPTHLEDVTFRDN
KLKNEFAMREIASTAISTAAKRTVISTEEGKREYGPKAATPNDACGFLESGMEYNLPHSP

FSSPTKASNVAATAHTSNISSSSSPHKVASSLFPPTCTLELPPTTHASFKRQGKALTDHT
*
>11673.m04802|LOC_Os07g48410.1|genepair1552-2
MDAYEATKVVFSRIQALDPDHAAKIMGFLLIQDHGEKEMIRLAFGPEALLHTVMAKARKE
LGLLPASGPGTPTSVAAAAAAAHSPFMLSRQNSGRCGTAPSPLSVSSPSSWAPPPVFSRN
NSISNGAGEEMVGLGDELISPANGGGPPSPFFGGDPLMDELQLQDQLAFLNEGGVPAGHQ
MPMFDGGECRSPGGGDGGLFSYNLGWANGGPGHRRSASVSELCLGGADGLGWKPCLYYAR
GYCKNGSACRFVHGGLPDDAAGKMDPSAVEQQCQDFLIRSKSQRLAAAAFPYSPTGSLPG
SPSAATKCLSLLLQQQQQQNESQRAAAAAALMLGGDEAHKFMGRPRLERADFASMMNPGS
RQIYLTFPADSTFREEDVSNYFSIYGPVHDVRIPYQQKRMFGFVTFVYPETVKLILAKGN
PHFICDARVLVKPYKEKGKVPDKYRKQHQPGERVDFSSCTTPTGLDARDPFDMHQLGARM
LQHSNSANEMLLRRKLEEQQQAAELQQAIELHSRRLMGLQLLDFKSRAAAAPTPIGNPFS
ASQTAANATGESPPDSGELGKGSGFLLAHKKAVNGADKEESTGESSSPNTDSDQSVEHNL
PDSPFASPTKSAGFARDPFAPTEAEISATASTGCSATYVGINNGASNGGTNHLLPSALDM
PSPKPYFFPMSRLASDHGAIGM*
>11686.m00216|LOC_Os12g02980.2|genepair1553-1
MAADHLVVAMLLLLALSPPAVADDTAVLGRKGGVVEGQAAGPGRYAVILDAGSTGTRVHV
FRFDNKLDLLKVGDNIELFAKVDPGLSSYAGRPQDAANSILPLLDKANTVVPARLMNKTP
LKLGATAGLRLIGDEKANQILEAVRDVVHTKSKYQYNPNWINVLEGSQEGSYIWVALNYL
LDKLGGDYSKTVGVVDLGGGSVQMAYAISSNTAATAPKVPEGKDPYVVKEYLKGKDYNIY
VHSYLHYGGFASRAHILERKDGPFSNCMLRGFSGNFTYNGKQYDATAAPQGADYHKCREE
VVKLLKVNAPCETKNCSFNGVWNGGGGAGQDDLYVASAFYYIASHVGFINSDAPSAKSTP
ATFKAVAEKVCKLSVKEAKVEYPNVRDHAYLCMDLIYEYSLLVDGFGLHPSKEITLVDKV
KHGEYYIDAAWPLGTAIEAVSPKKRLREIYK*
>11673.m04807|LOC_Os07g48430.2|genepair1553-2
MSCPIVPNQVTRQRQSPRRADPPLSALDSAEQEAVKPSPPPPGRIRYRTPSSPDLLLAPP
SDADAKNMRRYSALPGGGARPDTLADRLHRYRGVLLVILAPLALVSLVLLLMPRSPASSS
AAAGRRWGPLDANKYAVIFDAGSSGSRVHVFRFDANLDLLHIGDQIELFVQKKPGLSEYA
NNPQEAAKSLVSLLEDAKRVVPVELRGQTPVRVGATAGLRALGAEKSEEILQAVRDLLRE
KSSFKTQPDWVTVLDGPQEGAYEWVTINYLLGKLGKTYADTVGVVDLGGGSVQMAYAIAE
KDAVKAPKPSEGEDSYVKKLFLKGTTYYLYVHSYLHYGLLAARAEILKAGNGKGYSYCTL
EGHQGQYKYGNGKFEASASPSGASYSKCRDDVVKALKVDQACTHMKCSFGGIWNGGGGAG
QKNLFVASFFFDRAAEAGFVNPKAPVAKVKPSDFEKAAKRACKLNLKDAEAAYPGVQKDN
IPYICMDLVYQYTLLVDGFGVGSHQEMTLVKKVPYSNAFVEAAWPLGSAIEVAS*
>11686.m00222|LOC_Os12g03040.1|genepair1554-1
MPSSGGAMPALPPGFRFHPTDEELIVHYLMNQAASIKCPVPIIAEVNIYKCNPWDLPGKA
LFGENEWYFFSPRDRKYPNGARPNRAAGSGYWKATGTDKSILSTPTSDNIGVKKALVFYK
GKPPKGVKTDWIMHEYRLTGTSANNTTTTKQRRASSMTMRLDDWVLCRIHKKSNDFNSSD
QHDQEPEGSTVDEQLEDIHDNNSSSQQPPAPPDMNNQQSDFQPMTAMSMSKSCSLTDLLN
NLDCAALSQFLLDGSSDAIAELPAPPSPLIYPNQTLNYNINNNMPHAFESRLDHHDGYVN
NYNVNGLRRKRMMACSATSFDDGSSSSSSDFLHVAKKPLLLPSDSRGSGFGGGYCNQQLS
ETATGFQFQNGNMLSHPFPLNQQLLLNNHLQMQ*
>11673.m04809|LOC_Os07g48450.1|genepair1554-2
MEMTMSSAATSLPPGFRFHPTDEELILHYLRSRATAGQCPVPIIADVDIYKFDPWDLPSK
AVYGESEWYFFSPRDRKYPNGIRPNRAAGSGYWKATGTDKPIHDSATGESVGVKKALVFY
RGRPPKGTKTSWIMHEYRLAADPLAAAANTYKPSSSSRFRNVSMRLDDWVLCRIYKKSGQ
ASPMMPPLAADYDHDEPSGVLDDAYSFYAPPMISTTLIPKLPKIPSISELFDEHALAQIF
DAAADPPADHHQHALAVHPSLNQLLGVGDNFLAECYPSTASTATVAGGKRKASPAGDYAG
GGHTPAKRLNGSCFDVAPQSVVGGLQATPSSVLAGLNHQMLPPQLF*
>11668.m03453|LOC_Os02g36300.1|genepair1555-1
MGRRARCLCLLLLLLLVVGGGFHVANAQASPAPPQTGTRAVNRAVSTVITVVIGVFFVLV
FVCVIVNQCCDCDSSAGAGAGQGQSSAARRRRGLDPAAVAAIPVVPYAEVRKHRSGGGGA
LECAVCLTVFDDGDDLRLLPQCSHAFHPDCIDPWLEGHVTCPLCRANLEKQPAPSPPAVE
FSSSPAAAAAAAAEESRTPEAAAVRVEEVAEASDEEETRRREEEAVELESLRAVRRAARMP
RSNSTGHSLCALPAPRAPGPGDGGDHERFTVRLPPHVREEVLKSRRLRHATSLVLGIRGS
SREGSSRGGGGSWHGARRWPSFLARTVSWARGGVGADTSAKETPCRDAV*
>11670.m03565|LOC_Os04g37730.1|genepair1555-2
MGTRELLVVLAVVVMAAVGAGAQSSSASPAPATSQAPTVRQQTPFGRTMSTVITVSISVF
FFLLFFCAYINQCRLAEAGDARAAAAAAAGGGAGGGPSRRGKRGLDPAVVATFPIVPYRE
VVKHKIGKSVLECAVCLTSFDDGDDLRLLPHCSHAFHPECIDPWLESRVTCPLCRANLEK
PPPPPPPPPAAAAASPSPERSPRCQPSPPPPPPPHALVIPVEDEEDDSDEDDRKEEAVEL
EMLRSERRAARLPRSHSTGHSLVASAAAAAESGDHERFTLRLPQHVRDEVLRSLRLRHAA
SLVNLSDMSSEGSSRGGRRALGLAFGNGGGSSHGGRRWQAFLARTVSWARGGGDGSVRRG
WDGSTRRGKDDAESSRKGATSPAAGRP*
>11668.m03456|LOC_Os02g36330.1|genepair1556-1
MAPATTVPLLLLLLLLPTRVTTAAWRQQRARREALLAPPPSDAAGGGGAPGAPVAAGDGAV
GGAGEGEGEGEVLHHAWHIRTVGLDEAAIESIALARYRAGAGMLGATDCPVCLGEFRDGE
LLRLLPKCGHAFHVPCIDAWLRAHVNCPLCRAHVLHPADADADAGDGERVPPPAGANGGG
GGGGGAATDQASSPTDQTADQENPGQQQQGEQHELRVQIDRRDQPSSPEPPRRSPDPRSA
QSFRRVASMGSRSPPAPSEDAPEDEQTTTQSSKEKQGSGGDSACCGKAPSGSGRLHHMRR
SFSGGGGRRSLPSRHGRTSSSMLPL*
>11670.m03566|LOC_Os04g37740.1|genepair1556-2
MPLHHRRALHSNDCDDGGYGCSSWPLSPPPPSVILTPFASPSPAPWACPPAFPAPSPSPL
HGAAGGRRDQGGYHGSPPGGGGDADEHRRRIINLIVVGAAALAFLSMILLVVIVAVRRRR

LRRRRQRQQALLAPAAPADAVAVNVEDGGDDDAEGGGGGGGGGGVVHHIWYIRTVGLDEA
AIDSIAATRYRAGAGLLGAADCSVCLGEFQDGELVRLLPKCGHAFHVPCIDTWLRAHVNC
PLCRSDVLGPAATATESGGGDTGSMPQADPVANTIAAAQQAAAPGDAILERQEEEEEAEQ
EDQGAPPHMEENRQEQSSSPDPLPPPRNVRRAASMDAAIVSTAAEVAALERLPEAAPEEE
QSGGGGGGDKRGGAPGASCLKVSSSGRLSNLGAAERLPRSFFSRHCRARSSVLPL*
>11668.m03459|LOC_Os02g36350.1|genepair1557-1
MLPSRPRSGSWGGGTRSSPVSTPRSRHERSKSVATIFSPSSPESYGGVVMEMEEKKTSCT
LQEVDGLRQSDGGVDKEVVGGGAACSDDRVRLLEREVATAKATEMNMLESLIQQTKEMEQ
AKIALEEAKLEVATLRQQQGRAPAAEPAAAAQWSVMDLMFGGVDEEINGLRAKLRAAVQA
EEKSRKAADDLTAALSAVTMEAKQVKAWLSDAQAELEDANAEVDRLRESLHAAEAELWST
TEQLDGLTSDWKEAAVSWRAREKVLLARVRAAEDEAHAAGQENVELAELHRVVDDENGSL
RRALERAVEEVNAANESLELATGENSKLQDAVAEKESAMEALRQENESLKASEAEARGRA
KELDGQLAAARKAADHGHGAGELAAAADPLSSLHKWRGDMHGKLSATFLDSNRVMAGRKD
RMFASLSNIAELKSAAAAAAMDDFDYEFDHFDGGQYGDLDHAMKQKKRRSILRKFGDFFR
RRSLYKRNLAPVIHY*
>11670.m03571|LOC_Os04g37790.1|genepair1557-2
MRQKVSEMEEELRKEKDAAEKVQLLQREVEKAKESERKMLESLIYQTKQLEQTKISLEEA
KLEITTLQQANASLEASAAAAVVSRRGGAVEQRSVKDLVFGGGDDEIRALRCELRTAMQG
EEKSRKALDDLSVALSDVTMEAKQVKMWLSEAQAELEAANAEAERLRHELDAAEARLRDV
SDEHDRCRLDAEECAAAWGDKERVLLDCVRASEEEVNRERQENTKLVESQRVIRDENARL
RDILKQAVAEANVVKESLELARGENARLNDVVAEKDGALQSLRQEYECIKVSEAAAQGSL
KELNSLLAATTTTTATACSTPASARTASVADYGFDQHLPSVRLVSSAKGTPEAASHRWTA
ADKSRTPSSRRYSIGEPAKFKGGFSQSARMGNLNHKDRVFASLSNIADLKSAAEAAMSDF
DDEFDHVDESHYDSMDQSMKQKKKRPILRKFGDLFRRKSFYKANLAPVHT*
>11668.m03462|LOC_Os02g36380.1|genepair1558-1
MSASPSSMSGAGAGEAGVRTVVWFRRDLRVEDNPALAAAARAAGEVVPVYVWAPEEDGPY
YPGRVSRWWLSQSLKHLDASLRRLGASRLVTRRSADAVVALIELVRSIGATHLFFNHLYG
SIDPEFQIDPLSLVRDHRVKALLTAEGIAVQSFNADLLYEPWEVVDDDGCPFTMFAPFWD
RCLCMPDPAAPLLPPKRIAPGELPARRCPSDELVFEDESERGSNALLARAWSPGWQNADK
ALAAFLNGPLMDYSVNRKKADSASTSLLSPYLHFGELSVRKVFHQVRMKQLMWSNEGNHA
GDESCVLFLRSIGLREYSRYLTFNHPCSLEKPLLAHLRFFPWVVDEVYFKVWRQGRTGYP
LVDAGMRELWATGWLHDRIRVVVSSFFVKVLQLPWRWGMKYFWDTLLDADLESDALGWQY
ISGSLPDGRELDRIDNPQLEGYKFDPHGEYVRRWLPELARLPTEWIHHPWDAPESVLQAA
GIELGSNYPLPIVELDAAKTRLQDALSEMWELEAASRAAMENGMEEGLGDSSDVPPIAFP
PELQMEVDRAPAQPTVHGPTTAGRRREDQMVPSMTSSLVRAETELSADFDNSMDSRPEVP
SQVLFQPRMEREETVDGGGGGGMVGRSNGGGHQGQHQQQQHNFQTTIHRARGVAPSTSEA
SSNWTGREGGVVPVWSPPAASGPSDHYAADEADITSRSYLDRHPQSHTLMNWSQLSQSLT
TGWEVEN*
>11670.m03589|LOC_Os04g37920.5|genepair1558-2
MSVSSSSMGGGGGGDAGGRTVVWFRRDLRVEDNPALAAAARAGGEVVPAYVWAPEEDGPY
YPGRVSRWWLSQSLKHLDASLRRLGAGKLVTRRSADAVVALLQLVRDTGATRLFFNHLYD
PISLVRDHRLKEMMAAEGIIVQSFNADLLYEPWEVVDDEGQSFTMFAPFWNRCLSMPYDP
AAPLLPPKRINSGDLSMCPSDDLIFEDDSERGSNALLARAWSPGWQNADKALTAFLNGPL
IHYSVNRKKADSASTSLLSPYLHFGELSVRKVFHLVRMKQLVWSNEGNRAAEESCTLFLR
SIGLREYSRYLSFNHPCSHEKPLLAHLRFFPWVINECYFKIWRQGRTGYPLVDAGMRELW
ATGWLHDRIRVVVSSFFVKVLQLPWRWGMKYFWDTLLDADLESDALGWQYISGSLPDGRE
LDRIDNPQLEGYKFDPHGEYVRRWLPELARLPTEWIHHPWDAPASVLQAAGVELGSNYPL
PIVGLDAANARLQEALSEMWQLEAASRAAMDNGMEEGLGDSSEVPPIEFPRELQMEVDRE
PARVTANVLTTARRREDQMVPTMTSSLNRAETEISADFMNSVDSRAEVPTRVNFEPRTER
EENFRTTAGNVARTNGIHEHNNFQQPQHRMRNVLAPSVSEASSGWTGREGGVVPVWSPPA
ASDHSETFASDEADISSRSYLDRHPQSHRLMNWSQLSQSLTTGREVENSMQPNWIG*
>11668.m03464|LOC_Os02g36400.1|genepair1559-1
MARTARLLAAEDPPPPPAAAAERPRPAAAGSGLRSLSSAASGLWDRLSVLGAGVSKLEKA
LGDHFPEGARYFGLENFGNTCYCNSVLQALYHCIPFREQLLEYYATYKNTEDSEDNLLTC
LADLFSQITLAKKRTGVLAPKRFVQRVRKQNELFRSYMHQDAHEFWNFLVNDIIDILEED
CRTANSSPETTPEEVSNGAANALANGARERPLVTLVHRTFQGILTNETKCLMCDTITAKD
ETFFDLSIDVEQNSSLTSCLKSFFSTEILNGEDKFFCDKCSSLQEAHKRMKIKKAPHVLV
IHLKRFKYVEQLSRHKKLSYRVVYPLELKLGSMSEDADCEYSLFAVVVHVGSSPNHGHYV
SQIKSHGNWLSFDDDTVQISEESTLQTFYGSSREHCGGNTDHGYILFYERLGGKS*
>11670.m03593|LOC_Os04g37950.1|genepair1559-2
MVMGASGSKLEKALGDQFPEGERYFGLENFGNTCYCNSVLQALYFCIPFREKLLEYYANN
KTPGDAEENLLTCLADLFMQVSQSKKKTGVIAPKRFVQRVKKQNELFRSYMHQDAHEFLN
FLLNELVDILEKESNAAKDSPQSSSPEKVPNGPVQPLANGVRKEPPVTLVHKNFQGILTN
ETRCLRCETVTARDETFFDLSVDIEQNSSITSCLKNFCSTETLNAEDKFFCDKCCSLQEA
QKRMKIKKAPHILVIHLKRFKYIEQLGRYKKLSYRVVFPMELKLSTSDDVDTEYSLFAVV
VHVGSGPNHGHYVSLVKSHNHWLFFDDENVEMVEESTLQTFFGSSHEYSGNTDHGYILFY
EGLGGKS*
>11668.m03466|LOC_Os02g36420.1|genepair1560-1
MQSFLEAFFPDIWAKMNNAEQDAYCIFDSQVLTTFVSSLYLAGVFACLIAGHVTRRVGRR
NSMLIGASLFFVGAILNCAAVNIAMLVIGRILLGFAVGFTNQSAPVYLAEIAPARWRGAF
TSIFHFFLNVGMFVADLVNYRANTIPVWGWRLSLGVAVVPAAVILVGAAFIPDTPNSLVL
RGKLDEARASLRRIRGAAANIDAELKDIARAAEEDRQHHTGAFRRIVRREYRPHLVMAIA
IPVFFELTGMIVVTLFTPLLFYTVGFSSQKAILGSIITDVVSLASIAAAALTVDRYGRRT
LFMVGGGVLLVCLTGMAWTYGARLGSDGGKAMPRGYAVAVVALVCLYDAGFGISWGPLKW

IIPSEIFPLEVRSAGQSMSEAISLALTFAQTQSFLRMLCSFKFGAFAYNAAWVVVMTAFV
ALLLPETKGVPIESLGAVWAQHWYWKRFVKPPPPPPSTAETKQADGAPA*
>11670.m03596|LOC_Os04g37980.1|genepair1560-2
MAGGVIVANDGDGSAVDHGGRLTFSVVITCLVAASGGLIFGYDVGISGGVSTMEPFLRRF
FPGVVRRMAEARPGNEYCVYDSQALTAFTSSLYVAGLVASLVASRVTRAMGRQAVMVMGG
ALFFAGGAVTGFAVNIAMLIVGRMLLGFGVGFTNQAAPLFLAEMAPTRWRGSLTAGFQFF
LAVGVVIATVTNYFASRVPWGWRLSLGLAGAPAVVIFLGALFLTDTPSSLVMRGDTARAR
AALLRVRGAGADVEAELKGIVRAVEVARQGEDGAFRRMAARREYRPYLVFAVAMPMFFQL
TGVIVISFFSPLVFRTVGFGSNAALMGNVILGAVNLVCLMLSTLVIDRYGRKVLFMVGGA
IMIIAQVGVAWIMGAQVGKNGSEAMARPYAVAVVAFTCLHTAGFGWSWGPLGWVIPGEIF
PVDIRSAGQAMNVSIGLGLTFVQTQSFLAMLCRFRYGTFAYYAAWVAVMTVFIAVFLPET
KGVPLESMATVWARHWYWKRFAREQPKTSADEPTGTY*
>11668.m03469|LOC_Os02g36450.1|genepair1561-1
MAGGGSIANDGEAAAGGNGGGDEVTFTVVMSCLTAGAVGLLLGYDIGVTGGLTQMESFLQ
AFFPEVLRKMSSAKQDAYCIFDSQVLNAFVSSFYLSTMVASLVAGHLTKTLGRRNSLLIA
GVLFFAGTLLNLAAVNISMLIIGRILLGVAVGFSSLAAPVYLAEISPARWRGAFTSSIGL
FANFGFLMADMINYRATTMARWGWRLSLGAGIVPALIVIVGAASIPDTPNSLALRGRLDE
ARDSLRRIRGAGVAAADVDAELKDIVRAAEEDRRYESGALRRLLRREYRPHLVMAVLITV
FYEMTGGVVVSIFTPLLFYTVGFTSQKAILGSIITDVVSISSVAVAAVVVDRRGRRTLFM
VGGAVLILCQVAMAWIFGAELGTDGGRAMPRGYAVAMVAVVCMYAAGLCVSWVPLSSVVT
SEIFPLEVRSAALGLGGAISSALTFMQSQSFLEMLCSFKYGAFAYYAGWLVMMTAFVAAF
LPETKGVPIESMGAVWAQHWYWKRFVKLAPAKQADGPE*
>11670.m03597|LOC_Os04g37990.1|genepair1561-2
MAAGTEAARDYGGGVTASVVVTCLIAASCGLIFGYDIGVSGGVTQMQSFLTKFFPEVVKG
MRGAKRDAYCRYDNQVLTAFTSSLYIAGAVASLVASRVTRMVGRQAIMLTGGALFLAGSA
FNAGAVNIAMLIIGRILLGVGVGFTTQAAPLYLAETAPARWRGAFTAAYHIFLVIGTVAA
TAANYFTDRIPGWGWRVSLGLAAVPATVIVVGALFVPDTPASLVLRGHTEKARASLQRVR
GADADVDAEFKDIIRAVEEARRNDEGAFRRLRGRGYRHYLVMVVAIPTFFDLTGMVVIAV
FSPVLFRTLGFNSQRAILASIVLTLVNLCAVVVSSFTVDRVGRRFLFLAGGTAMLLCQVA
VAWILAEHLGRSHAAATMAKSYAAGVVALMCVYTASLGLSWGPLKWVVPSEIYPVEVRSA
GQALGLSVSLTLSFAQTQVFMSMLCAMKYAIFLFYAGWVLAMTAFIALFLPETKGVPLEA
MRAVWAKHWYWKRFAMDAKLDAQVNCL*
>11668.m03473|LOC_Os02g36490.1|genepair1562-1
MAFFPNIPEAKQEIQSLYRNYAHLFDMSSDNAGVDSRIMRRQPAMLIDRSVFLTGSAIWT
IALHYKAHIYRWLLQKFSAIQTTSSQRNAAVVGNIVIVLVNSFGILGSDFTTKHHGREVT
FTVSAILMVFCQITIPLLVEAQIGLGGGTRILTGYTTATFLLTCVVSYGLSWSWGSLFCT
IPGMKIQSAGQVIGMGLCFGLCFVQMQYFLLMLCRLKNAILAYYAMWIWS*
>11670.m03602|LOC_Os04g38040.1|genepair1562-2
MGRAPLVLIGRAVPIIGGAIPIIAINYGILVACWMLHMFDVVQSKLICRAMALHFSWRST
SPFLDTVCSSVYDLYNSSAIHRASCGEQLVVNGTNCDLGDYWQHSPTPDFLDSDRIYAVL
VLGCVCSSGGCGRGEAHAKQGHDVSGEKRPSRRDGLGRSFWWIMVARKYEWCVTYAVVVA
TLQLFLRLTGANVTTLFLPMLSQATGCGKAALAGHAVLVLANAGGVLGSALAARTYGREV
MCVIGGVLIVFCQVVIPVAMEMHGGGGAYAAAAATFFVACAASGGCGWSWGALFWAVPGE
GVRSAGDAVGAALGFALGFAQTHCFLLMLRQLKHAALAYYAVWIWSSVTTLSASELFAYL
DSDTVNQYDDDFNILNWWHEHNHTYPILSILARDVLTVPVSTISSESAFSLTGRIIEERR
RRLAPDMVQALALIKDWEQADAKMQHTMENLELINSFDNLFLDEVTTAIGTG*
>11668.m03474|LOC_Os02g36500.1|genepair1563-1
MERAVPVRKTHASTAGLLSWSESPGPDNAAAAAGAAAPPSSRPSLKPAGGITPAMFGAPV
TDLEAEDLTKRKMCSGSKMKEMTGSGIFSAQGANGDSETGSGDSNPPSKTSLRMYQQTVT
GISQISFSAEGSVSPKKPSSLPEVAKQRELSGTLESEADAKLKKQNSEAKSKELSGSDIF
GPPPEIPSRPLAARNLELQGNLDFALPQPRSIHTSVKVSNPAGGPSNIMFGEEPAVKTAK
KIHNQKFHELTGNNIFKEEAPPGSAEKSLSTAKLKEMSGSNIFADGKVASRDYFGGVRKP
PGGGSSIALV*
>11670.m03629|LOC_Os04g38310.1|genepair1563-2
MERAVPVRKPHTSTADLLTWSATGPDAAAAAASPVASSRPSLKPAGGITPAMFGAPVSEK
EAEDLSKSERKFCSGSKLKEMTGSGIFAENSENDDSEASNPANKTSVRMYQQTVTGISQI
SFSADGSVSPKKPSSLPEVAKQRELSGTLETDAEAKMNKQHSEAKSKELSGSDIFGPPPE
IPARPLAARNMELQGNLDFALPQPRSVHTSVKVSNPAGGPSNITFSEEPVVKTSKKIHNQ
KFQELTGNNIFKEDATPASAEKSLSSAKLKEMSGSDIFADGAAAPRDYLGGVRKPPGGES
SIALI*
>11668.m03475|LOC_Os02g36510.1|genepair1564-1
MASRSPTNKQISSEDRVRGARGKRHHHSSDEWSMEHAEEHEHGRGGGERDDAAEPEAEQP
ELEQEELSDSESGAESIEISDLKKRMWKDQMLLMKLEGGRPGGGGGRGGGGLAAAGAGTS
SEGQLEEETPEARCRRKAMLRAQDGVLRHMLKMMEACNARGFVYGVIDEAGQPMSGSSDS
LRGWWKDNVSFDRAGPMALIGPAAAGDSPQAGGGGLHRLQDIQDSTLGSVLSALIQHCEP
PQRSFPLERGLAPPWWPTGEEPWWGTQGETQAHQGAPPYRKPHDLKKAWKVSLLSAVIKH
MSPRFDQMRKLVWQSKRLQQKMSAKESDTWSKVIRQEEALDRRLKTSLHITLLDADGGEE
DDSDGLEDVVRGAAAQDKRKREYTRSGSGSSSGNSGGGKFPRGGSGGADHHQLAVMLPEL
AAAADQEGRSPINELMKLYYSCLQQEEGAADGGEAGGEGCDVAAAALAVPPEVLAGVDEV
AQDVLFDLIGSYPEVDDVLHFMDE*
>11670.m03638|LOC_Os04g38400.1|genepair1564-2
MDDKGKAKAADAAAAAEAAPEQEEFFSDSESGSESIEIADLKKRMWKDQMLLMKLEGRSG
HEGALAAQDHRVVRREEEAAEEEPPEARYRRKAMLRAQDGVLRHMLKMMEACNARGFVYG
IVDESGVPVSGSSDSLRGWWKDDVAFDRAGPTALSGRGGRGSPRSPAAAASFLHGLLDIQ

DSTLGSLLSALIQHCEPPQRSFPLDRGLPPPWWPTGGEAWWGLQGEAQASQGPPPYRKPH
DLKKAWKISLLRASAVIKHLSPRFDQIRKLVWQSKRLQHKMSARDADTWSRVITQEEALS
RHALRSLHITPLDDDDDEPNEGPTPRESHADKRKREVGGGGGGGGEEMQLSLPADIDVVP
EADRSSIDELMKLYYSCLQGTDTDGGGGEQGKDVAAGAGGDGSVAPETVHVDDDDMLEGL
LGVAQVVDMSDFPDSPICHWGSSSD*
>11668.m03484|LOC_Os02g36600.1|genepair1565-1
MARAVLTLTVLAFLCLVALGLSGRANANATAGRKMVGVYELKKGDFSIRVTNWGATIMSV
ILPDSKGNLADVVLGYDTVAEYVNGTAYFGGLIGRVANRIANARFTLDGKTYRLFRNDGN
NSLHGGHRGFSKVIWTVKEHVAGGDSPRITLYYHSFDGEQGFPGDLDVFVTYELPRPYVL
AVRMNATARGKATPVNLAHHAYWNLAGEGSGAASVLAETVRIHASRYTPVDAATLIPTGR
VAPVAGTPYDFLAGAPVGARIVGAAVPGGAVSGYDTNYAVDGAAADGERRRLRPVAEVRD
GATGRAMEVWADQPGVQFYTSNGLAGVRGKGGRVYGRYGALCLETQGFPDAVNHPSFPSQ
IVRPGQVYEHNMVFKFTF*
>11670.m03651|LOC_Os04g38530.1|genepair1565-2
MARPAPLLLLLAAVCLVAAASAGGADAAERKATTVGVYELRKGDFSIRVTNWGAVIMSVV
LPDSRGKLDDVVLGYDTIAEYVNSSTYFGALVGRVANRIAKARFVLDGKAYHLYPNDGKN
TLHGGHRGFSNVTWTVKEHVGGGDAPYITLYYHSFDGEQGFPGALDVYVTYQLSGPYVLS
VHMNATAAGKATPVNLAQHSYWNLGGTGSGDILGNTVQLFASRYTPVDAELIPTGQVAPV
TGTPCDLRAPTPVGARVHLVTGGLSKTGATIYGFDTNYAVDGGDDVDAHAHALRRVTVVR
DGKSGRSLELWANQPGVQFYTGNFLTADVKGKGGKAYGQYGALCLETQGFPDAVNHPNFP
SVIVRPGQVYKHEMVYKFSF*
>11668.m03495|LOC_Os02g36710.1|genepair1566-1
MTAPADKGKKAKTDADGGAAEENEQIDGALVLSIEKLQEIQDELEKVNEEASDKVLEVEQ
KYSEIRRPVYLRRSDVIQTIPDFWLTAFLSHPLLSELLTEEDQKMFKYLESVDVDDSKDV
KSGYSITLTFSENPYFEDKELTKTYAFADDGTTTINATSIKWKEGMEIANGNAKKKGSKR
PLVEESFFTWFTDTEHKSLADGVQDEEAEELGEDDDEEGSDADEGEEDEEEEN*
>11670.m03662|LOC_Os04g38620.1|genepair1566-2
MAAAEQKGKKPRTDGAEAEPVDAALLQSIEKLQEIQDEIEKVNEEACDKVLELEQKYNEV
RRPVYVRRNKIIKQIPDFWLTAFLSHPMLGELLTEDDQKIFKHLESIDVDDSEDIKSGYS
ITLTFSPNPYFEDTKLTKTYSFSDDEAVKVKATSIRWKKGMDIANDRAYTKKGDKRILID
ESFFTWFNSEKNRSFAHGAMDEVADVIKEDLWPNPLKYFNNEFEEELELLDDDDEVSDDD
DEEEDDEDQGEGEEDGEEN*
>11668.m03497|LOC_Os02g36730.1|genepair1567-1
MGAHCHQLAILVVLVLLASTPEVLAVRSLGVLAQTSSANASSAEQPRKLAEGNAAVAVTA
AAAAAAARFDTSTEKNTAATGSSSPSTVFDPDRMSKRRVRRGSDPIHNKC*
>11670.m03665|LOC_Os04g38650.1|genepair1567-2
MRGLNLALAVLVLVVVLLASFSDEVLAVRTPAVFAASRRSASPPTERPRELVEGGNAVAA
TATFDASVKAAAATATATGSSPSKVFDPDRMSKRRVRRGSDPIHNKC*
>11668.m03508|LOC_Os02g36840.1|genepair1568-1
MQPHAVCLPFPAQGHITPMMKLAKILHSRGFHVTFVSTEYNHRRLVRSRGAAAAAGIPGF
RFATIPDGLPPSDADATQDPPSLSYSTMTTCLPHFRKLLADLNNRLAPDDDDAAPPVTCV
VADHLMGFSLDAAAELGVPCALFWTASACGYMGYRNFRLLIDMGIIPLKGEEQLTNGFMD
MAVDWAPGMSKHMRLKDFPTFLRTTDRNDILMTFQLRQVERAEEADAVVLNTFDELERPA
LDAMRAITPAIYTVGPLAFLTEQIPPGGPLDDISPSLWREDDACLRWLDGRNPRSVVYVN
YGSVTVMSGHELEEFAWGLAGSGHDFLWIVRPDVVTRTAAATAAEAALPREFTEATKGRG
LVASWCDQEAVLRHPAVGLFLTHSGWNSTVEALSGGVPMLCWPFFAEQQTNCRYKCVEWG
VAMEVGDSVRREAVEGRIREAMGGGEKGKEMRRRAAEWKEAAARARGRSLANLERLIGDV
LLSGKKDRLIKEIENTNGSHILITEFLEASLRIPDLTLLKNNMQSFCYPTSPPTLDVLMQ
AILLIEADVALMDVSATMWSVQSRLTRCPRPWSSAEAVFQVPEEMKRMLGSFYGRCENKI
IGNRKAQGRRFTTMEAGEEIEDGEPSTPTYKAHHPPPHLPPPMRSSGVSLVLSVADLVLR
FVAIGGTAGSAIAMATTSETLPFAAPFVRFRAEYSDLPTLMFFVVASSVVCAYLVLSLPA
SVVHVVRPGARSSRAILAFLDTVMLALLTASASAAAAIVYLAHRGSARANWLGICQQFTS
FCQRITASLVGSFAAAVVLVALVFLSALSLARRA*
>11670.m03669|LOC_Os04g38690.1|genepair1568-2
MTKDGSVMEHGEISSKAPLVAPVAAGVNRAVAVVDTFLRFIAIIGTIGSAIAMGTTNETL
PFFTQFIQFEAKYSDLPSFTFFVAANAVVCTYLVLSIPLSIVHILRPRARYSRLFLVFFD
TAMLALLTAGASAAAAIVYLAHKGNVRANWFSICQQFDSFCERISGSLIGSFAAMVLLVV
LITLSAFALARRH*
>11668.m03512|LOC_Os02g36880.1|genepair1569-1
MRLARQQQQVVVAATMEHDVHHHRQMMQQQQQQEMDLPPGFRFHPTDEELITHYLLRKAA
DPAGFAARAVGEADLNKCEPWDLPSRATMGEKEWYFFCVKDRKYPTGLRTNRATESGYWK
ATGKDREIFRGKALVGMKKTLVFYTGRAPRGGKTGWVMHEYRIHGKHAAANSKQDQEWVL
CRVFKKSLELAPAAAAAVGRRGAGAGTDVGPSSMPMADDVVGLAPCALPPLMDVSGGGGG
AGTTSLSATAGAAAAPPPAHVTCFSNALEGQFLDTPYLLPAADPADHLAMSSASPFLEAL
QMQYVQDAAAAGGAGMVHELLMGGGWYCNKGERERLSGASQDTGLTSSEVNPGEISSSSR
QQRMDHHDASLWAY*
>11670.m03673|LOC_Os04g38720.2|genepair1569-2
MEQHQGQAGMDLPPGFRFHPTDEELITHYLAKKVADARFAALAVAEADLNKCEPWDLPSL
AKMGEKEWYFFCLKDRKYPTGLRTNRATESGYWKATGKDKDIFRRKALVGMKKTLVFYTG
RAPKGEKSGWVMHEYRLHGKLHAAALGFLHGKPASSKNEWVLCRVFKKSLVEVGAAGGKK
AAVVTMEMARGGSTSSSVADEIAMSSVVLPPLMDMSGAGAGAVDPATTAHVTCFSNALEG
QFFNPTAVHGHGGGDSSPFMASFTQYGQLHHGVSLVQLLESCNGYGGLVDMAASGSQLQP
AACGGERERLSASQDTGLTSDVNPEISSSSGQKFDHEAALWGY*
>11668.m03514|LOC_Os02g36890.1|genepair1570-1

MGRSPCCEKEAGLKKGPWTPEEDQKLLAYIEQHGHGCWRSLPTKAGLRRCGKSCRLRWTN
YLRPDIKRGKFTLQEEQTIIQLHALLGNRWSAIATHLPKRTDNEIKNYWNTHLKKRLARM
GIDPVTHKPRVDADADVAAGGGAAGGGARSRAAAHLSHTAQWESARLEAEARLAREAKLR
ALASPPPATAALSGVDSPTSTLSFSESALFGAGSAAPDIHGAARAAAVQAVQSSYGEACQ
EHHFGGATAETSFAGAGTLAGVLLDCSVTGADQRFAERTEACSGELQGEDDDDKGYWNSI
LNMVNSSMSSSSSSLTSEVVTDTEMFLPATAAAAASATPVEF*
>11670.m03675|LOC_Os04g38740.1|genepair1570-2
MGRSPCCEKEGLKKGPWTPEEDQKLLAYIEQHGHGCWRSLPSKAGLQRCGKSCRLRWTNY
LRPDIKRGKFSLQEEQTIIQLHALLGNRWSAIATHLPKRTDNEIKNYWNTHLKKRLAKMG
IDPVTHKPRSDVAGAGGGGGGAAGGAAGAQHAKAAAHLSHTAQWESARLEAEARLAREAK
LRALAASATPGAPHLPAPPASAAAAAAAHGLDSPTSTLSFSESAVLATVLEAHGAAAAAA
ARAAMQPMQAYDEACKDQHWGDVDAADVGFPGAGAGFTGLLLEGSLNQIPRPAGRDAEAD
GEFQETEEEKNYWNSILNLVNSSSAPMSTAVVVPASHAYSPAPDF*
>11668.m03517|LOC_Os02g36920.1|genepair1571-1
MGRGKIVIRRIDNSTSRQVTFSKRRNGIFKKAKELAILCDAEVGLMIFSSTGRLYEYSST
SMKSVIDRYGKSKDEQQAVANPNSELKI*
>11670.m03678|LOC_Os04g38770.1|genepair1571-2
MGRGKIVIRRIDNSTSRQVTFSKRRNGIFKKAKELAILCDAEVGLVIFSSTGRLYEYAST
SMKSVIDRYGRAKEEQQHVANPNSELKEFCSVFIYITEN*
>11668.m03518|LOC_Os02g36930.1|genepair1572-1
MGEDLSGLNVKELQSLENQLEISLRSVRTKKDHVLIDEIHELNRKGSLVHQENMELYKKI
SLIRQENAELYKKIYETEGPSEVNRDSPTPYNFAVIEKTNVPVQLGLSTLPQHSDAEQST
APKLG*
>11670.m03679|LOC_Os04g38780.1|genepair1572-2
MRKTTKRAIFNPPNTLQDNSYTKLAVLAKGGSKLETTTAQLARKSSKNRTEHPSKADRPN
LQRRVNITFSSPVLSAWQLMGQDLSGLGVKELQTLENQLEMSIRCIRTKKDQLMIDEIHE
LNRKGSLIHQENMELYRKIDDFTGYTLHYMMTANLSPQLYETGAENEANRDSTTPYNFAV
IEEANTPARLELNPPSQQNDAEQTTPPKLGLQLHP*
>11668.m03519|LOC_Os02g36940.1|genepair1573-1
MYSKPEDVGGGVTTAFAMQGKVPLAAWSTGLFNCFDDCGNCCVTCLCPCITFGQIAEIID
RGSSSCGTSGALYALVMLLTGCNCVYSCFYRAKMRSQYGLQEKPCADCPVHFFCEPCALS
QEYRELKKRGFDMNLGWHANMERQGHKPAMTMPPHMFPGMTR*
>11670.m03680|LOC_Os04g38790.1|genepair1573-2
MATGFACAYSCCYRSRLHQQYGLQEKPCGDCCVHWCCGPCALCQEYRELKSRGFDMSLGW
QGNMERMGKGVATAPPQMHPGMTR*
>11668.m03524|LOC_Os02g36980.1|genepair1574-1
MSQPAELSREENVYMAKLAEQAERYEEMVEFMEKVAKTVDSEELTVEERNLLSVAYKNVI
GARRASWRIISSIEQKEESRGNEDRCTLIKEYRGKIETELSKICDGILKLLDSHLVPSST
APESKVFYLKMKGDYYRYLAEFKTGAERKDAAENTMVAYKAAQDIALAELPPTHPIRLGL
ALNFSVFYYEILNSPDRACNLAKQAFDEAISELDTLSEESYKDSTLIMQLLRDNLTLWTS
DISEALPIYVVLGNLAADSLLGWWILELHFLWGEPQGGTFMSLIGSIVFGMSVVSLISRV
HSPAKCPA*
>11670.m03688|LOC_Os04g38870.1|genepair1574-2
MSAQAELSREENVYMAKLAEQAERYEEMVEFMEKVAKTVDSEELTVEERNLLSVAYKNVI
GARRASWRIISSIEQKEESRGNEDRVTLIKDYRGKIETELTKICDGILKLLESHLVPSST
APESKVFYLKMKGDYYRYLAEFKTGAERKDAAENTMVAYKAAQDIALAELPPTHPIRLGL
ALNFSVFYYEILNSPDRACNLAKQAFDEAISELDTLSEESYKDSTLIMQLLRDNLTLWTS
DISEDTAEEIREAPKRDSSEGQ*
>11668.m03525|LOC_Os02g36990.1|genepair1575-1
MDPASEELEQRSRYLSSLIRRTKLHAAPALAPPPPTPPPEPETKLQLEMEPQPERVEEAA
KKPAAVAAVVEKREVKGGGGGGGQAGKGKGKEKEMEKGKEERKVSVRVRAADMPLAMQRR
AVRLAFDAVAAMPRLDSKRLALALKKEFDATYGPAWHCIVGTGFGSYVTHSVGGFLYFSV
DKVYVLLFRTAVEPLGHPQ*
>11670.m03690|LOC_Os04g38880.1|genepair1575-2
MDRASEELERRSRYLSSLVRRTKLADPPEPEPEPEPEPEPERERERERVAAKESGGGEGKGGK
VVEEKEVKAAKEKKEKEKEAKVPRGEGGNGEEKKVAVRVRAADMPPALQRRAIRVALEAT
AAMPRIDSKRLALALKKEFDTTYGPAWHCIVGTSFGSYVTHSLGGFLYFSVDKLYILLFR
TAVEPLSYQR*
>11668.m03526|LOC_Os02g37000.1|genepair1576-1
MAGGGQAAASLLTKLAQAAAGLGIAASAASTALYTVDGGQRAVIFDRFRGVLPETSSEGT
HFIVPWLQKPFIFDIRTRPHSFSSTSGTKDLQMVSLTLRVLARPDIDRLPDIFTSLGLEY
DEKVLPSIGNEVLKAVVAQFNADQLLTERPHVSALVRDSLIRRAAEFNIVLDDVAITHLA
YGPEFSQAVEKKQVAQQEAERSRFLVARAEQERRAAIVRAEGESEAARLISEATAAAGTG
LIELRRIEAAKEIAGELARSPNVSYIPAGDSSQMLLGLSGAR*
>11670.m03692|LOC_Os04g38900.1|genepair1576-2
MAGGPAAVSFLTNIAKAAAGLGAAASLLSASLYTVDGGERAVIFDRFRGVLPETVGEGTH
FLVPWLQKPFVFDIRTRPHNFSSNSGTKGPCRWVYPHPSGLLSPPPTSVPFPTIFTSLGL
EYDDKVLPSIGNEVLKAVVAQFNADQLLTERPHVSALVRDALIRRAREFNIILDDVAITH
LSYGIEFSQAVEKKQVAQQEAERSKFLVAKAEQERRAAIVRAEGESESARLISEATAAAG
TGLIELRRIEAAREIAAELARSPNVAYVPAGDNGRMLLGLNAAGFGR*
>11668.m03529|LOC_Os02g37030.1|genepair1577-1
MAHDASSPSTARDAKKKRIRGNRSAKLKQSKLDVRREQWLSQESDPSSVWIGLVKDGKEV
KAVVSPGAAAGANSGSPILASPHPPLPRRRAEIRTREGDPEDFKEDSVGASQDVGSSDHE
SPLHSPVSYNPPIGCLQQKHCSGNGGGRSFSSGSSAWSSSRSVTDSDDDTGGSPENDDDG

VLDDWEAVADALSVDDNHNHQDPVPADPPVVPASCPVPANAATRQEPIKSSTRAWSPDDA
FRPQSLPSLSKQVSFPASMGNCWVAMGIGSAQKGVPSKPTSCPICYEDLDPTDSSFLPCP
CGFHLCLFCHKRILEADGRCPACRKQYISASSGGETVGSEREMGNLRLSRSCSMGPRY*
>11670.m03700|LOC_Os04g38970.1|genepair1577-2
MGHVDASAAAAAARDAKKKRGNRSSAKLKQCKLDARREQWLSQVKDGKEAKASTSPTGTE
PNAGSMTVPSPHPPLPRRRLDVRSKGGDPEEDREERGAARQELGSSYLDSPVHSPSSDNS
GSVGGMHRKHYNNGGGLNLSSSSSVWSSSRSVSEAEDDDTGGPEEENGVLDDWEAVADAD
ALTVDDCHSHQSSGHVAPPAAPNVCTAPANQTGRQDPIQRTKAWAPDDIFRPQSLPSISR
QVSFPASIGNGWMGAAQQANLSTPLTCPICCEDLDLTDSSFCPCPCKFCLCLFCHNKILE
ADGRCPGCRKEYVAARLSRSCSMGPRY*
>11668.m03530|LOC_Os02g37040.1|genepair1578-1
MENEWMHARLEKLRRSGRQCISAAEENGNINNACLCCICACSRMHVAAGGGHGGGADDGM
VVDYRGNPVDKSKTGGWLGAGLILGTELAERVCVMGISMNLVTYLVGDLHLSNAKSANIV
TNFMGTLNLLALVGGFLADAKLGRYLTIAISATIAATVRSRSRTHRTTHLRSSSRGAGIS
LRSAAMQGVSLLTVDTMVPGMRPPPCADARGAGAHLRCEPARGGQLAMLYAALYTVAAGA
GGLKANVSGFGSDQFDGGDPREERAMVFFFNRFYFCISLGSLFAVTVLVYVQDNVGRGWG
YGVSAVAMVLAVAVLVAGTPKYRYRRPEGSPLTVIGRVLATAWRKRRLPLPADAGELHGY
HTSKVAYTDRLRVYVTRCALLIVQYCCCQPVTVKAEMDTSYREEAEDDNSVRPLANTARK
SKSCMVLDEMNEWSKCLDRAAIMEADLAASPAKTNQTSAAPAATVTEVEEVKMVVKLLPI
WSTCILFWTVYSQMTTFSVEQATRMDRHLRPGAAPGGFAIPAGSLSVFLFLSILLFTSLN
ERVLVPAARRLTRRPQGLTSLQRVGAGLVLATVAMATSALVEKKRRDAANDGGGGGMISA
FWLVPQFFLVGAGEAFAYVGQLEFFIREAPERMKSMSTGLFLVTLSMGFFLSSFLVFAVD
AATRGAWIRNDLDAGRLDLFYWMLAVLGVANFAVFLVFARRHEYKQPAGTAAVVVAPAAA
KDGGAGEEKEMDDFVVVKEAVEGMDV*
>11670.m03706|LOC_Os04g39030.1|genepair1578-2
MVSAGVHGGDDGVVVDFRGNPVDKDRTGGWLGAGLILGTELAERVCVVGISMNLVTYLVG
DLHLSNARSANIVTNFLGTLNLLALLGGFLADAVLGRYLTVAVSATIAAIGVSLLAASTV
VPGMRPPPCGDAVAAAAAAESGGCVAASGGQMAMLYAALYTAAAGAGGLKANVSGFGSDQ
FDGRDRREGKAMLFFFNRFYFCISLGSVLAVTALVYVQEDVGRGWGYGASAAAMVAAVAV
FAAGTPRYRYRRPQGSPLTAIGRVLWAAWRKRRMPFPADAGELHGFHKAKVPHTNRLRCL
DKAAIVEADLAAATPPEQPVAALTVTEVEEAKMVVKLLPIWSTSILFWTVYSQMTTFSVE
QASHMDRRAGGFAVPAGSFSVFLFLSILLFTSASERLLVPLARRLMITRRPQGLTSLQRV
GAGLVLATLAMAVSALVEKKRRDASGGAGGGGVAMISAFWLVPQFFLVGAGEAFAYVGQL
EFFIREAPERMKSMSTGLFLATLAMGFFLSSLLVSAVDAATRGAWIRDGLDDGRLDLFYW
MLAALGVANFAAFLVFASRHQYRPAILPAADSPPDDEGAVREAATTVKGMDF*
>11668.m03538|LOC_Os02g37120.1|genepair1579-1
MVEASNPTQDMIDITGHVVHDDVSYDKDVLEIKLPDTVVTSDYGGNFVKDVCIDEGVLPH
RKISAEKKLDEKSPPNFDFLMIDTNSDLTYGGKGDAKKYAHGQKPKTVLLPVGFAADDNT
EKQCDLESRDTASDISEKKISLHELLKLESAEESKERLKLESAEESQTQHQSTTSAINEK
NMPPVHEEAIAQVSTNDCNDVATASKTDELITSNVSSINNTNGSSATISDRHDATAALDK
PMSTAETTDGLIGSKQFNEVGTAEAMPDALTSSSSSEVQPSEKSNDHPESFTSEPIADPQ
DENAVATSSSPHVVESSDANRQMNNKNSDNDGATDVHDFNQTDSESCADATNDGRISTSS
TDAQKDSTHVDELDVPDNNAKGKSLIGNGYPLEPCSLGPSIMCNPVSTSGHIGNISIRSD
SSTTSTRSFAFPVLQWDWNSSPVRMARAERRRNKRRRGWNKGFLCWKF*
>11670.m03730|LOC_Os04g39240.1|genepair1579-2
MQIMKIDDGRPYHSNVFHELVSNGGPKVDGEIERETKQHILLDTMVQQTNPSEYSFMKAG
QQNVDKAIQIRPEDVSYDKDVVEIKLPDIMVSSNYGVQFVKDVCIDEGVLADQKAIAEKV
SLNLNSSKGDTNGIRIKETADEPAKSVNDLKSQIVVLPEACVTDGDTVEQNHPCKLHDLE
GNSSVDGLTVVNVEKSTPKQLVSNDNAEYCQQMGADVSESSENHGPNLNGEPVDQVPSND
SHETGASIASAITNVNGLSVESTADGHSGVVTEDGVSGVALNKTEINQINHYNPFIAYGS
LEDTWEPKYSLPTIVDDVYSVPCPVEKTDSFSDIVYGALRGFNFLETGESIAEDSTLDSV
VENSSMTDVQASEEKDEARSDDILSDERKIPVDQRSPVENSDSLSDPVDRALSSTETDGA
RNEDSRLDSTEASPSRSYVQPSEDRNDQVDNFVYGIRTDAAHGTSSGTSPLTGKTEPIDA
KSENDPKCEIDSVQDGHDFNPREANDGTNISEDNKDSKSSTRQTGPVTEQNEPDSAKMTM
QTEPVAQRNEPDSAKVTMQTESVAQPNEADSAKVTARNVIRNPFESSFSGPSIISGPLTP
SGHIPYSGNISLRSDSSTTSTRSFAFPVLQTEWNSSPVKMAKADRRRLRRDRGWGYRILC
CKF*
>11668.m03543|LOC_Os02g37160.1|genepair1580-1
MKKEIIIRISVKTDKCQKKAMKEAATVSGVQSVTLAGGDRNLLLVIGEGVDTNKLTKKLK
RKVGSGEIVELRTVDTFEAAAAMFPGGMIMPPPAPGSKDAAAARAMATTRASPYHYQPSP
YGPYHQQQQWQPPYAMAPSPYAYPYQYHPSPMAMAAGGGGYGYGGSSYSRAVALSHPAIY
SPLVEKHDYHPMNHSTTTKKKTTTTTTTTGAGTGRAAAAAAGGKTFKAVSRSRRHHESDS
NACCIL*
>11670.m03737|LOC_Os04g39290.1|genepair1580-2
MKEIIIRMRPDSDKCHHKALKVAAAVSGVESVTVAGRDRDLLLVIGDGVDESKLTKKLRR
EVGEAEILELRTLDAGGSRGGGGAASLQLMTAAGARNGKGGGAVVFAQSSPYHGWHGHPA
TPGRSVPGVGRIMYPVTTTTTATAASPGAARWPGGEQYRSSSPQAALYYPRNPPNAYYYG
GLGVRDGLAVARSHPANYSPMVERHDHGAVGRGGRRRRAGRRPSCCSIL*
>11668.m03545|LOC_Os02g37180.1|genepair1581-1
MRTEMLIRMQASSEKNGNAKAMKVAAAMDGVESVTLAGEGRNLLRVVGSGVDSNHLTSRL
RRKVGHADIVELRTLHDTYPRGAAAGSYAATSTSGRLGSSNGGYYYSSQLSAGRGGAYSS
GGHQLYGGGYDSPYYHQAPQHPYDGGYYPSPYGAAAVQHEYYTTSSNDDPNGCSIM*
>11670.m03738|LOC_Os04g39300.1|genepair1581-2
MRLATNPFNWLLVIKQLYICITSMRTEILIWIQLSSERCRSKALKVASTVNGVQSVTVAG

EERNLLLVIGDGVVDASRLTRRLRNHVGYAEIVELTTSSSTAVPPVDVAAAAVTEDAVRP
RYHGLVGGGGGLPWFARVGCPVTAHSVVASHAAPAAALWPGAGEVGGSWAASYSAHPSPC
YRSSPLAGGYTLDVARSHAANYSPLIERHAGRGGHYPAHSCCSRRKLLRRSVPSCCTIQ*
>11668.m03557|LOC_Os02g37300.1|genepair1582-1
MAKQKIVIKVEMSCDKCRSKAMALVAATGGVDSVALAGDGKDQVVVVGDGVDSIKLTAAL
RKKVGHATLVTVGEVKKEEKKPEPAAAAVEYPWSYHPAYTYAPPAQHVFYQQYPASSPWW
C*
>11670.m03747|LOC_Os04g39350.1|genepair1582-2
MKQKIVIKVSMPCEKSRSKAMKLVVMASGVSSVEVTGDGKDRLQVVGDGVDAACLVTCLR
KKIGHAELVQVEEVKEKKPEEKKPEEKKPEPCYCPHPCYYHHHYGGIPVAVGDQPSDPCS
IM*
>11668.m03569|LOC_Os02g37420.1|genepair1583-1
MSYAYLFKYIIIGDTGVGKSCLLLQFTDKRFQPVHDLTIGVEFGARMITIDNKPIKLQIW
DTAGQESFRSITRSYYRGAAGALLVYDITRRETFNHLASWLEDARQHANANMTVMLIGNK
CDLSHRRAVSYEEGEQFAKEHGLVFMEASAKTAQNVEEAFIKTAGTIYKKIQDGVFDVSN
ESYGIKVGYAVPNASGGGAGSSSQGGGCCG*
>11670.m03757|LOC_Os04g39440.1|genepair1583-2
MSYAYLFKYIIIGDTGVGKSCLLLQFTDKRFQPVHDLTIGVEFGARMITVDSRPVKLQIW
DTAGQESFRSITRSYYRGAAGALLVYDITRRETFNHLASWLEDARQHANANMTIMLVGNK
SDLSHRRAVSYEEGEQFAKEHGLIFMEASAKTAQNVEEAFVKTAGAIYKKIQDGVFDLSN
EANGIKLGYTVPGQSGGAGSSSSQGGGCCSS*
>11668.m03570|LOC_Os02g37430.1|genepair1584-1
MKLVRFLMKLNNETVTIELKNGTTVHGTITGVDISMNTHLKTVKLTLKGKNPVTFDHLSV
RGNNIRYYILPDSLNLETLLVEDTPRVKAKKPTAGKPLGRGRGRGRGRGRGRGR*
>11670.m03760|LOC_Os04g39450.1|genepair1584-2
MALAAQLYGSAAAAAYRRLPLYGVPSSCRWPRTPLAALPKLSISTGGMGMNPFGVGAKVS
IKCTNGTTQVDELNFRSNQTEELVEGDADTVTQKRSAKIHDFCLGIPFGLTTLFLGTLSL
KFWRSGKSSFIFILGQAAISAVLAWKYSHAYILTNRILPWAFYASLRFLMKLNNETVTIE
LKNGTVVHGTITGVDISMNTHLKTVKLTLKGKNPVTLDHLSVRGNNIRYYILPDSLNLET
LLVEDTPRVKAKKPTAGRAAWAVRRERGAAVQWRGMCKLKFNRERVGCYLLVILVVALLI
GVLFGLGVFRHGYERFKDLGRNHTCYDCNTG*
>11668.m03586|LOC_Os02g37590.1|genepair1585-1
MGRGSHGCSVLGSSLLLLFCLGSAAAQKASTWKTLSGNPPAIIAKGGFSGLFPDSSDFAY
GFVAAASSPDTALWCDVQLTKDGAGICLPDIRMDNCTNIANVYPKGKKTYSVNGVSTPGW
FSVDYDSTGLSKVNLVQSLFSRVPYYDGTLPILPVESVFANYKAPAVWLNVQHDSFYSQF
NLSMRSYILSVSKQYIADYISSPEVNFLTSLSGRVNKKTKLVFRLLNELAVEPSTNQTYG
SMLKNLTFIKTFASGILVPKNYIWPVTQDNYLQPSTSVVGDAHKAGLEVYAADFANDFLL
SYNYSYDPLTEYLNFIDNGAFSVDGVLTDFPITPSEAIGCFSNLNNSKTDNAKPLIISHN
GASGDYPDCTDLAYQKAVTDGADVIDCPVQVTKDGIPICMSSIDLMDVTTVSTSQFSSQT
TVIKDIKNGAGVYSFNLTWDDIAKNLKPKISNPMTTFDVYRNPRNKNAGSFMRLSDFLAF
AKGKELSGVMISIEHAAFMAEKLGFGVVDAVIKALDDSGYSKQTAQKVMIQSTNSSVLVK
FKEQTKYNLVYMLEEDVRDAAPSSLADIKKFANAVSVRTTSIYPESKHYLINQTSHIVQT
LQSAGLPVYVYVLMNEFVSQPNDFFADATTQINTYVQKKGAGVDGIITDFPATVHRYRLS
PCTSKESNLPTFMLPVQPGGLSGTIIDPAAQPPAMAPMPLLTDSDVAESPLPPVKNVTAP
APGASRAIKMRTDASIIVALLVLCASLII*
>11670.m03777|LOC_Os04g39610.1|genepair1585-2
MRGSHVCSLVSSLVFLWLGVAAAQKASSWKTLSVRSYAMHVYKANGQLTISGNAPAIIAK
GGFSGIFPDSSEFAYQFALIASSPDTILYCDVRLTKDGLGICLPDIKMDNCTNIPDFYQQ
GRKSYLVNGVSTAGWFSVDYNGTELGQVSLKQSIFSRSPRFDPSFFPILAVEDIASKFKP
PGMWLNVQHDSFYSQFNLSMSNYIFSVSKRVIVDYISSPEVSFLTKVSGKLSNNTRLVFR
FLDESTIEPSTKQTYGSMLKNLTFVKTFASGIIVPKKYIWPVSPDNYLEPHTSVVDDAHK
AGLEIYAADFANDFMFSYNHSYDPLAEYLSFIDNGCFTNLKKSKTDHGKPLIISHNGASG
DYPACTDLAYQKAVDDGADVIDCPVQLTKDGIPICMSSINLMDDTTVAKSQFASQTAVIK
DIESVLGVFTFNLTWDDIVKNLRPKISTPFSSFKLDRNPRYRNAGNFMRLSDFLDFTKDK
DLSGIMISVEHAAFVAEELGFDMVDSVIKTLDAAGYSNQTAQKVMIQSSNSSVLVKFKQQ
TKYDLVYMINEEVKDAAPSSLAAIKKFADAVSVEGNSIFPENRHFTTYQTNLVESLQNAG
LPVYVYTLMNEFASQPYDFFSDATAQINAYVQGAGVNGVITDFPATARRYKLNTCMHMGN
NTPSFMAPARPGDLLQIISKPAQPPAMSPMPLLTGSDVAEPPLPPARTAQAPSLASRMQA
HAAIVVTLAMLLACHPLV*
>11668.m03613|LOC_Os02g37850.1|genepair1586-1
MPEVRNSGGRAALADPSGGGFFIRRTTSPPGAVAVKPLARRALPPTSNKENVPPSWAVTV
RATPKRRSPLPEWYPRSPLRDITSVVKVMNHSRRLCGKFSEITGLNFECSSDAVERKSRL
GNAAVRQQIQLSEDSSRSVDPATPVQKEEGVPQSTPTPPTQKALDAAAPCPGSTQAVAST
STAYLAEGKPKASSSSPSDCSFQTPSRPNDPALADLMEKELSSSIEQIEKMVRKNLKRAP
KAAQPSKVTIQKRTLLSMR*
>11670.m03783|LOC_Os04g39670.1|genepair1586-2
MVSGFLRFGGPLFCPFFDELFAGCSDASVCLCRCRSGEVFESEQEAEEDFRGIGGEGTLA
RIHTSRNYSNMPEMRDSKRTALGELSGGGGFFIRRVASPGALAARGPGKPLARRFIRPSN
NKENVPPVWAVKATATKRRSPLPDWYPRTPLRDITAIAKAIQRSRLRIAAAQQRSQTPEQ
NTPHCTEVRDSLDVEPGINSTQIVATPASSLAKDSLKIFSSPSETSLVTPSKPMDPVLLD
DMEKKLSSSIEQIEKMVRRNLKRTPKAAAAQPSKRAIQRRTLMLTIAWCSARGPSSRINS
WKCQNILKYSKNAQYLLPIVKHQNCHEEDWGVPEKERKTGVAVADKA*
>11668.m03614|LOC_Os02g37860.1|genepair1587-1
MAPTSKLSQGIKRASRSHTYHRRGLWAIKAKNGGTFPKAGKPAAAAEPKFYPADDVKPRA

PSTRKANPTKLRSTITPGTVLILLAGRYMGKRVVFLKQLKSGLLLITGPFKINGVPIRRV
NQAYVIATSTKVDISGVKVDKFDDKYFARDKKAKAKKTEGELFETEKEATKNLPDFKKDD
QKAVDAELIKAIEVVPDLKSYLGARNSRWPISQQNKEYEVYRFKYANDSEESNEDISGIE
VVYKTETGNGGSSDGWLYVLHEEREMSIIQFYICLFWDRVSNDDISGIEVPFLRRRKSKP
SFDDSFPKLSPYQMEKFLVAAPPPSGDAPAAPVPPRRHRWSRVAAELDGRIDARFRHRES
VRLRDSFSEMNRVVNDSVLGFQAVREGISAMEFDKKGIYLASVTASGCLTVHDFETLYCS
TYGPSRGLPDESSNYLLHISNSMPLCAVRWNPANQDEIVCVSRQTDMVLLFDIGCVSSTP
TEILRKGRSRYPVLSEFRKGLTDVAFSSDDKSWLFASGLDGAVFMWDMRLSKKHCLELIG
HPESQFSSVKLNIDNRTVFAATKEGTVHAWDLRGGRASAAFQSHNEVQQLSSVKISTLLG
KIPSLKDQTNIVSSEILSIDFNPSCSYQLAFHLDNGWSGALNINTLSVSHLHCPPPDWLE
HMNFMWQKLHRKPTWLPTSSIYAVGSASNTVGMHLLDFHPDTSSACHVDYNEEIRGSDEK
KPAANKFIPSSQRVVSCAAHPFCHTILAGTQCQHVSCAVDAAADPDRGCPGLLAGGAVGV
AVSKGGGVGDACSPSQEALDSSHRRRQRWHRDFDRLSRAGISQYSAQASVSVL*
>11670.m03786|LOC_Os04g39700.1|genepair1587-2
MAPTSKLSQGIKKASRSHTYHRRGLWAIKAKHGGAFPKAEKPAAAAAAAAPKFYPADDVK
PRQPSTRKPNPTKLRSSITPGTVLILLAGRFMGKRVVFLKQLKSGLLLVTGPFKINGVPI
RRVNQPYVIATSTKVDISGVNVEKFDDKYFSRDKKQKAKKTEGELFETEKEATKNLPEFK
KEDQKVVDAELIKAIEAVPDLKTYLGARFSLRDGDKPHEMVF*
>11668.m03617|LOC_Os02g37890.1|genepair1588-1
MGVYVFTDRLAFAAADWCLRLHGWPIMPPLLGAYVFTDRLAFAAADWCPRLHGWPIMPPL
LGAYVFTDRLAFAAADWCPRLHGWPIMPPLLGAYVFTDRLAFAAADWCPRLHGWPIMPPL
LGAYVFTDRLAFAAADWCPRLHGWPIMPPLLGAYVFTDRLAFAAADWCPRLHGWPIMPPL
LGAYVFTDRLAFVAADWCPRLHGWPIMPPLLGAYVFTDRLAFVAADWCPRLHGWPIMPPL
LGAYVFTDRLAFAAADWEAIRSYAEKAHELVVDVAAKLATSLGLDCSFGDWPCQFRINRY
NYTPDTVGKTGVQVHTDSGFLTVLQEDDRVGGLEVADPDTGEFAPVDPLPGTFLVNLGDV
ATAWSNGELHNVRHRVRCVAGVQRVSIALFLLAPKDDVVRAPEAFVSAERPRRFRDFGYD
DYRRLRQSTGEHAGEALARLAA*
>11670.m03816|LOC_Os04g39980.1|genepair1588-2
MVEIPAIDLRLAGGGGGAEETARLRDACARLGCFRVSGHGVPPGLQAEMKAAVRALFDLP
DDAKRRNADIIPGSGYVPPGTANPLYEAFGLCDAAAPADVDAFCARLDAPPHVRETVKAY
AERMHSLIVDVAGKVAASLGLHGASFQDWPCQFRMNRYNYTQDSVGSPGVQVHTDSGFLT
VLQEDECVGGLEVLDPAAGEFVPVDPLPGSFVVNVGDVGQAWSNGRLHNVKHRVQCVAAV
PRVSIAMFLLAPKDDTVSAPGELVDGEHPRRYREFKYDDYRRLRLSTGERAGEALARLAA
*
>11668.m03630|LOC_Os02g38010.1|genepair1589-1
MGAAEGCCGGAAIAAAAGVIEEAETVVPPPATAIASSKGIRIMTRTQKSHPLDPLSAAEI
SVAVATVRASGRTPEDAMEYAECEATVKSHPPFIEAMKKRGVDDMDLVMVDPWCAGYYSD
ADAPNRRIAKPLIFCRTESDSPMENGYARPVEGIHIVIDVQNNTVIEFEDRKFVPLPPPD
HLRNYTPGETRGGVDRSDLKTLIINQPDGPSFRVNGYFVEWQKWNFRIGFTPKEGLVIHS
VAYVDGNRGRRPIAHRLSFVEMVVPYGDPNEPHYRKNAFDAGEDGLGKNAHSLKKVIFVV
PC*
>11670.m03822|LOC_Os04g40040.1|genepair1589-2
MAATQEKAPVVVCCGGGGAAAARRVDGGPGSSSRGAIVAAPAAAAAAGKVSMVSGDDPRV
AAAAGGGGGAVMEEIAAAVQPTTAKVSSKGKRVKCLKWITMPVQRWTNRLVDYVNHGAIS
NMESALYLYAPLGTGRIPIMTRAQRSHPLDPLSAAEIAVAIATVRAAGKSPEERDGMRFV
EVVLLEPEKNVVALADAYFFPPFQPSLLPRTKGSAVIPSRLPPRRAKLTVYNRQSNETSI
WIVELSEVHAATRGGHHRGKVISSEVVPEVQPAMDAMEYAECEATVKSYPPFIEAMKRRG
VDDMELVMVDAWCAGYYSDADAPSRRLGKPLIFCRTDSDSPMENGYARPVEGIHVIVDMQ
NNVVIEFEDRKLVPLPPPDHLRNYTPGETRGGVDRSDLKPLIINQPEGPSFRINGYFVEW
QKWNFRIGFTPKEGLVIYSVAYVDGSRGRRPIAHRLSFVEMVVPYGDPNEPHYRKNAFDA
GEDGLGKNAHSLKRGCDCLGFIKYFDAHFTNFTGGVETIENCVCLHEEDHGILWKHQDWR
TGLAEVRRSRRLTVSFICTVANYEYGFYWHFYQDGKIEAEVKLTGILSLGALMPGESRKY
GTTIAPGLYAPVHQHFFVARMDMAVDCKPNEAHNQVVEVNVKVENAGTHNVHNNAFYAEE
KLLKSELQAMRDCDPSSARHWIVRNTRAVNRTGQPTGYRLVPGSNCLPLALPEAKFLRRA
GFLKHNLWVTQYKSDEVFPGGEFPNQNPRIHEGLATWVKKDRPLEETDIVLWYVFGLTHI
PRLEDWPVMPVERIGFMLMPHGFFNCSPAVDVPPGSSDADIKEAESPKAIQNGLISKL*
>11668.m03634|LOC_Os02g38040.1|genepair1590-1
MDPTPQSHPILAYVLSRLPSLLPVSPSLSTPRARDIEQPSPRAPSGAAEFDLVSRMPGLR
HPSVLSAMTRAVADVSSARDALRLLGPRPDHELVDSARAFLRSHAAEEAEEEEEDEKVAK
SREVVRLDEAHESYGGLLREAEERLDRVYRTAMRGRDMQVVAAAHGGGGEEEAGVVDDEV
VRVLRDAEEGKAVERLLLADRQLRHLPEQLGRIRGLLVLDVSRNQLKNVPDAIGGLEHLE
ELRLASNALVSLPDSIGLLTSLKILDVSGNKLRSLPDSISKCRSLVELDVSYNVLSYLPT
GIGQEMARLEKLWVHLNKLRSLPSSVCEMRSLRLLDAHFNQLRGLPAGIGRLAALESLNL
SSNFSDMRDLPASFGDLLGLRELDLSNNQIHALPDCFGRLQRLERLRLDQNPLAVPPKEV
VAGGVGAVKEYMARRWRDARAEEERRGSAVAESPRVSTPKEWLVRSVSSLGSWVSDVTRY
GAGQDKAAAEEEEDAYLQQNL*
>11670.m03826|LOC_Os04g40080.1|genepair1590-2
MPGLRHPSVLRAMTRAVADVSAARSALQVLGPRPDHELVDSSRAIVAATDAEAGGSRRVP
EGDLEACRAVVRLEETHDAYEALLQEAEGRLEAVYRSAMEGKDLEEPDGRDESAAAAAGD
DAAVQEEVIAVLRQAEEGKPVESVRLVDRQLRHLPEAFGRIQGLRVLDVSRNQLEVIPDA
IGGLDHLEELRLASNALISLPDSIGLLLNLRILNVGSNRLRSLPDSISKCRSLIELDASY
NGLAYLPTNIGYELVNLRKLWVHMNKLRSLPSSICEMRSLYLLDAHFNELCGLPSAIGKL
SSLEILNLSSNFSDLKDLPASFGDLLNLRELDLSNNQIHALPDNFGRLDKLEKLNLEQNP
LSMPPMEIVNKGVDAVKEYMLQRWLDILLEEERKSIAAAESPQAPTTPSAWLARSVSWVS

DVSGSLVGYLSGENKTEKDAYLDQQY*
>11668.m03637|LOC_Os02g38050.3|genepair1591-1
MSGRSSPMYEGLASRPDEWDVVLKVKYGETLKRFGGYVQGPQFSLNLSALRSKIASAFKF
GSDVDFILTYTDEDGDIVMLDDDDDLHDAAIHQKLNPLRINVQLNNSHTAAPQAKQQDSD
NIPLRSTTTEDPLAHIKSVIDEVLKPISMKSIQEPVPETLAKLSHEVLEAASPQLAELIK
PFVKLVTPSNNNPSNGHADGSCSSSTGLPQTQVDPKTNDEPKIDTSLGSQPLDTQNSKSS
GARGLKTLSVEAPATSGVKSSQGQQASLYPSIEELLFSPFLPNSGDDKSASKGISDAQSK
GKSVMTSATPPTPPAAPAFRPAPPIPSLNDWSQPPARGSTFYPSIWQSEADPKANSDSRW
RVPLCRAGHPFQPHAPLSRPPPPMPAPMSYGPSPHFPYPGRLLSSGHLHGDLGNNIENSP
ARTFHRWIQCDGCGVQPIVGPRYKSKTKEDYDLCDACFHRMGNEVEYTRIDKPLLPQRLL
RDPTLCRKIHSRAAMKSKREKLESRFILDVTVLDGTLMAPSTPFTKIWRMHNNGSIMWPL
GTQLIWVGGDQFALQTYVPLEIPVDGFPVDQEIDVAVDFVAPARPGRYISYWRLASPSGQ
KFGQRVWVHIQVEDPSFVSNNRTAAINLNLPPESNITNTSNLIDVNIEPVDQVFNQHVNS
TNKELLEHLIHHQIDEPKNPEPAPLPVPIVSSTTSLHPIIDVDVPSSSTAAAFVPVFDEP
APEPAVTPVPPTVNVPAGNAPASVGASSSDHHGIDNLTEEKLLKELEEMGFRQVDLNKEI
LRQNKYNLEQSVDDLCGVSEWDPLLEELQEMVRSHVPYSGQCSQPHRHECIGKVLKLAVV
SPFQGFEDTEINKEMLEKNGGSIKRAVMDLIAREKKDQ*
>11670.m03827|LOC_Os04g40090.1|genepair1591-2
MSRRRDAAPTAREGERDLVVKVKFGGTLKRFTAFVNGPHFDLNLAALRSKIASAFKFNPD
TEFVLTYTDEDGDVVILDDDSDLCDAAISQRLNPLRINVELKSSSDGVHQTKQQVLDSIS
VMSTALEDQLAQVKLAIDEALKFVPEQVPTVLAKISHDLRSKAASSAPSLADLLDRLAKL
MAPKSKMQSSSGSADGSSGSSSGRGQTLGSLNIKNDTELMAVSASNPLDMHNSGSTKSLG
LKGVLLDDIKAQAEHVSGYPYYVDTLSGWVKVDNKGSTNAQSKGKSVTSSAVPQVTSIGH
GAPTVHSAPASDCGEGLRSDLFWTQLGLSSESFGPNGQIGGDLNSTCPPPPLFPRYPLQS
LRADKSSIKGGCSYPPCICKSSTSKPENLSHYPVQSLQADRSLKGGHYFPPCTCKSNTSK
PDNLSPVGLYGPYSEGSSCNRCPYRDLSDKHESMAQHTLHRWIQCDGCGVTPIAGSRYKS
NIKDDYDLCNTCFSRMGNVNEYTRIDRPSFGSRRCRDLNQNQMLFPHLRQLHDCRFIKDV
TVPDGTVMAPSTPFTKIWRIHNNGSSMWPYGTCLTWVGGHLFARNSSVKLGISVDGFPID
QEIDVGVDFVTPAKPGGYVSYWRLASPTGQMFGQRVWVFIQVEHPVKTSSNKQSAAINLN
MPPEGSNTEWKHSVDANIQSADIVGKYSGSTITDPLAHALYHEATKPMEPELVSSAVPSV
PRAFESVLVPATDLLTSSAGAEKASKPAATPGPAPQAVPLPKPVSIPASGPAPAPVSATT
AAPVGAAAAPISEPTAPAAAIGMPSATARAASCLPTEPSSDHISAVEDNMLRELGQMGFG
QVDLNKEIIRRNEYNLEQSIDELCGILEWDALHDELHELGI*
>11668.m03646|LOC_Os02g38120.1|genepair1592-1
MKFMKLGTRPDTFFSNESVRSVCTEVATDLQILVGDCLYQLHKFPLLSKCLLLQALCAES
GCGGNGGDVIELPGFPGGVEAFDACAKFCYGITVTVSARNLVPLRCAAAHLGMSEAADRG
NLAAKLDAFLASCLLRRWKDALAVLNSTRHCAPLCEDIGLTSRCVDAVAALIASPAALPA
HSSSASPWWAHDVAELGVDLFWRIMVAVKATGAVHEKTVGDALKAYARRWLPNVAKDGIV
VGADQPFDGVGNGGDGGNASVKQIATRHRLLLEKIVSLIPAERDAVSCSFLLKLLKAANI
LSASATSRAELVRRVAWQLEEATVGDLLIPSLSCVSETLYDVDAVAAILDEFALRHAAAP
PPPVALAVSPDDDDDSPARSGGHRRSRSAESVGFDGAARRSSSAAPVSPDALVRVGRLVD
GFLIEVARDPNMPLDKLLAMAEAVPDTARPEHDGLYKVVDTYLKVHSEMSKSARKRLCRV
INCRKLSDKACAHAAQNELLPLRVVVQVLFFEHARAAAMAGGAHAAAELPGSIRALLQSK
SSGSDQEDDAADRVDEQRLRALAAGASPGDDWSVEGLRRAASKIATLRMKLEEDDDHDGG
GGDDEEFARRQQAGLARSASLRFRAFCAIPAARPKRMLSKLWPLARGVTTERH*
>11670.m03828|LOC_Os04g40100.1|genepair1592-2
MRVMKLGNRPDTFFSSGPVRSVSTDLATDMQILVDGCLFRLHKFPLLSKCMWLQALCVES
GDGGGAVELPAFPGGAEAFEACAKFCYGVAVTIGPHNVVAVRCAAARLGMSEAADRGNLA
AKLDAFLSSCLLRRWKDALAVLHSTRRYAALCEELGVTSRCVDAVAALAVGDASGAVPAG
SSSSSPPWWARDISELGVDLYWRVMVAVKATGTVHAKAIGDTLKAYARRWLPIAAKNHHA
AERTAGGGGGGAANAERATKNHRLLVEKIVSLLPAERNAVSCGFLLKLLKAANILGASPA
SKAELTRRVASQLEDANVSDLLIPAPPPCAGGVLYDVDAVVTILEEFALRQAAASGSPKG
SPARAGRHRRSMSAESGELEGARRSTSMAAVSHGAMVRVGRLVDGFLAMVATKDARTPLD
KMIAVAEAVPDFARPEHDDLYRAIDTYLRAHPEMDKSSRKKLCRVLNCRKLSEKASMHAA
QNELLPLRVVVQVLFFENARAAGLSSGHGNRVAARFPGDGGDVSALLGTGRPRTTEENGK
DGQSPAAAGSVAADGDWSVEGLRRAASRVATLKMRLEEEDGEDAGDEAFVHRTRAGLARS
ASSRVTAAAGRSKRMLSRLWPTSRTFT*
>11668.m03647|LOC_Os02g38130.1|genepair1593-1
MARSWLITGRGVAKKIRNAPHCSSRPISELGAEAQMECPNCKHVIDNSDVAIQWPGLPAG
VKFDPSDLELLEHLEQKIGLGGSKPHTFIDEFIPTIDNDEGICYSHPENLPGMKKDGTSG
HFFHRVSNAYGCGQRKRRKISNCDHVVSVEHVRWHKTGKSKAIVEKGVTKGWKKIMVLYK
SSQRGAKPDKANWVMHQYHLGAEEDEKDGELVVSKISYQLHGKQIDKSETGNADEESDAF
AARVGPKTPKSNTPQPCRLKNSPCETENYDPILEDQDEEESNIPIVSLKDDAGNPAWCAG
ETQAAREAVQACPNLDESLRCHEVLDSFYHETLLPSDRPILSQGGNEILDRNLNAVYGLP
DLYNVDLGTPPDFQLADLQFGSQESIGNWLDSI*
>11670.m03832|LOC_Os04g40140.1|genepair1593-2
MAQNWKIQQIYHNGVLKGWKKILVLYKGSKKNKIVQANWVMHQYNLGVEEGGEDGELVVS
KVFYQLSSKQTGTPEMDSVTEEASDALTIRSDPITPITNPPLPRCLMNSPCDTEQNGTIS
HDQEGECSTSTLRPMVEAGNRAGCSAGASTAGDFNEDLLQRCEFPEDPVPTLDDTLPFLY
TDETDLFSWEDFQFGSQESFGWVDGDHT*
>11668.m03648|LOC_Os02g38140.1|genepair1594-1
MEAAGYYNCKKNDGGICGGVCGGSEHGSKAILSMSRLKCALRGFDLRALLILLIGVPALI
FIIYVHGQKVTYFLRPIWEKPPKPFNVLPHYYHENVSMANLCKLHGWKVRETPRRVFDAV
LFSNELDILDIRWHELSPYVSEFVLLESNSTFTGLKKDLHFKENRQRFEFAESRLTYGMI

GGRFVKGENPFVEESYQRVALDQLIKIAGITDDDLLIMSDVDEIPSGHTINLLRWCDDTP
EVLHLQLRNYLYSFQFLLDDKSWRASIHRYRAGKTRYAHFRQTDDLLADSGWHCSFCFRH
INDFVFKMQAYSHVDRIRFKYFLNPKRIQHVICQGADLFDMLPEEYTFQEIIAKLGPIPS
TFSAVHLPAYLLEKMDQYRYLLPGNCMRESG*
>11670.m03833|LOC_Os04g40150.1|genepair1594-2
MPETGPYGHKKTDGICDGVCGEPASKAVLTMSRLRCALRGFDFRALLALLIGVPILILMI
YAHGQKVTYFLRPIWESPPKPFKTIPHYYHENVTMAKLCKLHGWKVRETPRRVFDAVLFS
NELDILEIRWNELSPYVSEFVLLESNSTFTGLKKPLHFKENRHRFGFAESRLTYGMIGGR
FVKGENPFVEESYQRVALDQLIKIAKIEDDDLLIMSDVDEIPSGHTIDLLRWCDDIPEIL
HLQLRNYLYSFEFFLDDKSWRASIHRYRSGKTRYAHFRQTDELLADSGWHCSFCFRYISD
FAFKMQAYSHVDRIRFKYFLNPERIQDVICRGADLFDMLPEEYTFQEIIAKLGPIPSTFS
AVHLPSYLLQNVDRYRYLLPGYCRRESG*
>11668.m03654|LOC_Os02g38200.1|genepair1595-1
MARRSAPLLQRLLSPTPSPSPSPPHPLAAAAVSRRTVTYMPRPGDGAPRAVTLIPGDGIG
PLVTGAVRQVMEAMHAPVYFESYEVRGDMPTVPPEVIDSIRRNKVCLKGGLATPVGGGVS
SLNVQLRKELDLYASLVNCFNLPGLPTRHDNVDIVVIRENTEGEYSGLEHEVVPGVVESL
KVITKFCSERIAKYAFEYAYLNNRKKVTAVHKANIMKLADGLFLESCREVATKYPGIQYN
EIIVDNCCMQLVAKPEQFDVMVTPNLYGNLVANTAAGIAGGTGVMPGGNVGQDHAVFEQG
ASAGNVGNVKVVEQKKANPVALLLSSAMMLRHLQFPSFADRLETAVKRVIAEGKYRTKDL
GGSSTTQEVTDAVIAHLD*
>11670.m03851|LOC_Os04g40310.1|genepair1595-2
MPRPGDGNPRAVTLIPGDGIGPLVTGAVQQVMEVMHAPVYFETYEVRGDMPTVPPAVIES
IRRNKVCLKGGLATPVGGGVSSLNMQLRKELDLYASLVNCSNFPGLPTRHQDVDIVVIRE
NTEGEYSGLEHEVVPGVVESLKVITKFCSERIAKYAFEYAYLNNRKKVTAVHKANIMKLA
DGLFLESCREVASKYPGIQYNEIIVDNCCMQLVAKPEQFDVMVTPNLYGNLVANTAAGIA
GGTGVMPGGNVGQDHAVFEQGASAGNVGNENILEQKKANPIALLLSSAMMLRHLQFPSFA
DRLETAVKRVIAEGKYRTKDLGGTSTTQEVTDAVIANLD*
>11668.m03656|LOC_Os02g38220.1|genepair1596-1
MSKAWGGLGGAGAWALDAERAEEEERESAAAPAPAAGFPSLREAAAGAAAGKSKKKKGTT
LSLSEFTTYGAAAGRRPAAAAAEPKGLTPQEMMMLPTGPRERSTEELDRSRLGGGFRSYG
SGERRGGFDDDGRRGGPGRDADLDMPSRADESGNWSLNKKSFTPSPADSGARSRYGSLGG
GGGGAPAASSFGRADDDSDWSRGKKPMPMPSRYPSLGSGGGGGGFRDSPTSTDSDRWSRA
APLPPHNGERERPRLVLDPPKRDASATPTPPPAEAARSRPSPFGAARPREDILAEKGLDW
RKMETEIDHKTSRPTSSQSSRPGSAHSSLPGSPGSQTSAVGSEGVPRARPKVNPFGDAKP
REVVLQEKGKDWRKIDLELEHRRIDRPETNEEKDLKEQINLLRVDLKETEANISDEDKKG
LSEKLSQMERELERLTVELDNKVRFGQRPGSGSGKVTAHLSNSPDESQITESMEQPRSRS
SIDQNPKPAEERWGFQGNRDRGSFGGNRNTDRSLTGQRW*
>11670.m03861|LOC_Os04g40400.1|genepair1596-2
MAKPWGGVGAWALDAERAEEEEREQAAAFPAPEPPAAAGGAASFPSLREAAAAAGGGKQK
KKNKGTTLSLSEFSGYGAQGQRRGGGGAAPVDPRGLTPEEMMMLPTGPRERSAEELDRSR
GFRSYGGGFGAGGGDRRGGFDDDRRGPGRSSDLDMPSRADEADNWGTNKRFTPALGDSGR
RDRFGGPSPAGRSDDIDDWSRDKKPMPSRYPSLGSGGGGGGGFRESPGFRDSPGPSDSDR
WSRGGSFAPMPHNGERERPRLNLDPPKRDPLATATPPAEVARNRPSPFGAARPREEVLAE
KGLDWRKMETEIEQKTSRPTSSQSSRPNSAHSSRPGSPGSQVSAVGSEGAPRSRPKVNPF
GNAKPREVVLQEKGKDWRKIDLELEHRAVNRPETNEERILKEEINLLKEKLKESEANKTD
GPDQASPEDPEDLSEKITQMEKQLELLTIELDDKVRFGQRPGSGAGRVSAVPPAIAEEPQ
IVVSIVDRPRSRGGMEPFPKPAEERWGFQGSRERGSFGGSRSSDRPMTRQRW*
>11668.m03657|LOC_Os02g38230.1|genepair1597-1
MARLAGVAALSLVLVLLGAGVPRPAAAAAAKTQVFLSKLPKALVVGVSPKHGEVVHAGEN
TVTVTWSLNTSEPAGADAAFKSVKVKLCYAPASRTDRGWRKASDDLHKDKACQFKVTVQP
YAAGAGRFDYVVARDIPTASYFVRAYAVDASGTEVAYGQSSPDAAFDVAGITGIHASLKV
AAGVFSTFSIAALAFFFVVEKRKKDK*
>11670.m03862|LOC_Os04g40410.1|genepair1597-2
MARFGAVIHRVFLPLLLLLVVLGACHVTPAAAAAGARLSALAKALVVEASPRAGQVLHAG
EDAITVTWSLNATAAAAAAGADAGYKAVKVTLCYAPASQVGRGWRKAHDDLSKDKACQFK
IAQQPYDGAGKFEYTVARDVPTASYYVRAYALDASGARVAYGETAPSASFAVAGITGVTA
SIEVAAGVLSAFSVAALAVFLVLENKKKNK*
>11668.m03660|LOC_Os02g38260.1|genepair1598-1
MRRRVALCLVLFAFAGLHAAAVEAVTLSTSSRWIVDDEAGGRRVKLACVNWPSHLEPVVT
EGLGMQPVDAISKKVASLGFNCVRLTYPIALATNASLSSLTVRRSLLAHGLAGAVAGVEA
NNPGLLDLTLIESFRAVVDSLGESGVMVILDNHVSRPGWCCADDDGNGFFGDRHFDPDAW
VRGLGAMAALFAGVPNVVGMSLRNELRGPRQNADDWYRYMQMGAEAVHAANPAALVIMGG
LGYDTDLSFLAARPVDVSFAAAERGKLVFELHWYSFADARAWESEDANEVCGRVARGVAR
RGGFLLDAGFPLFLSEFGADTRGGSRKDDRYLPCAAAVAAELDLDWALWALQGSYALRQG
VAGADEVYGVLDWSWSKPRNATALSRIQSLQRPLRGPGYDEARPYTVLFHPLTGRCVVRR
AADDAAAAAATLELGRCEDTDAWAYTQPASTLAMRGAGRGSPPLCLRAEGSGRPARLATS
DAGGCRGDALSTWRLVSGSTMHVAVNATTTTTPSRDGGGGLLCLDVGDDGRSVVTNPCRC
LDDAAAGECDPETQWFKLVTSTRSPATGAAAAATVARGLIAA*
>11670.m03873|LOC_Os04g40510.1|genepair1598-2
MRLVVVWLAAVAVLGLASHGRPAAAAAATTLSTASRWIVDEGGNRVKLACVNWPSHLEPM
LAEGLGKQPVGAIAKDVDAMGFNCVRLTWATFMVTNASYSSLTVAQSFQRLNLTESLAAI
RAVVSSLGENGVMVILDNHVSKPGWCCGNNDGNGFFGDAYFDPDVWVDGLTKMATMFAAV
PSVVAMSLRNELRGPRQNSADWYKYMQRGAEAVHAANPRVVVILSGMSFDNDLAFLNSRQ
VNVSFAGKVAFEVHWYGFSDGQAWRAGNANQVCARVAASVSRRALYLLDQGWPVFLSEFG

VDNRGGNVNDNRYYGCVAAVAADLDLDWALWTLQGSYYLREGVLGLDEVYGVLDWAWCKP
RNDTALTRLHALQRPFRGPGLAEAAPYTVMFHPTTGRCVVRRSSSVVQTTLELGSCGEAE
AWAYTASQQRLSPRDSPLLCLRAEGAGRPARLGLSCGDELARWSLTSDSKLHLAVNASSS
SSSPETSNGGMLCLDVGDDGRSLVTNPCRCLSADNSCDPESQWFKLVTSTRSVAATNTML
AQLPPKLRSWKIRSL*
>11668.m03666|LOC_Os02g38320.1|genepair1599-1
MALSTAFSALPTSPADVRVVTADGSGIRAHSSVLASASPVLERMIEQAPRGGVVPIAGAS
TGAVVVFLRFLYAASVRGAAAAAAAAEWEEAALAEHGAALMALAHAYRVAGPLKRRAEEA
VAARVAAEGAVDAMKLAALCDAPRLYLWCARLAGRELAAVRESEGWRFAARHDAALRADL
LQLIRDADQRKERWGRERGSQGVYLQLSDAMAALERVFARAAHGSPPPLPPPRTGQCCRM
ASPCAHRRGLLQLARHFFAGCGRRVAGGCTPCRRFFLLLRLHSSVCDKSDDDSCGVPLCS
NFKTNMEKGKVDKTWKLLVKKVMRARVMSAWAKRPVPAPEIVQKSWAKYNSSSRSRAARF
R*
>11670.m03886|LOC_Os04g40630.1|genepair1599-2
MTQWADLDALRPAVAADVQVVTSDGKSIAAHSFVLGTASPVLERMIERARRGWNAECTIR
VLGVSSDAVFAFLQLLYASRVTPEDEEVVTAHGPQLLALSHAYRIGWLKRAAEASVTARL
TPEHAVDMLKLARLCDAPRLYLRCARLAAKDFAAVERSEGWRFARRHDAALELEILQLLE
DADQRRERWARERASREAYRQLGEAMDSLEHIFSDDGCSCADADADADTDAPPCRGLRLL
MRHYATCGARKAAPGGGCTRCKRMVQLFRLHASVCDRAAPHDDGDRPCRVPLCSHFKGKM
RAEKADKTWRLLVKKVTRARAMSRLAAGREREVVPEVVAASWARYSSSGGAARLR*
>11668.m03667|LOC_Os02g38330.1|genepair1600-1
MRFLGLLSLVALIFLLSFRSLIHQQVLVGEGAAASGFLHGSGDDGRRQHAREWEEERKKM
RWFMVRDYAHARRHEPRNNRLDP*
>11670.m03887|LOC_Os04g40640.1|genepair1600-2
MRLLPASGIVRLVALVFLLIFSSSLQQQAGVGAIRLHDRKQHGEQWEEERTQMRSFMTMD
YSSVRRRRPIHN*
>11668.m03676|LOC_Os02g38410.1|genepair1601-1
MIKLRYSKRLFKRSCSSSKATACVGGGGGGHGNAVAAGGGGGGAGEIGWEVRPGGMLVQK
REGRGGEEVILVRVSTGFAWHDVSIAATSTFGELKVRLSMVTGLEPREQRLLFRGKERED
TDHLHMVGVRDKDKVLLLEDPALKDMKVRAALAAARVMQSPCQPFIQV*
>11670.m03893|LOC_Os04g40680.1|genepair1601-2
MIKLRYSKRLFRRSSSKGSTDSSSSSSSSSDGDVGGRSGGGGSGEIEWEVRPGGMLVQKR
DGRGGVEVITVRVATGFSWHDVSIGATCTFGELKTVVSIVTGLEPREQRLLFRGKEREDS
DHLHMVGVRDKDKVLLLEDPALKDMKLRAALAARATVQSPYQPFIQV*
>11668.m03678|LOC_Os02g38430.1|genepair1602-1
MEPPSGIRASAWSFFKFLPFFLGLLLLGIIKGALLFPWAWLIMMIGISALVLGLWPMHVI
WTYYCIIRSKLVGPVVKLLLLVAASVILVLWLIVGIVGSVLIGVVYGFLAPVMATFDAVG
EGKERPLYHCFVDGTWSTITGSCTVVRDLKDLLLHSYFSIMDDLRFHAPPGGEPYEIRVL
DIPGALFAAACGFLVDGIMFTLIAFYKFPVMLFKGWKRLIEDLVGREGPFLETACVPFAG
LAILLWPFAVFGAFLASIISSIPLGAFAAVVVYQESSLIMGLNYVISSVAIFDEYTNDVL
DMAPGSCFPRFKYRKNEASTEGGSLSRPASFKDKQDGKKAPSRVTSFKGSFDEFNPFKLL
DHLFEECKHRGEVLVAEGVITPKDIEETKSGKIGIGVLNVGLPAYVILHALIRSAKANSD
GLILSDGSEITSDNRPKNTIFDWFFDPLMVIKEQIKAQNFTEEEEAYLKKRVLLTSDPKR
LKEVVPHLPSSLNERKQAEIDALSRRLQGITRSISRYPTAKRRFDDLVRSLSEELERTMG
GSQSGSVSQMQKLRSGISRMLSQRSMGKRTSNRGDDREAQLTIDP*
>11670.m03896|LOC_Os04g40700.1|genepair1602-2
MEPPSGFWASLGSFLKFLPYFCGLLILGVIKGILICPWACLIMAIGLSALILGLWPMHVI
WTYYCIIRTKLVGPVVKLLLLIAATATLILWLIVGIPGSILAGLVYGFLAPIMATFGAVG
KGKEKPFVHCFVDGTWSTITGSCTIVRDVKDLLFHSYFSIMDDLRLQAPPDNKPYEIRLL
DIPGALLSAACGLILDGIMFTLIAIYKCPVMLFKGWKRLIQDMIGREGPFLETACVPFAG
LAILLWPFAVVGAVLASILSSIPLGAFGAVVAYQESSLKMGLSYVVSSVSIFDEYTNDVL
DMAPGSCFPRLKYRKREDSSHGGSLSRPTSFNKEKQEGKKPPARVTSFKNSIEDFNPFKL
LEHLFVECKHQGETLVNEGVITMKDIEETKSGKVGTGVLNVGLPAYVILNALLRSAKANS
VGLLLSDGSEITSDNRPKHTLYEWFFDPLLVIKEQIKAENFTEEEEKYLKMRVLLIGGPD
RVKGSLPDVPSLDERKKAEIDAFARRLQGITKSISRYPTAKRRFDILVKQLLSELERTVG
GGQSTNGSQSQSLRGGIARMLSQKSMGKAANVRDEDPEAQMTSHDRTP*
>11668.m03679|LOC_Os02g38440.1|genepair1603-1
MAGTCAHAEFFRAQPAWALALAGVGLLAAARAALRLALWLYAAFLRPGKPLRRRYGAWAV
VTGATDGIGRAMAFRLAASGLGLVLVGRSPDKLASVSEEIRGRYPRVEVRTFVLDFAADG
LAAGVEGLREAIRGLEVGVLVNNAGVSYPYARYLHEVDEELMRTLIRVNVEGLTRVTHAV
LPAMVERKRGAIVNIGSGSSSVMPSDPLYSVYAATKAYVDQFSRCLYVEYKSKGIDVQCQ
VPLYVATKMASIRKSSFFVPSADTYARAAIRHIGYEPRCTPYWPHSVMWFLISILPESLI
DSIRLGMCIKIRKKGQAKDAKKKAQ*
>11670.m03899|LOC_Os04g40730.1|genepair1603-2
MDALSAQPAWALALAGVGLMVAATASARLARWLYAAFLRPGKPLRRRYGEWAVVTGATDG
IGRALAFRFAGAGMSLVLVGRSPDKLAAVSGEIRGKHPRAEVRTFVLDFAAEGLAAKVAA
LGDSIRGLDVGVLVNSAGMSYPYARYFHEVDEELMRNLIRLNVEALTRVTHAVLPGMVER
KRGAIVNIGSGASSILPSYPLYSVYAATKAYVDQFSRCLYVEYKNKGIDVQCQVPLYAAT
KMASIKKASFFAPSPETYARAAVRYIGYEPRCTPYWPHAVLWFLISAFPEPIVDRLLLNM
SVGIRKRGMAKDARKKTQ*
>11668.m03704|LOC_Os02g38690.1|genepair1604-1
MNSFLFSALFFFWLLFCSCKASSAMGNSLPVESKFTFEEENDRIKYVVSSMQGWGEKMED
AHAAILNLDDTTSTSFFGVYDGHGGAEVALYCAKQFHIELCNHEDYHNDLINALDNVFLS
MDENLQQSDAWRELVIPHDNGCMYFLKAGVCAKPFPQATYTGPAYEGSTACVVVIRGNQM

IVGHVGDSRCVLSRQGGLAIDLSFDHKPCTRTESERERVQNAGGRSLGLRCEQVMGNYVV
KEQWVLGDFGGGDFAFKKNKDLDREKQMLVCDPDILADDITDDMEFLVIASQGLWSCVDS
ADVVSYIHDRLSVEGAELRVICEEVVEFGLASGENTTVILVQFKPGAFQYQLVDPAGFGT
AVSNIASTSAAPAGASDTSDEGVDDAATARPTVMGYDADSSTGSADATVDSDEVDPNATA
DSYNPRGHAEIVASHTDDEVYTSGSARVESGELAVPTPSANNTVADEVKVDAAVVAGGST
TAMAADEATVVSLLSTIVDNYYSINTSEEPARRLIPLPLSQPTTRFININAENARMTY
IVKRREYFLVKSSCRCGGRHQQREVTSGGAGVEHGSIGDVPPSIRRAGRWMEKGRRRGRR
AGSKYAKKEEEEGASCHAGCRHFVPEIGPWSEATIPENPRLPNSDENDMDAGGEEAKQER
HLVLAHELFLLSRPDLDDLANVALRSDALDAVKSDGMAPLFESLATAGVLLKPDDAAARR
DARADRRGGPQARREVSGFFLSSILFLAPQKRGRPLLCCVCLQ*
>11670.m03912|LOC_Os04g40850.1|genepair1604-2
MDGGVGEEGKQQPHLVLAHKLFLLSHPDVDDLAKVDLRADVLAAVKSDDMASLYESLGAG
GVLETDAALLAEMRGRIEEEIRKLDEKIADAEENLGESEVREAHLAKSLYFIRVGEKEKA
LEQLKVTEGKTVAVGQKMDLVFHTLQIGFFYMDFDLISKSIDKAKKLFEEGGDWERKNRL
KVYEGLYCMATRNFKKAASLFLDSISTFTTYELFPYDTFIFYTVLTSVISLDRVSLKAKV
VDAPEILAVIGKVPHLSEFLNSLYNCQYKSFFAAFSGLTEQIKLDRYLQPHFRYYMREVR
TVVYSQFLESYKSVTMEAMASAFGVTVDFIDLELSRFIAAGKLHCKIDKVACVLETNRPD
ARNAFYQATIKQGDFLLNRIQKLSRVIDL*
>11668.m03725|LOC_Os02g38900.1|genepair1605-1
MENGGGGGGDGGKSDVPADANEHCPGTQSEEAGKADACAGCPNQQICATAPKGPDPDLVG
IVERMATVKHKILVLSGKGGVGKSTFSAQLSFALAEMDCQVGLLDIDICGPSIPKMLGLE
GQDIHQSNLGWSPVYVESNLGVMSIGFMLPNPDDAVIWRGPRKNGLIKQFLKDVDWGEID
YLVVDAPPGTSDEHISIVQYLQATGIDGAIIVTTPQQVSLIDVRKEINFCKKVGVPILGV
VENMSGLRQVLSDFRFVKQGEGGEMDATEWALNYIKEKAPELLTMVACSEVFDSSKGGAE
KMCNEMGVPFLGKVPMDPQLCKAAEEGRSCFVDQKCSASAPALKSIVKKLIENQD*
>11670.m03915|LOC_Os04g40880.1|genepair1605-2
MENGGGDVPENANDHCPGTQSEAAGKADACAGCPNQQICATAPKGPDPGAITHLLAIIER
MNTVKHKILVLSGKGGVGKSTFSAQLSFALAEMDHQVGLLDIDICGPSIPKMLGLEGQDI
HQSNLGWSPVYVESNLGVMSIGFMLPNPDDAVIWRGPRKNGLIKQFLKDVDWGEIDYLVV
DAPPGTSDEHISIVQYLQIAGIDGAIIVTTPQQVSLIDVKKEINFCKKVGVPVLGVVENM
SGLRQAFSDMKFVKPSEAGETDATEWALNYIKEKAPELLSVVACSEVFDSSKGGAEKMCQ
EMEVPFLGKVPMDPQLCKAAEEGRSCFTDQKCSASAPALKSIIKKLVKTK*
>11668.m03727|LOC_Os02g38920.1|genepair1606-1
MGKIKIGINGFGRIGRLVARVALQSEDVELVAVNDPFITTEYMTYMFKYDTVHGQWKHHE
VKVKDSKTLIFGTKEVAVFGCRNPEEIPWAAAGAEYVVESTGVFTDKDKAAAHLKGGAKK
VVISAPSKDAPMFVVGVNEKEYKSDVNIVSNASCTTNCLAPLAKVINDRFGIVEGLMTTV
HAITATQKTVDGPSMKDWRGGRAASFNIIPSSTGAAKAVGKVLPALNGKLTGMAFRVPTV
DVSVVDLTVRLEKPASYDQIKAAIKEEAEGKLKGILGYVEEDLVSTDFQGDSRSSIFDAK
AGIALSDTFVKLVSWYDNEWGYSTRVIDLIRHMHSTN*
>11670.m03922|LOC_Os04g40950.1|genepair1606-2
MAKIKIGINGFGRIGRLVARVALQSDDVELVAVNDPFITTDYMTYMFKYDTVHGQWKHHE
VKVKDSKTLLFGEKEVTVFGCRNPEEIPWGETGAEFVVESTGVFTDKDKAAAHLKGGAKK
VVISAPSKDAPMFVVGVNEKEYKPDIDIVSNASCTTNCLAPLAKVINDRFGIVEGLMTTV
HAITATQKTVDGPSSKDWRGGRAASFNIIPSSTGAAKAVGKVLPALNGKLTGMAFRVPTV
DVSVVDLTVRLEKPASYDQIKAAIKEESEGKLKGILGYVEEDLVSTDFQGDNRSSIFDAK
AGIALNDNFVKLVSWYDNEWGYSSRVVDLIRHMYNTQ*
>11668.m03737|LOC_Os02g39010.1|genepair1607-1
MAAGSHGGYRGYEVAREREHDVGVSRRSKEHYHHRHPSRHRDSERRRDGGRSGGRELSNG
YSHRRDSPRPPPRRRPSEGRTEDREPGEVSGGSGSERSGERPMKTGEPRENGVTRVSKEE
AKMSPSKKRKQSPVIWDRNGSKRQARDPVRGIREVDAVVAEIIMHQSHSLPVMSSLSSIG
DGHSPMILDVSVDKVQEYEKNRIVDEEEGYPTMRNILTSRWADAGDEEENVFVPKKKKS
VSPVDSIERGSTKKVTSPESGEVLVYNSVRSSSRSSDSGVLQGSANRDLEVEKGDNIDVE
EAADDDYPAGHLLDSDFEGEDCRSETPECTRSPRRCINMLQGCRSVDEFERLNTINEGTY
GVVFRVRDKRTGEIVALKKVKMEKEREGFPLTSLREMNILLSFHHPSIVEVKEVVVGSND
RDIFMVMEYMEHDLKGVMETMKQPYSQSEVKCLMLQLLEGVKYLHDNWVLHRDLKTSNLL
LNNRGELKICDFGLSRQYGSPLKPYTQLVVTLWYRAPELLLGAKDYSTAIDMWSLGCIMG
ELLSKGPLFNGKSEIDQLDKIFRTLGTPDENIWPGYSKLPGATVKFGKQTHNRLRDKFRA
VSFTGGPMLSEAGFDLLNRLLTYDPEKRISAEDALNHEWFRELPLPRSKDFMPTFPALNE
QDRRFKKHMKSPDPLEEQRMKEQGNNGDRGLFG*
>11670.m03939|LOC_Os04g41100.1|genepair1607-2
MAAGRHGGYRDYEARERELDAEASRRSKEQQHHHHPSGRHQRGDSDPRCEADRRRDGGRS
RGGRELSNGYGHRRSPPPRSRLSARLGDREPGEVLSGSASDDSGGRPHRARENGVSSSSR
DGESVVAASASSPSKKRKFSPIIWDRDSPKPMHSDVAKGKKAVDSVPTELPLPPPPPLPP
QDHIPERLAVEKSPMDVEPAVASESPEQLQEHAESRVMEEEEEYSTMRNISTSRWAGAND
DEEEGAPHRKKKSASPADSAELGQRKKALSPELGEVVASDISGGRTMSRSSDSGRLGADE
NEDLEVDKDDYMDVDRDDDGNSDIANHQSGMDSEYEVRRSETPEPVKPPHRCINMLQGCR
SVDEFERLNKINEGTYGVVYRARDKKTGEIVALKKVKMEKEREGFPLTSLREINILLSFH
HPSIVDVKEVVVGSSLDSIFMVMEYMEHDLKGVMEAMKQPYSQSEVKCLMLQLLEGVKYL
HDNWVLHRDLKTSNLLLNNRGELKICDFGLSRQYGSPLKPYTQLVVTLWYRAPELLLGTK
EYSTAIDMWSVGCIMAELLAKEPLFNGKTEFEQLDKIFRTLGTPNEKIWPGYAKLPGVKV
NFVKQPYNRLRDKFPAASFSGRPILSEAGFDLLNNLLTYDPEKRLSADAALQHEWFREVP
LPKSKDFMPTFPALNELDRRTKRYLKSPDPLEEQRLKELQGNIGNRGLFG*
>11668.m03745|LOC_Os02g39090.1|genepair1608-1
MVSMPPSLPPLPLPPQLSLADLKALSVLGRGARGVVFHVVPAGGAAAAVSATADEDPMAL

KAISRAAARHKCAEVAGGPGGDGHRRIWFERDVLLALRHPLLPSLRGVVATDSVVGFAID
RCAGGDLNALRRRQAGRVFSVAAIRFYAAELVLALEHLHGLGVVYRDLKPENVLIQDSGH
IMLVDFDLSTTLPPPPPPPPPDTAPPPQTARSRGGRRDSTKAAAAVFGCFSSRRAAASRP
SPSSSSSSRSPPSTSRTASSSSSSTRCSSAAAKSNSFVGTEDYVAPEIVAGSGHDHAVDW
WGLGVVLYEMLYGRTPFRGRSRRETFHRVLAARPDMPGEPTPLRDLIGLLLEKDPGRRLG
AHGVKRHAFFRGVDWDRVLHVARPPFIPTPDDDDAGAAAEALDVEKVLHEAFAASTAAAA
GETAAVETAAPEAGSDRGRDEDFSVFF*
>11670.m03950|LOC_Os04g41160.1|genepair1608-2
MAAAAMAPSPPEPALPRELSLGDLRAVSMLGRGAKGVVFHVVPAAAGEEEASMALKAVSR
EAARHKKNGSGGEDGHRRIWFERDVLMSLRHPLLPSLRGVLATDAVVGFAIDRCGGGDLN
SLRRRQTEKMFSDSVIRFYAAELVLALDYLHSLGIVYRDLKPENVLIQDSGHIMLVDFDL
STRLPTPPPPPEEQDATIADSMPEPPPSSPSPNRAKGKRQPGAASCFPFCSVGATKPAAS
ADSPSPTSTSRTASASSSSSSSTATTASSSTAAGVRSPAKSNSFVGTEDYVAPEIIAGSG
HDFSVDWWGLGVVLYEMLYGRTPFRGLNRKETFYRVLSKQPELVGEKTPLRDLIARLLEK
DPEKRIGARGIKAHPFFNGVDWDRILRVARPPFIPPPPEDEDEAGEVLDVEKVVNEVFAA
NDGGAAAGVVEKPSPEAGGTLAVGDGEQRRDPSKEGDFSVFF*
>11668.m03750|LOC_Os02g39140.1|genepair1609-1
MAGSGSGSSSGGRKMKSQLSFTAGPPHLSHIAEDGAFPDRAGAEASVPRTFSAGGSSGGG
GFSIVGPWEESRDIISTLGGYESQFGGMASTSALEMAGMDRYLQLQHDQVPFKVRAKRGC
ATHPRSIAERERRTRISEKLRKLQELVPNMDKQTSTADMLDLAVEHIKGLQSQLQALKHE
QEKCTCCSRP*
>11670.m03958|LOC_Os04g41240.1|genepair1609-2
MSSSSNNNKKMKAPLSFASSRQGSGGLSQISEDGIPDLTDSIHGAAHHHGRSEENVSTHD
HVVRSFSSGGFSIGSWEDSNSIVFSTSTGKSGAHGNDDIIATLSNYESQLVAPREMAGVE
KYLQMQHDQVPFRVRAKRGCATHPRSIAERERRTRISEKLRKLQALVPNMDKQTSTSDML
DLAVDHIKGLQSQLQIIRHYPENLQCWSGVATCMGPWKGRFTTLAGRSVLVQSVLFSIPV
HESMAIGLPVWVIKAIDNKRRAFLWTALRAPLLGWPKGPSLADRLRHVRGIHFLCAGKWF
FLWFAFQRRCWTADLIQKRGIDSHLGCPFCVQDLDTANHILFDCMFARWFQDWWSSFRAC
LPEHLYDSFDSLVFLVSWRLWKERNSRVFDSALSSISVVLEFIHSEGHMWSLAGVVAFGD
LLGV*
>11668.m03756|LOC_Os02g39200.1|genepair1610-1
MQRNGVVECSVCRSRLVVPSPRSVSRAYDKHRSKISSKFRALNVLLVVGDCILVGLQPIL
VFMSKVDGKFQFSPISVNFLTEVTKVVFAIVMLIIQSRKQKVGEKPLLARSTFIQAARNN
ALLAVPALLYAINNYLKFIMQLYFNPSTVKMLSNLKVLVIAVLLKFIMKRRFSVIQWEAL
ALLLIGISINQLRTVPAGNTAFGLPVTAIAYIYTLIFVTVPSLASVYNEYALKSQYDTSI
YLQNLFLYGYGAIFNFLGILGTALFQGPESFNILRGHSRATMFLICNNAAQGILSSFFFK
YADTILKKYSSTVATIFTGLASAAFLGHTLTINFLLGISVVFISMHQFFSPLAKAKDDKP
AELLELEDTQNHRSSESSFVNMTAGAAEDASHRIGTDERQPLLPT*
>11670.m03966|LOC_Os04g41320.1|genepair1610-2
MQRNGVMECSVCHSKVVAPSPRSVSRAYDKHRSKISSKYRALNFLLVSGDCILVGLQPIL
VFMSKVDGKFQFSPISVNFLTEVTKVIFAIVMLIIQSRKQKVGEKPLLSLSTFVQAARNN
ALLAVPALLYAINNYLKFIMQLYFSPATVKMLSNLKVLVIAILLKFIMRRKFSIIQWEAL
ALLLIGISVNQLSSIPDGTKSFGLAVTTIAYIYTLIFVTVPSLASVYNEYALKSQFDTSI
YLQNLFLYGYGAIFNFLGILGTVIFQGPESFDILRGHSRATMFLICNNAAQGILSSFFFK
YADTILKKYSSTVATIFTGLASAAFLGHTLTVNFLLGISIVFISMHQFFSPLAKVKDDKP
AGALEPEDAQNHRSSDSSFVNMTAGAADDCLLDENVTLLQL*
>11668.m03770|LOC_Os02g39340.1|genepair1611-1
MSYFTCKPNSGLIVDRPVAGLGRTGRLLSHPQYSLTTHSVRFPKLQKQVYPRLVLVAASQ
KKLPPLCASSGKVNPEAENDPFMESLKKAMDDAKKPRPIQDLLKEQIAKLREQGSGGGGG
NRNRRGGSGDSGGPEDESFKESLDELVQVILATVAFILVYIHIIRGEELYRLARDYTRYL
VTGKRTARLKRAMQKWRNFSESFMQSEGSQEDQYERAATSKPTWWQQPQKFVHLMEELCR
GNWRPHAQES*
>11670.m03995|LOC_Os04g41580.1|genepair1611-2
MSYFQATTYKPHNGIIVDKVAIGLGSTCKLLHERAKCSYSNRFIKLQEQVYPRLLLVAAC
HNRIGPVYASSGKGNSERVNDPFSMESLNKAIAGTKKQWPIQDMLIDQISKIRGSGSGGN
GGGNKNSHEGSGGGSEDESLTESLYEMVQVLLATIAFILMYIHIIRGEELYRLARDYTRY
LVTGKRTSRLKRAMLNWHNFCEGITNKDSVQESTFERSTSEPMWWQQPLKFVHRIEELYR
GYFRPHAQES*
>11668.m03785|LOC_Os02g39490.1|genepair1612-1
MDLSSSKEATPPPEAWWTGETVAVVTGANRGIGHALAARLAEQGLAVVLTARDGARGEAA
AAALRARGLRSVRFRRLDVSDPASVAAFASWLRDELGGLDILVNNAAVSFNEIDTNSVEH
AETVLRTNFYGAKMLIEALLPLFRRSAANSRILNISSQLGLLNKVRDPSLRSMLLDEASL
TEGKIERMASRFLAEVKDGTWSAPGRGWPAVWTDYAVSKLALNAYSRVLAARLARGGDRV
AVNCFCPGFTRTDMTRGWGTRTAEEAGRVAAGLALLPPGDLPTGKFFKWCTPQLYSKL*
>11670.m04026|LOC_Os04g41870.1|genepair1612-2
MAAVRATGGTQGGVKEGTQMGIGNRAWWTRETVAVVTGANRGIGLALAARLGEHGITVVL
TARDAERGEAAAAALRARGLHVVFHRLDVADPASVQAFAAWLRDAIGGLDILVNNAAVSF
NEIDTNSVEHAETVLRTNFYGAKMLTEALLPLFRRSPATSRILNISSQLGLLNKVSDPEL
KRLLQDEERLTEAEVEGMASRFLAQVKDGTWRGQGWPKVWTDYSVSKLALNAYARVLARR
LQARGDRVSVNCFCPGFTRTDMTRGWGKRTAEEAAEIGARLALLPPGELPTGTFFKWCTP
QLYSKL*
>11668.m03792|LOC_Os02g39550.1|genepair1613-1
MASRAVIRRRKYLLDHVNAPTLSLSPFSTFQHGRSGSEDESRIGQRFLEQSSGDSKWEQG
QYGVKLIKGDLLALGNGLLRRPAHGISLPAYGIGRKEFGLPMGARHLLQSVRTASTATAG

QPKLDIEDEQSEDQKQNKRKKEASPEECDQAVEGLSSAKAKAKAKQVQESVKAGQSIVRK
FWARLLGIGPALRAVASMSRADWAAKLKHWKDEFVSTLQHYWLGTKLLWADVRISSRLLV
KLAGGKNLSRRERQQLTRTTADIFRLVPFAVFIIVPFMEFLLPVFLKLFPNMLPSTFQDK
MKEEEALKRKLKARMEYAKFLQDTAKEMAKEVQTSRSGEIKQTAEDLDEFLNKVRRGEHV
SNDEILNFAKLFNDELTLDNMSRPRLVNMCKYMGIRPFGTDHYLRFMLRKKLQDIKNDDK
MIQAEGVESLSEEELRQACRERGHLGLLSTEEMRQQLRDWLDLSLNHAVPSSLLILSRAF
TVSGKMKPEEAVVATLSSLPDEVVDTVGTVLPSEDSVSERRRKLEFLEMQEELIKEEEKK
KEKEEKAKQEKEEKAKLKEPKAAEEDLALKEMTGPTAREEEELREAKQHDKEKLCNFSRA
LAVLASASSVSKERQEFLSLVNKEIELYNSMLEKEGTEGEEEAKKAYMAAREESDKAAEV
DEEEKVSSALIEKVDAMLQKLEKEIDDVDAQIGNRWQILDRDLDGKVTPEEVASAAAYLK
DTIGKEGVQELVSNLSKDKDGKIRVEDIVKLASQTDENNEDEEEGRQ*
>11670.m04034|LOC_Os04g41950.1|genepair1613-2
MASRAIIRRRKYALEHTNIPVLLRHSSISTFGQEKFGCEIEQSTASQNSRESNHEKAQYI
LRKQGLLGLSILCHPTRGASLASYESKPQTFGFPLGARYFLQSVRPTSSTAGQPKVGILD
ERSENQNQNQGKKEASPEECDQAVEGLSTAKAKAKAKLVQEVQKSDQSIIHKFWAILLGI
GPALRAVASMSRADWAAKLKHWKDEFVSTLQHYWLGTKLLWADVRISSRLLVKLAGGKSL
TRRERQQLTRTTADLFRLVPFAVFIIVPFMEFLLPVFLKMFPNMLPSTFQDKMKEEEALK
RKLKARMEYARFLQDTAKEMAKEVQTSRSGEMKQTAEDLDEFLNKVRKGGHVSNEEILSF
AKLFNDELTLDNMNRARLVNMCKYMGIQPFGTDHYLTFMLRKKLQEIKNDDKMIQAEGVE
SLSEEELRQACRERGHLGLLSTEEMQNQLRDWLDLSLNHSVPSSLLILSRAFTMSGKMKP
EEAVVATLSSLPDEVVDTVGTVLPSEDSVSDRKRKLEFLEMQEELIKEEEKRQEKEDKAK
LEVPKATEEDVALKEMTEPTAREEKELKKAKVEHDRKEQLCDISQALAVLASASSVAKER
QEFLNLVNKEIELYNTMLEKEGTKGEEEARRAYKAAREESDHAAEIAAGEKVSSALIERV
DAMLQKLEKEIDDVDARIGNRWQLLDSDRDGKVTPDEVAAAANYLKDTIGKEGVQELISN
LSKDKDGKILVEDIVKLASQTGESNEQEETPRQ*
>11668.m03800|LOC_Os02g39620.1|genepair1614-1
MATKYIIGSVAASFAFAYVCEIYIAEGKLLGGTTTRTMATDEWGKETDKKFQAWPRTAGP
PVVMNPVRRQNFIVKSSE*
>11670.m04038|LOC_Os04g41980.1|genepair1614-2
MRYHFCRAINQDMATKYIVGSVTASFAFAYVCGVYFADKKVLGGTTPRTVADKEWGKVTE
EKLDAWPRVAGKPVSMNPVTRQNYVLVKKKKASGSKKASEP*
>11668.m03809|LOC_Os02g39700.2|genepair1615-1
MAAATAAPSIDEAKAKSVLRQVEFYFSDSNLPRDKFLRETVEQREDGLVSLALICSFARM
KSHLGLDAAVKPETVPEETVLAVAEVLRRSQMLRISEDGKMVGRASELLKADEIIKQVDS
RTVAASPLPYNVKLEDVQSFFAQYAKVNSVRLPRHISNKKHFCGTALVEFSEEDEAKIVL
ENNLFFAGANLEIKLKKEFDAETESKKEAYEKAHPKKDEQNEGYPKGLIVAFKLKRITVD
GGVQQNCADNDTATEEETPKSMKKTSTGESEERTTVNSDMEEQKSSDDMTEAKEVNAGEA
TESGDKCTVDALLESEKKGDNETSIKDDRGLSGKANSPISREDLKEAFKKFGTVRYVDFS
IGDESGYLRFEDSKAAEKARMSAVLADEGGLIVKDHIVTLEPVTGEAEKDYWNTIRGIQG
KYKDNRSYKGRTGKSYRGGKQFNGKRGRHSDSSEKGANKTQKVEAAA*
>11670.m04041|LOC_Os04g42010.1|genepair1615-2
MAAAATAAVPLDEAKAKEVLRQVEFYFSDSNLPRDNFLRKTVEESEDGLVSLALICSFSR
MKKHLGLDLDVKQETMPEETVLAVAEVLRRSSALRVTEDGKKVGRSIELSKLDEIMEQVD
SRTIAASPFPYNVKLEDVQSFFAQYGKVNSVRLPRHIADKRHFCGTALVEFSEEEEANAV
LKNTLVFAEADLEIKPKKEFDTEREAKKEAYEKSQPTKNGHDEGYPKGLIVAFKLKIIQI
DGGMAENGGDKEGETDDANKSRTGHDEKIPENSDIKEDLSDDVEKSKEAAAQSVKKGESP
SENADDPISREDFKEEFGKFGTVRYVDFSIGEDSGYIRFEDSKAAEKARALAAISDEGGL
IMKGHLVTLEPVSGQAEKDYWSAIKGGQGKYRDNRSNRGRDWKNNRGGRHFGGKRGRHSD
GHERANKARKVDAAA*
>11668.m03810|LOC_Os02g39710.1|genepair1616-1
MEAVEDKAMVGVGGAVAAGYSSSSWGLGTRACDSCGGEAARLYCRADGAFLCARCDARAH
GAGSRHARVWLCEVCEHAPAAVTCRADAAALCAACDADIHSANPLARRHERLPVAPFFGP
LADAPQPFPFSQAAADAAAAREEDADDDRSNEAEAASWLLPEPDDNSHEDSAAAADAFFA
DTGAYLGVDLDFARSMDGIKAIGVPVAPPELDLTAGSLFYPEHSMAHSLSSSEVAIVPDA
LSAGSAAPPMVVVVASKGKEREARLMRYREKRKNRRFDKTIRYASRKAYAETRPRIKGRF
AKRTADADDDDEAPCSPAFSALAASDGVVPSF*
>11670.m04042|LOC_Os04g42020.1|genepair1616-2
MEGDDKSAVVGGAYWGLAARACDACGGEAARLFCRADAAFLCAGCDARAHGPGSRHARVW
LCEVCEHAPAAVTCRADAAALCAACDADIHSANPLARRHERLPVAPFFGALADAPKPGSG
AHGGDAAAADDDGSNDAEAASWLLPEPDHGQKDGAVGATDELYADSDPYLDLDFARSMDD
IKAIGVQNGPPELDITGGKLFYSDHSMNHSVSSSEAAVVPDAAAGGGAPMPVVSRGRERE
ARLMRYREKRKSRRFEKTIRYASRKAYAETRPRIKGRFAKRTKGGAGADADADADADGED
EEMYSSAAAAVAALMAPGGSDADYGVDGVVPTF*
>11668.m03823|LOC_Os02g39790.1|genepair1617-1
MAVLSVADSPPVSAIGFEGYEKRLEITFSEAPVFADPNGRGLRALSRAQIDSVLDLARCT
IVSELSNEVFDSYVLSESSLFVYPYKIVIKTCGTTKLLLAIPRILELAEELSLPLEAVKY
SRGTFIFPEAQPSPHKNFSEEVAVLNRYFGGLKSGGNAYVIGDPAKPGQKWHVYYATQHP
EQPVVTLEMCMTGLDKKKASVFFKTSADGHTTYAKEMTKLSGISDIIPEMEVCDFDFEPC
GYSMNAIHGPAFSTIHVTPEDGFSYASYEVMGFNPASLAYGDLVKRVLRCFGPLEFSVAV
TIFGGRNHAGTWAKGLDVGAYSCSNMVEQELPSGGLLIYQSFTATAEIATGSPRSVLHCF
ADENTEKAGKMEALYWEDDAVEEIDGTEGKKMRSC*
>11670.m04049|LOC_Os04g42090.1|genepair1617-2
MESKGGKKKSSSSRSLMYEAPLGYSIEDVRPAGGVKKFQSAAYSNCSDQPAPMGVLSAAD
PPPVSAIGFEGYEKRLEITFSEAPVFADPDGRGLRALSRAQIDSVLDLARCTIVSELSNK

DFDSYVLSESSLFIYSDKIVIKTCGTTKLLLTIPRILELAEGLSMPLAAVKYSRGMFIFP
SAQPAPHRSFSEEVAVLNRYFGHLKSGGNAYVIGDPAKPGQKWHIYYATQHPEQPMVTLE
MCMTGLDKEKASVFFKTSADGHTSCAKEMTKLSGISDIIPEMEICDFDFEPCGYSMNAIH
GSAFSTIHVTPEDGFSYASYEVVGFDASTLAYGDLVKRVLRCFGPSEFSVAVTIFGGHGH
AGTWAKELNADAYKCNNMVEQELPCGGLLIYQSFDATEDVPVAVGSPKSVLHCFEAENMV
NPAPVKEGKLGNLLPWGEDALEENDGVFDE*
>11668.m03828|LOC_Os02g39840.1|genepair1618-1
MTQADQAVISLRPGGGGGIGGPRAGRLFPFGASTGSLDFLRPRGGASSGFAAKLGDLRFE
PLERVRYTRDQLVELHEIIDIPENILKLKQDIDIELHGEDEPWINNDSSVQTQSYNRYAE
TDNRDWRSRIEQPVQTPAIGGEEKSWDKFREAKESYISSGKQDQFNNQDKLSSQFSAKAQ
VGPAPALVKAEVPWSIQRGNLSNKERVLKTVKGILNKLTPEKFDLLKGQLIEAGITTADI
LKDVISLIFEKAVLEPTFCPMYAQLCFDLNEKLPSFPSEEPGGKEITFKRVLLNNCQEAF
EGADNLRSEVNKLTGLDQEMERRDKERLVKLRTLGNIRLVGELLKQKMVPEKIVHHIVQE
LLGSESNRCPAEENVEAICQFFNTIGKQLDENPKSRRFNDVYFNRLKDLTTNSQLASRLR
FMARDVLDLRSNQWVPRREEMKAKKISEIHREAENNLGLRPGSTASIRTGRTGTGGGGPL
SPGAFSMNQPGIVGMLPGMPGARKMPGMPGLGSDDWEVPHSRSKPRADPVRNLTPSLANK
PSPNNSRLLPQGSAALISGKTSALVGSGGPLSHGLVVTPSQTTGPPKSLIPAPSVDPIVE
QPAAAPKPSSTELQKKTISLLKEYFHILLLHEAQQCIEELKSPDYYPEVVKEAINLALDK
GTNSIDPLLRLLEHLYNKNVFKATDLETGCLLYSSLLDELAIDLPKAPVHFGEVIGRLVL
SHCLSIEVVEDTLKKIEDSFFRAAVFEAMMKIMKANPSGQAILGSHVAKIDACSKLLSSE
*
>11670.m04054|LOC_Os04g42140.1|genepair1618-2
MEKDHQPVISLRPGGGGGGPRPGRLFSPAFAAAASGSGDLLRSHVGGASKIGDPNFEVRE
RVRYTRDQLLELREIVDIPEAILRINQEIDIELHGEDQIWGRPESDVQVQTQTQAQPHNR
YGETDNRDWRARTVQPPAANEEKSWDNIREAKAAHASSGRQQEQVNRQDQLNHQFASKAQ
VGPTPALIKAEVPWSARRGNLSEKDRVLKTVKGILNKLTPEKFDLLKGQLMESGITTADI
LKDVISLIFEKAVFEPTFCPMYAQLCSDLNEKLPSFPSEEPGGKEITFKRVLLNNCQEAF
EGAESLRAEIAKLTGPDQEMERRDKERIVKLRTLGNIRLIGELLKQKMVPEKIVHHIVQE
LLGSGPDKKACPEEENVEAICQFFNTIGKQLDENPKSRRINDTYFIQMKELTTNLQLAPR
LRFMVRDVVDLRSNNWVPRREEIKAKTISEIHDEAMKTLGLRPGATGLTRNGRNAPGGPL
SPGGFPMNRPGTGGMMPGMPGTPGMPGSRKMPGMPGLDNDNWEVPRSKSMPRGDSLRNQG
PLLNKPSSINKPSSINSRLLPHGSGALIGKSALLGSGGPPSRPSSLMASLTHTPAQTAPS
PKPVSAAPAVVPVTDKAAGSSHEMPAAVQKKTVSLLEEYFGIRILDEAQQCIEELQCPEY
YSEIVKEAINLALDKGPNFIDPLVRLLEHLHAKKIFKTEDLKTGCLLYAALLEDIGIDLP
LAPALFGEVVARLSLSCGLSFEVVEEILKAVEDTYFRKGIFDAVMKTMGGNSSGILKFVF
PNRHYILLKLSNIGFVGRRGGGRVCWSRAASAAGALFARNMAGRHTIILMQPSQNRATRT
FMDFNSVNHALDGICGLYERKIRDINPMARDLTYDINDLYNFIDGLTDISALVFDRSLHA
FLPYDRRWIKQEMFQHLKRLAQQ*
>11668.m03829|LOC_Os02g39850.1|genepair1619-1
MKINVRGSTMVRPAEETPRVRLWNSSLDLVVPRFHTPSVYFFRRGEAAAAEGGSYFDGER
MRRALAEALVPFYPMAGRLAHDEDGRVEIDCNGEGVLFVEADAPGATVDDFGDFAPTMDL
KRLIPTVDYTDGISSFPILVLQVLQLYIFCLGSIYDPIIEFRQVTHFKCGGVALGVGMQH
HVADGFSGLHFINSWADLCRGVPIAVMPFIDRTLVRARDPPAPSHPHVEYQPAPAMLAPE
PPQALTAKPAPPPTAVDIFKLSRSDLGRLRSQLPRGEGAPRYSTYAVLAAHVWRCASLAR
GLPAEQPTKLYCATDGRQRLQPSLPDGYFGNVIFTATPLAEAGRVTGSLADGAATIQSAL
DRMDSGYCRSALDYLELQPDLSALVRGAHTFRCPNLGLTSWVRLPIHDADFGWGRPVFMG
PGGIAYEGLAFVLPSASGDGSLSVAISLQAEHMEKFRKMIFDF*
>11670.m04065|LOC_Os04g42250.1|genepair1619-2
MAITVRRSTMVRPAWETPRVRLWNSNLDLVVPRFHTPSVYFYRRGPEGGGAPEGFFDGER
MRRALAEALVPFYPMAGRLARDEDGRVEIDCNGEGVLFVEADAPDASVDDYGDFAPTMEL
KRLIPAVDYTDDISSFSLLVLQVTYFKCGGVSLGVGMQHHVADGMSGLHFINSWSDLCRG
TQIAIMPFIDRTLLRARDPPTPSYPHVEYQPAPAMLSSVPQSVTANKTTPPPTAVDIFKL
TRSDLGRLRSQLPSGEGAPRFSTYAVLAAHVWRCVSLARGLPSEQPTKLYCATDGRQRLQ
PPLPEGYFGNVIFTATPLAEAGKVTSGLADGAAVIQEALDRMNDSYCRSALDYLELQPDL
SALVRGAHTFRCPNLGLTSWVRLPIHDADFGWGRPVFMGPGGIAYEGLAFVLPSANKDGS
LSIAISLQAEHMEKFRKLIFEV*
>11668.m03835|LOC_Os02g39910.1|genepair1620-1
MKGKRGRVRLNVGGRVFETMASTLASAGRDTMLGAMIDASWNHGGGGDGDGEGGADEYFI
DRDPECFAVLLDLLRTGGLHVPPHVADGVLCREALYYGLLDRVRAARWGPFDGDRLRLAA
SVAGSAAGDGTAVRAAPDGGCCVAHGGAVRVYNWVMEERRAVHLDHAPVNDAAYLDEATL
LVAARERPGTGRRDGGVAAFSALTGDLRHRFRVAHDRHVRSYTPGALAFDSRCKVFASCK
GRFNEYGIGVWDCTTGEQADFFYEPPGCALGDADKLQWLDGTSTLMAATMFPRTDSSFIS
LLDFRDKKNVAWSWSDVGTPASLEDKHVLHAIAMEDGRSLCVINQYDDLGFLDVRSSGGA
GGVRWRSRSKLAARKKKAAPRGEETCYPKLAAHGGQLFASTNDTISVFSGPDHVLTSTLR
GSDAGAICDFSIGGDRLFALHNEENVVDVWETSPPPII*
>11670.m04072|LOC_Os04g42310.1|genepair1620-2
MGGGRVRFNVGGQVFETTTTTLANAGRESMLGALLDSSWNLAPTAGGGGGGGGGGGGGGV
AEYFIDRNPACFAVLLDLLRTGSLHVPPQLPEKLLYREALYYGLLDHVRAARWGAFDGDR
LRLAASVPGRAPGDGTAIRAAPDGGCCVAHGGAVHVYNWMLDERRPVSLDHSQVNDVAYL
DEATLLIAARERLGKCDGGMAAFSAVSGDLRHRFRVAHDRQAKSFTAGALAFDQDSSIFA
SCKGRLNEYGIGVWDRATGEQADFFYEPPGCALGDADKLQWLDATNALMVATLFPKTDNC
FIGLLDFRDKNVAWSWSDAGMAASLDDKRVLHAIAMEDERSVCVINQYDDLGFLDLRSNA
GGVRWSSRSKFMNRKVPSEESCYPKLATHGGQLFSSMNDSISVFSGPECVLTSTLRRSHG
GAICDFSIGGDRLFALHNEENVFDVWETPPPPII*

>11668.m03836|LOC_Os02g39920.1|genepair1621-1
MAELGELEGKLRDVGEKLQSPPDDVDALLKLIHEAEIYILKVEQAPSESMISAITPAMKA
LIKKELLDNSSYEVKLSVVSCISEITRITAPDTPYDDDVMKDVFSIMVGSFEKLDDMENP
LFRRIVAILETVAKVRLCVVMLDLECEDLILQMFHNFFTTVKPNHPENVTNCMTTIMILV
IEEDDEVEIPIAECLLKHAKSELKETSAASFELAEKVIGACSEKLKPVFLQLLKGTSLNE
YDNIIATICEDSSDVKEDMDADPSGKDVVDDGKLSERTISDELPQEPAKLEQDVTQTTAI
GSGATPVDNGTESAAANPKELSNPDSEKKDGVKQSAKVANGASAETSERVDGSPAMVKSK
RGRPPGLKSLEKKAAGKKVLGLKKVEETTDSTGKLSKQSSKDDSKSSTRKASGAGSSKKQ
QKISLKQKDETDSKEDTAKDLSLKEMVSPKSVSKGSAKTKGSQGQDNNGSKRKRSQEDEQ
ETPRSRKNKGLDASLVGARIQVWWPDDKKFYKGIVDSFDTASKRHKIAYDDGDVEVLLLR
DEKWEFVSEEQDKTPDVASEISPKPRGRGRKGRGSSVQLKEGNAETPKSGGGDLPKKRGR
PKGSSNGTPKSNISATSSKSKGKAARKDENETPKVGSDLKKEAEEGSEDKATKSTEKTKD
DLPEDGSNKSASKPKEASSGGKDLKGESKPSEGRAKPGRKPKVAGAAVAGEESKANVSAE
KEKQKEAEGEAAAEVEQGGSAGGASTGGKKRRRKA*
>11670.m04073|LOC_Os04g42320.1|genepair1621-2
MASDGAQMEVERRLRDIGARFTSLPDADDELLRLIEEAETWLARVDQSPPESMHKALRPT
MSALIKKELLDHSVPDIKLAVASCLTEVTRITAPEAPYDDDVMKDVFTRVVEAFEKLDDM
ESPSYARRVAMLETVAKVRSCVLMLDLDCDDLIRDMFHHFFRTISNTHQENVITSMETVM
KFVIDESEDVQQDMPSCLLQDLASYLLKNLKKEEKETLPASFELAEKVINKCYEKLKPVF
TPLLRGTPLDEYSEVVTSLFEDALDAGVADNSDAPGKDMVADGKLSHKIVSDESAQESSK
LEQDANCPGKDGTPPNNTSTSAVSNGCALIDRVKSPSGPSSSDKKAELPSDDNQAKDTDD
LISGAKEIPEPITTEPEKPSDHNLKKSHKLDTSTDSEVVDHSKAVNNNEDILVSRELSPE
TDDGDNKLPPETGNRAADDKSKHVDNTPAGKGKRGRPPASKSHEKKNVGKGKVSGLESKK
ADAVSDSGGRATRRLAKDDDIKSSFKKTGEGESSKKKQKENLKQQEDTPPDEDTDEDLSL
KDIVSPKSSAKTGKNKGQAGDSGGSKRKRAQEAEETPQPKKNKILKGNLVGSRIKVWWPD
DRKFYKGVVESFDVASKKHKVVYDDGDVERLHLKNEKWEFIDEGRDNNPDASSDMPHGRR
GRVSLGEQTKEGKIETPSSGKHRGTDVADPPKKRGRPKGVRSSNSSQNDDSPLKGKSAEN
DDEDISKTPKSGSALKNEGGRSSRSTGKTKDGLLKGSNKDETGNTKSASKSKNDGGSKHK
DSKDEAKSSGSNPKGASTPKAADGSKTNGLSTKRKQKEKEGESSEEEEHVSAKISTGKKR
RRKAHN*
>11668.m03839|LOC_Os02g39950.1|genepair1622-1
MAAALTRSSSVRSAIRRLGSSRAFSASAAAAPRRDARGAAAAAVAVAAGSGLGIWLLPPS
PRPLADSGQAGNEVAAFGDVAEEEEREEKRRFLFGDSYRRRVFFNYEKRIRTRSPPEKIF
EYFASIRNPEGEVYMLPADLMRAVVPVFPPSESKIVREGSLRGERNPGELHCAPSEFFML
FDTNGDGLISFAEYIFFVTLLSIPESSFNIAFKMFDLDHSGEIDKEEFKKVMALMRSYHR
QGAAHRDGLRFGLKVGQSVENGGLVEYFFGKDGNEQLRYDKFSNFLKQLHDEIVRLEFSH
YDVKSSKTISVKDFALSMVASADMNHINKLLDRVDDFDDYPDLKDLRITFEEFKAFADLR
RKLEPFAMAIFSYGKVNGLLTKQDLKRAATHVCEVDLTDKVVDVIFLVFDANRDGSLSAD
EFLRALQRRESDIRQPASSGLMGVFTCLLNCTKCSLQQTVI*
>11670.m04086|LOC_Os04g42430.1|genepair1622-2
MAPPLLPRGAALLLLLRSAARRPAAASSRGFLSSAAAGGREGALAAAAVAVVGSGLGLWL
VPPSLADSGEAVADAPAVQISVGSGSGAVGAVEERGRKRRFLLGDSFRRRVFFNYEKRIR
LLSPPEKIFEYFASVRNPEGEVFMLPADLMRAVVPVFPPSESNIVREGRLRGERNPGELH
CAPSEFFMLFDTNGDRLISFAEYIFFVTLLSIPESSFSAAFKMFDVDHSGEIDKEEFKKV
MALMRSYNRQGANHRDGLRTGLKVGQPVENGGVVEYFFGNDGNEPLHYDKFTNFLKGLHD
EIIRLEFSHYDVKSSNTIPAKDFALSMVASADMNHINKLLDRVDNLGNDPDLKGVRITFE
EFKSFADLRRRLEPLAMAIFTYGKVNGLLTKQDLKRAAHHVCGVDLTDRVVDIIFHVFDT
NHDGNLSSEEFVRALQRRETDVRQPATPGSMGLLSYWLNFNKCSSLTQMLLK*
>11668.m03841|LOC_Os02g39970.1|genepair1623-1
MGAVCSRKRSQLVHEDDSFQTSSRFSKTSSLKWLLLTLPRSNSDVSRKGQGKGPGRCPSL
MELCVAKACEDINKYSSFSVLPRDLSQQIFNELVASNRLTETLLETFRDCALQDIDLGEY
PGVNDAWMEVVASQRHSLLSVDISCSEITDSGLYLLRDCPNMQSLACNYCDMISEHGLGI
LSGLSNLTSLSFKSSDGITAEAMEAFANLVNLVNLDLERCLKIHGGLVHLKGLRNLESLN
MRYCNNIADSDIKYLSDLTNLKELQLACCRITDLGVSYLRGLSKLTQLNLEGCPVTAACL
EAISGLASLVVLNLSRCGIYGEGCENFQGLKKLKVLNLGFNNITDDCLAHLKELINLESL
NLDSCKVGDEGLLHLRGLMLLKSLELSDTEVGSSGLQHLSGLRNLESINLSFTLVTDTGM
KKISALNSLKSVNLDNRQITDVGLAALTSLTGLTHLDLFGARITDYGTSCFRFFKNLESL
EVCGGLITDAGVKNIKDLKALKQLNLSQNVNLTDKTLELISGLTALVSLNVSNTRVSNAG
LRHLKDLQNLRSLSLDSCRVTTSEVKKLQATVLPNLISVRPE*
>11670.m04095|LOC_Os04g42470.4|genepair1623-2
MGSACSRKRGQLLVDEEDLYSARFSKSSSFKWLLHTLPRSGSDVHRKVQGPVPVRCPSLM
ELCVAKVREDIGKYSDFSLLPRDLSQQVFNELVEWNILTEELLGAFRDCALQDICLADYP
GVRDAWMEVAASQGQSLLSVDISCSDVTDGGLNQLKDCINLQSLSCNYCDQISEHGLKTL
SGLSNVTSLSFKKCSAVTAEGAKAFANMVNLGSLDLERCPKIHGGLVHLKGLRKLEKLNL
RYCNGITDSDMKHLSDLTNLRELQLSCCKISDLGVSYLRGLSKLAHLNLEGCAVTAACLE
VISGLASLVLLNLSRCGVYDEGCEHLEGLVKLKVLNLGFNYITDACLVHLKELINLECLN
LDSCKIGDEGLAHLKGLLKLRSLELSDTEVGSNGLRHLSGLRNLQSINLSFTLVTDIGLK
KISGLNSLRSLNLDNRQITDNGLAALTCLTGLTHLDLFGARITDAGTNCLKYFKNLQSLE
VCGGLITDAGVKNIKDLKALTLLNLSQNGNLTDKSLELISRMSFALVRNESIQHTIYLFI
CISSMCTGLTALVSLNVSNSRVSNSGLHHLKPLQNLRSLSLESCKVTAIEIKKLQLAALP
NLVSVRPE*
>11668.m03845|LOC_Os02g40000.1|genepair1624-1
MASTACFVIVSKNDIPIYEAEVGSAPKREDQAYQHQFILHAALDVVQDLAWATNTMFLKS
VDRFDDLVVSVYVTAV*

>11670.m04100|LOC_Os04g42500.1|genepair1624-2
MASTACFAIVSKNDIPIYEAEVGSAPKKEDLAYQHQFILHAALDVVQDLAWSTNAMFLKS
VDRFNDLVVSVYFYLYNNIVFFNLMTHARFMLLHDSRSEDGIKSFFQEVHELYIKIFLNP
LYLPGSRITSSHFDTKVRALARSSLPVNSNLININSDRCVCGVMVRTANISRQLIGGRRR
SRERFWEHTSSDEPIAACQVNQEMRCYTTKHTLARLITVT*
>11668.m03847|LOC_Os02g40010.2|genepair1625-1
MGEEANCNVAMESAAPKENGHTTGGAAAEAKAAAAWAEIAVTDAAAVPKPTPPPAAVAVD
PRLQGISDAIRVVPHFPKQGIMFNDITPLLLRPGVFKDAVDIFVERYRGMAIAAVAGIEA
RGFIFGPAIALAIGAKFIPLRKPKKLPGEVISETYVLEYGTDCLQMHVGAIEPGERVLIV
DDLVATGGTLCAAIRLLERAGADVVECACLIGLPKFKDFYKLNGKPVYVLVESREYEK*
>11670.m04102|LOC_Os04g42520.1|genepair1625-2
MGEEDISNDSKSSCGCEDGTVEAPAAAAPKENGRAADPRLQAISDAIRVVPHFPKPGIMF
NDITALLLRPAAFKDAVDMFVERYRGMRIAAVAGIEARGFIFGPAIALAIGAKFIPLRKP
KKLPGEVISETYILEYGTDCLEMHVGATEPGERVVVVDDLVATGGTLCAAIKLLERAGAD
VVECACLIGLPKYKKDSQLHSFARARWRNWIGIGWQEYLSPVLRSPDKGVVRYGRGDSRR
ETCLHVWRSLLSRAPSIPPGSKGRPLLRFCLPLQFYYCCPYRVRLASVYLKQSHINSAHH
SLR*
>11668.m03853|LOC_Os02g40070.1|genepair1626-1
MASANNWLGFSLSGQENPQPHQDSSPPAAIDVSGAGDFYGLPTSQPTAADAHLGVAGHHH
NASYGIMEAFNRGAQEAQDWNMRGLDYNGGASELSMLVGSSGGKRAAAVEETEPKLEDFL
GGNSFVSEQDHHAAGGFLFSGVPMASSTNSNSGSNTMELSMIKTWLRNNGQVPAGHQPQQ
QQPAAAAAAAQQQAHEAAEMSTDASASSFGCSSDAMGRSNNGGAVSAAAGGTSSQSLALS
MSTGSHSHLPIVVAGGGNASGGAAESTSSENKRASGAMDSPGGGAIEAVPRKSIDTFGQR
TSIYRGVTRHRWTGRYEAHLWDNSCRREGQSRKGRQGGYDKEDKAARAYDLAALKYWGTT
TTTNFPISNYEKELDEMKHMTRQEYIAYLRRNSSGFSRGASKYRGVTRHHQHGRWQARIG
RVAGNKDLYLGTFSTEEEAAEAYDIAAIKFRGLNAVTNFDMSRYDVKSILESSTLPVGGA
ARRLKEAADHAEAAGATIWRAADMDGAGVISGLADVGMGAYAASYHHHHHHGWPTIAFQQ
PPPLAVHYPYGQAPAAPSRGWCKPEQDAAVAAAAHSLQDLQQLHLGSAAAAHNFFQASSSS
TVYNGGGGGYQGLGGNAFLMPASTVVADQGHSSTATNHGNTCSYGNEEQGKLIGYDAMAM
ASGAAGGGYQLSQGSASTVSIARANGYSANWSSPFNGAMG*
>11670.m04107|LOC_Os04g42570.1|genepair1626-2
MASADNWLGFSLSGQGNPQHHQNGSPSAAGDAAIDISGSGDFYGLPTPDAHHIGMAGEDA
PYGVMDAFNRGTHETQDWAMRGLDYGGGSSDLSMLVGSSGGGRRTVAGDGVGEAPKLENF
LDGNSFSDVHGQAAGGYLYSGSAVGGAGGYSNGGCGGGTIELSMIKTWLRSNQSQQQPSP
PQHADQGMSTDASASSYACSDVLVGSCGGGGAGGTASSHGQGLALSMSTGSVAAAGGGGA
VVAAESSSSENKRVDSPGGAVDGAVPRKSIDTFGQRTSIYRGIDGQEDMKLICGIIAVGE
KAKVARGDRMSNYEKELEEMKHMTRQEYIAHLRRNSSGFSRGASKYRGVTRHHQHGRWQA
RIGRVAGNKDIYLGTFSTEEEAAEAYDIAAIKFRGLNAVTNFDMSRYDVKSILDSSTLPV
GGAARRLKEAEVAAAAAGGGVIVSHLADGGVGGYYYGCGPTIAFGGGGQQPAPLAVHYPS
YGQASGWCKPEQDAVIAAGHCATDLQHLHLGSGGAAATHNFFQQPASSSAVYGNGGGGGG
NAFMMPMGAVVAAADHGGQSSAYGGGDESGRLVVGYDGVVDPYAAMRSAYELSQGSSSSS
VSVAKAANGYPDNWSSPFNGMG*
>11668.m03858|LOC_Os02g40100.1|genepair1627-1
MSEMEGALRSCMEQLLIAREEREQIIVEAASEISSEKKKARELQRKLDAATKKAAKLAAE
NSSLAKALDAKDAAIGELRESKSASDGELAGARARLDAAQKQAASLQYEVRMLQKELEIR
GQEREYDLQSVDASRRQQAESQKKIALLEGECQRLRAMVRKRLPGPAAIAKMRDEVDQPA
TPRRSRSVAPMSPRSVAPAAPMTPMSTSARPMTPTMSARPMTPMSARPMTPRRAAAAEHE
TPAAAAKLRAVEDENKALKQTLAKRDAELQFVQMKYADEACKLSVLQRQLSELTEENKQL
SDAHGQTESWASALISELEQFRAAKLQGAAASEMSLLDDFAEIERLEMASGGQGLRSPKN
AHSEAISSEKNDKDTVLENGISISNGQPEWVQDMCKLVMQKHETSGENIDTILEEITRAL
DQSANNQKGDDLNGSYDWSIMKEMVFSLTEKITSVIGISEEGNVASSQKLLLDRSEFCAR
LEHLVHVCHDLLHGKTDLEKFVHEVCLVLEYIINQYKNISFQEQPDTVNNNTENLDGEES
FGNMNGGCDIKSPKSSSAPDIQTEALEESIQSVEGRKTDHILVNQEESQLDEELTRVILD
QDEKISQENSASCEIESPHDHPSAETLAEKEEKHLASSSEISAAAEKLAECQETITNLSR
QLRALKSPAVSGNLDSPMSNSRPSSSDYKPQSLACILAEGEDSSTEDAISPATKEVHSKK
EPDAASRKSVAQDGSVNAALKAVEEELTQTVVHPIFPEPSQEIISADLKKKRRSPSLLGR
IMFRKKVEGS*
>11670.m04111|LOC_Os04g42610.1|genepair1627-2
MTEMEDSLRSCTEQLLRVREEKERLIIEAADKISLEQKKVWSLQQKLEDANKRFAKVTTE
NYNLRNIVNSKDKVITELSESAALLNQKLIDATARLEFTHKQCGSLQYEVRILQKELEIR
NKEREFDLKSIDAAQKHQQESTKKITALETECQRLRTMVQKRLPGPAALAKMKDEVERRG
SNCVENRRRKPRSSAQSSPQVVTPRHPVSEGYLVKMQELDDENRHLRQLLAKKENDLQFV
QLQYADEASKLSVVQGQLKELVGSHELDDDNRPEPWANSSLVSKGEHFRVGKQHASHSRG
RRIAGSDMQLLVDISEIEKLEMISRPSSAPHQCVPDASDTESKTALTETVCQDRILEDGL
SDKYPEWIQDVLELIIKKHQVLKISVDIIIDEIRSALRTEISDKGNDAANVTYDQAVIDS
MVATLVERVSCMIERSSGNNVLSSQSFLHEKSELTCHLEHLICVCSDVLDGKANLRKFIE
EVCLTLEWTVNQYIYCVDALETVDCITNDFDGNVSLRSLNMQEKQQMQSTNPKVAVGVQQ
EVQKEPFLIPGDPVENHSQVQFVTCKLDKELLAVRQDHGDNCQEKQSVHYKEESATADGS
MQLLPEEEGKQLTNSAISAAADKLAECQETITSLSKKLQALKCPANADAVDKRKSDNLHL
LVANQNFSSPPSIEAACKKENDERVTTEKNLLQEQDVGTGHKVDNNGSTQIAPRPVIPKS
PLTTVSVDMKKRKKKKQGGSLLSRLIFGKKA*
>11668.m03877|LOC_Os02g40260.1|genepair1628-1
MACHLRLFPFTQLLVLVLYKIAAVGADPANAELSTKITQHSPLYNPQPQDFPNEELYRNY
LVIQRFKKTITSDPKNITATWTGHDICGNTTYLGFYCAALPGRTKKPTVTVVIFNGYGLR

APKLEGFIDHLPDLALFHAASNDFGGDIPYLTGLAYNYKINVDSNLQIQDDFSRDMVGSH
VTTKVHGYCVNLDLDITFHLRPGKDDKKGRSIPGATDSKALLLNYNHLSGPLPVNIGFSK
LSYLALANNRLTGAIPPSILHLQDSLLEVLLLNNQLSGCLPHELGMLTKAAVFDAGMNQL
TGPIPSSFSCLTSVEQLNLGGNRLYGEIPDALCKLAAVPAGRLANLTLSSNYFTSVGPAC
LSLIKDGVLNVRNNCIPGFANQRRPAECASFHSQPKACPAASATHVACPAGAANAAAAPA
DRVVKDYSSYVTYATLHE*
>11670.m04112|LOC_Os04g42620.1|genepair1628-2
MAGSLSAALCLTLFCLLGACIAPGARANGRHSRRDLDINLGNGNGGGISIGIGGSGGGGG
GGSSGGSSGGGGDLRPCDFENERLYKAYKVIQKFRRTVTCDPQNIISSWSGADLCSTYKG
FFCERPPNITDRTIASVDFNGYNLQASSLKEFVDALPDLALFHANSNNFGGAVPDLSRLQ
YFYELDLSNNKLSPATFPTDVLKLKNATFVDIRFNSFYGELPGGVFCSFPQVQAIFVNNN
QFSGSLPDNIGDSPVNYLSLANNNFTGEIPKSIARMANTLFEVLFLNNKLSGCLPYELGL
LAKATVIDAGTNQLTGPIPASFACLRKVEQLNLADNLLYGEVPNALCELAFSWSGRLRNL
TLSNNYFTSLGSCCWDLIKEGKLNVDRNCIPYAPNQRSHDECAAFFHRTKTSACPCNTYV
PCGHNKHSAAGAGSEQDTAAAEEDKYRTYSALHP*
>11668.m03893|LOC_Os02g40410.1|genepair1629-1
MPSSALPRLLLLLVVAVAVAVGGEGFGFSGRVSGARGSLNFGPFLCRDSHGMGCDVMCLV
GFGLCWMENASILFHLVELWIPYSRRLSCSYLLPEGNQFVEGCLEQSQLWVKLKEMIYSY
LEKATDSDKFHADRKPAPCDGSSRRKSNATSLKVLLAKEMTKEVELKRKPPGVVARLMGL
EEDLPANGPVLHQAKSGFRKSQSCNQLKALNKDLKQQEQHRLIKSTTQDIHPFCQEAVQY
NDVYEVSEAQTRMSYFQDKISQKGGSSGNTSNRVDILRGSVMEKKCLGMAEKPLHSKDLQ
EALRVVSSNKDLFLKFLEEPNSIFSRQLVGSHTNLAPPQKKRITVLKPLGSFESDGTRQT
GTEQIQEQNGAAMREFHQSSNFKEENPSLPSRIVLLRPTPGKPSLTNAKLTPRTTPFLSI
NPSDFRVALDGNGATLRSTKVEPGIIHNQQDGCHQGDESFLSSSYSNGYGGDESSLGDSE
IDQNSDSDIDYIEDEGDSFSDSGGCSPVSKRTWHYTKKHGNPYLGSSFSKISHFAESSVT
KEAKQRLSERWATVTCDEISQEQVELPRSTCTLGEMLSLQDVKNDNFINELPSVSTSRCY
DRENELPTQAKYATACRKDEKNGERPMGLPRSTSVPLIPSTLNNMVANVKTSNHQGHERT
KHIVASNKEKSFFRGRVSDFFFPRSKRTTRQISANHTSDLSSGNTEDCGGDSQQDANHDL
DGNEKSTICEDILDICAVQSTSTSEGTTALTDVPASLDCRSGNLNKLGLNEVLNSTRDQP
SPTSVLDAPSEDSSCNEPESSASTTSKNAKAVSRSSAIEAVACSLSWDDTTSESALPGTR
GQSSFLPDVDDDESECHVLVQNIMSSAGLDDAQSSMLFTGWHLPDCPLDPILFNKVLELR
EQSSYQRLLFDCVNVALVEIGENTLLSTFPWSKAHSRTWMDASSPALGIEVWSILKDWIY
GARMFVVSRRDNAGIMIERVVKQEVEGTGWVKTMRTQLVDITEQIEGGVWEELVGEAVLD
FVPACQR*
>11670.m04127|LOC_Os04g42770.1|genepair1629-2
MEMSKGVESKRKPPSVVARLMGLDDDLPAKEPALQSSRRNLRRSHSLDNLAATNRPQQQQ
EQHYSRTTPNIHIGPKETVEFKDVYEVSEDPLKRHHILGQNFPWERSSGNKSDTRIEAVR
QKFMEAKRLATNENLIHSKQFQEALEVLSSNRELFLKFLEEPSPAFLKQLDGLDTTPAPP
PTKRITVLKPIKSVENNGIRETRTHQVINEENELVMGKTHQRSYSADDNFSKSTRIVVLK
PSPGKPNRTGARLTARAAPSEQTRRIDFHGGLQDDASILGSRELLHGSVQHMPESRHRRD
ESLISSTYSNGYGGDESSFSGSEVDYIDEGGSPSDSDAVSPMSRHSWDYIRRHNSPHSAS
TFSRAHSHSPESSVIREAKKRLSERWAMVSYNEINQEQVPLRRSSTTLGEMLSLQVAKKE
EAVAGIISVSSNRSSGTENELAMKDACKSTLREYDENGKSSPRNLAKSKSVPVSSSIFDN
VAVNAQSANSEGTPKVFTKSGRAKLSFTGKISSFFFPGNKRPTKEKTSLSSDSSGEIFGC
IGHMVPQSDHNLGPDEQMAFCKDEADNSTNHAPCSTKDAGSIEVPVSSDCVSGDVDEVKS
NGDLKSIHDETSPTSILDTVFEDSNSNEPESSRRTSCTERVALRCPAIDSVARSFSWEDT
NSGSPLLGGLKHSNVDDADYDDDELKCYSLVQEIVSSAGLCHLQLSMVFTGWYLPESPLD
PALCDKFLDRKEEDAKSRERRSHQKLIFDCVNMALVEIGQDTLLCSYPWSRACLRTWREK
LSETLGEEVWNIVSDWLYGDGSFAANKDDNAGIILERIMQEEVEGKGWIKLLTMETDEIT
EQIASEVLEDIVTDSVEHLSICCSEHGISMPVANL*
>11668.m03895|LOC_Os02g40430.1|genepair1630-1
MEAALEAARAKDTKQRLAGVERLHEALEAAARRGLTSAEVTSLVDACMDLTKDGNFRVAQ
GGLQALSAAAVLAGDHFKIHLNALVPAAVERLGDGKQPVRDAARQLLVTLMEVSSPTIIV
ERAGSYAWTHKSWRVREEFVRTVATAVGLFASTELPLQRVLLSPVLQLLNDLNQSVRDAA
ISCIEEMYRNMGSQFHEELQRHNLPSYMLKDINSRLDKIEPKARSSDGARMQYKVIERSR
SLADATCTQGDSDITEKPVEPIRVHSEKELLREMEKIASALDPEKDWSIRIAAMQRIEAL
VYGGAIDYPSFLTLLKQLVPPLSAQLSDRRSSIVKQVLFKLVVITVLVIAESADNCIKTI
LRNCKVSRILPLIADTAKNDRSAILRARCSEYAILILEYWADAPEIQRASDIYEDLIKCC
VADAMSEVRATARSCYRMFTKTWPERSRRLFMSFDPAVQRIINDEDGGLQKRYPSPSLRE
KGVQLSHASSHASGTHLAGYSTSAIVAMDKSAAISSESSLSSRSLLSQSKKIGRTAERSI
ESVLSSSKQKVSAIESLLKGVSGRQNFSAMRSTSLDLGVDPPSSRDPPIPLAATASDHLS
LQNSILLDSSLPSINNTRNGGSRLVDTVNPHVANKERSRSPYLSSLSSESISGSSLPYAR
SSSGRSPYGSTMEESNDTWSTRRMPQMQMDRHYLDMTYRDASHRNLHNHQVPHFQRPLRK
QVASRTSASSRHSFDDGHISSNDMSRYTDGPTSISDALSGGLSASSDWVARVTAFNFIQT
LLQQGQKGIQEVMQNFEKVMKLFFRYLDDPHHKVAQAAFSTLADIIPACKKQFESYVERI
LPYVFSRLIDPKELVRQPCSSTLEVVGRTYPIDTLLPALVRSLDEQRSPKAKLAVLEFAN
KSFSRYKVDSEGYSNSGFLKLWLSKLAPLIHEKNAKLKETSISGIIAVYSHFDSTAVLNF
ILNLSIEEQNLVRRALKQYTPRIEVDLVNYLQSKKERSRPKSYDQVDFGNSSEDGYALTP
KSSYAFGRFSASSLDNASGKKMNMVHGSIFLDISTGRTSSDVSIDNVKQCFKPEAEVLAT
SRESKNIARTVVEAARSWTDYPGKSDATIDDENSTGTPRLEFGRLAVSDGRGAVISTSVE
DAQEGNPLVELSSVKITPHTSNGPSIPQLIHQISNVSEVTSLDKREALQQLVTASTNNDN
SIWTKYFNQILTTILEVLDDSDSSIRELSLSLVAEMLHNQKDPMEESIEIVLEKLLHVTK
DVVAKVSNEANQCLNVVLAKYDPFRCLAVIVPLLVSDDEKMLVVCTNCLTKLVGRLSEEE
LMTQLPSFLPALFDAFNNQSPDVRKTVVFCLVDIYIMLGKAFVPYLEGLNSTQLRLVTIY

ANRISQARSGAPIDANH*
>11670.m04134|LOC_Os04g42840.1|genepair1630-2
MEAALEAARAKDTKERLAGVERLHEALDAAARQRGLTAGEVTALVDTCMDLIRDANFRVA
QGGLQALSAAAVVAGDHFKIHLNALVPAAVERLGDGKQPVREAARQLLITLMEVSSPTII
VERAGSYAWTHKSWRVREEFVRTVATAVGLFASTELPLQRVLLSPVLQLMNDSNQSVRDA
AIYCIEEMYTHMGSQFHEELQRHNLPPYMLREINSRLERIEPKVPTSDGNIMQYKAVESR
SVSVNPKRGSPRTKSTPRESTLFGGDTDITEKPVEPVRVHSEKELLREFEKIAATLVPEK
DWSVRIAAMQRIEALVYGGAIDYPSFLMLLKQLVPPLSTQLSDRRSSIVKQACHLLNVLS
KELLGDFEPCAELFIPMLFKLVVITVLVIAESADTCIKTILRNCKISRILPRIADTAKND
RSAVLRARCCEYALLILEYWADAPEIQRSADLYEDLIKCCVADAMSEVRATARSCYRLFA
KTWPERSRRLFMSFDPAIQRTINDEDGGVHKRYASPSLRERVVQPSRSLSHASGTSALGY
GTSAIVAMDKTAAISSDSSFSSNTLRLSQSKTVGRSSERSLESVLNSSKEKVSAIESLLK
GNSALLDSSVPSTINASARNGGSRLLESMTTQLGTRERSRSPYLGNISSESMTSLSLPFP
RRSLERPQEGGRMDEGSDIRSTRRFPQTQNYVDMPYRDAIHRDSHNNHVPNFQRPLLRKQ
VMSRASASIRHSFDDSQVQSGDVSGYTDALASLSDALSEGLSPSSDWVVRVSAFEFIRNL
LQQGQRGIQEITQNFEKVMKLFFRHLDDPHHKVAQAAFSTLAELIPACKKPFESYVERIL
PYVFSRLIDPKELVKKPCSSTLDVVGRTYAIDMLLPALVRSLDEQRSPKAKLAVLEFANK
SFSKYTVDSEGYSNSGFLKLWLSKLAPLVHEKNAKLKEASISGIISVYSHFDSTAVLNFI
LNLSVEEQNLLRRALKQYTPRIEVDLVNYLQSKKDRPRPKSYDQADYGTSSEDGYALASK
KSYPFGRYSSSSLDAEGGKWMNSVQESTPRNAPMARTTSDMSIDHTSQSIELDTGSEVLL
TRSRESKNNTSSLVETARSWPNYPEKTDAPLDDETAISTPRLDLSHRAASDGHNAVGSTA
EENVQEGDIAVKLSSIKTTLHADNELSIPQLLHQISNGTEVSSLEKREALQQLVKASVDN
DISIWAKYFNQILTAVLEVLDDSDSSTREIALSLVAEMLNNQSGAMEESIEIVLEKLLHV
TKDMVAKISNEANQCLNVLLAKYDPFRCLAVVVPLLVSDDEKTLVVCINSLTKLVGRLSE
EELMNQLPTFLPALFDAFSNQSPDVRKTVVFCLVDIYIMLGKAFVPYLEGLNSTQLRLVT
IYANRISQARCEFSVVSLVLGFALLMEVPI*
>11668.m03896|LOC_Os02g40440.1|genepair1631-1
MVAALVVPLLVGLLAVAAAPAPASAERAFFVFGDSLVDNGNNNYLITTARADAPPYGIDY
PTHQATGRFSNGLNIPDIISEHLGAEPALPYLSPELRGDKLLVGANFASAGVGILNDTGV
QFVNIIRIGDQLQYFREYQRKLRALVGEEQAKRIVNGALVLITLGGNDFVNNYYLVPMSV
RSRQYAIQDYVPFIISEYRKILSRLYELGARRVIVTGTGPLGCVPAELALHSRRGECAAE
LTRAVDLYNPQLVNMVRGLNRAIGAEVFVTANTNRMNFDYISNPQNYGFTNVQVACCGQG
PYNGIGLCTAASNVCDDREAFAFWDAFHPTEKANRIVVGQFMHGSTEYMHPMNLSTILAV
DDEERRL*
>11670.m04136|LOC_Os04g42860.1|genepair1631-2
MGASSLVGFLMAAVAALLVAAATPAAAAPRAFFVFGDSLVDNGNNNYLMTTARADAPPYG
IDFPTHMPTGRFSNGLNIPDIISEYLGSQPALPYLSPDLRGENLLVGANFASAGVGILND
TGIQFVNIIRIGQQLDNFENYQRNLAAFVGEDAARQVVQQSLVLITLGGNDFVNNYYLVP
FSVRSRQFAIQDYVPYLISEYRKILTRLHDLGPRRVIVTGTGMIGCVPAELAMHSIDGEC
ATDLTRAADLFNPQLERMLAELNSELGGHVFIAANTNKISFDFMFNPQDYGFVTAKVACC
GQGPYNGIGLCTPASNVCANRDVYAYWDAFHPTERANRLIVAQIMHGSTDHISPMNLSTI
LAMDERRN*
>11668.m03902|LOC_Os02g40500.1|genepair1632-1
MGMAQSSSSSSRPSDSEQLEEPSKPVMALDKAKEIVASSPVVVFSKTYCPFCARVKRLLA
ELAASYKAVELDVESDGSELQSALADWTGQRTVPCVFIKGKHIGGCDDTMAMHKGGNLVP
LLTEAGAIATPSL*
>11670.m04144|LOC_Os04g42930.2|genepair1632-2
MGIASSSSSTPESRKMALAKAKETVASAPVVVYSKSYCPFCVRVKKLFEQLGATFKAIEL
DGESDGSELQSALAEWTGQRTVPNVFINGKHIGGCDDTLALNNEGKLVPLLTEAGAIASS
AKTTITA*
>11668.m03905|LOC_Os02g40530.1|genepair1633-1
MEGQQFAWGREEGGWRKGPWTAQEDKLLVEYVRQHGEGRWNSVAKITGLKRSGKSCRLRW
VNYLRPDLKRGKITPQEESVILELHALWGNRWSTIARSLPGRTDNEIKNYWRTHFKKGKP
SKNIERARARFLKQRREMQQQSQLMQTGQQQQLGQDDDATSAVVDDNLAEVAPPAATSLT
HDGELQIMQEMAPDMDDLLYYHPGDMSPYSYDDLLGSGGGECGAVAASAGAAASTSEGSS
EELDGGAATWGSLWNLDDVVHDMMIDCAAGAGCCWGSFPPLQDKGLAFY*
>11670.m04146|LOC_Os04g42950.1|genepair1633-2
MAAAEVQSAAGWGRQLQQDGGGWRKGPWTSQEDALLVEHVRQHGEGRWNSVSKLTGLKRS
GKSCRLRWVNYLRPDLKRGKITPQEESIIVQLHALWGNRWSTIARSLPGRTDNEIKNYWR
THFKKGKPSKNIERARARFLKQRREMQQSQQLQQPTLMPTPTPQSKDIIVAETGDARTDD
DAGGAAAAVAPSSSSSSLSMGGGEAEDLIMHQDAMDDLMMCPAMSTTSSSTAPPSPGIS*
>11668.m03907|LOC_Os02g40550.1|genepair1634-1
MHVRSPARSMDTSLSIAVAAAAVVLLLLVRGADAEIRTTLIVSDARPLILFEQFGFERGG
KATISIRRSFWNLRRGSRRTAVDPSLMGFVLISGTQFPKINNASAYAAADPGDNGDDGGG
SYCVLTSEYALPVLRLGDVPPGGVTTTVSIDDPDQYAVVFSNCQDGVEVTVDVYTEMYNV
RDGISDGPRDYLPVGLRPLPTIYTVVSEVYFAFLALWACVCVRHRATVERIHAVMGALLL
FKALKMACAAEDSWYVERTGTPHGWDVAFYVFGFFKGVLLFTVIILIGTGWSILKPYLQE
REKNMLMIVIPLQVVENLLLVVIGETGPTGQDWVVWNQVFLLVDVICCCAVFFPIIWSIR
SMREASKTDGKAALNLQKLTLFKRFYLVVVGYLYFTRIIASAFLALLSYKYQWGVNVAIE
AASLAFYLFVFYNFQPVAKNPYLYIGDTVEDAAVEREMDDEGRF*
>11670.m04147|LOC_Os04g42960.1|genepair1634-2
MAASVSLAVAATAVVALLLALPVALAEIKTTPIVADSRPVILFEEFGFKPGGVSAVSVRG
VSWRVAEGSKLQAADPGLMGFILISNSLFFQINNESDYAEATGGAFCPLTSKYVLPLFRL
KDIAPDGNGKGSVTIDDDDQYTVLFSSCQDGVEVTMEVRTEMYNVRPGGGRGVREYLPVG

LLPLPGIFAAASAVYFVFLGAWAWACARHRATAGQIHAVMGALLLFKALKLACAAEDAWY
VERTGTPHGWDVAFYVFGFFKGVLLFTVIVLIGTGWSFLKPYLQEREKKVLMIVIPLQVV
ENIASAVIGETGPAGRDWLAWNQIFLLVDVICCCAVFFPIIWSIRNLREASKTDGKAARN
LKKLTLFKQFYLVVVGYLYFTRIAVSAFAAVLSYRYQWVVTVAMEAASLAFYIFVFYNFK
PVENNPYLYVGEDEEEEASGQLEMEGTFEI*
>11668.m03914|LOC_Os02g40620.1|genepair1635-1
MSAVIAPPAEHELPVHWAVAEPDNTQRRGVLGGVRLRIDTVCLVMVCMILIAVAFLAMMI
VAIVKDWTQPASYSVAIDSVAGLDPETDLPRDTLNPEFNLTLRLASQRADMGVCFEAGTT
VAVYYGGVLLAGAAVPALCAGPRPSAEEESVVAWGRGVPVPRLARDRLAGDLRGGGAAEF
DVTLTVQRYTYAESWDVVLCSGKVGDAAALITPCSLYDENVQEPSLEPGYGGYSSQPESP
PETGDDG*
>11670.m04148|LOC_Os04g42970.1|genepair1635-2
MGSRSPRDDGRREVARAAGVAAALIAASLFCLFIALVLQSTSTSGGGGDGGGVREECGRA
AYSAAVTVLSGLHPSNDLLHIGTLFPVFNLTVHVEVPPGGKAGGVCLGGHSVAAVVSYGG
AFLGEGSVGRVCVEPQQQEGDVAATAWGRDVWMPWVLRRRLAQEMKRGEAELEVAVPMRG
GDVLVCKAKIGGDLSPCTLEEASN*
>11668.m03918|LOC_Os02g40660.1|genepair1636-1
MKSGHGQALFTAECSHMFHFHCISSSVKHGNYVCPVCRAKWKEIPFNRSLSSIVPRGRSG
LNVNQARLPQQGTYMALLRQVPSHHREASGSHTSEPVDFNDDEPLQLIESGDSRDARCSR
AVEIKTYPEFSAIPQSSSEDDFAVLIHLKAPCANPEQVTGRPFNATSIGYPTSRAPVDLV
TVLDVSGSMAGTKLALLKRAMGFVIQHLGPSDRLSVIAFSSTARRLFHLRRMSHSGRQQA
LQAVNLLGAGGGTNIADALKKAAKVIEDRNYKNPVCSIILLSDGQDTYNISSNVRGTRPD
YRSLVPSSILNHTICTVPVHGFGFGADHDSDALHSIAESSGGTFSFIEDESVIQDAFAQC
IGGLLSVVVQDMRLTVECVHPSVQLHTIKSGSYLSKVAGDGRNGSIEVGHLYADEERDFL
LSLSFPQSRDQTMLLKVACAYRDSVTNEAIKIHADEVKILRPKSPTSEPVCMEVDRERNR
VRAADAIEAARAAAERGALSDAVAILEHCRRILSESFSRKSGDRLCISLDAELKEMQDRM
ASRQRYEASGRAYLLSGLSSHSWQRATTRGDSTDSTTLVYSYQTPSMVQMLQRSQNQCPS
PPGLRPQLRQTRSLLEKPHPRMAVYRKWGGELGGKYVFGGILEKVNGGLVLLIAGVLLSP
CRELQVLSTTCTSLLFRIGHIC*
>11670.m04149|LOC_Os04g42980.1|genepair1636-2
MEDAWRKAKRALGLGLCVHVPEAEEGEREDCSSERRRRLPAAASGARCRGEAAVTVGPES
VPAPSDVPVPLPLPGVVRRSKSGSSRSSSKRKCAICFDSMRHGNGQALFTAECSHMFHFH
CISSNVKHGNYFCPICRAKWKEIPFNRSLSSNIPHGRIGVSRARLTQQDANMALLHQVPN
HHQRVRRPHTSEPADFNDDEPLQQPEVFDNLNVRSTKTAEINTYPEFSTIPQSSSKDDFA
ILIHLKAPSANPDQGTGKLANESSAGSSRNRAPVDLVTVLDVSGSMAGTKLALLKRAMGF
VVQHLGPSDRLSVIAFSSSARRLFHLQRISHHGRQQALQAINSLGASGGTNIADALKKAM
KVIEDRSYKNSVCSIILLSDGQDTYNISSSVQGASPDYKSLVPSSIINDARHTVPLHAFG
FGADHDSDSLHSIAQASGGTFSFIEDEGVMQDAFAQCIGGLLSIVIQEMRVSMECVHPGV
QLSSIKSGSYPSKVARDGRNGSVDIGHLYADEERDILLSVNIPQSRHQTSALKVSCAYRD
PVTGETIKIQGDEVKINRPTTSNISEHVSIEVDRERNRIQAAESIECARAAAEKGALSEA
VAILEDCRRTLSQSFASRSGDRLSLSLDAELREMQERMANRQLYESSGRAYMLSGLSSHS
WQRATARGDSTDSSTVIYSYQTPSMVEMLQHSQNHLPSPQGRQRPRPR*
>11668.m03922|LOC_Os02g40700.1|genepair1637-1
MASSSNPDTMDTDPPGGGGTLSIAVERNPPESRLLQLGVKSWPKWGCPTGKFPVKFDARE
TCYLVKGKVRAHIKGSSECVEFGAGDLVVFPKGLSCTWDVLAAVDKYYKFDSS*
>11670.m04158|LOC_Os04g43060.1|genepair1637-2
MGTTSSPDTMAAAAGPSLSITVEKNPPEARLLQLGIKSWPKWGCPPGKFPLKFDARLTCY
LLKGRVRASVKGTGRCVEFGAGDLVVFPKGLSCTWDVVVGIDKHYNFDS*
>11668.m03925|LOC_Os02g40730.1|genepair1638-1
MATCADTLGPLLGTAAANATDYLCNQFADTTSAVDSTYLLFSAYLVFAMQLGFAMLCAGS
VRAKNTMNIMLTNVLDAAAGALFYYLFGFAFAFGAPSNGFIGKHFFGLKQVPQVGFDYSF
FLFQWAFAIAAAGITSGSIAERTQFVAYLIYSAFLTGFVYPVVSHWIWSADGWASASRTS
GSLLFGSGVIDFAGSGVVHMVGGVAGLWGALIEGPRIGRFDHAGRSVALRGHSASLVVLG
SFLLWFGWYGFNPGSFLTILKSYGPPGSIHGQWSAVGRTAVTTTLAGSTAALTTLFGKRL
QTGHWNVIDVCNGLLGGFAAITAGCSVVDPWAAIICGFVSAWVLIGLNALAARLKFDDPL
EAAQLHGGCGAWGVIFTALFARKEYVDQIFGQPGRPYGLFMGGGGRLLGAHIVVILVIAA
WVSFTMAPLFLVLNKLGLLRISAEDEMAGMDQTRHGGFAYAYHDDDASGKPDRSVGGFML
KSAHGTQVAAEMGGHV*
>11670.m04159|LOC_Os04g43070.1|genepair1638-2
MATCAADLAPLLGPVAANATDYLCNRFADTTSAVDATYLLFSAYLVFAMQLGFAMLCAGS
VRAKNTMNIMLTNVLDAAAGALFYYLFGFAFAFGTPSNGFIGKQFFGLKHMPQTGFDYDF
FLFQWAFAIAAAGITSGSIAERTQFVAYLIYSAFLTGFVYPVVSHWIWSADGWASASRTS
GPLLFGSGVIDFAGSGVVHMVGGVAGLWGALIEGPRIGRFDHAGRSVALKGHSASLVVLG
TFLLWFGWYGFNPGSFTTILKTYGPAGGINGQWSGVGRTAVTTTLAGSVAALTTLFGKRL
QTGHWNVVDVCNGLLGGFAAITAGCSVVDPWAAIICGFVSAWVLIGLNALAARLKFDDPL
EAAQLHGGCGAWGILFTALFARQKYVEEIYGAGRPYGLFMGGGGKLLAAHVIQILVIFGW
VSCTMGPLFYGLKKLGLLRISAEDETSGMDLTRHGGFAYVYHDEDEHDKSGVGGFMLRSA
QTRVEPAAAAASNSNNQV*
>11668.m03930|LOC_Os02g40780.1|genepair1639-1
MGIVGYIAYIDFMNNMGHCNFELVPEWIFQIFPPLKYLIYTPSFHSLHHTQFRTNYSLFM
PFYDYIYNTMDKSSDELYESSLKGTEETPDLVHLTHMTNLQSAYHLRIGIASIASKPYSD
SAWYMWTLWPLAWLSMVLAWIYGSSAFVVERIKLNKMKMQTWALPRYNFQYGLTWEREPI
NDLIEKAILDADMKGVKVISLGLLNQAKQLNGNGELFRQKYPKLGVRIIDGSGLATAVVL
KSIPSDAKKVFLRTGTSKIARAIAIALCDRGVQVIMNEKEVYHMLKSQIPENRASYLKLS

SDNVPQLWIVHNIDDNEQKMAPKGTIFIPISQFPLKKLRKDCTYMSTPAMRIPEEMKNIH
SCENWLPRRVMSAWHIAGILHALEGWNMHECGDEMMDIEKSWSAAIRHGFLPLTKA*
>11670.m04182|LOC_Os04g43270.1|genepair1639-2
MDSSSDELYERSLKGTEETPDIVHLTHMTSLKSTYHLRIGITSISSKPCNDSVWYMWMLW
PVAWLSMVLAWIYGSSAFVVERLKLKKFSMQVWALPRYNFQVMDSSAAEQLNGSGELFAK
KYPRLRVRLIDGSGLATAVVLNSIPFGTKQVFLCGSNSKVTRATAIALCQRGVQVILNQE
KEYGMLKSRVPESRAIYLKFSNDETPQIWIGDSIDDAQGRAPKGTIFIPTSQFPLKKARK
DCTYLSNPAMKIPETMQNVHTCENWLPRRVMSAWRIAGILHALEGWEMHECGDDMMTIEK
TWSAAIKHGFKPLTKPCSLNSGTDL*
>11668.m03931|LOC_Os02g40790.1|genepair1640-1
MATRPGPLTEWPWHRLGNFKYVVMAPVVAHGARRVMRNGWGDLDIAFSLILPSLLLRMIH
NQIWISLSRYQTARSKHRIVDRGIEFDQVDRERGWDDQILFNGLVFYAGYLAMPSVRRMP
VWRTDGAVVTALVHTGPVEFLYYWFHRALHHHFLYSRYHSHHHASIVTEPITYIAVAITD
TEWKTENKHRLRVGLVHAKIRSLIDIGTM*
>11670.m04183|LOC_Os04g43280.1|genepair1640-2
MATRPGPLTEWPWQWMGGYKYLVLAPVAMHTAHRLATKGWGDFDPAYTFMLPTLLLRMIH
NQIWISLSRYQTARRKHLIVDRSLDFEQVDRVLYLAWARAGMIRSS*
>11668.m03938|LOC_Os02g40860.1|genepair1641-1
MEHVIGGKFKLGRKIGSGSFGELYLGVNIQSSEEVAIKLESVKSRHPQLHYESKLYMLLQ
GGTGIPHLKWFGVEGEYNVMVIDLLGPSLEDLFNYCNRKFSLKTVLMLADQMINRVEYMH
TRGFLHRDIKPDNFLMGLGRKASQVYVIDYGLAKKYRDLQTHKHIPYRENKNLTGTARYA
SVNTHLGVEQSRRDDLESLGYVLMYFLRGSLPWQGLKAGTKKQKYDKISEKKMLTPVEVL
CKSYPTEFISYFHYCRSLRFEDKPDYSYLKRLFRDLFIREGYQLDYIFDWTKQGSESNRL
RSSGRTSGLVGPSAERTERAAARQDVPDRFSGTVDPFARRTGSGSGHYGEHTKHRNILDS
LLAPKTAVDLDKRRPTSSSRNGSTSRKALLSSSRPSSGDPIDPNRSNLIPTSSGSSRPST
MQRLHQSTGLETRSSLTKTARNVHDDPTLRTFERLSISADRRK*
>11670.m04206|LOC_Os04g43490.1|genepair1641-2
MERVIGGKFKLGKKIGSGSFGELYLAVNIQNSEEVAVKLESVKSRHPQLHYESKLYMLLQ
GGTGIPHLKWFGVEGEYNVMVIDLLGPSLEDLFNYCNRKLSLKSVLMLADQMIARVEYMH
TRGFLHRDIKPDNFLMGLGRKANQVYVIDYGLAKKYRDLQTHKHIPYRENKNLTGTARYA
SVNTHLGVEQSRRDDLESLGYVLMYFLRGSLPWQGLKAGTKKQKYDRISEKKMLTPVEVL
CKSYPSEFVSYFHYCRSLRFEDKPDYSYLKKLFRDLFIREGYQLDYVFDWTMLKYPQIRD
NKLRPSGKTSGLVGRSAERTERTTGEALARRTGSGSGRNGEPTKHRTLLDSLMSSKATAD
TDKTRPTSLSRNGSTSRRAVVSSSKPNCGDPSDTNRTSRLFSSSSSRPSAAQRALQSAGA
ELRSSSLSKTRKSSRDDPTIRSFEMLSLSADRRK*
>11668.m03941|LOC_Os02g40880.1|genepair1642-1
MPFKRFVEIGRVALVNYGKDYGRLVVIVDVVDQNRALVDAPDMVRCQINFKRLSLTDIKI
DIKRVPKKTTLIKAMEEADVKNKWENSSWGKKLIVQKRRASLNDFDRFKVMLAKIKRGGA
IRQELAKLKKTAAA*
>11670.m04211|LOC_Os04g43540.1|genepair1642-2
MVGSCREILPVVSSPFKRFVEIGRVALVNYGKDYGRLVVIVDVVDQNRALVDAPDMVRCQ
INFKRLSLTDIKIDIKRVPKKTTLIKAMEEADVKNKWENSSWGKKLIVQKRRASLNDFDR
FKVMLAKIKVILRSTLSNSTFIEGFVHTPSNTLIGI*
>11668.m03951|LOC_Os02g41460.1|genepair1643-1
MRGEEEPAVAAAAYTTASKAGLLMELSPPNHESSPPTAGGGGGGGGDGAGGSSSAGASSS
AGGGAATPQTPSRYEAQKRRDWNTFGQYLRNHRPPLGLAQCSGAHVLEFLRYLDQFGKTK
VHTAACPFFGHPNPPAPCPCPLRQAWGSLDALVGRLRAAFEENGGRPESNPFAVRAVRLY
LREVREHQARARGVSYEKKKRKKPQPADTSGGGGHPHPPPPPPPPPSAGAAC*
>11670.m04215|LOC_Os04g43580.1|genepair1643-2
MDLSPNPDSPPSGGGNGGGGGSSSSNSSPSMGAGAPQSPSRYEAQKRRDWNTFGQYLRNH
RPPLSLAQCSGAHVLEFLRYLDQFGKTKVHTAACPFFGHPSPPAPCPCPLRQAWGSLDAL
VGRLRAAFEENGGRPESNPFAARAVRLYLREVREHQARARGVSYEKKKRKKPQQQQLQGG
DSSGLHGHQHHPPPPPPAGAAC*
>11668.m03956|LOC_Os02g41510.1|genepair1644-1
MGRAPCCEKMGLKRGPWTAEEDRILVAHIERHGHSNWRALPRQAGLLRCGKSCRLRWINY
LRPDIKRGNFTREEEDAIIHLHDLLGNRWSAIAARLPGRTDNEIKNVWHTHLKKRLEPKP
SSGREAAAPKRKATKKAAAVAVAIDVPTTVPVSPEQSLSTTTTSAATTEEYSYSMASSAD
HNTTDSFTSEEEFQIDDSFWSETLAMTVDSTDSGMEMSGGDPLGAGGASPSSSNDDDMDD
FWLKLFIQAGGMQNLPQI*
>11670.m04225|LOC_Os04g43680.1|genepair1644-2
MGRAPCCEKMGLKKGPWTPEEDKVLVAHIQRHGHGNWRALPKQAGLLRCGKSCRLRWINY
LRPDIKRGNFSKEEEDTIIHLHELLGNRWSAIAARLPGRTDNEIKNVWHTHLKKRLDAPA
QGGHVAASGGKKHKKPKSAKKPAAAAAAPPASPERSASSSVTESSMASSVAEEHGNAGIS
SASASVCAKEESSFTSASEEFQIDDSFWSETLSMPLDGYDVSMEPGDAFVAPPSADDMDY
WLGVFMESGEAQDLPQI*
>11668.m03957|LOC_Os02g41520.1|genepair1645-1
MWRRRGAPSGIGLWAALLVAAAVLAAGGGAAAAAAKDEAYVTLLYGDEFVLGVRVLGKSI
RDTDTSRDLVVLVSDGVSEYSRKLLEADGFIVKHITLLANPNQVRPTRFWGVYTKLKIFN
MTSYKKVAYLDADTIVVKSIEDIFNCGKFCANLKHSERMNSGVMVVEPSETLFNDMMDKV
NSLPSYTGGDQGFLNSYYADFANSRVYEPNKPTTPEPETQRLSTLYNADVGLYMLANKWM
VDEKELRVIHYTLGPLKPWDWWTAWLVKPVAVWQDIRKNLEESLPGTGGGRNPHDQSVVK
ILFSLPLFMLICGYYGSCFQTNKELLCIRSLCAFARRARYKYKSEEALPSYSTIGAASSS
FGISHQKSHNGAHLKLPSYFGAITVLVCFISALISLAFAFIIIPRQVMPWTGLLLMYEWT
SVTFFLLFGSYLRVVYNWGSSSANHVGHNNLDSSENHAGAGLQRNTSDCDTDAAFYWSGM

AILSSIALLSPTVLGITALFAKLGLMVAGGVVLASFMTYASEHLAISAFVKGQRDRNASR
GSICFMC*
>11670.m04228|LOC_Os04g43700.1|genepair1645-2
MRSPTPARLALALVAALAAAALLGGAAAAAATEEAYVTLLYGDEFVLGVRVLGKSIRDTG
TRRDLVVLVSDGVSDYSRKLLQADGWIVSHITLLANPNQVRPKRFWGVYTKLKIFNMTSY
RKVVYLDADTVVVKSIEDLFKCGKFCGNLKHSERMNSGVMVVEPSETVFKDMMRQIDTLP
SYTGGCNSVECLYSDQGFLNSYYADFANSHVYEPEKPYTPEPETQRLSTLYNADVGLYML
ANKTCSINLLQEKVQEKGEFGWLMKRNLESFTTRWVPLNTGTGGQLWLVKPVGVWQDVRQ
TLEESLPGTGGGRSPHDQLVVKVLFILPVLLLTCGYNQSCSQFSNGMHSKLPSYFGALTV
LACFMSAGVSFAFAFAIIPKQIMPWTGLLLMFEWTFVSFFLLFGSYLRFVYRWGSLDANH
VGHAVVLIHQKNTWLQARIDGAGGVVLASFMTYASEHIAISAFSRAER*
>11668.m03966|LOC_Os02g41580.1|genepair1646-1
MEFTPKITGKTTSMSGELKRDYEIGEEIGRGRFGVVHRCTSRATGEAFAVKSVDRSQLAD
DLDRELAELEPKLAQLAAAGNPGVVQVHAVYEDDAWTHMVMDLCSGPDLLDWIRLRRGAP
VPEPVAAAVVAQLAEALAHCHRRGVAHRDVKPDNILLDVVDDGIDGGGTPRARLADFGSA
AWVGESGGSAEGLVGTPHYVAPEVVAGGEYGEKADVWSAGVVLYVLLSGGALPFGGETAA
EVLASVLRGSVRFPPRLFAGVSPAAKDLMRRMMCRDTWRRFSAEQVLGHPWIVSGGGARA
MEQPT*
>11670.m04229|LOC_Os04g43710.1|genepair1646-2
MSEELKRDFEIGEEIGRGRFGVVHRCASRSTGEAYAVKSVDRSRLSDDLDRSLAALEPKL
ARLAAAGNPGVVQVHAVYEDDDWTHTVMDLCTRPDILDWVRLRCGKPVPEPDATAVVAQI
AEALALCHRRGVAHRDVKPDNVLLDATGDGPPRVRLADFGSAAWVGDGISAEGARGDSRT
TSRPRWSPGASTGRRPTCGARGWSCTCSSPAARSRSAARRPPTCSPHVLRGNLRFPPRLF
SGVSPAAKDLMRRMMCRDVYRRFSAEQVLRHPWIVSGGGARDVQPT*
>11668.m03967|LOC_Os02g41590.1|genepair1647-1
MASEGVLLGMGNPLLDISAVVDDAFLTKYDVKLNNAILAEEKHLPMYDELASKGNVEYIA
GGATQNSIRVAQWMLQTPGATSYMGCIGKDKFGEEMKKNAQAAGVTAHYYEDEAAPTGTC
AVCVVGGERSLVANLSAANCYKSEHLKKPENWALVEKAKYIYIAGFFLTVSPDSIQLVAE
HAAANNKVFLMNLSAPFICEFFRDAQEKVLPFVDYIFGNETEARIFAKVRGWETENVEEI
ALKISQLPLASGKQKRIAVITQGADPVVVAEDGQVKTFPVILLPKEKLVDTNGAGDAFVG
GFLSQLVQQKSIEDSVKAGCYAANVIIQRSGCTYPEKPDFN*
>11670.m04233|LOC_Os04g43750.1|genepair1647-2
MESGSYEGVLLGMGNPLLDISAVVDEAFLAKYDIKPGNAILAEEKHLPMYNELASKVNVE
YIAGGSTQNSIRVAQWMLQIPGATSYMGCIGKDKFGEEMKKDAQTAGVNAHYYEDDNAPT
GTCAVCIVGGERSLVANLSAANCYRSEHLKRPENWTLVEKAKYIYIAGFFLTVSPDSIQL
VAEHAAATNKVFMMNLSAPFICEFFRDAQEKALPYADYIFGNETEARTFAKVRGWETENT
EEIALKISQLPKASGAHKRITVITQGCDPVVVADDGKVKTFPVIVLPKEKLVDTNGAGDA
FVGGFLSQLVQEKSIDECVRAACYAANVIIQRSGCTYPEKPDFN*
>11668.m03971|LOC_Os02g41630.1|genepair1648-1
MAGNGPINKEDPLNWGAAAAEMAGSHLDEVKRMVAQFREPLVKIQGATLRVGQVAAVAQA
KDAAGVAVELDEEARPRVKASSEWILNCIAHGGDIYGVTTGFGGTSHRRTKDGPALQVEL
LRHLNAGIFGTGSDGHTLPSETVRAAMLVRINTLLQGYSGIRFEILEAITKLLNTGVTPC
LPLRGTITASGDLVPLSYIAGLITGRPNAQAISPDGRKVDAAEAFKLAGIEGGFFTLNPK
EGLAIVNGTSVGSALAATVMFDANILAVLSEVLSAVFCEVMNGKPEYTDHLTHKLKHHPG
SIEAAAIMEHILAGSSFMSHAKKVNEMDPLLKPKQDRYALRTSPQWLGPQIEVIRAATKS
IEREVNSVNDNPVIDVHRGKALHGGNFQGTPIGVSMDNARLAIANIGKLMFAQFSELVNE
FYNNGLTSNLAGSRNPSLDYGFKGTEIAMASYCSELQYLANPITNHVQSAEQHNQDVNSL
GLVSARKTLEAVDILKLMTSTYIVALCQAVDLRHLEENIKSSVKNCVTQVAKKVLTMNPT
GDLSSARFSEKNLLTAIDREAVFSYADDPCSANYPLMQKLRAVLVEHALTSGDAEPEASV
FSKITKFEEELRSALPREIEAARVAVANGTAPVANRIVESRSFPLYRFVREELGCVFLTG
EKLKSPGEECNKVFLGISQGKLIDPMLDCLKEWNGEPLPIN*
>11670.m04234|LOC_Os04g43760.1|genepair1648-2
MASQTADATGFVASDPLSWGKAALEMTGSHLDEVKRMVAQSREAVVKIEGSSLRVGQVAA
VSAAKDASGVVVELDEEARPRVKASSEWILNCIAHGGDIYGVTTGFGGTSHRRTKDGQAL
QVELLRHLNAGIFGNGSDGNSLPSEVSRAAMLVRINTLLQGYSGIRFEILEAITKLINTG
VSPCLPLRGTITASGDLVPLSYIAGLITGRPNAQAVTVDGKKVDAAEAFKIAGIQGGFFR
LEPKEGLAIVNGTSVGSALAAMVLYDANVLAVLSEVLSAVFCEVMNGKPEYTDHLTHKLK
HHPGSIEAAAIMEHILAGSAFMPHAQKVNEVDPLLKPKQDRYALRTSPQWLGPQIEVIRA
ATKSIEREVNSVNDNPVIDVHRGKALHGGNFQGTPIGVSMDNTRLAIANIGKLMFAQFSE
LVNEFYNNGLTSNLAGSRNPSLDYGFKGTEIAMASYCSELQFLANPVTNHVQSAEQHNQD
VNSLGLVSARKTAEAVDILKLMSSTYLVALCQAVDLRHLEENLKSAVKNCVTTVAKKVLT
TGPAGGLHSARFSEKALLTAIDREAVYSYADDPCSANYPLMTKIRAVLVEHALANGPAEK
DDGSSVFSKITAFEEELREALPREMEAARVAFETGTAPITNRIKESRSFPLYRFVREELG
CVYLTGEKLKSPGEECNKVFLAISERKLIDPMLECLKEWNGEPLPIC*
>11668.m03973|LOC_Os02g41650.1|genepair1649-1
MECETGLVRSLNGDGLCMSSVSAPPRADPLNWGKAADELAGSHLDEVKRMVEDFRQPLVK
IEGASLTIAQVAAVAAGAGDARVELDESARGRVKASSDWVMNSMMNGTDSYGVTTGFGAT
SHRRTKEGGALQRELIRFLNAGAFGTGTDGHVLPAEATRAAMLVRINTLLQGYSGIRFEI
LEAITKLLNANVTPCLPLRGTITASGDLVPLSYIAGLITGRQNSVAVAPDGRKVTAAEAF
KIAGIEHGFFELQPKEGLAMVNGTAVGSGLASTVLFEANVLAILAEVLSAVFCEVMTGKP
EYTDHLTHKLKHHPGQIEAAAIMEHILEGSSYMKLAKKLGELDPLMKPKQDRYALRTSPQ
WLGPQIEVIRFATKSIEREINSVNDNPLIDVSRGKALHGGNFQGTPIGVSMDNTRLALAA
IGKLMFAQFSELVNDFYNNGLPSNLSGGRNPSLDYGFKGAEIAMASYCSELQFLGNPVTN
HVQSAEQHNQDVNSLGLISSRKTAEAIDILKLMSSTFLIALCQAIDLRHLEENMKTAVKN

CVMQVAKKSLSMNHMGGLHIARFCEKDLLTAIDREAVFAYADDPCSANYPLMQKLRAVLI
EHALANGDAERVLETSIFAKVAEFEQHVRAALPKEVEAARAAVENGTPLVPNRIKECRSY
PLYRFVREEVGTEYLTGEKTRSPGEELNKVLVAINERKHIDPLLECLKEWNGAPLPLC*
>11670.m04238|LOC_Os04g43800.1|genepair1649-2
MECENGHVAAAANGSSLCVAKPRADPLNWGKAAEELSGSHLDAVKRMVEEYRRPVVTIEG
ASLTIAQVAAVASAGAARVELDESARGRVKASSDWVMNSMMNGTDSYGVTTGFGATSHRR
TKEGGALQRELIRFLNAGAFGNGDDGHVLPAAATRAAMLVRINTLLQGYSGIRFEILETI
ATLLNANVTPCLPLRGTITASGDLVPLSYIAGLVTGRPNSVAVTPDGRKVDAAEAFKIAG
IQHGFFELQPKEGLAMVNGTAVGSGLASMVLFEANVLGVLAEVLSAVFCEVMNGKPEYTD
HLTHKLKHHPGQIEAAAIMEHILEGSSYMMLAKKLGELDPLMKPKQDRYALRTSPQWLGP
QIEVIRAATKSIEREINSVNDNPLIDVSRGKALHGGNFQGTPIGVSMDNTRLAIAAIGKL
MFAQFSELVNDFYNNGLPSNLSGGRNPSLDYGFKGAEIAMASYCSELQFLANPVTNHVQS
AEQHNQDVNSLGLISSRKTAEAIDVLKLMSSTFLIALCQAIDLRHLEENVRSAVKGCVTT
VARKTLSTSATGDLHKARFCEKDLLQAIDREAVFAYADDPCSANYPLMQKMRAVLIEHAL
ANGEAERNVDTSVFAKVATFEEELRVALPREVEAARAAVENGTAAKANRITECRSYPLYR
FVREELGTEYLTGEKTRSPGEEVNKVFVAMNQGKHIDALLECLKEWNGEPLPIC*
>11668.m03987|LOC_Os02g41770.1|genepair1650-1
MEIKDEETTAEVAMVVQSRFRRVCVFCGSSHGKKKIYQDAAIELGKELVARNIDLVYGGG
SVGLMGLVSQAVHNGGRHVIGVIPKTLMPREISGETVGEVKAVSDMHQRKAEMARQSDAF
IALPGGYGTLEELLEVIAWAQLGIHDKPVGLLNVDGYYNPLLSFIDKAVEEGFIRPSARH
IIVLAPTPKELIEKLEEYSPQHEKVVSKMKWEMEQMSYPQNYDIPRPKEGKMIIEAQRGS
RLWISDPKRMESEPVPDFQSEETVNSEISARENASLGLHNHKDQGTSSL*
>11670.m04242|LOC_Os04g43840.1|genepair1650-2
MMDTDHTEIIKEGEAVVEAMALLQSRFRRICVFCGSSQGKKKSYQDAAVELGKELVARNI
DLVYGGGSVGLMGLVSQAVYNGGRHVIGVIPKTLMPREITGETVGEVKAVADMHQRKAEM
ARQSDAFIALPGGYGTLEELLEVIAWAQLGIHDKPVGLLNVDGYYNSLLSFIDKAVEEEF
ISPSARHIIVLAPTPKELLEKLEAYSPRHDKVVPKMQWEMEKMSYCKSCEIPGLKEGNKA
TIQAQRGSML*
>11668.m03990|LOC_Os02g41800.1|genepair1651-1
MITFADLAEPAPGAERCVDRQLWLACAGGMCTVPPVGAAVYYFPQGHAEHALGLAAPELS
AARVPALVPCRVASVRYMADPDTDEVFARIRLVPLRAAEDGDVEEDGAAAGEEHEKPASF
AKTLTQSDANNGGGFSVPRYCAETIFPRLDYAADPPVQTVVAKDVHGVAWNFRHIYRGTP
RRHLLTTGWSTFVNQKKLVAGDSIVFLRGDGGDLHVGIRRAKRGFCGGGGGAEEASLPGW
DQYGGLMRGNASPCAAAKGRGKVRAEDLVEAARLANGGQPFEVVYYPRASTPEFCVRAAA
VRAAMRVQWCPGMRFKMAFETEDSSRISWFMGTVASVQVADPIRWPQSPWRLLQVTWDEP
DLLQNVKRVSPWLVELVSSMPAINLSSFSPPRKKPRILAYPEFPFEGQLLNPAFPPNPLA
HGHHHYHHNHPSFFPFPDVSAPAGIQGARHAQFGPSLSDLHLTHLQSSLMYPGLRRPDHV
GPTSIPPPRISTDLTMGSSPPARALSMGAKKPDDAKPPGLMLFGQRILTERQMSLSGTTS
PAATGNSSLNWNTEKGASEGSGSGVIQNSPTDNTSSERLQWFRENSTVSELGLEPGQCKV
FIESDTVGRNLDLSSLASFEQLYGRLSEMFCIDSAELRSRVLYRGATGEVRHAGDEPFSR
DFNKKAQSAQVSYCSPVSFFIIINNDQLRVLVDSRVLRCKNPVTTHQRLECRGPLAQGTS
ACSMQTQTQRCAISDLSGGLTSRLQPPGEAIPLGSLALAASSLTHDDEICDLHRAAYPWL
VES*
>11670.m04249|LOC_Os04g43910.1|genepair1651-2
MELAGPTEGDGGGSVDSQLWAACAGSMSSVPPVGAAVYYFPQGHAEQASAAVDLSSARVP
PLVPCRVVAVRFMADAESDEVFAKIRLVPLRPGDAVVDVGEAAAAEARREEENSRPRPTS
FAKTLTQSDANNGGGFSVPRFCAETIFPELDYSSEPPVQSVCAKDVHGVEWTFRHIYRGT
PRRHLLTTGWSPFVNKKQLTAGDSIVFMRDEGGNIHVGLRRAKRGFCSIGGDDESLSSIP
GWDQYRGLMRRNATATATGGRTPPKGKVPPENVLTAATRATTGQPFEVLYYPRASTPEFC
VRAAAVRTAMAVQWCPGMRFKMAFETEDSSRISWFMGTVAGVQASDPVRWPQSPWRLLQV
TWDEPELLQNVKRVCPWLVELVSSMPNLHLPSFSPPRKKPRNPPYAELPLEGQIFTGPVF
PPNPMAHDHHHHHGFPFLPFPDSSAQPAGIQGARHAQFASPFPEFHIGNLQPNLMLYAGI
RLPPADRAAPAPRPPRIIISTDLTIGSPGKPDDAACSPSSGGKKIDDTKPRGFLLFGQAI
LTEEQIKNGNSDGRPASPNWDAEKAPNTSEGSDSGVTQGSPTKNTTPSWSLPYFGGNNIS
RASEYELNPGQCKVFVESETVGRSLDLSALSSFEELYACLSDMFSIGSDELRSHLVYRSP
AGEVKHAGDEPFCAFVKSARKLRILTDAGSDNLGD*
>11668.m03994|LOC_Os02g41840.1|genepair1652-1
MARSTRSAATEQAYSRFAPTAASSRGRGVGGGNGFDEFDESDIWGSFEPAAEVAESPRAA
RHQVPAARPPGRKAAAAASKPAAHGSLPVNIPDWSKILGDEYRGHHAGDWEADDVDDDDI
DAASAVAVLPPHELAWRRRAASLSVHEDGMGIGRTLKVRDAVWKKTGFQA*
>11670.m04257|LOC_Os04g43990.1|genepair1652-2
MAGSARSAAAKHAYRMFAPSRGAAARCPGSPGADEFDESDVWGSYGAAGVESSPAELGAR
GRAIPSARAGRKAPLDRAAGSLPVNIPDWQKILGVEYRDHQAAAAEWELQGDGDDDYEYG
KVAGVGGVVIPPHELAWRGRAASLSVHEGIGRTLKGRDLSRVRDAVWKKTGFED*
>11668.m03998|LOC_Os02g41860.3|genepair1653-1
MAKDIEASAPEGGEFSAKDYTDPPPAPLIDVEELTKWSLYRAVIAEFIATLLFLYITVAT
VIGYKHQSDATVNTTDAACSGVGILGIAWAFGGMIFILVYCTAGISGGHINPAVTFGLFL
ARKVSLIRAVLYIIAQCLGAICGVGLVKGFQSSYYARYGGGANELSDGYSKGTGLGAEII
GTFVLVYTVFSATDPKRNARDSHIPVLAPLPIGFAVFMVHLATIPITGTGINPARSLGTA
VIYNKDKAWDDQWIFWVGPLIGAAIAAAYHQYVLRASAAKLGSYRSNA*
>11670.m04264|LOC_Os04g44060.1|genepair1653-2
MAKDIEAAAAAEGGEYMAKDYSDPPPAPLIDAEELTKWSLYRAVIAEFVATLLFLYITVA
TVIGYKHQSDPGANAADAACSGVGILGIAWAFGGMIFILVYCTAGVSGGHINPAVTFGLF
LARKVSLVRAVLYIVAQSLGAICGVGLVKGFQSAFYVRYGGGANELSDGYSKGTGLAAEI

IGTFVLVYTVFSATDPKRNARDSHVPVLAPLPIGFAVFMVHLATIPITGTGINPARSLGA
AVIYNQHKAWHDHWIFWVGPLIGAAIAAAYHQYVLRASAAKLGSSSSFRG*
>11668.m04003|LOC_Os02g41910.1|genepair1654-1
MAPSRRMVASAFLLLAILVATEMGTTKVAEARHCLSQSHRFKGMCVSSNNCANVCRTESF
PDGECKSHGLERKCFCKKPCVNSQMAGEAILFSDDIIANILAWLPPKNAARMRLVCKQWH
AVTSEHHFMHTNFSRSRDGHSVAGFFLSNELHKKFSYNPLRDSSATHPAAPDLSFVPESG
STVPRKINVTSSCNGLLLCRRPMDSSVASGARWCCYYVCNPATKRFVEIPTPPDGRGRHL
NLAYDPSRSPVYKVVALGLAGVHVYSSQARSWRAALRYERGSNPFAGIHHSRGVHWNGSL
VWVTSRSRSLLRFAVDDGEGELSSLPMPPARHLQPENRWICGYLGVGESAGAGRGHLRMI
GYTEEEKLAARFDVVGMAGDCREWRVLYRVDLTRMKELYPDIQRKTRKHHLIWPRRARLV
DCLDLWPLHVAEHGSLLLFGIPGKIMAYGMEDQAISVVWEDAAPPQPRFFRYAWFDFYPY
TAGLFAV*
>11670.m04271|LOC_Os04g44130.1|genepair1654-2
MAPISRRIAPLLFLMLLILVASEMGTTRVAEARHCVSQSHRFVGACMRKSNCEHVCMTEG
FPWGECRFHGIERKCFCKKRC*
>11668.m04007|LOC_Os02g41950.1|genepair1655-1
MSRLARALARVLAESLLGHAAGERFPEGCDDATCFLRLNRYPPCPFPPDDAFGLVPHTDS
DFLTVLCQDHVGGLQLMKGSRWVAVKPIPGALIVNIGDLFQAWSNNRYKSVEHRVMTNAT
TERYSVAYFLCPSYDSPIGTCREPSPYKAFTFGEYRRRVQEDVKKTGKKTGLSNFLV*
>11670.m04273|LOC_Os04g44150.1|genepair1655-2
MPAFADIAIDPPLADSYRALALLRRDRDGGIAPPAVQMVGSGGAVLERDLPMVDLERLTR
GGAGERKACAGAMARAASEWGFFQLTNHGVGRELMEEMRREQARLFRLPFETKEKAGLLN
GSYRWGNPTATSLRHLSWSEAFHVPLASISGADCDFGDLTSLRGVMQEVAEAMSRVANTV
AAALAEELTGRGGGGASAAPWFPAGCDETTCFLRLNRYPACPFAADTFGLVPHTDSDFLT
VLCQDQVGGLHLMKDSRWVAVRPRPDALVVNIGDLFQAWSNNRYKSVEHKVVANAKTDRL
SVAYFLCPSYDSLVGTCGEPSPYRAFTFGEYRKKVQEDVRTTGKKIGLPNFFKHSSVQ*
>11668.m04011|LOC_Os02g41990.1|genepair1656-1
MASSPEAAAVGEEEKGKGKKEEGRRGGGVLGRMWRALFGGREDYEKRLQYLSKEEAAVHA
RMRRRTQFSRTAVRNLIVLSVLAEVVAIVYAIMTTRNEHITWEMRAIRVLPMFVLPAVSS
VIYSTVVKFTRMLERKDQKTLEKLRAERKAKIDELKERTNYYLTQQLIQKYDLDPAAKAA
AASVLASKLGEETGLKVHVGEEPKLDSAVARSNDVEISPSEGLRNRKQSNARGSRTGGTT
AAQNPAQGAESSLTSSSGLEQPPMVVEHFQGSGASDGGWIAKIAALLVGEDPSQSYALIC
GNCHMHNGLARKEDYPHITYYCPHCHALNTSKQSLGQHSGSNSGRSTPVAPADGISASSS
VVESEVSNMTTIQELKNEENTEKQEVQAS*
>11670.m04276|LOC_Os04g44180.1|genepair1656-2
MASTAEVAAAAASASAAAAAAETKGKGEEGKRKGMGDTGDDLAGSVFAGRGDDYERWLQY
LSKEEAAVHARLRRSPAVASATSSSSPSSARQSFCLVSQEVHCSFITHSSVKIFKHGLGD
TKLEQKDQKLLERLREERKAKIDELKERTNYYLTQKLIQKYDLDPAAKAAAASVLATKLG
ADSGLKVSEAVGDHLEAMEPSRVVGHYQSSGLARKEDFPHVTYCCPHCHALNMSNQTIGR
WSGSNSGQLTSSAQVSGTNPVADNELGNQTEGQEIYVEENSGEGQGIYVEANSG*
>11668.m04014|LOC_Os02g42020.1|genepair1657-1
MQMSTNPNHYPVFPHSFRNQHVVSFQTSTIANGSGAIPVCPASSGGMNSDMTLLNTTPST
IVSTSSPNMLADSNQSLKYAAPMAVDWSYPELQLLNDGLLKYANEPGIMKYIKIAAMLPE
KTVRDVAMRCQWMAAKKENTRRRKTDEHYLGKKTRDRKDKMVESSWATNRQVQTTDIRSP
STLACNTVRDNQFQSGGWCVTYEVAV*
>11670.m04280|LOC_Os04g44210.2|genepair1657-2
MADDPSLDFGEFPQPFCSQQVVSFQPSVTTSGSGGMPVYLDCSSGMDSNTVMLSTTPSVV
VSTTSSNTVADPGQNLKYGGPLAADWSRLELDVLKDGLQKYGNEQGIMKYIKIAASLPSK
TVRDVAMKCQWLGKRENSRRRKSEDHHTGRKMKERKAKMAEPSLWGTNHPVQTDTRVPSF
VSHNAIQNNQILTGATGIDRAMQHLLVPNDRLLDQIEANMLACQPQNNIELFHRTRRNIN
GLLQTMNQMPGIMSKMPPLPVSVNENLASFVLPGLTVPQFLGGSQLKEEPRGW*
>11668.m04016|LOC_Os02g42040.1|genepair1658-1
MENTQNENSKEAKQDDETRQNKQDDEEARLEEYKKLVDQKTALRRSNLNSERPDANYLRT
LDSSIKRNTTVIKKLKTINDEQKDGLMDELKSVNLSKFVSEAVSYICEAKLRSADIQAAV
QVCSLLHQRYKDFSPCLTQGLLKVFFPGKSGEDLDADKNSRAMKKRSTLKLLIELYFVGI
VEDASIFVNIIKDLTSLEHLKDRETTQANLSLLSAFARQGRFFIGLQSHGQEAYDELFKD
LNVTADQKKFFKKALNTYYDAVAELLQSEHASLRLMEAENAKVLTAKGELSDENTASYEK
LRKSFDHLQRGVSSLAEALDMQPPVMPDDGNTTRVTTGSDVAPSTAKEPSALEPIWDDED
TKAFYESLPDLRAFVPAVLLGEAEPKLNEKGREQSEPVAEQDTDVHDNPQTSSITEYHLE
GKADDGVKDSEEKDKDKGKGVDKEKSKEKDFDRKTEREKEKIRAVDGASLDNLLQRLPGC
VSRDLIDQLTVEFCYLNSKASRKKLVRTLFNVPRTSLELLPYYSRLVATLSTCMKDVPSM
LLSMLEEEFNFLINKKDQINIETKIKNIRFIGELCKFKIAPAALVFSCLKSCLDDFSHHN
IDVACNLLETCGRFLYRSPETTIRMANMLEILMRLKNVKNLDPRHSTLVENAYYLCKPPE
RSARVSKVRPPLHQYIRKLLFSDLDKSSVEHVLRQLRKLPWVECQQYLIKCFLKVHKGKY
SHVHLIALLTAGLSRHHDDFAVSVVDEVLEETRVGLELNDYGMQQRRLAHMRFLGELYSY
KHIDSSVVFETLYLIIVFGHGTPEQDVLDPPEDCFRIRLIITLLQTCGHYFNRGSSKRKL
DKFLLHFQRYIISKGPLPLDIEFDVQDLFAELRPNMARYSSVEELDAALLELEEESERAAS
VEKPENEKLSDSESQKVQPHDTAFSANGRSSANGAEENGKDHEGADSESYSDSGSIDGHE
DEEDLMFEDKSNDASENEGDDEDDGIPAGSDEDEGVEVRHKVVQVDPKELEDFDRELKAL
LQESLESRKSEVRPRATLNMMVPMNVLEGSKDPRAVESESGEETVDEEGGSAGGGSKVRV
KVLMKKGHKQQTKQMFVPGDCSLVQSTKQQEAAELEEKQSIKRRILEYNEREEEEMNGGS
SQMGNWGQGGSNTGSSIRSGGRGIWDGSIRGGRQRHHIAGGFYHSYGRRR*
>11670.m04282|LOC_Os04g44220.1|genepair1658-2
MLVALSYALLLLLGVPRAGSSRGALTDGGRGWGDGRRMAPRLTQWKALHDCPHLAGGMKW

GRRKMRTGRKMMNRRIIAKKMIRENDAVRNGDLERAKARVTEGANLDTLIQKLPGCSSRD
LIDQLTVEFCYLNSKANRKKLAWALFNVPRTSLELLTYYSRLVATLSTYMKDLPSITLSM
LEDEFNFLINKKPSSSDCSTAGLSHYHEDFAVAVVDEVLEEIRVGLELNDYSMQQQRLAH
MQFFGELYNYEHIGSSIIFQTLYLIIVFGHKTPEPLGYKFNISGEELDLFAHLGSNMTRY
SSMEELSVALIELEANGYVASAEKCGNEWHSGSKEQTKQSDYVSFDANHKSSRDRIDENG
NDNEELAVRAIQMEASIRMDMKTLIFQAKGDPMEDLRRMTMAMITCLSKDLRATELENGG
ENASVSINDGDGGKVCIKVLVKKGHKQQIKEMFIPGDCSLVQSTKQQEAAELEEKQSIKN
*
>11668.m04018|LOC_Os02g42060.1|genepair1659-1
MSSIGAGAGGAVVGAAVAAVAVGGGAPPHVLAVDDSSVDRAVIAGILRSSRFRVTAVDSG
KRALELLGSVAVICSRNFLMFLWLAHHLAGTKCEHDNHGLLDAGDDGIRAPEESQGVIQA
EEDPRGDHVLGECANKNQQITEHLTIVLPSLLCSIGLLTRCLEEGAEDFLVKPVRPSDVS
RLFSRVLP*
>11670.m04288|LOC_Os04g44280.1|genepair1659-2
MATCRSRGVERGGAPHVLAVDDSSVDRAVISGILRSSQFRVTAVDSGKRALELLGSEPNV
SMIITDYWMPEMTGYELLKKVKESSRLKEIPVVIMSSENVSTRINRCLEEGAEDFLLKPV
QPSDVSRLCSRVLR*
>11668.m04040|LOC_Os02g42280.1|genepair1660-1
MGTGRKPVDGDGDGAASAVVFLVPFPAQGHVTPMLHLARALAARGDVAPTVVLPDFIHRR
VVRAGGNGGGGGVALASFPSGIPGGDDGDGDPGFASIVHAMENRMPAHLERVLMLMRDDD
DDRLAAAGRRAACLTVVVDVLASWAVPVATRCGVAAVGFWPAMLASYRVVAAIPELIDRG
LVSEYGIPILANGFNKNQGQVKANLQAEIISLFPEELELSTTDLPWLVGDAATQKSRFAF
WLRTMERVKTLRCILVNSFPGEAIAAGADQQQPLPQDQQILQVGPLLATIVTDRAKGNSN
LRCSPMKTTKNDTSTCQADDRTSCMEWLDQQRPGSVAYVSFGTWVAPIAPGEITELAVGL
EATGRPFLWVLKDDPSWRAGLPAGYTDQYSGRGKIVAWAPQEDVLAHGAVGCYLTHCGWN
STLEAIRHGVRMLCYPVAGDQFINCAYIVRAWGIGIRLRSADRGEVVDCVGRIMEGEDGR
RLREKLDELRERVMAGEALCVAKRNIEEFIRGISGQRLQ*
>11670.m04295|LOC_Os04g44350.1|genepair1660-2
MPQLHGTPAVSSNQLDDKDGREEEQVVRGLGILPAQLELSTKELPWLVGDSATQRSRFAF
WLQTLRRARGFRSVLVNSFPGEAVTGTAAAEDDDGPQRQAACPRVLPVGPLLVLAGCNVE
RAKGAGDDGGVAATNINNHPQPCSKNPSMWQADSTCIRWLDAQPAASVVYVSFGSWVGPI
GHDKIRELALGLEATGRPFLWAIKDDPSWRAGLPAGYAGSVAGRGKLVDWAPQDDVLGHA
AVGCYLTHCGWNSTVEAIQHGVRMLCCPVSGDQFINCAYITRVWEVGLKLGSVRRDVVRD
CIERIMGGAEGTRLQEKMDALRQRAVTAEARCLAQGNLRSFVNEIKRDHPLLTQIYNIL*
>11668.m04041|LOC_Os02g42290.1|genepair1661-1
MTPSALAHLASASPLPAFSPKPRARPGSAAGPALRRLAVAAPAPRAYFSSSPMPYQPQPQ
QPAGYSSHQAFGLVPMVIETTSRGERAYDIFSRLLKERIVLIHGPIADETASLVVAQLLF
LESENPLKPVHLYINSPGGVVTAGLAIYDTMQYIRCPVTTLCIGQAASMGSLLLAAGARG
ERRALPNARVMIHQPSGGAQGQATDIAIQAKEILKLRDRLNKIYQKHTGQEIDKIEQCME
RDLFMDPEEARDWGLIDEVIENRPASLIPEGATGVDLPHHSAAGVGGRGRDVEEPSAV*
>11670.m04300|LOC_Os04g44400.1|genepair1661-2
MLPVAPTRQPLTASSWPRLAAAGPTPPRPNLPLNPAPPPPPPPPNPSPAAMLRRLAAAAP
RAFFSSSTPHAPPPPAGYTQRREYGLVPMVIEHTSRGERAYDIFSRLLKERIVCIHGPIT
DDTASLVVAQLLFLESENPAKPVHLYINSPGGVVTAGLAIYDTMQYIRSPVTTLCIGQAA
SMASLLLAAGARGERRALPNARVMIHQPSGGASGQASDIAIHAKEILKVRDRLNKIYAKH
TSQAIDRIEQCMERDMFMDPEEAHDWGLIDEVIEHRPVSLVSDAVGSDLPNLGGGGDGAN
KATDEPSPA*
>11668.m04044|LOC_Os02g42310.1|genepair1662-1
MFLESPVGVGFSYTNTSSDLQQLGDKITADDAYIFLLNWFKRFPQYKSHDFYIAGESYAG
HYVPQLSEKIFDGNKQGPKENYINFKGFMIGNALMDDETDQTGMIDYAWDHAVISDRVYA
DVKKYCNFSMENVTDACDSALTEYFAVYRLIDMYSLYTPVCTEVSSSAAFGQRQVAVHGA
APKIFSKYHGWYMRPAGYDPCTSDHAEVYFNRADVQEALHANVTNIGYNWTHCSDVIGKW
RDAPFSTLPIIRKLVAGGIRVWVFSGDTDGRIPVTSTRLTLNKLGLKTVQEWTPWYDHQQ
VGGWTILYEGLTFVTIRGAGHEVPLHAPRQALSLFSHFLADKKMPPTAFP*
>11670.m04301|LOC_Os04g44410.1|genepair1662-2
MSMALLSLSLAVAFLAAASAAGATGASRSMRRPEEDLVAGLPGQPDVRFRHYAGYVGVGN
GKALFYWFFEAEKEPEKKPLLLWLNGAVNLLFLEAPVGVGFSYTNRTSDLRRLGDRVTAQ
DSYSFLLNWLNKFPEFKNRDFYIAGESYAGHYVPQLAELIYDGNKGASRDRVINIKGFMI
GNAVLNDATDQMGMVEYAWSHAIISDELYSAVRRECDSFKEEEDGGKPSKGCSPAVRAFL
RAYDDIDIYSIYTPTCLSSSSSSPASASPRRSSPGLVAAPRLFSKHEAWRRMQRVPAGYD
PCTEEYVKGYFNREDVQRALHANRTGLSYPYSPCSEAISKWNDSPSTVLPILKKLMGAGL
RIWVYSGDTDGRVPVTSTRYSLNTMKLRPRLMRKTAGDGAGEESEWGGWRAWYDRQQVGG
WAVEYEEGLTLVTVRGAGHQVPLFAPRRSLAMLYHFLRGSSLPASRSR*
>11668.m04051|LOC_Os02g42380.1|genepair1663-1
MSSRDAAATFHVYQPVQIPTATVAPAAAVSAAPAEAVAQLVPAPSKKAAGAAGGKDRHSK
VNGRGRRVRMPIVCAARVFQLTRELGLKSDGQTIEWLLRQAEPSILAATGTGTTPAAFVS
SSAPSTSSSHQHTLLGKRQRQESAAADAVSVAGAASAFWAALPAPGRPDAWGFSPLDAQP
TYVPMAQAHHHHLNLLAALSGAARRAEEESR*
>11670.m04304|LOC_Os04g44440.1|genepair1663-2
MASRDAATFQVYRPMAMPTPAALPPSSQQITMPFTAALVDAVLPAPRKAAATQGGKDRHS
KVNGRGRRVRMPIVCAARVFQLTRELGLKSDGQTIEWLLRQAEPSILAATGSGTTPAVFS
CSSAPSTASSSFLLGKRPRQEDHEAPTFWEALQQQPRPAVSSWGALVSPSQEAQAYASSV
AQVHHLNLLSALSGAATRRPAQEESR*
>11668.m04056|LOC_Os02g42430.1|genepair1664-1

MTKSSCAHVVGVPVTSKAYAIEEATTARDGGKKVDGDRLAVSLTHPSPYTSFGYKHSSKL
QVIHWVNKLGRRAQGFRDHVTLGPKLSETVRGKLSLGARILQAGGVERVFRQAFSAEKGE
RLVKALQCYLYTTGGPIAGMLFVSNRKIAFRSDRSLAVTSPAGDVVARVPYKVVVPLRRI
KRVRPSENADKPEQKYIHVATVDGFEFWFMGFVSYQRCCKYMQQVISEL*
>11670.m04310|LOC_Os04g44500.1|genepair1664-2
MRKSSINGVHVIGVPVTAKAFGIEEEVSLARGQSFRKADGDHLAVSLSHPSPYTSFGYKH
SSKGQVIHWVSKLSRRAQGFREHVTLGPKLSETVKGKLSLGAKILQAGGIERVFRKAFSA
EKGERLVKALQCYLYTTGGPIAGMLFVSTKKVAFRSDRPVTVTSAKGDVARVPYKVVVPL
RRIAQVRPSENADKPEEKYIHVVTVDGFEFWFMGFVSYQRSCKYMQQAISELQ*
>11668.m04057|LOC_Os02g42440.1|genepair1665-1
MEGSTSQDHVIGIPVSNTAYGIEEPDFAAEETTTPDHAGFVVGSFQFNNDANSPTTTTTT
TDRASKYGRKGDKIAQGIKEHVTLGPKLSETVKGKLTLGARILQAGGVEKVFRQWFSVDK
NEKLLRASQCYLSTTAGPIAGMLFVSTERVAFRSDRPLAVSAPGGDKVRVPYKVTIPLRK
VKAAKPSENKHKPEQKYIEVVTNDGFEFWFMGFVSYHRSLHHLEQAVAQARR*
>11670.m04311|LOC_Os04g44510.1|genepair1665-2
MEKAACNEHVIGIPVSNRAFGIEEPDFPSEGAAAYHAEAKSSATARTSSRFGRTGDRLAQ
GIKEHVTLGPKLYETMKGKLTLGARILQAGGVEKVFRRWFAVGKGEKLLRASQCYLSTTA
GPIAGMLFISTERIAFRSDRSLALTTPSGDTVRVPYKVAIPLRRVKTAKPSENKHRPEQK
YVQVVTDDGFEFWFMGFVSFQVTLKNLELAVAQAQ*
>11668.m04073|LOC_Os02g42560.1|genepair1666-1
MGLCCSKTQKETTPPHGGAEATTSPQVKRKSGKIAADDKGKKTRAGRAVEAAGDKKAVFV
VKTKSGAVNVEERRPVVVVPTMPVRTSSCTKEEVDAILIQCGRLSRSSSGRAASSETGAG
HRRSKRSYDFDQERRPGCGGGGDEERDWERHGGAVSRPSPHRGSPQRKRSGSRERSSGGG
SRRASRSPGRRAEGVSPAPAPAGSGGGGGGGERVVRQQPGKMVSVPAREKARAPSPAAAS
GKRCASPRSSSPARMVAAGNENAGGGQMTAAQTPSLSRSSSPYRRSPMAEIDENSLRNNN
GANHHKKISENALAIAAAPQKATERSKEKPKVVEETVLVAAAPPASKTTATRTASATAES
LNTKARSRRASRDFDQNTNSYATQLLEDIQSYHQQNTTSVAATAATLPSFSLPACVSKA
CSILDAVADLNSSSSDSHSCEPDRSANDRGSVNAPLGGGMDDLAEPGVHKRHATAPRGDI
RGGGGETEPQESAGSNSVSGNPWTPSWEPNSVESTDRTWSASRSTNNGDEVVEQGSSSHA
GARSPLNRSRQSSKQRAAQPEHSVRSRAGSSGGNSNNVVHRGRGAHRSGGGGGGSVASGR
SGVRAVSAMS*
>11670.m04327|LOC_Os04g44640.1|genepair1666-2
MGLCFSKKQQVRRRREEQQQPPCHEARKAGGGKKAGAKEVAIVPEAAKKAPPPRKAVPKA
EEPAADKRTVFVVKAAAAAAAAEVAASASGEAADEEAKRPAPEEEEAKPVVVSRVPVRTS
SCTKEEVDAILIQCGRLSRSSSSSGKVASGEGGGGHRRYSGSKRSYDFDRERRGGGGGGV
DDDCDWERQGAAVSRPSPRRRTPERKRSGSHERSGGSGSRRVSRSPGRRADSVPATASGE
RASRQQPGKMVSVPAREKGRAPSPVPAASGKRYPSPRSNSPARAGAAGNENAAAQLAHGP
SLSRSSSRKAEHSPYRRNPMAELDENTLGNHHSCNNNGRPQKKPTESGGALPQKVAEWAK
DQVAASRTAAKEKQEIVEVPVASSDTKGGNSGRMKATHSVSIVAESVVNQKGRSSRRSSH
DFDNNGNSYASLLLEDIQNYHQQSTGSAAAPAPAFSLPACVSKACSILEAVADLNSSSSE
NKSFELDRSANDKCSANGRYGDGKVAGGGTLVVESEVVVKDDLMEPSLHKYVSVRDIRGE
AEPQESAGSNSFAGNAWTPSWEPSSVDSTDRTWTASQSNNGDEVEQLSSGAVSPLELSWQ
GKQKLPSQEPSGGGRSRVGPTGNAQRGRSAHRGGGGAVNARSDVRAAPVPAQGDVASLFR
SMSRSQSTCRSDLLTGKMPRVQTSRLGKNPIEVDSDGGEDFHVDYEVGNDVDEGPEHGEI
DGLLSVVLRKMIVQEVKKNLVDTSSVNFHNPIDKKKSKVHSKTEPSYSRFSTKYFSTVLS
SLSPDQKRIIGDYGFNSLLMFDNAYVPNKFASWIANHVDVKSSQIVLKDKVIAINKECVH
HILGLPIGGMEFPTDCDAGKSFILSKFGKSALPSETLSDDEVITSFLIVAMACFLCPNSS
LVPSTKYLTVFENVDELRSYDWSKFVYEWMMTSIKKIQKFSTLGGCWFLWAVLYLDYVEF
GDKNVPIGKNVPIGFPRISHWKNNMITLYSNLDKVDEENFGLRPIKDFNDTTYFKVVPPE
NRINTFRDKLESAIGTMLPAFIKEKICSMVVSHCSANHIVDSESCEDIAISIMLLLCEHA
GSRYGGDPDENLIFDDINPGFQPNYAIDVENEIPCDAHNNGSTNKSVASKANETSFRHTP
SHDKELGVNDDSVIVSKSAVRFSLSPNFEKDQGLLTPEVGYANNSNSRFEHFRSASGPTV
SAVAAVRNVANKIKSRLSQLNNDDKRGPLFPDLIDSCEDEAVGYAKSLSCQKEYLNPRYV
TSSSSQPGISLHCLDNSPVQVIGINNNEGTRRTHCIQNVKKRRFDDVINSPDVEYFGHST
FPDCCKVLCTQTDNLYNAKNMLKSNNLDLSSTGGKLPPHGPRRVLVPSRKFSDPYVLSVR
RRFLVSDQEKRHYNAICKLSESSKWHSYDAVDIDNVRAKFSSFGQSLMKGGTVLSYVINV
FCRVLFNNNHPSNSKRHYFFSSIGELLLTDLSCADLSKVKRSSLGAASARKLHLSDMLFF
PIEHLEHWFLFVVDIKDRMLVLLDSLHEKGDPYFEDIECLLINNLQTFWDSYYGSSIDFT
TLKKVYPCVPKQRSCFDSGIFVMKCMELWSPRVVLPNEFSSDDIDNIRMLYANQLFFHPT
NKMLQTEVEEVVLNWFNPDQFAGEG*
>11668.m04074|LOC_Os02g42570.1|genepair1667-1
MATTTAALAPPSTSTSSVLLPSAAAAAAARCLGPLPRRARLRTARHVALTPRDLSAEDVA
AEEAAVAPKIGKRVRVTAPVRVYHVMKAPDLDIKGMEGVVKQYVAVWKGKRITANFPFKV
EFHLSVEGQDKPVRFFVHLREDEFEFIDE*
>11670.m04328|LOC_Os04g44650.1|genepair1667-2
MKFPTRPPTVPMAPATATAVASPSTSSRLLHRSLLSPTPTAARCLRPPLCRGRLRTVRQV
VANGDVSSPSSDVAAEESAAAPKIGKRVRVTAPVRVHHVSKAPDLDICGMEGVVKQYVGI
WKGKRITANLPFKVEFELRVDGQDKPVRFFAHLREDEFELVEDE*
>11668.m04075|LOC_Os02g42580.1|genepair1668-1
MDAVDRGGGGGGGGARGHGRRWKGKGVSAAISSSAAETQQPVPVLEDAPAAAALLRPQKK
IRSPDRRLQRSISSLSSAPASPDSSSVSNPMSPPAMSLPNQPPSSRHIFPFAYDPSPGAA
APRLLPLLQYSSLYPQPLLPQQQSPLQNQQMISFGSSQQQQQQPQFGAASPLFPPQFLP
PEEQQRLLLRYWSEALNLSPPGVRGGALPPSLYQHLLRAPGPPKLYRGVRQRHWGKWVAE
IRLPRNRTRLWLGTFDTAEDAAMAYDREAFKLRGENARLNFPDLFLGKGRTGGSGRTSAS

AAASCSSSSSSAPPTPDESHTQQAQPQPQQPTEESSNTEPKPLLFVAEQDGIPEPELNPQ
LQTAEQHGSDGNTAMFQPSVTSGGIWGPADEAWFSAWGPGSSVWDYDMDSAHGLLLQSRL
AGEQTGMDYAYTAPEVLVAPASPSPSPVAAVKAKRRAMSRPPSPMLSVPEKKTAATELFR
DRHFFNSAFFTDLREARASLSAPSSQATTQDAASRRALLLRYHRLLASARDDPCDFDDDL
AFTWHDAFRPHLRRTAASLRFEKAAVVFNVGAASSRIAAAVDRAAVGGVKAACGEFQRAA
GAFRAAGEMMEGEEEDTEDTVDMGPEASAMLERLMLAQAQECCFERALAAGTSSAACSKV
AKQAALYYEEAYASLVIPPLQNHFERSWVAHILLKAALFNAEASYHYAIELHEKTEIGEE
IARLQFGINAIVDAKKAVRGAPGSLYDAASRLEQDMNQNLERALNENNRIYLLRVPAAKL
LAPLPAASLVRSASLSEILDVKTETGNQSSQHGWFIYMPLNILQEYYEVNVMKNN*
>11670.m04330|LOC_Os04g44670.1|genepair1668-2
MDAPSGESGGGGGGGGGRRWKGKGVTPIQPRRQLGTVLEDSSAALLRPLKKIGRSPDRLL
RSASSLSTSSSAPPSPRSSSASDAPVRVISSSPSSPSPPSARHIFPFAYEASTTTVGGSP
RLHPLSWQQSSMSQPASPQQQQQQPLQHQQMISFGASPPCSTTQFVVPENAQQQQMLLRY
WSEALNLSPRGGPGGVPPWLYQQLLRVPPPPQKLYRGVRQRHWGKWVAEIRLPRNRTRLW
LGTFDTAEDAAMAYDREAFKLRGENARLNFPDRFLGKGRAGGKGRTSVSSSAAAAASCSS
SSLSPPETPDDANTQQQAPQQREQRDTAGVSMEKKQPQPPAPTSRQEGCSGGDAAAPYPA
EMLHAPAACGGMWVAPDESWFSTWGPGSSFWDDYDMDSARGLFLHPRFTGDETSMDHSGT
QATVPAVAATAAGMSMPCDDVPVTSSSSDLPPQGTPQTPTFMWKED*
>11668.m04076|LOC_Os02g42590.1|genepair1669-1
MSVEEEVVGEEEEEELFYESLDRILSSSGSSTSASDDDGDHPRRRRGYDAGAAAAAAALD
LWTSEPAPIQERRRRLLQLMGLSGDPSLARFERGRSAPCDAVGPLPSSPVARSRSSGATP
ASAAKPPLGGGRLRGASSDVSDATLEAVEEDPRCLIRNLDDGSEFLVREEFGLREVGTGR
QLTMEEFELFIGRSPIVQELMRRQSVVNSNSNSNSQSGASTPIERSSSGSSNGGARYKRR
NSWLRSLRSAAGSMVTYTRDRRSSDEKDTSSDKGGHRSSSATDDSQDGVANHGPDRVKVR
QYGKSYKELSGLFMNQDIKAHSGSIWSIKFSPDGHFLASAGEDCVIHVWEVLEWKMIEEK
GLEENGVFDPESMLVSTASEGSHREKKLRAKAVHNQRSVSSDRLMVPEHVFALSEKPVIT
FAGHSEDVLDLSWSKSQLKTYEAIAKLAVPMSHLTLRSHLDLCYPSTPYLHFSISVQYLL
SSSMDKTVRLWHVSSTYCLKTFSHSDYVTCIQFNPVNDKYFISGSLDKKGALVGSHKGKC
HVYDISDNMLKHKKQIDLHIKKRKSSQKKITGIQFVPGSSSKVIITSADSRIRVIDSFEL
VCKFKGFRNTNSQISACSAVKGRYLISASEDSHVYMWRCNDDSEPNTKKGIVSITNTHEH
FHCEGVTVAVSWPCASSTMTSREWPENGLSGSELDKDQEMPDAQHQSNIIRNTNPNHNGD
TSATWSEELMTPTKQSPRSSASHPMEGDQAPSHSAWGLVIVTAGHEGHIRTFQNFGFPVR
V*
>11670.m04333|LOC_Os04g44700.1|genepair1669-2
MRRGTGALLLLLLPTSASDDDDHPRRGRDAAAAAAAAAAAALDVWTSEPAPVQERRRRLL
QMMGLSGDPSLARLEMGRSASYDGPIRPETVSPISRSRSDGSVPASATKPPLAARSRQTS
SDSSEATPGGDDADPRCLIRNLDDGSEFVVKEESALREVGTGRQLTMEEFDLCVGRSPIV
QELMRRQNVASSGSSNGASALIQRSSSDSSNGATRHRRRGSWLRSIRNVAGSMVVGSRDR
RSSDEKDTSSEKGGRRSSSATDDSQESASAVRRGPERVKVRQYGKTCKELSGLFMNQDIQ
AHNGSIWSIKFSHDGRYLASAGEDCVIHVWEVSELERKREGNGVCNQLVAVVCNGSPEPI
LALASVDGSCWEKKHRARILETRKSASSDRLMFPEHVFALSEKPVKTFEGHSEDVLDLCW
SKSQYLLSSSMDKTVKLWHMSRTSCLKTFSHSDYVTCIQFNPVDDRYFISGSLDEKVRIW
SIPGREIVDWNDLHEMVTAACYTPDGQRALVGSHKGSCHIYDTSGISHPLMILISYECIS
CCTGIQVYRVMLGSHNRFSKYQQPNISLPVFEREGRSKAVVSVTNSYEHFHCQDVTVAVA
LPSAGSAMTSRTNSRKTEEQDSIPQQHTQPDKQQDSSDFQCLSGNGLSTSSNHSGDRTSA
TWPEELMTPTRRIRGQFAQRSGIGSGYLLPRCTKLRSSTNLLRPGHTRCVDRSCKKTPSS
HIRPLRLKESRTTDHGSKKKPSLIFGKYSRNNESKAAGMRCRN*
>11668.m04077|LOC_Os02g42600.1|genepair1670-1
MVYFGNTSIGEVEVWPSGDASLAAAAWAREIRVDRLSPPSERCQPLAVMHTVAVGARCLV
MESRLPKAADEPPQPLVAMHAACLKENKTAVVPLGEEELHLVAMTSRRNLTNHACFWGYK
VPFGLYNSCLTMLNLRCLGIVFDLDETLIVANTTRSFEDRIDALQRKLSNETDPQRINGM
IAEIKRYQDDKSILKQYIEGDQVYDDGKMYKVQPELVPPLSDNHQPMTRPVIRLQEKNII
LTRINPLIRDTSVLVRLRPAWEDLRSYLIARGRKRFEVYVCTMAERDYALEMWRLLDPDS
RLINSVQLNDRMVCVKSGLRKSLLNVFHDGSCHPGMALVIDDRLKVWDEKDQSRVHVVPA
FTPYYAPQAEANCSIPVLCVARNVACNVRGGFFKDFDEGLLPRISNVFYEDEINEIPSAP
DVGNYLISEDENVAAVNGNRDPLAFDGMADAEVERRMKEASGNAQAFTTTAANFVMPVLP
GQNFVSSSVAPVAPSLGMVPLSNNQGPPPFTQPVAQLSLSDPLQGSPAREEGEVPESELD
PDTRRRLLILQHGQDTRDPTPPLPAVPPVQVPVPPVQPHGNWFPVEDGMNPNNLNRGSAG
FPLESETMHYDKKQLPHPFFHGGENPISSDRFSYQNQRYPSQLPHSEDHRVLQNHAPSRY
RSFPGEELATRHVSSSQRNNQIVPGQHFARHAGSSAGILEEIAMKCGSKVEYRSALCDTA
DLQFSIEVWIVGEKVGEGIGRTRKEAQCQAAEISLRNLANKYLSSDPNKMTDMKENGFGS
NTNIFGYPGNSRDDVLPIASTSEETRFVKMGENNSRKAGGSIAALKELCTAEGYNLVFQA
RTSPDSSVGKEAYAEVEVCGQILGNGVGITWEDAKLQAADEALGMLRSMLGPLAQKRSSS
PRSLAPSFDKRFKPDFPRAVQRVPYGRYSRIEGHVP*
>11670.m04334|LOC_Os04g44710.1|genepair1670-2
MESRPTATADEPPPPLVAMHAACLRDGKTAVFPLGAEEIHLVAMTSKRNLPNLACFWGYK
VPSGLYNSCLSMLNLRCLGIVFDLDETLLVANTTRSFEDRIDALQRKLSNEIDPQHISGM
SAEIKRYQEDKSILKQYIENDQVIDGGKVYKVQTEVIPPLPDNHQPMTRPIIRLQEKNII
LTRINPLIRDTSVLVRLRPAWEDLRSYLIARGRKRFEVYVCTMAERDYALEMWRLLDPDS
RLINSVQLTDRLVCVKSGSRKSLLNVFHDGSCHPEMALVIDDRLKVWDEKDQCRVHVVPA
FSPYYSPQAEANFSVPVLCFARNVACNVRGGFFKEFDEVLLPRISEIHYEDEINDFPSAP
DVGNYLITEDENAAILNVNKDPLAFDGMADAEVERRLKEVSCSVQAVNPIPTNADVMPVA
PNQQLITSSVPEAPSLGMIPLNNDQGPQPPSSWPVAQSAPVDPSQSSPAREEGEVPESEL
DPDTRRRLLILQHGQDTRDPAPPCPAGSPVQTSVLPVQSHGNWSHVEDEMNPRSLNRTST

GFHLESDDINYDKKQPHNPPYFPDEDNLITSDRYNRRIHRYPSQLPHSEDHHMLNRSSIA
YRSFPGEDMGNRFGPSNHRSSKIEPGHQFVQNAETSAGVLEEIAVECGFKVEYQSTLCST
AELQFSIEVRILGEKVGEGIGKTRKAAKRQAVNMSLRNLAEKFLTSDPDKMMILKENGFS
SNSNSFRYSGGSRDDTSPVASTSNESRYMGERVDTLRKPAGSVAALKELCTVEGYNLVFQ
EQPSRPRGSSGKEAYAQVEIGGQILGKGVGATWEQAKLQAADEALGNLKSMLGIFAHKSS
GFQRSSVSNFNRFKPDFQRSLQTIPSGWDSRNNGRVL*
>11668.m04082|LOC_Os02g42650.1|genepair1671-1
MAAFSSSSSAPMLIRSVLFVSLLSAAFVFDSGEAGAAHRVVDPEWHPATATWYGSADGDG
SDGGACGYGTLVDVVPMKTRVGAVSPVLFKGGEGCGACYKVRCLDASICSRRAVTVIVTD
ECPGGVCAFGRTHFDLSGAAFARLAVAGHGGQLQNRGEISVVYRRTACKYGGKNIAFHVN
EGSTTFWLSLLVEFEDGDGDIGSMQLKQANSAQWQDMKHIWGATWSLTPGPLVGPFSVRL
TTLTTRQTLSAQDVIPKNWTPKATYTSRLNFA*
>11670.m04342|LOC_Os04g44780.1|genepair1671-2
MAAASSRSFSLCVLLLLLLLAPPISASFLFDGGKSKSAAAAAAVDMEWRPATATWYGDAE
GDGSTGGACGYGSLVDVVPMKARVGSVSPVLFKDGEGCGACYKVKCLDHGICSRRAVTVI
VTDECPGGLCAFGRTHFDLSGAAFSRMAVAGAGGHLRDRGQLSVVYRRTACKYGGKNIAF
RVNEGSTNFWLSLLVEFEDGQGDIGSMQIKQDLVLCSNTSLHKRREHLRDRIANSVEWLD
MKHVWGATWCLVRGPLVGPFSVRLTTLSAQKALTARDVIPRNWKPTATYTSRLNFEAAL*
>11668.m04085|LOC_Os02g42690.1|genepair1672-1
MDQLCTSGGSDSDKSLAAGDEPAVRVRGEVEAEAAAAAAAAEAQGGGCFDCNICLDFATE
PVVTLCGHLYCWPCIYEWLHPGGDDDGSNGDASSTRRRPCPVCKAAVSPDTLVPLYGRGR
GGSSKRARSGSAIPRRPIVHREPVERQSDRLGDDDGGHRHGSTGSSPPVRSPWHANHHAA
AASTPAPAPARLFDVVYPPPPAAVGGVGMFHSTTTTTGGMLGGMALAVLPWVSRGQSPAT
AAASAYYTSPYHMSPRLRRQHMEVERSLHQIWFFLVVFAVLCLLLF*
>11670.m04346|LOC_Os04g44820.1|genepair1672-2
MDQIYMAAVNNKTSLPDDEPMKKISGDMPVTAGNACFDCNICLDFAAEPVVTLCGHLYCW
PCIYEWLCPGVGSTASNNSSLARRQCPVCKATLSPDMLVPLYGRGGSLKKSLNGVPIPRR
PTVQREAVEHQNTHNNIDDRHHENMEPSPPPQPLRHSSHHSSATEFDFIYPPSPIGRGLI
HSTAGGVLGGMAVAVLPWAFRGQVPPSMFMSPHYVTAHNMSSRARRHQMEVERSLHQIWF
FLFVFVVLCLLLF*
>11668.m04088|LOC_Os02g42700.1|genepair1673-1
MASSALAVSVAKPAASPPVAVAAVTPQRRLLPQCRGVRAAPVVRLRSGRARGVSVVCAAQ
GQETSFQGHSDLSQQQMNFFPLISLVAANKPSIAWNCSFKEQTPIMKVPDVTKSTWQSLV
VESELPVLVEFWASWCGPCKMIDPVIGKLSKEYEGKLNCYKLNTDENPDIATQFGIRSIP
TMMIFKNGEKKDAVIGAVPESTLVSSIDKYIGRETSTQDLAEQVVDVEINWLPPQYMEPD
WISSPDPLRIQILCIGEANAEGGISFNPIRPSSSNGCSFFFPSILVLETNKSSSPVTTAM
QAQQRQ*
>11670.m04347|LOC_Os04g44830.1|genepair1673-2
MASALAASTAVCSSPLASASASASSARRLRAVPPSRGIRYQALRADSGFAGNRRGGGRGA
SVVCAVQGQDTSIQVPEVTKSTWQSLVMESELPVLVGYWATWCGPCKMIDPVVGKLSKEY
EGKLKCYKLNTDENPDIASQYGVRSIPTMMIFKNGEKKDAVIGAVPESTLIASIEKFVER
*
>11668.m04089|LOC_Os02g42710.1|genepair1674-1
MDPYAILKCRSQEQRSSIASGKGSNPEWNENFVFTVSDKATELLIKLLDSDTGSADDFVG
EATIPLEAVYTEGSIPPTLYNVVKDEHYCGEIKVGLTFTPEDVRQRGLPEDFGGWKQSR*
>11670.m04351|LOC_Os04g44870.1|genepair1674-2
MVQGTLEVLLVGAKGLENTDYLCNMDPYAVLKCRSQEQKSSVASGKGSDPEWNETFMFSV
THNATELIIKLMDSDSGTDDDFVGEATISLEAIYTEGSIPPTVYNVVKEEEYRGEIKVGL
TFTPEDDRDRGLSEEDIGGWKQSS*
>11668.m04096|LOC_Os02g42780.1|genepair1675-1
MRRPELIMRSLPLILFLSLGSFHLAAAAVDDQFTFDGFAGVNLTLDGTAVVTPGGLLMLT
NGTTLLKGHAFYPSPLRFFHEATSGGGSSTVRSFSTAFVFGIVSEYADLSSPGLAFVVAK
SRDFSSALQSQYMGLANARNNGNASNHFLAVELDTIVNAEFGDMSDNHVGIDVDGLASAA
ADDAGYHDDRTGAFVNMSLLSRAAARVWVDFDARTSLVNVTMAPLELPKPTTPLLSAAVN
LSAVIEDEAYVGFSSSTGVVASRHYVLAWSFKMDGPAPSLNVSKLPALPVTIARAPSNVL
KILLPIASAALVSALAIAVLVIHRRRRRYAELKEEWEVAFGPHRFSYKDLFRATNGFSDE
RLLGFGGFGRVYKGVLLVSRVEIAVKKVSHESRQGMKEFIAEVVSIGQLRHRNLVQLLGY
CRQKGELLLVYDYMPNGSLDKYLYAENSKILSWAQRFRIIKGIASSILYLHEDWEQVVLH
RDIKASNVLLDAEMNCRLGDFGLARLYDRGTDPHTTHVVGTIGYLAPELGHTGRPSKASD
IFAFGVFMLEVTCGRRPVLQDTNGGQLLLVDMVLEHWRQGTVTDAVDPRLQGDFAVEEAS
LVLKLCLLCSHPLPSARPGIRQVVQLLDGAMPLPELSQAHLSCNMLALMQNQMGNSCSVA
SSVAGNISDIPRAR*
>11670.m04356|LOC_Os04g44900.1|genepair1675-2
MVLPKPEMPFFVLLLFLGLGCLRPAAATDERFVFNGFTGANLSFDGMATVTSNGLLMLTN
GTNQLKGHAFFPSPLQFQRGPNSTAMQSFSTAFVIGIIGAFEDLSSHGMAFIIAKSKNLT
SALPGQFMGLVNSANNGNATNHLFAVEFDTILNSEFNDMSGNHVGIDVNGLNSVDADNAG
YYDDGTGDFKNMSLVSRRPMQVWVDFDGQTMQVNVTMAPLEVARPKKPLLSKIVNISSVI
DDTAYVGFSSATGILFCRHYVLGWSFKMNGAAPALNISSLPSLPVTFPKPRSKTLEIVLP
IASAVLVFAVAAAVFVFMRRRRMFSELKEEWEVTFGPHRFSYKDLFHATDGFSDKRLLGI
GGFGRVYRGVLPSSKAEVAVKKVAHGSRQGMREFVAEVVSIGRLRHRNLVQLLGYCRRKG
ELLLVYDYMPNGSLDKQLYDQGKITLRWAQRFRIIRGVASGLLYLHEDWEQVVVHRDIKA
SNVLLDADMNGRLGDFGLARLYDHGTDPHTTHVVGTMGYLAPELGHTGKASKASDVFAFG
AFMLEVACGRKPVAQDARDNRVVLVDWVLDRWRAGAITDTVDPRLHGDFVESEASLVLRL
GLLCSHPLPGARPGTRQLVQYLEGDVPLPELSPTYQSFNMLALMQDQGFDPYVMSYPMTS

TSAGTFSDLSGGR*
>11668.m04097|LOC_Os02g42790.1|genepair1676-1
MALLIVRQTFIRVHGSLHVSPVAMETRVAVVTGGNRGVGLEICRQLASNGILVVLTARDE
KKGSQAVKALEQSGLSGVIFHQLDVTDRSSIMLLVEFIRTKFGKFNILVNNAAIGGTTID
PERLRELLEQDPKASFQEDLMGFLNSYMGSLQQNYEMAKECLEINFYGTKDVTDCLMPLL
LLSNSGKVINLTSKISQLQFISNEGVIKVLSDIDNLSDEKLKDVASIFLKDFKDGNLEAH
GWQPVVSAYAVSKTLVNAYSRLLAKRHPSLEVCCVNPGFVKTDMNYGIGLISVEEGANAP
VRLALQEACSDSCLYFEQCEISEF*
>11670.m04364|LOC_Os04g44980.1|genepair1676-2
MEETIFSSTHTSLVADARIAVVTGGNKGIGLEVNNAAVGGIVPVDDPSFGLLPTEEKFSG
MDGHQRIEWMWKNCRQTYDAAKAGLKTNYYGTKNVTEALLPLLQSSSDGRIVNVASSFGL
LRTADTKDCMQFFTNEELKRELNDADSLSEERLDELLGMFVRDFEAGAVAERGWPTEFSA
YKVAKAAMSAYARILARKRPALRVNCVDPGYVKTDLTRNSGLLTPEEGASRVVAVALLPA
GGPTGALFDGGKEASFV*
>11668.m04099|LOC_Os02g42810.1|genepair1677-1
MGPGSNNSFPAEKRVAVVTGGNKGLGLEICKQLAANGVTVVLTARSEERGAGAAAALRQL
GLSEVLFHQFDVSEPSSAAGLADFIKHKFGKLDILVNNAGILGVTFDFGNLDLNKAIEGK
SANETLEWLMQHTVETAENAEECLKINYHGNKKTIQALLPLLQSSPDGRIVTVSSVFGQL
SFFSGEKLKEELNDFSKLSEERIDELAELFVRDFKDGELESRGWPARADAFAAYKTSKAL
QHAYTRVLARKHASSSSSPLRVNCVHPGYVKTDMTLGTGELTVEEGAAGPVALALSPPGG
ATGVFFIQTEPASFV*
>11670.m04366|LOC_Os04g45000.1|genepair1677-2
MEGATSSLPSQSARVAVVTGGNKEIGLEVCRQLAADGITVVLTARDETRGVEAAERLRGM
GLSSVVFHQLEVTDSSSVARLADFLKTRFGKLDILASSPSPCSIDTGIQQLLLAYRYSAS
DLTSDREEMCSVLQVNNAAVGGMEYAQGVDNNEEQFVGMDVLQRLQWMRKQGRETYDTAK
NGVQTNYYGAKHVIQGLLPLLLSSSEGKIVNVSSALGLLRFLGNEDLRKELDDIDNLTEE
RLDEVLASFLKDFEAGELEAHGWPMGSAAYKVAKVAMNAYTRISARKHPALRINCAHPGY
VKTDLTINSGFLTPEEGARNVVTVALLPDGGPTGAFFDEGKEASFV*
>11668.m04100|LOC_Os02g42820.1|genepair1678-1
MSFTGTQDKCKACDKTVHFIDLLTADGVSYHKTCFKCSHCKGTLSMCNYSSMDGVLYCKT
HFEQLFKETGSFSKKFSQGGKSSEKSDQGRAPSKLSSAFSGTQDKCAACQKTVYPLEKLT
LEGESYHKSCFKCSHGGCILTTSSYAALNGILYCKIHFSQLFKEKGSYNHLIQTAQSKQK
ESEEAAPVAATEASEKEQEVPPQDAT*
>11670.m04367|LOC_Os04g45010.1|genepair1678-2
MSFTGTQDKCTACDKTVHFIDLLTADGVPYHKTCFKCSHCKGILSMCSYSSMDGVLYCKT
HFEQLFKETGSFSKKFAPGCRSTDKELARAPSKICSAFSGTQDKCAACQKTVYPLEKLTL
EGESYHKSCFKCSHGGCILTTSSYAALNGVLYCKIHFGQLFMEKGSYNHMKKKSESQEVL
PEVVPEEQPAPPPPDENREDN*
>11668.m04103|LOC_Os02g42850.1|genepair1679-1
MGCKSCEKPRPNYRKGLWSPEEDQKLRDYILRHGHGCWSALPANAGLQRNGKSCRLRWIN
YLRPGLKHGVFSPEEEETVMSLHAALGNKWSRIARHLPGRTDNEVKNYWNSYLKKRVESG
GGKTSQGPPTTPASAASSPADSDDSHSLQQKPHEPANSDSSEPAHESSSASADSSCLTVT
TDHPPVSRPHAAVTPKVMFADWLDMEYICGQVAAAPGLDAAGFAVVGGAAGDQQQQQQQV
MSQDGSVHQADGPSCGVDDSSLQQQQQEGFGGNGGCWDFQEQFDSIDQMQASGGGGGFCD
LLSMSDFDLWAELERTA*
>11670.m04368|LOC_Os04g45020.1|genepair1679-2
MGCKACQKPKVHYRKGLWSPEEDQKLRDFILRYGHGCWSAVPVKAGLQRNGKSCRLRWIN
YLRPGLKHGMFSREEEETVMNLHATMGNKWSQIARHLPGRTDNEVKNYWNSYLKKRVEGA
EAAARKSAEPADVVTGSPNRSETGQERVAADRPASSESSGPVESSSADDSSSLTEPAAGL
AAVRPHAPVIPKVMFADWFDMDYGTSLAGTAPGLSYQGSSSVQVDVPCGGAVDSLHGLGD
GGFCWDFDDAADHMQGGGGLCDLLSMSEFLGIN*
>11668.m04104|LOC_Os02g42860.1|genepair1680-1
MDPRARYPPGIGNGRGGNPNYYNRGPPLQQQHNHHQQQQTSAPHHQQYVQRQPQQHHHHN
HHQQHQQQQQQWLRRNQIAREAAGTDRNSEPKAVAQSPAVDGIDSSSQDWKAQLKLPPQD
TRYRTEDVTATKGNEFEDYFLKRELLMGIYEKGFERPSPIQEESIPIALTGSDILARAKN
GTGKTAAFCIPALEKIDQEKNAIQVVILVPTRELALQTSQVCKELGKHLKIQVMVTTGGT
SLKDDIIRLYQPVHLLVGTPGRILDLTKKGICILKDCSMLIMDEADKLLSPEFQPSVEQL
IRYLPASRQILMFSATFPVTVKEFKDKYLPKPYVINLMDELTLKGITQFYAFVEERQKVH
CLNTLFSKLQINQSIIFCNSVNRVELLAKKITELGYSCFYIHAKMLQDHRNRVFHDFRNG
ACRNLVCTDLFTRGIDIQAVNVVINFDFPKTAETYLHRVGRSGRFGHLGLAVNLITYEDR
FNLLTKLYIANNLTEFQRVFTFSKGNKKTILVNTSSSAHTSEFDLEVEKRGFIMDLLWHM
GTFIFGVLFEMRQTTLPMDTLCLYHFG*
>11670.m04370|LOC_Os04g45040.1|genepair1680-2
MDPRARYPPGIGNGRGGNPNYYGRGPPPSQHQQHQHQHQQPPHPHHHQYVQRQPQPQQTP
HNSQHQQWLRRNQIAAEAAGASEQKAPPVADGIDSSSQDWKAQLKLPPQDTRYRTEDVTA
TKGNEFEDYFLKRELLMGIYEKGFERPSPIQEESIPIALTGSDILARAKNGTGKTAAFCI
PALEKIDQDKNAIQVVILVPTRELALQTSQVCKELGKHLKIQVMVTTGGTSLKDDIVRLY
QPVHLLVGTPGRILDLTKKGVCVLKNCSMLVMDEADKLLSPEFQPSIQELIRYLPSNRQI
LMFSATFPVTVKEFKDKYLPKPYVINLMDELTLKGITQFYAFVEERQKVHCLNTLFSKLQ
INQSIIFCNSVNRVELLAKKITELGYSCFYIHAKMLQDHRNRVFHDFRNGACRNLVCTDL
FTRGIDIQAVNVVINFDFPKSAETYLHRVGRSGRFGHLGLAVNLITYEDRFNLYRIEQEL
GTEIKPIPPQIDRAIYCQ*
>11668.m04105|LOC_Os02g42870.1|genepair1681-1
MGRAPCCDKKGLKKGPWTPEEDKLLVDYIQANGHGSWRLLPKLAGLNRCGKSCRLRWTNY

LRPDIKRGPFTAEEQKSIVQLHGIVGNKWSMIAAQLPGRTDNEIKNYWNTHLKKQLRRMG
LDDPPPGPAAGCPAARHMAQWETARLEAEARLSLLSSSGAAATTTITAATTTTSASSSST
TAGPVAAAATSPADVFLRLWNSSIGDSFRKLAVVAAGSSSPSRADVTKDAVKQEEEAAPA
GDDSSAASNEVEAATMAVDEYQMFLDFAGEELGLFHGRYGGFSLFPPVDLLEASLETAFK
*
>11670.m04373|LOC_Os04g45060.1|genepair1681-2
MGRTPCCDSKVLKKGPWTPDEDKLLVDYVQANGSGNWRLLPKLAGLNRCGKSCRLRWTNY
LRPDIKRGPFTPEEHKSILQLHAIVGNKWSMIAAQLPGRTDNEIKNYWNTNVKKQLRQGQ
AAAVGEQAALASLGGGAASCPAARHMAQWETARLEAEARLSLLSGTTSVATASVAASSSS
SSTAAAGGAEAPPDIYLRLWNSEVGDSFRKSARSAAREDQEPANASDEAAPVSATFARPG
DDSSAASNVTAAAAADEYQVFLDLAAEDFELFHGRHGGFPLFPAVDMLGETSLYTAFD*
>11668.m04106|LOC_Os02g42880.1|genepair1682-1
MAGEALKEAGATPAAANAGEEKAVIPAASTSPVISKTDDDTEPPADDSKALVVFVEKVAD
KPHAEKATATATPTRTSNDRDIALAKVETDKRESLIKAWEENEKAKAENRASKKLLDIIS
WENTKKAVIKTQLKKKEEELERKKAEYAEKAKNKEAIVHKEAEEKRAMVMARRGEEVIKA
EEIAAKYRATGVTPKKHIGCFGA*
>11670.m04374|LOC_Os04g45070.1|genepair1682-2
MAEEEAKKVEVEVTEAPPAAAAAAETEPAAKDVAEEKAVIPAPAPPAEEEKPPVDDSKAL
AIVEKVADEPPAEKPAQGGSNDRDVALARVETEKRNSLIKAWEENEKTKAENKASKKLSA
ILSWENTKKANIEAQLKKIEEQLEKKKAEYAEKMKNKVAIVHKEAEEKRAMVEAKRGEEV
LKAEEMAAKYRATGHAPKKLIGCFGA*
>11668.m04107|LOC_Os02g42890.1|genepair1683-1
MGLGVRAAPFTYVAHALAVAAATMVLVWCIHFRGGLAFEATNKNLIFNVHPVLMLIGYII
LGSEAIMVYKVLPTWKHDTTKLIHLILHAIALVFGAVGIYCAFKFHNESGIANLYSLHSW
LGIGTICLYGIQWIFGFVAFFFPRASPSVRKGVLPWHILFGLFVYILALATAELGFLEKL
TFLQSSGLDKYGAEAFLVNFTALIVVLFGASVVVAAVSPARVEEPHEYAPIPES*
>11670.m04377|LOC_Os04g45090.1|genepair1683-2
MAAGLGVKAAPFTYVAHALAVAAAVMVLVWCISFRGGLAFEADNKNLIFNVHPVLMLIGY
IILGSEAIMIYKIFPKLNHDTTKLIHLILHAIAIVLGAVGIYCAFKFHNESGIANLYSLH
SWLGIGTISLYGIQWIFGFVAFFYPGAAPHVRRGALPWHVLFGLFVYVLTLATAELGLLE
KLTFLQSSGLDKYGAEAFLVNFTGLVVALFGAAVVVAAVAPAHVEEPEGYAPIPVN*
>11668.m04110|LOC_Os02g42920.1|genepair1684-1
MDTKSSKPLFSNGRAAGAHVLVGSIRRFLVKIYNTTTTPCQSQKTQNASSYRGFVVQGLP
FLSVRPRPPGGLQNPYQEQKANSTSQPEADLAEEAAANNARTASTIEKAEEGEGGAGGRR
VGGAAMASAAAVFPDGRRWCKGGSSSSSPVTTAIFLFFFVVVVGVLVSARWITTTSHLSI
TNLDEWRTKTAILTSTQATSIPGTPTAPPPPRRAYSISCSSPPLRRDPAVPKNISQTLYL
ALSSRPACARVPEPQPLPPTTTNSSCPAYFRFIHEDLHPWRAAGGITRRMLERARDTANF
RLVVLRGRAYVERIAPAFQTRDLFTIWGILQLLRRYPGRVPDLDLMFDCVDWPVVQADRY
QGENATAMPPLFRYCGDDETLDVVFPDWSFWGWPEINIKPWDALQKDLDIGNKRVKWVDR
EPYAYWKGNPDVATKRKELDWIKESKAGYKQSDLASQCTHRYKIYIEGSAWSVSEKYILA
CNSMTLVVTPKYYDFFSRVLMPTQHYWPVRDDNKCSSIKHAVDWGNSNKKKAQKIGKQAS
NFIQQELSMDYIYDYMFHLLTEYAKLLRFKPTKPPEAIEICPELLACQAIGRERKFMEDS
MVKSANDAGPCDLPPPFSPEEFKELQQRKEKSMKQVETWQQKASQT*
>11670.m04382|LOC_Os04g45140.1|genepair1684-2
MEAAAKVGRLTRSSLQAASCPAVNGGVVVFFAAVVAGALVSASWMSTGARVTSIPMIATR
NMAQHAAASPEPEPTLPRLGNLAPPPRQPPPPAPAPAPDAAAAASPSSSCPAYFRWIHED
LRPWRDAGITREAVDGAARRYGAKFRVTVVAGRLHVARYGRCFQTRDMFTQWGVLQLLRR
YQGRVPDLDLMFDCQDLPVVNAGDRRGRTSSSPPPLFGYCGSEPTLDIAFPDWSFWGWPE
LNIKPWETLRGEIADGNAAVNWTGRAPYAYWKGNPTVGADRRNLLRCNASGKRDWNARIY
EQDWRKEVRDGFRESDLAKQCTHRYKIYIEGRGWSVSEKYILACDAVALIVRPRYHDFFS
RGLMPLQHYWPIPGGGRGMCRSIKFAVDWGNAHADKAQEIAGNATRFIQEDLTMDRVYDY
MFHLLTEYAKLLKYKPTVPDRAVEVTVESMTRGRRGLERQFMVDTMVEAGSGTGEPCELP
PPFSSEELETLRRRQADAVRQVETWEKR*
>11668.m04112|LOC_Os02g42940.1|genepair1685-1
MQEAKKDSIFGGLASGAPVGADRFPFDPREYSPAMSSDSRELLGIDPPELIFPFELKKQI
SCSLHLTNKTDEYVTFKVKTTSPKKYCVRPNNGIVAPQSTSNVLVTMQAQREAPPDMQCK
DKFLVQSAIVTQELTPKDITGDMFTKESGNVVDEVKLKVVYTQPHPTSLNGGSEEGLGSL
SYQEATKGSRESETVTSEPLALISKLKEEKSSAIQQNMKLREELDLLRRQMGSQHGGFSL
VFVLVIAILGILLGFLIKR*
>11670.m04388|LOC_Os04g45190.2|genepair1685-2
MGSDSKELLGIEPLELRFSFETKKQISCSMQLTNRTDDYIAFKVKTTSPKKYCVRPNSGI
VPPRSTSDVIVTMQAQREAPADMQCKDKFLVQSVVVTEGTTTKDVTGEMFNKGSGNVVDE
RKLKVVYVQPPRPPSPVREGSEEGSSPRASLSEGGNLNYQDTTRESDEPPLLSATRAHKD
AEDVTSDSETSALISRLTEERNSAIQQNNKLLEEMDLVRREISKQNGGFPFVFVVVVALL
GILLGYIMKR*
>11668.m04113|LOC_Os02g42950.1|genepair1686-1
MSSSSSSSAVFPLDHLAAPSPTEQLCYVHCNCCDTILAVGVPCSSLFKTVTVRCGHCANL
LSVNLRGLLLPAPAPAPANQLHFGPSLLSPTSPHGLLDEVAFQTPSLLMEQAASASLSSI
TGRSSSSCASNAPAMQMPPAKPVQQEPELPKNAPASANRPPEKRQRVPSAYNRFIKDEIQ
RIKAGNPDISHREAFSAAAKNWAHFPHIHFGLMPDQGFKKTFKPQDGSEDILLKDSLYAA
AAAAAAAAANMGVTPF*
>11670.m04404|LOC_Os04g45330.1|genepair1686-2
MMSSAPETFSLDHLSQHQQQPPPLAEQEQLCYVHCNFCDTILAVGVPCSSLFKTVTVRC
GHCANLLSVNLRGLLLPAAASTANQLPFGQALLSPTSPHGLLDEVPSFQAPASLMTEQAS

PNVSSITSSNSSCANNAPATSMASAANKATQREPQQPKNAPSANRTSEKRQRVPSAYNRF
IKDEIQRIKASNPDITHREAFSAAAKNWAHFPHIHFGLMPDQGLKKTGIQSQDGAGECML
FKDGLYAAAAAAAAATAASSMGVTPF*
>11668.m04115|LOC_Os02g42970.1|genepair1687-1
MDTFSHVPPGFRFHPTDEELVDYYLRKKVASKKIDLDVIKDVDLYKIEPWDLQEKCKIGM
EEQNDWYFFSHKDKKYPTGTRTNRATGAGFWKATGRDKPIYARSCLVGMRKTLVFYKGRA
PNGQKSDWIMHEYRLETNENGTTPEEGWVVCRVFKKRVATVRRMADGSPCWFDDHGAVGA
FMPDLSSPRQLLPHHHHHHPGSSAALYHGHHHQQLQQMYGHCKPELEYHHLLPQEAFLQH
LPQLESPKPPPPPPAAAAYIGGHLGSSSSTALTTHDDEASGSAAQQQPPSLEAVYMAGAG
VGIGVDASVTDWRLLDKFVASQLLSKESMSSYGSHPAQVFQAADGGKHEEALDYASTSAG
SGGGEADLWK*
>11670.m04405|LOC_Os04g45340.1|genepair1687-2
MEVEARIHLLLIKLVFYRSAEERESASVHINLLPVRELYFDPRKKMDAFSHVPPGFRFHP
TDEELVDYYLRKKVALKKIDLDVIKDIDLYKIEPWDLQEQCKIGNEEQNEWYFFSHKDKK
YPTGTRTNRATTAGFWKATGRDKPIYVKNCLVGMRKTLVFYRGRAPNGQKSDWIMHEYRL
ETNEYGAPQEEGWVVCRVFKKRVAAVQRAAGDGGDSPFWFNEHVAFMAPAPGLDSPYHGH
RQSHPCKLEVEYHHHLLPQEAAPFMHLPRLESPKLPAADIIVATAASSALQPCGHTTAQQ
LQLQIEPVYVTADASAADWRDLDKLVASQFGHGDSTAKEPSYCNPVQVFQVEGKQEDSLD
YVSTSASCGGEEDLWK*
>11668.m04118|LOC_Os02g43000.1|genepair1688-1
MHLLSGCVVATAVVLVLISLGAAPPAAVDAATATFIYAGCSPSKYEPNTAFQSNLNSLLS
SIASTASSGAAYNSFTAGGGAGPDPAAGTAAYGLYQCRGDLSPGDCVACVRQTVARLGAV
CANAYAASLQVDGCYVRYDAADFIGRADTTTAYRKCSSSTSRDGAFLSSRDGVLGELQAA
AGYKLSTSGTVQGVAQCLGDVPANDCTACLAEAVGQLKGACGTALAADVYLAQCYVRYWA
NGYYFRPNSDNSGDDVGRTVAIIIGILAGLALLVVFISFLRKAFGLQSAKKKRLICV*
>11670.m04417|LOC_Os04g45460.1|genepair1688-2
MHQLDRLCYAVVVLVVIGATVAEAATGTFIYAGCSPSKYQPGTPFEGNLNSLLASIANAA
PNGGYNSFTAGSNGTGDGAAAYGLYQCRGDLGNADCAACVRDAVGQLNEVCAAAYAASLQ
LEGCYVRYDSSNFVGQPDNAMVYRKCSTSTSGDGDFLKNRDAVLAALQGGLANGYKVSSS
GNVQGVSQCLGDLAAGDCTTCLAQAVGQLKGTCGTSLAADVYLAQCYVRYWANGFYFRPS
QDYSQDDVGRTVAIIVGILAGLAILVVFISFLRKSC*
>11668.m04119|LOC_Os02g43010.1|genepair1689-1
MAARWCFALLLALSAAAAGAGAKRTWEPVIRMPGEVVEEEVATVPRGSEGTEEEEKDGVG
TRWAVLVAGSSGYGNYRHQADVCHAYQILRKGGLKEENIVVFMYDDIANNILNPRPGVIV
NHPQGEDVYAGVPKDYTGDEVTAKNFYAVLLGNKTAVTGGSRKVIDSKPNDHIFIFYSDH
GGPGVLGMPNLPYLYAADFMKVLQEKHASNTYAKMVIYVEACESGSIFEGLMPEDLNIYV
TTASNAEESSWGTYCPGMEPSPPSEYITCLGDLYSVSWMEDSETHNLKEESIKKQYEVVK
KRTSDMNSYGAGSHVMEYGDRTFKDDKLYLYQGFDPANAEVKNKLSWEGPKAAVNQRDAD
LLFLWRRYELLHDKSEEKLKALREISDTVMHRKLLDSSVDLVGKLLFGFGNGPSVLQAVR
PSGQPLVDDWDCLKRMVRIFESHCGPLTQYGMKHMRAFANICNNGISGASMKEASIATCS
SHNSGRWSSLVQGYSA*
>11670.m04418|LOC_Os04g45470.1|genepair1689-2
MAARCWVWGFVVALLAVAAAADGEEEEGKWEPLIRMPTEEGDDAEAAAPAPAPAAADYGG
TRWAVLVAGSSGYGNYRHQADVCHAYQILQKGGVKEENIVVFMYDDIAHNILNPRPGTII
NHPKGGDVYAGVPKDYTGHQVTTENFFAVLLGNKTAVTGGSGKVIDSKPEDHIFIYYSDH
GGPGVLGMPNLPYLYAGDFIKVLQKKHASNSYSKMVIYVEACESGSIFEGLMPENLNIYV
TTASNAVENSWGTYCPGEEPSPPPEYITCLGDMYSVAWMEDSETHNLKKETIEDQYELVK
KRTSNANKLNEGSHVMEYGDKTFKDEKLFLYQGFNPANGNITNELIWPVPKATVNQRDAD
LLFMWKRYEQLNGVSEDKLRALREIEDTIAHRKHLDSSIDFIGKLVFGFENGPLALEAAR
SSGQPLVDNWDCLKKMVRIFESQCGSLTQYGMKYMRAFANICNNGVSEAKMMEASINACG
RYNSARWSPMTEGGHSA*
>11668.m04120|LOC_Os02g43020.1|genepair1690-1
MADEAKAKGNAAFSAGRYEEAARHFTDAIALAPGNHVLYSNRSAALASVHRYSEALADAE
KTVELKPDWAKGYSRLGAAHLGLGDAASAVAAYEKGLALDPTNEGLKAGLADAKKAAAAP
PRRPPPSGVDGIGQMFQGPELWTKIASDPTTRAYLEQPDFMQMLRDVQRNPSSLNMYLSD
PRMMQVLGLMLNIKIQRPEASESSQPSSSPPSQPQEQPEAKAREVEPEPEPEPMEVTDEE
KERKERKSSAQKEKEAGNAAYKKKDFETAIQHYTKAMELDDEDISYLTNRAAVYIEMGKY
DECIKDCDKAVERGRELRADFKMISRALTRKGTALAKLAKTSKDYDIAIETFQKALTEHR
NPDTLKKLNEAERAKKELEQQEYYDPKLADEEREKGNQLFKEQKYPDAVKHYTEAIRRNP
KDPKVYSNRAACYTKLGAMPEGLKDAEKCIELDPTFSKGYTRKGAIQFFMKEYDKAMETY
QAGLKHDPNNPELLDGVKRCIEQINKANRGDLTQEEIQERQNKAMQDPEIQNILTDPIMR
QVLVDLQENPRASQEHLKNPGVMQKIQKLVSAGIVQMR*
>11670.m04420|LOC_Os04g45480.2|genepair1690-2
MADEAKAKGNAAFSAGRFEEAAAHFTDAIALAPDNHVLYSNRSAAYASLHRYPEALADAE
RTVALRPDWAKGCSRLGAARLGLGDAAGAVAAYEKGLALEPSNGALKDGLAHARQARRPA
PASGAGAIGKVFQGPELWSRMAADPTTRPYLDQPDFMRMLRDVQRNPSSLNNYLSDPRMV
QVLSLMLNLRLPNNDAPPRPPAQSTPPPPPQQQQHQPETKAREPEPEPEPMEVTEEEKER
KERKAAAQEEKEAGNAAYKKDFETAIQHYTKAMELDDEDVSYLTNRAAVYLEMGKYDECI
NDCDKAVERGRELHADFKIISRALTRKGTALAKIAKCFKDYDVAIETYQKALTEHRNPDT
LKKLNDAEIAKKELEQQEYYDPKIADEEREKGNEFFKQQKYPEAVKHYSEALRRNPKDPR
VYSNRAACYTKLGAMPEGLKDAEKCIELDPTFSKGYTRKGAIQFFMKEYDKALETYQAGL
KHDPNNQELLDGVRRCVQRINKASRGELSQEELQERQNKAMQDPEIQNILKDPIMQQVLT
DFQENPKAAQAHLKNPGVMQKIQKLLPPVSPQRARSGCAVGRRRTWRAASCKTRAPWSFL
SAC*

>11668.m04121|LOC_Os02g43030.1|genepair1691-1
MAIDLGCHVGCASPETKQEETADPTAAPVVVDDVEAAAGGRRPGDGGGVNYVARAQWLRA
AVLGANDGLVSVASLMVGVGAANGTRRAMLVSGLAGLVAGACSMAIGEFVSVYAQCDIQA
AQIERARGGKDADGGEEEEELPSPTMAAVASALSFAAGAALPLLAGGFVRPWAARVAAVC
AASSLGLAGFGVASAYLGGAGVARSGVRMLVGGWLAMAVTYGVLKLFGMHGV*
>11670.m04424|LOC_Os04g45520.1|genepair1691-2
MARAQWLRAAVLGANDGLVSVASLMIGIGAVNENNKAMLVSGLAGLVAGACSMAIGEFVS
VYAQYDIEVTQIERDGDIDGADAAAAREKLPSPTQAAFASALAFAIGGLLPLLTSGFIKP
WGPRVGVVCAASSVGLAGFGAAGGYLGGANMVRSGTRVLLGGWLAMLITYAVLRLFATIF
HGMNISSSA*
>11668.m04122|LOC_Os02g43040.1|genepair1692-1
MATLSDSAGGGGGGRAGAELMVPQFHLKALHAILAVRAPRPLAAAPAPAASFRRRDRWFH
LPLHAPPPPASAEHLPEPSPGEPLVVDVYLTPSGGGGGAEAVVERWTVSCEPWSAGARGG
GGAAASGEGLAVNRAYKRCITLLRSVYTALRLLPAYRVFRLLCASGQAYNYEMGFRVGSF
AAPFTRAEEAAMSTRRFAPVETQLGRLVVSVQYLPSLAAFNLEICSLAPAMLITDYVGSP
AADPMRAFPASLTEAASSAPAFPPRRPNSWAPSPAPWPYTPGQQAKFSPPPALYASPTPS
PPTFAGGYLQSRLSGETAPMIIPGGGRGPVHNRNMSDPVRGFMLPPPSPKNIRGDSGGHE
TPMETGRTGIRMADLYTNLPSVPKIKIKDSRDESGRFSGVFSSSGSPRLGFSRSSSRLSM
QDDTDDLDFPFAVDDVDTPDSRPGSSGGKDVGDQASSSSHKSQDAAVGYLVHMLKSARPL
RDSSNSPLTSRVESVEGGNVSSFMSRRTSDALEELESFKEIKENLLARSRSRMQESLDKS
LRHS*
>11670.m04429|LOC_Os04g45570.1|genepair1692-2
MSSMSDSGTGGRGGAELMVEQFHLKVLHAVLAVRGPRPLQPAASASFRRRDRWFHLPLHD
PQPPPAAEGVEAPEAGEPLVVDILLAHAAAGGGGGGGAGGEVVERWTVVCEPWPDAAAGE
GIPVNRAYKRCMTMLRSVYATLRFLPAYRVFRLLCANQSYNYEMVHRVGSFAVPLSRDEE
AAMRSYQFVPVETQHGRLVVSVQYLPSLAAFNLEISSLSPSMLIADYVGSPAAEPMRAFP
ASLTGATGSAFPQALSNQPQRPHSWATPALWPQAPRQQARFSPPHLLNASPTPSPPNFPS
GYLQSRPKGGSAPMSIPQVGDRRSPIHRPITLPPTSPRRVGETGTSSAQQSPSERCPSFG
RADGFRIMDPYASLSPGRKGKDTKDESGRFSALSSCDSPRQDDIDDADYPFAVDDVDTPS
SQPGSSDGKEARDQASSSSHKSQDAAVGSLVHLLKTARPLRNSNCPSQASAVESSEAAST
SSVVSRRKSDALEELQSFKEIKERLMSRSRAKQQEPPEKP*
>11668.m04123|LOC_Os02g43050.1|genepair1693-1
MSNTPAVSTYCGYRELNAYNRIFLGTDVEKTPIGRSRCSPLHHQTKQEWSGRLVNSAKTV
ESSAREFLGKDKSTTLVASANFVDLAGSERASQALSAGTRLKEGCHINRSLLALGTVIRK
LSMGSNAHIPYRDSKLTRILQPSLGGNARTAIICTLSPATSHIEQSRNTLLFGSCAKEVV
TNAQVNVVMSDKALVKHLQKELARLESELRHPVQSSSLETLLKEKDNQIRKMEKEIKELK
SQRDLAQSRLQDLLQSVGDHDLNRQVQGKHSVRSPPSVGMPPSVSRDDSSQVSHDDSDLY
KEVRCIESNRTGGNDQLDLSAGESSSPQDSNMNSGLHGNDSNASVNSRHSRPSGEAPITL
EEHLENIRRPFVSLAKDLGSSTRNSSNLRVIGRSRSCRSLTGSTMFDDMEMDDCTPLNRS
LVEFPGRPVESHRRGSALHYDAETDTLSRAGSMSSEISTFKDAKTNGSVACDTEFTGIGE
FVAELKEMAQVHYQKQLGDQNANGKSIGLDPIEGVSQSPSRWPLEFEKKQQEIIELWQAC
SISLVHRTYFFLLFKGEAADSIYMEVELRRLSFLRDTYSRGSTPSNAIVGSLSTSPVASA
KKLQREREMLARQMQKRLSTEEREHTYTKWGVSLDSKRRKLQVARRLWTETKDLEHVRES
ASLVAKLIGLQEPGQVLKEMFGLSFAPQQQPTRRRSSNGWRYGIPSFA*
>11670.m04430|LOC_Os04g45580.1|genepair1693-2
MGAIGGDEVVQWDKMDGGEVVNGGGGGGVGKLERILVSVRLRPLSDKEIARGDPSEWECI
NDTTIISRSTFPDRPSAPTAYSFDRVFRSDCDTNEVYKQGAKEVALSVVSGINSSIFAYG
QTSSGKTYTMTGITEYTVADIYDYIGKHEERAFVLKFSAIEIYNEVVRDLLSAENTPLRL
WDDAEKGTYVENLTEVVLRDWNHLKELISVCEAQRKTGETYLNENSSRSHQILKLTIESS
AREFLGKDKSTTLVASVNFVDLAGSERASQALSAGARLKEGCHINRSLLTLGTVIRKLSK
VRNGHIPYRDSKLTRILQPSLGGNARTAIICTMSPARSHMEQSRNTLLFASCAKEVVTNA
QVNVVMSDKALVKQLQKELARLESELRCPASYSSLESLVKEKDNQIRKMEKEIKELKLQR
DLAQSRLQDLLQVVGDNHVHVSKQSSVSGRNFTFDVPQTCEDEQSTTESSEVVDSVQNFR
FQGRRVAQREHKPQQAENNVQFTTPSRYSVSSPPFSGMLPTNRSDHLSQISNEDSDDICK
EVRCIETNETGGNECLESSAVGSNSLQDPNAGSSMHINNDSNSSMNSRLRDESPVTLEQH
LENVRKPFANIVKDLGSSTRNSSSSKVLGRSRSCRSLTGSSLFEDLEKDDCTPPNRSFID
FAGRPQNCQRRGSALNYDAESETLSRAGSMLSEITTTRDGLKANSSVAGDTEFTGIGEFV
AELKEMAQVQYQKQLGHSGNGDLAEGTIRSVGLDPITDALQSPSRWPLEFEKKQQEIIDF
WHACNVSLVHRTYFFLLFKGDPADSIYMEVELRRLSFLKDTYSNGAIASIPNTSLVSSAK
KLQREREMLCRQMQRRLSIEERESMYTKWGVSLASKRRRLQVARCLWTETKDLEHVRESA
SLVARLIGLLEPGKALREMFGLSFAPQQFTRRSYNSWRYGRSSLN*
>11668.m04133|LOC_Os02g43150.1|genepair1694-1
MSSFAHHHHGSLVEKDGRMSALRSSLRPYEAAEEMAAAAAAGGPAAAWGAVERGAGMMGD
GFSVEDLLDLEELCEVDRDGGEQGEAAAAAAAAVEKERSSDSHGSSVVSYEPMPLLPPVM
DLPAHDVEELEWVSRIMDDSLAELPLPQLPAAAAALAACGKPQHRRPHEGAASALLDPMR
TPTICALSTEALVPVKSRRSKRSRASVWSLSGAPLSDSTSSSSTATTSSCSSSASFSPFL
QYVDFPALVASDLLDEQPRSKKSKHGKNGKQKPKKRGRKPKHQQPPHLAAAAGGGAALPA
TGDRRCSHCGVQKTPQWRAGPEGAKTLCNACGVRYKSGRLLPEYRPACSPTFVSSLHSNS
HRKVLEMRRKKETPVIVAAAAPAVASF*
>11670.m04437|LOC_Os04g45650.1|genepair1694-2
MPSYAHHHSSLDGKMDALKSSCRSEEAADEGAAAAPSAWGMVERDGFSVEDLLDLEEFCE
AEKDAAEENEQALALVAAPEEEKSKDDSQPSSVVTYELVAPPPPPPEIVDLPAHDVEELE
WVSRIMDDSLSELPPPPQPPASVVASLAARPPQPRQLQRRPQDGAYRALPPASYPVRTPT
ICALSTEALVPVKAKRSKRSRATAWSLSGAPPFSDSTSSSSTTTTSSCSSSASFSSFSPL

LKFEWHPLGGTSDLPDDHLLPPGEEVQAR*
>11668.m04135|LOC_Os02g43170.1|genepair1695-1
MKVQCDVCAAEAASVFCCADEAALCDACDHRVHRANKLAGKHRRFSLLNPSASGRSPTST
TAPLCDICQEKRGFLFCKEDRAILCRECDVPVHTASELTMRHSRYLLTGVRLSSEPAASP
APPSEEENSSSFCCSADDAVPAPAAPATSHGGSSGSSSISEYLTTLPGWHVEDFLVDDAT
AEAAAAAAATSSGISANGPCQGVTRIGGLQESAGYPAWMAQQQLCCDGLVAGDASPASRE
RWVPQMYADQLAAGSKRSRTSTASSYSYW*
>11670.m04442|LOC_Os04g45690.1|genepair1695-2
MKVQCDVCAAEAASVFCCADEAALCDACDRRVHSANKLAGKHRRFSLLQPLASSSSAQKP
PLCDICQEKRGFLFCKEDRAILCRECDVTVHTTSELTRRHGRFLLTGVRLSSAPMDSPAP
SEEEEEEAGEDYSCSPSSVAGTAAGSASDGSSISEYLTKTLPGWHVEDFLVDEATAASSS
SDGLFQGGLLAQIGGVPDGYAAWAGREQLHSGVAVAADERASRERWVPQMNAEWGAGSKR
PRASPPCLYW*
>11668.m04137|LOC_Os02g43190.1|genepair1696-1
MSMTIIHITKHSCQLRGSLHGLALEPLSGAIAAGNAVVLKPSEFAPSTAAFLAANIPKYL
DANAVKVVQGGAEVGEELMEHRWDKVLFTGNARVGRIIMTKAAKHLTPVALELGSKCPCI
VDCLDSKRECQIELLKSTLKRFFTEPEYMARILNEKHFHRLTNLLEDDQVKSSIVHGGNA
DPKTLWIEPTIVLNPPFDSDIMMEEIFGPLLPIITVKKTEDCIAFLKSKPKPLAIYAFTN
NEKLKQRIVAETSSGSVLFNDAIVQRRAHDAPSGRKEAVGSAGPRPGWAVLGRKDRKDPN
RKYGLDSVPFGGIGESGFGQYHGKYTFELFSHRKAVVRRSLLVEFMFRYPPWDEYKMGML
RRVFRFDYVSLVLALLAFWLLGIRR*
>11670.m04445|LOC_Os04g45720.1|genepair1696-2
MGRVAPSVEEVGGEQPPPALGPGETVSGTVAELRAAYESGRTRSLEWRQSQLRGLLRLLA
EEEAAAFRALREDLGKHQAEAYRDEIGVLVKSANAALREVGKWMAPEKVWVPLIAFPARA
QLEPQPLGVILVFSCWNVPLGLSLEPLVGALAAGNAVALKPSELAPATAKFLGDNVGKYM
DATAVKVIQGGPEVGEQLMEHRWDKVLFTGSPRIARVVMAAAAKHLTPVALELGGKCPCI
FDTIGGSARDLQTAVNRVVGGKWSSCAGQACLAIDYVLVEERFVPVLIKALKSTLKKFFA
DSDHMARIVNARHFQRLSDLLKDKSVAASVLHGGTLDAKNLCIEPTILLNPPLDSAIMTE
EIFGPLLPIITVKKIEDSIAFVRARPRPLAVYAFTKNAALRRRIVEETSSGSVTFNDAVV
QYGIDSLPFGGVGESGFGQYHGKYSFEMFSHKKAVLTRGYLIELTARYPPWDDSKISMMR
QLYRYNYVGFVLTFLGLKK*
>11668.m04147|LOC_Os02g43290.1|genepair1697-1
MLDGCGLLAFARCCCVGGGEMESDARVSDEPGCGGGGGGGAGSVVEKGGGVVARRFGWAE
IESVTGGFSSRVIGQGGFSTVYLASLTSSRLGAVKVQRSSERLHRAFCQERDVLLSLRHP
HVVRLLGYCDERDEGVLVFEYAPNGDLHERLHHRSGGKSRTVLPWARRMAVAFQVAMALE
YLHESRDPAVIHGDIKSSNVLLDANHDAKLCDFGFAHVGFSAAVRPPSGATRAWGRPVMG
SPGYADPHFLRSGVATKKSDVYSFGVLLLELVTGREAICAQTGHRLTAAVGPSIGEGKLA
DVVDRRLGGDYDVEEAATVAALALRCVSDGTGLRPSMAEVVRELQEKTTALISAVGSKPS
DKMVS*
>11670.m04446|LOC_Os04g45730.1|genepair1697-2
MFTGCGLFACVRRCDGGDVRKRGEAGAMSSRVAADPAGVEEEGSCKNVAAASARQLAWAD
VESVTGGFSSRVIGHGGFSTVYLASLSSSRLGAVKVHCSSERLHRAFRQELEVLLSLRHP
HIVRLLGYCDERDEGVLVFEYAPNGDLHERLHCSEVAGGVASVLPWARRVAIAFQVAMAL
EYLHESRHPAVIHGDIKASNVLLDANMNAKLCDFGFAHVGFSATVGCRPSARAVMGSPGY
VDPHLIRSGVATKKSDVYSFGVLLLELVTGKEAVCRDTGRRLTAAVGPMLSEGKVADVVD
RRLGGEHDGAEAAVMAELAMQCIGDSPGLRPSMADVVRALQEKTSALASAVGSRLDRKMM
F*
>11668.m04148|LOC_Os02g43300.1|genepair1698-1
MAAAFDLPPVTTPAPAAPSDVLLPTQPQVSGPEEFPAAAVNSNDDDMMMVDDVVVAGGVG
GSGSTGNRWPREETLALIRIRSEMDAAFRNATLKAPVWEELSRRLAELGYQRSGKKCKEK
FENVDKYYKRTKEGRTGRQDGKSYRFFSQLEALHAAAPPPPPQQRQGMPVEDPQPLAMAW
MMLPGAADLGFLSMSSESESDDESDEEEEEEEAVAPGGGGREGLGDDGDGDGEGGSSTRK
LMAMFEGMMRQVTEKQDAMQRVFLETLEKWEAERTEREEAWRRKEVARINREREQLSKER
AAAASRDAALIAFLQRVGGAGGEPVRLSPSSAGATRHDAAAAGLQLVPVPAPRAKAEDAW
AAAGGDGSGTTAPSRWPKEEVQALIDLRMEKEEQYNDMGPKGPLWEEIAAGMQRIGYNRS
AKRCKEKWENINKYFKKVKESNKRRPEDSKTCPYFHQLDAIYRKKHFAGRGGGGGGVTIA
ASHSSLAIVTVSEQDNPSQRELEGKSSNDVGNVQLAVPLLVHNAPDKKVEGSEGEPNVTA
AAEETDSDEMCGEYTDDGDDDDKMQYKIEFQKPTAGGGGDGNDAPVPATTAAATSSAPTS
NTSFLAVQ*
>11670.m04448|LOC_Os04g45750.1|genepair1698-2
MQQHQGGGSQYGAVPPDMGPFSPTHHASAPAPLPLSSRPPPAALSQPPPPQQQQQQPRTS
YDELAAATSAGAGGFPDDDMLGDAGGSGGGGGSGAAGNRWPREETLALIRIRSEMDATFR
DATLKGPLWEEVSRKLAELGYKRSAKKCKEKFENVHKYYKRTKEGRAGRQDGKSYRFFTE
LEALHAAAPQTPQPQQQQQQQLPPVTSSAPAMHAFAPPVPAPPPMSAMPPPPGPIQPAPI
SSAAPAVPLELPPQPPINLQGLSFSSMSGSESDDESEDDEMTAETGGSQDRLGKRKRGAG
GKRLATFFEGLIKQVVDRQEEMQRRFLETMEKREAERTAREEAWRRQEVARLNREQEQLA
QERAAAASRDAAIISFLQRIGGQSVQVPPAATVIQMPTPVQLQTPPPVKQPARQHQPQPT
PPPPQAAPIPAAPLQQQPPQPQHKETIHHEAVTPRRAPPTSGSSLELVPAAEQHVESGLG
GGEGGSASSSRWPKTEVQALIQLRMELDMRYQETGPKGPLWEEISSGMRRLGYNRSSKRC
KEKWENINKYFKKVKESNKKRPEDSKTCPYFHQLDVIYRRKHLTGGGGGGASAANVAATA
IEHQNPNRHEIEGKNINDNDKRKNGGGGGAQVPTSNGDTAPTTATFDVDSGMKKRFKLNM
RMFVRKLTGNPLAVKPEDIVRELSEQPPREFTTDETDSDDMGDDYTDDGEEGEDDGKMQY
RIQFQRPNPGGANTAPPPATTPASAVPTSTPTSTFLAMVQ*
>11668.m04151|LOC_Os02g43330.1|genepair1699-1

MESDCQFLVAPPQPHMYYDTAAAAVDEAQFLRQMVAAADHHAAAAGRGGGDGDGGGGGGG
GGERKRRFTEEQVRSLETTFHARRAKLEPREKAELARELGLQPRQVAIWFQNKRARWRSK
QIEHDYAALRAQYDALHARVESLRQEKLALADQVDELRGKLNERQDQSGSCDGGGAEGDD
DDKRNSVMNASSSGLVEEDYVSCLAVPVVDVSEDGSAACGGSSYEYDHHLDYLGGGQLPD
PFCGMPDLWEIWPMVEWNAVA*
>11670.m04454|LOC_Os04g45810.1|genepair1699-2
MDRGDHHHLQQQHQFLMPPPAPVVPPQLCMPAMMADEQYMDLGGGGAAAAPGRGGAGERK
RRFTEEQIRSLESMFHAHHAKLEPREKAELARELGLQPRQVAIWFQNKRARWRSKQLEHD
YAALRSKYDALHSRVESLKQEKLALTVQLHELRERLREREERSGNGGAATTAASSSSCNG
SGSEEVDDDDDKRNAAAGCLDLEPPESCVLGGATCATPADVSVESDQCDDQLDYDEGLFP
ESFCATPELWEPWPLVEWNAVA*
>11668.m04162|LOC_Os02g43430.1|genepair1700-1
MGCFGRVRDMIKKRKGKKRPPPHEGPAPPPPPPSALAGSSHSTSTVSSSAVATSQSTEDS
SAAAARPAAGWSRSSGSVSSARSIPELYEERGASSLQEFGLRELQAATRDFSRLLKIGEG
GFGSVYKGVVRLPGGPAGGTEVAIKKLNPNSRQGHKQWLTEVQFLGVVEHPNLVKLIGYC
AAQSERGPQRLLVYEFMSNKTLDDHLFNKAYPVLPWDIRLKIALGAAEGLLYLHEGLEVQ
VIYRDFKASNVLLDEEFRPKLSDFGLAREGPTADNTHVSTAVMGTYGYAAPDYVETGHLT
NKSDVWSFGVVLYEILTGRRSMERNRPKNEQKLLEWVRQYPVETKRFSRIIDIRLRHNYS
KQGTREIAKLANSCLAKHAKDRPTMREVVESIKQVMQHNELDGDVEASGESSPPHEVPGK
PTADDVAVAAARRRMLHLAALGENANNIARRRFMLMRAAAAPTPT*
>11670.m04466|LOC_Os04g45920.1|genepair1700-2
MAERGRQRCPAAGRVEMASRRSGEAVFVKQRRPGSMAGSPGGALDGWRWRLGSKAATMDG
RPMAPWRKGEAAPSADVTGAQSKLRRGCRAKGYHTCYSDRILCKRMDRQSWYLLVPGTSW
YLKNECRLAGNRVCVLLASSKSKKDKEVGRGAGLLPPRNGLHVPLPGQAQEQETAGGGGA
GAGTGSALPECRCRRFHRCVLERVRASRRAEHVHVVVGEHEAVGELLGGEHPGAGHKEWL
AEVQLLGVVEHPNLVNLLGYCAAQTERGPQRLLVYEFVPNKTLDDHLFDRSHPVLPWGVR
LQIALGAAEGLLYLHEGLEFQIIYRDFKAANVLLDDEFRPKLSDFGLAREGPSEGQTHVS
TAVMGTYGYAAPDYVRTGHLTTKSDVWSFGVVLYEILAGRRSIDKSRPKDEQKLLEWVRR
HPAGSPRFGRIMDGRLQGRYSVRAAREVAELAAGCLAKHGKDRPAMAEVVERLRRATRHA
ELDGEVYDDAGEESSSSPAAAAVEDDVAVAAAAARRRMLHLAALGENASASAHARRRLML
MRAAAAATAAT*
>11668.m04166|LOC_Os02g43470.1|genepair1701-1
MNALAATSRNFRQAARLLGLDSKLQKSLLIPLREIKVECTIPKDDGTLATFVGFRVQHDN
SRGPMKGGIRYHPEVDPDEVNALAQLMTWKTAVAAVPYGGAKGGIGCTPGELSRSELERL
TRVFTQKIHDLIGINTDVPAPDMGTNAQTMAWILDEYSKFHGHSPAVVTGKPIDLGGSLG
RDAATGRGVMYATEALLTEYSESISGSTFVIQGLGNVGSWAAKLIHQKGGKIVAVGDVTG
AIRNKSGIDIPALLKHRSEGGSLEDFYGAEVMDAAELLVHECDVLVPCALGGVLNRENAA
EVKARFIIEGANHPTDTEADEILAKKGVIVLPDIYANSGGVVVSYFEWVQNIQGFMWDEE
KVNRELQKYMKNAFQNIKDMCKSQNCNLRMGAFTLGVNRVAKATLLRGWEA*
>11670.m04472|LOC_Os04g45970.1|genepair1701-2
MNALAATSRNFRQAARLLGLDSKLEKSLLIPFREIKVECTIPKDDGTLASFIGFRVQHDN
ARGPMKGGIRYHPEVDPDEVNALAQLMTWKTAVAAIPYGGAKGGIGCAPGELSTSELERL
TRVFTQKIHDLIGAHTDVPAPDMGTNSQTMAWILDEYSKFHGHSPAVVTGKPIVSLGGSL
GRDAATGRGVMYATEALLAEHGKSISGSTFVIQGFGNVGSWAARIIHEKGGKVIALGDVT
GSIRNKNGLDIPALMKHRNEGGALKDFHDAEVMDSSELLVHECDVLIPCALGGVLNRENA
PDVKAKFIIEAANHPTDPEADEILAKKGVTILPDIYANSGGVIVSYFEWVQNIQGFMWDE
EKVNMELHKYMNNSFQHIKAMCKSHDCNLRMGAFTLGVNRVARATLLRGWEA*
>11668.m04175|LOC_Os02g43550.1|genepair1702-1
MAEGAELVGESAHQDMPRVVAALAGILERVAGRNDAAATPAELAAAPASPFRATAKPGIS
VRAYAARIARFAGCSPACYVVAYIYLDRLLRRGRRCLALALAVDSYSVHRLLITAVLSAV
KFMDDICYNNAYFAKVGGISLAEMNYLEVDFLFGVGFDLNVTPETFADYCAVLQSEMLCA
APPTRLHYCCLSEDDAGSSSSSSLREAAMEAS*
>11670.m04475|LOC_Os04g46000.1|genepair1702-2
MAEEEDLADMPRVVGVLAALLERVTERNDAAAAELELAVAGAPAASAFRATTKPDITVRA
YMARIARFAGCSPACYVVAYIYLDRLLRRRRRACAFSVDSYSVHRLLITAVLAAVKFMDD
ICYNNAYFAKVGGVSLPEMNYLEVDFLFGVGFDLNVSPETFGHYCAVLQSEMLCLELEPP
PSPSPAPAARLHCFLSEDDTSSSGSTQHQLAA*
>11668.m04176|LOC_Os02g43560.1|genepair1703-1
MVNFLKLSDIQSTDAESVDVGFISSVHKTTRKKGFTTCLLGRNSILFGKTTVVIGEKVEH
CCRWSQEEEITGIAGVDSCYGEPGGVLSGRLGVNGEKGSTQFQISYGVSSAFGFRCDFGT
GFITHGMGDHHGMPLPPPLQLPCHPKLLQMPFDQEDQPGIHGVMLSSDHCGLYPLPALPL
SNSAAAAAATVALGKHSAAAGSMPNIGGAEEVATTVTKAGNESTTCNGSTTWWRGSTMAA
MGEKGKMKIRRKMREPRFCFQTRSEVDVLDDGYKWRKYGQKVVKNSLHPRSYFRCTHSNC
RVKKRVERLSTDCRMVITTYEGRHTHSPCDDNSSGEHTNCFSSF*
>11670.m04483|LOC_Os04g46060.1|genepair1703-2
MALKKRLFFAVPLCRCNDKNSLAPSCELWLNPETSARAANSFAKLLARFHVQLEWVAVQL
CMHIRWMVMIQEKEQCGCACATVHVCKAFSGECPTIIEQFQGAKGSQLETACLPAALYAP
LCPYTPPSPPSFLAPLPSLQHKLPQLPQLVHDHAAATGTNHGVMFSSDHGCLYPLLPGIP
FCLDSGCGAAACDDDKPAGFAHLGSAEADTSAAAARVDSEIAAAATATTCHGPNSWWKGT
EKGKMKVRRKMREPRFCFQTRSDVDVLDDGYKWRKYGQKVVKNSLHPRFLQELCKRNVQM
H*
>11668.m04179|LOC_Os02g43590.1|genepair1704-1
MDANAGSFVAVRRLAGSERAAGAAAFHHSSSAEVVTGSTAWIGKGLSCVCAQRRDSDARL
SFDLTPVQEECLQRLQNRLEVQYDSSNSDHQEALKDLWRASFPGAELRGLISEQWKEMGW

QGKDPSTDFRGGGFISLENLVYFSKNFPKSFQELLRKQNGDRAIWEYPFAVAGVNITFML
IQMLDLQSVKPRSFIGAVFLKLLSENDQAFDILYCITFKLMDQQWLDMHATYMDFNTVMK
STRRQLERELLLEDIQRIEDMPSYKLLAR*
>11670.m04486|LOC_Os04g46090.1|genepair1704-2
MDRNAGSFVAMRRLSGGSSCHDPSPNTYAEVVAGSTAWIGRGFSCVCAQRRDSDQRISFD
LSPAQECCLQRLQNRIEVPYDGSNGEHQEALKTLWHVSFPGTELLGLVSDQWKEMGWQGK
DPSTDFRGGGFISLENLLYFAKNYTKSFQELLCKQNGDRALWEYPFAVAGVNITFMLIQM
LDLQAAKPRSLIGSVFLNLLLGTV*
>11668.m04187|LOC_Os02g43660.1|genepair1705-1
MAGVHGLAAAGLVVLLLAAVAPAFAVDYTVGDTSGWSSGVDYDTWAKSKTFSVGDSLVFQ
YSMMHTVAEVSSADYSACSASNSIQSYSDQNTKIALTKPGTRYFICGTSGHCSGGMKLAV
TVSAAAATTPTPTASSSPPSTATPATPSSDPGMDTPSSTPDATTTPTTTTTKSTGSTGGA
SGSEARSVMGLLVGAVGLAMMG*
>11670.m04490|LOC_Os04g46130.1|genepair1705-2
MQKHTGTSRQMAGLLPGVLVAVLLAAAAAPASAKDYTVGDSSGWTTGVDYTAWARGKTFN
IGDTLLFQYTSAGHSVVEVSEADHTSCSAANPLRSYKDGTTIVTLTRSGTRYFICGSTGH
CGAGMKLTVTVATLSGSAAGGTRLAKPSSSDADPTTTTTTRTSSATGGATGSWAPRTATW
LLFFAAVGALL*
>11668.m04188|LOC_Os02g43670.1|genepair1706-1
MAATTPAAAAAAAHVVHIEAVQTAVPTRVVEPGRTRLVAVAAPPLPAPALQRRVRAVLYY
RGAGGAAPGAWEDGVWVKESLSEALADHPEMAGRLRRRADGSWEVKLNDTGVRLLQATVD
ATLDEFLAGKGALARREAALAPWTDVNADDPDMCPPFFMQLTRFQGDGGYAVGVSCALVL
ADPLTLARFLLSWARTHARIRAQGKATPLPMAQYLAYFQRPETTRKRVRSVPIDSFAGDG
DGDAETVLFRAGTAAAAAADGGHGDHRALAAACVDKASEALGKKDKVSRLSVVVVDAAGG
DDSLAGKTTIETCTASPGGGAAAGGASLEAVQWSELGLEELVLRGSKPVHVSCSIVTGGG
GDEGLVVVMPDDGAGSLLVMATLPK*
>11670.m04491|LOC_Os04g46140.1|genepair1706-2
MAAMTATMIHVESMQTAVPTRITGAGRTLPVAVSGGEAPPPLTAASLQRRFRAVLYYRGI
EQLQAEEEEEERAVWVKESLSASLADHPEMAGRLRRRDDDDGGVRGPWEVRLNDNGVRLV
QASVDMPMSAFLEAKDLARREAALTLWTDVDVHEPEFCAPFFMQLTRFQDGGYAIGASCS
LLLADPLSLVDFLKAWARTHAEMRARGKPVAPPAVIQYARYLQSPGAAAAAVVRRLKSVP
LDSCSAAAATTVLFRAAAGAQVDRHALAAACVDQAVETLGGNARKPPRLTVLAAGGSGEL
RVEACGCGDGEETTTPPPPSRGHHALRAAYWGDLGLGEIALDGSEPVHVSCTVVSPCADE
GLVVVMAPAGGAELLISVTVPNY*
>11668.m04197|LOC_Os02g43740.1|genepair1707-1
MMEVADKIVEPKGPLVATAQLPNNVELLKSVVLNPSREGGSSQEQYEKDLLAEGEESFRS
EESSDEGGRSSFSGVSHPPEPIDVDLMSTVYVAIDEEKPEPPECLMRGLSVKGASMEDLS
VHAMDAKPDVIVCTVNVNGLVEERKVHCAVVAPDAVEAVSSTQASEEKDCVWDASFPPSG
NVSPHSSSDSTGAVAATSIRGRSTSTCRSGVLTSESILTVEKTCESAKDSTRGNSMESTK
TSMSRASDSSGVSDDSNWSNITGSANKPHKGNDPRWRAILAVRGRGNVLGMSHFRLLKRL
GCGDIGSVYLSELSGTRCYFAMKVMDKASLASRKKLNRAQTEREILQLLDHPFLPTLYTH
FETDRFSCLVMEFCPGGDLHTLRQRQPRKHFSEYAARFYAAEVLLALEYLHMLGVVYRDL
KPENVLVRDDGHIMLSDFDLSLRCAVSPTLIRASASDSDPRRAGGSFCVQPACMEPSSVC
IQPACFMPKLFGQRSKKQGRRPRSELGQGGGAALPELVAEPTSARSMSFVGTHEYLAPEI
IKGEGHGSAVDWWTFGIFLHELLYGKTPFKGSGNRATLFNVVGQQLRFPESPSTSYAGRD
LIRGLLVKEPQQRLGVKRGAAEIKQHPFFEGVNWALIRCSTPPEVPRHVEAELPAKYGVA
EPVASGGGGGKRVVGAEVRSGGKYLDFEFF*
>11670.m04495|LOC_Os04g46180.1|genepair1707-2
MEIVDKIAEPKEPLMVTGRKVQSLEAPIPIKASWKGKSSQQQQQQQDEKDFPADGEESF
LSLDSSDEGGRSSFSGASHPLEPIDMDLMKTVYVAIDEEKSEQPVCLVRGVSAKGPFIDD
LSICVTGMKANAVVGAGSADGLAEEMKVPGAAVPSLATARSSQATEAVSLPPDSEEKDCV
WDASLPPSGNVSPHSSIDSMGVVTAMNTMSSCTSTYKSEAVSSEPVPTMERNCGSVKGSV
RGDSLESAKTSMSRASDSSGVSDDSSWSNITGGASKPHKGNDPRWKAIHAVRTRDGVLGM
SHFRLLKRLGCGDIGSVYLSELSGTRCYFAMKVMDKASLASRKKLNRAQTEREILQLLDH
PFLPTLYTHFETDRFSCLVMEFCPGGDLHTLRQRQAGKHFSEYAARFYAAEVLLALEYLH
MLGVVYRDLKPENVLVRDDGHIMLSDFDLSLRCAVSPTLIRASAFDSDPRRAGGSFCVQP
VCMEPTSVCIQPACFMPKLFGQKSKKKTKKTRSELGPSATTMPELVAEPTSARSMSFVGT
HEYLAPEIIKGEGHGSAVDWWTFGIFLHELLYGKTPFKGSGNRATLFNVVGQQLRFPESP
STSYASRDLIKGLLVKEPQHRLGVKRGATEIKQHPFFEGVNWALIRCSTPPDVPRPVEAE
LPVKYGVAEAIGSNSKRMVGADMKSGGKYLDFEFF*
>11668.m04199|LOC_Os02g43760.1|genepair1708-1
MGKRWIPLEANPDVMNQFMWGLGVAEGEAQFCDVYGLDDELLAMVPQPVLAVLFLYPLTS
LDDDEEESGAAATSTAGDKDLSKRVYFTKQTVGNACGTVGVIHAIGNAASKLKLVEGSYF
DRFYKQTVDMDPVQRAAFLEEDDEMEDAHSVAASAGDTDANVEVNEHFVCFSCVDGELYE
LDGRKSQPICHGPSSPDTLLQDAAKVIKARIASNPDSMNFNVMALSKVV*
>11670.m04496|LOC_Os04g46190.1|genepair1708-2
MGKRWLPLEANPEVMNQFMRGLGVPAEAGFCDVYGLDDEMLAMVPQPVLAVILLYPQDRK
KESVASPSSTVESKKLSKNVYFTKQTIGNACGTVGIIHAIGNALSRIKLVEGSYFDRFYK
QTADMDPAQRASFLEEDEEMEKAHSVAVSAGDTEAKDGVIEHYVCFSCVDDEIFELDGGN
SQPISHGPSSPDSLLQDAAKVIKARIAQYPGSLNFNVMALSKQ*
>11668.m04202|LOC_Os02g43790.1|genepair1709-1
MLLNPASREVAALDSIRHHLLEEEEETPATAPAPTRRPVYCRSSSFGSLVADQWSESLPF
RPNDAEDMVVYGALRDAFSSGWLPDGSFAAVKPESQDSYDGSSIGSFLASSSSEAGTPGE
VTSTEATVTPGIREGEGEAVAVASRGKHYRGVRQRPWGKFAAEIRDPAKNGARVWLGTFD

SAEEAAVAYDRAAYRMRGSRALLNFPLRIGSEIAAAAAAAAAGNKRPYPDPASSGSSSPS
SSSSSSSSSSSGSPKRRKRGEAAAASMAMALVPPPPPPAQAPVQLALPAQPWFAAGPIQQ
LVS*
>11670.m04500|LOC_Os04g46220.1|genepair1709-2
MTARSMLRNHPEASVLDTIRQHLLEEPRGGGGGEAAEASFGSLVADMWSDSLPFRDDDAD
DMVVFGAMRDAFSCGWLPDGVFAEVKPEPLLSPDSSSYDGSSCCFGFADVSEPVTPSDAA
SGAAEAAAAAAAATAEHGKEEEAAAAVARGKHYRGVRQRPWGKFAAEIRDPAKNGARVWL
GTFDTAEDAALAYDRAAYRMRGSRALLNFPLRIGSEIAAAAAAAAAAAGDKRPSPEPAT
SESSFSSSSSCTTTTTSSSTSSSGSPKRRKRGEAAAASMSMPLVPPPSQLNWPVQAWYPA
AAPVEQVAITPRVEQLVI*
>11668.m04205|LOC_Os02g43820.1|genepair1710-1
MDFSGDIDDLISLQLLHDQLLGVEADACLPVAVHHDGVAAFAEHQQGFHPAAFLPQQPMT
MTPAGYVDMANDQYLGAHAAAGEAEAVYRAAAAEPVMIRFGGEVSPVSDPRRPPLTISLP
PTSHAWAAAEAVHPAALLQAQTAAAAADPNDFRKYRGVRQRPWGKFAAEIRDPKKRGSRV
WLGTYDTAIEAARAYDRAAFRMRGAKAILNFPNEVGSRGADFLAPPPPPPPPTTSTHGKR
KRHETAAADPDVEVIGESSKSVKTETYTSPASSSLASTTTSTVTSSSTSPSPSSEAAACG
GGGGELFVPPMPSSWSWDQLEGFFGILSPLSPHPQMGFPEVAVN*
>11670.m04502|LOC_Os04g46240.1|genepair1710-2
MDFPGDAEDFALEFIREHLLGGDAPVLPPAAVPAAAAYLPTSTMFLPQQQRGYAGLTPQE
YVVDSAPAADQAAFRDDQPDPAADVMIMFGGERFPAVKPSSSSSPSLTVTVPPSSFGSWA
PAAVPAVAATAAAVEDFRKYRGVRQRPWGKFAAEIRDPKRRGSRVWLGTYDTPVEAARAY
DRAAFRMRGAKAILNFPNEVGTRGAELWATPPPTNKRKRQPEDDTAAADDVEVIGVANKA
VKTEAPTSAYSSSSLSSMSRDTTATTSSAGTSTGSSEPTSFPVVTPSSWSWDQYWDGLPP
LSPLSPHPALGFPQLTVS*
>11668.m04207|LOC_Os02g43840.1|genepair1711-1
MDTGNKGTATPVLLNVYDLTPANDYLYWLGFGVFHSGIEVHGMEYGFGAHDFPSSGVFEV
ESKSCPGFIYRKTVWLGTTDMSHGEYRSFIEKLAGKYHGNSYHLVSKNCNHFTDDVCKNL
TGKPIPSWVNRLARVGSFFDCLLPESVQVSPVGRVPTLRPVADDDLDSISTVSDNNEEDK
HLLPAPSNDLHSVDVPLKLAKDVL*
>11670.m04507|LOC_Os04g46290.1|genepair1711-2
MAVQNGGGDGSGSGAASVVVNVYDLTPMNNYLYWFGLGIFHSGIEVHGVEYGFGAHEFPT
SGVFEVEPKNCPGFVYRRSVRMGTTGMSRAEFRSFIEKLTGKYNDDVSKNLTGKPIPGWV
NRLARVGSFFNYLLPKSIQVSAVRHVPTHPAFSVALSC*
>11668.m04215|LOC_Os02g43870.1|genepair1712-1
MAESTAATAPPGDEPEAKGRSNSILLPILGILLVYLLYRYLRPRLRGLRLDRLTSRLPAC
LRRSRTANTMLPYFAPIADRLGALQPYLAPIADRLGVGAQGGVGAGAYGRADALVKFPGG
EALSVAAILEAPGEVVAKSSHSTLYRAAMRSGEAAVLLRFVRPACALTSDEASAAARRIG
AVSHPNLVPLRAFYVGPRGEKLLVHPFYAAGSLRRFLQEGIVDSQRWNIICKLSISIVKG
LDYLHTGLEKPIIHGNIKTNNILLDANYECKISDFGLYLLLNPAGAQEMLETSAAQGYKA
PELIKMRDATRETDIYSLGVVLLEMLAQKESAKDNTPNPRDILLPVSFKNLVLERKISDA
FSSDLVRQSKKSGKEKNLNAFFELATACCSPSPSLRPNTKFILKKLEEIAK*
>11670.m04510|LOC_Os04g46320.1|genepair1712-2
MRTRVINAVLFTVLAVLVALVLGYFVVRCQRRQRRRRRRGAVLPSHGARADRFQSGGGTS
GYGAGCAGGAEEALVRFPGGEGLTVAAILEAPGEVVAKSGHSTLYRAGLSAGEAVALLRF
VRPVCAAAADEATAAARLLGALQHPNLVPIRALYVGPRGEMLLVHPFYAAGSLRRFLQEG
INVSQKWGIICKLSIGIVKGLDHLHSGSQKPIVHGNLKTNNIMLDADFQPRISDFGLYLL
LNPAAAQQMLEASAMQGYKAPELIKMREATRESDIYSLGVILLEMLAQKEAANSSSPNAR
DIHLPSSFKDLVLERKISDAFSSELVKQSKNSGKEQNLNAFFELATACCNPSPSLRPDTK
RILKMLEEISR*
>11668.m04218|LOC_Os02g43900.1|genepair1713-1
MADDVVEAAASSSPSTQGGGGGGIHVVAKNAMNSSKQFSSALAPVAVLLAVAVGAVSLLP
SLAQAVWEVPHLFLLGLVVSYGVFAQKISTGGSGGGGGGGGGDDGARAWNSRYLSDDPLV
VVADNAASDDGGGGGASGRPLSLPVRRLKPPPPAPTMPGIESEAGDASDDGIGAETDSSA
STAGFWGAAPSPPSVLDAVCRSRKPAATATAAAPSAMSKGFPSYISPAPRCDQSFSDDGE
VTDWDDDDDEKADVQDEMAASPQRSYVDHDDYNGDGDELSELAAEVEDEVDRKADEFIAK
FREQIRLQRL*
>11670.m04511|LOC_Os04g46330.1|genepair1713-2
MADSAPAPEARVSGGDVPARLQQALALLFPTNLAAKAVLFAVVVALLPLLPTSQAPRIWE
LPHILLLGLIISYGVFGQRNADSEVAAVAATKTVDDESVESYVTQMMHGPLVFEENDGGG
EADAAGKEGVQAWSSQYFPDDPLVVVADAGAGSNTGKGDEREKPLLLPVRKLKPATEESA
TLTESFSDGAIEEEEEEEEEETEFLLRKARYGGVREHAIPSPSSVLDADLTLSPCSPPLL
PPPPPPPPPPPFLDHDRPALRKAKARSFNDYGRVGLQTAAGCGGGGHNFRSKSAIQASRS
TFPTSPFDDHDLEEKVAASDISSFSSDDVVTDDGEDGDNHKEIYNYEEEEGDVDRLDDDD
GSCDEELFELATRLAPEEEEVVEDEVDRKADEFIAKFREQIRMQRVVEPGRR*
>11668.m04225|LOC_Os02g43930.2|genepair1714-1
MYGRMPKKSNNTKYYEVLGVSKTATQDELKKAYRKAAIKNHPDKGGDPEKFKELAQAYEV
LNDPEKREIYDQYGEDALKEGMGGGSSSDFHSPFDLFEQIFQNRGGFGVGRGHRQKRGED
VVHTMKVSLEDLYNGTTKKLSLSRNALCTKCKGKGSKSGAAATCHGCHGAGMRTITRQIG
LGMIQQMNTVCPECRGSGEMISDKDKCPSCKGNKVVQQKKVLEVHVEKGMQHGQKIVFQG
EADEAPDTVTGDIVFVLQLKDHPKFKRKFDDLFTEHTISLTEALCGFQFVLTHLDGRQLL
IKSNPGEVIKPGQHKAINDEGMPQHGRPFMKGRLFVEFNVEFPEPGALTPGQCRSLEKIL
PPRPRNQLSDMELDQCEETTMHDVNIEEEMRRRQQHRRQEAYDEDDDEDAGAGPRVQCAQ
Q*
>11670.m04517|LOC_Os04g46390.1|genepair1714-2

MARAPGGVRRRSGRRGAGGGGAGGGGEALRKGPWMAEEDEVLLEHVRTHGPMDWSSIRSK
GLLPRTGKSCRLRWVNKLRPNLKSGCKFTAEEERVVIELQAQFGNKWARIATYLQGRTDN
DVKNFWSTRQKRLARLLRGPLPAARPNKHNSGKGKAPSSSSLDSQTATFHQSSASLDQAS
LEGNSLGWQCREAAPFMGYDQACSGFFAFEGPLPLQLLPPADGEASSSNAAQSAPPPLLF
DQPPYPLINFPGWPERYVDVGHGFVDAGAMDGLAYQELLPMVQSVPMIMPFFGMESNNTK
YYEVLGVPKTASKDELKKAYRKAAIKNHPDKGGDPEKFKELSQAYEVLTDPEKRDIYDQY
GEDALKDGMGGGSDFHNPFDIFEQFFGGGAFGGSSSRVRRQRRGEDVAHTLKVSLEDVYN
GSMKKLSLSRNILCPKCKGKGTKSEAPATCYGCHGVGMRNIMRQIGLGMIQHMQTVCPEC
RGSGEIISDRDKCTNCRASKVIQEKKVLEVHIEKGMQHGQKIVFQGEADEAPDTVTGDIV
FILQVKVHPRFKRKYDDLFIERTISLTEALCGFQFILTHLDSRQLLIKANPGEIIKPGQH
KAINDEGMPHHGRPFMKGRLFVEFNVEFPESGVLSRDQCRALEMILPPKPGHQLSDMDLD
QCEETTMHDVNIEEEMRRKQYQRKQEAYDEDEEEDAPRVQCAQQ*
>11668.m04226|LOC_Os02g43940.1|genepair1715-1
MEAAAIHAPTTTTSSDSGSCVTNGTREKREISKHKQLKRKRSTSPAPPPGCGGGQGQEAA
VETDADAGAAGEEESSSCGGAGAGEGERKRGDAGRHPSYRGVRRRSWGKWVSEIREPRKK
SRIWLGTFPTAEMAARAHDVAALAIKGRAAHLNFPSLAHTLPRPASTSPSDIQAAAALAA
AAAATDQCESSSSAVAAATATDAEAAESTSSAAASPCAATSSVEENALFDLPDLLLDLSD
GLWCSPVWTTAPADQYDAGDDGDDAAAPLLWAEQCWMDAPAAPVQPD*
>11670.m04518|LOC_Os04g46400.1|genepair1715-2
MEADASHTPTTSSSVSVSFSSSSLSTSSSTSSLVDNGAQDRPKSSKPKHAAKKRKRAAAE
EPANAAHGAGEDTSSCSTDDNAAASGKAQAGGGGGGVDSSSTCTAASAPRSGFKHPSYRG
VRRRSWGKWVSEIREPRKKSRIWLGTFPTAEMAARAHDVAALAIKGRNAHLNFPDSAHEL
PRPESTSPADIQAAAAKAAAEVRCEEESSPSSSPTAEQPEEEAACPDTVHADGGQDNALF
DLPDLLLDLRDGLWWSPVWPAALAAEEYDGGDAVVLNEPLLWAE*
>11668.m04229|LOC_Os02g43970.1|genepair1716-1
MDDSSFGSEPTTSSSGGEAPASPPSTASSSSDGAGGKKKRPRKDGHHPTYRGVRMRSWGK
WVSEIREPRKKSRIWLGTFATAEMAARAHDVAALAIKGRAAHLNFPDLAHELPRPATAAP
KDVQAAAALAAAADFPASSANAGASNNPDGSDDASAGSASPPPPPDAADDALFDLPDLLL
DLRYGPPSSGLSCASSWEDEVGLISGAGAAAAGVFRLEEPLLWEY*
>11670.m04522|LOC_Os04g46440.1|genepair1716-2
MDDSHDLASPTSPDTASSSSSSTSTSSSSATVAPKKRPRNDGRHPTYRGVRMRSWGKWVS
EIREPRKKSRIWLGTFATAEMAARAHDVAALAIKGRTAHLNFPDLAHLLPRPATAAPKDV
QAAALLAAAAADFPSVSVDANAKSPDTCSVASAASPQPPPPDAEADPDSTLFDLPDLLLD
LRYETSSSLSCGASWAVDDDVAGGVVFRLEEPMLWDY*
>11668.m04232|LOC_Os02g44000.1|genepair1717-1
MKSPELQSSVIRDLVLLSCVGLRPVLVHGGGPEINSWLGRVGVEPQFRNGLRVTDALTME
VVEMVLVGKVNKQLVSLISVAGATAVGLCGKDARLLTARPSPDAAALGFVGEVTRVNPSV
LHPIIESGHIPVIATVAADETGQAYNINADTAAGEIAAALGAEKLLLLTDVSGILADRND
PGSLVKEIDIAGVRQMVADGKVGGGMIPKVECCVRALAQGVHTASIIDGRVPHSLLLEIL
TDEGTGTMITG*
>11670.m04524|LOC_Os04g46460.1|genepair1717-2
MLLAKPHLSSSSFLPSTRVSSPAPGPNHAKPIAASPAPRRCLRLAVTSAAAPAASSAEAA
AALSRVDVLSEALPFIQRFKGKTVVVKYGGAAMKSPELQASVIRDLVLLSCVGLHPVLVH
GGGPEINSWLLRVGVEPQFRNGLRVTDALTMEVVEMVLVGKVNKHLVSLINLAGGTAVGL
CGKDARLLTARPSPNAAALGFVGEVSRVDATVLHPIIASGHIPVIATVAADETGQAYNIN
ADTAAGEIAAVGAEKLLLLTDVSGILADRNDPGSLVKEIDIAGVRQMVADGQVAGGMIP
KVECCVRALAQGVHTASIIDGRVPHSLLLEILTDEGTGTMITG*
>11668.m04233|LOC_Os02g44010.1|genepair1718-1
MRNPPGRHLLRVATRAVRSSSALGSGGGGGGASTSATSPVAASSSSGGRPRTRSGGRLLR
ATSPPPPSAIAAAACWESRTMRREGEEEWEEVVAGAEEAVHGAPVPVAEEEEEYRVVFWS
PPTGDEVRAAFTSIEEVFGDPFRAHSYETEEQSALSTSVHSSSGNSSGSDDWIEPAAYAL
NSTALLTREHRNVLDAFRLLQKDPNVQKMVMSLSCDRAVWDAVMNNEAVQEFRRSFQDDK
EAAGRKGNPGGPAGVLKWILGNTQAKIMEFMNNIMKIVNMLFHSDDDEAKPDLYTDAVKV
SFMLSVFIFIVVAIARINFEPWDFKVW*
>11670.m04527|LOC_Os04g46480.1|genepair1718-2
MVVHVVYRRRESSWRGGETSARLHQGAAAMSNPAGGKHLVRLAGSSSLRGGAALSPAVSI
SSGSRPATRAGARALRAASPPPACSIASVGCWESRALRLDGDEDWEVVVAQGDDAVGADS
GAFDAVQEAADEHAEAFGAPPTDQEVRAAVASIQEVFENHPGLDSDAPAQALALSPISGL
PPSGMFVNYFSEGSTPSDIKIEDSTPSDVKIDQLASLEHSTPDTASEECIEPAMLVLNST
ALLTREHRNVLDAFHLLQVDSSVQKMVMALSTDKSVWDAVMKNEVVQEFRKSFQDAKEAD
PNGSSSASPGVMKWVMETTQAKIKEFLESILKLVNMLFQAQSEDYDLYDDTVRMSFMLAV
FVFIVVTVARIK*
>11668.m04240|LOC_Os02g44080.1|genepair1719-1
MVKLAFGSLGDSFSATSVKAYVAEFIATLLFVFAGVGSAIAYGQLTNGGALDPAGLVAIA
IAHALALFVGVSVAANISGGHLNPAVTFGLAVGGHITILTGLFYWIAQLLGASIACLLLK
FVTHGKAIPTHGVAGISELEGVVMEIVITFALVYTVYATAADPKKGSLGTIAPIAIGFIV
GANILAAGPFSGGSMNPARSFGPAVAAGNFAGNWVYWVGPLIGGGLAGLVYGDVFIGSYQ
PVADQDYA*
>11670.m04529|LOC_Os04g46490.1|genepair1719-2
MANICANMKRCFSPPALRAYFAEFFSTFLFVFIAVGSTISARMLTPDETSDASSLMATAV
AQAFGLFAAVFIAADVSGGHVNPAVTFAYAIGGHITVPSAIFYWASQMLGSTFACLVLHY
ISAGQAVPTTRIAVEMTGFGAGILEGVLTFMVVYTVHVAGDPRGGGFGGRKGPAATALGA
LVVGAVTGACVLAAGSLTGASMNPARSFGPAVVSGHYSNQAVYWAGPMVGAAVAALVHQA
LVFPTVPEPAPAPATNESARHGSVQTVVV*

>11668.m04241|LOC_Os02g44090.1|genepair1720-1
MAMASGGTVCSMCGDVGFPDKLFRCARCRRRFQHSYCTNYYGDGAPVEAGAGVCDWCLSD
AAVVAGKKGPSSEGNEEPFSREYRGRSKQAASTGGGEQEGGGGGGRRVSKAGAVRRYKLL
KDVLC*
>11670.m04540|LOC_Os04g46600.1|genepair1720-2
MAGAGTVCSMCGDVGFPDKLFQCARCRYRFQHSYCTNYYGDAAPAPAGADMCDWCLSDVA
GKARYSSAAGKQQGAGSQESSTTTSSSSAGRGGGGKPGAGEQESGRRGTKAAGRSFGAGE
LRSNWPSRTLHAAKPSHVDVGVNESYPKTQSGRQRHAPTQTSSSARPPLYE*
>11668.m04245|LOC_Os02g44120.1|genepair1721-1
MAMVVAGSHRPPPPPQSLRLVPPPPPQPPPPPLTYRHHCKVCKKGFMCGRALGGHMRAHG
IGDDNDTMDDDDGRDDDHSLSPCDGGGEPSEAAGSPTTTTTKRMYALRTNPGRPRNCRTC
ENCGKEFTSWKTLLDHGRCGLDEEDGRLDVSLRSPPLHDGGDENDGEEEEEGDDLTLAAG
GWSKGKRSRRAKVMAVGTGSVSELQLPAPSTEEEDLANFLVMLSSSSSSSSRVAQPAIVV
DDADQESCASGSKDEERNRFLVPQPISMAAPMMAQMTVIAPQVVPQHISTVPRGMFECKA
CKKVFTSHQALGGHRASHKKVKGCFAAKLESSRNETSQTQTQQQHVSAAPHDNTRATTSH
VITSDISMDANTIGASADADGKAAASGVGAGEIVLAGASSTDMAMMMSVEDFAPTPLAPS
AVSPFKKKGKVHECSICHRVFTSGQALGGHKRCHWLTSGATDPLTKLQPVAQDHAMMAAM
CHQLTLGRPIFDPTDQRILDLNVPTNPLAEAVAARQQQQQQVAALNDGALCLNAAASVYL
QSWTGHSNGSHVNKTTATSSRINDAAGGVTTEDDEADSTSAKRAKIGDLKDMKVAGESLP
WLQVGIGISSESKEKNTQE*
>11670.m04547|LOC_Os04g46670.1|genepair1721-2
MCESSPSLHHGQMQPSPSSPRAPPPTAAQASGYKHFCRVCNKGFTCGSALGGHMRAHGVG
DGDGLGADDDDDDDDDSLGDEAVRRARGGADDPWNAGGPSSSGAATHVYELRTNPNRVTR
SRQVCKNCGKEFTSWEHFLEHGKCSSGEDDDDEDDVDHSLQPWSPSPEADGEEDSAPAAG
WLKGKRSRRCKGTGVDLSPTPSACAAGEEEDLANCLVMLSSSKVDQAGVTEAEQRSSSSA
SKEHKRLITFMEPTTYVLDTVMALPPPAPAPQYVSTVPRGMFECKACKKVFSSHQALGGH
RASHKKVKGCFAAKLESNAAEVAEPSHHAEVADRSEDNPAKATSDARRNVHASIDGDGNA
GTSDAAAELSMAIVPIEPPVAALAAAPLKKKGKMHECSVCHRLVTSGQALGGHKRCHWLT
SSSADHTASVPPLADDLVPLSFRPMLDAPEPALDLSIAANPPLLASAATVRPKVGGSSFH
LDAPPPVYIPSSPAIPSQRNKATATTGSQNANDAVGLSTAAAEDEADSTTVKRARLSDLK
DVSMAGETTPWLQVGIGSSSRGGADDNDKE*
>11668.m04246|LOC_Os02g44130.1|genepair1722-1
MAKNACKLCYRRFASPRALAGHMRSHSVAAANAAAAAAAAAAAKLQISSASSASTSFTAA
DEEEEEEEEEEDVGFKKPLSIYALRENPKRSLRVSEYAFSDRESEAESTPTPAAKGLRAG
GGGGGGDGEPMSSLSYAGTPEEEVALALMMLSRDTWPSVERGGGGGEYSDDGSDDGYALP
PPSPAPAPAPVPEKRTRFQCPACKKVFRSYQALGGHRASHVRGGRGGCCAPPVAPPPQPH
PQPPLPEHDAGEEDMDGKAPPHECPYCYRVFASGQALGGHKKSHVCSAAAAAAHAQTPGG
GAPPPQPKILGMIDLNFAPPVDEVELSAVSDPHFPSNPPGP*
>11670.m04548|LOC_Os04g46680.1|genepair1722-2
MAKNTCKLCYRRFGNPRALAGHMRSHSVAASRSQISSTSSASTSVAVGDDDGGGDAKRPI
QGYVLREKPKRRVRLAESDFSDRESETEYYSSPPHGKRANTGSGDVEQVSSVSDATSEED
VALSLMMLSRDTWPATPPPPPPYRLRGAGYDDGSDGGDAPPAPAAAAAQKRTRFQCPACK
KVFRSYQALGGHRASHMRGGRGGCCAPPPNPPPSPATPLQPLPECDGGEEEGAKPHPHEC
PYCFRVFASGQALGGHKRSQLCSAAAAAASGDDIPAMIKSNGFIDLNLPAPFDDVELSAV
SDPFLSSKPGS*
>11668.m04253|LOC_Os02g44200.1|genepair1723-1
MQQPSVSLAPNTSCHPQHAGSAAGSSRRSHGHLGVLLHVDHSDGRRAGALYAATNLRSLE
AIPATGPTLRSLEEAIAAPNLLSHEAVISANNTYQDAKPRARKFFELEMTVQDCDLDQYG
VVNNTVYPSYIERAREELISGLGMSRTSIACTGNAMALTELNIKYFTPLKRGEKFVVRLS
LGRIKGARIYAEQYIERLPDRKLVVESTATIICLNRKHRPTRVWPELSSKLLDYFSSQED
*
>11670.m04552|LOC_Os04g46710.1|genepair1723-2
MQQQQLCSSHCLPARAGSIASPGSGRRVVPLGRRRASLGKVTAYAYPTTRRVVDAAKSSL
LQDVHVAASNPSLQLLQDYAPAKKSAKQQNGSRTKDGFYEVEMTVQDDELDEYGVVNNAI
YASYIHSGRDVFLENVGVGVDYWTSTGNALALSELNLKFYTPLRKDDRFVVRMKVVKIKG
VRIIVEHLIETLPDRKLVVDAKATAVCLDNKYRPTRVFPELSTKLHQFFLS*
>11668.m04256|LOC_Os02g44230.1|genepair1724-1
MANSDVLVVALLSSQFSVMPGGYSSSGMNVGVSRLKIEEVLVNGLLDAMKSSSPRRRLNV
AFGEDNSSEEEDPAYSAWMAKCPSALASFKQIVASAQGKKIAVFLDYDGTLSPIVDDPDK
AVMSPVMRAAVRNVAKYFPTAIVSGRSRNKVFEFVKLKELYYAGSHGMDIMAPSANHEHS
AEKVTKSLLQVVSGIEGATVENNKFCVSVHYRNVAEKDWKLVARLVNEVLEAFPRLKVTN
GRMVLEVRPVIDWDKGKAVEFLLQSLGLNDSENVIPIYIGDDRTDEDAFKVLRQRNCGYG
ILVSQVPKETEAFYSLRDPSEVMEFLNFLVRWKKHSV*
>11670.m04559|LOC_Os04g46760.1|genepair1724-2
MKISANFLLNNCARTYTKKKTLKKCKRELVEVVDGLVGVMMTSSNREKPDIESGYDGSSD
EDSTENSRAEICPSALCFFDQIVASAQDKKVVLFLDYDGTLSPIVNDPEKAFMSSEMRAT
VKSVAKHFPTAIVSGRSRDKVFDFVKLTEIYYAGSHGMDILASFADSDSTIEKTKETKLF
QPANEFLTMITEVSKSLIEVTKAIKGATVENNKFCVSVHYRNVDKKNWKLVAQVVNNVLK
DFPSLKVSTGRKVLEVRPMINWDKGKAVEFLLRSLELDDSETVLPIYIGDDKTDEDAFKV
LRERKNGCGILVSQVPKKSEAFFMLRGPSEVVILPVMLNFFAALLIMPS*
>11668.m04264|LOC_Os02g44300.1|genepair1725-1
MSNTLLRIYPSELKIPFELKRQNSGILELTNKTDHHVAFKVKTTNPRKYSVRPTTGIVLP
RGSCGITISMQPPKEIPTDYNCKDKFLIQSVVVEDGTTQKDIHSDMFSKEAGKVVEEFKL
RVVYIPANPPSPVPEEEEDEIDSLDSDVDHEVQMPSTFDAASRKGYTSGSQASHDEGVSL

TKAVLSKYVDENQKLQQELDLLKKKRSSSDGGFTALFVPFVFAFFVFIGYLMAGSNV*
>11670.m04562|LOC_Os04g46780.2|genepair1725-2
MSNTLLRVHPSELKIPYEYKRKRSCCMQLTNKTNQYVAFKVKTTNPRKYSVRHACGILPP
RSSCDITVTMQAPVEMLSDYHCKDKFLVQSVAVGYGATMRDFVPELFTKAPGRVIEEFKL
RVVYVAANPPSPVPEEEEEEEEDASPQSEVMSHGVKMTSVFDAVTVSTLTDRSADKVSSA
EGVSVESMLVAEREYPVEENQKLQQQMELLRAARSSQQGFSAMFVLLVFMSSVCIGHFMK
QIKV*
>11668.m04268|LOC_Os02g44310.1|genepair1726-1
MASKAFALFLAVNLVVLGVASACGGSPSCPTPTPSTPTPSTPTPTPSAFGRCPRDALKLG
VCANVLGLIKAKVGVPPAEPCCPLLEGLVDLEAAVCLCTAIRGNILGINLNLPIDLSLIL
NYCGKTVPTGFKC*
>11670.m04565|LOC_Os04g46810.1|genepair1726-2
MAGKASIALFLAVNLVVFSLASACGGRCPTPTPSTPSTPTPTPAAFGKCPRDALKLGVCA
NVLGLIKAKVGVPPAEPCCPLLEGLVDLEAAVCLCTAIRGNILGINLNLPVDLSLILNYC
GKRVPTGFKC*
>11668.m04269|LOC_Os02g44320.1|genepair1727-1
MASRAFLLVALNLVLFFTVASACGKYCPTPSTPSTTPSTPSYNTKCPKNALKFAACADVL
GLVSAEVGQPPYEPCCGVLGGLADLEAAVCLCTAIKANVLGITLDIPVKLSLLVNYCGKN
VPSGFICA*
>11670.m04567|LOC_Os04g46830.1|genepair1727-2
MASKILVFLLAINLLFFTTANACGCACGKCPTPPPPALPPPPPPTPTTPSYHNKCPVNTL
KFGACADVLGAISGEVGQVPAQPCCSLISGLADLEAAVCLCTAIKANVLGVVVNIPVKLS
LLVNYCGKCVPSGYTCA*
>11668.m04274|LOC_Os02g44370.1|genepair1728-1
MRAALFGAERSGVVDLGGIGGGNRGLFWPAGKGGLVVVEPRSVLDCTRSPSPRNSTSTLS
SSQGGGGADSTGVAAVSESSAAAAEATKWGAPGEHGGGGGGGGGGGKEDWSSGCELPPIP
GTLDVGLVGGEGWDTMLGNAAAAAAGQDQSFLNWIIGAAGDLEQPGPPLLDNAGFGIPAV
DPLGFSLDHSLSGVASDLSSSGAHTATGGAGGGKASLGFGLFSPEATSLEQPPPPMLFHE
GIDTKPPLLGAQPPGLLNHYHHQPPNPAATFFMPHPSFPEHNHQSPLLQPPPKRHHSMPD
DIYLARNQLPPAAAAAQGLPFSPLHASVPFQLQPSPPPIRGAMKTTAAEAAQQQLLDELA
AAAKATEAGNSVGAREILARLNQQLPPLGKPFLRSASYLREALLLALADSHHGVSSVTTP
LDVALKLAAYKSFSDLSPVLQFANFTATQALLDEIGGTATSCIHVIDFDLGVGGQWASFL
QELAHRRAAGGVTLPLLKLTAFVSTASHHPLELHLTQDNLSQFAADLGIPFEFNAVSLDA
FNPGELISSTGDEVVAVSLPVGCSARAPPLPAILRLVKQLSPKIVVAIDHGADRADLSFS
QHFLNCFQSCVFLLDSLDAAGIDADSACKIERFLIQPRVHDMVLGRHKVHKAIAWRSVFA
AAGFKPVPPSNLAEAQADCLLKRVQVRGFHVEKCGAALTLYWQRGELVSISSWRC*
>11670.m04570|LOC_Os04g46860.1|genepair1728-2
MRAAPFSADGNGAAELAGSIAALLWPEDKGGGGGGGGGSLLVEPRSVLDCRGSPSPPNST
STLSSSHGSGAADSISTGVAAVSESSAAAAEATRWAAPGEHGGGGGGELPPIPGALDVGF
VAEESWDAMLGDAAAAAGQEQTFLNWIMAAPGDMEPQAPGLSQQQLLANAAGFGFPLQHH
PGGVSSPAALASDLSSSGGRSLTSSSGSNSKATSAFGLLSPEAALQPPPATTAPFHNGAD
MKPPLLGLPSPTLLLNQHQPTPASTLFMPFPSFSDHQQQPLLQPPPKRHHSVPDNLFLLH
NQPQPPPPAPAQCLPFPTLHSAVPFQLQPSMQHPRNAMKSTAAAAAAQQQHLLDELAAAA
KATEVGNSIGAREILARLNQQLPPIGKPFLRSASYLKDALLLALADGHHAATRLTSPLDV
ALKLTAYKSFSDLSPVLQFANFTVTQALLDEIASTTASCIRVIDFDLGVGGQWASFLQEL
AHRCGSGGVSLPMLKLTAFVSAASHHPLELHLTQDNLSQFAADLGIPFEFNAINLDAFDP
MELIAPTADEVVAVSLPVGCSARTPLPAMLQLVKQLAPKIVVAIDYGSDRSDLPFSQHFL
NCLQSCLCLLESLDAAGTDADAVSKIERFLIQPRVEDAVLGRRRADKAIAWRTVLTSAGF
APQPLSNLAEAQADCLLKRVQVRGFHVEKRGAGLALYWQRGELVSVSAWRC*
>11668.m04275|LOC_Os02g44380.1|genepair1729-1
MGEEDAPEMPDASPRLPWEPGGVEHFDAALLGDAAYIVDEEEVSDVEMSEGSPVAPSESS
ALPSPPSLRKRLAPVVPSDVPEEVVRAVDAVIMGGGLERLREMVSGEDGELSHFVVDVLM
LTMGGVDGLDEGAGDGAVTLPSIMSSSRAAAIAAELLPYIPCGVEPSPRTRMARALLATL
SSCTRNRTMCTSSGLLAILLDAAEKLFVGMGQRSKWDGAPLVQCIQMLGGHSVSVKDLHS
WLLLIKKTLGTCWATSLTLALEKAVGCKEAKGPAVTFELGGEGSGLLAPAESRWPFSNGF
GFATWIYVESFSDSLNTGMATAAIAAAAASTSGKSSPSAAAAAASTLAGEGTKHMPRLFC
FLTMDNHGVEAYFHGQFLVVESGAGKGKKASLHFTYEFRPQCWYFVGLEYTSKQALLGKV
ESELRLYVDGELHESCPFELPRILKPLAFCCIGTNPPPTIAGLQQRRRQCPLFAEMGPIY
IFTESIGPERMSRIASRGGDALPSFSNGSGLPWKATNAHIRHIAEDNYTLDIEIGGSLHL
LYHPSLLNGRFCPDASPSGSTGTHRRPAEVLGMVHLSYRVRPAESLWALAYGGPMALLPL
TVSNVEMDNMEPILGDLSSSLATASLSVPIFRIISLATQHPGNKEELCRAHGPELLSQVL
HYLLETLSRLESGEKEILSDEELVAAIASLCQSQKNDHGLKVQLFSTLLLDLKMWSSCNY
VLQKKLLSSLADMVFAESACMHDANALQMLLDGCRRCYWVIHEADSIDTFTFTGTERPLE
KVNSLVDELLVVIELLLGAVSSTLASDDVRSLVGFVVDCPQPNQVARVLLLIYRLIVHPN
TSRANVFAQSFISRGGVEALLVLLQREAKSGDNNISDSCIVPQNSLWNAGSDPKSISSDL
DLKTTAGEANCNGHKTQPLEHHEPPCHEGSTEPGFSSKWCLLKNQFLKNLGGIDFLNIAD
NVQNDIYNIDNGDGVLVGIVHVLGALVASGHLKFNLPAAKPKLPSSFLTTSNGEGNSMFE
DRVSLLLFAMQKAFQAAPRRLMTRNVYKALFSAVINVPSANGSLKLHDSGHRVKHTPLLS
VLLRSLPFASRAFQAHAIQDLLYLASTNNENRIALTSIAEWPEWILEVLISNHEDVEATI
HCAEWLSMVGGSSTGDQRIRREESLPIFKRRLLGNLLEFSAQELQVQNSKIIFPDLSFSL
GRSIALPNKHRDKQVSENSSSITHEFILRDIAHIVSMKQTEGINAAAAGVAEEGMMPKET
KIQADKATHLSLALAENAIVLLMLVEDHLRSRSQHYFLSCSIDSAASPASGASSAATRSN
SLSRTGSEPLDAGGSRQSLSSDAGGLPVDVLASMADANGQISSEVMERVTAAAAAEPYGS
VRHAFVSYGTCISDLSEGWKYRSRLWYGVCIPPKSNIFGGGGSGWESWKSVLEKDSNGSW

IELPLVKKSIAMLQALLLDSQLGGGLGIGGGSGAGMDAMSALNQLLDSDQPFFCILRLTL
ASIREDDNGEDDIFMRNISMKNVISEGLGCQTGSMMIHDDNSCSPSRKPQSALLWRVLGP
ILNMPVSESKRQRVLVASSIIYSEVWHAVSSDRKPLRKKYVGLIMPPFVAVLKRYRSVLA
GIHELTSSDGQNPLVVDDCALAADTLPIEAAVSMISPGWAAAFASPPVAMALAMIAAGTS
GTETIAPPTNKLRRRDTSLLERRSAKLHTFSSFQKPIDATPNLPTSAPKDKAAAKAAALA
AARDLERSAKIGSRRGLSAVAMATSGQRRAAGDIERAQRWNTSEAMCAAWLECLQSADSK
SVSGRDFSALSYKYVALLVSSFALARNLQRVEELLTCEYTIEATFGHADGEANTVNKRKK
NILAVNTMHAFFSYRHLDLLYYFSTQIFWKLDSTETSSRMRRFMKRNYKWSEHLSMANNY
EENMLLCDGAESNYSHREDADSLFANALRTNSSIIVADAISVDGGHGNAKQTETETIYSS
VDDSTSSDFSSVHNLVRSTVVAPCYSSSKNNERIIVELPSLMVRPFKVVRGTFQVTSKMI
NFIIDEHMSDSDSYMDDAASTSGQYDQQDKDRSWFISSLHKIYSRRYLLRRSALELFMVD
RSNFFFDFEDTEACKHAYRAIVHTKPPYLNSIFLATQRPEKILKKSQLTERWARWELFMQ
YPVFPWVLADYHSKTLDLEDPSAYRDLSEPIGALNPARLKKFQEHYSSFNDPTIPKFHYS
SHYSCPGTVLYYLVRIEPFTALSIQQQGGRFGQDDHMFSCINKTWNSVLEDMNDVKELVP
EMFYLPELFTNVNSVDLGSTQLTGKLSSVQLPPWAENPVDFICKHRKALESDYVSAHLHE
WIDLIFGHKQRGKDAVMANNVFPYATYEGMVDIDQITNPVQRRSMQDQICNFGQTPSQLL
TIPHTKRRPLADILKLQTIFWNPTEVRSYVLPNPENCNVPASAMLVSDDSIVVVGANVPA
AHLALHHWQPNTSNGPGTPFLFHHGRNAINLSGGAIMRIFKGSAGSVDDYQFPRAIAFAA
SAIQNSSVVVVTCEREVITGGHADNSVKLISPDGARTIETAFGHLAPVTCVALSPDSNYL
VTGSRDTTVILWRIRQIGSLRKKSTPEPPPSTPTTPNDSASGDSSTSNSSKVLETSRRRR
IEGPMHVLRGHLGEVTCCSVSSALGLVASSSSTSGVLLHNLRTGRLIRKLDKQEAHLMCL
SSEGIILIWNESEKRLSTFTVNGIPIGTSVLTPFSGRVSCIEISVDGKFALIGTCLSNSC
NRDGFSAIDDYELDKPNGDDDLPELNETKPSVHVPSVCFIDLYKLELSSKIGDRMLQEGN
GVLES*
>11670.m04574|LOC_Os04g46900.1|genepair1729-2
MAEEPRESLDSSPEPPPPGQDSDEQFSAVPLADEVRAEGSELDPDTSAGTSVAVTPARSE
PSPPPPRRRRPRPLGVPPDAPQEVVRAVDDAIMVGGGAAAAAGVNRLHEMVSEEQGELPH
TVVDVLLGTMGGADGLDEVEDKTGTGAPPSIMFNSRAAVVAAELLPYLPCGDEPSPRTRM
AVGIHATLRACTRNRAMCSSSGLLPVLLDSAEKLLIGMGRASSWDGTPLLHCIQLLGGHS
LSVKDLHSWLGLVKKALGTSWATPLMLALEKAMGSEEARGPAATFEFDGESSGLLGPGDS
RWPFSNGYGFATWIYIESFSDTLSTATAAAAIAAAAAATSGKSSAMSAAAAASALAGEGT
THMPRLFSFLSSDNQGVEAYFHGQFLVVESGGGRGKKASLHFTYAFKPQRWYFVGLEHTN
KHGLLGKGDSELRLYVDGSLHESRPFEFPRISKPLAFCCIGTNPPPTIAGLQRRRRQCPL
FAEMGPIYIFREPIGPERMGRLASRGGDVLPSFGNGAGLPWRATNDHVKNMAEESFTLNQ
QIGGCLHLLYHPSLLNGRLCPDASPSGSAGTHRRPAEVLGLVHVSSRVRPAESLWALAYG
GPMALLPLTISNVQMDSLEPMLGELSIATASLSAPIFRIISLAIQHPGNNEELCRTCSPE
ILSRVLHYQLQAFPKMEGGEGEAVTDEELVDAIVSLCQSQRNNHELKVQLFSTLLLDLKM
WSSCTYGLQKKLLSSLADMIFTEAACMRDANALQMLLDSCRRCYWAIREPNSIDNFALTG
TKRSLGEINALIDELLVVVELLLGSASSTAASDDVRCLIGFVVDCPQPNQVARVLHLIYR
LIVQPNISRANMFAQSFISCGGVDALLVLLQREAKAGNNSILDNSDALLSENDFLRNDDS
DTKAASGEAKSQDDQIQSVELEQHESILHEEHTELGSTSTNDVPCEILGSSIGRKLSSSE
NQLLKNLGGINFSITADNVRNNVYNVDKGDGIVVGIIHILGALVASGHLKFASRAANPNL
PGGLLTTVHEEGNTMSEDRVSLLLFALQKAFQAAPRRLMTVNVYMALISAAINVSSVDEN
LNLYDCGHRFEHIQLLLVLLRSLPYASRSFQSRAIQDLLFLACSHPENRTTMTSISEWPE
WISEVLIYNHEMGAKKYADGISIGDIEDLIHNFLIIMLEHSMRQKDGWKDVEATIHCAEW
LSMVGGSSTGDQRIRREESLPILKRRLLGGLLDFSARELQVQTEVIAAAAAGVAAEGLSP
EEAKTQAENAAHLSVALAENAIVILMLVEDHLRSQGQHFCTSRSLDSAVPSASMVSSAAS
RSNSLCRSGNEPMDAGTSRRSSLSTDAGGLPLDVLTSMADSNGQISAAVMERLTSAAAAE
PYESVKHAFVSYGSCIADLAESWKYRSRLWYGVGIPSKSDTFGGGGSGWEFWKSVLEKDS
NGTWVDLPLVKKSVAVLQALLLDDSGLGGGLGIGGGSGPGMGVMTALYQLLDSDQPFLCM
LRMTLVSMREDDNGEGDAFTGNISIKDVISEGLGHQAGSMMPLDSNNRSSTRKPRSALLW
SVLGPILNMPINESKRQRVLVASSILYSEVWHAIGRDRSPLRKQYIELILPPFIAILRRW
RPLLAGIHELTSSDGQNPLIADDRALAADALPIEAALSMISPGWAAAFASPPVALALAMM
AAGASGTEAIAPPRTLNRRDTSVPERKAAPKLQSFTSFQKPIETAPNKHGSTPKDKAAVK
AAALAATRDLERTAKIGSGRGLSAVAMATSGQRRSAGDIERAKRWNTSEAMSAAWMECLQ
SVDSKSVSGRDFSALSYKYVAILVSGFALARNLQRVEIERQTQADVLNRHRVSTGVRAWR
HLLHCLTEMDRLYGPFGEPLCAPDRIFWKLDFTESSSRMRRFMKRNHKGSDHLGAAADYE
ERKLSNVAQSNECNPEGTEPLVTDTLPSTAPIITAEAMSVDDRNEDNEQLESDTTQSSVD
DRLQQADQQSVKGSIDSRGSGISADRNLVRSTVIAPGYVPSDADERIIVELPSSMVRPLK
VVRGTFQVTSKRINFIIDESASESNMDDHASTSGQCDQQDKDRSWLISSLHQIYSRRYLL
RRSALELFMDMEARKNAYRAIVHSKPPNLNDIFLATQRAEQILKRTQLTERWANWEISNF
EYLMELNTLAGRSYNDITQYPVFPWIIADYRSEILNLDDPCTYRDLSKPIGALNPERLEK
FQERYSTFEDPIIPKFHYGSHYSSAGTVLYYLFRVEPYTTLSIQLQGGKFDHADRMFSDL
SGTWDSVLEDMSDVKELVPEMFYLPEVFTNINSIDFGTTQLGGKLDSVNLPPWAEDPVDF
VHKHRKALESEHVSAHLHEWIDLIFGFKQRGKEAVMANNVFFYITYEGTVDIDKITDPVQ
RQAMQDQIAYFGQTPSQLLTIPHMRRKPLAEVLHLQTIFRNPSELKSYLLPNPDRCNVPA
STIWPRDTLSISSW*
>11668.m04284|LOC_Os02g44470.1|genepair1730-1
MSNSASGMAVCDECKLKFLELKAKRSFRFIVFKINEKVQQVVVDRLGQPGESYDDFTACL
PADECRYAVFDFDFVTDENCQKSKIFFISWAPDTSRVRSKMLYASSKDRFKRELDGIQVE
LQATDPSEMSMDIVKSRAL*
>11670.m04575|LOC_Os04g46910.1|genepair1730-2
MANSASGMAVGDECKLKFQELKSKRSFRFITFKIDERTQQVVVDRLGQPGDTYDDFTASM
PASECRYAVFDFDFVTDENCQKSKIFFISWSPDTSKVRSKMLYASSKDRFKRELDGIQVE

LQATDPSEMSMDIVKARAL*
>11668.m04287|LOC_Os02g44500.1|genepair1731-1
MPSRTAPFISRLRLLRTAAALSPFSSSAPPRHSPRILRAIPVGAPHPPRVSAAVSPLVRP
VAAGFALFSMATAASSAASVHDFTVKDASGKDVDLSTFKGKVLLIVNVASQCGLTNSNYT
ELSQLYEKYKDQGFEILAFPCNQFGGQEPGTNEEIVQFACTRFKAEYPIFDKVDVNGDNT
APIYKFLKSSKGGLFGDNIKWNFSKFLVDKEGRVVERYAPTTSPLSMEKDIKKLLGSS*
>11670.m04581|LOC_Os04g46960.1|genepair1731-2
MAAAPSATSVHDFTVKDASGKDVNLSTYKGKVLLIVNVASQCGLTNSNYTELSQLYEKYK
VQGFEILAFPCNQFGGQEPGSNEEIVQFACTRFKAEYPIFDKVDVNGNNAAPLYKYLKSN
KGGLFGDSIKWNFSKFLVDKEGRVVDRYAPTTSPLSIEKDIKKLLGSS*
>11668.m04291|LOC_Os02g44540.1|genepair1732-1
MAELTTAADVTAAPPPPPGPTPPPEPVVTATVVAEQKVSPTAPPLDVPAPAPPPPKKRKV
EEAGFHNSAYYKIRATVADLRVRFVQVYEATDFRNSDAAREILKADCGDNQVAEKPCAGE
PCTNGRRGEKQTEIKVVMELAKKMRHDLGATFEPAKPPEKPLAGVVKDGPVEPPPSAENN
HAPQTGETTVCSNIVNNIVPTNPNSDGAAEKMGETPRSEIAQGSCVTGGSPIGWNFLVWP
GGEVVYYGRTKEVFRAGQAEN*
>11670.m04586|LOC_Os04g47010.1|genepair1732-2
MVAPTTTTPPPPPPPPSESTPTSDPKPPPPPPTSSTAAPKKRKLEEVGFHHSPYYNIRAA
VANLRGRFIQLCKGTDTQKKDAALEILKEIKVLMELSKEMRLDLPTAAGPVKLMDEPTSR
DARNMPAGKIPPGEKNQVRPADQAASFMHSSGEKVPLNPVDIKHDAKPSVTDSTKKSGQC
LQGSYIVGGSPIGWNFLMWPGSSTRYCGLTRSEWLARQSAK*
>11668.m04297|LOC_Os02g44590.1|genepair1733-1
MAHLGAVLCIRVALVVVLLPSLLATVAVAHNDTGEHKNYLIIVRKPYEYDHNVYKTVSSW
HASLLASVCDTAKEELATDPGAETRLIYSYRNVVNGFCARVTREEVYEMAKKDWFVKAIP
EKTYKLMTTYTPKMVGLTGAPAAYHGGLWNRSNMGEGMIIGVLDDGIAAGHPSFDAAGMG
PPPARWKGRCDFNSSVCNNKLIGARSFFESAKWKWRGVDDPVLPVYELAHGTHTSSTAGG
NFVPGANVMGNGFGTAAGMAPRAHLALYQVCSEDRGCDRDDILAAMDDAVDEGVDVLSIS
LGDDEAGDFAGDPVALGAYTAIMRGVFVSSSAGNNGPNPLTVSNEAPWLLTVAASTTGRK
FVATVKLGTGVEFDGEALYQPPNFPSTQWPLIADTRGDGTCSDEHLMKEHVAGKLVVCNQ
GGNLTGLRKGSYLHDAGAAGMVLIGPEFMGSMVQPKSHILPVAQIVYLSGEELKAYMKST
KSPTAALIYKGTVFGDRKTPEVAPFSSRGPSRQNQGILKPDITGPGVNIIAGVPVTSGLA
TPPNPLAAKFDIMSGTSMAAPHLSGIAALIKKAHPKWSPAAIKSAMMTTADTLDRRRRPI
TDQKGNNANMFGLGAGFINPTKAMNPGLVYDLTAQDYVPFLCGLGYSDHEVSSIIHPAPS
VSCKQLPAVEQKDLNYPSITVFLDREPYVVSVSRAVTNVGPRGKAVYAAKVDMPATVLVT
VTPDTLRFKKVNQVRKFTVTFRGANGGPMKGGVAEGQLRWVSPDHVVRSPIVVSAQKFLN
GNTSSSDHAGH*
>11670.m04600|LOC_Os04g47150.1|genepair1733-2
MDNRNHGRCSLPRLAVGAAVLLLAVSLAATPAASHAGHDDTGLHSNYLVIVRKPYAYDTN
LYKNVSSWHASLVASVCDMAKEALERDPSSVSRLIYSYRNVVNGFAARLTPEEVEEMSKN
DWFIRADPEKTYQLQTTHTPQLLGLMGGARRGGVWNTSNMGEGIIIGILDDGIYAGHPSF
DGAGMKPPPAKWSGRCDFNKTVCNNKLIGARSYFESAKWKWKGLRDPVLPINEGQHGTHT
SSTAAGSFVPGANVSGYAVGTAGGMAPRAHIAFYQVCYVEKGCDRDDILAAVDDALEDGV
DILSLSLGDEQAGDFSDDPVSLGGYSAAMHGVLVSAAGGNTGPGPSTVVNEAPWVITVGA
GTTDRRFVATVKLGSGVSLDGESLSEPKDFGAEMRPLVHDVGDGMCTTESVLRAMNVTGK
IIICDAGGDVSVAKAKLVLRSGAAGMIVIAPQVYGSVIVPRPHVLPTVQMPFMIGQKIKA
YIRSTPSPTANFIFKGTVFKAKSPVAAPFSSRGPNRRSRGILKPDIIGPGVNILAGVPKI
EDLALGAEEVMPKFDIKSGTSMAAPHISGVAALIKNAHPTWSPAAIKSAMMTTADYTDNL
RKPITDVDGAPATYYAIGAGYVNARKAIDPGLVYNLSSLDYIPYLCGLGYKDQKVNSIIH
PGPAVECAKMPKVDQKDLNYPSITAVLDMEPYEVSINRSATNVGAATSTYAVEVDVPATL
AVEVNPAKLEFRALNEVLNYTVTVKTASGKAPASTIEGQLKWVSGKKYVVRSPILVCAGT
GGKSAASMGAAPA*
>11668.m04303|LOC_Os02g44630.3|genepair1734-1
MEGKEEDVRLGANRYSERQPIGTAAQGAGDDKDYKEPPPAPLFEPGELKSWSFYRAGIAE
FVATFLFLYITILTVMGVSKSSSKCATVGIQGIAWSFGGMIFALVYCTAGISGGHINPAV
TFGLFLARKLSLTRAIFYIVMQCLGAICGAGVVKGFQQGLYMGNGGGANVVASGYTKGDG
LGAEIVGTFILVYTVFSATDAKRNARDSHVPILAPLPIGFAVFLVHLATIPITGTGINPA
RSLGAAIIYNKDHAWNDHWIFWVGPFVGAALAAIYHQVIIRAIPFKSRS*
>11670.m04609|LOC_Os04g47220.1|genepair1734-2
MSGWEPTSSRRGSRSARRRRAPTTRTTRSRRRRRCSSQGSSSRGPSTAQGSPSSWPPSSS
SYITALNAMGVNNSTSKCATVGIQGIAWSFGGMIFALVYCTAGISGAHINPAVTFGLFLA
RKLSLTRALFYMVMQCLGAICGAGVVKGFQKGLYETTGGGANVVAPGYTKGDGLGAEIVG
TFILVYTVFSATDAKRNARDSHVPILAPLPIGFAVFLVHLATIPITGTGINPARSLGAAI
IYNRGHAWDDHWIFWVGPFIGAALAAIYHQVVIRAIPFKSRS*
>11668.m04305|LOC_Os02g44640.1|genepair1735-1
MTTQEHTIFVNEVDDKLYIYLEYVSGGSIHKLLQEYGQFGEPAIRSYTKQILLGLAYLHA
KNTVHRDIKGANILVDPNGRVKLADFGMAKHINGQQCAFSFKGSPYWMAPEVIKNSNGCN
LAVDIWSLGCTVLEMATSKPPWSQYEGIAAVFKIGNSKELPPIPDHLSEEGRDFIRQCLQ
RNPSSRPTAVDLLQHSFIRNASPLEKSLSDPLLQLSTTSCKPDLKVVGHARNMSSLGLEG
QSIYQRRAAKFSSVHSDIHVRSYISCPVSPCGSPHLRSRSPQHQNGIMSPSPISSPRTTS
GASTPLTGGNGAIPFNHARHLAYNNEGFTITSRCLDEPLPNQPPDPVLGRFVRVKQPSLG
FQERAVPEADILSPQFGRMGHVSVWNLHDKPLPSEHASQKGFEDRVKLKPPLDLRSGPPH
LGCNHGH*
>11670.m04610|LOC_Os04g47240.1|genepair1735-2
MPPWWGKSFSKDAKKTTKENLIDTFHRLISPNDQKGSTKSKRSCRRGNDSSVEKSCRSTT

VSRPTSPSKEVSRCQSFSADRPHAHPLPIPGVRPPVTRTVSDITESKPILEKRGKPPLLL
PLPKPNRPPRRHGNSEVVSEIVVASPSSNCSDSDDHGDSQLQSPVGNDAENATLVTLKNK
SSNARKECPGPITAKNMKEIHRPANQVHGSHILSTSPRGVAADSYQSNLQNPRPLVLDSA
PNSLMSSPSRSPRRICPDHIPTSAFWAVKPHTDVTFVGSGQCSSPGSGQTSGHNSVGGDM
LAQLFWQPSRSSPECSPIPSPRMTSPGPSSRVHSGSVSPLHPRSGGMAPESPTNRHDDGK
KKQTHKLPLPPLSISHSSFHPNNSTPTSPISVPRSPGRTENPPSPGSRWKKGKLIGRGTF
GHVYVGFNSDSGEMCAMKEVTLFLDDPKSKESAKQLGQEISLLSRLQHPNIVQYYGSETV
DDKLYIYLEYVSGGSIHKLLQEYGQLGEQAIRSYTQQILSGLAYLHAKNTVHRDIKGANI
LVDPSGRVKLADFGMAKHINGQQCPFSFKGSPYWMAPEVIKNSNGCNLAVDIWSLGCTVL
EMATSKPPWSQYEGIAAMFKIGNSKELPPIPDHLSEPGKDFIRKCLQRDPSQRPTAMELL
QHPFVQKAVSLEKSVLSEPLEHLAVISCRSSAKMAAHTRNISSLGLEGQTIYQRRGAKFS
SKHSDIRIRSNISCPVSPCGSPLLKSRSPQHSNGRMSPSPISSPRTTSGTSTPLSGGNGA
IPFNHLKQSTYSNEGFAIPSRSPDDLFASRPTDPDLGQFIRVHQVSQGLQERVVSEADIL
SPQFGKRLGNVFDLRDKLSPSERFTHHAFVDHVKLNPSLDLTSGSPHLGLKHGN*
>11668.m04307|LOC_Os02g44660.1|genepair1736-1
MSRGLSGPRVWPVVGSLPGLVQHAENMHEWIAANLRRAGGTYQTCIFAVPGVARRGGLVT
VTCDPRNLEHVLKSRFDNYPKGPFWHAVFRDLLGDGIFNSDGETWVAQRKTAALEFTTRT
LRTAMSRWVSRSIHHRLLPILDDAAAGKAHVDLQDLLLRLTFDNICGLAFGKDPETLAKG
LPENAFASAFDRATEATLNRFIFPEYLWRCKKWLGLGMETTLASSVAHVDQYLAAVIKAR
KLELAGNGKCDTVAMHDDLLSRFMRKGSYSDESLQHVALNFILAGRDTSSVALSWFFWLV
STHPAVERKVVHELCAVLAASRGAHDPALWLAAPFTFEELDSLVYLKAALSETLRLYPSV
PEDSKHVVADDYLPDGTFVPAGSSVTYSIYSAGRMKTVWGEDCLEFRPERWLSADGSKFE
PHDSYKFVAFNAGPRICLGKDLAYLQMKNIAGSVLLRHRLAVAQGHRVEQKMSLTLFMKN
GLRMEMNPKGKRSSNCYFYLQLPETKDTCVISKFQCVISSVKSCPTCFIFNMPPWWGKSS
SKEVKKTAKENLIDTFHRLLSPNEQKGRTKSRGNRRHSKDPTAEKGCWSTAQSRSASPSK
EVSRCQSFAAARAHAQPLPLPRSRAMVARTASDITESKVVLEKRGKGQQLPLPTTNWVKE
RPETTEPVAELSTASISSHGSIDSDDPGDLRLQGPVANDTDNVAKVATTGNSSVVHKECS
SAITRKGTKEVTMPTNAFLSNQILSTSPRGTVVADSYQSNLQNSRKVVLDSAPNSVMSSP
SRSPRILCPDQIPSSAFWAVKPHTDVTFVGSAQCSSPGSGQTSGHNSVGGDMLAQLFWQP
SRGSPECSPIPSPRMTSPGPSSRVHSGSVSPLHPRAGGMAPESPTRRLDEGKRKQTHRLP
LPPLSICNNSTFLPNNSTPTSPISHSPGRVENPTSPGSRWKKGKLVGRGTFGHVYIGFNS
DKGEMCAMKEVTLFSDDPKSKESAKQLCQEILLLNRLQHPNIVRYYGSEMV*
>11670.m04613|LOC_Os04g47250.1|genepair1736-2
MEAGTWAVVVAAVAAYMAWFWRMSRGLSGPRVWPVVGSLPGLVRHAEDMHEWIAANLRRT
RGTYQTCIFAVPGLARRGGLVTVTCDPRNLEHVLKSRFDNYPKGPFWHGVFGDLLGDGIF
NSDGETWVAQRKTAALEFTTRTLRTAMSRWVSRSIHSRLLPILSDAAAAGGGGGGATVDL
QDLLLRLTFDNICGLAFGKDPETLARGLPENDFASAFDRATEATLNRFIFPECVWRFKKW
MGLGMETTLARSVQHVDRYLSAVIKARKLELAAGNGKGDASSATPHDDLLSRFMRKGTYS
DESLQHVALNFILAGRDTSSVALSWFFWLVSTHPAVERKIVRELCTVLAASRGADDPALW
LAAPLNFEELDQLVYLKAALSETLRLYPSVPEDSKHVVADDVLPDGTFVPAGSSVTYSIY
SAGRMKTVWGDDCLEFRPERWLSADGTKFEPHDSFRFVAFNAGPRICLGKDLAYLQMRNI
AGSVLLRHRLAVAPGHRVEQKMSLTLFMKHGLRMEVRPRDLAPIVDELRGAGEYAAAARA
TAACA*
>11668.m04318|LOC_Os02g44770.1|genepair1737-1
MEQVRRKSSLRSHGSGKSSPRVGSGVFDERSGLAADRDGGEEGEVVVRIDGEGPCREPFA
FPGQDGGGAGNVSPNTDSTASTPRGASRTWSSWTNSPRSPPKVRRDGSVEFWKNDGGGDG
GSDGGGGRGGGGGGGGGRRACEAFSFKNRAPPRPSSSQASSPSLSPQPAHAAGAVDGGED
PPTRLIGNFLRKQKASGAELSLDLDMEELGRPSQLHAQPSFSNSLEREARVSFQPKRRVA
SSSDSDSDTGGSRRRGGDDGEVVRCTSSSTAAGHLMRAKTRSRLMDPPPQPQPASAVPPV
GDEERRSSVLRTPTKSGQFISGLMTGKSGQIAKSGPLEEEEEDPFMDEDIPDEFKRGKLD
AITILQWLSLVLIIAALACSLTIKALSGKKVWGLHLWKWELLVFVLICGRLVSGWVIRIA
VFCVERNFLLRKRVLYFVYGVRSAVQNALWLGLVLSSWHFMFDKNVQRETNSPVLPYVQK
ILFCFLVATLIRLVKTLLLKVLASSFHVNTYFDRIQEALFNQFVIETLSGPPLVDENQFL
AEMHELQRAGATIPAELRSTVPTKNLSGQRSIRMSGVIPKGEGSKQLSKEKGEHQIEEGI
TIDKLHKLNQKNISAWNMKRLMRIVRFGTLTTMDEQIQQATGEGDESATQIRSEYEAKIA
AKKIFHNVAKPGSKYIYLSDLLRFMRQEEAIKTMDLFEGAQEHSRVSKRSLKNWVVNAFR
ERKALALTLNDTKTAVNKLNQMVNVVVGIIVFALWLLILGIATTHFFVFLSSQVLVAVFV
FGNTLKTIFEAIVFLFVMHPYDVGDRCEIEDCQVVVEEMNIMTTVFLRYDNLKIYYPNSV
LATKPIMNYYRSPDMGEGIDFSVHVATPVEKLALMKERLLRYIDNKKDHWYPGAMIVLRD
VDDTNKLKVSIWLRHTLNWQDMGMRFVRRELVLQEMIKVLKDLDIEYRMLPLDVNVRNAP
PIQSTRMPTTWNYS*
>11670.m04620|LOC_Os04g47320.1|genepair1737-2
MDPRGKGSITSHASDKSSRSGSFDFEHDQDPDRDRRHDDAHRREVVVKIEPEAHVPVDLH
AGGSHAANAPGAGGVAVGGVVPGSGSVSSASSSPGGGGNGESFSFKNRPPQSPASPAMSV
GGEGSDDPPTRLIGSFLRKQAAAGGELALDPDLEMEEMRRPPRAPTSMNASRELRVSFQD
PHKRFSPSTSSASTSSYAGDSRNQACSTAEAAEVIRCTSMSTGNNLLARSKTRSRLMDPP
PPTISHPTEAERNDRKSFVGKGPPKSGQLRSGLIGKSGLIGLSGPIGKPGAFDDDDDDPF
VDEGLAADLKRDTVDCLLILEWVGLIVIMGALVCSLSIRSLANKKLSGLHLWKWELLVFV
LICGRLVSGWVIRICVFFVERNFLLRKKVLYFVYGVRRAVRNVLWLGLALISWHLLFDKD
AKRDSHTLVLPYVTKVLCCLLVATVIRLVKTLLLKVLASSFHVSTYFDRIQDALFNQYVI
ETLSGPPLVDESRMLAEVQRLQSAGINIPSELEATAMPSKPPMPAKSGRLTVNPSKRGGA
GGGANKQLQKQKSDRHCDDGITIDQLHRLSQKNISAWSMKRLMKIVRYGALTTMDEQIKH
ATGEDELATQIHSEYEAKVAAKRIFHNVAKPHSKHIYLSDLMRFMRQEEALKAMDLFEGA
QEHNRVSKRSLKNWVVSAFRERKALALTLNDTKTAVNKLHQMANVVVVVIVIALWLSILG

IATSRFFVFISSQLLVAVFMFGNTLKTIFEAIVFLFVMHPFDVGDRCEVDGMQVTILFIV
LSALYDCFTLSDLVLNCCEIQVVVEEMNIMTTIFLRYDNLKVYYPNSQLAIQPIMNYYRS
PDMGDAVDFSVHVATPVEKLALMKERLMHYLDNKKEHWYPGSMVVLRDVDDTNKLKVSIW
CRHTINFQDMGMRFERRELLLQEMIKILKDLDIEYRMLPLDINVRNAPMIQSLRMPSTWI
TY*
>11668.m04324|LOC_Os02g44820.1|genepair1738-1
MAEAELLLERSRAITLNGRDKRGRALVRIVGKYFPGKLSSASLVRSPQFRCFCRRKSDPV
SFVAAARALGGRAEAALRGYVRRRVLPEIGEREFVVVYVHSLVDRGDNFPGVAAIRAAYE
ALPAAAKERLRAVYFVHPGFQARLFFATLGRFLFSSGLYEKLRYMSRLEYLWEHVSKGEM
EVPECARRHDEELERRPLMDYGIEATDRRCMFDAASMDTSASLHSLRCIS*
>11670.m04621|LOC_Os04g47330.1|genepair1738-2
MTKQEILLEKKRVITVQGRDKAGRPIVRIVGKNFPARELGGGGHAEAALKGYVRRRVTPA
IGDAEFVVVYMHSGVDRRENFPGVGAVRTAYESMPAAVRERLHAVYFLHPGLQSRLFFST
LGRFLFSSGLYGKLRYVSRLEYLWAHVRKGELDVPEAVRRHDDELEQRPLMDYGIEASER
TSKNGPKPHCTPPSPPKHRRHLNHMNMARSNLLCCLGTRREDNARQRQTRPTPRPRLRRL
MSGMKRVFGRSPPCGQTAVAPDSGIVVVEPRRQTAARRVGKGARDGGVNNREEISREEAA
AATIQAGFRGHLARRAFRALRSLVKLQALARGSYVRKQAGVAIRFMKVLVRLQVRVRARQ
LLHRSKDQ*
>11668.m04327|LOC_Os02g44850.1|genepair1739-1
MGSHSFSYVLVALCLLGVAAEATQLAPAVFVFGDSTVDVGNNNYLNITKQARANYPKHGV
DFTGSTPTGRFSNGYNLADQLAQQLGFPMSPPAYLSLTAKTIVSQMYKGINFASGGSGLG
DKTGQGAGDVIPMFQQVQYFSKVVAMMQKLSGSRTTNTLLSKSIFLISTGSNDMFEYSLS
GGNGDDREFLLGFAAAYRSYVRALYRLGARKFSVVSITPLGCTPSQRARRLSEDGTRGCY
GPINTLSLRSYPTLAASLRDLADELPSMAYSLSDSFAMVSFIFANPRTNAWSFTELESGC
CGSGPFGALGCDETAPLCNNRDDHLFWDANHPTQAASAIAAQTLFTGNRTFVSPVNVREL
ALL*
>11670.m04628|LOC_Os04g47390.1|genepair1739-2
MASRRGAFALVAALCLLELARRGLAEEPAVPAMFVFGDSTVDVGNNNFLANCKANCKANY
PRYGVDYPFQSPTGRFSNGYNLADQLAQKLGFDKSPPPYLSLPDVTIISQMSKGINFASG
GSGLIDSTGWKVCTEVFNMSAQVQSFTSAVQKMGNGTADLISRSLIFINTGSNDLFEYTD
FPSNTTRNDTEFLQSLVASYKGHLKDLYGAGARKFSVVSPSLVGCCPSQRAVAHDTNDLD
FHGCSRAANGLSRQLYPMLGSMLRGLAADLPGMHYSLGDSVGMAELVLNGTVLPGANFTV
LDRPCCGGGVGGCNGTAPLCLDRGSYLFWDNFHPTAAASNVFARELFFDPGAFVHPMNVH
ELAELRP*
>11668.m04330|LOC_Os02g44880.1|genepair1740-1
MVVGFRRTISFPAPKAATAAKGEAYRVRSASLPCRFHPLVVQLDEDVATMRELVGRLASA
ASAGSVAGAAEQLGRVLVSLSELLHHPQAQEPLRRLGRSPFAERLLDNFLRLADAHGSFR
AALVALSALQAEARAALRREDPARLASAARALRRSGRDLPRIASSARAVAAKPPPPPPAG
LPADGTALAAAIADATAAVASASAAVFSGVSSLSIAAATARVEVAATPCWMPSPARFTTP
SATPRHHIITTKPSSLRIWWVADLMRWMSRAKRRSASKQHADSDASSSSTSSAATARPQP
NVAVDPDERERKAAFERLDNLGRCIADVESIGEKVFRALGMAFYEIKPRVQKGALRVHSS
CGRSRVSTLCVCEPEVPGSPDVAMYGIEFDRLQSRSASTRFRAERGPRPCGFEISRPSGG
PSPGVPVALILGGRKLPSCHAVVALEYDHASVQKERWLAGCPTGCVPPVPLAADYGCFHS
VSQLVYYTSCLYILLVRARQGHIPYLYGLWRNAQLFRAGSMGSKQCYLKVR*
>11670.m04636|LOC_Os04g47450.1|genepair1740-2
MAGYRRTISFPAPRPAAFAIGATARDKLAAAYHVRSASLPCRFHPLVVQLDDDVAALRLV
IGQSPPAAPSASSVSAAASQVGRVLVSLSELLHHPQAQEPLRRLGRSTFAERLLDDFLRL
ADAHGSFREALVGLSALQAETRAALRRGDPARLASAARAQRRAGRDLPRLAAAARAVVSK
SPAQLPEDLPADTAAIAAAVADATIAVASGSAAVFSGLSSLSNSAAAARVEVASTPCWVT
APARLTASSDEPSTSHHRIWWVADLVRWMSRAKRRSAKKQNDGGGDDGESSTVQLRSESR
MKPEEKARRAAFERHENLERCIASVDSSGEKVFRALVNTRVSLLNILSPSF*
>11668.m04332|LOC_Os02g44900.1|genepair1741-1
MGCKGSKLEQQEAVALCRGRADLLAAAVRHRYALAEAHAALADSLASMSASLHLVLAPAV
AAGTAVALPSARKDVDAAAEAAASPPHSSSHINFAPSSGSESGSVSSSPSRRVAAGHEQL
YQPSALPFPHYAYGYGYAPEPPFGYPPGSLQLYYARSRPPPPSVAVEQRAPASERVYFGS
FEPAQYHPYGGETRRADRAAAPPPSPPRASSWDFFNVFDNYEVYDNYCYDAPGTGATTPA
PYTPSRSSREVREEEGIPELEEDDAVVKEVSSEYSAHGSGGARSRRSSIGGVSSSIAEVD
EEENPVVDKGVVGGGVARQQTPAHGNVAASVPTPRRAADGADVAGEIKAQFVRAADAVRA
LAPILEVGRRSYHPRSSVYHVSSRMVSVIALPHSGYGGSDLLDVGGGEKVVGARNLSLTL
QKLYIWEKKLYDEVKAEEKMRLLLAKNSKRLKFLDQKGAEAPKIDATRNLVRKLSTKIRI
AVRVIAKVSKKINRSTCVVCLSESNCLNSEDILAFHIIFKSSLCQKKSLIAIFYRFVKMW
QDKLNCYHIQCQAISEAKNLDSIISGGTSRDLAMELELELIKWIVNFSSWVNEQRSFIKA
LNGWLALCLNYQQEETADGVPPYSPGRVGAPLVFVICNSWSQAMDRISEKEVITSMQALV
SSVRSLWEKQNVEQTEQLIAIREREKWNKILERKTLEINKEADTLNRKLALVPGRQSLLP
TAQTYQVHFLEANSVQVSLKRVLEALESYSSNSLRALDETLRHAEEERLSRERAKVS*
>11670.m04639|LOC_Os04g47480.1|genepair1741-2
MGCKGSKLEDQEAVALCRGRAELLAAAVRHRYALADAHGALADSLHSMAAPLHRLLLLQL
QASSPQLTLPTARKGGRPRTAAAAATLSLPHGRSAHLDDLGSPSGSETASPADSPLRAFP
EQQLPYPHYAYGYGTGPAFAYPPPPASSLQFYYARSRPPPPSVGVAQRAPVSTERVYYGS
FDPTSGYPQYYANGGVPATAAPQRMAAPAPPRSPPRESSWAFLNVFANYEPYDNYYYDST
AAAASAAAYTPSRSSREVREEEGIPELEEDEDDCVFKEVASGGYSAGSGGHRSRRSSIGS
LSSVAEQENAVIDNDVVASTSEIYRRPLAHRNVAMRALAQAAQRVAGNGGNVDVAGEIKA
QLVRAAEATRELAPLLEVGKPSYQEHSHASSRLMSSIPVPNLGCKGVDLVDIRGGGVMVD
SKSLSLTLEKLYFWERKLYGEVKFYAIMTCCRLVQKKFGPGCRQAEEKMRLLLAKNSKRL

KLLDQRGAEAHKIDATRNLLRKLSTKIKIAVRVIAKVSTKINKVRDEELGPQVNALIQGF
IKMWQYKLHSYHTQFQVISEAKNLVSVVSRENGPDLAMELELELIKWIINFSSWVNAHRN
FVRALNGWLALCLNYETGETTYGEPPYSPGRIGAPLVFIICNRWSQAMDQISEKDVVNAM
KALVSSVQHLWEQQNQEEGEERILAIRERERWMKMLEKKTLEVKREADELNKKLALVLRR
QSLHQRPTMQTYEAHCVEASSVHINLRLVLQALENFAANSLQAFQEILRQSDS*
>11668.m04333|LOC_Os02g44910.1|genepair1742-1
MRVNPALFLPLMAEYAAPTWAILISGFFMLLSVSLSMYLIFQHLSAYNNPEEQKFVLGVI
LMVPCYAVESYVSLVNPDTSVYCGILRDAYEAFAMYCFGRYITACLGGEERTIAFLKREG
GGDSGEPLLHGASEKGIIHHHFPVNYILKPWRMGVRFYQIIKFGIFQYVIIKTLTASLSL
ILQPFGAYCDGEFNLRCGYPYFAAVLNFSQYWALYCLVEWYTATKDELAHIKPLAKFLSF
KSIVFLTWWQGIMIAIMYSLGLMGIASVVHLYVFPAKPYSLLGNHRSPENISVLGDYAAT
DPVDPDEIKDISRPTKLRLPQLEPDEIIVTNVKESVRDFVIGSGEYVIKDLKFTMKQAVR
PVGKRFEKLMKKKGKFGQSRDDNWVSTSTPQRAIHGIDDPLICGSSSDSGIGRGKRHRRD
VRSEYCKCRIPQIVPCFKGPSVIEFLYRSARYCTRTQVHNSLCNFVKVADTNWMYAKLLG
STP*
>11670.m04645|LOC_Os04g47530.1|genepair1742-2
MRVNLRLLMPLLAQYTTPTWATLVAGFFVLLSLSLSIYLIFEHLSAYNNPEEQKFVLGVI
LMVPCYAIESYVSLINPNTSVYCGILRDGYEAFAMYCFGRYPYFAAVLNFSQYWALYCLV
EWYTATKDELAHIKPLAKFLSFKSIVFLTWWQGVVIAIMYSLGLLRSPLAQSLELKSSIQ
DFIICIEMGIASIVHLYVFPAKPYELQANQSPGNVSVLGDYVSSDPVDPFEIKESNRPAK
LKLPQLEPDERSTTNIKESVRDFVVGSGEYVIKDFKFTVNQAVRPVEKRFDKLMKKNKKS
QDDNWVSAVSPDRPVRGIDDPLLGGSTSDSGFTKGKKHRRAVSTVAAADSWGGGDLASDG
YEIRGRRWAVKN*
>11668.m04334|LOC_Os02g44920.1|genepair1743-1
MGNCFGSDDGEVEAVKVMARHALPQAAMARPVMVAIAQPNARVAMSPGRPPTGKLPSQAT
ATSTGGGRSVAGGGGRTNAGGDASAEGRILEAPNLRIFTFAELRAATRNFKADTVLGEGG
FGRVHKGWVDERTMSPARSGSGMAVAVKKLDPESLQGVQEWQSEVNFLGRLSHPNLVRLL
GYCWEDKELLLVYEYMAQGSLENHLFRSEPRKGGSASPQQPLSWSLRLRIAIGAARGLAF
LHSSEKHVIYRDFKASNILLDTQFHAKLSDFGLAKDGPAGGSSHVTTRVMGTYGYAAPEY
VATGHLYVKSDVYGFGVVLLELLTGLRALDAGRPSGQHHLVDWAKPFLSDRRKLARLMDP
RLEGQYSSRGAQRAAQLTLRCLAADHKNRPSMREVVAVLEEIESMSRGGSGGGAPGSASP
RPAARGGGGGGAHGYGQSPRPGSDWAGPAAGHPSPRVR*
>11670.m04654|LOC_Os04g47620.1|genepair1743-2
MGNCFGSEEAEAAATVRAPAQGHHGRRQDSDQISNLAAEAAAPANRRSPIMAPPKALASM
SSAAGHHSGMRSSSSMSSITTRSSSSSSNLPLGAAGGADVGVAGALYPEPEGRILEVPNL
RIFTFAELRAATRNFKPDSVLGEGGFGRVYKGWVDERTMSPARSGTGMVIAVKKLNPESV
QGLQEWQSEINFLGRLSHPNLVRLIGYCVEDRELLLVYEFMAKGSLENHLFRKGSAYQPI
SWNLCLRIAIGAARGLAFLHSSERQIIYRDFKASNILLDTHYNAKLSDFGLAKNGPTAGE
SHVTTRVMGTYGYAAPEYVATGHLYVKSDVYGFGVVLLEMLTGMRALDTGRPAPQHSLVE
WAKPYLADRRKLARLVDPRLEGQYPSRAAQQAAQLTLRCLSGDPRSRPSMAEVVQALVEI
ERIRSRPKAASSREDASPRVPTRSGGHGHHHHHHSSRPRSGSDGARSGYPSPRVR*
>11668.m04335|LOC_Os02g44930.1|genepair1744-1
MKGKADASKKGEGRLKAAGGAGKRKKAAASGKPKRPPSAFFVFMSEFRQEYQAAHPDNKS
VAAVSKAAGEKWRAMSEQEKAPYVDKAGQKKQDYEKTKANFDKKESTSSKKAKTHDDGEG
SDKSKSEVDDDQDGGSDEENEDDEE*
>11670.m04662|LOC_Os04g47690.1|genepair1744-2
MGKADADAEFKAAGKRKKAGGAGKPKRGLTPFFAFLAEFRPQYMEKHPNTKGVAAVTKAA
GEKWRAMSDEEKAQYGGKKPDGESKPAAASKKKESTSSKKAKTDGAEQEGEGSDKSKSDV
EDDENDGSGEDE*
>11668.m04340|LOC_Os02g44980.1|genepair1745-1
MGLGNEASSSSSRLLDPAPLLPHHGGDGAGKLSSQPKTFANVFIAVVGAGVLGLPYTFSR
TGWAAGSILLLSVAALTFYCMMLLVACRRRLADEHPKIASFGDLGDAVFRGPGRLAVDTM
LVLSQASFCVGYLIFISNTMAHLYPVFAPSSNALLSPKALFIWAMLPFQLGLNSIKTLTL
LAPLSIFADVVDLGAMGVVLGEDVSVWLAKPPPVFAFGGLSAILYGIGVSVYAFEGIGMV
LPLEAEAANKKKFGTTLGLSMGFIAVMYGLFGAMGYIAFGDATRDIITTNLGTGWLSAAV
QLGLCINLFFTMPVMMHPVYEVAERLLHGKRYCWWLRWLLVLAVGLSAMYVPNFTDFLAL
VGSSVCVLLGFVLPASFHLKVFGAEMSWSGVLSDVLLVLLGLSLAVFGTYTSLLQIFHSS
SA*
>11670.m04672|LOC_Os04g47780.1|genepair1745-2
MELKPQVASRPHAPKKWQLPSHSQFEPRIIVHLVHKPIQSDVTTPHKYATYKVVSREPGP
RCFFRGKVHEASWLRASPYYKRGTRTRRATRFPHLSDSELRRRLCAAAAAALVVCFPNSL
IPQRAITMGLHKEASSSSSRLDAAPLLPHHGHGGGGAGHHLSSQPKTFANVFIAVVGSGV
LGLPYTFSRTGWVAGSVLLLAVAALTFHCMMLLVACRRRLAYDHPKIASFGDLGAAVCGP
AGRHVVDAMLVLSQASFCVGYLIFISNTMAHLYPVGDSSPSSPLLTAKAIFIWVMLPFQL
GLNSIKTLTLLAPLSIFADVVDLGAMGVVLGQDVSTWLANKPPVFASAGPTEILYGLGVA
VYAFEGIGMVLPLEAEAADKRKFGGTLALSMAFIAVMYGLFGAMGYLAFGAATRDIITTN
LGTGWLSVTVQLGLCINLFFTMPVMMNPVYEVAERLLCRKRYAWWLRWLLVMVVGLMAML
VPNFADFLSLVGSSVCVLLGFVLPAAFHLKVFGAEVGWPGLAGDVAVIVVGTALAVSGTW
TSLAQIFSSSDVAMICIGPRIIFVLGLPLDGRVEEEDGWRCRRHRISWERVDGSGRQERC
VVTGEEKLEDRRTHPRPPQLPPAAHALRLSRGEPCLPLRIARQGLSRRDTYTSESLVVLE
AHEAAVPACLDKTEAVLSTDPPLTDFQGTLQIDVWFHGRRRAGASWRHANGSSSPFGKRL
VPHCHHVGRPIPPHA*
>11668.m04343|LOC_Os02g45010.1|genepair1746-1
MFEEVPASEHYNVAPAGTTLTTTARSFDHHARSDVSPIKYEIAGSPLYLGGTNTVLQVQD

MTMMSSTPACYGEHHHHHHHQLTKEGSCNHQQQEQHELAISPMASFLQQISSGSASVGVH
NSSLDYSGLGDQPDKICCQDGREMEASPFGMRSLPDLGSFAGYTPAIESTSVQPYMRCAN
SSDSNRQEQETVPARSSSSGSGAAATDRKKRKSEERQESTVKKSKQEASKASPPKQPVPK
VKLGEKITALQQIVSPFGKTDTASVLFETIKYIKFLHEQVQLLSEPYTNSSRSNKQGNSV
PWGDQAEASKGETMEHDLRNRGLCLVPVSWTPEVYRDGNAMDYWTPAYRGCLYR*
>11670.m04675|LOC_Os04g47810.1|genepair1746-2
MHGCALHPAAYRRIFHNPRSTRSHGLVAAASPPLHQPAAACGETHRIIQMHALFAVARDT
AFSSSPAADEHLPNEHPGGHLWNQSVLSREEEHGSNDDSFRESLVSLLDNTRDYVMAPEV
FEGVPVACDYLKGMDSDGMAASAVAAYSLDNNGQHASVSSIEHGIASSPLLAYQLGKNSA
VVQRSIQQQEVGSPMAAFLQQLIPTSVLDQSGIGFGGVCLDGSALEASFCMRTSPDVSSF
SGHRSATAEELMSTDTREQEITRLARSCSSSGSDRNKKKLSEVRGGGKAKKFKSETSHST
SSPKHQSPKVKLGEKITALQQIVSPFGKTDTASVLLETITYIKFLHEQIQLFSQPYMTNS
TNKGHIHWGGEGKRKAGLEHDLRGRGLCLVPVSWTSQEYCDSILPECWAPAYRNYFYR*
>11668.m04350|LOC_Os02g45070.1|genepair1747-1
MRPGKGTFGDRCIVKANHFFAELPDKDLHQYDVSITPEVPSRGVNRAVIGEIVTQYRQSH
LGGRLPVYDGRKSLYTAGPLPFTSRTFDVILQDEEESLAVGQGAQRRERPFKVVIKFAAR
ADLHHLAMFLAGRQADAPQEALQVLDIVLRELPTARYSPVARSFYSPNLGRRQQLGEGLE
SWRGFYQSIRPTQMGLSLNIDMSSTAFIEPLPVIDFVAQLLNRDISVRPLSDADRVKIKK
ALRGVKVEVTHRGNMRRKYRISGLTSQATRELSFPIDNHGTVKTVVQYFQETYGFNIKHT
TLPCLQVGNQQRPNYLPMEVCKIVEGQRYSKRLNEKQITALLKVTCQRPQERELDILQTV
HHNAYHQDPYAQEFGIRIDERLASVEARVLPPPWLKYHDSGREKDVLPRIGQWNMMNKKM
VNGGRVNNWTCINFSRHVQDNAARSFCRELAIMCQISGMDFSIDPVVPLVTARPEHVERA
LKARYQEAMNILKPQGGELDLLIAILPDNNGSLYGDLKRICETDLGLVSQCCLTKHVFKM
SKQYLANVALKINVKVGGRNTVLVDALTRRIPLVSDRPTIIFGADVTHPHPGEDSSPSIA
AVVASQDWPEVTKYAGLVSAQAHRQELIQDLFKVWKDPQRGTVSGGMIRELLISFKRATG
QKPQRIIFYRDGVSEGQFYQVLFYELDAIRKACASLEADYQPPVTFVVVQKRHHTRLFAN
NHKDQRTVDRSGNILPGTVVDSKICHPTEFDFYLCSHAGIQGTSRPAHYHVLWDENKFTA
DGLQTLTNNLCYTYARCTRSVSIVPPAYYAHLAAFRARFYMEPDTSDSGSMASGAHTRGG
GPLPGARSTKPAGNVAVRPLPDLKENVKRVMFYC*
>11670.m04685|LOC_Os04g47870.1|genepair1747-2
MVKKKRTGSGSTGESSGEAPGAPGHGSSQRAERGPQQHGGGRGWVPQHGGRGGGQYQGRG
GHYQGRGGQGSHHPGGGPPEYQGRGGPGSHHPGGGPPDYQGRGGSGSHHPGGGPPEYQPR
DYQGRGGPRPRGGMPQPYYGGPRGSGGRSVPSGSSRTVPELHQAPHVQYQAPMVSPTPSG
AGSSSQPAAEVSSGQVQQQFQQLATRDQSSTSQAIQIAPPSSKSVRFPLRPGKGTYGDRC
IVKANHFFAELPDKDLHQYDVSITPEVTSRGVNRAVMFELVTLYRYSHLGGRLPAYDGRK
SLYTAGPLPFASRTFEITLQDEEDSLGGGQGTQRRERLFRVVIKFAARADLHHLAMFLAG
RQADAPQEALQVLDIVLRELPTTRYSPVGRSFYSPNLGRRQQLGEGLESWRGFYQSIRPT
QMGLSLNIDMSSTAFIEPLPVIDFVAQLLNRDISVRPLSDSDRVKIKKALRGVKVEVTHR
GNMRRKYRISGLTSQATRELSFPVDDRGTVKTVVQYFLETYGFSIQHTTLPCLQVGNQQR
PNYLPMEVCKIVEGQRYSKRLNEKQITALLKVTCQRPQERELDILRTVSHNAYHEDQYAQ
EFGIKIDERLASVEARVLPPPRLKYHDSGREKDVLPRVGQWNMMNKKMVNGGRVNNWACI
NFSRNVQDSAARGFCHELAIMCQISGMDFALEPVLPPLTARPEHVERALKARYQDAMNML
RPQGRELDLLIVILPDNNGSLYGDLKRICETDLGLVSQCCLTKHVFKMSKQYLANVALKI
NVKVGGRNTVLVDALTRRIPLVSDRPTIIFGADVTHPHPGEDSSPSIAAVVASQDWPEVT
KYAGLVSAQAHRQELIQDLFKVWQDPHRGTVTGGMIKELLISFKRATGQKPQRIIFYRDG
VSEGQFYQVLLYELDAIRKACASLEPNYQPPVTFVVVQKRHHTRLFANNHNDQRTVDRSG
NILPGTVVDSKICHPTEFDFYLCSHAGIQGTSRPAHYHVLWDENKFTADELQTLTNNLCY
TYARCTRSVSIVPPAYYAHLAAFRARFYMEPETSDSGSMASGAATSRGLPPGVRSARVAG
NVAVRPLPALKENVKRVMFYC*
>11668.m04351|LOC_Os02g45080.1|genepair1748-1
MGISGERKGAARQYNRSKVPRLRWTAELHRSFVRAIDCLGGQQKATPKLILQLMDVRGLT
ISHVKSHLQMYRGTRHGIGQNDMQPQLHLKQHSFGSDEQSPKEFMCPPIKRAKVGTEASG
KYRCMEGSSDMRSSAPPAGTRYFIDDCMRLQEVSMDRRRSDQHDAAAAARARAPAAAASS
LHQALGFWVQGRREEPFMVHQISKPKAHQLNHMVRNMKISCKENHESRFFMSLVMVLLLI
SADLVSRETPSKL*
>11670.m04687|LOC_Os04g47890.1|genepair1748-2
MGSGGGGCGRNGAVRQYIRSKVPRLRWTGELHCSFVQAIEFLGGQDKATPKLILQLMGVK
GLTISHVKSHLQMYRCSRLGSHGTGRRSEMQPQLQRKHSCGADEQVPREFLCPPLKRTRM
GTEATYKGMQGSQGISEMRTTGTQYCIDDYMQAMAMERRIKEEGLRWQRDAAAAAAADGG
AAASNLQTVGCSVQESDPFKIIKPEVHHLGPVLKLQCSKVENSGFISSSTGTAARDQPEP
PPLEKCSLSLSLGPDPKCMPAIASSPSESSCILSSSSRSFSDCSGNSGCLVAPGVNLELS
MSICGS*
>11668.m04353|LOC_Os02g45100.1|genepair1749-1
MSRAVDAQQEPTEGRRFYSPDRPEGLAFPTSYRALYDLPTSPECLFEEDKFRQTRTWGEN
LTFYTGVSYLAGATSGALVGLRRAAAEAERGESAKLRINRALNQSGSVGRAFGNRFGIVA
MLFAGTESFVRDQRDGADDWVNTVAAGASAGALYRIASGPRSMIVAGILGGVLSGAAVAG
KPMLQRFAPKLSARLDYLR*
>11670.m04688|LOC_Os04g47900.1|genepair1749-2
MAAADQPAYGDRRPSRRTYKPDQPEGLTISFRELYDLPTSPEFLFHEEALRSRRTCGEDL
TFYTGCGYLVGRAAGAAAGLKRAAEEAERGESMKLRGQPRPQPVRLPRARVRQPARRRRA
ALRGDREHRGGPPRRRRLGQHRRRRDRYRRALPRGCRPAGGDRRQLRRGAHGRRGGRGEA
SADEIRA*
>11668.m04359|LOC_Os02g45160.1|genepair1750-1
MDAAAREAQVQGSLEWRVTVPEGSSVTVEHEAGVAERAWAWVVRMLVAVRAAVAGFARKV

WKIGADDPRRAVHSLKVGLALTLVSIVYYTRPVYDGVGGNAMWAVMTVVVVFEYTVGGCM
YKGFNRAVATASAGLLALGVNWVADKSGDKLEPFILSGSLFLLAAAATFSRFIPTVKARF
DYGVTIFILTFSLVAVSGYRVDQLLDLAQQRMSTIGIGIVICLAVCVVIWPVWAGQELHL
LTVRNMEKLAGAVEGCVEDYFAAKPAAAKSEGYKCVLNSKASEDSQANLARWEPPHGRFG
FRHPYAQYTKVGAAMRHCAYCVEALNSCVRAEVQAPEHVKRLLGDVCTRLASQCARVLRE
ASTSVAAMTSPKTLDFAVADMNTAVHELQGDLRALPPVLALEPAAEMSLMDAMPLFTVAS
LLIEISARIEGVVDAVETLASLASFKQVEDDDDKKGQTEMKVHPLNVPDDHDASTHESQT
TTKHPEQV*
>11670.m04691|LOC_Os04g47930.1|genepair1750-2
MAAVANGGAAPEWRVTVPEGATVTVEREAGRCRRAAVVWAWQLVSCVAALGSRASGLAGR
VWKIGADDPRRAVHGVKVGLALALVSVFYYTRPLYDGVGGAAMWAVMTVVVVFEFTVGGC
VYKGFNRATATVSAGAVALGVHWIASKSGDKLEPVVRSGSVFLLAAAATFSRFIPTVKAR
FDYGVTIFILTYSLVAVSGYRVDALVAMAQQRVSTIAIGIFICLAVCLLICPVWAGQELH
RLTARNMDKLAGAVEACVEGYFVAGEEEAAGPEYKRRPAAAAAAEGYKCVLNSKASEDAQ
ANLARWEPAHGRFGFRHPYAQYKAVGAAMRHCAYCVEALSGCIRSAEAQSPEGVNRHLAG
ASTRVATRCAAVLREASSSVAAMTTPSRGLDFAVADMNTAVQELQSEVRELPSKLAAAAG
EPAAAQQLMDAVQLLTVTSLLIEVSARIEGVVDAVDTLATLAAFRSADDDDDDDDEKPTT
EADAKLQTVSDHVTVEPEAAAAAARTTKNIEQV*
>11668.m04361|LOC_Os02g45180.1|genepair1751-1
MAKLYVQAVPPPDLNRNTEWFMYPGVWTTYICILFFSWLLVLSVFGCTPGMAWTVVNLFH
FAITYHFFHWKKGTPFADDQGMYNTLTWWEQMDNGKQLTRNRKFLVVVPVVLYLIASHTT
DYQHPMLFLNTLAVAVLVVAKLPNMHKVRIFGINAGN*
>11670.m04696|LOC_Os04g47970.2|genepair1751-2
MAKLYVQAVQPADLNKNTEWFMYPGVWTTYILILFFSWLLVLSVFGCTPGMAWTFVNLAH
FAMTYHFFHWKKGTPFADDQGMYNRLTWWEQMDNGKQLTRNRKFLTVVPLVLYLIALHTT
DYQHPMLFLNTIAVVVLVVAKLPNMHKVRIFGINAGN*
>11668.m04363|LOC_Os02g45200.2|genepair1752-1
MWGLGLVKPMEEMLMGANPNPNGSSNQPPPPPSSAASAQRPIAPPAAGAAAGAGAAGAGA
GTERRARPQKEKALNCPRCNSTNTKFCYYNNYSLQQPRYFCKTCRRYWTEGGSLRNVPVG
GGSRKNKRSSSSVVPSAAASASTSAAVSGSVPVGLAAKNPKLMHEGAQDLNLAFPHHHGR
ALQPPEFTAFPSLESSSVCNPGGNLAAANGAGGRGSVGAFSAMELLRSTGCYVPLPQMAP
LGMPAEYAAAGFHLGEFRMPPPPQQQQQQAQTVLGFSLDTHGAGAGGGSGVFGACSAGL
QESAAGRLLFPFEDLKPVVSAAAGDANSGGDHQYDHGKNQGGGGGVIGGHEAPGFWNSSM
IGNGSSNGGGGGGSW*
>11670.m04698|LOC_Os04g47990.2|genepair1752-2
MDAAHWHQGLGLVKPMEEMLMAANAAAGANPNPAATAPSSVTGGALRGGGGGGAPPVAGG
AGAGSTERRARPQKEKALNCPRCNSTNTKFCYYNNYSLQQPRYFCKTCRRYWTEGGSLRN
VPVGGGSRKNKRSSSSAASASPASASTANSVVTSASMSMSMASTGGGASKNPKLVHEGAQ
DLNLAFPHHGGLQAPGEFPAFPSLESSSVCNPGGPMGTNGRGGGALSAMELLRSTGCYMP
LQVPMQMPAEYATPGFALGEFRAPPPPPQSSQSLLGFSLDAHGSVGGPSAAGFGSSAGLQ
GVPESTGRLLFPFEDLKPTVSSGTGGGGASGGGAGVDGGHQFDHGKEQQAGGGGGGPGGH
DTPGFWNGMIGGGSGTSW*
>11668.m04368|LOC_Os02g45240.1|genepair1753-1
MATPRSPSPRGDARLDSAPLLGGGGGGGGGRRHGGALRRPSLRGAARFLRRSGRRAMREPS
LLVREAAADQLEERQADWAYSRPVVALDLLWNLAFILVAAAVLALSREESPSMPLRLWIV
GYAVQCVLHMACVAIEYRMRRGQSGESPMAADEETGTDGSSSSSDEDAGERAPRGRNGDY
VRIAKHLESANTMFSFIWWIIGFYWVSAGGQVLTHDAPQLYWLCIVFLAFDVFFVVFCVA
LACIIGIAVCCCLPCIIAILYAVSDQEGASEDDIRQIPRYKFRRMDEPEKQSVNMTGSSG
GIMIECGTNQPIEKVLAAEDAECCICLSAYDDGAELRELPCGHHFHCVCIDKWLHINATC
PLCKFNVRKNSSSSGSEEV*
>11670.m04706|LOC_Os04g48050.1|genepair1753-2
MREPSVLVREAAAEHLEERQADWAYSRPVVALDLLWNLAFITVAAVVLVLSRGEDSPMPL
RTWVAGYALQCVVHMVCVAIEYRMRRGQRDRAPASADEERGSDGSSSSSDDDVTEDDRRG
SCTDCVSIAKHLESANTMFSFIWWIIGFYWISAGGEDVIRDAPQLYWLCIVFLAFDVFFV
VFCVALACIIGIAVCCCLPCIIAILYAVSDQEGASEDDIRQIPRYKFRRTDEPEKQTADE
TGPFGGIMTECGTNQPIEKVLAPEDAECCICLSAYDDGAELRELPCGHHFHCACIDKWLH
INATCPLCKFNIRKSGSSSGSEEV*
>11668.m04369|LOC_Os02g45250.1|genepair1754-1
MSFGGLFDGGGGGGMQFPFASGFASSPALSLALDNAGGGIGGRMLGGGAGAGSSAGGAMT
RDTEAENDSRSGSDHLDAISAAGEDDVEDAEPSNSRKRKKRYHRHTPQQIQELEALFKEC
PHPDEKQRAELSRRLSLDARQVKFWFQNRRTQMKTQLERHENALLKQENDKLRAENMTIR
EAMRSPMCGSCGSPAMLGEVSLEEQHLRIENARLKDELNRVCALATKFLGKPISLLSPPP
LLQPHLSLPMPNSSLELAIGGIGGLGSLGTLPGCMNEFAGGVSSPMGTVITPARATGAAI
PSLVGNIDRSVFLELAISAMDELVKMAQMDDPLWVPALPGSPSKEVLNFEEYLHSFLPCI
GMKPAGYVSEASRESGLVIIDNSLALVETLMDERRWSDMFSCMIAKATVLEEVSTGIAGS
RNGALLLMKAELQVLSPLVPIREVTFLRFCKQLAEGAWAVVDVSIDGLVRDHNSGTAPTG
GNVKCRRVPSGCVMQDTPNGYCKVTWVEHTEYDEASVHQLYRPLLRSGLAFGARRWLATL
QRQCECLAILMSSATVTANDSTAISQEGKRSMLKLARRMTENFCAGVSASSAREWSKLDG
ATGSIGEDVRVMARKSVSEPGEPPGVVLSAATSVWVPVAPEKLFNFLRDEQLRAEWDILS
NGGPMQEMTQIAKGQRDGNSVSLLRASAVSANQSSMLILQETCTDASGSIVVYAPVDIPA
MQLVMNGGDSTYVALLPSGFAILPDGPRIGATGYETGGSLLTVAFQILVNNQPTAKLTVE
SVETVNNLISCTIKKIKTALQCDA*
>11670.m04708|LOC_Os04g48070.1|genepair1754-2
MQFPFSGAGPGVFTSSPALSLALADAVAGRNSGGGGKMVTAAHGGVGGGGGGGRAKARDA

LEVENEMSRSGSDHLDVVSCGDAGGGGGDDDDDEDAEHGNPPKRKKRYHRHTPQQIQELE
AMFKECPHPDEKQRAELSKRLGLEPRQVKFWFQNRRTQMKMQLERHENSLLKQENDKLRS
ENLSIREATSNAVCVGCGGPAMLGEVSLEEHHLRVENARLKDELSRVCALAAKFLGKSIS
VMAPPQMHQPHPVPGSSLELAVGGIGSMPSATMPISTITDFAGAMSSSMGTVITPMKSEA
EPSAMAGIDKSLFLELAMSAMDELVKMAQMGDPLWIPGASVPSSPAKESLNFEEYLNTFP
PCIGVKPEGYVSEASRESGIVIIDDGAALVETLMDERRWSDMFSCMIAKASTTEEISTGV
AGSRNGALLLVSDEHSVMQAELQVLSPLVPIREVKFLRFSKQLADGVWAVVDVSADELMR
DQGITSASSTANMNCRRLPSGCVLQDTPNGFVKVTWVEHTEYDEASVHPLYRPLLRSGLA
LGAGRWIATLQRQCECLALLMSSIALPENDSSAIHPEGKRSMLKLARRMTDNFCAGVSTS
STREWSKLVGLTGNIGEDVHVMARKSVDEPGTPPGVVLSAATSVWMPVMPERLFNFLHNK
GLRAEWDILSNGGPMQEVTSIAKGQQNGNTVCLLKASPTKDKQNSMLILQETCADASGSM
VVYAPVDIPAMHLVMSGGDSSCVALLPSGFAILPAGPSIGADHKMGGSLLTVAFQILANS
QPSAKLTVESVETVSNLISCTIKKIKTALHCDV*
>11668.m04372|LOC_Os02g45280.1|genepair1755-1
METLVCVAVWAVAMAMVVASVMWAYRWSHPRANGRLPPGSLGLPLLGETLQFFAPNTTCD
ISPFVKERLNRYGSIFKTSVVGRPVVVTADPEMNYYVFQQEGKLFESWYPDTFTEIFGRD
NVGSLHGFMYKYLKSLVLRLYGQENLRAVLLDETDRACRTSLASWAAQPSVELKDSISAM
IFDLTAKKLISYEPSKSSENLRKNFVAFIRGLISFPVDIPGTAYHECMKGRRNAMKVLKK
MMRERMEEPGRQCEDFFDVLIEELGREKPVLTEGIALDLMFVLLFASFETTSLALTLGVR
LLAENPTVLDALTEEHEAIVRGRKEGCDAAGLTWADYKSMTFTSQVTLEMVRLANIVPGI
FRKALQDIEFKGYTIPAGWGVMVCPPAVHLNPEIYEDPLAFNPWRWQMQLTLVVLMLSGL
SVGLIPKIYRVQQDKVEITGGSKHFMAFGGGLRFCVGTDLSKVLIATFIHHLVTKYRWKT
VKGGNIVRTPGLSFPDGFHVQFFPKN*
>11670.m04721|LOC_Os04g48170.1|genepair1755-2
MQPYLQLASLRLATTIPLAPRLYDANLLAASGAAMASSMAYIALLCAALAAVVALLRWAY
RWSHPRSNGRLPPGSLGLPVIGETLQFFAPNPTCDLSPFVKERIKRYGSIFKTSVVGRPV
VVSADPEMNYYVFQQEGKLFESWYPDTFTEIFGRDNVGSLHGFMYKYLKTLVLRLYGQEN
LKSVLLAETDAACRGSLASWASQPSVELKEGISTMIFDLTAKKLIGYDPSKPSQVNLRKN
FGAFICGLISFPLNIPGTAYHECMEGRKNAMKVLRGMMKERMAEPERPCEDFFDHVIQEL
RREKPLLTETIALDLMFVLLFASFETTALALTIGVKLLTENPKVVDALREEHEAIIRNRK
DPNSGVTWAEYKSMTFTSQVIMEIVRLANIVPGIFRKALQDVEIKGYTIPAGWGIMVCPP
AVHLNPEIYEDPLAFNPWRWQGKPEITGGTKHFMAFGGGLRFCVGTDLSKVLMATFIHSL
VTKYSWRTVKGGNIVRTPGLSFPDGFHIQLFPKN*
>11668.m04375|LOC_Os02g45310.1|genepair1756-1
MGVRPAATKLNISPAAARRPSSFLPIATVALLCSASYFLGAWQHGGFSSPSASPSSVSVA
TAVACTTTTTATTRSATRPRKRTPAGQGQALDFSAHHAAAADGAVLSSSGDSAATRRYQA
CPARYSEYTPCEDVKRSLRYPRERLVYRERHCPTGRERLRCLVPAPSGYRNPFPWPASRD
VAWFANVPHKELTVEKAVQNWIRVDGDKFRFPGGGTMFPHGADAYIDDIGKLIPLHDGSV
RTALDTGCGVASWGAYLLSRDILAMSFAPRDSHEAQVQFALERGVPAMIGVLASNRLTYP
ARAFDMAHCSRCLIPWHLYDGLYLIEVDRVLRPGGYWILSGPPINWKKYWKGWERTKEDL
NAEQQAIEAVARSLCWKKIKEAGDIAVWQKPANHASCKASRKSPPFCSHKNPDAAWYDKM
EACVTPLPEVSDASEVAGGALKKWPQRLTAVPPRISRGSIKGVTSKAFVQDTELWRKRIQ
HYKGVINQFEQKGRYRNVLDMNAGLGGFAAALASDPLWVMNMVPTVGNSSTLGVVYERGL
IGSYQDWCEGMSTYPRTYDLIHADSVFTLYKNRCEMDIILLEMDRILRPEGTVIIRDDVD
MLVKVKSAADGMRWDSQIVDHEDGPLVREKILLVVKTYWTAKEQDQ*
>11670.m04727|LOC_Os04g48230.1|genepair1756-2
MAVGATATKLHMPSAGGRRPSLFHLAAVAVLCTVSYLIGIWHHGGFSASPAGGVASSVSI
ATTASVSCVSPTPTLLGGGGGGGDSSSSAPLDFAAHHTAEGMEVASGQVHRTYEACPAKY
SEYTPCEDVERSLRFPRDRLVYRERHCPSEGERLRCLVPAPQGYRNPFPWPTSRDVAWFA
NVPHKELTVEKAVQNWIRVEGEKFRFPGGGTMFPHGAGAYIDDIGKIIPLHDGSIRTALD
TGCGVASWGAYLLSRNILAMSFAPRDSHEAQVQFALERGVPAMIGVLSSNRLTYPARAFD
MAHCSRCLIPWQLYDGLYLAEVDRILRPGGYWILSGPPINWKKHWKGWQRTKEDLNAEQQ
AIEAVAKSLCWKKITLKEVGDIAIWQKPTNHIHCKASRKVVKSPPFCSNKNPDAAWYDKM
EACITPLPEVSDIKEIAGGQLKKWPERLTAVPPRIASGSIEGVTDEMFVEDTKLWQKRVG
HYKSVISQFGQKGRYRNLLDMNARFGGFAAALVDDPVWVMNMVPTVGNSTTLGVIYERGL
IGSYQDWCEGMSTYPRTYDLIHADSVFTLYKDRCQMDNILLEMDRILRPEGTVIIRDDVD
MLVKIKSITDGMRWNSQIVDHEDGPLVREKLLLVVKTYWTLGEEKE*
>11668.m04377|LOC_Os02g45320.1|genepair1757-1
MPESCVAAVLLYLDPPEICQVARLNRAFRGAASADCVWAGKLPVNYRYLLAFAAAADDEG
GDGGHGNGKRSSPSSKKDIFARLCRPTPFDFGNKEFWIDKNKGGICLSISSKAMVITGID
DRRYWSQLATEESRFHHIAYLQQIWWLEVDGELDFCFPAGSYSIFFRLHLGRPYRRMGRR
ICGTEQVHGWEAKPTRFQLSTSDEQHATSEYYLEQEGSWILYHVGDFVVLNSDELMKLKF
SMLQIDCTHTKGGLCVDSVLIYPKGYRHEKANIVHM*
>11670.m04732|LOC_Os04g48270.1|genepair1757-2
MGAGASSMMGPEGYGRGWGQTSLGDMPESCVAAVLLYLDPPEICKVARLNRAFRGAASAD
CVWAAKLPANYRYLAALAAAADDDSGGDGATEGNGSRCSSAAMIKKEIYARLCRPTPFDG
GTKEFWMEKNKGGLCISISSKAMAITGIDDRRYWSHLSTEESRFHHVAYLQQIWWLEVAG
EIDFCFPAGSYSLFFRLQLGRPHKYMGRRVYGYESIHGWNIKPTRFQLSTSDDQQATSQY
YLNEPGNWILYHVGDFVVSSSDQLTNLKFSMMQIDCTHTKGGLCVDSVFIYPKGHRHEDC
TICK*
>11668.m04383|LOC_Os02g45380.1|genepair1758-1
MSSPRRDGRGAVDDLTASLLHKGDGGEAVFVVVVVPPVAEEEEPPPVLTCKPPGRFARAV
KEAWSVPFPMMPSMSAGAAGAEARSILGLALPMILTGLLLYLRSMISMLFLGRLGGLALA
GGSLAIGFANITGYSVLSGLAMGMEPICGQAFGAGHYDLLGVTMQRTVLLLVAASVPIAG

LWVHMRPLLLLCGQDAAIAAVAETYILASLPDLLLQAFLHPVRIYLRTQSINLPLTVCAA
LAIALHLPINYVAVSVLGLGIKGVALASVLANLNLVLFLFGYIWFKGVHKRTGGFALSAD
CLRGWGELVSLALPSCISVCLEWWWYEIMILLCGLLANPQATVASMGILIQTTSLIYIFP
SSLGFGVSTRVSNELGANRPERACRAATVGLMLGFAFGGVASAFACHVRGAWATMFTADP
AIVALTASVLPILGACELGNCPQTTGCGVLRGSARPKDAASINLRSFYLVGTPVALILAF
WYHYDFRGLWLGLLAAQATCVVRMLLVIGETDWTAEAKRAQQLTGAADIKDCGGKGDHVA
VIEQPDEQC*
>11670.m04735|LOC_Os04g48290.1|genepair1758-2
MTTCADDQTGCAFFAPLLSSKGAEVVILVAGDEAEEQQPAPVLTSKPPGRLAKAVNEAWS
VSLGVAFPVTPSMFTCSARGEARSILGLAFPMILTGLLLYLRSMISMLFLGHLGGLALAG
GSLAIGFANITGYSVLSGLAMGMEPICGQAFGAGNYALLGVTMQRTVLLLIAAAIPIGGL
WVQMRPLLLFCGQDAAIAAVAETYIFASLPDLVLQAFLHPVRIYLRTQSINLPLTVCAGL
AIAIHLPINYVLVVVLGLGVKAVALASVLANLNLVLFLLAYIFLKGVHKRTGGFLLSAES
FRGWGELISLALPSCVSVCLEWWWYEIMILLCGLLLNPQATVASMGILIQTTSLIYIFPS
SLSFGVSTRVSNELGAGQPEEASRAATVGLVLGFGFGAFASAFAFLVRNVWASMFTADPA
IVALTASVLPILGLCELGNCPQTTGCGVLRGSARPKDAASINLRSFYLVGTPVALVMAFW
FHLDFRGLWFGLLAAQATCTVRMLLVIGRTDWAAEAKRSKQLTGAGAANMESDDRVAADE
KSRLPVDTDVERSSDHTDRC*
>11668.m04384|LOC_Os02g45390.1|genepair1759-1
MLGSGLNLVTTVIGFGMSATFIVFVCARLICGRAARADAEADAAEGRVAAARAMAAGPAP
FDFDVEFRATDLDRTIEHTRSGLEPFVVAAIPTMKYSYEAFQSKDDAQCSICLGEYNEKE
ILRIMPKCRHNFHLSCIDVWLQKQTTCPICRISLKDLPSGKPAESPVRSLPQLFSHPESS
KRRAFFQQQGSTYTALAQNCKEFVYDRTGF*
>11670.m04737|LOC_Os04g48310.1|genepair1759-2
MLGSGLNLVSAALGFGMTAAFVAFVCARFVCCRARRADASASRPHPSPVDFDADFPSDFD
RPIEHSRSGLEPLAVAAIPTMKYNCEAFHSEDDTQCSICLSEYKEKDILRIVPICHHNFH
LYCLDAWLLKQTTCPICRISLKELPDGKSTVSSAPTMSQPPTLPESSVNPTSHFLPVHQE
HRSHQDGPDMPESVEVVIEIRQ*
>11668.m04387|LOC_Os02g45420.1|genepair1760-1
MADLTEHPSPTAPPQVQTAGLPAAASPGPASPHSPSEQGDKTAPGAATASTMTTASSGEP
SPRSSGKHAFYRGIRCRSGKWVSEIREPRKARRIWLGTYPTAEMAAAAYDVAARALRGAD
AVLNFPGATASRPVPASASPADIRAAAAAAAAAAHLERPHGPTGTAYPATAAAEHHQQQ
QQQQYGSGSPAADDVSGYPPMEGGIGNDDFMDEEAIFELPQLLRNMAAGMMMSPPRLSPT
TSDVSPEPSEAGESLWSYRDP*
>11670.m04739|LOC_Os04g48330.1|genepair1760-2
MAAVAYDVAARALRGPDAALNFPDLAASRTAPPASSSADDIRAAAAAAAAASLQHDRAGGG
IAPAASGSAHQQRGGSSAAARTTAGSGGAQQEGSSGAGAGSHQYFLDEEALFETPQFLRN
MAAGMMMSPPRLSPSSSDDSPDPSEAGGSLWSYRDP*
>11668.m04390|LOC_Os02g45450.1|genepair1761-1
MDVSAALSSDYSSGTPSPVAADADDGSSAYMTVSSAPPKRRAGRTKFKETRHPVFKGVRR
RNPGRWVCEVREPHGKQRIWLGTFETAEMAARAHDVAALALRGRAACLNFADSPRRLRVP
PIGASHDDIRRAAAEAAEAFRPPPDESNAATEVAAAASGATNSNAEQFASHPYYEVMDDG
LDLGMQGYLDMAQGMLIDPPPMAGDPAVGSGEDDNDGEVQLWSY*
>11670.m04741|LOC_Os04g48350.1|genepair1761-2
MEWAYYGSGYSSSGTPSPVGGDGDEDSYMTVSSAPPKRRAGRTKFKETRHPVYKGVRSRN
PGRWVCEVREPHGKQRIWLGTFETAEMAARAHDVAAMALRGRAACLNFADSPRRLRVPPL
GAGHEEIRRAAVEAAELFRPAPGQHNAAAEAAAAVAAQATAASAELFADFPCYPMDGLEF
EMQGYLDMAQGMLIEPPPLAGQSTWAEEDYDCEVNLWSY*
>11668.m04394|LOC_Os02g45480.2|genepair1762-1
MDIETDGRFGNKRVHHRLGPANGAASSSTSGKVCIHWRAGRCNRFPCPYLHSELPEATAK
RPSQSGGGGNVWRNPHSGGGGGRGAGGAGGPNKWGRGPGGADGGPRHKVPDRPCRYFLAG
DCSYGEKCRYPHSYSMSDSITMLTPLQGHEKVVTGIALPAGSDKLYSGSKDGTVRMWDCQ
TGQCAGVINMGREIGCMISEGPWLFVGIPDAVKVWNMQTQAEMNLTGPTGQVYALAVGNE
LLFAATQDGRILAWRFSAATNGFEPAASLVGHQLAVVSLVVGAMRLYSASMDKTIRVWDL
ATLQCIQTLSDHTGVVMSVLCWDQFLLSCSLDQTIKVWAATESGSLEVTYTHKEEHGALA
LSGMPDAQSKPVLLCSLNDNTVRLYDLPSFSDRGRIFSKQEIRAIQVGPSGLFFTGDGTG
ELKVWQWVIDGSQTK*
>11670.m04743|LOC_Os04g48370.1|genepair1762-2
MAVRPNDSRPSDVRPPFSQIPRFRGGRKGQHQMEEPSLEVNNPVAELNAIKFSLMTSSDM
EKLSSATIIEMCDVTNAKLGLPNGAPQCATCGSRSIRDCDGKKKLTGKLLGHFGVIKLAA
TVHNSYFIEEVVQLLNQICPGCLTLKQNGDTKKADGTTIQGTCKYCSKDGSKLYPSIIFK
MLTSPRVTLSRSKLHRNTSVMDKMSIIAEVAGGVAHKSKNKAPHETLPQDFWDFIPDDNQ
PPIFNVTKKILSPYQVFHMLKKLDPELINQDDRTKAYKRMVDLYSKKSDDESSASTDTYG
TKWLKDIILSKRSDNAFRSIMVGDPKINLNEIGIPMGLALNLVVSEQVSSYNFETINLKC
NLHLLTKEVLLVRRNGNLIFVRKANQLEIGDIAYRLLQDGDLVLVNSPPSVHQHSLIALS
AKLLSTQSAVSINPLCCDPFKGDFDGDCLHGYIPQCLQSRIELEELVGLSGQLLNQQDGR
SLVSLTHDSLAAAHQLTNADVFLEKAEFQQLQMLSSSISLTPMPSVFKSTNSQGPLWTDL
YLFSDHYSRRKLSEEVHLALDEAEEAFQIKQILLNSVSIPNLKYYDGGDDRSNTDEQSGF
TQVSLPIIRSSMTSFKSVFNDLLKMVQQYVSKDNSMMTMINSGSKGSVLKFVQQTACVGL
QLPASKFPFRIPSQLSCVSWNRHKSRNCEITDGTSECVGGQDMYAVVRNSFLDGLNPLEC
LLHAISGRANFFSENADVPGTLTRKLMYHLRDTYVAYDGTVRSSYGQQIVRFSYDTADGM
YSDHDLEGEPGAPVGSWAACSISEAAYGALDHPVNSLEDSPLMNLQEVLKCHKGTNSLDH
TGLLFLSKHLRKYRYGFEYASLEVKDHLERVDFSDMVDTETMKIKRLRLEFIVREIIDQY
NTLRKQLNNAIPSVSISNSETLHLKMENKSGKLGKNLGTGNECVKNQTCCVTMVVQVEIN

SMSQLDVIKERVIPSILATLLKGFLEFKNVKVQCQEDNELVLKVVCKIHNRCYWKEYPST
ALTGCGRLPICEWAVPWAKQSRFKAAKDLRPAHSFINAAKRDSVDNLSGTLDAIAWGKEP
CAGSSGPFKILYSGKSHETKQNEHIYDFLHNPEVQALEKNVMDTYRKRTEKTSKRRSALN
SEGNATINGGAISFNQKFLNAKVGIWENIIDMRTSLQNMLREYTLNEVVTEQDKSCLMEA
LKFHPRGYDKIGVGIREIKIGVNPGHPSSRCFIVLRNDDTTADFSYNKFQGVCSRGGCGV
LHIGGSGPMLLAGRRYGDECRCYLHAGSINDGFSLLTPLRGHQKEPLLFVGIPDAVKAWD
LAKLQWTHPFWSYRSYNALALLGSISIILFSGSNNQGLGCYGKLETGSLAVTYTHNEDHG
ALALAGMQDAQLNPILLWSTNYNIVHLYELPSMEEQVRKAVFLNRETFGSQFALAISRIP
YSVVEEYTSTGLEELFADVGTWKKQVGDTSLSRGLETVGTKHTLAQVQENGLSDLFLHFK
ITGSPWKRKNKHLNQQQMYDYQAMTLRPIQEMMRNNTEHYGFNFFLFEKLGQQTGTCTSD
HLNKKVAVCSNLGMEINRNELGYTHLLLASRLPLDVGAYLERTCNGVSSKRFVGNHFTAH
IPLRESSADRRPRENKRRERKSKATMPVSTLVTVLHITYSAIPS*
>11668.m04398|LOC_Os02g45520.1|genepair1763-1
MVNFGKRLMADQLEEWKEYYINYKMMKKKVKQYVQQTQNGGRNREQVLKEFSRMLDDQIE
KIVLFLLQQQGHLASRIEKLGEERALLMEQADASQISELREAYREVGIDLMKLLRFVDMN
ATGIRKILKKFDKRFGYKFTDYYVSTRANHPCSQLQQIFKQVGIVAVVGALSRNLAFLQD
HQGNFPSIYDHPSITLKDPIIEQINHSVQKLTHATNLLQFIGQHALIIPEDMHSGSEDLV
DDQSYHFMSLLLNLANTFLYMVNTYIIVPTADDYSVSLGAAATVCGVIIGSMAVAQVFSS
VYFSAWSNKSYFRPLVFSSIMLFLGNLLYALAYDVNSLTVLIVGRLLCGLGSARAVNRRY
ISDCVPLKTRLQASAGFVSASALGMACGPALAEPDHIVRENSVNTPSSDSGHRRNSNLED
GLAQPFLIDAKESLDENGEDNDENEEDPEDSHKPATSLAAAYRLLTPSVKVQLLIYFMLK
FAMEILLSESSVVTTFYFNWSTSTVAMFLAVLGLTVLPVNVIVGSYVTNLFQDRQILVAS
EIMVLIGIAMSFRFTSHYSVPQYVSSALITFVFAEVLEGVNLSLLSRVMSSRLSRGTYNG
GLLSTEAGTLARVAADMTITAAGYLGQNSLLNVTLLPSFVICVASIVATFCTYNSLY*
>11670.m04745|LOC_Os04g48390.1|genepair1763-2
MVNFGKKLMADQVEEWKGYYINYKLMKKMLKQYVQQTQLGGKDREQVLKEFSRILDEQIE
RIVLFLLQQQGHLANRIEELGEQRAALLEQHDISQVFQLREAYREVGRDLIKLLRFVDMN
ATGIRKILKKFDKRFGYRFTDYYVTTRANHPYSQLQQVFKQVGIVAVVGALSRNLAYLQD
HEGSVLSIYDHPSVTLKDPIIDQVNHAVQKLTHATSFLQFLGQHALIIQEDVQSGSEDLV
DDQSYHFMSLILNLVNTFLYMVNTYIIVPTADDYAVSLGAAATVCGVIIGSMAVAQVFSS
VYFSAWSNRSYFRPLVFSSIMLFAGNLLYALAYDLNSLTVLLIGRLLCGLGSARAVNRRY
ISDCVPLKIRLQASAGFVSASALGMACGPALAGLLQTRFKIYSLTFDQSTLPGWVMCIAW
LVYLLWLWISFKEPGHFAKSSDTAQPAESGHQVNANLEEGLAQPLLTGSEEGQDQNAEDN
DDNEEESKNSHGPATSISSAYKLLTPSVKVQLLIYFMLKYAMEILLSESSVITTYYFNWS
TSAVAIFLAILGCTVLPVNAIVGSYITNLFEDRQILVASEIMVLIGIIMSFRYTPHYSVP
QYVLSALITFVFAEVLEGVNLSLLSRVMSSRLARGTYNGGLLSTEAGTLARVVADATITA
AGYLGPDLLLNITLLPPLVICIASLVATFCTYNTLY*
>11668.m04399|LOC_Os02g45530.1|genepair1764-1
MAFLVATTLFLFGQLLLCFSQQVRGVNYTFMREAVEAPVMAYYDYIIIGGGTAGCPLAAT
LSERYRVLLLERGGSPYDDARVLNMAHFADVLADTSGASPSQRFVSEDGVINARPRVLGG
GSCINAGFFTRAGPGYVRALGWDPKEVVSAYQWVEDVVAFQPELGPWQAALRRGLLEIGV
VPDNGFTYDHILGTKVGGSIFDAQGRRHTAADLLRYSRPDGIDVFLRARVARIVFSRKGT
KPVARGVLYHDARGGSHMAYLNHGARNEIILSAGALGSPQLLMLSGVGPADHLEEFGISL
VLDHPGVGQGMSDNPMNAIYVPSPSPVELSLIQVVGITRFGSYIEGASGSDWNSRTSGAA
AAQVRSFGMFSPQTGQLATVPPKQRTPEAIARAVEAMSQVPDAALRGGFILEKVLGPQST
GRLALRNLDPDDNPTVSFNYFSHPDDLRRCAAGIATIERVIRSRAFSRFAYPNFAFPATI
NVTAEFPANLMRMRGGSDPRALEQFCRDTVMTIWHYHGGCQVGRVVDRDYRVLGIEALRV
IDGSTFNASPGTNPQATVMMLGRLKPPITLKSVTLCMDLQGSLSSRALAEYLSLWDELQS
VAVQEGRIWKEQNARAFDQKEKAVESLVADIKEDVMVWRAAGKETGDASVGFIFKDWRGL
VLLADHKALHHYGSAAQAE*
>11670.m04746|LOC_Os04g48400.1|genepair1764-2
MASDMDTTRALAILAATSFVAMLACVQAAGDESYTFMKDAVQSPQVSYYDYIIVGGGTAG
CPLAATLSQRFRVLLLERGGSPYDDERIGNMTRFADTLSDTSPSSPAQRFVSEDGVINSR
PRVLGGGSCINAGFYTRASDEYVRGLGWDLEATTAAYRWVEDVVAFQPELGPWQSALERG
LLEAGIAPQNGFTFDHLGGTKVGGSIFDAEGRRHTAADLLRYARTDGIDVLLRARVAKIL
FNVRAGRRPVAHGVVFHDSEGQMHRAYLSNGRGNEIILSAGAMGSPQLLMLSGVGPADHL
RSFGITLVLNQPAVGQGMSDNPMNAIYVPSPSPVEVSLIQVVGITEVGSYIEGASGANWG
VRRSGSGGDRPHRNFGMFSPQTGQLATVPPKQRTPEAIARAAEAMSQLDDTAFRGGFILE
KILGPLSTGHLELRNRNPDDNPSVTFNYFAHPEDLRRCVAGVSVIERVIRSEAFANFTYP
YFSVETLLNMTAGFPVNLRPRHDNDSTSLEQFCKDTVMTIWHYHGGCQVNRVVDAEYRVI
GVDALRVIDGSTFNASPGTNPQATVMMLGRYMGVKIQNERLGNEGLGRRNL*
>11668.m04403|LOC_Os02g45570.1|genepair1765-1
MDEEKEADSPQPPSKLPRLSGADPNAGVVTMAAPPPPVGLGLGLGLGGDSRGERDVEASA
AAAHKATALTFMQQQELEHQVLIYRYFAAGAPVPVHLVLPIWKSVASSSFGPHRFPSLMG
LGNLCFDYRSSMEPDPGRCRRTDGKKWRCSRDVVPGHKYCERHVHRGRGRSRKPVEASAA
ATPANNGGGGGIVFSPTSVLLAHGTARAT*
>11670.m04758|LOC_Os04g48510.1|genepair1765-2
MLAEGRQVYLPPPPPSKLPRLSGTDPTDGVVTMAAPSPLVLGLGLGLGGSGSDSSGSDAE
ASAATVREARPPSALTFMQRQELEQQVLIYRYFAAGAPVPVHLVLPIWKSIAAASSFGPQ
SFPSLTGLGSLCFDYRSSMEPEPGRCRRTDGKKWRCSRDVVPGHKYCERHVHRGRGRSRK
PMEASAAVAPTYLPVRPALHTVATLATSAPSLSHLGFSSASKVLLAHTTTGTTRAT*
>11668.m04406|LOC_Os02g45600.1|genepair1766-1
MAPSQIRFGHQIPFSRPDSDEEEEEDDEDEEVEEEEEEEEEEEYEGEEEEMEGEVPVSSP
LMLPAARGGGGGGVSVVETVAAALRRSLLLCSSVRAAEDEGAAAAAAAAAGMQIGRPTDV

RHVSHVTFDRFVGFLGLPADLEPDVPRPAPSASVSVFGVSPTSMQCSYDNRGNSVPTILL
TMQKKLYQLGGLQAEGIFRINADNSQELHVREQLNMGVVPDGVDMHCLTGLIKAWFRELP
SGVLDSLTPEQVMHCNTEEECALLASTLPPVEAALLDWAINLMADVVEHENYNKMNARNI
AMVFAPNMTQMADPLTALIHAVQVMNFLKTLILKTVKGREETAMPSSAFPSSSGSPSDKD
EPQALEHLDKPTICSTQQNNDFPMISGATLDHFLFRAEPLRHNDAQGSAGRPKKRDNKDH
DNSSREFSPIDSDSSSQASNSASKFSNDNVEGLFDRFKFRKGVGRLCRHPVFQLSRSMKK
SGEAGQACV*
>11670.m04789|LOC_Os04g48790.1|genepair1766-2
MAPFQLRFGLRMSPSRSSDEEEEDDEDEEGFEYEEILSDDGTDSPPPLMMQAEKGGGGLV
GAVVGALRRSLVMCSAGKVGEEEDSEDEEEEGMEIGRPTDVRHVSHVTFDRFGGFLGLPA
DLEPEVPSPTPSASVNVFGVSPTSLQCSFDHKGNSVPTILLMMQRKLYEREGLKIEGIFR
INAENSQEICVRKQLNSGVVPDEVDLHCLAGLIKAWFRELPTGVLDSLTPEQVMHCNTEE
DCALLASMLPPVEAALLDWAINLMADVVEHENYNKMNARNIAMVFAPNMTQMADPLTALI
HAVQVMNFLKTLILKTLKEREAAGTPKTTEPCSGSPNGQDKPPTPENLERPIICSDQKGI
DKPMFDMATCDQLLFGPKQFLDHRENNKFEGPEKHDIGQPKRHSEASPLGNDSNNQVSSP
GKEFGNRNVEGLFDKFSFRKGVERLCRHPVFQLSRSMKKSADVVVFDAPGEARQAWGLI*
>11668.m04408|LOC_Os02g45620.1|genepair1767-1
MSPGVSAKKRHAGAGFTLGCGCKDAKSVSVSASAAGTPSTTATRRRSAGTNPSGSTTTDT
LTMTSASSSFLWEHSVVEFDHDGGGGCGPESFSGLLRELSELEQSVASWGRKSHHQHHDK
KHSPAPSSPLPPQEDRKEKNGGNGDATDKPGDCRDGGGGGDGVGVALDGSVAVVKQSDDP
LGDFRQSMLQMIVENGIVAGEDLREMLRRFLTLNAPHHHDVILRAFAEIWDGVFAATASL
VHHHHPPPSSRREPVAPAARPPAPRTPPRHRHPSPRAWRV*
>11670.m04796|LOC_Os04g48830.1|genepair1767-2
MSPSAAAKMRLGGGGGGGGFMLGCGCRDAKAVAVAVSATSPCSAATETSTATTATWRRAR
THPSASASASTGTLTVPSASSSFLWDDADAEADGEEVDFKRESSATTPSFSGLLRQLNEL
EQSVMTWGWKSPRRGNHFSPPPPPPPPPPLPLRPVVLHRAVDAGGKRSNKEDDAKFSSPP
PSSHCPTTQLHRKVKSVDQRNREDDEAHFAPPAPPPLPLPPQQLRNVKGVDKGGSKEDSK
HCPPPPQAPKHRKTKSCDNNDGFTAGKLDGSLAVVKQSEDPRGDFRRSMLNMIVENRIVT
GDELRELLHRFLELNAPHHHDAILRAFAEIWDEVFAGPDEPRHGPPRPPPRQRTPPRRRH
PLPAWRL*
>11668.m04415|LOC_Os02g45690.1|genepair1768-1
MSRTAIILHRFRQAAASQSLVETSLQSCPYFGVPLRWLSCTEQTSKWETSTSYQIDDVDQ
YSPISSVAKICTHPLSSHVNHCYHHSRSLGFSSVSSSRRMYSSDARAKPEDYKNAMAKVS
STETSEVGATDHSGNTWIDILDSARHSTIDATAAALKKLKAMTDPIVPCIQELYATYPDL
QRMVIPLGGTLMGTAVAWFVMPIVLRKLHKYTSENPLITLEGESTKKYMSYQTSLWSALE
DPAKCIITFMAFSQMAAIVVPSISVYLPQAWRGTFVVSLLWFLQKWKTNFIANIMTNQSA
IGMDRDRLLTFDKVSSLALIALGGMALAEACGVPVQSILTVGGVGGVATAFAARDVLGNI
LSGLSLQFSKPFLVGDNIKAGSIEGKVIEIGLTSTLLINPENLPVVVPNSLFSSQIIVNK
SRAVWRARVVKIPVIIEDLEKIPTISEEIKVKLRSNPNIDAPYCYLSRLESSHGELTIGC
NIKSMRRDEWTTVEQDILLKAASIVKQYES*
>11670.m04810|LOC_Os04g48940.1|genepair1768-2
MSMIAATLRRSSRVTGSQNIMEICLGPCVSSGASSRWFSSCTKHSNTSILNQIKAVDRYS
PVNGMSMISRVPLSAHMDTNWLSTSNPRFNALPGFLGASSICRAYSSDTGIKAEVPQNTV
SNVPSTETVALGTSDGGSSWIDIFDNARKCTLDATTDAGKKVKELTDAITPHVQQFFDAN
PNLEKVVVPLGGTIFGTMMAWFVMPIVLRRIHKYSIQSPISALLGSSTKNDVSYETSLWS
ALEDPAKYLITFMAFSEMAGFTAPSISAYLPQAWRGAIVLSFVWFLHRWKTNFITKVAAS
SIDQTRLSAFDKISSLGLIALGVMALAEACGVAAQSILTVGGVGGVATAFAARDVLGNML
SGFSLQFSSPFKAGEYIKAGSIEGKVIEIGLTSTELMNPEQLPVTVPNSLFSSQVIVNRS
RAKWRSNVTKIPIRIEDIEKVPAISEEIKVMLRSNPKVVLDSEAPAPYCYLSRLESSYGE
LTIGCNLTKMTKDECLSTTQGILLEPAKIIKLHGVELGSTTQCC*
>11668.m04416|LOC_Os02g45700.1|genepair1769-1
MKAAAATGGGGKETLAATLLRYLIILIVPFTVLYILYTLHAILSSTPSCPLDRPIVTSSV
SLSQLSTTRNHTPSSSSLSTPPPAPVSMAATTLQHVVFGIAASARLWEKRKDYIKIWWRP
NAGMRGFVWMDQPVRESGVPDGLPPIKISSNTSGFPYKNRRGHRSAIRISRIVSETFRLG
LSGVRWYVMGDDDTVFLPDNLVAVLQKLDHRQPYYIGYPSESHLQNIFFSYGMAFGGGGF
AISQPLAARLERMQDACIHRYPSLYGSDDRIHACMAELGVPLTRHPGFHQYDVYGDLLGL
LAAHPVAPLVSLHHLDVVRPLFPNARSRPAALRRLFEGPVALDSAGAVQQSICYDARNRW
TVSVSWGFVVMASRGMISAREMELPARTFLNWYKRADYKAHAFNTRPLARRPCEKPSFYY
LSSARRTVARDGETTVTTYQRWRHRNDMRPPCRWKIADPDALLDTVVVLKKPDPGLWDRS
PMRNCCRVLSSPKGQEGNKTMTIDVGVCKDWEFSQV*
>11670.m04812|LOC_Os04g48950.1|genepair1769-2
MKGGGGGGKEAVTASILRFLLLLLLPLTALYFFYTLHLLLASAASSSSSSCPPDAASSSS
SVRLSTNGTSAGAAAVTVAAGKKAPAAASTETTLQHVVFGIAASSRFWDKRKEYIKVWWR
PRGAMRGYVWLDREVRESNMSTARTGLPAIRISSDTSGFPYTHRRGHRSAIRISRIVSET
FRLGLPGVRWFVMGDDDTVFFPDNLLTVLNKFDHRQPYYIGSLSESHLQNIYFSYGMAYG
GGGFAISRPLAEALARMQDGCIRRYPALYGSDDRIQACMAELGVPLTKHPGFHQYDVYGD
LLGLLAAHPVAPIVTLHHLDVVQPLFPNAKSRPAAVRRLFDGPIELDPAGIMQQSICYDG
GNRWTVSVAWGFAVLVSRGVMSPREMEMPARTFLNWYRRADYTAYAFNTRPLARSPCQKP
AVYYLSSARRAAALRGGDTTVTRYERWRRANETRPACRWNIADPDAHLDHIVVLKKPDPG
LWDRSPRRNCCRVLSSPKEGKKGGDKTMTIDVGVCRDGEFSQVV*
>11668.m04417|LOC_Os02g45710.1|genepair1770-1
MGFPVGYSELLLPRLLLQVLLLLGHLHRFLLWAFHAVGLGDLIDNPPGLAATEQDLMLQG
RGGGMAEGWASSSALQHRRPEFRAIPPMAIEEALPVVRFDELVASAPAAVCGGGDCAVCL
SGICGRDEVRRLSNCRHVFHRGCLDRWMAHEQRTCPLCRAPLIPDELLPAASGLPDPSDY

DLSYYPSPLPLAPTPTLLRPHELLLNGLGGFQ*
>11670.m04817|LOC_Os04g49000.1|genepair1770-2
MGFPVCCYSELLLPKQLLHLLLLLGYIRRFLLWAFHAVGLGDLLDLGDEQQAVLQDHARE
HRAPAQALPPQQQHRRAEFRTVPAMVIEEVLPVVRFDELVAAAPAVCGGGDCAVCLSGIG
GGDEVRRLSNCRHVFHRGCLDRWMEHDDQRTCPLCRAPLIPDEMASALWAAAGVPDASDF
DFSYFGAPLTPMPTPTLLRPHELLLTGLGGYQ*
>11668.m04422|LOC_Os02g45760.1|genepair1771-1
MMVAALSSRVGTGPSQHLADLYSGEHRAACQLLQDVSPCFGLALHGANLAILDAVAGHRA
IHLVDFDVSAAQHVALIKALADRRVPATSLKVTVVADPTSPFTPAMTQSLAATCERLKKL
AQQAGIDFRFRAVSCRAPEIEASKLGCEPGEALAVNLAFTLSRVPDESVSPANPRDELLR
RVRALGPRVVTLVEQELNTNTAPMAARFSDASAHYGAVLESLDATLGRDSADRTRAEAAL
ASKVANAVGREGPDRVERCEVFGKWRARFGMAGFRAVAIGEDIGGRVRARLGPALPAFDV
KLDNGRLGVGWMGRVVTVASAWR*
>11670.m04828|LOC_Os04g49110.1|genepair1771-2
MEPGAPWRDPRQGYLYGVGSAVQMPMQQRSDAAAAGGVLKRSLGDMERWQQHQHQQRQIA
MQQQLYLRTVRQRTAAASAAVSPLTSADIAAVLGGPPSQPLVLSGSSMGGAFGSPSSTLS
SITTASRAVAMPLMQPQLQRQQQVTYMASSPQVQAFGTARALPPAPATSDLSILQELEKQ
LLGDDDEVEAAMSGTGSAVTGSEWEEQLNSITAAPSPPLTAATTPNNNNNAVGMTRSPSN
SSTSTASSSASCSPPTSATTSRQLLSEAAAAIADGHNETAATHLTALKRAANSRGDVEQR
LVAMMVAALSSRIGQTASVPDICGGETRAGSQLLHDISPCFRLALHAANVAIVDAVGDHR
AIHLVDFDVSAPQHADLIRCLAARRLPGTSLKVTAVTDPASPFTQSVTATLHLQKLAERA
GIDYRFKMVSCRAGEIEASKLGCEAGEALAVNLAFALSHVPDESVSPANPRDEILRRVRA
LGPQVVALVEQELNSNTAPLTTRFTDACAHYGAILESLDATIPRESAERARAEAALGGRA
ANAVAREGADRLERCEVFGKWRSRFGMAGFRPVALGPGIADQVLARQGPVAAGFAVKAEN
GVLRLGWMGRVVTVASAWR*
>11668.m04423|LOC_Os02g45770.1|genepair1772-1
MGRGRVELKRIENKINRQVTFSKRRNGLLKKAYELSVLCDAEVALIIFSSRGKLYEFGSA
GCFVLAQLGDLFIICLVGGITKTLERYQHCCYNAQDSNNALSETQSWYHEMSKLKAKFEA
LQRTQRHLLGEDLGPLSVKELQQLEKQLECALSQARQRKTQLMMEQVEELRRKERQLGEI
NRQLKHKLEVEGSTSNYRAMQQASWAQGAVVENGAAYVQPPPHSAAMDSEPTLQIGYPHQ
FVPAEANTIQRSTAPAGAENNFMLGWVL*
>11670.m04832|LOC_Os04g49150.1|genepair1772-2
MDRSEMGRGRVELKRIENKINRQVTFSKRRNGLLKKAYELSVLCDAEVALIIFSSRGKLY
EFGSAGINKTLEKYNSCCYNAQGSNSALAGGEHQSWYQEMSRLKTKLECLQRSQRHMLGE
DLGPLSIKELQQLEKQLEYSLSQARQRKTQIMMEQVDDLRRKERQLGELNKQLKNKLEAE
ADSSNCRSAIQDSWVHGTVVSGGRVLNAQPPPDIDCEPTLQIGYYQFVRPEAANPRSNGG
GGDQNNNFVMGWPL*
>11668.m04424|LOC_Os02g45780.1|genepair1773-1
MGFPLVCYCMAIPKPLIALAKLLAAIREALQLMLFVVGICHHPERSGRPAAVDAPLPDEV
KDRLPPLEFAQLLAASEHGCHGCDDDEAVAGCIVCLERLEADDVVRRLGNCAHAFHRGCI
DRWIDLGRLTCPLCRSTLLPRARPAAGPRGRLGRLATRLTGVVW*
>11670.m04833|LOC_Os04g49160.1|genepair1773-2
MSFPLVCFCRQIPRPIVALFKLLQAVALAFVLILCFLGLYEFPYTVEDHAPLIHGRRRDP
LGDDGLQPEAVKRGLPLVEYMQLADLSADCHDGESGYPATCRVCLERLEATDEVRRLGNC
THAFHIGCIDRWIDLGEVTCPLCRSHLLPRQRRGLLGSRRFG*
>11668.m04428|LOC_Os02g45820.1|genepair1774-1
MDSFSSSSGSPPNTEALMDQIKAQLAQAYAQEFLETVGNKCFAKCVTKPGSSLSGSESSC
ISRCVDRYIEATGIVSRALFSSTR*
>11670.m04839|LOC_Os04g49220.1|genepair1774-2
MDSFSSPSSAGSTASTEHLMEQIKAQLAQAYAQEFLEVRLLEPSSDRRYYQFVGDQDHQS
TQGNAGVVGRIYHKVPPLFYRVAIAPFDHLETVGNKCFAKCVTKPGTSLSGSESSCISRS
YLLREIFATQTQFTKVEREDLGKHKDGRVTRVLGDMEIICSRLELEKCLFEGYLNLAAAP
DVVPHDLHQLLGDAEHAEPVLGAQHVHHGELEHLPHLLVRRRQLLPGEERRVDDLMKPPF
PQLGRNRVERMLVPQPRQQLQRTAGEVLVHRVAGVGAEADGAGDVPLDLGAPRLVRAAQR
PELDGGARWAVGRRGPTQGGDAACPGRRTVDPTPCGRIWPPHARRDGRGSEAARMSRHGA
CAGGALRPSARRPSAVGRRPRVSVAWPGQTAAVAFPEGVARTWPMVGVMRHGRSDGNGEV
VRPEAAVWPFGAAVVALGCTWQFVCPEAGCKGSDGSANGASGGGSSSSLPVCTLTLLGVP
PLLFGEFLCWIEAAVG*
>11668.m04431|LOC_Os02g45850.1|genepair1775-1
MEFTTSSRFSKEEEDEEQDEAGRREIPFMTATAEAAPAPTSSSSSPAHHAASASASASAS
GSSTPFRSDDGAGASGSGGGGGGGGGEAEVVEKEHMFDKVVTPSDVGKLNRLVIPKQYAEK
YFPLDAAANEKGLLLNFEDRAGKPWRFRYSYWNSSQSYVMTKGWSRFVKEKRLDAGDTVS
FSRGIGDEAARHRLFIDWKRRADTRDPLRLPRGLPLPMPLTSHYAPWGIGGGGGFFVQPS
PPATLYEHRLRQGLDFRAFNPAAAMGRQVLLFGSARIPPQAPLLARAPSPLHHHYTLQPS
GDGVRAAGSPVVLDSVPVIESPTTAAKRVRLFGVNLDNPHAGGGGGAAAGESSNHGNALS
LQTPAWMRRDPTLRLLELPPHHHHGAESSAASSPSSSSSSKRDAHSALDLDL*
>11670.m04840|LOC_Os04g49230.1|genepair1775-2
MEQEQDEEEEEAEASPREIPFMTSAAAAATASSSSPTSVSPSATASAAASTSASGSPFRS
SDGAGASGSGGGGGGEDVEVIEKEHMFDKVVTPSDVGKLNRLVIPKQHAEKYFPLDSAAN
EKGLLLSFEDRTGKLWRFRYSYWNSSQSYVMTKGWSRFVKEKRLDAGDTVSFCRGAAEAT
RDRLFIDWKRRADVRDPHRFQRLPLPMTSPYGPWGGGAGASSCRPRRPPRSTSITAFARA
STSATSTPLCRRGSSSSSAPQGRGFISTRPCHRRRRHLRLLTNSTLRCTTRAP*
>11668.m04436|LOC_Os02g45900.1|genepair1776-1
MTQALHMLKFFGKLVEPKSEQSLKTLFKLSPDGSISSLFQNFVTYVQDSVSTELKDARAQ

CLQKLKHLSDHFELPNVFDKRSIEDFLVRNAKSILCTASSSSRLHYLPEASPFDLLVVDE
AAQLKECESLIPLQLPGVRHAVLIGYEFQLPALVKSRVCEDAEFGRSLFERLSSLGHPKH
LLDVQYRMHPGISKFPVSSFYENKISDGENVLHRDYERKPLAGPMYGSYSFINVDAGKES
KGKHDKSLMNPIEVAAVTRIVQRLFKGTHCIMPLHSGMASLSSDSLVCAESVDTGRKLCV
GVVSPYKGQVRAIQERLGKAYETHGGFTVKVRSVDGFQGAEEDIIIFSAVRSNTTGSVGF
LSNVNRTNVALTRAKHCLWILGNANTLASSKDHLAAVIELDEVDDLLEMDSLRLSGSRFG
VTCKKRVDVHPRD*
>11670.m04844|LOC_Os04g49270.1|genepair1776-2
MAHFDLLLARLQSSEVLRRDRTMDGTSGHLRLRPRPVGREVASAPGWLTPGRRSNCHLQS
ATRWLEARTEFENRRRSGRDHILEGAHGGWQAGSWRIRCGGDMGDASRIDPSCGSGWRWR
GDSHHKALWSSLDDGGDAEGSGPDGPADGAATWGRRSSEGGGRRASGAGGGELGDERRML
TFQLLKLVKRIPETFTSSSNYFNSFTYPLLEETHVDVFSSLDGYSHQNFISVARMKELLH
DDETTFFCFEVANPAKDEKSKETYAPCEGDIIVLTSRKPKQVSDLTRNMTSYILGSIVKG
GEDDDDLPDNCFIARLSSVLPVETDSSTNEPKEPLFAVILINMKTYDRIWDCLHKGNSHI
VDTVWRYKSKFQLPIALTVAFATKEVDEAMSSSSQLSQRFAARSAVDLNLEKYMLNNSQL
NAVADCVLVSEKISSPIKLIWGPPRTGHRTLTCAPTNTAVLEVASRIVKLVHESPASSGQ
YLSDIVLFGNKKRMKIGHGDSDPLKWPDLLASVHTDVCNKSKIRKARLLCVRILRYLKIN
LKLPDWDRLSLSDDDRKREIRVYLLQRTKCILCTVSSSYVLHNVSMDDRSECLKPLELLV
VDEAAQLKECETLIPMQLPGIKQAVFIGDECQLPALVKSKISDNADFGRSVFERLSSLGY
NKHLLNIQYRMRPEISKFPVASFYDGKISDGPNVVSKNYKRNILPGKMFGPYSFINVDGG
HETTEKHGRSLKNTIEVAAVLWIVRRLFEESVFLGSKLTVGVVSPYNAQVRAIQEKIGKT
YDMYDDFSVKVKSVDGFQGAEEDVIIISTVRSNRAGSVGFLTNLQRTNMALTRAKHCLWI
VGNGTTLSNSRSVWQKVVNDAKHRGCFFEASEYKHLSNAIVNAVIELDDAENLVKMDSLQ
ITNPRFQRAGSRYRA*
>11668.m04437|LOC_Os02g45910.1|genepair1777-1
MSYYYDSDDDDVVLVQQGAGTRSARAKDGRYSSWSQSELEKQMFSWSLKDVLNKDLLKKK
VKKIPTIFSSLKEYMGSFTVPLIEETRADLCSALEGIKHAPAAEVTRIKLCSDEQLIYSF
FANKADPKDIFQEVYAPKEADTLLLTDRKPRHISDLGRGEKPLVIASVLKAEDAEGNTVV
RLSSKHVEQQFGLESSLFAVFLINMTTYNRIWSELDAVVASVRNTDIIRMIVNCNPKVGQ
ECSYSSELPLHLPDRALGGLEDFKLNKSQKVAVLDCVSAMQQRSSSVRLIWGPPGTGKTK
TISTLLWAMLVKNHRTLTCAPTNTAVVEVASRVLNLLEDPSAGSGKACFLSDVVLFGNED
RMNVDGNLTKIFLEKRARRLQKCLSPGSGWVHSLSSMIRILEQPLVQYDSYVEQIEREIE
EDLAEKKRNKNKNKENDKKQVKEDVPKVIPIMPSIYYILKVH*
>11670.m04849|LOC_Os04g49320.1|genepair1777-2
MQMKRIPSTFSDLKSYLESYTSPLLEEMRTEMSSSLEAISTMPSTKISWIEQKKNNKVYD
IVFDADSQNSKACNRPESYVPSVGDIIILSDVKPEHISDITRNGRPYIVAFVTEGGDEDD
DSPPVKYVIISSGKIDSEDGKCQDRKEIKLFAAYLLNIVTYIRIWRCLDYNTAVRRNQSL
IQEMVHYPLVADIVQKQKKDHSIDSMEIWSKLSTMDLNNSQNDAILNCISSMHSNNSSSS
FSLIWGPPGTGKTKTISVLLWLMREMDHGTLTCAPTNLAVKQVASRFLKVIKESSDRACL
GDVLLCGNKQRMCVDGNLKEIYLHDRVRTLLGCFVPMTGWRHRLSSLSDLFENGYSQYQK
YLEDQKEGDSLTFYSYTRKRFNATYPELRRCFKEVLFHVPKSTILEVNYNNIISLLELLE
DFNKKFMNKNIEDEVKGIFLYNDDQSDSSVSSLTKFSKTAISLGKIRIRCLELLNMLLSS
LKLPITSSKRTIREFCMESASIVFCTVSSSSKISNKKLQLLVVDEAAQLKECEGLIPLRL
PTLKHAILIGDECQLPATVKSKVCEDASFGRSLFERLSSLGHEKHLLNMQYRMHPSISIF
PNISFYDRKLLDAPNVKQKEHRKKYLPGLMFGPYSFFNIEDAHSKTKNKVTVGVICPYTA
QVLAIQQKLGKMKFDPVIVKINSGDGFQGGEEDIITLSTVRSNSDGAVGFLVKPAAHKCV
TYTSKVLPLGFWVMQQPYLGLALSGQI*
>11668.m04439|LOC_Os02g45930.1|genepair1778-1
MDAYAWCRRGGAADCEEQEEDIGSPSTSAGSSARSSGSSSELADDASSSSSSSSAERRFE
MSDLMTQLPFKRGLSRFFDGKSQSFASLAAVASLEDLAKPPRKRLKPSQSCGGGLDAHRG
RVLSPRRHCPKAVVAGAKKATARAALSMLAASPRRPPLAAPARPEGVAAKFLVVN*
>11670.m04854|LOC_Os04g49370.1|genepair1778-2
MEAYMLFSRREGMIRCDEQEEDIGCPSESELSLSSSSEGMELADDASSSGSSSSAAGHFE
MSSLMTELPLKRGLSKFFDGKSQSFASLAAVGGLEDMAKPMRKRLKTSRSCGGRARAAGR
APAWPPLAGAALRQCQRRVLQEGVQGRTAVRARREQEDAFAGDGGDLAEAGRDARASSVI
CLGDLPARFVYALMSSIIIISTKQY*
>11668.m04440|LOC_Os02g45940.1|genepair1779-1
MSGRGKGGKGLGKGGAKRHRKVLRDNIQGITKPAIRRLARRGGVKRISGLIYEETRGVLK
IFLENVIRDAVTYTEHARRKTVTAMDVVYALKRQGRTLYGFGG*
>11670.m04860|LOC_Os04g49420.1|genepair1779-2
MSGRGKGGKGLGKGGAKRHRKVLRDNIQGITKPAIRRLARRGGVKRISGLIYEETRGVLK
IFLENVIRDAVTYTEHARRKTVTAMDVVYALKRQGRTLYGFGG*
>11668.m04444|LOC_Os02g45980.1|genepair1780-1
MAGSFEGRSPAARGVEQDIALVALKKGAHLLKCGKRGKPKLCPFRLSSDEKMLMWYSKDR
EKRLNLSSVSSVVLGHKTVHSIYGSPRLMQKNVLQSNLDFSEPFFSPRQRTWSDLDYYME
KVTPDVVNRVKHSCRDIKVADKLNEQIITQLPKQKSSEGLHVAYGATSLKDIFVWGEVPG
NVLDHGDVSKANVSLPRLLNTTHIIDVQSVACGEKHAAIVTKQGEVFSWGVDNGGRLGHK
VSVSVSDPKIIESLASTRVKAIAFGAKHTCAVSISGELYEWGEGTHCLGLWGDQYQRSQW
FPHKLFGPLDGISILKIACGHWHTAIISSAGQLFAYGDGTFGVLGHGDTLTVARPKQVES
LKGVRAKAIACGPWHTAAIVERMGTVKSNAPSGKLFTWGDADRGKLGHADKKMKLVPTCV
EPLNDFDFAQVSCAKAQTIVLTITGVVFTIGSKEHGRLGNPLSEDTSICLIEGPLKTEFV
REISSGSSHIAVLTMNGKVFTWGKGTEGQLGLGDYVDRSYPTLVEALEDKQVHSIACGFN
FTMAICLHRPLSSKDQSVCSNCQLTFGFTRKKHNCYNCGSMFCNSCSNNKVSRAALAPDR
SKRYRVCDACFSQMQKVEEHRKLDPQQKIQKDEVCPIEIRSYTPKLARIFKEANAIMEKM

ASAQSPHQRSQNLAVPDHVRTLRWGLVECPSQFRCVRESIPYCSTLNKQTVSGSIVRVMN
ETMAPKPASSLLKSANDSKAELDLMENILLEEVKQLQEQVTTLAKQCRQRSLKVQLYKRK
VEETWLIAKDEAAKCKAAKDIIKVLIDQHDFLSKNLLAGEKLDNSRIMPSHIASAKSLKA
ELPDPPDKNVFTSEFQQSKSNRDHHNSRQVDRECTQPSIASMANYSVTHQNCRRTSNGST
RCTEGTDATTAPTDSNGVIEQIERGVYATVVTSPGGKKCIKRIRFSRKHFGEDQAQKWWE
ANESMIFGKYSSMEQTVG*
>11670.m04861|LOC_Os04g49430.1|genepair1780-2
MAGSFDGRMPTRGVEQAIVALKKGAHLLKFGKRGKPKFCPFRLSSDEKTLVWYSKDREKH
LSLNSVSTVVLGQKTICRDRDQAECWFLGLTALTSSPYSPLPLAGSKSGRQINSCAISPR
SYLQRKCRLSAALDTPRHTQVYPSYGSPKIQKKIFGGNLDSSEALFYPRHPTSCDIDSYI
EKLNPKIATPVKHGLKDIMVANRRAQNITHTPKLKTFEGPRVACRLDSLKDVFLWGSVLG
SMLGSEDIPKSLPRLVGSAKMLDVQSIACGENHAAIITKQGEVYTWGSYSSGRLDQKVNV
NASCPKMVESLASVHVKAVAFGSKHTCAITVSGELFEWGEGAHSLGLMNDWYGRNQWFPH
KFLGPMDNISVSKIACGEWHTAIITSSGQLFTYGDGTFGVLGHGDTQGVARPKEVESLKG
SRVKSVACGPWHTAAIVEVMSSFKGNAPSGKLFTWGDADRGKLGHADRKMKLLPTCVDSL
TAYDLIQVSCGTAVTVVLTVTGMVFTIGSSRHGQLGNPKADGESICSVEGPLKTEFVREI
SSGSSHVAVLTMSGKVFTWGKGAEGQLGLGDYSNRSSPTLVEALEGRDVESVVCGFNFTA
AICLHKTMSMKDQSVCSSCQMVFGFTRKEHNCYNCGSMFCSSCTSNKITMAALAPDTNRR
YRVCDACFYQLQKVVDSSKDKSRLKISKGEMFKLELKAYTPKLSRLFKEANFFAEKMASV
QGTNQRNEEPATAVQTKTERWGQVECPALFISAQDSFQTQPVSNSEGCAISFSQRMHDSA
VLKSGVSLRFTDAQREEIEMMQTELQGQVKQLHSQVAVLTEQCQQKSVMVQLYKQKLDDT
WLVVRDEAARCKAAKDIIKVLTDQCNALSEKLSIGQLSENSKITPNSIPMQPTKIELQGN
TGRNLVTRKLGQLNNTQNHGSSIQTEKECVPLSNAVVPEDVPFSQQNGARKFSSDGYTTE
APLAAPPTDSVTEQIEHGVYVTFSTSSCGKKDIKRIRFSRKHFGEKEAQHWWEHNKTRVY
DNYKVEQMATPSVTLSCSSTR*
>11668.m04449|LOC_Os02g46030.1|genepair1781-1
MEMACLPGNAMATDENGADDRAGGESTVDHLRSHMNYGDMDLSGEEHVPKARKPYTITKQ
REKWTDEEHRLFLEALQLHGRAWRRIQEHIGTKTAVQIRSHAQKFFSKVVRESSGSNTGS
GGASAAAAAAAIQIPPPRPKRKPAHPYPRKVDGAAKKHVPALRQLEKPPLWMQSLSEQEE
GSPTSVLTAAQIGTEALGGGFSNNSSGSGSLAPSAAGTDEHVDGGGSPASSVDREDGCLS
PSIPTAELAMQAPNTKMSIATTDAKEASSEASVFRLFGKSVVVKDSDQLHLLNGSNIATS
GSVERATRNILVPSFAAAPEGSSSNPWPSSMQQFLYFLPRSDGFAAQPVMPWLSYNGSLP
CALFYPAAAAAANQQCHRDSEGVEFRVSQREGSLTGSNTASSVVLGSSAAVPAAAAAAQN
SDVAESRGQGNSREAAASPRLTKCESSASVTLLQRGFMPYKRCAAESELLRSEAAGGEEA
VADGELTRLCL*
>11670.m04863|LOC_Os04g49450.1|genepair1781-2
MARFQETKARNDQGPVADHVGHQNLMENLTDPLDSSGMDMMDEARIPKARKPYTITKQRE
KWTEDEHKLFLEALQLHGRAWRRIQEHIGTKTAVQIRSHAQKFFSKVIKESSGDNCNSLG
AASSIQIPPPRPKRKPVHPYPRNLGSTASKNVPALKQLEKPQLQVQSLYDQDNGSPTSVL
TVPQIRADTLGSESGGSPTSTIDIEERCPTPSIATAELAMELPPTNDEEVKGNGDHEEVT
CDRSGVPVLRLFGKRVMVNDLHQMSAPDAGNLQTVADMEVDASAETPTSGTGKFSSHGAA
EANTWNPWLTNTQQFLYYLPNGQIFSVHSALPCFTYHNEGVTCTQFSNPQVVASDQQHQH
QTSEAVDYKGIQREGSWTESNTSSSSVPETATHNSETTESYRNGNRNEDEMVPSPDSRKC
VSPGSNCRRGFVPYKRCVADSEALLKSQAPQEEADGEMTRLCL*
>11668.m04454|LOC_Os02g46080.1|genepair1782-1
MFSWLLRIASACLGPARRYARTRKDEDGGDNGGGVADGLLWSRDLGRHAAGEFSFAVVQA
NEALEDHSQVETGSAATFVGVYDGHGGADAARFISDHLFAHLIRLARESETVSEEVVRGA
FSATEEGFLTLVRRTQFLKPMIAAVGSCCLVGIIWRGVLYVANLGDSRAVVGYLGRTNKI
TAEQITRDHNACKEEVRQELISRHPDDSQIVVLKHGVWRIKGIIQVSRTIGDAYLKRREF
ALDPSITRFRLSEPLRRPVLTAEPSICTRVLSLQDQFVIFASDGLWEHLTNQQAVDIVYK
NPRAGIAKRLVNTALKEAARKREMRFVDLKKVEKGVRRFFHDDITVVVVYIDHELLQEKN
VSVPELSVRGFVDSANDGVSVRYHHVGILYHYLCEPLLKRLGFYQVSQMVGMSVDRFLIG
ALVERWRPETNTFHLPVEEMTVSLQDVSCLWGLPIQGEPIIGKLEGPWVDEIETLLGVTP
GKQVMKQKKRKADGDLESGGNMTSSNYSISLKKLRDQFKEMPENATKEQIEHYTRAFILD
ILGSMIFPDTSGDGVPAMYLQFLQDLDKPKEYNWGATTLAVLYRQLSFGAERERLEMAGP
LVLLQHWCWSRLRPGRPVDQDRNPISWGEPDPESCPTFGAKWCGQELYPLPHNSGVAFYQ
NLLDQVQDNEVSWEPYNGLLMSMPRRVRAERAFWYSRVPLIHFWIIEFHYPDRVMRQLGR
KQTIPPPPPHDESKLRQLHKINHTGKPRDWRKLHAEYVNQYNRIWQSMVDEEQHFDQASL
PQYRHWFQQYGMYTVFLDARSAQGLWDPIPYPLDNFDWTGYMPSGPPLSRLDINMEAKLS
SMLSESGLPLCVDDIRSDDSASPFSTPKPIKPEEINMDVMDDWLYSNRGFTRYLSLGTDS
DVLESQDKH*
>11670.m04867|LOC_Os04g49490.1|genepair1782-2
MWPWLERIASACWDRVRRYALTRRDEEDGSGSGGDADDLLLWSRDLVRHAAGEFSFAVVQ
ANDVLEDHSQVETGAAATFIGVYDGHGGAEASRFISNHLAAHLVRLAQERGTISEDIVRN
AFSATEEGFLSLVRRTHLIKPSIASIGSCCLVGIIWKGTLYLANLGDSRAVVGCLTGSNK
IVAEQLTRDHNASMEEVRQELRSLHPDDSQIVVLKNGVWRIKGIIQVSRSIGDAYLKKQE
FALDPSMTRFHLSEPLRRPVLTSEPSIYTRVLHSQDSFFIFASDGLWEHLTNQQAVEIVH
NNPREGIARRLVKAALKEAARKREMKYNDIKKLEKGVRRFFHDDITVVVVFIDHELLQDG
DESTPEISVRGFVDSGGPSSFSGLNGIS*
>11668.m04455|LOC_Os02g46090.1|genepair1783-1
MGNTCGVTLRSKYFASFRGASQRHDEAGYAPVATSAAAAAAADEPAGKKAPRGSAAAADA
PHAASMKRGAPAPAELTANVLGHPTPSLSEHYALGRKLGQGQFGTTYLCTDLATGVDYAC
KSIAKRKLITKEDVEDVRREIQIMHHLAGHRNVVAIKGAYEDPQYVHIVMELCAGGELFD
RIIERGQFSERKAAELTRIIVGVIEACHSLGVIHRDLKPENFLLANKDDDLSLKAIDFGL

SVFFKPGQVFTDVVGSPYYVAPEVLRKCYGPEADVWTAGVILYILLSGVPPFWAETQQGI
FDAVLKGVIDFDSDPWPVISDSAKDLIRRMLNPRPKERLTAHEVLCHPWICDHGVAPDRP
LDPAVLSRIKQFSAMNKLKKMALRVIAESLSEEEIAGLKEMFKAMDTDNSGAITYDELKE
GMRKYGSTLKDTEIRDLMEAADVDNSGTIDYIEFIAATLHLNKLEREEHLVAAFSYFDKD
GSGYITVDELQQACKEHNMPDAFLDDVIKEADQDNDGRIDYGEFVAMMTKGNMGVGRRTM
RNSLNISMR*
>11670.m04870|LOC_Os04g49510.2|genepair1783-2
MGNACGGSLRSKYLSFKQTASQRHDTDDNNNAAAADSPKKPSRPPAAAKTDDHPVSASAP
AAAMRRGQAPADLGSVLGHPTPNLRDLYAMGRKLGQGQFGTTYLCTELSTGVDYACKSIS
KRKLITKEDIEDVRREIQIMHHLSGHKNVVAIKGAYEDQLYVHIVMELCAGGELFDRIIQ
RGHYSERKAAELTRIIVGVVEACHSLGVMHRDLKPENFLLANKDDDLSLKAIDFGLSVFF
KPGQTFTDVVGSPYYVAPEVLLKHYGPEADVWTAGVILYILLSGVPPFWAETQQGIFDAV
LKGFIDFDSDPWPVISESAKDLITKMLNPRPKERLTAHEVLCHPWIRDHGVAPDRPLDPA
VLSRIKQFSAMNKLKKMALRVIAESLSEEEIAGLKEMFQTMDADNSGAITYDELKEGLRK
YGSTLKDTEIRDLMDAADIDNSGTIDYIEFIAATLHLNKLEREEHLVAAFSYFDKDGSGY
ITVDELQQACKEHNMPDAFLDDVINEADQDNDGRIDYGEFVAMMTKGNMGVGRRTMRNSL
NISMRDAPGAL*
>11668.m04458|LOC_Os02g46120.1|genepair1784-1
MARPLPNQTVARTSATSLRHFPGHSKPSLPLSAPALRKAAAALLLAAAVALPCAVLYRAA
VLDAVQPVQVGWDRGPWWERGQPPPAVVVPEEDGDVDPAAADDLDSDDLKLEQVLQEASM
DNKTIILTTLNAAWASSGSVIDLFIDSFRRGVRTSSLLRHLVIITFDWKAYKRCMKIHAY
CFALATENVDFSQEKRFLTAGYLDMMWKRLDFLRLVLEKGYSFIFSDADITWFRNPFPHF
YPDGDFQIACDHYVGNATDLGNIANGGFNYVRSNNQSIEFYKFWYSSRLRYPGYHDQDVF
NFIKHDPYITDIGLKIKFLSTTYFGGICEPSRDLNKVCTMHANCCIGLQSKLHDLRVIME
DWRNYMSMPPSLKRFGALSWGVPQNCSLSLLNQ*
>11670.m04879|LOC_Os04g49590.1|genepair1784-2
MERPPRRPSAARPRPRSLQRAAAALFLAIFVALPFAVLYRGPVSRSLHDSWEWDPLPSLD
ASEEDGAARDDDLDSQDLKLEQVLQKASMGDNTVILTTLNSAWASPGSVIDLFIDSFRSG
VRTSSLINHLVIIAFDWNAYKQCLKIHPYCFALGTDGVDFSEEKRFLTSGYLEMMWKRID
FLRLVLESGFSFIFSDADIMWFRSPFPYFYPDGDFQIACDHYFGNATDLRNIANGGFNYV
KSNERSIEFYSFWYSSRLRYPGLHDQDVFNVIKHDPYVSDIGLKIKFLSTSYFGGFCEPS
RDLNKVCTMHANCCIGLQSKVPDLRVMMEDWRSYLSLPPSLKRLSALAWRVPQNCSLSWS
HQ*
>11668.m04464|LOC_Os02g46180.1|genepair1785-1
MDFTSSYFHAFGNPDFAAVFSGGGSAQAIRPGTTTSSSGGAKAVNVGRGGAARQGAPSVF
CVQDAEVEEAHHFLDECTLCRKGLAGDIFMYRGDTPFCSEECRREQIEMDRNRHRRKKQQ
YSPTAQAAAHHHRSERAPQRQLQPQR*
>11670.m04885|LOC_Os04g49650.1|genepair1785-2
MKGSGAAMPSSMFYVHEADVAQIHHFLEECSLCGKSLSGDIFMYRGDTPFCSEECRQQQI
EVDRAKHRRKKRAAAHAVSVRKEHRNHHHHHRHHHQQPQPRPAIDANPWGTARGPALRV*
>11668.m04465|LOC_Os02g46190.1|genepair1786-1
MEDYFYFSATLELEPVGNLESVSPSPSPRRTTSRDVDVAGELRGRHHHYLDACFLCGRML
AGNKDIFMYRGDTPFCSEECRQRQIDADDASEMMKKRAKMQPAAARGEQQPQRRQSPHGI
PVWAR*
>11670.m04887|LOC_Os04g49660.1|genepair1786-2
MARYHPTSIHDDIEAGFSGHSASPVKPAASPRRPGGRLFCDPCDDADDLLGHHHYLDICF
RCRRPLGGNRDIFMYRGDMPFCSEECRQEQIEIDEAREQRSKQTGRAEQERQRQQKASHP
RIPVWAW*
>11668.m04467|LOC_Os02g46210.1|genepair1787-1
MASASSSSSSFFDIEPLDGGEACLSGHAMDACSLCRKPLTRNCDIFMYRGNTPFCSEECR
DHQMEMDEAAVRVSATNARERAARNEQRHRLDAGSVAVAANVPVLS*
>11670.m04889|LOC_Os04g49680.1|genepair1787-2
MAERNSRAVAGCGRSRRRASPTVERRQRSAAAERRLRAEQPRAVAAVVVGSPPSSSPWAR
LHGAAVRRHRPGVAVAVHRWFKVPADDAPQSLTLLLCRGSGSGGGGSVASLIPWSSRTPS
SRSCSRSVSTLGVPSASWTVVGAGAVTLVVFADLAVALDVVAHTHIRRDATSAKPGKNTA
EGPPVNGFSESRGSLACAFFFDAEPVGETGRHALDACALCTKPLRRDSDIFMYRGDTPFC
SEECRYEQMHLDAACARQAASARRKQQQQGQRSRHETAPAAPVSRKAGVSVASC*
>11668.m04481|LOC_Os02g46340.1|genepair1788-1
MASLPPPAPPAIGGDPLAATIPPSLPSPAPSSSSLNLSPSLLIITALLAFVFFASVSIHF
LLRCLARPSHPAPSPLPRASAAAQRATTASAVEAGEATAASAVGRSHEGEAAAGGGEEVD
DEKERLIASLPLFTMASALAALPKSSPDCAVCLSPFAPEAELRLLPACRHAFHASCVDAW
LRTTPSCPLCRATVSLPHPPLPTAAAAAASNAAQQDPLDSRSSNNSRSFRVEIGSISNRR
SSAAADDRRTYSLGSFDYRVDEEVEAVVSRIARPAAAKSTTGSVTPAPGEALAEAAGSRG
WLREYVDRLASSASSLSGRWSGRWSARWSQSHHSNRQEDSWRWDPEAAVMSAPRGVDDDE
PGFVTLYRWIVGV*
>11670.m04891|LOC_Os04g49700.1|genepair1788-2
MASTLSSPSPPSADPVTGSSDAASSFLPSLLIIAALLAFVLLASVSIHLLLRLLSRSSPP
PPPPPPLPRTRREVHNVEAADASPVRRNGVCEGKKEVVGDEKQRLIESLPLFTMASSLAA
LPKSSPDCAVCLSPFTLDAELRLLPACRHAFHAACVDAWLRTTPSCPLCRATVTLPHPSI
SAILAAEQPPPPEPRSRDRSRSFRVEMGSVSNRSASTATGGNARPTYSLGTFDYHIDEEV
EAVVSRAAPMTTRSAAAVKEDKPAAEQSPPPPGEAVAEAAGATRGWLREYVERLATSASS
LSSFSGRWSSRWSQSYQSHHSHSQEEPWLWDAEAVRMSPPGTEEEETAFMVLYRWIAGV*
>11668.m04483|LOC_Os02g46360.1|genepair1789-1
MRRLLVPVLLLMLGLSTCESSVLQDTCKSVAAGHKYVTYNDCIKAFQADSASATAADARG

LAAIAARIAEKAANATSARIAALRAAEKDARRKDRLGVCAEVYSDAVDQLGETAEDIARG
GDEATQDAVTQLSAALDAPGTCEDAFGEADDASPLAPEDAEFAKLATIALAVAASLSPPP
STPATMD*
>11670.m04894|LOC_Os04g49730.1|genepair1789-2
MKLVRSFSPLIVFSLLLLLTSSTTSRASLVDDACTSFAASHADIGYAYCVRFFQSDEGSA
TADRYGLAAIAVKISAATARGTAKRIADLQDLERDKRRKDCLSACGEVYDSAVDSLDEAA
KGIASRSADGLRDAVTVLSAALDTPDTCEDGFRELGLASPLVAEDEEFSKESAIALGVTS
ALSPPS*
>11668.m04486|LOC_Os02g46380.2|genepair1790-1
MAGDSGNSDGGSNRDEEVQIQIADSSKAATSSTHEVPIQNSPVKSWQWWLMVGVNMFFLI
AGQTASTLLGRFYYNQGGNSKWMSTFVQTAGFPILFIALFLFHSKTSSTQTVTSSPAPTI
SIPKITLIYVVLGLIIAADDLMYSYGLLYLPVSTYSLICASQLAFNAVFSYFLNAQKFTP
LIFNSVVLLTFSASLLGVDEDSQGTTSISHGKYILGFLLTLGASATYSLILSLMQVTFEK
VIKRETFSVVLNMQIYTALVATLASLVGLFASGEWMTLQGEMHAFQSGKLSYVMTLLWTA
ISWQVASVGVVGLIFVVSSLFSNVISTLALPIIPVFAVIFFHDKMDGVKIIAMLMAIWGF
MSYGHQLYVDGKKGRKTTVSVEETS*
>11670.m04895|LOC_Os04g49740.1|genepair1790-2
MADSNAGGNNSSGAANNAEVQIPIPAPSKAEAAAAPETPAGKPFRWWAMVAVDVFFLIAG
QTSATLLGRYYYTQGGRSKWISAFVRTAGFPILFFTLFFFPSKSPSSCTNTPMAKLAVIY
IVLGLIIAADDMMYTGGLKYLPASTYSLICASQLAFNVVFSYVLNSQKVTPLIFNSVVLL
TMSASLIGVSKESQGVTGVSGGKYLLGFVLTLGASCTYSLILALMQLTFETIIKKHTFSA
VLNMQIYTALVATAASVVGLFASGEWRSLRGEMNAFRSGQFSYLMTLLWAAVSWQVANIG
VLGLIFEVSALFSNVISTVSLPVIPFFAVVVFHDRMNGVKIVAMLIAIWGFISYLFQHYL
DGKKAKKASSGDSVRGQEDVVAESDKSTSRADGTAAAAAAAAPLPPVSSQRLRWWAVVLA
NIVFVLGGQSVATLLGRIYYDQGGGSLWLATVVQSCGAPLAVPLLLYFRRPEASPVARPP
LLKIAAIYAGLGVLLAGDNLMYSYALLYLPLSTYSLVCATQLCFNAVFSYFLNKERFTAL
VLNSVVLLTFSAALVGVSHSSEETNSSVPEGKFALGFVLALSASAAFALILSLMQLTFDT
VLRSNAAHAVLELQLWSNAAASCVSVAGLFISGEWSSLTAEMDGYKKGEVAYGMTLAWTA
ISWQLATMGMVGLVATVSSLFTNVISTVGMPLSPIMAVIFLGDRMDGAKVIAMLIGIWGF
LSYVYQHYLDDAKSKNTAGSADVTQTSEAKL*
>11668.m04499|LOC_Os02g46500.1|genepair1791-1
MAAMDEPPQLFLCPISMELMEDPVTVSTGVTYDRRSIEEWLFVYGRTTCPATMQPLSNFD
LTPNHTLKRVISSWLDRGSSSSSSSSPSTSTLSSPIHELATPLSRALEQERLLAALAELE
ETPFKVTKLKSMRARMAGDVAMQGEFVASGGVRVVGRVMAQALAESGGDFSAFAACEEAA
AVLAALPLSDEASVRVVLAPECIRPVMALLQRGGAEARLHAMDILTKISSSGSGGDWTAG
VDIDDVIKSLLELLSDEASTRLSSRALDVLLDVVERARGARAKAVEVGAVHVLVELLADA
DDRRVTERVLLLLKRLCKCPEGRLAFAEHDLSVAAVARTMLRVSELSTQLAVKVLWLVSV
VAPSEKVLEDMMLTGAVAKLLGLLHVESSPSTKQKTVRMVRIHGVVWRQYACFPTDFRDY
LRLLD*
>11670.m04918|LOC_Os04g49970.1|genepair1791-2
MAMEPPPLFLCPISMELMEDPVTVATGVTYDRRSIERWFFKYGKTTCPATMQRLASFDFT
PNHTLKRVISSWLDRASSSSSPSTAGSPATTSSSSSSNAMERERLPSVLAGIEETPFKAT
ALKNLKSCMAGDEAAREDFVACGGIQVLGRVMTQALEESSAGGDFSAFRTCEEAAAVLAT
LPLSDDASVELLLKPECIRPVSVLVQRGSAEARLHAMSMISKISRASVRDWTAEVDVDDM
VKALLELLSDGASAKLSSRALDVLLDVTARYSRGARRAKAVELGAVRVLAELLLDADRHV
AERSLLLLKRMCKCPEGRLAFAEHGLAVAAVARAVLRVSGLATRLAVNVLWLVACAPAPA
ERVLEDMVVGGAVAKLLALMQVESSPSTKDKAVKMLRAHGAFWRQYPCFPTDLKDYLKSL
N*
>11668.m04505|LOC_Os02g46560.1|genepair1792-1
MSTLVDALCAPCSDTALIYDTFNASAAASFLFDNAAAFCDADILGATATGEKEATSSAAA
AAAEAAPPRKKRRRRAKSCKSREETETQRMTHIAVERNRRRQMNEYLAILRSLMPEPYVQ
RGDQASIVGGAIEFVKELEQQLQSLEAQKRTLLPHHKARCDDATPMHNASGSNVGAGGCM
EPTTTTSNCSSSVTEDAPSADAPPFAQFFAYPQYVWCHSPRDSTTTTTAASASASASASS
SSPATVAAALQSEHRSGLADIEVSLVETHASVRVMSPRRPGQLLKMIAGLQALRLTVLHL
NVTTLDSLVLYTLSVKVEEGCSLTTVDDIAAAVHHVLCIVDAEAAASEHLLAAGQLATTA
TAVAKRELATYMY*
>11670.m04932|LOC_Os04g50090.1|genepair1792-2
MALDTLCSAGGDVLIYDTFNASAAAAAAVVPPASFLFGNNNAGGAAGTETRVQVAAGAVP
EVDQLLKQAQQPGRRKRRRRARSCKSREDAESQRMTHIAVERNRRRQMNEYLAVLRSLMP
ESYVHRGDQASIVGGAIDFVKELEQLLQSLEAQKRTLLMQPPPPPQQQREPKCDAADSTS
AADQETPAAAAAAAAADGPPFARFFTYPQYVWCHNPAQDGGGGGGAAAENRAGVADIEVS
LVETHASIRVMAARRPGQLLKMVAGLQALRLTVLHLNVTALGSLALYSISVKRFHGGKAI
ASLGPLGVFFLCALYLWYSTTVVEEGCGMATVDDIAAAVHHVLCIIDAEAASQMLLAGEA
SG*
>11668.m04509|LOC_Os02g46600.1|genepair1793-1
MASSPPTTPNLGSQPTWVPYEPTRDCSQGLCSMYCPQWCYFIFPPPPPAFDITGSSSDDS
SGPTFSPLVIAIIGVLASAFLLVSYYTIISKYCGTFSSLRNRLLGSSAHRGSGGGADGGD
NSRSQEPWSVALSDGMDETLINKITVCKYRRGDGFVDSTDCSVCLGEFREGESLRLLPKC
SHAFHVPCIDTWLKSHSNCPLCRCNIAFVTVGMVSPEPEARVPREDRRDNHELVLTIDNP
EHVREEPQNVVTGVAVGNGGRNHEAKDGPGRSEDANGTAEIREDGALMPPTRAPSSLSDT
HREGRMSIADVLQASLEDELMVARESGLLAGSSGSSRRCHGEHSKDGGGRSGRALPDGAN
MKRLAPAGRSCFSSRSGRGKDSVLPM*
>11670.m04933|LOC_Os04g50100.1|genepair1793-2
MASSAPAWVPYEPTRDCSQGLCSMYCPQWCYFIFPPPPPFDVAGTSADDSSGPVFSPLVI

AIIGVLASAFLLVSYYTFISKYCGTVSSLRGRVFGSSSGGAAYGGGAGSGGRHGHGQSRS
HESWNVSPPSGLDETLINKITVCKYRRGDGFVHTTDCSVCLGEFSDGESLRLLPRCSHAF
HQQCIDTWLKSHSNCPLCRANITFVTVGLASPEPEGCAPGETGGDNTHEVVVVMDGLENL
CEEQQEAVSRASTADDDHDAKDVAEGMEEANGAAEIREEGSPPKRGASSFDLHRDNRMCI
ADVLQESMEDELTAARESGLLAGGAGTSRRCHGENSKGRGGRSRRALQLQDAMEALPGKR
LPSGGRSCFSSKSGRGKDSDHPM*
>11668.m04510|LOC_Os02g46610.1|genepair1794-1
MAIDHASPFSLKNRGDMGGRGYEEEEVENQRWPPWLKPLLSTSFFVQCRIHADAHKSECN
MYCLDCMNGALCSLCLSHHRDHHAIQIRRSSYHDVIRVSEIQKVLDITGVQTYIINSARV
VFLNERPQPRPGKGVTNTCEVCERSLLDSFRFCSLGCKIVGTSGGYRPRKKHGGCGGGGG
GGDGGKKKKKRAALKDARYESEDSCTSTSGGSSDKSSVVQSFTPLTPPPTSASYRTGNKR
RKGVPHRSPFGSLIVEF*
>11670.m04935|LOC_Os04g50120.1|genepair1794-2
MAIDDESPLRINTTRGGAMGGGGECDGAENQRWPPWLKPLLATSFFGQCKLHADSHKSEC
NMYCLDCMNGALCSQCLSYHRDHHAIQIRRSSYHDVIRVSEIQKVLDITGVQTYIINSAR
VVFLNERPQPRPGKGVTNTCEVCERSLLDTFRFCSLGCKIVGTSGDYRGRKRHAGGGIKK
TKKLHKGAAAVPSDSDDSSTTTSGGSDKSSVVQSFTPSTPPATANSYRTGKRRKGVPHRS
PFGSLMVEF*
>11668.m04521|LOC_Os02g46720.1|genepair1795-1
MAAGGSSSNPMSREASSSAAAAAAGVAVRDVGDDKPLPSAEVDITYWAAQEEAAALLESM
AARARGEDDLPEEQLQANNQLQEDEVIALQAIFGDDMVILENKDNLRFIQIFVHYTLPDS
IRVFLNLRRSGAMVGTDDSENHNGGELYHLAAKWLDEPKVSYLCAALDEVWTELPGQEVI
YRWVDWLNSSSWSSIALNDEIVLDPDKTLKIGDERAIARRILVESTIPLMQSYSEKRSHK
IFLESLLCMESHCKIHVKEGNLMQLACPDTNCRNPLPPSVLKSLLRDDGYAQWESFALQK
LLDAMPDLVYCPRCSAACLEVDNDAQCPGCFFTFCTLCKRRRHVGDTCITPEEKIRILKG
LTRMSSMRVGPRPQVVSPERQKLYSIPEEQLLKEKREIDELINIQEALRDSKQCPRCKMA
ISKIEGCNKMTCGNCGRFFCYRCNKAIGGYDHFWNGNCDMFEREQDENPQQQDDENFGGD
PDEDAELLEPEWVLLTYPCPNCGRRNEKSYMYRLLL*
>11670.m04948|LOC_Os04g50740.1|genepair1795-2
MESPKRKLDDESGGEAAAAGLHLLLHEMLLRARREGEEPDLLPDEQLRSNDQLQQDEDKI
FCYSASGLRKLPMLALEAIYGDNIGIFSAKAGLWCFQSSALSHLGFDDGILIQQPGSMMG
PVDVRAVAEIASVESVAQWLISYNEEQCHESFLSGLHDCMICFTEYGGIDFITLPCQHYF
CRRCMETYSRMHVTEGTVLKLLCPNDKCGGIIPPSLLKRLLGDTDFERWERLILQKTLDS
MSDLAYCPRCGAACLEDEENNAQCPKCFFSFCARCRDRRHIGEKCMTIEEKLNSLQDRTV
VPFLSKDSFASKMNLSNEISSIKEVLRSSVRCPHCGTAISRVSGCNHMLCSNCRQSFCYG
CGKAENHGHSSEPCRYQENLATKKNPTVLIEEVKKELEGELSRQHPCPNCRQPNPKIVED
NFSHIICTFHPFAVIWQMGNNSHMFCWACQVHYCAQCHRMVRKSSEHYGPRGCKQHSVDP
EIPLRFKANKNDDSGS*
>11668.m04522|LOC_Os02g46730.1|genepair1796-1
MSSEASSSSSAAVAVRDLGDDNPSASPEVDTTYWAAQEEATALLESMAARVRGEEELSEE
QMQANDQLQEDEVIALEAIFGGDMVILENKDSLRFIQIFVHYSLPDGIRVFLNLRRSGAL
VGTGDNENHNGGEVCYACRLQHLPPVVLTCLLPRFTVRRDLTRYFCKAFLNAEFVLVRML
CMESHCKIHVKERNLTQLTCPDTNCRSPLPPSLLKSLLRDDGYAQWESFALKKLLDAMPD
LVYCPRVALSLQSFCYTQLCYFFHKKKDENDIYLMERQKLHSMPAEQLLKERRELEELMN
IQEALRSSKQCPHCKMAISKIEGCNKMICVNCGGYFCYRCNQAIKGYEHFWGGNCVLFGT
HAHYQIRNPQQQRDENPGDHAELLEQRVQLTYPCPNCGSRNEKYPKLELSYSSTTLDLQ*
>11670.m04950|LOC_Os04g50760.1|genepair1796-2
MAAASAVASSSSSPALEPHPDLSPVFSSPSAASSSSSSSRDEGARGVSCEGGGGDDVFDL
DAPWVAAAEAESRLEEAVTAAAAARVGLCCTEEKGKGKGEKEEDEIRNNRQRQEDEIYIH
YDLNDGAEVCAKLSSANEKNPKDGRCCVGIEGHGDEPEDFSYTCNFEYLPPLVLTCLLPL
SYPSKEPPYFTVTVKWMDGPNVSQLCKMLDTIWAELPGQEVVYRWVESLRNSSRSYLWFD
GKITLGPDTPMQKGDNRAISRSLSLESVIPSMLSYSSKKRYQAFLEDLHMCMICLSQSKG
SNFIRLPCQHLFCVKCLGTLCRMHVKEGSVFQLVCPDTKCNASIPPYVLKRLLTEDEFER
WDRLTLEKALDSMSDVVYCPRCVIGCLEDEDNNAQCPKCSFFFCSFCKEPCHPRRQCLTP
EEKLQRRQASGRMSEREVAQEILNIKALYNDVRLCPKCRMAISKTAGCNKMVCGNCGQFF
CFRCGKAIKGYDHFSECKLFAPRDISAWERQMEEQYGNHVRLSLRPVGGTIRCPKCRERN
FKDDEKYIFCWACRANYCTMCRREVQDKRGHFGSPECVGLEDF*
>11668.m04529|LOC_Os02g46780.1|genepair1797-1
MGRGRAPCCAKVGLNRGSWTPQEDMRLIAYIQKHGHANWRALPKQAGLLRCGKSCRLRWI
NYLRPDLKRGNFTADEEDTIIKLHGLLGNKWSKIASCLPGRTDNEIKNVWNTHLKKRVSQ
REKPGDTKKKGKAADASDDADAHSPSSSASSSTTTAANNNNSGDTAGEQCGTSKEPENVD
VSFFEQDIDISDMLVDAPTEAPLVAAPMPPSPCSSSSLTTTTCVGAVSDELLDLPEIDIE
PDIWSIIDGYGGDEPGDGDATVPCTASPGEEGAEWWVENLEKELGLWGPMDESLAHPDPP
GQVCYPGPLTETEGDPVSTYFQSGPTASPLQEIASPAVLS*
>11670.m04951|LOC_Os04g50770.1|genepair1797-2
MGKGRAPCCAKVGLNKGSWTPEEDMRLVAYIQKYGHANWRALPKQAGLLRCGKSCRLRWI
NYLRPDLKRGNFTAEEEETIIKLHGLLGNKWSKIASCLPGRTDNEIKNVWNTHLKKRVSP
EQKKGGGKSKKKTTCTDVLVPSPSPSSSTTTTTNCSSGDSAGEQSNTSKEEEEETDKIEI
PMLELDPCCFDFDMLVDPVVPDTYCPAVSASASASAPTSPCSSTSPSCARAGVDPLLDLP
EIVDLGPELWSIMDGGAGDGCTEAPPPAWSNAAAAAAANATVATTTSLEEEEGKEWWLED
LEKELGLWGPTDDYHCHPGPQGQPGRAGPPPSAVVEDPVSCYFQAGPTAAATWQGHEPSA
VITSNPMDYYV*
>11668.m04534|LOC_Os02g46830.1|genepair1798-1
MHRQLSLSPGPKQQQHDDGGNIGIGSDAAEVMAVPEESSAAKGRSVREERTIHLIPLLTF

LCFLLLFLFSHDPSSADMSSFRDGGNGGNRRLRML*
>11670.m04952|LOC_Os04g50780.1|genepair1798-2
MPRLYQEEEDGDDLEAKPEKAPAARPSAKERSVHLIPLLTALCFVILFLFSHDPSASEMS
SFGGKVGNRKHKLF*
>11668.m04536|LOC_Os02g46850.1|genepair1799-1
MTSYLIFTHLNVCLRALHEKDELPHGSRQISRSKFFLVALICSFAWYAVPGYLFPTLTSI
SWVCWVFSKSVTAQQLGSGLKGLGVGAFSLDWTAISSFLFSPLISPFFATANIFVGFVLF
LYVLVPIAYWGFDLYNAKTFPIFSSHLFMSNGTSYDITAIVNDKFELDIDAYNKLGRINL
SVFFALAYGLSFATIASTVTHVGLFYGKEIYHRFRASQKEKPDIHTRLMKKYDDIPVWWF
YSLMALSMTVALILCTVLKHEVQLPWWGLLFACGMAFIFTLPISIISATTNQTPGLNVIT
EYAIGLIIPGHPIANVCFKVYGYMSMSQAIAFLSDFKLGHYMKIPPKSMFLVQFIGTIVA
GTVNLGVAWWLLGSIHDICQDSLPADSPWTCPNDRVFFDASVIWGLIGPIRIFGPHGNYS
ALNWFFLIGAAGPVIVYIFHKMFPNKKWITLTNLPVLIGATASMPPATAVNYNSWLLFGT
IFNFFVFRYRKKWWERYNYILSAALDAGVAFMAVLLYFSLSMENRSIDWWGTAGEHCPLA
TCPTAKGVDLGPTSVCPVF*
>11670.m04957|LOC_Os04g50820.1|genepair1799-2
MEIEHLGARAAAAAGEDDQSPVEQVRLTVPTTDDPSLPVWTFRMWTIGLLSCAMLSYINQ
FFSYRSEPIVITQITVQVAALPIGHFLARVLPKRKFTVFGRECSLNPGPFNVKEHVLISI
FANAGAAFGNGGAYAIDIINIIKAFYHRSISFPTSLLLVITTQVLGYGWAGLMRKYVVEP
AHMWWPQSLVQVSLLRALHEKENLRMTRAKFFLIALICSAAWYVVPGYLFPTVGAVSWLC
WAFPRSVTMQQIGSGMSGLGVGAFTLDWATVVSFLGSPLVYPFFAIVNVWVGFVLLVYVM
LPIAYWVLNLYQASTFPFFSASLFDHTGEEYRISEIVNDRFELDTDAYARQGKIHLSLFF
ATSYGLGFATIAATLSHVTLFYGTEMYRRFRQAARENPDVHTRLMRRYDDIPNWWFYGML
ALAMVAALLLCTVFKDEVQLPWWALLCAVAVAAFFTLPISVITATTNTTPGLNIITEYVM
GLIMPGKPIANVCFKVYGYISMNQAVSFLTDFKLGHYMKIPPRSMFLVQFIGTIVAGTVN
MSVAWWLLSTVPHICDKKHLPEGSPWTCPGSRVFFDASVIWGLVGPRRIFGPLGYYGALN
WFFLGGLAGPAVVWLLARALPRHAGWIRLIHLPVLLGATANMPPASTLNYTAWCSVGAVF
NYLVFRRRKAWWQRYNYVLSAAMDAGVAIMGVLIYFCLSSRGITPDWWGNSDINIDHCDL
STCPTAKGVIVEGCPVF*
>11668.m04554|LOC_Os02g47020.1|genepair1800-1
MAISSLHATTSLHSPCTTNTSFRQNQVIFFTTRSNRRGSTRYGGARTFQVSCSVDKPVVI
GLAADSGCGKSTFMRRLTSVFGGAAEPPKGGNPDSNTLISDTTTVICLDDYHSLDRTGRK
EKGVTALDPRANDFDLMYEQVKAIKEGKAIEKPIYNHVTGLLDPPELIQPPKIFVIEGLH
PMFDERVRDLLDFSIYLDISDEVKFAWKIQRDMAERGHSLESIKASIEARKPDFDAFIDP
QKQYADAVIEVLPTQLIPDDNEGKVLRVKLIMKEGVKNFNPVYLFDEGSSITWVPCGRKL
TCSYPGIKFAYGPDTYFGHEVSVLEMDGQFDRLDELIYVESHLSNLSTKFYGEVTQQMLK
HADFPGSNNGTGLFQTIVGLKIRDLYEQIIAERAGAPTEAAKV*
>11670.m04963|LOC_Os04g50880.1|genepair1800-2
MAARSFSSASTTTILLRSGRWRRTMRAAAFPFRVSCSAAAAAAAGGTVVIGLAADSGCGK
TTFVRRLTSVLGAGTAAAAAPPWGGNPGSNTLLGDAATVICLDDYHSLDRAGRKERGVTA
LDPRANDFDLMYRQLKAIKEGRAVAKPIYNHATGLLDPPELITPPKILVVEGLHPMYDER
VRGLLDFSIYLDISSDIKFAWKIQRDMAERGHSLESIKASIEARKPDFDAYIDPQKQYAD
AVIEVLPTRLIAAADDDGDEGKVLRVKLIMKEGVEHFAPAYLFDEGSTISWIPCGRKLSC
SYPGIKFSYFPDTYFGHEVSVLEMDGKFDKLDELIYVESHLSNLSTKYYGEVTQQMLKHA
DFPGSNNGTGLFQTIVGLKIRDLYEQPEDELLSLLQSLADMDITYKALQETDIGRHVNGL
RKHPSGEVRLLAISSSSSSSASPWTGSSAAATAASWRESGSGSAMGSTTVVAVAGSTGIS
ATSCFSLPWNPSPRW*
>11668.m04558|LOC_Os02g47060.1|genepair1801-1
MCDYFLQRMEGEQAAGDLADIVLRAGGAAAAAVAGGGIPSTEWQLPPAEEEEEEPGLFPL
PPSSSDGSGMSGADAFGDPFAGLPDPFGGDYPSSGGAAAAADFFDAVVAKAGFVDVGVLG
GGGGGGCDGGGVDGGGGGSSLLGMSKPILPRAAMQLPSVSPRAIRPYPVMAGDTVKLGAP
MAGGPCAFDGAAAAGLHMSSSPRGAVGGIKRRKNQARKVVCIPAPAAAGGRTSGEVVPSD
LWAWRKYGQKPIKGSPYPRGYYRCSSSKGCSARKQVERSRTDPNMLVITYTSEHNHPWPT
QRNALAGSTRSHHAKNSSSNSSSSGASSASKNNSSHSGYHHHHHQKPLVKAEPNDQSAAA
TTAATVPVKEEAAMVGTSSEALAKTTQKSMEDAAAAASATAAAVEHSDLMQQMFSQSYRP
MIPEAAAGGHHDDFFADLAELESDPMSLIFSKEYMATNYKPAGDPAGKEMNAVDKGLDPA
YMLDWSSTTVVTRAGGSSFMQGEGGL*
>11670.m04967|LOC_Os04g50920.1|genepair1801-2
MPPCVWGSHTPLSLLSPPAAWAGERMEGDQAGGDLTDIVRAGGGAMPGSVVVDLPSTAAE
WQLPAEPMLFPPPPSLSSTTDGCGAGGAAGADIFGGGGGDLFSGLVDPFSSDYSSGADFL
DAMPDAMAKVGFDTAVGGGCGGGGGGGGGSGGHLLDMSRKPLLPRGMPMAAVGGLAAPRV
MPSPLSPRAIRPYPPISAGDMMKLGITAGQAAGCAIDAAVAGMQMSSPRSGGIKRRKNQA
RKVVCIPAPTAAGGRPSGEVVPSDLWAWRKYGQKPIKGSPYPRGYYRCSSSKGCSARKQV
ERSRTDPNMLVITYTSEHNHPWPTQRNALAGSTRSHHSKNSGGGGGSGSKGSQNDKSQQQ
PSVKEEQKDQATTATTTTTSTITTTNSASPVVVKEEEAALAGSSEALELERVMDTTAAGV
VDHSELMDHVFSESYKPMIPETGQPDDFFADLAELESDPMSLIFSKEYMEAKPSGGDHAQ
EKAMAKELDPFDMLDWSTTTNSSAGSSFEQGKRG*
>11668.m04561|LOC_Os02g47090.1|genepair1802-1
MDAGDAMESGQRAALLPESHGPKTEDDSLQVPLLKDKKRGGSKAPAIVLGFECLESTAFN
GISTNLVVYLETVLHGSNLASASNVTTWYGTSYLTPIFGAIVADTFLGNYNTILISLAVY
LLGMMLVTFSAFLPATAALCAAGATCGTGAAAAQTVAFVGLYLVAVGSGGVRSSLLPFGA
EQFDDDSAADRERKAAFFSWFYLCVDFGLIVSGVLLVWIQQNVSWGLGFGIATACIAVAF
AAFVLATPMYKRRLPTGTPLKSLAQVVVAAFRKVGMKLPADAELLYEVSDKVDSQPKIAH
TSEFTFLDKAAVVSESDLEERPEAASSWKLCTVTQVEELKILLRLLPIWATSIIVSAAYS

QMSTTFIQQGSAMDMHIFSVPVPAASLSSFQVLCVLTWVILYSKVIVPALRGFSSSGAAG
EPSQLQRMGAGRLLMALAMAVAALVETKRLNAAASGEAINIAWQMPQYFFLAGAEVFCYI
AQLEFFFGEAPDTMKSTCTSLALLTIALGSYLSSLIYAVVEAFTATAGGHGWISDDLNQG
HLDYFFWMLAAMCTLNFVVYSGFAKNYKLKTVLS*
>11670.m04969|LOC_Os04g50940.1|genepair1802-2
MDAGDAMERGQQRLPESWNPKLQDDVSLTVPLIQDKKSGSKAPAVVLGFECLESTAFNGI
ATNLVVYLETVLHGSSLASASNVTTWFGTSYLTPVFGAIIADTFFGNYNTILVSLVFYLL
GMVLVTFSAFLPTTALCAVAGSTSCQQPVFGAQTIAFVGLYLVAFGSGGVRAALLPFGAE
QFDDDNAVDRERKMSFFSWFYMCVDFGMIVSGLFIVWIQQNVSWGLGFGIATVCVAIAFG
GFVLATPMYKRSMPTGTPLKSLAQVVVAACRKVSLRVPADAALLYEVHDKIDQPKITHTD
EFSFLDKAAVIVQSDLEEDSNDASAAAAGSWRLCTVTQVEELKILMRLLPIWATSIVLSA
AYAQLNTTFVQQGAAMNMRIMSFTIPAASMVSFEVFCVLAWVLVYGSVIVPLLRSFSPAN
GEPSQLRRMGAGRLLIAVAMAIAALVEMVRLDAAARGESLSIAWQMPQYFMLAGGEVFCY
IAQLEFFYSEAPESMKSICTSLALLTVALGSYMSSFIYAVVNAFTAVDGRPGWISDNLNE
GHLDYFFWVMSALCTLNFVVYSAFARNYKVKTVVS*
>11668.m04565|LOC_Os02g47130.1|genepair1803-1
MEVEAATAAAMDFHALSRRELQALCKRNGVRANMTNAAMADALQSLPTVDGVDEIGTAAL
CLPTPSRSTMKSALKAAAAIGEEQQHGSPLPRGRRVSVMSPEAIRLDVEEGEDEMKRDLV
KEIVRTPGVALRSTSRRARATPAPIPTPATTRRTAAARKVEEAAPTPATLRRSQRTAARK
AAAPVVEEVTATKTTTRRSARSKVMVDLEQEVEDMAVALQEVKVQEEDPKDVASDEKCDE
EEEATKILGGNSKEVESEEGEEVVSSAAPTELAVISVMSCDDPKEEEIVATGEEPAKTQE
VMEDSPILGVLSKPEPVEPLSEKIEDASVGDGLGFGKLSALKEITGEMNDKEVDADEVPE
EKLPADVTDDKTSEEDDLNEVEKLSAVEIPQADLTGDKTSEEEDLNEVKEGSAYENPQAD
RIDAESSEEDDLDGDYSEESDIDEESNEEGMLDEEFAAEEYASSEETDDESDPSEVATDS
DEVEVEKLQVAMEDGLTAEANQVDDEEDDFSGDLPSDFDNADNFSDDETESDATVVISSA
SKAAVVKTLDDSSVTEASSEEEVSQQEVEASVNSIVKSLDEFTFTVEGTQKDELTEEMKS
TDDAEDVGAKELKKEKKKKKPTVQELNATSMRKLKTMLKEELIAKAAAGEGKRLALAELD
DNAGGVDC*
>11670.m04971|LOC_Os04g50960.1|genepair1803-2
MDFHALPRRDLQALCKRNGVRANMTNAAMADALAKLATVDGIEEFVKQAVALPPAPATKP
AVKAVAEEDPREKKGSPLPRGRRVTVSSPEVVNLDDSDEEAPGQSKDAPLPRGRRGTVRS
SKLIRPDDGEEEGKEDENRGENAPVHGEGRRGASRRARAEPVVAPTTRRRAAPTSKIETG
DVAVEAPPAPTTRRRPQTSTEAAPAPTTRRRAQSTVAASAEEKVPRGRRTTRRAAAKKPD
MLEEETREPLAPEQNSAHEEPVQEEQGIEVEVPAETVEAVAQECLPDPDAVVEEKPVQEE
EVGRSDLSVPVRLVNSGIEVEAPAETVEAVAQECSPDAVVEDEQAAVEAEQTISQDDSPI
FGIVTGTIVGASEEAPVCNSECPKNMATEESSDTVSEEKEAVPADEVPLVTVTCGEATRD
AELPTNIGNAKEEDDDEMEAAHEAGFAVEVDGSETVDELIGTLTEHADNAIQLNFSAELS
CADEEAGVFATDDLQQSSATVKTMVADSEANEEEDALEAENEVGFAVEEKEVRTGDEPHE
TLSNDADGAIQLGEDALEAANEAGCAVEEKGVETVDEPHDTETNAAENAQEEGVVASEDL
LQISETEQDEFNSDICHAVEHNERDNVESVSSEREDVSMENAFTGDLTLKFDGPGDLGDR
NTSLLEEGARTLPLSTETPNNVTDAVVTAAEEMVSETMGVSNKKSTELAAMEDGNEVKVV
EKQKEDPVELVKLSLRTLRAKLKEQLTKHKVMTPDLIYAL*
>11668.m04566|LOC_Os02g47140.1|genepair1804-1
MPPKLDPTQVVDVFVRVTGGEVGAASSLAPKIGPLGLSPKKIGEDIAKETAKDWKGLRVT
VKLTVQNRQAKVSVVPSAAALVIKALKEPERDRKKVKNIKHSGNISLDDVIEIARVMRPR
SMAKEMAGTVKEILGTCVSVGCTVDGKDPKDLQQEISDGEVEIPSA*
>11670.m04974|LOC_Os04g50990.1|genepair1804-2
MPPKLDPTQVVDVFVRVTGGEVGAASSLAPKIGPLGLSPKKIGEDIAKETAKDWKGLRVT
VKLTVQNRQAKVSVVPSAAALVIKALKEPERDRKKVKNIKHSGNISLDDVIEIARIMRNR
SMAKEMAGTVKEILGTCVSVGCTVDGKDPKDLQQEISDGEVEIPSA*
>11668.m04568|LOC_Os02g47160.1|genepair1805-1
MSGAKVPPCNDNPKLMAYVEYYSPTGAIIALPVTITTTASCIYWSFKKRERNRKRAELFK
KNGGLLLQQRFAAFTSQGMMDLSARLFGAEELKVATDNYSENRILGRDSYDNCSRNNWIP
YSQLTEKSDVYSIGVVLAELLTTEKPVSFARPEDLRKLAMYYLVMLVNKGCILQAVKPII
LAEAREEQLYDVAHLSIMCLSLKGEQSTMKEVASVLNGLRRSLAKDKAIKGKEVYPQNKN
EEEEYLLPGSGDAVSRVSTAQILHSSGYTAAEPAALRALSDIAGRYVASLGRAASAIAEA
RGRTEPNLADLTLALEDHALGGFPGASDPARPVLRSGALSELAGFVRVVREVPFPKPVPR
RGGAPRGKAWESFAAAGKEPPPKHVPRWLPRFPDKPEPEPEPKAAYDEATARWEARVRHE
EEANAEEAVVLKPSVDGGGERRGVVPEKRGKVSFRVRAERKKRRVGLDQQ*
>11670.m04978|LOC_Os04g51030.1|genepair1805-2
MVCRRASIPAATEAAAVASWPLGPLALALALAILSAMPHVALSQNLTCKDVPFPFREERS
GATSVPGFMVTCGRNNEAMLRIGEHIYKIDKVSVPEHFITIRAGPIQQVCYDRSRRPKQI
KPITRVDPAPAPTSLQATPFTFSRRNKLVATGCNYRFFANFTSSSGGGDGTPTSCDTWCN
DGSSDTIFNGSCADKACCTTEMQMDGAQEFNLTLKPSGNVEASTCSAVFFLDKDEQVFTS
AGDGRNMALKEALVPLGYRKMFLDWAIPGNCEQNSASQYPCGSMSTCNDVYNGTSYICRC
NEGYEGNPYEQNGCSGLDVCAHPERNPCMYPEYCKDEQGVTSCACPEGRNGDGRKKGSGC
KRHFPLDTALGVGLALTVTLATTLLCYYWTMKKRKVARKRAELFRKNGGLLLQQRFLMIT
SQGEESSAKIFSAEELKNATDNYSDGRILGRGANGTVYKGILPNRTTIAIKKSILFDESH
VEQFVNEITILSQIDHPNVVKLLGCCLETKVPLLVYEFIPNGTLFQHIHNKRTLTWEDCL
RIAEETAGALAYLHSTSSTPIIHRDIKSSNILLDENFVAKIADFGASRSVPSDHTHVTTL
IQGTIGYLDPEYFQTSQLTEKSDVYSFGVVLAELLTRQKPISVGRPEESCNLAMYIVILL
NERRLLQEIEPQILVEAGEEQIYAVAQLSARCLNVKGEERPVMREVASVLHGLRESFDEE
QIIRSDESIQIINEQESVHSEARPISSLQSSDETSTTQYSLPSEILSASHLER*
>11668.m04569|LOC_Os02g47170.1|genepair1806-1

MAGCSEGGGGDGEVACCGVGDTSPGTIVWVRRRNGSWWPGRILGPDELPPSQIMSPRSGT
PVKLLGREDASVDWYNIEKSKRVKAFRCGEFDACIEKAEATEGTSVKKREKYARREDAIL
HALELEKKLLASKHQTQGSRPANVSVCSKHNKDLGSTRYKSKKSKKRKGITASSDIKKEA
EQYVLHAGSKRNFQDSPTRGISDNLFGNHLGDISHVRHIQAGENLDSKEKITTAEKIRSD
GSDFDESIEKCDRRQPLVQILHSSPKLPHQSQHNDGYGDVLTQGEMDRSPANYRAKRSRY
VFLPTDSGETHSHSDLPSVQVASTGGDFETESYLHHPAFSEEQTSSDLVEKHIYESSERE
CSESETEDDAELLQCSDLILHPASHAHDPYFLPASDKFRHANIDADADELTYSSYMCQVN
ESEEDGSSELGVSQWHMKGKRNSRNAPKRSDMADGNPWLDKSDGFMEGSPYKINGRNPRE
GSMQIPNQQLLGQNFYQNEEVNYDSEETDFFEDTGHSEVNLYHGRTYSSCLKATRDFSRS
YSYFNDYGNDSSKVSPLNRDSDKIFHFDRNAYWSGPSFYQKYSSRLRGRGPMLFDVDLKV
QASYQGEHVPLVSLMSRLNGKAIVGHPVQIEILEDGSTDHLVFCGDVSLEGRTGGQPAWC
TGRRTAMQRIPRSNPSGALDCDDEGTLAYPDWEMKPDFRKYSNSDFRKYSNSNHQVKVDK
KSISNVRRPSASKSQKKQSKKASLSSQKVRTLSSISTGKKHHGVGGQAKAHKQSGIFGGL
IKPGGIPLVTCVPAKVAFTRILEAVGRPPLAVAHRVRMASPALRDPS*
>11670.m04981|LOC_Os04g51060.1|genepair1806-2
MGRSSGGGGGGGGGEGEGCGEAETGITDCSPGIIVWVRRRNGSWWPGRILGPDELPASQV
MSPKTGTPVKLLGREDASVDWYNLEKSKRVKAFRCGEFDACIEKALTSRGTPVKRREKYA
RREDAILHALELERKQLASKYQNQGFRSDDISSVPFADMRREFDNSSTEYYSRNNTQKPQ
FPLGNSASQQCKDLSSTRYKSKKSKKRKGDSSNLPGKTKGLEQNFPYAGSKRDFSESLAL
EGAENTLSNRNNGSSHLGHMQAGPNLGSDGKNTPLTKKISEESVFEESLVKKHDRCRPLA
QVVQSSLKLPHSFQRDDDSGPVLIEEGNDPLTTIYQAQQGWSTYMPNDSGETNNHGDIPP
TQITSMGAHFETEGYLKQPDSFSAEQKISEFAEKQRSDSCERECSETETEDDAELLQSRI
FMNSKSPGQIYMYYFSMLILYKGYAKRQSPGSDACDPYSIQASKKSRHVDGDVADDMVAF
STGIPQQNVLKEEDGSSELGVSQWHMKGKRNQRSALKRPMGKTDGNISLDRSNSSLKGSL
YRVNESNPNMESTGASSHQYFGRSFYQTQELDYDYDNADLTNKARGHAEVRYYGKDYPPS
LTPTRDLEQSYTSFNNTETYCKTSPPNKNGDQMSSLGRKACLEGASLYRQNYSSQLGYMG
PMLFNVDLNVQAGYQGEHVPLVSLMSRLNGKAIVGHPIQIEILEDGSTDHLVLASDDFLE
HSTSASPAWRTGRRTAMPRIPRSNSTRVTLDDGDDEGLWDMNPPFSRSSTPFNQQFRLSK
RSNTSFRSPLSHRSQKKPSNSKKGSSSSQKVRALSSISIGKRHHREGRQAKLHNILGDLI
KPEGAIPLVTCVPAKVVFSRIMEAVGRPSLSIAHRARVASPAIRDAQR*
>11668.m04570|LOC_Os02g47180.1|genepair1807-1
MDAGSHSISSEKSHGLAPRPPLQEAGSRPYMPSLSTASRNPSAKCYGDRFIPDRSAMDMD
MAHYLLTEPKKDKENAAASPSKEVYRRLLAEKLLNNRTRILAFRNKPPEPENVSAADTAS
THQAKPAKQRRYIPQSAERTLDAPDLVDDYYLNLLDWGSKNVLSIALGDTVYLWDASSGS
TSELVTVDEDSGPITSVSWAPDGQHVAVGLNSSDIQLWDTSSNRLLRTLRGVHESRVGSL
AWNNNILTTGGMDGNIVNNDVRIRNHVVQTYQGHSQEVCGLKWSGSGQQLASGGNDNLLH
IWDVSMASSVPSAGRNQWLHRLEDHTAAVKALAWCPFQSNLLATGGGGSDRCIKFWNTHT
GACLNSVDTGSQVCALLWNKNERELLSSHGFTQNQLTLWKYPSMVKMAELTGHTSRVLFM
AQSPDGCTVASAAADETLRFWNVFGSPEAPKPAAKASHTGMFNSFNHLR*
>11670.m04986|LOC_Os04g51110.1|genepair1807-2
MDAGSHSISSEKSSRYVAPRQPLQEAGSRPYMPSLSTASRNPSAKCYGDRFIPDRSAMDM
DMAHYLLTEPRKDKENAAASPAKEAYRKLLAEKILNNRTRILSFRNKPPEPESILTELRA
DAASIQAKPAKQRRYIPQSAERTLDAPELVDDYYLNLLDWGSSNVLSIALGNSVYLWDAT
NSSTSELVTVDEDNGPVTSVSWAPDGRHIAVGLNSSDVQLWDTSSNRLLRTMRGVHDSRV
GSLAWNNNILTTGGMDGKIVNNDVRIRNHVVQTYQGHQQEVCGLKWSGSGQQLASGGNDN
LLHIWDVSMASSMPSAGRTQWLHRLEDHLAAVKALAWCPFQSNLLASGGGGSDRCIKFWN
THTGACLNSIDTGSQVCSLVWNKNERELLSSHGFAQNQLTLWKYPSMVKMAELTGHTSRV
LFTAQVIFCSLYLPFKLTNIALNRLLITCLVVILQSPDGLTVASAAADETLRFWNVFGAP
EAPKTATKGSHTGMFNNSNHIHIR*
>11668.m04571|LOC_Os02g47190.1|genepair1808-1
MERAGYGVGVGGAGAVGAGVVLSRDPKPRLRWTPDLHERFVEAVTKLGGPDKATPKSVLR
LMGMKGLTLYHLKSHLQKYRLGKQNKKDTGLEASRGAFAAHGISFASAAPPTIPSAENNN
AGETPLADALRYQIEVQRKLHEQLEVQKKLQMRIEAQGKYLQTILEKAQNNLSYDATGTA
NLEATRTQLTDFNLALSGFMNNVSQVCEQNNGELAKAISEDNLRTTNLGFQLYHGIQDSD
DVKCSQDEGLLLLDLNIKGGGYDHLSSNAMRGGESGLKISQHRR*
>11670.m04990|LOC_Os04g51130.1|genepair1808-2
MFEGCYGGVGSAAAAAARDPKPRLRWTPDLHERFVDAVTRLGGPDKATPKSVLRLMGMKG
LTLYHLKSHLQKYRLGRQSKKSAGLELAVADSGDALKYQVEVQRKLQEQLEVQKKLQMRI
EAQGRYLKEILEKAQKNISLDANGSANLSSTRSQITDINLALSGFMDNATQVQEENNELM
KPISDDNLKVNNLGFQLYHLGSQESKDVKCTPKTEELLLLDLNIQGGYELSSRGMQGCEL
DLKINQQRR*
>11668.m04575|LOC_Os02g47230.1|genepair1809-1
MVLKFSGSGRHQYKSGGSPSLRTSRFHRSSRLAAYPGIIDESGFTSDGAGEAYTYMRTTT
ASAGARAAPSTWDLPPKVNHRSFQPRVIRSPSASGVPSIGEEDYDDDDDDDEETVLLEE
DRVPREWTAQVEPGVQITFVSIPGGAGNDLKRIRFSREMFNKWEAQRWWGENYDRVVELY
NVQTFSRQQGFSTPTSSVDEAMQRDSFYSRVGSTRESPAMMMPPPPPLPSSGAGREHPIS
RTASSKAQLSSSSSVAAARPPFYPSTAVPDPSDHVWAHHFNLLNSAAAGPAAPYDPSRGT
TSSRDEASVSISNASDLEATEWVEQDEPGVSITIREFGDGTRELRRVRFRQATPQSTHTS
RKR*
>11670.m04994|LOC_Os04g51170.1|genepair1809-2
MAWHVWSKCSIHKEREADRERGSEKMLACIACSSKEGGEDGSRGAATPHGRDAVKSLTSQ
LKDMVLKFSGSNKHQHYKAATAGSPSFRSRSYRRPYPGFIDDSAFMTTTRPGGEAYMYTR
AAPPPPVRAASTSMATWDMTRSKSNRGWQQDAGRSPGGTTWIQSIEEEAGADDVTVVEDA
VPREWTAQVEPGVQITFVTLPGGGNDLKRIRFSREIFNKWEAQRWWGENYDRIVELYNVQ

TFSGRQQGVSTPTSSVDDSILRESSFCSRGGSTRESPVVTPATSSSLAKEPIARSMSCKA
MAASASNYAAAAASTRAACYPSVAVPDPSDHVWAHHFNMLNSAAAGPSAAGGGVPSLYDP
SRGTTSSRDEASVSISNASDMEATEWIEQDEPGVCLTIRELGDGTRELRRIRFSRERFGE
DRAKCKEEPAPQVAAAEGAEPRRRRPGAAVRGALGVAAPFAASFLFSFVVGLAGLVLGGL
SSTASVSMPSTCRILSTGVDLRSSKVCELGLLNYRAKHVFYPSSNRKFRCHDDYYWASVF
EVEYTEYFSGQTSYAVAEAPKEALPLNCRPDFGVAWSTTLKFKVNESYSCRYTLGSSKAD
IYSDRLFNCTSEEPSTIEMLKRIFVLLSKSYMLEDFNSIGMLGYVMAGVVAGILSALLIT
ILLRSLRGLVLAVVGSSYSKMIGLKELLLDYEIMERFL*
>11668.m04580|LOC_Os02g47280.1|genepair1810-1
MAMPYASLSPAVADHRSSPAAATASLLPFCRSTPLSAGGGGVAMGEDAPMTARWPPAAAA
RLPPFTAAQYEELEQQALIYKYLVAGVPVPPDLVLPIRRGLDSLAARFYNHPALGYGPYF
GKKLDPEPGRCRRTDGKKWRCSKEAAPDSKYCERHMHRGRNRSRKPVETQLVAQSQPPSS
VVGSAAAPLAAASNGSSFQNHSLYPAIAGSNGGGGGRNMPSSFGSALGSQLHMDNAAPYA
AVGGGTGKDLRYTAYGTRSLADEQSQLITEAINTSIENPWRLLPSQNSPFPLSSYSQLGA
LSDLGQNTPSSLSKVQRQPLSFFGNDYAAVDSVKQENQTLRPFFDEWPKGRDSWSDLADE
NANLSSFSGTQLSISIPMASSDFSAASSRSTNGD*
>11670.m04997|LOC_Os04g51190.2|genepair1810-2
MAMPFASLSPAADHRPSFIFPFCRSSPLSAVGEEAQQHMMGARWAAAVARPPPFTAAQYE
ELEQQALIYKYLVAGVPVPADLLLPIRRGLDSLASRFYHHPVLGYGSYFGKKLDPEPGRC
RRTDGKKWRCSKEAAPDSKYCERHMHRGRNRSRKPVEAQLVAPHSQPPATAPAAAVTSTA
FQNHSLYPAIANGGGANGGGGGGGGGGSAPGSFALGSNTQLHMDNAASYSTVAAGAGNKD
FRYSAYGVRPLADEHSPLITGAMDTSIDNSWCLLPSQTSTFSVSSYPMLGNLSELDQNTI
CSLPKVEREPLSFFGSDYVTVDSGKQENQTLRPFFDEWPKARDSWPDLADDNSLATFSAT
QLSISIPMATSDFSTTSSRSHNGIYSR*
>11668.m04584|LOC_Os02g47320.1|genepair1811-1
MDANRRQGGIQQLLAAEQEAQQIVNAARSVPRKLSEKHAFLLLFFCTLTKAKEEAEREIA
EYRAQMEAEFQRKVAESSGDSGANVKRLEQETDTKIAQLKEQAANVSPEVIQMLLRHMEP
FFCLAIFVNYGAGKWVILKDDESETATSMDADCRRLRDRSTTRRAADRRLPDCAGACPCR
ASFGSPCGAGGVVSAPRHRRRMAVRRRADIVTLLHC*
>11670.m05006|LOC_Os04g51270.1|genepair1811-2
MDANRRQSGIQQLLAAEQEAQQIVNAARAAKSARLRQAKEEAEREIAEYRAQMEAEFQRK
VAESSGDSGANVKRLEQETAEKIAQLKQQAASISPEVIQMLLRHVTTVKN*
>11668.m04589|LOC_Os02g47370.1|genepair1812-1
MRAAGKPTSGGSGTSRSSNIRGLLQDFLEQQHRLDVRRQEALERHAQERAAIEQQWRQSM
QALERERLMLEQAWMEREEQRRVREEARAERRDELLTTLLNRLLQDDDL*
>11670.m05011|LOC_Os04g51320.1|genepair1812-2
MEPGCGEGGGGGGGRDERVPQWGAQETRELIAARGEMERESAAAAAARRSAKTLWEAVSA
RLRERGYRRTAEQCKCKWKNLVNRYKGKETSDPENGRQCPFFDELHAVFTERARTMQQQL
LESESGPSVKKKLKRPSGDLSSEDSDDEEDGSGDSGDEKPIRSRKRKIADKRQQSQRMAE
KSRTSISSIHELLQDFLVQQQRMDIQWHEMMERRSQERIVFEQEWRQSMQKLEQERLMLE
HTWMEREEQRRMREEARAEKRDALLTTLLNKVLQEDL*
>11668.m04592|LOC_Os02g47400.1|genepair1813-1
MANGGVCLSCSALVCALVFLTVDGDFVDITYVASAVAKGAVCLDGSPPAYHLARGFGSGV
NSWLVHFEGGGWCSNVTTCLQRKRTRLGSSKQMAKQIAFSGILSNTPDYNPDFYNWNKVK
VRYCDGSSFTGDVEKVDPATKLHYRGARVWQAVMDDLLAKGMNSANNGSAKNLPSACTSR
LSPGMCFFPQNEVKQIQTPLFILNAAYDSWQVRNILVPGFADPHGKWHSCKHDIDQCPAS
QLQILQGFRDDFLKALKEQGTPSTRGLFINSCFVHCQSETQETWFASGSPMLETKTIADA
VGDWFYDRNPFQKIDCPYPCDSTCHNRIYDDPSEA*
>11670.m05013|LOC_Os04g51340.1|genepair1813-2
MPRYCRHPALSSLPFIASSLPPSLFTAQHAATIYATPSLPAVVTGRASHSQPEAHLLSVI
FSVFFSSTLAMAASSGEWLSRAAMVLVLGLVVASSAKAGDVDMVFLKSAVSTGAVCLDGS
PPVYHFSPGSGSGANNWLVHMEGGGWCRNAQECSVRQGNFRGSSKFMRPLSFSGIIGGNQ
RNNPDFYNWNRIKVRYCDGSSFTGDVETVETSTNLHFRGARVWNAIIEDLLAKGMSKAQN
ALLSGCSAGGLAAILHCDQFRDLLPATANVKCFSDAGFFVDGKDITGNDFVRTFYKDVVN
LHGSAKNLPSSCTSKMSPDLCFFPQNVVPTLRTPLFILNAAYDAWQIKNVLAPSAADKKK
TWAKCKLDITACSSSQLTTLQNFRTDFLAALPKPEQSPANLSIFIDSCYAHCQSGSQDTW
LAQGSPVVEKTQIGKAVGDWFHDREVSRRIDCPYPCNPTCKNRDDD*
>11668.m04594|LOC_Os02g47410.2|genepair1814-1
MEVSWEVDAAGPPWRPSESTAFLPFAAAAAAGDRAGASLSGRRNGLAARSSNLSSVRKRP
FVARLTTDIVQTFGKCNPEFKYSDSLNPKRFLTNPSVPAHNDGLDNANSDLILYVNLELV
NKKSDRRYVIKEMLGQGTFGQVAKCWDGETNSYVAVKVIKNQPAFYQQAIMEVSLLSMLN
EKYDPDDQHHIVRMLDFFLYQNHLCIAFEMLGHNLYELLKRNSLRGLQLKYVRTFSRQIL
DALVVMKDAGIIHCDLKPENILITPNVKTAAGVKVIDFGSACLEGKTIYSYIQSRYYRSP
EVLLGYPYTTAIDMWSFGCIVAELYIGLPLFPGASEYDVLCRMIEILGGQPPDDLLREAK
NTGRFFKQVGSIYPGIEMQNGPISAYRILTEEEIETRESKKPKVGRWYFPRGRLDKLIYT
YPWKNLNGENLPETEKTDRLALVDFLRGLVEFDPNKRWSPLQASYHPFITGEAFTGPYEP
IQETPRIPVGRVAAVDHNPGGGHWLAAGLSPQVGSINRGLPFNNAFAPKIPFSYGSSCGS
FGSHGSFNDNVGLASSYGSYDVNSVNMYHSPLGPSGFNLHSQAGGTFLGSSPDIRRRSYL
YHGGGIRLSPGCPGPMSLGASPSQFTPPNSQMQIPSTATGKYGSTSPARSSHGSLGKAAA
VGQYNRRRNLGHPPISMPPHEYTSQLTQGHHGDGTISNHFDGYARGHSGYPQSALPNPGH
FSWRPHTCAGSGLSTDTSNHGSFPPSRYGGFPPTHSSNVSADTLASTSSIPDPADWDPNY
SEESLLQEDTSLSDALSDLHLKDASGQTNQSSRLAHIQSHAIANSNSLSMNQRGDRLFHA
STLTESSASTGHVTYDGYHNANYSQLNFQSRHGQPFQRYNHMTASYLRPMGNHHNGQPVW
PNYGMAEPPPATMADGMPWGGRPGHSFTAGGLPSSFAGKDFGRIF*

>11670.m05016|LOC_Os04g51370.1|genepair1814-2
MEEKAAAAPPWEPSVGTVFRRLAGAGDSGRSPEASLPSPSSSGNGVATRISNLHGVKRKP
FVARLTADIIQTFVRCNPAFKYSESLNPKIFLTNPSTPAHNDGLDNANWDLILYVNLELV
NRTSNRRFVVKEMLGQGTFGQVVKCLDTETNDYVAVKVIKNQPAFYHQALVEVSLLRALN
QTHDPDDQYNIVRMLDYLLFQNHLCIAFEMLGQNLYELLKRNSFRGLKMKFVRAFSKQIL
DAMVVMRGARIIHCDLKPENILLTPSVTTDAAVKVIDFGSACLEGKTVYSYIQSRYYRSP
EVILGYPYNTAIDMWSFGCIVAELFLGLPLFPGASEYDVLQRMVKILGGQPPDYMLREAK
NSAKFFKHVGSIYRGNEVHDGIGSSYRLLTEEEIEVRESEKPKVVKWYFPQLRLDQLICS
YPWKNSELTETEKAERVILVDFLKGLLKFDPNERWSPLQASCHPFITGEPFTGPYEPIPE
TPKIPIGRAAAVEHNPGGGHWLAAGLSPQVGSVNRSLPPNNPYPPKIPYSYGSSYGSFGS
HGSYVGNAGHASSYGSFGDGNAVNMYYSPLGPGFKQIESSPDVRLRPRFSHDRGIRLSPG
STGPMSLGASPSQFTPPNYQMQIPSNSTGMHGSGSPASGGIHGSPLGKTPSSYSKRRGLP
MPPHEYPSQHGQGRHGDGVGFSHSDANVRGHPVYSQNSIPSSGYSSWRPQIGSGSFSLEA
SSSHGPSQTFHSHFAPRLQTLDNLSDSSAASTLDNAYWDPYFSDESLLHEDNSLSADLSS
SLHLGDSANPTSGSARTANVQSHIFMGSNPLPAGESYRADHFFHASSRGSLGGNTHSAVP
VNYGGYNPVNHPQQNPQIRHGQPYIQHRYNQATSTHNHPRGSHHNRQPAWPMADGTPWGG
TSGHPFTTTGLPSSLPRKDYGSIF*
>11668.m03043|LOC_Os02g32350.1|genepair1815-1
MASATGASGWLRGKVKGVTSGDCLLIMGSTKADVPPPEKSITLSYLMAPRLARRGGVDEP
FAWESREFLRKLCIGKEVTFRVDYTAPNVGREFGTVYLGDKNVAYSIIAAGWARVKEQGP
KGGEPSPYLTELLRLEEVAKQQGLGRWSKEPGAAEESIRDLPPSAIGEASGFDAKGFAVA
NKGKSLEAIVEQVRDGSTVRVYLLPSFQFVQIYVAGVQSPSMGRRPPNPTVVAAAESTAD
GATNGGDSEEAPAPLTTAQRLAAAAVSTEIPPDRFGIEAKHFTETRVLNRDVRIVVEGTD
SFSNIIGSVYYSDGDTLKDLALELVENGLAKYVEWSANMMDVDAKIKLKNAELQAKKDQL
RIWTGFKPPVTNSKPIHDQKFTGKVVEVVSGDCIIVADDAAPYGSPSAERRVNLSSIRAP
KMGNPRRDEKPDNFAREAKEFLRTRLIGKQVTVEMEYSRRISTVDGQPTTNTADARVLDY
GSVFLGSPSQADGDDVSSIPSSGNQPGINIAETLLSRGFARTSKHRDYEERSHYFDLLLA
AESRAEKAKKGVHSAKESPVMHITDLTTVSAKKARDFLPFLQRNRRHSAIVEYVFSGHRF
KLTIPKETCSIAFSFSGVRCPGKDEPYSNEAIALMRRRILQRDVEIEVEAVDRTGTFLGS
LWESKTNMASVLLEAGLAKLSSFGLDRIPDANVLMRAEQSAKQQKLKIWENYVEGEEVSN
GSASESKQKEILKVVVTEVLGGGKFYVQTVGDHRVASIQQQLASLKLKDAPVIGAFNPVK
GEIVLAQFSADNSWNRAMIVNGPRGAVSSQDDKFEVFYIDYGNQEVVPYSRIRPADPSIS
SSPALAQLCSLAFIKVPNLEDDFGHEAAVYLNDCLLNSQKQYRAMIEERDTSGGKSKGQG
TGTILIVTLVDAETETSINATMLEEGLARLERSKRWDTRERKAALQNLEQFQEKAKKERL
QIWQYGDVESDEEEQAPAARRTGGRR*
>11670.m03158|LOC_Os04g32960.1|genepair1815-2
MVTGGAVPAAAPVWKGKVKSVPSGDTVVIMDTSKAEEVIPPPEMSVTLSCIIAPNLARRG
GMDEPFAWESREYLRRLLIGQDVRFRVEYTASPSGRKFGMVFFAEKNVACMVVAAGLAKV
KEQGQKGEISPYVAELLRLETIARDQGLGRWSKLPGALESSIRDLPPSTIGDGRSFDAKG
FVAENKGKSLEAIVEHVRDGSTIRVHLIPSFLYVQVYVAGVQAPSMGRRATPPPNAQAGV
GNGAANGEASTTPAPMAAAQKLLASADIYSEVPPDRFGQEAKHFTETRVLNREVRIVMEG
TDNFNNIFGSVYYSDGDVVKDLALDLVQNGLAKYVEWSANVLDPQLKTKLRNADLQVKKE
QLRIWTGFKPPVTNTKPIHNQKFTGKVIEVVNGYCLVIADDAEPYGSPSAERRVNLSSIR
PPKFEKPSEENKSSEQFARTAKEFLRTRLIGKQVNVSMEYSRRINIADGQIAGPRTNSTE
TRVLEYGSVFLPSSSHADGETATSSSDSSNNQLGINVAALLVSRGLADITRHRDYEDRSH
HYDALIAAHARAEKTKKGYHSKKECPPIHMTDLTRVPKKAKEFLHLLQRSRRHSAIVEYV
FSGHRFKVTIPKETCTIAFALSGVRCPGRDEPYSDEAITMMRRRILQRNVEIEINTVDRT
GTFLGSLWESNINVASVLLEAGLAKISSFAVDKMPDAQVLLKTEKIAKQKKLKVWENYEE
VEVSNVSLYDNKETLKVIVTEVLGAGMFYVQALADEHVEFVRHQLASLDIKDDPAEALEV
KELETSKEVATLTKDLPETLDAEDPSSDVAKDESVTSKDIDPLPDDSNTAPFTPMKGEMV
LALFRCDNSWNRAMIIGECQGVEGPEFEVFYIDYGNQELVPHSCLRPINLSISSIPPLAK
LCSLAFVKVPSLNDYLGQEAAMYLNSILLDNGREFEAIVEERDAASGGKLQGQGTGEILG
VTLLDSETDNSINAEMLERGYGQLERRRWDSRERRAAIKKLEEFQEVARKEQLGVWCPKN
ARKQGMDENEYPVLARAPPPPKKGYDLIKFIASRSD*
>11668.m03050|LOC_Os02g32420.1|genepair1816-1
MMAVQAQYVAHASRSSSSAAYAIRPALENAAPSSGASALFLDEAVSAALLQQQLVVAAAA
VGGGGNNNNTAVFSDLRSELTCSQRRFDDFGGGFVPRKRARVGGEGEAAAGLLMSSSVME
GGGHRALLPPPPPQVTPQAFGDVHKSSSRVVGSGAASTSGRPVCGGGLLLSHLYRQSVEV
DALVRFEVRATDRAFIYRHGRWSSVSFCLLSSLEWAFDSICTYLQNERLRAGLEEARRRH
LRAVVSAVDRAAARRLHAAEAELERALGRNAELDERLRQMGAEGQAWLGIAKSHEAAAAG
LRATLDQLLQSPCAAAAAAAEGEGDAEDAQSCCFVQAPDGGAAEVSGGGNGRRACRACGE
ADACVLLLPCRHLCLCRGCEAAADACPVCAATKNASLHVLLP*
>11670.m03165|LOC_Os04g33030.1|genepair1816-2
MAVQAQYLSHASFPHDLYGLRALEGATAAGSLFLDDHGGCAPATPAAAAAGIGHTVLSDL
PRSELTCNDNNGAGYGFVPRKRARLDADESAGALMAAAAAQQQRMVLPPHGLVFPGDVQS
RAVGCGAASTSGRAGNAAGLSQGLLSQLYHQGVEIDALVRLESERMRAGLEEARRRHVRA
VVSTVERAAAGRLRAAEAELERARCRNMELEERLRQMTAEGQAWLSVAKSHEAVAAGLRA
TLDQLLQSPCAALAVAGAAGAGGAEGDAEDAQSCCYETPCGGDNAGADDAASKTPAAALC
KACGAGEASMLLLPCRHLCLCRGCEAAVDACPVCAATKNASLHVLLS*
>11668.m03057|LOC_Os02g32490.1|genepair1817-1
MGTASGDQPAGASSDKLRHVESMSELPSGAGRISGINAVVLGESLAAEEHDLVYPSAEFS
ADALVPSPKKYQKMYERSINDPAGFWSEIADAFYWKEKWNPSEVCSENLDVTKGPVQISW
FKGGKTNICYNAVDRNVEAGNGDKIAMYWEGNEPGQDGKLTYSELLDRVCQLANYLKSVG
VGKGDAVIIYLPMLLELPIAMLACARIGAVHSVVFAGFSADSLAQRIVDCKPKLVITCNA

VKRGVKPILLKDIVDAGLAESEKQGVAVGLCLTYENQSAMKREDTKWQAGRDVWWQDVVT
SFPTKCDVEWVDAEDPLFLLYTSGSTGKPKGVLHTTGGYMVYSATTFKYAFDYKPSDIYW
CTADCGWITGHSYVTYGPLLNGATVLVFEGTPNYPDSGRCWDIVDKYKVTIFYTAPTLVR
SLMRDGTEYVTRHSRKSLRVLGSVGEPINPSAWRWFYNIVGDSRCPISDTWWQTETGGFM
ITPLPGAWPQKPGSATFPFFGVQPVIVDEKGKEIEGECSGYLCIKKSWPGAFRTLYGDHD
RYETTYFKPFAGYYFTGDGCSRDKDGYHWLTGRVDDVINVSGHRIGTAEVESALVSHPKC
AEAAVVAVEHEVKGQGIYAFVTLVDGVPYSEELRKSLILTVRNQIGAFAAPDKIHWAPGL
PKTRSGKIMRRILRKIASKQLDELGDTSTLADPGVVDQLIALKDC*
>11670.m03182|LOC_Os04g33190.1|genepair1817-2
MAQLHIYASCGCARARAAIPLPAQAQASAAALPWVMSVTSPPLLRRGGWAMGASDHAAVA
AAASVFRVFLHMAGDQYQEMYRRSIDDPAGFWSEIAETFYWKHKWNPDEVCTENLDVTKG
PIKIEWFKGGKTNICYNAVDRNVEAGDGEKIAMYWEGNEPGQDAKLTYSELLNKVCQLAN
YLKSVGVGKGDAVVIYLPMLMELPIAMLACARIGAVHSVVFAGFSADALAQRIIDCKPKV
VITCNAVKRGKKLIALKDIVDVSLADSAKNGVDVGISLTYENQSAMKREDTKWTSGRDVW
WQDVVPNFPTKCDVEWVDAEDPLFLLYTSGSTGKPKAPNYPDPSRCWDVVDKYGVTIFYT
APTLIRALMRDGTEYVTRYSRKSLRVLGSVGEPINPTAWRWFYDIIGDARCPISDTWWQT
ETGGFMITPLPGAWPLKPGSATFPFFGVQPVIVDEKGKEMEGECSGYLCIKKSWPGAFRT
LYGDKDRYETTYFKPFAGYYFSGDGCSRITGCLHTGKCNLNARDKDGYHFLTGRVDDVIN
VSGHRIGTAEVESALVSHPKCAEAAVVGIDHEVKGQGIYAFVTLVDGVPYSDELRKSLVM
TVRSQIGAFAAPDKIHWAPGLPKTRSGKIMRRILRKIASRQLDELGDTSTLAEPGVVDQL
IALADS*
>11668.m03058|LOC_Os02g32500.1|genepair1818-1
MSNSTNKRKRRKDTQKSRMCAASMRTVLVRSPSSDKILKIFVWLFQLMIIVDDAGAFLPA
LNHSPWDGVTIADFVMPFFLFMVGISLTLAYKRVPDKLEATKKAVLRALKLFCLGLVLQG
GFFHGVRSLTFGVDITKIRLMGILQRIAIAYLLAAICEIWLKGDDDVDCGLDVIRRYRYQ
LVVALLLSTMYTVILNGVYVPDWEYQISGPGSTEKSFSVRCGVRGDTGPACNAVGMLDRT
ILGIDHLYRRPVYARTKQCSINYPQNGPLPPDAPSWCQAPFDPEGLLSSVMAIVTCLIGL
QFGHIIIHFEKHKGRIINWLIPSFSMLALAFSMDFIGIRMNKPLYTISYALATSGAAGLL
FAGIYTLVDVYGFRKLTIPMEWMGKHALMIYVLVACNILPIFIHGFYWREPKNNLLKFIG
VGA*
>11670.m03183|LOC_Os04g33200.1|genepair1818-2
MGGSYELVRTDDAAGHVLDLEAGRCAAVYPKGGGGGGGIAPPPVPSPPSSPASARQRLVS
LDVFRGITVLLMILVDDAGAFLPAINHSPWDGVTLADFVMPFFLFIVGVALALAYKRVPN
KLEATRKAILRALKLFCVGLVLQGGFFHGVRSLTFGIDMEKIRLMGILQRIAIAYIVTAL
CEIWLKGDDDVDSGFDLLKRNRYQLFIGLIVMITYMGFLYGTYVPDWEYRISVPGSTEKS
FFVKCSVRGDTGPGCNAVGMIDRKILGIQHLYCRPVYARSKQCSINSPQNGPLRPDAPSW
CQAPFDPEGLLSSVMAIVTCLIGLQYGHVIVHFQKHKERIMKWLIPSFSMLILAFSLDFF
GMHMNKPLYTVSYALATAGAAGLLFAGIYALVDMYGHRRPTAVMEWMGTHALMIYVLIAC
NILPIFIHGFYWREPKNNLLRLIGVGA*
>11668.m03060|LOC_Os02g32520.1|genepair1819-1
MEVCCCSTSSAVPGRRFAAAGAAAAAVAARWGAVGVGRAVVLAHPLRPAPRGGHAHAQQA
GARRARRAVVRAVFERFTERAVKAVVLSQREAKGLGEGAVAPRHLLLGLIAEDRSAGGFL
SSGINIERAREECRGIGARDLTPGAPSPSGSGLEMDIPFSGSCKRVFEVAVEFSRNMGCS
FISPEHLALALFTLDDPTTNSLLRSLGADPSQLASVALTRLQAELAKDCREPAGASSFKV
PKKSPAGAGRSAFSKSLNSKKEKGALDQFCLDLTTQASGGFIDPIIGREEEIERVVQIIC
RRTKNNPILLGEAGVGKTAIAEGLALRIANGDVPIYLVAKRIMSLDVGLLIAGAKERGEL
ESRVTSLIREVREAGDVILFIDEVHNLIGSGTVGKGKGAGLDIGNLLKPPLARGELQCIA
ATTLDEHRMHFEKDKALARRFQPVLVEEPSQDDAVKILLGLREKYETYHKCKFTLEAINA
AVYLSARYIPDRQLPDKAIDLIDEAGSRARMESFNRKKEGQSSILLKSPDEYWQEIRAAQ
NMHEVVSSNQMKYSPRQENGSAAIKAPSEDMNELTSELQVEEPIVVGTEEIARVASLWSG
IPVQQLTADDRKLLVGLDGELRKRVIGQDDAVMAISRAVKRSRVGLNDPDRPIATLLFCG
PTGVGKTELTKALAASYFGSESAMLRLDMSEYMERHTVSKLIGSPPGYIGYGETGTLTEA
VRRKPFTVVLLDEIEKAHPDIFNILLQIFEDGHLSDSQGRRVSFKNTLIVMTSNIGSTSI
SKGRRSMGFMTEDTESSSYVAMKSLVMEELKAFFRPELLNRIDEMVVFRPLEKTQMLAIL
DIILQEVKGRLLALGIGLEVSDAMKDLICEEGYDKSYGARPLRRAVTHLIEDVISEAILF
GEYKPGDTILMDIDAGGKLCMSHLNEKVVQLSDPTRTF*
>11670.m03184|LOC_Os04g33210.1|genepair1819-2
MEACCCSSSSVPSASILATGAGLRRRFSPAGAGGGGRAVAVAAGRPIRASAALLAAPAPR
RRGGVVVRAVFERFTERAVKAVVFSQREARGMGDETVAPHHLLLGLVAEDRSPLGFLASG
VRVERAREACRAAVGKEGLAQAPVGLATDVPFSGASKRVFEAAVEFSRNMGCNFISPEHI
ALGLFNLNDPTTNNVLKSLGVDSSQLAKQALTRVQGELAKDGREPVGLSSFKVREKFTPG
GGKSAIVKYSNKNKEKSALALFCLDLTMRASGGLIDPVIGRKDEIERVVQIICRRTKNNP
ILLGEAGVGKTAIAEGLAHKIANGDVPIFLVGKRILSLDVALLMAGAKERGELEARVTSL
IREVRKAGDVILFIDEVHTLIGSGIAGRGSKGAGLDIANLLKPALARGELQCIASTTLDE
HRLHFDKDKALARRFQPVLVNEPSQEDAVKILLGLREKYETYHKCKYTLESINAAVYLSA
RYIADRHLPDKAIDLIDEAGSRARMESFKRKKEEQCSILSKSPDEYWQEIRAVQNMHEVA
LTNKVKYSLNQNDQEDAVDIELVGEDKTSPASMLSTSTDKKSATVRLDMSEYMERHAVSK
LIGSPPGYMGFGEGGTLTEAVRRKPFTVVLLDEIEKAHPDIFNILLQIFEDGHLTDSQGR
RVSFKNTLIVMTSNVGSTSISNGKRSIGFQTQTDTEEKSYAAMKSLVMEELKAFFRPELL
NRIDEVVVFHPLEKTQACCFYRYCTCILPCTFVNTMLAILNIMLQEVKGRILALGIGLEV
SDSMKDLISQHGYDKSYGARPLRRAVTQLVEDVISEAILSGQFKPGDTIMVDTDATGKPC
LSRLNDQTVQLSDPTPTL*
>11668.m03061|LOC_Os02g32530.1|genepair1820-1
MAMASSSSASAAAARGGSLVEEWSGRVKALEAGFRKWMAEQPTHIEAAVTTAVGAVQGAA

LGGLMGTLTADGGSPFPTPPPPPPNANPQAMASFKQAQALAGGPLVQARNFAVMTGANAG
ISCVMRRIRGGEDIQGSMAAAFGSGALFSIVSGMGTPNPVANAITTGAAFAIFQGGFFMI
GQKFSKPQSEDLYYSRARSMLQKLGLEKYEKNFKKGLLTDQTLPLLTDRQVIVFVCNQHH
F*
>11670.m03185|LOC_Os04g33220.1|genepair1820-2
MEGMGQQKRRPLVVMASAQAAARGGANPLAELTDRVKTLEAGLRAWLAKQPTHVEAAVAT
AVGAVQGGALGGLMGTLAPDGGSPFPVPQPPPGADPNALASFKQAQALAGGPLVQARNFA
VMTGANAGISCVMRRIRGVEDVQGSMAAAFGSGALFSIVSGMGSPNPVANAITTGVAFAV
FQGGFFMIGQKFSQPPSEDIYYSRGRNMLKQLGLQNYEKNFKKGLLTDQTLPLLNDSALR
DVKIPPGPRLLILDHIQRDPELTKST*
>11668.m03063|LOC_Os02g32550.1|genepair1821-1
MGEKVSEAAAGVEDQDGGVAAAGMDGIQYCSEHPYRPGAAAAAVAGGGICAFCLQEKLGR
LVSSSKSSPFFPLGGHPPPSASPSSPPSFRRAAAVVAEPPPPLRSSSGASRKLIPFGRRK
ASSSSSSSSSASVALAGGGLKRSKSVAPRPEEHYSSSASSVTAESPRKKSFWSFLYLSS
SPYTHQAVTSTSYGANGGAAAAARRKSVSVASAAWASRGGSAAGAHEQQQPRAAATSSVS
GRRLEAIGEPESPSQVSSSSSFGRKVARSRSVGCGSRSFSGDFLERISNGFGDCTLRRVE
SQREPKPNKMRALGHLGGGGGGGADDDDDDDDVYQHQHRIKCAGFFGGMGPTPSYWLSAA
EGAATAGGVGGARKLGGRSHRSWAWTALASPMRALRPTSSTTTTTTKTITAVHSSHVVAH
SNGSTPAAALSISSPVPASSTAATD*
>11670.m03189|LOC_Os04g33260.1|genepair1821-2
MEEKVGETVAAAAVAVAPVVAGDQDGVAYCSEHPYPPGAAAAAGVAAGGGICAFCLQEKL
GMLVSSSKSSPFHPPPASASSSTPTSHVAAESSSSSLPLHPSAAAAARKVIPASAAGGLK
RSKSVAPRPEETTPVTAPAPSAVTADSPRKKSFWSFLYSSSSSSSGGHQGSASMANGGGG
GGASVRRKSVSVASASSASLGRRLEAIVEPDSPGRRSEGSSSSSFGRKVARSRSVGCGSR
SFSGDFLERLSTGFGDCALRRVESHREPKPKSSAGALAHLGGNHSAAGNGDDDEYESTQQ
HRIKCAGFFGGLGAAPPPTSSSYWLSAADGATAAAPSARTHGARSHRSWAWALASPMRAL
RPTSSSSSKSIMAAPHNRGGVSGNGGMAMAALACPQLAQAQAHGDGGGASGQLRLRATCI
VGYGWGFSVAAGHNEDSKQCAIENRPQVQLLVVCVTAQPRLCCPPAMVAGREALACLPIA
QAIMMAWRLIFFSLQVPN*
>11668.m03066|LOC_Os02g32580.1|genepair1822-1
MEHKDRQRHAAAAVAKPRQRSASFHGRGEAEQRHSLLKQRPRTQPDLLAGLRGQSFRRGG
GEGRAPAGPSRVLLTVAVRQSMWPLHVMARAEWSVADLVAAAVELYIREGRRPLLPSADP
AAFGLHFSQFSLQSLNPEEKLMELGSRSFFLCPKAAAAAVAAVSSGEDTGGLSGEDEANS
AKKPSVLAPWLGFLHFWPLL*
>11670.m03195|LOC_Os04g33310.1|genepair1822-2
MPPLTPHIEKGYAGGRQSRQQEASAAAAAAAAAAGGQLLLQRGPGQRSASFHGRGTEPWH
QLARQRPKTQPDLLAGVRGRATAASFGPAAAAGGGEQLEPEAAGRRTPSKVLVSVAVQRS
LWPLHVMASAAWSVADLVAAAVALYVKEGRRPPLPSADPSDFGLHYSQFSLESLDPREKV
MELGSRSFFLCPKSSAAVHAPSPSCSSDEASRIRDRDAPAAARAGAAPAWVSYMQFWPMM
*
>11668.m03074|LOC_Os02g32650.1|genepair1823-1
MECGGGGREQQMQIVCVRGGGGGGGGGGGGEEDGSSEWDQSSSRSALSLFKEKEEEIERKK
LEVREKVFSMLGRVEEETKRLAFIRQELEVMSDPTRREVETIRKRIDKVNRQLKPLGKNC
LKKEKEYKACLEAYNEKSNEKATLVNRLMELVGESEQLRMKKLEELNKTVESLY*
>11670.m03213|LOC_Os04g33450.1|genepair1823-2
MQGEVDQPMQMVLRVKHPSSLGGGGGGGEEEAGEASSRSALSVFKAKEEQIERKKMEVRE
KVFAQLGRVEEESKRLAFIRQELEGMADPTRKEVEVIRKRIDVVNRQLKPLGKTCVKKEK
EYKEILEAYNEKNKEKALLVNRLIELVSESERMRMKKLEELNKTVDSLY*
>11668.m03075|LOC_Os02g32660.1|genepair1824-1
MAAPASAVPGSAAGLRAGAVRFPVPAGARSWRAAAELPTSRSLLSGRRFPGAVRVGGSGG
RVAVRAAGASGEVMIPEGESDGMPVSAGSDDLQLPALDDELSTEVGAEVEIESSGASDVE
GVKRVVEELAAEQKPRVVPPTGDGQKIFQMDSMLNGYKYHLEYRYSLYRRLRSDIDQYEG
GLETFSRGYEKFGFNHSAEGVTYREWAPGAHSAALVGDFNNWNPNADRMSKNEFGVWEIF
LPNNADGSSPIPHGSRVKVRMETPSGIKDSIPAWIKYSVQAAGEIPYNGIYYDPPEEEKY
IFKHPQPKRPKSLRIYETHVGMSSTEPKINTYANFRDEVLPRIKKLGYNAVQIMAIQEHA
YYGSFGYHVTNFFAPSSRFGTPEDLKSLIDKAHELGLVVLMDVVHSHASNNTLDGLNGFD
GTDTHYFHSGSRGHHWMWDSRLFNYGNWEVLRFLLSNARWWLEEYKFDGFRFDGVTSMMY
THHGLQVAFTGNYSEYFGFATDADAVVYLMLVNDLIHGLYPEAITIGEDVSGMPTFALPV
QDGGVGFDYRLHMAVPDKWIELLKQSDESWKMGDIVHTLTNRRWSEKCVTYAESHDQALV
GDKTIAFWLMDKDMYDFMALDRPATPSIDRGIALHKMIRLITMGLGGEGYLNFMGNEFGH
PEWIDFPRAPQVLPNGKFIPGNNNSYDKCRRRFDLGDADYLRYRGMLEFDRAMQSLEEKY
GFMTSDHQYISRKHEEDKMIIFEKGDLVFVFNFHWSNSYFDYRVGCLKPGKYKVVLDSDA
GLFGGFGRIHHTAEHFTADCSHDNRPYSFSVYSPSRTCVVYAPAE*
>11670.m03214|LOC_Os04g33460.1|genepair1824-2
MASFAVSGARLGVVRAGGGGGGGGGPAARSGGVDLPSVLFRRKDSFSRGVVSCAGAPGKV
LVPGGGSDDLLSSAEPDVETQEQPEESQIPDDNKVKPFEEEEEIPAVAEASIKVVAEDKL
ESSEVIQDIEENVTEGVIKDADEPTVEDKPRVIPPPGDGQKIYQIDPMLEGFRNHLDYRY
SEYKRMRAAIDQHEGGLDAFSRGYEKLGFTRSAEGITYREWAPGAQSAALVGDFNNWNPN
ADTMTRNEYGVWEISLPNNADGSPAIPHGSRVKIRMDTPSGVKDSIPAWIKFAVQAPGEI
PYNGIYYDPPEEEKYVFQHPQPKRPNSLRIYESHIGMSSPVCX*
>11668.m03082|LOC_Os02g32730.1|genepair1825-1
MGIAEVALHSMPGAFAAHSPASNLPLAADAARGRRRRSANSLHSSRALQGPVRFPGLRAA
VECQCQRIDDLARVTEGNGAWVKDAVDKASHALGDVRVPGQAVGGNGSVNGSAAKPPPQR
RKASSVEDEAWELLRESVVYYCGSPVGTIAANDPNDANPMNYDQVFIRDFIPSGIAFLLK

GEYEIVRNFILHTLQLQSWEKTMDCHSPGQGLMPASFKVRTIPLDGDEDATEEVLDPDFG
EAAIGRVAPVDSGLWWIILLRAYGKCSGDLTVQERIDVQTGIKMILKLCLADGFDMFPTL
LVTDGSCMIDRRMGIHGHPLEIQALFYSALLCAREMLTPEDGSADLIRALNNRLIALSFH
IREYYWVDMQKLNEIYRYKTEEYSYDAVNKFNIYPDQVSPWLVEWIPPKGGYFIGNLQPA
HMDFRFFSLGNLWSIVSSLATTHQSHAILDLIESKWSDLVAEMPLKICYPALENQEWKII
TGSDPKNTPWSYHNGGSWPTLLWQLTVASIKMNRPEIAAKAVEVAERRIAIDKWPEYYDT
KRARFIGKQSRLYQTWSIAGYLVAKQLLDKPDAARILSNDEDSEILNALSTNRKRGKKVL
KKTFIV*
>11670.m03217|LOC_Os04g33490.1|genepair1825-2
MEIARVVAPPLRLAWCPASRRWGRRKGRPPCPSSLPGPRHDHAVKRPGGEAAGGRSVNGA
APAPAPAPAEAPAKAPQRRQRRGPHDVEDEAWGLLRESVVRYCGSPVGTIAACDPNDASP
LNYDQVFIRDFVPSGIAFLLKGDYEIVRNFILHTLQLQSWEKTMDCHSPGQGLMPASFKV
RVVPLDGDDDVTEEVLDPDFGEAAIGRVAPVDSGLWWIILLRAYGKCSGDLSVQERIDVQ
TGIKMILKLCLADGFDMFPTLLVTDGSCMIDRRMGIHGHPLEIQALFYSALLCAREMLTP
EDGSADLIRALNSRLIALSFHIREYYWLDKRKLNEIYRYKTEEYSYDAVNKFNIYPDQIP
PWLVEWIPPKGGYFIGNLQPAHMDFRFFSLGNLWSIVSSLATSHQSDAILDLVEAKWSDL
VADMPMKICYPALEDQEWKFITGSDPKNTAWSYHNGGSWPTLLWQLTVACIKVDRSEIAA
KAVEVAERRIANDKWPEYYDTKRARFIGKQSRLFQTWTIAGFLVAKQLLENPDKSRILWN
NEDEEILNAMNRMTDASNLKRRRGRKGLKKTYIV*
>11668.m03094|LOC_Os02g32850.1|genepair1826-1
MPLPAMDSLNTKLYLQNCYMLKENERLRKAAVLLNQENQALLSELKHRLARSPSPAAAAP
GVANDSKNAAAAAGRHAGPPPVQDKSASKSK*
>11670.m03226|LOC_Os04g33560.1|genepair1826-2
MDRLNAKLYLQNCYIMKENERLRKKALLLNQENQALLTELKQRLAKTKAAAAAAAATKAN
GNGNMPAGGGRASLPDLNSAPPAHGHDKAVPKSKKTAAK*
>11668.m03104|LOC_Os02g32950.1|genepair1827-1
MSRVLEPLIVGKVIGEVLDNFNPTVKMTATYGANKQVFNGHEFFPSAVAGKPRVEVQGGD
LRSFFTLVMTDPDVPGPSDPYLREHLHWIVTDIPGTTDASFGREVVSYESPRPNIGIHRF
ILVLFRQKRRQAVSPPPSRDRFSTRQFAEDNDLGLPVAAVYFNAQRETAARRR*
>11670.m03227|LOC_Os04g33570.1|genepair1827-2
MSRVLEPLVVGKVIGEVIDNFNPTVKMTATYSSNKQVFNGHELFPSAVVSKPRVEVQGGD
LRSFFTLVMTDPDVPGPSDPYLREHLHWIVTDIPGTTDASFGREVVSYESPKPNIGIHRF
VLVLFKQKRRQAVTPPSSRDYFSTRRFAADNDLGLPVAAVYFNAQRETAARRR*
>11668.m03106|LOC_Os02g32970.1|genepair1828-1
MADSGKKRDGSANGAEKKASPTPKPPSSSTSGFSKNVPLLVFVLLLGLLYRQLQPPVPKI
CGTPGGPPVTGPRLQLKDGRHLAYHEYGVPKDQAKHKIIFVHGFDSCRYDALQVSPELAE
ELGVYMVSFDRPGYGESDPHPGRTEDSIAFDIEGLADGLQLGPKFYLIGYSMGGEIMWSC
LKNIPHRLAGVSILGPVGNYWWSGYPSNVSTEAWYVQLPQDQWAVRVAHHAPWLAYWWNT
QKLFPASSVISFNPAILSREDLTVIPKFAYRTYAGQVRQQGEHESLHRDMLVGFGKWGWS
PLEMENPFPAGEAAVHLWHGAEDLIVPVQLSRHIAQRLPWVRYHELPTAGHLFPITEGMP
DLIVRSMLLTDE*
>11670.m03229|LOC_Os04g33590.1|genepair1828-2
MVRKLILALAVFLPALVYQQLQPPPPKICGSPGGPPVTGTRTQLKDGRHLAYLESGVPKD
QAKYKIIFVHGFDSCRYDALPISPELAQELGIYQLSFDRPGYAESDPNPASTEKSIALDV
EELADNLQLGPKFYLMGFSMGGEIMWSCLKHISHRLAGVAILGPVGNYWWSGLPSNVSWH
AWNQQLPQDKWAVWVSHHLPWLTYWWNSQKLFPASSVIAYNPALLSEEDKLIMPKFAFRT
YMPQIRQQGEYSCLHRDMTVGFGKWSWSPLELEDPFAGGKGKVHLWHGAEDLIVPVSLSR
YLSEKLPWVVYHELPKSGHMFPLADGMADTIVKSLLLGDQPPQA*
>11668.m03108|LOC_Os02g32990.1|genepair1829-1
MCKRSCMAPSESCAARPADERPAVCSCGGAGGGQAAAGTSSDRHHQLVLLQAEAVEKKKG
GRGAAAPDEAAMADGGGGGGGAGDHHQQAALLAPLPVSRRPAPSSVAAGEERESARERLK
RHRTEMAGRVRIPEMWGQERLLKDWVDCAVFDRPLAATRGLLTARDALVAECAAPARRPP
HGPTARPLRVQNGCS*
>11670.m03231|LOC_Os04g33610.1|genepair1829-2
MATSGDVEEVPEPGMDHPAEPCMGVGGDQLVPTTEEISLPLAAETTSDHHEAAQLEQSAE
TSTSESESEEVAAKTTSDSSEAAAVIPKHAAEGSSTASEEEQVAKKELKEAEEDDGLQGE
SARERLKRHRREMAGRVWVPDMWGQEKLLKDWVDCAAFDRPLVPPDLLTARRALVAECCA
RRPDRTTTPPGPDDDAARQIQPSPGAKKLLLIIELDY*
>11668.m03109|LOC_Os02g33000.1|genepair1830-1
MATAARAASCTPAACAVLVIPILVLIMAGQVRVAEALSIGVNYGQIANNLPSPSRVSWLL
RSLKISKVKLFDADPHVLRAFLGTGVEFVVGIGNEAVPAMASPAAAESWLQLHVVPHLRA
GARITCITVGNEVFKGNDTALQASLLPALRSVHQALGALGLQGRVNVTTAHSLDIMGVSY
PPSAGAFHPSAAPHLQPFLAFLSAARAPFLINCYPYFAYKDDPARVPLEYVLFQPNAGVV
DPRTRLVYDNMLYAQVDAVYAAIQAMGHTDIDVKVSETGWPSRGDPDEAGATPENAGTYI
GNLLRRIEMKQGTPLRPQAPIDVYVFALFNENLKPGPASERNYGLFYPDGTPVYNVGLRG
YLPPMASHEAATQVIHWFLLIATASVVFALS*
>11670.m03234|LOC_Os04g33640.1|genepair1830-2
MAAGEQPGTGSPPQPSTRLFLLVLAVILTDQVLAASAQGMSIGINYGQIADNLPSPTRVS
GLLRSMQISKVKLYDADQNVLSAFLDTGVEFVVGIGNENVSAMVDPAAAQAWVQQHVRPY
LPSARITCITVGNEVFKGNDTALKANLLPAMQSVYNAVVALGLQGQVNVTTAHSLDIMGS
SYPPSAGAFRPDAVPYIQPLLNFLSMAGSPFLINCYPYFAYKADPGSVPLEYVLFQPNAG
VTDPNTKLNYDNMLYAQIDSVYAAMQALGHTDVDVKISETGWPSRGDPDEAGATPEYAGI
YIGNLLRRIEMKQGTPLRPSSPIDVYVFALFNENLKPGPASERNYGLFYPDGTPVYDVGL
RGYLPPMDESKSARKAVSVLALIAIASITLILS*

>11668.m03112|LOC_Os02g33030.1|genepair1831-1
MGTAKKSAIFVALLCTHILVNHACDSDVVFGYSGSTGPEHWGSLSPNFTTCSKGTYQSPI
NILKDDAVYNPKLEPLEMDYTAANTTIVDNVFNIALRYNDTAGTVKVDGKKYKLRQLHWH
SPSEHTINGQRFAVELHMVHHSDDGNITVIAVLYRHGKPDPFLFQIKDKLAALYLEGCKA
EKGEPLPVGLVDMRELKKGADRYFRYVGSLTTPPCTENVIWNIFGEIREMTKEQAAALRA
PLHGSYRHNRRPTQPLNGRTVQLNYM*
>11670.m03237|LOC_Os04g33660.1|genepair1831-2
MAASHGNAIFVLLLCTLFLPSLACDSGGVKFGYTGSIGPDFWGNLSADFTRCSNGKQQSP
IDIDTNNLVHELNMEPLHRNYTAANATLVDNIFNVALRYEEAAGVLSINGVKYTLKQMHW
HSPSEHTINGFRFPLELHMVHTNENGNITVLAFLYRFGRPDPFFEQDKLAALNAEGCKAE
KGSPVPAGSVSLLTMRQHVHIYYRYVGSLTTPPCAENVIWNIPAMPREMTPQQAADLMAP
LDEGYRRNSRPTQQMNGRTVQLYHRFWGKKKRRSSP*
>11668.m03115|LOC_Os02g33060.1|genepair1832-1
MPPLTLDSLLHGGGGEPEDECEDEFSGSDDDGEDGGGGSEEWGGDVDGEYDPYSPAESLW
LRIGEDIDWSEVGAVLEREDSTKGASNPKSAAACSCAGAPAARMPTCAGGGGTAKAVVIA
GLPAAARKASREHERRRRLGRARARARVFAGDAVEVAEPGSPKVSCLGGVRSRARAQQPC
CPAAAAAGRRRWWCAPWLVSAACRRCWSAPPPRV*
>11670.m03238|LOC_Os04g33670.1|genepair1832-2
MPQINLESLFCGGGEAGSRVACETIALPGCSDAPAESRCVRIGDGAIWAELAGGAVLERD
GSTKGSSNPKAAAASGKGKKGGPRPSSAESRRLPVTGKAAVVICGLPAGKMVAQKKRLSP
CLGRGWRRAPAAAGARVFASEAVETDPGSPKVSCFGAVRSERSPATAAAAPAPPVEDEER
NGGCWASVAATLRHLCRSSSNPLEGELETNEWNATATSSPTVAALSPPRPVAVGLGEMKR
LASRRWPETMAVAGQGPVSAA*
>11668.m03120|LOC_Os02g33090.1|genepair1833-1
MPRPPPPLFGQAGQDQRRRYLGLCTEGLGSESSESSGGDVDLGTGGGDDTGNDGVGRALP
CKRQHRPIDDEEEEEEKTVVPAALAPPLPAWTRAAFPPPISVIGAGGKPWLYLRAHRGDG
RLVLREVRIPSRELLHGRREDGRFKLHFAHPDEQLQQQLLLLADDQDPAEKNE*
>11670.m03242|LOC_Os04g33710.1|genepair1833-2
MAPPPPPPPLHCYKAPEEQRLGPRPATQSRPPPHLLPYSGGLDLLTEALGAESFDPDDDD
DATAASPAMEDVGAAVAAVDFLAPPCKRPHHVLLSSSSEGVGHDDDDNQHAVMVLRRTRS
GRAFPPPISVIGKGGRPWLSLRAHREAGRLVLREMRLPSQELLQPCKEDGRFKLLIHPEA
GRRSGGAGAGPRVGSGREGHGALES*
>11668.m03122|LOC_Os02g33110.1|genepair1834-1
MGTRLLALAPWLLLLLLQLAGASHVVHRSLEAEQAPSSVPASIVSPLLRTGYHFQPPMNW
INGPLYYKGWYHLFYQYNPKGAVWGNIVWAHSVSQDLINWIALEPAIKPDIPSDQYGCWS
GSATILPDGTPAILYTGIDRPNINYQVQNIAFPKNASDPLLREWVKPAYNPVATPEPGMN
ATQFRDPTTAWYADGHWRMLVGGLKGARLGLAYLYRSRDFKTWVRAKHPLHSALTGMWEC
PDFFPLQAPGLQAGLDTSVPSSKYVLKNSLDLTRYDYYTVGIYNKVTERYVPDNPAGDYH
RLRYDYGNFYASKTFFDPVKHRRILLGWANESDSVTYDKAKGWAGIHAIPRKVWLDPSGK
QLLQWPIEELETLRGKSVSVFDKVVKPGEHFQVTGLGTYQADVEVSLEVSGLEKAEALDP
AFGDDAERLCGAKGADVRGGVVFGLWVLASAGLEEKTAVFFRVFKPAGHGAKPVVLMCTD
PTKSSLSPDLYKPTFAGFVDTDISSGKISLRSLIDRSVVESFGAGGKTCILSRVYPSMAI
GDKAHLYVFNNGEADIKISHLKAWEMKKPLMNGA*
>11670.m03245|LOC_Os04g33740.1|genepair1834-2
MGVLGSRVAWAWLVQLLLLQQLAGASHVVYDDLELQAAATTADGVPPSIVDSELRTGYHF
QPPKNWINDPNAPMYYKGWYHLFYQYNPKGAVWGNIVWAHSVSRDLINWVALKPAIEPSI
RADKYGCWSGSATMMADGTPVIMYTGVNRPDVNYQVQNVALPRNGSDPLLREWVKPGHNP
VIVPEGGINATQFRDPTTAWRGADGHWRLLVGSLAGQSRGVAYVYRSRDFRRWTRAAQPL
HSAPTGMWECPDFYPVTADGRREGVDTSSAVVDAAASARVKYVLKNSLDLRRYDYYTVGT
YDRKAERYVPDDPAGDEHHIRYDYGNFYASKTFYDPAKRRRILWGWANESDTAADDVAKG
WAGIQAIPRKVWLDPSGKQLLQWPIEEVERLRGKWPVILKDRVVKPGEHVEVTGLQTAQA
DVEVSFEVGSLEAAERLDPAMAYDAQRLCSARGADARGGVGPFGLWVLASAGLEEKTAVF
FRVFRPAARGGGAGKPVVLMCTDPTKSSRNPNMYQPTFAGFVDTDITNGKISLRSLIDRS
VVESFGAGGKACILSRVYPSLAIGKNARLYVFNNGKAEIKVSQLTAWEMKKPVMMNGA*
>11668.m03125|LOC_Os02g33140.1|genepair1835-1
MSGRKKTREPKEENVTLGPTVREGEYVFGVAHIFASFNDTFIHVTDLSGRETLVRITGGM
KVKADRDESSPYAAMLASQDVAQRCKELGITALHIKLRATGGNKTKTPGPGAQSALRALA
RSGMKIGRIEDVTPVPTDSTRRKGGRRGRRL*
>11670.m03246|LOC_Os04g33750.1|genepair1835-2
MSGRKKTREPKEENVTLGPTVREGEYVFGVAHIFASFNDTFIHVTDLSGRETLVRITGGM
KVKADRDESSPYAAMLASQDVAQRCKELGITALHIKLRATGGNKTKTPGPGAQSALRALA
RSGMKIGRIEDVTPVPTDSTRRKGGRRGRRL*
>11668.m03135|LOC_Os02g33230.1|genepair1836-1
METPEIFTGAAAATVVVRRAVVIGNGSPGAENQCLGLVRALGLADHLTLYRVTRPQGGIN
EWLHFLPVSLHKLIDQVLRQFFRNTRLAPVVEGRKHYRVPNGGSVGVGLSSVLEADAKKI
VAVARDTFEKEGPTLIVACGWDTISYSSSIRHLASGNVFVIQIQHPRSRLDRFDLVVTPR
HDYYALIAGGQEEIPRLFRRWITPQEPPGRNVVLTVGALHQADSAALRLAAIAWHDELAP
LPKPLLIVNIGGPTRNCKYGVDLARQLIASTYNVLDSCGSVRVSFSRRTPRKVSDIISKE
FAGHPKIYIWDGEEPNPHMGHLAWADAFVVTADSISMLSEACSTGKPVYVIGTEYCKWKF
SAFHKTLRERGVVRPFTGLEDISNSWSYPPLNDAIEVATRVREAIAERGWSVG*
>11670.m03248|LOC_Os04g33770.1|genepair1836-2
MEPLGGGGGGPAGAPEIFAGGTGARGSVRRAVVIGNGCAGAENQCLGLLRALGLADRLTL
YRAIRPTGGINKWLHFLPISLHKLVDQVLRQMFSSNKFATLFQGAKMAQYTVCNGQSLGL
SSVLEADTKRIVTMVNDTFEKEGLALVVACGRDTISYASSIRCLAPDNVFVIQIQHPRYR

LDRFDLVVLTVGALHQADSAALRTAASDWHDELANSPKPLVVVNIGGPTRNCNYDVGLAK
KLISSLHNVLKTCGSVRVSFSRRTPHKVSDLILKEFSTHPKVYIWNGEGPNPHLGHLAWA
DAFVITADSISMLSEACSTGKPVYVVVGTEHCRWKFSDFHNRLHERGAVRPFTGLEDMSDN
WSYPPLNDAIDVAAREFLSSHRLQEEVLPFQKENQQVVLVLTSSLVHLGCATGQERENVQ
SDDFCVGWSFHAQTGCRGLVQSCADSLGKGCPGGSDRSAPAADEAKRHAQNRRVDELAKL
KHSLLKCRLPLPWNAATILEQLLRNHHRATLAAMAMECHWLPQSSSSSNMGARSCFAAVA
RGP*
>11668.m03136|LOC_Os02g33240.1|genepair1837-1
MAATTTTVEDLPGDVLACALRRLDGPSLAAAGCATSGLRALADDPDTWRALCLSRWPSLA
AAEQRCVLSAAGAVSPRRLFADAFPFPCVDDAAAAAPLDGDDQRLPGELVSAVDVYHGGA
AVVSRVVETSTSSSWFLASPFRVDAVEGKSPAPAPASVASSWSPAELELSWILLDPSTGR
AVNVSSRRPVAVERHWYTGDTLVRYAVVLAGCKFEATVSCSEEAGQITEVSLAADDADGA
AISGEGCLRLLAAAMAGPRKGGRGQEGEAKRRYDEFVRRKRGRKESKARREVLVDLCCSA
VSAVAVISFLAAVVLR*
>11670.m03253|LOC_Os04g33820.1|genepair1837-2
MAPGREEEKMVATTTIEDLHADVLARALRRLDGRSLAAASCATAGLRALAADPETWRALC
LAEWPSMAGHPRLLSVVPPRRLFADAFPFPRPDAGELGGGGGGPLPSELVSAVDVYYRGA
PLLSRVVETPASSPWFLGSPFRVEAVECKKPAAEAALSPAELELSWVVVDPARGRAVNVS
SRRAVAVDRHWYTGETLVRFAVVLGGCKFETTVTCSEGAGNISEVSLAVQDADGAAASGE
RSLRLLAAAMEEQRIGGGRERDEAKRRYDEFVKSRKGRKESKARREALIDLCCSAASAMA
VLSFVAAVVLR*
>11668.m03146|LOC_Os02g33330.1|genepair1838-1
MSGGGGGGRGMTRGLDLKLNLSLPAVARAVSPAADDESSPSSCLSSESELRQQHGGGGGQ
LQWSDSPEATSMVLAACPRCFLYVMLAEADPRCPKCRSPVILDFLHAGGGGGINADGRRH
RRG*
>11670.m03257|LOC_Os04g33860.1|genepair1838-2
MSRSNKKSSRGIDLKLNLSLPARGDSSSRRAMAADEESSPSSCLSSENEHGLQWSNSPEA
TSMVLAACPRCFIYVMLPQDDPRCPQCKSPVILDFLQQDNGNNNANSNSSRKTRRG*
>11668.m03156|LOC_Os02g33430.1|genepair1839-1
MAGSGRPPAAQKKILQSLRPPLPFAASSRSPFAAPNDYHRFPAGGAAAAAASGSGGIGAG
GAGGGGDIEEGLVIRTPQKRKAPEESDVAESSDCMITSPGFAVSPMLTPVSGKAVKTSKS
KTKNNKAGPQTPTSNVGSPLNPPTPVGTCRYDSSLGLLTKKFINLLKQAPDGILDLNNAA
ETLEVQKRRIYDITNVLEGIGLIEKTLKNRIRWKGLDDSGVELDNGLSALQAEVENLSLK
EQALDERISDMREKLRGLTEDENNQRWLYVTEDDIKGLPCFQNETLIAIKAPHGTTLEVP
DPDEAGDYLQRRYRIVLRSTMGPIDVYLVSQFDEKFEDLGGGATPSGHANVPKHQPTEVF
NTTNAGVGQCSNSVAVDNNIQHSQTIPQDPSASHDFGGMTRIIPSDIDTDADYWLISEGD
VSITDMWKTAPDVQWDESLDTDVFLSEDVRTPSSHNQQPSAVGGPQMQVSDMHKP*
>11670.m03266|LOC_Os04g33950.1|genepair1839-2
MSSGGGRPPAAQHIVRSVRQRFVPLPPPLARAPFAAAPGDYHRFAAASRGGEIEEGIVIR
RTPLKRKTPCGESEAAESSERMMTSPGFTEGVGSPLMTPVSGKTSRTTKSMAKFNKAGPQ
TPISNAGSPGNPSTPASSRYDNSLGLLTRKFINLLKQTQDGILDLNDAAKILDVRKRRIY
DITNVLEGTGLIEKKLKNRIRWRGSDDSGTNLDSDISCLKTEVENLYIQEQALDRSISEI
REKMEELTEDESNHRWLFVTEDDIKGLPCFQNEALIAIKGPRGTTVEVPDPDEAGDYLQR
RYRILLRSTMGPIDIYLVR*
>11668.m03158|LOC_Os02g33450.1|genepair1840-1
MAACCSSLATAVSSSSAKPLAGIPPAAPHSLSLPRAPAARPLRLSASSSRSARASSFVAR
AGGVDDAPLVGNKAPDFDAEAVFDQEFINVKLSDYIGKKYVILFFYPLDFTFVCPTEITA
FSDRYDEFEKLNTEILGVSIDSVFSHLAWVQTDRKSGGLGDLKYPLISDVTKSISKSFGV
LIPDQGIALRGLFIIDKEGVIQHSTINNLAIGRSVDETMRTLQALQYVQDNPDEVCPAGW
KPGDKSMKPDPKGSKEYFAAI*
>11670.m03268|LOC_Os04g33970.1|genepair1840-2
MACAFSVSSAAAPLASPKGDLPLVGNKAPDFEAEAMFDQGFIKSKCMFVSSAEITAFSDR
YEEFEKINTEVLGVSIDSVISKSFGVLIPDQGIALRGLFIIDKEGVIQHSTINNLAIGRS
VDETLRTLQALQYVQENPDEVCPAGWKPGEKSMKPDPKDSKEYFASI*
>11668.m03168|LOC_Os02g33550.1|genepair1841-1
MSKEKHHKREHHLRRCCGGMAACILALVLVVGFIALVVYLALRPSKPSFYLQDLQLRSVD
LGDPSLSATAQVTLASRNPNDHVGVHYRRLDVFVTYRDEPVTVPVSLPPTYQGHRDVTIW
SPVLSGESVPVAGFVADALRQDVAAGYVALQVKVDGRVKWKVGSWVSGSYHLFVSCPAML
ASAGPGGVGPMPLGGASAAVVNGTGAGAVASLRFTQPTGCSVEV*
>11670.m03270|LOC_Os04g33990.1|genepair1841-2
MGKDKHHRDWILRRCCGSIAACILTLAVLVGFIVLVIYLAIHPSKPSFYLQDVQLRNIDL
SDPAISLNLQVTIASRNPNDRVGVYYKTLHVFTTYREEPITVPVELPAIYQGHKDVSVWS
PVMSGESVPVGQYVADAMRQDIAAGYVLLHVKVDGRVKWKVGSWVSGGYHLFVTCPALLA
ASGGNVGGAFAMSATAGGGAGGNATVSLKFAQAADCTVDV*
>11668.m03171|LOC_Os02g33580.1|genepair1842-1
MEARASPSPSPQRPASMGRGGGGGGGGGDASAALSFIYKGWREVRDSASADLRLMRARAD
SLRTLADRELEHLLVSASTTVAAPAPPVAAGAPIAEVEFVRNRIQPKISELRRQYAASGD
WELGLGRRVLEGWVAPPPPRGATTARVDLSGITAIRNALVPEVAGGGGASTAWWSGDEME
EEEEKEWEVVRMIRGGLKELERRSQSSGEILGGIPGPSELVEKFKSRLKSFNMEPLGSKE
VPPLDLTEIMANLVRQSGPFLDQLGLRRELRDKLVETLYSRQNHSLSADSSLLGDDNSTD
ELDLRIASVLQSTGYHTDDGLWNEPSKYEVSDNKRHVAIVTTASLPWMTGTAVNPLFRAA
YLARNSKQDVTLVVPWLCKSDQELVYPNSMTFSSPEEQETYIKKWLEERLGFESNFKISF
YPGKFSKERRSIIPAGDTSQFISSREADIAILEEPEHLNWYHHGNRWTDKFNHVVGVVHT
NYLEYIKREKNGALQAFLVKHINNWVTRAYCDKVLRLSAATQDLPKSIICNVHGVNPKFL

KIGDKIMADRENGQQSFSKGAYFLGKMVWAKGYRELLDLLDKRKSDLQGFKLDVYGSGED
SQEVQSTAKKLNLNLNFFKGRDHADDSLHGYKVFINPSISDVLCTATAEALAMGKFVICA
EHPSNEFFMSFPNCLTYRTPEEFVARVNEAMAREPQPLTPEQRYNLSWEAATERFMEYSD
LDKVLSQPVTEGVHRSKTRRTIQSNLSDAMDGGLAFAHHCLTGSEVLRLATGAIPGTRDY
DKQHCVDMGLLPPQVQHPVYGW*
>11670.m03271|LOC_Os04g34000.1|genepair1842-2
MGGERPPPISGGGAFAFISKGWREVRDSASADLRQMRARADRELEHLLASASALAGPPLP
PVAAGAPIAEVEFVRKRIQPKIMELRRQYSSTVRDAGWAPKAAGASLRVDLSGITAIRNA
IVAEGGGGGGGGGRWGLVRWKGHADDEGRKEWEVVRMIRSGLKEFERRSLSSEVFGGFRG
RGEFVEKFKLSLKSLNKESQESKEVLPLDLTEILAYLVRQSGPFLDQLGIRRDLCDKIVE
TLYSKHNGRLIYHSLSADRSLIGNENMTDELDLRIARVLESTGHHTEESFWKDHAKYKLS
DNRRHVAIVTTASLPWMTGTAINPLFRAAYLARSTKQKVTLVVPWLCKSDQELVYPNNIT
FSSPEEQENYIRNWLQERLGFEANFKISFYPGKFSKERRSIIPAGDTSQFISSSEADIAI
LEEPEHLNWYHHGKRWTDKFKHVIGVVHTNYLEYIKREKNGALQAFLVKHINNWVTRAYC
HKVLRLSAATQDLPRSVVCNVHGVNPKFLKVGEKIAADKEHGQQSFTKGAYFLGKMVWAK
GYRELIDLLSKHKSDLEGFNVDVYGNGEDSQAVQMAARKLNLSLNFFKGRDHADSSLHGY
KVFINPSVSDVLCTATAEALAMGKFVICADHPSNEFFKSFPNCLTYKTSEEFVARVKEAM
ASEPSPLTPEQRYSLSWEAATERFMEYSELDKVLNNKIGYSGQDGKRSKVRKIPLLPRLS
EVVDGGLAFAHHCLTGNEILRLATGAIPGTRDYDKQQCMDLNLLPPQVQHPVYGW*
>11668.m03173|LOC_Os02g33590.1|genepair1843-1
MEEAAAEVPSYFLCPISLEIMRDPVTLATGITYDRSSIERWMFGGGGGDGGKGTCPVTRR
QLAPAEREATPNHTLRRLIQAWCAAHAVERFPTPRPPVDSCRVAALVDEGTTTMLGGGGR
QRQLAALREIKAIAAESDRNKRCVEATPGAVEFLVSVVVQSHAAASTSASSDDDDLFDSV
IDSPMSTSSPEEEALGVLYSLKPSEPTLRRVLGKDNGVGFLDTLASVLRRPSYRSRAYAI
LLLKAVTSAMPPERLMAVSPELVEEVVRVVSDGVSSKAVKAALHVLCRLCPWGRNRVKAV
EAGAVAALVELLLDEEGGGGRRRAAELAVVAIDHLCGCAEGRSELVAHPAGLAVVSKRAM
RVSPAATESAVRALHAVARNAATPAVLQEMLAVGVVAKLLLVLQADGGERARARAREMLR
ANARVWKDSPCLQAHLKASYPS*
>11670.m03274|LOC_Os04g34030.1|genepair1843-2
MGEESSPAAAPAVEVLSYFVCPISLEIMRDPVTLSTGITYDRESIERWVFTDGHGECPVT
KQRLAPADREPTPNHTLRRLIQGWCAVHAVERFPTPRPPVDAARVAAIVDAARPLLRRRR
QREELMASLRELADIVAESDRNRRCVQGASGAVEFLLSVVKERASVAGVDDATSAKPEET
TCGGVHDPAKASSPEEAALSILHSLKLSEESFKRVLEGSGGEDFLETMACVLRRPSYLSR
MQGIHLLKSALPAMAPARLTSARAALVDGVVGVVADRPSAKAVKVALHVLCRLCPWARNR
VKAVDAGAVSALVRLLLDEGCGGGGGDRRACELAAVAIDHICRLRGGAPGSGGAPGGAQR
QVACSADRCPHTAGAESAVRALHAVARHSATSAVLQEMLPVGVVARLLFLVQVGASGERT
RARAREMLKMHARVWRDSPCLASHLNASYPR*
>11668.m03174|LOC_Os02g33600.1|genepair1844-1
MDASDGRPSPRSRQLQGPRPPRLAVSKDSHKVRKPPVVPQPRGGGGVVAGPSRPQQQQQP
RAPVIIYDASPKVIHTRPSEFMALVQRLTGPGSAAVAAVAAPVAAGFHTEASSSSSASAS
ALPPQFQLPQEFMLSPTAALSPAARYAAIERSVRPLPPTTAHYYTADADDPILLDVDGDA
AAFAAALGPARPSILSPVPSALPPAASSGLFSPLDQASLSWLSDLSPFLHSAGAAAAPPP
PFAPSPRSLLLSTPTMPSPATFSVMEFFSSNFPDL*
>11670.m03276|LOC_Os04g34050.1|genepair1844-2
MEFPSSTSPSPSPSSGQHQQQPTTPRRQLQGPRPPRLNVRMESHAIKKPSSGAAAAAAQA
QAHQLPAQAQARREQQQPPPRAPVIIYDASPKIIHAKPNEFMALVQRLTGPGSGPPAPPH
QGEAQAQDYPMMDEAAAQQFFPPELLLSPSAAMSPAARLATIERSVRPMPEPAPEYVDIT
NGGGGGGVDDGGLAAILGSIRPGILSPLPSSLPPAAVPGQFSPLPFDASCISWLNELSPI
LRAASAGAASSGSGGGGSGGNTSNGGAARPPPSYYADPFVPSPRHLLATPTVPSPATCAE
LFSNLPDL*
>11668.m03177|LOC_Os02g33630.1|genepair1845-1
MPRSDYSDDGEDDVFFDAFDDDDDDDDDKNRSSTEISTSEAGYELWAGEPMSVRERRHRF
LKGMGFLEPGPTGTAFPQWLAEIATTDCCSFHDFEERISSICSSFRSCFSDSILAATDNT
NDSADNCTRDVDYNSSGRRSTTSHDQGQHDVLSEIVEEAGTSSDEMVTPNAPEIVPGFSK
LMRKLLRIRFGHGPKRNEFKSLWEIFMRKKVSDRVLSMDDVHVQPRGLNSGTLYRTKVHQ
QNKKWMDFTAVYMCQEIQAHEGLIRVMKFSSSGWHLASGGEDCVVRVWQITEVESSPDLY
GRDVPEDMNKKKDVKIKPLAIIPKKVFSITETPLHEFQGHTSDVLDLAWSKSDFLLSSSK
DTTIRMWKVGCYDCLAVFRHGDYVTCVQFNPVDERYFISGSIDGKVRVWDVSDKRVVDWD
DTKYIITAISHRPDGKGFVVGSVKGRCRFYDQSGLFPLGRNIERNKLMRIKRRRCAANKI
TNIQFSQGHPSRMIITSKDHKIRVSEGHKITQKFQGQWRSKVLVPPSLTPDGRYLISAGR
DSKIRIWNFDGGGRRRRVVSSRELFFSEGVTAVAPWARAMGGGGGGADAPTLCYDRERCS
FGTWFVVPDGAAAAAATTWPEERLLPSLRYVNCAGMDDCRSQVPAAWNMVVVTGSRGGAI
RAFHNYGLPVRL*
>11670.m03279|LOC_Os04g34080.1|genepair1845-2
MPSSESDRDDIFFDAFDDVTSTREPSLSDDCSTSDEGLASRRFEYDIWGNEPMSVEERRQ
RFLKGMGFDEFLATRVDFSQPQGEITTVGPFADLGLEESTTSDISSVNSSVPENESVSDA
SCCIGDIDSGERYTVQNDGYGELTSMLKDVASHKVVSLLEFDGVPGLSQSVQKLLRKVYS
SSMEEKKNVFNKKKGVKSLWKSFMKNRSFGGICKHDVNVKNCTIGIPSRTKVQHRKKKTM
EFSAVHLGQEIQAHKGLIKVMKFSPSGWYLATGGEDCIVRIWQIMEVEASSKLHGGDNPQ
NYDDKITIIKTELGRGKNHALAVVPKKGFRISETPLHEFQGHTDDILDMAWSESDYLLTS
SKDKMVRLWKVGCDGCLGLFKHKDYVTCVQFNPIDERYFISGSIDGKVRVWDALDKRVVD
WTDTRKIITALSYQPDGKGFIVGTTSGECRFYDQSGENIQLDKELFMQGKKSAVHRVNSL
QSRSSDSSRITITSTGSKIRVADGVDIIQKFEGPWNLKALSSPSLTSDGRYLISAGLDSN
VYIWNFDIPSVADHKGEAKSVRSCEKFFSKDVTTAVPWPGLHQERQQVKNSSSLTEESVS

SPILHRHGERRSPAARCFADGMKGTPTWPEEKLPPAKAADAPRLSDCLSTISPAWNTVIV
TASRDGVIRSFHNYGLPVRL*
>11668.m03182|LOC_Os02g33680.1|genepair1846-1
MDPSSSSSSSPFSTPFHSISSTSTLRPQLAIAIVATPSVCGRNEIEQQPSMVRKEMGGG
GGRLAAEYQGLEVKVPTFFRCPISLDVMRSPVSLCTGVTYDRASIQRWIDSGNTTCPATM
LPLPSTDLVPNLTLRRLIALWASTAAPSSPAAPSAVGPTPAAAAAELLRRVAAPGVDPCP
ALRKLAAFLSDDDVDEFDKNALARAGGAAETVASVLRRRGKGDDDDGGVEAVEAAVRVLA
VLATSDCIEEENRRRVAAALAAGGAAPSVAASLARVMRSGSGLEARVDAARLVESLLRDG
ARGATAPGVRAAVAESEELVAELIRLVGPTDEKGSLDAQAVDAGLSCLAAIVAATRRARA
EMVRLGAVPAAVRVLATDHHGGSHAQALRVLEAAVGCAEGRAAVCEVAEAAIPAVVSRMM
RCGGMGGAEAAVSVLWAVCHRYRDRRAVEAAAASEGGLTKLLLLMQSGCSPAARQMASEL
LKMFKVNAKSCLAGYDSKTTHIMPF*
>11670.m03285|LOC_Os04g34140.1|genepair1846-2
MGRKESSTTTTTAAAAAAATRSMRLPPQHQALEVKIPSFFRCPISLDVMRSPVSLCTGV
TYDRASIQRWLDSGNTTCPATMLPLPSTDLVPNLTLRSLISHWSSSSPATSGDASVTSSP
AGLVRQVASPDADPSAALRQLAAYLSDDDVDEFEKNALVGAGGAAEAVASVLRRKGEREV
GVEGCEAAVRVLAAVVAMDGVEDANKRRVAAGLAADAAASAASLARVMRGASGLEARVDA
ARLVEFLLANAADEAREAVAESAELVAELVRLVGPADEKGSLDARAVGAGLSCLATISRS
RRAARAEMVRAGTVRAAARALRATAADPAASARALRVLESAVGCAEGRAALCEDAEQAVP
AVVGRMMKAGRDGAEAAEAVLWAVCHKYRDRRDADAAEASEGGLTRLLLLLQSGCSPAAR
QMALELLKIYKVNAKSCLAGYDSKTTHIMPF*
>11668.m03186|LOC_Os02g33720.1|genepair1847-1
MLLAPAYGHGDSSAAAAAAGASQVIAGQEAPAGGDVVAGPAGTSSFDANVVMILAVLLCA
LICALGLNSIVRCALRCSSGGRMMMSSSSSAAAGDDGELGPSAAAQAAGVRRKALRAMPT
MVYSAAGGPSPACAICLADLEPGERVRVLPKCNHGFHVRCVDRWLLARSTCPTCRQPLFA
TPPVRPFLAPLRPEGFVTPYDF*
>11670.m03296|LOC_Os04g34230.1|genepair1847-2
MARDAPRPTARCRRAMAVAPPDPDVGRPNPAKEVPDPPPPQAAPPSSPLVVVVDVVSPTP
SSSHQGKGCCSGRPRRRLPGSPLTSSGGGEEEQGRPTQLGEPKIFSCRIPKKAFVHTPRV
EMYSRRILLHTPFSGQPSGPSQPVSGATIVEGGSPGSNFDANIVMILAVLLCALICALGL
NSIVRCALRCSSRMVVDPEPSRVTRLAQSGLRRKALRSMPILLYSTGLKLNTVSPMCAIC
LSDFEDGEHVRVLPKCNHGFHVRCIDRWLLARSTCPTCRQSLFGAPQKASGCSESEGSQA
EPAPARPVLAPLRPEGLVTPYDF*
>11668.m03242|LOC_Os02g34270.1|genepair1848-1
MAALFEAAETAAIVAALTRVIADGGRGGSGAGVPPPAPSLVVPPLAGTGGGRRVDVAREE
EMVGVVSAGDHTGEASVAAAGVVVAAPATARRYRGVRRRPWGKWAAEIRDPRKAARVWLG
TFRTTEDAARAYDAAVLRFRGRRAKLNFPEEASRPRRPWKGHDVDHMSCSPPSIANARFL
GSWIFGPPPPSRSVAAATTTLLGGSHGSNGADNGRE*
>11670.m03373|LOC_Os04g34970.1|genepair1848-2
MALSEPDLAAETEAIVSALTHVVAYGGGGPPPSEETAASAVTRTAPWRADGARQGAVPAA
RKYRGVRRRPWGKWAAEIRDPHRAARVWLGTFATAEDAARAYDAAALRFRGGRARLNFPE
DAAAADERRATDAAAAAAAASSAPAALLESQPGDVADCLDYSRILAGAPERPTSSTVPDG
FFGGGGNGRFLHSWSIGTSPSPSGSGSGGAGGGGGGGGGGAPVRPLFHGGNGWEQRGDSA
YNGF*
>11668.m03247|LOC_Os02g34320.1|genepair1849-1
MAQEGTSSNAPPAAASMGSGDGDNKEGTGESGNNQLLLPAIAASADKGKGVVAGTGNVDA
KGKTTAMAPAASSTNAPNNQGGGGGGGGRSRERMHIFAERERRRKIKNMFTDLRDLVPSL
TNKADKATIVGEAISFIRSLEETVADLERRKRERNSLAARCARLGLGGSSSSSAPPPPPP
PAAADDTAAVMPPAPAVPPPDAAAVTAGPEPAPAPAPGTLMVWSGPSVVLNLCGGDQAFI
NVSVARRPGVLTMIVDVLERHSIDVVTAQIASDLSRSLFTIHTSVDRERGMFMDTATAEE
IYQLAVSEIM*
>11670.m03379|LOC_Os04g35010.1|genepair1849-2
MSQEGADLSQDVDESPYHTAVVTTNNLVRSIKAEKSNSSSSSGKPVETDIGLKVASPTMF
GFNTKIEGTGKNMAVKREEGEGGGRPGVSSGVSTRDTNGKGKNAMDMEHALHIWTERERR
KKMKNMFSTLHGLLPKIPGKTDKASIVGEAIGYIKTLEDVVQKLETIKTERVRAHQWAAA
AAAAVAANGGGEGSSHSHSQPPRHATAVTVAVAEPAPVAAAVNAQAPQKKAAAAAAPTLQ
TWSAPNITLTMAGVDAFINMCLPRQRASFTTVAFVLEKHQIDVVTSTISADHDKSLFSVH
VRLNEASLQSTEGLTPEAKYKLAVSELMVRLAE*
>11668.m03258|LOC_Os02g34430.1|genepair1850-1
MSDSELSQSTVVFGLRMWVLVGVAVGAAFVLFLVLLSVLCLLASRRRRRRGPNTSPVQQ
LPVSAPPKNPQKVKAPKDIQEVPAQATAAAAAKTPLAQVLQMPAPPPPPPPMAAAAPPPE
TVQIATGKEHRITYPEPPHRSGSSSHGSGEAPSVPEVSHLGWGHWYTLKELEAATEMFAD
ENVIGEGGYGIVYHGVLENGTQVAVKNLLNNRGQAEKEFKVEVEAIGRVRHKNLVRLLGY
CAEGNQRMLVYEYVDNGNLEQWLHGEVGPVSPLSWDSRVKIILGTAKGLMYLHEGLEPKV
VHRDVKSSNILLDKHWNAKLSDFGLAKLLGSERSYVTTRVMGTFGYVAPEYAGTGMLNET
SDVYSFGILIMEIISGRVPVDYNRPPGEVNLVDWLKTMVSTRNSEGVVDPKMPQKPTSRA
LKKALLVALRCVDPDARKRPKIGHVIHMLEVDDFPYRDERRGARAPVQARVADKPVAIEA
GDRESDSSGNNSARQTEPFRWRNPES*
>11670.m03386|LOC_Os04g35080.1|genepair1850-2
MSDSELSQSTVVFGLHLWELVGIGVGAAFVLLLVLLSLLCLVASRRRRRRGVAVATPVL
HLATAVAPPKHPGKPPKDIQEVPSRAAAAAAAPKAQPAQVIQAPPPQPPPSESIQIETGK
EHRITFREQQHQPPQPPPYHQRSGGPSSRGGSGESRGGGGGGGGGGAEPGVPEVSHLGWG
HWYTLKELEDATAMFADEKVIGEGGYGIVYHGVLEDGTQVAVKNLLNNRGQAEREFKVEV
EAIGRVRHKNLVRLLGYCAEGNQRMLVYEYVNNGNLEQWLHGDVGPVSPLTWDMRMKIIL

GTAKGLMYLHEGLEPKVVHRDVKSSNILLDKTWNAKLSDFGLAKLLGSERSYVTTRVMGT
FGYVAPEYAGTGMLNETSDVYSFGILIMEIISGRVPVDYNRPPGEVNLVEWLKTMVSNRN
SEGVLDPKMTEKPTSRALKKALLVALRCVDPEARKRPKIGHVIHMLEVDDFPYRDERRGG
KAPGQVKSGEIPPVEAGDSSGNNTPKETPKGQPKDEPFKWRNQAAA*
>11668.m03261|LOC_Os02g34460.1|genepair1851-1
MIIPKKNRNEICKYLFQEGVLYAKKDYNLAKHPQIDVPNLQVIKLMQSFKSKEYVRETFS
WQYYYWYLTNDGIEHLRNYLNLPSEIVPATLKKSARPPGRPFGSGPPGDRPRGPPRFEGD
RPRFGDRDGYRGGPRGAPGDFGGEKGGAPAEFQPSFRSSGGRPGFGRGGGGGFGAGPTSS
SME*
>11670.m03387|LOC_Os04g35090.1|genepair1851-2
MIIPKKNRNEICKYLFQEGVLYAKKDYNLAKHPQIDVPNLQVIKLMQSFKSKEYVRETFS
WQYYYWYLTNDGIEHLRNYLNLPSEIVPATLKKSARPPGRPFGSGPPGDRPRGPPRFEGD
RPRFGDRDGYRGGPRGAPGDFGGEKGGAPAEFQPSFRSSGGRPGFGRGGGGGFGAGPTSS
SME*
>11668.m03265|LOC_Os02g34500.1|genepair1852-1
MQYHQHRSRLPPPPPPPPFGRGGGGAGYPRGHKQLYAPPPPPPPQHQQQRRYEVLMEAGR
LAAEYLVAKGVLPPSSLQRGVGAWGAPSPPPPPAAAGAAPHPPQQQREDPAFYGRRRYDD
EYSNNSSARPRRNSSNSSSSSSSRDDYSGGSYNGRGKRKYGDYRRGYSDWGRDREKERGR
PGSNGRRYEENDDEDGAPGFRRERRGSGGNDDARSSVADVYREATPLMRKELGDLEMNGT
ESRAANPSGEVKEADAPQMVQSEENEEGEMEEDGMVLNSEPEVVELRMDTNDDVNNASVG
VDMETELQRSPNGNVPGEKAEDDDKVLVESALDSIALDDEVANTENNLHGDERNLLKYCE
YAKAPTKRRSSRPQRNAASVQIEPAVSETTDQISIGEASQIVPGEVANEISVTNLKSENR
EDQIYRENTDFSTSCNGTLEPILLEENNESAATGNIIEEKNDVQLHVVKEPEEEVNVSAF
APSHKDSLMQETDLSPSTASHKDSLLEGNLPLLTDSHNSLIEETGPPLTHSHEDSMVEET
NLSSLTTSHKGNLKQETDLSQTISSHENNLKLQFKESCGIDMLPQDVDLIELSGQRKSVG
GELFSNVGAEAASKMEDENLEQPNPFKICDQNLIGSSEVSVIHNNPGLAQCSTEGSCTES
QKNQHQDFVTTSGDIAGSTNNMCQLPLDNKGVQVIDIEDDTPIEVGGFDSSKAKSDMICS
NMDNMMGPVVHSGDLPGIQDGYNLAISDYLGGDIPCYPSMQSDLHGGIGANDSEGITVMD
DPIYGSLTDIGNCD*
>11670.m03395|LOC_Os04g35170.1|genepair1852-2
MEAGRLAAEYLVAKGVLPPASLQRRGVGGGGWVQLPPPPPPPPPPQGTLAFYGAQNGRRR
LDDDDDGNPNPRSRRNRGGENNNDDSSSSYNGRGKRKFGAYSRHSDWGRDKGRSRGNSDS
RSYDDEDDDGPPGYRRERRGGGRFDDAGSSMSGVAASKTEAMGESELEDTGSKVGSSSNF
RKDVDPPQEVEGVDKLNKINEESNPSNSEVVEQMTNGESTSNNASCIVIDEEQTKAKYLP
VPSDDKVSDEKPDDSSVLNEKIEDDETLAEKAEDDKTSDERVPGVKNNLRDDCNNLLSYC
SYPNVPTRRRSIVAHRNAAPAHREVAVAEQIDLVSSEDETHMATDVTGHGSSLTNTQEGN
KDGLACLEHTDTSTTCNQMVEPVRFQTEETQIGTDDLRGQKNIEQHYAVHESREENMLPP
KVGVQQQVEEGMQIYNVDTPPQDEDLIASADKEKVAGVALLPSIKAEAVVAKEEDKFGQS
SSFKICDLNLVGSPEVAELRNDPGLGQFSTAGCSMEPQNQQQEFRNTGNSADDTNMHAQI
PLHNKVVQVIDLEDDSPIEAGACDTSKAKEENMANPAVTTDVLPGIQDGYNFAISDYLGA
DIPCYQPMQTDLPNGMSLNDSEGITVMDDSIYGSLSDIGFMEVWDQQPQDYEKFF*
>11668.m03271|LOC_Os02g34560.1|genepair1853-1
MELAVGAGGMRRSASHTSLSESDDFDLSRLLNKPRINVERQRSFDDRSLSDVSYSGGGHG
GTRGGFDGMYSPGGGLRSLVGTPASSALHSFEPHPIVGDAWEALRRSLVFFRGQPLGTIA
AFDHASEEVLNYDQVFVRDFVPSALAFLMNGEPEIVRHFLLKTLLLQGWEKKVDRFKLGE
GAMPASFKVLHDSKKGVDTLHADFGESAIGRVAPVDSGFWWIILLRAYTKSTGDLTLAET
PECQKGMRLILSLCLSEGFDTFPTLLCADGCCMIDRRMGVYGYPIEIQALFFMALRCALQ
LLKHDNEGKEFVERIATRLHALSYHMRSYYWLDFQQLNDIYRYKTEEYSHTAVNKFNVIP
DSIPDWLFDFMPCQGGFFIGNVSPARMDFRWFALGNMIAILSSLATPEQSTAIMDLIEER
WEELIGEMPLKICYPAIENHEWRIVTGCDPKNTRWSYHNGGSWPVLLWLLTAACIKTGRP
QIARRAIDLAERRLLKDGWPEYYDGKLGRYVGKQARKFQTWSIAGYLVAKMMLEDPSHLG
MISLEEDKAMKPVLKRSASWTN*
>11670.m03408|LOC_Os04g35280.1|genepair1853-2
MEVAGMRKASSHASMAAAAADPDDFDLTRMLNHRPRINVDRQRSFDDRSLAELSISGTAS
RGGGGGGYPAMMESYESMYSPGGGLRSLCGTPASSTRLSFDPHPLVFDAWDALRRSLVCF
RGQPLGTIAAVDHSSDEVLNYDQVFVRDFVPSALAFLMNGEPEIVKNFLLKTLLLQGWEK
RIDRFKLGEGAMPASFKVLKDAKRGGAERLVADFGESAIGRVAPVDSGFWWIILLRAYTK
STGDLSLAETAECQRGIRLIMNQCLAEGFDTFPTLLCADGCCMIDRRMGVYGYPIEIQAL
FFMALRCALLMLKPDAPEGKETMDRVATRLHALTYHMRSYFWLDFQQLNDVYRYRTEEYS
HTAVNKFNVIPESIPDWVFDFMPSRGGYFVGNVSPARMDFRWFALGNFVAILASMATPEQ
AAAIMDLIEERWEDLIGEMPLKISFPAIESHEWEFVTGCDPKNTRWSYHNGGSWPVLLWL
LTAACIKTGRLKIARRAIELAEARLARDGWPEYYDGKLGRYVGKQARKLQTWSVAGYLVA
KMMVEDPSHLGMISLEEDRAMMKPVLKRSASWTV*
>11668.m03281|LOC_Os02g34640.1|genepair1854-1
MPSSSSPPPPPPPATTATPRRRRRRRLLPSSAGGGGSSSSFSSTNSSSSSPFVSFLPPPT
SPSPFHRFLPSPLRASSVPFSWEHRPGIPKTPARSSRSSSKQPPPGVVGGGKHYPSAPPL
PLPPSLLSRAASDPYASAVVPAEYAASGAMPPHPPPGYYYPAGAKAARRLRLRRRRPRLA
DALAEWLSVLSLYRSCKRVAACFAAKAKPPPPAPAP*
>11670.m03419|LOC_Os04g35390.1|genepair1854-2
MPTDAAAAAAASSPSPLLPPAPPSAARRRRRRLLTSPNPSVSSTSTSSSSSSSSSSSSSL
SFPFAPFSPAPSPFHHRFLSPLRASAVPFSWEHRPGIPKTPARQHQHHRGGCGGGKVYST
PLPLPPSLLSSKVVVADEDRGGADRLAVSDDDDDAKAARRRSRRRHRRRLLQRPRRPAAL
AAALTDWLAVLSLYRSCTRFLEVVVVEAKSWVFRLLQIFTNCFS*
>11668.m03282|LOC_Os02g34650.1|genepair1855-1

MQQLLTLVRHTADLYHELHALDRFEQDYRRKLEEEKKSVAFERGDTVQIIRQELKSQRRH
VKSLKKKSLWNKMLEDVMDKLVDIVHFLHVEIQESFGTYDGALQLNQPSESRQTLGSAGL
SLHYANIISQIDNIVSRSTVPPQSTRDALYQGLPPTIKSALRKKLHNCPQPQEVPITEIR
SSMERTLQWIIPIANNTARAHHGFGWVGEWANTGNDAMRRAAGQPDVIKIETFYHADKAK
TEACILDLVLWLHHLISYSRPSNGGRSRSPSRSPVRSPPLTPPHQVPTTTSSSSPPPPAV
ARPSGGTGGGLTREDRAMLQDVYAGRRRRAPCHSRSQELSSARGGGGAGGDSSSAVAAAA
QLSKNDRLSKSSNDAPARSGGGGGGGGKLFPLSRRPSSAVVSPAVDFDIDGIKALADAQKR
Q*
>11670.m03421|LOC_Os04g35410.1|genepair1855-2
MDCYMLKSRRLVGVADAWSVDDGADGISGFPDAMSAAAHKQMVTGAEEAMLGKLELDSQS
NLFSNSDDFLLSGSSKLGISASEDIDLPGKQGLPESTTDRPGSNGSSRVPRLRVLGTAGM
AGFGKAVDILDTIGCLVTTSLSTDGGFISRAKTKGCPISILAFEVANTILKGATIMQSLS
EDTVTYFKQVVLPSEGVQNLISSDMSVLMRIVANDKREELKIFSQEIVRFGNRCKDPQWH
NLDRYFVKLESENPPQKQLKETAVAEMQKLMDLVHRTTDLYHELHALDRFEQDYRCKLTV
KGNSYQKDNLPGENIEVVRIELKSQRNYVKSLKKRSLWSKTLEDIVEKLVDIVQYLHFEI
NASFGSSDEGELSSESTEDCQRLGPAGLALHYANIIIQIYSVVSRSGYIPPNTRDALYQG
LPPRVRSALPNRLRTSSVPQELNIDQIRATMDKTLKWLVPMAINTTCARGFLRFSEWARS
GTERVGRRPGQPDVVETLYHADKAKTEDYILDLVVWLHHLVNQSNRPANAKDKDKEQSTS
PLTKSDLDKAQQE*
>11668.m03298|LOC_Os02g34810.1|genepair1856-1
MAERIAASLLPAASPSPAPSPPPPRPRVSAAAAASFPCCSTSAGGLRLRSRPSRFPQKAA
TTRSGRAGAGARAVVRCMAAAAVAASDAAQLKSAREDIREILKTTYCHPIMVRLGWHDSG
TYDKNIEEWPQRGGADGSLRFDAELSHGANAGLINALKLIQPIKDKYPGITYADLFQLAS
ATAIEEAGGPKIPMKYGRVDVTAAEQCPPEGRLPDAGPRVPADHLREVFYRMGLDDKEIV
ALSGAHTLGRSRPDRSGWGKPETKYTKDGPGEPGGQSWTVEWLKFDNSYFKDIKEQRDQD
LLVLPTDAALFEDPSFKVYAEKYAEDQEAFFKDYAEAHAKLSDLGAKFDPPEGFSLDDEP
AVEEKDPEPAPAPAAAPPPPPVEEKKEAEPTPVPVTVGAAVASSPADDNNGAAPQPEPFV
AAKYSYGKKELSDSMKQKIRAEYEGFGGSPDKPLQSNYFLNIMLLIGGLAFLTSLLGS*
>11670.m03433|LOC_Os04g35520.1|genepair1856-2
MAAQRLAALHAAAPSAFSSTSSASHGRPAARSSTTALLPVALPRASATLRAAPSRLLPQE
AKAAGSGRSVMCMASASASAASAAVASGAAELKAAREDIRELLKTTHCHPILVRLGWHDS
GTYDKNIKEWPQRGGANGSLRFDVELKHGANAGLVNALKLVQPIKDKYPNISYADLFQLA
SATAIEEAGGPKIPMTYGRIDVTGPEQCPPEGKLPDAGPSAPADHLRKVFYRMGLDDKEI
VVLSGAHTLGRSRPERSGWGKPETKYTKNGPGAPGGQSWTAEWLKFDNSYFKEIKEKRDQ
DLLVLPTDAALFEDPTFKVYAEKYAEDQEAFFKDYAGAHAKLSNLGAKFNPPELYQPDAP
PTTGAADLQTAAHTPQGPGHE*
>11668.m03303|LOC_Os02g34860.1|genepair1857-1
MEMEVEVAGDDEAVPEAPERSVVLISAGASHSVALLSGGVVCSWGRGEDGQLGHGDAEDR
PVPTVLTAAFDDAPGGVASVVICGADHTTAYSDEELQLYSWGWGDFGRLGHGNSSDVFNP
QPIQALQGVRITQIACGDSHCLAVTVAGHVHSWGRNQNGQLGLGNTEDSLLPQKIQAFEG
VRVKMIAAGAEHTAAVTEDGDLYGWGWGRYGNLGLGDRDDRLIPEKVSSVNGQKMVLVAC
GWRHTITVSSSGSIYTYGWSKYGQLGHGDFEDHLVPHKLEALKDTTISQISGGWRHTMAL
AADGKLYGWGWNKFGQVGVGDNEDHCSPVQVNFPNEQKVVQVACGWRHTLALTEAKNVFS
WGRGTSGQLGHGEIVDRNIPKMIDALSSDGSACKQLESSKAVPMSAKVWVSPSERYAIVP
DEKAGKGIPAGNGTETHVPQGDVKRMRV*
>11670.m03439|LOC_Os04g35570.1|genepair1857-2
MDAVMSAADDAGAASGREDPPPAVVLVSAGASHSVALLAGNVLCSWGRGEDGQLGHGDAE
DRLVPTVLSGFDAAAPGITSVICGADHTTAYSEDEQQVYSWGWGDFGRLGHGNSSDVFTP
QPVKALQGIKIKQIACGDSHCLAVTMAGEVQSWGRNQNGQLGLGTTEDSLLPQKIQSFEG
VCVKMIAAGAEHTAAVTEDGDLYGWGWGRYGNLGLGDRNDRLVPEKVSSVEGEKMVLIAC
GWRHTITVSSSGSLYTYGWSKYGQLGHGDFEDHLVPHKLEALKDSSISQISGGWRHTMAL
TSDGKLYGWGWNKFGQVGVGDTDDHCFPVQVKFPEDQKVAQVACGWRHTLAFTEKKNVFS
WGRGTSGQLGHGEIVDRNKPVMIDALSPDGPGCKKLEPSTAVPFAAKVWVSPSERYAIVP
DEKVPNSGEGTARGNGADANVPENDVKRMRVHS*
>11668.m03310|LOC_Os02g34930.1|genepair1858-1
MEDDDDDFTFPAPAATATATAVAAAATSSLAPSSQGGLLWPFSSTMATADDDDAASGGQA
VAATAPVAAARRVDEEEEEERMDQLWERDRDARAGDEERMDLLWEDFNDELLLQLRRRQQ
QRAAAGTPPSPSPSPAAAADDDDDEETPSSSPPGGGGGLYGCAPTMLRASSRAGAVGQFY
GGRRGGGGGSRATTGWELLLRLFRKLFAVDKSSPSPAPPCRHHHRRHGSIYVP*
>11670.m03440|LOC_Os04g35580.1|genepair1858-2
MDDFTFPITAAAAAAAADTAAAAAAAAEPLRLHHHHHHRRRGRLHFAASPLWFPSSCPV
AAAPPDVPVADDADTADAAVASAVKDVDVVVFVRDAMEGKEQEQEDEEGGGGGQEEATSD
AGRREEEEGKKEEEATAAAAATGELSRGDGDEDGGDHGGGGAARDKEEKMDQLWENFNEE
LRQALHQRVGSCPRADARAAAAAGMELSPETSDAESEPAAAAALRGHIGCAPMLRPSSRA
GAGGYRRTATSWVLLMKIFRRLFVIEKTISSSSSAAASASGRHGSARR*
>11668.m03311|LOC_Os02g34940.1|genepair1859-1
MASSTAFAAAFALLLLASSAAAEGEAVLTLDAGNFTEVVGAHDFIVVEFYAPWCGHCNQL
APEYEAAAAALRSHDPPVVLAKVDASADLNRGLAGEHGVQGYPTIRILRDRGARSHNYAG
PRDAAGIVAYLKRQAGPASVEIAASASPPAADSIANDGVVVVGVFPELSGSEFESFMAVA
EKMRADYDFRHTTDAGVLPRGDRTVRGPLVRLFKPFDELFVDSQDFDRDALEKFIESSGF
PTVVTFDTSPANQKYLLKYFDNAGTKAMLFLSFSDDRAEEFRTQFHEAANQYSANNISFL
IGDVTASQGAFQYFGLKESEVPLVFILASKSKYIKPTVEPDQILPYLKEFTEGTLAPHVK
SEPIPEVNDQPVKTVVADNLREVVFNSGKNVLLEFYAPWCGHCQKLAPILEEVAVSLKDD
EDVVIAKMDGTANDVPSDFAVEGYPSMYFYSSGGNLLPYDGRTAEEIIDFITKNKGSRPG

EATTTESVKDEL*
>11670.m03442|LOC_Os04g35600.1|genepair1859-2
MAVNLVLSFALAILISSSPTAVGVDATEELKEAVLTLDAGNFSEVVAKHPFIVVKFYAPW
CGHCKQLAPEVTDYFDTFSSQHHGRFNIKYEKAASILRKNELPVVLAKVDAYNERNKELK
DKYGVYSYPTIKIMKNGGSDVRGYGGPREADGIVEYLKRQVGPASLKLESAEEAAHSVVD
KGVILVGVFPEFAGMEYENFMVVAEKMRADYDFFHTSDASILPRGDQSVKGPIVRLFKPF
DELFVDSEDFGKDALEKFIEVSGFPMVVTYDADPTNHKFLERYYSTPSSKAMLFVSFGDD
RIESFKSQIHEAARKFSGNNISFLIGDVADADRVFQGTSCPLISNYGNLTPYVKSEPIPK
VNDQPVKVVVADNIDDIVFNSGKNVLLEFYAPWCGHCRKFALILEEIAVSLQDDQDIVIA
KMDGTVNDIPTDFTVEGYPTIYFYSSSGNLLSYDGARTAEEIISFINENRGPKAGAAAAV
DEKTQIDAVEEEDTDGLYTAYNRDY*
>11668.m03313|LOC_Os02g34960.1|genepair1860-1
MRRLLTPPWPAAAAAAPASAAAASAVTVEAVLALLERAISAGDVRRLGAAVHALLVKTAL
THHTLLSNRLVALYALLPTPAASLAAFDDLPHKNAHSYNSLLAALARGRGTLPDALRLLD
GMPPASRNVVSYNTVISSLARHGRESEALRVFAQLARDRGLGQQQVAIDRFTVVSAASAC
AGLRDARHLRELHGAVVVSGMEVTVIMANAMVDAYSKAGRVEDARGVFDQMTIRDSVSWT
SMIAGYCRASMLDDAVQVFDMMPAQDAIAWTALISGHEQNGEEEIALELFERMTGEGVVP
TPFALVSCLGACAKVGLVARGKEVHGFILRRSIGSDPFNVFIHNALIDMYSKCGDMVAAM
AVFDRMLERDIISWNSMVTGFSHNGQGKQSLAVFERMLKDEVQPTYVTFLAVLTACSHAG
LVSDGRRILESMQDHGVEPRAEHYAAFIDALGRNRQLEEASEFIKGLSSKIGLGTTGSWG
ALLGACHVHGNIEIAEEVAEALFQLEPENSGRYVMLSNIYSAAGQWDDARQVRALMKGKG
LRKDQAYSWIEVQRAKHMFVADDTSHHEANEIYEMLDKLFHHMFIIGGNVEELIVG*
>11670.m03443|LOC_Os04g35610.1|genepair1860-2
MARPHHTGIHLVSHLRASAPLADLLRSAPGLRAARAAHARALRSPFAGETFLLNTLLSAY
ARLGSLHDARRVFDGMPHRNTFSYNALLSACARLGRADDALALFGAIPDPDQCSYNAVVA
ALAQHGRGGDALRFLAAMHADDFVLNAYSFASALSACASEKASRTGEQVHALVTKSSHGS
DVYIGTALVDMYAKCERPEEAQKVFDAMPERNIVSWNSLITCYEQNGPVDEALALFVRMM
KDGFVPDEVTLASVMSACAGLAAGREGRQVHTRMVKSDRFREDMVLNNALVDMYAKCGRT
WEAKCVFDRMAIRSVVSETSMITGYAKSANVGDAQAVFLQMVEKNVVAWNVLIATYAHNS
EEEEALRLFVRLKRESVWPTHYTYGNVLNACANLANLQLGQQAHVHVLKEGFRFDSGPES
DVFVGNSLVDMYLKTGSISDGAKVFERMAARDNVSWNAMIVGYAQNGRAKDALLLFERML
CSNERPDSVTMIGVLSACGHSGLVKEGRRYFQSMTEDHGIIPTRDHYTCMIDLLGRAGHL
KEVEELIENMPMEPDAVLWASLLGACRLHKNIDMGEWAAGKLFELDPDNSGPYVLLSNMY
AELGKWADVFRVRRSMKHRGVSKQPGCSWIEIGRKVNVFLARDNIHPCRNEIHDTLRIIQ
MQMSRMSIDAEIADDLMNFSSEACG*
>11668.m03314|LOC_Os02g34970.1|genepair1861-1
MGGATNLPPGFHFFPSDEELVVHFLRRKVSLLPCHPDIIPTLLPHRYNPWELNGKALQAG
NQWYFFCHLTQSRTSSNGHWSPIGVDETVRSGGRNVGLKKTLLFSIGEPSEGIRTNWIMH
EYHLLDGDCVAGGSSNLTSSSSNRRSHRKRGHSSMESNNWVLCRVFESSCGSQVSFHGEG
TELSCLDEVFLSLDDYDELLATSNYLRICMDIVFYHHKEKRKVGGAYAIIWPSMLSMEIM
HACVEIALR*
>11670.m03448|LOC_Os04g35660.1|genepair1861-2
MAGASNLPPGFHFFPSDEELIIHFLRRKASLLPCQPDIVPTLILNLYDPWELNGKALQSG
NQWYFFSHATQTRTSPNGHWKPIADETVISGGCNVGLKKTLIFFIGEPFEAIKTNWVMHE
YHLMDGSTNCSSSSTSSSSSKRSHKKKGHSDTESKNWVICRVFESSYDSQVSFHEEGTEL
SCLDEVFLSLDDYDEVSFAK*
>11668.m03318|LOC_Os02g35010.1|genepair1862-1
MPLGWLRRKWRRRRDKPAGDAAAASTTATSPRDSVDLGGTSAYPSACASPSSTTPTPTRW
GAGASAVPPRCPGPQDQHHGLPLPRPVSKSAPMPLASPAAAPPSPSPPACASAAESVSGG
SSSDDEADHRNYRYTDPVVHTSGRTVLPDGHNGMVEEKRFVSCGILQEHQKFFEVPIANV
NEVHHMQIFEPSTSESSYSRGRMLPEDTFAVRPRSHSPGPRGHAYSACCARDFGFTPRSP
VKRMDDPRSPSQPLPLPPVPVASSSIPSSSITSSQFQSQWKRGKLLGSGTFGQVYLGFNS
ENGQFCAIKEVQVFLDDSHSKERLRQLNQEIDMLKQLSHQNIVQYYGSELFSLSFGLPNL
NNFEQADEALSIYLEYVSGGSIHKLLREYGPFKEPVIRNYTRQILSGLAYLHGRNTVHRD
IKGANILVGPNGEVKLADFGMAKHVTSFAEIRSFRGSPYWMAPEVVMNNKGYNLAVDIWS
LGCTIIEMATAKHPWYPYEDVAAIFKIANSKDIPEIPDCFSKEGKDFLSLCLKRDPVQRP
SAASLLGHPFVHDHQAVRAPTCNGTQLRNGISSPAASHRKPNRESSSRRNIAPLHGIAGL
SAREFAGFSTAYPSPHNTSSSPTAVRANMSLPVSPCSSPLRQFKQSNWSCLPSPTHPALS
PGLSAAAYPNNPLQNQSRRSAAVPDPWLELSQPRPPSPYGSPKRF*
>11670.m03452|LOC_Os04g35700.1|genepair1862-2
MPAWWPRKSRSKAKGVGKPPGAGAASAASSPRKSLDLVDLSASPSVTPRAREKARSLDSP
SAAAPRHGGGRCGGGFGEVGYKLPVPVGDAGPGGQGQGQEPELEPAPVRFEENGDVVGGV
AGDASSEELSVCSHDSSDEATDQQVCRSTDPASFVRGRNMPSDSHKILNEDNHFMSYSMP
REHRKFFEVPVTNMGELHLHCDDLSTSETSSRGRMLPEDFLAPRTRSLSPGPKGHTFAVN
NVNSREFGFSPRSPVKMMDGLKSPPHPLPLPPGPATCSPLPPSPTAYSPHPLGPTTCLQS
ESQWKKGKLLGSGTFGQVYLGFNSENGQFCAIKEVQVISDDPHSKERLKQLNQEIDMLRQ
LSHPNIVQYYGSEMTDDALSIYLEFVSGGSIHKLLREYGPFKEPVIRNYTGQILSGLAYL
HGRNTVHRDIKGANILVGPNGEVKLADFGMAKHISSFAEIRSFKGSPYWMAPEVIMNGRG
YHLPVDIWSLGCTIIEMATAKPPWHKYEGVAAIFKIANSKEIPEIPDSFSEEGKSFLQMC
LKRDPASRFTATQLMDHPFVQDHPAVRAAKSGALRNAFAPADGTHTTSNREFSRKSITPL
KDIGVSARDFTGFSTAVPSPHTASPISVVRTNMSLPVSPCSSPLRQFKQSNWSCLPSPPH
PTFSNGATSYNLSSYMMNETRRIPTISDTWQDISQLKVQSPYGSPKRF*
>11668.m03325|LOC_Os02g35080.1|genepair1863-1
MGVPEAVALEIPAAAEAEEEVEEEEGSPSPPVVARVPPRIRRRLLLRHRGGAPATAEEIE

AKLRDADLRRQQFHEALSCKARCTVRYPSCPSQEEDPKKRLEAKLVAAEQKRLSLLAKEQ
SRLAKLDELRQAAKNDAELRFKKEREELGMKVESRVRQAEEKRTQLMHARSQRRAALEER
TTKYLVQRVAWENKYRERVHSAILQKRTAAEKRRTGLLEGEKRRAQGRFSQVQLAARTLS
CQREADRSKLKEQLEDKLQRAKRQRAEYLKQRGSTHSFTYTASVKHGDFLSRNLARCWRR
FITSRKTTVVLARAFDMLRINEESVKPMPFEKLALCIESPTVLQTTRAFLDRLESRFTLS
QSSSPSSPENIDHLLKHLGSPKRTLSKSGGRTRVTPTKAARNSDVSKLPRYSPRIVLCAY
MILGHPSVVFNERGEREKLLVESAENFVKEFELLIKTILDGSSGACILKQPILDDLSPGS
SNYQESSAVVADRKKFRSQLASFDKAWCAYLYHFVVWKAKDAKSLEEDLVRAACKLELSM
MQTCKFTAEGQPENLNDNLKAIQKEVMVDQTLLREKVRHLGGEAGIERMEVALSEARTKF
FEAKGNRSPLATTIKNVAATCSSGESPISDMKENSNINDKRPSQAVQSMFRVPSSPSESN
TAGITMSNPMTVSSTLSEKRPTENEQMVNEILHGFLADSSSNIGTVEGGFKEKVRETMEK
AFWDVVVDSLRGDMPDYSYLVQLVKEVRDTLYEMVPKGWKEEIINNIDLEILLQVLESGT
QDMQYLGQILQYSLGMLRKLSSPAKEDEMKRSHDKLLGELTEHSECNNSGSNSFVIAVIK
GLRFTMEELKALKTEVSRARIQLLEPIIKGSGGVEYLQKAFADRYGFPSNASVALRSTAQ
WISTSKDTVEVEWNEHAQPFVATLRSGHGVPDQRQSTIPVSDDTGLPECTGQRLDQLIRI
GLLQLISGIEGVQMQSVTETFKLNWLRLSMLVQRQVLATDDPNITPTELESATSQLFNTL
AELLDNFPDVSTAKIMEVMLRSSSSSSSGSTTGSPSDERTESRKQILARVFLKSLQTDDP
VFKKVSRSVYCAFRAITLGGSGARGRKLADAALRRIGATKLTDRVVRSAEILIRAASISQ
QVHGPWYNHLV*
>11670.m03467|LOC_Os04g35840.1|genepair1863-2
MPPRIRRRLLEGSRVGGGAPTSAEEIEAKLKEAELRRQQFHEWVSCKARKKPRSPSWSSQ
EEDQGQRLEAKLQAAEQKRLSLLAKAQNRLAKLDELRQAAKNIVEMRIEKEREELGTRVE
SRVRQAEANRMRLLHAHMQKRAAMKERTARSLVRKQTSERKYTERVKSLILQKRNAAEKK
RLALLEAEKRKAQARILHIQRAAKTVCSKRESERRQLQEQLESKLQKAKRQRAEYLKQRV
SPRSSAHADYIKHAEFLSTKLARCWKRFLKSNKTTYALVQAYDALGINEMSVKSMPFENL
AMLMESPTTLQTTKAVLDRFEKRLLLSQPTGSSSAENIDHLLKRLGSPKRKAPASRSRVA
AKKPAKGSETSKLSRYSLRVVLCSYMILAHPGAVLSGQGEKENLLMESAENFVKEFELLV
KTVLDRPGGASMQSTDAASQKKFRTQLAAFDKAWCAYLYHFVVWKLKDAKSLEQDLVRAA
CKLELSMMQTCKLSSDGQSHDLSHDMKAIQKQVTDDQKLLREKIQHLSGDAGIERMNSAL
SDTRSKFFEAKENGNPLATSVANVSTPLSINSSGQVPNPTSKPTVEGSSFTAQSLPGAAS
SSSSTSPMKPPTDNEQMVNEMLHEDDVSFARNSDNVSSAEKDFQAKVKATMEKAFWDLVT
DSMRGDKPDYSQLINLVKEVRNSLHELASNELKEEILENIDLEILSQVLQSGSQDTRYLG
QILQYSLDMVRKLSAPAKEDDMKRSHEKLLNELAASSEVNDNGISSFVIAVIKGLRFTLE
EIKQLQTEVSKARIQLMQPIIKGSAGVEYLQKAFTDRYGPPANASVSLPITKQWVSATKS
IVEQEWSSHLESLQALPADHAQHVVPVLRAGHGAPAPQASSSAASSSGLPECKGEKIDKL
TRVGLLQLISNVEGLNMQSTPETFQINLLRLRAVQDQFQKVIVIATSMLVLHQVLMLKIA
PPELQNTISELYDALVKLLDNNADASTKEIVEAMTRSLASVGSLPEEQIQDTTELATKML
LKSLQAGDIVFGKVSRAVYFAFRGVVLGGGAKGKKLAEAPLRRLGAAKLADRVVKAGEVL
IKMAVISEKVHGQWYKALAL*
>11668.m03327|LOC_Os02g35100.1|genepair1864-1
MPLFKRKPFSLLEPPKDLDSKEKVFQIRFTREIFRDYQDYINRLNLYRQRVWSCKISGKS
NLTFEEALVSEHHAVSKAQKLPTELMAPVLRMIQYSTLGLYELVEKIYASLQEAVFEGLE
LYAKQDGLEAACRILKILGSDGTKMYEVGWLLRDKTIISTSVIKGEDLIHRRPPVSRNTL
KIFIRDATSQNAPWVIHENLAKRYGIPIEPPNDMMFGEGLQKKGRKRREDGPMGDPKKKM
KNDEEHINVPIKYPIDDLLVQPSADDHALLKRPPLATDFRVPKYSVGDLLMVWDFCLSFG
RVLNLSPFSLVDLENAICHKESNALLVEIHTAIFHLLIKDEGDYFTILRTKKRKLKVTLV
TWAEYLCDFLEMTKTEELTRNIATVRKGYYSLIDTDIKLKILRELVEEAITTSPVREKLS
ERVDQRQALAATKRESTRKAKDEQNSSIDGLQDDNESVDEQGKGKEEKDKNNISRSKTEG
KRHGVQHLETEIEKLSIRSSPLGKDRHYNRYWFFRREGRLFVESADSKEWGYYSTKEELD
VLMSSLNVKGLRERALKRQLDKLYSKISNALEKRSKEITHKLLLEEAVLRRSTRVRAQPR
DNPSMSFLKYVNKWKDN*
>11670.m03471|LOC_Os04g35880.1|genepair1864-2
MYEVGWIGQDNAETNTSVLQADDLVIRKKARASRSMLKIYIRESTSQNSPWIIHANLAKK
YGIPTEPPKDLLNGQGLPKLRRGLENGTTDDVRKKLKKGEPIDDLLLRPTADDPSLSKRR
PLSTDFRVPVDSVGDLLMVWDFCMTYGRILCLSPFSLSDLENAICHKESNLVLLVELHAA
LFHLLIKDGGGYFMFLQNKRRKLKVTLVTWAEYLCDFLEMTSKEEFSSSLSTVRRGHYGL
VHTAVKLKILRELVDEAITTSAVRQNIDEKIDQQQAIAASKRELARNKKEEHKLAMEGVT
EKEMSQTDAAENVNGNVNGQVVEKEGKEKKNIYANKMGEGKVHPVRTYTLLHVNLHMHVH
SYICKCMSLLKLSMTAGASLVANLCFFCIADVRISMVTLIEKRMALEMGQFSGFLHSLNT
KAMSSQRGLDTYL*
>11668.m03330|LOC_Os02g35130.1|genepair1865-1
MGGSEYDDDWELPSADITVVLCGKLGCGKSATGNSIVGREAFVSEYSHASVTSTCQLAST
ALKDGRTLNVIDTPGLFEMTITSEDAGKEIVKCMSMAKDGIHAVLMVFSATSRFTREDSS
TIETIKEFFGEKIVDHMILVFTYGDLVGENKLKSMLNNAPEYLQKTVELCKNRVVLFDNM
TKDRWLQEKQLENLLDVVDSVNTNNGGKPFSDQMLACIKEAHAREQEVHDAIGYTEEQIS
ELKKEIQRTRDEQLANITNMVEEKLNITVDKLQQQLMEEQNARLEAERLAAEARLRSDEE
IRKLKKRLEKAQQENEEFRKMASQHKCSIL*
>11670.m03487|LOC_Os04g36030.1|genepair1865-2
MEEVYWLDDLELIMGYTVKLARTGEAHHAANLFAVSDSTLSSFRGKSLSAACINHVLLHS
PSSSSLLPTRKFPVSLSVSATPVISQLSIDPIMGGGGGENRVDNHDDDDDDWELAAGAAL
ADVTLVLVGKVGSGKSATANSILGDEAFESKCSYAGVTQTCQKKSTTVQDGCLIRTINVI
DTPGLFDMDIKAEDVRREIVKCMDMAKDGIHAMLMVFSATSRFSCEDEKTIETLKSFFGD
KILDHMILVFTRGDEVGGETSWKNMLSDSAPTYLQDILKLFENRVVLFENKTSSTQDRQA
QRKKMLDAVDFVVSSNHGKPFSNQLFTQIQEVHHRQKDANSEVYSSMQETDSYISLITKM

VEEKLNGTILRMEQQLLKEQEARLDIQNEMTKAILRSEEDIRRLRLSLEKAEQESNNARE
ENKRFRESEKASKEQEKQTEAEIQKLKEKMEKDREEREEEIRRLRDDLEKEREERQKQSG
CIIL*
>11668.m03331|LOC_Os02g35140.1|genepair1866-1
MAGSVVAAAAAAGGGTGSSCDALYRELWHACAGPLVTVPRQGELVYYFPQGHMEQLEAST
DQQLDQHLPLFNLPSKILCKVVNVELRAETDSDEVYAQIMLQPEADQNELTSPKPEPHEP
EKCNVHSFCKTLTASDTSTHGGFSVLRRHAEECLPPLDMTQNPPWQELVARDLHGNEWHF
RHIFRGSPHHSLILGQPRRHLLTTGWSVFVSSKRLVAGDAFIFLRGENGELRVGVRRLMR
QLNNMPSSVISSHSMHLGVLATASHAISTGTLFSVFYKPRTSQSEFVVSANKYLEAKNSK
ISVGMRFKMRFEGDEAPERRFSGTIIGVGSMSTSPWANSDWRSLKVQWDEPSVVPRPDRV
SPWELEPLAVSNSQPSPQPPARNKRARPPASNSIAPELPPVFGLWKSSAESTQGFSFSGL
QRTQELYPSSPNPIFSTSLNVGFSTKNEPSALSNKHFYWPMRETRANSYSASISKVPSEK
KQEPSSAGCRLFGIEISSAVEATSPLAAVSGVGQDQPAASVDAESDQLSQPSHANKSDAP
AASSEPSPHETQSRQVRSCTKVIMQGMAVGRAVDLTRLHGYDDLRCKLEEMFDIQGELSA
SLKKWKVVYTDDEDDMMLVGDDPWPLMRWTLTTLLQCLILLSRVLVNAALLILYIQRAVL
FNGLCFQIHWINAGGFGGWKEGYSTKRASGVCREPVVDAIDVEGPQGIDMLAIALRKHSL
NVAQGSNLEALPFITELIIEILVVQTQRKTYIEVLS*
>11670.m03490|LOC_Os04g36060.1|genepair1866-2
MEMAANPGGSGTCSDALFRELWHACAGPLVTVPKRGERVYYFPQGHMEQLEASTNQQLDQ
YLPMFNLPSKILCSVVNVELRAEADSDEVYAQIMLQPEADQSELTSLDPELQDLEKCTAH
SFCKTLTASDTSTHGGFSVLRRHAEECLPQLDMSQNPPCQELVAKDLHGTEWHFRHIFRG
QPRRHLLTTGWSVFVSSKRLVAGDAFIFLRGESGELRVGVRRLMRQVNNMPSSVISSHSM
HLGVLATASHAISTGTLFSVFYKPRTSRSEFVVSVNKYLEAKKQNLSVGMRFKMRFEGDE
APERRFSGTIIGIGSVPAMSKSPWADSDWKSLKVQWDEPSAIVRPDRVSPWELEPLDASN
PQPPQPPLRNKRARPPASPSVVAELPPSFDVDSDQISQPSNGNKSDAPGTSSERSPLESQ
SRQVRSCTKVIMQGMAVGRAVDLTKLNGYGDLRSKLEEMFDIQGDLCPTLKRWQVVYTDD
EDDMMLVGDDPWEKFQCFFITASCAEDATPFCLELWWYLNCKCQEVPKTGKTAWKRHSGK
CMPFQIVLCFGRSLGVLVWCPDLGDSVGGGAERRRTGGVERRRREPVTRRRGGKERRRPS
WRALAANRRRGEAANQRRGEAATQRGGSDPAGGLRRRGEAAKRRHGEAANRWREEAVRRG
GDPSERWRTGGVERRRREPATRRTGSSGVGFFHASSSSLIAGANGGSDGGGGARTLSWGT
WWRRLGRLAPTVALGWCGRWSTREEGIIDVGKRIHVGPPFGEGNRRRSTLVLPHVRSAPF
HAVVPGAVGGQRRQIVAAAVAEADGNENGNKHFTCHRENANYGLKSASENKIELLRGAHS
WADSNLIEDVLASVNNDVGEASALLKAMASPCFPIREDGLPDQLSSEINKTHGLPSGNGT
AENNLVNDSQLLPLPMNMSSVPIEPEVEELDDDYFNHRKDALKIMRAATKHSQAASNAFL
RGDHAAAKELSLRAQEERSAAEELNKKAAKEIFRLRNSNNSIWKLDMHGLHASEAVEVLE
RHLHRIEFQPPGNNAASSDEVARSEPRVSGPSIEPGPGKVVFVRPIQAILEVITGIGKHS
KGQASLPVAVRGFLIENGYRFDELRPGHKLLTSQPEVKNHKGIKGHSPSNLGFRRSGLQL
KVTAIFGWIKGDTRTRELNPSAESYTLTGSASEADTKPREVSVAVVSSIMDIPSADWDAC
AVDSVDPDNFNPFLTHAFLSSLEESGSAVKETGWLPLHVVARDENETVVGVVPLYLKSHS
RGEFVFDQSWAEAYYSYGLEYYPKLQSCVPFTPVTGQRILVRDTPYRDQVFEALVKELKS
LTTKLKVSSLHITFPSEGEFSKLKDSEFLQRIGMQYHWRNRDYRSFDEFLMDLKQPKRKN
IRQERKKIPAQNLKMKRLRGDEIKDYFLCAFSWGRPYLTREFFHRLGEKMGDKVMLIVAE
HDDKHVAGALNLIGGDTLFGRLWGCLPDAYFPNLHFEACYYQNIHTTQAIEAAIELNLSK
VEAGAQGEHKIQRGYLPVTTYSCHYFLDPGFGAAIGNYLVHETAQVRSCHRRKERARKKL
SECHIDISAKIPTKKDIRYCLIMLFVQGYKDWFFLPPLINQRPRRTKLQCRKLSQTRKSF
ASCSLPLHLDCTGCFIRQAHHRLRKQPFDHLCSSSPRHRMAYTSRMNLKRKGK*
>11668.m03336|LOC_Os02g35180.1|genepair1867-1
MGAEAVRVLVVDDSPVDRRVVELLLRAHCGGGGGAAAGEAAPFHVTAVDSGKKAMELLGR
RRGDRDHLTPSSPAAAAAANDQAIDIVLTDYCMPEMTGYDLLKAIKALGSPNPIPVVVMS
SENEPQRISRCLTAGAEDFILKPLKMNDVQRLRKCSGATRPKSAVAGDDDRCCNTAKKAA
AAAAATPEQQQQQQRSSHLAGLAMVMNASSFEVSHYFQLIFKLILLAYAVLCLSQLLHRW
SNGSSLLSLWCA*
>11670.m03491|LOC_Os04g36070.1|genepair1867-2
MEGGRGVTRVLLVDDSPVDRRVVQLLLSSSACAGSFHVIAVDSAKKAMEFLGLKEEGKEQ
AIDMVLTDYCMPEMTGYELLKAIKALSPLKPIPVIVMSSENEPQRISRCMNAGAEDFIVK
PLQSKDVQRLRNCSPANTQCCDAGSDGKPPLLLLPSDHVVVDATAASPPPPPSRRRAHFA
GVAMVLHSSSVELSHYFPFLFKFILLVYAILCLGELLHRWSNGCFLNLWCA*
>11668.m03343|LOC_Os02g35240.1|genepair1868-1
MAAGGGGGPQYRGVRRRKWGKWVSEIRQPGTKVRIWLGSFDSAEMAAVAHDVAALRLRGR
DGAQLNFPGSVGWLPQPATTDPVDIRAAAAEAAERVRREPALVSAAASAAPRRLELGGGD
GEFDEMESPRLWAEMAEAMLLDPPKWGPDGSDGADGSSQSWAHGSLWDGC*
>11670.m03500|LOC_Os04g36640.1|genepair1868-2
MASAAAAAGGGCQYRGVRRRKWGKWVSEIRQPGTKTRIWLGSFESAEMAAVAHDVAALRL
RGRDAQLNFPGSVDRLPRPASSRPGDIRAAAAEAADRVRREPALVVVRGAAAAAGELAAV
RWAGLEVEVEQQLGGSDEEFEVDSPRLWAEMAEAMLLDPPVWAVDVSEMEGPHCWAHGSL
WDAC*
>11668.m03366|LOC_Os02g35460.1|genepair1869-1
MPDESSEKQPLASLLLPSDHLFIPFVGAPEFMGEQMDNEELNLSLSLQPSYPSRFQTEFS
CCYCPKRFQSSQALGGHQNAHKLQRNLAKRNREAFLSISQRKGANAGIKDGSSALSAESI
CKISSGKKHHKEAWQVMQGSCGSSSSGTVMHKSIEQDVEDEDLSNGTIDLSLKL*
>11670.m03501|LOC_Os04g36650.1|genepair1869-2
MEFGMVEDAAAAAGEEGLTLSLSLQPSPPRFQALFSCCYCPRKFRSSQALGGHQNAHKLQ
RNLARRGREAAASLAAAAAAAAAAAASSGDQQQQGRTTAAAAAVLAGGESAPPAAARAAA
DLDGAGVWGGAGMRGRPAHHHRLMQGGYSSGGSSAAGGRGNGELADEMIDLSLKL*

>11668.m03368|LOC_Os02g35470.2|genepair1870-1
MQRQGRSLQRSGSKRVLDPTGGGGGDDDDHAAKRPRVPALASVIVEALKVDSLQKLCSSL
EPILRRVVSEEVERALAKLGPARIQGRSSPKRIEGPDGRNLQLKFTTRLSLPLFTGGKVE
GEQGAAIHVVLLDANTGVAVTSGPESCAKLDVLVLEGDFNNEEDEDWTEEEFESHIVKER
EGKRPLLTGDLQVTLKEGVGTIGELIFTDNSSWIRSRKFRLGLRVAPGSFEGIRVREAKT
EAFTVKDHRGELYKKHYPPALKDDVWRLEKIGKDGAFHKKLNASGIYTVEDFLQLLVKDQ
QRLRSILGSGMSNKMWESLVEHAKTCVLSGKHYVYYAIDSRNVGAIFNNIYEFTGLIADD
QFISAENLTDNQKIYADGLVKKAYEDWMHVVEYDGKALLSFKQKKKSVTTRSDTAAAATN
SPVSYGSSNTHKQLSQPAKAGQTSTGTTSEADGSTSAYNGNQAGRYAVNSQSIPANVTTQ
YERSSLTPESQFNGSALQNQVSRGSNILALGPPQQQHQQNFEFSALGGQSMQPTGLNPFD
DWSQPQENRSGVDDYLMEEIRMRSHEILENEEMQQMLRILSMGGASTNLTEDGFAFPNYM
PSTPPNFNFGDDRARPPGKAVVGWLKIKAAMRWGIFVRKKAAERRAQLVELED*
>11670.m03502|LOC_Os04g36660.1|genepair1870-2
MQRQGRHLERSGSKRALDAGGGGGDDDDRAPKRPRVPALASVIVEALKVDSLQKLCSSLE
PILRRVVSEEVERALAKLGPAATPARIQGRSSPKRIEGPSGINLQLQFRSRLSLPLFTGG
KVEGEQGAAIHVVLLDANTGRVVTSGPESFAKLDVLVLEGDFNKEQDEDWTEEEFENHIV
KEREGKRPLLTGDLQVTLKEGVGTIGELIFTDNSSWIRSRKFRLGLRVSSGFCEGVRVKE
AKTEAFTVKDHRGELYKKHYPPALKDDVWRLEKIGKDGAFHKKLNSNGIYTVEHFLQLLV
RDQQKLRTILGSNMSNKMWESLVEHAKTCVLSGKHYIYYSSDARSVGAIFNNIYEFTGLI
ADDQYISAENLSENQRLFADTLVKQAYDDWINVVEYDGKELLRFKQKKKSVTTRSDTAKA
STSYPSSYGSTHSHKQLTGGPVNIEQSSMSSMSEDGTRNMSNGSQAARYAANPQDISQSI
TMPYDMSSLRPEEQFAGSSIQTQASRSSNMLALGPTQQQNFEFSALGQSMQPSPLNPFDD
WSRLQENRGGVDDYLMEEIRVRSHEILENEEDMQQMLRILSMGGSSANMNHGDGFSPFMP
SPAPAFNYEDDRARPSGKAVVGWLKIKAAMRWGIFVRKKAAERRAQLVELED*
>11668.m03370|LOC_Os02g35490.1|genepair1871-1
MAGGGGKAAAGGGEAPAITLEHTPTWIVSAVCFVIVIISLLFERLLHRLGKRLKKSRKKP
LYEGLLKVKEELMLLGFISLLLNVFQGLTQKICVKASVMDHLQPCKLDFSGAKTAKTTAH
LAAAGVRRLLAGGGAKSDYCEKKGLVPILSVEAIHQLHIFIFVLAVSHVVLSAVTVLLGI
AQTRKWQHWENKIQASDENGMMKEHSPLGKWIIGMKLSGPQMIKHVQEFPFIKNHFKGHG
KRWKTFGWLRSFLKQFYGSVTEEDYVTMRLGFIMKHCRGNPKFNFYKYMIRALEDDFKKV
VGISWYLWAMLIIFLLLNVQGWYVYIWISAVPFVMLLVVGTKLEHIITELAHQVAEKHTA
IEGELVVSPSDELFWFHRPKIVLLLIHIVLFQNAFEIAFFFWLLVTYGFKSCIMGNKGYV
IARLVISVISQLLCGYSTLPLYAIVSHMGTSFKKVIFDENVVEGLANWAQNARRRNARAA
RTQNVGDSPVDESNVGEVQMTSPPTKSVQQGTARLI*
>11670.m03504|LOC_Os04g36680.1|genepair1871-2
MAATEATTIEDTPTWIVAAVCSAIVLISFAFERSLHYLGKALERRRRTLYEALLKLKEEL
MLLGFISLLLVVFQEPIQRICIAESLMGHWLPCRSDGKASSHHGVAAASAAVVSGAGARR
LLGEGTAGSGHCSSKGKVPLLSLHAIEQIHIFIFVLAITHVVLSAVTVLLGLLQMRRWRH
WENAIKADGDFGPKMINRAQQFKFIQDRYKGFDKVTMVIIWMRSFFKQFYGSVTKDDYTA
MRLGFVMEHFRGHPKFNFYDYMIKALEKDYKRVVGIKWYLWIFVMIFLLLNITGWHSYFW
ISLIPLVLLLIGTKLEHIITQLAYEVATKHTAVEGDIAVSPSDNLFWFHSPRLVLALLR
FILFQNAFEFAYFIWTMATFGFNSCIMDRLPYRVSRIVICVVVQVLCSYSTLPLYAIVSH
MGSSFKSAVFSDDVADNLRKWADEARRRTGRAAAGVGCLGAAAGSSRREGIHIQNM*
>11668.m03382|LOC_Os02g35600.1|genepair1872-1
MMNEKENLCLYGLPNETWDVTLPAEEVPPELPEPALGINFARDGMIEKDWLSLVAVHSDA
WLLSVAFYFGARFGFDKEARRRLFTMINGLPTVYEVVTGIAKKQTKVSNGSSKSNKSNPK
PSKQSNSNSKPAKPPQPKDEEDSGPEGTEDEDQAYMCGACGETYANGEFWICCDVCEKWF
HGKCVRITPAKAEHIKQYKCPGCSSKRSRE*
>11670.m03509|LOC_Os04g36730.1|genepair1872-2
MDGGYGSVTIVHDARSPEDVFQDFCGRRSGIVKALTIEVEKFYKQCDPEKENLCLYGLPN
GTWAVTLPADEVPPELPEPALGINFARDGMQEKDWLSLIAVHSDSWLLSVAFYFGARFGF
DKKARERLFMMTSSLPTVFEVVSGGVNTQSKTANGSSKNKSGSKPPKRPNSDSKPQKQVQ
AKYEEENGGRGNGGDEDQAETICGACGEAYANGEFWICCDICETWFHGKCVRITPAKAEH
IKHYKCPGCSNKRTRE*
>11668.m03391|LOC_Os02g35690.1|genepair1873-1
MDDDGGASPSSSLSPSRSPSPLPVADPVTVAAAPPGHLALAIPIPKPGSSSGGGGGGGGR
EDAWSDGATSTLIDAWGERFVALGRGSLRHPQWQEVAEVVSSRDGYSKQPKSDVQCKNRI
DTLKKKYKVEKAKPDSSWPYFHRLDTLLAPVHKPAGAYPAAAAAGAAGAGNSGSNSAAAA
TAARSTAPMAPRVNFPQRTRTQFLPSSGVKRRMPSPPQVSASSESSDGFPPEPPMAAANG
KRRREVEEEVNGADSGHRTQGLRELAQAIRRFGEVYERVELAKREQELRMERDRLEAARE
LEDQRVQFFLKMQMELSKANNAGASAAAAAVGAVATAIAAADGNGTRRTAMATDVGTSSN
HHVRYRFQDSRHCHAAPQQPQHQYNENNAAEAARGTGNGSDTDNKEDEDEAEDEEDESQ*
>11670.m03515|LOC_Os04g36790.1|genepair1873-2
MDDDDAAASASPSPSPSPVASALPVADPVTVAAGPPSGLLALALPIQKQQHAASPNPGGG
GGGREDAWSEGATAALIDAWGERFVALGRGSLRHPQWQEVADAVSSREGYAKAPKSDVQC
KNRIDTLKKKYKIERAKPASSWQFFGRLDDLLAPTFNQKPGGNGGGGVGASVNGRNPVPA
ALRVGFPQRSRTPLMPAPVSAVKRRAPSPEPSASSESSDGFPPERQPAFPPLPLPPPPNG
KRSRADEGRGGGAGGGGDRAQGLRELAQAIRRFGEAYERVETAKLEQSAEMERRRLDFAS
ELESQRVQFFLNTQMELSQVKDHSSSPANAAAPPGATGGAGGTSRRMASVNDASASGNYH
RRYRVSDGGRHRHHPQPPPSRPHYQYHENNIAVAAAAAASDGEQSSDEEDDEEEESQ*
>11668.m03397|LOC_Os02g35750.1|genepair1874-1
MAAPAPTASPPPPPAMSGLLSFASSRPYPPLPAPRPAAAAPRPRLRIAGSAAAAPNAVSH
RASSSFSSGDRLRSLVRRGELDEALRLVGSARRPDAGTCAALIKKLSASGRTAEARRVLA
ACGPDVMAYNAMVAGYCGAGQLDAARRLVAEMPVEPDAYTYNTLIRGLCGRGRTANALAV

LDEMLRRRCVPDVVTYTILLEATCKRSGYKQAMKLLDEMRDKGCTPDIVTYNVVVNGICQ
EGRVDDAIEFLKNLPSYGCEPNTVSYNIVLKGLCTAERWEDAEELMGEMGQKGCPPNVVT
FNMLISFLCRKGLVEPALEVLEQIPKYGCTPNSLSYNPLLHAFCKQKKMDKAMAFLDLMV
SRGCYPDIVSYNTLLTALCRSGEVDVAVELLHQLKDKGCAPVLISYNTVIDGLTKAGKTK
EALELLNEMVSKGLQPDIITYSTIAAGLCREDRIEDAIRAFGKVQDMGIRPNTVLYNAII
LGLCKRRETHSAIDLFAYMIGNGCMPNESTYTILIEGLAYEGLIKEARDLLDELCSRAGE
EILLDVHLIDVAFLTTFLPCSNANGGPFLRLPPEGSPVYDALL*
>11670.m03520|LOC_Os04g36840.1|genepair1874-2
MTTPAAAAAAAASSSSASSPPNPPPPSLSLMPSRLHALLCIYLPHPRPLARLLHYSAAGP
PSQQQQLPPSSSPPSPSHASAELWIAKALASAALLRPRHLPGFRRIDPSPLAAAAALRLA
PCASSALAVFTALHCSPLSITPSAHSCQQIIVVLCRSGRQADALQLFDQMTTHYGYSPDA
RFLSFLVSSCTCANLLDASATLLSKASEFGCRVEAYAYNKLMSSLIGRGRVHDVVALFER
WIQDRVYSPDVWSFNVVIKGVCRVGQVQKALELVERMNEFGCSPDTVTHNILVDGLCRTN
EVSRGHEVLRRLQRDGVCMPNVVTFTSVISGYCKAGKLEDAMAVYNDMVASGIMPNTVTY
NVLINGYGKVGDLGSAVEVYQQMTRLRCPPDVVTFSSLIDGYCRCGQLDDALRIWSDMAQ
HRIQPNVYTFSIIIHSLCKQNRSDEAIGLLNELNLRPDIAPQAFIYNPVIDVLCKCGKVD
EANLIRKGMEEKGCRPDKYTYTILIIGYCMKSRISEAIMFFHEMVEAGCSPDSITVNCFI
SCLLKAGMPNEVDHVMRLASGGASSIQEVPSPVRQRLDISVAL*
>11668.m03405|LOC_Os02g35830.1|genepair1875-1
MAEDAPPSSLPLLLPRKSLSSSSAQRKQQYARCVSHAGDELHSFRSCLAWMCVDHSTRAR
GAASWAAFLLLAVAAPSAATLALPSPVGGGGSPFDGQVQVSLTLAAALAYLTLTALLQGR
GLRRLLYLDRLRDDSEEVRSGYIEELAGSFRVLACFLLPCTLAEAAYKAYWYLAAPPFRS
PWWSAAACAVEVASWAYRTAVFFMVCVLFRTICYLQILRMKGFAREFCRFADVAAVLESH
RRIRKQLHRISHRYRRFILCCLVLVTTSQFAALLATTRPHAQINLATAGELALCSLSLVA
GLLVCLQSAAKITHKTQAITSVAAGWHADATINAFDNDQEDPNPDLPRIVGYLVPVNAYW
MASGESSSDSSSSSSSSDDDDSGHPKSKYIPFQNNHCFQQRQALVTYLENNRAGITVYGF
VVDRTWLHALFMIEFSLVMWLLGKTVGIS*
>11670.m03539|LOC_Os04g37520.1|genepair1875-2
MADTASPAAAREAMIEPASTPLLRRRGSYTRSMSHARDELGSFRSCLRWMCVEHSDGSSA
VASWLVFTLLAVAVPAAARAALPRRAYDGQVQASLTLSAALAYLTLSRLVRRRGLRRLLY
LDRLRHDSQDVRAGYTVELAGSFRLLACFVLPCFLADAAYKVYWYCANRPFPLWWSAAAC
ALEMASWMYRTAMFFMACVLFRIICFLQILRMTGFARDFGQCADVADVLRQHRRIREQLR
RISHRYRKFIVSCLLLVTASQFSALLAATRPHAQVNIATSGELALCSLSLVTGLLICLHS
AAKITHKTQAITSVAAQWHADATINSQERDHENPRTPIKASSYLHAAGPVVPQPAPNASS
SGDESEDETSPSDDGLDGTKIVSFHATHISFQKRQALVTYLENNRAGITVFGFVVDRTWL
HALFMIEFSLVMWLLGKTIGIS*
>11668.m03406|LOC_Os02g35840.1|genepair1876-1
MGRSPRGRNLPARRRGSSSSSSDLPSCCWKMKGTCEQNDIALVSEKKEWKGASCPVCLEH
PHDAVLLLCTSHHKGCRPYMCGTNHQHSNCLEHFKEAYAKEKLAHSVLIESSPGLSLSLN
SQPASKQQCAMELACPLCRGDVKGWTVVEPARQYLNRKKRACMHDGCSFIGSYKELCKHV
NSKHPSAKPREVDPAHADEWKKFECERERQDAISTIRSMTPGAVIMGDYVVEFNGGSNNN
LLSDGDDLEERLNFFTSLDRTLNERLDFYESSDSSLDDSIDFLASLFGHGRRIASGDSYT
RAYRRYRERPRRNVTASSVAASDIQHDSANTRRGRVSGIRAIGRTSRRYHPVVTHVRSTH
GI*
>11670.m03540|LOC_Os04g37530.1|genepair1876-2
MARAPRDRTLSSRRASSATSAVPSYYQKTKKASKENGLQLTSEKKDWKRATCSICLEHPH
KAVLLLCSSHSKGCRPYMCDTNRQHSNCLEQFKNAYSRGKPACELSGAVAQASKKPQEME
LVCPICRGDVKGWTVVEPARRFLNRKRRTCMHEGCSFGGSYRKLRNHVRSNHPSSNPREI
DSASLAEWKELEYEKDRQDAISIITALNPGSTIMGDYFIDPNSDSNDSFDYSSDSLTFSD
SDPGTQLNGSTSSRRPARIIPNARVRY*
>11668.m03408|LOC_Os02g35860.1|genepair1877-1
MEQDLPALKPQWLVQGQVTTTGAANLWTVASPRPGPDNQGRGGSSRNHSSSGHHRDQNSR
TSSSRISSNGPRKHDRDRDGMGKSRGYASFGRNREREREKEFDSRDRESRSITADGFGSF
STCRPERDRLNRSRSRTDSWNKGVVSPNNCNTSRNNTGTGGSFEREFPQLPFDDKRQDIN
RVPSPASPIQRTVAPDRWNSLLADVPGSSEPKKNLGVSSVLRPAPSKQPEAAPNSGISLS
MAETVMQVPLSVGPQLSMEAQKMEEISLRQNTLRPMTSPAIKSSVTNSSKTKGVRNGDPS
GPIKAHQSLIPSTNGSARAPVKTDLSKVSHAGNLKILTREQNCTIHTAKDCPDNPMSPPP
APVASVEPLKKPCVSQKLKVATHDIPLSILQSAYVDKKLNARDKHRFFESLRIKSSNGSS
STAESGCPSPSSVADVKQDSCLNVGKDVSLYHSGKDISLYHSGTKCMGNGKCSCEEANSS
DGSQRHLSDNENNSSLDHTADGVSQNLLVESRSISSSEPSDRGDEFRVFLSNNTEGSSSS
APADSDDGYKRSQSGSEEASSSSETTEPGDEEHPAEDSLPADFVAFMISLGWEKDKKVEP
LGLEEIAVTVRANEELEQKLLSMEDNANIKIVLLYIYSGRGLDKELMKPNAGDKNNA*
>11670.m03541|LOC_Os04g37540.1|genepair1877-2
MDRGEPSLKPEWLVRGHGAVAATSLWTGTSSPRADDQGRSISSRNQSSGRDRERSSQQSI
SRRSSGSIGPRRHDRDGTAKSRGYASFGRSNRDRGGEKDSESRNWESRLGPPDDPLYDGF
KPFSSCRPERDRLNHTRLKVDTLNQAVGESLDNGVRSVSRKVSGGVSFEREFPHLGSDDK
NGKQDVGRVPSPGISTPIQSMPLGTALDGRSSVLAEVPVLSGPTNCPVPSSLLRTGSSKQ
MEVPNCGTALSMAETVMQAPLKISTTPQLSIDTHKIEERTMKQCILRPRTPSSNKISVSS
SSDKLKSKGARAGDSNGPVKGTMQLPLQLSGSFIRAPVKHELVKPTQSGSFQVLSREQNG
TVNTAKESTSNPASPVLGRSYSVEPLRKPIVNQKLKGVANGLPLQLQGSFGERKSSAKDK
HKFFELLRSKSLNGSCTSTVSSSTLLDEQNNSCLELFDSGVKCMEHGSSSCEEANSCEGS
QQHLSDNEEINPPWEPHDVFDEGMQEVLSDNRDFNSSSEIADTQDVYMKPHTNNAGSSPS
IIPAEIYDGSMGSNCSDDETVMLFEPIGTGEEESYPAQDRPSPEEMAFLVSLGWKEDEIV
PPLKQEEIADCVSVSYVLLISAIIFYSLPIHNLHV*

>11668.m03413|LOC_Os02g35910.1|genepair1878-1
MSTRSKSVPVPAGGGAATVPLAVLLRREVVSEKTAAERPELQVGLFSQAKKGEDYTFLKP
DCERLPGVPSSSFSAFGLFDGHNGNGAAIYTKENLLSNILTAIPADLNREDWLAALPRAM
VAAFVKTDKDFQTKARSSGTTVTFVIIDGLFITVASVGDSRCVLEAEGSIYHLSADHRFD
ASKEEVDRVTESGGDVGRLNVVGGAEIGPLRCWPGGLCLSRSIGDQDVGQFIVPVPYVKQ
VKLSTAGGRLIISSDGVWDVLTAEVAFNCSRTLPPEAAAEQIVKEAVQQKGLRDDTTCIV
VDILPDKANLTMPHTKKQPGMGVFKNMFRKKTPSDSSSHTDREYMDPDIVEEIFEDGCAF
LSKRLDSEYPVRNMFKLFICAICQVELKPSQGISVHEDSSQPGNLRRWDGPFLCQGCQEK
KEAMEGKRRSRDSSSRNSGSSE*
>11670.m03554|LOC_Os04g37660.1|genepair1878-2
MAAAAAAATVEAVGVAGGRRRRSGSVALGDLLRREASAERASASASAGAGGRERERRPSV
AAGQACRAKKGEDFALLKPACERLPAGGAPFSAFALFDGHNGSGAAVYAKENILSNVMCC
VPADLSGDEWLAALPRALVAGFVKTDKDFQTRAHSSGTTVTFVIIDGYVVTVASVGDSRC
VLEAEGTIYHLSADHRFDASEEEVGRVTECGGEVGRLNVVGGAEIGPLRCWPGGLCLSRS
IGDQDVGEFIIPVPYVKQIKLSSAGGRIIISSDGVWDALTVDTAFSCARGLPPEAAADQI
VKEAIASKGLRDDTTCIVIDIIPPEKISPTVQPAKKAGKGLFKNIFYKKATSDSPCHADK
DQCTQPDLVEEVFEDGCPSLSRRLDSEYPVRNMFKLFICAICQVELESGQGISIHEGLSK
SGKLRPWDGPFLCHSCQEKKEAMEGKRHSRDSSSRNSGSSE*
>11668.m03416|LOC_Os02g35940.1|genepair1879-1
MASSTAADGDDEVVREFGPILRVYKSGRLERPLVAPPVGPGHDAATGVHSRDVHLGDYSA
RLYLPPPAAAAERLPVVVYVHGGGFVAESAASPSYHLFLNRLAAACPALCVSVDYRLAPE
HPLPAGYDDCLAALRWVLSAADPWVAARGDLDRVFLAGDSAGGNICHHLAMHHHHDAPPR
RRLRGAVLIHPWFWGSEAVGEEAPDPEGRARGAGLWVYACPGTTGMDDPRMNPMAPGAPP
LGRMACDRVMVCAAEGDFLRWRAHAYAAAVAAAKGGAAVEVLETAGAGHVFHLFDPDGDK
AKELLDRMVTFVNGAGADAA*
>11670.m03557|LOC_Os04g37680.1|genepair1879-2
MSSDAGDDDDVVLDFRPLIVVYKSGRLERPLATPPVPPGTDAATGVASRDVRLSAASFVR
LYLPPPCAAVAGGERLPVVVYFHGGGFVIGSAASPAYHRCLNDLAAACPAVAVSVDYRLA
PEHPLPAAYEDSAAALAWVLSAADPWLAVHGDLSRVFLAGDSAGGNICHHLAMRHGLTSQ
HPPHRLKGIVLIHPWFWGKEPIGGEAAAGEQKGLWEFVCPDAADGADDPRMNPTAAGAPG
LENLACEKVMVCVAEGDTLRWRGRAYAEAVVRARGGEAAAVELLESEGVGHVFYLFEPGH
EKADELLRRIAAFISAK*
>11668.m03417|LOC_Os02g35950.1|genepair1880-1
MQMAAAAAAASTEAPAVAAAAPHHHHPHPHAHGMHPHAHHHIQAQPRWVVIPYPPPHGMV
AAAPPPPPPQFVKHFAPPASVTPPPPQQAQQAQAAAAAAGGGSGGGNGDENRTIWVGDLQ
YWMDEGYLHNCFGPSGEVVTIKVIRNRHSGVSEGYGFVEFFSHASAEKALQNFSGHVMPN
TDRAFKLNWASYSMGEKRAELASDHSIFVGDLAVDVTDEMLMDLFAKKYRSVKGAKVIID
ANTGRSRGYGFVRFGDDNDKTHAMTEMNGAYCSTRPIRIGPATPRRSSGDSGSSPPRQSD
SDSTNRTIYVGGLDPNATEDELRKAFAKYGDLASVKIPVGKQCGFVQFVNRPDAEEALQG
LNGSTIGKQAVRLSWGRSPASKQVGTFDSFLKLLTLTLIDMFKT*
>11670.m03560|LOC_Os04g37690.3|genepair1880-2
MAQARWVVLPYPPPPPPMVAAPPPPPPQYAKHFAAGPPPAAAAGRRTPTPPAPAGSGGNG
CEENKTIWVGDLQYWMDENYLHSCFGPSGEVVTIKVIRNRQTGQSEGYGFVEFYSHGSAE
KALQNFTGHVMPNTDRPFKLNWASYSMGEKRSEVASDYSIFVGDLAADVTDEMLMELFAN
KYRSVKGAKVIIDANTGRSRGYGFVRFGDDNDKSHAMTEMNGAYCSTRPIRIGPATPRRS
SGDSGSSTPGHSDGDSTNRTVYVGGLDPNVSEDELRKAFAKYGDVASVKIPLGKQCGFVQ
FVSRTDAEEALQGLNGSVIGKQAVRLSWGRSPSHKQSRADSGSRRNNMYYGTPFYGGYGY
ASPVPHPNMYAAAYGAYPVYGSQQLVS*
>11668.m03422|LOC_Os02g36000.1|genepair1881-1
MELLVAGGGGGGAVCCMCGDHGLPRELFRCGHCHHRLQHRYCSELYPRVAAYRRCNWCLR
EGRRRGGGGGSPATATAAAKRRMSAALETSTGDSNKVDKSSRRGGGGGCSRSAFCAEPG
KPVKKPKAAASDDDDGDRVVVMPVDETAATATATALERKPPARKARFRVKVRRYKLLAEV
LSC*
>11670.m03562|LOC_Os04g37700.1|genepair1881-2
MASASPSSNSVVGGAGAGAGDVCCMCGDRGLPEELFRCRLCRVRLQHRYCSDLYPRATAY
RRCNWCLVREPAPAAAAGAAGHVHAMVDKPTTVRRKTASSSSPPPSSSMTDQETSPTTMS
SEAERQRRLQEAAGWSASRRAPDTAGLGRPVKKQKAAADDDEGEEAPGARAAPAAKGNNG
GNKEEMQAAGKKTGVKVRVRRYKLLAEVISC*
>11668.m02853|LOC_Os02g30050.1|genepair1882-1
MARIKVDELRGKNKAELQAQLKDLKAELSLLRVAKVTGGAPNKLSKIKVVRTSIARVLTV
ISQKQRAALREAYKKKSLLPLDLRPKKTRAIRRRLTKHQLSLKTEREKKREKYFPMRKYA
IKA*
>11670.m02918|LOC_Os04g30730.1|genepair1882-2
MARIKVHELRGKNKAELQAQLKDLKAELSLLRVAKVTGGAPNKLSKIKVVRTSIARVLTV
ISQKQKAALREAYKKKSLLPLDLRPKKTRAIRRRLTKHQLSLKTEREKKREKYFPMRKYA
VKA*
>11668.m02854|LOC_Os02g30060.1|genepair1883-1
MGTITAAKAAAAAAVFQSPCSPAPAASFPARSVRPDRRRAVSLSVSGVRTHVAAVEQAVV
QDAIAQSEAPVVVVTGASRGIGKAIALAFGKAGCKVLVNYARSSTDAEEVCKEIEGFGGQ
AITFRGDVSNEADVDSMIKAAVDTWGTIDVLVNNAGITRDTLLLRMKKSQWQDVVDLNLT
GVFLCTQAATKVMMKKKKGRVINIASVVGLTGNLGQVNYAAAKAAVIGLTKTTAREFASR
NITVNAVAPGFISSDMTSQLGEEIEKKNLITIPLGRYGEPEEVADLVEFLALSPGGSYIT
GQVSYSRVTIQVLTIDGGMVM*
>11670.m02921|LOC_Os04g30760.1|genepair1883-2

MATSATAGAAAAVASPAVAPRGAAVAAVARRGFVSFGAAAAARSRAVRSGGFSGVQTHVA
AVEQALVQDATKLEAPVVIVTGASRGIGKATALALGKAGCKVLVNYARSSKEAEEVSKEI
EACGGQAITFGGDVSKEADVDSMMKAALDKWGTIDVLVNNAGITRDTLLMRMKKSQWQDV
IDLNLTGVFLCTQAATKIMMKKKKGKIINIASVVGLVGNIGQANYSAAKAGVIGLTKTVA
REYASRNINVNAIAPGFIASDMTAELGEDLEKKILSTIPLGRYGKPEEVAGLVEFLALNP
AANYITGQVLTIDGGMVM*
>11668.m02863|LOC_Os02g30150.1|genepair1884-1
MAGVAAVFAGKAIATSVITYVINKAFDYLKDNKEAGGLKPTRERLEKLLPQIKVVLDAVD
MEHIGDQSDALDAWLWQLRDAVELAKDALDELEYYKLEREAKKIQAGSKVSGSLHQYKGK
IVQRFNHTFNTGSLKRLKNAVKALADVASGVERFIQVLNQFGNKVNFKQEVEFKNLRETS
SLPHSLVLGREEESNIVVQWLTKRENSASEQIVGNIPIFCIVGLGGIGKTTLAQVICNDN
KVKDYFDLFVWVCVSHIFDVETLTRKILQGVTRTEIGMIGLDALHKALQEKLSSRTFLLV
LDDVWNDESLRGWETLVSPLRYGKTGSKILLTTRMESVANLAARAMQGECQSLSLSGLKE
TELLLLLERHAFFGVNPDDYRNLQHISKKMVSKLSGSPLAAKVLGGLLNNKRDSNTWNRI
LASSVHNIQQGKEGIMTVLKLSYQHLPTHLQSCFRYCSLFHKDYEFTKKELVYLWMGSGL
IQQSVDGMTPEDVGMGYLDALTRKSFFEIKSRPRSSRDIKCRLFEEYYEERFVVHDLLHE
LARSASVNECARVSISSEKIPNTIRHLCLDVISLTVVEQISQSKKLRTLIMHFQEQDQAE
QEHMLKKVLAVTKSLRVLSLTANYPFKLPDAVGDLVHLRYLSLSLMWGEGNTTHSCWFPQ
VVYNLYHLQTMKFNNPRPAVPMEGQMEGMCKLVNLRHLHLTLVIRPMIPFIGKLTSLHEL
YGFSIQQKVGYTIVELKNLRDIHHLHVSGLENVCNIEEAAEIMLDQKEHLSAVTLVWAPG
SSDSCDPSKADAILDKLQPHSNTSKLQLEGYPGSRPPFWLQDLILINLTYIYLRDCQSMQ
CLPYLGHLPSLQYLYIVNMKSVECVDSSFYGSGEKPSGLQSLKVLEIENMPVCTEWVGLE
GENLFPRLETLAVRDCQELRRLPTLPTSIRQIEIDHAGLQAMPTFFVSSDGSSSSMFNLS
LSKLMISNCPYITTLWHGCSLYALEELSIQQCASLSCLPEDSFSSCSSLKTLEIVKCPNL
IARQIMLPHTMRTITFGLCANAELALLDSLTGLKYLKRIFLDGCAMSKLPLQLFAGLIGL
THMVLNACSIAHLPTVEAFARLINLEYLFIWDCKELVSLIGIQGLASLMSLTIASCDKLV
EDSSILSPEDADSSGLSLNLSELDIDHPSILLREPLRSVTTIKRLQISGGPNLALLPEEY
LLHNCHALEELVLTNASHLQCLPQAVTTLTSLQSMHINNAVKIQTLPDMPASLTSLHIYG
CSSELKKRCQKHVGHDWVKIAHISDADIR*
>11670.m02938|LOC_Os04g30930.1|genepair1884-2
MALAFAGKSVAVSAISMIVRKSFDYLEKYAKAEGMKSVQERLERTLPQVQVVFDAIDMER
IRDQSEALDAWLWQLRDAVEEAEDVLDEVEYYKLEKKVKTRGNKVSSSLYKCKRVVVQQF
NSTFKAGTFKRLLDAIRKLDEIVVGVERFVLLVDRLDSCTSRHVCHQEVSNPRETSSFSV
DEIVIGRDTERVKIVEWLIEQDNVHDHDVCAVNAFSIVGIGGMGKTTLAQAIYNDQRVKQ
CFDQAMWICVSNDFDVPALMKKIIQEITREGTNVTNFNTLQEIVRENLKSKKFLLVFDDV
WNDERRPDWEKLVAPLKFGQKGSKILLTTRMESVVDIVERVLGGRTKSLRLEGLHDKDLL
AIFNRHAFFEVNPDDYFNLQEIGKKITRKLSGCPLAAKIMGGLLNNSLDSIYWNRMLREN
ISNIEHNSEGIMKILRLSYHHLAPHLQACFRYCGMFREDYWFRKDELINFWMGSGLIQLS
ANENQRPEDIGEFYLGILTKKSFFELQLNKSTNLYEGYDLHEVNVPPKCGFIASELMDLK
DLRYLCIRCLENVNADEATLAKLGEKENLIMLSLTWKNSQQESDTEERVLNNLQPHMNLT
KLKIKGYNGSRSPCWLGNTTIINLTYLYISNCSYWQHLPPLGELPSLKYLYLICLNSVKR
IDSSFYGCERPFGFPSLEYLFIEHLPALEEWVEMEGEHLFPRLKALVVRHCKELRNVPTL
PSTVNYLEMDSVGLTTLHEPYVPNENAEPQKPSLSRLKICHCPYLETLEQLNQFLSLEEL
HIEHCENLVQLPMDHLQMLSFLKHMTVLGCPKLMVPPATIRLPLPTKKLHVGSCGTYETC
LVNSLCGLTSLTTLMLYGCDIAALPPVEVCKSLIALSCLEIVSCHELADLNGMEELTSLT
ELKVIGCNKLEELPVVSSQRFQASEHNQVVTACTSYLRKLKRLQISDPFVLQWAPLRSVT
SVTNMTINSCRCLPEEWLMQNCNNLQRIGVRDASHLEFLPSIMASLTSLESLEFTRVMLI
QSLPELPSSLRRLQILGCNPVLMRRCRKSRGRDWHKIAHIPDLRIVEDIPSSYSWNSYL*
>11668.m02867|LOC_Os02g30190.1|genepair1885-1
MALSHCQRQDLLLPPLLLLMSVFVYIPLAGAAEWSRNVPSLIDFKHQQFSPTGSVFGSNL
KWMTSQGGVPTFGNWSAAGDTPYTQKFENLRRSKKTATGVYSNPNEVITETPDQPPPPLR
SPLHPSSHDALNQRQRYRSAGMQTPDRKASSSDGRVPVTPGRSRLKQGGRGFEPALDEVT
VPPFGDWDDANAASGEKYTGIFNRVRRDKLTPNSSVKQQPPSSPSGGRRQEHKVQQIYVY
RYIAK*
>11670.m02948|LOC_Os04g31030.1|genepair1885-2
MAQPDIPAFGNWDTTGNTPYTQKFENARKNKKAGISSHPNDPRRHPEPPSKSPLHPAYTP
DAQGQSPMNPQHGRRQEADPHRRHSLSQQREVGGGIGSAPRSPYRMVHGSASPAQPNNPS
KPKHRSSGMQTPERRASSEGHGQHTPRRSRDKQGGRGYDAPEDDVAVPPFGEWDEGNAAS
GEKFTGIFNRVRDDKLSPNTSTRQPDTNRSQENKVKQTCPCCIL*
>11668.m02869|LOC_Os02g30210.1|genepair1886-1
MLTLVGTRDSFAQSQAQLCAGMQLKAPTRAKYSQGFMPIGESDAYCALIPGLPEDLAKIC
LALVPRSQFPVMGSVSKRWMSFLESKEFIAVRKEVGKLEEWVYVLTADAGSKGSHWEVLG
CSGQKHSPLPPMPGPTKAGFGVVVLDGKLFVIAGYAADHGKECVSDEVYRYDSCLNRWVE
LSKMNVARCDFACAEVNGMIYVAGGFGPNGDSLSSVEVYDAEQNKWTLIESLRRPRWGCF
ACSFEGKLYVMGGRSRFTIGNTRFVDVYNPNDNSWGEVKNGCVMVTAHAVLDKKLFCIEW
KNQRSLAVFNPADNSWQKVPVPLTGSSSTRFCFGIHDGKLLLFSLDEEPCYKTLMYDPAA
PTGSEWFTSELKPPGLCLCSVTIRA*
>11670.m02958|LOC_Os04g31120.1|genepair1886-2
MLTLVGAREPFVKAQTNLPATMQLKFPTRTQGDSYGALIPGLPEDLAKVCLALVPRSYFP
VMGAVSKSWMSFIGSKEFIAVRKEVGRLEERIYALITGDGGKGPYWEVLGSLEQQNRMLP
PMPGLTKAGFSVVVLDGKLLVMAGYGVDYGKECVSDEVYQYDARLNRWAALAKMNVARRD
FACAEVNGAVYVAGGFGSDGDGLSSVEVYDPQRNKWTIIESLRRPRWGSFACSFNGKLYI
MGGRSSFTIGNSRFIDVYDPILHSWTEIKKGCVMVTSHAVINKRLFCIEWKNQRSLAIFN
PSDSSWQKIPVPLTGSSATLFSLGVLDGKLLLFSQEEEPGYQTLMYDPTAPAGSEWHTST

LKPSGLCLCSVTIES*
>11668.m02871|LOC_Os02g30230.1|genepair1887-1
MSAAPPPPPPESPVAAAAGGGDKVLAAAQHIVKSLATSKNAADDMIRILSGFDNRLSQIT
SDLFPSPDLAADSDERGNPLWGQISAAAFDAAEQLIQVWDGTPEALVFEATEDEVAEYLS
AVDVAIEHLARGSGGGAGGAGSSSSSTAGRAGVAVQLAMARLEEELRHLMVRHAVPLDPT
GLFFSLRRLSLGSMDDLDTSSEFDAATPHSIDVAPETARGGPLVNPFEDQVFDPVRPEAV
DDLRAIADRMARAGYSRELADAYCGIRRDLLDEYLSALGVERLSIDEVQRIEWKHLNDKM
KKWVQAVKTVVRVLLAGERRLCDQVLSVSDELREECFIESTKGCIMQILSFGDAVAVCPR
SPEKLSRILDMYEALAEVIPEMKDLCLGSSGDGVISDVQANLDRLGDAIRGTLFEFGKVL
QLESSRRAMTAGEIHPMTRYVMNYLRLLVVYSDTLDALLDDNADDQIDLARAEDQDQEHL
ESMTPLGKRLLKLISYLEANLEEKSKLYEDSALECIFSMNNLLYIVQKVRDSELGKILGD
HWVKRRNGKIRQYSKSYLRISWMKVLSFLKDDGHGSGSGSSSGSGSGHSSSRMSIKEKFK
NFNLAFEEIYRNQTTWKVPDPQLREELKISISENVIPAYRAFLGRYGSQVDGGRNSGKYI
KYTPEDLESQLSDLFEGAPGPANHSRRRT*
>11670.m02984|LOC_Os04g31330.1|genepair1887-2
MSAPPAPPHPQPEELEVAEEPPAAAGGVGNDKVLAAAQHIVKSLATSKNAADDMIRILSG
FDHRFSSITADLFPSPLPSSGAGPTPPPPPPPRGAFEAAERLIRQWDATSELLVFEGPEG
DVADYLEAVDVAVDQLLSGVGAAAADAEAEAAGVVVQLAMARMEEELRHLMVRHAVPLDA
SGLFCSLRRLSLESMDDLDTSSEFDPITPHSLEGGPDTARSASLVGNPFDDQVFDLVRPE
AIDDLRSIAQRMDRAGYASELEQVYCGVRRDLLDECLAVLGVERLSIDEVQRMEWKLLND
KMKKWVHGVKTVVRSLLTGERRICDQVLAVSDELRDECFVESTKGCIMQILNFGDAVAVC
SRSPEKLSRILDMYEALAEVIPELKELFFGNSGNDVICDLEGVLERLGDAVKGTLLEFGK
VLQQESSRRPMMAGEIHPMTRYVMNYLRLLVVYSDTLDKLLGDDSAGDVDHSDTHRGGDD
EEEYLESLSPLGRHLVKLISYLEANLEEKSKLYEDGALQCIFSMNNILYIVQKVKDSELG
RILGDHWIRRRRGKIRQNSKNYLRISWTKVLSFLKDDAHGGRSGSGSGSGNSSRIKEKFK
NFNLAFDEIYRSQTLWKVPDPQLREELKISISENVIPAYRAFLGRYGSLVDSGRNSGRYI
KYTPEDLENQLSDLFEGSLGPANHSRRR*
>11668.m02873|LOC_Os02g30240.1|genepair1888-1
MKKASRFLKGLLSAIVAAVKARSAAVRAKTSAVRTRLIVLGILRNKKLLLSAINRKIHAI
VSSGGGGGSSHGEYGGGGESYGGEQQQHLSGIHLVGGGGYRKAAVLHSLPSFVVEQERSA
VVLLSSLPSFAMDRDVYGGGGEAEAEEEQGVGGKQQQQSVIELARGAAAAEAGGGAEFRL
EDEIDHVADVFIRRFHEQMKLQKLESFKRLCEMLDRS*
>11670.m02986|LOC_Os04g31350.1|genepair1888-2
MRSKASMFLKQMVSTIVAVVKAKSTAVRAKTSAMKTRLLIFGVLRNRKLLATAINHKIHA
IMGGAAAQDTTNDGGVAGVEDDDGGGSKKAVVLYNTAPSFLTERGYYDHAGEEEEEEEDS
DEYLTHSLFQEEDDEDDELVNAPGSVIDLVRDAKEGEGGEFRLEDEIDHVADVFIRRIHK
QLKLQKLDSFKRFCEMLERSA*
>11668.m02877|LOC_Os02g30280.1|genepair1889-1
MAPHLKEVCSSLAASKELVKALAGIWGPGDGALNPSTASSLLSALRAELDLARAHARRLA
KEDRRRGDEAARARARLAEDAREWGRRQREKAAAVRVAAAELDGERRSRRRAERVNAKL
GRALADAERELAASRRELERERRSRERLEKVCDELVRGGLVAAGGGGRGGEEEVEEMRRE
AERAQEELEKEREMLRLADELREERVQMKLLEARLQFEEKNAVVEQLRDELEAFLGSKKD
RQQQEEPPPPDADDHRRRRPDGHQFQSILVAVNKNGDHEDDNDGDEEDDGGGRGECVAED
SDGSEMHSIELNVDGNSKDYSWSYTTASKDMTTTARSKNAAAIDRRSQEGAGEEDRWDDG
GCSERSKDLDEEDAERYEAIKNLREQMLAGHGFVLVSQEWGQC*
>11670.m02991|LOC_Os04g31400.1|genepair1889-2
MSHPLLLLLLLLLLLLLLLIPSFKAVVSKGSRRSRSKILEADVKGSWHGGGHGHWLSSTDV
MSNATAMEIVTCSQDDVSRCPQEKTVNLHDLHNSLIASKELVRVLAHIWGPGELNPSTTS
LISALRSEIDLARSHVRKLIKEQKSEGIESLKKQLVQEMESWKSKQKEKVANALQYIVSE
LDSEKKSRRRAERINKKLGMALANTEASLQAATKELERERKSKGRVEKICTELIRGIGED
KAEVEALKKETEKAQEELQKEREMLQLADEWREQRVQMKLLEARLQFEEKNAAINQLHDE
LQAYLDTKKEHGQSNDQMTLLRASENGREIADNIQKNSGECDDEDEDDDDDDSASEGSDM
HSIELNVDGNSKSYTWSYTPTSKDRKRNASFSQRGMDSGSSCGFDRKFQETGEELLEGDW
AEGCSNGMLNFEHDEERYLAIKNLREQMLAGSGFIVSQGREHAESEFCGL*
>11668.m02879|LOC_Os02g30300.1|genepair1890-1
MVAKRGAGACALSWDEEGEVSRGEKRRRADGDGSSDVGGGGGIGAFDALHDELVVSILAD
VAASAGSPADLAAAMLTCRRFRELGKHGLVLARASPSAVAVRAAAWCDDAHRFLVRCAEA
GNVEASYLLGMIMFYCFENRKLGAELLGAAARRGHGEALYSMAIIQFNGSGLPKDGRNLQ
AGAQLCARAASRGHTDALRELGHCVSDGYGVRRSLSGGRRLLIQANFRELCAAVANGGAR
FAAALGRSGECKPPGPHMCLLSDYGCHVAGAAGRRAHAANAFLAGWYASRPLASGAGAAA
LRMCSQPTCGRPETRKHEFRRCSVCSGVIYCSRACQAMHWKVAHKSACVPMAHWLVAANA
GAGNAVGAAAAAAAQMAMP*
>11670.m03012|LOC_Os04g31610.1|genepair1890-2
MRTRRGACYSPASCQDGRRKRRRIAGGGGEGSAAAAAAVAGGAEGPANDMFEELPDDLVV
SILADVAASARSPGDLAGAMLTCKRFRELGQSKVVLARASPRCLAVRAKAWSDAAHRFLQ
RCADAGNLDACYLLGMIRFYCLGSRGSGAALMAAAAVGGHREALYSLAVIQFNGSGGSKD
DRDLRAGAALCARAASLGHVDALRELGHCLQDGYGVRRSVLDGRRLLIQANARELAAAVA
ASASLLRAATGKPAAAASRRHSCLLSDFGCHAAAPKAGGEAHAANRFLVDWFASRPLAGS
TAAAAAAPTPGSAAEDEAAGLRLCSHALCGRPETRRHEFRRCSVCGVVNYCSRACQALHW
KTAHKAECTPMDRWLDNAAAGAANPNAAAMAAPAP*
>11668.m02890|LOC_Os02g30410.1|genepair1891-1
MMACRALALLPLPAPSRLPRPRAASLARLDAARRRASASLLVRCCANPAGPGGQEDPPQD
AVLKAISQVANSKGRVAQTTNVVMGGTVTDDATDEWLVLDKQVNTYPTVRGFTAIGTGGD
DFVQAMVVAVESVLEEQIPKAQISHKVSSKGKYVSVKIGPIPVVSSEQVQAVYNAMKKDE

RMKFFL*
>11670.m03020|LOC_Os04g31690.1|genepair1891-2
MACCRALALRSLLVPDHPRAAAAAAARPAAGRRAPRRRSRHLRCCSGGGDPGQPPQEAVL
EAISKVARSKGRVALTTNMVLGGTVTDDASDEWLVLDQKVNSYPTNRGFTAIGTGGDDFV
QSMVVAVESVLQEPIPKGQVSHKCHLGGSMFL*
>11668.m02896|LOC_Os02g30470.1|genepair1892-1
MADWGPVVIATVLFVLLTPGLLFQLPAHGRIVGFGTMHTSGVAVLVHAVIYFALITIFLI
AIGVHIYAG*
>11670.m03034|LOC_Os04g31820.1|genepair1892-2
MADWGPVVVATVLFVLLTPGLLCTVPGRGRVAEFGSFHTSGLAIIVHAVLYFALLTIFLI
AIGVHIYAG*
>11668.m02911|LOC_Os02g30620.1|genepair1893-1
MDVELNSRALMDEALKARDAAERKFHARDVKGARRSAIKAQNLCPSLDGISQMVSTLEVL
LASESKVDGENDWYRILSLSASADEEEVKKQYRKLALQLHPDKNKSVGAEGAFKLISEAW
AVLSDKSRKMQYDQKRKDHPVTNGANGLYTYDKKAHKRARKNAAASAAAAAAAAAAAAEA
TTRPVGLDTFWTSCNRCRMQYEYLRIYLNHNLLCPNCHHAFMAVETGYPCNGTSSSFSWS
TKQQQQNHKHSYSSASRTSGVPGTGHGVYQQENTYETYNNQSFQWNQYSKTNNSAGTNAY
SSTASEKPKRKHEESYIYNYSSSGNEFGQERPTSGRGRFSKRRQNINNGYASVDCNGDNK
ETVAATAGTTVLADVGRVNGTSVEKFRSAVSGRRANVMREIFQLDTRGLLIEKAKAAIRE
KLQDLNISATRHIAAKGKAERKNHVDHDVKGNGILPHNPSHKFKICNSKGADVENPATDE
NNLEQKRVPVSIDVPDPDFYDFDKDRTERTFDNDQVWATYDSEDGMPRLYAMVQKVISRK
PFRIRMSFLNSKSNIELSPINWVASGFSKTCGDFRVGRYQIFETVNIFSHRVSWSKGPRG
IIKIVPKKGDTWALYRNWSSDWNELTPDDVIYKYEIVEVIDDFTDEQGVTVIPLLKVAGF
KAVFHRRTDSDVVRRIPKEELFRFSHRVPSRLLTGEEGNNAPKGCHELDPAATPVDLLKV
ITEVKEVATTEISE*
>11670.m03046|LOC_Os04g31940.1|genepair1893-2
MYTYDKKANKRARKNAAAAAAAAAAAAAAAEATTRPAGVDTFWTSCNRCRMQYEYLRIYL
NHNLLCPNCHHAFLAVETGFPCNGSSSSFSWSTKQQPQNNNSTKHSYGSTSRTSSIPGTG
HGGYQQDGTYDSYNNQSFQWNQYSKTTPAAGTNAYGTQALEKPKRKHEESYSYNYSATGN
SYGHERTNSRRGRFSKRRRHSNDGYTTMDFGGDNRETVAASTETTAFTDVAVAQVNGTSG
EKLRSAVSGRRANVLREISQIDTRALLIEKAKAAIQEKLQEWNITSSSRLAERGKSQGKV
YPSDNNIKQNGGLSDKHVKGLKQCSSRSVDTQAPTVDEKNPEQRRVPVSIDVPDPDFHDF
DKDRTERAFDSDQVWATYDSEDGMPRLYAMVQKVLSMRPFRIRMSFLNSKSNSELAPISW
VASGFQKTCGDFRVGRYQISETVNIFSHKVSWTKGPRGIIRIVPQKGDTWALYRNWSPDW
NELTPDDVIYKYEIVEIIDDFTDEQGLTVIPLLKVAGFKAVFHRHMDPKEARRIPKEELF
RFSHRVPSRLLTGEEGNNAPKGCHELDPAATPVDLLKVITEVTEDTATQPAK*
>11668.m02913|LOC_Os02g30630.1|genepair1894-1
MATTAAAAAAALSAAATAKTGRKNHQRHHVLPARGRVGAAAVRCSAVSPVTPPSPAPPAT
PLRPWGPAEPRKGADILVEALERCGVSDVFAYPGGASMEIHQALTRSPVITNHLFRHEQG
EAFAASGYARASGRVGVCVATSGPGATNLVSALADALLDSVPMVAITGQVPRRMIGTDAF
QETPIVEVTRSITKHNYLVLDVEDIPRVIQEAFFLASSGRPGPVLVDIPKDIQQQMAVPV
WDTSMNLPGYIARLPKPPATELLEQVLRLVGESRRPILYVGGGCSASGDELRWFVELTGI
PVTTTLMGLGNFPSDDPLSLRMLGMHGTVYANYAVDKADLLLAFGVRFDDRVTGKIEAFA
SRAKIVHIDIDPAEIGKNKQPHVSICADVKLALQGLNALLQQSTTKTSSDFSAWHNELDQ
QKREFPLGYKTFGEEIPPQYAIQVLDELTKGEAIIATGVGQHQMWAAQYYTYKRPRQWLS
SAGLGAMGFGLPAAAGASVANPGVTVVDIDGDGSFLMNIQELALIRIENLPVKVMVLNNQ
HLGMVVQWEDRFYKANRAHTYLGNPECESEIYPDFVTIAKGFNIPAVRVTKKSEVRAAIK
KMLETPGPYLLDIIVPHQEHVLPMIPSGGAFKDMILDGDGRTVY*
>11670.m03053|LOC_Os04g32010.1|genepair1894-2
MAAAAAAASLSVSDAAAKLPKPGGQVQRRRDRDRPRVDAAACTRDSRRPTRERCSTTVSL
AATATATTATPVRAPVRTRAPMGQRKGADIVVEALERCGVRDVFEYPGGASMEIHQALTR
SPVIRNHLLRHEQGEAFAASGYARSSGRPGVCVATSGPGATNLVSALADAHLDSVPLVAI
TGQAPRRMIGTDAFQETPIVEFTRSITKHNYLILDVDDIPRVINEAFFLASTGRPGPVLV
DIPKDIQQQMAVPSWDAPMRLPGYISRLPKPPAANLLDEVIRLVGDAERPVLYVGGGCSA
SGYELRRFVELTGIPVTTTLMGIGNFPSDDPLSLRMLGMHGTVYANYAVDNADLLLALGV
RFDDRVTGKVEAFASRAKIVHVDIDPSELGKNKQPHVSICADVKLALQGMNAMLEEQSAA
AARKNLDFSAWRSELEKKKVEFPLGYRTFGEEIPPQYAIQVLDEVTNGEAIVATGVGQHQ
MWATQHYTYRRPRQWLSSAGLGAMGFGLPAAAGAAVANPGATVVDIDGDGSLLMNIQELA
MVRVEDLPVKVMVLNNQHLGMVVQWEDRFYDANRAHTYLGNPAANGGGEVYPDFVTIAGG
FGIPAARVTRKGEVRAAVEEMMAAPGPYLLDVVVPHQEHVLPMIPSNGAFKDIIVDGDGR
SSY*
>11668.m02915|LOC_Os02g30650.1|genepair1895-1
MGEEEKVKEEAAAEKGKEAAVAEEKEAAAAGEEKKEDAPPPPPPPEEVVMRVFMHCEGCA
RKVKKILRGFDGVEDVVADSKAHKVIVKGKKAAADPMKVVHRVQKKTGRKVELLSPMPPP
VEEKKEEEKKEEPEPPKPEEKEPTVIAVVLKVHMHCEACAQVIRKKILKMKGVQSAEPDM
KASQVTVKGVFEESKLTDYVHKRIGKNAAVVKSEPAPPPENAGDANAKDDKKAAEGGEEK
DESKEEKKEGDDEKEKEKEKDDSNAAEVEEKDKEKDPSALAAANLYMHYPRFSNPGGYGV
PGYAYPYAPQLFSDENPNACVVM*
>11670.m03055|LOC_Os04g32030.1|genepair1895-2
MGEEEKKEAAKAKEEEKPKQEEKPKEQEEGKKEEKPKEEGEKGKDGGGEEKKDGEAAAA
ADAPPPPPPPEEVVMRVYMHCEGCARKVRKILKGFDGVEDVIADSKAHKVLVKGKKAAAD
PMKVVERVQKKTGRKVELLSPMPPPPEEKKEEEKKEEPEPPKPEEKKEPPVIAVVLKVHM
HCEACAQGIRKRILKMKGVQSAEPDLKASEVTVKGVFEEAKLAEYVHKRTGKHAAIIKSE
PVAPPEKVAAEGGDEKKAAEGGDEKKDEKEEKKDGKEEGGNGGGDEKKEEKEKEKEGGNA

DGEEKDKDKEKDPAAIAAANLYLHYPRFAFPAGYYPPGPGYAYPPPYPPSYPPPYQPSYP
PYPSHPSHHPSQIFSDENPNACSVM*
>11668.m02923|LOC_Os02g30720.1|genepair1896-1
MPLRPARSPLLLPPGPPPHPHPSRGSLRARWLVDAVDLATGRAERLKTTADWVEGGNSSG
LHNWEELAYQYAAQGACLALVARRKKALEGVAAAALERGSPDVLVLPADVSDADQSRRAV
EETVAHFGKLNHLVANAGIWSSCSFDEVTNITAFTKMMDVNFWGSVYPTYYALPHLKASK
GKLVVSCSAAGTVGTSRMSFYNATKAAQLRFYETLRAELGSEVGITVLTPGYVESEITKG
KGIQSGGDVAVNEEARDEQIGVFPVGRVAELGEVAMDGIRAGDWYVTWPSLFRPLQLVAC
LAPGVLDWACRALYGTRKGARPPLGKRIMEATGMKRLFPEALRRNPAIKTEDEEYCDGEE
GYGAADDAAAAYLLQCRKGLRAAHTYIRCIFSLSDSIL*
>11670.m03060|LOC_Os04g32070.1|genepair1896-2
METLSMLKVGYTLLRSETPATDLVNAFMDWAARRSLLLLAVFVPPYLAYRLASSALAAAS
PEDVAGKVVLVTGASSGIGEQVAYRYARRGARLALVARREASLGEVAARARALGSPDVLA
VPGDVARPDDCRRFVQATVEHFGRLDHLVNNAGLANVCWFEEVPDVANFKQVLDVNFWGT
VHPTHAALPHLKASRGKIFVNSSASAVLAMPRMSFYNASKAAVHNFAETLRMELHGEVGV
TVATPGWVDSEMTKGKHLSSHGAMEVDQDTRDAQVGVFPVERGERCAEAIVDAVARGRRR
VTSPAWYGALFLWRTMAPEVADACQRVFYHRRSSAAGGGGRARAALEATGAKAVLQPPSL
RSSEIKVE*
>11668.m02937|LOC_Os02g30850.1|genepair1897-1
MQYGAAAEQAWYMPAAAPAPMVESAVARVERLASESAVVVFSVSSCCMCHAVKRLFCGMG
VHPTVHELDLDPRGRELERALARLVGYGGPAAASPPVVPVVFIGGKLVGAMDRVMAAHIN
GSLVPLLKEAGALWL*
>11670.m03085|LOC_Os04g32300.1|genepair1897-2
MQYGAAAAEQAWYMPAAAMVVAAAAETAAERVERLASESAVVVFSVSSCCMCHAVKRLFC
GMGVHPAVHELDLDPRGRDLERALARLVGAGGAAAAAVPVVFIGGKLVGAMDRVMAAHIN
GSLVPLLKEAGALWL*
>11668.m02942|LOC_Os02g30900.1|genepair1898-1
MGCFSCFDSPAEEQLNPKVGGPYGGGSSSSAAAAAYGGGGGSSAGRHGERGGGYPDLHHH
HQQQQLPMAAPRVEKLSAGAEKTRVKSNAILREPSAPKDANGNVISAQTFTFRELATATR
NFRPECFLGEGGFGRVYKGRLESTGQVVAIKQLNRDGLQGNREFLVEVLMLSLLHHQNLV
NLIGYCADGDQRLLVYEYMHFGSLEDHLHDLPPDKEALDWNTRMKIAAGAAKGLEYLHDK
ANPPVIYRDFKSSNILLDESFHPKLSDFGLAKLGPVGDKSHVSTRVMGTYGYCAPEYAMT
GQLTVKSDVYSFGVVLLELITGRRAIDSTRPHGEQNLVSWARPLFNDRRKLPKMADPRLE
GRYPMRGLYQALAVASMCIQSEAASRPLIADVVTALSYLASQSYDPNAAHASRKPGGDQR
SKVGENGRVVSRNDEASSSGHKSPNKDREDSPKEPPGILNKDFDRERMVAEAKMWGDRER
MVAEAKMWGDRERMVAEAKMWGENWRDKRRAIENGQGSLDSPTENG*
>11670.m03086|LOC_Os04g32310.1|genepair1898-2
MGEEEEEELYYGKGGARGGGNGGGALSAAAAAASSSSGVGGGARGRGNATVKKELSALKD
ANGNVISAQTFTFRQLAAATRNFREECFIGEGGFGRVYKGRLDGTGQIVAIKQLNRDGTQ
GNKEFLVEVLMLSLLHHQNLVNLVGYCADGDQRLLVYEYMPLGSLEDHLHDLPPDKVPLD
WNTRMKIAAGAAKGLEYLHDKAQPPVIYRDFKSSNILLGEDFHPKLSDFGLAKLGPVGDK
SHVSTRVMGTYGYCAPEYAMTGQLTVKSDVYSFGVVLLELITGRKAIDSTRPHVEPNLVS
WHLIILTSGIYTVLTSAITFFKRFIGKVDVLIIVRTKCRARPLFNDRRKLPKMADPGLEG
RYPMRGLYQALAVASMCIQSEAASRPLIADVVTALSYLASQKYDPNTTPSSKKAGGGEAG
RALSRNDEAGSSGHKSPSSKDSPREQQLPGILNDRERMVAEAKMWGENWREKRRAAATTS
SNAQEQICRTSVICSDVRQKERKEDVVQLTTDRASTNVEALGAAVGPAAALVGVPVGSGG
GGGGEEEAEAEHIIGVARAKGVSGDDGEADGGECEAARRASVLGEDTAGGKQVEESLDDG
GVQVARVGASDQSLVGTPRRMMAVSAMGTEIMFASSIITRRASMGRIYRLGRLLRPFSKV
SP*
>11668.m02955|LOC_Os02g31030.1|genepair1899-1
MALDVLAAGAPATSAILAEVDAAGARSAAAGGGGGGGGQRFAVIGHRGKGMNALASPDRR
MQEVKENSLRSFNEAARFPVDYVEFDVQVTKDGCPVIFHDNFIFTKEDGKILDKRVTDLQ
LEDFLLYGPQNEQGKGGKPLLRKLKDGRIVNWNVQSDDPLCTLQEAFEKVNPRLGFNIEL
KFDDNLEYQEEELTCILQAILKVVFEYAKDRPIIFSSFQPDAAQVMRKLQSTYPVYFLTN
GGTEIYADVRRNSLEEAIKLCLASGMQGIVSEARGIFRHPAAVPKIKEANLSLLTYGTLN
NVPEAVYMQHLMGVNGVIVDLVQEITEAVSELITVPEPDLNADNLSNGAAKDAATPHFSQ
CEISFLLRLIPELVQ*
>11670.m03087|LOC_Os04g32320.1|genepair1899-2
MAQPKAASAAAQLSLAASACIAGAEAAAMVGREGAPAMVAAAAAAAMVVIGHRGKGMNAL
GSADPRLREVKENSLRSFHAAARVAGVSYVEFDVQTGASTIVQLNLNQMVTKDGYPVIFH
DDFIFTEQDGEICGRRVTDLRLDEFLSYGPQKDQSKAGKPLFRKLNDGRVLRWDVQSDDA
LCTLQEALDGVDRRVGFNVELKFDDDVVFEHAKERPIFFSSFQPDAARIMRKLQDRYPVY
FLTKGGTQVFADERRNSLEAAVKLCVAGSLRGIVSEARAVLRQPSAIGRIKEAGLSLLTY
GQLNNVPKAVYLQQLMGVDGVIVDLVAEIAAAVSEFAAAAAAAVPVPERDSSSSYMDGGG
DVGLLEMTSPAARTTASFSRREDVSFLLRLTPELVQ*
>11668.m02956|LOC_Os02g31040.1|genepair1900-1
MQGTWNARPHSGSRRRSSPSRNSPRQTGQSVAPSMPAPYVAVPREGNGFDRGLIELLLLV
LVPTVDEAVDFLNDDEAVDGRVRGAATRRRRPCRCWPSHYASYGVINMSDISALSYLKVF
MDAHCQLGDLVDAVVPFMGESITDGTLATFLKKPGDRVEADEPIAQIETDKVTMDVASPE
AGIIEKFVASEGGIVTPGVKVAIISKSAAQSKTHTQSSEDTSQKHSTKPPSTKENKVEAK
PPKVESSTTHESKLTSSSEPQLPPKERERRVPMPRLRKRIANRLKDSQNTFAMLITFNEV
DMTNLMKLLSDYKDQFVEKHGVKLGLMSCFVKAAVSALQNQPIVNAVIDGDDIIYREYID
ISVAVGTSKGLVVLVIHDIDAMNFADIEKGINNLAKKATEGAQSINNMAGGTFTISNGGV
YGSLISTPIINSPQSSILGMHSIVQRLVVVNGSVLARPMMYLALMYDHRLIDGREAVLFL

RRIKDVVEDPRRLLLDI*
>11670.m03088|LOC_Os04g32330.1|genepair1900-2
MASRIASRLLRRSNATLGLIRSYSHARNYSSQLSALIPIGSQSSKLTRRRYYLPNASPYQ
LWSRSFASDNGDQVEAVVPFMGESVTDGTLANFLKKPGDRVEADEPIAQIETDKVTIDVA
SPEAGVIEKFIASEGDTVTPGTKVAIISKSAAPAETHVAPSEDSTPKETPPKAEETKPKL
EEKSPKAEPPKMPLPPKTSPTEPQLPPKERERRVPMPRLRKRIANRLKDSQNTFAMLTTF
NEVDMTNLMKLRSDYKDEFVTKHGVKLGLMSCFVKAAVTALQNQPIVNAVIDGDDIIYRD
YVDISVAVGTSKGLVVPVIRDADNMNFADIEKGINALAKKATEGALSIDEMAGGTFTISN
GGVYGSLISTPIINPPQSAILGMHSIVQRPVVVDGNILARPMMYLALTYDHRLIDGREAV
YFLRRIKDVVEDPRRLLLDI*
>11668.m02997|LOC_Os02g31910.1|genepair1901-1
MSVQEDGAARPEPDVLRRHDSLYGDAEKVSNNKRHGAGGSWARTLQLAFQSIGVVYGDVG
TSPLYVYSSTFPNGIKHPDDLVGVLSLILYTLILIPMVKYVFIVLYANDNGDGGTFALYS
LISRHAKIRMIPNDQTEDANVSNYSIEAPSSQLRRAEWVKQKLESSNAAKIALFTITILG
TSMVMGDGTLTPAISVLSAVSGIREKAPNLTQSQVVWISVAILFVLFSMQRFGTDKVGYT
FAPVISVWFLLIAGIGMYNLTVHEITILRAFNPKYIVDYFRRNGKEAWVSLGGVVLCITG
TEAMFADLGHFNIRAIQLSFTCVLFPSVALCYMGQAAYLRKFPENVGDTFYRSIPAPLFW
PVFVVAIMGAIIASQAMLSGAFAILSKALSLGCFPRVEVVHTSNKYEGQVYIPEVNFLIG
AASVAVTLAFQTTANIGNAYGICVVTVFSITTHLMTVVMLLIWKVRLPFIAAFYAAFGLA
EFLYLSSILSKFAEGGYLPFCFSLVLMALMATWHYVHVKRYWYELDRVVPAAETTALLAR
RDVRRVPGVGLLYSELVQGIPPVFPRLVDKIPSVHAVFVFMSIKHLPVPRVAPAERFIFR
RVVGADAGAGHRLFRCVARYGYTDQLEGAKEFAAFLLDRLKVFVHEESVFACSRGDNDDD
DAMRRAQAMAEEEKRVIDAEAERGVVYLMGEANVTAAAGSSVMKRIVVNYVYTLLRKNLR
EGHKALSVPKDQLLKVGITYEI*
>11670.m03151|LOC_Os04g32920.1|genepair1901-2
MSSALEVEGSGSPGVEPAATATASRLKRHDSLFGDAEKVSGGKHHGGSAVSWAVTLHLAF
QSVGIIYGDIGTSPLYVYSSTFPDGIGHRDDLVGVLSLILYTLIIIPMLKYVFIVLYAND
NGDGGTFALYSLISRYAKIRMIPNQQAEDAMVSNYSIEAPSSQLRRAQWVKHKLESSRAA
KMALFFLTILGTSMVMGDGTLTPAISVLSAVSGIREKAPNLTQTQVVLISVAILFMLFSV
QRFGTDKVGYTFAPIISVWFLLIAGIGLYNLVVHEITILKAFNPWYIVQYFRRNGKKGWV
SLGGVVLCVTGTEGMFADLGHFNIRAVQISFNCILFPSVALCYIGQAAYLRKFPENVSDT
FYKSIPGKYRDRLNFGPLFWPTFIVAILAAIIASQAMLSGAFAILSKALSLGCLPRVRVI
HTSKKYEGQVYIPEVNFMMGLASIIVTIAFRTTTSIGNAYGICVVTTFMVTTHLMTVVML
LIWKKHLVFILLFYCVFGFTEVVYLSSILSKFVDGGYLPFCFAMVLMTMMATWHYVHVRR
YWYELDHIVPTAELASLLEENGGVRRVPGVGLLYTELVQGIPPLFPRLVRKIPSVHAVFV
FISIKHLPIPHVAAAERFLFRQVGPRARRVFRCVARYGYTDALEEPREFAAFLVDGLKMF
IQEESAFAPHQEMIDAAADDDDEAAARPRRSTSSAVHSEEAIQAASSGRTTASSVQLQAG
GEPPAAMDVEEEKRLIDREVGRGVVYLMGEANVSAGPNSSILKRIAVNYIYTFLRKNLTE
GHRALAIPNDQLLKVGITYEI*
>11668.m03010|LOC_Os02g32040.1|genepair1902-1
MDFYFFSSSAPAPEKKTRRQQQQQQREQEGGGGGNEARYLGVRRRPWGRYAAEIRDPAT
KERHWLGTFDTAEEAAVAYDRAARTIRGAAARTNFAYPDLPPGSSLTPYLSPDLSTNDLH
RHYYGAGAGADTQTAAALPAPAQPAHGGDAQEMAYGGGGGQNVGGVFDVVGGGGGAAWCD
ASELEFGGYDDAGASAAAAAVYFEEGYVHSPMFSPMPAADEVAADGFQLGGSSSSSYYY*
>11670.m03137|LOC_Os04g32790.1|genepair1902-2
MMNFSSYFYSSSAAAAGGGGGGGEKKSSSSSASKKKQQQAAAAEGGNNQTRYLGVRRRPW
GRYAAEIRDPATKERHWLGTFDTAEEAAVAYDRAARSLRGARARTNFAYPDLPPGSSVTP
YLSPDLSADASDQLLQPFYANPSAAAALPTPAAVMAGGGGVEFGGEYMYGGGVDMSSLMD
DIAAMPDDLPPSVTGGGGGFASSDEYSSGGGGMVDDVSMYCGGNGGGSSWCDASDFASYS
SSSPAAAAAAGSHGMYFEEGYVHSPLFSPMPAVDDAGADGFQLGGSSSSYYY*
>11668.m03012|LOC_Os02g32060.1|genepair1903-1
MSSPDKVVARTGRLRQRYDNEYRLVAGCVPYRVKKDEANPRILGDVPGQVEVLMVSTPNR
ADMVFPKGGWEDDEEVYEAASREAMEEAGVKGIVNRTTLGHWVFKSKSSQNSSSPRGACK
GYIFAMEVTEELESWPEQATHGRRWVSPGEAYQLCRYEWMREALTALLERLSMIEPVPSA
QELSDQTSMYMMLQASSDSAVALC*
>11670.m03132|LOC_Os04g32740.1|genepair1903-2
MAASSSKKKEEEEVAVVAARKGRLRQRYDGEYRLVAGCVPYRVVAAGGGGGGGELEVLMV
STPNRADLVFPKGGWEDDEDVYEAACREAMEEAGVKGNINVMSLYRNNVTTITITDIRAG
CNLLLSYTSVSVMLIKIFKLKQIQLILSSPKDKEIFDYKFEYCEYISIYRVSLGMWVMRS
KSSQSGGGGEASRSPRGGACKGYMFELEVTEEMDRWPEQATHGRRWLPPADAFRLSRYGW
MREALAALLDRRCLLLLPPPQPEPSEHAGVYGLAMLKAAAAAAADRAVALC*
>11668.m03018|LOC_Os02g32110.1|genepair1904-1
MVGARAGRVPAAAAAAAAVLIVAACVFSSLAGAAAAAEVVGGAAQGNTERISGSAGDVLE
DNPVGRLKVFVYDLPSKYNKRIVAKDPRCLNHMFAAEIFMHRFLLSSAVRTLNPEQADWF
YAPVYTTCDLTHAGLPLPFKSPRMMRSAIQFLSRKWPFWNRTDGADHFFVVPHDFGACFH
YQEEKAIERGILPLLRRATLVQTFGQKNHVCLKEGSITIPPYAPPQKMQAHLIPPDTPRS
IFVYFRGLFYDNGNDPEGGYYARGARASLWENFKNNPLFDISTEHPATYYEDMQRSVFCL
CPLGWAPWSPRLVEAVVFGCIPVIIADDIVLPFADAIPWDEIGVFVDEEDVPRLDSILTS
IPIDDILRKQRLLANPSMKQAMLFPQPAQPRDAFHQILNGLARKLPHPDSVYLKPGEKHL
NWTAGPVADLKPWK*
>11670.m03125|LOC_Os04g32670.1|genepair1904-2
MGSRTVGWWLLAAAVVLAAAAADSGEAERAAEQHSERISGSAGDVLEDNPVGRLKVFIYD
LPRKYNKKMVNKDPRCLNHMFAAEIFMHRFLLSSAVRTLNPKEADWFYTPVYTTCDLTPA
GLPLPFKSPRVMRSAIQYISHKWPFWNRTDGADHFFVVPHDFGACFHYQEEKAIERGILP

LLQRATLVQTFGQENHVCLKEGSITIPPYAPPQKMQAHLIPPDTPRSIFVYFRGLFYDTG
NDPEGGYYARGARASLWENFKNNPLFDISTDHPPTYYEDMQRAVFCLCPLGWAPWSPRLV
EAVVFGCIPVIIADDIVLPFADAIPWEEIGVFVEEKDVPKLDTILTSMPIDDILRKQRLL
ANPSMKQAMLFPQPAQPRDAFHQILNGLARKLPHPEGVYLQPSDKRLNWTAGPVGDLKAW
*
>11668.m03019|LOC_Os02g32120.1|genepair1905-1
MLRVAGRRLTTALAWRPAAAAGARGPLAGGSLPGDDEFSREPQRPRFAVDSPFFAASRGF
SSETLVPRNQDVSLTELPATVSAVKNPSAKIVYDEYNHERYQPGDPSKRAFAYFVLSGGR
FIYASLLRLLVLKFVLSMSASKDVLALASLEVDLSSIEPGTTVTVKWRGKPVFIRRRTEE
DINLANSVDIGSLRDPQQDAERVKNPEWLVVIGVCTHLGCIPLPNAGDFGGWFCPCHGSH
YDISGRIRKGPAPYNLEVPTYSFLEENKLLIG*
>11670.m03124|LOC_Os04g32660.1|genepair1905-2
MLRVAGRRLSSSLSWRPAATAAAAAGGPRGGPLAGKNDDDDNNGNGRVQPRFSIESPFFA
AARGFSSSETLVPRNQDTGLAELPATVAALKNPNPKVVYDEYNHERHAPGDPSKRAFAYF
VLSGGRFIYASLLRLLVLKFVLSMSASKDVLALASLEVDLSSIEPGTTVTVKWRGKPVFI
RRRTEDDIALANSVDVGSLRHPQQDAERVKNPEWLVVIGVCTHLGCIPLPNAGDFGGWFC
PCHGSHYDISGRIRKGPAPFNLEVPTYSFLEENKLLIG*
>11668.m03021|LOC_Os02g32140.1|genepair1906-1
MSSRQPIGSGGGGAPPDWHDVEPQWQPVDDVVTVAAAAAYLAAPAGATLSSESADRRWRP
VGPSAAAAAAAALQAVEAQGAAAAERHYRGVRRRPWGKWAAEIRDPNKAARVWLGTFDTAE
AAAAAYDDAALRFKGAKAKLNFPERVRGRTGQGGFLVSPAVPRPPPHGVPAPAPAVAPAP
FPDLIQYARLLRSGEDAAAAAAVAGIAATAAPAAQILDFAAQRLVGVSPAMAPRPPSTLP
TTTTAASSPSAWPHGGGHGS*
>11670.m03120|LOC_Os04g32620.1|genepair1906-2
MVTALAHVIRAAPDLHLPHHPSSSASAAAHPQQASSFYPTAAAAASSPSDQLAAAAAAEE
QGRRRHYRGVRQRPWGKWAAEIRDPKKAARVWLGTFDTAEDAAIAYDEAALRFKGTKAKL
NFPERVQGRTDLGFLVTRGIPPAATHGGGYYPSSSPAAGACPPPRQQQTVVPYPDLMRYA
QLLQGGVGGSYMPFGGAATMSSSTVSSSSAPQILDFSTQQLIRAGPPSPMPSSGSGSATA
AASSTTSASSPGAWPYGGSERKKKDSSS*
>11668.m04328|LOC_Os02g44860.1|genepair1907-1
MAGQMLPPIALVAVAICITAAAAAKVPAIYVFGDSTADVGNNNYLTGAAVPRANFPHNGI
DFPTSRPTGRFSNGYNGVDFLGHAIAFLSVGTVWDGDWEIMPKYDNDHNILQPLSALNMG
FRRSPPPFLAVANKTSNPLFRGLQGTNFASAGSGILDSTGQSIIPMSKQVQQFAAVQRNI
SARISQQAADTVLSRSLFLISTGGNDIFAFFSANSTPSSAEMQRFVTNLVSLYTNHVKDL
YVLGARKFAVIDVPPIGCCPYPRSLQPLGACIDVLNELARGLNKGVKDAMHGLSVSFSGF
KYSIGSSHAVVQSIMKHPQRLGFKEVTTACCGSGKFNGESGCTPNATLCDNRHDYLFWDL
LHPTHATSKIAAAAIYNGSVRFAAPINFRQLVDDQH*
>11680.m05080|LOC_Os06g50940.1|genepair1907-2
MVGMKKKSVGLGRLSLMISMVQVLGAVGGGGVHPSKMRLVPAVYVLGDSTLDVGNNNHLP
GKDVPRANKPYYGIDFPGSKPTGRFSNGFNAADYVAKNLGFDKSPPAYLVLKARNYLVPA
ALVMGVNYASAGAGILDSTNTGRSIPLSKQVVYLNSTRAEMVAKAGSGAVSDLLAKSFFL
FGVGSNDMFAFAAAQQKLNRSATPSEVEAFYTSLISNYSAAITELYGMGARKFGIINVGP
VGCVPSVRVANATGGCNDGMNQLAAGFDAALRGHMSGLAARLPGLAYSIADSYALTQLTF
ADPGAAGYANADSACCGGGRLGAEGPCQRGAALCGDRDRFVFWDSVHPSQQANKLGAKAY
FHGPPQFTSPINFNQLANYNS*
>11668.m04330|LOC_Os02g44880.1|genepair1908-1
MVVGFRRTISFPAPKAATAAKGEAYRVRSASLPCRFHPLVVQLDEDVATMRELVGRLASA
ASAGSVAGAAEQLGRVLVSLSELLHHPQAQEPLRRLGRSPFAERLLDNFLRLADAHGSFR
AALVALSALQAEARAALRREDPARLASAARALRRSGRDLPRIASSARAVAAKPPPPPPAG
LPADGTALAAAIADATAAVASASAAVFSGVSSLSIAAATARVEVAATPCWMPSPARFTTP
SATPRHHIITTKPSSLRIWWVADLMRWMSRAKRRSASKQHADSDASSSSTSSAATARPQP
NVAVDPDERERKAAFERLDNLGRCIADVESIGEKVFRALGMAFYEIKPRVQKGALRVHSS
CGRSRVSTLCVCEPEVPGSPDVAMYGIEFDRLQSRSASTRFRAERGPRPCGFEISRPSGG
PSPGVPVALILGGRKLPSCHAVVALEYDHASVQKERWLAGCPTGCVPPVPLAADYGCFHS
VSQLVYYTSCLYILLVRARQGHIPYLYGLWRNAQLFRAGSMGSKQCYLKVR*
>11680.m05084|LOC_Os06g50980.1|genepair1908-2
MAPSFARSISFPLSPSRSSSKHSSPPATPGYHARSISLPCRSHPILAHLHTHIRAVRSWA
HDPTSVASGLAHLDALHAALGELLDLPEAQAALSAANDRLLDAFLRLADAHGSFQETVVA
LKQDVAEALAAIRRRDGARLASAVRSQRKAGKELARLAAAARDGARPSRLGLGGSAAEVE
VTGLLMESAAVTAAASATLFNTVASMSASASAAACSCRKTAALVCLIKKTSASSEEEKET
MALVERLEELEECIDELDNGSDKVFRSLVQTRVALLNIHTHIF*
>11668.m04333|LOC_Os02g44910.1|genepair1909-1
MRVNPALFLPLMAEYAAPTWAILISGFFMLLSVSLSMYLIFQHLSAYNNPEEQKFVLGVI
LMVPCYAVESYVSLVNPDTSVYCGILRDAYEAFAMYCFGRYITACLGGEERTIAFLKREG
GGDSGEPLLHGASEKGIIHHHFPVNYILKPWRMGVRFYQIIKFGIFQYVIIKTLTASLSL
ILQPFGAYCDGEFNLRCGYPYFAAVLNFSQYWALYCLVEWYTATKDELAHIKPLAKFLSF
KSIVFLTWWQGIMIAIMYSLGLMGIASVVHLYVFPAKPYSLLGNHRSPENISVLGDYAAT
DPVDPDEIKDISRPTKLRLPQLEPDEIIVTNVKESVRDFVIGSGEYVIKDLKFTMKQAVR
PVGKRFEKLMKKKGKFGQSRDDNWVSTSTPQRAIHGIDDPLICGSSSDSGIGRGKRHRRD
VRSEYCKCRIPQIVPCFKGPSVIEFLYRSARYCTRTQVHNSLCNFVKVADTNWMYAKLLG
STP*
>11680.m05096|LOC_Os06g51100.1|genepair1909-2
MELAEQLYSVFRSYAPPIWASITAGIFVITSLSLSLFLLFNHLSAYKNPEEQKFLVGVIL
MVPCYAVESYISLVNPSISVDIEILRDGYEAFAMYCFGRYLVACLGGEDRTIEFLKREGS

SGSDVPLLDHETGQRYVNHPFPMNYMLKPWPLGEWFYLVIKFGLVQYVIIKTICAILAVI
LESFGVYCEGEFKWNCGYSYTAVVLNFSQSWALYCLVQFYAAIKDELAHIKPLAKFLTFK
SIVFLTWWQGVVIALLYNWGLLRGPIAQELQFKSSIQDFIICIEMGVASIAHLYVFPAKP
YEMMGDRFIGGVSVLGDYASVDCPLDPDEVKDSERPTKTRLPQPGDRVRCSTGIKESVRD
VVLGGGEYIVNDLKFTVNHAVEPINEKLHRISQNIKKHEKEKKKTNDDSCINSQQSLSRV
ISGIDDPLLNGSLSDNSGQKKSRKHRRKSGYGSAESGGESSDQGLGGYEIRGHRWITRE*
>11668.m04334|LOC_Os02g44920.1|genepair1910-1
MGNCFGSDDGEVEAVKVMARHALPQAAMARPVMVAIAQPNARVAMSPGRPPTGKLPSQAT
ATSTGGGRSVAGGGGRTNAGGDASAEGRILEAPNLRIFTFAELRAATRNFKADTVLGEGG
FGRVHKGWVDERTMSPARSGSGMAVAVKKLDPESLQGVQEWQSEVNFLGRLSHPNLVRLL
GYCWEDKELLLVYEYMAQGSLENHLFRSEPRKGGSASPQQPLSWSLRLRIAIGAARGLAF
LHSSEKHVIYRDFKASNILLDTQFHAKLSDFGLAKDGPAGGSSHVTTRVMGTYGYAAPEY
VATGHLYVKSDVYGFGVVLLELLTGLRALDAGRPSGQHHLVDWAKPFLSDRRKLARLMDP
RLEGQYSSRGAQRAAQLTLRCLAADHKNRPSMREVVAVLEEIESMSRGGSGGGAPGSASP
RPAARGGGGGGAHGYGQSPRPGSDWAGPAAGHPSPRVR*
>11680.m05104|LOC_Os06g51170.1|genepair1910-2
MGNCASAIDSFFFTKRANNENDDDDAAPGMSASKRTTSSTTTGKLSTLSNSTFIPSTISG
VSTDDAYPDGQILESRNLRIFTFAELKNATKNFRTDTVLGEGGFGKVYKGWVDERTMNPS
KSSTGVVVAVKKLNPESVQGTEQWESEVNFLGRISHPNLVKLLGYCKDNDELLLVYEFMA
KGSLENHLFRRGAVYEPLPWSLRLKILIGAARGLAFLHSSERQIIYRDFKASNILLDSNF
NAKLSDFGLAKHGPDGGLSHVTTRVMGTYGYAAPEYVATGHLYVKSDVYGFGVVLLEMLS
GLRALDPSRPSGKLNLVDWAKPLLADRRKLSQLMDSRLEGQYHSRGALQAAQLTLKCLSG
DPKSRPSMKEVVEALEKIKLIKSKSREPRNSSSLVRGQGNSPRSDSARTSSKGR*
>11668.m04335|LOC_Os02g44930.1|genepair1911-1
MKGKADASKKGEGRLKAAGGAGKRKKAAASGKPKRPPSAFFVFMSEFRQEYQAAHPDNKS
VAAVSKAAGEKWRAMSEQEKAPYVDKAGQKKQDYEKTKANFDKKESTSSKKAKTHDDGEG
SDKSKSEVDDDQDGGSDEENEDDEE*
>11680.m05112|LOC_Os06g51220.4|genepair1911-2
MKGAKSKGAAKPDAKLAVKSKGAEKPAAKGRKGKAGKDPNKPKRAPSAFFVFMEEFRKEF
KEKNPKNKSVAAVGKAAGDRWKSLTEADKAPYVAKANKLKAEYNKAIAAYNKGESTAKKA
PAKEEEEDDEEESDKSKSEVNDEDDDEGSEEDEDDDESLSIIIIVY*
>11668.m04350|LOC_Os02g45070.1|genepair1912-1
MRPGKGTFGDRCIVKANHFFAELPDKDLHQYDVSITPEVPSRGVNRAVIGEIVTQYRQSH
LGGRLPVYDGRKSLYTAGPLPFTSRTFDVILQDEEESLAVGQGAQRRERPFKVVIKFAAR
ADLHHLAMFLAGRQADAPQEALQVLDIVLRELPTARYSPVARSFYSPNLGRRQQLGEGLE
SWRGFYQSIRPTQMGLSLNIDMSSTAFIEPLPVIDFVAQLLNRDISVRPLSDADRVKIKK
ALRGVKVEVTHRGNMRRKYRISGLTSQATRELSFPIDNHGTVKTVVQYFQETYGFNIKHT
TLPCLQVGNQQRPNYLPMEVCKIVEGQRYSKRLNEKQITALLKVTCQRPQERELDILQTV
HHNAYHQDPYAQEFGIRIDERLASVEARVLPPPWLKYHDSGREKDVLPRIGQWNMMNKKM
VNGGRVNNWTCINFSRHVQDNAARSFCRELAIMCQISGMDFSIDPVVPLVTARPEHVERA
LKARYQEAMNILKPQGGELDLLIAILPDNNGSLYGDLKRICETDLGLVSQCCLTKHVFKM
SKQYLANVALKINVKVGGRNTVLVDALTRRIPLVSDRPTIIFGADVTHPHPGEDSSPSIA
AVVASQDWPEVTKYAGLVSAQAHRQELIQDLFKVWKDPQRGTVSGGMIRELLISFKRATG
QKPQRIIFYRDGVSEGQFYQVLFYELDAIRKACASLEADYQPPVTFVVVQKRHHTRLFAN
NHKDQRTVDRSGNILPGTVVDSKICHPTEFDFYLCSHAGIQGTSRPAHYHVLWDENKFTA
DGLQTLTNNLCYTYARCTRSVSIVPPAYYAHLAAFRARFYMEPDTSDSGSMASGAHTRGG
GPLPGARSTKPAGNVAVRPLPDLKENVKRVMFYC*
>11680.m05125|LOC_Os06g51310.1|genepair1912-2
MGSRRPRLPGFGEDCEPRGGGRGGGGRGRGSYYPQAQQYHPQGHGGRGGAGYYHGAAPQP
RGAMVVQQWRPATAAAEHLGHQQPYNSSVRPQHYYGPSAIAPELLQAMDAPHEPPANVSS
PEAASPEASSPRSLALEVTEQLQDLSVQYQLSESQEEIVQHVPVSTKSFKFPHRPGSGSI
GTRCLVKANHFFAQLPDKDLHQYDVSITPELTSRIRSRAVMEELVRLHKMSYLGGRLPAY
DGRKSLYTAGPLPFTSKEFRISLLEEDDGSGSERRQKTYNVVIKFAARADLHRLEQFLAG
RQAEAPQEALQVLDIVLRELPTARYAPFGRSFFSPDLGRRRSLGEGLETWRGFYQSIRPT
QMGLSLNIDMSATAFFEPLPVIDFVIQLLNTDIRSRPLSDAERVKIKKALRGVKVGVTHR
GNMRRKYRISGLTSQATRELTFPVDQGGTVKSVVQYFQETYGFAIQHTYLPCLQVGNQQR
PNYLPMEVCKIVEGQRYSKRLNQNQIRALLEETCQRPHDRERDIIQMVNHNSYHEDPYAK
EFGIKISERLALVEARILPAPRLKYNETGREKDCLPRVGQWNMMNKKMVNGGRVRSWICV
NFARNVQESVASGFCRELARMCQASGMDFALEPVLPSMYARPDQVERALKARFHDAMNIL
GPQHKELDLLIGLLPDNNGSLYGDLKRICEIDLGLVSQCCCTKQVFKMNKQILANLALKI
NVKVGGRNTVLVDAVSRRIPLVTDRPTIIFGADVTHPHPGEDSSPSIAAVVASQDWPEVT
KYAGLVSAQSHRQELIDDLYNITHDPHRGPICGGMVRELLISFKRSTGQKPQRIIFYRDG
VSEGQFYQVLLHELDAIRKACASLEANYQPQVTFIVVQKRHHTRLFAHNHNDQNSVDRSG
NILPGTVVDSKICHPTEFDFFLCSHAGIKGTSRPAHYHVLWDENNFTADALQTLTNNLCY
TYARCTRSVSIVPPAYYAHLAAFRARFYMESDSSDSGSMASGRGGGSSTSRSTRAAGGGA
VRPLPALKDSVKNVMFYC*
>11668.m04941|LOC_Os02g50710.1|genepair1913-1
MERSSSFGTSWADQWDYGGDPSPRAAARRDGHGGGKKQGGVEKTKAAAATGLRKVKEGTA
HGFQWIKDKCQKKNAGGGKKQQADEESGIAGY*
>11680.m01296|LOC_Os06g13190.1|genepair1913-2
MSSSSYGTSWADQWDYGSDPPPPSSSSGKRSGGGGGGGGKKGMEKTKAAAASGLRKVKEG
TAHGFQWIKDKYQQKKSGGKKHGDQQSSEIAGY*
>11668.m04943|LOC_Os02g50730.1|genepair1914-1
MGASTSPRLALLVVVAAAAFVFVSPAMAFPMGLPATANPFPNPWSAFQNLSGCHAGEERE

GLGRLKDYLSHFGYLPPPPSSSPYSDAFDDSLEAAIAAYQRNFGLNATGELDTDTVDQMV
APRCGVADVINGTSTMDRNSSAAALRGRHLYSYFPGGPMWPPFRRNLRYAITATSATSID
RATLSAVFARAFSRWAAATRLQFTEVSSASNADITIGFYSGDHGDGEAFDGPLGTLAHAF
SPTDGRFHLDAAEAWVASGDVSTSSSFGTAVDLESVAVHEIGHLLGLGHSSVPDSIMYPT
IRTGTRKVDLESDDVLGIQSLYGTNPNFKGVTPTSPSTSSREMDGSAAAAGIRPWSGFVG
LVVPAVVLLLAP*
>11680.m01295|LOC_Os06g13180.1|genepair1914-2
MATTTSPLVVVLLAVVAAIAVSLVQPAFALPAGLPDIKSLTNPWSAFKNLSGCHFGDERQ
GLGKLKDYLWHFGYLSYPSSSSLSPSFNDLFDADMELAIKMYQGNFGLDVTGDLDAATVS
QMMAPRCGVADVVNGTSTMGGGGGVRGRGLYSYFPGSPRWPRSRTTLRYAITATSQTSID
RATLSKVFASAFARWSAATTLNFTEAASAADADITIGFYGGDHGDGEAFDGPLGTLAHAF
SPTNGRLHLDASEAWVAGGDVTRASSNAAVDLESVAVHEIGHILGLGHSSAADSIMFPTL
TSRTKKVNLATDDVAGIQGLYGNNPNFKGVTPPATSSREMDSAGAGELSRPWRRLLDGAA
GLLVGLSLAWL*
>11668.m04944|LOC_Os02g50740.1|genepair1915-1
MLSGAMSLASQPSLRSLPSLDVHDLNTSPSLHQFIATIKGHSSASAYVSALAVDGDSLYI
ASSDGSIRLWALDGARRSQEEQQQDDGCSSSSSSTTVADTDSSVKSLLATGNGGLLLSSH
QDGKIRAWRAGSRRRDGETRPQLVLRAVLPTAVDRLRTCLLPWSYVEIRRHRRCTWVHHV
DAVTALAVSPDGALLYSASWDRSIKVWSLPGFRCVESIAAAHDDAINALAVSPDGRVYTG
SADKKIKAWTRGPGQRKHALVGTMERHRSAVNALALGANGKVLYSGACDRSVVVWESAGG
GDGGMEATGTLRGHARAILCLAAAGELVCSGSADRTVRVWRRGGAENNGYTCLAVMESHG
AAVKSLALVRGGRDDDGSCSSEGSSALVCSGALDGDVKIWSVFIPCL*
>11680.m01291|LOC_Os06g13140.1|genepair1915-2
MSPNSEEGIISQTISSSSSSSCHYQCIATLSGNSSYVSGLAVDGDSLYVASSDGHIRLWP
LDMAMAMVREESTSSSSQGEVSRSTVAVTGSPVKCLAATGDGLVSSHQDGTIRVWRHAGG
RRRLALRAVLPTAADCLRALLLPGGGYVEVRRHKRRAWVHHVDAVTALALSPDGESMYSV
SWDRSLKAWRLPGLRCAESVAAAHDDAINAVVAAPDGHVYTASADGTVKAWRRRTGQKKL
SLVCVMERHGAAVNALALGGGGRVLYSGACDRSVVAWENSAGAGAGGADVRMVATATLRG
HARAVLCLAADGDVVCSGSADRTVRVWRRGATAAYTCLAVLDGHGGAVKSLALARGGAGC
DRCCACHVEESSSCSCAALVCSGSLDCDVKLWRVTVSEAIKGSKVHHVGRLLGLIFHLFV
WLSNFVEFKGAGYIYIKGIRSTGLWYQSKQSRYQEVDTQALGTYGYQAFALMRATRRREM
RQGKHRDEMATLLTN*
>11668.m04946|LOC_Os02g50760.1|genepair1916-1
MAGSKFGSFKSEKGSSAGAAAGAGAAAQRRDPYEVLGVGRNATEQEIKSAFRRMALKYHP
DKNADDPVASDKFQEATFSYNILSDPDKRRQYDSSGFEAIEADSQELELDLSSLNTVNTV
FAALFSKLGVPIKTTVSATVLEEALNGSVGISQLELGQSVFRKVEKQSAHFYSVDITDKE
AKMGLVCRVQSTAKSKFKLLYFEPEENGGLSLALQEDSVKTGKVTSAGMFFLGFPVYRFE
QNNSEKLRCVEAKILAKRSELSKFESEYREVLAKFTEMTSRYAQEMQTIDELLKERNAIH
ASYTNNTTLQRSSSSNKGKTSSKESKSDDDQTVKKEKKSKSKSMEGSRSDDDGPRKEKKP
KERLRRKKWFNIHLKVDKRRPC*
>11680.m01282|LOC_Os06g13060.1|genepair1916-2
MAGSRFGSFKSEKGDPAATAAQRRDPYEVLGVGRNATDQEIKSAFRRMALKYHPDKNGDD
PVASDMFQEVTFSYNILSDPDKRRQYDTSGFEAIEADSQELELDLSSLNTVNTVFAALFS
KLGVPIKTTVSATVLEEALNGSVMVSQLQLGNSVHRKVEKQSAHFYSVDITEKEAKMGLV
CRVKSTDRSKFKLLYFELEENGGLSLALQEDSVKTGKVTSAGMYFLGFPVYRFEQNNLCQ
LLKTVRIPLNSTLHSGDNFFRSVNYTIEVVCGESFPAEKEKLQSVEAKILTKRAELSKFE
TEYREIDNLLKERNEIHASYTNNSPLKRSSSRSKAKSPSKFSKGEEENSQRKEKKVKDQP
TGGCRSADEDSNEKKTKERFPKKKWLNIPFKIDRRKPC*
>11668.m04947|LOC_Os02g50770.1|genepair1917-1
MRPSFLLLLVVFAGVLAGGARAGPPLAGENLPGQLLPGEKPPGQPLPGQPLPGQPLPGQS
LPGQPLPGQPLPGQSLPGQPLVGEKPPGQPLPGQPLPGQSLPGQPLPGQPLVGEKPPGQP
LVPGQPLVGEKPPGQPLGGDKLSPDYYAQTCPRAERIVAEVVQSKQMANPTTAAGVLRLF
FHDCFVSGCDASVLVAATAFEKSEQSAEINHSLPGDAFDAVVRAKLALELECPEVVSCAD
ILALAARVLITMTGGPRYPISFGRKDSLTSSPTAPDKEMPQSNFTMDQVIKLFQDKGFTV
QEMVALSGGHTLGFSHCKEFAQRIYDYQGKPGNVDPTMNPVLSKGLQTACKEYLKDPTIA
AFNDVMTPGKFDNMYFVNLERGLGLLATDEEMWSDKRTQPFVKLYASNPTAFFDDFSRAI
DKLSLFGVKTGAAGEIRRRCDTYNHGPMPK*
>11680.m01281|LOC_Os06g13050.1|genepair1917-2
MDTHLVVVLALLAAAAAAEAKMSADYYSKTCPRADRIIADVLAQKQISNPTTAAGVLRLF
FHDCFVGGCDASVLVASTAAARSERDADVNLSLPGDAFDALARAKAALEVECPGVVSCAD
LLAVAARDLVTMTGGPYYPLRLGRKDGLSSSPSAPDAEIPHANLTVSRLVAVFAAKGFTV
QDLVALSGAHTLGFSHCKEFAARIYGGGGGGADPTMNPALAKRLQEACRDYRRGPTIAAF
NDVMTPGRFDNMYFVNLRRGLGLLATDQELYGDARTRPHVERYAANETAFFADFARAARR
LSHHGVKNGANGEIGGGVNLPDLWVMWLGLAARGTTRWSGDDVVQRTVIWRVVQNLLPGK
TKDQSSGESTPSGIMWSFAAGSNLSTSASFNAEKESRKNLNKFYKEIRTLKNVNMAGRQF
GDEGLFFLAESLAYNKSAEEVDFSGNGITAVGIEAFDGILQINTALKSLNLSGNAIGDEG
AKCLSDILVENVGIQKLLLNSTNIGDEGAKAISDMLKKNKTIRTLQLSNNTIEYSGFASI
AEALLENNVLRSLFVNGNYGGPLGASSLAKGILGNKTLRELHLHGNGFGNEGVRALMSAL
SAHKGKITVLDIGNNNITSEGSLHVAEFIKRTKSLLWLSLYMNDISDEGAEKVADALKQN
KTISTVDLGGNNIHSKGVSAIAETLKDNSVVTTLELSYNPIGPEGVKALCDVLKFNGKIQ
TLKLGWCQIGVSGAEFVADCLKYNTTLSTLDLRANGLGDDGAICLARSFKIINESLTSLD
LGFNEIRDDGAFALAQALKANEDLAVTSLNLANNFFTKFGQVALSEARDHVYEMSEKEID
IFF*
>11668.m04952|LOC_Os02g50810.1|genepair1918-1

MGNCQAAEAAAVVIQHPGGRVERLCWSTSAAEVMRANPGHYIALVTLRVAEERQDGDGGA
RRTVRLTRVKLLKPKETLLLGHAYRLITTHEVTKAVQARKEEKVRKAQQQLEESRQKLQS
KARAAASAAAEVDEAAEENDNDSDNFDDEAALDASLDQVGLHGSEW*
>11680.m01272|LOC_Os06g12960.1|genepair1918-2
MGNCQAAEAATVVVQHPGGRVERLYWATTAAEVMRANPGHYVALVTLRVAEEKRPPPPPP
PPPARAERRGTGTGTVRVTRVKLLKPRDTLLLGQAYRLITVDEVTRALQAKKEEKSRRAA
AQHHHLESKPAAAAAVAAGVRINSGGDDHTQLDENLDQHDRDGQRSSSATHSRHRQWRPS
LHSIAEVRS*
>11668.m04958|LOC_Os02g50860.1|genepair1919-1
MASSASRFIKCVTVGDGAVGKTCMLICYTSNKFPTDYIPTVFDNFSANVVVDSTTVNLGL
WDTAGQEDYNRLRPLSYRGADVFVLAFSLVSRASYENIMKKWIPELQHYAPGVPIVLVGT
KLDLREDKHYLLDHPGMIPVTTAQGEELRKQIGAAYYIECSSKTQQNVKGVFDAAIKVVI
QPPTKQREKKKKKSRQGCSMMNMFRGRKMSCFKS*
>11680.m01255|LOC_Os06g12790.1|genepair1919-2
MASSASRFIKCVTVGDGAVGKTCMLICYTSNKFPTDYVPTVFDNFSANVVVDGTTVNLGL
WDTAGQEDYNRLRPLSYRGADVFVLAFSLVSRASYENVMKKWLPELQHYAPGVPIVLVGT
KLDLREDKHYLLDHPSLVPVTTAQGEELRKHIGATCYIECSSKTQQNVKAVFDAAIKVVI
KPPTKQRDRKKKKTRRGCSFFCKGVMSRRRLVCFK*
>11668.m04960|LOC_Os02g50880.1|genepair1920-1
MDDPSSTSKGKRKRGRKHKAAAENHAPASPVASTAADNPAPAAAGRRGRKSRRHEAPADA
DGSRPPSPPRRGEAKPVANGGGDAVVEAGGPVGWDEVARVVPSMDAVVKVFCVHTEPNFS
LPWQRKRQYSSSSSGFIIGGRRVLTNAHSVEHYTQVKLKKRGSDTKYLATVLAIGTECDI
ALLTVDDDEFWEGVLPVEFGSLPALQDAVTVVGYPIGGDTISVTSGVVSRIEILSYVHGS
TELLGLQIDAAINSGNSGGPAFNDRGKCVGIAFQSLKHEDAENIGYVIPTPVIMHFIQDY
EKSGEYTGFPILGIEWQKMENPDLRKAMGMKPDQKGVRVRRVEPTAPESGCLQPSDIILS
FDGIDIANDGTVPFRHGERIGFSYLVSQKYTGEKALVKVLRNSKVHEFKIKLATHKRLVA
AHVKGRPPSYYIVAGFVFAAVSVPYLRSEYGKDYEYDAPVKLLVKHLHAMAQSPDEQLVV
VSQVLVADINIGYEEIVNTQVLAFNGQPVKNLKNLVSMVENCKDEFLKFDLEYDQIVVLE
TKTAKAATQDILTTHCIPSAMSDDLKT*
>11680.m01254|LOC_Os06g12780.1|genepair1920-2
MDNHELASTSSPKRKPGRRPGRKPKPPPAPSPAAAPAPAAENGTHDPASGQKRKRGRKPK
PPAAAAAAASSDGHHHPSSPLAAAVSASDSPDPASSPAPRGRGRKSRRGRPEPPSDAGAA
PHAPPSPPRRGAKKGAAANAKKAAAEVPVVEPLRWEQVAKVMPSMDAVVKVFCVHTEPNF
SLPWQRKRQYSSSSSGFIIGGHRVLTNAHSVEHYTQVKLKKRGSDTKYLATVLAIGTECD
IAMLTVEDDEFWKGVSPLEFGSLPALQDAVTVVGYPIGGDTISVTSGVVSRIEILSYVHG
STELLGLQIDAAINSGNSGGPAFNDKGKCVGIAFQSLKHEDVENIGYVIPTPVINHFIQD
YEKSGEYTGFPILGIEWQKMENPDLRKAMGMKSDQKGVRVRRVEPTAPESGCLQPSDIIL
SFDGIDIANDGTVPFRHGERIGFSYLISQKYTGEKAHVKILRNSKVLEFNIKLATHKRLI
PAHIKGRPPSYYIVAGFVFMVVSVPYLRSEYGKDYEYDAPVKLLDKHLHAMAQSPDEQLV
VVSQVLVADINIGYEEIVNIQVLSFNGKPVKNLKHLATMVEDCNEEYLKFDMDYDQVVCA
IHIPFVPVALIQIVLVLTGAISV*
>11668.m04962|LOC_Os02g50900.1|genepair1921-1
MEEQLNPLAVTQLLQHTLRGLCTQGDSQWVYAVFWRILPRNYPPPKWDLQGGVYDRSRGN
RRNWILAWEDGFCNFAASACDQEDTPAAAGYTDYAAAGHEVKGLQPELFFKMSHDIYNYG
EGLVGKVAADHGHKWVSQEANEHEINLVTSWNNPADSHPRTWEAQFQSGIKTIALIAVRE
GVVQLGSMKKVAEDLSYVVALRRKFGYLESIPGVLLPHPSSAAFPGAGGLQDAAWAPSPT
MDLYDPYYGAHAAAAQMHHIVPSMSSLEALLSKLPSVGPTAAPGAIRGAIGGGSVAKEEL
DDAMDAAGNGGGESTSAATTPLVPYYVDVAKPDEGF*
>11680.m01250|LOC_Os06g12740.1|genepair1921-2
MDEQLSPVAVTHLLQHTLRSLCTSGDDSQWVYAVFWRILPRNYPPPKILAWEDGFCNFAA
TSAACGDGAAAAYAAAECEETKQVGVAGGGLQPELFFKMSHDIYNYGEGLIGKVAADHSH
KWVFKEPQEQEINLISSWNNPADSHPRTWEAQFQSGIQTIALIAVREGVVQLGSMKKVAE
DLSYVVALRRKFGYLESIPGVLLPHPSSAAAAFPGGPPDAAGWPAGMMVSPPVPPELYVD
PYGGAAAGAVPPPSMQIMPSMSSLEALLSKLPSVVPAAAAPSPPPGSSSMPPTGAAAASS
APPKEEAAEDDYVHCHGMDMATSSTNGGGESTGGAPLPSSYFVNVGVKPSEGF*
>11668.m04965|LOC_Os02g50930.1|genepair1922-1
MVAAVAASLRSLAPLSAYRSPSHGIHAVVRDSSAYTTRPPPPPPTADGGGNGGRISPAVL
FIIVILAVIFFISGLLHLLVRLLMKKQHRRGGAENAAPSPHSRHVGRDAAMDRQLQQLFH
LHDSGLDQAFIDALPVFAYRDIVGGDKEPFDCAVCLCEFDGEDRLRLLPVCGHAFHLHCI
DTWLLSNSTCPLCRGTLYVPGLTIESLMFDFDERLEEGRLSEECEDGFQSSRQKKPMDEE
QTVTEKRVFPVRLGKFKNVGNTGVGGVDNGNAAGIVSREPGESSSSSLDTRRCFSMGTYQ
YVLGASELRVALQPGRNKNGVGSRLKGRATGISSVNAEIMEGKRICAKSKGESFSMSKIW
QWSNVKGKLPAGSDNCSETASFPWMKRDATGDKSNM*
>11680.m01242|LOC_Os06g12680.1|genepair1922-2
MEALSRTERVGGGGGAMVEVAFASSAGSSAAAPRRLRGELVVRDAIPYAGVAPPPPALPL
PQPQLQPQVQATTSGGGGGGKISPAVLFIIVILAVVFFISGLLHLLVRLLMKKQHRRGGG
GGAAAGVSRSAAGDDAGGGGDAALQRQLQQLFHLHDSGLDQAFIDALPVFAYREIVVGGG
GDGDKEPFDCAVCLCEFDAEDRLRLLPLCGHAFHLHCIDTWLLSNSTCPLCRGVLFVPGL
TENNPMFDFDEGLEEGRLSEDCDNGFGYPGHKATEGMQTPGTEKRVFPVRLGKFKNVGTQ
GAVEGGGIGNANGAVLRREEGESSSSSLDARRCFSMGTYQYVLGTSELRVSLQPDRIRNG
GGGVTRARPTGLSSVNAEIMEGKKICARNKGESFSVSKIWQWSNLKGKLPTGSDECSEAG
SLPWMKRGGIGDTSNM*
>11668.m04966|LOC_Os02g50940.1|genepair1923-1
MDTDLGGGGGASGMDEAEAAFFARRGRRCCCFPWPSSASSHQRVGGAEEESWWQRAVDAV

LKVREWSELVAGPRWKTFIRRFGRGGGGGGGGGGPRPHNYGRKLNYDALSYALNFDEGHG
ASPEGDYTGYRDFSARFAAPPASAKSSMDLGGRDAPPLFNPPPPHDGAGRA*
>11680.m01240|LOC_Os06g12660.1|genepair1923-2
MAVSAAAAGMDEADAAFFSRRGNRCCCFWGPWASSSYSRAGGPAAAAEEEWWHRVGGGGG
ERRRWWRRGVDALMKVREWSELVAGPRWKTFIRRFRRSPRHHHHGGGGGGGGGGRKLNYD
PLSYALNFDEGHGGACSPEGDYAGYRDFSTRFVAPPPPAAASAKSSMDFGGRDAPPLFHH
PPPQQPHPHPHPPSPSAARG*
>11668.m04968|LOC_Os02g50960.1|genepair1924-1
MITAADFYHVMTAMVPLYVAMILAYGSVKWWRIFTPDQCSGINRFVALFAVPLLSFHFIS
TNNPYTMNLRFIAADTLQKLMVLAMLTAWSHLSRRGSLEWTITLFSLSTLPNTLVMGIPL
LKGMYGEFSGSLMVQIVVLQCIIWYTLMLFMFEYRGARMLITEQFPDTAANIASIVVDPD
VVSLDGRRDAIETETEVKEDGRIHVTVRRSNASRSDIYSRRSMGFSSTTPRPSNLTNAEI
YSLQSSRNPTPRGSSFNHTDFYSMVGRSSNFGAADAFGVRTGATPRPSNYEDDASKPKYP
LPASNAAPMAGHYPAPNPAVSSAPKGAKKAATNGQAKGEDLHMFVWSSSASPVSDVFGGG
APDYNDAAAVKSPRKMDGAKDREDYVERDDFSFGNRGVMDRDAEAGDEKAAAAAGADPSK
AMAAPTAMPPTSVMTRLILIMVWRKLIRNPNTYSSLIGLIWSLVCFRWNFEMPAIVLKSI
SILSDAGLGMAMFSLGLFMALQPHIIACGNKVATYAMAVRFLAGPAVMAAASFAVGLRGT
LLHVAIVQAALPQGIVPFVFAKEYSVHPSILSTAVIFGMLIALPITLVYYILLGL*
>11680.m01235|LOC_Os06g12610.1|genepair1924-2
MITGADFYHVMTAMVPLYVAMILAYGSVKWWRIFTPDQCSGINRFVALFAVPLLSFHFIS
TNNPYTMNLRFIAADTLQKLIVLALLTLWSHLSRRGSLEWTITLFSLSTLPNTLVMGIPL
LKGMYGEFSGSLMVQIVVLQCIIWYTLMLFMFEYRGARILITEQFPDTAGAIASIVVDAD
VVSLDGRRDMIETEAEVKEDGKIHVTVRRSNASRSDVYSRRSMGFSSTTPRPSNLTNAEI
YSLQSSRNPTPRGSSFNHTDFYSMVGRSSNFAAGDAFGVRTGATPRPSNYEEDAAAPNKA
GSKYGQYPAPNPAMAAPPKPKKAANGQAKGEDGKDLHMFVWSSSASPVSDVFGNGAEYND
AAAVKEVRMAVASPRKADGVERDDFSFGNRGVAERDAEAGDEKSVAAAVSGEHGKPGLTP
APTAMPPTSVMTRLILIMVWRKLIRNPNTYSSLIGLIWSLVCFRWNFEMPAIILKSISIL
SDAGLGMAMFSLGLFMALQPRIIACGNKVATFAMAVRFLTGPAVMAAASIAVGLRGTLLH
VAIVQAALPQGIVPFVFAKEYSVHPDILSTAVIFGMLIALPITLVYYILLGL*
>11668.m04970|LOC_Os02g50970.1|genepair1925-1
MKNFFRKLHIGEGSGDGASSSPPPPPSSRKGSGGVGGNHHLHAEQRQPSASAVSSWLDSV
PGRPQPPTPSTPSEAEGSPFSSSVGSGAEERRQSVAAERRRSQEEEWERRRSQEEEAVRE
MRRSQEEDEVEERVIRESSEAEERKRVREKEDDDLEEFQLQLVLEMSARDNPEEMEIEVA
KQISLGFCPPQSSTAEALAARYWNFNALGYDDRISDGFYDLYVTGNGPASITMPSLKDLR
AQSLSHRVNWEAVLVHRGEDPELMKLDQTALIMSLELRESKPSEFVGNDLVQKLAGLVAR
HMGGTFFDSEGMLVKYQKMMRYLRTSIGSVVVPLGQLKIGLARHRALLFKVLADNIGIPC
RLLKGRQYTGSDDGALNIVKFDDGREFIVDLVADPGTLIPSDGAVLSTEFEESSFSNNHH
FNKDNDIRQLGSSNSLSNSACSSFECELLDRRSTWINVGPSDSDGATTSQTSKNNQQNTL
SDSFGILSVSTFTSENRPITNESRSTDDIAAAKNKERSSVTINSSSTSPSPSSPEVGSTP
AVRRMKVKDISEYMINAAKENPQLAQKIHEVLLENGVVAPPDLFSEDSMEEPKDLIVYDT
TLFQSKDEMKKRMNELGSREYADRGHGPLLPHHPGHELPSKVPHRAPLDSLKPVEGLGID
HPPDIQDNTSFISQYEPSAPPQEASSQLTKQLPVTAAAVATAAVVASSMVVAAAKSNNDV
NFDVPVAAAATVTAAAVVATTAAVSKQYEHLEPGNQLHSLPSPSEGNESIEKSADEFWDK
QNFEIDHGQDNTLDQEKDSAEVRQDAERTSDKSSGTESAKSEITLDDVAEFEIQWEEITI
GERIGLGSFGEVYRGEWHGTEVAVKKFLQQDISSDALEEFRTEVRIIKRLRHPNVVLFMG
AITRVPNLSIVTEFLPRGSLFRLIHRPNNQLDERKRLRMALDVARGMNYLHNCTPVIVHR
DLKSPNLLVDKNWVVKVCDFGLSKMKNKTFLSSRSTAGTAEWMAPEVLRNEPSDEKCDVF
SYGVILWELCTLLQPWEGMNAMQVVGAVGFQNRRLDIPDNTDPAIAEIIAKCWQTDPKLR
PSFADIMASLKPLLKNMTAQAPRQRVQQTDE*
>11680.m01233|LOC_Os06g12590.1|genepair1925-2
MKNFLRKLHIGDSAGDGASSLAPPPPVSKKGGGGGGGGGGGGAQHEHKHGSGISSWLSSV
TGRPQTQPSPSPPFAADAVVEAEAAALASSVEVRRLEVEEEEEKARRESREESVRKREME
KEKQEAELEEYHMQLALEMSAREDPEATQIEVAKQISLGSCPLQSSPAEVVAFRYWSFSA
LSYDDKILDGFYDIFVIGDEPTLPTIPSLTELHQQPFSHASKTEAVLVNRAQDTKLVQLE
QKALIMAVEVRSKTPEFVGHNLVQRLATLVSDYMGGPVIDPESFLSKYQNVSSSLRASIR
SAVMPLGELTIGLARHRALLFKVLADSLAVPCRLVKGRQYTGSDDGALSIVKFNDGREYI
VDLMSDPGTLIPSDGAGLGREFEDSLFADSHHVNKDDCNTQLGSSFSEVSSSMYGSFENE
SLEKVSTPSNFGHSDPYGITTGQTGSQGSAVSGSFGELSISTSTSENLPVIHESRNTDHT
MSTQSKDKSSAANNSSSSSPSSSEVGGAPAVRRMKVKDVSEYMISAAKENPQIAERIHAV
LLENGVVPPPDLFSEESREQPKDLIVYDTSLFQTKDEMIKRMNELESTTNADFCHGPSVP
HPPGHELQTKAVPYRIPLDLKPIQGLGTYHPSDSRNSTGSSHMYEPSAPPQEDPLQLIKQ
MPVAAAAVATAAVVASSMVVAAAKSNSDIKLDVPVAAAATAAAVVATTAAVNKQYEYLEP
GCQLLSLPSSSGANELIPKGRHDFWDNQLEIDHGQTSVPEKEKDLVEVPQEAERVSDKSV
GTESSRSDIALDGVAEFEIQWEEITLGERVGLGSFGEVYKGEWHGTEVAVKKFLQQDISS
DALDEFRTEFQIMKRLRHPNVVLFMGAVTRVPNLSIVTEFLPRGSLFRLIHRPNNQLDER
RRLRMALDVARGMNYLHNCSPVVVHRDLKSPNLLVDKNWVVKVCDFGLSRMKNSTFLSSR
STAGTAEWMAPEVLRNEPSDEKCDVFSYGVILWELFTLLQPWEGMNPMQVVGAVGFQQRR
LDIPAHVDPTIAEIIRRCWQTDPKMRPSFSEIMSSLKPLLKNTLANQPQRQRVQRADG*
>11668.m04972|LOC_Os02g50980.1|genepair1926-1
MASVFWGSGHPADEVADFDEYDPTPYGGGYDIALTFGRALPPSDEICHPISTASSSSSSY
DRPQQGRRPPAEETHLSAGHGRRPDDDEATHGGGYRKPKPAYGDDEQQRRHTSGGGRKKH
GGDDDDGSGDERKPRYKKHDDDDDGERKPRYKKRDDDDDDSDGERKQRYEKNNRRRHDYD
D*
>11680.m01232|LOC_Os06g12580.1|genepair1926-2

MATGYYRGGGGARDHADEPDDFDEYDPTPYGGGYDLFITFGRPLPPSDETCYPCSAPSTS
YDAPHYSADEPSPYAHHSKPQPAYGFRPQHEQQQQPSYASSGYRPQHEQQQSYGSSGYGS
KPQPAYGFRPQAEEENTYGSGYGSGYGGGGRKQQEEESYGSGYGRKPQVEESYGSGYGTK
PQQEESYGSGYGSGYGTKPQQEESYGSGYGRKPQQEESYGSEYGSGYGRKPQAESYGSGY
GSRPQQGGEEYGSGGYGRKAQEESYGSAGYGGRKTEEESYGGGSGYGYGKKAQEESEGTY
GSGGYPKPKPYSQEETQGSYGYGYGEKPAYESGGYNKPSYGGGDEYQGGYGRKKHDDNDS
DDEKKQRYQKHHHHRRQEYDD*
>11668.m04973|LOC_Os02g50990.1|genepair1927-1
MEVAAAEDALEESGVAVYLPRLLAGVISGALTGLFALAGALTGAVTGALAGRASDSGVLR
GAGLGAFAGAVLSIEVLEASRAYWCSDRLGSHGTSSMADFIEQLLQARFVQEQIVPSGYA
THRWQVSISDFGHDDLYDIFGDCSSKGLSRESLNKLPHYVVTDQTRNSFGEDLSCTICLQ
VHLSKLALCCRQTCLAALHNFAHGLGSTTIIKAKQHPFHDCRQYSECLVSIFELSPFWQS
LLRKSRSPVI*
>11680.m01230|LOC_Os06g12560.1|genepair1927-2
MEVAPAAAMTRAEEESRRRAATRLPRLLRGVVSGMLTGIFAVAGGLTGAVTGALAGRASD
GGVLRGAGLGTFAGAVLSIEILEASRAYWCQDRSSSPGSLSMGDFVKQLIHARFVQEQNE
ASGHITYRWQVGIADVVNGAVHEILGDVPSGEGLSKYSLMKLPYHVVIDHNNGSIGESLS
CPVCLQDVVAGQTVRRLPKCSHTFHQPCVDKWLVGHGSCPMY*
>11668.m04977|LOC_Os02g51000.1|genepair1928-1
MVVTIDLREVTIDLREEDEAAAAAAMKAIAGRSDEEVLAAVLARKGGPVPFLQAAIDAAA
AAATKAIAGRSDEEVLAAVLARKGGPVPFLQAAIDVAQRRSDLFLDPSAPGVVAEMAVEA
QAKAEAEERRKRAKGEPRKAEEMLKEEEPMKAEEMLKEEEPMKAEEMLKEEPRTPMREAG
RDKVERAAVVERVRDPKPNAGNGLDLEKYSWTQERPEVTITIPVPQGTKSSLVTYEIMKN
HLKVGLKGCSFIIDGELFEPVKVNDCLWTIEDGNTLSILLTKENQKEWWTSVIKGDPELD
PRDMKVPELRDCDVEAKETIVRILSHGLPKRAMDPATSDDIQQEELLKNAQYSHMDS*
>11680.m01226|LOC_Os06g12530.1|genepair1928-2
MAIISDFQEEEAPPRQQQQPASVAAAAGSGDEVLAAELERRGGAIPFLQAAIDVARRRSD
LFRDPSAVSRVTSMASAARAVVEAEERKAREAKRKAEEAERKAAEAERKAKAPAEPKPES
SAGKDSMEVDKKEEGNVRKPNAGNGLDLEKYSWIQQLPEVTITVPVPQGTKSRFVVCDIK
KNHLKVGLKGQPPIIDGELFKPVKVDDCFWSIEDGKSLSILLTKQNQMEWWKSVVKGDPE
VDTQKVEPENSKLADLDPETRQTVEKMMFDQRQKQMGLPTSDEMQKQDMLKKFMAQHPEM
DFSNAKIA*
>11668.m04979|LOC_Os02g51010.1|genepair1929-1
MEGKAAVTTSTEHGDGEASRTAARTVVSGSSRGGAASRALSVADLILRVVAVVAIVDSAI
AMGTTNQTLPFFTQFLRFKAQYSDLPTLTLFVVANSAVTAYLVLSIPLSVVHIIRSRASY
SRLVLIFLDSVMLALVAAVASASAAIVYLAHKGNVRANWFAVCQQFDSFCERISGPLIGS
FAAMAVLLLLVLLSAAALARR*
>11680.m01223|LOC_Os06g12500.1|genepair1929-2
MEGSEEHGETSKAPLSRGVSKGVSILDVILRFVAIIGTLASAIAMGTTNQTLPFFTQFIR
FKAQYSDLPTLTFFVVANSIVSAYLILSLPLSIVHVIRSRAKYSRLILIFFDAAMLALVT
AGASAAAAIVYLAHKGNARANWLAICQQFDSFCERISGSLIGSFAAMVVLVLLIFLSAIA
LARR*
>11668.m04985|LOC_Os02g51070.1|genepair1930-1
MGVCGMNTQNIKDFTFFQRHLLETPSFKSYIIKGFGFSTPRFLEIRFLRGLFGSARQWLY
SRSAAVVLVQIYLVDPVGVAAAQPLLALPNTAIIKDFVGFCHIATTSSSPKTGQGHEPTT
CFRPVLLAPSPLCLTRGPHTITHQIEAPQADHVEDSVSSPKYVKPAVAKQNGEVVSRATK
SDAPVSKPKVDPSVPASKAEADGNAQAVESKAALDKKEDVGVAEPLEAKADAGGDAGAVS
SADDSENKESGPLAGPNVMNVIVVASECSPFCKTGGLGDVVGALPKALARRGHRVMVVIP
RYGEYAEAKDLGVRKRYRVAGQDSEVSYFHAFIDGVDFVFLEAPPFRHRHNDIYGGERFD
VLKRMILFCKAAVEVPWFAPCGGSIYGDGNLVFIANDWHTALLPVYLKAYYRDNGLMQYT
RSVLVIHNIAHQGRGPVDDFATMDLPEHYIDHFRLYDPVGGEHSNVFAAGLKMADRAVTV
SHGYLWEIKTMDGGWGLHEIINHNDWKLQGIVNGIDMAEWNPEVDEHLQSDGYANYTFET
LDTGKKQCKEALQRQLGLQVRDDVPLIGFIGRLDHQKGVDIIGDAMPWIAGQDVQVVMLG
TGRPDLEEMLRRFESEHNDKVRGWVGFSVQLAHRITAGADVLLMPSRFEPCGLNQLYAMA
YGTVPVVHAVGGLRDTVAPFDPFADTGLGWTFDRAEANRMIDALGHCLNTYRNYKESWRG
LQARGMAQDLSWDHAAELYEDVLVKAKYQW*
>11680.m01218|LOC_Os06g12450.1|genepair1930-2
MSSAVVASSTTFLVALASSASRGGPRRGRVVGVAAPPALLYDGRAGRLALRAPPPPRPRP
RRRDAGVVRRADDGENEAAVERAGEDDDEEEEFSSGAWQPPRSRRGGVGKVLKRRGTVPP
VGRYGSGGDAARVRGAAAPAPAPTQDAASSKNGALLSGRDDDTPASRNGSVVTGADKPAA
ATPPVTITKLPAPDSPVILPSVDKPQPEFVIPDATAPAPPPPGSNPRSSAPLPKPDNSEF
AEDKSAKVVESAPKPKATRSSPIPAVEEETWDFKKYFDLNEPDAAEDGDDDDDWADSDAS
DSEIDQDDDSGPLAGENVMNVIVVAAECSPWCKTGGLGDVAGALPKALARRGHRVMVVVP
RYGDYAEAQDVGIRKYYKAAGQDLEVKYFHAFIDGVDFVFIDAPLFRHRQDDIYGGNRQE
IMKRMILFCKAAVEVPWHVPCGGVPYGDGNLVFLANDWHTALLPVYLKAYYRDNGMMQYT
RSVLVIHNIAYQGRGPVDEFPYMELPEHYLDHFKLYDPVGGEHANIFGAGLKMADRVVTV
SPGYLWELKTTEGGWGLHDIIRENDWKMNGIVNGIDYREWNPEVDVHLQSDGYANYTVAS
LDSSKPRCKAALQRELGLEVRDDVPLIGFIGRLDGQKGVDIIGDAMPWIAGQDVQLVLLG
SGRRDLEVMLQRFEAQHNSKVRGWVGFSVKMAHRITAGADVLVMPSRFEPCGLNQLYAMA
YGTVPVVHAVGGLRDTMSAFDPFEDTGLGWTFDRAEPHKLIEALGHCLETYRKYKESWRG
LQVRGMSQDLSWDHAAELYEEVLVKAKYQW*
>11668.m04987|LOC_Os02g51090.1|genepair1931-1
MEKWRFYGGHQFQSYSFHEEENLFQDWSLDYLLLGEDEPFFTHHFSTSVHSNFVQDELYT
LFDGDILSIWGDMKEDAYHRSDKDGGEKEEKLDHEKAMELQLQRLPSGRQSGEKTLTFEL

VSQYFCLPIKQAAQELNVGLTLLKRRCRVLGIPRWPHRKVKSLETIIKNVQELGMETGQD
EDNTRNAVEMLQQTKKLIEQSPDAKLDDWTKMLRQACFKENYKRRRLLAIEG*
>11680.m01209|LOC_Os06g12360.1|genepair1931-2
MRHNRSLAALLRAGRYGAARRLFDALPARSVVTWNSLLAGLARRPDARAAREFFDAMPVR
DAVSWNTLLAAYSASPHPDHLAAARRLFDEMPQRDVVTWNTLLGAYARRGLMDEARRLFD
EMPQRNAASWNTMVTGFFAAGQVVKALDVFDAMPAKDSASLSTMVSGFTKNGMLHEAEEL
LTKRLSVTDMDKAVDAYNTLIVAYGQAGRFSDAKRLFDMIPKGQYQHNMLKRKGFERNVV
SWNSMMICYIKAGDVCSARALFNEMPDKDLVSWNTMISGYTQASDMKESEKLFWEMPDPD
TVSWNLIIQGFMQKGEAEHARGFFDRMPERGTISWNTMISGYEKNGNYISSVKLFSKMLE
VGEIPDRHTFSSVLAACASIPMLGLGAQIHQLVEKSFVPDTAISNALITMYSRCGALNDA
EAIFKQMHTKKDLVSWNALIGCYEHHGRATKALQLFKEMRRAKVMPTHITFVSLLSACVN
AGLVSEGRMVFDTMVHEYGIVARIEHYAALVNLIGRHGQLDDALEVINSMPMAPDRSVWG
AFLGACTAKKNEPLAQMAAKELSTINPDSSAPYVLIHNLHAHEGKWGSAAVVREEMERQG
IYKQPGYSWIDLEGKMHVFISGDTWHPNAQEIFSVLEDWQWHNPMSLEMNQLTQVKLIAK
RNNQIMDKQATATKMHKATNIRHIYSKFKVDEQQNTSWWPYCTSLWPDSYLLEEEALFSS
LSFPSFHPQPVYSTVMQSNVLQDELGVIFEDDVLKYWDEMEQSENKVEKSEKGLPLLYYG
DENGAASKIMRDDVRSEEKALTFELVSQYFYMPITQAARELNVGLTLLKKKCRELGIPRW
PHRKMKSLQTLINNVQVLQEASKANNEEQLRMLVEMLQEERRLLEQKPYVQLEEKTKRLR
QACFKANYKKRRLLALEAGEP*
>11668.m04989|LOC_Os02g51110.1|genepair1932-1
MASNNSRTNSRANYSNEIHDLSTVQNGTMPTMYYGEKAIADFFPPHLLKKVVSEVVATFL
LVFMTCGAAGISGSDLSRISQLGQSIAGGLIVTVMIYAVGHISGAHMNPAVTLAFAVFRH
FPWIQVPFYWAAQFTGAICASFVLKAVIHPVDVIGTTTPVGPHWHSLVVEVIVTFNMMFV
TLAVATDTRAVGELAGLAVGSAVCITSIFAGAISGGSMNPARTLGPALASNKFDGLWIYF
LGPVMGTLSGAWTYTFIRFEDTPKEGSSQKLSSFKLRRLRSQQSIAADDVDEMENIQV*
>11680.m01203|LOC_Os06g12310.1|genepair1932-2
MASTTAPSRTNSRVNYSNEIHDLSTVQSVSAVPSVYYPEKSFADIFPPNLLKKVISEVVA
TFLLVFVTCGAASIYGEDMKRISQLGQSVVGGLIVTVMIYATGHISGAHMNPAVTLSFAF
FRHFPWIQVPFYWAAQFTGAMCAAFVLRAVLYPIEVLGTTTPTGPHWHALVIEIVVTFNM
MFVTCAVATDSRAVGELAGLAVGSAVCITSIFAGPVSGGSMNPARTLAPAVASNVYTGLW
IYFLGPVVGTLSGAWVYTYIRFEEAPAAAGGAAPQKLSSFKLRRLQSQSMAADEFDNV*
>11668.m04991|LOC_Os02g51130.1|genepair1933-1
MAGGGGGGRASAQRRAALAALITLLLLASLAFLLSATGTASAPNSAPFRLAAIRRHAEDH
AAVLAAYAAQARKLSAASASQTESFLSISGHLSSLSSRISLSTVALLEKETRGQIKRARS
LAGAAKEAFDTQSKIQKLSDTVFAVDQQLLRARRAGLLNSRIAAGSTPKSLHCLVMRLLE
ARLANASAIPDDPPVPPPQFTDPALYHYAIFSDNVLAVSVVVASAARAAAEPARHVFHVV
TAPMYLPAFRVWFARRPPPLGTHVQLLAVSDFPFLNASASPVIRQIEDGNRDVPLLDYLR
FYLPEMFPALRRVVLLEDDVVVQRDLAGLWRVDLGGKVNAALETCFGGFRRYGKHINFSD
PAVQERFNPRACAWSYGLNVFDLQAWRRDQCTQRFHQLMEMNENGTLWDPASVLPAGLMT
FYGNTRPLDKSWHVMGLGYNPHIRPEDIKGAAVIHFNGNMKPWLDVAFNQYKHLWTKYVD
TEMEFLTLCNFGL*
>11680.m01200|LOC_Os06g12280.1|genepair1933-2
MAGGRAFRPSAPRRAAFAALLTLLLLATLSFLLSSPPPTHASHRSSYLGASPPSRLAAIR
RHAADHAAVLAAYAAHARRLKEASAAQSLSFATMSSDLSALSSRLASHLSLPEDAVKPLE
KEARDRIKLARLLAADAKEGFDTQSKIQKLSDTVFAVGEHLARARRAGRMSSRIAAGSTP
KSLHCLAMRLLEARLAKPSAFADDPDPSPEFDDPSLYHYAVFSDNVLAVSVVVASAARAA
ADPSRHVFHVVTAPMYLPAFRVWFARRPPPLGVHVQLLAYSDFPFLNETSSPVLRQIEAG
KRDVALLDYLRFYLPDMFPALQRVVLLEDDVVVQKDLAGLWHLDLDGKVNGAVEMCFGGF
RRYSKYLNFTQAIVQERFDPGACAWAYGVNVYDLEAWRRDGCTELFHQYMEMNEDGVLWD
PTSVLPAGLMTFYGNTKPLDKSWHVMGLGYNPSISPEVIAGAAVIHFNGNMKPWLDVALN
QYKALWTKYVDTEMEFLTLCNFGL*
>11668.m04992|LOC_Os02g51140.1|genepair1934-1
MVKTSANPNAKGKAAASSSGLPSPKTTPRGVRPKAFKKKAKADPEMQKVAAAEAAATAET
ASAPPLKPAEVSPAAAVAKENGGQRMSRKEKTKMKEGDQMKEDKKGRIKGKEKKDDKARE
RKGEAGFIFMCSAKTKPECFQNGVFGLPKGKIDVVEKIQPGAKLFLYDFDLKLLYGIYKA
KTKGGLDLVRGAFHGKFPAQVKFKVDKDCLPLPESSFKHAIKENYNSKGKFTQELSLKQV
HRLLELFKPISLPQSSIQYVKERHRRLDVFEGRLPHYVEGRRLPRHVEEMHHLRHVEERR
LPYDHEERRLPYDHEERRRPRYVEDIRHPQFLKERHAITDSLHDPFRSRHVTHLPELQHA
PPTYYHHVAHTFDERYHQPQVDIVYERSAPRAIVEATDREAFLARDYRVPEEIVARSDHV
DELYRSYRLATRAMDLHQGPSYVTAAYENPGPAYSESIHQMPVSSTRPNVPGAPVSSLYS
FAGAPAYR*
>11680.m01198|LOC_Os06g12260.1|genepair1934-2
MKKPNVAAKVKAAAAGGADGGSSKGSPSTPASLRVTKLVKGKAKVKAKASREKAAAPAAA
AGKEAASLGDAGGNADASPAAPALRPAAVADADGASKGSPSTPASVKKTSRLNKVKAKAK
AAAAAAASGSPSVGAAGGNADASPAAPAPRSATVADGSAARVISTPAAATAEASTPKRRL
KLKPKLAEANANANAVVATKNGVGADNNDGDAIKKRKREIAGERTSNVKERRKEEEGSKK
EERLDSKGGGLIFMCNAQTKPECFQSRLFGYPRGKIGIVEKIRPGMRLFLYDFDLKLLYG
VYKAVSKGGLDLVRDAFSGKFPAQVKFKIDKDCLPLPESRFKDAIRENYSAKSKFNPELN
SRQVHRLIALFESVSVPQPAPQKPLEEMHHYEGKTQPHQYEERRSSLPVMHVPPPKDLYR
ATRFDPHPVDYRIDHSLSNAHDGPHIHYQQTLVARESQRVPLDIEPRLVPHALEPRHGPS
IPEIQHVPHAYYRHLAPSDVPYYRSQVDPLPDRVAARTVADPFLSRDYTAVPATRSDGAA
RVEELRRIGDIASLGARVEELYRPGELAARGSRVEELYRPGEIVARGSRVEELYRPREVA
AHSARMEDLYRPGEIPIRGARVEDLYRPGEISARAVRVEDLYRSDQRITHAVDLPLHAPY
PTAHYEVPNPAYSDISQRIWYKYCPPDSNLISSLSKKKAIYVISLG*

>11668.m04993|LOC_Os02g51150.1|genepair1935-1
MGIGASDELLGTFVPIAVYWLYSGLYLALDGVERLDVYRLHPREEEAAKNVVSRGTVVRG
VLVQQAFQVAVSLTLFAVIGDESDIEQKQPSALVILLQFAIAMFVMDTWQYFMHRYMHIN
KFLYKHIHSKHHTLVVPYSFGALYNHPLEGLILDTIGGALSFLVSGMTPRTSIFFFSFAT
IKTVDDHCGLWLPGNILHALFNNNSAYHDIHHQLYGNKYNFSQPFFVIWDKILGTYMPYS
IEHRKGGGFESRPVKLNIAEQTKTD*
>11680.m01197|LOC_Os06g12250.1|genepair1935-2
MAFAVSDELLGTFVPIAVYWLYSGLYIVLDAMGMDDYRLHPKGEEATKNVVSKWTVVKGV
LVQQSFQIAVSLLLFTIIGDESGTVRKQPPALVIALQFIIAMFVMDTWQYFMHRYMHVNK
FLYKHVHSKHHTLVVPYAFGALYNHPLEGLILDTIGGALSFLIAGMTPKTAIFFFSFATI
KTVDDHCGLWLPGNILHVFFSNNSAYHDIHHQLYGNKYNFSQPFFVMWDKILGTYMPYTL
ENRKGGGFEARPIKLNVAEQSKTD*
>11668.m05006|LOC_Os02g51280.1|genepair1936-1
MTMDVAGDAGGGRRPNFPLQLLEKKEEQPCSSSAAGGGTGPSSAGGNGNNGSGPGGAGGE
MQLRKAAPKRSSTKDRHTKVEGRGRRIRMPALCAARVFQLTRELGHKTDGETIEWLLQQA
EPAVIAATGTGTIPANFTSLNISLRSSGSSLSAPAHLRALPSPAAAARFGSRADAWDRVV
SLGFPSEGPASSSSSPSPLLLNFHSGSVGLDVQPSPSAAAAAADLSRKRRWEQEMQQQQQ
QQQQQQQQQQQQYQQQMAGYTQSQMPGTVWMVPSNSTQGGGPSGGGGGGGGGSGESIWTF
PQMSSAAAAAAVYRGSVPSGLHFMNFPAPMALLPGQQLGLGPVGGGGGGGGGGEGHMGIL
AALNAYRTQAATDAAGQQGGGGGGGGGSSQQQHGGGGGGGERHQSISTSDS*
>11680.m01195|LOC_Os06g12230.1|genepair1936-2
MDVTGDGGGGGQRPNFPLQLLGKKEEQTCSTSQTAGAGGGGVVGANGSAAAAPPKRTSTK
DRHTKVDGRGRRIRMPAICAARVFQLTRELGHKTDGETIEWLLQQAEPAVIAATGTGTIP
ANFTSLNISLRSSGSSLSIPSHLRLAGLAGPRFGGGARAADAWDRVVGLGFGGAADAPSS
ATSSSSSPLLLSFHSGSVGLDVSPPSASTSPAAADLSRKRRWEQEMQQQQQYQQQMAGYT
QSQIPAGTVWMVPSSNAQAAGGGAPPGGGGESIWTFPQSGSGGGGGAATVYRGVPSGLHF
MNFPATPMALLPGGQQLGLAGAGGGGEGHPGILAALNAYRAQAAQPDAGAAAQNGAQGSS
QHRQHQHHGGGGGGGDERHESMSASDS*
>11668.m05007|LOC_Os02g51290.1|genepair1937-1
MGSGSGSFLKVVVKNLDVLAGPIVSLAYPLYASVRAIETKSAVDDQQWLTYWVLYSFITL
FELTFSPVLEWLPLWSYAKLFFNCWLVLPYFNGAAHVYEHFVRPMVVNQQIVNIWYIPRK
DESDRPDDVISAAQRYIEQNGSRAFESLVNKFKASNTRRSILEEVEAERRAKAELEAEAR
DENPFFNQNYRY*
>11680.m01194|LOC_Os06g12220.1|genepair1937-2
MGSGSFLKLLANNFDVLAGPLVSLAYPLYASVRAIETKSPVDDQQWLTYWVLYSFITLFE
LTFAPVIEWLPFWSYAKLFFNCWLVLPCFHGAAYVYDHFVRPMFVNRQIVNVWYVPRKEN
LSKPDDVLSAAERYIEQNGPEAFEKLISKSTRPSTSKRSTKQSILEEVESEHMARAERES
WGENPFYDKNYRC*
>11668.m05010|LOC_Os02g51320.1|genepair1938-1
MSSRRSSRGSISEEEINELISKLQSLLPNSRRRGSSQASTTKLLKETCNYIKSLHREVDD
LSDRLSDLMATMDHNSPGAEIIRSILRS*
>11680.m01193|LOC_Os06g12210.1|genepair1938-2
MSSRRSSRSSVSEEEINELISKLQSLLPSSRRRGANQASTTKLLKETCSYIKSLHREVDD
LSDRLSDLMAGMDHNSPGAEIIRSLLR*
>11668.m05015|LOC_Os02g51370.1|genepair1939-1
MGCTGSRHAFRGGVRGGKTAYARSRSGPAAVHHTVSLKSSTLGSLSLERDRDEEMMKWRD
DGGAAKTTPPPQQMARRQRQLVLATTAPAKTPAREPEVINVWELMEGLDDKDEEGDVRGE
ERRGQSTPGSPEFDPDIIAAFRKALDEVPAAGECPGDEVCVKKREIQRFPGIVRERVSAF
QKRIDAKLAKMAPPPPSPSPPPEPEPQLPPPPPDSDRKVVLYLTSLRGIRKTYEDCWATK
SILQGYGVLVDERDLSMHAGFKEELHAALGAPGSLPQVFADGRHLGGAEEVRRMHESGEL
SKALGDCEMAPPAAAGKGIALDACSGCGGVRFVPCEECSGSCKVFLEELDTFRRCPDCNE
NGLVRCPLC*
>11680.m01191|LOC_Os06g12190.1|genepair1939-2
MGCKGSKHALHGGVPAAAAAAAERRMCSSRRSVAVRSAAVSFAAGVGDGVMLVKGGGGGG
VGYTSATVGQEKRCRRAPPRTPTKTPLRAPEEINVWELMAGLDYDDEEEEEEEEVVVDGH
GGERQVKSAPGSPAFDPDVLAAFRKAVEELPPESPPRDAAAAADDDDNKKGEIQKFPGVV
RARIILFQKEIDAKLAKKAPPPPPPESARRVVVYLTSLRGIRQTYEDCCATASILRSYGV
RVDERDLSLHAGYKDELRAALGDGAGGGGGVPGQGRPLPQVFVDGCHVGGAEDVRRMHES
GELTGTLLKACDTAAAAVAAVGKGGRQLAPPSEPCGGCGGVRFVPCDACSGSCKVFVDDD
EDGGAFRRCPECNENGLVRCPVC*
>11668.m05018|LOC_Os02g51400.1|genepair1940-1
MRLRCLLTRPPVSLAISASAGGGEGCFARRFGAAVAPRPWGAGRRLCRFYGSSKGGVGSA
EARSAAAAEGSSGRCSEQEHAKLGERDQQEWLSGERFLTDCKRRESPFLTRRERFRNEFM
RRVVPWEKGNLTWQNFPYYVKYDARTTCSVLCLRTELYRERLVRALAHELQVPLLVLDSS
VLAPYDYGDDYSESDEEDEHDESEDEESDIEDEGDEDWTSNGEAKTDESDDEDALKSVEE
LKKSVDRLRKLVPCTLEEFAKRVAGAEEGTTSESSESPESSEEDKRPYQRGDRVKYVGSS
EAFEADQRIILGKVPTKDGSRNAYTFISGRTLSKGQRGEVYEINGDQVAVIFDPLAEKLH
DGDNDATSKEENTEASIYWVDSQDIVHDHDTESEDWHIAIEALCEVLPSLQPAIVYFPDS
SQWLSRAVPKSNRREFIQKVEEMFDKLTGPVVMICGQNILEAEPKDKDKEPPALMFHNLS
RLSSLPSSLKRLVGGRPKYSRSSGISKLFTNSLIVPLPEEDEQRRIFNNQIEEDRKIIIS
RHNLVELHKVLQEHELSCVELLHVKSDGVVLTRQKAEKVVGWARSHYLSSAVLPNIKGDR
LIIPRESLDVAIERLKEQGIKTKRPSQNIKNLAKDEYERNFISAVVPPDEIGVKFDDIGA
LEDVKRTLDELVTLPMRRPELFSHGNLLRNGDVTLTQVVLRWPVAISGENEIVKKCLPCK
GVLLFGPPGTGKTLLAKALATEAGANFISITGSTLTSKWFGDAEKLTKALFSFASRLAPV

IIFVDEVDSLLGARGGAFEHEATRRMRNEFMAAWDGLRSKESQRILILGATNRPFDLDDA
VIRRLPRRIYVDLPDAQNRMKILKILLAKENLESDFRFDELANSTEGYSGSDLKNLCIAS
AYRPVHELLEEEKKGGPCSQNTGLRPLRLDDFIQAKAKVSPSVSYDATSMNELRKWNEHD
WPILKTSKLNVRLLKPGNRFPHMRGCFGERDSSWEIVITIDWSPTPIFKAKDEIKSGKAN
RDGYGGGGRGIDRGGRDDGCSCAWARLALLRRMEASSAGRGERGKKGNLKGRRQGRRDHE
PCLEKGSGGARSFNGGRASLQGSGDGKWTAFAKTRVEDTIAHALMSKVSGVRTRDHSSKA
FILASHRMVGIAIRKIEHDVINVMQLEN*
>11680.m01188|LOC_Os06g12160.1|genepair1940-2
MRLRGLLRPQLLRTHETGGALVVGLGEPGGSAVARRPPPLPFGDGRRRPSSRFYCSKGGV
GSVEAAVGSGGGGSSSSSSEQEHARLGERDQKEWLSGERFVTGCRRRESPFLTKRERFRD
QFLRRVVPWEKATLSWRSFPYYVDEDARQLLSDCVAAHLRHKDVALEYGSRLQSSGGRIL
LQSLSGTELYRERLVKALAHELRVPLLVLDSSVLAPYDFGEDCSESEEEDDHAESEDEGS
VSEVEDEGDDDEEKSGESDDDDAIKSVEDLKKLVPCTLEEFAKRVASAQGSSSTSESSDT
AESPEDGKRPLQKGDRVKYVGASVLVEADHRINLGQIPTQEGGTNAYTSINGRTLSNGQR
GEVYEINGDQAAVIFDPSEDKLSDDKKDEASKEHLAKPAVCWVDTQDIELDHDIQAEDWH
IAIEALREVLPSLQPAIVYFPDSSQWLSRAVPRSNRREFVEKVEEVFDQLTGSLVLICGQ
NITEAAPKEKEPKTLVFHNLARLSPLTSSLKRLVGGLKARKPSKSNDISKLFRNKFFIPL
PKDDEQLRVFNNQIEEDRKIIISRHNLVEMHKVLEEHELSCEDLLHVKLEGIILTKQRAE
KVIGWARSHYLSSVTCPSIKGDRLIIPRESLDLAIGRLKAQEASSRKSSEKIKILAKDEF
ERNFISAVVPPNEIGVKFDDIGALEDVKKTLDELVTLPMRRPELFSHGNLLRPCKGILLF
GPPGTGKTLLAKALATEAGANFISITGSNLTSKWFGDAEKLTKALFSFASRLAPVIIFVD
EVDSLLGARGGAFEHEATRRMRNEFMAAWDGLRSKENQRILILGATNRPFDLDDAVIRRL
PRRIYVDLPDSQNRMKILKILLAKENLESDFRFDELANATEGYSGSDLKNLCIAAAYRPV
HELLEEEKGGVSGTKISLRPLKLEDFVQAKAKVSPSVAFDATSMNELRKWNEQYGEGGSR
SKSPFGFGS*
>11668.m05019|LOC_Os02g51410.1|genepair1941-1
MEAGVGLALQSRAAGFGGSDRRRSALYGGEGRARIGSLRVAEPAVAKAAVWARGSKPVAP
LRAKKSSGGHETLHNSVDEALLLKRKSEEVLFYLNGRCIYLVGMMGSGKSTVGKIMSEVL
GYSFFDSDKLVEQAVGMPSVAQIFKVHSEAFFRDNESSVLRDLSSMKRLVVATGGGAVIR
PVNWKYMKKGLSVWLDVPLDALARRIAKVGTASRPLLDQPSGDPYTMAFSKLSMLAEQRG
DAYANADVRVSLEEIASKQGHDDVSKLTPTDIAIESFHKIENFVIEHTVDNPLNILVPKD
EDLHLRSQKNTKCHRRFYRNPEAETNWALAFSIVHSPSSSMRRGIRQRPAACSCLGIVKP
LEALPAPVLYSTQFTRRLLQIGVAILPFIGFFAVTIFFFNSTIPIILKICGLNASDMWGN
NAEAQPLAFDLGHVGCSNPYLRLP*
>11680.m01187|LOC_Os06g12150.1|genepair1941-2
MMGSGKSTVAKILAEVLGYSFFDSDKLVEQAVGMPSVAQIFKEHSEAFFRDNESSVLRDL
SSMRRLVVATGGGAVIRPVNWKYMKKGLSVWLDVPLDALARRIAQVGTASRPLLDQPSSD
PYTAAFSKLSMLAEQRGDAYANADARVSLEEIAAKQGHDDVSKLTPTDIAIEALLKIENF
VTEHSTSSGPVGDLIVDSQNRRTKAL*
>11668.m05024|LOC_Os02g51450.1|genepair1942-1
MGAAMLLAPMPLLLLASTPLAAVHLPTRCRLRLQLLSRAAPEAASATTTTTTAPDNHFSV
EEYLISNCNLTQPQAHKASKSIAHLKSRSNPDAVLAFLADFGLSPKEVAAIVASNPRILC
ARIDRSLAPICSELRAVGLSPSQIARLAQITGRYFLCRSFVSKVRFWLPLFGSSERLLQA
SDWNYWLLTSDLEKVVEPNVSFLKECGLSARDISKLLVAAPRLVTMHPDYVKDAVRRAIQ
LGVAPGSQMFRHALSTAGCIGQDKIDAKVAVLKESLGWSQEEVNLAVSKAPRILVASEER
LRRNAEFLIDEVGLQPQYVARRSVLLMYSLERRLVPRHLVVKLLKERGLIEQDRCFFNAV
APTEEKFLEKFVVPFEGCVPGLADAYESACAGKTPVQAE*
>11680.m01182|LOC_Os06g12100.1|genepair1942-2
MLHLQKHLLLLSLPPRATASTLLSLRHHGLFSLTRFSAAAAAAKSAGHFAVEEYLVATCH
LTPDQATKASKSISHLKSPSRPDAVVAFLAGLGLSAADIAAAVAYDPRLLCAEVDRTLAP
RLAELAGLGLSPSQIARLVLVDPARFRRPTVISKLQYYVPLFGSFETLLQALKNNSYLLS
SDLEKVVKPNVALLRECGLGACDIAKLCIPLPRLLTTSPERVRDMVAQAENVGVRRGSKM
FRHAILAVAYISEEKIAAKMQFLMKTLKWSDAEARIAVSKLPVVLRSSEDKLSRVSEFLI
SEVGLEPAYIAYRPAMLTYSLERRLMPRHCVLKYLKDNGLIESDKSYYSAVQVTEEVFVE
KYISPYEDTAPHLAEDYAAVSSVKIPTRFRLKGPKTGHASAQTA*
>11668.m05029|LOC_Os02g51500.1|genepair1943-1
MAGGKEPIEVKFRLFDGTDIGPSKYDPSTTVSALKEFILARWPQDKEITPKTVNDLKLIN
AGRILENNRTLAESRVPVGEVPGGVITMHVVVRPPQPDKNSEKQLANSPKQNRCGCTIL*
>11680.m01172|LOC_Os06g12020.1|genepair1943-2
MASGGGGGGGMEAVEVRFRLDDGSDIGPSMHDQATTVTALKEFVLARWPQGKEIAPRTVN
DVTIINAGQVLENNRTLAESRNLAAESPEGPITMHVVVRRSRPERRVKQPPKARPPERIG
CGCTIL*
>11668.m05035|LOC_Os02g51550.1|genepair1944-1
MASVPAAAKQGRRHVVEGPPRAPPASLLAGGGPATAAAADHGGVETAAFLKNCALCGRDL
GPGKDTYIYRGEVAFCSKECRECVIEYYERKERNCSLTSIKDTPAVSGASGSDQSGASGS
ETVAAA*
>11680.m01168|LOC_Os06g11980.1|genepair1944-2
MTTLGKRHRNNSSMRRTTSMSGFAVAEEEEQQGRQPPARAARGGGGGGSAAPSPGWGAMQ
RRHSGDFAVAETAAFLKACGICNRRLGPGRDTFIYMGEVAFCSHECRQQQMNLDELNEKK
CFQREGGGGSDKSGNSGAVAAA*
>11668.m05039|LOC_Os02g51590.1|genepair1945-1
MAASTSQHALVSVKSLCTGANFGFEKRTSKVRFVLVGRCCSGTRKLGLVCASNSHSSVME
PAQLPLSPESGNTPKKSSESALILIRHGESLWNEKNLFTGCVDVPLTPKGVDEAIEAGKR
ICNIPVDVIYTSSLIRAQMTAMLAMMQHRRKKVPIVVHSESEQAHRWSKIYSEETKKQSI

PVITAWQLNERMYGELQGLNKQETADRFGKEQVHEWRRSYDIPPPNGESLEMCAERAVAY
FKDQIVPQLVAGKHVMIAAHGNSLRSIIMHLDKLTSQEVISLELSTGIPMLYIFKEGKFI
RRGSPAGPSEAGVYAYTRSLAQYRQKLDNMFQ*
>11680.m01166|LOC_Os06g11960.1|genepair1945-2
MRVSKHTCEVRYGELQGLNKQETADRFGNEQVHKWRRSYDIPPPNGESLEMCAERAVAYF
KEHVVPQLTAGKHVMIAAHGNSLRSIIMQLDKLTSQEVISLELSTGIPMLYIFKEGKFIR
RGSPVGPSEASVCAYTRKLAQYRQKLDSMV*
>11668.m05040|LOC_Os02g51600.1|genepair1946-1
MSNHNSPCDIPKPASVDEFVKNGKKKKSFMSSIFRKKGRSGTGSSDKKLLSRRDIVFGLD
EKCDDRSELLDSSPAVRKSFSDRHCATKIESLTLSCLDSPHRQFDTREYRVFVGTWNVAG
KPPNSSLNLEDFLQIEGLPDIYVLGFQEIVPLNAGNVLVIEDNEPAAKWLGLIYQALNKP
QDQSSGDELSPPETSDSRQGGGSGSRDSIPKSSSGGMLFFQKPSLKMLSKNYRVDSALVK
TCTCLTDPSTMQRRAREMREFLYRIEASPPPSLASAAAAADEDGGPDAGGELARSSVNYC
LIASKQMVGIFLSVWVRRELVQYIGHLRVDSVGRGIMGRLGNKGCIAMSMTLHQTSVCFV
CSHLASGEKEGDEVRRNSDVAEIIKSTQFPRICKVPGQRIPDKILDHDRVIWLGDLNYRV
ALSYDETKTLMGENDWDTLLEKDQLMIERQAGRVFKGWKEGKIYFAPTYKYKQNSDSYAG
ETAKSKKKRRTPAWCDRILWHGQGIEQLQYIRGESRFSDHRPVCSVFVIEADVDNGSMIR
KGYSTLDSRIHFESPIPQRHSFYDDF*
>11680.m01162|LOC_Os06g11920.1|genepair1946-2
MSSNMFGKKGWDSNGMDTSGSVCRSSSDINYINQRARLKSASLNCVGSPPRKNNNATQYR
MFVATWNVGGRTPNKRLNLQDFLQVEESPDIYVLGFQEIVPLTAGNVLVLEDNEPAARWL
ALIHQALNMPQEPADGDEPSPLTPPPSSSTTTSESSNGARTRRRDAVSRSASGNLFFHTP
SLKMLSNSYRVDSALVKTCNCSPEHSSVRRRAAEVRESVYLADAPAPAGETAAPAADEDD
APTTEAQCEAGCGGGGGMSYCLIASKQMVGLFLSVWVRKELVEHVGHLRVDCVGRGIMGW
LGNKGCIAISMTLHHTSLCFVCSHLASGEKEGDELRRNADVAEILKSAHFPRACRPAPAA
ARRVPERILDHDRMIWLGDLNYRMSLSYDETRTLLEDNDWDALLEKDQLLIEREAGRVFR
GWNEGKICFAPTYKYTHNSDAYAGETAKSKKKRRTPAWCDRILWQGDGIEQLQYLRGESR
FSDHRPVCGVFAVEVDGGDGDGGGAAGKIMKGYYSLNARIGGDRSQCHQGDVS*
>11668.m05048|LOC_Os02g51670.1|genepair1947-1
MAAAIDMYKYNTSTHQIASSDQELMKALEPFIRSASSSSASSPCHHYYSSSPSMSQDSYM
PTPSYPTSSITTAAATTTSSFSQLPPLYSSQYHAASPAASATNGPMGLTHLGPAQIQQIQ
AQFLAQQQQQRALAGAFLRPRGQPMKQSGSPPRAGPFAAVAGAAQSKLYRGVRQRHWGKW
VAEIRLPKNRTRLWLGTFDTAEDAALAYDKAAFRLRGDLARLNFPTLRRGGAHLAGPLHA
SVDAKLTAICQSLATSSSKNTPAESAASAAEPESPKCSASTEGEDSVSAGSPPPPTPLSP
PVPEMEKLDFTEAPWDESETFHLRKYPSWEIDWDSILS*
>11680.m01156|LOC_Os06g11860.1|genepair1947-2
MAAIDLYKYQLSSSSSSSSSDQELMKALEPFIRSASPTSTSTSTPLFYSSSSISTTTTTP
FSYSSPLPQESYYLPASSSYAAIVPPPTTTTNTTTSFSELPPLPPSSSSFASPANAAAVG
LAHLGPEQIQQIQVQFLMQQQLQQRGMAASASASAAASYLGPRAQPMKQAGAAAAAAAGG
KMYRGVRQRHWGKWVAEIRLPKNRTRLWLGTFDTAEDAALAYDKAAFRLRGDAARLNFPT
LRRGGAHLAGPLHASIDAKLTAICHSLAAAPPASSKKAAAAAAHPDSPKGSASTTTTTSE
GDESAISACSPPLPPPPPPPPAALPEMANLDFTEAPWDESDAFHLYKCPSWEIDWDSILS
*
>11668.m05049|LOC_Os02g51680.1|genepair1948-1
MTNQDVVVSEMGIAAGAALPGGPAGPAGGLFACRSAAASMRQTYLDLAAAAVAARSASCT
SWADAMRASSPTRSSRSASDVDEFTAWVRKHPSALSKFEEIAAKSRGKKIVMFMDYDGTL
SPIVADPDTAYMSDAMRAAVREVAKTFPTAIVSGRCRDKVRNFVGLSDLYYAGSHGMDIK
GPSSNPESALCQPASEFLPMIDEVYKTLVEKTKSTPGAKVENNKFCLSVHFRCVDEKRWN
ALGEQVKAVIKEYPKLKLTQGRKVLEIRPSIEWDKGKALEFLLESLGFANCGDVMPVYIG
DDRTDEDAFKVLRKRGQGLGILVSKCPKDTNASYSLQDPTEVMEFLLRLVEWKRKSSSSS
LMIRPRV*
>11680.m01154|LOC_Os06g11840.1|genepair1948-2
MPGSSGRAPLFACRGAAAVSASSMLGGGGAAYQAAVVAHVAPVPAIRPCASWVVEAMRAS
SPTRPAAAAVDAEYDAWTQRKHPSALGSFEQVAAAASGKRVVVFLDYDGTLSPIVADPDM
AFMSDEMRAAVRDVAEHFPAAIVTGRCVDKVQSFVGLPELYYAGSHGMDIKGPSSNEEED
TKILLQPAREFLPVINKAYKALMEKTKSTPGARVENNKFCLSVHFRCVDEKRWNPLAEQV
KAVLRDYPELKLTQGRKVLEIRPSIMWDKGKAVEFLLKSLGFDDDRRDVLPVYIGDDRTD
EDAFKVLRKRGQGLGILVSKCAKETDASYSLQDPAEKYTNAGAHVFVTMLLTVVFTAAVA
LALVNAVNSHDFAAHLAGVDCRMGLAGPVRCPASGFVELLVLALHVVRCVLAILDRLHAC
LMSPSLQLSIASPCHGVPCSIGAVEASAIIVSDAPEGLISTLTTDHIYYMTVFPAKLALT
SEEV*
>11668.m05052|LOC_Os02g51710.1|genepair1949-1
MANLAASLLFLLAVASSATVPAHGRRDLLPTRIKLVRGADAGAGAVAGGDKMECVYTVYI
RTGSIWKAGTDANITLELAGADGNGVGITDLPSWGGLMGEGHSYFERGNLDIFSGRGPCM
AAPPCWMRVASDGTGPHHGWYCNYVEVTVTGAHKGCAQQLFTVEQWLATDAAPYKLEAIR
DKCSAAGAGAAAA*
>11680.m01151|LOC_Os06g11810.1|genepair1949-2
MAPKIPIFFFLLALAAGVQGETGGVGGGGGNVEYNCVYTVFVRTGSAWKGGTDSTIGVEF
AGADGRGVRIADLERWGGLMGAGHDYYERGNLDVFSGRGPCLPAAPCWMNLTSDGAGAHH
GWYCNYVEVTATGPHRGCAQRRFDVEQWLATDASPYRLTAVRDQCRGHAAA*
>11668.m05056|LOC_Os02g51750.1|genepair1950-1
MATLTVPAAVPPVAEDCEQLRKAFKGWGTNEKLIISILAHRDAAQRRAIRRAYAEAYGEE
LLRALNDEIHGKFERAVIQWTLDPAERDAVLANEEARKWHPGGRALVEIACTRTPSQLFA
AKQAYHERFKRSLEEDVAAHITGDYRKLLVPLVTVYRYDGPEVNTSLAHSEAKILHEKIH

DKAYSDDEIIRILTTRSKAQLLATFNSYNDQFGHPITKDLKADPKDEFLGTLRAIIRCFT
CPDRYFEKVIRLALGGMGTDENSLTRIITTRAEVDLKLIKEAYQKRNSVPLERAVAKDTT
RDYEDILLALLGAE*
>11680.m01150|LOC_Os06g11800.1|genepair1950-2
MATLTVPSAVPPVADDCDQLRKAFQGWGTNEALIISILAHRDAAQRRAIRRAYADTYGEE
LLRSITDEISGDFERAVILWTLDPAERDAVLANEVARKWYPGSGSRVLVEIACARGPAQL
FAVRQAYHERFKRSLEEDVAAHATGDFRKLLVPLISAYRYEGPEVNTKLAHSEAKILHEK
IQHKAYGDDEIIRILTTRSKAQLIATFNRYNDEYGHPINKDLKADPKDEFLSTLRAIIRC
FCCPDRYFEKVIRLAIAGMGTDENSLTRIITTRAEVDLKLITEAYQKRNSVPLERAVAGD
TSGDYERMLLALLGQEQ*
>11668.m05060|LOC_Os02g51770.3|genepair1951-1
MGYLPSSLGSKAAHFVSDLTTVILNPISEREPSSPLPEVDKDEEKSEDDKDSEQNSDTPD
GPDTSSFRAFLISFLSSSGSSNGSMEIIPDQNGELGYPTLTPMGKSKKGKSGLLSRGKHS
IGKIISKAARIGGFKQNVEPKIDREVVDHVESVSPVLELEESKEVASFINLPAMSEPSVL
LSEVMRFNIYASFPVLAKGMNWVLLYSTWRHGISLSTLYRRSMLCPGYSLLVVGDKEGAV
FGGLVEAPLQPTSAKKYQGSNSCFVFTNLHSNPSIYRPTGANNYFTVCSTDYLALGGGGH
FALYLDADLLSGSSSNSETFNNMCLSHSPDFAVKDVELWGFVYPSKYDETLALCRTEKPG
ICRW*
>11680.m01149|LOC_Os06g11790.1|genepair1951-2
MGYLPSLGGKAAHLVSDLATVILNPVSERESQRHPSHLPEATEVQENIYEDDDDDNSVKN
SEIPNGPDTSSFRAFLMSFMSSSNSSSDSIEIIPEHNMNMEYPTLTPVGKGSNGRKGLFS
RGKHSIGKIINKAGRIGGFRQKPSYSIDGETVQTEYDAPGLELKGSKESASHDKLPAMSE
PSMLLSETMRTVLYTSLPVLVQGRNWMLVYSTWRHGISLSTLYRRSMLCAGYSLLIVGDR
KGAVFGGLVEAPLQPLIKKKYQGTNNCFVFTNIAGRPVIYRPTGANNYFTFCSTDYLAMG
GGGHFALYLDGDLLNGSSSTSETFNNPCLSRSREFEVKDVELWGFVNASKYDEMLTICRT
EKQGIWNL*
>11668.m05063|LOC_Os02g51800.1|genepair1952-1
MGRSPCCDENGLKKGPWTTEEDEKLMEYIQKNGHGSWRALPKLAGLNRCGKSCRLRWTNY
LRPDIKRGKFTSAEKDTILQLHAVLGNKWSAIAKHLPGRTDNEIKNYWNTHLKKDLIQKG
IDPTTHRPRTDFFAALPQLIALANLRQLVGQLPWDDPAAAAAGLQQAEAAQAAKFGQYLH
LQALLQPPPPSAATSPRSGCIGGAAAAMPGGDMEQMGAGLLSPPAMSSAALSPMSTSSLS
PLPSPMSSTALSPLPPSSFFGNGGQGSFLAGQLPNIQMHGSSLFDQSAAIINDANHNQDY
AASTGEREIINGATKTTTTTLLPEGSLPPLADYPTISNLGDVYSTPSCDGNITEFPLLPD
ALFDELMRDYISNCFLEDWKNTDSKNLDVSDILQIQVAVKILPLASLSGIGPEPHLSVRE
SAAQCQKA*
>11680.m01148|LOC_Os06g11780.1|genepair1952-2
MGRPPCCDENGLKKGPWTPEEDEKLMSYIQKHGHASWRVLPELAGLNRCGKSCRLRWTNY
LKPDIKRGNFSREEEQTILQLHSILGNKWSAIAKHLPGRTDNEIKNFWNTHLRKKLIKMG
IDPMTHCPRTDFFQSLPQLIALANLRQIIERQPWDGHIEGLQTVAVQAAKLEYMQSLLHS
AVSIVTSPTTTTTTSSLNTFTTELEQTNHLCPPQVPSSSVPELAVQVPHSQMPSTSFDQE
IGKTNLFSNNIVNGNEWCSMEADNSSQKSLLVPENSIPPLIDMPVQNFCNTISTPNCDGG
NSIPLPSWSEILLDEELMGEFA*
>11668.m05068|LOC_Os02g51850.1|genepair1953-1
MATRLLCWTALLLPIIAATAAASPLPEACPVPTAAEEILGPGGTCTTLDRRGDPVGVIEG
DEVTLAKAITLLHMNKDDYIAVLFYASWCPFSQECKPNFEILASLFPSIRHFAFEESSIR
PSIISRYGIHGFPTLFLLNSTMRVRYHGPRTVKSLAAFYRDVSGFDVSMTSEAVLHSVDG
IELKKDAEQENCPFWWARSPEKILQQDTYLALATAFVILRLLYLLFPKIGSFAKRAWRRH
TLFPNLVGVHEYFFTYLEQARHKFFRLYPSKRGNLQEGARNATAWASKSLASVSIGEPST
IGRTNSTNELR*
>11680.m01144|LOC_Os06g11740.1|genepair1953-2
MRWWPALPLLLLAVAVAGAGDAAPVCTRPSAAEAIVGSPEACRSPLRRPLGVTEGDDAIL
ARAVNLLHANKEDFAAVLFYASWCPFSQECRLRFEKLACIFPTIRHLAIEESTVRLRTRY
RYGIHGYPTLFLINSTVRVRYHGPRTVKSLAAFYNDVSGINPSMDPAVGDDNIEPKRDCE
QEKCLFWSARTPENILQPDTYLTLAASFVILRLLYLFYPKITAFVKRTWSRRTLFTCLEQ
GKHKFNRVYPSKQGNLHDGARHATAWASKSLASVSIGEPSTS*
>11668.m05073|LOC_Os02g51890.1|genepair1954-1
MAAASSSTSSAGSAAPAHAPAAGGAGGGGSSGVPNHRTRFGDTTLTKVFVGGLAWETPSK
GLQDHFQQYGEILEAVVITDRETSRSKGYGFVTFREPESAREAVRNPNPTIGGRRANCNI
ASMGPPRPSPSRGRAPRGSLFPDQPHMGPQPYMGGRLPPQHMTAPPQQMYYHPQFGYWYP
QDYPYQQHAVYNSQALQHYYPQLYGPTSPSTPSYQFMGYMPGALGPRAGFSPMQQQAPRP
PFIQQPALQFDGGSFPPGPSLPPDFRLQLPPHALSRQPDETTGAQSAPPVSASAAATPTT
DSKEASKTVESNSDLNTSN*
>11680.m01143|LOC_Os06g11730.1|genepair1954-2
MAAAAAAPSSSTSGSPGAAAAAARPTMPYRSRFGDTTLTKVFVGGLAWETPSEGLRRHFE
QYGEILEAVVIADRLTGRSKGYGFVTFREAEAARRAVQDPNPMIAGRRANCNIASLGPPR
PAQPPRGRASPGAQFQAPPPAFQGPPYIARGTPPPPAQMMPQPQHHGPPATIYHPSQSWY
WYPPDYQYQQGLMNSQVLQSYYAAALQAQAQAQAQLYGMAASPTAPSPYQYLGYMPAPAP
AVTPTAVLPPAQQQITGPPPPFVQQPAQHVTAPPPFVHHPTAAAQIQGSFVPLPSLPHNF
RLQLPPNAMSILPPTPTALQPADLQAAPAATGATNPNNTPTGA*
>11668.m05074|LOC_Os02g51900.1|genepair1955-1
MGSNSRLHAVLIPYPAQGHVTPLLHLAKVLHSRGFHVTFVNSEYNHHRLLRSRGTGALAG
LDDFRFETIPDGLPPPSESDNDDVTQDIPTVCTSFLTHGPAAFGALLARLNSEPGTPPVS
CVIPDGVMSFAQRVASDMGILAPAFWTTSACGFMGYLHYAELIDRGYVPLKDESYLTNGY
LDTVLDWVPGMPGIRLRDMPSFIRTTDRDEFMLNFDSGEAQNARHAQGLILNTFDAVEHD

VVDALRRIFPRVYTVGPLLTFAGAAAARRPEVGAIGGNLWKEDASCLRWLDAQQPGSVVY
VNFGSITVMSPAHLAEFAWGLARCGRPFLWVIRPDLVASEKAMLPEEFVSETKERGIFLS
WCPQEQVLEHPATGLFLTHSGWNSTLESISAGVPMICWPFFAEQMTNCRYACTKWDIGLE
IDTDVKREEVARLVQEAMDGEKSKDMRAKAMAWKEKAVAATEEGGTSSAGIDRLVEFLLA
RGDHAS*
>11680.m01142|LOC_Os06g11720.1|genepair1955-2
MCDSPSSSSCSSLSLALAMGERMRRAAHAMLFPFPCSGHINPTLKLAELLHSRGVHVTFV
NTEHNHERLLRRRGGGALRGREGFRFEAVPDGLRDDERAAPDSTVRLYLSLRRSCGAPL
VEVARRVASGGGVPPVTCVVLSGLVSFALDVAEELGVPAFVLWGTSACGFACTLRLRQLR
QRGYTPLKDESYLTNGYLDTPIDWIAGVPTVRLGDVSSFVRTLDPTSFALRVEEDEANSC
ARAQGLILNTFDDLESDVLDALRDEFPRVYTVGPLAADRANGGLSLWEEDAACMAWLDAQ
PAGSVLYVSFGSLTVMSPEELAELAWGLADTRRTFLWVIRPGLIAGAGAGDHDVVTNALP
DGFVAETKGRCFIAEWCAQEEVLRHRAVGGFLTHSGWNSTTESICAGVPMICWPGFADQY
INSRYVRDEWGIGLRLDEELRREQVAAHVEKLMGGGGGGGDRGKEMRRNAARWKAAAEAA
TAKGGSSYGGLDKLVEQLRLGQ*
>11668.m05085|LOC_Os02g52010.1|genepair1956-1
MASDRRVCTVHHMSHAAIVLVSVLLLCSAHPSAGARRLMELYKPPPSEQLTYHNGTVLRG
DIPVSVVWYGRFTPAQKAVVSDFLLLLTVASPAPTPSVSQWWNTINQLYLSKAAAQGKNG
GGGGKITTQVRLAGQLTDDQCSLGKSLKLSQLPALAARAKPKKGGIALVLTAQDVSVEGF
CMSRCGTHASNAKARTAYVWVGNSATQCPGQCAWPFHQPVYGPQTPALVPPSGDVGMDGM
VMNIASMVAGVVTNPFGDGFYQGPKEAPLEAATACPGVYGSGAYPGYAGNLAVDPATGAS
YNANGAHGRKYLLPALFDPATSTCSTLV*
>11680.m01136|LOC_Os06g11660.1|genepair1956-2
MAKKQSLLLAMMLVVAGLAVSAMADRKLMSLVKPQPNQLTYHNGAVLSGDIPVSILWYGR
FTPAQKAVVTDFVLSLAAPLQAAPAPSVSQWWGSIHRLYLSKAVAVGKNGGAHGGGGGGR
AKNARVVLSGQVSDEGCSLGKSLKLSQLPTLAARARPGKGGVALVLTAQDVAVEGFCMSR
CGTHGPVSRAGAAYAWVGNSATQCPGQCAWPFHQPVYGPQAAPLVPPSGDVGMDGMVINV
ASMVAGAVTNPFGDGFYQGERGAALEAATACTGVYGKGAYPGYAGALLVDKATGASYNAH
GAHGRKYLLPALFDPDTSACSTLV*
>11668.m05097|LOC_Os02g52130.1|genepair1957-1
MGECGGGEYRCWEELLPDALGLVFRNLPLREVLTVVPRVCKSWSRVVAGPYCWQEIDIEE
WRQQQGKPEQLVRMVEMLVARSCGSCRRISVSGLPGDPLFSFIGDHARALRTLEIPRSEI
SDAAVESVAPRLPNVTFLDISSCTKIGARALEAFGKNCKSLVGLRRVMHPTDVAGKACQR
DEARAIACTMPRLRHLEMGYMVIATDAVLDILARCRDLRFLDLRGCWAVDDKFLQERHPG
LRVLGPGVDDCFENSYLEECSDYSDDSSIYSWELMEDDDDDYYAVGSDDEAIWDDGQGLE
NLEVRFYGGGFSESYAGFDWPPSP*
>11680.m01133|LOC_Os06g11630.1|genepair1957-2
MGECSEHRCWDELLPDALGLIFRKLSLKDVLTVVPRVCKSWGRVVAGPYCWQEIDIQEWS
QQQSKPDQLKRMVRMLVARSGGSFHRISVSGLPGDPLFTFIGDHARSLKTMELPRSDISD
SLVENVAPRLSNVTFLDISSCTKIGARALEAFGKHCKSLIGLRRVMHPTDVVGRASQHDE
ARAIACNMPKLRHLEIGYMLIATKAVVEIASQCHDLKFLDLRGCWNVDDKLLQESYPGLK
VVGPYVDDCYENSFWEECSDDSDDSIYWELMDDDYYAAGSDDEGIWDDGQGLEGLEVRFY
GGGFSESHAGFDWPPSP*
>11668.m05098|LOC_Os02g52140.1|genepair1958-1
MDEEEHEVYGQEIPEDGDMDGADVDMASGGDDAAKLQELDQMKRRLKEMEEEAAALRDMQ
AKVAKEMQGGPPGGDPSASTAEAKEQVDARSVYVGNVDYACTPEEVQQHFQACGTVNRVT
ILTDKFGQPKGFAYVEFLEQEAVQEALNLNESELHGRQIKVAPKRTNVPGMKQRPPRGYN
PYHGYPYRSYGAPYFPPYGYGRVPRFRRPMRYRPYF*
>11680.m01132|LOC_Os06g11620.1|genepair1958-2
MADEEHEVYGQEIPLDGEDVDMGAPGDEAAKMQELDEMKRRLKEMEEEANALREMQTKVA
KEMQGLDPNASSSESKEEMDARSVYVGNVDYACTPEEVQQHFNSCGTVNRVTILTDKFGQ
PKGFAYVEFLEVEAVQEAVKLNESELHGRQIKVAPKRTNVPGMKQPRGGRGFGGHPYMRP
YGAPFYNPYGYGYPRFRRPRRPYF*
>11668.m05100|LOC_Os02g52150.2|genepair1959-1
MASIVASKRIPLFRLVEQLLAASPAQGAASALRPVAVAGGSRAYNTGAQLRRHERDESDD
DSGRGYDTRRPTRDATMPAFFSGNVFRDPFSAPQSLGRLLSLMDDLATPAGRAGAATLRR
GWNAKESEEALHLRVDMPGLGKEHVKVWAEQNSLVIKGEGEKEAGEDEGAAPARYSGRIE
LAPEVYRMDQIKAEMKNGVLKVVVPKVKEEQRRDVFQVNVE*
>11680.m01131|LOC_Os06g11610.1|genepair1959-2
MASTVALKGRPLATLLRQLLAADAPPAATGRPVAAAPAASGKPVTAPAAATATNAASRRL
YNTEGAPLRRYDVVDESGTDSGDEYDATDDGRRLTVPFFFSASDVLDPFGAPTSLGRLLA
LMEDAAVATAAAPGTNGLATAAARRGGWWVAKEDDDAVHLKVSMPGLGKEHVKVWAEQNS
LVIKGEGEKDPEDDADAAPPRYTRRIELPADAFKMDKIKAEMKNGVLRVAVPKLKEEERK
DVFQVNVE*
>11668.m05101|LOC_Os02g52160.1|genepair1960-1
MWVFYLISLPLTLGMVTVTLRYFAGPGVPRYVIATVGYAWFCSLSFIILVPADIWTTLTG
REKGGIGFFWSWSYWSTFILTWAVVPTIQGYEDAGDFTVKERLKTSIHMNLLFYSIVGAI
GLFGLILLLVMHRAWDGGIVGFAMACSNTFGLVTGAFLLGFGLSEIPRNIWKNADWTHRQ
KVLSHRVAKMAVKLDNAHQEYSNAIVVAQATSNQMSKRDLLRPYMDIIDKMLAQMLREDP
SFKPSGGRLGENDMDYDTDDKTMATLRRQLRRAHEEYYRCKSEYMTYVMEALELEDTIKN
YERRDANGWKFVSSFRESRPGTLGSLLDTMEFIWRCVLRKQLQKGFAIVLGCMSAAILLA
EATLLPSGVDLSLFSILVKSVGKQEVLVQVAAFVPLMYMCICTYYSLFQIGMLMFYSLTP
RQTSSVSLLMICSMVARYAPPISYNFLNLIRLGGDAKTTFEKRMGNIDDAVPFFGRGFNR
IYPLFMVVYTLLVASNFFGRLINFFGSWKRFKFQREEENMDGFDPSGMIILQKERSWIEQ

GCKVGEQVIPLARNFNNVNTDVESGKVPLVENTLEMKSGATSSRADGRVGQSKYANNRET
IATKYSAIREQSRQAVKPAKKEISSTSVSLLEEGSSEQWSNTGAPVGSSAGISQTWATMK
IGFQNFKANMGSKKFIPLRQDPGFAPHSNVSSPESLDEIFQKLKRRPADMPVDYLDDDDD
NTGDMDPTFPGSTR*
>11680.m01122|LOC_Os06g11520.1|genepair1960-2
MWAFYLLSLPLTVGMVVATLRYFAGPAVPLHVLATVGYAWLCSLSFIVLVPADISTTITG
SQEGDVGFFWSWTYWSTFFLSWSIVPTLQGYEDAGDFTVKERLKTSIHKNLVYYKIIGSI
GLVGVILIITMRHDWAGGIMGFAMACSNTFGLVTGAFLLGFGLSEIPKNIWKTADWTRRQ
KFLYHRIANMAGKFDNAHQEYCHAIAVVQATSKQMTKREPLRPFMDIIDDMLAQMLRDDP
LFKPSGGKLGEDDMDYDTDENTMASLRRQLRRANEEYYRCKSKYTSYVMEALELEDTIKN
YEQRDANEWKYVSGLRESRSCTLGSFLDFIEFIWRCILKKQLLKVLAVILGCISAAILLA
EATLLPSDVDLSLFSVLTNVVGKQEVLVQVVAFIPLMYMCICTYYSLFRIGMMVVYSLTP
RQTSSVSLLMICSMVARYAAPISYNFLNLIHLGGNSKTTFEKRMGNIDDVVPFFGRSFNR
IYPLIMVVYTLLVAGNFFGYVLEFFGSWKRFRFWTEQEEDHTDGFDPSGVLILQKERCWI
EQGHKVGELVAPLARNFTGIYKDVESGNVQQDEETAGMKATTLPSKKEGRLQSKYASNVA
LKYSSIREQNSSHQAVKQAQTETQSTSVVPETGNSETPSSVSKEPDSSAGIASRWTLMKT
GFQNFKANMSSKKFLPLSLSSTQSSSSGSLDEIFEGLKRHSSNASVDYLDDDDGI*
>11668.m05103|LOC_Os02g52180.1|genepair1961-1
MASCGASCLTIAALLLAACASSAAATSYTVGDASGWTIGVDYTSWAGSKSFKVGDSLVFK
YASGAHTVVEVSAAGYLACAAANALGSDSSGSTTVALKTPGKHYFICTIAGHCAGGMKME
VDVSGSSSSSSGGGGGGGGGGSTPSSPSSPTPTTPNPSTPTPTTPYPSTPMPTTPYPSTP
MTTPTTPYTTPTSPACSGGAGATPVTPVTPGTVPFMSYNGAGGLGPVALATIGMVCFVVF
VQLGLL*
>11680.m01119|LOC_Os06g11490.1|genepair1961-2
MASSLALVALLLVSCAVVAAAATKYTVGDTSGWAMGADYTTWASDKKFKMGDTLVFNYAG
GAHSVDEVSAADYAACTASNALQSDSSGTTTVTLKTAGKHYFICGIAGHCSNGMKLVVDV
AAASPAPAPKAPSTTPTTPSTTPATPASPGTSSGLTPTTPATVLAPPAKQSAGAAGLRAR
SWAMLGLAGLAAVQLGLF*
>11668.m05106|LOC_Os02g52210.1|genepair1962-1
MPSCRARMHAHQHQHALLAALLACALAASSSTAGAQPAGQQGYAYGDVSGQQVHVSTTMI
VLLAAVVGVFLFIAISTIYLRHCTGYDPATEGGGVGGSRSMILPANSFVSRRQRRPRGLD
SSVVRMFPTMKYAEAKALRVGKVAGAALECAVCLSEFEDDEMLRFLPKCSHAFHPDCIGQ
WLASHVTCPVCRRNLDPNKDTTEEVIIPAAAAADPNSTSSEIVVIRQEDGAHPAAVVIDV
VTEEDDEERRKEELELQAIGTQLRAMRSRSGLRPKTSAAKLPRSHSTGHSLAVRLDGDLE
RYTLRLPEHVHREMVAAGEQSVRRGRRLGEGVGMGARCSPRFSRSGRWSSFLSNSLSGKL
SFLSPSSRRTPDSTQVEVSSSSSSSVTKVKGKRVAAVDVADDGSAHGTAQYPGCTVASSA
AAAAVDVEKAATRQVRT*
>11680.m01115|LOC_Os06g11450.1|genepair1962-2
MPTPRPRHRHHALPLALALAAPLLLLAAADGQPNESRDKNNGGGGGGFMAPSGGGGGMGS
QSPSFSAPMVVLLVALIAAFFFIGFFSIYIRRCGGEASTGPTIPAAALAALSRQEQRSRR
QRGLDPAVVESFPTMKYAEARELRDGGKDAVLECAVCLSEFDDDEELRLLPKCSHAFHPD
CIGEWLAGHVTCPVCRCNLAPDAAAAEANVVSGEVDGEQQEEEVVVAIDVDREGEEEDEE
RRREAMELERIGSQRRAVRSRSGRPLPLPRSHSTGHSLATTRLGDAGDLERFTLRLPEHV
RREMVAAAGEESLRRTAVREGRVGGGARSARIGRSDRWPSFIARTFSSRIPFWSASRRVL
DAAEAGADAAATTTTTTPTSTARTKRDKTAAAADGSVSSAKGSVRFDCLGGGGGGGGPSN
RVVAFANDDEEDDDEKPIARQV*
>11668.m05111|LOC_Os02g52250.1|genepair1963-1
MASLKELLPTPKAAASTFYDHSSDPWFKERYGGESAQSDAAAAAAKPSGPAKPVPPYGKR
GGFVPRRPEDFGDGGAFPEIHVAQYPLGMGRRDEKGGSKILALTVDAKGSVAFDAVVKQG
ENASKIVYSKHSDLVPKIATADSEATADDEEYQKQIEETTERTKAALEKVVNVRLSAAQP
KNVPTHDSESKFIKYKPSQQSAAFNSGAKERIIRMSEMAQDPLEPPKFKHKRVPRASGSP
PVPVMHSPPRPVTVKDQQDWKIPPCISNWKNPKGYTIPLDKRLAADGRGLQEVQINDNFA
KLSEALYVAEQKAREAVQMRSKVQRELQLKEKERKEQELRALAQKARMERTGAPPAPTGV
PAGGGRGAVDDREEDMDLEQPREQRRESREEREARIERDRIREERRRERERERRLEARDA
AMGKKSKLTRDRDRDVSEKIALGMASTGGAKGGEVMYDQRLFNQDKGMDSGFATDDQYNI
YSKGLFTAQPTLSTLYRPKKDGDSDVYGDADEQLEKVMKTDRFKPDKGFSGASERSGKRD
RPVEFDKQEENDPFGLDQFLTEVKKGKKAVEKIGSGGAMRASGGSSMRDDYEGGGSGRSR
INFERGR*
>11680.m01112|LOC_Os06g11420.1|genepair1963-2
MVLRLPDPSHGGGAPPHDHTEDEWFKERYGGGGGGGDAPRSSRAVNPVPPYGRRSALAPR
RKEDFGDGGAFPEVHVAQYPLDMGRRGGDGDGEQRGSSGGVLSLTVDGSGGRVEFDAVVR
QGENAGKTVYSSPGDVLPKINAAAADADDDEQAAVEETTARTSAALRAIVEKRLSAVQPS
NTLASNHDPEFIKYTPARQTSAFNSGAAERIIRMGETQQDPLEPPKFKHKRVPAPAGSPP
VPVLRSPPRPPSQKDHDDWKVPPSISSWKNPKGYSIPLDKRAALDGRGLHDVQVSDAFAA
LAEALYAAEQKAREAVETRAKVHTEMKMREKEKAEQHLLQLATKARAEMLGAAPPAPSER
SKAAAERDAIREERRRERRLEARAAAAAASKKSAATRDRDRDVSERIALGMANTGGGGGE
VTYDQRLFNQEKGMGSGFAGDDQYNVYSGRLFAAQPALSTLYKPSKHGEEDPDAYGDADE
HLGKIAKTRRFVPDKAFTGAPASVAAGKRERPVEFDGPEMEEDPFHLDQFLTQMKKGKHQ
*
>11668.m05112|LOC_Os02g52260.1|genepair1964-1
MELFVSTVASARATAPLCFASSFHHRRAAPPAVAAAATLRRSNRHLPTRGWRCASAAAPD
PVPSEEPASASASTVVVTEDKPDPPPAEEKSEEVAAVSNGGSLETVAAAPVSSGAAEEDG
GLDDILSKLDIQVTPTLVLYGSGALVVLWVLSSVVSAIDSIPLVPKVLELIGTGYSIWFT
SRYLLFKESRDKLFAKFEDLKERII*

>11680.m01110|LOC_Os06g11400.1|genepair1964-2
MDALVCTNAFATAIAAGRFPLPRGRSPPYAAAAVPALRSRRCLPTRGLLRLRCARGVDWT
DPSFVAVAEKPDAGAEAWKALASAGGGGIEEEEDGPFEAINGDGGYSVEESVVLPPFEQS
LVAAVADSVEDDALSQALSSKLDFKETSTFVMYGSGAFIAGWILSAVVSAIDSIPLFPKI
LQIVGLGYTIWFSTRYLLFKENRDELFVKVDDLKRKITGYATDQAWEFMDSPEEERGDRR
WRKKKEEHGGLVAVLSSRRSASTAAFPLSSLAPSLYSLSTEEDGGAIRVVDAVIGPLIDR
GSRACGDAVAVETDMEVRGLSLASSSSPSSFRQQVAPDPPLLSLPPSPPPTPTVGEHELM
VQLPCYLQELHLLREIVSVKLELPFPKRLLVLDQAAATLHKMTPPHAGGSDEVHLSRSGR
RR*
>11668.m05121|LOC_Os02g52340.1|genepair1965-1
MARERREIKRIESAAARQVTFSKRRRGLFKKAEELSVLCDADVALIVFSSTGKLSHFASS
SMNEIIDKYNTHSNNLGKAEQPSLDLNLEHSKYAHLNEQLAEASLRLRQMRGEELEGLSI
DELQQLEKNLEAGLHRVMLTKDQQFMEQISELQRKSSQLAEENMQLRNQVSQISPAEKQV
VDTENFVTEGQSSESVMTALHSGSSQSQDNDDGSDVSLKLGCTATQQKR*
>11680.m01103|LOC_Os06g11330.1|genepair1965-2
MARERREIRRIESAAARQVTFSKRRRGLFKKAEELAVLCDADVALVVFSSTGKLSQFASS
NMNEIIDKYTTHSKNLGKTDKQPSIDLNMRGEELEGLSVEELQQMEKNLEAGLQRVLCTK
DQQFMQEISELQRKGIQLAEENMRLRDQMPQVPTAGLAVPDTENVLTEDGQSSESVMTAL
NSGSSQDNDDGSDISLKLG*
>11668.m05123|LOC_Os02g52360.1|genepair1966-1
MEGGGEGTTTAAAAAAVAAGKEEEAEVVVVRNPRCYLDVSIGGDMEGRIVVELYASVAPR
TAENFRALCTGEKGVSAATGVPLHYKISLKAASVCTRWTKGSCIHRIVKGFMVQGGDITA
GDGTGGESIYGLNFEDENFVLKHERKGMLSMANAGPDTNGSQFFITTTRTPHLDGKHVVF
GRVIKGMGVVRSMEHVSVGESDRPITDIVIVDCGELPEGASDGVVNFFSDGDMYPDWPND
LEEKPAEISWWMTAVDSAKSFGNEYFKKKDYKTALKKYRKAMRYLDLCWEKEEIDEEKSS
ALRKTKSIILTNSSACKLKLGDLKGALLDADFALREGEGNPKAFFRQGQARIALNDIDAA
VESFKHALQLEPNDGGIKRELAAAKKKIADRRDQERKAFSRMFQPSGGSEKIDEENN*
>11680.m01102|LOC_Os06g11320.1|genepair1966-2
MEGGGEGAESAAVASAAAAEVEVKNPRCFMDVSIGGEIEGRIVIELYASVVPRTAENFRA
LCTGEKGVGAVTGKHLHYKGSCFHRVIKGFMVQGGDITAGDGTGGESIYGLKFEDENFVL
KHERKGMLSMANSGPNTNGSQFFITTTRTPHLDGKHVVFGRVIKGMGVVRSVEHAPVGEA
DRPTSDVEIVDCGELPEGADDGVVNFFNDGDTYPDWPNDLDEKPMEVSWWMDAVESAKAF
GNNNFKKQDYKAALRKYRKALRYLDVCWEKEDIDEEKSSALRKTKSIILTNSSACKLKLG
DLKGALLDADFALRESEGNAKAFFRQGQAHIALNDIDAAVESFKHALELEPSDGGIKREL
AAAKKKIADRRNQERKAFARMFQPSGKSDKDNEESK*
>11668.m05125|LOC_Os02g52380.1|genepair1967-1
MELTTALPASPPPPPPQEDFRFDGPAFSAFPEGVASAGTNPFFSADAMDSNPFLATAVTA
PPSPNPFELNHQSASPGAADPFDLFQHFTSAPASPARAAAIYAQFDGGVGDGNGADHDMA
VVGDDDDDFQPRASYSSGTATSTVPFDWEEKPGKPKPKSELATCAAAATSANVGEVDDAD
FDFGVLLDKSVQVPELTTADELFDKGKIRPLKPPPGLLDGGSVASSPRSPISKSPMWSPR
LRGKVGSGVDFDPFSTALAKAAKGPSPLGAGAKDTADAGTASSPKKPDPVSVTSPRCIPP
ATMINGGRKKWRLSDMLLFRRSAAKARAAGANISKEPVFKYSPVQQLGTPVKKATAGQSA
AANGDVSAGKHKKQSKKATAAEDGMASPHRQSVMGCVRLNPGLHRLAKGFNGSSLHFGHR
RAAARSVMNR*
>11680.m01085|LOC_Os06g11150.1|genepair1967-2
MEVMVRPASPPPRHHDFRFDSPAASPYATALSSPRGRLATATFLTAPPSPDPFEAVMAAQ
QQPETPRLTHANPFDLFQHFSSAPASPRRAAAIYAHFAEGGNGGGRDDGEDEEEEDDDDD
EGFRPRASYTVNASSVPFDWEERPGTPKAGLGGGGGGAAWDTDFEFGTVVDKAAPEENLT
TADELFEKGKIRPLKAPLPKTADELFDKGKVRPLKPPPGLLDGGSVASSPRSPMSRGGGM
WSPRRRSRVGSGVDFDPFAAALLEATKAPSPSPSPLGVAASGSPAKKADQFTTRPASKSA
GWRRWRLSDLLLFRSSSEHGRVTKDPIFKSSPARHPDSPVKKASARPTTTPGKANGKADT
ASKPRKHAGDKNAAAAAEGILGSVRLSPLQRLARGLRGSSWYHGHGGMAKLGTKG*
>11668.m05141|LOC_Os02g52480.1|genepair1968-1
MGKYMRKFRGATGEELAAMEVTQVVGVRTRSRSAAAAGATTTKVKAASAASTRRRKALLP
TAVVGTTRRDGGSCYLQLRSRMLFMAPPRPAPAARAPVVAEAAGSGNGAAAHAAAGLSRC
SSTASSVDAAAQDRSLACRSDVAEAGSEHVPEGSASDSASGRDRERRETTPSSFLPGEVS
DLESDLAGGQKRSRPLPSAATASAQQATRPKIPPAAEIEAFFAAAEEAEAKRFAAKYNFD
VVRGVPLDAGRFEWTPVVSSRS*
>11680.m01075|LOC_Os06g11050.1|genepair1968-2
MGKKKKRDGAAARRQARVVVGGVRTRAAVTARRVVASAEEGCGLVGRGGGGGSGGDDGEG
GCYLRLRSRRLPFVAAAVVSSRREEALGDSVAEAASSSSSRAVELLGCSGEEEAMAEKVC
TQAGEDHDEESSVGDSGCGRERSATTPSSRRPPGDADSSDAESNQEAKQQMCRRSSTTSA
AAFHAGATTRSFRMMAPPAAAAEIEEFLAAAERSEAERFAAKYNFDVVRGVPLDAGGAGR
FEWTARFDSDSNWAALGQRVTRCDAESSRGPIKRVPDLAVLADRHSGELPGVDVFVTTVD
PVDEPILYTVNTFLSILTTDYPVDSCKPRLRLAASAENPVDFSGVDVRLPMLVYISREKP
CAGKRQCSNPAAASPDLAGASTCDSTHTRDVTWLLASESPKNSARRLRVH*
>11668.m05147|LOC_Os02g52540.1|genepair1969-1
MAFMRYHRALPQGETTVEEFRAWLSQFDANGDGRIGREELERALRSLNLWFAWWKAREAM
READANRNGVVDRDEMVRLYAFAQRHLHLKMNDLDDVASY*
>11680.m01073|LOC_Os06g11030.1|genepair1969-2
MAFRRYRALPQGEVTVEEFRAWLGQFDADGDGRISRDELQRALRSLNLWFAWWKARAGVR
AADANRDGAVAGDDEVATLFAFAQRHLNVKIAELGASYY*
>11668.m05149|LOC_Os02g52560.1|genepair1970-1
MDVKRARSPRAPGVDADDDKKRAAEWRGAVRPHMVLVGFLITLPVLVFVFGGRWGSFQTT

SAPNVGGRHVVPGGVTTTQKNEAPKNVSVPATATKSLPQPQDKLLGGLLSAAFEESSCQS
RYKSSLYRKKSPFPLSPYLVQKLRKYEAYHKKCGPGTKRYRKAIEQLKAGRNADNAECKY
VVWFPCNGLGNRMLTIASTFLYALISNRVLLMHVAAEQEGLFCEPFPGSSWVLPGDFPHN
NPQGLHIGAPESYVNMLKNNVVRNDDPGSVSASSLPPYVYLHVEQFRLKLSDNIFCDEDQ
LILNKFNWMILKSDSYFAPALFMTPMYEKELEKMFPQKESVFHHLGRYLFHPTNKVWGIV
SRYYEAYLARVDEKIGFQIRIFPEKPIKFENMYDQLTRCIREQRLLPELGTAEPANTTAE
AGKVKAVLIASLYSGYYEKIRGMYYENPTKTGEIVAVYQPSHEEQQQYTSNEHNQKALAE
IYLLSYCDKIAMSAWSTFGYVAYSFAGVKPWILLRPDWDKERSEVACVRSTSVEPCLHSP
PILSCRAKKEVDAATVKPYVRHCEDVGFGLKLFDS*
>11680.m01068|LOC_Os06g10980.1|genepair1970-2
MDTDKLGEAAAAHPPEAEKRRGVAAPGAATVLVLVALPLMLVSYFFGDLAADTVVRLHRF
KESSLSSSSPAAAADRLLGGLLSPEFDEASCLSRYEASSRWKPSPFRVSPYLVERLRRYE
ANHRRCGPGTARYRDAVARLRSGDGDGDAECRYVVWLPIQGLGNRMLSLVSTFLYALLTG
RVVLVHEPPEMEGLFCEPFPGTSWLLPPDFPYKGGFSAASNESYVNMLKNGVVRHDGDGG
ALPPYVYLHLEQIHLRLQNHTFCEEDHRVLDRFNWMVLRSDSYFAVALFLVPAYRAELDR
MFPAKGSVFHHLGRYLFHPGNRAWGIVERFYDGYLAGADERLGIQVRIVPQMAVPFDVMY
EQILRCTREHGLLPQVTSTSESAGGRPPPPPTATATKVKAVLVVSLKREYYDKLHGAYYT
NATASGEVVAVYQPSHDGDQHTEARAHNERALAEIYLLSFSDAVVTTAWSTFGYVAHALA
GVRPWQLAPLDWGKMRADVACARPASVEPCLHSPPPLVCRARRDRDPAAHLPFLRHCEDV
PAGLKLFD*
>11668.m05157|LOC_Os02g52640.1|genepair1971-1
MDLKERIRRSPTPPTQGEELLAASPAGPRGGRKGRAVVLPLSAAALVACAVVLLLLAGGS
AARRGQFVGADPTVLPSRGGGVGDLHLSQSKSNDGENVTIASSEVVNDKLLGGLLTTGFD
EQSCLSRYQSVLYRKASSHFPSAYLLERLREHEALQKKCGPHTESYKKAIEQLKAGQGAK
VMECNYLVWVAYSGLGNRILTMASAFLYAILTRRVLLVDSDKGTADLFCEPFPETSWLLP
PKFPIKQFKNFSNGSPESYGNMLKNKAIRSNPAFLYLHMAHDYSDYDKLFFCEDNQQYLR
NIPWLILKSDNYFVPSLFLIPAYQEELTRLFPQRDSVFHHLGRYLFHPSNVVWGMVTRYY
DSYLARADERLGIQIRVFDPEPGPFQHVLDQVLACTLKENLLPAINSKQPIVSTRHSRLK
SVLITSLNSGYYEKIRSMYWEHPTTNGEMISFHQPSHEEHQNSDKKMHNMKAWAEIYLLS
LSDVMVTSAWSTFGYVAQGLSGLRPWLLFKPENRIAPDPPCRQVLSMEPCFHAPPFYDCK
AKRGADTGKFVPYVTLYLSYRKAIFDMVNTNAVV*
>11680.m01067|LOC_Os06g10970.1|genepair1971-2
MNSEGGIGAGITNRPSQQCSAAAAEDGDDYERPCPWIPGKKKKKKITCLAICLIASPILI
LLVSRRGSPFPSMSGWASPSRMYASKGSKRDVLMGGLLVPGLDERTCASRYSSAMYRKNT
ARSPCRHLVKRLREQEALQRRCGPGTAAYWRAAERLGSRRNGTAGADDEGCKYLVLVPYR
GLGNRMLAMASAFLYAMLTGRALLVDRGESLADLFCEPFPGTSWLLPPEFPIKNLQDLTG
EAPESYRNLVQSDRPATSVSELPYVFVDLDHGCTYHDKLFYCDDERHFLHRAPWLLMRTD
GYFPPALFLNPAYQDELDRLFPRKDSVFYLLAHYLLHPTNKVWGLITRFYDSYLRDSDER
LGIQVRVFDGDTPFKHILDQITACTSQERLLPEVVEQEPSSFPAPAAAATAARSKAVLMT
GLNSWYYDNIRSRYWQSPTATGEVVRVHQPSHEEHQLSGSTTHDMKAMAEMYLLSMTDAI
VTSGWSTFGYVGHGLGGLSPWVMFKPENLTTPDPPCRRAVSMEPCLHGPPFYDCRVKRGA
DTGKLVPHVRHCEDMSWGLKLVHPE*
>11668.m05165|LOC_Os02g52720.1|genepair1972-1
MEGSNLKSAALLEQLRVHLASGAGKELVEKIGFVYQLNISPKKLAFDEEVFVVDLKKGVV
SKGPYEGKPDATFSFTDDDFLAISSGKLNPQMAFIMGKLKIKGSISAAQKFTPDIFPKPS
KL*
>11680.m01059|LOC_Os06g10890.1|genepair1972-2
MDGSSLKSAQLLEQMRLHMATDAGKDIAKKVGLVYQFNIAPKKIGVDEEIFVVDLKKGEV
TKGPYEGKPDATFSFTDSDFLSIATGKMNPQIAFIRFCRWLMNYENCVQRRDKDQGEHKR
GAEVHPGYLPQAFQTVEEWGEKTGNRNNTRVPDVMAGNRISLWVILVLEFDGVMLG*
>11668.m05171|LOC_Os02g52780.1|genepair1973-1
MDFPGGSGRQQQLPPMTPLPLARQGSVYSLTFDEFQSTLGGVGKDFGSMNMDELLRSIWT
AEESHAVGAATTTTATTASVAAAEHAAVGAPPVQRQGSLTLPRTLSQKTVDEVWRDMMCF
GGGGASTAPAAAEPPPPAHRQQTLGEITLEEFLVRAGVVREDMSVPPVPPAPTPTAAAVP
PPPPPQQQTPMLFGQSNVFPPMVPPLSLGNGLVSGAVGHGGGGAASLVSPVRPVSSNGFG
KMEGGDLSSLSPSPVPYVFKGGLRGRKAPGIEKVVERRQRRMIKNRESAARSRQRKQAYM
MELEAEVAKLKELNDELQKKQDEMLEQQKNEVLERMSRQVGPTAKRICLRRTLTGPW*
>11680.m01058|LOC_Os06g10880.1|genepair1973-2
MELPADGSALARQGSIYSLTFDEFQSALGSAEKDFGSMNMDELLRNIWTAEESQAIAPAA
AAASAAAVVGDAQQQQQPIQRQGSLTLPRTLSQKTVDEVWRDIMGLGGSDDEDPAAAAAA
AAPAQRQPTLGEMTLEEFLVRAGVVREDMGQTIVLPPQAQALFPGSNVVAPAMQLANGML
PGVVGVAPGAAAAMTVAAPATPVVLNGLGKVEGGDLSSLSPVPYPFDTALRVRKGPTVEK
VVERRQRRMIKNRESAARSRARKQAYIMELEAEVAKLKEQKAELQKKQVEMIQKQNDEVM
ERITQQLGPKAKRFCLRRTLTGPC*
>11668.m05177|LOC_Os02g52830.1|genepair1974-1
MAGAGEYGGGGGGGGGFGGDFMVLRPDKGGIGDLFHLLWSCKVAENAAVDCPIGTEIAER
RRRWALFVSLVAQMLLLWTKKPMALLGGGTEYWMNLLNENGGSVFMLITNALQGKVKMPD
KSSINYRSCIGLLDTRIDLDEKIKPEDRNYHAALSIMAAKLAYENELVVRTVVQNHWQMN
FVGFYNCWNEFQEDYTTQAFMVSDKAEDASLAVVAFCGTKPFDTEQWCADVDFSWYEIPG
VGKVHGGFMKALGLQRNGGWPEQPTGAGDDGGSDKKPFAYYVIRERLRAFLAENPRARFV
VAGHSLGGALAILFPTVLALHGEEDMLARLHGVYTFGQPRVGDEGLCRFMDGHLATPVSR
YFRFVYCNDIVPRVPYDDTALLFKHFGTCLYFDSFYKGHVTEEEPNKNYFSLLTVVPKYA
NAAWELARSFVIGYVDGPEYGEGWLMRIARAAGLLLPGLPPHAPQDYVNATRLGAASLEP
LR*

>11680.m01055|LOC_Os06g10850.1|genepair1974-2
MDDDDDCRRRDVTLGDEFMVLQPENGGARSLADLLLSCKVGENKAVRCGRPGAEVALPWH
RWIIAVSLLAQMLLRLSKGVMAKVGRAVEYWMNLVSENDNVLGLIRNALHGKVKTPDRNS
PNYRSFIGLLDTRIDLDEKIKPGDSNYHAALCIMAAKLAYENELVIKDAVEKNWKMTFLE
FFNCWNDFQNDYTTQAFMFADKPEDAELVVVAFRGTQPFDMEQWSTDVDISWYEIPGVGK
VHGGFMKALGLQNNAAAGKKPSWPAEIAPPSAAKNSEKTKSFAYYAIRARLRAFLAANPR
ARLVVTGHSLGGALAALFPVVLALHGGEADAAALGRLDGVYTYGQPRVGDAALGEWVAAA
SSLEGKHLRFVYCNDVVPRVPYDDAAFLFRHFGRCVYFDAAYRARAMAEEPNKNYFSPAF
ALAKHANAAWELARGLAIGRVAGGEYAEGWAMRAARVVGLVFPGLPPHAPQDYVNATRLA
GASLRKLLD*
>11668.m05180|LOC_Os02g52860.1|genepair1975-1
MALSDRSRESLLPSFLYTSSAARSFATGATRFPSPVSPASPAPGAVGGGAPISIQAPREK
IEMYSPAFYAACTAGGIASCGLTHMAVTPLDLVKCNMQIDPAKYKSITSGFGVLLKEQGP
RGFFRGWVPTLLGYSAQGACKFGFYEFFKKYYSDIAGPEYAQKYKTLIYLAGSASAEVIA
DVALCPFEAVKVRVQTQPGFARGLSDGLPKFVRSEGALGLYKGIVPLWGRQIPYTMMKFA
SFETIVEMIYKHAVPVPKSECSKSFQLGISFAGGYIAGVFCAIVSHPADNLVSFLNNAKG
ATVGDAVKKLGLWGLFTRGLPLRIVMIGTLTGAQWGIYDAFKVMVGLPTTGGVTPAPGAA
EKALQASA*
>11680.m01051|LOC_Os06g10810.1|genepair1975-2
MALSDRSRESLLPSFLYSTAGARPYTAGGGGAAFRLPAAAAPGGVGGGAPIEIQAPREKI
EMYSPAFYAACTAGGIASCGLTHMAVTPLDLVKCNMQIDPAKYKSITSGFGILAKEQGVR
GFFRGWVPTLLGYSAQGACKFGFYEFFKKYYSDIAGPEYAQKYKTLIYLAGSASAEVIAD
VALCPFEAVKVRVQTQPGFARGLGDGLPKFIKSEGALGLYKGIVPLWGRQIPYTMMKFAS
FETIVEQIYKHAVPVPKSECSKSFQLGISFAGGYIAGVFCAIVSHPADNLVSFLNNAKGA
TVGDAVKKLGLWGLFTRGLPLRIVMIGTLTGAQWGIYDAFKVMVGLPTTGGVTPAPSTSD
AGLKAVSA*
>11668.m05181|LOC_Os02g52870.1|genepair1976-1
MSSTMPAQGGVRHHRTCRMYWCYQCGRAIRIISYPSTDVFCPRCFGRFLHEIDPPPRPAP
PPPHFFPQPYHPHYDGHPRRWLIYGGEAPPVAAPGRAFRQPAPAVPGRAFRQPGPAPAPS
PAPAPPRRRMPSPPPVARRPSTPPAIDPGNYFNGPNLNNLIEELTQNDRPGPAPAPSSAI
DSLPTVQITGAHLSDGSQCPVCKEDFELGEAARQMPCKHVYHSDCIVPWLRLHNSCPVCR
YQLLSSAAAGSNANSRARRGSANNGGGGGGGDGRDREQTIVRWGPFSWMWPPRGLEDPDD
GWEYGRRGRPEAGDAGGRMCNDHGNFRKLVTMMDG*
>11680.m01050|LOC_Os06g10800.1|genepair1976-2
MSTGGNGGGVGVRRRRWNLYWCYVCRRALRVVVPSATSDVYCPRCFGRFLHEIDLPVPRV
SPPAEDQFFQPPFLPYDGPRRWVLYTGGGGGGDYGGADVTARRRRLPSPPPAPGTRRQDG
AGDGDPPPPAPAIDPGEYFAGPDLNALIDALTQDDRPGPPPAPESAIESLPTVHISPDHL
PADGGSECPVCKEEFELGEAARELPCKHAYHSDCIVPWLRLHNSCPVCRQEVPPPPEPDG
ESPGIDGGCDDGVGGGGEPAEPPRPAMAGWDPIALLAIALRPDLNGWENSHGRSESEADD
DEVAGGGVSTTAMIHSFFVVAACFLFISFLV*
>11668.m05182|LOC_Os02g52880.1|genepair1977-1
MVKSGQEAAPGSGGNAAAAAARQGGGGGGRGRQYKGVRMRSWGSWVSEIRAPNQKTRIWL
GSYSTAEAAARAYDAALLCLKGSAADLNFPVHLPFHIPAAAMSPKSIQRVAAAAAANATS
PLQHHSGAPSFATATGGYNPAAAPTTPPCSYGDMSSCSAVSSPETANYYGADHDMVARED
DVDYAALADIDAFFQSPKCMDYSMMDPCSTFFSPAPESLAAEWEDEASHPNPRGRDSLAF
ALLVSFLPSAAAAAAARAMGYTKEQLLARLQELNIEFSCYDHPVVLTVEEQAKHVGHLGG
ALSKNLLLKDKKHRLYVVSALAGTKVDMKILSQRLGLGKGGLRMAPEENLLEVLQVPLGC
VTPFALLNESASFFHGYAIAFLTDSYNCVFASSAVSLLLDQGFKSKQSCYFHPLTNDVTI
ALSSSNLDKFLMSIGRQPAYVDLEASPVVGKDNPPDLADFVPSGVPNSAEPIEKVTPTNV
PRQNDVPKEKTCLPEVKAKPKVQNKGAEKTQSKIPTNGANVEKFVNDVFDIMSPLFLSEV
SKKLNVKQEELSSIFDGFKEQATIDLESVTTSLKNAAYTAGFEAGFETMLNSGLKGQASR
K*
>11680.m01048|LOC_Os06g10780.1|genepair1977-2
MEGVQHMEATSKCQTRHTHTEQQQQQQHQTARHNERSLAFQAIKRFDMVKTAASNGAAA
ARRVGGGGDGKRAAYKGVRMRSWGSWVSEIRAPSQKTRIWLGSYSTAEAAARAYDAALLC
LKGSAAADLNFPVRLPFDLPAAAMSPKSIQRVAAAAAANANANASSSCSAAVFAGVDDSG
GASASEASTPACSSSDGAASPSPVSSPETVISDVDVDYSLLADIEAFFQSPKCMEYAMMD
PCSAFFAPPPPPAMAMEEECGWEEEGDIALWSFSSLD*
>11668.m05187|LOC_Os02g52920.1|genepair1978-1
MAAVAATSPALTLAVAAAVIAAACLLLCAEAVWLDLPQSGTKCVSEEIQSNVVVLADYAL
MYESHPSSHPTIAVKVTSPYGNTLHHNENATVGQFAFTTSEAGNYLACFWIDSAEKGSGI
SINLDWKIGIAAKDWDVIAKKEKIEGVELELRKLEVAVQSIHQNMIYLKAREAEMRTVSE
KTNARVAWFSILSLSVCIVVSILQLWHLQGYFQKKKLI*
>11680.m01046|LOC_Os06g10760.1|genepair1978-2
MAAVAAMVVVVALLLGGGAVEAVWLDLPPTGTKCVSEEIQPNVVVLADYALMYESHPTAH
PTVAVKVTSPYGNTVHHNENATTGQFAFTTSEAGNYLACFWLDSPEKGSGVSLNLDWKIG
IAAKDWDTVAKKEKIEGVELELRKLEAAVESIHHNLLYLKAREAEMRTVSEKTNSRVAWF
SILSLGVCIVVSVLQLWHLQGFFRKKKLI*
>11668.m05188|LOC_Os02g52930.1|genepair1979-1
MGVGRVMNDAKPYLAMILLQVGFAGMYVVAVASLKRGMSHFVLVVYRNLFATAVMAPFAL
WFERRVRPRLTLIIFLKIMGLAILEPVLDQNLYYMGANLTSAGFASALINVLPAVTFVMA
LVLRMEKVKLKSVHSQAKIAGTLFTVAGAVLMVLYHGPVVQFPWTKGQHHDGGSGAGGAA
GGGFLQGTIFIVVACVCWSGFFVLQSNTLQSYPAELSLTTLICLMGSVLSGTVALVAERH
NTHAWLIGFDTRLFTCVYAGIVCSGVAYYVQGIVSRQRGPVFVTAFNPLCMIITAIMGSI

ILKEEINLGSVIGAVIIVIGLYALIWGKGADKVEQTDAGAAAAGSNKGGGELPLTAVPNG
HGSKHGNGGHVYDVETPPAANGHY*
>11680.m01045|LOC_Os06g10750.1|genepair1979-2
MGVGRALSDAKPYVAMVLLQVGFAGMYIVSVASLKRGMNHFVLVVYRNLVATVLMAPFAL
LLERGVRPKMTLRIFLKIMGLAILEPVLDQNLYYMGAKLTSAGFASALVNILPAVTFLLA
VLLRMEKVRLRSLHSQAKIAGTVFTVAGAVLMIMYHGPVVQFPWSSSASGSAGHHVDGAA
AAAATASSASSWLNGTVMLVGSCVCWSGFFILQNNTLQSYPAELSLTALICVLGSAMSGA
VALVAERRDMSVWVIGFDTRLFTAVYSGIVCSGVAYYVQGLVTRARGPVFVTAFQPLCMI
ITAVLGSTILKEEITLGSVIGAVIIVVGLYALIWGKGGDHADNGKPPAAAAAAPEKGLPL
TTLQANGDGDGKLAVLVADVEMPAVKDVY*
>11668.m05191|LOC_Os02g52950.1|genepair1980-1
MDAGAAPASPSPPRSSKKRSSRPKPRAGDAARHPAPNPSPPPAAAAPASSRSRERERKRR
QRGAFADPAAVTAPAAGGQHGGAVQKLWGDADEVALLAGAAAFRARAGHVPRLPDMGALF
DSIRGSLSPHIDQAKVYYKLKRLKGKYLHAAPGASAGPHERRVRDLCASVWGADLEPLAE
GDDERAAAAAAAAAADQPRTVPDAAAMLPVLTEMLDEYWKTDGRALSSVSLAKGLSLLGT
EEARFIEGKWRRQLDSEIQTQMRRHDLAKEVYALLMDAIKALGP*
>11680.m01041|LOC_Os06g10710.1|genepair1980-2
MSSSRRPPARAAATEAAADAALPLHPSSPRSKKRSSSSRRAAAGDRRPAARAPNPSLSPR
GGGGAPSRKSERRRRPRSLAMAVHGHASTSGGPGLVWNDADEVALLTAAVAFRARNGFAP
RLPDMGALFESLRGSISSHIDQAKVYYKLKRNKSKFLHAPPQATTTTPHDRRVRALSAEL
WGSELAPPAVEGDADAAEAADERDAEEGYIGGNLHVSVRLPVVSEVLGDYWRKNGRVLSG
VSLERGLALVGPEEGRMAEAKWKRQLEVETQTQGRRHDLAKEVCAMLIDAVRGLGP*
>11668.m05192|LOC_Os02g52960.1|genepair1981-1
MARAEGLPEGAAPGVGVDLYAQARKALSVRTPFEGEGTAPRVPTLPARLVNWSGQSDARK
KHKKIQPQDVADVELPPQPATEPSAKTGVWEQFEAYFRPVNLDDIDMLMPKFPFGYGGLD
SCILIPFVGSGKELMDTAETFDVAVAETSSYLGLGGEERVSNKEHSERSERSEQSVEQGI
HEVIVRQFVGNKERGEQSVEQGIHEVVVQQENWPLEVEQATSSAGIVSSKCEEEGESSLN
WLLGSKERFVLTSERPNKKRKLLGVDAGLEQLVLLPRSGAEASSVCDVCCLGESSTVSNS
MLNCNRCKVTVHQKCYGLHVVPDGQWLCTWCKDLESLQSLKKDADNTLSMPCVLCPKEKG
ALKPVKGEPGQTAHGGNLKFVHLFCSLWTPGALVEDMESMEPVTNVGSVQENQWKLVCSI
CKVKHGVCVRCSHGTCRTPFHPICARESKHQMEIWGKFGYPNVELRAFCSKHSTIGYANS
LERSNCASHQSPTEARLKDANLITGKVPKLRFTRKNKDKFMNYEATSFNSSNLIKVETIE
QASLPHTVRSSDSLAIQGMEVDTDNLSVGGNLMRNSADVALVLRKLIDQGKVSVGDVASE
VGISSESLEAALVGETTTFSHGLKLKIIKWLQNSAHIPAAQAKILKGGPMVVHNSKPGRS
EDTNSVNMKNSLVPNDEKGTTAYLSDSAVMKSSSTRSKDNNKIMRDNTAVCATGVTTLLQ
NGIKKMAEAGAERECSSPAEDCAKGTPKEEHGGLISNNISGNTQFGTSMAIPNENKGTSP
GKKRYNLTEAEPGSELEGVSSLNQYFPPGDNVKHELNLVENGVGNNHDCNADHVPGQPFS
NFNDSHYYIHPLIKEKMTQLWDNTFKQDKLAPCHPEDPLCYPDERRRVGSSIKLTETTDV
MDQVSRAKSLGILEHSPDDEVEGEMLYLQARLLDTAAFLKHRYEDLIAKVVQNLSRELDA
FSRRKWDFIFVNQFLRDVREAKKRGRKEKRHKEAQAILAAAAAAVVASSRNSTVRKDAND
DVVPAKQENSPKFGTGPPNVGQRTSSLLRLKDLSKPPNNKISQDNNRSTFHMPNYSKENA
LYCDVCMRSETVLNRIFVCSRCKAAVHIGCYRNIENISGPWKCELCEDISPEDTCVGDQS
DCNGTNLSLVQCDLCHGTSGAFRKTADGQWIHAFCAEWLLETEYVRGQDSPVKGMESLVK
DKDTCCVCLHTVGACLKCNNGDCQTTFHPYCARHAGFYMNTKGSGGILQHKAYCSKHSIE
QKEADMQQYGLEEFNNMKRMRVELEKLRLLCERIIKREKVKRERVLCDHDILAKTKDTLV
FSYLTHGASSESATTSVNNKSYSGTMQRSDDVTVDSTISGKKAIRFSLNNRDAEINTADS
SRTLISFKRKFSERGSLAGKQLPRRPVTSQKLEAGEKKTKDKKNRETFQKELFMTSDQAS
TQNQRLPKGYAYVPRDSLSKDKLRNRNTQAHEPQEPGG*
>11680.m01038|LOC_Os06g10690.2|genepair1981-2
MLHCSSCKVSVHQKCYGVHVVPDQSWLCAWCKSIRSARRQTRSDAGRTVLMPCVLCPKEK
GALKPVKRDSGQIADGGNLKFVHLFCSLWTPEVVVEDLNSMEPVTNVGDIQENRTKLVCS
LCKVMHGACIRCSHGACRACFHPICARESKHQMEIWGKTGNTNVEMRAFCLKHSTVQETI
SIQNDRICAEEDTSQIELDDASLATQKIQQLRLTRNNKDKFTSSMIASSCSSSLKQTTEL
ATSPSTARSVESQETQITDMAVDRPIGDRCLVSNSGDVSTALRKLIDQGMVNVGDIESEL
GVSSESLEAALVPETSTFSPGLKLKIIKLLQNTIRVPSVQEKCSKEGSLALQGTVFTGES
KSLTDTQICSELEEGISSFDHCCPEGDNTNKDWADSVENGFHNCGEDCISGKCFLNQDGS
RCYVHPFIERKLRILWDHIFKQNKHPIHCHEQSTCDPHDRIAGSSSTKLEQLADIAVADQ
VSKAKSSGILEHSPHDEIEGELLHLQSRLLDDVGGAKQRYEDLVLKIVQSLSHDLDSFNK
RKWDHIIANQFLRDLREAKKRGNTERRHKEAQAIMAAAARCILPTSRNAPVRKVAECDVL
SAKQESVPVAVPAKQEVHSPKQESIPKFNTGSSRVSQLISVQQANDSSPNSKVSADANIG
SFDLAKFSKKNALPCDICMRSETVLNRIFVCSSCKAAVHLDCYRSVTNPTGPWKCELCQE
MPSDVVAGSQSDCDGSKPCLLQCDLCHGTSGAFRKTIKGRCIHAFCAEWLLESTFTRGQY
NAVDGMESLPKDKDTCAICHRNVGSCLKCSTVDCQITFHPTCARDAGFYMDTKTIGSTLE
HKAYCGKHGIEQRKADLLQLHGPEEVKNMKQMRVDLEVLRLICERVVKREKLKKDLVVCG
HDTLAARRNSIAYSTRTSYCGSGPGASSESATTSVNNSYSGLMQRTDDVAVDSIISRKPT
VRFSLNNSDADRNTADSSTSSISYKQKLDDRESLADKNLPKKPATAMQISEEGETKSSDK
KNQRPPKSIVYTRRSALSKKRQLSQNVEGPGG*
>11668.m05196|LOC_Os02g53000.1|genepair1982-1
MAGVCGSVAAAAAMVVVVMVSSLPGGVEAKTTIEPCTGSDSCSALLGYTLYADMKVSEVA
ALFGTDPAALLAANALDFGAPGAAHRILPMGLFVRVPTRCSCTDGVRKSVSVRYAARPAD
TLATVADGVFAGLAFADQIRNANAVASADPDAPLDPGQKLVVPLPCVCFNSSDNNLPAVY
LSYVVQVGDTVPAIAASYETTVTDVMNVNAMGSPIAAPGDILAIPLPDHGLIVANGTYAL
TAGNCVQCSCGPGNLNLYCTPASLTGSCPSMQCSNSNVLLGCNVSSCSYGGFVNGTITTL
LSTGLQSRCPGPHQFPELTEPPTTVNHDSTFLPPLSAPGPAEAGGAIPPPNSGPSVQGG

SFTLPKVSTANGPAGSVSEAPWMNKPHQILSSFILCLLLLYSQM*
>11680.m01034|LOC_Os06g10660.1|genepair1982-2
MGSPIVAPGDILAIPLPACASMFPNSASDYGLLVANGTYALTAGNCVQCSCGPGDLKLYC
TPASLTASCSSMQCPNSNLMLGNVTAQSTSGGCNVSSCSYAGLVNGTIATSLSSGLQPTC
PGPHQFPPLRATPIAVNQGSYLAPSPAPGAGEAGGDIPGFPGSSNVSPANGPSGSVSQAA
SVNRPHQIVALILSVALYFQM*
>11668.m05214|LOC_Os02g53160.1|genepair1983-1
MQLEISPRQRSQQQKEEEGEHQQRAGEEAVGAVFSIEPWVDAAAVLVPPLNFAEVNDGIF
RSGFPAADNFAFLLSLKLRSIVYLCPEPYPEENTRFLEQNGIKLHQFGIDGSKELLVNIP
EEKIREALKVILDVRNQPVLIHCKRGKECAYVQQQKIFFWCKNYLLTGWIALNTRYPSLL
CPDF*
>11680.m01033|LOC_Os06g10650.1|genepair1983-2
MKLEVMPKQRAMEAEQREEAMEMSGLELWKHEKPASMVVFLPPPPPPPPLVPAAAAAAAA
ACGEEATLVPPLNFAMVDDGIFRSGFPAAANFRFLKSLNLRSIVYLCPEPYPETNAEFLA
KNGIKLHQFGIEGRKEPFVNIPDDKIREALKVVLDVKNQPLLIHCKRGKHRTGCVVGCLR
KLQKWCLSSVFDEYQRFAAAKARSTDQRFMELFDISSLKHLTASHC*
>11668.m05223|LOC_Os02g53230.1|genepair1984-1
MSRGSREEEDEEEVYDSEEEEEEEGEVEERGGKRSRGGGGGGKWSGVESFIDDAASEDED
EEEEEDDDEDYVGGGGGRARKRKRASILIDDMAQVDDEEEEEEDGEFEDGFIDDTRADDP
DQDVGRSSRRHPSSMLDEEEDVDALVKLIHDRYIIPSSHFVDDDDGVTEVEQQALLPSVK
DPKLWMVKCAIGHERETAICLMQKSIDTPDLQIKSVLALDHLKNYIYVEAYKEAHVKEAC
KGVRNIFASRKVTLVPIREVADVLSVQSKSTDISINTWVRMKLGAYKGDLAKVVDVDDVH
QKVTVKLIPRIDLQALANKFDGLKVVKEKKSFVPPPKLFSANEAREPDNDINEDAASLST
LFTNRKKGHFMKGDSVIVIKGDLKNLEGYVEKAEDATVHIRSKLPGLLNTLVFNEGDLCK
CFNPGDHVKVVSGVQEGATGLVVKVEGHVLIILSDTTKEHIRVFADHVVESSEVTTGLTR
IGDYELHDLVLLGESKGLWNIYTEEYFLFMIVTTLNIQALSVQAHNHASLLGDQLGMGTV
DPRFGAFRSSARILQSPGRLPPKAPHTNYGRRFGGRDHGGKGHDTLVNRCIKIKSGPYKG
YRGRVKEMTGALVRVELDSLMKVVAVKREDIADTATVATPFGESHNSWGNETPVHPSRTP
LRPFQTPLRDPGATPVPNGMRTPMPSRAWAPMSPPRLALGWQSMPGTPVPQPHEAPTPGS
GWAVTPGVSFGDASGKNPSSYATPTPSGQPMTPNPASYLPSTPGGQPMTLGYIEMDIMSP
AIGEEGGRNWLLPDVLVNVLREGYDTTCGVVKEVLPDGSCRVALGSSGSGDEITAFPNEF
EVVKPKKNDKLKIMSGSWRGLTGKLLGVDGSDGIVKVDGLETTDQTKILDTAILGKLAA*
>11680.m01030|LOC_Os06g10620.1|genepair1984-2
MKLGIYKGDLAKVVDVDNVRQRVTVKLIPRIDLQALASKLEGREAVKKKAFVPPPRFFNI
DEAREMHIRVERRRDKDSGEYFEMIDGLMFKDGFLYKTVSIKSISTQNIQPSFDELEKFR
KPGDDMNGDMSSLSTLFANRKKGHFMKGDAVIVIKGDLKNLEGWVEKVEDETVHIRPKIS
DLPKTLAFNEKELCKYFKPGDHVKVVSGVQEGATGMVVKVEGHVLIILSDTTKEHIRVFA
DHVVESSEITTGITRIGDYELHDLVLLDNLSFGVIIRVETEAFQVLKGVPDRPEVVLVKL
REIKSKIDRRTSAKDRSNNMISSKDVVRVVEGACKGKQGPVEHIHKGILFIYDRHHLEHA
GFICAKAQSCLLVGGSAGGRRGNGMDTSDPRLGALRSPASILQSPGRLPPRGPHMNFGGR
FGGGGRGGRGHDALVGKCIKIKSGPYKGYRGRVKEVTGVLVRVELDSLMKIVTVKRDDIA
DTPTVATPFREPRYPLGGETPMHPSRTPLHPYQTPMRDPGATPIHDGMRTPMRRGWAPMS
PPRDNWEEGNPATWGSSPAYQPGTPPARPYEAPTPGSGWANTPGVSYNDAPTPRESNYGN
APSPYVPSTPVGQPMTPNSASYLPGTPGGQPMTPGNVGMDIMSPIIGGEGEGNWLLPDVL
VNVLRAGDDGPGVVREVLADGSCRVALGSSGNGEIVTVLPTELEVIRPKKSDKIKIMNGN
FRGYSGKLIGIDGSDGIVKLDDTYEVKILDMVILAKLAS*
>11668.m05235|LOC_Os02g53340.1|genepair1985-1
MSDQERDDIPMLLRNVELPTFPPRSTSMCIPVRDDEYEEDTFVPHTGPLFVQPPTQTAAA
GIPFTNTPDMPPRPPQGKQVNKPHAIMPEEIGGNRWSYSGNVPKNEHLMMSGPLGQCDDP
DCVNCPPACKNKRHFHRGSSTLDSKFHNFLCEHGGGWKKEIERFLSRIPVMNPHAKVVQQ
WNQFFVISCLVAIFIDPLFFFLLSVQKDNKCIVLNWHFATALAVVRSVTDAIYFLHMLLQ
FRLAYVAPESRVVGAGDLVDEPKKIAVRYLRGYFLLDFFVVLPLPQVMILLVIPKYVGLS
TANYAKNLLRITVLLQYVPRIIRFVPLLGGQSDSSANGFIFESAWANFVINLLMFVLAGH
VVGSCWYLFGLQRVNQCLRNACSASKIPSCDGFIDCGRGINIGKQNQLSRQQWFNDSAST
ACFDTGDNGFHYGIYEQAVLLTTEDNAVKRYIYSLFWGFQQISTLAGNLVPSYFAWEVLF
TMAIIGLGLLLFALLIGNMQNFLQALGRRRLEMQLRRRDVEQWMSHRRLPEDLRRRVRRA
ERFTWAATQGVNEEELLSNLPEDIQRDIRRHFFRFLNKVRLFTLMDWPILDAICDKLRQN
LYISGSDILYQGGPVEKMVFIVRGKLESISADGSKAPLHEGDVCGEELLTWYLEHSSANR
DGGRMRFHGMRLVAIRTVRCLTNVEAFVLRASDLEEVTSQFSRFLRNPRVQGAIRYESPY
WRTIAATRIQVAWRYRNRRLKRAGMSKLNDQSYNSALERGARECDARQHGRV*
>11680.m01026|LOC_Os06g10580.1|genepair1985-2
MSGQERDDVPMLELQRFPTRSVSMCIPVRDDIYEDSIISHSGPIFTPAPTQYTSVAIPSG
NRDMLDKLPRPKVKSKPHVVTPEEVGISNWPYDQHVPKNKHLMMYSEPLGLCDNPDCVDC
PRACKNKRHFQRSLAPFDNKFHNILYGYGDRWKKKAGHYLSYIPIMKPHDKAVHRWNQFF
VISCLLAIFNDPLFFFLLSVDKDYKCIVFNWNFAIALAVGRSVTDAIYFLHMLLQFRLAY
VAPESRVVGTGDLVDEPMKIAMRYLRGFFVLDLFVVLPLPQVMILLVIPKYVGLSSANYA
KNLLRATVLLQYVPRIIRFVPLLGGQSTNGFIFESAWSTFVINLLMFVLAGHVVGSCWYL
FGLQRVNQCLRDSCAASNISKALCNNCTDCGITGINRTNWLNNSDLTGCFDTKSGNFPYG
IYQQAVLLTTEPGLKRYIYSLFWGFQQISTLAGNLIPSYFVWEVIFTMAIIGLGLLLFAL
LIGSMQNFLQALGKRRLEMQLRRRDVEQWMSHRRLPEDLRRRVRSAERFSWVATRGVNEE
ELLSNLPEDIQRGIRRHFFGFLKKVRLFNLMDNATWDAICDKLRQNLYITGSDILYQGGP
VEKMVFIVRGRLESISADGNKSPLQEGDVCGEELLSWYLEQSSVNRDGGKIKLHGMRLVA
IRTVRCLTNVEAFVLRARDLEEVTSQFSRFLRNPLVLGTIRYESPYWKNLAANRIQVAWR
YRKRRLKRAEMQRLQ*

>11668.m05237|LOC_Os02g53360.1|genepair1986-1
MRIRRRPQSLQALSSLHPSSDPSTAPQPPPRNHGRYWLSVDKVDEEEKKSERLHLHPNAD
LADDDSSAAMRAAAALPLFPQDNAVVECSKIRPRGGAQQGAADGHRSLENGHYSKPDPAI
RSTTGERLVNGVVRAMPVAANAKEETKNDGGGGGGAKKRRGPAVLMEGSRCSRVNGRGWR
CSQPTLVGYSLCEHHLGKGRMRSVTGGRGGAGQLGRTEPRKNTAAAAVAAAPKAAAAVAE
PPVVRPC*
>11680.m01025|LOC_Os06g10570.1|genepair1986-2
MRIRRRPQCVSLQSSDPSTSTAATQNAAARSREGGGGDGVGRRLHQLHHHGNVDLGKKSS
GVARRRLALLQQENGVDCSDSKGPGEEHGGAGDAHRSVPLPCTGGEVGSKSEPAAAVAPA
VIVDVKEEEKSVGNGGGGGGAKKRRGGGAPAVLMEGSRCSRVNGRGWRCSQPTLVGYALC
EHHLGKGRMRSVTGGGGGRGGASQLGRTEHRPPATARNPAAAAAPPPKADEPGPNHIAHH
*
>11668.m05239|LOC_Os02g53380.1|genepair1987-1
MKGGAWKQSGVADHVGYLGGGGGALVGRARRARLCLYGLALAFGAFAAFLAFAPSLPAPP
PSSPAAAWFDGLLASASPYRAQVSGYFSSLFPANSTSPEPPGGAATNRRGSSGGGGFSAT
GGQAGTNGSSTVVAGEQGRGVEVSSSNAGGVPSGNSPSGNATAAMQSNPPPNDQAGGGAA
ANNSTTGSAGEAAVPSRSSARNGTMTKDGAPDRINGTDVIASSSGDVTAVKANARNAAGS
THQLGGASAIVDSSNGTAAPSINKTGNAAVATDGNGAAPQRGGAPGKNQTVPNPPAALDQ
NKSGSRAAASGGSNSTMDATPQGIASNTTEAAVDAGGKKKKTHWIEAMASCDMFYGNWVR
DDSYPLYPEGSCPHIDESFNCPLNGRPDNAYQRLRWQPSGCSIPRLNPSDMLERLRGKRL
VFVGDSLNRNMWESLVCILRNSVKDKRKVFEVSGRQQFRAEGSYSFLFQDYNCSVEFFRS
PFLVQEWEFPVRKGLTKETLRLDMISNSFPRYKDADIIIFNTGHWWTHEKTSLGKDYYQE
GNRVYSELNVDDAFQKALITWAKWVDSSVNPKKTTVFFRGYSSSHFSGGQWNSGGSCDKE
TEPITNEKFLTPYPRKMSILEDVLSGMKTPVVYLNITRMTDYRKEAHPSVYRKQKLTEEE
KKSPQIYQDCSHWCLPGVPDSWNELLYAQIMVKQHQMLHQ*
>11680.m01024|LOC_Os06g10560.1|genepair1987-2
MKGLWKQSGLAGMACAADVGAAGAGRRARLAVYVLAVAFAAFTAYVAVSSSSPPPAGEGA
SWFGGVYASTAPYRAQVSGFFSSIFPTGSSTPSPEQQPPPPRRGEGGGQVSSHGIDEHAR
VRSGAAHSVPVDPAASTKHSGSGGGGGAASNNGGGGSAPPPGNLAGSGTPPAKGSGGDGG
GAPANNSTSGGAPANSAVEQSSPAGDGGGSPSTASSSAGKSSSANTGEESVDKSNKQSGS
GGEAPSNGDAVSDKKNSTAKADTEVAVKASSDNSTGTGSSAKGESNVGSNSSAGSGNGVA
SSVSSAAVNSTAVKTDAKDVVVATSTDSAGSVSDVKADLSNRSDTPPASGSGHSNHTSDV
TVSPAKGNAKDGGADTNKASGNVASTSNQTASTAMVAKKAGGSPSKNQTSVASTNSKNQN
QTSAGVASGGSSGTTSKQEETTSQGSVGSSKDHPAQAINSKTSNYSEVLVKGNGSSTKQA
SQKQPDKKVDWIKEMASCDMFHGNWVRDESYPLYPEGSCPHIDEPFDCYLNGRPDRAYQK
LRWQPSSCNIPRLNPTDMLERLRGKRLVFVGDSLNRNMWESLVCILRNSVKDKRKVFEAS
GRHEFKTEGSYSFLFTDYNCSVEFFRSPFLVQEWEMKVSNGKKKETLRLDIVEQSSPKYK
DADFLIFNTGHWWTHEKTSLGKDYYQEGNHVYSELNVVDAFHKALVTWSRWIDANVNPKK
TTVLFRGYSASHFSGGQWNSGGSCDKETEPIRNEQYLSTYPPKMSILEDVIHKMKTPVVY
LNITRMTDYRKDAHPSIYRKRNLTEDERRSPERYQDCSHWCLPGVPDSWNELLYAQLLIK
QHQMLQQ*
>11668.m05253|LOC_Os02g53510.1|genepair1988-1
MASLGYYDDRGAPSSYRYDARQAADGAGTSFHLFIFLATASLLGAASLYSRCESAVESLF
DQVRVVVVLSPLLLLLAMQYWAAAAGGGGRRSGLSSLLMAPLVGEHSGGGGGWYDQRRDG
SSPWGVALALALVLLLISYQSCFQDWWFPLVNRRR*
>11680.m01019|LOC_Os06g10510.1|genepair1988-2
MSMASSGGYYSYYNGHQPAPYYYGYAQPARVAGGGGVGGSQRPSAHALLLVATLLLVAVT
TLYARCEEAVESLLDQLRVLLILSPLLLIVAVQVWAASAAAAAADRRGAGGGLMYLLAQL
MGMGDGGGSPYGRWHGGGGGASSSPWGVALVLVLVLFLVSYQSSFQSWWFPLLSRR*
>11668.m05254|LOC_Os02g53520.1|genepair1989-1
MATRPASRQRRASSAAAAVAVVRSSPQPQQQQQQQLPIPQSGSPTSTTTTTTSSSRLTPE
LSLDGPASPLFAGLDEDPAPKENVTVTVRFRPLSPREIRQGEEVAWYADGDTVVRSEQNP
SVAYAYDRVFAPTTTTRQVYDVAAQHVVSGAMEGVNGTIFAYGVTSSGKTHTMHGDQRSP
GIIPLAVKDAFSIIQETPNREFLLRVSYLEIYNEVVNDLLNPAGQNLRIREDPQGTFVEG
IKEEVVLSPAHALSLIAAGEEHRHVGSTNFNLLSSRSHTIFTLTVESSPCGESNEGEAVT
FSQLNLIDLAGSESSRAETTGVRRKEGSYINKSLLTLGTVISKLTDGKATHIPFRDSKLT
RLLQSSLSGQGRVSLICTVTPASSNSEETHNTLKFAHRAKRIEVQASQNKIIDEKSLIKK
YQNEIRRLKEELEQLKMGIITGTPVKDAGEDNIILWKQKLEDGNVKLQSRLEQEEEAKAA
LLARIQRLTKLILVSTKATQTSRFSPHPGPRRRHSFGEEELAYLPYKRRDIVLDNESNEL
LSPVEGLGMTLEDSKEEKKNRKGILNWFKLRKREGGASILTSSEGDKSSLTKSTAPSTPI
GESVNFPSEPRISNSLVGESASVDLFSIGHGEFATDSLHGEETPLASRKTIDHVDLLREQ
LKILSGEVALHTSVLKRLTEEAGRSPNNEKIQMEMKKVNDEIKGKKHQIASLERQIPHSI
SNNQGMADKLELTPSYAELLEQLNEKSFDLEVKAADNRVIQDQLNEKTTECMELQEEVAH
LKEQLYQTLQAKDSLSNSIMMQKNAGINHETDNHADQELSVPREVPGETSPKEPQSVEID
ELKQKVCELIEVKAQLETRNQKLLEESTYAKGLASAAGVELKALSEEVTKLMNQNEKLAS
ELASVRSPTPRRANSGLRGTRRDSISRRHEPAPRRDNNAGYEREKALEAVLMEKEQKEAE
LQRRIEESKQKEAFLESELANMWVLVAKLKKSQGHDLEDFDTKYIGS*
>11680.m01018|LOC_Os06g10500.1|genepair1989-2
MECLSPKNLIDLAGSESSRVETAGVHQKEGSYINKSLLTLGKLICTVTPASSNSEETHNT
LKFAHRAKHIEIQATQNKIMDARSLIKKYQNEIRQLKEELEQLRRSIRTGTPIEDTMQKK
HHLLETKGDFGLIALLVLRGFVGFGEKFKSSPKEETENQKGHLNWLNLRKCDSGSTNLTS
SDGENPSSTKSLPALSTPLGIGFFNVTSEQRMSDYMLAENVPANLLCVGHREFPSDSLPV
QETPLVSRKTSDHVDILREQFNILSGEVALHQSVLKRLSEEAGKNAMNEQIEMEMKVVND
EVKLNKQKIASLERRISNSMSNSRGMHDNLELSLPYIEIPEQLNEKAFQLEWYNVVVLLH

HAVSTGGYGIIGMKAQKASECQEFLLSERTTFQHNTGIVQETGSQAHKGKPLPSDVSDEF
LKKASQAEIDELKQRVSELTEAKSQLDSCNHKLLEESTYAKGLASVTSVELKALSVKVTK
LMKQNERLSSELASGRNQRRGSHGPRGARRESHTKRYEPARRGDMNALEAMLKEKDQRQA
ELHTKIEESKQKEAFLEKELANMWTVLANLKKTRGIDQEDFDSKYNGSWA*
>11668.m05255|LOC_Os02g53530.1|genepair1990-1
MAKPQDMRSVDSFSQLPFIRPAPPPQQQARDTIRLFGCEFSNDLQLRPTEAGAGSPDAAN
GSTVTSEGSNGGDGGTKNGGAATAAAERKFECHYCCRNFPTSQALGGHQNAHKRERQHAK
RAHLQASLAMHRYMPGHMYGLFNYHHHIGGRFDHHPPPPPPPPPPAHYPMWTSAAPGAFA
GPGSMAQPINGSPVQAGLWSVPPPTENFGSTAGRQGADKLATTVAGTPAAGEVACKDEMV
PMSLLSSSPSLSSCSSTSPEMLGRCELGQKEGVSLDLHL*
>11680.m01015|LOC_Os06g10470.1|genepair1990-2
MAAPHGMHGVAAAAPDATIRLFGRDVVSNDDAVVVVVDGQLPKEEAEEEAGGGAAAAAGE
TRRFECHYCRRNFPTSQALGGHQNAHKRERQHARRAHLEASLAAAHYLGQSAHLVYGGAA
LFGYGGHAAAVSPQYGPVWASSAVAPPGLYATSMGMARPAAYGAGVDVSALWRASSSSSS
SPPMMGSGGGGAFGTVAGGGRHGEAAAAALVGCRAGKDENVVMSVVTSLPSLPSWQLPAP
EKMGRSELGQEAGVVSLELRL*
>11668.m05272|LOC_Os02g53690.1|genepair1991-1
MMMMSGRPSGGAGGGRYPFTASQWQELEHQALIYKYMASGTPIPSDLILPLRRSFLLDSA
LATSPSLAFPPQPSLGWGCFGMGFGRKAEDPEPGRCRRTDGKKWRCSKEAYPDSKYCEKH
MHRGKNRSRKPVEMSLATPPPPSSSATSAASNTSAGVAPTTTTTSSPAPSYSRPAPHDAA
PYQALYGGPYAAATARTPAAAAYHAQVSPFHLQLDTTHPHPPPSYYSMDHKEYAYGHATK
EVHGEHAFFSDGTEREHHHAAAGHGQWQFKQLGMEPKQSTTPLFPGAGYGHTAASPYAID
LSKEDDDEKERRQQQQQQQQQHCFLLGADLRLEKPAGHDHAAAAQKPLRHFFDEWPHEKN
SKGSWMGLEGETQLSMSIPMAANDLPITTTSRYHNDD*
>11680.m00999|LOC_Os06g10310.1|genepair1991-2
MAAGAPVPPDLLLHLRHRAAAAAAADVDTVPSLAFPPHHLGWGCYGAAAAQYGRRVEDPE
PGRCRRTDGKKWRCSREAYGESKYCEKHMHRGKNRSRKPVEMPPPAAAAVYRPSALSISP
PPHDADAPSYGAGAGAPLQLHLDSFHASTSPPPSYHRYAHTSSAPLFPSSAAGYGGGWSL
SKEHCLTLGGAAADLSLDKPADHHHDATSATTEKPLRRFFDEWPRSDDGRTPWDGTQLSI
SIPTAAAASPDLAIAGAASRYHSNALVYIRLMLTPYFC*
>11668.m05275|LOC_Os02g53720.1|genepair1992-1
MTTTTTTRLLLAAILLAVAAADDDGQTLLEIKKSFRNVDNVLYDWAGDGAPRRYCSWRGV
LCDNVTFAVAALDLKSNELSGQIPDEIGDCTSLKTLILKNNQLVGMIPSTLSQLPNLKIL
DLAQNKLNGEIPRLIYWNEVLQYLRLNICNVGQIVRGLRSNNLEGSLSPEMCQLTGLWYL
DLSYNRLTGEIPFNIGFLQVATLSLQGNNFSGPIPSVIGLMQALAVLDLSFNQLSGPIPS
ILGNLTYTEKLYLQGNRLTGSIPPELGNMSTLHYLNLANNNLEGPIPDNISSCMNLISLN
LSSNYLSGAIPIELAKMKNLDTLDLSCNMVAGPIPSAIGSLEHLLRLNFSNNNLVGYIPA
EFGNLRSIMEIFLFLCFSDLSSNHLGGLIPQEVGMLQNLILLKLESNNITGDVSSLINCF
SLNVLNVSYNNLAGIVPTDNNFSRFSPDSFLGNPGLCGYWLGSSCYSTSHVQRSSVSRSA
ILGIAVAGLVILLMILAAACWPHWAQVPKDVSLCKPDIHALPSSNVPPKLVILHMNMAFL
VYEDIMRMTENLSEKYIIGYGASSTVYKCVLKNCKPVAIKKLYAHYPQSLKEFETELETV
GSIKHRNLVSLQGYSLSPAGNLLFYDYLENGSLWDVLHGSSKKQKLDWEARLRIALGAAQ
GLAYLHHDCNPRIIHRDVKSKNILLDKDYEAHLADFGIAKSLCTSKTHTSTYVMGTIGYI
DPEYACTSRLNEKSDVYSYGIVLLELLTGKKPVDNECNLHHLILSKAADNTVMEMVDPDI
ADTCKDLGEVKKVFQLALLCSKRQPSDRPTMHEVVRVLDCLVYPDPPSKPALPPALPQSS
TVPSYVNEYVSLRGGSTLSCENSSSASDAELFLKFGEVISQNTE*
>11680.m00990|LOC_Os06g10230.1|genepair1992-2
MTPAPAAASYRALVALLLVAVAVADDGSTLLEIKKSFRNVDNVLYDWAGGDYCSWRGVLC
DNVTFAVAALNLSGLNLGGEISPAVGRLKGIVSIDLKSNGLSGQIPDEIGDCSSLKTLDL
SFNSLDGDIPFSVSKLKHIESLILKNNQLIGVIPSTLSQLPNLKILDLAQNKLSGEIPRL
IYWNEVLQYLGLRGNNLEGSISPDICQLTGLWYFDVKNNSLTGPIPETIGNCTSFQVLDL
SYNKLSGSIPFNIGFLQVATLSLQGNMFTGPIPSVIGLMQALAVLDLSYNQLSGPIPSIL
GNLTYTEKLYMQGNKLTGPIPPELGNMSTLHYLELNDNQLSGFIPPEFGKLTGLFDLNLA
NNNFEGPIPDNISSCVNLNSFNAYGNRLNGTIPPSLHKLESMTYLNLSSNFLSGSIPIEL
SRINNLDTLDLSCNMITGPIPSTIGSLEHLLRLNLSNNGLVGFIPAEIGNLRSIMEIDMS
NNHLGGLIPQELGMLQNLMLLNLKNNNITGDVSSLMNCFSLNILNVSYNNLAGVVPTDNN
FSRFSPDSFLGNPGLCGYWLGSSCRSSGHQQKPLISKAAILGIAVGGLVILLMILVAVCR
PHSPPVFKDVSVSKPVSNVPPKLVILHMNLSLLVYEDIMTMTENLSEKYIIGYGASSTVY
KCVSKNRKPVAVKKLYAHYPQSFKEFETELETVGSIKHRNLVSLQGYSLSPVGNLLFYDY
MENGSLWDVLHEGPTKKKKLDWETRLRIALGAAQGLAYLHHDCSPRIIHRDVKSKNILLD
KDYEAHLTDFGIAKSLCVSKTHTSTYVMGTIGYIDPEYARTSRLNEKSDVYSYGIVLLEL
LTGKKPVDNECNLHHLILSKTANNAVMETVDPDIADTCKDLGEVKKVFQLALLCTKRQPS
DRPTMHEVVRVLDCLVRPDPPPKSAQQLAMPQRPAVPSYINEYVSLRGTSVLSCANSSCT
SDAELFLKFGEVISQNTE*
>11668.m05279|LOC_Os02g53750.1|genepair1993-1
MRRMLRCFFGGGGDETGGDEEKKASAAAVVKNKKAVRRMRSATGRLRSLSLEDLSRTLAQ
SGLQAFTLAELKAATRSFSGSNFIGEGGFGPVYKGFIDAKLRPGLLQPQHVAVKYLDGEG
DQGHREWLAEVVYLGMLSHPHLVKLIGYCCQDDHRMLVYEYMARGSLEHHLFKNLLSSLP
WATRLKIAVGAAKGLAFLHDADTPVIYRDFKASNILLDSDYTAKLSDFGLAKEGPQGDAT
HVTTRVMGTHGYAAPEYILTGHLTAKSDVYSFGVVLLELLTGRRSVDKRRRGREQNLVDW
ARPYLRRPERLHRVMDPSLEGGYSDKAAGKAAMVAYHCLHSVPKSRPHMRDVVAALEPLL
QTSCGDVLAGPFVYTVPSAAAVVVAAKDDGKKAAAAAGEDGEEEVAAAAKAKRRYVASAV
HAEGAMRKGEHRYASSVSGSPRQSRDRGG*
>11680.m00982|LOC_Os06g10160.1|genepair1993-2

MARGLFACFGRGGEEAEEEAGKRPALRRRRTVNLRSLSLEDLSRTLAKTNLHAFTLDELK
AATKNFSTSNFLGEGGFGPVYKGFVDGELRPGALESQHVAVKYLDSDGVQGHREWLAEVV
YLGMLSHPHLVKLVGFCNQDDHRMLVYEYMPRGSLENHLFKNLLASLPWSTRLKIAVGAA
KGLAFLHEAETPVIYRDFKASNILLDKDYTAKLSDFGLAKEGPQGDATHVTTRVMGTHGY
AAPEYILTGHLTARSDVYSFGVVLLELLTGRRSVDKRRRGREQNLVDWARPYLRRADRLH
RIMDPSLELQYSARAAHAAAKVAHQCLQSVPKSRPCMRDVVDALEPLLAVDDDVPMGPFV
FTVGGEEAAAAAAGSSAAGDAGDDEPARGSRRGKKHVTSAVHAESPLRDGRYASRVKRPE
SPPSVI*
>11668.m05305|LOC_Os02g54000.1|genepair1994-1
MAVPRSAGLGAVGGWLICKKMSSHGFSTKGGDAGSKLPSTVTTLNPNAAEFVPSTFRSPF
GSRTVADVSKPDFRGLSGKTNLGRSESSKSNNSDDETHQFWHRQLPDDIIPDFSDMEKVE
QQHGELSFSGLSLNAPPFFGTAASNLSREHHGLLSQAGKNLDLGHNDLYYDENSGSNSGK
QNHADNLCYTNGKLDLLYDHDPLEYLASQFPGFSVESLAELYCANGCDFDLTVEILTQLE
MQVDASSCQNLNLAPNTPNIGTGNFPVLPGTEDLNCLFEGNVGAHGITNRHNSSTMSRTG
DFVSAIQRLALQDAGHMKFKNESPKYANGLLSIVARKQYGCDTRSSLGNKFLKASSNVHS
APVSLKTGDAMASMCSESRGEAGDFARIRNTCFEQATQAYMMGNKALAKELSMKGQLYNL
QMKAAHETAREAIYQQRNPFSSEQGQDRLIDLHGLQVSEAIQVVKAELALLMGATRLTVV
LSTNVYVLIGIATCVAHEFGVEVCDLYCDGGVDTDKWGDAQIGHYIGIGRIIHTSLSLSM
FDRYPVELRLFDRYSIEPQMFDRTKYQKNVEASHNKGLKTVPNSIRSENYVITFEVEEEK
FPFFGKKYQLKFANESMFENHCLVHFPSFMRTQFAPLLGGYGSSLVDPRGKLVARSFDIL
GDVAFSLQDSLKVPVYSLPDIISAGLYSTFVFQKPDPDAMPPIVTPELHDPENDQEEEWL
WTQQASMDDGRVSRTDILPPADNEKGILGPGPADMRL*
>11680.m00952|LOC_Os06g09890.1|genepair1994-2
MTSLNKVVSNGDTRSMLPNKVTALNPNAAEFVPSCIRPSFESSAVSDVSKADLRASGKTI
LDRSESSKSNNSDDEAHQFWRKQLPDDIIPDFSFEKIEQEPEELSLAGLSLNAPPFYGTT
ASRFSREHDLSSQANKSLELGLTSLLYEDNSQASFPTMGSSNWEQNFVGDLHFTNGNQGL
HYDSESAAGFSDSFASDYAAATDDVLDPLAYLASQFPDFSSESLAELYYANGCDFNHTIE
ILTQLEMQVDATSNPTLTPRTPNFSTGDFPALPTVEDQNGFSKGNADILSIFNGRSSPSV
STGTGDFVSAVRKLASQNSGHWKYKKGPEYGNGVSTVSVPKQYSSTTKTSSGNKFQSVSS
ARAAPWLETGDAVANMYSESRGEARDFARIRNACFEQARQAYLIGNKALAKELSMKGQTY
NTQMKASHEKAREAIYRQRNPSSQRGSDRLIDLHGLHVNEAIHILKVELGTLKSTARATG
ERMQVMICVGTGHHTKGSRTARLPIAVEQFLLEEGLHYTQAQPGLLRVVVF*
>11668.m05310|LOC_Os02g54050.1|genepair1995-1
MEKKYKGVRRRKWGKWVSEIRLPNSRDRIWLGSYDSPEKAARAFDAAFVTLRGHGAAGAD
LNFPDSPPSCDARSSDPRQVQAAALSHANRAHVTPQQAAAALMSPPSLSPPPGFATGSEV
VAPAVRADGSIDWRPVMAHPPPLYSPPGWGGGHAYDFLQPPPPSPPLPSCDDDMVDVVDE
SSASLWSFDTRDSYFRY*
>11680.m00944|LOC_Os06g09810.1|genepair1995-2
MEPVSFMQKSAAAAADGGSAAQAAAERRKYKGVRLRQWGKWAAEIRLPSSCERIWLGSYD
TPEKAARAFDAAFICLRGVQAIAGLNFPESPPPPTAARTGDLREVYAFAVSHANRPSAEA
APADIVVPAQVATEESDGVVRGNAAPPPVQVAAGSLDWSQFMANPPPMYSPTATAGSQAM
WPVTAPAAEADGEDDELATTCRWSFDA*
>11668.m05311|LOC_Os02g54060.1|genepair1996-1
MAPVQFSAAGVGAVAFATKGMASRDALRLPPPAAVRVLRQAPRPSRGLVVRAAAASVAPK
YTTLKPLADRVLVKIKSAEQKTTGGILLPSAAQSKPQGGEVVAIGEGRTVGDNKVEVSIQ
VGSQVVYSKYAGTEVELNDSNHLILKEDDIIGILETDDAKDMKPLSDRVLIKVAEAEDKT
PGGLLLTETTKEKPSIGTVVAVGPGPLDEEGKRIPLSVSAGSTVLYSKYAGSEFKGSDGT
SYIVLRVSDLMAVLS*
>11680.m00930|LOC_Os06g09670.1|genepair1996-2
MSSVQLSGAGVAAVAFTKNGASSFDGLRLAPPSVRVCSSRRPSRSLVVKAATVVTPKYTS
LKPLGDRVLVKLGAAEEKTVGGILLPSTAQSKPQGGEVVAVGEGRTIGDKKVEVSLQIGA
EVVYSKYAGTEVQFNDTKHLILKEDDIIGVLETDDVKDMKPLNDRVLIKVAEAEDKTAGG
LILTETTKEKPSIGTVVAVGPGPLDDEGKRQPLSVSAGSTVMYSKYAGSEFKGADGTNYI
VLRVSDVMAVLS*
>11668.m05319|LOC_Os02g54100.1|genepair1997-1
MAAELERQFAGYKQRRAERSGAPRGDDDGADARGGGGEEEEEEEGDGDGGDVRGRRYEAY
TRRRDERLREREGWRARMERKEAEVRALWAQLERRAAGCATATATATDDDGGGAAGVREK
AGKDGEKRRRSDVAAPASRISGKKHARTRSFSSTATKSSLPDAGARRALSQEPPPPPTSE
RPTTAGAGSHRVARVTGGGATTTAPKPRVFSGHRSSTAKEHGSSSAKGGTTKPKPPRSLP
RRSSSGGLENLKEAVLSNTCAAVAPAQSCSTEQATVHGETGNASPPSPFAGAAAANARAA
SPDSDCGEAVDGGSYDREAEAKRVGEHDAEEVTVSPQKLANGEITSDSDTEPSYVYVKKD
DVEGEEDAMARRSEALAVSDAKPAELEVEKNNSDAAARGEETTAPPSDAVAAESATTIVA
EEAPARESSDESSSSSSSSFSGIRSGRGSPPSSAPASYISRAPSIERLLEEDAALLRKKR
QQSADKLALMAMTTTTMSTPPARVSGAARSRGFKSFLSFGKKNRRGKDVTVIDCTSPSVP
SVADDDSGSGGWPSGETIKPRMASSDAASDDMDHGYAIAASPQGCSLQSLVVASPAKSEL
HEIDPQEKSPKAHRSFFSFRSFNCGRS*
>11680.m00925|LOC_Os06g09620.1|genepair1997-2
MRGGGGDDDLWAKAAELERQFEGYKRRVAERRSSSSAAAADRHDGDGDGGAVEVVAVGKG
RRYDAYVRRRDEKLRQGWRARMERKEAEMKALWARLDVDRRRDGDLAAGNGKQQKPGNLE
ARPAASPATPRSSSATKATLSRPRTTPRTTTPSPAGAAASPRLSSSNPDARRRAPPQPEP
PSTPRKENRVPSAAAASTAAATATPRLRALSRSRSSLKESASSVRDSPRRAPPPPRRSHD
GDAGDRPKQQPEPVHAATTTADDAVAPAARSCQSQQQVVLAEIKAAAAFRLRRSGNGAAQ
GRQPAASPRPVITRQLDGRRKPSDRNSDVEAKNFNLDEGIGEDDDDDTAQSSVEIGSLKI
TGDSDTEPSYVYITKDIDDEAMNTSQPQPLAASDSNAEEPESLAPHQSEKETRHLEETAM

AASSEATAKERPATDREDDSPQSSDQSFYSNVDSSFSHRSELELAASATDSPLHGSPSST
GPSTEQLLEADAAMLRKKREEEEEEEEEDEAAAGEINSLLIPSTTTSSSSSVACPVTVQSP
MEAVAGFKRFLTFGKKNAAAAVAPPADDSGVGHGWPSGDSGVRQRICSSDAASDDSDNSY
VIPAHVTEMVMCSSILAKLCALFSCKACTTEGAHFIGKIPTSTSFVLLVLIVQVQRVLML
AIGDAVNRDNRC*
>11668.m05324|LOC_Os02g54130.2|genepair1998-1
MADGGEKCRDAAGEGGGGGDLYAVLGLKKECSDADLKLAYRKLAMRWHPDKCSSSSSAKH
MEEAKEKFQEIQGAYSVLSDSNKRFLYDVGVYDDDDNDDDNLQGMGDFIGEMAQMMSQAR
PTRQESFKELQQLFVDMFQADLDSGFCNGPSKCYHTQAQSQTRTSSTSPSMSPSPPPPVA
TEAESPSCNGINKRGSSAMDSGKPPRASEVGSGQSQSGFCFGQKSDAKQAAKTRSGNTAS
RRRNGRKQKVSSKHDVSSEDEMPGSQWHGVA*
>11680.m00918|LOC_Os06g09560.1|genepair1998-2
MARGGGGGGGADADLYAVLGLSRECTDADLRLAYRKLAMIWHPDRCSVAGGSASAAGVDE
AKERFQEIQGAYSVLSDSNKRFLYDVGVYDGNDGDDDDEADLSGMGDFLGEMAQMMSQA
TPAESFEELQQLFVDMFQDDIDAGLCQSTPPPPSWPSPPAAANARSPAAAATSRKGVNKR
CSPAAMDMDSGLSSLLGISGFCFEAPWTSQDASTAAGGGGGKRRKQRPPPASHNV*
>11668.m05326|LOC_Os02g54150.1|genepair1999-1
MAAAVAVEQEAACLQSFELYESKSRFYIFGTNTGKTHWRLLKINRSEPSDLDLHECCTVY
TQSEYHELLKNLDEDHRLTGGVKFVTKFYGIIGFIKFVGPFYMLIITEQRKIGEIFDHPV
YQVIKTSMVELANSKTRSRFLNSKDENRYKKILNTLDLRKDFFFSYSYHIMRSLQKNLSD
PQEGWNIYESTFVWNEFLTQGIRNFLGSTLWTVALVYGFFKQDKISISGKDIMFTLIARR
SRHFAGTRYLKRGVNEKGRVANDVETEQIVYGAGPRPTEVSSVVQNRGSIPLFWSQETSK
MNIKPDIILHQKGENYEATRLHFENLRRRYGDPIIILNLIKKRERRESILRREFDRAIRI
INKNIPEENHLRFLHWDLHENSRGKPTNVLDVLLKVAFRALRLTEFFYCQLAPSTGSDTA
HHWPSLLSGLDPFLCEENSNSDNTDCTEIVGDISQEDISGSSDSSCNGTTEDKAENNESP
PLKPPKFQKGVLRTNCIDCLDRTNVAQYAYGLAALGHQLHVLGSVESPELGLDDPLAHHL
MHFYERMGDTLAVQYSGSAAHNKIFSAKRGHLKLFIRSQEFFRTLQRHYSNTCIDANKQA
AINLFLGYFQPQEGKPALWELESSSVDHIAVHARTIKRVRSDGSILYGSNTSISGCSGCH
NEDKELLNAAPLDVKSGSQFPVLESDSVHGNEISLTCESEVSNLRYTPMIPQTHHVPGGV
ETESSIHSGDSNFLDLEWLSTSGNSSDERSIAISTPDVNLSAENVISGINSETMENQDAD
IYTQNLPEHFVQWVNHGDTFWY*
>11680.m00916|LOC_Os06g09540.1|genepair1999-2
MAVEAEAEVEVEVEAGGCLLQSFELYEAESVSASRPNRNGCCPCRVVVLPPDFCCADAWC
ALLDSGKDGASFEKFYILGTNTDKTSWKLLKIDRIEPSELNIDESSTVYSHSGYLDLLKV
LDEDHRSTGGVKFVTKCFGIIGFIKFLGPYYMLIITEQRKIGAIFGHPVYQVTRTAMIEL
SNSESRAKFLNSKDEDRYKKLLQTIDLRKDFFFSHSYNIMRSFQKNFNDPKEGWDLYDTM
FVWNEFLTRGVRNILKSTIWTVALVYGFFKQDKLAISGKDIMLTLVARRSRHYAGTRYLK
RGVNDEGSVANDVETEQIIFEDMLGPKQISSVVQNRGSIPLFWSQETSKLNLKPDIILHE
KDKNYEATRLHFENLRIRYGNPIIILNLIKKRERRPRESILRSEFDKAIKIINNDLPGEN
HLRFLHWDLHKNSQRKSTNALQMLLKVAFEALNLTEFFYYQVPPARRAESSFNLHAPLKN
GFGPHECDDSNNDDITDCIDNIDDMSQEDTCGSSDTSGNGTAEDIAEGNGSISVKPPKFQ
KGVLRTNCIDCLDRTNVAQYAYGLAALGHQLHALGSIESPELDLDSPLAHHLMHFYERMG
DTLAVQYGGSAAHNKIFSAKRGHLKFAIQSQEFFRTLQRYYSNAYMDAYKQAAINLFLGY
FQPCEGEPALWELEPVAGEGVLGENASSSDAINEISSAPDNTVTVSKSRYTPTEPHVKHV
SCELDYCNGSGDSNFLDIDWLSSSDNERPTTISTPDVNASADSVSAGVSSRRTEDHAAEI
QAQGLSEHFVQWIDQGETFWF*
>11668.m05327|LOC_Os02g54160.1|genepair2000-1
MCGGAIIHHLKGHPEGSRRATEGLLWPEKKKPRWGGGGRRHFGGFVEEDDEDFEADFEEF
EVDSGDSDLELGEEDDDDVVEIKPAAFKRALSRDNLSTITTAGFDGPAAKSAKRKRKNQF
RGIRQRPWGKWAAEIRDPRKGVRVWLGTFNSAEEAARAYDAEARRIRGKKAKVNFPEAPT
TAQKRRAGSTTAKAPKSSVEQKPTVKPAFNNLANANAFVYPSANFTSNKPFVQPDNMPFV
PAMNSAAPIEDPIINSDQGSNSFGCSDFGWENDTKTPDITSIAPISTIAEVDESAFIKSS
TNPMVPPVMENSAVDLPDLEPYMRFLLDDGAGDSIDSLLNLDGSQDVVSNMDLWSFDDMP
VSDFY*
>11680.m00900|LOC_Os06g09390.1|genepair2000-2
MCGGAILSDLIPPPRRVTAGDLWLEKTKKQQQKKKNKGARRLPLRQEEEDDFEADFEEF
EVDSGEWEVESDADEAKPLAAPRSGFAKGGLKNTTVAGADGPAARSAKRKRKNQFRGIRQ
RPWGKWAAEIRDPRKGVRVWLGTFNSPEEAARAYDAEARRIRGKKAKVNFPDGAPVASQR
SHAEPSSMNMPAFSIEEKPAVMSAGNKTMYNTNAYAYPAVEYTLQEPFVQIQNVSFVPAM
NAIEDTFVNLSSDQGSNSFGCSDFSQENDIKTPDITSMLAPTMTGVDDSAFLQNNASDAM
VPPVMGNASIDLADLEPYMKFLIDGGSDESIDTLLSSDGSQDVASSMDLWSFDDMPVSAE
FY*
>11668.m05329|LOC_Os02g54180.1|genepair2001-1
MNQMAAMSSCHHLSSSISTPRFGTRKATMAKFVRAPVHSCSCSNAHLSQTAAPAKMVFED
QVRGVVCYRDDKGNMICEGYDEGPRLGMRLPEKACFPWPMGVQVTDFIELSTLRVFEDVD
SLQPRKDQKGKL*
>11680.m00894|LOC_Os06g09350.1|genepair2001-2
MAARSSLHPSSMASIPRLIGTRKAALRFRAPAHGGTSSSQLSIAAATANKKVFEDQLRGI
VCYRDDKGEMVCEGYDEGPRLGMRLPEKACFPWPMEVQITDFIELATFRVFEDADVLQIK
NDQKRQI*
>11668.m05346|LOC_Os02g54340.1|genepair2002-1
MAPEPEDDIMNEKNPRPLDEDDIALLKTYGLGPYSTSIKKVEKEIKEMAKKINDLCGIKE
SDTGLAPPSQWDLVSDKQMMQEEQPLQVARCTKIISPNTDDAKYVINVKQIAKFVVGLGD
KVSPTDIEEGMRVGVDRNKYQIQIPLPPKIDPSVTMMTVEEKPDVTYNDVGGCKEQIEKM

REVVELPMLHPEKFVKLGIDPPKGVLCYGPPGTGKTLLARAVANRTDACFIRVIGSELVQ
KYVGEGARMVRELFQMARSKKACIVFFDEVDAIGGARFDDGVGGDNEVQRTMLEIVNQLD
GFDARGNIKVLMATNRPDTLDPALLRPGRLDRKVEFGLPDLEGRTQIFKIHTRTMNCERD
IRFELLARLCPNSTGADIRSVCTEAGMYAIRARRKTVTEKDFLDAVNKVIKGYQKFSATP
KYMVYN*
>11680.m00888|LOC_Os06g09290.1|genepair2002-2
MAPEPEDDIMNEKNPRPLDEDDIALLKTYGLGPYSTSIKKVEKEIKEMAKKINDLCGIKE
SDTGLAPPSQWDLVSDKQMMQEEQPLQVARCTKIISPNTDDAKYVINVKQIAKFVVGLGD
KVSPTDIEEGMRVGVDRNKYQIQIPLPPKIDPSVTMMTVEEKPDVTYNDVGGCKEQIEKM
REVVELPMLHPEKFVKLGIDPPKGVLCYGPPGTGKTLLARAVANRTDACFIRVIGSELVQ
KYVGEGARMVRELFQMARSKKACIVFFDEVDAIGGARFDDGVGGDNEVQRTMLEIVNQLD
GFDARGNIKVLMATNRPDTLDPALLRPGRLDRKVEFGLPDLEGRTQIFKIHTRTMNCERD
IRFELLARLCPNSTGADIRSVCTEAGMYAIRARRKTVTEKDFLDAVNKVIKGYQKFSATP
KYMVYN*
>11668.m05373|LOC_Os02g54590.1|genepair2003-1
MGKDGGDPGGGGYPLVAVCIDKDKNSQNALKYATESLAHKGQTIVLVHVHTKGSSGGVED
AAGYKQPSDPQMKDLFLPFRCFCTRKDIHCKDVVLDDHDVSKAIVEFAAHAAIEKLVVGA
TARGGFVRFKAEISSSISKTAPDFSTVYVVSKGGKVTSVRQAVRQAPAVSPLRTMIQGPK
PDNVSTQKWTPPPPPSTTRPDIAGTPKIQDNFIMSPFARGANTSVRKAFPDYSMPESSDI
SFISSGPRRSLDLYPPRLSSGSDAHDHHSFEATRPPSMWGGDSFGSDSQSSNSSFASSLP
MEDMEAEMKRLRLELKQTMDMYSTACKEALTAKQKAMELQRWKTDEEQRSHETRLTEESA
MALIEQEKAKARAAIEAAEASQRLAEMEAQKRISAEMKALKETEERLKSMGGGGSRGAVR
YRKYTIEEIELATEHFADGRKIGEGGYGPVYKGHLDHTPVAIKVLRPDAAQGRSQFNQEV
EVLSCIRHPNMVLLLGACPEYGCLVYEYMANGSLDDCLFRRGGGPVIPWQHRFRIAAEIA
TGLLFLHQTKPEPLVHRDLKPGNILLDRNYVSKISDVGLARLVPPSVADSVTQCHMTSAA
GTFCYIDPEYQQTGMLGVKSDVYSLGVMLLQIVTAKPPMGLTHHVARALDHGTIVDMLDP
AVHDWPVDEARCFAEISIRCCELRRKDRPDLATVVLPELNRLRALGEDNMQLCNTMSGGG
RSSMHSSPYNSNSSMHQPHRQTDMAIEHSVGRSSYDADTSQQAMQGRRLNYN*
>11680.m00882|LOC_Os06g09230.1|genepair2003-2
MERYHDGKDLDTSSYPLVAVCIDKDKNSQNALKWAIDTLVQKGQIIVLVHVNTKGTSGKW
SGGRLRLQAADGPAHEGSLPHFPLLLHAQRRKWRSRCSIKCGSHTDPSIFALAGVCSITE
FCAVAAIEKLVVGATARGGFRFKADIPTTISKGAPDFCTVYVINKGKVSSVRNSTRQAPR
VSPLRSQIQNMAAAAAKPEPATAMAPTPQKWSSSSRGHDHLETPKVDSYIRSPFARGPMG
GATRKSYADLSHLSMPDSADISFVSSGRRSVEHNPVPARLSAASAESYDHSFETSRTPWG
GDSFGGNDHTSFSQSSTSSFCSIGMDDVETEMKRLRLELKQTMDMYSTACKEALNAKQKA
MELQRWKAEEEQRTHDARLTEESAMALIEREKAKAKAAMDAAEASQRIAELEVQKRITAE
KKLLKEAEDRKNRGGGGGGMSHEIRYRRYSIEEIEHATDRFNDARKIGEGGYGPVYKGHL
DHTAVAIKVLRPDAAQGRSQFQQEVEVLSCIRHPNMVLLLGACPEYGCLVYEYMANGSLD
DCLFRRGGGGGGGGGPVIPWQHRFRIAAEIATGLLFLHQTKPEPLVHRDLKPGNILLDRN
YVSKISDVGLARLVPPNVADNVTQYRMTSTAGTFCYIDPEYQQTGMLGVKSDVYSLGVML
LQIITAKPPMGLTHHVGRAMERGALADMLDPAVPDWPVEEAQCLAEMALRCCELRRKDRP
DLGSAVLPELNRLRALGEDNMQFCGAIRGGVGGGLYSSSLLSTPSRSQAAVTGLTFLPPL
NSFNSEEF*
>11668.m05374|LOC_Os02g54600.1|genepair2004-1
MRPGGPPSLRAGLQQQQQQQPGTPGRSRRRPDLTLPLPQRDLTSLAVPLPLPLPPSSAPS
STSSSGSSSLGGVPTPPNSVGSAPPAPPPLSELERVRRIGSGAGGTVWMVRHRPTGRPYA
LKVLYGNHDDAVRRQITREIAILRTAEHPAVVRCHGMYEQAGELQILLEYMDGGSLEGRR
IASEAFLADVARQVLSGIAYLHRRHIVHRDIKPSNLLIDSGRRVKIADFGVGRILNQTMD
PCNSSVGTIAYMSPERINTDLNDGAYDGYAGDIWSFGLSILEFYMGRFPLGENLGKQGDW
AALMCAICYSDSPAPPPNASPEFKSFISCCLQKNPARRPSAAQLLQHRFVAGPQQQQQPQ
PQPLAPPPS*
>11680.m00877|LOC_Os06g09180.1|genepair2004-2
MRAGDMPGRGARRRPDLTLPMPQRDAPTSLAVPLPLPPAATTTTSAPPAGGAMHPLASAG
AAPPPPLEELERVRRVGSGAGGTVWMVRHRGTGKEYALKVLYGNHDDAVRRQIAREIAIL
RTAEHPAVVRCHDMYERGGELQILLEYMDGGSLDGRRIADERFLADVARQVLSGIAYLHR
RHIVHRDIKPSNLLIDSARRVKIADFGVGRILNQTMDPCNSSVGTIAYMSPERINTDLND
GAYDGYAGDIWSFGLSILEFYMGKFPFGENLGKQGDWAALMCAICYSDPPEPPAAVSPEF
RSFVGYCLQKNPAKRPSAAQLMQHPFVAGPQPQPLAAPPPSS*
>11668.m05378|LOC_Os02g54640.1|genepair2005-1
MARRSCFFFLLPLLVAALAGSPVVTAQRNALPAAAAAAASVRVGVILNLTSAVGVRRRVG
IQMAVEDYYAANPGSATRVELHFRDSAGDVLPAASAAVDLIKNVQVQAMIGPPSSAATEF
VAHIGSHSRVPVLSYSATSPSLSPAQTPFFVRAAVNDSFQAAPVAAVLDAFRWRAAAVVY
EDSPYGSGILPALADALQGAGAKIMDRTAVPVDATDDRLDALLYRLRAMPTRVFVVHMLH
NVAGRLFRRAKMLGMMSDGYIWVATDGVATFMDRFSPEEVDAMQGVVSLRPYVQETDAVK
NFSARFKARLRRDHPTVDDVREPTVLRFWAYDTAWAIAAAAESAGVAGPAFQTPQTSAPL
TDLDRLGVSATGTALLNAVLSTTFDGLAGKFRLVDGQLQPPAYEVVNIIGKGARTVGFWT
PEFGITQDLNAGSAKTLRQILWPGEPRDTPRGWTVSPSGLPLRVSVPTKRGFTQFVDVGN
VTATGRRNITGYCIDVFDEVMKIMPYPVSYVYDPYPDSPESYEKLVDQVSSQKADAVVGD
VTITASRMEEVDFTMPFTESGWSMVVAVQKETSTSMWIFLQPLTTSLWLASLAFFCFTGF
VVWVIEHRINEEFRGTPWQQFGLIFYFSFSTLVFSHKEKLESNLSRFVVIIWVFVVLILT
SSYTASLTSMLTVQKLQPTVTDVRELLRRGDYIGFQEGTFIVPVLEKMGFEGRMRSYSTV
DQYADALSKGSANGGVAAIFDEIPYLKLFLSQYCNGYTMVGPIYKTDGFGFVFPRGSPMV
ADVSRAILTLAEGEKMAQIEKKWFGEPGACQSQGSAVGSSNLSFRSFGGLFLITGVVTSA
MLLIYLAVFFYRERDELRAAEAAAAASGSGSGSGSRSLRRLRAWARHYDQKDLKSPTFKR

RWSDESVRNGSEYAASRTPRWGDESPCNVAGAADADAGRIPEEVVGGMSPFSISTSSEER
NGAVSPAAAEFDNSSDRAAVVAGTSQPR*
>11680.m00872|LOC_Os06g09130.1|genepair2005-2
MTMQTRVFIVHMLPARASRLFARAKALGMMTKGYVWIVTDSIGIVLDVLPQHSIESMEGI
VGFRPYIAESTRITDFSSRFTTLFRTKYHPNTDIRMAKPTIFQLWAYDVAWAVATATEKV
HRTRSLNPTFHPPGNIGKNLVDDLPALPAGPELLNSILQGEFDGLAGQFRLIDRHLQVPT
YEIVNVIGEKTRVIGFYSPDSGLTMSVNSRIIHGDAKFSTSSSDLENIVWPGDSTTVPKG
WDFPVNAKILQIGVPVRRDFKTFVNVETNPNTNRSTVSGYSIDMFEAAVKKLPYALRYEY
IPYDCAVSYDLLVSQVFYKKFDAAVGDVTIIANRTRYVDFTMPYTESGVSMLVLSKSDDE
PTTWIFLQPLAKDLWIATMIFIFFTGLVVWVIERPINRDFQGSKWKQCITAFYFAFSTLT
FSHGQKIQSIQSKIVVVIWCLVLMILVQSYTASLSSMLTAERLQPSVTDLKQLLANGDSV
GHQSGSFVQSILKKLKFDDHKIKVYSTQEEYAKALRMGSKHGGVSAIFDEIPYLNSFCSK
YGREFQMVGPIDRTSGFGFVLPKGSPLVPDLSEAILSLTEEPERLKIEKTWFMDSSLDYY
GSHSKGSSRISFQSFQGLFIIVGCLLGAVLLINFSKFLYDKCKEMRGFGSDRVHSGERVV
CYGEAQPQPPQIVMVDRRSCAC*
>11668.m05391|LOC_Os02g54760.1|genepair2006-1
MRVFGRGDLITDPKEIAWQYLRSDFVVDAVAALPLPQILIWFVIPAIKYSTDEHNNNILV
LIVLAQYFPRLYLIFPLTYEIVKTTGVVAKTAWQGAAYNMLLYMIASHVCCYGQTITVST
YIGETLYCIFLAVLGLVLFAHLIGNVQTYLQSITVRVEEWRLKQRDTEEWMRHRQLPHEL
RERVRRFIQYKWLATRGVNEESILQALPADLRRDIKRHLCLGLVRRVPFFSQMDNQLLDA
ICERLVSSLCTQGTYIVREGDPVTEMLFIIRGKLESSTTNGGRTGFFNSTTLKSGDFCGE
ELLGWALVPKPTVNLPSSTRTVKALIEVEAFALQAEDLKFVANQFRRLHSKRLQHTFRYY
SHHWRTWASCFIQAAWRRYKRRKMARDLSMRESFCSMRSDDSNGEDDSPPKQNLAMKIMS
GSRKGPQNMKELPKLRKPDEPDFSAEPCE*
>11680.m00843|LOC_Os06g08850.1|genepair2006-2
MELRKQRTVRFHEERAKPTIPTHQKQAGLATSKLGLGISEKNKIFLAGNELWYKKIIDPS
SDFILTWNYVLRIACFVALFMDPLYFYVPKIYYGTPNSCIGRDTRLAIIVTVFRSITDLF
YVLQIIIKFRTAYINPSSTLGVFSRGDLVTDPGNIAKHYLRSSFVVDLVASLPLPQLKRA
SALLYLKFEIIIWSVIPSVKYSLSEHDDDILLLIALFQYVLRLYLVFSLNSKIVEVTGAF
SKTAWQGAAYNLLLYMIASHVLGALWYLLSVDRQTACWEKYCSKEAGCQNRYLACDIQSD
SNWKISTAIFNKCDATNKTIDFDFGMFTPLLSNQAPDQGFLKKFFYCLWWGLQNLSCYGQ
TLTVSTYIGETLYAIFLAVLGLVLFAHLIGNVQTYLQSITARVEEWRIKQRDTEEWMRHR
QLPQKLRERVRRFVHYKWLATRGVDEESILKALPADLRRDIKRHLCLDLVCRVISLPFIT
TFEWTKEVDTRKINRRGNEKNDRIGTCTFSFFPFLASYTLDTGSFLVTGQLQLPVNVSLM
DGQLLDAICERLVSSLSTVGTYIVREGDPVTEMLFIIRGKLESSTTDGGRTGFFNSITLK
TGDFCGEELLGWALVPKPTVNLPSSTRTVKTIVEVEAFALRAEDLKFVASQFRRLHSRKL
QHTFRYYSHHWRTWAACFIQAAWRRYKRRRLAKDLSIRESFFSRRSFEDDGSPEHSLVLN
AVRKGAHIIKELPKFRKPSEPDFSAEHDD*
>11668.m05400|LOC_Os02g54830.1|genepair2007-1
MSNSTWQPSPPQTPAAAASSASGIDGVENKISPSIVFIVAVLAIVFFVCGLLHLLVRHLL
RLHRQRRAREDAESATAFEGQLQQLFHLHDAGVDQAFIDALPVFLYRNVVGVGGEDGKDP
FDCAVCLCEFAADDQLRLLPKCSHAFHLECIDTWLLSHSTCPLCRRSLLAELSPTCTPVV
MVLESESSRDMVHAADDEPADVGGEDAPGAEEVVEVKLGKFMCVEGNVFFNVNAIAGEGD
RAGTSSNGNGDANAKAGGLGQRRCHSMGSYEYVMDAHASLRVSIKPPRKKPAAAASKSRR
RGAMSECEFGASKRGESSLRLPPFRATPRKNPDDDAAAAAGAKLAKDSFSVSKIWMVPSK
KEPGAAAERRAVSFRWPAAKDWDVEAGSCGGNSAVSSVAAEERPSFARRTLLWVVGGSRQ
LSRVGSCS*
>11680.m00840|LOC_Os06g08820.1|genepair2007-2
MDTSASTSPPPPRSVLSTIEDKMSPGVLLIIAILAVVFFLFGLLNLLIQNLLRMRRARRR
RRRVGDGGGGVGMGSPTAFQGQLQQLFHLHDAGVDQTFIDALPVFVYRAVVGAGLRKDDP
FDCAVCLCEFAGDDKLRLLPTCGHAFHVPCIDAWLLSHSTCPICRGSVLAAAAAADDDDD
SSASTPVARRVLDSESLGETFANNGGGDSEGSSPKAAAAEEEEVVEVKLGKLKCIDGNGN
AGDLAVVKGTTTSNDGDIGGGGRGDLGQRRCFSMGSYEYVMDEHAALRVAVRTPKRRPPA
RSRSRRRLALSECDFAGSASKKGAWEAAVIEAASADAAAARLNRDSFSVSKIWMTSATRK
EDVRTAAELAGGGRRAASFRWPAMAEASKKHGGINDERRDVEAGGNGDSSLADERPSLAR
TALQYIVGGGGGGRQQSSRVGSHS*
>11668.m05407|LOC_Os02g54890.1|genepair2008-1
MRVLEDDMFPSTPGKVKIERATAMNRQLHRCFASTSTMFLWALFLVAMTASYLSFQSFVD
TSSKYFAASWGGLHWERQIRASAAPRRPPGSAAGAGMSVLVTGAAGFVGTHCSLALRKRG
DGVVGIDNFNSYYDPSLKKARRSLLASHGVFVIEGDINDGRLLAKLFDVVPFTHVLHLAA
QAGVRYAMENPASYVHSNIAGLVTLLEACKDADPQPAIVWASSSSVYGLNDKVPFTESDR
TDQPASLYAATKKAGEEITHTYNHIYGLSITGLRFFTVYGPWGRPDMAYFSFTRNILQGK
PITVYRGKNRVDLARDFTYIDDIVKGCLGSLDTAGKSTGTGGKKRGPAPYRIFNLGNTSP
VTVPNLVSILEKHLRVKAKKNVVEMPGNGDVPFTHANISLARQQLGYKPTTNLDVGLKKF
VKWYLSYYGYTRGSKNL*
>11680.m00839|LOC_Os06g08810.1|genepair2008-2
MRVVVEEEEAEVMYPSTPGKVKVEQRSSAAMSRQVHRCFASTGTMFLWALFLVAMTATYL
SFRSLAGDAAASSSRYFPAASWGGLHWERQIRASASPRRPPGSAEGAGLSVLVTGAAGFV
GAHCSLALRKRGDGVVGIDNYNSYYDPSLKKARRALLGSHGVFVVDGDINDGRLLAKLFD
VVPFTHVLHLAAQAGVRYAMENPSSYVRSNVAGLVSLLESCKDADPQPAVVWASSSSVYG
LNDAVPFSEAHRTDKPASLYAATKKAGEAITHTYNHIYGLSITGLRFFTVYGPWGRPDMA
YFFFTRNILQGKPVTVYRGRDHVDIARDFTYIDDIVRGCLAALDTAGRSTGGGGRKRGAA
PYRIFNLGNTSPVTVPALVAMLERCLMVKARRHVVEMPGNGDVPFTHANISLAREQLGYK
PTTSLEMGLKKFVRWYLSYYGYNRGTHAFRSRL*

>11668.m05427|LOC_Os02g55070.1|genepair2009-1
MVQHQYVPRKRRAERSLAPAAPAAIFNVEAEEGRAGDVPLHKRVKEELQLQPPPPSLQLW
QQMDLFPNKQKKNILIELLALILGDRLKWDMHILDGSSPLGLRLRKSPSLLELIQMKLAM
ENTKKEDIKSRSLIASERVKASNFAADFLKIGTWECTSQYEGDLVAKCYFAKHKLVWEVL
DAGLKRKIEIQWSDIIALKATCPENGIGTLDLVLARPPTFFKETDPQPRKHTLWQVASDF
TGGQASIKRRHILQCQSSLLSKNFEKLIQCDQRLNYLSLQPYMIDSPVFRPKTEGSIFEN
PNKSKSYHGFSYLEGEHESHLSKYIDHVSPCDFPLMSKKDGMKDDIANQQQSFSRPINWG
ASDVDLQVDVSQELKSPHPNSLSQARSLSIDDLLSHLDDCIVEQKPAGNNPSLPISEASS
NELLEKITQQLLSDSHVAPASDEKRVMARVGSLLSLLQKDAVPANLPKFEPNDSGKIGVV
EVGISSALDMGIANGTNPPGISRKDSYEELLSNLFNISEDFDD*
>11680.m00832|LOC_Os06g08740.2|genepair2009-2
MVQLPSSGKRPAEPAMAAASAAGATVKLEADEMLHGGEEDGGPLSKRAKAGVQMPAPPPP
PPPPQQQDMYHNVLDEPSPLGLRLRKSPSLLDLIQMRLSQANSTAGQSSADDCSSEPPKK
KDLKSGTSTAGERLKASNFPANILRIGTWEYISRYEGDLVAKCYFAKHKLVWEVLEGGLK
SKIEIQWSDITALKASCPENGQGTLDVVLARPPLFFKETDPQPRKHTLWQATSDFTGGQA
SMNRRHFLQCPSSLLSKNFEKLLQCDQRLNQLSQQPDIILDSPVFEPRCSIFEDPVESKC
QGFTNLKDEHELSGFSGSLSPCAGSSMSAKIEVNDSIATQAGFLAQPGNPGPSAVNVQGV
SRNVNGAPELNIPSWWSQLKVPGLRPSMSVDDLVNHLGNCISEQITSVNPTLPSNEVPTK
ETLEEIAQYLLGDAQGPPASTSDERSLMARVDSLCCLIQKDTPPVAQPKPEPNDSDSIGG
DGTEGSDEEFSSAASTVKTTGPAQPPAMSRKDSFGDLLMNLPRIASLPQFLFKIPEDSEN
*
>11668.m05433|LOC_Os02g55130.1|genepair2010-1
MSCPGCSSIAYGASEEIGPFRIKTNGTGLYLNKYSWNREANLLFLESPAGVGFSYSNTTS
DLKTSGDERTAQDALQFLISWMSRFPQYRHRDFYIAGESYAGHYVPQLARKIVEFNKASP
YPFINLKGILVGNGVTDNYYDNIGTVTYWWTHAMISDTTYKAIMSSCNFTSANVSRLCNR
AMSYAMNHEFGDIDQYSIYTPSCAAAAAANATGRRRGKAAVLRFKDTFLRRRSFGYDPCT
ETYAEKYYNRPDVQKAMHANITGIPYRWTACSDVLIKTWRDSEFSMLPTYKLLMKAGLRI
WVFSGDTDSVVPVTATRFALSHLGLKTKIRWYPWYSAGQVGGWSEVYEGLTFASVRGAGH
EVPLFQPRRAFRMFQSFLAGEPLPKS*
>11680.m00828|LOC_Os06g08720.1|genepair2010-2
MTTTTTTSAIGSLLVLLLVVVVGGVCSASVGGGGGGGEAWRAEQERDRVARVPGQDFDVG
FAQYAGYVAVSEERGASLFYWFFEAADDPASKPLVLWLNGGPGCSSIAYGVAEEVGPFHV
NADGQGVHLNPYSWNQVANILFLDSPVGVGYSYSNASDDILNNGDARTANDSLTFLTKWI
ERFPQYKGREFYVTGESYAGHYVPQLAQAIKRHHEATGDKSINLKGYMAGNALFDDFHDH
LGIFQFMWTNGLISDQTYRLLNVFCDYESFVHTSSQCNKILDIASDEAGNIDSYSIFTPT
CHASFASSRNKVMKRLHSVGKMGERYDPCTEKHSTVYFNLAEVQKALHVSPIINKSKWET
CSDVVNTNWKDCERSVLHIYHELIQYGLRIWVFSGDTDAVLPVTSTRYSINALKLPTVTP
WNAWYDDDGEVGGWTQGYKGLNFVTVRGAGHEVPLHRPKQALILIKSFLAGSPMPSVQDF
YSDV*
>11668.m05437|LOC_Os02g55170.1|genepair2011-1
MALFRKFFFKKPPDGLLLITDNIYVFDHCFSMKEMEEDHFEAHIRGVAAHLLDNFGDHSF
MISNFGIRDEESPIYHILSEYGMTVLDYPGHYEGCPLLTMEMVHCILKSSESWLSLGQRN
FLIMHCEQGCWPILAFMLAALLIYLGQYSDEQKTLDMLYKQSPVELLEMFSPLNPMPSQL
RYLRYVSMRNVVPEWPPADRALTLDSVILRMVPDFHGQGGFRPIFRIYGPDPLMPTDQTP
KVLFSTPKRSNVVRFYSQADELVKINLQCHVQGDVVLECINLYEDLDREDMVFRIMFNTG
FIRSNILMLNRDQIDILWNTQDQFPKDFRAEASVLYQVAN*
>11680.m00809|LOC_Os06g08540.1|genepair2011-2
MSLFRRLFHRRPPQGLVEISDNIFVFNHCFSTDFFEEDELKPYIGGILKQLIGRYSIDSF
MVFNFEGSKKDNQIACIFSDFDMSVMGYPRNYEGCPLLTMEMIHHFVRSSESWLSLGQDN
FLLIHSEQGGWPVLAFALAALLLYLRRYNNERKALEMVYKQAPPGLVEHFSPLNPAPSQM
RYLKYVSRRHMSPEQWPPADRMINLNCVIIRGVPNFDGKGGCRPILQIYGPDPFAPNDKS
TKVLFSTPKTSDSVQLYTQEDSEIIKFNARCPVQGDVVMECISLDENFEHEVMVFRVMFN
MAFIEDNLLLLDRDQIDILWDTKLRFPVDFRVEVIFSEMDTITSLNSSQLSSEDKENFSR
VEDAFSHLDWSTKSDDVTTNATEQNGSNNEHDGFDAVSLQETENSNSTSEHSLLGTRSVQ
VIQMETEHNHSSAPTFDIVKDPVADTHSLPETYSLALESQEHKLFEDSSPRELPKLDTTK
NNPNSDLPSANSRDSEAAGDAAVAEWSDTNTDTFLSDTPSSSSPSSPQKFDEDSMEAGIV
EIQTQTAEPQRC*
>11668.m05439|LOC_Os02g55180.2|genepair2012-1
MMGEAEEGLMHRRIEFHAARRPPRAVEGAGGRFWVEILSPDADKAAVVAAARSEGLVRGL
EKGEGSGGGIDPELRVARMYLRRIGAGLQNFGNTCYLNSVLQCLTYTEPFVAYLQSGEHM
SSCRTIGFCALCALQRHVNSALQSTGKILRPVHIVRNLRCISRSFRISRQEDAHELMVSL
LESMHKCCLPSGVPSGSPSAYEKSLVHRIFGGLLRSQVRCTTCSHCSNKFDPFLDLSLEI
ANAATLVKALQHFTAEELLDGGEKQYNCEHCRQKVVAKKRFMIEKAPSVLTIHLKRFSPF
NPRHKIDKKVQFQPTLNLKPYVSNPEGMEFKYSLYGVLVHAGWNTQSGHYYCFVRTSSGI
WHNLDDNKVYQVREADVLRQKAYMLFYVRDRTRSSVMYSDNCTVNLSVNKMISEKITCMN
GSIKKDTVETKTLRVPSLVKEDVNLKKQNSENGQSSNISNAPQDQCSKSHSNTEVLEAAA
SPNNDPASTQKASCIRPDTAAVNLPMKTEQTAPDNRREITSPAQADVSVLHNASFNQKLY
EKQLQEHQLETDDALTDSRKDAPAALCTYGVGDGLLGRNGQSSEPHIGPCPAALPIHNGG
EGLLGANGQASEAHSGPCSSAFPIHNGGEGLLGASGQSSEPHTGPCPAALPIHDGGQGLL
GPNGQASEPHTDSCPAAFPIHSGGEGLLGANGQASELHTDPCPAAFPIHSGGEGLLGANG
QASEPHTDPCPAAFPIHSAGEGLLGANGQDSEHRTGPIPAAFPVWNGTGVILEKYGQVSG
PADPFCKPTPTISDTVSIAQIIPTEHAAVSNGTVSSSDDLTGNTEANESSEFVKNYGEQV
MVRDLSAETSGDRANADEQTSMQNNTLEVGKDVAKDTDNVANAEEQVLNHPLAEQVKSEK
QIYPGISTTLICSEDTTQLIDKDTGSGKLNKKMNCKSKRQVKYPAVRMFFGPKQLLLASV

KLHKKRKHKRSKKHHALSVHIESIITDQQTSTSETVFSKIISHKSRGQKRSCASASSEDG
TQLFNKKQHIEGTTNSVPMDNNDTKLASADSNDAKLASAELPSSCTNSLVNQTDSRNNVN
ANERGPWHFNLLTRGLREITVPRWDDTEIKNTKETEILHPRTRSIGYVLDEWDEEYDRGK
RKKIRKPKHGFSGPNPFQETANIRSRQRMRLQSDQTKSGNQPLRI*
>11680.m00808|LOC_Os06g08530.2|genepair2012-2
METLNPDAGDRAAAGAAQGVGVAAGGEGEARRAEKGEVVGGGLDPELSVARIYLGRIGAG
LQNLGNTCYLNSVLQCLTYTEPFAAYLQSGKHKSSCRTAGFCALCALQNHVKTALQSTGK
IVTPSQIVKNLRCISRSFRNSRQEDAHELMVNLLESMHKCCLPSGVPSESPSAYEKSLVH
KIFGGRLRSQVKCTQCSHCSNKFDPFLDLSLDIGKATSLVRALQNFTAEELLDGGEKQYQ
CQRCRKKVVAKKKFTIDKAPYVLTIHLKRFSPFNPREKIDKKVDFQPMLDLKPFISDSKG
ADFKYSLYGVLVHAGWNTQSGHYYCFVRTSSGMWHNLDDNQVRQVREADVLRQKAYMLFY
VRDRVGNPTPRKDNITANMPARRTIPEKISGLSDMIQSGVIEAKLNGSSSPYGDKRLHGI
SNGNSIKTSREHYLKKDGKTEAPKASENNGLASTQKASAPQIDGATLSAQSKQITSTGHR
EVSSSDRSASLTHVIVNQAVAMVPSQELQPKVDGLTDTSSLGNGNAILSERNKQTSQHQN
PFSMPASHGKDTGAGLAAQTFPTKDAIVSNGVVPSSRDPISSEKVCGLQKSIKQDDKTVK
ELPISENNIVSGLERVNARKQTSSEVSMKVAAADSCNSNTPKRVDLKSKKLVRYPVMNMW
LGPRQVMLGSLKVQKKKCNRTRRRSVVCEDMANATCSGNNTSEQQASTSTTTSSETVQC
TPRGRKRAYDSDSPKNNNQKQNKQDVIGADTGSGELNMDKRNVISETAASAELPKLGPGS
SANQEHSRNNVHAKLGVPRHFTVLTRDLAEVTVPCWDDVAVSNAEARESKHSESKSIGYV
LDEWDEEYDRGKTKKIRNSKEDYGGPNPFQEEANYISQRNMKQRTYQPKSWKKHAHVRR*
>11668.m05446|LOC_Os02g55250.1|genepair2013-1
MQPNARQMRGGGGGGGGGQDDFFDQMLSTLPAVWSELGSGKPAWDLTAGAVGGGGGASDD
HSAAAFDDSALLASRLRQHQIDGGGDKPIMLQLSDLHRHHGLAAGDDSGGAAGFLPLSLF
ADRSQDDIDAAFKSPNGARGDHALYNGFGAAGMHGAAAMQPPPFGQGGSMPAQSFGGGAA
ASGGGGGGGSASAAAAAGASSGGGAAAPPRQRQRARRGQATDPHSIAERVYHSPTTFPFSP
PFFIASMPCCLRRERIAERMKALQELVPNANKTDKASMLDEIIDYVKFLQLQVKVLSMSR
LGGASAVAPLVANMSSESNGNGNATSSSGNGEAANGSSNGDNNGGGTLRVTEQQVAKLME
EDMGSAMQYLQGKGLCLMPISLATAISSATSSSLLPRTGGGAGGSLHEGGNGTSPPLVNG
TATGCDDAGVFFSVKFVVELSFLLLNEDCRGKEESKLLVQKGP*
>11680.m00804|LOC_Os06g08500.1|genepair2013-2
MQPSSRDTVAGGGGEGTQDDFFDQMLSTLPSAWADLGGGGGGAAGKSPWEVDPAAAAAAS
QVFDESALLASRLRHHQIGGAGGGGGEKPVMLQLSELHRQAGGGEEDGSGAFSPLPLFTD
RTNVPPREEMEGGFKSPNAAAGGEHALFNGFGVHGGSGGAGQPPFGQLRRERIAERMKSL
QELVPNANKTDKASMLDEIIDYVKFLQLQVKVLSMSRLGGAAGMAPLVASMSSEGNSNGS
SNGGGGKASKGGTGGEGGGGGGGGGGGGTGGGMRVTEQQVAKMMEEDMGTAMQYLQGKGL
CLMPISLASAISSATSSASLLSRPSIRHAGAPPQTMLDAAGPTSPAAMSNGDDPRHAKAD
GGAGGTQ*
>11668.m05453|LOC_Os02g55320.1|genepair2014-1
MRAAEERKGVVPAARRRDQFPVGMRVLAVDDDPVCLKVLETLLLRCQYHVTTTNQAAIAL
KMLRENRDMFDLVISDVHMPDMDGFKLLELVGLEMDLPVIMLSVNGETKTVLKGITHGAC
DYLLKPVRIEELRNIWQHVIRRKFSTRDRANLDFYEECNKPPNADSDHVHGHVTCGSPDQ
SGRPSKKRKEYCSEEEDEGEVNTQDIDDPSAPKKPRVVWSVELHRKFVAAVNQLGIDKAV
PKRILELMNVEKLTRENVASHLQARNSFHYLLFISMYCPRNNPAALGTQGVPASKSIQTM
SGSHTLSHSINDANKYHLSLPGNQKGNLGQGLATSLGQTQMQQKWIHEETDDLSTILSGN
GLSNGMSGTLQSVTSSPLLPQELAECTQAKIVSQPSIRTSSVSSEHIEGAVGVSSGLLES
RVSQQSTIPLSGFSANGLLIHGSFNNTCANKLGGTSSSCAPARSSNDLMVARDTKGGASS
FGGAMLLPPDTEQKYLNFGGGNGLKQKFDDRTADSLFDLKFVWSSVPSSQLASNIGAHHA
MSQRWNNSSSNSSNIGARMIGQATSSGSTVIPQMKTDFLVSGDMAMPKNASDLSIPKLQS
ELSSSSCSFDGLLNSIVKVEKDDVTFSDDLGCGDFYSLGACI*
>11680.m00798|LOC_Os06g08440.1|genepair2014-2
MLLGALRMEERKGLMGRERDQFPVGMRVLAVDDDPVCLKVLETLLRRCQYHVTSTNQAIT
ALKLLRENRDMFDLVISDVHMPDMDGFKLLELVGLEMDLPVIMLSVNGETKTVMKGITHG
ACDYLLKPVRIEELRNIWQHVVRRKFGNRERNNLDFSKECNKPQSADTDHGPYQPTCGSS
DQNGRSSRKRKELHGEDDDEGDDNDYQENDEPSAAKKPRVVWSVELHRKFVAAVNQLGID
KAVPKRILELMNVEKLTRENVASHLQKYRLYLKRLGAVASQQASIVAAFGGRDPSFLHIG
AFEGLQSYQPFAPSAALPSFNPHGLLTRTSAAAAFGLQELAAPSSTIQTSTGNVTVGHCL
EENQQANLAQGLTAAIGQPQLQQNWIHQEGNGLSDVFSGSSLTNTLSSTLQRVPSSSLPP
QELLECKQAKVSMPPSIRIPPSSSALLERTLGVSTNLGDSSISQQGALPIDGGFSADRLP
LHSSFDGAVATKLDTSLAASQREIGQQGKFSVSMLVSPSDNLALAKNAKTGASSSGSTII
LPLDTARHSDYLQFGGASNSLQKMDGQKQDHIQSSNIIWSSMPSTQLPSDTQIHNTQNQR
LDSGSFNHNIGAHLADQTNASASILPQMKFDTRISEEKMKQKNTYDLGSSKLQGGFNSSG
CNFDGLLNSIIKVEKDDLPFMDNELGCDLFPLGACI*
>11668.m05459|LOC_Os02g55380.1|genepair2015-1
MARPQQRYRGVRQRHWGSWVSEIRHPLLKTRIWLGTFETAEDAARAYDEAARIMCGPRAR
TNFPLADATAAAAAAAASSSFLSAALVAKLHRFNLASVQATQRQREAAATAAAASSASAT
TPPLGNAAAADDDARTTTTYGAEWSGRFLEEQHVEQMIDELLDSNFSMEICY*
>11680.m00787|LOC_Os06g08340.1|genepair2015-2
MARPQQRYRGVRQRHWGSWVSEIRHPLLKTRIWLGTFETAEDAARAYDEAARIMCGPRVR
TNFPHDVADEAAPPPPPHSAAAASSSFLSAALVAKLHRFNLASVQAAQRGNSNDDDSTTS
SSAAASSRAVIPSLPAAAGALGNAAATAEWSGGFLEEQYVDQMIEELLDSNFSMEISC*
>11668.m05461|LOC_Os02g55400.1|genepair2016-1
MWNPLSWVMEAAAIMAIVLANGGGRPPDWQDFVGIVVLLVINSTISFIEENNAGNAAAAL
MAGLAPKTKVLRDGKWQEQDASILVPGDIISIKLGDIIPADARLLEGDPLKVDQAALTGE
SMPVNKHAGQGVFSGSTVKQGEIEAVVIATGVHTFFGKAAHLVDSTNNIGHFQLVLTAIG

NFCIISIGVGMIIEIIVMYPIQHRAYRDGIDNLLVLLIGGIPIAMPTVLSVTMAIGSHRL
SQQGAITKRMTAIEEMAGMDVLCSDKTGTLTLNKLTVDKTLIEVYGRGLDKDSVLLYAAR
ASRVENQDAIDTCIVGMLADPKEARAGIKEVHFLPFNPVEKRTAITYIDGNGEWHRISKG
APEQIIELCKMSKDAEKKVHTLIDQYADRGLRSLGVSYQKVPEKSKESEGEPWQFVGLLP
LFDPPRHDSAETIRRALHLGVNVKMITGDQLAIGKETARRLGMGTNMYPSTTLLGDKSSE
MSGLPIDELIEKADGFAGVFPEHKYEIVKRLQDRKHICGMTGDGVNDAPALKKADIGIAV
DDATDAARSASDIVLTEPGLSVIVSAVLTSRAIFQRMKNYTIYAVSITIRIVLGFMLVAL
LWKFDFAPFMVLIIAILNDGTIMTISKDRVKPSPTPDSWKLKEIFATGIVLGTYMALITA
LFFYLAHDTDFFTETFGVRSIKTNEKEMMAALYLQVSIISQALIFVTRSRSWSFVERPGA
LLVIAFLAAQLVATCIAVYAEWEFCKMQGIGWGWGGAIWAFSVVTYFPLDVLKFIIRYAL
SGRAWNNINNKTAFVNKNDYGKGEREAQWATAQRTLHGLNQSSTSSDLFNDKTGYRELSE
IAEQAAKRAEVARLRELHTLKGHVESVVKLKGLDIDTIQQSYTV*
>11680.m00784|LOC_Os06g08310.2|genepair2016-2
MASSLSLDDINDDSVDLSKAPVAEVFQKLKCDRKGLTGAEGESRLRLYGPNKLEEKKESK
LLKFLGFMWNPLSWVMEIAAIMAIVLANGGGRPPDWQDFVGIVSLLIINSTISYIEEANA
GDAAAALMAGLAPKTKLLRDGRWEEQEAAILVPGDIISIKLGDIIPADARLLEGDPLKID
QSALTGESLPVNKHPGQEVFSGSTVKQGEIEAVVIATGVRTFFGKAAHLVDSTNNVGHFQ
QVLTAIGNFCIISIGAGMAVEVLVMYPIQHRAYRDGIDNLLVLLIGGIPIAMPTVLSVTM
AIGSHRLSQQGAITKRMTAIEEMAGMDVLCSDKTGTLTLNKLTVDKTLIEVCSKGVDKDM
VLLYAARASRVENQDAIDTCIVNMLDDPKEARAGIQEVHFLPFNPVDKRTAITYIDGNGD
WHRVSKGAPEQIIELCNMAADAEKKVHALIDSYADRGLRSLGVSYQQVPEKSKDSGGDPW
QFIGLLPLFDPPRHDSAETIRRALHLGVNVKMITGDQLAIAKETGRRLGMGTNMYPSTTL
LGDKNSQVNGLPIDELIERADGFAGVFPEHKYEIVKRLQEMSHICGMTGDGVNDAPALKK
ADIGIAVDDATDAARSASDIVLTEPGLSVIVSAVLTSRAIFQRMKNYTIYAVSITIRIVL
GFLLIAIIWKFDFAPFMVLIIAILNDGTIMTISKDRVKPSPTPDCWKLNEIFLTGVVLGT
YMALVTVLFFYLAHDTNFFTDVFGVTSIRESERELMAALYLQVSIISQALIFVTRSRSWS
FVERPGFLLLFAFFAAQMVATAIAVYARWDFCRIQGIGWRWGGAVWQFSVVTYLPLDVLK
FIIRYALTGGKAGDSAQKKASSPPPTSQP*
>11668.m05469|LOC_Os02g55480.1|genepair2017-1
MRCPFSILNSVRGMNPRFSSPSILVSATTQNWIVRRLIQQGSQLVWVGATNNAEWQPQPG
CSKVAGPCLVRHEHEMHWRKALLIETAHKHIMEDQKRLRDFDLLQALSVVLFVEKDMRIR
LEVDRSVDGTDGRDASRSGVRAAATSPAGRRGGAVRDAAAVIVPDNHRSDIEFEIAVFGI
GIMLQQQRSACEEQQPGWWTLDSNVREAKERLDQKLRSQRESAVVVVKRHNKAQVAGTSS
DGGEQSTATTAAPQWEVYTRKEGRRRMWFRRLGRRPTPEEEEECAVCLEELRAGEAVAHL
PCTHRFHWGCAVPWVQTASRCPVCRAAVYLTSPAPAASNNYN*
>11680.m00777|LOC_Os06g08250.1|genepair2017-2
MAAMLPGVECARRRRLRQGGGAGADAAAAGGGTRRSSFCVHAAGHGGGQTCGGAAANHSG
KQRSSVMELIHGWSLDSNAREAKERLDQKLRSQRESVIKRHHSTGSIKLNRGATGGGGGG
GRSTATAAMGVQREVYSRKGVMRRLMRWSRLRWDAAEQAECAMLDIISVDRCQICVTSST
ESTDMKKSSTDTVHEEVDGVTRWLPNGPMFTCNFISLLLLMAHSDIATPPDFMAHQFSEH
VAAKRAFLSHLHNEGLASFEN*
>11668.m05478|LOC_Os02g55560.1|genepair2018-1
MCVEELEGAERLDFGGVAELETTPADFEMEKVCENTVSLDFKQARSSSFVPVIRSGDWSD
IGGRDYMEDAHVCISDLANNFGHNSVDDEIISFYGVFDGHGGKDAAHYVRDNLPRVIVED
ADFPLELEKVVRRSFVQTDSQFAERCSHQNALSSGTTALTAMIFGRSLLVANAGDCRAVL
SRRGTAIEMSKDHRTCCLNERKRIESLGGYVDDGYLNGQLAVTRALGDWHLEGLKEVGEP
GGPLSAEPELKMITLTKEDEFLIIGSDGIWDFFSNQNAVDFTRKRLQEHNDLRLCCKQIV
EEAIRRGASDNLTAVMVSFHQEAPPQLRVNRTGRVERSISAEGLHSLRVLLEGQ*
>11680.m00766|LOC_Os06g08140.1|genepair2018-2
MCVEESEGAERLDFGEPAAAAADAGKSKSKSPDELPSPRMERVCENTTAADFKQNKSGNF
VPNIRSGDWSDIGGRQYMEDTHVCITDLAKNFGYQSVDNEAISFYGVFDGHGGKDAAHFV
RDNLPRIIVEDADFPLELEKVVRRSFVHADNQFAKTTLSSGTTALTAMIFGRTLLIANAG
DCRAVLSRCGTAIEMSVDHRPCSLSEKLRVESLGGYVDDGYLNGLLGVTRALGDWHLEGM
KEAGNPGGPLSAEPELKMITLTKDDEFLIIGSDGIWDVFSNQNVVDFARRRLQEHNDVKS
CCREIVEEAIKRGATDNLTAVLVSFHLEAPPQVRVSRPGRVARSISAEGLNSLRTLLRNQ
*
>11668.m05480|LOC_Os02g55580.1|genepair2019-1
MRMSKLSMATAVILLLVVMAMEVEGIRLDAETRAATSNQMVNKSTENVPKDSGDSLGEAK
RSIAGNEVRAVAHKLPEFHEDYYGPSDHTPRHH*
>11680.m00765|LOC_Os06g08130.1|genepair2019-2
MKTSLPLVAAVLALFLMAATVQSIRLDAESHSAFSKQTVNNTSGDNVVAVVVAKTDGEPS
GEMEKAISEEKSRVGHELPEIHVDYYGPRGHNPRHH*
>11668.m05509|LOC_Os02g55870.1|genepair2020-1
MAAGLLLKLVGFCFWAVFWLGGAATVSTNAGGEAAAAAVVVDARSAIAVTDEDFVCATLD
WWPPDKCDYGTCSWGLATLLNMDLSNKILLNAVKAFSPLKLRLGGSLQDVLIYDTGDPRQ
PCTPFTKNSSAMFGFTQGCLPLHRWDELNAFFQKSGAKIIFGLNALNGRVPMSDDSLGGP
WNYTNAASFIRYTVSKGYDIHGWELGNELSGSGVGARVDADQYAQDVLALKQIIDNSYQG
HASKPLVIAPGGFFDAAWFTELISRTKPNQMDVMTHHIYNLGPGVDTHLIDKILDPSYLD
GEAGTFSSLQGILKSAGTSTVAWVGEAGGAYNSGHHLVTDAFVFSFWYLDQLGMSSKYDT
KTYCRQTLIGGNYGLLNTTTFEPNPDYYSALLWNRLMGTKVLSATFNGTNKIRAYAHCAR
DSRGITLLLINLSGNNTNHIYVTSEGVQPYSTKKEGRRRFGRIPGLGEEAQLIREEYHLT
PKDGSLQSQHVLLNGNVLAPDANGEIPKLEPVQVEGTQPITVAPYSIVFAHIPRFYAPAC
R*
>11680.m00760|LOC_Os06g08090.1|genepair2020-2

MEMRRFLGGLCFLALLRLGGAAEAVVGVAGVDGRRAIAATDEDFVCATMDWWPPDKCDYG
TCSWGLASLLNLDLSNKILLNAIRGTFSPLKLRLGGSLQDKLVYGTGDGGGPCAPFVKNT
SEMFGFTQGCLPLHRWDELNAFFQKSGARIVFGLNALNGRVPLPDGSMGGPWDYTNAASL
IRYTASKGYKIHGWELGNELSGSGVGTKVGADQYAADVIALKSLVDTIYQGNPSKPLVLA
PGGFFDAGWFTEVIVKTRPNLLNVVTHHIYNLGPGVDTHLIEKILNPSYLDGMVSTFSNL
QGILKSAGTSAVAWVGESGGAYNSGRHLVTDSFVFSFWFLDQLGMSAKYDTKSYCRQSLI
GGNYGLLNKETFQPNPDYYSALLWHRLMGTKVLSATFNGTNMIRTYAHCAKDSPGITLLL
INLSGNTTSQVSVTSEGAHANTVKKHSRKTRHLAAGSMREEYHLTAKDGSLQSQVMLLNG
RALVADENGEIPRLEPVKVDAAQPIAVAPYSIVFAHIHNFPAPACS*
>11668.m05511|LOC_Os02g55890.1|genepair2021-1
MAILSDVATEVLIPIAAIIGIGFSIAQWVLVARVKLAPSQPGASRSKDGYGDSLIEEEEG
LNDHNVVAKCAEIQNAIAEGATSFLFTEYQYVGVFMSIFAVVIFLFLGSVEGFSTKTHPC
TYSKDKECKPALFNALFSTVSFLLGAITSVVSGFLGMKIATYANARTTLEARKGVGKAFI
TAFRSGAVMGFLLASNGLLVLYIAINLFKMYYGDDWEGLFESITGYGLGGSSMALFGRVG
GGIYTKAADVGADLVGKVERNIPEDDPRNPAVIADNVGDNVGDIAGMGSDLFGSYAESSC
AALVVASISSFGINHDFTGMCYPLLVSSMGIIVCLITTLFATDFFEIKAVKEIEPSLKKQ
LIISTALMTVGIALVSWLALPYKFTIFNFGEQKEVTNWGLFLCVSIGLWAGLIIGYVTEY
YTSNAYSPVQDVADACRTGAATNVIFGLALGYKSVIIPIFAIALGIYVSFTIAAMYGIAV
AALGMLSTIATGLSIDAYGPISDNAGGIAEMAGMSHRIRERTDALDAAGNTTAAIGKGFA
IGSAALVSLALFGAFVSRAGVKVVDVLSPKVIIGLIVGAMLPYWFSAMTMKSVGSAALKM
VEEVRRQFNTIPGLMEGTGKPDYANCVKISTDASIKQMIPPGALVMLTPLIVGTLFGVQT
LSGVLAGALVSGVQVAISASNTGGAWDNAKKYIEAGASEHARSLGPKGSDCHKAAVIGDT
IGDPLKDTSGPSLNILIKLMAVESLVFAPFFATHGGILFKLF*
>11680.m00758|LOC_Os06g08080.1|genepair2021-2
MAILSALGTEVFIPVAAAVGVAFAVAQWLLVARVKVNPAHAAAAAASGGSKNGGYGDYLI
EEEEGLNDHNVVVKCHEIQTAISEGATSFLFTEYQYVGIFMSIFAVVIFLFLGSVEGFST
KSQPCTYSKDKYCKPALFNALFSTASFLLGAITSLVSGYLGMKIATFANARTTLEARKGV
GKAFIIAFRSGAVMGFLLASSGLVVLYIAINVFKLYYGDDWEGLFESITGYGLGGSSMAL
FGRVGGGIYTKAADVGADLVGKVERNIPEDDPRNPAVIADNVGDNVGDIAGMGSDLFGSY
AESSCAALVVASISSFGINHDFTGMCYPLLVSSVGIIVCLITTLFATDFFEIKAVKEIEP
ALKKQLIISTALMTVGIAIISWLALPAKFTIFNFGAQKEVTNWGLFFCVAIGLWAGLIIG
FVTEYYTSNAYSPVQDVADSCRTGAATNVIFGLALGYKSVIIPIFAIAVSIYVSFSIAAM
YGIAVAALGMLSTIATGLAIDAYGPISDNAGGIAEMAGMSHRIRERTDALDAAGNTTAAI
GKGFAIGSAALVSLALFGAFVSRAGVKVVDVLSPKVFIGLIVGAMLPYWFSAMTMKSVGS
AALKMVEEVRRQFNSIPGLMEGTGKPDYATCVKISTDASIKEMIPPGALVMLTPLIVGTL
FGVETLSGVLAGALVSGVQIAISASNTGGAWDNAKKYIEAGASEHARTLGPKGSDCHKAA
VIGDTIGDPLKDTSGPSLNILIKLMAVESLVFAPFFATHGGLLFKWF*
>11668.m05516|LOC_Os02g55940.1|genepair2022-1
MALRRINLRKKPLLLFPRPFSSSSSPNPPFPPPPPPPNDRDDASPKPSEGEGRNPAASLF
QDLRDRLMSTPSHLPSRRIPTAPPPRPSGNAEPVASIDDIRRQLESYRGSLAARGAPPGS
SPDGAAPSLLDLVRSTSSPTSPQGPNSGHFSSLAESLRNLPSGRQPQQRRQPRSTTPFLS
PTAHPIFGRELGENARKAEGKEENSAIELKKEYSYAELGKKLGQLRPSGAGNDGKEWFSL
EELQGRIAKLANLDIADDMRLGGQYVALRKSLLGIQADQKTKDDIKKTRSMQGLSFLANI
GGPATPQYLQHPPQEELLERYFHPDHMSSEEKMKLELQRVRDEFKMSENDCGSARVQIAQ
LTVKIKHLSTVLHKKDKHSRKGLQDMVQRRKKYLKYLRRTDWDSYCLVLSKLGLRDVPEY
KAPDYKNKSTSKAKSKKSKSKKRKMKA*
>11680.m00738|LOC_Os06g07900.1|genepair2022-2
MALLLLRRRIHRGHKTLLPRAFSSSSGEGMFPPPTSDPASADQRTTKLSSHFAEIRGHLN
ATPPSSPPRRIPESPPPDDVRRSLHLFRNPHPSSGATSAAAANPSPSFADVFRARPAPPT
SRATGADAFPFSALRESLNKNLGTSPTASAVPLPGATASSPDWSSILSSRQRHDGKPLPE
SVFGRETRGEARRGRDGKVEEQQFIRLYSDNELGKKLSELRPPVGKDGKEWFSVEELSRR
LKKLREMDREERALQSGLGTDVLRDAIVTLQTKDLKTNNFAIGECLMKLMDNLFVTDDAV
VTFGLLVAAAQSMSALMAFGSQATPAYLLGKPQQELVERYFHPDHMSSAEKMKQELQSVR
DEFKMSENDCGSARVQVAQLTTKIKHLSTTLHKKDKHSRKGLQEMVQRRKKYLKYLRRTD
WDSYCLVLSKLGLRDVPEYKPPDYKSKRSSSGKTKAKRKMKRKMKA*
>11668.m05520|LOC_Os02g55970.1|genepair2023-1
MAGGGTSIRKYVGALKDTTTVSIAKVNSDYKDLDIAIVKATNHVENLPKEKYIRDIFYHL
SAGRARADVAYCIRALGRRLSKTRNWAVALKTLIVIHRALREVDPTFRDELISYGRSSTH
MLHLSYFKDDSSAEAWDYSAWVRNYALYLEERLESFRVLKYDVEKDPPRTRDLDTVGLLE
QLPALQQLLFRLLGCQPQGSSSYNNIIQHALSMVALESVRIHTAINDGILNLVDKFFEMQ
RDDALRALDLFKRAINQAGQLSEFYEMCKTIHIGRGERFLKIELPPTSFLQAMEEYVRDA
PLASINQRNQAVLAIEYKRKPEDEESSSSAPLPPPPVSTSESEPEPEPEPEPVKEVSPVHEP
TDLLGMNEPTPDVSKIDQKNSLALAIVQPDNTPKAAAPTTENVATSWELALVAAPSSNGN
AATSNKLAGGLDLLTLDSLYNEAHRQAQQNASYNPWEAAAPASSGPMMQQPMQNPFYASN
AIAPPLNVQMAAMAQQQQHMFMLQQQQQQYDQQQQQMMMMMGRQPYDQQQGSSSNPFASP
YMSAGVHPYGPGMQLHAGNSYTYSGTGMM*
>11680.m00731|LOC_Os06g07830.1|genepair2023-2
MAGVGTQPTSLRKYLGALKDTTTVSLAKVNSDYKELDIAIVKATNHVERPSKEKYIREIF
YSISASRPRADVAYCIHALARRLSKTRNWAVALKTLIVIHRALREVDPTFREELINYGRS
RSHMLNLAYFKDDSSAGAWDFSAWIRTYALYLEERLECFRVLKYDVETDPPKTRDLETGD
LLDHLPALQQLLFRLLACQPQGASSYNVIIQHALSMVALESVKIYTAISDGTINLVDKFF
EMQRSDAVRALDIYKRATNQAERLSEFYEVCKTIHIGRGEKFLKIEQPPASFLVTMEEYV
TEAPTVAQKDKVLAIEYKKEAEEEEKPASPPPAPEPEPEQEPEPEPEPVKEEAPKEEPTD
LLGLNEPNPAAAEIEEKNALALAIVPIDDVPKVAPAQNGVTGWELALVTTPSSNETAITS

SKKLAGGLDLLTLDSLYDDANRRASQPTSYNPWDVNPGVAGAGAAPMMQQPMMHDPFYAS
SGYAAPHNVQMTAMAQQQQAFMLQQQMMMAAAAAAAAAPPPQVFHHHQQQQQHLQANPAN
PFGNPFAAAAAAHHPYGAAAAAGNGYTGLI*
>11668.m05522|LOC_Os02g55990.1|genepair2024-1
MGRIQYAAVARGAVVMAEHGDAAFPNAGAVARQILDRLSAGDGGGGGDCNISYTQDLHVF
HVKRTDGVTALCMADDAAGRRIPFAFLEDIHGRFVKTYGRAALTALAYAMNDEFSRVLGQ
QMDYYSNDPNADRISRMRGEMDQVRNVMIDNIDKVLERGDRLELLVDKTATMQGNTMRFK
RQARRFRNTVWWRNVKLTAASVFILLVIIYIVLVYMCHGFTLPSCI*
>11680.m00725|LOC_Os06g07780.1|genepair2024-2
MAILYAVVARGTVVLAEHSAAATNAGAVARQVLERLPGGGADSHVSYTQDRYVFHAKRTD
GITALCMADEAAGRRIPFAFLEDIHGRFVKTYGRAALTSLAYGMNDEFSRVLSQQMDYYS
NDPNADRINRMRGEISQVRTVMIDNIDKVLERGDRLDMLVDKTANMQGNTIRFKRQARRF
RNTTWWRNVKLTIALIFLLTVIIYVVLVFMCHGFTLPTCIR*
>11668.m05523|LOC_Os02g56000.1|genepair2025-1
MSSPPPAAAAAMAVDDADDDQLASMSTEDIVRATRLLDNETRVLKDELQRTNLEVESYKE
KIKENQEKIKLNKQLPYLVGNIVEILEMNPEDEAEEDGANIDLDSQRKGKCVVLKTSTRQ
TIFLPVIGLVDPEKLKPGDLVGVNKDSYLILDTLPSEYDSRVKAMEVDEKPTEDYNDIGG
LEKQIQELVEAIVLPMTHKDRFQKLGIRPPKGVLLYGPPGTGKTLMARACAAQTNATFLK
LAGPQLVQMFIGDGAKLVRDAFQLAKEKSPCIIFIDEIDAIGTKRFDSEVSGDREVQRTM
LELLNQLDGFSSDERIKVIAATNRADILDPALMRSGRLDRKIEFPHPSEEARARILQIHS
RKMNVNPDVNFEELARSTDDFNGAQLKAVCVEAGMLALRRDATEVTHEDFNEGIIQVQAK
KKSSLNYYA*
>11680.m00710|LOC_Os06g07630.1|genepair2025-2
MTSSGAPPPAAMAVDDAEDDQLASMSTEDIVRASRLLDNEIRVLKDELQRTNLELESFKE
KIKENQEKIKLNKQLPYLVGNIVEILEMNPEDEAEEDGANIDLDSQRKGKCVVLKTSTRQ
TIFLPVVGLVDPDKLKPGDLVGVNKDSYLILDTLPSEYDSRVKAMEVDEKPTEDYNDIGG
LEKQIQELVEAIVLPMTHKDRFQKLGIRPPKGVLLYGPPGTGKTLMARACAAQTNATFLK
LAGPQLVQMFIGDGAKLVRDAFQLAKEKAPCIIFIDEIDAIGTKRFDSEVSGDREVQRTM
LELLNQLDGFSSDERIKVIAATNRADILDPALMRSGRLDRKIEFPHPSEEARARILQIHS
RKMNVNPDVNFEELARSTDDFNGAQLKAVCVEAGMLALRRDATEVTHEDFNEGIIQVQAK
KKSSLNYYA*
>11668.m00355|LOC_Os02g04230.1|genepair2026-1
MAGGGVVDGVRRWLRRRRGRTASAYQPLRSSSLARHPGGDGGLEVVPGPFALPAAIRVPA
RNSPPPPDAQLRNEVVVVAQQNNSFKFSGSKYYKVASGVGILQQDDLFSRSACAFQVLEY
ILTVPWQKNYSVELSIVGVRTWELRSFMKDEIFVHDRSNDSNMEARDDMNQWNCAMRTSD
MEFFTEYGEASQYQIQEVVGKGSYGVVAAAVDTHTGERVAIKKINDVFEHVSDAIRILRE
IKVLRLLRHPDIVVIKHIMLPPTRREFRDIYVVFELMESDLHQVIEANHDLSPEHHRFFL
YQLLCALKYIHSANVFHRDLKPKNILANSDCKLKICDFGLARVAFNDSPSTIFWTDYVAT
RWYRAPELCGSFFSKYTPAIDIWSIGCIFAEILTGRPLFPGRNVVHQLDLITDLLGTPSS
ETLSRIRNENARGYLTGMQRKHPIPFSHKFHNADPLALRLLERLLAFDPKDRPTAEEALA
DPYFRGISKLSREPSRLPVSKFEFEFERRKLTKDDVREMIYREILEYHPQMLQEYIRGGE
QISFLYPSGVDRFKRQFAHLEENYSRGERSTPLRRQHASLPRERVCSSVDSNNQDSDNEE
RRAISSIARTMISPPRSQEKGKNRASAYPNGIINLNSNPKIYLKSASISASTCIIRGNKG
PKENGISEDMEEVVYELSDNVTRMLS*
>11680.m04906|LOC_Os06g49430.1|genepair2026-2
MGGGGTLVDGFRRLFHRRTASGSNQSSNAGEEAASSDLEVADDPDLVALRSIRIRVPKRK
MPLPVESHKKNTVEMEFFTEYGEASQYQIQEVIGKGSYGVVAAAVDTRTGERVAIKKIND
VFEHVSDATRILREIKLLRLLRHPDIVEIKHIMLPPSRREFQDIYVVFELMESDLHQVIR
ANDDLTPEHYQFFLYQLLRALKYIHAANVFHRDLKPKNILANSDCKLKICDFGLARASFN
DAPSAIFWTDYVATRWYRAPELCGSFFSKYTPAIDIWSIGCIFAELLTGRPLFPGKNVVH
QLDIITDLLGTPSSETLSRIRNEKARRYLSTMRKKHAVPFSQKFRNTDPLALRLLERLLA
FDPKDRSSAEEALADPYFASLANVEREPSRHPISKLEFEFERRKLTKDDVRELIYREILE
YHPQMLQEYMKGGEQISFLYPSGVDRFKRQFAHLEENYSKGERGSPLQRKHASLPRERVG
VSKDGYNQQNTNDQERSADSVARTTVSPPMSQDAQQHGSAGQNGVTSTDLSSRSYLKSAS
ISASKCVAVKDNKEPEDDYISEEMEGSVDGLSEQVSRMHS*
>11668.m00357|LOC_Os02g04250.1|genepair2027-1
MKQSMRSRQEPRRVSNGVIIAAMLLSLCVLSIVKARYCSTPFVKPDDQLQEQMNSSIRME
TDEPATMAAGEQEEDEEEESSGGGGAEPEVSATPAVVVTAAAGGGGGKRKPTCRMTSKRS
ERCEARGDIRVEGNASTIYIGGIDKEWKTKPYARYHDPVAMAVVREFTLKPVTESSPACT
RNHSVPAFVFSNGGFSGNLYHDYTDVLVPLFLSTHQFKGQVQFLLSGLKPWWVNKFNLFF
RQLTKYDILDIDNDKDVHCFPRIVVGATFHKDMGVDPKRSPGHVSVVDFKRALRRAFGLE
RVAASRGGATGNGKPRLLIISRKNSRRFLNEREMAQAAAAVGFEVRIAEPDQHTDMSTFA
QLVNSADVMIGVHGAGLTNMVFLPRGAVLIQVVPFGGLEWLTTVTFKNPAKDMEVTYMDY
NVQLEESSLIDQYPRNHQVLTDPYAVHKQGWDALKTAYLDKQNIKMDMDRFKKTLQEALD
RLPPA*
>11680.m04894|LOC_Os06g49320.1|genepair2027-2
MKHHHVARGRAEPRRMGNAAMVITMLLSLCVLTYIKARYCSTPFPKAAEEMEVVEIDEDY
DSTRYKMTGPIGEEDFDPTRPTCYVTSKRSERCAAVGDIRVDGNHSKIYINPLDKEWRTK
PYARLHDAVAMDDVREFTLVPFGGANHTAVPPLCTRNHSVPAFLFSSGGFAGNLYHDYTD
VLVPLFTSTNHFGGEVQFLLSGIKDWWLDKFTPLFRQLSRYDVIDVDNDQEVHCFPRIFI
GATFHRAMGIDPARSPGGVTVADFKRLLRRTFRLERAVASRTGAPRRDKPRLLIISRKSS
RRFLNERAMAHAAALARFDVRIAEPDNHTDMPNFARLVNSADVMMGVHGAGLTNMVFLPS
RAVLIQVVPFGGLEWLTRVTFKDPAKDMDVNYMEYNVSFDESSLRELYPRDHFYIQHPYD
VHKKGWDAIKTVYLDKQNVELNLTKLTNTLERARDFLPEP*

>11668.m00366|LOC_Os02g04330.1|genepair2028-1
MERSSRLNPNATPFVPPPRPSFEESLSKRKASEKQVGDTEKDENADKSAEYELPDSLSLD
DYAESLGKLNISAESSSKGEATNSAFDPSQYEQNGVDNHLAVVESLSKMFPDVSADFIVE
ALKAHEFDTELTIDMLADLCEGDDYGHPAEVSAKEIAMECLTTSFTRNPGREYNLICPSE
ALSEKQRIQRRVLCYFPASTNSRRCRKFTTMAYPVSPIAGRRSNWRSFAASLNLEDGPAS
SDSTSSPSEQTSDGGEVYGDPSENLNSRKLKSDELKSLLADSERSKLLKKLSEANQYNRF
LKRQLQMKDNDVVKFKSELAVMELELQALVALAEEIANFDVPSGSRKINGKYIQSHLLTR
LEAVHDKVMEQIKDVDSLKHQEISVFWVGIAENVQIMGSFDGWSQGEAMSMEYSGYQARF
SATLNLRPGRYEIKFLVDGEWRLSLEYPIDGEGSMQNNILVVN*
>11680.m04880|LOC_Os06g49200.1|genepair2028-2
MEGRSQLNPDASPFIPSSLSLFTYNDSKRQAESSSMNDPSARACRPSMCEENDMDPLALT
KSVLLMFPNISEEFIDELLQANEFDICLTVDMLHELNSQNMLHGDAIMGFPTFPDVKKFH
GNLGLPDGDLSESNSSLDQSLQKGMSLTTSGAKSASAMIPDNISLHDKLGVQKDDKPAIA
STTN*
>11668.m00385|LOC_Os02g04490.1|genepair2029-1
MSGQAPQTNQVGGSGVGGADGLPQQMQDVVGLGGLDTQFLLMRNTMRDRIFEYIGRKQSS
TDWRRRLPELAKRLEEILYRKFLNKADYLNMMRGPVEPQLQFAIKTLSAQNQQNQQNQQM
PRQMASSSGYGTMIPTPGITQSATGNSRMPYVTDNTGLPSSGATMVPQGANTGTSLPGSM
SNGYQHLTTSVPLNSTTSSIPSTMGPVGIQRQVTHMIPTPGFNNQQNVPVNPDFSNGAGY
FNGEPTVTSQMQQQKQFPSNQNSHQIQHIGGHSNSGMHSNMLENSSAYGLSDGHVNGGMG
VHGSNMQLTNRSAASEAYINISTYGNSPKPVQQQFNQHPPQRIPTPVDISGSGNFYNTGS
SALTAANNHSMGATNLPSRSRMNSMLHTNQLNMQSIQPQPQIKTEVLDQPEKMNFQSSQL
THEQLIRQQHSMQQHQMQPSSQFVQNQYHLNQQQPNSQHQQSILRSNSLKQPQLSSSHSM
QLSEQGALPHTELISSQATEHADIPIYQGQYQQRSAHDNVKGGQVFGHLSSSQNFHSNAS
HDSQQLLPTNQQLDDSSNDVSYVLKGSQPEQMHQAQWRPQTMEKAPVTNDSSLEKQIQAD
LCQRTMSQDGAQQPFSSDWRLPGCTVTPADPALPKLPSGGLEQAAGNIYYFRQMKWLLLL
FHAKSCLTPVGSCKFHRCFQCPVCSNAKKLLQRSAELASKQKPPEPRKIAQQNTAQRIMN
GVEGDIMDIDLVSDEIFDSQPSVPKRLKMQPVSPSTAEREVSMPSNAGLILQETHSELPD
QNNKVGQLKMDVKIDPRPLQKPAKIGYGTDGNVPTARHNVAPGGSNEIKTHVKQEIMPID
KETSETAPEVKNEANDSTDITVSKSGKPKIKGVSMTELFTPEQIQEHINSLRLWVGQSKA
KAEKNQLMGHNENENSCQLCKVEKLTFEPPPIYCSPCGARIKRNAPYYTVGTGDTRHFFC
IPCYNESRGDTIEVEGQNFLKARFEKKRNDEETEEWWVQCDKCECWQHQICALFNGRRND
GGQAEYTCPNCYVEEVKRGLRMPLPQSAVLGAKDLPRTVLSDHIEDRLFKRLKQERQDRA
AQERKSIEEVPGAEGLVVRVVSSVDKKLEVKPRFLEIFQEDNYPTEFPYKSKAVLLFQKI
EGVEVCLFGMYVQEFGAECSYPNQRRVYLSYLDSVKYFRPEIRTVSGEALRTFVYHEILI
GYLEYCKQRGFTSCYIWACPPLKGEDYILYCHPEIQKTPKSDKLREWYLSMLRKATKEEI
VVELTNLYDHFFITMGECKAKVTASRLPYFDGDYWPGAAEDMINQLRQEEDDRKQQKKGK
TKKIITKRALKAAGHTDLSGNASKDAMLMHKLGETIYPMKEDFIMVHLQYSCSHCCTLMV
SGKRWVCHQCRSFYICDKCYDAEQQLEDRERHPSNSRDTHTLHPVDIVGLPKDTKDRDDI
LESEFFDTRQAFLSLCQGNHYQYDTLRRAKHSSMMVLYHLHNPTAPAFVTTCNVCCHDIE
TGQGWRCEVCPDFDVCNSCYQKGAVNHAHKLTNHPSAADRDAQNKEARQMRVQQLRKMLD
LLVHASTCRSGSCQYPNCRKVKGLFRHGMQCKTRASGGCVLCKKMWYMLQLHARACRDSG
CNVPRCRDLKEHLRRLQQQSDSRRRAAVNEMMRQRAAEVAANE*
>11680.m04872|LOC_Os06g49130.1|genepair2029-2
MKQGQGAHLSGQRIGHHPTAQMNPGDGDGNGRHQVASGHASADPELMNLRIRMTNRLIWE
LLSREPKLQTRPRKLVSDLAKRFEAVIYKKNPNKAAYYSILNGEIFPHLQHALSTHMAQH
QQGQQMLQQLTSSSSYGTTIPIPDVVQNASGNTRALYEMDNTSGPMSNGHHHFSANFPLH
STTKGASLEMSAVSMQEGKITHMIPTPGSSNQQSLPGNFHYSTGTGYLNGKSNVMAQMQE
QQAPFASKINCCPVQRDLGGYAGSGVHSDILNNSSPYGVSEAHMIDGMGLHRSNVQVINR
TVVPETFINPSPYGISPNKPLQRHVNPSTRSTPTPADIAASTSFNGTGSSALSTTSYLDM
TTVNSLPKSRMDSGLIMSQPTIQSFQTEYYIQTEGLDLQEKISLEQLHQQVNQLHLIQPH
SQYAQNQCSLKLQQQNSLHHLVMSRGNVLTQCHLGSDHAEKLLDKRNQLHSELVSSQINE
HVGLTNLQGHYEQTQYHDNYKKGQMSASSQNLGIPAPHDLLPPQQQFDDGSYRLSCFLKE
TYTKPLQPHCKSKPMKEVIMTSLLSGKIQDGFCQKKMARDREHHPIISGWHSAGCAATSF
GSEEVMENTKQYHAQARWLLFLFHAKSCTSPPGSCKSSYCDRVRELVVHLTDCQIKDCSY
RHCRESKMVSDHYKNCINEHCHVCCKAKEMLRRSSELAHKQNPAEPILITQHNMNQRSAD
RVHGDRMDIDQAVETFDDQPPAAKRPKLQLVSPDASENVPVCQKNPGFMLQEAHPRQLDQ
NKKMVPDQEVDVGLDIRHPQVTLVSCHGSDEKIGAAQNTVIPGALNKIHCHVQQETVVAD
KESVTVVDVKKKTGSVDVTISKTGKPKVKGVSLMELFTPEQIHEHINSLRQWIGQWVQCD
KCECWQHQICALFNARRNDVEEAEYTCFKCYIEEFKRGLRMPLPESVVRGAKDLPRTLLS
DHIEERLFKRLREERQERANKLKTSLDEVPGADGLVVRVVSSVDKKLEVKPHFFKILQED
NYPAEFPYKSKAILLFQKIEGVEVCLFGMYVQEYGAECKFPNQRRVYLSYLDSVKYFRPD
IETVSGQALRTYVYHEILIGYLEYYKQRGFTSCYIWACPPVKGEDYILYCHPEIQKTPKS
DKLRQWYLSMLQKAIKENIVVELTNLYDQFFVTAKECKIKVSAARLPYFDGDYWPGAAED
IINQLQLEGDGKLLKKGRVNKIITKRALKAAGHTDLSGNASKEAMLMQKLGEIICPIKDD
LIMVHLQYSCSHCCTFMVSGRRWVCNECKSFYICDRCYNAEQRLEEKERHPSNSKCLHIL
HPVEIVGVSEDTKDRDIILENEIFDTRQAFLSFCQGYHYQYDTLRRAKHSTMMMLYHLHN
PTGPAFVATCNVCNCDIENGQGWYCKDCPDFDMCASCYQKHEVPIIITS*
>11668.m00387|LOC_Os02g04510.1|genepair2030-1
MAAMSSRCAASSSSSAFLRAPLPGGRHHQGSCGFVAAGGGAKTTVRVAAALDTEATAKAG
KSSSRARRARKDGGEPGGSSSTALLAPTHPDGGGGGGVALDDVIVNPVGLGRRSRQIFDE
VWRKFSRLGQMSTVASPEQEEQEGVLFRGGGPMCDFAVPGAQDTTVLVVGATSRIGRIVV
RKLMLRGYNVKALVRRNDQEVIDMLPRSVDIVVGDVGDASSVQAAVSGCNKVIYCATARS
TITGDLNRVDNQGVRNVTKAFQDYYNQLAQSRAGKSSKSKLSIAKFKSAKSLQGWEVNQG

SYFQDIYPSRFDEGTDASFEISENGQAVFSGFVFTRGGYVEVSKRLSLPLGSTLDRYDGL
LLSVGGNGRSYVIILETGPLADTSQSKKYFARMNTKVGFCRVRVPFSDFRPVNPQDPPLD
PFLVHTLTIRFEPKKQRPGDSSQGASDPRNFQLIMEFIKALPTGQETDIVLVSCTGSGIE
PNRREQVLKAKKAGEDALRRSGLGYTIVCPGPLQEEPGGQRALIFDQGNRISQGISCADV
ADICVKALHDSTARNKSFDVCYEHVSKQGDELYELVAHLPDKANNYLAPALSVLEKNT*
>11680.m04870|LOC_Os06g49120.1|genepair2030-2
MAAAIVAARAPPGGGGAAAVIAYHHCASPSRALPLAAGPSAGGGVVVGRRYHHHGGACCF
AAKPTQVAAEVDQGGAAEPGVASANAAVEAKPRKKARSRRGRKGKRSSSSETRTTAVLEE
EEEVEVKEKKTEEDGEEGKRKEKAAGLDLDEVMAVSPVGLGRRSRQIFDEVWRKFSRLGQ
MSSASSTALAEEEQAVLIRGGPMCEFTVPGAQDTTVLVVGATSRIGRIVVRKLMLRGYNV
KALVRRNDAEVIDMLPRSVDIVVGDVGDPSTVKSAVSGCSKIIYCATARSTITGDLNRVD
NQGVRNVSKAFQDYYNELAQLRAGKSSKSKLLIAKFKSPKSLNGWEVDQGSYFPNTFASR
FDEGIDASFDFSEAGQAVFSGFVFTRGGYVEISKRLSLPLGSTLDRYDGLLFSVGGNGRS
YVVILETGPLADTSQSKKYFARMTTKVGFCRVRVPFSAFRPVNPQDPPLDPFLVHTLTIR
FEPKRQRPGDGSQSATDPRNFELILEYIKALPTGQETDFILVSCSGSGIEPNRREQVLKA
KKAGEDALRRSGLGYTIVRPGPLQEEPGGQRALIFDQGNRISQGISCADVADICVKALHD
STARNKSFDVCYEYVAKQGNELYELVAHLPDKANNYLTPALSVLEKNT*
>11668.m00392|LOC_Os02g04550.1|genepair2031-1
MLQAPAVVALAAVLAASTSPATAAAAAADPAPQEEAAAEGGAASTLCNVPPTLSGEDKQA
EKIKHPKSASAMRCTSKCVSTCVLGGAGSPGVDGPFNIRR*
>11680.m04864|LOC_Os06g49070.1|genepair2031-2
MNGGGLARAAAPAVVALALAAILSTPPPQPETFSNIPQTLSGGDGKQQVRIKRPKSAKAL
QCTSKCVATCIRGGEGPLNASGSFQGTIPQPAVLSARMSAISSRMEKMGNEESFDKEKQQ
QFKPRQGMDSRMVTD*
>11668.m00401|LOC_Os02g04640.1|genepair2032-1
MSTQSVIAVKQFSGPDKIAQAYTVPQPSAHVLSNANYDYDLCGSTNSTSLSCAIQSSNIK
TESISSSSLPKILPFSTDSNGESSLSRMSQAEFSDPILSSSSTFCTSLYTSSPMNSGSCR
KTGYLPFLPQPPKCEQQQNSAGQSSSSLMLLDADLRNSGHADDEHTDDLKDFLNLSSDCS
FHGKCSAMAYNEQMEFQFLSEQLGIAISNNEESPRLDDIYDRPPQLMSLPVSSCSDQEDL
QDARSPAKVQLSSSRSSSGTASCNKPRLRWTPELHERFVDAVNKLEGPEKATPKGVLKLM
KVEGLTIYHIKSHLQKYRLAKYLPETKEDKKQEEKKTKSVANGNDHAKKKSAQMAEALRM
QMEVQKQLHEQLEVQRQLQLRIEEHARYLQKILEEQQKARESISSMTSTTEGESPEFAPM
EKTEDKAETSSAPLSKCRITDTDAECHSKVDNKKTKPQADLEMVHDE*
>11680.m04861|LOC_Os06g49040.1|genepair2032-2
MSSQSVVAVKQITAPDKIVETCPSTKHSAHKLFDVKPDFQGLIDDNLSSSSQSSSIKIEL
IRSSSLPNILPFQKRSSEPEPESPLSHVSHPNVSEPVYSNSSTFCTSLFSSSSMETEPCR
QLGTLPFLPHPPKCEQQVSAGHSSSSSLLVPGGDGDIGNAHDEPEQSDDLKDFLNLSGGD
ASDGSFHGENNAMAFAEQMEFQFLSEQLGIAITDNEESPRLDDIYGTPPQLSSLPVSSCS
NQSVQKAGSPVKVQLSSPRSSSGSATTNKARLRWTLELHERFVEAVNKLDGPEKATPKGV
LKLMKVEGLTIYHVKSHLQKYRLAKYLPETKEDKKASSEDKKSQSGSSGNDSVKKKNLQV
AEALRMQMEVQKQLHEQLEVQRQLQLRIEEHARYLQRILEEQHKVSISSNSLSLKPPAES
QPESPKPTSEKKEAESEAGAATSAQPSSEDKSPDAECKSSPPVGSKRARVHVGDEHQCS*
>11668.m00402|LOC_Os02g04650.1|genepair2033-1
MEETATAAAAAEKASSYRYWVREATGDAAPLPVPRKLDATAAAAANGNGNPPPLGSVWNQ
AGTWEEKNLNSWANSRIKEMLGSLDSLEFPTGKASLDGVSKCIGDAFLVTVRNKKRVGYT
YELSLKFKGEWLIKEEKMKVKGHLDIPEFSFGELEDLEVEVRFTDDKGLASDDKRQICKD
LKSFLSPIREKLRAFEEELKDR*
>11680.m04860|LOC_Os06g49030.1|genepair2033-2
MEETAAAAAEKATSYSYWVREATGDAAPLPAPRKIDAADLAAKPAPTTLGSVWNKAGTWE
EKNLNSWANGRIKDLLGSLDPLEFSTGKASVYEVSKCSGDAFLVTVRNKKRVGYTYELGL
KFKGEWLIKEENKKVKGYLDIPEFSFGELEDLEVQISFTDIKDLSSDNKAQISKDLKSFL
APIREKLRKFEEELKDR*
>11668.m00405|LOC_Os02g04680.1|genepair2034-1
MGSFGMDWNQKSSVLWDWENMPPIGNSANENPKNVMLAESKLAGVGVDIGHESGHSSGGT
FSSSSEIGYGSSKSSISASIDSPSKVGNTIELNFASAEEHDKNMDKGKSKVDDTGTSRSP
VVAANRVEPLIGLKLGKRTYFEDVCGGQNVKSSPSGVSVATPSPGLAKKVKVAQQNTQNP
HCQVEGCNVDLSSAKPYHRKHRVCEPHSKTLKVIVAGLERRFCQQCSRFHGLAEFDQKKR
SCRRRLHDHNARRRKPQPEAISLSSSRLSTLLYGDARQQASFLFGQAPYGQMGSCASSWD
NPVPGGFKFTATKAPWSRPTIAAGVDGTHVSNQQASGNVLPHGAHHSFDGLMAFKETNAK
VLNQGMEASAVASGSARGPDFEHALSLLSIDSVGAANLQPGSQIHPGVTAIAGTSNPVMM
PSPAIWQGGLSLDQQAQFQAFDRLGNDDDEDHLQLPKPSYDNSHYDQMN*
>11680.m04857|LOC_Os06g49010.1|genepair2034-2
MASFGMNWNQKSPVFWDWENPAPFGPNTMENPKSIPHPEPRGVVVAAANHGSTNSSGGTF
TSSSELANGSSKSSLSASFDSSSKLGNSLEFRFASVKGHGKNMCKDGEAGRVEDSGTSPA
VAVSHGEPVIGLKLGKRTYFENVCGGQNVKSSSAASGVTCPSTVVKKMKVSQQSTQSSYC
QVEGCKVDLSSAREYHRKHKVCEAHSKAPKVIVSGLERRFCQQCSRFHGLAEFDQKKKSC
RRRLSDHNARRRKPQQEAISFGSSRLATMFYDARQQTDIYFGQSPFGQVRSNAISSCDNL
GGFKFTEAKLPWMKPMKTIGLEDLNFSTLQMPGNVVSHTVHHHDFDGLIPFKGNTPKVLN
QGVDPACAVVSSNSNGAPDLRRALSLLSSDSWGPADVQAGSQVHPGGVMPPLAVAAATVT
APTNPVSVMHALHPSTGGGGFWQDGDDPPPLDHASQAQAFMHPGNGSSSGYGHLH*
>11668.m00418|LOC_Os02g04800.1|genepair2035-1
MARLPLRSRAAHLRRRLQPRVVPAEQPVFPPPRESDSHRRRTDDGKGMAEEDGVERGEEE
LATDEVHSGGGGEEGLSLETQHTRYSILRWYQVPDTPYIIISSTGYGTFRYQAGLGTRYQ
AGLGTRYLIPQVLDALYQIWYLWDSCRSRYQVPNAQYQIWHLWDSCRSRYQVPCYPRYQA

GLGTRYMIPQVPDAQYQIWYLWDSCRSRYQALMPMGAMRKREKRRGKHKNKMAIFLGGGR
TLFLLVAILDSDRRCYILDTHVTMMVMDIACDKLHLDGGEHHEQLRAITVFVVGLQEPGD
AGTCRRACNPEEEGCDHTGGLPASSQRFSIKGRVYPAILPVDGKEVSGKVFKGITDRELN
VLDIFEDEEYVKRTVEISLTDTSEKLLAYAYIWGNQDDPDLYGEWDFEEWKRVHLEDYVK
MTQEFMEELEQLEPKTET*
>11680.m04851|LOC_Os06g48960.1|genepair2035-2
MAPPPAAAAAVDGVGVGVGAHSVFVYGSLMQDEVVRTIIKRVPPSSPALLPNYHRFNIKG
RIYPAILPVQSKKVAGKVITGVTDAELQILDEFEDVEYVRTRVEISLTDTSETMLADTYV
WADAEDPNLYGEWDFEEWKRLHMKDFLAMTHGFMDGLEQPESKSRVETYQSFMQEIQQPG
TTTTTTQVEI*
>11668.m00419|LOC_Os02g04810.1|genepair2036-1
MASMKQQQTPASSAVTAAAAASSSATAAVAACEGERKAAAINSELWHACAGPLVSLPPVG
SLVVYFPQGHSEQLLITVLVHYQSGWEHWAYWLPFSVALLVWSIDSLGRTNPAIIRAFLF
RKMRKKVYKSNIILRNAFSMLLETAEYLLNLSFLFQVAASMQKDVDAHVPSYPNLPSKLI
CLLHGVNLHADPDTDEVYAQMTLQPVNTYGKEALQISELALKQARPQMEFFCKTLTASDT
STHGGFSVPRRAAEKIFPPLDFSMQPPAQELQARDIHDNVWTFRHIYRGQPKRHLLTTGW
SLFVSGKRLFAGDSVIVVRASPTEFVIPFAKYQKALYSNQISLGMRFRMMFETEELGTRR
YMGTITGISDLDPVGWDESAAGERRNRVSIWEIEPVAAPFFLCPQPFFGVKRPRQLDDES
EMENLFKRAMPWLGEEVCIKDTQNQNSTAPGLSLVQWMNMNRQQSSSLANTAAQSEYLQA
LGNPAMQNLAADELARQLYVQNNLLQQNCIQFNSPKLPQQMQTMNDLSKAAIPLNQLGAI
INPQDQKQDAVNHQRQQNSIQVIPLSQAQSNLVQAQVIVQNQMQQQKPSPTQNPQRINGQ
RLLLSHQQKDQNLQLQQQLLLQQKQQLQQQQQQQQQNQQQLNKSLGQLVNLASQQSKLFD
EELQLQILQKLQQQSLMSQSTSTLSQPPLIQEQQKLITDMQKQLSNSHSLAQQQMMPQQE
IKPSLQATPLLPTVQQEQQQKLLQKQVSLADVSGVAFQPISSTNVIPKTGGAMIISGATQ
SVVTEEMPSCSTSPSTANGNHFTQSTKNRHCINTERLPPSTAPMLIPTSIDAVTATPLMT
KELPKPNNNVKQSVVNSKLPNVAPGPQNCINHALQTDNLETSSSATSLCPSRTDGLVHQG
FPSSNFNQHQMFKDALPDVEMEGVDPSNSGLFGINNDNLLGFPIETEDLLINALDSVKYQ
NHISTDVENNYPMQKDALQEISTSMVSQSFGQSDMAFNSIDSAINDGAFLNKNSWPAAPL
LQRMRTFTKVYKRGAVGRSIDIGRYSGYEELKHALARMFGIEGQLEDRQRIGWKLVYKDH
EDDILLLGDDPWE*
>11680.m04850|LOC_Os06g48950.1|genepair2036-2
MAAAAAAAVVGSGCEGEKTKAPAINSELWHACAGPLVSLPPAGSLVVYFPQGHSEQADPE
TDEVYAQMTLQPVTSYGKEALQLSELALKQARPQTEFFCKTLTASDTSTHGGFSVPRRAA
EKIFPPLDFSMQPPAQELQARDLHDNVWTFRHIYRGQPKRHLLTTGWSLFVSGKRLFAGD
SVIFVRDEKQQLLLGIRRANRQPTNISSSVLSSDSMHIGILAAAAHAAANNSPFTIFYNP
RASPTEFVIPFAKYQKAVYGNQISLGMRFRMMFETEELGTRRYMGTITGISDLDPVRWKN
SQWRNLQVGWDESAAGERRNRVSIWEIEPVAAPFFICPPPFFGAKRPRQLDDESSEMENL
LKRAMPWLGEEICIKDPQTQNTIMPGLSLVQWMNMNMQQSSSFANTAMQSEYLRSLSNPN
MQNLGAADLSRQLCLQNQLLQQNNIQFNTPKLSQQMQPVNELAKAGIPLNQLGVSTKPQE
QIHDASNLQRQQPSMNHMLPLSQAQTNLGQAQVLVQNQMQQQHASSTQGQQPATSQPLLL
PQQQQQQQQQQQQQQQQQQQQQKLLQQQQQQLLLQQQQQLSKMPAQLSSLANQQFQLTDQQ
LQLQLLQKLQQQQQSLLSQPAVTLAQLPLIQEQQKLLLDMQQQLSNSQTLSQQQMMPQQS
TKVPSQNTPLPLPVQQEPQQKLLQKQAMLADTSEAAVPPTTSVNVISTTGSPLMTTGATH
SVLTEEIPSCSTSPSTANGNHLLQPILGRNKHCSMINTEKVPQSAAPMSVPSSLEAVTAT
PRMMKDSPKLNHNVKQSVVASKLANAGTGSQNYVNNPPPTDYLETASSATSVWLSQNDGL
LHQNFPMSNFNQPQMFKDAPPDAEIHAANTSNNALFGINGDGPLGFPIGLGTDDFLSNGI
DAAKYENHISTEIDNSYRIPKDAQQEISSSMVSQSFGASDMAFNSIDSTINDGGFLNRSS
WPPAAPLKRMRTFTKVYKRGAVGRSIDMSQFSGYDELKHALARMFSIEGQLEERQRIGWK
LVYKDHEDDILLLGDDPWEEFVGCVKCIRILSPQEVQQMSLEGCDLGNNIPPNQACSSSD
GGNAWRARCDQNSEAILKISMMKSKVEDVRYWNTA*
>11668.m00422|LOC_Os02g04840.1|genepair2037-1
MRIRGDGGGDEEEAAMAVVSAPPQHLVFAYYITGHGFGHATRALEVVRHLIAAGHDVHVV
TGAPEFVFTTEISSPNLHIRKVLLDCGAVQADALTVDRLASLEKYHQTAVMPRESILRTE
VEWLNTIKADLVVSDVVPVACRAAADAGIRSVCVTNFSWDFIYAEYVVVAGHHHRSIVWQ
IAEDYSHCEFLLRLPGYCPMPAFRDVIDVPLVVRRLHKSRSEVRKELGIKDDVKVVIFNF
GGQPAGWKLKKEWLPDGWLCLVCGASETQELPPNFIKLAKDAYTPDLMAASDCMLGKIGY
GTVSEALAYKLPFVFVRRDYFNEEPFLRNMLEHYQCGVEMVRRDLLTGHWKPYLQRAMTL
HPCYDGPINGGEVAAHILQDTAVGKKYISGKLSGARRLRDAIVLGYQLQRAPGRDVGIPD
WYSVSEKEIGVRPAPTYHEVNGSAESSFEDFEILHGDIQGLTDTMAFLTSLSGLVGNDPR
SPEKQSRERVAASVFFDWEEEIYVARAPGRLDVMGGIADYSGSLVLQMPIREACHVAIQR
SNPMKQKLWKHTQARQLANGRAVPLLQIVSFGSELSNRAPTFDMDLSDFMDGDKPISYDK
AKEYFSQDPSQKWAAYVAGTILVLMTELGVVFTDSMSILVSSSVPEGKGVSSSASVEVAS
MSAIAAAYGLNIPPRDLAILCQKVENRIVGAPCGVMDQMTSACGEANKLLAMICQPAEVK
ELVSIPTHIRFWGLDSGIRHSVGGTDYGSVRVGTYMGRKMIKCAASDLLSESLPSCPPIQ
SGNTNSDEYEEHGVDLLKSEASLEYLCNLPPHRYEAVYARDIPEIITGDAFLEKYGDHND
AVTTVDPKRSYCVKAPTRHPIYENFRVEAFKALLTAAKTVEQLSALGELMYQCHYSYNAC
GLGSDGTDRLVNMVQEVQHRKTSQDGGPSLFGAKITGGGSGGSVCVIGKNCLKSSEEIFE
IQKRYKAATGYLPIVFEGSSPGAGKFGYLKIRRRST*
>11680.m04849|LOC_Os06g48940.1|genepair2037-2
MPPAGDAAHAPPPPHRRLVFAFYLTGHGFGHATRAIEVVRHLIAAGHEVHVATAVPEFVF
TAELPRSPSSQGLLHIRRAILDCGAVQTDALTVDPLASLLKYHETAVVPRESILRTEAEW
LTSINADLVISDVVPVACRVAADVGIPSVCIGNFSWDYIYAEYIVASGDHHRSIVWQIAE
DYSHCDILLRLPGYCPMPAFRDVTDVPLVVRGLRKSRSEVRKELGIAENAKVVVFNFGGQ
PAGWNLKQEWLPDGWICLVCGASDSQEVPPNFIKLAKDAYTPDAMAASDCMLGKIGYGTA

SEALAYKLPFIFVRRDYFNEEPFLRNLLEHYQSSIEMTRRDFLHGHWKPYLLRALTLQPC
YDGPTNGGEVAARILEDTAVGKKCISDKFNGARRLQDAIVLGYQLQRAPGRDVAIPDWYS
LSETEVGACPISENIKTKESTESCFEDFEILHGDLQGLPDTMSFLKSLSEFNQSELKSNG
KQPQERIAASVLFDWEEEMYIARAPGRLDVMGGIGDYSGSLVLQLPLREACHVAVQRNHP
SNQKLWENTQARRLENGGMEPVVQIVSFGSELSNRSPTFDMKLSDLMDVDKPISYEKARE
FFCRNPSQKWAAYVAGTILVLMTELDVKFTDSMSILVSSDVPEGKGVSSSASVEVATMAA
IAAAYGLNIAPRDLALLCQKVENHVVGAPCGVMDQMTSACGEANKLLAMVCQPAEVKELV
AIPNHMRFWGLDSGIRHSVGGGDYGSVRVGTYMGRKMIKCAASDLASESSVSDAPVQSND
YKQNAIELLKSEASLEYLCNIPPHRYEAIYAKDIPEVITGDAFLKKYGDHDDTVTAIDPK
RSYNVKAPTRHPIYENFRVETFKALLEAANTDEQLSALGELMYQCHYSYNACGLGSDGTD
LLVNLVQEMQHRNMSKGESPSLFGAKITGGGSGGSVCVMGKNCLKSSEEIIEIQQRYKAA
TGYLPILFDGSSPGAAKFGYLKIRRRPSSPIAAK*
>11668.m00443|LOC_Os02g05050.1|genepair2038-1
MGYFRAPRRKAAAAAEGESVRAALLVGGGGEEAAVPKGYFAVYVGAEARRFVVPTSYLRQ
PAFRGLMELAADEFGFAQEGGLRLPCREEDFQATVAALDARRRPASGGAIMSTMVKARSL
*
>11680.m04841|LOC_Os06g48860.1|genepair2038-2
MMVMGYLLAPKKGGRRRRGKDQPSAAAHHGDNDDGLRETLLEQQQQPASSSSPTAGGGGV
PKGYFAVYVGEEARRFVVPTGYLREPAFRDLMERAADEFGFAQAGGLRVPCGEDDFEDLL
RRLRRKNGGAAAAKAKKAIS*
>11668.m00444|LOC_Os02g05060.1|genepair2039-1
MGEQGGRASSNKIRDIVRLHQLLKRWKRAALAPKAGKNNNGGGASVPKGFFAVCVGEEMR
RFVIPTEYLGHWAFEQLLRKAEEEFGFQHEGALRIPCDVEVFEGILRLVGRKDEKAAMCY
SSSEHEILCR*
>11680.m04840|LOC_Os06g48850.1|genepair2039-2
MEDHQGGGVGRASNKIRDIVRLQQLLKRWKKLATMAPGGRSGVPKGSFAVYVGEEMRRFV
IPTEYLGHWAFERLLRDAEEEFGFRHQGALRIPCDVAAFEATLRLVAAGNGNAKAKDDAA
AMCSCSSDTEILCR*
>11668.m00471|LOC_Os02g05330.1|genepair2040-1
MAGMAPEGSQFDAKHYDSKMQELLHQGDNEEFFTSYDEVFESFDDMGLQENLLRGIYAYG
FEKPSAIQQRGIVPFCKGLDVIQQAQSGTGKTATFCSGILQQLDYGLVECQSLVLAPTRE
LAQQIEKVMRALGDYLGVKVHACVGGTSVREDQRILASGVHVVVGTPGRVFDMLRRQSLR
PDHIKMFVLDEADEMLSRGFKDQIYDIFQLLPPKIQVGVFSATMPPEALEITRKFMNKPV
RILVKRDELTLEGIKQFYVNVEKEDWKLDTLCDLYETLAITQSVIFVNTRRKVDWLTDKM
RSRDHTVSATHGDMDQNTRDIIMREFRSGSSRVLITTDLLARGIDVQQVSLVINYDLPTQ
PENYLHRIGRSGRFGRKGVAINFVTRDDERMLFDIQRFYNVTIEELPANVADLL*
>11680.m04829|LOC_Os06g48750.1|genepair2040-2
MAGMAPEGSQFDAKHYDSKMQELLNQGETEEFFTSYDEVHESFDDMGLQENLLRGIYAYG
FEKPSAIQQRGIVPFCKGLDVIQQAQSGTGKTATFCSGILQQLDYAVVECQALVLAPTRE
LAQQIEKVMRALGDYLGVKVHACVGGTSVREDQRILASGVHVVVGTPGRVFDMLRRQSLR
PDYIKMFVLDEADEMLSRGFKDQIYDIFQLLPSKIQVGVFSATMPPEALEITRKFMNKPV
RILVKRDELTLEGIKQFYVNVEKEEWKLDTLCDLYETLAITQSVIFVNTRRKVDWLTDKM
RGRDHTVSATHGDMDQNTRDIIMREFRSGSSRVLITTDLLARGIDVQQVSLVINYDLPTQ
PENYLHRIGRSGRFGRKGVAINFVTRDDERMLFDIQRFYNVVIEELPANVADLL*
>11668.m00472|LOC_Os02g05340.1|genepair2041-1
MAPPENPNGAGPAAPSDPAQPQPQPPAKSKWKKKDEKKDDDLSEEDLALKEQLELYVVRA
QDADPGVQKLALESMRQEIRSATSSMTSVPKPLKFLRPHYGTLKSYFETMPESSDLKRYM
ADILSVLALTMSAEGERESLKYRLLGSEGDIGSWGHEYVRNLAGEIAQEFQKRQDDDMPI
DALMELVQQIVSFHMKHNAEPEAVDLLMEVEDLDLLVEHVDSTNYKRTCLYLTSSSKYLP
APDDMLALDIAYTIYMKFEDLTSALRIALLLDNKSMQYVKQVYTATEDLQLKKQFSFIIA
RHGLAMEIDDEIAADGNDKEGLQEIVNNSKLSEGYLTLARDIEVMEAKSPEDIYKVHLID
GRGASSSLDSARQNLAATFVNAFVNAGFGQDKLMTAPSDSSSSGASGNWLFKNKEHGKAS
AAASLGMILLWDTDSGLAQLDKYLHSNDAHVVAGALLGIGIVTCGVKNDCDPALAILMEY
INKDDTNIRIGAILGLGIAYAGSQKEELKSYLSTVLGDSEKSTLEVLIFSAIALGLVFVG
SCNEEIAQSIIFALMERSEAELAEPIIRLLPVALGLLYLGKQESVEATAEVSKTFDEKIR
KYCDVTLMSLAYAGTGNVLKVQKLLGICSQHLEKGETHQGPAVLGIALIAMAEELGAEMA
VRSLERLLQYGEQNIRRAVPLALGILCISNPKVNVMDTLSRLSHDADADVSMAAIISLGF
IGAGTNNARIAGMLRNLSSYYYKEAAHLFCVRIAQGLVHLGKGLLTLSPYHSDRFLLSPM
ALGGLVTVLHACLDMKSTILGKYHYILYIIVLAMQPRMLLTVDEDLKPLSVPVRVGQAVD
VVGQAGRPKTITGFQTHSTPVLLAAGERAELATEKYIPLTSVLEGFVILKKNPEYNEE*
>11680.m04817|LOC_Os06g48640.1|genepair2041-2
MPPREDPNGDASSASPSDPVPPPPQQPQPQPAKGKGKKKDEKKDDDLSEEDQALKEQLEL
YVVRAQDTDPGVQKLALESMRQEIRSATSSMTSVPKPLKFLRPHYGTLKAYFETMPESDL
KKYMADILSVLALTMSAEGERESLNYRMIGSEGDIGSWGHEYVRNLAGEIAQEFQKRQDD
GMPTDSLKELVEQIVSFHMKHNAEPEAVDLLMEVEKLDLLVKHVDSTNYKRTCLYLTSSS
KYLPAPDDMSALKIAFEIYMKFGDFANALRIALLLYDKSLELKPIFTATDDFQLKKQFAF
IIARHGLSIEIDDDIAADENEKEALQDIISNIKLSEGYLTLARDIEVMEPKSPEDIYKVH
LIDGRGATSSSLDSARQNLAATFVNAFVNAGFGQDKLMTTPSDSSSSGSSGNWLFKNKEH
GKASAAASLGMILLWDSDSGLAQLDKYLHSNDIHVVAGALLGIGIVSCGVKSDCDPAFAL
ISEYFSRDESIIRIGAILGLGIAYAGSQKEEVRENLTAFLTDSQVPLEVLVFSAISLGLV
FVGSCNEEVAQTIICVLMERSEPELAEPIMRLLPVALGLLYLGKQESVEATAEVSKTFDE
KIRNYCDVTLMSLAYAGTGNVLKVQKLLGICSEHLEKGETHQGPAVLGISLIAMAEELGA
EMAVRSLERLLQYGEQNIRRAVPLALGLLCISNPKVNVMDTMSRLSHDADAEVSMAAIIS
LGLIGAGTNNARIAGMLRNLSSYYYKEAGHLFCVRIAQGLVHLGKGLLTLSPYHSDRFLL

SPIALAGLVTVLHACLDMKSIILGKYHYMLYILTLAMQPRMLLTVDEDLKPLSVPVRVGQ
AVDVVGQAGRPKTITGFQTHSTPVLLAAGERAELATEKYLPLTPVLEGFVILRKNPEYHE
D*
>11668.m00486|LOC_Os02g05470.2|genepair2042-1
MSSSCIPTGLRLDLDIVKAATSPGAHSSPLRPAHSSPSSTLSEASNTSSSSATSVSLKRA
RAPRKRPNQAYNEAAALLASIHPSVFPVKKSPKTASRPPTRQLSGLSAVFDPSSDLLPPL
PVLADAAFLLRDTPPTPRQNCSSPAPVSSAFREFRDPAPLSPASPHTTDEPGELDFDDDC
FLDVDEAAADGAAGGIDGIMGSLTVETNTAAATTSDDSILSSSGIHPYLRSLMVVGLAGR
FELGLGSRHGARPNLNRALKRRDDDGAWWMWPAVPVKDITVAPPPPTPTPTPPRPQGTAS
NTTAAMPSPPSAPEKKKSKKKKVVKVEKVMAREKEEEESPPDARCKEEEAADGTVDAGNG
DGAPTMAPKTGLGLKLDADDVLKAWSGKGSMFADGGAPESPKSAAEVRAKLADIDLFPEN
GNGAGGIREASVMRYKEKRRTRLFSKKIRYQVRKVNADCRPRMKGRFVRSPSLLQQVLEE
ES*
>11680.m04814|LOC_Os06g48610.1|genepair2042-2
MASSCIPTGLRLDLEMVKAAAPPGGSSAAAHSSASSTLSEASNSSSSSVASLSLKRARTP
RKRPNQTYNEAAALLASMYPSVFPVIKGAGTAPPRLLGLATALADDPSSSDLLPPFPVLG
NGSAAHLLRDTPPPPTPRCPAPIKSCSSPAPVSSVFREFRDAAPSPGTPDADADVTDDDY
LGELDFEDEDGFDADSFLAVDDGVAEGIDRIMGKLSMEKNSAAASRADAVLSSAAIHPYI
RSLMVLGLGFRQGRLNAGQALKRHDDESDWWMCPAIPVKEIAAPPAPSVAMPVPVPMPLQ
ASDKKKKKSKKKSLKEIERDNAIAVGECKFGDEGTLGFDHGDAGISALKDPKIGLGLNLN
TEEVLKAWCNSGSVFAGCDALESPRSSSDLHAKLADIDLFLDNNTSGVIREGSMLKLRHK
QKQCTPLLSNKTRYQSRKVNAECRPRVKGKYVSQASLLQQASEKER*
>11668.m00487|LOC_Os02g05480.1|genepair2043-1
MAIMVDPPNGMGNQGKYYYSMWQTLFEIDTKYVPIKPIGRGAYGIVCSSINRETNEKVAI
KKIHNVFDNRVDALRTLRELKLLRHLRHENVIALKDIMMPVHRRSFKDVYLVYELMDTDL
HQIIKSPQGLSNDHCQYFLFQLLRGLKYLHSAEILHRDLKPGNLLVNANCDLKICDFGLA
RTNSSKGQFMTEYVVTRWYRAPELLLCCDNYGTSIDVWSVGCIFAELLGRKPIFPGTECL
NQLKLIVNVLGTMSESDLEFIDNPKARRYIKSLPYTPGVPLASMYPHAHPLAIDLLQKML
IFDPTKRISVTEALEHPYMSPLYDPSANPPAQVPIDLDIDENISADMIREMMWHEMLHYH
PEVVAAMSAR*
>11680.m04811|LOC_Os06g48590.1|genepair2043-2
MAMMVDPPNGMGNQGKHYYTMWQTLFEIDTKYVPIKPIGRGAYGIVCSSINRATNEKVAI
KKINNVFDNRVDALRTLRELKLLRHLRHENVIALKDIMMPVHRRSFKDVYLVYELMDTDL
HQIIKSSQPLSNDHCQYFLFQLLRGLKYLHSAGILHRDLKPGNLLVNANCDLKICDFGLA
RTNNTKGQFMTEYVVTRWYRAPELLLCCDNYGTSIDVWSVGCIFAELLGRKPIFPGTECL
NQLKLIVNVLGTMSEADIEFIDNPKARKYIKTLPYTPGIPLTSMYPQAHPLAIDLLQKML
VFDPSKRISVTEALEHPYMSPLYDPSANPPAQVPIDLDIDENLGVDMIREMMWQEMLHYH
PEVVAGVNM*
>11668.m00502|LOC_Os02g05610.1|genepair2044-1
MADVLLPELRSPPPPPPPPPPTNDDRARAVDLPSPTPECDDRSPRRELGRSPEDHGVPLP
PPPPLGSSRPERLASDRPEEGASAAAQPCGGRSESPTARFGFEHERGRERSMNTSRRAPD
CLDSGCDAPYNGQSNTRRKGLMTYKQFTQKLEDDVSPGEAESRYQEYKTSYITSQKQDYF
DHHKNEDRLKDMYHPTNLLSVIERRNELCKAAAKNLILDLRSGTLDLGPGMTAGTASKSG
NDSDGIPADDEDYHNKRRRHHRGPLEETELVSVAPKAHPVSSHYRRIQTDIHQTLALVKK
LDEEKGIVGNILTTGDHTKSNGDKSYAGSTGPLVIVRGLSTVKGLDGFELLDTLLTYLWH
VHGIDYYGMSESTNAKGLRHVRADTKNANMDKSSAADWEKKLDYFWQERLTNGKDPLVAL
TAKDKIDASADKVLESYVTKVKDDNYGWTYGCGAKGCIKVFHAPDFVLKHLNLKHPDLVS
KLTSRVQEDIYFQNYMNDPNAPGGTPVMQQQSPEQQGPTPSELTPGAFGGQGSFVEMPTP
PVLIPVPGAGPLGPFVPAPPEVVMQMMRPVMPMYPPRPPNPRRLRSYKDLDAPDDEVTLV
DYRSL*
>11680.m04805|LOC_Os06g48530.1|genepair2044-2
MADVIDPASTEAPRARRPPPPPPDSPEGRSPPLPPPPPGGPPQPAATRKRSRSPPPPPPP
PSLPPPPPLGSSRPERYRDNHHRGGGGGRGGGSSSPPPYRSGRRHSPSRRSPSPPFKRSR
RDDGYDRRGGRGSPPPRYGYGDRRYGYDHERGGGRGGYDDDRYHGRYQNRAADWADSGFG
ASNDGPGITQREGLMTYKQFIQVLEDDISPAEAEKRYQEYRTEYITTQKRAYFDLNKNDD
RLKDKYHPTNLSSVIDRRNDSCKATAKDFFHDLQNGTLDLGPGITAAAASGSDGNSDDDG
DSDKRRKHGRGSSKETDPLSGAPVAHPVSSESRRVQVDIEQALALVRKLDTEKGIVGNIL
SSGDHDKSDVDKSHIGSMGPIIIIRGLTTVKGLEGVELLDTLLTYLWRIHGVDYYGMSET
NEAKGSRHVRADNKTSNTTNINAADWEKKVDTFWQERLRGQDPMVILAAKDKIDAAAVEV
LEPYVRKIRDEKYGWKYGCGAKGCTKLFHAPEFVHKHLRLKHPELVLELTSKVREDLYFQ
NYMNDPNAPGGTPVMQQSAPDKSRQRPGMDNRLRYDRANRREYDRAERDGSRYGRGDRSP
SLDGADDQMFDAFRGRGPNAPFVPELPAPPILMPIPGAGPLGPFVPAPPEIAMHMLREQG
PPPPFEPNGPPHANPGVLGPMMGGPAPIITMPPSFRQDPRRLRSYNDLDAPDEEVTVLDY
RSL*
>11668.m00503|LOC_Os02g05620.1|genepair2045-1
MESVAAAARSLRAGVEKSRALGQALARAGPRVEEIQLALPALEAAVRPIRAPRAELAAAG
PHIDRAVGPAAAVLKVFDAVHGLEPPLLAAGAVAGDLAGYLAVLGRLEEALRFLSDNSGL
AAQWLADIVEYLGDHDLADPRFLADLAVTLEGLKKPSGDLDGGLLAAALDMLEAEFRRLL
ADHSAPLPLAMQQLNTTSASTAAPSLIPAATVHKLSLILDRLIANGRQDRCLSVYADARG
GVVSASLRALGLDYLRNPVDVAQALGPGVELWGRHLEFVVRCLLESERQLCNKVFGQRKD
DASACFADVAAHAGILDFLSFGRAAADAKKDPIKLLRLLEVFDSLNKLRLDFNRLFGGKK
ACVEIQNQTRDLVKLLVDGAVEIFEELLVQVELQRHMPPPPDGGVPRLVSFVVEYCNRLL
SEKYRPVLAQVLTIHRSWRKETFSDKMLVNAVLNIVKALEANFDVWSKAYDNVTLSYLFM
MNTHWHFFKNLKATRLGELLGDAWLQEHEQFKDYYLTVFMRDSWGVVSPLLNREGLILFS

KGRATAKDLVKQRLKTFNASFDEMFRKQSAWVIPEKDLREKTCGLVVQAIVPAYRSYMQN
YGPLVEQDVSASKYVKYTVDGLEKMLSALFIPRPRRAGSFQIRHTNDKITSAMTGFYRSA
STVK*
>11680.m04784|LOC_Os06g48330.1|genepair2045-2
MGVGEEEGRGMASLLAARRALRAGVERSRALSHALARAGPRLGEIRARLPPMEAAVRPIR
APRDALEGAGECVDRAVGPAAAVLKVFDAVHGLEPPLLAASAAADDLPGYLAVLSRLEEA
LHFLSDNCGIASQWLADIVEYLGDRSLADPRFVSDLAEALSHLKTPSANLDGGLLAAALD
ILEAEFRRLLTEHSAPLAMKEPNNSSDPGSITPPRIPASAVHKLSLILDRLAANGRLGTC
TAAYADARGDTVSASLRALGLDYLHDPAEDAQVLTPNVEHWGRHLEFAVRHLLEAERKLC
VAVFERRPEAASSCFAEIASRAGILDFLKFGRAICDARKDPIKLLRLLDVFDSLSKLRMD
FNRLFGGKACVEIQTRTRELVKRVVDGSVEIFEELLVQVELQRNMPPPADGGVPRIVSFV
AKYCNQLLGDPYRSVLTQVLVIHRSWRKETFNDKMLVDAVLNIVKALEANFEAWSKAYED
VTLSYLFMMNTHWHFFKHLKGTKMGEILGDEWLREHEQYKDYYSAVFLRESWGTLAPLLS
REGIILFSKGRATARDLVKQRLKSFNANFDEMYQKQSAWIISDRDLQQKTCHLVVQAIVP
VYRSFMQNYGPLVDQQDASANKYVKFTAEGLDKMLSTLFLPKPRRAGSFQIRHSNGKITS
AMTGLYRSSSTLK*
>11668.m00505|LOC_Os02g05630.2|genepair2046-1
MHGRPSRPLASSSSSSSSRVFSFFLAPRVFLFLVVVVVVVFLPGRSSCWWLEGTEELEEE
MGFAGDCSPVSGGGLSENGKFSYGYASAPGKRASMEDFYETRIDGVDGETIGLFGVFDGH
GGARAAEYVKQHLFSNLIKHPKFISDIKSAIAETYNHTDSEFLKAESSHTRDAGSTASTA
ILVGDRLLVANVGDSRAVVCRGGDAIAVSRDHKPDQSDERQRIEDAGGFVMWAGTWRVGG
VLAVSRAFGDKLLKQYVVADPEIKEEIVDSSLEFLILASDGLWDVVSNKEAVDMVRPIQD
PEQAAKRLLQEAYQRGSADNITVVIVRFLEGTTTGGGPSREAASDQNS*
>11680.m04781|LOC_Os06g48300.1|genepair2046-2
MREVLLLGSLVVLALLSLFPCCSCLSQGAEEEEDDGEVRLMGLAGEAAGSPGSGGGFSAN
GKFSYGYASSPGKRSSMEDFYDTRIDGVDGETVGLFGVFDGHGGARAAEFVKQNLFTNLI
KHPKLFSDTKSAIAETYTSTDSELLKAETSHNRDAGSTASTAILVGDRLLVANVGDSRAV
ICRGGDAIAVSRDHKPDQSDERQRIEDAGGFVMWAGTWRVGGVLAVSRAFGDKLLKQYVV
ADPEIKEEVVDSSLEFLILASDGLWDVVTNEEAVAMVKPILDSEQAAKKLLQEASQRGSA
DNITCLVVRFLEQENHLPERPTNDQAS*
>11668.m00507|LOC_Os02g05640.1|genepair2047-1
MDSVVDGGLDTRLSLAVGCCPPRRRPVLLFGEVLPSPEKKVAAAAVVAAGKRGREQRGEA
EAEATTTRQRRSCKKGRRGRGDDDDDDGDRRSPSGGGGDEEGASRKKLRLTGEQATLLED
SFRAHNILSHAEKQELAGKLGLSARQVEVWFQNRRARTKLKQTEADCDLLRRWCDHLAAD
NARLRRDLAELRRSSSSPPVSGLAVATPVVCPSCAHDDKRRLAFATAAAAAGDMASN*
>11680.m04780|LOC_Os06g48290.1|genepair2047-2
MEGEDLGSWLGLGIGGGGYAYGGDDCRRSPSSPSPVQMLFSQHVKEEITRGYDHGRDEEQ
ASGSKIMKGERGARLRVMRSIRNSGGDGSRSRVLSLGDDGGDGGSGGGGGGGTRKKLQLT
KEQSTLLEDSFRVHNILSHAQKHELARQLKLKPRQVEVWFQNRRARTKLKQTEVDCEFLK
RCCESLTEENKQLKHELMELRRLASPAAAAAGSQLYVQFPRAAAAAMVNVCPSCEKVTVM
GGGGGETGKSSSSYSS*
>11668.m00510|LOC_Os02g05670.1|genepair2048-1
MKVTTQLKPMASSSTSPSPSRLPRSASGKAPTSPPSSSNASRRHHQQPPRASASTPATPA
PSRHHLRSLSISCMTIRTDDDSPPPAATHKDKAATAKPLSYYSSMLSPRKLMQRASRAFR
RSKSSRRRKSKDDVIVGVGGGGGDISASVNGKGSESSASVPSLDAITTTDDDVHGGGARQ
DQQEVVPEKIIHEANTPVVIAVAAVEEEEPNTIKSPEPEKEIATTATTIIEEEEVVDDDE
PKKGDAAATPVPTDSPAAASSTEEDKFVVVVKEEDKFFAVVKEDDKFVAVVKEAIKKQRD
DDADGDELVRRFKGSRVKTAMEKRSEEEQPRRREMARRSNDVIEEARSKLLEKRQCSRVK
ALVGAFETVMDAKPAGDGAAAKPQHYHPRR*
>11680.m04754|LOC_Os06g48050.1|genepair2048-2
MATRPSPRSRDSARPKSTSGRPPSSPRSSDTTRRSSFTAASSSDKPVPSFLRPTVSSSLH
SSSSSSPSSSSLLSSSPSSKGTGTTPRRSADKAPSASAASSRPITPKDKAPAVATSRPIT
PKDKAKAAAASTSRWSAVSPRQLMQKASNAFTRGSSSKSRAAKKDKEAATPAATSAAAKD
AATPASTSAVRKKAATPATSAAGKAVAGASPGPTRAQPTEEHHQQPTEAPPAEPSPAAAA
VQEEAVTSRAQLTEEHHQQPPEAPAEPSPAAVAVQEEAAAAETEAEQEKQPDETPQEVVA
AVEEKVQDEQVSTEAAEEAIAVEEDAAAVKTDAPEPEETQTQTGAIAESETEFQRSSEDE
PASDAIVEEAAIESATQNEPDEPESTTVEEMAVEEEIKTDESQQEETPKPEERMENSETS
VISEDPKEMDAISEDQKVEEPVVAEEQEELSEVVKMAVEPSISSEPATPLEEEDRDDVET
EQANSSEPATTVEEEEDRDDVETEQASSSDPATPLNEEAINEEGTSTETGKTKKVAFKGS
KVKTAMERRPEGEQPRKKDVARSNDVIEETKRTLMMKRKSKVKALVGAFETVMDTPSPGK
SS*
>11668.m00513|LOC_Os02g05690.1|genepair2049-1
MAEDHDAEGVRMTGEGAADGCDAHSLAGGEEEIGDQRRRRRASSTHRPPPPPRGPRRGRR
PSSTSPPLGPSTPAHEGLAAEDERRGAPDAPPAGGERGGASRVLDGEAGDAEVVGESANA
VLAVIALGGGKRGAVAGGGMAEEPPEAASDLEAALDVARTAASPSTEVAMKVVSLSHDAE
QGMRQFVTEVVSVGRFRHRNVVPLLGYCRRRGELLLVYDYMPNGGLDQWLHGQVHLRRRC
PVLRATIYLQRCLGIASSPLQRVAGMLGDNAWERIQLLWNFQEDVQDMKSKMTDLKVALS
YADKHSRETDDDALVLRHWLNKYKSVAYDMEDTLDELLTNAMIWENNPCTVKLFFSSINP
LIVRITMSSKMRNIRMKLDKIAEDQKKFPSMQLANPTGQDSTDKWRETFIGHTDEIEMVG
RAREKKEILIKVLQNDGGQEISIIPVVGLGGMGKTTLAKAVYTDKETHMFDVKAWVHVSM
EFQLNKIVSGIISHVEGSTPANIADLQYLKSQLDRILCNKLYLIILDDLWEEGWSKLEKL
MEMLQSGKKGSKIIVTTRSEKVVNTLSTIRLSYFHTVDPIKLVGMSIDECWFIMKPRNME
NCEFSDLVDIGKEIAQRCSGVPLVAKALGYVMQKHRTREEWMEIKNSNILDTKDDEEGIL
KGFIQDTDGQPLQKVAMEYVNELLGMSFLTIFTSPTVLASRMLFKPTLKLHMHDMVHELA

RHVAENRNTKRDNLNFHYHLLLNQNETSSAYKSLATKVRALHFRGCDKMHLPKQAFSHTL
CLRVLDLGGRQVSELPSSVYKLKLLRYLDASSLRISSFSKSFNHLLNLQALILSNTYLKT
LPTNIGCLQKLQYFDLSGCANLNELPTSFGDLSSLLFLNLASCHELEALPMSFGNLNRLQ
FLSLSDCYKLNSLPESCCQLHDLAHLDLSDCYNLGKLPDCIDQLSKLEYLNMTSCSKVQA
LPESLCKLTMLRHLNLSYCLRLENLPSCIGDLQLQSLDIQGSFLLRDLPNSIFNMSTLKT
VDGTFTYLVSSKVEKLRENLKLEGCCKLDGGSTDLCSRITELKKTHCHELEIQGLEDFKH
LEGIEHAILLNSLKLTKLIFSWQPKQYTNETAHHKTVLGMLVPPRSVHHLAIKGYCGIEL
PKWMLDIRSYLPHLTTIFLHGLMECNRLPPLGCLPHLRALLMTKMPKIKSVGPEFYGDYG
SCQKLRMIILASMDNLEEWWTTRSSKQDNELSLIPDLQMLWASDCPKLKFLPYPPRSLTW
FIENSNHVLPEHGFGNLTSATYPLHLSIERAPNSREMWRRAQHLSSIESLTLMSIAGLRA
LPEAIQCFTSLWRLSILGCGELETLPEWLGDYFTCLEEISIDTCPMLSSLPESIRRLTKL
KKLRITNCPVLSEKCQGEDRHKIAHILEPIFLLADTVSRVLVTANWRSKPLDFRWHLSLL
KKRKGWLSCRSSLSVAAYQAGLNLDICLSVRQVSRWGSEKFRTGGGAHIRESVPPNHMED
YPSGRSKAAIGFLRRGGGFSSRNQSTEERTIQNYDGPGITTRLNPMKTRLSDNQERPRYL
RDSFRSSTSMAIHGSSSKVPLRKFGDEKRRQTLLAGVDIAESSSRNAGGKHLEGSNKRIV
VDDRSSDVLHTETEGLATEQDQLIAPNAGVSDSASSSDISEHAVESLVRSAAPSSRTRRQ
KDKELNLGQSGVCSSSCTNRPTISRYAPADVKRPCNHASGVQQHGHNNLDCTSVPNFLPS
GCSSGSVYSRRFDAMRKRTSDGGSFSRSRGLSGTASLDDSPPAYPAIAGPRIRTTTTERA
SQQNALRSRRNFQDSAVSVRTRRPPWGARFRISEEREDGMISQRDSSIGNQQSDQVHSSS
EEASTESSSRPFSAELPHAIYSSRGEGSNAFTARRRRSSSLYEERPPQTFHDLFRERNGR
RHISIEGIAEVLLALDRIEQEAELTYEQLMVLETNILLGAFTSHDQHSDMRMDIDNMSYE
ELLALGDRIGSVSTALSEEQFVKCLRRSIYIPVATKANAQVVDDIKCSICQGVYCY*
>11680.m04753|LOC_Os06g48040.1|genepair2049-2
MEEDSGRSISTTGFLRRGSSVSLKDQGNEERPNKTKLNPMNARWADSKEKPRYLREPFRS
SGTKAACPSSSKAPVRKYFEEKQGRTFLGEADNAESSSRRTEANRLQCSKKAVVEEDVHP
YGQQDEPEDLLSTSTTEDQPAELDPELLDSSVSSGVSAHAIGSVVRNAALRSKSRQQKGK
EELCQIRPQTASAFVNRSTIPRNSTNGVKSSNAAGPGVQRRTLKNLGCTSISDVLPSGCS
SANSIHNKRAEVMRNRAFDGESSSRPRGLNGHSSLGHSPAMYSGITGPRVRTAEQSASQQ
TRTSSRSIQESADSSRIRRPSTQHARVRVPNEREDSVFALRETLARDRQPEWAHFSLGEA
APRRSMRPFSMELPHEIYSSSRQGSSNQTARSRSSYRPDESPPQMFHGLLVERENYRRIN
MEGIAEVLLALDRIEQDDELTYEQLLVLETNLFLSGLGLHDQHRDMRMDIDNMSYEELLA
LEERIGSVSTALSDEQLVKCLKRNVYKLPNSDLEANRAVLDDAKCSICQEEYIEGEEVGR
MQCEHQYHVSCIHEWLRQKNWCPICKTSAIPSEMDKGGT*
>11668.m00514|LOC_Os02g05700.1|genepair2050-1
MEWDSDSEGSGDEEEEEEEEEEEGVEVGGGGDGGVGVGVGGGFALAIEGVLGACGLVVSD
ALEPDFPIIYVNRGFEDATGYRAEEVLGRNCRFLQCRGPFAKRRHPLVDTTVVTDIRRCL
EEGTVFQGDLLNFRKDGSPFMAKLQLTPIYGDDETITHYMGMQFFNDSNVDLGPLSVSTT
KEIVRSTLITPDNTIRPSPMGKGFCSEHSDLFLLSDEVLCQKILSRLSPRDIASVNSVCK
RLYHLTRNDDLWRMVCQNAWGSEATQVLETVAGTRSLAWGRLARELTTLEAVTWRKLTVG
GAVEPSRCNFSACAAGNRVVLFGGEGVNMQPMNDTFVLDLNASKPEWRHINVRSAPPGRW
GHTLSCLNGSRLVLFGGCGRQGLLNDVFMLDLDAQQPTWREIPGLAPPVPRSWHSSCTLD
GTKLVVSGGCADSGVLLSDTYLLDVTMERPVWREIPASWTPPCRLGHSLSVYDGRKILMF
GGLAKSGPLRLRSNDVFTLDLSENKPCWRCITGSGMPGASNPAGVGPPPRLDHVAVSLPG
GRILIFGGSVAGLHSASKLYLLDPTEEKPTWRILNVPGRPPRFAWGHSTCVVGGTKAIVL
GGQTGEEWTLTELHELSLMFPTLNQKDLELYSWKL*
>11680.m04735|LOC_Os06g47890.1|genepair2050-2
MEWDSESDGAGSIGAGEEEEEEEEEEEEGGFGGGGGGGGGGGGMFSFAIEGMLRASGPCGL
VVTDALEPDCPIIYVNCGFEEATGYRAEEVLGRNCRFLQCRGPFAQRRHPLVDAMVVSEI
RKCIDNGTEFRGDLLNFRKDGSPLMNKLHLTPIYGDDETITHYMGIQFFTNANVDLGPLP
GSLTKEPVRSTRFTPDNFFRPISTGPGQSNFCREYSSLFQLTDEVLCQSILSRLSPRDIA
SVSSVCRRLYLLTRNEDLWRMVCQNAWGSETTRALETVPAAKRLGWGRLARELTTLEAVA
WRKLTVGGAVEPSRCNFSACAVGNRVVLFGGEGVNMQPMNDTFVLDLNASNPEWRHVNVS
SAPPGRWGHTLSCLNGSLLVVFGGCGRQGLLNDVFTLDLDAKQPTWREIPGVAPPVPRSW
HSSCTLDGTKLVVSGGCADSGVLLSDTYLLDVTMDKPVWREVPASWTPPSRLGHSMSVYG
GRKILMFGGLAKSGPLRLRSSDVFTMDLSEEEPCWRCLTGSGMPGAGNPAGAGPPPRLDH
VAVSLPGGRVLIFGGSVAGLHSASQLYLLDPTEEKPTWRILNVPGRPPRFAWGHSTCVVG
GTKAIVLGGQTGEEWMLTEIHELSLASSTV*
>11668.m00515|LOC_Os02g05710.1|genepair2051-1
MGSGMVKKKVVKAGSFDLDAKLDKSWMEDITCPICLDFPHNAVLLRCTSYEKGCRPFICD
TDQSRSNCLERFKGAHGLPTNMKVPSFNGAPLDSIHIISSNTTDRPACPLCRGDVIGWVV
IDEARLHLNQKKRCCEESCCSYVGNFHELQKHTQQKHPNSRPSEIDPARRVDWENFQQSS
DIIDVLSTIHAQVPNGIVLGDYVIEYGDDDAGDDYEVYHRVRGNWWTSCIFCKSFCRSSG
GRSRARARERRSSGRRSSNRSSQESFTIEVPSGSVDIREIRFDEIDDEYIVTGAMPGIAA
SRRIASHYRDPRYGRRRSYY*
>11680.m04731|LOC_Os06g47860.1|genepair2051-2
MGSGNLMMKKVVRPSSFDFDIQLDKSWTEDVTCPICLDYPHNAVLLRCTSYEKGCRPFVC
DTDQTRSNCLERFKGAYELPANMKVSTIAVAPLDSIHIVAPNVNNRPSCPLCRGDVIGWI
VIGEARLHLNQKKRCCEEDCCSFVGNFNELQKHTQQKHPDSRPSEIDPARQVDWENFQQS
SDIVDVLSTIHAQVPNGIVLGDYVIEYGDDETGEEYEVFRRVRRHWWSFMFFRGFSRSSR
RRRRARARERRGSGRRNSNQAHLESFNLEVPTQSVDLREIRFDEIDDEYIVTGAIPSIAT
PGRMASFHYRSVETVYC*
>11668.m00535|LOC_Os02g05900.1|genepair2052-1
MPCARAEDALPSAAIAALEMPSSAAAAAAAGGLIQKVGGGRRSGGSGRALHRSAHLSAED
DGHAPPPASCSKDDKIGGGGKREGSQRSRMRKYRSQLEQEVKKLQRQLEEEIDLHVALAD

AVMQNAGPVLNSSVKIPYKAQELLTNIASLESAVSKLEKDLNELYYQLCHERNERLLAES
KPGCLPSTSPDHSLSTCTCTWEEHISSLRDLKFGGSESMRSTQQDLYSELEYEQDVGEDP
EERQMVSLNRLFEKHRDVSLNRLLEKHRDEEMQESCSTDKEGEEDEKIDTLSFEQSILKI
TSMKLGNLWNNPNQLSEEMVRCMRNIFLRLSESSKMLPKESSDCSSSSAERLSGSTLASF
SDSSIIPSMLRSPSVDSNRNDEMTTEASNFDPYKVNGKESRRDIGNYHSAAEVSWMSVGK
EQLEYASEALKKFRFLVEQLSKVNPNSMNCDERLAFWINLYNALIMHAYLAYGVPRNDIK
LFSLMQKSLMLALNKFKITEEHKKYSIDGTEPLVLFGLSCGMFSSPAVRIFSAANVRQEL
QESLRDYVQASVGISDRGKLLIPKLLQSYAKGNVEDSLLADWICHHLTPDQVAVIRDSSS
QRKQRLLGARSFTVVAFDSKFRYLFLPDSSGSQKPEPKRTS*
>11680.m04721|LOC_Os06g47770.1|genepair2052-2
MRSVTLGGSIQRVMRRIGGGGGGRRSAGSRGAPQRAECLGAGSGDASASCSGDDSSNGTG
KRDGSRRVRMRRYRSELEQEVKKLQRQLEEEIDLQLALTDAITNNATLILEPSAKLPNKA
QELIISIASLENTVSKLEKDLNDLCYQLCHLRNNTRLAENNSRYLETLAEENNSRGLLST
SLQYQPPSTCKCTGEEDISTLRDIKLGESESMQENLFPGLEDQQNIQKESEGREIVSQDG
LLEEHQDVPSNRLLEKHWNEEMQESYPMENGGREYQIIDALSFDQSHQRKSSINGNVWNG
NPNKLSEEMVRCMRDIFLRLSDSSSEISPKGSSVNSISSTERLSGCTLTSVSDSSLMASV
MQSPSVDSNHDSIDEVRYFDPYNVNGKEVRRDIGNYCSVAEVSWMYVGKEQLAYASEALK
NFRNLVEQLSKVDPTCMTCAERLAFWINLYNTLIMHAYLAYGVPENDIKLFSLMQKACYI
VGGQSFSAAEIEFVILKMKTPVHRPQLSLMLALHKFRVTEEHKKYSIDDAEPLVLFGLSC
GMFSSPAVRIFSAGNVRQELQESMRDYIRASVGINDSGKLIVPKLLQSYAKGTVEDSLLA
DWICRHLTPNQVAAVQDTSSSRKQRLLGVRSFSVVPFDSKFRYLFLPDNSSSQH*
>11668.m00539|LOC_Os02g05940.1|genepair2053-1
MHPLHSPCKKHSNKFPIPVLALALVLLINLASLTSSCTEQDRSSLLRFLRELSQDGGLAA
SWQDGTDCCKWDGITCSQDSTVTDVSLASRSLQGRISPSLGNLPGLLRLNLSHNLLSGAL
PKELLSSSSLITIDVSFNRLDGDLDELPSSTPARPLQVLNISSNLLAGQFPSSTWVVMKN
MVALNVSNNSFSGHIPANFCTNSPYLSVLELSYNQLSGSIPPGFGSCSRLRVLKAGHNNL
SGTIPDEIFNATSLECLSFPNNDFQGTLEWANVVKLSKLATLDLGENNFSGNISESIGQL
NRLEELHLNNNKMFGSIPSNLSNCTSLKIIDLNNNNFSGELIYVNFSNLPNLKTLDLMRN
NFSGEIPESIYTCSNLTALRVSSNKLHGQLSKGLGNLKSLSFLSLAGNCLTNIANALQIL
SSSSNLTTLLIGHNFMNERMPDGSIDGFENLQVLSLSECSLSGKIPRWLSKLSRLEVLEL
DNNRLTGPIPDWISSLNFLFYLDISNNSLTGEIPMSLLQMPMLRSDRAAAQLDRRAFQLP
IYISASLLQYRKASAFPKVLNLGKNEFTGLIPPEIGLLKVLLSLNLSFNKLYGDIPQSIC
NLTDLLVLDLSSNNLTGTIPAALNNLNFLSEFNISYNDLEGPIPTGGQLDTFTNSSFYGN
PKLCGPMLVRHCSSADGHLISKKQQNKKVILAIVFGVFFGAIVILMLSGYLLWSISGMSF
RTKNRCSNDYTEALSSNISSEHLLVMLQQGKEAEDKITFTGIMEATNNFNREHIIGCGGY
GLVYRAELPDGSKLAIKKLNGEMCLMEREFSAEVETLSMAQHDNLVPLLGYCIQGNSRLL
IYSYMENGSLDDWLHNKDDGTSTILDWPRRLKIAKGASHGLSYIHNICKPRIVHRDIKSS
NILLDKEFKAYIADFGLSRLILPNKTHVTTELVGTLGYIPPEYGQAWVATLKGDVYSFGV
VLLELLTGRRPVPILSTSKELVPWVQEMISEGKQIEVLDSTLQGTGCEEQMLKVLETACK
CVDGNPLMRPTMMEVVASLDSIDPDLKMQ*
>11680.m04720|LOC_Os06g47760.1|genepair2053-2
MSWVKRTDCCKWEGITCSSDGTVTDVLLAAKGLQGHISPLLGNLTGLLHLNLSHNLLNGN
LPMELLFSRSIIVLDVSFNRLDGSLPELQSSSGGFPLQVLNISSNLFTGQFSSKLWEAMK
NIVALNASNNSFTGQIPSSICINSPSLAILDLSYNQFSGSIPPELGNCSKLREFKAGYNN
FNGALPEELFSATSLEHLSLPSNDLQGVLDGSDILKLVKLTVLDLGSTGLSGNIPDSIGQ
LSTLEELRLDNNNMSGELPSAVGNCTNLRYLSLRNNKFVGDLSKVNFTRLNLRIADFSIN
NFTGTVPESIYSCSNLIALRLAFNKFHGQLSPRMGNLKSMSFFSIADNHLTNITNALQIL
KSCKNLTAVLIGTNFKGETISKSETIDGFENLRVLTIDSCGLVGQIPTWISKLKKLEVLD
LSNNMLSGKIPFWISDLPVLFYLDITNNSLTGDIPTALMNTPMLQLGKNAAQLDPNFLEL
PVYWTRSRQYRLLNAFPNALNLGNNGFTGVIPPEIGRLKMLDGFNISFNRLSGEIPQQIC
NLTNLQLLDLSSNQLTGELPSALTDMHFLSKFNVSNNELEGPVPTGGQFDTFLNSSYSGN
SKLCGAVLSNLCSPTTRKENFTSQRRNLRCALVTGITLGALVALALLACFLIGRLVYDDH
TESLTHLQMRYRR*
>11668.m00570|LOC_Os02g06240.1|genepair2054-1
MSGELPSALGNCTNLKIINLKYNSFRGELLKLSPRIGNLKSLLFLSLSNNAFVNIANVIH
ALKNSRKINTLIIGTNFKGETMPEDIPITDGFQSLQVLSIPSCSLSGKIPLWLSKLAKLE
VLDLSNNQLTGPIPDWIHDLNFLYFIDISNNKLTGDLPTAIMLMPMLQPDKVATQLDPRA
FEQPVYAGPSLTYGKNNALPAMLNLANNELTGAIPSEVGQLKSLTLLNLSFNSLSGQIPQ
QLFDLTNLQVVDLSNNHLSGSIPPGLNNLHFLTTFDASNNDLEGWIPAGVQSSYPYDFSG
NPKLCDPTLTRTCDSAEAPPVSTLTGEETTPKIVFAIAFGAFFCLGVVYDQAVLSRFFG*
>11680.m04709|LOC_Os06g47650.1|genepair2054-2
MQLPNLPCSSSSSTTTTTTTKLSVAFFRLLVILLLSFASPTSSCTEQEESSLIGFLEGLL
PGHNGSLSTSWVKGIDCCKWEGINCSSDGTVTDVSLASKGLQGRISPSLGNLTGLLHLNL
SHNLLNGYLPMELLFSRSIIVLDVSFNRLDGSLPELESPSGGSPLQVLNISSNSFTGQFS
SKQWEVMKNIVALNVSNNSFTGQIPPSICINSPSFAILDLCYNQFSGSISSGLGNCSKMR
EFKAGYNNFSGALPEELFSATSLEHLSLPNNDLQGVLDGSHIVKLVKLTVLDLGSTGLSG
NIPDSIGQLSTLEELRLDNNNMSGELPSALGNCTNLRYLSLRNNKFVGDLSKVNFTWLNL
RIADFSINNFTGTVPESIFSCSNLIALRLAFNKFHGQLSPRMGTLKSLSFFSISDNHFTN
ITNALQILRSCKNLTSLLIGTNFKGETIPQDETVDGFENLRVLTIDSCGAMGQIPPWISK
LKKLEVLDLSNNMLIGEIPFWIRDMPVLFYLDITNNSLTGDIPVALMNLPMLQSGKNAAQ
LDPNFLELPVYWTPSRQYRLLNAFPNALNLGNNSFTGVIPPEIGQLKMLDGFNVSFNRLS
GEIPQQICNLTNLQLLDLSSNQLTGELPAALTNLHFLSKFNVSNNELEGPVPTGRQFDTF
LNSSYSGNPKLCGPMLSNLCDSVPTHASSMKQRNKKAIIALALGVFFGGIAILFLLGRFL
ISIRRTSSVHQNKSSNNGDIEAASLSSVSEHLHDMIKGTILVMVPQGKGGSNNLKFKDIL

KATNNFDQQNIIGCGGNGLVYKAELPNGSKLAIKKLNGEMCLMEREFTAEVEALSMAQHD
NLVPLWGYCIQGNSRLLIYSYMENGSLDDWLHNRDNGRPLLDWPTRLKIAQGASRGLSYI
HNICKPHIVHRDIKSSNILLDREFRACVADFGLARLILPYDTHVTTELIGTLGYIPPEYS
QAWVATLRGDIYSFGVVLLELLTGKRPVQVLSKSKELVQWTREMRSHGKDTEVLDPALRG
RGHEEQMLKVLDVACKCISHNPCKRPTIQEVVSCLDNVDADLQVQM*
>11668.m00579|LOC_Os02g06330.1|genepair2055-1
MAGVGVGEGEVRYRGVRRRPSGRYAAEIRDPAKKTPIWLGTFDSAEAAARAYDAAARSLR
GAAARTNFPPSTSSSAPPPPPPQHQPPQHLPPAAAAATSSHSSTVESWGVTVAAAAASAG
ALGLPPPRNGGSNPAADEDCRSYCGSSSSVLCEDGASGAGDEAAAPRCSPLPFDLNLPAS
SPSAAAAAEADEMDWRCDTLLHL*
>11680.m04703|LOC_Os06g47590.1|genepair2055-2
MRKSKQPQPQPSPEIRYRGVRKRPSGRYAAEIRDPAKKTPIWLGTFDSAEVAARAYDDAA
RSLRGPTARTNFPLAAPSAPPPRPPAAAAAAATSSHSSTVESWSGGAPRAAASALARSAA
PMEATQEEDCHSYCGSSSSVLCEDGSDDAAASRTPLPFDLNMPPPEEELDMAAVADQMGI
RYDTLLRL*
>11668.m00580|LOC_Os02g06340.1|genepair2056-1
MELARSACSKEHHKIYAGWFAVADPDGDGRVTGADATKFFAMSGLSRADLKQVWAIADTK
RQGFLGFGEFVAAMQLVSLAQAGEEITQDSLKREDLSSLDPPVMEGLDELLARSKAGVKR
VHPEENGAPQVQVHSANSWFSSKSAKKMQVPLTAVTSVIDGLKRLYVEKLKPLEVAYRFN
DFASPLLTSSDFDAKPMVMLLGQYSTGKTTFIKHLLQTSYPGAHVGPEPTTDRFVVVMSG
SDERTIPGNTIAVQADMPFTGLTTFGGAFLSKFECSQMPHPLLDHITFVDTPGVLSGEKQ
RTQRSYDFTGITSWFAAKCDLILLLFDPHKLDISDEFKRVIASLRGHDDKIRVVLNKADQ
VDTQQLMRVYGALMWSLGKVLNTPEVARVYIGSFNDKPVNESAVGPIGKDLFEKEQDDLL
CDLKDIPKKACDRRVNEFVKRARAAKIHAYIIGHLKKEMPAMMGKAKAQQRLIDNLGDEF
AKVQREYHLPAGDFPDVEHFKEVLGGYNIDKFEKMKPKLVQAVDDMLAYDIPELLKNFRN
PYE*
>11680.m04677|LOC_Os06g47330.1|genepair2056-2
MEIGQHPASGYSKEHQKTYQEWFAFADSDGDGRITGPDAIKFFAMSKLPRADLKQVWAIA
DSKRQGYLGFSEFVTAMQLVSLAQAGDEITQDSLKRDDLGSLNPPTMDGLDALLVKSKHH
AKRVDPDIDGFPQAQSPATSQWFSSKSSKKIPLNAVTSVIDGLKKLYIEKLKPLEVTYKF
NDFVSPLLTNSDFDAKPMVMLLGQYSTGKTTFIKHLLRTSYPGAHIGPEPTTDRFVVVMS
GPDERTIPGNTIAVQADMPFSGLTTFGTAFLSKFECSQMPHPLLEHITFVDTPGVLSGEK
QRTQRSYDFTGVTSWFAAKCDLILLLFDPHKLDISDEFKRVIGSLRGHDDKIRVVLNKAD
QVDTQQLMRVYGALMWSLGKVLNTPEVARVYIGSFNDKPVNESAVGPIGKELFEREQDDL
LSDLKDIPKKACDRRINEFVKRARAAKIHAHIIGHLKKEMPAMMGKAKAQQRLIDNLENE
FAKVQREQHLPAGDFPYVEHFRDVLGGYSIDKFEKIKPKMVQAVDDMLGYDIPELLKNFR
NPYE*
>11668.m00585|LOC_Os02g06380.1|genepair2057-1
MQLSFTPSTHSRTQAQTVCVSVRQGGRRRGAKNCGGGGGGERRKEMKGMSGPKLLVVHPA
SKGYNGVVSLSGAASPGPSAASMPLFLGSRRRCCAWLVALLLALLTCVSLLTVFSTARAA
SEGAALPQRARLTVAGAAGAAAASAAVGAAGGLPAYVFDALVQYAAAAGANATASMPEED
VRAIASVLRRRAPCRLLVFGLGAETPLWRALNHGGRTVFLDENPFYVAHVEGALPGLEAY
DVSYATAVREFPDLLDAARAAQSADCRPVQNLLFSDCRLAINDLPNQLYDVSWDVILVDG
PSGFTEGSPGRMSAIFSAAVMARTKGSETEVLVHDYQREVEIACAREFLCPENRVEATAT
PSLGHFLVRGGAAANRDAFCGGAAGATTKKAN*
>11680.m04675|LOC_Os06g47310.1|genepair2057-2
MAVLGSRRRMWVVLFLAGFACVSLGTMLCAARDHPPPPVAARRLAAAEAQAATLAVSARG
GGAGGGGGGLPGYVFDALVQYASAGGNSTASMPGGDVRAIAAVVKRRAPCNFLVFGLGGE
TPLWRALNHGGRTVFLDENQYYVVSHLEGRHPGLEAYDVVYTTTVREFPDLLDAARAARSA
ECRPVQNLLYSDCRLAINDLPNQLYDVAWDIILVDGPRGYTAASPGRMSAIFTAGVMARS
RAEKGAETDVLVHDYEREVERACSREFLCEENRVEETSTRSLAHFVVPGGRDLRRETFCA
GGGGSGAST*
>11668.m00587|LOC_Os02g06400.1|genepair2058-1
MAELRHSTVAAAAARSSNSPAKRDSDASAASSPFASTSSARGRGGGDDDDGKDAHRSSPL
LPHHHKRLGLPSPLRSLLALEDPRSPSASSSYRILFALLAFLLLAATFSATSVWSRLNAP
YLCQKDGITLHCPETKEAPSLWENPRAATTSWKPCAERRSNEPSDVPPENETSGYIFIHA
EGGLNQQRIAICNAVAIAKIMKATLILPVLKQDQIWKDQTKFEDIFDVDHFINYLKDDVR
IVRDIPDWFTEKDELFTSIKRTVKNIPKYASAQFYIDNVLPRIKEKKIMSIKPFVDRLGY
DNVPMEINRLRCRVNYHALKFLPDIEEMADKLAARMRNRTGSVNPYMALHLRFEKGMVGL
SFCDFAGTREEKEMMAAYRQKEWPRRYKNGSHLWPLALQKRKEGRCPLEPGEIAIILRAL
GYTRGTQIYVASGQVYGGKNRMAPLRNMFPNLVTKEELASAAEMAPFRKHVTSLAALDFL
VCLRSDAFVMTHGGNFAKLIMGARRYGGRHRLKSIKPDKGLMSKSLGDPHMGWAAFSDDV
VITHQTRAGLPEPTFPNYDLWENPLTPCMCPTA*
>11680.m04670|LOC_Os06g47290.1|genepair2058-2
MMTKPPQSRGGGGGGGGALGRRGFAALLAAAVIALALLCLFYGAAFAPTLRSRRLPLQRR
FEAVPADLALSSLPVCDARYSELIPCLDRGLHNQLRLRLNLSLMQHYERHCPPAHRRLNC
LIPPPAGYRVPIRWPRSRDEVWKANIPHTHLASEKSDQRWMVVNGDKINFPGGGTHFHTG
ADKYIVHLAQMLNFPNGKLNNGGNIRNVLDVGCGVASFGAYLLPLDIIAMSLAPNDVHEN
QIQFALERGIPSTLGVLGTRRLPYPSHSFELAHCSRCRIDWLQRDGILLLEVDRVLRPGG
YFVYSSPEAYAMDPINRNIWRKMSDLARRMCWQIASKEDQTVIWIKPLTNECYMKREPGT
LPNMCDRDDDPDAAWNVPMKACVTPYSERVHKVKGSNLLPWPQRLTAPPPRLEELGISSN
NFSDDSEIWHFRVIQYWKLMKSEIQKDSFRNVMDMNANLGGFAASLRKKDVWVMNVVPST
ESGKLKIIYDRGLLGTIHNWCESFSTYPRTYDLVHAWLLFSEIEKQGCSVEDLLIEMDRI
MRPQGYAIIRDKVAVINHIKKLLPAACWAVRPQPENLAQEAQRSQSNDTPVTADSGMAEL

RHATAAAAATRASSSPAKRDAESSASSSPFVASSSSPRGGGGGDGKDGAPRSSAPLHHQK
YHLPSPLRSLLALEDPRSPTASASYRILVAVVACLFVAALVSAPSVWSRIVNERESLWEN
PHAAAASWKPCAERHLVPENETSGYIFIHAEGGLNQQRIAICNAVAIAKIMNATLILPVL
KQDQIWKDQTKFEDIFDVDYFINYLKDDVRIVRDIPEWFTEKDELFTSIKRTVKNIPKYA
SAQFYIDNVLPRIKDKKIMSIKPFVDRLGYDNVPMEINRLRCRVNYHALKFLPEIEEMAE
KLATRMRNRTGNVNPYMALHLRFEKGMVGLSFCDFAGTREEKAMMADYRQKQWPRRFKNG
SHLWSLALEKRKEGRCPLEPGEIGIILRAMGYTKETQIYVASGQVYGGSNRMAPLRNMFP
NLVTKEDLASKEEIEHFKKHVTSLAALDFLVCLKSDVFVMTHGGNFAKLIIGFRRYMGRH
RLKSIKPDKGLMSKFFGDPYMPWATFVEDVMITHQTRTGLPESTFPHYDLWENPLTPCMC
RA*
>11668.m00590|LOC_Os02g06430.1|genepair2059-1
MFDSLLNSKFYNKCKHAIKCTRTRLDLVRRKKQAMVKFMKKDVADLIGNRLESHAFGRME
ALIVEMNQASCYDMIEQYCEYIVKQLNNLQKQNECPQEALEAVSTLIFATARFPELPELC
DLRHMFTERYGSFVEPFVSSEFVQKLQNKSFTNEEKLQVMQSIAEEFSVPFNSKALERKI
SGVPQNKHDLQNKSSFKRVEVEASACNELKVDRHAVHERKSKVTPEVHERKQEMPVKPKD
IHVIPDAIGKVGEKSRKNRSDIPYDVPPSDLKQINDQELKKDHKKHSHHQRELRNADKSA
PPYAEPKEDVAEKSDGKGYHVHRSRMAGGLDHNWGHADLGLKTLGLEKQGIEPASSLDGK
TLNKAPPYSKPYKASDEYGQSVQDRQKMPEKAVNMRPPYVKPNSTNQAVDDYKHAGTGEI
GHQRDGLADDNTLQPVSVRRKSAKPPTHGDRYDDEAKMTSQTPGGRRRHSSRRNGSDDDY
DQRVGYMLPPDDDAVNNARHFKRMSERRKHGSRQGGSASGNDYESDEDETNSVIDFGNLL
PRAPSSHRKHRSRSAHPRSGGRDDEERVMDKLLMHYSKKGIDREEHKTRTKSRTPRPRAD
QPADGVGERSNREVAPQHPPERTVSLPSDSGNLGVKPKAPARSISMQPDKSRGIVHPSMP
DFDELAARISALRKE*
>11680.m04666|LOC_Os06g47250.1|genepair2059-2
MPWVTPDNRGGREKKKKKKKKLGGGEEWGKMFGSLLNSKFYNKCKHAFKCIRTRLALIR
RKKQAMIRFMKKDIADLLTNGLDTHAFGRMDGLIIEMNHASCYDMIEQFCEYIGKQLNSL
QKQGDCPQETREAVSTLIFAAARFPDLPELCDLRHIFTERYGHFLEPFVSLEFVQKLDNK
VFTNEEKIQAMQSVSEELLVDFDIKAFKIKLWATPETKHDLPAKDSKKQVELAVPLSSKK
GDDAAPSGRKSEAATLGHKNKLEASLKQQKDVHPVADGIDRLRENTRRQHADKSDGKGHV
EKPVSDSEMKRRNIQKEVQKANKKDVRPCEKELMEAVELDLNGLPKKEFGSLKVPEAESK
KTFALNVKPKKDNDLEKENESNLGHHHRSHIPCAADHADSGLRTLGLDKQGLQSVNPLNG
NTKNRMPPYSKLDGSTGKKCSEKEENTGCLNARPHHLADKGNPVQDRQPVPERAAYVRPP
YIKPKLNMETVNDDPAERAASDYSKRAIPEQTDHLSDKDPLRPVSVRSKYAKPPAPAAVY
DEAPANEKVSSRTPSSHRRHTSRQNAVNDGSARRDGSRQPHGGKGMDDVNGENVQRTPSS
RPRHSGRRNGALYTEDYDGFMQRHKSEEDEAAIDFGNLLPRTGNGHRRHKSRNTDARSGV
DEEERMMDKLLRHYSKKGLDAEINPAPTNKAEEQSEQKGSMHPPGRAISLPGESVCRDED
VKFPARSTSLQPDCPKTVHVHPKMPDFDELAARVRALRKA*
>11668.m00600|LOC_Os02g06520.1|genepair2060-1
MVGDQEGRRQRRRGWGEEEEDRISDLPDALRLQILSLLPLKSAIRTGALSSRWRGLWEQR
WPDPSSLDVRLPSGGGAASASVAALRAEHLAGIDRRGRRRMDLFSLAFHAGQLAPPELKR
FIEYAAACDVEEVRLRLDGGGGRGARGGTRRPGALAVHFPIGSKLLARLSVRGLHLTASA
NAMVATLEVIHFHSVSLTDAALRRVVSACPRLRELELRYCRHLRRIDFTVGASNLKSLTV
VDCSRATELRVPSAPLLRSFRFSGAFLCSNIFGSTADCVEHLYLCSGGPETGLPRTNLPS
AVPRLSNLTVLTLCSIALQYVSASVATPNVEKSLNSLRELQLLMFGMANSNLADIYSFLK
ACRCPQLERLFVQLPTNTYDSFTTNYLEVAEEEPPEGGLENLRLVKMTNFKGYRNELRLV
DFLLRKASRLNKLFLIAPKEVHPQGLRKVHSEALPHFLKTDVLHLERASAIAQIIFDESV
SPQILPLHSEVFVRV*
>11680.m04652|LOC_Os06g47120.1|genepair2060-2
MVKRQREEEPATASQSGDDDSNSDEWKDFLNLDGGEEAAEVDDDCNLAPPEKDAEAAKAS
QSGDDDSNTDQWKGYLNLEDGEEAAEVDDDRNLDQPPEKDAEAAKVDVDRKLDQLPEKDA
EAAKVDGDRITALQEKARVRILDFLPIKSVILMGSLCKRWREMYGLYWRDVAVDVELPTD
GDALSKLEERAGQQEPKRRLRYFFLLVVERKNVQREYFNSCLEYAGKCSPEVIHISDRGG
AGRKFKMHLTSKQLVRLSIIGVALGHFQGKFCEGVSFPTLEEIHIKNSTINKMDDLKNLV
GACPILRVLDLRGCKTITQIDVDTAGEHLMSLTVMDCERVRRLTAGKHLRSFRYSGNFLT
SLSLPDNDSLADLFIGFPRSQSTTPGPENSFKRLPDLSNLTFLTLCSTSLRAVTVAGNTI
QTNLRSLRELQLLMFKLEPINLSDVRRFLNTCGYYPQLTKLFVQLPERDCTYTENTSSEN
VEGEQQDGFEKLDVVKMTNFKYDWNEIRLLQFLFKKAKLLQKLILVRPIPVPVDRPFRLQ
VPANVQLTDCADDSTVKSFHSELLTSKTN*
>11668.m00601|LOC_Os02g06530.1|genepair2061-1
MEVAECSEKLQILKASASASMAYSIVQFPVKWQSIKYKLQQLCSNLNAPGDDGSCNENVI
LVQFLQTATATVSHIQAIASQCSDESYNGGRLRLRSDLDSISSKLDIHLKDLKEMVSSRI
SVRSQAVIATRPAIGASLSNKRFYINDLFLRVRIGDLAQRKQALVTIGELLSEDIEYVKI
VALDIDNSITLLISFLESGDACIQEQAARIVSLIAGYDSYRGMLVKAGVVAPLVQLLDSP
SCTSTTVSSRERAAHALRELTSNSDNVWAVCAQGGVTVLLIVCANVDSRGKLVSSAFAVL
KNLSRVEEVKMFMVEQGAVMELVKLSRQKEEERKVGSVELLHYMALADANVRQAAISMGM
IQSLTQLMNPDLPYSSKAREVALSAIAFFCLPSKALTDDLISSNFLSWLQTYLNNVDYAV
LECTLNILVRLTRISEEYSKMVGRAGFMTALVSSLGAKSCQVREMAAQVLCNLLLLHSNR
VVFIQDGDNLNRLLQSLEHGDGKTMAKDLAISCLVSLAETSAGRKKIISSQHFVSLKGLA
DSGDLPAKKIVKKLCANKLQSILTRIRIT*
>11680.m04640|LOC_Os06g47000.1|genepair2061-2
MGFFFFASRAAARFLGEARIIHPGVSTAALLVAAASGGGLVAYADSAGENASSETSQEAP
RKKKVVVLGTGWAGTSFLKDLDCSKYEVKVISPRNYFAFTPLLPSVTCGTVEARSIVEPI
RKMLEKKRKDVAFYEAECFKIDASKKAVHCRSAVGTNFDGNGDFMVDYDYLVVALGATVN
TFNTPGVMENCYFLKEVEDAQKIRRNVIDCFEKASLPNISEEEKRKILHFVIIGGGPTGV

EFAAEMHDFLVEDLVKLYPAIQDFVKITIIQSGEHILNMFDQRIATFAEMKFQRDGIEVN
TGFRVVKVSDDLITMKSKSLGEVSVPYGMAVWSAGIGTRPVIMDFMQQIGQTNRRVLATN
EWLRVHECDNIYAIGDCASITQRKIMDDISTVFKMADKDNSGTLTLKEINDVLEDICIRY
PQVELYMKSMHMLDIRDLIKDAIGDSHKESMVVNIEEFKKALSHVDSQVKSIPATAQVAA
QQGHYLAECFNKMDQCKEHPEGTSILGNLHPWVESKLLQSSQATGFPWAIALSGSGTLYM
QVLFAPTATWLSLTVTERCSSALRQTIRIRDVFHTEIRLMLGAQQQEELLFQMPGHGIHA
CKIPMEEESIKSGGVTRGQIKGDEMGEAAEEGAVDGSCHGEAAAEALEVLRSMAMASSSL
TCSIPQFPAKWQSIKDKLRQLCSGLDSLCGSVGFGVDVGGDEERHGVLVQLVASASATVR
SIQAMASQCGDGTYKGGRLRLRSDLDNLSSKLEAHMKQLREMASSGMPSPSQAIVAVRPS
ADAGAGEKMFYMRDLFSRVRIGGSVQRSQSLATIGELLAEDEVCVKIVAVDIDDGVALLT
GFLESSDARLQEEAAGAVAMVASFDSYRGMLVKAGVIAPLVQLLDDAAATAAVAAGGGAT
AVAKERAAQALRELTENSDNVWAVCAHGGLTTLLHACGDAGSGGKLVASSFAVLRNLSRV
EEVKVFMAEQGVVTELVKLSQKKEEARKLGAVELLHAMALDDADVREEAVSMGVIQSLLQ
LIYPDLPYSYKAREVALAAIWFFCFSSVNSIDDLISSDVLGWLLFYLNNGDYAVLECTLK
ILRHLSEVSEEYNRMMGRAGYLSALSSLLGAKSCRVREMAAQVLSSLLLLHPNRVIFIQD
GDNLNRLLQLLDPAEGKLVAKDLILSAILSLADTNSGRKKIISSEHFSSLKELADTGDFD
AKKVVKKLGTNRFQTIFSKIWSV*
>11668.m00604|LOC_Os02g06560.1|genepair2062-1
MAICGAKAAAPSLVVAASMGAVEALKDQAGLCRWDYALRSLYLRAAASSPRLRSPLSNTA
AAAARPPAEEAADVRLRKVHHLVCWGPN*
>11680.m04637|LOC_Os06g46970.1|genepair2062-2
MSSSSLSKKASSFVVTASMSAVEALKDQAGLCRWDYALRSLYQRAAAAKQVTGRAVPVSL
SSQTGGAAASSSPAAACGRAARSKRSEEEKMQKAYHLVCWGPN*
>11668.m00607|LOC_Os02g06590.1|genepair2063-1
MLGPPRRGPAYKTKLCALWQRGNCNRDTCSFAHGHGDIRRPPSSRGAFTHHPDSADMLTQ
LLPEDSMTFFINSSLGISTICVDDNACAVPEGELGEEIIEPVISEAGLIGGFHLGGGIPL
EESPGVIVPFMIEGHLLEKEILAIHDLLAGRECERRHEKKTDDGETNSSRSLSLSDNNDE
KKKDKFSSGDEKEDHEKQLKQIRLDMEALRDDKTQMEVILDEKIDEVRKISSKVNDLEVQ
LRREKDECHRMTSKMKKFIKAHARFLKAQEEVKRSQARFERLGDLLASDILKRGANEEGS
SVNEDLNERSPNTAATKKRSIPYSTSEEAKAVKKRRERDSDTMTRSDKYRSDVTDFDKTS
KGTEATKSLYLKKKLWEDEKSKYFVNLQVKGSPVRHVLPSTGMAAHAIDDLNEAIELEDR
HESIDALLENDADDKTRSPAIPLQPPPVVQNAYEQSGFEHISFDIPFGLSIYCAKFPLTQ
LAGSTQATRFAAARQIGEIAKSHPQELNVLLKKDICIIIILRRKRVSPYLRSKNWDTRVA
AAHAIGAIAENVKHTSVKDLFASAEAEKHASGLSGIGDVGSTLRHADTTATSELAFGSFD
INRVLEFGSPLLASGGQEYDIANDNGKNPAERLARQKQNLRRRLGLDVCEQFMDFNDVIK
DEDLLAQKNYWGANMQNNGFYSFNTGQNIQHLVASMVPSAIALEHSVGMALSSICDDLIT
PLTFFQDTVDAITDEDNLEYSENGRWPFQQFVDQLIHDMFDPIWEVRHGTIMALREILTH
QGACAGVYFPDLNSPFADLDDKNNLDSLKRAHGIDLNEDIDSGQLEPVLKRQKKEESNPE
VMDIQLDKEPSNGDYSKTEASLSTEPTVSSGEPNLAHAKVESPFQVDGSANPSKVDPYCT
PPHETLNSMPKLSSTHLPENSKFIKLMKLANYSAVKNWEFLQDCAIRFLCVLSLDRFGDY
VSDQVVAPVRETCAQALGAVLKYMHPTLVCHTLNILLQMQRRQEWEVRHGSLLGIKYLVA
VRQEMLKDLLNYVLHACKAGLEDPDDDVRAVAAEALIPAAASLVRLNDQMLHSIVMLLWD
ILLDLDDLSPSTSSVMNLLAEIYSQPEMVPKMLGTTAIGGDNEFDLNSVTLVAGEEKMGS
NDNPYVLAALTPRLWPFMRHSITSVRRSAVRTLERLLEVGNTRNSAKLWLASILGDALQV
VFQNLLLESNDEIIRSSERAWKLLLQCPTEDLESAASSYFSNWVQLATTPYGTALDSAKM
FLPVALPRGSRSRAAAKIKSARLEHENTRMISFGSTGENTSQEKHSEASLSVSKIIVGSD
SDKSVTHTRVLTSMALGLFASKLPEGSWQVVLGPLASDLMSLSGVQRQVASMVIVSWFKD
LRKSDPAAVGTLLAFLSSLKGWMLDLLACSDPSFPTKDSPLPYAELARTYRKMRNEANNL
FQSIESCALLKEYTSNLNFEADMLSVDDAINFTSKLLLPSEPDFSLDSDKIVLNNIESAK
QGLLSTSGYLKCVQNNLHVTVSSLVASAVVWMAGLPSKLNPVILPLMAAVKREQEEILQD
KAADALAELIFSCVGRKPGPNDKLTKNLCTLTCTDASETPQAAVINSIQVIEDQNLLSIG
KRFSNHKSRGQMTSGGESKSEGFISRRGSELAFKHLCEKFGASLFEKLPKLWDCLTEFLK
PVKTGDDLMKEDPSIAQLGRSCEDKEPQSLINNIQVVRSVTPHLAEPLRPQLLSLLPCIL
GCVRHPHVAVRLAAARCITSMAKSLTANVMVIVIENVIPMLSDSSSVCARQGAGMLLSLL
VQGLAVELVPYAPFLVVPLLRCMSDPDGSVRQTVTHSFAALVPLLPLAKGSLLPDGLSER
LSSSAEDAQFLEQLLDNSQIDDYKLSIDLSVELRRYQQEGINWLAFLRRFKLHGILCDDM
GLGKTLQASAIVAADIAESRARNDEQDPKSLIICPSTLVAHWEYEIEKYIDSSIMKPLQY
IGSSQDRIILRSQFDKFNVIITSYDIIRKDIDFLENVFWNYCVLDEGHIIKNSRSKITSA
VKQLKAQHRLILSGTPIQNNVLELWSLFDFLMPGFLGTEKQFQATYGKPLLAAKDPKCSA
KDAEAGILAMEALHKQVMPFLLRRTKDEVLSDLPEKIIQDRYCNLSLLQLKLYDKFSNSN
AKQEISTIVKENELDQSTSQPKATRHVFQALQYLLKLCSHPLLVTGESPPDYLVDLLKEI
GMGTGDELHDLHHSPKLVALQEILQECGIGSEISSPDASAAIGQHRVLIFAQHKALLDII
EKDLFQSHMRSVTYLRLDGSVEPEKRFEIVKAFNSDPTIDVLLLTTHVGGLGLNLTSADT
LVFMEHDWNPMKDLQAMDRAHRLGQRKVVNVHRLIMRGTLEEKVMSLQRFKVSVANAVIN
AENASLKTMNTDQLLDLFASTPASRKASVLPSSSGGDQSKDSKGKSGGKGLKSILNGLDE
LWDQSQYADEYDLNQFLAKLNG*
>11680.m04629|LOC_Os06g46890.1|genepair2063-2
MPGIICSVITDRALEIEALVIQDLPSGRDRKKDDRFNKADDKHDNEAQLQRIQLDMEALH
EEKSSLEVILNGKINEASKLSSRIADLESQLNDEKEACERSQARFERFADLLASDTLKPC
SKDQGSSAAKEDPYNAYEMSPSDQRQNHETTRKRSVALSTSEEGRNGKKRRESYDNRDPM
SEKYRPEDALEPFQSSKGTEAKELLSVKKNLGDGDNNEKGNVVSSANVFTDRMEIDKDFS
SKE*
>11668.m00611|LOC_Os02g06630.1|genepair2064-1
MASTRRRRAGGVPMAVIRLGVAAIVVLGAGVIGGARAQLRMGFYDESCPAAERIVGEYVR

QHVGRVPTVAAALLRLHYHDCFVRGCDASILLNSTGNGGAAEKDAAPNQTLRGFDLIDRV
KGLVEAACPGVVSCADVLALAARDAVAAIGGPSWRVPTGRRDGTVSSMQEALAEIPSPAM
SFPELAGLFATKGLSVRDLVWLSGAHTIGIAHCSSFADRLYNGGGGAGNANGNNTDPPPL
DAAYAANLRERKCRTAGDGVVEMDPGSHLTFDLGYYRAVLRHRGLLRSDAALVTDAAARA
DIAGAVASPPEVFFQVFGRSMATLGAVQVKTGSDGEIRRNCAVVNSGH*
>11680.m04618|LOC_Os06g46790.1|genepair2064-2
MAMAAPLTLVVVAAAVAVVFVGGGVEGWVLKEGFYEQSCPRAEALVKHYVEQHVPLAPSV
AATLIRTHFHDCFVRGCDASVLLNGTDGAEAEKDAAPNLTLRGFAFIDRIKSVVESECPG
VVSCADILALATRDAISVIGGPFWRVATGRRDGRVSIKQEALDQIPAPTMNFTDLLSSFQ
SKGLDLADLIWLSGKLTIHSSIY*
>11668.m00612|LOC_Os02g06640.1|genepair2065-1
MQIFVKTLTGKTITLEVESSDTIDNVKAKIQDKEGIPPDQQRLIFAGKQLEDGRTLADYN
IQKESTLHLVLRLRGGMQIFVKTLTGKTITLEVESSDTIDNVKAKIQDKEGIPPDQQRLI
FAGKQLEDGRTLADYNIQKESTLHLVLRLRGGMQIFVKTLTGKTITLEVESSDTIDNVKA
KIQDKEGIPPDQQRLIFAGKQLEDGRTLADYNIQKESTLHLVLRLRGGMQIFVKTLTGKT
ITLEVESSDTIDNVKAKIQDKEGIPPDQQRLIFAGKQLEDGRTLADYNIQKESTLHLVLR
LRGGMQIFVKTLTGKTITLEVESSDTIDNVKAKIQDKEGIPPDQQRLIFAGKQLEDGRTL
[truncated: 1,029,832 more chars]
