# Supplementary material for: Intron gain and loss in segmentally duplicated genes in rice
Source: Genome Biol. 2006 May 23;7(5):R41. doi: 10.1186/gb-2006-7-5-r41 (PMC1779517; doi:10.1186/gb-2006-7-5-r41)
Supplement: Additional File 3 — The ClustalW alignment of the two rice duplicated genes and their orthologous gene from Arabidopsis. [file gb-2006-7-5-r41-S3.pdf]

Additional Data File 3. ClustalW alignment of the two rice duplicated genes and their orthologous gene from Arabidopsis (bottom one). The phases of introns (0,1,2) are highlighted. The intron loss and intron gain are highlighted in red and green respectively. The “~” indicates missing of intron at that position.

```
CLUSTAL W (1.83) Multiple Sequence Alignments

Sequence format is Pearson
Sequence 1: LOC_Os01g13130.1      256 aa
Sequence 2: LOC_Os05g14240.1      251 aa
Sequence 3: At2g25810.1           249 aa
Start of Pairwise alignments
Aligning...
Sequences (1:2) Aligned. Score:  60
Sequences (1:3) Aligned. Score:  53
Sequences (2:3) Aligned. Score:  56
Guide tree        file created:   [/tmp/3MLr1SjSp3/6kTga9wjJ5.dnd]
Start of Multiple Alignment
There are 2 groups
Aligning...
Group 1: Sequences:   2      Score:4325
Group 2: Sequences:   3      Score:4113
Alignment Score 2629
GCG-Alignment file created      [/tmp/3MLr1SjSp3/P5W730m2jT]

MPLLPMTKLELGHRGEAWEPGCLRAVAGELLFTFLFVFIGVASTITA1G--KAAGGAGEA
----MAKEVDPCDHGEVVDAGCVRAVLAELVLTFVFVFTGVAATMAA1GVPEVAGAAMPM
-----MKKIELGHHSEAAKPDCIKALIVEFITTFLFVFAGVGSAMAT1D--SLVG--NTL
      .:::  .:.*. ...*::*:  *:: **:*** **.::::: .  . .*

AAVTAAAMAQALVVAVLATAGFHVSGGHLNPAVTLSLAVGGHITLFRSALYVAAQLAGSS
AALAGVAIATALAAGVLVTAGFHVSGGHLNPAVTVALLARGHITAFRSALYVAAQLLASS
VGLFAVAVAHAFVVAVMISAG-HISGGHLNPAVTLGLLLGGHISVFRAFLYWIDQLLASS
..: ..*:* *:...*: :** *:**********:.*   ***: **: **   ** .**

LACLLLRCLTGGA~ATPVHALADGVGPVQGVAAEAVFTFTLLLVICATILDPRRAAPPGT
LACILLRYLTGGM0ATPVHTLGSGIGPMQGLVMEIILTFSLLFVVYATILDPR-SSVPGF
AACFLLSYLTGGM0GTPVHTLASGVSYTQGIIWEIILTFSLLFTVYATIVDPKKGSLDGF
 **:**  ****  .****:*..*:.  **:  * ::**:**:.: ***:**: .:  *

GPLLTGLLVGANTVAGGALTGASMNPARSFGPALATGEWAHHWVYWVGPLAGGPLAVVAY
GPLLTGLIVGANTIAGGNFSGASMNPARSFGPALATGVWTHHWIYWLGPLIGGPLAGLVY
GPLLTGFVVGANILAGGAFSGASMNPARSFGPALVSGNWTDHWVYWVGPLIGGGLAGFIY
******::**** :*** ::**************.:* *:.**:**:*** ** ** . *

ELLFMDVEDA--GGAHQPLPQE--
ESLFLVKR------THEPLLDNSF
ENVLIDRPHVPVADDEQPLLN---
* :::          .:** :


 CLUSTAL W (1.83) Multiple Sequence Alignments

Sequence format is Pearson
Sequence 1: LOC_Os01g61080.1      555 aa
```

```
Sequence 2: LOC_Os05g39720.1      572 aa
Sequence 3: At2g38470.1           519 aa
Start of Pairwise alignments
Aligning...
Sequences (1:2) Aligned. Score:  51
Sequences (1:3) Aligned. Score:  39
Sequences (2:3) Aligned. Score:  35
Guide tree        file created:   [/tmp/3MLr1SjSp3/RsPCY9qnDU.dnd]
Start of Multiple Alignment
There are 2 groups
Aligning...
Group 1: Sequences:   2      Score:8719
Group 2: Sequences:   3      Score:7220
Alignment Score 3762
GCG-Alignment file created      [/tmp/3MLr1SjSp3/P5W730m2jT]

MTTSSSGSVETSANSRLGTFSFAS--ASFTDLLGGNAGAGGGGVSRYKAMTPPSLPLSPP
-MTAAPGSLP-LVNSRPVSLSLAASRSSFSSLLSG--GAG----SSLNLMTPPS---SLP
----MAASFLTMDNSRTRQNMNGSANWSQQSGRTSTSSLEDLEIPKFRSFAPSSISISPS
     ..*.    ***      .:   *  .   .  .      .  . ::*.*   * .

PVSPSSFFNSPIGMNQADFLGSPVLLTSS0--~IFPSPTTG--AFASQHFDWRPEVAAAQ
PSSPSSYFG---GVSSSGFLDSPILLTPS0--~LFPSPTTTGALFSWITTATATAAIAPE
LVSPSTCFS------PSLFLDSPAFVSSS~AN0VLASPTTG---------ALITNVTNQK
  ***: *.       : **.** :::.*    ::.****             . .   :

SADQGGKDEQRNS-YSDFSFQ----TAPASEEAVRTTT-----FQPPVPPAPL0GDEAYR
SQVQGGVKDEQQQ-YSDFTFLPTASTAPATTMAGATATTSNSFMQDSMLMAPL0GGDPYN
GINEGDKSNNNNFNLFDFSFH----TQSSGVSAPTTTT------TTTTTTTTT~NSSIFQ
.  :*. .::.:    **:*     * .:   *  *:*        .   :.  ... :.

SQ~QQQQPWGYQQQPAGMDAG----ANAASFGAAPFQATSSEMAPQVQGGGGYSQPQSQR
GE~QQQ-PWSYQEPTMDADTRPAEFTSSAAAGDVAGNGSYSQVAAPAAAGG---FRQQSR
SQ0EQQ-KKNQSEQWSQTETR-------PNNQAVSYNG------------------REQ
.: :**   . .:     ::        .    .. :.                    .:

RSSDDGYNWRKYGQKQVKGSENPRSYYKCTFPNCPTKKKVERSLDGQITEIVYKGTHNHA
RSSDDGYNWRKYGQKQMKGSENPRSYYKCTFPGCPTKKKVEQSPDGQVTEIVYKGAHSHP
RKGEDGYNWRKYGQKQVKGSENPRSYYKCTFPNCPTKKKVERSLEGQITEIVYKGSHNHP
*..:************:***************.********:* :**:*******:*.*.

KPQNTRRNSGSSAAQVLQSGGDMSEHSFGGMSGT--AATPENSSASFGDDEIR---VGSP
KPPQNGRGRGGSGYALHGGAASDAYSSADALSGTP-VATPENSSASFGDDEAVNGVSSSL
KPQSTRRSSSSSSTFHSAVYNASLDHNRQASSDQPNSNNSFHQSDSFGMQQEDNTTSDSV
** .. *. ..*.             .  . *.     .. :.* *** ::      .*

RAGN--GGGDEFDDDE------PDSKR2WRKDG-DGEGIS-MAGNRTVREPRVVVQTMSD
RVASSVGGGEDLDDDE------PDSKR~WRRDGGDGEGVSLVAGNRTVREPRVVVQTMSD
GDDEFEQGSSIVSRDEEDCGSEPEAKR2WKGDNETNGGNG--GGSKTVREPRIVVQTTSD
   .   *.. .. **      *::** *: *.  . * .  .*.:******:**** **

IDILDDGYRWRKYGQKVVKGNPNP2RSYYKCTTAGCPVRKHVERASHDLRAVITTYEGKH
IDILDDGYRWRKYGQKVVKGNPNP2RSYYKCTTAGCPVRKHVERASNDLRAVITTYEGKH
IDILDDGYRWRKYGQKVVKGNPNP2RSYYKCTTIGCPVRKHVERASHDMRAVITTYEGKH
************************ ******** ************:*:***********

NHDVPAARG--SAALYRPAPPAAAATSSHPYLPNQPPPMSYQPTGPQPYALRPDGFGGQG
```

```
NHDVPAARGSAAAALYRATPPPQASNAGMMPTTAQPSSYLQGGGGVLPAGGYGASYGGAP
NHDVPAARG--SGYATNRAPQDSSSVPIRPAAIAGHSNYTTSSQAPYTLQMLHNNNTNTG
*********  :.   . :*   :: .         .       .  .      .  .

PFGGVVGGSSFGGFSG-FDDARG--SYMSQHQQQQRQNDAMH-ASRAKEEPGDD---MFF
TTTQPANGGGFAALSGRFDDDATGASYSYTSQQQQQPNDAVYYASRAKDEPRDDGIMSFF
PFGYAMNNN-----------------NNNSNLQTQQNFVGGGFSRAKEEPNEE--TSFF
.     ...                      : * : * .    ****:** ::    **

QNSLY-
EQPLLF
DSFMP-
:. :


 CLUSTAL W (1.83) Multiple Sequence Alignments

Sequence format is Pearson
Sequence 1: LOC_Os01g62490.1      577 aa
Sequence 2: LOC_Os05g38420.1      574 aa
Sequence 3: At5g60020.1           577 aa
Start of Pairwise alignments
Aligning...
Sequences (1:2) Aligned. Score:  79
Sequences (1:3) Aligned. Score:  69
Sequences (2:3) Aligned. Score:  69
Guide tree        file created:   [/tmp/3MLr1SjSp3/bAGOqqcFug.dnd]
Start of Multiple Alignment
There are 2 groups
Aligning...
Group 1: Sequences:   2      Score:11378
Group 2: Sequences:   3      Score:10714
Alignment Score 8267
GCG-Alignment file created      [/tmp/3MLr1SjSp3/P5W730m2jT]

MAISYLLRSSILAVAALLLFSVNLAKGDIREYQFD0VKTTNVTRLCSSKSIVTVNGQFPG
MAAASSVLRCCLLVAALMTLSAMGAEAITRQYLFD0VQTTSVTRLCSTKSIVTVNGQYPG
-----MALQLLLAVFSCVLLLPQPAFGITRHYTLE0IKMQNVTRLCHTKSLVSVNGQFPG
           * * : : :    * .  *.* :: ::  .***** :**:*:****:**

PTVFAREGDLVVIRVINHSPYNMSIH2WHGIRQLRSGWADGPAYITQCPIQPGGSYVYKY
PTLFAREGDHVEVTVVNHSPYNMSIH2WHGIRQLLSGWADGPSYITQCPIQPGGSYVYRF
PKLIAREGDQVLIKVVNQVPNNISLH2WHGIRQLRSGWADGPAYITQCPIQTGQSYVYNY
*.::***** * : *:*: * *:*:* ******* *******:********.* ****.:

TITGQRGTLWWHAHISWLRATVYGPIIILPKAGVPYPFPAPDKEVPVVF1GEWWKADTEA
TITGQRGTLWWHAHISWLRATVHGPMVILPPAGVGYPFPAPHEEVPIMF~GEWWNNDTEA
TIVGQRGTLWYHAHISWLRSTVYGPLIILPKRGVPYPFAKPHKEVPMIF1GEWFNADTEA
**.*******:********:**:**::***  ** ***. *.:***::* ***:: ****

VISQATQTGGGPNVSDAFTINGLPGPLYNCSAK1DTFKLKVEAGKTYMLRLINAALNDEL
VISQALQTGGGPNISDAYTLNGLPGPLYNCSAQ1DTFKLKVKPGKTYMLRLINAALNDEL
IIRQATQTGGGPNVSDAYTINGLPGPLYNCSAK1DTFRLRVKPGKTYLLRLINAALNDEL
:* ** *******:***:*:************: ***:*:*:.****:************

FFSIAGHTLTVVDVDAVYVKPFTVDTLLITPGQTTNVLLTTKPSYPGATFYMLAAPYSTA
FFSIANHTLTVVDVDALYVKPFTVDTLIIAPGQTSNVLLTAKPTYPGASYYMLARPYTTT
```

```
FFSIANHTVTVVEADAIYVKPFETETILIAPGQTTNVLLKTKSSYPSASFFMTARPYVTG
*****.**:***:.**:***** .:*::*:****:****.:*.:**.*:::* * ** *

MSGTFDNTTVAGILEYEDPS---SHSTAAFNKNLPVLRPTLPQINDTSFVSNYTAKLRSF
Q-GTFDNTTVAGVLEYDDP-----CPTTAAGKIVPIFSPTLPQINDTNAVSNFTAKLRSL
Q-GTFDNSTVAGILEYEPPKQTKGAHSRTSIKNLQLFKPILPALNDTNFATKFSNKLRSL
  *****:****:***: *       : :  * : :: * ** :***. .:::: ****:

ATAEYPANVPQQVDTRFFFTVGLGTHPCAVNG--TCQGP-NGSRFAAAVNNVSFVLPSTA
ASAGYPAAVPQQVDHRFFFTVGLGTHPCAVNG--TCQGP-NGSRFAASINNVSFVLPATA
NSKNFPANVPLNVDRKFFFTVGLGTNPCNHKNNQTCQGPTNTTMFAASISNISFTMPTKA
 :  :** ** :** :*********:**  :.  ***** * : ***::.*:**.:*:.*

LLQSHYTGRSNGVYASNFPAMPLSPFNYTGTPPNNTNVSNGTRLVVLPYGASVELVMQGT
LLQSHFAGKSKGVYASNFPYYPLNPFNYTGTPPNNTNVMNGTKVLVLPYGANVELVMQDT
LLQSHYSGQSHGVYSPKFPWSPIVPFNYTGTPPNNTMVSNGTNLMVLPYNTSVELVMQDT
*****::*:*:***..:**  *: ************ * ***.::****.:.******.*

SVLGAESHPFHLHGFNFFVVGQGFGNFDPVNDPAKYNLVDPVERNTVGVPAAGWVAIRFL
SILGAESHPLHLHGFNFFVVGQGFGNFDPINDPAKFNLYDPVERNTVGVPAGGWVAIRFH
SILGAESHPLHLHGFNFFVVGQGFGNFDPNKDPRNFNLVDPIERNTVGVPSGGWAAIRFL
*:*******:****************** :** ::** **:********:.**.****

VDNP1GVWFMHCHLEVHVSWGLKMAWVVQDGSLPNQKILPPPSDLPKC
ADNP1GVWFMHCHLEVHMSWGLKMAWLVLDGSRPDQKLPPPPLDLPKC
ADNP1GVWFMHCHLEVHTSWGLRMAWLVLDGDKPDQKLLPPPADLPKC
.*** *********** ****:***:* **. *:**: *** *****


 CLUSTAL W (1.83) Multiple Sequence Alignments

Sequence format is Pearson
Sequence 1: LOC_Os01g50760.1      353 aa
Sequence 2: LOC_Os05g46580.1      355 aa
Sequence 3: At5g47770.1           384 aa
Start of Pairwise alignments
Aligning...
Sequences (1:2) Aligned. Score:  79
Sequences (1:3) Aligned. Score:  70
Sequences (2:3) Aligned. Score:  66
Guide tree        file created:   [/tmp/3MLr1SjSp3/snNL4gLRCj.dnd]
Start of Multiple Alignment
There are 2 groups
Aligning...
Group 1: Sequences:   2      Score:7017
Group 2: Sequences:   3      Score:6616
Alignment Score 4976
GCG-Alignment file created      [/tmp/3MLr1SjSp3/P5W730m2jT]

MAAA--VVANGASGDSS-----------------------------KAAFAEIYSRLKEE
MAAANGSACGGGGVDKE-----------------------------KEEFKQIYGVLKEE
MSVSCCCRNLGKTIKKAIPSHHLHLRSLGGSLYRRRIQSSSMETDLKSTFLNVYSVLKSD
*:.:      *   ..                              *  * ::*. **.:

MLEDPAFEFTDESLQWIDR0MLDYNVLG~GKCNRGISVIDSFKMLKGTDVLNKEETFLAC
LLRDPAFEFTDSSRQWIDR0MLDYNVPG1GKCNRGLSVVDSYKLLKGTNVLSQEDMFLAS
LLHDPSFEFTNESRLWVDR0MLDYNVRG1GKLNRGLSVVDSFKLLKQGNDLTEQEVFLSC
```

```
:*.**:****:.*  *:** ****** * ** ***:**:**:*:**  : *.::: **:.

TLGWCIEW0LQAYFLVLDDIMDNSQTRRGQPCWFRVPQ0VGLIAVNDGIILRNHISRILQ
TLGWCVEW0LQAYFLVLDDIMDDSHTRRGQPCWFRVPQ0VGSIAINDGIILRNHITRMLR
ALGWCIEW0LQAYFLVLDDIMDNSVTRRGQPCWFRVPQ0VGMVAINDGILLRNHIHRILK
:****:** *************:* ************* ** :*:****:***** *:*:

RHFKGKLYYVDLIDLFNE0VEFKTASGQLLDLITTHEGEKDLTKYNLT2VHRRIVQYKTA
LHFRGKLYYADLLDLFNE0VEFKTASGQLLDLITTHEGEKDLNKYNIG2VHRRIVQYKTS
KHFRDKPYYVDLVDLFNE0VELQTACGQMIDLITTFEGEKDLAKYSLS2IHRRIVQYKTA
 **:.* **.**:***** **::**.**::*****.****** **.:  :*********:

YYSFYLP0VACALLLSGENLDNFGDVKNILVEMGTYFQVQ0DDYLDCYGDPEFIGK0IGT
YYSFYLP0VACALLLSGEDLTKYGAVEDILVKMGIYFQVQ0DDYLDCYGDPKFIGK0IGT
YYSFYLP0VACALLMAGENLENHIDVKNVLVDMGIYFQVQ0DDYLDCFADPETLGK0IGT
******* ******::**:* :.  *:::**.** ***** ******:.**: :** ***

DIEDYKCSWLVVQALERADENQKHILF0ENYGKPDPECVAKVKDLYKELNLE0AVFHEYE
DIEDYKCSWLVVQALERADESQKSVLF0ENYGKKDPACVAKVKSLYRELNLE0AVFLDYE
DIEDFKCSWLVVKALERCSEEQTKILY0ENYGKPDPSNVAKVKDLYKELDLE0GVFMEYE
****:*******:****..*.*. :*: ***** **  *****.**:**:** .** :**

RESYNKLIADIEAHPNKAVQNVLKSFLHKIYKRQK
NESYKKLIADIEAQPSIAVQNVLKSFLHKIYKRQK
SKSYEKLTGAIEGHQSKAIQAVLKSFLAKIYKRQK
 :**:** . **.: . *:* ****** *******


 CLUSTAL W (1.83) Multiple Sequence Alignments

Sequence format is Pearson
Sequence 1: LOC_Os01g18400.1      290 aa
Sequence 2: LOC_Os05g04690.1      295 aa
Sequence 3: At1g02360.1           272 aa
Start of Pairwise alignments
Aligning...
Sequences (1:2) Aligned. Score:  64
Sequences (1:3) Aligned. Score:  61
Sequences (2:3) Aligned. Score:  61
Guide tree        file created:   [/tmp/3MLr1SjSp3/W3DjYI0Ycn.dnd]
Start of Multiple Alignment
There are 2 groups
Aligning...
Group 1: Sequences:   2      Score:5178
Group 2: Sequences:   3      Score:4839
Alignment Score 3524
GCG-Alignment file created      [/tmp/3MLr1SjSp3/P5W730m2jT]

MAKPTPAPRATPFLLAAVLSIVVVAASGAEARWYGGGGGGGYSPSPSPVSSIVSEQLY-A
MAGRRRRPFAAPVINYLLLALWLAASSSFAVAVAVAGHGRGRR---SHVSSIVTEEMYNK
MAQQH-----SFLLLCFFLSISYLLSSAQTEATS--------------IERLVPRDLY-N
**        : .:   .*::    :*.                    :. :*..::*

SLFLHKDDAACPARGFYTYASFVRAATRFPRFAATGCADARKREVAAFLAQISHETTGGW
SLFIHKDDAACPARNFYTYAAFLRAADQYPSFGGAGGRDTRRREVAAFLAQVSHETTGGW
KIFIHKDNTACPANGFYTYESFVQATRRFPRFGSVGSPVTQRLEVAAFLAQISHETTGGW
.:*:***::****..**** :*::*: ::* *...*   ::: ********:********
```

```
ATAPDGPYAWGLCYKEEINPQSSYCD-ATDKQWPCYPGKSYHGRGPIQIS2WNFNYGPAG
ATAPDGPYTWGLCFKEELKPASNYCDAAVAARWPCFPGKSYHGRGPIQLS~WNFNYGPAG
ATAPDGPYAWGLCFKEEVSPQSTYCD-SSDTQWPCFPNKTYQGRGPIQLS2WNYNYGPAG
********:****:***:.* *.*** :   :***:*.*:*:******:* **:******

QALGFDGLRNPEIVANCSDIAFQTALWFWMTPR-DTKP-SCHQVMVGEYRPGPADVAANR
EAVGFDGLREPEVVAGDAVVAFKTALWFWMTPRPPSKPYSCHDVMTGRYRPSRADAAANR
RALGFDGLRNPETVSNNSVIAFQTALWFWMTPQ-SPKP-SCHDVMIGKYRPTAADLAANR
.*:******:** *:. : :**:*********:  .** ***:** *.***  ** ****

TAG-FGLVTNIVNGGLECN--RAGDARVNNRIGFYRRYCQVLGVDVGPNLDCEHQQPF-
TAAGFGLTTNIINGGLECNNRTGGDPRVEDRIGFFRRYCGALGVDVGDNLDCAHQLPYS
TGG-FGLTTNIINGGLECG--IPGDGRVNDRIGFFQRYTGLFKVATGPNLDCENQRPYA
*.. ***.***:******.    ** **::****::**   : * .* **** :* *:


 CLUSTAL W (1.83) Multiple Sequence Alignments

Sequence format is Pearson
Sequence 1: LOC_Os01g55240.1      327 aa
Sequence 2: LOC_Os05g48700.1      353 aa
Sequence 3: At1g30040.1           341 aa
Start of Pairwise alignments
Aligning...
Sequences (1:2) Aligned. Score:  59
Sequences (1:3) Aligned. Score:  50
Sequences (2:3) Aligned. Score:  46
Guide tree        file created:   [/tmp/3MLr1SjSp3/99yTDExg5u.dnd]
Start of Multiple Alignment
There are 2 groups
Aligning...
Group 1: Sequences:   2      Score:5528
Group 2: Sequences:   3      Score:5272
Alignment Score 3115
GCG-Alignment file created      [/tmp/3MLr1SjSp3/P5W730m2jT]

MVVLAGPPAVD-HIPLLRSPD--PGDVFSGVPVVDLGSPGAARAVVDACERYGFFKVVNH
MVAITAPSSIE-HIPLVRCPKGANAGPQAVIPCIDLSAPGAAAAVADACRTLGFFKATNH
MVVLPQPVTLDNHISLIPTYKPVPVLTSHSIPVVNLADPEAKTRIVKACEEFGFFKVVNH
**.:. * ::: **.*:   .         :* ::*. * *   :..**.  ****..**

GVATDTMDKAESEAVRFFSQTQPDK-DRSGPAYPFGYGSKRIGFNGDMGWLEYLLLALD-
GVPAGLADALESSAMAFFALPHQEKLDMSGPARPLGYGSKSIGSNGDVGWLEYLLLSAG-
GVRPELMTRLEQEAIGFFGLPQSLK-NRAGPPEPYGYGNKRIGPNGDVGWIEYLLLNANP
** .      *..*: **. .:  * : :**. * ***.* ** ***:**:*****  .

--DASLADACTVPSCAVF2RAALNEYISGVRKVAVRVMEAMSEGLGIAQAD----ALSAL
--AASSGGAALP---AAL~RAAVEAYTGAVRGVGCRVMELMAEGLGLGASEEGRCVLRRM
QLSSPKTSAVFRQTPQIF2RESVEEYMKEIKEVSYKVLEMVAEELGIEPRD----TLSKM
   :.  .*        : * ::: *   :: *. :*:* ::* **:   :    .*  :

VT-AEGSDQVFRVNHYPPCRALQGLG---CSVTGFGEHTDPQLVSVLRSNGTSGLQIALR
VVGCEGSDEMLRVNHYPPCLLPPGRDRDECGVTGFGEHTDPQIISVLRSNCTAGLQILLR
LR-DEKSDSCLRLNHYPAAEEEAEKM----VKVGFGEHTDPQIISVLRSNNTAGLQICVK
:   * **. :*:****..             .*********::****** *:**** ::
```

```
-----DGQWVSVPSDRDSFFVNVGDSLQ0VLTNGRFKSVKHRVVAN-SLKSRVSFIYFGG
GDYSSPARWVPVPPDPDSFFVNVGDSLQ0VLTNGRFRSVKHRVLAPEGEESRLSVIYFGG
-----DGSWVAVPPDHSSFFINVGDALQ0VMTNGRFKSVKHRVLAD-TRRSRISMIYFGG
      . **.**.* .***:****:** *:*****:******:*    .**:*.*****

PPLAQRIAPLPQLLGEGEQSLYKEFTWDEYKKAAYKSRLGDNRLAQFEKK----------
PAASQRIAPLEQVMREGEQSLYREFTWGEYKKAAYKTRLGDNRLGPYELQHAAANDEAAT
PPLSQKIAPLPCLVPEQDDWLYKEFTWSQYKSSAYKSKLGDYRLGLFEKQPLLNHKTLV-
*. :*:****  :: * :: **:****.:**.:***::*** **. :* :

--
KK
--


 CLUSTAL W (1.83) Multiple Sequence Alignments

Sequence format is Pearson
Sequence 1: LOC_Os10g39810.1      301 aa
Sequence 2: LOC_Os03g01820.1      300 aa
Sequence 3: At4g12110.1           298 aa
Start of Pairwise alignments
Aligning...
Sequences (1:2) Aligned. Score:  65
Sequences (1:3) Aligned. Score:  64
Sequences (2:3) Aligned. Score:  54
Guide tree        file created:   [/tmp/3MLr1SjSp3/gszx61aBcw.dnd]
Start of Multiple Alignment
There are 2 groups
Aligning...
Group 1: Sequences:   2      Score:5546
Group 2: Sequences:   3      Score:5307
Alignment Score 3822
GCG-Alignment file created      [/tmp/3MLr1SjSp3/P5W730m2jT]

MLPYATAAEAEAALGRAMTAAESLWFRYSAGIPDYVLFWHNILFLFVVFTLAPLPVALLE
MLPYATAAEAEAAVGRGLTWAEAAWFRYSAAIPDYCLYCHNVPILLLVYTLAPLPLALLE
MIPYATVEEASIALGRNLTRLETLWFDYSATKSDYYLYCHNILFLFLVFSLVPLPLVFVE
*:****. **. *:** :*  *: ** ***  .** *: **: :*::*::*.***:.::*

LRA--PAAVGPFKLQPKVRLSREEFFRCYRDVMRLFFLVIGPLQLVSYPTVK0MVGIHTG
LRR--HLPL-PHKLQPGVRHPPAAFLRCYAATARVLLLAVGPVQLASFPAVR~AVGIRTG
LARSASGLFNRYKIQPKVNYSLSDMFKCYKDVMTMFILVVGPLQLVSYPSIQ0MIEIRSG
*       .  .*:** *. .   :::**  .  :::*.:**:**.*:*:::  : *::*

LPLPSLGEMAAQLLVYFLVEDYLNYWIHRLLHGEWGYEKIHRVHHEFTAPIGFAAPYAHW
LPLPSAGETAAQVAVYLLVEDYLGYWIHRLLHTPWAYHHIHRVHHEFTAPMGYAAPYAHW
LPLPTITEMLSQLVVYFLIEDYTNYWVHRFFHSKWGYDKIHRVHHEYTAPIGYAAPYAHW
****:  *  :*: **:*:*** .**:**::*  *.*.:*******:***:*:*******

AEVLILGIPSFVGPALAPGHMITFWLWIVLRQMEAIETHS2GFDFPFNLTKYIPFYGGAE
AEILILGFPAFAGPAIVPCHMTTFWLWFVLRHLEAIHIHS2GFKLPFDPTKYIPLYGGVE
AEVLLLGIPTFMGPAIAPGHMITFWLWIALRQMEAIETHS2GYDFPWSPTKYIPFYGGAE
**:*:**:*:* ***:.* ** *****:.**::***. ** *:.:*:. *****:***.*

YHDYHHYVGRQSQSNFASVFTYCDYLYGTDK0GYRYHKAYQAK0MKALGQTEGEKADSNG
YHDYHHFVGGHSQSNFSSVFTFCDYIYGTDR0GYRYHKASLSK0LKEMAGNHVEKGDENG
```

```
YHDYHHYVGGQSQSNFASVFTYCDYIYGTDK0GYRFQKKLLEQ0IKESSKKSNKHN----
******:** :*****:****:***:****: ***::*    : :*  . .  ::

LSYAKLD
FGNGKQD
-GGIKSD
 .  * *
 CLUSTAL W (1.83) Multiple Sequence Alignments

Sequence format is Pearson
Sequence 1: LOC_Os02g46780.1      340 aa
Sequence 2: LOC_Os04g50770.1      371 aa
Sequence 3: At1g79180.1           294 aa
Start of Pairwise alignments
Aligning...
Sequences (1:2) Aligned. Score:  60
Sequences (1:3) Aligned. Score:  40
Sequences (2:3) Aligned. Score:  41
Guide tree        file created:   [/tmp/3MLr1SjSp3/0W6fShDXYL.dnd]
Start of Multiple Alignment
There are 2 groups
Aligning...
Group 1: Sequences:   2      Score:5814
Group 2: Sequences:   3      Score:4331
Alignment Score 2613
GCG-Alignment file created      [/tmp/3MLr1SjSp3/P5W730m2jT]

MGRGRAPCCAKVGLNRGSWTPQEDMRLIAYIQKHGHANWRALPKQA~GLLRCGKSCRLRW
MGKGRAPCCAKVGLNKGSWTPEEDMRLVAYIQKYGHANWRALPKQA1GLLRCGKSCRLRW
MGKGRAPCCDKTKVKRGPWSPEEDIKLISFIQKFGHENWRSLPKQS1GLLRCGKSCRLRW
**:****** *. :::*.*:*:**::*:::***.** ***:****: *************

INYLRPDLKRGNFTADEEDTIIKLHGLLGN2KWSKIASCLPGRTDNEIKNVWNTHLKKRV
INYLRPDLKRGNFTAEEEETIIKLHGLLGN2KWSKIASCLPGRTDNEIKNVWNTHLKKRV
INYLRPDLKRGNFTSEEEETIIKLHHNYGN2KWSKIASQLPGRTDNEIKNVWHTHLKKRL
**************::**:******   ** ******* *************:******:

SQREKPGDTKKKGKAADASDDADAHSPSSSASSSTTTAANNNNSGDTAGEQCGTSKEPE-
SPEQKKG----GGKSKKKTTCTDVLVPSPSPSSSTTTTTN-CSSGDSAGEQSNTSKEEEE
AQ-------------SSGTADEPASPCSSDSVSRGKDDKSSHVEDSLNRETNHRNELS-
:               .     :   *..* * *  .  :     *: ..: .  :* .

---NVDVSFFEQDIDIS--DMLVD---APTEAPLVAAPMPPS-PCSSSSLTTTTCVGAVS
ETDKIEIPMLELDPCCFDFDMLVDPVVPDTYCPAVSASASASAPTSPCSSTSPSCARAGV
------TSMSSGGSNQQ-------------DDPKIDELRFEYIEEAYSEFNDIIIQEVDK
       .: . .                   * :           : .. .       .

DELLDLPEI-DIEPDIWSIIDGYGGD-------------EPGDGDATVPCTASPGEE-G
DPLLDLPEIVDLGPELWSIMDGGAGDGCTEAPPPAWSNAAAAAAANATVATTTSLEEEEG
PDLLEIPFD--SDPDIWSFLDTSNSFQQS----------TANENSSGSRATTEEESDEDE
  **::*      *::**::*   .                  ...: . * .  :*

AEWWVENLEKELGLWGPMDESLAHPDPPGQVCYPG-PLTETEGDPVSTYFQSGPTASP-L
KEWWLEDLEKELGLWGPTDDYHCHPGPQGQPGRAGPPPSAVVEDPVSCYFQAGPTAAATW
VKKWFKHLESELGLE--------------------------EDDNQQQYKEEESSSSLL
 : *.:.**.****                            *  . * :  .:::.
```

```
QEIASPAVLS--------
QGHEPSAVITSNPMDYYV
KNYELMIH----------
:


CLUSTAL W (1.83) Multiple Sequence Alignments

Sequence format is Pearson
Sequence 1: LOC_Os02g52830.1      482 aa
Sequence 2: LOC_Os06g10850.1      489 aa
Sequence 3: At5g67050.1           477 aa
Start of Pairwise alignments
Aligning...
Sequences (1:2) Aligned. Score:  61
Sequences (1:3) Aligned. Score:  45
Sequences (2:3) Aligned. Score:  41
Guide tree        file created:   [/tmp/3MLr1SjSp3/RMLx7X2LxF.dnd]
Start of Multiple Alignment
There are 2 groups
Aligning...
Group 1: Sequences:   2      Score:8388
Group 2: Sequences:   3      Score:7254
Alignment Score 4404
GCG-Alignment file created      [/tmp/3MLr1SjSp3/P5W730m2jT]

-MAGAGEYGGGGGGGGGFGGDFMVLRPDKGGIGDLFHLLWSCKVAENAAVDCP-IGTEIA
-MDDDDDCRRRD---VTLGDEFMVLQPENGGARSLADLLLSCKVGENKAVRCGRPGAEVA
MMNSDDDDEPRG---------YLILRPEELRPWELVRLLFSGDIEKPRSVDSS--ETEEH
 * . .:    .         :::*:*::    .*  ** * .: :  :* .    :*

ERRRRWALFVSLVAQMLLLWTKKPMALLGGGTEYWMNLLNENGGSVFMLITNALQ1GKVK
LPWHRWIIAVSLLAQMLLRLSKGVMAKVGRAVEYWMNLVSEND-NVLGLIRNALH1GKVK
SFRHRWLIFVSLVLLKLLRFFSKLLALVGSALEFSLNFLSNNS-----FSGLFLR1GEVV
   :** : ***:   **   .  :* :* . *: :*::.:*.     :    *: *:*

MPDKSSINYRSCIGLLDTRIDLDEKIKPED-RNYHAALSIMAAKLAYENELVVRTVVQNH
TPDRNSPNYRSFIGLLDTRIDLDEKIKPGD-SNYHAALCIMAAKLAYENELVIKDAVEKN
MPQRTSENYQSFIGHLDTRVSLDMTLNREDGEKYYAALSIMASKIAYENAARIKHVVENH
 *::.* **:* ** ****:.** .::  *  :*:***.***:*:****   :: .*:::

WQ0MNFVGFYNCWN1EFQEDYTTQAFMVSDKAE------DASLAVVAFCGTKPFDTEQWC
WK0MTFLEFFNCWN1DFQNDYTTQAFMFADKPE------DAELVVVAFRGTQPFDMEQWS
WN~MKYLGLVDYWN1EYQEKETTQAFIMSTDETTTRSNGQETTVVVAFRGTELFNSEDWC
*: *.:: : : ** ::*:. *****:.: .        :   .**** **: *: *:*.

ADVDFSWYEIPGVGKVHGGFMKALGLQRNGG------WPEQPTGAGDDGGSDK-KPFAYY
TDVDISWYEIPGVGKVHGGFMKALGLQNNAAAGKKPSWPAEIAPPSAAKNSEKTKSFAYY
SDFDITWFELPNIGNIHGGFMKALGLQNNCS------WPKEPLS-----NPDRKSPLAYY
:*.*::*:*:*.:*::***********.* .      ** :        ..:: ..:***

VIRERLRAFLAENPRARFVVAGHSLGGALAILFPTVLALHG---EEDMLARLHGVYTFGQ
AIRARLRAFLAANPRARLVVTGHSLGGALAALFPVVLALHGGEADAAALGRLDGVYTYGQ
SIRDSLKTLIAQNKNTKFVLTGHSLGGALAILFTAVLVIHH---ETELLERIQGVYTYGQ
 **  *::::* * .:::*::********* **..**.:*    :   * *:.****:**

PRVGDEGLCRFMDGHLATPVSRYFRFVYCNDIVPRVPYDDTALLFKHFGTCLYFDSFYKG
```

```
PRVGDAALGEWVA-AASSLEGKHLRFVYCNDVVPRVPYDDAAFLFRHFGRCVYFDAAYRA
PRVGDSKFGEFMEKKLEKYNIKYYRFVYNNDIVPRLPYDDKDLMFKHFGTCIYYDQNYQA
*****  : .::     .   :: **** **:***:****  ::*:*** *:*:*  *:.

H0VTEEEPNKNYFSLLTVVPKYANAAWELARSFVIGYVDGPEYGEGWLMRIARAAGLLLP
R~AMAEEPNKNYFSPAFALAKHANAAWELARGLAIGRVAGGEYAEGWAMRAARVVGLVFP
K0VMREQSDENFFLLRGIIKMMWSAILEFIRSFTIVAEKGSEYSEGWLLKGGRALGIIVP
: .  *:.::*:*     :    .*  *: *.:.*    * **.*** :: .*. *::.*

GLPPHAPQDYVNATRLGAASLEPLR------
GLPPHAPQDYVNATRLAGASLRKLLD-----
GVSNHTPQDYVNATRLTLPCVFQVYRDVSIT
*:. *:**********  ..:  :


 CLUSTAL W (1.83) Multiple Sequence Alignments

Sequence format is Pearson
Sequence 1: LOC_Os02g08230.1      628 aa
Sequence 2: LOC_Os06g44300.1      627 aa
Sequence 3: At5g57800.1           632 aa
Start of Pairwise alignments
Aligning...
Sequences (1:2) Aligned. Score:  79
Sequences (1:3) Aligned. Score:  60
Sequences (2:3) Aligned. Score:  62
Guide tree        file created:   [/tmp/3MLr1SjSp3/R1C7qduBFc.dnd]
Start of Multiple Alignment
There are 2 groups
Aligning...
Group 1: Sequences:   2      Score:12366
Group 2: Sequences:   3      Score:11099
Alignment Score 8430
GCG-Alignment file created      [/tmp/3MLr1SjSp3/P5W730m2jT]

---MAAPPLSSWPWASLGSYK0YVLYGAVVWKVAEEWRQQGAAPVG-SWWLHLLLLFAAR
MAISMASPLSSWPWAFLGSYK0YLLYGPVVGKVVQEWREQGRLPLGTSWCLHLILLLALR
----MVAFLSAWPWENFGNLK0YLLYAPLAAQVVYSWVYEEDISKV-LWCIHILIICGLK
     .. **:***  :*. * *:**..:. :*. .*  :   .    * :*:::: . :

GLTYQFWFSYGNMLFFTRRRRVVPDSVDFRQVDAEWD2WDNFLLLQTLIGATLVGSPAVA
SLTYQLWFSYGNMLFFTRRRRVVDDGVDFRQIDTEWD2WDNMVIMQTLIAAVLVTSRVFP
ALVHELWSVFNNMLFVTRTLRINPKGIDFKQIDHEWH2WDNYIILQAIIVSLICYMSPPL
.*.:::*  :.****.**  *:  ..:**:*:* **. *** :::*::* : :

RQQLLLPSLKQAWDPRGWAIALLLHVLVAEPLFYWAHRALHRA-PLFSRYHAAHHHASVT
-----ATSDLSAWDLRGWAIAVVLHVAVSEPAFYWAHRALHLG-PLFSRYHSLHHSFQAT
---MMMINSLPLWNTKGLIALIVLHVTFSEPLYYFLHRSFHRNNYFFTHYHSFHHSSPVP
       .    *: :*    ::*** .:** :*: **::*    :*::**: **   ..

TPLT1AGFGTPLESLLLTVVIGVPLAGAFLMGVGSVGLVYGHVLLFDFLRSMGYSNVEVI
QALT1AGFVTPLESLILTLVAWAPLAGAFMAGHGSVSLVYGHILLFDYLRSMGYSNVEVI
HPMT1AGNATLLENIILCVVAGVPLIGCCLFGVGSLSAIYGYAVMFDFMRCLGHCNVEIF
 .:* **  * **.::* :*  .** *. : * **:. :**: ::**::*.:*:.***::

SPRVFQAVPLLRYLIYTP2TYLSLHHREKDSNFCLFMPIFDLLGGTLNHKSWELQKEVYL
SHKTFQDFPFLRYLIYTP2SYLSLHHREKDSNFCLFMPLFDALGGTLNPKSWQLQKEVDL
```

```
SHKLFEILPVLRYLIYTP2TYHSLHHQEMGTNFCLFMPLFDVLGDTQNPNSWELQKKIRL
* : *: .*.******** :* ****:* .:*******:** **.* * :**:***:: *

1--~GKNDQAPDFVFLAHVVDIMASMHVPFVLRSCSSTPFANHFVLLPFWPVAFGFMLLM
1--~GKNHRVPDFVFLVHVVDVVSSMHVPFAFRACSSLPFATHLVLLPLWPIAFGFMLLQ
~SA1GERKRVPEFVFLAHGVDVMSAMHAPFVFRSFASMPYTTRIFLLPMWPFTFCVMLGM
    *:..:.*:****.* **::::**.**.:*: :* *::.::.***:**.:* .**

WCCSKTFLVSSYRLRGNLHQMWTVPRYGFQ0YFIPAAKKGINEQIELAILRADRMGVKVL
WFCSKTFTVSFYKLRGFLHQTWSVPRYGFQ0YFIPSAKKGINEMIELAILRADKMGVKVL
WAWSKTFLFSFYTLRNNLCQTWGVPRFGFQ0YFLPFATKGINDQIEAAILRADKIGVKVI
*  **** .* * **. * * * ***:*** **:* *.****: ** ******::****:

SLAALNK0NEALNGGGTLFVNKHPELRVRVVHGNTLTAAVILNEIPSNVKDVFLTGATSK
SLAALNK0NEALNGGGTLFVRKHPDLRVRVVHGNTLTAAVILNEIPGDVAEVFLTGATSK
SLAALNK0NEALNGGGTLFVNKHPDLRVRVVHGNTLTAAVILYEIPKDVNEVFLTGATSK
******* ************.***:***************** *** :* :*********

LGRAIALYLCRKKIRVL0MLTLSSERFLKIQREAPAEFQQYLVQVTKYQPAQNCK0TWLV
LGRAIALYLCRKKIRVL0MLTLSTERFMNIQREAPAEFQQYLVQVTKYQAAQNCK0TWIV
LGRAIALYLCRRGVRVL0MLTLSMERFQKIQKEAPVEFQNNLVQVTKYNAAQHCK0TWIV
***********: :*** ***** *** :**:***.***: *******:.**:** **:*

GKWLSPREQRWAPAGTHFHQFVVPPIIGFRRDCTYGKLAAMRLPKDVQGLGYCE0YTMER
GKWLSPREQRWAPAGTHFHQFVVPPIIGFRRDCTYGKLAAMRLPEDVEGLGTCE~YTMGR
GKWLTPREQSWAPAGTHFHQFVVPPILKFRRNCTYGDLAAMKLPKDVEGLGTCE0YTMER
****:**** ****************: ***:****.****:**:**:*** ** *** *

GVVHACHAGGVVHFLEGWEHHEVGAIDVDRIDVVWKAALKHGLTPA-----
GVVHACHAGGVVHFLEGWDHHEVGAIDVDRIDAVWNAALRHGLTPA-----
GVVHACHAGGVVHMLEGWKHHEVGAIDVDRIDLVWEAAMKYGLSAVSSLTN
*************:****.************* **:**:::**:..


 CLUSTAL W (1.83) Multiple Sequence Alignments

Sequence format is Pearson
Sequence 1: LOC_Os02g14430.1      327 aa
Sequence 2: LOC_Os06g35480.1      318 aa
Sequence 3: At5g58400.1           325 aa
Start of Pairwise alignments
Aligning...
Sequences (1:2) Aligned. Score:  60
Sequences (1:3) Aligned. Score:  58
Sequences (2:3) Aligned. Score:  52
Guide tree        file created:   [/tmp/3MLr1SjSp3/9QCkQp43uH.dnd]
Start of Multiple Alignment
There are 2 groups
Aligning...
Group 1: Sequences:   2      Score:5595
Group 2: Sequences:   3      Score:5362
Alignment Score 3362
GCG-Alignment file created      [/tmp/3MLr1SjSp3/P5W730m2jT]

MASR-SSWHCCLLAFFLLSSAAGAAYGQQLSTTFYAASCPTLQVVVRATVLGALLAERRM
MAT---QWVLVVVAVMAVLFAGGAAGG-QLSTRYYDGKCPNVQSIVRAGMAQAVAAEPRM
MECYEQSRQRAAFVVLLFIVMLGSQAQAQLRTDFYSDSCPSLLPTVRRVVQREVAKERRI
```

```
*     .     ...: .    *:    ** * :*  .**.:   **  :   :  * *:

GASLVRLFFHDCFVQ~GCDASILLDDVPATSFVGEKTAFPNVNSVRGYDVIDQIKRNVEL
GASILRMFFHDCFVN0GCDASILLDDT--ANFTGEKNAGPNANSVRGYEVIDAIKTQVEA
AASLLRLFFHDCFVN0GCDASILLDDT--RSFLGEKTAGPNNNSVRGYEVIDAIKSRVER
.**::*:*******: **********.   .* ***.* ** ******:*** ** .**

LCPGVVSCADIVALAARDSTAL0LGGPSWAVPLGRRDSTTASLSAAN-SDLPAPSSDLAT
SCNATVSCADILALAARDAVNL0LGGPTWTVQLGRRDALTASQSAAN-GNLPGPGSDLAT
LCPGVVSCADILAITARDSVLL0MGGRGWSVKLGRRDSITASFSTANSGVLPPPTSTLDN
 * ..******:*::***:. * :**  *:* *****: *** *:** . ** * * * .

LIAGFGNKGLSPRDMTALSGAHTIGFSQCANFRDRVYNDTNIDPAFAALRRRGCPAAPGS
LVTMFGNKGLSPRDMTALSGAHTLGQARCATFRSRIFGDGNVDAAFAALRQQACPQS--G
LINLFRANGLSPRDMVALSGAHTIGQARCVTFRSRIYNSTNIDLSFALSRRRSCPAATGS
*:  *  :*******.*******:* ::*..**.*::.. *:* :**  *::.** :  .

GDSSLAPLDAQTQNVFDNAYYRNLLAQRGLLHSDQELFNGGSQDALVQQYSSNPALFAAD
GDTTLAPIDVQTPDAFDNAYYANLVKKQGLFHSDQELFNGGSQDALVRKYAGNAGMFAAD
GDNNAAILDLRTPEKFDGSYFMQLVNHRGLLTSDQVLFNGGSTDSIVVSYSRSVQAFYRD
**.. * :* :* : **.:*: :*: ::**: *** ****** *::* .*: .   *  *

FAAAMIKMGNIKPLTGAAGQIRRSCRAVNSS
FAKAMVRMGALLPAAGTPTEVRLNCRKVN--
FVAAMIKMGDISPLTGSNGQIRRSCRRPN--
*. **::** : * :*:  ::* .**  *


 CLUSTAL W (1.83) Multiple Sequence Alignments

Sequence format is Pearson
Sequence 1: LOC_Os03g16960.1      265 aa
Sequence 2: LOC_Os05g02200.1      272 aa
Sequence 3: At5g48540.1           263 aa
Start of Pairwise alignments
Aligning...
Sequences (1:2) Aligned. Score:  37
Sequences (1:3) Aligned. Score:  38
Sequences (2:3) Aligned. Score:  32
Guide tree        file created:   [/tmp/3MLr1SjSp3/7e4E6208Ok.dnd]
Start of Multiple Alignment
There are 2 groups
Aligning...
Group 1: Sequences:   2      Score:3968
Group 2: Sequences:   3      Score:3126
Alignment Score 1841
GCG-Alignment file created      [/tmp/3MLr1SjSp3/P5W730m2jT]

-----MAIISSKACRCLLLVSFALLPLSMAMDPLGSYCSGNS-LAGSSKAVASINSVLTD
MAMASSCSPLVAVLVALLLVAATVPAPCAAVDPVNTYCARNL---SGAPAQASVAQVLSE
--------MKTLVVKCFLLL--ALVCSCRAADSIWQLCNTNSNISASSQVSKNIDSLLAT
            .  .:**:  ::   . * *.:   *  *    ..: .  .: ..*:

LVTKGSTGVGFATSTAGKGNN--VIYGLVQCRGDVSTSDCQACLASAANQILTSCNYQSD
LVPRASA-GYYATATAGRGGDGSAIWGLAQCRGDIPAPDCALCASAAARQLAGACRGRAD
LVSKTPS-KGFKTTTSSSYNNKEKVYGLAQCRGDISNTDCSTCIQDAAKKIREVCQNQSD
**.: .:   : *:*:.  .:   ::**.*****:. .**  *   **.::   *. ::*
```

```
SRI2WYDYCFMRFENENFFGQADTDNGVIMENVQAMDN--AKAFQKAVGKVMSKATAQVS
ARV~WYDYCFARYDDADFVGLPDTGYALILLNTQNATD--PEAFEKAQRKVMARVAADAG
SRI~LYDFCFLRYSQENFIGKLDTGAGLIYFNVANVTEIDPKKFDNELGALFDKIRSEAV
:*:  **:** *:.: :*.*  **. .:*  *.    :  .: *::    :: :  ::.

QAGSGGLGRVKDQYTPFINIYGFAQCTRDLSPLTCAQCLSTAVSRFDQYCGAQQGCRILY
DAGGGGLARETARFKDGVTIYGLGWCTRDITAADCGLCVAQAVAEMPNYCRFRRGCRVLY
LPKNKGLGKGKTKLTPFVTLNGLVQCTRDLSELDCAQCFATAVGSFMTTCHNKKGCRVLY
 . . **.: . : .  :.: *:  ****::   *. *.: **. :   *  ::***:**

SSCMVRYEIYPFYFPLAT---SSTATTDMTKYTKTIVHH
SSCMARYETYPFFFPLDGGQSADASASAAGDYDRVVLNP
SSCYVRYEFYPFYFPLDP---AKTGPS-VGRISSVHLSP
*** .*** ***:***     :.:..:       . :


 CLUSTAL W (1.83) Multiple Sequence Alignments

Sequence format is Pearson
Sequence 1: LOC_Os03g21820.1      259 aa
Sequence 2: LOC_Os05g39990.1      246 aa
Sequence 3: At2g40610.1           253 aa
Start of Pairwise alignments
Aligning...
Sequences (1:2) Aligned. Score:  75
Sequences (1:3) Aligned. Score:  64
Sequences (2:3) Aligned. Score:  71
Guide tree        file created:   [/tmp/3MLr1SjSp3/gg0HAWZdW5.dnd]
Start of Multiple Alignment
There are 2 groups
Aligning...
Group 1: Sequences:   2      Score:4764
Group 2: Sequences:   3      Score:4623
Alignment Score 3486
GCG-Alignment file created      [/tmp/3MLr1SjSp3/P5W730m2jT]

MAPP--LLLLLASLLLVAARRALGLGLGQWQPGHATFYGGGDASGTM1GGACGYGNLYSQ
------MAIAGVLFLLFLARQASAAGYGGWQSAHATFYGGGDASGTM1GGACGYGNLYSQ
MYTPSYLKYSIISIISVLFLQGTHGDDGGWQGGHATFYGGEDASGTM1GGACGYGNLYGQ
      :      :: .   :.   . * ** .******* ****** **********.*

GYGTSTAALSTALFNRGLSCGSCYELRCAGDHRRSCLPGGATVTVTATNFCPPNYALPSD
GYGTNTAALSTALFNDGAACGSCYELRCD-NAGSSCLPG--SITVTATNFCPPNYGLPSD
GYGTNTAALSTALFNNGLTCGACYEMKCN-DDPRWCLGS--TITVTATNFCPPNPGLSND
****.********** * :**:***::*  :    ** .  ::*********** .*..*

GGGWCNPPRRHFDLAEPAFLRIARHAAGIVPVSFR~RVACARKGGVRFTVNGHAYFNLVL
DGGWCNPPRPHFDMAEPAFLHIAQYRAGIVPVSFR2RVPCVKKGGVRFTVNGHSYFNLVL
NGGWCNPPLQHFDLAEPAFLQIAQYRAGIVPVSFR2RVPCMKKGGIRFTINGHSYFNLVL
.*******  ***:******:**:: ********* **.* :***:***:***:******

VTNVGGAGDVRSLAVKGSGSGSRVGGRWQPMSRNWGQNWQSNAYLDGKALSFRVTAGDGR
VTNVAGAGDVRSVSIKGSRTG------WQPMSRNWGQNWQSNAFLDGQSLSFQVTASDGR
ISNVGGAGDVHAVSIKGSKTQS-----WQAMSRNWGQNWQSNSYMNDQSLSFQVTTSDGR
::**.*****::::*** :       **.************::::.::***:**:.***
```

```
SLTCADVAPAGWQFGQTFEGRQF
TVTSNNVAHPGWQFGQTFEGGQF
TLVSNDVAPSNWQFGQTYQGGQF
::.. :** ..******::* **


 CLUSTAL W (1.83) Multiple Sequence Alignments

Sequence format is Pearson
Sequence 1: LOC_Os03g04060.1      256 aa
Sequence 2: LOC_Os06g51050.1      320 aa
Sequence 3: At3g12500.1           322 aa
Start of Pairwise alignments
Aligning...
Sequences (1:2) Aligned. Score:  57
Sequences (1:3) Aligned. Score:  55
Sequences (2:3) Aligned. Score:  62
Guide tree        file created:   [/tmp/3MLr1SjSp3/Y0PTzIy5Nm.dnd]
Start of Multiple Alignment
There are 2 groups
Aligning...
Group 1: Sequences:   2      Score:5796
Group 2: Sequences:   3      Score:4440
Alignment Score 3385
GCG-Alignment file created      [/tmp/3MLr1SjSp3/P5W730m2jT]

MRR--LLPLAGATLLIAAAGGASGQQAG--------------------------------
MRA--LALAVVAMAVVAVRGEQCGSQAGGALCPNCLCCSQYGWCGSTSDYCG-AGCQSQC
MKTNLFLFLIFSLLLSLSSAEQCGRQAGGALCPNGLCCSEFGWCGNTEPYCKQPGCQSQC
*:   :     :  :    .  .* ***

------------------VGSIITRAMFESMLSHRGDQGCQG-AFYTYDAFIKAAGDFPR
SGGCGGGPTPPSSGGGSGVASIISPSLFDQMLLHRNDQACAAKGFYTYDAFVAAANAYPD
TP--GG--TPPGPTG--DLSGIISSSQFDDMLKHRNDAACPARGFYTYNAFITAAKSFPG
                  :..**: : *:.** **.* .* . .****:**: **  :*

FGTTGNDETRRRELAAFFGQTSHETT1GGWATAPDGPFAWGYCRVNEIT-----------
FATTGDADTCKREVAAFLAQTSHETT~GGWPTAPDGPYSWGYCFKEENNGNAPTYCEPKP
FGTTGDTATRKKEVAAFFGQTSHETT1GGWATAPDGPYSWGYCFKQEQN-PASDYCEPSA
*.***:  * ::*:***:.******* ***.******::****  :* .

----PSDPPYYGRGPIQLT2HKYNYQLAGDALGLDLVNNPDLVSSDPVVAFRTAIWFWMT
EWPCAAGKKYYGRGPIQIT~YNYNYGPAGQAIGSDLLNNPDLVASDATVSFKTAFWFWMT
TWPCASGKRYYGRGPMQLS~WNYNYGLCGRAIGVDLLNNPDLVANDAVIAFKAAIWFWMT
    .:.  ******:*::  :***  .* *:* **:******:.*..::*::*:*****

AQSPKPSCHDVITNQWTPSGDDRSSGRLPGYGMATNIINGGEECGKGYSTDNAKDRVGYY
PQSPKPSCHAVITGQWTPSADDQAAGRVPGYGEITNIINGGVECGHG-ADDKVADRIGFY
AQPPKPSCHAVIAGQWQPSDADRAAGRLPGYGVITNIINGGLECGRG-QDGRVADRIGFY
.*.****** **:.** **  *:::**:****  ******* ***:*   ... **:*:*

KRYCDMFRVGYGDNIACRDQKPYGGG------
KRYCDMLGVSYGDNLDCYNQRPYPPS------
QRYCNIFGVNPGGNLDCYNQRSFVNGLLEAAI
:***::: *. *.*: * :*:.:  .
```

```
 CLUSTAL W (1.83) Multiple Sequence Alignments

Sequence format is Pearson
Sequence 1: LOC_Os03g55420.1      324 aa
Sequence 2: LOC_Os07g02440.1      330 aa
Sequence 3: At4g37520.1           329 aa
Start of Pairwise alignments
Aligning...
Sequences (1:2) Aligned. Score:  73
Sequences (1:3) Aligned. Score:  60
Sequences (2:3) Aligned. Score:  62
Guide tree        file created:   [/tmp/3MLr1SjSp3/nQGP0Eh4wP.dnd]
Start of Multiple Alignment
There are 2 groups
Aligning...
Group 1: Sequences:   2      Score:6132
Group 2: Sequences:   3      Score:5751
Alignment Score 4043
GCG-Alignment file created      [/tmp/3MLr1SjSp3/P5W730m2jT]

-MGAGIR------ILVVMLAVAAAGSGVVAQLRRDYYASVCPDVETIVRDAVTKKVQETS
MMVVVMRRRMAAAAMLVLVAMAGGATVCAAQLRRNYYAGVCPNVESIVRGAVARKVQETF
-MVVVNKTN----LLLLLLSLCLTLDLSSAQLRRNFYAGSCPNVEQIVRNAVQKKVQQTF
 * .  :       :::::::.       *****::**. **:** ***.** :***:*

VAVGATVRLFFHDCFVE0GCDASVIVVSSGNNTAEKDHPNNLSLAGDGFDTVIKARAAVD
ATVGATVRLFFHDCFVD~GCDASVVVASAGNNTAEKDHPNNLSLAGDGFDTVIKAKAAVD
TTIPATLRLYFHDCFVN0GCDASVMIASTNNNKAEKDHEENLSLAGDGFDTVIKAKEALD
.:: **:**:******: ******::.*:.**.***** :***************: *:*

AVPQCTNQVSCADILVMATRDVIAL0AGGPSYAVELGRLDGLSSTASSVDGKLPPPSFNL
AVPGCRDRVSCADILAMATRDAIAL0AGGPSYAVELGRLDGLRSTASSVNGRLPPPTFNL
AVPNCRNKVSCADILTMATRDVVNL0AGGPQYDVELGRLDGLSSTAASVGGKLPHPTDDV
*** * ::*******.*****.: * ****.* ********* ***:**.*:** *: ::

DQLTSLFAANNLSQTDMIALS1AAHTVGFAHCGTFASRIQ----PSAVDPTMDAGYASQL
DQLTALFAANGLSQADMIALS1AGHTVGFAHCNTFLGRIR----GSSVDPTMSPRYAAQL
NKLTSLFAKNGLSLNDMIALS1GAHTLGFAHCTKVFNRIYTFNKTTKVDPTVNKDYVTEL
::**:*** *.**  ****** ..**:***** .. .**      : ****:.  *.::*

QAACPAGVDPNIALELDPVTPRAFDNQYFVNLQKGMGLFTSDQVLYSDDRSRPTVDAWAA
QRSCPPNVDPRIAVTMDPVTPRAFDNQYFKNLQNGMGLLGSDQVLYSDPRSRPIVDSWAQ
KASCPRNIDPRVAINMDPTTPRQFDNVYYKNLQQGKGLFTSDQVLFTDRRSKPTVDLWAN
: :** .:**.:*: :**.*** *** *: ***:* **: *****::* **:* ** **

NSSDFELAFVAAMTNLGRVGVKTDPSQGNIRRDCAMLI
SSAAFNQAFVTAMTKLGRVGVKTG-SQGNIRRNCAVLN
NGQLFNQAFINSMIKLGRVGVKTG-SNGNIRRDCGAFN
..  *: **: :* :********. *:*****:*. :


 CLUSTAL W (1.83) Multiple Sequence Alignments

Sequence format is Pearson
Sequence 1: LOC_Os03g60080.1      316 aa
Sequence 2: LOC_Os07g12340.1      276 aa
Sequence 3: At5g08790.1           283 aa
```

```
Start of Pairwise alignments
Aligning...
Sequences (1:2) Aligned. Score:  63
Sequences (1:3) Aligned. Score:  45
Sequences (2:3) Aligned. Score:  43
Guide tree        file created:   [/tmp/3MLr1SjSp3/mrU8SdWzr0.dnd]
Start of Multiple Alignment
There are 2 groups
Aligning...
Group 1: Sequences:   2      Score:4970
Group 2: Sequences:   3      Score:4221
Alignment Score 2711
GCG-Alignment file created      [/tmp/3MLr1SjSp3/P5W730m2jT]

MGMGMRRERDAEAELNLPPGFRFHPTDDELVEHYLCRKAAGQRLPVPIIAEVDLYKFDPW
MAAAKRRVRDAEADLNLPPGFRFHPTDEELVAHYLCPRAAGRAAPVPIIAELDLYRHDPW
----------MKSELNLPAGFRFHPTDEELVKFYLCRKCASEQISAPVIAEIDLYKFNPW
           :::****.********:*** .*** :.*..  ..*:***:***:.:**

DLP~ERALFGAREWYFFTPRDRKYPNGSRPNRAAGNGYWKATGADKPVAPRGRTLGIKKA
DLP~HRALFGRREWYFFTPRDRKYPNGSRPNRAAASGYWKATGADKPVLHNGRTAGIKKA
ELP1EMSLYGEKEWYFFSPRDRKYPNGSRPNRAAGTGYWKATGADKPIG-KPKTLGIKKA
:** . :*:* :*****:****************..***********:  . :* *****

LVFYAGKAPRGVKTDWIMHEYRLADAGRAAAGAKKGSLR0LDDWVLCRLYNKKNEWEKMQ
LVFYHGKPPRGVKTEWIMHEYRLAKKGGAAAAAGAGALR~LDDWVLCRLYNKKNEWEKMQ
LVFYAGKAPKGIKTNWIMHEYRLANVDRSASVNKKNNLR0LDDWVLCRIYNKKGTMEKYF
**** **.*:*:**:*********. . :*:    . ** ********:****.  **

QGKEVKEEASDMVTSQSHSHTHSWGETRTPESEIVDNDPFPELDSFPAFQPAPPPATAMM
SRK---EEEEAMAAAQS------WGETRTPESEVVDSDAFPEMD----YSLPAASFDDAL
PAD---EKPRTTTMAEQSSSPFDTSDSTYPTLQEDDSSSSGGHG----HVVSPDVLEVQS
  .   *:    . ::.       .::  *  :  *...    .    .  ..

VPKKESMDDATAAAAAAATIPRNNSSLFVDLSYDDIQGMYSGLDMLPPGDDFYSSLFASP
LPKEEARDDD----------------WLMGMSLDDLQGLGS----LLQADDL--SMLAPP
EPKWGELEDALEAFDT----------SMFGSSMELLQPDAFVPQFLYQSDYF--TSFQDP
 **    :*                  :.. * : :*        *  .* :  : :  *

RVKGTTPRAGAGMGMVPF-
PAAKTEP-----LGAPFF-
PEQKPFLN----WSFAPQG
    .        .


 CLUSTAL W (1.83) Multiple Sequence Alignments

Sequence format is Pearson
Sequence 1: LOC_Os03g18140.1      558 aa
Sequence 2: LOC_Os07g49280.1      605 aa
Sequence 3: At3g55990.1           487 aa
Start of Pairwise alignments
Aligning...
Sequences (1:2) Aligned. Score:  54
Sequences (1:3) Aligned. Score:  55
Sequences (2:3) Aligned. Score:  48
Guide tree        file created:   [/tmp/3MLr1SjSp3/JKoVtWUlcR.dnd]
```

```
Start of Multiple Alignment
There are 2 groups
Aligning...
Group 1: Sequences:   2      Score:8369
Group 2: Sequences:   3      Score:8112
Alignment Score 4945
GCG-Alignment file created      [/tmp/3MLr1SjSp3/P5W730m2jT]

MQQRRKSVFASA--------PFAMKQAALGAGVAAR-RNGAPLSLAAVVFALFVF---AT
MKKKKNGMGAAADRGRLLALAHHDKLNPTKPSEAQRRFKPSILLLLGSSLPRLVPPLPSS
MQPWRR------------------KFPLFETGVTMKQRKNSNLSIFVVVFSVFLF---GI
*:  :.                  *     .. : :  : : * :    :. ::    .

FLYNEDIKSIADFPFGAGALRAKSPDLHVLQETVGAAHLAAGSIAKRGEEVIVRVLD---
FLPVVIKQTEFHQRWLVGDLNPPPPPCHLLPIQGQGQMQMQQRRKPPPAAAPVAAKQPSP
FMYNEDVKSIAEFPFST----SKPHDVH--------------------------------
*:     ::  .  : .    . .   *

--APASTAMAAAAGSSSNNSTIEVAKANANANANAADAGVKVDEGQERERDVTLPSVKEG
RRTPGPLSFAGALLSLLVVATFLYINDHGNMMPPHASPDPDLRLLQEAAHQKVNSILLSR
----------DEATPITEITTLPVQESIKNSDP-IQESIKNADSVQDSVKDVAEP-----
              .    :*:   :   *  .   ..  .    *:  :: . .

GADEARRREDEEAAEKES-------------SAKAAAATAALRTVVSVPDT---------
HAPAPPPRTNTNTSSSDQHLRLINIPMSSDLDLELGGNSTSSSGVEIQFEQQQQQEEKNL
------VQEEVSKTEEVK-------------KIELFAATEDEEDVELPPEE---------
       : : . :.. .             . :  . :     *    :

--CDLYRGNWVYDEVN--APVYKESQCEFLTEQVTCMRNGRRDDSYQKWRWQPTDCDLP2
RGCELYKGRWVYDAAGREAPLYRESECGFLTEQVTCMRNGRRDDSYQRWRWQPEGCDLP~
--CDLFTGEWVFDNET--HPLYKEDQCEFLTAQVTCMRNGRRDSLYQNWRWQPRDCSLP2
  *:*: *.**:*      *:*:*.:* *** ***********. **.***** .*.**

RFDARLLLERLRNKRLMFVGDSLNRNQWESMVCLVQSVIPKGKKTLTKFVNGGNSNIFYA
SFDARALLERLRNKRMMFVGDSLNRNQWESMVCLVQSAIPYGQKTLTKFVNNGSLNVFRA
KFKAKLLLEKLRNKRMMFVGDSLNRNQWESMVCLVQSVVPPGRKSLN---KTGSLSVFRV
 *.*: ***:*****:*********************.:* *:*:*.   : *. .:* .

H0EYNATVEFYWAPFLVESNSDNPQVHSVPDRVIQWHSIAKHAHNWLGVDYLIFNTYIWW
H0EYNATVEFYWAPFLVQSNSDDPQVHSVRDRVIAWRSIAKHAANWKGVHYLVFNTYIWW
E0DYNATVEFYWAPFLVESNSDDPNMHSILNRIIMPESIEKHGVNWKGVDFLVFNTYIWW
. :**************:****:*:.**: :*:*  .** **. ** **.:*:*******

LNTLDMKV2LKG---SFDQG---ATEYVEVDRPVAYKEVLKTWAKWVDRNIDPNRTTVFF
LNNFQIKV2LKSRGAPFAGSGGWSSRYALVDRAIAYREVLKTWAKWVDRRIDPNKTHVFF
MNTFAMKV2LRG---SFDKG---DTEYEEIERPVAYRRVMRTWGDWVERNIDPLRTTVFF
:*.: :** *:.   .*  .    :.*  ::*.:**:.*::**..**:*.*** :* ***

MSMSPNHI2TPEAWG-NYGGIKCAMETLPITNRTTS-LDVGTDWRLYAGAQEVLQTFRRV
MAMSPNHF2MPEAWGGSAGAVKCAMETQPIVNRTSGGLDIGTDWRLHGVARGVLRSMRRV
ASMSPLHI2KSLDWE-NPDGIKCALETTPILNMSMP-FSVGTDYRLFSVAENVTHSLN-V
 :*** *:  .  *  . ..:***:** ** * :   :.:***:**.. *. * :::. *

PVHLVDITALSELRKDAHTSVHTLRQGKLLTPEQQSDPKTYADCIHWCLPGLPDTWNQFL
GVRFVDITALSELRKDAHTSVHTLRQGKLLTPEQQADPRTYADCIHWCLPGLPDTWNHFL
PVYFLNITKLSEYRKDAHTSVHTIRQGKMLTPEQQADPNTYADCIHWCLPGLPDTWNEFL
 * :.:** *** **********:****:******:**.******************.**
```

```
YARIASAPWSSDQ
YAHIVAHAA----
YTRIISRS-----
*::* : .


 CLUSTAL W (1.83) Multiple Sequence Alignments

Sequence format is Pearson
Sequence 1: LOC_Os03g18690.1      450 aa
Sequence 2: LOC_Os07g49150.1      448 aa
Sequence 3: At4g29040.1           443 aa
Start of Pairwise alignments
Aligning...
Sequences (1:2) Aligned. Score:  98
Sequences (1:3) Aligned. Score:  91
Sequences (2:3) Aligned. Score:  92
Guide tree        file created:   [/tmp/3MLr1SjSp3/pNSWckggam.dnd]
Start of Multiple Alignment
There are 2 groups
Aligning...
Group 1: Sequences:   2      Score:9562
Group 2: Sequences:   3      Score:9237
Alignment Score 7656
GCG-Alignment file created      [/tmp/3MLr1SjSp3/P5W730m2jT]

MGQGTPGGMGKQGGLPGDRKPGDGGAGDKKDRKFEPPAAPSRVGRKQRKQKGPEAAARLP
MGQGTPGGMGKQGGAPGDRKPG--GDGDKKDRKFEPPAAPSRVGRKQRKQKGPEAAARLP
MGQGPSGGLNRQG----DRKPD---GGDKKEKKFEPAAPPARVGRKQRKQKGPEAAARLP
****..**:.:**    ****.    ****::****.*.*:*******************

AVAPLSKCRLRLLKLERVKDYLLMEEEFVVSQERLRPSEDKTEEDRSKVDDLRGTPMSVG
NVAPLSKCRLRLLKLERVKDYLLMEEEFVAAQERLRPTEDKTEEDRSKVDDLRGTPMSVG
TVTPSTKCKLRLLKLERIKDYLLMEEEFVANQERLKPQEEKAEEDRSKVDDLRGTPMSVG
 *:* :**:********:***********. ****:* *:*:******************

SLEEIIDESHAIVSSSVGPEYYVGILSFVDKDQLEPGCAILMHNK0VLSVVGILQDEVDP
SLEEIIDESHAIVSSSVGPEYYVGILSFVDKDQLEPGCSILMHNK0VLSVVGILQDEVDP
NLEELIDENHAIVSSSVGPEYYVGILSFVDKDQLEPGCSILMHNK0VLSVVGILQDEVDP
.***:***.*****************************:****** **************

MVSVMKVEKAPLESYADIGGLDAQIQEIKEAVELPLTHPELYEDIGIRPPKGVILYGEPG
MVSVMKVEKAPLESYADIGGLDAQIQEIKEAVELPLTHPELYEDIGIRPPKGVILYGEPG
MVSVMKVEKAPLESYADIGGLEAQIQEIKEAVELPLTHPELYEDIGIKPPKGVILYGEPG
*********************:*************************:************

TGKTLLAK0AVANSTSATFLRVVGSELIQKYLGDGPKLVRELFRVADDLSPSIVFIDEID
TGKTLLAK0AVANSTSATFLRVVGSELIQKYLGDGPKLVRELFRVADELSPSIVFIDEID
TGKTLLAK0AVANSTSATFLRVVGSELIQKYLGDGPKLVRELFRVADDLSPSIVFIDEID
******** **************************************:************

AVGTK2RYDAHSGGEREIQRTMLELLNQLDGFDSRGDVKVILATNRIESLDPALLRPGRI
AVGTK~RYDAHSGGEREIQRTMLELLNQLDGFDSRGDVKVILATNRIESLDPALLRPGRI
AVGTK2RYDAHSGGEREIQRTMLELLNQLDGFDSRGDVKVILATNRIESLDPALLRPGRI
***** ******************************************************
```

```
DRKIEFPLPDIKTRRRIFQ0IHTSKMTLADDVNLEEFVMTKDEFSGADIKAICTEAGLLA
DRKIEFPLPDIKTRRRIFQ0IHTSKMTLADDVNLEEFVMTKDEFSGADIKAICTEAGLLA
DRKIEFPLPDIKTRRRIFQ0IHTSKMTLSEDVNLEEFVMTKDEFSGADIKAICTEAGLLA
******************* ********::******************************

LRERRMK0VTHADFKKAKEKVMFKKKEGVPEGLYM
LRERRMK0VTHADFKKAKEKVMFKKKEGVPEGLYM
LRERRMK0VTHPDFKKAKEKVMFKKKEGVPEGLYM
******* ***.***********************


 CLUSTAL W (1.83) Multiple Sequence Alignments

Sequence format is Pearson
Sequence 1: LOC_Os03g19200.1      668 aa
Sequence 2: LOC_Os07g49000.1      603 aa
Sequence 3: At4g02100.1           546 aa
Start of Pairwise alignments
Aligning...
Sequences (1:2) Aligned. Score:  70
Sequences (1:3) Aligned. Score:  49
Sequences (2:3) Aligned. Score:  49
Guide tree        file created:   [/tmp/3MLr1SjSp3/R8JVidYg6w.dnd]
Start of Multiple Alignment
There are 2 groups
Aligning...
Group 1: Sequences:   2      Score:10978
Group 2: Sequences:   3      Score:8827
Alignment Score 5936
GCG-Alignment file created      [/tmp/3MLr1SjSp3/P5W730m2jT]

MAVSSPTSAPEKKRKWLLSNRK0-VIDKYLREARAILATAPEA-----GG--GDAVAALG
MAVSS----PDRRRNWLRGHRK0QIIGDYLAEARAALAAAAAAPLDGEGGEHSAATAALG
MAVYG------EKKHWWLRNKK0-IVDKYMKEAKSLIASQDPN----------DVKSALN
*** .      .:::*   ::*  ::..*: **:: :*:               . :**.

LVDAALELSPRMESALELRGRALLSLRRYRDVAEMLRDYIPSCAKTCSGDDTLSSSTSSS
LVEAVLEMSPRMEAALELRARSLLALRRYRAVADMLRDYIPSCTKPCSADDTSSSSSSSS
LLESALSVSPRYELALELKARSLLYLRRYKDVADMLQDYIPSLKLAGGGEDSGIGSSELS
*:::.*.:*** * ****:.*:** ****: **:**:*****   . ..:*:  .*:. *

LSSSGSGDLGTISRAKLLS--PDRHRSDAAAEPGAAVARSFRCFDISELKRRVLASLSKN
SCSSAS-------RTDLLS--PARDRSDAAS----AASRFLCCFDISDLKHRVLAGFSKN
FTHSSR------ESVKLLNDLPSHHHHHDSS---------FKCFSVSDLKKKVMAGLTKN
   *.         ..**.  * :.: . ::         : **.:*:**::*:*.::**

PNTDTQW2RYLVLGQACFHLGLIEDAMVLLQTGRRLASAAFRRESVCLSEDSFS-SSSPA
TSADTQW~RYLVLGQACFHLGLMEDAAALLQTGRRLASAAFRRESVCWSEDSFSPSNLTA
CDEQGQW2RYLVLGQACCHLGLMEDAMVLLQTGKRLATAAFRRQSICWSDDSFILFSSED
 . : ** ********* ****:*** .*****:***:*****:*:* *:***   .

AAVAPIPSGNTTKSGSAFIIPAMESEAVSQLLAHVKLLLRRRTAAMAALDAGLPAEAVRH
NAISAPASRRASKSG----AAGSEAESVSQLLAHVKLLLRRRAAAVAALDADLPAEAVRH
GGSSPPSSVVVTSAS------QPRSESIAHVLSHIKLLLRRRAAALAALDAGLYTESIRH
 . :. .*  .:.:.        .:*:::::*:*:*******:**:*****.* :*::**

FSKILEARRGVLPHPFAAACLVGRAAAFQAGGRPADAIADCNRALALDPAYIPALRARAD
```

```
FSKVLDARRGVLPHPFATACLVGRAEALRSSGRAADAIADCNRALALDPAFIPALRSRAD
FSKILDSRRG-APQGFLAQCFMHRASAYRSAGRIAESIADCNKTLALDPSCLQALETRAA
***:*::***  *: * : *:: ** * ::.** *::*****::*****: : **.:**

LLQSVGALADCLRDLDHLKLLYDAALRDGKLPGPRWRPQGGVRYREIAGAHRKLTARIQG
LLESVGALSDCLRDLEHLKLLYDAALRDGKLPGPTWRPQGGVRFSEIAGAHRALTPRIQQ
LLESVRCFPDSLHDLEHLKLLYNSILRDRKLPGPVWKRHN-VRYREIPGKLCVLTTKIQQ
**:** .:.*.*:**:******:: *** ***** *: :. **: **.*    **.:**

LRSRVAAGEACNIDYYALLGVRRGCTRSELERAHLLLTLKLKPDRAVVFGERLELVDEHR
LRGRVAGGEACSVDYYALLGVRRGCTRSELERAHLLLTLKLRPDRCASFAERLELVDEHR
LKQKIANGEIGNVDYYALMGIRRDCSRSELDRAYLLLNLKHKPERSMSFIDRFELTDDEE
*: ::* **  .:*****:*:**.*:****:**:***.** :*:*.  * :*:**.*:..

DLEAVRDQARMSALLLYRMLQKGYSFIMSAVIDEEAAERQRAREAAAAAAAAAAAAAAAA
DLEAVRDQARMSALSLYRMLQKGYSFIMSVVQDEEAAERQRAKDAAAAT---AAAAAAAA
ELDSVKDRARMSTLLLYRLIQKGYSVVTSNIA---AVEKQRK----------------AI
:*::*:*:****:* ***::*****.: * :    *.*:**                 *

AAALAVKQEATKQELAAPPMPEKPRQTESAPCAKRSMASKAKPKAKPAATVPSMMSRKAA
AAALAREQEET------AAVPEKAR--------------------ISSVSVPSTN-----
AAAVATESHRS-------------------------------------------------
***:* :.. :

TTTTTAKAAMSKAAVTAPKAAAATAVAATAVAAAATAASTSTAAPVYQGVFCRDMAVVGT
--------------------VQVQVTQAAAMPTAAMAAAAAMGSPVFQGVFCRDMAVVGT
------------------------NIIETPIRAVAVAVNSNNNTNVVKGVFCRDLTVVGS
                            :.: :.* *. :   : * :******::***:

LLSRGGFVDRAMPVKCEAMSC
LLSRGGF-DRPIPVKCEAMSC
LIARTGF-NQPIPVKYEALSC
*::* ** ::.:*** **:**


 CLUSTAL W (1.83) Multiple Sequence Alignments

Sequence format is Pearson
Sequence 1: LOC_Os08g39370.1      548 aa
Sequence 2: LOC_Os09g31130.1      543 aa
Sequence 3: At5g47560.1           540 aa
Start of Pairwise alignments
Aligning...
Sequences (1:2) Aligned. Score:  76
Sequences (1:3) Aligned. Score:  60
Sequences (2:3) Aligned. Score:  63
Guide tree        file created:   [/tmp/3MLr1SjSp3/MwHzm6VzGx.dnd]
Start of Multiple Alignment
There are 2 groups
Aligning...
Group 1: Sequences:   2      Score:10402
Group 2: Sequences:   3      Score:9516
Alignment Score 6826
GCG-Alignment file created      [/tmp/3MLr1SjSp3/P5W730m2jT]

MDKNGSSYGESSSDDVEAPLLLPAARGGTMAKGDRRRPASSAAAAWVRALLAHKYPAIAA
MDPR-RSHWESSSEDVTRPLLPLHDDDG--AAGRR-------SCAALRSLLANKYLAVAS
```

```
--MNGGDVTVAGSDDLKSPLLPVVHNDEPFERQTVG--------QQLRTIFTPKNCYIAL
   .  .   :.*:*:  ***     .                   :*:::: *   :*

GPAACAAVCAAVDLGD-GHGEARNMLGVLAWVFLWWVTGAVPLAVASMAPLFLFPALGIS
GPVACALICGLVDLG--GHRAARNMLGVLAWVFLWWITDAVPLAVASMAPLFLFPVFGIS
GPLLCAVVCLCVDLGGDETTTARNMLGVLVWMFAWWLTEAVPMPITSMTPLFLFPLFGIS
**  ** :*  ****      ********.*:* **:* ***:.::**:****** :***

SSDDVARAYMGDVISLVLGSFILALAVDHHRIHRRLALN~VLSLFCGDPVRPSLLLLGVT
SSDAVAKAYMDDVISLVLGSFILALAIEHYNIHRRLALN0ITSLFCGDPVKPPLLLLGIC
AADDVANSYMDDVISLVLGSFILALAVEHYNIHRRLALN0ITLVFCVEPLNAPLLLLGIC
::* **.:**.***************::*:.******** :  :** :*:...*****:

GTTALVSMWIHNTACTVMMMPVATGILQRFPRGD------IDDGGGQEVRRFSKAVVLGV
GTTMFISMWIHNTPCTVMMMPVATGILQRFPRVDGASSSASSAADAREVQRFSKAVVLGV
ATTAFVSMWMHNVAAAVMMMPVATGILQRLPSSS-----STTEVVHPAVGKFSRAVVLGV
.** ::***:**...:*************:*  .              * :**:******

VYASAIGGMATLTGTGVNIILVGMWSSYFPEQRPITFSSWMSFGLPMAIILFLALWLTLC
VYASAIGGMATLTGTGVNIILVGMWSTYFPEQPPITFSSWMSFGLPLALVLFVALWATLC
IYSAAVGGMSTLTGTGVNLILVGMWKSYFPEADPISFSQWFFFGFPLALCIFVVLWCVLC
:*::*:***:********:******.:****  **:**.*: **:*:*: :*:.** .**

LMYCSNNTAKALSAYLDRSHLRRELSLL1GPMAFAEKMVLAVFG0GLVVLWMSRNITDNI
VLYCSKNTGRALSAYLDRSHLRRELSLL1GPMAFAEKMVLAVFG0GLIVLWMTRSLTDDI
VMYCPKGAGQALSPYLHKSHLRRELDLL~GPMNFAEKMVLAVFG~GLVVLWMTRNITDDI
::**.:.:.:***.**.:*******.** *** *********** **:****:*.:**:*

PGWGVLFHNKVGDGTVT0IMMATLLFIIPSGKREGEKLMDWNKCKKIQWNIILLLGAGFA
PGWGSLFHGEVGDGTVT0IMMATLLFIIPSGKNDGEKLMDWGKCRRLQWNIILLLGAGFA
PGWGRIFAGRAGDGTVS~VMMATLLFIIPSNIKKGEKLMDWNKCKKLPWNIVLLLGAGFA
**** :* ...*****: :***********. ..*******.**::: ***:********

IADGFKTSGLTDILSNGLRFLKGAPTLVIVPVACVFSGIMTEFTSDDSTTTLVLPLFAEL
IADGFRASGLTDILSEGLGFLRGAPALAIAPVACVFSGAITEFTSDDATATLVLPLLAEL
IADGVRTSGLAEVLSKGLVFLETAPYWAIAPTVCLIAATITEFTSNNATTTLLVPLLIEI
****.::***:::**:** **. **  .*.*..*:::. :*****:::*:**::**: *:

AKSIEVHPALLMVSGAIGAQLSYLLPTGSPSNVVGFSTGYITIKDLVATGLPLKIVAIAA
GKSIGVHPLLLMVPGAVGAQLSYLLPTGSPGNVVGFSTGYISIKDMVIAGTPLKIVGVAA
AKNMGIHPLLLMVPGAIGAQFAFLLPTGTPSNVVGFTTGHIEIKDMIKTGLPLKIAGTIF
.*.: :** ****.**:***:::*****:*.*****:**:* ***:: :* ****..

LTVLLPTL1GSTIFGMDIKS
LTILLPTL1GSVVFGMDQKL
LSILMPTL1GAYVFASMGGV
*::*:*** *: :*.
```

CLUSTAL W (1.83) Multiple Sequence Alignments

Sequence format is Pearson
Sequence 1: LOC_Os08g41730.1   219 aa
Sequence 2: LOC_Os09g32800.1   221 aa
Sequence 3: At3g60820.1   223 aa
Start of Pairwise alignments
Aligning...

```
Sequences (1:2) Aligned. Score:  86
Sequences (1:3) Aligned. Score:  73
Sequences (2:3) Aligned. Score:  75
Guide tree        file created:   [/tmp/3MLr1SjSp3/dw2JJkNhWY.dnd]
Start of Multiple Alignment
There are 2 groups
Aligning...
Group 1: Sequences:   2      Score:4481
Group 2: Sequences:   3      Score:4231
Alignment Score 3240
GCG-Alignment file created      [/tmp/3MLr1SjSp3/P5W730m2jT]

MAR----FEVYENNG~GTCVAVAGADYCVVAADTRLSVGYSILSRDSSKIAH2LADKCVL
MSRR--GDWVYENNG2GTCVAIAGADYCVVAADTRLSVGYNILTRDHSKICE2LADKCAL
MTKQHANWSPYDNNG2GTCVAIAGSDYCVIAADTRMSTGYSILSRDYSKIHK2LADRAVL
*::       *:*** *****:**:****:*****:*.**.**:** *** . ***:..*

ASSGFQGDVKALQRNLTAKEL0LYEHNHNKKMSCPAMAQLLSNTLYYKRFFPYYAFNVLG
ASSGFQGDIKALHKNLAAREL0LYQHQHNKRMSCPAMAQLLSNTLYYKRFFPYYAFNVLG
SSSGFQADVKALQKVLKSRHL0IYQHQHNKQMSCPAMAQLLSNTLYFKRFFPYYAFNVLG
:*****.*:***:: * ::.* :*:*:***:***************:*************

GLDSE1GKGCVFTYDAVGSYERTGYSAQGTGAALMMPVLDNQLKSPSPLLLPAR0DAVTP
GLDSE1GKGCVFTYDAVGSYERTGYSAQGTGSALIMPVLDNQLKSPSPLLLPAR0DAVTP
GLDEE1GKGCVFTYDAVGSYERVGYGAQGSGSTLIMPFLDNQLKSPSPLLLPKQ0DSNTP
***.* ****************.**.***:*::*:**.************** : *: **

LSQSDAVDLVKDVFASATERDIYT0GDKLEIVVINSAGSHREFIELRKD
LSETEAVDLVKDVFASATERDIYT0GDKLEIVVINKAGTKREYIDLRKD
LSEAEAVDLVKTVFASATERDIYT0GDKLEIMILKADGIKTELMDLRKD
**:::****** ************ ******::::  * : * ::****


 CLUSTAL W (1.83) Multiple Sequence Alignments

Sequence format is Pearson
Sequence 1: LOC_Os08g41880.1      623 aa
Sequence 2: LOC_Os09g32840.1      630 aa
Sequence 3: At5g50400.1           611 aa
Start of Pairwise alignments
Aligning...
Sequences (1:2) Aligned. Score:  69
Sequences (1:3) Aligned. Score:  64
Sequences (2:3) Aligned. Score:  64
Guide tree        file created:   [/tmp/3MLr1SjSp3/UZsZ6RdDIh.dnd]
Start of Multiple Alignment
There are 2 groups
Aligning...
Group 1: Sequences:   2      Score:11695
Group 2: Sequences:   3      Score:11250
Alignment Score 8366
GCG-Alignment file created      [/tmp/3MLr1SjSp3/P5W730m2jT]

MVSRKRGGGGGVAMAVAMLLAAASA-------------SRPSSSLEGFQPLSKIAVHKAT
MAMP----LGGILLLFLVLLAAAAAGGGGGVWAFSSSSSSSSYSRIGEQPLSLIGIHRAT
MARN-------FLLVLLWFIVQVSS---------------SHENGRGDQALSQIDIYAIN
*.         . : .  ::. .::               .  .  * *.** * ::  .
```

```
VDLHGSAFVSATPALLGDQ0GEDTEWVTVKYGWANPSADDWIAVFSPADF2ISGSCPNPS
VGIDAAASVQASPRLLGVK0GEDTAWVTVDFAAPHASDGDWIGVFSPSNF2NASTCPGPS
LAQHHSAFIHVSPLVLGSQ0GQDTEWVNVVISNPEPSSDDWVGVFSPAKF2DSSSCAPTD
:  . :* : .:* :** : *:** **.*  . ...* .**:.****:.*  :.:*. ..

RYPDEPLLCTAPIK0YQFANYSANYVYWGKGSIRFQLINQRYDFSFALFTGGLEN0PKLV
GSDSGPVICSAPIK0YQLANYSSDYGKTGKGTLKFQLINQRQDFSFALFTGGLSN0PKLI
DKEIAPFICSAPVK0YMYAKSSPDYMKTGNAVLKFMLINQRADFSFALFTGGLSN0PTLV
     *.:*:**:* *  *: *.:*   *:. ::* ***** ***********.* *.*:

AVSEAISFKNPKAPVYPRLAQGKSYDE~MTVTWTSGYDISEAYPFVEWGMVVAGAAAPTR
AVSNKIAFANPKAPVYPRLAQGKSWNE0MTVTWTSGYDIKEAYPFVEWGMKWS---PPTR
SVSNHVSFINPKAPVYPRLALGKKWDE0MTVTWTSGYNIGEAVPFVEWSRKGT---RSRR
:**: ::* *********** **.::* *********:* ** *****.   :    . *

TAAGTLTFNRGSMC1GEPARTVGWRDPGFIHTAFLRDLWPNK2EYYYKIGHELSDGSIVW
TAAGTVTFDRESLC1GEPARTVGWRDPGFIHTAFLTDLWPNK2EYYYKIGHMLPDGKIVW
SPAGTLTFTRNSMC1GAPARTVGWRDPGFIHTASLKDLWPNL2KYTYRMGHELMNGSIVW
:.***:** * *:* * **************** * *****  :* *::** * :*.***

GKQYTFRAPPFPGQNSLQRIIVFGDMGK0AERDGSNEFANYQPGSLNTTDRLVEDLDNYD
GKFYSFKAPPFPGQKSLQRVVIFGDMGK0AERDGSNEYSNYQPGSLNTTDTLIKDLDNID
SKNFTFKSSPYPGQDSLQRVIIFGDMGK0GERDGSNEYNDYQPGSLNTTDQLIKDLKNID
.* ::*::.*:***.****:::****** .*******: :********** *::**.* *

IVFHIGDLPYANGYISQWDQFTAQVAPITAKKPYMIA2SGNHERDWPNTGGFFDVKDSGG
IVFHIGDITYANGYISQWDQFTQQVEPITARVPYMIA2SGNHERDWPNSGSFFNGTDSGG
IVFHIGDITYANGYISQWDQFTAQVEPIASTVPYMVA2SGNHERDWPDSGSFYGGKDSGG
*******:.************* ** **::  ***:* *********::*.*:. .****

ECGVPAETMYYYPAENRANF2WYKVDYGMFRFCIADSEHDWREGTDQYKFIEQCLSTVDR
ECGVLAETMYYTPTENRANY2WYKTDYGMFRFCVADSEHDWREGTEQYAFIESCLATVDR
ECGVPAETMFDFPAENKAKF~WYSADYGMFRFCVADTEHDWREGSEQYQFIERCLASVDR
**** ****:  *:**:*:: **..********:**:*******::** *** **::***

KHQPWLIFAAHRVLGYSSNWWYADQGSFEEPEGRESLQRLWQRHRVDVAFFGHVHNYERT
KKQPWLVFIAHRVLGYSSGFFYGAGGAFAEPTARQSLQRLWQRHRVDLAFYGHVHNYERT
RAQPWLIFIAHRVLGYSTNDWYGQEGSFEEPMGRESLQKLWQKYKVDIAFYGHVHNYERT
: ****:* ********:. :*.  *:* ** .*:***:***:::**:**:*********

CPMYQSQCVSGERRRYSGTMNGTIFVVAGGGGSHLSDYTSAIPKWSVFRDRDFGFVKLTA
CPVYDGRCASPERSRYSGAVGGTIHAVVGGGGSHLSNFTAEAPPWSVYREMDYGFVKLTA
CPIYQNQCMDNEKSHYSGAFKGTIHVVVGGAGSHLSSFSSLKPKWSIFRDYDYGFVKLTA
**:*:.:* . *: :***:. ***..*.**.*****.:::  * **::*: *:*******

FNHSSLLFEYKKSSDGKVYDSFTVERDYRDVLSCVHDSCLPTTLAS-
FNYTSLLYEYRRSSDGEVHDSFTVHREYRDVLACVADSCPPTIPPAT
FDHSSLLFEYKKSSNGAVHDSFTIFREYRDVLACVRDSCEPTTLAS-
*:::***:**::**:* *:****: *:*****:** *** **  .:


 CLUSTAL W (1.83) Multiple Sequence Alignments

Sequence format is Pearson
Sequence 1: LOC_Os08g44590.1      383 aa
Sequence 2: LOC_Os09g39720.1      378 aa
```

```
Sequence 3: At2g36690.1           366 aa
Start of Pairwise alignments
Aligning...
Sequences (1:2) Aligned. Score:  57
Sequences (1:3) Aligned. Score:  51
Sequences (2:3) Aligned. Score:  45
Guide tree        file created:   [/tmp/3MLr1SjSp3/kWYGFDm1S3.dnd]
Start of Multiple Alignment
There are 2 groups
Aligning...
Group 1: Sequences:   2      Score:6210
Group 2: Sequences:   3      Score:5796
Alignment Score 3625
GCG-Alignment file created      [/tmp/3MLr1SjSp3/P5W730m2jT]

MAIVDLVNAGEQQQMGSKRAAAEDGDGGVDDSREYYCRRGVRHLCDSGITRLPGNYVLPA
MAILERA---EEANIG-------EGSG----SSE--WELGVRQLCDSGITTLPARYVLPP
MSPSMIAPVMEEENKK--------------------YQKGVKHLCENGLTKVPTKYIWPE
*:    .   *: :                       . **::**:.*:* :* .*: *

SDRPGQAAGAAAAAGG--SVKLPVVDLSRLRVPSERGAVLRTLDAACREYGFFQ~VVNHG
ADRP------ARYVTP--PALLPVVDLAALRARDP--CQLAALHAACRDYGFFQ~LLNHG
PDRP-ILTKSDKLIKPNKNLKLPLIDFAELLGPNRP-HVLRTIAEACKTYGFFQ0VVNHG
.***                 **::*:: *   .     * ::  **: ***** ::***

VGGEVVGGMLDVARR-FFELPQPERERYMSADVRAPVRYGTSFNQVRDAVLCWRDFLKLA
VP---PDAMLYAARRFFFDLPLPARKRYMSADIRAAVRYGTSFNQLNDAVLSWRDFLKLL
MEGDVSKNMIDVCKR-FFELPYEERSKYMSSDMSAPVRYGTSFNQIKDNVFCWRDFLKLY
:       *: ..:* **:**   *.:***:*: *.*********:.* *:.*******

CMP---LAAVVESWPTSPADL~REVASRYAEANQRVFMEVMEAALEALGVG--GGGVMED
IRDTRRLADVLPSWPDAPDDL~RPAAAAYATACQRLFRELMEAALDALGIVRCRRQLLEE
AHP---LPDYLPHWPSSPSDF2RSSAATYAKETKEMFEMMVKAILESLEIDG-SDEAAKE
      *.  :  ** :* *: *  *: **   :.:*  :::* *::* :        ::

LAAGTQMMTVNCYPECPQPELTLGMPPHSDYGFLTLVLQDEVAGLQVMH--AGEWLTVDP
CDAGSQMMMVNCFPACPEPELTLGMPPHSDYGLLTILLQDEVRGLEVSYGDGGGWAVVEP
LEEGSQVVVVNCYPPCPEPELTLGMPPHSDYGFLTLLLQDEVEGLQILY--RDEWVTVDP
   *:*:: ***:* **:**************:**::***** **:: :   . * .*:*

LPGSFVVNVGDHLE0ILSNGRYRSVLHRVKVNSRRLRVSVASFHSVAPERVVSPAPELID
LPGAVVVNVGDHLE~ILSNGLYRSVLHRVRVNGRRARVSVASLHSLAAERVIGPAAELVD
IPGSFVVNVGDHLE0IFSNGRYKSVLHRVLVNSTKPRISVASLHSFPLTSVVKPSPKLVD
:**:.********* *:*** *:****** **. : *:****:**..   *: *:.:*:*

DR--HPRRYMDTDLATFLAYLASAAGNHKSFLHSRRLY------------
EQRGRPRRYMDTDMAAFLAYLASAEGNHKSFLHSRRINTISSSGLTQPSN
KH--NPSQYMDTDFTTFLQYITSREPKWKNFLESRKIHHV----------
.:  .* :*****:::** *::*   : *.**.**::


 CLUSTAL W (1.83) Multiple Sequence Alignments

Sequence format is Pearson
Sequence 1: LOC_Os08g34910.1      664 aa
Sequence 2: LOC_Os09g26360.1      617 aa
Sequence 3: At2g26450.1           614 aa
```

```
Start of Pairwise alignments
Aligning...
Sequences (1:2) Aligned. Score:  64
Sequences (1:3) Aligned. Score:  42
Sequences (2:3) Aligned. Score:  42
Guide tree        file created:   [/tmp/3MLr1SjSp3/TC2FU2P9Ws.dnd]
Start of Multiple Alignment
There are 2 groups
Aligning...
Group 1: Sequences:   2      Score:11029
Group 2: Sequences:   3      Score:9035
Alignment Score 5606
GCG-Alignment file created      [/tmp/3MLr1SjSp3/P5W730m2jT]

MSTTFSDFDPITERRHVERQRQERRRVMVAAGAASVILIIIVMGGAAVAYN---ASFGDG
MSSAFGDFGPLTERRRAEKARQQRRRIMIALGTVSIIIILIVMGAAAITYSGKKSEEDEG
--MAFQDFDKIQERVNANRKRKFRKRIIVGTVSLLVVVAAIVGGAFAYVAYEKRNEQQQQ
   :* **. : ** ..:: *: *:*:::.  :  :::  ** *. * .      .  :

GSSSSSGSAS------------GGGAQPSLHGVS--------------KIIKAMCAQTDY
GSKGSSKGKSKGGGGGDDEDGGGGGGKADLRAVS--------------KSIKMMCAQTDF
QQQAKNHNKSGSGNNVVKDSDKKSPSPPTPSQKAPVSAAQSVKPGQGDKIIQTLCSSTLY
 ..... . *             . . .     :              * *: :*:.* :

KDTCEKSLAKAAANASASSSSSSPKDVVRASVAVIGDAIEKAFDKSSVIVSDDPRVKAAV
ADSCATSIGKAAN-----ASVSSPKDIIRTAVDVIGGAVDQAFDRADLIMSNDPRVKAAV
MQICEKTLKNRTDKG---FALDNPTTFLKSAIEAVNEDLDLVLEKVLSLKTENQDDKDAI
 : * .:: : :       : ..*. .::::: .:.  :: .:::   : :::   * *:

ADCKEIYENAKDDLDRTLAGIDAGGVDGLTKGGYQLRVWLSAVIAHQETCIDGFPDGDLK
ADCKELFDDAKDDLNCTLKGID--GKDGLKQG-FQLRVWLSAVIANMETCIDGFPDGEFR
EQCKLLVEDAKEETVASLNKINVTEVNSFEKVVPDLESWLSAVMSYQETCLDGFEEGNLK
 :** : ::**::   :*  *:    :.: :   :*. *****::  ***:*** :*:::

DKMRDAMESGKELTSNALALIGKASSFLAALHLPASSAASHRRLLSFAFDEDVTKQPEVN
DKVKESFNNGREFTSNALALIEKASSFLSALK------GSQRRLL---------------
SEVKTSVNSSQVLTSNSLALIKTFTENLSPVMK-----VVERHLLD--------------
.::: :.:..: :***:**** . :. *:.:         .*:**

RSSGNSLRRLLSFAFDEDATKQPEVNRSSGNSLRRLLSFAFDENAPKQPKGNDDDVLVWV
-------------AGEEDN---------GGGAADPHLALAEDG------------IPEWV
---------------------------------------------------------DIPSWV
                                                          :  **

NRQERRLLKAK-FQNKLKPNVVVAKDGSGKFKTINDALAAMPKKYTG2RYVIYVKEGVYE
PDGDRRVLKGGGFKNNLTPNVIVAKDGSGKFKTINEALAAMPKTYSG-RYVIYVKEGVYA
SNDDRRMLRAVDVK-ALKPNATVAKDGSGDFTTINDALRAMPEKYEG2RYIIYVKQGIYD
   :**:*:.  .:  *.**. *******.*.***:** ***:.* * **:****:*:*

EYVTITKKMANVTMYGDGAKKTIITGNRNFVDGLTTYKTATF1NAQGDGFMGVALGFRNT
EYVTITKKMASVTMYGDGSRKSIVTGSKNFADGLTTFKTATF1AAQGDGFMAIGMGFQNT
EYVTVDKKKANLTMVGDGSQKTIVTGNKSHAKKIRTFLTATF1VAQGEGFMAQSMGFRNT
****: ** *.:** ***::*:*:**.:.... : *: ****  ***:***. .:**:**

ARAAKHQAVALLVQSDKSIFLNCRMEGHQDTLYAHSKAQFYRNCVISGTVDFIFGDAAAV
AGAAKHQAVALLVQSDKSVFLNCWMDGFQDTLYAHSKAQFYRNCVITGTIDFVFGDAAAV
AGPEGHQAVAIRVQSDRSIFLNCRFEGYQDTLYAYTHRQYYRSCVIVGTIDFIFGDAAAI
```

```
*  .  *****: ****:*:**** ::*.******::: *:**.*** **:**:******:

FQNCVIVLRRPLDNQQNIATAQGRADRREATGFVLQHYRFAAESALGDASRPAVRSYLAR
FQNCVLTLRRPMDNQQNIATAQGRADGREATGFVLQKCEFNAEPALTDAKLPPIRNYLGR
FQNCNIFIRKGLPGQKNTVTAQGRVDKFQTTGFVVHNCKIAANEDLKPVK-EEYKSYLGR
**** : :*: : .*:* .*****.*  ::****::: .: *:  *  ..    :.**.*

PWREYSRTLIMNSDIPAFVDKAGYLPWSG-DFGLKTLWYAEYGNKGAGAATAGRVSWPGY
PWREFSRTVIMESDIPAIIDKAGYMPWNG-EFALKTLYYAEYANKGPGADTAGRVAWPGY
PWKNYSRTIIMESKIENVIDPVGWLRWQETDFAIDTLYYAEYNNKGSSGDTTSRVKWPGF
**:::***:**:*.*  .:* .*:: *.  :*.:.**:**** ***... *:.** ***:

KKVISKKEATKFTVQNFLHAEPWIKPTGTPVKYGMWA-
KKVISKADATKFTVDNFLHAKPWIDPTGTPVKYDFFT-
K-VINKEEALNYTVGPFLQGD-WISASGSPVKLGLYDA
* **.* :* ::**  **:.. **..:*:*** .::


 CLUSTAL W (1.83) Multiple Sequence Alignments

Sequence format is Pearson
Sequence 1: LOC_Os02g51600.1      566 aa
Sequence 2: LOC_Os06g11920.1      533 aa
Sequence 3: At5g65090.1           529 aa
Start of Pairwise alignments
Aligning...
Sequences (1:2) Aligned. Score:  58
Sequences (1:3) Aligned. Score:  55
Sequences (2:3) Aligned. Score:  51
Guide tree        file created:   [/tmp/3MLr1SjSp3/mpEDgHxD23.dnd]
Start of Multiple Alignment
There are 2 groups
Aligning...
Group 1: Sequences:   2      Score:9051
Group 2: Sequences:   3      Score:8511
Alignment Score 5492
GCG-Alignment file created      [/tmp/3MLr1SjSp3/P5W730m2jT]

MSNHNSPCDIPKPASVDEFVKNGKKKK0SFMSSIFR~KKGRSGT~GSSDKKLLSRRDIVF
MS-------------------------~---SNMFG~KKGWDSN1G--------------
MNN-----------------RGNNDDL~DHHYGVFN1DFERRMT~SRK------------
*.                              .:*  .     . .

1GLDEKCDDRSELLDSSPAVRKSFS1DR---HCATKIESLTLSCLDSPHR-QFDTREY2R
~------------MDTSGSVCRSSS1DINYINQRARLKSASLNCVGSPPRKNNNATQY2R
~--------KSVLDNTSPMIWKTVS1ER---KSSPGIEGLNLSSFDRPMAPTTEIREL2R
              ::*  : :: * :    :  . ::. .*.... *     :  :  *

VFVGTWNVAGKPPNSSLNLEDFLQIEGLPDIYVL2GFQEIVPLNAGNVLVIEDNEPAAKW
MFVATWNVGGRTPNKRLNLQDFLQVEESPDIYVL2GFQEIVPLTAGNVLVLEDNEPAARW
VFLATWNVGGRTPNNDLNLEDFLLVEGTADLYIC~GFQEIVPLSAGNVLVVEDNEPAAKW
:*:.****.*:.**. ***:*** :*  .*:*:  ********.******:*******:*

LGLIYQALNKPQDQSSGDE---LSPP-----ETSDSRQGGGSGSRDSIPKSSSGGMLFFQ
LALIHQALNMPQEPADGDEPSPLTPPPSSSTTTSESSNGARTRRRDAVSRSASG-NLFFH
LALISQALNKPKQESVYSN---------AAYSASRTTTCSSSSCGSEESRAPSS-LSFFQ
*.** **** *:: :  .:             :* :   . :   .  .::.*.   **:
```

```
KPSLKMLSKNYRVDSALVKTCTCLTDPSTMQRRAREMREFLYRIEASPPPSLASAAAAAD
TPSLKMLSNSYRVDSALVKTCNCSPEHSSVRRRAAEVRESVY-LADAPAPAGETAAPAAD
RPNLKVLSRNYRVDSSLLKTCNCPVIDTSVGWEARRSKRFSDPSTDSSNNVEPENFRVHE
 *.**:**..*****:*:***.*    :::  .* . :.       :.         . :

EDGGPDAGGELAR-----SSVNYCLIASKQMVGIFLSVWVRRELVQYIGHLRVDSVGRGI
EDDAPTTEAQCEAGCGGGGGMSYCLIASKQMVGLFLSVWVRKELVEHVGHLRVDCVGRGI
NFLFDDVPATTKMP----GQMSYRLIASKQMVGLFLSVWARRELIPHISHLRLDSVGRGI
:     . .         . :.* *********:*****.*:**: ::.***:*.*****

MGRLGNK0GCIAMSMTLHQTSVCFVCSHLASGEKEGDEVRRNSDVAEIIKSTQFPRICK-
MGWLGNK~GCIAISMTLHHTSLCFVCSHLASGEKEGDELRRNADVAEILKSAHFPRACRP
MGRLGNK0GCIAISMSLHQTSFCFVCSHLASGEKEGDELRRNADVAEILKHTQFPKLTK-
** **** ****:**:**:**.****************:***:*****:* ::**:  :

--VPGQRIPDKILDH2DRVIWLGDLNYRVALSYDETKTLMGENDWDTLLEKDQ0LMIERQ
APAAARRVPERILDH2DRMIWLGDLNYRMSLSYDETRTLLEDNDWDALLEKDQ0LLIERE
--NPNCHAPERIIDH2DRVLWLGDLNYRVALTYEETRVLLEDNDWDTLLERDQ0LNMERG
   .  : *::*:** **::********::*:*:**:.*: :****:***:** * :**

AGRVFKGWKEGKIYFAPTYKYKQNSDSYAGETAKSKKKRRTPA2WCDRILWHGQGIEQLQ
AGRVFRGWNEGKICFAPTYKYTHNSDAYAGETAKSKKKRRTPA2WCDRILWQGDGIEQLQ
AGRVFSGFQEGQIFFAPTYKYSQNSDAYAGEMTKSKKKRRTPA2WCDRILWKGEGIEQLS
***** *::**:* *******.:***:**** :********** *******:*:*****.

YIRGESRFSDHRPVCSVFVIEADVDNGS------MIRKGYSTLDSRIHFESPIPQRHSFY
YLRGESRFSDHRPVCGVFAVEVDGGDGDGGGAAGKIMKGYYSLNARIGGDRSQCHQGDVS
YIRGESRFSDHRPVCAIFAVEVDVKSLN----KGRFRKGYSCAAVRLVEDVAIPQRHSFY
*:*************.:*.:*.*  . .       : ***     *:  :  .  :: ..

DDF
---
D--


 CLUSTAL W (1.83) Multiple Sequence Alignments

Sequence format is Pearson
Sequence 1: LOC_Os01g48540.1      624 aa
Sequence 2: LOC_Os05g48520.1      622 aa
Sequence 3: At5g19580.1           594 aa
Start of Pairwise alignments
Aligning...
Sequences (1:2) Aligned. Score:  68
Sequences (1:3) Aligned. Score:  42
Sequences (2:3) Aligned. Score:  40
Guide tree        file created:   [/tmp/3MLr1SjSp3/ferO9Qb8Z4.dnd]
Start of Multiple Alignment
There are 2 groups
Aligning...
Group 1: Sequences:   2      Score:11431
Group 2: Sequences:   3      Score:9061
Alignment Score 5824
GCG-Alignment file created      [/tmp/3MLr1SjSp3/P5W730m2jT]

MWP-LLLRAAVVYAALLAAGEADG--SHDVLDIFGTRSESDYYRNAFQGKQGQAVPLPRG
```

```
MGSSSLPRAAVLAVALLLLLADSGEAFFDLFSIFRPRSDSDYFP--FDGSPGQAKRKPK-
--MKASTRVIWTISVLMLAAVSEAIFPLPFLPFLPGFNNG------FRDNEAVKVAKPQ-
       *.     .*:     ..     .: ::   .:.      * .. .     *:

GGLRREQQELGAAGPGGSGLSKAPPRSAPSKVALDSLKLPVDTSAGFAGGWNLVSENSGV
----IEQEEDGAAPATATGLTKVPPLGAPSKAALDTIVLPVDDSAGHAGSWTIVSENSGV
-----------------------PAGAVGGKAARRQRGGGLDAQTTWGGKWELFLENSGV
                       *. .. .*.*       :* .:  .* * :. *****

SAMHLVVMQHG-KAIMFDTCTTGRSLMRLPPGRCRPDPRSKQPGAMDCWAHAVEFDYNTG
SAMHLAVMRHG-KAIMFDTSTTGRSLMRLPMNNCRADPRAKREGTMDCWAHAVEFDYSTG
SGMHAILMPVINKVQYYDATIWRISKIKLPPG-VPCHVVDAKTNKVDCWAHSILMDVNTG
*.**  :*    *.  :*:     * ::** .    .    : . :*****:: :* .**

ALRSLK0IVTDTWCSSGAFDADGNMVQTGGFFEGDKSVRYLSACGTCDWKEFPKSLADGR
ALRSLK~TATDTWCSSGAFDADGNLIQTGGYFEGDKAVRRLDACDTCDWREYPNSFAEGR
ALKPLG0LSTDTWCSSGGLTVNGTLVSTGGYGGGANTARYLSSCENCKWEEYPQALAAKR
**:.*    ********.: .:*.::.***:  * ::.* *.:* .*.*.*:*::*   *

2WYGTQLVLPDGSFIVIGGRRAFSYEFVPAAGRANARATPLRLLRDTTDDVENNLYPFVN
~WYATQQVLPDGRFIVFGGRRAFSYEFVPQPGMTNGQSIKFPLLRETTDDVENNLYPFVN
~WYSTQATLPDGKFFVIGGRDALNYEYIPEEGQNNRKLFDSLLLRQTDDPEENNLYPFVW
 **.** .**** *:*:*** *:.**::*  *  * :     ***:* *  ********

LLPDGTLFIFANDRSIVFNYRTGQVVRELPILPGGSRNYPASAMSTLLPLDLR---KGAG
LLPDGNLFVFANDRSVVFDHRTGKVVRELPKLAGGGRNHPASAMSAMLPLDLRNLTRGAD
LNTDGNLFIFANNRSILLSPKTNQVIKEFPQLPGGARNYPGSGSSALLPIQLYVK-NPKV
* .**.**:***:**:::. :*.:*::*:* *.**.**:*.*. *::**::*    .

LSAEVIICGGATKNAFKLGETSTFPPALRDCARINPSKPGARWALDQMPSGRVMGDVLIL
PEPEVIVCGGALKTAFRLGENNTYQPTLRDCARINLGKIDAVWAVEAMPVGRVMGDLLVL
IPAEVLVCGGSKQDAYYKAGKKIYEPALQDCARIRINSAKPRWKTEMMPTPRIMSDTVIL
  .**::***: : *:  . .. : *:*:*****. ..  . *  : **  *:*.* ::*

PTGDLLMLNGAAKGCSGWGFGRQALLSPVLYSPYLRRGKRFRVLNPSNIPRMYHSTSALL
PTGDLLMLNGAAKGSSGWGFARQPILSPILYSPRHPEGSRFRPLAASTVARMYHSTSAVL
PNGDILLVNGAKRGCSGWGYGKDPAFAPLLYKPHAARGKRFRQLKPTTIPRMYHSSAIIL
*.**:*::*** :*.****:.::. ::*:**.*   .*.*** * .:.:.*****:: :*

PDATVLVAGSNTNSAYNFSGVDFPTEVRVERFTPPYLSPQLSPNRPAIDAASVPGDGMRY
PDATVLVAGGNTNAAYNFSGVDFPTEVRVERFAPPYLSRELTGNRAVIDVASVPAGGMRY
PDGKVLVGGSNTNDGYKYN-VEFPTELRVEKFSPPYLDPALANIRPKIVTTGTP-KQVKY
**..***.*.*** .*:.. *:****:***:*:****.  *: *.  * .:..*   ::*

GARFTFRFTTPAQGVGQGDFKVTMYAPPFTTHGYSMNQRLLILPVTAFAAQGQRHTVTVD
GTKFTFRFHTPVAAVEWGDVRVTMYAPPFTTHGYSMNQRLLVLPVAGFSAQGQMYELTVD
GQFFNVKVDLKEKGATKGNLKVTMLAPAFTTHSISMNMRMLILGVNNVKPAGAGYDIQAV
*  *..:.     ..  *:.:*** **.****. *** *:*:* *  . . *  : : .

APPKPELAPPGYYMVYVVAKGVPSKAAWVKMHK
TPRKPELAPPGYYLVYVVSKDVPSEAAWVKIQ-
APPNGNIAPPGYYLIFAIYKGVPSTGEWIQVV-
:* : ::******:::.: *.*** . *:::
```

CLUSTAL W (1.83) Multiple Sequence Alignments

```
Sequence format is Pearson
Sequence 1: LOC_Os04g59540.1     1381 aa
Sequence 2: LOC_Os08g01390.1     1610 aa
Sequence 3: At3g14270.1          1791 aa
Start of Pairwise alignments
Aligning...
Sequences (1:2) Aligned. Score:  57
Sequences (1:3) Aligned. Score:  33
Sequences (2:3) Aligned. Score:  32
Guide tree        file created:   [/tmp/3MLr1SjSp3/rvKkMRRs7R.dnd]
Start of Multiple Alignment
There are 2 groups
Aligning...
Group 1: Sequences:   2      Score:23443
Group 2: Sequences:   3      Score:15923
Alignment Score 9820
GCG-Alignment file created      [/tmp/3MLr1SjSp3/P5W730m2jT]

------------------------------------------------------------
------------------------------------------------------------
MGTRDSNNRTFSEIVGLIKSWLPWRSEPATVSRDFWMPDQSCRVCYECDCQFTLINRRHH


------------------------------------------------------------
------------------------------------------------------------
CRHCGRVFCGKCTANSIPFAPSDLRTPREDWERIRVCNYCFRQWEQGDGGPHVSNITELS


------------------------------------------------------------
------------------------------------------------------------
TSPSETSLLSSKTSTTANSSSFALGSMPGLIGLNQRVHHGSDVSLHGVSSMETSVTKQGK


-----------------~-----~------------------------------------
-----MSCAQQDMDPPT2RDALS~EALDGNRSDHS-----------------VYDAEHSG
ETSRRSSFIATDVEDPS~RFALN2SIRSDDEYDEYGAYQTDIETSHSPRANEYYGPMEYN


---MNSVVEIMAISYPPEDGRYYCS-------------------SVAAGDDSPAAHHTN-
QNGIGDTKKRTSSSDLSTDDNFSSSALQSKHEHMNRDALSIDDRSVKSGDESDGAESTSG
GMGIDDVPCKHLGGETADQKSLSGSPLIHQCLESLIREGSEQFQKKSEHDGRDECEASSP
   :...      .  . :     *                   .    *    .. :.

-------AHDDWDW-----DWVPPPPAD--------AAAAAAADDDDDDGDPTPAGSPGS
KSGSIDSTCTENDS-----IWIPPEAADKEYEADSVSGKIAYADDDDDYSDGIKWGRSSF
ADISDDQVVEPVDFENNGLLWVPPEPENEEDERE--SALFDEEDNEGDASGEWGYLRPST
       .    *       *:** . :        :.     *::.* ..      ..

RGEDEEEERQR-------AQMVSAMNGQLNMLASRFLASAGV-------EEEWLEVVTAL
PATNEEQEVSHNTRDERESAMLDAMNGQLKILVSRFLASAGISFSKGESGESWLDILTSL
SFGSGEYRGEDRTTEEHKKAMKNVVDGHFRALLAQLLQVENISVSDEEGKESWLEIITSL
   . * . .          * ..::*::. * :::*   .:        *.**:::*:*

SWEAALLIQTHACTAGNDMDPASHVKIKCVASGRRRQ~SQVVRGLVFRKNAAHKHMPTKC
SWEAALLIKPDAS-KGKEMDPGSYIKVKCIASGTRRQ2SEVIKGLVFKKNAAHKHMPTSC
SWEAANLLKPDMS-KSGGMDPGGYVKVKCLASGFRHD2SMVVKGVVCKKNVVNRRMSTKI
***** *::.. .  .  ***..::*:**:*** *:: * *::*:* :**..:::*.*.
```

```
HRPTLLLLHGALGLDSHLG-FSSFDSMEQ~D~KLILRASISHIIHTCSPNVVMVEKTVSR
HNPRLLLLKGVLG-HSDVG-LSSFNSMDQ0E~KDHLERAISKMMEICSPNVILVEKTVSR
EKARLLILGGGLEYQRVSNQLSSFDTLLQ~Q0EKDHLKMAVAKIHAERPNILLVEKSVSR
... **:* * *  .   . :***::: * : :          :.   **:::***:***

DIQELLLHHGVTLLLDMKLHRLQRIARCSGAPLLSFSQLLHDCPNHLKHCDYFHIDKFFE
DIQELLLKEGVTLIFDMKLNRLERIARCTGSPIISFSEVLDKP--KLKRCDSFHIEKFIE
FAQEYLLAKDISLVLNIKRPLLDRIARCTGAQIIPSVDHLSSQ--KLGYCENFRVDRYPE
  ** ** ..::*::::*   *:*****:*: ::.  : * .   :*  *: *::::: *

DHNTTTTTSAAALNKPSKTLMFLEGFSNPLGCT0ILLRGASTQELKKIKQVLHYTIFAAY
EHN----SASDGGKRLSKTLMFLEGFPKPLGCT0ILLRGANSEELKKVKQVMHYTVFAAY
EHG----STGQVGKKVVKTLMYFEHCPKPLGFT0ILLRGANEDELKKVKHVVQYGVFAAY
:*.    ::.   ::  ****::*  .:*** * ******. :****:*:*::* :****

HLVVETSFFEDQRVFLNDTNVDGTPQITHQT---------SIVSNRSLPTD--------Y
HLILETSFFEDQRIFLNDQNASRENSVTAMAGPSANGYDPSVLCASDFPSRDDSPALRLY
HLALETSFLADEGASPELPLNSPITVALPDK---------STSIERSISTVP--------
** :****: *:    :    .                  *     .:.:

DVTCTSRGSLLEYHDGDHKATVPFTNKPDSYTQDEGTAIHCEAPP---SENLLSSVSGSL
HATSNGYTDVKKSLSSSTKVDAPSSITNSSSSVGEDASIRYDSKPPLHSQRLPSPVPGTL
GFTVSTYEKSPTMLSCAEPQRANSVPVSELLSTTTNLSIQKDIPPIPYGSGWQAREINPS
  * .   .     .      .      .  :   . :*: :  *   ..   :   ..

RRFIDIFRYQNIYLPVTSSQDTTGHQNEQDTETSQETASD------------TLTKDHSC
RKYVGMLSHQNIYLPVTSLQETSATQIEGEVESGKEIVSNGFHVGSKVEEPAVSTANVDC
FVFSRHNISLNLPDRVIESRNSDLSGRSVPVDTPADKSNP----------IVVADETTNN
  :       *:   * . :::     .  .::  :  .             .     .

EYMDQLND-LQEQVFAKTNQKMSQPDPFGTEKHQQNVEQYRAGENINSDTDEADDVMDSQ
SQDHQRQERIQDIMPTNSTHDKREESPVMVEDGEQHSTIDIISKEKTTNEDQADDALDTH
SLHLSGQGFVRKSSQIGTSIMVENQDNGSELTIAQQQNNEKPKETQSQKEEFPPSPSDHQ
.   . :  ::.     :.    : .        *:       :  . . : . .  * :

SILILLSSQCVTKQVVCEESHLYRINYYGNFDVSLGRYLQDILQ-----NQ0NLSCSSCG
SILILMSSQCITKQVICEQSHLSRIKYYGNFDVSLGRYLQDILQNPVLQNQ0KLSCSSCG
SILVSLSSRSVWKGTVCERSHLFRIKYYGSFDKPLGRFLRDHLF-----DQ0SYRCRSCE
***: :**:.: * .:**.*** **:***.** .***:*:* *      :* .  * **

EPPDAHMYSYTHRNGNLTINVRRLLPQHHLPGESEGKIWMWTRCLRCEHERGISKSSRRV
EFPESHLYSYTHRNGNLTVRVKHLAPQHHLPGESEGKIWMWTRCLRCENEHGMSKSTPRV
MPSEAHVHCYTHRQGSLTISVKKLQ-DYLLPGEKEGKIWMWHRCLRCPRLNGFPPATLRV
  .::*::.****:*.**: *::*  :: ****.******* ***** . .*:. :: **

LISTEARNLSFGKFLELSFSSHSAARRLSVCGHLVNRDCLRYF2GLGSKVAKFQYSSVEI
LISSEARSLSFGKFLELSFSSHSAARRLSICGHLVNRDCLRFF2GLGSKVAMFRYSSVEI
VMSDAAWGLSFGKFLELSFSNHAAASRVACCGHSLHRDCLRFY2GFGNMVACFRYATIDV
::*  * .************.*:** *:: *** ::*****:: *:*. ** *:*:::::

YTACKPQRTLEFHNPDMREWFEQEGRN0VLARGVKLFYEVSSLIQHMK--IFSEVAINCG
YTTCKPQTTLQFDNPCRQDWFKEERRY0VLARGIKLFSEVASMLQPLKDQLLDVTTTNCT
HSVYLPPSILSFN-YENQDWIQRETDE0VIERAELLFSEVLNAISQIAEKGFRRRIG---
::.  *   *.*.    ::*::.*    *: *.  ** ** . :. :    :

DSLPVKEVSQLEEMLIEEKAQFV0DSLVKAVDESGMSSSSVNEILGVNCLYQDLLIRLYV
```

```
GSLPVKDFSELEELLIKEKAFFE0DSLEKTINQNENLSASVHELVDINWSYQDLLLELYL
---------ELEEVLQKEKAEFE0ENMQKILHREVNEGQPLVDILELYRIHRQLLFQSYM
         :***:* :*** *  :.: * :...   . .: ::: :   :::**:. *:

WDRRFHQIVECKSGRMANCVGKKE------------------AAEFAGEP----------
WDRRLDQLTKCVSAGQERVVSCKDPFDTVVKNIRVNQEIENKADELTCDRTT--------
WDHRLINASTLHKLENSDDTKREENEKPPLAKSQTLPEMNAGTNSLLTGSEVNLNPDGDS
**:*: :     .      .  ::                  : .:

-------AATGESAVPFENGYIKEMQYSSET-------------LTDENSRREEQ-----
----SVLSAVGLTECPSNRNYIDHQSVDIEAPVLTENQGAGCAQFSSTGGRNDEDSYTAP
TGDTGSLNNVQKEADTNSDLYQEKDDGGEVSPSKTLPDTSYPLENKVDVRRTQSDGQIVM
         .     . .  * .. . .  :              .    * :.:

---------HITKVPSFRVLEG-TDTQLINPECGDNR-----------------ETWIWS
CQLEVDSMAQTKEVPSFEISEVQGDGIVVHPISLDQEPSNAPNHFRKIPDWDTGEGWIWN
KNLSATLDAAWIGERQTSVEIPTNNKVSLPPSTMSNSSTFPPISEGLMPIDLPEQQNEFK
               .  :     :   : *   .:                  :   :.

PLHELRESYRHELQAGYLERFELVNNYSPSHLSPLHKQ----SSAEFIVGPGGNVLCISE
SFHECQLAYRKDIQNEILDKFEIVNRYSPSHMSPLFEQHEEVSSPQFTVGPGGNILSVLE
VAYPVSPALPSKNYENSEDSVSWLSVPFLNFYRSINKNFLLSSQKLDTFGEHSPIYISSF
  :    :   .      : .. :.    ..  .: ::    *.    .*  . :

DEISSIISRALAISEERRHLLLDALMVEGEAAYSRGSESSKMEKSYSSLSEASSASSSWS
DEISSIIARALAVSGEHRHL------VENETEGARLEHAKTMEKSYSFMSGSSLDSSPWS
REAELQGGPRLLLPVGLNDIVVPVYDDEPTSMIAYALMSPEYQRQTSAEGESLVSYPSEL
 * .   .  * :.   ..:       *  :  :    :   ::. *  . :    ..

SIGSSDSDASFS----------------SDDLFSSYDSSLLSSLLHPEVSVNGKSSL-KG
SIGSLDSEASFLSLGSS----------VSSDDLSGYDSLPLFSSIHPEVAVNGKVAL-RG
NIPRPVDDTIFDPSRSNGSVDESILSISSSRSTSLLDPLSYTKALHARVSYGEDGTLGKV
.*    .:: *                 *.   *  *.    . :*..*: . . :* :

KYSVICVHANQFYTLRKKCCPSELAYITSLSRCMKWDAQGGKSKAFFAKTLDDRFIIKQI
KYSVTSIYAKQFQDLRKKCCPSELAYITSLSRCKKWDAQGGKSKAFFAKTVDDRFIIKQI
KYTVTCYYAKRFEALRGICLPSELEYIRSLSRCKKWGAQGGKSNVFFAKTLDDRFIIKQV
**:* . :*::*  **  * **** ** ***** **.******:.*****:********:

KKTEFESFIEFAPDYFKHVYHSLDTGSQTCLAKILGIYQ0VK--QIRHGKEVKLDLMVME
KKTEFESFIKFAPDYFKHVYHSLDTGSQTCLAKILGIYQ0VK--QTRHGKEIKIDLLVME
TKTELESFIKFAPAYFKYLSESISTKSPTCLAKILGIYQ0VATKQLKSGKETKMDVLIME
.***:****:*** ***:: .*:.* * *********** *   * : *** *:*:::**

NLLFGHKLSRIYDLKGVVFSRHVSDSNDHGTVYLDQNFVDDMRVSPIYVGGRTKHLLQRA
NLLFGHNISRIYDLKGAIFSRHVAHSNDRNTVYLDQNYVEDMRVSPIYIGGRTKHLLQRA
NLLFGRTVKRLYDLKGSSRARYNPDSSGSNKVLLDQNLIEAMPTSPIFVGNKAKRLLERA
*****:.:.*:*****   :*: ..*.. ..* **** :: * .***::*.::*:**:**

IWNDTAFLT0SINVMDYSLLVGVDKEKHEFVFGIIDYLRQYTWDKQLETWVKTS--LVVP
IWNDTSFLT0SVNVMDYSLLVGVDKEKHELVFGIIDYLRQYTWDKQLETWVKSS--LVVP
VWNDTAFLA0LGDVMDYSLLVGVDEEKNELVLGIIDFLRQYTWDKHLESWVKFTGILGGP
:****:**:   :***********:**:*:*:****:********:**:*** :  *  *

KNASPTVISPKEYKKRFRKFMAKYFLTVPDDWST--------------------------
KNVSPTVVSPKEYKKRFRKFMAKHFLTVPDTWSSENSSGPSKSFGHSNNMLVEVHSDDNL
KNEAPTVISPKQYKRRFRKAMTTYFLMVPDQWSPPNVVANNSKSDQPEETSQAGTQAE--
```

```
** :***:***:**:**** *:.:** *** **.

-----------
LQHPIEAETVV
-----------


 CLUSTAL W (1.83) Multiple Sequence Alignments

Sequence format is Pearson
Sequence 1: LOC_Os11g01820.1      801 aa
Sequence 2: LOC_Os05g39600.1      834 aa
Sequence 3: At2g13620.1           821 aa
Start of Pairwise alignments
Aligning...
Sequences (1:2) Aligned. Score:  34
Sequences (1:3) Aligned. Score:  35
Sequences (2:3) Aligned. Score:  38
Guide tree        file created:   [/tmp/3MLr1SjSp3/hixpyoEVK3.dnd]
Start of Multiple Alignment
There are 2 groups
Aligning...
Group 1: Sequences:   2      Score:11864
Group 2: Sequences:   3      Score:8884
Alignment Score 4887
GCG-Alignment file created      [/tmp/3MLr1SjSp3/P5W730m2jT]

MAGANASTVKPVVAACYDNNLVNSQG--MFLGDEPLRFALPLLLVQVSIILILSAAAHHV
----------MDKIDCYVVPQTTGTGRNIFQGGSPLSASLPLLGVQLVLIVAVTRVLYFL
-MATSEEPSTDASIICYAPSMITTNG--VWQGDNPLDFSLPLFVLQLTLVVVVTRFFVFI
               **     .  *  :: *..**  :***: :*: ::: ::    .:

LRRLGQCRFVTHML~VGIFLGPSVLGRNPHLRTALFSERGTYILESVSLVALILFLFSMA
LKPLKQPRVVSEIM0GGIILGPSVLSRHAAFREVVFPARGEPVLNTVATFGLMYVIFLIG
LKPFRQPRVISEIL0GGIVLGPSVLGRSTKFAHTIFPQRSVMVLETMANVGLLYFLFLVG
*: : * *.::.::  **.******.* . :  .:*. *.  :*:::: ..*: .:* :.

VKTDLTLLRRPTARALAVGLAGSLVPLAVTLPVFHALSPSLPADLRG-SSLITELAVRLS
VRMDPRLVVRSGRKGVVIGLSGFLLPLAMTGAGSSGEAMATEPDVSRRSTFLFALATSLS
VEMDIMVVRKTGKRALTIAIGGMVLPFLIGAAFS--FSMHRSEDHLGQGTYILFLGVALS
*. *  :: :.  :.:.:.:.* ::*: :  .     :     *    .: :  *.. **

LSSFPVVADALAELDLLNSELGRVALNASLITDVTSWFLRACFAAAFLITQAKSPLFTAK
VTSFAVLSPILSELSLLNSDLGRIAMSASMTTDGIAWIIMVVYILAEAF--LVSPATSIW
VTAFPVLARILAELKLINTEIGRISMSAALVNDMFAWILLALAIALAES--DKTSFASLW
:::*.*::  *:**.*:*:::**:::.*:: .*  :*:: .            :.  :

VLASFAAFVLFVFFVARPAGRYIARKRTPPGDLLSEGSFVLVVIAALLSALVTDVIGFKF
AFLSLAVLAAFILFVVRPVALRVIER-TPPGKPVEETYVFVFLLVVLLVGFYSDVIGTNS
VMISSAVFIAVCVFVVRPGIAWIIRK-TPEGENFSEFHICLILTGVMISGFITDAIGTHS
.: * *.:  . .**.**    : .: ** *. ..*  . :.:  .:: .: :*.** :

MIGPMMLGLALPGGMPIGATLTERLDSFFIALFLPVYMALAGYRTDLAELSLIGVSAEHE
FHGALMLGLAIPDGPPLGTALGEKIEAMVSGLILPLYYAMTGLSTDVWRMH---------
VFGAFVFGLVIPNGP-LGLTLIEKLEDFVSGLLLPLFFAISGLKTNIAAIQGP-------
. *.:::**.:*.*  :* :* *::: :. .*:**:: *::*  *::  :
```

```
EKFCALELFVALCVAGKMVGCVAAGLFFSMPFREATVLALMLNIRGIVEVAAINNWGDTM
--WGRLQVVMFLAWVGKLVGVMVSSLYLEIPLRDAVSLSLFMNSKGIVEVITFNFFLTNK
ATWLTLFLVIFLACAGKVIGTVIVAFFHGMPVREGITLGLLLNTKGLVEMIVLNVGKDQK
  :  * :.: *. .**::* :  .::  :*.*:.  *.*::* :*:**: .:*

~KATAEHYSTLTLSMVVITAVATPLIKLLYDPSGRFARAKRRTMEESRPNAELRVMACLF
0LIGKNTFSILICLSVAITAVSVPVAGWLYDPARRYAVYKRRTVQHLKADADLRILACVH
~VLDDETFATMVLVALVMTGVITPIVTILYKPVKKSVSYKRRTIQQTKPDSELRVLVCVH
     : :: :    :.:*.* .*:   **.*  : .  ****::. :.:::**::.*:.

SEDHAAPLLDLIEASGSSRDAPVSLIVLHLTELVGHAASVLKPHRKSRSSCG--------
DQSHVPGTLAVLEASNATPQTPLSLYLLQLVEIAGRSAPVFIPHKLRHSASRAGAAAASA
TPRNVPTIINLLEASHPTKRSPICIYVLHLVELTGRASAMLIVHNTRKSGR-----PALN
   :..  : ::*** .:  :*:.: :*:*.*:.*::::::  *.  :*.

-NPTPSDRIVNAFRYFEQQAPLGAVTVSPYVVASPYSSMQHDVCLLAHSRKANLILLPFH
APPTDSDRIINAFFQYELKHPEGAVSVHPFTTISPYSSMHDEVCRLAVEKRTSLILLHYH
RTQAQSDHIINAFENYEQHA--AFVAVQPLTAISPYSTMHEDVCSLAEDKRVSFIIIPFH
   : **:*:***  :* :   . *:* * .. ****:*:.:** ** .::..:*:: :*

K--SSDGARSTANNAIRGINRSVMQYAPCSVGILIDH------------------GVAAG
KRHMLAGGVR-AAVGLRVVNRKVLEVAPCSVAVFVDRNAGSVGLCAFIPGPVQDQSTSSR
KQQTVDGGMESTNPAYRLVNQNLLENSPCSVGILVDR------------------GLNGA
*     *.   :  . * :*:.::: :****.:::*:                  .  .

SACATASNS-----TLQRVALYFLGGADDREALAYVARMAECGLVAVTVVRLKLRDWVGM
SRSGGHAASGSGQGFKAAVASLFFGGGDDREALAYATRMARHEGVAVGVIRFLPARGIKD
TRLNSNTVS-------LQVAVLFFGGPDDREALAYAWRMAQHPGITLTVLRFIHDEDEAD
:     : *         **  *:** ********. ***.   ::: *:*:

GGRD----------------EMRDEEALQEFWQRYSCAGAERVAYVEKTVEDGEGTASVV
EPSD------------------RRIDNRAIEEVKALAARSRNIKVQEELVGDMERIVQVL
TASTRATNDSDLKIPKMDHRKQRQLDDDYINLFRAENAEYESIVYIEKLVSNGEETVAAV
                      *  :       :   *  . :   *: * : *  . .:

RAMSDK-FDLLVVGRREGGGDGAEGSSAAALTSGLSEWSEFPELGVLGDMLASADFAAKV
RGLDEAGYDLVLVGMRHR-------WYPVMPANGMSDWSECPELGVIGDLLASSDFDTPY
RSMDSS-HDLFIVGRGEG-------MSSPLTA-GLTDWSECPELGAIGDLLASSDFAATV
*.:..  .**.:**  .          .   : *:::*** ****.:**:***:** :

SILVVQQQAATRNDADY-------------------------------
SVLIMKQQD-QGGLNAAVPGAQDVWHVNDGGAEDAPAPPRNMSTAGS
SVLVVQQYVGSWAQEDDMDFPESPVHSHETKVTYGLENPR-------
*:*:::*


 CLUSTAL W (1.83) Multiple Sequence Alignments

Sequence format is Pearson
Sequence 1: LOC_Os03g25480.1      518 aa
Sequence 2: LOC_Os07g44140.1      517 aa
Sequence 3: At2g46950.1           572 aa
Start of Pairwise alignments
Aligning...
Sequences (1:2) Aligned. Score:  52
Sequences (1:3) Aligned. Score:  47
Sequences (2:3) Aligned. Score:  47
```

```
Guide tree        file created:   [/tmp/3MLr1SjSp3/8ZBgAdpD4W.dnd]
Start of Multiple Alignment
There are 2 groups
Aligning...
Group 1: Sequences:   2      Score:8619
Group 2: Sequences:   3      Score:8247
Alignment Score 4963
GCG-Alignment file created      [/tmp/3MLr1SjSp3/P5W730m2jT]

----------------------------------------------------------MA
----------------------------------------------------------MG
MLFCSVRILFIISSKQLVLISETEVTTTLTLHTTHKIESPTANPPQSKKNLFVIRMELLS
                                                          :.

TLLLLAVAAAAAAWVWWG-----RYAWRARAVARRLAAQGVRGPRRGGLLRGCNDEVRRR
NLGWMVAAAVAAVVASWAFDAVVKLVWRPRAITRRLRAQGVGGPGYR-FFSGNLGEIRR-
TINLLAIALVLLVVPKIYG-ACRILVWRPWMLSRRFKKQGISGPKYR-ILYGNLREIRK-
.:  :. * .  .            .**.  ::**:  **: **    :: *   *:*:

KAEAEADGVAMDVGDHDYLRRVVPHFVAWKELY1GTPFLYWFGPQPRICVSDYNLVKQIL
-LRDEGAGVVLDVSSHDFVPIVQPHFRKWIPLY1GKTFMYWFGARPTICLADVSMVRQVL
-MKNEAKLMVLDPNSNDIVPRVLPHLQQWKSQY1GETFLYWQGTDPRLCISDHELAKQIL
  . *.  :.:* ..:* :  * **:  *   * * .*:** *. * :*::* .:.:*:*

SKKYGHFVKNDAHPAILSMIGKGLVLVEGADWVRHRRVLTPAFTMDKLK0VMTKTMASCA
SDRTGMYPKNVSNPYFARLLGKGLVLTDGDEWKRHRKVVHPAFNMDKLK~MMTVTMSDCA
SNKFVFFSKSKTKPEILKLSGNGLIFVNGLDWVRHRRILNPAFSMDKLK0LMTQLMVDCT
*.:   : *. ::* :  : *:**::.:* :* ***::: ***.***** :**  * .*:

ECLIQGWLDHASNSKSIEIEVEFSKQFQDLTADVICRTAFGSNSEKGKEVFHAQKQLQAI
QSMISEWESELG-TKGDIVEIELSRRFEELTADVISHTAFGSSYKEGKQVFLAQRELQFL
FRMFLEWKKQRN-GVETEQFVLISREFKRLTADIIATAAFGSSYAEGIEVFKSQLELQKC
  ::  *  .. .        : :*:.*: ****:*. :****.  :* :** :* :**

AIATILNLQLPGF2KYLPTKRNRCKWKLENKLRNTLMQIIQSRITS-EGNGYGDDLLGVM
AFSTFLSIQIPGS~SYLPTKKNLKTWSVDKKVRSMLTDIIKSRLNNKDVAGYGNDLLGLM
CAAALTDLYFPGI2QYLPTPSNLQIWKLDMKVNSSIKRIIDARLTS-ESKDYGNDLLGIM
. ::: .: :**  .****  *   *.:: *:.. :  **.:*:.. :  .**:****:*

LNACFSTEQGEKRDELILCVDEIIDECKTFFFAGHETTSHLLTWTMFLLSVYPEWQDRLR
LEACAP-EHGE--SQPQLSMDEIIAECKTFFFAGHDTTSHLLTWTMFLLSTHPEWQEKLR
LTAASS---NE--SEKKMSIDEIIEECKTFFFAGHETTANLLTWSTMLLSLHQDWQEKLR
* *. .   .*  .:  :.:**** **********:**::****: :*** : :**::**

EEVLRECRK-ENPNADMLSKLKE0MTMVLLETLRLYPPVIFMFRKPITDMQLGRLHLPRG
EEVATECDG-KVPTGDMLNKLKL~VNMFLLETLRLYGPVAFIQRRVNAELELGGITVPEG
EEVFNECGKDKIPDAETCSKLKL0MNTVFMESLRLYGPVLNLLRLASEDMKLGNLEIPKG
***  **   : * .:  .***  :. .::*:**** **  : *    :::** : :*.*

TAIVIPIPILHRDKEVWGDDADEFNPLRFANGVTRAAKIPHAHLGFSIGPRSCIGQNFAM
TVLSIPIATIHRDKEVWGEDADIFKPERFKNGVSKAGKYPNALLSFSSGPRACIGQNFAM
TTIILPIAKMHRDKAVWGSDADKFNPMRFANGLSRAANHPNALLAFSMGPRACIGQNFAI
*.: :**. :**** ***.*** *:* ** **:::*.: *:* *.** ***:*******:

LEAKLVMAMILQKFSFALSPKYVHAPADLITLQPKFGLPILLKALDA
IEAKAVIAMILQRFSFTLSPKYVHVPTDVITLRPKYGLPMILKSLKV
MEAKTVLAMILQRFRLNLSADYKHAPADHLTLQPQYDLPVILEPIDG
```

```
:*** *:*****:* : **..* *.*:* :**:*::.**::*:.:.


 CLUSTAL W (1.83) Multiple Sequence Alignments

Sequence format is Pearson
Sequence 1: LOC_Os11g45720.1      485 aa
Sequence 2: LOC_Os12g37660.1      414 aa
Sequence 3: At5g61680.1           338 aa
Start of Pairwise alignments
Aligning...
Sequences (1:2) Aligned. Score:  57
Sequences (1:3) Aligned. Score:  39
Sequences (2:3) Aligned. Score:  39
Guide tree        file created:   [/tmp/3MLr1SjSp3/Gv40Pd0E66.dnd]
Start of Multiple Alignment
There are 2 groups
Aligning...
Group 1: Sequences:   2      Score:6814
Group 2: Sequences:   3      Score:5011
Alignment Score 2974
GCG-Alignment file created      [/tmp/3MLr1SjSp3/P5W730m2jT]

-----------MDRPNLAVAVVGLLAVVAATLPAPS--WQFFDLFLPAGPSHRSSGG--G
MEIISPSSSSNNNSPVLATFLVVLVVLLASSRPASSQNQQSFTINPGGAAAARPGGGKGG
-----------MGYNYVSLIVTILLVVITSPVVFGN----------DAAPIPENKGR---
            .   ::  :. *:.::::.    .           ...  .  *

FGKWVLMNHEEYVEKK~SLYAMKAAGDIGG--KTIDASLSAAEEAKVTWVVDPKGTPGDT
GGGGGPGSFSDFLTQN~VQHYVLSEQKYAGKVKALDAELSAAEAGAARYVVSGDGKG---
--------IEQWFNTN0--------------------------------VKQNGRG---
         .::.  :                                  *. .*

TFTTIAAALEKVPEGNTKRVILDLKPGAEF~REKLLLNITKPYITFKSDPANPAVIAWND
KFRTITEAIKAVPEYNKKRVILDIRPG-TY2KEKLLIPFTKPFITFVGNPRSPPTIMWDD
HFKTITEAINSVRAGNTRRVIIKIGPG-VY~KEKVTIDRSKPFITLYGHPNAMPVLTFDG
 * **: *:: *   *.:***:.: **  : :**: :  :**:**: ..*   ..: ::.

MAATRGKDGKPVGTVGSTTVAVESDYFMAYGVVFK~NDAPLAKPGAEGGQAVALRLFGTK
RAATHGKDGQPMGTMLSATVAVEADYFMASSIIFK0NNAPMAAPGAHGGQAVALRVFGSK
TAAQYG-------TVDSATLIVLSDYFMAVNIILK0NSAPMPDGKRKGAQALSMRISGNK
 **  *       *: *:*: * :***** .::*  *.**:.    .*.**:::*: *.*

AAIYNCTIDGGQDTLYDHKGLHYIKDSLIMGSVDFIFGFGRSLYE~GCTIVSVTKEVSVL
VAMYNCTIDGGQDTLYDHKGLHYFKNCLIRGSVDFIFGFGRSLYA0DCTIESVTKEVAVV
AAFYNCKFYGYQDTICDDTGNHFFKDCYIEGTFDFIFGSGRSLYL0GTQLNVVGDGIRVI
.*:***.: * ***: *..* *::*:. * *:.***** *****  .  :  * . : *:

TAQQRTKTIEGAIESGFSFKNCSIKGQGQ-IYLGRAWGDSSRVVYSYTDMSKEVVPIGWD
TAQQRSKNIAEAIDTGFSFLRCKISGIGQ-IYLGRAWGDSSRVVYSYTTMGKEVVPIGWD
TAHAG---KSAAEKSGYSFVHCKVTGTGTGIYLGRSWMSHPKVVYAYTDMSSVVNPSGWQ
**:        * .:*:** .*.:.* *  *****:* . .:***:** *.. * * **:

GWNIAKPE~SSGIYYGEFKCTGPGSDAKKRVGWALDLTADQAKPFIGTHYIYGDSWILPP
GWEVQKPE2HSGIYYGEYKCSGPGALPSKRIGWSLVLSDIQAKPFTGSHFVYGDSWILPP
ENREAGRD2KT-VFYGEYKCTGTGSHKEKRVKYTQDIDDIEAKYFISLGYIQGSSWLLPP
  .    :  : ::***:**:*.*:  .**: ::  :   :** * .  :: *.**:***
```

```
P2DGKSAASTSTASKSTASAIPRNSTAPATATESNSTAPATPSNSTAPVTASNSTAPATA
P~--KSM-----------------------------------------------------
P~---SF-----------------------------------------------------
*    *

SSSNPPATKSYSGPPATPSASSTPAKASR
-----------------------------
-----------------------------


 CLUSTAL W (1.83) Multiple Sequence Alignments

Sequence format is Pearson
Sequence 1: LOC_Os01g74320.1      446 aa
Sequence 2: LOC_Os05g02130.1      450 aa
Sequence 3: At2g28840.1           456 aa
Start of Pairwise alignments
Aligning...
Sequences (1:2) Aligned. Score:  64
Sequences (1:3) Aligned. Score:  53
Sequences (2:3) Aligned. Score:  63
Guide tree        file created:   [/tmp/3MLr1SjSp3/ZJz3WE9EjV.dnd]
Start of Multiple Alignment
There are 2 groups
Aligning...
Group 1: Sequences:   2      Score:7901
Group 2: Sequences:   3      Score:7437
Alignment Score 5223
GCG-Alignment file created      [/tmp/3MLr1SjSp3/P5W730m2jT]

MGHGLSCSRDTDEYDLFRAAQLGDIHALSALLAADPALARRATVYDRFTALHIAAANGRL
MGHGVSCARTGDEHDFFRAAQLGDLDALAALLAADPSLARRATLYDRLSVLHIAAANGRI
MGQSMSCG-SRPEHGIFASVQCGDIITIRRVMATEPSLLNQTTPYDRHSVLHVAAANGQI
**:.:**.    *:.:* :.* **: ::  ::*::*:* .::* *** :.**:*****::

Q~VLSMLLDRDGDVDVLSRKKQ~TPLMVAAMRGNTECVVRLLRGGAN~VLTFDSPRARTC
E0VLSMFLDRGAPPDAVNRHKQ0TPLMLAAMHGKIDCVLKLLQADAN0ILMFDSVHARTC
E0ILSLLLERFTNPDLLNRHKQ0TPLMLAAMYGRISCVKKLAEVGAN0ILMFDSVNRRTC
: :**::*:*    * :.*:** ****:*** *. .** :* . .** :* *** . ***

LHHAAYYGHAECLQAILGAAAQAQGPVAAS~WGFARFVNVRDERGATPLHLAARHARASC
LHHAAYYGHVDCLQAIL-AAAQTT-PVADS2WGFARFVNVRDDHGATPLHLAARQGRPGC
LHYAAYYGHANCVQAIL-SAAQSS-PVAVH2WGYARFVNIRDDKGATPLHLAARQRRPEC
**:******.:*:**** :***:  ***   **:*****:**::**********: *. *

VRLLLDKGAIVSAPTAVY~GFPGSTALHLAARAGSMECIRELLAWGADRLQRDSA~GRIA
VQVLLENGAIVSALTGSY2GFPGSTSLHLAARSGNLDCIRKLLAWGADRLQRDSA2GRIP
VNVLLDSGSLVCASTSVY2GSPGSTPLHLAARSGSIDCVRKLLAWGADRLQRDAS2GRIP
*.:**:.*::*.* *. * * ****.******:*.::*:*:************:: ***.

YAVAMRRGHRACAALLNPAAAEPIVWPSPLKFIGELEADAKALLEAALMEANREREKRIL
YSVALKRNHGACAALLNPTSAEPMVWPSPLKFISELEPEAKALLEAALMEANREREKKIL
YVVAMKHKHGACGALLNPSSAEPLVWPSPLKFISELNDEAKLLLEQALMEANREREKTIL
* **::: * **.*****::***:*********.**: :** *** *********** **

HGSDINIKGGDEEEESEDEE----~----EACNICFEQACSMEVKECGHQMCAACTLAIC
```

```
NGTKYSLPSPSPGDDSADDDACSE0VSDTELCCICFDQACTIEVQDCGHQMCAPCTLALC
KGTAYSLPSPSFSDTDDNMS---E0VSDTELCCICFEQVCTIEVKDCGHQMCAQCTLALC
:*:  .: . .  : . : .         * * ***:*.*::**::******* ****:*

CHSKPNPKTLLLHPPACPFCRTTISRLVVATTNS----------------NKTN----S
CHNKPNPTTLTPPSPACPFCRGSISRLVVAQTRSA------------CDPDKPSSLQLT
CHNKPNPTTSTVTPPVCPFCRSTIACLVVAQNNNNNNEKSKSLDDVVVVDREAGDVSSSK
**.****.*    .*.***** :*: **** ...                 :  .    .

RRRSR------SRSSSFKGGLSSAMGSFSRI-GRGSGRLVVDGSSVGELADKPDHDFSSV
RKRSRRSHNLSEGSSSFKG-LPSAMGSFSKL-GRGSSRMADSDSSN---LDKPEHDL---
FRKHRRSINLGEESSSFMG--LSTIGSFGRITGRGSGRIAAENELM----DKPIL-----
 :: *      . **** *   *::***.:: ****.*:. ...      ***

AAAAAICDT
---------
---------


 CLUSTAL W (1.83) Multiple Sequence Alignments

Sequence format is Pearson
Sequence 1: LOC_Os01g09540.1      303 aa
Sequence 2: LOC_Os05g10210.1      265 aa
Sequence 3: At4g25150.1           260 aa
Start of Pairwise alignments
Aligning...
Sequences (1:2) Aligned. Score:  46
Sequences (1:3) Aligned. Score:  33
Sequences (2:3) Aligned. Score:  38
Guide tree        file created:   [/tmp/3MLr1SjSp3/iSOFXUpygq.dnd]
Start of Multiple Alignment
There are 2 groups
Aligning...
Group 1: Sequences:   2      Score:4129
Group 2: Sequences:   3      Score:3749
Alignment Score 1815
GCG-Alignment file created      [/tmp/3MLr1SjSp3/P5W730m2jT]

MAPKRLVCFLAVAAALATTCHGWGAGAGDVVSSSAAALSFVDRLRQMMIPAAVGDGDYCD
MATARLILLLTVAAAAAGSCC-FCSAQEVIVGGVGEQLATAP----PAAPAPSPPPPYCG
---MRILVNLILFSLIPLAFSNENSSSYLIARPLIFETQLKN------INDNVN--LHCT
    *::  * : :  . :     :.   :.                          :*

SWRVGVEANNVRGWTAAPRKCDNYVENYMRGHHYRRDSKVVVDEAAAYAEAAVLSGDPAA
SVRTAVEAHNIIGWKTVPADCAEYVSDYLTGERYGRDSDVVINEAIAYAESLKLSG----
SWRFAAETNNLAPWKTIPAECADYVKDYLMGEGYVVDVERVSEEAKVYASSFESNG----
* *  ..*::*:  *.: * .* :**.:*: *. *  * . * :** .**.:   .*

DANATWVFDVDETALSHVKFYKKHGF~GYHRTDEPAFMEWLIAGRASALPNTVTLYKKLL
HGKEIWVFDVDETALSTLPYQAKHGY2GTKPYDHASFVQYVAGGSAPALQGTLRLYRRLL
DGKDIWIFDIDETLLSNLPYYMEHGC2GLEVFDHSKFDMWVEKGIAPAIAPSLKLYQKVI
..:  *:**:*** ** : :  :**  * .  *.. *  ::  * *.*:  :: **::::

LLGVKIVFLSDRPDTPELRNATATNLIKEGFDCWDELIL2RSENSTATGSVVEYKSGERK
QLGIKPVFLTDR--TEDQRAVTTHNLLSQGYYSWEKLLL~QPVGLQTT--TQAFKTGERQ
HLGYKVILLTGR--RENHRVITVENLRNAGFHNWDKLIL2RSLDDRNK-TATMYKSEKRE
```

```
 ** * ::*:.*    : *  *. ** . *:  *::*:* :. .   .  .  :*: :*:

KLEEEKGMVIIGNIGDQWSDLLGSPEGRRTFKLPNPAYYIDNYKRAGAAVRAAVAITASS
KLVS-AGYVIVGNIGDQWSDILGSPEGYRTFKYPNPIYYVA-------------------
EMVK-EGYRIRGNSGDQWSDLLGSAMSERSFKLPNPMYYIP-------------------
:: .  *  * ** ******:***. . *:** *** **:

SSSSS
-----
-----



 CLUSTAL W (1.83) Multiple Sequence Alignments

Sequence format is Pearson
Sequence 1: LOC_Os11g09020.1      476 aa
Sequence 2: LOC_Os12g08090.1      475 aa
Sequence 3: At5g63850.1           466 aa
Start of Pairwise alignments
Aligning...
Sequences (1:2) Aligned. Score:  83
Sequences (1:3) Aligned. Score:  68
Sequences (2:3) Aligned. Score:  66
Guide tree        file created:   [/tmp/3MLr1SjSp3/lNRKwQFQFI.dnd]
Start of Multiple Alignment
There are 2 groups
Aligning...
Group 1: Sequences:   2      Score:9610
Group 2: Sequences:   3      Score:8545
Alignment Score 6650
GCG-Alignment file created      [/tmp/3MLr1SjSp3/P5W730m2jT]

MGKAAAMEVSASAAAEAGMMVGHGEWRDDDGRARRM~GTVWTASAHIITAVIGSGVLSLA
MASGQKVVKPMEVSVEAG-NAGEAAWLDDDGRARRT~GTFWTASAHIITAVIGSGVLSLA
----------MDVPRPAF------KCFDDDGRLKRS1GTVWTASAHIITAVIGSGVLSLA
           ...  *          ***** :*  **.*******************

WAIAQLGWVAGPAVMLLFAFVIYYTSTLLAECYRSGDPCTGKRNYTYMDAVRANL~GGSK
WAIAQLGWVAGPAVMLLFAFVIYYTSTLLAECYRTGDPATGKRNYTYMDAVRANL~GGAK
WAIGQLGWIAGPTVMLLFSFVTYYSSTLLSDCYRTGDPVSGKRNYTYMDAVRSIL1GGFR
***.****:***:*****:** **:****::***:*** :************: * ** :

VRLCGVIQYANLFGVAIGYTIAASISM2LAIKRADCFHEKGHKNPCRSSSNPYMILFGVV
VTFCGVIQYANLVGVAIGYTIASSISM~RAIRRAGCFHHNGHGDPCRSSSNPYMILFGVV
FKICGLIQYLNLFGITVGYTIAASISM2MAIKRSNCFHESGGKNPCHMSSNPYMIMFGVT
.  :**:*** **.*:::*****:****  **:*:.***..*  :**: *******:***.

QIVFSQIPDFDQIWWLSIVAAIMSFTYSTIGLSLGIAQTV~ANGGFMGSLTGISVGTGVT
QIVFSQIPDFDQIWWLSIVAAVMSFTYSGIGLSLGIVQTI1SNGGIQGSLTGISIGVGVS
EILLSQIKDFDQIWWLSIVAAIMSFTYSAIGLALGIIQVA1ANGVVKGSLTGISIG-AVT
:*::*** *************:****** ***:*** *.  :** .  *******:* .*:

SMQKVWRSLQAFGDIAFAYSYSIILIEIQ0DTIKAPPPSEAKVMKRATMVSVATTTVFYM
STQKVWRSLQAFGDIAFAYSFSNILIEIQ0DTIKAPPPSEAKVMKSATRLSVATTTVFYM
QTQKIWRTFQALGDIAFAYSYSVVLIEIQ0DTVRSPP-AESKTMKIATRISIAVTTTFYM
. **:**::**:********:* :***** **:::** :*:*.** ** :*:*.**.***
```

```
LCGCMGYAAFGDKSPDNLLTGFGFYEPFWLLDVANAAIVVHLVGAYQVFVQPIFAFVERW
LCGCMGYAAFGDAAPDNLLTGFGFYEPFWLLDVANVAIVVHLVGAYQVFVQPIFAFVERW
LCGCMGYAAFGDKAPGNLLTGFGFYNPFWLLDVANAAIVIHLVGAYQVFAQPIFAFIEKQ
************ :*.*********:*********.***:*********.******:*:

AAARWPDGGFISRELRV------GPFSLSVFRLTWRTAFVCATTVVSMLLPFFGDVVGLL
ASRRWPDSAFIAKELRV------GPFALSLFRLTWRSAFVCLTTVVAMLLPFFGNVVGLL
AAARFPDSDLVTKEYEIRIPGFRSPYKVNVFRAVYRSGFVVLTTVISMLMPFFNDVVGIL
*: *:**. ::::* .:      .*: :.:** .:*:.**  ***::**:***.:***:*

GAVSFWPLTVYFPVEMYIAQRGVRRGSARWLCLKVLSAACLVVSVAAAAGSIADVVDALK
GAVSFWPLTVYFPVEMYIAQRGVPRGSARWVSLKTLSACCLVVSIAAAAGSIADVIDALK
GALGFWPLTVYFPVEMYIRQRKVERWSMKWVCLQMLSCGCLMITLVAGVGSIAGVMLDLK
**:.************** ** * * * :*:.*: **. **::::.*..****.*:  **

VYRPFSG--
VYRPFSG--
VYKPFKTTY
**:**.


 CLUSTAL W (1.83) Multiple Sequence Alignments

Sequence format is Pearson
Sequence 1: LOC_Os12g02840.1      839 aa
Sequence 2: LOC_Os05g40650.1      874 aa
Sequence 3: At2g13620.1           821 aa
Start of Pairwise alignments
Aligning...
Sequences (1:2) Aligned. Score:  39
Sequences (1:3) Aligned. Score:  40
Sequences (2:3) Aligned. Score:  55
Guide tree        file created:   [/tmp/3MLr1SjSp3/MAal24iJfY.dnd]
Start of Multiple Alignment
There are 2 groups
Aligning...
Group 1: Sequences:   2      Score:13924
Group 2: Sequences:   3      Score:12341
Alignment Score 6918
GCG-Alignment file created      [/tmp/3MLr1SjSp3/P5W730m2jT]

-----------------MAAHTVTDPLEELWNHTMSMDKTHLMCFYPSKITMGGVWTGD
MAPIMSGAAAAAGGTGGAVPLIKNATSASQMSRGKAGTGAGAVVCYSPMMVTAYGIWQGA
------------------------------MATSEEPSTDASIICYAPSMITTNGVWQGD
                              :           ::*: *  :*  *:* *

NPLDFSIPLLLFQILLITSTTRAATLLLSPLRLPTYISQIL~AGFLLGPSVLGHLPHFSN
SPLDFSLPLFLLQVAIIVATTRLLVILLKPFRQPRVIAEIL0AGVILGPSVMGQVSTWAV
NPLDFSLPLFVLQLTLVVVVTRFFVFILKPFRQPRVISEIL0GGIVLGPSVLGRSTKFAH
.*****:**:::*: ::. .**  .::*.*:* *  *::** .*.:*****:*: . ::

LVFPVRSLFVLESMALLGLVYYTFIVGVEIEVSAITRAGIRSFGFAIGCTLPPFLVGALT
KVFPERSLLTLETVAHLGLLYFLFLVGLEMDVNTIRRSGKKALIIAVAGMALPFCIGTAT
TIFPQRSVMVLETMANVGLLYFLFLVGVEMDIMVVRKTGKRALTIAIGGMVLPFLIGAAF
 :** **::.**::* :**:*: *:**:*::: .: ::* ::: :*:.    ** :*:
```

```
GYVALSTDDKHKGDTFLNKLSFPIFLGSTFSSTAFAVLARNIAELKLAGTDVGQLTLSAS
SFIFR-----HQVSKNVHQASFLLFLGVALSVTAFPVLARILAEVKLLNSDLGRIAMSAA
SFSM------HRSEDHLGQGTYILFLGVALSVTAFPVLARILAELKLINTEIGRISMSAA
.:        *: .  : : :: :*** ::* ***.**** :**:** .:::*::::**:

LINDTFAWTGLTVATVLGHSRCTITQTTWTLTSGVVIFGASYLLLRPMLLRLARRAAEGE
IVNDMCAWILLALAIAISEVNSSAFSSLWVLIAGVAFVLACFYVVRPLMWWIVRRVPEGE
LVNDMFAWILLALAIALAESDKTSFASLWVMISSAVFIAVCVFVVRPGIAWIIRKTPEGE
::**  **  *::* .:..   :   : *.: :...:. ..  ::** :  : *:..***

AVGEDRECWILIGVMVAALVADAGGTHAIFGAFVFGLAVPNGPVGVALVEKVEDFVVGAL
AIGDVHITLILTGVMVAGVCTDAIGIHSVFGAFVYGLVMPSGPLGVVLIEKLEDFVTGLL
NFSEFHICLILTGVMISGFITDAIGTHSVFGAFVFGLVIPNGPLGLTLIEKLEDFVSGLL
 ..: :   ** ***::.. :** * *::*****:**.:*.**:*:.*:**:**** * *

LPLFFALSGLRTDTAKITNMHSAVLLMVAAMVAAVLKVVAAIGVAGVFGMPLGDGTSIGL
LPLFFAISGLRTNVTKVRDPITVGLLVLVFVMASFAKIMGTILIAVSYTMTFRDGVALGF
LPLFFAISGLKTNIAAIQGPATWLTLFLVIFLACAGKVIGTVIVAFFHGMPVREGITLGL
******:***:*: : : .  :   *.:. .:*.  *::.:: :*  . *.. :* ::*:

LLNTKGIIELVILNIARNKG0IMSDQSFTVLVFVSALITAMVSPFLGMVVKPARRLVFYK
LMNTRGLVEMIVLNIGRDKE~VLDDESFAVMVLVSVAMTALVTPVVTTVYRPARRLVGYK
LLNTKGLVEMIVLNVGKDQK~VLDDETFATMVLVALVMTGVITPIVTILYKPVKKSVSYK
*:**:*::*:::**:.:::  ::.*::*:.:*:*:  :*.:::*.:  : :*.:: * **

RRTVAWAHPESELRVLACVHVPRDVPALLTLLDVVTPSSRSPVGVLALHLIEFVGRSSAL
RRNLQRSKHDAELRMLACVHTTRNVPSIISLLELSNPTKRSPIFIYALHLVELTGRASNM
RRTIQQTKPDSELRVLVCVHTPRNVPTIINLLEASHPTKRSPICIYVLHLVELTGRASAM
**.:  :: ::***:*.***..*:**:::.**:   *:.***: : .***:*:.**:* :

LLINASAPSSSSYDASVHGRSHTEMQFKHISHAFMAYEEQSVGVSARTMAAVSPYASMHE
LAAHHSASN-------PGGAS------DHIFNAFESYEEMVGGVSVQALTAVSPYQTMHE
LIVHNTRKSGRPALNRTQAQS------DHIINAFENYEQHAAFVAVQPLTAISPYSTMHE
*  : :  .         . *      .** :**  **:    *:.:.::*:*** :***

DITSAAENQHSALILLPFHKYRSVDGGLEVSHPAIQPLNCSVQSFSPCTVGILVDRGLAA
DVCVLAEDKHVSLIVLPFHKQQTVDGGMEPINASLRGFNESILASAPCSVGILVDRGLSA
DVCSLAEDKRVSFIIIPFHKQQTVDGGMESTNPAYRLVNQNLLENSPCSVGILVDRGLNG
*:   **::: ::*::**** ::****:*  :.: : .* .:   :**:********* .

VPGG-----GYRVVALFFGGSDDREVAALATRMVRNPTIDLTLLRFVQKG----------
AAAR--MAAVHHVALLFFGGPDDREGLAYAWRMVENPGVCLTIVRLIPPGYTAPAISPPQ
ATRLNSNTVSLQVAVLFFGGPDDREALAYAWRMAQHPGITLTVLRFIHDEDEADTASTR-
..         :*. *****.****  * * **..:* : **::*::

--------GSFTASEFDALKERKADEGCLRDFLERANEGGGATVEYRERGVFNASEMVGE
PPMPAAHSRAINVVPEVAKSERQMDEEYLNEFRSRN--LGNDAILYVEQVVANSEETVAA
----ATNDSDLKIPKMDHRKQRQLDDDYINLFRAEN--AEYESIVYIEKLVSNGEETVAA
          :.       .:*: *:  :. *  .       :: * *: * *..* *.

IQSVEAMGNKDLFVVGKVPGGSG--LTAGMAEWSESPELGPIGDLLASKDFQTTASVLVL
IRSQLDNAH-ELYIVGRHPGEASSPLTSALAEWMESPELGPIGDLLVSSEFSKMASVLVM
VRS-MDSSH-DLFIVGRGEGMSS-PLTAGLTDWSECPELGAIGDLLASSDFAATVSVLVV
::*    .: :*::**:  * :.  **:.:::* *.****.*****.*.:*   .****:

QAYGRPAAVVGAGAGAMSVDFGGDSVAMAERTASGRRPWARPGV----------------
QQYVITAPLPPP--VALAGPPTDDPVRQYLTNANQRPSVAIGGNQMGAAGRGGWSGGAGG
```

```
QQYVGSWAQEDD--MDFP----ESPVHSHETKVTYGLENPR------------------
* *  . .        :.     ..*     ...     .

-
Y
-


 CLUSTAL W (1.83) Multiple Sequence Alignments

Sequence format is Pearson
Sequence 1: LOC_Os02g50810.1      166 aa
Sequence 2: LOC_Os06g12960.1      189 aa
Sequence 3: At5g50090.1           159 aa
Start of Pairwise alignments
Aligning...
Sequences (1:2) Aligned. Score:  57
Sequences (1:3) Aligned. Score:  33
Sequences (2:3) Aligned. Score:  38
Guide tree        file created:   [/tmp/3MLr1SjSp3/s97BPyTjpM.dnd]
Start of Multiple Alignment
There are 2 groups
Aligning...
Group 1: Sequences:   2      Score:2734
Group 2: Sequences:   3      Score:2313
Alignment Score 1143
GCG-Alignment file created      [/tmp/3MLr1SjSp3/P5W730m2jT]

MGNCQAAEAAAVVIQHPGGRVERLCWSTSAAEVMRANPGHYIALVTLRVAEE--------
MGNCQAAEAATVVVQHPGGRVERLYWATTAAEVMRANPGHYVALVTLRVAEEKRPPPPPP
MGNCQAVDTARVVIQHPNGKEEKLSCPVSASYVMKMNPGHCVSLLISTTALS--------
******.::* **:***.*: *:*  ..:*: **: **** ::*:   .* .

----RQDGDGGARRTVRLTRVKLLKPKETLLLGHAYRLITTH1EVTKAVQARKEEKVRKA
PPPARAERRGTGTGTVRVTRVKLLKPRDTLLLGQAYRLITVD~EVTRALQAKKEEKSRRA
-----SASSGHG-GPLRLTRIKLLRPTDTLVLGHVYRLITTK1EVMKGLMAKKCSKLKKE
         * .  .:*:**:***:* :**:**:.*****.. ** :.: *:* .* ::

QQQLEESRQKLQSKARAAASA--------AAE~VDEAAEE~NDND-----SDNFDDEAAL
AAQHHHLESKPAAAAAVAAGVRINSGGDDHTQ~LDENLDQ0HDRDGQRSSSATHSRHRQW
SKGSDDKLEMVKAINSTKLDN--------EDQ0LQMKKQE~KERS---------RISRSW
    ..  .   :   .  .           : ::   :: ::..

DASLDQVGLHGSEW
RPSLHSIAEVRS--
QPSLQSISEGGSS-
 .**..:.   *
 CLUSTAL W (1.83) Multiple Sequence Alignments

Sequence format is Pearson
Sequence 1: LOC_Os02g54640.1      988 aa
Sequence 2: LOC_Os09g26160.1      950 aa
Sequence 3: At2g24710.1           895 aa
Start of Pairwise alignments
Aligning...
Sequences (1:2) Aligned. Score:  37
Sequences (1:3) Aligned. Score:  37
```

```
Sequences (2:3) Aligned. Score:  34
Guide tree        file created:   [/tmp/3MLr1SjSp3/S8BXCLM0Ml.dnd]
Start of Multiple Alignment
There are 2 groups
Aligning...
Group 1: Sequences:   2      Score:14068
Group 2: Sequences:   3      Score:12801
Alignment Score 6051
GCG-Alignment file created      [/tmp/3MLr1SjSp3/P5W730m2jT]

MARRSCFFFLLPLLVAALAGSP-------VVTAQRNALPAAAAAAASVRVGVILNLTSAV
MEAGARLAFVMPLVVVLLLMIFSLGVRGVDVVVDGGGGGAAAARRRRVEVGVILDRRTWL
MRTEKLFFCILLVFFFCL--EFN---------------RGQNNGKTLVDVGVVTDVDTSH
*     :  :: :..  *                      .       * ***: :  :

GVRRRVGIQMAVEDYYAANPGS--ATRVELHFRDSAGDVLPAASA1AVDLIKNVQVQAMI
GNISWACMELAVEDFYADEERASYTTALRLHLRDTRLDAVDAASA1GVDLLKNVHVQAIV
SKVVMLCINMSISDFYSSNPQF--ETRLVVNVGDSKSDVVGAAIA1ALDLIKNKQVKAIL
.      :::::.*:*: :      * : ::. *:  *.: ** * .:**:** :*:*::

GPPSSAATEFVAHIGSHSRVPVLSYSATSPSLSPAQTPFFVRAAVNDSFQAAPVAAVLDA
GPQTSAQAKFLAELGEKSSVPVVSFSANSPCRTASQTPYFIRTAWNDSSQAEAIASLVQR
GPWTSMQAHFLIEIGQKSRVPIVSYSATSPILTSLRSPYFLRATYEDSFQVQPIKAIIKL
** :*  :.*: .:*.:* **::*:**.**  :. ::*:*:*:: :** *. .: :::.

FRWRAAAVVYEDSPYGSGILPALADALQGAGAKIMDRTAVPVDATDDRLDALLYRLRAMP
FNWRDVIPVIEDDDSNTRFIPDLVDALRNAEIRVTHRCKIHPSAGADDIKKVVLSLKEKW
FGWREVVPVYIDNTFGEGIMPRLTDALQDINVRIPYRSVIAINATDHEISVELLKMMNMP
* ** .  *  *.  .  ::* *.***:.   ::  *  :  .*  . :.  :  :

TRVFVVHMLHNVAGRLFRRAKMLGMMSDGYIWVATDGVATFMDRFSPEEVDAMQGVVSLR
TSVFVVRMSYQLALSFFKHAKDEGMMGQGFVWIAAYGLTDIFDVVGSPAFDVMQGVIGMK
TRVFLVHMYYDLASRFFIKAKELGLMEPGYVWILTNGVIDDLSLINETAVEAMEGVLGIK
* **:*:* :::*  :* :**  *:*  *::*: : *:   :. ..   .:.*:**:.::

PYVQETDAVKNFSARFKARLRRDHPTVDDVREPTVLRFWAYDTAWAIAAAAESAGVAGPA
PYVNDTKQLQNFRQRWRKMYKSENP-GTTLSEPTLSGLYAYDTVWALALAAEKAGYVNSD
TYIPKSPDLEKFRSRWRSLFP--------RVELSVYGLWAYDATTALAVAIEEAGTNNMT
.*: .:  :::*  *::              * ::  ::***:. *:* * *.**  .

F-QTPQTSAPLTDLDRLGVSATGTALLNAVLSTTFDGLAGKFRLVDGQLQPPAYEVVNII
F-LLSEKNNGSTDFDRINTSNAAKKLQSTLLNIDFQGMSGKFQFQDMHLLSMTYEIINIV
FSKVVDTGRNVSELEALGLSQFGPKLLQTLLTVQFRGLAGEFRFFRGQLQPSVFEIVNII
*    :..   :::: :. *  .  * .::*.  * *::*:*::   :* . .:*::**:

GKGARTVGFWTPEFGITQDLNAGS---------AKTLRQILWPGEPRDTPRGWTVSPSGL
GEEQRVVGFWTPEFNISRGLNT-----------KADVNEIIWPGGETTVPRGWLF-PMNK
NTGEKSIGFWKEGNGLVKKLDQQASSISALSTWKDHLKHIVWPGEADSVPKGWQIPTKGK
.   : :***.   .: : *:                :..*:***    .*:** . . .

PLRVSVPTKRGFTQFVDVGNVTATGRRNITGYCIDVFDEVMKIMPYPVSYVYDPYP----
TLKIGVPAKPGFSGFIKK------EKYNFTGLCIEVFEEVLNGLPYKIPHDYVEFGNGKG
KLRIGVPKRTGYTDLVKVTRDPITNSTVVTGFCIDFFEAVIRELPYDVSYEFIPFEKPDG
 *::.** : *:: ::.            .** **:.*: *:. :** :.: :  :

DSPESYEKLVDQVSSQ0KADAVVGDVTITASRMEEVDFTMPFTESGWSMVVAVQKETSTS
ESNGTYDELIYKVYQN0DFDAAVGDITILANRSLYVDFTLPYTESGVRMLVPVQDQRQKT
```

```
KTAGNYNDLVYQVYLG0RYDAVVGDTTILVNRSSYVDFTFPFIKSGVGLIVEMTDPVKRD
.:  .*:.*: :*      **.*** ** ..*   ****:*: :**  ::* : .  .

MWIFLQPLTTSLWLASLAFFCFTGFVVWVIEHRINEEFRGTPWQQFGLIFYFSFSTLVFS
AWTFLQPLTADLWLGTAAFFVLTGFVVWFIEHRTNEDFRGPPVNQIGSVFYFAFSTLVFA
YILFMKPLSWKLWLTSFISFFLVGCTVWVLEYKRNPDFSGPPRFQASTICWFAFSTMVFA
   *::**: .*** :   * :.* .**.:*:: * :* *.*  * . : :*:***:**:

H1KEKLESNLSRFVVIIWVFVVLILTSSYTASLTSMLTVQKLQPTVTDVRELLRRGDYIG
H~RQKIVNNLSRVLLVIWLFVVLILQRSYTASLSSILTVEQLQPTVTNLDEVIRKGANVG
P1RERVFSFWARALVIAWYFLVLVLTQSYTASLASLLTSQKLNPTITSMSSLLEKGETVG
  :::: .  :* ::: * *:**:*  ******:*:** ::*:**:*.: .::.:*  :*

FQEGTFIVPVLEKMGFEG-RMRSYSTVDQYADALSKGSANGGVAAIFDEIPYLKLFLSQY
YLNDSFMPELLKRLKIDESKLIALDSPDEYNEALSTG----RVAVVVDEIPYLKVFLSKY
YQRTSFILGKLKERGFPQSSLVPFDTAEECDELLSKGPKKGGVSGAFLEIPYLRLFLGQF
: . :*:   *:.  :    : . .: ::  : **.*     *:  . *****::**.::

CNGYTMVGPIYKTDGFGF~VFPRGSPMVADVSRAILTLAEGEKMAQIEKKWFGEPGACQS
CHNYTMVGPTYKFDGFGF0AFPLGSPLTAEISRGILNFTSSNRMAQLERELYNN-RTCPD
CNTYKMVEEPFNVDGFGF0VFPIGSPLVADVSRAILKVAESPKAMELERAWFKKKEQSCP
*: *.**   :: ***** .** ***:.*::**.**..:.. :  ::*:  : :   .

QGSAVGSSNLSFRSFGGLFLITGVVTSAMLLIYLAVFFYRERDELRAAEAAAAASGSGSG
KDDSQTSSSLTLRSFLGLFIITGASSLLALFLHVVITLYNHRHDL-----SSASSSQSSW
DPITNPDPNPSFTSRQ-----LDIDSFLFLFVGVLLVCVMA-------------------
.  :  ... :: *         .  :   *:: : :

SGSRSLRRLRAWARHYDQKDLKSPTFKRRWSDESVRNGSEYAASRTPRWGDESPCNVAGA
CG-----WFAILLKIFHEGDRPN---APQLDEPAVSNANTTADTPWSTPDHHIIENVDSG
--------LGNFTYCFLAKDQVS-----YLDKVEMSPCSSSQQMPVKR---KTQLNMSQV
        :      :   *  .       ..   :   .            .   *:

ADADAGRIP--EEVVGGMSPFSISTSSEERNGAVSPAAAEFDNSSDRAAVVAGTSQPR
SDVESVREEDREDFVQGPDPPSFAYMHSERGQ--------------------------
HDQDSL----------------------------------------------------
 * ::
```
