# Supplementary material for: Intron gain and loss in segmentally duplicated genes in rice
Source: Genome Biol. 2006 May 23;7(5):R41. doi: 10.1186/gb-2006-7-5-r41 (PMC1779517; doi:10.1186/gb-2006-7-5-r41)
Supplement: Additional File 6 — The ClustalW alignment of the two rice duplicated proteins with putative orthologous proteins from Arabidopsis, poplar, maize and sorghum. [file gb-2006-7-5-r41-S6.pdf]

Additional Data File 6. ClustalW alignment of the two rice duplicated genes and their homologous genes from Arabidopsis (Atxgxxxxx.x), maize, sorghum and poplar. The phases of introns (0,1,2) are highlighted. The gained introns are highlighted in green. The “~” indicates a missing intron at that position. The “^” indicates no amino acid at that position.

```
CLUSTAL W (1.83) multiple sequence alignment


maize_                   --MESSRMRCCMLLVVSLALLLPLG---MAADSIGSYCSGSS--YAGSSKAVANINSVLA
sorghum_                 --MKFSTMRCCVLLVS--LALLPLG---MAADSIGSYCSGSR--YAGSNKAVTSINSVLA
LOC_Os03g16960.1         MAIISSKACRCLLLVS--FALLPLS---MAMDPLGSYCSGNS--LAGSSKAVASINSVLT
LOC_Os05g02200.1         MAMASSCSPLVAVLVALLLVAATVPAPCAAVDPVNTYCARN---LSGAP-AQASVAQVLS
At5g48540.1              --MKTLVVKCFLLLA----LVCSCR----AADSIWQLCNTNSN-ISASSQVSKNIDSLLA
poplar_                  --MSSSRFTSFLYLLTLSLLLQNVLG----VDPLYSSCSGNEKSTANYGSYKTSLDVLMS
                           :          *                 *.:   *  .    :       .:  :::

maize_                   DLVASASSTGGYATSTAGKGNNS-IIYGLAQCRGDVSASDCASCLADAAKQLPSTCSYSS
sorghum_                 DLVATASTGGGYATSTAGKGNN--IIYGLAQCRGDVSASDCAACLADAAKQLPSTCSYSS
LOC_Os03g16960.1         DLVTKGSTGVGFATSTAGKGNN--VIYGLVQCRGDVSTSDCQACLASAANQILTSCNYQS
LOC_Os05g02200.1         ELVPRASAG-YYATATAGRGGDGSAIWGLAQCRGDIPAPDCALCASAAARQLAGACRGRA
At5g48540.1              TLVSKTPSK-GFKTTTSSSYNNKEKVYGLAQCRGDISNTDCSTCIQDAAKKIREVCQNQS
poplar_                  SFYQLAPAKEGFALGSLG-QKNLDRPYGLVLCRGDVSSPDCSACVADATREIRKRCPYGK
                          :    .:   :   : .   :    :**. ****:. .**  *   *:.::   *

maize_                   DARI2WYDYCFMRYENANFFGQADTDAGVILVNVQAMD--NPKAFEKAVGKVMGKATAQAS
sorghum_                 DARI2WYDYCFMRYENADFFGQADTGAGVILVNVQAMD--NPKAFEKAVGKVIGKATAQAS
LOC_Os03g16960.1         DSRI2WYDYCFMRFENENFFGQADTDNGVIMENVQAMD--NAKAFQKAVGKVMSKATAQVS
LOC_Os05g02200.1         DARV~WYDYCFARYDDADFVGLPDTGYALILLNTQNAT--DPEAFEKAQRKVMARVAADAG
At5g48540.1              DSRI~LYDFCFLRYSQENFIGKLDTGAGLIYFNVANVTEIDPKKFDNELGALFDKIRSEAV
poplar_                  SGFI~AYDNCLLKYSNKDFFGQIDSQNKIYLYNVRNVS--NPVVFNQKTKDLLSQLANKAY
                         .. : ** *: ::.: :*.*  *:   :   *.      :.  *::    :: :   ..

maize_                   AAGSAGLGRDKEQYTPFVSIYGLAQCTRDLAPLTCAQCLSTALSRFGDYCGAQQGCQINY
sorghum_                 AAGSAGLGRDKDQYTPFVSIYGLAQCTRDLAPLTCAQCLSTAVSRFGDYCGAQQGCQINY
LOC_Os03g16960.1         QAGSGGLGRVKDQYTPFINIYGFAQCTRDLSPLTCAQCLSTAVSRFDQYCGAQQGCRILY
LOC_Os05g02200.1         DAGGGGLARETARFKDGVTIYGLGWCTRDITAADCGLCVAQAVAEMPNYCRFRRGCRVLY
At5g48540.1              LPKNKGLGKGKTKLTPFVTLNGLVQCTRDLSELDCAQCFATAVGSFMTTCHNKKGCRVLY
poplar_                  IAR-KMYATGELGLGGSKKLYGMAQCTRDLSSANCKKCLDGAISELQGFAGGKEGGRVTG
                          .     .          .:  *:  ****::   *  *.  *:. :   .  :.* ::

maize_                   SSCRVRYEIYPFYFPLAGKG--GGLATTDMTKNTKIVVRP
sorghum_                 SSCRVRYEIYPFYFPLAGNGGAGGRATTDMTKNTKIIVHP
LOC_Os03g16960.1         SSCMVRYEIYPFYFPLATSS----TATTDMTKYTKTIVHH
LOC_Os05g02200.1         SSCMARYETYPFFFPLDGGQ-SADASASAAGDYDRVVLNP
At5g48540.1              SSCYVRYEFYPFYFPLDPAK-----TGPSVGRISSVHLSP
poplar_                  GSCTVRYEIYPFVKA-------------------------
                         .** .*** ***  .
```

```
CLUSTAL W (1.83) multiple sequence alignment


LOC_Os06g51050.1      ---MRALALA----VVAMAVVAVRGEQCGSQAGGALCPNCLCCSQYGWCGSTSDYCG-AG
sorghum_              --MMRALAVLAM--VALFAASSARAEQCGTQAGGALCPNCLCCSKFGWCGSTSDYCG-SG
maize_                MAMTRALAMVAMLATAALFFMSARAQQCGTQAGGALCPDCLCCSQWGYCGSTPDYCT-DG
At3g12500.1           ---MKTNLFLFL--IFSLLLSLSSAEQCGRQAGGALCPNGLCCSEFGWCGNTEPYCKQPG
LOC_Os03g04060.1      ---MRRLLPLAG----ATLLIAAAGGASGQQAG---------------------------
poplar_               ------------------------------------------------------------


LOC_Os06g51050.1      CQSQCSG-GCGGGPTP----PSSGGGSGVASIISPSLFDQMLLHRNDQACAAKGFYTYDA
sorghum_              CQSQCTG-SCGSTPSTPTPTPSSGGGGSVASIISESLFNQMLLHRNDAACPANGFYTYSA
maize_                CQSQCFGSGCGGGGGT----PATPPSGPVSEIISESLFNEMLLHRNDVACPAIGFYTYDA
At3g12500.1           CQSQCTP---GG--------TPPGPTGDLSGIISSSQFDDMLKHRNDAACPARGFYTYNA
LOC_Os03g04060.1      ----------------------------VGSIITRAMFESMLSHRGDQGCQG-AFYTYDA
poplar_               ------------------------------------------------------------


LOC_Os06g51050.1      FVAAANAYPDFATTGDADTCKREVAAFLAQTSHETTGGWPTAPDGPYSWGYCFKEEN-NG
sorghum_              FIAAANAFPGFGTTGGADTQKRELAAFLAQTSHETTGGWATAPDGAYAWGYCFKEEQGAA
maize_                FIAAANAFPGFGTTGGADTQKRELAAFLAQTSHETTGGWDTAPDGPYTWGYCFKEEVGGV
At3g12500.1           FITAAKSFPGFGTTGDTATRKKEVAAFFGQTSHETTGGWATAPDGPYSWGYCFKQEQ--N
LOC_Os03g04060.1      FIKAAGDFPRFGTTGNDETRRRELAAFFGQTSHETTGGWATAPDGPFAWGYCRVNEITPS
poplar_               --------------------------------------WNLTASGPRPN-----------
                                                            *  :..*. .

LOC_Os06g51050.1      NAPTYCEPKPEWPCAAGKKYYGRGPIQIT~YNYNYGPAGQAIGSDLLNNPDLVASDATVSF
sorghum_              SGPDYCEPSTQWPCAAGKKYYGRGPIQIS~YNYNYGAAGQAIGAGILANPDLVASDPTVSF
maize_                WGPDYCQPSPQWPCADGQKYYGRGPIQLS~WNYNYGPAGEAIGQDLLGNPGLVAADATVSF
At3g12500.1           PASDYCEPSATWPCASGKRYYGRGPMQLS~WNYNYGLCGRAIGVDLLNNPDLVANDAVIAF
LOC_Os03g04060.1      DPP----------------YYGRGPIQLT2HKYNYQLAGDALGLDLVNNPDLVSSDPVVAF
poplar_               ---------------------PQG------SYHYGLIKTSRTITLANSGPIINRKQRYAV
                                            :*      .*:*     :    :  .  ::  .   :.

LOC_Os06g51050.1      KTAFWFWMTPQSPKPSCHAVITGQWTPSADDQAAGRVPGYGEITNIINGGVECGHG-ADD
sorghum_              ETAVWFWMTPQSPKPSCHAVMTGQWTPSGADTAAGRLPGYGVVTNIINGGLECGKG-ADS
maize_                ETALWYWMTPQPPKPSCHDVITGQWAPSPADVAAGRLPGYGVLTNIINGGLECGHG-ADA
At3g12500.1           KAAIWFWMTAQPPKPSCHAVIAGQWQPSDADRAAGRLPGYGVITNIINGGLECGRG-QDG
LOC_Os03g04060.1      RTAIWFWMTAQSPKPSCHDVITNQWTPSGDDRSSGRLPGYGMATNIINGGEECGKGYSTD
poplar_               N-GVSYIPADTPLKIADYFNIPGVFSLGSMPSS----PSWGNNAYLQTAVMSAN------
                      . .. :  :  . * : :  :.. :  .    :    *.:*  : : ..  ...

LOC_Os06g51050.1      KVADRIGFYKRYCDMLGVSYGDNLDCYNQRPYPPS------
sorghum_              RVADRIGFYKRYCDLLGVSYGDNLDCANQKPFNS-------
maize_                RVASRIGFYKRYCDMF-------------------------
At3g12500.1           RVADRIGFYQRYCNIFGVNPGGNLDCYNQRSFVNGLLEAAI
LOC_Os03g04060.1      NAKDRVGYYKRYCDMFRVGYGDNIACRDQKPYGGG------
poplar_               --------FREFIEIVFQNWEDTVQSWHID-----------
                              ::.: ::.
```

CLUSTAL W (1.83) multiple sequence alignment

```
maize_                   ------------------------------------------------------------
sorghum_                 --MGPLLRAAVLSVALLALAAVGGEAHSASDFLNIFKPRNEHDYFHNANQGQEEDVMPR-
LOC_Os01g48540.1         -MWPLLLRAAVVYAALLAAG----EADGSHDVLDIFGTRSESDYYRNAFQGKQGQAVPLP
LOC_Os05g48520.1         MGSSSLPRAAVLAVALLLLLAD--SGEAFFDLFSIFRPRSDSDYFP--FDGSPGQAKRKP
At5g19580.1              --MKASTRVIWTISVLMLAAVS--EAIFPLPFLPFLPG------FNNGFRDNEAVKVAKP


maize_                   ------------------------------------------------------------
sorghum_                 -----ASDQQNLITAPVSSTGLMNVPPRSAPTAVAQDTVVLPVDNAAGFPGAWSMISENA
LOC_Os01g48540.1         RGGGLRREQQELGAAGPGGSGLSKAPPRSAPSKVALDSLKLPVDTSAGFAGGWNLVSENS
LOC_Os05g48520.1         -----KIEQEEDGAAPATATGLTKVPPLGAPSKAALDTIVLPVDDSAGHAGSWTIVSENS
At5g19580.1              --------------QPAGAVG----------GKAARRQRGGGLDAQTTWGGKWELFLENS


maize_                   ------------------------------------------------------------
sorghum_                 GVSAMHMVIMHSD-KAIMFDTVTTGPSLLRLPKGNCRLDLRSKEVGAQDCAAHAVEFDYA
LOC_Os01g48540.1         GVSAMHLVVMQHG-KAIMFDTCTTGRSLMRLPPGRCRPDPRSKQPGAMDCWAHAVEFDYN
LOC_Os05g48520.1         GVSAMHLAVMRHG-KAIMFDTSTTGRSLMRLPMNNCRADPRAKREGTMDCWAHAVEFDYS
At5g19580.1              GVSGMHAILMPVINKVQYYDATIWRISKIKLPPG-VPCHVVDAKTNKVDCWAHSILMDVN


maize_                   -----------------------------------------------------------M
sorghum_                 TNGVRALKILTDVWCSSGALDAEGNLVQTGGYFEGEKVVRYLSPCGNCDWREFPGSLAEG
LOC_Os01g48540.1         TGALRSLKIVTDTWCSSGAFDADGNMVQTGGFFEGDKSVRYLSACGTCDWKEFPKSLADG
LOC_Os05g48520.1         TGALRSLKTATDTWCSSGAFDADGNLIQTGGYFEGDKAVRRLDACDTCDWREYPNSFAEG
At5g19580.1              TGALKPLGLSTDTWCSSGGLTVNGTLVSTGGYGGGANTARYLSSCENCKWEEYPQALAAK


maize_                   R~RYGTQQLLPDGRSIVLGGRRAFSYEFVPAEGQSNAQAIPLQILRDTTDDVENNLYPFVH
sorghum_                 R~WYGTQQILPDGRSIVLGGRRAFSYEFVPAEGQSNAQANPLQILRDTTDDVENNLYPFVH
LOC_Os01g48540.1         R2WYGTQLVLPDGSFIVIGGRRAFSYEFVPAAGRANARATPLRLLRDTTDDVENNLYPFVN
LOC_Os05g48520.1         R~WYATQQVLPDGRFIVFGGRRAFSYEFVPQPGMTNGQSIKFPLLRETTDDVENNLYPFVN
At5g19580.1              R~WYSTQATLPDGKFFVIGGRDALNYEYIPEEGQNNRKLFDSLLLRQTDDPEENNLYPFVW
                         * *.**  ****  :*:*** *:.**::*  *  * :     :**:* *  ********

maize_                   LLPDGTLFIFANDRSILFDPRNGQVVREFPVLPGGGRNYPASGMSALLPLDLR---RGDV
sorghum_                 LLPDGTLFIFANDRSILFDPRNGQVVRELPVLPGGGRNYPASGMSALLPLDLR---RGDV
LOC_Os01g48540.1         LLPDGTLFIFANDRSIVFNYRTGQVVRELPILPGGSRNYPASAMSTLLPLDLR---KGAG
LOC_Os05g48520.1         LLPDGNLFVFANDRSVVFDHRTGKVVRELPKLAGGGRNHPASAMSAMLPLDLRNLTRGAD
At5g19580.1              LNTDGNLFIFANNRSILLSPKTNQVIKEFPQLPGGARNYPGSGSSALLPIQLYVK-NPKV
                         * .**.**:***:**:::. :..:*::*:* *.**.**:*.*. *::**::*    .

maize_                   LSPEVIVCGGSPKNAFTLGESNTFPPALKDCARINPLKPDARWALDQMPVARTMGDLLIL
sorghum_                 LSPEVIVCGGAPKNAFKLGEANTFNAALKDCARINPLKPGARWATDQMPVPRTMGDLLVL
LOC_Os01g48540.1         LSAEVIICGGATKNAFKLGETSTFPPALRDCARINPSKPGARWALDQMPSGRVMGDVLIL
LOC_Os05g48520.1         PEPEVIVCGGALKTAFRLGENNTYQPTLRDCARINLGKIDAVWAVEAMPVGRVMGDLLVL
At5g19580.1              IPAEVLVCGGSKQDAYYKAGKKIYEPALQDCARIRINSAKPRWKTEMMPTPRIMSDTVIL
                           .**::***: : *:  .  . : .:*:*****.  .  . *  : **  * *.* ::*

maize_                   PTGDLLILNGAAKGCSGWGFGRQPVLSPLLYSPRQARGSRFRALAPTTIPRMYHATSAVL
sorghum_                 PTGDLLMLNGAAKGCSGWGFGRQPVLSPLLYTPRLKRGSRFRALAPTTIPRMYHASSAVL
LOC_Os01g48540.1         PTGDLLMLNGAAKGCSGWGFGRQALLSPVLYSPYLRRGKRFRVLNPSNIPRMYHSTSALL
LOC_Os05g48520.1         PTGDLLMLNGAAKGSSGWGFARQPILSPILYSPRHPEGSRFRPLAASTVARMYHSTSAVL
At5g19580.1              PNGDILLVNGAKRGCSGWGYGKDPAFAPLLYKPHAARGKRFRQLKPTTIPRMYHSSAIIL
```

```
                      *.**:*::*** :*.****:.::. ::*:**.*   .*.*** * .:.:.****::: :*

maize_                PDATVLVAGSNTNSAYNFSGVDFQTEVRVERFTPPYLAPERAANRPAIDVATVPGDGMAY
sorghum_              PDATVIVAGSNTNSAYNFSGVDFQTEVRVERFTPPYLSPELAANRPVIDVGTVPGDGMAY
LOC_Os01g48540.1      PDATVLVAGSNTNSAYNFSGVDFPTEVRVERFTPPYLSPQLSPNRPAIDAASVPGDGMRY
LOC_Os05g48520.1      PDATVLVAGGNTNAAYNFSGVDFPTEVRVERFAPPYLSRELTGNRAVIDVASVPAGGMRY
At5g19580.1           PDGKVLVGGSNTNDGYKYN-VEFPTELRVEKFSPPYLDPALANIRPKIVTTGTP-KQVKY
                      **..*:*.*.*** .*::. *:* **:***:*:****    :  *. * .  .*   : *

maize_                GAKFTFQFSTPVQAVAEPDLKVTMYAPPFTTHGYSMNQRLLVLSVTAFAANGQRYTITVD
sorghum_              GAKFTLQFSTPGQAVVQDDVKVTLYAPPFTTHGYSMNQRLLVLSVTTFTADGQRHTVTVD
LOC_Os01g48540.1      GARFTFRFTTPAQGVGQGDFKVTMYAPPFTTHGYSMNQRLLILPVTAFAAQGQRHTVTVD
LOC_Os05g48520.1      GTKFTFRFHTPVAAVEWGDVRVTMYAPPFTTHGYSMNQRLLVLPVAGFSAQGQMYELTVD
At5g19580.1           GQFFNVKVDLKEKGATKGNLKVTMLAPAFTTHSISMNMRMLILGVNNVKPAGAGYDIQAV
                      *  *..:.     ..   :.:**: **.****. *** *:*:* *  . . *  : : .

maize_                APGKPELAPPGYYLLYVIAKGVPSKAAWVKVHK
sorghum_              APGKPELAPPGYYMLYVIAKGVPSKAAWVKVHK
LOC_Os01g48540.1      APPKPELAPPGYYMVYVVAKGVPSKAAWVKMHK
LOC_Os05g48520.1      TPRKPELAPPGYYLVYVVSKDVPSEAAWVKIQ-
At5g19580.1           APPNGNIAPPGYYLIFAIYKGVPSTGEWIQVV-
                      :* : ::******:::.: *.*** . *:::
```

```
CLUSTAL W (1.83) multiple sequence alignment


LOC_Os11g45720.1      --------------MDRPNLAVAVVGLLAVVAATLP-------APS--WQFFDLFLPAGP
maize_                ------------------------------------------------------------
LOC_Os12g37660.1      ---MEIISPSSSSNNNSPVLATFLVVLVVLLASSRP-------ASSQNQQSFTINPGGAA
sorghum_              MARQNCHRPSGVALVAIAITATTLLLPCLVSVASSPEEEPLPSSSSSSDDSVAGNGGYGA
At5g61680.1           ----------------MGYNYVSLIVTILLVVITSP-------------VVFGNDAAPIP


LOC_Os11g45720.1      SHRSSGG--GFGKWVLMNHEEYVEKKSLYAMKAAGD-----IGG--KTIDASLSAAEEAK
maize_                ------------------------------------------------------------
LOC_Os12g37660.1      AARPGGGKGGGGGGGPGSFSDFLTQNVQHYVLSEQK-----YAGKVKALDAELSAAEAGA
sorghum_              TNNNNIKMPAADGGAAGSFTEFVTENVELYSNVSSEQHKYGAGAGGKVWDPELLAAQGMA
At5g61680.1           ENK-------------GRIEQWFNTN----------------------------------


LOC_Os11g45720.1      VTWVVDPKGTPGDTTFTTIAAALEKVPEGNTKRVILDLKPGAEF~REKLLLNITKPYITFK
maize_                --------------------------------------------^----------------
LOC_Os12g37660.1      ARYVVSGDGKG---KFRTITEAIKAVPEYNKKRVILDIRPG-TY2KEKLLIPFTKPFITFV
sorghum_              LRYVVSPDGKG---KFRSINEAIKAVPDGNKRRVILDIRTA-TY~KEKVVVPYMKPFVTFS
At5g61680.1           ----VKQNGRG---HFKTITEAINSVRAGNTRRVIIKIGPG-VY~KEKVTIDRSKPFITLY


LOC_Os11g45720.1      SDPANPAVIAWNDMAATRGKDGKPVGTVGSTTVAVESDYFMAYGVVFKNDAPLAKPGAEG
maize_                -----------------------PVGTVGSATLAVESDYFTAYGVVFRNDAPLAKPGAKG
LOC_Os12g37660.1      GNPRSPPTIMWDDRAATHGKDGQPMGTMLSATVAVEADYFMASSIIFKNNAPMAAPGAHG
sorghum_              GNPKNPPVIMWDDRAATRGKDGKPVGTYGSATVAVESDYFMASGVHFKNAAPLAAPGTEG
At5g61680.1           GHPNAMPVLTFDGTAAQYG-------TVDSATLIVLSDYFMAVNIILKNSAPMPDGKRKG
                                                *  *:*: * :*** * .: ::* **:.    .*
```

```
LOC_Os11g45720.1      GQAVALRLFGTKAAIYNCTIDGGQDTLYDHKGLHYIKDSLIMGSVDFIFGFGRSLYEGCT
maize_                GQAVAVRLFGTKTQIYNCTIDGGQDTLYDHKGLHYFKGCLIRGSVDFIFGFGRSFYEDCR
LOC_Os12g37660.1      GQAVALRVFGSKVAMYNCTIDGGQDTLYDHKGLHYFKNCLIRGSVDFIFGFGRSLYADCT
sorghum_              GQAVAVRVYGNKAAFYDCTFDGGQDTLYDHRGLHYFKSCHIQGTVDFIFGFGRSLYEDCA
At5g61680.1           AQALSMRISGNKAAFYNCKFYGYQDTICDDTGNHFFKDCYIEGTFDFIFGSGRSLYLGTQ
                      .**:::*: *.*. :*:*.: * ***: *. * *::*.. * *:.***** ***:* .

LOC_Os11g45720.1      IVSVTKEVSVLTAQQRTKTIEGAIESGFSFKNCSIKGQ---GQIYLGRAWGDSSRVVYSY
maize_                IESVVKEVAVLTAQQRSKSIEGAIDTGFSFKNCSIGGVK-GGQIYLGRAWGDSSRVVYSY
LOC_Os12g37660.1      IESVTKEVAVVTAQQRSKNIAEAIDTGFSFLRCKISGI---GQIYLGRAWGDSSRVVYSY
sorghum_              ITSVTKDVAIVTAQQRTRSIADALETGFSFLRCRIGSSTGAGQIYLGRAWGDSSRVVYAY
At5g61680.1           LNVVGDGIRVITAHAG---KSAAEKSGYSFVHCKVTGTG--TGIYLGRSWMSHPKVVYAY
                      :  * . : ::**:        * .:*:** .* : .      *****:* . .:***:*

LOC_Os11g45720.1      TDMSKEVVPIGWDGWNIAKPESSGIYYGEFKCTGPGSDAKK--RVGWALDLTADQAKPFI
maize_                TKMGEEVVPVGWDGWQIAKPESSGIYYGEFKCFGPGADAKKKKRVGWALDLTEAQAKPFV
LOC_Os12g37660.1      TTMGKEVVPIGWDGWEVQKPEHSGIYYGEYKCSGPGALPSK--RIGWSLVLSDIQAKPFT
sorghum_              TTMGKEVVPVGWDKWTVQKPEHSGIYYGEYQCSGPGALPHK--R----------------
At5g61680.1           TDMSSVVNPSGWQENREAGRDKT-VFYGEYKCTGTGSHKEK--RVKYTQDIDDIEAKYFI
                      * *.. * * **:       : : ::***::* *.*:   *  *

LOC_Os11g45720.1      GTHYIYGDSWILPPPDGKSAASTSTASKSTASAIPRNSTAPATATESNSTAPATPSNSTA
maize_                GTHYVLGDTWIQPPPK--------------------------------------------
LOC_Os12g37660.1      GSHFVYGDSWILPPPKSM------------------------------------------
sorghum_              ------------------------------------------------------------
At5g61680.1           SLGYIQGSSWLLPPPSF-------------------------------------------


LOC_Os11g45720.1      PVTASNSTAPATASSSNPPATKSYSGPPATPSASSTPAKASR
maize_                ------------------------------------------
LOC_Os12g37660.1      ------------------------------------------
sorghum_              ------------------------------------------
At5g61680.1           ------------------------------------------

CLUSTAL W (1.83) multiple sequence alignment


maize_                ------------------------------------------------------------
sorghum_              ------------------------------------------------------------
LOC_Os12g02840.1      ------------------MAAHTVTDPLEELWNHTMSMDKTHLMCFYPSKITMGGVWTGD
LOC_Os05g40650.1      MAPIMSGAAAAAGGTGGAVPLIKNATSASQMSRGKAGTGAGAVVCYSPMMVTAYGIWQGA
At2g13620.1           ------------------------------MATSEEPSTDASIICYAPSMITTNGVWQGD


maize_                ------------------------------------------------------------
sorghum_              ------------------------------------------------------------
LOC_Os12g02840.1      NPLDFSIPLLLFQILLITSTTRAATLLLSPLRLPTYISQILAGFLLGPSVLGHLPHFSNL
LOC_Os05g40650.1      SPLDFSLPLFLLQVAIIVATTRLLVILLKPFRQPRVIAEILAGVILGPSVMGQVSTWAVK
At2g13620.1           NPLDFSLPLFVLQLTLVVVVTRFFVFILKPFRQPRVISEILGGIVLGPSVLGRSTKFAHT


maize_                ------------------------------------------------------------
sorghum_              ------------------------------------------------------------
LOC_Os12g02840.1      VFPVRSLFVLESMALLGLVYYTFIVGVEIEVSAITRAGIRSFGFAIGCTLPPFLVGALTG
LOC_Os05g40650.1      VFPERSLLTLETVAHLGLLYFLFLVGLEMDVNTIRRSGKKALIIAVAGMALPFCIGTATS
At2g13620.1           IFPQRSVMVLETMANVGLLYFLFLVGVEMDIMVVRKTGKRALTIAIGGMVLPFLIGAAFS
```

```
maize_                 ------------------------------------------------------------
sorghum_               ------------------------------------------------------------
LOC_Os12g02840.1       YVALSTDDKHKGDTFLNKLSFPIFLGSTFSSTAFAVLARNIAELKLAGTDVGQLTLSASL
LOC_Os05g40650.1       FIFR-----HQVSKNVHQASFLLFLGVALSVTAFPVLARILAEVKLLNSDLGRIAMSAAI
At2g13620.1            FSM------HRSEDHLGQGTYILFLGVALSVTAFPVLARILAELKLINTEIGRISMSAAL


maize_                 ------------------------------------------------------------
sorghum_               ------------------------------------------------------------
LOC_Os12g02840.1       INDTFAWTGLTVATVLGHSRCTITQTTWTLTSGVVIFGASYLLLRPMLLRLARRAAEGEA
LOC_Os05g40650.1       VNDMCAWILLALAIAISEVNSSAFSSLWVLIAGVAFVLACFYVVRPLMWWIVRRVPEGEA
At2g13620.1            VNDMFAWILLALAIALAESDKTSFASLWVMISSAVFIAVCVFVVRPGIAWIIRKTPEGEN


maize_                 ------------------------------------------------------------
sorghum_               ------------------------------------------------------------
LOC_Os12g02840.1       VGEDRECWILIGVMVAALVADAGGTHAIFGAFVFGLAVPNGPVGVALVEKVEDFVVGALL
LOC_Os05g40650.1       IGDVHITLILTGVMVAGVCTDAIGIHSVFGAFVYGLVMPSGPLGVVLIEKLEDFVTGLLL
At2g13620.1            FSEFHICLILTGVMISGFITDAIGTHSVFGAFVFGLVIPNGPLGLTLIEKLEDFVSGLLL


maize_                 ------------------------------------------------------------
sorghum_               ------------------------------------------------MPLLDGTSIGLL
LOC_Os12g02840.1       PLFFALSGLRTDTAKITNMHSAVLLMVAAMVAAVLKVVAAIGVAGVFGMPLGDGTSIGLL
LOC_Os05g40650.1       PLFFAISGLRTNVTKVRDPITVGLLVLVFVMASFAKIMGTILIAVSYTMTFRDGVALGFL
At2g13620.1            PLFFAISGLKTNIAAIQGPATWLTLFLVIFLACAGKVIGTVIVAFFHGMPVREGITLGLL


maize_                 -------------------^-MSDQSFTVLVFMSALITALVTPLLALVVKPARRLVFYKRR
sorghum_               LNTKGIIELIILNIGRNKR~IMSDQSFTVLVFMSALITALVTPLLALVVKPARRLVFYKRR
LOC_Os12g02840.1       LNTKGIIELVILNIARNKG0IMSDQSFTVLVFVSALITAMVSPFLGMVVKPARRLVFYKRR
LOC_Os05g40650.1       MNTRGLVEMIVLNIGRDKE~VLDDESFAVMVLVSVAMTALVTPVVTTVYRPARRLVGYKRR
At2g13620.1            LNTKGLVEMIVLNVGKDQK~VLDDETFATMVLVALVMTGVITPIVTILYKPVKKSVSYKRR
                                           :.*::*:.:*:::  :*.:::*.:  : :*.:: * ****

maize_                 TIAWPQLEAEFHVLVCVHMPRDVPALLTLLDVASPSDRSPVAVQALHLIEFAGRSSALLL
sorghum_               TIAWPQPDAEFHVLACVHMPRDVPAVLTLLDVASPSDRSPVALQALHLIEFAGRSSAMLL
LOC_Os12g02840.1       TVAWAHPESELRVLACVHVPRDVPALLTLLDVVTPSSRSPVGVLALHLIEFVGRSSALLL
LOC_Os05g40650.1       NLQRSKHDAELRMLACVHTTRNVPSIISLLELSNPTKRSPIFIYALHLVELTGRASNMLA
At2g13620.1            TIQQTKPDSELRVLVCVHTPRNVPTIINLLEASHPTKRSPICIYVLHLVELTGRASAMLI
                       .:  .: ::*:::*.*** .*:**:::.**:   *:.***: : .***:*:.**:* :*

maize_                 INASAPSSS-FEHSVHRRSQVELQFKHIAHAFMAYEENVAGVLARTMAAVSPYATMHDDV
sorghum_               INASAPSSS-FEHSAHRRSQVELQFKHIAHAFMAYEENVAGVSTRTMAAVSPYATMHDDV
LOC_Os12g02840.1       INASAPSSSSYDASVHGRSHTEMQFKHISHAFMAYEEQSVGVSARTMAAVSPYASMHEDI
LOC_Os05g40650.1       AHHSASN-------------PGGASDHIFNAFESYEEMVGGVSVQALTAVSPYQTMHEDV
At2g13620.1            VHNTRKSGR------PALNRTQAQSDHIINAFENYEQHAAFVAVQPLTAISPYSTMHEDV
                        : :  .                  .** :**  **:    * .:.::*:*** :**:*:

maize_                 TSAAEEQHSALILLPFHKHRSVDGGLEVFHPEIQALNQSIQRFSPCTVGILVDRGLGGVP
sorghum_               TAAAEEQHSALILLPFHKYRSVDGGMEVSHPAIQPLNQSIQLFSPCTVGILVDRGLGGVP
LOC_Os12g02840.1       TSAAENQHSALILLPFHKYRSVDGGLEVSHPAIQPLNCSVQSFSPCTVGILVDRGLAAVP
LOC_Os05g40650.1       CVLAEDKHVSLIVLPFHKQQTVDGGMEPINASLRGFNESILASAPCSVGILVDRGLSAAA
At2g13620.1            CSLAEDKRVSFIIIPFHKQQTVDGGMESTNPAYRLVNQNLLENSPCSVGILVDRGLNGAT
                          **::: ::*::**** ::****:*  :.  : .* .:   :**:********* ...

maize_                 GA-----GCRVAALFFGGRDDREVAALAIRMVSNPAIDLTLLRFAQKG------------
sorghum_               GA-----GCRVAALFFGGRDDREVAALAIRMVYNPAVDLTLLRFAQKG------------
LOC_Os12g02840.1       GG-----GYRVVALFFGGSDDREVAALATRMVRNPTIDLTLLRFVQKG------------
```

```
LOC_Os05g40650.1      AR--MAAVHHVALLFFGGPDDREGLAYAWRMVENPGVCLTIVRLIPPGYTAPAISPPQPP
At2g13620.1           RLNSNTVSLQVAVLFFGGPDDREALAYAWRMAQHPGITLTVLRFIHDEDEADTASTR---
                               :*. ***** ****  * * **. :* : **::*:

maize_                ------GSFTGTEFDALKERKADDGILREFLDRANSVS--GGGGAGVEYRERGVFNASEM
sorghum_              ------GSFTGTEFDALKERKADDAILREFLDRANSVSAGGGGGAGVEYRERGVFNASEM
LOC_Os12g02840.1      ------GSFTASEFDALKERKADEGCLRDFLERAN-----EGGGATVEYRERGVFNASEM
LOC_Os05g40650.1      MPAAHSRAINVVPEVAKSERQMDEEYLNEFRSRNL-------GNDAILYVEQVVANSEET
At2g13620.1           --ATNDSDLKIPKMDHRKQRQLDDDYINLFRAENA-------EYESIVYIEKLVSNGEET
                              :.       .:*: *:  :. *  .             : * *: * *..*

maize_                VAQIREVEALG-KDLFVVGKVPGLPA--LTAGMAEWSECPELGPIGDLLSSKDFQTTASV
sorghum_              VAQIREVEALG-KDLFVVGKVPGLPA--LTAGMAEWSECPELGPIGDLLSSRDFHTMASV
LOC_Os12g02840.1      VGEIQSVEAMGNKDLFVVGKVPGGSG--LTAGMAEWSESPELGPIGDLLASKDFQTTASV
LOC_Os05g40650.1      VAAIRSQLDNA-HELYIVGRHPGEASSPLTSALAEWMESPELGPIGDLLVSSEFSKMASV
At2g13620.1           VAAVRS-MDSS-HDLFIVGRGEGMSS-PLTAGLTDWSECPELGAIGDLLASSDFAATVSV
                      *. ::.    . ::*::**:  * ..  **:.:::* *.****.***** * :*   .**

maize_                LVILYYLRH---------------------------------------------------
sorghum_              LVLQSYARPSAGG--MSAELGLGVGGDGVPAAGRPPRPDQIRRGSLGNRS----------
LOC_Os12g02840.1      LVLQAYGRPAAVVGAGAGAMSVDFGGDSVAMAERTASGRRPWARPGV-------------
LOC_Os05g40650.1      LVMQQYVITAPLPPPVALAGPPTDDPVRQYLTNANQRPSVAIGGNQMGAAGRGGWSGGAG
At2g13620.1           LVVQQYVGSWAQEDDMDFP----ESPVHSHETKVTYGLENPR------------------
                      **:  *

maize_                --
sorghum_              --
LOC_Os12g02840.1      --
LOC_Os05g40650.1      GY
At2g13620.1           --
```
